# Discovery of Two Structurally Distinct Classes of Inhibitors Targeting the Nuclease MUS81 and Enhancing Efficacy of Chemotherapy in Cancer Cells

**DOI:** 10.1021/acs.jmedchem.5c02096

**Published:** 2026-02-25

**Authors:** Jana Prochazkova, Benoit Carbain, Victoria Marini, Fedor Nikulenkov, Stepan Havel, Naresh Akavaram, Prashant Khirsariya, Alexandra Sisakova, Jakub Cibulka, Michala Boudova, Magdalena Zacpalova, Magdalena Kalovska, Joana Rodrigues, Lukas Daniel, Jan Brezovsky, Petr Bartunek, Claus Azzalin, Kamil Paruch, Lumir Krejci

**Affiliations:** † Department of Biology, Faculty of Medicine, 37748 Masaryk University, 62500 Brno, Czech Republic; ‡ Department of Chemistry, Faculty of Science, Masaryk University, 62500 Brno, Czech Republic; § International Clinical Research Center, St. Anne’s University Hospital, 656 91 Brno, Czech Republic; ∥ NCBR, Faculty of Science, Masaryk University, 62500 Brno, Czech Republic; ⊥ 705996 GIMM - Gulbenkian Institute for Molecular Medicine, 1649-035 Lisbon, Portugal; # Loschmidt Laboratories, Department of Experimental Biology and RECETOX, Faculty of Science, Masaryk University, 62500 Brno, Czech Republic; ¶ Laboratory of Biomolecular Interactions and Transport, Faculty of Biology, Department of Gene Expression, Institute of Molecular Biology and Biotechnology, Faculty of Biology, Adam Mickiewicz University, 61-614 Poznan, Poland; ∇ Institute of Molecular Genetics of the Czech Academy of Sciences, 14220 Prague, Czech Republic; ○ Faculty of Medicine, University of Lisbon, 1649-028 Lisbon, Portugal

## Abstract

Nucleases are promising pharmacological targets due to their essential role in maintaining genomic stability. They are crucial for regulation of cell viability, and their modulation is exploitable in disease prevention and treatment, including cancer. The conserved structure-specific endonuclease MUS81 resolves branched DNA intermediates during replication, repair, and recombination. Aberrant MUS81 activity causes DNA damage, chromosomal abnormalities, and genome instability, contributing to oncogenesis. Thus, pharmacological targeting of MUS81 is an attractive yet underexplored therapeutic strategy. We describe the discovery of two chemically distinct small-molecule classes of MUS81 inhibitors, exemplified by compounds MU262 and MU876. Both compounds effectively inhibit MUS81 in vitro and in cells, sensitizing cancer cells to DNA-damaging agents by impairing DNA repair. These inhibitors can also serve as chemical biology tools for a deeper study of MUS81 function and as leads for drug discovery aimed at therapies exploiting DNA repair vulnerabilities in cancer treatment.

## Graphical abstract

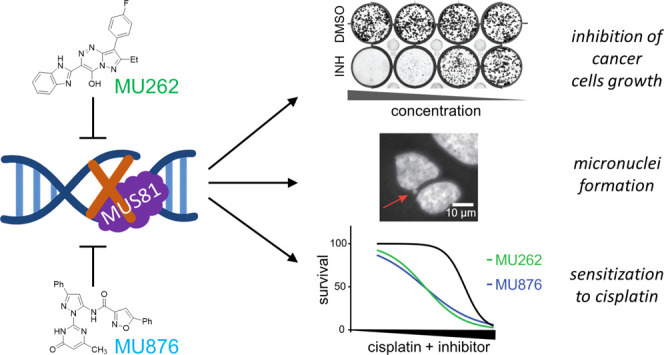

## Introduction

Genomic integrity is fundamental for cell survival and the prevention of diseases associated with uncontrolled proliferation, such as cancer. To maintain genomic stability, cells rely on various DNA repair pathways, including homologous recombination (HR), in which nucleases play critical roles. Among these, the structure-specific endonuclease MUS81 of the conserved XPF/MUS81 family emerges as key players in maintaining genome integrity.^1^ MUS81 forms a heterodimer with either EME1 or EME2, catalytically inactive partners, which together function in DNA repair and replication fork processing.^2–4^ The importance of MUS81 for cellular homeostasis is manifested by the increased tumor incidence in MUS81-knockout mice, demonstrating its role as a tumor suppressor.^5,6^ Clinically, reduced MUS81 expression correlates with poor prognosis in hepatocellular and colorectal cancers,^7,8^ while elevated levels are associated with increased migration and metastasis in gastric and ovarian cancers,^9,10^ highlighting its context-dependent dual role in tumorigenesis. EME1 is also upregulated in cancer types, and its depletion leads to cell cycle arrest and apoptosis,^11^ further confirming the reliance of some cancers on MUS81-EME1 activity. Collectively, these findings establish MUS81 as an attractive therapeutic target.^7,12–17^

At the molecular level, MUS81 preferentially cleaves branched DNA structures, such as 3′flap, replication forks, and nicked Holliday junctions, intermediates frequently formed during recombination or replication. By resolving these structures, MUS81 ensures accurate chromosome segregation during mitosis.^18–21^ Its dysfunction leads to hallmark features of genome instability, including chromosomal abnormalities including micronuclei, anaphase and ultrafine bridges, and sister chromatin exchanges.^19,22–24^ MUS81 also plays a critical role in processing stalled replication forks.^25–27^ When forks stall due to obstacles or lesions, MUS81-mediated cleavage enables their restart or resolution,^23,28–34^ often through the generation of transient double-strand breaks (DSBs) that are subsequently repaired by break-induced replication (BIR).^31,35^ Consistent with this role, MUS81-deficient cells are hypersensitive to replication stress-inducing agents such as hydroxyurea (HU) or camptothecin (CPT).^24,31^ Dysregulated fork processing, including replication–transcription collisions, is increasingly recognized as a major driver of genomic instability and cancer development,^36,37^ with MUS81 facilitating replication restart in these contexts.^38,39^

Given its essential functions in DNA repair and replication fork processing, MUS81 has emerged as a promising target for cancer therapy. Selective inhibition of MUS81 may induce synthetic lethality in cancer cells with compromised DNA repair pathways^40–43^ and enhance the efficacy of existing chemotherapies and radiotherapies.^15,44–46^ Despite its therapeutic potential, the development of small-molecule MUS81 inhibitors remains underexplored. To date, only two reports have described small-molecule inhibitors, Dyngo-4a and compound **23** (Figure 1).^47,48^ While these compounds demonstrated biochemical inhibition of MUS81, their mechanisms of action remain undefined, and evidence of cellular efficacy is minimal. Currently, no MUS81 inhibitors with well-characterized cellular activity and translational potential are available. This represents a significant unmet need in the field and highlights the importance of discovering and characterizing novel MUS81 inhibitors with defined mechanisms of action and validated cellular activity.

**Figure 1:**
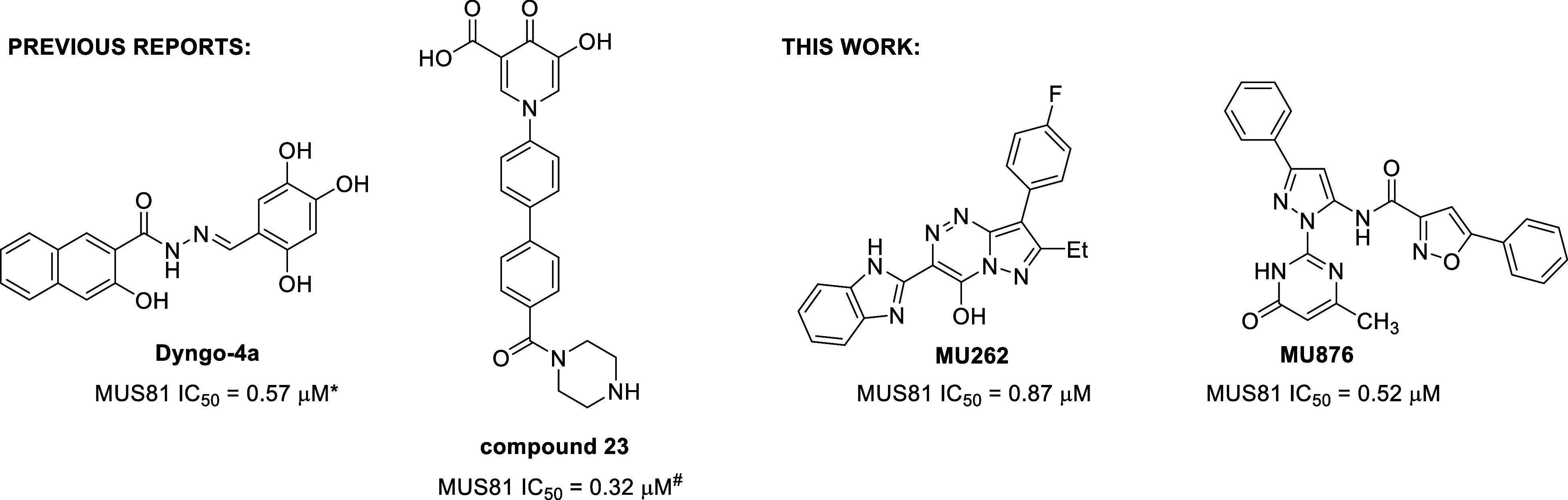
Chemical structures of previously reported MUS81 inhibitors (**Dyngo-4a** and compound **23**) and the newly identified MUS81 inhibitors **MU262** and **MU876** described in this study and their respective IC_50_ values. Note: IC_50_ values for the previously reported inhibitors were determined using the corresponding published assays (*FRET-based DNA cleavage assay; ^#^differential scanning fluorimetry assay) and may therefore not be directly comparable to those reported here.

Our study identifies two classes of novel small-molecule inhibitors of MUS81, exemplified by compounds **MU262** and **MU876** (Figure 1), using two complementary high-throughput screening approaches. We describe structure–activity relationship (SAR) development in both series, leading to the identification of the most potent compounds. In addition, we assessed the compounds’ impact on MUS81 nuclease activity in vitro and elucidated the mechanistic aspects of MUS81 inhibition, including the compounds’ binding to the target protein and a possible mode of action. We also show the impact of the newly discovered inhibitors on MUS81-dependent cellular processes such as recombination-dependent repair pathways and formation of chromosomal aberrations. Importantly, the inhibitors reported herein potentiate the effects of the chemotherapeutic drug cisplatin and prevent cancer cell proliferation. In conclusion, the MUS81 inhibitors described in this study can be used as both molecular biology tools for the elucidation of the biological functions of MUS81 in genomic maintenance and as lead compounds for developing targeted cancer therapies that exploit the inherent genomic instability of tumor cells.

## Results

### Identification of Small-Molecule Inhibitors of MUS81

To identify the small-molecule inhibitors of MUS81, we used two complementary screening approaches. The first involved a structure-based virtual screening, where over 140,000 small molecules were docked into the active site of MUS81 ^49^ using AutoDock Vina software.^50^ The predicted binding energies of these compounds ranged from −9.6 to −3.0 kcal mol^–1^. Compounds with a binding energy lower than −8.0 kcal mol^–1^ were selected for further analysis, yielding a set of 9074 candidates. These compounds were grouped into 27 clusters based on their predicted interactions with MUS81 using AuposSOM tool.^51^ Each cluster represented a unique type of binding mode within the enzyme’s active site. Visual inspection of the top candidates using PyMol^52^ confirmed their potential to efficiently block the catalytic site of MUS81. From this initial screening, 99 commercially available molecules, representing these clusters proportionally to the cluster sizes, were selected for further evaluation based on their predicted dissociation constants (*K*_D_) using neural network scoring function NNScore 2.0 ^53^ prioritizing compounds with values below 1 μM. A final set of 20 compounds (Supplementary Table 1) were purchased and evaluated in an in vitro nuclease assay using a 3′flap substrate, a well-established and relevant substrate for MUS81.^18^ The cleavage by purified MUS81-EME1 was monitored by gel electrophoresis, and the compound activity was quantified by measuring the reduction in cleavage product intensity. Several compounds showed inhibitory activity with IC_50_ below 30 μM. Among them, the compound **1** (Scheme 1) exhibited the most potent inhibition, with IC_50_ ∼5 μM and was selected for further optimization and characterization.

**Scheme 1:**
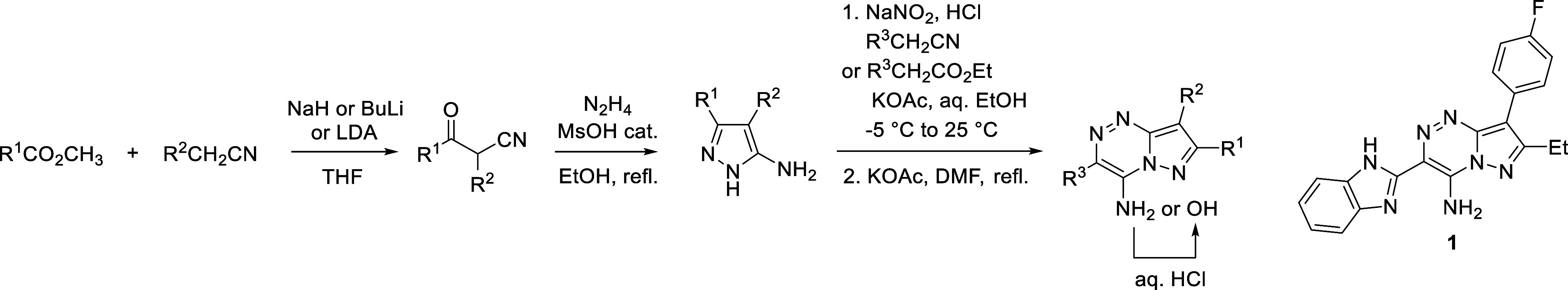
Synthetic Route Used for the Preparation of the Compound **1** and Its Analogues

The second approach included a high-throughput in vitro screening of approximately 100,000 small molecules from the UCLA Molecular Screening Shared Resource compound libraries. For this, we designed a fluorogenic assay specific for MUS81-EME1, consisting of a 3′flap DNA substrate labeled with fluorescein (FAM) and a Black Hole Quencher (BHQ1; Figure S1). Upon endonucleolytic cleavage by recombinant MUS81-EME1, the fluorophore is separated from the quencher, resulting in a significant fluorescence increase, which provides a direct and quantitative readout of the enzymatic activity (Figure S1). Inhibitor activity is reflected by a corresponding reduction in the fluorescent signal. From the initial screen, several hundred compounds showing reduced fluorescence were selected for validation in a dose–response assay. Of these, 72 compounds were confirmed to inhibit MUS81-EME1 nuclease activity. Based on the potency (IC_50_ values) and availability, 23 hits were purchased for further testing (Supplementary Table 2). Among these, compound **2** (Scheme 2) was selected as the lead candidate for further development based on its favorable in vitro activity profile.

**Scheme 2:**
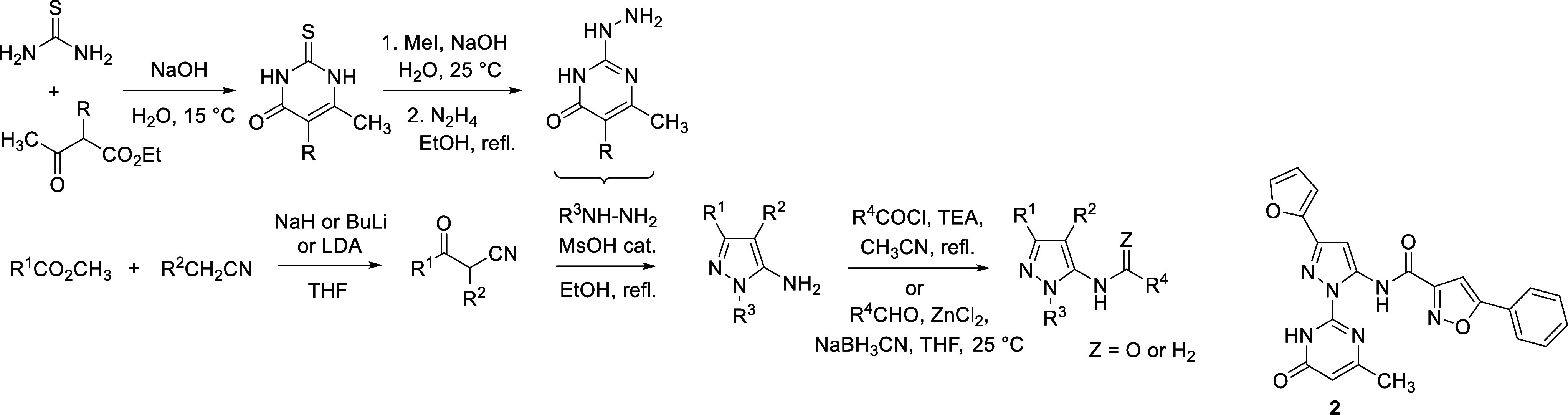
Synthesis of the Compound **2** and Its Analogues

The candidate compounds **1** and **2** from both screens are both relatively small (MW = 373 and 428 g/mol, respectively), providing opportunities for further chemical modification and optimization of biological and physicochemical properties (e.g., activity, selectivity, and aqueous solubility). These candidate compounds were thus subjected to medicinal chemistry optimization and SAR development, as described below.

### SAR Optimization of the Lead Compounds from the In Silico Screening

The hit **1** was first resynthesized using the route depicted in Scheme 1. Specifically, condensation of properly substituted β-ketonitrile with hydrazine, followed by diazotization, reaction with cyanomethylbenzimidazole, and final cyclization, provided the target compound (Scheme 1). Upon confirmation of the activity of the resynthesized compound **1**, the methodology was used for the preparation of additional analogues and development of the SAR in the series.

The SAR development in this series consisted of changing all four substituents around the pyrazolo­[5,1-*c*]­[1,2,4]­triazine scaffold (Table 1 and Supplementary Table 1). We took into account the binding mode of the compound **1** docked in the active site of MUS81 (Figure S2), which suggested the N atoms of the benzimidazole motif and the amino group at position 4 of the scaffold to be important for interaction with the Mg^2+^ ion of the active site. On the other hand, the structure pointed to the possibility of modifying the ethyl group and the fluorophenyl substituent. However, the observed SAR trends were found to be rather empirical, as described below. Rather unpredictably, deletion of the substituents R^1^ (ethyl in **1**) led to a significant decrease of MUS81 inhibitory activity, as illustrated by the compound **4** (Table 1). Less potent were also the analogues with removed or alternatively substituted R^2^ phenyls (compounds **3**, **5**, **6**, **7**, **8**, and **9**), cyclohexyl analogue **10**, and the compound **11** with R^1^ ethyl replaced with isopropyl (Table 1). Interestingly, the lack of 4-fluorophenyl motif could be compensated by larger substituents R^1^, exemplified by compounds **12, 13**, **14**, and **15** (Table 1). However, these compounds were found to be only sparingly soluble in aqueous DMSO.

**Table 1:**
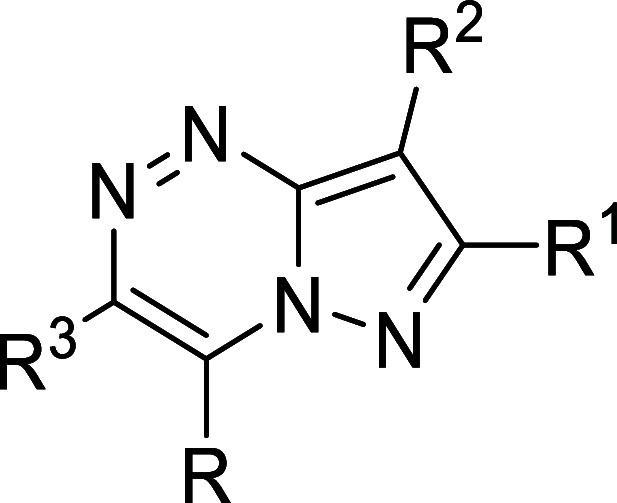

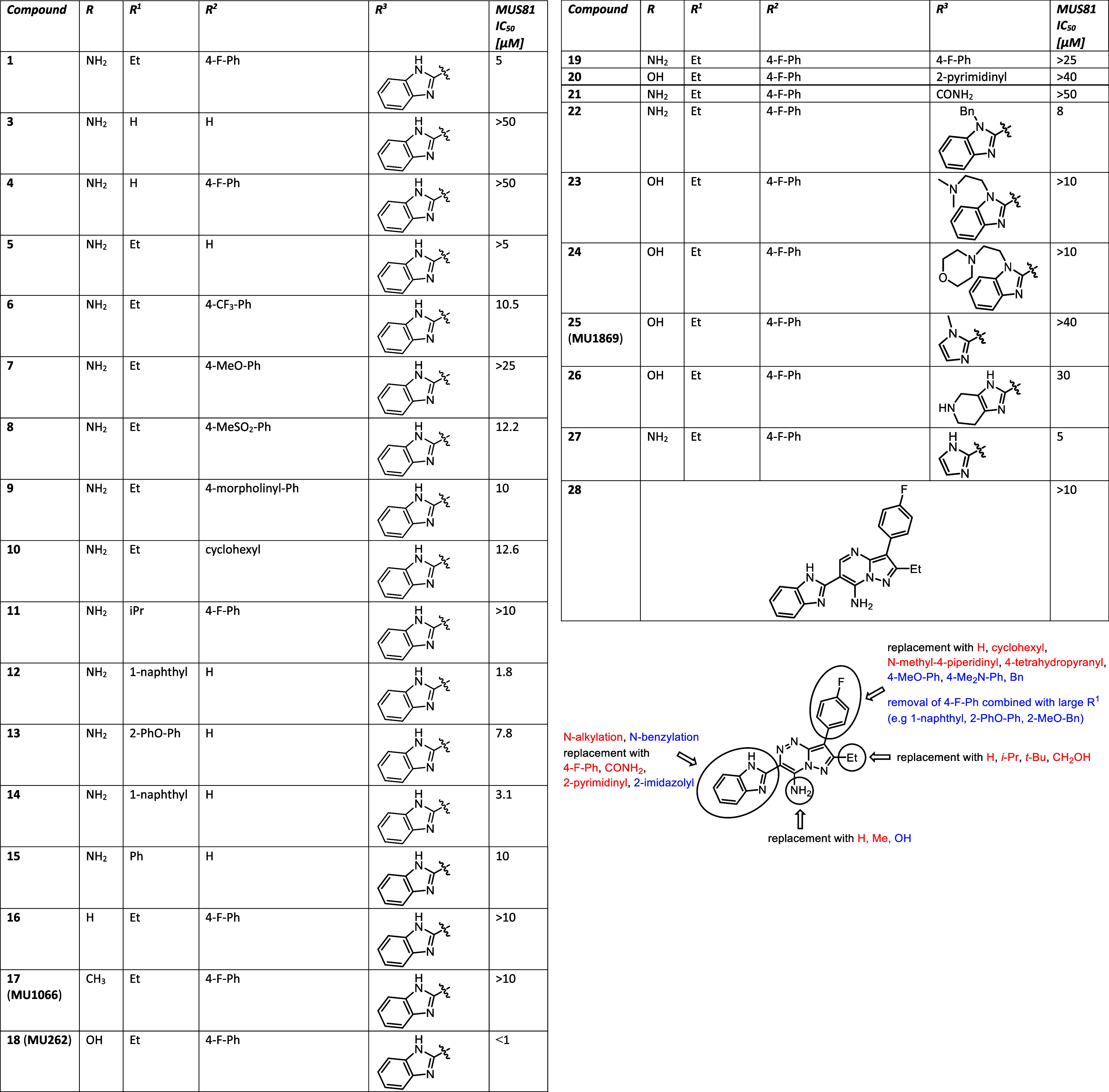
Structures and MUS81 In Vitro IC_50_ Values of the Compound **1** and Its Selected Analogues^a^ ^b^

a Table including the complete set is included in Supporting Information (Supplementary table 3) and graphical summary of SAR around compound **1**.

b Graphical summary of SAR around compound **1** with tolerated modifications in blue and those that led to significant loss of activity in red.

In accordance with the model (Figure S2), deletion of the amino group in **1** led to the significantly less active compound **16** (Table 1). The methylated analogue **17** was also similarly inactive. In contrast, the replacement by a hydroxy group led to the analogue **18** (**MU262**) (Table 1) that showed significantly improved activity in the primary biochemical nuclease assay.

Our attempts to replace the benzimidazole motif (i.e., substituent R^3^) were only partly successful, as illustrated by the compounds **19**, **20**, and **21** in Table 1. Similarly, numerous compounds with modified benzimidazole motif were significantly less potent—e.g., **22**, **23**, **24**, **25**, and **26** (Table 1); except for the imidazole analogue **27**, which was however less soluble in aqueous DMSO. Finally, replacement of the pyrazolo­[5,1-*c*]­[1,2,4]­triazine core in **1** by the isosteric pyrazolo­[1,5-*a*]­pyridine scaffold resulted in the comparatively less potent compound **28** (Table 1).

### SAR Optimization of the Lead Compounds from In Vitro Screening

In the second series, hit **2** served as the starting point in SAR development (Table 2). Along this line, we synthesized 53 analogues with the preserved central aminopyrazole scaffold (Table 2 and Supplementary Table 2) as this motif was present also in several other HTS hits. Majority of the target compounds were prepared via condensation of properly substituted β-ketonitriles with heterocyclic hydrazines (typically possessing pyrimidine substituents), followed by acylation or alkylation of the NH_2_ group, as shown in Scheme 2.

**Table 2:**
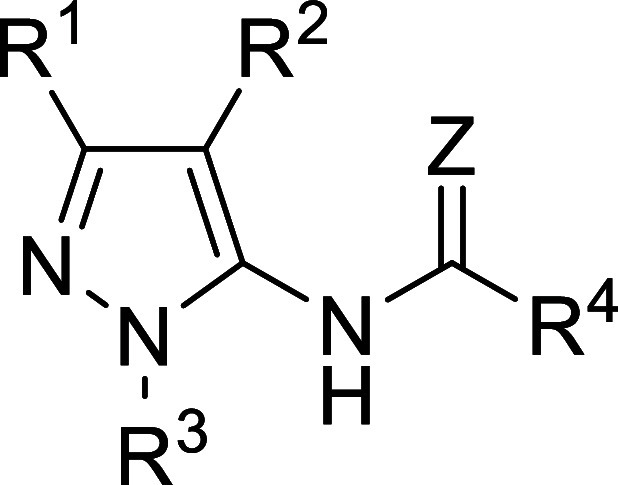

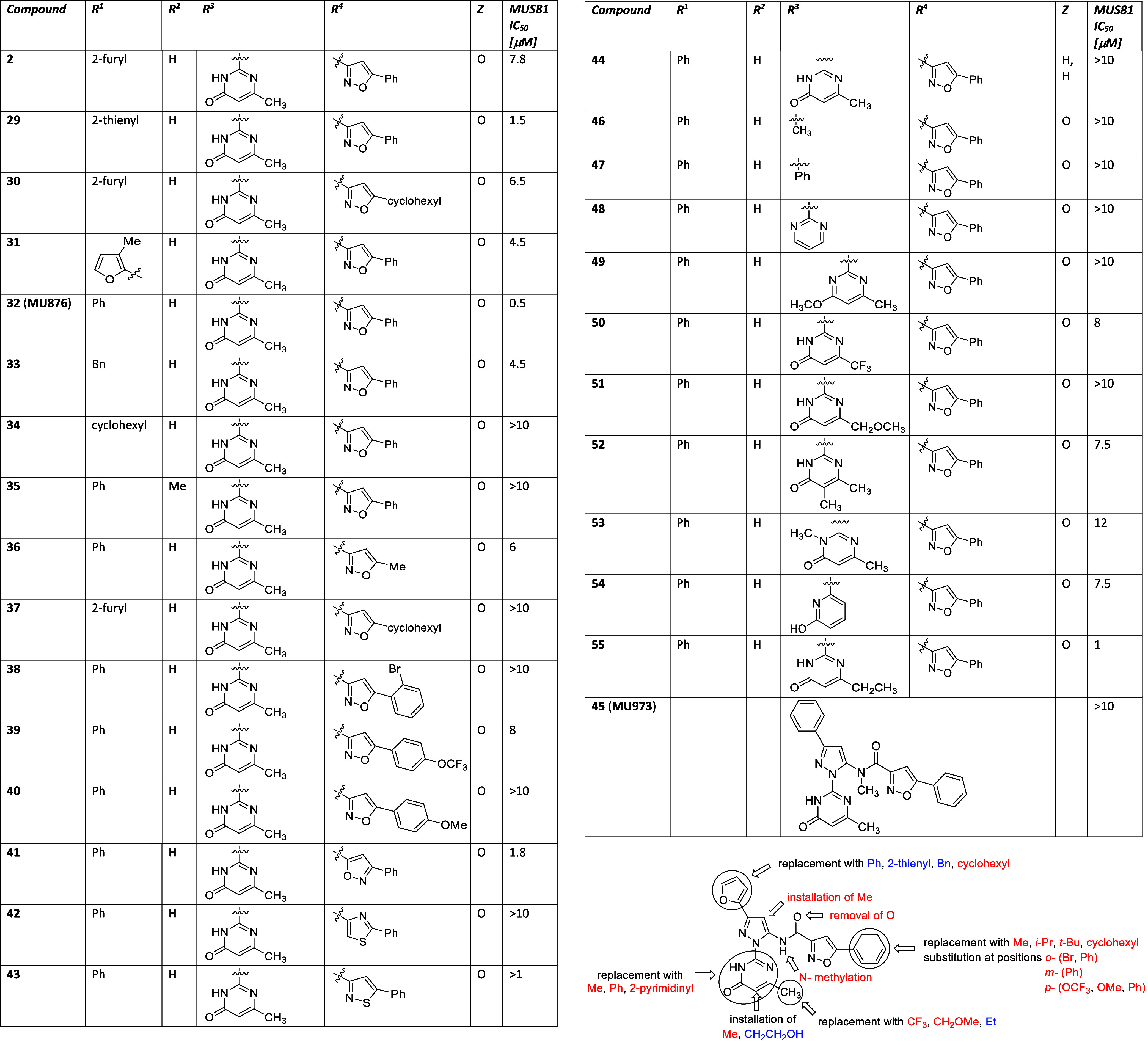
Structures and MUS81 IC_50_ Values of the Compound **2** and Its Selected Analogues^a^ ^b^

a Table including the complete set is included in Supporting Information (Supplementary table 4) and graphical summary of SAR around compound **2**.

b Graphical summary of SAR around compound **2** with tolerated modifications in blue and those that led to significant loss of activity in red.

As in the former series, the SAR mapping consisted of gradual structural changes of the individual substituents in the starting compound **2**. Modification of R^1^ afforded several analogues with activity superior to that of **2**, namely, compounds **29**, **30**, **31**, **32** (**MU876**), and **33** with the IC_50_ values 1.5, 4.5, 4.5, 0.5, and 4.5 μM, respectively (Table 2). In contrast, variation of the substituent R^4^ (typically substituted isoxazole) was less productive, with nearly all corresponding analogues exhibiting lower potency than the leads **2** and **32** (**MU876**) (Table 2). Less potent were also compounds with isosteres of the isoxazole motif, exemplified by the compounds **41**, **42**, and **43** in Table 2, and the analogues **44** and **45** lacking the amidic carbonyl group and possessing methylated amidic nitrogen, respectively. Replacement/elaboration of the methylpyrimidinone motif R^3^ demonstrated that this moiety also needs to be preserved, and even minor modifications or substitutions resulted in a significant decrease in the activity (**49**, **50**, **51**, **52, MU1003** = **53**). Of this subset, only the ethylated analogue **55** showed activity comparable to that of **32** (**MU876**) (Table 2).

### Two Chemically Diverse Compounds, **18** (**MU262**) and **32** (**MU876**), Inhibit MUS81-Mediated DNA Repair Pathways in Cells

To extend the SAR findings into a cellular context, selected derivatives from both chemical series that showed sufficient in vitro activity were tested in cell-based assays. Due to the current lack of assays that allow for direct and selective monitoring of MUS81 activity in cells, we used green fluorescent protein (GFP)-based reporter cell lines that detect DSB repair by HR or BIR, two pathways in which MUS81 plays an important role^54–56^ (Supplementary Tables 3 and 4).

These reporter assays enabled functional validation of MUS81 inhibition within a cellular setting and guided compound prioritization based on biological efficacy. Of the tested compounds, **18** (**MU262**) and **32** (**MU876**) represented the most potent analogues within their respective chemical series in both in vitro as well as in cellulo settings (Tables 1 and 2, Supplementary Tables 3 and 4) and were selected for further profiling (Figure 2A and D). Both compounds showed potent and dose-dependent inhibition of MUS81 with IC_50_ = 0.87 μM for **MU262** and 0.52 μM for **MU876** in the in vitro assay (Figure 2F–I). The robustness of the biochemical nuclease assay was confirmed using two independent controls: (i) EDTA-mediated metal chelation, which fully abolishes enzyme activity, consistent with prior reports;^48^ and (ii) inclusion of Dyngo-4a, a recently described MUS81 inhibitor^48^ (Figure S3A). For each active compound, a structurally similar negative control was also selected, **17** (**MU1066**) and **25** (**MU1869**) for **18**, and **45** (**MU973**) for **32** (Figure 2B,C and 22E). These compounds show no activity in the in vitro assay (Figures 2G and I, S3B–D).

**Figure 2:**
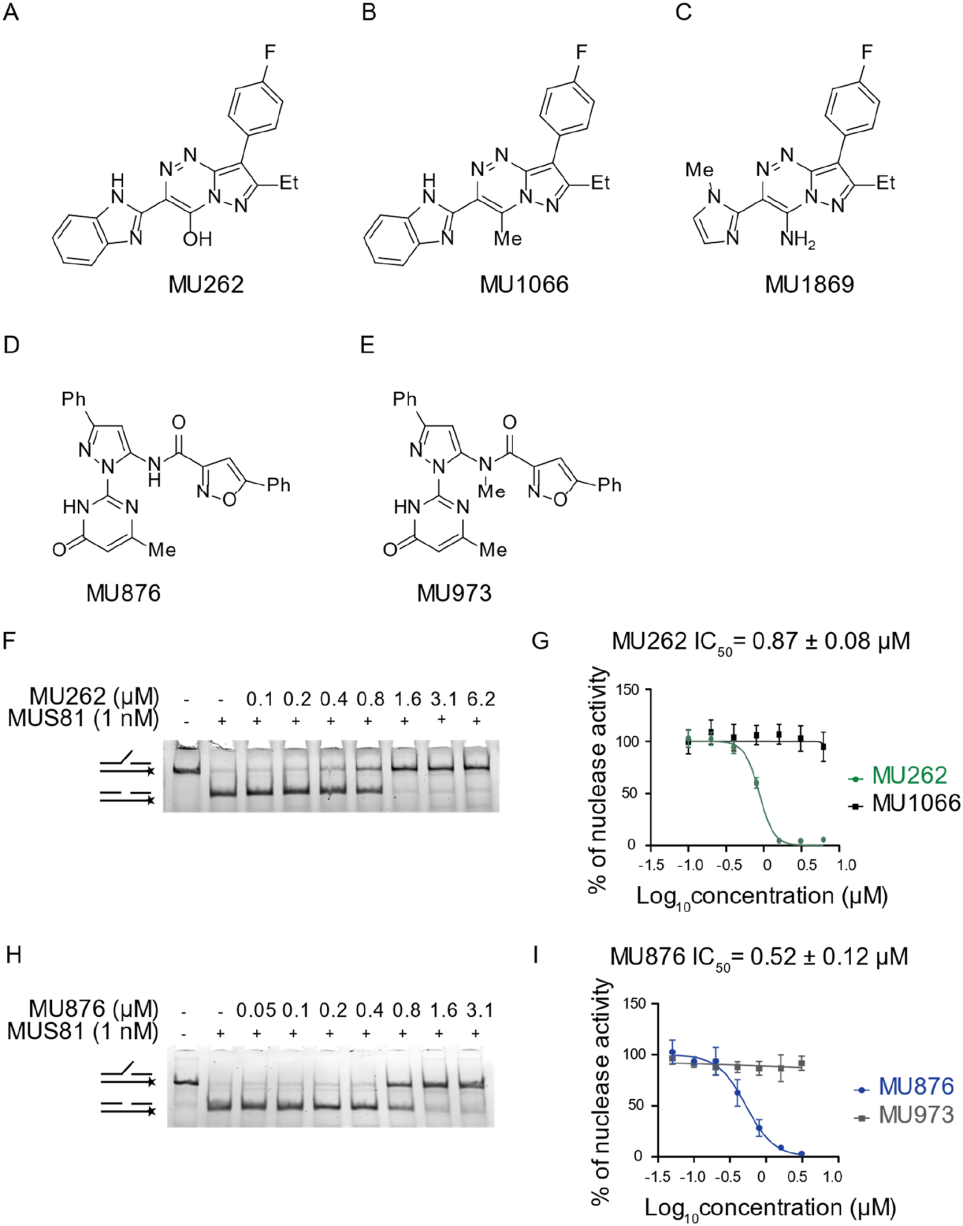
Identification of two chemically diverse small molecules inhibiting MUS81–EME1 nuclease activity in vitro. (A) Chemical structure of **MU262** = **18**. (B) Chemical structure of **MU1066** = **17**. (C) Chemical structure of **MU1869** = **25**. (D) Chemical structure of **MU876** = **32**. (E) Chemical structure of **MU973** = **45**. (F) Purified MUS81–EME1 (hereafter labeled MUS81, 1 nM) was incubated with increasing concentrations of **MU262**, followed by the addition of 3 nM fluorescently labeled 3′ flap DNA. The reaction products were resolved on a native PAGE. Schematics on the side of the gel represent substrate and expected product of reaction. *n* = 3. (G) Quantification curve of **MU262** and its negative control compound **MU1066**. 3′ flap DNA substrate processed by MUS81 was quantified using Multi Gauge software. Nonlinear regression fitting model was used to calculate IC_50_ (mean ± s.e.), *n* = 3 or more. (H) Purified MUS81–EME1 (1 nM) was incubated with increasing concentrations of **MU876**, followed by the addition of 3 nM fluorescently labeled 3′ flap DNA. The reaction products were resolved on a native PAGE gel. Schematics on the side of the gel represent substrate and expected product of reaction. *n* = 3. (I) Quantification curve of MU876 and its negative control compound MU973. 3′ flap DNA substrate processed by MUS81 was quantified using Multi Gauge software. Nonlinear regression fitting model was used to calculate IC_50_ (mean ± s.e.), *n* = 3 or more.

In cell-based assays assessing BIR, we have first validated MUS81 dependency by using two different siRNAs to deplete MUS81 and observed a marked reduction in BIR efficiency (Figures 3A and S4A). Notably, both compounds decrease BIR to the same extent as the siRNA-mediated depletion of MUS81, with an IC_50_ of 1.53 μM for **MU262** and 0.24 μM for **MU876**, respectively, while inactive control compounds showed no measurable effects (Figure 3A,B). In addition to inhibiting BIR, both **MU262** and **MU876** significantly reduced the efficiency of HR repair, with IC_50_ values in the range of 0.5–1 μM (Figure 3C). This inhibition was again absent with their respective control analogues, further supporting the specificity of these inhibitors toward MUS81-dependent repair pathways.

**Figure 3:**
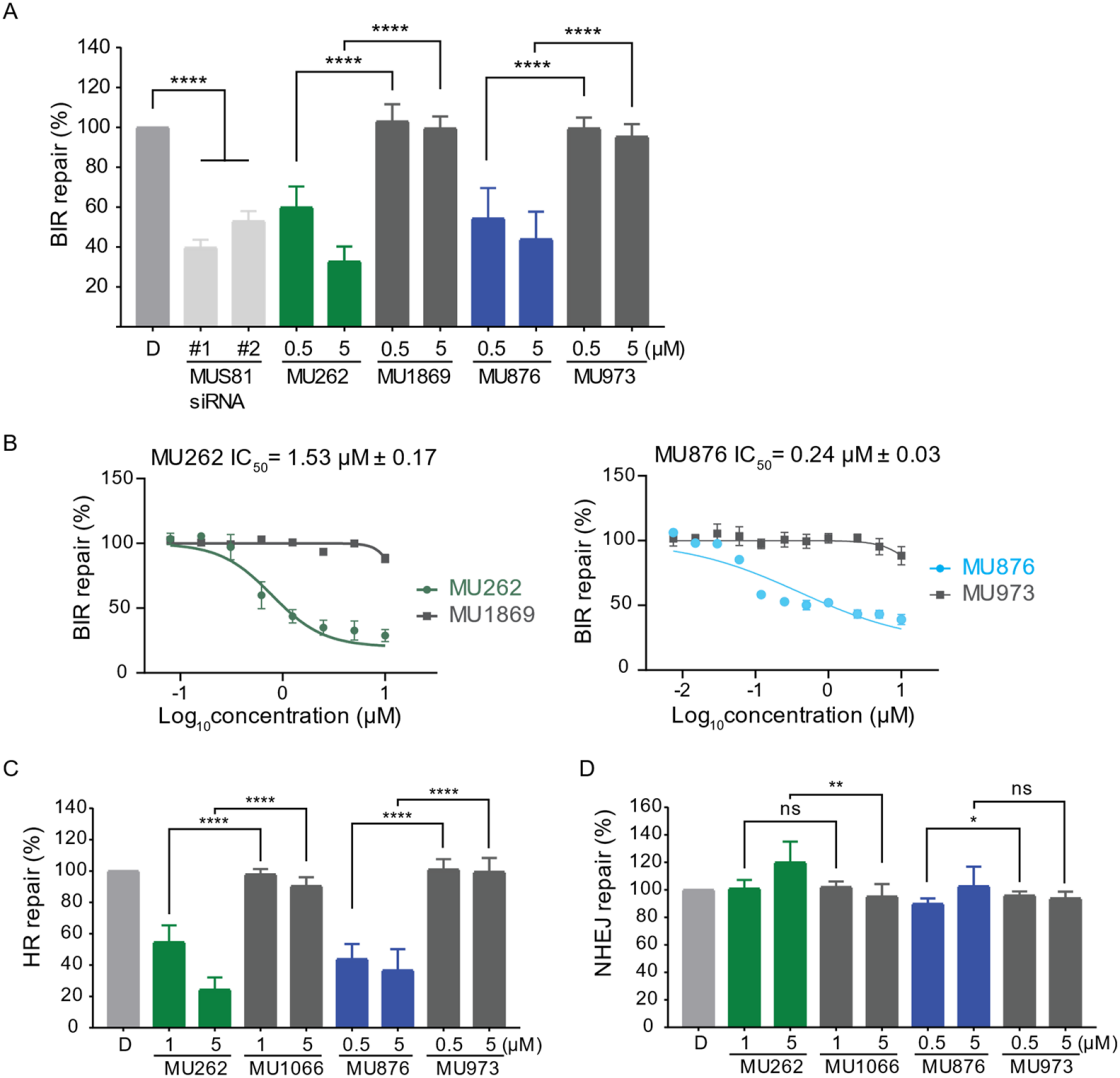
MUS81 inhibitors suppress HR and BIR. (A) I-SceI-based BIR repair efficiency was measured in U2OS BIR-GFP cells treated with DMSO, two different siRNAs targeting MUS81, or the indicated concentrations of **MU262** and **MU876**, and their control analogues for 72 h. The percentage of repair was normalized to the DMSO-treated control (D). *n* = 3. Error bars represent standard deviation (s.d.); ****p <0.0001 (unpaired, two-tailed *t*-test). (B) Experiment was done as in (A), except a concentration range was wider to determine the IC_50_ value for **MU262** and **MU876** via a nonlinear regression fitting model. *n* = 3. (C,D) I-SceI-based HR (C) and NHEJ (D) repair efficiency was measured using U2OS DR-GFP and EJ5-GFP cells, respectively, following treatment with DMSO, **MU262**,**MU876**, or their control analogues for 72 h. The percentage of repair was normalized to the DMSO-treated control. *n* = 3. Error bars represent s.d.; *p <0.05, **p <0.01, ****p <0.0001 (unpaired, two-tailed *t*-test).

To investigate their potential off-target effects, both compounds were also profiled in the MUS81-independent cell-based assay monitoring a nonhomologous end joining (NHEJ) pathway. In accordance with previous reports showing that inhibition of HR factors enhances NHEJ as a compensatory mechanism,^57,58^ we observed that siRNA-depletion of BRCA2 or MUS81 modestly increased NHEJ activity (Figure S4B). Correspondingly, the treatment with MUS81 inhibitors **MU262** and **MU876** had no negative effect on NHEJ (Figure 3D), indicating that they do not broadly suppress DNA repair but impair only HR/BIR pathways. Therefore, both compounds can be used as chemically orthogonal chemical probes for targeting the nuclease MUS81 in the cellular context.

### Inhibition of MUS81 Does Not Induce Immediate Cytotoxicity or Major Disruption of Cell Cycle Progression

Given these promising findings, we further investigated the specificity of **MU262** and **MU876** and potential short-term cytotoxicity. To mirror the treatment duration used in BIR and HR assays, U2OS cells were exposed to the inhibitors or siRNA for 3 days. Annexin V staining followed by flow cytometry showed no increase in apoptosis upon treatment with either **MU262** or **MU876**, nor with MUS81 depletion by siRNA. In contrast, cells treated with CPT displayed robust apoptosis (Figure S5A,B). Similarly, cell cycle profiling by propidium iodide (PI) staining combined with 5-Ethynyl-2′-deoxyuridine (EdU) incorporation revealed no major alterations. While CPT reduced the proportion of S phase cells, due to its antiproliferative effect, MUS81 depletion only modestly increased the proportion of cells in the G1 phase (Figure S5C). Treatment with 1 μM **MU262** and **MU876** showed a mild increase in the S phase (Figure S5C), potentially reflecting a mild defect in recombination-dependent repair during replication. Importantly, the control compounds elicited no effect on the cell cycle at this concentration, and neither **MU262** and **MU876** affected the cell cycle progression at lower concentration (0.5 μM; Figure S5C). These observations align with previous studies reporting that short-term depletion of MUS81 does not severely affect cell cycle arrest under unchallenged conditions.^40,59,60^

To evaluate the long-term effects of the inhibitors on the cellular viability, we treated cells twice a week for almost 3 weeks with low doses of **MU262** and **MU876**. Under these conditions, **MU262** caused a significant reduction in viability at concentrations of 1–3 μM, while **MU876** reduced proliferation at 0.25 and 0.5 μM (Figure S6A,B). These long-term effects closely mirror the phenotypes observed following siRNA-mediated depletion of MUS81 (Figure S6C,D), supporting the notion that prolonged MUS81 inhibition leads to accumulation of DNA repair defects over multiple cell cycles, ultimately resulting in reduced proliferation, as reported previously.^6,34^

### **MU262** and **MU876** Inhibit MUS81 via a Different Mode of Action

To elucidate the mechanism underlying the inhibitory activity of **MU262** and **MU876**, we performed several in vitro interaction assays. Using an electrophoretic mobility shift assay (EMSA), we examined how these compounds affect the binding of purified MUS81–EME1 complex to a 3′ flap DNA substrate. As expected, MUS81–EME1 efficiently bound to the 3′flap DNA substrate (Figure 4A). However, **MU876** disrupted this interaction in a concentration-dependent manner, effectively dissociating MUS81–EME1 from the DNA. In contrast, control compound **MU973** had no significant effect (Figures 4A and S7). Interestingly, neither **MU262** nor its inactive analogue **MU1066** disrupted the MUS81–DNA interaction (Figures 4B and S7), suggesting a different mechanism of action for **MU262** compared to **MU876**. This suggests that these two structurally different compounds interact with MUS81 via different binding modes. A previous study has shown that multiple MUS81 domains, including the nuclease active site, contribute to DNA substrate binding,^61^ offering the possibility that **MU876** may block the binding by targeting the protein–DNA interface.

**Figure 4:**
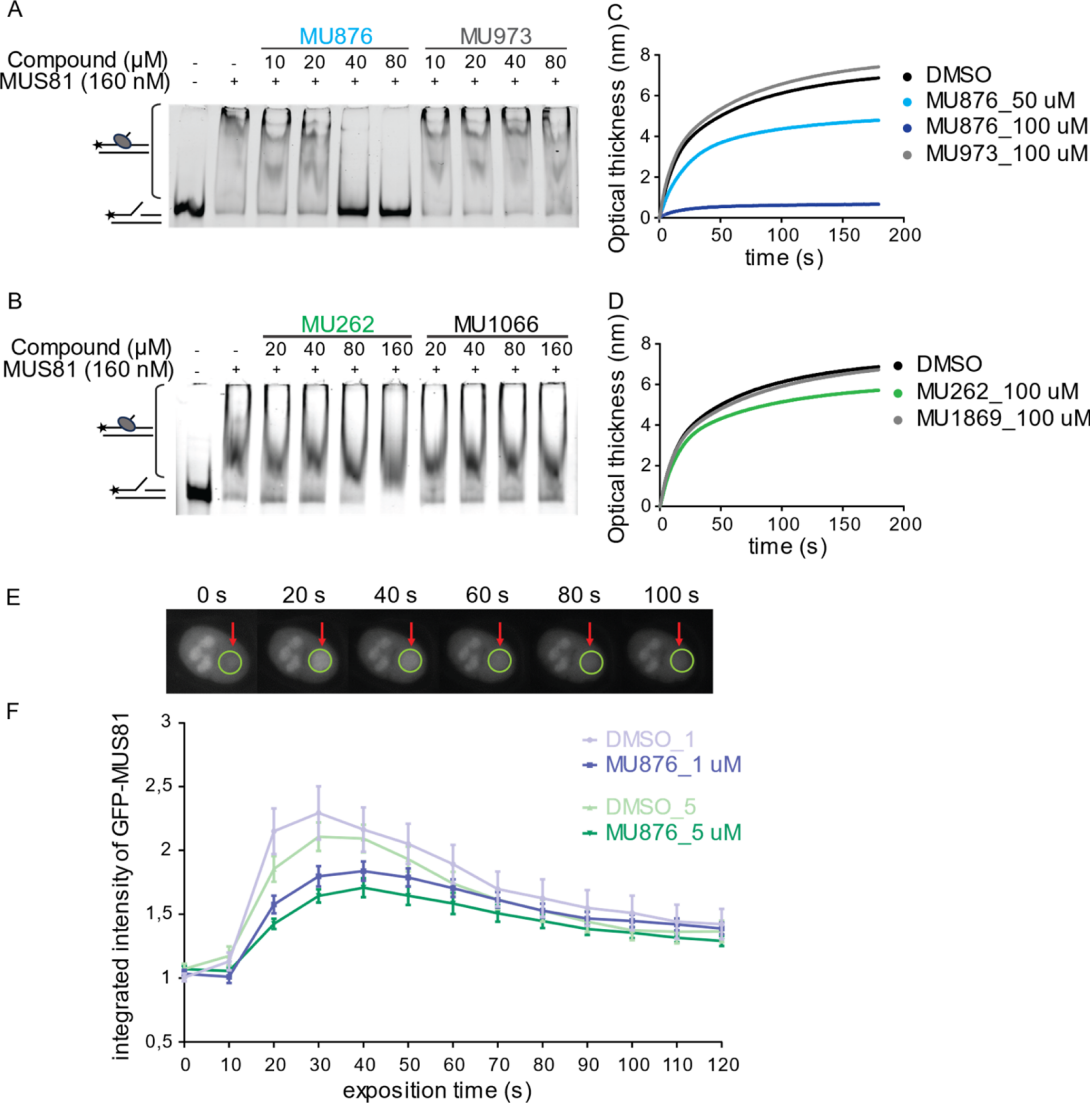
**MU262** and **MU876** inhibit MUS81 by distinct mechanisms of action. (A,B) Purified MUS81–EME1 (160 nM) was incubated with increasing concentrations of **MU262**, **MU876**, or their respective control analogues, followed by the addition of 3 nM fluorescently labeled 3′ flap DNA substrate. MgCl_2_ was omitted from the reaction buffer to prevent substrate cleavage. The reaction products were resolved on a native PAGE gel. The lower band represents a free DNA substrate, while the upper smear corresponds to the MUS81–DNA complex. *n* = 3. (C,D) A 3′ flap cleavage-resistant DNA substrate was immobilized on a streptavidin-coated (SAX) sensor and incubated with truncated MUS81 (200 nM) alone, or with MUS81 premixed with DMSO, **MU876**, **MU262**, or their respective control analogues. The real-time binding kinetics were measured as a change in optical thickness over time. Representative plots shown from two independent experiments. (E) Representative images of a DMSO-treated cell within 100 s after microirradiation. GFP-MUS81 U2OS cells were preincubated with DMSO or **MU876** at 1 and 5 μM final concentration for 2 h. Localized DNA damage was induced using a 355 nm laser microirradiation at specific sites in individual nuclei. The recruitment and retention of GFP-MUS81 at damage sites was traced by a sequential live-cell imaging for 120 s after microirradiation. (F) The integrated intensity of a GFP-MUS81 signal accumulation ± SE from (E). *n* = 3.

To further confirm these findings, we employed biolayer interferometry (BLI) to monitor the binding kinetics of MUS81 to the immobilized DNA substrate in the presence of the inhibitors. Consistent with the results obtained by the EMSA assay, **MU876** impaired MUS81–DNA binding in a dose-dependent manner (Figure 4C), whereas **MU262** had only negligible effect on this interaction (Figure 4D).

To provide structural context for the distinct inhibitory mechanisms of MU262 and MU876, we performed molecular docking studies using a MUS81 structure containing a complete active site, including the catalytic loop and both Mg^2+^ ions. For MU876, both cis and trans amide conformers were considered, as the preferred solution-state conformation is not known. Docking resulted in multiple plausible binding poses for each compound, which were subsequently rescored to identify the most favorable configurations (Supplementary Table 5).

The predicted binding mode of MU262 closely resembles that previously observed for compound 1 (Figure S2) and was compatible with simultaneous DNA binding, consistent with its biochemical profile (Figure S8A–C). In contrast, both conformers of MU876 were predicted to extend further above the catalytic magnesium ions and potentially sterically interfere with DNA binding (Figure S8A–C). Indeed, the superposition of the docking models with DNA-bound MUS81 structures revealed substantial overlap between MU876 and the DNA substrate, whereas MU262 could be accommodated without major steric clashes.

In summary, these data provide a structural and biochemical rationale for the distinct mechanisms of action of the two inhibitors. While **MU876** appears to inhibit the nuclease MUS81 via interfering with DNA binding, **MU262** likely impairs the MUS81 enzymatic activity through an alternative, DNA binding-independent mechanism.

### Direct Engagement of **MU876** with MUS81 in Cells

To confirm that **MU876** directly targets MUS81 in a cellular setting, we generated HEK293 cells expressing GFP-tagged MUS81. As expected, GFP-MUS81 localized to the nucleus, with increased accumulation in nucleoli, in accordance with the previously observed nucleolar retention which is even enhanced upon UV treatment.^62^ To assess DNA damage recruitment dynamics, we used laser microirradiation. GFP-MUS81 rapidly accumulated at damage sites, forming transient foci that disappeared shortly after recruitment (Figure 4E). Treatment with **MU876** led to a concentration-dependent reduction of MUS81 recruitment to damage sites, however not affecting the nuclease retention once recruited (Figure 4F). This corresponded to our in vitro experiments and supported the concept that **MU876** impairs the binding of MUS81 to damaged DNA in cells.

### **MU262** and **MU876** Phenocopy siRNA-Mediated MUS81 Depletion

Next, we explored the phenotypic consequences of MUS81 inhibition by **MU262** and **MU876** in cells. One phenotype linked to MUS81 depletion is the formation of micronuclei, a hallmark of chromosomal aberrations.^24^ Accordingly, we observed an increased number of micronuclei upon depletion of MUS81 by siRNA in U2OS cells (Figure 5A). The treatment with **MU262** and **MU876** also significantly increased micronuclei formation, similarly to the effect of MUS81 depletion (Figure 5A,B). The inactive control compounds had no effect on the formation of micronuclei (Figure 5A), confirming the specificity of the observed phenotype.

**Figure 5:**
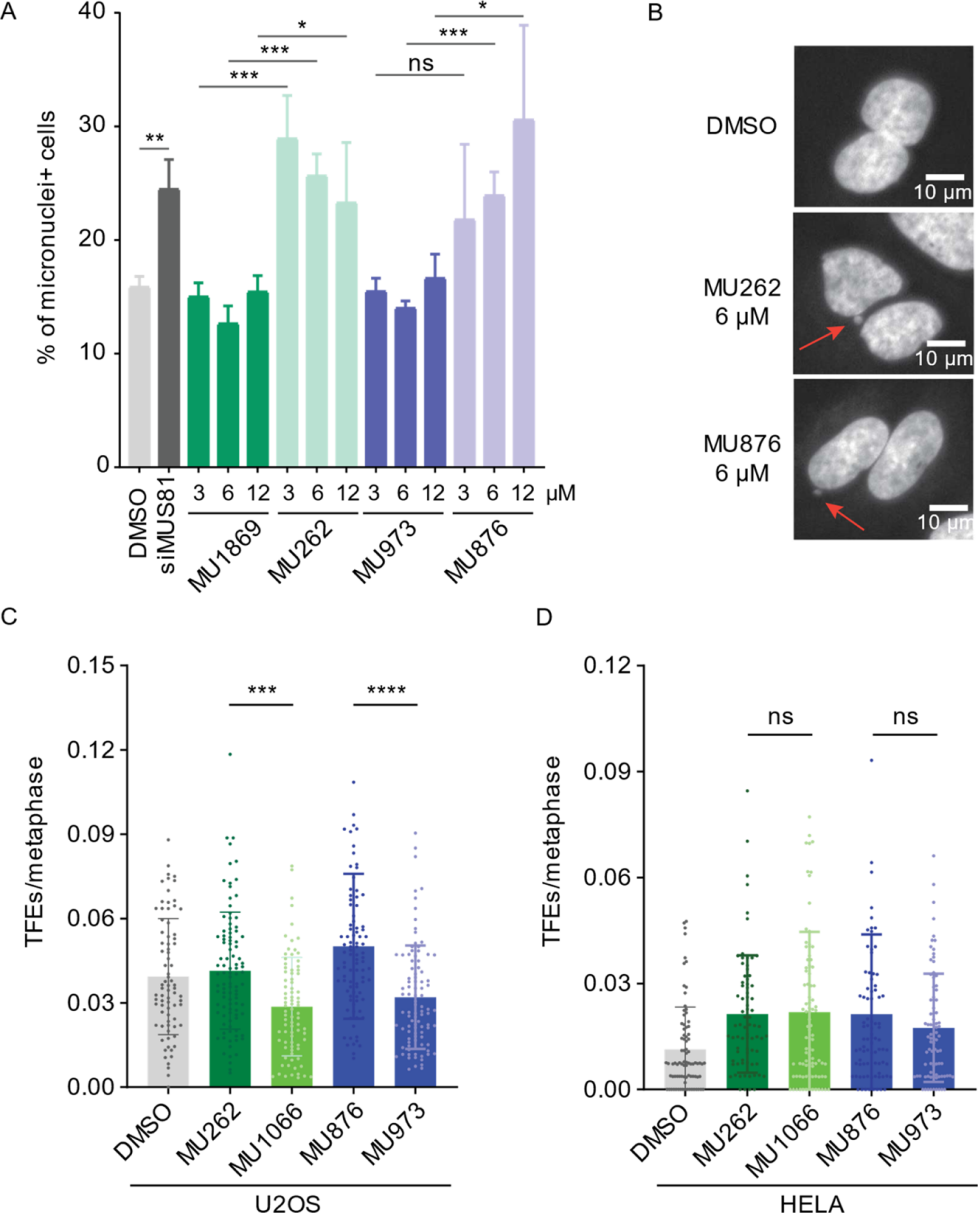
Small-molecule inhibitors of MUS81 mimic the effect of siRNA-mediated MUS81 depletion in cells. (A) U2OS cells were treated with DMSO, MUS81-targeting siRNA, **MU262**, **MU876**, or their respective control analogues for 48 h. Cytochalasin B (1.25 μg/mL) was added 16 h before harvest to prevent cytokinesis. The graph shows the percentage of binucleated cells with micronuclei. *n* = 3; error bars represent s.d.; *p <0.05, **p <0.01 ***p <0.001 (unpaired, two-tailed *t*-test). (B) Representative images corresponding to the selected conditions from (A). (C) U2OS and (D) HeLa cells were incubated with DMSO, the indicated inhibitors, and their respective control analogues for 48 h. Nocodazole (200 ng/mL) was added for 5 h prior to the end of treatment to arrest cells in mitosis. Mitotic cells were collected by shake-off, and telomere fragility events (TFEs) were quantified manually for each metaphase spread. At least 70 metaphase spreads were quantified per condition. *n* = 3; error bars represent s.d.; ***p < 0.001, ****p < 0.0001 (ordinary One-way ANOVA).

In addition to its role in DNA repair, MUS81 has been implicated in telomere maintenance in cells that use alternative lengthening of telomere (ALT) pathway, where depletion of MUS81 reduces telomere recombination and increases telomere loss.^26,63,64^ Using fluorescence in situ hybridization (FISH) on metaphase spreads, we demonstrated that both **MU262** and **MU876** significantly increased the presence of telomere-free ends (TFEs) (unlike the negative control compounds) in ALT-positive U2OS cells, but not in telomerase-positive and ALT-negative HeLa cells (Figure 5C,D). This confirmed that MUS81 inhibition impairs ALT-dependent telomere maintenance and further verified the ability of the compounds to inhibit MUS81 activity in the cell.

MUS81 plays a critical role in DNA repair, especially during replication and cell division.^26^ Persistent DNA lesions are marked by phosphorylation of histone H2AX (γH2AX), a widely used marker of DNA damage that can be visualized by fluorescence microscopy.^65^ To evaluate the impact of the MUS81 inhibitors **MU262** and **MU876** on DNA damage accumulation, we treated U2OS cells with them for 72 h and quantified γH2AX foci. Similarly to the cisplatin treatment, both inhibitors significantly increased the number of DNA damage foci in a concentration-dependent manner (Figures 6A,B and S9A,B), indicating the accumulation of unresolved DNA damage. In comparison, siRNA-mediated MUS81 depletion resulted in only a modest increase in γH2AX foci, likely due to the limited time of the protein absence upon the knock-down compared to a three-day treatment with the inhibitors (Figure S9A,B).

**Figure 6:**
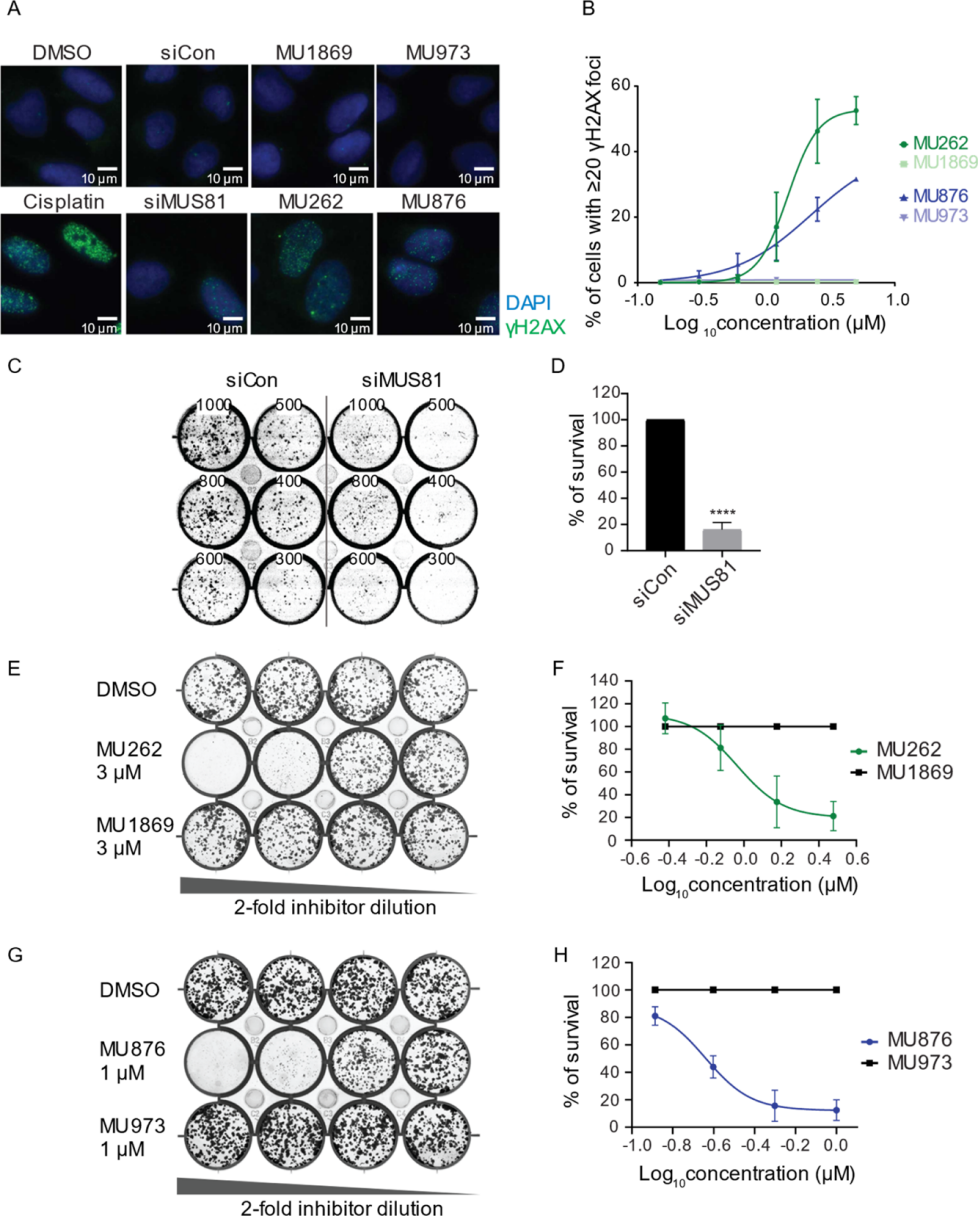
MUS81 inhibition induces persistent DNA damage leading to reduced cell survival. (A) U2OS cells were treated with DMSO, cisplatin as a positive control, control or MUS81-targeting siRNA, **MU262**, **MU876**, and their negative control counterparts at the indicated concentrations for 72 h. Representative images show staining of a DNA damage marker γH2AX in green with counterstaining of DAPI in blue. The concentration of all inhibitors shown in (A) is 2.5 μM. (B) DNA damage was assessed by quantifying γH2AX foci per nucleus from (A) using CellProfiller software. The graph represents percentage (%) of cells with ≥20 γH2AX foci per nucleus across different concentrations of inhibitors. *n* = 3; error bars represent s.d. (C) Indicated number of U2OS cells was transfected with a nontargeting siRNA (siCon) or MUS81-targeting siRNA (siMUS81) seeded for a CFA. Cells were grown for 10–12 days, and colonies were visualized by crystal violet staining after harvest. *n* = 3, representative picture is shown. (D) Quantification of (C), error bars represent s.d.; ****p <0.0001 (unpaired, two-tailed *t*-test). (E) U2OS cells were treated with **MU262** and its negative control **MU1869** and seeded for a CFA. Cells were grown for 10–12 days, and colonies were visualized by crystal violet staining after harvest. *n* = 3, representative picture is shown. (F) Quantification of (E) using a nonlinear regression fitting model. *n* = 3. (G) U2OS cells was treated with **MU876** and its negative control **MU973** and seeded for a CFA. Cells were grown for 10–12 days, and colonies were visualized by crystal violet staining after harvest. *n* = 3, representative picture is shown. (H) Quantification of (G) using a nonlinear regression fitting model. *n* = 3.

To determine whether this persistent damage translates to reduced viability, we used a colony formation assay (CFA). As expected, MUS81 depletion reduced the clonogenic survival of U2OS cells (Figure 6C,D). Similarly, **MU262** and **MU876** significantly decreased cell survival, whereas the inactive control compounds had no effect (Figure 6E–H). These results demonstrate that inhibition of MUS81 impairs the resolution of DNA lesions and leads to increased DNA damage, chromosomal aberrations, and reduced survival. These observations correlate with the previous report on impaired viability of MUS81-depleted cells under replication stress.^40^

### MUS81 Inhibitors **MU262** and **MU876** Potentiate the Effect of Chemotherapy and Impair DNA Repair

Despite the severe side effects associated with radio- and chemotherapy, cisplatin remains the primary option for numerous cancer patients worldwide.^66^ Since MUS81-depleted cells are more sensitive to cisplatin,^67^ we explored whether MUS81 inhibitors could potentiate its cytotoxic effect. We treated various cancer cell lines, including U2OS, HEK293, and CAL51, with increasing concentrations of **MU262** and **MU876** in combination with cisplatin and monitored cell survival 5 days after the treatment. As expected, cisplatin induced a dose-dependent decrease of viability, which was further enhanced by cotreatment with both MUS81 inhibitors in a dose-dependent manner (Figures 7A,B and S10A–D). In contrast, no additional sensitization was observed in CAL51 MUS81^–/–^ cells (Figure S10E,F), supporting the on-target activity of the MUS81 inhibitors.

**Figure 7:**
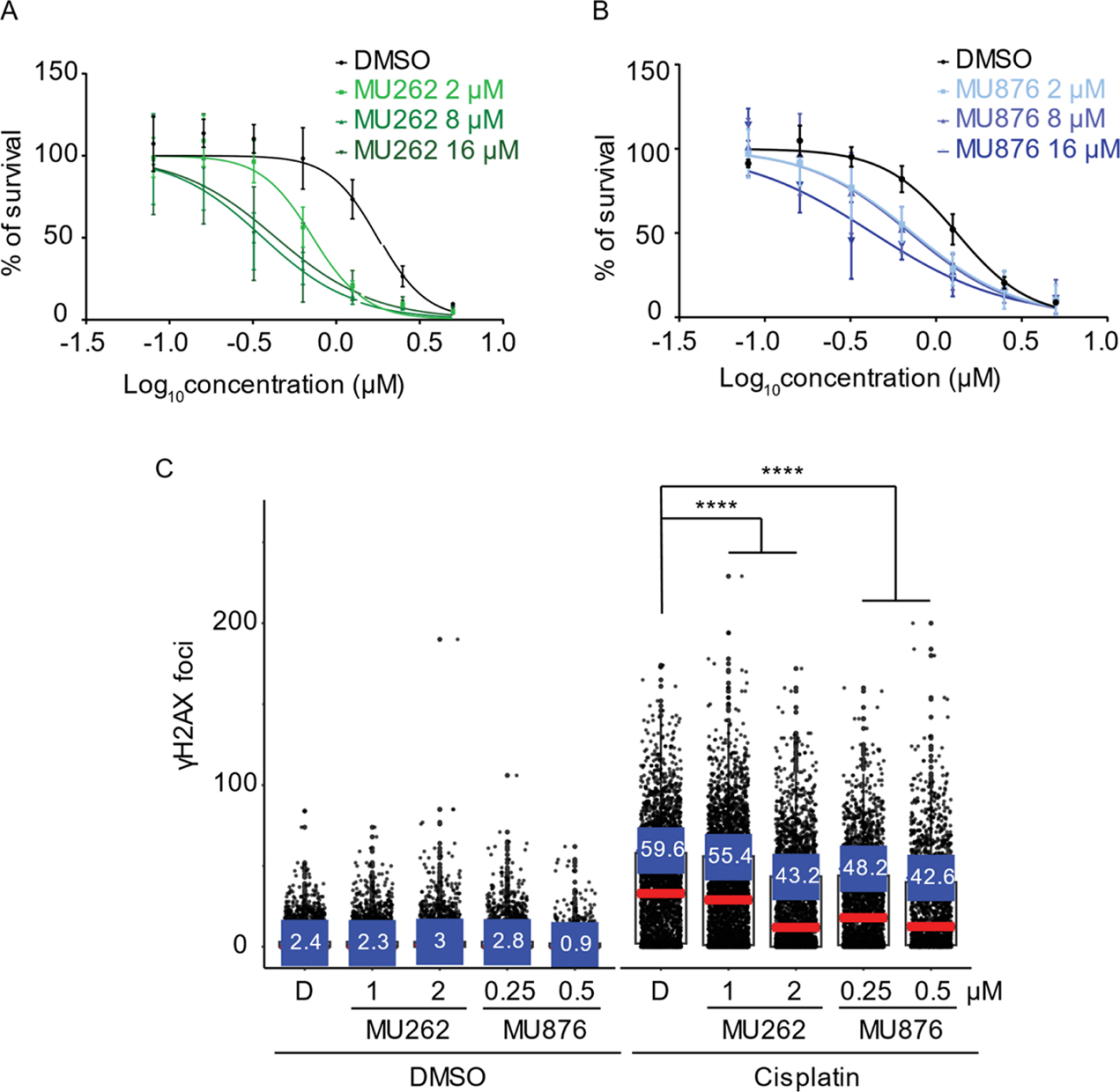
MUS81 inhibitors synergize with cisplatin to reduce cancer cell viability. (A,B) U2OS cells were treated with DMSO, **MU262**, or **MU876** at the indicated concentrations in combination with a range of cisplatin (0–5 μM) for 96–120 h. Cell viability was assessed by the Cy-Quant assay. The survival was normalized to the DMSO-treated control. Graphs were generated in Graphpad Prism using the nonlinear regression fitting model. *n* ≥ 3, error bars represent s.d. (C) CAL51 WT cells were treated with DMSO, **MU262**, or **MU876** at the indicated concentrations at each passage over 2 weeks. Cisplatin (12 μM) was added for the last 24 h before harvest. DNA damage was assessed by quantifying γH2AX foci using fluorescence microscopy and CellProfiller software. Data were visualized plotted in R software. Red markers represent the median of γH2AX foci number per nucleus; blue markers represent the percentage of cells with ≥20 γH2AX foci per nucleus. *n* = 3, error bars represent s.d.; ****p <0.0001 (unpaired, two-tailed *t*-test).

Mechanistically, the nuclease activity of MUS81 is critical for the repair of DNA damage induced by cross-linking agents such as mitomycin C (MMC) and cisplatin.^5^ To assess whether our MUS81 inhibitors impact DNA repair induced by cisplatin treatment, we examined γH2AX foci formation following the cisplatin treatment in the presence or absence of **MU262** and **MU876**. Consistent with siRNA-mediated MUS81 depletion in U2OS (Figure S11A) and genetic deletion in CAL51 cells (Figure S11B), both compounds significantly reduced the number of γH2AX foci in a concentration-dependent manner compared to the DMSO controls (Figures 7C and S11C). These findings suggest that MUS81 activity is required for cisplatin-induced DNA breaks and inhibition of MUS81 mimics genetic loss of function, effectively impairing cellular DNA damage response and enhancing the efficacy of cisplatin.

## Discussion and Conclusions

Genomic instability is a hallmark of cancer and other diseases, often arising from defects in the DNA repair pathways. While this vulnerability can be therapeutically exploited, as demonstrated by PARP inhibition in BRCA-mutated tumors,^68^ the number of clinically used agents that directly target DNA repair proteins remains limited.^69^ In this respect, DNA nucleases, in particular, those involved in the processing of stalled or collapsed replication forks, represent a promising yet underexplored class of targets. Among these, the endonuclease MUS81 plays a crucial role in safeguarding genome stability, especially during the S phase and mitosis.

In our study, we have identified and characterized two distinct small-molecule inhibitors of MUS81 endonuclease, **MU262**, and **MU876**. These compounds efficiently inhibit the in vitro nuclease activity of a purified MUS81–EME1 complex at submicromolar concentrations. Importantly, both inhibitors phenocopy the effects of MUS81 depletion in cell-based assays, establishing their functional relevance and validating MUS81 as a druggable target. The comprehensive set of cell-based assays used in this study includes also detection of direct engagement of **MU876** with the nuclease MUS81 in living cells via recruitment assays.

Our characterization of **MU262** and **MU876** represents a significant advancement over previously published studies on MUS81 inhibition, which lacked in-depth cellular validation.^47,48^ In this study, we used a comprehensive set of biochemical, biophysical, and cell-based analyses, demonstrating that inhibitors **MU262** and **MU876** can effectively modulate MUS81-dependent processes, including the BIR and HR pathways. Notably, treatment with either compound led to the accumulation of chromosomal aberrations such as formation of micronuclei and loss of telomeric DNA, i.e., phenotypes that closely mirror those observed in this study and previously also in MUS81-deficient mouse or human cells.^22,24,70^ The structurally orthogonal MUS81 inhibitors reported herein also provide a unique opportunity to target the nuclease through different modes of inhibition: while **MU876** directly disrupts MUS81–DNA binding both in vitro and in cells, the precise mode of action of MU262 has yet to be fully defined. Of note, both compounds can be docked into the active site of the human MUS81–EME1 complex adopting similar binding modes (Figure S8A–C), with MU876 being more likely to interfere with DNA binding (Figure S8C). This represents a good opportunity for future studies using structural and biochemical approaches to further elucidate MU262’s interaction with MUS81.

Despite their mild toxicity under short-term treatment, prolonged exposure to either **MU262** or **MU876** led to a marked reduction in cell proliferation and survival, closely mirroring the effect observed in this study but also previously upon MUS81 knockdown or knockout.^34,60,71^ This likely reflects the requirement for multiple rounds of DNA replication before the cumulative burden of unresolved DNA lesions, checkpoint activation, and chromosomal abnormalities manifests as a measurable growth phenotype. Such delayed phenotypes have been consistently observed in MUS81-deficient models, including transgenic mice, and underscore the importance of considering time-dependent consequences when evaluating nuclease inhibition.^5^ We therefore propose that MUS81 inhibition produces only subtle effects under unchallenged conditions but becomes functionally significant during prolonged proliferation or under replication stress. This model supports the potential therapeutic value of MUS81 inhibition, particularly in combination with DNA-damaging agents. Importantly, the cellular phenotypes observed with **MU262** or **MU876**, including micronuclei formation, telomere fragility, and increased γH2AX foci, closely recapitulate those reported upon the genetic loss of MUS81, supporting an on-target mechanism of action. Nonetheless, we acknowledge that the inhibitors were tested at micromolar concentrations where off-target effects may occur. To address this, we included structurally closely related negative control compounds, which failed to reproduce the observed phenotypes, strengthening the evidence for compound specificity. Still, we cannot fully rule out the contribution of off-target activity to some of the observed effects.

Given the structural similarity and functional redundancy among human nucleases, dual inhibition can be both mechanistically plausible and therapeutically advantageous. For instance, synthetic lethality has been demonstrated in HEK 293 cells lacking both MUS81 and GEN1.^72^ Thus, future efforts focused on exploration of combined nuclease inhibition may lead to new therapeutic options with increased efficacy and lower risk of acquired resistance.^73^

Importantly, we demonstrate that MUS81 inhibition sensitizes cells to cisplatin, a routinely used chemotherapeutic agent whose efficacy is often limited by acquired resistance.^74–76^ Both **MU262** and **MU876** enhance cisplatin-induced cytotoxicity across multiple cancer cell lines, underscoring the therapeutic potential of MUS81 inhibition in combination with chemotherapy. This effect was absent in MUS81-knockout cells and confirmed the specificity of the compounds. Mechanistically, we show that these inhibitors reduce the formation of γH2AX foci following cisplatin treatment, which is consistent with the model where MUS81 facilitates the generation of DSBs during interstrand cross-link (ICL) repair.^67^ This further validates MUS81 as a critical component of the DNA damage response and suggests that its inhibition may potentiate the effects of genotoxic therapies. In contrast to single-agent therapies that are frequently associated with acquired resistance,^77^ combination therapies including MUS81 inhibitors may provide a more robust and durable clinical outcome. Notably, both compounds **MU262** and **MU876** exhibit good microsomal stability and do not significantly inhibit major cytochrome P450 isoforms (Supplementary Table 6–8), making them suitable candidates for further in vivo evaluation. Future studies will aim to assess their therapeutic efficacy in mouse tumor models, particularly in combination with cisplatin, to establish their potential beyond cellular systems.

Collectively, the data described in this report define newly discovered compounds **MU262** and **MU876** as first-in-class inhibitors of the nuclease MUS81 with confirmed cellular activity that can be used as valuable tools in molecular biology studies focused on this nuclease. The findings reported herein also provide a foundation for further development of new single-agent or combination anticancer therapies that would utilize pharmacological targeting of MUS81.

## Experimental Section

### In Silico Ligand-Based Screening for MUS81–EME1 Inhibitors

The three-dimensional structures of almost 150,000 molecules for virtual screening were downloaded from the clean drug-like subset of the ZINC database.^78^ Only structures matching the selection criteria (xlogP ≤ 5, molecular weight ≤ 500 g/mol, number of H-bond donors ≤ 5, and number of H-bond acceptors ≤ 10) were selected for the screening. Furthermore, only molecules with a similarity lower than 0.8 (Tanimoto coefficient) were selected. Input files in Sybyl mol2 format were converted into AutoDock compliant format by MGLTools.^79^

The crystal structure of human MUS81–EME1 was not described at the time of the initial screen. Therefore, the crystal structure of a chimerical complex of zebrafish MUS81/human EME1 was used as a template for homology modeling (PDB ID: 1J25;^61^). The chimeric complex was crystallized with truncated MUS81 (aa 303-612) and EME1 (aa 246-570) which preserves the nuclease activity of the complex. Since MUS81 nuclease activity is known to be dependent on the presence of a bivalent metal ion, the Mn^2+^ was added into the active site of the MUS81 protein based on superposition with the structure of the Hef nuclease domain (PDB-ID:1J25 ^80^). Corresponding amino acid sequences of MUS81 (residues 246-551) and EME1 (residues 246-570) were downloaded in FASTA format from the UniProtKB/Swiss-Prot database (MUS81 ID-Q96NY9, EME1 ID-Q96AY2). The model of the MUS81–EME1 complex was built by using SWISS-MODEL^81,82^ and was verified by I-TASSER web server.^82^ The active site of MUS81 included in this structure was used for molecular docking using the AutoDock Vina.^50^ The region of the active site selected for molecular docking was set to 33 × 27 × 30 Å centered at the Mn^2+^ ion.

The docked conformations were scored by the AutoDock Vina software and rescored using NNScore 2.0 software.^53^ A consensus score for each conformation was calculated by averaging the ranks obtained with the AutoDock Vina score and the final NNScore 2.0 score. The common features in the binding modes of the docked conformations were used as a ground for molecule clustering. The clustering analysis was performed by AuposSOM^51^ using default parameters where the map size was changed to 6 × 5 to increase the maximal number of clusters.

### In Vitro High-Throughput Screening

The HTS was performed at the Molecular Screening Shared Resource (University of California Los Angeles) with approximately 100,000 compounds from their drug discovery libraries. All compounds were dissolved in DMSO. The compound transfer was done using BioMek FX (Beckman Coulter) equipped with a 0.5 μL pin tool, and other solutions were added by a BioTek EL406 dispenser. The different components of the reaction were dispensed to black low-volume and flat-bottom 384-well plates (Polystyrene NBS; Corning 3820) in this order: (1) 4 μL of master mix, (2) 50 nL of compounds, and (3) 1 μL of recombinant MUS81–EME1. After a 30 min preincubation at room temperature, the reaction was initiated by adding 1 μL of 3′flap DNA substrate labeled with 6-carboxyfluorescein (FAM) and a Black Hole Quencher (BHQ1) (Figure S1). The plates were then incubated at room temperature for 1 h before measuring fluorescence with the Acquest 384-1536 plate reader (Molecular Devices). The final concentrations in the reaction were: 50 mM Tris–HCl pH 7.5, 1 mM DTT, 5 mM MgCl_2_, 0.1 mg/mL BSA, 100 mM KCl, 17 nM MUS81–EME1, 40 nM DNA, 8.3 μM compound (0.83% DMSO).

### Dose Response Assay (CZ OPENSCREEN IMG CAS)

The dose–response assay was performed in triplicate with 10 concentration points ranging from 5 nM to 10 μM. The reaction was done similarly to the primary HTS, with some modifications. The reaction volume was 5 μL and samples were prepared in 1536-well plates. The compounds were transferred with an Echo 550 liquid handler (Labcyte, Beckman Coulter). Other reaction components were dispensed by Multidrop. Plates were scanned in an EnVision reader (PerkinElmer).

### Expression and Purification of Human MUS81

The MUS81–EME1 full-length expression plasmid was obtained from Stephen C. West (The Francis Crick Institute, US) and purified as previously described.^83^ Briefly, 8 g of *Escherichia coli* cell pellet was sonicated in 40 mL of buffer C (50 mM Tris–HCl pH 7.5, 10% sucrose, 10 mM EDTA, 1 mM DTT, 0.01% NP40, and protease inhibitors) containing 150 mM KCl and clarified by centrifugation (100,000*g*, 60 min). The cleared lysate was applied sequentially onto a 7 mL Q Sepharose and SP Sepharose columns (GE Healthcare Life Sciences). The SP Sepharose column was developed with a 70 mL gradient of 100–800 mM KCl in buffer K (20 mM K_2_HPO_4_ pH 7.5, 10% sucrose, 10 mM EDTA, 1 mM β-mercaptoethanol, and 0.01% NP40). The peak fractions were pooled and mixed with 1.5 mL of His-Select Nickel Affinity Gel (Sigma-Aldrich). The beads were washed with 10 column volumes of buffer K containing 150 mM KCl and 5 mM imidazole and bound proteins were eluted using 10–1000 mM imidazole in buffer K containing 150 mM KCl. The imidazole fractions were pooled and further fractionated using 0.5 mL heparin (GE Healthcare Life Sciences) with a 10 mL gradient of 250–900 mM KCl in buffer K. The fractions containing purified MUS81 were pooled, concentrated using a Vivaspin (Sartorius Stedim Biotech) concentrator, and stored in 5 μL aliquots at −80 °C.

A truncated version of the MUS81–EME1 complex, encompassing amino acids 246-551 of MUS81 and a 178-570 of EME1, was generated. Plasmid coexpressing both proteins was transformed into bacteria and purified as described above.

### DNA Substrates

All oligonucleotides used in this study were purchased from Eurofins Genomics and are listed in Supplementary Table 9. The fluorescently labeled DNA substrates were prepared as previously described.^84^ In short, equimolar amounts of individual oligonucleotides were annealed in hybridization buffer H (50 mM Tris–HCl pH 7.5, 100 mM NaCl, 10 mM MgCl_2_). The mixture was heated to 70 °C for 3 min and cooled slowly to room temperature. The annealed DNA substrates were purified by fractionation on a 1 mL Mono Q column (GE Healthcare Life Sciences) with a 20 mL gradient of 50–1000 mM NaCl in 10 mM Tris–HCl pH 7.5. Fractions containing the DNA substrate were concentrated using a Vivaspin concentrator (Sartorius Stedim Biotech) with a 5 kDa cutoff and washed with lower salt buffer. The concentration of the DNA substrates was determined by absorbance measurement at 260 nm.

### Gel-Based Nuclease Assay

Nuclease assays were performed in reaction buffer containing 50 mM Tris pH 7.5, 10 mM MgCl_2_, 1 mM DTT, 85 mM KCl, and 20% glycerol. Purified MUS81–EME1 (1 nM) was preincubated in 9 μL reaction with indicated concentrations of inhibitors for 15 min at room temperature, followed by the addition of 3 nM of a 3′flap DNA substrate (listed in Supplementary Table 9) and a further incubation for 15 min at 37 °C. The final concentration of DMSO in the reactions was 2%. The reactions were stopped by the addition of 0.1% SDS and 500 μg/mL of proteinase K, followed by incubation for 5 min at 37 °C. Each reaction was then mixed with 1/5 volume of loading buffer (60% glycerol, 10 mM Tris–HCl pH 7.5, 60 mM EDTA, and 0.1% Orange G) and loaded on a native PAGE gel (12% acrylamide gel in 1xTBE). The fluorescent DNA was visualized using a FLA-9000 (Fujifilm). Bands corresponding to cleaved substrates were selected for quantification using the MultiGauge 3.2 software. Final data represent integrated density of these bands with background subtraction. Fitting of the data with sigmoid curves was performed in OriginPro (OriginLab).

### Electrophoretic Mobility Shift Assay

Purified MUS81–EME1 (160 nM) was incubated in a modified assay buffer without MgCl_2_ (50 mM Tris–HCl pH 7.4, 150 mM NaCl, and 0.05% Tween-20) with 15 nM of a 3′flap DNA substrate (listed in Supplementary Table 5) in the absence or presence of selected inhibitors at RT for 10 min, followed by an incubation at 25 °C for 10 min. The reaction was stopped on ice and products were resolved using native PAGE (7.5% acrylamide gel in 0.5% TBE) at 4 °C for 50 min (6.5 V/cm). Gel images were captured using a FLA-9000 (Fujifilm) and Typhoon (Amersham) scanners and quantified with Multi Gauge V3.2 software (Fujifilm).

### Biolayer Interferometry Measurements (BLI)

Measurements were performed on a BLItz instrument (ForteBio) in a buffer containing 50 mM Tris–HCl pH 7.5, 50 mM KCl, 10 mM MgCl_2_, and 0.05% Tween 20. A concentration of 15 nM biotinylated 3′-flap (blocked) DNA substrate was immobilized on streptavidin-coated (SAX) sensors and incubated with 200 nM MUS81–EME1 fragment alone or premixed with indicated amount of inhibitors/DMSO. The real-time kinetics of protein association were measured as changes in optical thickness. The data were plotted in GraphPad Prism (GraphPad Software).

### Cell Lines and Culture Conditions

U2OS WT cells were obtained from the European Collection of Authenticated Cell Cultures; U2OS-DR and U2OS-EJ5 cells were a kind gift from Dr. Jeremy Stark (City of Hope National Medical Center), while BIR-GFP cells were generously provided by Dr. Thanos D Halazonetis (University of Geneva). The U2OS GFP-MUS81 stable cell line was established using GFP-MUS81 WT or nuclease-dead (ND) DNA cloned into a pAIO plasmid^85^ and cotransfected with a Flp-In recombinase plasmid. CAL51 cells were a kind gift from Dr. Martin Mistrik (Palacky University, Olomouc) and were used to generate the CAL51 MUS81^–/–^ cell line by CRISPR-Cas9 as described previously.^86^

All cells were cultured in Dulbecco’s modified Eagle’s medium (DMEM, Gibco) supplemented with 10% (v/v) fetal bovine serum (FBS, Gibco), 100 U/mL penicillin, and 100 μg/mL streptomycin (both from Biosera). All cells were grown at 37 °C in a humidified atmosphere in 5% CO2.

### RNAi

Cells were reverse-transfected with Lipofectamine RNAiMax (Life Technologies) and 30 nM of siRNA according to manufacturer’s instructions. Control siRNA used as a negative control (XWNeg9) and MUS81 siRNA (s37038) were obtained from ThermoFisher Scientific.

### Cell Proliferation Assay

CAL51 WT, CAL51 MUS81^–/–^, U2OS, and HEK293 cells were seeded in a 96-well plate and treated with the respective concentration of selected inhibitors. After incubation for 4–5 days, cells were harvested while the untreated control was still subconfluent. The plate was then incubated at −80 °C to achieve cell lysis. Cell proliferation was assessed using the CyQUANT Cell Proliferation Assay kit (Thermo Fisher Scientific) according to manufacturer’s instructions. Absorbance was measured using a plate reader (Infinite F500, Tecan Austria GmbH).

### Clonogenic Survival Assays

Cells were trypsinized, plated at the desired density in 6-well plates for colony forming assay, and then incubated for 10 days. Inhibitors at varying concentrations were added to the media after 24 h. After incubation, colonies were fixed and stained with 0.5% crystal violet before scanning the plates. The colonies were then solubilized using 10% acetic acid solution, and absorbance was measured with a plate reader. The percentage of survival of colonies was plotted with GraphPad Prism (GraphPad Software).

### Immunoblotting

To prepare whole cell extracts, cells were harvested by trypsinization, washed with cold PBS, and resuspended in SDS-PAGE loading buffer. The samples were then sonicated and boiled at 70 °C for 10 min. Equal amounts of protein (50-100 μg) were separated on a 10% SDS-PAGE at 100 V, followed by transfer of proteins to nitrocellulose membrane using the semidry Trans-blot turbo Transfer system (1704150; Biorad). After transfer, membranes were blocked in 5% milk/PBST for 1 hour at room temperature and then incubated at 4 °C on a rocker overnight with the corresponding primary antibodies (Actin—ab184220, Abcam; MUS81—ab14387, Abcam). The next day, the membranes were washed with PBST and incubated with the corresponding secondary antibodies (Anti-Rabbit IgG, A6154, Sigma-Aldrich; Anti-Mouse IgG, A0168, Sigma-Aldrich) for 1 hour at room temperature. Finally, the blots were developed by the Immobilon Western Chemiluminescent horseradish peroxidase (HRP) Substrate (WBKLS0500; MERCK Millipore), and images were acquired using the Luminescent Image Analyzer (ImageQuant LAS 4000; Fujifilm).

### HR, BIR, and NHEJ Assays

Reporter DR-GFP and EJ2-GFP U2OS cells^87^ along with BIR-GFP cells^88^ were transfected with 2.5 μg of I-SceI-expressing pCAGGS vector and subsequently treated with MUS81 inhibitors at the indicated concentrations. After transfection (72 h), cells were trypsinized and resuspended in 3% BSA in PBS. GFP fluorescence detection was carried out using a BD FACSVerse flow cytometer, and the data were analyzed with FlowJo software (BD Life Sciences). Data presented in the graph were normalized to the DMSO-treated sample.

### EdU Cell Cycle Assay

U2OS WT cells (300,000) were seeded in a 6-well plate; a subset of them was treated with either control siRNA or siRNA targeting MUS81. After adhesion, cells were treated with the respective inhibitors for 72 h during which CPT (100 μM, Sigma-Aldrich) was added for the last 16 h and 10 μM EdU (Sigma-Aldrich) was added for the last 1 h. Cells were then harvested and stained by using the Click-iT EdU Alexa Fluor 647 Flow Cytometry Assay Kit (Thermo Fisher Scientific). Finally, cells were resuspended in 1xPBS containing 5 μg/mL of propidium iodide (PI) and analyzed using a BD FACSVerse flow cytometer (Becton Dickinson) and FlowJo software (BD Life Sciences). At least 20,000 cells were used for each measurement, and experiments were performed in duplicates.

### Annexin V Apoptotic Assay

U2OS WT cells (300,000) were seeded in a 6-well plate, a subset of them was treated with either control siRNA or siRNA targeting MUS81. After adhesion, cells were treated with the respective inhibitors for 72 h during which CPT (100 μM, Sigma-Aldrich) was added for the last 2 h. Cells were harvested, washed in ice-cold PBS, and collected by centrifugation at 500*g* for 5 min. Cells were simultaneously stained with FITC-labeled annexin V antibody (556419, Becton Dickinson) and PI (5 μL) at room temperature for 15 min, protected from light. Stained cells were analyzed using a BD FACSVerse flow cytometer (Becton Dickinson) and FlowJo software (BD Life Sciences). At least 20,000 cells were used for each measurement, and experiments were performed in duplicates.

### Immunofluorescence

U2OS, CAL51 WT, and CAL51 MUS81^–/–^ cells (10,000) were seeded in a 96-well plate 24 h before the treatment. Cells were treated with the respective MUS81 inhibitor according to the assay scheme for either 2 weeks or 24 h. In certain settings, cells were treated with cisplatin (12 μM, Sigma-Aldrich) for the last 24 h. After washing, cells were fixed with 3% PFA for 10 min at room temperature and permeabilized with 0.5% Triton X-100 (Sigma-Aldrich). Subsequently, cells were blocked with 5% bovine serum albumin (BSA, Sigma) and incubated with γH2AX antibody (05-636, MERCK Millipore) and Alexa Fluor 488 antimouse secondary antibodies (Thermo Fischer, 1:1000). Images of the cells were taken using Nikon Eclipse microscope with nuclear staining by DAPI. The γH2AX foci were detected and quantified using CellProfiler (Broad Institute of MIT and Harvard).^89^ The analyzed data were plotted in RStudio software using R programming language.^90^

### Laser Microirradiation

HEK293 cells with GFP-MUS81 stable expression (cloned into a pAIO-based vector; a kind gift from Josef Jiricny) were grown in 35 mm ibidi’s μ-Dishes and preincubated with MU876 = 32 at 1 and 5 μM final concentration for 2 h. Damage was induced using a 355 nm laser line (UGA-42 Firefly, Rapp OptoElectronic, 20% output power, 3 iterations) connected to a Delta Vision Elite Pro microscope (GE Healthcare). Recruitment and retention of the GFP-MUS81 signal at the damage site was traced with a 100*x*/1.4 (Olympus) objective at the indicated times after irradiation. GFP-MUS81 accumulation at the damage site was compared with an undamaged region within the same microirradiated cell. The average accumulation ± SD of GFP-MUS81 of the three biological replicates is plotted.

### Solubility

Analysis of the aqueous solubility was provided by Bienta Enamine Biology Services. Kinetic solubility assay was performed according to Enamine’s aqueous solubility SOP. Briefly, using a 20 mM stock solution of the compound in 100% DMSO, dilutions were prepared to a theoretical concentration of 400 μM in duplicates in PBS pH 7.4 (138 mM NaCl, 2.7 mM KCl, 10 mM K-phosphate) with 2% final DMSO. The experimental compound dilutions in PBS were further allowed to equilibrate at 25 °C in a thermostatic shaker for 2 h and filtered through HTS filter plates using a vacuum manifold. The filtrates of test compounds were diluted 2-fold with acetonitrile with 2% DMSO. In parallel, compound dilutions in 50% acetonitrile/PBS were prepared to theoretical concentrations of 0.1, 1, 50, 100, and 200 μM with 2% final DMSO to generate calibration curves. Ondansetron was used as a reference compound to control proper assay performance. All 22 samples were diluted 100-fold with 50% acetonitrile/water (v/v) mixes before LC-MS/MS measurement. The effective range of this assay is 0.2–400 μM and can be changed depending on the chemical nature of the compounds.

The thermodynamic solubility assay was performed according to Enamine’s aqueous solubility SOP. Briefly, the dry powder forms of the test compounds were mixed with phosphate buffer (138 mM NaCl, 2.7 mM KCl, 10 mM K-phosphate, pH 7.4) to the theoretical concentration of 4 mM and further allowed to equilibrate at 25 °C in a thermostatic shaker. After 4 and 24 h shaking, incubation mixtures were filtered through HTS filter plates using a vacuum manifold. The filtrates of the test compounds were diluted 2-fold with acetonitrile. In parallel, using a 20 mM stock solution, compound dilutions in 50% acetonitrile/PBS mixes were prepared to theoretical concentrations of 1, 25, 100, and 200 μM to generate calibration curves. Ondansetron was used as a reference compound to control proper assay performance. All samples were diluted 100-fold with 50% methanol/water (v/v) mixes before the LC-MS/MS measurement. The effective range of this assay is 2–400 μM and can be changed depending on the chemical nature of the compounds.

### Analysis of Micronuclei

CAL51 WT and CAL51 MUS81^–/–^ cells were seeded in a 96-well plate 24 h before the treatment. Alternatively, cells were transfected with 30 nM siRNA (control or MUS81) for 48 h before treatment. Subsequently, cells were treated with respective inhibitors for 48 h. During the last 16 h, cytochalasin B (Sigma-Aldrich) was added to the cells at a concentration of 1.25 μg/mL. At the end of the treatment, cells were washed in 1XPBS, fixed with 4% formaldehyde, and washed again. DAPI (5 μg/mL, Panreac AppliChem) was added for nuclear staining. Images were acquired using a Nikon Eclipse fluorescence microscope and analyzed using ImageJ software.^91^

### Cell Survival Assays

CAL51 WT cells were treated with DMSO, MU262 = 18, MU876 = 32, nontargeting siRNA, or siRNA targeting MUS81. Cells were trypsinized, counted, and replated twice a week and retreated with inhibitors or siRNA with every passage. The cell number was plotted at each time point. Data were plotted in Graphpad Prism (GraphPad Software) and a 2-way ANOVA test was used to assess significance.

### Detection of TFEs by DNA Fluorescence In Situ Hybridization (FISH)

U2OS and HeLa cells were seeded in 10 cm dishes. The next day, the medium was changed, and compounds were added at desired concentrations. This process was repeated after 43 h except that compounds were added together with 200 ng/mL of nocodazole. Cells were collected 5 h later by shake-off and incubated in 75 mM KCl at 37 °C for 10 min. Chromosomes were fixed in ice-cold methanol/acetic acid (3:1) and spread on glass slides. Slides were then treated with 20 μg/mL RNase A (Sigma-Aldrich), in 1x PBS at 37 °C for 1 h, fixed in 4% formaldehyde (Sigma-Aldrich) in 1x PBS for 2 min, and treated with 70 μg/mL pepsin (Sigma-Aldrich) in 2 mM glycine, pH 2 (Sigma-Aldrich) at 37 °C for 5 min. Slides were fixed again with 4% formaldehyde in 1x PBS for 2 min, incubated subsequently in 70%, 90%, and 100% ethanol for 5 min each, and air-dried. A C-rich telomeric PNA probe (5′-AF568-OO-*p*4CCCTAACCCTAACCCTAA-3′; Panagene) diluted in hybridization solution (10 mM Tris–HCl pH 7.2, 70% formamide, 0.5% blocking solution (Roche)) was applied onto the slides followed by incubation at 80 °C for 5 min and at room temperature for 2 h. Slides were washed twice in 10 mM Tris–HCl pH 7.2, 70% formamide, and 0.1% BSA and three times in 100 mM Tris–HCl pH 7.2, 150 mM NaCl, and 0.08% Tween-20 at room temperature for 10 min each. DNA was counterstained with 100 ng/mL DAPI (Sigma-Aldrich) in 1x PBS and slides were mounted in Vectashield (Vector Labs). Images were acquired with a Zeiss Cell Observer equipped with a cooled Axiocam 506 m camera and a 63*X*/1.4NA oil DIC M27 PlanApo N objective. Image analysis was performed using ImageJ^91^ and Photoshop software (Adobe Inc.). TFEs were identified as chromatid ends lacking a detectable telomeric signal, and the number of TFEs per metaphase is displayed.

### Synthesis

All commercially available reagents were used as supplied without further purification. The reaction solvents were purchased anhydrous and were stored under nitrogen. Unless noted otherwise, the reactions were carried out in oven-dried glassware under an atmosphere of nitrogen. Analytical thin-layer chromatography (TLC) was performed using aluminum plates precoated with silica gel (silica gel 60 F_254_, Merck). TLC plates were visualized by exposure to ultraviolet light (λ = 254 nm) and/or by submersion in aqueous ceric ammonium molybdate (CAM). All solutions were concentrated by rotary evaporation at 40 °C unless noted otherwise. As indicated, either manual column chromatography was carried out using silica gel (pore size 60 Å, 230–400 mesh particle size, 40–63 μm particle size), or column chromatography was carried out using the Biotage Selekt purification system. Purification by preparative thin layer chromatography was performed using plates from Merck (PLC Silica gel 60 F_254_, 1 mm). Reverse-phase column chromatography was carried out using C_18_-reversed phase silica gel (pore size of 90 Å, 230–400 mesh particle size, 40–63 μm particle size). NMR spectra were obtained in indicated deuterated solvents; chemical shifts are quoted in parts per million (δ) referenced to the appropriate deuterated solvent employed. Multiplicities are indicated by s (singlet), d (doublet), t (triplet), q (quartet), p (pentet), quin (quintet), sept (septet), m (multiplet), or (br) broad, or combinations thereof. Coupling constant values are given in Hz. HPLC separations were performed on Ultimate 3000 LC Systems (Thermo Scientific) with UV detection. IR spectra (4000–400 cm^–1^) were collected on ALPHA ATR spectrometer (BRUKER); solid samples were measured neat and oily samples as films. High-resolution mass spectra were obtained on Agilent 6224 Accurate-Mass TOF LC-MS with dual electrospray/chemical ionization mode or on MALDI-TOF Ultraflextreme (Bruker Daltonics) with positive ion detection. Melting points were determined with a Stuart SMP40 automatic melting point apparatus. The purity of the synthesized target compounds was determined by HPLC analysis with UV detection (Ultimate 3000 LC analytical Systems, Thermo Scientific) and ^1^H NMR. All final compounds reported herein were >95% pure (unless stated otherwise).

#### 2-(4-Fluorophenyl)-3-oxopentanenitrile

NaH (60% suspension in mineral oil; 667 mg, 16.66 mmol; 2 equiv) was added to a solution of 4-fluorophenylacetonitrile (1 mL, 8.33 mmol; 1 equiv) in anhydrous THF (25 mL) at 25 °C under a nitrogen atmosphere and stirred for 15 min. Methyl propionate (0.8 mL, 8.33 mmol, 1 equiv) was added, and the reaction mixture was stirred at 25 °C for an additional 1 h 30 min. The reaction mixture was cooled to 0 °C and saturated aqueous solution of NH_4_Cl (50 mL) was added. The mixture was extracted with EtOAc (3 × 75 mL). The organic extracts were dried over MgSO_4_, filtered, and the solvent was evaporated in vacuo. The residue obtained after the workup was purified by column chromatography on silica gel (hexane:EtOAc, gradient 1:0 to 1:1). The product was obtained as an orange oil (2.92 g, 92%). ^1^H NMR (500 MHz, Chloroform-*d*) δ (ppm): 7.40–7.34 (m, 2H), 7.15–7.09 (m, 2H), 4.67 (s, 1H), 2.74–2.52 (m, 2H), 1.05 (t, *J* = 7.2 Hz, 3H). ^13^C NMR (126 MHz, Chloroform-*d*) δ (ppm): 199.35, 163.24 (d, *J* = 249.8 Hz), 129.96 (d, *J* = 8.2 Hz), 125.92 (d, *J* = 3.5 Hz), 116.81 (d, *J* = 21.9 Hz), 116.36, 49.80, 33.40, 7.71. HRMS (APCI): calcd for C_11_H_9_FNO [M – H]^−^ = 190.0674, found [M – H]^−^ = 190.0672.

#### 3-Ethyl-4-(4-fluorophenyl)-1*H*-pyrazol-5-amine

N_2_H_4_·H_2_O (64% in H_2_O, 0.48 mL, 9.88 mmol; 1 equiv) and CH_3_SO_3_H (64 μL, 0.99 mmol, 0.1 equiv) were added to a solution of 2-(4-fluorophenyl)-3-oxopentanenitrile (1.89 g, 9.88 mmol; 1 equiv) in absolute EtOH (20 mL) at 25 °C under a nitrogen atmosphere. Then, the reaction mixture was refluxed for 45 min. The solvent was evaporated in vacuo, and the residue was quenched with saturated aqueous solution of NaHCO_3_ (80 mL) and extracted with EtOAc (2 × 100 mL). The organic extracts were washed with brine (2 × 80 mL), dried over MgSO_4_, filtered, and the solvent was evaporated in vacuo. The residue was sonicated in EtOH (10 mL), and the precipitate was collected by filtration to afford the product as a white crystalline solid (1.1 g, 54%). The filtrate was concentrated in vacuo, and the residue was purified by column chromatography on silica gel (hexane:EtOAc, gradient 2:1 to 0:1) to afford an additional product as a white solid (727 mg, 36%). ^1^H NMR (500 MHz, DMSO-*d*_6_) δ (ppm): 11.42 (s, 1H), 7.36–7.28 (m, 2H), 7.22–7.13 (m, 2H), 4.38 (s, 2H), 2.53 (q, *J* = 7.6 Hz, 2H), 1.09 (t, *J* = 7.6 Hz, 3H). ^13^C NMR (126 MHz, DMSO-*d*_6_) δ (ppm): 160.12 (d, *J* = 242.2 Hz), 150.84, 143.06, 130.42 (d, *J* = 3.1 Hz), 129.92 (d, *J* = 7.9 Hz), 115.10, 102.76, 18.37, 13.24.

^19^F NMR (471 MHz, DMSO-*d*_6_) δ (ppm): −117.79. HRMS (APCI): calcd for C_11_H_11_FN_3_ [M – H]^−^ = 204.0942, we found [M – H]^−^ = 204.0948. mp = 163–166 °C.

#### 3-(1*H*-Benzo­[*d*]­imidazole-2-yl)-7-ethyl-8-(4-fluorophenyl)­pyrazolo­[5,1-*c*]­[1,2,4]­triazin-4-ol (MU262)

Aqueous HCl (35%, 0.34 mL, 4.0 mmol; 4 equiv) and H_2_O (5 mL) were added to a solution of 3-ethyl-4-(4-fluorophenyl)-1*H*-pyrazol-5-amine (205 mg, 1.0 mmol; 1 equiv) in EtOH (5 mL). The reaction mixture was cooled to −10 °C and a precooled solution (0 °C) of NaNO_2_ (136 mg, 2.0 mmol, 2 equiv) in H_2_O (1 mL) was added. The reaction mixture turned yellow and was stirred for 30 min at −5 °C, then a precooled solution (−5 °C) of methyl 2-(1*H*-benzo­[*d*]­imidazole-2-yl)­acetate (209 mg, 1.10 mmol, 1.1 equiv) in EtOH (5 mL) and KOAc (560 mg, 6.0 mmol, 6 equiv) in H_2_O (5 mL) was added. The resulting mixture was allowed to warm to 25 °C and stirred for 16 h. Then, cold water (5 mL) was added, and the precipitate was collected by filtration and washed with additional water (2.5 mL). The product was dried under a vacuum to yield a yellow solid (290 mg, 0.74 mmol), which was used directly in the next step without additional purification. The yellow solids (290 mg, 0.74 mmol) were dissolved in anhydrous DMF (5 mL) and KOAc (8 mg, 0.08 mmol, 0.1 equiv) was added and refluxed for 2 h. Then, the reaction mixture was poured into water (5 mL), and the precipitate was collected by filtration. The solids were dissolved in dioxane (2.5 mL) at 50 °C, and the solution was poured into water (10 mL). The precipitate was collected by filtration, washed with water (5 mL), then with Et_2_O (2.5 mL), and dried in vacuo. The compound was purified by reversed-phase column chromatography using a Biotage Selekt purification system (water:MeOH:7 M NH_3_ in methanol, gradient 80:20:2 to 30:70:2). The product was obtained as a yellow solid (210 mg, 56%). ^1^H NMR (500 MHz, DMSO-*d*_6_) δ (ppm): 14.03 (s, 2H), 7.84–7.79 (m, 2H), 7.78–7.73 (m, 2H), 7.47–7.42 (m, 2H), 7.35–7.29 (m, 2H), 2.94 (q, *J* = 7.5 Hz, 2H), 1.28 (t, *J* = 7.5 Hz, 3H). ^13^C NMR (126 MHz, DMSO-*d*_6_) δ (ppm): 160.84 (d, *J* = 243.7 Hz), 155.65, 149.28, 148.89, 148.34, 131.07, 130.74 (d, *J* = 7.9 Hz), 128.37 (d, *J* = 3.2 Hz), 124.67, 119.14, 115.22 (d, *J* = 21.2 Hz), 113.32, 108.22, 20.77, 13.06. ^19^F NMR (471 MHz, DMSO-*d*_6_) δ (ppm): −114.60. HRMS (APCI): calcd for C_20_H_14_FN_6_O [M – H]^−^ = 373.1219, we found [M – H]^−^ = 373.1217.

#### 6-Methyl-2-thioxo-2,3-dihydropyrimidin-4­(1*H*)-one

Thiourea (10 g, 130 mmol) was added to a solution of sodium hydroxide (10.9 g, 272 mmol) in H_2_O (205 mL) at 15 °C and stirred for 10 min. Ethyl acetoacetate (20.8 g, 160 mmol) was added dropwise at 15 °C and the reaction mixture was allowed to warm to room temperature and stirred for 3 h. The pH was adjusted to 4–5 by the careful addition of conc. hydrochloric acid (3.5 mL). The precipitate was collected by filtration, washed with cold water (20 mL), and dried in vacuo. The product was obtained as an off-white solid (7.18 g, 39%). ^1^H NMR (300 MHz, DMSO-*d*_6_) δ (ppm): 12.23 (s, 2H), 5.67 (d, *J* = 1.1 Hz, 1H), 2.06 (s, 3H). HRMS (APCI): calcd for C_5_H_5_N_2_OS [M – H]^−^ = 141.0128, [M – H]^−^ = 141.0128. mp > 265 °C (dec.).

#### 6-Methyl-2-(methylthio)­pyrimidin-4­(3*H*)-one

6-Methyl-2-thioxo-2,3-dihydropyrimidin-4­(1*H*)-one (7.18 g, 50.5 mmol) was added to a solution of NaOH (2.08 g, 52.01 mmol) in water (68 mL) and the reaction mixture was stirred at 25 °C for 20 min. Iodomethane (3.92 mL, 63.12 mmol) was added dropwise, and the mixture was stirred at room temperature for an additional 4 h. The precipitate was collected by filtration, washed with ice cold water (2 × 20 mL), and dried in vacuo. The product was obtained as a white solid (7.8 g, 99%). ^1^H NMR (300 MHz, DMSO-*d*_6_) δ (ppm): 12.40 (s, 1H), 5.96 (s, 1H), 2.47 (s, 3H), 2.17 (s, 3H). HRMS (APCI): calcd for C_6_H_9_N_2_OS [M + H]^+^ = 157.0430, [M + H]^+^ = 157.0428. mp = 225–226 °C.

#### 2-Hydrazinyl-6-methylpyrimidin-4­(3*H*)-one

Hydrazine hydrate (64% aq solution, 10.18 g, 203 mmol) was added to a solution of 6-methyl-2-(methylthio)­pyrimidin-4­(3*H*)-one (7.8 g, 49.93 mmol) in EtOH (20 mL) and stirred at 80 °C for 6 h. Then, the reaction mixture was cooled to room temperature, and the resulting precipitates were collected by filtration, washed with water (2 mL), and dried in vacuo. The product was obtained as an off-white solid (4.15 g, 59%). ^1^H NMR (300 MHz, DMSO-*d*_6_) δ (ppm): 8.89 (s, 2H), 5.37 (s, 1H), 4.70 (s, 1H), 2.00 (s, 3H). HRMS (APCI): calcd for C_5_H_9_N_4_O [M + H]^+^ = 141.0771, [M + H]^+^ = 141.0770. mp = 231–233 °C.

#### 2-(5-Amino-3-phenyl-1*H*-pyrazol-1-yl)-6-methylpyrimidin-4­(3*H*)-one

Methanesulfonic acid (21 mg, 0.21 mmol) was added to a solution of 2-hydrazinyl-6-methylpyrimidin-4­(3*H*)-one (300 mg, 2.14 mmol) and 3-oxo-3-phenylpropanenitrile (311 mg, 2.14 mmol) in EtOH (6 mL) under a nitrogen atmosphere at 25 °C. The reaction mixture was refluxed for 4 h, then cooled to room temperature, and concentrated in vacuo. The crude product was purified by column chromatography on silica gel (7 M NH_3_ in MeOH:MeOH:dichloromethane, 3:7:90). The product was obtained as a pale-yellow solid (540 mg, 94%). ^1^H NMR (500 MHz, DMSO-*d*_6_) δ (ppm): 11.87 (s, 1H), 8.06–7.84 (m, 2H), 7.52–7.29 (m, 3H), 7.04 (s, 2H), 6.09 (s, 1H), 5.88 (s, 1H), 2.28 (s, 3H). ^13^C NMR (126 MHz, DMSO-*d*_6_) δ (ppm): 164.3, 153.2, 151.7, 132.7, 129.2, 128.9, 126.6, 107.5, 85.7, 23.7. HRMS (APCI): calcd for C_14_H_14_N_5_O [M + H]^+^ = 268.1193, found [M + H]^+^ = 268.1193. mp = 239–241 °C.

#### *N*-(1-(4-Methyl-6-oxo-1,6-dihydropyrimidin-2-yl)-3-phenyl-1*H*-pyrazol-5-yl)-5-phenylisoxazole-3-carboxamide (MU876)

Et_3_N (16 μL, 0.11 mmol) was added to a solution of 2-(5-amino-3-phenyl-1*H*-pyrazol-1-yl)-6-methylpyrimidin-4­(3*H*)-one (30 mg, 0.11 mmol) in acetonitrile (1 mL). The reaction mixture was heated to reflux, and a solution of 5-phenylisoxazole-3-carbonyl chloride (11 mg, 0.11 mmol) in acetonitrile (0.5 mL) was added dropwise. The resulting mixture was refluxed for an additional 4 h. The reaction mixture was cooled to room temperature, diluted with saturated aqueous NaHCO_3_ solution (5 mL), and filtered. The obtained solids were washed with water (5 mL) and then with EtOAc (3 mL). The compound was purified by reversed-phase column chromatography using a Biotage Selekt purification system (water:MeOH:0.7 M NH_3_ in methanol, gradient 90:10:10 to 0:70:30). The product was obtained as a white solid (16 mg, 58%). ^1^H NMR (500 MHz, DMSO-*d*_6_) δ (ppm): 15.46 (s, 1H), 8.05–8.01 (m, 2H), 7.93 (d, *J* = 7.6 Hz, 2H), 7.83 (s, 1H), 7.62–7.55 (m, 3H), 7.47 (t, *J* = 7.6 Hz, 2H), 7.38 (t, *J* = 7.3 Hz, 1H), 7.25 (s, 1H), 5.82 (s, 1H), 2.27 (s, 3H). ^13^C NMR (126 MHz, DMSO-*d*_6_) δ (ppm): 172.7, 162.7, 150.9, 154.8, 141.7, 130.9, 129.3, 128.7, 126.3, 125.8, 125.7, 107.2, 99.9, 93.9, 22.9. HRMS (APCI): calcd for C_24_H_17_N_6_O_3_ [M – H]^−^ = 437.1368, found [M – H]^−^ = 437.1365. mp > 320 °C (dec.).

## Supplementary Material











## Notes

Additional data are provided, including supplementary figures and tables, detailed materials and methods, synthetic procedures with NMR and IR spectra for all compounds, and HPLC analysis of compound 18 and 32 (PDF)
Molecular formula strings (CSV)
Molecular docking structures 1 (PDB)
Molecular docking structures 2 (PDB)
Molecular docking structures 3 (PDB)

## Abbreviations Used

ALT: alternative lengthening of telomeres
BHQ1: black hole quencher
BIR: break-induced replication
BLI: biolayer interferometry
CAM: ceric ammonium molybdate
CFA: colony formation assay
CPT: camptothecin
EdU: 5-Ethynyl-2′-deoxyuridine
EMSA: electrophoretic mobility shift assay
FAM: 6-carboxyfluorescein
FISH: fluorescence in situ hybridization
GFP: green fluorescent protein
HR: homologous recombination
HRP: horseradish peroxidase
HU: hydroxyurea
ICL: interstrand cross-link
MMC: mitomycin C
NHEJ: nonhomologous end joining
PI: propidium iodide
SAR: structure–activity relationship
TFEs: telomere-free ends
TLC: thin-layer chromatography.
