# Supplementary material for: Discovery of Two Structurally Distinct Classes of Inhibitors Targeting the Nuclease MUS81 and Enhancing Efficacy of Chemotherapy in Cancer Cells
Source: J Med Chem. 2026 Feb 25;69(5):5350–69. doi: 10.1021/acs.jmedchem.5c02096 (PMC12990037; doi:10.1021/acs.jmedchem.5c02096)
Supplement: Supplementary file 1 [file jm5c02096_si_001.pdf]

Supporting information

Supplementary Figures, and Tables and Methods containing additional biological and chemical data

Discovery of two structurally distinct classes of inhibitors targeting the nuclease MUS81 and enhancing efficacy of chemotherapy in cancer cells

Jana Prochazkova[1], Benoit Carbain[2,3], Victoria Marini[1], Fedor Nikulenkov[1], Stepan Havel[2,3], Naresh Akavaram[2], Prashant Khirsariya[2,3], Alexandra Sisakova[4], Jakub Cibulka[1], Michala Boudova[1], Magdalena Zacpalova[1], Magdalena Kalovska[1], Joana Rodrigues[5], Lukas Daniel[6], Jan Brezovsky[6,7], Petr Bartunek[8], Claus Azzalin [5,9], Kamil Paruch[2,3,*], Lumir Krejci[1,4,*]

1 Department of Biology, Faculty of Medicine, Masaryk University, 62500 Brno, Czech Republic
2 Department of Chemistry, Faculty of Science, Masaryk University, 62500 Brno, Czech Republic
3 International Clinical Research Center, St. Anne's University Hospital, Brno 656 91, Czech Republic
4 NCBR, Faculty of Science, Masaryk University, 62500 Brno, Czech Republic
5 GIMM - Gulbenkian Institute for Molecular Medicine, 1649-035 Lisbon, Portugal
6 Loschmidt Laboratories, Department of Experimental Biology and RECETOX, Faculty of Science, Masaryk University, 62500 Brno, Czech Republic
7 Laboratory of Biomolecular Interactions and Transport, Faculty of Biology, Department of Gene Expression, Institute of Molecular Biology and Biotechnology, Faculty of Biology, Adam Mickiewicz University, 61-614 Poznan, Poland
8 Institute of Molecular Genetics of the Czech Academy of Sciences, Prague, Czech Republic
9 Faculty of Medicine, University of Lisbon, 1649-028 Lisbon, Portugal

*Correspondence: lkrejci@chemi.muni.cz (L.K.), paruch@chemi.muni.cz (K.P.)

Table of Contents

Supplementary Figures 4

Supplementary Tables 16

Supplementary Methods 41

References: 43

Experimental procedures for all intermediates and final compounds 44

General information 44

General procedure A1: Formation of ketonitrile by deprotonation of phenylacetonitrile with NaH 44

General procedure A2: Formation of ketonitrile by deprotonation of CH_3CN with NaH 45

General procedure A3: Formation of ketonitrile by deprotonation of CH_3CN with *n*-BuLi 45

General procedure A4: Formation of ketonitrile by deprotonation of alkylacetonitrile with LDA 45

General procedure A5: Formation of ketonitrile by deprotonation of CH_3CN with NaH 46

General procedure B1: Formation of aminopyrazole using hydrazine hydrate and methanesulfonic acid 46

General procedure B2: Formation of aminopyrazole using arylhydrazine and methanesulfonic acid 46

General procedure C: Diazotization of aminopyrazoles and cyclization to pyrazolotriazines 46

General procedure D: Suzuki reaction 47

General procedure E: Preparation of diketo ester using *t*-BuOK and diethyl oxalate 47

General procedure F: Preparation of isoxazole esters 48

General procedure G: Hydrolysis of isoxazole and isothiazole esters to corresponding carboxylic acids 48

General procedure H: Preparation of acid chlorides 48

General procedure I: Preparation of amides using acid chlorides 48

Experimental procedures for compound 1 and its analogs 49

Preparation of target compound 1 and its analogs 93

Experimental procedures for compound 2 and its analogs 156

Preparation of target compound 2 and its analogs 195

^{1}H NMR, ^{13}C NMR and HRMS spectra of compound S1-S108 232

^{1}H NMR, ^{13}C NMR and HRMS spectra of compound 1 and 3-28 362

^{1}H NMR, ^{13}C NMR and HRMS spectra of compound 56-117 416

^{1}H NMR, ^{13}C NMR, HRMS and IR spectra of compound S109-S188 539

^{1}H NMR, ^{13}C NMR, HRMS and IR spectra of compound 2, S190 and 29-55 678

^{1}H NMR, ^{13}C NMR, HRMS and IR spectra of compound 118-141 736

^{1}H NMR, ^{13}C NMR, HRMS and IR spectra of compound S191-S192 786

^{1}H and ^{13}C NMR HRMS and IR spectra of compound 142 789

^{1}H and ^{13}C NMR HRMS and IR spectra of compound S193-S194 791

^{1}H and ^{13}C NMR, HRMS and IR spectra of compound 143 795

HPLC analytical chromatogram of compound 18 797

HPLC analytical chromatogram of compound 32 798

Supplementary Figures

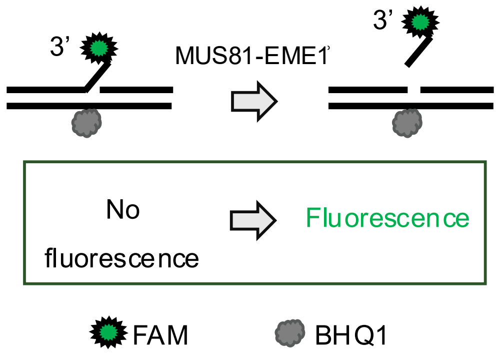

Figure S1: A schematic representation of a fluorogenic assay specific for MUS81-EME1. The assay consists of a 3'flap DNA substrate labelled with fluorescein (FAM) and a Black Hole Quencher. Upon endonucleolytic cleavage of the substrate by recombinant MUS81-EME1, FAM separates from BHQ1, resulting in a significant fluorescence increase, providing a direct readout of the enzymatic activity.

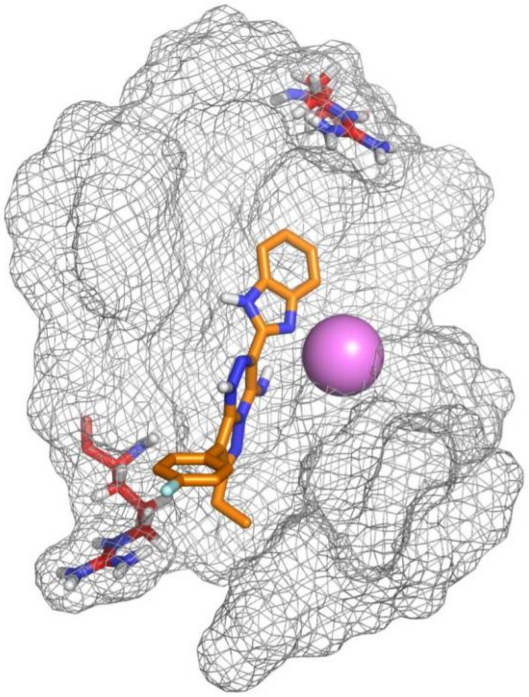

Figure S2: Docking model of compound 1 in the MUS81 active site. Compound **1** was docked into the active site of the MUS81 nuclease based on the published crystal structure[1]. Note: the imine tautomer of compound **1** was used for the docking, consistent with the structure provided by the supplier.

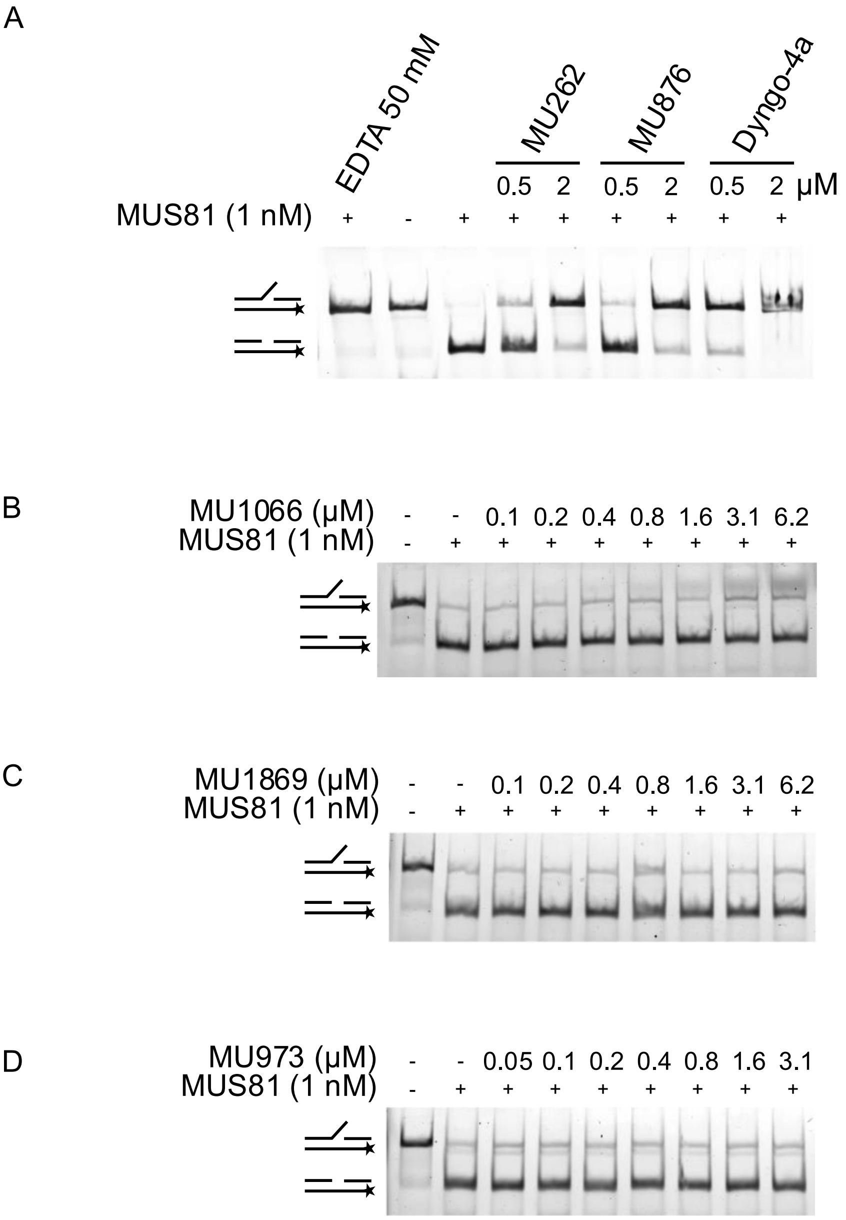

Figure S3. Validation of MUS81–EME1 nuclease inhibition using inactive and positive control compounds.

Purified human MUS81-EME1 (1 nM) was incubated with (A) indicated concentrations of MU262=18, MU876=32, Dyngo-4a and EDTA; or with increasing concentrations of MU1066=17 (B), MU1869=25 (C) or MU973=45 (D), followed by the addition of 3 nm of fluorescently labelled 3’flap DNA. The reaction products were resolved on a native PAGE gel. The lower band corresponds to the cleaved DNA. Representative picture shown, n = 3.

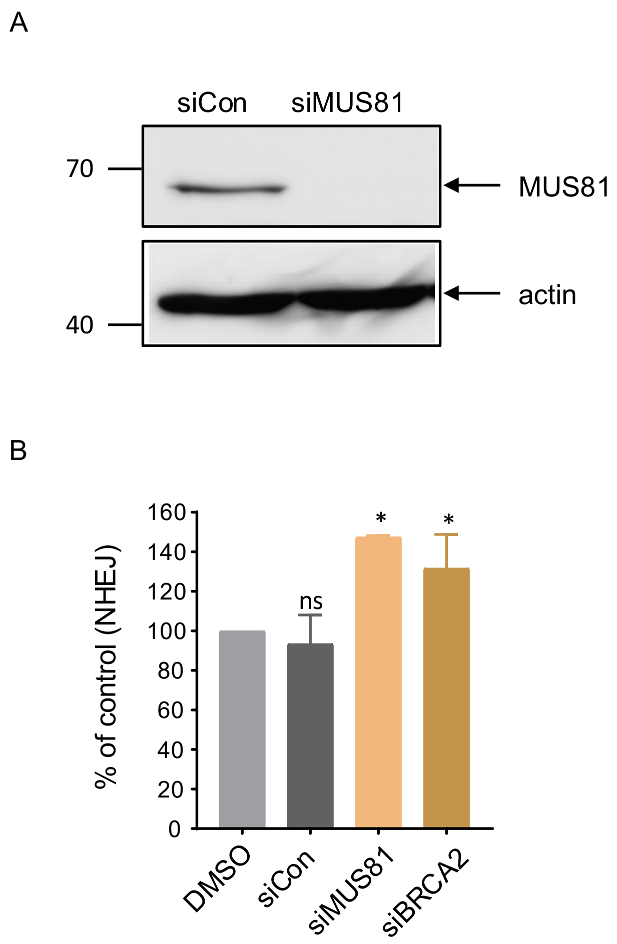

Figure S4: MUS81 depletion has no detectable effect on NHEJ. (A) U2OS BIR-GFP cells were treated with non-targeting siRNA (siCon) or siRNA against MUS81. Cells were harvested after three days, and WB was done using actin as a loading control.

(B) I-SceI-based NHEJ repair efficiency was measured using U2OS EJ5-GFP cells, treated with DMSO or siRNAs targeting MUS81 and BRCA2, respectively for 72 hours. The percentage of repair was normalised to the DMSO treated control. *n* = at least 2. error bars, s.d., **, $p < 0.01$ (unpaired, two-tailed *t*-test).

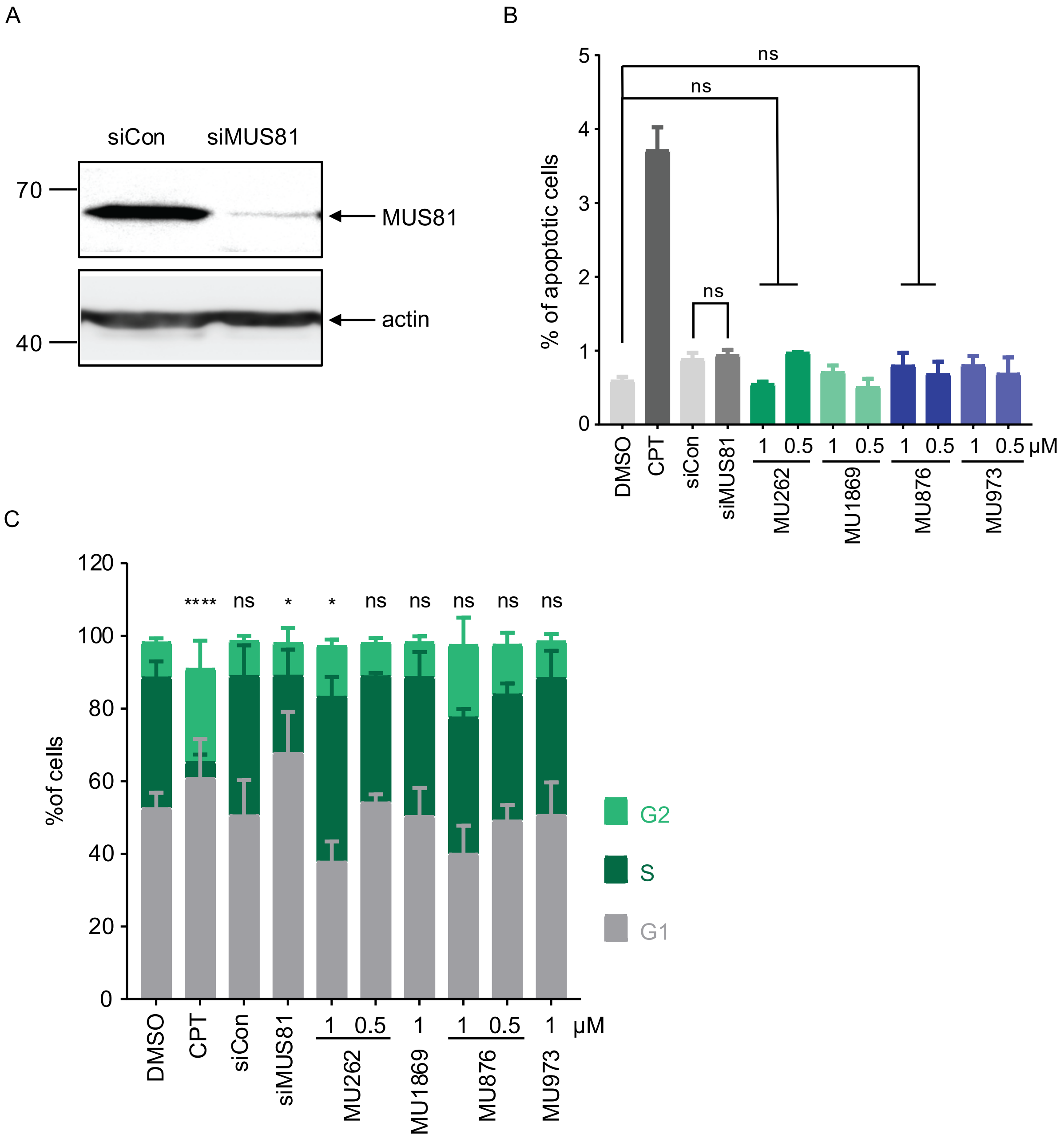

Figure S5: Short-term MUS81 inhibition does not induce apoptosis or major cell cycle defects. (A) U2OS WT cells were treated with non-targeting siRNA (siCon) or siRNA against MUS81. Cells were harvested after three, days and WB was done using actin as a loading control.
(B) U2OS WT cells were treated with DMSO, CPT, control or MUS81-targetting siRNA or the indicated concentrations of **MU262=18**, **MU1869=25**, **MU876=32** and **MU973=45** for 72 hours. Apoptotic cells were determined by Annexin V staining and measured by flow cytometry. n = at least 2. error bars, s.d. (unpaired, two-tailed *t*-test).
(C) U2OS WT cells were treated with DMSO, CPT, control or MUS81-targetting siRNA or the indicated concentrations of **MU262=18**, **MU1869=25**, **MU876=32** and **MU973=45** for 72 hours. Cells were labelled with EdU 30 minutes before harvest. Cell cycle was analysed using EdU and PI staining. Data were acquired by flow cytometry. n = 3. Error bars represent comparison to DMSO, s.d., *, $p < 0.05$ (unpaired, two-tailed *t*-test).

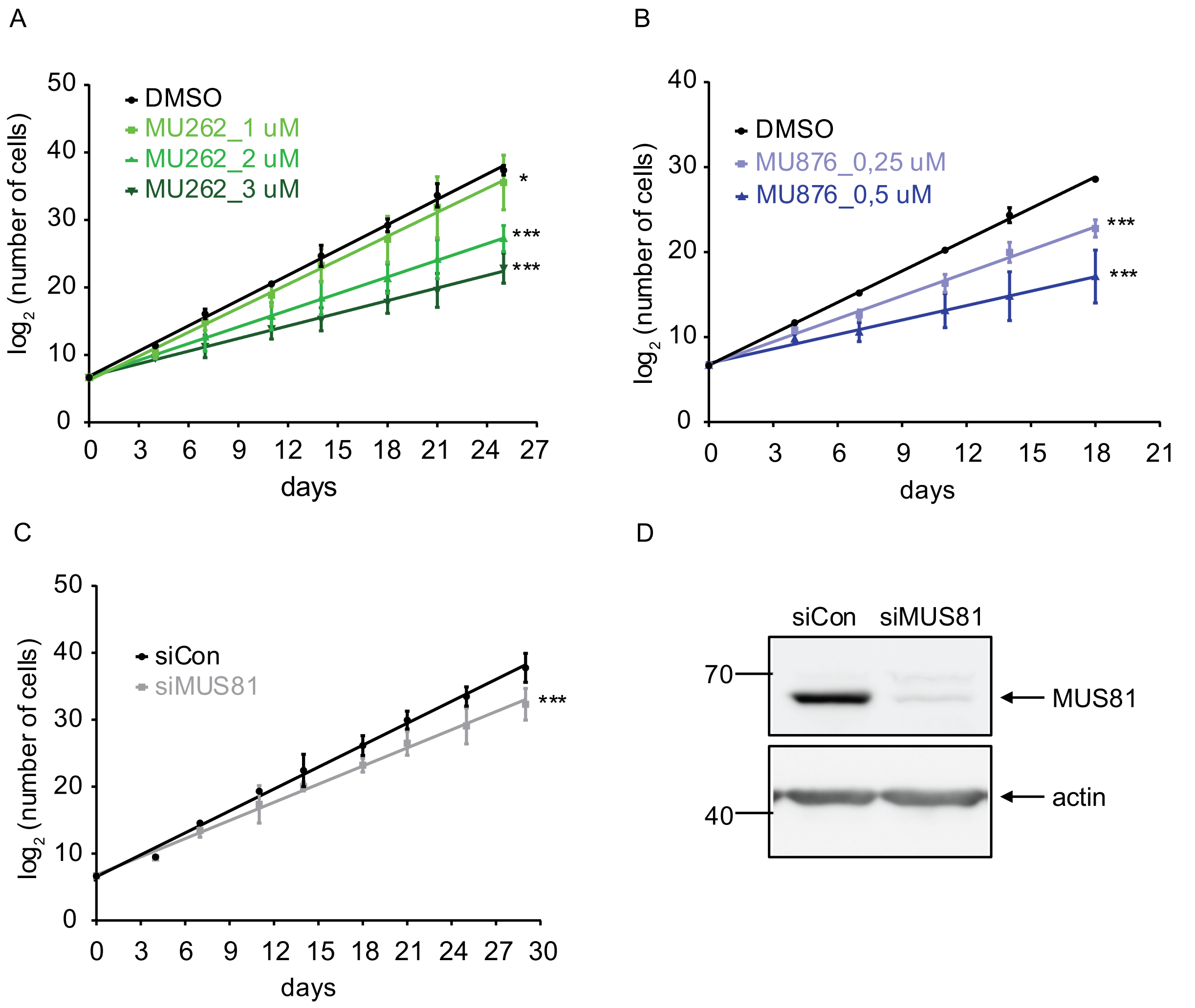

Figure S6: Long-term MUS81 depletion or inhibition reduces cell proliferation. (A) CAL51 WT cells were treated with DMSO and the indicated concentrations of MU262. Cells were left to grow for the indicated time and counted after each passage. The plots show non-linear fit of the exponential cell growth. n = at least 3. *, $p < 0.05$, ***, $p < 0.001$ (two-way ANOVA test).
(B) CAL51 WT cells were treated with DMSO and the indicated concentrations of MU876. Cells were left to grow for the indicated time and counted after each passage. The plots show non-linear fit of the exponential cell growth. n = 2. ***, $p < 0.001$ (two-way ANOVA test).
(C) CAL51 WT cells were treated with the non-targeting siRNA (siCon) and siRNA targeting MUS81. Cells were left to grow for the indicated time and counted after each passage. The plots show non-linear fit of the exponential cell growth. n = 2. ***, $p < 0.001$ (two-way ANOVA test).
(D) CAL51 WT cells were treated with control siRNA or siRNA against MUS81. Cells were harvested after three days, and WB was done using actin as a loading control.

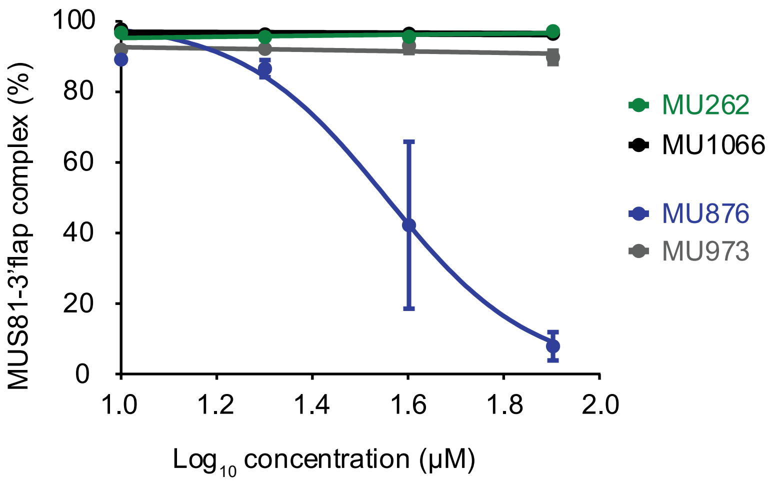

Figure S7: Quantitative analysis of MUS81-DNA binding inhibition by MU262 and MU876. Quantification of Fig. 4 A-B. Purified MUS81 (160 nM) was incubated with increasing concentrations of **MU262**, **MU876**, or their respective control analogues, followed by the addition of 3 nM fluorescently labelled 3' flap DNA substrate. $MgCl_2$ was omitted from the reaction buffer to prevent substrate cleavage. The reaction products were resolved on a native PAGE gel. The lower DNA band corresponding to the free DNA was used for quantification. Values were normalised to the negative control containing no enzyme. The graph represents % of 3'flap DNA substrate bound to MUS81 fitted to a sigmoid curve. n = 3.

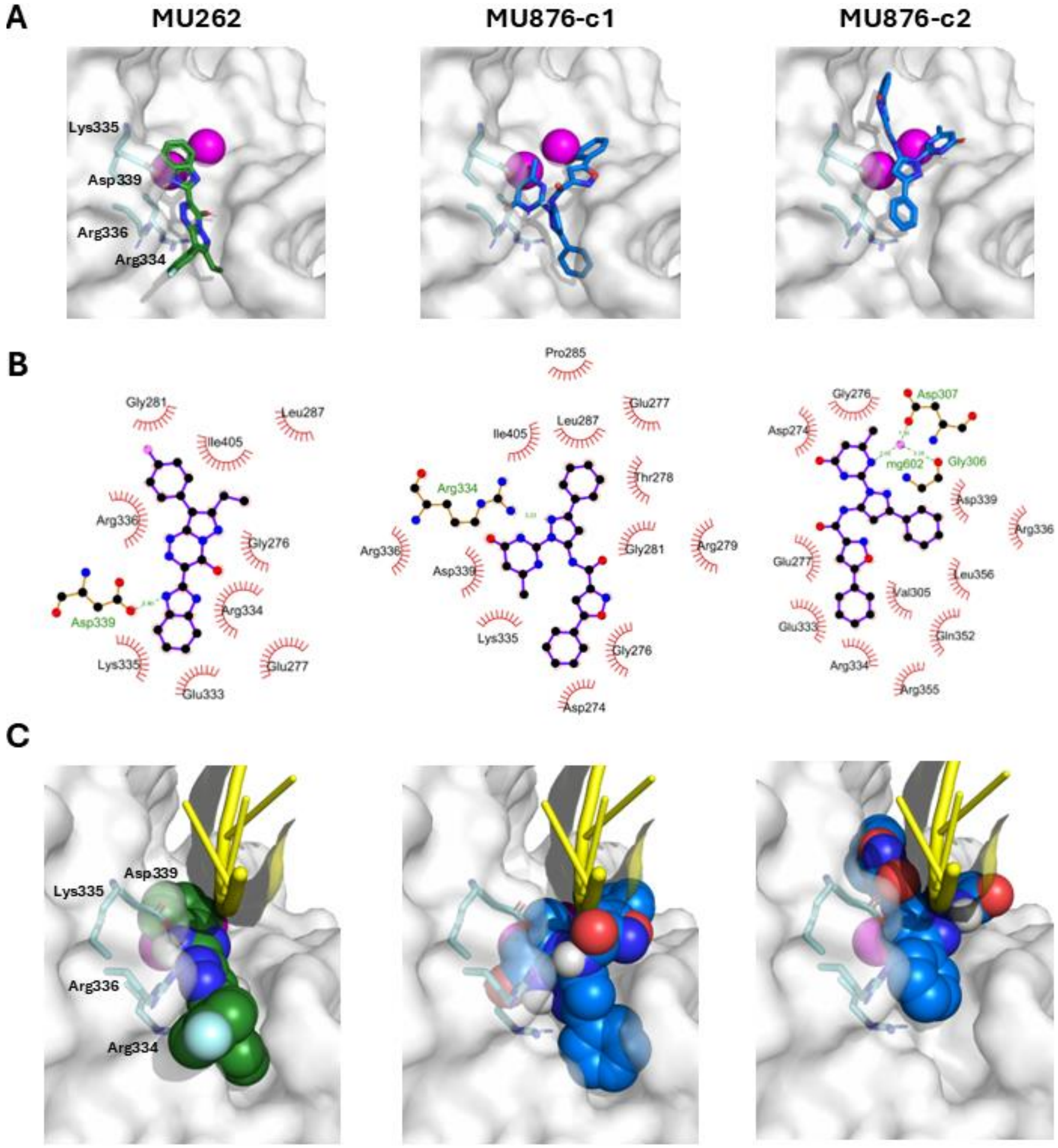

Figure S8. Molecular docking analysis of MU262 and two amide bond conformers of MU876 bound to human MUS81 endonuclease. (A) Top view of the most favorable binding modes of **MU262** (green sticks) and two conformers (c1 and c2) of **MU876** (blue sticks) obtained with AutoDock Vina 1.2.5 (see Table S5)[2]. The inhibitors were docked to MUS81-EME1 structure (PDB-ID: 9F99) with missing residues 371–377 modeled from PDB-ID: 9F98[3]. The active site of human MUS81 is represented by white surface, Mg^{2+} ions as purple spheres, and interacting charged residues as cyan sticks. (B) Diagrams of interactions between MUS81 and the inhibitors generated with LigPlot+[4]. (C) Evaluation of steric clashes between bound inhibitors (blue and green spheres) and 32-bp dsDNA with 3-nt 3′ flap (yellow backbone and surface), superimposed from PDB-ID: 4P0R[1]. The active site of human MUS81 is represented by white surface, Mg^{2+} ions as purple spheres, and interacting charged residues as cyan sticks.

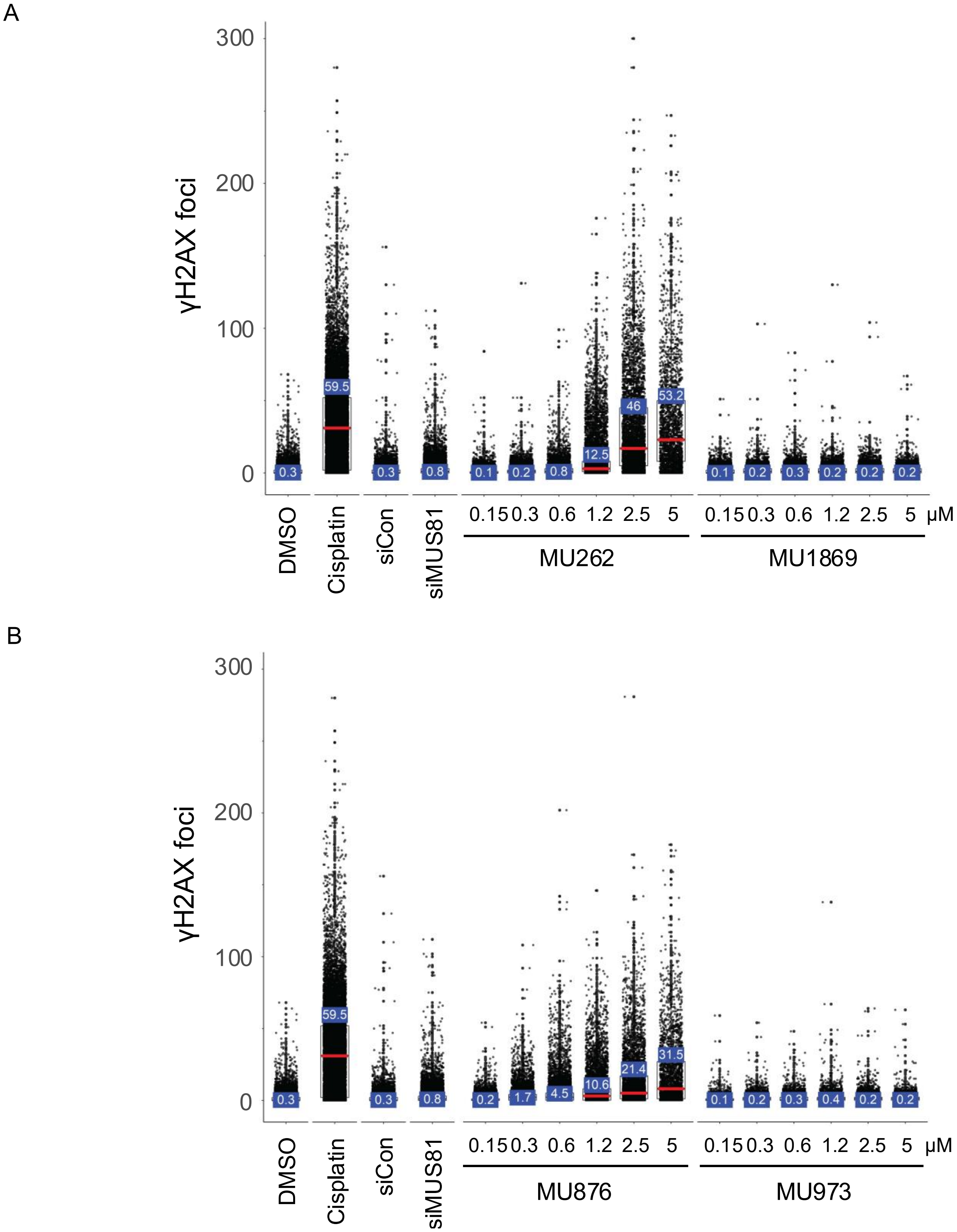

Figure S9: MUS81 inhibition induces persistent DNA damage. (A)U2OS cells were treated with DMSO, cisplatin as a positive control, non-targeting siRNA (siCon) or MUS81-targeting siRNA, **MU262**, and its negative control counterpart at the indicated concentrations for 72 hours. (B) U2OS cells were treated with DMSO, cisplatin as a positive control, non-targeting siRNA (siCon)

or MUS81-targeting siRNA, **MU876**, and its negative control counterpart at the indicated concentrations for 72 hours. The number of γH2AX foci was assessed by fluorescence microscopy and quantified by a CellProfiller software. Data are plotted in R software. Red marker represents median of γH2AX foci number per nucleus. n = 3.

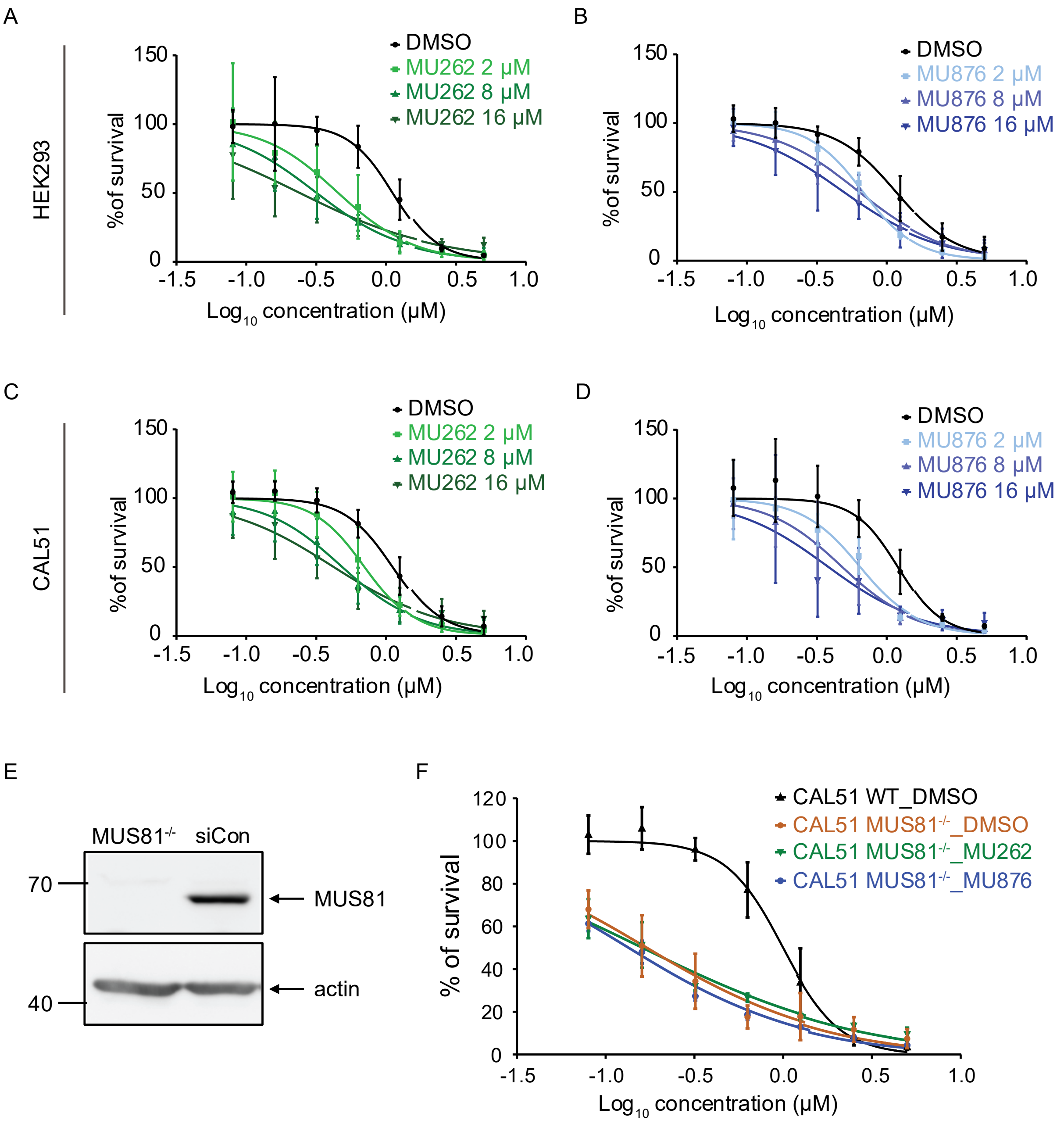

Figure S10: MUS81 inhibition or depletion enhance cellular sensitivity to cisplatin. HEK293 cells (A-B) and CAL51 cells (C-D) were treated with DMSO, **MU262**, or **MU876** at the indicated concentrations in combination with range of cisplatin (0 – 5 µM) for 96 – 120 hours. Cell viability was assessed by the Cy-Quant assay. The survival was normalised to the DMSO-treated control. Graphs were generated in Graphpad Prism using the non-linear regression fitting model. n ≥ 3, error bars represent s.d. (E) CAL51 MUS81$^{-/-}$ cells and CAL51 cells treated with control siRNA. Cells were harvested after three days and WB was done using actin as a loading control. (F) CAL51 WT and MUS81-/- cells were treated with DMSO, **MU262** and **MU876** at 16 µM concentration together with the indicated concentration of cisplatin for 96 – 120 hours. Cell viability was assessed by the Cy-Quant assay. The survival rate of cells was normalised to the DMSO control. Graphs are plotted in Graphpad Prism using the non-linear regression fitting model. n = 3.

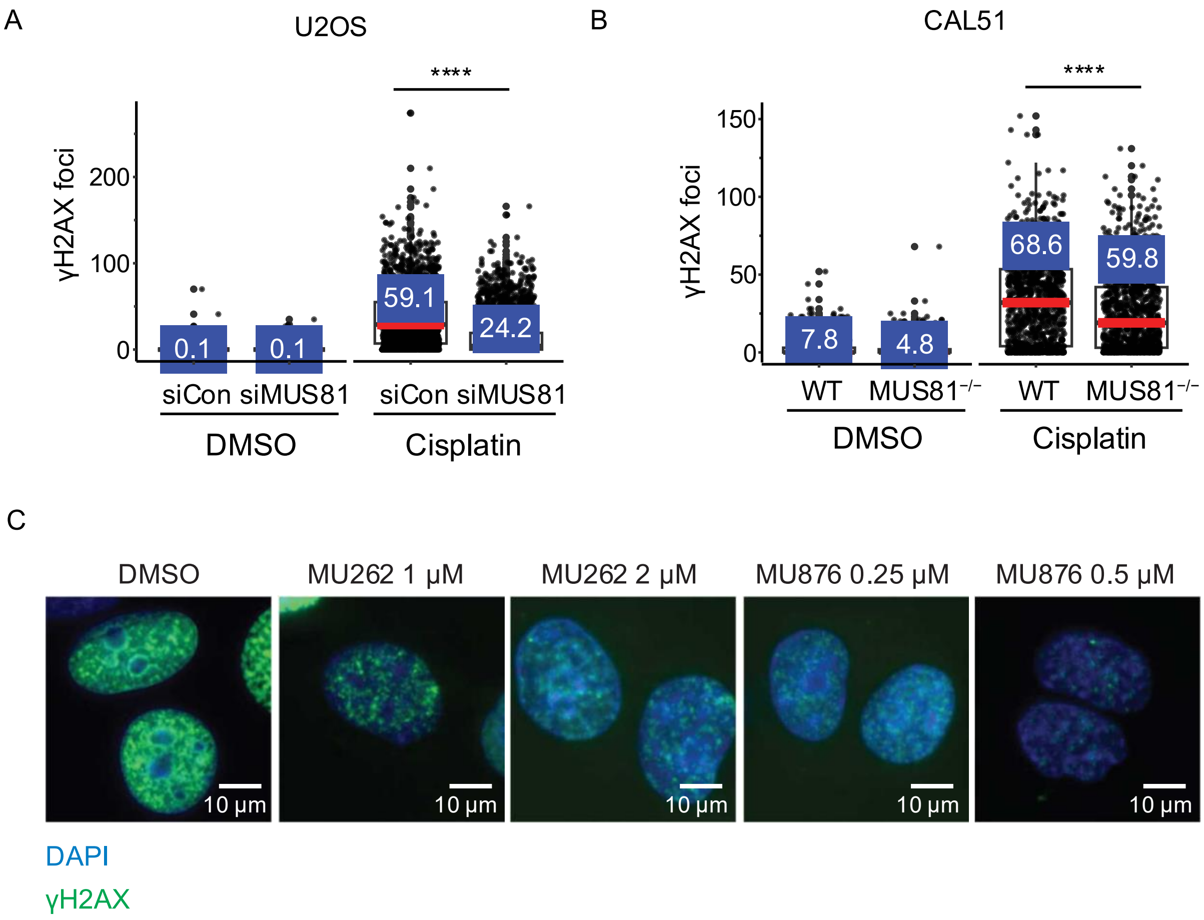

Figure S11: MUS81 depletion or inhibition impair cisplatin-induced DNA damage signalling. (A) U2OS cells were treated with DMSO, non-targeting siRNA (siCon) or siRNA targeting MUS81 with every passage for a total length of two weeks. After that cisplatin (12 µM) was added for the last 24 hours before harvest. The number of foci of a DNA damage marker γH2AX was assessed by fluorescence microscopy and quantified by a CellProfiller software. Data are plotted in R software. Red marker represents median of γH2AX foci number per nucleus. n = 1., error bars, s.d., *, $p < 0.05$, **, $p < 0.01$ ***, $p < 0.001$ (unpaired, two-tailed *t*-test). (B) CAL51 WT and MUS81$^{-/-}$ cells were passaged for a total length of two weeks. After that cisplatin (12 µM) was added for the last 24 hours before harvest. The number of foci of a DNA damage marker γH2AX was assessed by fluorescence microscopy and quantified by a CellProfiller software. Data are plotted in R software. Red marker represents median of γH2AX foci number per nucleus. n = 1., error bars, s.d., *, $p < 0.05$, **, $p < 0.01$ ***, $p < 0.001$ (unpaired, two-tailed *t*-test). (C) CAL51 WT cells were treated with DMSO, **MU262**, or **MU876** at the indicated concentrations at each passage over two weeks. Cisplatin (12 µM) was added for the last 24 hours before harvest. DNA damage was assessed by visualising γH2AX foci using fluorescence microscopy, representative pictures are shown.

Supplementary Tables

Supplementary table 1: List of compounds identified and shortlisted in the *in silico* screening
ZINC code of the shortlisted compounds that were selected from in the silico screening. The values of the predicted kD and predicted binding energy are shown.

Cluster	ZINC code	Predicted kD	Predicted binding energy (kcal/mol)
4	ZINC06880332	534.85 nM	-8.8
8	ZINC32892790	172.09 nM	-8.6
11	ZINC12880025	383.02 nM	-8.6
12	ZINC16045112	147.41 nM	-8.7
12	ZINC32906093	750.98 nM	-8.8
12	ZINC09660573	118.64 nM	-8.4
13	ZINC08013256	276.73 nM	-8.6
15	ZINC11694577	263.81 nM	-8.0
17	ZINC12305064	274.44 nM	-8.6
18	ZINC12517277	125.85 nM	-8.7
18	ZINC24032934	531.23 nM	-8.5
19	ZINC04783831	33.46 nM	-8.7
22	ZINC12793119	5.6 nM	-8.3
23	ZINC13135663	359.29 nM	-8.3
25	ZINC08762198	39.02 nM	-8.6
26	ZINC09421470	75.7 nM	-8.7
26	ZINC08783076	604.88 nM	-8.9
27	ZINC12030683	322.91 nM	-9.0
27	ZINC23074683	80.25 nM	-8.5
27	ZINC12540449	400.19 nM	-8.6

Supplementary table 2: List of compounds identified and shortlisted in the *in vitro* screening
Compound ID of the shortlisted compounds that were identified in the high-throughput screening. The compounds were purchased and a dose-response curve of the activity was assessed. The IC_{50} values are shown.

Ranking	Compound ID	Provider	Dose-response IC_{50} (µM)
1	F3225-3251	Life Chemicals	1.05
2	F1838-5493	Life Chemicals	1.87
3	F2575-0781	Life Chemicals	2.42
4	F1838-5596	Life Chemicals	2.65
5	F1762-0073	Life Chemicals	2.86
6	F3225-3505	Life Chemicals	3.3
7	F3225-3301	Life Chemicals	3.31
8	F3225-2696	Life Chemicals	3.89
9	F3225-2548	Life Chemicals	4.02
10	F5772-6567	Life Chemicals	4.09
11	F1838-5483	Life Chemicals	4.9
12	F1838-5548	Life Chemicals	5.37
13	F1322-0083	Life Chemicals	6.83
14	F3023-0495	Life Chemicals	7.02
15	ASN 05099309	ASINEX	7.21
16	ASN 00851028	ASINEX	7.26
17	F2493-3378	Life Chemicals	7.6
18	F5297-0151	Life Chemicals	7.8
19	F5297-0927	Life Chemicals	7.9
20	F5297-0683	Life Chemicals	8.01
21	41210186	Chembridge	8.31
22	F2825-0006	Life Chemicals	12.21
23	F2493-3430	Life Chemicals	14.14

Supplementary table 3: Structures and estimates of *in vitro* and cell-based activities of compound 1 and its analogues. The preliminary estimates of IC_{50} threshold are based on triple-point assays.

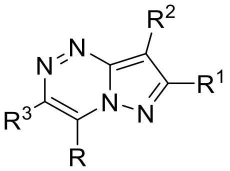

compound numbers	R	R^1	R^2	R^3	In vitro IC_{50} threshold (µM)	Cell based IC_{50} threshold (µM)
**1**	NH_2	Et	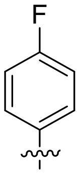	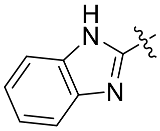	5-10	>25
**3**	NH_2	H	H	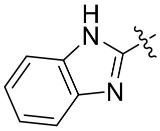	>20	N.A.
**4**	NH_2	H	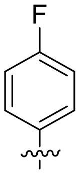	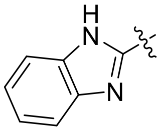	>20	N.A.
**5**	NH_2	Et	H	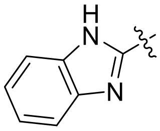	10-20	N.A.
**6**	NH_2	Et	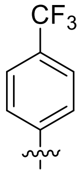	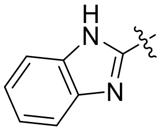	1-5	>25
**7**	NH_2	Et	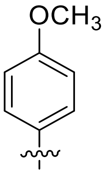	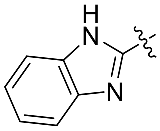	>20	>25

**8**	NH_2	Et	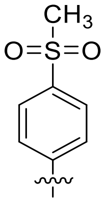	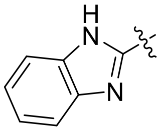	>20	5-25
**9**	NH_2	Et	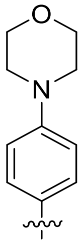	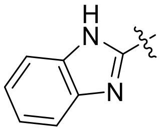	>5	>25
**10**	NH_2	Et	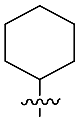	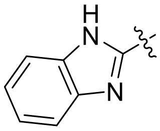	10-20	>25
**11**	NH_2	iPr	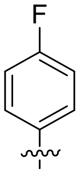	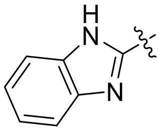	10-20	N.A.
**12**	NH_2	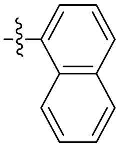	H	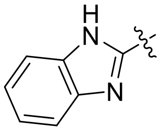	1-5	>25
**13**	NH_2	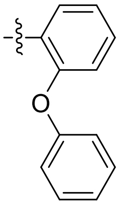	H	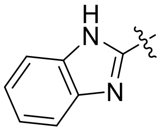	5-10	>25
**14**	NH_2	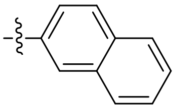	H	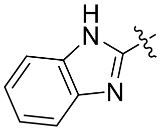	1-5	>25
**15**	NH_2	Ph	H	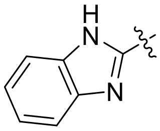	10-20	>25
**16**	H	Et	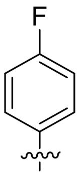	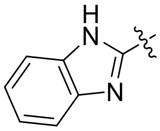	>10	5-25

**17**	CH_3	Et	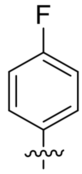	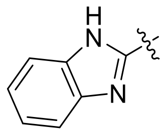	>20	>25
**18**	OH	Et	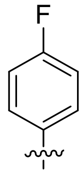	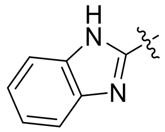	<1	<5
**19**	NH_2	Et	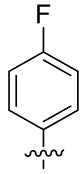	CN	>20	N.A.
**20**	OH	Et	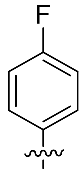	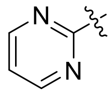	>20	5-25
**21**	NH_2	Et	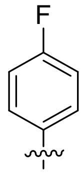	$CONH_2$	>20	N.A.
**22**	NH_2	Et	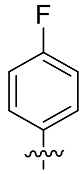	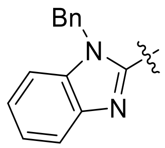	1-5	>25
**23**	OH	Et	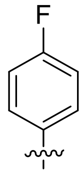	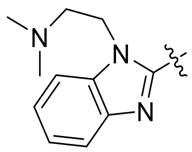	>10	5-25
**24**	OH	Et	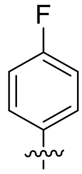	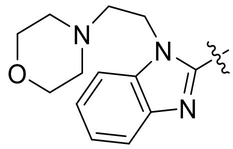	>10	5-25
**25**	OH	Et	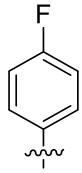	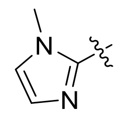	>20	>25

**26**	OH	Et	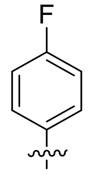	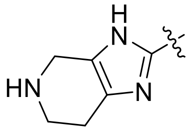	>20	N.A.
**27**	NH_2	Et	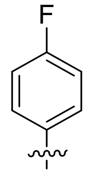	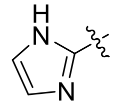	5-10	>25
**56**	NH_2	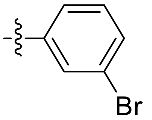	H	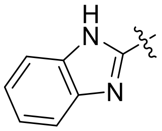	5-10	5-25
**57**	NH_2	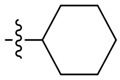	H	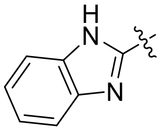	>20	N.A.
**58**	NH_2	iPr	H	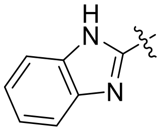	>20	N.A.
**59**	NH_2	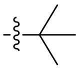	H	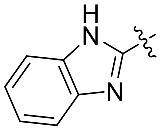	>20	N.A.
**60**	NH_2	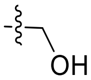	H	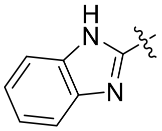	>20	N.A.
**61**	NH_2	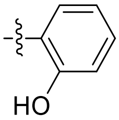	H	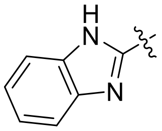	10-20	>25
**62**	NH_2	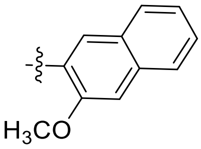	H	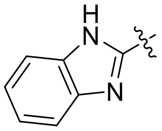	1-5	>25
**63**	NH_2	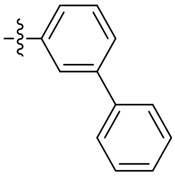	H	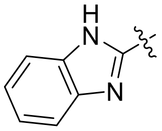	>20	N.A.
**64**	NH_2	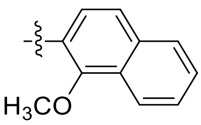	H	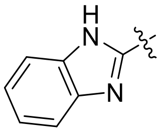	>10	N.A.

**65**	NH_2	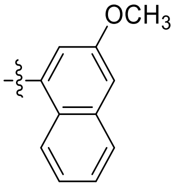	H	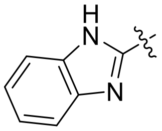	1-5	>25
**66**	NH_2	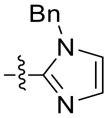	H	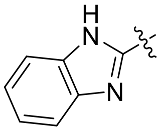	10-20	>25
**67**	NH_2	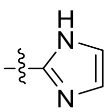	H	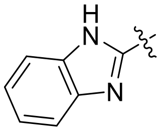	>10	N.A.
**68**	NH_2	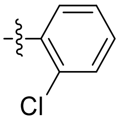	H	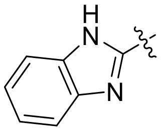	10-20	N.A.
**69**	NH_2	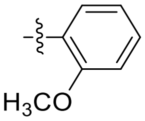	H	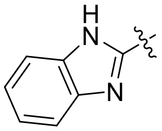	1-5	>25
**70**	NH_2	Et	H	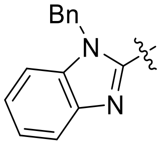	>20	N.A.
**71**	NH_2	Et	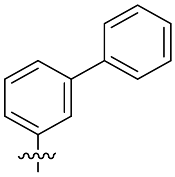	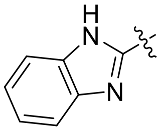	5-10	>25
**72**	NH_2	Et	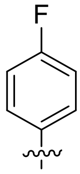	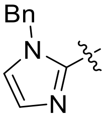	>5	>25
**73**	NH_2	Et	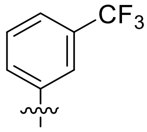	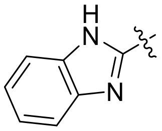	5-10	5-25
**74**	NH_2	Et	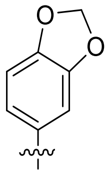	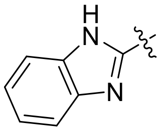	>20	>25

**75**	NH_2	Et	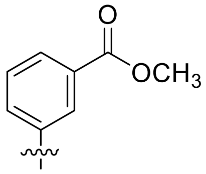	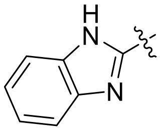	1-5	5-25
**76**	NH_2	Et	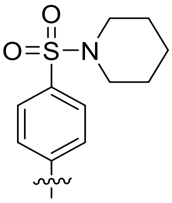	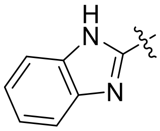	>5	5-25
**77**	NH_2	Et	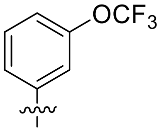	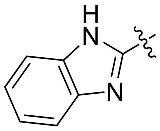	1-5	>25
**78**	NH_2	Et	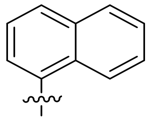	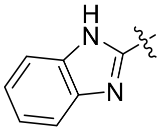	1-5	>25
**79**	OH	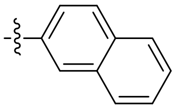	H	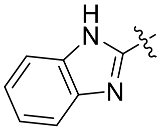	5-10	>25
**80**	OH	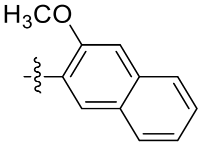	H	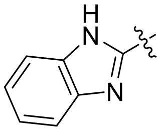	1-5	>25
**81**	OH	Et	H	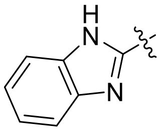	>10	N.A.
**82**	OH	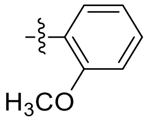	H	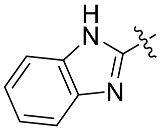	5-10	5-25
**83**	OH	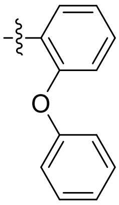	H	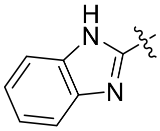	1-5	<5
**84**	OH	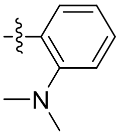	H	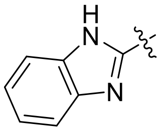	<1	5-25

**85**	OH	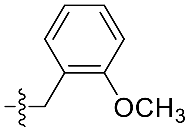	H	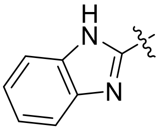	1-5	>25
**86**	OH	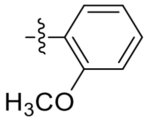	H	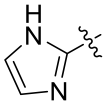	>20	>25
**87**	OH	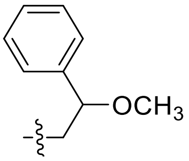	H	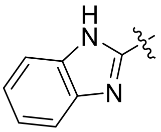	>20	5-25
**88**	OH	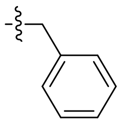	H	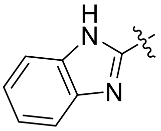	1-5	5-25
**89**	OH	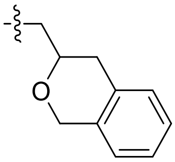	H	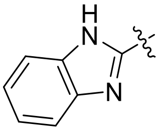	1-5	5-25
**90**	OH	H	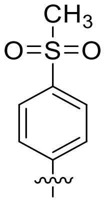	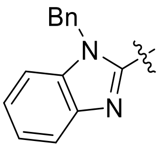	1-5	5-25
**91**	OH	H	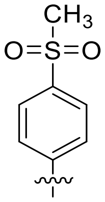	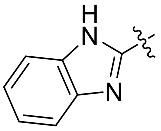	5-10	>25
**92**	OH	H	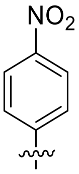	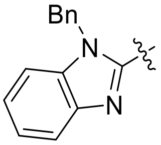	1-5	5-25
**93**	OH	Et	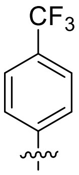	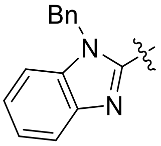	10-20	5-25
**94**	OH	Et	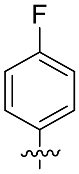	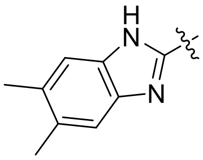	1-5	<5

**95**	OH	Et	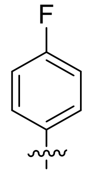	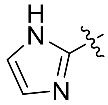	>10	N.A.
**96**	OH	Et	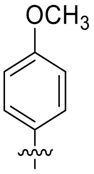	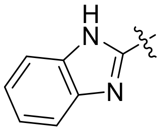	1-5	5-25
**97**	OH	Et	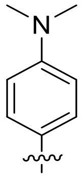	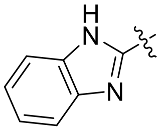	1-5	<5
**98**	NH_2	Et	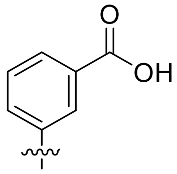	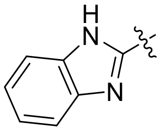	1-5	N.A.
**99**	OH	Et	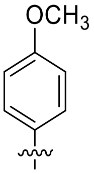	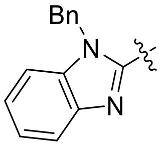	1-5	5-25
**100**	OH	Et	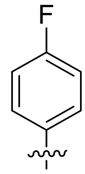	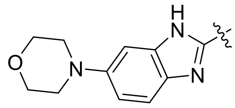	1-5	5-25
**101**	OH	Et	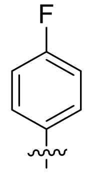	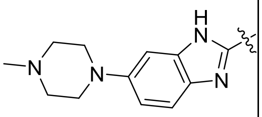	>20	N.A.
**102**	OH	Et	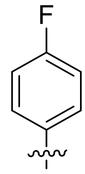	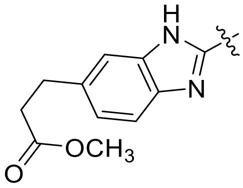	1-5	<5
**103**	OH	Et	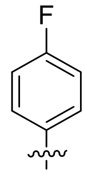	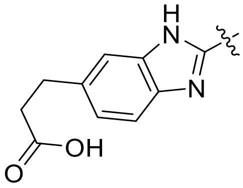	>20	N.A.

**104**	OH	Et	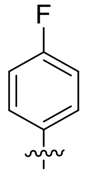	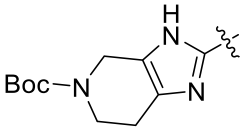	1-5	<5
**105**	OH	Et	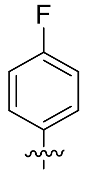	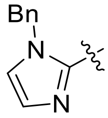	10-20	>25
**106**	OH	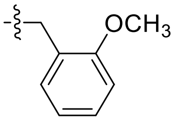	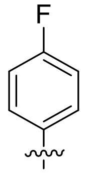	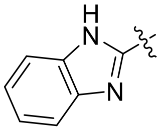	1-5	5-25
**108**	OH	Et	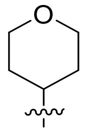	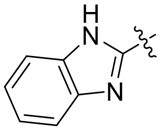	>20	>25
**109**	OH	Et	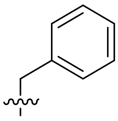	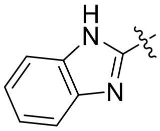	<1	<5
**110**	OH	Et	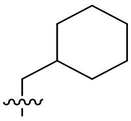	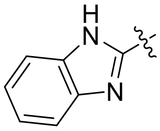	<1	<5
**111**	OH	Et	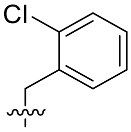	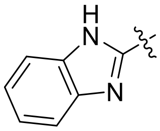	<1	5-25
**112**	OH	Et	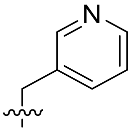	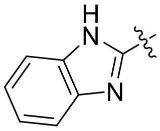	>20	N.A.
**113**	OH	Et	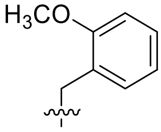	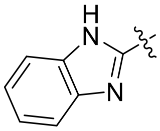	<1	<5
**114**	OH	Et	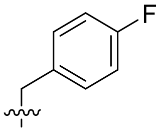	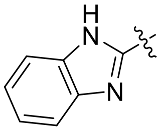	<1	<5
**115**	OH	Et	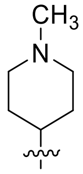	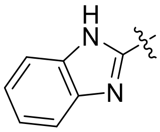	>20	N.A.

**116**	OH	Et	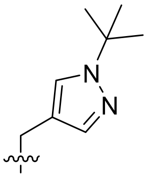	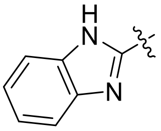	>20	N.A.

Miscellaneous targets:

**28**	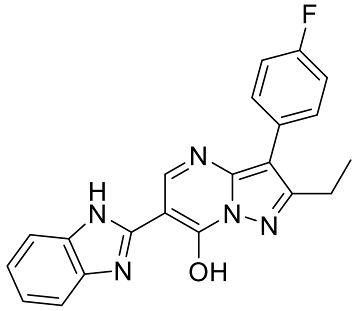	>20	>25
**117**	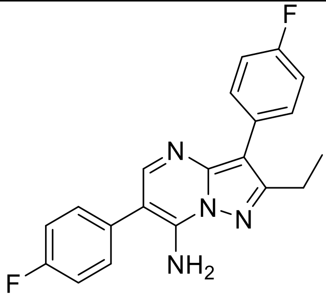	>20	N.A.
**107**	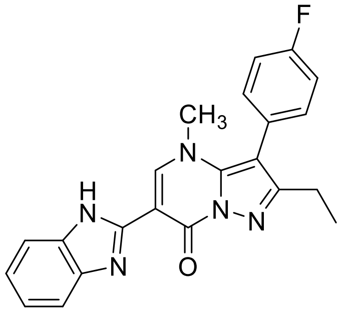	10-20	>25

*N.A. = not assessed

Supplementary table 4: Structures and estimates of *in vitro* and cell-based activities of compound 2 and its analogues. The preliminary estimates of IC_{50} threshold are based on triple-point assays.

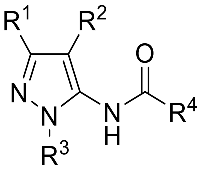

compound numbers	R^1	R^2	R^3	R^4	In vitro IC_{50} threshold (µM)	Cell based IC_{50} threshold (µM)
**36**	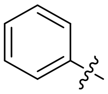	H	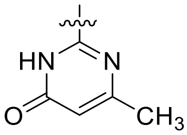	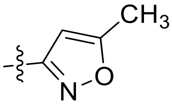	5-10	<5
**32**		H		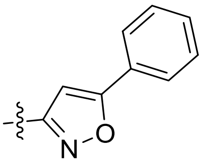	<1	<1
**35**		CH_3			>10	<5
**39**		H		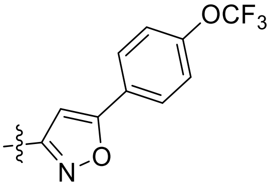	5-10	5-25
**38**		H		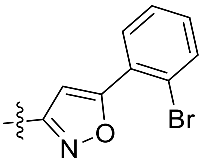	>10	<5
**118**		H		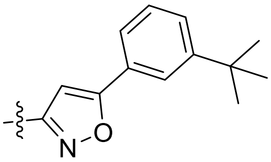	5-10	<5
**37**		H		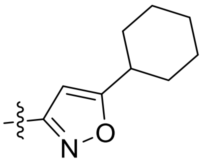	>10	5-25

**40**		H		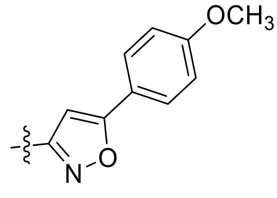	>10	5-25
**41**		H		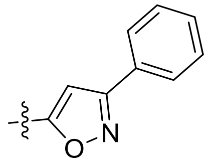	1-5	<5
**42**		H		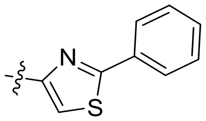	5-10	<5
**43**		H		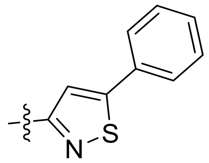	<1	<5
**44**		H		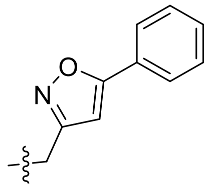	>10	5-25
**119**		H		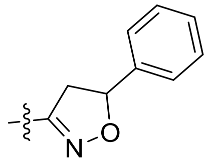	>10	<5
**120**		H		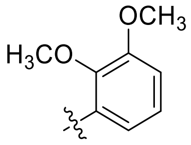	>10	N.A.
**121**		H		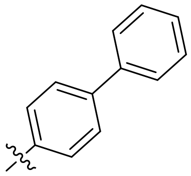	>10	N.A.
**122**		H		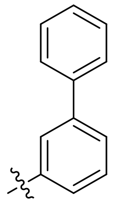	>10	<5
**123**		H		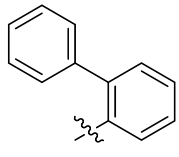	>10	N.A.

**140**		H		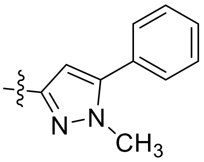	>10	N.A.
**2**	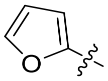	H	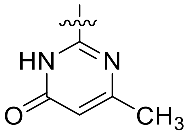	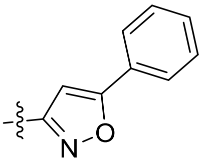	<1	5-25
**124**		H		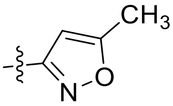	>10	5-25
**30**		H		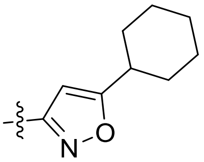	5-10	<5
**125**		H		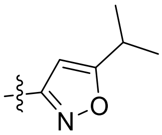	>10	N.A.
**126**		H		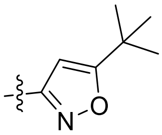	>10	>25
**127**		H		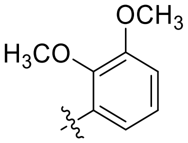	>10	>25
**128**		H		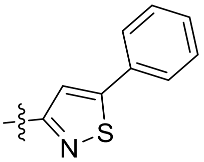	<1	5-25
**29**	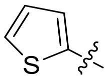	H	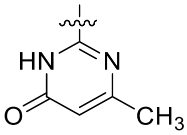	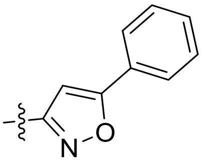	1-5	5-25
**129**	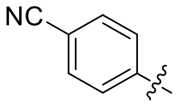	H			1-5	>25
**34**	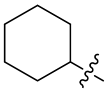	H			>10	<5
**130**	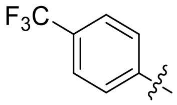	H			>10	N.A.

**131**	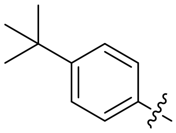	H			1-5	<5
**132**	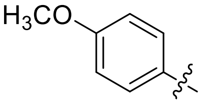	H			>10	<5
**33**	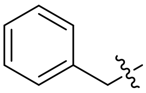	H			1-5	<5
**31**	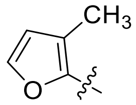	H			1-5	5-25
**133**	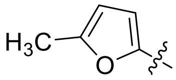	H			1-5	5-25
**134**	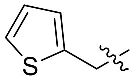	H			1-5	<5
**142**		H	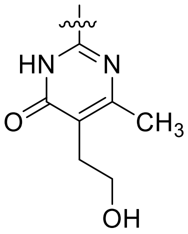		1-5	<5
**48**		H	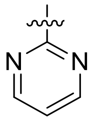		>10	5-25
**49**	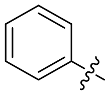	H	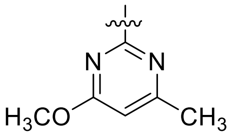		>10	5-25
**51**		H	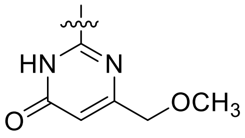	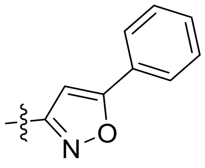	>10	N.A.
**46**		H	Methyl		>10	>25
**50**		H	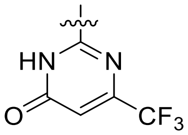		5-10	<5
**55**		H	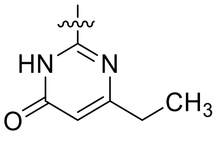		<1	<5

**135**		H	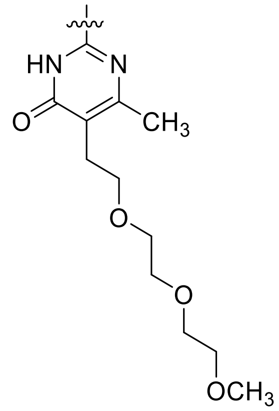		>10	<5
**52**		H	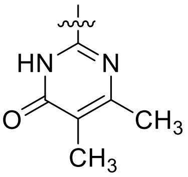		5-10	<5
**136**		H	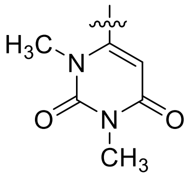		>10	>25
**53**		H	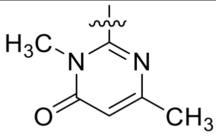		10-20	5-25
**137**		H	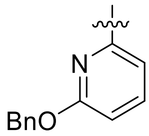		>10	>25
**54**		H	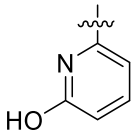		5-10	5-25
**47**		H	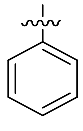		>10	N.A.
**139**	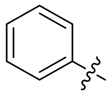	H	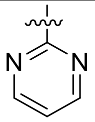	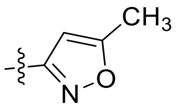	>10	>25
**138**	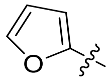	H	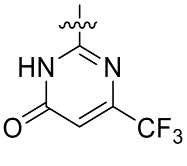	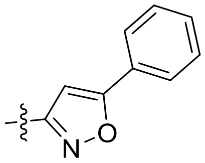	>10	>25

**143**	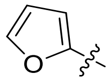	H	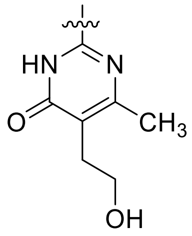	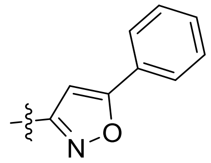	>10	>25

Miscellaneous targets:

**141**	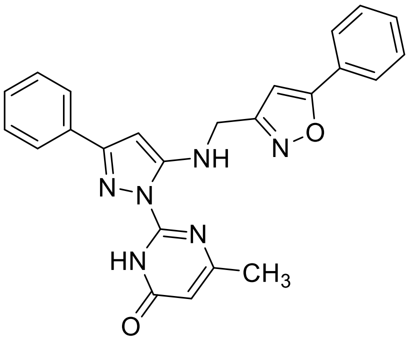	>10	<5
**45**	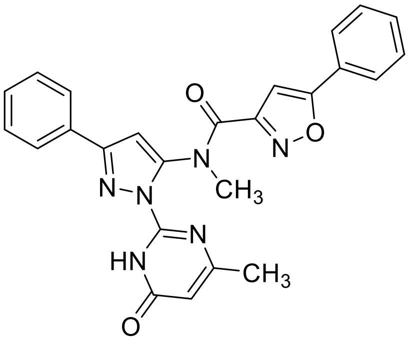	>20	>25

*N.A. = not assessed

Supplementary table 5: Predicted binding modes of MU262 and two conformers of MU876 docked into the active site of human MUS81-EME1 heterodimer. The structure of the complex was obtained from PDB-ID: 9F99, chains A and B with missing residues 371–377 modeled from PDB-ID: 9F98[3]. The receptor was protonated at pH 7.5 using H++ server[5] and prepared with AutoDockTools 1.4[6]. Ligand geometries were optimized using the MMFF94 force field in Avogadro 1.99[7], considering two possible amide bond conformations for MU876 (c1 and c2). Docking was performed with AutoDock Vina 1.2.5[2], using a 35 × 35 × 35 Å grid box centered on the catalytic Mg^{2+} ion 602 with exhaustiveness of 50. modes were rescored with five Smina scoring functions and ranked by consensus[8]. The most favorable mode for each compound is highlighted in bold.

Cmpd	Pose	AD4_scoring [kcal/mol]	Rank	dkoes_fast [kcal/mol]	Rank	dkoes_scoring [kcal/mol]	Rank	Vinardo [kcal/mol]	Rank	Vina [kcal/mol]	Rank	Consensus rank
**MU262**	1	-25.41911	4	-5.1378	4	-4.72602	5	-6.15653	3	-8.38287	3	4
	**2**	**-30.2821**	**2**	**-5.51563**	**2**	**-5.4897**	**2**	**-6.12995**	**5**	**-8.42936**	**2**	**1**
	3	-23.66753	6	-5.1011	6	-4.67017	7	-6.24083	2	-8.44415	1	5
	4	-24.33264	5	-4.90731	7	-4.71567	6	-5.97107	6	-8.26048	4	6
	5	-21.39956	8	-4.83296	8	-4.87154	4	-5.76961	7	-8.05147	6	7
	6	-29.69219	3	-5.23515	3	-5.81413	1	-6.80068	1	-8.18989	5	2
	7	-33.44172	1	-6.34691	1	-5.45068	3	-6.14997	4	-7.91538	7	3
	8	-23.03284	7	-5.13335	5	-4.59022	8	-5.37094	8	-7.86637	8	8
**MU876-c1**	1	-35.01838	4	-6.53128	4	-4.99042	6	-6.54903	3	-8.99991	1	3
	2	-35.28483	3	-6.55384	3	-5.01116	5	-6.57585	2	-8.96889	2	2
	**3**	**-40.01431**	**1**	**-6.63314**	**1**	**-5.99042**	**1**	**-6.98891**	**1**	**-8.48544**	**4**	**1**
	4	-27.94102	8	-5.20256	9	-5.08551	3	-6.30243	7	-8.24145	6	7
	5	-37.80954	2	-6.56132	2	-5.85859	2	-6.34014	5	-7.99669	9	4
	6	-30.28411	7	-6.03752	6	-4.81627	9	-6.19351	9	-8.48599	3	8
	7	-31.58779	6	-6.34527	5	-4.90206	8	-6.43916	4	-8.01001	8	6
	8	-31.96342	5	-5.7724	7	-5.03197	4	-6.32285	6	-8.14385	7	5
	9	-25.95711	9	-5.5034	8	-4.99032	7	-6.19659	8	-8.31315	5	9
**MU876-c2**	**1**	**-34.40833**	**1**	**-6.37204**	**2**	**-5.21783**	**3**	**-6.61404**	**4**	**-8.85436**	**2**	**1**
	2	-33.28092	4	-6.20346	5	-5.48245	2	-6.73676	3	-8.89164	1	2
	3	-33.11997	5	-6.36377	3	-5.15385	4	-6.4995	6	-8.72655	3	3
	4	-34.01735	2	-6.68132	1	-5.00969	8	-6.34975	8	-8.60626	4	5
	5	-32.7976	6	-6.09576	7	-5.14352	5	-6.98639	2	-8.48387	5	6
	6	-29.24328	9	-5.70468	8	-5.12822	6	-6.45787	7	-8.29016	7	8
	7	-32.5932	7	-6.32229	4	-5.00105	9	-6.54693	5	-8.32614	6	7
	8	-33.42875	3	-5.31687	9	-6.15317	1	-7.15915	1	-8.22195	8	4
	9	-31.55825	8	-6.10898	6	-5.08852	7	-6.27136	9	-8.13922	9	9

Supplementary table 6: Human microsomal stability data for MU262, MU876 and reference compounds

Compound ID	Time, min	Analyte Peak Area		Analyte Peak Area, Mean of 2	% Remaining, Mean of 2	R^2	k_{el}, min^{-1}	$t_{1/2}$, min	Cl_{int}, µl/min/mg	% Remaining without cofactor, Mean of 2
		Inc. 1	Inc. 2							
Diclofenac human	0	4.40E-01	4.65E-01	4.53E-01	100	0.997	0.079	8.7	191	100
	7	3.15E-01	3.14E-01	3.15E-01	70	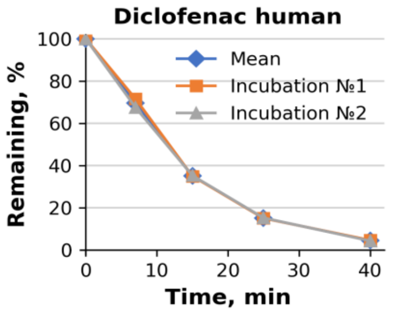				
	15	1.54E-01	1.64E-01	1.59E-01	35					
	25	6.62E-02	7.09E-02	6.85E-02	15					
	40	2.06E-02	2.02E-02	2.04E-02	5					107
Propranolol human	0	3.05E-01	3.13E-01	3.09E-01	100	0.957	0.008	82.6	20	100
	7	3.13E-01	3.15E-01	3.14E-01	102	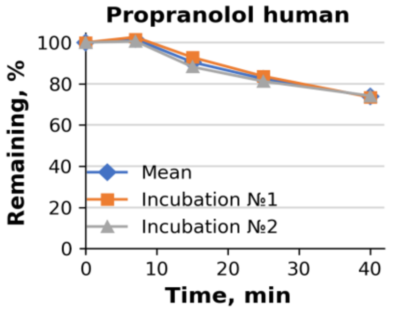				
	15	2.83E-01	2.76E-01	2.79E-01	90					
	25	2.55E-01	2.54E-01	2.55E-01	82					
	40	2.24E-01	2.32E-01	2.28E-01	74					111
**MU262 human**	0	7.35E-02	7.16E-02	7.26E-02	100	0.741	0.008	86.3	19	100
	7	6.29E-02	5.92E-02	6.11E-02	84	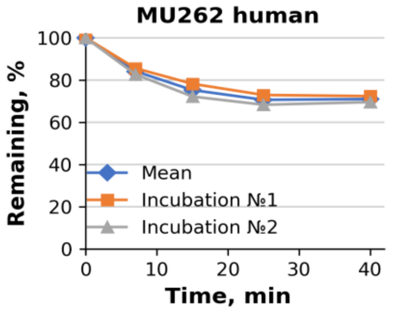				
	15	5.75E-02	5.17E-02	5.46E-02	75					
	25	5.36E-02	4.89E-02	5.13E-02	71					
	40	5.32E-02	4.98E-02	5.15E-02	71					83

Compound ID	Time, min	Analyte Peak Area		Analyte Peak Area, Mean of 2	% Remaining, Mean of 2	R^2	k_{el}, min^{-1}	$t_{1/2}$, min	Cl_{int}, µl/min/mg	% Remaining without cofactor, Mean of 2
		Inc. 1	Inc. 2							
MU876 human	0	5.72E-02	6.25E-02	5.99E-02	100	0.671	0.012	58.9	28	100
	7	6.14E-02	8.53E-02	7.33E-02	123					
	15	5.79E-02	7.16E-02	6.48E-02	108					
	25	5.07E-02	6.48E-02	5.77E-02	96					
	40	3.63E-02	4.40E-02	4.01E-02	67					112

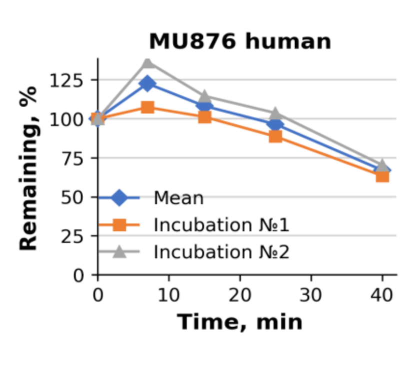

Supplementary table 7: Mouse microsomal stability data for MU262, MU876 and reference compounds

Compound ID	Time, min	Analyte Peak Area		Analyte Peak Area, Mean of 2	% Remaining, Mean of 2	R^2	k_{el}, min^{-1}	$t_{1/2}$, min	Cl_{int}, µl/min/mg	% Remaining without cofactor, Mean of 2
		Inc. 1	Inc. 2							
Imipramine mouse	0	1.84E+00	1.75E+00	1.79E+00	100	0.996	0.087	7.9	211	100
	7	1.18E+00	1.13E+00	1.15E+00	64	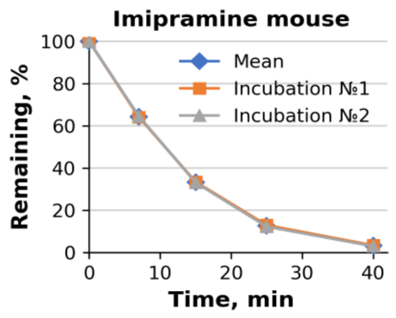				
	15	6.16E-01	5.80E-01	5.98E-01	33					
	25	2.39E-01	2.15E-01	2.27E-01	13					
	40	6.31E-02	5.27E-02	5.79E-02	3					103
Propranolol mouse	0	2.93E-01	2.94E-01	2.93E-01	100	0.894	0.021	32.7	51	100
	7	2.04E-01	1.95E-01	2.00E-01	68	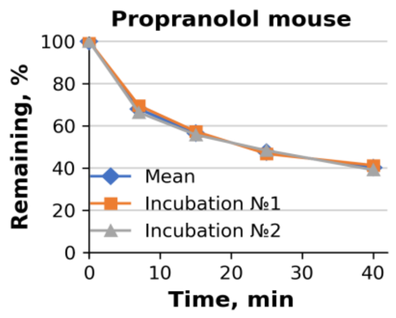				
	15	1.68E-01	1.64E-01	1.66E-01	57					
	25	1.37E-01	1.42E-01	1.40E-01	48					
	40	1.21E-01	1.15E-01	1.18E-01	40					96
**MU262 mouse**	0	8.21E-02	7.53E-02	7.87E-02	100	0.858	0.013	55.4	30	100
	7	6.20E-02	5.55E-02	5.87E-02	75	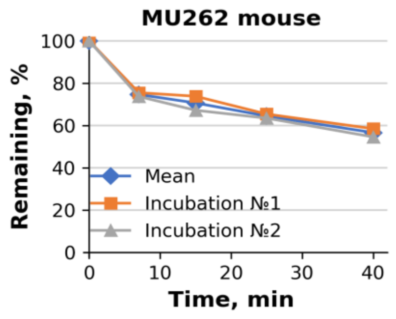				
	15	6.06E-02	5.06E-02	5.56E-02	71					
	25	5.37E-02	4.78E-02	5.08E-02	64					
	40	4.80E-02	4.11E-02	4.45E-02	57					*74*

Compound ID	Time, min	Analyte Peak Area		Analyte Peak Area, Mean of 2	% Remaining, Mean of 2	R^2	k_{el}, min^{-1}	$t_{1/2}$, min	Cl_{int}, µl/min/mg	% Remaining without cofactor, Mean of 2
		Inc. 1	Inc. 2							
MU876 mouse	0	4.69E-02	5.03E-02	4.86E-02	100	0.932	0.018	38.8	43	100
	7	4.68E-02	5.65E-02	5.17E-02	106	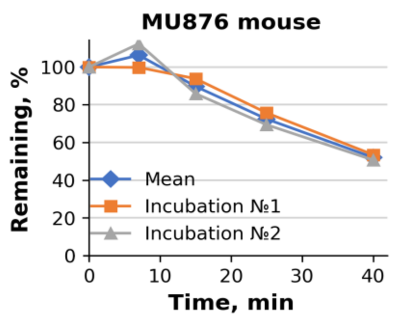				
	15	4.40E-02	4.32E-02	4.36E-02	90					
	25	3.55E-02	3.49E-02	3.52E-02	72					
	40	2.51E-02	2.55E-02	2.53E-02	52					87

Supplementary table 8: CYP450 inhibition by MU262 and MU876 (10 µM)

CYP	% Inhibition		% Inhibition, Mean	SD	SE
MU262					
3A4	4.4	5.9	5.1	1.1	0.8
2D6	2.5	1.8	2.1	0.5	0.4
1A2	5.3	11.2	8.2	4.2	3.0
2C19	9.4	8.6	9.0	0.6	0.4
2C9	0.8	-1.9	-0.5	1.9	1.3
MU876					
3A4	5.9	10.2	8.0	3.0	2.1
2D6	-2.5	0.4	-1.1	2.0	1.4
1A2	7.2	1.8	4.5	3.8	2.7
2C19	4.3	4.3	4.3	0.0	0.0
2C9	0.8	-0.8	0.0	1.1	0.8

Supplementary table 9: List of DNA substrates used in this study.
DNA substate used for *in vitro* nuclease assay, EMSA and BLI.

<table>
<tr><th>MUS81 substrates</th><th>Substrate structure</th></tr>
<tr><td>in vitro nuclease assay and EMSA
3' flap (FITC-labeled)</td><td rowspan="4">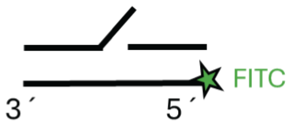
</td></tr>
<tr><td>5' - AGCTATGACCATGATTACGAATTGCTTGGAATCCTGACGAACTGTAG - 3'</td></tr>
<tr><td>5' - AATTCGTGCAGGCATGGTAGCT - 3'</td></tr>
<tr><td>5' - FITC - AGCTACCATGCCTGCACGAATTAAGCAATTCGTAATCATGGTCATAGCT - 3'</td></tr>
<tr><td>Biolayer Interferometry (BLI)
blocked 3' flap (FITC-, biotin-labeled)</td><td rowspan="5">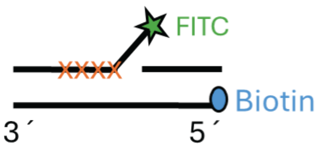
</td></tr>
<tr><td>5' - AGCTATGACCATGATTACGAAT*T*G*C*TTGGAATCCTGA - FITC - 3'</td></tr>
<tr><td>5' - AATTCGTGCAGGCATGGTAGCT - 3'</td></tr>
<tr><td>5' - biotin - AGCTACCATGCCTGCACGAATTAAGCAATTCGTAATCATGGTCATAGCT - 3'</td></tr>
<tr><td>* thio bond</td></tr>
</table>

Supplementary Methods

Assessment of Metabolic Stability in Human and Mouse Liver Microsomes for MU262 and MU876 (outsourced to Bienta Enamine Biology Services)

Microsomal incubations were carried out in 96-well plates in 5 aliquots of 30 µL each (one for each time point). Liver microsomal incubation medium comprised of phosphate buffer (100 mM, pH 7.4), $MgCl_2$ (3.3 mM), NADPH (3 mM), glucose-6-phosphate (5.3 mM), glucose-6-phosphate dehydrogenase (0.67 units/ml) with 0.42 mg of liver microsomal protein per ml. In the control reactions, the NADPH-cofactor system was substituted with phosphate buffer. Test compounds (2 µM, final acetonitrile concentration 1.6 %) were incubated with microsomes at 37°C, shaking at 100 rpm. Five time points over 40 minutes were analyzed. The reactions were stopped by adding 5 volumes of acetonitrile with internal standard to incubation aliquots, followed by protein sedimentation by centrifuging at 5500 rpm for 5 minutes. Each reaction was performed in duplicates. Supernatants were analyzed using the HPLC system coupled with a tandem mass spectrometer.

The elimination constant (k_{el}), half-life ($t_{1/2}$), and intrinsic clearance (Cl_{int}) were determined in a plot of ln(AUC) versus time, using linear regression analysis:

$$k_{el} = -slope \qquad t_{1/2} = \frac{0.693}{k} \qquad Cl_{int} = \frac{0.693}{t_{1/2}} \times \frac{\mu l_{incubation}}{mg_{microsomes}}$$

Determination of *in vitro* CYP450 Inhibition (panel of 5: CYP3A4, 2D6, 1A2, 2C19, and 2C9) in Human Liver Microsomes for MU262 and MU876 (outsourced to Bienta Enamine Biology Services)

The potential for CYP450 inhibition of 5 major cytochromes 3A4, 2D6, 1A2, 2C19, and 2C9 was assessed using LC-MS/MS based assay, in which biotransformations of the CYP450 specific substrates (Table A, below) were used as markers to quantify the enzymatic activity (Walsky et al, Drug metabolism and disposition, 2004, p647-660). The enzymatic reactions were performed under linear conditions with substrate concentration below Km values and conversion of substrate below 20%.

Table A. Isoform-specific CYP450 substrates and metabolites

CYP450	Substrate	Km of substrate, µM	Metabolite
3A4	Testosterone	56	6β-Hydroxytestosterone
2D6	Dextromethorphan	5.6	Dextrorphan
1A2	Phenacetin	60	Acetaminophen
2C19	S-Mephenytoin	48	4-Hydroxymephenytoin
2C9	Diclofenac	12	4'-Hydroxydiclofenac

The incubations medium contained test compounds (final DMSO concentration 0.3-0.45%), 50 or 100 mM phosphate buffer (pH 7.4), 3.3 mM $MgCl_2$, 1.3 mM NADPH and human liver microsomes (0.1 mg/ml). Control incubations were performed without NADPH. Test compounds were preincubated with enzyme and substrate at 37 °C during 10 min. The enzymatic reactions were initiated by addition of NADPH, the mixtures were incubated at 37°C

during 10-40 min shaking at 100 rpm. Incubations were performed in duplicate. Incubations were terminated by addition of 1 volume of acetonitrile containing internal standard, followed by protein sedimentation by centrifuging at 5500 rpm for 5 min. Supernatants were analyzed using the HPLC system coupled with tandem mass spectrometer.
Quantification of the decrease in metabolite formation in the presence of a specific inhibitor was used to assess CYP450 inhibition. Reference inhibitors specific for each CYP enzyme were used to assess inhibition in the control experiments for every batch of tested compounds. Test concentrations of the reference compounds corresponded to approximately 5x fold of IC_{50} values for each CYP450, which is expected to produce 80-100% inhibition in the properly performing assay (Table B, below).

Table B. Isoform-specific CYP450 inhibition by reference inhibitors

CYP450	Reference inhibitor	5x IC_{50}, µM	CYP inhibition, %
3A4	Ketoconazole	0.105	88.9
2D6	Quinidine	0.225	82.6
1A2	Furafylline	15	83.0
2C19	Tranylcypromine	50	90.4
2C9	Sulphaphenazole	1.15	80.3

Docking of MU inhibitors into active site of MUS81

Protein Structure Preparation. The MUS81-EME1 heterodimer structure was obtained from the Protein Data Bank[9] (PDB-ID: 9F99)[3], selecting chains A (MUS81) and B (EME1) as the most complete chains. Missing residues 371–377 in the MUS81 chain were modeled using the corresponding region from an aligned structure (PDB-ID: 9F98)[3]. The receptor was protonated with H++ server[5] at pH of 7.5 and prepared for docking using AutoDockTools 1.4.[6].

Ligand Preparation. Four compounds were evaluated: Compound 1, MU262, and MU876 in two amide bond conformations. Ligand three-dimensional coordinates were generated and geometry optimized using the MMFF94 force field[10] as implemented in Avogadro 1.99 package[7]. Subsequently, ligands were prepared for docking using AutoDockTools 1.4.[6].

Molecular Docking. Docking calculations were performed using AutoDock Vina 1.2.5.[2]. The search space was defined as a cubic box (35 × 35 × 35 Å) centered on the catalytic Mg^{2+} ion (residue 602; coordinates: x = 5.184, y= 5.259, z = −46.887 Å). An exhaustiveness parameter of 50 was used to ensure adequate conformational sampling. To improve the reliability of pose selection, docked poses were rescored with five scoring functions available in Smina software[8]: AutoDock4 (ad4_scoring), Vina, Vinardo, and the machine learning–based dkoes_fast and dkoes_scoring functions. Poses were ranked independently by each scoring function, and the average rank across all five functions was used to identify the most probable binding mode for each ligand.

Interaction Analysis. Protein–ligand interactions for the consensus-selected poses were analysed and visualized using LigPlot+ software[4].

References:

(1) Gwon, G. H.; Jo, A.; Baek, K.; Jin, K. S.; Fu, Y.; Lee, J. B.; Kim, Y.; Cho, Y. Crystal Structures of the Structure-Selective Nuclease Mus81-Eme1 Bound to Flap DNA Substrates. *EMBO J*, 2014, *33*, 1061–1072.

(2) Trott, O.; Olson, A. J. AutoDock Vina: Improving the Speed and Accuracy of Docking with a New Scoring Function, Efficient Optimization, and Multithreading. *J Comput Chem*, 2010, *31*, 455–461.

(3) Collie, G. W.; Börjesson, U.; Chen, Y.; Dong, Z.; Di Fruscia, P.; Gohlke, A.; Hoyle, A.; Hunt, T. A.; Jesani, M. H.; Luo, H.; Luptak, J.; Milbradt, A. G.; Narasimhan, P.; Packer, M.; Patel, S.; Qiao, J.; Storer, R. I.; Stubbs, C. J.; Tart, J.; Truman, C.; Wang, A. T.; Wheeler, M. G.; Winter-Holt, J. Fragment-Based Discovery of Novel MUS81 Inhibitors. *ACS Med Chem Lett*, 2024, *15*, 1151–1158.

(4) Laskowski, R. A.; Swindells, M. B. LigPlot+: Multiple Ligand–Protein Interaction Diagrams for Drug Discovery. *J. Chem. Inf. Model.* **2011**, *51* (10), 2778–2786. https://doi.org/10.1021/ci200227u.

(5) Gordon, J. C.; Myers, J. B.; Folta, T.; Shoja, V.; Heath, L. S.; Onufriev, A. H++: A Server for Estimating p Ka s and Adding Missing Hydrogens to Macromolecules. *Nucleic Acids Res* **2005**, *33* (suppl_2), W368–W371. https://doi.org/10.1093/nar/gki464.

(6) Morris, G. M.; Huey, R.; Lindstrom, W.; Sanner, M. F.; Belew, R. K.; Goodsell, D. S.; Olson, A. J. AutoDock4 and AutoDockTools4: Automated Docking with Selective Receptor Flexibility. *Journal of Computational Chemistry* **2009**, *30* (16), 2785–2791. https://doi.org/10.1002/jcc.21256.

(7) Hanwell, M. D.; Curtis, D. E.; Lonie, D. C.; Vandermeersch, T.; Zurek, E.; Hutchison, G. R. Avogadro: An Advanced Semantic Chemical Editor, Visualization, and Analysis Platform. *J Cheminform* **2012**, *4* (1), 17. https://doi.org/10.1186/1758-2946-4-17.

(8) Koes, D. R.; Baumgartner, M. P.; Camacho, C. J. Lessons Learned in Empirical Scoring with Smina from the CSAR 2011 Benchmarking Exercise. *J. Chem. Inf. Model.* **2013**, *53* (8), 1893–1904. https://doi.org/10.1021/ci300604z.

(9) Berman, H. M.; Westbrook, J.; Feng, Z.; Gilliland, G.; Bhat, T. N.; Weissig, H.; Shindyalov, I. N.; Bourne, P. E. The Protein Data Bank. *Nucleic Acids Res* **2000**, *28* (1), 235–242. https://doi.org/10.1093/nar/28.1.235.

(10) Halgren, T. A. Merck Molecular Force Field. I. Basis, Form, Scope, Parameterization, and Performance of MMFF94. *Journal of Computational Chemistry* **1996**, *17* (5–6), 490–519. https://doi.org/10.1002/(SICI)1096-987X(199604)17:5/6%253C490::AID-JCC1%253E3.0.CO;2-P.

Experimental procedures for all intermediates and final compounds

General information

All commercially available reagents were used as supplied without further purification. The reaction solvents were purchased anhydrous and were stored under nitrogen. Unless noted otherwise, the reactions were carried out in oven-dried glassware under the atmosphere of nitrogen. Analytical thin-layer chromatography (TLC) was performed using aluminum plates pre-coated with silica gel (silica gel 60 F_{254}, Merck). TLC plates were visualized by exposure to ultraviolet light (λ = 254 nm) and/or by submersion in aqueous ceric ammonium molybdate (CAM). All solutions were concentrated by rotary evaporation at 40 °C, unless noted otherwise. As indicated, either manual column chromatography was carried out using silica gel (pore size 60 Å, 230-400 mesh particle size, 40-63 μm particle size) or column chromatography was carried out using the Biotage Selekt purification system. Purification by preparative thin layer chromatography was performed using plates from Merck (PLC Silica gel 60 F_{254}, 1 mm). Reverse phase column chromatography was carried out using C_{18}-reversed phase silica gel (pore size 90 Å, 230-400 mesh particle size, 40-63 μm particle size). Nuclear magnetic resonance spectra were recorded using Bruker Avance 500 MHz and 300 MHz instruments at 30 °C. NMR spectra were obtained in indicated deuterated solvents; chemical shifts are quoted in parts per million (δ) referenced to the appropriate deuterated solvent employed. Multiplicities are indicated by s (singlet), d (doublet), t (triplet), q (quartet), p (pentet), quin (quintet), sept (septet), m (multiplet) or (br) broad, or combinations thereof. Homonuclear and heteronuclear two dimensional NMR experiments were used where appropriate to facilitate assignment of chemical shifts. Coupling constant values are given in Hz. IR spectra (4000-400 cm^{-1}) were collected on Alpha Bruker FT-IR Spectrometer (Platinum ATR); solid samples were measured neat and oily samples as films. High-resolution mass spectra were obtained on Agilent 6224 Accurate-Mass TOF LC-MS with dual electrospray/chemical ionization mode or on MALDI-TOF Ultraflextreme (Bruker Daltonics) with positive ions detection. Melting points were determined with Stuart SMP40 automatic melting point apparatus. Microwave reactions were carried out using a CEM Discover SP microwave reactor equipped with a single-mode cavity and a pressure- and temperature-controlled system. Reactions were performed in sealed microwave tubes (10 mL or appropriate size) under magnetic stirring. The purity of the synthesized target compounds were determined by HPLC analysis with UV detection (Ultimate 3000 LC Analytical Systems, Thermo Scientific) using Agilent ZORBAX Eclipse Plus C18 column and ^{1}H-NMR. All final compounds reported herein were >95% pure (unless stated otherwise).

General procedure A1: Formation of ketonitrile by deprotonation of phenylacetonitrile with NaH

A solution of the appropriate phenylacetonitrile (1 eq.; unless stated otherwise) in anhydrous THF (3 mL per 1 mmol of phenylacetonitrile) was added under N_2 to NaH (60% suspension in mineral oil, 2 eq.;

unless stated otherwise). The mixture was stirred for 20 min, then the appropriate ester (1.1 eq.; unless stated otherwise) was added and the reaction mixture was stirred at 25 °C for additional 2 h. The mixture was cooled to 0 °C and saturated aqueous solution of NH_4Cl (6 mL per 1 mmol of phenylacetonitrile) was added. The mixture was extracted with EtOAc (3 × 9 mL per 1 mmol of phenylacetonitrile). The organic extracts were combined, dried over $MgSO_4$, filtered, and the solvent was evaporated. The residue obtained after the workup was purified using column chromatography or preparative TLC (unless stated otherwise).

General procedure A2: Formation of ketonitrile by deprotonation of CH_3CN with NaH

A solution of the appropriate ester (1 eq.; unless stated otherwise) and CH_3CN (3 eq.; unless stated otherwise) in anhydrous THF (1 mL per 1 mmol of ester) was added under nitrogen to NaH (60% suspension in mineral oil, 3 eq.; unless stated otherwise) and the mixture was refluxed for 4 h. The reaction mixture was cooled to 0 °C, quenched with saturated aqueous solution of NH_4Cl (2 mL per 1 mmol of ester), and extracted with EtOAc (3 ×2 mL per 1 mmol of ester). The organic extracts were combined, dried over $MgSO_4$, filtered, and the solvent was evaporated. The residue obtained after the workup was purified using column chromatography or preparative TLC (unless stated otherwise).

General procedure A3: Formation of ketonitrile by deprotonation of CH_3CN with *n*-BuLi

n-BuLi solution in hexane (2.7 M, 1.05 eq.; unless stated otherwise) was added under nitrogen to a solution of CH_3CN (1 eq.; unless stated otherwise) in anhydrous THF (3 mL per 1 mmol of ester) at -78 °C. The reaction mixture was stirred at -78 °C for 30 min, then a solution of the appropriate ester (1 eq.; unless stated otherwise) in anhydrous THF (6 mL per 1 mmol of ester) was added dropwise and the mixture was stirred at -78 °C for 2 h. The reaction mixture was quenched with saturated aqueous solution of NH_4Cl (6 mL per 1 mmol of ester) and extracted with dichloromethane (3 × 50 mL per 1 mmol of ester). The organic extracts were combined, dried over $MgSO_4$, filtered, and the solvent was evaporated. The residue obtained after the workup was purified using column chromatography or preparative TLC (unless stated otherwise).

General procedure A4: Formation of ketonitrile by deprotonation of alkylacetonitrile with LDA

n-BuLi solution in hexane (2.5 M, 1.2 eq.; unless stated otherwise) was added under nitrogen to a solution of diisopropylamine (1.3 eq.; unless stated otherwise) in anhydrous THF (1.5 mL per 1 mmol of alkylacetonitrile) at -78 °C. The reaction mixture was stirred at -78 °C for 30 min and then added to a solution of alkylacetonitrile (1 eq.; unless stated otherwise) in anhydrous THF (1.5 mL per 1 mmol of alkylacetonitrile) at -78 °C. The reaction mixture was stirred at -78 °C for 30 min and the appropriate acylchloride (1.6 eq.; unless stated otherwise) was added at -78 °C. The mixture was allowed to warm to 25 °C and stirred for 18 h. The reaction mixture was quenched with saturated aqueous solution of NH_4Cl (15 mL per 1 mmol of alkylacetonitrile) and extracted with EtOAc (3 × 15 mL per 1 mmol of alkylacetonitrile). The organic extracts were combined, dried over $MgSO_4$, filtered, and the solvent was

evaporated. The residue obtained after the workup was purified using column chromatography or preparative TLC (unless stated otherwise).

General procedure A5: Formation of ketonitrile by deprotonation of CH_3CN with NaH

NaH (60% suspension in mineral oil, 2 eq) was added to a solution of acetonitrile (1.2 eq) in anhydrous THF (10 mL per 0.62 mmol of ester) under nitrogen and stirred at room temperature for 15 min. Then a solution of appropriate ester (1.0 eq) in THF (5.0 mL per 0.62 mmol of ester) was added and the reaction mixture was refluxed for 2 to 48 h. The reaction mixture was cooled to 0 °C, quenched with 1 N aqueous HCl solution (20 mL per 6.20 mmol of ester) and extracted with EtOAc (3 × 15 mL per 6.2 mmol of ester). The organic extracts were washed with brine (10 mL per 6.20 mmol of ester), dried over $MgSO_4$, filtered, and the solvent was evaporated *in vacuo*. The crude material was purified by column chromatography on silica gel.

General procedure B1: Formation of aminopyrazole using hydrazine hydrate and methanesulfonic acid

A mixture of appropriate ketonitrile (1 eq; unless stated otherwise), $N_2H_4.H_2O$ (64% in H_2O, 1.3 eq.; unless stated otherwise) and CH_3SO_3H (0.1 eq.; unless stated otherwise) in absolute EtOH (5 mL per 1 mmol of the substrate) was refluxed for 4 h (unless stated otherwise). The solvent was evaporated *in vacuo* and the residue was quenched with saturated aqueous solution of $NaHCO_3$ (20 mL per 1 mmol of the substrate) and extracted with EtOAc (20 mL per 1 mmol of the substrate) (unless stated otherwise). The organic extracts were washed with brine (2 × 20 mL per 1 mmol of the substrate), dried over $MgSO_4$, filtered, and the solvent was removed *in vacuo*. The crude material was purified by flash column chromatography on silica gel.

General procedure B2: Formation of aminopyrazole using arylhydrazine and methanesulfonic acid

A mixture of appropriate ketonitrile (1 to 1.4 eq) and arylhydrazine (1.0 eq; unless stated otherwise) and CH_3SO_3H (0.1 eq.; unless stated otherwise) in absolute EtOH (2 mL per 0.29 mmol) was refluxed for 4 (unless stated otherwise). The reaction mixture was cooled to 25 °C, concentrated *in vacuo* (unless mentioned otherwise), and the residue was purified by flash column chromatography on silica gel (unless mentioned otherwise).

General procedure C: Diazotization of aminopyrazoles and cyclization to pyrazolotriazines

Diazotization step:

Appropriate aminopyrazole (1 eq.; unless stated otherwise) was dissolved in EtOH (5 mL per 1 mmol of aminopyrazole) and H_2O (1.67 mL per 1 mmol of aminopyrazole), and 35% aqueous HCl (4 eq.; unless stated otherwise) was added. The solution was cooled to -10 °C and a pre-cooled solution (0 °C) of $NaNO_2$ (2 eq.; unless stated otherwise) in H_2O (1 mL per 1 mmol of aminopyrazole) was added. The reaction mixture turned yellow and was stirred for 20 min at -5 °C, then a pre-cooled solution (- 5 °C)

of KOAc (8 eq.; unless stated otherwise) and 2-(cyanomethyl)-benzimidazole (or alkyl benzimidazole-2-acetate) (1.05 eq.; unless stated otherwise) in EtOH (5 mL per 1 mmol of aminopyrazole) and H_2O (1.67 mL per 1 mmol of aminopyrazole) was added. The resulting mixture was allowed to warm up to 25 °C and stirred from 1-16 h. Cold H_2O (5 mL per 1 mmol of aminopyrazole) was added and the precipitate was filtered and washed with water (2.5 mL per 1 mmol of aminopyrazole). The product was dried under vacuum to yield a solid, which was used directly in the next step without additional purification.

Cyclization step:

The dried solid was dissolved in anhydrous DMF (5 mL per 1 mmol of aminopyrazole), KOAc (0.05 eq.; unless stated otherwise) was added and the mixture was refluxed under N_2 for 3 h, unless stated otherwise. The solution was poured into water (5 mL per 1 mmol of aminopyrazole), the precipitate was collected by filtration, dissolved in dioxane (2.5 mL per 1 mmol of aminopyrazole) at 50 °C, and the solution was poured into water (10 mL per 1 mmol of aminopyrazole). The precipitate was collected by filtration, washed with water (5 mL per 1 mmol of aminopyrazole), then with Et_2O (2.5 mL per 1 mmol of aminopyrazole) and dried under vacuum to yield the product.

General procedure D: Suzuki reaction

Degassed dioxane (4 mL per 0.1 mmol of arylhalide) and H_2O (1 mL per 0.1 mmol of arylhalide) were added to a mixture of arylhalide (1 eq.; unless stated otherwise), appropriate boronic acid or ester (1.2 eq.; unless stated otherwise), Pd(dppf)Cl_2 (0.05 eq.; unless stated otherwise) and K_3PO_4 (4 eq.: unless stated otherwise). The mixture was stirred under N_2 at 120 °C for 4 h (unless stated otherwise). The reaction mixture was quenched with saturated aqueous solution of NH_4Cl (10 mL per 0.1 mmol of arylhalide) and extracted with EtOAc (3 × 10 mL per 0.1 mmol of arylhalide). The organic extracts were combined, dried over $MgSO_4$, filtered, and the solvent was evaporated. The residue obtained after the workup was purified using column chromatography or preparative TLC (unless stated otherwise).

General procedure E: Preparation of diketo ester using *t*-BuOK and diethyl oxalate

t-BuOK (1.0 M in THF, 1.2 eq) was added dropwise to a solution of appropriate ketone (1.0 eq) and diethyl oxalate (1.1 eq) in anhydrous toluene (10 mL per 7.92 mmol of ketone) at room temperature under nitrogen atmosphere. The reaction mixture was stirred at room temperature for 16 h. The reaction mixture was acidified (pH~3-4) by the careful addition of aqueous 1 N HCl (20 mL per 7.92 mmol of ketone) and extracted with EtOAc (3 × 20 mL per 7.92 mmol of ketone). The organic extracts were washed with water (3 × 15 mL per 7.92 mmol of ketone) followed by brine (3 × 15 mL per 7.92 mmol of ketone), dried over $MgSO_4$, filtered, and the solvent was evaporated *in vacuo*. The crude material was purified by column chromatography on silica gel (unless mentioned otherwise).

General procedure F: Preparation of isoxazole esters

NH_2OH•HCl (1.2 to 1.5 eq) was added to a solution of appropriate diketoester (1.0 eq) in EtOH/methanol (16 mL/ 6.93 mmol of diketo ester) and the mixture was stirred at 50 °C for 16 h. The solvent was evaporated *in vacuo* and the residue was purified by column chromatography on silica gel.

General procedure G: Hydrolysis of isoxazole and isothiazole esters to corresponding carboxylic acids

NaOH (2.0 M in H_2O, 1.5 eq) was added to a solution of appropriate isoxazole ester (1.0 eq) in EtOH (15 mL per 6.76 mmol of isoxazole ester) at room temperature and the mixture was stirred for 1 h. The reaction mixture was acidified (pH~3-4) by the careful addition of aqueous 1 N HCl and extracted with EtOAc (3 × 20 mL). The combined organic extracts were washed with water (3 × 20 mL), brine (20 mL), dried over $MgSO_4$, filtered, and the solvent was evaporated *in* vacuo to provide corresponding isoxazole acid.

General procedure H: Preparation of acid chlorides

$SOCl_2$ (2.0 eq to 10 volumes) and appropriate acid (1.0 eq) were mixed and under nitrogen atmosphere and the mixture was refluxed for 20-240 minutes. The excess of $SOCl_2$ was evaporated *in vacuo*. The obtained residue was treated with toluene (2 × 2 mL/0.53 mmol of acid), the solvents were evaporated and the resulting product was dried *in vacuo*.

General procedure I: Preparation of amides using acid chlorides

Et_3N (1.0 to 2 eq.; unless stated otherwise) was added to a mixture of appropriate amine (1.0 eq; unless stated otherwise) in acetonitrile (0.5 mL to 1.0 mL per 0.074 mmol of amine reacted). The reaction mixture was heated to reflux, a solution of appropriate acid chloride (1.0 to 2.0 eq; unless stated otherwise) in acetonitrile (0.4 to 0.5 mL 0.074 mmol of acid chloride) was added dropwise, and the resulting mixture was refluxed for additional 2-5 h. The reaction mixture was cooled to room temperature, diluted with saturated aqueous $NaHCO_3$ solution (3 mL per 0.074 mmol of amine reacted) and filtered. The obtained solid was washed with water (3 mL per 0.074 mmol of amine reacted) and then with a mixture of EtOAc:hexane (1:4, 2 mL per 0.074 mmol of amine reacted) to obtained pure compound (unless stated otherwise).

Experimental procedures for compound 1 and its analogs

2-(4-Fluorophenyl)-3-oxopentanenitrile **(S1)**

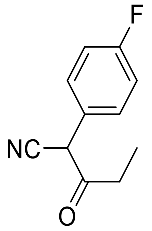

The compound was prepared according to General procedure A1 using 4-fluorophenylacetonitrile (1 mL, 8.33 mmol; 1 eq.), NaH (60% suspension in mineral oil; 667 mg, 16.66 mmol; 2 eq.), methyl propionate (0.8 mL, 8.33 mmol, 1 eq.) and THF (24 mL). Reaction time: 15 min for the formation of the sodium salt, then additional 1 h 30 min for the alkylation step. The residue obtained after the workup was purified by column chromatography on silica gel (hexane:EtOAc, gradient 1:0 to 1:1). The product was obtained as an orange oil (2.92 g, 92%).

^{1}H NMR (500 MHz, Chloroform-*d*) *δ* (ppm) 7.40 – 7.34 (m, 2H), 7.15 – 7.09 (m, 2H), 4.67 (s, 1H), 2.74 – 2.52 (m, 2H), 1.05 (t, *J* = 7.2 Hz, 3H).

^{13}C NMR (126 MHz, Chloroform-*d*) *δ* (ppm) 199.35, 163.24 (d, *J* = 249.8 Hz), 129.96 (d, *J* = 8.2 Hz), 125.92 (d, *J* = 3.5 Hz), 116.81 (d, *J* = 21.9 Hz), 116.36, 49.80, 33.40, 7.71.

HRMS (APCI): calcd. for $C_{11}H_9FNO$ $[M-H]^-$ = 190.0674, found $[M-H]^-$ = 190.0672.

3-(3-Bromophenyl)-3-oxopropanenitrile **(S2)**

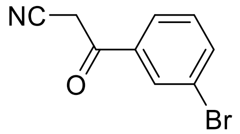

The compound was prepared according to General procedure A3 using *n*-BuLi solution in hexane (2.36 M, 2.27 mL, 5.35 mmol, 2 eq.), CH_3CN (0.42 mL, 8.02 mmol, 3 eq.) and THF (5 mL) (reaction time: 30 min for the formation of the lithium salt) and then using methyl 3-bromobenzoate (575 mg, 2.67 mmol; 1 eq.) and THF (5 mL) (reaction time: 2 h for the alkylation step). The residue obtained after the workup was purified by column chromatography on silica gel (hexane:EtOAc, gradient 5:1 to 1:3). The product was obtained as a beige solid (590 mg, 99%).

^{1}H NMR (500 MHz, Chloroform-*d*) *δ* (ppm) 8.05 (dd, *J* = 1.9 Hz, 1H), 7.84 (ddd, *J* = 7.8, 1.8, 1.0 Hz, 1H), 7.79 (ddd, *J* = 8.0, 2.0, 1.0 Hz, 1H), 7.42 (dd, *J* = 7.9 Hz, 1H), 4.06 (s, 2H).

^{13}C NMR (126 MHz, Chloroform-*d*) *δ* (ppm) 186.03, 137.76, 136.07, 131.61, 130.86, 127.11, 123.68, 113.37, 29.58.

3-Cyclohexyl-3-oxopropanenitrile **(S3)**

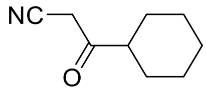

The compound was prepared according to General procedure A3 using *n*-BuLi (2.36 M, 2.57 mL, 6.08 mmol, 2 eq.), CH_3CN (0.48 mL, 9.11 mmol, 3 eq.) in THF (5 mL) and methyl cyclohexanecarboxylate (432 mg, 3.04 mmol; 1 eq.) in THF (5 mL). Reaction time: 30 min (deprotonation step) and 1 h (alkylation step). The residue obtained after workup was purified by column chromatography on silica gel (hexane:EtOAc, gradient 1:0 to 1:1). The product was obtained as a yellow oil (460 mg, 99%).

^{1}H NMR (500 MHz, Chloroform-*d*) δ (ppm) 3.48 (s, 2H), 2.55 (tt, J = 11.1, 3.4 Hz, 1H), 1.94 – 1.86 (m, 2H), 1.84 – 1.77 (m, 2H), 1.72 – 1.65 (m, 1H), 1.44 – 1.15 (m, 5H).

^{13}C NMR (126 MHz, Chloroform-*d*) δ (ppm) 200.49, 114.05, 50.13, 30.38, 28.29, 25.65, 25.38.

HRMS (APCI): calcd. for $C_9H_{17}N_2O$ $[M+NH_4]^+$ = 169.1335, found $[M+NH_4]^+$ = 169.1340.

HRMS (APCI): calcd. for $C_9H_{12}NO$ $[M-H]^-$ = 150.0924, found $[M-H]^-$ = 150.0923.

<u>3-Oxo-3-(2-phenoxyphenyl)propanenitrile</u> **(S4)**

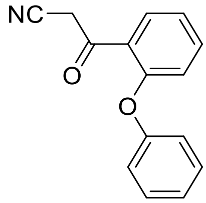

The compound was prepared according to General procedure A3 using *n*-BuLi (2.36 M, 1.92 mL, 4.52 mmol, 2 eq.), CH_3CN (0.35 mL, 6.78 mmol, 3 eq.) in THF (5 mL) and methyl 2-phenoxybenzoate (516 mg, 2.26 mmol; 1 eq.) in THF (5 mL). Reaction time: 30 min (deprotonation step) and 1 h (alkylation step). The residue obtained after workup was purified by column chromatography on silica gel (hexane:EtOAc, gradient 1:0 to 1:1). The product was obtained as a yellow solid (528 mg, 99%).

^{1}H NMR (500 MHz, Chloroform-*d*) δ (ppm) 7.96 (dd, J = 7.9, 1.8 Hz, 1H), 7.49 (ddd, J = 8.4, 7.3, 1.8 Hz, 1H), 7.46 – 7.41 (m, 2H), 7.29 – 7.22 (m, 1H), 7.19 (ddd, J = 8.1, 7.3, 1.1 Hz, 1H), 7.12 – 7.07 (m, 2H), 6.85 (dd, J = 8.4, 1.0 Hz, 1H), 4.16 (s, 2H).

^{13}C NMR (126 MHz, Chloroform-*d*) δ (ppm) 187.80, 157.69, 154.89, 135.63, 131.58, 130.57, 126.26, 125.37, 123.51, 120.09, 117.96, 114.40, 34.15.

HRMS (APCI): calcd. for $C_{15}H_{12}NO_2$ $[M+H]^+$ = 238.0863, found $[M+H]^+$ = 238.0865.

<u>3-(Naphthalen-2-yl)-3-oxopropanenitrile</u> **(S5)**

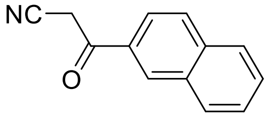

The compound was prepared according to General procedure A3 using *n*-BuLi (2.36 M, 2.37 mL, 5.61 mmol, 2 eq.), CH_3CN (0.44 mL, 8.41 mmol, 3 eq.) in THF (5 mL) and methyl 2-naphthoate (522 mg,

2.80 mmol; 1 eq.) in THF (10 mL). Reaction time: 30 min (deprotonation step) and 1 h (alkylation step). The residue obtained after workup was purified by column chromatography on silica gel (hexane:EtOAc, gradient 1:0 to 1:1). The product was obtained as an off-white solid (540 mg, 99%).

^{1}H NMR (300 MHz, Chloroform-*d*) *δ* (ppm)) δ 8.18 (t, *J* = 1.8 Hz, 2H), 7.98 (t, *J* = 1.3 Hz, 1H), 7.96 (t, *J* = 1.3 Hz, 1H), 7.70 (dd, *J* = 2.0, 1.1 Hz, 1H), 7.67 (dd, *J* = 2.1, 1.1 Hz, 1H), 7.32 (t, *J* = 7.9 Hz, 2H).

^{13}C NMR (126 MHz, Chloroform-*d*) *δ* (ppm) 187.09, 136.34, 132.46, 131.83, 130.87, 129.89, 129.68, 129.38, 128.10, 127.58, 123.56, 113.97, 29.56.

HRMS (APCI): calcd. for $C_{13}H_8NO$ $[M-H]^-$ = 194.0611, found $[M-H]^-$ = 194.0605.

<u>2-([1,1'-Biphenyl]-3-yl)-3-oxopentanenitrile</u> **(S6)**

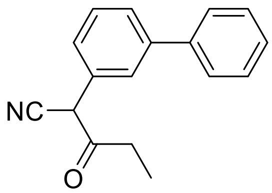

This compound was synthesized according to the procedure reported in *J. Org. Chem.* 2018, 83, 24, 15380–15405.

<u>4-Methyl-3-oxopentanenitrile</u> **(S7)**

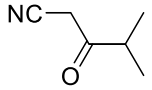

The compound was prepared according to General procedure A2 using sodium hydride (60% suspension in mineral oil, 1.40 g, 39.42 mmol, 2 eq.), CH_3CN (2.7 mL, 59.13 mmol, 3 eq.) in THF (25 mL) and methyl isobutyrate (2.25 mL, 19.71 mmol; 1 eq.). Reaction time: 6 h. The residue obtained after workup was purified by column chromatography on silica gel (hexane:EtOAc, 4:1). The product was obtained as yellow oil (1.89 g, 99%).

^{1}H NMR (500 MHz, Chloroform-*d*) *δ* (ppm) 3.51 (s, 2H), 2.81 (hept, *J* = 6.9 Hz, 1H), 1.18 (d, *J* = 6.9 Hz, 6H).

^{13}C NMR (126 MHz, Chloroform-*d*) *δ* (ppm) 201.23, 113.98, 40.68, 30.18, 17.98.

HRMS (APCI): calcd. for C_6H_8NO $[M-H]^-$ = 110.0611, found $[M-H]^-$ = 110.1618.

<u>4,4-Dimethyl-3-oxopentanenitrile</u> **(S8)**

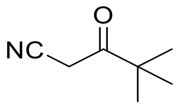

The compound was prepared according to General procedure A2 using sodium hydride (60% suspension in mineral oil, 1.40 g, 33.82 mmol, 2 eq.), CH_3CN (2.7 mL, 50.73 mmol, 3 eq.) in THF (25 mL) and methyl pivalate (2.25 mL, 16.91 mmol; 1 eq.). Reaction time: 6 h. The residue obtained after workup

was purified by column chromatography on silica gel (hexane:EtOAc, 4:1). The product was obtained as a white solid (2.40 g, 93%).

^{1}H NMR (500 MHz, Chloroform-*d*) δ (ppm) 3.62 (s, 2H), 1.19 (s, 9H).

^{13}C NMR (126 MHz, Chloroform-*d*) δ 202.87, 114.24, 44.79, 27.58, 26.26.

HRMS (APCI): calcd. for $C_7H_{10}NO$ [M-H]$^-$ = 124.0768, found [M-H]$^-$ = 124.0774.

<u>2-(4-Fluorophenyl)-4-methyl-3-oxopentanenitrile</u> **(S9)**

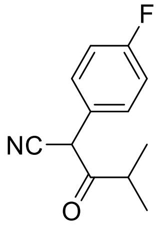

The compound was prepared according to General procedure A1 using 4-fluorophenylacetonitrile (0.2 mL, 1.31 mmol; 1 eq.), NaH (60% suspension in mineral oil; 105 mg, 2.62 mmol; 2 eq.), methyl isobutyrate (0.18 mL, 1.57 mmol, 1.2 eq.) and THF (4 mL). Reaction time: 15 min for the formation of the sodium salt, then additional 1.5 h for the alkylation step. The residue obtained after the workup was purified by column chromatography on silica gel (hexane:EtOAc, gradient 1:0 to 4:1). The product was obtained as a white solid (266 mg, 83%).

^{1}H NMR (500 MHz, Chloroform-*d*) δ (ppm) 7.42 – 7.33 (m, 2H), 7.16 – 7.09 (m, 2H), 4.77 (s, 1H), 2.94 (hept, J = 6.8 Hz, 1H), 1.11 (dd, J = 6.9, 4.5 Hz, 6H).

^{13}C NMR (126 MHz, Chloroform-*d*) δ (ppm) 202.47, 163.23 (d, J = 249.6 Hz), 130.16 (d, J = 8.5 Hz), 125.83 (d, J = 3.7 Hz), 116.79 (d, J = 22.1 Hz), 116.33, 48.40, 38.92, 18.97, 18.70.

HRMS (APCI): calcd. for $C_{12}H_{11}FNO$ [M-H]$^-$ = 204.0830, found [M-H]$^-$ = 204.0838.

<u>3-(3-Methoxynaphthalen-2-yl)-3-oxopropanenitrile</u> **(S10)**

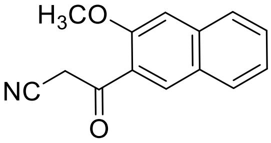

The compound was prepared according to General procedure A2 using sodium hydride (60% suspension in mineral oil, 136 mg, 3.40 mmol, 2 eq.), CH_3CN (0.25 mL, 5.10 mmol, 3 eq.) in THF (5 mL) and methyl 3-methoxy-2-naphthoate (365 mg, 1.70 mmol; 1 eq.). Reaction time: 6 h. The residue obtained after workup was purified by column chromatography on silica gel (hexane:EtOAc, 5:2). The product was obtained as yellow oil (100 mg, 26%).

^{1}H NMR (500 MHz, Chloroform-*d*) δ (ppm) 8.37 (s, 1H), 7.88 (d, J = 8.2 Hz, 1H), 7.76 (d, J = 8.3 Hz, 1H), 7.57 (ddd, J = 8.2, 6.9, 1.2 Hz, 1H), 7.41 (ddd, J = 8.1, 6.9, 1.1 Hz, 1H), 7.23 (s, 1H), 4.16 (s, 2H), 4.06 (s, 3H).

^{13}C NMR (126 MHz, Chloroform-*d*) δ (ppm) 188.79, 155.26, 137.19, 133.51, 129.84, 129.64, 128.08, 126.65, 126.22, 125.20, 114.61, 107.01, 55.95, 34.14.

<u>3-([1,1'-Biphenyl]-3-yl)-3-oxopropanenitrile</u> **(S11)**

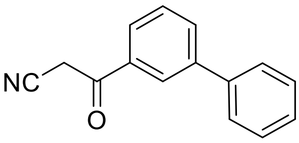

The compound was prepared according to General procedure A2 using sodium hydride (60% suspension in mineral oil, 188 mg, 4.72 mmol, 2 eq.), CH_3CN (0.25 mL, 4.72 mmol, 2 eq.) in THF (5 mL) and methyl[1,1'-biphenyl]-3-carboxylate (500 mg, 2.36 mmol; 1 eq.). Reaction time: 16 h. The residue obtained after workup was purified by column chromatography on silica gel (hexane:EtOAc, 4:1). The product was obtained as yellow oil (0.44 g, 76%).
^{1}H NMR (500 MHz, Chloroform-*d*) δ (ppm) ^{1}H NMR (500 MHz, $CDCl_3$) δ 7.65 – 7.58 (m, 2H), 7.54 – 7.44 (m, 5H), 7.39 – 7.34 (m, 2H), 3.20 (s, 2H).
^{13}C NMR (126 MHz, Chloroform-*d*) δ (ppm) 193.68, 140.99, 139.69, 137.54, 132.43, 130.62, 129.54, 129.02, 128.10, 113.75, 32.25.
HRMS (APCI): calcd. for $C_{15}H_{10}NO$ $[M-H]^-$ = 220.0768, found $[M- H]^-$ = 220.0765.

<u>3-(1-Methoxynaphthalen-2-yl)-3-oxopropanenitrile</u> **(S12)**

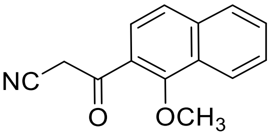

The compound was prepared according to General procedure A3 using *n*-BuLi (2.7 M, 1.03 mL, 7.76 mmol, 1.2 eq.), CH_3CN (0.25 mL, 4.6 mmol, 2 eq.) in THF (4 mL) and methyl 1-methoxy-2-naphthoate (500 mg, 2.30 mmol; 1 eq.) in THF (4 mL). Reaction time: 30 min (deprotonation step) and 1 h (alkylation step). The residue obtained after workup was purified by column chromatography on silica gel (hexane:EtOAc, 5:1). The product was obtained as a yellow oil (260 mg, 50%).
^{1}H NMR (500 MHz, Chloroform-*d*) δ (ppm) 8.20 (d, J = 8.1 Hz, 1H), 7.89 (d, J = 7.5 Hz, 1H), 7.78 (d, J = 8.7 Hz, 1H), 7.67 (d, J = 8.5 Hz, 1H), 7.66 – 7.57 (m, 2H), 4.27 (s, 2H), 4.06 (s, 3H).
^{13}C NMR (126 MHz, Chloroform-*d*) δ (ppm) 188.79, 158.84, 137.94, 129.45, 128.54, 127.64, 127.27, 125.23, 125.11, 125.07, 123.64, 114.47, 64.53, 33.18.

<u>3-(3-Methoxynaphthalen-1-yl)-3-oxopropanenitrile</u> **(S13)**

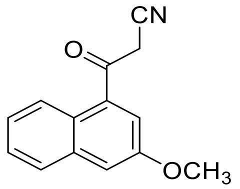

The compound was prepared according to General procedure A3 using *n*-BuLi (2.7 M, 1 mL, 2.87 mmol, 1.05 eq.), CH_3CN (0.28 mL, 5.46 mmol, 2 eq.) in THF (4 mL) and methyl 3-methoxy-1-naphthoate (590 mg, 2.73 mmol; 1 eq.) in THF (4 mL). Reaction time: 30 min (deprotonation step) and 1 h (alkylation step). The residue obtained after workup was purified by column chromatography on silica gel (hexane:EtOAc, 5:2). The product was obtained as a yellow oil (210 mg, 36%).

^{1}H NMR (500 MHz, Chloroform-*d*) δ (ppm) 8.00 (d, J = 9.0 Hz, 1H), 7.97 – 7.93 (m, 1H), 7.81 (d, J = 8.2 Hz, 1H), 7.54 (ddd, J = 8.5, 6.9, 1.4 Hz, 1H), 7.41 (ddd, J = 8.0, 6.8, 1.1 Hz, 1H), 7.30 (d, J = 9.1 Hz, 1H), 4.05 (s, 3H), 4.03 (s, 2H).

^{13}C NMR (126 MHz, Chloroform-*d*) δ (ppm) 192.52, 156.41, 134.48, 130.95, 129.11, 128.97, 128.54, 124.85, 123.67, 120.33, 114.65, 112.25, 56.64, 34.39.

Methyl 2-((tert-butyldiphenylsilyl)oxy)acetate **(S14)**

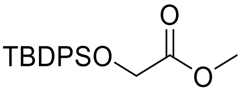

Under nitrogen atmosphere, methyl 2-hydroxyacetate (2.3 g, 25.70 mmol; 1eq.) was dissolved in dry dichloromethane (30 mL). To the solution was added Et_3N (7.2 mL, 51.4 mmol; 2 eq.), DMAP (1.7 g, 12.50 mmol; 0.5 eq.) and TBDPSCl (7.3 mL, 28.27 mmol; 1.1 eq.), and the reaction mixture was stirred for 16 h at room temperature. The mixture was poured into water (50 mL) and extracted with dichloromethane (3 ×25 mL). The combined organic extracts were washed with hydrochloric acid (10%, 2 × 75 mL), brine (75 mL) and dried over $MgSO_4$. The solvent was evaporated, and the product was dried under *in vacuo*. The obtained colorless oil (8.2 g, 98%) was used in the next step without further purification.

^{1}H NMR (500 MHz, Chloroform-*d*) δ (ppm) 7.71 – 7.68 (m, 4H), 7.46 – 7.41 (m, 2H), 7.42 – 7.37 (m, 4H), 4.25 (s, 2H), 3.69 (s, 3H), 1.10 (s, 9H).

^{13}C NMR (126 MHz, Chloroform-*d*) δ (ppm) 171.80, 135.74, 130.04, 127.93, 62.30, 51.77, 26.83, 19.41.

4-((*tert*-Butyldiphenylsilyl)oxy)-3-oxobutanenitrile **(S15)**

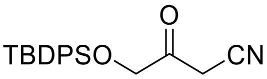

The compound was prepared according to General procedure A3 using *n*-BuLi (2.7 M, 0.6 mL, 1.6 mmol, 1.1 eq.), CH_3CN (0.12 mL, 2.28 mmol, 1.5 eq.) in THF (5 mL) and methyl 2-((*tert*-butyldiphenylsilyl)oxy)acetate (**S14**, 500 mg, 1.52 mmol; 1 eq.) in THF (5 mL). Reaction time: 30 min

(deprotonation step) and 1 h (alkylation step). The residue obtained after workup was purified by column chromatography on silica gel (hexane:EtOAc, 5:1). The product was obtained as a colorless oil (370 mg, 71%).

^{1}H NMR (500 MHz, Chloroform-*d*) δ (ppm) 7.65 – 7.60 (m, 4H), 7.50 – 7.46 (m, 2H), 7.45 – 7.40 (m, 4H), 4.27 (s, 2H), 3.73 (s, 2H), 1.12 (s, 9H).

^{13}C NMR (126 MHz, Chloroform-*d*) δ (ppm) 198.24, 135.57, 131.78, 130.56, 128.28, 113.45, 69.15, 29.50, 26.88, 19.28.

HRMS (APCI): calcd. for $C_{20}H_{22}NO_2Si$ [M-H]$^-$ = 336.1425, found [M-H]$^-$ = 336.1429.

3-(1-Benzyl-1*H*-imidazol-2-yl)-3-oxopropanenitrile **(S16)**

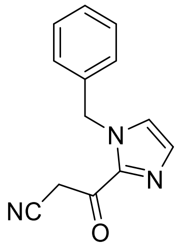

The compound was prepared according to General procedure A2 using sodium hydride (60%, 105 mg, 2.62 mmol, 2 eq.), CH_3CN (0.2 mL, 3.9 mmol, 3 eq.) in THF (5 mL) and methyl 1-benzyl-1*H*-imidazole-2-carboxylate (280 mg, 1.30 mmol; 1 eq.). Reaction time: 2 h. The residue obtained after workup was purified by column chromatography on silica gel (hexane:EtOAc, 0:1 to 1:0). The product was obtained as yellow oil (220 mg, 76%).

^{1}H NMR (500 MHz, Chloroform-*d*) δ (ppm) 7.42 – 7.28 (m, 3H), 7.23 – 7.16 (m, 4H), 5.60 (s, 2H), 4.30 (d, J = 0.8 Hz, 2H).

3-(2-chlorophenyl)-3-oxopropanenitrile **(S17)**

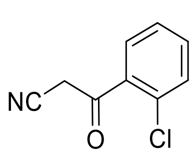

The compound was prepared according to General procedure A2 using sodium hydride (60%, 1.2 g, 30 mmol, 2 eq.), CH_3CN (2.4 mL, 45 mmol, 3 eq.) in THF (25 mL) and methyl 2-chlorobenzoate (2.55 g, 15 mmol; 1 eq.). Reaction time: 4 h. The residue obtained after workup was purified by column chromatography on silica gel (hexane:EtOAc, gradient 4:1 to 2:1). The product was obtained as a yellow solid (2.26 g, 84%).

^{1}H NMR (500 MHz, Chloroform-*d*) δ (ppm) 7.64 (dd, J = 7.7, 1.4 Hz, 1H), 7.54 – 7.45 (m, 2H), 7.44 – 7.30 (m, 1H), 4.14 (s, 2H).

^{13}C NMR (126 MHz, Chloroform-*d*) δ (ppm) 189.50, 135.86, 133.88, 131.85, 131.14, 130.57, 127.63, 113.45, 33.05.

HRMS (APCI): calcd. for C_9H_5ClON $[M-H]^-$ = 178.0065, found $[M-H]^-$ = 178.0067.

3-(Naphthalen-1-yl)-3-oxopropanenitrile **(S18)**

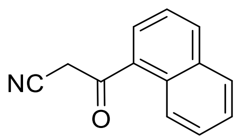

The compound was prepared according to General procedure A3 using *n*-BuLi (2.7 M, 1 mL, 2.82 mmol, 1.05 eq.), CH_3CN (0.18 mL, 3.50 mmol, 1.3 eq.) in THF (4 mL) and methyl 1-naphthoate (500 mg, 2.69 mmol; 1 eq.) in THF (4 mL). Reaction time: 30 min (deprotonation step) and 1 h (alkylation step). The residue obtained after workup was purified by column chromatography on silica gel (hexane:EtOAc, 2:1). The product was obtained as a yellow solid (310 mg, 59%).

^{1}H NMR (500 MHz, Chloroform-*d*) δ (ppm) 8.81 (d, J = 8.7 Hz, 1H), 8.11 (d, J = 8.2 Hz, 1H), 7.91 (t, J = 8.2 Hz, 1H), 7.71 – 7.65 (m, 1H), 7.63 – 7.57 (m, 1H), 7.56 – 7.52 (m, 1H), 4.19 (s, 1H).

^{13}C NMR (126 MHz, Chloroform-*d*) δ (ppm) 189.60, 135.34, 134.26, 131.70, 130.51, 129.63, 129.32, 128.85, 127.29, 125.77, 124.31, 114.16, 31.90.

HRMS (APCI): calcd. for $C_{13}H_8NO$ $[M-H]^-$ = 194.0611, found $[M-H]^-$ = 194.0608.

3-(2-(Dimethylamino)phenyl)-3-oxopropanenitrile **(S19)**

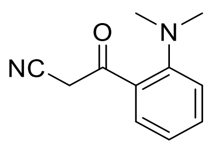

The compound was prepared according to General procedure A2 using sodium hydride (60%, 222 mg, 5.58 mmol, 2 eq.), CH_3CN (0.44 mL, 8.37 mmol, 3 eq.) in THF (5 mL) and methyl 2-(dimethylamino)benzoate (500 mg, 2.79 mmol; 1 eq.). Reaction time: 4 h. The residue obtained after the workup was purified by column chromatography on silica gel (hexane:EtOAc, gradient 9:1 to 1:1). The product was obtained as a yellow oil (340 mg, 33%).

^{1}H NMR (300 MHz, Chloroform-*d*) δ (ppm) 7.59 – 7.39 (m, 2H), 7.23 – 7.05 (m, 2H), 4.18 (s, 2H), 2.85 (s, 6H).

4-(2-Methoxyphenyl)-3-oxobutanenitrile **(S20)**

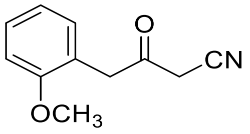

The compound was prepared according to General procedure A3 using *n*-BuLi solution in hexanes (2.5 M, 4.8 mL, 12.10 mmol, 1.3 eq.), CH_3CN (0.73 mL, 13.98 mmol, 1.5 eq.) and THF (15 mL) (reaction time: 30 min for the formation of the lithium salt) and then using methyl 2-(2-methoxyphenyl)acetate

(1.68 g, 9.32 mmol; 1 eq.) and THF (5 mL) (reaction time: 2 h for the alkylation step). The residue obtained after the workup (1.30 g, 74%) was used in the next step without further purification.

5-Methoxy-3-oxo-5-phenylpentanenitrile **(S21)**

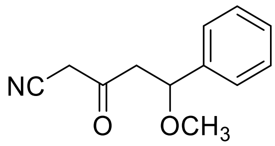

The compound was prepared according to General procedure A3 using *n*-BuLi solution in hexanes (2.5 M, 1.2 mL, 3.12 mmol, 1.3 eq.), CH_3CN (0.19 mL, 3.60 mmol, 1.5 eq.) and THF (5 mL) (reaction time: 30 min for the formation of the lithium salt) and then using ethyl 3-methoxy-3-phenylpropanoate (500 mg, 2.40 mmol; 1 eq.) and THF (3 mL) (reaction time: 2 h for the alkylation step). The residue obtained after the workup was purified by column chromatography on silica gel (cyclohexane:EtOAc, gradient 9:1 to 1:1). The product was obtained as a colorless oil (290 mg, 60%).

^{1}H NMR (500 MHz, Chloroform-*d*) δ (ppm) 7.41 – 7.36 (m, 2H), 7.35 – 7.29 (m, 3H), 4.61 (dd, J = 9.5, 3.8 Hz, 1H), 3.60 (d, J = 19.4 Hz, 1H), 3.49 (d, J = 19.5 Hz, 1H), 3.20 (s, 3H), 3.05 (dd, J = 15.1, 9.5 Hz, 1H), 2.70 (dd, J = 15.1, 3.9 Hz, 1H).

^{13}C NMR (126 MHz, Chloroform-*d*) δ (ppm) 195.99, 139.88, 129.00, 128.58, 126.52, 113.68, 79.96, 56.97, 50.49, 33.55.

3-Oxo-4-phenylbutanenitrile **(S22)**

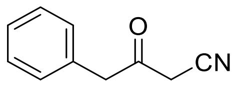

The compound was prepared according to General procedure A3 using *n*-BuLi (2.5 M, 5.68 mL, 14.23 mmol, 1.67 eq.), CH_3CN (1.11 mL, 21.31 mmol, 2.5 eq.) in THF (12 mL) and methyl 2-phenylacetate (1.28 g, 8.52 mmol; 1 eq.) in THF (30 mL). Reaction time: 30 min (deprotonation step) and 2 h (alkylation step). The residue obtained after workup was loaded on ISOLUTE® HM-N and purified by column chromatography on silica gel using Biotage Selekt purification system (cyclohexane:EtOAc, gradient 1:0 to 1:1). The product was obtained as a pale-yellow oil (1.22 g, 90%).

^{1}H NMR (500 MHz, Chloroform-*d*) δ (ppm) 7.40 – 7.35 (m, 2H), 7.35 – 7.30 (m, 1H), 7.24 – 7.20 (m, 2H), 3.86 (s, 2H), 3.46 (s, 2H).

^{13}C NMR (126 MHz, Chloroform-*d*) δ (ppm) 195.33, 132.07, 129.55, 129.38, 128.09, 113.70, 49.30, 31.25.

Methyl 2-(isochroman-3-yl)acetate **(S23)**

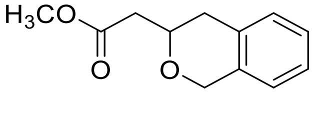

To a solution of 2-(isochroman-1-yl)acetic acid (600 mg, 3.12 mmol, 1 eq.) in MeOH (30 mL) was added conc. H_2SO_4 (34 µL, 0.62 mmol, 96%, 0.2 eq.) and the mixture was refluxed for 16 h. The mixture was poured into H_2O (30 mL) and the resulting solution was extracted with dichloromethane (4 × 60 mL). The organic extracts were combined, dried over $MgSO_4$, filtered, and the solvent was evaporated *in vacuo*. The residue was loaded on ISOLUTE® HM-N and purified by column chromatography using Biotage Selekt purification system (cyclohexane:EtOAc, gradient 95:5 to 0:1). The product was obtained as a colorless oil (567 mg, 88%).

^{1}H NMR (500 MHz, Chloroform-*d*) δ (ppm) 7.21 – 7.16 (m, 2H), 7.14 – 7.10 (m, 1H), 7.07 – 7.02 (m, 1H), 5.25 (dd, J = 9.7, 3.5 Hz, 1H), 4.13 (ddd, J = 11.4, 5.3, 4.3 Hz, 1H), 3.82 (ddd, J = 11.4, 9.0, 3.9 Hz, 1H), 3.75 (s, 3H), 3.03 – 2.94 (m, 1H), 2.89 (dd, J = 15.2, 3.5 Hz, 1H), 2.77 (dd, J = 15.2, 9.7 Hz, 1H), 2.75 – 2.69 (m, 1H).

^{13}C NMR (126 MHz, Chloroform-*d*) δ (ppm) 171.87, 136.82, 134.09, 129.24, 126.87, 126.43, 124.62, 73.07, 63.26, 52.00, 41.72, 28.95.

HRMS (APCI): calcd. for $C_{12}H_{15}O_3$ $[M+H]^+$ = 207.1016, found $[M+H]^+$ = 207.1013.

4-(Isochroman-3-yl)-3-oxobutanenitrile **(S24)**

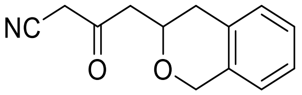

The compound was prepared according to General procedure A3 using *n*-BuLi (2.5 M, 1.36 mL, 3.40 mmol, 1.3 eq.), CH_3CN (0.274 mL, 5.24 mmol, 2 eq.) in THF (6 mL) and methyl 2-(isochroman-3-yl)acetate (540 mg, 2.62 mmol; 1 eq.) in THF (18 mL). Reaction time: 30 min (deprotonation step) and 2 h (alkylation step). The residue obtained after the workup was loaded on ISOLUTE® HM-N and purified by column chromatography on silica gel using Biotage Selekt purification system (cyclohexane:EtOAc, gradient 1:0 to 1:1). The product was obtained as a white solid (516 mg, 92%).

^{1}H NMR (500 MHz, Chloroform-*d*) δ (ppm) 7.23 – 7.18 (m, 2H), 7.14 (dd, J = 5.3, 3.8 Hz, 1H), 7.06 – 6.99 (m, 1H), 5.20 (t, J = 6.4 Hz, 1H), 4.15 (ddd, J = 11.3, 5.6, 3.0 Hz, 1H), 3.78 (ddd, J = 11.4, 10.3, 3.6 Hz, 1H), 3.67 (d, J = 19.5 Hz, 1H), 3.56 (d, J = 19.5 Hz, 1H), 3.11 – 2.97 (m, 3H), 2.70 (dt, J = 16.4, 3.4 Hz, 1H).

^{13}C NMR (126 MHz, Chloroform-*d*) δ (ppm) 196.42, 135.80, 133.86, 129.44, 127.31, 126.73, 124.43, 113.83, 73.22, 64.04, 48.51, 33.50, 28.80.

HRMS (APCI): calcd. for $C_{13}H_{14}NO_2$ $[M+H]^+$ = 216.1019, found $[M+H]^+$ = 216.1020.

2-(4-Fluorophenyl)-4-(2-methoxyphenyl)-3-oxobutanenitrile **(S25)**

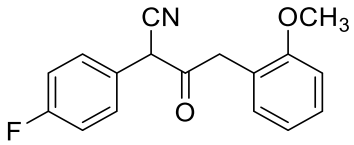

The compound was prepared according to General procedure A1 using 4-fluorophenylacetonitrile (0.82 mL, 6.84 mmol; 1.1 eq.), NaH (60% suspension in mineral oil; 497 mg, 12.43 mmol; 2 eq.), methyl 2-(2-methoxyphenyl)acetate (1 mL, 6.22 mmol, 1 eq.) and THF (10 mL). Reaction time: 15 min for the formation of the sodium salt, then an additional 1.5 h for the alkylation step. The residue obtained after the workup (1.5 g, 85%) was used in the next step without further purification.

2-Cyclohexyl-3-oxopentanenitrile **(S26)**

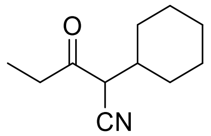

The compound was prepared according to General procedure A4 using *n*-BuLi (2.5 M, 1.15 mL, 2.88 mmol, 1.2 eq.), diisopropylamine (0.437 mL, 3.12 mmol, 1.3 eq.) and THF (3 mL) (reaction time: 30 min for the formation of LDA), then deprotonation of cyclohexylacetonitrile (295 mg, 2.40 mmol; 1 eq.) in THF (3 mL) (reaction time: 30 min) and final addition of propionyl chloride (0.346 mL, 3.84 mmol, 1.6 eq.) (reaction time: 16 h). The residue obtained after workup was loaded on ISOLUTE® HM-N and purified by column chromatography on silica gel using Biotage Selekt purification system (hexane:EtOAc, gradient 1:0 to 4:1). The product was obtained as a colorless oil (319 mg, 74%) containing ca. 10 % impurities (by ^{1}H NMR), was used as such in the next step.

^{1}H NMR (500 MHz, Chloroform-*d*) δ (ppm) 2.61 (q, J = 7.6 Hz, 1H), 2.51 (q, J = 7.6 Hz, 1H), 2.27 – 2.20 (m, 1H), 1.87 – 1.59 (m, 5H), 1.44 – 1.30 (m, 1H), 1.31 – 1.13 (m, 5H), 1.12 – 1.02 (m, 2H).

HRMS (APCI): calcd. for $C_{11}H_{18}NO$ $[M+H]^+$ = 180.1383, found $[M+H]^+$ = 180.1380.

3-Oxo-2-(tetrahydro-2*H*-pyran-4-yl)pentanenitrile **(S27)**

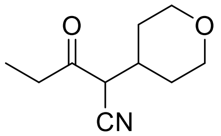

The compound was prepared according to General procedure A4 using *n*-BuLi (2.5 M, 1.15 mL, 2.88 mmol, 1.2 eq.), diisopropylamine (437 µL, 3.12 mmol, 1.3 eq.) and THF (3 mL) (reaction time: 30 min for the formation of LDA), then deprotonation of 2-(tetrahydro-2*H*-pyran-4-yl)acetonitrile (300 mg, 2.40 mmol; 1 eq.) in THF (3 mL) (reaction time: 30 min) and final addition of propionyl chloride (0.346 mL, 3.84 mmol, 1.6 eq.) (reaction time: 16 h). The residue obtained after workup was loaded on ISOLUTE® HM-N and purified by column chromatography on silica gel using Biotage Selekt purification system (hexane:EtOAc, gradient 1:0 to 1:1). The product was obtained as a colorless oil (471 mg, quantitative) containing ca. 20 % impurities (by ^{1}H NMR), was used as such in the next step.

^{1}H NMR (500 MHz, Chloroform-*d*) δ (ppm) 3.99 (dd, J = 11.9, 4.7 Hz, 0H), 3.36 (td, J = 12.0, 2.1 Hz, 1H), 2.63 (q, J = 7.6 Hz, 1H), 2.52 (q, J = 7.6 Hz, 1H), 2.31 (q, J = 7.4 Hz, 1H), 1.78 – 1.70 (m, 1H),

1.55 – 1.48 (m, 1H), 1.38 (t, J = 7.0 Hz, 2H), 1.24 (t, J = 7.6 Hz, 1H), 1.19 (qd, J = 5.2, 4.6, 2.7 Hz, 2H), 1.10 (dt, J = 14.5, 7.5 Hz, 3H).

2-Benzyl-3-oxopentanenitrile **(S28)**

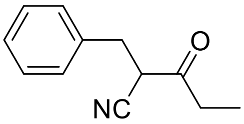

The compound was prepared according to General procedure A4 using *n*-BuLi (2.5 M, 2.3 mL, 5.75 mmol, 1.2 eq.), diisopropylamine (0.874 mL, 6.23 mmol, 1.3 eq.) and THF (3 mL) (reaction time: 30 min for the formation of LDA), then deprotonation of 3-phenylpropanenitrile (629 mg, 4.79 mmol; 1 eq.) in THF (3 mL) (reaction time: 30 min) and final addition of propionyl chloride (0.67 mL, 7.67 mmol, 1.6 eq.) Reaction time: 16 h. The residue obtained after workup was loaded on ISOLUTE® HM-N and purified by column chromatography on silica gel using Biotage Selekt purification system (hexane:EtOAc, gradient 1:0 to 1:1). The product was obtained as a colorless oil (315 mg, 35%) containing ca. 10 % impurities (by ^{1}H NMR), was used as such in the next step.

^{1}H NMR (500 MHz, Chloroform-*d*) δ 7.39 – 7.16 (m, 5H), 3.43 (s, 1H), 2.96 (t, J = 7.4 Hz, 1H), 2.71 (q, J = 7.5 Hz, 1H), 2.62 (t, J = 7.4 Hz, 1H), 2.52 (q, J = 7.5 Hz, 1H), 1.18 (dt, J = 46.4, 7.6 Hz, 3H).

2-(Cyclohexylmethyl)-3-oxopentanenitrile **(S29)**

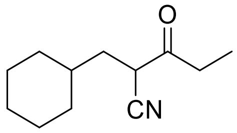

The compound was prepared according to General procedure A4 using *n*-BuLi (2.5 M, 2.6 mL, 6.57 mmol, 1.2 eq.), diisopropylamine (1.7 mL, 7.11 mmol, 1.3 eq.) and THF (3 mL) (reaction time: 30 min for the formation of LDA), then deprotonation of 3-cyclohexylpropanenitrile (750 mg, 5.47 mmol; 1 eq.) in THF (3 mL) (reaction time: 30 min) and final addition of propionyl chloride (57 µL, 6.56 mmol, 1.20 eq.) (reaction time: 16 h). The crude product obtained after the workup (1.10 g, quantitative) was used in the next step without further purification.

2-(2-Chlorobenzyl)-3-oxopentanenitrile **(S30)**

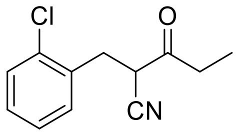

The compound was prepared according to General procedure A4 using *n*-BuLi (2.5 M, 1.4 mL, 3.62 mmol, 1.2 eq.), diisopropylamine (0.56 mL, 3.93 mmol, 1.3 eq.) and THF (3 mL) (reaction time: 30 min for the formation of LDA), then deprotonation of 3-(2-chlorophenyl)propanenitrile (500 mg, 3.01 mmol; 1 eq.) in THF (3 mL) (reaction time: 30 min) and final addition of propionyl chloride (0.32 mL, 3.62

mmol, 1.2 eq.) (reaction time: 16 h). The crude product obtained after the workup (670 mg, quantitative) was used in the next step without further purification.

3-Oxo-2-(pyridin-3-ylmethyl)pentanenitrile **(S31)**

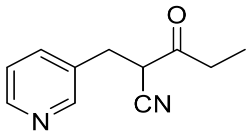

The compound was prepared according to General procedure A4 using *n*-BuLi (2.5 M, 1.5 mL, 3.64 mmol, 1.2 eq.), diisopropylamine (0.56 mL, 3.94 mmol, 1.3 eq.) and THF (3 mL) (reaction time: 30 min for the formation of LDA), then deprotonation of 3-(pyridin-3-yl)propanenitrile (400 mg, 3.03 mmol; 1 eq.) in THF (3 mL) (reaction time: 30 min) and final addition of propionyl chloride (0.32 mL, 3.64 mmol, 1.2 eq.) (reaction time: 16 h). The crude product obtained after the workup (560 mg, quantitative) was used in the next step without further purification.

2-(2-Methoxybenzyl)-3-oxopentanenitrile **(S32)**

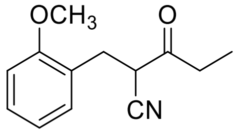

The compound was prepared according to General procedure A4 using *n*-BuLi (2.5 M, 1.5 mL, 3.72 mmol, 1.2 eq.), diisopropylamine (0.57 mL, 4.03 mmol, 1.3 eq.) and THF (3 mL) (reaction time: 30 min for the formation of LDA), then deprotonation of 3-(2-methoxyphenyl)propanenitrile (500 mg, 3.01 mmol; 1 eq.) in THF (3 mL) (reaction time: 30 min) and final addition of propionyl chloride (0.33 mL, 3.44 mmol, 1.2 eq.) (reaction time: 16 h). The crude product obtained after the workup (670 mg, quantitative) was used in the next step without further purification.

2-(4-Fluorobenzyl)-3-oxopentanenitrile **(S33)**

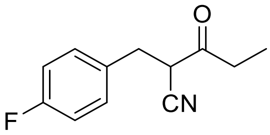

The compound was prepared according to General procedure A4 using *n*-BuLi (2.5 M, 1.9 mL, 4.83 mmol, 1.2 eq.), diisopropylamine (0.74 mL, 5.23 mmol, 1.3 eq.) and THF (3 mL) (reaction time: 30 min for the formation of LDA), then deprotonation of 3-(4-fluorophenyl)propanenitrile (600 mg, 4.02 mmol; 1 eq.) in THF (3 mL) (reaction time: 30 min) and final addition of propionyl chloride (0.42 mL, 4.83 mmol, 1.2 eq.) (reaction time: 16 h). The crude product obtained after the workup (820 mg, quantitative) was used in the next step without further purification.

2-(1-Methylpiperidin-4-yl)-3-oxopentanenitrile **(S34)**

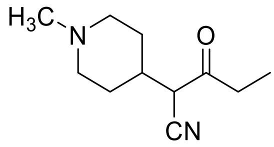

The compound was prepared according to General procedure A4 using *n*-BuLi (2.5 M, 1.7 mL, 4.34 mmol, 1.2 eq.), diisopropylamine (0.67 mL, 4.7 mmol, 1.3 eq.) and THF (3 mL) (reaction time: 30 min for the formation of LDA), then deprotonation of 2-(1-methylpiperidin-4-yl)acetonitrile (500 mg, 3.62 mmol; 1 eq.) in THF (3 mL) (reaction time: 30 min) and final addition of propionyl chloride (0.38 mL, 4.34 mmol, 1.2 eq.) (reaction time: 16 h). The crude product obtained after the workup (700 mg, quantitative) was used in the next step without further purification.

3-(1-(*tert*-Butyl)-1*H*-pyrazol-4-yl)acrylonitrile **(S35)**

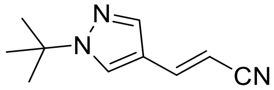

Diethyl cyanomethylphosphonate (0.45 mL, 2.76 mmol, 1.05 eq.) was slowly added at 0 °C to a stirred mixture sodium hydride in mineral oil (60%, 116 mg, 2.89 mmol, 1.1 eq.) in THF (8 mL). After 30 min, 1-*tert*-butyl-1*H*-pyrazole-4-carbaldehyde (400 mg, 2.63 mmol, 1 eq.) was added and the mixture was stirred at 23 °C for additional 16 h. The mixture was poured into water (30 mL) and extracted with EtOAc (3 × 20 mL). The combined organic extracts were washed with brine (30 mL), dried over $MgSO_4$, and the solvent was evaporated. The residue was purified by column chromatography (hexane:EtOAc, gradient 9:1 to 3:7). The product was obtained as a colorless solid (350 mg, 76%) – as a mixture of *E* and *Z* isomers (5:1).

The NMR spectra of *E*-isomer:

^{1}H NMR (500 MHz, Chloroform-*d*) δ (ppm) 7.68 (s, 1H), 7.66 (s, 1H), 7.24 (d, *J* = 16.6 Hz, 1H), 5.54 (d, *J* = 16.5 Hz, 1H), 1.58 (s, 9H).

^{13}C NMR (126 MHz, Chloroform-*d*) δ (ppm) 141.49, 137.60, 126.17, 118.84, 117.26, 92.78, 59.41, 29.74.

The NMR spectra of *Z*-isomer:

^{1}H NMR (500 MHz, Chloroform-*d*) δ (ppm) 8.17 (s, 1H), 7.86 (s, 1H), 7.01 (d, *J* = 11.6 Hz, 1H), 5.12 (d, *J* = 11.6 Hz, 1H), 1.60 (s, 9H).

^{13}C NMR (126 MHz, Chloroform-*d*) δ (ppm) 140.19, 137.60, 126.84, 118.72, 116.85, 90.64, 59.41, 29.74.

HRMS (APCI): calcd. for $C_{10}H_{14}N_3$ $[M+H]^+$ =176.1182, found $[M+H]^+$ = 176.1184.

3-(1-(*tert*-Butyl)-1*H*-pyrazol-4-yl)propanenitrile **(S36)**

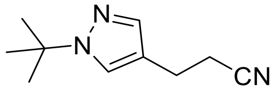

A mixture of ethyl 3-(1-(*tert*-butyl)-1*H*-pyrazol-4-yl)acrylonitrile (**S35**, 350 mg, 2.0 mmol, 1eq.) and Pd/C (50 mg) in Methanol (5 mL) was stirred in a pressure vessel at 23 °C under hydrogen atmosphere (15 bar) for 3 h. The mixture was filtered through a micro HPLC filter, and the filtrate was concentrated under vacuum. The crude product was dried under vacuum and used in the next step without further purification. The product was obtained as a colorless oil (350 mg, 99%).

^{1}H NMR (126 MHz, DMSO-d_6) δ (ppm) 7.44 (s, 1H), 7.40 (s, 1H), 2.83 (t, J = 7.2 Hz, 2H), 2.55 (t, J = 7.2 Hz, 2H), 1.57 (s, 9H).

^{13}C NMR (126 MHz, DMSO-d_6) δ (ppm) 137.85, 124.42, 119.54, 116.99, 58.54, 29.94, 21.02, 19.62.

HRMS (APCI): calcd. for $C_{10}H_{16}N_3$ [M+H]$^+$ = 178.1339, found [M+H]$^+$ = 178.1342.

2-((1-(*tert*-Butyl)-1*H*-pyrazol-4-yl)methyl)-3-oxopentanenitrile **(S37)**

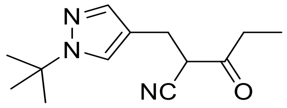

The compound was prepared according to General procedure A4 using *n*-BuLi (2.5 M, 0.92 mL, 2.3 mmol, 1.2 eq.), diisopropylamine (0.35 mL, 2.49 mmol, 1.3 eq.) and THF (3 mL) (reaction time: 30 min for the formation of LDA), then deprotonation of 3-(1-(*tert*-butyl)-1*H*-pyrazol-4-yl)propanenitrile (**S36**, 0.34 g, 1.92 mmol; 1 eq.) in THF (3 mL) (reaction time: 30 min) and final addition of propionyl chloride (0.2 mL, 2.3 mmol, 1.2 eq.) (reaction time: 16 h). The crude product obtained after the workup (450 mg, quantitative) was used in the next step without further purification.

3-Ethyl-4-(4-fluorophenyl)-1*H*-pyrazol-5-amine **(S38)**

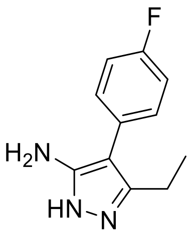

The compound was prepared according to General procedure B1 using 2-(4-fluorophenyl)-3-oxopentanenitrile (**S1**, 1.89 g, 9.88 mmol; 1 eq.), $N_2H_4.H_2O$ (64% in H_2O, 0.48 mL, 9.88 mmol; 1 eq.), CH_3SO_3H (64 µL, 0.99 mmol, 0.1 eq.) and EtOH (20 mL). Reaction time: 45 min at reflux. The residue was sonicated in EtOH (10 mL) and the precipitate was collected by filtration to afford the product as a white crystalline solid (1.1 g, 54%). The filtrate was concentrated in a vacuum and the residue was purified by column chromatography on silica gel (hexane:EtOAc, gradient 2:1 to 0:1) to afford additional product as a white solid (727 mg, 36%).

^{1}H NMR (500 MHz, DMSO-d_6) δ (ppm) 11.42 (s, 1H), 7.36 – 7.28 (m, 2H), 7.22 – 7.13 (m, 2H), 4.38 (s, 2H), 2.53 (q, J = 7.6 Hz, 2H), 1.09 (t, J = 7.6 Hz, 3H).

^{13}C NMR (126 MHz, DMSO-d_6) δ (ppm) 160.12 (d, J = 242.2 Hz), 150.84, 143.06, 130.42 (d, J = 3.1 Hz), 129.92 (d, J = 7.9 Hz), 115.10, 102.76, 18.37, 13.24.
^{19}F NMR (471 MHz, DMSO-d_6) δ (ppm) -117.79.
HRMS (APCI): calcd. for $C_{11}H_{11}FN_3$ [M-H]$^-$ = 204.0942, found [M-H]$^-$ = 204.0948
Mp = 163-166 °C.

4-(4-Fluorophenyl)-1*H*-pyrazol-5-amine **(S39)**

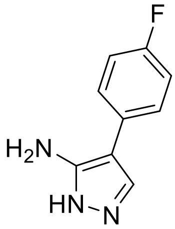

The compound was prepared according to General procedure B1 using 2-(4-fluorophenyl)-3-oxopropanenitrile (382 mg, 2.34 mmol; 1 eq. CAS: 398-42-5), $N_2H_4.H_2O$ (64% in H_2O, 0.114 mL, 2.34 mmol; 1 eq.), CH_3SO_3H (15 µL, 0.23 mmol, 0.1 eq.) and EtOH (10 mL). Reaction time: 1 h at reflux. The residue was sonicated in EtOH (5 mL) and the precipitate was collected by filtration and dried under vacuum to afford the product as a yellow solid (222 mg, 54 %). The filtrate was concentrated in a vacuum and the residue was purified by column chromatography on silica gel (hexane:EtOAc, gradient 2:1 to 0:1) to afford additional product as a yellow solid (136 mg, 33%).
^{1}H NMR (500 MHz, DMSO-d_6) δ (ppm) 7.63 (s, 1H), 7.55 – 7.48 (m, 2H), 7.17 – 7.10 (m, 2H), 4.71 (s, 2H).
^{13}C NMR (126 MHz, DMSO-d_6) δ (ppm) 159.76 (d, J = 241.3 Hz), 130.48 (d, J = 3.3 Hz), 127.18 (d, J = 7.5 Hz), 115.14 (d, J = 21.0 Hz).
HRMS (APCI): calcd. for $C_9H_7FN_3$ [M-H]$^-$ = 176.0629, found [M-H]$^-$ = 176.0627.

3-(3-Bromophenyl)-1*H*-pyrazol-5-amine **(S40)**

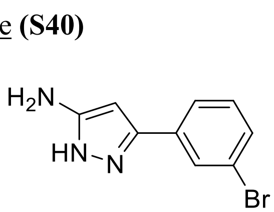

The compound was prepared according to General procedure B1 using 3-(3-bromophenyl)-3-oxopropanenitrile (**S2**, 575 mg, 2.57 mmol; 1 eq.), $N_2H_4.H_2O$ (64% in H_2O, 0.39 mL, 7.7 mmol; 3 eq.), CH_3SO_3H (17 µL, 0.26 mmol, 0.1 eq.) and EtOH (13 mL). Reaction time: 2 h at reflux. The residue obtained after the workup was purified by column chromatography on silica gel (hexane:EtOAc:MeOH, gradient 1:1:0 to 0:8:1). The product was obtained as an off-white solid (504 mg, 82%).
^{1}H NMR (500 MHz, DMSO-d_6) δ (ppm) 11.66 (s, 1H), 7.84 (t, J = 1.8 Hz, 1H), 7.65 (dt, J = 7.8, 1.3 Hz, 1H), 7.43 (d, J = 7.8 Hz, 1H), 7.32 (t, J = 7.9 Hz, 1H), 5.78 (s, 1H), 4.89 (s, 2H).

^{13}C NMR (126 MHz, DMSO-d_6) δ (ppm) 130.69, 129.64, 127.01, 123.67, 121.97.
HRMS (APCI): calcd. For $C_9H_9BrN_3$ $[M+H]^+ = 237.9974$, found $[M+H]^+ = 237.9979$.

3-Cyclohexyl-1*H*-pyrazol-5-amine **(S41)**

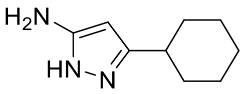

The compound was prepared according to General procedure B1 using 3-cyclohexyl-3-oxopropanenitrile (**S3**, 355 mg, 2.35 mmol; 1 eq.), $N_2H_4.H_2O$ (64% in H_2O, 0.23 mL, 4.70 mmol; 2 eq.), CH_3SO_3H (15 µL, 0.24 mmol, 0.1 eq.) and EtOH (12 mL). Reaction time: 2 h at reflux. The residue obtained after the workup was purified by column chromatography on silica gel (hexane:EtOAc:MeOH, gradient 1:1:0 to 0:9:1). The product was obtained as a pink wax (386 mg, 99%).
^1H NMR (500 MHz, Chloroform-*d*) δ (ppm) 5.42 (s, 1H), 2.61 – 2.44 (m, 1H), 1.98 – 1.88 (m, 2H), 1.82 – 1.73 (m, 2H), 1.73 – 1.64 (m, 1H), 1.43 – 1.28 (m, 4H), 1.28 – 1.17 (m, 1H).
^{13}C NMR (126 MHz, Chloroform-*d*) δ (ppm) 154.56, 151.23, 90.07, 35.78, 32.71, 26.15, 26.03.
HRMS (APCI): calcd. for $C_9H_{16}N_3$ $[M+H]^+ = 166.1339$, found $[M+H]^+ = 166.1335$.

3-(2-phenoxyphenyl)-1H-pyrazol-5-amine **(S42)**

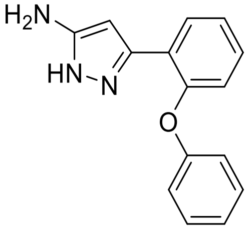

The compound was prepared according to General procedure B1 using 3-oxo-3-(2-phenoxyphenyl)propanenitrile (**S4**, 461 mg, 1.94 mmol; 1 eq.), $N_2H_4.H_2O$ (64% in H_2O, 0.189 mL, 3.89 mmol; 2 eq.), CH_3SO_3H (13 µL, 0.19 mmol, 0.1 eq.) and EtOH (10 mL). Reaction time: 2 h at reflux. The residue obtained after the workup was purified by column chromatography on silica gel (hexane:EtOAc:MeOH, gradient 1:1:0 to 0:9:1). The product was obtained as an off-white solid (392 mg, 80%).
^1H NMR (500 MHz, DMSO-d_6) δ (ppm) 11.69 (s, 1H), 7.86 (s, 1H), 7.40 – 7.32 (m, 2H), 7.29 (td, J = 7.7, 1.7 Hz, 1H), 7.21 (td, J = 7.5, 1.3 Hz, 1H), 7.08 (t, J = 7.4 Hz, 1H), 6.98 – 6.88 (m, 3H), 5.78 (s, 1H), 4.66 (s, 2H).
^{13}C NMR (126 MHz, DMSO-d_6) δ (ppm) 157.16, 152.10, 129.91, 128.66, 127.75, 124.23, 122.73, 120.62, 117.44.
HRMS (APCI): calcd. for $C_{15}H_{14}N_3O$ $[M+H]^+ = 252.1131$, found $[M+H]^+ = 252.1138$.

3-(Naphthalen-2-yl)-1*H*-pyrazol-5-amine **(S43)**

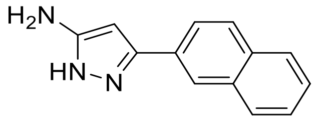

The compound was prepared according to General procedure B1 using 3-(naphthalen-2-yl)-3-oxopropanenitrile (**S5**, 472 mg, 2.42 mmol; 1 eq.), $N_2H_4.H_2O$ (64% in H_2O, 0.24 mL, 4.83 mmol; 2 eq.), CH_3SO_3H (16 µL, 0.24 mmol, 0.1 eq.) and EtOH (12 mL). Reaction time: 2 h at reflux. The residue obtained after the workup was purified by column chromatography on silica gel (hexane:EtOAc:MeOH, gradient 1:1:0 to 0:9:1). The product was obtained as a beige solid (390 mg, 77%).

^{1}H NMR (500 MHz, DMSO-d_6) δ (ppm) 12.35 – 11.35 (m, 1H), 8.15 (d, J = 1.4 Hz, 1H), 7.95 – 7.81 (m, 4H), 7.58 – 7.43 (m, 2H), 5.90 (s, 1H), 4.83 (s, 2H).

^{13}C NMR (126 MHz, DMSO-d_6) δ (ppm) 133.13, 132.22, 127.97, 127.81, 127.53, 126.31, 125.68, 123.46, 122.79.

HRMS (APCI): calcd. for $C_{13}H_{12}N_3$ $[M+H]^+$ = 210.1026, found $[M+H]^+$ = 210.1031.

3-Isopropyl-1*H*-pyrazol-5-amine **(S44)**

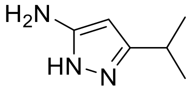

The compound was prepared according to General procedure B1 using 4-methyl-3-oxopentanenitrile (**S7**, 2.46 g, 22 mmol; 1 eq.), $N_2H_4.H_2O$ (64% in H_2O, 3.4 mL, 44 mmol; 2 eq.), CH_3SO_3H (150 µL, 2.2 mmol, 0.1 eq.) and EtOH (50 mL). Reaction time: 2 h at reflux. The residue obtained after workup was purified by column chromatography on silica gel (hexane:EtOAc:MeOH, gradient 1:1:0 to 0:9:1). The product was obtained as a dark red wax (2.42 g, 98%).

^{1}H NMR (500 MHz, Chloroform-d) δ (ppm) 5.43 (s, 1H), 2.87 (hept, J = 6.9 Hz, 1H), 1.23 (d, J = 6.9 Hz, 6H).

^{13}C NMR (126 MHz, Chloroform-d) δ (ppm) 154.53, 152.19, 89.94, 26.27, 22.31.

3-(*tert*-butyl)-1*H*-pyrazol-5-amine **(S45)**

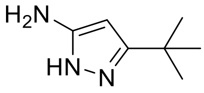

The compound was prepared according to General procedure B1 using 4,4-dimethyl-3-oxopentanenitrile (**S8**, 2.6 g, 20.8 mmol; 1 eq.), $N_2H_4.H_2O$ (64% in H_2O, 3.2 mL, 41.6 mmol; 2 eq.), CH_3SO_3H (130 µL, 2.0 mmol, 0.1 eq.) and EtOH (25 mL). Reaction time: 2 h at reflux. The residue obtained after the workup was purified by column chromatography on silica gel (hexane:EtOAc:MeOH, gradient 1:1:0 to 0:9:1). The product was obtained as a dark red wax (1.63 g, 56%).

^{1}H NMR (500 MHz, DMSO-d_6) δ (ppm) 5.18 (s, 1H), 1.18 (s, 9H).

^{13}C NMR (126 MHz, DMSO-d_6) δ (ppm) 154.04, 153.31, 87.37, 30.64, 30.05.

HRMS (APCI): calcd. for $C_7H_{14}N_3$ $[M+H]^+$ = 140.1182, found $[M+H]^+$ = 140.1191.

4-(4-Fluorophenyl)-3-isopropyl-1*H*-pyrazol-5-amine **(S46)**

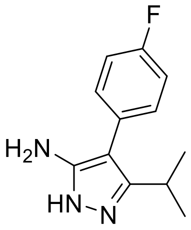

The compound was prepared according to General procedure B1 using 2-(4-fluorophenyl)-4-methyl-3-oxopentanenitrile (**S9**, 205 mg, 0.73 mmol; 1 eq.), $N_2H_4.H_2O$ (64% in H_2O, 0.17 mL, 2.19 mmol; 3 eq.), CH_3SO_3H (6 µL, 0.07 mmol, 0.1 eq.) and EtOH (2 mL). Reaction time: 45 min at reflux. The residue obtained after the workup was purified by column chromatography on silica gel (hexane:EtOAc:MeOH, gradient 1:1:0 to 0:9:1). The product was obtained as a white solid (80 mg, 50%).

^{1}H NMR (500 MHz, chloroform-*d*) δ (ppm) 7.30 – 7.26 (m, 2H), 7.14 – 7.09 (m, 2H), 4.97 (s, 2H), 3.04 (hept, J = 7.0 Hz, 1H), 1.24 (d, J = 7.0 Hz, 6H).

^{13}C NMR (126 MHz, chloroform-*d*) δ (ppm) 162.01 (d, J = 246.9 Hz), 151.59, 148.37, 131.25 (d, J = 8.1 Hz), 128.34 (d, J = 2.8 Hz), 116.06 (d, J = 21.5 Hz), 104.47, 25.30, 22.07.

3-(((*tert*-Butyldiphenylsilyl)oxy)methyl)-1*H*-pyrazol-5-amine **(S47)**

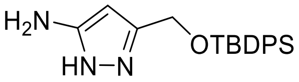

The compound was prepared according to General procedure B1 using 4-((*tert*-butyldiphenylsilyl)oxy)-3-oxobutanenitrile (**S15**, 350 mg, 1.0 mmol; 1 eq.), $N_2H_4.H_2O$ (64% in H_2O, 0.24 mL, 3.0 mmol; 3 eq.), CH_3SO_3H (7 µL, 0.1 mmol, 0.1 eq.) and EtOH (5 mL). Reaction time: 45 min at reflux. The residue obtained after the workup was purified by column chromatography on silica gel (hexane:EtOAc:MeOH, gradient 1:1:0 to 0:9:1). The product was obtained as a colorless oil (360 mg, 98%).

^{1}H NMR (500 MHz, chloroform-*d*) δ (ppm) 11.27 (s, 1H), 7.69 – 7.61 (m, 4H), 7.50 – 7.40 (m, 6H), 5.34 (s, 1H), 4.65 (s, 2H), 4.54 (s, 2H), 1.00 (s, 9H).

^{13}C NMR (126 MHz, chloroform-*d*) δ (ppm) 135.00, 133.00, 129.80, 127.82, 59.68, 26.57, 18.77.

HRMS (APCI): calcd. for $C_{20}H_{26}N_3OSi$ $[M+H]^+$ = 352.1840, found $[M+H]^+$ = 352.1843

3-(3-Methoxynaphthalen-2-yl)-1*H*-pyrazol-5-amine **(S48)**

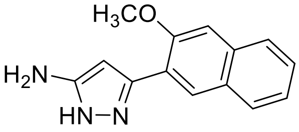

The compound was prepared according to General procedure B1 using 3-(3-methoxynaphthalen-2-yl)-3-oxopropanenitrile (**S10**, 100 mg, 0.44 mmol; 1 eq.), $N_2H_4.H_2O$ (64% in H_2O, 70 µL, 0.88 mmol; 2 eq.), CH_3SO_3H (7 µL, 0.1 mmol, 0.4 eq.) and EtOH (5 mL). Reaction time: 45 min at reflux. The residue

obtained after the workup was purified by column chromatography on silica gel (hexane:EtOAc:MeOH, gradient 1:1:0 to 0:9:1). The product was obtained as a colorless wax (93 mg, 88%).

^{1}H NMR (500 MHz, chloroform-*d*) δ (ppm) 11.71 (s, 1H), 8.16 (s, 1H), 7.81 (t, J = 8.6 Hz, 2H), 7.47 – 7.41 (m, 1H), 7.41 (s, 1H), 7.38 – 7.32 (m, 1H), 5.99 (s, 1H), 4.63 (s, 2H), 3.97 (s, J = 21.0 Hz, 3H).

^{13}C NMR (126 MHz, chloroform-*d*) δ (ppm) 135.00, 133.00, 129.80, 127.82, 59.68, 26.57, 18.77.

3-([1,1'-Biphenyl]-3-yl)-1H-pyrazol-5-amine **(S49)**

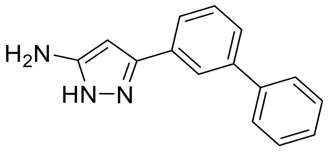

The compound was prepared according to General procedure B1 using 3-([1,1'-biphenyl]-3-yl)-3-oxopropanenitrile (**S11**, 424 mg, 1.92 mmol; 1 eq.), $N_2H_4.H_2O$ (64% in H_2O, 300 µL, 3.84 mmol; 2 eq.), CH_3SO_3H (14 µL, 0.2 mmol, 0.1 eq.) and EtOH (5 mL). Reaction time: 45 min at reflux. The residue obtained after the workup was purified by column chromatography on silica gel (hexane:EtOAc:MeOH, gradient 1:1:0 to 0:9:1). The product was obtained as a black wax (350 mg, 77%).

^{1}H NMR (500 MHz, DMSO-*d*$_6$) δ (ppm) 11.42 (s, 1H), 7.58 (s, 1H), 7.44 – 7.25 (m, 6H), 7.21 (t, J = 7.4 Hz, 3H), 4.75 (s, 1H), 4.49 (s, 1H).

^{13}C NMR (126 MHz, DMSO-*d*$_6$) δ (ppm) 147.03, 141.41, 139.91, 137.80, 130.40, 128.92, 128.78, 127.92, 127.49, 127.24, 126.85, 91.00.

HRMS (APCI): calcd. for $C_{15}H_{14}N_3$ $[M+H]^+$ = 236.1182, found $[M+H]^+$ = 236.1184

3-(1-Methoxynaphthalen-2-yl)-1*H*-pyrazol-5-amine **(S50)**

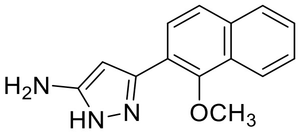

The compound was prepared according to General procedure B1 using 3-(1-methoxynaphthalen-2-yl)-3-oxopropanenitrile (**S12**, 260 mg, 1.16 mmol; 1 eq.), $N_2H_4.H_2O$ (64% in H_2O, 110 µL, 2.32 mmol; 2 eq.), CH_3SO_3H (7 µL, 0.1 mmol, 0.1 eq.) and EtOH (5 mL). Reaction time: 45 min at reflux. The residue obtained after the workup was purified by column chromatography on silica gel (hexane:EtOAc:MeOH, gradient 1:1:0 to 0:9:1). The product was obtained as a yellow wax (186 mg, 67%).

^{1}H NMR (500 MHz, DMSO-*d*$_6$) δ (ppm) 11.42 (s, 1H), 7.58 (s, 1H), 7.44 – 7.25 (m, 4H), 7.24 – 7.16 (m, 2H), 4.75 (s, 1H), 4.49 (s, 1H), 3.31 (s, 3H).

^{13}C NMR (126 MHz, DMSO-*d*$_6$) δ (ppm) 147.03, 141.41, 139.91, 137.80, 130.39, 128.91, 128.78, 127.91, 127.48, 127.24, 126.85, 91.00, 59.69.

3-(3-Methoxynaphthalen-1-yl)-1*H*-pyrazol-5-amine **(S51)**

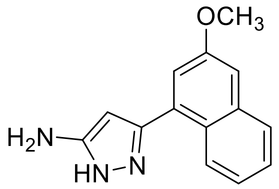

The compound was prepared according to General procedure B1 using 3-(3-methoxynaphthalen-1-yl)-3-oxopropanenitrile (**S13**, 220 mg, 0.97 mmol; 1 eq.), $N_2H_4.H_2O$ (64% in H_2O, 62 µL, 1.94 mmol; 2 eq.), CH_3SO_3H (7 µL, 0.1 mmol, 0.1 eq.) and EtOH (5 mL). Reaction time: 45 min at reflux. The residue obtained after the workup was purified by column chromatography on silica gel (hexane:EtOAc:MeOH, gradient 1:1:0 to 0:9:1). The product was obtained as a yellow wax (200 mg, 86%).

^{1}H NMR (500 MHz, DMSO-d_6) δ (ppm) 11.50 (s, 1H), 7.98 (d, J = 9.0 Hz, 1H), 7.88 (d, J = 8.0 Hz, 1H), 7.70 (d, J = 8.3 Hz, 1H), 7.49 (d, J = 9.1 Hz, 1H), 7.44 – 7.39 (m, 1H), 7.35 (t, J = 7.4 Hz, 1H), 5.51 (s, 1H), 4.61 (s, J = 99.3 Hz, 1H), 3.84 (s, 3H).

^{13}C NMR (126 MHz, DMSO-d_6) δ (ppm) 155.28, 133.27, 129.87, 128.32, 127.75, 126.50, 124.67, 123.69, 123.46, 113.99, 56.86.

<u>3-(1-benzyl-1*H*-imidazol-2-yl)-1*H*-pyrazol-5-amine</u> **(S52)**

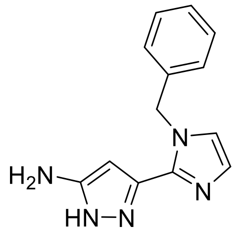

The compound was prepared according to General procedure B1 using 3-(1-benzyl-1*H*-imidazol-2-yl)-3-oxopropanenitrile (**S16**, 200 mg, 0.88 mmol; 1 eq.), $N_2H_4.H_2O$ (64% in H_2O, 60 µL, 1.7 mmol; 2 eq.), CH_3SO_3H (7 µL, 0.1 mmol, 0.1 eq.) and EtOH (5 mL). Reaction time: 45 min at reflux. The residue obtained after the workup was purified by column chromatography on silica gel (hexane:EtOAc:MeOH, gradient 1:1:0 to 0:9:1). The product was obtained as a yellow wax (84 mg, 40%).

^{1}H NMR (500 MHz, DMSO-d_6) δ (ppm) 11.67 (s, 1H), 7.76 (s, 1H), 7.32 – 7.27 (m, 2H), 7.19 – 7.13 (m, 3H), 6.95 – 6.92 (m, 1H), 5.66 (s, 2H), 5.19 (s, 1H).

^{13}C NMR (126 MHz, DMSO-d_6) δ (ppm) 137.84, 129.13, 129.00, 128.92, 128.17, 127.92, 127.70, 127.48, 119.58, 113.33, 49.36.

<u>3-(2-chlorophenyl)-1*H*-pyrazol-5-amine</u> **(S53)**

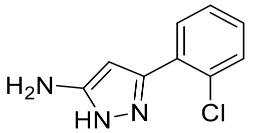

The compound was prepared according to General procedure B1 using 3-(2-chlorophenyl)-3-oxopropanenitrile (**S17**, 735 mg, 4.2 mmol; 1 eq.), $N_2H_4.H_2O$ (64% in H_2O, 0.54 mL, 8.4 mmol; 2 eq.),

CH_3SO_3H (10 µL, 0.13 mmol, 0.03 eq.) and EtOH (10 mL). Reaction time: 45 min at reflux. The residue obtained after the workup was purified by column chromatography on silica gel (hexane:EtOAc:MeOH, gradient 1:1:0 to 0:9:1). The product was obtained as a yellow wax (760 mg, 93%).

^{1}H NMR (500 MHz, DMSO-d_6) δ (ppm) 11.74 (s, 1H), 7.67 (d, J = 6.0 Hz, 1H), 7.48 (d, J = 7.7 Hz, 1H), 7.38 – 7.33 (m, 1H), 7.33 – 7.29 (m, 1H), 5.84 (s, 1H), 4.82 (s, 2H).

^{13}C NMR (126 MHz, DMSO-d_6) δ (ppm) 159.06, 143.81, 130.64, 130.23, 129.89, 128.82, 127.15, 92.73.

HRMS (APCI): calcd. for $C_9H_9ClN_3$ $[M+H]^+$ = 194.0480, found $[M+H]^+$ = 194.0476.

3-(Naphthalen-1-yl)-1*H*-pyrazol-5-amine **(S54)**

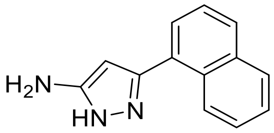

The compound was prepared according to General procedure B1 using 3-(naphthalen-1-yl)-3-oxopropanenitrile (**S18**, 280 mg, 1.43 mmol; 1 eq.), $N_2H_4.H_2O$ (64% in H_2O, 0.23 mL, 2.9 mmol; 2 eq.), CH_3SO_3H (10 µL, 0.14 mmol, 0.1 eq.) and EtOH (5 mL). Reaction time: 45 min at reflux. The residue obtained after the workup was purified by column chromatography on silica gel (hexane:EtOAc:MeOH, gradient 1:1:0 to 0:9:1). The product was obtained as a yellow wax (185 mg, 62%).

^{1}H NMR (500 MHz, DMSO-d_6) δ (ppm) 11.75 (s, 1H), 8.45 (s, 1H), 7.95 (dd, J = 6.0, 3.4 Hz, 1H), 7.90 (d, J = 8.0 Hz, 1H), 7.60 – 7.56 (m, 1H), 7.56 – 7.51 (m, 3H), 5.71 (s, 1H), 4.83 (s, 2H).

^{13}C NMR (126 MHz, DMSO-d_6) δ (ppm) 133.46, 130.54, 128.19, 127.78, 126.25, 126.18, 125.82, 125.41.

HRMS (APCI): calcd. for $C_{13}H_{12}N_3$ $[M+H]^+$ = 210.1026, found $[M+H]^+$ = 210.1028.

3-(2-(Dimethylamino)phenyl)-1*H*-pyrazol-5-amine **(S55)**

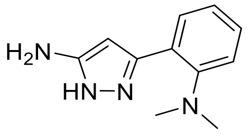

The compound was prepared according to General procedure B1 using 3-(2-(dimethylamino)phenyl)-3-oxopropanenitrile (**S19**, 280 mg, 1.5 mmol; 1 eq.), $N_2H_4.H_2O$ (64% in H_2O, 0.23 mL, 3.0 mmol; 2 eq.), CH_3SO_3H (10 µL, 0.14 mmol, 0.1 eq.) and EtOH (5 mL). Reaction time: 45 min at reflux. The residue obtained after the workup was purified by column chromatography on silica gel (hexane:EtOAc:MeOH, gradient 1:1:0 to 0:9:1). The product was obtained as a yellow wax (215 mg, 70%).

^{1}H NMR (500 MHz, DMSO-d_6) δ (ppm) 11.75 (s, 1H), 7.98 – 7.94 (m, 1H), 7.90 (d, J = 8.1 Hz, 1H), 7.60 – 7.56 (m, 1H), 7.55 – 7.51 (m, 1H), 5.71 (s, 1H), 4.83 (s, 2H), 3.32 (s, 6H).

^{13}C NMR (126 MHz, DMSO-d_6) δ (ppm) 133.45, 130.53, 128.18, 127.77, 126.25, 125.82, 125.40, 59.70.

3-(2-Methoxybenzyl)-1*H*-pyrazol-5-amine **(S56)**

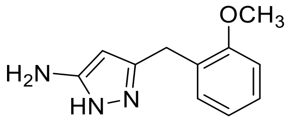

The compound was prepared according to General procedure B1 using 4-(2-methoxyphenyl)-3-oxobutanenitrile (**S20**, 1.3 g, 6.87 mmol; 1 eq.), $N_2H_4.H_2O$ (64% in H_2O, 1.7 mL, 27.48 mmol; 4 eq.), CH_3SO_3H (45 µL, 0.7 mmol, 0.1 eq.) and EtOH (10 mL). Reaction time: 45 min at reflux. The residue obtained after the workup was purified by column chromatography on silica gel (dichloromethane:MeOH, gradient 19:1 to 9:1). The product was obtained as a colorless wax (300 mg, 22%).

^{1}H NMR (500 MHz, Chloroform-*d*) δ (ppm) 7.23 – 7.17 (m, 1H), 7.11 (dd, *J* = 7.4, 1.7 Hz, 1H), 6.89 – 6.83 (m, 2H), 5.77 (s, 2H), 5.42 (s, 1H), 3.84 (s, 2H), 3.80 (s, 3H).

^{13}C NMR (126 MHz, Chloroform-*d*) δ (ppm) 157.14, 154.29, 144.27, 130.17, 128.08, 126.86, 120.78, 110.63, 92.03, 55.46, 27.15.

3-(2-Methoxy-2-phenylethyl)-1*H*-pyrazol-5-amine **(S57)**

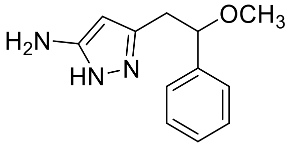

The compound was prepared according to General procedure B1 using 5-methoxy-3-oxo-5-phenylpentanenitrile (**S21**, 350 mg, 1.72 mmol; 1 eq.), $N_2H_4.H_2O$ (64% in H_2O, 0.42 mL, 6.89 mmol; 4 eq.), CH_3SO_3H (11 µL, 0.17 mmol, 0.1 eq.) and EtOH (5 mL). Reaction time: 45 min at reflux. The residue obtained after the workup was purified by column chromatography on silica gel (dichloromethane:MeOH, gradient 19:1 to 9:1). The product was obtained as a colorless wax (350 mg, 94%).

^{1}H NMR (500 MHz, Chloroform-*d*) δ (ppm7.40 – 7.27 (m, 5H), 5.42 (s, 1H), 4.35 (dd, *J* = 8.6, 4.0 Hz, 1H), 3.25 (s, 3H), 3.02 – 2.81 (m, 2H).

^{13}C NMR (126 MHz, Chloroform-*d*) δ (ppm) 154.37, 142.31, 140.96, 128.68, 128.15, 126.62, 92.72, 83.27, 56.80, 34.98.

3-Benzyl-1*H*-pyrazol-5-amine **(S58)**

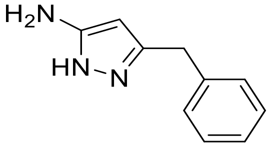

The compound was prepared according to General procedure B1 using 3-oxo-4-phenylbutanenitrile (**S22**, 619 mg, 3.89 mmol; 1 eq.), $N_2H_4.H_2O$ (64% in H_2O, 0.403 mL, 7.78 mmol; 2 eq.), CH_3SO_3H (25 µL, 0.23 mmol, 0.1 eq.) and EtOH (19 mL). Reaction time: 2 h at reflux. The residue obtained after the

workup was loaded on ISOLUTE® HM-N and purified by column chromatography on silica gel using Biotage Selekt purification system (hexane:EtOAc:MeOH, gradient 1:1:0 to 0:9:1). The product was obtained as a pale-yellow wax (203 mg, 30%).

^{1}H NMR (500 MHz, Chloroform-*d*) δ (ppm) 7.32 – 7.27 (m, 2H), 7.25 – 7.18 (m, 3H), 5.45 (s, 1H), 3.88 (s, 2H).

^{13}C NMR (126 MHz, Chloroform-*d*) δ (ppm) 154.55, 144.46, 137.92, 128.84, 126.90, 92.65, 32.73.

HRMS (APCI): calcd. for $C_{10}H_{12}N_3$ $[M+H]^+$ = 174.1026, found $[M+H]^+$ = 174.1023.

<u>3-(Isochroman-3-ylmethyl)-1*H*-pyrazol-5-amine</u> **(S59)**

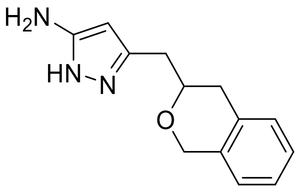

The compound was prepared according to General procedure B1 using 4-(isochroman-3-yl)-3-oxobutanenitrile (**S24**, 494 mg, 2.30 mmol; 1 eq.), $N_2H_4.H_2O$ (64% in H_2O, 0.238 mL, 4.59 mmol; 2 eq.), CH_3SO_3H (15 µL, 0.23 mmol, 0.1 eq.) and EtOH (11 mL). Reaction time: 2 h at reflux. The residue obtained after the workup was loaded on ISOLUTE® HM-N and purified by column chromatography on silica gel using Biotage Selekt purification system (hexane:EtOAc:MeOH, gradient 1:1:0 to 0:9:1). The product was obtained as a pale-yellow wax (523 mg, 99%).

^{1}H NMR (500 MHz, Chloroform-*d*) δ (ppm) 7.21 – 7.13 (m, 2H), 7.13 – 7.05 (m, 2H), 5.44 (s, 1H), 5.21 (bs, 3H), 4.99 (ddd, J = 8.3, 3.2, 1.4 Hz, 1H), 4.18 (ddd, J = 11.3, 5.5, 3.2 Hz, 1H), 3.79 (ddd, J = 11.3, 10.1, 3.6 Hz, 1H), 3.18 (dd, J = 15.6, 3.2 Hz, 1H), 3.07 – 2.92 (m, 2H), 2.67 (dt, J = 16.3, 3.5 Hz, 1H).

^{13}C NMR (126 MHz, Chloroform-*d*) δ (ppm) 154.30, 142.17, 136.64, 134.15, 129.18, 126.83, 126.40, 124.73, 92.94, 75.37, 63.72, 32.50, 29.05.

HRMS (APCI): calcd. for $C_{13}H_{16}N_3O$ $[M+H]^+$ = 230.1288, found $[M+H]^+$ = 230.1290.

<u>4-(4-Fluorophenyl)-3-(2-methoxybenzyl)-1*H*-pyrazol-5-amine</u> **(S60)**

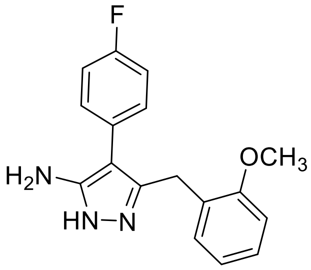

The compound was prepared according to General procedure B1 using 2-(4-fluorophenyl)-4-(2-methoxyphenyl)-3-oxobutanenitrile (**S25**, 580 mg, 2.05 mmol; 1 eq.), $N_2H_4.H_2O$ (64% in H_2O, 0.23 mL, 4.1 mmol; 2 eq.), CH_3SO_3H (15 µL, 0.23 mmol, 0.1 eq.) and EtOH (11 mL). Reaction time: 2 h at reflux. The residue obtained after the workup was loaded on ISOLUTE® HM-N and purified by column

chromatography on silica gel using Biotage Selekt purification system (hexane:EtOAc:MeOH, gradient 1:1:0 to 0:4:1). The product was obtained as an off-white foam (0.2 g, 33%).

^{1}H NMR (500 MHz, Chloroform-*d*) δ (ppm) 7.17 – 7.07 (m, 2H), 7.01 (td, J = 7.8, 1.8 Hz, 1H), 6.93 – 6.87 (m, 2H), 6.85 (dd, J = 7.7, 1.8 Hz, 1H), 6.73 – 6.63 (m, 2H), 5.90 (s, 3H), 3.71 (s, 2H), 3.61 (s, 3H).

^{13}C NMR (126 MHz, Chloroform-*d*) δ (ppm) 161.46 (d, J = 245.3 Hz), 157.18, 152.00, 140.15, 130.70 (d, J = 7.5 Hz), 130.06, 129.06, 128.16, 126.36, 120.77, 115.62 (d, J = 21.6 Hz), 110.59, 105.58, 55.39, 25.88.

^{19}F NMR (471 MHz, Chloroform-*d*) δ (ppm) -116.08.

4-Cyclohexyl-3-ethyl-1*H*-pyrazol-5-amine **(S61)**

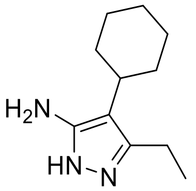

The compound was prepared according to General procedure B1 using 2-cyclohexyl-3-oxopentanenitrile (**S26**, 304 mg, 1.70 mmol; 1 eq.), $N_2H_4.H_2O$ (64% in H_2O, 0.134 mL, 2.21 mmol; 1.3 eq.), CH_3SO_3H (33 µL, 0.51 mmol, 0.3 eq.) and EtOH (7 mL). Reaction time: 4 h at reflux. The residue obtained after the workup was loaded on ISOLUTE® HM-N and purified twice by column chromatography on silica gel using Biotage Selekt purification system (cyclohexane:EtOAc:MeOH, gradient 1:2:0 to 0:9:1). The product was obtained as a pale pink solid (146 mg, 45%).

^{1}H NMR (500 MHz, Chloroform-*d*) δ (ppm) 5.62 (s, 2H), 2.59 (q, J = 7.5 Hz, 2H), 2.38 – 2.29 (m, 1H), 1.82 (dt, J = 12.8, 3.0 Hz, 2H), 1.77 – 1.70 (m, 3H), 1.53 (qd, J = 12.7, 3.3 Hz, 2H), 1.33 (qt, J = 12.5, 3.2 Hz, 2H), 1.27 – 1.18 (m, 4H).

^{13}C NMR (126 MHz, Chloroform-*d*) δ (ppm) 152.30, 142.96, 108.52, 34.50, 32.79, 27.28, 26.28, 18.82, 13.80.

HRMS (APCI): calcd. for $C_{11}H_{20}N_3$ $[M+H]^+$ = 194.1652, found $[M+H]^+$ = 194.1654.

3-Ethyl-4-(tetrahydro-2*H*-pyran-4-yl)-1*H*-pyrazol-5-amine **(S62)**

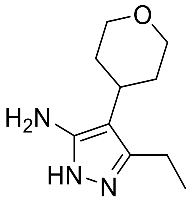

The compound was prepared according to General procedure B1 using 3-oxo-2-(tetrahydro-2*H*-pyran-4-yl)pentanenitrile (**S27**, 454 g, 2.51 mmol; 1 eq.), $N_2H_4.H_2O$ (64% in H_2O, 0.214 mL, 3.5 mmol; 1.4 eq.), CH_3SO_3H (49 µL, 0.75 mmol, 0.3 eq.) and EtOH (10 mL). Reaction time: 4 h at reflux. The residue obtained after the workup was loaded on ISOLUTE® HM-N and purified twice by column

chromatography on silica gel using Biotage Selekt purification system (EtOAc:MeOH, gradient 1:0 to 9:1). The product was obtained as a white solid (160 mg, 33%).

^{1}H NMR (500 MHz, Chloroform-*d*) δ (ppm) 5.25 (s, 2H), 4.06 (dd, J = 11.7, 4.7 Hz, 2H), 3.47 (td, J = 11.9, 2.0 Hz, 2H), 2.62 (q, J = 7.5 Hz, 3H), 2.02 – 1.88 (m, 2H), 1.64 – 1.58 (m, 2H), 1.23 (t, J = 7.6 Hz, 3H).

^{13}C NMR (126 MHz, Chloroform-*d*) δ (ppm) 152.28, 143.29, 106.72, 68.80, 32.28, 31.62, 18.77, 13.82.

HRMS (APCI): calcd. for $C_{10}H_{18}N_3O$ $[M+H]^+$ = 196.1444, found $[M+H]^+$ = 196.1442

4-Benzyl-3-ethyl-1*H*-pyrazol-5-amine **(S63)**

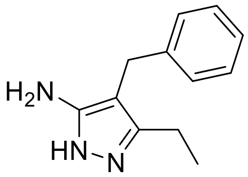

The compound was prepared according to General procedure B1 using 2-benzyl-3-oxopentanenitrile (**S28**, 302 mg, 1.61 mmol; 1 eq.), $N_2H_4.H_2O$ (64% in H_2O, 0.147 mL, 2.42 mmol; 1.5 eq.), CH_3SO_3H (21 µL, 0.33 mmol, 0.2 eq.) and EtOH (7 mL). Reaction time: 3 h at reflux. The residue obtained after the workup was loaded on ISOLUTE® HM-N and purified by column chromatography on silica gel using Biotage Selekt purification system (cyclohexane:EtOAc:MeOH, gradient 1:1:0 to 0:9:1). The product was obtained as a yellow solid (131 mg, 40%).

^{1}H NMR (500 MHz, Chloroform-*d*) δ (ppm) 7.30 – 7.24 (m, 2H), 7.21 – 7.16 (m, 3H), 5.29 (bs, 2H), 3.70 (s, 2H), 2.55 (q, J = 7.6 Hz, 2H), 1.18 (t, J = 7.6 Hz, 3H).

^{13}C NMR (126 MHz, Chloroform-*d*) δ (ppm) 153.46, 144.06, 140.32, 128.68, 128.24, 126.26, 101.87, 28.44, 18.42, 13.38.

HRMS (APCI): calcd. for $C_{12}H_{16}N_3$ $[M+H]^+$ = 202.1339, found $[M+H]^+$ = 202.1340.

4-(Cyclohexylmethyl)-3-ethyl-1*H*-pyrazol-5-amine **(S64)**

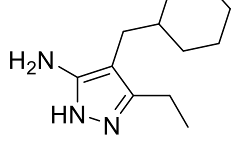

The compound was prepared according to General procedure B1 using 2-(cyclohexylmethyl)-3-oxopentanenitrile (**S29**, 1.1 g, 5.47 mmol; 1 eq.), $N_2H_4.H_2O$ (60 % in H_2O, 1.7 mL, 7.1 mmol; 1.3 eq.), CH_3SO_3H (35 µL, 0.55 mmol, 0.1 eq.) and EtOH (10 mL). Reaction time: 4 h at reflux. The residue obtained after workup was loaded on ISOLUTE® HM-N and purified by column chromatography on silica gel using Biotage Selekt purification system (dichloromethane:MeOH, gradient 19:1 to 9:1). The product was obtained as a yellow oil (330 mg, 29%).

^{1}H NMR (500 MHz, Chloroform-*d*) δ (ppm) 2.55 – 2.46 (m, 2H), 2.14 (dd, *J* = 7.2, 2.1 Hz, 2H), 1.73 – 1.59 (m, 5H), 1.40 – 1.32 (m, 1H), 1.22 – 1.10 (m, 6H), 0.95 – 0.84 (m, 2H).
^{13}C NMR (126 MHz, Chloroform-*d*) δ (ppm) 153.24, 143.78, 102.31, 39.05, 33.49, 30.45, 26.63, 26.41, 18.40, 13.22.
HRMS (APCI): calcd. for $C_{12}H_{22}N_3$ $[M+H]^+$ = 208.1808, found $[M+H]^+$ = 208.1808.

4-(2-Chlorobenzyl)-3-ethyl-1H-pyrazol-5-amine **(S65)**

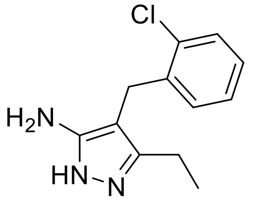

The compound was prepared according to General procedure B1 using 2-(2-chlorobenzyl)-3-oxopentanenitrile (**S30**, 670 mg, 3.01 mmol; 1 eq.), $N_2H_4.H_2O$ (80 % in H_2O, 0.37 mL, 6.03 mmol; 2 eq.), CH_3SO_3H (20 µL, 0.30 mmol, 0.1 eq.) and EtOH (10 mL). Reaction time: 4 h at reflux. The residue obtained after the workup was loaded on ISOLUTE® HM-N and purified by column chromatography on silica gel using Biotage Selekt purification system (dichloromethane:MeOH, gradient 19:1 to 9:1). The product was obtained as a colorless wax (180 mg, 25%).
^{1}H NMR (500 MHz, Chloroform-*d*) δ (ppm) 7.38 – 7.31 (m, 1H), 7.16 – 7.09 (m, 2H), 7.08 – 7.03 (m, 1H), 3.75 (s, 2H), 2.50 (q, *J* = 7.6 Hz, 2H), 1.12 (t, *J* = 7.6 Hz, 3H).
^{13}C NMR (126 MHz, Chloroform-*d*) δ (ppm) 152.81, 144.97, 137.72, 133.97, 129.52, 129.33, 127.47, 126.87, 99.65, 25.97, 18.47, 13.31.
HRMS (APCI): calcd. for $C_{12}H_{15}ClN_3$ $[M+H]^+$ = 236.0949, found $[M+H]^+$ = 236.0952.

3-Ethyl-4-(pyridin-3-ylmethyl)-1*H*-pyrazol-5-amine **(S66)**

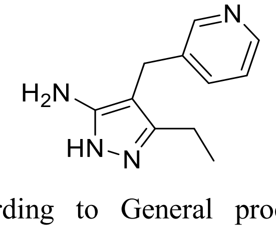

The compound was prepared according to General procedure B1 using 3-oxo-2-(pyridin-3-ylmethyl)pentanenitrile (**S31**, 560 mg, 3.0 mmol; 1 eq.), $N_2H_4.H_2O$ (80 % in H_2O, 0.37 mL, 6.05 mmol; 2 eq.), CH_3SO_3H (20 µL, 0.30 mmol, 0.1 eq.) and EtOH (10 mL). Reaction time: 4 h at reflux. The residue obtained after the workup was loaded on ISOLUTE® HM-N and purified by column chromatography on silica gel using Biotage Selekt purification system (dichloromethane:MeOH, gradient 19:1 to 9:1). The product was obtained as colorless wax (200 mg, 33%).
^{1}H NMR (500 MHz, Chloroform-*d*) δ (ppm) 7.38 – 7.31 (m, 1H), 7.16 – 7.09 (m, 2H), 7.08 – 7.04 (m, 1H), 3.75 (s, 2H), 2.50 (q, *J* = 7.6 Hz, 2H), 1.12 (t, *J* = 7.6 Hz, 3H).

^{13}C NMR (126 MHz, Chloroform-*d*) δ (ppm) 152.81, 144.97, 137.72, 133.97, 129.52, 129.33, 127.47, 126.87, 99.65, 25.97, 18.47, 13.31.

HRMS (APCI): calcd. for $C_{11}H_{15}N_4$ $[M+H]^+$ = 203.1291, found $[M+H]^+$ = 203.1292.

3-Ethyl-4-(2-methoxybenzyl)-1*H*-pyrazol-5-amine **(S67)**

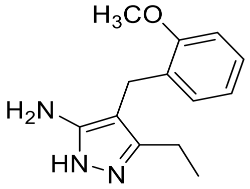

The compound was prepared according to General procedure B1 using 2-(2-methoxybenzyl)-3-oxopentanenitrile (**S32**, 670 mg, 3.01 mmol; 1 eq.), $N_2H_4.H_2O$ (80 % in H_2O, 0.37 mL, 6.0 mmol; 2 eq.), CH_3SO_3H (20 µL, 0.30 mmol, 0.1 eq.) and EtOH (10 mL). Reaction time: 4 h at reflux. The residue obtained after the workup was loaded on ISOLUTE® HM-N and purified by column chromatography on silica gel using Biotage Selekt purification system (dichloromethane:MeOH, gradient 19:1 to 9:1). The product was obtained as colorless wax (200 mg, 28%).

^{1}H NMR (500 MHz, Chloroform-*d*) δ (ppm) 7.17 (td, *J* = 7.8, 1.8 Hz, 1H), 7.09 – 7.02 (m, 1H), 6.89 – 6.83 (m, 2H), 3.85 (s, 3H), 3.63 (s, 2H), 2.57 (q, *J* = 7.6 Hz, 2H), 1.17 (t, *J* = 7.6 Hz, 3H).

^{13}C NMR (126 MHz, Chloroform-*d*) δ (ppm) 157.29, 153.76, 144.00, 129.30, 128.85, 127.40, 120.75, 110.40, 101.71, 55.44, 22.71, 18.41, 13.22.

3-Ethyl-4-(4-fluorobenzyl)-1*H*-pyrazol-5-amine **(S68)**

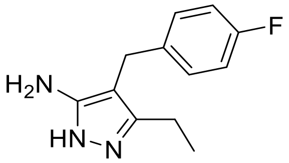

The compound was prepared according to General procedure B1 using 2-(4-fluorobenzyl)-3-oxopentanenitrile (**S33**, 820 mg, 4.02 mmol; 1 eq.), $N_2H_4.H_2O$ (80 % in H_2O, 0.49 mL, 8.1 mmol; 2 eq.), CH_3SO_3H (25 µL, 0.40 mmol, 0.1 eq.) and EtOH (10 mL). Reaction time: 4 h at reflux. The residue obtained after the workup was loaded on ISOLUTE® HM-N and purified by column chromatography on silica gel using Biotage Selekt purification system (dichloromethane:MeOH, gradient 19:1 to 9:1). The product was obtained as colorless wax (200 mg, 23%).

^{1}H NMR (500 MHz, Chloroform-*d*) δ (ppm) 7.16 – 7.08 (m, 2H), 6.99 – 6.91 (m, 2H), 3.66 (s, 2H), 2.53 (q, *J* = 7.6 Hz, 2H), 1.17 (t, *J* = 7.6 Hz, 3H).

^{13}C NMR (126 MHz, Chloroform-*d*) δ (ppm) 161.56 (d, *J* = 243.7 Hz), 153.50, 143.98, 135.95, 129.56 (d, *J* = 8.2 Hz), 115.40 (d, *J* = 21.1 Hz), 101.81, 27.65, 18.42, 13.39.

^{19}F NMR (471 MHz, Chloroform-*d*) δ (ppm) -117.30.

HRMS (APCI): calcd. for $C_{12}H_{15}FN_3$ $[M+H]^+$ = 220.1245, found $[M+H]^+$ = 220.1245.

3-Ethyl-4-(1-methylpiperidin-4-yl)-1*H*-pyrazol-5-amine **(S69)**

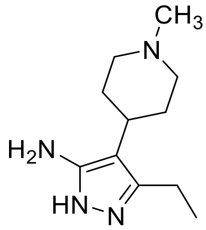

The compound was prepared according to General procedure B1 using 2-(1-methylpiperidin-4-yl)-3-oxopentanenitrile (**S34**, 700 mg, 3.62 mmol; 1 eq.), $N_2H_4.H_2O$ (80 % in H_2O, 0.44 mL, 7.2 mmol; 2 eq.), CH_3SO_3H (25 µL, 0.4 mmol, 0.1 eq.) and EtOH (10 mL). Reaction time: 4 h at reflux. The residue obtained after the workup was loaded on ISOLUTE® HM-N and purified by column chromatography on silica gel using Biotage Selekt purification system (dichloromethane:MeOH, gradient 19:1 to 4:1). The product was obtained as a pale yellow foam (160 mg, 23%).

^{1}H NMR (500 MHz, Chloroform-*d*) δ (ppm) 4.00 (s, 2H), 3.13 – 3.02 (m, 2H), 2.59 (q, J = 7.6 Hz, 2H), 2.39 (s, 3H), 2.31 – 2.24 (m, 1H), 2.20 – 2.04 (m, 4H), 1.79 – 1.66 (m, 2H), 1.20 (t, J = 7.6 Hz, 3H).

^{13}C NMR (126 MHz, Chloroform-*d*) δ (ppm) 152.88, 142.89, 106.59, 56.71, 46.42, 31.79, 31.36, 18.78, 13.89.

4-((1-(*tert*-Butyl)-1*H*-pyrazol-4-yl)methyl)-3-ethyl-1*H*-pyrazol-5-amine **(S70)**

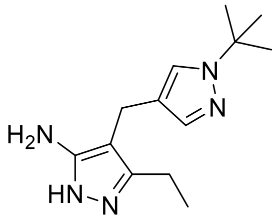

The compound was prepared according to General procedure B1 using 2-((1-(*tert*-butyl)-1*H*-pyrazol-4-yl)methyl)-3-oxopentanenitrile (**S37**, 450 mg, 1.92 mmol; 1 eq.), $N_2H_4.H_2O$ (80 % in H_2O, 0.23 mL, 3.8 mmol; 2 eq.), CH_3SO_3H (25 µL, 0.4 mmol, 0.1 eq.) and EtOH (6 mL). Reaction time: 4 h at reflux. The residue obtained after the workup was loaded on ISOLUTE® HM-N and purified by column chromatography on silica gel using Biotage Selekt purification system (dichloromethane:MeOH, gradient 19:1 to 4:1). The product was obtained as a pale yellow foam (200 mg, 42%).

^{1}H NMR (500 MHz, Chloroform-*d*) δ (ppm) 7.34 (s, 1H), 7.24 (s, 1H), 3.52 (s, 2H), 2.56 (q, J = 7.6 Hz, 2H), 1.55 (s, 9H), 1.19 (t, J = 7.6 Hz, 3H).

^{13}C NMR (126 MHz, Chloroform-*d*) δ (ppm) 153.21, 143.33, 137.89, 124.13, 119.16, 102.02, 58.13, 29.83, 18.24, 17.46, 13.32.

4-([1,1'-Biphenyl]-3-yl)-3-ethyl-1*H*-pyrazol-5-amine **(S71)**

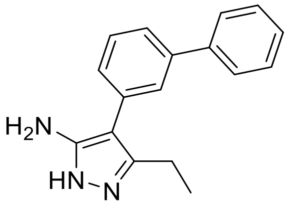

This compound was synthesized according to the procedure reported in *J. Org. Chem.* **2018**, *83*, 24, 15380–15405.

3-Ethyl-4-(4-(trifluoromethyl)phenyl)-1*H*-pyrazol-5-amine **(S72)**

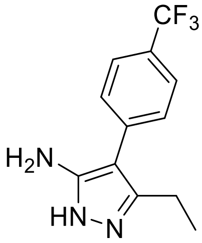

This compound was synthesized according to the procedure reported in *J. Org. Chem.* **2018**, *83*, 24, 15380–15405.

3-Ethyl-4-(4-methoxyphenyl)-1*H*-pyrazol-5-amine **(S73)**

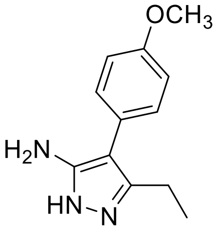

This compound was synthesized according to the procedure reported in *J. Org. Chem.* **2018**, *83*, 24, 15380–15405.

3-Ethyl-4-(3-(trifluoromethyl)phenyl)-1*H*-pyrazol-5-amine **(S74)**

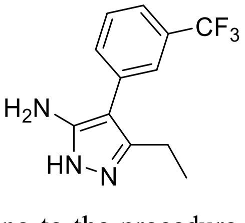

This compound was synthesized according to the procedure reported in *J. Org. Chem.* **2018**, *83*, 24, 15380–15405.

4-(Benzo[*d*][1,3]dioxol-5-yl)-3-ethyl-1*H*-pyrazol-5-amine **(S75)**

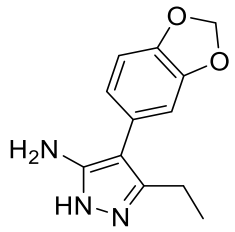

This compound was synthesized according to the procedure reported in *J. Org. Chem.* **2018**, *83*, 24, 15380–15405.

Methyl 3-(5-amino-3-ethyl-1*H*-pyrazol-4-yl)benzoate **(S76)**

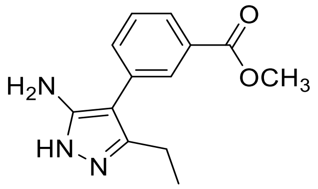

This compound was synthesized according to the procedure reported in *J. Org. Chem.* **2018**, *83*, 24, 15380–15405.

3-Ethyl-4-(4-(methylsulfonyl)phenyl)-1*H*-pyrazol-5-amine **(S77)**

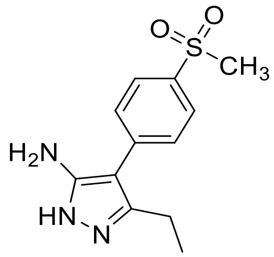

This compound was synthesized according to the procedure reported in *J. Org. Chem.* **2018**, *83*, 24, 15380–15405.

3-Ethyl-4-(4-(piperidin-1-ylsulfonyl)phenyl)-1*H*-pyrazol-5-amine **(S78)**

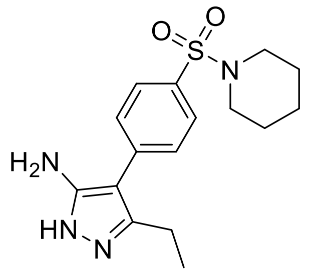

This compound was synthesized according to the procedure reported in *J. Org. Chem.* **2018**, *83*, 24, 15380–15405.

3-Ethyl-4-(3-(trifluoromethoxy)phenyl)-1*H*-pyrazol-5-amine **(S79)**

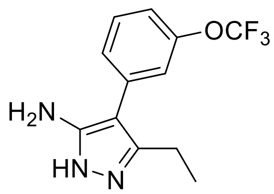

This compound was synthesized according to the procedure reported in *J. Org. Chem.* **2018**, *83*, 24, 15380–15405.

<u>3-Ethyl-4-(naphthalen-1-yl)-1*H*-pyrazol-5-amine</u> **(S80)**

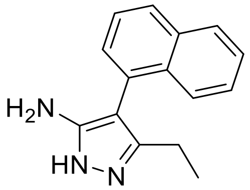

This compound was synthesized according to the procedure reported in *J. Org. Chem.* **2018**, *83*, 24, 15380–15405.

<u>3-Ethyl-4-(4-morpholinophenyl)-1*H*-pyrazol-5-amine</u> **(S81)**

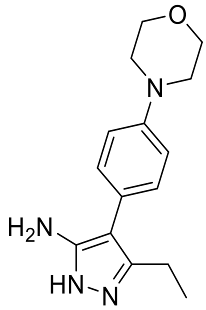

This compound was synthesized according to the procedure reported in *J. Org. Chem.* **2018**, 83, 24, 15380–15405.

<u>*N*-(3-ethyl-4-(4-fluorophenyl)-1*H*-pyrazol-5-yl)formamide</u> **(S82)**

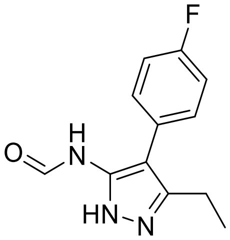

3-Ethyl-4-(4-fluorophenyl)-1*H*-pyrazol-5-amine (**S38**, 400 mg, 1.95 mmol; 1 eq.) was mixed with formic acid (4 mL). The mixture was stirred in the microwave reactor at 110 °C for 3 h, then poured into water (50 mL) and extracted with EtOAc (3 × 25 mL). The combined organic extracts were washed with brine (50 mL), dried over $MgSO_4$, filtered, and the solvent was evaporated. The residue was purified by

column chromatography on silica gel (hexane:EtOAc, gradient 1:0 to 0:1). The product was obtained as a white solid (250 mg, 55%).

^{1}H NMR (500 MHz, DMSO-d_6) δ (ppm) 12.53 (s, 1H), 9.69 (d, J = 10.9 Hz, 1H), 8.69 – 7.85 (m, 1H), 7.37 – 7.16 (m, 4H), 2.61 (q, J = 7.6 Hz, 2H), 1.13 (t, J = 7.6 Hz, 3H).

^{13}C NMR (126 MHz, DMSO-d_6) δ (ppm) 163.35, 160.81 (d, J = 242.9 Hz), 143.10 (d, J = 88.9 Hz), 130.97 (d, J = 8.1 Hz), 130.42 (d, J = 8.1 Hz), 128.24 (d, J = 3.2 Hz), 115.31 (d, J = 21.2 Hz), 115.03, 17.72, 13.27 (d, J = 8.1 Hz).

^{19}F NMR (471 MHz, DMSO-d_6) δ (ppm) -116.23.

3-Ethyl-4-(4-fluorophenyl)-*N*-methyl-1*H*-pyrazol-5-amine **(S83)**

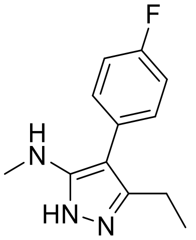

N-(3-Ethyl-4-(4-fluorophenyl)-1*H*-pyrazol-5-yl)formamide (**S82**, 500 mg, 2.14 mmol, 1 eq.) was dissolved in THF (6 mL) and the solution was cooled to 0 °C. Solution of $LiAlH_4$ in THF (2 M, 1.1 mL, 1 eq.) was added and the mixture was stirred under reflux for 16 h. The mixture was cooled to 23 °C, quenched with aqueous solution of sodium hydroxide (1 M, 50 mL) and extracted with EtOAc (3 × 30 mL). The combined organic extracts were washed with brine (50 mL), dried over $MgSO_4$, filtered, and the solvent was evaporated. The residue was purified by column chromatography on silica gel (dichloromethane:MeOH, gradient 9:1 to 5:1). The product was obtained as a white solid (300 mg, 64%).

^{1}H NMR (500 MHz, DMSO-d_6) δ (ppm) 11.45 (s, 1H), 7.33 – 7.26 (m, 2H), 7.21 – 7.14 (m, 2H), 4.60 (s, 1H), 2.66 (s, 1H), 2.52 (q, J = 7.5 Hz, 2H), 1.09 (t, q, J = 7.5 Hz, 3H).

^{13}C NMR (126 MHz, DMSO-d_6) δ (ppm) 160.13 (d, J = 241.7 Hz), 154.39, 141.79*, 130.22 (d, J = 3.1 Hz), 130.05 (d, J = 7.9 Hz), 115.17 (d, J = 21.1 Hz), 102.35, 30.58, 17.97, 13.35.

* - detected by HMBC

^{19}F NMR (471 MHz, DMSO-d_6) δ (ppm) -117.66.

2-((2-Methyl-1,3-dioxolan-2-yl)methyl)-1*H*-benzo[*d*]imidazole **(S84)**

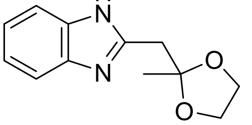

Potassium ethoxide (980 mg, 11.5 mmol, 1 eq.) was added to a solution of benzene-1,2-diamine (2 g, 18.5 mmol, 1.65 eq.) and ethyl 2-(2-methyl-1,3-dioxolan-2-yl)acetate (2 g, 11.2 mmol, 1eq.) in anhydrous EtOH (10 mL). The reaction mixture was refluxed for 72 h. The solvent was evaporated and

the residue was purified by column flash chromatography on silica gel (hexane:EtOAc:MeOH, gradient 1:1:0 to 2:4:1) to afford the product as a brown solid (840 mg, 34%).

^{1}H NMR (500 MHz, DMSO-d_6) δ (ppm) 11.97 (s, 1H), 7.53 (d, J = 7.5 Hz, 1H), 7.44 (d, J = 7.5 Hz, 1H), 7.16 – 7.07 (m, 2H), 3.91 – 3.89 (m, 4H), 3.10 (s, 2H), 1.35 (s, 3H).

^{13}C NMR (126 MHz, DMSO-d_6) δ (ppm) 150.32, 143.14, 134.44, 121.43, 120.71, 118.16, 110.92, 107.96, 64.15, 38.42, 24.07.

1-(1*H*-benzo[*d*]imidazol-2-yl)propan-2-one **(S85)**

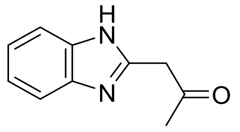

Water (0.5 mL) and hydrochloric acid (35%, 0.5 mL) were added to a solution of 2-((2-methyl-1,3-dioxolan-2-yl)methyl)-1*H*-benzo[*d*]imidazole (**S84**, 230 mg, 1.06 mmol, 1.0 eq.) in THF (5 mL). The reaction mixture was refluxed for 3 h. The solvent was evaporated and the crude product (183 mg) was used in the next step without further purification.

Ethyl 2-(6-morpholino-1*H*-benzo[*d*]imidazol-2-yl)acetate **(S86)**

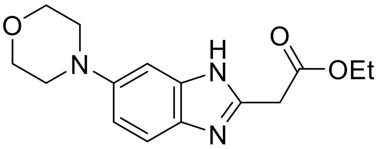

Pd/C (10%, 10 mg) was added to a solution of 5-morpholino-2-nitroaniline (350 mg, 1.57 mmol, 1 eq.) in EtOH (5 mL), and the mixture was bubbled with H_2 for 5 min. The mixture was then stirred at reflux under an atmosphere of H_2 for 1 h. The mixture was filtered through a micro HPLC filter and the solvent was evaporated. The crude diamine was used in the next step without further purification.

Ethyl 3-ethoxy-3-iminopropanoate hydrochloride (306 mg, 1.57 mmol, 1 eq.) was dissolved in EtOH (5 mL), the solution was cooled to 0 °C and a solution of the diamine in EtOH (5 mL) was slowly added. The mixture was stirred at 0 °C for 45 min, then under reflux for additional 2 h. The mixture was poured into water (20 mL) neutralised with saturated solution of sodium bicarbonate (10 mL) and extracted with EtOAc (3×10 mL). The combined organic extracts were washed with brine (30 mL), dried over $MgSO_4$, and the solvent was evaporated. The crude product was purified by column flash chromatography on silica gel (dichloromethane:MeOH, 10:1) to afford the products as a pale brown foam (320 mg, 70%).

^{1}H NMR (500 MHz, DMSO-d_6) δ (ppm) 12.06 (s, 1H), 7.36 (d, J = 8.7 Hz, 1H), 6.95 (s, 1H), 6.90 (dd, J = 8.7, 2.2 Hz, 1H), 4.16 – 4.08 (m, 2H), 3.89 (s, 2H), 3.79 – 3.73 (m, 4H), 3.08 – 3.03 (m, 4H), 1.20 (t, J = 7.1 Hz, 3H).

^{13}C NMR (126 MHz, DMSO-d_6) δ (ppm) 168.79, 147.43, 146.83, 112.89, 66.25, 60.68, 50.41, 35.09, 14.00.

Methyl-3-(4-amino-3-nitrophenyl)acrylate **(S87)**

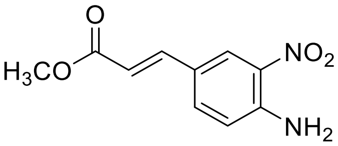

A mixture of 4-bromo-2-nitroaniline (2 g, 9.20 mmol, 1 eq.), triethylamine (3.8 mL, 27.60 mmol, 3 eq.), methyl acrylate (1.65 mL, 18.40 mmol, 2 eq.) and Pd(dppf)Cl_2 (0.32 g, 0.46 mmol, 0.05 eq.) in degassed DMF (12 mL) and water (0.2 mL) was stirred at 100 °C for 16 h. The mixture was poured into brine solution (100 mL) and the solid was collected by filtration. The solid was suspended in dichloromethane:MeOH (10:1 mL), the mixture was filtered, and the solid was dried under *vacuum*. The product was obtained as a pale brown solid (1.18 g, 57%).

^{1}H NMR (500 MHz, DMSO-d_6) δ (ppm) 8.23 (d, J = 2.1 Hz, 1H), 7.85 – 7.80 (m, 3H), 7.58 (d, J = 16.0 Hz, 1H), 7.04 (d, J = 8.8 Hz, 1H), 6.44 (d, J = 16.0 Hz, 1H), 3.70 (s, 3H).

^{13}C NMR (126 MHz, DMSO-d_6) δ (ppm) 166.77, 147.29, 143.18, 133.52, 129.90, 127.40, 121.64, 119.87, 114.89, 51.23.

Methyl 3-(2-(2-ethoxy-2-oxoethyl)-1*H*-benzo[*d*]imidazol-6-yl)propanoate **(S88)**

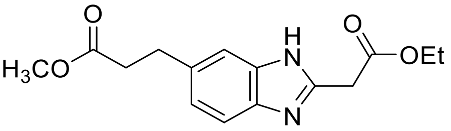

Pd/C (10%, 10 mg) was added to a solution of methyl 3-(4-amino-3-nitrophenyl)acrylate (**S87**, 400 mg, 1.78 mmol, 1 eq.) in EtOH (10 mL), and the mixture was bubbled with H_2 for 5 min. The mixture was then stirred for 1 h at reflux under an atmosphere of H_2. The mixture was filtered through an HPLC filter and the solvent was evaporated. The crude diamine was used in the next step without further purification. Ethyl 3-ethoxy-3-iminopropanoate hydrochloride (350 mg, 1.78 mmol, 1 eq.) was dissolved in EtOH (5 mL) and the solution was cooled to 0 °C. A solution of the diamine in EtOH (5 mL) was slowly added and the mixture was stirred at 0 °C for 45 min, then under reflux for additional 2 h. The mixture was poured into water (20 mL), neutralized with saturated solution of sodium bicarbonate (10 mL) and extracted with EtOAc (3 × 10 mL). The combined organic extracts were washed with brine (30 mL), dried over $MgSO_4$, and the solvent was evaporated. The residue was purified by column flash chromatography on silica gel (dichloromethane:MeOH, 20:1 to 10:1) to afford the products as a white solid (430 mg, 87%).

^{1}H NMR (500 MHz, DMSO-d_6) δ (ppm) 7.50 (d, J = 8.3 Hz, 1H), 7.40 (d, J = 1.5 Hz, 1H), 7.10 (dd, J = 8.3, 1.7 Hz, 1H), 4.24 (q, J = 7.1 Hz, 2H), 4.10 (s, 2H), 3.66 (s, 3H), 3.06 (t, J = 7.8 Hz, 2H), 2.72 – 2.63 (m, 2H), 1.30 (t, J = 7.2 Hz, 3H).

Ethyl 2-(1-benzyl-1*H*-imidazol-2-yl)acetate **(S89)**

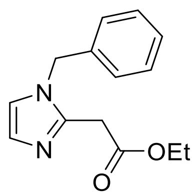

1-Benzyl-2-methylimidazole (3.32 g, 17.32 mmol; 1eq.) was dissolved in dry THF (30 mL). To the solution was added TEA (7.2 mL, 51.97 mmol; 3 eq.) and ethyl chloroformate (4.6 mL, 48.5 mmol; 2.8 eq.). The reaction mixture was stirred at 23 °C for 24 h, then poured into water (50 mL) and extracted with EtOAc (3 × 25 mL). The combined organic extracts were washed with brine (50 mL), dried over $MgSO_4$, and the solvent was evaporated. The residue was purified by column flash chromatography (EtOAc:dichloromethane:MeOH, 1:1:0.5). The product was obtained as a yellow wax (1.0 g, 24%).

^{1}H NMR (500 MHz, Chloroform-*d*) δ (ppm) 7.37 – 7.26 (m, 3H), 7.12 – 7.06 (m, 2H), 7.02 (d, J = 1.4 Hz, 1H), 6.86 (d, J = 1.3 Hz, 1H), 5.13 (s, 2H), 4.11 (q, J = 7.1 Hz, 2H), 3.75 (s, 2H), 1.22 (t, J = 7.2 Hz, 3H).

^{13}C NMR (126 MHz, Chloroform-*d*) δ (ppm) 169.01, 141.49, 136.06, 129.10, 128.27, 128.02, 127.13, 121.11, 61.55, 50.17, 34.00, 14.21.

tert-Butyl 2-(2-ethoxy-2-oxoethyl)-3,4,6,7-tetrahydro-5*H*-imidazo[4,5-*c*]pyridine-5-carboxylate **(S90)**

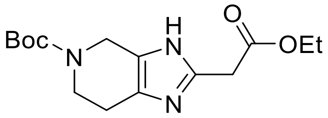

To a cold (0 °C) solution of 2-(4,5,6,7-tetrahydro-3*H*-imidazo[4,5-*c*]pyridine-2-yl)acetate dihydrochloride (262 mg, 0.93 mmol) in dichloromethane (5 mL) was added triethylamine (0.388 mL, 2.79 mmol, 3 eq.) followed by Boc_2O (203 mg, 0.93 mmol, 1 eq.). The mixture was stirred at 0 °C for 30 min, then at 25 °C for additional 4 h. Saturated aqueous solution of NH_4Cl (10 mL) was added and the mixture was extracted with dichloromethane (3 × 30 mL). The combined organic extracts were washed with brine (20 mL), dried over $MgSO_4$, filtered, and the solvent was evaporated *in vacuo*. The residue was purified by column chromatography on silica gel (hexane:EtOAc:MeOH, gradient 2:1:0 to 0:1:0 to 0:20:1). The product was obtained as a yellow wax (177 mg, 62%).

^{1}H NMR (500 MHz, Chloroform-*d*) δ (ppm) 10.24 (s, 1H), 4.39 (s, 2H), 4.13 (q, J = 7.1 Hz, 2H), 3.75 (s, 2H), 3.65 (t, J = 5.9 Hz, 2H), 2.59 (t, J = 5.6 Hz, 2H), 1.42 (s, 9H), 1.22 (t, J = 7.2 Hz, 3H).

^{13}C NMR (126 MHz, Chloroform-*d*) δ (ppm) 170.17, 155.17, 139.77, 80.02, 77.36, 61.50, 43.03, 42.16, 40.99, 34.29, 28.46, 22.31, 14.11.

HRMS (APCI): calcd. for $C_{15}H_{24}N_3O_4$ $[M+H]^+$ = 310.1761, found $[M+H]^+$ = 310.1758.

N-(2-morpholinoethyl)-2-nitroaniline **(S91)**

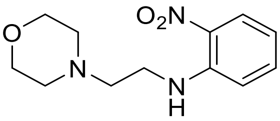

Sodium hydride (60%, 580 mg, 14.50 mmol, 2 eq.) was added to a solution of 2-nitroaniline (1 g, 7.25 mmol, 1 eq.) in anhydrou THF (50 mL) at 0 °C and the mixture was stirred for 30 min at that temperature. TBAI (130 mg, 0.36 mmol, 0.05 eq.) and 4-(2-chloroethyl)morpholine hydrochloride (1.34 g, 7.2 mmol, 1eq) were added and the mixture was stirred at 23 °C for 16 h. The mixture was poured into water (50 mL) and extracted with EtOAc (3 × 50 mL). The combined organic extracts were washed with brine (50 mL), dried over $MgSO_4$, filtered, and the solvent was evaporated. The crude product (1.76 g, 97%) was used in the next step without further purification.

^{1}H NMR (300 MHz, Chloroform-*d*) δ (ppm) 8.46 (s, 1H), 8.17 (dd, *J* = 8.6, 1.6 Hz, 1H), 7.49 – 7.36 (m, 1H), 6.82 (d, *J* = 1.2 Hz, 1H), 6.70 – 6.58 (m, 1H), 3.82 – 3.68 (m, 4H), 3.59 (t, *J* = 6.9 Hz, 2H), 2.72 (t, *J* = 6.7 Hz, 2H), 2.54 – 2.49 (m, 4H).

Ethyl 2-(1-(2-morpholinoethyl)-1*H*-benzo[*d*]imidazol-2-yl)acetate **(S92)**

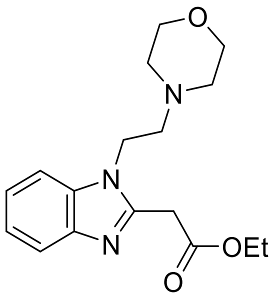

N-(2-morpholinoethyl)-2-nitroaniline (**S91**, 1.76 g, 7.0 mmol, 1 eq.) was dissolved in EtOH (20 mL), Pd/C (10%, 20 mg) was added, and the mixture was then stirred in a high-pressure apparatus at 60 °C under hydrogen (25 bar) for 16 h. The mixture was filtered through an HPLC filter and the solvent was evaporated. The crude diamine was used in the next step without further purification.

Ethyl 3-ethoxy-3-iminopropanoate hydrochloride (1.37 g, 7.0 mmol, 1 eq.) was dissolved in EtOH (5 mL), the solution was cooled to 0 °C and a solution of the diamine in EtOH (5 mL) was slowly added. The mixture was stirred for 45 min at 0 °C, then under reflux for additional 2 h. The mixture was poured into water (20 mL) neutralised with saturated solution of sodium bicarbonate (10 mL) and extracted with EtOAc (3 × 20 mL). The combined organic extracts were washed with brine (30 mL), dried over $MgSO_4$, and the solvent was evaporated. The residue was purified by column flash chromatography on silica gel (dichloromethane:MeOH, 10:1) to afford the product as a light brown foam (340 mg, 15%).

^{1}H NMR (300 MHz, Chloroform-*d*) δ (ppm) 7.77 – 7.69 (m, 1H), 7.37 – 7.31 (m, 1H), 7.30 – 7.22 (m, 1H), 4.28 (t, *J* = 6.6 Hz, 2H), 4.20 (q, *J* = 7.1 Hz, 2H), 4.13 (s, 2H), 3.73 – 3.63 (m, 4H), 2.73 (t, *J* = 6.6 Hz, 2H), 2.55 – 2.44 (m, 4H), 1.26 (t, *J* = 7.1 Hz, 3H).

^{13}C NMR (75 MHz, Chloroform-*d*) δ (ppm) 168.60, 148.07, 142.66, 135.18, 122.86, 122.40, 119.94, 109.55, 66.96, 61.83, 57.66, 54.20, 42.19, 34.81, 14.26.

<u>N^1,N^1-dimethyl-N^2-(2-nitrophenyl)ethane-1,2-diamine</u> **(S93)**

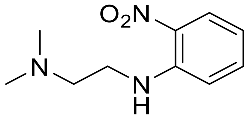

2-Nitroaniline (1 g, 7.25 mmol, 1 eq.) was dissolved in anhydrous THF (30 mL). The solution was cooled to 0 °C, sodium hydride (60%, 0.58 g, 14.5 mmol, 2 eq.) was added and the mixture was stirred at 0 °C for 30 min. Tetrabutylammonium iodide (130 mg, 0.36 mmol, 0.05 eq.) and N^1,N^1-dimethylethane-1,2-diamine hydrochloride (1.04 g, 7.2 mmol, 1eq) were added and the mixture was stirred at 23 °C for 16 h. The mixture was poured into water (50 mL) and extracted with EtOAc (3 × 30 mL). The combined organic extracts were washed with brine (50 mL), dried over $MgSO_4$, filtered, and the solvent was evaporated. The crude product (690 mg, 46%) was used in the next step without further purification.

^{1}H NMR (300 MHz, Chloroform-*d*) δ (ppm) 8.28 (s, 1H), 8.16 (dd, J = 8.6, 1.6 Hz, 1H), 7.49 – 7.38 (m, 1H), 6.84 (dd, J = 8.7, 1.2 Hz, 1H), 6.69 – 6.58 (m, 1H), 3.44 – 3.31 (m, 2H), 2.66 (t, J = 6.3 Hz, 2H), 2.33 (s, 6H).

^{13}C NMR (75 MHz, Chloroform-*d*) δ (ppm) 145.46, 136.27, 132.24, 127.05, 115.28, 114.03, 57.54, 45.35, 40.78.

<u>Ethyl 2-(1-(2-(dimethylamino)ethyl)-1*H*-benzo[*d*]imidazol-2-yl)acetate</u> **(S94)**

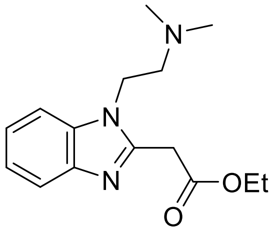

N^1,N^1-dimethyl-N^2-(2-nitrophenyl)ethane-1,2-diamine (**S93**, 680 mg, 3.20 mmol, 1 eq.) was dissolved in EtOH (20 mL), Pd/C (10%, 20 mg) was added, and the mixture was stirred in a high-pressure apparatus at 60 °C under hydrogen (25 bar) for 16 h. The mixture was filtered through and HPLC filter and the solvent was evaporated. The crude diamine was used in the next step without further purification. The diamine was dissolved in diethyl malonate (8 mL) and stirred for 3 h at 150 °C. The crude mixture was purified by column flash chromatography on silica gel (dichloromethane:MeOH, 10:1) to afford the product as a light brown foam (320 mg, 36%).

^{1}H NMR (300 MHz, Chloroform-*d*) δ (ppm) 7.76 – 7.68 (m, 1H), 7.39 – 7.31 (m, 1H), 7.28 – 7.22 (m, 2H), 4.27 (t, J = 7.0 Hz, 2H), 4.20 (q, J = 7.2 Hz, 2H), 4.08 (s, 2H), 2.68 (t, J = 7.0 Hz, 2H), 2.30 (s, 6H), 1.26 (t, J = 7.1 Hz, 3H).

^{13}C NMR (75 MHz, Chloroform-*d*) δ (ppm) 168.62, 148.06, 142.82, 135.27, 122.77, 122.24, 119.93, 109.54, 61.75, 58.34, 45.89, 42.76, 34.76, 14.24.

(*E*)-2-(2-(piperidin-1-yl)vinyl)-1*H*-benzo[*d*]imidazole **(S95)**

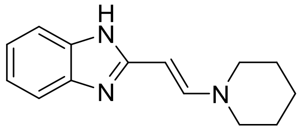

A mixture of 2-ethynyl-1*H*-benzimidazole (60 mg, 0.42 mmol, 1eq.) and piperidine (41 µL, 0.42 mmol, 1 eq.) in THF (5 mL) was stirred at 23 °C for 48 h. The solvent was evaporated and the crude product (95 mg, quantitative) was used in the next step without further purification.

^{1}H NMR (300 MHz, chloroform-*d*) δ (ppm) 7.58 (d, J = 13.6 Hz, 1H), 7.47 – 7.35 (m, 2H), 7.15 – 7.03 (m, 2H), 5.16 (d, J = 13.5 Hz, 1H), 3.22 – 3.05 (m, 4H), 1.67 – 1.50 (m, 6H).

N^1-benzylbenzene-1,2-diamine **(S96)**

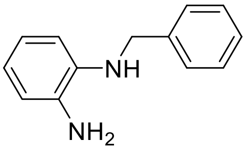

Benzyl bromide (2.7 g, 16.0 mmol) was added to a mixture *o*-diaminobenzene (8 g, 74.0 mmol) and K_2CO_3 (6.0 g, 44.0 mmol) in anhydrous MeOH (40 mL). The reaction mixture was stirred under N_2 at 25 °C for 20 hrs, the solvent was evaporated and the residue was purified by column flash chromatography on silica gel (hexane:EtOAc, 2:1) to afford the product a dark red wax (2.58 g, 81%).

^{1}H NMR (500 MHz, Chloroform-*d*) δ (ppm) δ 7.42 (d, J = 7.1 Hz, 2H), 7.37 (t, J = 7.6 Hz, 2H), 7.34 – 7.28 (m, 1H), 6.86 – 6.79 (m, 1H), 6.78 – 6.67 (m, 3H), 4.33 (s, 2H).

^{13}C NMR (126 MHz, Chloroform-*d*) δ (ppm) 139.56, 137.82, 134.36, 128.74, 127.93, 127.40, 120.89, 119.03, 116.71, 112.24, 48.82.

1-Benzyl-2-methyl-1*H*-benzo[d]imidazole **(S97)**

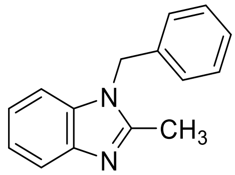

N^1-benzylbenzene-1,2-diamine (**S96**, 387 mg, 1.95 mmol) was dissolved in acetic acid (2 mL) and EtOH (2 mL). The reaction mixture was refluxed for 18 h, then poured into saturated aqueous solution of sodium bicarbonate (25 mL) and water (25 mL) and extracted with EtOAc (3 × 25 mL). The combined organic layers were washed with brine (25 mL), dried over $MgSO_4$, and the solvent was evaporated under reduced pressure. The residue was purified by flash column chromatography (hexane:EtOAc, gradient 1:0 to 1:1). The product was obtained as a red solid (256 mg, 60%).

^{1}H NMR (500 MHz, Chloroform-*d*) δ (ppm) 2.59 (s, 3H), 5.34 (s, 2H), 7.09-7.06 (m, 2H), 7.85 – 7.69 (m, 3H), 7.36 – 7.19 (m, 3H), 7.75 (s, 1H).

^{13}C NMR (126 MHz, Chloroform-*d*) δ (ppm) 14.07, 47.27, 109.49, 119.28, 122.23, 122.48, 126.39, 128.08, 129.17, 135.5, 135.98, 142.67, 152.02.

Methyl 2-(1-benzyl-1*H*-benzo[*d*]imidazol-2-yl)acetate **(S98)**

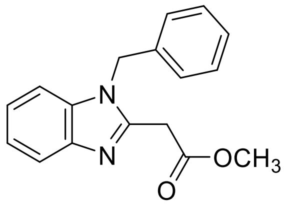

1-Benzyl-2-methyl-1*H*-benzo[*d*]imidazole (**S97**, 610 mg, 2.74 mmol; 1eq.) was dissolved in dry THF (5 mL). To solution was added DIPEA (1.5 mL, 8.22 mmol; 3 eq.) and methyl chloroformate (0.45 mL, 5.76 mmol; 2.1 eq.). The reaction mixture was stirred at room temperature for 18 h, then poured into water (50 mL) and extracted with EtOAc (3 × 25 mL). The combined organic extracts were washed with brine (25 mL), dried over $MgSO_4$, and the solvent was evaporated *in vacuo*. The residue was purified by column flash chromatography (100% EtOAc). The product was obtained as a red solid (657 mg, 86%).

^{1}H NMR (500 MHz, Chloroform-*d*) δ (ppm) 7.70 (d, J = 7.7 Hz, 1H), 7.32 – 7.09 (m, 6H), 6.97 (d, J = 6.4 Hz, 2H), 5.32 (s, 2H), 3.88 (s, 2H), 3.55 (s, 3H).

^{13}C NMR (126 MHz, Chloroform-*d*) δ (ppm) 168.69, 147.93, 142.73, 135.82, 135.68, 129.13, 128.13, 126.44, 123.15, 122.50, 120.00, 110.00, 52.65, 47.53, 34.68.

HRMS (APCI): calcd. for $C_{18}H_{19}N_2O_2$ $[M+H]^+$ = 281.1285, found $[M+H]^+$ = 281.1291.

2-(1-Benzyl-1*H*-benzo[*d*]imidazol-2-yl)acetonitrile **(S99)**

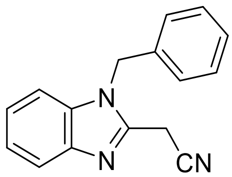

Ethyl 2-cyanoacetate (2.20 g, 20.0 mmol; 1 eq.) followed by methanesulfonic acid (0.1 mL) were added to a solution of N^1-benzylbenzene-1,2-diamine (**S96**, 2.58 g, 13.0 mmol; 1.54 eq.) in ethylene glycol (15 mL). The solution was refluxed for 4 hrs under N_2, poured into a mixture of water (100 mL) with saturated aqueous $NaHCO_3$ (25 mL), and extracted with EtOAc (3 × 50 mL). The organic extracts were washed with water (100 mL), brine (25 mL), then dried over $MgSO_4$, filtered, and the solvent was evaporated. The residue was purified by flash chromatography on silica gel (hexane:EtOAc, 10:1) to yield the product as a white crystalline solid (2.43 g, 75%).

MP = 135.0 – 136.0 ºC.

^{1}H NMR (500 MHz, Chloroform-*d*) δ (ppm) 7.86 – 7.78 (m, 1H), 7.38 – 7.28 (m, 6H), 7.12 – 7.04 (m, 2H), 5.45 (s, 2H), 3.92 (s, 2H).

13C NMR (126 MHz, Chloroform-*d*) *δ* (ppm) 143.42, 142.19, 136.04, 134.83, 129.50, 128.70, 126.49, 124.07, 123.17, 120.36, 114.21, 109.95, 47.61, 18.43.

HRMS (APCI): calcd. for $C_{16}H_{12}N_3$ $[M-H]^-$ = 246.1037, found $[M-H]^-$ = 246.1037.

1-Benzyl-5,6-dimethyl-1*H*-benzo[*d*]imidazole **(S100)**

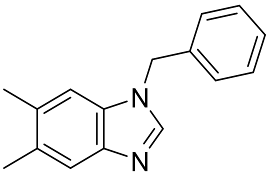

Sodium hydride (60 %, 910 mg, 22.50 mmol, 1.1 eq.) was slowly added to a stirred solution of 5,6-dimethyl-1*H*-benzo[*d*]imidazole (3 g, 20.50 mmol, 1eq.) in DMF (20 mL) at 0 °C. After 30 min was added benzyl bromide (2.7 mL, 22.50 mmol, 1.1 eq.) and the mixture was stirred at 23 °C for 16 h. The the mixture was poured into water (50 mL), the precipitate was collected by filtration, washed with water (10 mL) and diethyl ether (2 ×25 mL). The product obtained as a white solid (4.52 g, 93%), was used in the next step without further purification.

^{1}H NMR (500 MHz, Chloroform-*d*) *δ* (ppm) δ 8.23 (s, 1H), 7.42 (s, *J* = 22.5 Hz, 1H), 7.33 (t, *J* = 7.3 Hz, 2H), 7.30 – 7.22 (m, 4H), 5.43 (s, 2H), 2.28 (s, *J* = 2.0 Hz, 3H), 2.27 (s, 3H).

^{13}C NMR (126 MHz, Chloroform-*d*) *δ* (ppm) 143.29, 142.18, 137.16, 132.22, 130.94, 129.84, 128.60, 127.55, 127.11, 119.48, 110.51, 47.43, 20.03, 19.76.

1-Benzyl-2,5,6-trimethyl-1*H*-benzo[*d*]imidazole **(S101)**

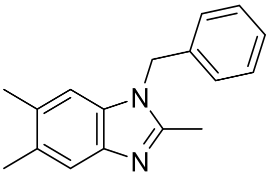

A suspension of 1-benzyl-5,6-dimethyl-1*H*-benzo[*d*]imidazole (**S100**, 237 mg, 1.10 mmol, 1eq.) in dioxane/THF (2+2 mL) was cooled to -40 °C and a solution of *n*-BuLi (2.7 M, 0.47 mL, 1.26 mmol, 1.2 eq.) was added. The mixture was stirred at -40 °C for 40 min, MeI was added (80 µL, 1.26 mmol, 1.2 eq), and the mixture was stirred at 23 °C for 16 h. The reaction mixture was poured into water (25 mL) and saturated solution of NH_4Cl (25 mL) and extracted with EtOAc (3 × 25 mL). The combined organic layers were washed with brine (50 mL) and dried over $MgSO_4$. The solvent was removed under reduced pressure and the residue was purified by column chromatography on silica gel (eluent: EtOAc). The product was obtained as a white solid (156 mg, 59%).

^{1}H NMR (500 MHz, $CDCl_3$) δ 7.40 (s, 1H), 7.23 – 7.16 (m, 3H), 7.00 – 6.92 (m, 2H), 6.90 (s, 1H), 5.18 (s, 2H), 2.43 (s, 3H), 2.28 (s, 3H), 2.24 (s, 3H).

^{13}C NMR (126 MHz, $CDCl_3$) δ 151.07, 141.40, 136.32, 134.19, 131.30, 130.78, 129.08, 127.88, 126.26, 119.46, 109.71, 47.08, 20.59, 20.30, 14.03.

Methyl 2-(1-benzyl-5,6-dimethyl-1*H*-benzo[*d*]imidazol-2-yl)acetate **(S102)**

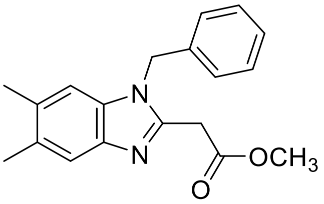

Methyl chloroformate (0.18 mL, 2.38 mmol; 2.5 eq.) was added to a stirred solution of 1-benzyl-2,5,6-trimethyl-1*H*-benzo[*d*]imidazole (**S101**, 250 mg, 0.95 mmol; 1eq.) and DIPEA (0.5 mL, 2.85 mmol; 3 eq.) was in dry THF (5 mL) and the reaction mixture was stirred at 23 °C for 18 h. The mixture was poured into water (50 mL) and extracted with EtOAc (3 × 25 mL). The combined organic extracts were washed with brine (50 mL), dried over $MgSO_4$, and the solvent was evaporated. The residue was purified by column flash chromatography (100% EtOAc). The product was obtained as a yellow solid (219 mg, 72%).

^{1}H NMR (500 MHz, Chloroform-*d*) δ (ppm) ^{1}H NMR (500 MHz, $CDCl_3$) δ 7.45 (s, 1H), 7.22 – 7.17 (m, 3H), 6.98 – 6.93 (m, 2H), 6.92 (s, 1H), 5.26 (s, 2H), 3.83 (s, 2H), 3.51 (s, 3H), 2.28 (s, 3H), 2.24 (s, 3H).

^{13}C NMR (126 MHz, Chloroform-*d*) δ (ppm) 168.79, 146.97, 141.25, 134.36, 132.24, 131.29, 129.04, 127.96, 126.32, 119.98, 110.10, 52.53, 47.33, 34.59, 20.62, 20.29.

Methyl 2-(5,6-dimethyl-1*H*-benzo[*d*]imidazol-2-yl)acetate **(S103)**

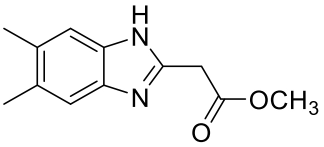

A mixture of methyl 2-(1-benzyl-5,6-dimethyl-1*H*-benzo[*d*]imidazol-2-yl)acetate (**S102**, 160 mg, 0.52 mmol, 1eq.) and $Pd(OH)_2/C$ (10 %, 35 mg, 0.03 mmol, 5%) in degassed EtOH (5 mL) was refluxed under a hydrogen atmosphere (1 bar) for 3 h. The mixture was filtered through an HPLC filter and the solvent was evaporated. The crude product was dried under vacuum and used in the next step without further purification. The product was obtained as a white solid (96 mg, 85%).

^{1}H NMR (126 MHz, DMSO-d_6) δ (ppm) 12.06 (s, 1H), 7.26 (s, 2H), 3.92 (s, 2H), 3.66 (s, 2H), 2.29 (s, 3H).

^{13}C NMR (126 MHz, DMSO-d_6) δ (ppm) 169.23, 146.58, 129.72, 51.96, 34.88, 19.85.

Ethyl-2-(1*H*-benzo[*d*]imidazol-2-yl)-3-(dimethylamino)acrylate **(S104)**

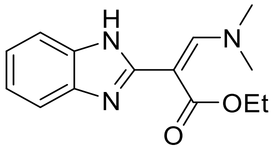

Ethyl 2-(1*H*-benzo[*d*]imidazol-2-yl)acetate (533 mg, 2.61 mmol, 1 eq.) and *t*-butoxybis(dimethylamino)methane (0.65 mL, 3.13 mmol, 1.2 eq.) were dissolved in toluene (10 mL) and the mixture was stirred at 23 °C for 5 h. The resulting precipitate was collected by filtration, washed with toluene (5 mL), diethyl ether (10 mL) and dried under *vacuum*. The product obtained as a yellow solid (500 mg, 74%), was used in the next step without further purification.

^{1}H NMR (500 MHz, DMSO-d_6) δ (ppm) 12.09 (s, 1H), 7.69 (s, 1H), 7.54 (d, J = 7.6 Hz, 1H), 7.40 (d, J = 7.5 Hz, 1H), 7.15 – 7.06 (m, 2H), 4.05 (q, J = 7.1 Hz, 2H), 2.73 (s, 6H), 1.13 (t, J = 7.1 Hz, 3H).

^{13}C NMR (126 MHz, DMSO-d_6) δ (ppm) 167.72, 152.41, 148.61, 142.91, 134.32, 121.36, 120.54, 118.21, 110.65, 87.81, 58.76, 14.54.

Ethyl 4-hydroxypyrazolo[5,1-*c*][1,2,4]triazine-3-carboxylate **(S105)**

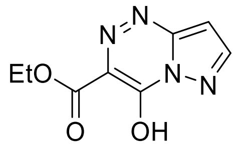

The compound was prepared according to General procedure C using:

Diazotization step:

3-aminopyrazole (303 mg, 3.65 mmol; 1 eq.), 35% aqueous HCl (1.29 mL, 14.59 mmol; 4 eq.) in EtOH (2 mL) and H_2O (2 mL), $NaNO_2$ (302 mg, 4.38 mmol, 1.2 eq.) in EtOH (2 mL) and H_2O (2 mL) (reaction time: 15 min), diethylmalonate (0.66 mL, 4.38 mmol, 1.2 eq.) in EtOH (3 mL) and H_2O (3 mL) and KOAc (2.15 g, 21.88 mmol, 6 eq.). Reaction time: 6 h. EtOAc (30 mL) was added followed by water (20 mL). The organic phase was separated, washed with brine (20 mL), dried over $MgSO_4$, filtered, and the solvent was evaporated. The residue was purified by column chromatography on silica gel (hexane:EtOAc, gradient 2:1; 1:1; 1:2; 0:1) to give the product (diethyl 2-(2-(1*H*-pyrazol-5-yl)hydrazono)malonate) as a yellow solid (565 mg, 61%).

Cyclization step:

the yellow solid (25 mg, 0.983 mmol) was dissolved in glacial acetic acid (10 mL) and the mixture was refluxed under N_2 for 3 h. The solvent was evaporated, to the residue was added dichloromethane (5 mL) and the precipitate was collected by filtration and washed with cold dichloromethane (5 mL). The solid was dried *in vacuo* to afford the product as a yellow solid (190 mg, 93%).

^{1}H NMR (500 MHz, DMSO-d_6) δ (ppm) 14.58 (s, 1H), 8.11 (d, J = 2.1 Hz, 1H), 6.48 (d, J = 2.1 Hz, 1H), 4.32 (q, J = 7.1 Hz, 2H), 1.31 (t, J = 7.1 Hz, 3H).

^{13}C NMR (125 MHz, DMSO-d_6) δ (ppm) 161.48, 147.12, 144.93, 142.39, 127.15, 89.98, 61.04, 14.06.

HRMS (APCI): calcd. for $C_8H_9N_4O_3$ [M+H]$^+$ = 209.0669, found [M+H]$^{+\cdot}$= 209.0670.

MP > 250 °C (dec.).

3-(1-Benzyl-1*H*-benzo[*d*]imidazol-2-yl)pyrazolo[5,1-*c*][1,2,4]triazin-4-ol **(S106)**

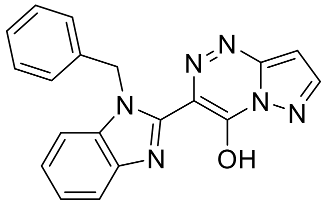

A mixture of ethyl 4-hydroxypyrazolo[5,1-*c*][1,2,4]triazine-3-carboxylate (**S105**, 532 mg, 2.55 mmol, 1.05 eq.) and N^1-benzylbenzene-1,2-diamine (**S96**, 250 mg, 2.43 mmol, 1 eq.) in EtOH (2 mL) and AcOH (2 mL) was refluxed for 4 h. After cooling to 25 °C, a precipitate formed. EtOH (4 mL) was added and the solid was collected by filtration and washed on filter with EtOH (10 mL). The solid was dried under vacuum to afford the product as a pale yellow solid (45 mg, 54%).

^{1}H NMR (500 MHz, DMSO-d_6) δ (ppm) 8.10 (d, J = 2.1 Hz, 1H), 7.86 (dd, J = 6.9, 2.0 Hz, 1H), 7.61 (d, J = 7.6 Hz, 1H), 7.43 – 7.34 (m, 2H), 7.29 – 7.21 (m, 5H), 6.63 (d, J = 2.2 Hz, 1H), 5.99 (s, 2H).

^{13}C NMR (126 MHz, DMSO-d_6) δ (ppm) 149.51, 146.64, 144.49, 136.38, 133.57, 128.51, 127.49, 126.93, 124.09, 123.89, 116.92, 111.64, 93.57, 48.64.

HRMS (APCI): calcd. for $C_{19}H_{15}N_6O$ [M+H]$^+$ = 343.1302, found [M+H]$^{+\cdot}$= 343.1299.

3-(1-Benzyl-1*H*-benzo[*d*]imidazol-2-yl)-8-bromopyrazolo[5,1-*c*][1,2,4]triazin-4-ol **(S107)**

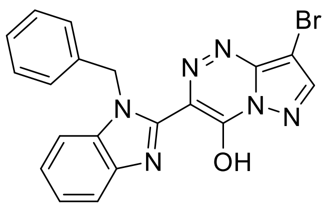

A solution of *N*-bromosuccinimide (55 mg, 0.31 mmol, 1.05 eq.) in DMF (2 mL) was added dropwise to a stirred mixture of 3-(1-benzyl-1*H*-benzo[*d*]imidazol-2-yl)pyrazolo[5,1-*c*][1,2,4]triazin-4-ol (**S106**, 100 mg, 0.29 mmol, 1 eq.) in DMF (20 mL) and acetonitrile (20 mL) at 0 °C. The reaction mixture was stirred at 0 °C for 10 min, then at 25 °C for 30 min. The solvent was evaporated, and the solid residue was triturated with cold EtOH (*10* mL), collected by filtration, and then washed with EtOH (10 mL) and dried under vacuum. The product was obtained as a yellow solid (114 mg, 93%).

^{1}H NMR (500 MHz, DMSO-d$_6$) δ (ppm) 14.18 (s, 1H), 8.21 (s, 1H), 7.95 (d, J = 7.5 Hz, 1H), 7.75 (d, J = 7.8 Hz, 1H), 7.49 (p, J = 7.3 Hz, 2H), 7.31 (d, J = 4.4 Hz, 4H), 7.29 – 7.23 (m, 1H), 6.29 (s, 2H).

^{13}C NMR (126 MHz, DMSO-d$_6$) δ (ppm) 149.12, 146.25, 144.14, 135.86, 132.66, 128.66, 127.66, 126.95, 125.16, 112.14, 49.58.

HRMS (APCI): calcd. for $C_{19}H_{14}BrN_6O$ [M+H]$^+$ = 421.0407.1245, found [M+H]$^{+\cdot}$= 421.0407.

3-(1*H*-benzo[*d*]imidazol-2-yl)-7-(((*tert*-butyldiphenylsilyl)oxy)methyl)pyrazolo[5,1-*c*][1,2,4]triazin-4-amine **(S108)**

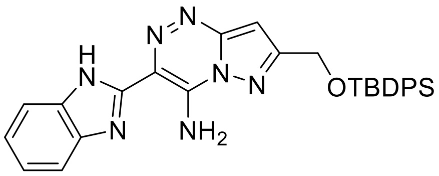

The compound was prepared according to General procedure C using:

Diazotization step:

3-(((*tert*-butyldiphenylsilyl)oxy)methyl)-1*H*-pyrazol-5-amine (**S47**, 163 mg, 0.5 mmol; 1 eq.), 35% aqueous HCl (0.2 mL, 2.0 mmol; 4 eq.) in EtOH (4 mL) and H_2O (4 mL), $NaNO_2$ (68 mg, 1.0 mmol, 2 eq.) in H_2O (1 mL) (reaction time: 30 min), 2-(cyanomethyl)-benzimidazole (94 mg, 0.6 mmol, 1.2 eq.) in EtOH (1 mL) and KOAc (300 mg, 3.0 mmol, 6 eq.) in H_2O (1 mL). Reaction time: 16 h. The yellow solid (210 mg, 0.39 mmol) obtained after the filtration was used in the next step without further purification.

Cyclization step:

the dried, filtered yellow solid (210 mg, 0.39 mmol), DMF (3 mL), and KOAc (4 mg, 0.04 mmol, 0.1 eq.). Reaction time: 2 h. The product was obtained as a yellow solid (114 mg, 44%).

^{1}H NMR (500 MHz, DMSO-d_6) δ (ppm) 13.45 (s, 1H), 9.94 (s, 1H), 9.34 (s, 1H), 7.74 (d, J = 7.7 Hz, 1H), 7.72 – 7.66 (m, 4H), 7.54 (d, J = 7.7 Hz, 1H), 7.49 – 7.43 (m, 6H), 7.29 – 7.19 (m, 2H), 6.97 (s, 1H), 5.02 (s, 2H), 1.07 (s, 9H).

^{13}C NMR (126 MHz, DMSO-d_6) δ (ppm) 158.76, 150.00, 149.51, 138.95, 135.05, 134.42, 132.52, 129.99, 127.96, 127.44, 121.81, 118.77, 118.29, 111.45, 94.51, 60.35, 26.61, 18.82.

HRMS (APCI): calcd. for $C_{29}H_{30}N_7OSi$ $[M+H]^+$ = 520.2276, found $[M+H]^+$ = 520.2273.

Preparation of target compound 1 and its analogs

<u>3-(1*H*-benzo[*d*]imidazol-2-yl)-7-ethyl-8-(4-fluorophenyl)pyrazolo[5,1-*c*][1,2,4]triazin-4-amine</u> **(1)**

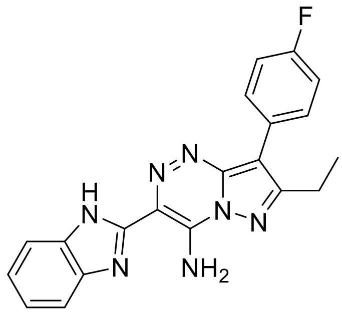

The compound was prepared according to General procedure C using:

Diazotization step:

3-ethyl-4-(4-fluorophenyl)-1*H*-pyrazol-5-amine (**S38**, 200 mg, 0.98 mmol; 1 eq.), 35% aqueous HCl (0.344 mL, 3.90 mmol; 4 eq.) in EtOH (3 mL) and H_2O (3 mL), $NaNO_2$ (135 mg, 1.95 mmol, 2 eq.) in EtOH (2 mL) and H_2O (2 mL) (reaction time: 15 min), 2-(cyanomethyl)-benzimidazole (184 mg, 1.17 mmol, 1.2 eq.) in EtOH (3 mL) and H_2O (3 mL) and KOAc (765 mg, 7.8 mmol, 8 eq.). Reaction time: 1 h. The yellow solid (213 mg, 0.57 mmol) obtained after the filtration was used in the next step without further purification.

Cyclization step:

the dried yellow solid (213 mg, 0.57 mmol), DMF (2 mL), and KOAc (5 mg, 0.05 mmol, 0.05 eq.). Reaction time: 1 h. The product was obtained as a yellow solid (69 mg, 19% (2 steps)).

^{1}H NMR (500 MHz, DMSO-d_6) δ (ppm) 13.35 (s, 1H), 9.95 (s, 1H), 9.34 (s, 1H), 7.89 – 7.84 (m, 2H), 7.74 (d, J = 7.7 Hz, 1H), 7.56 (d, J = 7.6 Hz, 1H), 7.39 – 7.34 (m, 2H), 7.30 – 7.20 (m, 1H), 3.05 (q, J = 7.6 Hz, 2H), 1.35 (t, J = 7.5 Hz, 3H).

^{13}C NMR (126 MHz, DMSO-d_6) δ (ppm) 161.11 (d, J = 244.3 Hz), 150.08, 146.36, 142.74, 138.73, 133.76, 130.91 (d, J = 8.1 Hz), 127.73, 122.97, 121.93, 119.24, 118.34, 115.52 (d, J = 21.7 Hz), 111.54, 107.28, 20.89, 13.13.

HRMS (APCI): calcd. for $C_{20}H_{17}FN_7$ $[M+H]^+$ = 374.1524, found $[M+H]^+$ = 374.1529.

Mp > 260 °C (decomp.).

<u>3-(1*H*-benzo[*d*]imidazol-2-yl)pyrazolo[5,1-*c*][1,2,4]triazin-4-amine</u> **(3)**

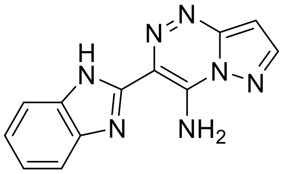

The compound was prepared according to General procedure C using:

Diazotization step:

3-aminopyrazole (250 mg, 3 mmol; 1 eq.), 35% aqueous HCl (1.06 mL, 12.03 mmol; 4 eq.) in EtOH (3 mL) and H_2O (3 mL), $NaNO_2$ (415 mg, 6.02 mmol, 2 eq.) in EtOH (2 mL) and H_2O (2 mL) (reaction time: 20 min), 2-(cyanomethyl)-benzimidazole (567 mg, 3.61 mmol, 1.2 eq.) in EtOH (3 mL) and H_2O

(3 mL) and KOAc (1.77 g, 18 mmol, 6 eq.). Reaction time: 2 h. The yellow solid (768 mg, 3.06 mmol) obtained after the filtration was used in the next step without further purification.

Cyclization step:

the dried yellow solid (99 mg, 0.39 mmol), DMF (1 mL), and KOAc (2 mg, 0.02 mmol, 0.05 eq.). Reaction time: 1 h. The product was obtained as an orange solid (76 mg, 75% (2 steps)).

^{1}H NMR (500 MHz, DMSO-*d*$_6$) δ (ppm) 13.35 (s, 1H), 9.95 (s, 1H), 9.34 (s, 1H), 7.89 – 7.84 (m, 2H), 7.74 (d, *J* = 7.7 Hz, 1H), 7.56 (d, *J* = 7.6 Hz, 1H), 7.39 – 7.34 (m, 2H), 7.30 – 7.20 (m, 1H), 3.05 (q, *J* = 7.6 Hz, 2H), 1.35 (t, *J* = 7.5 Hz, 3H).

^{13}C NMR (126 MHz, DMSO-*d*$_6$) δ (ppm) 161.11 (d, *J* = 244.3 Hz), 150.08, 146.36, 142.74, 138.73, 133.76, 130.91 (d, *J* = 8.1 Hz), 127.73, 122.97, 121.93, 119.24, 118.34, 115.52 (d, *J* = 21.7 Hz), 111.54, 107.28, 20.89, 13.13.

HRMS (APCI): calcd. for $C_{12}H_8N_7$ [M-H]$^-$ = 250.0846, found [M-H]$^-$ = 250.0844.

3-(1*H*-benzo[*d*]imidazol-2-yl)-8-(4-fluorophenyl)pyrazolo[5,1-*c*][1,2,4]triazin-4-amine **(4)**

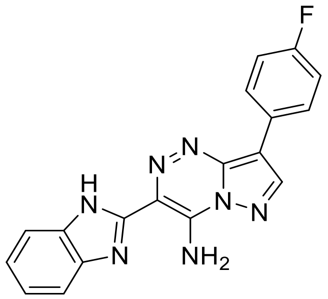

The compound was prepared according to General procedure C using:

Diazotization step:

4-(4-fluorophenyl)-1*H*-pyrazol-5-amine (**S39**, 222 mg, 1.25 mmol; 1 eq.), 35% aqueous HCl (0.44 mL, 5.01 mmol; 4 eq.) in EtOH (3 mL) and H_2O (3 mL), $NaNO_2$ (173 mg, 2.50 mmol, 2 eq.) in EtOH (2 mL) and H_2O (2 mL) (reaction time: 20 min), 2-(cyanomethyl)-benzimidazole (236 mg, 1.50 mmol, 1.2 eq.) in EtOH (3 mL) and H_2O (3 mL) and KOAc (738 mg, 7.52 mmol, 6 eq.) Reaction time: 2 h. The yellow solid (247 mg, 0.72 mmol) obtained after the filtration was used in the next step without further purification.

Cyclization step:

the dried yellow solid (238 mg, 0.69 mmol), DMF (3 mL), and KOAc (3 mg, 0.03 mmol, 0.05 eq.). Reaction time: 2 h. The product was obtained as a yellow solid (235 mg, 57% (2 steps)).

^{1}H NMR (500 MHz, DMSO-*d*$_6$) δ (ppm) 13.45 (s, 1H), 10.04 (s, 1H), 9.59 (s, 1H), 8.95 (s, 1H), 8.40 – 8.32 (m, 2H), 7.75 (s, 1H), 7.59 (s, 1H), 7.40 – 7.32 (m, 2H), 7.26 (s, 2H).

^{13}C NMR (126 MHz, DMSO-*d*$_6$) δ (ppm) 160.92 (d, *J* = 245.0 Hz), 149.92, 145.05, 143.63, 139.39, 127.92 (d, *J* = 7.4 Hz), 119.29, 115.66 (d, *J* = 21.7 Hz), 108.94.

^{19}F NMR (282 MHz, DMSO-*d*$_6$) δ (ppm) -115.55.

HRMS (APCI): calcd. for $C_{18}H_{11}FN_7$ [M-H]$^-$ = 344.1065, found [M-H]$^-$ = 344.1069.

3-(1*H*-benzo[*d*]imidazol-2-yl)-7-ethylpyrazolo[5,1-*c*][1,2,4]triazin-4-amine **(5)**

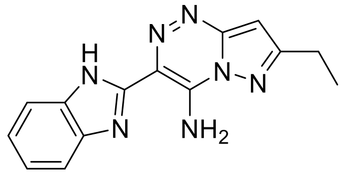

The compound was prepared according to General procedure C using:

Diazotization step:

3-ethyl-1*H*-pyrazol-5-amine (850 mg, 7.65 mmol; 1 eq.), 35% aqueous HCl (3.0 mL, 30.60 mmol; 4 eq.) in EtOH (20 mL) and H_2O (20 mL), $NaNO_2$ (1.10 g, 15.30 mmol, 2 eq.) in H_2O (3 mL) (reaction time: 30 min), 2-(cyanomethyl)-benzimidazole (1.50 g, 9.18 mmol, 1.2 eq.) in EtOH (3 mL) and KOAc (4.50 g, 45.90 mmol, 6 eq.) in H_2O (3 mL). Reaction time: 16 h. The yellow solid (1.90 g, 6.50 mmol) obtained after the filtration was used in the next step without further purification.

Cyclization step:

the dried yellow solid (1.9 g, 6.50 mmol), DMF (10 mL), and KOAc (32 mg, 0.30 mmol, 0.05 eq.). Reaction time: 2 h. The product was obtained as a yellow solid (1.25 g, 59%).

^{1}H NMR (500 MHz, DMSO-d_6) δ (ppm) 13.40 (s, 1H), 9.89 (s, 1H), 9.29 (s, 1H), 7.73 (d, J = 7.7 Hz, 1H), 7.56 – 7.51 (m, 1H), 7.29 – 7.19 (m, 2H), 6.88 (s, 1H), 2.90 (q, J = 7.6 Hz, 2H), 1.36 (t, J = 7.6 Hz, 3H).

^{13}C NMR (126 MHz, DMSO-d_6) δ (ppm) 161.59, 150.17, 149.62, 142.65, 138.70, 133.69, 122.78, 121.75, 118.34, 118.22, 111.39, 94.53, 21.75, 13.37.

HRMS (APCI): calcd. for $C_{14}H_{12}N_7$ [M-H]$^-$ = 278.1160, found [M-H]$^-$ = 278.1159.

3-(1*H*-benzo[*d*]imidazol-2-yl)-7-ethyl-8-(4-(trifluoromethyl)phenyl)pyrazolo[5,1-*c*][1,2,4]triazin-4-amine **(6)**

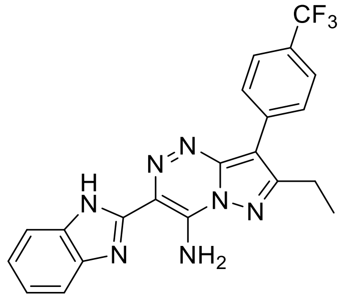

The compound was prepared according to General procedure C using:

Diazotization step:

3-ethyl-4-(4-(trifluoromethyl)phenyl)-1*H*-pyrazol-5-amine (**S72**, 40 mg, 0.16 mmol; 1 eq.), 35% aqueous HCl (0.06 mL, 0.64 mmol; 4 eq.) in EtOH (3 mL) and H_2O (3 mL), $NaNO_2$ (24 mg, 0.32 mmol, 2 eq.) in H_2O (1 mL) (reaction time: 30 min), 2-(cyanomethyl)-benzimidazole (30 mg, 0.19 mmol, 1.2

eq.) in EtOH (1 mL) and KOAc (95 mg, 0.96 mmol, 6 eq.) in H_2O (1 mL). Reaction time: 16 h. The yellow solid (66 mg, 0.15 mmol) obtained after the filtration was used in the next step without further purification.

Cyclization step:

the dried yellow solid (66 mg, 0.15 mmol), DMF (3 mL), and KOAc (5 mg, 0.05 mmol, 0.5 eq.). Reaction time: 2 h. After the reaction completion, the reaction mixture was poured into water (20 mL) and extracted with EtOAc (3 × 20 mL). The combined organic extracts were washed with brine (50 mL) and dried over $MgSO_4$. The solvent was evaporated and the residue was purified by preparative TLC (dichloromethane:MeOH, 9:1). The product was obtained as a yellow solid (20 mg, 30%).

^{1}H NMR (500 MHz, DMSO-d_6) δ (ppm) 13.40 (s, 1H), 10.00 (s, 1H), 9.43 (s, 1H), 8.11 (d, J = 8.0 Hz, 2H), 7.87 (d, J = 8.1 Hz, 2H), 7.75 (d, J = 7.7 Hz, 1H), 7.56 (d, J = 7.7 Hz, 1H), 7.30 – 7.20 (m, 2H), 3.10 (q, J = 7.5 Hz, 2H), 1.37 (t, J = 7.5 Hz, 3H).

^{13}C NMR (126 MHz, DMSO-d_6) δ (ppm) 158.33, 146.56, 142.66, 138.71, 135.82, 133.70, 129.11, 126.64 (q, J = 31.8 Hz), 125.36 (q, J = 3.8 Hz), 124.40 (q, J = 272.0 Hz), 122.96, 121.87, 119.85, 118.32, 111.48, 106.31, 21.10, 12.96.

^{19}F NMR (471 MHz, DMSO-d_6) δ (ppm) -60.81.

HRMS (APCI): calcd. for $C_{21}H_{17}F_3N_7$ $[M+H]^+$ = 424.1492, found $[M+H]^+$ = 424.1490.

<u>3-(1*H*-benzo[*d*]imidazol-2-yl)-7-ethyl-8-(4-methoxyphenyl)pyrazolo[5,1-*c*][1,2,4]triazin-4-amine</u> **(7)**

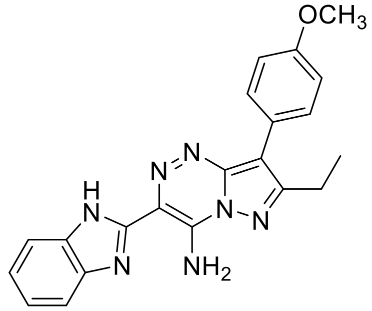

The compound was prepared according to General procedure C using:

Diazotization step:

3-ethyl-4-(4-methoxyphenyl)-1*H*-pyrazol-5-amine (**S73**, 55 mg, 0.25 mmol; 1 eq.), 35% aqueous HCl (0.1 mL, 1.0 mmol; 4 eq.) in EtOH (3 mL) and H_2O (3 mL), $NaNO_2$ (35 mg, 0.50 mmol, 2 eq.) in H_2O (1 mL) (reaction time: 30 min), 2-(cyanomethyl)-benzimidazole (47 mg, 0.30 mmol, 1.2 eq.) in EtOH (1 mL) and KOAc (147 mg, 1.50 mmol, 6 eq.) in H_2O (1 mL). Reaction time: 16 h. The yellow solid (100 mg, 0.24 mmol) obtained after the filtration was used in the next step without further purification.

Cyclization step:

the dried yellow solid (100 mg, 0.24 mmol), DMF (3 mL), and KOAc (2 mg, 0.02 mmol, 0.1 eq.). Reaction time: 2 h. The product was obtained as a yellow solid (45 mg, 46%).

^{1}H NMR (500 MHz, DMSO-d_6) δ (ppm) ^{1}H NMR (500 MHz, DMSO) δ 13.34 (s, 1H), 9.92 (s, 1H), 9.27 (s, 1H), 7.78 – 7.71 (m, 3H), 7.55 (dd, J = 8.0, 1.3 Hz, 1H), 7.31 – 7.19 (m, 2H), 7.13 – 7.06 (m, 2H), 3.83 (s, 3H), 3.03 (q, J = 7.7 Hz, 2H), 1.34 (t, J = 7.5 Hz, 3H).

^{13}C NMR (126 MHz, DMSO-d_6) δ (ppm) 158.14, 157.86, 150.15, 146.16, 142.71, 138.62, 133.71, 130.15, 123.46, 122.79, 121.78, 118.75, 118.21, 114.06, 111.43, 108.19, 55.11, 20.86, 13.17.

HRMS (APCI): calcd. for $C_{21}H_{20}N_7O$ $[M+H]^+$ = 386.1724, found $[M+H]^+$ = 386.1726.

3-(1*H*-benzo[*d*]imidazol-2-yl)-7-ethyl-8-(4-(methylsulfonyl)phenyl)pyrazolo[5,1-*c*][1,2,4]triazin-4-amine **(8)**

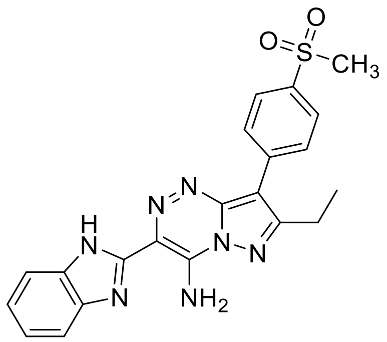

The compound was prepared according to General procedure C using:

Diazotization step:

3-ethyl-4-(4-(methylsulfonyl)phenyl)-1*H*-pyrazol-5-amine (**S77**, 53 mg, 0.20 mmol; 1 eq.), 35% aqueous HCl (75 µL, 0.80 mmol; 4 eq.) in EtOH (3 mL) and H_2O (3 mL), $NaNO_2$ (30 mg, 0.40 mmol, 2 eq.) in H_2O (1 mL) (reaction time: 30 min), 2-(cyanomethyl)-benzimidazole (37 mg, 0.22 mmol, 1.2 eq.) in EtOH (1 mL) and KOAc (125 mg, 1.20 mmol, 6 eq.) in H_2O (1 mL). Reaction time: 16 h. The yellow solid (72 mg, 0.16 mmol) obtained after the filtration was used in the next step without further purification.

Cyclization step:

the dried yellow solid (72 mg, 0.16 mmol), DMF (3 mL), and KOAc (5 mg, 0.06 mmol, 0.4 eq.). Reaction time: 2 h. The product was obtained as a yellow solid (56 mg, 65%).

^{1}H NMR (500 MHz, DMSO-d_6) δ (ppm) 13.41 (s, 1H), 10.03 (s, 1H), 9.48 (s, 1H), 8.24 – 8.19 (m, 2H), 7.91 – 7.86 (m, 2H), 7.77 (d, J = 7.7 Hz, 1H), 7.57 (d, J = 7.7 Hz, 1H), 7.32 – 7.22 (m, 2H), 3.67 (t, J = 4.8 Hz, 4H), 3.15 (q, J = 7.5 Hz, 2H), 2.97 (t, J = 4.7 Hz, 4H), 1.40 (t, J = 7.5 Hz, 3H).

^{13}C NMR (126 MHz, DMSO-d_6) δ (ppm) 158.99, 150.33, 147.17, 143.17, 139.27, 138.80, 137.39, 134.23, 129.55, 127.76, 123.54, 122.43, 120.59, 118.88, 112.03, 106.62, 44.15, 21.71, 13.46.

HRMS (ESI): calcd. for $C_{21}H_{18}N_7O_2S$ $[M-H]^-$= 432.1248, found $[M-H]^-$ = 432.1249.

3-(1*H*-benzo[*d*]imidazol-2-yl)-7-ethyl-8-(4-morpholinophenyl)pyrazolo[5,1-*c*][1,2,4]triazin-4-amine **(9)**

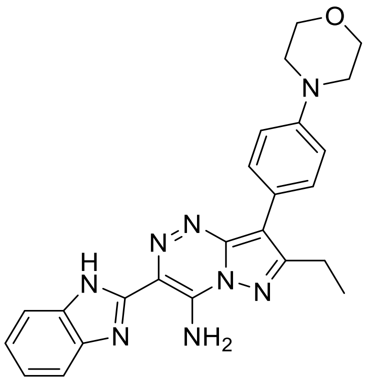

The compound was prepared according to General procedure C using:

Diazotization step:

3-ethyl-4-(4-morpholinophenyl)-1*H*-pyrazol-5-amine (**S81**, 143 mg, 0.53 mmol; 1 eq.), 35% aqueous HCl (0.185 mL, 2.1 mmol; 4 eq.) in EtOH (2 mL) and H_2O (2 mL), $NaNO_2$ (44 mg, 0.63 mmol, 1.2 eq.) in EtOH (1 mL) and H_2O (1 mL) (reaction time: 15 min), 2-(cyanomethyl)-benzimidazole (99 mg, 0.63 mmol, 1.2 eq.) in EtOH (3 mL) and H_2O (2 mL) and NaOAc (345 mg, 4.20 mmol, 8 eq.). Reaction time: 16 h. The mixture was poured into brine (20 mL) and extracted with EtOAc (2 × 50 mL). The combined organic extracts were dried over $MgSO_4$, filtered, and the solvent was evaporated *in vacuo*. The residue was obtained as a yellow solid (296 mg, quant.) and used in the next step without further purification.

Cyclization step:

the dried yellow solid (296 mg), DMF (2 mL), and NaOAc (4 mg, 0.05 mmol, 0.1 eq.). Reaction time: 2 h. The mixture was poured into brine (20 mL) and extracted with EtOAc (3 × 50 mL). The combined organic extracts were dried over $MgSO_4$, filtered, and the solvent was evaporated *in vacuo*. The residue was dissolved in boiling MeOH (25 mL) and the solution was allowed to cool to 25 °C. The resulting precipitate was collected by filtration and dried under vacuum. The product was obtained as an orange solid (47 mg, 20%).

^{1}H NMR (500 MHz, DMSO-*d*$_6$) δ (ppm) 13.34 (s, 1H), 9.90 (s, 1H), 9.25 (s, 1H), 7.76 – 7.72 (m, 1H), 7.70 (d, *J* = 8.8 Hz, 2H), 7.55 (d, *J* = 7.4 Hz, 1H), 7.25 (s, 2H), 7.09 (d, *J* = 8.9 Hz, 2H), 3.82 – 3.73 (m, 4H), 3.24 – 3.15 (m, 4H), 3.04 (q, *J* = 7.5 Hz, 2H), 1.35 (t, *J* = 7.6 Hz, 3H).

^{13}C NMR (126 MHz, DMSO-*d*$_6$) δ (ppm) 157.80, 150.20, 149.69, 146.13, 138.62, 133.72, 129.61, 122.78, 121.75, 118.59, 118.22, 114.94, 111.42, 108.52, 66.08, 48.19, 20.92, 13.20.

HRMS (APCI): calcd. for $C_{24}H_{23}N_8O$ [M-H]$^-$ = 439.2000, found [M-H]$^-$ = 439.2002.

3-(1*H*-benzo[d]imidazol-2-yl)-8-cyclohexyl-7-ethylpyrazolo[5,1-*c*][1,2,4]triazin-4-amine **(10)**

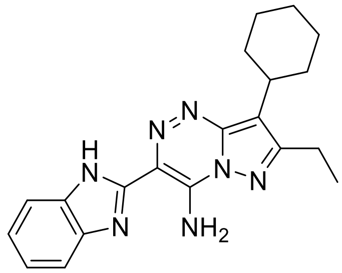

The compound was prepared according to General procedure C using:

Diazotization step:

4-cyclohexyl-3-ethyl-1*H*-pyrazol-5-amine (**S61**, 117 mg, 0.61 mmol; 1 eq.), 35% aqueous HCl (0.214 mL, 2.42 mmol; 4 eq.) in EtOH (1 mL) and H_2O (3 mL), $NaNO_2$ (84 mg, 1.21 mmol, 2 eq.) in EtOH (1 mL) and H_2O (1 mL) (reaction time: 20 min), 2-(cyanomethyl)-benzimidazole (0.1 g, 0.70 mmol, 1.05 eq.) in EtOH (2 mL) and H_2O (1 mL) and KOAc (475 mg, 4.84 mmol, 8 eq.). Reaction time: 16 h. The yellow solid (215 mg, 0.60 mmol) obtained after the filtration was used in the next step without further purification.

Cyclization step:

the dried yellow solid (215 mg, 0.60 mmol), DMF (3 mL), and KOAc (3 mg, 0.03 mmol, 0.05 eq.). Reaction time: 2 h. The product was obtained as a yellow solid (149 mg, 68% (2 steps)).

^{1}H NMR (500 MHz, DMSO-d_6) δ (ppm) 13.24 (s, 1H), 9.76 (s, 1H), 9.08 (s, 1H), 7.72 (d, J = 7.0 Hz, 1H), 7.55 (d, J = 7.0 Hz, 1H), 7.35 – 7.13 (m, 2H), 2.89 (q, J = 7.5 Hz, 3H), 2.20 – 2.07 (m, 2H), 1.90 – 1.72 (m, 5H), 1.50 – 1.36 (m, 3H), 1.33 (t, J = 7.6 Hz, 3H).

^{13}C NMR (126 MHz, DMSO-d_6) δ (ppm) 158.17, 150.43, 146.74, 142.77, 138.54, 133.68, 122.66, 121.70, 118.14, 117.32, 113.14, 111.35, 34.11, 32.65, 26.54, 25.71, 20.28, 14.03.

HRMS (APCI): calcd. for $C_{20}H_{24}N_7$ $[M+H]^+$ = 362.2088, found $[M+H]^+$ = 362.2091.

<u>3-(1*H*-benzo[*d*]imidazol-2-yl)-8-(4-fluorophenyl)-7-isopropylpyrazolo[5,1-*c*][1,2,4]triazin-4-amine</u> **(11)**

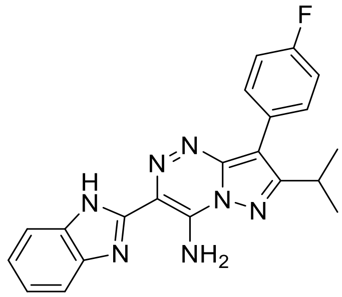

The compound was prepared according to General procedure C using:

Diazotization step:

4-(4-fluorophenyl)-3-isopropyl-1*H*-pyrazol-5-amine (**S46**, 60 mg, 0.27 mmol; 1 eq.), 35% aqueous HCl (0.1 mL, 1.1 mmol; 4 eq.) in EtOH (2 mL) and H_2O (2 mL), $NaNO_2$ (38 mg, 0.54 mmol, 2 eq.) in H_2O

(1 mL) (reaction time: 30 min), 2-(cyanomethyl)-benzimidazole (50 mg, 0.32 mmol, 1.2 eq.) in EtOH (1 mL) and KOAc (160 mg, 1.62 mmol, 6 eq.) in H_2O (1 mL). Reaction time: 16 h. The yellow solid (50 mg, 0.13 mmol) obtained after the filtration was used in the next step without further purification.
Cyclization step:
the dried yellow solid (40 mg, 0.1 mmol), DMF (1 mL), and KOAc (1 mg, 0.01 mmol, 0.1 eq.). Reaction time: 2 h. The product was obtained as a yellow solid (22 mg, 44%).
^{1}H NMR (500 MHz, DMSO-*d*$_6$) δ (ppm) 13.36 (s, 1H), 9.95 (s, 1H), 9.21 (s, 1H), 7.83 – 7.72 (m, 3H), 7.57 (d, *J* = 7.0 Hz, 1H), 7.42 – 7.35 (m, 2H), 7.31 – 7.22 (m, 2H), 3.46 (hept, *J* = 6.9 Hz, 1H), 1.41 (d, *J* = 6.9 Hz, 6H).
^{13}C NMR (126 MHz, DMSO-*d*$_6$) δ (ppm) 161.92, 161.18 (d, *J* = 244.1 Hz), 150.57, 146.34, 138.68, 133.72, 131.40 (d, *J* = 7.9 Hz), 127.64 (d, *J* = 3.6 Hz), 122.87, 121.84, 119.06, 118.26, 115.46 (d, *J* = 21.1 Hz), 111.46, 107.03, 26.32, 22.35.
HRMS (ESI): calcd. for $C_{21}H_{17}FN_7$ $[M-H]^-$ = 386.1535, found $[M-H]^-$ = 386.1541.

3-(1*H*-benzo[*d*]imidazol-2-yl)-7-(naphthalen-1-yl)pyrazolo[5,1-*c*][1,2,4]triazin-4-amine **(12)**

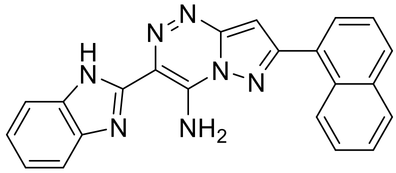

The compound was prepared according to General procedure C using:
Diazotization step:
3-(naphthalen-1-yl)-1*H*-pyrazol-5-amine (**S54**, 120 mg, 0.57 mmol; 1 eq.), 35% aqueous HCl (0.65 mL, 2.3 mmol; 4 eq.) in EtOH (3 mL) and H_2O (3 mL), $NaNO_2$ (80 mg, 1.14 mmol, 2 eq.) in H_2O (1 mL) (reaction time: 30 min), 2-(cyanomethyl)-benzimidazole (107 mg, 0.70 mmol, 1.2 eq.) in EtOH (1 mL) and KOAc (337 mg, 3.40 mmol, 6 eq.) in H_2O (1 mL). Reaction time: 16 h. The yellow solid (198 mg, 0.5 mmol) obtained after the filtration was used in the next step without further purification.
Cyclization step:
the dried yellow solid (198 mg, 0.5 mmol), DMF (3 mL), and KOAc (5 mg, 0.05 mmol, 0.1 eq.). Reaction time: 2 h. The product was obtained as a yellow solid (144 mg, 67%).
^{1}H NMR (500 MHz, DMSO-*d*$_6$) δ (ppm) 13.51 (s, 1H), 10.03 (s, 1H), 9.52 (s, 1H), 8.71 – 8.64 (m, 1H), 8.10 (d, *J* = 8.2 Hz, 1H), 8.08 – 8.04 (m, 1H), 7.97 (dd, *J* = 7.1, 1.1 Hz, 1H), 7.76 (s, 1H), 7.71 – 7.66 (m, 1H), 7.65 – 7.61 (m, 2H), 7.58 (s, 1H), 7.44 (s, 1H), 7.27 (s, 2H).
^{13}C NMR (126 MHz, DMSO-*d*$_6$) δ (ppm) 157.34, 150.61, 150.07, 143.22, 139.50, 133.96, 131.11, 130.30, 130.18, 128.94, 128.91, 127.53, 126.72, 126.36, 125.90, 123.42, 122.36, 119.50, 118.85, 112.01, 97.78.
HRMS (APCI): calcd. for $C_{22}H_{16}N_7$ $[M+H]^+$ = 378.1462, found $[M+H]^+$ = 378.1459.

3-(1*H*-benzo[*d*]imidazol-2-yl)-7-(2-phenoxyphenyl)pyrazolo[5,1-*c*][1,2,4]triazin-4-amine **(13)**

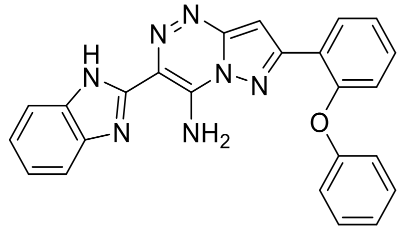

The compound was prepared according to General procedure C using:

Diazotization step:

3-(2-phenoxyphenyl)-1*H*-pyrazol-5-amine (**S42**, 213 mg, 0.85 mmol; 1 eq.), 35% aqueous HCl (0.30 mL, 3.39 mmol; 4 eq.) in EtOH (2 mL) and H_2O (2 mL), $NaNO_2$ (117 mg, 1.69 mmol, 2 eq.) in EtOH (1 mL) and H_2O (2 mL) (reaction time: 20 min), 2-(cyanomethyl)-benzimidazole (160 mg, 1.02 mmol, 1.2 eq.) in EtOH (3 mL) and H_2O (3 mL) and KOAc (666 mg, 6.78 mmol, 8 eq.). Reaction time: 2 h. The yellow solid (355 mg, 0.85 mmol) obtained after the filtration was used in the next step without further purification.

Cyclization step:

the dried yellow solid (345 mg, 0.82 mmol), DMF (6 mL), and KOAc (4 mg, 0.04 mmol, 0.05 eq.). Reaction time: 2 h. The product was obtained as a yellow solid (248 mg, 70%).

^{1}H NMR (500 MHz, DMSO-d_6) δ (ppm) 13.46 (s, 1H), 9.96 (s, 1H), 9.37 (s, 1H), 8.39 (d, J = 7.8 Hz, 1H), 7.75 (d, J = 7.5 Hz, 1H), 7.58 – 7.49 (m, 2H), 7.40 (t, J = 7.8 Hz, 3H), 7.29 (s, 1H), 7.25 (t, J = 8.1 Hz, 2H), 7.13 (d, J = 7.7 Hz, 2H), 7.06 (d, J = 7.9 Hz, 2H).

^{13}C NMR (126 MHz, DMSO-d_6) δ (ppm) 156.77, 153.82, 152.36, 149.99, 149.84, 142.69, 138.70, 133.74, 131.24, 130.12, 129.64, 124.41, 123.79, 123.20, 122.90, 121.83, 120.67, 118.93, 118.30, 117.53, 111.47, 96.63.

HRMS (APCI): calcd. for $C_{24}H_{18}N_7O$ $[M+H]^+$ = 420.1567, found $[M+H]^+$ = 420.1565.

3-(1*H*-benzo[*d*]imidazol-2-yl)-7-(naphthalen-2-yl)pyrazolo[5,1-*c*][1,2,4]triazin-4-amine **(14)**

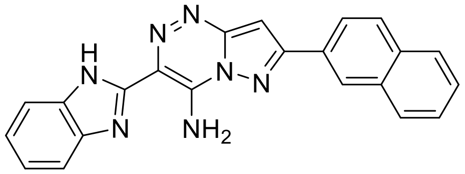

The compound was prepared according to General procedure C using:

Diazotization step:

3-(naphthalen-2-yl)-1*H*-pyrazol-5-amine (**S43**, 206 mg, 0.98 mmol; 1 eq.), 35% aqueous HCl (0.35 mL, 3.94 mmol; 4 eq.) in EtOH (2 mL) and H_2O (2 mL), $NaNO_2$ (136 mg, 1.97 mmol, 2 eq.) in EtOH (1 mL) and H_2O (2 mL) (reaction time: 20 min), 2-(cyanomethyl)-benzimidazole (186 mg, 1.18 mmol, 1.2 eq.) in EtOH (3 mL) and H_2O (3 mL) and KOAc (773 mg, 7.87 mmol, 8 eq.). Reaction time: 2 h. The yellow

solid (367 mg, 0.97 mmol) obtained after the filtration was used in the next step without further purification.

Cyclization step:

the dried yellow solid (367 mg, 0.97 mmol), DMF (6 mL), and KOAc (5 mg, 0.05 mmol, 0.05 eq.). Reaction time: 2 h. The product was obtained as an orange solid (195 mg, 52%).

^{1}H NMR (500 MHz, DMSO-*d*$_6$) δ (ppm) 13.47 (s, 1H), 9.99 (s, 1H), 9.40 (s, 1H), 8.75 (s, 1H), 8.33 (dd, *J* = 8.5, 1.8 Hz, 1H), 8.10 (d, *J* = 8.6 Hz, 1H), 8.05 (d, *J* = 7.1 Hz, 1H), 8.00 (d, *J* = 6.4 Hz, 1H), 7.77 (d, *J* = 7.6 Hz, 1H), 7.68 (s, 1H), 7.64 – 7.51 (m, 3H), 7.31 – 7.23 (m, 2H).

^{13}C NMR (126 MHz, DMSO-*d*$_6$) δ (ppm) 156.25, 150.36, 150.05, 142.72, 138.86, 133.76, 133.45, 132.95, 129.23, 128.48, 128.28, 127.76, 126.91, 126.81, 125.88, 124.21, 122.91, 121.85, 119.04, 118.31, 111.49, 93.72.

HRMS (APCI): calcd. for $C_{22}H_{16}N_7$ [M+H]$^+$ = 378.1462, found [M+H]$^+$ = 378.1449.

3-(1*H*-benzo[*d*]imidazol-2-yl)-7-phenylpyrazolo[5,1-*c*][1,2,4]triazin-4-amine **(15)**

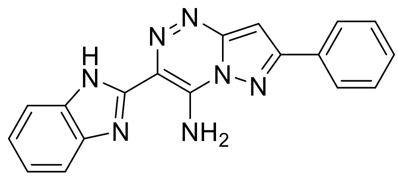

The compound was prepared according to General procedure C using:

Diazotization step:

3-phenyl-1*H*-pyrazol-5-amine (2 g, 12.50 mmol; 1 eq.), 35% aqueous HCl (4.3 mL, 50 mmol; 4 eq.) in EtOH (20 mL) and H_2O (20 mL), $NaNO_2$ (1.73 g, 25 mmol, 2 eq.) in H_2O (3 mL) (reaction time: 30 min), 2-(cyanomethyl)-benzimidazole (2.30 g, 15 mmol, 1.2 eq.) in EtOH (10 mL) and KOAc (7.4 g, 75 mmol, 6 eq.) in H_2O (8 mL). Reaction time: 16 h. The yellow solid (3.1 g, 9 mmol) obtained after the filtration was used in the next step without further purification.

Cyclization step:

the dried yellow solid (3.10 g, 9 mmol), DMF (10 mL), and KOAc (10 mg, 0.10 mmol, 0.01 eq.). Reaction time: 2 h. The product was obtained as a yellow solid (2.50 g, 60%).

^{1}H NMR (500 MHz, DMSO-*d*$_6$) δ (ppm) 13.46 (s, 1H), 9.95 (s, 1H), 9.34 (s, 1H), 8.19 (d, *J* = 7.4 Hz, 2H), 7.75 (s, 1H), 7.60 – 7.48 (m, 5H), 7.26 (s, 2H).

^{13}C NMR (126 MHz, DMSO-*d*$_6$) δ (ppm) 149.89, 149.70, 149.15, 142.67, 139.60, 138.88, 133.74, 129.73, 122.94, 121.87, 119.22, 118.70, 118.29, 111.51, 93.59.

HRMS (APCI): calcd. for $C_{18}H_{14}N_7$ [M+H]$^+$ = 328.1305, found [M+H]$^+$ = 328.1309.

3-(1*H*-benzo[*d*]imidazol-2-yl)-7-ethyl-8-(4-fluorophenyl)pyrazolo[5,1-*c*][1,2,4]triazine **(16)**

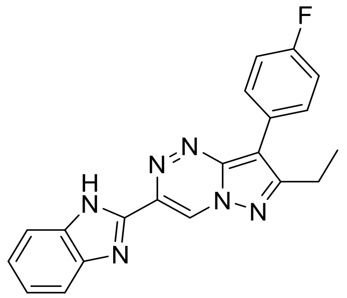

35% aqueous HCl (0.2 mL, 2.16 mmol; 4 eq.) was added to a solution of 3-ethyl-4-(4-fluorophenyl)-1*H*-pyrazol-5-amine (**S38**, 110 mg, 0.54 mmol; 1 eq.) in a mixture of EtOH (2 mL) and H_2O (2 mL). The solution was cooled to -10 °C and a pre-cooled solution (0 °C) of $NaNO_2$ (68 mg, 1.08 mmol, 2 eq.) in H_2O (0.5 mL) was added. The reaction mixture turned yellow and was stirred for 30 min at -5 °C, then a pre-cooled solution (-5 °C) of KOAc (300 mg, 3 mmol, 6 eq.) in H_2O (0.5 mL) and (*E*)-2-(2-(piperidin-1-yl)vinyl)-1*H*-benzo[*d*]imidazole (**S95**, 95 mg, 0.42 mmol, 0.85 eq.) in EtOH (0.5 mL) were added. The resulting mixture was allowed to warm up to 25 °C and stirred for 16 h. The mixture was poured into water (25 mL), the solid part was collected by filtration. The compound was purified by column chromatography on silica gel (hexane:EtOAc, 1:1) and then (dichloromethane:MeOH, gradient 9:1 to 5:1). The product was obtained as a red solid (90 mg, 50%).

^{1}H NMR (300 MHz, DMSO-d_6) δ (ppm) 13.54 (s, 1H), 9.71 (s, 1H), 7.95 – 7.82 (m, 2H), 7.70 – 7.64 (m, 2H), 7.49 – 7.35 (m, 2H), 7.33 – 7.21 (m, 2H), 3.09 (q, J = 7.5 Hz, 2H), 1.35 (t, J = 7.5 Hz, 3H).

^{13}C NMR (126 MHz, DMSO-d_6) δ (ppm) 162.62 (d, J = 248.2 Hz), 160.92, 148.11, 146.82, 135.63, 131.35 (d, J = 8.0 Hz), 126.19 (d, J = 3.6 Hz), 124.39, 123.37, 121.13, 120.09, 116.10 (d, J = 22.0 Hz), 111.70, 21.47, 13.45.

^{19}F NMR (282 MHz, DMSO) δ (ppm) -114.36.

HRMS (APCI): calcd. for $C_{20}H_{16}FN_6$ $[M+H]^+$= 359.1415, found $[M+H]^+$ = 359.1418.

3-(1*H*-benzo[*d*]imidazol-2-yl)-7-ethyl-8-(4-fluorophenyl)-4-methylpyrazolo[5,1-*c*][1,2,4]triazine **(17)**

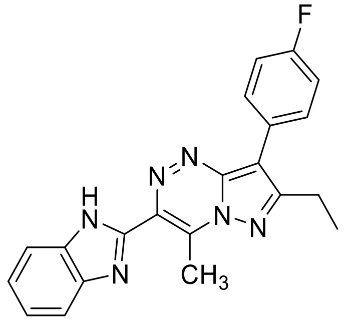

The compound was prepared according to General procedure C using:

Diazotization step:

3-ethyl-4-(4-fluorophenyl)-1*H*-pyrazol-5-amine (**S38**, 178 mg, 0.87 mmol; 1 eq.), 35% aqueous HCl (0.3 mL, 3.48 mmol; 4 eq.) in EtOH (5 mL) and H_2O (5 mL), $NaNO_2$ (120 mg, 1.74 mmol, 2 eq.) in EtOH (1 mL) (reaction time: 15 min), 1-(1*H*-benzo[*d*]imidazol-2-yl)propan-2-one (183 mg, 1.06 mmol,

1 eq.) in EtOH (2 mL) and KOAc (0.68 g, 6.96 mmol, 8 eq.). Reaction time: 16 h. The yellow solid (255 mg, 0.65 mmol) obtained after the filtration was used in the next step without further purification.

Cyclization step:

the dried yellow solid (255 mg), was dissolved in EtOH (5 mL) and CH_3SO_3H (20 µL) was added and the mixture was stirred under reflux for 4 h. The solvent was evaporated and the residue was purified by column flash chromatography on silica gel (hexane:EtOAc, gradient 1:0 to 3:2). The product was obtained as a yellow solid (151 mg, 47%).

^{1}H NMR (500 MHz, DMSO-d_6) δ (ppm) (s, 1H), 7.92 – 7.86 (m, 2H), 7.79 – 7.74 (m, 1H), 7.63 – 7.58 (m, 1H), 7.46 – 7.38 (m, 2H), 7.32 – 7.21 (m, 2H), 3.47 (s, 3H), 3.11 (q, J = 7.6 Hz, 2H), 1.36 (t, J = 7.5 Hz, 3H).

^{13}C NMR (126 MHz, DMSO-d_6) δ (ppm) 161.44 (d, J = 244.9 Hz), 158.49, 148.39, 146.35, 143.74, 135.81, 134.20, 134.11, 131.15 (d, J = 8.2 Hz), 126.82 (d, J = 3.2 Hz), 123.17, 121.90, 119.15, 115.69 (d, J = 21.5 Hz), 111.80, 109.44, 20.82, 13.53, 12.89.

^{19}F NMR (282 MHz, DMSO-d_6) δ (ppm) -114.59.

HRMS (APCI): calcd. for $C_{21}H_{18}FN_6$ $[M+H]^+$= 373.1571, found $[M+H]^+$ = 373.1570.

3-(1*H*-benzo[*d*]imidazol-2-yl)-7-ethyl-8-(4-fluorophenyl)pyrazolo[5,1-*c*][1,2,4]triazin-4-ol **(18)**

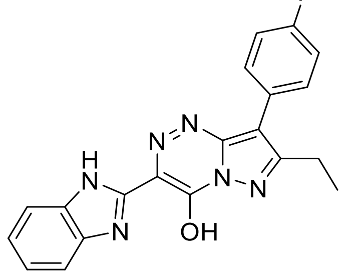

The compound was prepared according to General procedure C using:

Diazotization step:

3-ethyl-4-(4-fluorophenyl)-1*H*-pyrazol-5-amine (**S38**, 205 mg, 1.0 mmol; 1 eq.), 35% aqueous HCl (0.34 mL, 4.0 mmol; 4 eq.) in EtOH (5 mL) and H_2O (5 mL), $NaNO_2$ (136 mg, 2.0 mmol, 2 eq.) in H_2O (1 mL) (reaction time: 30 min), methyl 2-(1*H*-benzo[*d*]imidazol-2-yl)acetate (209 mg, 1.10 mmol, 1.1 eq.) in EtOH (5 mL) and KOAc (560 mg, 6.0 mmol, 6 eq.) in H_2O (5 mL). Reaction time: 16 h. The yellow solid (290 mg, 0.74 mmol) obtained after the filtration was used in the next step without further purification.

Cyclization step:

the dried yellow solid (290 mg, 0.74 mmol), DMF (5 mL), and KOAc (8 mg, 0.08 mmol, 0.1 eq.). Reaction time: 2 h. The compound was purified by reversed phase column chromatography using Biotage Selekt purification system (water:MeOH:7 M NH_3 in methanol, gradient 80:20:2 to 30:70:2). The product was obtained as a yellow solid (210 mg, 56%).

^{1}H NMR (500 MHz, DMSO-d_6) δ (ppm) 14.03 (s, 2H), 7.84 – 7.79 (m, 2H), 7.78 – 7.73 (m, 2H), 7.47 – 7.42 (m, 2H), 7.35 – 7.29 (m, 2H), 2.94 (q, J = 7.5 Hz, 2H), 1.28 (t, J = 7.5 Hz, 3H).

^{13}C NMR (126 MHz, DMSO-d_6) δ (ppm) 160.84 (d, J = 243.7 Hz), 155.65, 149.28, 148.89, 148.34, 131.07, 130.74 (d, J = 7.9 Hz), 128.37 (d, J = 3.2 Hz), 124.67, 119.14, 115.22 (d, J = 21.2 Hz), 113.32, 108.22, 20.77, 13.06.

^{19}F NMR (471 MHz, DMSO-d_6) δ (ppm) -114.60.

HRMS (APCI): calcd. for $C_{20}H_{14}FN_6O$ [M-H]$^-$ = 373.1219, found [M-H]$^-$ = 373.1217.

4-Amino-7-ethyl-8-(4-fluorophenyl)pyrazolo[5,1-*c*][1,2,4]triazine-3-carbonitrile **(19)**

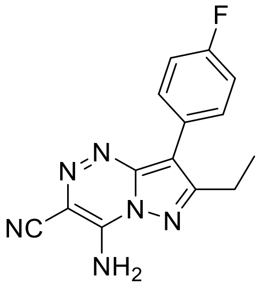

Diazotization step (only):

The compound was prepared according to General procedure C using 3-ethyl-4-(4-fluorophenyl)-1*H*-pyrazol-5-amine (**S38**, 207 mg, 1.01 mmol; 1 eq.), 35% aqueous HCl (0.344 mL, 3.90 mmol; 4 eq.) in EtOH (2 mL) and H_2O (2 mL), $NaNO_2$ (139 mg, 2.02 mmol, 2 eq.) in EtOH (2 mL) and H_2O (2 mL) (reaction time: 20 min), malononitrile (138 mg, 1.21 mmol, 1.2 eq.) in EtOH (2 mL) and H_2O (2 mL) and KOAc (765 mg, 7.80 mmol, 8 eq.). Reaction time: 16 h. The mixture was poured into H_2O (20 mL) and extracted with EtOAc (2 × 50 mL). The combined organic extracts were washed with brine (30 mL), dried over $MgSO_4$, filtered, and the solvent was evaporated *in vacuo*. The residue was purified using column chromatography (hexane:EtOAc, gradient 10:1 to 1:2). The product was obtained as a yellow solid (188 mg, 66%).

^{1}H NMR (500 MHz, DMSO-d_6) δ (ppm) 9.29 (s, 2H), 7.85 – 7.74 (m, 2H), 7.37 (t, J = 8.9 Hz, 2H), 3.02 (q, J = 7.6 Hz, 2H), 1.31 (t, J = 7.5 Hz, 3H).

^{13}C NMR (126 MHz, DMSO-d_6) δ (ppm) 161.34 (d, J = 244.4 Hz), 158.44, 145.48, 142.53, 131.07 (d, J = 8.2 Hz), 126.86 (d, J = 3.6 Hz), 115.86, 115.54 (d, J = 21.7 Hz), 109.56, 105.30, 20.68, 12.81.

HRMS (APCI): calcd. for $C_{14}H_{10}FN_6$ [M-H]$^-$ = 281.0956, found [M-H]$^-$ = 281.0956.

7-Ethyl-8-(4-fluorophenyl)-3-(pyrimidin-2-yl)pyrazolo[5,1-*c*][1,2,4]triazin-4-ol **(20)**

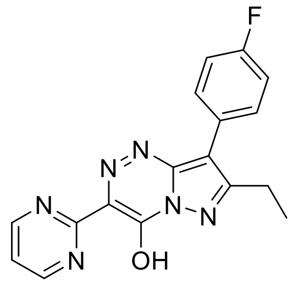

The compound was prepared according to General procedure C using:
Diazotization step:
3-ethyl-4-(4-fluorophenyl)-1*H*-pyrazol-5-amine (**S38**, 200 mg, 0.97 mmol; 1 eq.), 35% aqueous HCl (0.34 mL, 3.9 mmol; 4 eq.) in EtOH (3 mL) and H_2O (3 mL), $NaNO_2$ (134 mg, 1.95 mmol, 2 eq.) in H_2O (1 mL) (reaction time: 30 min), ethyl 2-(pyrimidin-2-yl)acetate (0.19 g, 1.17 mmol, 1 eq.) in EtOH (2 mL) and KOAc (574 mg, 5.85 mmol, 6 eq.). Reaction time: 2 h. The mixture was poured into water (20 mL) and extracted with EtOAc (2 × 50 mL). The yellow solid (194 mg, 0.55 mmol) obtained after the filtration was used in the next step without further purification.
Cyclization step:
the dried yellow solid (194 mg, 0.55 mmol), DMF (3 mL), and KOAc (5 mg, 0.05 mmol, 0.1 eq.). Reaction time: 2 h. After the filtration, the solid part was suspended in hot dioxane (10 mL) and the solution was poured into water (30 mL). The precipitate was collected by filtration, washed with water (10 mL), diethyl ether (10 mL) and dried under *vacuum*. The product was obtained as a yellow solid (50 mg, 15%).
^{1}H NMR (500 MHz, DMSO-d_6) δ (ppm) 14.24 (s, 1H), 8.97 (d, J = 4.9 Hz, 2H), 7.60 (t, J = 4.9 Hz, 1H), 7.57 – 7.51 (m, 2H), 7.39 – 7.31 (m, 2H), 2.78 (q, J = 7.5 Hz, 2H), 1.20 (t, J = 7.5 Hz, 3H).
^{13}C NMR (126 MHz, DMSO-d_6) δ (ppm) 161.56 (d, J = 237.5 Hz), 160.56, 157.50, 157.29, 147.80, 140.42, 134.94, 131.63 (d, J = 8.2 Hz), 125.87 (d, J = 3.0 Hz), 120.84, 115.71 (d, J = 21.6 Hz), 101.49, 20.11, 12.52.
HRMS (APCI): calcd. for $C_{17}H_{14}FN_6O$ $[M+H]^+$ = 337.1208, found $[M+H]^+$ = 337.1211.

<u>4-Amino-7-ethyl-8-(4-fluorophenyl)pyrazolo[5,1-*c*][1,2,4]triazine-3-carboxamide</u> **(21)**

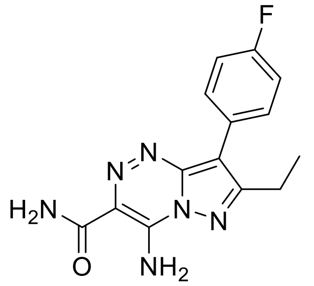

To a solution of 4-amino-7-ethyl-8-(4-fluorophenyl)pyrazolo[5,1-*c*][1,2,4]triazine-3-carbonitrile (**19**, 75 mg, 0.27 mmol, 1 eq.) in EtOH (1 mL) was added KOH (60 mg, 1.06 mmol, 4 eq.) and the mixture was refluxed for 24 h. The solvent was removed *in vacuo*, H_2O (10 mL) was added to the residue and the

mixture was extracted with EtOAc (3 × 10 mL). The combined organic extracts were washed with brine (10 mL), dried over $MgSO_4$, filtered and concentrated *in vacuo*. The residue was purified by column chromatography (hexane:EtOAc, gradient 10:1 to 1:2). The isolated product was further purified by reverse phase column chromatography (H_2O:CH_3CN:HCOOH, gradient 2:1:0.1% to 0:1:0.1%). The product was obtained as an orange solid (50 mg, 63%).

^{1}H NMR (500 MHz, DMSO-*d*$_6$) *δ* (ppm) 9.24 (s, 1H), 8.96 (s, 1H), 8.34 (s, 1H), 7.91 – 7.77 (m, 2H), 7.69 (s, 1H), 7.45 – 7.27 (m, 2H), 3.04 (q, *J* = 7.5 Hz, 2H), 1.33 (t, *J* = 7.5 Hz, 3H).

^{13}C NMR (126 MHz, DMSO-*d*$_6$) *δ* (ppm) 168.28, 161.10 (d, *J* = 244.0 Hz), 158.08, 146.58, 140.55, 130.84 (d, *J* = 8.2 Hz), 127.45, 119.28, 115.44 (d, *J* = 21.7 Hz), 107.89, 20.81, 12.88.

HRMS (APCI): calcd. for $C_{14}H_{14}FN_6O$ $[M+H]^+$ = 301.1208, found $[M+H]^+$ = 301.1209.

3-(1-Benzyl-1*H*-benzo[*d*]imidazol-2-yl)-7-ethyl-8-(4-fluorophenyl)pyrazolo[5,1-*c*][1,2,4]triazin-4-amine **(22)**

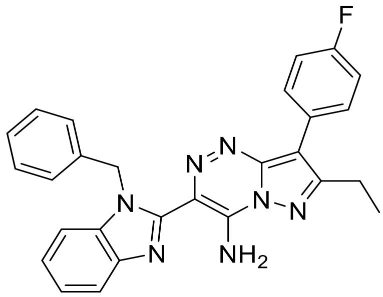

The compound was prepared according to General procedure C using:

Diazotization step:

3-ethyl-4-(4-fluorophenyl)-1*H*-pyrazol-5-amine (**S38**, 205 mg, 1.0 mmol; 1 eq.), 35% aqueous HCl (0.4 mL, 4.0 mmol; 4 eq.) in EtOH (2 mL) and H_2O (2 mL), $NaNO_2$ (140 mg, 2.0 mmol, 2 eq.) in H_2O (1 mL) (reaction time: 20 min), 2-(1-benzyl-1*H*-benzo[*d*]imidazol-2-yl)acetonitrile (**S99**, 247 mg, 1.0 mmol, 1 eq.) in EtOH (1 mL) and KOAc (0.58 g, 6.0 mmol, 6 eq.) in H_2O (1 mL). Reaction time: 16 h. The yellow solid (190 mg, 0.51 mmol) obtained after the filtration was used in the next step without further purification.

Cyclization step:

the dried yellow solid (19 mg, 0.51 mmol), DMF (3 mL), and KOAc (5 mg, 0.049 mmol, 0.1 eq.). Reaction time: 2 h. The product was poured into water (20 mL) and extracted with EtOAc (3 × 20 mL). The combined organic layers were washed with brine (30 mL), dried over $MgSO_4$, the solvent was evaporated, and the residue was purified by column flash chromatography on silica gel (hexane:EtOAc, 1:1). The product was obtained as a yellow solid (60 mg, 13% (2 steps)).

^{1}H NMR (500 MHz, DMSO-*d*$_6$) *δ* (ppm) 10.37 (s, 1H), 9.37 (s, 1H), 7.86 – 7.78 (m, 3H), 7.61 – 7.55 (m, 1H), 7.36 – 7.31 (m, 2H), 7.32 – 7.28 (m, 2H), 7.27 – 7.23 (m, 2H), 7.20 (d, *J* = 7.3 Hz, 3H), 6.30 (s, 2H), 3.05 (q, *J* = 7.5 Hz, 2H), 1.34 (t, *J* = 7.5 Hz, 3H).

^{13}C NMR (126 MHz, DMSO-d_6) δ (ppm) 161.01 (d, J = 244.3 Hz), 158.00, 148.02, 145.52, 141.07, 139.52, 137.57, 135.31, 130.81 (d, J = 8.1 Hz), 127.51 (d, J = 3.0 Hz), 127.12, 126.62, 122.95 (d, J = 67.8 Hz), 120.55, 118.64, 115.41 (d, J = 21.6 Hz), 110.78, 107.13, 48.88, 20.82, 13.09.

HRMS (APCI): calcd. for $C_{27}H_{23}N_7F$ $[M+H]^+$ = 464.1993, found $[M+H]^+$ = 464.1997.

<u>3-(1-(2-(Dimethylamino)ethyl)-1*H*-benzo[*d*]imidazol-2-yl)-7-ethyl-8-(4-fluorophenyl)pyrazolo[5,1-*c*][1,2,4]triazin-4-ol</u> **(23)**

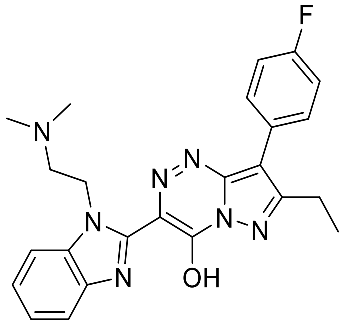

The compound was prepared according to General procedure C using:

Diazotization step:

3-ethyl-4-(4-fluorophenyl)-1*H*-pyrazol-5-amine (**S38**, 110 mg, 0.49 mmol; 1 eq.), 35% aqueous HCl (190 µL, 2 mmol; 4 eq.) in EtOH (3 mL) and H_2O (3 mL), $NaNO_2$ (68 mg, 1 mmol, 2 eq.) in EtOH (1 mL) (reaction time: 15 min), ethyl 2-(1-(2-(dimethylamino)ethyl)-1*H*-benzo[*d*]imidazol-2-yl)acetate (**S94**, 145 mg, 0.53 mmol, 1.2 eq.) in EtOH (2 mL) and KOAc (300 mg, 3.0 mmol, 6 eq.). Reaction time: 16 h. The yellow solid (185 mg, 0.40 mmol) obtained after the filtration was used in the next step without further purification.

Cyclization step:

the dried yellow solid (185 mg, 0.4 mmol), DMF (3 mL), and KOAc (4 mg, 0.04 mmol, 0.1 eq.). Reaction time: 2 h. After the filtration, the solid part was suspended in hot dioxane (5 mL) and the mixture was poured into water (20 mL). The precipitate was collected by filtration, washed with water (10 mL) and diethyl ether (10 mL), and dried under *vacuum*. The product was obtained as a yellow solid (105 mg, 48%).

^{1}H NMR (500 MHz, DMSO-d_6) δ (ppm) 8.09 – 8.05 (m, 1H), 7.97 – 7.92 (m, 1H), 7.79 – 7.73 (m, 2H), 7.59 – 7.50 (m, 2H), 7.42 – 7.27 (m, 2H), 5.28 (s, 2H), 3.63 (t, J = 7.0 Hz, 2H), 3.03 – 2.89 (m, 2H), 2.87 (s, 6H), 1.27 (t, J = 7.5 Hz, 3H).

^{13}C NMR (126 MHz, DMSO-d_6) δ (ppm) 130.95, 127.88, 124.86, 115.49, 115.32, 111.58, 54.42, 42.69, 20.65, 13.00.

^{19}F NMR (471 MHz, DMSO-d_6) δ (ppm) -115.61.

HRMS (APCI): calcd. for $C_{24}H_{25}FN_7O$ $[M+H]^+$= 446.2099, found $[M+H]^+$ = 446.2102.

7-Ethyl-8-(4-fluorophenyl)-3-(1-(2-morpholinoethyl)-1*H*-benzo[*d*]imidazol-2-yl)pyrazolo[5,1-*c*][1,2,4]triazin-4-ol **(24)**

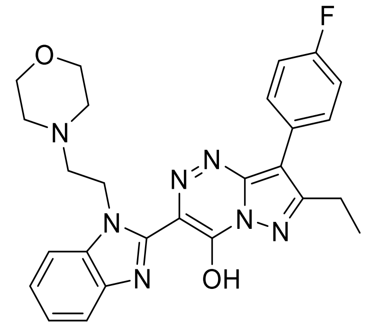

The compound was prepared according to General procedure C using:

Diazotization step:

3-Ethyl-4-(4-fluorophenyl)-1*H*-pyrazol-5-amine (**S38**, 100 mg, 0.49 mmol; 1 eq.), 35% aqueous HCl (190 µL, 2 mmol; 4 eq.) in EtOH (3 mL) and H_2O (3 mL), $NaNO_2$ (68 mg, 1 mmol, 2 eq.) in EtOH (1 mL) (reaction time: 15 min), ethyl 2-(1-(2-morpholinoethyl)-1*H*-benzo[*d*]imidazol-2-yl)acetate (**S92**, 170 mg, 0.53 mmol, 1.2 eq.) in EtOH (2 mL) and KOAc (300 mg, 3.0 mmol, 6 eq.). Reaction time: 16 h. The yellow solid (177 mg, 0.35 mmol) obtained after the filtration was used in the next step without further purification.

Cyclization step:

the dried yellow solid (177 mg, 0.35 mmol), DMF (3 mL), and KOAc (3 mg, 0.03 mmol, 0.1 eq.). Reaction time: 2 h. After the filtration, the solid part was suspended in hot dioxane (5 mL) and the mixture was poured into water (20 mL). The solid precipitate was collected by filtration, washed with water (10 mL), diethyl ether (10 mL) and dried under *vacuum*. The product was obtained as a yellow solid (153 mg, 65%).

^{1}H NMR (500 MHz, DMSO-d_6) δ (ppm) 7.94 – 7.86 (m, 2H), 7.78 (dd, J = 8.5, 5.5 Hz, 2H), 7.55 – 7.45 (m, 2H), 7.37 – 7.30 (m, 2H), 5.04 (s, 2H), 3.39 (t, J = 4.5 Hz, 4H), 2.93 (q, J = 7.5 Hz, 2H), 2.90 – 2.79 (m, 2H), 2.47 (s, 4H), 1.27 (t, J = 7.5 Hz, 3H).

^{13}C NMR (126 MHz, DMSO-d_6) δ (ppm) 161.43 (d, J = 243.6 Hz), 156.39, 149.84, 147.20, 133.53, 131.33 (d, J = 8.0 Hz), 128.63, 125.34, 125.08, 115.85 (d, J = 21.3 Hz), 115.57, 112.46, 66.36, 57.30, 53.85, 44.14, 21.28, 13.54.

^{19}F NMR (282 MHz, DMSO-d_6) δ (ppm) -115.78.

HRMS (APCI): calcd. for $C_{26}H_{27}FN_7O_2$ $[M+H]^+$= 488.2205, found $[M+H]^+$ = 488.2206.

7-Ethyl-8-(4-fluorophenyl)-3-(1-methyl-1*H*-imidazol-2-yl)pyrazolo[5,1-*c*][1,2,4]triazin-4-ol **(25)**

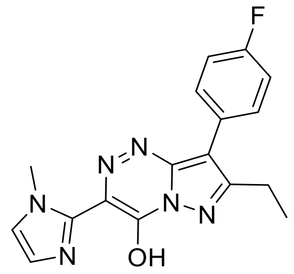

The compound was prepared according to General procedure C using:

Diazotization step:

3-ethyl-4-(4-fluorophenyl)-1*H*-pyrazol-5-amine (**S38**, 200 mg, 0.97 mmol; 1 eq.), 35% aqueous HCl (336 µL, 3.9 mmol; 4 eq.) in EtOH (10 mL) and H_2O (10 mL), $NaNO_2$ (135 mg, 1.95 mmol, 2 eq.) in EtOH (1 mL) (reaction time: 15 min), ethyl 2-(1-methyl-1*H*-imidazol-2-yl)acetate hydrochloride (238 mg, 1.17 mmol, 1.2 eq.) in EtOH (2 mL) and KOAc (570 mg, 4.14 mmol, 6 eq.). Reaction time: 16 h. The yellow solid (214 mg, 0.6 mmol) obtained after the filtration was used in the next step without further purification.

Cyclization step:

the dried yellow solid (214 mg, 0.6 mmol), DMF (3 mL), and KOAc (7 mg, 0.07 mmol, 0.1 eq.). Reaction time: 2 h. After the filtration, the solid part was suspended in hot dioxane (5 mL) and the mixture was poured into water (30 mL). The solid precipitate was collected by filtration, washed with water (10 mL), diethyl ether (10 mL) and dried under *vacuum*. The product was obtained as a yellow solid (125 mg, 38%).

^{1}H NMR (500 MHz, DMSO-d_6) δ (ppm) 13.51 (s, 1H), 7.83 – 7.76 (m, 2H), 7.61 (s, 1H), 7.48 (s, 1H), 7.31 – 7.24 (m, 2H), 4.11 (s, 3H), 2.92 (q, J = 7.5 Hz, 2H), 1.28 (t, J = 7.4 Hz, 3H).

^{13}C NMR (126 MHz, DMSO-d_6) δ (ppm) 160.45 (d, J = 242.9 Hz), 155.08, 148.63, 148.52, 141.68, 130.31 (d, J = 7.6 Hz), 128.49 (d, J = 3.9 Hz), 123.41, 121.25, 117.36, 114.78 (d, J = 21.1 Hz), 105.87, 37.11, 20.58, 12.68.

HRMS (APCI): calcd. for $C_{17}H_{16}FN_6O$ $[M+H]^+$ = 339.1364, found $[M+H]^+$ = 339.1362.

7-Ethyl-8-(4-fluorophenyl)-3-(4,5,6,7-tetrahydro-3*H*-imidazo[4,5-*c*]pyridin-2-yl)pyrazolo[5,1-*c*][1,2,4]triazin-4-ol **(26)**

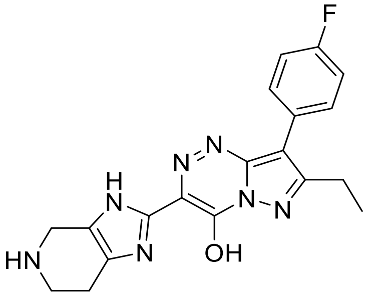

To a cold (0 °C) solution of *tert*-butyl 2-(7-ethyl-8-(4-fluorophenyl)-4-hydroxypyrazolo[5,1-*c*][1,2,4]triazin-3-yl)-3,4,6,7-tetrahydro-5*H*-imidazo[4,5-*c*]pyridine-5-carboxylate (**104**, 121 mg, 0.25

mmol, 1 eq.) in dichloromethane (2 mL) was added TFA (1 mL) dropwise. The mixture was stirred at 0 °C for 15 min, then at 25 °C for 2 h. The solvent was removed *in vacuo*, the residue was quenched with saturated aqueous solution of $NaHCO_3$ (10 mL), and the mixture was extracted with EtOAc (6 × 20 mL). The combined organic extracts were dried over $MgSO_4$, filtered, and the solvent was evaporated *in vacuo*. The residue was purified by reverse phase column chromatography using Biotage Selekt purification system (H_2O:MeOH: 7 M NH_3 in MeOH, gradient 4:1:0.1% to 0:1:0.1%). The product was obtained as an orange solid (41 mg, 43%).

^{1}H NMR (500 MHz, Methanol-*d*$_4$) δ (ppm) 7.69 – 7.61 (m, 2H), 7.22 – 7.14 (m, 2H), 3.92 – 3.80 (m, 2H), 3.13 – 3.04 (m, 2H), 2.93 (q, J = 7.6 Hz, 2H), 2.82 – 2.71 (m, 2H), 1.30 (t, J = 7.6 Hz, 3H).

^{13}C NMR (126 MHz, Methanol-*d*$_4$) δ (ppm) 162.96 (d, J = 244.3 Hz), 157.70, 150.89, 150.57, 144.82, 132.32 (d, J = 8.1 Hz), 130.28 (d, J = 3.4 Hz), 127.85, 116.14 (d, J = 21.7 Hz), 106.26, 44.30, 43.90, 24.23, 21.84, 14.21.

^{19}F NMR (471 MHz, Methanol-*d*$_4$) δ (ppm) -118.65.

HRMS (APCI): calcd. for $C_{19}H_{17}FN_7O$ $[M-H]^-$ = 378.1484, found $[M-H]^-$ = 378.1482.

7-Ethyl-8-(4-fluorophenyl)-3-(1*H*-imidazol-2-yl)pyrazolo[5,1-*c*][1,2,4]triazin-4-amine **(27)**

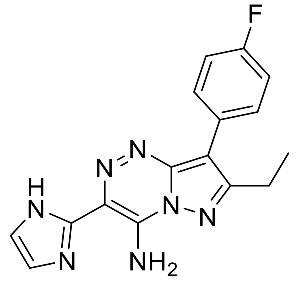

A mixture of 3-(1-benzyl-1*H*-imidazol-2-yl)-7-ethyl-8-(4-fluorophenyl)pyrazolo[5,1-*c*][1,2,4]triazin-4-amine (**72**, 50 mg, 0.12 mmol, 1eq.) and $Pd(OH)_2$/C (10%, 17 mg, 0.01mmol, 10%) in degassed EtOH (5 mL) was refluxed under hydrogen atmosphere (1 bar) for 3 h. The solvent was evaporated and the residue was purified by column chromatography on silica gel (EtOAc:MeOH, 9:1). The product was obtained as a yellow solid (22 mg, 57%).

^{1}H NMR (126 MHz, DMSO-*d*$_6$) δ (ppm) 13.09 (s, 1H), 9.77 (s, 1H), 9.04 (s, 1H), 7.91 – 7.79 (m, 2H), 7.38 – 7.32 (m, 2H), 7.30 (s, 1H), 7.19 (s, 1H), 3.04 (q, J = 7.6 Hz, 2H), 1.35 (t, J = 7.5 Hz, 3H).

^{13}C NMR (126 MHz, DMSO-*d*$_6$) δ (ppm) 160.86 (d, J = 243.8 Hz), 157.53, 146.29, 143.93, 137.13, 130.64 (d, J = 7.8 Hz), 127.97 (d, J = 2.69 Hz), 127.75, 120.31, 117.46, 115.35 (d, J = 21.4 Hz), 106.06, 20.83, 13.07.

^{19}F NMR (471 MHz, DMSO-*d*$_6$) δ (ppm) -115.77.

HRMS (APCI): calcd. for $C_{16}H_{15}FN_7$ $[M+H]^+$ = 324.1367, found $[M+H]^+$ = 324.1370.

6-(1*H*-benzo[*d*]imidazol-2-yl)-2-ethyl-3-(4-fluorophenyl)pyrazolo[1,5-*a*]pyrimidin-7-ol **(28)**

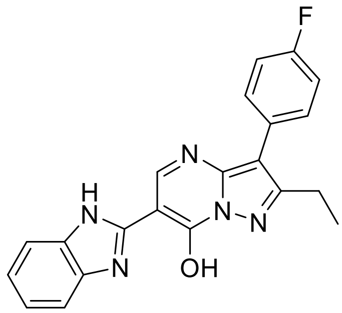

A mixture of 3-ethyl-4-(4-fluorophenyl)-1*H*-pyrazol-5-amine (**S38**, 75 mg, 0.37 mmol; 1 eq.) and ethyl-2-(1*H*-benzo[*d*]imidazol-2-yl)-3-(dimethylamino)acrylate (**S104**, 100 mg, 0.38 mmol, 1.05 eq.) in EtOH (3 mL) was stirred in the microwave reactor at 130 °C for 1 h. The precipitate was collected by filtration, washed with EtOH (5 mL), diethyl ether (5 mL) and dried under *vacuum*. The product was obtained as a white solid (15 mg, 11%)

^{1}H NMR (500 MHz, DMSO-d_6) δ (ppm) 13.29 (s, 2H), 8.69 (s, 1H), 7.75 – 7.70 (m, 2H), 7.69 – 7.61 (m, 2H), 7.39 – 7.33 (m, 2H), 7.31 – 7.22 (m, 2H), 2.87 (q, J = 7.6 Hz, 2H), 1.26 (t, J = 7.5 Hz, 3H).

^{13}C NMR (126 MHz, DMSO-d_6) δ (ppm) 161.23 (d, J = 243.6 Hz), 156.61, 155.59, 149.80, 131.13 (d, J = 7.9 Hz), 129.56, 124.16, 115.50 (d, J = 21.1 Hz), 113.56, 21.38, 13.48.

HRMS (APCI): calcd. for $C_{21}H_{17}FN_5O$ $[M+H]^+$= 374.1412, found $[M+H]^+$ = 374.1416.

3-(1*H*-benzo[*d*]imidazol-2-yl)-7-(3-bromophenyl)pyrazolo[5,1-*c*][1,2,4]triazin-4-amine **(56)**

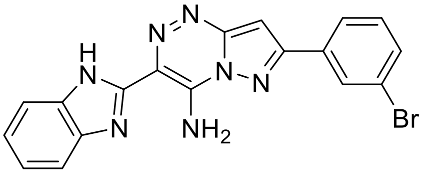

The compound was prepared according to General procedure C using:

Diazotization step:

3-(3-bromophenyl)-1*H*-pyrazol-5-amine (**S40**, 298 mg, 1.25 mmol; 1 eq.), 35% aqueous HCl (0.44 mL, 5.01 mmol; 4 eq.) in EtOH (2 mL) and H_2O (2 mL), $NaNO_2$ (173 mg, 2.5 mmol, 2 eq.) in EtOH (1 mL) and H_2O (2 mL) (reaction time: 20 min), 2-(cyanomethyl)-benzimidazole (236 mg, 1.5 mmol, 1.2 eq.) in EtOH (3 mL) and H_2O (3 mL) and KOAc (982 g, 10.01 mmol, 8 eq.). Reaction time: 2 h. The yellow solid (471 mg, 1.16 mmol) obtained after the filtration was used in the next step without further purification.

Cyclization step:

the dried yellow solid (465 mg, 1.14 mmol), DMF (6 mL), and KOAc (6 mg, 0.06 mmol, 0.05 eq.). Reaction time: 2 h. The product was obtained as a brown solid (336 mg, 66%).

^{1}H NMR (500 MHz, DMSO-d_6) δ (ppm) 13.47 (s, 1H), 9.97 (s, 1H), 9.42 (s, 1H), 8.42 (t, J = 1.9 Hz, 1H), 8.18 (d, J = 7.8 Hz, 1H), 7.76 (s, 1H), 7.69 (dd, J = 8.2, 2.0 Hz, 1H), 7.63 (s, 1H), 7.59 – 7.49 (m, 2H), 7.26 (d, J = 7.2 Hz, 2H).

^{13}C NMR (126 MHz, DMSO-d_6) δ (ppm) 154.59, 150.28, 149.95, 142.75, 138.86, 134.11, 133.75, 132.26, 131.11, 128.89, 125.66, 122.93, 122.35, 121.88, 119.17, 118.34, 111.50, 93.81.
HRMS (APCI): calcd. for $C_{18}H_{13}BrN_7$ $[M+H]^+ = 406.0410$, found $[M+H]^+ = 406.0412$.

3-(1*H*-benzo[*d*]imidazol-2-yl)-7-cyclohexylpyrazolo[5,1-*c*][1,2,4]triazin-4-amine **(57)**

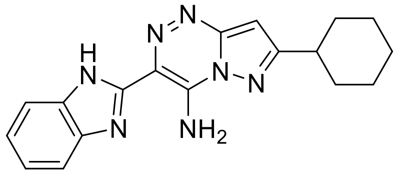

The compound was prepared according to General procedure C using:
Diazotization step:
3-cyclohexyl-1*H*-pyrazol-5-amine (**S41**, 316 mg, 1.91 mmol; 1 eq.), 35% aqueous HCl (0.675 mL, 7.65 mmol; 4 eq.) in EtOH (2 mL) and H_2O (2 mL), $NaNO_2$ (264 mg, 3.82 mmol, 2 eq.) in EtOH (2 mL) and H_2O (3 mL) (reaction time: 20 min), 2-(cyanomethyl)-benzimidazole (361 mg, 2.29 mmol, 1.2 eq.) in EtOH (4 mL) and H_2O (4 mL) and KOAc (1.501 g, 15.30 mmol, 8 eq.). Reaction time: 2 h. The yellow solid (606 mg, 1.82 mmol) obtained after the filtration was used in the next step without further purification.
Cyclization step:
the dried yellow solid (600 mg, 1.80 mmol), DMF (10 mL), and KOAc (9 mg, 0.09 mmol, 0.05 eq.). Reaction time: 2 h. The product was obtained as a brown solid (497 mg, 78%).
^1H NMR (500 MHz, DMSO-d_6) δ (ppm) 13.40 (s, 1H), 9.87 (s, 1H), 9.19 (s, 1H), 7.73 (d, J = 7.6 Hz, 1H), 7.53 (d, J = 7.6 Hz, 1H), 7.30 – 7.16 (m, 2H), 6.88 (s, 1H), 2.89 (tt, J = 11.6, 3.6 Hz, 1H), 2.06 (d, J = 10.6 Hz, 2H), 1.83 (dt, J = 13.1, 3.5 Hz, 2H), 1.76 – 1.70 (m, 1H), 1.60 (qd, J = 12.5, 3.4 Hz, 2H), 1.43 (qt, J = 12.6, 3.4 Hz, 2H), 1.35 – 1.24 (m, 1H).
^{13}C NMR (126 MHz, DMSO-d_6) δ (ppm) 164.86, 150.21, 149.50, 142.68, 138.75, 133.71, 122.79, 121.76, 118.30, 118.22, 111.40, 93.48, 37.70, 32.42, 25.69, 25.54.
HRMS (APCI): calcd. for $C_{18}H_{20}N_7$ $[M+H]^+ = 334.1775$, found $[M+H]^+ = 334.1765$.

3-(1*H*-benzo[*d*]imidazol-2-yl)-7-isopropylpyrazolo[5,1-*c*][1,2,4]triazin-4-amine **(58)**

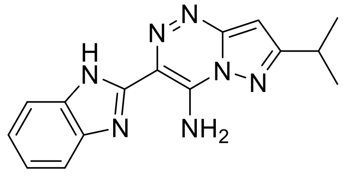

The compound was prepared according to General procedure C using:
Diazotization step:
3-isopropyl-1*H*-pyrazol-5-amine (**S44**, 200 mg, 1.60 mmol; 1 eq.), 35% aqueous HCl (0.6 mL, 6.4 mmol; 4 eq.) in EtOH (6 mL) and H_2O (6 mL), $NaNO_2$ (220 mg, 3.20 mmol, 2 eq.) in H_2O (2 mL)

(reaction time: 30 min), 2-(cyanomethyl)-benzimidazole (300 mg, 1.92 mmol, 1.2 eq.) in EtOH (6 mL) and KOAc (942 mg, 9.6 mmol, 6 eq.). Reaction time: 16 h. The yellow solid (280 mg, 1.34 mmol) obtained after the filtration was used in the next step without further purification.

Cyclization step:

the dried yellow solid (280 mg, 1.34 mmol), DMF (5 mL), and KOAc (7 mg, 0.07 mmol, 0.05 eq.). Reaction time: 2 h. The product was obtained as an orange solid (22 mg, 22%).

^{1}H NMR (500 MHz, DMSO-d_6) δ (ppm) 13.40 (s, 1H), 9.88 (s, 1H), 9.22 (s, 1H), 7.73 (d, J = 7.6 Hz, 1H), 7.53 (d, J = 7.2 Hz, 1H), 7.29 – 7.19 (m, 2H), 6.91 (s, 1H), 3.22 (hept, J = 6.9 Hz, 1H), 1.39 (d, J = 6.9 Hz, 6H).

^{13}C NMR (126 MHz, DMSO-d_6) δ (ppm) 165.93, 150.19, 149.56, 142.66, 138.75, 133.69, 122.79, 121.76, 118.31, 118.22, 111.39, 93.24, 28.16, 22.41.

HRMS (APCI): calcd. for $C_{15}H_{16}N_7$ $[M+H]^+$ = 294.1462, found $[M+H]^+$ = 294.1450.

3-(1*H*-benzo[*d*]imidazol-2-yl)-7-(*tert*-butyl)pyrazolo[5,1-*c*][1,2,4]triazin-4-amine **(59)**

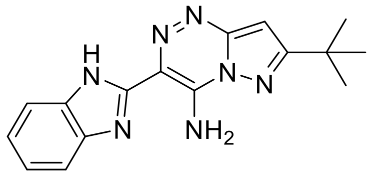

The compound was prepared according to General procedure C using:

Diazotization step:

3-(*tert*-butyl)-1*H*-pyrazol-5-amine (**S45**, 222 mg, 1.60 mmol; 1 eq.), 35% aqueous HCl (0.6 mL, 6.4 mmol; 4 eq.) in EtOH (6 mL) and H_2O (6 mL), $NaNO_2$ (220 mg, 3.2 mmol, 2 eq.) in H_2O (2 mL) (reaction time: 30 min), 2-(cyanomethyl)-benzimidazole (300 mg, 1.92 mmol, 1.2 eq.) in EtOH (6 mL) and KOAc (942 mg, 9.60 mmol, 6 eq.). Reaction time: 16 h. The yellow solid (425 mg, 1.38 mmol) obtained after the filtration was used in the next step without further purification.

Cyclization step:

the dried yellow solid (210 mg, 0.7 mmol), DMF (5 mL), and KOAc (7 mg, 0.07 mmol, 0.05 eq.). Reaction time: 2 h. The product was obtained as a yellow solid (106 mg, 22%).

^{1}H NMR (500 MHz, DMSO-d_6) δ (ppm) 13.39 (s, 1H), 9.85 (s, 1H), 9.05 (s, 1H), 7.74 (d, J = 7.6 Hz, 1H), 7.53 (d, J = 7.2 Hz, 1H), 7.29 – 7.19 (m, 2H), 6.95 (s, 1H), 1.45 (s, 9H).

^{13}C NMR (126 MHz, DMSO-d_6) δ (ppm) 168.63, 150.21, 149.53, 142.67, 138.71, 133.70, 122.77, 121.76, 118.22, 118.20, 111.39, 92.98, 32.72, 30.11.

HRMS (ESI): calcd. for $C_{16}H_{16}N_7$ $[M-H]^-$ = 306.1473, found $[M-H]^-$ = 306.1478.

(4-Amino-3-(1*H*-benzo[*d*]imidazol-2-yl)pyrazolo[5,1-*c*][1,2,4]triazin-7-yl)methanol **(60)**

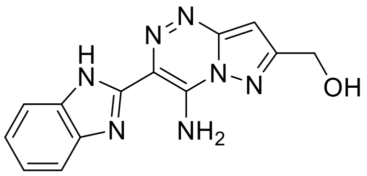

To solution of 3-(1*H*-benzo[*d*]imidazol-2-yl)-7-(((*tert*-butyldiphenylsilyl)oxy)methyl)pyrazolo[5,1-*c*][1,2,4]triazin-4-amine (**S108**, 130 mg, 0.25 mmol; 1 eq.) in THF (5 mL) was added TBAF (1M in THF, 0.5 mL, 0.5 mmol) and the mixture was stirred at 23 °C for 2 h. The solvent was evaporated and the residue was purified by column chromatography on silica gel (dichloromethane:MeOH:7M NH_3 in MeOH, gradient 5:3:0 to 10:0:1). The product was obtained as an orange solid (52 mg, 57%).

^{1}H NMR (500 MHz, DMSO-d_6) δ (ppm) 13.43 (s, 1H), 9.93 (s, 1H), 9.42 (s, 1H), 7.73 (d, J = 7.2 Hz, 1H), 7.57 – 7.51 (m, 2H), 7.30 – 7.18 (m, 2H), 6.94 (s, 1H), 5.54 (t, J = 5.8 Hz, 1H), 4.77 (d, J = 5.8 Hz, 2H).

^{13}C NMR (126 MHz, DMSO-d_6) δ (ppm) 160.83, 150.09, 149.51, 142.64, 138.93, 133.69, 122.83, 121.79, 118.48, 118.25, 111.43, 94.54, 57.68.

HRMS (APCI): calcd. for $C_{13}H_{12}N_7O$ [M+H]$^+$ = 282.1098, found [M+H]$^+$ = 282.1102

2-(4-Amino-3-(1*H*-benzo[d]imidazol-2-yl)pyrazolo[5,1-*c*][1,2,4]triazin-7-yl)phenol **(61)**

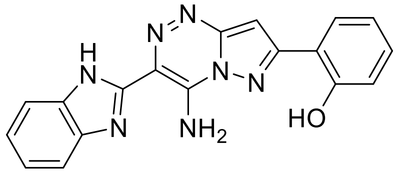

The compound was prepared according to General procedure C using:

Diazotization step:

2-(3-Amino-1*H*-pyrazol-5-yl)phenol (117 mg, 0.67 mmol; 1 eq. CAS: 10523-64-5), 35% aqueous HCl (0.2 mL, 2.0 mmol; 4 eq.) in EtOH (3 mL) and H_2O (3 mL), $NaNO_2$ (100 mg, 1.34 mmol, 2 eq.) in H_2O (1 mL) (reaction time: 30 min), 2-(cyanomethyl)-benzimidazole (130 mg, 0.80 mmol, 1.2 eq.) in EtOH (1 mL) and KOAc (400 mg, 4.02 mmol, 6 eq.) in H_2O (1 mL). Reaction time: 16 h. The yellow solid (126 mg, 0.36 mmol) obtained after the filtration was used in the next step without further purification.

Cyclization step:

the dried yellow solid (126 mg, 0.36 mmol), DMF (3 mL), and KOAc (4 mg, 0.04 mmol, 0.1 eq.). Reaction time: 2 h. The product was obtained as a yellow solid (33 mg, 14%).

^{1}H NMR (500 MHz, DMSO-d_6) δ (ppm) 13.46 (s, 1H), 10.31 (s, 1H), 9.98 (s, 1H), 9.64 (s, 1H), 8.13 (dd, J = 7.8, 1.6 Hz, 1H), 7.76 (d, J = 7.5 Hz, 1H), 7.57 (s, 1H), 7.56 (d, J = 6.6 Hz, 1H), 7.37 – 7.32 (m, 1H), 7.30 – 7.22 (m, 2H), 7.08 – 7.04 (m, 1H), 7.03 – 6.98 (m, 1H).

^{13}C NMR (126 MHz, DMSO-d_6) δ (ppm) 155.90, 155.04, 150.01, 149.40, 142.71, 138.63, 133.75, 130.89, 128.61, 122.89, 121.82, 119.39, 119.12, 118.31, 117.12, 116.59, 111.46, 94.71.

HRMS (APCI): calcd. for $C_{18}H_{14}N_7O$ [M+H]$^+$ = 344.1254, found [M+H]$^+$ = 344.1256.

3-(1*H*-benzo[*d*]imidazol-2-yl)-7-(3-methoxynaphthalen-2-yl)pyrazolo[5,1-*c*][1,2,4]triazin-4-amine **(62)**

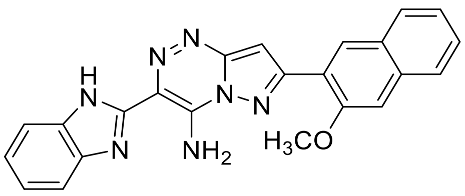

The compound was prepared according to General procedure C using:

Diazotization step:

3-(3-methoxynaphthalen-2-yl)-1*H*-pyrazol-5-amine (**S48**, 90 mg, 0.38 mmol; 1 eq.), 35% aqueous HCl (0.15 mL, 1.52 mmol; 4 eq.) in EtOH (3 mL) and H_2O (3 mL), $NaNO_2$ (60 mg, 0.76 mmol, 2 eq.) in H_2O (1 mL) (reaction time: 30 min), 2-(cyanomethyl)-benzimidazole (73 mg, 0.46 mmol, 1.2 eq.) in EtOH (1 mL) and KOAc (223 mg, 2.28 mmol, 6 eq.) in H_2O (1 mL). Reaction time: 16 h. The yellow solid (103 mg, 0.25 mmol) obtained after the filtration was used in the next step without further purification.

Cyclization step:

the dried yellow solid (103 mg, 0.25 mmol), DMF (3 mL), and KOAc (2 mg, 0.02 mmol, 0.1 eq.). Reaction time: 2 h. The product was obtained as a yellow solid (40 mg, 25%).

^{1}H NMR (500 MHz, DMSO-d_6) δ (ppm) 13.48 (s, 1H), 9.97 (s, 1H), 9.43 (s, 1H), 8.77 (s, 1H), 7.93 (dd, J = 20.4, 8.1 Hz, 2H), 7.76 (s, 1H), 7.66 – 7.49 (m, 4H), 7.44 (t, J = 7.1 Hz, 1H), 7.27 (s, 2H), 4.09 (s, 3H).

^{13}C NMR (126 MHz, DMSO-d_6) δ (ppm) 155.20, 153.18, 150.10, 149.79, 138.68, 134.60, 128.83, 127.95, 127.85, 127.26, 126.53, 124.34, 121.86, 118.79, 106.65, 97.86, 55.75.

HRMS (APCI): calcd. for $C_{23}H_{18}N_7O$ $[M+H]^+$= 408.1567, found $[M+H]^+$ = 408.1566.

7-([1,1'-Biphenyl]-3-yl)-3-(1*H*-benzo[*d*]imidazol-2-yl)pyrazolo[5,1-*c*][1,2,4]triazin-4-amine **(63)**

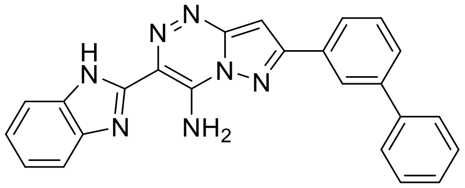

The compound was prepared according to General procedure C using:

Diazotization step:

3-([1,1'-Biphenyl]-3-yl)-1*H*-pyrazol-5-amine (**S49**, 320 mg, 1.50 mmol; 1 eq.), 35% aqueous HCl (0.6 mL, 6.0 mmol; 4 eq.) in EtOH (3 mL) and H_2O (3 mL), $NaNO_2$ (228 mg, 3.0 mmol, 2 eq.) in H_2O (1 mL) (reaction time: 30 min), 2-(cyanomethyl)-benzimidazole (130 mg, 1.8 mmol, 1.2 eq.) in EtOH (1 mL) and KOAc (880 mg, 9.0 mmol, 6 eq.) in H_2O (1 mL). Reaction time: 16 h. The yellow solid (600 mg, 1.5 mmol) obtained after the filtration was used in the next step without further purification.

Cyclization step:

the dried yellow solid (600 mg, 1.50 mmol), DMF (3 mL), and KOAc (15 mg, 0.15 mmol, 0.1 eq.). Reaction time: 2 h. The product was obtained as a yellow solid (327 mg, 54%).

^{1}H NMR (500 MHz, DMSO-d_6) δ (ppm) 13.43 (s, 1H), 9.97 (s, 1H), 9.41 (s, 1H), 8.07 – 8.00 (m, 1H), 7.74 (s, 1H), 7.62 – 7.51 (m, 3H), 7.50 – 7.43 (m, 1H), 7.43 – 7.35 (m, 3H), 7.34 – 7.26 (m, 2H), 7.24 (d, J = 6.5 Hz, 2H), 6.11 (s, 1H).

^{13}C NMR (126 MHz, DMSO-d_6) δ (ppm) 156.68, 149.95, 148.99, 142.61, 141.26, 140.64, 138.74, 134.11, 133.78, 130.63, 130.54, 130.00, 129.33, 129.20, 128.32, 127.56, 127.39, 122.86, 121.78, 118.73, 118.28, 111.43, 109.46, 96.72.

HRMS (APCI): calcd. for $C_{24}H_{18}N_7$ $[M+H]^+$ = 404.1618, found $[M+H]^+$ = 404.1615.

<u>3-(1*H*-benzo[*d*]imidazol-2-yl)-7-(1-methoxynaphthalen-2-yl)pyrazolo[5,1-*c*][1,2,4]triazin-4-amine</u>
(64)

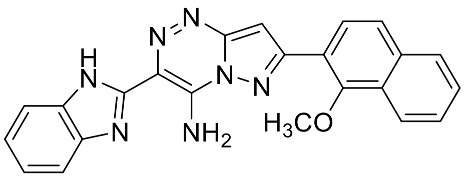

The compound was prepared according to General procedure C using:

Diazotization step:

3-(1-methoxynaphthalen-2-yl)-1*H*-pyrazol-5-amine (**S50**, 180 mg, 0.75 mmol; 1 eq.), 35% aqueous HCl (0.3 mL, 3.0 mmol; 4 eq.) in EtOH (3 mL) and H_2O (3 mL), $NaNO_2$ (103 mg, 1.50 mmol, 2 eq.) in H_2O (1 mL) (reaction time: 30 min), 2-(cyanomethyl)-benzimidazole (138 mg, 0.90 mmol, 1.2 eq.) in EtOH (1 mL) and KOAc (450 mg, 4.5 mmol, 6 eq.) in H_2O (1 mL). Reaction time: 16 h. The yellow solid (200 mg, 0.47 mmol) obtained after the filtration was used in the next step without further purification.

Cyclization step:

the dried yellow solid (200 mg, 0.47 mmol), DMF (3 mL), and KOAc (5 mg, 0.05 mmol, 0.1 eq.). Reaction time: 2 h. The product was obtained as a yellow solid (150 mg, 49%).

^{1}H NMR (500 MHz, DMSO-d_6) δ (ppm) 13.50 (s, 1H), 9.99 (s, J = 46.1 Hz, 1H), 9.42 (s, 1H), 8.43 (d, J = 8.6 Hz, 1H), 8.24 (d, J = 8.0 Hz, 1H), 8.02 (d, J = 7.6 Hz, 1H), 7.89 (t, J = 9.7 Hz, 1H), 7.76 (s, 1H), 7.70 – 7.62 (m, 2H), 7.61 (s, 1H), 7.57 (s, 1H), 7.26 (s, 2H), 3.96 (s, 3H).

^{13}C NMR (126 MHz, DMSO-d_6) δ (ppm) 154.78, 153.01, 150.22, 150.05, 142.71, 138.73, 135.01, 133.76, 128.05, 127.91, 127.29, 126.74, 125.97, 125.75, 124.12, 122.87, 122.53, 122.33, 121.84, 120.30, 118.90, 118.30, 111.47, 96.59, 66.30, 61.79.

HRMS (APCI): calcd. for $C_{23}H_{18}N_7O$ $[M+H]^+$= 408.1567, found $[M+H]^+$ = 408.1567.

<u>3-(1*H*-benzo[*d*]imidazol-2-yl)-7-(3-methoxynaphthalen-1-yl)pyrazolo[5,1-*c*][1,2,4]triazin-4-amine</u>
(65)

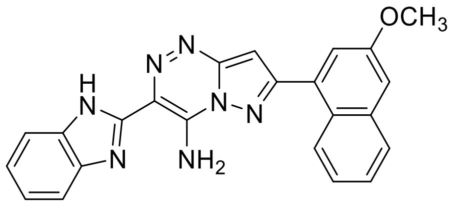

The compound was prepared according to General procedure C using:

Diazotization step:

3-(3-methoxynaphthalen-1-yl)-1*H*-pyrazol-5-amine (**S51**, 180 mg, 0.75 mmol; 1 eq.), 35% aqueous HCl (0.3 mL, 3.0 mmol; 4 eq.) in EtOH (3 mL) and H_2O (3 mL), $NaNO_2$ (103 mg, 1.50 mmol, 2 eq.) in H_2O (1 mL) (reaction time: 30 min), 2-(cyanomethyl)-benzimidazole (138 mg, 0.90 mmol, 1.2 eq.) in EtOH (1 mL) and KOAc (450 mg, 4.50 mmol, 6 eq.) in H_2O (1 mL). Reaction time: 16 h. The yellow solid (300 mg, 0.71 mmol) obtained after the filtration was used in the next step without further purification.

Cyclization step:

the dried yellow solid (300 mg, 0.71 mmol), DMF (3 mL), and KOAc (7 mg, 0.07 mmol, 0.1 eq.). Reaction time: 2 h. The product was obtained as a yellow solid (30 mg, 10%).

^{1}H NMR (500 MHz, DMSO-d_6) δ (ppm) 13.50 (s, 1H), 9.99 (s, 1H), 9.51 (s, 1H), 8.14 (d, J = 9.1 Hz, 1H), 7.99 – 7.93 (m, 1H), 7.76 (d, J = 6.5 Hz, 1H), 7.68 – 7.63 (m, 1H), 7.62 (d, J = 9.2 Hz, 1H), 7.57 (d, J = 6.4 Hz, 1H), 7.46 – 7.37 (m, 1H), 7.31 – 7.22 (m, 1H), 7.17 (s, 1H), 3.89 (s, 1H).

^{13}C NMR (126 MHz, DMSO-d_6) δ (ppm) 155.12, 153.41, 150.19, 149.50, 138.95, 133.02, 131.04, 128.20, 127.88, 126.97, 124.71, 123.68, 122.88, 121.81, 118.57, 118.29, 117.95, 115.33, 113.71, 111.45, 99.05, 56.40.

HRMS (APCI): calcd. for $C_{23}H_{18}N_7O$ $[M+H]^+$ = 408.1567, found $[M+H]^+$ = 408.1568.

<u>3-(1*H*-benzo[*d*]imidazol-2-yl)-7-(1-benzyl-1*H*-imidazol-2-yl)pyrazolo[5,1-*c*][1,2,4]triazin-4-amine</u> **(66)**

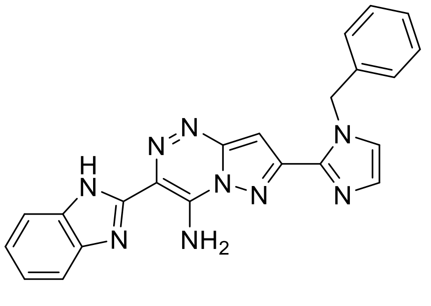

The compound was prepared according to General procedure C using:

Diazotization step:

3-(1-benzyl-1*H*-imidazol-2-yl)-1*H*-pyrazol-5-amine (**S52**, 70 mg, 0.30 mmol; 1 eq.), 35% aqueous HCl (0.1 mL, 1.2 mmol; 4 eq.) in EtOH (4 mL) and H_2O (4 mL), $NaNO_2$ (45 mg, 0.60 mmol, 2 eq.) in H_2O (1 mL) (reaction time: 30 min), 2-(cyanomethyl)-benzimidazole (60 mg, 0.36 mmol, 1.2 eq.) in EtOH

(1 mL) and KOAc (180 mg, 1.8 mmol, 6 eq.) in H_2O (1 mL). Reaction time: 16 h. The yellow solid (106 mg, 0.25 mmol) obtained after the filtration was used in the next step without further purification.

Cyclization step:

the dried yellow solid (106 mg, 0.25 mmol), DMF (3 mL), and KOAc (10 mg, 0.10 mmol, 0.1 eq.). Reaction time: 2 h. The product was obtained as a yellow solid (80 mg, 66%).

^{1}H NMR (500 MHz, DMSO-d_6) δ (ppm) 13.47 (s, 1H), 9.99 (s, 1H), 9.44 (s, 1H), 7.75 (d, J = 7.7 Hz, 1H), 7.55 (d, J = 7.4 Hz, 1H), 7.48 (s, 1H), 7.30 – 7.18 (m, 8H), 7.16 (s, 1H), 6.04 (s, 2H).

^{13}C NMR (126 MHz, DMSO-d_6) δ (ppm) 159.89, 159.49, 159.04, 152.70, 148.85, 148.81, 148.15, 143.78, 139.22, 138.47, 137.53, 137.40, 134.07, 132.93, 131.85, 129.25, 128.34, 121.54, 105.09, 60.16.

3-(1*H*-benzo[*d*]imidazol-2-yl)-7-(1*H*-imidazol-2-yl)pyrazolo[5,1-*c*][1,2,4]triazin-4-amine **(67)**

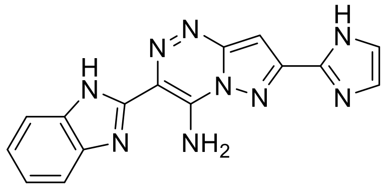

A mixture of 3-(1*H*-benzo[*d*]imidazol-2-yl)-7-(1-benzyl-1*H*-imidazol-2-yl)pyrazolo[5,1-*c*][1,2,4]triazin-4-amine (**66**, 40 mg, 0.10 mmol, 1eq.) and $Pd(OH)_2/C$ (10%, 14 mg, 0.01 mmol) in degassed EtOH (5 mL) was refluxed under a hydrogen atmosphere (1 bar) for 3 h. The solvent was evaporated, and the residue was purified by column chromatography on silica gel (EtOAc:MeOH, 9:1). The product was obtained as a yellow solid (22 mg, 57%).

^{1}H NMR (126 MHz, DMSO-d_6) δ (ppm) 13.48 (s, 1H), 12.88 (s, 1H), 10.00 (s, 1H), 9.02 (s, 1H), 7.95 (s, 1H), 7.75 (d, J = 7.7 Hz, 1H), 7.56 (d, J = 7.6 Hz, 1H), 7.38 (s, 1H), 7.33 (s, 1H), 7.26 (p, J = 6.9 Hz, 2H), 7.16 (s, 1H).

^{13}C NMR (126 MHz, DMSO-d_6) δ (ppm) 150.42, 150.23, 149.68, 143.19, 140.13, 139.41, 134.27, 130.26, 123.47, 122.40, 119.75, 119.22, 118.82, 112.04, 94.12.

HRMS (APCI): calcd. for $C_{15}H_{12}N_9$ $[M+H]^+$ = 318.1210, found $[M+H]^+$ = 318.1213.

3-(1*H*-benzo[*d*]imidazol-2-yl)-7-(2-chlorophenyl)pyrazolo[5,1-*c*][1,2,4]triazin-4-amine **(68)**

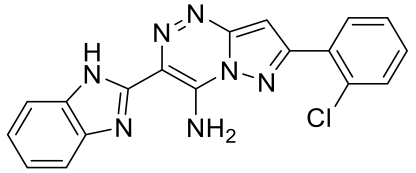

The compound was prepared according to General procedure C using:

Diazotization step:

3-(2-chlorophenyl)-1*H*-pyrazol-5-amine (**S53**, 350 mg, 1.81 mmol; 1 eq.), 35% aqueous HCl (0.6 mL, 7.24 mmol; 4 eq.) in EtOH (10 mL) and H_2O (10 mL), $NaNO_2$ (250 mg, 3.62 mmol, 2 eq.) in H_2O (1

mL) (reaction time: 30 min), 2-(cyanomethyl)-benzimidazole (350 mg, 3.60 mmol, 1.2 eq.) in EtOH (1 mL) and KOAc (1.1 g, 10.90 mmol, 6 eq.) in H_2O (1 mL). Reaction time: 16 h. The yellow solid (540 mg, 1.42 mmol) obtained after the filtration was used in the next step without further purification.

Cyclization step:

the dried yellow solid (540 mg, 1.42 mmol), DMF (5 mL), and KOAc (13 mg, 0.14 mmol, 0.1 eq.). Reaction time: 2 h. The product was obtained as a yellow solid (391 mg, 60%).

^{1}H NMR (500 MHz, DMSO-d_6) δ (ppm) 13.50 (s, 1H), 10.01 (s, 1H), 9.50 (s, 1H), 8.07 – 7.99 (m, 1H), 7.76 (s, 1H), 7.72 – 7.64 (m, 1H), 7.59 – 7.51 (m, 3H), 7.46 (s, 1H), 7.31 – 7.22 (m, 2H).

^{13}C NMR (126 MHz, DMSO-d_6) δ (ppm) 159.89, 159.49, 159.04, 152.70, 148.85, 148.81, 148.15, 143.78, 139.22, 138.47, 137.53, 137.40, 134.07, 132.93, 131.85, 129.25, 128.34, 121.54, 105.09, 60.16.

HRMS (APCI): calcd. for $C_{18}H_{13}ClN_7$ $[M+H]^+ = 362.0915$, found $[M+H]^+ = 362.0916$.

3-(1*H*-benzo[*d*]imidazol-2-yl)-7-(2-methoxyphenyl)pyrazolo[5,1-*c*][1,2,4]triazin-4-amine **(69)**

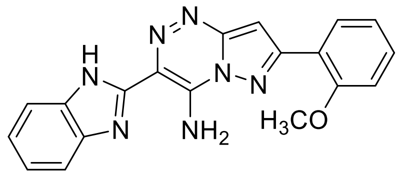

The compound was prepared according to General procedure C using:

Diazotization step:

3-(2-methoxyphenyl)-1*H*-pyrazol-5-amine (350 mg, 1.85 mmol; 1 eq. CAS: 909861-26-3), 35% aqueous HCl (0.65 mL, 7.4 mmol; 4 eq.) in EtOH (10 mL) and H_2O (10 mL), $NaNO_2$ (250 mg, 3.70 mmol, 2 eq.) in H_2O (1 mL) (reaction time: 30 min), 2-(cyanomethyl)-benzimidazole (350 mg, 3.70 mmol, 1.2 eq.) in EtOH (1 mL) and KOAc (1.1 g, 10.90 mmol, 6 eq.) in H_2O (1 mL). Reaction time: 16 h. The yellow solid (550 mg, 1.5 mmol) obtained after filtration was used in the next step without further purification.

Cyclization step:

the dried, filtered yellow solid (550 mg, 1.50 mmol), DMF (5 mL), and KOAc (13 mg, 0.15 mmol, 0.1 eq.). Reaction time: 2 h. The product was obtained as a yellow solid (336 mg, 51%).

^{1}H NMR (500 MHz, DMSO-d_6) δ (ppm) 13.45 (s, 1H), 9.92 (s, 1H), 9.32 (s, 1H), 8.28 (dd, J = 7.7, 1.8 Hz, 1H), 7.76 (d, J = 7.6 Hz, 1H), 7.55 (d, J = 7.4 Hz, 1H), 7.52 – 7.46 (m, 2H), 7.30 – 7.21 (m, 3H), 7.14 (td, J = 7.6, 0.9 Hz, 1H), 3.99 (s, 3H).

^{13}C NMR (126 MHz, DMSO-d_6) δ (ppm) 157.45, 153.29, 150.13, 149.76, 142.73, 138.62, 133.75, 130.99, 128.88, 122.87, 121.83, 120.49, 120.04, 118.66, 118.30, 112.21, 111.46, 97.29, 55.65.

HRMS (APCI): calcd. for $C_{19}H_{16}N_7O$ $[M+H]^+ = 358.1411$, found $[M+H]^+ = 358.1412$.

3-(1-benzyl-1*H*-benzo[*d*]imidazol-2-yl)-7-ethylpyrazolo[5,1-*c*][1,2,4]triazin-4-amine **(70)**

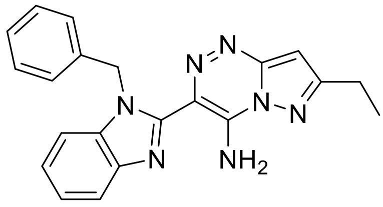

The compound was prepared according to General procedure C using:

Diazotization step:

3-ethyl-1*H*-pyrazol-5-amine (222 mg, 2.0 mmol; 1 eq.), 35% aqueous HCl (0.8 mL, 8.0 mmol; 4 eq.) in EtOH (5 mL) and H_2O (5 mL), $NaNO_2$ (140 mg, 2.0 mmol, 2 eq.) in H_2O (1 mL) (reaction time: 20 min), 2-(1-benzyl-1*H*-benzo[*d*]imidazol-2-yl)acetonitrile (**S99**, 590 mg, 2.40 mmol, 1.2 eq.) in EtOH (2 mL) and KOAc (1.17 g, 12 mmol, 6 eq.) in H_2O (2 mL). Reaction time: 16 h. The yellow solid (660 mg, 1.70 mmol) obtained after the filtration was used in the next step without further purification.

Cyclization step:

the dried yellow solid (660 mg, 1.70 mmol), DMF (5 mL), and KOAc (20 mg, 0.20 mmol, 0.1 eq.). Reaction time: 2 h. The reaction mixture was poured into water (20 mL) and extracted with EtOAc (3 × 20 mL). The combined organic layers were washed with brine (30 mL), dried over $MgSO_4$, and the solvent was evaporated. The product was purified by column flash chromatography on silica gel (EtOAc:MeOH, 10:1). The product was obtained as a yellow solid (300 mg, 40%).

^{1}H NMR (500 MHz, DMSO-d_6) δ (ppm) 10.26 (s, 1H), 9.29 (s, 1H), 7.84 – 7.76 (m, 1H), 7.67 – 7.56 (m, 1H), 7.33 – 7.28 (m, 2H), 7.27 – 7.23 (m, 2H), 7.22 – 7.17 (m, 3H), 6.82 (s, 1H), 6.27 (s, 2H), 2.89 (q, J = 7.6 Hz, 2H), 1.35 (t, J = 7.6 Hz, 3H).

^{13}C NMR (126 MHz, DMSO-d_6) δ (ppm) 161.67, 148.80, 148.15, 141.04, 139.53, 137.65, 135.36, 128.43, 127.12, 126.69, 123.16, 122.62, 119.65, 118.62, 110.75, 94.41, 48.87, 21.72, 13.37.

HRMS (APCI): calcd. for $C_{21}H_{20}N_7$ $[M+H]^+$ = 370.1775, found $[M+H]^+$ = 370.1776.

<u>8-([1,1'-Biphenyl]-3-yl)-3-(1*H*-benzo[*d*]imidazol-2-yl)-7-ethylpyrazolo[5,1-*c*][1,2,4]triazin-4-amine</u> **(71)**

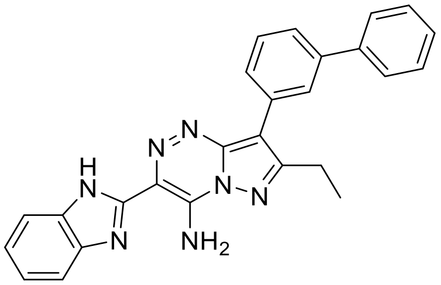

The compound was prepared according to General procedure C using:

Diazotization step:

4-([1,1'-biphenyl]-3-yl)-3-ethyl-1*H*-pyrazol-5-amine (**S71**, 138 mg, 0.52 mmol; 1 eq.), 35% aqueous HCl (0.093 mL, 1.05 mmol; 2 eq.) in EtOH (1 mL) and H_2O (1 mL), $NaNO_2$ (38 mg, 0.55 mmol, 1.05

eq.) in H_2O (1 mL) (reaction time: 30 min), 2-(cyanomethyl)-benzimidazole (87 mg, 0.55 mmol, 1.05 eq.) in EtOH (2 mL) and H_2O (0.5 mL) and NaOAc (172 mg, 2.1 mmol, 4 eq.). Reaction time: 16 h. The reaction mixture was poured into brine (20 mL) and extracted with EtOAc (2 × 50 mL). The combined organic extracts were dried over $MgSO_4$, filtered, and the solvent was evaporated *in vacuo*. The obtained yellow solid (126 mg, 56 %) was used in the next step without further purification.

Cyclization step:

the dried yellow solid (126 mg), DMF (2 mL) and KOAc (1.40 mg, 0.05 eq.). Reaction time: 2 h. The reaction mixture was poured into brine (20 mL) and extracted with EtOAc (3 × 50 mL). The combined organic extracts were dried over $MgSO_4$, filtered and concentrated *in vacuo*. The residue was purified by column chromatography on silica gel (dichloromethane:MeOH, gradient 1:0 to 10:1). The obtained solid was triturated by EtOH (2 mL), collected by filtration and dried in a vacuum. The product was obtained as a yellow solid (98 mg, 43%).

^{1}H NMR (500 MHz, DMSO-d_6) δ (ppm) 13.43 (s, 1H), 9.97 (s, 1H), 9.37 (s, 1H), 8.23 (t, J = 1.8 Hz, 1H), 7.81 (d, J = 7.5 Hz, 1H), 7.75 (dt, J = 8.0, 2.3 Hz, 3H), 7.69 – 7.65 (m, 1H), 7.63 (t, J = 7.6 Hz, 1H), 7.57 – 7.50 (m, 3H), 7.41 (t, J = 7.4 Hz, 1H), 7.30 – 7.22 (m, 2H), 3.14 (q, J = 7.5 Hz, 2H), 1.40 (t, J = 7.6 Hz, 3H).

^{13}C NMR (126 MHz, DMSO-d_6) δ (ppm) 158.16, 150.07, 146.51, 142.72, 140.44, 140.19, 138.71, 133.74, 131.98, 129.23, 129.02, 127.60, 127.55, 127.39, 126.69, 124.99, 122.90, 121.85, 119.26, 118.30, 111.46, 107.95, 21.10, 13.12.

HRMS (APCI): calcd. for $C_{26}H_{22}N_7$ $[M+H]^+$ = 432.1931, found $[M+H]^+$ = 432.1929.

3-(1-Benzyl-1*H*-imidazol-2-yl)-7-ethyl-8-(4-fluorophenyl)pyrazolo[5,1-*c*][1,2,4]triazin-4-amine **(72)**

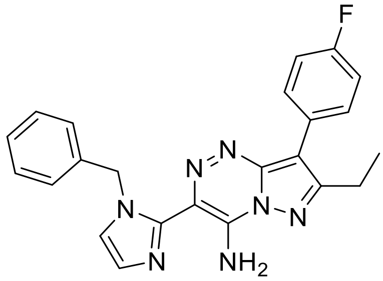

The compound was prepared according to General procedure C using:

Diazotization step:

3-Ethyl-4-(4-fluorophenyl)-1*H*-pyrazol-5-amine (**S38**, 150 mg, 0.73 mmol; 1 eq.), 35% aqueous HCl (0.26 mL, 2.9 mmol; 4 eq.) in EtOH (3 mL) and H_2O (3 mL), $NaNO_2$ (100 mg, 1.5 mmol, 2 eq.) in H_2O (1 mL) (reaction time: 30 min), 2-(1-benzyl-1*H*-imidazol-2-yl)acetonitrile (170 mg, 0.87 mmol, 1.2 eq.) in EtOH (1 mL) and KOAc (430 mg, 4.4 mmol, 6 eq.) in H_2O (1 mL). Reaction time: 16 h. The yellow solid (150 mg, 0.35 mmol) obtained after the filtration was used in the next step without further purification.

Cyclization step:

the dried yellow solid (150 mg, 0.35 mmol), DMF (3 mL), and KOAc (4 mg, 0.04 mmol, 0.1 eq.). Reaction time: 2 h. The product was obtained as a yellow solid (110 mg, 36%).

^{1}H NMR (500 MHz, DMSO-d_6) δ (ppm) 10.10 (s, 1H), 9.02 (s, 1H), 7.89 – 7.75 (m, 2H), 7.48 (d, J = 1.1 Hz, 1H), 7.36 – 7.30 (m, 2H), 7.28 (t, J = 7.3 Hz, 2H), 7.23 (d, J = 1.1 Hz, 1H), 7.20 (t, J = 6.5 Hz, 3H), 5.97 (s, 2H), 3.02 (q, J = 7.5 Hz, 2H), 1.32 (t, J = 7.5 Hz, 3H).

^{13}C NMR (126 MHz, DMSO-d_6) δ (ppm) 160.87 (d, J = 244.1 Hz), 157.60, 145.61, 141.66, 138.20, 138.02, 130.66 (d, J = 8.1 Hz), 128.42, 127.80 (d, J = 3.4 Hz), 127.20, 126.94, 126.78, 123.39, 121.39, 115.35 (d, J = 21.0 Hz), 106.05, 51.17, 20.80, 13.06.

^{19}F NMR (471 MHz, DMSO-d_6) δ (ppm) -115.72.

HRMS (APCI): calcd. for $C_{23}H_{21}FN_7$ $[M+H]^+$ = 414.1837, found $[M+H]^+$ = 414.1837

3-(1*H*-benzo[*d*]imidazol-2-yl)-7-ethyl-8-(3-(trifluoromethyl)phenyl)pyrazolo[5,1-*c*][1,2,4]triazin-4-amine **(73)**

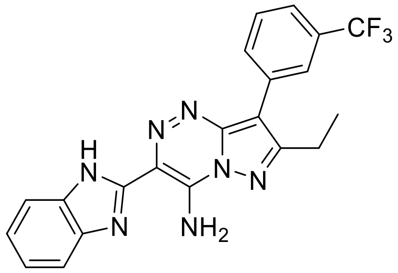

The compound was prepared according to General procedure C using:

Diazotization step:

3-ethyl-4-(3-(trifluoromethyl)phenyl)-1*H*-pyrazol-5-amine (**S74**, 40 mg, 0.16 mmol; 1 eq.), 35% aqueous HCl (0.65 mL, 0.64 mmol; 4 eq.) in EtOH (3 mL) and H_2O (3 mL), $NaNO_2$ (22 mg, 0.32 mmol, 2 eq.) in H_2O (1 mL) (reaction time: 30 min), 2-(cyanomethyl)-benzimidazole (30 mg, 0.19 mmol, 1.2 eq.) in EtOH (1 mL) and KOAc (94 mg, 0.96 mmol, 6 eq.) in H_2O (1 mL). Reaction time: 16 h. The yellow solid (66 mg, 0.15 mmol) obtained after the filtration was used in the next step without further purification.

Cyclization step:

the dried yellow solid (66 mg, 0.15 mmol), DMF (3 mL), and KOAc (5 mg, 0.06 mmol, 0.4 eq.). Reaction time: 2 h. The reaction mixture was poured into water (20 mL) and extracted with EtOAc (3 × 20 mL). The combined organic layers were washed with brine (30 mL), dried over $MgSO_4$, the solvent was evaporated, and the residue was purified by column flash chromatography on silica gel (EtOAc to dichloromethane:MeOH, gradient 1:0:0 to 0:10:1). The product was obtained as a yellow solid (30 mg, 45% (2 steps)).

^{1}H NMR (500 MHz, DMSO-d_6) δ (ppm) 13.47 (s, 1H), 9.99 (s, 1H), 9.46 (s, 1H), 8.39 (s, 1H), 8.12 (d, J = 7.7 Hz, 1H), 7.81 – 7.70 (m, 3H), 7.58 – 7.53 (m, 1H), 7.31 – 7.21 (m, 2H), 3.13 (q, J = 7.5 Hz, 2H), 1.39 (t, J = 7.5 Hz, 3H).

^{13}C NMR (126 MHz, DMSO-d_6) δ (ppm) 158.14, 149.91, 146.53, 142.68, 138.71, 133.71, 132.58, 131.94, 129.69, 129.40 (q, J = 31.4 Hz), 125.13 (q, J = 3.9 Hz), 124.31 (q, J = 272.5 Hz), 122.96, 122.89 (q, J = 4.2 Hz), 121.86, 119.77, 118.33, 111.45, 106.10, 21.14, 12.85.

^{19}F NMR (471 MHz, DMSO-d_6) δ (ppm) -61.18.

HRMS (APCI): calcd. for $C_{21}H_{17}F_3N_7$ $[M+H]^+$ = 424.1492, found $[M+H]^+$ = 424.1491.

8-(Benzo[*d*][1,3]dioxol-5-yl)-3-(1*H*-benzo[*d*]imidazol-2-yl)-7-ethylpyrazolo[5,1-*c*][1,2,4]triazin-4-amine **(74)**

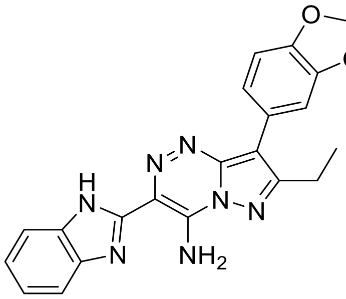

The compound was prepared according to General procedure C using:

Diazotization step:

4-(benzo[*d*][1,3]dioxol-5-yl)-3-ethyl-1*H*-pyrazol-5-amine (**S75**, 45 mg, 0.19 mmol; 1 eq.), 35% aqueous HCl (0.065 mL, 0.76 mmol; 4 eq.) in EtOH (3 mL) and H_2O (3 mL), $NaNO_2$ (26 mg, 0.38 mmol, 2 eq.) in H_2O (1 mL) (reaction time: 30 min), 2-(cyanomethyl)-benzimidazole (36 mg, 0.23 mmol, 1.2 eq.) in EtOH (1 mL) and KOAc (112 mg, 1.14 mmol, 6 eq.) in H_2O (1 mL). Reaction time: 16 h. The yellow solid (71 mg, 0.17 mmol) obtained after the filtration was used in the next step without further purification.

Cyclization step:

the dried yellow solid (71 mg, 0.17 mmol), DMF (3 mL), and KOAc (5 mg, 0.06 mmol, 0.4 eq.). Reaction time: 2 h. The reaction mixture was poured into water (20 mL) and extracted with EtOAc (3 × 20 mL). The combined organic layers were washed with brine (30 mL), dried over $MgSO_4$, the solvent was evaporated, and the residue was purified by column flash chromatography on silica gel (hexane:EtOAc, gradient10:1 to 0:1). The product was obtained as a yellow solid (35 mg, 46% (2 steps)).

^1H NMR (500 MHz, DMSO-d_6) δ (ppm) 13.35 (s, 1H), 9.93 (s, 1H), 9.30 (s, 1H), 7.74 (d, J = 7.7 Hz, 1H), 7.58 – 7.53 (m, 1H), 7.40 (d, J = 1.7 Hz, 1H), 7.31 – 7.20 (m, 3H), 7.07 (d, J = 8.0 Hz, 1H), 6.10 (s, 2H), 3.04 (q, J = 7.5 Hz, 2H), 1.35 (t, J = 7.5 Hz, 3H).

^{13}C NMR (126 MHz, DMSO-d_6) δ (ppm) 157.90, 150.08, 147.43, 146.18, 146.10, 142.68, 138.61, 133.70, 124.89, 122.82, 122.46, 121.80, 118.88, 118.23, 111.44, 109.39, 108.48, 108.14, 101.01, 20.87, 13.07.

HRMS (APCI): calcd. for $C_{21}H_{18}N_7O_2$ $[M+H]^+$ = 400.1516, found $[M+H]^+$ = 400.1516.

Methyl 3-(4-amino-3-(1*H*-benzo[*d*]imidazol-2-yl)-7-ethylpyrazolo[5,1-*c*][1,2,4]triazin-8-yl)benzoate **(75)**

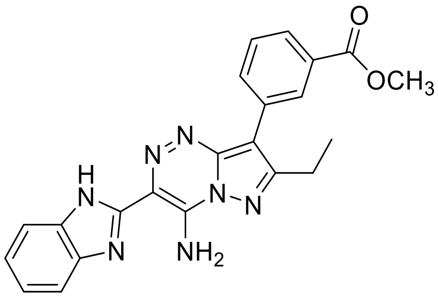

The compound was prepared according to General procedure C using:

Diazotization step:

methyl 3-(5-amino-3-ethyl-1*H*-pyrazol-4-yl)benzoate (**S76**, 85 mg, 0.35 mmol; 1 eq.), 35% aqueous HCl (0.12 mL, 1.40 mmol; 4 eq.) in EtOH (3 mL) and H_2O (3 mL), $NaNO_2$ (48 mg, 0.70 mmol, 2 eq.) in H_2O (1 mL) (reaction time: 30 min), 2-(cyanomethyl)-benzimidazole (64 mg, 0.42 mmol, 1.2 eq.) in EtOH (1 mL) and KOAc (206 mg, 2.10 mmol, 6 eq.) in H_2O (1 mL). Reaction time: 16 h. The yellow solid (150 mg, 0.35 mmol) obtained after the filtration was used in the next step without further purification.

Cyclization step:

the dried yellow solid (150 mg, 0.35 mmol), DMF (3 mL), and KOAc (4 mg, 0.04 mmol, 0.1 eq.). Reaction time: 2 h. Reaction time: 2 h. The product was obtained as a yellow solid (90 mg, 62%).

^{1}H NMR (500 MHz, DMSO-d_6) δ (ppm) ^{1}H NMR (500 MHz, DMSO) δ 13.42 (s, 1H), 9.97 (s, 1H), 9.41 (s, 1H), 8.59 (s, 1H), 8.10 (d, J = 7.9 Hz, 1H), 7.98 – 7.92 (m, 1H), 7.75 (d, J = 7.7 Hz, 1H), 7.68 (t, J = 7.9 Hz, 1H), 7.56 (d, J = 8.1 Hz, 1H), 7.31 – 7.20 (m, 2H), 3.91 (s, 3H), 3.09 (q, J = 7.6 Hz, 2H), 1.38 (t, J = 7.6 Hz, 3H).

^{13}C NMR (126 MHz, DMSO-d_6) δ (ppm) 166.22, 158.08, 149.97, 146.44, 142.68, 138.69, 133.70, 132.97, 131.96, 129.99, 129.47, 129.08, 127.11, 122.92, 121.84, 119.55, 118.30, 111.44, 106.74, 52.21, 21.06, 13.02.

HRMS (APCI): calcd. for $C_{22}H_{20}N_7O_2$ [M+H]$^+$= 414.1672, found [M+H]$^+$ = 414.1674.

3-(1*H*-benzo[*d*]imidazol-2-yl)-7-ethyl-8-(4-(piperidin-1-ylsulfonyl)phenyl)pyrazolo[5,1-*c*][1,2,4]triazin-4-amine **(76)**

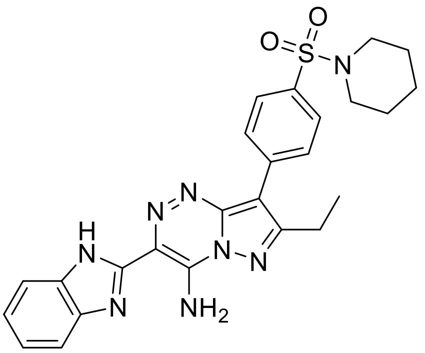

The compound was prepared according to General procedure C using:

Diazotization step:

3-ethyl-4-(4-(piperidin-1-ylsulfonyl)phenyl)-1*H*-pyrazol-5-amine (**S78**, 57 mg, 0.2 mmol; 1 eq.), 35% aqueous HCl (75 µL, 0.80 mmol; 4 eq.) in EtOH (3 mL) and H_2O (3 mL), $NaNO_2$ (30 mg, 0.4 mmol, 2 eq.) in H_2O (1 mL) (reaction time: 30 min), 2-(cyanomethyl)-benzimidazole (37 mg, 0.22 mmol, 1.2 eq.) in EtOH (1 mL) and KOAc (125 mg, 1.20 mmol, 6 eq.) in H_2O (1 mL). Reaction time: 16 h. The yellow solid (88 mg, 0.17 mmol) obtained after the filtration was used in the next step without further purification.

Cyclization step:

the dried yellow solid (88 mg, 0.17 mmol), DMF (3 mL), and KOAc (5 mg, 0.06 mmol, 0.4 eq.). Reaction time: 2 h. The residue obtained after the workup was purified by column chromatography on silica gel (dichloromethane:MeOH, 20:1). The product was obtained as a yellow solid (30 mg, 30%).

^{1}H NMR (500 MHz, DMSO-d_6) δ (ppm) 13.40 (s, 1H), 10.03 (s, 1H), 9.47 (s, 1H), 8.21 – 8.15 (m, 2H), 7.90 – 7.84 (m, 2H), 7.76 (s, 1H), 7.58 (s, 1H), 7.29 – 7.24 (m, 2H), 3.14 (q, J = 7.5 Hz, 2H), 3.01 – 2.95 (m, 4H), 1.62 – 1.54 (m, 4H), 1.45 – 1.36 (m, 5H).

^{13}C NMR (126 MHz, DMSO-d_6) δ (ppm) 158.42, 149.80, 146.57, 142.71, 138.74, 136.26, 133.07, 128.86, 127.73, 120.03, 118.35, 111.63, 106.06, 46.57, 24.71, 22.80, 21.26, 12.91.

HRMS (APCI): calcd. for $C_{25}H_{27}N_8O_2S$ $[M+H]^+$ = 503.1972, found $[M+H]^+$ = 503.1975

<u>3-(1*H*-benzo[*d*]imidazol-2-yl)-7-ethyl-8-(3-(trifluoromethoxy)phenyl)pyrazolo[5,1-*c*][1,2,4]triazin-4-amine</u> **(77)**

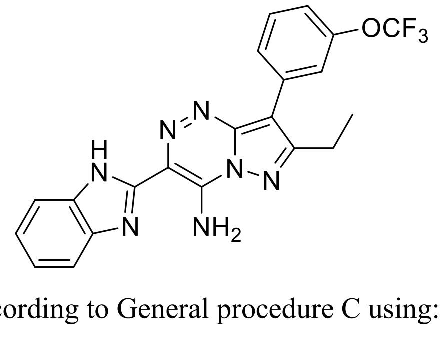

The compound was prepared according to General procedure C using:

Diazotization step:

3-ethyl-4-(3-(trifluoromethoxy)phenyl)-1*H*-pyrazol-5-amine (**S79**, 55 mg, 0.20 mmol; 1 eq.), 35% aqueous HCl (75 µL, 0.80 mmol; 4 eq.) in EtOH (3 mL) and H_2O (3 mL), $NaNO_2$ (30 mg, 0.4 mmol, 2 eq.) in H_2O (1 mL) (reaction time: 30 min), 2-(cyanomethyl)-benzimidazole (37 mg, 0.22 mmol, 1.2 eq.) in EtOH (1 mL) and KOAc (125 mg, 1.20 mmol, 6 eq.) in H_2O (1 mL). Reaction time: 16 h. The yellow solid (46 mg, 0.10 mmol) obtained after the filtration was used in the next step without further purification.

Cyclization step:

the dried yellow solid (46 mg, 0.10 mmol), DMF (3 mL), and KOAc (5 mg, 0.06 mmol, 0.6 eq.). Reaction time: 2 h. The product was obtained as a yellow solid (18 mg, 20%).

^{1}H NMR (500 MHz, DMSO-d_6) δ (ppm) 13.47 (s, 1H), 10.01 (s, 1H), 9.45 (s, 1H), 7.98 (s, 1H), 7.88 (d, J = 7.8 Hz, 1H), 7.79 – 7.74 (m, 1H), 7.68 (t, J = 8.0 Hz, 1H), 7.58 – 7.54 (m, 1H), 7.37 (d, J = 8.3 Hz, 1H), 7.29 – 7.25 (m, 2H), 3.12 (q, J = 7.6 Hz, 2H), 1.39 (t, J = 7.5 Hz, 3H).

^{13}C NMR (126 MHz, DMSO-d_6) δ (ppm) 158.13, 149.89, 148.61, 146.44, 138.71, 133.72, 130.49, 127.25, 122.96, 121.89, 120.84, 120.16 (q, J = 256.3 Hz), 119.73, 118.88, 118.32, 111.49, 106.11, 21.10, 12.84.

^{19}F NMR (471 MHz, DMSO-d_6) δ (ppm) -56.57.

HRMS (APCI): calcd. for $C_{21}H_{17}F_3N_7O$ $[M+H]^+$ = 440.1441, found $[M+H]^+$ = 440.1441.

<u>3-(1*H*-benzo[*d*]imidazol-2-yl)-7-ethyl-8-(naphthalen-1-yl)pyrazolo[5,1-*c*][1,2,4]triazin-4-amine</u> **(78)**

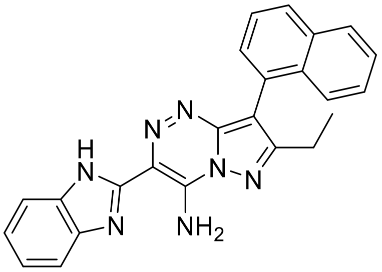

The compound was prepared according to General procedure C using:

Diazotization step:

3-ethyl-4-(naphthalen-1-yl)-1*H*-pyrazol-5-amine (**S80**, 48 mg, 0.200 mmol; 1 eq.), 35% aqueous HCl (75 µL, 0.8 mmol; 4 eq.) in EtOH (3 mL) and H_2O (3 mL), $NaNO_2$ (30 mg, 0.40 mmol, 2 eq.) in H_2O (1 mL) (reaction time: 30 min), 2-(cyanomethyl)-benzimidazole (37 mg, 0.22 mmol, 1.2 eq.) in EtOH (1 mL) and KOAc (125 mg, 1.20 mmol, 6 eq.) in H_2O (1 mL). Reaction time: 16 h. The yellow solid (76 mg, 0.18 mmol) obtained after the filtration was used in the next step without further purification.

Cyclization step:

the dried yellow solid (76 mg, 0.18 mmol), DMF (3 mL), and KOAc (5 mg, 0.08 mmol, 0.3 eq.). Reaction time: 2 h. The product was obtained as a yellow solid (34 mg, 42%).

^{1}H NMR (500 MHz, DMSO-d_6) δ (ppm) 13.31 (s, 1H), 10.02 (s, 1H), 9.43 (s, 1H), 8.06 (dd, J = 8.1, 4.1 Hz, 2H), 7.82 – 7.74 (m, 1H), 7.72 – 7.63 (m, 3H), 7.61 – 7.52 (m, 2H), 7.50 – 7.43 (m, 1H), 7.31 – 7.21 (m, 2H), 2.88 – 2.70 (m, 2H), 1.18 (t, J = 7.5 Hz, 3H).

^{13}C NMR (126 MHz, DMSO-d_6) δ (ppm) 159.42, 150.13, 147.12, 142.68, 138.86, 133.72, 133.41, 132.32, 129.08, 128.67, 128.17, 128.11, 126.13, 125.98, 125.57, 122.81, 121.80, 118.87, 118.24, 111.46, 107.12, 20.58, 13.29.

HRMS (APCI): calcd. for $C_{24}H_{20}N_7$ $[M+H]^+$ = 406.1775, found $[M+H]^+$ = 406.1777.

3-(1*H*-benzo[*d*]imidazol-2-yl)-7-(naphthalen-2-yl)pyrazolo[5,1-*c*][1,2,4]triazin-4-ol **(79)**

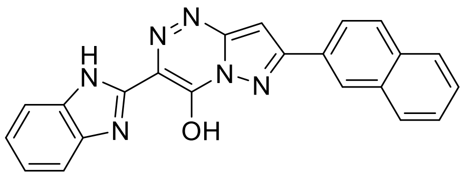

To 3-(1*H*-benzo[*d*]imidazol-2-yl)-7-(naphthalen-2-yl)pyrazolo[5,1-*c*][1,2,4]triazin-4-amine (**14**, 73 mg, 0.19 mmol; 1 eq.) was added 23 % aqueous HCl (5 mL) and the mixture was refluxed for 1 h. The reaction mixture was poured into water (10 mL) and neutralized by addition of saturated aqueous solution of $NaHCO_3$ (until neutral pH, 10 mL). The precipitate was collected by filtration, washed with water (6 mL), then with Et_2O (5 mL) and dried under vacuum. The product was obtained as an orange solid (44 mg, 60%).

^{1}H NMR (500 MHz, DMSO-d_6) δ 8.69 (s, 1H), 8.25 (dd, J = 8.5, 1.7 Hz, 1H), 8.11 – 8.04 (m, 2H), 8.02 – 7.94 (m, 1H), 7.86 – 7.80 (m, 2H), 7.62 – 7.55 (m, 2H), 7.55 – 7.50 (m, 2H), 7.42 (s, 1H).

^{13}C NMR (126 MHz, DMSO-d_6) δ (ppm) 155.11, 148.61, 145.91, 133.37, 132.98, 131.19, 129.13, 128.45, 128.37, 127.68, 126.82, 126.66, 125.89, 125.51, 124.06, 120.55, 113.87, 91.92.

HRMS (APCI): calcd. for $C_{22}H_{15}N_6O$ $[M+H]^+$ = 379.1302, found $[M+H]^+$ = 379.1299.

3-(1*H*-benzo[*d*]imidazol-2-yl)-7-(3-methoxynaphthalen-2-yl)pyrazolo[5,1-*c*][1,2,4]triazin-4-ol **(80)**

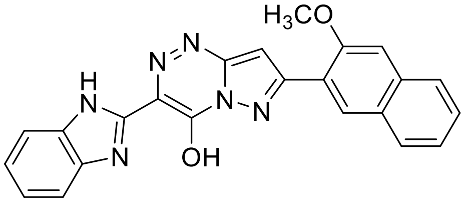

The compound was prepared according to General procedure C using:

Diazotization step:

3-(3-methoxynaphthalen-2-yl)-1*H*-pyrazol-5-amine (**S48**, 240 mg, 1.0 mmol; 1 eq.), 35% aqueous HCl (0.4 mL, 4.0 mmol; 4 eq.) in EtOH (3 mL) and H_2O (3 mL), $NaNO_2$ (136 mg, 2.0 mmol, 2 eq.) in H_2O (1 mL) (reaction time: 30 min), 2-(cyanomethyl)-benzimidazole (172 mg, 1.10 mmol, 1.2 eq.) in EtOH

(1 mL) and KOAc (590 mg, 6.0 mmol, 6 eq.) in H_2O (1 mL). Reaction time: 16 h. The yellow solid (340 mg, 0.80 mmol) obtained after the filtration was used in the next step without further purification.

Cyclization step:

the dried yellow solid (340 mg, 0.80 mmol), DMF (3 mL), and KOAc (8 mg, 0.08 mmol, 0.1 eq.). Reaction time: 2 h. The product was obtained as a yellow solid (224 mg, 55%).

^{1}H NMR (500 MHz, DMSO-d_6) δ (ppm) 14.07 (s, 2H), 8.64 (s, 1H), 8.02 (d, J = 8.1 Hz, 1H), 7.89 (d, J = 8.2 Hz, 1H), 7.80 – 7.72 (m, 2H), 7.54 – 7.50 (m, 2H), 7.47 – 7.44 (m, 2H), 7.43 – 7.38 (m, 1H), 7.36 (s, 1H), 4.06 (s, 3H).

^{13}C NMR (126 MHz, DMSO-d_6) δ (ppm) 155.24, 151.27, 149.07, 148.50, 134.31, 131.27, 128.35, 128.09, 127.98, 126.91, 126.36, 124.64, 124.05, 122.61, 113.40, 106.43, 98.91, 55.65.

HRMS (APCI): calcd. for $C_{23}H_{18}N_7O$ $[M+H]^+$= 408.1567, found $[M+H]^+$ = 408.1566.

3-(1*H*-benzo[*d*]imidazol-2-yl)-7-ethylpyrazolo[5,1-*c*][1,2,4]triazin-4-ol **(81)**

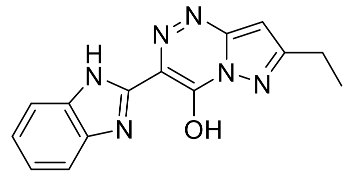

The compound was prepared according to General procedure C using:

Diazotization step:

3-ethyl-1*H*-pyrazol-5-amine (56 mg, 0.50 mmol; 1 eq.), 35% aqueous HCl (0.2 mL, 2.0 mmol; 4 eq.) in EtOH (2 mL) and H_2O (2 mL), $NaNO_2$ (68 mg, 1 mmol, 2 eq.) in H_2O (0.5 mL) (reaction time: 30 min), ethyl 2-(1*H*-benzo[*d*]imidazol-2-yl)acetate (100 mg, 0.50 mmol, 1 eq.) in EtOH (0.5 mL) and KOAc (300 mg, 3 mmol, 6 eq.) in H_2O (0.5 mL). Reaction time: 16 h. The yellow solid (120 mg, 0.40 mmol) obtained after the filtration was used in the next step without further purification.

Cyclization step:

the dried yellow solid (119 mg, 0.40 mmol), DMF (3 mL), and KOAc (4 mg, 0.04 mmol, 0.1 eq.). Reaction time: 2 h. The product was obtained as a yellow solid (91 mg, 65%).

^{1}H NMR (300 MHz, DMSO-d_6) δ (ppm) 14.00 (s, 2H), 7.73 (dd, J = 6.1, 3.2 Hz, 2H), 7.43 (dd, J = 6.1, 3.2 Hz, 2H), 6.66 (s, 1H), 2.78 (q, J = 7.6 Hz, 2H), 1.30 (t, J = 7.6 Hz, 3H).

HRMS (APCI): calcd. for $C_{14}H_{13}N_6O$ $[M+H]^+$= 281.1175, found $[M+H]^+$ = 281.1178.

3-(1*H*-benzo[*d*]imidazol-2-yl)-7-(2-methoxyphenyl)pyrazolo[5,1-*c*][1,2,4]triazin-4-ol **(82)**

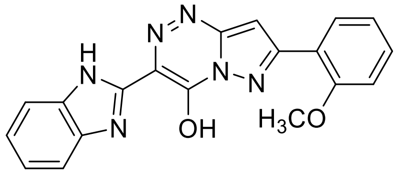

The compound was prepared according to General procedure C using:

Diazotization step:

3-(2-methoxyphenyl)-1*H*-pyrazol-5-amine (95 mg, 0.50 mmol; 1 eq. CAS: 909861-26-3), 35% aqueous HCl (0.2 mL, 2.0 mmol; 4 eq.) in EtOH (2 mL) and H_2O (2 mL), $NaNO_2$ (68 mg, 1 mmol, 2 eq.) in H_2O (0.50 mL) (reaction time: 30 min), ethyl 2-(1*H*-benzo[*d*]imidazol-2-yl)acetate (100 mg, 0.50 mmol, 1 eq.) in EtOH (0.5 mL) and KOAc (300 mg, 3 mmol, 6 eq.) in H_2O (0.5 mL). Reaction time: 16 h. The yellow solid (170 mg, 0.45 mmol) obtained after the filtration was used in the next step without further purification.

Cyclization step:

the dried yellow solid (170 mg, 0.45 mmol), DMF (3 mL), and KOAc (4 mg, 0.04 mmol, 0.1 eq.). Reaction time: 2 h. The product was obtained as a yellow solid (125 mg, 70%).

^{1}H NMR (500 MHz, DMSO-d_6) δ (ppm14.07 (s, 2H), 8.13 (dd, J = 7.7, 1.8 Hz, 1H), 7.79 – 7.72 (m, 2H), 7.48 – 7.40 (m, 3H), 7.28 (s, 1H), 7.19 (dd, J = 8.5, 1.1 Hz, 1H), 7.10 (td, J = 7.4, 1.1 Hz, 1H), 3.96 (s, 3H).

^{13}C NMR (126 MHz, DMSO-d_6) δ (ppm) 157.22, 152.76, 151.36, 149.05, 148.50, 131.17, 130.30, 128.59, 124.66, 120.84, 120.54, 118.53, 113.37, 112.05, 98.45, 55.56.

HRMS (APCI): calcd. for $C_{19}H_{15}N_6O_2$ $[M+H]^+$ = 359.1251, found $[M+H]^+$ = 359.1252.

3-(1*H*-benzo[*d*]imidazol-2-yl)-7-(2-phenoxyphenyl)pyrazolo[5,1-*c*][1,2,4]triazin-4-ol **(83)**

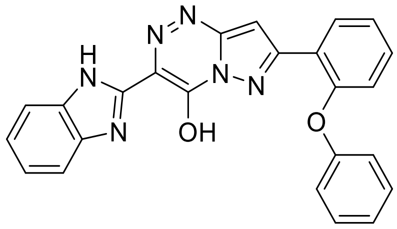

The compound was prepared according to General procedure C using:

Diazotization step:

3-(2-phenoxyphenyl)-1*H*-pyrazol-5-amine (**S42**, 125 mg, 0.5 mmol; 1 eq.), 35% aqueous HCl (0.2 mL, 2.0 mmol; 4 eq.) in EtOH (2 mL) and H_2O (2 mL), $NaNO_2$ (68 mg, 1 mmol, 2 eq.) in H_2O (0.5 mL) (reaction time: 30 min), ethyl 2-(1*H*-benzo[*d*]imidazol-2-yl)acetate (100 mg, 0.5 mmol, 1 eq.) in EtOH (0.5 mL) and KOAc (300 mg, 3 mmol, 6 eq.) in H_2O (0.5 mL). Reaction time: 16 h. The yellow solid (175 mg, 0.4 mmol) obtained after the filtration was used in the next step without further purification.

Cyclization step:

the dried yellow solid (170 mg, 0.45 mmol), DMF (3 mL), and KOAc (4 mg, 0.04 mmol, 0.1 eq.). Reaction time: 2 h. The product was obtained as a yellow solid (137 mg, 65%).

^{1}H NMR (500 MHz, DMSO-d_6) δ (ppm) 14.08 (s, 2H), 8.27 (dd, J = 7.8, 1.7 Hz, 1H), 7.80 – 7.71 (m, 2H), 7.55 – 7.47 (m, 1H), 7.46 – 7.43 (m, 2H), 7.41 – 7.33 (m, 3H), 7.14 – 7.07 (m, 3H), 7.06 – 6.98 (m, 2H).

^{13}C NMR (126 MHz, DMSO-d_6) δ (ppm) 157.04, 153.40, 152.98, 150.45, 148.93, 148.35, 131.17, 130.64, 130.05, 129.41, 124.75, 124.68, 124.61, 122.92, 120.88, 118.79, 117.25, 113.39, 97.70.
HRMS (APCI): calcd. for $C_{24}H_{17}N_6O_2$ $[M+H]^+ = 421.1408$, found $[M+H]^+ = 421.1410$.

3-(1*H*-benzo[*d*]imidazol-2-yl)-7-(2-(dimethylamino)phenyl)pyrazolo[5,1-*c*][1,2,4]triazin-4-ol **(84)**

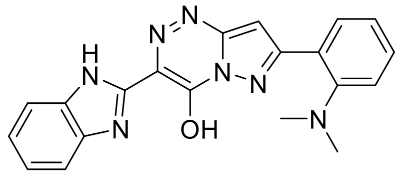

The compound was prepared according to General procedure C using:
Diazotization step:
3-(2-(dimethylamino)phenyl)-1*H*-pyrazol-5-amine (**S55**, 101 mg, 0.50 mmol; 1 eq.), 35% aqueous HCl (0.2 mL, 2.0 mmol; 4 eq.) in EtOH (2 mL) and H_2O (2 mL), $NaNO_2$ (68 mg, 1 mmol, 2 eq.) in H_2O (0.5 mL) (reaction time: 30 min), ethyl 2-(1*H*-benzo[*d*]imidazol-2-yl)acetate (100 mg, 0.50 mmol, 1 eq.) in EtOH (0.5 mL) and KOAc (300 mg, 3 mmol, 6 eq.) in H_2O (0.5 mL). Reaction time: 16 h. The yellow solid (175 mg, 0.45 mmol) obtained after the filtration was used in the next step without further purification.
Cyclization step:
the dried yellow solid (175 mg, 0.45 mmol), DMF (3 mL), and KOAc (4 mg, 0.04 mmol, 0.1 eq.). Reaction time: 2 h. The product was obtained as a yellow solid (102 mg, 55%).
^1H NMR (500 MHz, DMSO-d_6) δ (ppm) 14.06 (s, 2H), 7.86 – 7.80 (m, 1H), 7.78 – 7.73 (m, 2H), 7.48 – 7.40 (m, 2H), 7.39 – 7.34 (m, 1H), 7.28 (s, 1H), 7.18 (d, J = 8.1 Hz, 1H), 7.09 (t, J = 7.4 Hz, 1H), 2.65 (s, 6H).
^{13}C NMR (126 MHz, DMSO-d_6) δ (ppm) 154.40, 152.87, 152.24, 149.10, 148.51, 131.20, 130.34, 129.54, 125.58, 124.63, 121.67, 118.41, 113.36, 97.07, 43.93.
HRMS (APCI): calcd. for $C_{14}H_{12}N_7$ $[M+H]^+= 372.1567$, found $[M+H]^+ = 372.1569$.

3-(1*H*-benzo[*d*]imidazol-2-yl)-7-(2-methoxybenzyl)pyrazolo[5,1-*c*][1,2,4]triazin-4-ol **(85)**

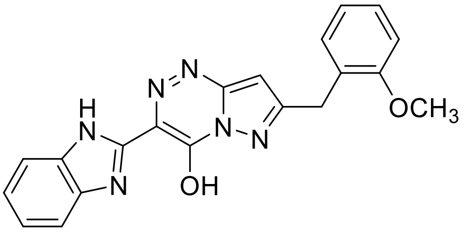

The compound was prepared according to General procedure C using:
Diazotization step:
3-(2-methoxybenzyl)-1*H*-pyrazol-5-amine (**S56**, 300 mg, 1.48 mmol; 1 eq.), 35% aqueous HCl (510 µL, 5.9 mmol; 4 eq.) in EtOH (5 mL) and H_2O (5 mL), $NaNO_2$ (203 mg, 2.98 mmol, 2 eq.) in EtOH (1 mL)

(reaction time: 15 min), ethyl 2-(1*H*-1,3-benzodiazol-2-yl)acetate (362 mg, 1.77 mmol, 1.2 eq.) in EtOH (2 mL) and KOAc (870 mg, 8.86 mmol, 6 eq.). Reaction time: 16 h. The yellow solid (526 mg, 1.35 mmol) obtained after the filtration was used in the next step without further purification.

Cyclization step:

the dried yellow solid (526 mg, 1.35 mmol), DMF (5 mL), and KOAc (10 mg, 0.10 mmol, 0.1 eq.). Reaction time: 2 h. After the filtration, the solid part was suspended in hot dioxane (10 mL) and the mixture was poured into water (30 mL). The solid was collected by filtration, washed with water (10 mL), diethyl ether (10 mL) and dried under *vacuum*. The product was obtained as a yellow solid (320 mg, 58%).

^{1}H NMR (500 MHz, DMSO-d_6) δ (ppm) 14.01 (s, 2H), 7.77 – 7.70 (m, 2H), 7.47 – 7.40 (m, 2H), 7.27 – 7.21 (m, 1H), 7.18 (dd, J = 7.5, 1.8 Hz, 1H), 7.02 (dd, J = 8.2, 1.1 Hz, 1H), 6.89 (d, J = 1.1 Hz, 1H), 6.50 (s, 1H), 4.08 (s, 2H), 3.84 (s, 3H).

^{13}C NMR (126 MHz, DMSO-d_6) δ (ppm) 157.36, 156.88, 153.22, 149.59, 149.01, 131.75, 130.53, 128.31, 127.62, 125.11, 120.80, 113.89, 111.31, 97.07, 55.86, 29.45.

HRMS (APCI): calcd. for $C_{20}H_{17}N_6O_2$ $[M+H]^+$ = 373.1408., found $[M+H]^+$ = 373.1411.

<u>3-(1*H*-imidazol-2-yl)-7-(2-methoxyphenyl)pyrazolo[5,1-*c*][1,2,4]triazin-4-ol</u> **(86)**

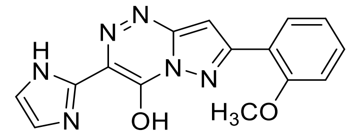

The compound was prepared according to General procedure C using:

Diazotization step:

3-(2-Methoxyphenyl)-1*H*-pyrazol-5-amine (230 mg, 1.22 mmol; 1 eq. CAS: 909861-26-3), 35% aqueous HCl (420 µL, 4.86 mmol; 4 eq.) in EtOH (5 mL) and H_2O (5 mL), $NaNO_2$ (168 mg, 2.43 mmol, 2 eq.) in EtOH (1 mL) (reaction time: 15 min), ethyl 2-(1*H*-imidazol-2-yl)acetate (224 mg, 1.46 mmol, 1.2 eq.) in EtOH (2 mL) and KOAc (720 mg, 7.29 mmol, 6 eq.). Reaction time: 16 h. The yellow solid (397 mg, 1.22 mmol) obtained after the filtration was used in the next step without further purification.

Cyclization step:

the dried yellow solid (397 mg, 1.22 mmol), DMF (5 mL), and KOAc (10 mg, 0.10 mmol, 0.1 eq.). Reaction time: 2 h. After the filtration, the solid part was suspended in hot dioxane (10 mL), and the mixture was poured into water (30 mL). The solid was collected by filtration, washed with water (10 mL), diethyl ether (10 mL) and dried under *vacuum*. The product was obtained as a yellow solid (300 mg, 80%).

^{1}H NMR (500 MHz, DMSO-d_6) δ (ppm) 8.14 – 8.09 (m, 2H), 7.46 (s, 1H), 7.44 – 7.37 (m, 2H), 7.20 – 7.15 (m, 2H), 7.08 (t, J = 7.5 Hz, 1H), 3.94 (s, 3H).

^{13}C NMR (126 MHz, DMSO-d_6) δ (ppm) 157.18, 152.94, 151.02, 148.39, 143.35, 130.09, 128.55, 121.09, 120.49, 119.55, 118.11, 112.01, 97.04, 55.53.

HRMS (APCI): calcd. for $C_{15}H_{13}N_6O_2$ $[M+H]^+$ = 309.1095., found $[M+H]^+$ = 309.1094.

3-(1*H*-benzo[*d*]imidazol-2-yl)-7-(2-methoxy-2-phenylethyl)pyrazolo[5,1-*c*][1,2,4]triazin-4-ol **(87)**

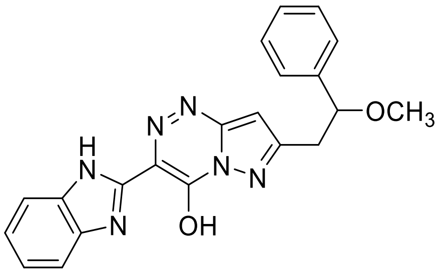

The compound was prepared according to General procedure C using:

Diazotization step:

3-(2-methoxy-2-phenylethyl)-1*H*-pyrazol-5-amine (**S57**, 150 mg, 0.69 mmol; 1 eq.), 35% aqueous HCl (240 µL, 2.76 mmol; 4 eq.) in EtOH (5 mL) and H_2O (5 mL), $NaNO_2$ (95 mg, 1.38 mmol, 2 eq.) in EtOH (1 mL) (reaction time: 15 min), ethyl 2-(1*H*-1,3-benzodiazol-2-yl)acetate (169 mg, 0.83 mmol, 1.2 eq.) in EtOH (2 mL) and KOAc (0.41 g, 4.14 mmol, 6 eq.). Reaction time: 16 h. The yellow solid (222 mg, 0.55 mmol) obtained after the filtration was used in the next step without further purification.

Cyclization step:

the dried yellow solid (222 mg, 0.55 mmol), DMF (5 mL), and KOAc (5 mg, 0.05 mmol, 0.1 eq.). Reaction time: 2 h. After the filtration, the solid part was suspended in hot dioxane (10 mL), and the mixture was poured into water (30 mL). The solid was collected by filtration, washed with water (10 mL), diethyl ether (10 mL) and dried under *vacuum*. The product was obtained as a yellow solid (150 mg, 56%).

^1H NMR (500 MHz, DMSO-d_6) δ (ppm14.01 (s, 2H), 7.73 (dd, J = 6.0, 3.2 Hz, 2H), 7.45 – 7.40 (m, 2H), 7.40 – 7.33 (m, 4H), 7.34 – 7.26 (m, 1H), 6.57 (s, 1H), 4.63 (dd, J = 8.0, 5.7 Hz, 1H), 3.23 (dd, J = 14.5, 8.1 Hz, 1H), 3.14 (s, 3H), 3.07 (dd, J = 14.5, 5.7 Hz, 1H).

^{13}C NMR (126 MHz, DMSO-d_6) δ (ppm) 154.48, 149.01, 148.48, 141.34, 131.14, 128.30, 127.63, 126.73, 124.59, 113.33, 96.86, 82.17, 56.03, 37.09.

HRMS (APCI): calcd. for $C_{21}H_{19}N_6O_2$ $[M-H]^-$ = 387.1564, found $[M-H]^-$ = 387.1563.

3-(1*H*-benzo[*d*]imidazol-2-yl)-7-benzylpyrazolo[5,1-*c*][1,2,4]triazin-4-ol **(88)**

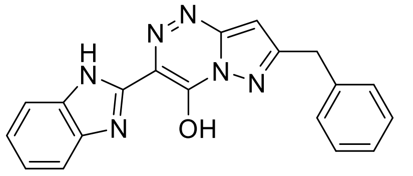

The compound was prepared according to General procedure C using:

Diazotization step:

3-benzyl-1*H*-pyrazol-5-amine (**S58**, 165 mg, 0.95 mmol; 1 eq.), 35% aqueous HCl (0.336 mL, 3.81 mmol; 4 eq.) in EtOH (2 mL) and H_2O (2 mL), $NaNO_2$ (0.131 g, 1.91 mmol, 2 eq.) in EtOH (1 mL) and H_2O (2 mL) (reaction time: 30 min), ethyl 2-(1*H*-benzo[*d*]imidazol-2-yl)acetate (214 mg, 1.05 mmol, 1.1 eq.) in EtOH (3 mL) and H_2O (3 mL) and KOAc (748 mg, 7.62 mmol, 8 eq.). Reaction time: 2 h. The yellow solid (384 mg, quant.) obtained after the filtration was used in the next step without further purification.

Cyclization step:

the dried yellow solid (384 mg), DMF (4 mL), and KOAc (9 mg, 0.1 mmol, 0.1 eq.). Reaction time: 2 h. The product was obtained as a yellow solid (272 mg, 83%).

^{1}H NMR (500 MHz, DMSO-d_6) δ (ppm) 14.02 (s, 2H), 7.78 – 7.70 (m, 2H), 7.47 – 7.40 (m, 2H), 7.36 – 7.28 (m, 4H), 7.25 – 7.18 (m, 1H), 6.61 (s, 1H), 4.13 (s, 2H).

^{13}C NMR (126 MHz, DMSO-d_6) δ (ppm) 156.59, 152.73, 149.08, 148.44, 139.34, 131.20, 128.68, 128.39, 126.18, 124.63, 118.61, 113.38, 96.47, 34.64.

HRMS (APCI): calcd. for $C_{19}H_{15}N_6O$ $[M+H]^+ = 343.1302$, found $[M+H]^+ = 343.1304$.

3-(1*H*-benzo[*d*]imidazol-2-yl)-7-(isochroman-3-ylmethyl)pyrazolo[5,1-*c*][1,2,4]triazin-4-ol **(89)**

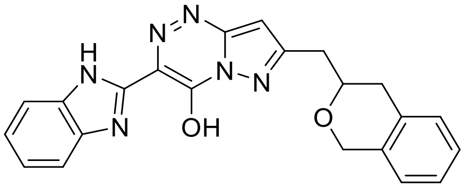

The compound was prepared according to General procedure C using:

Diazotization step:

3-(isochroman-3-ylmethyl)-1*H*-pyrazol-5-amine (**S59**, 202 mg, 0.88 mmol; 1 eq.), 35% aqueous HCl (0.311 mL, 3.52 mmol; 4 eq.) in EtOH (2 mL) and H_2O (2 mL), $NaNO_2$ (122 mg, 1.76 mmol, 2 eq.) in EtOH (1 mL) and H_2O (2 mL) (reaction time: 30 min), ethyl 2-(1*H*-benzo[*d*]imidazol-2-yl)acetate (198 mg, 0.97 mmol, 1.1 eq.) in EtOH (3 mL) and H_2O (3 mL) and KOAc (692 mg, 7.05 mmol, 8 eq.). Reaction time: 2 h. The yellow solid (215 mg, quant.) obtained after the filtration was used in the next step without further purification.

Cyclization step:

the dried yellow solid (215 mg), DMF (5 mL), and KOAc (9 mg, 0.09 mmol, 0.1 eq.). Reaction time: 2 h. The product was obtained as a yellow solid (245 mg, 70% (2 steps)).

^{1}H NMR (500 MHz, DMSO-d_6) δ (ppm) 14.00 (s, 2H), 7.78 – 7.70 (m, 2H), 7.47 – 7.40 (m, 2H), 7.33 – 7.28 (m, 1H), 7.24 – 7.11 (m, 3H), 6.62 (s, 1H), 5.16 (dd, J = 8.8, 3.6 Hz, 1H), 4.09 (dt, J = 10.3, 4.8 Hz, 1H), 3.72 (ddd, J = 11.8, 8.7, 4.1 Hz, 1H), 3.41 (dd, J = 15.0, 3.7 Hz, 1H), 3.22 (dd, J = 15.0, 8.7 Hz, 1H), 2.93 – 2.84 (m, 1H), 2.75 – 2.65 (m, 1H).

^{13}C NMR (126 MHz, DMSO-d_6) δ (ppm) 155.00, 149.09, 148.54, 137.51, 133.80, 131.23, 128.80, 126.39, 126.02, 125.03, 124.62, 113.38, 97.03, 74.65, 62.10, 35.05, 28.35 ^{19}F NMR (471 MHz, Methanol-d_4) δ (ppm) -116.85.

HRMS (APCI): calcd. for $C_{22}H_{19}N_6O_2$ $[M+H]^+$ = 399.1564, found $[M+H]^+$ = 399.1562.

3-(1-Benzyl-1*H*-benzo[*d*]imidazol-2-yl)-8-(4-(methylsulfonyl)phenyl)pyrazolo[5,1-*c*][1,2,4]triazin-4-ol **(90)**

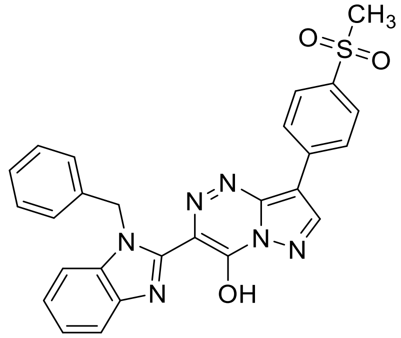

The compound was prepared according to General procedure D using 3-(1-benzyl-1*H*-benzo[*d*]imidazol-2-yl)-8-bromopyrazolo[5,1-*c*][1,2,4]triazin-4-ol (**S107**, 38 mg, 0.09 mmol, 1 eq.), (4-(methylsulfonyl)phenyl)boronic acid (22 mg, 0.11 mmol, 1.2 eq.), K_3PO_4 (77 mg, 0.36 mmol, 4 eq.), Pd(dppf)Cl_2 (3 mg, 0.005 mmol, 0.05 eq.), dioxane (4 mL) and H_2O (1 mL). Reaction time: 4 h at 120 °C. The residue obtained after the workup was purified using column chromatography on silica gel (EtOAc:MeOH, gradient 1:0 to 9:1). The product was obtained as a yellow solid (44 mg, 98%).

^{1}H NMR (500 MHz, DMSO-d_6) δ (ppm)) 14.23 (s, 1H), 8.76 (s, 1H), 8.50 (d, J = 8.2 Hz, 2H), 8.01 – 7.93 (m, 3H), 7.74 (d, J = 8.0 Hz, 1H), 7.50 (dt, J = 18.9, 7.3 Hz, 2H), 7.37 – 7.29 (m, 4H), 7.28 – 7.24 (m, 1H), 6.36 (s, 2H), 3.22 (s, 3H).

^{13}C NMR (126 MHz, DMSO-d_6) δ (ppm) 142.68, 128.69, 127.70, 127.45, 126.97, 126.12, 124.84,* 112.15, 49.57, 43.72.

*assigned by HSQC

HRMS (ESI): calcd. for $C_{26}H_{21}N_6O_3S$ $[M+H]^+$ = 497.1390, found $[M-H]^-$= 497.1387.

3-(1*H*-benzo[*d*]imidazol-2-yl)-8-(4-(methylsulfonyl)phenyl)pyrazolo[5,1-*c*][1,2,4]triazin-4-ol **(91)**

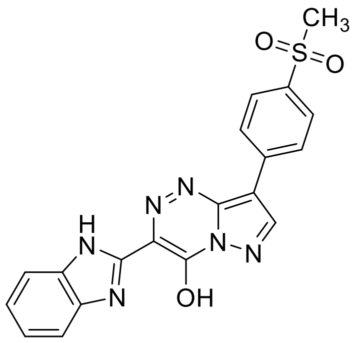

To a solution of 3-(1-benzyl-1*H*-benzo[*d*]imidazol-2-yl)-8-(4-(methylsulfonyl)phenyl)pyrazolo[5,1-*c*][1,2,4]triazin-4-ol (**90**, 25 mg, 0.05 mmol, 1 eq.) in MeOH (5 mL) was added 10% Pd/C (3 mg) and the mixture was bubbled with H_2 for 5 min. The mixture was then stirred at reflux under an atmosphere of H_2 for 16 h. The mixture was filtered through a Silia*MetS*® Thiol (Si-Thiol, 500 mg) metal scavenger cartridge, and the solvent was removed *in vacuo*. The residue was triturated with dichloromethane (1 mL), the solid was collected by filtration and the trituration was repeated with EtOH (1 mL). The product was obtained as a yellow solid (10 mg, 50%).

^{1}H NMR (500 MHz, DMSO-d_6) δ (ppm) 14.21 (s, 1H), 8.76 (s, 1H), 8.59 – 8.53 (m, 2H), 7.99 (d, J = 8.6 Hz, 2H), 7.81 – 7.75 (m, 2H), 7.51 – 7.41 (m, 2H), 3.24 (s, 3H).

^{13}C NMR (126 MHz, DMSO-d_6) δ (ppm) 142.54, 127.46, 126.10, 124.82,* 113.57.*

*assigned by HSQC

HRMS (APCI): calcd. for $C_{19}H_{15}N_6O_3S$ [M+H]$^+$ = 407.0775, found [M+H]$^+$ = 407.0772.

<u>3-(1-Benzyl-1*H*-benzo[*d*]imidazol-2-yl)-8-(4-nitrophenyl)pyrazolo[5,1-*c*][1,2,4]triazin-4-ol</u> **(92)**

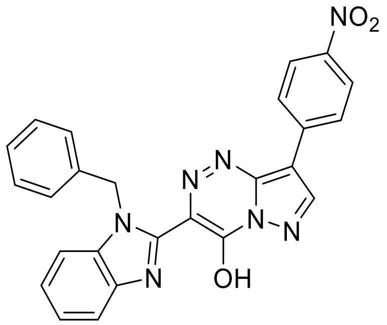

The compound was prepared according to General procedure D using 3-(1-benzyl-1*H*-benzo[*d*]imidazol-2-yl)-8-bromopyrazolo[5,1-*c*][1,2,4]triazin-4-ol (**S107**, 26 mg, 0.06 mmol, 1 eq.), 4-nitrophenylboronic acid (13 mg, 0.007 mmol, 1.2 eq.), K_3PO_4 (52 mg, 0.25 mmol, 4 eq.), Pd(dppf)Cl_2 (2 mg, 0.003 mmol, 0.05 eq.), dioxane (4 mL) and H_2O (1 mL). Reaction time: 4 h at 120 °C. The residue obtained after the workup was purified using column chromatography on silica gel (EtOAc:MeOH, gradient 1:0 to 9:1). The product was obtained as an orange solid (18 mg, 63%).

^{1}H NMR (500 MHz, DMSO-d_6) δ (ppm) 8.69 (s, 1H), 8.53 (d, J = 8.5 Hz, 2H), 8.27 (d, J = 8.4 Hz, 2H), 7.79 (s, 1H), 7.51 (d, J = 5.9 Hz, 1H), 7.34 – 7.15 (m, 7H), 5.78 (s, 2H).

^{13}C NMR (126 MHz, DMSO-d_6) δ (ppm) 128.45, 127.33, 126.87, 125.34, 124.08, 111.03.

HRMS (ESI): calcd. for $C_{25}H_{18}N_7O_3$ [M+H]$^+$ = 464.1466, found [M+H]$^+$ = 464.1462.

<u>3-(1-Benzyl-1*H*-benzo[*d*]imidazol-2-yl)-7-ethyl-8-(4-(trifluoromethyl)phenyl)pyrazolo[5,1-*c*][1,2,4]triazin-4-amine</u> **(93)**

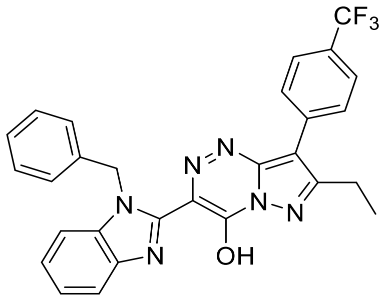

The compound was prepared according to General procedure C using:

Diazotization step:

3-ethyl-4-(4-(trifluoromethyl)phenyl)-1H-pyrazol-5-amine (**S72**, 40 mg, 0.16 mmol; 1 eq.), 35% aqueous HCl (0.06 mL, 0.64 mmol; 4 eq.) in EtOH (3 mL) and H_2O (3 mL), $NaNO_2$ (24 mg, 0.32 mmol, 2 eq.) in H_2O (1 mL) (reaction time: 30 min), ethyl 2-(1-benzyl-1*H*-benzo[*d*]imidazol-2-yl)acetate (40 mg, 0.19 mmol, 1.2 eq.) in EtOH (1 mL) and KOAc (95 mg, 0.96 mmol, 6 eq.) in H_2O (1 mL). Reaction time: 16 h. The yellow solid (75 mg, 0.14 mmol) obtained after the filtration was used in the next step without further purification.

Cyclization step:

the dried yellow solid (75 mg, 0.14 mmol), DMF (3 mL), and KOAc (5 mg, 0.05 mmol, 0.5 eq.). Reaction time: 2 h. The product was obtained as a yellow solid (32 mg, 40%).

^{1}H NMR (500 MHz, DMSO-d_6) δ (ppm) 14.15 (s, 1H), 8.04 (d, J = 8.1 Hz, 2H), 7.91 (d, J = 7.6 Hz, 1H), 7.81 (d, J = 8.1 Hz, 2H), 7.64 (s, 1H), 7.46 – 7.36 (m, 2H), 7.32 – 7.21 (m, 5H), 6.14 (s, 2H), 3.00 (q, J = 7.5 Hz, 2H), 1.30 (t, J = 7.4 Hz, 3H).

^{19}F NMR (471 MHz, DMSO-d_6) δ (ppm) -60.72.

HRMS (APCI): calcd. for $C_{28}H_{22}F_3N_6O$ $[M+H]^+$ = 515.1802, found $[M+H]^+$ = 515.1801

3-(5,6-Dimethyl-1*H*-benzo[*d*]imidazol-2-yl)-7-ethyl-8-(4-fluorophenyl)pyrazolo[5,1-*c*][1,2,4]triazin-4-ol **(94)**

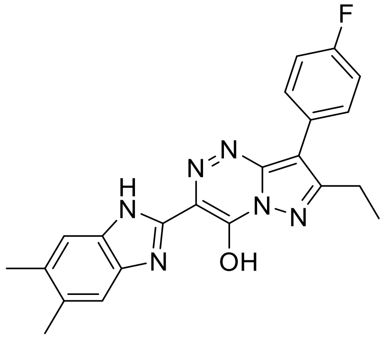

The compound was prepared according to General procedure C using:

Diazotization step:

3-ethyl-4-(4-fluorophenyl)-1*H*-pyrazol-5-amine (**S38**, 93 mg, 0.44 mmol; 1.1 eq.), 35% aqueous HCl (0.16 mL, 1.76 mmol; 4 eq.) in EtOH (3 mL) and H_2O (3 mL), $NaNO_2$ (60 mg, 0.88 mmol, 2 eq.) in H_2O

(1 mL) (reaction time: 30 min), methyl 2-(5,6-dimethyl-1*H*-benzo[*d*]imidazol-2-yl)acetate (**S103**, 90 mg, 0.4 mmol, 1 eq.) in EtOH (1 mL) and KOAc (240 mg, 2.40 mmol, 6 eq.) in H_2O (1 mL). Reaction time: 16 h. The yellow solid (147 mg, 0.35 mmol) obtained after the filtration was used in the next step without further purification.

Cyclization step:

the dried yellow solid (147 mg, 0.35 mmol), DMF (3 mL), and KOAc (4 mg, 0.04 mmol, 0.1 eq.). Reaction time: 2 h. The product was obtained as a yellow solid (91 mg, 57%).

^{1}H NMR (500 MHz, DMSO-d_6) δ (ppm) 13.82 (s, 2H), 7.86 – 7.79 (m, 2H), 7.51 (s, 2H), 7.36 – 7.28 (m, 2H), 2.95 (q, J = 7.5 Hz, 2H), 2.37 (s, 6H), 1.29 (t, J = 7.5 Hz, 3H).

^{13}C NMR (126 MHz, DMSO-d_6) δ (ppm) 160.77 (d, J = 243.5 Hz), 155.51, 149.33, 148.77, 147.48, 133.51, 130.67 (d, J = 8.0 Hz), 129.84, 128.53 (d, J = 3.2 Hz), 119.68, 115.21 (d, J = 21.2 Hz), 113.30, 107.75, 20.82, 19.91, 13.09.

^{19}F NMR (471 MHz, DMSO-d_6) δ (ppm) -116.07.

HRMS (APCI): calcd. for $C_{22}H_{20}FN_6O$ [M+H]$^+$ = 403.1677, found [M+H]$^+$ = 403.1677

<u>7-Ethyl-8-(4-fluorophenyl)-3-(1*H*-imidazol-2-yl)pyrazolo[5,1-*c*][1,2,4]triazin-4-ol</u> **(95)**

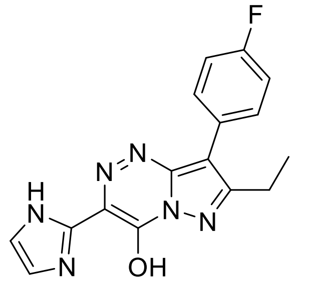

The compound was prepared according to General procedure C using:

Diazotization step:

3-ethyl-4-(4-fluorophenyl)-1*H*-pyrazol-5-amine (**S38**, 117 mg, 0.50 mmol; 1 eq.), 35% aqueous HCl (0.2 mL, 2.0 mmol; 4 eq.) in EtOH (2 mL) and H_2O (2 mL), $NaNO_2$ (68 mg, 1 mmol, 2 eq.) in H_2O (0.5 mL) (reaction time: 30 min), ethyl 2-(1*H*-imidazol-2-yl)acetate hydrobromide (100 mg, 0.50 mmol, 1 eq.) in EtOH (0.50 mL) and KOAc (300 mg, 3 mmol, 6 eq.) in H_2O (0.5 mL). Reaction time: 16 h. The yellow solid (102 mg, 0.30 mmol) obtained after the filtration was used in the next step without further purification.

Cyclization step:

the dried yellow solid (102 mg, 0.30 mmol), DMF (3 mL), and KOAc (3 mg, 0.03 mmol, 0.1 eq.). Reaction time: 2 h. The product was obtained as a yellow solid (85 mg, 52%).

^{1}H NMR (500 MHz, DMSO-d_6) δ (ppm) 13.48 (s, 2H), 7.84 – 7.77 (m, 2H), 7.42 (s, 2H), 7.33 – 7.23 (m, 2H), 2.93 (q, J = 7.5 Hz, 2H), 1.29 (t, J = 7.5 Hz, 3H).

^{13}C NMR (126 MHz, DMSO-d_6) δ (ppm) 160.39 (d, J = 243.5 Hz), 155.01, 148.06, 143.24, 130.24 (d, J = 7.9 Hz), 128.51, 119.79, 117.61, 114.64 (d, J = 21.3 Hz), 106.41, 20.45, 12.57.

HRMS (APCI): calcd. for $C_{16}H_{14}FN_6O$ $[M+H]^+ = 325.1208$, found $[M+H]^+ = 325.1212$.

3-(1*H*-benzo[*d*]imidazol-2-yl)-7-ethyl-8-(4-methoxyphenyl)pyrazolo[5,1-*c*][1,2,4]triazin-4-ol **(96)**

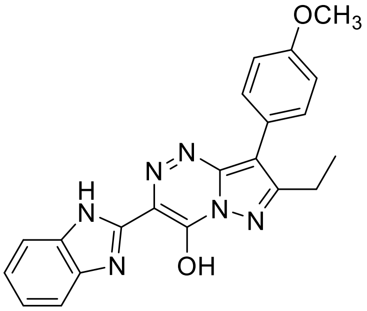

The compound was prepared according to General procedure C using:

Diazotization step:

3-ethyl-4-(4-methoxyphenyl)-1*H*-pyrazol-5-amine (**S73**, 100 mg, 0.50 mmol; 1 eq.), 35% aqueous HCl (0.2 mL, 2.0 mmol; 4 eq.) in EtOH (2 mL) and H_2O (2 mL), $NaNO_2$ (68 mg, 1 mmol, 2 eq.) in H_2O (0.5 mL) (reaction time: 30 min), ethyl 2-(1*H*-benzo[*d*]imidazol-2-yl)acetate (100 mg, 0.5 mmol, 1 eq.) in EtOH (0.5 mL) and KOAc (300 mg, 3 mmol, 6 eq.) in H_2O (0.5 mL). Reaction time: 16 h. The yellow solid (182 mg, 0.45 mmol) obtained after the filtration was used in the next step without further purification.

Cyclization step:

the dried yellow solid (182 mg, 0.45 mmol), DMF (3 mL), and KOAc (4 mg, 0.04 mmol, 0.1 eq.). Reaction time: 2 h. The product was obtained as a yellow solid (80 mg, 41%).

^{1}H NMR (500 MHz, DMSO-d_6) δ (ppm) 13.99 (s, 1H), 7.78 – 7.72 (m, 2H), 7.72 – 7.68 (m, 2H), 7.47 – 7.40 (m, 2H), 7.10 – 7.03 (m, 2H), 3.82 (s, 3H), 2.93 (q, $J = 7.5$ Hz, 2H), 1.28 (t, $J = 7.6$ Hz, 3H).

^{13}C NMR (126 MHz, DMSO-d_6) δ (ppm) 157.90, 155.60, 149.03, 148.53, 131.23, 130.10, 124.56, 124.21, 118.81, 113.92, 113.31, 109.17, 55.08, 20.82, 13.18.

HRMS (APCI): calcd. for $C_{21}H_{19}N_6O_2$ $[M+H]^+ = 387.1564$, found $[M+H]^+= 387.1565$.

3-(1*H*-benzo[*d*]imidazol-2-yl)-8-(4-(dimethylamino)phenyl)-7-ethylpyrazolo[5,1-*c*][1,2,4]triazin-4-ol **(97)**

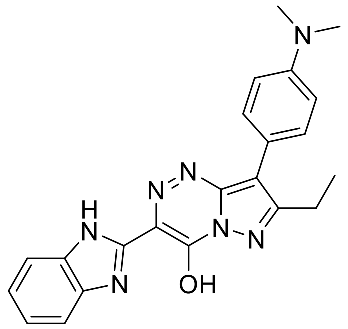

The compound was prepared according to General procedure C using:

Diazotization step:

4-(4-(dimethylamino)phenyl)-3-ethyl-1*H*-pyrazol-5-amine (115 mg, 0.50 mmol; 1 eq.), 35% aqueous HCl (0.2 mL, 2.0 mmol; 4 eq.) in EtOH (2 mL) and H_2O (2 mL), $NaNO_2$ (68 mg, 1 mmol, 2 eq.) in H_2O (0.5 mL) (reaction time: 30 min), ethyl 2-(1*H*-benzo[*d*]imidazol-2-yl)acetate (100 mg, 0.50 mmol, 1 eq.) in EtOH (0.5 mL) and KOAc (300 mg, 3 mmol, 6 eq.) in H_2O (0.5 mL). Reaction time: 16 h. The yellow solid (179 mg, 0.43 mmol) obtained after the filtration was used in the next step without further purification.

Cyclization step:

the dried yellow solid (179 mg, 0.43 mmol), DMF (3 mL), and KOAc (4 mg, 0.04 mmol, 0.1 eq.). Reaction time: 2 h. The product was obtained as a yellow solid (120 mg, 60%).

^{1}H NMR (500 MHz, DMSO-d_6) δ (ppm) 13.96 (s, 2H), 7.79 – 7.70 (m, 2H), 7.64 – 7.58 (m, 2H), 7.48 – 7.39 (m, 2H), 6.88 – 6.82 (m, 2H), 2.96 (s, 6H), 2.92 (q, J = 7.6 Hz, 2H), 1.28 (t, J = 7.5 Hz, 3H).

^{13}C NMR (126 MHz, DMSO-d_6) δ (ppm) 162.77, 156.05, 149.69, 149.66, 149.53, 149.15, 149.10, 131.68, 130.25, 130.13, 125.06, 120.05, 113.77, 112.84, 112.10, 40.61, 21.44, 13.78.

HRMS (APCI): calcd. for $C_{22}H_{22}N_7O$ $[M+H]^+$ = 400.1880, found $[M+H]^+$ = 400.1878.

3-(4-amino-3-(1*H*-benzo[*d*]imidazol-2-yl)-7-ethylpyrazolo[5,1-*c*][1,2,4]triazin-8-yl)benzoic acid **(98)**

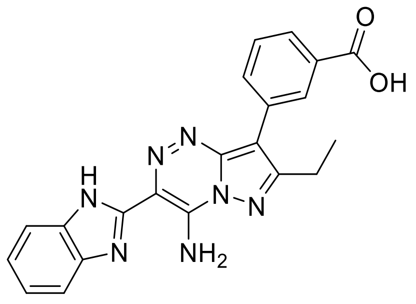

Methyl 3-(4-amino-3-(1*H*-benzo[*d*]imidazol-2-yl)-7-ethylpyrazolo[5,1-*c*][1,2,4]triazin-8-yl)benzoate (**75**, 45 mg, 0.11 mmol, 1 eq.) was dissolved in methanol (3 mL) and an aqueous solution of sodium hydroxide (3 M, 1.50 mL) was added. The mixture was stirred at 23 °C for 16 h, diluted with water (25 mL), neutralized with aqueous solution of potassium hydrogen sulfate (3 M, 1.50 mL). The mixture was extracted with EtOAc (3 × 25 mL), the combined organic extracts were washed with brine (50 mL), dried over $MgSO_4$, filtered, and the solvent was evaporated. To the residue was added EtOAc (1 mL) and the solid was collected by filtration and dried under vacuum. The product was obtained as a yellow solid (10 mg, 23%).

1H NMR (500 MHz, DMSO-d_6) δ (ppm) 13.41 (s, 1H), 9.96 (s, 1H), 9.40 (s, 1H), 8.59 – 8.55 (m, 1H), 8.08 (dt, J = 7.8, 1.5 Hz, 1H), 7.98 – 7.91 (m, 1H), 7.78 – 7.73 (m, 1H), 7.66 (t, J = 7.7 Hz, 1H), 7.62 – 7.53 (m, 1H), 7.31 – 7.21 (m, 2H), 3.10 (q, J = 7.5 Hz, 2H), 1.38 (t, J = 7.5 Hz, 3H).

^{13}C NMR (126 MHz, DMSO-d_6) δ (ppm) 167.32, 158.08, 150.01, 146.45, 142.70, 138.69, 133.70, 132.60, 131.71, 131.31, 129.71, 128.85, 127.30, 122.92, 121.85, 119.46, 118.31, 111.44, 107.02, 21.06, 13.07.

HRMS (APCI): calcd. for $C_{21}H_{18}N_7O_2$ $[M+H]^+$= 400.1516, found $[M+H]^+$ = 400.1519.

<u>3-(1-Benzyl-1*H*-benzo[*d*]imidazol-2-yl)-7-ethyl-8-(4-methoxyphenyl)pyrazolo[5,1-*c*][1,2,4]triazin-4-ol</u> **(99)**

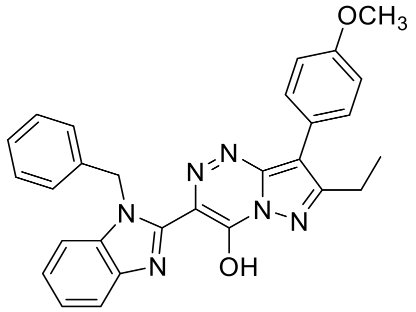

The compound was prepared according to General procedure C using:

Diazotization step:

3-ethyl-4-(4-methoxyphenyl)-1*H*-pyrazol-5-amine (**S73**, 55 mg, 0.25 mmol; 1 eq.), 35% aqueous HCl (0.1 mL, 1.0 mmol; 4 eq.) in EtOH (3 mL) and H_2O (3 mL), $NaNO_2$ (35 mg, 0.5 mmol, 2 eq.) in H_2O (1 mL) (reaction time: 30 min), methyl 2-(1-benzyl-1*H*-benzo[*d*]imidazol-2-yl)acetate (**S98**, 63 mg, 0.30 mmol, 1.2 eq.) in EtOH (1 mL) and KOAc (147 mg, 1.50 mmol, 6 eq.) in H_2O (1 mL). Reaction time: 16 h. The yellow solid (120 mg, 0.24 mmol) obtained after the filtration was used in the next step without further purification.

Cyclization step:

the dried yellow solid (120 mg, 0.24 mmol), DMF (3 mL), and KOAc (2 mg, 0.02 mmol, 0.1 eq.). Reaction time: 2 h. The product was obtained as a yellow solid (90 mg, 76%).

1H NMR (500 MHz, DMSO-*d6*) δ (ppm) 14.09 (s, 1H), 7.91 – 7.88 (m, 1H), 7.64 – 7.61 (m, 3H), 7.43 – 7.39 (m, 2H), 7.30 – 7.23 (m, 5H), 7.04 (d, J = 8.3 Hz, 2H), 6.33 – 6.08 (m, 2H), 3.80 (s, 3H), 2.90 (d, J = 7.9 Hz, 2H), 1.26 (t, J = 7.5 Hz, 3H).

^{13}C NMR (126 MHz, DMSO-d_6) δ (ppm) 130.16, 128.88, 128.54, 127.50, 126.89, 125.18, 113.95, 111.66, 55.07, 20.71, 13.06.

HRMS (APCI): calcd. for $C_{28}H_{25}N_6O_2$ $[M+H]^+$ = 477.2034, found $[M+H]^+$ = 477.2033.

<u>7-Ethyl-8-(4-fluorophenyl)-3-(6-morpholino-1*H*-benzo[*d*]imidazol-2-yl)pyrazolo[5,1-*c*][1,2,4]triazin-4-ol</u> **(100)**

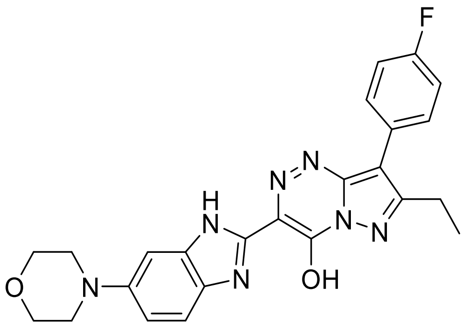

The compound was prepared according to General procedure C using:

Diazotization step:

3-ethyl-4-(4-fluorophenyl)-1*H*-pyrazol-5-amine (**S38**, 70 mg, 0.35 mmol; 1 eq.), 35% aqueous HCl (125 µL, 1.4 mmol; 4 eq.) in EtOH (3 mL) and H_2O (3 mL), $NaNO_2$ (36 mg, 0.52 mmol, 1.5 eq.) in EtOH (1 mL) (reaction time: 15 min), ethyl 2-(6-morpholino-1*H*-benzo[*d*]imidazol-2-yl)acetate (**S86**, 100 mg, 0.35 mmol, 1 eq.) in EtOH (2 mL) and KOAc (210 mg, 2.10 mmol, 6 eq.). Reaction time: 16 h. The yellow solid (143 mg, 0.30 mmol) obtained after the filtration was used in the next step without further purification.

Cyclization step:

the dried yellow solid (143 mg, 0.30 mmol), DMF (3 mL), and KOAc (3 mg, 0.03 mmol, 0.1 eq.). Reaction time: 2 h. After the filtration, the solid part was suspended in dichloromethane:MeOH (3:0.6 mL), the solid was collected by filtration and dried under vacuum. The product was obtained as a yellow solid (80 mg, 50%).

^{1}H NMR (500 MHz, DMSO-d_6) δ (ppm) 13.83 (s, 1H), 13.76 (s, 1H), 7.85 – 7.78 (m, 2H), 7.60 (d, J = 8.8 Hz, 1H), 7.36 – 7.27 (m, 2H), 7.18 (dd, J = 9.0, 2.2 Hz, 1H), 7.14 (d, J = 2.2 Hz, 1H), 3.81 – 3.76 (m, 4H), 3.18 – 3.12 (m, 4H), 2.94 (q, J = 7.5 Hz, 2H), 1.28 (t, J = 7.5 Hz, 3H).

^{13}C NMR (126 MHz, DMSO-d_6) δ (ppm) 160.79 (d, J = 243.7 Hz), 155.56, 149.47, 149.31, 148.74, 147.31, 132.19, 130.69 (d, J = 8.0 Hz), 128.48 (d, J = 3.2 Hz), 124.74, 119.45, 115.22 (d, J = 21.4 Hz), 115.05, 113.73, 98.06, 66.03, 49.25, 20.81, 13.08.

^{19}F NMR (282 MHz, DMSO-d_6) δ (ppm) -116.06.

HRMS (APCI): calcd. for $C_{24}H_{23}FN_7O_2$ $[M+H]^+$= 460.1892, found $[M+H]^+$ = 460.1893.

<u>7-Ethyl-8-(4-fluorophenyl)-3-(6-(4-methylpiperazin-1-yl)-1*H*-benzo[*d*]imidazol-2-yl)pyrazolo[5,1-*c*][1,2,4]triazin-4-ol</u> **(101)**

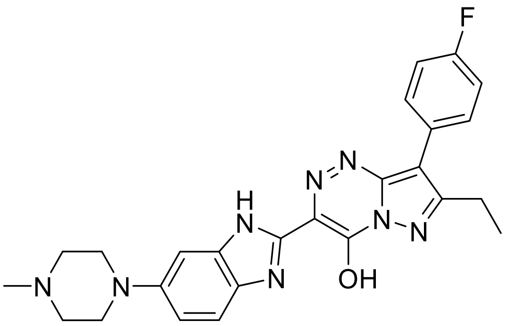

The compound was prepared according to General procedure C using:

Diazotization step:

3-ethyl-4-(4-fluorophenyl)-1*H*-pyrazol-5-amine (**S38**, 70 mg, 0.35 mmol; 1 eq.), 35% aqueous HCl (125 µL, 1.4 mmol; 4 eq.) in EtOH (3 mL) and H_2O (3 mL), $NaNO_2$ (36 mg, 0.52 mmol, 1.5 eq.) in EtOH (1 mL) (reaction time: 15 min), ethyl 2-(5-(4-methylpiperazin-1-yl)-1*H*-benzo[*d*]imidazol-2-yl)acetate (100 mg, 0.35 mmol, 1 eq.) in EtOH (2 mL) and KOAc (210 mg, 2.1 mmol, 6 eq.). Reaction time: 16 h. The yellow solid (123 mg, 0.25 mmol) obtained after the filtration was used in the next step without further purification.

Cyclization step:

the dried yellow solid (123 mg, 0.25 mmol), DMF (3 mL), and KOAc (3 mg, 0.03 mmol, 0.1 eq.). Reaction time: 2 h. After the reaction was completed, the solvent was evaporated, and the residue was suspended in EtOAc:MeOH (10:1 mL), filtered and the obtained solid was washed with water (3 mL), EtOAc (2 mL) and dried under vacuum. The product was obtained as a yellow solid (35 mg, 21%).

^{1}H NMR (500 MHz, DMSO-d_6) δ (ppm) 13.16 (s, 2H), 7.87 – 7.80 (m, 2H), 7.52 (d, J = 8.8 Hz, 1H), 7.30 (t, J = 8.7 Hz, 2H), 7.15 – 7.11 (m, 1H), 7.03 (d, J = 8.9 Hz, 1H), 3.18 – 3.13 (m, 4H), 2.93 (q, J = 7.5 Hz, 2H), 2.57 – 2.51 (m, 4H), 2.27 (s, 3H), 1.28 (t, J = 7.5 Hz, 3H).

^{13}C NMR (126 MHz, DMSO-d_6) δ (ppm) 160.99 (d, J = 242.9 Hz), 155.45, 150.03, 149.28 (d, J = 5.2 Hz), 148.71, 135.87, 130.87 (d, J = 7.7 Hz), 129.63 (d, J = 3.4 Hz), 123.26, 115.60 (d, J = 21.0 Hz), 114.93, 114.69, 106.37, 99.99, 55.11, 49.93, 46.03, 21.46, 13.65.

^{19}F NMR (282 MHz, DMSO-d_6) δ (ppm) -116.74.

HRMS (APCI): calcd. for $C_{25}H_{26}FN_8O$ $[M+H]^+$= 473.3223, found $[M+H]^+$ = 473.3234.

Methyl 3-(2-(7-ethyl-8-(4-fluorophenyl)-4-hydroxypyrazolo[5,1-*c*][1,2,4]triazin-3-yl)-1*H*-benzo[*d*]imidazol-6-yl)propanoate **(102)**

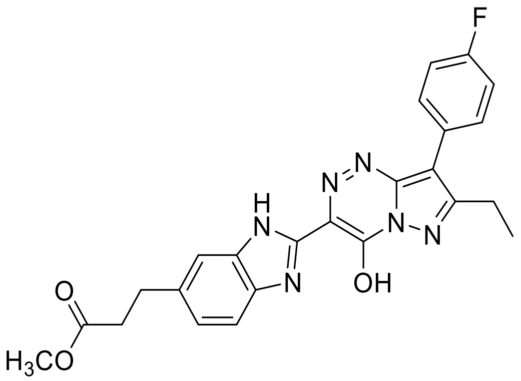

The compound was prepared according to General procedure C using:

Diazotization step:

3-ethyl-4-(4-fluorophenyl)-1*H*-pyrazol-5-amine (**S38**, 300 mg, 0.69 mmol; 1 eq.), 35% aqueous HCl (250 µL, 2.8 mmol; 4 eq.) in EtOH (5 mL) and H_2O (5 mL), $NaNO_2$ (72 mg, 1.04 mmol, 1.5 eq.) in EtOH (1 mL) (reaction time: 15 min), methyl 3-(2-(2-ethoxy-2-oxoethyl)-1*H*-benzo[*d*]imidazol-6-yl)propanoate (**S88**, 200 mg, 0.69 mmol, 1 eq.) in EtOH (2 mL) and KOAc (420 mg, 4.2 mmol, 6 eq.). Reaction time: 16 h. The yellow solid (310 mg, 0.65 mmol) obtained after the filtration was used in the next step without further purification.

Cyclization step:

the dried yellow solid (310 mg, 0.65 mmol), DMF (5 mL), and KOAc (6 mg, 0.06 mmol, 0.1 eq.). Reaction time: 2 h. After filtration, the residue was suspended in EtOAc:MeOH (20:0.5 mL), the solid was collected by filtration, washed with EtOAc (2 × 5 mL), and dried under vacuum. The product was obtained as a yellow solid (200 mg, 63%).

^{1}H NMR (500 MHz, DMSO-d_6) δ (ppm) 14.01 (s, 1H), 13.92 (s, 1H), 7.84 – 7.78 (m, 2H), 7.66 (d, J = 8.3 Hz, 1H), 7.57 (s, 1H), 7.36 – 7.29 (m, 3H), 3.60 (s, 3H), 3.02 (t, J = 7.5 Hz, 2H), 2.94 (q, J = 7.5 Hz, 2H), 2.70 (t, J = 7.5 Hz, 2H), 1.28 (t, J = 7.5 Hz, 3H).

^{13}C NMR (126 MHz, DMSO-d_6) δ (ppm) 172.46, 160.84 (d, J = 243.9 Hz), 155.66, 149.24, 148.83, 148.21, 137.65, 131.25, 130.75 (d, J = 7.9 Hz), 129.65, 128.79 – 128.13 (m), 125.34, 119.24, 115.24 (d, J = 21.3 Hz), 113.22, 112.48, 108.12, 51.27, 35.18, 30.31, 20.78, 13.07.

^{19}F NMR (282 MHz, DMSO-d_6) δ (ppm) -115.95.

HRMS (APCI): calcd. for $C_{24}H_{22}FN_6O_3$ $[M+H]^+$= 461.1732, found $[M+H]^+$ = 461.1734.

<u>3-(2-(7-Ethyl-8-(4-fluorophenyl)-4-hydroxypyrazolo[5,1-*c*][1,2,4]triazin-3-yl)-1*H*-benzo[*d*]imidazol-6-yl)propanoic acid</u> **(103)**

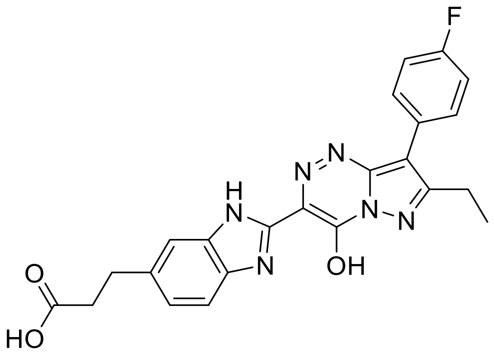

Methyl 3-(2-(7-ethyl-8-(4-fluorophenyl)-4-hydroxypyrazolo[5,1-*c*][1,2,4]triazin-3-yl)-1*H*-benzo[*d*]imidazol-6-yl)propanoate (**102**, 90 mg, 0.19 mmol, 1 eq.) was dissolved in THF:H_2O (8:2 mL, 4:1), LiOH (70 mg, 2.92 mmol, 15 eq.) was added. The mixture was stirred at 23 °C for 16 h, then neutralized with aqueous hydrochloric acid (2 M, 1.50 mL). The THF was evaporated, and the resulting solid was collected by filtration and washed on the filter with water (5 mL) and then with mixture of EtOAc:MeOH (1:0.2 mL) and dried under vacuum. The product was obtained as a yellow solid (30 mg, 35%).

^{1}H NMR (500 MHz, DMSO-d_6) δ (ppm) 14.02 (s, 1H), 13.92 (s, 1H), 7.85 – 7.77 (m, 2H), 7.66 (d, J = 8.3 Hz, 1H), 7.58 (s, 1H), 7.37 – 7.28 (m, 3H), 2.99 (t, J = 7.5 Hz, 2H), 2.94 (q, J = 7.5 Hz, 2H), 2.61 (t, J = 7.5 Hz, 2H), 1.28 (t, J = 7.5 Hz, 3H).

^{13}C NMR (126 MHz, DMSO-d_6) δ (ppm) 173.50, 160.85 (d, J = 243.7 Hz), 155.67, 148.82, 148.12, 138.06, 131.24, 130.76 (d, J = 7.9 Hz), 129.59, 128.35, 125.39, 119.30, 115.25 (d, J = 21.3 Hz), 113.19, 112.48, 108.08, 35.51, 30.40, 20.78, 13.07.

^{19}F NMR (282 MHz, DMSO-d_6) δ (ppm) -115.93.

HRMS (APCI): calcd. for $C_{23}H_{20}FN_6O_3$ $[M+H]^+$= 447.1575, found $[M+H]^+$ = 447.1577.

<u>*tert*-Butyl 2-(7-ethyl-8-(4-fluorophenyl)-4-hydroxypyrazolo[5,1-*c*][1,2,4]triazin-3-yl)-3,4,6,7-tetrahydro-5*H*-imidazo[4,5-*c*]pyridine-5-carboxylate</u> **(104)**

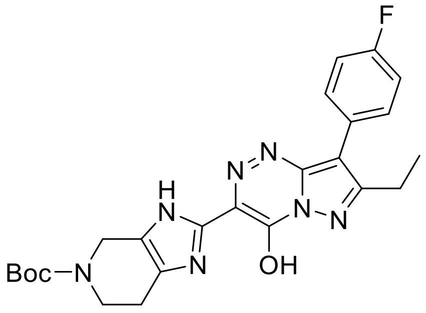

The compound was prepared according to General procedure C using:

Diazotization step:

3-ethyl-4-(4-fluorophenyl)-1*H*-pyrazol-5-amine (**S38**, 80 mg, 0.39 mmol; 1 eq.), 35% aqueous HCl (0.14 mL, 1.55 mmol; 4 eq.) in EtOH (1 mL) and H_2O (3 mL), $NaNO_2$ (0.054 g, 0.78 mmol, 2 eq.) in EtOH (1 mL) and H_2O (1 mL) (reaction time: 30 min), *tert*-butyl 2-(2-ethoxy-2-oxoethyl)-3,4,6,7-

tetrahydro-5*H*-imidazo[4,5-*c*]pyridine-5-carboxylate (**S90**, 120 mg, 0.39 mmol, 1 eq.) in EtOH (2 mL) and H_2O (1 mL) and KOAc (305 mg, 3.10 mmol, 8 eq.) Reaction time: 2 h. The reaction mixture was poured into water (20 mL) and extracted with EtOAc (2 × 50 mL). The combined organic extracts were washed with brine (35 mL), dried over $MgSO_4$, filtered, and the solvent was removed *in vacuo*. The resulting yellow solid (210 mg, quant.) was used in the next step without further purification.

Cyclization step:

the dried yellow solid (210 mg), DMF (2 mL), and KOAc (2 mg, 0.02 mmol, 0.1 eq.). Reaction time: 1.5 h. The product was obtained as an orange solid (144 mg, 77% (2 steps)).

^{1}H NMR (500 MHz, DMSO-d_6) δ (ppm) 13.71 (s, 1H), 13.49 (s, 1H), 7.79 (dd, J = 8.7, 5.7 Hz, 2H), 7.29 (dd, J = 8.9 Hz, 1H), 4.49 (s, 2H), 3.68 (t, J = 5.6 Hz, 2H), 2.92 (q, J = 7.6 Hz, 2H), 2.72 (q, J = 5.9, 5.4 Hz, 2H), 1.44 (s, 9H), 1.26 (t, J = 7.5 Hz, 3H).

^{13}C NMR (126 MHz, DMSO-d_6) δ (ppm) 160.64 (d, J = 243.4 Hz), 155.29, 148.06, 142.29, 130.54 (d, J = 7.5 Hz), 128.78, 124.78, 115.15 (d, J = 21.6 Hz), 79.62, 27.94, 20.81, 13.15.

^{19}F NMR (471 MHz, DMSO-d_6) δ (ppm) -116.38.

HRMS (APCI): calcd. for $C_{24}H_{27}FN_7O_3$ $[M+H]^+$ = 480.2154, found $[M+H]^+$ = 480.2152.

3-(1-Benzyl-1*H*-imidazol-2-yl)-7-ethyl-8-(4-fluorophenyl)pyrazolo[5,1-*c*][1,2,4]triazin-4-ol **(105)**

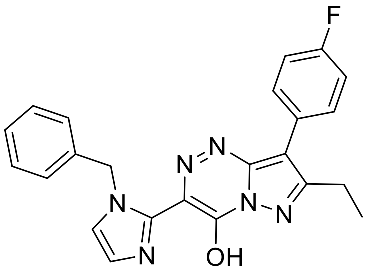

The compound was prepared according to General procedure C using:

Diazotization step:

3-ethyl-4-(4-fluorophenyl)-1*H*-pyrazol-5-amine (**S38**, 200 mg, 0.97 mmol; 1 eq.), 35% aqueous HCl (336 µL, 3.9 mmol; 4 eq.) in EtOH (10 mL) and H_2O (10 mL), $NaNO_2$ (135 mg, 1.95 mmol, 2 eq.) in EtOH (1 mL) (reaction time: 15 min), ethyl 2-(1-benzyl-1*H*-imidazol-2-yl)acetate (**S89**, 286 mg, 1.17 mmol, 1.2 eq.) in EtOH (2 mL) and KOAc (570 mg, 4.14 mmol, 6 eq.). Reaction time: 16 h. The yellow solid (302 mg, 0.7 mmol) obtained after the filtration was used in the next step without further purification.

Cyclization step:

the dried yellow solid (302 mg, 0.7 mmol), DMF (5 mL), and KOAc (7 mg, 0.07 mmol, 0.1 eq.). Reaction time: 2 h. After the filtration, the solid was suspended in hot dioxane (10 mL) and the mixture was poured into water (30 mL). The solid was collected by filtration, washed with water (10 mL), diethyl ether (10 mL) and dried under vacuum. The product was obtained as a yellow solid (200 mg, 50%).

^{1}H NMR (500 MHz, DMSO-d_6) δ (ppm) 13.70 (s, 1H), 7.79 – 7.74 (m, 2H), 7.74 – 7.70 (m, 1H), 7.55 (s, 1H), 7.37 – 7.24 (m, 7H), 5.93 (s, 2H), 2.91 (q, J = 7.5 Hz, 2H), 1.26 (t, J = 7.5 Hz, 2H).
^{13}C NMR (126 MHz, DMSO-d_6) δ (ppm) 160.68 (d, J = 243.1 Hz), 155.41, 148.72, 141.26, 136.17, 130.60 (d, J = 8.0 Hz), 128.66, 128.48, 127.89, 127.42, 122.81, 118.28, 115.18 (d, J = 21.2 Hz), 106.41, 51.84, 20.79, 13.02.
^{19}F NMR (471 MHz, DMSO-d_6) δ (ppm) -116.26.
HRMS (APCI): calcd. for $C_{23}H_{20}FN_6O$ $[M+H]^+$ = 415.1677, found $[M+H]^+$ = 415.1680.

3-(1*H*-benzo[*d*]imidazol-2-yl)-8-(4-fluorophenyl)-7-(2-methoxybenzyl)pyrazolo[5,1-*c*][1,2,4]triazin-4-ol **(106)**

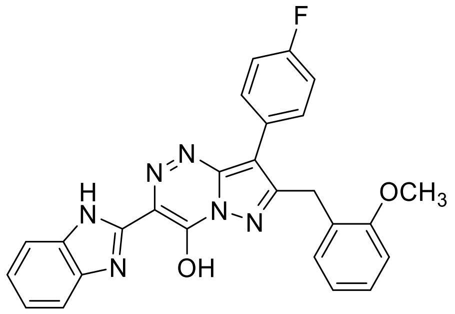

The compound was prepared according to General procedure C using:
Diazotization step:
4-(4-fluorophenyl)-3-(2-methoxybenzyl)-1*H*-pyrazol-5-amine (**S60**, 190 mg, 0.64 mmol; 1 eq.), 35% aqueous HCl (220 µL, 2.56 mmol; 4 eq.) in EtOH (5 mL) and H_2O (5 mL), $NaNO_2$ (88 mg, 1.28 mmol, 2 eq.) in EtOH (1 mL) (reaction time: 15 min), ethyl 2-(1*H*-1,3-benzodiazol-2-yl)acetate (157 mg, 0.77 mmol, 1.2 eq.) in EtOH (2 mL) and KOAc (380 mg, 3.8 mmol, 6 eq.). Reaction time: 16 h. The yellow solid (242 mg, 0.5 mmol) obtained after the filtration was used in the next step without further purification.
Cyclization step:
the dried yellow solid (242 mg, 0.50 mmol), DMF (5 mL), and KOAc (5 mg, 0.05 mmol, 0.1 eq.). Reaction time: 2 h. After the filtration, the solid part was suspended in hot dioxane (6 mL), and the mixture was poured into water (30 mL). The solid part was collected by filtration, washed with water (10 mL), diethyl ether (10 mL) and dried under vacuum. The product was obtained as a yellow solid (200 mg, 67%).
^{1}H NMR (500 MHz, DMSO-d_6) δ (ppm) 14.06 (s, 2H), 7.78 – 7.69 (m, 4H), 7.47 – 7.42 (m, 2H), 7.29 – 7.22 (m, 2H), 7.21 – 7.12 (m, 1H), 6.96 (dd, J = 24.0, 7.9 Hz, 2H), 6.84 – 6.78 (m, 1H), 4.20 (s, 2H), 3.78 (s, 3H).
^{13}C NMR (126 MHz, DMSO-d_6) δ (ppm) 160.86 (d, J = 243.6 Hz), 156.52, 152.67, 149.40, 148.85, 148.30, 131.10, 130.60 (d, J = 8.0 Hz), 129.27, 128.26 (d, J = 2.4 Hz), 127.54, 126.76, 124.72, 120.21, 119.34, 115.21, 115.04, 113.37, 110.60, 109.23, 55.31, 27.08.

^{19}F NMR (471 MHz, DMSO-d_6) δ (ppm) -115.82.

HRMS (APCI): calcd. for $C_{26}H_{20}FN_6O_2$ $[M+H]^+$ = 467.1626, found $[M+H]^+$ = 467.1630.

6-(1*H*-benzo[*d*]imidazol-2-yl)-2-ethyl-3-(4-fluorophenyl)-4-methylpyrazolo[1,5-*a*]pyrimidin-7(4*H*)-one **(107)**

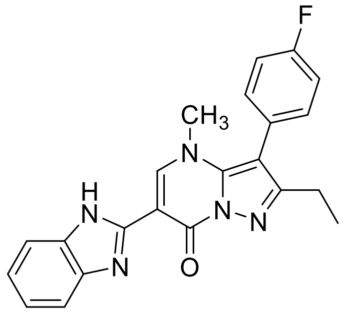

A mixture of 3-ethyl-4-(4-fluorophenyl)-*N*-methyl-1*H*-pyrazol-5-amine (**S83**, 75 mg, 0.34 mmol; 1 eq.) and ethyl-2-(1*H*-benzo[*d*]imidazol-2-yl)-3-(dimethylamino)acrylate (**S104**, 93 mg, 0.36 mmol, 1.05 eq.) in EtOH (3 mL) was stirred in the microwave reactor at 130 °C for 1 h. The precipitate was collected by filtration, washed with EtOH (5 mL), diethyl ether (5 mL) and dried under vacuum. The product was obtained as a white solid (20 mg, 15%)

^{1}H NMR (500 MHz, DMSO-d_6) δ (ppm) 12.31 (s, 1H), 8.94 (s, 1H), 7.69 – 7.63 (m, 1H), 7.59 – 7.45 (m, 3H), 7.36 – 7.27 (m, 2H), 7.19 – 7.12 (m, 2H), 3.50 (s, 3H), 2.55 (q, J = 7.6 Hz, 2H), 1.10 (t, J = 7.6 Hz, 3H).

^{13}C NMR (126 MHz, DMSO-d_6) δ (ppm) 161.91 (d, J = 245.1 Hz), 157.04, 154.57, 147.14, 144.81, 142.65, 138.38, 134.46, 133.57 (d, J = 8.3 Hz), 127.00 (d, J = 3.3 Hz), 121.47 (d, J = 10.5 Hz), 117.44, 115.09 (d, J = 21.4 Hz), 112.18, 104.72, 98.38, 41.51, 20.13, 13.04.

^{19}F NMR (471 MHz, DMSO-d_6) δ (ppm) -113.99.

HRMS (APCI): calcd. for $C_{22}H_{19}FN_5O$ $[M+H]^+$ = 388.1568, found $[M+H]^+$ = 388.1569.

3-(1*H*-benzo[*d*]imidazol-2-yl)-7-ethyl-8-(tetrahydro-2*H*-pyran-4-yl)pyrazolo[5,1-*c*][1,2,4]triazin-4-ol **(108)**

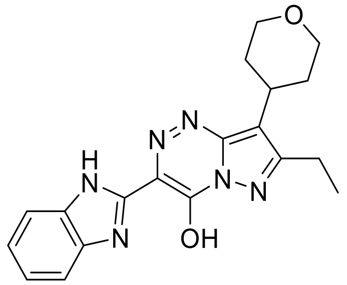

The compound was prepared according to General procedure C using:

Diazotization step:

3-ethyl-4-(tetrahydro-2*H*-pyran-4-yl)-1*H*-pyrazol-5-amine (**S62**, 114 mg, 0.58 mmol; 1 eq.), 35% aqueous HCl (200 µL, 2.33 mmol; 4 eq.) in EtOH (5 mL) and H_2O (5 mL), $NaNO_2$ (81 mg, 1.17 mmol, 2 eq.) in EtOH (1 mL) (reaction time: 15 min), ethyl 2-(1*H*-1,3-benzodiazol-2-yl)acetate (131 mg, 0.64 mmol, 1.1 eq.) in EtOH (2 mL) and KOAc (344 mg, 3.5 mmol, 6 eq.). Reaction time: 16 h. The yellow solid (190 mg, 0.5 mmol) obtained after the filtration was used in the next step without further purification.

Cyclization step:

the dried yellow solid (190 mg, 0.5 mmol), DMF (5 mL), and KOAc (5 mg, 0.05 mmol, 0.1 eq.). Reaction time: 2 h. After the filtration, the solid was suspended in hot dioxane (5 mL) and the mixture was poured into water (20 mL). The solid was collected by filtration, washed with water (10 mL), diethyl ether (10 mL) and dried under vacuum. The product was obtained as a yellow solid (130 mg, 61%).

^{1}H NMR (500 MHz, DMSO-d_6) δ (ppm) 13.90 (s, 2H), 7.73 (dd, J = 6.0, 3.2 Hz, 2H), 7.43 (dt, J = 6.0, 3.6 Hz, 2H), 3.98 (dd, J = 11.2, 4.2 Hz, 2H), 3.53 – 3.44 (m, 2H), 3.11 – 2.98 (m, 1H), 2.80 (q, J = 7.5 Hz, 2H), 2.42 – 2.30 (m, 2H), 1.70 – 1.63 (m, 2H), 1.28 (t, J = 7.5 Hz, 3H).

^{13}C NMR (126 MHz, DMSO-d_6) δ (ppm) 156.03, 149.81, 149.20, 148.85, 131.07, 124.50, 117.40, 113.20, 112.54, 67.65, 32.49, 31.52, 20.16, 13.99.

HRMS (APCI): calcd. for $C_{19}H_{21}N_6O_2$ $[M+H]^+$ = 365.1721, found $[M+H]^+$ = 365.1719.

<u>3-(1*H*-benzo[*d*]imidazol-2-yl)-8-benzyl-7-ethylpyrazolo[5,1-*c*][1,2,4]triazin-4-ol</u> **(109)**

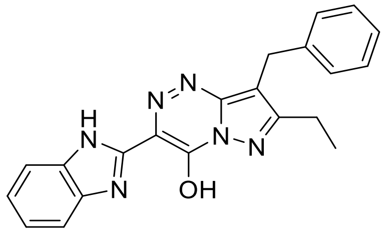

The compound was prepared according to General procedure C using:

Diazotization step:

4-benzyl-3-ethyl-1*H*-pyrazol-5-amine (**S63**, 121 mg, 0.60 mmol; 1 eq.), 35% aqueous HCl (207 µL, 2.4 mmol; 4 eq.) in EtOH (5 mL) and H_2O (5 mL), $NaNO_2$ (83 mg, 1.20 mmol, 2 eq.) in EtOH (1 mL) (reaction time: 15 min), ethyl 2-(1*H*-1,3-benzodiazol-2-yl)acetate (135 mg, 0.60 mmol, 1.1 eq.) in EtOH (2 mL) and KOAc (354 mg, 3.60 mmol, 6 eq.) Reaction time: 16 h. The yellow solid (213 mg, 0.55 mmol) obtained after the filtration was used in the next step without further purification.

Cyclization step:

the dried yellow solid (213 mg, 0.55 mmol), DMF (5 mL), and KOAc (6 mg, 0.06 mmol, 0.1 eq.). Reaction time: 2 h. After the filtration, the solid was suspended in hot dioxane (5 mL) and the mixture was poured into water (20 mL). The solid was collected by filtration, washed with water (10 mL), diethyl ether (10 mL) and dried under vacuum. The product was obtained as a yellow solid (165 mg, 74%).

^{1}H NMR (500 MHz, DMSO-d_6) δ (ppm) 13.94 (s, 1H), 7.77 – 7.70 (m, 2H), 7.46 – 7.40 (m, 2H), 7.33 – 7.23 (m, 4H), 7.20 – 7.12 (m, 1H), 4.16 (s, 2H), 2.69 (q, J = 7.6 Hz, 2H), 1.16 (t, J = 7.6 Hz, 3H).
^{13}C NMR (126 MHz, DMSO-d_6) δ (ppm) 156.93, 150.23, 149.18, 148.73, 140.81, 131.11, 128.29, 128.25, 128.15, 125.80, 124.50, 117.94, 113.24, 107.76, 27.77, 19.99, 13.14.
HRMS (APCI): calcd. for $C_{21}H_{18}N_6O$ $[M+H]^+$ = 371.1615, found $[M+H]^+$ = 371.1616.

3-(1*H*-benzo[*d*]imidazol-2-yl)-8-(cyclohexylmethyl)-7-ethylpyrazolo[5,1-*c*][1,2,4]triazin-4-ol **(110)**

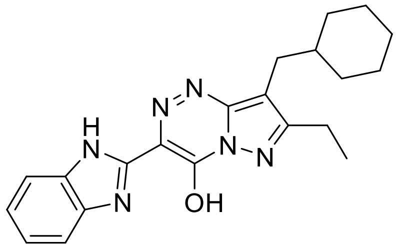

The compound was prepared according to General procedure C using:
Diazotization step:
4-(cyclohexylmethyl)-3-ethyl-1*H*-pyrazol-5-amine (**S64**, 160 mg, 0.77 mmol; 1 eq.), 35% aqueous HCl (270 µL, 3.1 mmol; 4 eq.) in EtOH (5 mL) and H_2O (5 mL), $NaNO_2$ (107 mg, 1.54 mmol, 2 eq.) in EtOH (1 mL) (reaction time: 15 min), ethyl 2-(1*H*-1,3-benzodiazol-2-yl)acetate (173 mg, 0.85 mmol, 1.1 eq.) in EtOH (2 mL) and KOAc (454 mg, 4.63 mmol, 6 eq.). Reaction time: 16 h. The yellow solid (264 mg, 0.67 mmol) obtained after the filtration was used in the next step without further purification.
Cyclization step:
the dried yellow solid (264 mg, 0.67 mmol), DMF (5 mL), and KOAc (7 mg, 0.07 mmol, 0.1 eq.). Reaction time: 2 h. After the filtration, the solid was suspended in hot dioxane (5 mL) and the mixture was poured into water (20 mL). The solid was collected by filtration, washed with water (10 mL), diethyl ether (10 mL) and dried under vacuum. The product was obtained as a yellow solid (220 mg, 76%).
^{1}H NMR (500 MHz, DMSO-d_6) δ (ppm) 13.87 (s, 2H), 7.76 – 7.69 (m, 2H), 7.45 – 7.38 (m, 2H), 2.75 (q, J = 7.6 Hz, 2H), 2.66 (d, J = 6.9 Hz, 2H), 1.77 – 1.54 (m, 6H), 1.28 (t, J = 7.6 Hz, 3H), 1.22 – 1.10 (m, 3H), 1.06 – 0.97 (m, 2H).
^{13}C NMR (126 MHz, DMSO-d_6) δ (ppm) 157.10, 150.26, 149.24, 148.86, 131.09, 124.42, 117.39, 113.19, 107.72, 66.29, 38.32, 32.73, 29.67, 26.03, 25.67, 19.94, 13.42.
HRMS (APCI): calcd. for $C_{21}H_{24}N_6O$ $[M+H]^+$ = 377.2084, found $[M+H]^+$ = 377.2082.

3-(1*H*-benzo[*d*]imidazol-2-yl)-8-(2-chlorobenzyl)-7-ethylpyrazolo[5,1-*c*][1,2,4]triazin-4-ol **(111)**

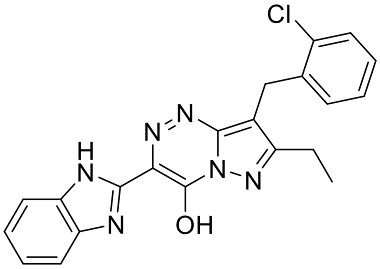

The compound was prepared according to General procedure C using:

Diazotization step:

4-(2-chlorobenzyl)-3-ethyl-1*H*-pyrazol-5-amine (**S65**, 130 mg, 0.55 mmol; 1 eq.), 35% aqueous HCl (190 μL, 2.2 mmol; 4 eq.) in EtOH (5 mL) and H_2O (5 mL), $NaNO_2$ (76 mg, 1.1 mmol, 2 eq.) in EtOH (1 mL) (reaction time: 15 min), ethyl 2-(1*H*-1,3-benzodiazol-2-yl)acetate (124 mg, 0.6 mmol, 1.1 eq.) in EtOH (2 mL) and KOAc (325 mg, 4.63 mmol, 6 eq.). Reaction time: 16 h. The yellow solid (190 mg, 0.45 mmol) obtained after the filtration was used in the next step without further purification.

Cyclization step:

the dried yellow solid (190 mg, 0.45 mmol), DMF (5 mL), and KOAc (5 mg, 0.05 mmol, 0.1 eq.). Reaction time: 2 h. After the filtration, the solid was suspended in hot dioxane (5 mL) and the mixture was poured into water (20 mL). The solid was collected by filtration, washed with water (10 mL), diethyl ether (10 mL) and dried under vacuum. The product was obtained as a yellow solid (150 mg, 67%).

^{1}H NMR (500 MHz, DMSO-*d*$_6$) δ (ppm) 13.95 (s, 1H), 7.77 – 7.70 (m, 2H), 7.48 – 7.39 (m, 3H), 7.28 – 7.18 (m, 3H), 4.25 (s, 2H), 2.68 (q, J = 7.5 Hz, 2H), 1.16 (t, J = 7.6 Hz, 3H).

^{13}C NMR (126 MHz, DMSO-*d*$_6$) δ (ppm) 157.09, 150.45, 149.12, 148.66, 137.64, 132.71, 131.08, 130.39, 129.04, 127.85, 127.07, 124.54, 118.13, 113.24, 105.57, 25.50, 20.08, 13.11.

HRMS (APCI): calcd. for $C_{21}H_{18}ClN_6O$ $[M+H]^+$ = 405.1225, found $[M+H]^+$ = 405.1229.

3-(1*H*-benzo[*d*]imidazol-2-yl)-7-ethyl-8-(pyridin-3-ylmethyl)pyrazolo[5,1-*c*][1,2,4]triazin-4-ol **(112)**

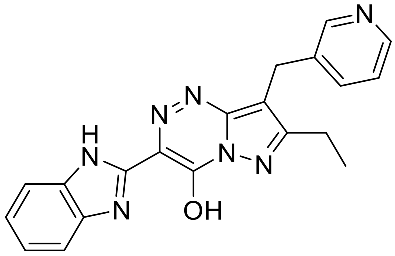

The compound was prepared according to General procedure C using:

Diazotization step:

3-ethyl-4-(pyridin-3-ylmethyl)-1*H*-pyrazol-5-amine (**S66**, 100 mg, 0.50 mmol; 1 eq.), 35% aqueous HCl (170 µL, 2.0 mmol; 4 eq.) in EtOH (5 mL) and H_2O (5 mL), $NaNO_2$ (68 mg, 1.0 mmol, 2 eq.) in EtOH (1 mL) (reaction time: 15 min), ethyl 2-(1*H*-1,3-benzodiazol-2-yl)acetate (111 mg, 0.54 mmol, 1.1 eq.) in EtOH (2 mL) and KOAc (290 mg, 2.97 mmol, 6 eq.). Reaction time: 16 h. The yellow solid (175 mg, 0.45 mmol) obtained after the filtration was used in the next step without further purification.

Cyclization step:

the dried yellow solid (175 mg, 0.45 mmol), DMF (5 mL), and KOAc (5 mg, 0.05 mmol, 0.1 eq.). Reaction time: 2 h. After the filtration, the solid was suspended in hot dioxane (5 mL) and the mixture was poured into water (20 mL). The solid was collected by filtration, washed with water (10 mL), diethyl ether (10 mL) and dried under vacuum. The product was obtained as a yellow solid (130 mg, 71%).

^{1}H NMR (500 MHz, DMSO-d_6) δ (ppm) 13.95 (s, 1H), 8.61 – 8.57 (m, 1H), 8.41 – 8.36 (m, 1H), 7.77 – 7.70 (m, 2H), 7.69 – 7.65 (m, 1H), 7.47 – 7.40 (m, 2H), 7.28 (dd, J = 7.9, 4.7 Hz, 1H), 4.18 (s, 2H), 2.73 (q, J = 7.6 Hz, 2H), 1.18 (t, J = 7.6 Hz, 3H).

^{13}C NMR (126 MHz, DMSO-d_6) δ (ppm) 156.79, 149.43, 149.10, 148.65, 147.19, 136.34, 135.69, 131.18, 124.53, 123.41, 118.34, 113.28, 106.67, 25.11, 19.90, 13.26.

HRMS (APCI): calcd. for $C_{20}H_{18}N_7O$ $[M+H]^+$ = 372.1567, found $[M+H]^+$ = 372.1570.

<u>3-(1*H*-benzo[*d*]imidazol-2-yl)-7-ethyl-8-(2-methoxybenzyl)pyrazolo[5,1-*c*][1,2,4]triazin-4-ol</u> **(113)**

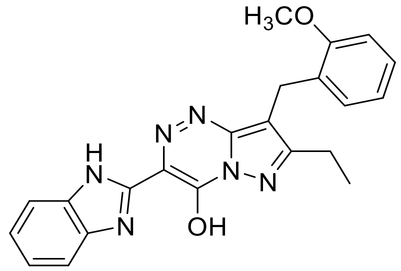

The compound was prepared according to General procedure C using:

Diazotization step:

3-ethyl-4-(2-methoxybenzyl)-1H-pyrazol-5-amine (**S67**, 110 mg, 0.48 mmol; 1 eq.), 35% aqueous HCl (160 µL, 1.9 mmol; 4 eq.) in EtOH (5 mL) and H_2O (5 mL), $NaNO_2$ (66 mg, 0.95 mmol, 2 eq.) in EtOH (1 mL) (reaction time: 15 min), ethyl 2-(1*H*-1,3-benzodiazol-2-yl)acetate (107 mg, 0.52 mmol, 1.1 eq.) in EtOH (2 mL) and KOAc (280 mg, 2.85 mmol, 6 eq.). Reaction time: 16 h. The yellow solid (167 mg, 0.4 mmol) obtained after the filtration was used in the next step without further purification.

Cyclization step:

the dried yellow solid (167 mg, 0.4 mmol), DMF (5 mL), and KOAc (4 mg, 0.04 mmol, 0.1 eq.). Reaction time: 2 h. After the filtration, the solid was suspended in hot dioxane (5 mL) and the mixture was poured into water (20 mL). The solid was collected by filtration, washed with water (10 mL), diethyl ether (10 mL) and dried under vacuum. The product was obtained as a yellow solid (100 mg, 53%).

^{1}H NMR (500 MHz, DMSO-d_6) δ (ppm) 13.79 (s, 2H), 7.77 – 7.71 (m, 2H), 7.44 – 7.39 (m, 2H), 7.20 – 7.13 (m, 1H), 7.07 (dd, J = 7.5, 1.8 Hz, 1H), 6.98 (d, J = 8.1 Hz, 1H), 6.81 (t, J = 7.4 Hz, 1H), 4.12 (s, 2H), 3.85 (s, 3H), 2.69 (q, J = 7.6 Hz, 2H), 1.19 (t, J = 7.6 Hz, 3H).

^{13}C NMR (126 MHz, DMSO-d_6) δ (ppm) 157.19, 156.63, 150.40, 149.21, 148.78, 131.12, 129.08, 128.23, 127.14, 124.46, 120.07, 117.81, 113.21, 110.40, 106.89, 55.25, 21.63, 19.92, 13.18.

HRMS (APCI): calcd. for $C_{22}H_{21}N_6O_2$ $[M+H]^+$ = 401.1721, found $[M+H]^+$ = 401.1722.

3-(1*H*-benzo[*d*]imidazol-2-yl)-7-ethyl-8-(4-fluorobenzyl)pyrazolo[5,1-*c*][1,2,4]triazin-4-ol **(114)**

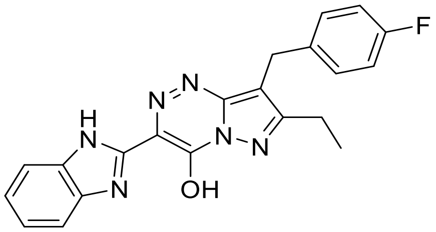

The compound was prepared according to General procedure C using:

Diazotization step:

3-ethyl-4-(4-fluorobenzyl)-1*H*-pyrazol-5-amine (**S68**, 100 mg, 0.46 mmol; 1 eq.), 35% aqueous HCl (160 µL, 1.8 mmol; 4 eq.) in EtOH (5 mL) and H_2O (5 mL), $NaNO_2$ (63 mg, 0.91 mmol, 2 eq.) in EtOH (1 mL) (reaction time: 15 min), ethyl 2-(1*H*-1,3-benzodiazol-2-yl)acetate (103 mg, 0.46 mmol, 1.1 eq.) in EtOH (2 mL) and KOAc (270 mg, 2.74 mmol, 6 eq.). Reaction time: 16 h. The yellow solid (170 mg, 0.42 mmol) obtained after the filtration was used in the next step without further purification.

Cyclization step:

the dried yellow solid (170 mg, 0.42 mmol), DMF (5 mL), and KOAc (4 mg, 0.04 mmol, 0.1 eq.). Reaction time: 2 h. After the filtration, the solid was suspended in hot dioxane (5 mL) and the mixture was poured into water (20 mL). The solid part was collected by filtration, washed with water (10 mL), diethyl ether (10 mL) and dried under vacuum. The product was obtained as a yellow solid (150 mg, 85%).

^1H NMR (500 MHz, DMSO-d_6) δ (ppm) 13.95 (s, 1H), 7.77 – 7.70 (m, 2H), 7.47 – 7.39 (m, 2H), 7.37 – 7.29 (m, 2H), 7.12 – 7.04 (m, 2H), 4.15 (s, 2H), 2.69 (q, J = 7.6 Hz, 2H), 1.16 (t, J = 7.6 Hz, 3H).

^{13}C NMR (126 MHz, DMSO-d_6) δ (ppm) 160.58 (d, J = 241.5 Hz), 156.88, 150.21, 149.15, 148.69, 136.97, 131.08, 129.89 (d, J = 8.0 Hz), 124.55, 118.01, 114.92 (d, J = 21.0 Hz), 113.25, 107.64, 26.96, 19.96, 13.20.

^{19}F NMR (471 MHz, DMSO-d_6) δ (ppm) -117.49.

HRMS (APCI): calcd. for $C_{21}H_{18}FN_6O$ $[M+H]^+$ 389.1521, found $[M+H]^+$ = 389.1525.

3-(1*H*-benzo[*d*]imidazol-2-yl)-7-ethyl-8-(1-methylpiperidin-4-yl)pyrazolo[5,1-*c*][1,2,4]triazin-4-ol **(115)**

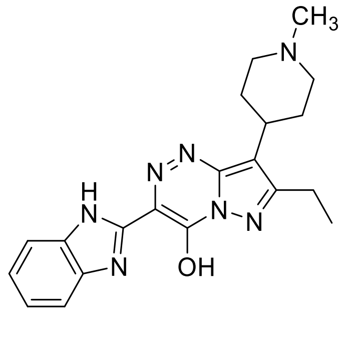

The compound was prepared according to General procedure C using:
Diazotization step:
3-ethyl-4-(1-methylpiperidin-4-yl)-1*H*-pyrazol-5-amine (**S69**, 110 mg, 0.53 mmol; 1 eq.), 35% aqueous HCl (180 µL, 2.11 mmol; 4 eq.) in EtOH (5 mL) and H_2O (5 mL), $NaNO_2$ (69 mg, 1.06 mmol, 2 eq.) in EtOH (1 mL) (reaction time: 15 min), ethyl 2-(1*H*-1,3-benzodiazol-2-yl)acetate (119 mg, 0.58 mmol, 1.1 eq.) in EtOH (2 mL) and KOAc (310 mg, 3.17 mmol, 6 eq.). Reaction time: 16 h. The yellow solid (160 mg, 0.40 mmol) obtained after the filtration was used in the next step without further purification.
Cyclization step:
the dried yellow solid (160 mg, 0.40 mmol), DMF (5 mL), and KOAc (4 mg, 0.04 mmol, 0.1 eq.). Reaction time: 2 h. After the filtration, the solid was suspended in hot dioxane (5 mL) and the mixture was poured into water (20 mL). The solid was collected by filtration, washed with water (10 mL), diethyl ether (10 mL) and dried under vacuum. The product was obtained as a yellow solid (120 mg, 60%).
^{1}H NMR (500 MHz, DMSO-d_6) δ (ppm) 7.71 – 7.64 (m, 2H), 7.32 – 7.25 (m, 2H), 3.23 – 3.13 (m, 2H), 2.95 – 2.86 (m, 1H), 2.78 (q, J = 7.6 Hz, 2H), 2.51 – 2.41 (m, 7H), 1.87 – 1.79 (m, 2H), 1.29 (t, J = 7.6 Hz, 3H).
^{13}C NMR (126 MHz, DMSO-d_6) δ (ppm) 155.19, 149.92, 149.62, 149.13, 134.00, 122.53, 119.78, 113.34, 109.66, 55.12, 44.51, 30.87, 30.35, 19.92, 13.48.
HRMS (APCI): calcd. for $C_{20}H_{24}N_7O$ $[M+H]^+$ 378.2037, found $[M+H]^+$ = 378.2039.

<u>3-(1*H*-Benzo[*d*]imidazol-2-yl)-8-((1-(*tert*-butyl)-1*H*-pyrazol-4-yl)methyl)-7-ethylpyrazolo[5,1-*c*][1,2,4]triazin-4-ol</u> **(116)**

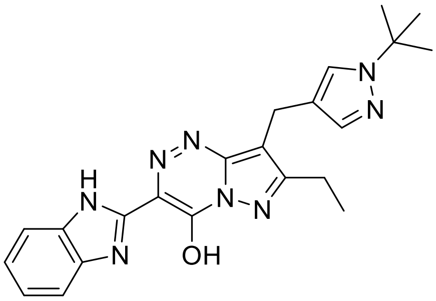

The compound was prepared according to General procedure C using:
Diazotization step:
4-((1-(*tert*-butyl)-1*H*-pyrazol-4-yl)methyl)-3-ethyl-1*H*-pyrazol-5-amine (**S70**, 100 mg, 0.40 mmol; 1 eq.), 35% aqueous HCl (140 µL, 1.62 mmol; 4 eq.) in EtOH (5 mL) and H_2O (5 mL), $NaNO_2$ (56 mg, 0.80 mmol, 2 eq.) in EtOH (1 mL) (reaction time: 15 min), ethyl 2-(1*H*-1,3-benzodiazol-2-yl)acetate (91 mg, 0.45 mmol, 1.1 eq.) in EtOH (2 mL) and KOAc (230 mg, 2.43 mmol, 6 eq.). Reaction time: 16 h. The yellow solid (130 mg, 0.30 mmol) obtained after the filtration was used in the next step without further purification.
Cyclization step:

the dried yellow solid (130 mg, 0.30 mmol), DMF (5 mL), and KOAc (3 mg, 0.03 mmol, 0.1 eq.). Reaction time: 2 h. After the filtration, the solid was suspended in hot dioxane (5 mL) and the mixture was poured into water (20 mL). The solid was collected by filtration, washed with water (10 mL), diethyl ether (10 mL) and dried under vacuum. The product was obtained as a yellow solid (80 mg, 48%).

^{1}H NMR (500 MHz, DMSO-d_6) δ (ppm) 13.93 (s, 1H), 7.76 – 7.71 (m, 2H), 7.56 (s, 1H), 7.46 – 7.39 (m, 2H), 7.28 (s, 1H), 3.96 (s, 2H), 2.71 (q, J = 7.6 Hz, 2H), 1.45 (s, 9H), 1.19 (t, J = 7.6 Hz, 3H).

^{13}C NMR (126 MHz, DMSO-d_6) δ (ppm) 156.69, 149.81, 149.23, 148.78, 137.24, 131.07, 124.48, 124.39, 119.15, 117.67, 113.20, 108.37, 57.52, 29.44, 19.95, 16.98, 13.24.

HRMS (APCI): calcd. for $C_{22}H_{25}N_8O$ [M+H]$^+$ 417.2146, found [M+H]$^+$ = 417.2148.

2-Ethyl-3,6-bis(4-fluorophenyl)pyrazolo[1,5-*a*]pyrimidin-7-amine **(117)**

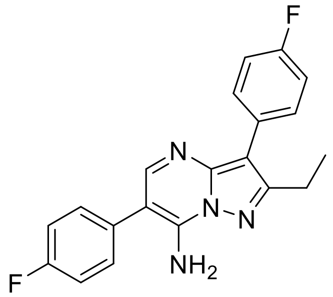

A mixture of 3-ethyl-4-(4-fluorophenyl)-1*H*-pyrazol-5-amine (**S38**, 41 mg, 0.20 mmol, 1eq.) and 2-(4-fluorophenyl)-3-oxopropanenitrile (33 mg, 0.20 mmol, 1 eq.) in DMF (2 mL) was refluxed for 1 h. The solvent was removed *in vacuo,* and the residue was purified twice by column chromatography on silica gel (hexane:EtOAc, gradient 3:1 to 0:1). The product was obtained as a white solid (26 mg, 37%).

^{1}H NMR (500 MHz, Chloroform-*d*) δ (ppm) 8.21 (s, 1H), 7.63 (dd, J = 8.8, 5.4 Hz, 2H), 7.46 (dd, J = 8.8, 5.3 Hz, 2H), 7.22 (t, J = 8.6 Hz, 2H), 7.16 (t, J = 8.7 Hz, 2H), 5.86 (s, 2H), 2.99 (d, J = 7.5 Hz, 2H), 1.36 (t, J = 7.5 Hz, 3H).

^{13}C NMR (126 MHz, Chloroform-*d*) δ (ppm) 162.68 (d, J = 249.0 Hz), 161.74 (d, J = 245.4 Hz), 157.70, 149.98, 145.77, 144.43, 131.14 (d, J = 8.2 Hz), 130.80 (d, J = 8.2 Hz), 129.82 (d, J = 3.1 Hz), 128.61 (d, J = 3.0 Hz), 116.81 (d, J = 21.2 Hz), 115.68 (d, J = 21.2 Hz), 107.44, 102.09, 21.50, 13.63.

^{19}F NMR (282 MHz, Chloroform-*d*) δ (ppm) -113.45, -116.52.

HRMS (APCI): calcd. for $C_{20}H_{17}N_4F_2$ [M+H]$^+$ = 351.1416, found [M+H]$^+$ = 351.1419.

Experimental procedures for compound 2 and its analogs

4-(2-Cyanoacetyl)benzonitrile (**S109**)

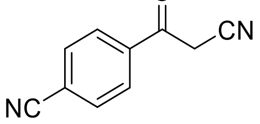

The compound was prepared according to General procedure A5 using NaH (60% suspension in mineral oil, 496 mg, 12.41 mmol), acetonitrile (0.388 mL, 7.45 mmol) in THF (10 mL) and methyl 4-cyanobenzoate (1 g, 6.21 mmol) in anhydrous THF (5 mL). The reaction mixture was refluxed for 2 h. The residue was purified by column chromatography (hexane:EtOAc, 8:2 to 7:3). The product was obtained as a white solid (732 mg, 69%).

^{1}H NMR (500 MHz, Chloroform-*d*) δ (ppm) 8.07 – 7.99 (m, 2H), 7.88 – 7.81 (m, 2H), 4.11 (s, 2H).

^{13}C NMR (126 MHz, Chloroform-*d*) δ (ppm) 186.2, 137.2, 133.1, 129.0, 118.2, 117.4, 113.1, 29.8.

HRMS (APCI): calcd. for $C_{10}H_5N_2O$ $[M-H]^-$ = 169.0407, found $[M-H]^-$ = 169.0406.

mp = 124−130 °C.

3-Oxo-3-(4-(trifluoromethyl)phenyl)propanenitrile **(S110)**

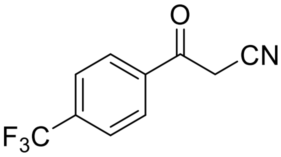

The compound was prepared according to General procedure A5 using NaH (60% suspension in mineral oil, 391 mg, 9.796 mmol), acetonitrile (0.306 mL, 5.878 mmol), THF (10 mL) and methyl 4-(trifluoromethyl)benzoate (1 g, 4.90 mmol) in anhydrous THF (5 mL). The reaction mixture was refluxed for 2 h. The residue was purified by column chromatography (hexane:EtOAc, 8:2 to 7:3). The product was obtained as a light brown solid (850 mg, 81%).

^{1}H NMR (500 MHz, Chloroform-*d*) δ (ppm) 8.05 (dp, J = 7.9, 0.9 Hz, 2H), 7.85 – 7.77 (m, 2H), 4.13 (s, 2H).

^{13}C NMR (126 MHz, Chloroform-*d*) δ (ppm) 186.55, 137.00, 136.08 (q, J = 33.1 Hz), 129.01, 126.40 (q, J = 3.6 Hz), 123.35 (J = 273.0 Hz), 113.34, 29.82.

^{19}F NMR (471 MHz, Chloroform-*d*) δ (ppm) -63.40.

HRMS (APCI): calcd. for $C_{10}H_5F_3NO$ $[M-H]^-$ = 212.0329, found $[M-H]^-$ = 212.0327.

mp = 43−45 °C.

3-(4-(*tert*-Butyl)phenyl)-3-oxopropanenitrile **(S111)**

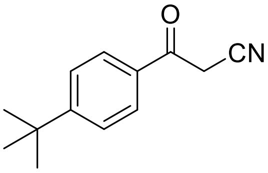

The compound was prepared according to General procedure A5 using NaH (60% suspension in mineral oil, 420 mg, 10.4 mmol), acetonitrile (0.33 mL, 6.2 mmol), THF (10 mL) and methyl 4-(trifluoromethyl)benzoate (1 g, 5.20 mmol) in anhydrous THF (5 mL). The reaction mixture was

refluxed for 2 h. The residue was purified by column chromatography (hexane:EtOAc, 8:2 to 7:3). The product was obtained as a yellow solid (740 mg, 70%).

^{1}H NMR (500 MHz, Chloroform-*d*) δ (ppm) 7.91 – 7.83 (m, 2H), 7.57 – 7.49 (m, 2H), 4.05 (s, 2H), 1.35 (s, 9H).

^{13}C NMR (126 MHz, Chloroform-*d*) δ (ppm) 186.7, 158.9, 131.8, 128.5, 126.1, 113.9, 35.4, 31.0, 29.2.

HRMS (APCI): calcd. for $C_{13}H_{14}NO$ $[M-H]^-$ = 200.1081, found $[M-H]^-$ = 200.1079.

mp = 71−75 °C.

3-(4-Methoxyphenyl)-3-oxopropanenitrile **(S112)**

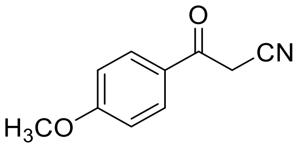

The compound was prepared according to General procedure A5 using NaH (60% suspension in mineral oil, 481 mg, 12.03 mmol), acetonitrile (0.377 mL, 7.72 mmol), anhydrous THF (10 mL) and methyl 4-methoxybenzoate (1 g, 6.02 mmol) in anhydrous THF (5 mL). The reaction mixture was refluxed for 48 h. The residue was purified by column chromatography (hexane:EtOAc, 8:2 to 7:3). The product was obtained as a yellow solid (781 mg, 74%).

^{1}H NMR (500 MHz, Chloroform-*d*) δ (ppm) 7.93 – 7.86 (m, 2H), 7.01 – 6.95 (m, 2H), 4.01 (s, 2H), 3.89 (s, 3H).

^{13}C NMR (126 MHz, Chloroform-*d*) δ (ppm) 185.58, 164.88, 131.06, 127.45, 114.48, 114.21, 55.79, 29.13.

HRMS (APCI): calcd. for $C_{10}H_8NO_2$ $[M-H]^-$ = 174.0561, found $[M-H]^-$ = 174.0559.

mp = 123−126 °C.

Methyl 2-phenylacetate **(S113)**

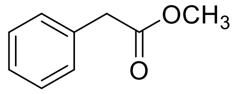

Conc. H_2SO_4 (96%, 30 µL) was added to a solution of 2-phenylacetic acid (1.50 g, 11.01 mmol) in methanol (15 mL) and the mixture was refluxed for 4 h. The reaction mixture was cooled to room temperature and the solvent was evaporated *in vacuo*. Water (15 mL) was added to the residue and the mixture was extracted with EtOAc (3 × 15 mL). The combined organic extracts were dried over $MgSO_4$, filtered, and the solvent was evaporated *in vacuo*. The product was obtained as a clear oil (1.25 g, 76%).

^{1}H NMR (300 MHz, Chloroform-*d*) δ (ppm) 7.37 – 7.23 (m, 5H), 3.70 (s, 3H), 3.63 (s, 2H).

^{13}C NMR (75 MHz, Chloroform-*d*) δ (ppm) 171.9, 134.0, 129.2, 128.5, 127.1, 51.9, 41.1.

HRMS (APCI): calcd. for $C_9H_{11}O_2$ $[M+H]^+$ = 151.0754, found $[M+H]^+$ = 151.0753.

3-(3-Methylfuran-2-yl)-3-oxopropanenitrile **(S114)**

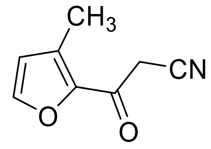

The compound was prepared according to General procedure A5 using NaH (60% suspension in mineral oil, 171 mg, 4.28 mmol), acetonitrile (0.134 mL, 2.57 mmol), anhydrous THF (3 mL) and methyl 3-methylfuran-2-carboxylate (300 mg, 2.14 mmol) in anhydrous THF (2 mL). The reaction mixture was refluxed for 48 h. The residue was purified by column chromatography (hexane:EtOAc, 7:3 to 8:4). The product was obtained as an off-white solid (171 mg, 54%).

^{1}H NMR (300 MHz, Chloroform-*d*) δ (ppm) 7.47 (d, J = 1.8 Hz, 1H), 6.47 (d, J = 1.7 Hz, 1H), 3.94 (s, 2H), 2.41 (s, 3H).

13C NMR (75 MHz, Chloroform-d) δ (ppm) 177.01, 146.53, 146.09 (d, J = 2.6 Hz), 134.12, 116.75, 113.72, 29.40, 11.80.

HRMS (APCI): calcd. for $C_8H_8NO_2$ $[M+H]^+$ = 150.0550, found $[M+H]^+$ = 150.0550.

mp = 92−95 °C.

3-(5-Methylfuran-2-yl)-3-oxopropanenitrile **(S115)**

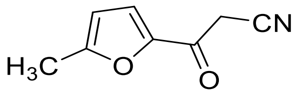

The compound was prepared according to General procedure A5 using NaH (60% suspension in mineral oil, 171 mg, 4.28 mmol), acetonitrile (0.134 mL, 2.57 mmol), anhydrous THF (3 mL) and methyl 3-methylfuran-2-carboxylate (300 mg, 2.14 mmol) in anhydrous THF (2 mL). The reaction mixture was refluxed for 48 h. The residue was purified by column chromatography (hexane:EtOAc, 7:3 to 8:4). The product was obtained as a pale yellow solid (191 mg, 60%).

^{1}H NMR (300 MHz, Chloroform-*d*) δ (ppm) 7.29 (d, J = 3.6 Hz, 1H), 6.30 – 6.19 (m, 1H), 3.89 (s, 2H), 2.42 (s, 3H).

^{13}C NMR (75 MHz, Chloroform-*d*) δ (ppm) 174.7, 159.6, 149.2, 121.3, 113.6, 110.2, 28.4, 14.1.

HRMS (APCI): calcd. for $C_8H_8NO_2$ $[M+H]^+$ = 150.0550, found $[M+H]^+$ = 150.0549.

mp = 97−100 °C.

3-Oxo-4-(thiophen-2-yl)butanenitrile **(S116)**

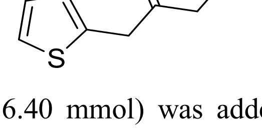

n-BuLi (2.7 M in heptane; 2.37 mL, 6.40 mmol) was added at -78 ºC dropwise to a solution of acetonitrile (334 µL, 6.40 mmol) in THF (5 mL) and the reaction mixture was stirred at -78 ºC for 1 h.

Then, a solution of the methyl 2-(thiophen-2-yl)acetate (500 mg, 3.20 mmol; CAS: 19432-68-9) in THF (3 mL) was added dropwise and the reaction mixture was stirred at -78 °C for 1 h and then at room temperature for additional 1 h. Aqueous saturated solution of NH_4Cl (15 mL) was added and the mixture was extracted with EtOAc (2 × 50 mL). The combined organic extracts were dried over $MgSO_4$, filtered, and the solvent was evaporated *in vacuo*. The residue was purified by column chromatography (hexane:EtOAc, 7:3). The product was obtained as an orange oil (360 mg, 68%).

^{1}H NMR (500 MHz, Chloroform-*d*) δ (ppm) 7.29 (dd, J = 5.1, 1.2 Hz, 1H), 7.02 (dd, J = 5.2, 3.5 Hz, 1H), 6.99 – 6.95 (m, 1H), 4.06 (s, 2H), 3.53 (s, 2H).

^{13}C NMR (126 MHz, Chloroform-*d*) δ (ppm) 194.00, 132.77, 128.14, 127.73, 126.37, 113.49, 43.01, 30.99 ppm.

HRMS (APCI): calcd. for C_8H_6NOS $[M-H]^-$ = 164.0176, found $[M-H]^-$ = 164.0178.

Preparation of hydazine derivatives

Scheme S1: synthesis of 2-hydrazinyl-6-methylpyrimidin-4(3*H*)-one **(S119)**

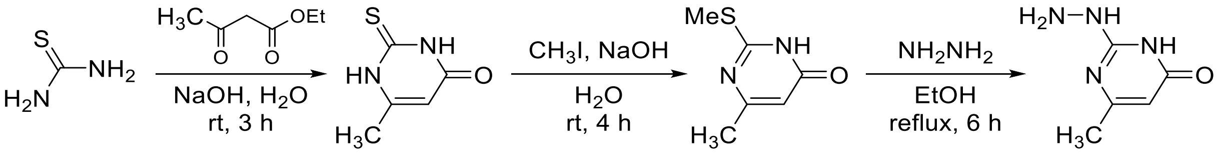

6-Methyl-2-thioxo-2,3-dihydropyrimidin-4(1*H*)-one **(S117)**

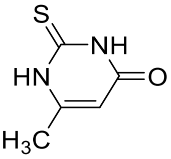

Thiourea (10 g, 130 mmol) was added to a solution of sodium hydroxide (10.9 g, 272 mmol) in H_2O (205 mL) at 15 °C. The reaction mixture was stirred at 15 °C for 10 min, ethyl acetoacetate (20.8 g, 160 mmol) was added dropwise at 15 °C. The mixture was allowed to warm to room temperature, and stirred for 3 h. The pH was adjusted to 4-5 by the careful addition of hydrochloric acid. The precipitate was collected by filtration, washed with cold water (20 mL) and dried *in vacuo*. The product was obtained as an off-white solid (7.18 g, 39%).

^{1}H NMR (300 MHz, DMSO-d_6) δ (ppm) 12.23 (s, 2H), 5.67 (d, J = 1.1 Hz, 1H), 2.06 (s, 3H).

HRMS (APCI): calcd. for $C_5H_5N_2OS$ $[M-H]^-$ = 141.0128, found $[M-H]^-$ = 141.0128.

mp >265 °C (dec.)

6-Methyl-2-(methylthio)pyrimidin-4(3*H*)-one **(S118)**

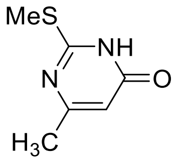

6-methyl-2-thioxo-2,3-dihydropyrimidin-4(1*H*)-one (**S117**, 7.18 g, 50.5 mmol) was added to a solution of NaOH (2.08 g, 52.01 mmol) in water (68 mL) and the reaction mixture was stirred at 25 °C for 20 min. Iodomethane (3.92 mL, 63.12 mmol) was added dropwise and the mixture was stirred at room temperature for additional 4 h. The precipitate was collected by filtration, washed with ice cold water (2 × 20 mL) and dried *in vacuo*. The product was obtained as a white solid (7.8 g, 99%).

^{1}H NMR (300 MHz, DMSO-d_6) δ (ppm) 12.40 (s, 1H), 5.96 (s, 1H), 2.47 (s, 3H), 2.17 (s, 3H).

HRMS (APCI): calcd. for $C_6H_9N_2OS$ $[M+H]^+ = 157.0430$, found $[M+H]^+ = 157.0428$.

mp = 225−226 °C.

<u>2-Hydrazinyl-6-methylpyrimidin-4(3*H*)-one</u> **(S119)**

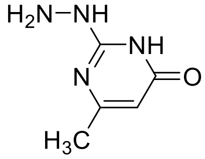

To a stirred solution of 6-methyl-2-(methylthio)pyrimidin-4(3*H*)-one (**S118**, 7.8 g, 49.93 mmol) in EtOH (20 mL) was added hydrazine hydrate (64% aq. solution, 10.18 g, 203 mmol) and the reaction mixture was refluxed for 6 h. Then mixture was cooled to the room temperature and the resulting precipitate was collected by vacuum filtration, washed with water (2 mL) and dried *in vacuo*. The product was obtained as an off-white solid (4.15 g, 59%).

^{1}H NMR (300 MHz, DMSO-d_6) δ (ppm) 8.89 (s, 2H), 5.37 (s, 1H), 4.70 (s, 1H), 2.00 (s, 3H).

HRMS (APCI): calcd. for $C_5H_9N_4O$ $[M+H]^+ = 141.0771$, found $[M+H]^+ = 141.0770$.

mp = 231−233 °C.

<u>2-Hydrazinyl-6-(trifluoromethyl)pyrimidin-4(3*H*)-one</u> **(S120)**

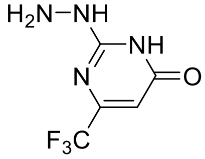

To a stirred solution of 2-(methylthio)-6-(trifluoromethyl)-3,4-dihydropyrimidin-4-ol (500 mg, 2.38 mmol, CAS: 16097-62-4) in *i*-PrOH (20 mL) was added hydrazine hydrate (64% aq. solution, 930 mg, 11.89 mmol) and the mixture was stirred at 80 °C for 16 h. The solvent was evaporated *in vacuo* and the residue was purified by column chromatography on silica gel (23% aqueous NH_3:methanol:dichloromethane; 2:8:90). The product was obtained as a white solid (171 mg, 37%).

^{1}H NMR (500 MHz, DMSO-d_6) δ (ppm) 9.25 (br s, 1H), 6.16 (br s, 1H), 5.89 (s, 1H).

^{13}C NMR (126 MHz, DMSO-d_6) δ (ppm) 161.8, 158.5, 153.5 (q, J = 33.3 Hz), 120.9 (q, J = 275.3 Hz), 98.5.

^{19}F NMR (471 MHz, DMSO-d_6) δ (ppm) -70.5.

HRMS (APCI): calcd. for $C_5H_6F_3N_4O$ [M+H]$^+$ = 195.0488, found [M+H]$^+$ = 195.0490.

mp = 221−224 °C.

<u>6-Ethyl-2-hydrazinylpyrimidin-4(3*H*)-one</u> **(S121)**

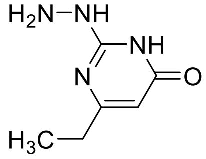

To a stirred solution of 6-ethyl-2-(methylthio)-3,4-dihydropyrimidin-4-ol (400 mg, 2.35 mmol, CAS: 70967-37-2) in EtOH (1.2 mL) was added hydrazine hydrate (64% aq. solution, 735 mg, 9.40 mol) and the reaction mixture was stirred at 80 °C for 6 h. The solvent was evaporated *in vacuo* and the residue was mixed with EtOAc:hexane (1:4, 3 mL). The solid was collected by filtration, washed with a mixture of H_2O:MeOH (5:95, 2 mL) and dried *in vacuo*. The product was obtained as a white solid (205 mg, 57%).

^{1}H NMR (300 MHz, DMSO-d_6) δ (ppm) 8.50 (s, 2H), 5.36 (s, 1H), 4.41 (s, 1H), 2.27 (q, J = 7.5 Hz, 2H), 1.08 (t, J = 7.6 Hz, 3H).

^{13}C NMR (75 MHz, DMSO-d_6) δ (ppm) 163.3, 157.7, 98.9, 30.4, 12.8.

HRMS (APCI): calcd. for $C_6H_{11}N_4O$ [M+H]$^+$ = 155.0927, found [M+H]$^+$ = 155.0928.

mp >180 °C (dec.)

Scheme S2: synthesis of 2-hydrazinyl-5-(2-(2-(2-methoxyethoxy)ethoxy)ethyl)-6-methylpyrimidin-4(3*H*)-one **(S126)**

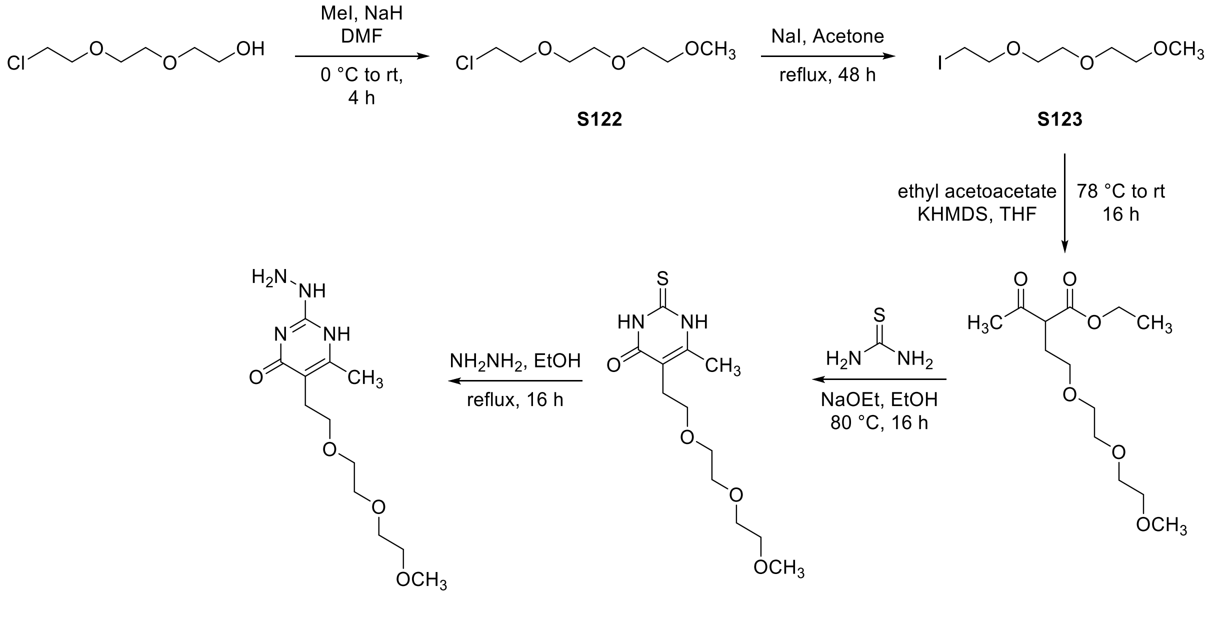

1-Chloro-2-(2-(2-methoxyethoxy)ethoxy)ethane **(S122)**

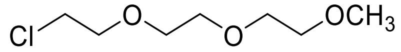

Iodomethane (2.21 mL, 35.58 mmol) was added to a solution of 2-(2-(2-chloroethoxy)ethoxy)ethan-1-ol (3 g, 17.79 mmol) in DMF (30 mL), the mixture was cooled to 0 °C and sodium hydride (60% suspension in mineral oil, 1.067 g, 26.68 mmol) was added portion wise. The reaction mixture was stirred at 65 °C for 4 h, then poured into ice cold water (100 mL) and extracted with EtOAc (4 × 30 mL). The combined organic extracts were dried over $MgSO_4$, filtered, and the solvent was evaporated *in vacuo*. The resulting yellow oil was purified by flash chromatography (hexane:EtOAc, 7:3). The product was obtained as a colorless oil (1.95 g, 60%).

^{1}H NMR (300 MHz, Chloroform-*d*) δ (ppm) 3.80 – 3.72 (m, 2H), 3.72 – 3.58 (m, 8H), 3.55 (m, 2H), 3.37 (s, 3H).

^{13}C NMR (75 MHz, Chloroform-*d*) δ (ppm) 72.1, 71.5, 70.8, 70.7, 70.7, 59.1, 42.8.

HRMS (APCI): calcd. for $C_7H_{16}ClO_3$ $[M+H]^+$ = 183.0782, found $[M+H]^+$ = 183.0782.

1-Iodo-2-(2-(2-methoxyethoxy)ethoxy)ethane **(S123)**

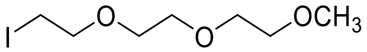

NaI (5.82 g, 38.87 mmol) was added to a solution of 1-chloro-2-(2-(2-methoxyethoxy)ethoxy)ethane (**S122**, 3.35 g, 19.43 mmol) in acetone (40 mL) and the reaction mixture was refluxed for 48 h. The solvent was evaporated *in vacuo*, the residue was mixed with EtOAc (50 mL), and the mixture was washed with 5% aqueous $Na_2S_2O_3$ solution (2× 20 mL) followed by water (2× 20 mL). The organic layer was dried over $MgSO_4$, filtered, and the solvent was evaporated *in vacuo*. The product was obtained as a light yellow oil (3.05 g, 63%).

^{1}H NMR (300 MHz, Chloroform-*d*) δ (ppm) 3.79 – 3.68 (m, 2H), 3.69 – 3.59 (m, 6H), 3.58 – 3.49 (m, 2H), 3.36 (s, 3H), 3.30 – 3.19 (m, 2H).

^{13}C NMR (75 MHz, Chloroform-*d*) δ (ppm) 72.1, 72.1, 70.7, 70.7, 70.3, 59.1, 3.0.

HRMS (APCI): calcd. for $C_7H_{16}IO_3$ $[M+H]^+$ = 275.0139, found $[M+H]^+$ = 275.0139.

Ethyl 2-acetyl-4-(2-(2-methoxyethoxy)ethoxy)butanoate **(S124)**

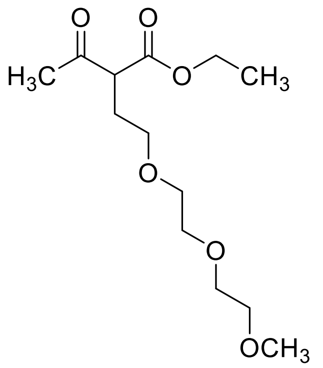

KHMDS (1M solution in THF, 2.01 mL, 2.01 mmol) was added dropwise at -78 °C to a stirred solution of ethylacetoacetate (237 mg, 1.82 mmol) in THF (5 mL) under nitrogen atmosphere over the period of 10 min. The reaction mixture was allowed to warm to room temperature and stirred for 30 min. A solution of 1-iodo-2-(2-(2-methoxyethoxy)ethoxy)ethane (**S123**, 500 mg, 1.82 mmol) in THF (4 mL) was added and the reaction mixture was stirred for 16 h. The solvents were evaporated *in vacuo* and the residue was purified by column chromatography on silica gel (hexane:EtOAc, 6:4). The product was obtained as a light brown oil (360 mg, 71%).

^{1}H NMR (300 MHz, Chloroform-*d*) δ (ppm) 4.27 – 4.06 (m, 2H), 3.73 – 3.41 (m, 11H), 3.37 (s, 3H), 2.25 (s, 3H), 2.21 – 2.04 (m, 2H), 1.26 (t, J = 7.1 Hz, 3H).

^{13}C NMR (75 MHz, Chloroform-*d*) δ (ppm) 203.2, 169.8, 72.1, 70.6, 70.3, 68.6, 61.4, 59.1, 56.6, 29.4, 28.3, 14.2.

HRMS (APCI): calcd. for $C_{13}H_{25}O_6$ $[M+H]^+$ = 277.1646, found $[M+H]^+$ = 277.1647.

5-(2-(2-(2-Methoxyethoxy)ethoxy)ethyl)-6-methyl-2-thioxo-2,3-dihydropyrimidin-4(1*H*)-one **(S125)**

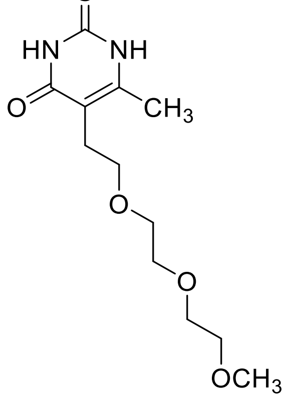

NaOEt (2.6 M solution in EtOH, 4.17 mL, 10.86 mmol) was added to a solution of ethyl 2-acetyl-4-(2-(2-methoxyethoxy)ethoxy)butanoate (**S124**, 750 mg, 2.71 mmol) and thiourea (207 mg, 2.71 mmol) in EtOH (8 mL) under nitrogen atmosphere and the mixture was refluxed for 16 h. The solvent was evaporated under *in vacuo* and the residue was purified by column chromatography (dichloromethane:MeOH, 96:4). The product was obtained as a light brown gum (360 mg, 46%).

^{1}H NMR (300 MHz, Chloroform-*d*) δ (ppm) 10.38 (s, 1H), 10.17 (s, 1H), 3.65 – 3.52 (m, 10H), 3.38 (s, 3H), 2.65 (t, J = 6.1 Hz, 2H), 2.26 (s, 3H).

^{13}C NMR (75 MHz, Chloroform-*d*) δ (ppm) 173.9, 161.5, 149.8, 113.2, 72.0, 70.6, 70.4, 69.3, 59.0, 25.6, 17.2.

HRMS (APCI): calcd. for $C_{12}H_{21}N_2O_4S$ $[M+H]^+$ = 289.1217, found $[M+H]^+$ = 289.1217.

2-Hydrazinyl-5-(2-(2-(2-methoxyethoxy)ethoxy)ethyl)-6-methylpyrimidin-4(3*H*)-one **(S126)**

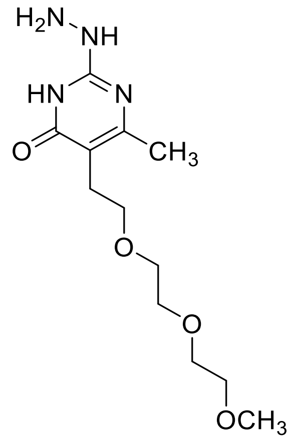

Hydrazine hydrate (64% aq. solution, 1.38 g, 17.69 mmol) was added to a solution of 5-(2-(2-(2-methoxyethoxy)ethoxy)ethyl)-6-methyl-2-thioxo-2,3-dihydropyrimidin-4(1*H*)-one (**S125**, 340 mg, 1.18 mmol) in EtOH (5 mL) and the mixture was stirred at 80 °C for 16 h. The solvent was evaporated *in vacuo*, and the residue was co-evaporated with toluene (2 × 3 mL). The product was obtained as an off-white gum (340 mg), was used into the next step without further purification.

^{1}H NMR (300 MHz, DMSO-*d*$_6$) δ (ppm) 3.51 – 3.46 (m, 6H), 3.45 – 3.39 (m, 2H), 3.35 (t, J = 6.7 Hz, 2H), 3.24 (s, 3H), 2.56 – 2.49 (m, 2H), 2.08 (d, J = 1.4 Hz, 3H).

HRMS (APCI): calcd. for $C_{12}H_{23}N_4O_4$ $[M+H]^+$ = 287.1714, found $[M+H]^+$ = 287.1714.

2-(Benzyloxy)-6-chloropyridine **(S127)**

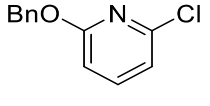

Benzyl bromide (458 µL, 3.86 mmol) and K_2CO_3 (1.07 g, 7.72 mmol) were added to a solution of 6-chloropyridin-2-ol (500 mg, 3.86 mmol) in DMF (5 mL) and the reaction mixture was stirred at 50 °C for 16 h. Water (35 mL) was added and the mixture was extracted with EtOAc (2 × 50 mL). The organic extracts were separated, dried over $MgSO_4$, filtered, and the solvent was evaporated *in vacuo*. The

residue was purified by column chromatography (hexane:EtOAc, 90:10). The product was obtained as a colorless oil (580 mg, 68%).

^{1}H NMR (500 MHz, Chloroform-*d*): δ (ppm) 7.53 (td, *J* = 7.7, 3.7 Hz, 1H), 7.50 – 7.43 (m, 2H), 7.43 – 7.37 (m, 2H), 7.36 – 7.30 (m, 1H), 6.92 (dd, *J* = 7.5, 4.0 Hz, 1H), 6.72 (dd, *J* = 8.3, 3.9 Hz, 1H), 5.38 (d, *J* = 3.9 Hz, 2H).

^{13}C NMR (126 MHz, Chloroform-*d*): δ (ppm) 163.43, 148.45, 140.81, 136.79, 128.65, 128.41, 128.20, 116.65, 109.56, 68.50.

2-(Benzyloxy)-6-hydrazinylpyridine **(S128)**

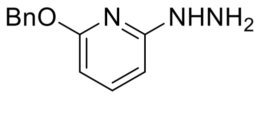

Hydrazine hydrate (64% aq. Solution, 794 µL, 25.49 mmol) was added to a solution of 2-(benzyloxy)-6-chloropyridine (**S127**, 560 mg, 2.55 mmol) in EtOH (6 mL) and the reaction mixture was stirred in the microwave reactor at 140 °C for 40 min. The solvents were evaporated *in vacuo* and the residue was purified by column chromatography (dichloromethane:MeOH, 90:10). The product was obtained as a pale yellow wax (150 mg, 27%).

^{1}H NMR (300 MHz, Chloroform-*d*) δ (ppm) 7.46 – 7.42 (m, 2H), 7.40 – 7.35 (m, 3H), 7.34 – 7.30 (m, 1H), 6.22 (dd, *J* = 13.9, 7.9 Hz, 2H), 5.35 – 5.26 (m, 3H), 3.28 (br s, 2H).

Preparation of aminopyrazole derivatives

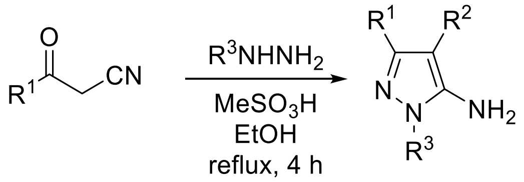

4-(5-Amino-1-(4-methyl-6-oxo-1,6-dihydropyrimidin-2-yl)-1*H*-pyrazol-3-yl)benzonitrile **(S129)**

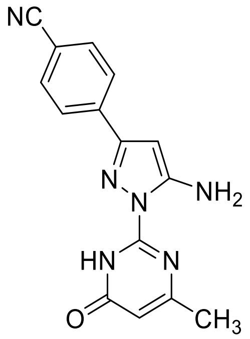

The compound was prepared according to General procedure B2 using methanesulfonic acid (2.7 mg, 0.029 mmol), 2-hydrazinyl-6-methylpyrimidin-4(3*H*)-one (**S119**, 40 mg, 0.29 mmol) and 4-(2-cyanoacetyl)benzonitrile (**S109**, 68 mg, 0.399 mmol) in EtOH (2 mL). The reaction mixture was refluxed for 4 h. The reaction mixture was cooled to room temperature, NH_3 (7 M solution in MeOH, 2 mL) was

added, followed by saturated aqueous $NaHCO_3$ solution (4 mL), and the mixture was stirred for 10 min. The precipitate was collected by filtration, washed with H_2O (2 × 3 mL), then with a mixture of EtOAc:hexanes (1:4, 2 mL) and dried *in vacuo*. The product was obtained as a white solid (64 mg, 65%).
^{1}H NMR (300 MHz, DMSO-d_6) δ (ppm) 12.14 (s, 1H), 8.15 (d, J = 8.4 Hz, 2H), 7.88 (d, J = 8.3 Hz, 2H), 7.12 (br s, 2H), 6.11 (s, 1H), 5.98 (s, 1H), 2.29 (s, 3H).
HRMS (APCI): calcd. for $C_{15}H_{11}N_6O$ $[M-H]^-$ = 291.1000, found $[M-H]^-$ = 291.1000.
mp >301 °C (dec.)

2-(5-Amino-3-cyclohexyl-1*H*-pyrazol-1-yl)-6-methylpyrimidin-4(3*H*)-one **(S130)**

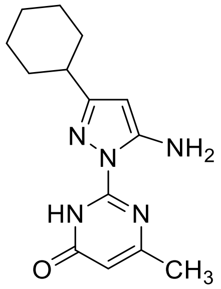

The compound was prepared according to General procedure B2 using methanesulfonic acid (2.7 mg, 0.029 mmol), 2-hydrazinyl-6-methylpyrimidin-4(3*H*)-one (**S119**, 40 mg, 0.29 mmol) and 3-cyclohexyl-3-oxopropanenitrile (**S3**, 43 mg, 0.29 mmol) in EtOH (2 mL). The reaction mixture was refluxed for 4 h. The crude product was purified by column chromatography on silica gel (7 NH_3 in MeOH:MeOH: dichloromethane, 3:7:90). The product was obtained as a white solid (67 mg, 86%).
^{1}H NMR (300 MHz, Chloroform-d) δ (ppm) 10.2 (s, 1H), 6.25 (s, 1H), 6.02 (s, 1H), 5.92 (s, 1H), 5.34 (s, 1H), 2.52 (t, J = 10.8 Hz, 1H), 2.28 (d, J = 0.9 Hz, 3H), 2.00 – 1.66 (m, 5H), 1.47 – 1.18 (m, 5H).
^{13}C NMR (75 MHz, Chloroform-d) δ (ppm) 164.4, 162.5, 161.5, 149.5, 148.8, 108.3, 88.1, 37.9, 32.3, 26.2, 26.2, 24.0.
HRMS (APCI): calcd. for $C_{14}H_{20}N_5O$ $[M+H]^+$ = 274.1662, found $[M+H]^+$ = 274.1660.
mp = 221−224 °C.

2-(5-Amino-3-(4-(trifluoromethyl)phenyl)-1*H*-pyrazol-1-yl)-6-methylpyrimidin-4(3*H*)-one **(S131)**

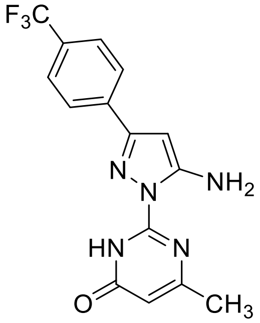

The compound was prepared according to General procedure B2 using methanesulfonic acid (2.7 mg, 0.029 mmol), 2-hydrazinyl-6-methylpyrimidin-4(3*H*)-one (**S119**, 40 mg, 0.29 mmol) and 3-oxo-3-(4-

(trifluoromethyl)phenyl)propanenitrile (**S110**, 61 mg, 0.29 mmol) in EtOH (2 mL). The reaction mixture was refluxed for 4 h. The reaction mixture was cooled to room temperature, NH_3 (7 M solution in MeOH, 2 mL) was added, followed by saturated aqueous $NaHCO_3$ solution (4 mL), and the mixture was stirred at room temperature for 10 min. The precipitated was collected by filtration, washed with H_2O (2 × 3 mL), then with a mixture of EtOAc:hexane (1:4, 2 mL), and dried *in vacuo*. The product was obtained as a white solid (69 mg, 72%).

^{1}H NMR (500 MHz, DMSO-d_6) δ (ppm) 12.18 (s, 1H), 8.17 (d, J = 8.1 Hz, 2H), 7.77 (d, J = 8.2 Hz, 2H), 7.11 (s, 2H), 6.09 (s, 1H), 5.96 (s, 1H), 2.28 (s, 3H).

^{13}C NMR (126 MHz, DMSO-d_6) δ (ppm) 151.5, 150.9, 136.4, 128.7, 128.4, 128.2, 126.5, 125.3, 125.3, 125.3, 123.2, 85.4, 22.9.

^{19}F NMR (282 MHz, DMSO-d_6) δ (ppm) -61.0.

HRMS (APCI): calcd. for $C_{15}H_{13}F_3N_5O$ $[M+H]^+$ = 336.1067, found $[M+H]^+$ = 336.1070.

mp = 284−288 °C.

2-(5-Amino-3-(4-(tert-butyl)phenyl)-1*H*-pyrazol-1-yl)-6-methylpyrimidin-4(3*H*)-one **(S132)**

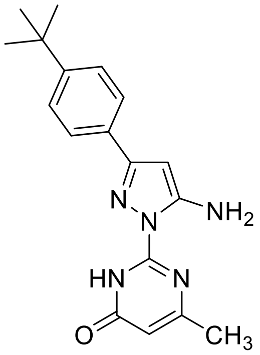

The compound was prepared according to General procedure B2 using methanesulfonic acid (2.7 mg, 0.029 mmol), 2-hydrazinyl-6-methylpyrimidin-4(3*H*)-one (**S119**, 40 mg, 0.29 mmol) and 3-(4-(*tert*-butyl)phenyl)-3-oxopropanenitrile (**S111**, 57 mg, 0.29 mmol) in EtOH (2 mL). The reaction mixture was refluxed for 4 h. The reaction mixture was cooled to room temperature, NH_3 (7 M solution in MeOH, 2 mL) was added, followed by saturated aqueous $NaHCO_3$ solution (4 mL), and the mixture was stirred for 10 min. The precipitate was collected by filtration, washed with H_2O (2 × 3 mL), then with a mixture of EtOAc:hexane (1:4, 2 mL), and dried *in vacuo*. The product was obtained as a white solid (64 mg, 69%).

^{1}H NMR (500 MHz, DMSO-d_6) δ (ppm) 7.77 (d, J = 8.4 Hz, 2H), 7.41 (d, J = 8.5 Hz, 2H), 7.02 (s, 2H), 5.83 (s, 1H), 5.76 (s, 1H), 2.18 (d, J = 0.8 Hz, 3H), 1.31 (s, 9H).

^{13}C NMR (126 MHz, DMSO-d_6) δ (ppm) 162.3, 154.5, 151.0, 150.9, 150.5, 130.3, 125.5, 125.0, 106.4, 84.9, 34.3, 31.1, 23.0.

HRMS (APCI): calcd. for $C_{28}H_{22}N_5O$ $[M+H]^+$ = 324.1819, found $[M+H]^+$ = 324.1817.

mp = 231−250 °C.

2-(5-Amino-3-(4-methoxyphenyl)-1*H*-pyrazol-1-yl)-6-methylpyrimidin-4(3*H*)-one **(S133)**

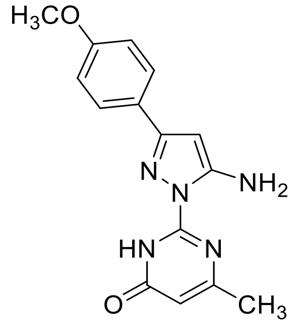

The compound was prepared according to General procedure B2 using methanesulfonic acid (2.7 mg, 0.029 mmol), 2-hydrazinyl-6-methylpyrimidin-4(3*H*)-one (**S119**, 40 mg, 0.29 mmol) and 3-(4-methoxyphenyl)-3-oxopropanenitrile (**S112**, 60 mg, 0.34 mmol) in EtOH (2 mL). The reaction mixture was refluxed for 16 h. The reaction mixture was cooled to room temperature, NH_3 (7 M solution in MeOH, 2 mL) was added, followed by saturated aqueous $NaHCO_3$ solution (4 mL), and the mixture was stirred for 10 min. The precipitate was collected by filtration and washed with H_2O (2 × 3 mL), then with a mixture of EtOAc:hexane (1:4, 2 mL), and dried *in vacuo*. The product was obtained as a white solid (65 mg, 77%).

^{1}H NMR (500 MHz, DMSO-d_6) δ (ppm) 11.77 (s, 1H), 7.88 (d, J = 8.3 Hz, 2H), 7.02 (s, 2H), 6.98 (d, J = 8.9 Hz, 2H), 6.04 (s, 1H), 5.81 (s, 1H), 3.80 (s, 3H), 2.27 (s, 3H).

^{13}C NMR (126 MHz, DMSO-d_6) δ (ppm) 159.8, 153.2, 151.1, 127.5, 124.7, 113.8, 84.8, 55.1, 23.2.

HRMS (APCI): calcd. for $C_{15}H_{16}N_5O_2$ $[M+H]^+$ = 298.1299, found $[M+H]^+$ = 298.1297.

mp = 289−293 °C.

2-(5-Amino-3-benzyl-1*H*-pyrazol-1-yl)-6-methylpyrimidin-4(3*H*)-one **(S134)**

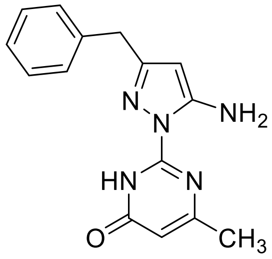

The compound was prepared according to General procedure B2 using methanesulfonic acid (6 mg, 0.063 mmol), 2-hydrazinyl-6-methylpyrimidin-4(3*H*)-one (**S119**, 88 mg, 0.63 mmol) and 3-oxo-4-phenylbutanenitrile (**S22**, 100 mg, 0.63 mmol) in EtOH (3 mL). The reaction mixture was refluxed for 4 h. The crude product was purified by column chromatography on silica gel (7M NH_3 in MeOH:MeOH: dichloromethane, 2:8:90). The product was obtained as a brown solid (98 mg, 56%).

^{1}H NMR (300 MHz, Chloroform-d) δ (ppm) 7.38 – 7.18 (m, 6H), 6.03 (s, 1H), 5.90 (s, 2H), 5.27 (s, 1H), 3.84 (s, 2H), 2.28 (s, 3H).

^{13}C NMR (75 MHz, DMSO-d_6) δ (ppm) 154.8, 150.7, 139.1, 128.6, 128.3, 128.0, 126.1, 105.9, 87.7, 34.4, 22.6.

HRMS (APCI): calcd. for $C_{15}H_{16}N_5O$ $[M+H]^+ = 282.1349$, found $[M+H]^+ = 282.1349$.

2-(5-Amino-3-(3-methylfuran-2-yl)-1*H*-pyrazol-1-yl)-6-methylpyrimidin-4(3*H*)-one **(S135)**

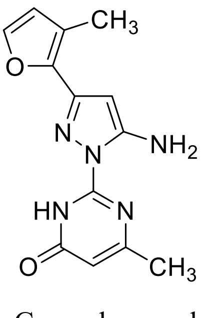

The compound was prepared according to General procedure B2 using methanesulfonic acid (8 mg, 0.081 mmol), 2-hydrazinyl-6-methylpyrimidin-4(3*H*)-one (**S119**, 113 mg, 0.81 mmol) and 3-(3-methylfuran-2-yl)-3-oxopropanenitrile (**S114**, 120 mg, 0.81 mmol) in EtOH (3 mL). The reaction mixture was refluxed for 16 h. The crude product was purified by column chromatography on silica gel (7M NH_3 in MeOH:MeOH:dichloromethane, 2:8:90). The product was obtained as a pale brown solid (53 mg, 24%).

^1H NMR (300 MHz, DMSO-d_6) δ (ppm) 7.56 (d, J = 1.7 Hz, 1H), 7.01 (s, 2H), 6.42 (d, J = 1.7 Hz, 1H), 5.72 (s, 1H), 5.56 (s, 1H), 2.27 (s, 3H), 2.13 (s, 3H).

^{13}C NMR (75 MHz, DMSO-d_6) δ (ppm) 170.7, 162.4, 156.2, 150.4, 143.9, 143.8, 141.4, 117.3, 114.6, 106.1, 85.0, 23.1, 10.7.

HRMS (APCI): calcd. for $C_{13}H_{14}N_5O_2$ $[M+H]^+ = 272.1142$, found $[M+H]^+ = 272.1143$.

mp >220 °C (dec.)

2-(5-Amino-3-(5-methylfuran-2-yl)-1*H*-pyrazol-1-yl)-6-methylpyrimidin-4(3*H*)-one **(S136)**

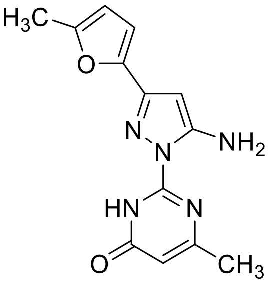

The compound was prepared according to General procedure B2 using methanesulfonic acid (8 mg, 0.081 mmol), 2-hydrazinyl-6-methylpyrimidin-4(3*H*)-one (**S119**, 113 mg, 0.81 mmol) and 3-(5-methylfuran-2-yl)-3-oxopropanenitrile (**S115**, 120 mg, 0.81 mmol) in EtOH (3 mL). The reaction mixture was refluxed for 16 h. The crude product was purified by column chromatography on silica gel (7M NH_3 in MeOH:MeOH:dichloromethane, 2:8:90). The product was obtained as a light yellow solid (130 mg, 60%).

^1H NMR (300 MHz, DMSO-d_6) δ (ppm) 11.42 (s, 1H), 7.03 (s, 2H), 6.75 (d, J = 3.2 Hz, 1H), 6.20 (d, J = 3.2 Hz, 1H), 6.13 (s, 1H), 5.64 (s, 1H), 2.33 (s, 3H), 2.29 (s, 3H).
^{13}C NMR (75 MHz, DMSO-d_6) δ (ppm) 152.1, 150.8, 146.2, 145.3, 109.2, 105.8, 107.8, 84.7, 13.3.
HRMS (APCI): calcd. for $C_{13}H_{14}N_5O_2$ $[M+H]^+$ = 272.1142, found $[M+H]^+$ = 272.1142.
mp = 240−243 °C.

2-(5-Amino-3-phenyl-1*H*-pyrazol-1-yl)-6-methylpyrimidin-4(3*H*)-one **(S137)**

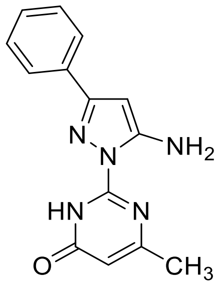

The compound was prepared according to General procedure B2 using methanesulfonic acid (21 mg, 0.21 mmol), 2-hydrazinyl-6-methylpyrimidin-4(3*H*)-one (**S119**, 300 mg, 2.14 mmol) and 3-oxo-3-phenylpropanenitrile (311 mg, 2.14 mmol) in EtOH (6 mL). The reaction mixture was refluxed for 4 h. The crude product was purified by column chromatography on silica gel (7M NH_3 in MeOH:MeOH: dichloromethane, 3:7:90). The product was obtained as a yellow solid (540 mg, 94%).
^1H NMR (500 MHz, DMSO-d_6) δ (ppm) 11.87 (s, 1H), 8.06 – 7.84 (m, 2H), 7.52 – 7.29 (m, 3H), 7.04 (s, 2H), 6.09 (s, 1H), 5.88 (s, 1H), 2.28 (s, 3H).
^{13}C NMR (126 MHz, DMSO-d_6) δ (ppm) 164.3, 153.2, 151.7, 132.7, 129.2, 128.9, 126.6, 107.5, 85.7, 23.7.
HRMS (APCI): calcd. for $C_{14}H_{14}N_5O$ $[M+H]^+$ = 268.1193, found $[M+H]^+$ = 268.1193.
mp = 239−241 °C.

2-(5-Amino-3-(furan-2-yl)-1*H*-pyrazol-1-yl)-6-methylpyrimidin-4(3*H*)-one **(S138)**

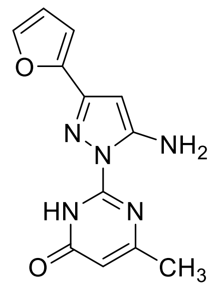

The compound was prepared according to General procedure B2 using methanesulfonic acid (14 mg, 0.14 mmol), 2-hydrazinyl-6-methylpyrimidin-4(3*H*)-one (**S119**, 193 mg, 1.43 mmol) and 3-(furan-2-yl)-3-oxopropanenitrile (200 mg, 1.43 mmol) in EtOH (6 mL). The reaction mixture was refluxed for 4 h. The crude product was purified by column chromatography on silica gel (7M solution NH_3 in

MeOH:MeOH:dichloromethane, 3:7:90). The product was obtained as a light brown solid (165 mg, 45%).

^{1}H NMR (500 MHz, DMSO-d_6) δ (ppm) 7.73 (d, J = 1.8 Hz, 1H), 7.06 (s, 2H), 6.86 (d, J = 3.3 Hz, 1H), 6.59 (dd, J = 3.3, 1.8 Hz, 1H), 6.07 (s, 1H), 5.67 (s, 1H), 2.27 (s, 3H).

^{13}C NMR (126 MHz, DMSO-d_6) δ (ppm) 150.8, 148.1, 144.6, 143.0, 111.6, 107.6, 105.8, 84.8, 22.9.

HRMS (APCI): calcd. for $C_{12}H_{12}N_5O_2$ $[M+H]^+$ = 258.0986, found $[M+H]^+$ = 258.0983.

mp >165 °C (dec).

2-(5-amino-3-(thiophen-2-yl)-1*H*-pyrazol-1-yl)-6-methylpyrimidin-4(3*H*)-one **(S139)**

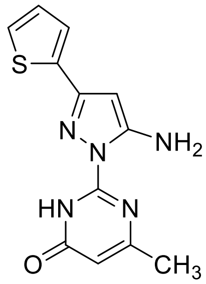

The compound was prepared by General Procedure B2 using methanesulfonic acid (6 mg, 0.06 mmol), 2-hydrazinyl-6-methylpyrimidin-4(3*H*)-one (**S119**, 90 mg, 0.6 mmol, 1 eq.) and 3-oxo-3-(thiophen-2-yl)propanenitrile (100 mg, 0.66 mmol, 1.1 eq.) in EtOH (3 mL). The reaction mixture was refluxed for 4 h. The reaction mixture was cooled to room temperature, and NH_3 (7 M solution in MeOH, 2 mL) was added, followed by saturated aqueous solution of $NaHCO_3$ (4 mL). The mixture was stirred at room temperature for 10 min, the precipitate was collected by filtration, washed with H_2O (2 × 3 mL), then with a mixture of EtOAc:hexane (1:4, 2 mL), and dried under vacuum. The product was obtained as a white solid (75 mg, 42%) and used directly in the next step.

2-(5-Amino-3-(thiophen-2-ylmethyl)-1*H*-pyrazol-1-yl)-6-methylpyrimidin-4(3*H*)-one **(S140)**

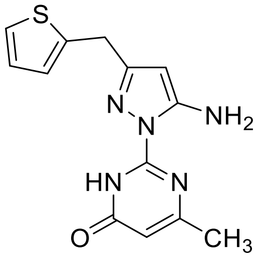

The compound was prepared according to General procedure B2 using methanesulfonic acid (4.63 µL, 0.71 mmol), 2-hydrazinyl-6-methylpyrimidin-4(3*H*)-one (**S119**, 100 mg, 0.71 mmol) and 3-oxo-4-(thiophen-2-yl)butanenitrile (**S116**, 123 mg, 0.75 mmol) in EtOH (5 mL). The reaction mixture was refluxed for 4 h. The solvent was evaporated *in vacuo* and the residue was dissolved in dichloromethane (2 mL). Hexane (7 mL) was added and the resulting precipitate was collected by filtration, washed with

hexane (5 mL), diethyl ether (3 mL) and dried *in vacuo*. The product was obtained as an off-white solid (130 mg, 63%).

^{1}H NMR (500 MHz, DMSO-d_6) δ (ppm) 7.75 (br s, 3H), 7.39 (dd, J = 5.1, 1.3 Hz, 1H), 7.03 – 6.99 (m, 1H), 6.98 (dd, J = 5.1, 3.4 Hz, 1H), 6.34 (s, 1H), 5.47 (s, 1H), 4.13 (s, 2H), 2.37 (s, 3H).

^{13}C NMR (126 MHz, DMSO-d_6) δ (ppm) 167.28, 165.61, 153.56, 151.78, 151.30, 139.67, 126.99, 126.19, 124.92, 104.90, 89.01, 27.36, 22.65.

HRMS (APCI): calcd. for $C_{13}H_{14}N_5OS$ $[M+H]^+$ = 288.0914, found $[M+H]^+$ = 288.0917.

2-(5-Amino-4-methyl-3-phenyl-1*H*-pyrazol-1-yl)-6-methylpyrimidin-4(3*H*)-one **(S141)**

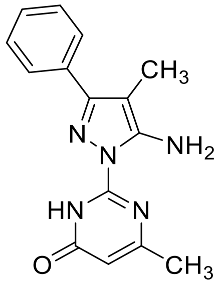

The compound was prepared according to General procedure B2 using methanesulfonic acid (9.28 µL, 0.14 mmol), 2-hydrazinyl-6-methylpyrimidin-4(3*H*)-one (**S119**, 200 mg, 1.43 mmol) and 2-methyl-3-oxo-3-phenylpropanenitrile (227 mg, 1.43 mmol) in EtOH (5 mL). The reaction mixture was refluxed for 6 h. The reaction mixture was cooled to room temperature, NH_3 (7 M solution in MeOH, 2 mL) was added and the mixture was stirred for 2 h. The mixture was concentrated *in vacuo* to the volume of ca. 3 mL, the precipitate was collected by filtration, washed with diethyl ether (10 mL) and dried *in vacuo*. The product was obtained as a pale yellow solid (370 mg, 92%).

^{1}H NMR (500 MHz, DMSO-d_6) δ (ppm) 11.74 (br s, 1H), 7.93 – 7.74 (m, 2H), 7.51 – 7.43 (m, 2H), 7.42 – 7.36 (m, 1H), 6.73 (br s, 2H), 6.09 (s, 1H), 2.28 (s, 3H), 2.05 (s, 3H).

^{13}C NMR (126 MHz, DMSO-d_6) δ (ppm) 164.29, 163.03, 152.19, 150.34, 147.94, 133.06, 128.29, 128.23, 127.60, 106.83, 94.12, 23.27, 8.18.

HRMS (APCI): calcd. for $C_{15}H_{16}N_5O$ $[M+H]^+$ = 282.1349, found $[M+H]^+$ = 282.1350.

2-(5-Amino-3-phenyl-1*H*-pyrazol-1-yl)-6-(trifluoromethyl)pyrimidin-4(3*H*)-one **(S142)**

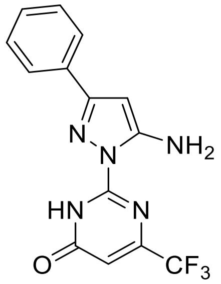

The compound was prepared according to General procedure B2 using methanesulfonic acid (3 mg, 0.03 mmol), 2-hydrazinyl-6-(trifluoromethyl)pyrimidin-4(3*H*)-one (**S120**, 50 mg, 0.26 mmol) and 3-oxo-3-

phenylpropanenitrile (37 mg, 0.26 mmol) in EtOH (2 mL). The reaction mixture was refluxed for 4 h. The crude product was purified by column chromatography on silica gel (7M NH_3 in MeOH:MeOH:dichloromethane, 3:7:90). The product was obtained as a light brown solid (72 mg, 87%).

^{1}H NMR (500 MHz, DMSO-d_6) δ (ppm) 7.91 – 7.99 (m, 2H), 7.40 – 7.47 (m, 2H), 7.34 – 7.40 (m, 1H), 6.59 (s, 1H), 5.91 (s, 1H).

^{13}C NMR (126 MHz, DMSO-d_6) δ (ppm) 152.9, 151.3, 132.2, 128.7, 128.4, 126.1, 121.9, 120.8 (d, J = 273.7 Hz), 106.96, 85.4.

^{19}F NMR (471 MHz, DMSO-d_6) δ (ppm) -69.9.

HRMS (APCI): calcd. for $C_{14}H_{11}F_3N_5O$ $[M+H]^+$ = 322.0910, found $[M+H]^+$ = 322.0909.

mp >295 °C (dec.)

2-(5-Amino-3-phenyl-1*H*-pyrazol-1-yl)-6-ethylpyrimidin-4(3*H*)-one **(S143)**

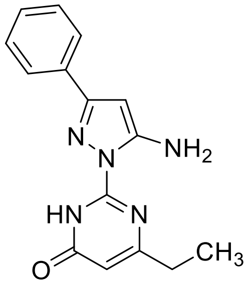

The compound was prepared according to General procedure B2 using methanesulfonic acid (6 mg, 0.07 mmol), 6-ethyl-2-hydrazinylpyrimidin-4(3*H*)-one (**S121**, 100 mg, 0.65 mmol) and 3-oxo-3-phenylpropanenitrile (94 mg, 0.65 mmol) in EtOH (2 mL). The reaction mixture was refluxed for 4 h. The solvent was evaporated *in vacuo* and saturated aqueous $NaHCO_3$ solution (4 mL) was added to the residue. The solid was collected by filtration, washed with H_2O (2 × 3 mL) and recrystallized from MeOH (4 mL). The product was obtained as a light brown solid (79 mg, 43%).

^{1}H NMR (300 MHz, DMSO-d_6) δ (ppm) 11.89 (s, 1H), 8.03 – 7.89 (m, 2H), 7.49 – 7.33 (m, 3H), 7.04 (s, 2H), 6.07 (s, 1H), 5.89 (s, 1H), 2.57 (q, J = 7.7 Hz, 2H), 1.19 (t, J = 7.5 Hz, 3H).

^{13}C NMR (75 MHz, DMSO-d_6) δ (ppm) 152.8, 151.2, 132.2, 128.7, 128.4, 126.0, 105.6, 85.2, 12.0.

HRMS (APCI): calcd. for $C_{15}H_{16}N_5O$ $[M+H]^+$ = 282.1349, found $[M+H]^+$ = 282.1348.

mp = 211−213 °C.

2-(5-Amino-3-phenyl-1*H*-pyrazol-1-yl)-5-(2-hydroxyethyl)-6-methylpyrimidin-4(3*H*)-one **(S144)**

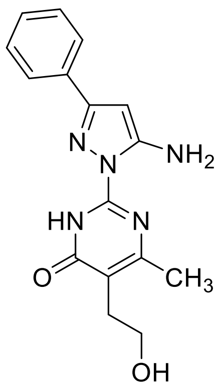

The compound was prepared according to General procedure B2 using methanesulfonic acid (5 mg, 0.05 mmol), 2-hydrazinyl-5-(2-hydroxyethyl)-6-methylpyrimidin-4(3*H*)-one (100 mg, 0.54 mmol, CAS: 74187-94-3) and 3-oxo-3-phenylpropanenitrile (79 mg, 0.54 mmol) in EtOH (3 mL). The reaction mixture was refluxed for 4 h. The solvent evaporated *in vacuo* and water (5 mL) was added to the residue. The solid was collected by filtration, washed with H_2O (3 mL), then with a mixture of EtOAc:hexane (1:4, 2 mL), and dried *in vacuo*. The product was obtained as an off-white solid (132 mg, 78%).

^{1}H NMR (500 MHz, DMSO-d_6) δ (ppm) 11.87 (s, 1H), 7.95 (d, J = 7.5 Hz, 2H), 7.42 (t, J = 7.4 Hz, 2H), 7.39 – 7.32 (m, 1H), 6.99 (s, 2H), 5.87 (s, 1H), 4.61 (t, J = 5.6 Hz, 1H), 3.50 (q, J = 6.6 Hz, 2H), 2.62 (t, J = 7.0 Hz, 2H), 2.33 (s, 3H).

^{13}C NMR (126 MHz, DMSO-d_6) δ (ppm) 160.3, 150.9. 132.3, 128.6, 128.4, 126.0, 116.0, 85.1, 59.3, 29.1, 21.4.

HRMS (APCI): calcd. for $C_{16}H_{18}N_5O_2$ $[M+H]^+$ = 312.1455, found $[M+H]^+$ = 312.1453.

mp = 221−223 °C.

3-Phenyl-1-(pyrimidin-2-yl)-1*H*-pyrazol-5-amine **(S145)**

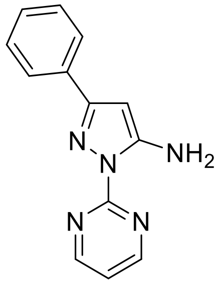

The compound was prepared according to General procedure B2 using methanesulfonic acid (7 mg, 0.069 mmol), 2-hydrazinylpyrimidine (76 mg, 0.69 mmol) and 3-oxo-3-phenylpropanenitrile (100 mg, 0.69 mmol) in EtOH (3 mL). The reaction mixture was refluxed for 6 h. The crude product was purified by column chromatography on silica gel (7M NH_3 in MeOH:MeOH:dichloromethane, 2:8:90). The product was obtained as a light brown solid (81 mg, 50%).

^{1}H NMR (500 MHz, Chloroform-d) δ (ppm) 8.76 (d, J = 4.8 Hz, 2H), 7.96 – 7.86 (m, 2H), 7.42 – 7.37 (m, 2H), 7.36 – 7.31 (m, 1H), 7.14 (t, J = 4.8 Hz, 1H), 5.91 (s, 1H).

^{13}C NMR (126 MHz, Chloroform-*d*) δ (ppm) 158.5, 158.1, 154.3, 150.5, 132.8, 128.7, 128.5, 126.6, 117.2, 87.9.
HRMS (APCI): calcd. for $C_{13}H_{12}N_5$ $[M+H]^+ = 238.1087$, found $[M+H]^+ = 238.1085$.
mp = 202−205 °C.

1-(4-Methoxy-6-methylpyrimidin-2-yl)-3-phenyl-1*H*-pyrazol-5-amine **(S146)**

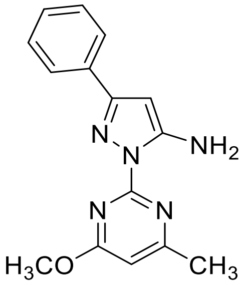

The compound was prepared according to General procedure B2 using methanesulfonic acid (2.4 mg, 0.026 mmol), 2-hydrazinyl-4-methoxy-6-methylpyrimidine (40 mg, 0.26 mmol) and 3-oxo-3-phenylpropanenitrile (38 mg, 0.26 mmol) in EtOH (2 mL). The reaction mixture was refluxed for 4 h. The crude product was purified by column chromatography on silica gel (7M NH_3 in MeOH:MeOH:dichloromethane, 2:8:90). The product was obtained as an off-white solid (63 mg, 87%).
^{1}H NMR (500 MHz, Chloroform-*d*) δ (ppm) 7.93 – 7.87 (m, 2H), 7.38 (td, J = 7.4, 1.6 Hz, 2H), 7.35 – 7.30 (m, 1H), 6.40 (s, 1H), 5.92 (s, 1H), 4.11 (s, 3H), 2.49 (s, 3H).
^{13}C NMR (126 MHz, Chloroform-*d*) δ (ppm) 171.1, 168.5, 157.2, 153.6, 150.6, 132.7, 128.7, 128.5, 126.5, 103.0, 87.8, 54.4, 24.1.
HRMS (APCI): calcd. for $C_{15}H_{16}N_5O$ $[M+H]^+ = 282.1349$, found $[M+H]^+ = 282.1351$.
mp = 177−180 °C.

2-(5-Amino-3-phenyl-1*H*-pyrazol-1-yl)-6-(methoxymethyl)pyrimidin-4(3*H*)-one **(S147)**

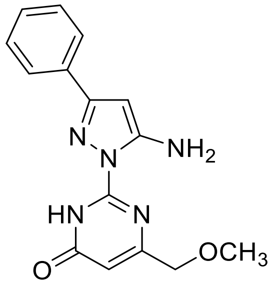

The compound was prepared according to General procedure B2 using methanesulfonic acid (2.3 mg, 0.024 mmol), 2-hydrazinyl-6-(methoxymethyl)pyrimidin-4(3*H*)-one (40 mg, 0.24 mmol, CAS: 1158794-33-2) and 3-oxo-3-phenylpropanenitrile (34 mg, 0.24 mmol) in EtOH (2 mL). The reaction mixture was refluxed for 4 h. The crude product was purified by column chromatography on silica gel (7M NH_3 in MeOH:MeOH:dichloromethane, 2:8:90). The product was obtained as a white solid (59 mg, 86%).

^{1}H NMR (500 MHz, DMSO-d_6) δ (ppm) 11.98 (s, 1H), 7.96 (d, J = 7.5 Hz, 2H), 7.50 – 7.31 (m, 3H), 7.01 (s, 2H), 6.13 (s, 1H), 5.88 (s, 1H), 4.34 (s, 2H), 3.40 (s, 3H).

^{13}C NMR (126 MHz, DMSO-d_6) δ (ppm) 152.9, 151.2, 132.1, 128.7, 128.4, 126.1, 104.8, 85.1, 72.7, 58.3.

HRMS (APCI): calcd. for $C_{15}H_{16}N_5O_2$ $[M+H]^+$ = 298.1299, found $[M+H]^+$ = 298.1298.

mp = 218−221 °C.

2-(5-Amino-3-phenyl-1*H*-pyrazol-1-yl)-5-(2-(2-(2-methoxyethoxy)ethoxy)ethyl)-6-methylpyrimidin-4(3*H*)-one **(S148)**

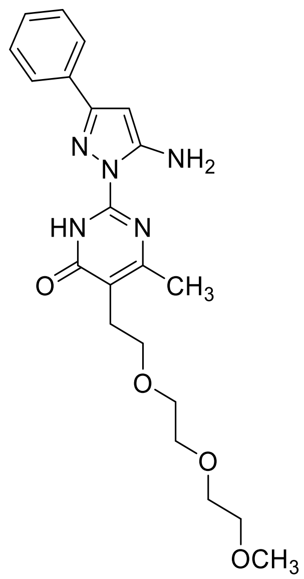

The compound was prepared according to General procedure B2 using methanesulfonic acid (11 mg, 0.12 mmol), 2-hydrazinyl-5-(2-(2-(2-methoxyethoxy)ethoxy)ethyl)-6-methylpyrimidin-4(3*H*)-one (**S126**, 330 mg (crude), 1.15 mmol) and 3-oxo-3-phenylpropanenitrile (167 mg, 1.15 mmol) in EtOH (5 mL). The reaction mixture was refluxed for 4 h. The crude product was purified by column chromatography on silica gel (7M NH_3 in MeOH:MeOH:dichloromethane, 2:8:90). The product was obtained as a light brown solid (205 mg, 43%).

^{1}H NMR (300 MHz, Chloroform-d) δ (ppm) 10.38 (s, 1H), 7.83 – 7.68 (m, 2H), 7.45 – 7.34 (m, 3H), 6.01 (s, 2H), 5.79 (s, 1H), 3.69 – 3.56 (m, 8H), 3.56 – 3.48 (m, 2H), 3.36 (s, 3H), 2.82 (t, J = 6.8 Hz, 2H), 2.34 (s, 3H).

^{13}C NMR (75 MHz, Chloroform-d) δ (ppm) 154.1, 150.1, 146.4, 131.9, 129.3, 128.7, 126.2, 117.5, 87.2, 72.1, 70.8, 70.6, 70.4, 69.5, 59.1, 26.5, 22.0.

HRMS (APCI): calcd. for $C_{21}H_{28}N_5O_4$ $[M+H]^+$ = 414.2136, found $[M+H]^+$ = 414.2136.

mp = 143−146 °C.

2-(5-Amino-3-phenyl-1*H*-pyrazol-1-yl)-5,6-dimethylpyrimidin-4(3*H*)-one **(S149)**

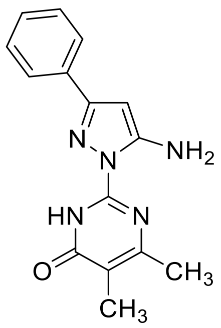

The compound was prepared according to General procedure B2 using methanesulfonic acid (6.2 mg, 0.065 mmol), 2-hydrazinyl-5,6-dimethylpyrimidin-4(3*H*)-one (100 mg, 0.65 mmol, CAS: 59224-10-1) and 3-oxo-3-phenylpropanenitrile (94 mg, 0.65 mmol) in EtOH (3 mL). The reaction mixture was refluxed for 4 h. The crude product was purified by column chromatography on silica gel (7M NH_3 in MeOH:MeOH:dichloromethane, 2:8:90). The product was obtained as a white solid (102 mg, 56%).

^{1}H NMR (300 MHz, DMSO-d_6) δ (ppm) 7.82 – 7.73 (m, 2H), 7.43 – 7.33 (m, 2H), 7.33 – 7.24 (m, 1H), 6.98 (s, 2H), 5.71 (s, 1H), 2.12 (s, 3H), 1.85 (s, 3H).

^{13}C NMR (75 MHz, DMSO-d_6) δ (ppm) 171.9, 156.6, 155.2, 150.8, 149.7, 133.6, 128.3, 127.6, 125.6, 112.4, 85.0, 21.2, 11.4.

HRMS (APCI): calcd. for $C_{15}H_{16}N_5O$ $[M+H]^+$ = 282.1349, found $[M+H]^+$ = 282.1348.

mp = 276–281 °C.

6-(5-Amino-3-phenyl-1*H*-pyrazol-1-yl)-1,3-dimethylpyrimidine-2,4(1*H*,3*H*)-dione **(S150)**

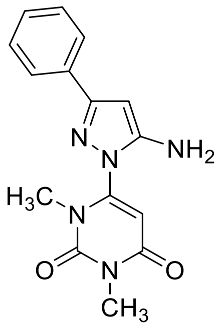

The compound was prepared according to General procedure B2 using methanesulfonic acid (6 mg, 0.0 mmol), 6-hydrazinyl-1,3-dimethylpyrimidine-2,4(1*H*,3*H*)-dione (100 mg, 0.59 mmol, CAS: 40012-14-4) and 3-oxo-3-phenylpropanenitrile (85 mg, 0.59 mmol) in EtOH (3 mL). The reaction mixture was refluxed for 4 h. The crude product was purified by column chromatography on silica gel (7M NH_3 in MeOH:MeOH:dichloromethane, 2:8:90). The product was obtained as a white solid (141 mg, 81%).

^{1}H NMR (300 MHz, Chloroform-*d*) δ (ppm) 7.82 – 7.66 (m, 2H), 7.48 – 7.29 (m, 3H), 5.91 (s, 1H), 5.90 (s, 1H), 4.14 (s, 2H), 3.37 (s, 3H), 3.28 (s, 3H).

^{13}C NMR (75 MHz, Chloroform-*d*) δ (ppm) 162.4, 154.5, 152.1, 147.9, 146.1, 132.3, 129.0, 128.8, 125.9, 99.4, 87.8, 32.5, 28.5.

HRMS (APCI): calcd. for $C_{15}H_{16}N_5O_2$ $[M+H]^+$ = 298.1299, found $[M+H]^+$ = 298.1300.

mp = 158−160 °C.

2-(5-Amino-3-phenyl-1*H*-pyrazol-1-yl)-3,6-dimethylpyrimidin-4(3*H*)-one **(S151)**

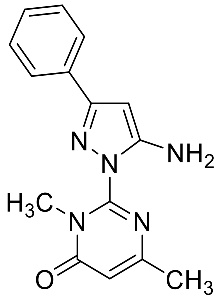

The compound was prepared according to General procedure B2 using methanesulfonic acid (9.3 µL, 0.097 mmol), 2-hydrazinyl-3,6-dimethylpyrimidin-4(3*H*)-one (150 mg, 0.97 mmol; CAS:3493-94-5) and 3-oxo-3-phenylpropanenitrile (141 mg, 0.97 mmol) in EtOH (5 mL). The reaction mixture was refluxed for 3 h and then quenched with addition of 7M NH_3 in MeOH (2 mL). The crude product was purified by column chromatography on silica gel (EtOAc:dichloromethane, 20:80). The product was obtained as a pale yellow solid (80 mg, 29%) containing traces of residual EtOAc, which was used directly in the next step.

^{1}H NMR (500 MHz, Chloroform-*d*) δ (ppm) 7.81 – 7.75 (m, 2H), 7.44 – 7.39 (m, 2H), 7.39 – 7.34 (m, 1H), 6.24 (d, J = 0.9 Hz, 1H), 5.91 (s, 1H), 5.09 (br s, 2H), 3.78 (s, 3H), 2.28 (s, 3H).

^{13}C NMR (126 MHz, Chloroform-*d*) δ (ppm) 163.50, 161.26, 153.21, 150.30, 132.42, 129.01, 128.80, 126.04, 110.05, 105.71, 88.34, 34.38, 23.49.

HRMS (APCI): calcd. for $C_{15}H_{16}N_5O$ $[M+H]^+$ = 282.1349, found $[M+H]^+$ = 282.1348.

1-(6-(Benzyloxy)pyridin-2-yl)-3-phenyl-1*H*-pyrazol-5-amine **(S152)**

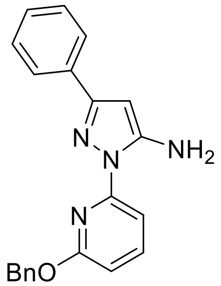

The compound was prepared according to General procedure B2 using methanesulfonic acid (4.5 µL, 0.07 mmol), 2-(benzyloxy)-6-hydrazinylpyridine (**S128**, 150 mg, 0.70 mmol) and 3-oxo-3-phenylpropanenitrile (101 mg, 0.70 mmol) in EtOH (5 mL). The reaction mixture was refluxed for 3 h. The crude product was purified by column chromatography on silica gel (EtOAc:hexane; 10:90) followed by trituration with hexanes (5 mL) and drying *in vacuo*. The product was obtained as an off-white solid (50 mg, 21%).

^{1}H NMR (500 MHz, DMSO-d_6) δ (ppm) 7.90 (t, J = 8.0 Hz, 1H), 7.84 – 7.78 (m, 2H), 7.54 (d, J = 7.9 Hz, 1H), 7.51 – 7.46 (m, 2H), 7.45 – 7.38 (m, 4H), 7.37 – 7.32 (m, 2H), 6.76 (d, J = 8.1 Hz, 1H), 6.54 (br s, 2H), 5.86 (br s, 1H), 5.38 (s, 2H).

^{13}C NMR (126 MHz, DMSO-d_6) δ (ppm) 161.41, 151.80, 151.20, 149.99, 141.84, 136.62, 132.96, 128.48, 128.44, 128.05, 127.88, 127.64, 125.37, 105.81, 105.50, 85.64, 67.69.

HRMS (APCI): calcd. for $C_{21}H_{19}N_4O$ $[M+H]^+$ = 343.1553, found $[M+H]^+$ = 343.1556.

2-(5-Amino-3-(furan-2-yl)-1*H*-pyrazol-1-yl)-6-(trifluoromethyl)pyrimidin-4(3*H*)-one **(S153)**

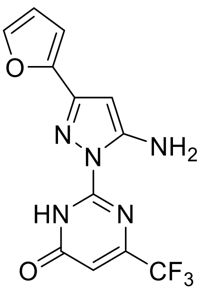

The compound was prepared according to General procedure B2 using methanesulfonic acid (3 mg, 0.026 mmol), 2-hydrazinyl-6-(trifluoromethyl)pyrimidin-4(3*H*)-one (**S120**, 50 mg, 0.26 mmol) and 3-(furan-2-yl)-3-oxopropanenitrile (35 mg, 0.26 mmol) in EtOH (3 mL). The reaction mixture was refluxed for 4 h. The crude product was purified by column chromatography on silica gel (7M NH_3 in MeOH:MeOH:dichloromethane, 5:5:90). The product was obtained as a white solid (25 mg, 31%).

^{1}H NMR (300 MHz, DMSO-d_6) δ (ppm) 7.77 (dd, J = 1.8, 0.8 Hz, 1H), 6.92 (dd, J = 3.4, 0.8 Hz, 1H), 6.74 (s, 1H), 6.61 (dd, J = 3.4, 1.8 Hz, 1H), 5.74 (s, 1H).

^{13}C NMR (126 MHz, DMSO-d_6) δ (ppm) 151.1, 147.6, 145.8, 143.4, 121.6, 119.4, 117.2, 111.7, 108.5, 85.2.

^{19}F NMR (282 MHz, DMSO-d_6) δ (ppm) -69.83.

HRMS (APCI): calcd. for $C_{12}H_9F_3N_5O_2$ $[M+H]^+$ = 312.0703, found $[M+H]^+$ = 312.0699.

mp >238 °C (dec.)

2-(5-Amino-3-(furan-2-yl)-1*H*-pyrazol-1-yl)-5-(2-hydroxyethyl)-6-methylpyrimidin-4(3*H*)-one **(S154)**

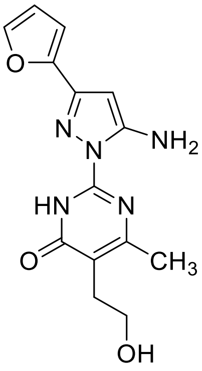

The compound was prepared according to General procedure B2 using methanesulfonic acid (3 mg, 0.027 mmol), 2-hydrazinyl-5-(2-hydroxyethyl)-6-methylpyrimidin-4(3*H*)-one (50 mg, 0.27 mmol, CAS: 74187-94-3) and 3-(furan-2-yl)-3-oxopropanenitrile (37 mg, 0.27 mmol) in EtOH (3 mL). The reaction mixture was refluxed for 4 h. The crude product was purified by column chromatography on silica gel (7M NH_3 in MeOH:MeOH:dichloromethane, 7.5:7.5:85). The product was obtained as a light brown solid (55 mg, 67%).

^{1}H NMR (300 MHz, DMSO-d_6) δ (ppm) 11.53 (s, 1H), 7.74 (d, J = 1.8 Hz, 1H), 7.00 (s, 2H), 6.87 (d, J = 3.4 Hz, 1H), 6.59 (dd, J = 3.4, 1.8 Hz, 1H), 5.69 (s, 1H), 4.61 (s, 1H), 3.49 (s, 2H), 2.63 (t, J = 7.0 Hz, 2H), 2.35 (s, 3H).

^{13}C NMR (126 MHz, DMSO-d_6) δ (ppm) 150.6, 148.9, 147.9, 143.1, 112.9, 111.6, 84.9, 59.3, 29.0, 21.4.

HRMS (APCI): calcd. for $C_{14}H_{16}N_5O_3$ $[M+H]^+$ = 302.1248, found $[M+H]^+$ = 302.1246.

mp >182 °C (dec.)

Preparation of acid chlorides

Scheme S3: synthesis of 5-cyclohexylisoxazole-3-carbonyl chloride **(S158)**

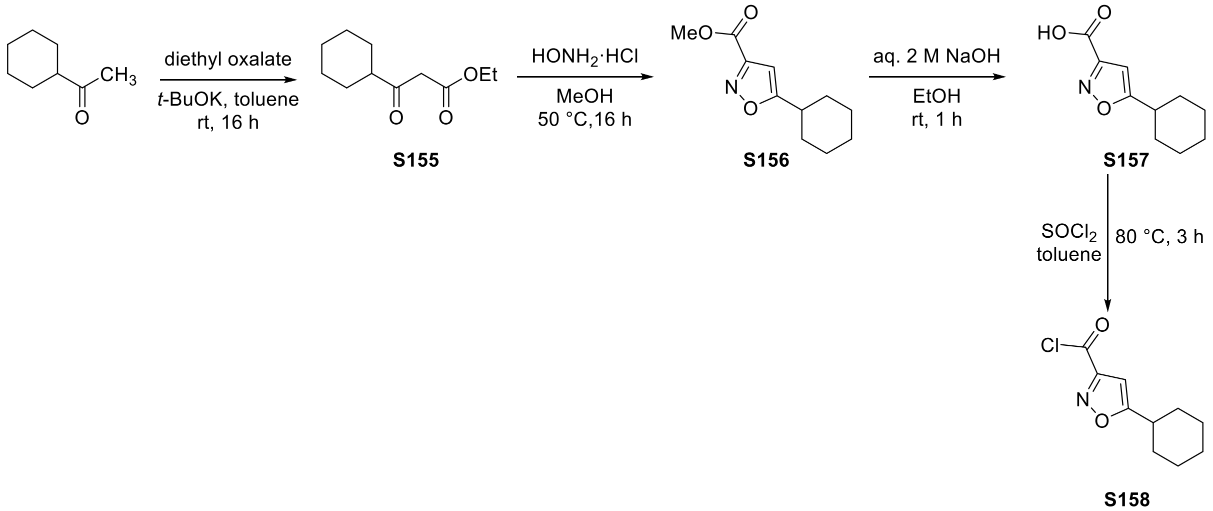

Ethyl 4-cyclohexyl-2,4-dioxobutanoate **(S155)**

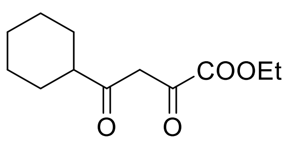

The compound was prepared according to General procedure E using 1-cyclohexylethan-1-one (1 g, 7.92 mmol), diethyl oxalate (1.27 g, 8.72 mmol), toluene (10 mL) and *t*-BuOK (1 M in THF, 9.5 mL, 9.5 mmol). The product was obtained as a light brown oil (1.57 g, 88%).

^{1}H NMR (500 MHz, Chloroform-*d*) δ (ppm) 14.64 (s, 1H), 6.38 (s, 1H), 4.34 (q, J = 7.1 Hz, 2H), 2.37 (tt, J = 11.5, 3.4 Hz, 1H), 1.94 – 1.85 (m, 2H), 1.82 (dt, J = 13.2, 3.7 Hz, 2H), 1.70 (dtd, J = 9.6, 3.0, 1.6 Hz, 1H), 1.46 – 1.33 (m, 6H), 1.32 – 1.19 (m, 5H). (keto-enol tautomer)

^{13}C NMR (126 MHz, Chloroform-*d*) δ (ppm) 205.9, 167.7, 162.2, 100.1, 62.4, 48.7, 28.9, 25.7, 25.5, 14.0.
HRMS (APCI): calcd. for $C_{12}H_{19}O_4$ $[M+H]^+$ = 227.1278, found $[M+H]^+$ = 227.1277.

Ethyl 3-cyclohexyl-3-oxopropanoate **(S156)**

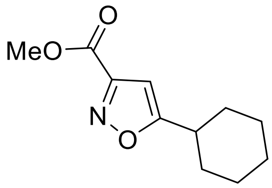

The compound was prepared according to General procedure F using ethyl 4-cyclohexyl-2,4-dioxobutanoate (**S155**, 1.57 g, 6.93 mmol), MeOH (16 mL) and $HONH_2$•HCl (723 mg, 10.4 mmol). The crude product was purified by column chromatography on silica gel (hexane:EtOAc, 9:1). The product was obtained as a white solid (1.51 g, 98%).
^1H NMR (500 MHz, Chloroform-*d*) δ (ppm) 6.37 (d, J = 0.8 Hz, 1H), 3.95 (s, 3H), 2.87 – 2.79 (m, 1H), 2.10 – 2.02 (m, 2H), 1.82 (dt, J = 12.7, 3.6 Hz, 2H), 1.76 – 1.69 (m, 1H), 1.51 – 1.34 (m, 4H), 1.32 – 1.23 (m, 1H).
^{13}C NMR (126 MHz, Chloroform-*d*) δ (ppm) 179.8, 160.8, 155.9, 99.8, 52.7, 36.3, 31.0, 25.7, 25.5.
HRMS (APCI): calcd. for $C_{11}H_{16}NO_3$ $[M+H]^+$ = 210.1125, found $[M+H]^+$ = 210.1126.
mp = 73−74 °C.

5-Cyclohexylisoxazole-3-carboxylic acid **(S157)**

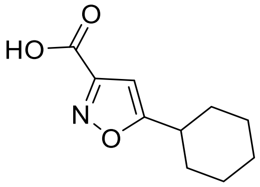

The compound was prepared according to General procedure G using ethyl 3-cyclohexyl-3-oxopropanoate (**S156**, 1.51 g, 6.76 mmol), EtOH (15 mL) and NaOH (2 M in H_2O, 5.07 mL, 10.14 mmol). The product was obtained as light brown solid (840 mg, 64%).
^1H NMR (500 MHz, Chloroform-*d*) δ (ppm) 10.17 (s, 1H), 6.43 (s, 1H), 2.85 (tt, J = 11.3, 3.7 Hz, 1H), 2.08 (dt, J = 12.6, 3.6 Hz, 2H), 1.82 (dq, J = 11.0, 3.6 Hz, 2H), 1.73 (dddd, J = 12.8, 5.2, 3.5, 1.6 Hz, 1H), 1.55 – 1.34 (m, 4H), 1.29 (tt, J = 12.5, 3.5 Hz, 1H).
^{13}C NMR (126 MHz, Chloroform-*d*) δ (ppm) 180.6, 164.2, 155.6, 100.2, 36.5, 31.1, 25.8, 25.6.
HRMS (APCI): calcd. for $C_{10}H_{14}NO_3$ $[M+H]^+$ = 196.0968, found $[M+H]^+$ = 196.0970.
mp = 130−131 °C.

5-Cyclohexylisoxazole-3-carbonyl chloride **(S158)**

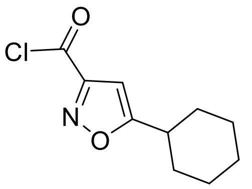

$SOCl_2$ (47 µL, 0.63 mmol) was added to a solution of 5-cyclohexylisoxazole-3-carboxylic acid (100 mg, 0.53 mmol) in toluene (2 mL) and the reaction mixture was stirred at 80 °C for 3 h. The excess $SOCl_2$ evaporated *in vacuo* and the residue was mixed with toluene (2 × 2 mL) and the solvent was evaporated. After drying *in vacuo*, the product was obtained as a clear gum (109 mg, quant.) and used directly in the next step.

Ethyl 4-(4-methoxyphenyl)-2,4-dioxobutanoate **(S159)**

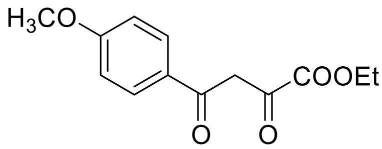

The compound was prepared according to General procedure E using 1-(4-methoxyphenyl)ethan-1-one (1.00 g, 6.66 mmol), diethyl oxalate (1.07 g, 7.32 mmol), toluene (10 mL) and *t*-BuOK (1 M in THF, 7.98 mL, 7.98 mmol). The product was obtained as light brown solid (1.47 g, 89%).

^{1}H NMR (500 MHz, Chloroform-*d*) δ (ppm) 15.44 (s, 1H), 7.98 (d, J = 9.0 Hz, 2H), 7.02 (s, 1H), 6.98 (d, J = 9.0 Hz, 2H), 4.39 (q, J = 7.1 Hz, 2H), 3.89 (s, 3H), 1.41 (t, J = 7.1 Hz, 3H).

^{13}C NMR (126 MHz, Chloroform-*d*) δ (ppm) 190.3, 168.2, 164.3, 162.5, 130.3, 127.8, 114.2, 97.7, 62.5, 55.6, 14.1.

HRMS (APCI): calcd. for $C_{13}H_{15}O_5$ $[M+H]^+$ = 251.0914, found $[M+H]^+$ = 251.0916.

Ethyl 5-(4-methoxyphenyl)isoxazole-3-carboxylate **(S160)**

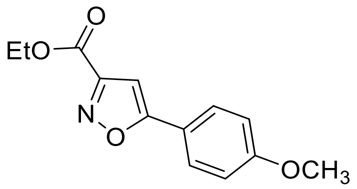

The compound was prepared according to General procedure F using ethyl 4-(4-methoxyphenyl)-2,4-dioxobutanoate (**S159**, 1.47 g, 5.87 mmol), EtOH (15 mL) and $HONH_2{\cdot}HCl$ (612 mg, 8.81 mmol). The crude product was purified by column chromatography on silica gel (hexane:EtOAc, 85:15). The product was obtained as a light brown solid (1.38 g, 95%).

^{1}H NMR (500 MHz, Chloroform-*d*) δ (ppm) 7.74 (d, J = 8.9 Hz, 2H), 6.99 (d, J = 8.9 Hz, 2H), 6.79 (s, 1H), 4.46 (d, J = 7.1 Hz, 2H), 3.87 (s, 3H), 1.44 (s, 3H).

^{13}C NMR (126 MHz, Chloroform-*d*) δ (ppm) 171.9, 161.7, 160.3, 157.1, 127.7, 119.6, 114.7, 98.7, 62.3, 55.6, 14.3.

HRMS (APCI): calcd. for $C_{13}H_{14}NO_4$ $[M+H]^+$ = 248.0917, found $[M+H]^+$ = 248.0914.
mp = 87−89 °C.

5-(4-Methoxyphenyl)isoxazole-3-carboxylic acid **(S161)**

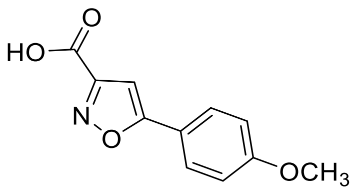

The compound was prepared according to General procedure G using ethyl 5-(4-methoxyphenyl)isoxazole-3-carboxylate (**S160**, 1.38 g, 5.58 mmol), EtOH (14 mL) and NaOH (2 M in H_2O, 4.19 mL, 8.37 mmol). The product was obtained as a yellow solid (580 mg, 47%).
^{1}H NMR (500 MHz, Chloroform-*d*) δ (ppm) 7.76 (d, J = 8.9 Hz, 2H), 7.01 (d, J = 8.9 Hz, 2H), 6.85 (s, 1H), 3.88 (s, 3H).
^{13}C NMR (126 MHz, Chloroform-*d*) δ (ppm) 172.7, 161.9, 161.4, 156.2, 127.8, 119.4, 114.8, 98.7, 55.6.
HRMS (APCI): calcd. for $C_{11}H_{10}NO_4$ $[M+H]^+$ = 220.0604, found $[M+H]^+$ = 220.0601.
mp > 148 °C (dec).

5-(4-Methoxyphenyl)isoxazole-3-carbonyl chloride **(S162)**

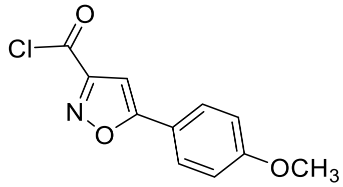

The compound was prepared according to General procedure H using 5-(4-methoxyphenyl)isoxazole-3-carboxylic acid (**S161**, 200 mg, 0.91 mmol) and $SOCl_2$ (331 µL, 4.56 mmol). The reaction mixture was refluxed for 4 h. The product was obtained as a light brown solid (216 mg, quant.) and used directly in the next step.

Ethyl 4-(2-bromophenyl)-2,4-dioxobutanoate **(S163)**

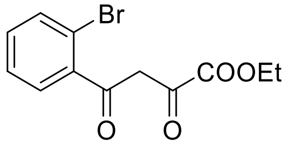

The compound was prepared according to General procedure E using 1-(2-bromophenyl)ethan-1-one (1.00 g, 5.02 mmol), diethyl oxalate (807 mg, 5.53 mmol), toluene (10 mL) and *t*-BuOK (1 M in THF, 6.03 mL, 6.03 mmol). The product was obtained as a dark red oil (1.31 g, 87%).

^{1}H NMR (500 MHz, Chloroform-*d*) δ (ppm) 14.49 (s, 1H), 7.67 (dd, J = 8.0, 1.1 Hz, 1H), 7.57 (dd, J = 7.6, 1.8 Hz, 1H), 7.42 (td, J = 7.6, 1.2 Hz, 1H), 7.35 (td, J = 7.7, 1.8 Hz, 1H), 6.89 (s, 1H), 4.38 (q, J = 7.2 Hz, 2H), 1.39 (t, J = 7.2 Hz, 3H).

^{13}C NMR (126 MHz, Chloroform-*d*) δ (ppm) 194.3, 167.6, 162.0, 138.2, 134.3, 132.8, 130.2, 127.7, 120.3, 103.1, 62.8, 14.2.

HRMS (APCI): calcd. for $C_{12}H_{12}BrO_4$ $[M+H]^+$ = 298.9913, found $[M+H]^+$ = 298.9911.

Ethyl 5-(2-bromophenyl)isoxazole-3-carboxylate **(S164)**

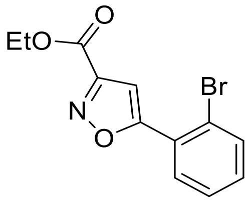

The compound was prepared according to General procedure F using ethyl 4-(2-bromophenyl)-2,4-dioxobutanoate (**S163**, 1.31 g, 4.38 mmol), EtOH (14 mL) and $HONH_2$•HCl (456 mg, 6.57 mmol). The crude product was purified by column chromatography on silica gel (hexane:EtOAc, 90:10). The product was obtained as a light brown oil (1.21 g, 93%).

^{1}H NMR (500 MHz, Chloroform-*d*) δ (ppm) 7.88 (dd, J = 7.9, 1.7 Hz, 1H), 7.73 (dd, J = 7.9, 1.2 Hz, 1H), 7.46 (td, J = 7.6, 1.2 Hz, 1H), 7.36 (s, 1H), 7.35 – 7.30 (m, 1H), 4.49 (q, J = 7.2 Hz, 2H), 1.45 (t, J = 7.1 Hz, 3H).

^{13}C NMR (126 MHz, Chloroform-*d*) δ (ppm) 169.6, 160.1, 156.8, 134.5, 131.7, 130.3, 128.0, 127.8, 121.4, 104.8, 62.4, 14.3.

HRMS (APCI): calcd. for $C_{12}H_{11}BrNO_3$ $[M+H]^+$ = 295.9917, found $[M+H]^+$ = 295.9915.

5-(2-Bromophenyl)isoxazole-3-carboxylic acid **(S165)**

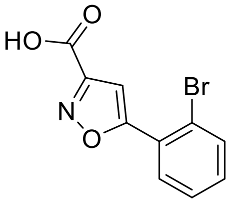

The compound was prepared according to General procedure G using ethyl 5-(2-bromophenyl)isoxazole-3-carboxylate (**S164**, 1.21 g, 4.09 mmol), EtOH (13 mL) and NaOH (2 M in H_2O, 3.06 mL, 6.13 mmol). The product was obtained as a light brown solid (930 mg, 85%).

^{1}H NMR (500 MHz, Chloroform-*d*) δ (ppm) 8.14 (s, 1H), 7.91 (dd, J = 7.9, 1.7 Hz, 1H), 7.75 (dd, J = 8.1, 1.2 Hz, 1H), 7.48 (td, J = 7.6, 1.2 Hz, 1H), 7.44 (s, 1H), 7.35 (td, J = 7.7, 1.7 Hz, 1H).

^{13}C NMR (126 MHz, Chloroform-*d*) δ (ppm) 170.1, 163.2, 155.9, 134.4, 131.8, 130.2, 127.9, 127.4, 121.4, 104.8.

HRMS (APCI): calcd. for $C_{10}H_7BrNO_3$ $[M+H]^+$ = 267.9604, found $[M+H]^+$ = 267.9603.

mp = 138−141 °C.

5-(2-Bromophenyl)isoxazole-3-carbonyl chloride **(S166)**

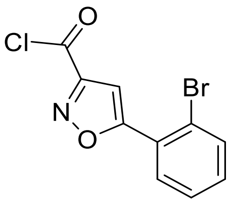

The compound was prepared according to General procedure H using 5-(2-bromophenyl)isoxazole-3-carboxylic acid (**S165**, 200 mg, 0.75 mmol) and $SOCl_2$ (271 µL, 3.73 mmol). The reaction mixture was refluxed for 4 h. The product was obtained as a light brown solid (213 mg, quant.) and used directly in the next step.

Ethyl 4-(3-(*tert*-butyl)phenyl)-2,4-dioxobutanoate **(S167)**

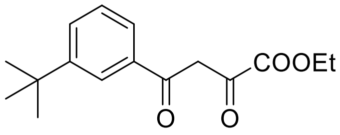

The compound was prepared according to General procedure E using 1-(3-(*tert*-butyl)phenyl)ethan-1-one (150 mg, 0.85 mmol), diethyl oxalate (137 mg, 0.94 mmol), toluene (4 mL) and *t*-BuOK (1 M in THF, 1.02 mL, 1.02 mmol). The product was obtained as a light brown gum (259 mg, crude) and used as such in the next step without further purification.

^{1}H NMR (500 MHz, Chloroform-*d*) δ (ppm) 15.38 (s, 1H), 8.02 (d, J = 1.9 Hz, 1H), 7.79 (dd, J = 7.8, 1.4 Hz, 1H), 7.69 – 7.61 (m, 1H), 7.43 (t, J = 7.8 Hz, 1H), 7.07 (s, 1H), 4.41 (q, J = 7.1 Hz, 2H), 1.42 (t, J = 7.1 Hz, 3H), 1.37 (s, 9H).

^{13}C NMR (126 MHz, Chloroform-*d*) δ (ppm) 191.5, 169.8, 162.5, 152.3, 134.9, 131.2, 128.8, 125.4, 124.8, 98.2, 62.7, 35.1, 31.4, 14.2, 14.1.

HRMS (APCI): calcd. for $C_{16}H_{21}O_4$ $[M+H]^+$ = 277.1434, found $[M+H]^+$ = 277.1432.

Ethyl 5-(3-(*tert*-butyl)phenyl)isoxazole-3-carboxylate **(S168)**

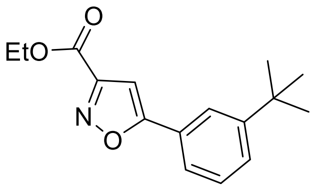

The compound was prepared according to General procedure F using ethyl 4-(3-(*tert*-butyl)phenyl)-2,4-dioxobutanoate (**S167**, 259 mg, 0.94 mmol), EtOH (5 mL) and $HONH_2{\cdot}HCl$ (78 mg, 1.12 mmol). The crude product was purified by column chromatography on silica gel (hexane:EtOAc, 9:1). The product was obtained as a colorless gum (175 mg, 75% over the 2 steps).

^{1}H NMR (300 MHz, Chloroform-*d*) δ (ppm) 7.85 – 7.79 (m, 1H), 7.61 (dt, *J* = 7.6, 1.5 Hz, 1H), 7.51 (ddd, *J* = 7.9, 2.0, 1.2 Hz, 1H), 7.46 – 7.37 (m, 1H), 6.92 (s, 1H), 4.48 (q, *J* = 7.2 Hz, 2H), 1.45 (t, *J* = 7.1 Hz, 3H), 1.37 (s, 9H).

^{13}C NMR (126 MHz, Chloroform-*d*) δ (ppm) 172.4, 160.2, 157.1, 152.4, 129.0, 128.1, 126.6, 123.4, 123.0, 99.9, 62.3, 35.0, 31.3, 14.3.

HRMS (APCI): calcd. for $C_{16}H_{20}NO_3$ $[M+H]^+$ = 274.1438, found $[M+H]^+$ = 274.1437.

5-(3-(*tert*-butyl)phenyl)isoxazole-3-carboxylic acid **(S169)**

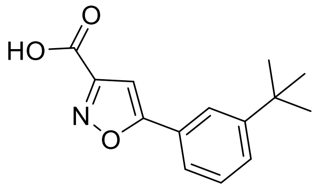

The compound was prepared according to General procedure G using ethyl 5-(3-(*tert*-butyl)phenyl)isoxazole-3-carboxylate (**S168**, 175 mg, 0.64 mmol), EtOH (2 mL) and NaOH (2 M in H_2O, 0.48 mL, 0.96 mmol). The product was obtained as off-white solid (134 mg, 85%).

^{1}H NMR (300 MHz, DMSO-*d*$_6$) δ (ppm) 13.99 (s, 1H), 7.92 (t, *J* = 1.9 Hz, 1H), 7.75 (dt, *J* = 7.5, 1.4 Hz, 1H), 7.62 – 7.53 (m, 1H), 7.49 (d, *J* = 7.7 Hz, 1H), 7.46 (s, 1H), 1.34 (s, 9H).

^{13}C NMR (126 MHz, DMSO-*d*$_6$) δ (ppm) 171.1, 157.9, 151.9, 131.2, 129.1, 127.8, 126.0, 123.0, 122.6, 100.8, 34.6, 30.9.

HRMS (APCI): calcd. for $C_{14}H_{16}NO_3$ $[M+H]^+$ = 246.1125, found $[M+H]^+$ = 246.1122.

mp = 127−129 °C.

5-(3-(*tert*-butyl)phenyl)isoxazole-3-carbonyl chloride **(S170)**

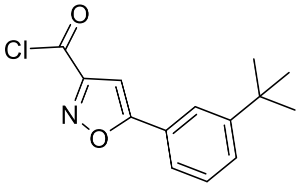

The compound was prepared according to General procedure H using 5-(3-(*tert*-butyl)phenyl)isoxazole-3-carboxylic acid (**S169**, 110 mg, 0.45 mmol) and $SOCl_2$ (163 µL, 2.24 mmol). The reaction mixture was refluxed for 4 h. The product was obtained as an off-white solid (118 mg, quant.) and used directly in the next step.

Ethyl 5,5-dimethyl-2,4-dioxohexanoate **(S171)**

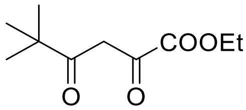

The compound was prepared according to General procedure E using 3,3-dimethylbutan-2-one (1 g, 9.98 mmol), diethyl oxalate (1.60 g, 10.98 mmol), toluene (10 mL) and *t*-BuOK (1 M in THF, 11.98 mL, 11.98 mmol). The product was obtained as a light brown oil (1.63 g, 82%).

^{1}H NMR (500 MHz, Chloroform-*d*) δ (ppm) 14.75 (s, 1H), 6.53 (s, 1H), 4.36 (q, J = 7.2 Hz, 2H), 1.38 (t, J = 7.1 Hz, 3H), 1.21 (s, 9H).

^{13}C NMR (126 MHz, Chloroform-*d*) δ (ppm) 209.3, 167.7, 162.5, 98.0, 62.6, 41.8, 26.9, 14.2.

HRMS (APCI): calcd. for $C_{10}H_{17}O_4$ [M+H]$^+$ = 201.1121, found [M+H]$^+$ = 201.1119.

Ethyl 5-(*tert*-butyl)isoxazole-3-carboxylate **(S172)**

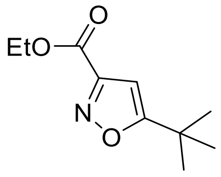

The compound was prepared according to General procedure F using ethyl 5,5-dimethyl-2,4-dioxohexanoate (**S171**, 1.63 g, 7.40 mmol), EtOH (17 mL) and $HONH_2$•HCl (771 mg, 11.1 mmol). The crude product was purified by column chromatography on silica gel (hexane:EtOAc, 9:1). The product was obtained as a light brown oil (1.40 g, 96%).

^{1}H NMR (500 MHz, Chloroform-*d*) δ (ppm) 6.36 (s, 1H), 4.42 (q, J = 7.1 Hz, 2H), 1.40 (t, J = 7.1 Hz, 3H), 1.36 (s, 9H).

^{13}C NMR (126 MHz, Chloroform-*d*) δ (ppm) 183.3, 160.5, 156.3, 99.2, 62.1, 33.1, 28.9, 14.3.

HRMS (APCI): calcd. for $C_{10}H_{16}NO_3$ [M+H]$^+$ = 198.1125, found [M+H]$^+$ = 198.1123.

5-(*tert*-Butyl)isoxazole-3-carboxylic acid **(S173)**

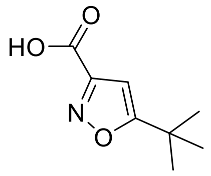

The compound was prepared according to General procedure G using ethyl 5-(*tert*-butyl)isoxazole-3-carboxylate (**S172**, 1.40 g, 7.1 mmol), EtOH (14 mL) and NaOH (2 M in H_2O, 5.32 mL, 10.65 mmol). The product was obtained as a light brown gum (1.20 g, quant.).

^{1}H NMR (500 MHz, Chloroform-*d*) δ (ppm) 6.43 (s, 1H), 1.39 (s, 9H).

^{13}C NMR (126 MHz, Chloroform-*d*) δ (ppm) 184.1, 163.1, 155.5, 99.5, 33.2, 28.9.

HRMS (APCI): calcd. for $C_8H_{12}NO_3$ [M+H]$^+$ = 170.0812, found [M+H]$^+$ = 170.0810.

Ethyl 2,4-dioxo-4-(4-(trifluoromethoxy)phenyl)butanoate **(S174)**

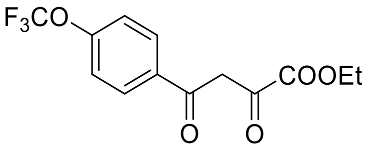

The compound was prepared according to General procedure E using 1-(4-(trifluoromethoxy)phenyl)ethan-1-one (700 mg, 3.43 mmol), diethyl oxalate (550 mg, 3.77 mmol), toluene (7 mL) and *t*-BuOK (1 M in THF, 4.11 mL, 4.11 mmol). The product was obtained as a yellow gum (975 mg, 93%).

^{1}H NMR (500 MHz, Chloroform-*d*) δ (ppm) 15.15 (s, 1H), 8.07 – 8.02 (m, 2H), 7.37 – 7.30 (m, 2H), 7.04 (s, 1H), 4.41 (q, J = 7.1 Hz, 2H), 1.42 (t, J = 7.1 Hz, 3H).

^{13}C NMR (126 MHz, Chloroform-*d*) δ (ppm) 189.2, 170.3, 162.2, 153.3, 133.4, 130.0, 120.8, 120.4 (q, J = 259.0 Hz), 98.0, 62.9, 14.2.

^{19}F NMR (282 MHz, Chloroform-*d*) δ (ppm) -57.6.

HRMS (APCI): calcd. for $C_{13}H_{12}F_3O_5$ $[M+H]^+$ = 305.0631, found $[M+H]^+$ = 305.0628.

Ethyl 5-(4-(trifluoromethoxy)phenyl)isoxazole-3-carboxylate **(S175)**

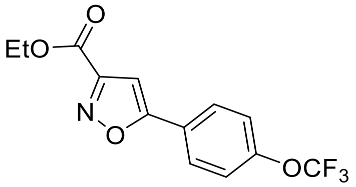

The compound was prepared according to General procedure F using ethyl 2,4-dioxo-4-(4-(trifluoromethoxy)phenyl)butanoate (**S174**, 975 mg, 3.20 mmol), EtOH (10 mL) and HONH$_2$•HCl (334 mg, 4.81 mmol). The crude product was purified by column chromatography on silica gel (hexane:EtOAc, 8:2). The product was obtained as a white solid (809 mg, 84%).

^{1}H NMR (500 MHz, Chloroform-*d*) δ (ppm) 7.80 – 7.89 (m, 2H), 7.34 (dt, J = 7.9, 1.1 Hz, 2H), 6.93 (s, 1H), 4.48 (q, J = 7.2 Hz, 2H), 1.45 (t, J = 7.1 Hz, 3H).

^{13}C NMR (126 MHz, Chloroform-*d*) δ (ppm) 170.4, 160.0, 157.3, 151.0, 127.8, 125.4, 121.6, 120.5 (q, J = 259.2 Hz), 100.5, 62.5, 14.3.

^{19}F NMR (471 MHz, Chloroform-*d*) δ (ppm) -57.8.

HRMS (APCI): calcd. for $C_{13}H_{11}F_3NO_4$ $[M+H]^+$ = 302.0635, found $[M+H]^+$ = 302.0634.

mp = 132−134 °C.

5-(4-(Trifluoromethoxy)phenyl)isoxazole-3-carboxylic acid **(S176)**

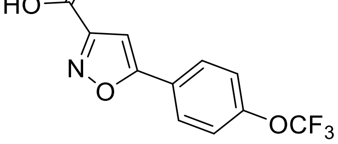

The compound was prepared according to General procedure G using ethyl 5-(4-(trifluoromethoxy)phenyl)isoxazole-3-carboxylate (**S175**, 809 mg, 2.69 mmol), EtOH (9 mL) and NaOH (2 M in H_2O, 2.01 mL, 4.03 mmol). The product was obtained as a white solid (705 mg, 96%).

^{1}H NMR (500 MHz, DMSO-d_6) δ (ppm) 14.11 (s, 1H), 8.12 – 8.04 (m, 2H), 7.59 – 7.52 (m, 2H), 7.48 (d, J = 1.2 Hz, 1H).

^{13}C NMR (126 MHz, DMSO-d_6) δ (ppm) 169.4, 160.7, 158.0, 149.7, 128.0, 125.4, 121.7, 119.9 (q, J = 257.8 Hz), 101.6.

^{19}F NMR (471 MHz, DMSO-d_6) δ (ppm) -56.80 – -56.54 (m).

HRMS (APCI): calcd. for $C_{11}H_7F_3NO_4$ $[M+H]^+$ = 274.0322, found $[M+H]^+$ = 274.0320.

mp = 183−184 °C.

5-Phenylisoxazole-3-carbonyl chloride **(S177)**

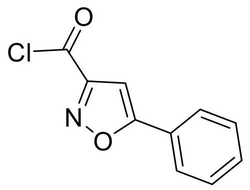

The compound was prepared according to General procedure H using 5-phenylisoxazole-3-carboxylic acid (2 g, 10.57 mmol) and $SOCl_2$ (3.83 mL, 52.86 mmol). The reaction mixture was refluxed for 4 h. The product was obtained as light brown solid (2.19 g, quant.) and used directly in the next step.

3-Phenylisoxazole-5-carbonyl chloride **(S178)**

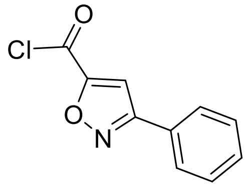

The compound was prepared according to General procedure H using 3-phenylisoxazole-5-carboxylic acid (500 mg, 2.64 mmol) and $SOCl_2$ (959 µL, 13.22 mmol). The reaction mixture was refluxed for 4 h. The product was obtained as a light brown solid (549 mg, quant.) and used directly in the next step.

2-Phenylthiazole-4-carbonyl chloride **(S179)**

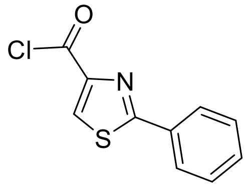

The compound was prepared according to General procedure H using 2-phenylthiazole-4-carboxylic acid (100 mg, 0.49 mmol) and $SOCl_2$ (177 µL, 2.44 mmol). The reaction mixture was refluxed for 4 h. The product was obtained as a white solid (109 mg, quant.) and used directly in the next step.

5-Phenyl-4,5-dihydroisoxazole-3-carbonyl chloride **(S180)**

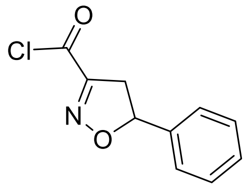

The compound was prepared according to General procedure H using 5-phenyl-4,5-dihydroisoxazole-3-carboxylic acid (60 mg, 0.31 mmol) and $SOCl_2$ (113 µL, 1.57 mmol). The reaction mixture was refluxed for 4 h. The product was obtained as a colorless oil (66 mg, quant.) and used directly in the next step.

2-(5-phenylisoxazol-3-yl)acetyl chloride **(S181)**

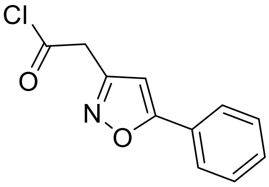

DMF (5 µL) was added to a solution of 2-(5-phenylisoxazol-3-yl)acetic acid (50 mg, 0.25 mmol) in dichloromethane (2 mL) under nitrogen atmosphere. The mixture was cooled to 0 °C, oxalyl chloride (42 µL, 0.49 mmol) was added dropwise, and the reaction mixture was stirred at room temperature for 2 h. The solvent was removed by purging nitrogen and the residue was dried *in vacuo*. The product was obtained as a brown gum (55 mg) and used directly in the next step.

[1,1'-Biphenyl]-4-carbonyl chloride **(S182)**

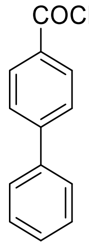

DMF (10 µL) was added to a solution of [1,1'-biphenyl]-4-carboxylic acid (500 mg, 2.52 mmol) in $SOCl_2$ (2.5 mL) under nitrogen atmosphere and the mixture was refluxed for 16 h. The excess of $SOCl_2$ was removed *in vacuo*, the residue mixed with toluene (5 mL), and the solvent was evaporated *in vacuo*. The addition and evaporation of toluene was repeated twice, and the residue was dried *in vacuo*. The product was obtained as a white solid (546 mg, quant.) and used directly in the next step.

[1,1'-Biphenyl]-3-carbonyl chloride **(S183)**

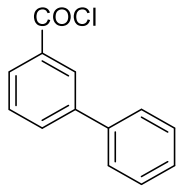

The compound was prepared according to General procedure H using [1,1'-biphenyl]-3-carboxylic acid (500 mg, 2.52 mmol) and $SOCl_2$ (6.6 mL). The reaction mixture was refluxed for 2 h. The product was obtained as a brown oil (546 mg, quant.) and used directly in the next step.

2,3-Dimethoxybenzoyl chloride **(S184)**

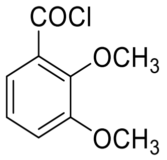

The compound was prepared according to General procedure H using 2,3-dimethoxybenzoic acid (1 g, 5.49 mmol) and $SOCl_2$ (800 µL, 10.97 mmol). The reaction mixture was refluxed for 20 min. The product was obtained as a white solid (1.10 g, quant.) and used directly in the next step.

Scheme S4: synthesis of 5-phenylisothiazole-3-carbonyl chloride **(S189)**

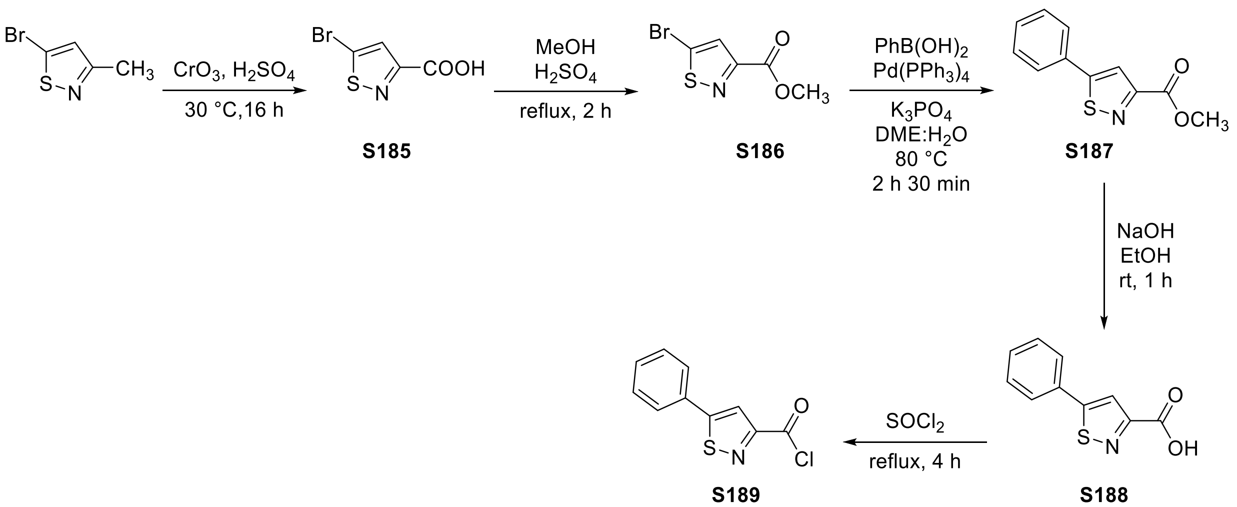

5-Bromoisothiazole-3-carboxylic acid **(S185)**

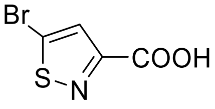

CrO_3 (2.60 g, 26.93 mmol) was added portionwise over the period of 4 h to a mixture of conc. H_2SO_4 (96%, 23 mL) and 5-bromo-3-methylisothiazole (1.0 g, 5.62 mmol) at room temperature. The reaction

mixture was stirred at room temperature for 16 h, then poured into ice-cold water (40 mL), and extracted with EtOAc (3 × 30 mL). The combined organic extracts were washed with water (3 × 20 mL), brine (20 mL), dried over $MgSO_4$, filtered, and the solvent was evaporated *in vacuo*. The product was obtained as a white solid (198 mg, 17%).
^{1}H NMR (300 MHz, DMSO-d_6) δ (ppm) 13.70 (s, 1H), 7.95 (s, 1H).
^{13}C NMR (75 MHz, DMSO-d_6) δ (ppm) 161.2, 160.6, 137.5, 129.3.
HRMS (APCI): calcd. for $C_4H_1BrNO_2S$ $[M-H]^-$ = 207.8896, found $[M-H]^-$ = 207.8900.
mp = 179−181 °C.

Methyl 5-bromoisothiazole-3-carboxylate **(S186)**

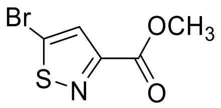

Conc. H_2SO_4 (96%, 10 µL) was added to a solution of 5-bromoisothiazole-3-carboxylic acid (**S185**, 190 mg, 0.91 mmol) in MeOH (2 mL) and the mixture was refluxed for 2 h. The solvent was evaporated *in vacuo*, the residue was mixed with H_2O (4 mL), and the mixture was extracted with EtOAc (3 × 15 mL). The combined organic extracts were washed with water (3 × 10 mL), saturated aqueous $NaHCO_3$ solution (10 mL), dried over $MgSO_4$, filtered, and the solvent was evaporated *in vacuo*. The product was obtained as a colorless oil (172 mg, 85%).
^{1}H NMR (300 MHz, Chloroform-*d*) δ (ppm) 7.79 (s, 1H), 3.97 (s, 3H).
^{13}C NMR (75 MHz, Chloroform-*d*) δ (ppm) 160.3, 159.7, 137.7, 129.2, 53.1.
HRMS (APCI): calcd. for $C_5H_5BrNO_2S$ $[M+H]^+$ = 223.9198, found $[M+H]^+$ = 223.9195.

Methyl 5-phenylisothiazole-3-carboxylate **(S187)**

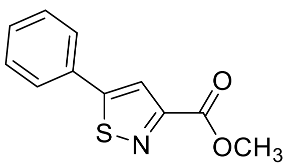

Phenyl boronic acid (98 mg, 0.80 mmol) and K_3PO_4 (2 M in H_2O, 0.75 mL, 1.46 mmol) were added to a solution of methyl 5-bromoisothiazole-3-carboxylate (**S186**, 162 mg, 0.73 mmol) in DME (3.0 mL). The reaction mixture was deoxygenated by argon purgin for 20 min, then $Pd(PPh_3)_4$ (42 mg, 0.04 mmol) was added, and the reaction mixture was stirred at 80 °C for 2.5 h. The reaction mixture was diluted with water (10 mL), and extracted with EtOAc (3 × 5 mL). The combined organic extracts were washed with water (3 × 10 mL), brine (15 mL), dried over $MgSO_4$, filtered, and the solvent was evaporated *in vacuo*. The residue was purified by column chromatography on silica gel (hexane:EtOAc, 9:1). The product methyl 5-phenylisothiazole-3-carboxylate was obtained as a white solid (66 mg, 41%).
The aqueous layer from the workup above was acidified by using aqueous 1 M HCl solution and extracted with EtOAc (3 × 5 mL). The combined organic extracts were washed with water (3 × 5 mL),

brine (5 mL), dried over $MgSO_4$, filtered, and the solvent was evaporated *in vacuo*. The side product 5-phenylisothiazole-3-carboxylic acid was obtained as an off-white solid (72 mg, 48%).

^{1}H NMR (300 MHz, Chloroform-*d*) δ (ppm) 4.00 (s, 3H), 7.41 – 7.52 (m, 3H), 7.57 – 7.67 (m, 2H), 7.97 (s, 1H).

^{13}C NMR (75 MHz, Chloroform-*d*) δ (ppm) 169.8, 161.4, 159.9, 130.3, 129.6, 126.8, 121.9, 52.9.

HRMS (APCI): calcd. for $C_{11}H_{10}NO_2S$ $[M+H]^+$ = 220.0427, found $[M+H]^+$ = 220.0425.

mp = 76−78 °C.

5-Phenylisothiazole-3-carboxylic acid **(S188)**

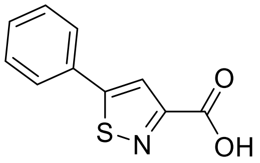

The compound was prepared according to General procedure G using methyl 5-phenylisothiazole-3-carboxylate (**S187**, 50 mg, 0.23 mmol), EtOH (1 mL) and NaOH (2 M in H_2O, 0.17 mL, 0.34 mmol). The product was obtained as a white solid (42 mg, 90%).

^{1}H NMR (300 MHz, Chloroform-*d*) δ (ppm) 8.01 (s, 1H), 7.62 (s, 2H), 7.56 – 7.41 (m, 3H).

^{13}C NMR (75 MHz, Chloroform-*d*) δ (ppm) 171.1, 161.6, 159.4, 130.6, 129.9, 129.7, 126.9, 121.4.

HRMS (APCI): calcd. for $C_{10}H_8NO_2S$ $[M+H]^+$ = 206.0270, found $[M+H]^+$ = 206.0270.

mp = 148−150 °C.

5-Phenylisothiazole-3-carbonyl chloride **(S189)**

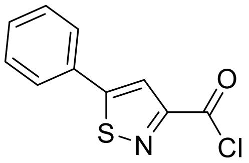

The compound was prepared according to General procedure H using 5-phenylisothiazole-3-carboxylic acid (**S188**, 40 mg, 0.19 mmol) and $SOCl_2$ (71 µL, 0.97 mmol). The product was obtained as an off-white solid (44 mg, quant.) and used directly in the next step.

Preparation of target compound 2 and its analogs

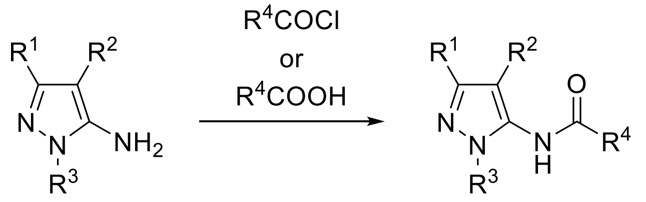

<u>*N*-(3-(furan-2-yl)-1-(4-methyl-6-oxo-1,6-dihydropyrimidin-2-yl)-1*H*-pyrazol-5-yl)-5-phenylisoxazole-3-carboxamide</u> **(2)**

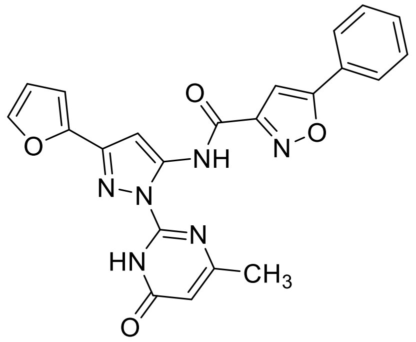

The compound was prepared by General procedure I using Et_3N (28 µL, 0.2 mmol), 2-(5-amino-3-(furan-2-yl)-1*H*-pyrazol-1-yl)-6-methylpyrimidin-4(3*H*)-one (**S138**, 50 mg, 0.2 mmol), acetonitrile (2 + 2 mL) and 5-phenylisoxazole-3-carbonyl chloride (**S177**, 40 mg, 0.2 mmol). The obtained solid was additionally washed with EtOAc (3 mL). The product was obtained as a white solid (30 mg, 35%).

^1H NMR (500 MHz, DMSO-d_6) δ (ppm) 13.56 (s, 1H), 8.04 – 7.99 (m, 2H), 7.82 (s, 1H), 7.61 (s, 1H), 7.60 – 7.53 (m, 3H), 7.22 (s, 1H), 7.02 (d, J = 3.3 Hz, 1H), 6.67 – 6.62 (m, 1H), 6.49 (s, 1H), 2.55 (s, 3H).

^{13}C NMR (126 MHz, DMSO-d_6) δ (ppm) 171.75, 158.85, 154.70, 147.05, 144.46, 143.74, 139.69, 131.10, 129.32, 126.00, 125.93, 111.86, 108.78, 99.98, 94.59, 22.82.

HRMS (APCI): calcd. for $C_{22}H_{17}N_6O_4$ $[M+H]^+$= 429.1306, found $[M+H]^+$ = 429.1310.

<u>*N*-(1-(4-methyl-6-oxo-1,6-dihydropyrimidin-2-yl)-3-(thiophen-2-yl)-1*H*-pyrazol-5-yl)-5-phenylisoxazole-3-carboxamide</u> **(29)**

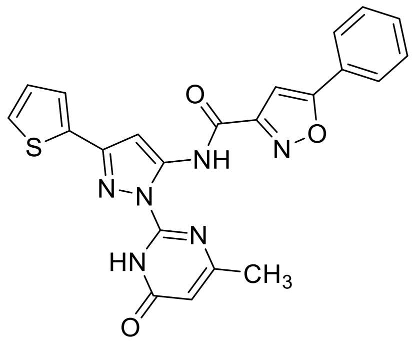

The compound was prepared by General procedure I using Et_3N (31 µL, 0.22 mmol), 2-(5-amino-3-(thiophen-2-yl)-1*H*-pyrazol-1-yl)-6-methylpyrimidin-4(3*H*)-one (**S139**, 60 mg, 0.22 mmol), acetonitrile (2 + 2 mL) and 5-phenylisoxazole-3-carbonyl chloride (**S177**, 46 mg, 0.22 mmol). The obtained solid was additionally washed with EtOAc (3 mL). The product was obtained as a white solid (33 mg, 34 %).

^{1}H NMR (500 MHz, DMSO-d_6) δ (ppm) 15.84 (s, 1H), 8.05 – 7.99 (m, 2H), 7.82 (s, 1H), 7.63 – 7.51 (m, 5H), 7.16 – 7.11 (m, 2H), 5.75 (s, 1H), 2.23 (s, 3H).

^{13}C NMR (126 MHz, DMSO-d_6) δ (ppm) 171.50, 155.51, 146.51, 142.52, 138.37, 136.70, 131.36, 129.84, 128.23, 126.88, 126.40, 126.28, 125.80, 100.40, 93.96, 23.58.

HRMS (APCI): calcd. for $C_{22}H_{17}N_6O_3S$ $[M+H]^+$= 445.1077, found $[M+H]^+$ = 445.1080.

<u>5-Cyclohexyl-*N*-(3-(furan-2-yl)-1-(4-methyl-6-oxo-1,6-dihydropyrimidin-2-yl)-1*H*-pyrazol-5-yl)isoxazole-3-carboxamide</u> **(30)**

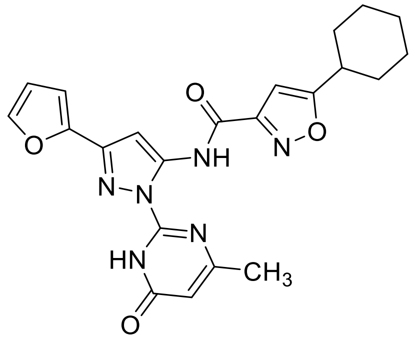

The compound was prepared according to General procedure I using Et_3N (16.1 µL, 0.12 mmol), 2-(5-amino-3-(furan-2-yl)-1*H*-pyrazol-1-yl)-6-methylpyrimidin-4(3*H*)-one (**S138**, 30 mg, 0.12 mmol), acetonitrile (1.0 + 0.5 mL) and 5-cyclohexylisoxazole-3-carbonyl chloride (**S158**, 25 mg, 0.116 mmol). The product was obtained as a white solid (31 mg, 61%).

^{1}H NMR (300 MHz, DMSO-d_6) δ (ppm) 14.93 (s, 1H), 7.77 (d, J = 1.7 Hz, 1H), 7.08 (s, 1H), 6.92 (d, J = 3.4 Hz, 1H), 6.89 (s, 1H), 6.62 (dd, J = 3.4, 1.8 Hz, 1H), 5.85 (s, 1H), 2.92 (td, J = 11.2, 9.2, 5.7 Hz, 1H), 2.25 (s, 3H), 2.03 (d, J = 12.3 Hz, 2H), 1.72 (dd, J = 25.9, 11.9 Hz, 3H), 1.57 – 1.16 (m, 5H).

^{13}C NMR (126 MHz, DMSO-d_6) δ (ppm) 179.7, 171.2, 158.5, 156.9, 155.1, 148.0, 143.0, 140.6, 111.7, 107.4, 106.8, 99.2, 93.5, 35.5, 30.4, 25.2, 25.0, 22.8.

HRMS (APCI): calcd. for $C_{22}H_{23}N_6O_4$ $[M+H]^+$= 435.1775, found $[M+H]^+$ = 435.1779.

mp >285 °C (dec.)

<u>*N*-(1-(4-Methyl-6-oxo-1,6-dihydropyrimidin-2-yl)-3-(3-methylfuran-2-yl)-1*H*-pyrazol-5-yl)-5-phenylisoxazole-3-carboxamide</u> **(31)**

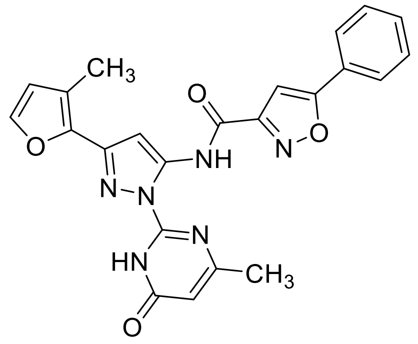

The compound was prepared according to General procedure I using Et_3N (22 µL, 0.16 mmol), 2-(5-amino-3-(3-methylfuran-2-yl)-1*H*-pyrazol-1-yl)-6-methylpyrimidin-4(3*H*)-one (**S135**, 30 mg, 0.11 mmol), acetonitrile (1.0 + 0.5 mL) and 5-phenylisoxazole-3-carbonyl chloride (**S177**, 32 mg, 0.16 mmol). The product was obtained as an off-white solid (30 mg, 61%).

^{1}H NMR (300 MHz, DMSO-d_6) δ (ppm) 15.73 (s, 1H), 8.07 – 7.97 (m, 2H), 7.84 (s, 1H), 7.65 (d, J = 1.7 Hz, 1H), 7.58 (d, J = 6.8 Hz, 3H), 7.03 (s, 1H), 6.49 (d, J = 1.8 Hz, 1H), 5.77 (s, 1H), 2.35 (s, 3H), 2.23 (s, 3H).

HRMS (APCI): calcd. for $C_{23}H_{19}N_6O_4$ $[M+H]^+$= 443.1462, found $[M+H]^+$ = 443.1462.

mp >310 °C (dec.)

N-(1-(4-Methyl-6-oxo-1,6-dihydropyrimidin-2-yl)-3-phenyl-1*H*-pyrazol-5-yl)-5-phenylisoxazole-3-carboxamide **(32)**

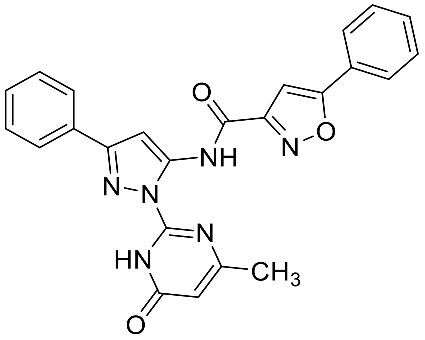

The compound was prepared according to General procedure I using Et_3N (16 µL, 0.11 mmol), 2-(5-amino-3-phenyl-1*H*-pyrazol-1-yl)-6-methylpyrimidin-4(3*H*)-one (**S137**, 30 mg, 0.11 mmol), acetonitrile (1.0 + 0.5 mL) and 5-phenylisoxazole-3-carbonyl chloride (**S177**, 11 mg, 0.11 mmol). The obtained solid was additionally washed with water (3 mL) and then with EtOAc (3 mL). The compound was purified by reversed phase column chromatography using Biotage Selekt purification system (water:MeOH:0.7 M NH_3 in methanol, gradient 90:10:10 to 0:70:30). The product was obtained as a white solid (16 mg, 58%).

^{1}H NMR (500 MHz, DMSO-d_6) δ (ppm) 15.46 (s, 1H), 8.05 – 8.01 (m, 2H), 7.93 (d, J = 7.6 Hz, 2H), 7.83 (s, 1H), 7.62 – 7.55 (m, 3H), 7.47 (t, J = 7.6 Hz, 2H), 7.38 (t, J = 7.3 Hz, 1H), 7.25 (s, 1H), 5.82 (s, 1H), 2.27 (s, 3H).

^{13}C NMR (126 MHz, DMSO-d_6) δ (ppm) 172.7, 162.7, 150.9, 154.8, 141.7, 130.9, 129.3, 128.7, 126.3, 125.8, 125.7, 107.2, 99.9, 93.9, 22.9.
HRMS (APCI): calcd. for $C_{24}H_{17}N_6O_3$ $[M-H]^-$= 437.1368, found $[M-H]^-$= 437.1365.
mp >320 °C (dec.)

N-(3-Benzyl-1-(4-methyl-6-oxo-1,6-dihydropyrimidin-2-yl)-1*H*-pyrazol-5-yl)-5-phenylisoxazole-3-carboxamide **(33)**

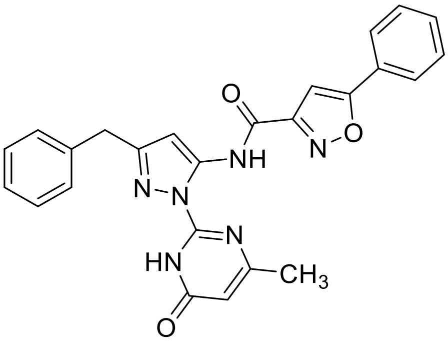

The compound was prepared according to General procedure I using Et_3N (21 μL, 0.15 mmol), 2-(5-amino-3-benzyl-1*H*-pyrazol-1-yl)-6-methylpyrimidin-4(3*H*)-one (**S134**, 30 mg, 0.11 mmol), acetonitrile (1.0 + 0.4 mL) and 5-phenylisoxazole-3-carbonyl chloride (**S177**, 31 mg, 0.15 mmol). The product was obtained as a light brown solid (36 mg, 75%).
^1H NMR (700 MHz, DMSO-d_6) δ (ppm) 14.35 (s, 1H), 8.02 – 7.97 (m, 2H), 7.70 – 7.62 (m, 1H), 7.61 – 7.37 (m, 4H), 7.32 (d, *J* = 4.5 Hz, 4H), 7.23 (h, *J* = 4.4 Hz, 1H), 6.66 (s, 1H), 6.11 (s, 1H), 3.98 (s, 2H), 2.36 (s, 3H).
^{13}C NMR (176 MHz, DMSO-d_6) δ (ppm) 171.39, 159.23, 154.57, 153.25, 139.92, 139.09, 130.97, 129.29, 128.69, 128.41, 127.63, 127.26, 126.22, 126.12, 125.84, 125.42, 105.81, 99.86, 96.72, 34.31, 22.79.
HRMS (APCI): calcd. for $C_{25}H_{21}N_6O_3$ $[M+H]^+$= 453.1670, found $[M+H]^+$= 453.1671.
mp >240 °C (dec.)

N-(3-Cyclohexyl-1-(4-methyl-6-oxo-1,6-dihydropyrimidin-2-yl)-1*H*-pyrazol-5-yl)-5-phenylisoxazole-3-carboxamide **(34)**

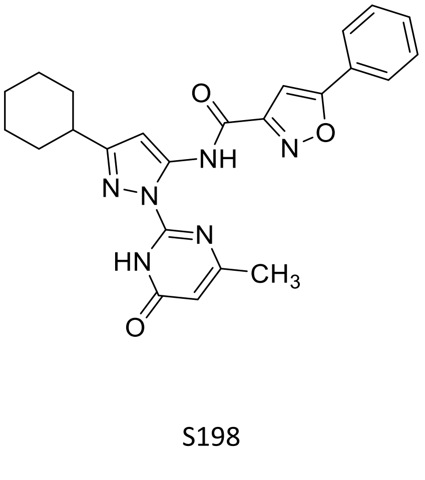

The compound was prepared according to General procedure I using Et_3N (10 µL, 0.07 mmol), 2-(5-amino-3-cyclohexyl-1*H*-pyrazol-1-yl)-6-methylpyrimidin-4(3*H*)-one (**S130**, 15 mg, 0.05 mmol) acetonitrile (0.8 + 0.3 mL) and 5-phenylisoxazole-3-carbonyl chloride (**S177**, 14 mg, 0.07 mmol). The product was obtained as an off white solid (21 mg, 87%).

^{1}H NMR (300 MHz, DMSO-d_6) δ (ppm) 13.55 (s, 1H), 12.56 (s, 1H), 8.01 (dd, J = 6.8, 2.9 Hz, 2H), 7.62 (s, 1H), 7.61 – 7.54 (m, 3H), 6.81 (s, 1H), 6.29 (s, 1H), 2.67 (q, J = 10.8 Hz, 1H), 2.45 (s, 3H), 1.94 (d, J = 12.2 Hz, 2H), 1.74 (dd, J = 27.2, 11.3 Hz, 3H), 1.56 – 1.16 (m, 5H).

HRMS (ESI): calcd. for $C_{24}H_{23}N_6O_3$ [M-H]$^-$= 443.1837, found [M-H]$^-$= 443.1834.

mp >276 °C (dec.)

N-(4-Methyl-1-(4-methyl-6-oxo-1,6-dihydropyrimidin-2-yl)-3-phenyl-1*H*-pyrazol-5-yl)-5-phenylisoxazole-3-carboxamide **(35)**

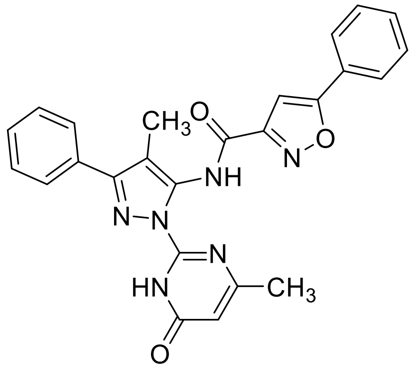

The compound was prepared according to General procedure I using Et_3N (50 µL, 0.34 mmol), 2-(5-amino-4-methyl-3-phenyl-1*H*-pyrazol-1-yl)-6-methylpyrimidin-4(3*H*)-one (**S141**, 50 mg, 0.18 mmol), acetonitrile (5 mL + 2 mL) and 5-phenylisoxazole-3-carbonyl chloride (**S177**, 48 mg, 0.23 mmol). The reaction mixture was refluxed for 5 h. Then reaction mixture was cooled to room temperature, 7M NH_3 in methanol (2 mL) was added and the mixture was stirred for 5 min. The solid was collected by filtration, washed with methanol (5 mL), water (10 mL), diethyl ether (15 mL), and dried *in vacuo.* The product was obtained as a white solid (40 mg, 50%).

^{1}H NMR (500 MHz, DMSO-d_6) δ (ppm) 12.32 (br s, 1H), 11.14 (br s, 1H), 8.05 – 7.93 (m, 2H), 7.90 – 7.79 (m, 2H), 7.62 – 7.55 (m, 3H), 7.55 – 7.49 (m, 2H), 7.49 – 7.42 (m, 2H), 6.27 (s, 1H), 2.25 (s, 3H), 2.21 (s, 3H).

^{13}C NMR (126 MHz, DMSO-d_6) δ (ppm) 170.87, 158.70, 156.59, 151.08, 135.04, 132.24, 130.63, 128.99, 128.21, 128.13, 127.31, 125.96, 125.60, 111.56, 106.23, 99.62, 22.49, 9.42.

HRMS (APCI): calcd. for $C_{25}H_{21}N_6O_3$ [M+H]$^+$= 453.1670, found [M+H]$^+$= 453.1671.

5-Methyl-*N*-(1-(4-methyl-6-oxo-1,6-dihydropyrimidin-2-yl)-3-phenyl-1*H*-pyrazol-5-yl)isoxazole-3-carboxamide **(36)**

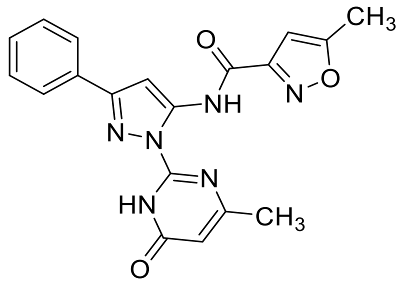

The compound was prepared according to General procedure I using Et_3N (10 µL, 0.07 mmol), 2-(5-amino-3-phenyl-1*H*-pyrazol-1-yl)-6-methylpyrimidin-4(3*H*)-one (**S137**, 20 mg, 0.07 mmol), acetonitrile (1 + 0.4 mL) and 5-methylisoxazole-3-carbonyl chloride (11 mg, 0.07 mmol). The obtained solid was additionally washed with EtOAc (3 mL). The product was obtained as a white solid (16 mg, 58%).

^{1}H NMR (500 MHz, DMSO-d_6) δ (ppm) 15.11 (s, 1H), 7.94 – 7.89 (m, 2H), 7.46 (t, J = 7.6 Hz, 2H), 7.37 (dd, J = 8.4, 6.3 Hz, 1H), 7.22 (s, 1H), 6.85 (s, 1H), 5.67 (s, 1H), 2.52 (s, 3H), 2.20 (s, 3H).

^{13}C NMR (126 MHz, DMSO-d_6) δ (ppm) 172.2, 172.0, 160.8, 158.9, 157.6, 155.0, 149.8, 140.7, 132.9, 128.6, 128.2, 125.6, 107.5, 101.4, 93.6, 22.7, 11.9.

^{1}H NMR (500 MHz, Methanol-d_4) δ (ppm) 8.01 – 7.86 (m, 2H), 7.42 (t, J = 7.5 Hz, 2H), 7.35 (t, J = 7.4 Hz, 1H), 7.29 (s, 1H), 6.68 (s, 1H), 6.04 (s, 1H), 2.54 (s, 3H), 2.38 (s, 3H).

HRMS (APCI): calcd. for $C_{19}H_{17}N_6O_3$ $[M+H]^+$= 377.1357, found $[M+H]^+$= 377.1359.

mp >285 °C (dec.)

5-Cyclohexyl-*N*-(1-(4-methyl-6-oxo-1,6-dihydropyrimidin-2-yl)-3-phenyl-1*H*-pyrazol-5-yl)isoxazole-3-carboxamide **(37)**

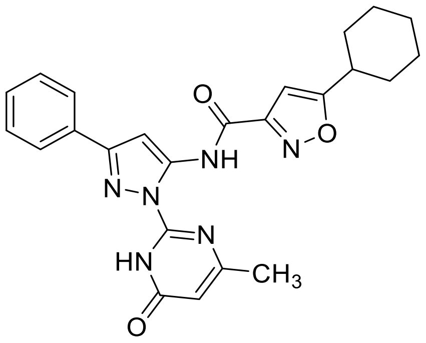

The compound was prepared according to General procedure I using Et_3N (31 µL, 0.22 mmol), 2-(5-amino-3-phenyl-1*H*-pyrazol-1-yl)-6-methylpyrimidin-4(3*H*)-one (**S137**, 30 mg, 0.11 mmol), acetonitrile (1 + 0.5 mL) and 5-cyclohexylisoxazole-3-carbonyl chloride (**S158**, 48 mg, 0.22 mmol). The product was obtained as a white solid (30 mg, 60%).

^{1}H NMR (300 MHz, trifluoroacetic acid-d) δ (ppm) 7.95 (dd, J = 7.5, 2.0 Hz, 2H), 7.57 (d, J = 6.9 Hz, 3H), 6.90 (s, 1H), 6.74 (d, J = 0.8 Hz, 1H), 2.99 (d, J = 15.2 Hz, 1H), 2.82 (s, 3H), 2.22 (d, J = 11.4 Hz, 2H), 1.91 (dd, J = 30.0, 11.5 Hz, 3H), 1.71 – 1.32 (m, 5H).

HRMS (APCI): calcd. for $C_{24}H_{23}N_6O_3$ $[M-H]^-$= 443.1837, found $[M-H]^-$= 443.1835.

mp >316 °C (dec.)

5-(2-Bromophenyl)-*N*-(1-(4-methyl-6-oxo-1,6-dihydropyrimidin-2-yl)-3-phenyl-1*H*-pyrazol-5-yl)isoxazole-3-carboxamide **(38)**

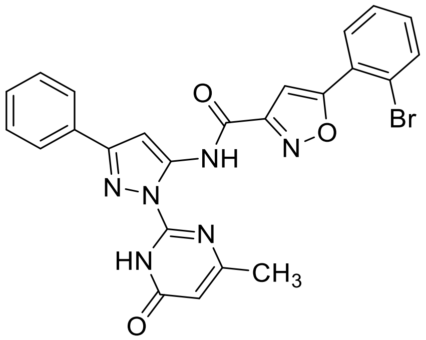

The compound was prepared according to General procedure I using Et_3N (13 µL, 0.09 mmol), 2-(5-amino-3-phenyl-1*H*-pyrazol-1-yl)-6-methylpyrimidin-4(3*H*)-one (**S137**, 20 mg, 0.08 mmol), acetonitrile (1 + 0.4 mL) and 5-(2-bromophenyl)isoxazole-3-carbonyl chloride (**S166**, 26 mg, 0.09 mmol). The product was obtained as a light brown solid (28 mg, 72%).

^{1}H NMR (300 MHz, DMSO-*d*$_6$) *δ* (ppm) 13.51 (s, 1H), 12.78 (s, 1H), 8.08 – 7.99 (m, 2H), 7.97 – 7.86 (m, 2H), 7.65 – 7.42 (m, 6H), 7.39 (s, 1H), 6.37 (s, 1H), 2.50 (s, 3H, overlapped with DMSO).

HRMS (APCI): calcd. for $C_{24}H_{16}BrN_6O_3$ [M-H]$^-$= 517.0456, found [M-H]$^-$= 517.0453.

mp >295 °C (dec.)

N-(1-(4-Methyl-6-oxo-1,6-dihydropyrimidin-2-yl)-3-phenyl-1*H*-pyrazol-5-yl)-5-(4-(trifluoromethoxy)phenyl)isoxazole-3-carboxamide **(39)**

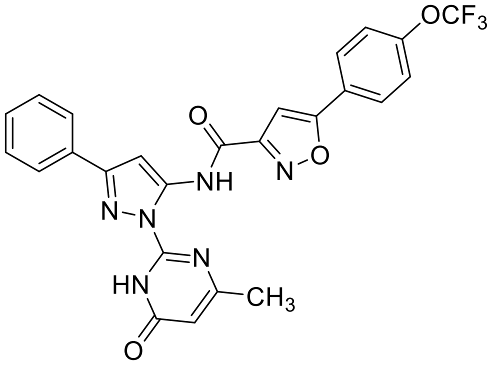

Et_3N (62.6 µL, 0.449 mmol) and T_3P/propylphosphonic anhydride (50% in EtOAc, 0.143 mL, 0.22 mmol) were added to a solution of 5-(4-(trifluoromethoxy)phenyl)isoxazole-3-carboxylic acid (**S176**, 20 mg, 0.75 mmol) in THF (1 mL) and the mixture was stirred at room temperature for 1 h. Then, a solution of 2-(5-amino-3-phenyl-1*H*-pyrazol-1-yl)-6-methylpyrimidin-4(3*H*)-one (**S137**, 20 mg, 0.075 mmol) in THF (0.4 mL) was added and the reaction mixture was refluxed for 16 h. The solvent was evaporated *in vacuo*, the residue was mixed with saturated aqueous $NaHCO_3$ solution (3 mL) and the mxiture was extracted with EtOAc (3 × 3 mL). The combined organic extracts were dried over $MgSO_4$,

filtered, and the solvent evaporated *in vacuo*. The residue was purified by preparative TLC (dichloromethane:MeOH, 93:7). The product was obtained as a white solid (14 mg, 44%).
^{1}H NMR (300 MHz, DMSO-d_6) δ (ppm) 13.91 (s, 2H), 8.18 (d, J = 8.7 Hz, 2H), 8.03 (d, J = 7.5 Hz, 2H), 7.77 (s, 1H), 7.60 (d, J = 8.4 Hz, 2H), 7.55 – 7.40 (m, 3H), 7.38 (s, 1H), 6.28 (s, 1H), 2.47 (s, 3H).
HRMS (APCI): calcd. for $C_{25}H_{16}F_3N_6O_4$ [M-H]$^-$= 521.1191, found [M-H]$^-$= 521.1189.
mp >297 °C (dec.)

<u>5-(4-Methoxyphenyl)-*N*-(1-(4-methyl-6-oxo-1,6-dihydropyrimidin-2-yl)-3-phenyl-1*H*-pyrazol-5-yl)isoxazole-3-carboxamide</u> **(40)**

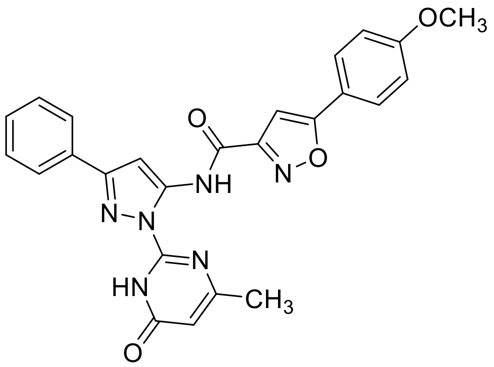

The compound was prepared according to General procedure I using Et_3N (13 µL, 0.09 mmol), 2-(5-amino-3-phenyl-1*H*-pyrazol-1-yl)-6-methylpyrimidin-4(3*H*)-one (**S137**, 20 mg, 0.08 mmol), acetonitrile (1 + 0.5 mL) and 5-(4-methoxyphenyl)isoxazole-3-carbonyl chloride (**S162**, 18 mg, 0.09 mmol). The crude product (15 mg) was mixed with DMSO (1.8 mL) and heated at 135 °C until a clear solution formed. The mixture was cooled to room temperature and aqueous saturated $NaHCO_3$ solution (0.2 mL) was added. The resulting precipitate was collected by filtration, washed with water (3 mL) and dried *in vacuo*. The product was obtained as a white solid (30 mg, 60%).
^{1}H NMR (300 MHz, DMSO-d_6) δ (ppm) 13.38 (s, 1H), 12.76 (s, 1H), 8.04 (d, J = 7.4 Hz, 2H), 7.96 (d, J = 8.8 Hz, 2H), 7.57 – 7.41 (m, 4H), 7.40 (s, 1H), 7.13 (d, J = 8.8 Hz, 2H), 6.39 (s, 1H), 3.86 (s, 3H).
HRMS (APCI): calcd. for $C_{25}H_{19}N_6O_4$ [M-H]$^-$= 467.1473, found [M-H]$^-$= 467.1470.
mp >304 °C (dec.)

<u>*N*-(1-(4-Methyl-6-oxo-1,6-dihydropyrimidin-2-yl)-3-phenyl-1*H*-pyrazol-5-yl)-3-phenylisoxazole-5-carboxamide</u> **(41)**

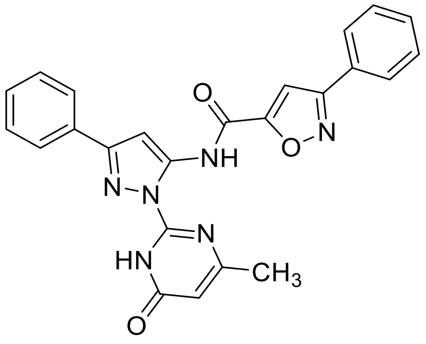

The compound was prepared according to General procedure I using Et_3N (13 µL, 0.09 mmol), 2-(5-amino-3-phenyl-1*H*-pyrazol-1-yl)-6-methylpyrimidin-4(3*H*)-one (**S137**, 20 mg, 0.08 mmol), acetonitrile (1 + 0.5 mL) and 3-phenylisoxazole-5-carbonyl chloride (**S178**, 19 mg, 0.090 mmol). The product was obtained as a white solid (13 mg, 40%).

^{1}H NMR (300 MHz, Trifluoroacetic Acid-*d*) δ (ppm) 7.94 (d, J = 7.6 Hz, 2H), 7.85 (d, J = 7.0 Hz, 2H), 7.76 (d, J = 5.3 Hz, 2H), 7.56 (dd, J = 17.1, 7.1 Hz, 6H), 6.90 (s, 1H), 2.82 (s, 3H).

HRMS (APCI): calcd. for $C_{24}H_{19}N_6O_3$ $[M+H]^+$= 439.1513, found $[M+H]^+$= 439.1512.

mp > 300°C (dec).

<u>*N*-(1-(4-Methyl-6-oxo-1,6-dihydropyrimidin-2-yl)-3-phenyl-1*H*-pyrazol-5-yl)-2-phenylthiazole-4-carboxamide</u> **(42)**

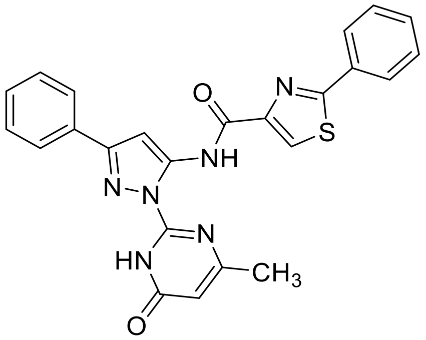

The compound was prepared according to General procedure I using Et_3N (13 µL, 0.09 mmol), 2-(5-amino-3-phenyl-1*H*-pyrazol-1-yl)-6-methylpyrimidin-4(3*H*)-one (**S137**, 20 mg, 0.08 mmol), acetonitrile (1 + 0.5 mL) and 2-phenylthiazole-4-carbonyl chloride (**S179**, 20 mg, 0.09 mmol). The product was obtained as an off-white solid (33 mg, 97%).

^{1}H NMR (700 MHz, DMSO-d_6) δ (ppm) 13.93 (br s, 1H), 8.62 – 8.52 (m, 1H), 8.37 – 8.24 (m, 2H), 8.21 – 8.09 (m, 1H), 7.96 (dd, J = 18.2, 6.7 Hz, 2H), 7.62 – 7.41 (m, 5H), 7.42 – 7.29 (m, 2H), 5.88 (s, 1H), 2.21 (s, 3H).

^{13}C NMR (176 MHz, DMSO-d_6) δ (ppm) 168.27, 157.16, 149.88, 140.24, 133.07, 132.75, 132.21, 130.80, 130.19, 129.12, 128.63, 128.23, 127.31, 126.32, 126.19, 125.66, 124.16, 107.27, 93.86, 23.18.

HRMS (APCI): calcd. for $C_{24}H_{19}N_6O_2S$ $[M+H]^+$= 455.1285, found $[M+H]^+$= 455.1284.

mp >308 °C (dec.)

<u>*N*-(1-(4-Methyl-6-oxo-1,6-dihydropyrimidin-2-yl)-3-phenyl-1*H*-pyrazol-5-yl)-5-phenylisothiazole-3-carboxamide</u> **(43)**

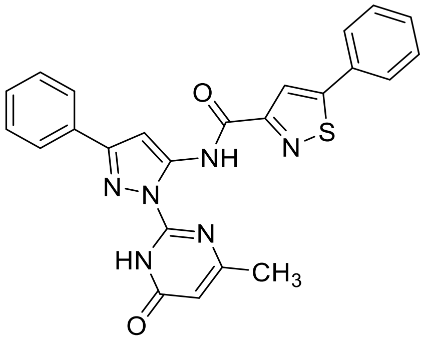

The compound was prepared according to General procedure I using Et_3N (15 µL, 0.11 mmol), 2-(5-amino-3-phenyl-1*H*-pyrazol-1-yl)-6-methylpyrimidin-4(3*H*)-one (**S137**, 20 mg, 0.08 mmol), acetonitrile (1 + 0.5 mL) and 5-phenylisothiazole-3-carbonyl chloride (**S189**, 23 mg, 0.11 mmol). The product was obtained as an off-white solid (19 mg, 50%).

^{1}H NMR (300 MHz, DMSO-*d*$_6$) *δ* (ppm) 14.81 (s, 1H), 8.41 (s, 1H), 8.01 – 7.87 (m, 4H), 7.63 – 7.42 (m, 5H), 7.42 – 7.34 (m, 1H), 7.28 (s, 1H), 5.79 (s, 1H), 2.29 (s, 3H).

HRMS (APCI): calcd. for $C_{24}H_{19}N_6O_2S$ $[M+H]^+$= 455.1285, found $[M+H]^+$= 455.1284.

mp >308 °C (dec.)

<u>6-Methyl-2-(3-phenyl-5-(((5-phenylisoxazol-3-yl)methyl)amino)-1*H*-pyrazol-1-yl)pyrimidin-4(3*H*)-one</u> **(44)**

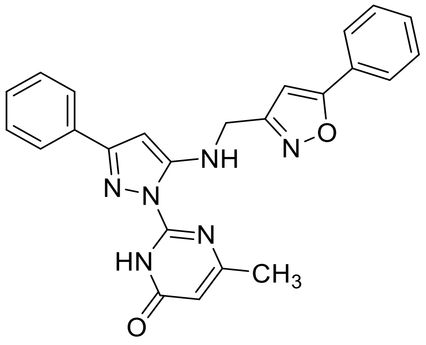

$ZnCl_2$ (0.5 M in THF, 1.49 mL, 0.75 mmol) and 5-phenylisoxazole-3-carbaldehyde (84 mg, 0.49 mmol) were added to a solution of 2-(5-amino-3-phenyl-1*H*-pyrazol-1-yl)-6-methylpyrimidin-4(3*H*)-one (**S137**, 100 mg, 0.37 mmol) in THF (3 mL) and the reaction mixture was stirred at room temperature for 16 h. Then, $NaBH_3CN$ (31 mg, 0.49 mmol) was added and reaction mixture was refluxed for 16 h. The solvent was evaporated *in vacuo* and the residue was purified by column chromatography on silica gel (dichloromethane:MeOH, 9:1). The product was obtained as an off-white solid (6 mg, 4%).

^{1}H NMR (300 MHz, Chloroform-*d*) *δ* (ppm) 10.34 (s, 1H), 8.25 (s, 1H), 8.03 – 7.69 (m, 4H), 7.53 – 7.35 (m, 6H), 6.55 (s, 1H), 6.06 (s, 1H), 5.84 (s, 1H), 4.61 (d, *J* = 5.2 Hz, 2H), 2.33 (s, 3H).

^{13}C NMR (126 MHz, Chloroform-*d*) *δ* (ppm) 171.0, 162.0, 130.6, 129.5, 129.2, 128.8, 127.3, 126.5, 126.0, 98.3, 41.2, 22.8.

HRMS (APCI): calcd. for $C_{24}H_{21}N_6O_2$ $[M+H]^+$= 425.1721, found $[M+H]^+$= 425.1719.
mp >216 °C (dec.)

N-(1-(4-methoxy-6-methylpyrimidin-2-yl)-3-phenyl-1*H*-pyrazol-5-yl)-*N*-methyl-5-phenylisoxazole-3-carboxamide **(S190)**

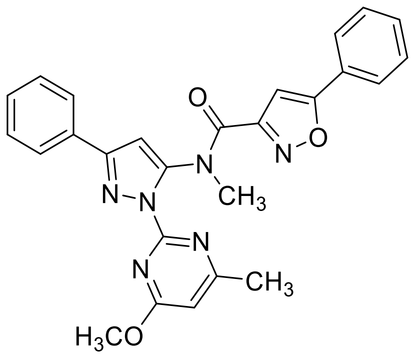

NaH (60%suspension in mineral oil, 17 mg, 0.43 mmol) was added to a cooled (0 °C) solution of *N*-(1-(4-methoxy-6-methylpyrimidin-2-yl)-3-phenyl-1*H*-pyrazol-5-yl)-5-phenylisoxazole-3-carboxamide (**49**, 130 mg, 0.29 mmol) in DMF (2 mL) and the mixture was stirred at room temperature for 20 min. Then, MeI (27 µL, 0.43 mmol) was added and thhe reaction mixture was stirred at room temperature for 2 h. The reaction mixture was poured into ice-cold water (10 mL) and extracted with EtOAc (3 × 20 mL). The combined organic extracts were washed with water (3 × 10 mL), brine (10 mL), dried over $MgSO_4$, filtered, and the solvent evaporated *in vacuo*. The residue was purified by preparative TLC (hexane:EtOAc, 6:4). The product was obtained as a white solid (66 mg, 49%).
^{1}H NMR (300 MHz, Chloroform-*d*) δ (ppm) 8.00 – 7.88 (m, 2H), 7.68 – 7.54 (m, 2H), 7.48 – 7.31 (m, 6H), 6.76 (s, 1H), 6.54 (s, 1H), 6.48 (s, 1H), 3.99 (s, 3H), 3.51 (s, 3H), 2.50 (s, 3H).
HRMS (APCI): calcd. for $C_{26}H_{23}N_6O_3$ $[M+H]^+$= 467.1826, found $[M+H]^+$= 467.1826.

N-Methyl-*N*-(1-(4-methyl-6-oxo-1,6-dihydropyrimidin-2-yl)-3-phenyl-1*H*-pyrazol-5-yl)-5-phenylisoxazole-3-carboxamide **(45)**

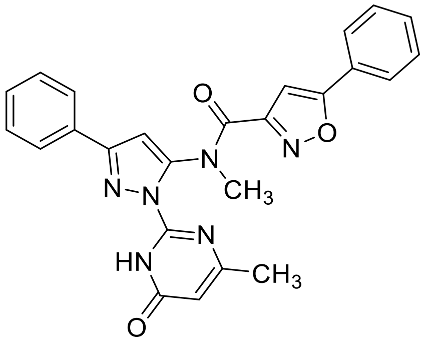

A mixture of *N*-(1-(4-methoxy-6-methylpyrimidin-2-yl)-3-phenyl-1*H*-pyrazol-5-yl)-N-methyl-5-phenylisoxazole-3-carboxamide (**S190**, 21 mg, 0.045 mmol) and pyridine•HCl (16 mg, 0.14 mmol) was heated at 150 °C for 1.5 h. The reaction mixture was cooled to room temperature, water (0.2 mL) was

added and the pH was neutralized by adding saturated aqueous solution of $NaHCO_3$. The solid was collected by filtration, washed with water (3 mL) and dried *in vacuo*. The resulting solid (18 mg) was mixed with MeOH (0.5 mL) and heated to reflux. The mixture was cooled to room temperature, the precipitate was collected by filtration, washed with MeOH (0.1 mL), and dried *in vacuo*. The product was obtained as an off-white solid (11 mg, 54%).

^{1}H NMR (300 MHz, Chloroform-*d*) δ (ppm) 7.86 (dd, J = 7.9, 1.8 Hz, 2H), 7.67 (dd, J = 6.7, 3.0 Hz, 2H), 7.53 – 7.36 (m, 7H), 6.80 (d, J = 2.3 Hz, 2H), 6.08 (d, J = 1.0 Hz, 1H), 3.48 (s, 3H), 2.24 (d, J = 0.9 Hz, 3H).

^{13}C NMR (75 MHz, Chloroform-*d*) δ (ppm) 170.6, 165.2, 161.5, 160.8, 159.1, 153.6, 146.1, 144.0, 131.0, 130.9, 130.1, 129.3, 129.2, 126.8, 126.4, 126.1, 110.4, 107.1, 100.5, 38.1, 24.1.

HRMS (APCI): calcd. for $C_{25}H_{21}N_6O_3$ $[M+H]^+$= 453.1670, found $[M+H]^+$= 453.1669.

mp >257 °C (dec.)

N-(1-Methyl-3-phenyl-1*H*-pyrazol-5-yl)-5-phenylisoxazole-3-carboxamide **(46)**

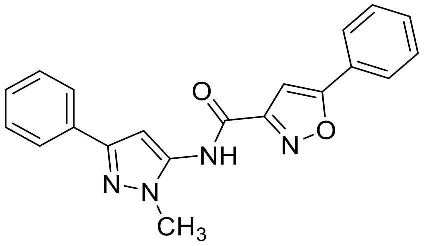

Et_3N (145 µL, 1.04 mmol) and T_3P/propylphosphonic anhydride (50% in EtOAc, 0.33 mL, 0.52 mmol) were added to a solution of 5-phenylisoxazole-3-carboxylic acid (33 mg, 0.17 mmol) in THF (5 mL) and the mixture was stirred at room temperature for 1 h. Then, 1-methyl-3-phenyl-1*H*-pyrazol-5-amine (30 mg, 0.17 mmol) was added and the reaction mixture was refluxed for 16 h. The reaction mixture was cooled to room temperature, quenched with saturated aqueous solution of $NaHCO_3$ (5 mL), and extracted with EtOAc (3 × 15 mL). The combined organic extracts were dried over $MgSO_4$, filtered, and the solvent evaporated *in vacuo*. The residue was purified by preparative TLC (hexanes:EtOAc, 6:4, run two times). The product was obtained as a white solid (25 mg, 42%).

^{1}H NMR (300 MHz, Chloroform-*d*) δ (ppm) 8.48 (s, 1H), 7.87 – 7.78 (m, 4H), 7.53 (dt, J = 4.6, 2.8 Hz, 3H), 7.41 (t, J = 7.4 Hz, 2H), 7.36 – 7.29 (m, 1H), 7.07 (s, 1H), 6.78 (s, 1H), 3.93 (s, 3H).

^{13}C NMR (126 MHz, Chloroform-*d*) δ (ppm) 172.6, 158.3, 156.6, 150.3, 135.1, 133.1, 131.1, 129.3, 128.6, 127.9, 126.4, 126.0, 125.5, 99.2, 97.4, 35.8.

HRMS (APCI): calcd. for $C_{20}H_{17}N_4O_2$ $[M+H]^+$= 345.1346, found $[M+H]^+$= 345.1344.

mp = 168−170 °C.

N-(1,3-Diphenyl-1*H*-pyrazol-5-yl)-5-phenylisoxazole-3-carboxamide **(47)**

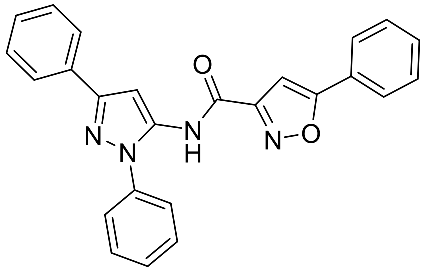

The compound was prepared according to General procedure I using Et_3N (83 µL, 0.6 mmol), 1,3-diphenyl-1*H*-pyrazol-5-amine (70 mg, 0.3 mmol, CAS: 5356-71-8), acetonitrile (5 mL) and 5-phenylisoxazole-3-carbonyl chloride (**S177**, 80 mg, 0.39 mmol). The reaction mixture was refluxed for 3 h. The crude product was purified by column chromatography on silica gel (hexane:EtOAc, 7:3). The product was obtained as a white solid (60 mg, 50%).

^{1}H NMR (500 MHz, Chloroform-*d*) δ (ppm) 8.87 (br s, 1H), 7.93 (d, J = 7.7 Hz, 2H), 7.87 – 7.75 (m, 2H), 7.71 – 7.57 (m, 4H), 7.57 – 7.47 (m, 4H), 7.44 (t, J = 7.5 Hz, 2H), 7.38 – 7.33 (m, 1H), 7.25 (s, 1H), 7.05 (s, 1H).

^{13}C NMR (126 MHz, Chloroform-*d*) δ (ppm) 172.67, 158.52, 155.27, 152.23, 137.87, 135.70, 133.08, 131.22, 130.28, 129.40, 128.91, 128.78, 128.37, 126.61, 126.15, 126.00, 124.85, 99.22, 96.00.

HRMS (APCI): calcd. for $C_{25}H_{19}N_4O_2$ $[M+H]^+$= 407.1503, found $[M+H]^+$ = 407.1502.

<u>5-Phenyl-*N*-(3-phenyl-1-(pyrimidin-2-yl)-1*H*-pyrazol-5-yl)isoxazole-3-carboxamide</u> **(48)**

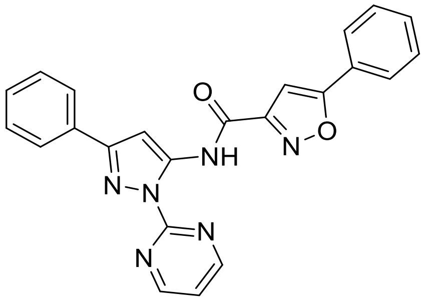

The compound was prepared according to General procedure I using Et_3N (23 µL, 0.17 mmol), 3-phenyl-1-(pyrimidin-2-yl)-1*H*-pyrazol-5-amine (**S145**, 20 mg, 0.084 mmol), acetonitrile (1 + 0.4 mL) and 5-phenylisoxazole-3-carbonyl chloride (**S177**, 35 mg, 0.17 mmol). The reaction mixture was cooled to room temperature and quenched with aqueous saturated solution of $NaHCO_3$ (3 mL). The resulting precipitate was collected by filtration, washed with water (3 mL), dried *in vacuo*, and purified by reversed phase column chromatography (100% CH_3CN). The product was obtained as a light brown solid (16 mg, 45%).

^{1}H NMR (500 MHz, Chloroform-*d*) δ (ppm) 13.13 (s, 1H), 8.93 (d, *J* = 4.9 Hz, 2H), 8.10 – 8.01 (m, 2H), 7.86 (dd, *J* = 7.6, 2.0 Hz, 2H), 7.58 – 7.49 (m, 4H), 7.46 (t, *J* = 7.3 Hz, 2H), 7.42 – 7.36 (m, 1H), 7.29 (d, *J* = 4.8 Hz, 1H), 7.10 (s, 1H).

^{13}C NMR (126 MHz, Chloroform-*d*) δ (ppm) 172.4, 159.2, 158.9, 157.8, 155.5, 154.7, 140.4, 132.2, 131.1, 129.4, 129.2, 128.7, 126.8, 126.8, 126.1, 118.1, 99.4, 96.8.

HRMS (APCI): calcd. for $C_{23}H_{17}N_6O_2$ $[M+H]^+$= 409.1408, found $[M+H]^+$= 409.1404.
mp >226 °C (dec.)

N-(1-(4-Methoxy-6-methylpyrimidin-2-yl)-3-phenyl-1*H*-pyrazol-5-yl)-5-phenylisoxazole-3-carboxamide **(49)**

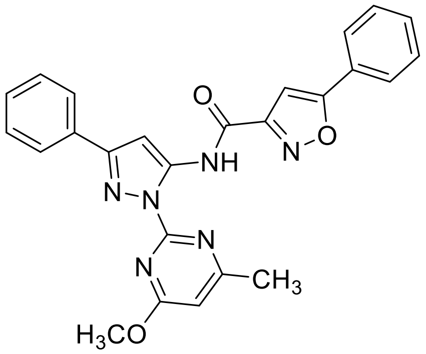

The compound was prepared according to General procedure I using Et_3N (13 µL, 0.09 mmol), 1-(4-methoxy-6-methylpyrimidin-2-yl)-3-phenyl-1*H*-pyrazol-5-amine (**S146**, 20 mg, 0.07 mmol), acetonitrile (1 + 0.4 mL) and 5-phenylisoxazole-3-carbonyl chloride (**S177**, 19 mg, 0.09 mmol). The reaction mixture was cooled to room temperature and quenched with aqeuous saturated solution of $NaHCO_3$ (3 mL). The resulting precipitate was collected by filtration, washed with water (3 mL), and dried *in vacuo*. The product was obtained as an off-white solid (15 mg, 47%).
^{1}H NMR (500 MHz, Chloroform-*d*) δ (ppm) 13.47 (s, 1H), 8.05 – 8.01 (m, 2H), 7.87 – 7.83 (m, 2H), 7.54 – 7.43 (m, 7H), 7.41 – 7.36 (m, 1H), 7.09 (s, 1H), 6.51 (s, 1H), 4.22 (s, 3H), 2.69 (s, 3H).
^{13}C NMR (126 MHz, Chloroform-*d*) δ (ppm) 172.3, 171.7, 168.3, 159.3, 157.2, 155.5, 153.8, 140.5, 132.5, 131.0, 129.3, 129.0, 128.7, 126.9, 126.6, 126.2, 103.9, 99.4, 96.3, 54.8, 23.7.
HRMS (APCI): calcd. for $C_{25}H_{21}N_6O_3$ $[M+H]^+$= 453.1670, found $[M+H]^+$= 453.1666.
mp >206 °C (dec.)

N-(1-(6-Oxo-4-(trifluoromethyl)-1,6-dihydropyrimidin-2-yl)-3-phenyl-1*H*-pyrazol-5-yl)-5-phenylisoxazole-3-carboxamide **(50)**

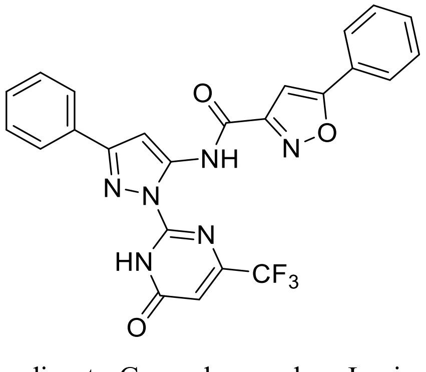

The compound was prepared according to General procedure I using Et_3N (10 µL, 0.08 mmol), 2-(5-amino-3-phenyl-1*H*-pyrazol-1-yl)-6-(trifluoromethyl)pyrimidin-4(3*H*)-one (**S142**, 20 mg, 0.06 mmol),

acetonitrile (1 + 0.3 mL) and 5-phenylisoxazole-3-carbonyl chloride (**S177**, 16 mg, 0.08 mmol). The product was obtained as an off-white solid (21 mg, 69%).

^{1}H NMR (300 MHz, DMSO-d_6) δ (ppm) 12.70 (s, 2H), 8.09 (d, J = 6.8 Hz, 2H), 8.05 – 7.97 (m, 2H), 7.66 (s, 1H), 7.59 (dd, J = 5.2, 2.0 Hz, 3H), 7.55 – 7.39 (m, 4H), 6.80 (s, 1H).

HRMS (ESI): calcd. for $C_{24}H_{14}F_3N_6O_3$ [M-H]$^-$= 491.1085, found [M-H]$^-$= 491.1081.

mp >250 °C (dec.)

N-(1-(4-(Methoxymethyl)-6-oxo-1,6-dihydropyrimidin-2-yl)-3-phenyl-1*H*-pyrazol-5-yl)-5-phenylisoxazole-3-carboxamide **(51)**

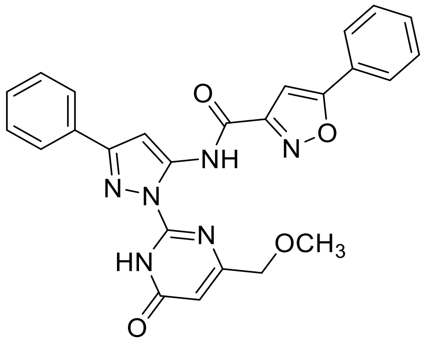

The compound was prepared according to General procedure I using Et_3N (11.2 µL, 0.08 mmol), 2-(5-amino-3-phenyl-1*H*-pyrazol-1-yl)-6-(methoxymethyl)pyrimidin-4(3*H*)-one (**S147**, 20 mg, 0.07 mmol), acetonitrile (1 + 0.4 mL) and 5-phenylisoxazole-3-carbonyl chloride (**S177**, 17 mg, 0.08 mmol). The reaction mixture was cooled to room temperature and quenched with aqueous saturated solution of $NaHCO_3$ (3 mL). The resulting precipitate was collected by filtration, washed with water (3 mL), and dried *in vacuo*. The product was obtained as an off-white solid (16 mg, 49%).

^{1}H NMR (300 MHz, DMSO-d_6) δ (ppm) 13.17 (s, 1H), 8.13 – 7.94 (m, 4H), 7.65 (s, 1H), 7.59 (dd, J = 5.1, 1.9 Hz, 3H), 7.55 – 7.42 (m, 3H), 7.40 (s, 1H), 6.40 (s, 1H), 4.54 (s, 2H), 3.48 (s, 3H).

HRMS (ESI): calcd. for $C_{25}H_{19}N_6O_4$ [M-H]$^-$= 467.1473, found [M-H]$^-$= 467.1470.

mp >309 °C (dec.)

N-(1-(4,5-Dimethyl-6-oxo-1,6-dihydropyrimidin-2-yl)-3-phenyl-1*H*-pyrazol-5-yl)-5-phenylisoxazole-3-carboxamide **(52)**

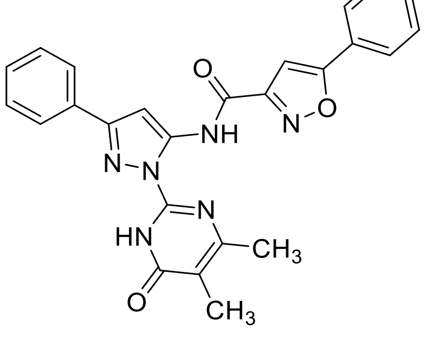

The compound was prepared according to General procedure I using Et_3N (21 µL, 0.15 mmol), 2-(5-amino-3-phenyl-1*H*-pyrazol-1-yl)-5,6-dimethylpyrimidin-4(3*H*)-one (**S149**, 30 mg, 0.11 mmol), acetonitrile (1+ 0.4 mL) and 5-phenylisoxazole-3-carbonyl chloride (**S177**, 31 mg, 0.15 mmol). The crude product (43 mg) was mixed with DMSO (1 mL) and heated to 135 °C until a clear solution obtained (10 min). Then cooled to room temperature, solid precipitated were filtered, washed with water (3 mL) and dried *in vacuo*. The product was obtained as a white solid (22 mg, 46%).

^{1}H NMR (300 MHz, DMSO-d_6) δ (ppm) 13.42 (s, 1H), 12.78 (s, 1H), 8.10 – 7.97 (m, 4H), 7.68 – 7.54 (m, 4H), 7.54 – 7.42 (m, 3H), 7.39 (s, 1H), 2.54 (s, 3H), 2.05 (s, 3H).

^{13}C NMR (75 MHz, Trifluoroacetic Acid-*d*) δ (ppm) 177.0, 172.9, 161.8, 160.3, 160.0, 159.5, 150.1, 141.6, 133.8, 133.4, 131.2, 131.1, 130.6, 128.5, 127.9, 127.4, 117.1, 100.5, 19.1, 10.8.

HRMS (ESI): calcd. for $C_{25}H_{19}N_6O_3$ $[M-H]^-$= 451.1524, found $[M-H]^-$= 451.1526.

mp >335 °C (dec.)

N-(1-(1,4-Dimethyl-6-oxo-1,6-dihydropyrimidin-2-yl)-3-phenyl-1*H*-pyrazol-5-yl)-5-phenylisoxazole-3-carboxamide **(53)**

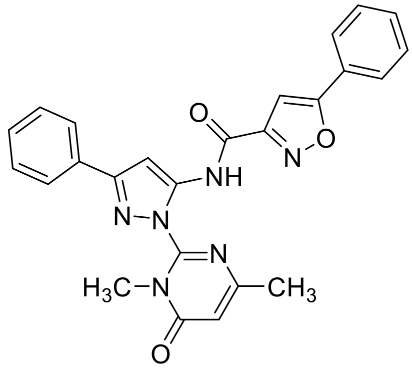

The compound was prepared according to General procedure I using Et_3N (79 µL, 0.57 mmol), 2-(5-amino-3-phenyl-1*H*-pyrazol-1-yl)-3,6-dimethylpyrimidin-4(3*H*)-one (**S151**, 80 mg, 0.28 mmol), acetonitrile (5 mL) and 5-phenylisoxazole-3-carbonyl chloride (**S177**, 77 mg, 0.37 mmol). The reaction mixture was refluxed for 3 h and then cooled to room temperature. The precipitate was collected by filtration, washed with diethyl ether (20 mL), water (20 mL), again with diethyl ether (10 mL), and dried *in vacuo*. The product was obtained as a white solid (50 mg, 39%).

^{1}H NMR (500 MHz, DMSO-d_6) δ (ppm) 11.64 (s, 1H), 8.02 – 7.95 (m, 2H), 7.93 (dt, J = 6.2, 1.4 Hz, 2H), 7.64 – 7.54 (m, 4H), 7.52 – 7.47 (m, 2H), 7.46 – 7.41 (m, 1H), 7.18 (s, 1H), 6.40 (s, 1H), 3.54 (s, 3H), 2.22 (s, 3H).

^{13}C NMR (126 MHz, DMSO-d_6) δ (ppm) 71.35, 162.21, 161.40, 158.64, 155.88, 152.25, 148.16, 139.26, 131.42, 131.10, 129.33, 129.07, 128.86, 125.97, 125.89, 125.77, 110.33, 100.09, 96.66, 32.37, 22.68 ppm.

HRMS (APCI): calcd. for $C_{25}H_{21}N_6O_3$ $[M+H]^+$= 453.1670, found $[M+H]^+$= 453.1669.

N-(1-(6-Hydroxypyridin-2-yl)-3-phenyl-1H-pyrazol-5-yl)-5-phenylisoxazole-3-carboxamide **(54)**

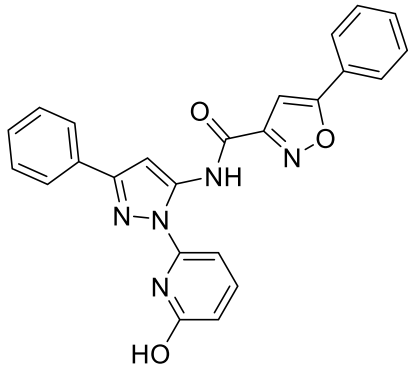

Palladium on activated charcoal (10% Pd basis, 11 mg, 0.01 mmol) was added to solution of *N*-(1-(6-(benzyloxy)pyridin-2-yl)-3-phenyl-1*H*-pyrazol-5-yl)-5-phenylisoxazole-3-carboxamide (**50**, 50 mg, 0.1 mmol) in dichloromethane:THF (1:1, 10 mL) at 23 °C and the mixture was purged with H_2 (1 bar) for 5 min. The reaction mixture was stirred at 50 °C under H_2 atmosphere for 4 h, filtered through Celite®, the filter plug was washed with dichloromethane (20 mL) and methanol (10 mL), the filtrate eas collected and the solvents were evaporated *in vacuo*. The residue was purified by column chromatography on silica gel (dichloromethane:MeOH, 95:5). The product was obtained as a white solid (29 mg, 71%).

^{1}H NMR (500 MHz, DMSO-d_6) δ (ppm) 13.00 (s, 1H), 11.40 (s, 1H), 8.07 – 7.99 (m, 2H), 7.99 – 7.94 (m, 2H), 7.93 – 7.90 (m, 1H), 7.66 – 7.56 (m, 4H), 7.57 – 7.53 (m, 1H), 7.52 – 7.46 (m, 2H), 7.45 – 7.40 (m, 1H), 7.38 (br s, 1H), 6.72 (d, J = 8.0 Hz, 1H).

^{13}C NMR (126 MHz, DMSO-d_6) δ (ppm) 171.38, 161.98, 158.79, 154.97, 151.60, 151.11, 142.71, 138.85, 131.96, 131.08, 129.36, 128.81, 128.72, 126.11, 125.87, 125.62, 106.60, 104.58, 99.90, 94.95.

HRMS (APCI): calcd. for $C_{24}H_{18}N_5O_3$ $[M+H]^+$= 424.1404, found $[M+H]^+$= 424.1406.

N-(1-(4-Ethyl-6-oxo-1,6-dihydropyrimidin-2-yl)-3-phenyl-1H-pyrazol-5-yl)-5-phenylisoxazole-3-carboxamide **(55)**

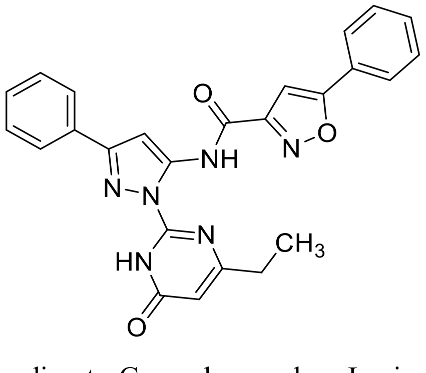

The compound was prepared according to General procedure I using Et_3N (21 µL, 0.15 mmol), 2-(5-amino-3-phenyl-1*H*-pyrazol-1-yl)-6-ethylpyrimidin-4(3*H*)-one (**S143**, 30 mg, 0.11 mmol), acetonitrile (1 + 0.4 mL) and 5-phenylisoxazole-3-carbonyl chloride (**S177**, 31 mg, 0.15 mmol). The product was obtained as a white solid (36 mg, 75%).

^{1}H NMR (300 MHz, DMSO-d_6) δ (ppm) 13.28 (s, 1H), 12.81 (s, 1H), 8.10 – 7.97 (m, 4H), 7.64 (s, 1H), 7.59 (dd, J = 5.1, 1.9 Hz, 3H), 7.55 – 7.42 (m, 3H), 7.42 (s, 1H), 6.37 (s, 1H), 2.78 (q, J = 7.6 Hz, 2H), 1.32 (t, J = 7.5 Hz, 3H).
HRMS (APCI): calcd. for $C_{25}H_{21}N_6O_3$ $[M+H]^+$= 453.1670, found $[M+H]^+$= 453.1671.
mp >290 °C (dec.)

<u>5-(3-(*tert*-Butyl)phenyl)-*N*-(1-(4-methyl-6-oxo-1,6-dihydropyrimidin-2-yl)-3-phenyl-1*H*-pyrazol-5-yl)isoxazole-3-carboxamide</u> **(118)**

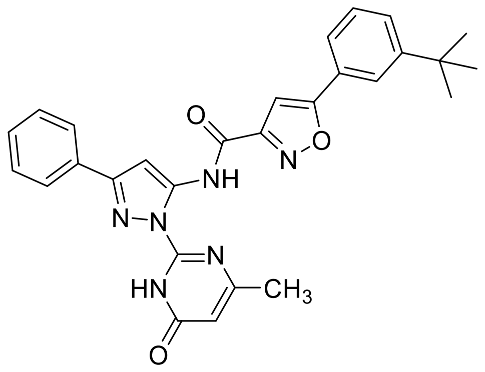

The compound was prepared according to General procedure I using Et_3N (10 µL, 0.07 mmol), 2-(5-amino-3-phenyl-1*H*-pyrazol-1-yl)-6-methylpyrimidin-4(3*H*)-one (**S137**, 15 mg, 0.06 mmol), acetonitrile (0.8 + 0.4 mL) and 5-(3-(*ter*t-butyl)phenyl)isoxazole-3-carbonyl chloride (**S170**, 18 mg, 0.07 mmol). The product was obtained as a white solid (21 mg, 74%).
^{1}H NMR (500 MHz, DMSO-d_6) δ (ppm) 13.31 (s, 1H), 12.38 (br s, 1H), 8.05 – 7.99 (m, 2H), 7.97 (t, J = 1.9 Hz, 1H), 7.79 (dt, J = 7.6, 1.4 Hz, 1H), 7.61 (dt, J = 8.1, 1.4 Hz, 1H), 7.58 – 7.55 (m, 1H), 7.55 – 7.46 (m, 3H), 7.46 – 7.41 (m, 1H), 7.37 (s, 1H), 6.32 (s, 1H), 2.50 (s, 3H, overlapped with DMSO), 1.38 (s, 9H).
^{13}C NMR (126 MHz, DMSO-d_6) δ (ppm) 171.88, 158.60, 154.54, 152.01, 151.80, 139.88, 131.44, 128.73, 128.61, 128.30, 127.62, 125.76, 125.66, 122.87, 122.43, 106.04, 99.42, 94.86, 34.26, 30.59, 22.31.
HRMS (APCI): calcd. for $C_{28}H_{27}N_6O_3$ $[M+H]^+$= 495.2139, found $[M+H]^+$= 495.2137.
mp >309 °C (dec.)

<u>*N*-(1-(4-Methyl-6-oxo-1,6-dihydropyrimidin-2-yl)-3-phenyl-1*H*-pyrazol-5-yl)-5-phenyl-4,5-dihydroisoxazole-3-carboxamide</u> **(119)**

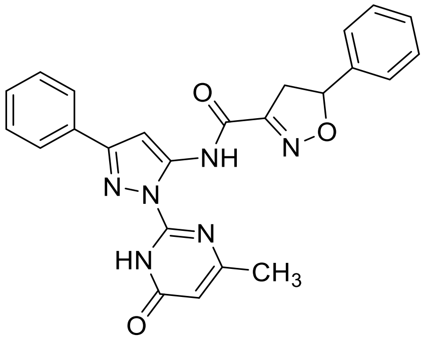

The compound was prepared according to General procedure I using Et_3N (19 µL, 0.14 mmol), 2-(5-amino-3-phenyl-1*H*-pyrazol-1-yl)-6-methylpyrimidin-4(3*H*)-one (**S137**, 30 mg, 0.11 mmol), acetonitrile (1 + 0.5 mL) and 5-phenyl-4,5-dihydroisoxazole-3-carbonyl chloride (**S180**, 28 mg, 0.14 mmol). The product was obtained as a white solid (35 mg, 71%).

^{1}H NMR (300 MHz, DMSO-d_6) δ (ppm) 13.16 (s, 1H), 12.76 (s, 1H), 8.08 – 7.96 (m, 2H), 7.57 – 7.34 (m, 8H), 7.29 (s, 1H), 6.34 (s, 1H), 5.92 (dd, *J* = 11.4, 9.4 Hz, 1H), 3.81 (dd, *J* = 17.7, 11.4 Hz, 1H), 3.36 – 3.22 (m, 1H), 2.38 (s, 3H).

HRMS (ESI): calcd. for $C_{24}H_{19}N_6O_3$ [M-H]$^-$= 439.1524, found [M-H]$^-$= 439.1526.

mp >294 °C (dec.)

<u>2,3-Dimethoxy-*N*-(1-(4-methyl-6-oxo-1,6-dihydropyrimidin-2-yl)-3-phenyl-1*H*-pyrazol-5-yl)benzamide</u> **(120)**

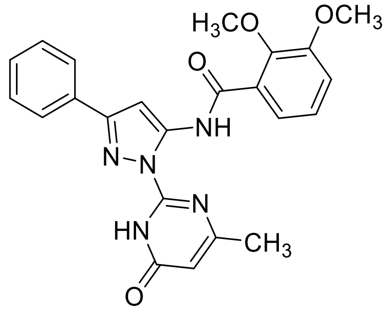

The compound was prepared according to General procedure I using Et_3N (13 µL, 0.09 mmol), 2-(5-amino-3-phenyl-1*H*-pyrazol-1-yl)-6-methylpyrimidin-4(3*H*)-one (**S137**, 20 mg, 0.08 mmol), acetonitrile (1 + 0.5 mL) and 2,3-dimethoxybenzoyl chloride (18 mg, 0.09 mmol). The product was obtained as a white solid (13 mg, 40%).

^{1}H NMR (300 MHz, DMSO-d_6) δ (ppm) 12.86 (s, 1H), 12.61 (s, 1H), 8.05 (d, *J* = 7.4 Hz, 2H), 7.61 – 7.39 (m, 5H), 7.39 – 7.23 (m, 2H), 6.30 (s, 1H), 3.90 (s, 3H), 3.82 (s, 3H), 2.38 (s, 3H).

HRMS (APCI): calcd. for $C_{23}H_{22}N_5O_4$ [M+H]$^+$= 432.1666, found [M+H]$^+$= 432.1662.

mp = 269−272 °C

<u>*N*-(1-(4-Methyl-6-oxo-1,6-dihydropyrimidin-2-yl)-3-phenyl-1*H*-pyrazol-5-yl)-[1,1'-biphenyl]-4-carboxamide</u> **(121)**

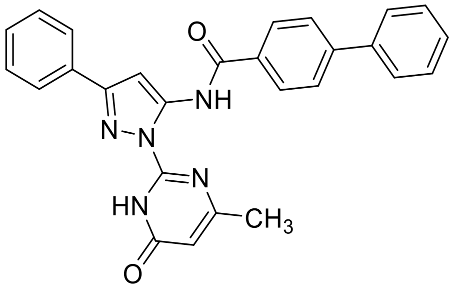

The compound was prepared according to General procedure I using Et_3N (13 µL, 0.09 mmol), 2-(5-amino-3-phenyl-1*H*-pyrazol-1-yl)-6-methylpyrimidin-4(3*H*)-one (**S137**, 20 mg, 0.08 mmol), acetonitrile (1 + 0.5 mL) and [1,1'-biphenyl]-4-carbonyl chloride (**S182**, 19 mg, 0.09 mmol). The crude product (24 mg) was combined with DMSO (2 mL) and heated at 135 °C until a clear solution formed. The solution was cooled to room temperature, the precipitate was collected by filtration, washed with water (1 mL) and dried *in vacuo*. The product was obtained as a white solid (12 mg, 36%).

^{1}H NMR (300 MHz, DMSO-d_6) δ (ppm) 12.89 (s, 2H), 8.09 (dd, J = 13.9, 7.8 Hz, 4H), 7.96 (d, J = 8.1 Hz, 2H), 7.80 (d, J = 7.6 Hz, 2H), 7.60 – 7.35 (m, 7H), 6.37 (s, 1H).

HRMS (APCI): calcd. for $C_{27}H_{22}N_5O_2$ $[M+H]^+$= 448.1768, found $[M+H]^+$= 448.1765.

mp >232 °C (dec.)

N-(1-(4-Methyl-6-oxo-1,6-dihydropyrimidin-2-yl)-3-phenyl-1*H*-pyrazol-5-yl)-[1,1'-biphenyl]-3-carboxamide **(122)**

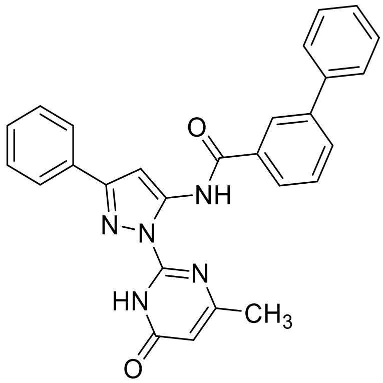

The compound was prepared according to General procedure I using Et_3N (13 µL, 0.09 mmol), 2-(5-amino-3-phenyl-1*H*-pyrazol-1-yl)-6-methylpyrimidin-4(3*H*)-one (**S137**, 20 mg, 0.08 mmol), acetonitrile (1 + 0.5 mL) and [1,1'-biphenyl]-3-carbonyl chloride (**S183**, 19 mg, 0.09 mmol). The product was obtained as a white solid (16 mg, 48%).

^{1}H NMR (300 MHz, DMSO-d_6) δ (ppm) 13.09 (s, 1H), 12.72 (s, 1H), 8.23 (s, 1H), 8.09 – 8.00 (m, 3H), 8.00 – 7.93 (m, 1H), 7.85 – 7.67 (m, 3H), 7.60 – 7.36 (m, 7H), 6.23 (s, 1H), 2.23 (s, 3H),

HRMS (APCI): calcd. for $C_{27}H_{22}N_5O_2$ $[M+H]^+$= 448.1768, found $[M+H]^+$= 448.1769.

mp >246 °C (dec.)

N-(1-(4-Methyl-6-oxo-1,6-dihydropyrimidin-2-yl)-3-phenyl-1*H*-pyrazol-5-yl)-[1,1'-biphenyl]-2-carboxamide **(123)**

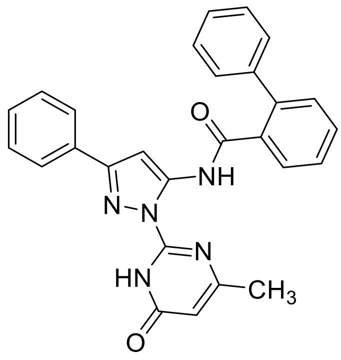

Et_3N (62 µL, 0.45 mmol) and T_3P/propylphosphonic anhydride (50% in EtOAc, 0.143 mL, 0.22 mmol) were added to a solution of [1,1'-biphenyl]-2-carboxylic acid (15 mg, 0.75 mmol) in THF (1.0 mL), and the mixture was stirred at room temperature for 1 h. Then, 2-(5-amino-3-phenyl-1*H*-pyrazol-1-yl)-6-methylpyrimidin-4(3*H*)-one (**S137**, 20 mg, 0.08 mmol) was added and the reaction mixture was refluxed for 48 h. The solvent was evaporated *in vacuo*, saturated aqueous solution of $NaHCO_3$ (3 mL) was added to the residue and the mixture was extracted with EtOAc (3 × 10 mL). The combined organic extracts were dried over $MgSO_4$, filtered, and the solvent evaporated *in vacuo*. The residue was purified by preparative TLC (dichloromethane:MeOH, 94:6). The product was obtained as a white solid (14 mg, 42%).

^{1}H NMR (500 MHz, Methanol-d_4) δ (ppm) 7.80 (d, J = 7.5 Hz, 1H), 7.70 (t, J = 7.5 Hz, 1H), 7.64 – 7.56 (m, 2H), 7.45 (d, J = 7.5 Hz, 2H), 7.36 (t, J = 7.5 Hz, 2H), 7.30 (d, J = 7.3 Hz, 1H), 7.23 – 7.14 (m, 3H), 7.05 (t, J = 7.6 Hz, 2H), 6.89 (s, 1H), 5.94 (s, 1H), 1.97 (s, 3H).

^{13}C NMR (126 MHz, Methanol-d_4) δ (ppm) 174.8, 168.2, 165.0, 155.3, 154.8, 136.1, 132.6, 132.1, 131.2, 130.2, 129.8, 129.6, 129.4, 129.3, 129.0, 128.9, 128.4, 110.1, 98.3, 23.0.

HRMS (APCI): calcd. for $C_{27}H_{22}N_5O_2$ $[M+H]^+$= 448.1768, found $[M+H]^+$= 448.1770.

mp >238 °C (dec.)

N-(3-(Furan-2-yl)-1-(4-methyl-6-oxo-1,6-dihydropyrimidin-2-yl)-1*H*-pyrazol-5-yl)-5-methylisoxazole-3-carboxamide **(124)**

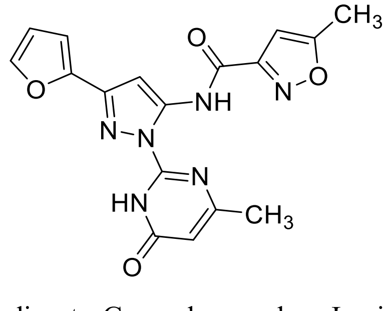

The compound was prepared according to General procedure I using Et_3N (16 µL, 0.12 mmol), 2-(5-amino-3-(furan-2-yl)-1*H*-pyrazol-1-yl)-6-methylpyrimidin-4(3*H*)-one (**S138**, 30 mg, 0.12 mmol),

acetonitrile (1 + 0.5 mL) and 5-methylisoxazole-3-carbonyl chloride (17 mg, 0.12 mmol). The obtained solid was washed with water (3 mL), then with EtOAc (2 mL), and dried *in vacuo*. The product was obtained as a white solid (32 mg, 75%).

^{1}H NMR (500 MHz, DMSO-d_6) δ (ppm) 15.15 (s, 1H), 7.76 (d, J = 1.6 Hz, 1H), 7.05 (s, 1H), 6.90 (d, J = 3.2 Hz, 1H), 6.85 (d, J = 1.1 Hz, 1H), 6.61 (dd, J = 3.3, 1.8 Hz, 1H), 5.68 (s, 1H), 2.51 (s, 3H), 2.19 (s, 3H).

^{13}C NMR (126 MHz, DMSO-d_6) δ (ppm) 172.0, 171.9, 160.9, 158.9, 157.5, 155.2, 148.3, 142.8, 142.6, 140.6, 111.7, 107.5, 107.0, 101.3, 93.2, 22.7, 11.9.

HRMS (ESI): calcd. for $C_{17}H_{13}N_6O_4$ [M-H]$^-$= 365.1004, found [M-H]$^-$= 365.1000.

mp >290 °C (dec.)

<u>*N*-(3-(Furan-2-yl)-1-(4-methyl-6-oxo-1,6-dihydropyrimidin-2-yl)-1*H*-pyrazol-5-yl)-5-isopropylisoxazole-3-carboxamide</u> **(125)**

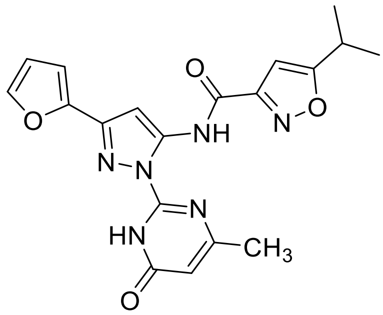

The compound was prepared according to General procedure I using Et_3N (14 µL, 0.1 mmol), 2-(5-amino-3-(furan-2-yl)-1*H*-pyrazol-1-yl)-6-methylpyrimidin-4(3*H*)-one (**S138**, 25 mg, 0.1 mmol), acetonitrile (1 + 0.5 mL) and 5-isopropylisoxazole-3-carbonyl chloride (17 mg, 0.1 mmol). The product was obtained as a light brown solid (26 mg, 68%).

^{1}H NMR (300 MHz, DMSO-d_6) δ (ppm) 13.50 (s, 1H), 12.90 (s, 1H), 7.82 (s, 1H), 7.19 (s, 1H), 7.02 (d, J = 3.4 Hz, 1H), 6.81 (s, 1H), 6.67 – 6.62 (m, 1H), 6.46 (s, 1H), 3.24 – 3.13 (m, 1H), 1.31 (d, J = 6.4 Hz, 6H),

^{13}C NMR (176 MHz, DMSO-d_6) δ (ppm) 181.24, 158.06, 154.97, 147.21, 144.23, 143.62, 139.89, 111.83, 108.58, 99.27, 94.33, 26.65, 22.73, 20.48.

HRMS (APCI): calcd. for $C_{19}H_{19}N_6O_4$ [M+H]$^+$= 395.1462, found [M+H]$^+$= 395.1460.

mp >250°C (dec.)

<u>5-(*tert*-Butyl)-N-(3-(furan-2-yl)-1-(4-methyl-6-oxo-1,6-dihydropyrimidin-2-yl)-1*H*-pyrazol-5-yl)isoxazole-3-carboxamide</u> **(126)**

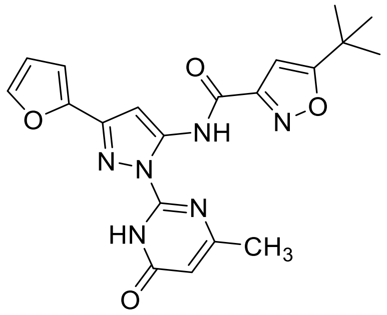

HOBt•H_2O (10 mg, 0.08 mmol), EDCI•HCl (22 mg, 0.12 mmol) and DIPEA (27 µL, 0.16 mmol) were added to a solution of 5-(*tert*-butyl)isoxazole-3-carboxylic acid (**S173**, 12 mg, 0.78 mmol) in DMF (1 mL) and the mixture was stirred at room temperature for 15 min. Then, 2-(5-amino-3-(furan-2-yl)-1*H*-pyrazol-1-yl)-6-methylpyrimidin-4(3*H*)-one (**S138**, 20 mg, 0.08 mmol) was added and the reaction mixture was stirred at room temperature for 48 h. H_2O (30 mL) was added and the mixture was stirred for 15 min. The precipitate was collected by filtration, washed with water (3 mL), and dried *in vacuo*. The product was obtained as a light brown solid (6 mg, 18%).

^{1}H NMR (300 MHz, DMSO-d_6) δ (ppm) 14.96 (s, 1H), 7.77 (s, 1H), 7.08 (s, 1H), 6.91 (d, J = 2.7 Hz, 2H), 6.62 (d, J = 2.5 Hz, 1H), 5.86 (s, 1H), 2.25 (s, 3H), 1.37 (s, 9H).

^{13}C NMR (126 MHz, DMSO-d_6) δ (ppm) 183.1, 158.5, 155.1, 148.0, 143.0, 111.7, 107.3, 98.6, 93.5, 32.6, 28.4, 22.9.

HRMS (APCI): calcd. for $C_{20}H_{21}N_6O_4$ $[M+H]^+$= 409.1619, found $[M+H]^+$= 409.1615.

mp >197 °C (dec.)

N-(3-(furan-2-yl)-1-(4-methyl-6-oxo-1,6-dihydropyrimidin-2-yl)-1*H*-pyrazol-5-yl)-2,3-dimethoxybenzamide **(127)**

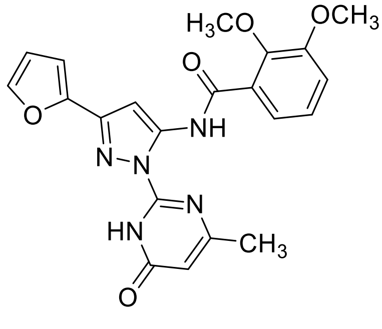

The compound was prepared according to General procedure I using Et_3N (20 µL, 0.14 mmol), 2-(5-amino-3-(furan-2-yl)-1*H*-pyrazol-1-yl)-6-methylpyrimidin-4(3*H*)-one (**S138**, 30 mg, 0.12 mmol), acetonitrile (1 + 0.5 mL) and 2,3-dimethoxybenzoyl chloride (28 mg, 0.14 mmol). The reaction mixture was cooled to room temperature, quenched with saturated aqueous solution of $NaHCO_3$ (3 mL) and extracted with EtOAc (3 × 5 mL). The combined organic extracts were washed with water (3 × 5 mL), brine (5 mL), dried over $MgSO_4$, filtered, and the solvent evaporated *in vacuo*. The residue was purified by preparative TLC (dichloromethane:MeOH, 94:6). The product was obtained as an off-white solid (12 mg, 24%).

^1H NMR (300 MHz, DMSO-d_6) δ (ppm) 13.11 (s, 1H), 12.66 (s, 1H), 7.77 (s, 1H), 7.56 – 7.47 (m, 1H), 7.29 (dt, J = 22.4, 8.0 Hz, 3H), 7.01 (s, 1H), 6.62 (s, 1H), 6.19 (s, 1H), 3.90 (s, 3H), 3.82 (s, 3H), 2.32 (s, 3H).
^{13}C NMR (75 MHz, DMSO-d_6) δ (ppm) 161.5, 152.8, 147.3, 143.5, 126.6, 124.5, 121.8, 116.8, 111.8, 94.9, 61.2, 56.3, 22.1.
HRMS (APCI): calcd. for $C_{21}H_{20}N_5O_5$ $[M+H]^+$= 422.1459, found $[M+H]^+$= 422.1459.
mp >210 °C (dec.)

N-(3-(furan-2-yl)-1-(4-methyl-6-oxo-1,6-dihydropyrimidin-2-yl)-1*H*-pyrazol-5-yl)-5-phenylisothiazole-3-carboxamide **(128)**

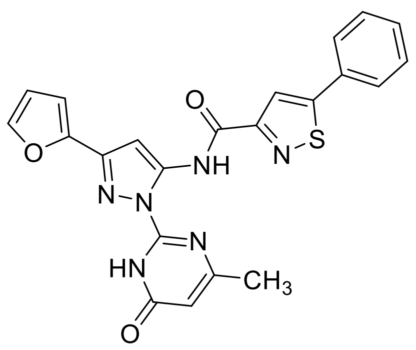

The compound was prepared according to General procedure I using Et_3N (20 µL, 0.14 mmol), 2-(5-amino-3-(furan-2-yl)-1*H*-pyrazol-1-yl)-6-methylpyrimidin-4(3*H*)-one (**S138**, 30 mg, 0.12 mmol), acetonitrile (1 + 0.5 mL) and 5-phenylisothiazole-3-carbonyl chloride (**S189**, 31 mg, 0.14 mmol). The crude product (30 mg) was mixed with DMSO (1.5 mL) and heated at 135 °C until a clear solution formed. The solution was cooled to room temperature and H_2O (0.2 mL) was added. The resulting precipitate was collceted by filtration, washed with water (2 mL), and dried *in vacuo*. The product was obtained as a light brown solid (19 mg, 37%).
^1H NMR (300 MHz, DMSO-d_6) δ (ppm) 14.16 (s, 1H), 12.83 (s, 1H), 8.38 (s, 1H), 7.90 (dd, J = 7.5, 2.1 Hz, 2H), 7.81 (d, J = 1.7 Hz, 1H), 7.53 (dd, J = 4.9, 2.4 Hz, 3H), 7.18 (s, 1H), 6.98 (d, J = 3.4 Hz, 1H), 6.64 (dd, J = 3.4, 1.8 Hz, 1H), 6.22 (s, 1H), 2.45 (s, 3H).
HRMS (APCI): calcd. for $C_{22}H_{17}N_6O_3S$ $[M+H]^+$= 445.1077, found $[M+H]^+$= 445.1077.
mp >289 °C (dec.)

N-(3-(4-cyanophenyl)-1-(4-methyl-6-oxo-1,6-dihydropyrimidin-2-yl)-1*H*-pyrazol-5-yl)-5-phenylisoxazole-3-carboxamide **(129)**

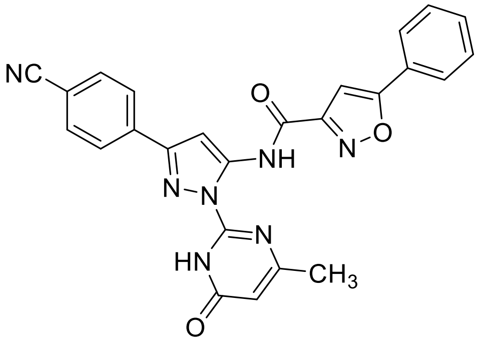

The compound was prepared according to General procedure I using Et_3N (9 µL, 0.06 mmol), 4-(5-amino-1-(4-methyl-6-oxo-1,6-dihydropyrimidin-2-yl)-1*H*-pyrazol-3-yl)benzonitrile (**S129**, 15 mg, 0.05 mmol), acetonitrile (0.8+ 0.3 mL) and 5-phenylisoxazole-3-carbonyl chloride (**S177**, 13 mg, 0.06 mmol). The product was obtained as a white solid (21 mg, 87%).

^{1}H NMR (300 MHz, DMSO-*d*$_6$) *δ* (ppm) 15.67 (s, 1H), 8.15 (d, *J* = 8.1 Hz, 2H), 8.10 – 7.97 (m, 2H), 7.91 (d, *J* = 8.2 Hz, 2H), 7.86 (s, 1H), 7.59 (d, *J* = 6.7 Hz, 3H), 7.36 (s, 1H), 5.79 (s, 1H), 2.25 (s, 3H).

HRMS (ESI): calcd. for $C_{25}H_{16}N_7O_3$ $[M-H]^-$= 462.1320, found $[M-H]^-$= 462.1317.

mp >330 °C (dec.)

N-(1-(4-methyl-6-oxo-1,6-dihydropyrimidin-2-yl)-3-(4-(trifluoromethyl)phenyl)-1*H*-pyrazol-5-yl)-5-phenylisoxazole-3-carboxamide **(130)**

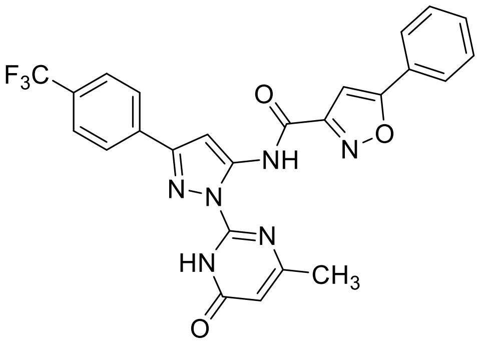

The compound was prepared according to General procedure I using Et_3N (8 µL, 0.54 mmol), 2-(5-amino-3-(4-(trifluoromethyl)phenyl)-1*H*-pyrazol-1-yl)-6-methylpyrimidin-4(3*H*)-one (**S131**, 15 mg, 0.05 mmol), acetonitrile (0.8 + 0.3 mL) and 5-phenylisoxazole-3-carbonyl chloride (**S177**, 11 mg, 0.05 mmol). The crude product (20 mg) was mixed with DMSO (1.5 mL) and heated at 135 °C until a clear solution formed. The solution was cooled to room temperature, the resulting precipitated was collected by filtration, washed with H_2O (1 mL), and dried *in vacuo*. The product was obtained as a white solid (12 mg, 53%).

^{1}H NMR (300 MHz, Trifluoroacetic Acid-*d*) *δ* (ppm) 8.11 (d, *J* = 8.1 Hz, 2H), 7.88 (dd, *J* = 6.8, 2.8 Hz, 2H), 7.79 (d, *J* = 8.2 Hz, 2H), 7.70 (s, 1H), 7.58 – 7.47 (m, 3H), 7.21 (s, 1H), 6.86 (s, 1H), 2.80 (s, 3H).

HRMS (ESI): calcd. for $C_{25}H_{16}F_3N_6O_3$ $[M-H]^-$= 505.1241, found $[M-H]^-$= 505.1243.

mp >350 °C (dec.)

<u>*N*-(3-(4-(t*ert*-butyl)phenyl)-1-(4-methyl-6-oxo-1,6-dihydropyrimidin-2-yl)-1*H*-pyrazol-5-yl)-5-phenylisoxazole-3-carboxamide</u> **(131)**

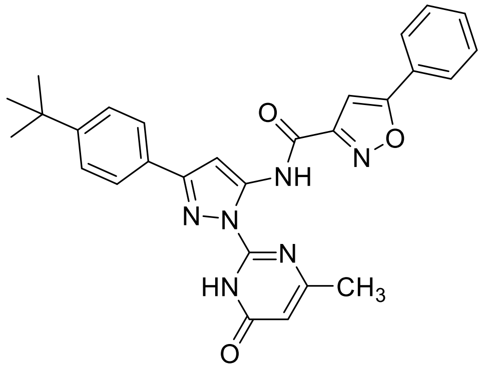

The compound was prepared according to General procedure I using Et_3N (12 µL, 0.09 mmol), 2-(5-amino-3-(4-(*tert*-butyl)phenyl)-1*H*-pyrazol-1-yl)-6-methylpyrimidin-4(3*H*)-one (**S132**, 20 mg, 0.06 mmol), acetonitrile (0.8 + 0.3 mL) and 5-phenylisoxazole-3-carbonyl chloride (**S177**, 18 mg, 0.09 mmol). The product was obtained as a white solid (11 mg, 36%).

^{1}H NMR (300 MHz, DMSO-d_6) δ (ppm) 13.93 (s, 1H), 12.78 (s, 1H), 8.03 (dd, J = 6.8, 2.8 Hz, 2H), 7.93 (d, J = 8.4 Hz, 2H), 7.68 (s, 1H), 7.59 (dd, J = 5.1, 1.9 Hz, 3H), 7.51 (d, J = 8.4 Hz, 2H), 7.33 (s, 1H), 6.25 (s, 1H), 2.45 (s, 3H), 1.33 (s, 9H).

HRMS (APCI): calcd. for $C_{28}H_{27}N_6O_3$ $[M+H]^+$= 495.2139, found $[M+H]^+$= 495.2139.

mp >309 °C (dec.)

<u>*N*-(3-(4-methoxyphenyl)-1-(4-methyl-6-oxo-1,6-dihydropyrimidin-2-yl)-1*H*-pyrazol-5-yl)-5-phenylisoxazole-3-carboxamide</u> **(132)**

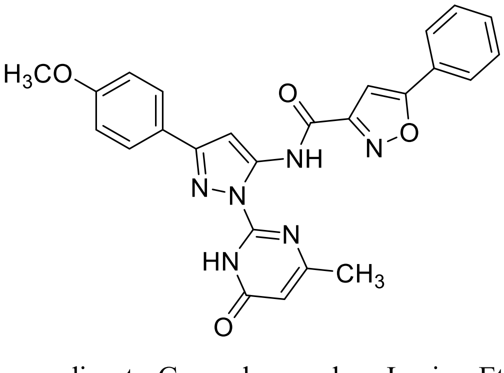

The compound was prepared according to General procedure I using Et_3N (13 µL, 0.09 mmol), 2-(5-amino-3-(4-methoxyphenyl)-1*H*-pyrazol-1-yl)-6-methylpyrimidin-4(3*H*)-one (**S133**, 20 mg, 0.07 mmol), acetonitrile (0.8 + 0.3 mL) and 5-phenylisoxazole-3-carbonyl chloride (**S177**, 20 mg, 0.09 mmol). The product was obtained as an off-white solid (24 mg, 76%).

^{1}H NMR (300 MHz, DMSO-d_6) δ (ppm) 14.01 (s, 1H), 12.68 (s, 1H), 8.06 – 7.98 (m, 2H), 7.94 (d, J = 8.7 Hz, 2H), 7.69 (s, 1H), 7.58 (dd, J = 5.2, 1.9 Hz, 3H), 7.28 (s, 1H), 7.04 (d, J = 8.8 Hz, 2H), 6.19 (s, 1H), 3.82 (s, 3H), 2.42 (s, 3H).

HRMS (APCI): calcd. for $C_{25}H_{21}N_6O_4$ $[M+H]^+$= 469.1619, found $[M+H]^+$= 469.1619.

mp >270 °C (dec.)

<u>*N*-(1-(4-methyl-6-oxo-1,6-dihydropyrimidin-2-yl)-3-(5-methylfuran-2-yl)-1*H*-pyrazol-5-yl)-5-phenylisoxazole-3-carboxamide</u> **(133)**

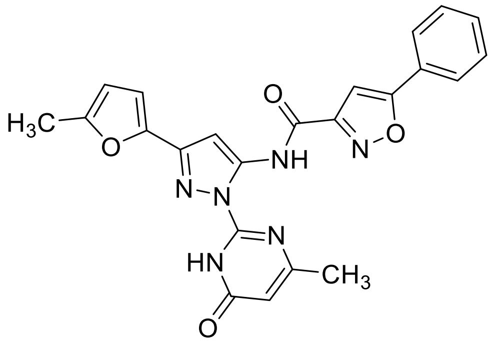

The compound was prepared according to General procedure I using Et_3N (22 µL, 0.16 mmol), 2-(5-amino-3-(5-methylfuran-2-yl)-1*H*-pyrazol-1-yl)-6-methylpyrimidin-4(3*H*)-one (**S136**, 30 mg, 0.11 mmol), acetonitrile (1 + 0.4 mL) and 5-phenylisoxazole-3-carbonyl chloride (**S177**, 32 mg, 0.16 mmol). The product was obtained as a light yellow solid (29 mg, 59%).

^{1}H NMR (300 MHz, DMSO-*d*$_6$) *δ* (ppm) 13.58 (s, 1H), 12.96 (s, 1H), 8.02 (dd, *J* = 6.6, 3.1 Hz, 2H), 7.63 (s, 1H), 7.58 (dd, *J* = 5.0, 1.9 Hz, 3H), 7.18 (s, 1H), 6.91 (d, *J* = 3.2 Hz, 1H), 6.48 (s, 1H), 6.26 (dd, *J* = 3.2, 1.2 Hz, 1H), 2.55 (s, 3H), 2.38 (s, 3H).

HRMS (APCI): calcd. for $C_{23}H_{19}N_6O_4$ $[M+H]^+$= 443.1462, found $[M+H]^+$= 443.1462.

mp >300 °C (dec.)

<u>*N*-(1-(4-methyl-6-oxo-1,6-dihydropyrimidin-2-yl)-3-(thiophen-2-ylmethyl)-1*H*-pyrazol-5-yl)-5-phenylisoxazole-3-carboxamide</u> **(134)**

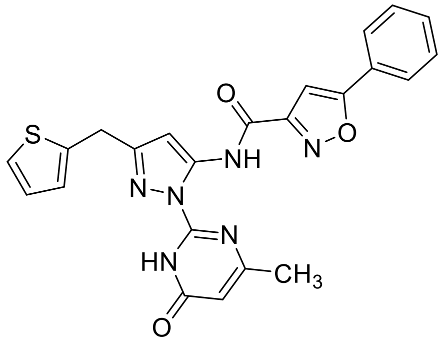

The compound was prepared according to General procedure I using Et_3N (78 µL, 0.56 mmol), 2-(5-amino-3-(thiophen-2-ylmethyl)-1*H*-pyrazol-1-yl)-6-methylpyrimidin-4(3*H*)-one (**S140**, 80 mg, 0.28 mmol), acetonitrile (5 mL) and 5-phenylisoxazole-3-carbonyl chloride (**S177**, 75 mg, 0.36 mmol). The reaction mixture was refluxed for 3 h, then cooled to room temperature. The resulting precipitate was collected by filtration, washed with water (5 mL), diethyl ether (15 mL), and dried *in vacuo*. The product was obtained as an off-white solid (50 mg, 39%).

^{1}H NMR (500 MHz, DMSO-d_6) δ (ppm) 13.32 (br s, 1H), 12.45 (br s, 1H), 8.10 – 7.89 (m, 2H), 7.68 – 7.52 (m, 3H), 7.49 (s, 1H), 7.36 (d, J = 5.1 Hz, 1H), 7.11 – 6.94 (m, 2H), 6.82 (s, 1H), 6.37 (s, 1H), 4.22 (s, 2H), 2.55 (s, 3H; mearged with DMSO signal).
^{13}C NMR (126 MHz, DMSO-d_6) δ (ppm) 171.48, 158.61, 154.36, 153.35, 140.53, 139.08, 130.72, 128.96, 126.64, 125.82, 125.66, 125.50, 124.29, 104.75, 99.49, 97.02, 28.41, 22.31.
HRMS (APCI): calcd. for $C_{23}H_{19}N_6O_3S$ $[M+H]^+$= 459.1234, found $[M+H]^+$= 459.1235.

N-(1-(5-(2-(2-(2-methoxyethoxy)ethoxy)ethyl)-4-methyl-6-oxo-1,6-dihydropyrimidin-2-yl)-3-phenyl-1*H*-pyrazol-5-yl)-5-phenylisoxazole-3-carboxamide **(135)**

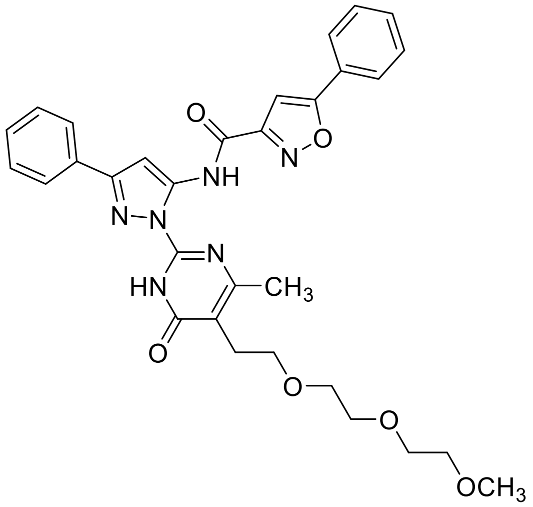

The compound was prepared according to General procedure I using using Et_3N (14 µL, 0.10 mmol), 2-(5-amino-3-phenyl-1*H*-pyrazol-1-yl)-5-(2-(2-(2-methoxyethoxy)ethoxy)ethyl)-6-methylpyrimidin-4(3*H*)-one (**S148**, 30 mg, 0.07 mmol), acetonitrile (1 + 0.4 mL) and 5-phenylisoxazole-3-carbonyl chloride (**S177**, 21 mg, 0.10 mmol). The product was obtained as a white solid (25 mg, 59%).
^{1}H NMR (300 MHz, DMSO-d_6) δ (ppm) 14.87 (s, 1H), 12.88 (s, 1H), 8.02 (dd, *J* = 7.6, 2.1 Hz, 2H), 8.00 – 7.92 (m, 2H), 7.71 (s, 1H), 7.65 – 7.53 (m, 3H), 7.48 (t, *J* = 7.3 Hz, 2H), 7.43 – 7.35 (m, 1H), 7.28 (s, 1H), 3.60 – 3.45 (m, 8H), 3.45 – 3.38 (m, 2H), 3.22 (s, 3H), 2.72 (t, *J* = 6.8 Hz, 2H), 2.41 (s, 3H).
^{13}C NMR (75 MHz, DMSO-d_6) δ (ppm) 171.2, 159.5, 154.8, 153.2, 150.6, 141.0, 132.4, 130.9, 129.3, 128.6, 128.4, 126.2, 125.8, 125.8, 115.0, 99.9, 94.0, 71.2, 69.8, 69.6, 69.5, 68.9, 57.9, 20.8.
HRMS (APCI): calcd. for $C_{31}H_{33}N_6O_6$ $[M+H]^+$= 585.2456, found $[M+H]^+$= 585.2455.
mp >195 °C (dec.)

N-(1-(1,3-dimethyl-2,6-dioxo-1,2,3,6-tetrahydropyrimidin-4-yl)-3-phenyl-1*H*-pyrazol-5-yl)-5-phenylisoxazole-3-carboxamide **(136)**

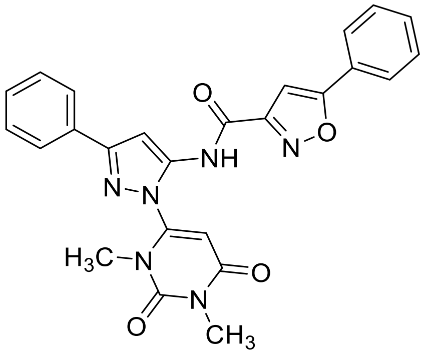

The compound was prepared according to General procedure I using Et_3N (20 µL, 0.14 mmol), 6-(5-amino-3-phenyl-1*H*-pyrazol-1-yl)-1,3-dimethylpyrimidine-2,4(1*H*,3*H*)-dione (**S150**, 30 mg, 0.10 mmol), acetonitrile (1 mL + 0.4 mL) and 5-phenylisoxazole-3-carbonyl chloride (**S177**, 29 mg, 0.14 mmol). The reaction mixture was cooled to room temperature, quenched with saturated aqueous solution of $NaHCO_3$ (3 mL) and extracted with EtOAc (3 × 15 mL). The combined organic extracts were washed with water (3 × 10 mL), brine (10 mL), dried over $MgSO_4$, filtered, and the solvent evaporated *in vacuo*. The residue was purified by preparative TLC (hexane:EtOAc, 7:3). The product was obtained as a white solid (10 mg, 21%).

^{1}H NMR (300 MHz, Chloroform-*d*) δ (ppm) 8.92 (s, 1H), 7.89 – 7.83 (m, 2H), 7.83 – 7.75 (m, 2H), 7.56 – 7.37 (m, 6H), 7.04 (s, 1H), 6.99 (s, 1H), 5.88 (s, 1H), 3.42 (s, 3H), 3.38 (s, 3H).

HRMS (APCI): calcd. for $C_{25}H_{21}N_6O_4$ $[M+H]^+$= 469.1619, found $[M+H]^+$= 469.1618.

N-(1-(6-(benzyloxy)pyridin-2-yl)-3-phenyl-1*H*-pyrazol-5-yl)-5-phenylisoxazole-3-carboxamide **(137)**

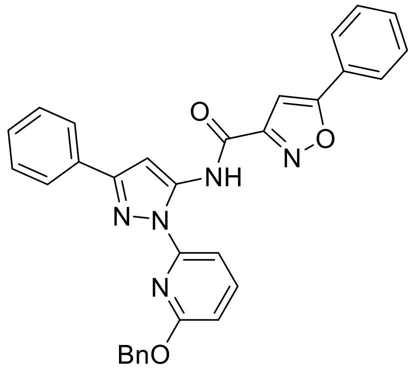

The compound was prepared according to General procedure I using Et_3N (41 µL, 0.29 mmol), 1-(6-(benzyloxy)pyridin-2-yl)-3-phenyl-1*H*-pyrazol-5-amine (**S152**, 50 mg, 0.15 mmol), acetonitrile (4 mL) and 5-phenylisoxazole-3-carbonyl chloride (**S177**, 39 mg, 0.19 mmol). The reaction mixture was refluxed for 3 h. The crude product was purified by column chromatography on silica gel (hexane:EtOAc, 8:2). The product was obtained as a white solid (50 mg, 67%).

^{1}H NMR (500 MHz, Chloroform-*d*) δ (ppm) 12.39 (s, 1H), 8.00 – 7.95 (m, 2H), 7.85 – 7.80 (m, 2H), 7.79 – 7.75 (m, 2H), 7.64 – 7.58 (m, 2H), 7.54 – 7.48 (m, 4H), 7.48 – 7.41 (m, 4H), 7.40 – 7.35 (m, 2H), 7.08 (s, 1H), 6.81 – 6.74 (m, 1H), 5.70 (s, 2H).

^{13}C NMR (126 MHz, Chloroform-*d*) δ (ppm) 172.17, 162.72, 159.05, 155.57, 152.57, 152.11, 141.70, 138.72, 136.77, 132.76, 131.07, 129.35, 128.80, 128.75, 128.62, 128.13, 128.11, 126.77, 126.22, 126.11, 108.25, 106.98, 99.53, 96.30, 69.53.

HRMS (APCI): calcd. for $C_{31}H_{24}N_5O_3$ [M+H]$^+$= 514.1874, found [M+H]$^+$= 514.1878.

N-(3-(furan-2-yl)-1-(6-oxo-4-(trifluoromethyl)-1,6-dihydropyrimidin-2-yl)-1*H*-pyrazol-5-yl)-5-phenylisoxazole-3-carboxamide **(138)**

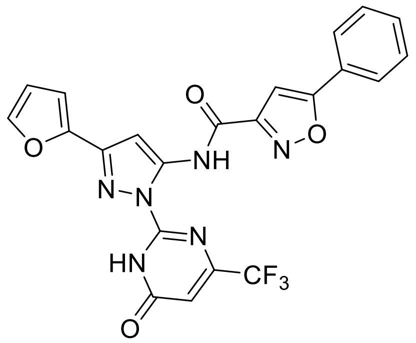

The compound was prepared according to General procedure I using Et_3N (8 µL, 0.06 mmol), 2-(5-amino-3-(furan-2-yl)-1*H*-pyrazol-1-yl)-6-(trifluoromethyl)pyrimidin-4(3*H*)-one (**S153**, 15 mg, 0.06 mmol), acetonitrile (0.8+ 0.3 mL) and 5-phenylisoxazole-3-carbonyl chloride (**S177**, 12 mg, 0.06 mmol). The product was obtained as an off-white solid (23 mg, 65%).

^{1}H NMR (300 MHz, DMSO-d_6) δ (ppm) 13.39 (s, 2H), 8.02 (dd, *J* = 7.2, 2.5 Hz, 2H), 7.83 (d, *J* = 1.7 Hz, 1H), 7.71 (s, 1H), 7.59 (dd, *J* = 5.2, 2.0 Hz, 3H), 7.22 (s, 1H), 7.05 (d, *J* = 3.4 Hz, 1H), 6.66 (dd, *J* = 3.4, 1.8 Hz, 2H).

^{13}C NMR (126 MHz, DMSO-d_6) δ (ppm) 171.2, 170.5, 159.0, 158.6, 154.5, 151.23, 151.49, 147.7, 143.2, 143.2, 139.9, 131.0, 129.4, 126.2, 125.8, 111.7, 107.8, 106.4, 99.6, 93.8.

^{19}F NMR (471 MHz, DMSO-d_6) δ (ppm) -69.13.

HRMS (ESI): calcd. for $C_{22}H_{12}F_3N_6O_4$ [M-H]$^-$= 481.0878, found [M-H]$^-$= 481.0875.

mp >280 °C (dec.)

5-Methyl-*N*-(3-phenyl-1-(pyrimidin-2-yl)-1*H*-pyrazol-5-yl)isoxazole-3-carboxamide **(139)**

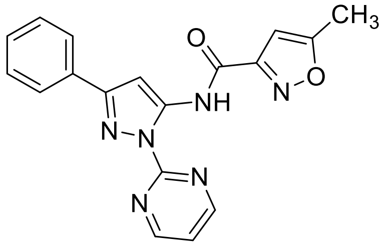

The compound was prepared according to General procedure I using Et_3N (12 µL, 0.08 mmol), 3-phenyl-1-(pyrimidin-2-yl)-1*H*-pyrazol-5-amine (**S145**, 20 mg, 0.08 mmol), acetonitrile (0.8+ 0.3 mL) and 5-methylisoxazole-3-carbonyl chloride (12 mg, 0.08 mmol). The reaction mixture was cooled to room

temperature, quenched with saturated aqueous solution of $NaHCO_3$ (1 mL) and the solvents were evaporated *in vacuo*. The residue was purified by column chromatography on silica gel. (dichloromethane:MeOH, 98:2). The product was obtained as a light brown solid (16 mg, 55%).

^{1}H NMR (500 MHz, Chloroform-*d*) δ (ppm) 13.02 (s, 1H), 8.90 (d, J = 4.8 Hz, 2H), 8.09 – 7.95 (m, 2H), 7.48 (s, 1H), 7.46 – 7.41 (m, 2H), 7.41 – 7.36 (m, 1H), 7.26 (t, 2H, merged with $CHCl_3$ solvent residual peak), 6.58 (d, J = 1.1 Hz, 1H), 2.54 (d, J = 0.9 Hz, 3H).

^{13}C NMR (126 MHz, Chloroform-*d*) δ (ppm) 172.0, 158.8, 157.8, 155.7, 154.6, 140.5, 132.2, 129.2, 128.7, 126.8, 118.0, 101.8, 96.7, 12.6.

HRMS (APCI): calcd. for $C_{18}H_{15}N_6O_2$ $[M+H]^+$= 347.1251, found $[M+H]^+$= 347.1255.

mp >218 °C (dec.)

<u>1-methyl-*N*-(1-(4-methyl-6-oxo-1,6-dihydropyrimidin-2-yl)-3-phenyl-1*H*-pyrazol-5-yl)-5-phenyl-1*H*-pyrazole-3-carboxamide</u> **(140)**

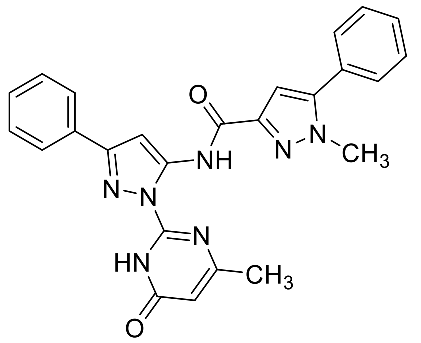

HATU (43 mg, 0.11 mmol) and DIPEA (39 µL, 0.22 mmol) were added to a solution of 1-methyl-5-phenyl-1*H*-pyrazole-3-carboxylic acid (25 mg, 0.11 mmol) in DMF (2 mL) and the mixture was stirred at room temperature for 20 min. Then, 2-(5-amino-3-phenyl-1*H*-pyrazol-1-yl)-6-methylpyrimidin-4(3*H*)-one (**S137**, 30 mg, 0.11 mmol) was added and the reaction mixture was stirred at room temperature for 16 h. The solvents were removed *in vacuo* and the residue was purified by column chromatography on silica gel (dichloromethane:MeOH, 98.5:1.5). The product was obtained as a white solid (4 mg, 8%).

^{1}H NMR (500 MHz, Chloroform-*d*) δ (ppm) 12.81 (s, 1H), 7.96 – 7.85 (m, 2H), 7.63 – 7.34 (m, 10H), 6.98 (s, 1H), 6.16 (s, 1H), 3.97 (s, 3H), 2.54 (s, 3H).

^{13}C NMR (126 MHz, Chloroform-*d*) δ (ppm) 159.0, 155.1, 148.6, 146.3, 144.8, 141.7, 131.3, 129.9, 129.7, 129.4, 129.1, 129.0, 126.6, 109.3, 107.6, 95.9, 38.3, 23.8.

HRMS (APCI): calcd. for $C_{25}H_{22}N_7O_2$ $[M+H]^+$= 452.1829, found $[M+H]^+$= 452.1826.

mp >215 °C (dec.)

<u>*N*-(1-(4-methyl-6-oxo-1,6-dihydropyrimidin-2-yl)-3-phenyl-1*H*-pyrazol-5-yl)-2-(5-phenylisoxazol-3-yl)acetamide</u> **(141)**

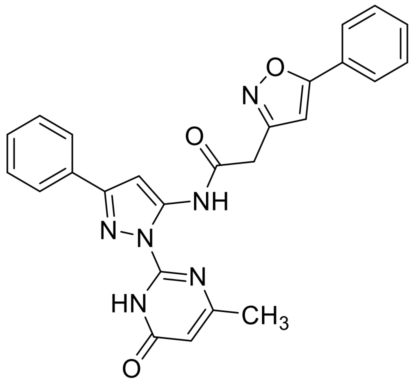

The compound was prepared according to General procedure I using Et_3N (15 µL, 0.11 mmol), 2-(5-amino-3-phenyl-1*H*-pyrazol-1-yl)-6-methylpyrimidin-4(3*H*)-one (**S137**, 20 mg, 0.08 mmol), acetonitrile (1 mL+ 0.5 mL), and 2-(5-phenylisoxazol-3-yl)acetyl chloride (**S181**, 23 mg, 0.11 mmol). The reaction mixture was refluxed for 2 h, and then cooled to room temperature and saturated aqueous $NaHCO_3$ solution (3 mL) was added. The resulting solid was collected by filtration, washed with water (3 mL), and purified by preparative TLC (dichloromethane:MeOH, 94:6). The product was obtained as a light brown solid (8 mg, 24%).

^{1}H NMR (300 MHz, DMSO-d_6) δ (ppm) 13.70 (s, 1H), 8.00 – 7.79 (m, 4H), 7.61 – 7.32 (m, 7H), 7.10 (s, 1H), 5.81 (s, 1H), 3.98 (s, 2H), 2.15 (s, 3H).

^{13}C NMR (176 MHz, DMSO-d_6) δ 169.25, 164.30, 158.85, 149.91, 140.66, 132.70, 130.30, 129.17, 128.59, 128.20, 126.86, 125.58, 107.19, 101.40, 93.36, 34.86, 23.00.

HRMS (APCI): calcd. for $C_{25}H_{21}N_6O_3$ $[M+H]^+$= 453.1670, found $[M+H]^+$= 453.1670.

mp = 185−188 °C.

Scheme S5: synthesis of **142**

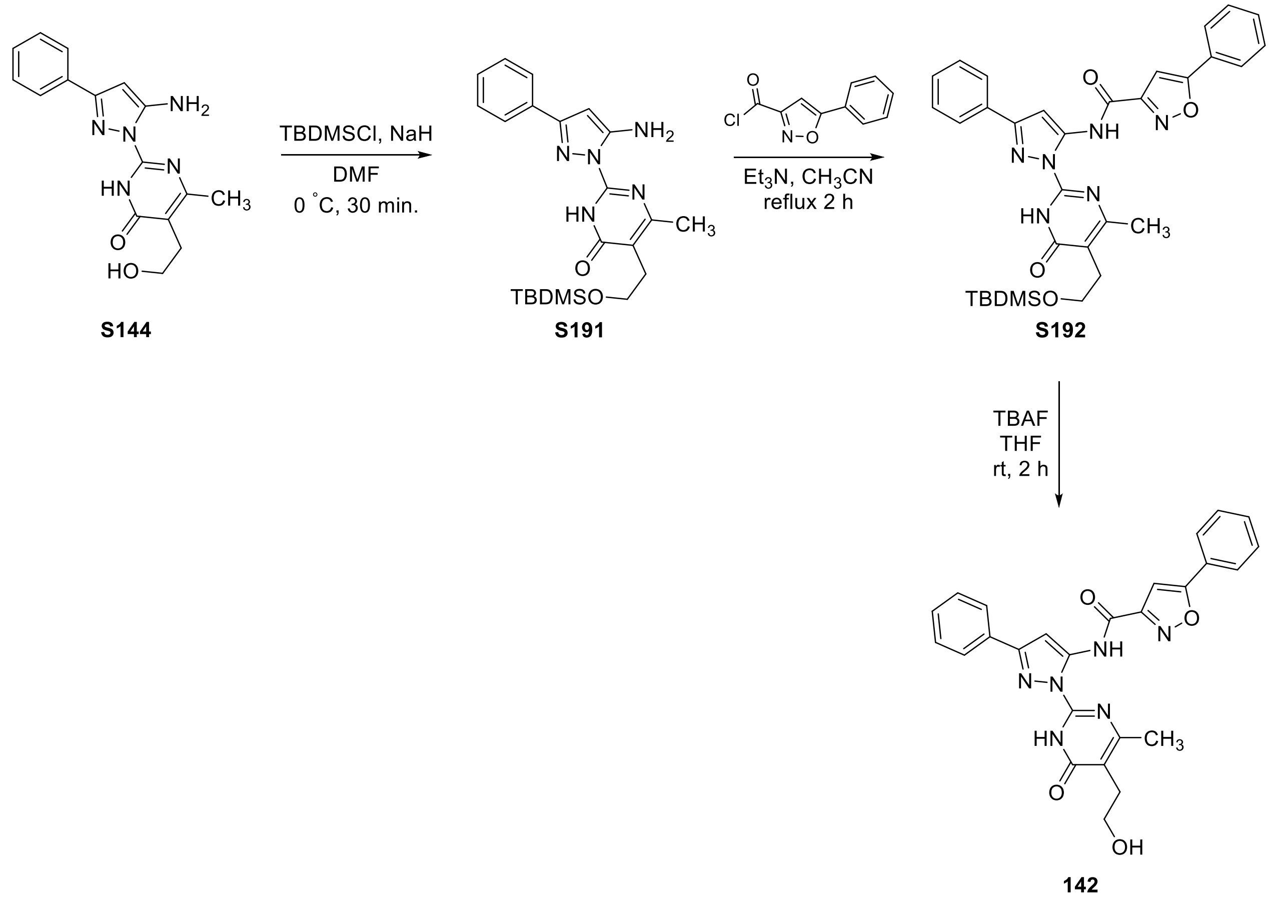

<u>2-(5-Amino-3-phenyl-1*H*-pyrazol-1-yl)-5-(2-((tert-butyldimethylsilyl)oxy)ethyl)-6-methylpyrimidin-4(3H)-one</u> **(S191)**

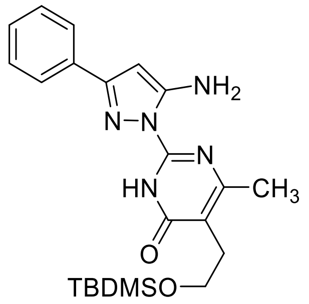

NaH (60% dispersion in mineral oil, 17 mg, 0.43 mmol) was added to a cooled (0 °C) solution of 2-(5-amino-3-phenyl-1*H*-pyrazol-1-yl)-5-(2-hydroxyethyl)-6-methylpyrimidin-4(3*H*)-one (**S144**, 30 mg, 0.1 mmol) in DMF (1 mL) and the reaction mixture was stirred at 0 °C for 15 min. Then, a solution of TBDMSCl (15 mg, 0.1 mmol) in DMF (0.5 mL) was added and the reaction mixture was stirred at 0 °C for 30 min. The reaction mixture was quenched with ice-cold water (10 mL) and extracted with EtOAc (3 × 10 mL). The combined organic extracts were washed with water (3 × 6 mL) brine (5 mL), dried over $MgSO_4$, filtered, and the solvent was evaporated *in vacuo*. The product was obtained as a light brown solid (30 mg, 73%) and used as such in the next step.

^{1}H NMR (500 MHz, Chloroform-*d*) δ (ppm) 7.78 (dt, J = 6.5, 1.5 Hz, 2H), 7.50 – 7.34 (m, 3H), 6.02 (s, 1H), 5.83 (s, 1H), 3.81 (t, J = 6.4 Hz, 2H), 2.78 (t, J = 6.4 Hz, 2H), 2.38 (s, 3H), 0.88 (s, 9H), 0.02 (s, 6H).

^{13}C NMR (126 MHz, Chloroform-*d*) δ (ppm) 129.4, 128.8, 126.3, 87.3, 61.7, 29.9, 29.5, 26.1, 18.5, -5.2.

HRMS (APCI) m/z: [M+H]$^+$ calcd for $C_{22}H_{32}N_5O_2Si$ 426.2320; found 426.2318.

N-(1-(5-(2-((*tert*-butyldimethylsilyl)oxy)ethyl)-4-methyl-6-oxo-1,6-dihydropyrimidin-2-yl)-3-phenyl-1*H*-pyrazol-5-yl)-5-phenylisoxazole-3-carboxamide (**S192**)

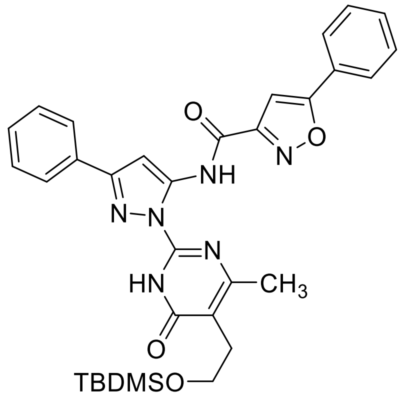

The compound was prepared according to General procedure I using Et_3N (7 µL, 0.05 mmol), 2-(5-amino-3-phenyl-1*H*-pyrazol-1-yl)-5-(2-((*tert*-butyldimethylsilyl)oxy)ethyl)-6-methylpyrimidin-4(3*H*)-one (**S191**, 20 mg, 0.05 mmol), acetonitrile (1 + 0.4 mL) and 5-phenylisoxazole-3-carbonyl chloride (**S177**, 10 mg, 0.05 mmol). The product was obtained as a white solid (17 mg) and used directly in the next step without further purification.

HRMS (APCI): calcd. for $C_{32}H_{37}N_6O_4Si$ [M+H]$^+$= 597.2640, found [M+H]$^+$= 597.2635.

N-(1-(5-(2-hydroxyethyl)-4-methyl-6-oxo-1,6-dihydropyrimidin-2-yl)-3-phenyl-1H-pyrazol-5-yl)-5-phenylisoxazole-3-carboxamide (**142**)

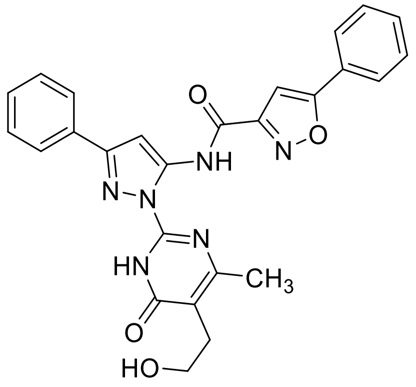

TBAF (1 M in THF, 0.14 mL, 0.14 mmol) was added to a solution of *N*-(1-(5-(2-((*tert*-butyldimethylsilyl)oxy)ethyl)-4-methyl-6-oxo-1,6-dihydropyrimidin-2-yl)-3-phenyl-1*H*-pyrazol-5-yl)-5-phenylisoxazole-3-carboxamide (**S192**, 15 mg, 0.03 mmol) in THF (0.5 mL) and the reaction mixture

was stirred at room temperature for 2 h. The solvent was evaporated *in vacuo* and the residue was purified by column chromatography on silica gel (dichloromethane:MeOH, 97:3). The product was obtained as a white solid (9 mg, 40%, over the 2 steps).

^{1}H NMR (500 MHz, DMSO-d_6) δ (ppm) 13.42 (s, 1H), 12.81 (s, 1H), 8.08 – 8.00 (m, 4H), 7.64 (s, 1H), 7.62 – 7.56 (m, 3H), 7.52 – 7.46 (m, 2H), 7.46 – 7.41 (m, 1H), 7.38 (s, 1H), 4.67 (s, 2H), 3.56 (t, J = 6.9 Hz, 2H), 2.69 (t, J = 6.9 Hz, 2H), 2.57 (s, 3H).

^{13}C NMR (126 MHz, DMSO-d_6) δ (ppm) 171.8, 158.8, 154.7, 152.0, 139.8, 131.6, 131.1, 129.3, 129.0, 128.7, 126.1, 126.0, 100.0, 95.0, 79.2, 78.9, 78.7, 59.2, 29.0.

HRMS (APCI): calcd. for $C_{26}H_{23}N_6O_4$ $[M+H]^+$= 483.1775, found $[M+H]^+$= 483.1773.

mp >255 °C (dec.)

Scheme S6: synthesis of 143

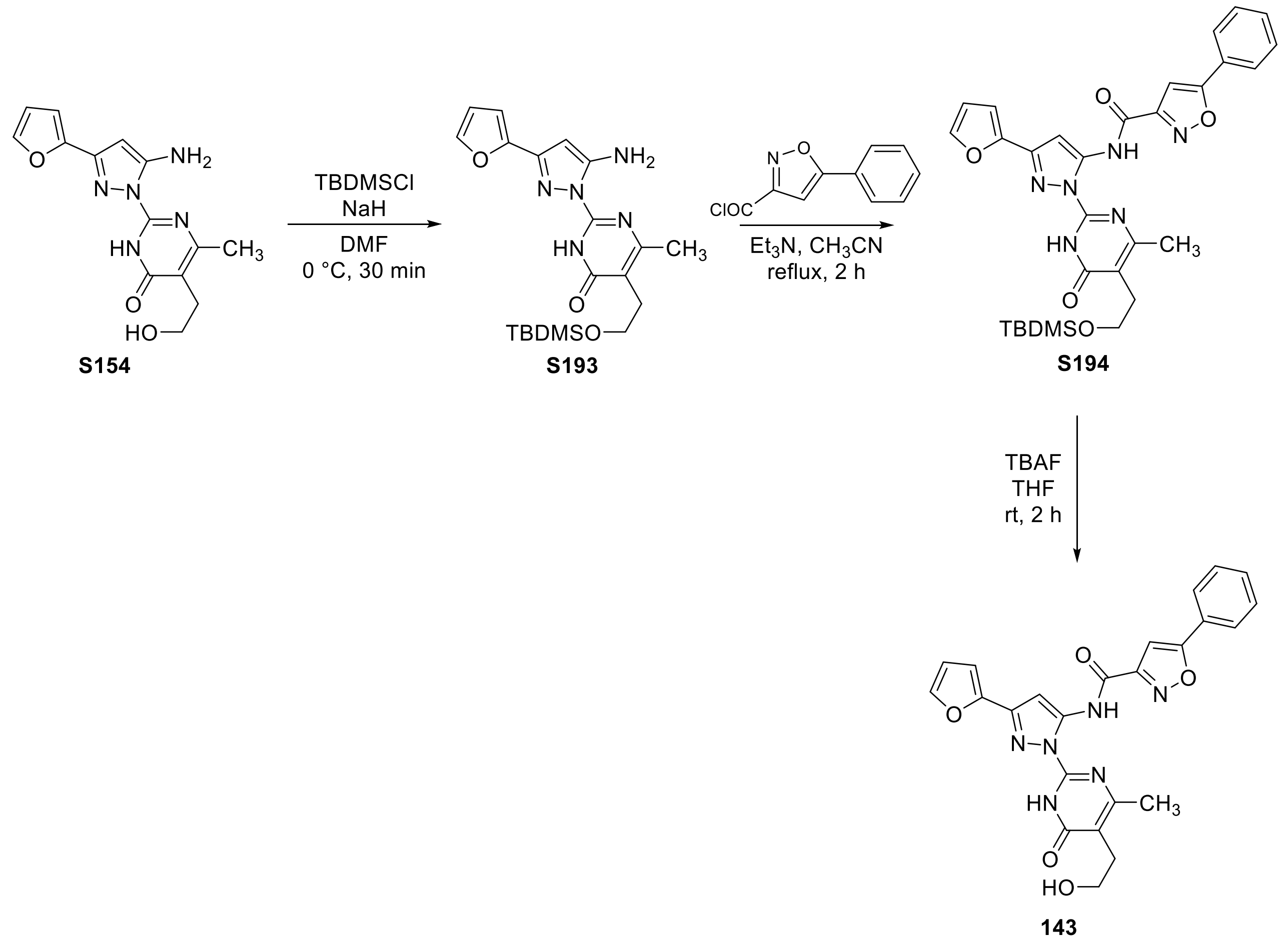

2-(5-Amino-3-(furan-2-yl)-1*H*-pyrazol-1-yl)-5-(2-((*tert*-butyldimethylsilyl)oxy)ethyl)-6-methylpyrimidin-4(3*H*)-one **(S193)**

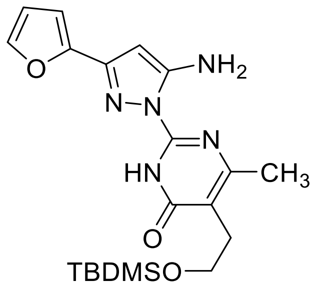

NaH (60% dispersion in mineral oil, 4 mg, 0.17 mmol) was added to a cooled (0 °C) solution of 2-(5-amino-3-(furan-2-yl)-1*H*-pyrazol-1-yl)-5-(2-hydroxyethyl)-6-methylpyrimidin-4(3*H*)-one (**S154**, 47 mg, 0.16 mmol) in DMF (2 mL) and the reaction mixture was stirred at 0 °C for 15 min. Then a solution of TBDMSCl (26 mg, 0.17 mmol) in DMF (0.4 mL) was added and the reaction mixture was stirred at 0 °C for 30 min. The reaction mixture was quenched with ice-cold water (5 mL) and extracted with EtOAc (3 × 10 mL). The combined organic extracts were washed with water (2 × 8 mL), brine (5 mL), dried over $MgSO_4$, filtered, and the solvent was evaporated *in vacuo*. The product was obtained as a light brown solid (31 mg, 48%).

^{1}H NMR (300 MHz, Chloroform-*d*) δ (ppm) 7.49 (dd, J = 1.8, 0.8 Hz, 1H), 6.79 (d, J = 3.4 Hz, 1H), 6.50 (dd, J = 3.4, 1.8 Hz, 1H), 5.76 (s, 1H), 3.81 (t, J = 6.3 Hz, 2H), 2.77 (t, J = 6.3 Hz, 2H), 2.38 (s, 3H), 0.87 (s, 9H), 0.01 (s, 6H).

^{13}C NMR (126 MHz, Chloroform-*d*) δ (ppm) 149.8, 147.7, 147.6, 146.6, 146.4, 143.2, 143.1, 111.8, 111.8, 108.7, 108.5, 87.0, 61.7, 29.5, 26.1, 22.2, 18.5, -5.2.

HRMS (APCI): calcd. for $C_{20}H_{30}N_5O_3Si$ $[M+H]^+$= 416.2112, found $[M+H]^+$= 416.2110.

mp = 169−171 °C.

N-(1-(5-(2-((*tert*-butyldimethylsilyl)oxy)ethyl)-4-methyl-6-oxo-1,6-dihydropyrimidin-2-yl)-3-(furan-2-yl)-1*H*-pyrazol-5-yl)-5-phenylisoxazole-3-carboxamide (**S194**)

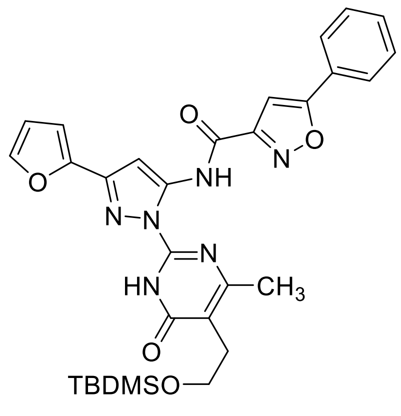

The compound was prepared according to General procedure I using Et_3N (8 µL, 0.06 mmol), 2-(5-amino-3-(furan-2-yl)-1*H*-pyrazol-1-yl)-5-(2-((*tert*-butyldimethylsilyl)oxy)ethyl)-6-methylpyrimidin-

4(3*H*)-one (**S193**, 20 mg, 0.05 mmol), acetonitrile (1 + 0.4 mL) and 5-phenylisoxazole-3-carbonyl chloride (12 mg, 0.06 mmol). The product was obtained as an off-white solid (18 mg, 64%).

^{1}H NMR (500 MHz, Chloroform-*d*) δ (ppm) 13.14 (s, 1H), 10.34 (s, 1H), 7.91 – 7.81 (m, 2H), 7.60 – 7.47 (m, 4H), 7.28 (s, 1H), 7.07 (s, 1H), 6.87 (d, *J* = 3.3 Hz, 1H), 6.53 (dd, *J* = 3.4, 1.8 Hz, 1H), 3.84 (t, *J* = 6.4 Hz, 2H), 2.82 (s, 2H), 2.60 (s, 3H), 0.88 (s, 9H), 0.04 (s, 6H).

^{13}C NMR (126 MHz, Chloroform-*d*) δ (ppm) 172.4, 161.2, 160.2, 158.6, 155.6, 146.8, 146.4, 146.0, 143.6, 140.0, 131.0, 129.2, 126.6, 126.0, 118.8, 111.8, 109.5, 99.1, 96.1, 61.4, 29.5, 26.0, 21.7, 18.3, -5.4.

HRMS (APCI): calcd. for $C_{30}H_{35}N_6O_5Si$ $[M+H]^+$= 587.2433, found $[M+H]^+$= 587.2429.

mp >220 °C (dec.)

<u>*N*-(3-(furan-2-yl)-1-(5-(2-hydroxyethyl)-4-methyl-6-oxo-1,6-dihydropyrimidin-2-yl)-1*H*-pyrazol-5-yl)-5-phenylisoxazole-3-carboxamide</u> (**143**)

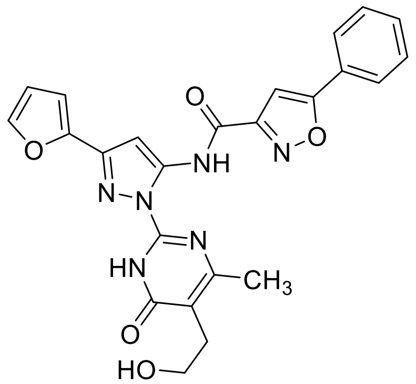

TBAF (1 M in THF, 128 µL, 0.128 mmol) was added to a solution of *N*-(1-(5-(2-((*tert*-butyldimethylsilyl)oxy)ethyl)-4-methyl-6-oxo-1,6-dihydropyrimidin-2-yl)-3-(furan-2-yl)-1*H*-pyrazol-5-yl)-5-phenylisoxazole-3-carboxamide (**S194**, 15 mg, 0.03 mmol) in THF (0.5 mL) and the reaction mixture was stirred at room temperature for 2 h. The solvent was evaporated *in vacuo* and the residue was purified by column chromatography on silica gel (dichloromethane:MeOH, 97:3). The product was obtained as a white solid (11 mg, 92%).

^{1}H NMR (300 MHz, DMSO-*d*$_6$) δ (ppm) 13.58 (s, 1H), 13.09 (s, 1H), 8.02 (dd, *J* = 6.6, 3.0 Hz, 2H), 7.83 (d, *J* = 1.7 Hz, 1H), 7.64 (s, 1H), 7.59 (dd, *J* = 5.0, 1.9 Hz, 3H), 7.22 (s, 1H), 7.02 (d, *J* = 3.4 Hz, 1H), 6.65 (dd, *J* = 3.4, 1.8 Hz, 1H), 4.69 (s, 1H), 3.57 (t, *J* = 6.8 Hz, 2H), 2.77 – 2.68 (m, 2H), 2.62 (s, 3H).

^{13}C NMR (75 MHz, DMSO-*d*$_6$) δ (ppm) 171.8, 158.8, 154.7, 147.1, 143.7, 139.5, 131.1, 129.3, 126.0, 125.9, 111.8, 108.7, 100.0, 94.5, 59.1.

HRMS (ESI): calcd. for $C_{24}H_{19}N_6O_5$ $[M-H]^-$= 471.1422, found $[M-H]^-$= 471.1419.

mp >265 °C (dec.)

^{1}H NMR, ^{13}C NMR and HRMS spectra of compound S1-S108

^{1}H (500 MHz) and ^{13}C NMR (126 MHz) spectra of **S1** in chloroform-*d*

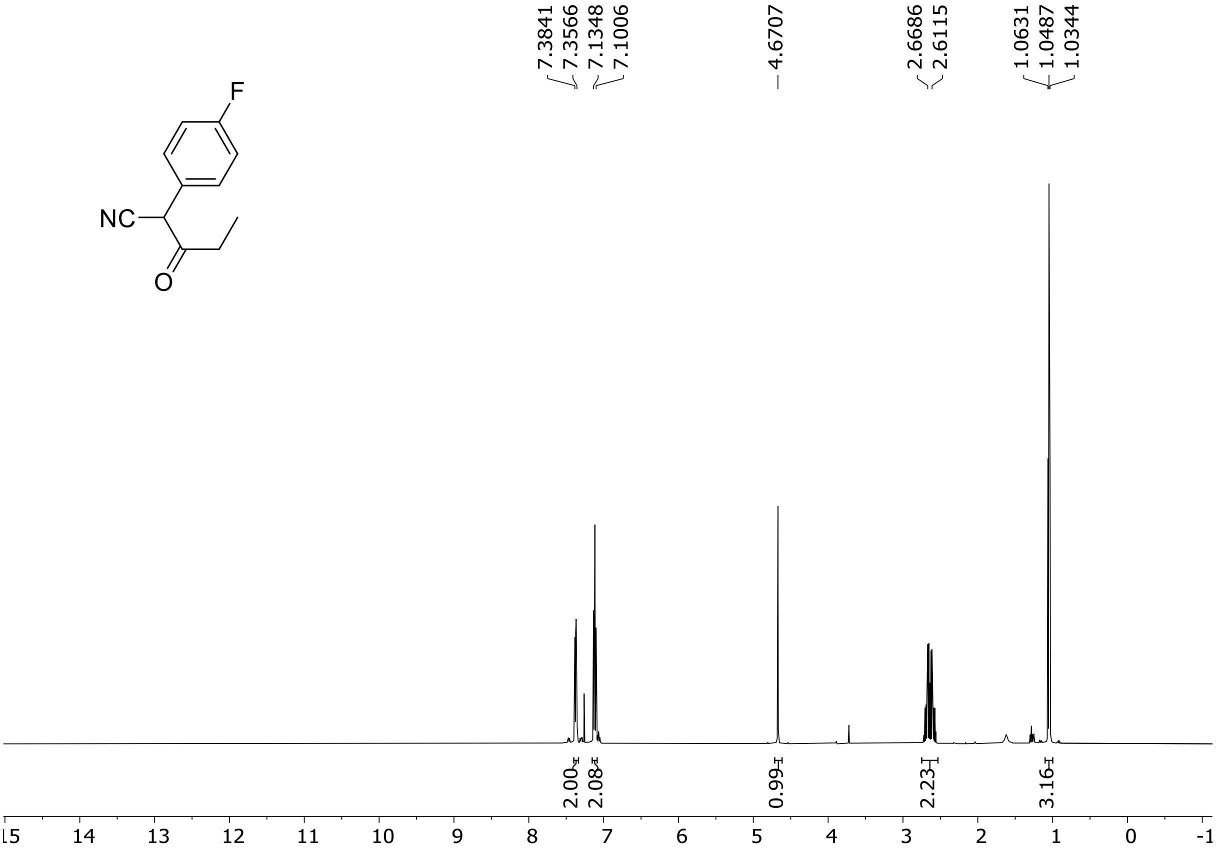

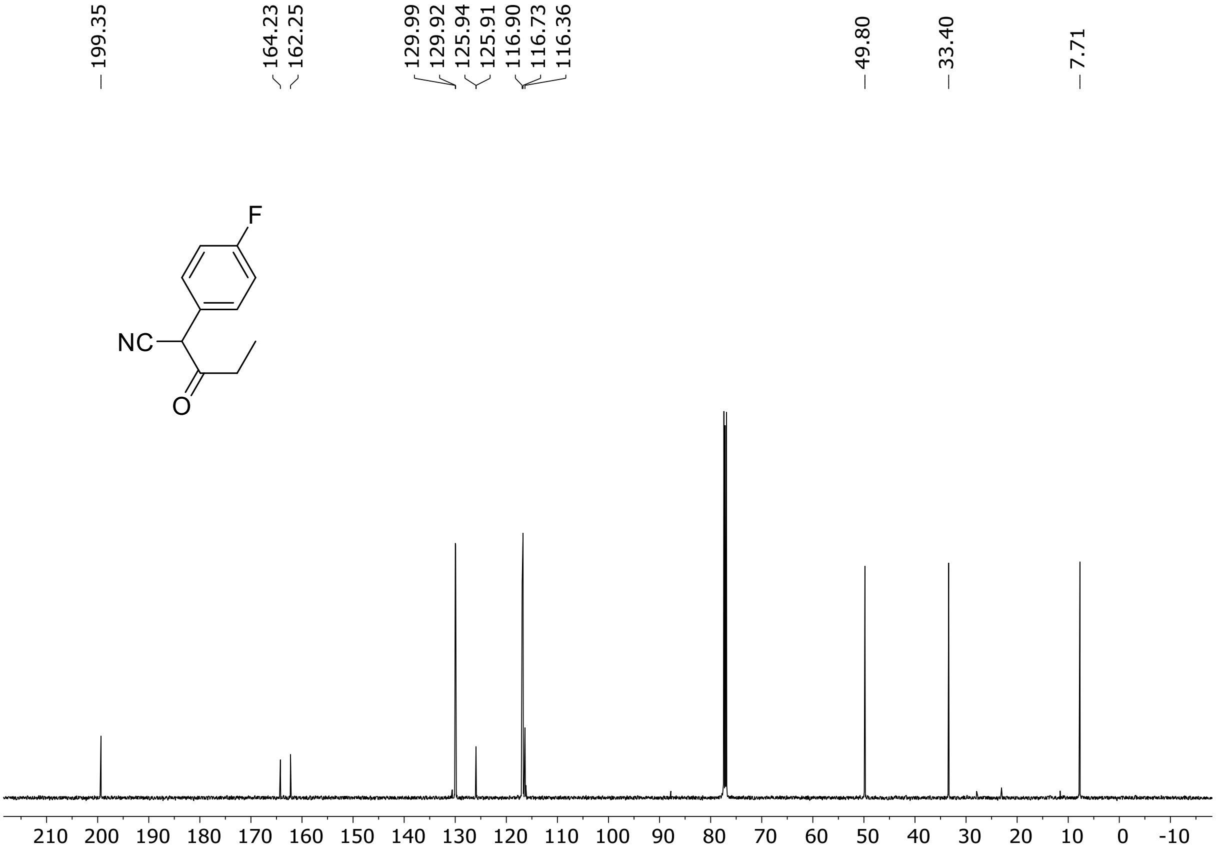

HRMS spectrum of **S1**

$C_{11}H_{10}FNO$ **mono m/z = 191,0746**

ESI - (MMI)
nitrogen flow 5 L/min, gas temperature 300°C, vaporizer 250°C,
nebulizer 45 psi, Vcap -2000 V, skimmer **65** V, fragmentor **40** V,
dissolved in MeOH

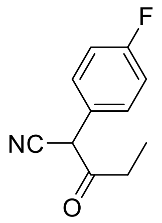

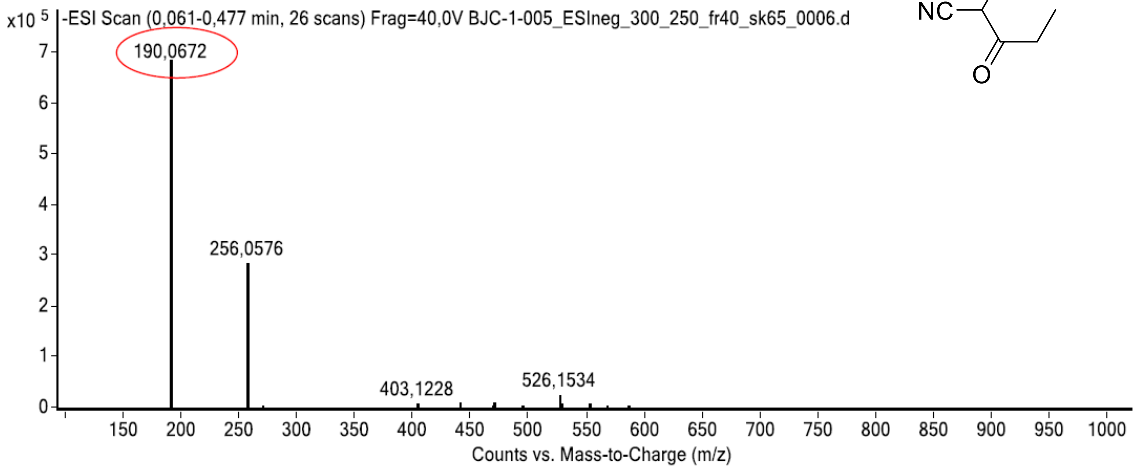

calculated mass:
[M-H]⁻ = 190,0674 observed: [M-H]⁻ = 190,0672 max. mass error = 1,1 ppm

^{1}H (500 MHz) and ^{13}C NMR (126 MHz) spectra of **S2** in chloroform-*d*

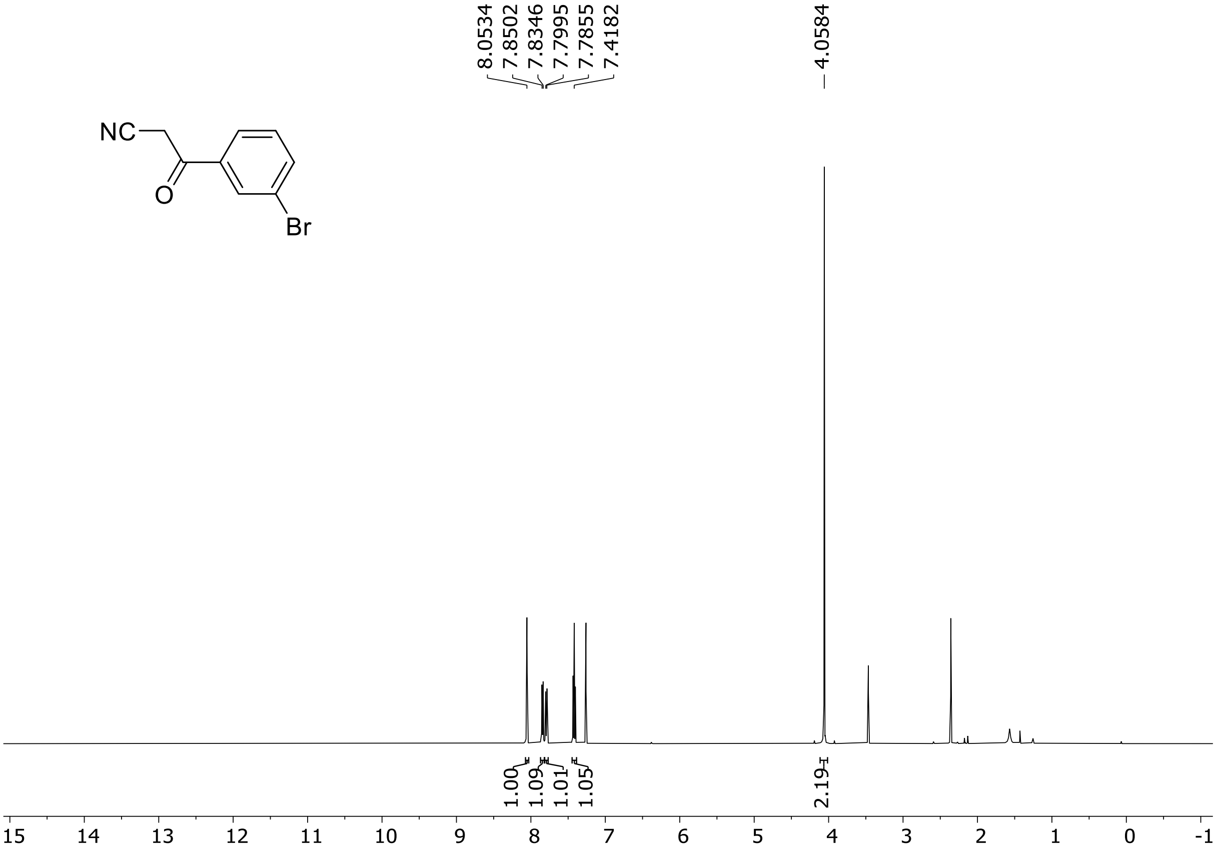

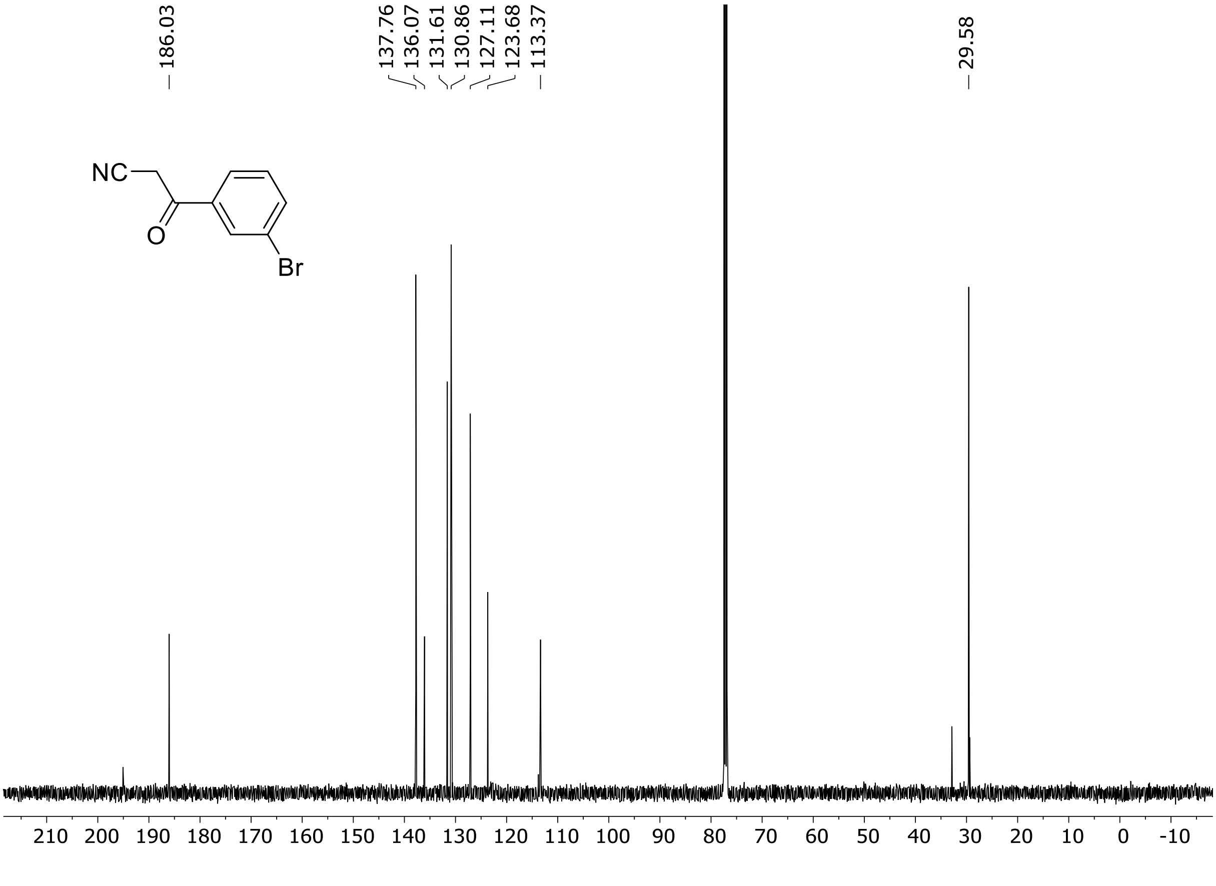

^{1}H (500 MHz) and ^{13}C NMR (126 MHz) spectra of **S3** in chloroform-*d*

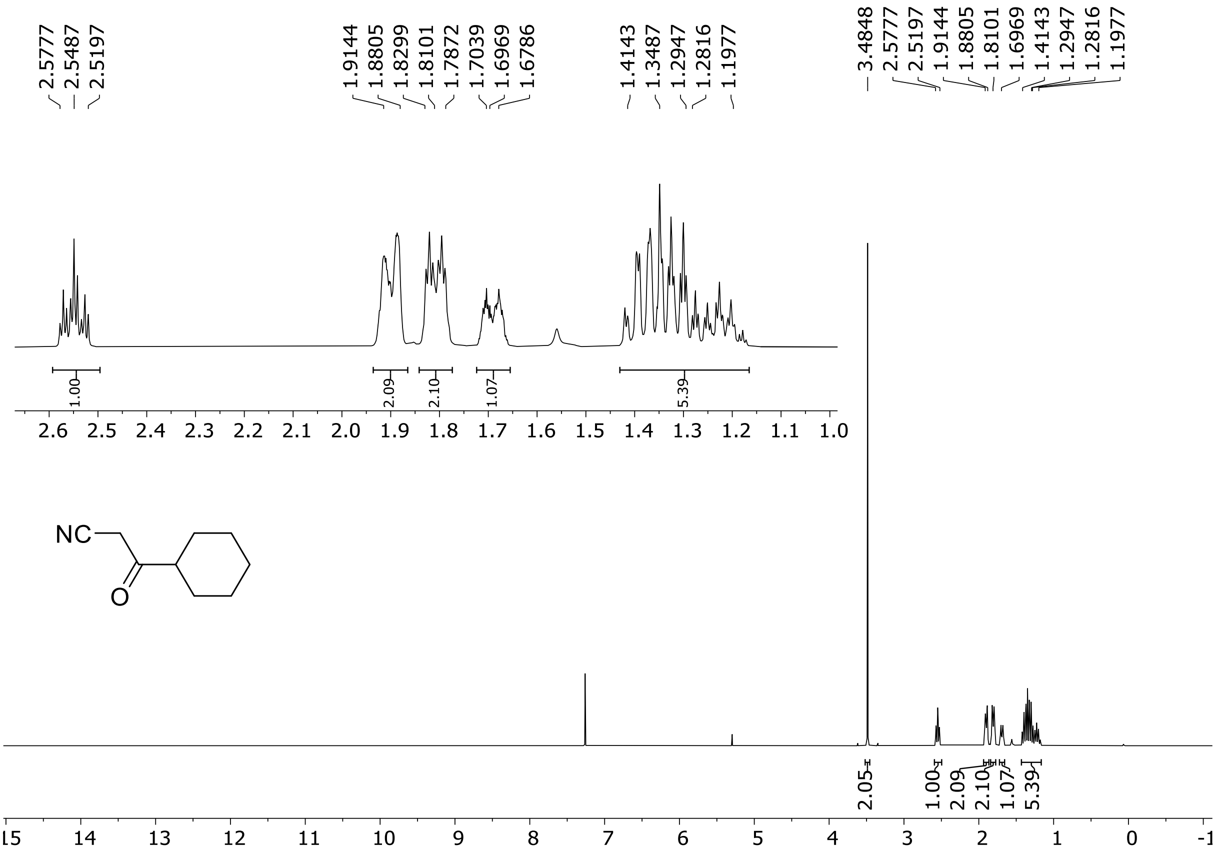

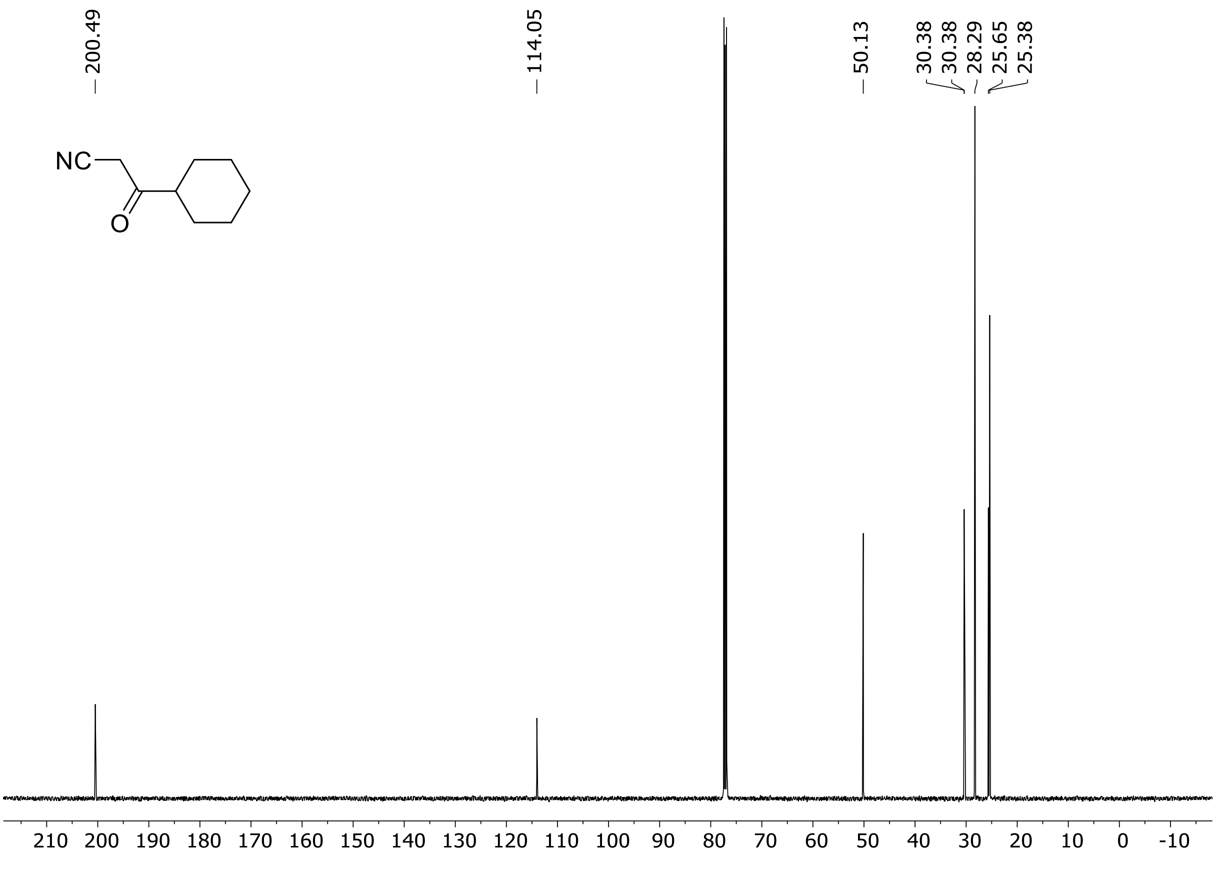

HRMS spectrum of **S3**

$C_9H_{13}NO$ **mono m/z = 151.0997**

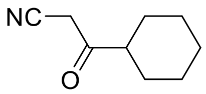

APCI + (MMI)
nitrogen flow 5 L/min, gas temperature 300°C, nebulizer 45 psi, skimmer 60 V, fragmentor 25 V, dissolved in methanol

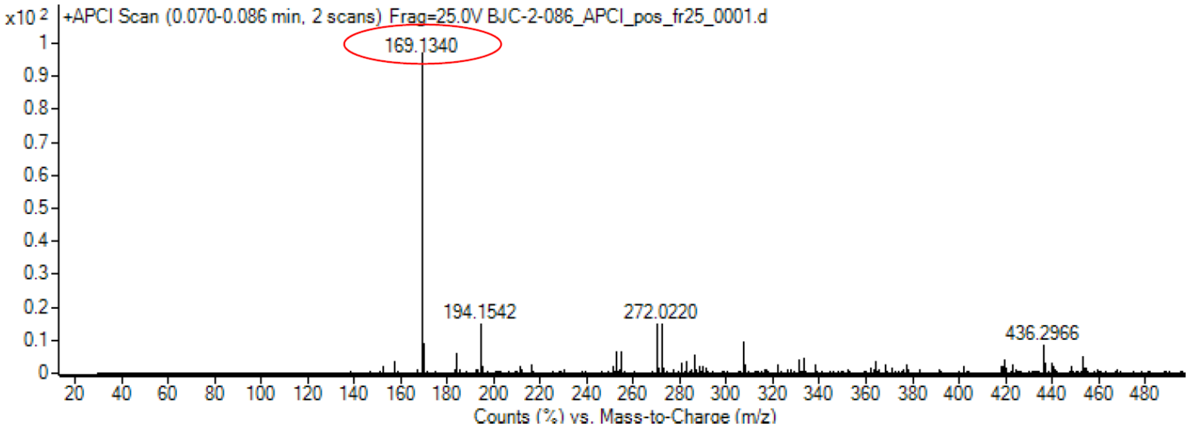

calculated mass: $[M+NH_4]^+$ = 169.1335 observed: $[M+H]^+$ = 169.1340 max. mass error = 2.9 ppm

FT-IR spectrum (neat) of **S3**

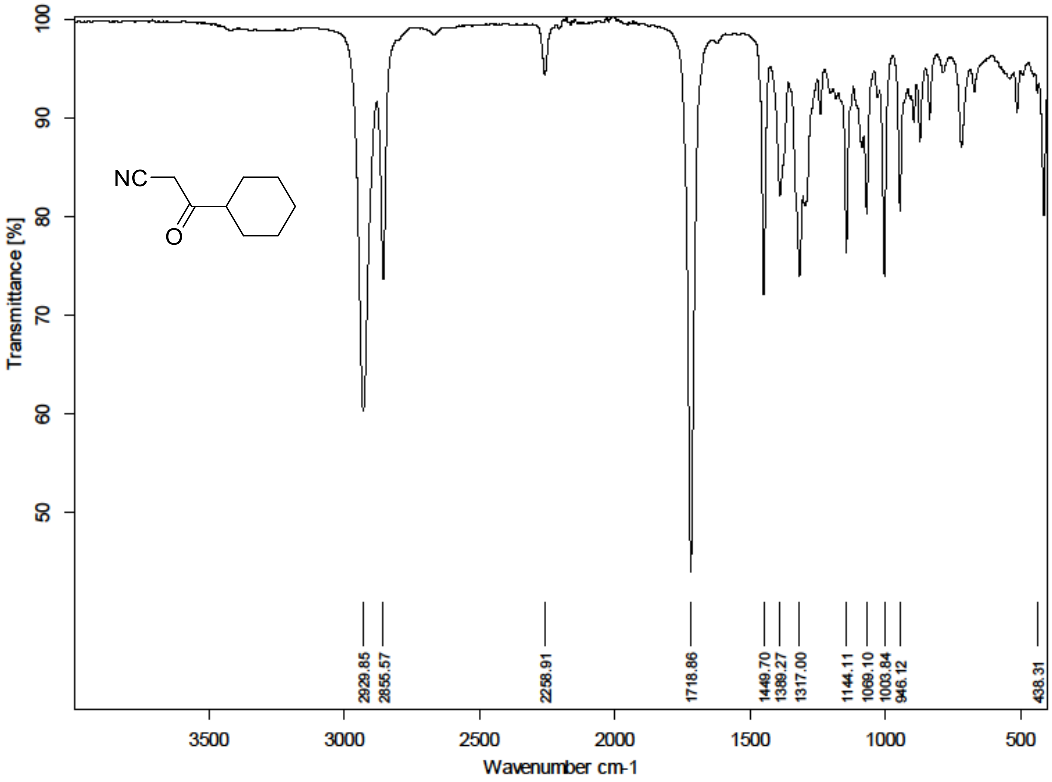

^{1}H (500 MHz) and ^{13}C NMR (126 MHz) spectra of **S4** in chloroform-*d*

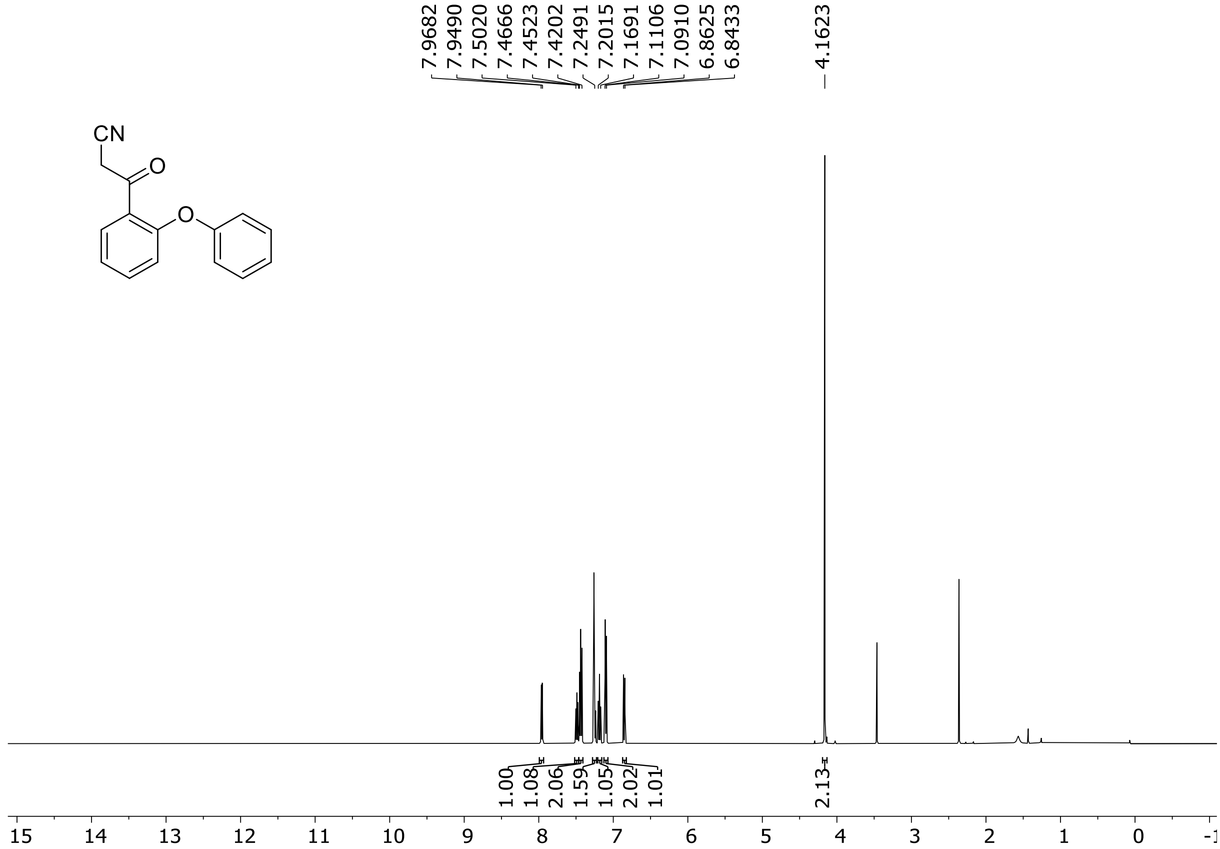

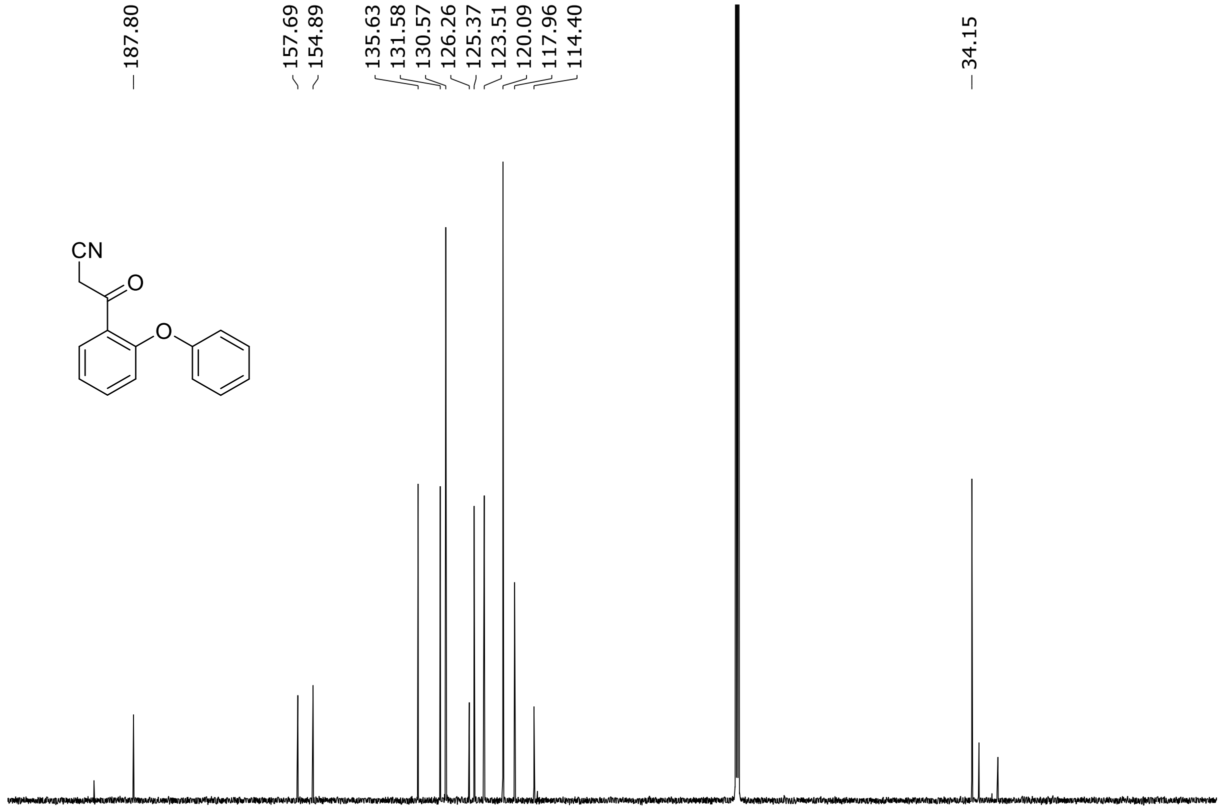

HRMS spectrum of **S4**

$C_{15}H_{11}NO_2$ **mono m/z = 237.079**

APCI + (MMI)
nitrogen flow 5 L/min, gas temperature 300°C, nebulizer 45 psi,
skimmer 60 V, fragmentor 23 V, dissolved in methanol

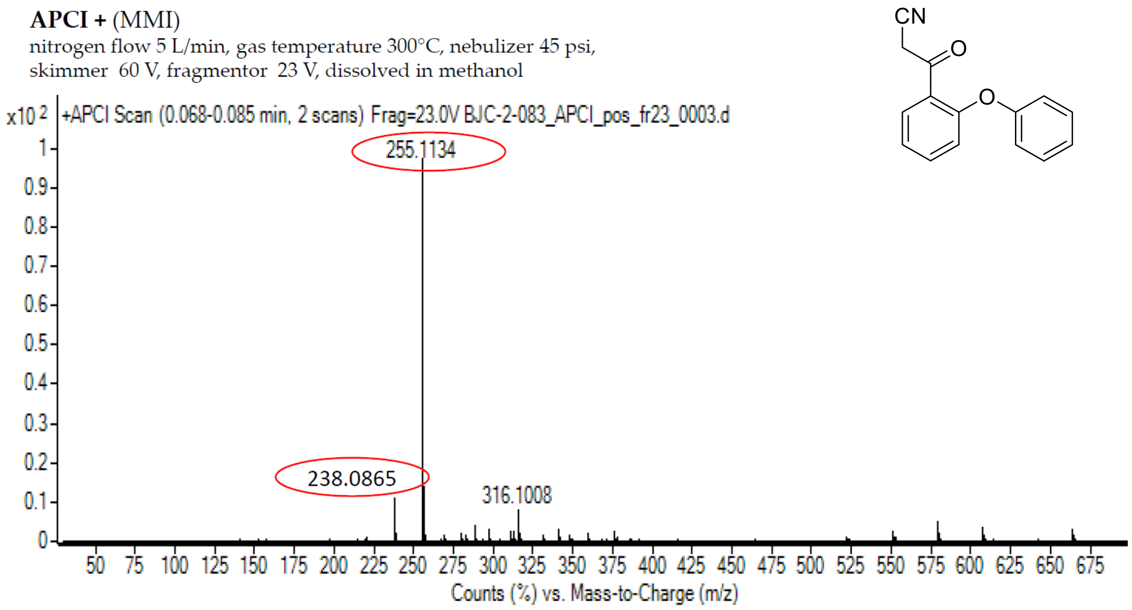

calculated mass: $[M+H]^+$ = 238.0863 observed: $[M+H]^+$ = 238.0865 max. mass error = 0.8 ppm

calculated mass: $[M+NH_4]^+$ = 255.1128 observed: $[M+NH_4]^+$ = 255.1134 max. mass error = 2.4 ppm

^{1}H (500 MHz) and ^{13}C NMR (126 MHz) spectra of **S5** in chloroform-*d*

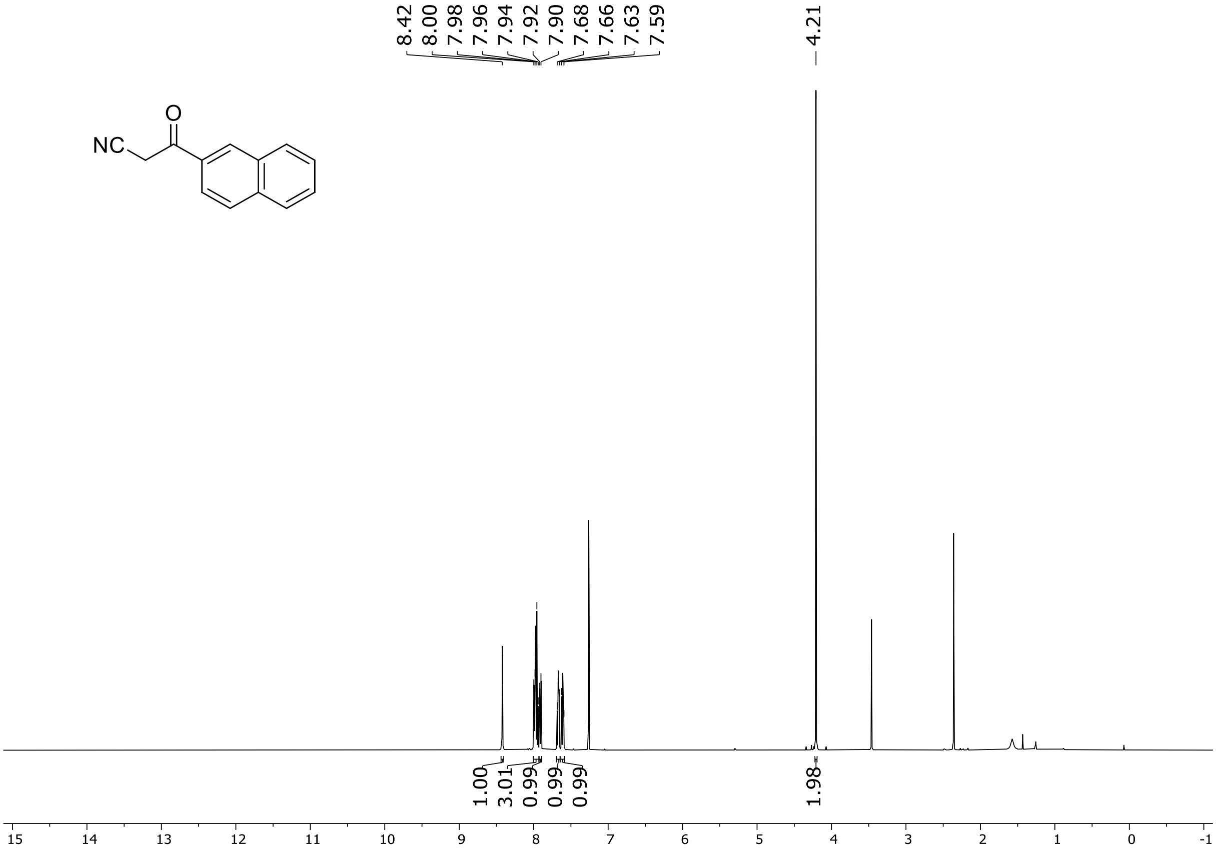

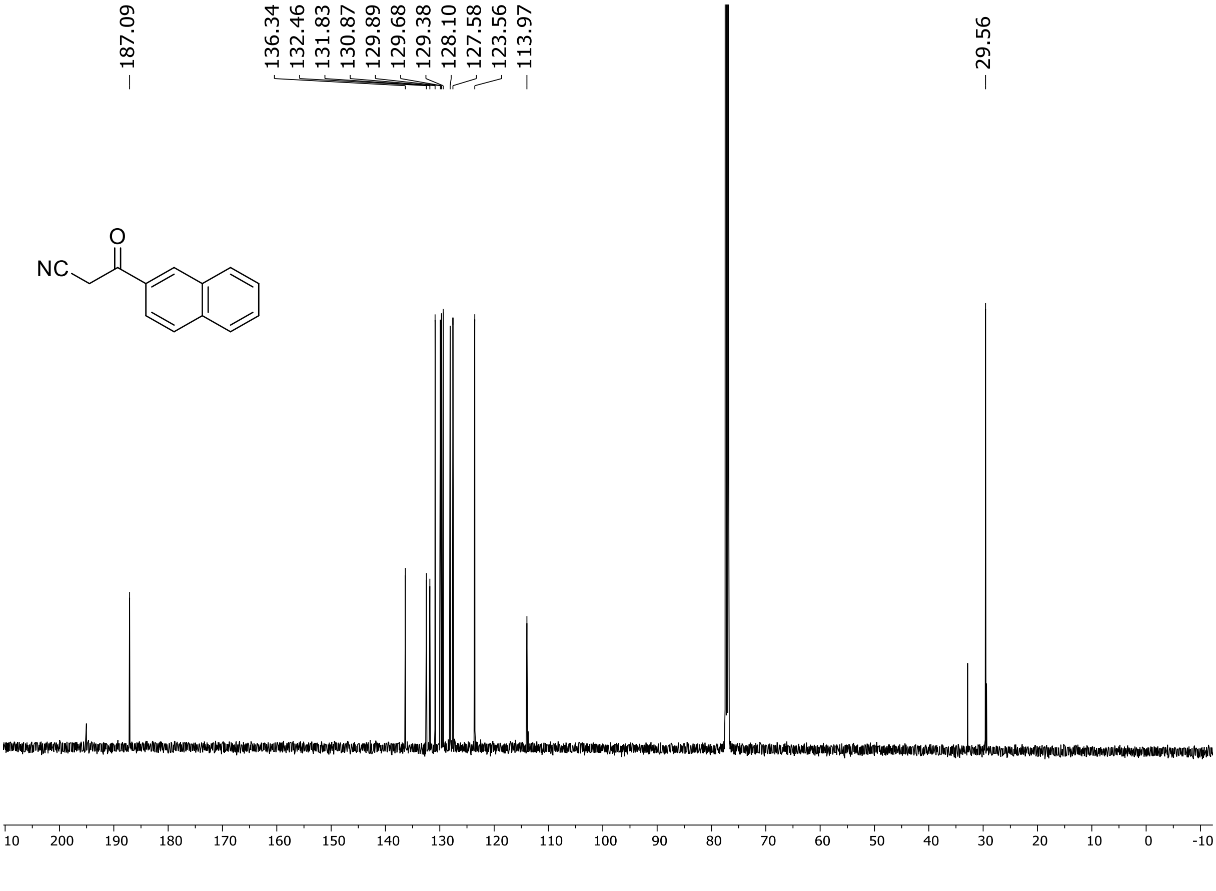

HRMS spectrum of **S5**

$C_{13}H_9NO$ mono m/z = 195.0684

APCI - (MMI)
nitrogen flow 5 L/min, gas temperature 300°C, nebulizer 45 psi,
skimmer 60 V, fragmentor 40 V, dissolved in methanol

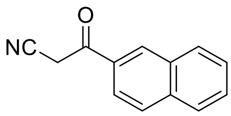

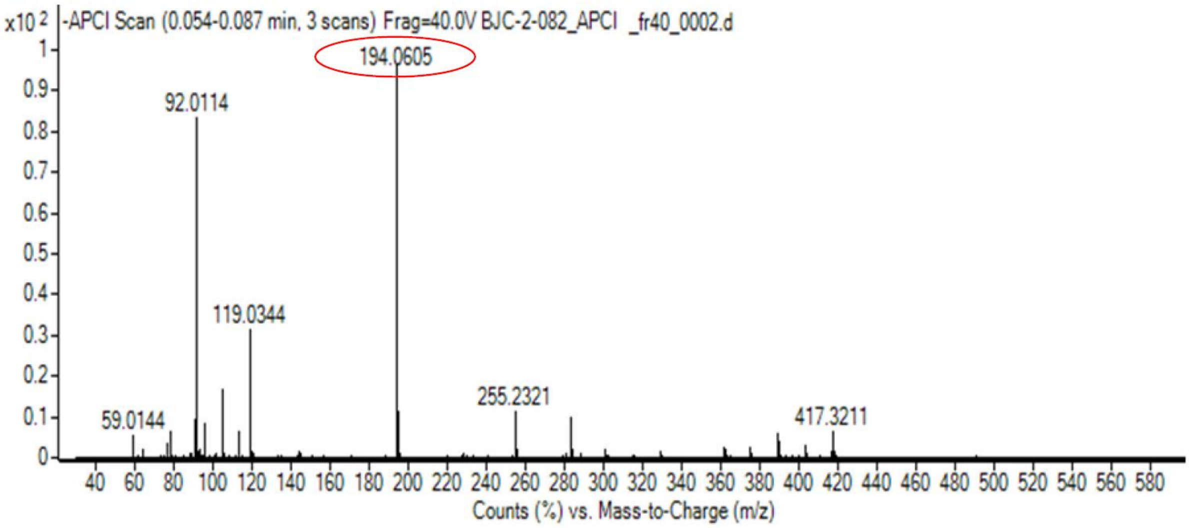

calculated mass: [M-H]$^-$ = 194.0611 observed: [M-H]$^-$ = 194.0605 max. mass error = 3.1 ppm

^{1}H (500 MHz) and ^{13}C NMR (126 MHz) spectra of **S7** in chloroform-*d*

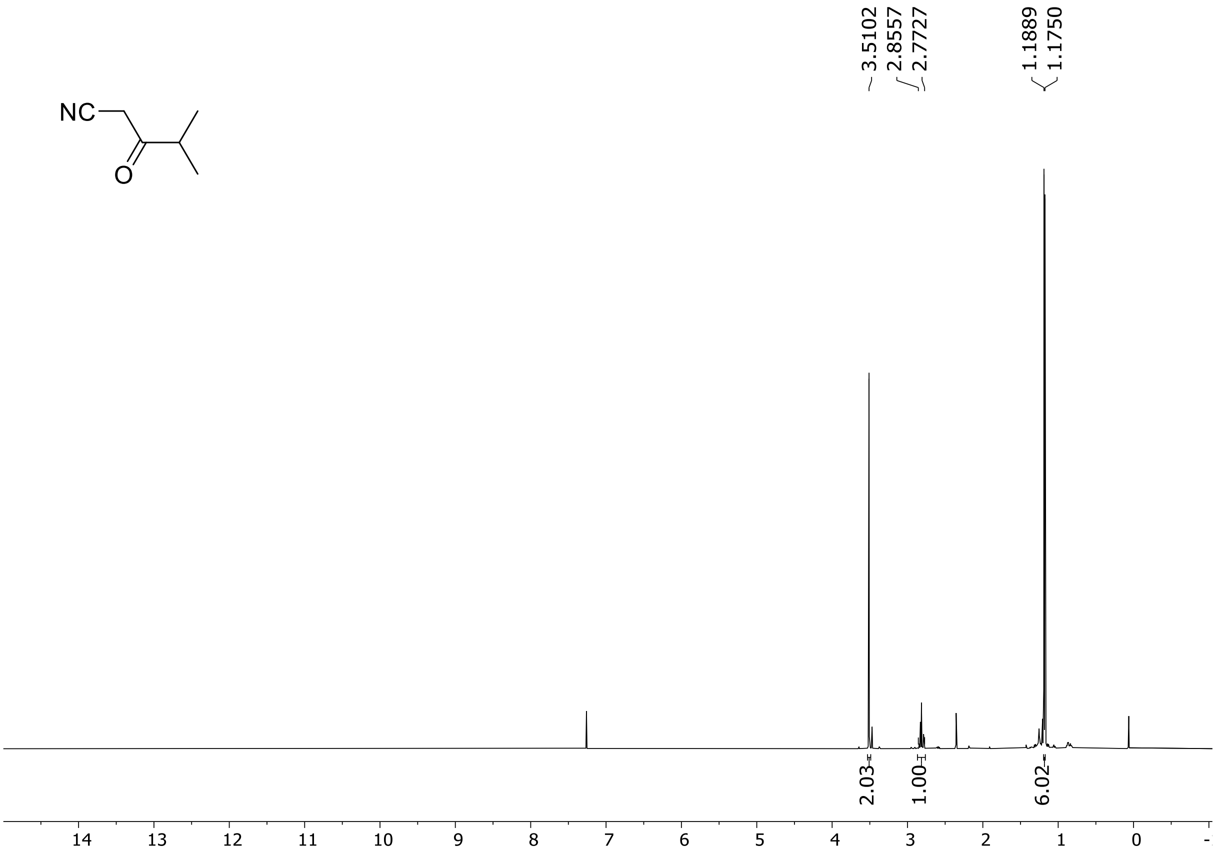

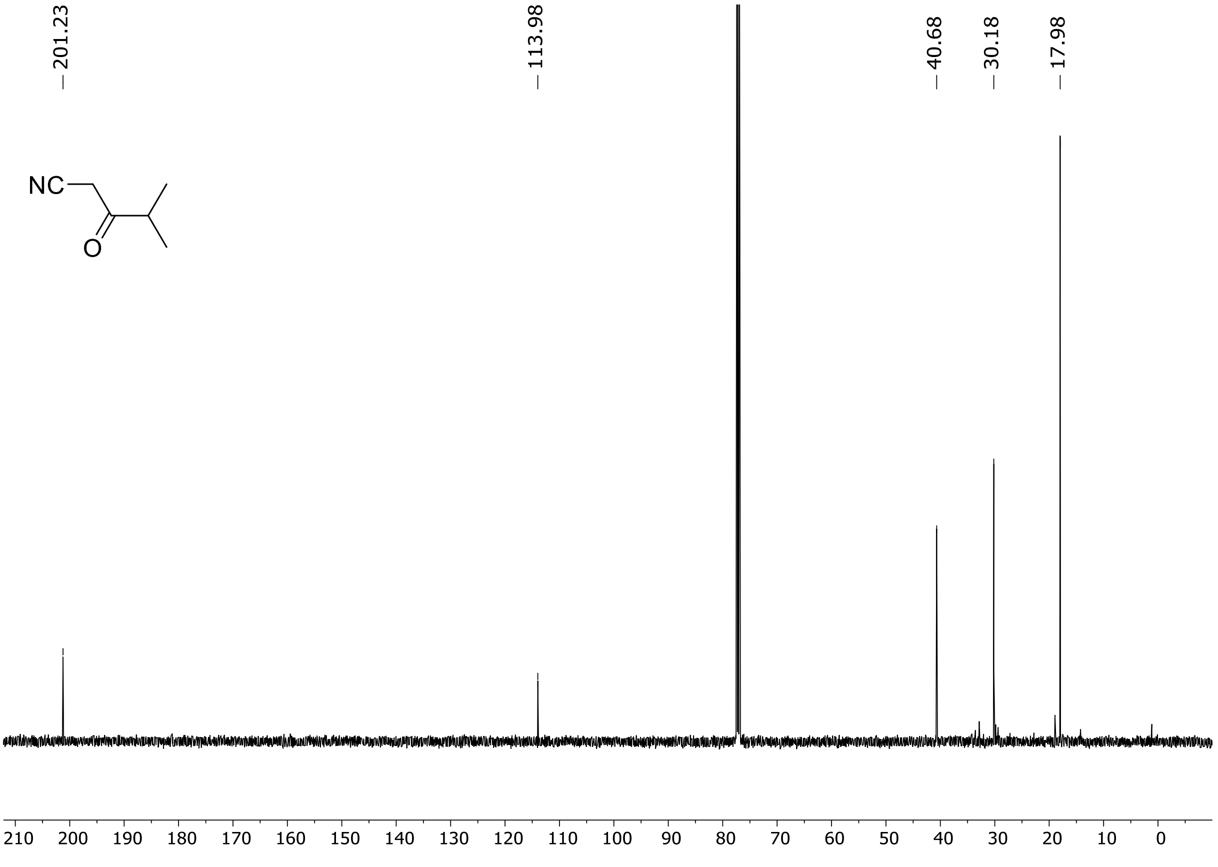

HRMS spectrum of **S7**

C_6H_9NO **mono m/z = 111.0684**

APCI - (MMI)
nitrogen flow 5 L/min, gas temperature 300°C, nebulizer 45 psi, vaporizer 200°C
skimmer **65** V, fragmentor **40** V, dissolved in methanol

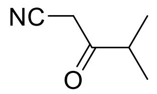

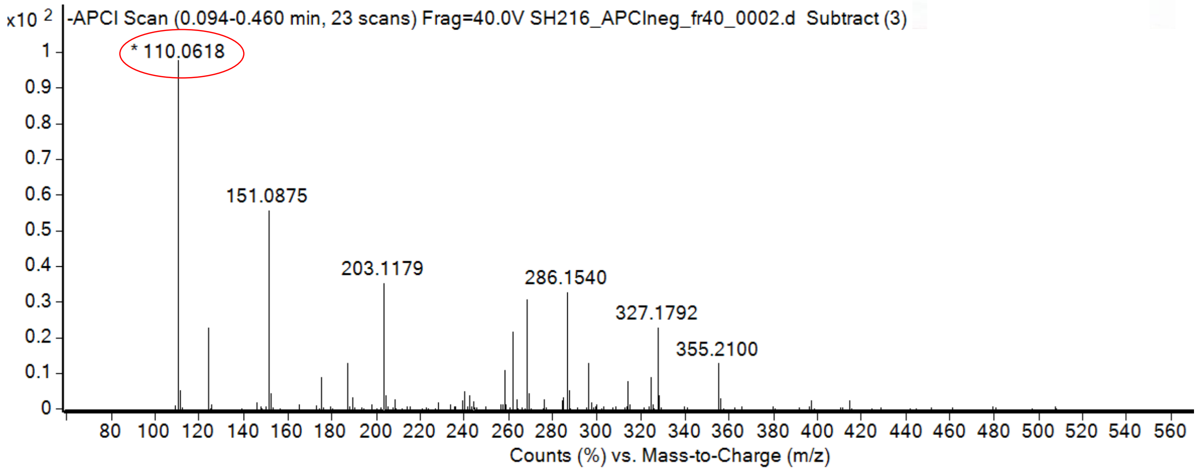

calculated mass: [M-H]- = 110.0611 observed: [M-H]- = 110.1618 max. mass error = 6.3 ppm

^{1}H (500 MHz) and ^{13}C NMR (126 MHz) spectra of **S8** in chloroform-*d*

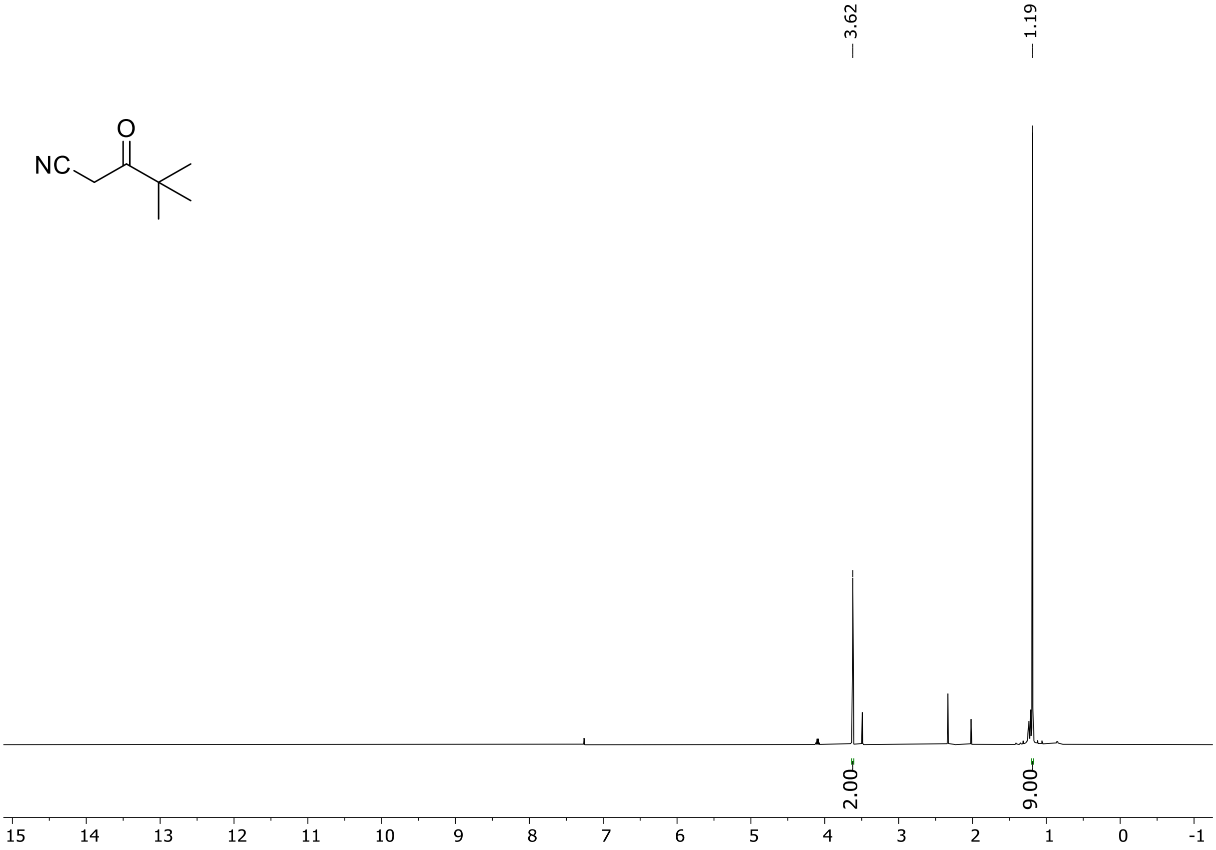

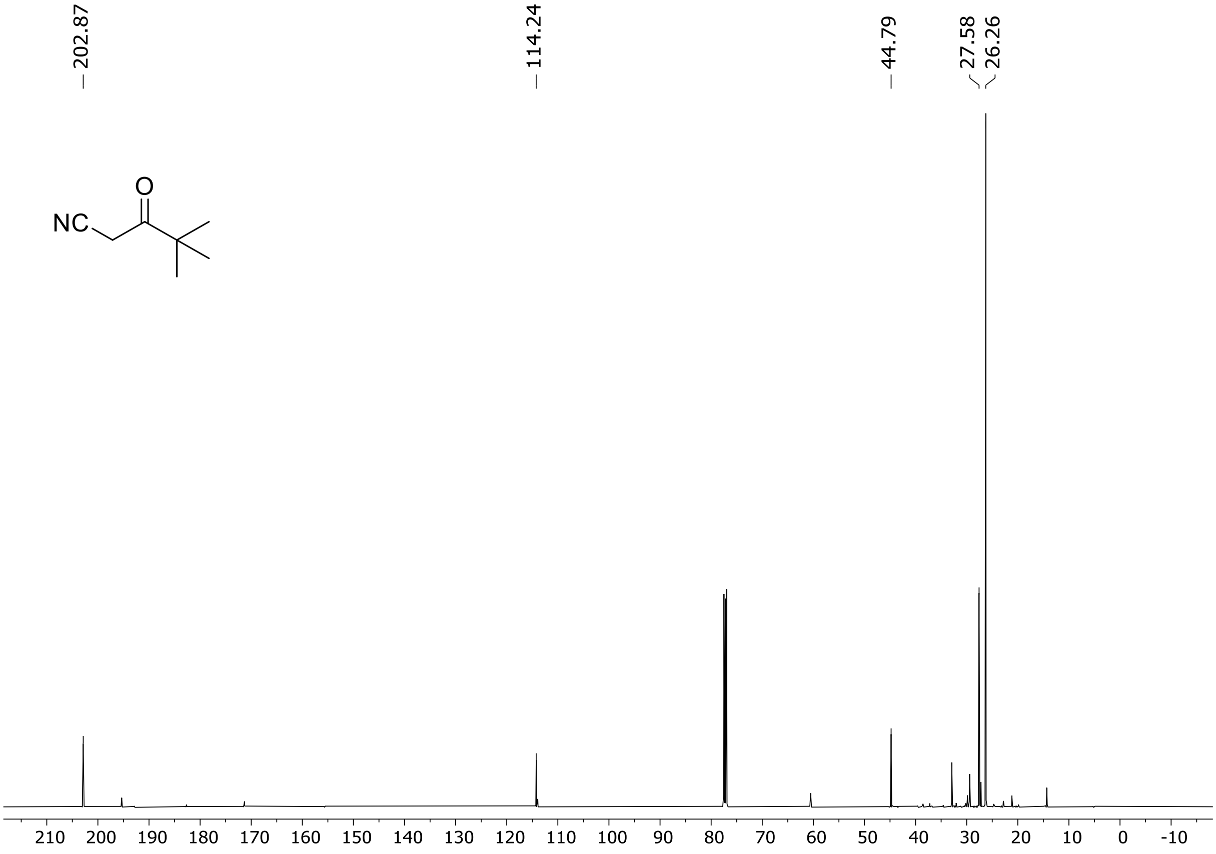

HRMS spectrum of **S8**

$C_7H_{11}NO$ **mono m/z = 125.0841**

APCI - (MMI)
nitrogen flow 5 L/min, gas temperature 300°C, nebulizer 45 psi, vaporizer 200°C
skimmer **65** V, fragmentor **27** V, dissolved in methanol

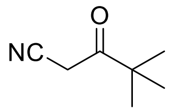

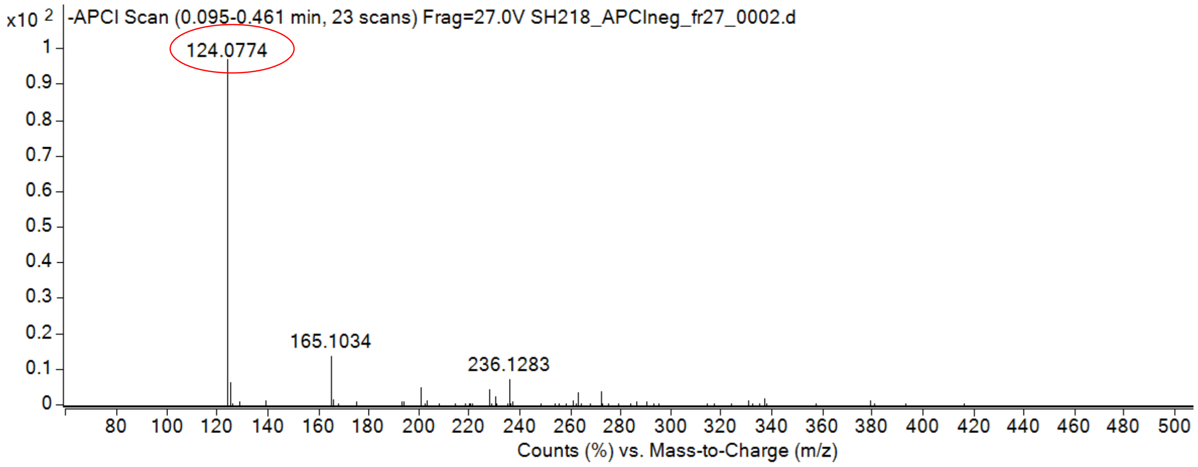

calculated mass: $[M\text{-}H]^-$ = 124.0768 observed: $[M\text{-}H]^-$ = 124.0774 max. mass error = 4.8 ppm

^{1}H (500 MHz) and ^{13}C NMR (126 MHz) spectra of **S9** in chloroform-*d*

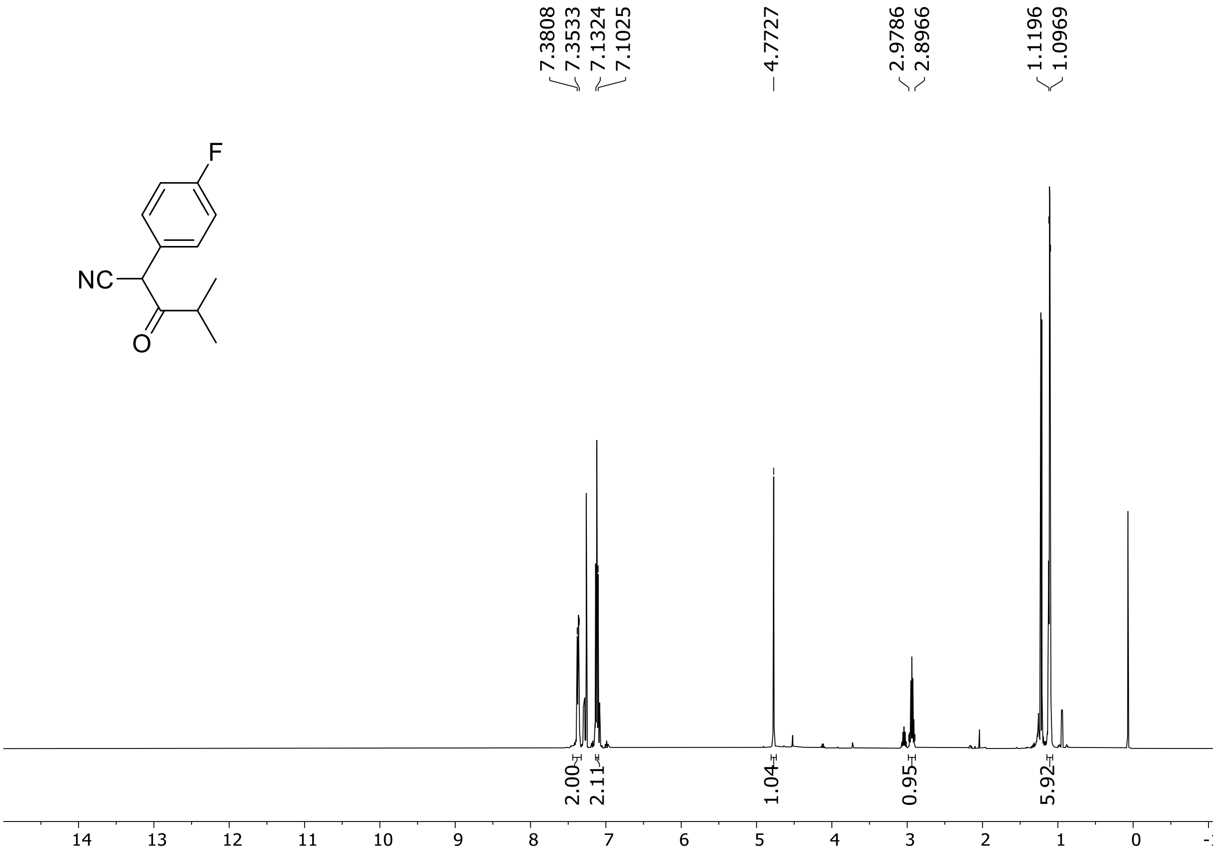

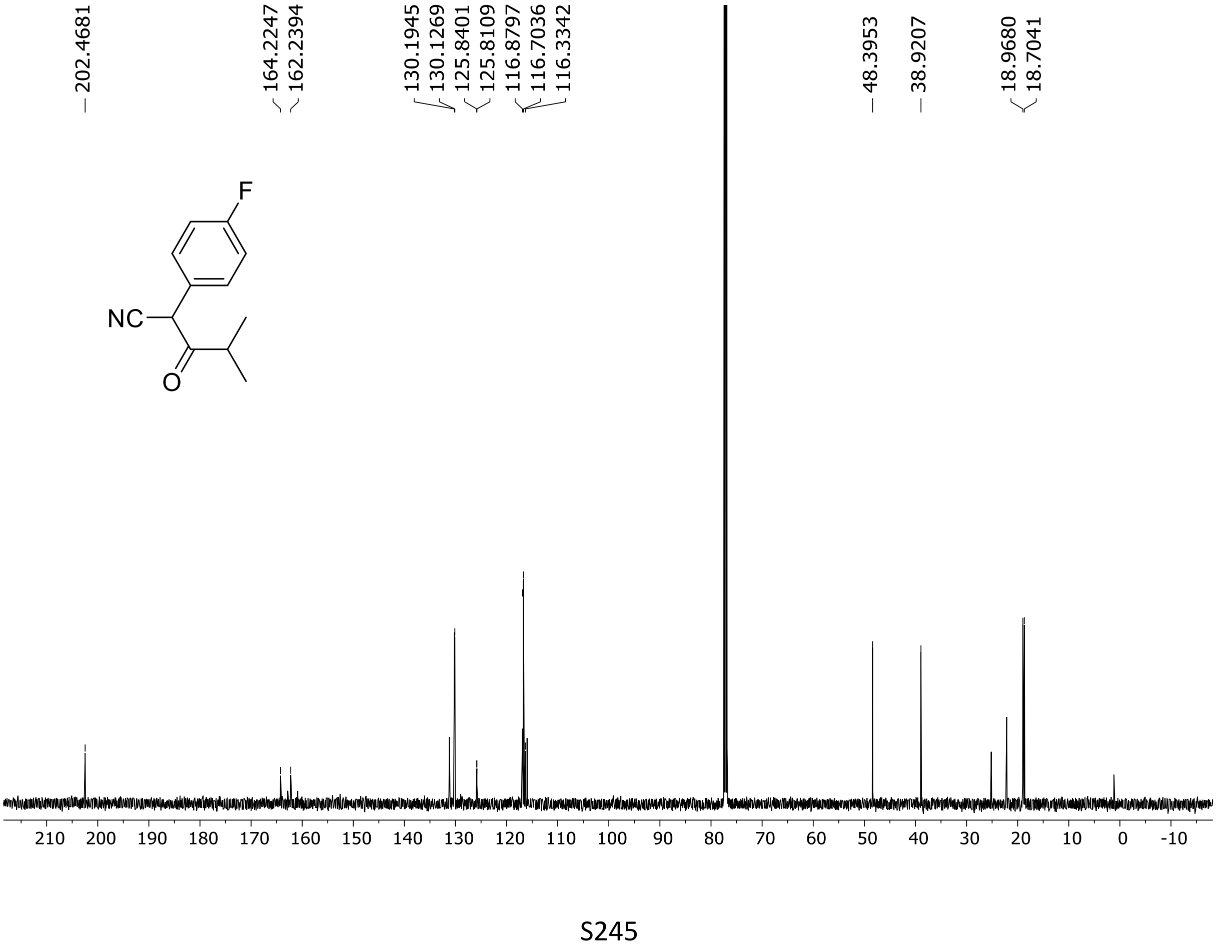

HRMS spectrum of **S9**

$C_{12}H_{12}NOF$ **mono m/z = 205.0903**

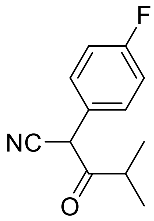

APCI - (MMI)
nitrogen flow 5 L/min, gas temperature 300°C, nebulizer 45 psi, vaporizer 200°C, skimmer **65** V, fragmentor **36** V, dissolved in methanol

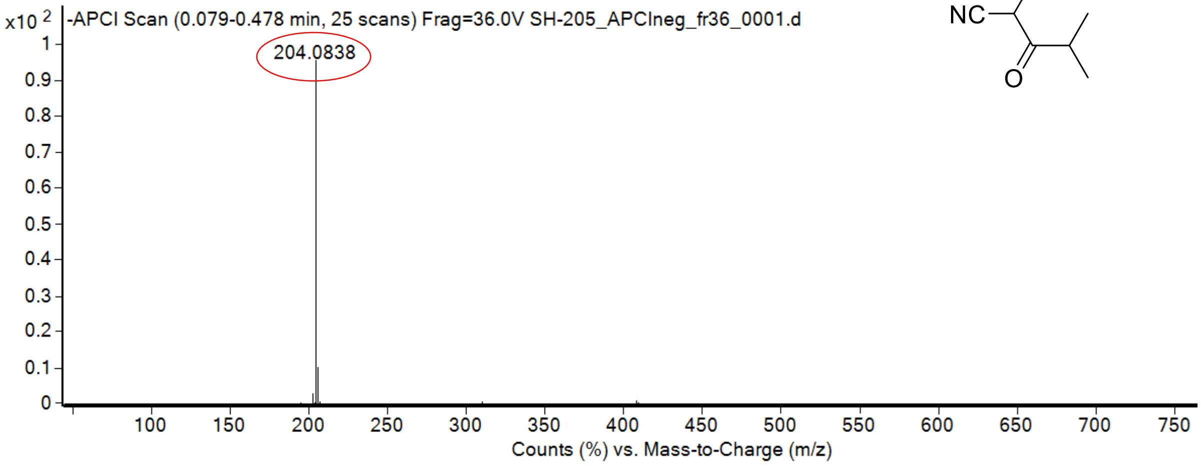

calculated mass: $[M-H]^-$ = 204.0830 observed: $[M+H]^+$ = 204.0838 max. mass error = 3.9 ppm

^{1}H (500 MHz) and ^{13}C NMR (126 MHz) spectra of **S10** in chloroform-*d*

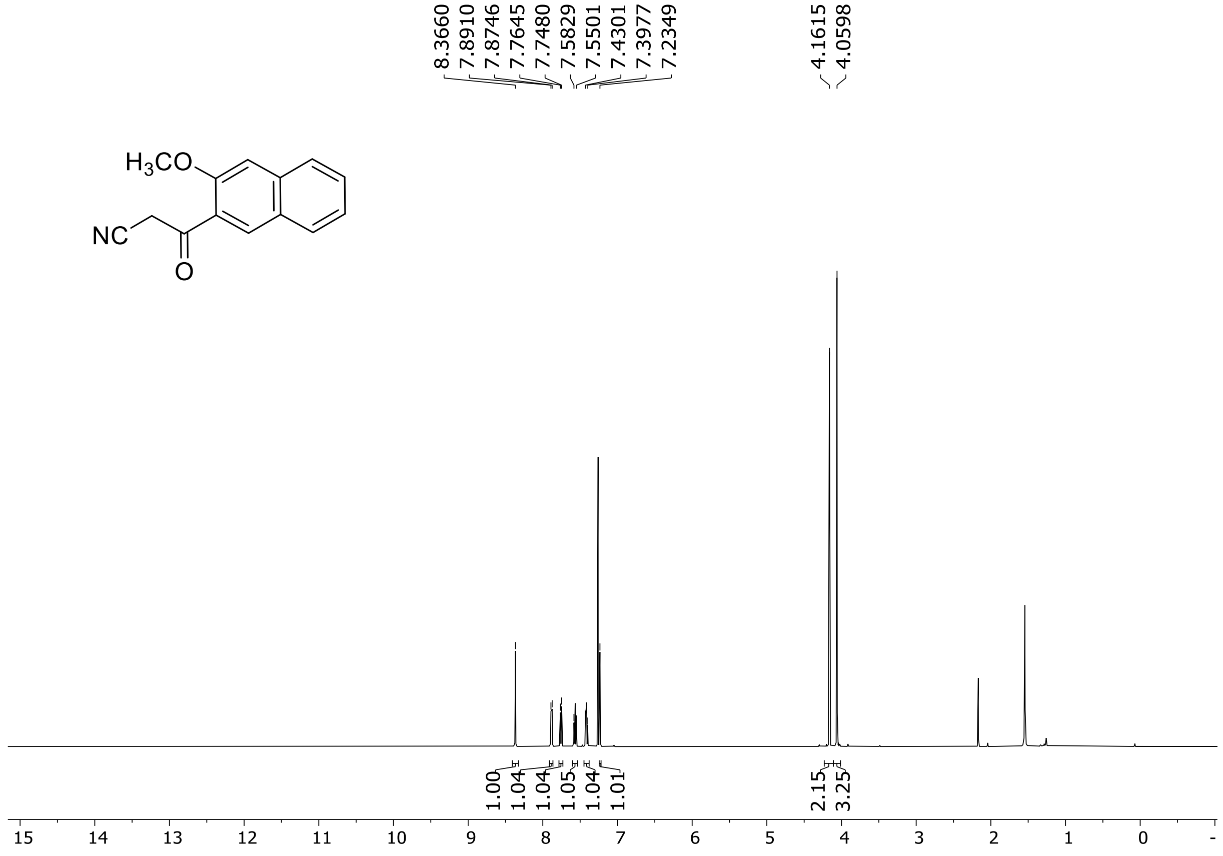

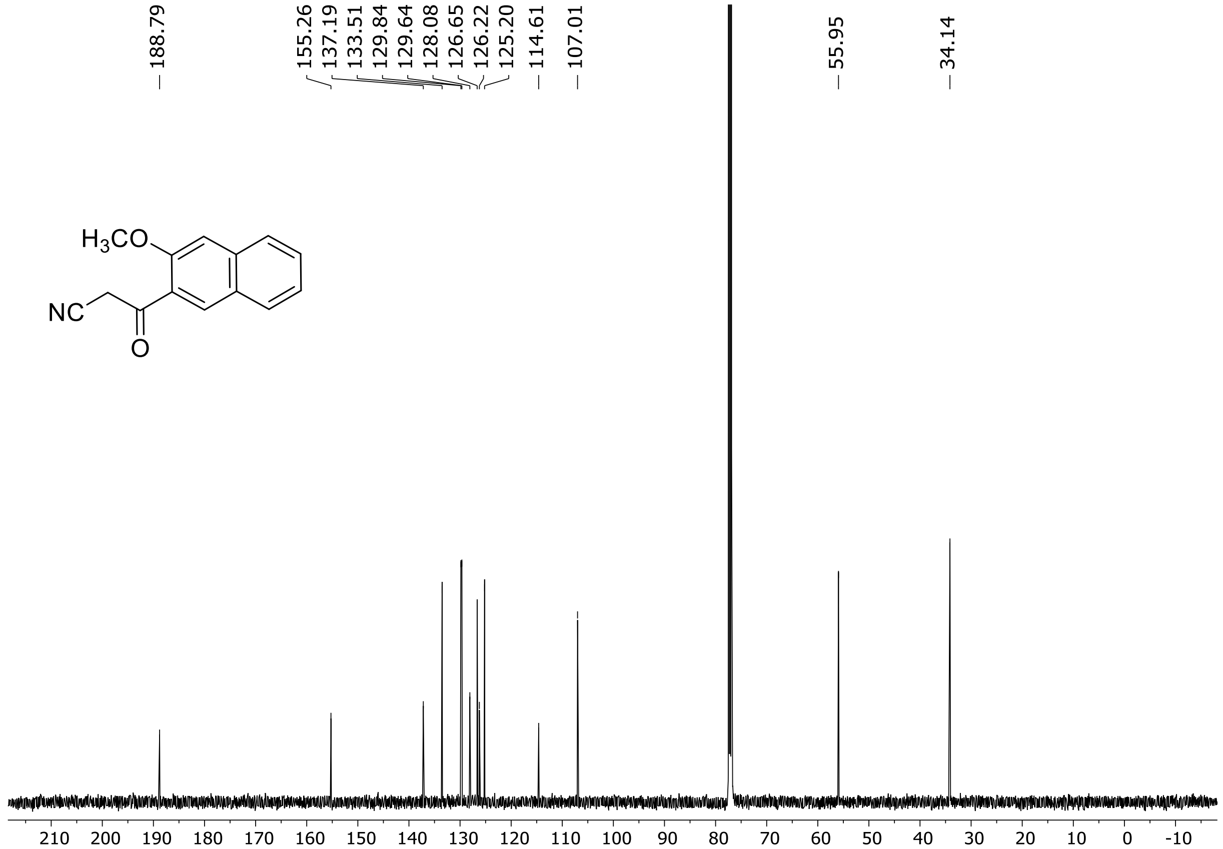

^{1}H (500 MHz) and ^{13}C NMR (126 MHz) spectra of **S11** in chloroform-*d*

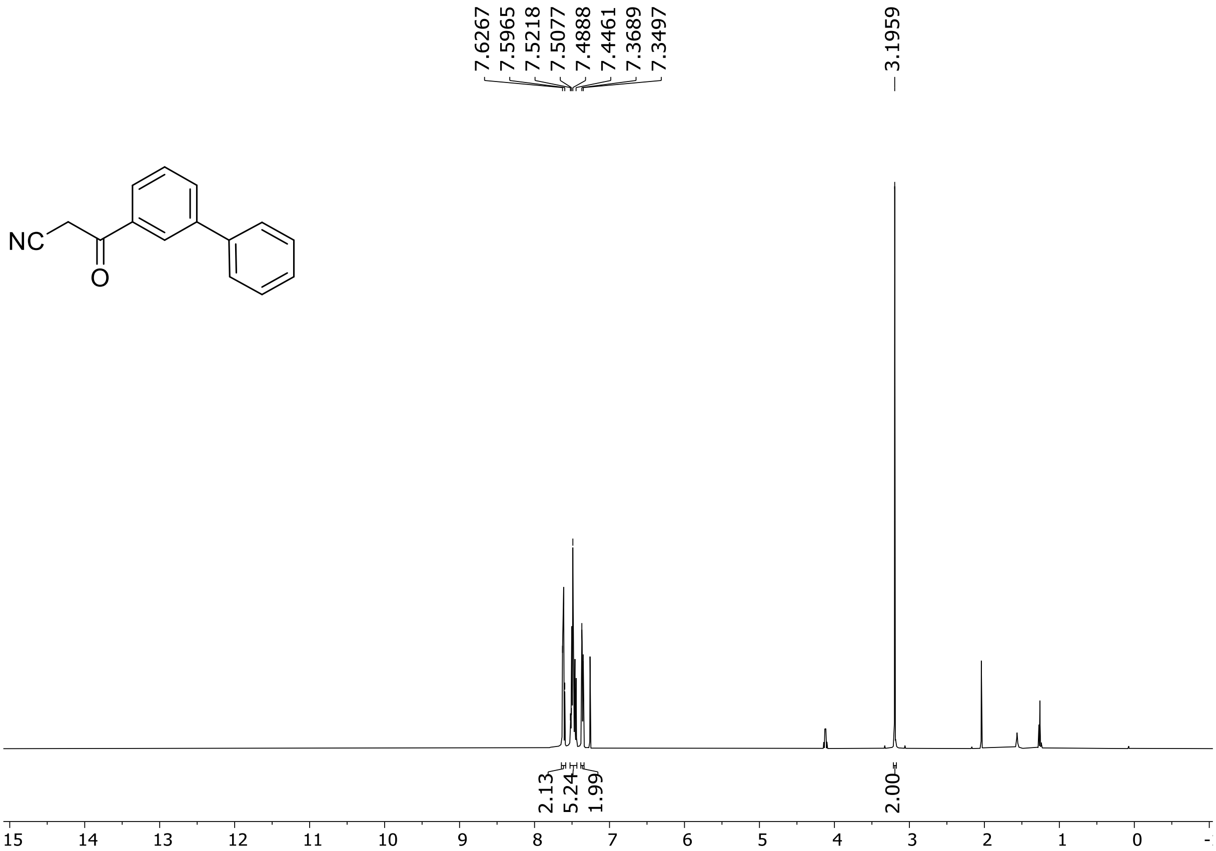

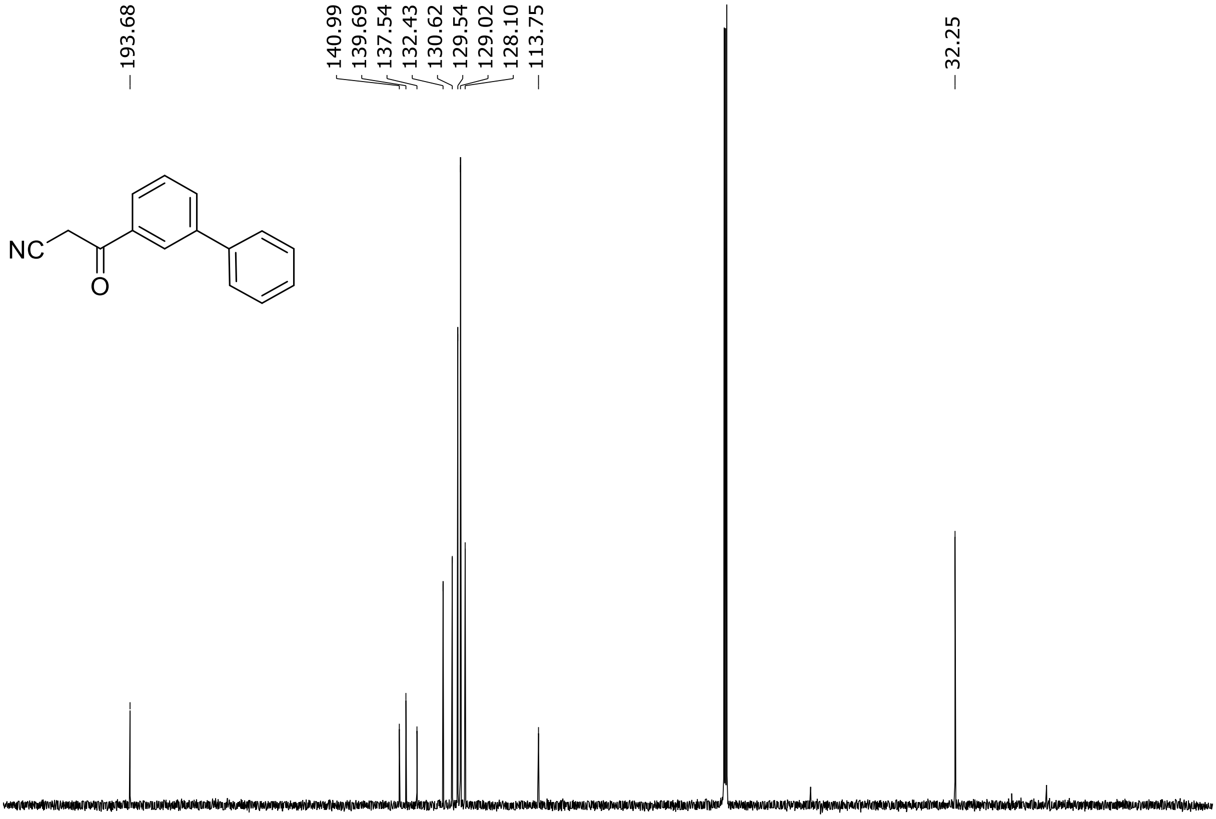

HRMS spectrum of **S11**

$C_{15}H_{11}NO$ **mono m/z = 221.0841**

APCI - (MMI)
nitrogen flow 5 L/min, gas temperature 300°C, nebulizer 45 psi, vaporizer 200°C
skimmer **65** V, fragmentor **15** V, dissolved in methanol

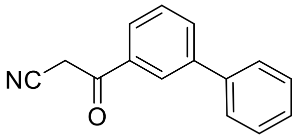

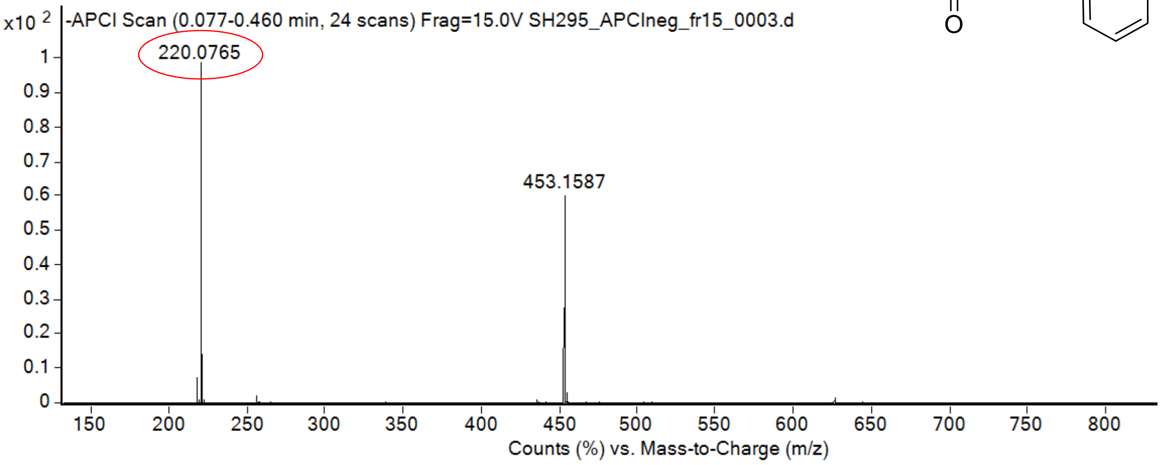

calculated mass: [M-H]$^-$ = 220.0768 observed: [M-H]$^-$ = 220.0765 max. mass error = 1.3 ppm

^{1}H (500 MHz) and ^{13}C NMR (126 MHz) spectra of **S12** in chloroform-*d*

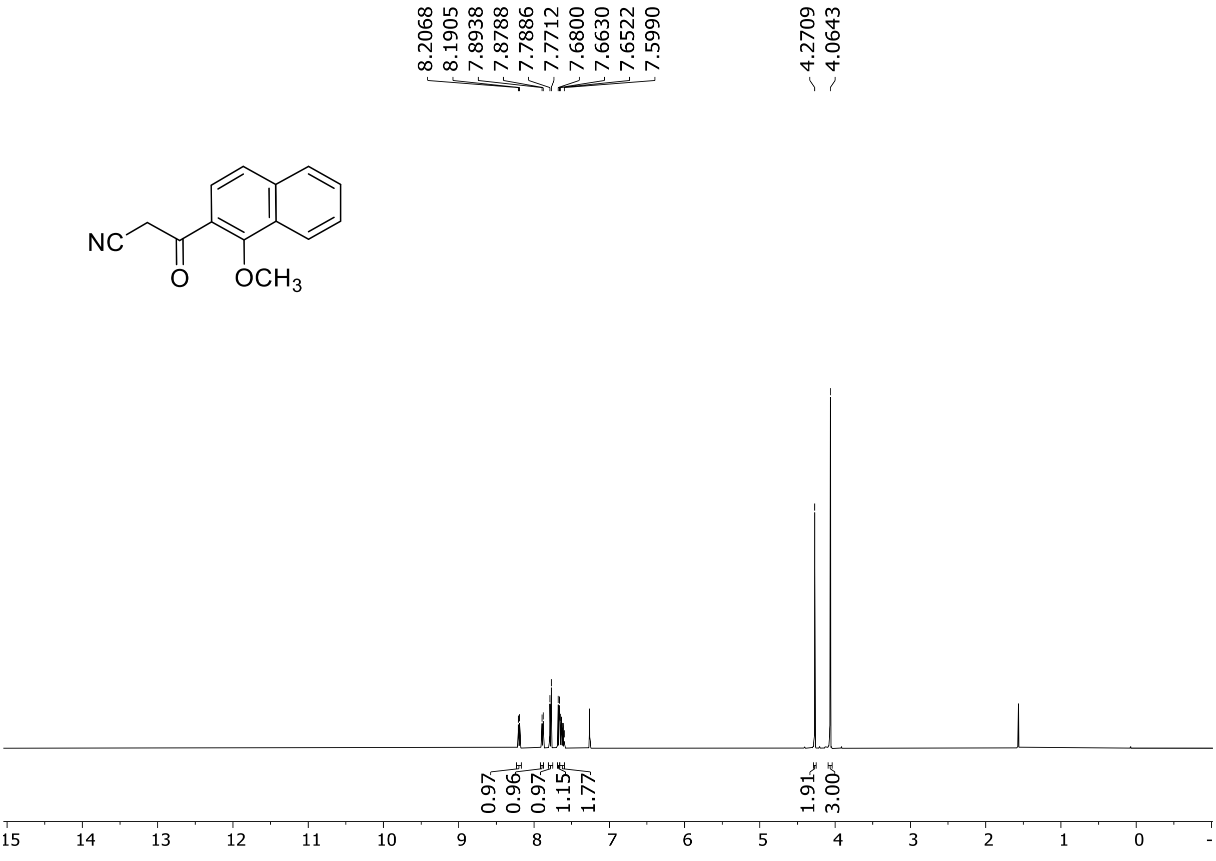

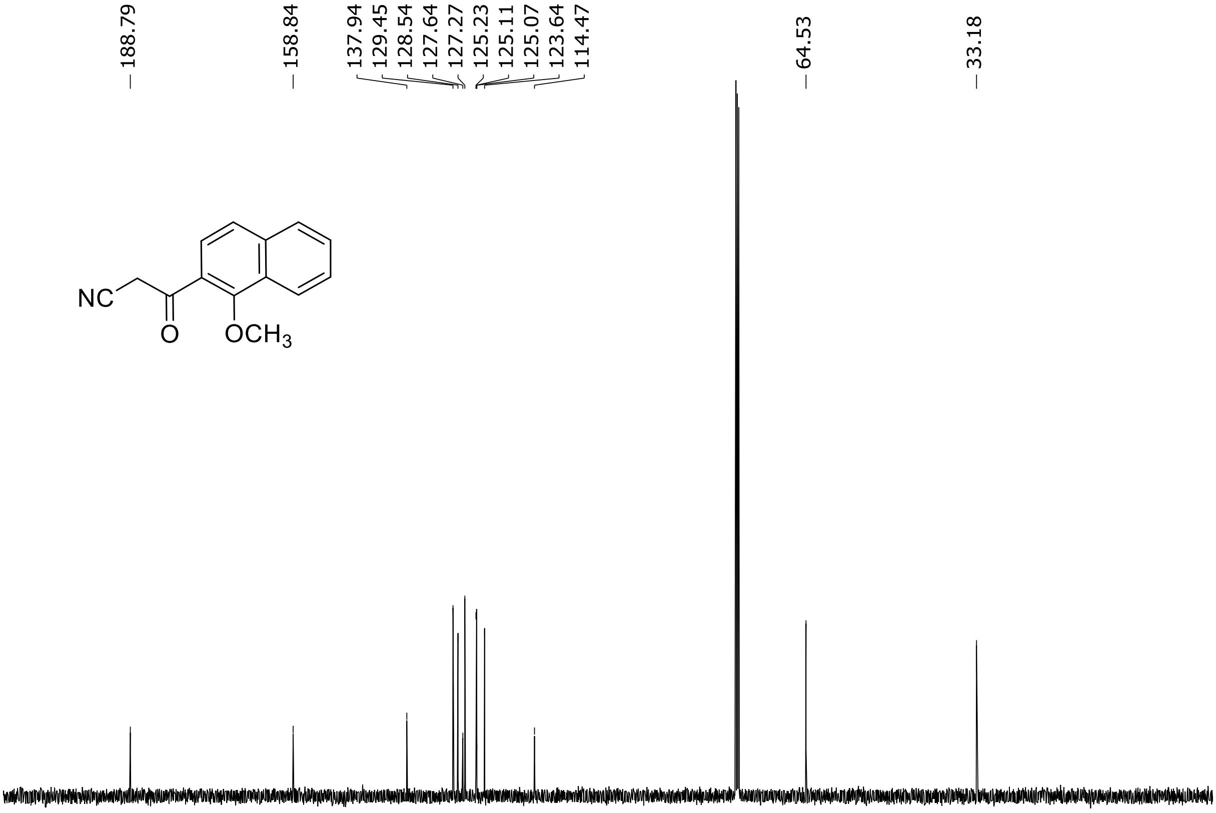

^{1}H (500 MHz) and ^{13}C NMR (126 MHz) spectra of **S13** in chloroform-*d*

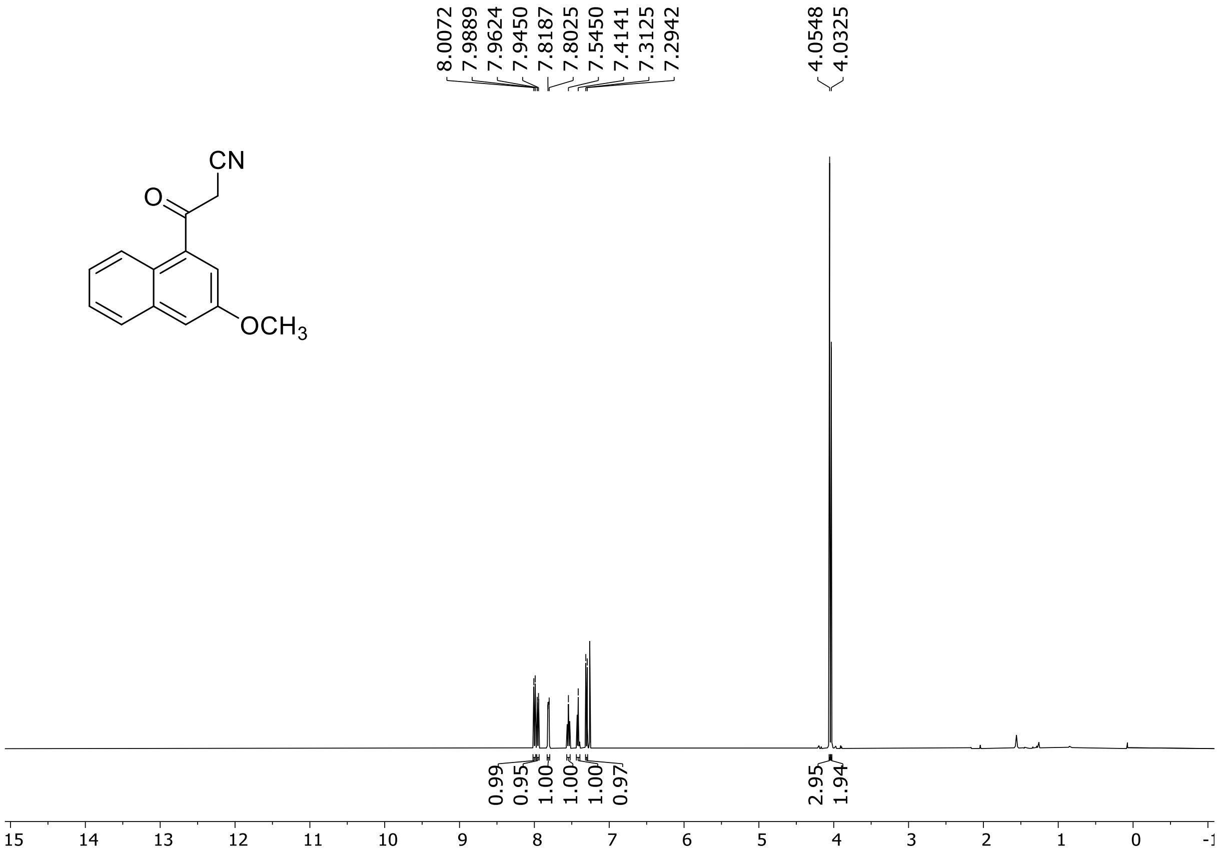

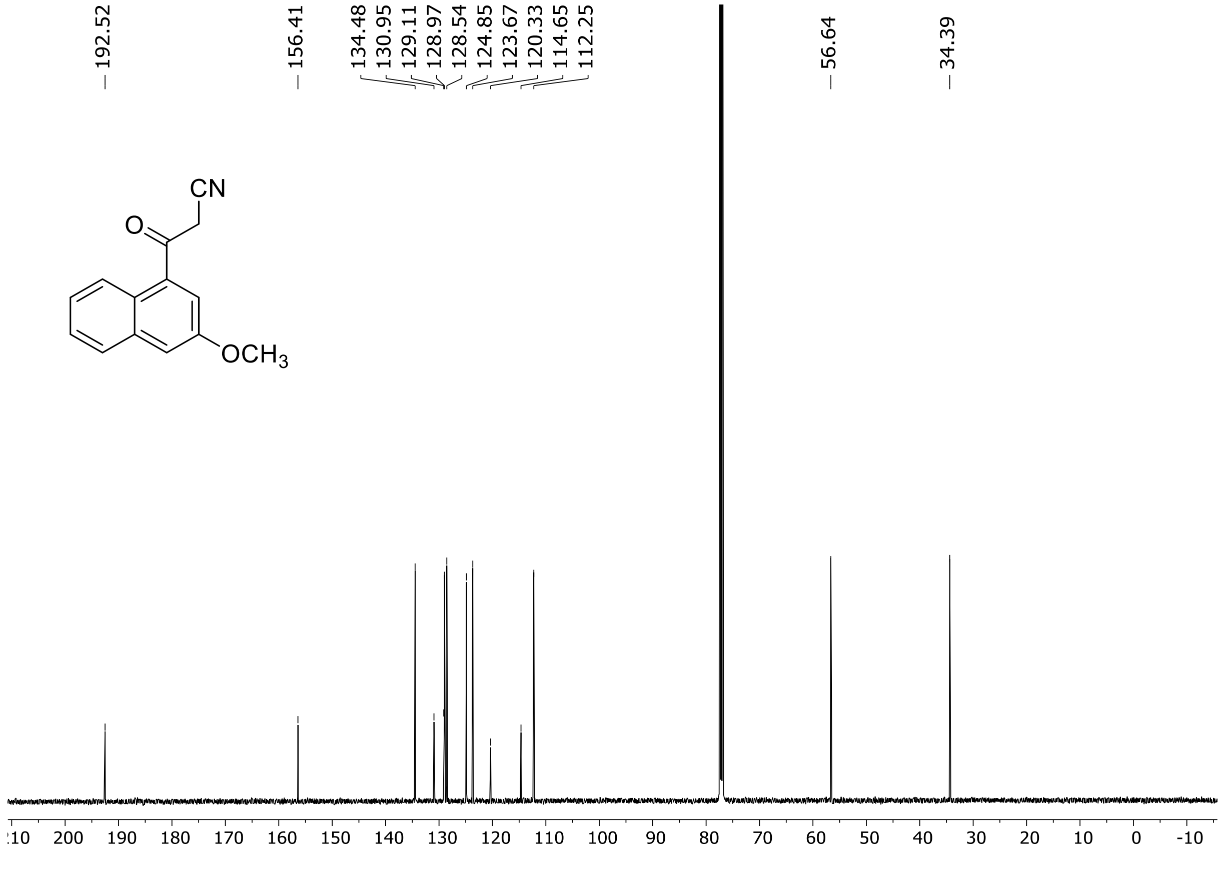

^{1}H (500 MHz) and ^{13}C NMR (126 MHz) spectra of **S14** in chloroform-*d*

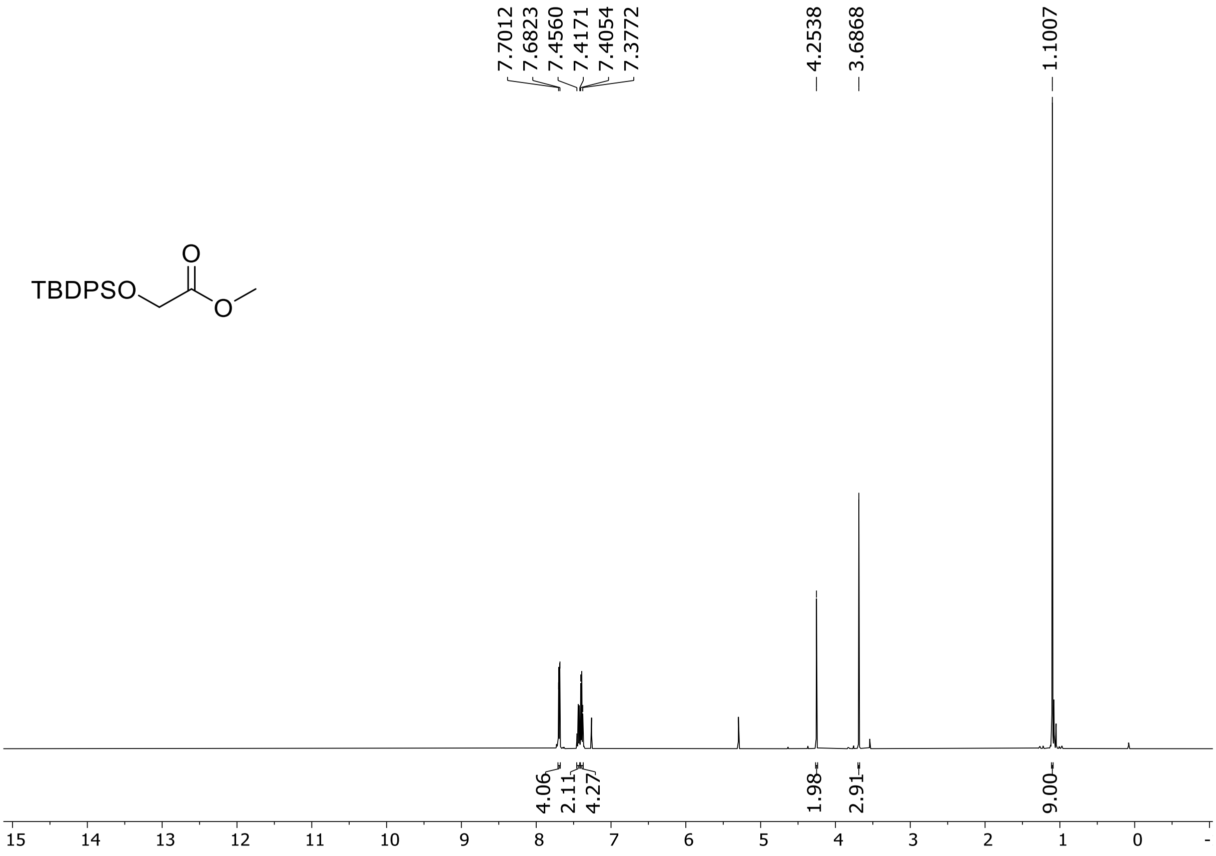

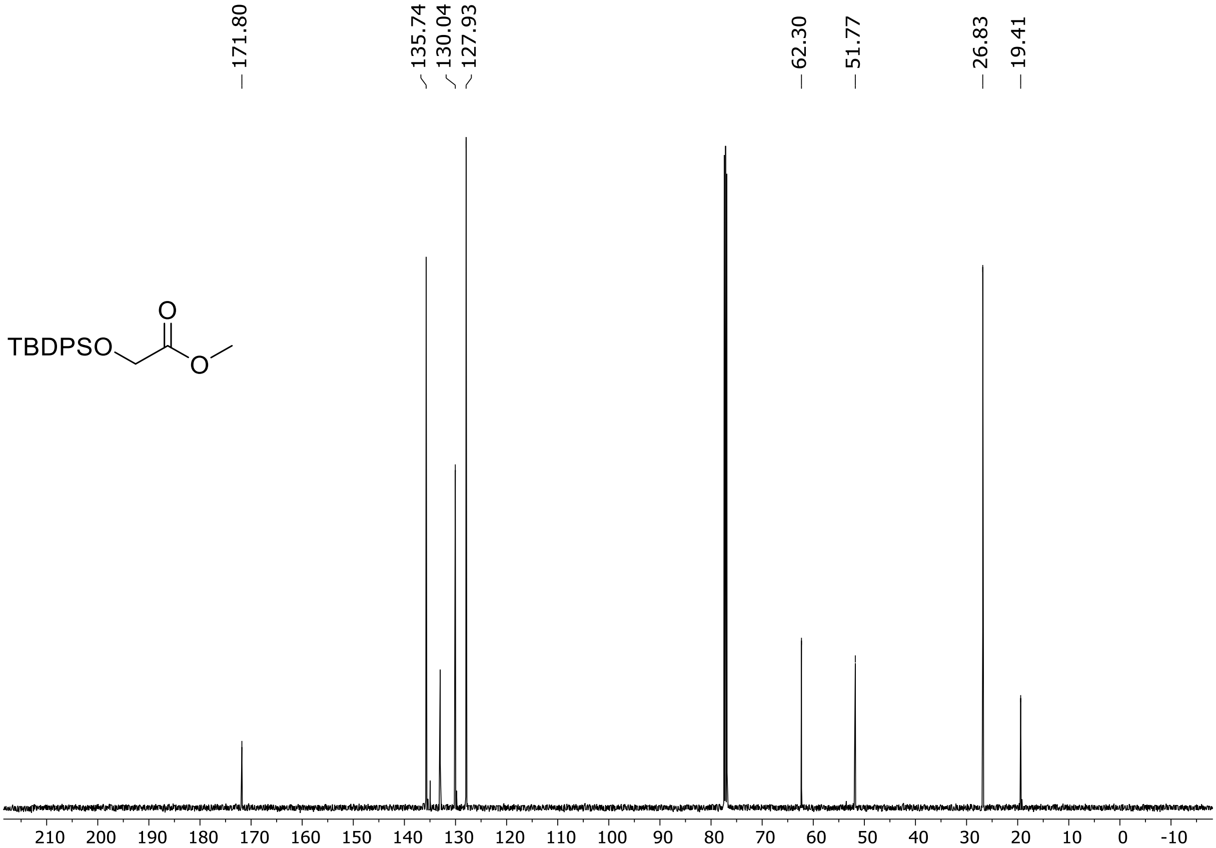

^{1}H (500 MHz) and ^{13}C NMR (126 MHz) spectra of **S15** in chloroform-*d*

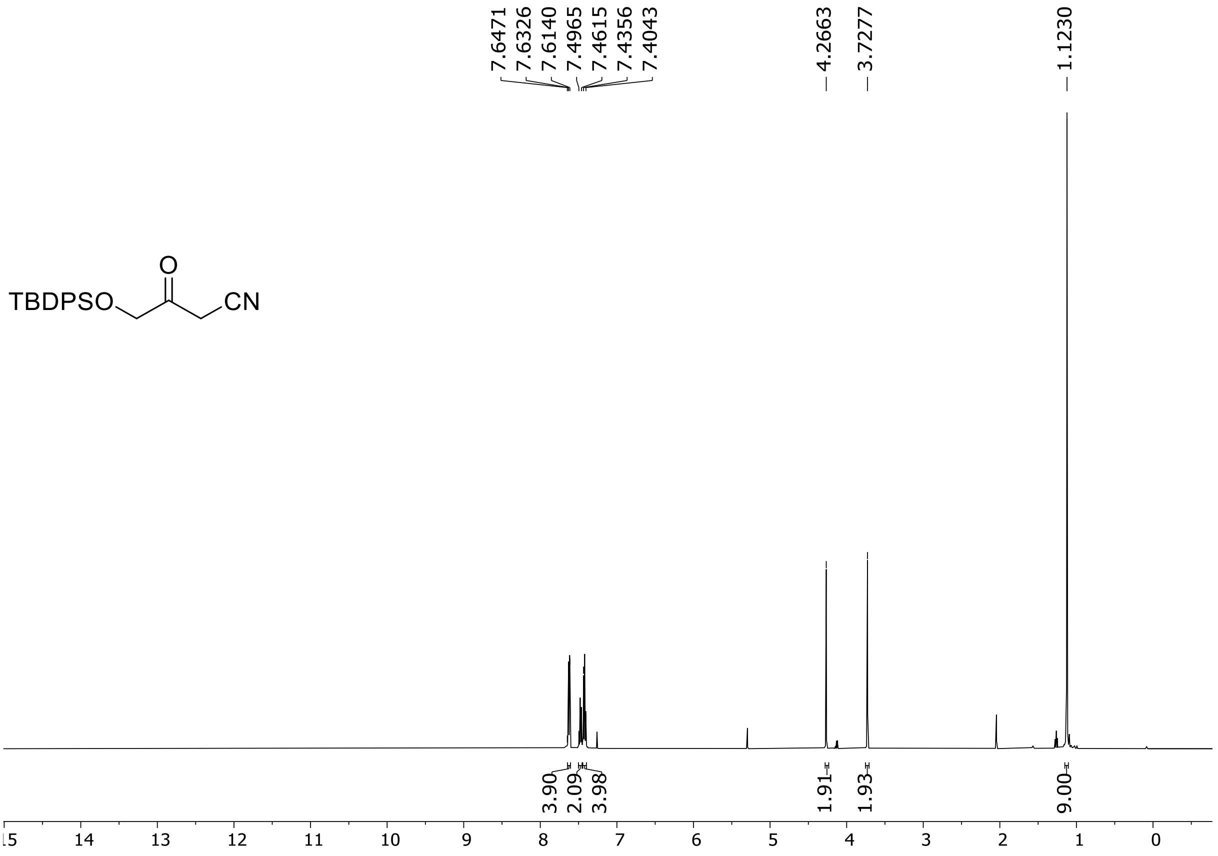

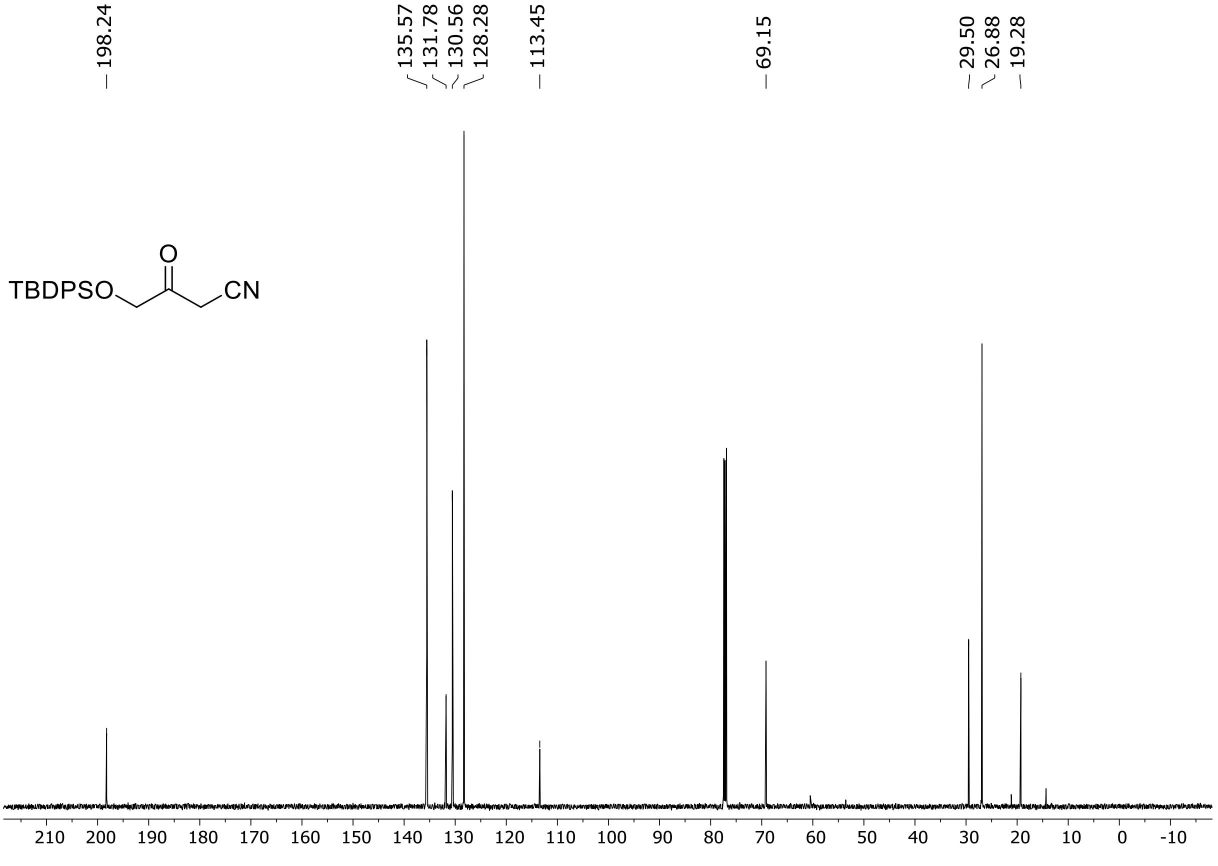

HRMS spectrum of **S15**

$C_{20}H_{23}NO_2Si$ **mono m/z = 337.1498**

APCI - (MMI)
nitrogen flow 5 L/min, gas temperature 300°C, nebulizer 45 psi, vaporizer 200°C
skimmer **65** V, fragmentor **18** V, dissolved in methanol

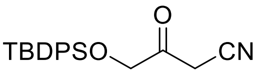

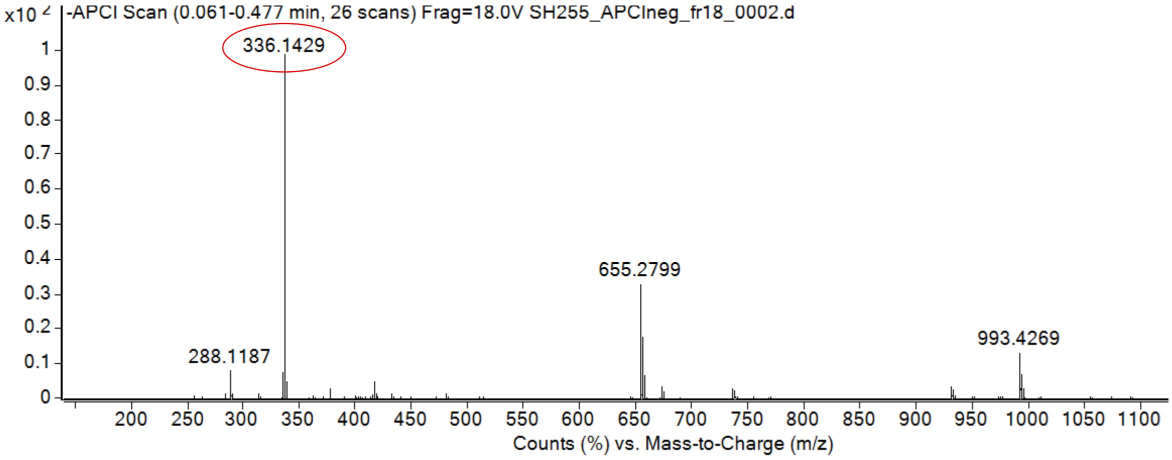

calculated mass: [M-H]$^-$ = 336.1425 observed: [M-H]$^-$ = 336.1429 max. mass error = 1.1 ppm

^{1}H (500 MHz) spectrum of **S16** in chloroform-*d*

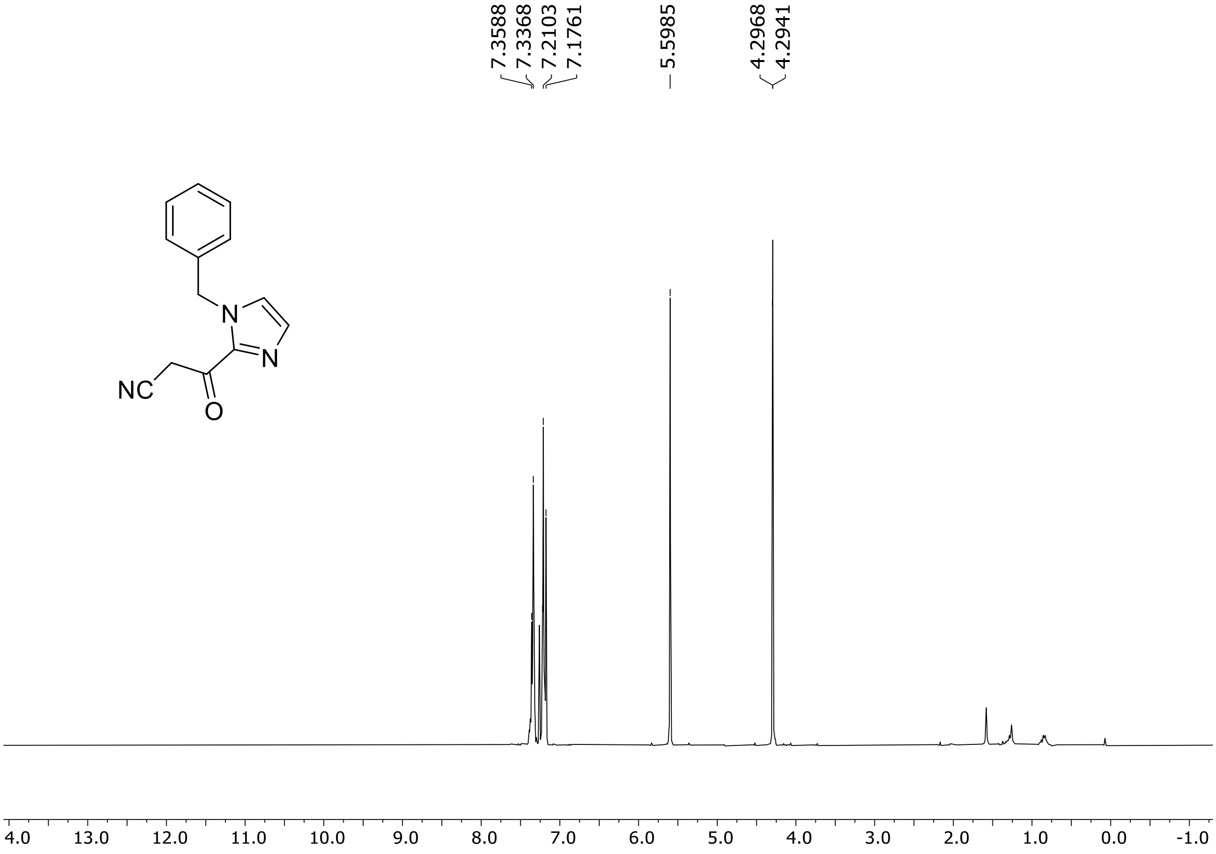

^{1}H (500 MHz) and ^{13}C NMR (126 MHz) spectra of **S17** in chloroform-*d*

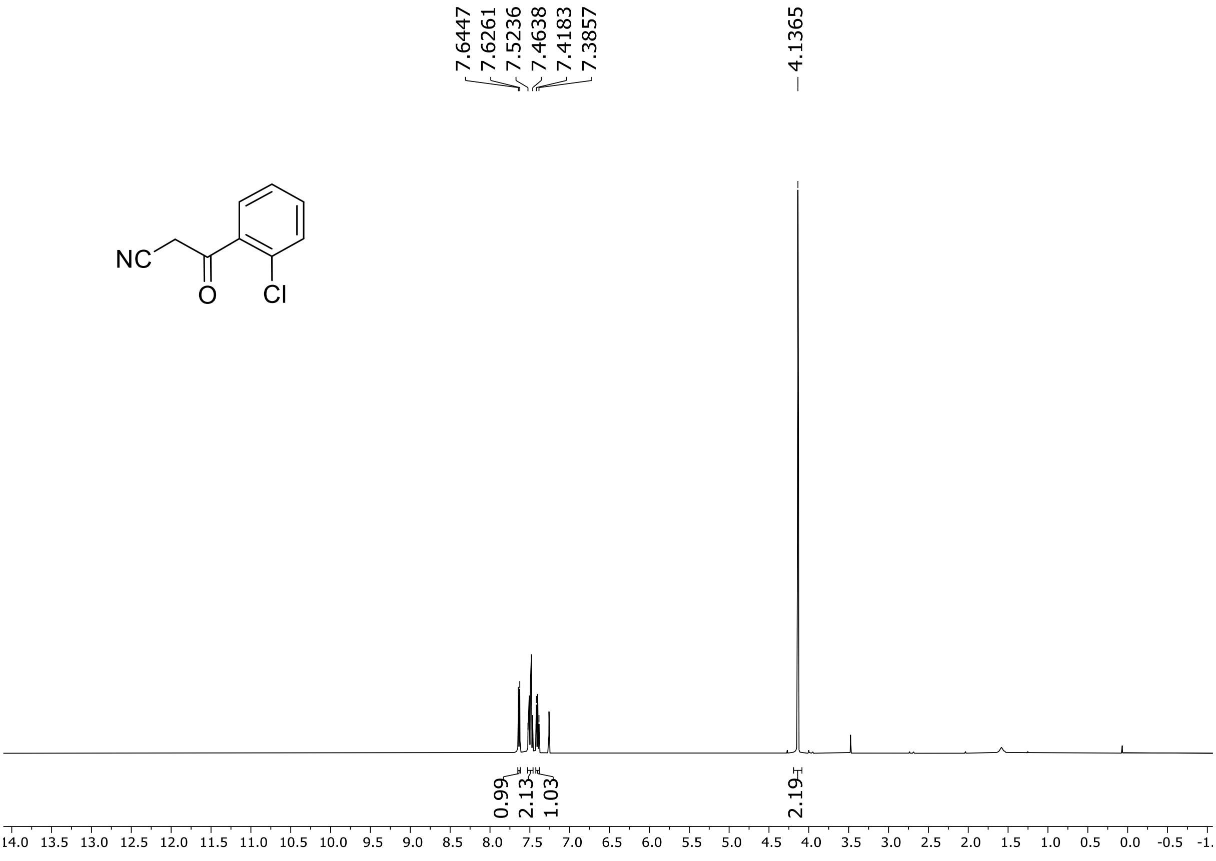

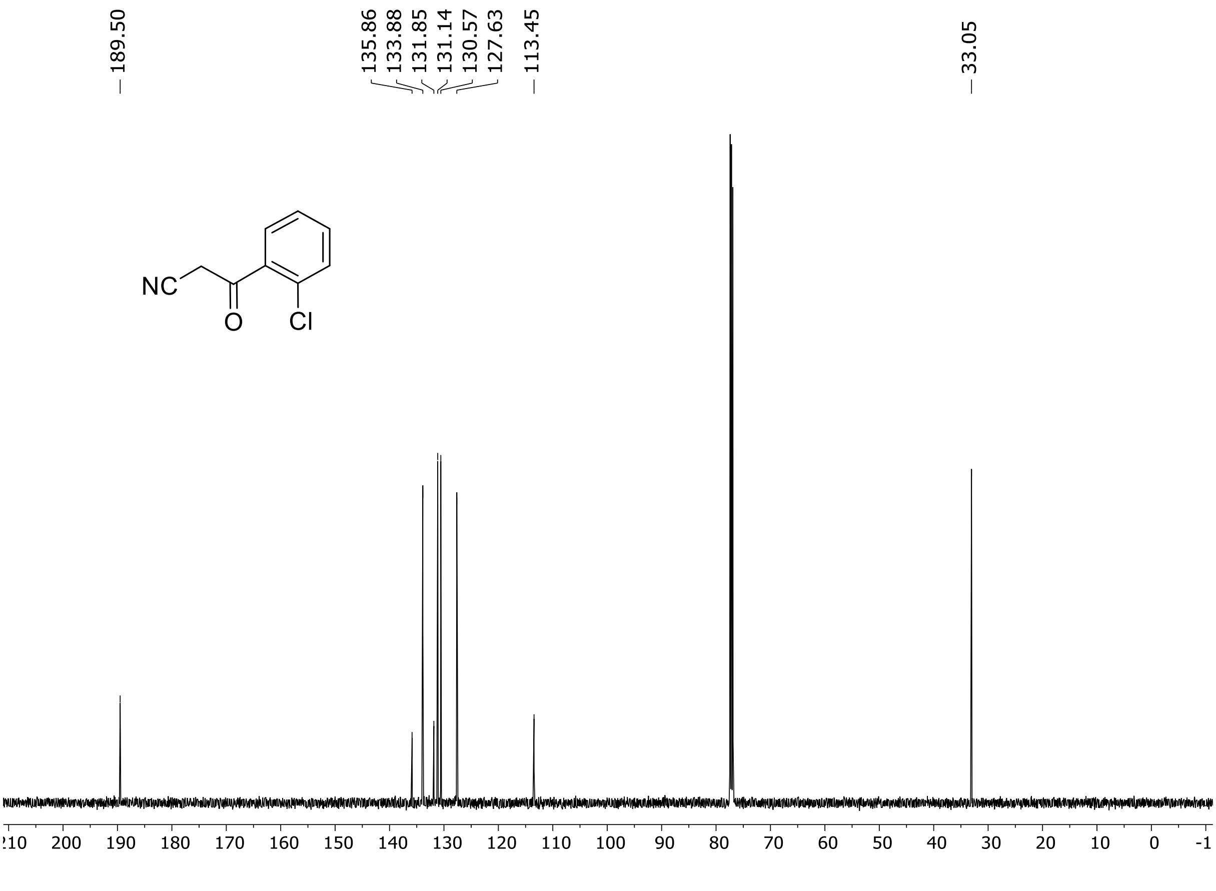

HRMS spectrum of **S17**

C_9H_6ClNO **mono m/z = 179.0138**

APCI - (MMI)
nitrogen flow 5 L/min, gas temperature 300°C, nebulizer 45 psi, vaporizer 200°C
skimmer **65** V, fragmentor **25** V, dissolved in methanol

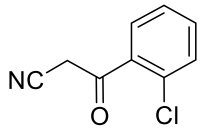

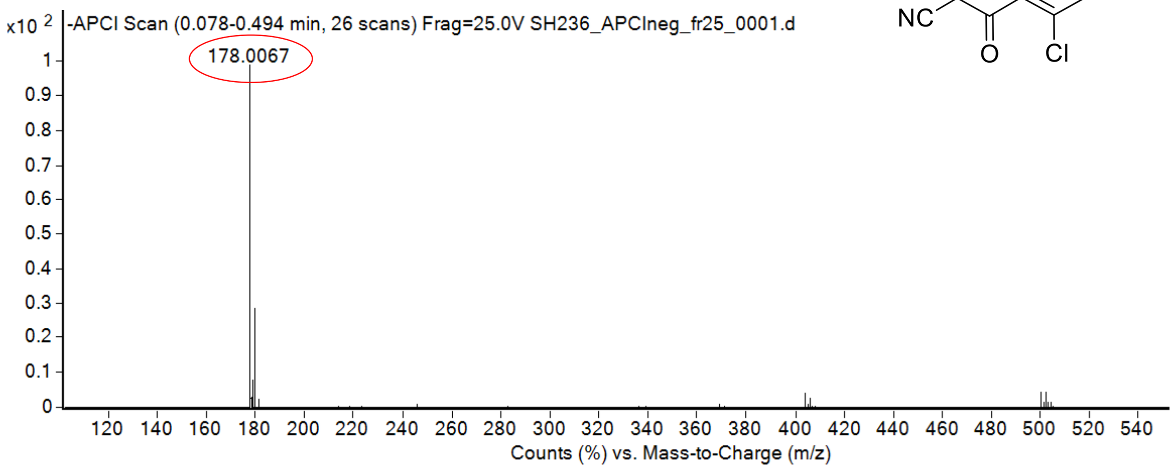

calculated mass: $[M-H]^-$ = 178.0065 observed: $[M-H]^-$ = 178.0067 max. mass error = 1.1 ppm

^{1}H (500 MHz) and ^{13}C NMR (126 MHz) spectra of **S18** in chloroform-*d*

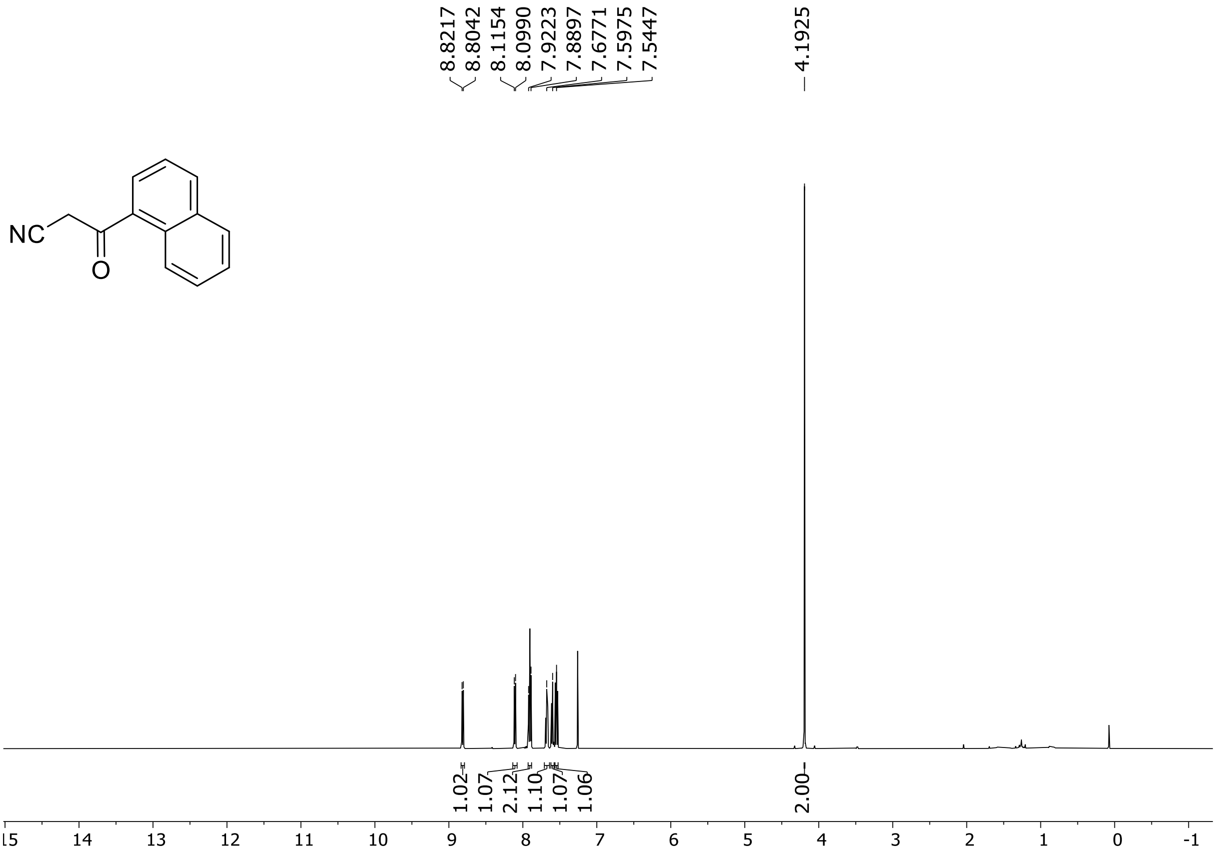

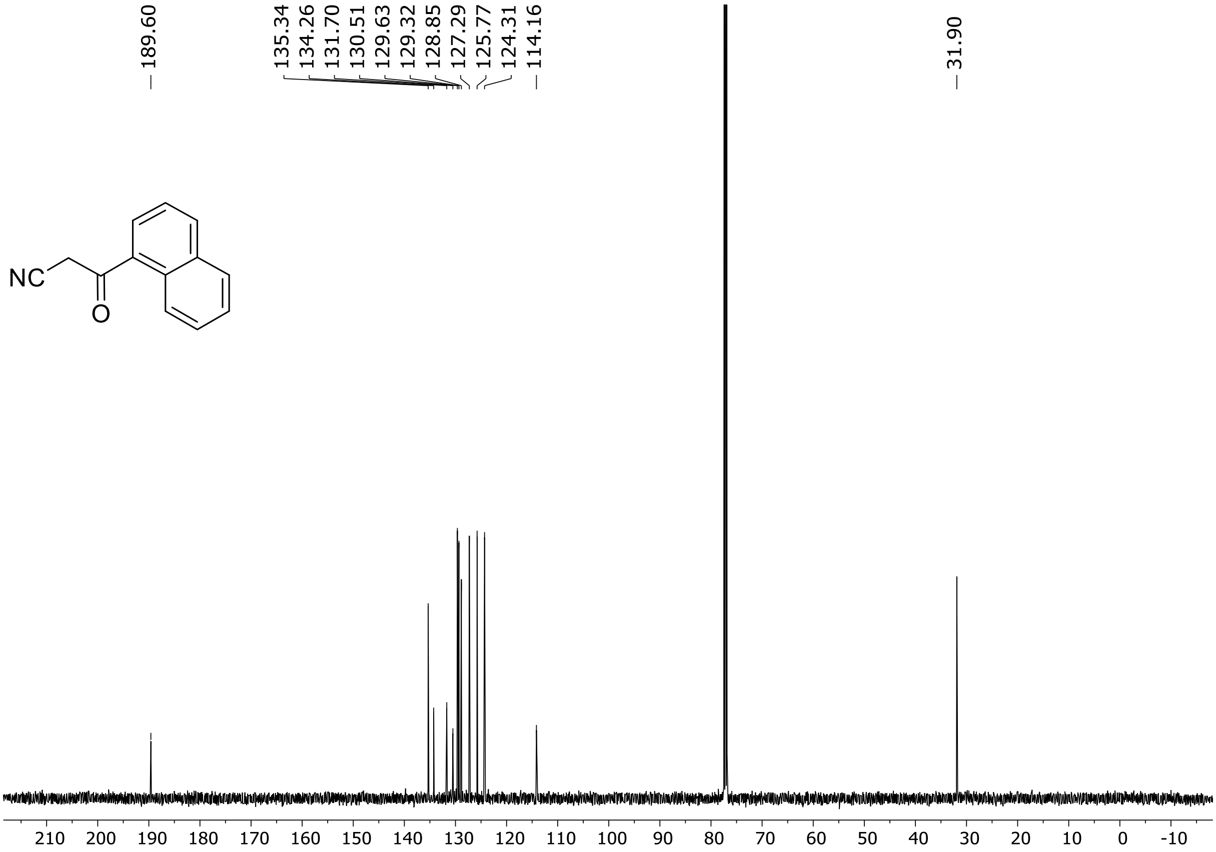

HRMS spectrum of **S18**

$C_{13}H_9NO$ mono m/z = 195.0684

APCI - (MMI)
nitrogen flow 5 L/min, gas temperature 300°C, nebulizer 45 psi, vaporizer 200°C
skimmer **65** V, fragmentor **20** V, dissolved in methanol

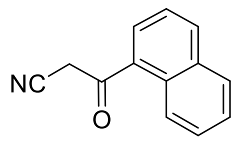

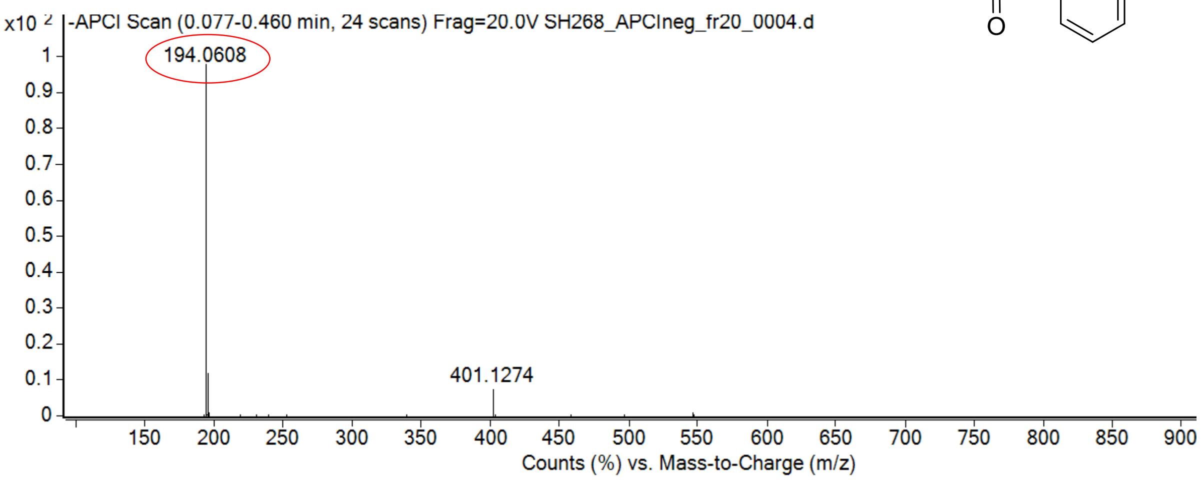

calculated mass: $[M\text{-}H]^-$ = 194.0611 observed: $[M\text{-}H]^-$ = 194.0608 max. mass error = 1.5 ppm

^{1}H (300 MHz) NMR spectrum of **S19** in DMSO-d_6

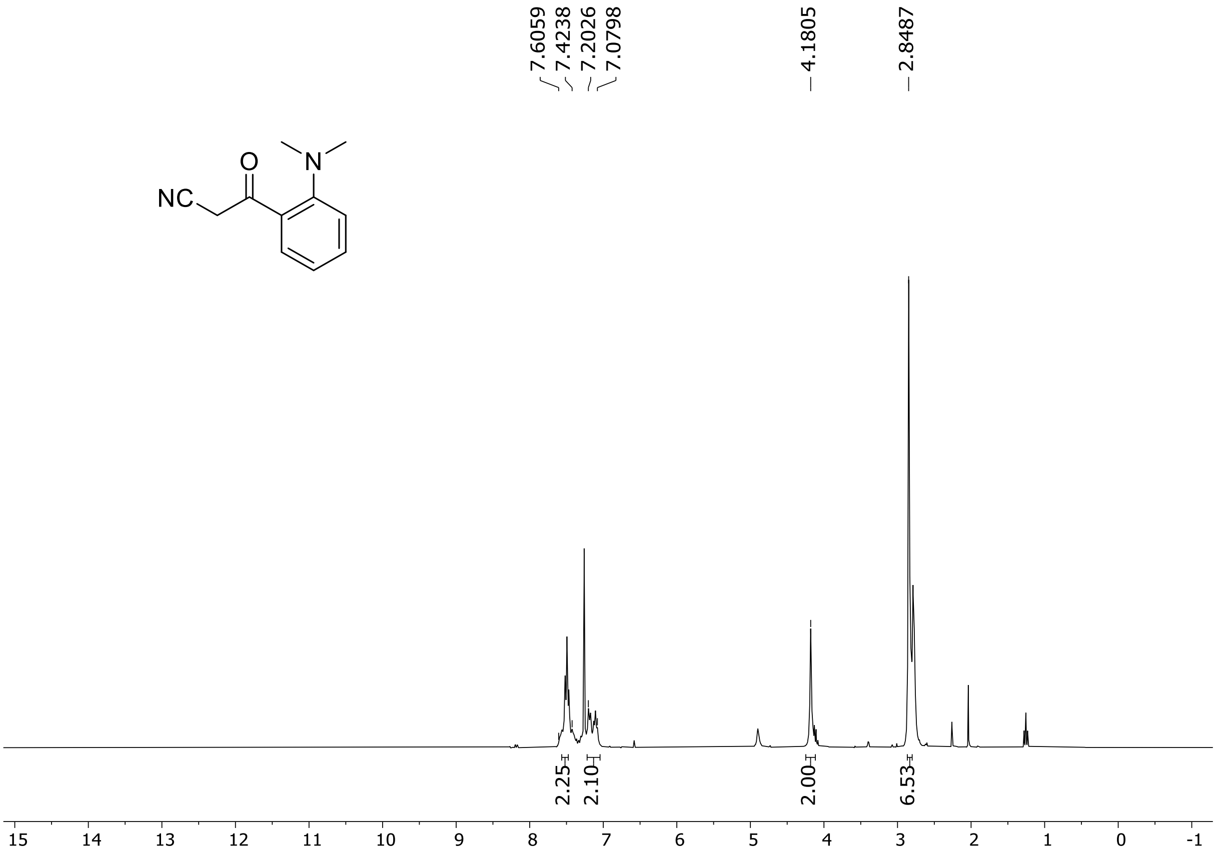

^{1}H (500 MHz) and ^{13}C NMR (126 MHz) spectra of **S21** in chloroform-*d*

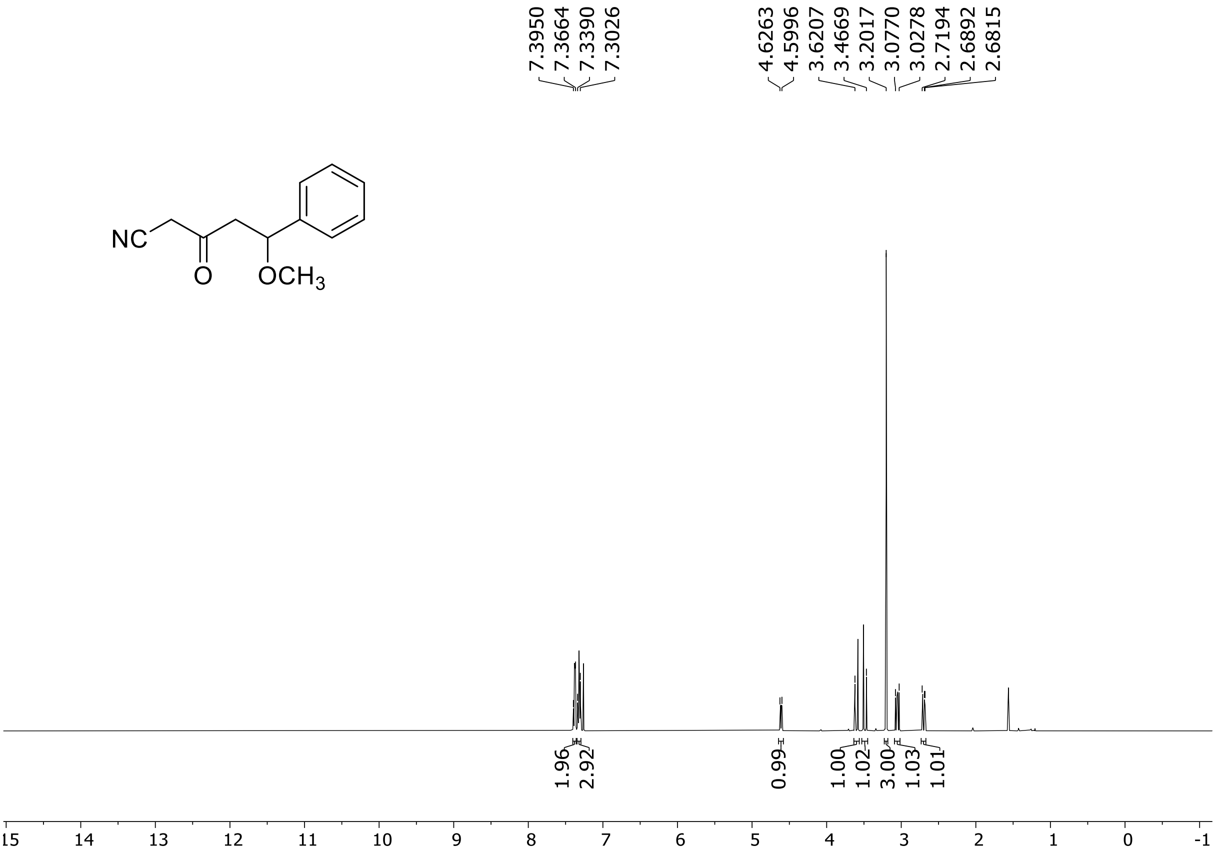

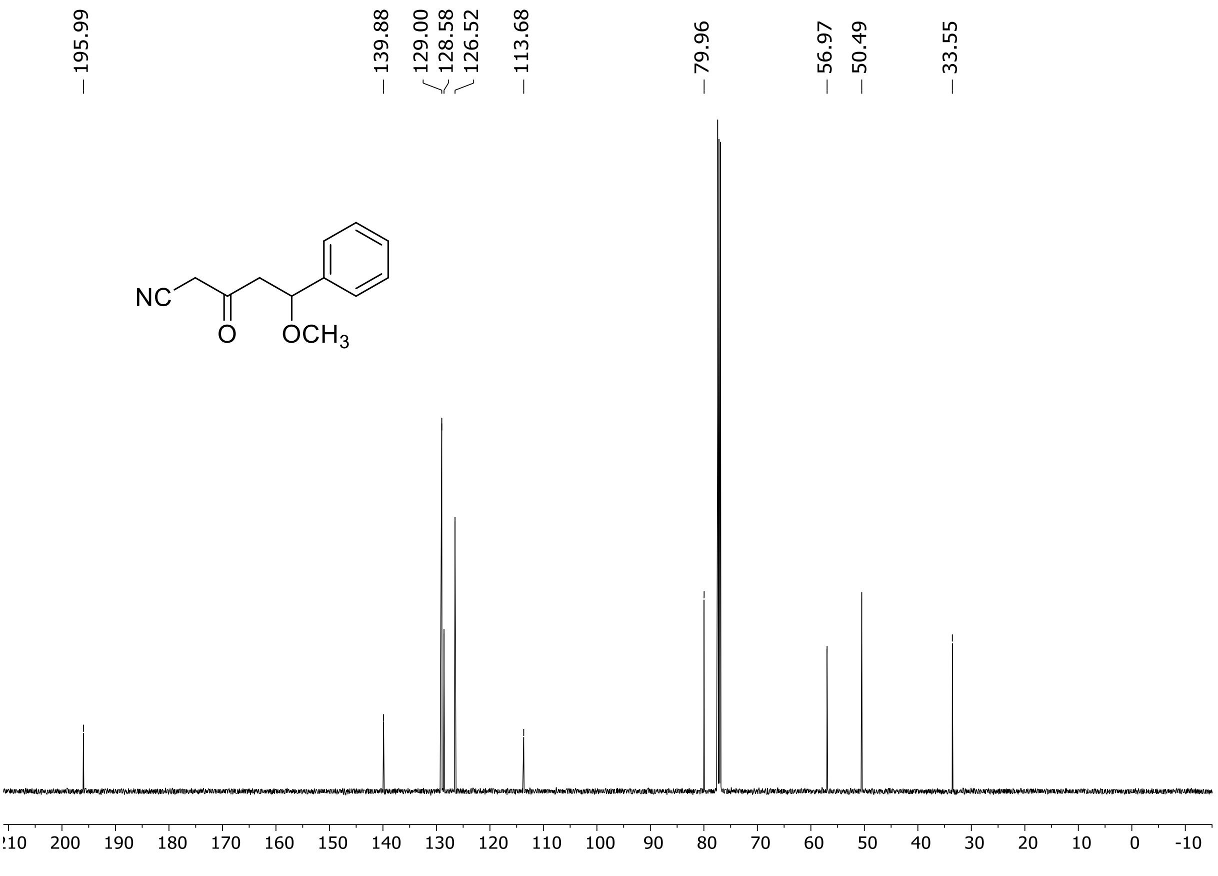

^{1}H (500 MHz) and ^{13}C NMR (126 MHz) spectra of **S22** in chloroform-*d*

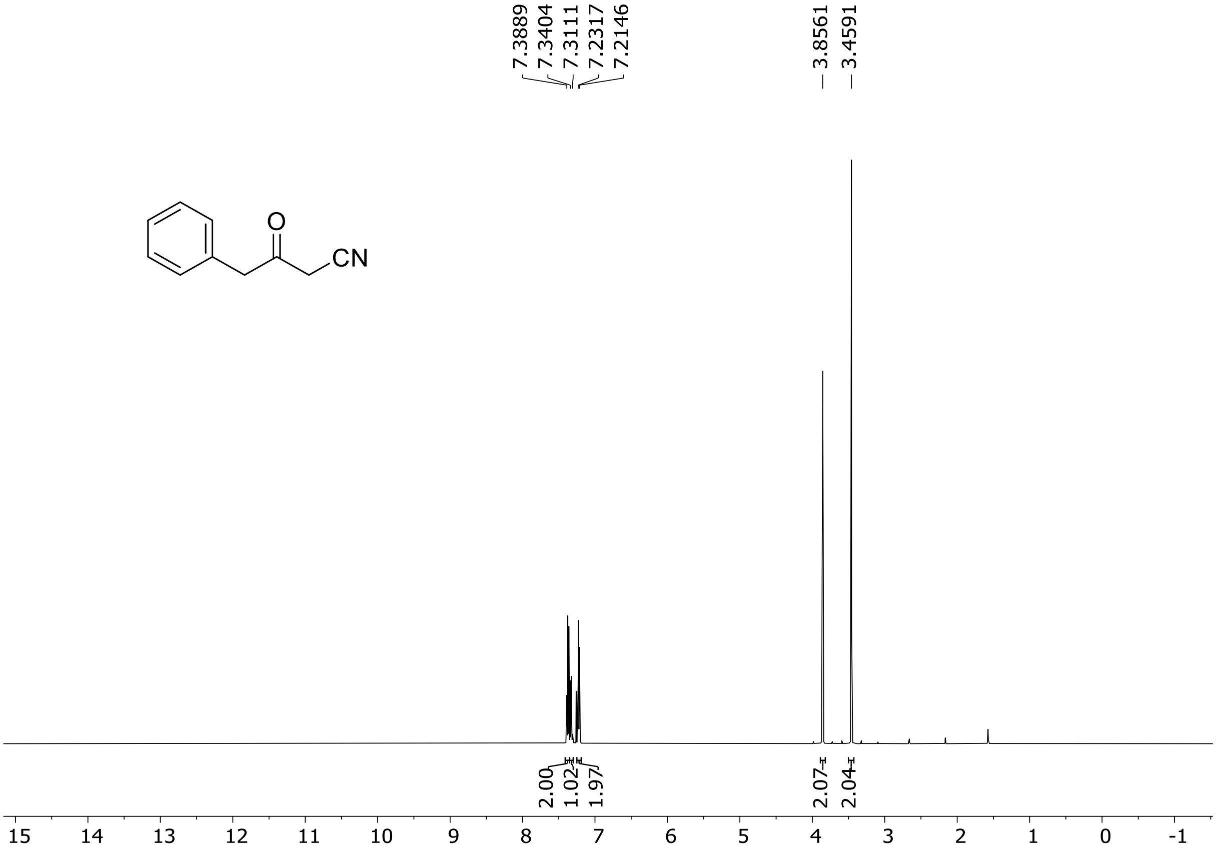

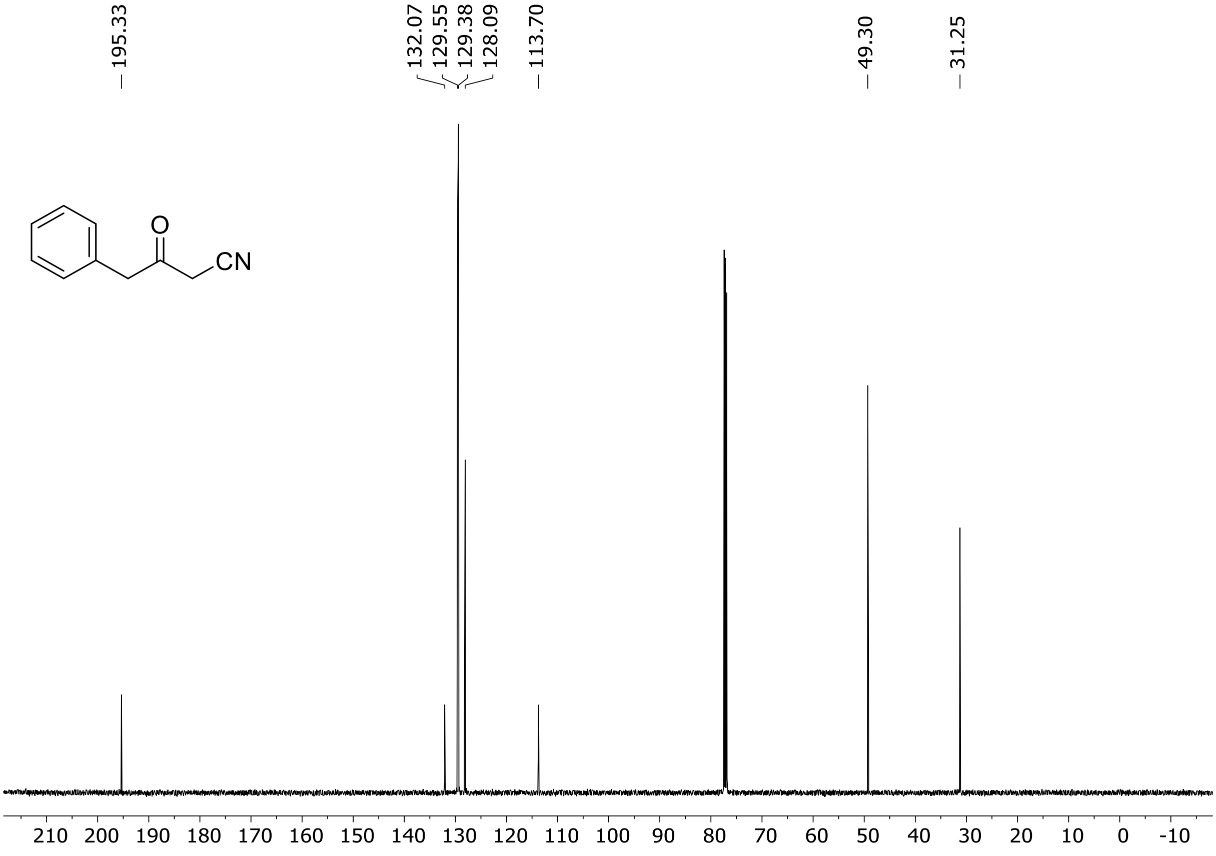

^{1}H (500 MHz) and ^{13}C NMR (126 MHz) spectra of **S23** in chloroform-*d*

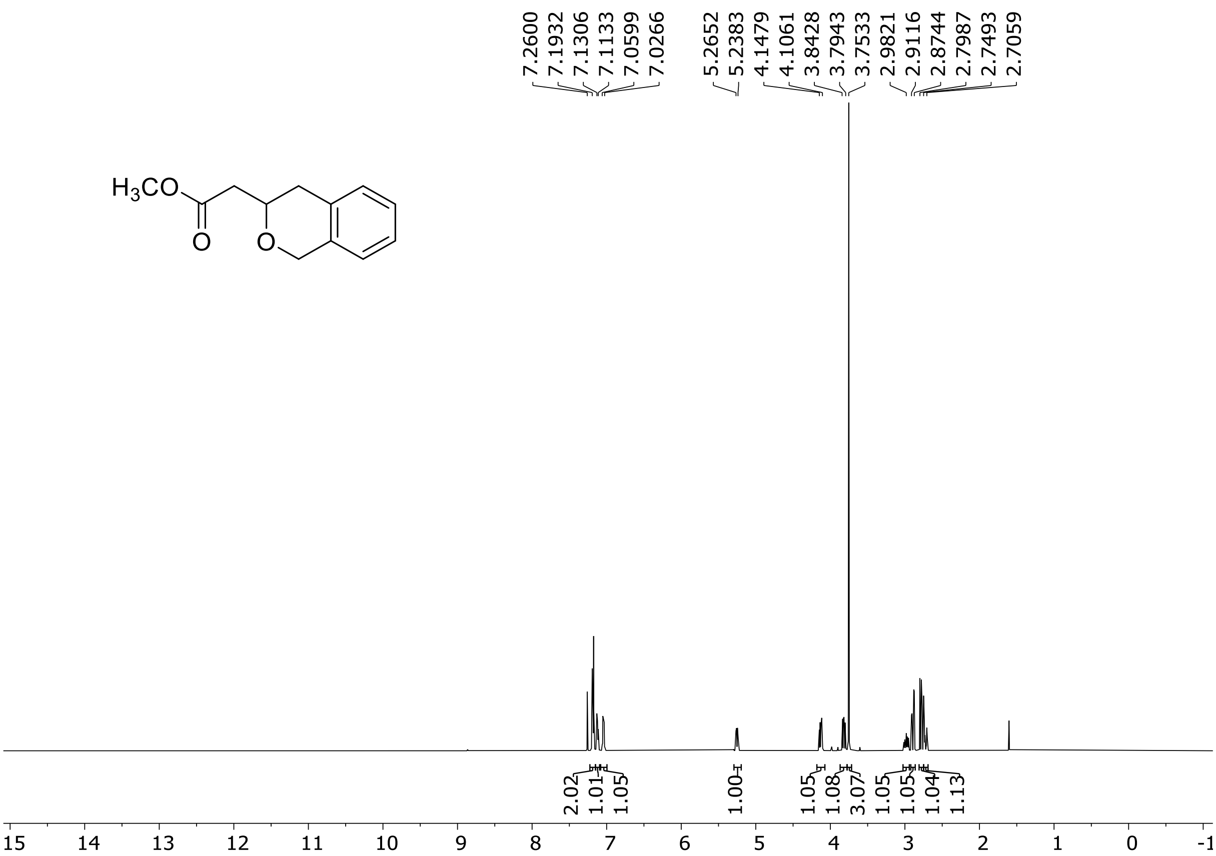

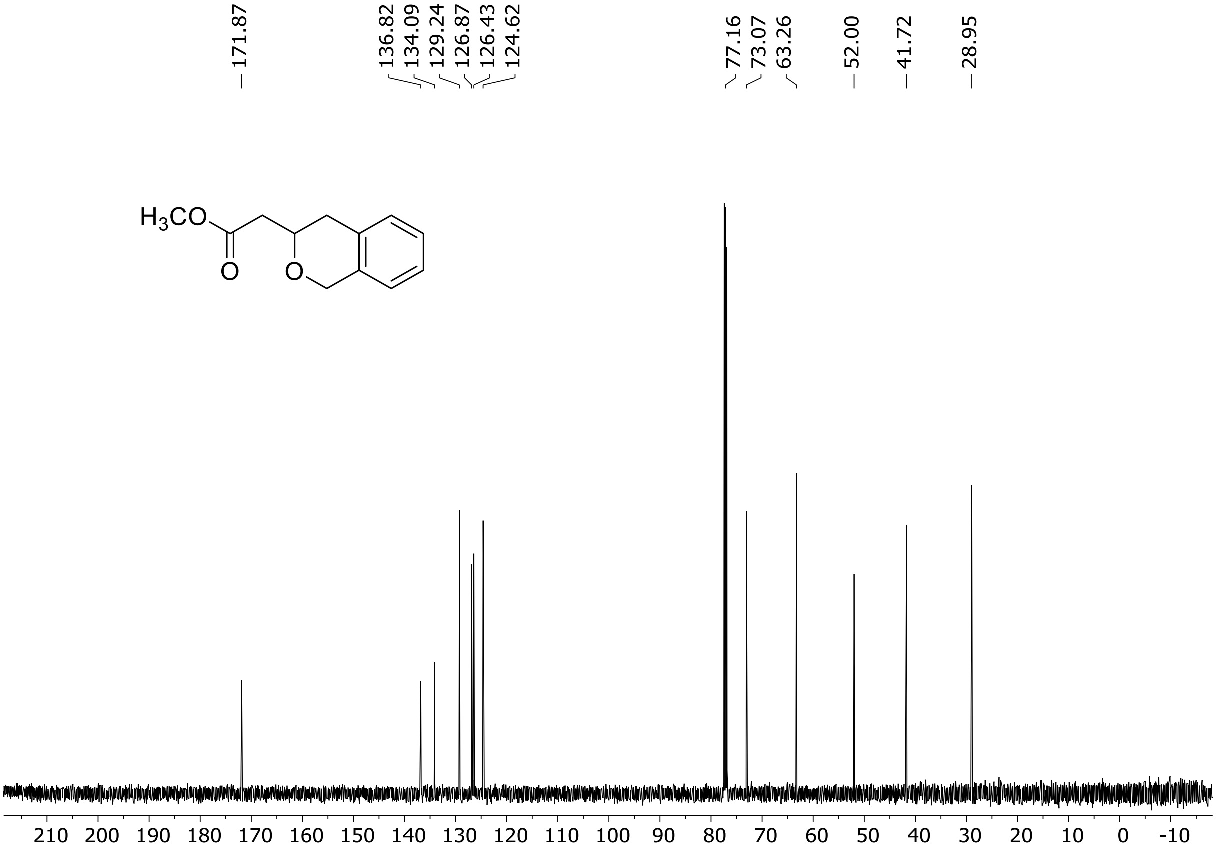

HRMS spectrum of **S23**

$C_{12}H_{14}O_3$ **exact mass: 206.0943**

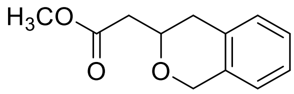

APCI + (MMI)
nitrogen flow 3 L/min, gas temperature 325°C, nebulizer 45 psig, skimmer 65 V, vaporizer 200°C, fragmentor 22 V, dissolved in methanol

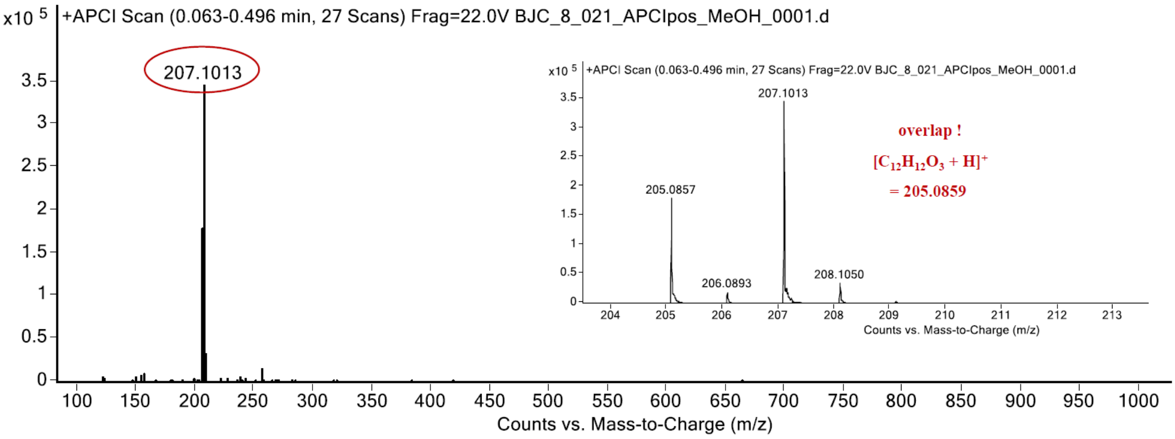

expected mass: $[M+H]^+$ = 207.1016 observed mass: $[M+H]^+$ = 207.1013 mass accuracy = - 1.4 ppm

^{1}H (500 MHz) and ^{13}C NMR (126 MHz) spectra of **S24** in chloroform-*d*

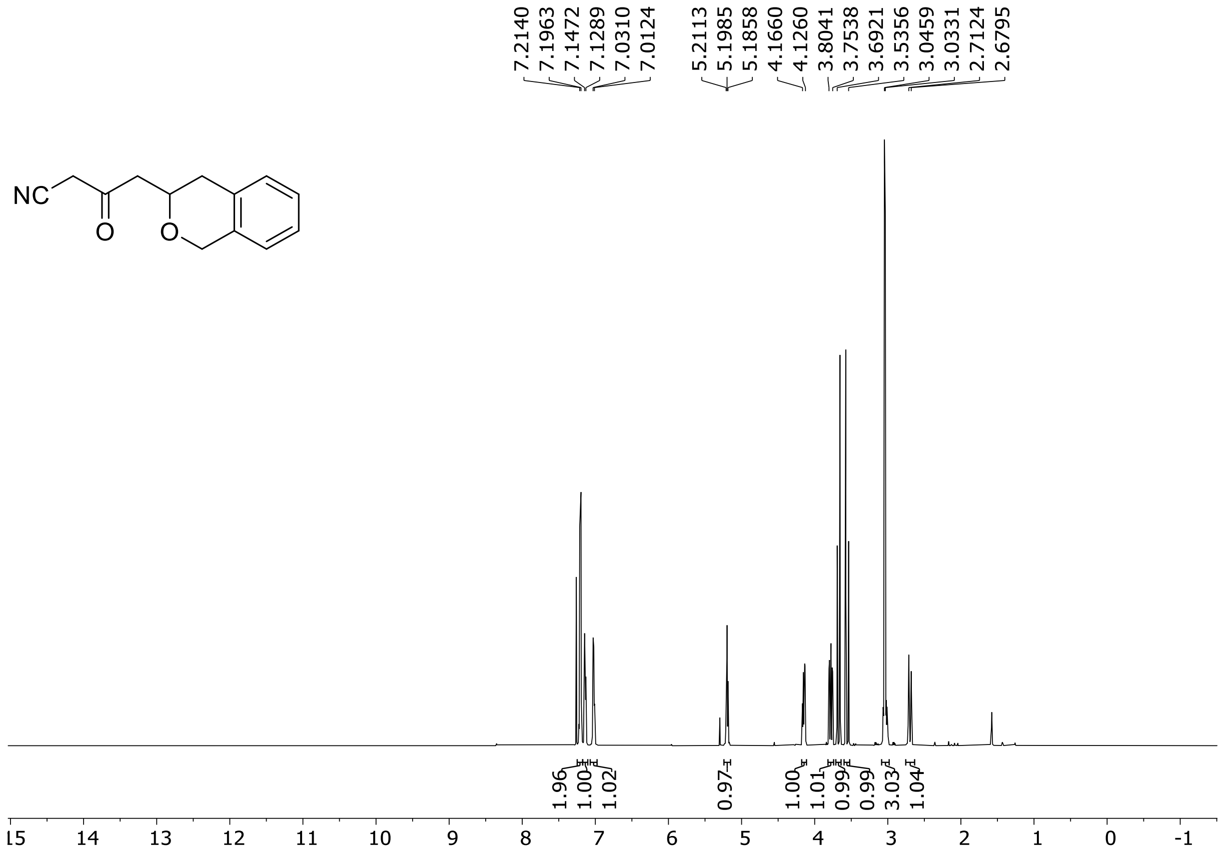

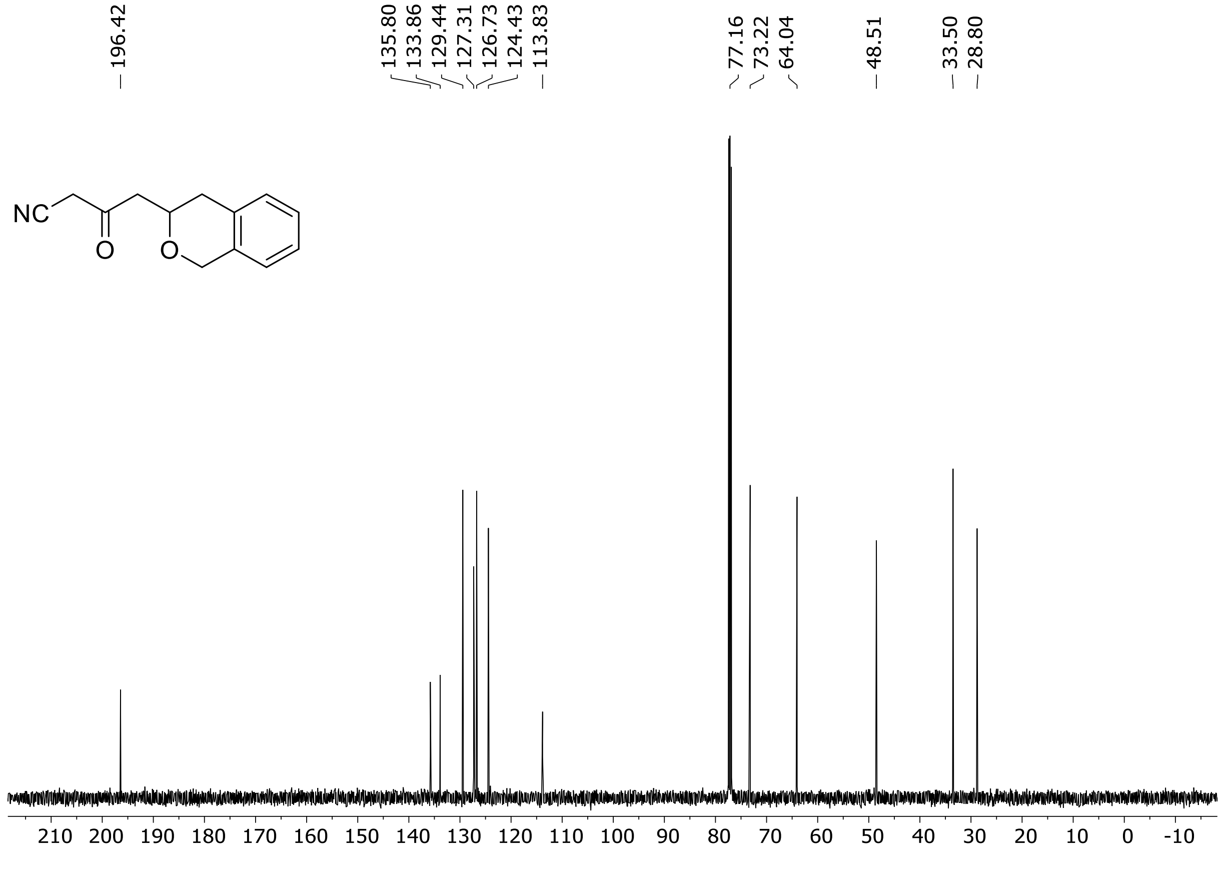

HRMS spectrum of **S24**

$C_{13}H_{13}NO_2$ **exact mass: 215.0946**

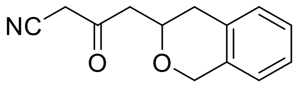

APCI + (MMI)
nitrogen flow 3 L/min, gas temperature 325°C, nebulizer 45 psig, skimmer 65 V, vaporizer 200°C, fragmentor 20 V, dissolved in methanol

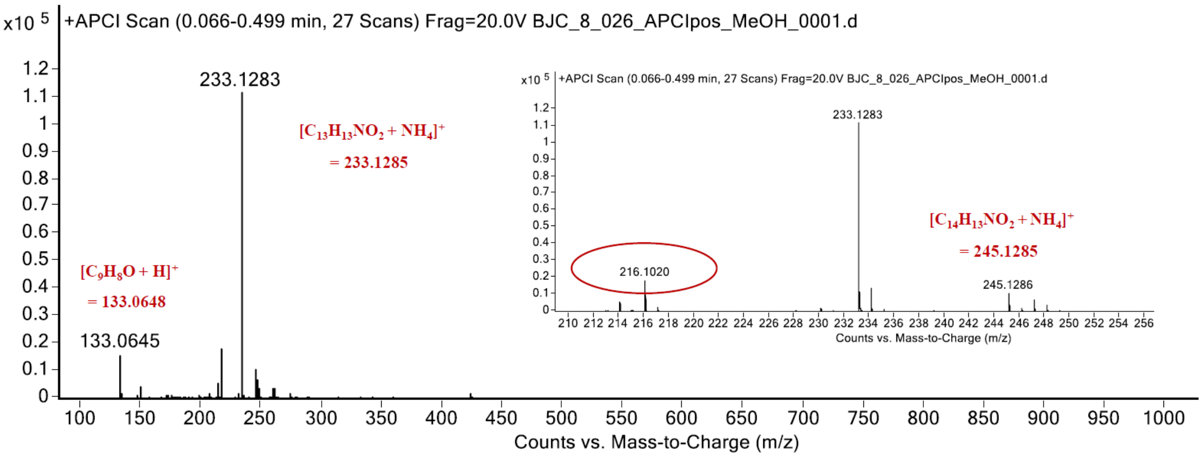

expected mass: $[M+H]^+ = 216.1019$ observed mass: $[M+H]^+ = 216.1020$ mass accuracy = 0.5 ppm

^{1}H (500 MHz) NMR spectrum of **S26** in chloroform-*d*

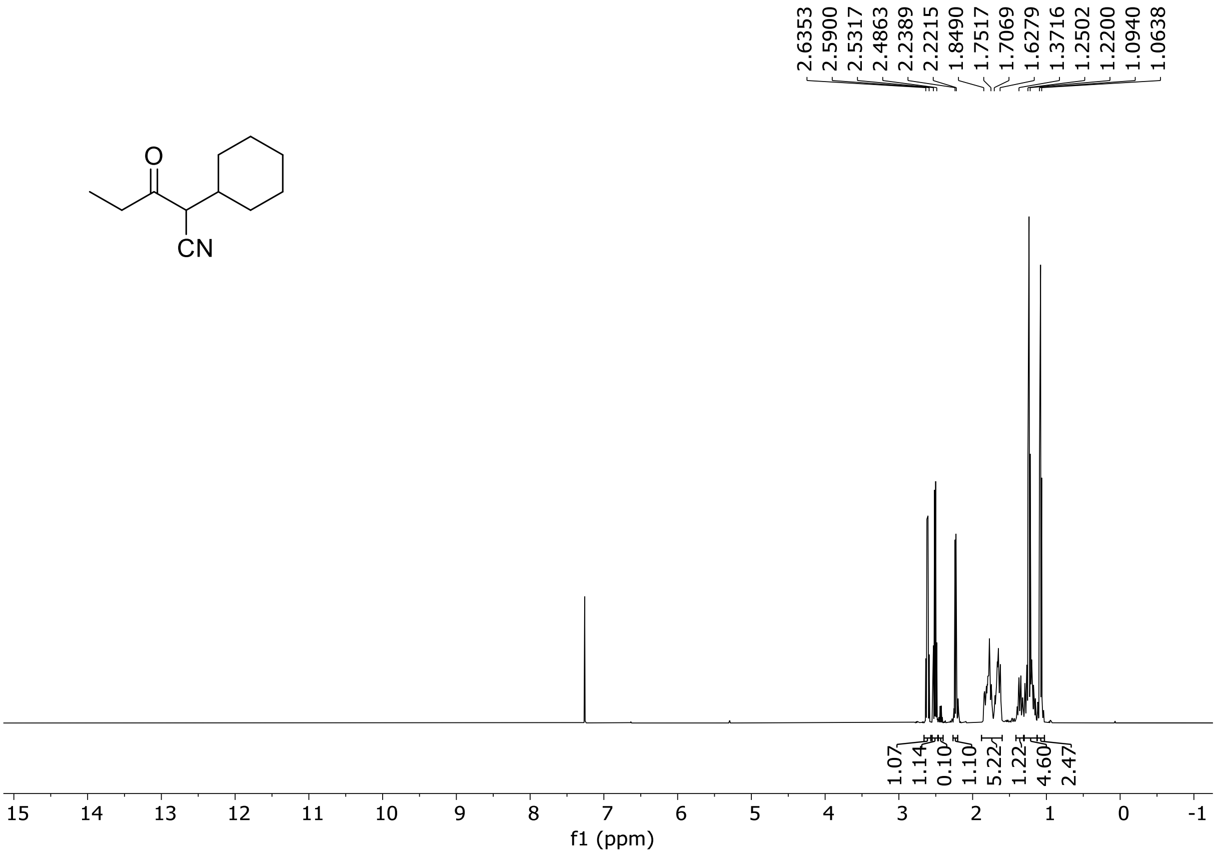

HRMS spectrum of **S26**

$C_{11}H_{17}NO$ **exact mass: 179.1310**

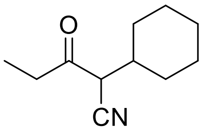

APCI + (MMI)
nitrogen flow 3 L/min, gas temperature 325°C, nebulizer 45 psig, skimmer 65 V, vaporizer 200°C, fragmentor 30 V, dissolved in methanol

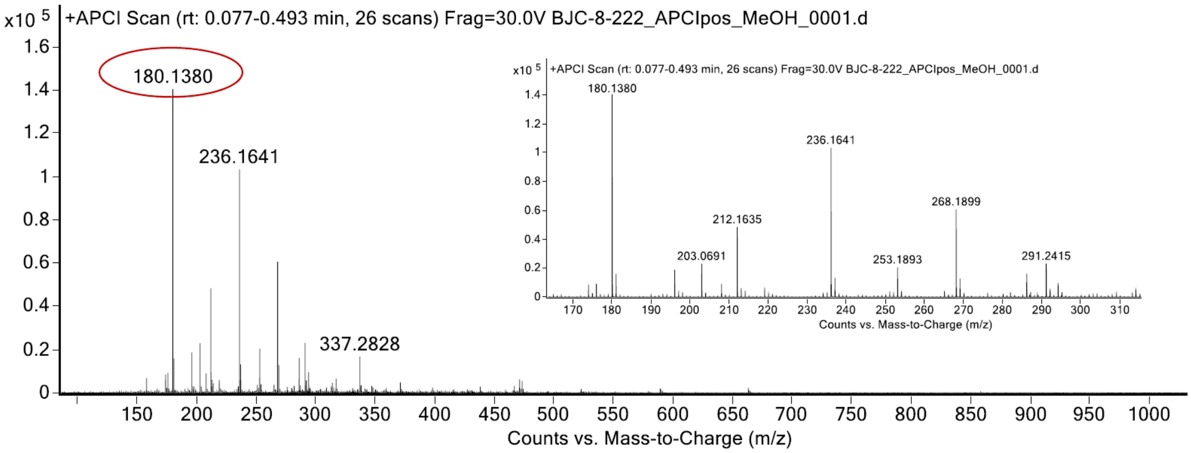

expected mass: [M+H]$^{+}$ = 180.1383 observed mass: [M+H]$^{+}$ = 180.1380 mass accuracy = - 1.7 ppm

^{1}H (500 MHz) NMR spectrum of **S27** in chloroform-*d*

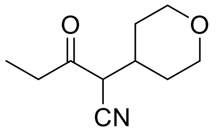

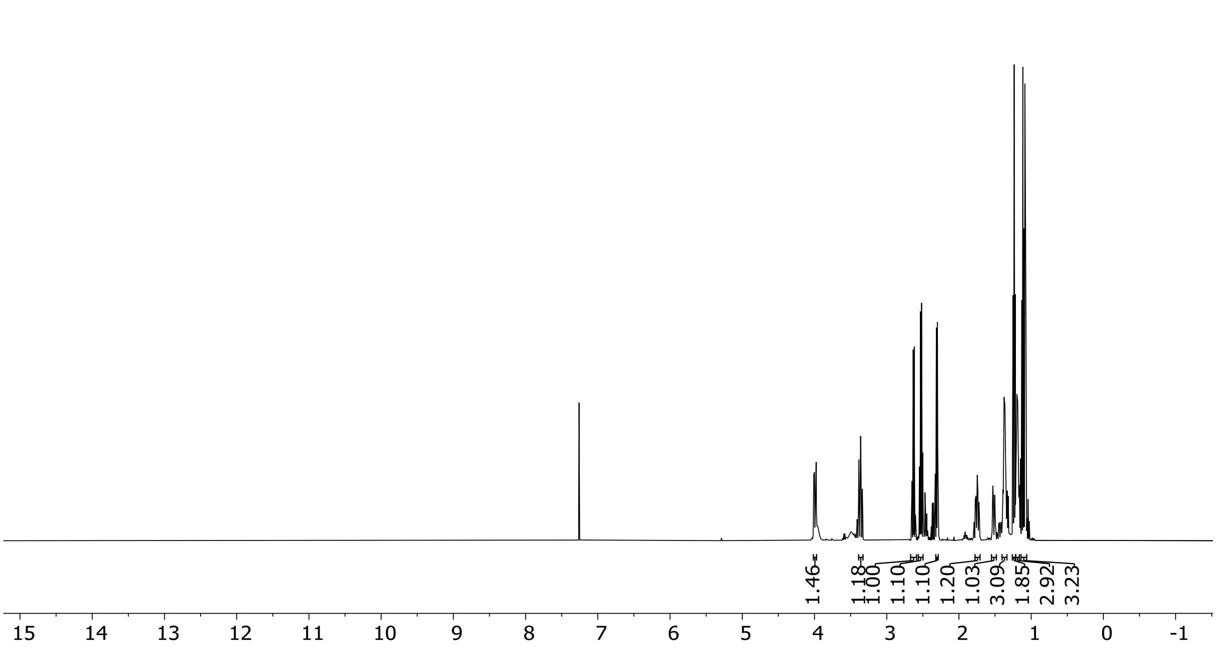

^{1}H (500 MHz) NMR spectrum of **S28** in chloroform-*d*

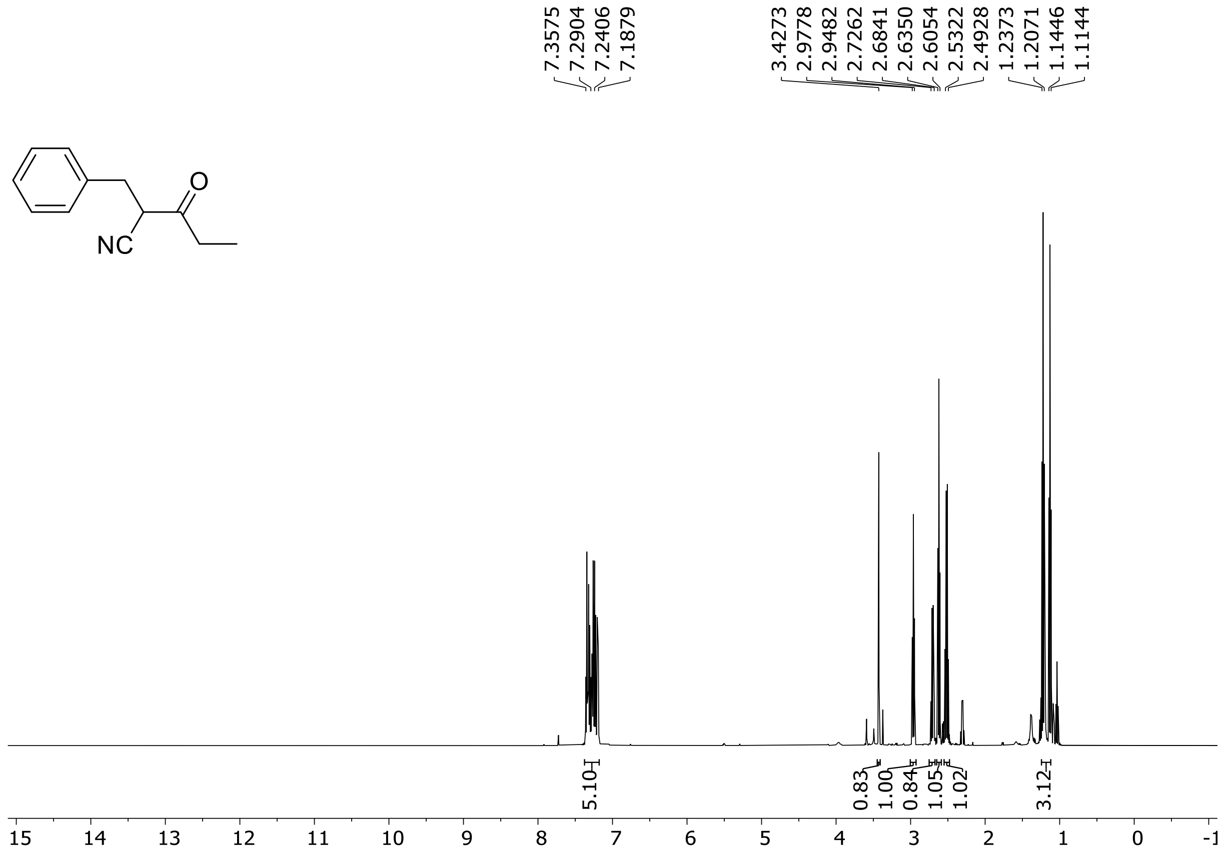

^{1}H (500 MHz) and ^{13}C NMR (126 MHz) spectra of **S35** in chloroform-*d*

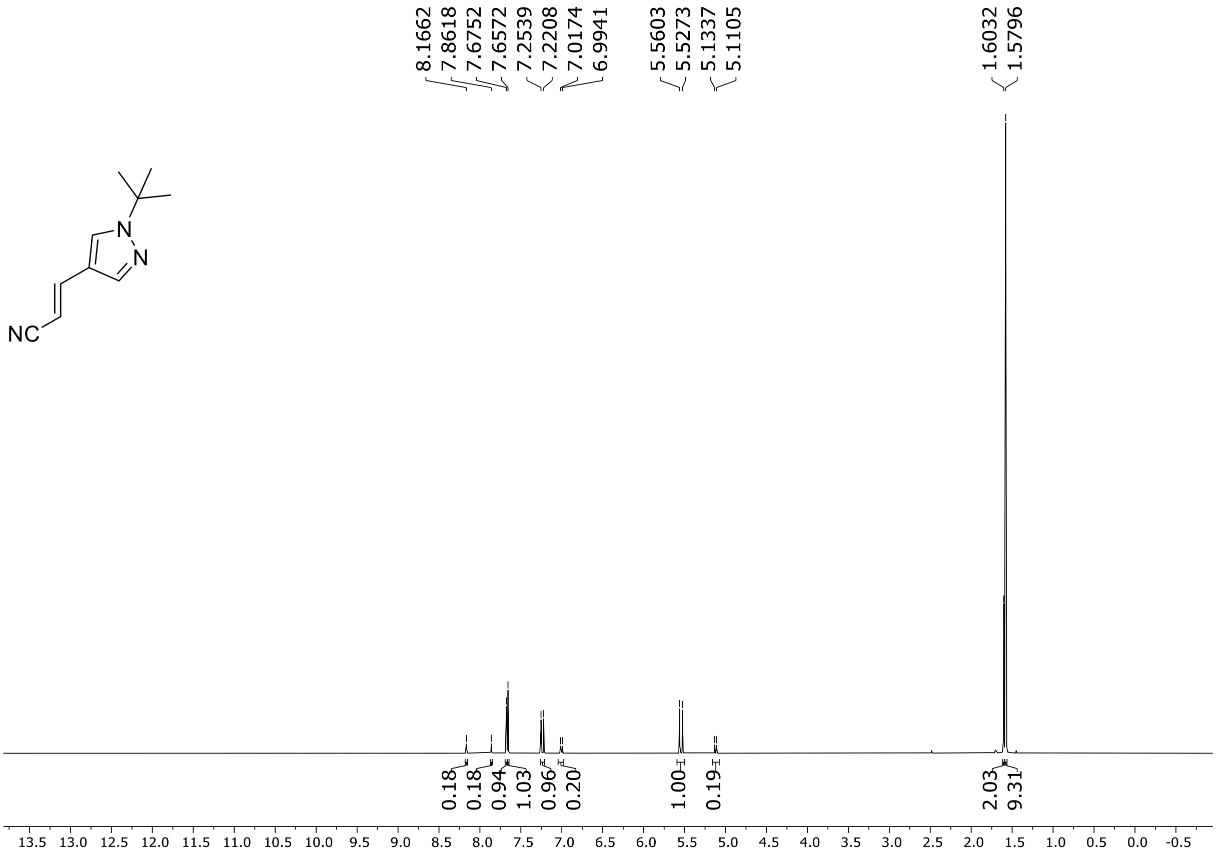

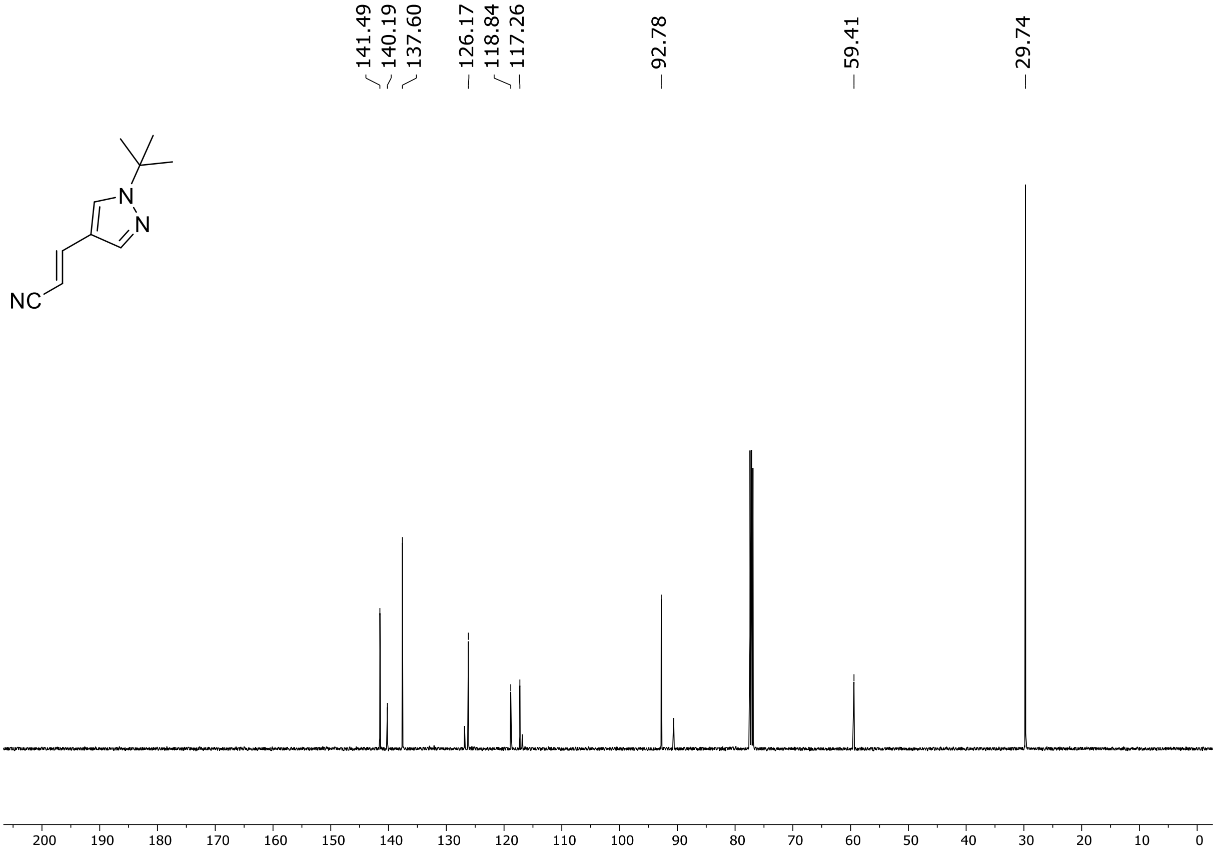

HRMS spectrum of **S35**

$C_{10}H_{13}N_3$ **exact mass: 175.1109**

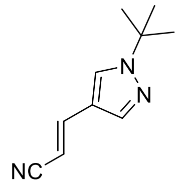

APCI + (MMI)
nitrogen flow 5 L/min, gas temperature 325°C, nebulizer 45 psig, skimmer 65 V, vaporizer 200°C, fragmentor 18 V, dissolved in methanol

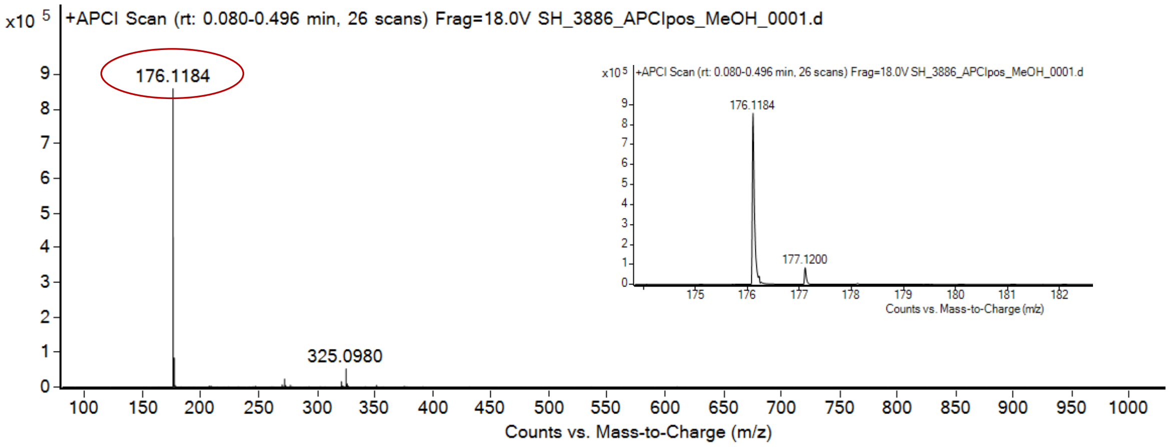

expected mass: $[M+H]^+$ = 176.1182 observed mass: $[M+H]^+$ = 176.1184 mass accuracy = 1.1 ppm

^{1}H (500 MHz) and ^{13}C NMR (126 MHz) spectra of **S36** in chloroform-*d*

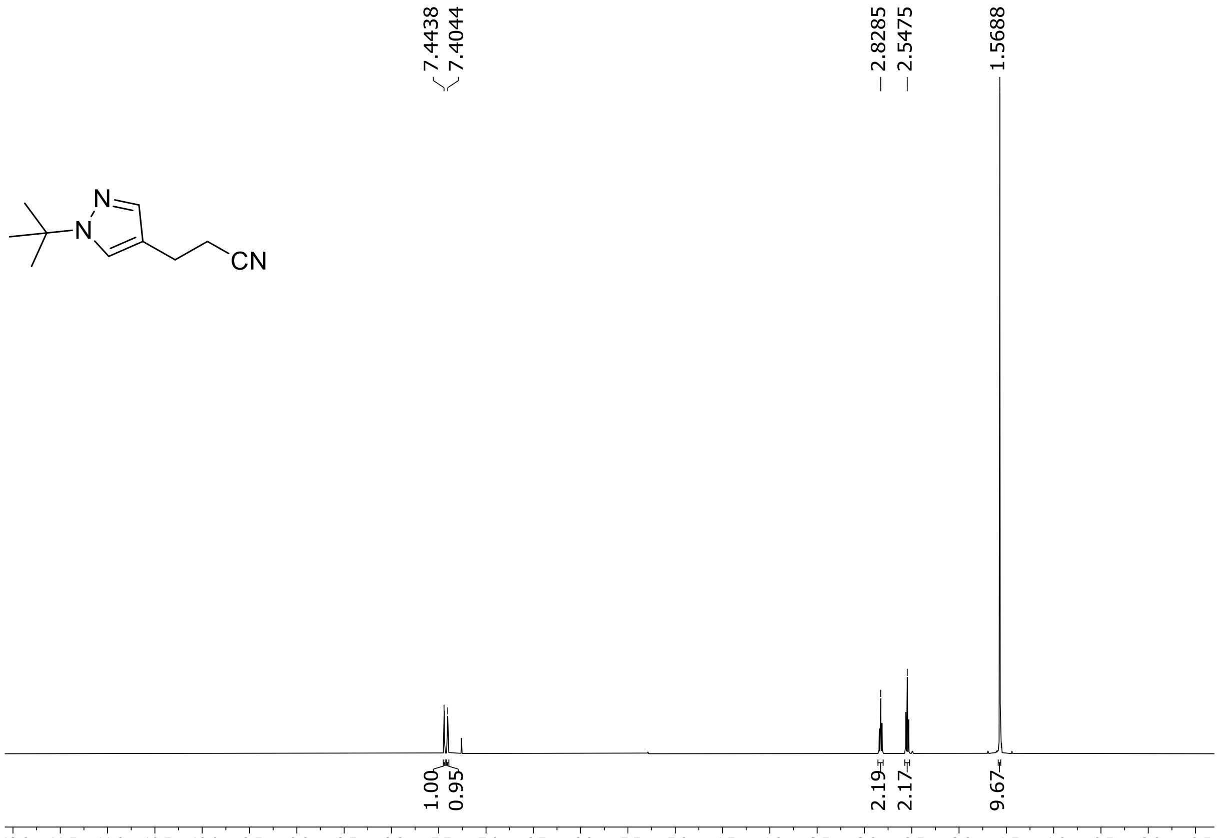

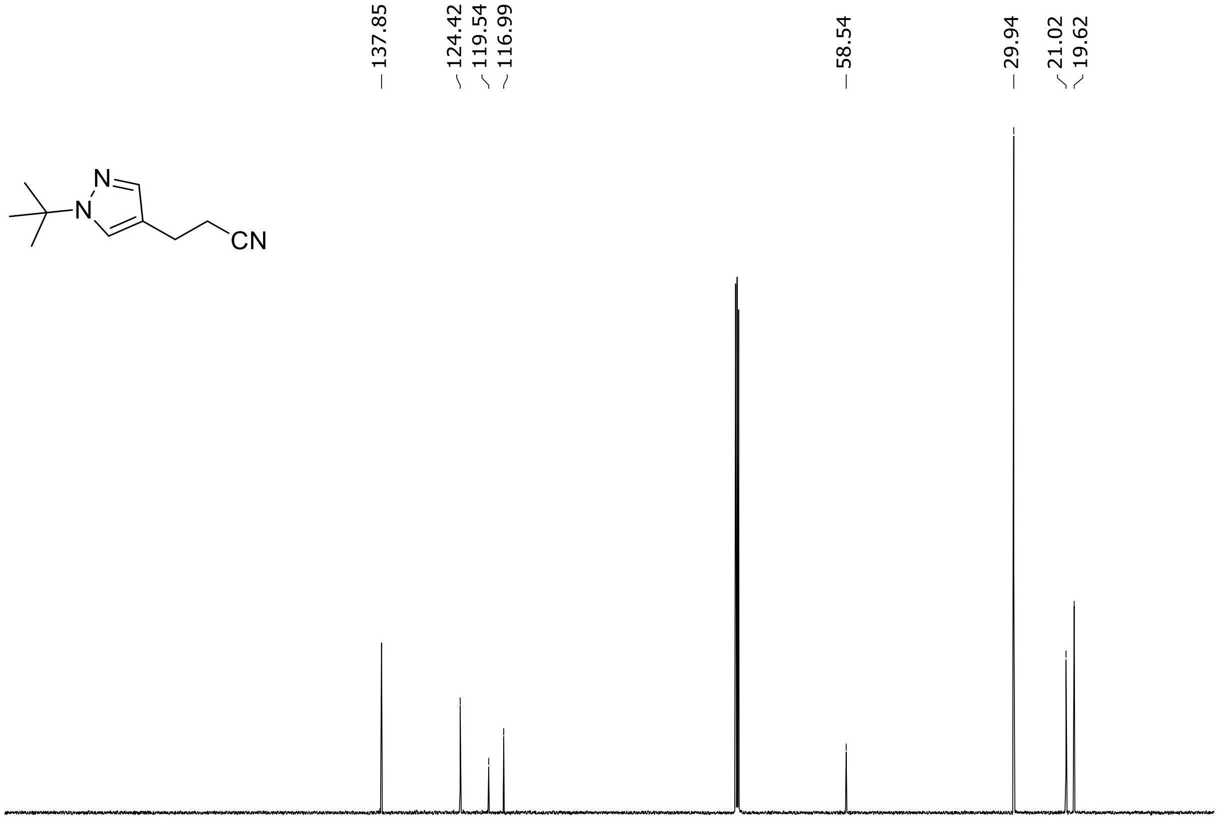

HRMS spectrum of **S36**

$C_{10}H_{15}N_3$ **exact mass: 177.1266**

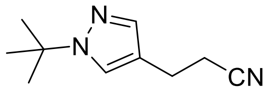

APCI + (MMI)
nitrogen flow 5 L/min, gas temperature 325°C, nebulizer 45 psig, skimmer 65 V, vaporizer 200°C, fragmentor 20 V, dissolved in methanol

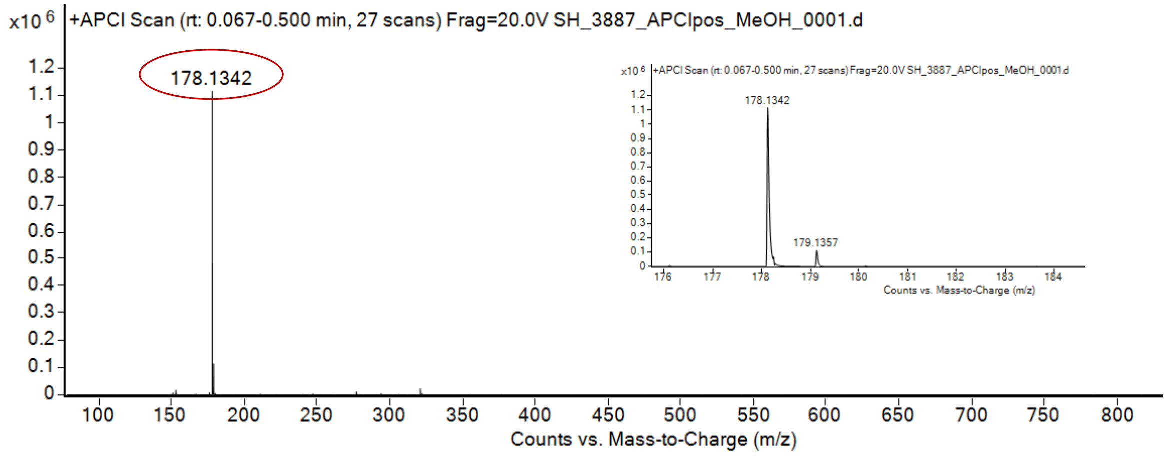

expected mass: $[M+H]^+$ = 178.1339 observed mass: $[M+H]^+$ = 178.1342 mass accuracy = - 1.7 ppm

^{1}H (500 MHz) and ^{13}C NMR (126 MHz) spectra of **S38** in DMSO-d_6

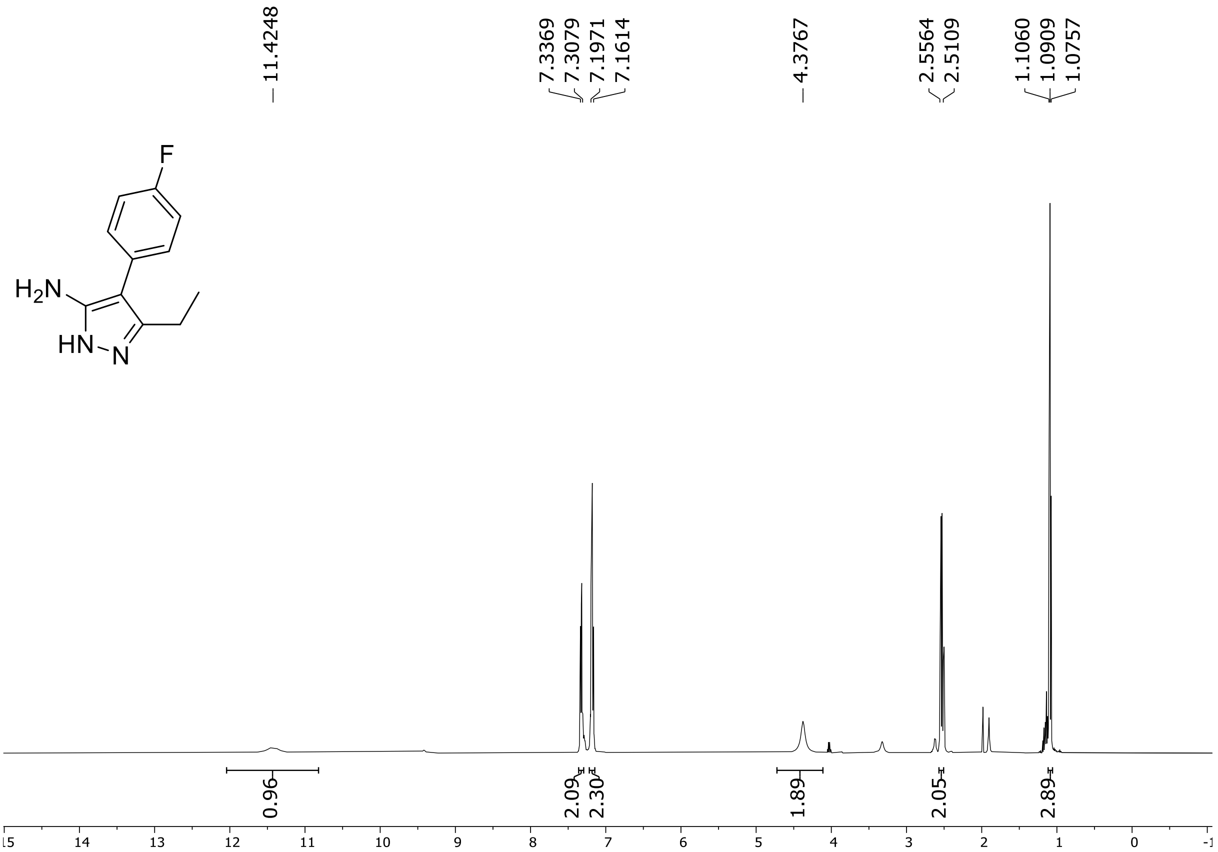

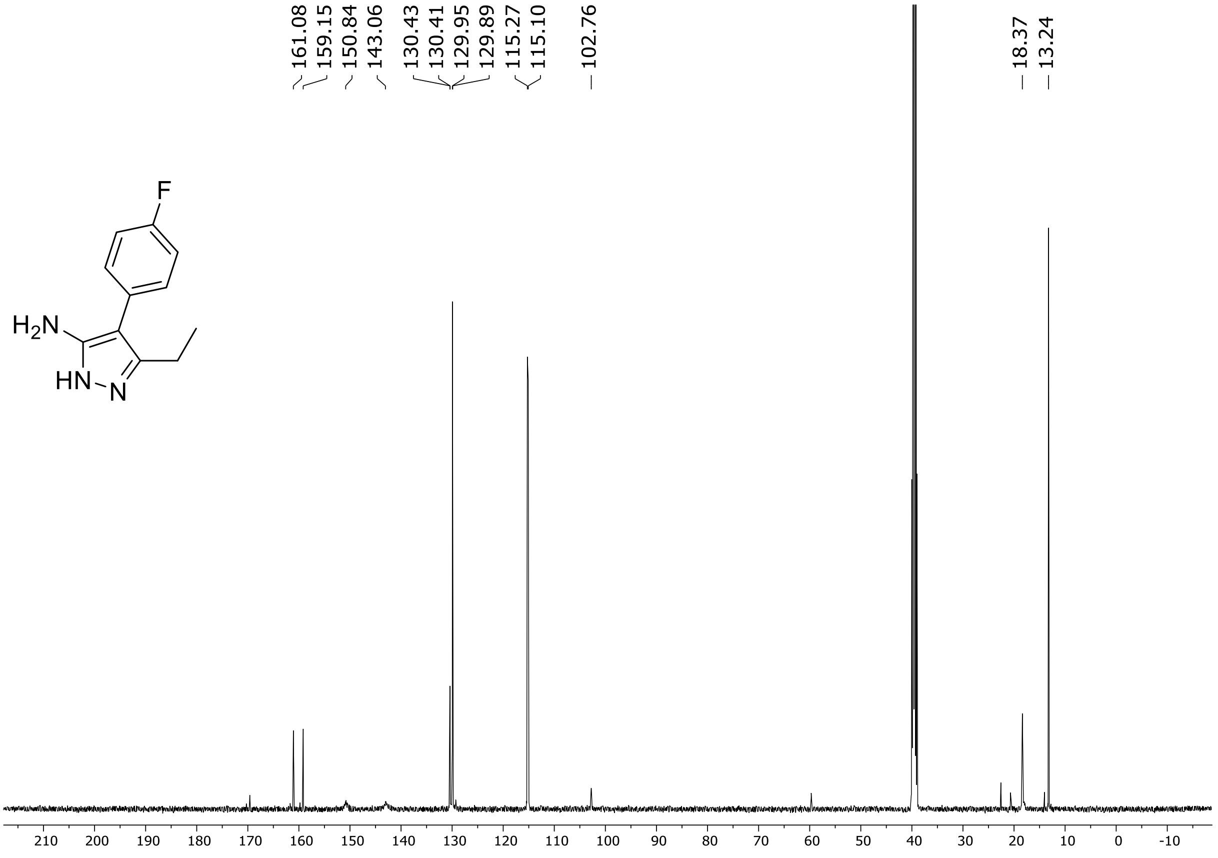

^{19}F (471 MHz) NMR spectrum of **S38** DMSO-d_6

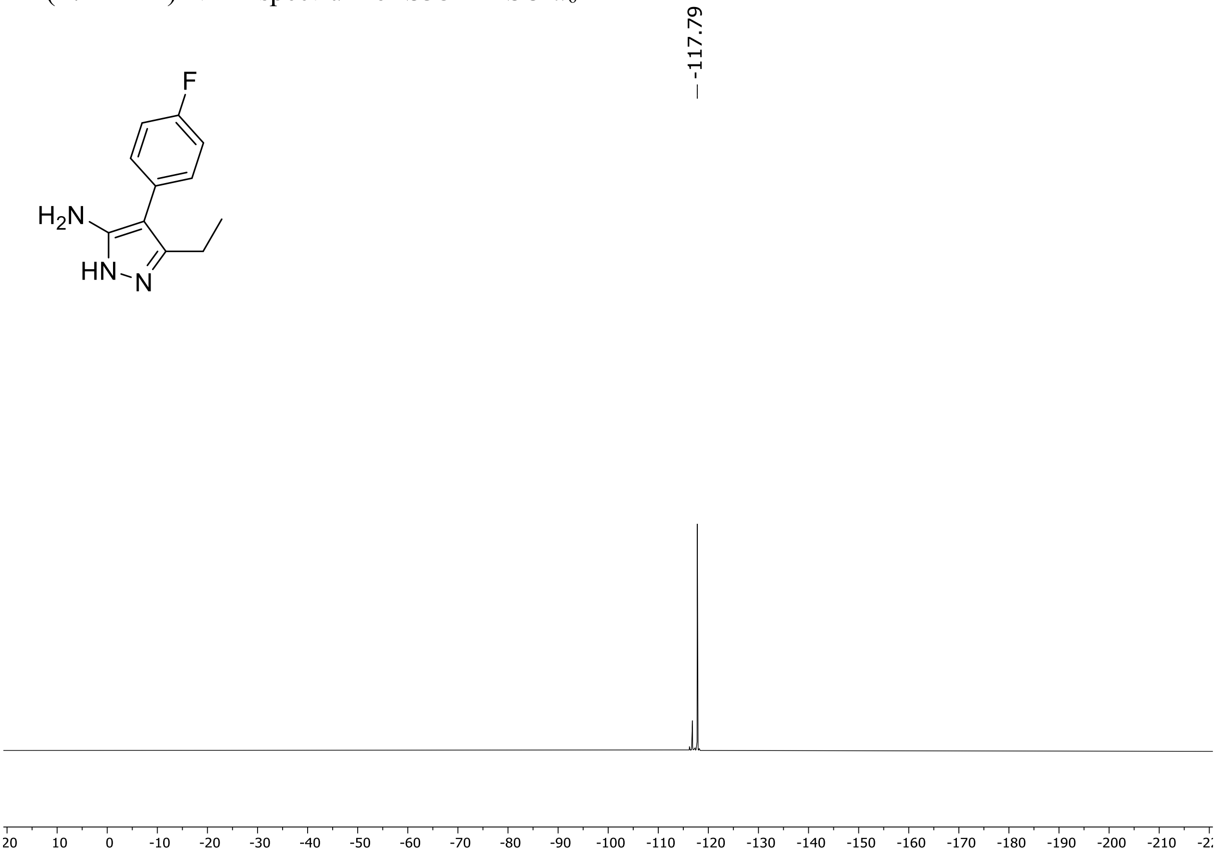

HRMS spectrum of **S38**

$C_{11}H_{12}FN_3$ **mono m/z = 205,1001**

ESI- (MMI)
nitrogen flow 5 L/min, gas temperature 300°C, vaporizer 250°C,
nebulizer 45 psi, corona current 4 uA, Vcap -2000 V,
skimmer **65** V, fragmentor **150** V, dissolved in methanol (CH3OH)

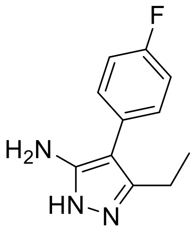

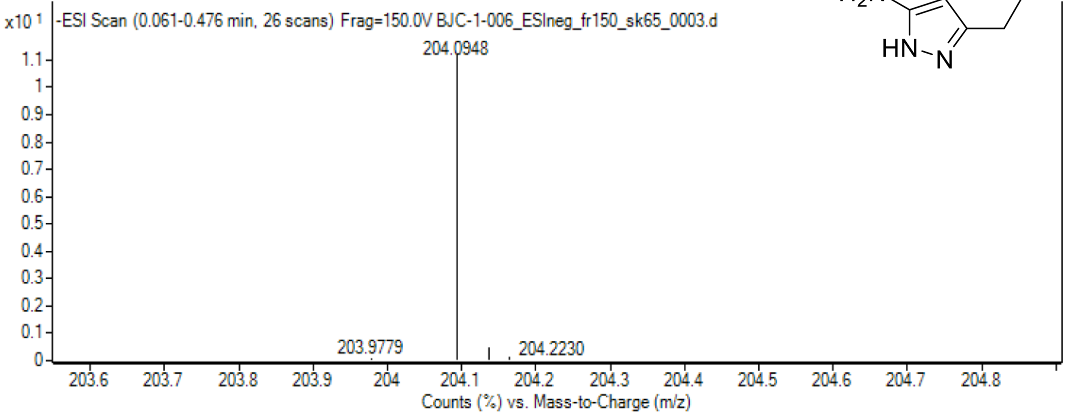

calculated mass: $[M-H]^-$ = 204,0942 observed: $[M-H]^-$ = 204,0948 max. mass error 2,9 ppm

^{1}H (500 MHz) and ^{13}C NMR (126 MHz) spectra of **S39** in DMSO-d_6

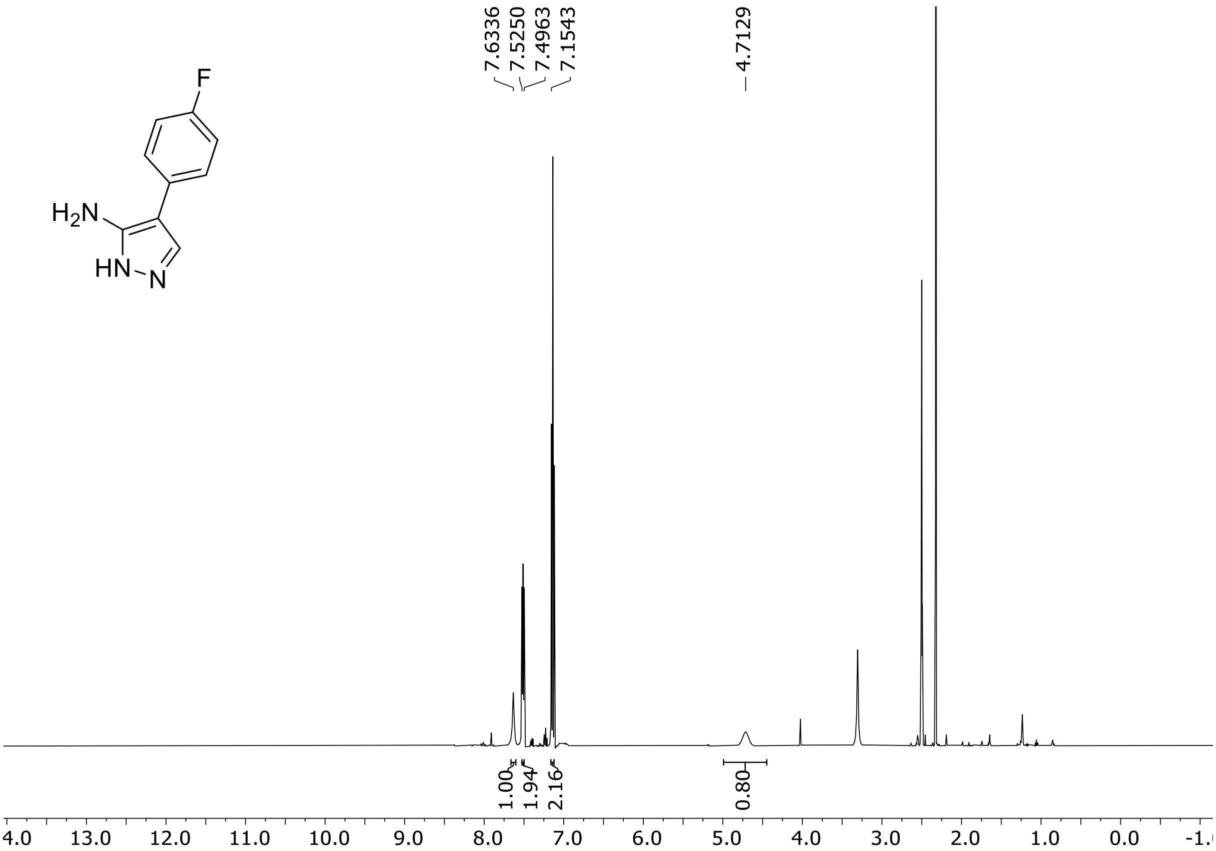

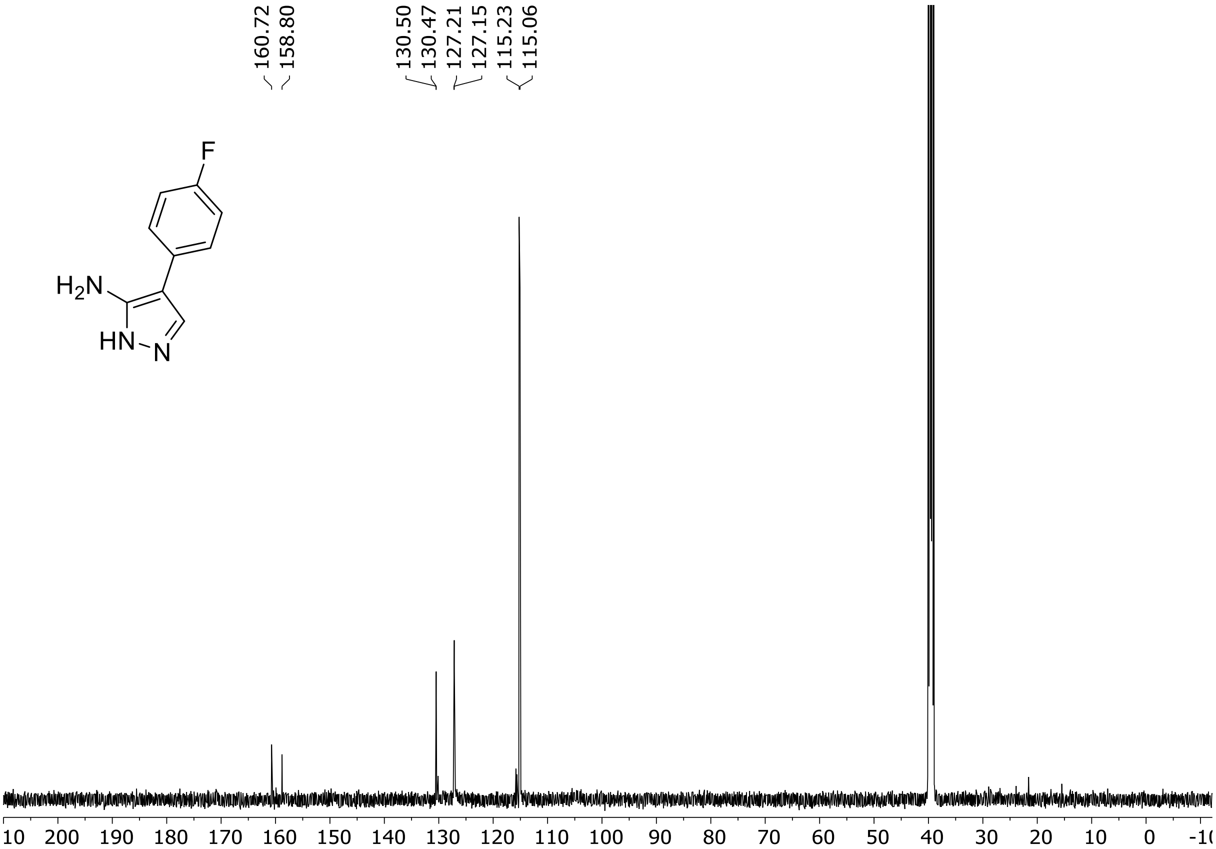

HRMS spectrum **S39**

$C_9H_8FN_3$ **mono m/z = 177,0702**

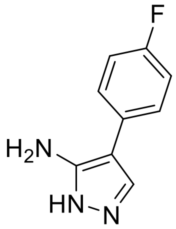

ESI - (MMI)
nitrogen flow 5 L/min, gas temperature 300°C, vaporizer 200°C, nebulizer 45 psi, Vcap -2000 V, skimmer **65** V, fragmentor **40**V, dissolved in DMSO

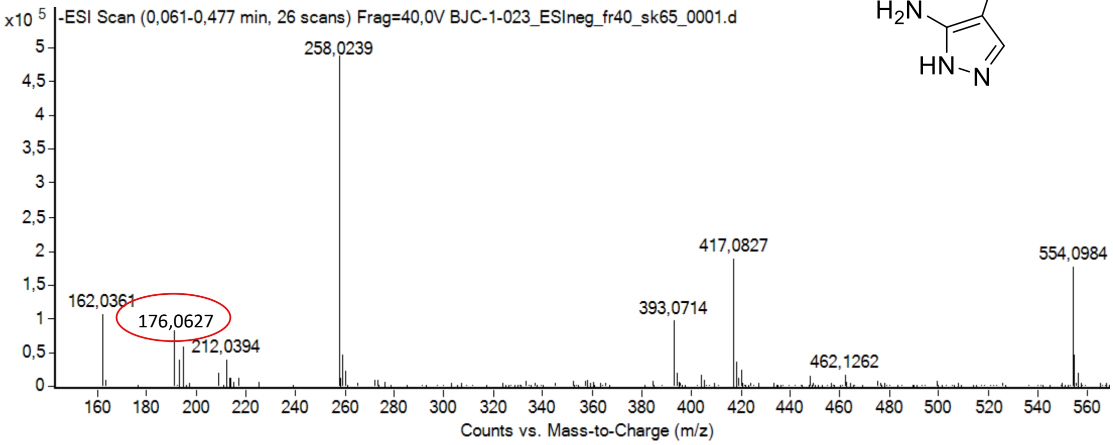

calculated mass:
$[M\text{-}H]^-$ = 176,0629 observed: $[M\text{-}H]^-$ = 176,0627 max. mass error = 1,1 ppm
$[M+Cl]^-$ = 212,0396 observed: $[M+Cl]^-$ = 212,0394 max. mass error = 0,9 ppm

^{1}H (500 MHz) and ^{13}C NMR (126 MHz) spectra of **S40** in DMSO-d_6

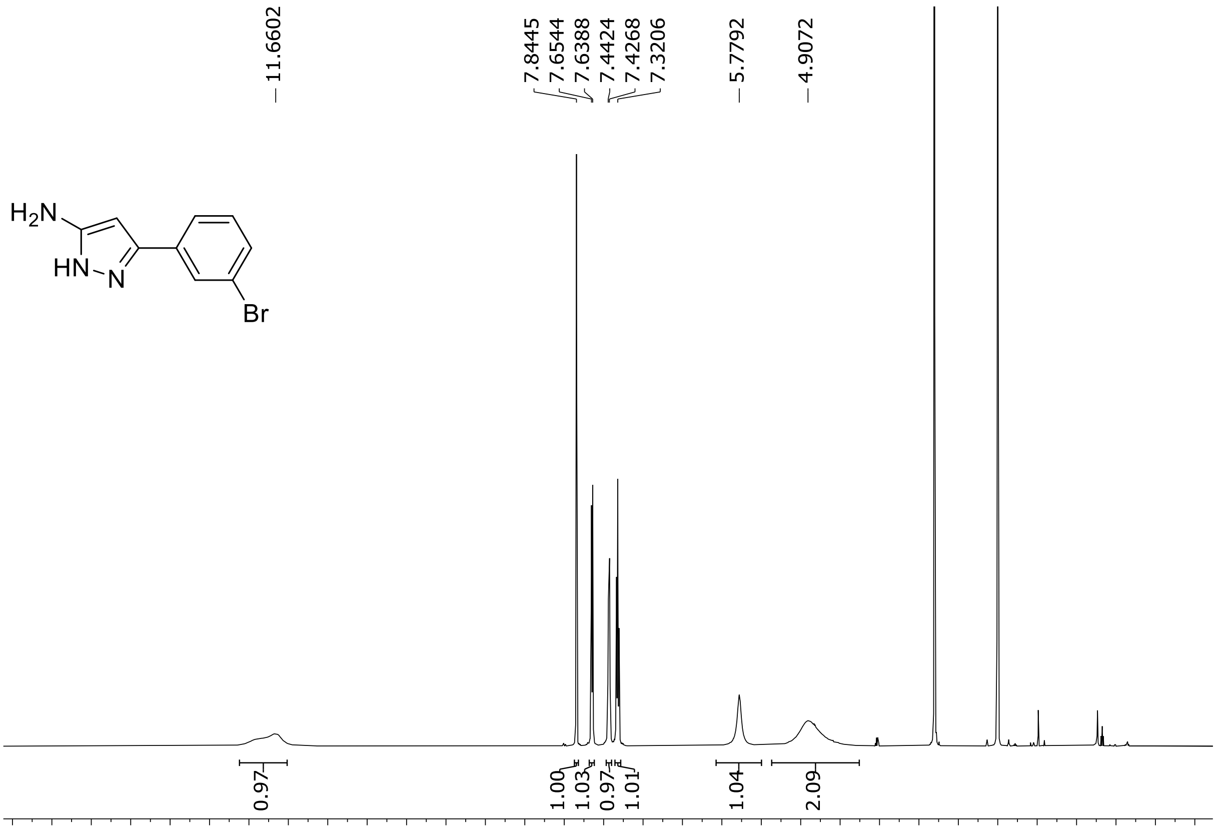

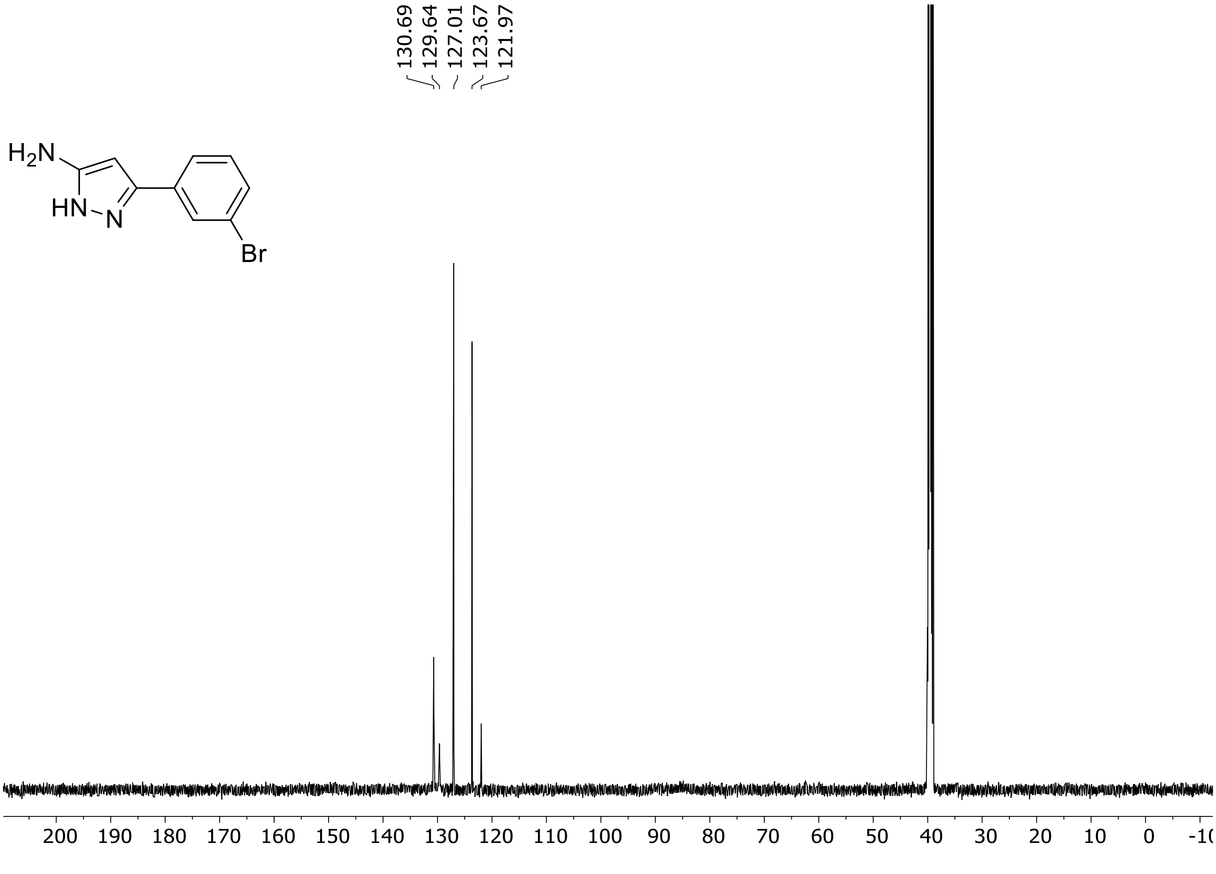

HRMS spectrum of **S40**

$C_9H_8BrN_3$ **mono m/z = 236.9902**

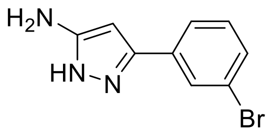

APCI + (MMI)
nitrogen flow 5 L/min, gas temperature 300°C, nebulizer 45 psi,
skimmer 60 V, fragmentor 32 V, dissolved in methanol

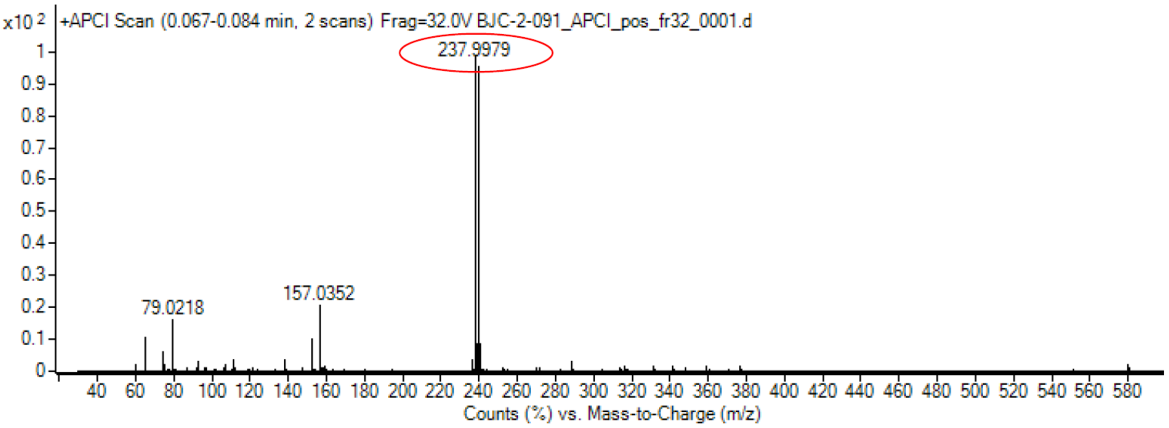

calculated mass: $[M+H]^+$ = 237.9974 observed: $[M+H]^+$ = 237.9979 max. mass error = 2.1 ppm

^{1}H (500 MHz) and ^{13}C NMR (126 MHz) spectra of **S41** in chloroform-*d*

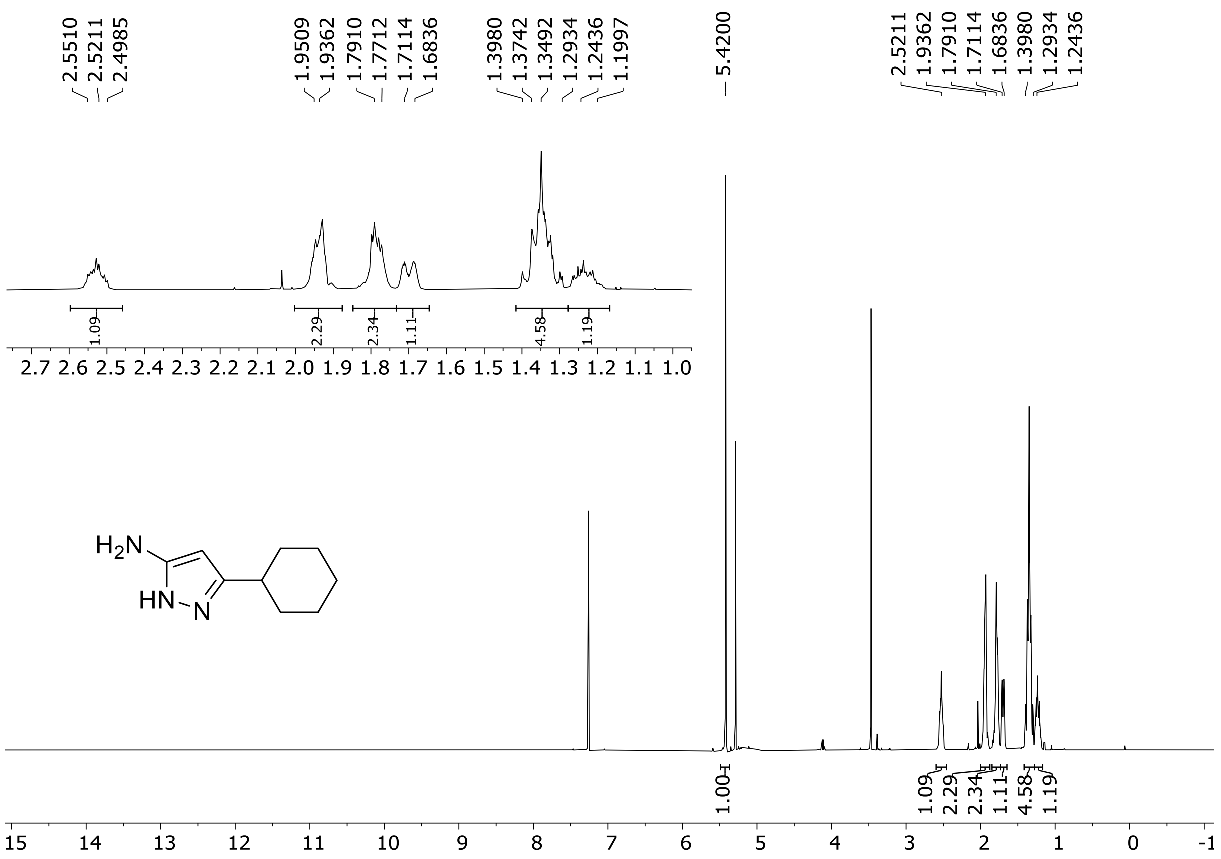

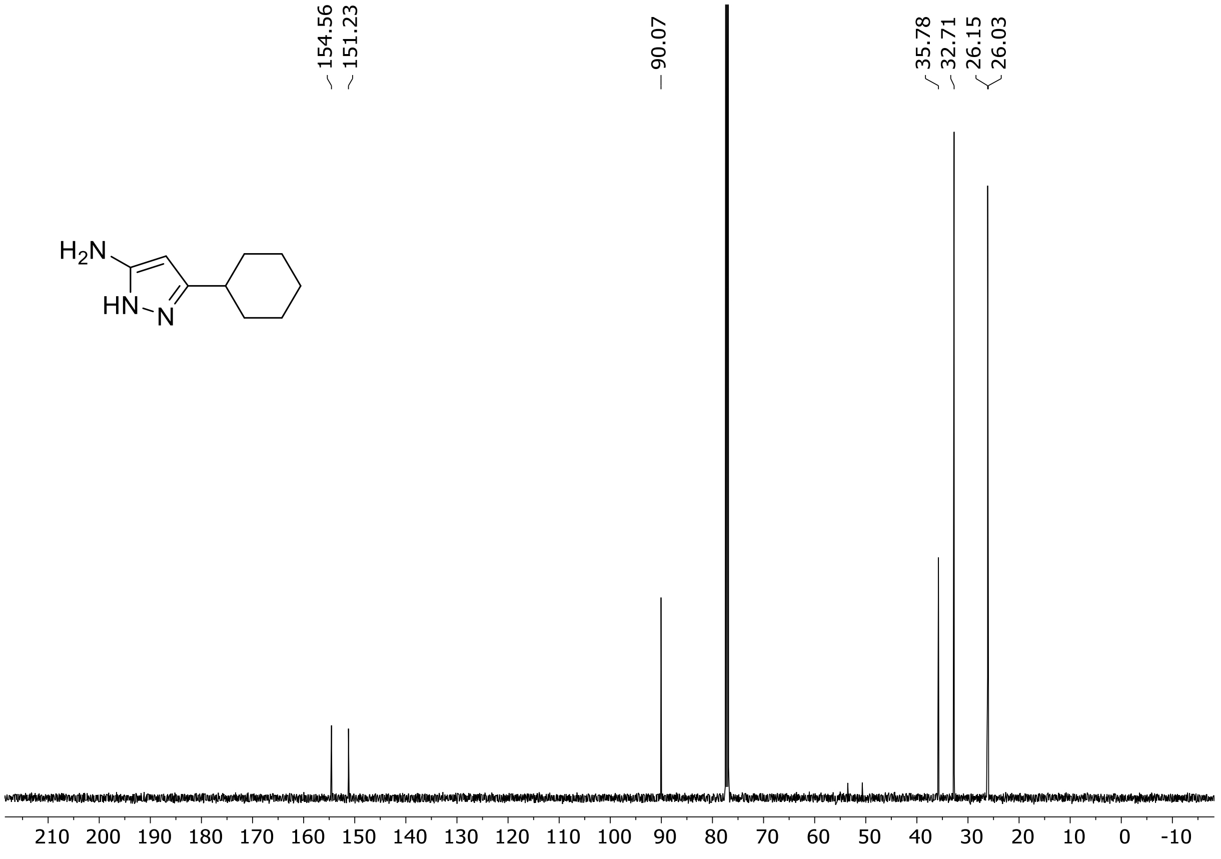

HRMS spectrum of **S41**

$C_9H_{15}N_3$ **mono m/z = 165.1266**

APCI + (MMI)
nitrogen flow 5 L/min, gas temperature 300°C, nebulizer 45 psi, skimmer 60 V, fragmentor 22 V, dissolved in methanol

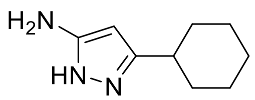

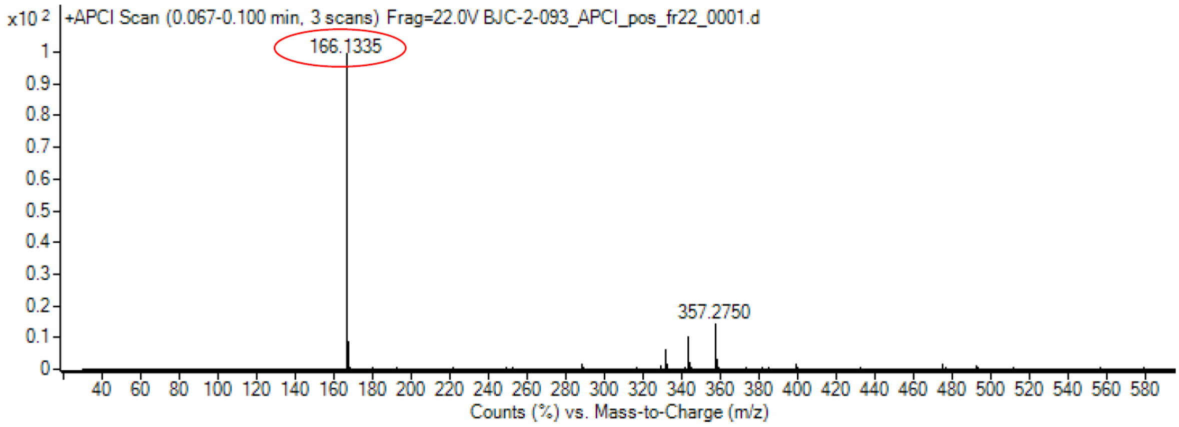

calculated mass: $[M+H]^+$= 166.1339 observed: $[M+H]^+$ = 166.1335 max. mass error = 2.4 ppm

^{1}H (500 MHz) and ^{13}C NMR (126 MHz) spectra of **S42** in DMSO-d_6

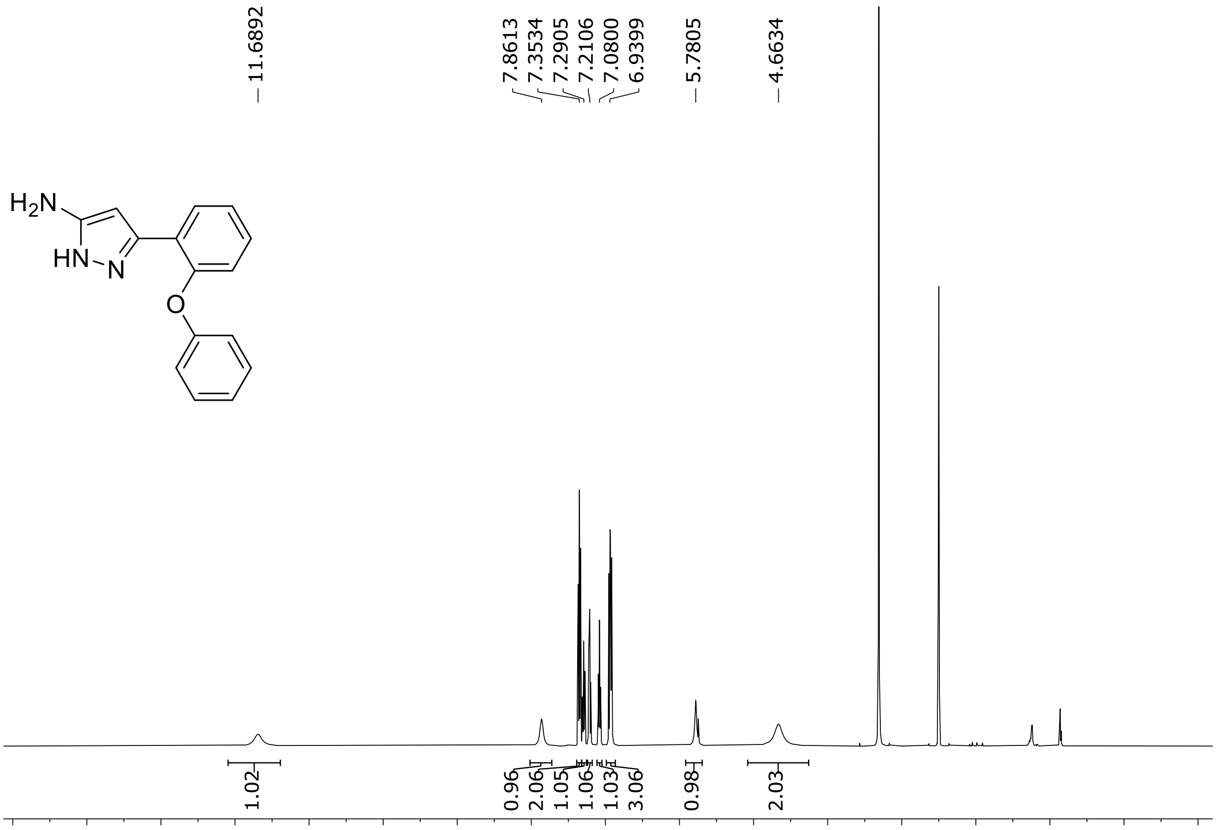

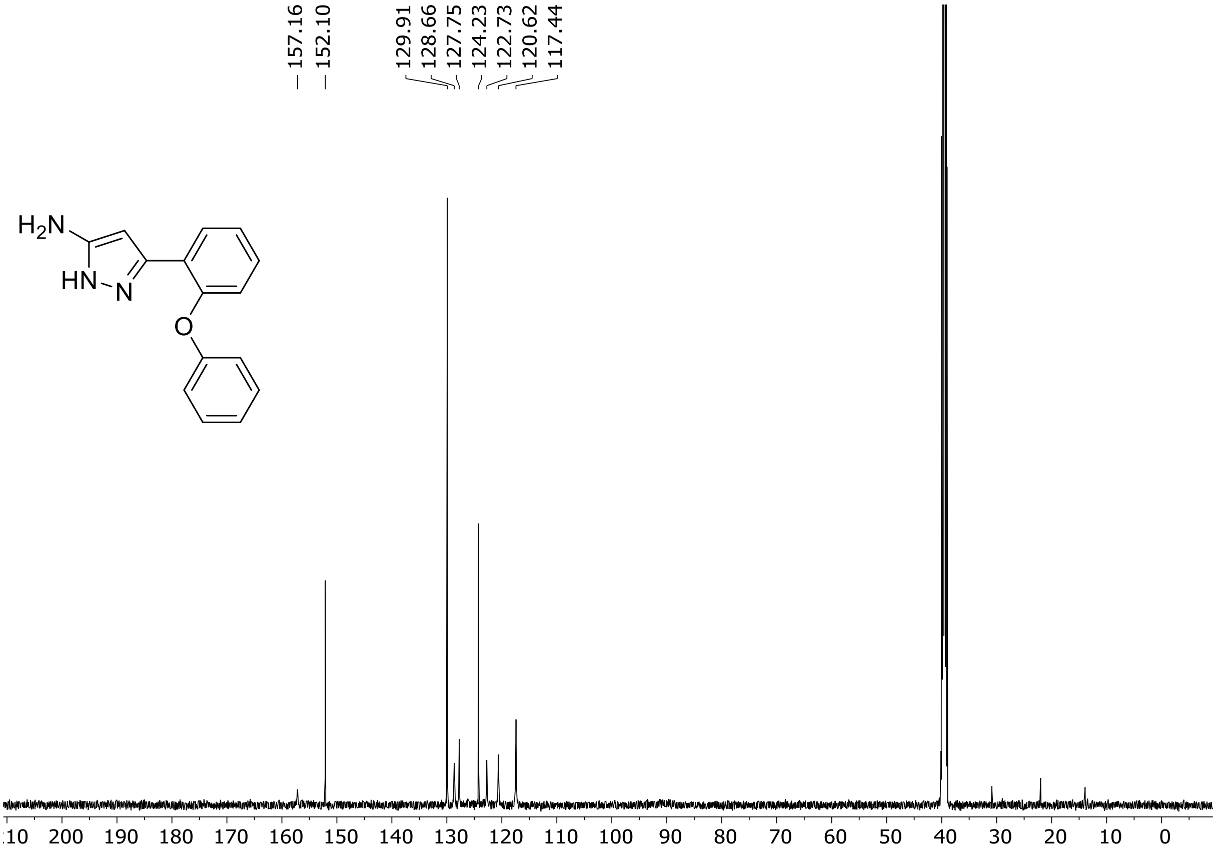

HRMS spectrum of **S42**

$C_{15}H_{13}N_3O$ mono m/z = 251.1059

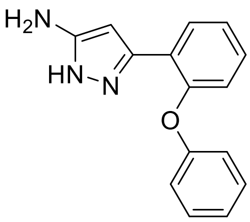

APCI + (MMI)
nitrogen flow 5 L/min, gas temperature 300°C, nebulizer 45 psi, skimmer 60 V, fragmentor 25 V, dissolved in methanol

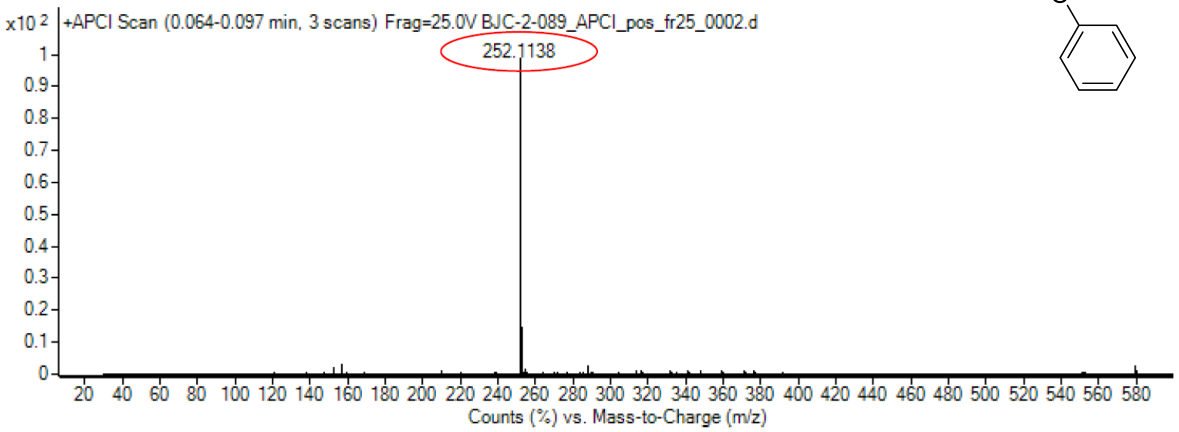

calculated mass: $[M+H]^+$ = 252.1131 observed: $[M+H]^+$ = 252.1138 max. mass error = 2.8 ppm

^{1}H (500 MHz) and ^{13}C NMR (126 MHz) spectra of **S43** in DMSO-d_6

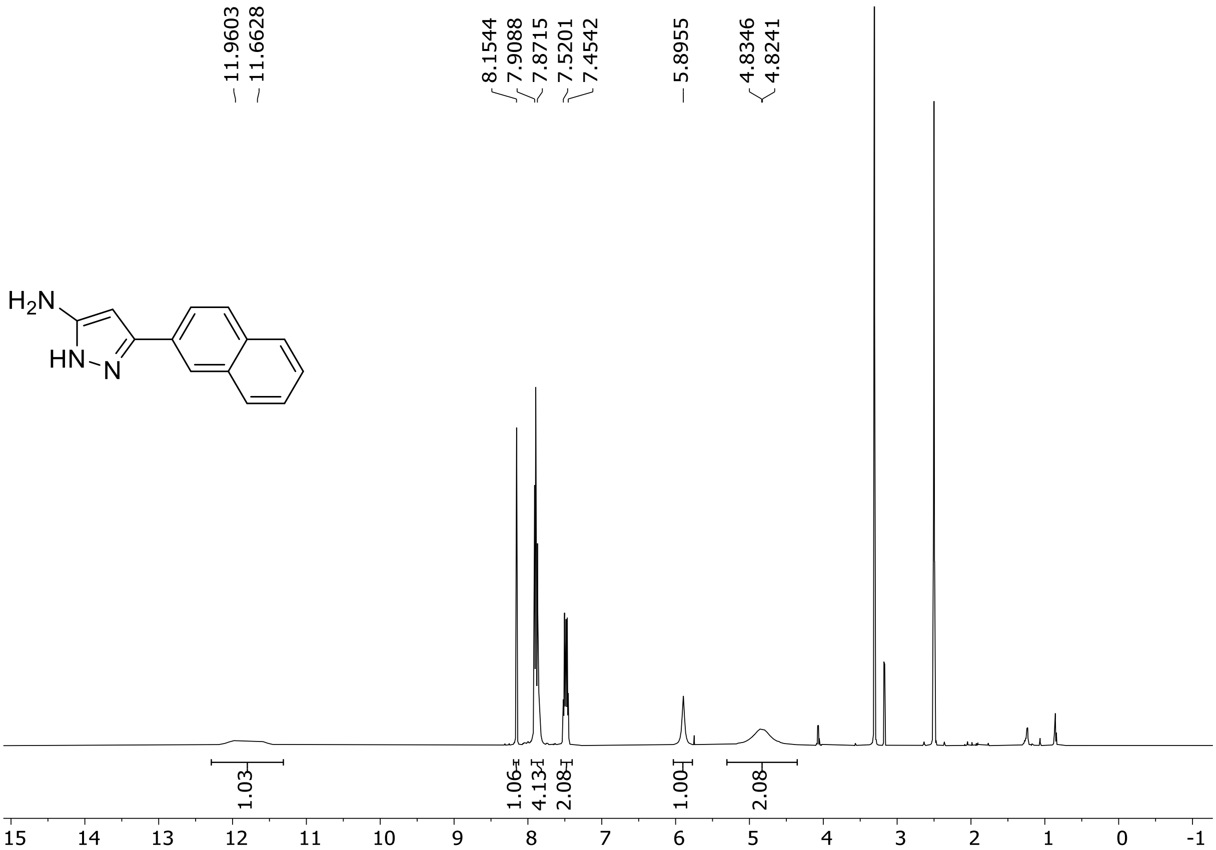

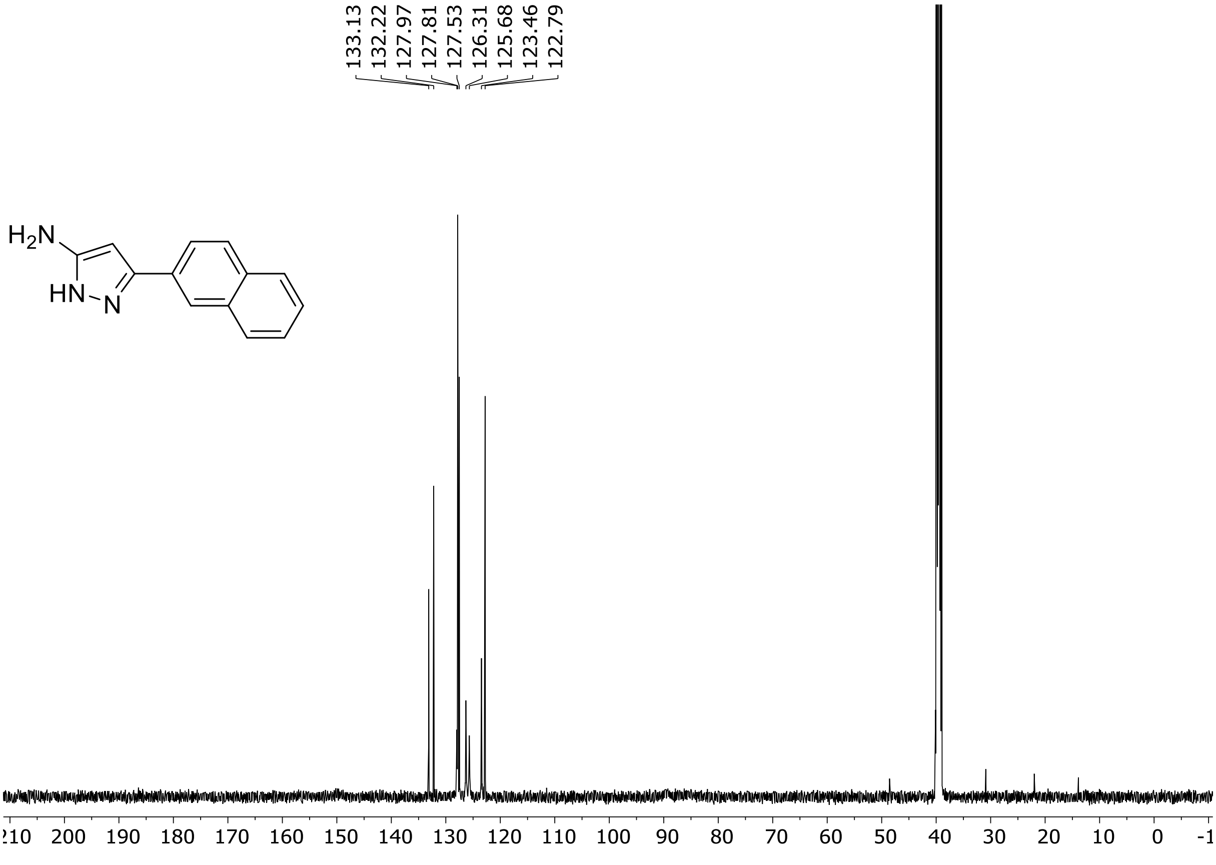

HRMS spectrum of **S43**

$C_{13}H_{11}N_3$ **mono m/z = 209.0953**

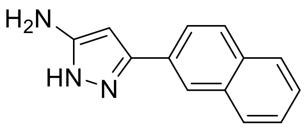

ESI + (MMI)
nitrogen flow 5 L/min, gas temperature 300°C, nebulizer 45 psi, skimmer 60 V, fragmentor 40 V, dissolved in methanol

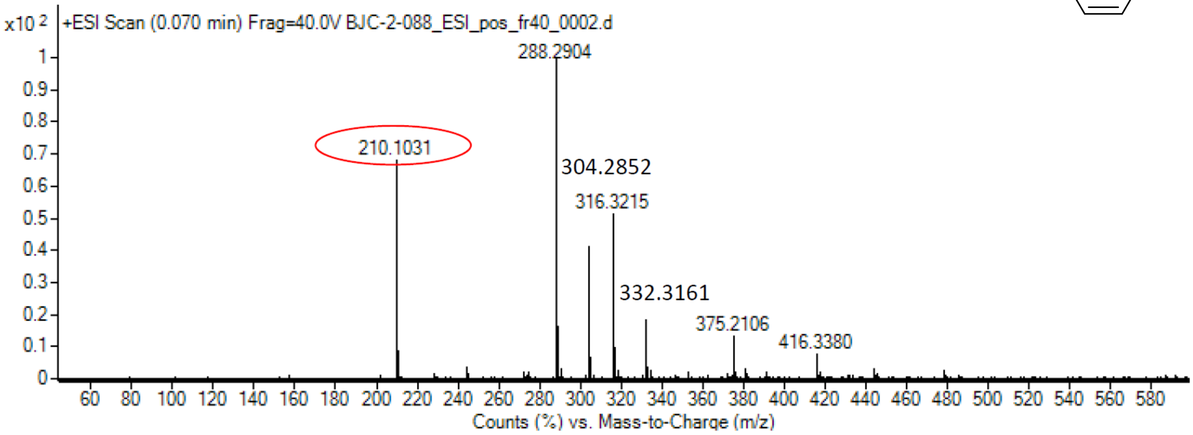

calculated mass: $[M+H]^+$ = 210.1026 observed: $[M+H]^+$ = 210.1031 max. mass error = 2.4 ppm

^{1}H (500 MHz) and ^{13}C NMR (126 MHz) spectra of **S44** in chloroform-*d*

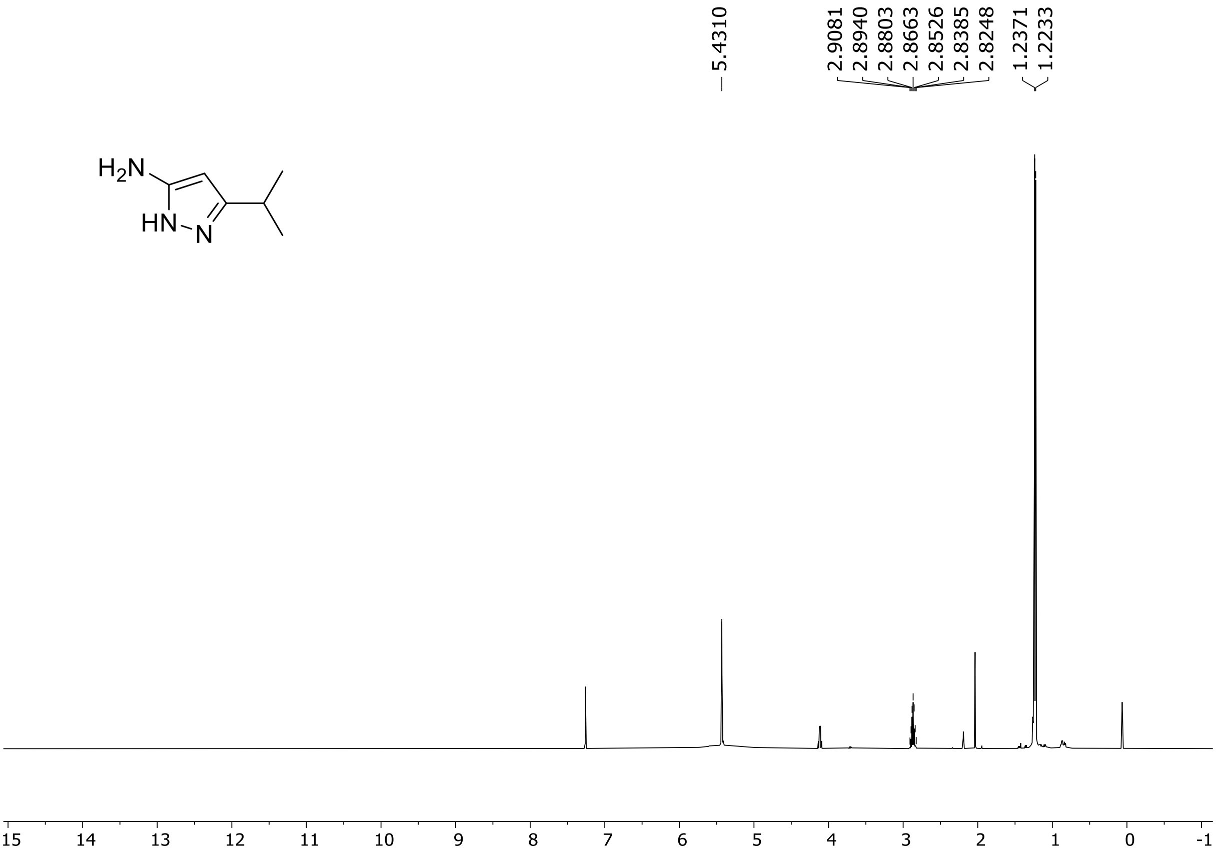

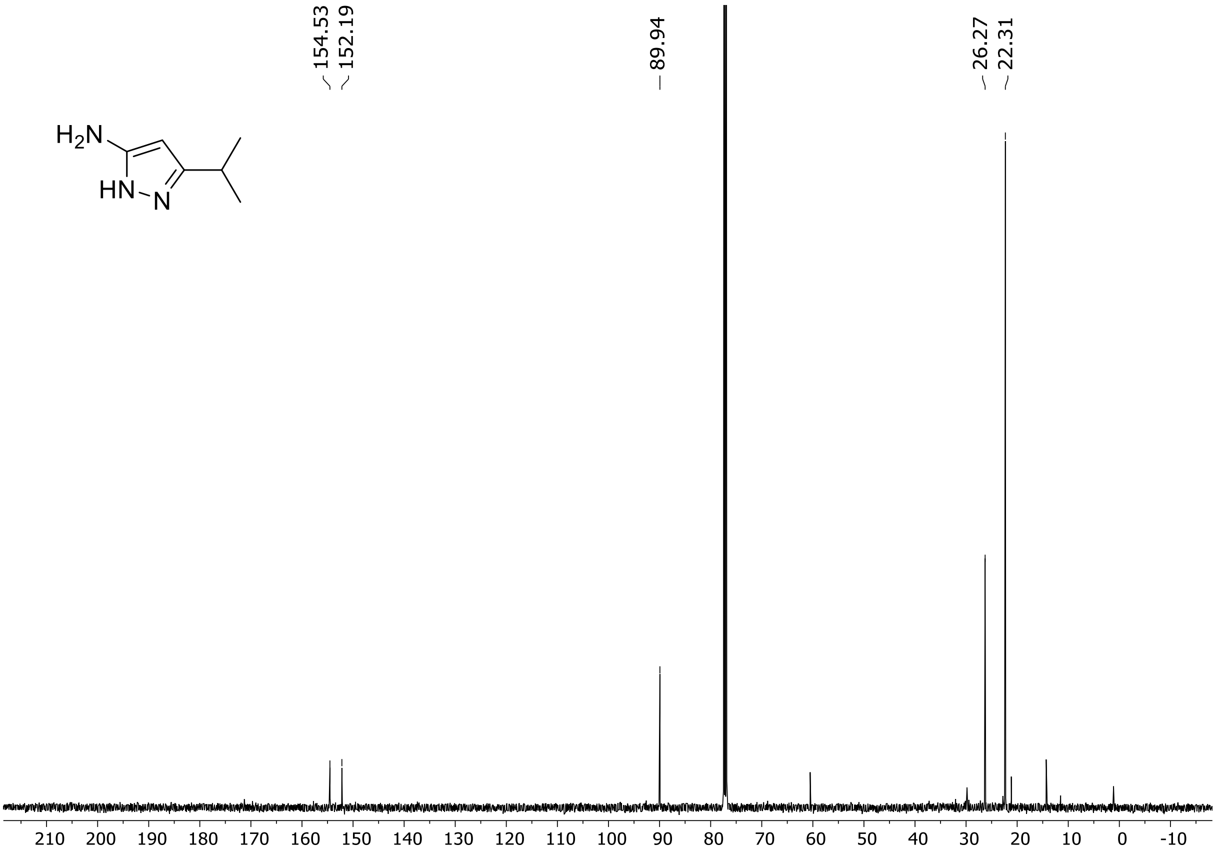

^{1}H (500 MHz) and ^{13}C NMR (126 MHz) spectra of **S45** in DMSO-d_6

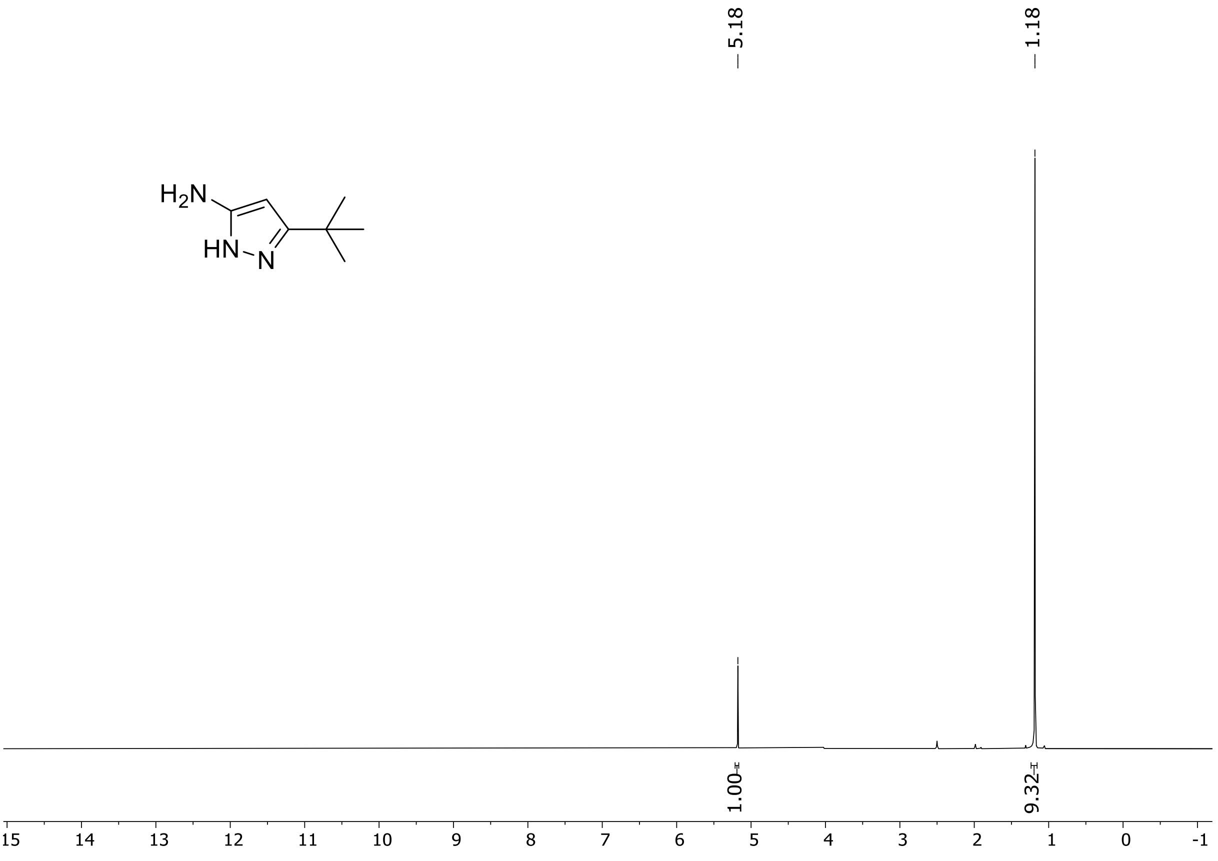

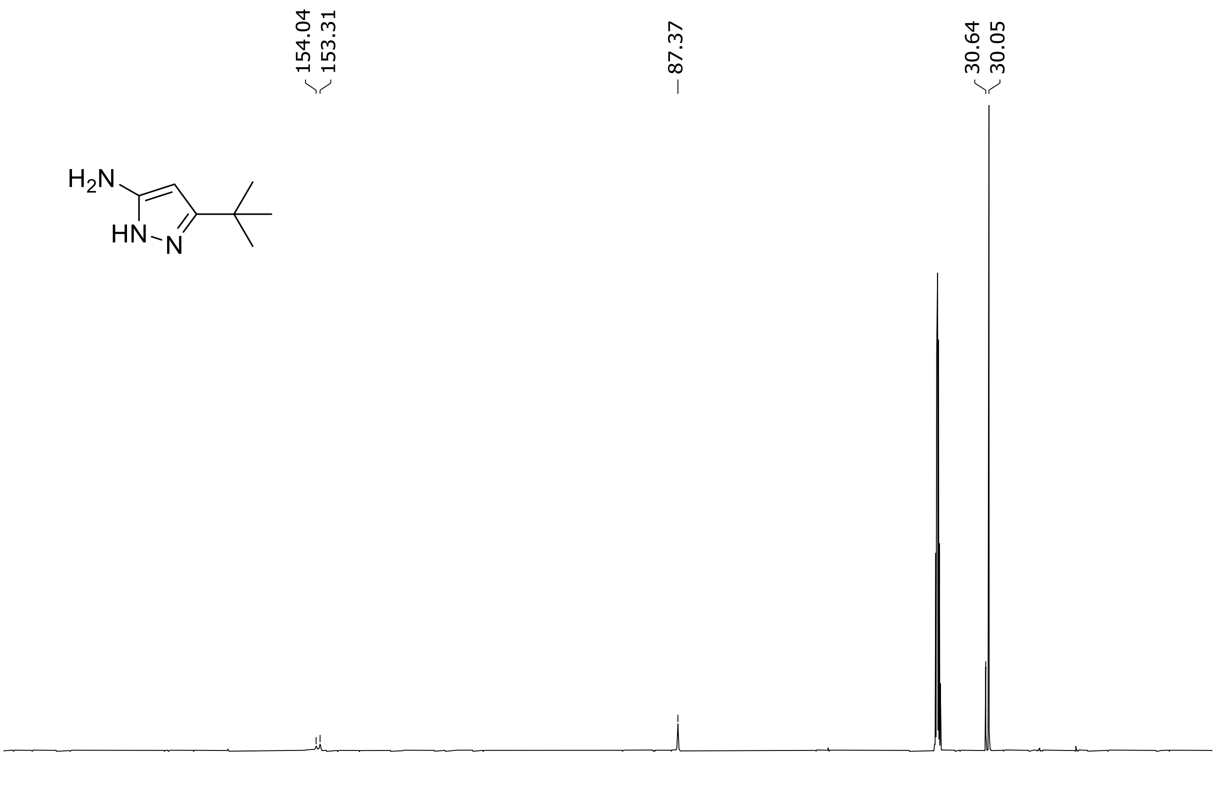

HRMS spectrum of **S45**

$C_7H_{13}N_3$ **mono m/z = 139.1109**

APCI + (MMI)
nitrogen flow 5 L/min, gas temperature 300°C, nebulizer 45 psi, vaporizer 200°C
skimmer **65** V, fragmentor **20** V, dissolved in methanol

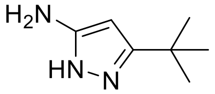

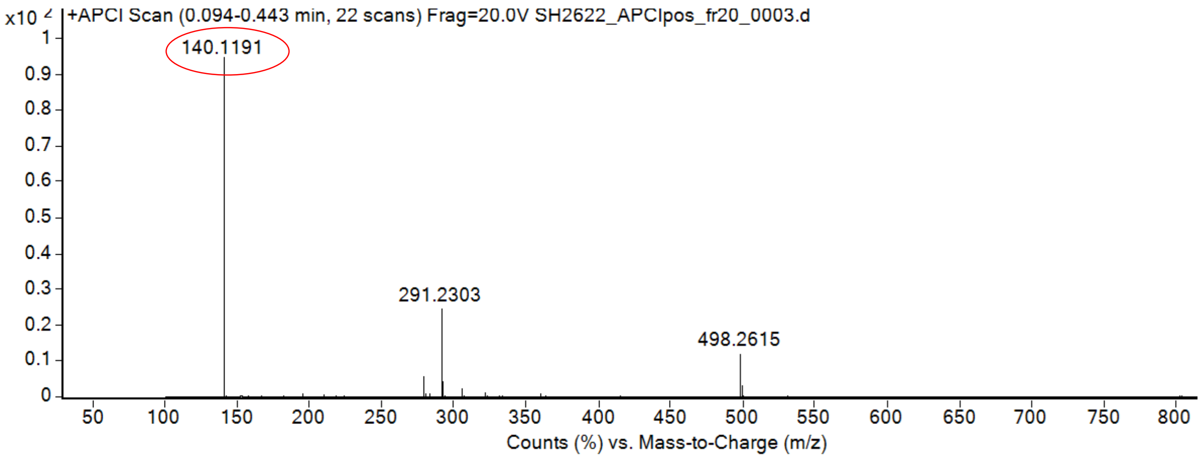

calculated mass: $[M+H]^+ = 140.1182$ observed: $[M+H]^+ = 140.1191$ max. mass error = 6.4 ppm

^{1}H (500 MHz) and ^{13}C NMR (126 MHz) spectra of **S46** in chloroform-*d*

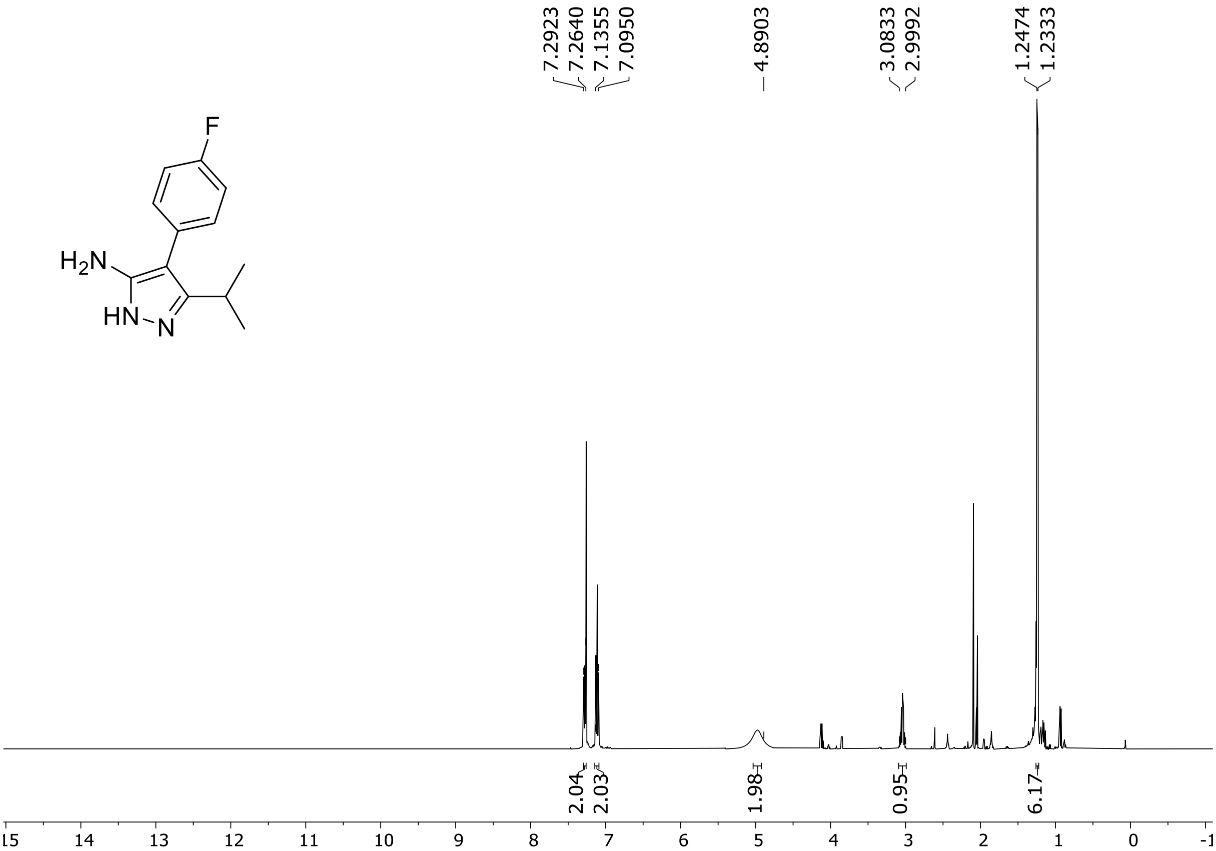

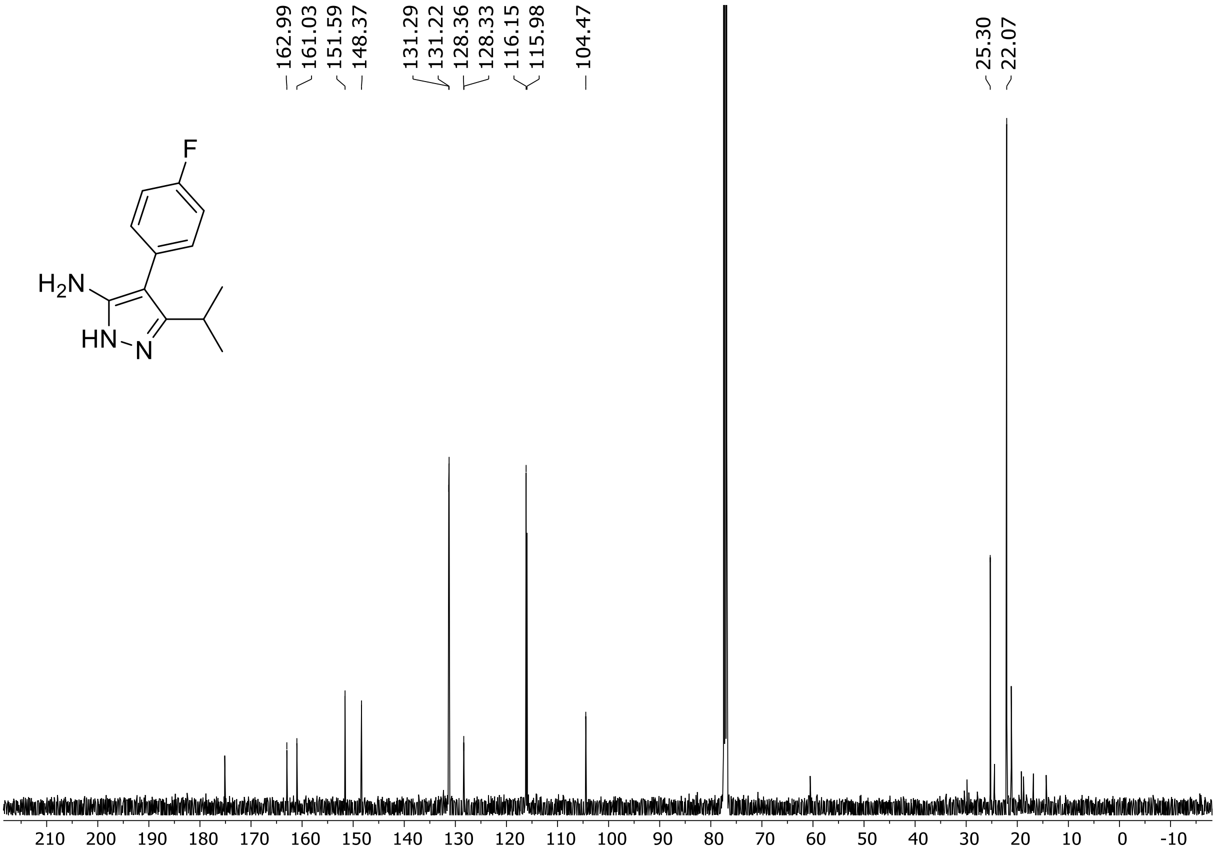

^{1}H (500 MHz) and ^{13}C NMR (126 MHz) spectra of **S47** in chloroform-*d*

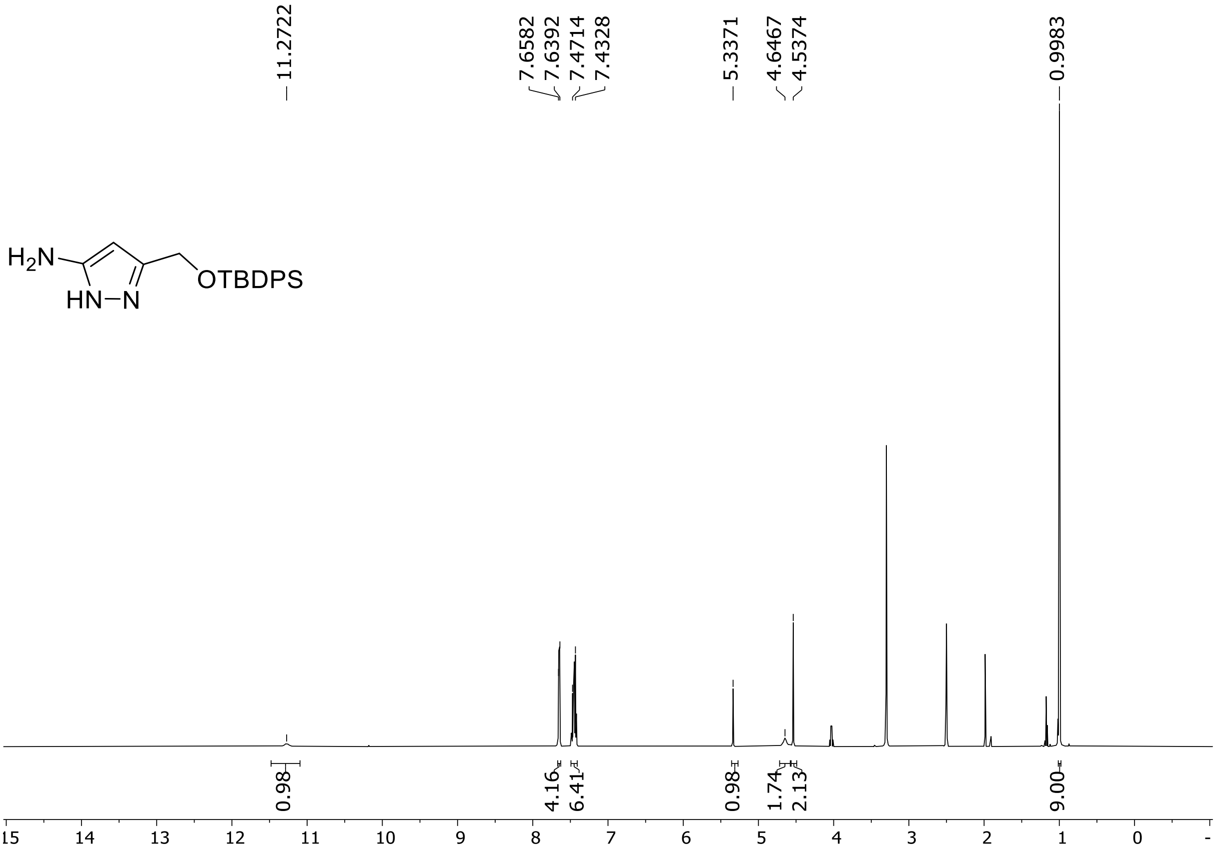

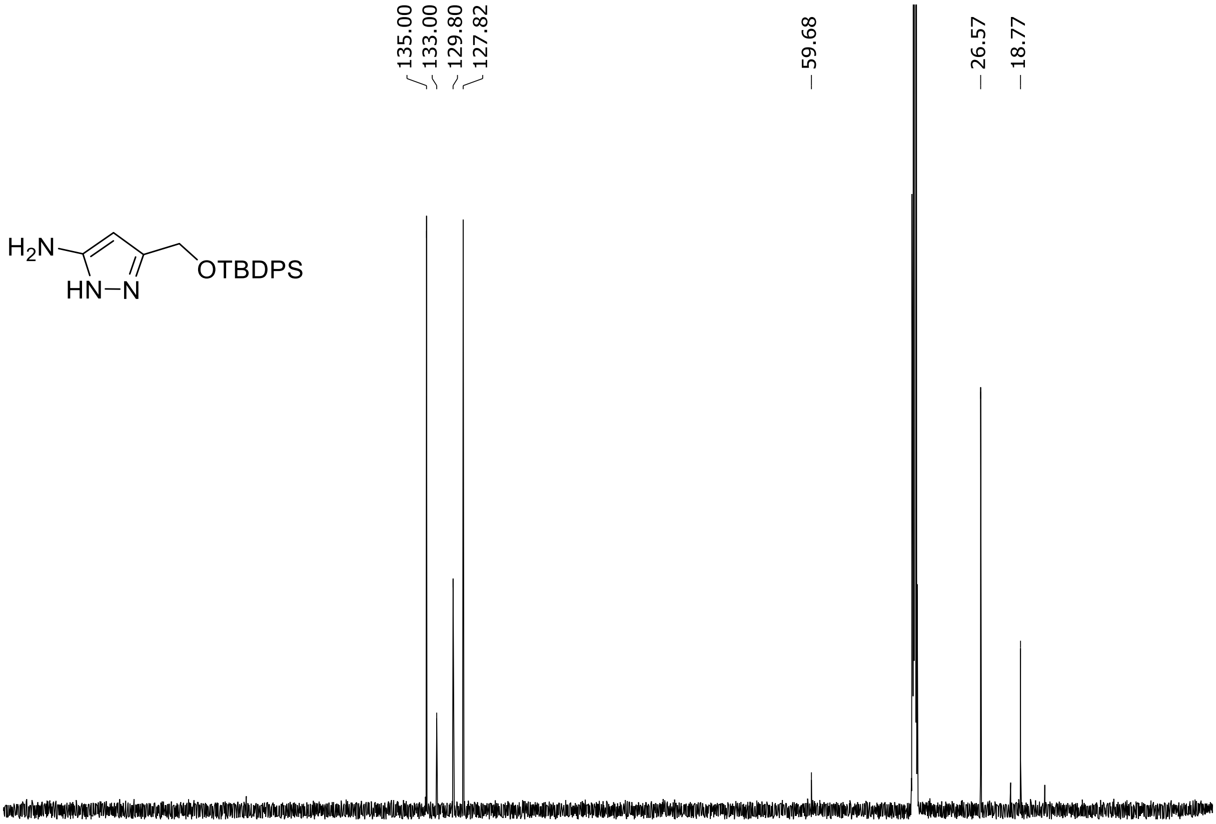

HRMS spectrum of **S47**

$C_{20}H_{25}N_3OSi$ **mono m/z = 351.1767**

APCI + (MMI)
nitrogen flow 5 L/min, gas temperature 300°C, nebulizer 45 psi, vaporizer 200°C
skimmer **65** V, fragmentor **5** V, dissolved in methanol

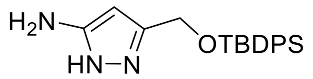

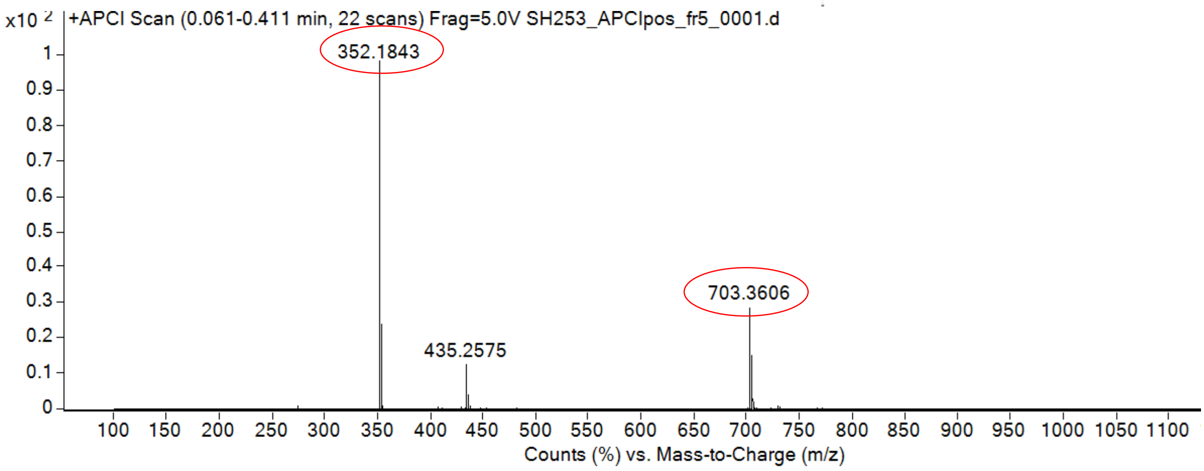

calculated mass: $[M+H]^+$ = 352.1840 observed: $[M+H]^+$ = 352.1843 max. mass error = 0.8 ppm

calculated mass: $[2xM+H]^+$ = 703.3607 observed: $[2xM+H]^+$ = 703.3606 max. mass error = 0.1 ppm

^{1}H (500 MHz) and ^{13}C NMR (126 MHz) spectra of **S48** in DMSO-d_6

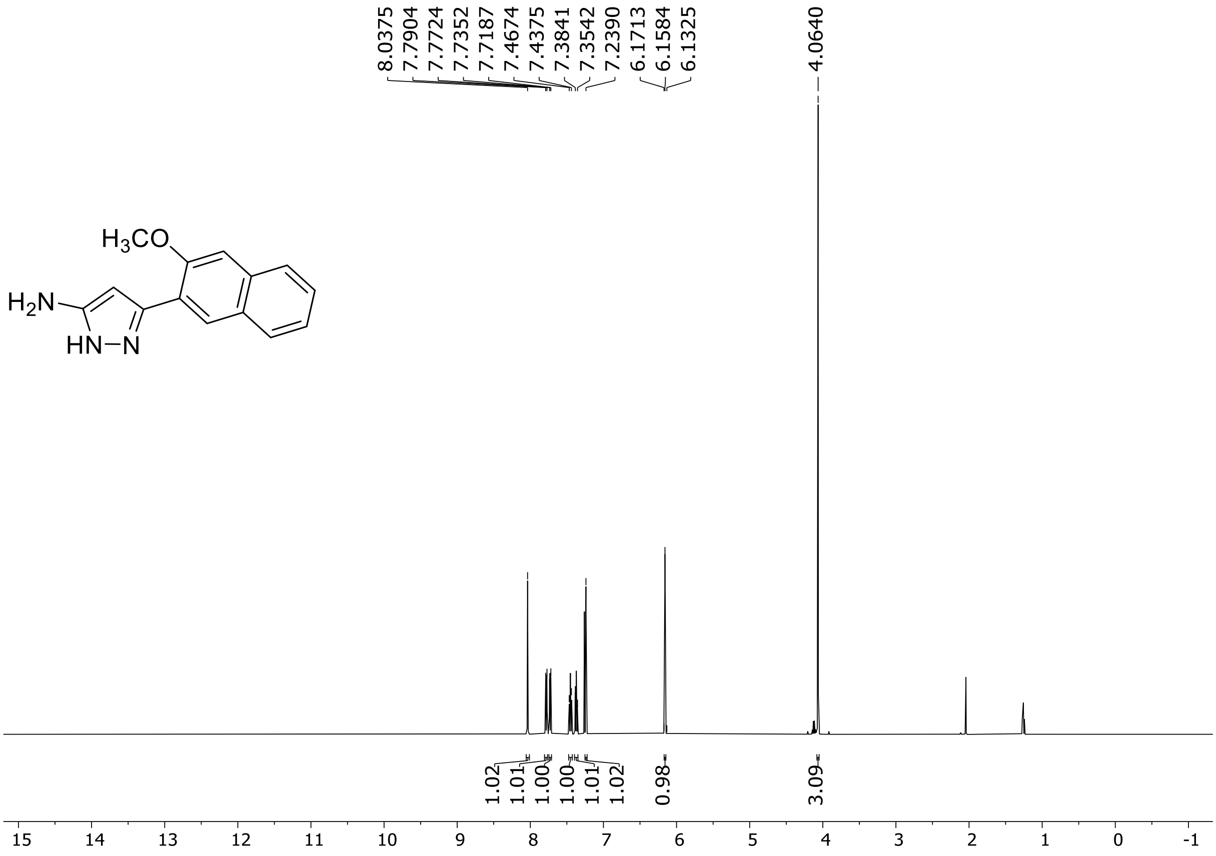

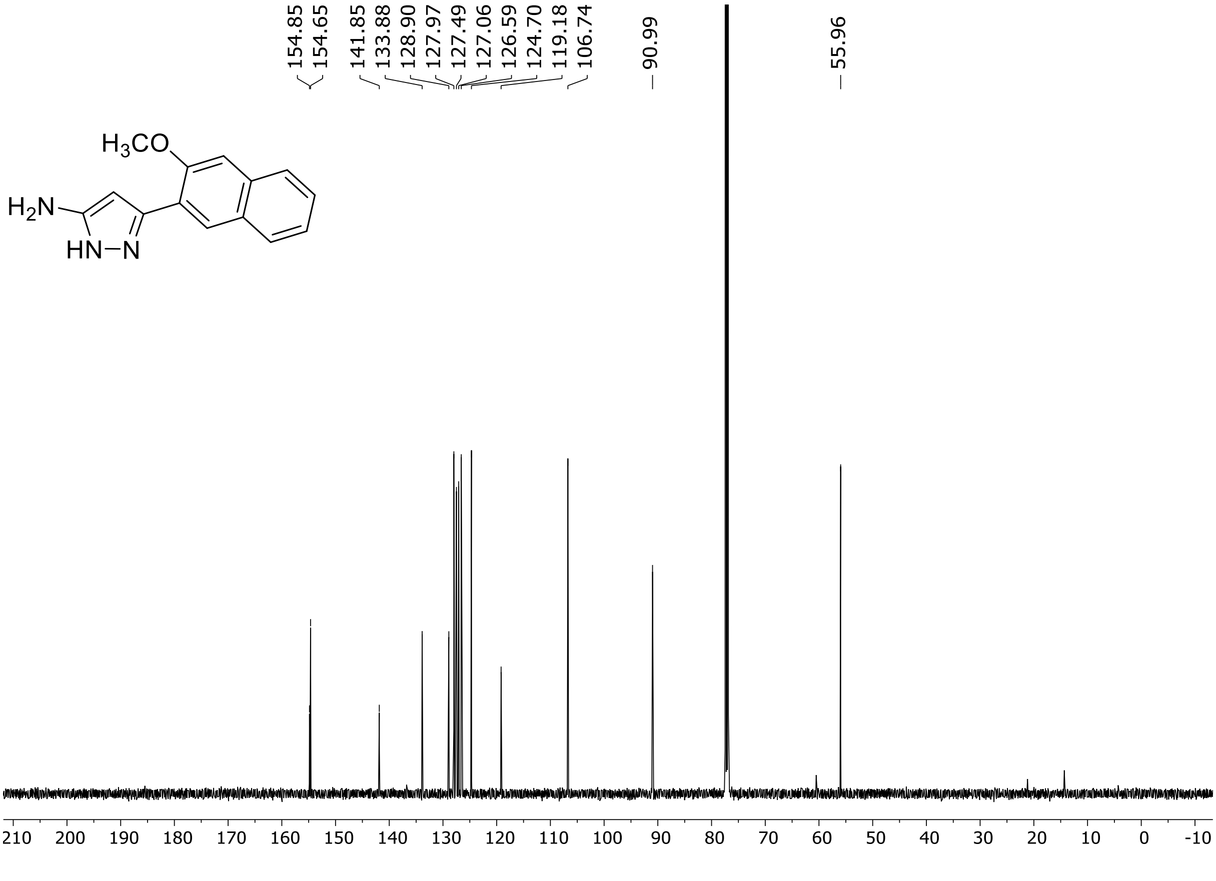

^{1}H (500 MHz) and ^{13}C NMR (126 MHz) spectra of **S49** in DMSO-d_6

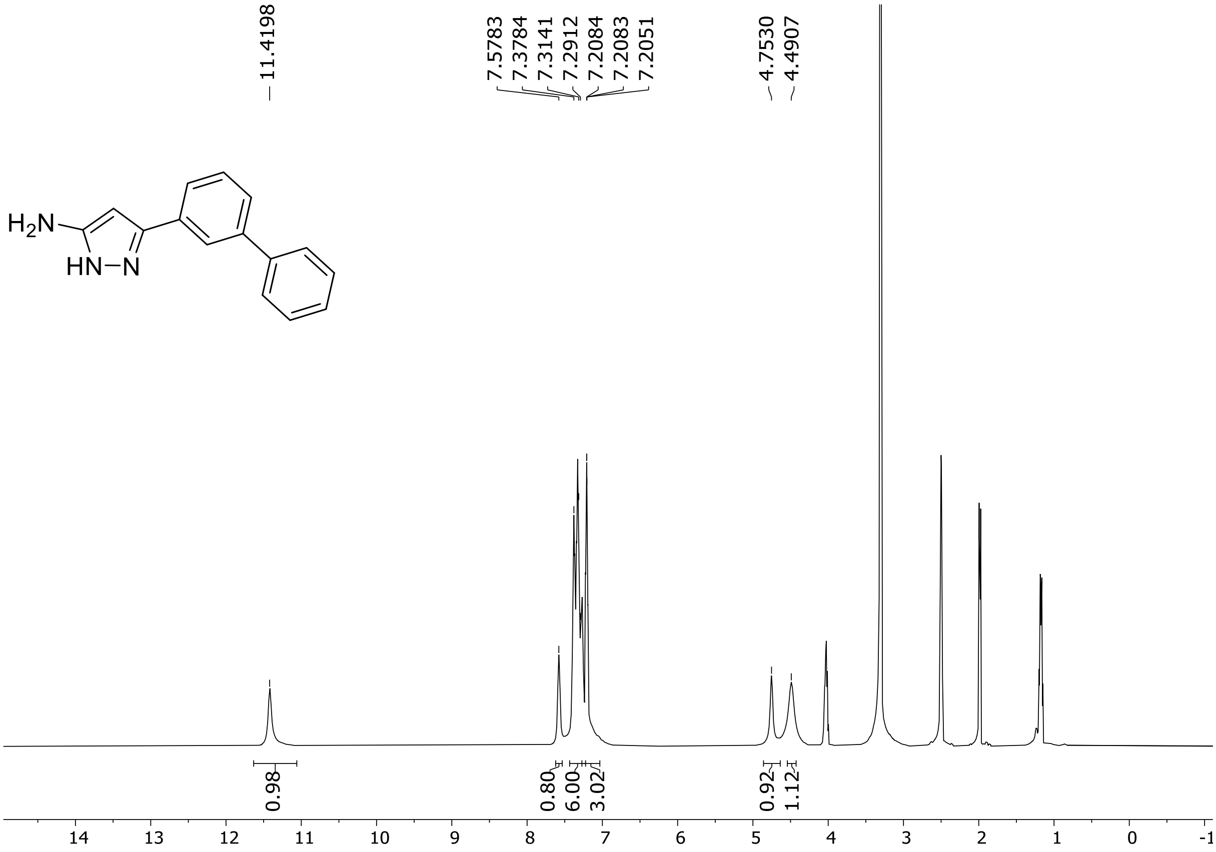

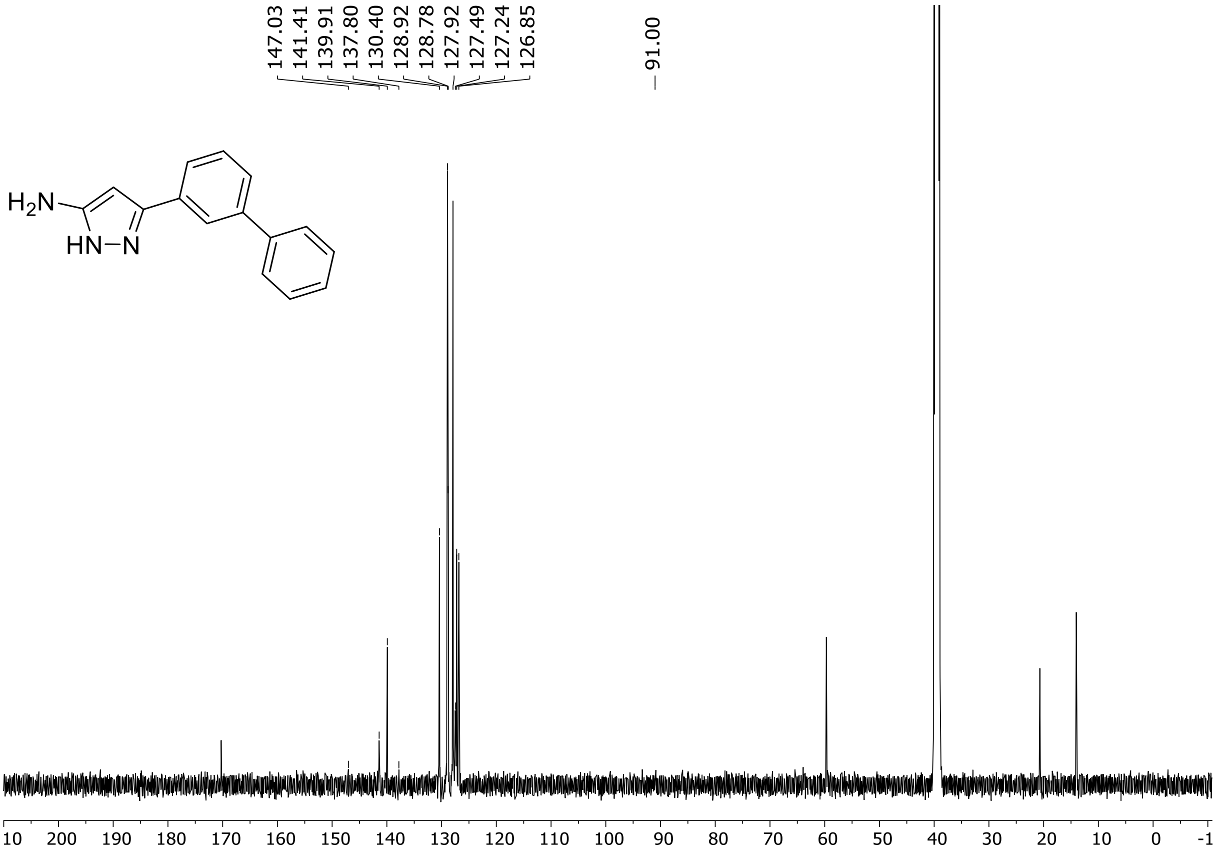

HRMS spectrum of **S49**

$C_{15}H_{13}N_3$ **mono m/z = 235.1109**

APCI + (MMI)
nitrogen flow 5 L/min, gas temperature 300°C, nebulizer 45 psi, vaporizer 200°C
skimmer **65** V, fragmentor **16** V, dissolved in methanol

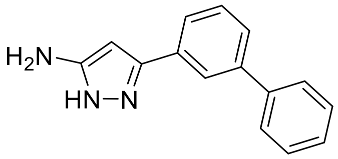

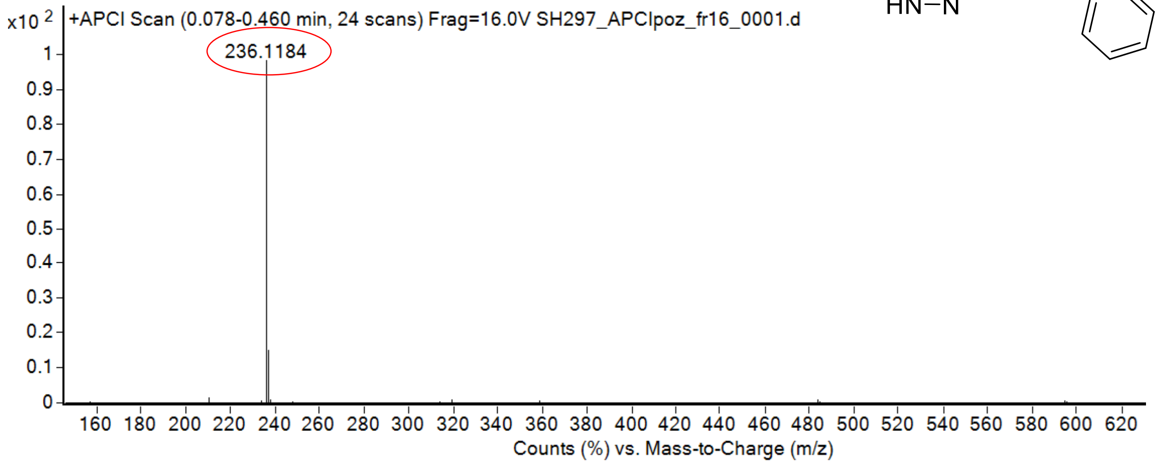

calculated mass: $[M+H]^+$ = 236.1182 observed: $[M+H]^+$ = 236.1184 max. mass error = 0.8 ppm

^{1}H (500 MHz) and ^{13}C NMR (126 MHz) spectra of **S50** in DMSO-d_6

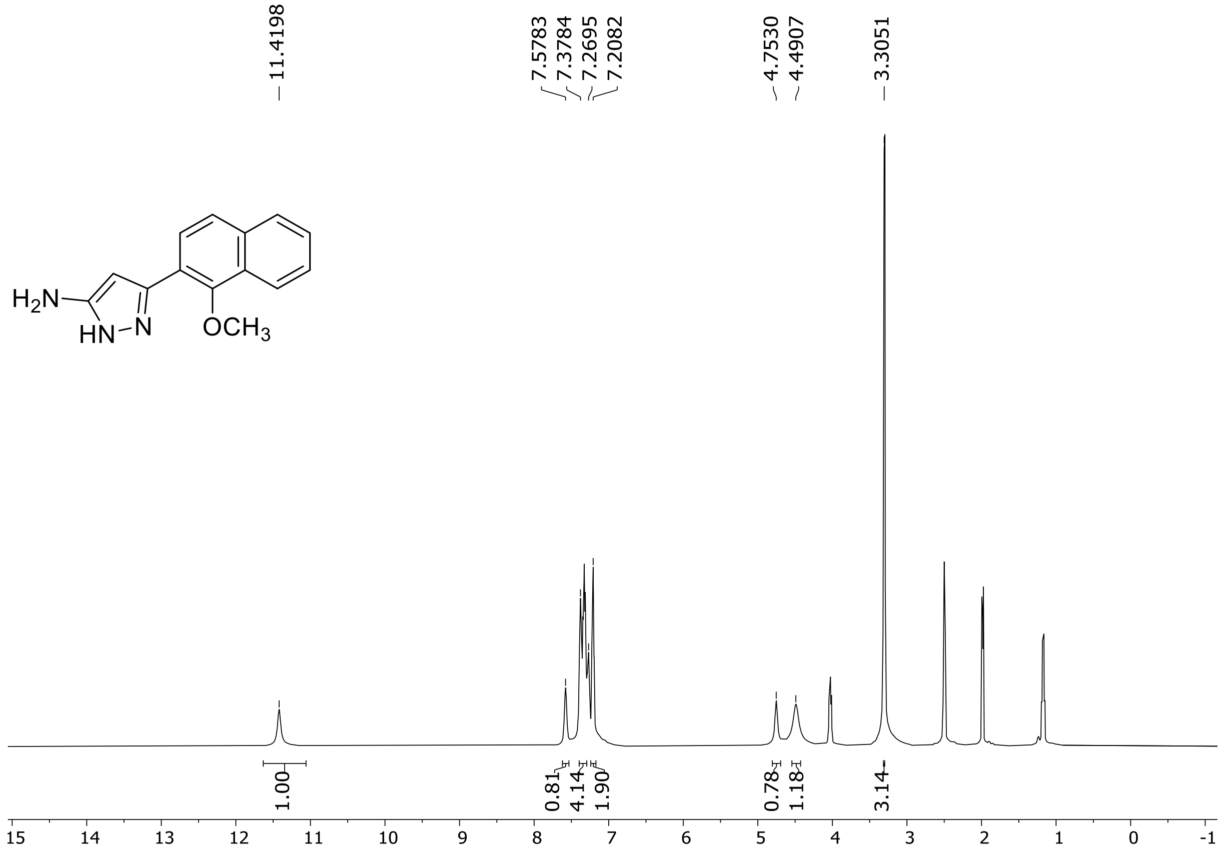

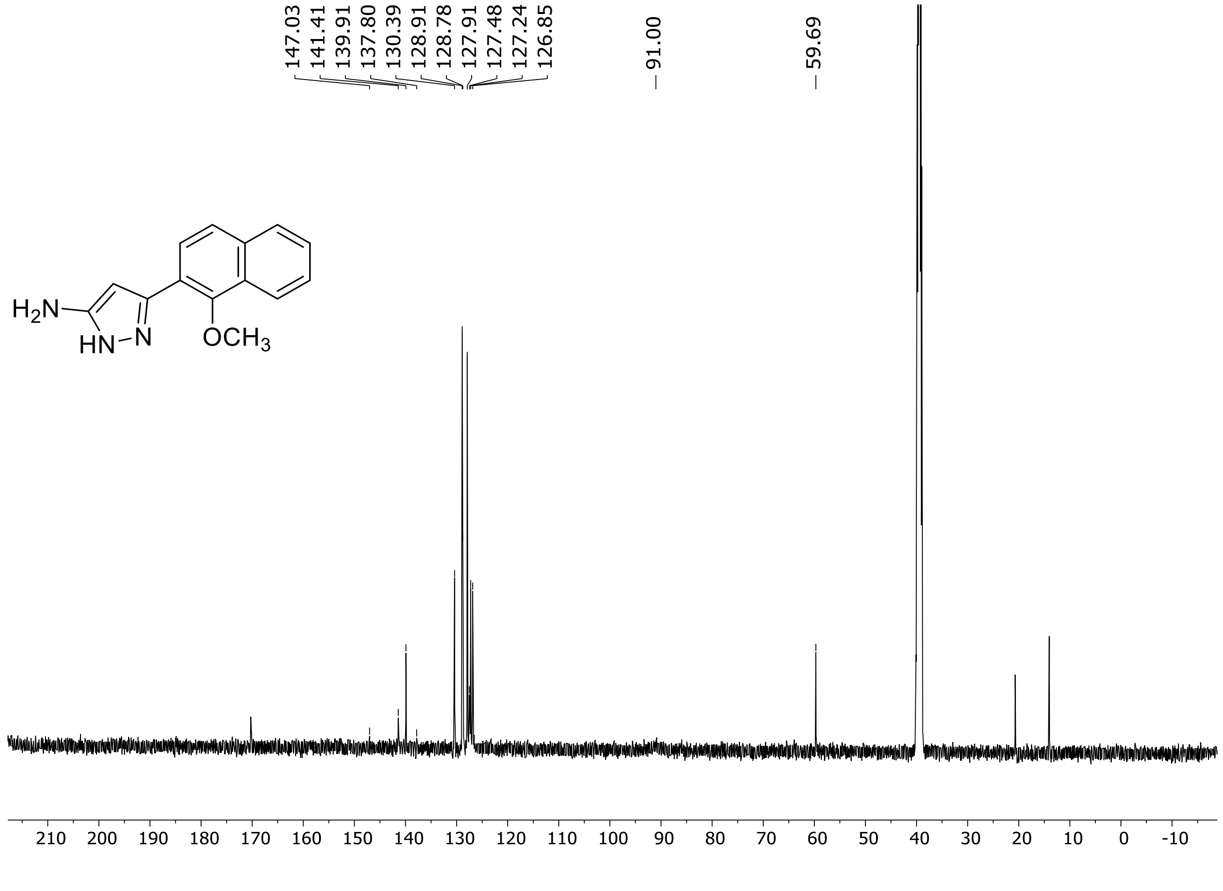

^{1}H (500 MHz) and ^{13}C NMR (126 MHz) spectra of **S51** in DMSO-d_6

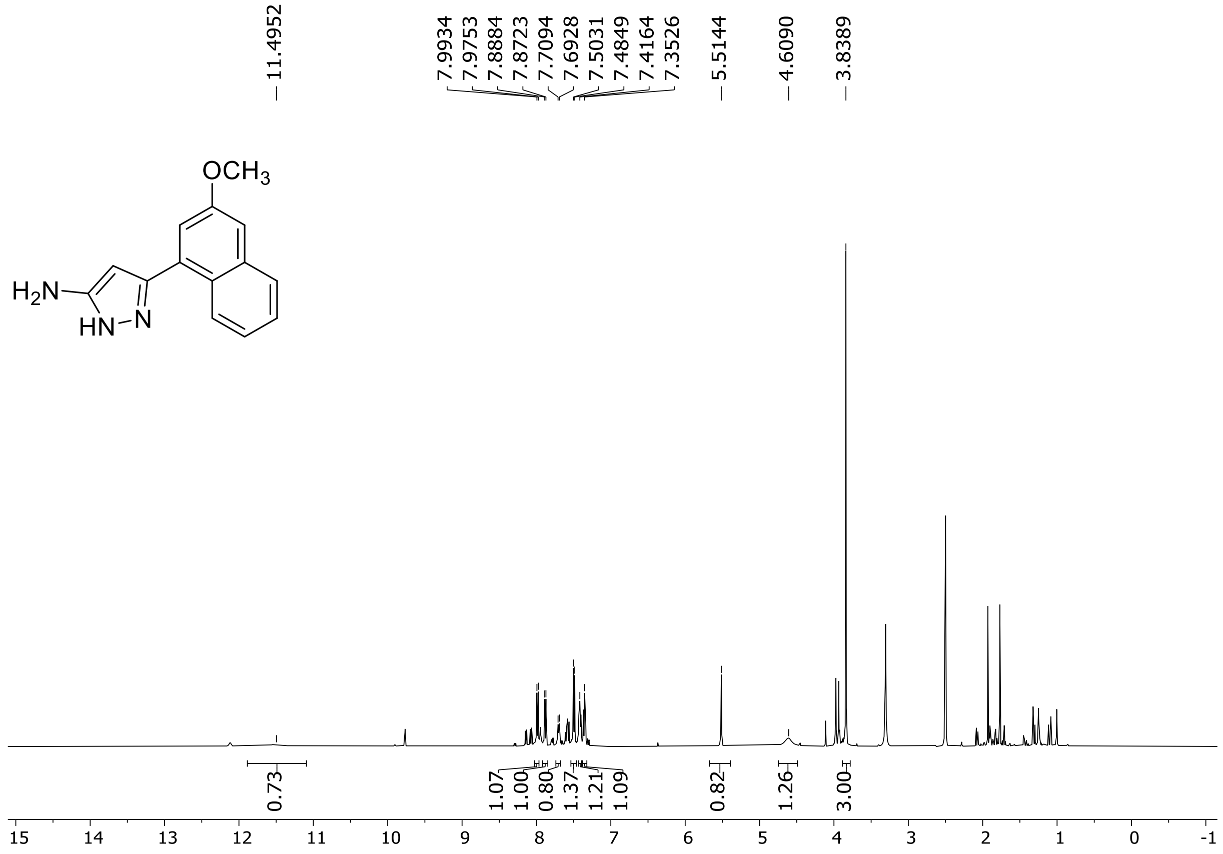

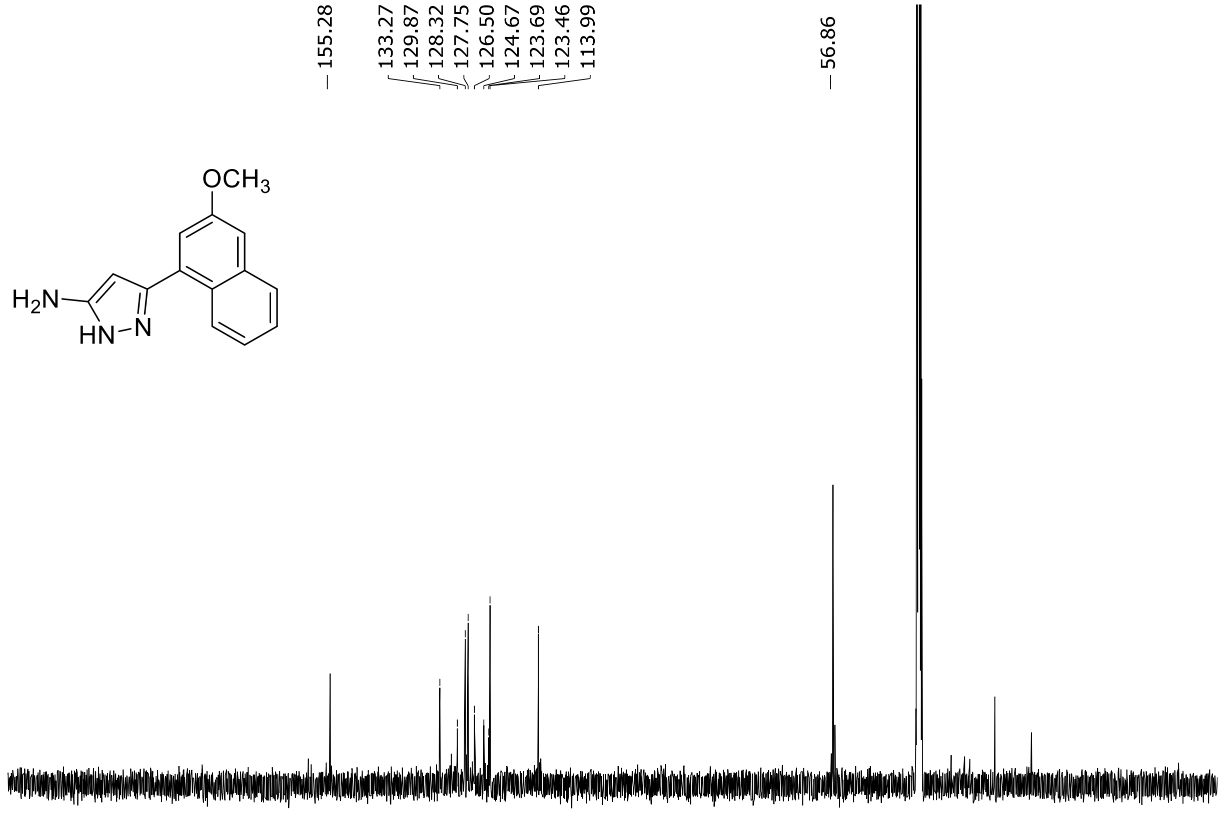

^{1}H (500 MHz) and ^{13}C NMR (126 MHz) spectra of **S52** in DMSO-d_6

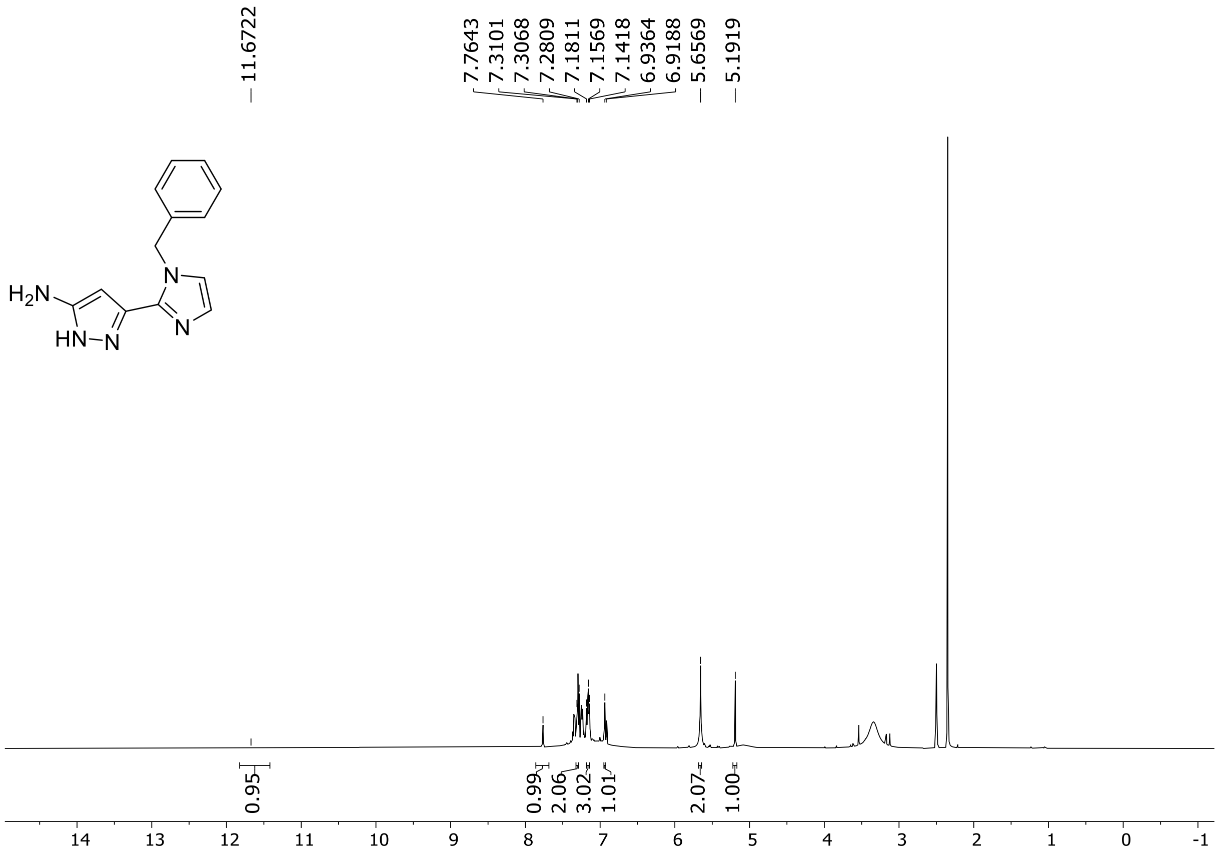

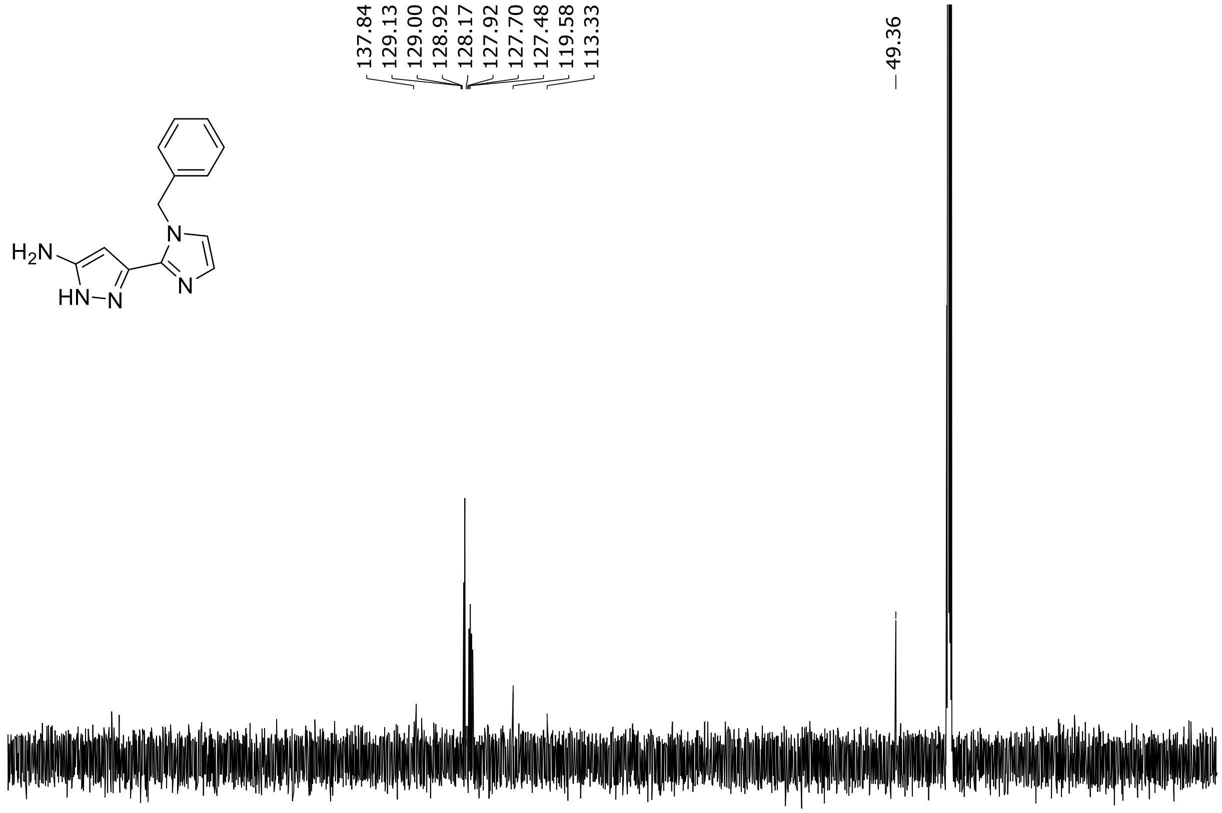

^{1}H (500 MHz) and ^{13}C NMR (126 MHz) spectra of **S53** in DMSO-d_6

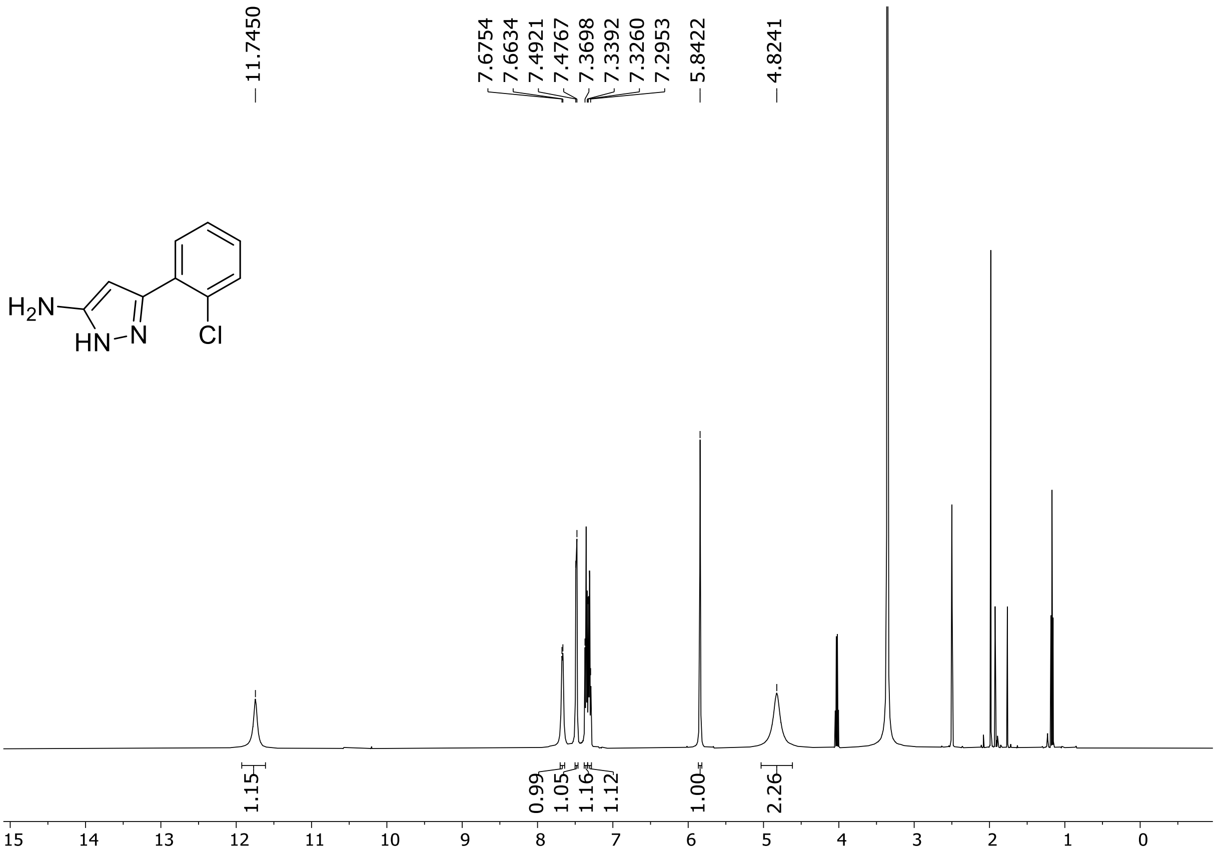

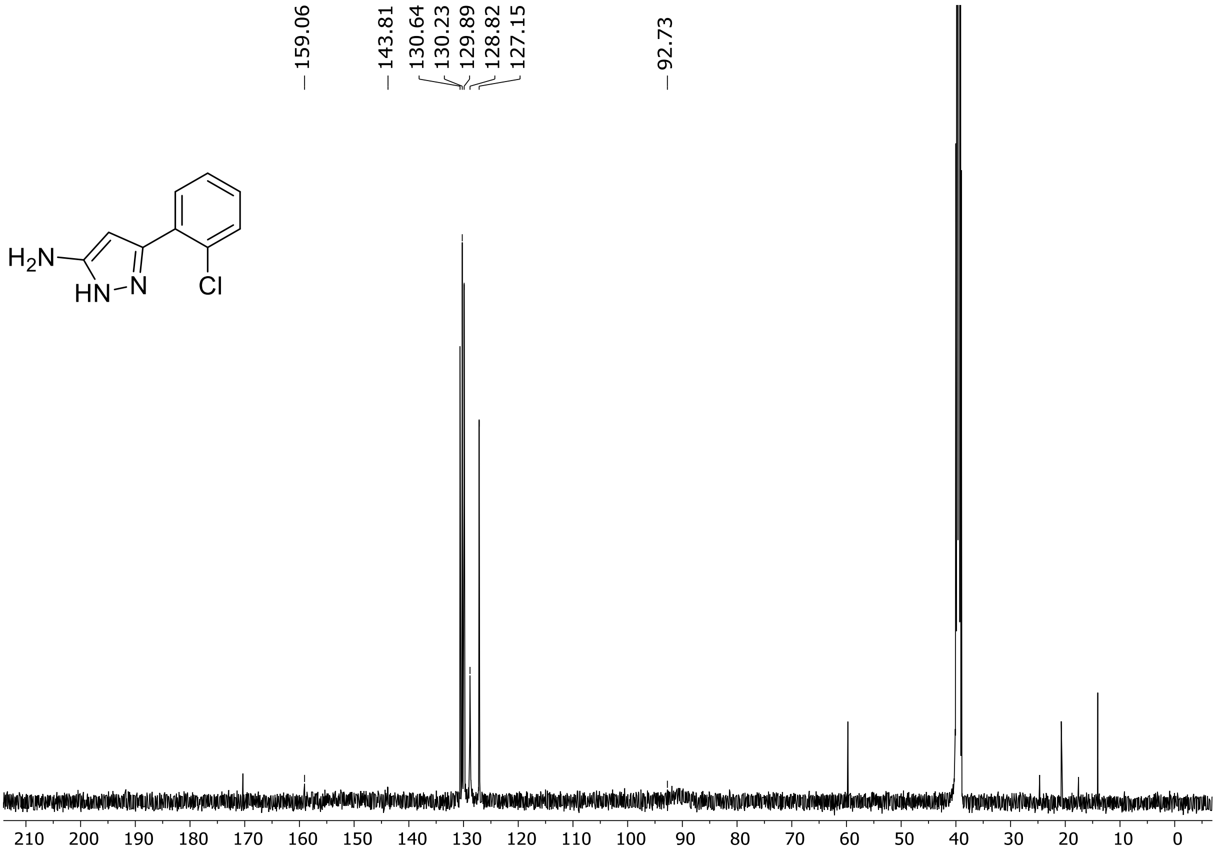

HRMS spectrum of **S53**

$C_9H_8ClN_3$ **mono m/z = 193.0407**

APCI + (MMI)
nitrogen flow 5 L/min, gas temperature 300°C, nebulizer 45 psi, vaporizer 200°C
skimmer **65** V, fragmentor **17** V, dissolved in methanol

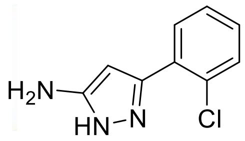

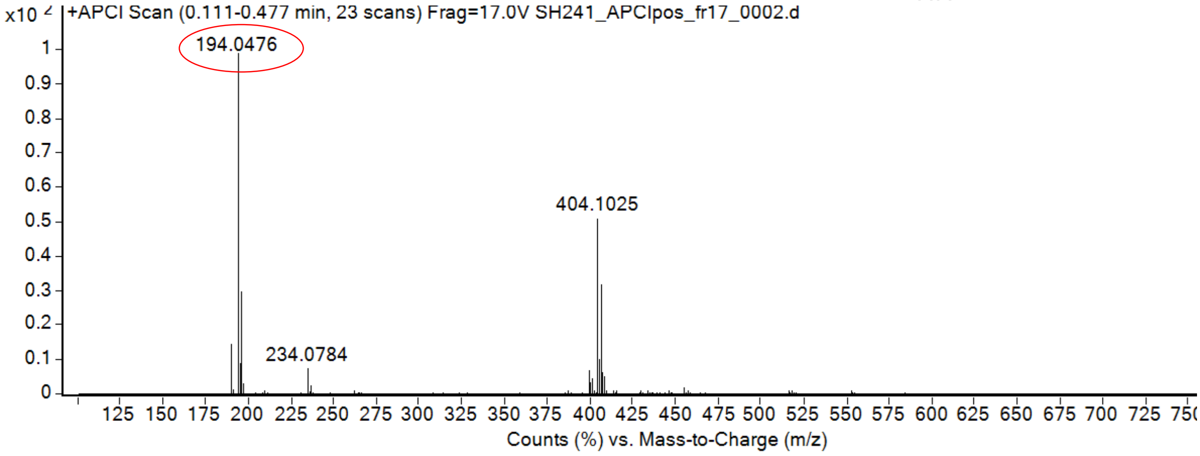

calculated mass: [M+H]$^+$ = 194.0480 observed: [M+H]$^+$ = 194.0476 max. mass error = 2 ppm

^{1}H (500 MHz) and ^{13}C NMR (126 MHz) spectra of **S54** in DMSO-d_6

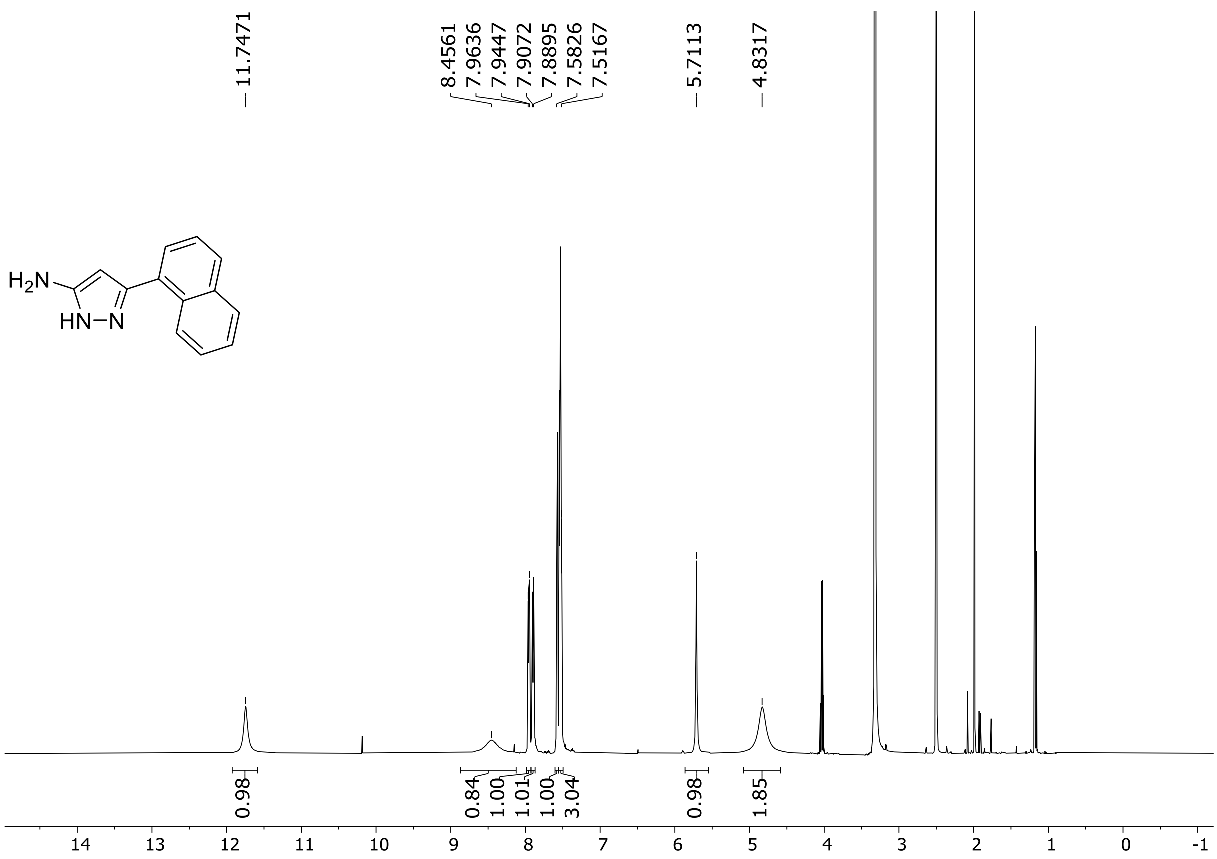

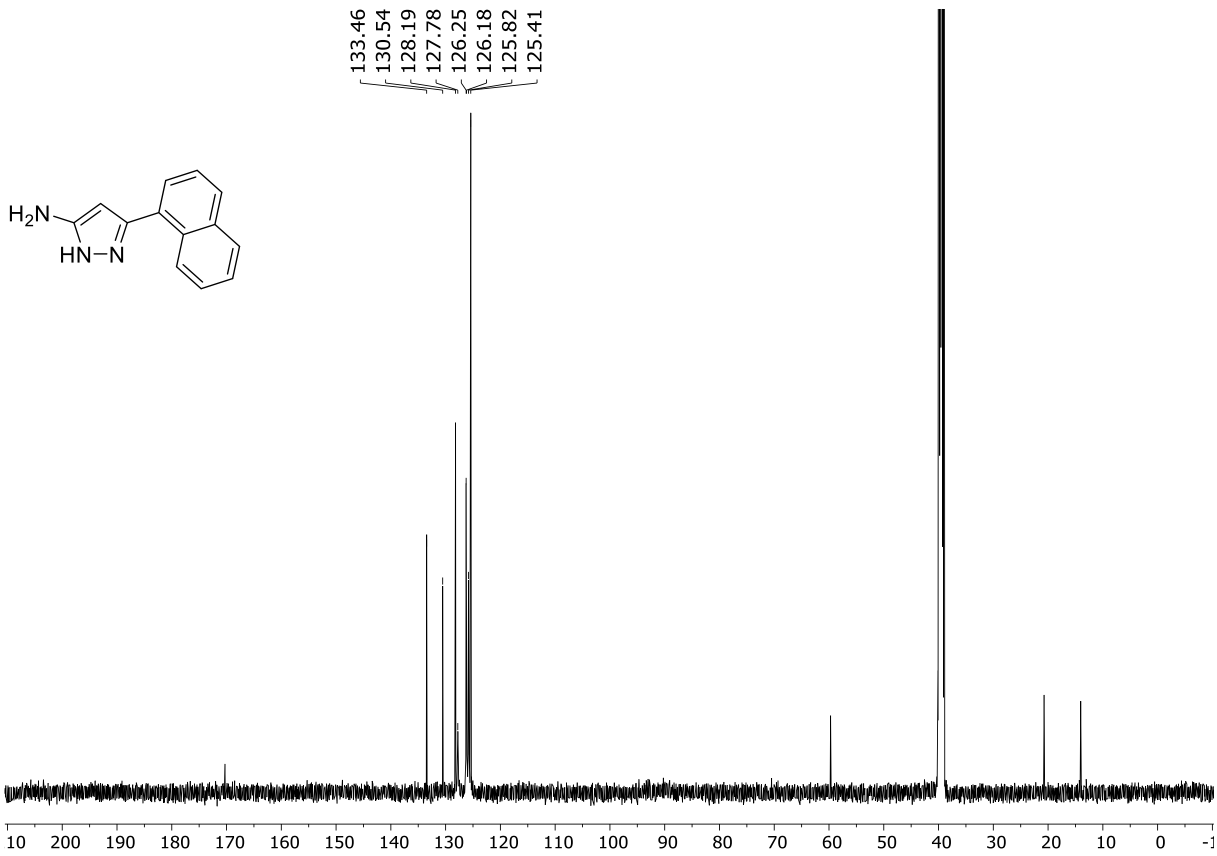

HRMS spectrum of **S54**

$C_{13}H_{11}N_3$ **mono m/z = 209.0953**

APCI + (MMI)
nitrogen flow 5 L/min, gas temperature 300°C, nebulizer 45 psi, vaporizer 200°C
skimmer **65** V, fragmentor **18** V, dissolved in methanol

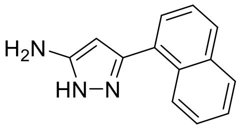

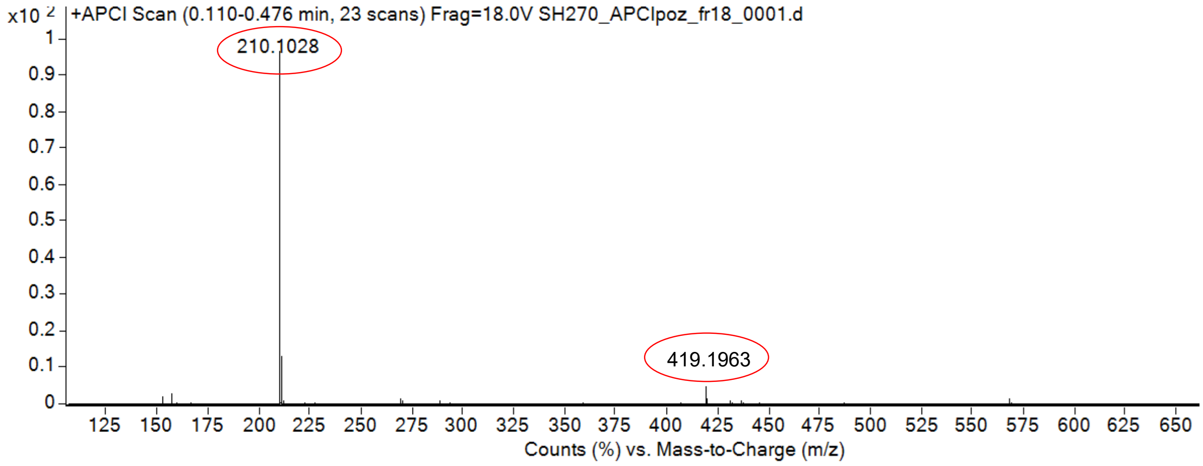

calculated mass: $[M+H]^+$ = 210.1026 observed: $[M+H]^+$ = 210.1028 max. mass error = 0.9 ppm

calculated mass: $[2xM+H]^+$ = 419.1979 observed: $[2xM+H]^+$ = 419.1963 max. mass error = 3.3 ppm

^{1}H (500 MHz) and ^{13}C NMR (126 MHz) spectra of **S55** in DMSO-d_6

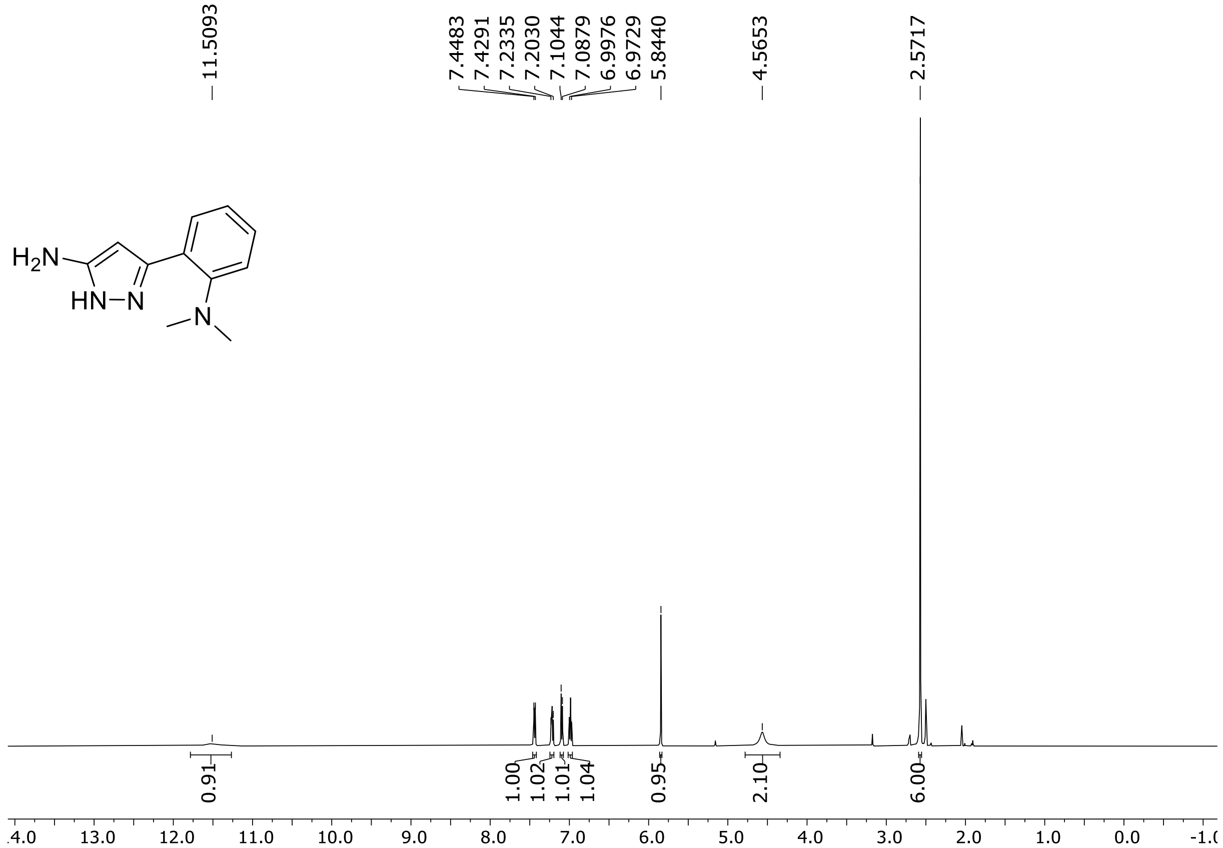

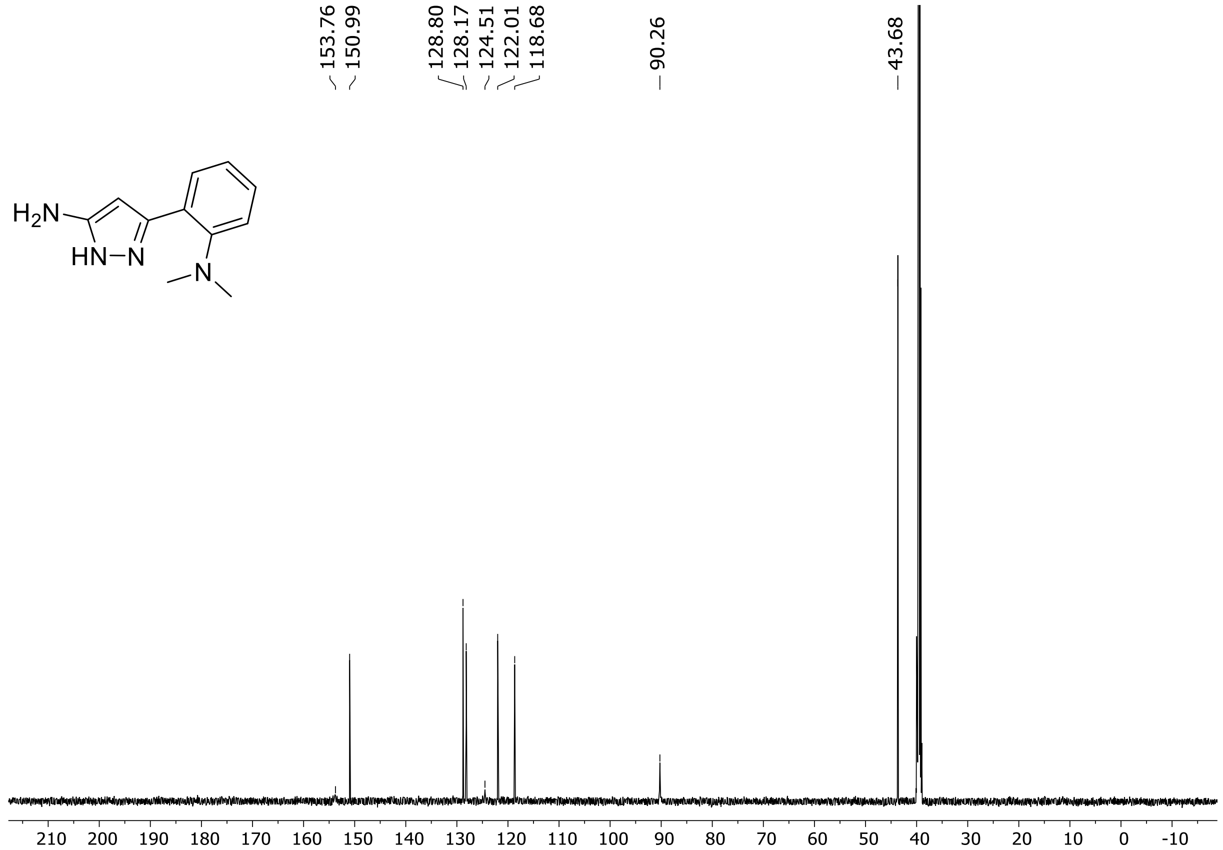

^{1}H (500 MHz) and ^{13}C NMR (126 MHz) spectra of **S56** in chloroform-*d*

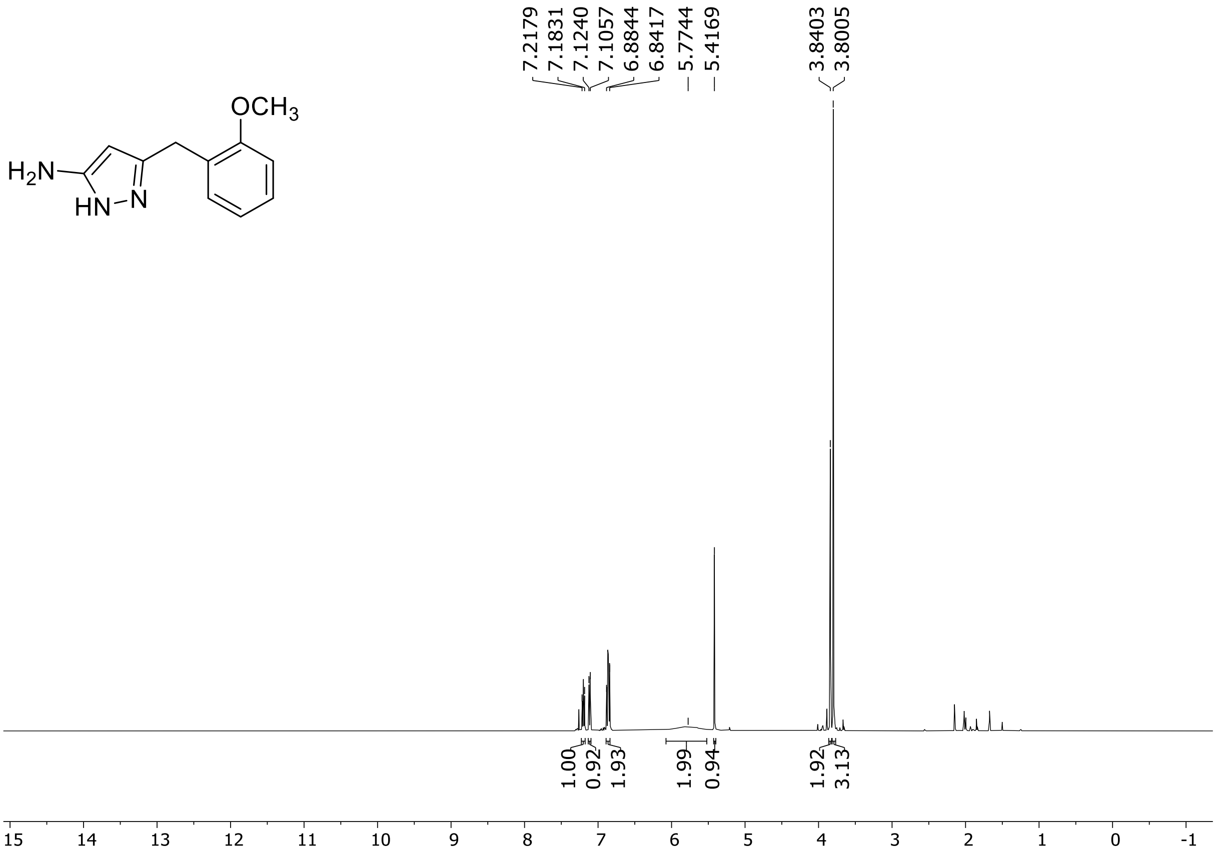

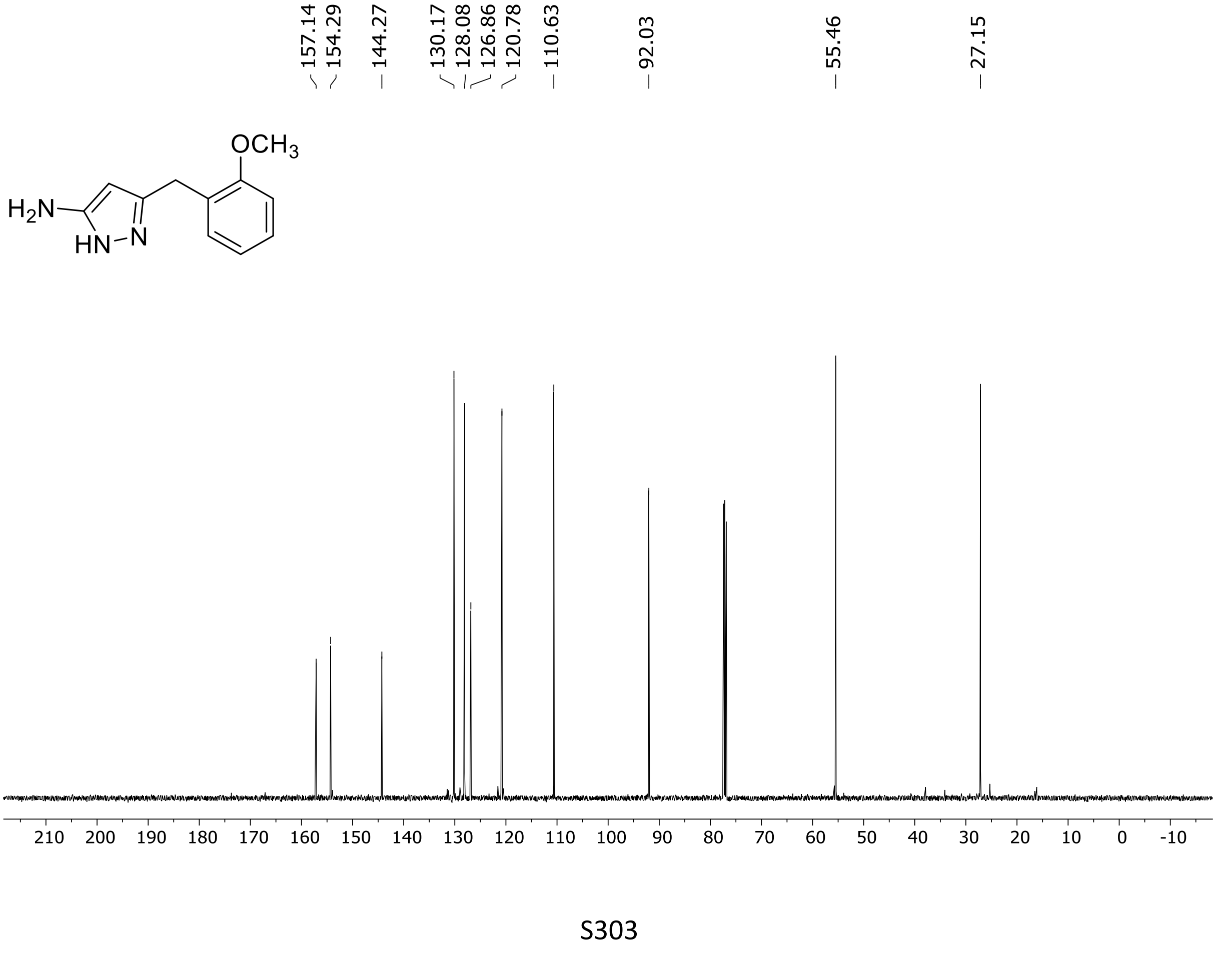

^{1}H (500 MHz) and ^{13}C NMR (126 MHz) spectra of **S57** in chloroform-*d*

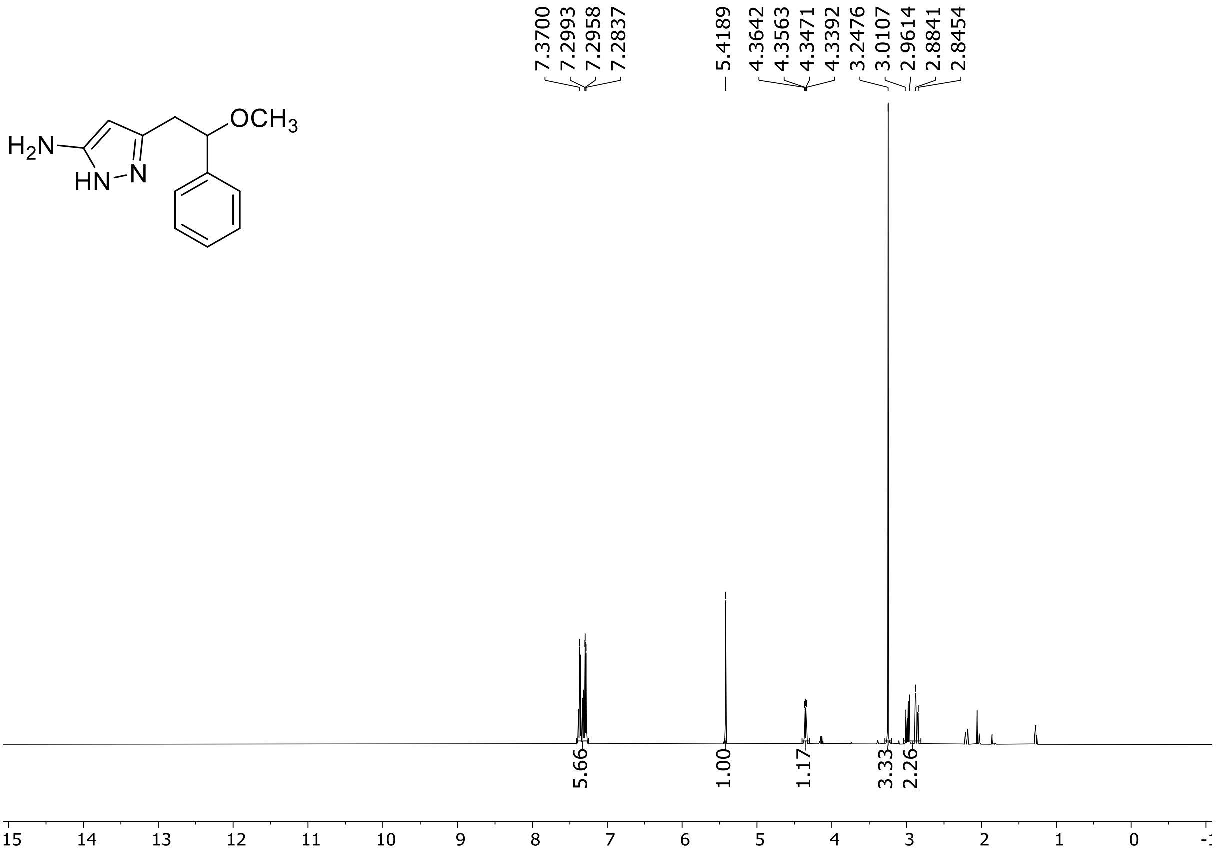

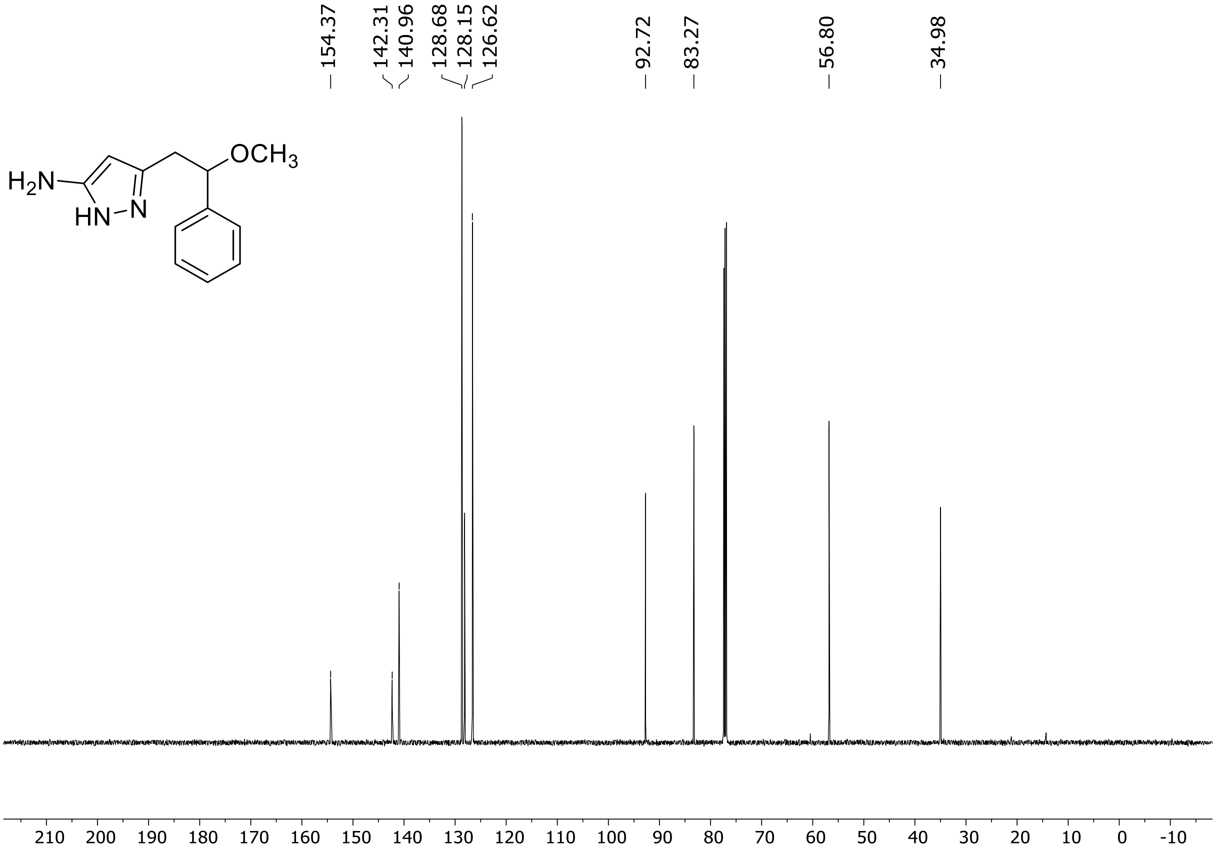

^{1}H (500 MHz) and ^{13}C NMR (126 MHz) spectra of **S58** in chloroform-*d*

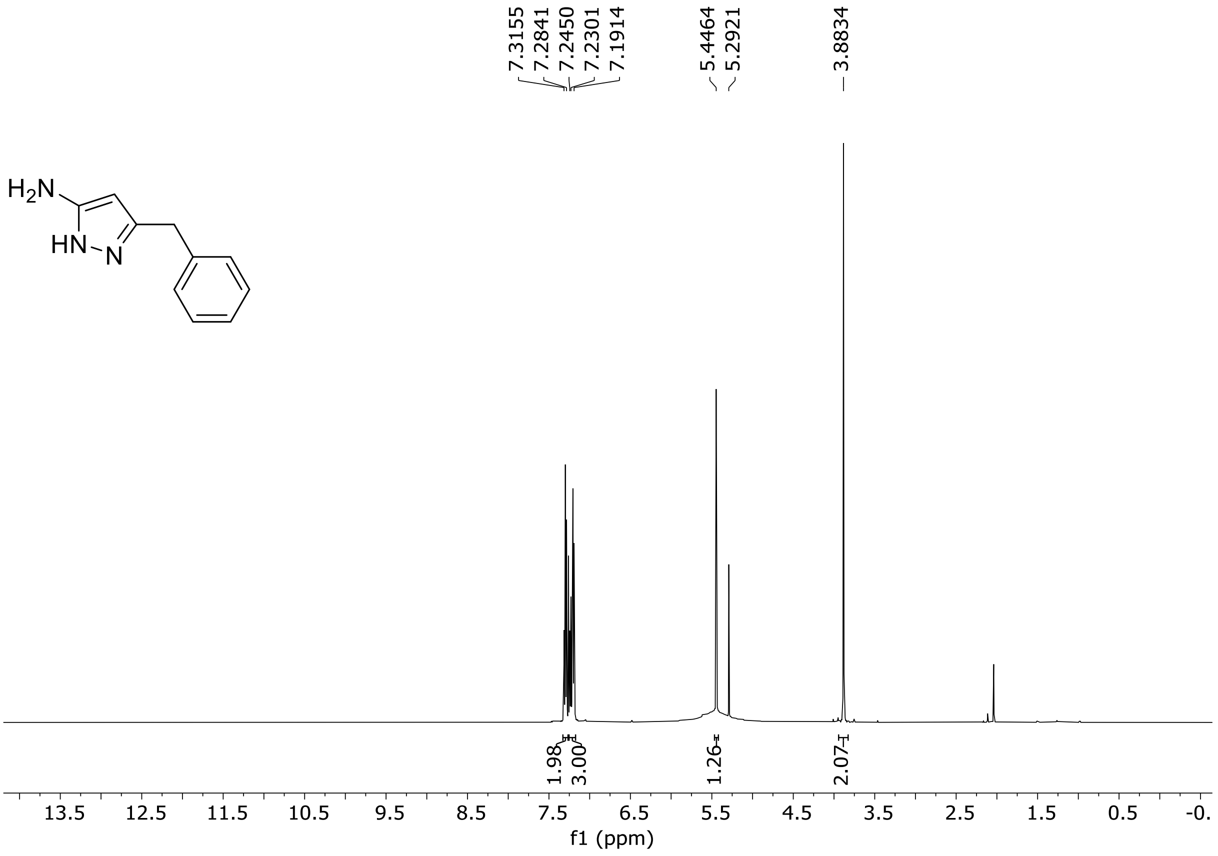

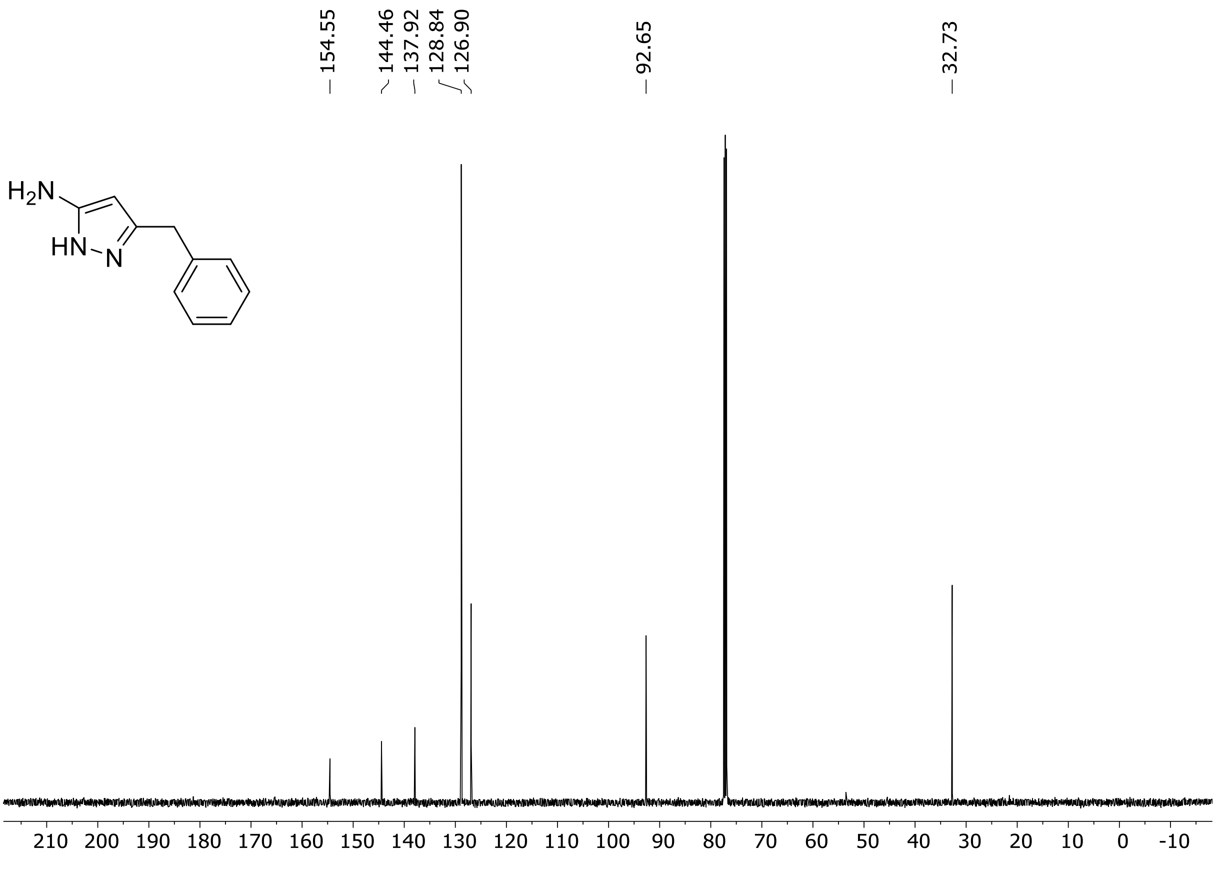

HRMS spectrum of **S58**

$C_{10}H_{11}N_3$ **exact mass: 173.0953**

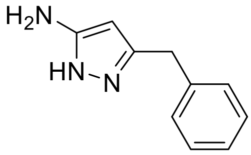

APCI + (MMI)
nitrogen flow 3 L/min, gas temperature 325°C, nebulizer 45 psig, skimmer 65 V, vaporizer 200°C, fragmentor 20 V, dissolved in methanol

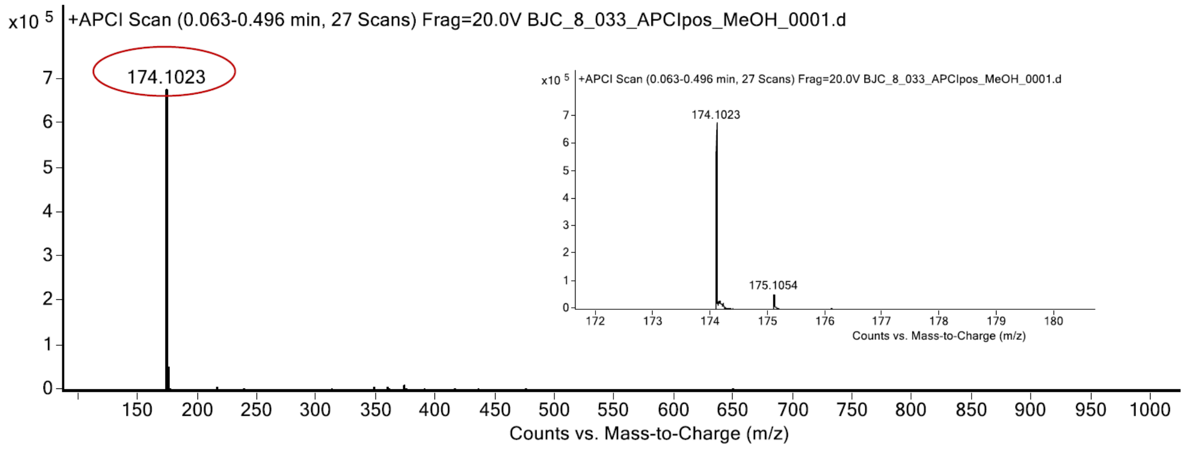

expected mass: $[M+H]^+$ = 174.1026 observed mass: $[M+H]^+$ = 174.1023 mass accuracy = - 1.7 ppm

^{1}H (500 MHz) and ^{13}C NMR (126 MHz) spectra of **S59** in chloroform-*d*

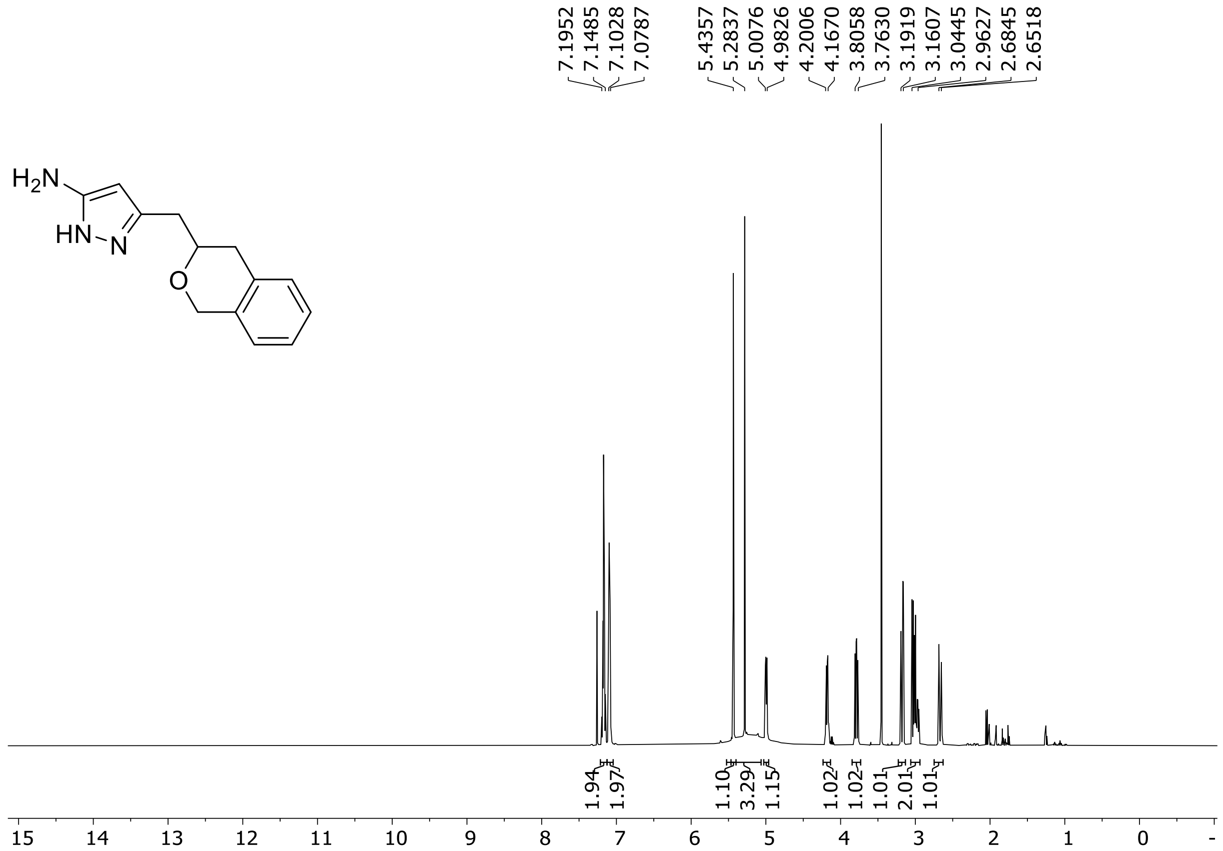

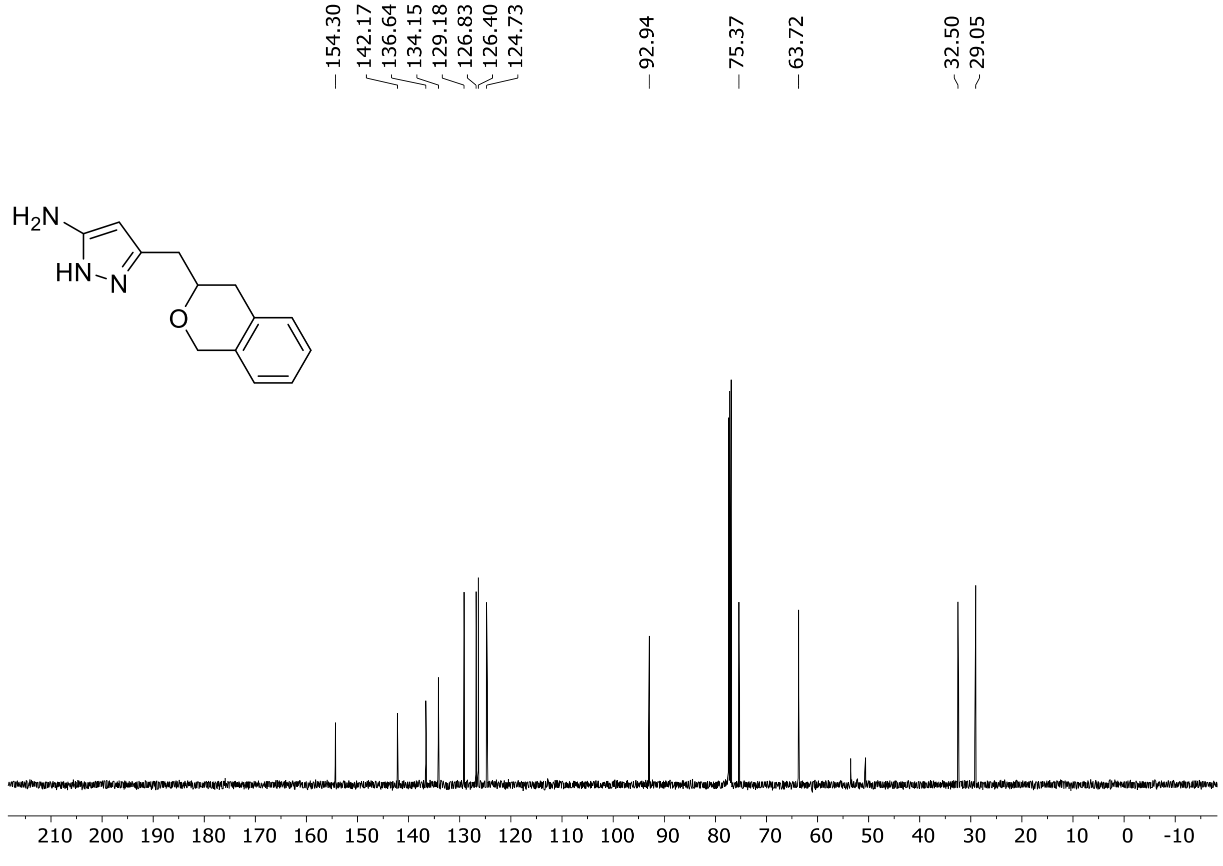

HRMS spectrum of **S59**

$C_{13}H_{15}N_3O$ **exact mass: 229.1215**

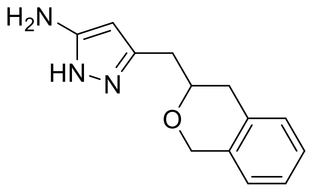

APCI + (MMI)
nitrogen flow 3 L/min, gas temperature 325°C, nebulizer 45 psig, skimmer 65 V, vaporizer 200°C, fragmentor 20 V, dissolved in methanol

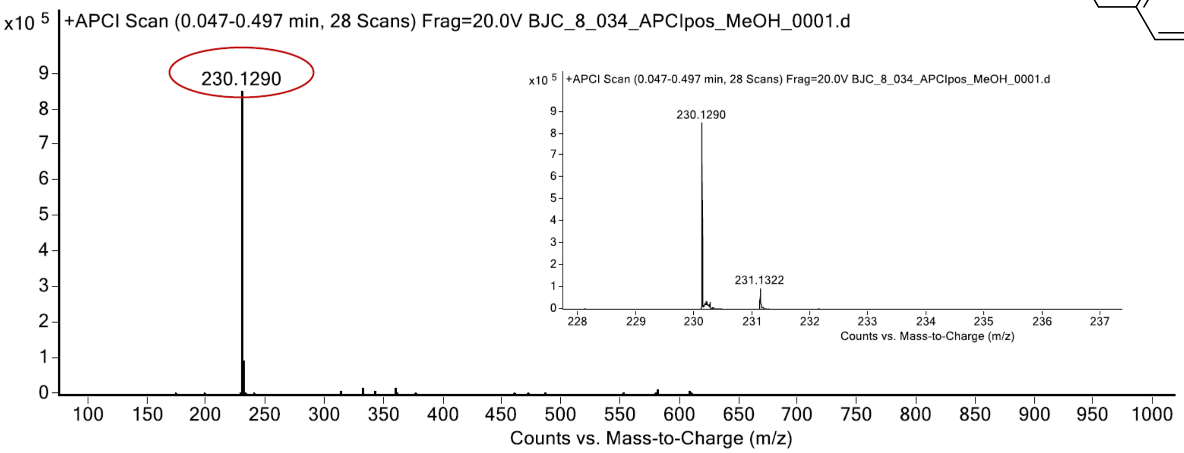

expected mass: $[M+H]^+$ = 230.1288 observed mass: $[M+H]^+$ = 230.1290 mass accuracy = 0.8 ppm

^{1}H (500 MHz) and ^{13}C NMR (126 MHz) spectra of **S60** in chloroform-*d*

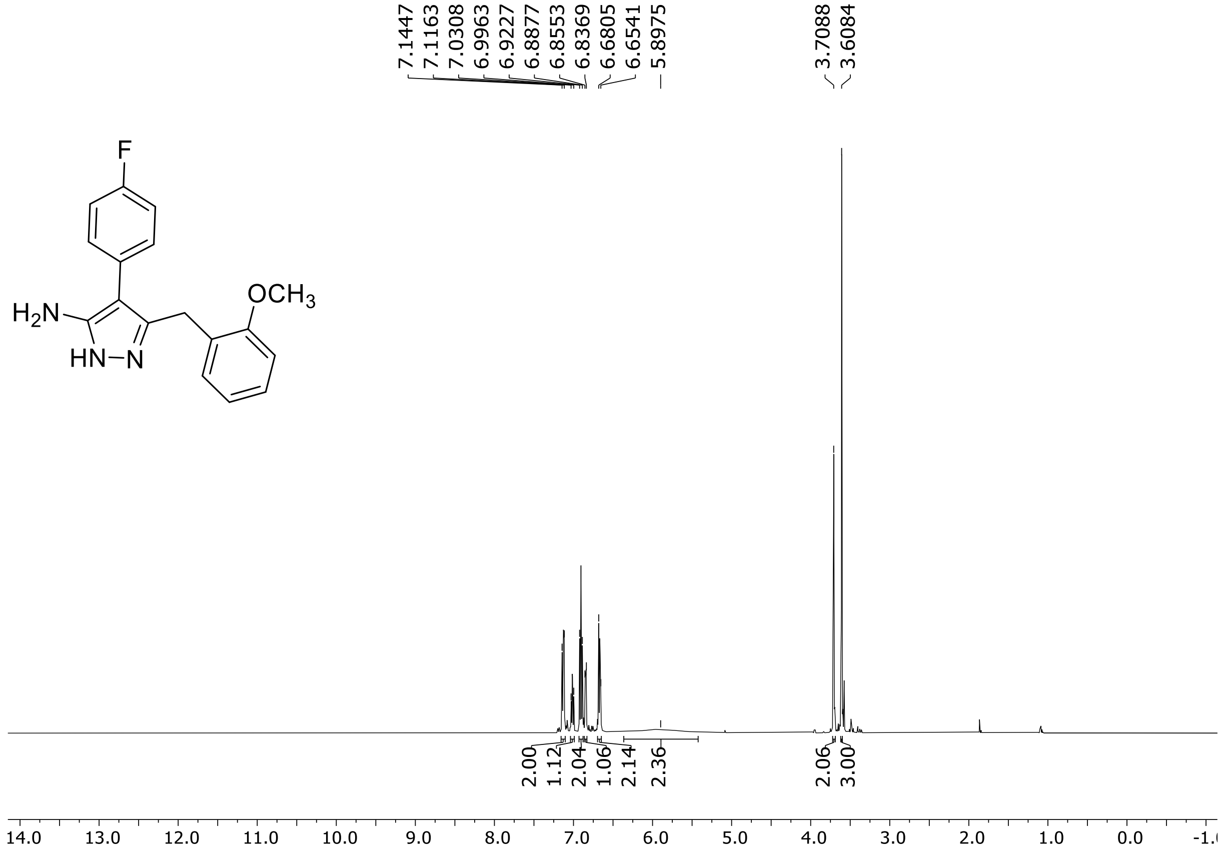

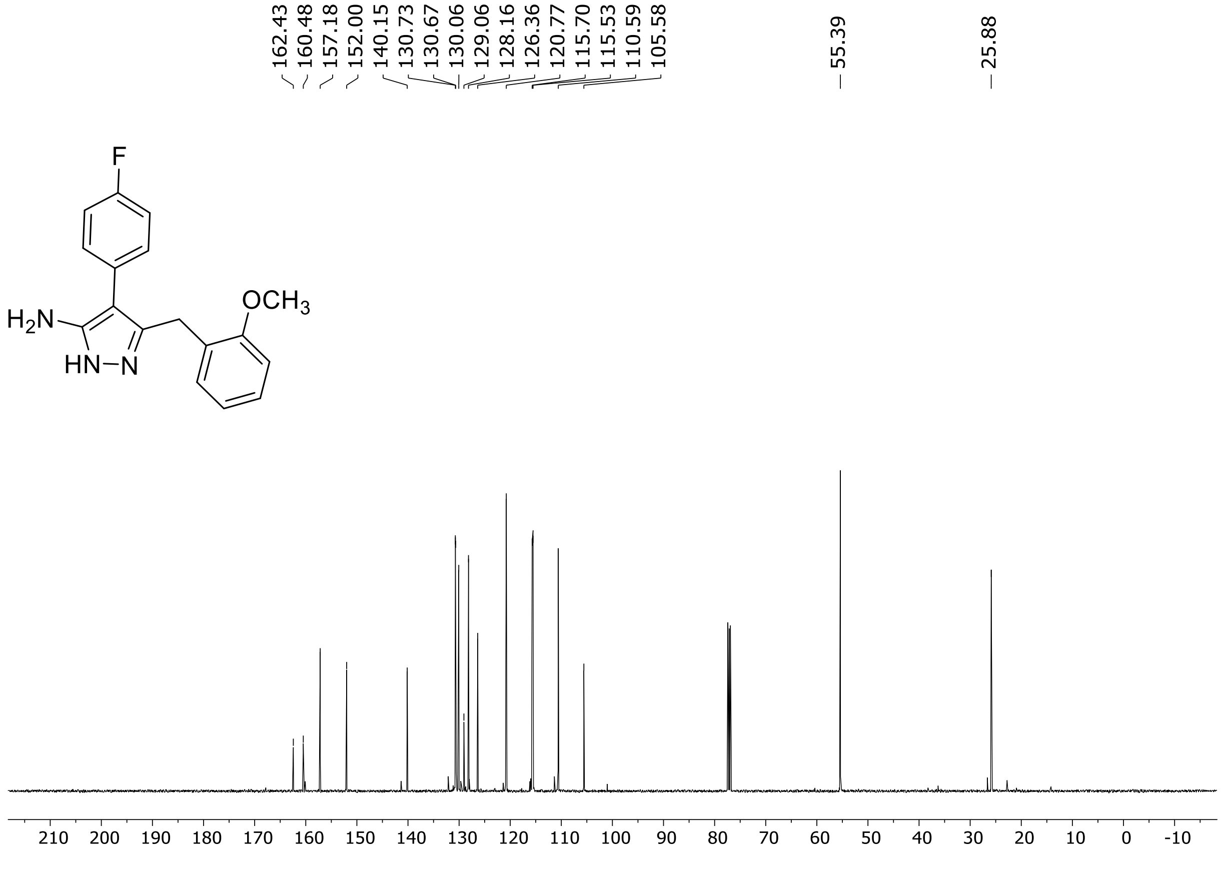

^{19}F (471 MHz) NMR spectrum of **S60** in chloroform-*d*

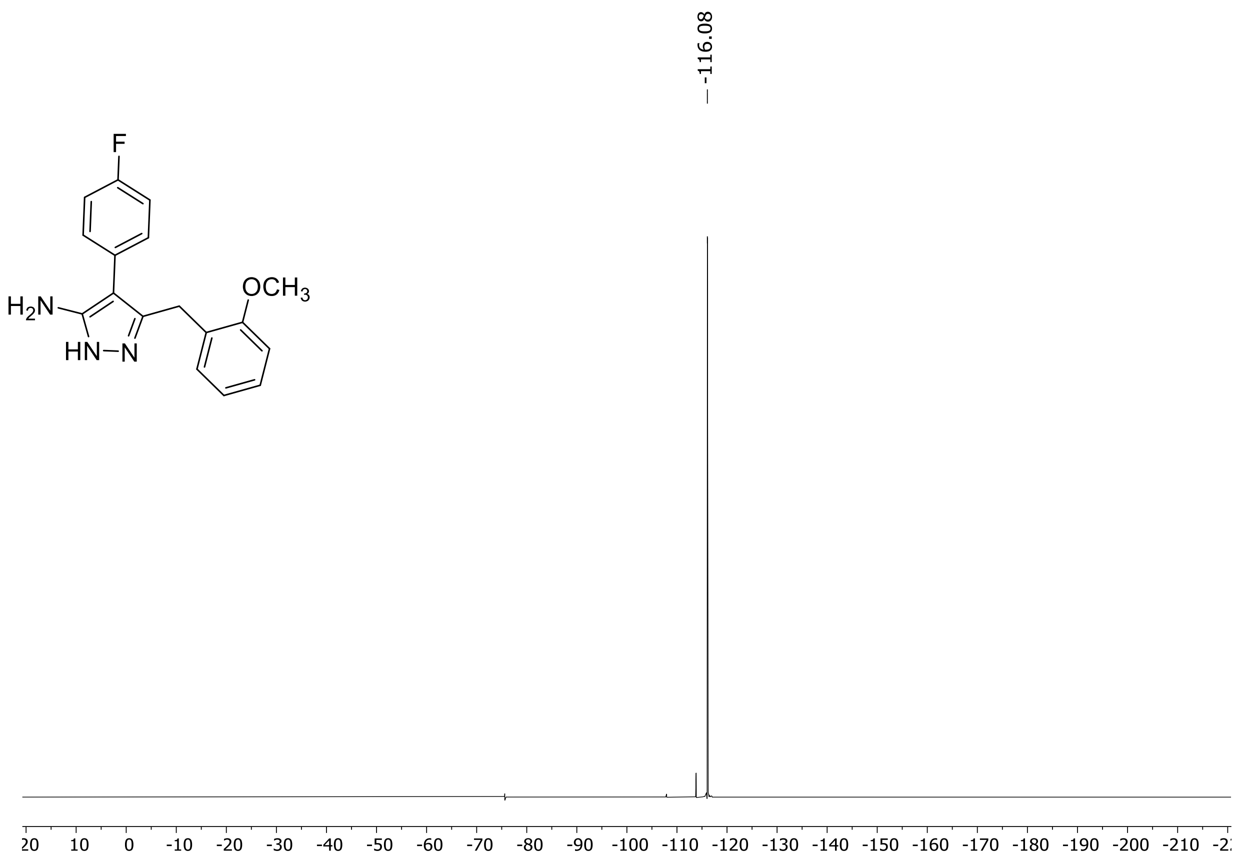

^{1}H (500 MHz) and ^{13}C NMR (126 MHz) spectra of **S61** in chloroform-*d*

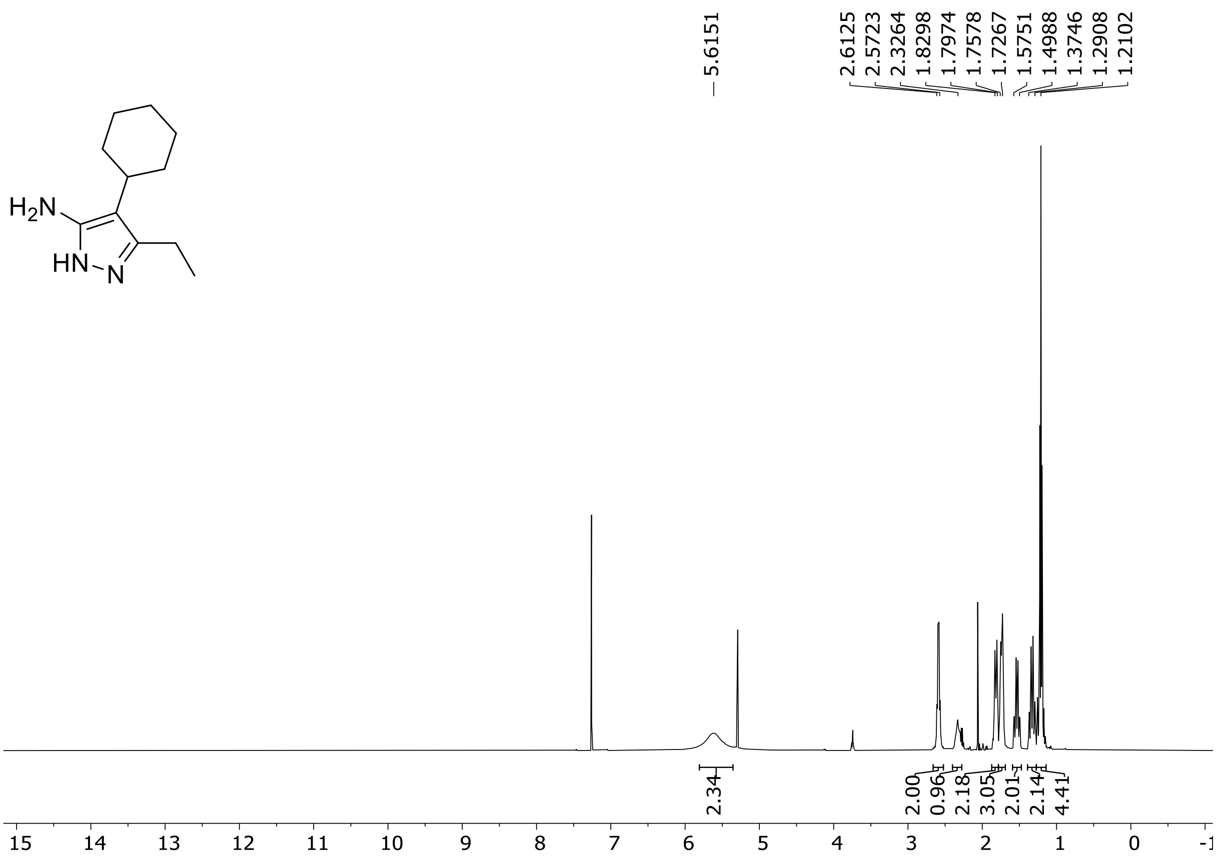

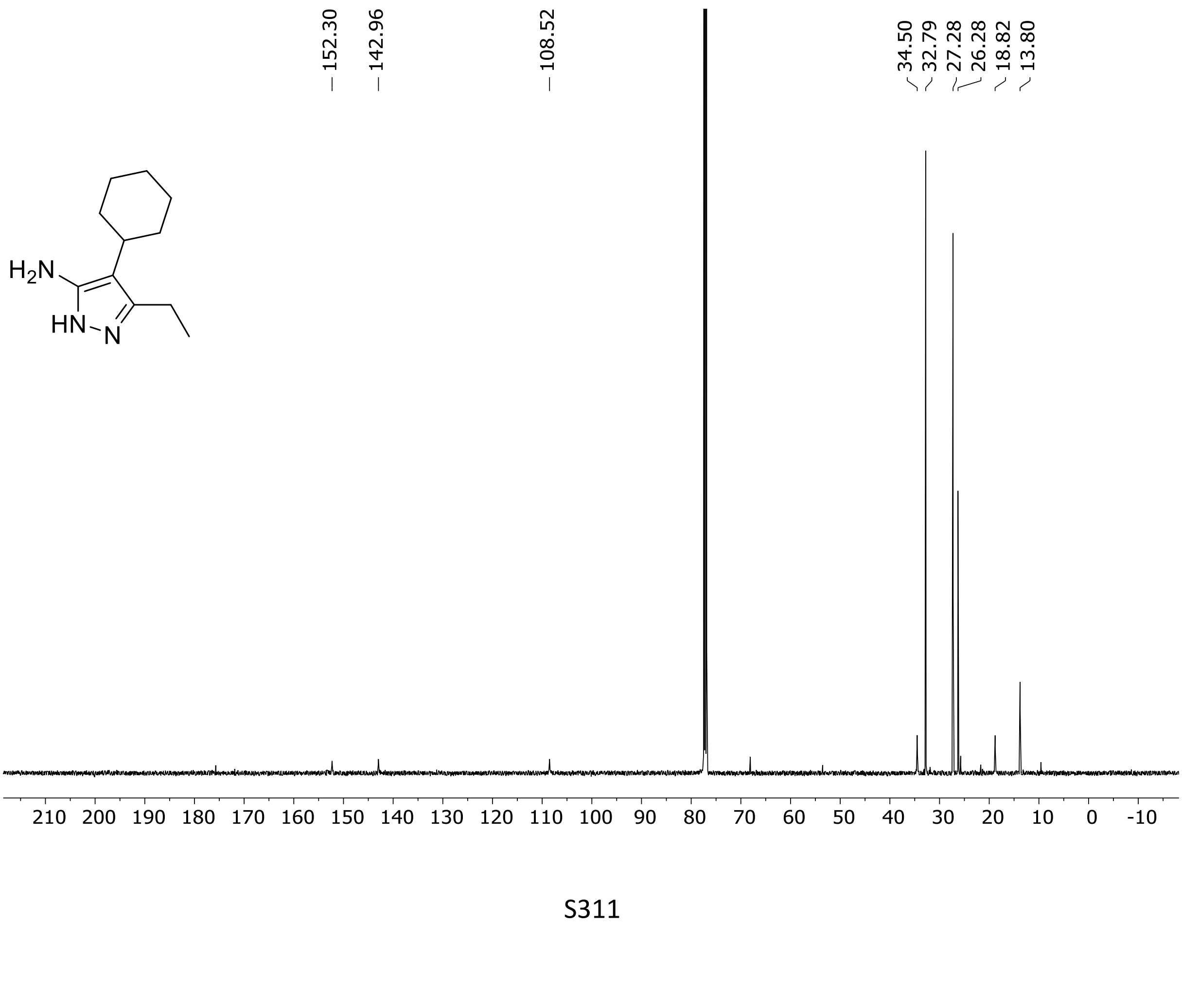

HRMS spectrum of **S61**

$C_{11}H_{19}N_3$ **exact mass: 193.1579**

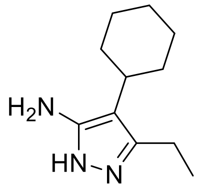

APCI + (MMI)
nitrogen flow 3 L/min, gas temperature 325°C, nebulizer 45 psig, skimmer 65 V, vaporizer 200°C, fragmentor 30 V, dissolved in methanol

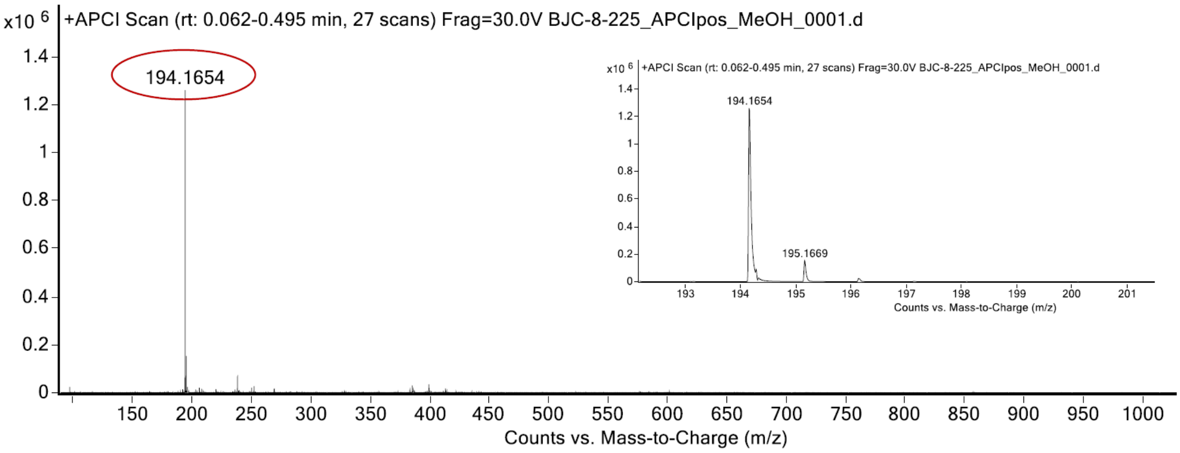

expected mass: $[M+H]^+$ = 194.1652 observed mass: $[M+H]^+$ = 194.1654 mass accuracy = 1.0 ppm

^{1}H (500 MHz) and ^{13}C NMR (126 MHz) spectra of **S62** in chloroform-*d*

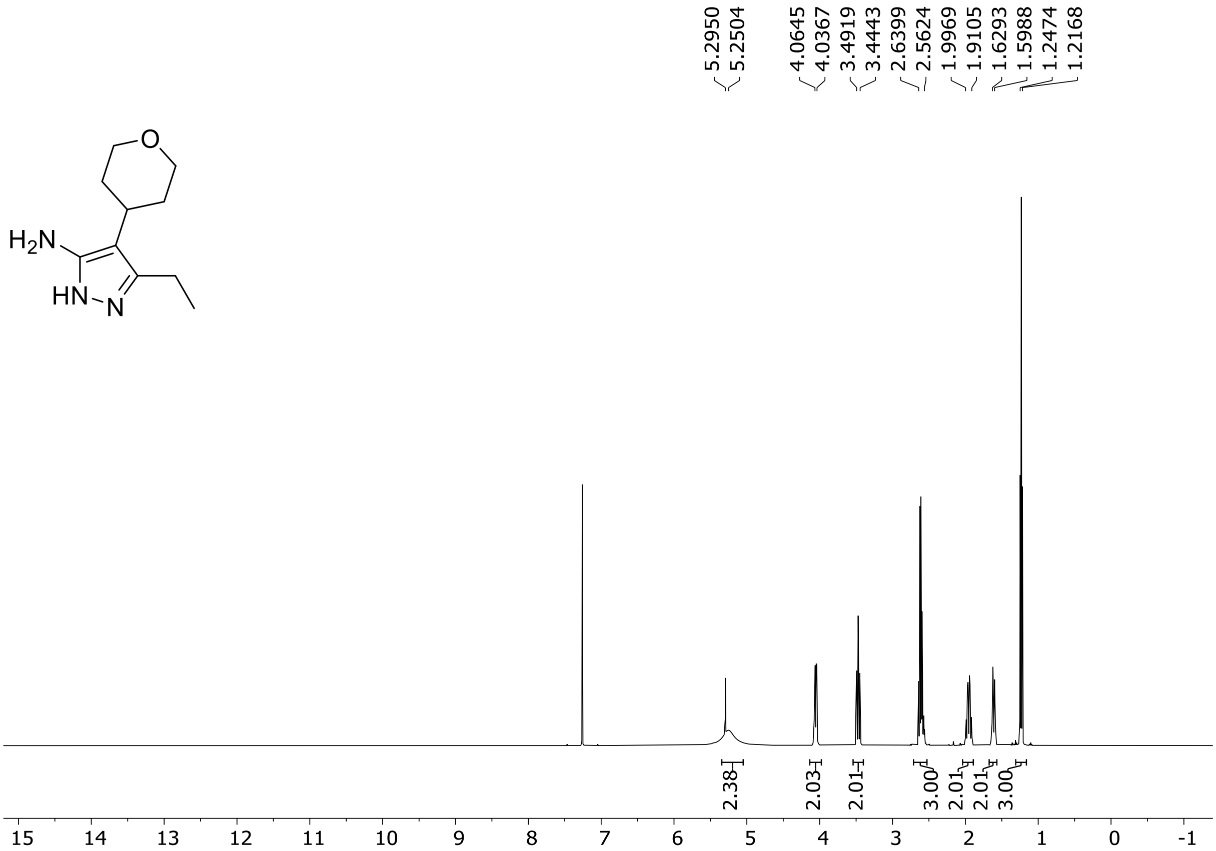

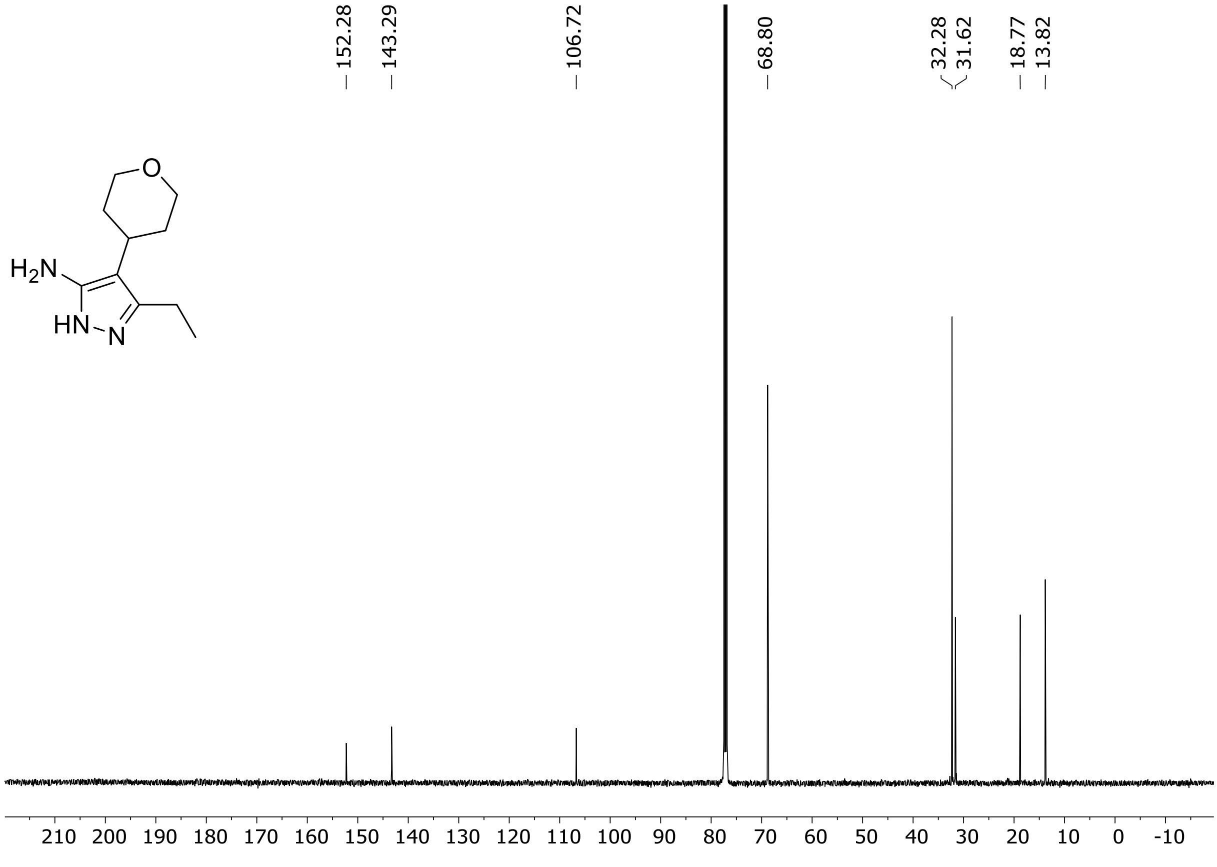

HRMS spectrum of **S62**

$C_{10}H_{17}N_3O$ **exact mass: 193.1579**

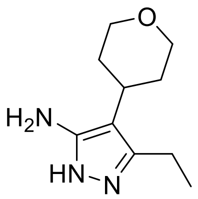

APCI + (MMI)
nitrogen flow 3 L/min, gas temperature 325°C, nebulizer 45 psig, skimmer 65 V, vaporizer 200°C, fragmentor 25 V, dissolved in methanol

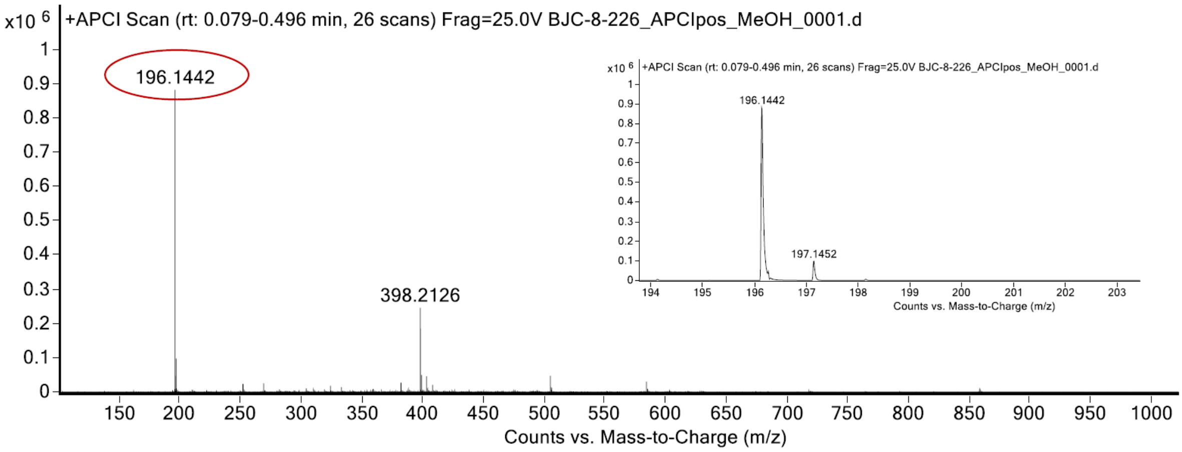

expected mass: $[M+H]^+$ = 196.1444 observed mass: $[M+H]^+$ = 196.1442 mass accuracy = - 1.0 ppm

^{1}H (500 MHz) and ^{13}C NMR (126 MHz) spectra of **S63** in chloroform-*d*

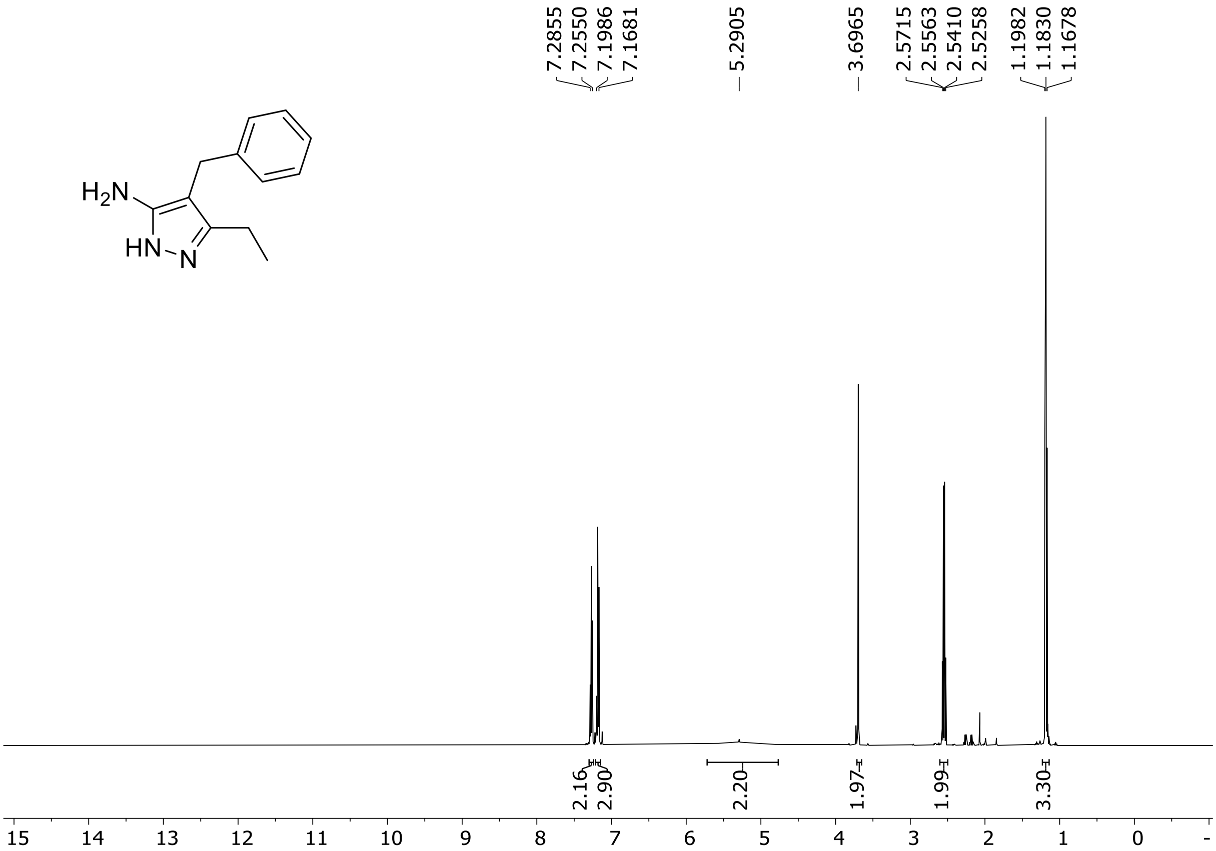

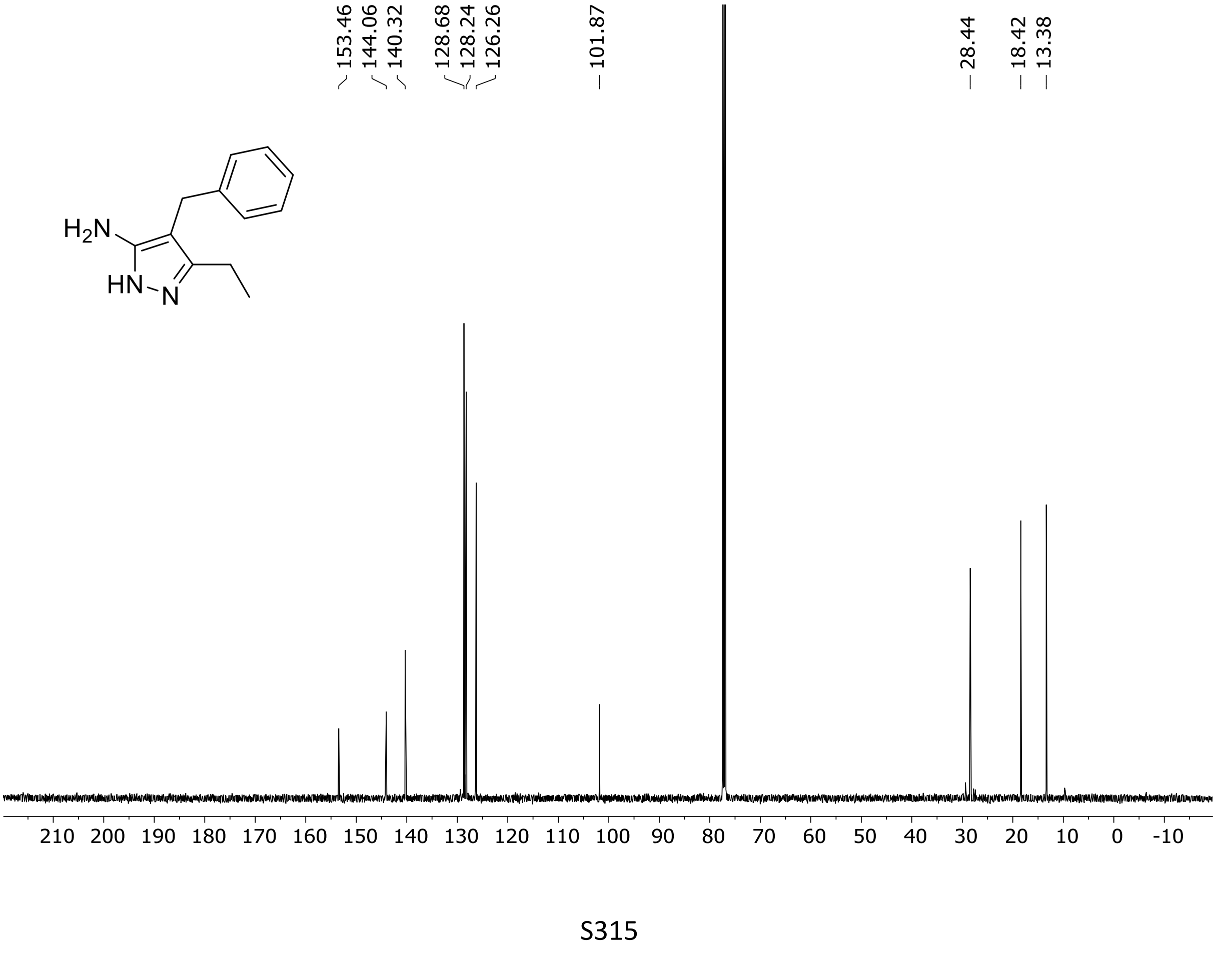

HRMS spectrum of **S63**

$C_{12}H_{15}N_3$ **exact mass: 201.1266**

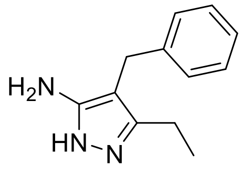

APCI + (MMI)
nitrogen flow 3 L/min, gas temperature 325°C, nebulizer 45 psig, skimmer 65 V, vaporizer 200°C, fragmentor 20 V, dissolved in methanol

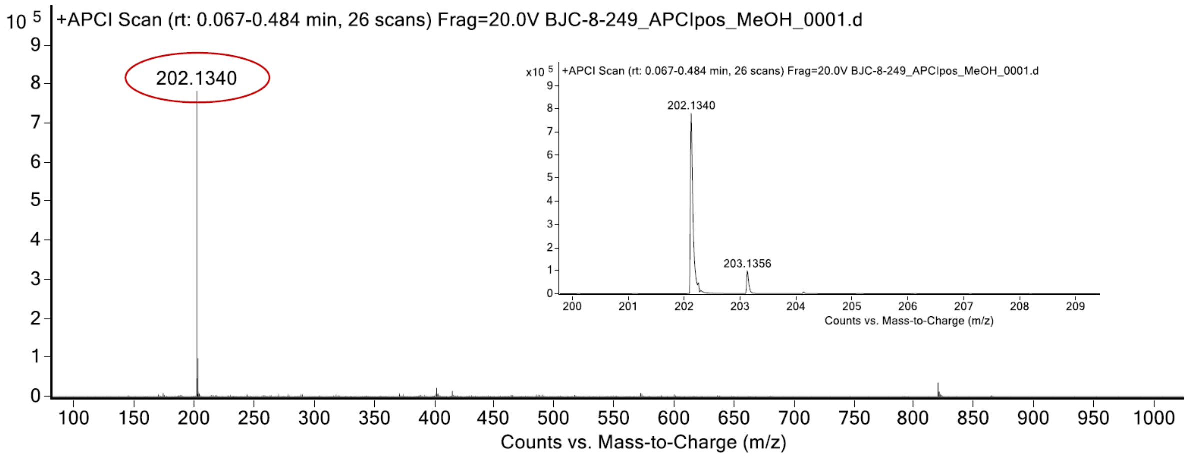

expected mass: $[M+H]^+$ = 202.1339 observed mass: $[M+H]^+$ = 202.1340 mass accuracy = 0.5 ppm

^{1}H (500 MHz) and ^{13}C NMR (126 MHz) spectra of **S64** in chloroform-*d*

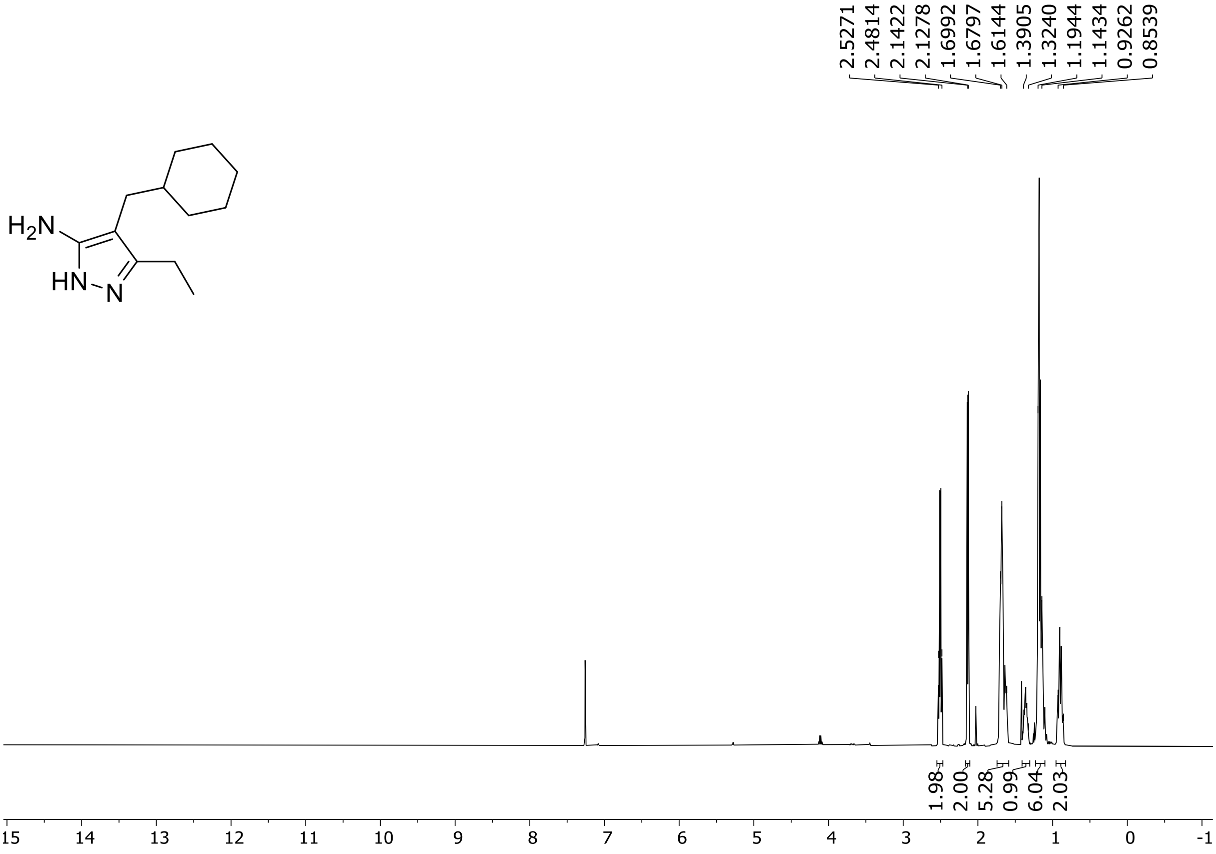

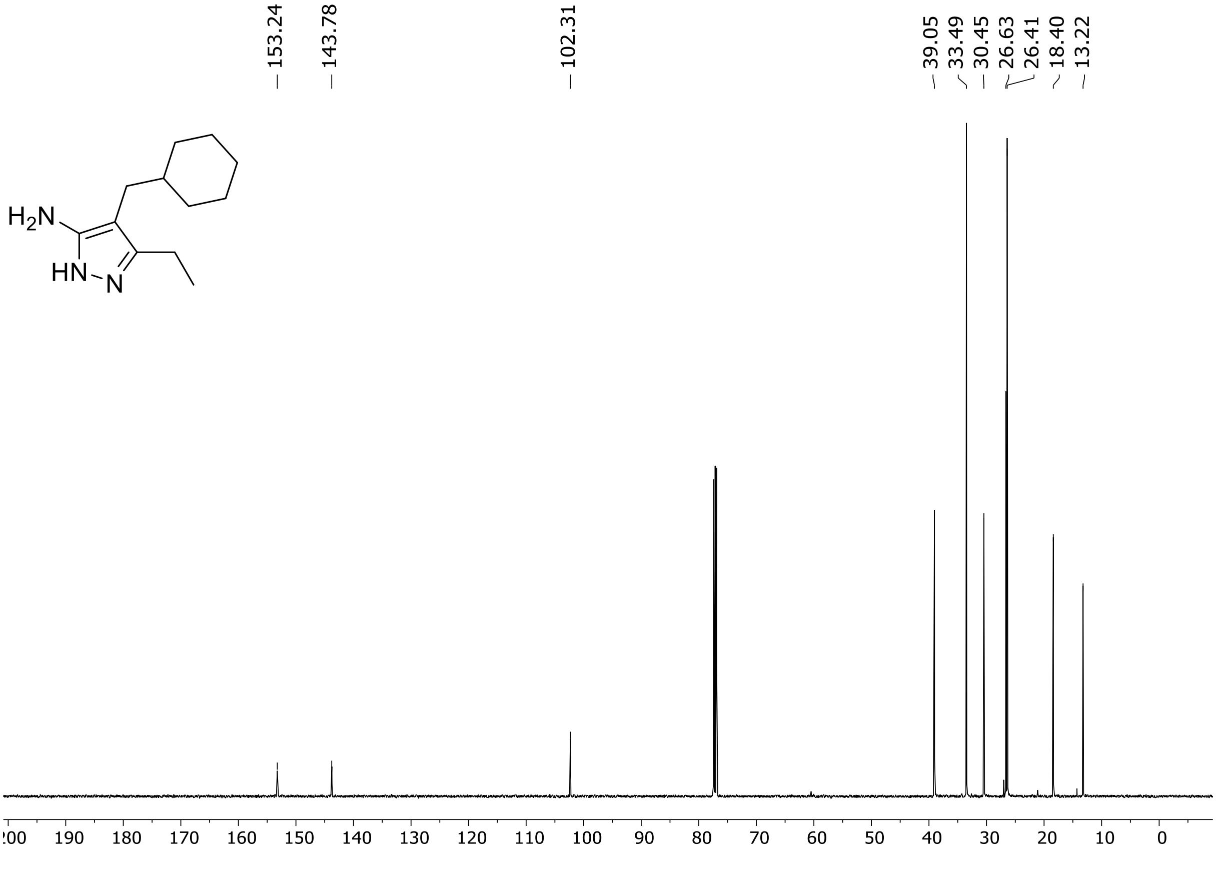

HRMS spectrum of **S64**

$C_{12}H_{21}N_3$ **exact mass: 207.1735**

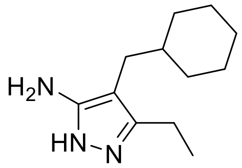

APCI + (MMI)
nitrogen flow 5 L/min, gas temperature 325°C, nebulizer 45 psig, skimmer 65 V, vaporizer 200°C, fragmentor 20 V, dissolved in methanol

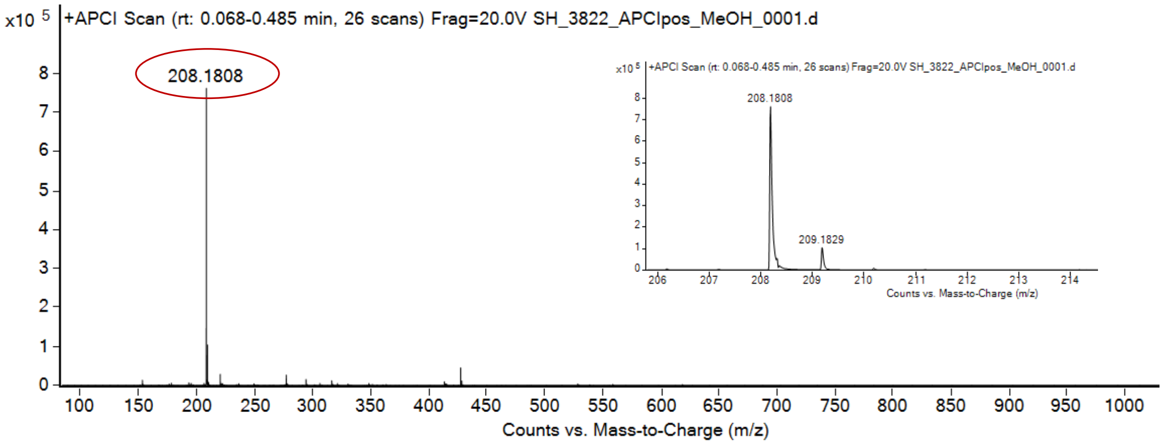

expected mass: $[M+H]^+$ = 208.1808 observed mass: $[M+H]^+$ = 208.1808 mass accuracy < 0.1 ppm

^{1}H (500 MHz) and ^{13}C NMR (126 MHz) spectra of **S65** in chloroform-*d*

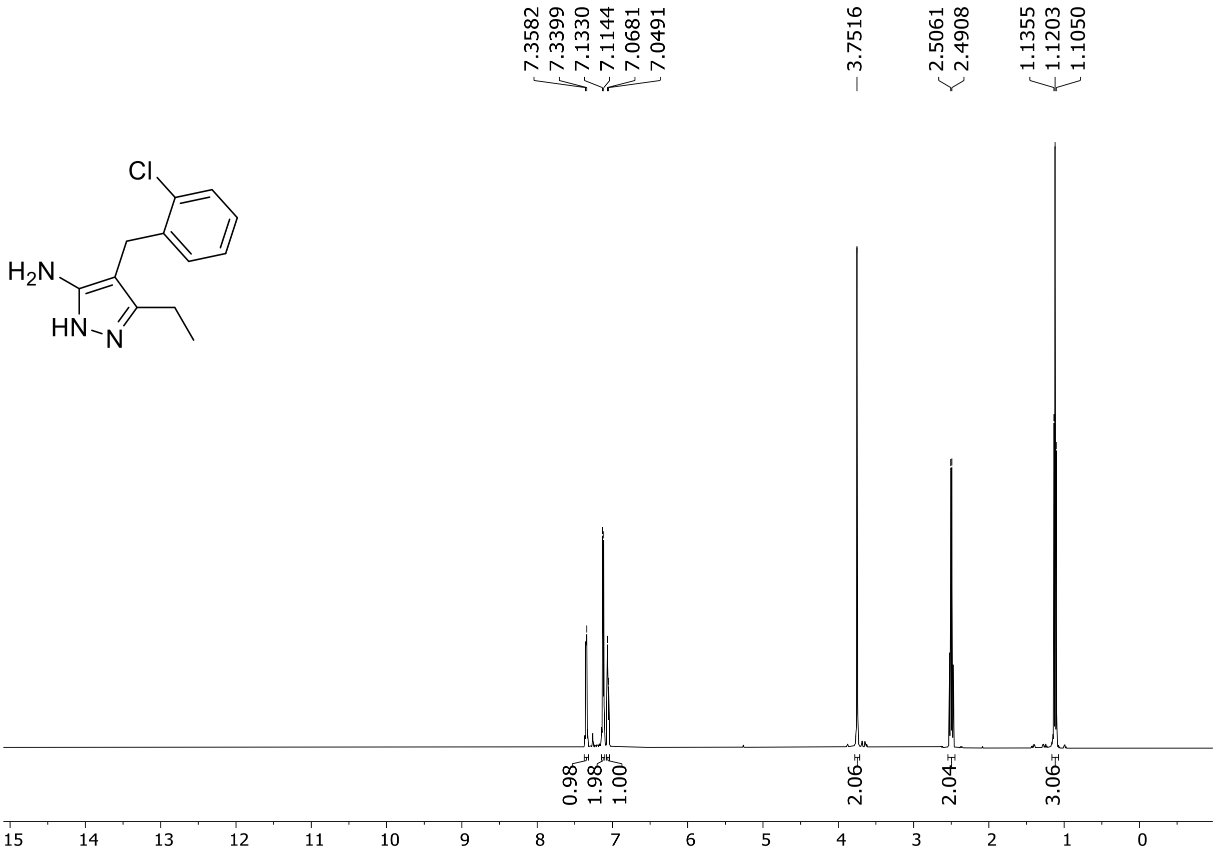

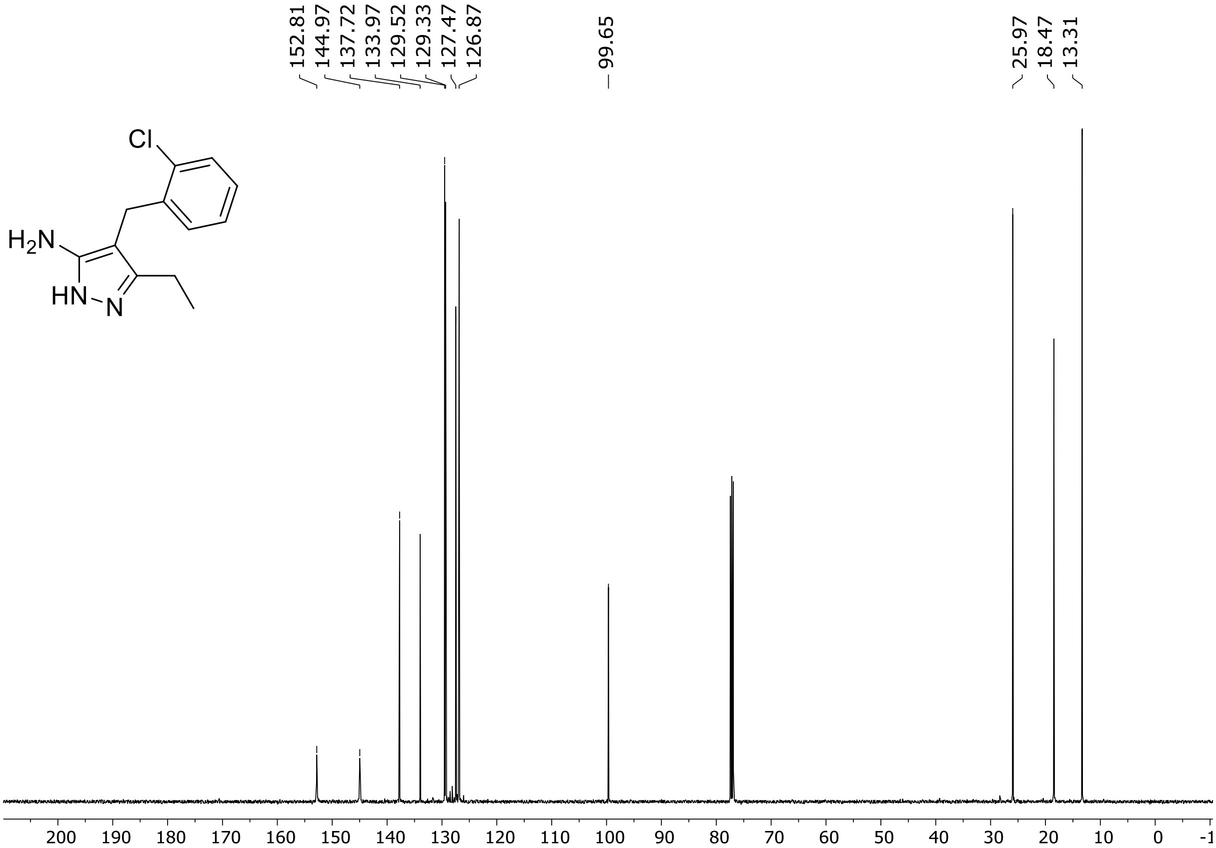

HRMS spectrum of **S65**

$C_{12}H_{14}ClN_3$ **exact mass: 235.0876**

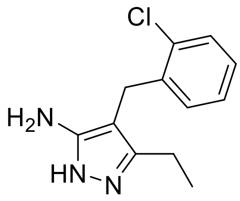

APCI + (MMI)
nitrogen flow 5 L/min, gas temperature 325°C, nebulizer 45 psig, skimmer 65 V, vaporizer 200°C, fragmentor 25 V, dissolved in methanol

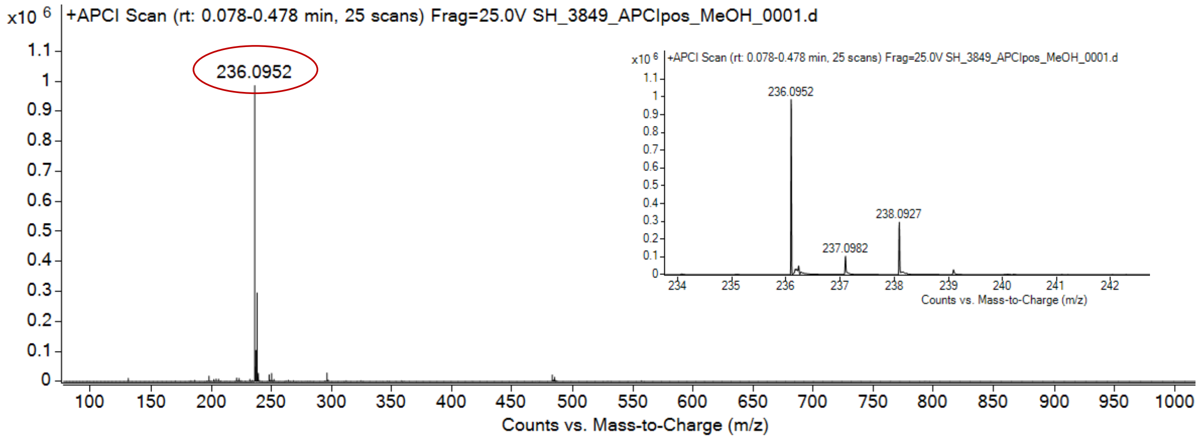

expected mass: $[M+H]^+$ = 236.0949 observed mass : $[M+H]^+$ = 236.0952 mass accuracy = 1.3 ppm

^{1}H (500 MHz) and ^{13}C NMR (126 MHz) spectra of **S66** in chloroform-*d*

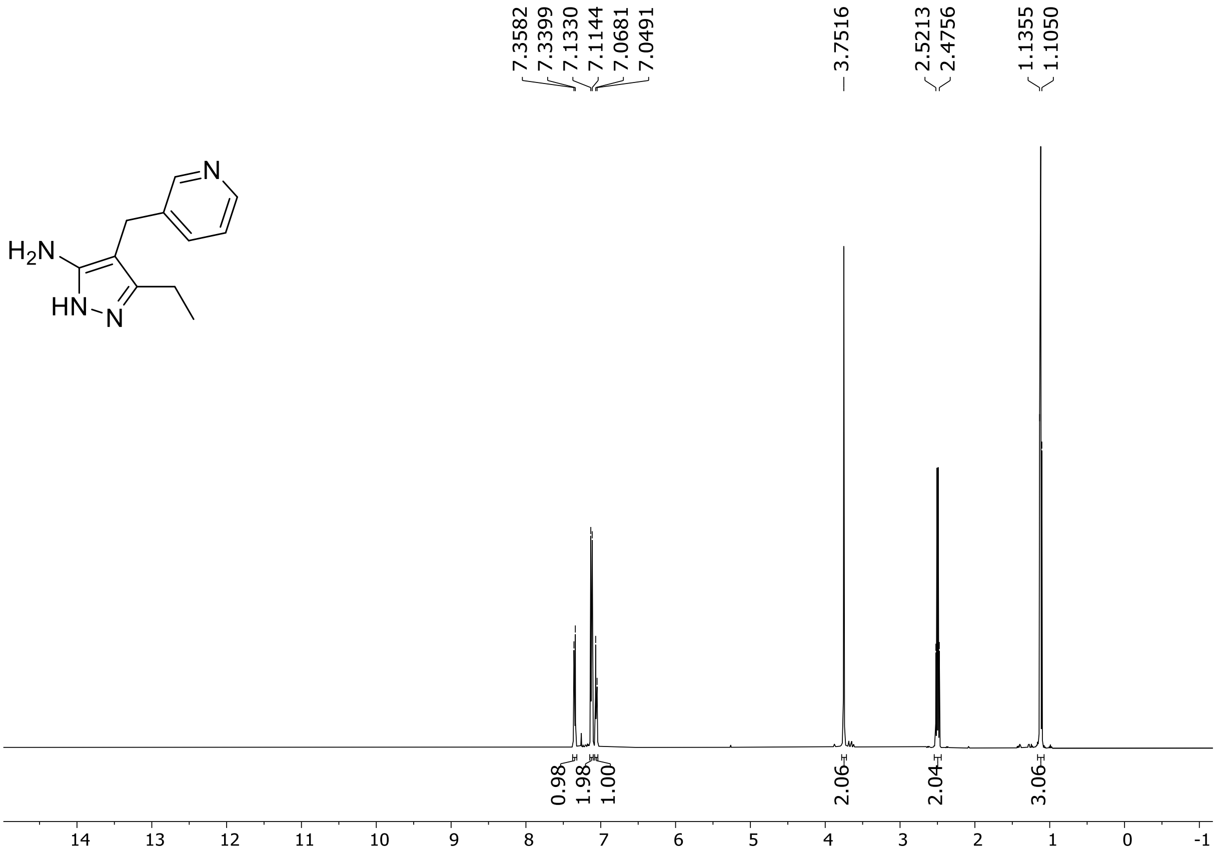

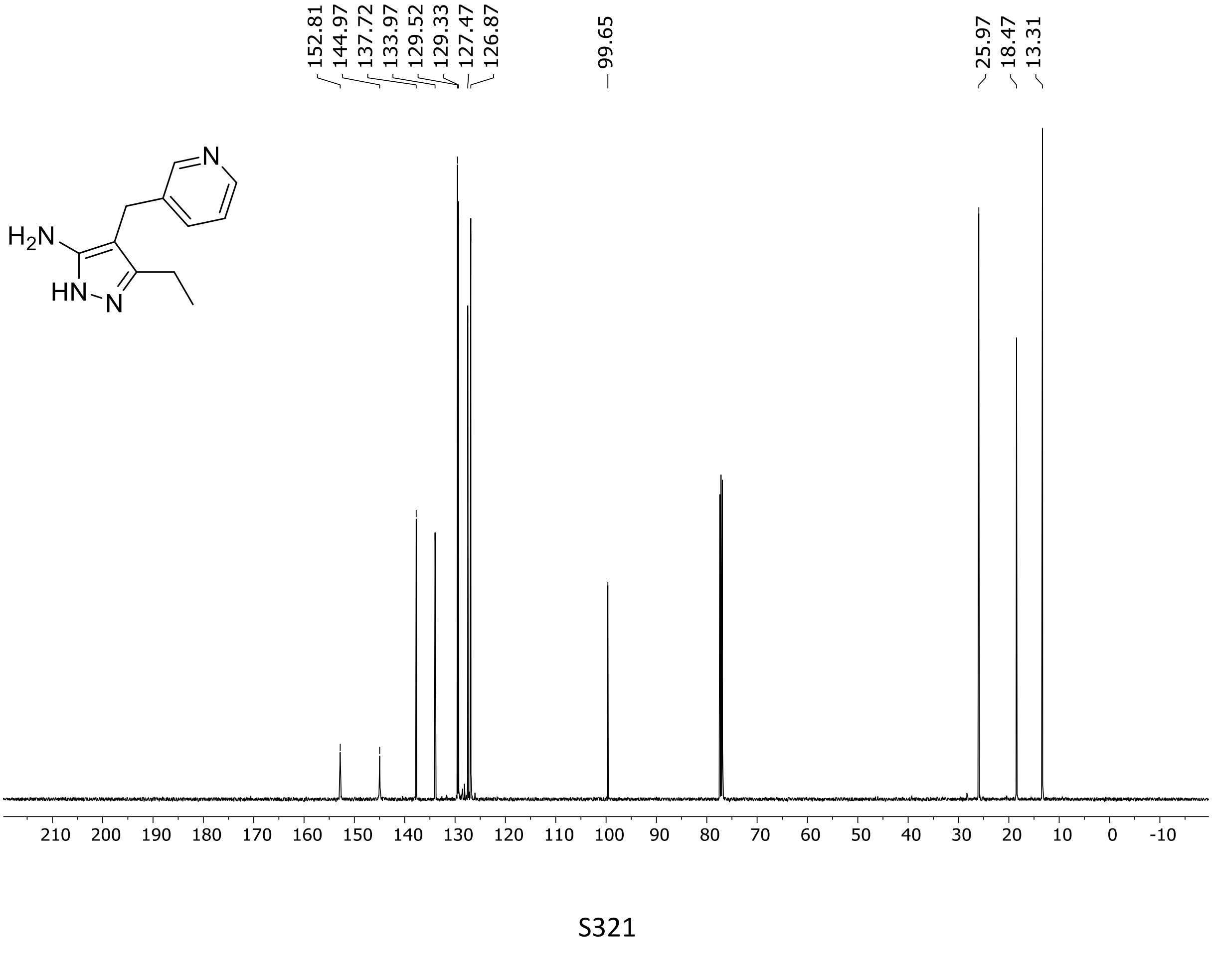

HRMS spectrum of **S66**

$C_{11}H_{14}N_4$ **exact mass: 202.1218**

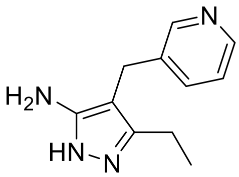

APCI + (MMI)
nitrogen flow 5 L/min, gas temperature 325°C, nebulizer 45 psig, skimmer 65 V, vaporizer 200°C, fragmentor 18 V, dissolved in methanol

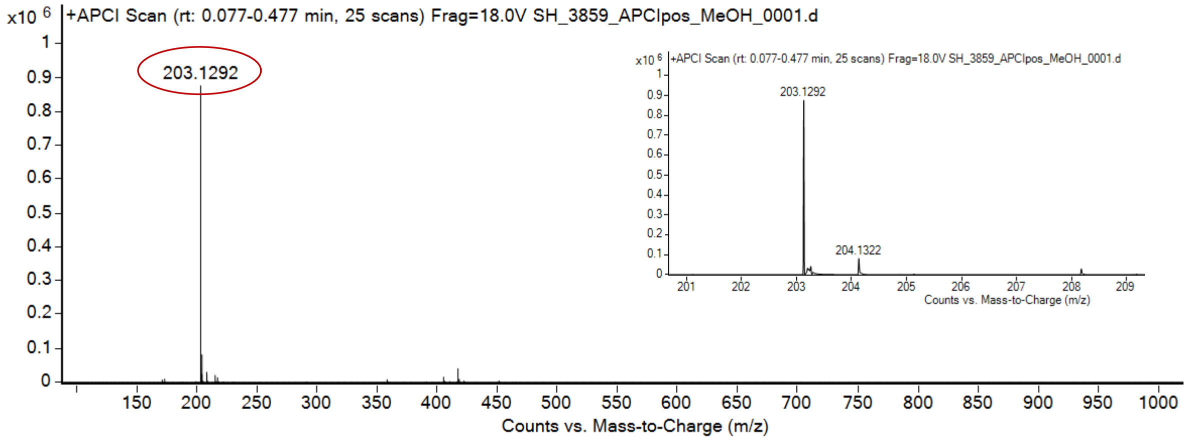

expected mass: $[M+H]^+$ = 203.1291 observed mass : $[M+H]^+$ = 203.1292 mass accuracy = 0.5 ppm

^{1}H (500 MHz) and ^{13}C NMR (126 MHz) spectra of **S67** in chloroform-*d*

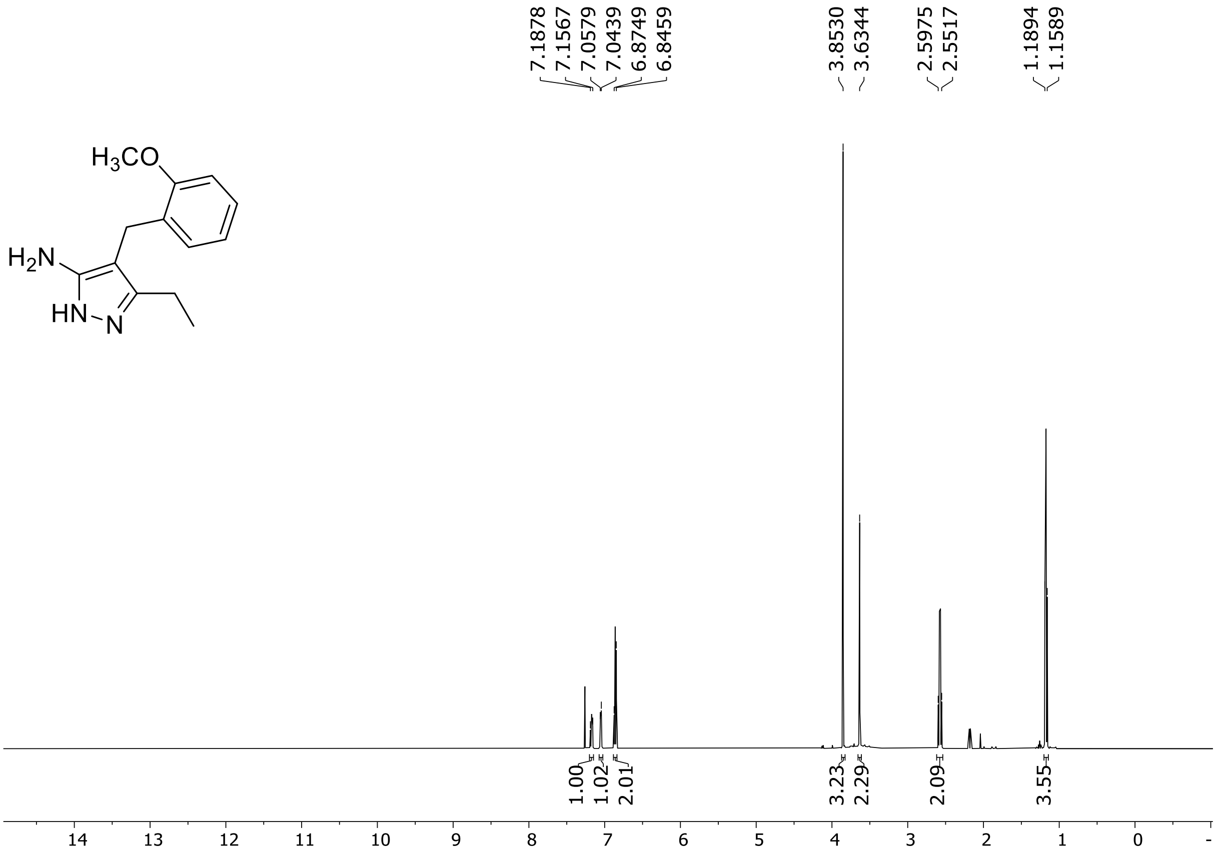

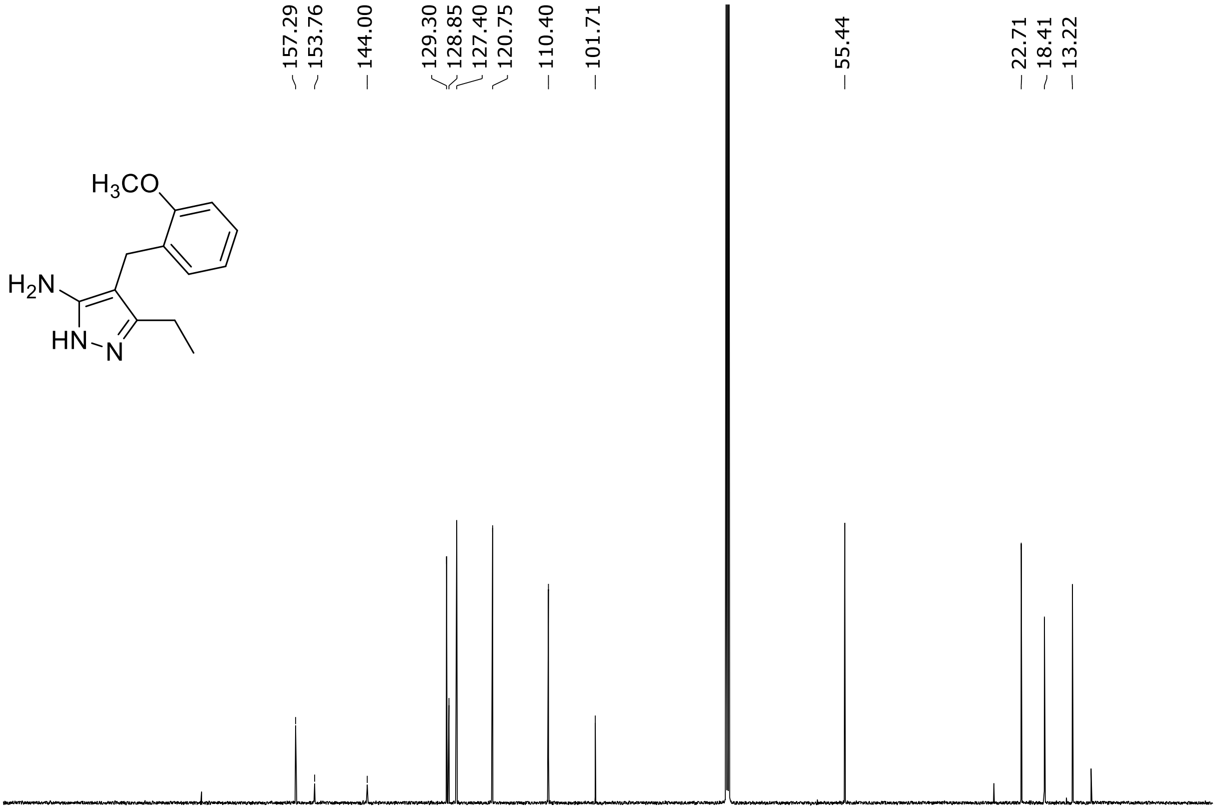

^{1}H (500 MHz) and ^{13}C NMR (126 MHz) spectra of **S68** in chloroform-*d*

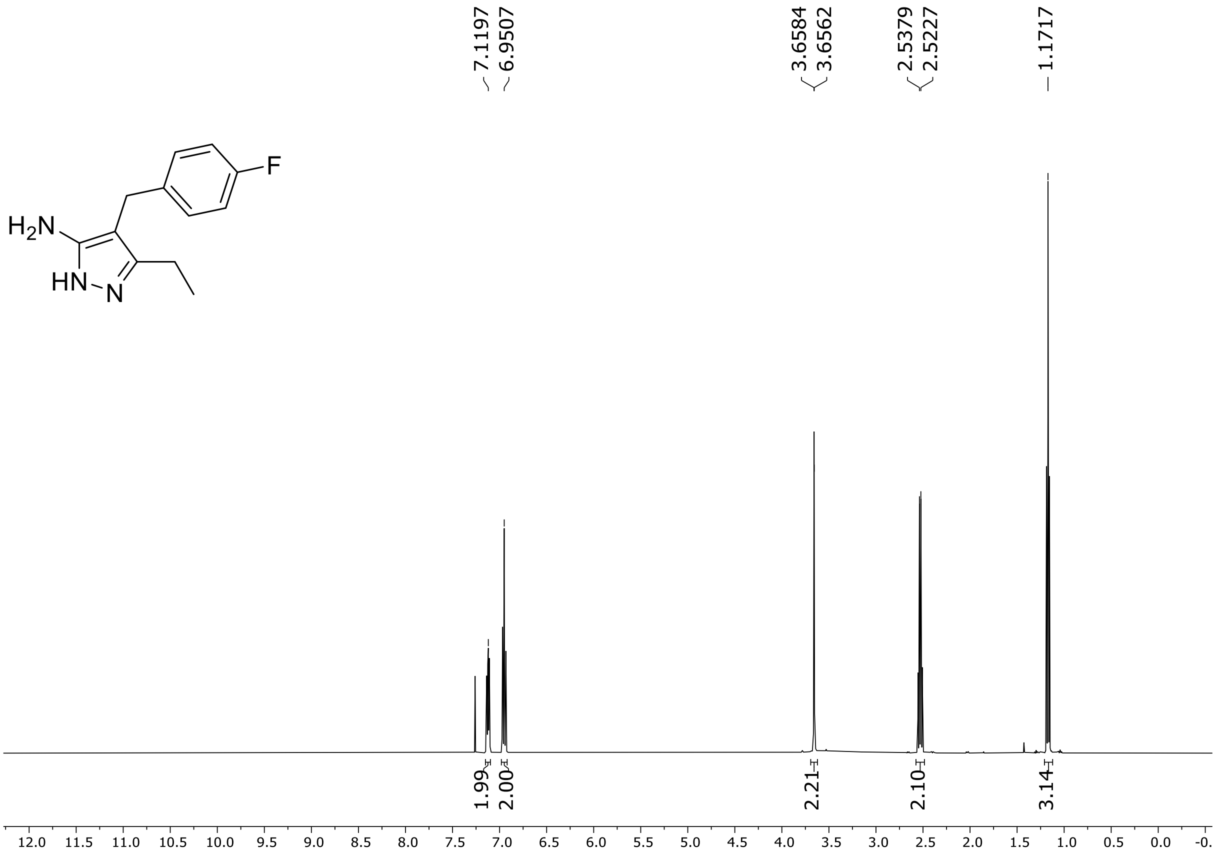

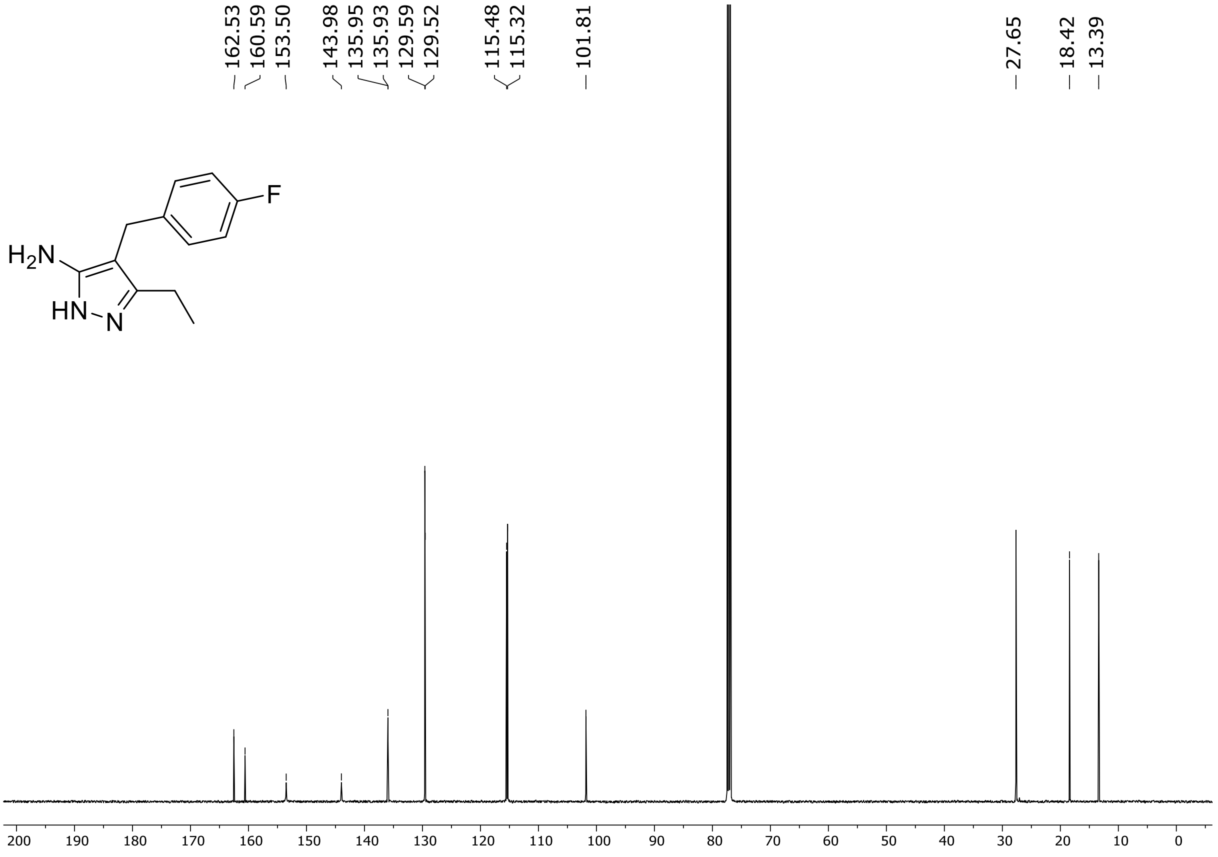

^{19}F (471 MHz) NMR spectrum of **S68** in chloroform-*d*

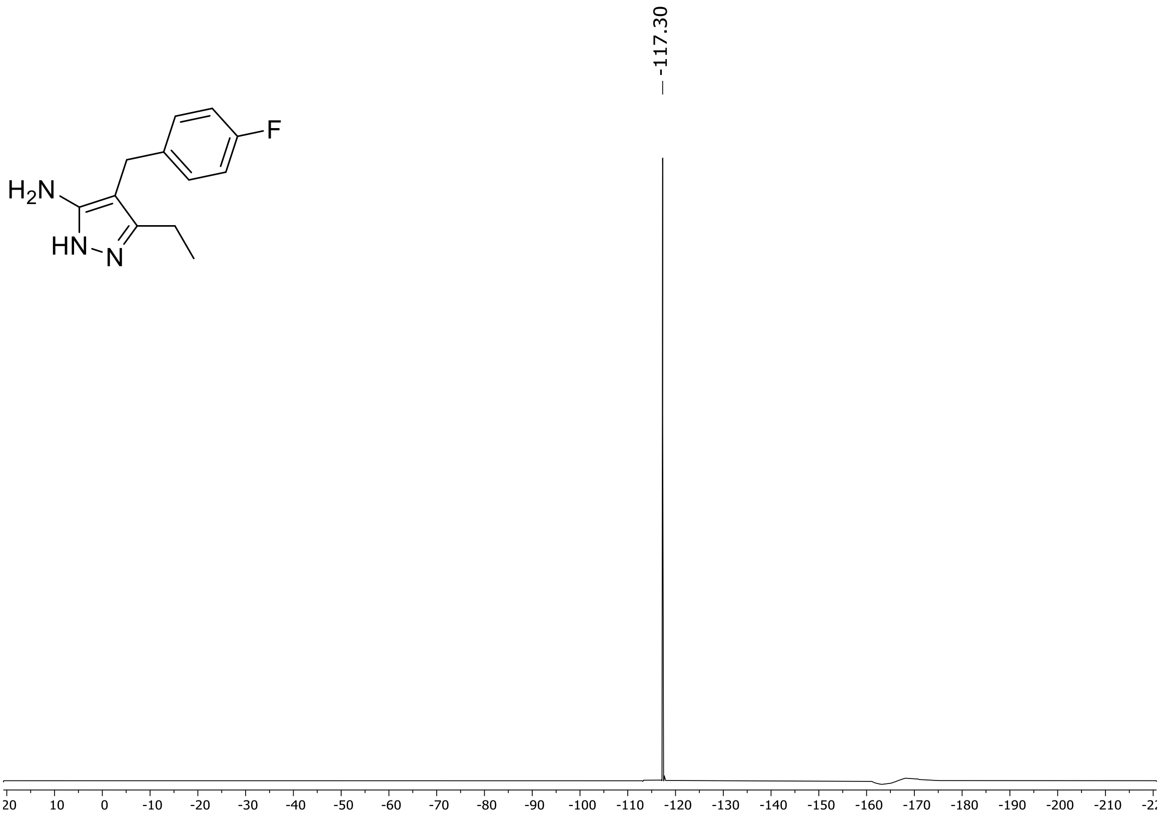

HRMS spectrum of **S68**

$C_{12}H_{14}FN_3$ **exact mass: 219.1172**

APCI + (MMI)
nitrogen flow 5 L/min, gas temperature 325°C, nebulizer 45 psig, skimmer 65 V, vaporizer 200°C, fragmentor 18 V, dissolved in methanol

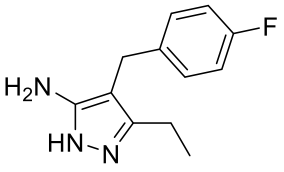

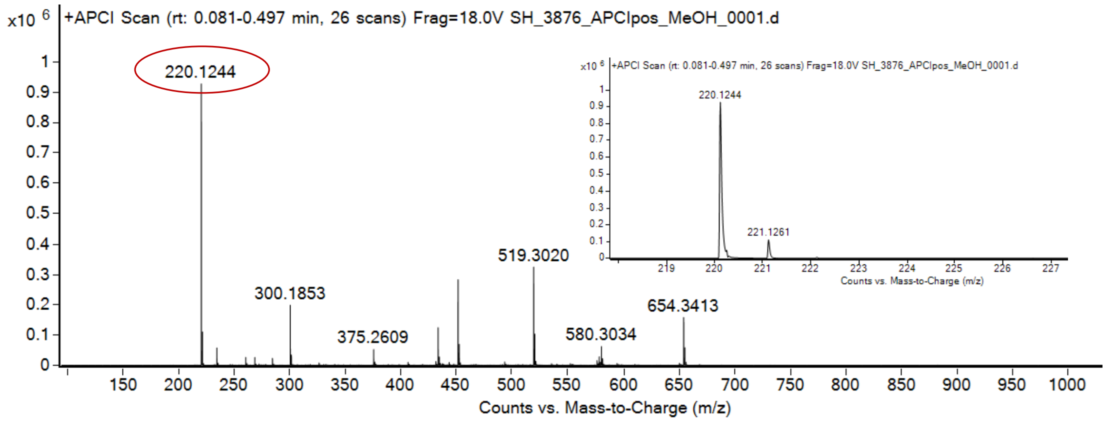

expected mass: [M+H]$^+$ = 220.1245 observed mass: [M+H]$^+$ = 220.1244 mass accuracy = - 0.5 ppm

^{1}H (500 MHz) and ^{13}C NMR (126 MHz) spectra of **S69** in chloroform-*d*

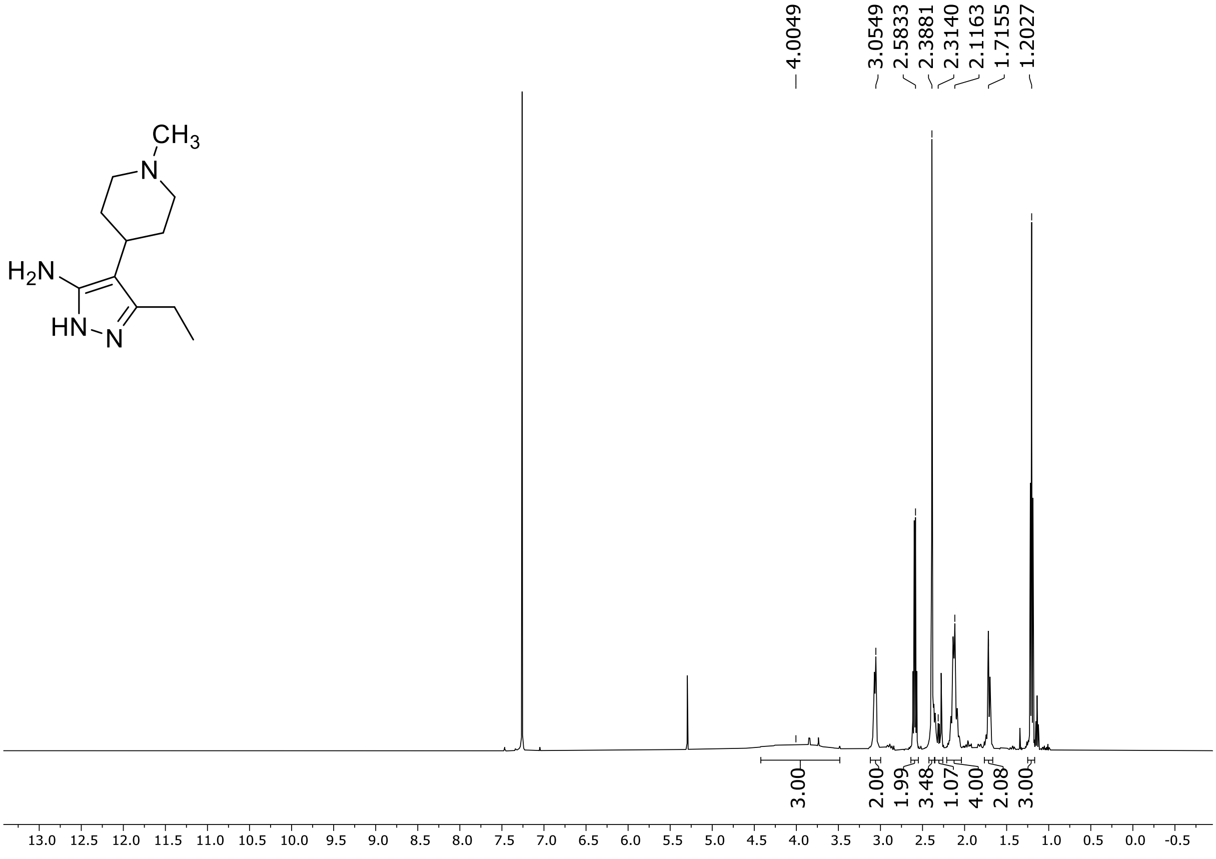

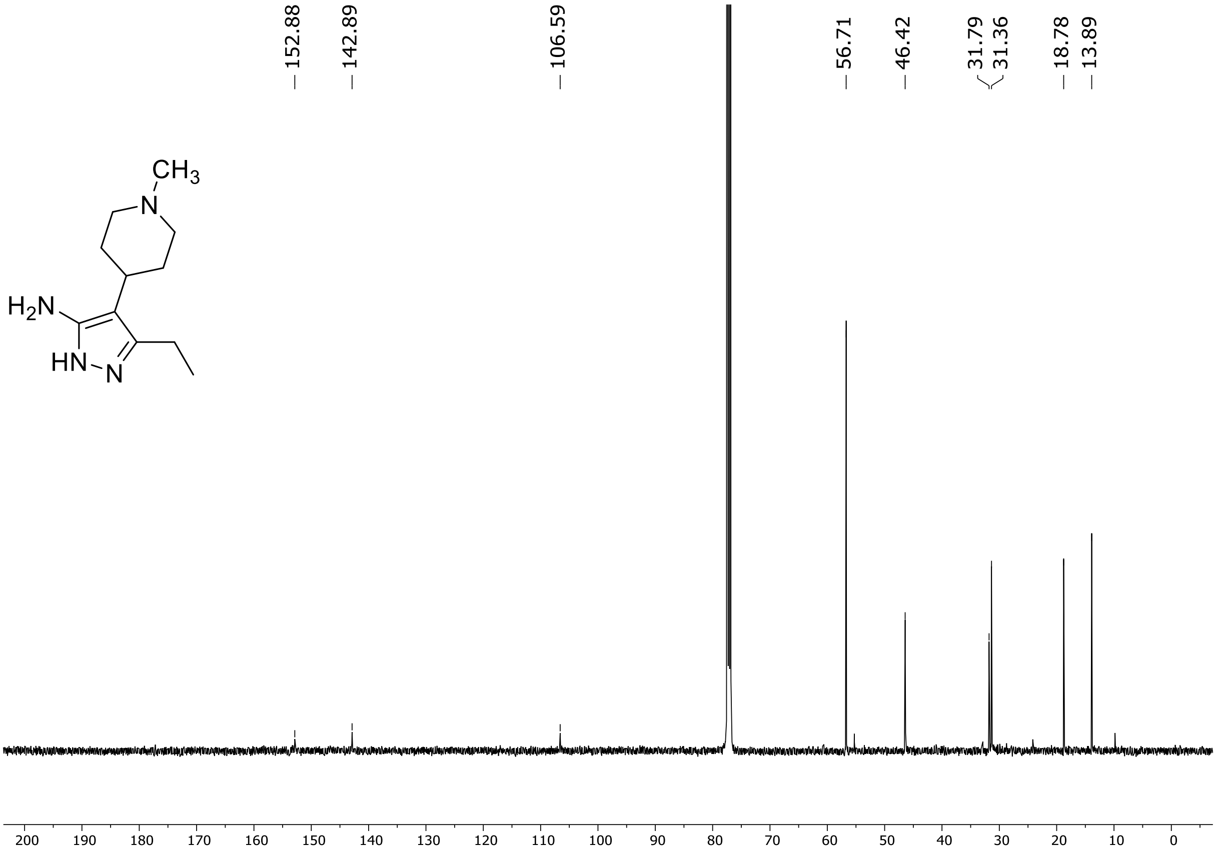

^{1}H (500 MHz) and ^{13}C NMR (126 MHz) spectra of **S70** in chloroform-*d*

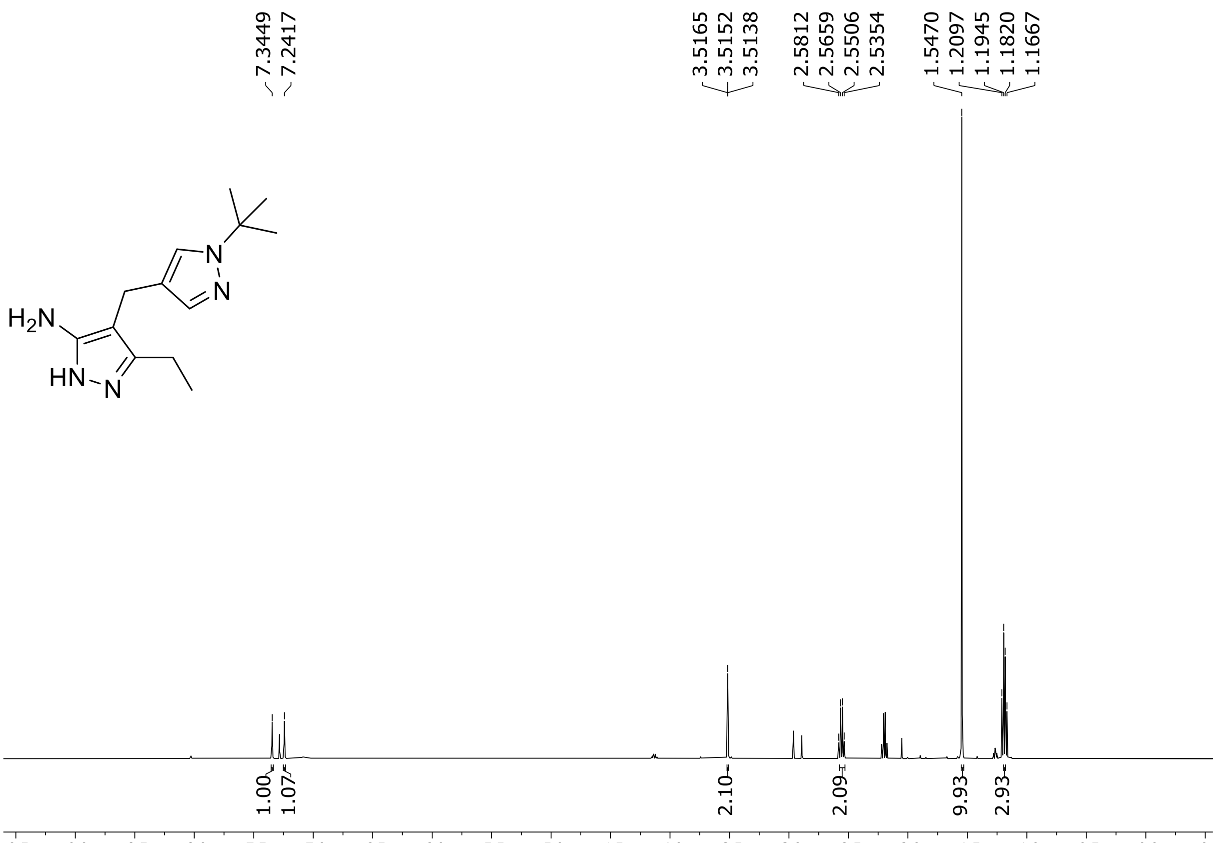

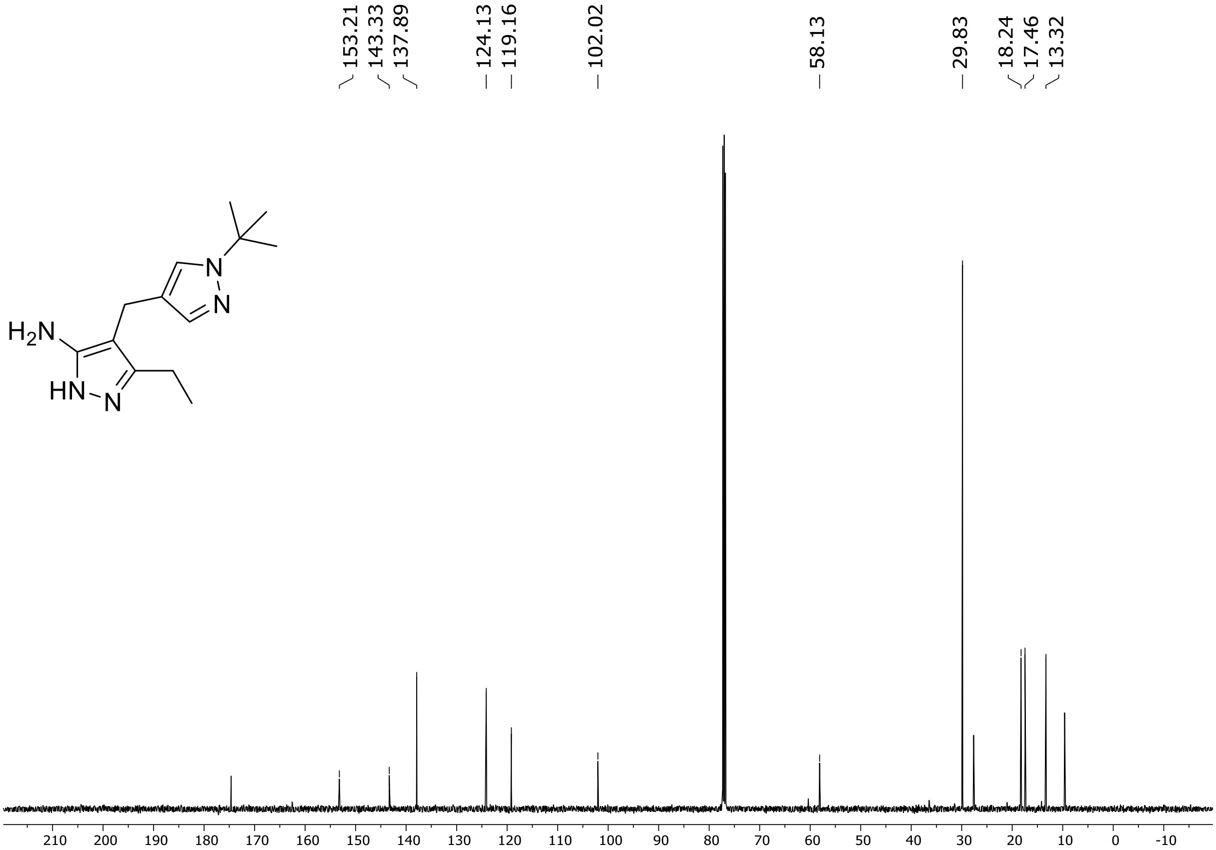

^{1}H (500 MHz) and ^{13}C NMR (126 MHz) spectra of **S82** in DMSO-d_6

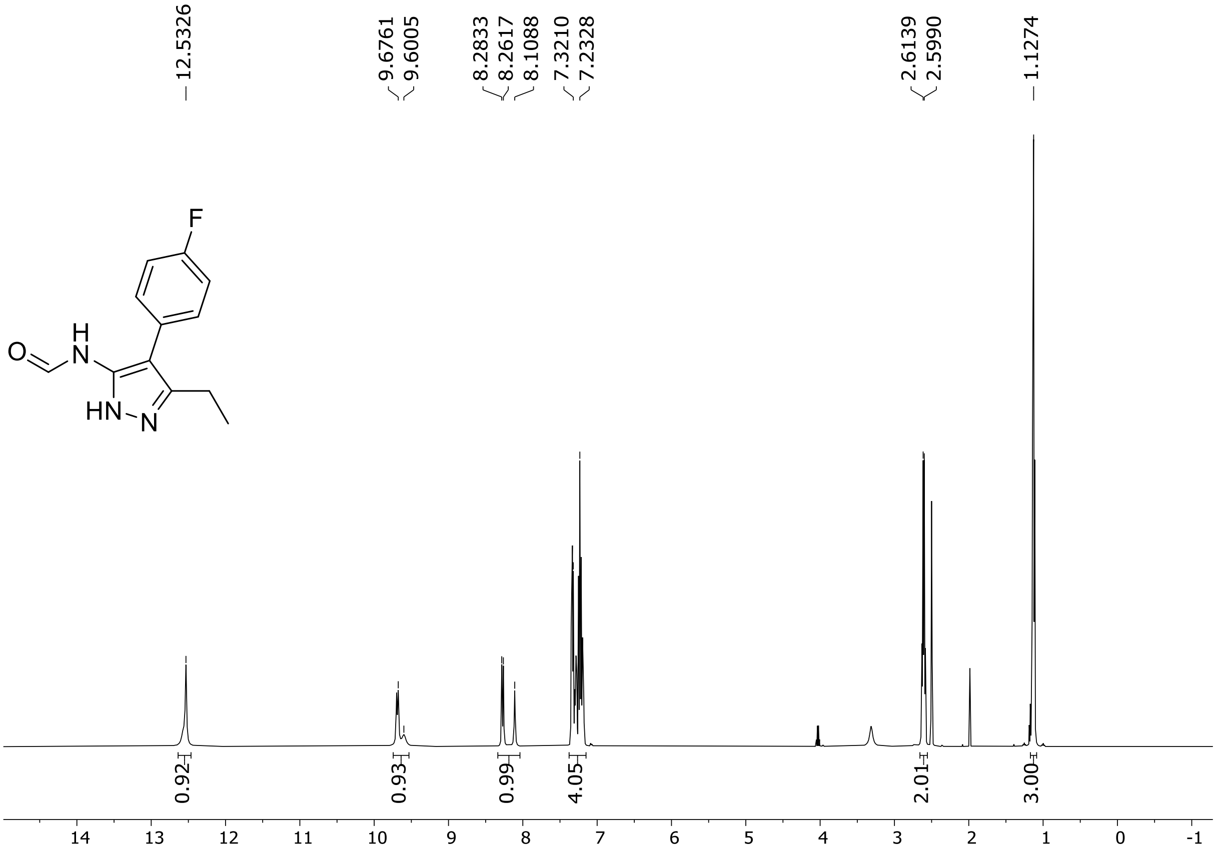

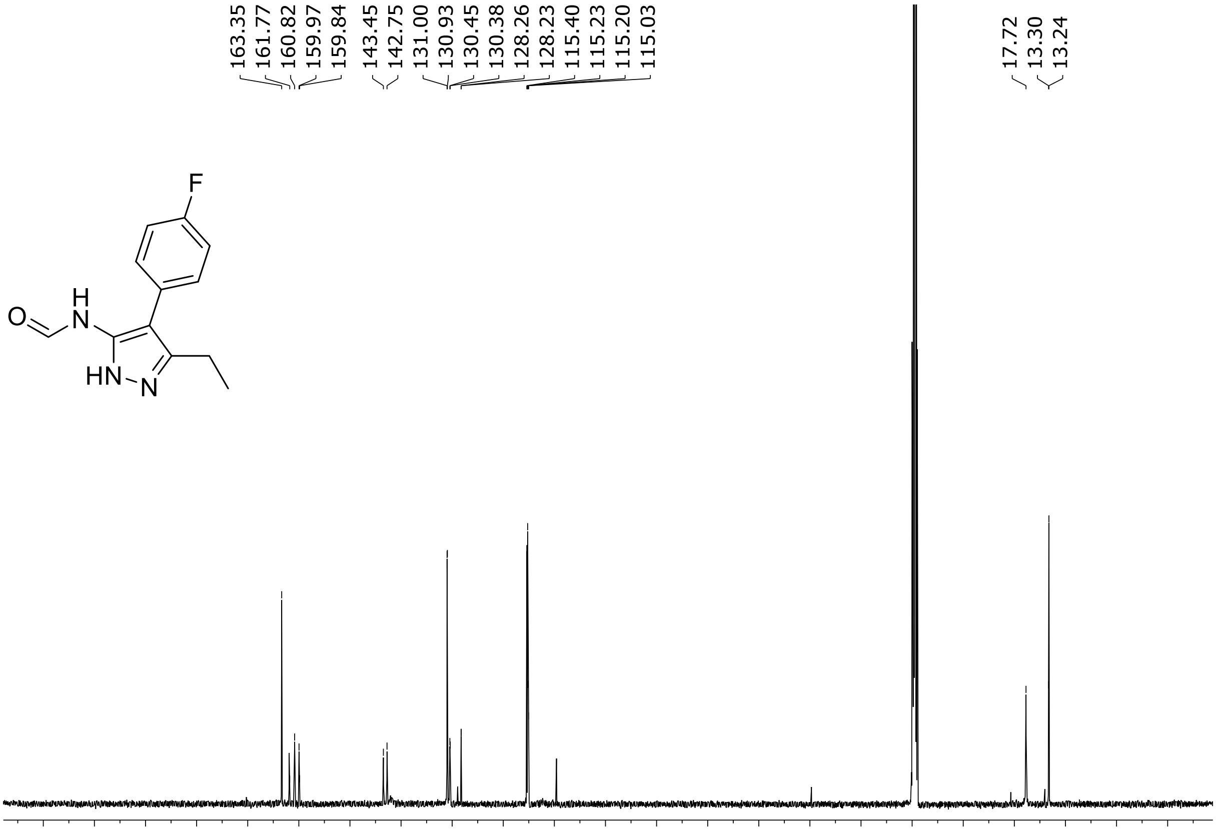

^{19}F (471 MHz) NMR spectrum of **S82** in DMSO-d_6

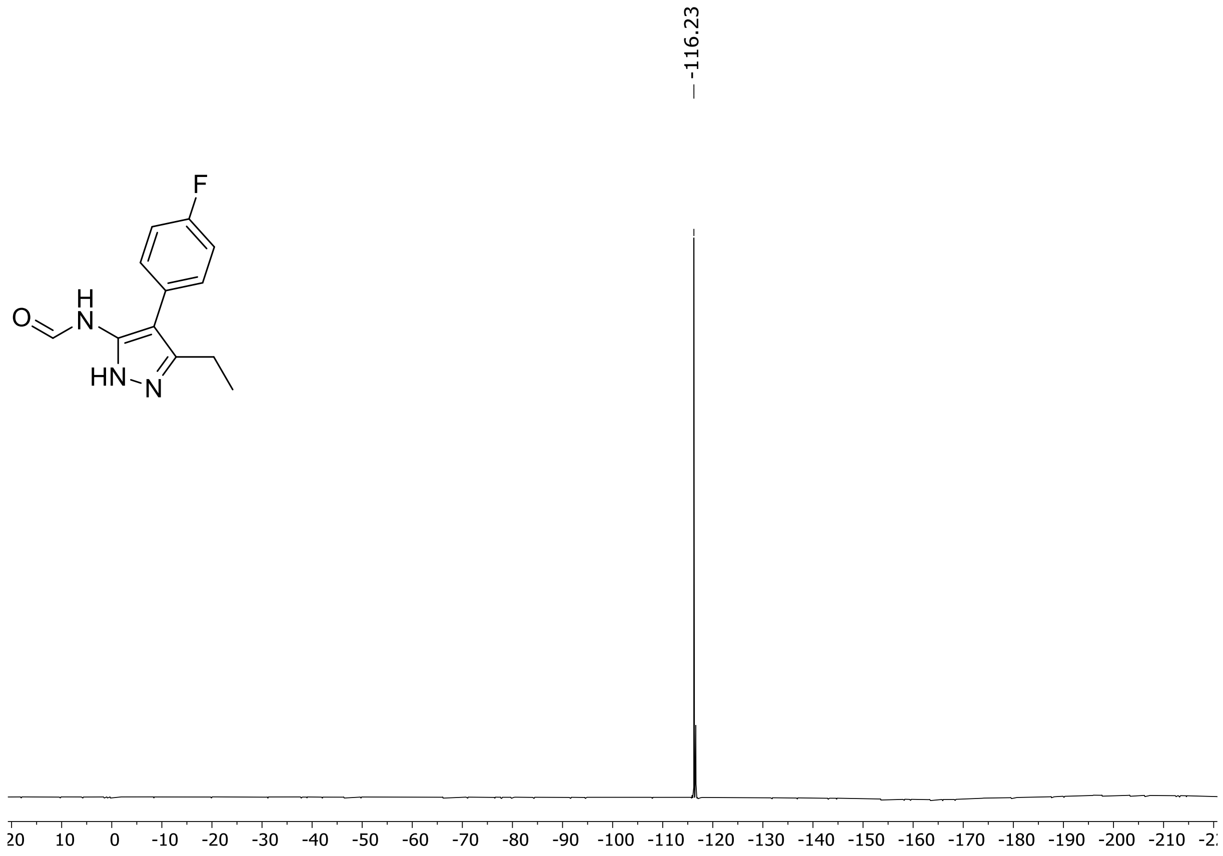

^{1}H (500 MHz) and ^{13}C NMR (126 MHz) spectra of **S83** in DMSO-d_6

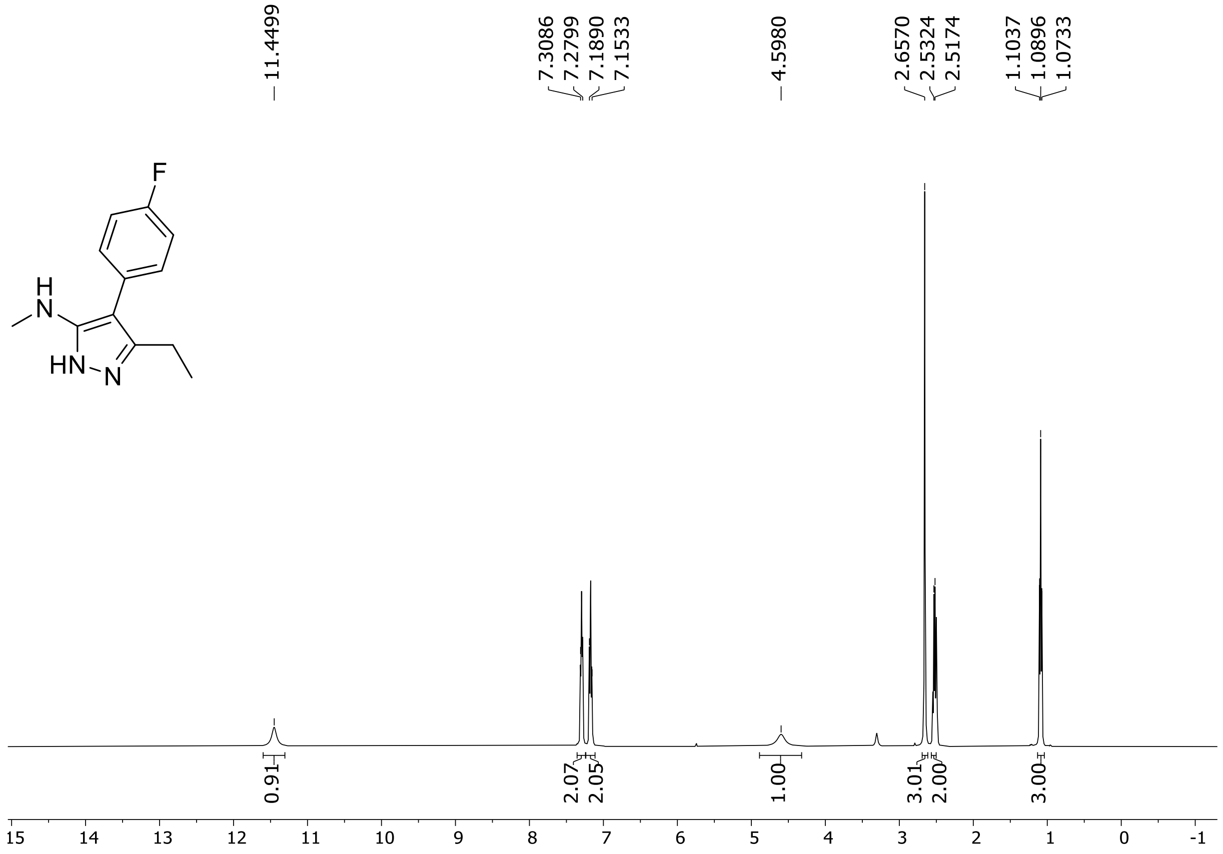

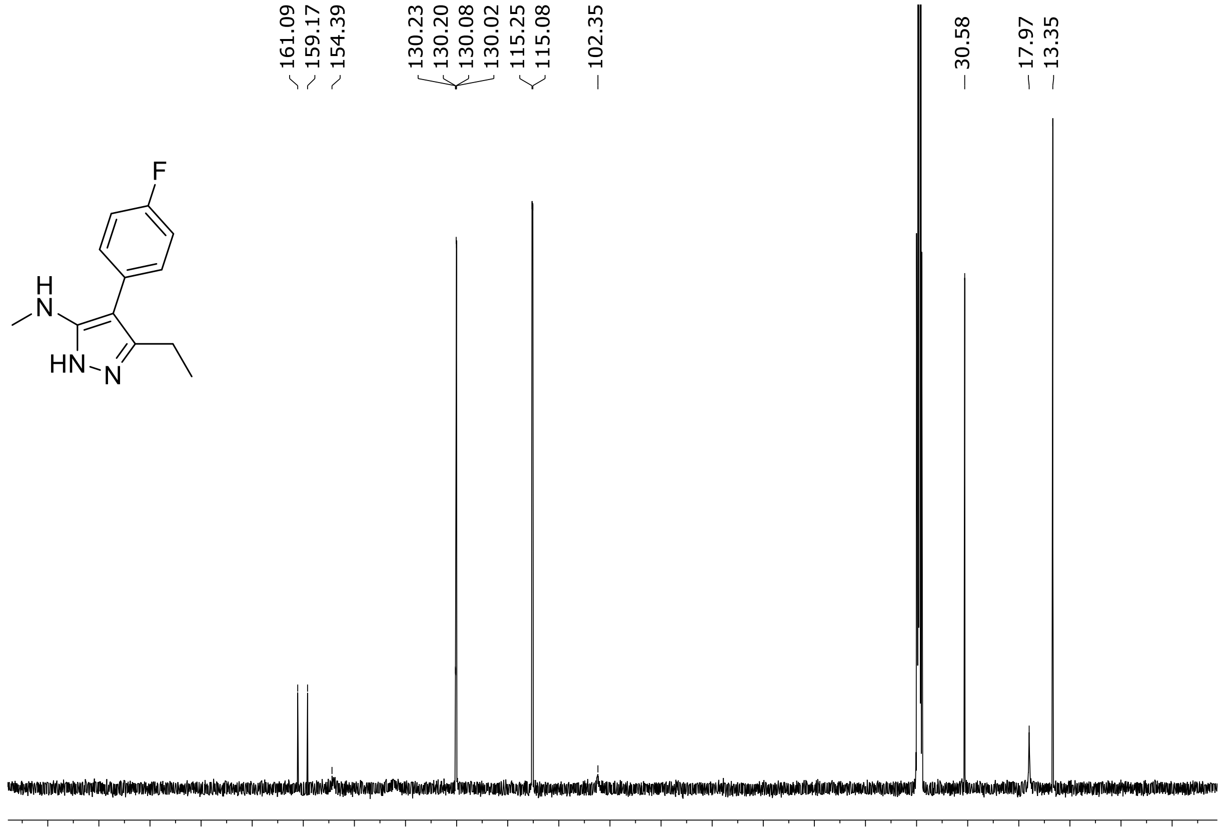

^{19}F (471 MHz) NMR spectrum of **S83** in DMSO-d_6

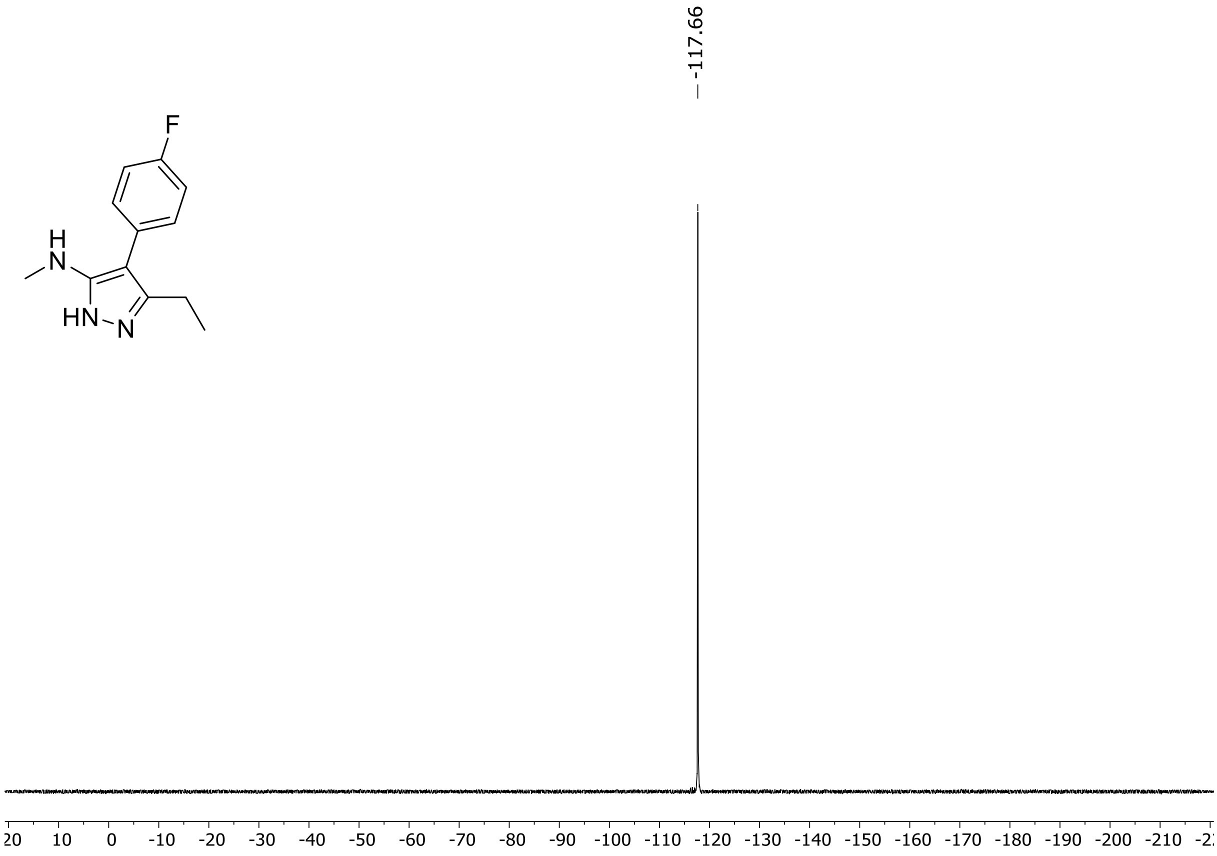

^1H (500 MHz) and ^{13}C NMR (126 MHz) spectra of **S84** in DMSO-d_6

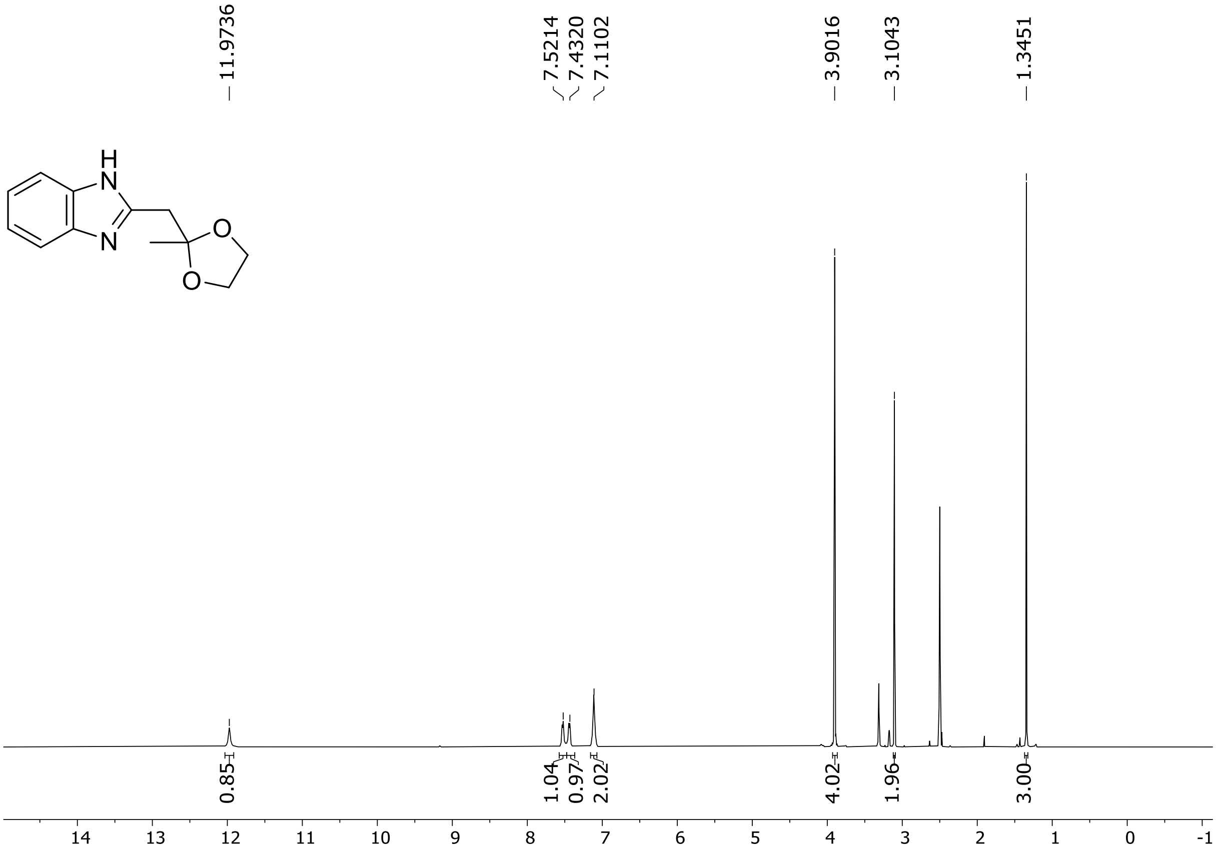

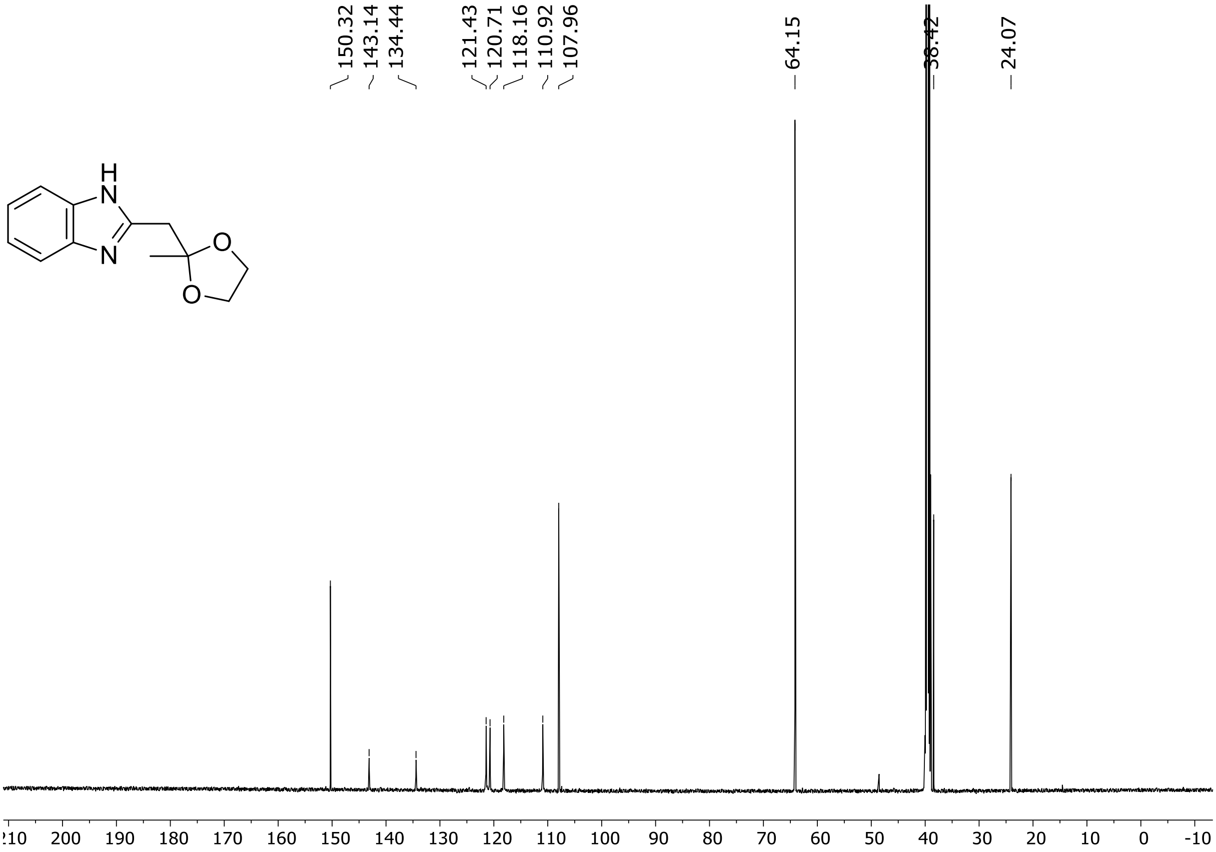

^{1}H (500 MHz) and ^{13}C NMR (126 MHz) spectra of **S86** in DMSO-d_6

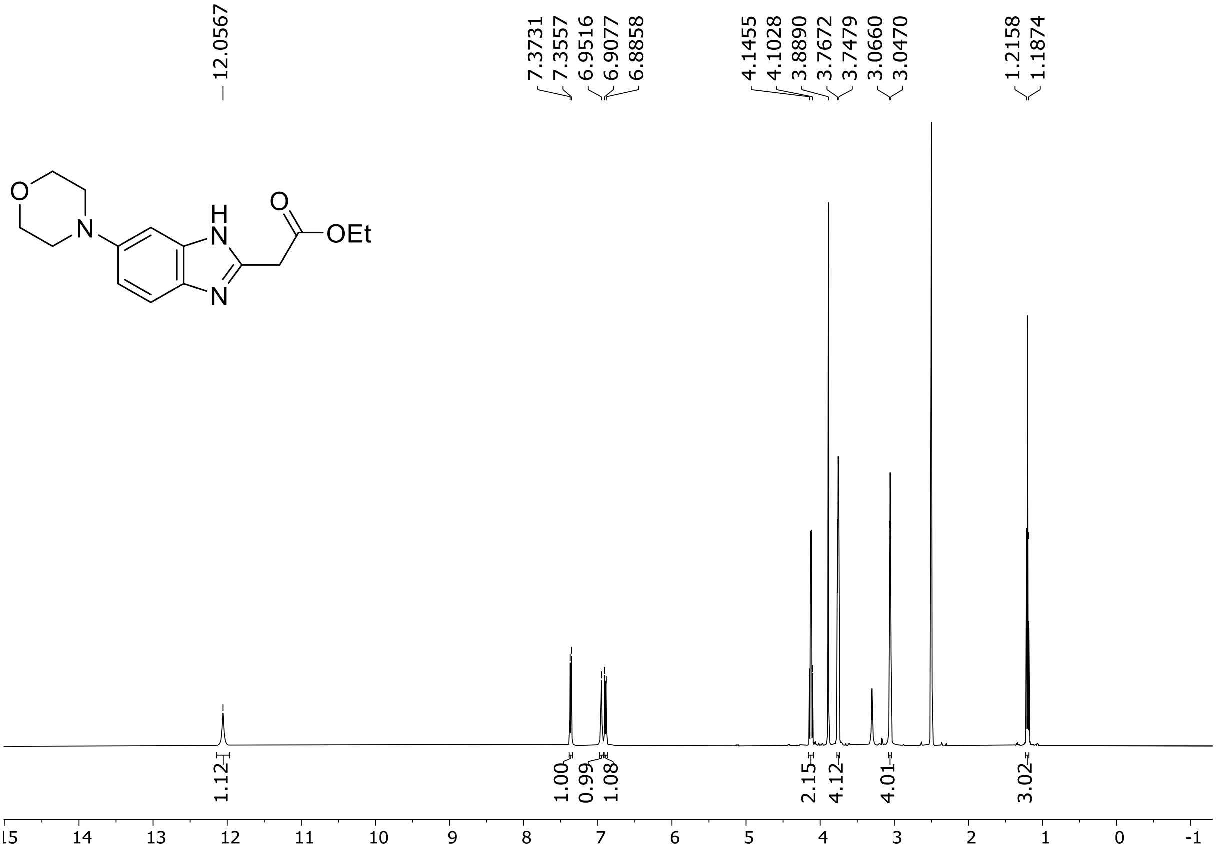

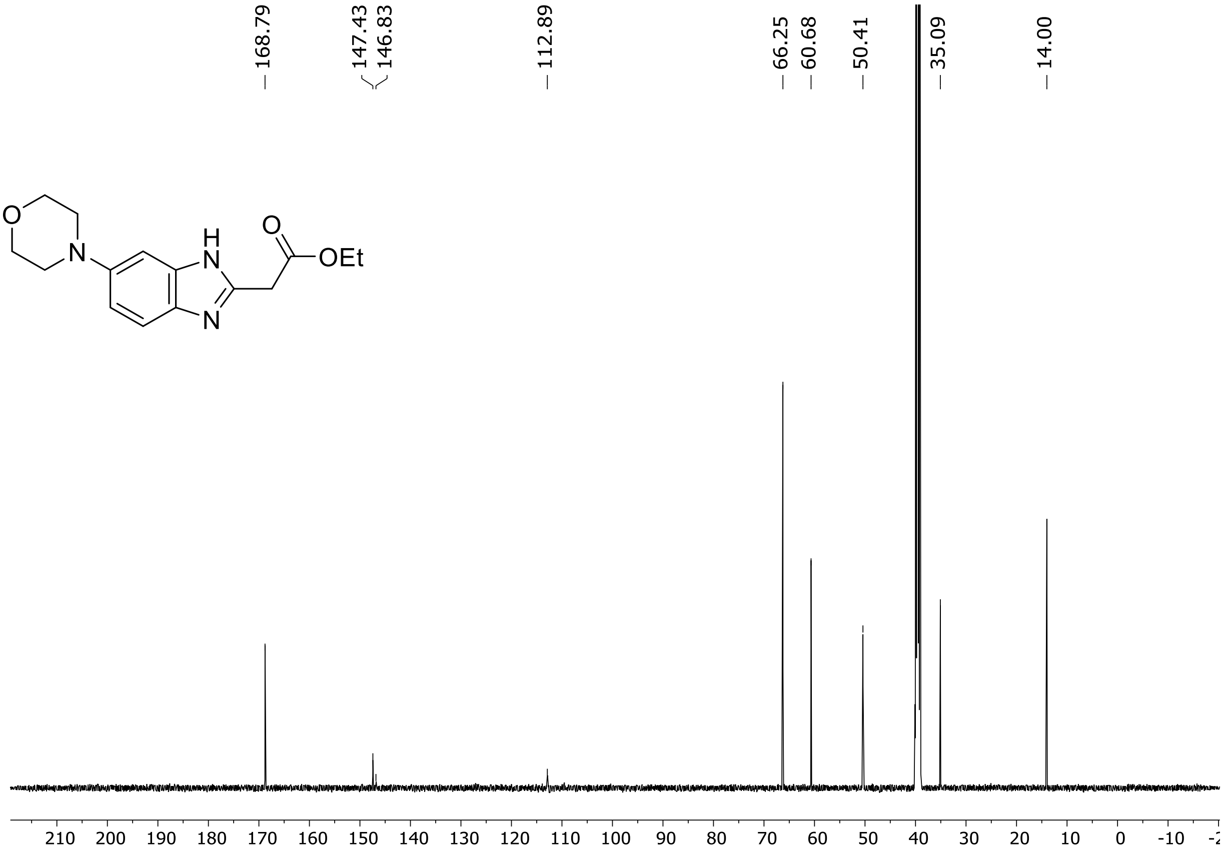

^{1}H (500 MHz) and ^{13}C NMR (126 MHz) spectra of **S87** in DMSO-d_6

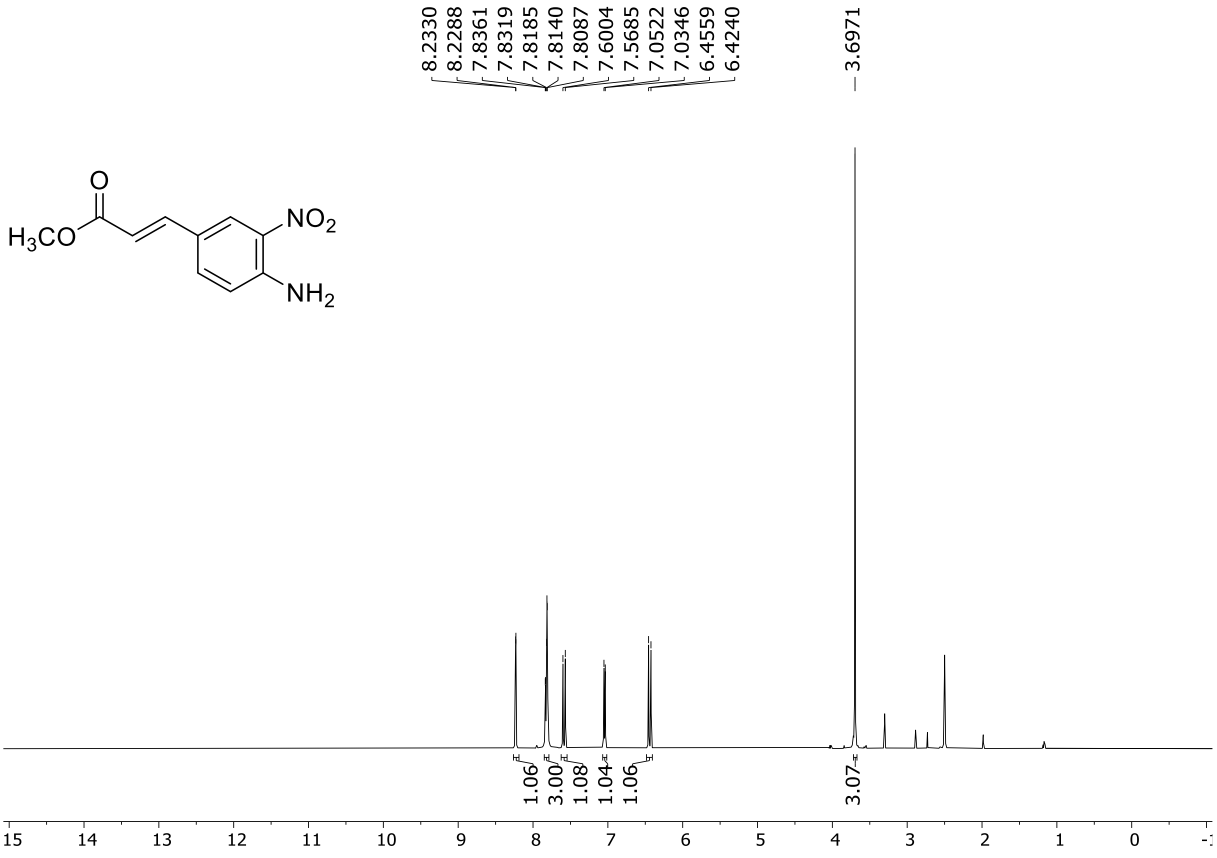

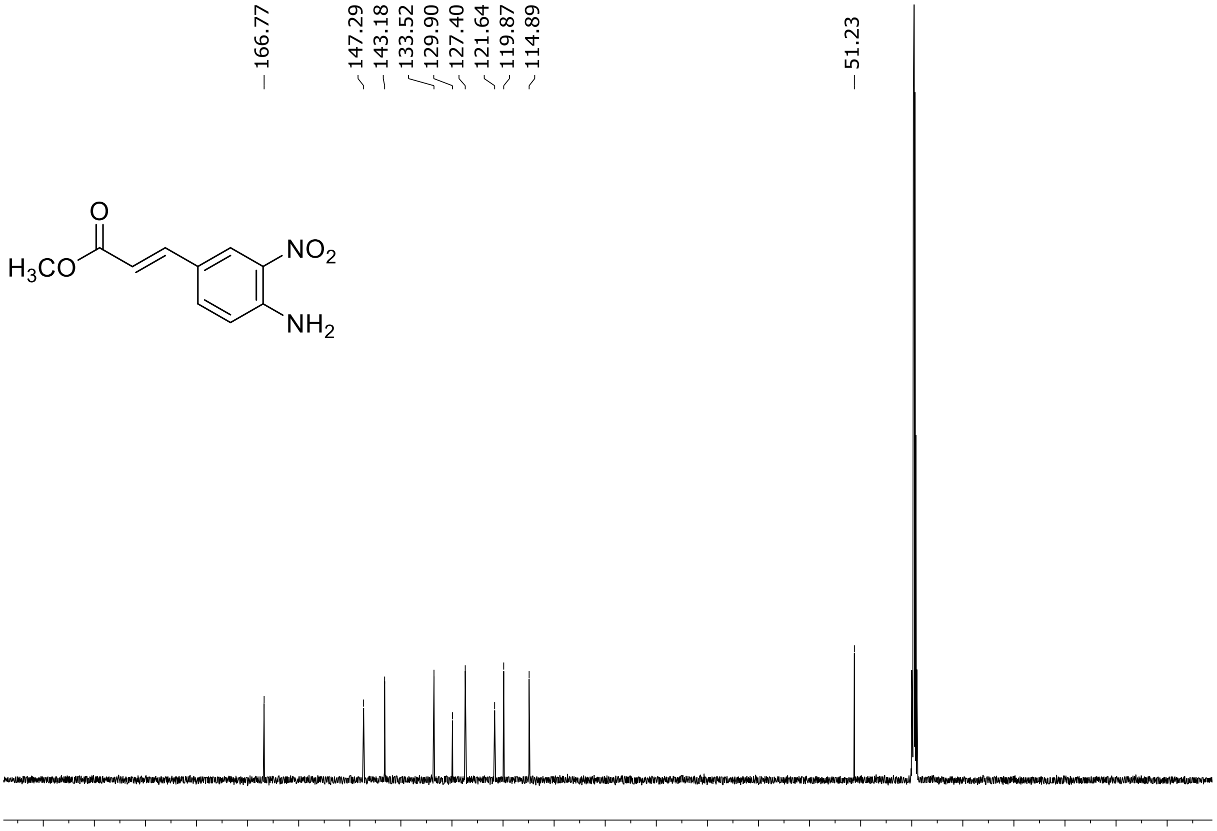

^{1}H (500 MHz) NMR spectrum of **S88** in DMSO-d_6

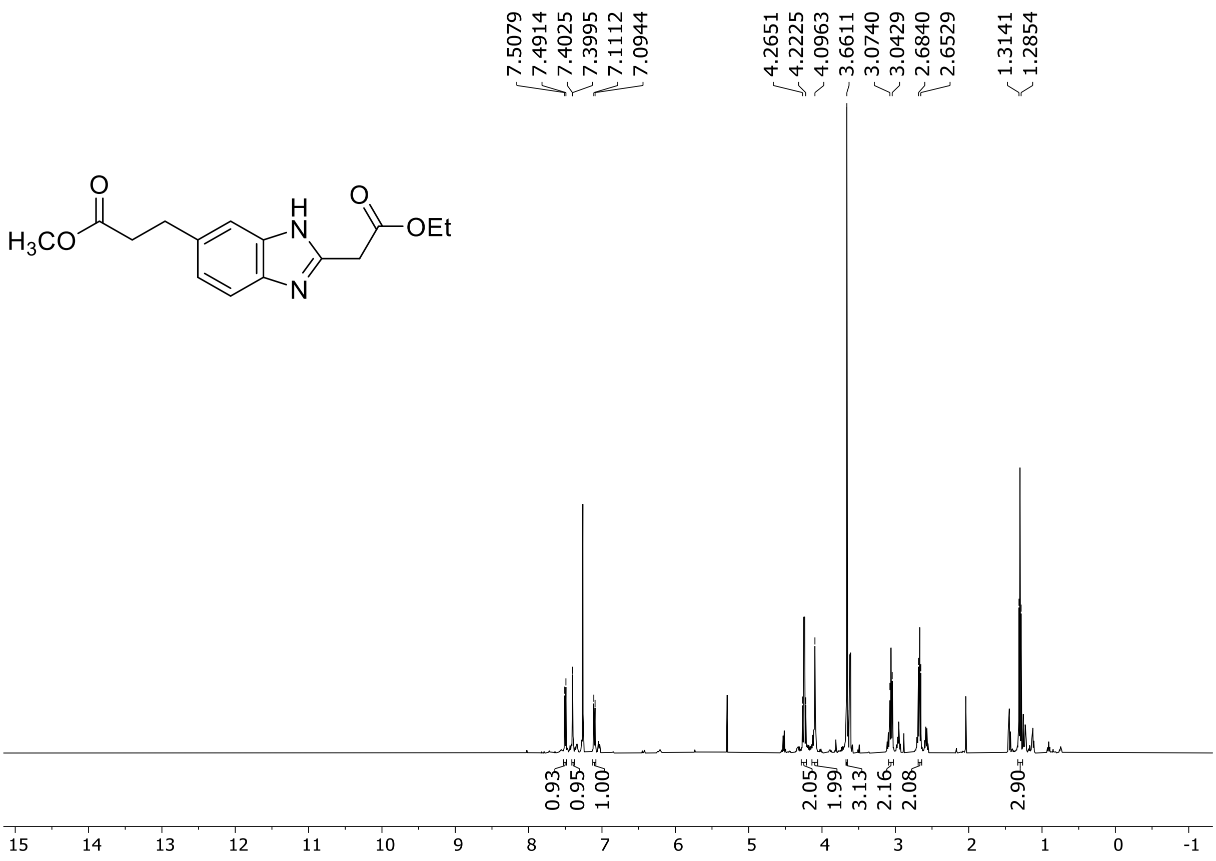

^{1}H (500 MHz) and ^{13}C NMR (126 MHz) spectra of **S89** in chloroform-*d*

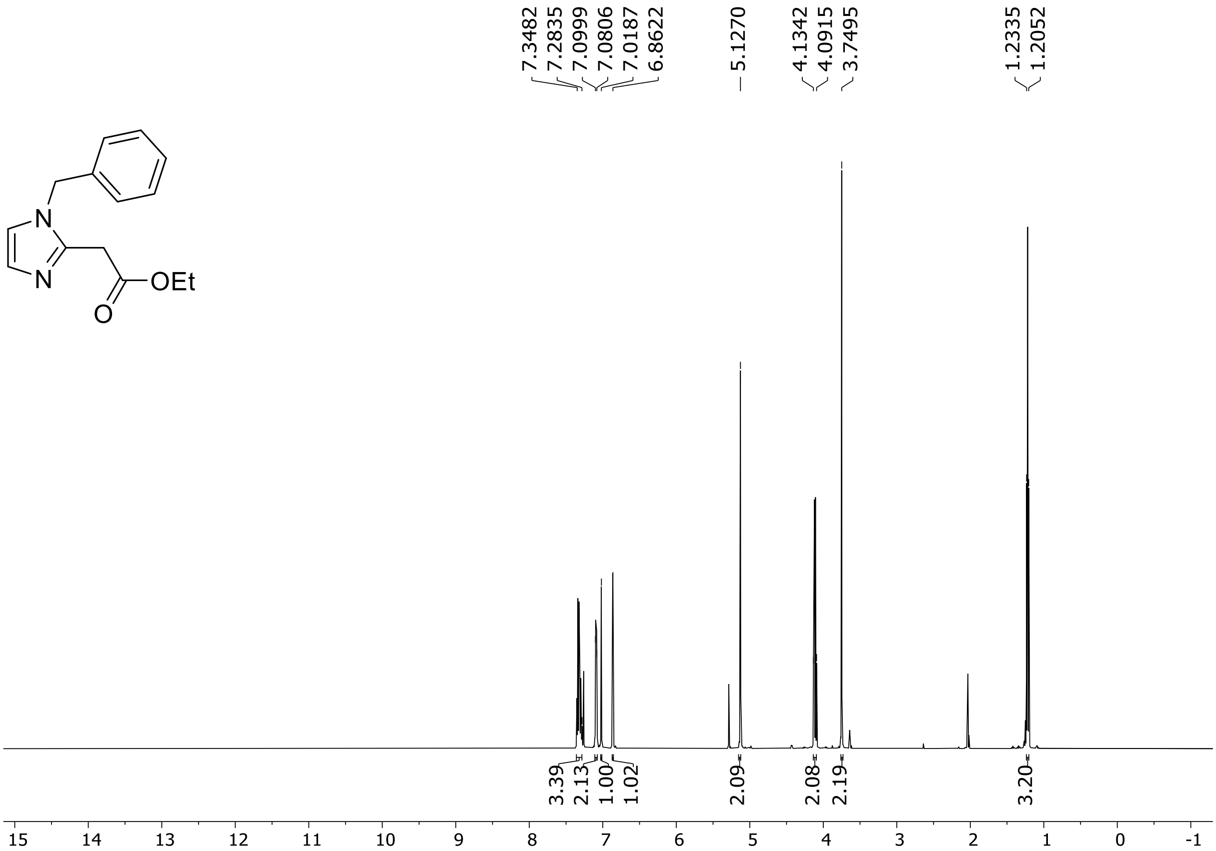

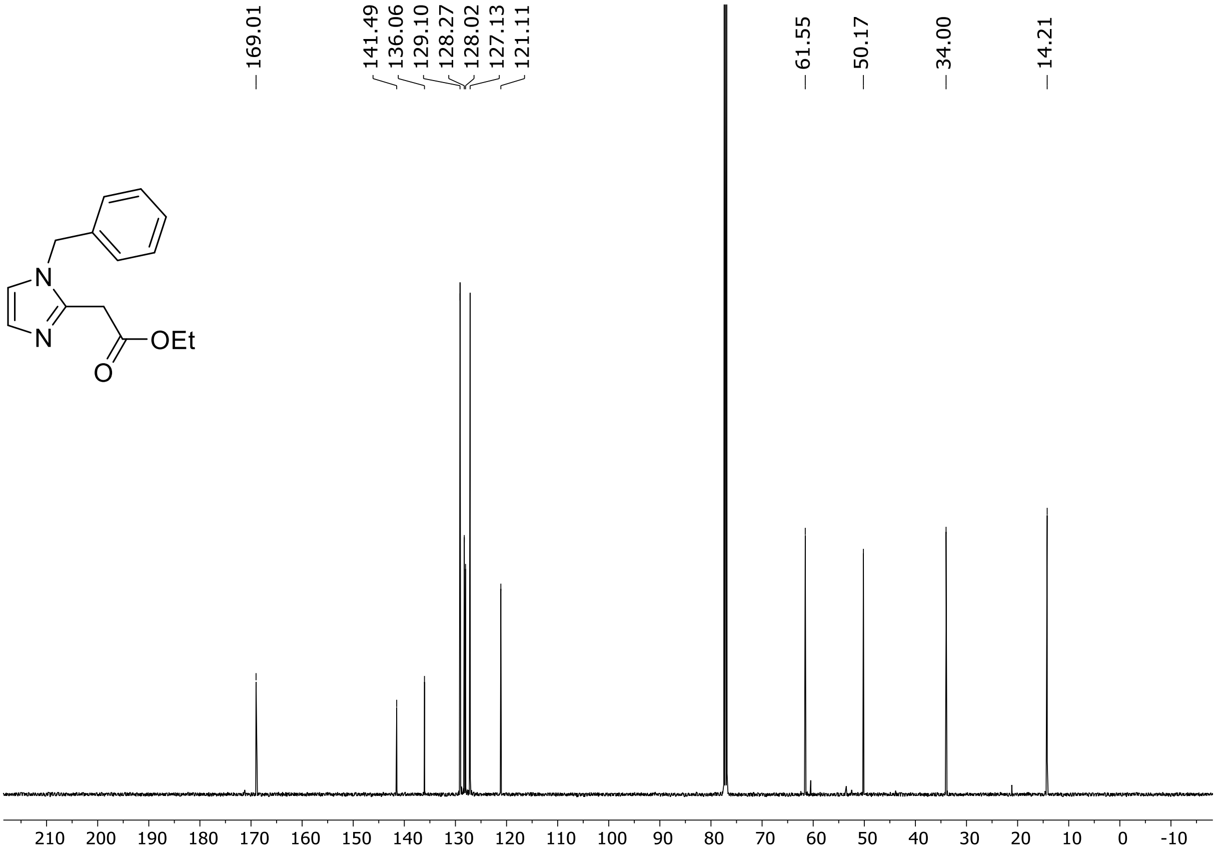

^{1}H (500 MHz) and ^{13}C NMR (126 MHz) spectra of **S90** in chloroform-*d*

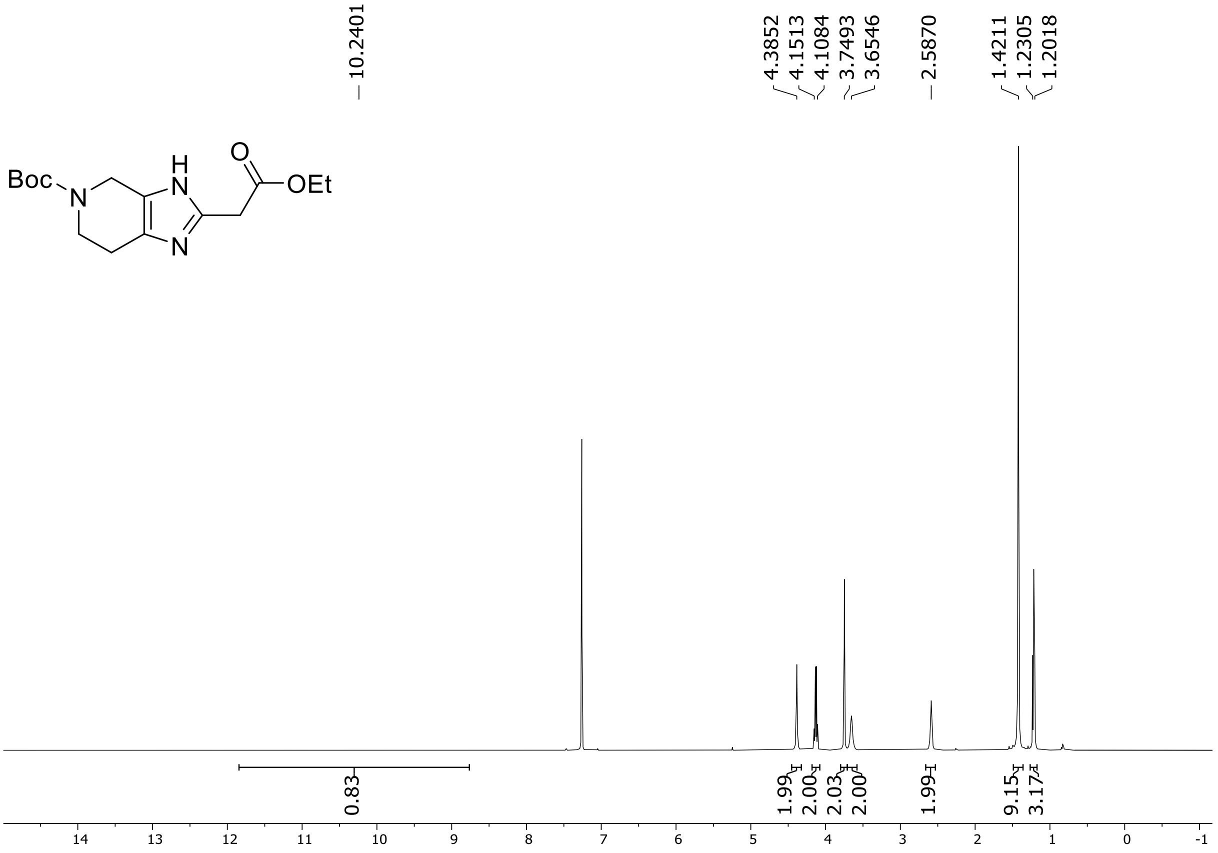

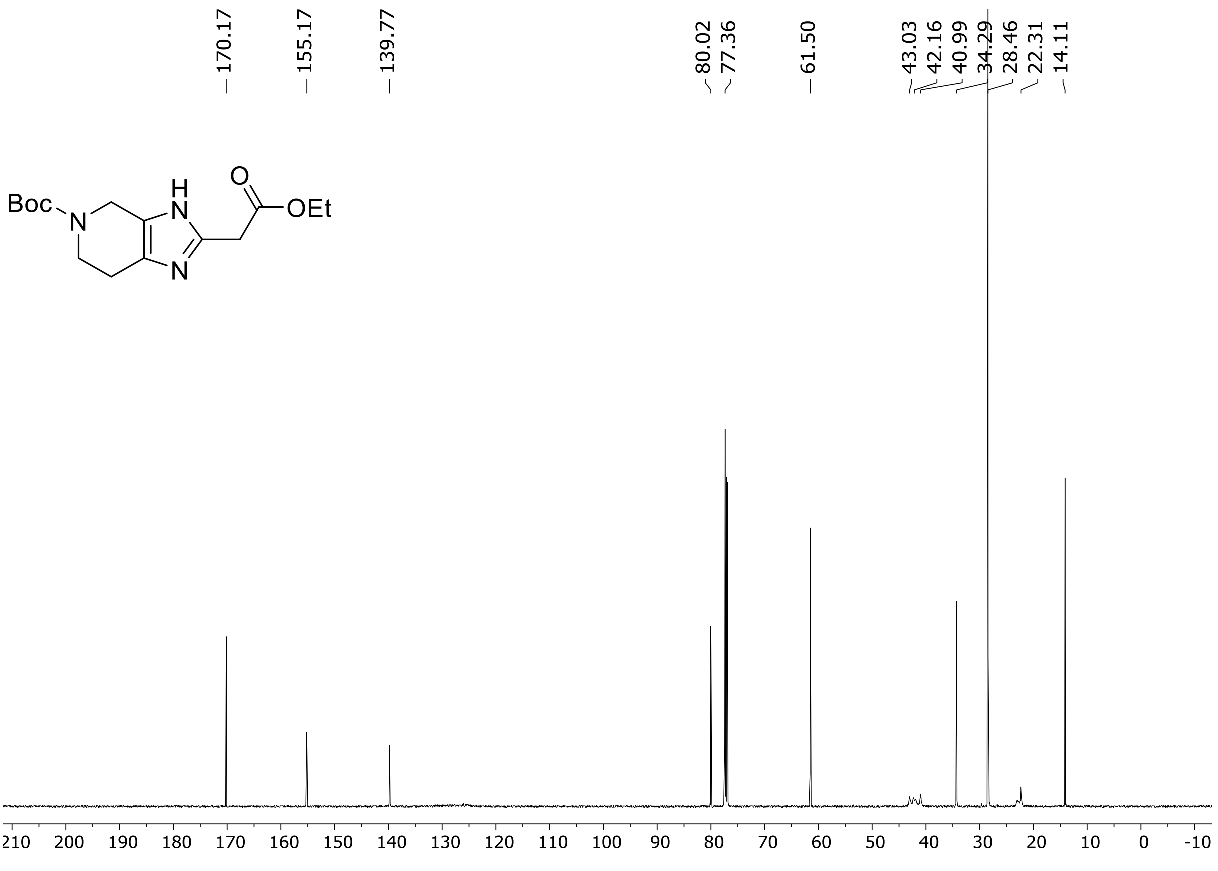

HRMS spectrum of **S90**

$C_{15}H_{23}N_3O_4$ **exact mass: 309.1689**

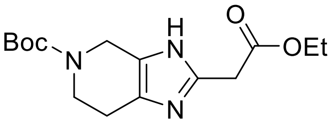

APCI + (MMI)
nitrogen flow 5 L/min, gas temperature 325°C, nebulizer 45 psig, skimmer 65 V, vaporizer 200°C, fragmentor 15 V, dissolved in methanol

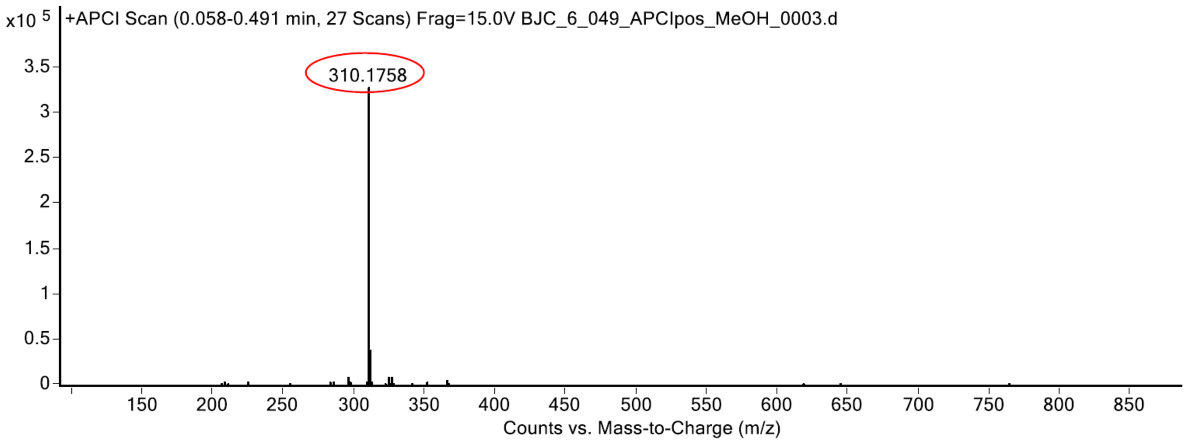

expected mass: [M+H]$^+$ = 310.1761 observed mass: [M+H]$^+$ = 310.1758 mass accuracy = - 1.0 ppm

^{1}H (500 MHz) spectrum of **S91** in chloroform-*d*

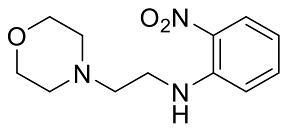

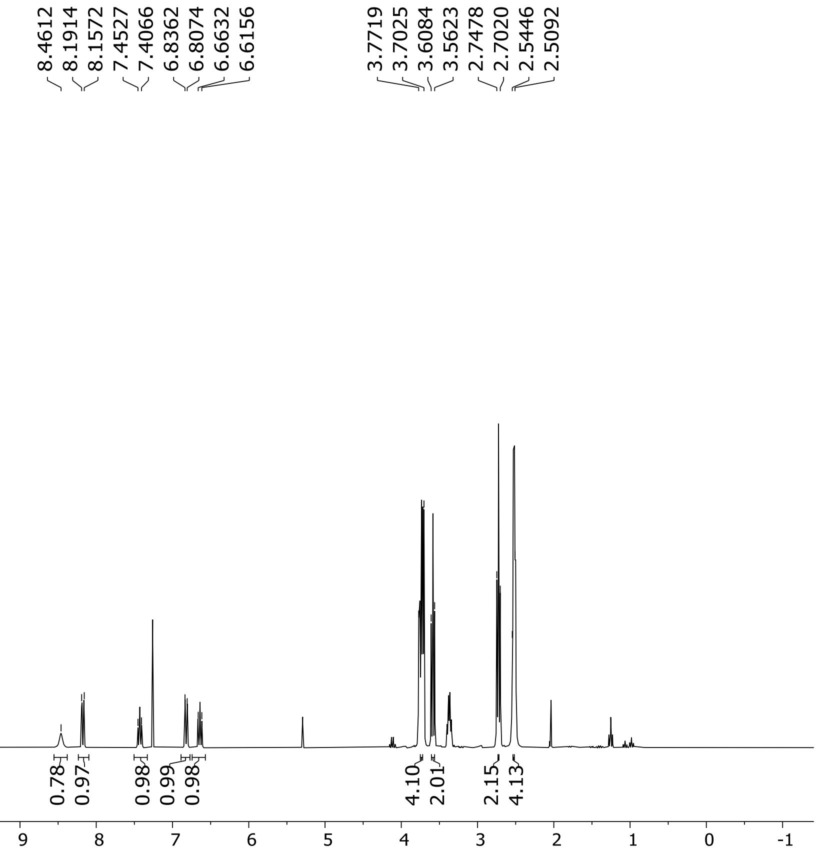

^{1}H (500 MHz) and ^{13}C NMR (126 MHz) spectra of **S92** in chloroform-*d*

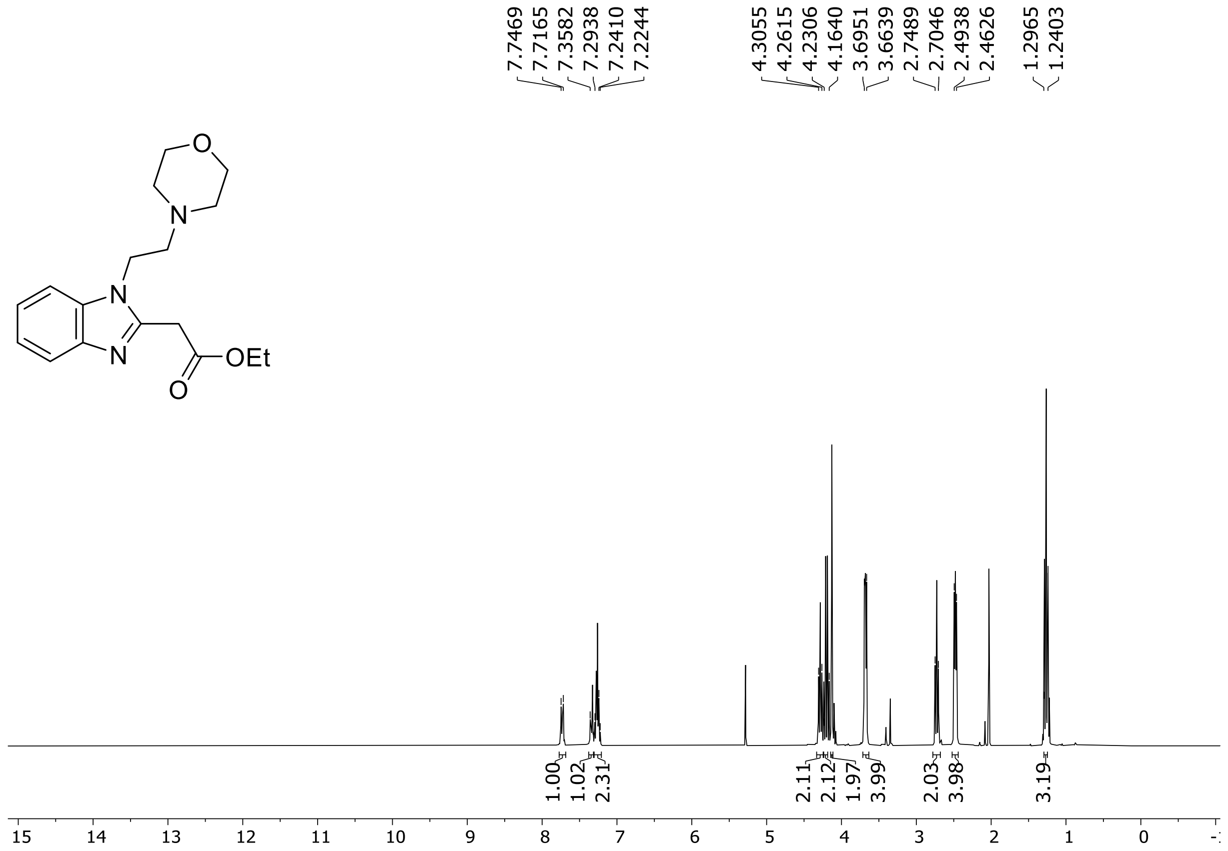

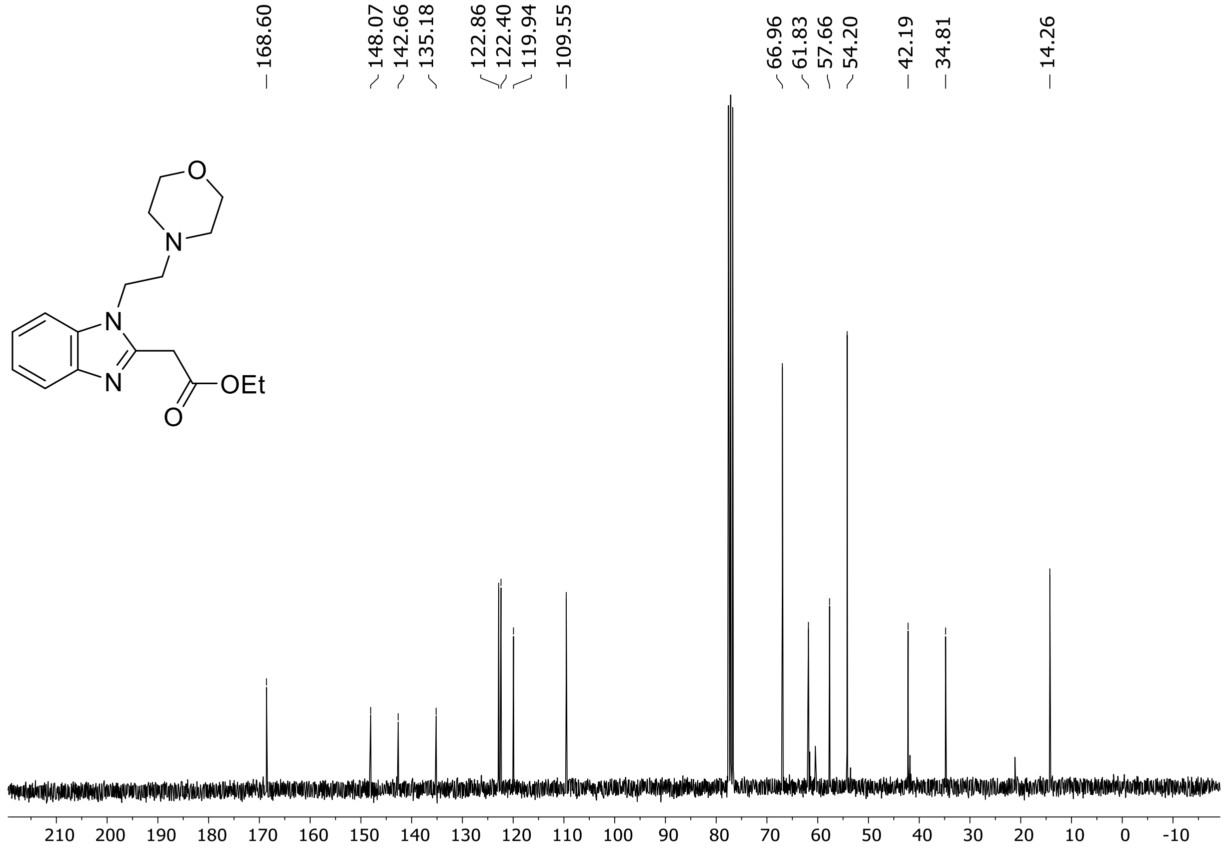

^{1}H (500 MHz) and ^{13}C NMR (126 MHz) spectra of **S93** in chloroform-*d*

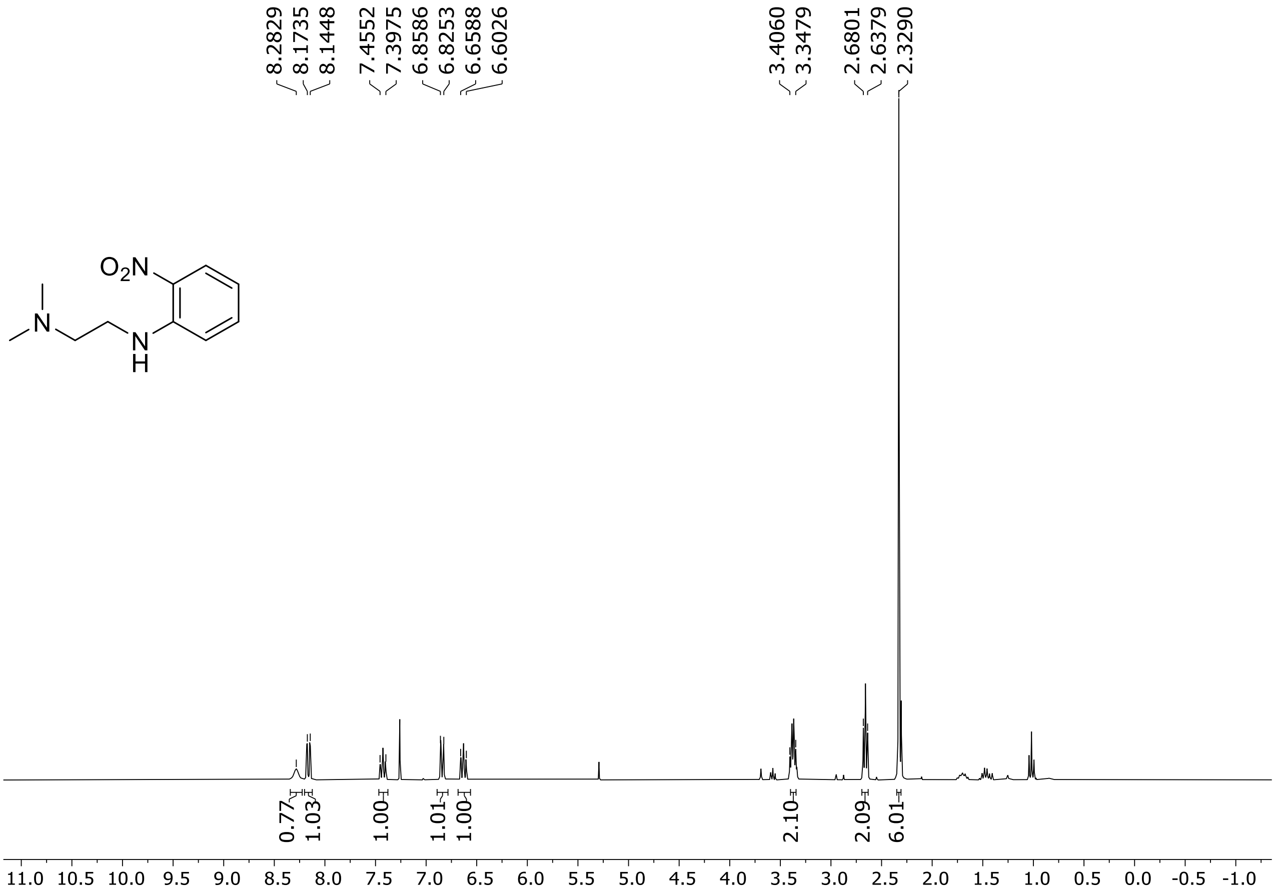

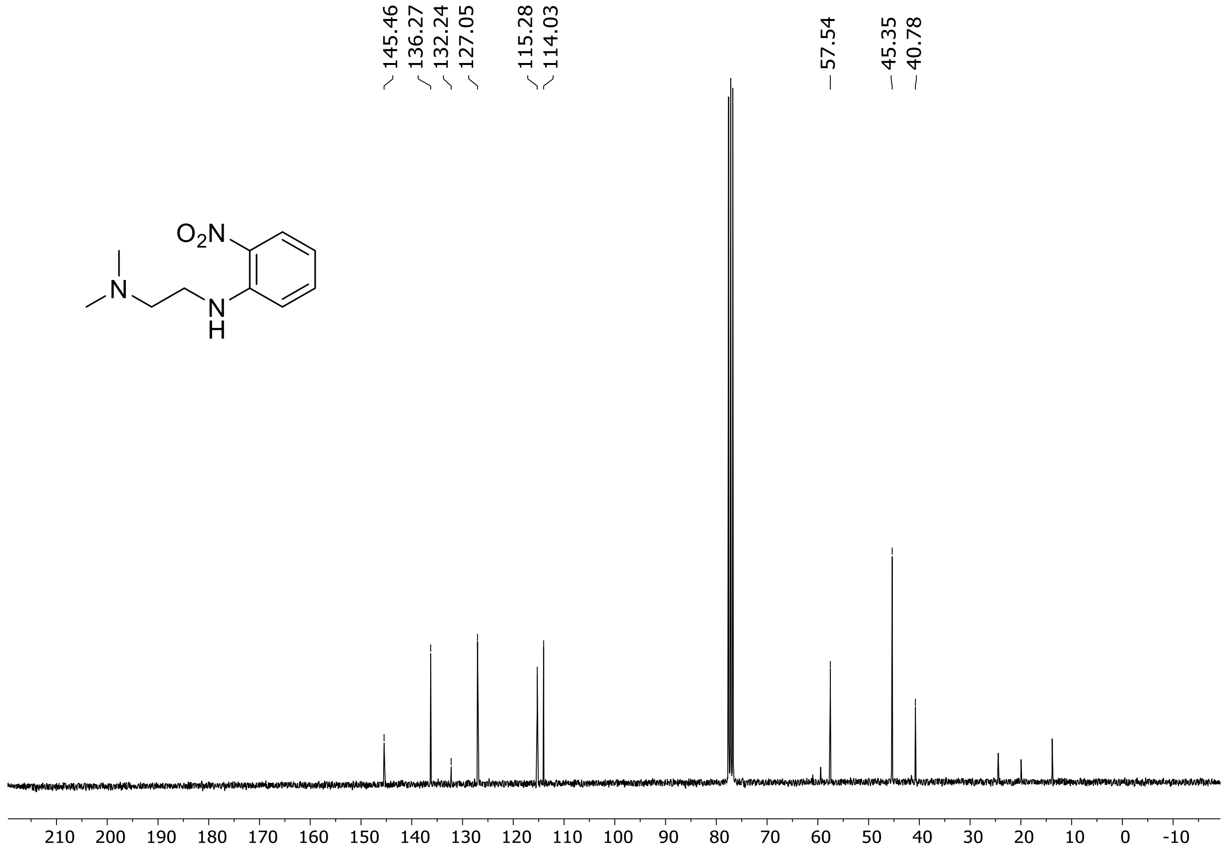

^{1}H (500 MHz) and ^{13}C NMR (126 MHz) spectra of **S94** in chloroform-*d*

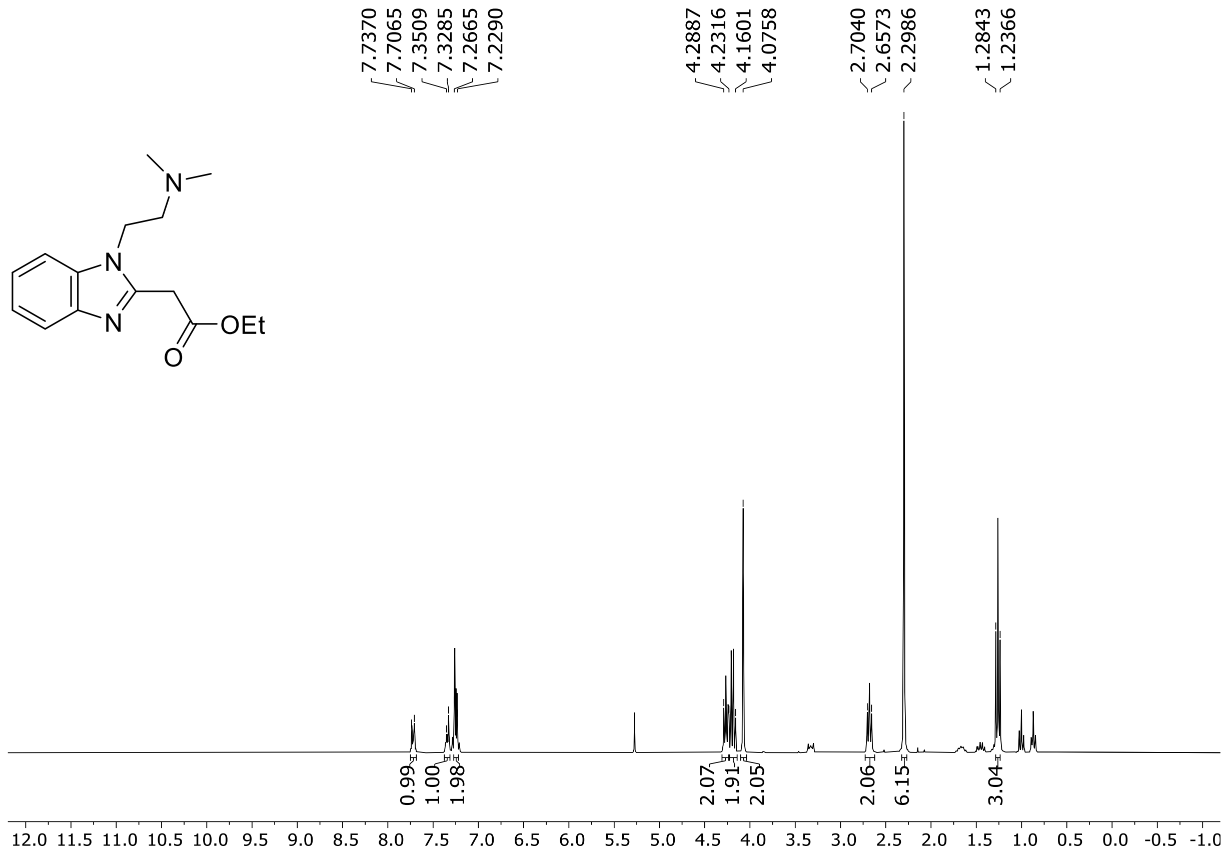

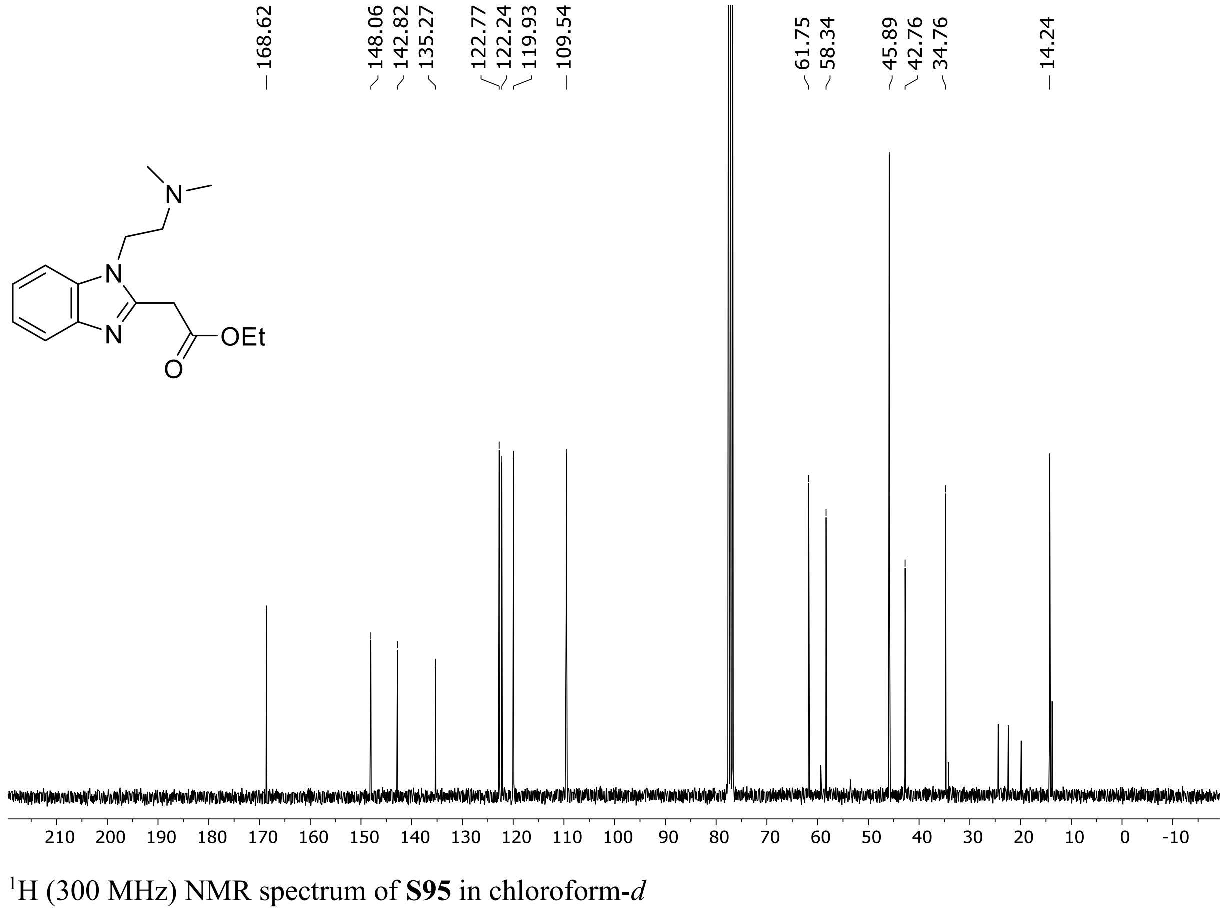

^{1}H (300 MHz) NMR spectrum of **S95** in chloroform-*d*

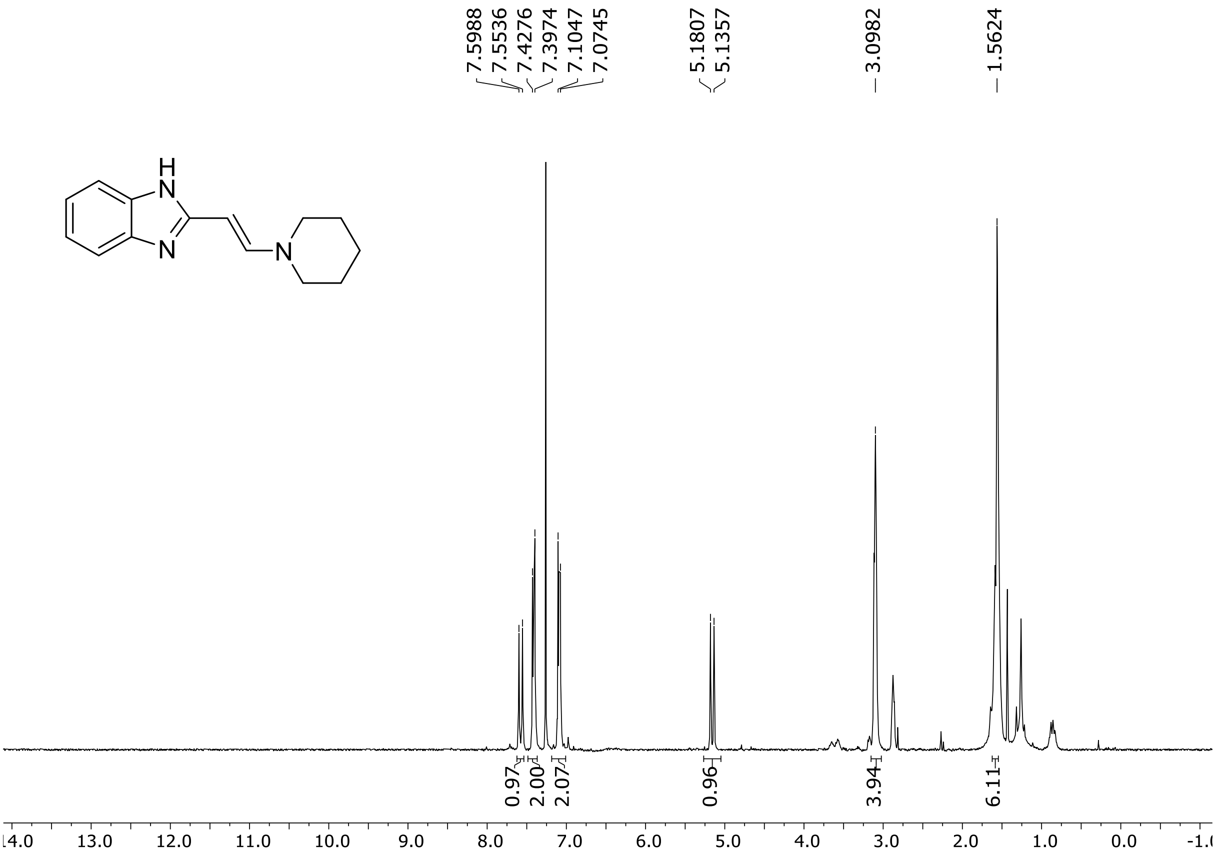

^{1}H (500 MHz) and ^{13}C NMR (126 MHz) spectra of **S96** in chloroform-*d*

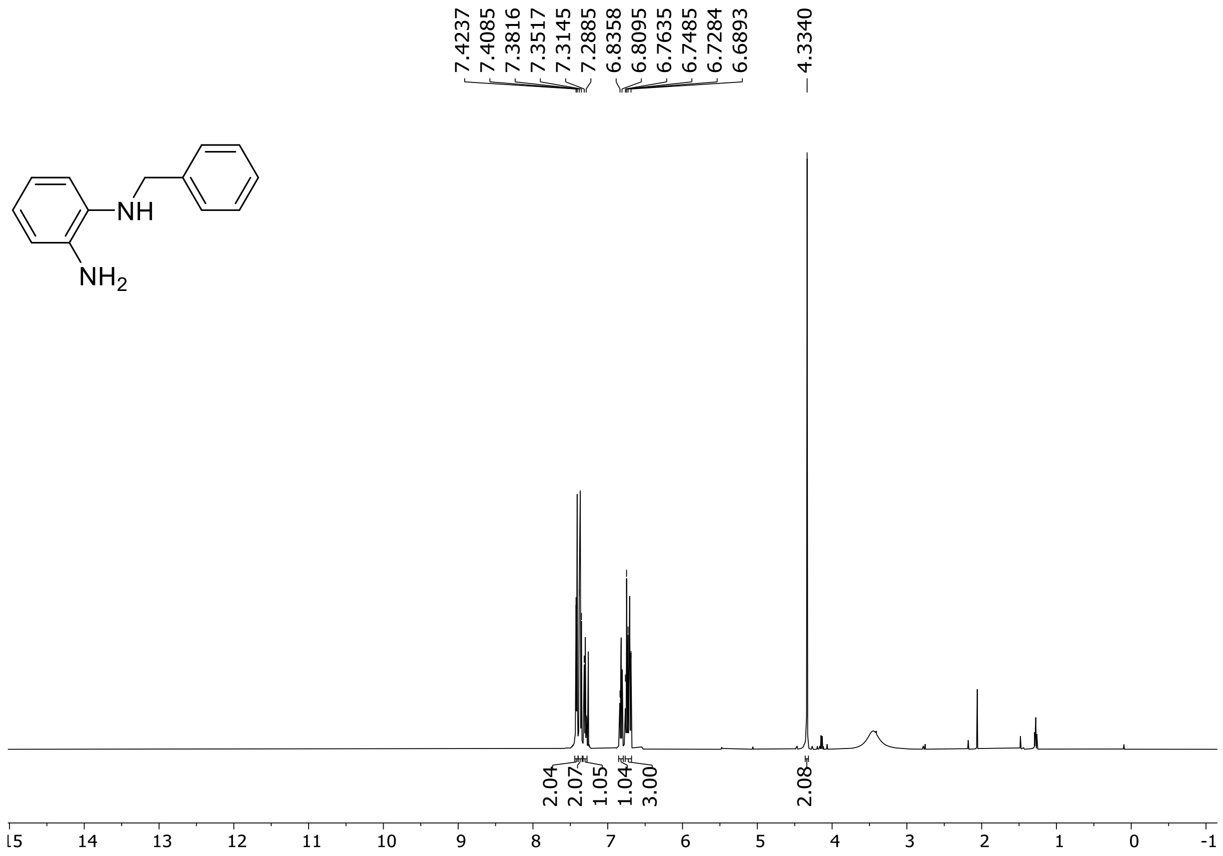

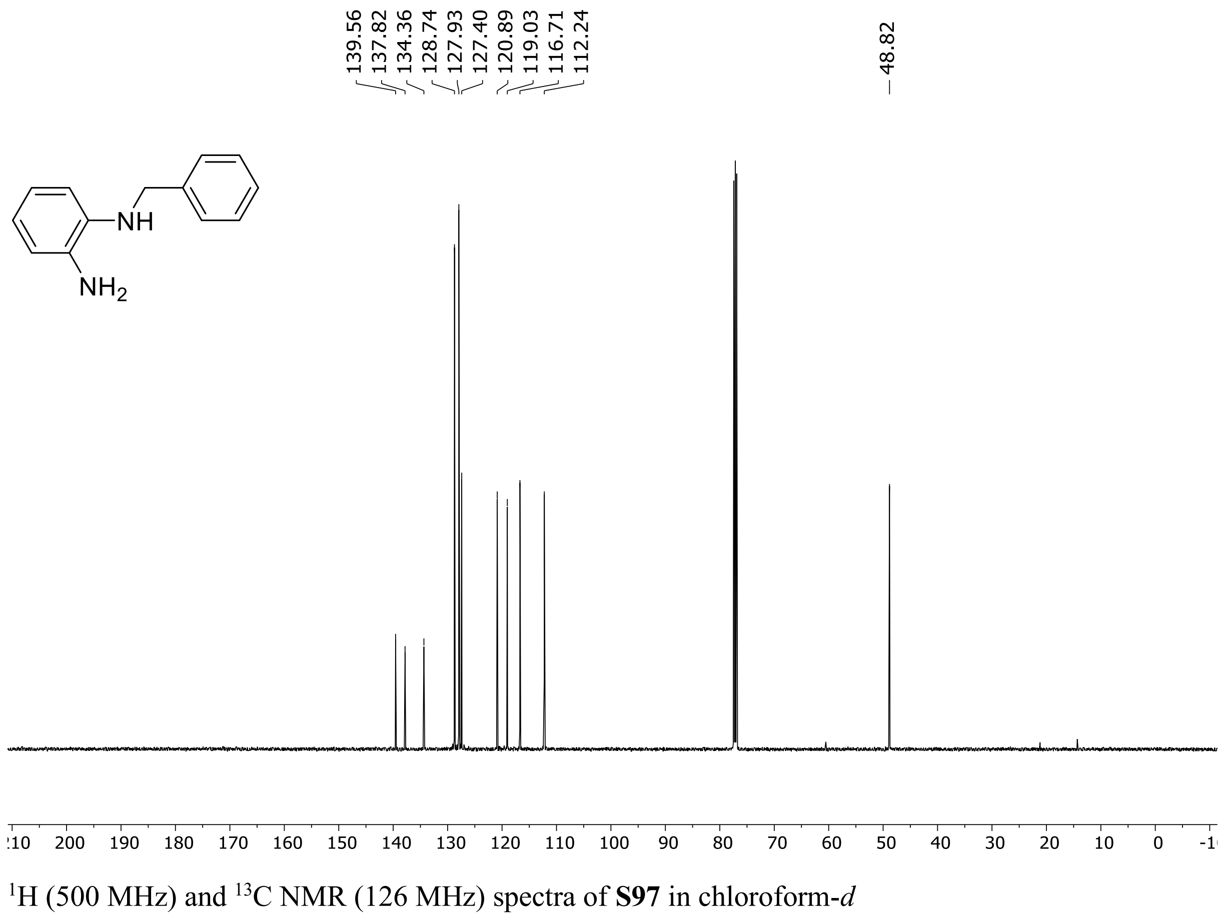

^{1}H (500 MHz) and ^{13}C NMR (126 MHz) spectra of **S97** in chloroform-*d*

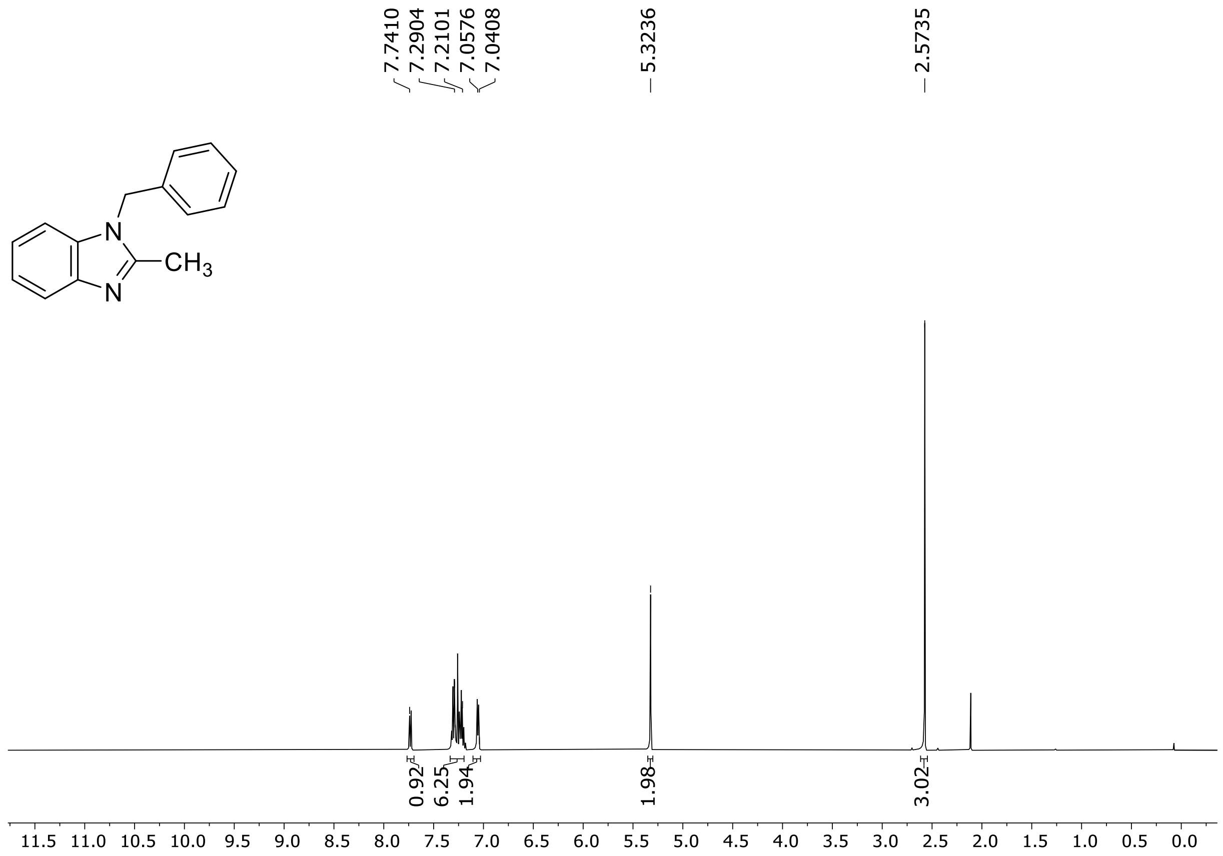

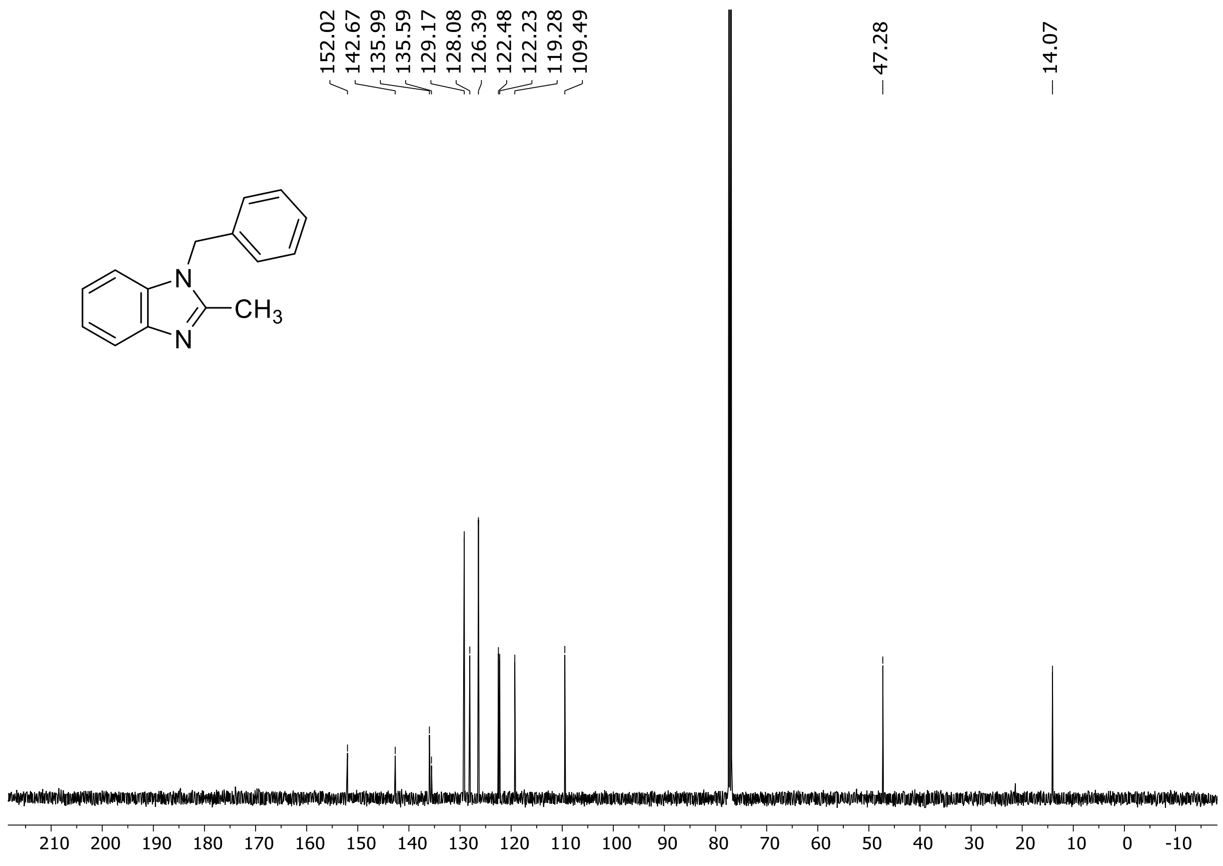

^{1}H (500 MHz) and ^{13}C NMR (126 MHz) spectra of **S98** in chloroform-*d*

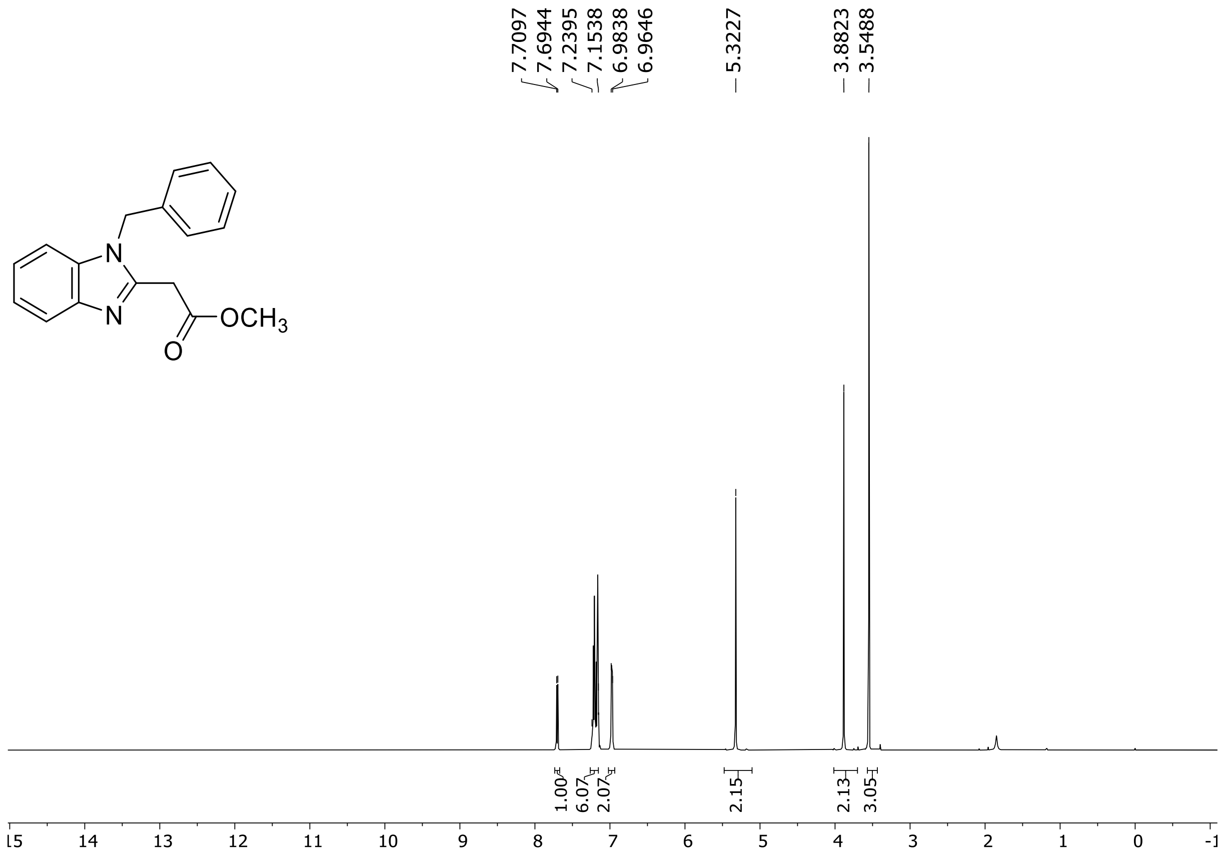

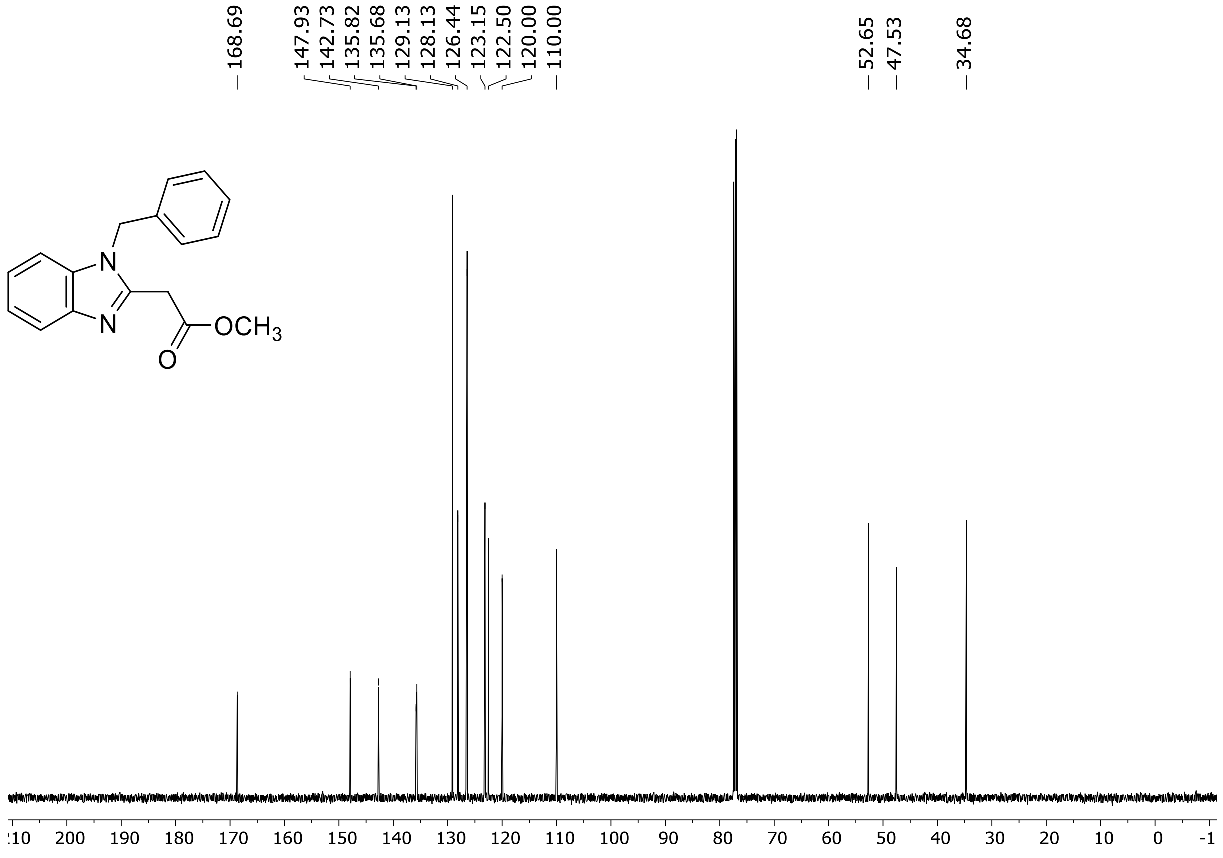

^{1}H (500 MHz) and ^{13}C NMR (126 MHz) spectra of **S99** in chloroform-*d*

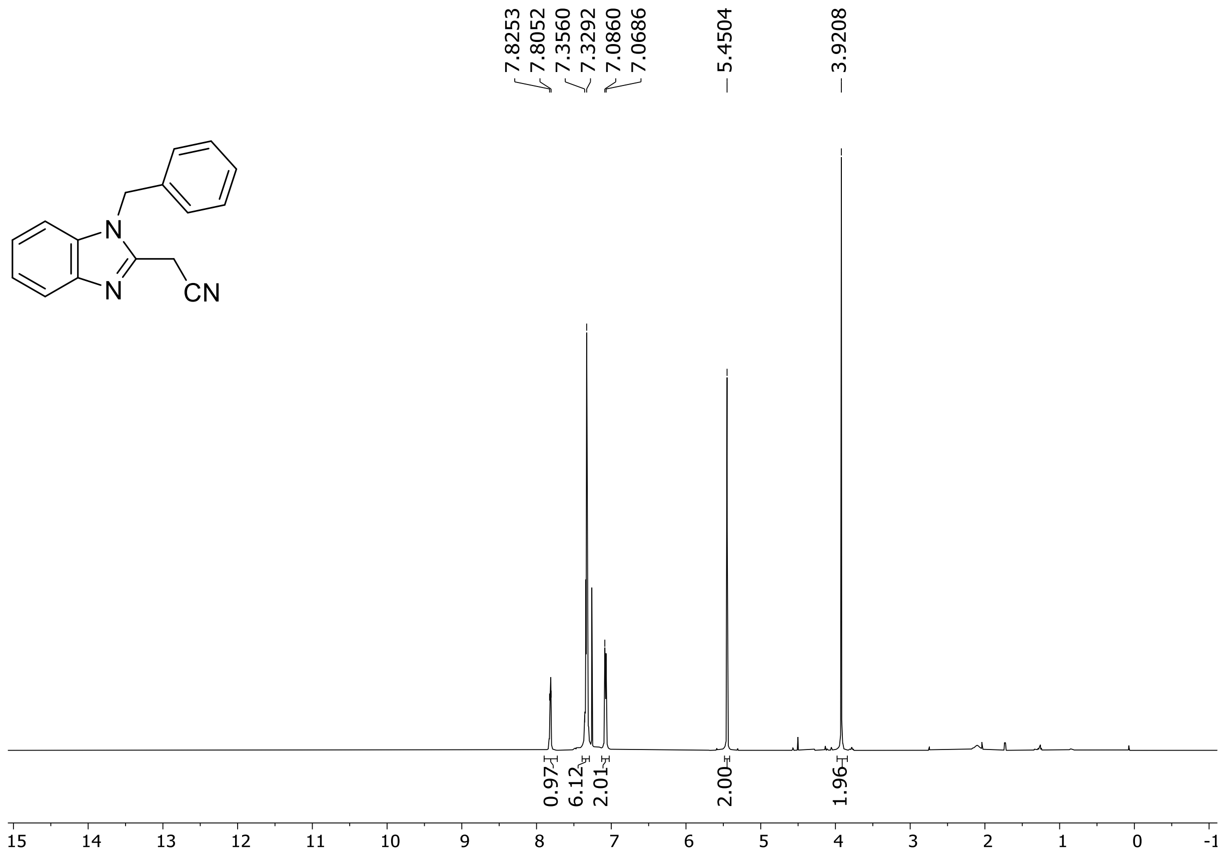

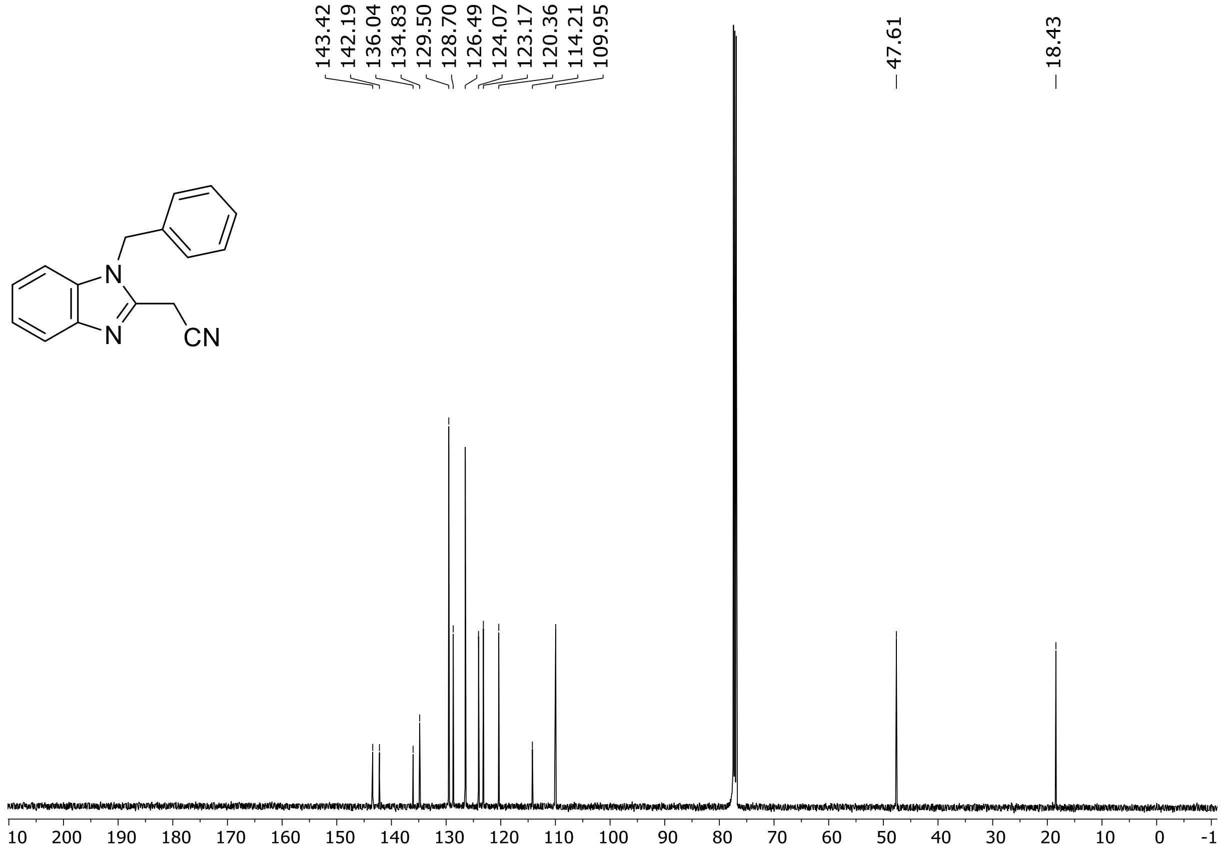

HRMS spectrum of **S99**

$C_{16}H_{13}N_3$ **mono m/z = 247.1109**

APCI - (MMI)
nitrogen flow 5 L/min, gas temperature 300°C, nebulizer 45 psi, vaporizer 200°C
skimmer **65** V, fragmentor **18** V, dissolved in methanol

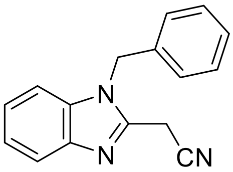

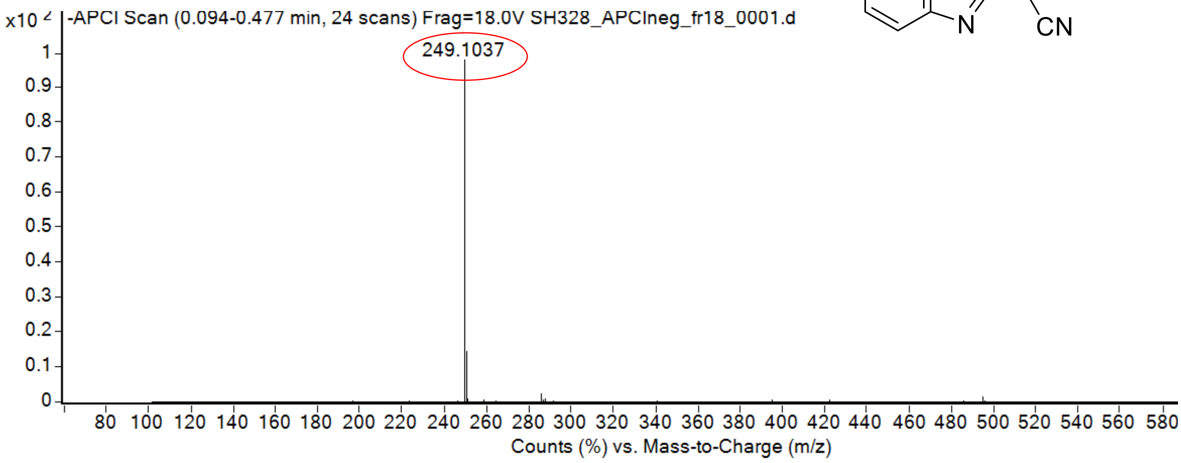

calculated mass: $[M\text{-}H]^-$ = 246.1037 observed: $[M\text{-}H]^-$ = 246.1037 max. mass error = < 0.1 ppm

^{1}H (500 MHz) and ^{13}C NMR (126 MHz) spectra of **S100** in chloroform-*d*

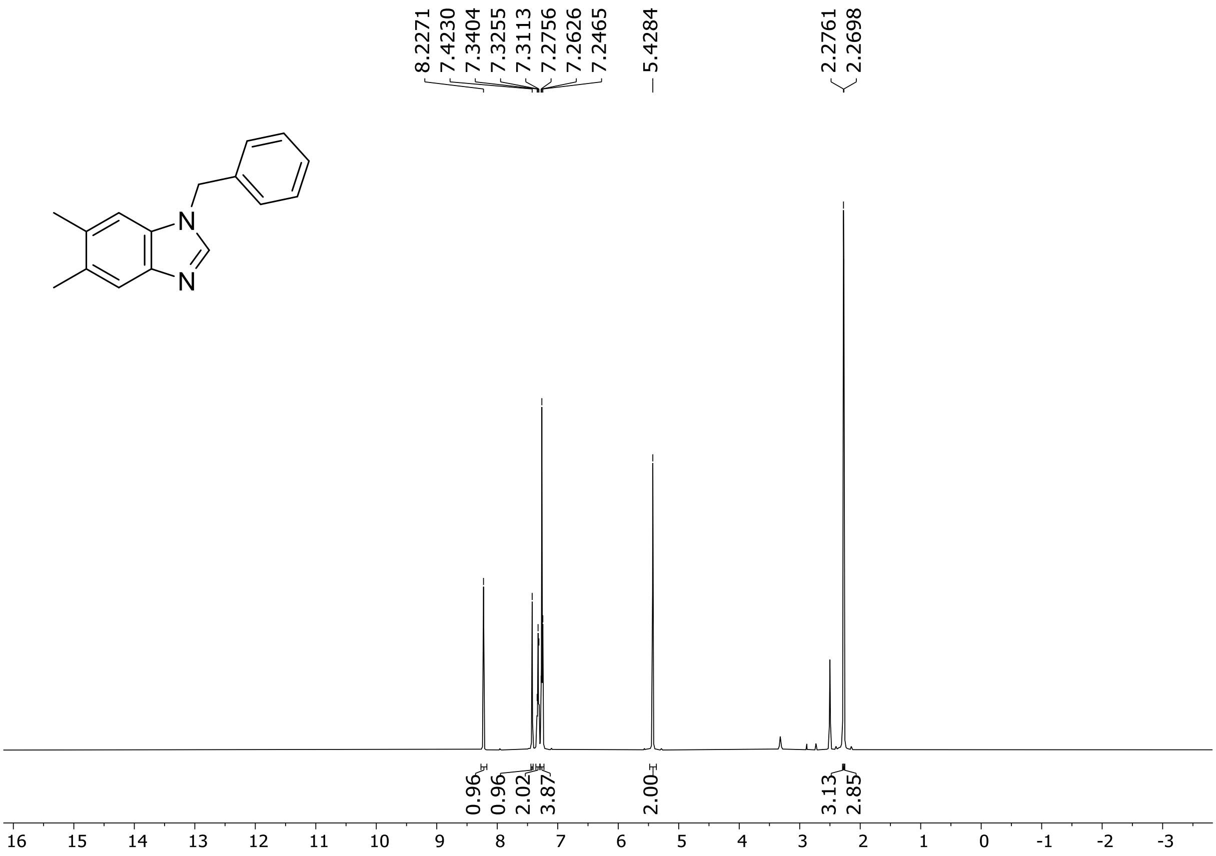
8.2271
7.4230
7.3404
7.3255
7.3113
7.2756
7.2626
7.2465
5.4284
2.2761
2.2698
N
N
0.96
0.96
2.02
3.87
2.00
3.13
2.85
16 15 14 13 12 11 10 9 8 7 6 5 4 3 2 1 0 -1 -2 -3

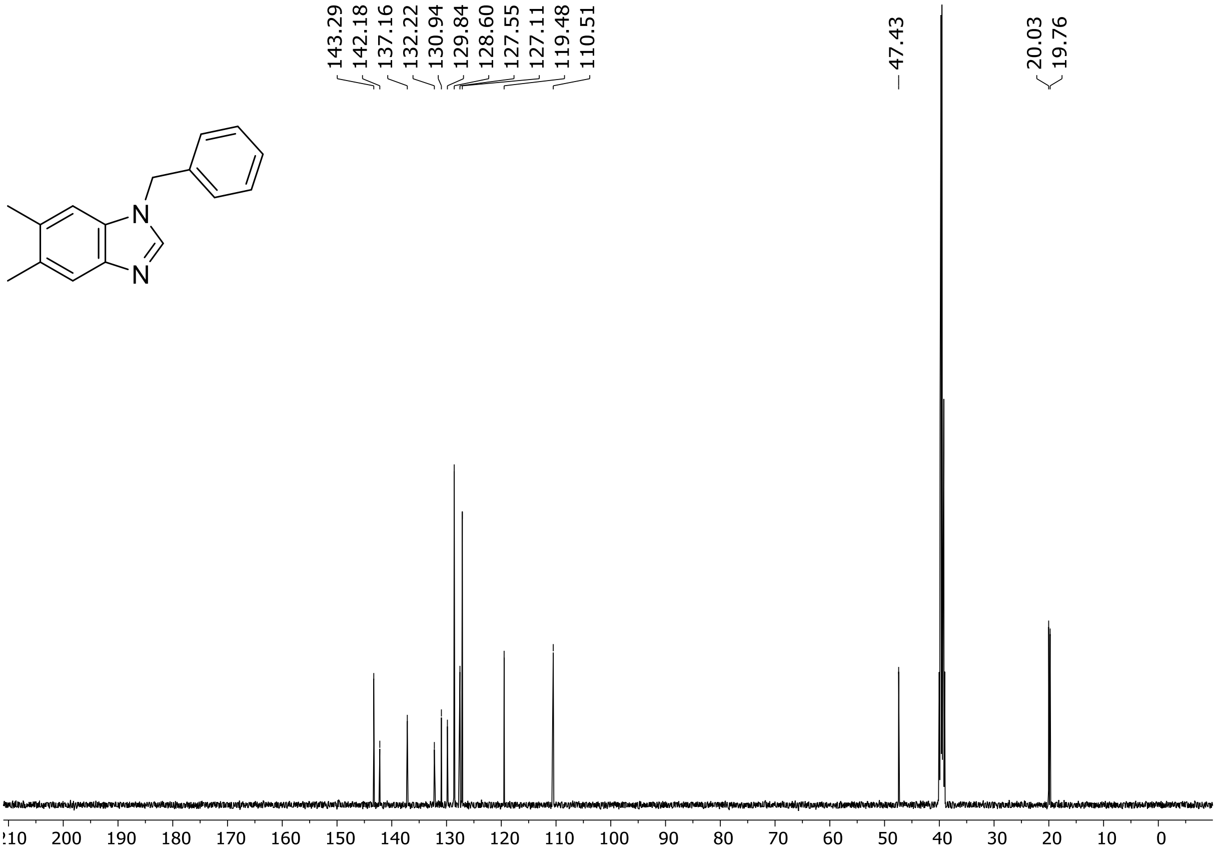
143.29
142.18
137.16
132.22
130.94
129.84
128.60
127.55
127.11
119.48
110.51
47.43
20.03
19.76
N
N
210 200 190 180 170 160 150 140 130 120 110 100 90 80 70 60 50 40 30 20 10 0

^{1}H (500 MHz) and ^{13}C NMR (126 MHz) spectra of **S101** in chloroform-*d*

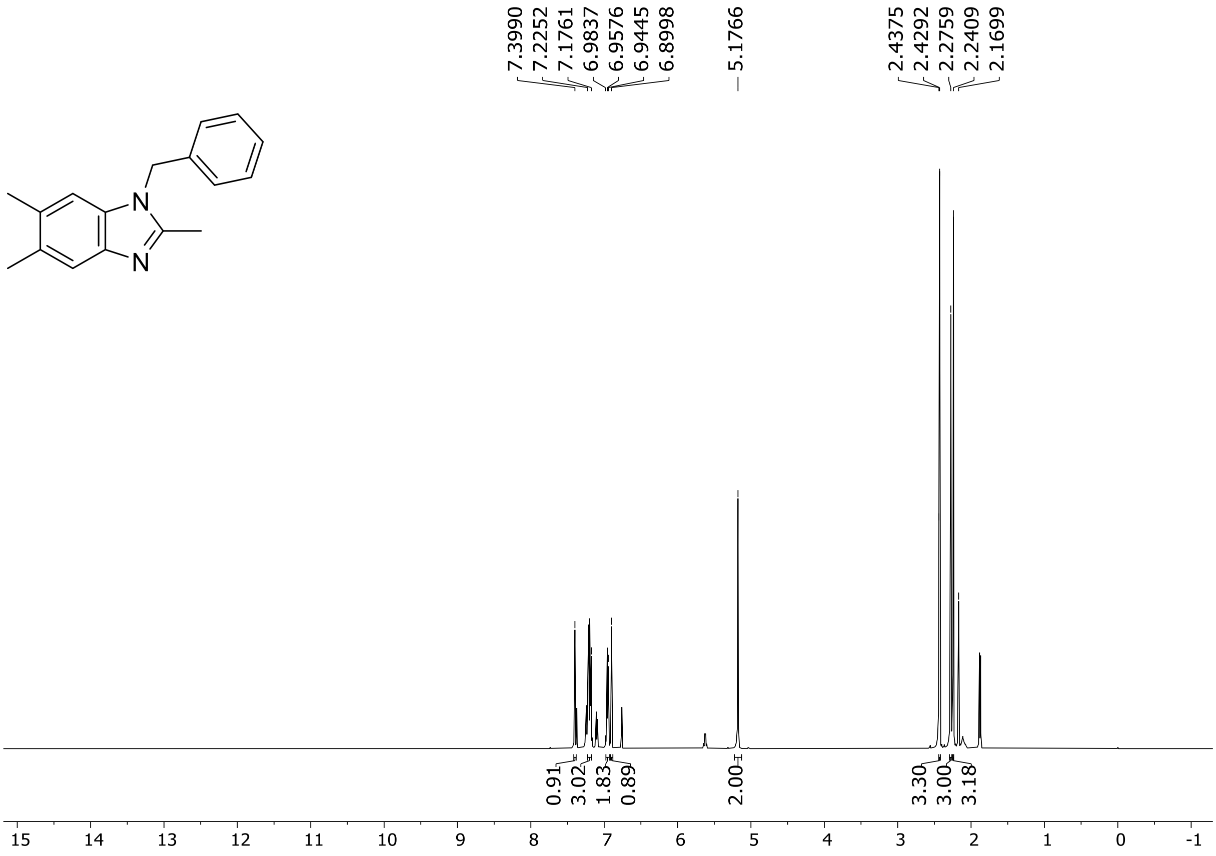

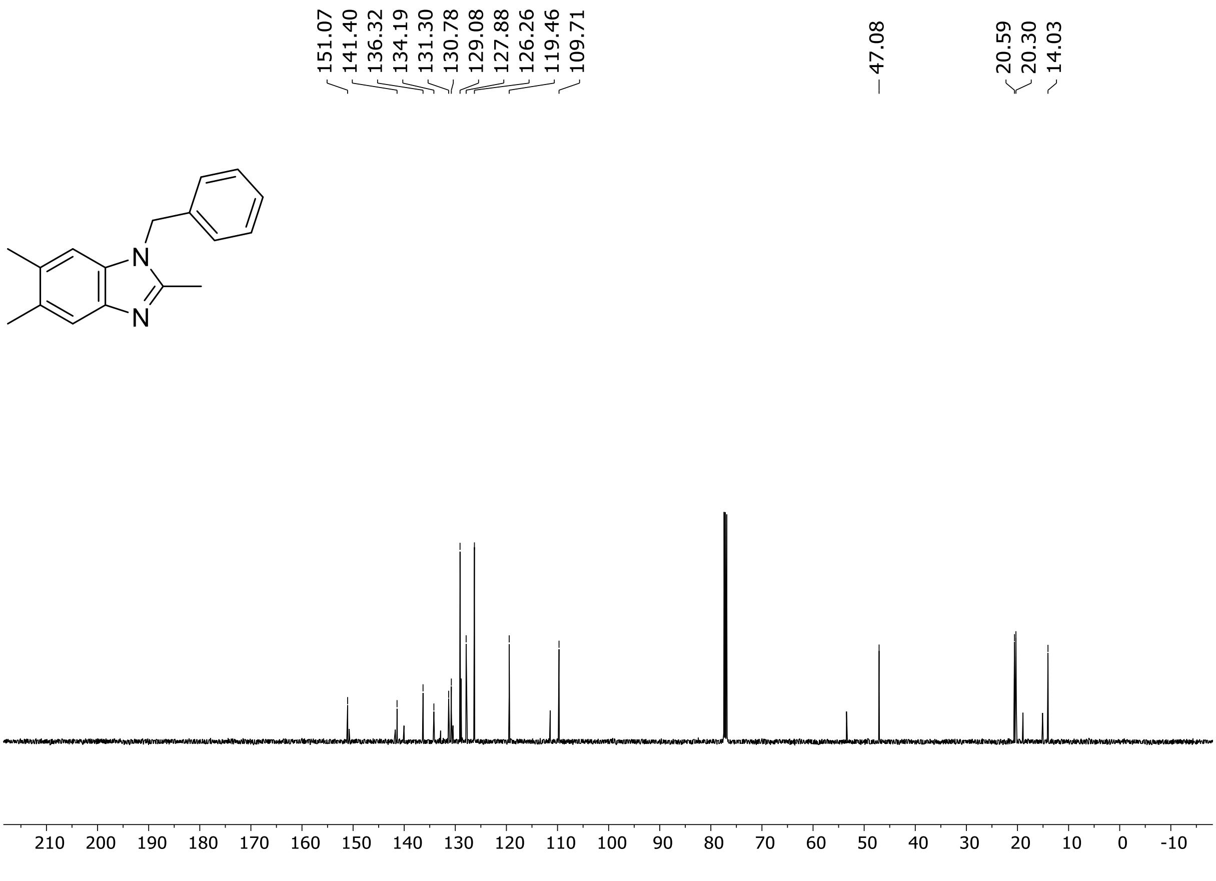

^{1}H (500 MHz) and ^{13}C NMR (126 MHz) spectra of **S102** in chloroform-*d*

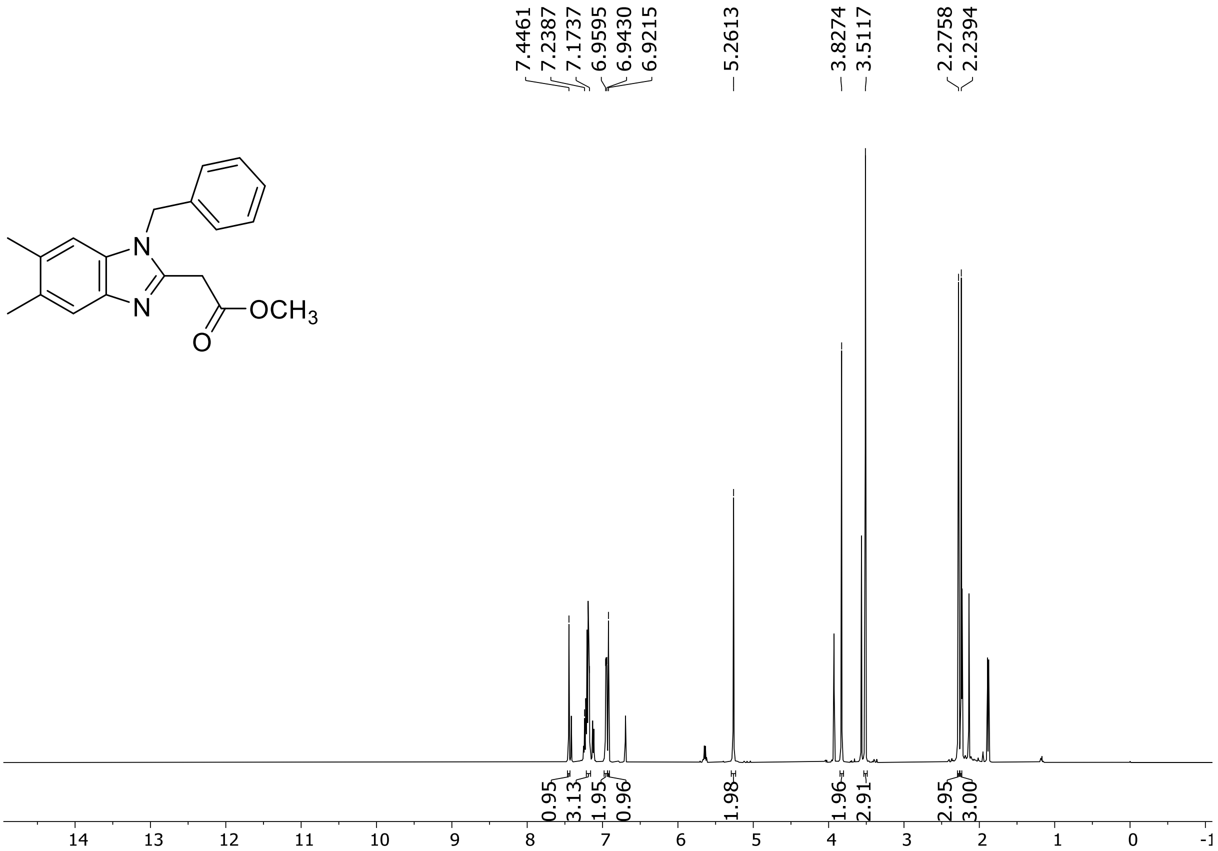

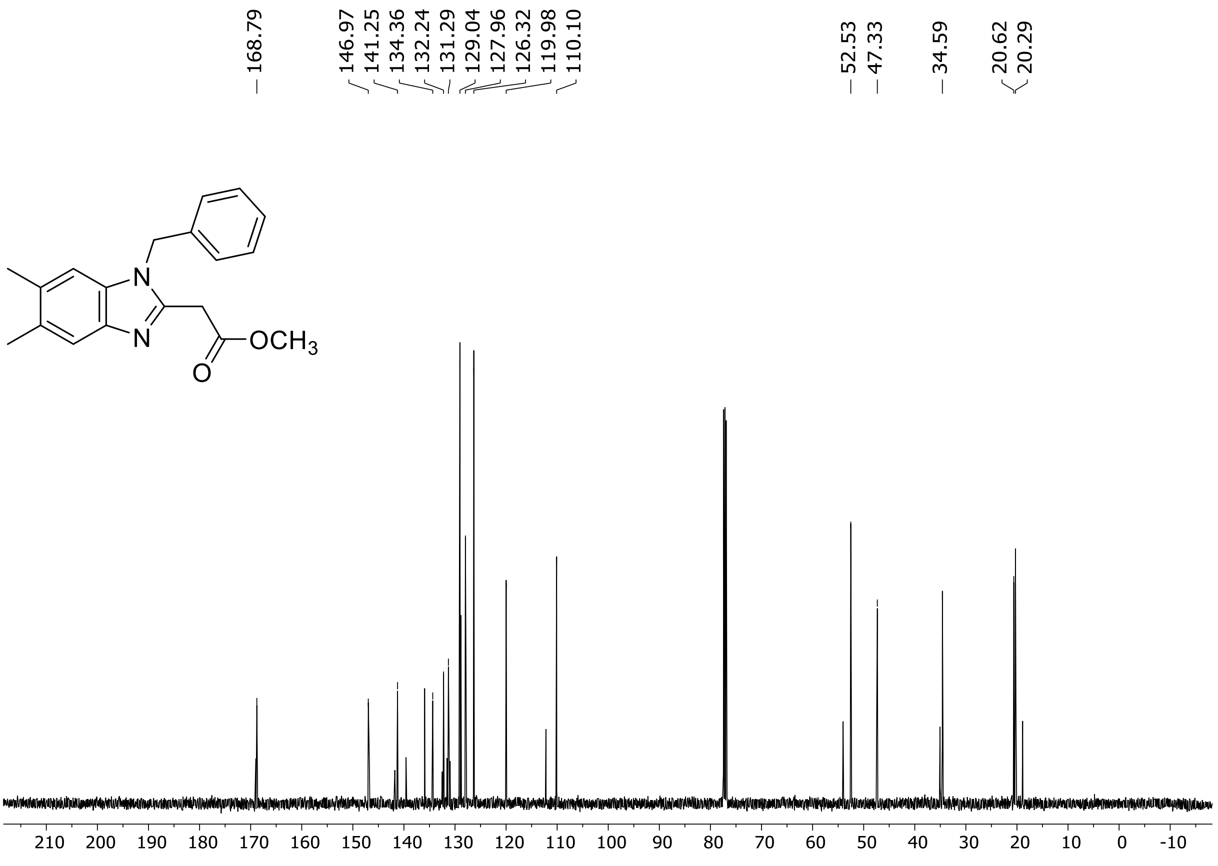

^{1}H (500 MHz) and ^{13}C NMR (126 MHz) spectra of **S103** in DMSO-d_6

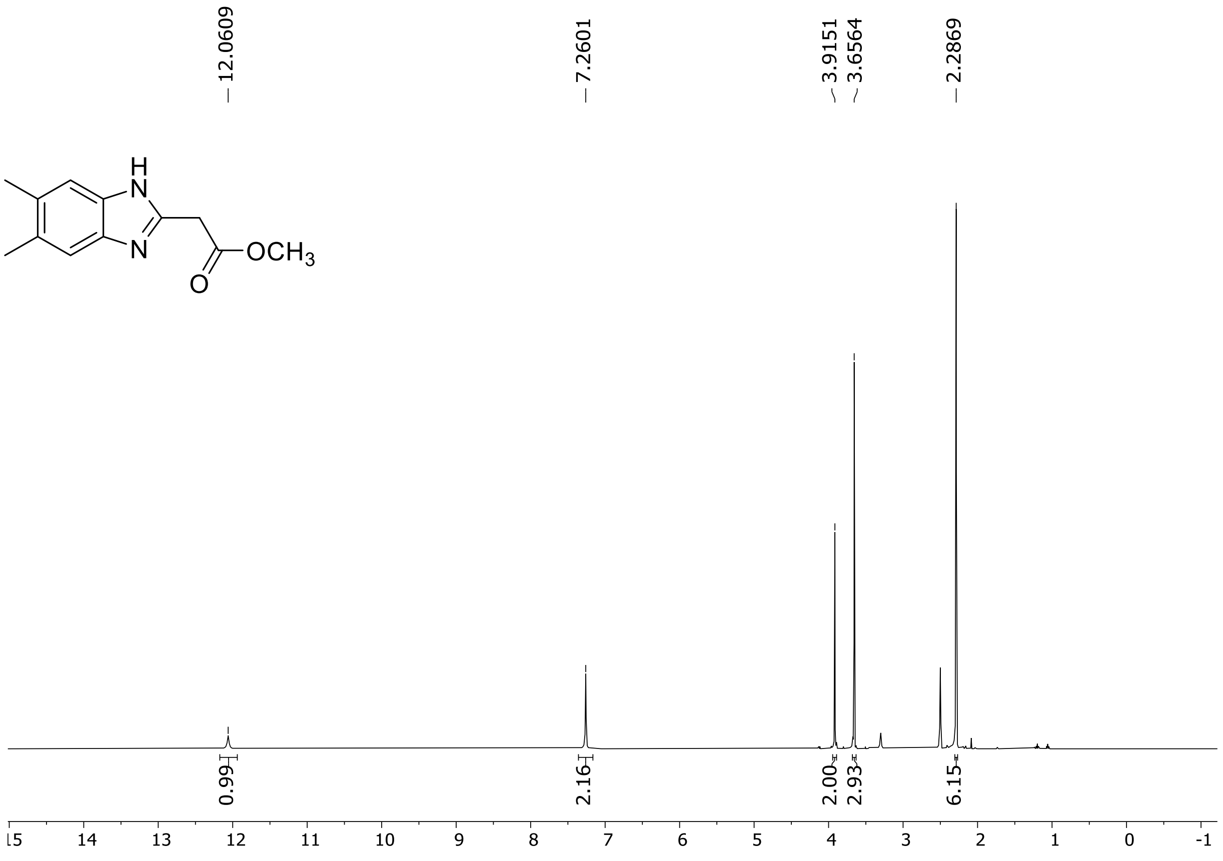

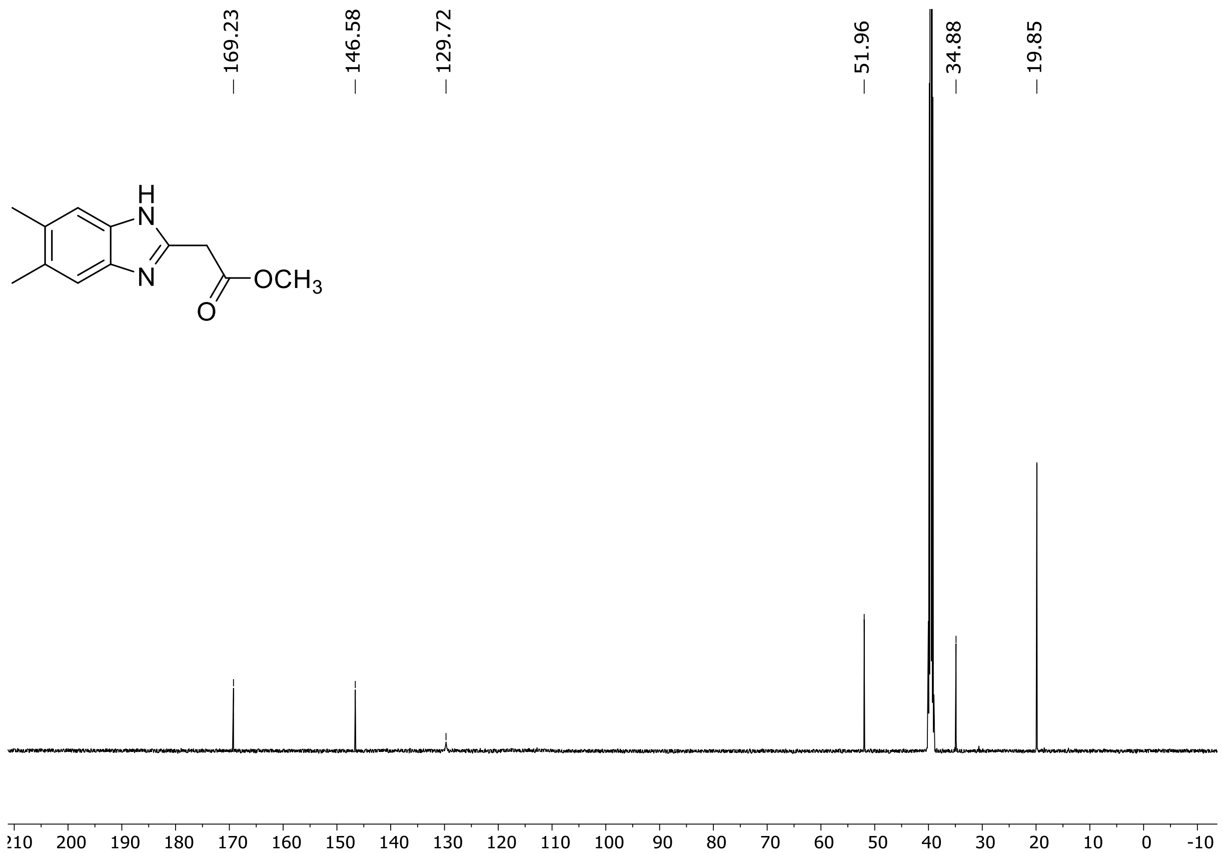

^{1}H (500 MHz) and ^{13}C NMR (126 MHz) spectra of **S104** in DMSO-d_6

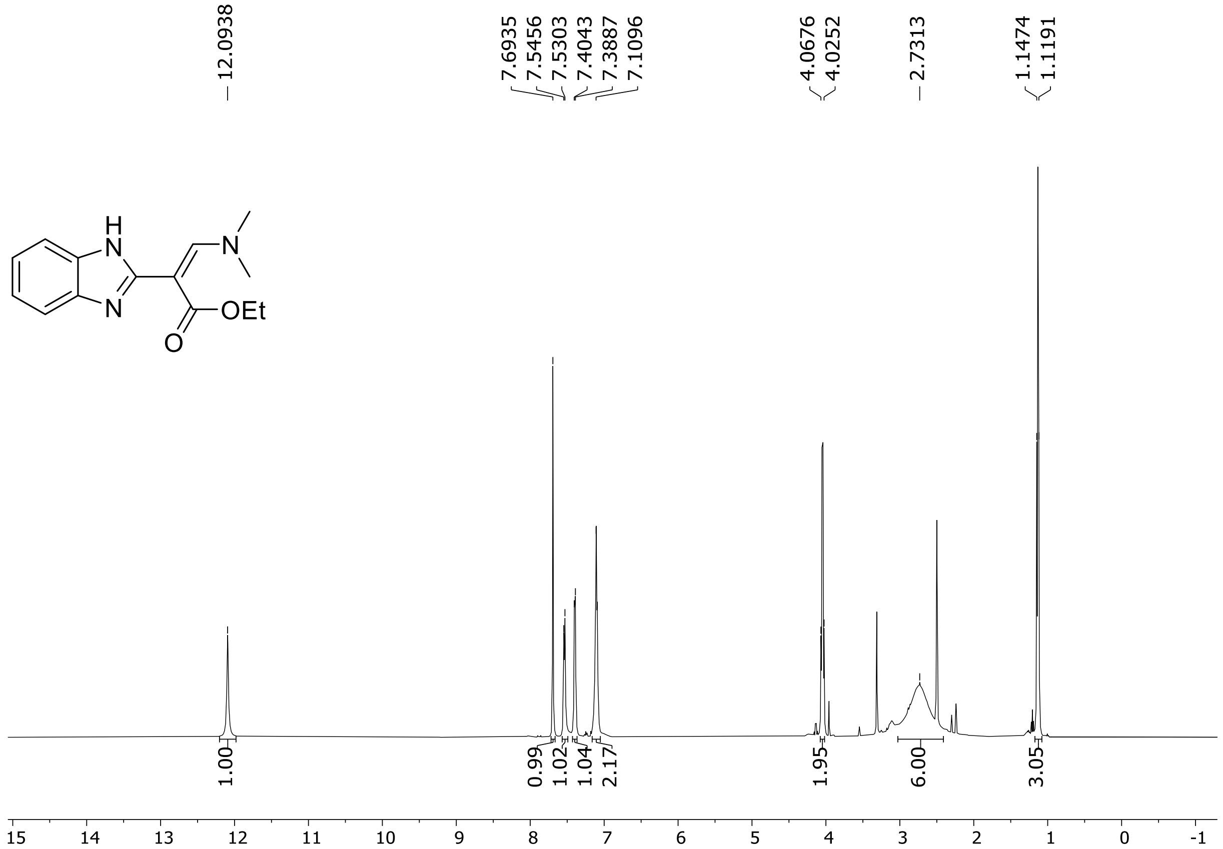

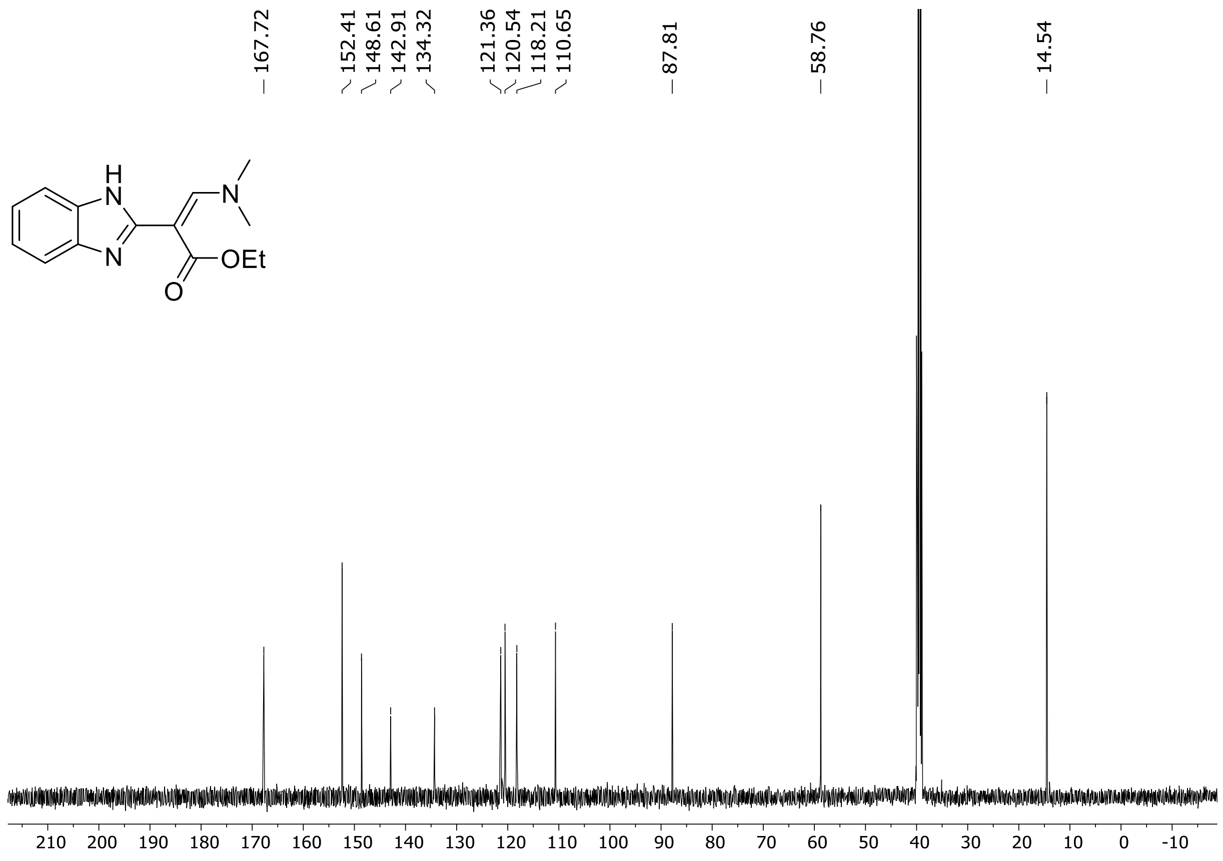

^{1}H (500 MHz) and ^{13}C NMR (126 MHz) spectra of **S105** in DMSO-d_6

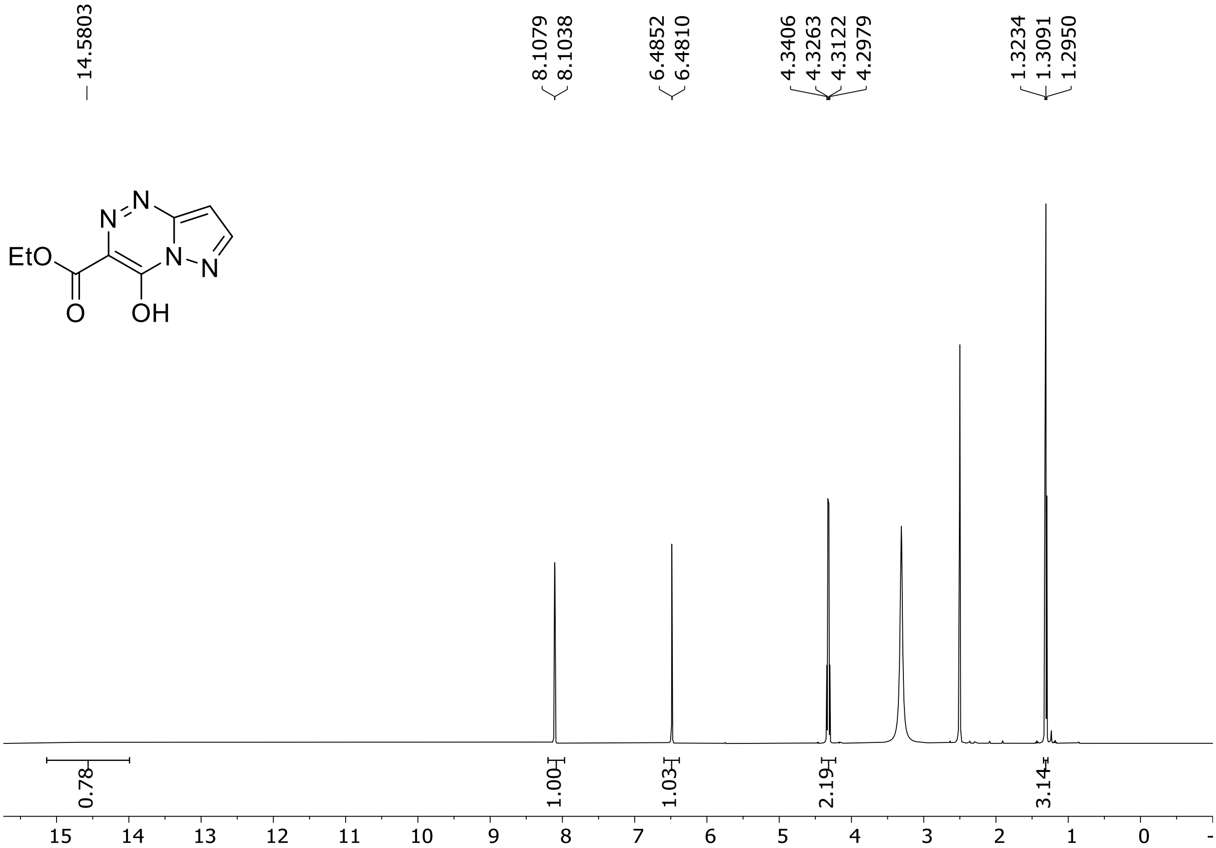

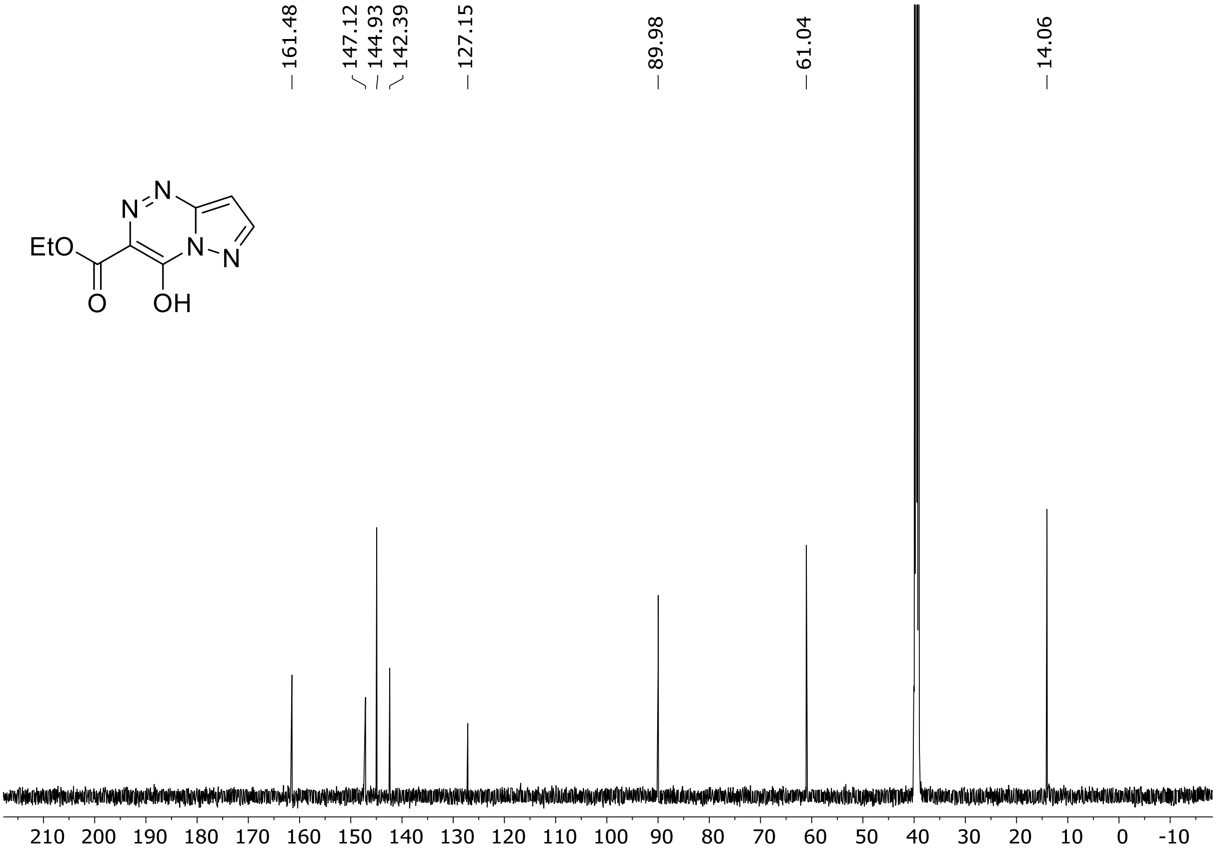

HRMS spectrum of **S105**

$C_8H_8N_4O_3$ **mono m/z = 208.17**

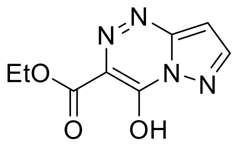

APCI + (MMI)

nitrogen flow 5 L/min, gas temperature 300°C, vaporizer 200°C, nebulizer 45 psi, skimmer **65** V, fragmentor 35 V, dissolved in MeOH

ZOOM – range of interest

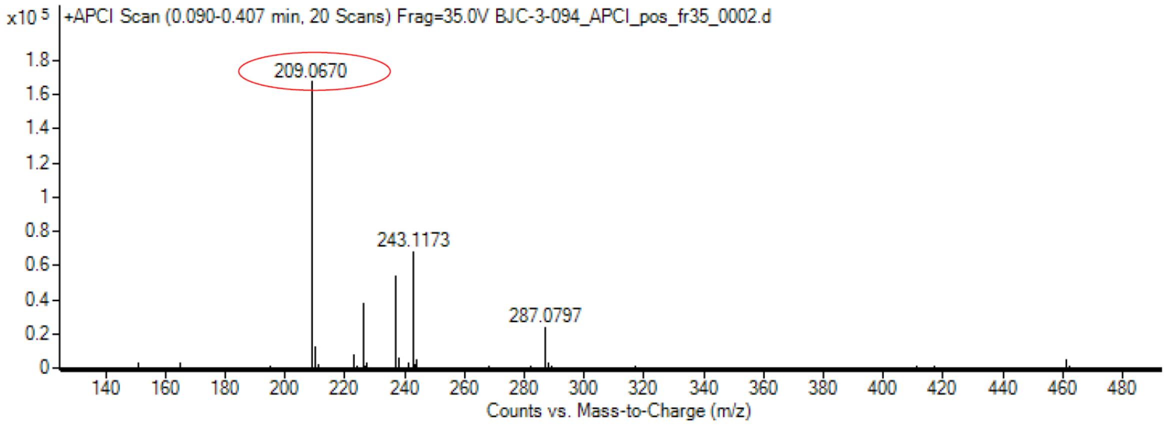

calculated mass: $[M+H]^+$ = 209.0669 observed: $[M+H]^+$ = 209.0670 max. mass error = 0.5 ppm

^{1}H (500 MHz) and ^{13}C NMR (126 MHz) spectra of **S106** in DMSO-d_6

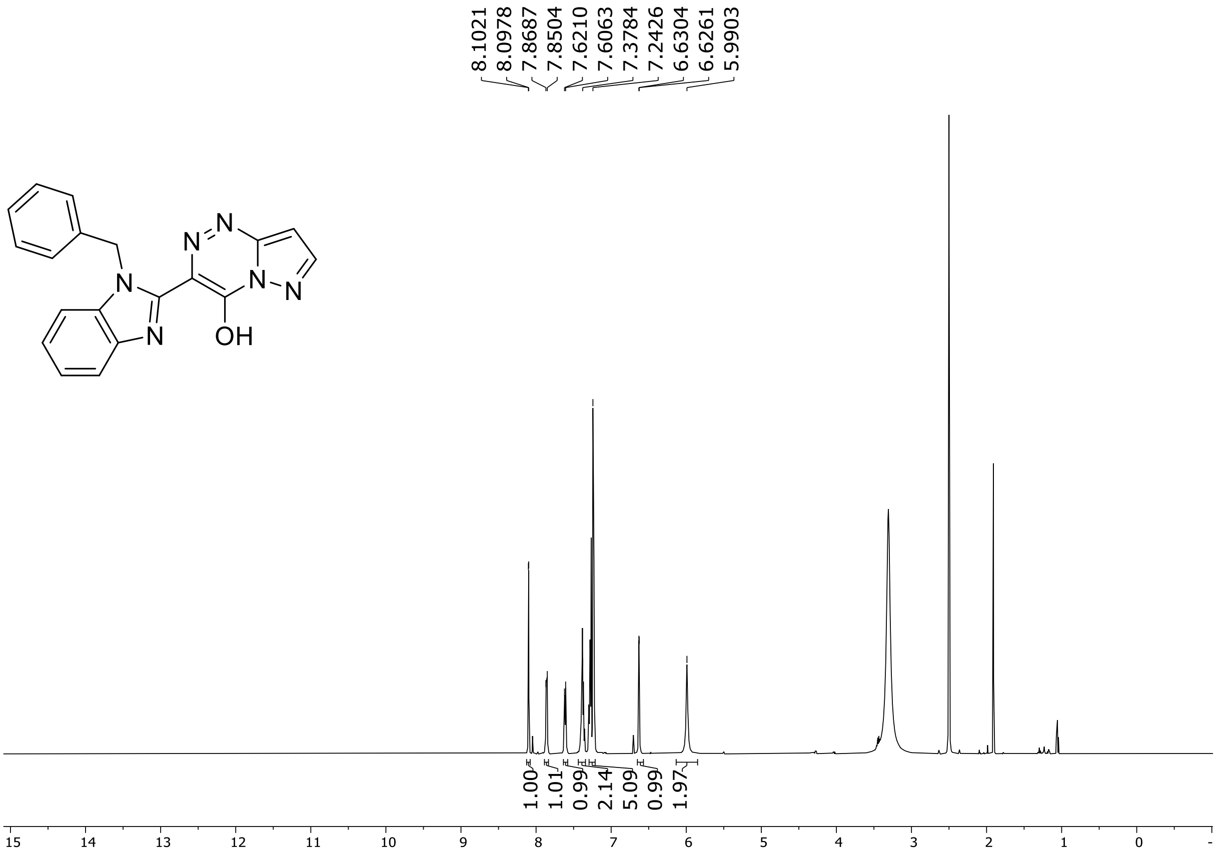

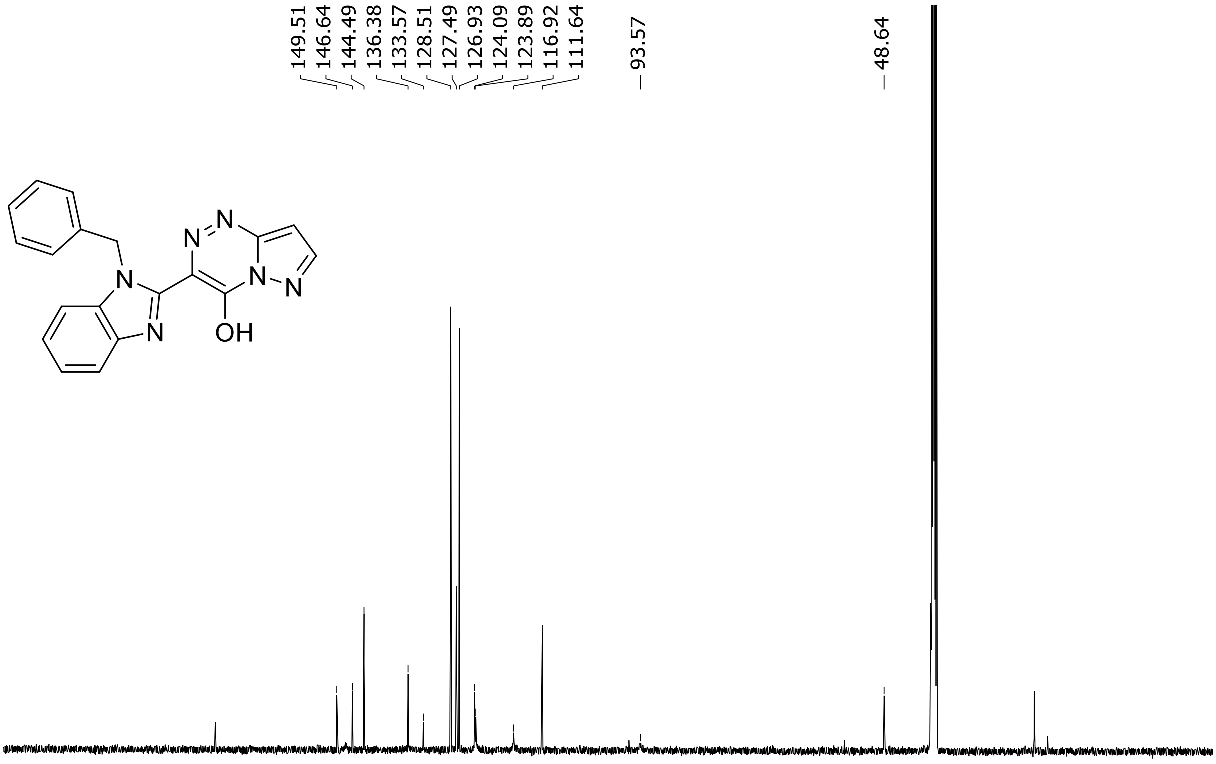

HRMS spectrum of **S106**

$C_{19}H_{14}N_6O$ mono m/z = 342.35

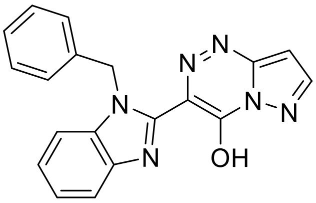

APCI + (MMI)

nitrogen flow 5 L/min, gas temperature 300°C, vaporizer 200°C, nebulizer 45 psi, skimmer **65** V, fragmentor 35 V, dissolved in MeOH

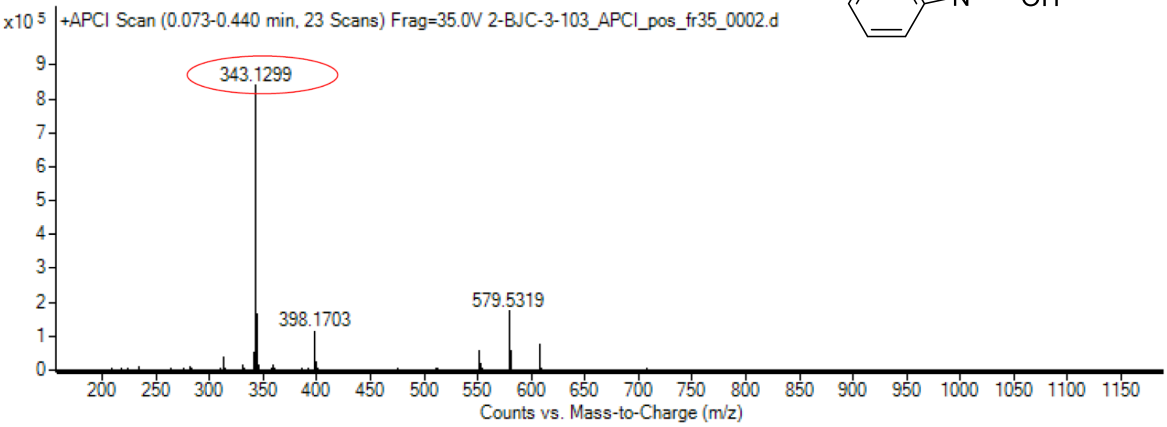

calculated mass: $[M+H]^+$ =343.1302 observed: $[M+H]^+$ = 343.1299 max. mass error = 0.8 ppm

^{1}H (500 MHz) and ^{13}C NMR (126 MHz) spectra of **S107** in DMSO-d_6

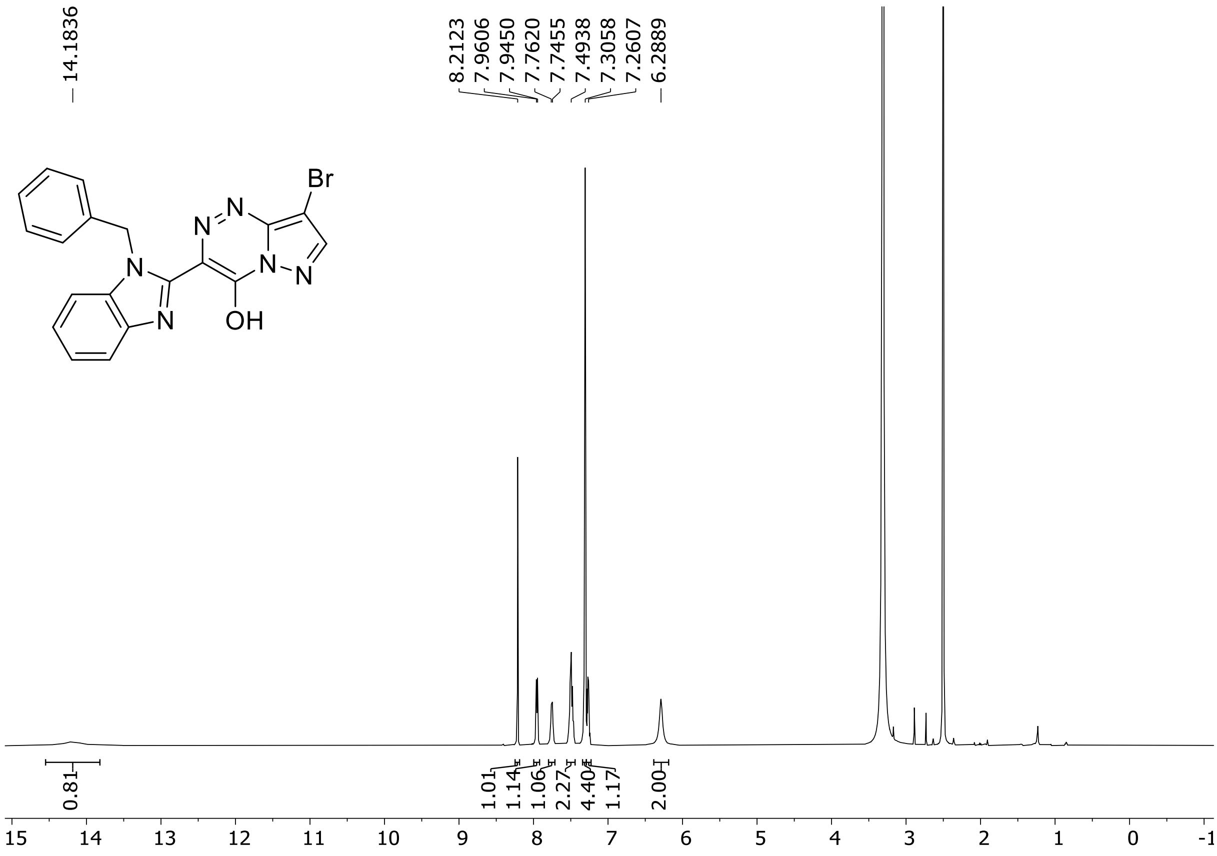

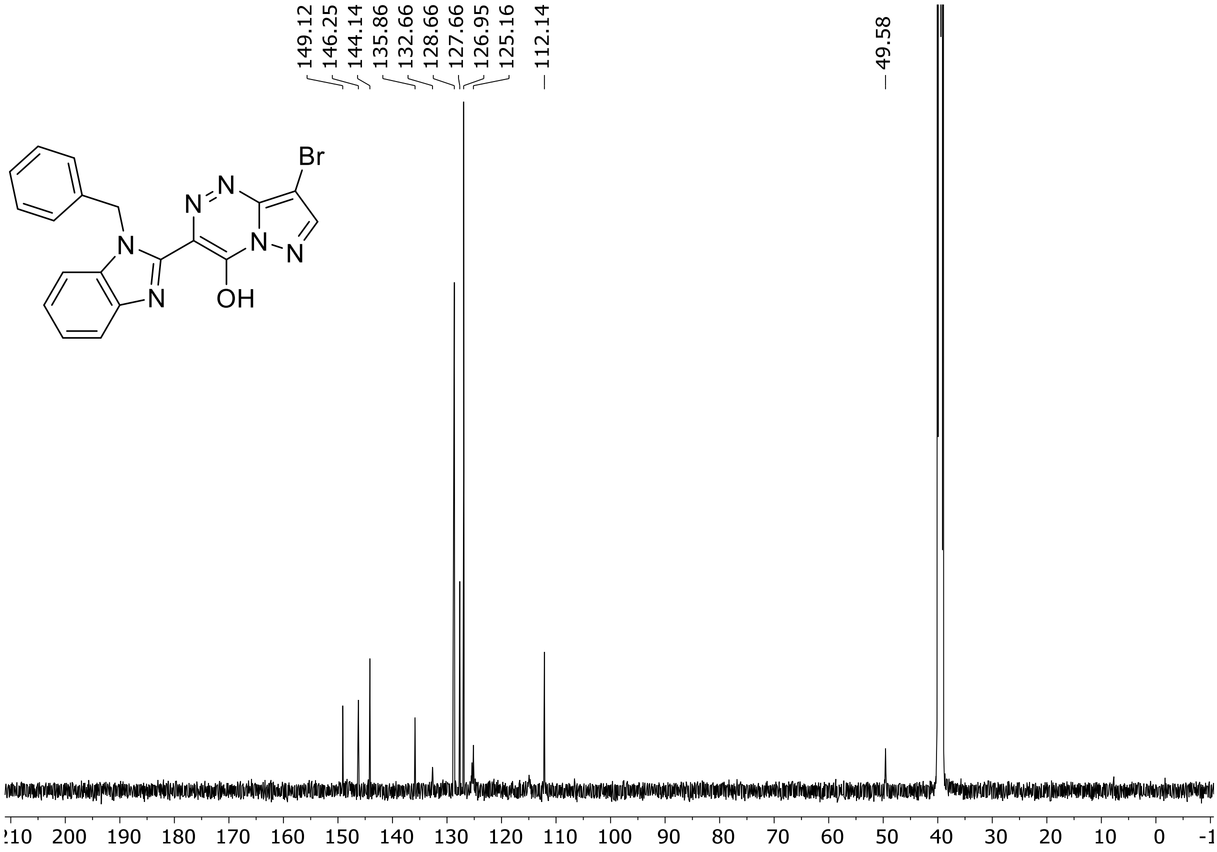

HRMS spectrum of **S107**

$C_{19}H_{13}BrN_6O$ mono m/z = 420.03

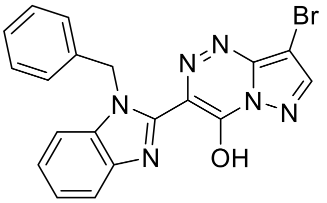

ESI + (MMI)
nitrogen flow 5 L/min, gas temperature 300°C, vaporizer 200°C, nebulizer 45 psi, skimmer **65** V, fragmentor 45 V, dissolved in MeOH

ZOOM – range of interest

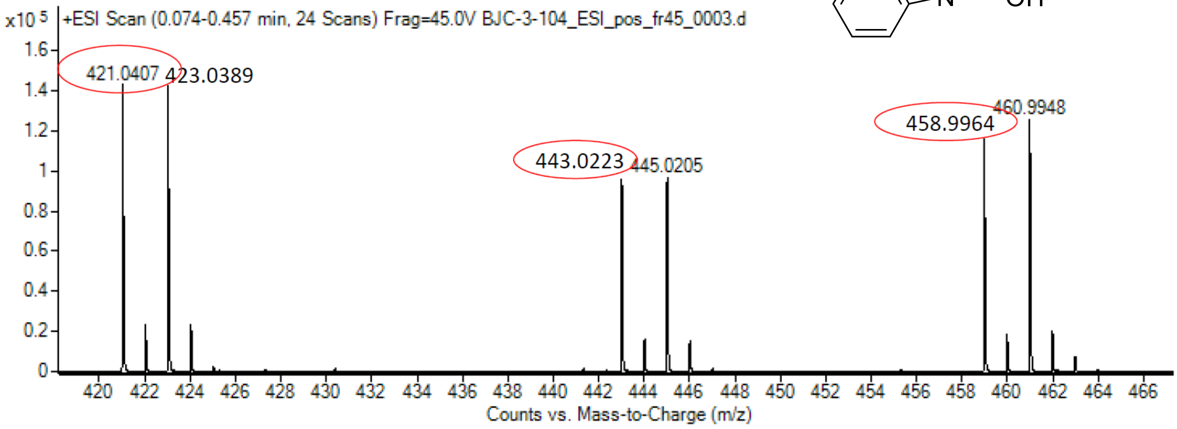

calculated mass: $[M+H]^+$ = 421.0407 observed: $[M+H]^+$ = 421.0407 max. mass error < 0.1 ppm

calculated mass: $[M+Na]^+$ = 443.0226 observed: $[M+Na]^+$ = 443.0223 max. mass error = 0.7 ppm

calculated mass: $[M+K]^+$ = 458.9966 observed: $[M+K]^+$ = 458.9964 max. mass error = 0.4 ppm

^{1}H (500 MHz) and ^{13}C NMR (126 MHz) spectra of **S108** in DMSO-d_6

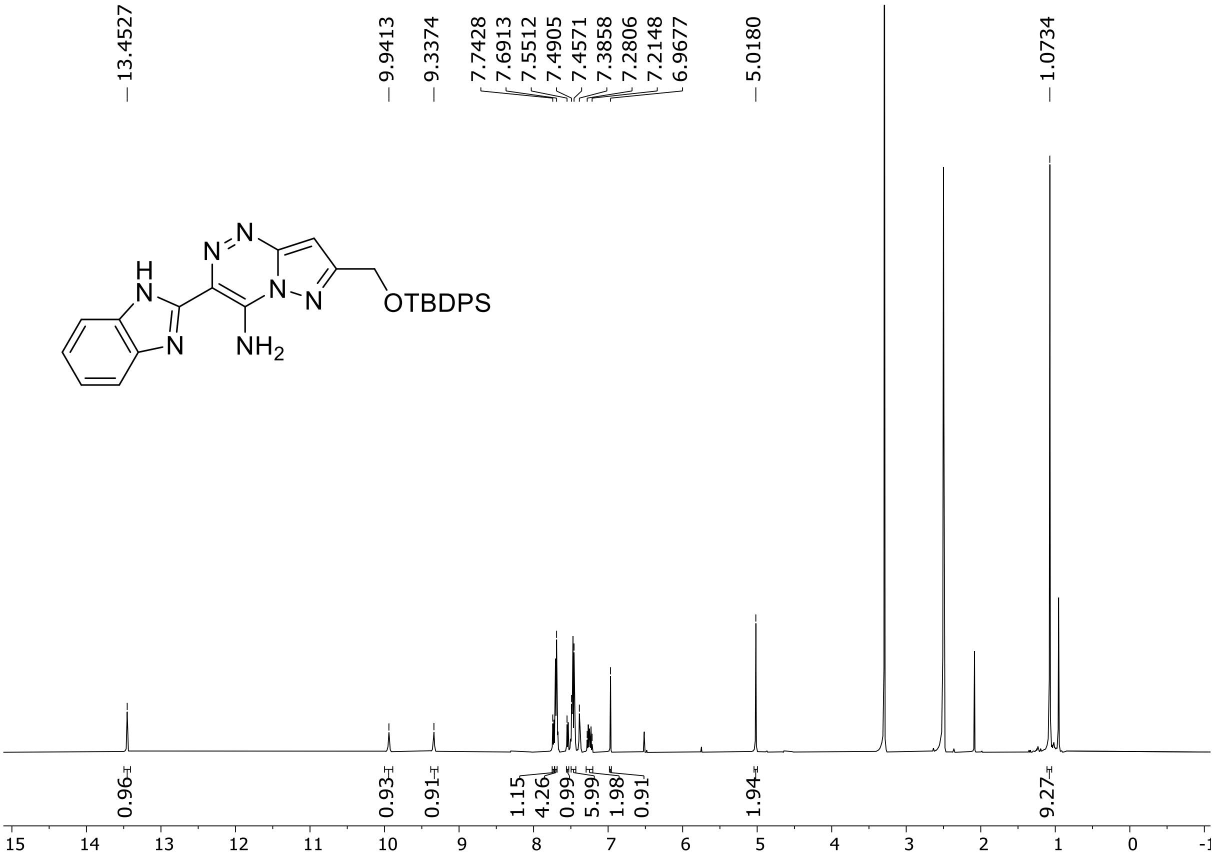

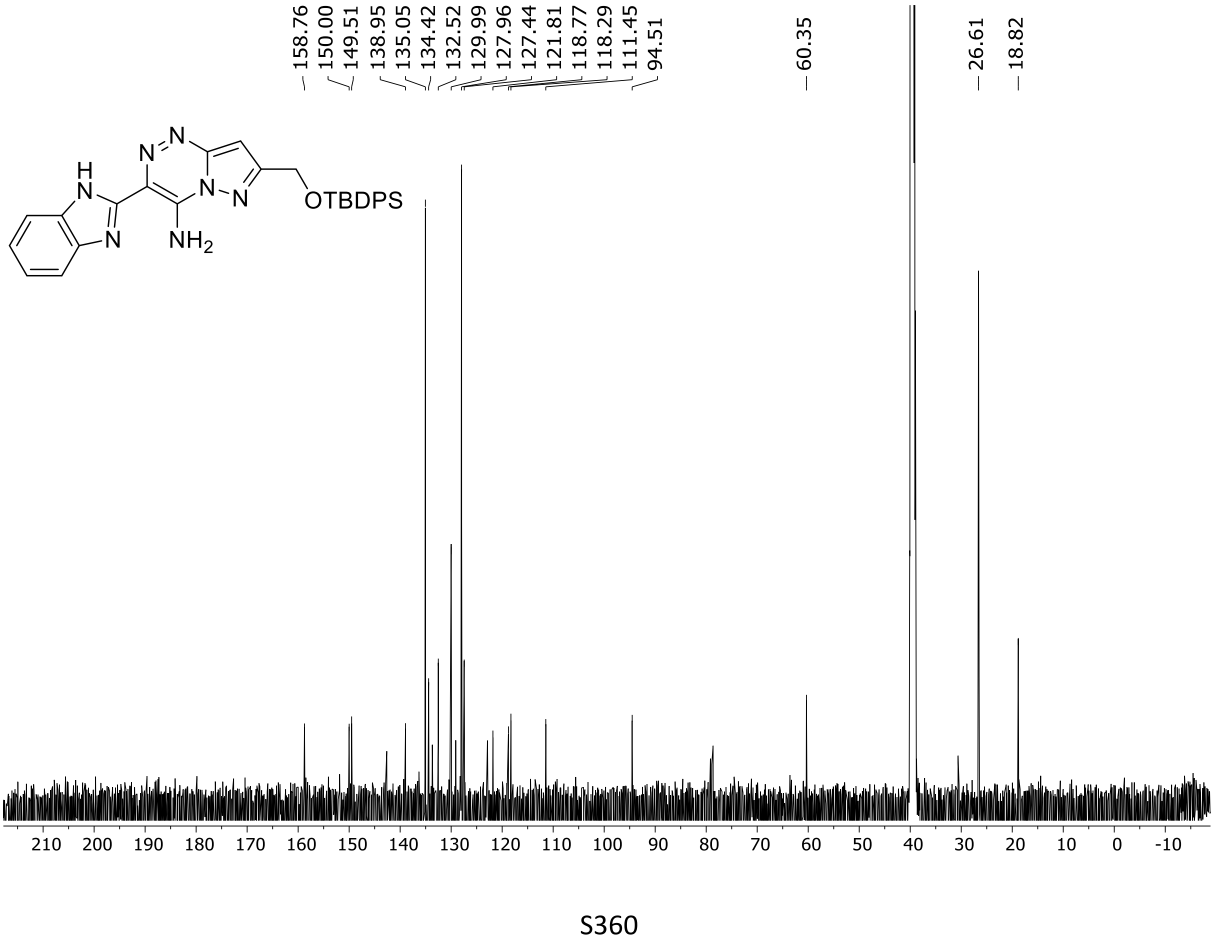

HRMS spectrum of **S108**

$C_{29}H_{29}N_7OSi$ **mono m/z = 519.2203**

APCI + (MMI)
nitrogen flow 5 L/min, gas temperature 300°C, nebulizer 45 psi, vaporizer 200°C
skimmer **65** V, fragmentor **20** V, dissolved in methanol

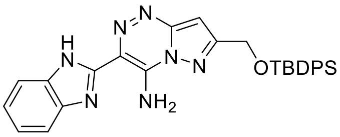

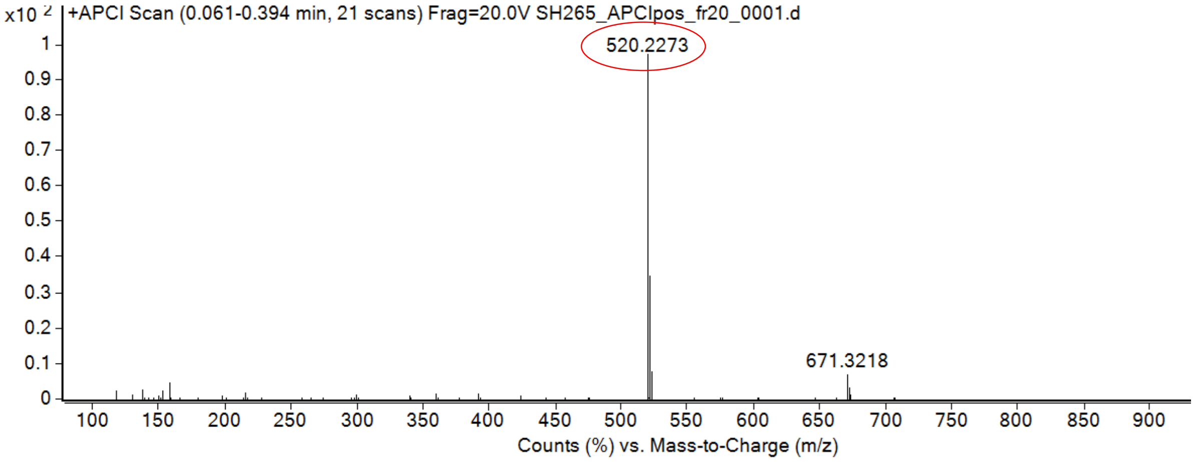

calculated mass: $[M+H]^+$ = 520.2276 observed: $[M+H]^+$ = 520.2273 max. mass error = 0.5 ppm

^{1}H NMR, ^{13}C NMR and HRMS spectra of compound 1 and 3-28

^{1}H (500 MHz) and ^{13}C NMR (126 MHz) spectra of **1** in DMSO-d_6

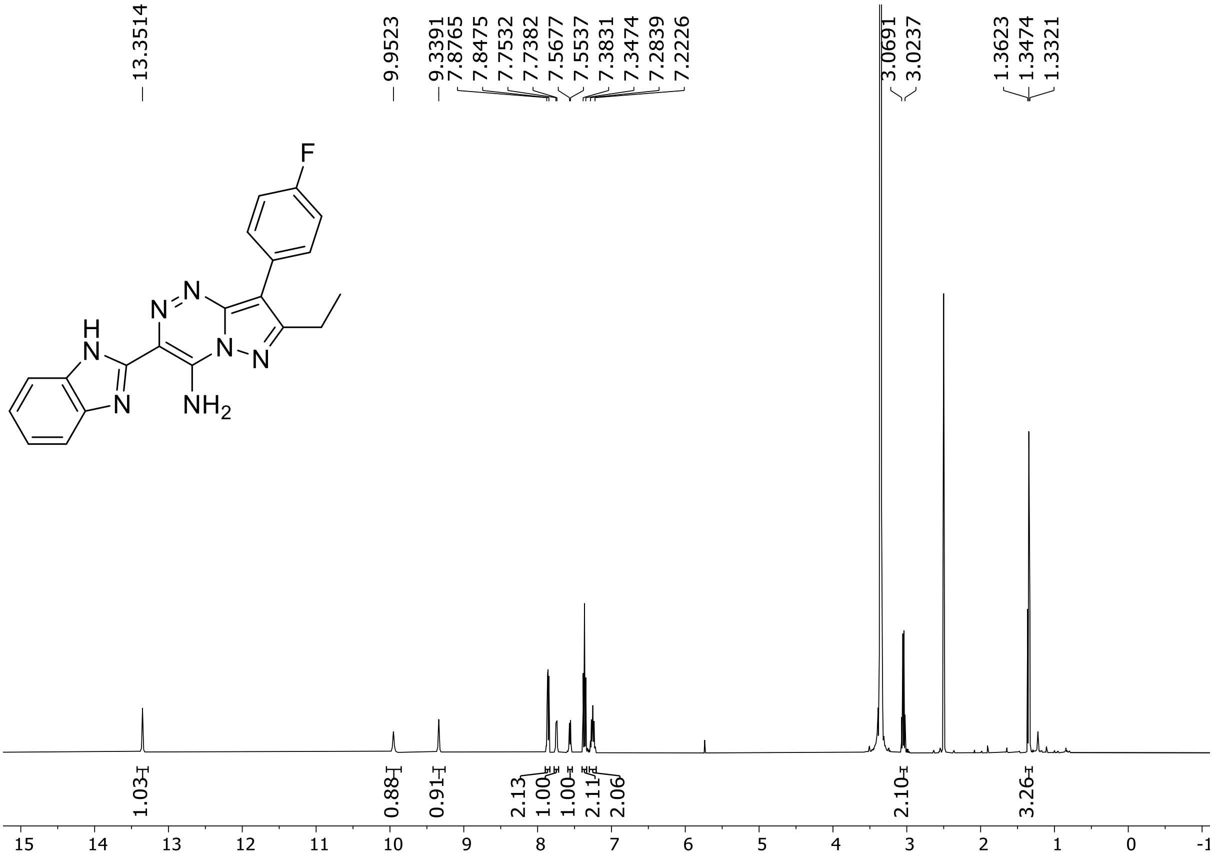

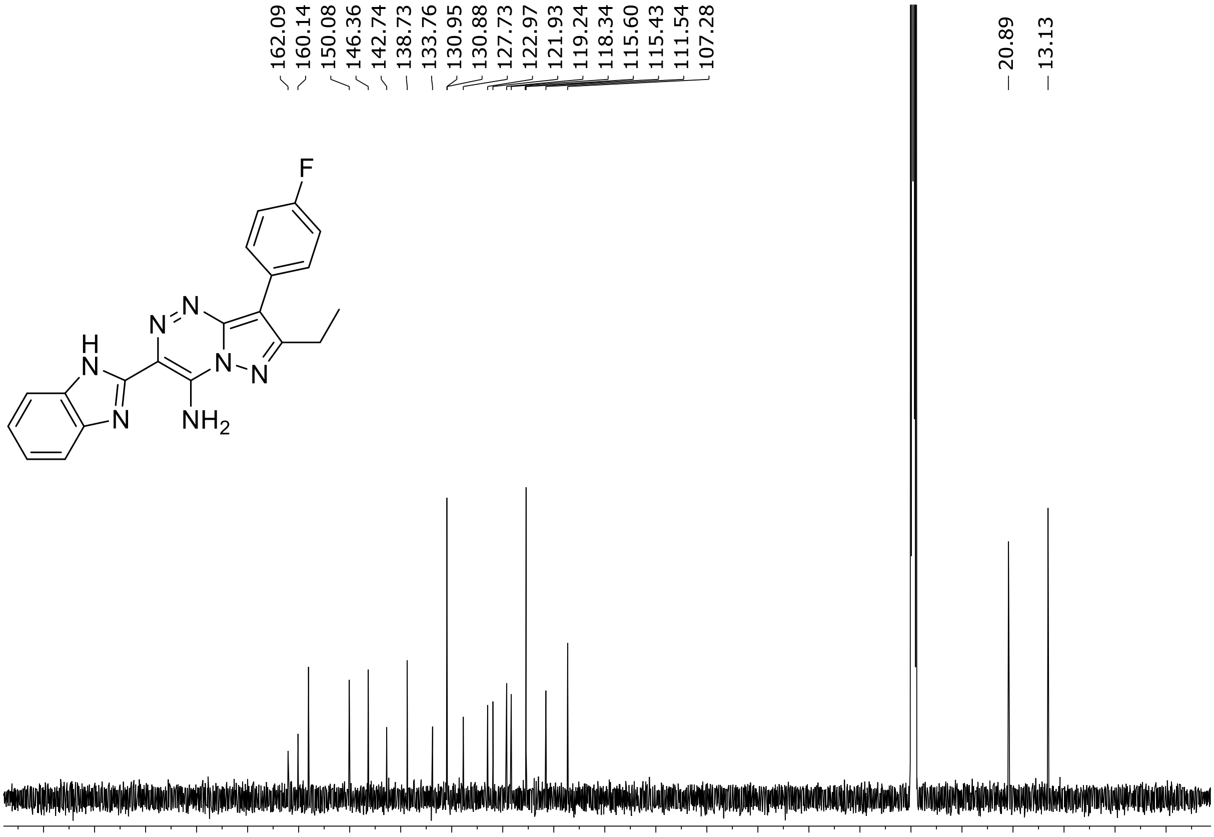

HRMS spectrum of **1**

$C_{20}H_{16}FN_7$ **mono m/z = 373,1446**

APCI + (MMI)
nitrogen flow 5 L/min, gas temperature 300°C, vaporizer 250°C,
nebulizer 45 psi, corona current 4 uA, Vcap -2000 V,
skimmer **65** V, fragmentor **35** V, dissolved in methanol (CH3OH)

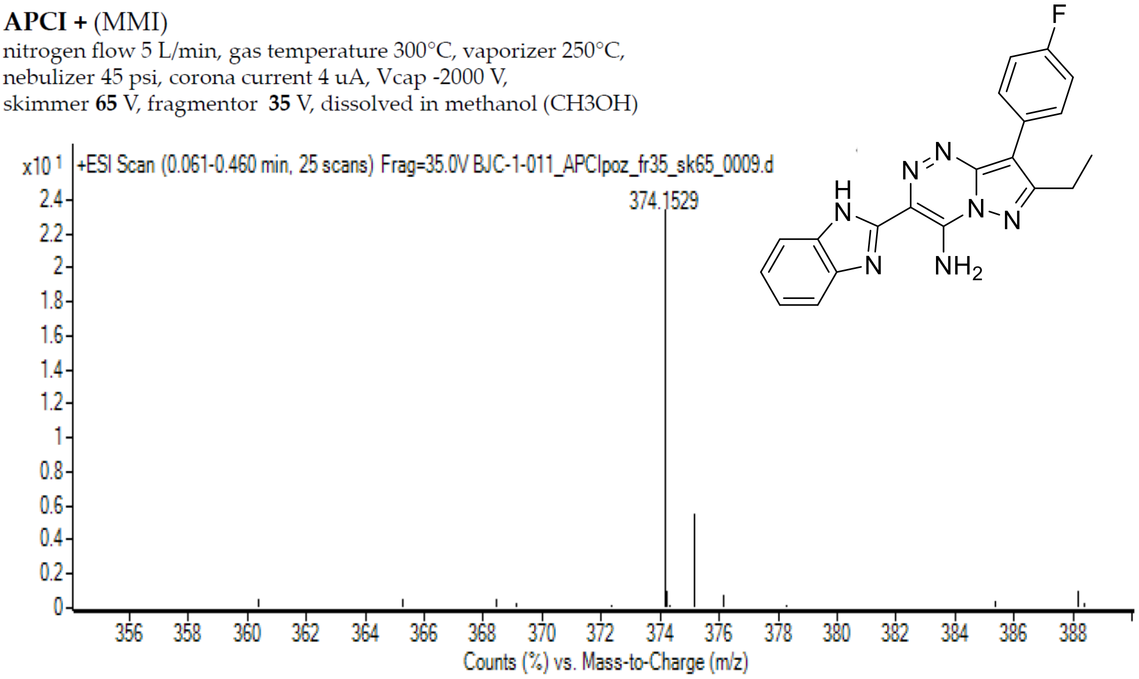

calculated mass: $[M+H]^+$ = 374,1524 observed: $[M+H]^+$ =374,1529 max. mass error 1,3 ppm

^{1}H (500 MHz) and ^{13}C NMR (126 MHz) spectra of **3** in DMSO-d_6

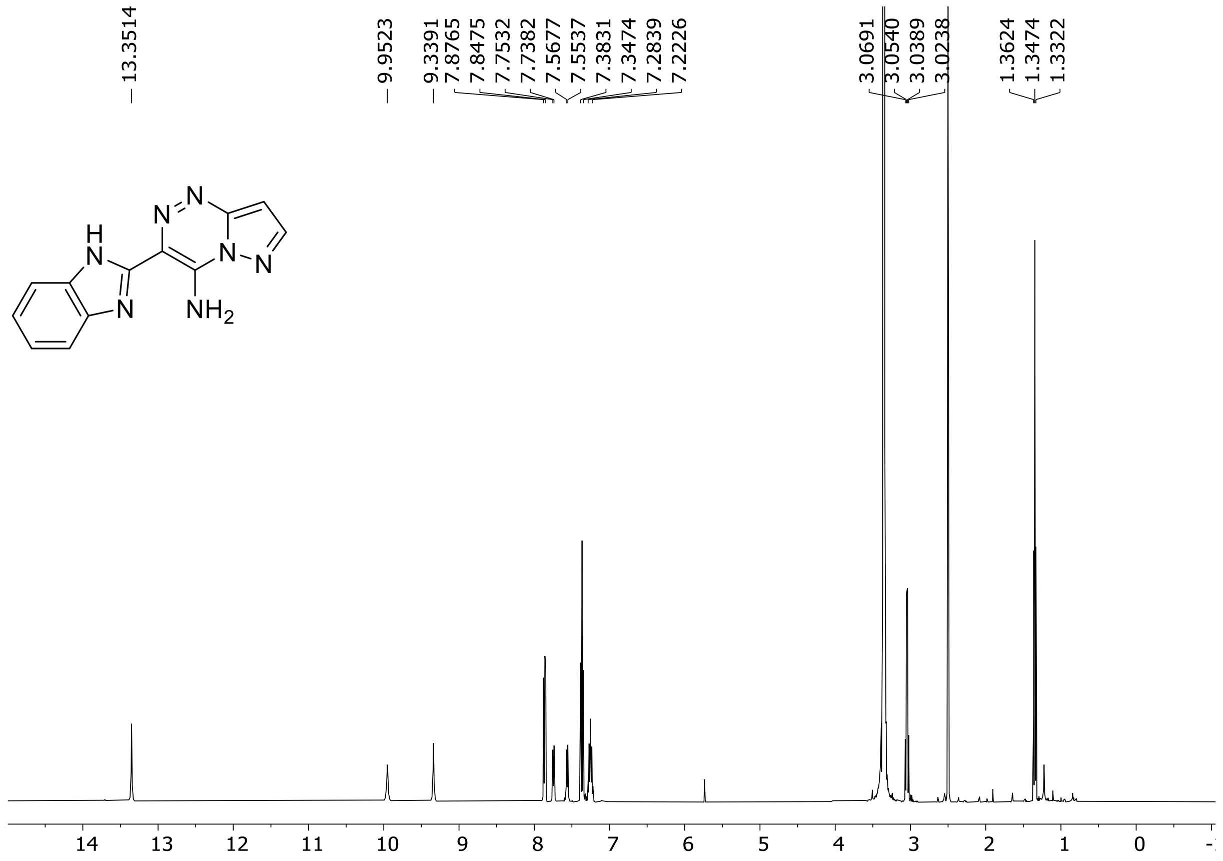

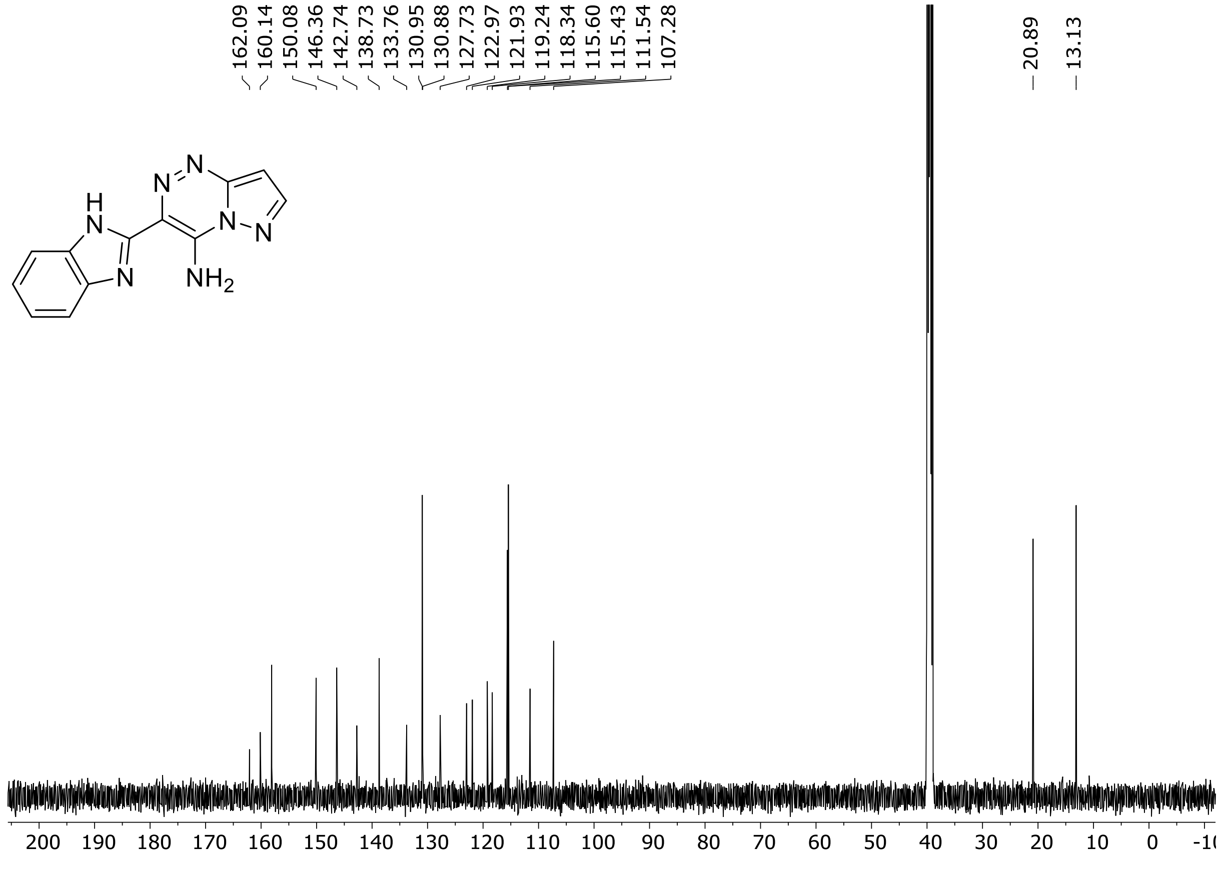

HRMS spectrum of **3**

$C_{12}H_9N_7$ **mono m/z = 251,2467**

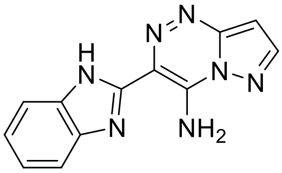

ESI - (MMI)
nitrogen flow 5 L/min, gas temperature 300°C, vaporizer 200°C, nebulizer 45 psi, Vcap -2000 V, skimmer **65** V, fragmentor **35** V, dissolved in DMSO

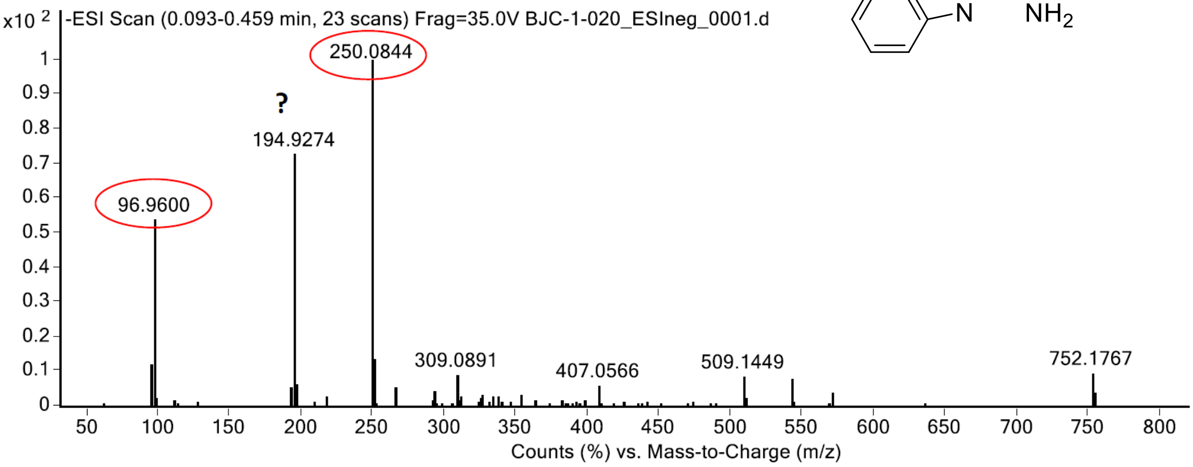

calculated mass: $[M-H]^-$ = 250,0846 observed: $[M-H]^-$ = 250,0844 max. mass error = 0,7 ppm

sulphuric acid
calculated mass: $[M-H]^-$ = 96,9601 observed: $[M-H]^-$ = 96,960 max. mass error = 1 ppm

^{1}H (500 MHz) and ^{13}C NMR (126 MHz) spectra of **4** in DMSO-d_6

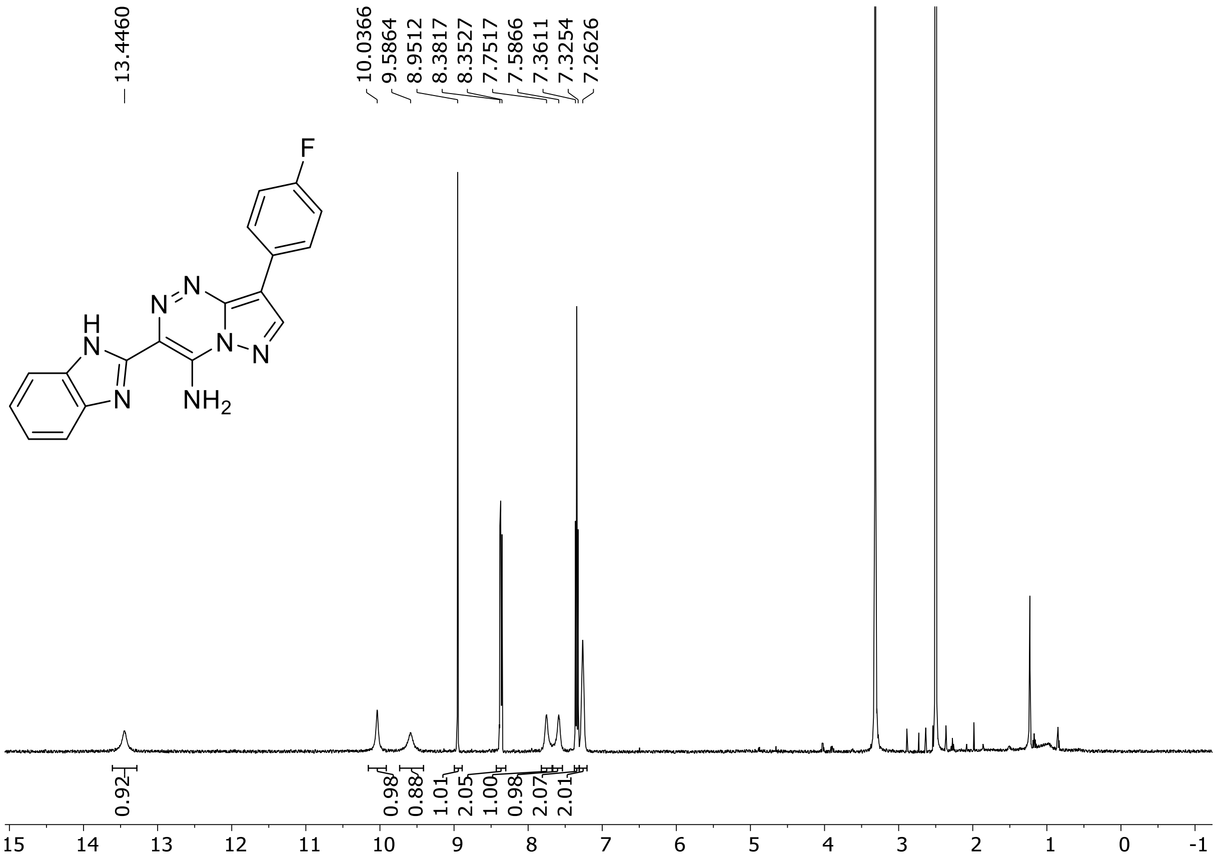

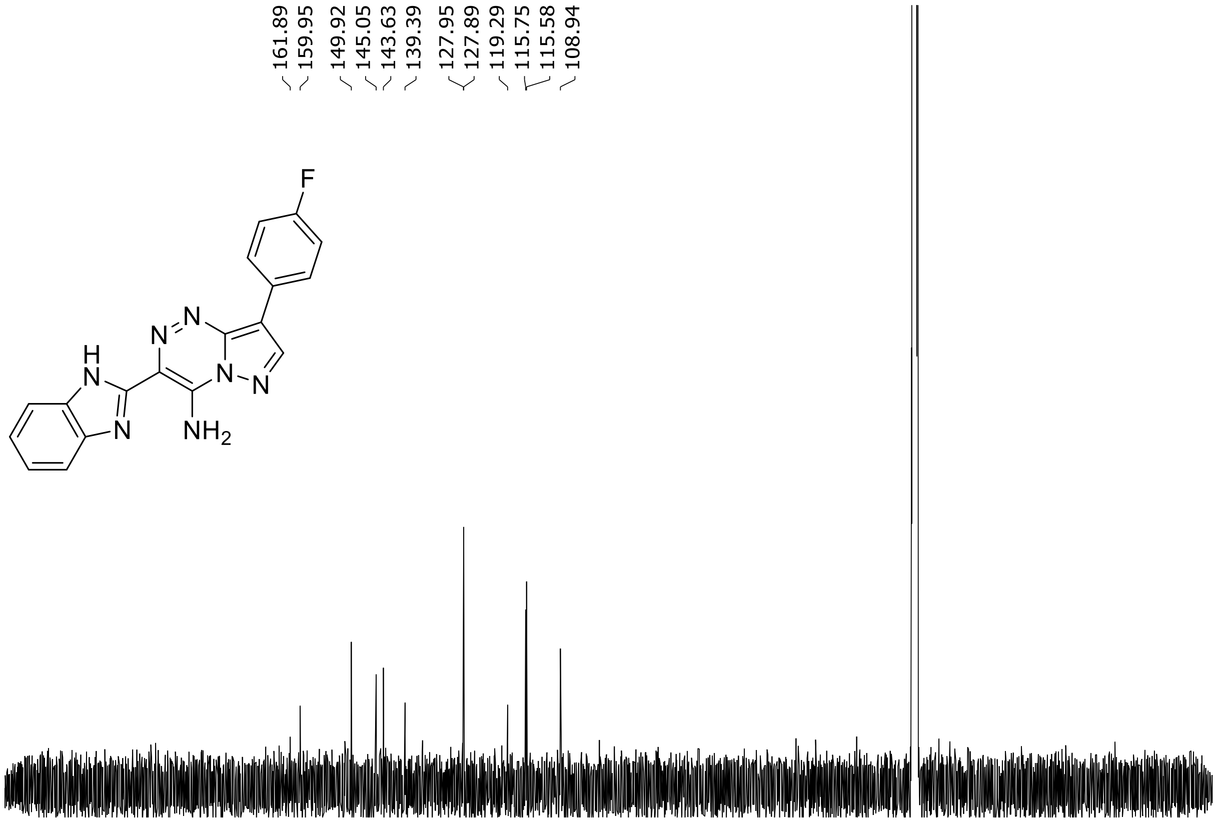

^{19}F (282 MHz) NMR spectrum of **4** DMSO-d_6

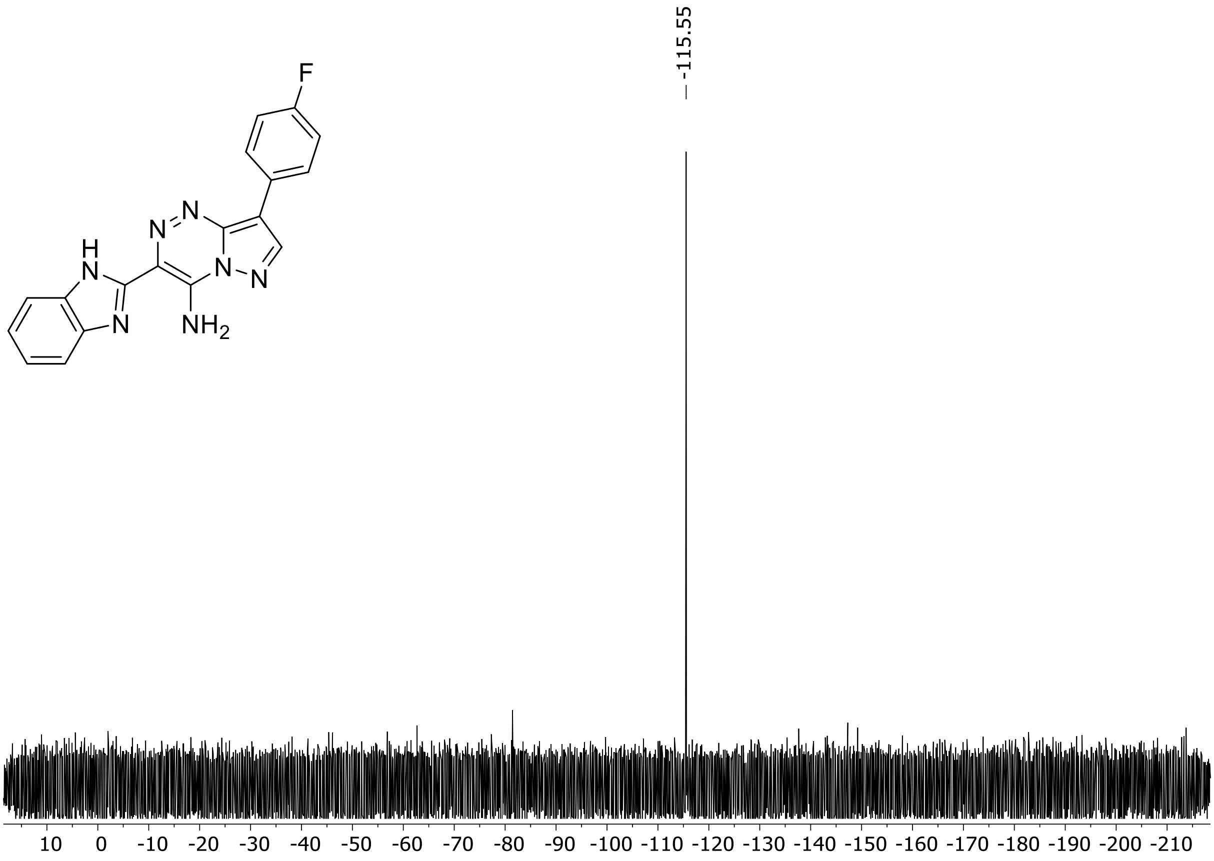

HRMS Spectra of **4**

$C_{18}H_{12}FN_7$ **mono m/z = 345,1138**

ESI - (MMI)
nitrogen flow 5 L/min, gas temperature 300°C, vaporizer 200°C,
nebulizer 30 psi, skimmer **40** V, fragmentor **60** V, dissolved in DMSO

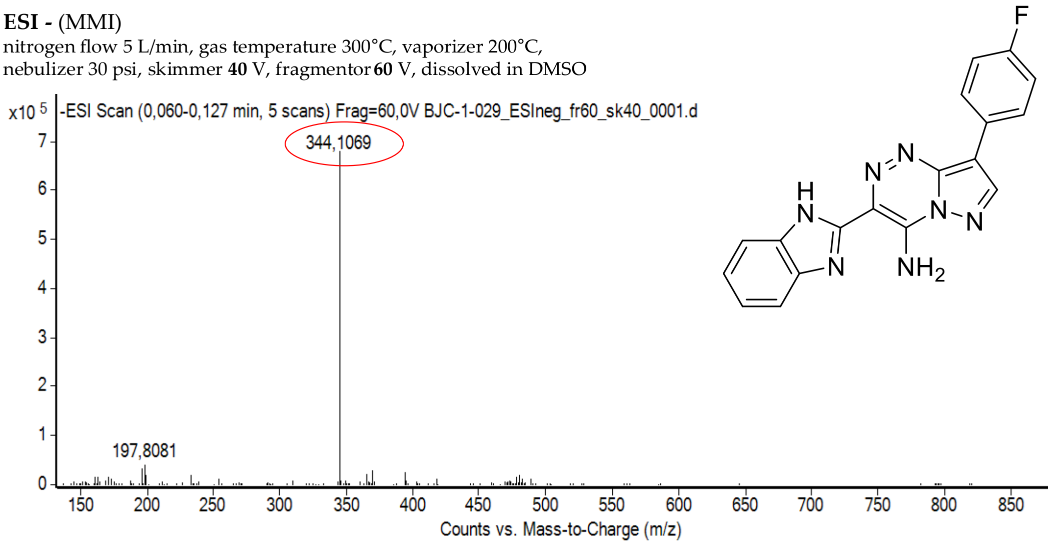

calculated mass: [M-H]$^-$ = 344,1065 observed: [M-H]$^-$ = 344,1069 max. mass error = 1,2 ppm

^{1}H (500 MHz) and ^{13}C NMR (126 MHz) spectra of **5** in DMSO-d_6

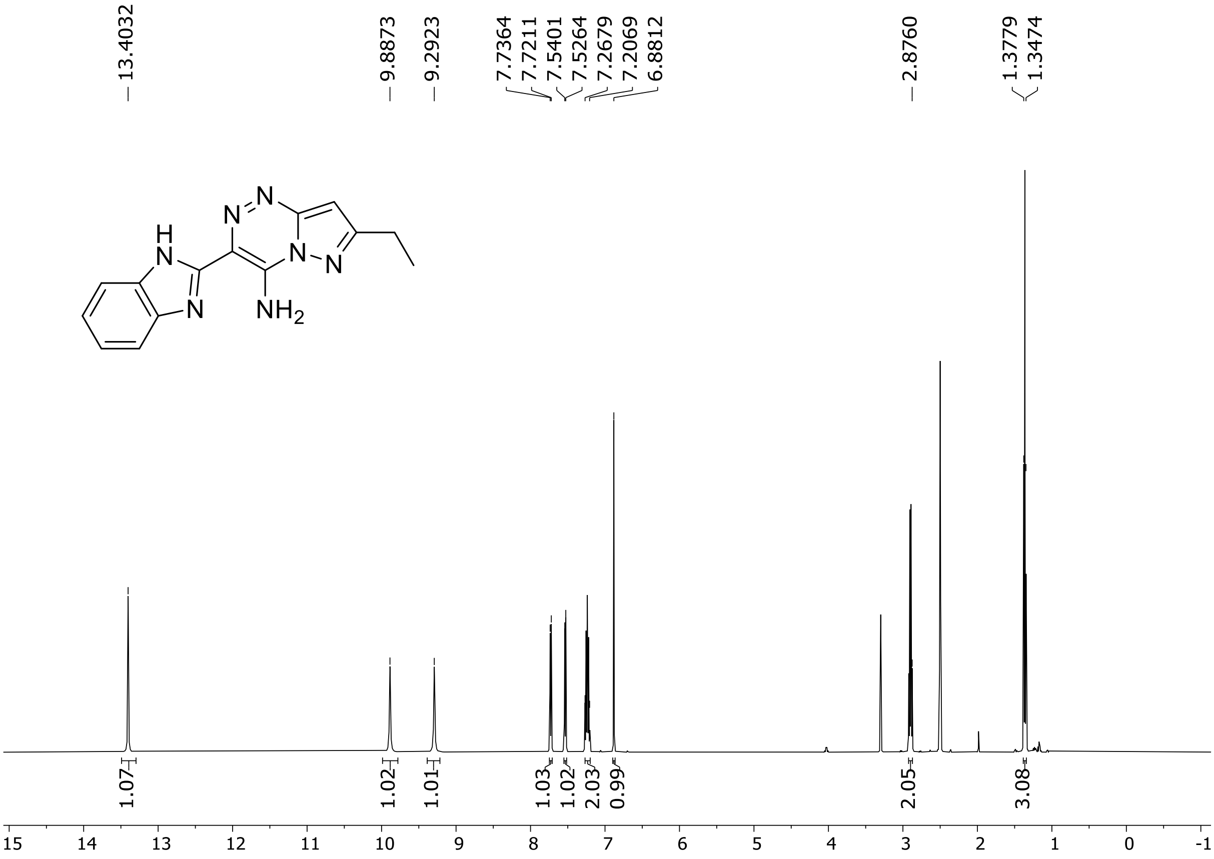

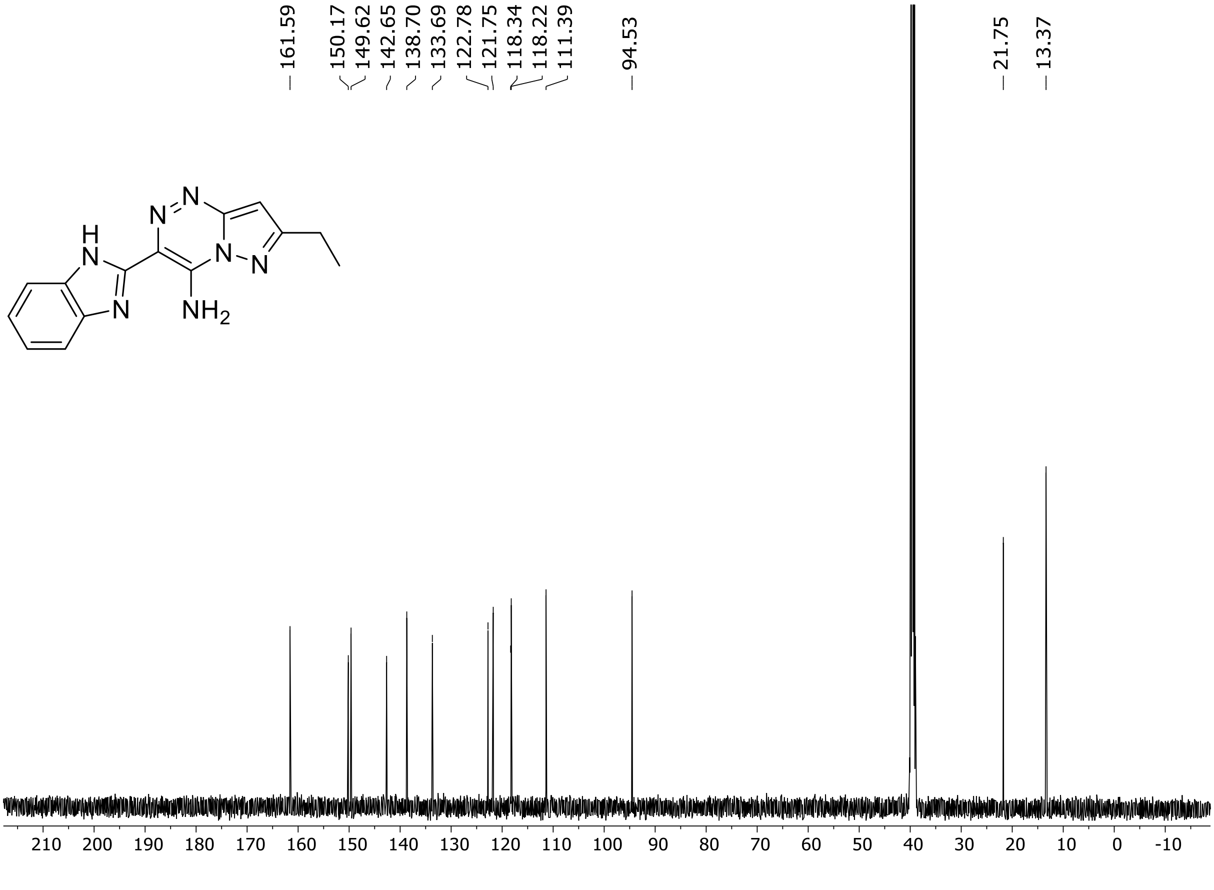

HRMS spectrum of **5**

$C_{14}H_{13}N_7$ **mono m/z = 279,1232**

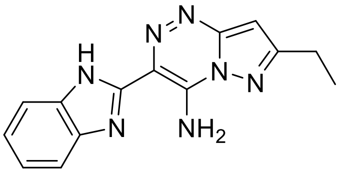

APCI - (MMI)
nitrogen flow 5 L/min, gas temperature 300°C, vaporizer 250°C,
nebulizer 45 psi, skimmer **65** V, fragmentor **30** V, dissolved in DMSO

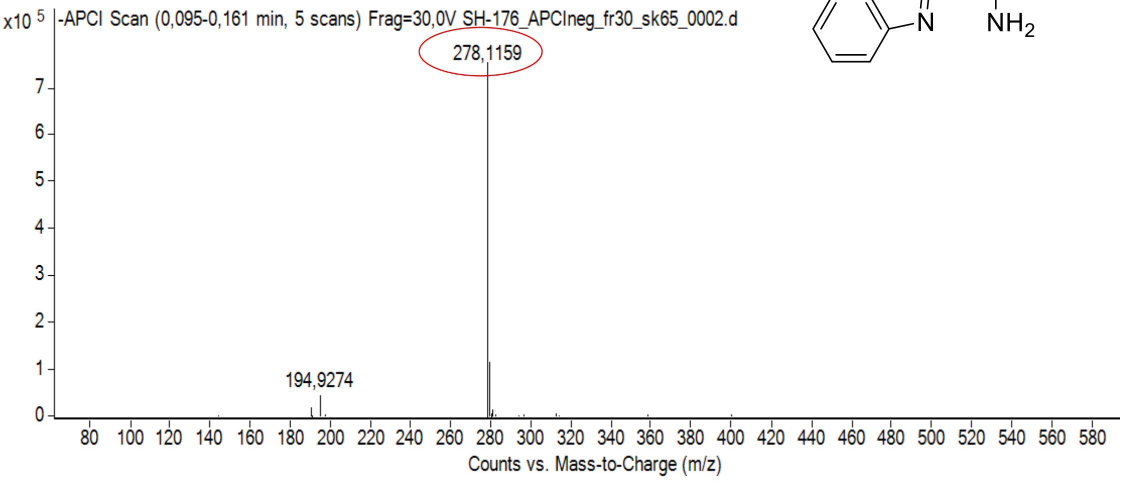

calculated mass: $[M\text{-}H]^-$ = 278,1160 observed: $[M\text{-}H]^-$ = 278,1159 max. mass error = 0,4 ppm

^{1}H (500 MHz) and ^{13}C NMR (126 MHz) spectra of **6** in DMSO-d_6

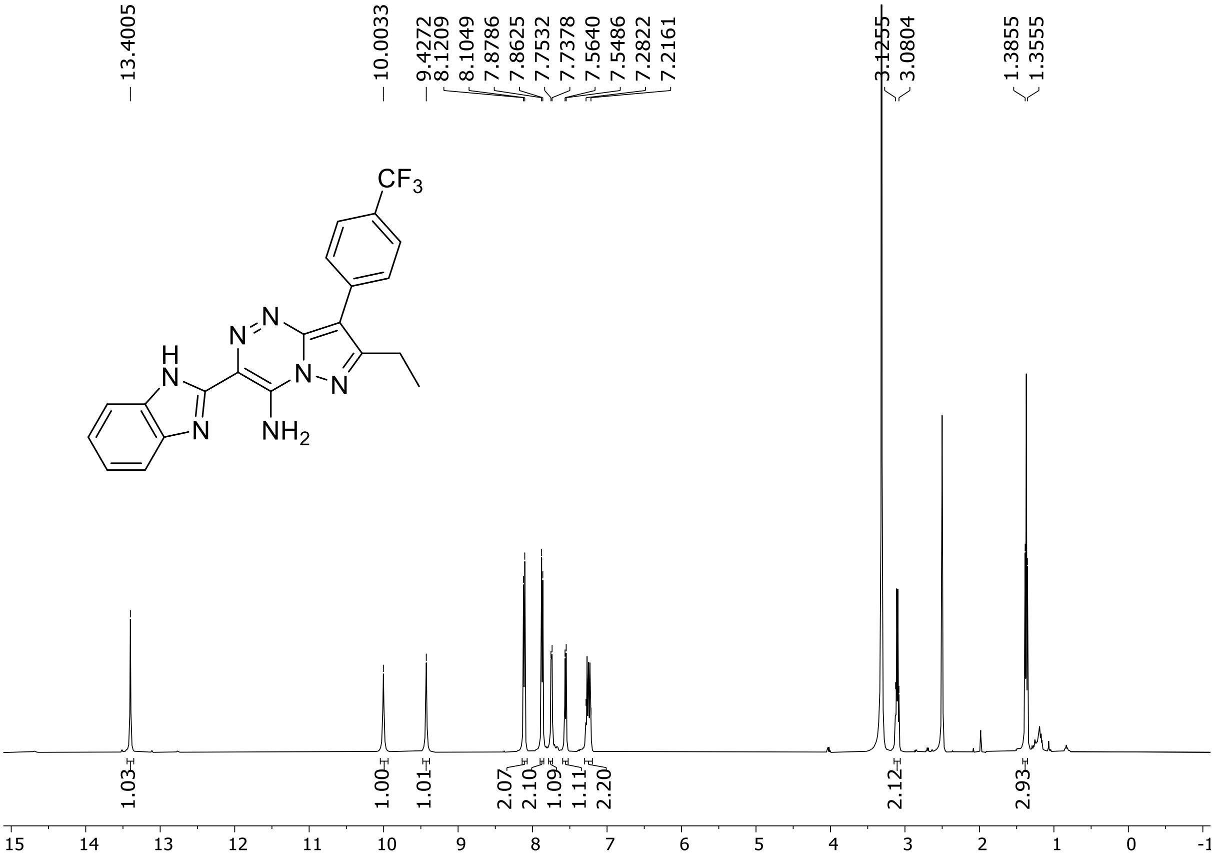

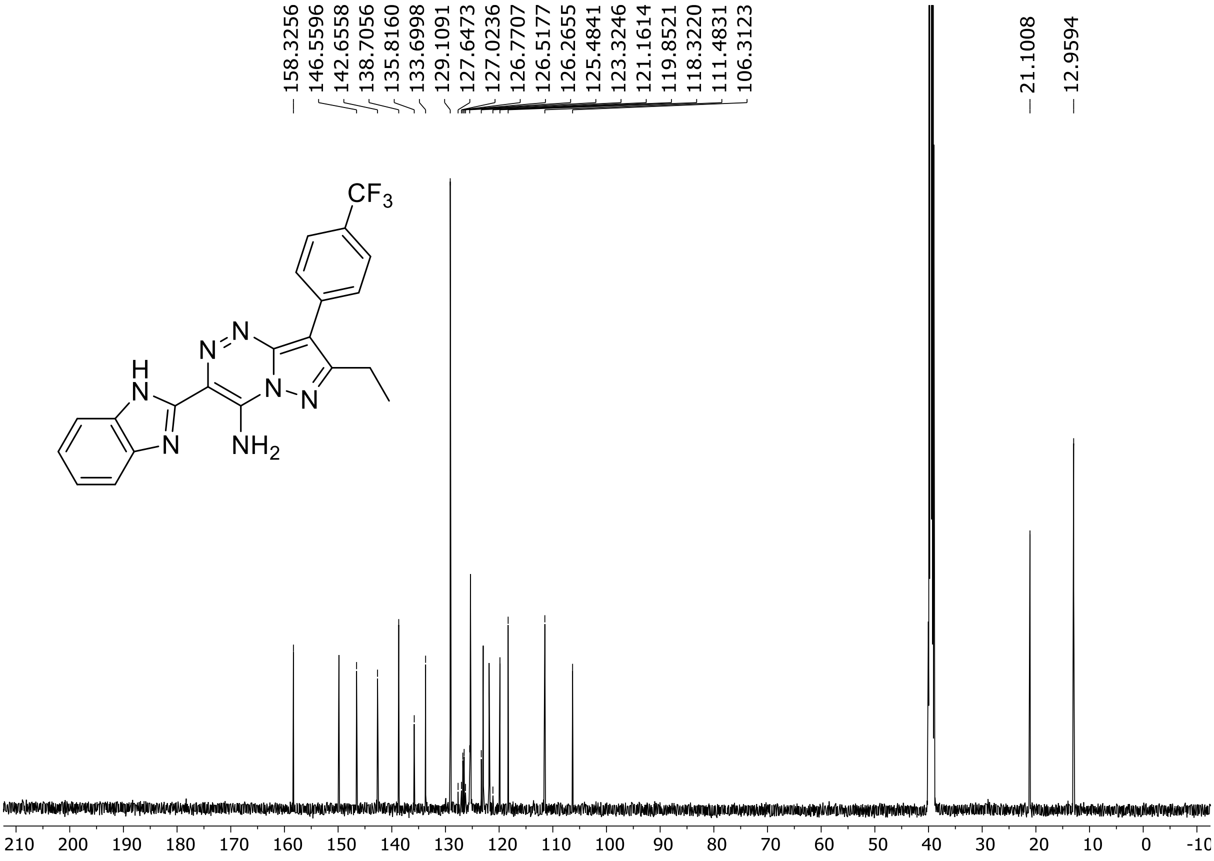

^{19}F (471 MHz) NMR spectrum of **6** DMSO-d_6

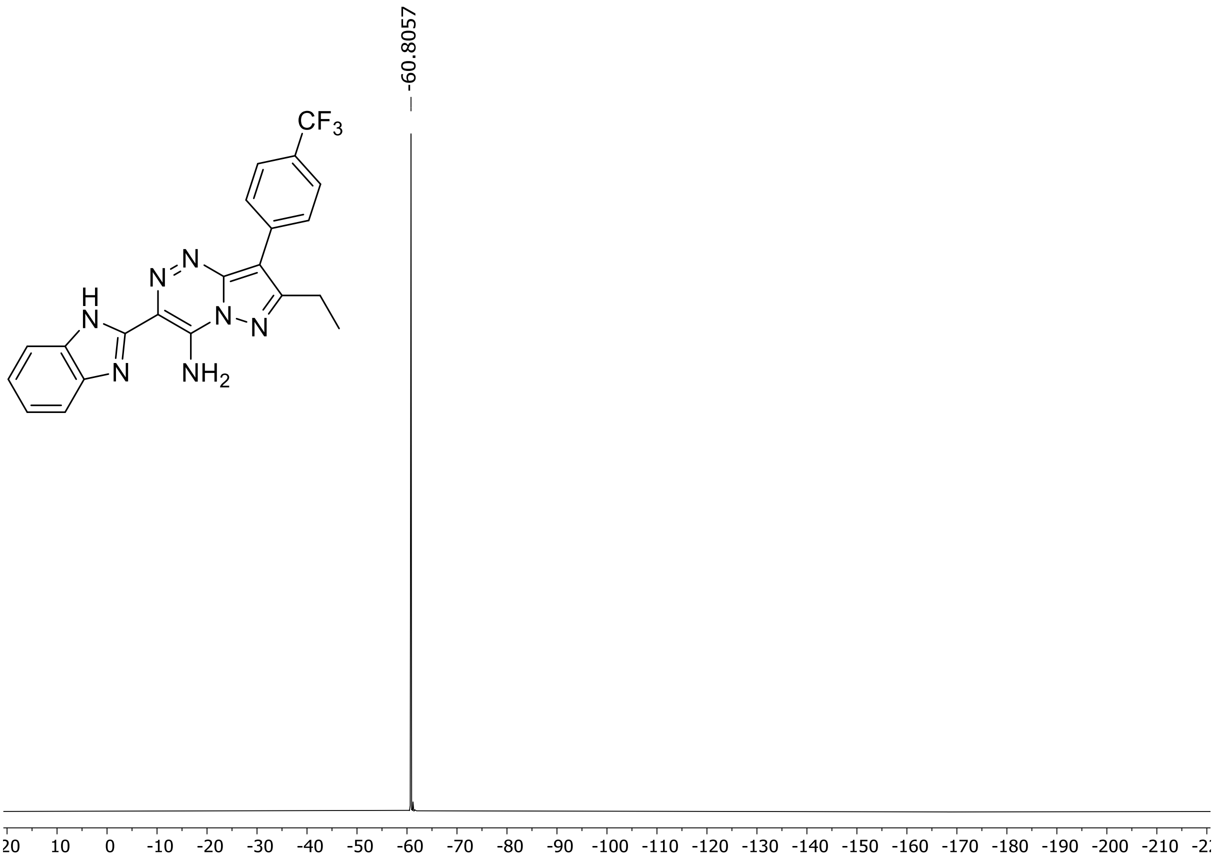

HRMS spectrum of **6**

$C_{21}H_{16}F_3N_7$ **mono *m/z* 423.1419**

APCI + (MMI)
nitrogen flow 5 L/min, gas temperature 325°C, nebulizer 45 psi, skimmer 65 V,
vaporizer 250°C, fragmentor 35 V, dissolved in methanol

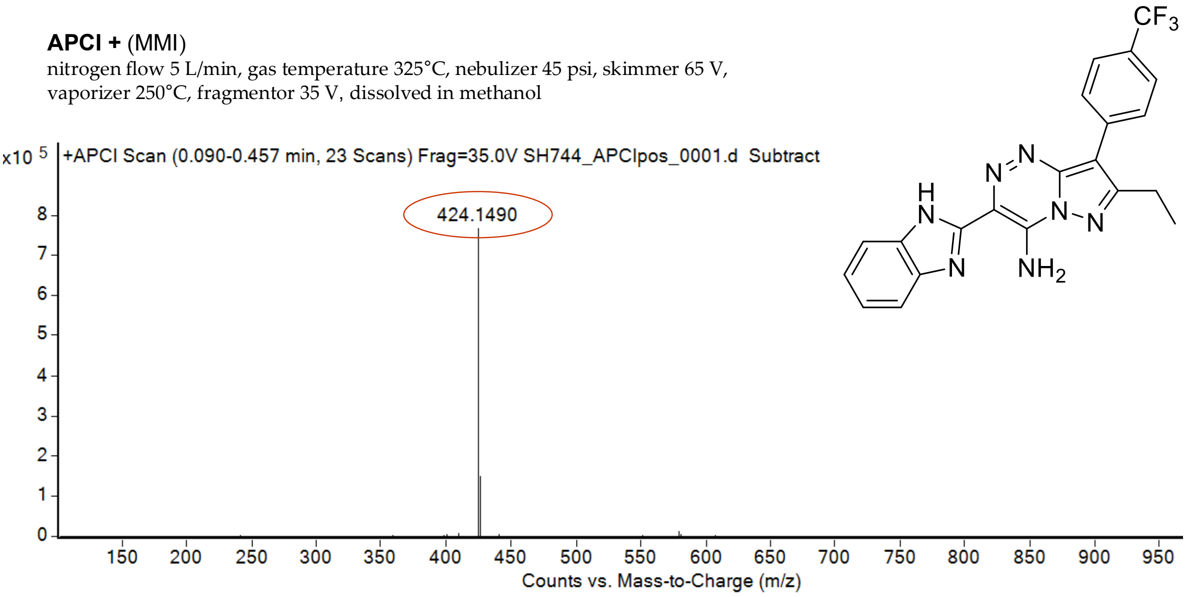

calculated mass: $[M+H]^+$ = 424.1492 observed: $[M+H]^+$ = 424.1490 mass accuracy = - 0.4 ppm

^{1}H (500 MHz) and ^{13}C NMR (126 MHz) spectra of **7** in DMSO-d_6

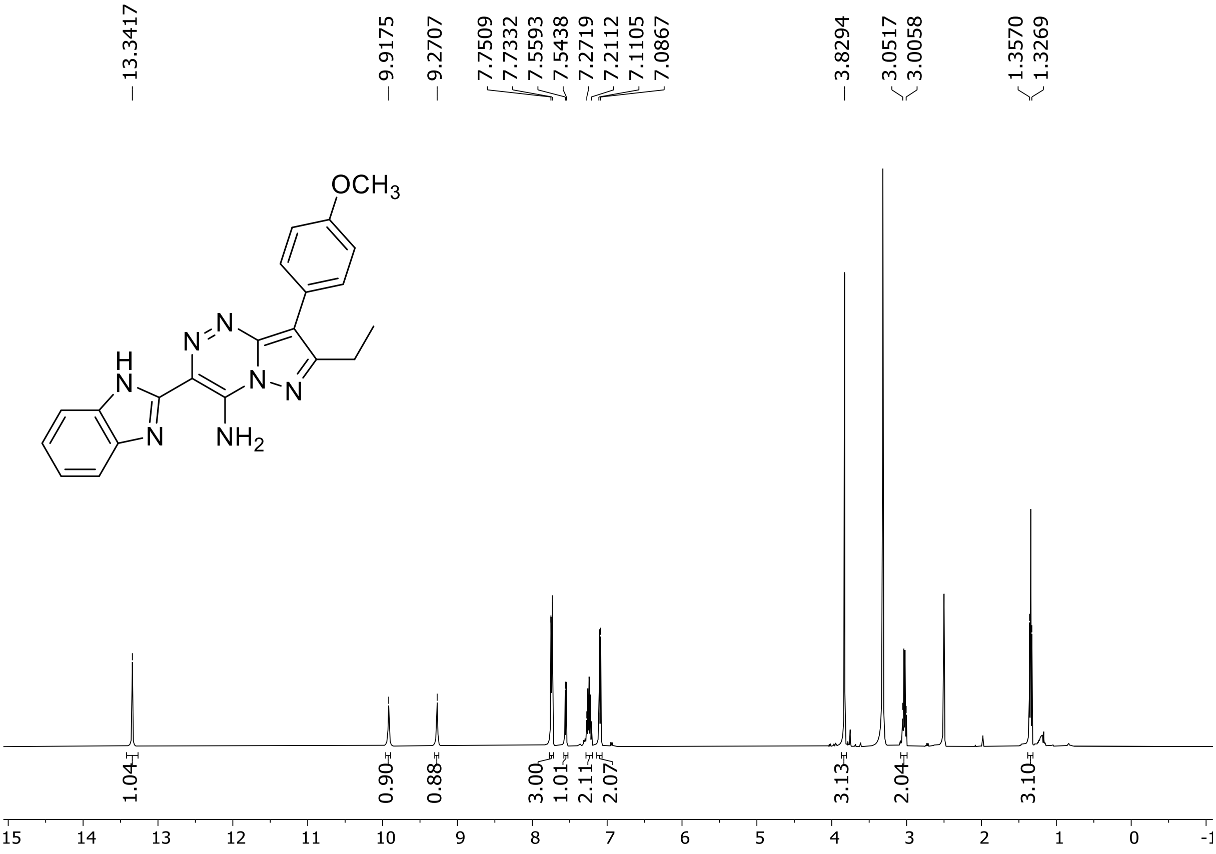

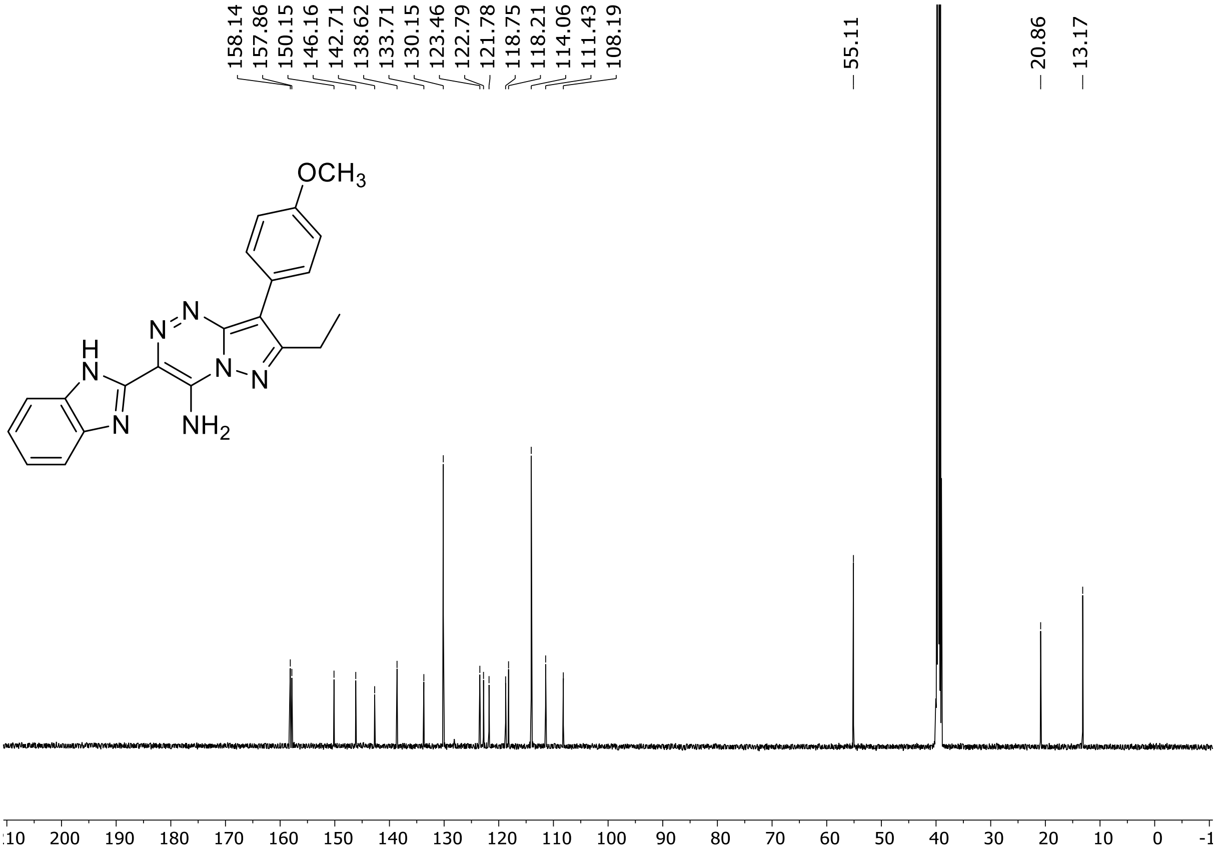

HRMS spectrum of **7**

$C_{21}H_{19}N_7O$ **mono *m/z* 385.1651**

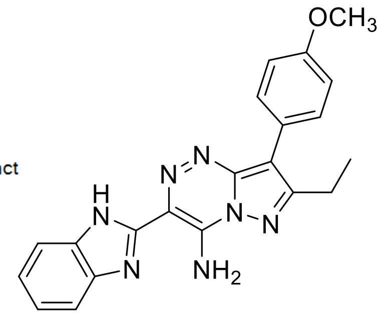

APCI + (MMI)
nitrogen flow 5 L/min, gas temperature 325°C, nebulizer 45 psi, skimmer 65 V,
vaporizer 250°C, fragmentor 45 V, dissolved in methanol

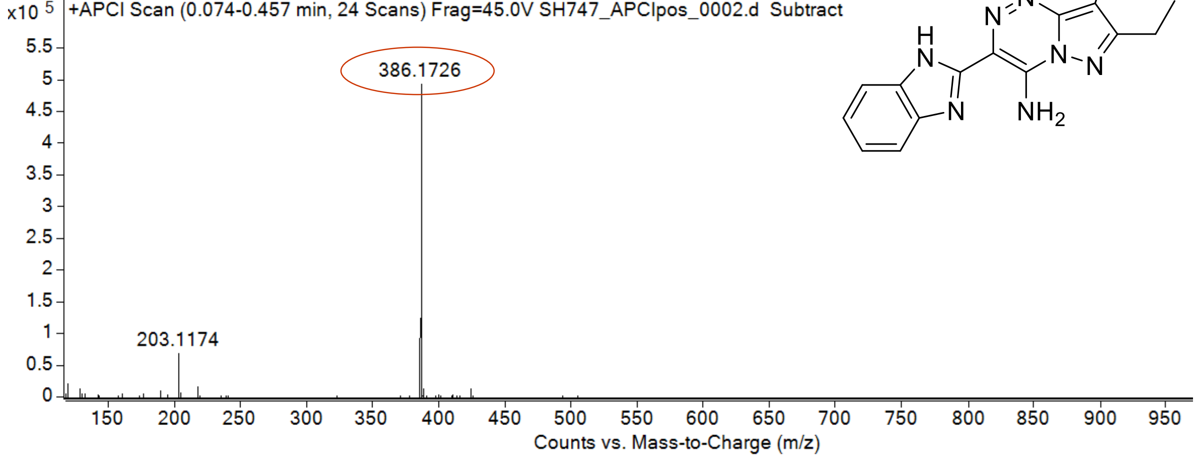

calculated mass: $[M+H]^+$ = 386.1724 observed: $[M+H]^+$ = 386.1726 mass accuracy = 0.5 ppm

^{1}H (500 MHz) and ^{13}C NMR (126 MHz) spectra of **8** in DMSO-d_6

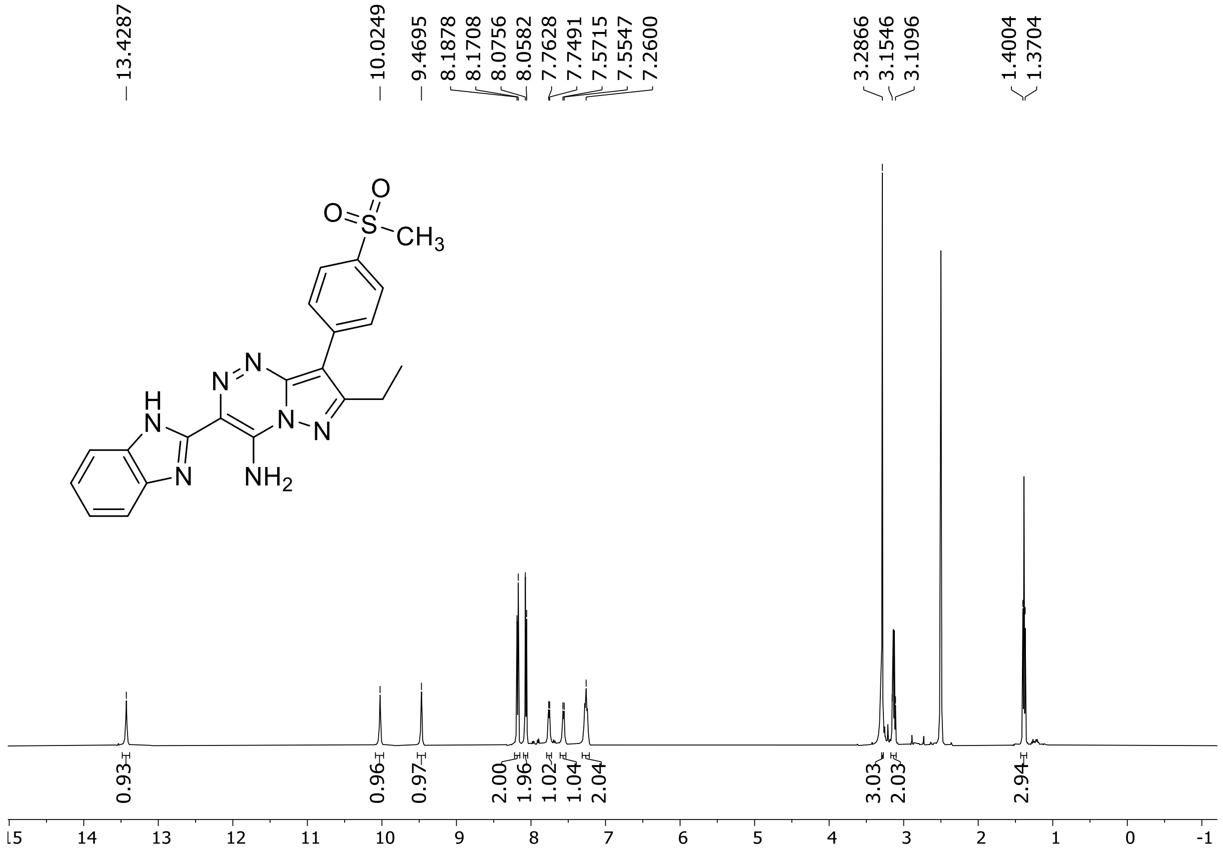

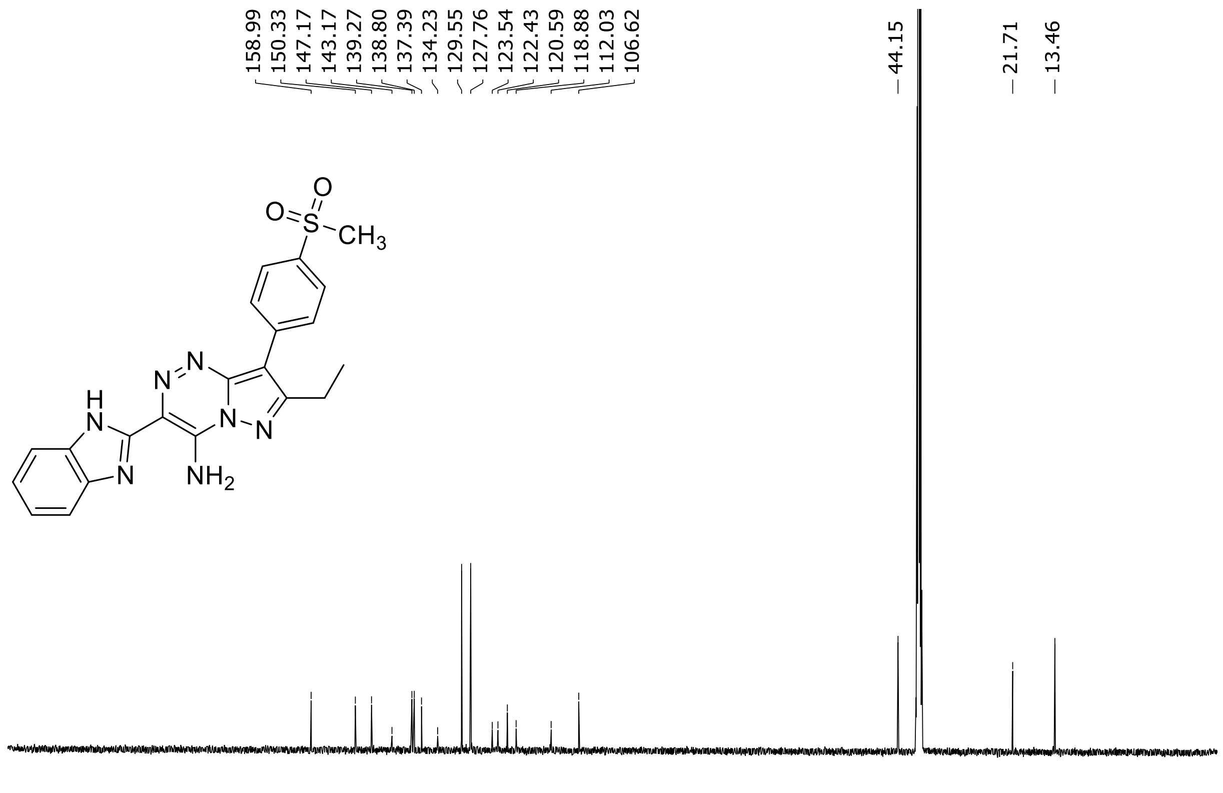

HRMS spectrum of **8**

$C_{21}H_{19}N_7O_2S$ mono *m/z* 433.1321

ESI - (MMI)
nitrogen flow 5 L/min, gas temperature 325°C, nebulizer 45 psi, skimmer 65 V, capillary voltage 2500V, fragmentor 200 V, dissolved in methanol

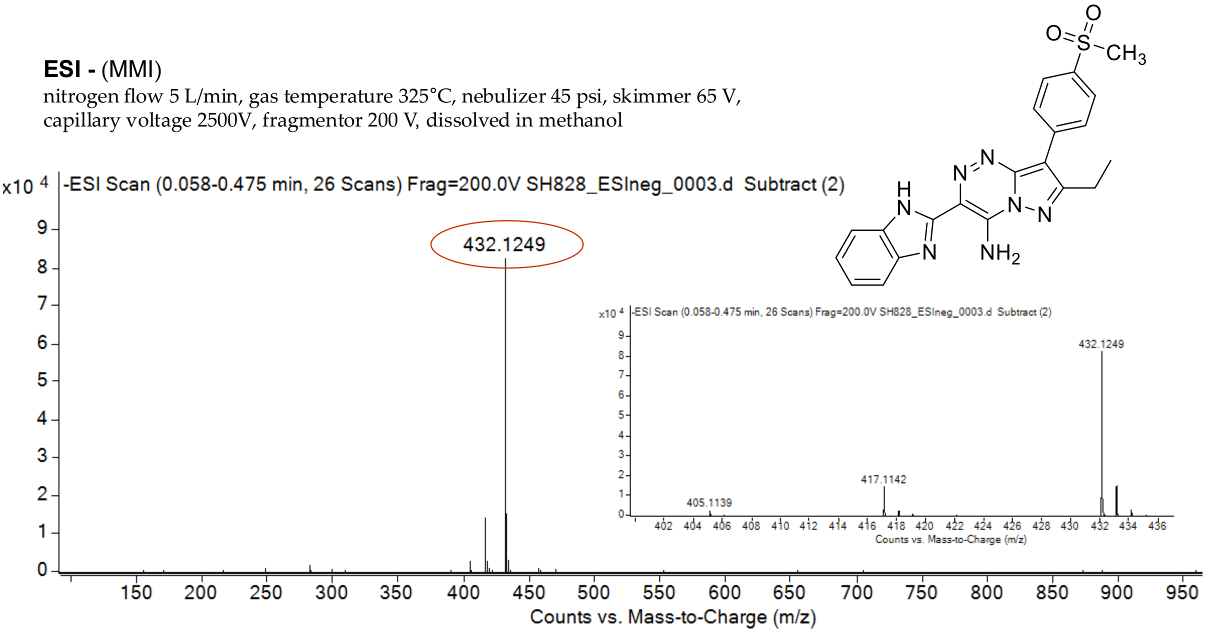

calculated mass: $[M-H]^-$ = 432.1248 observed: $[M-H]^-$ = 432.1249 mass accuracy = 0.2 ppm

^{1}H (500 MHz) and ^{13}C NMR (126 MHz) spectra of **9** in DMSO-d_6

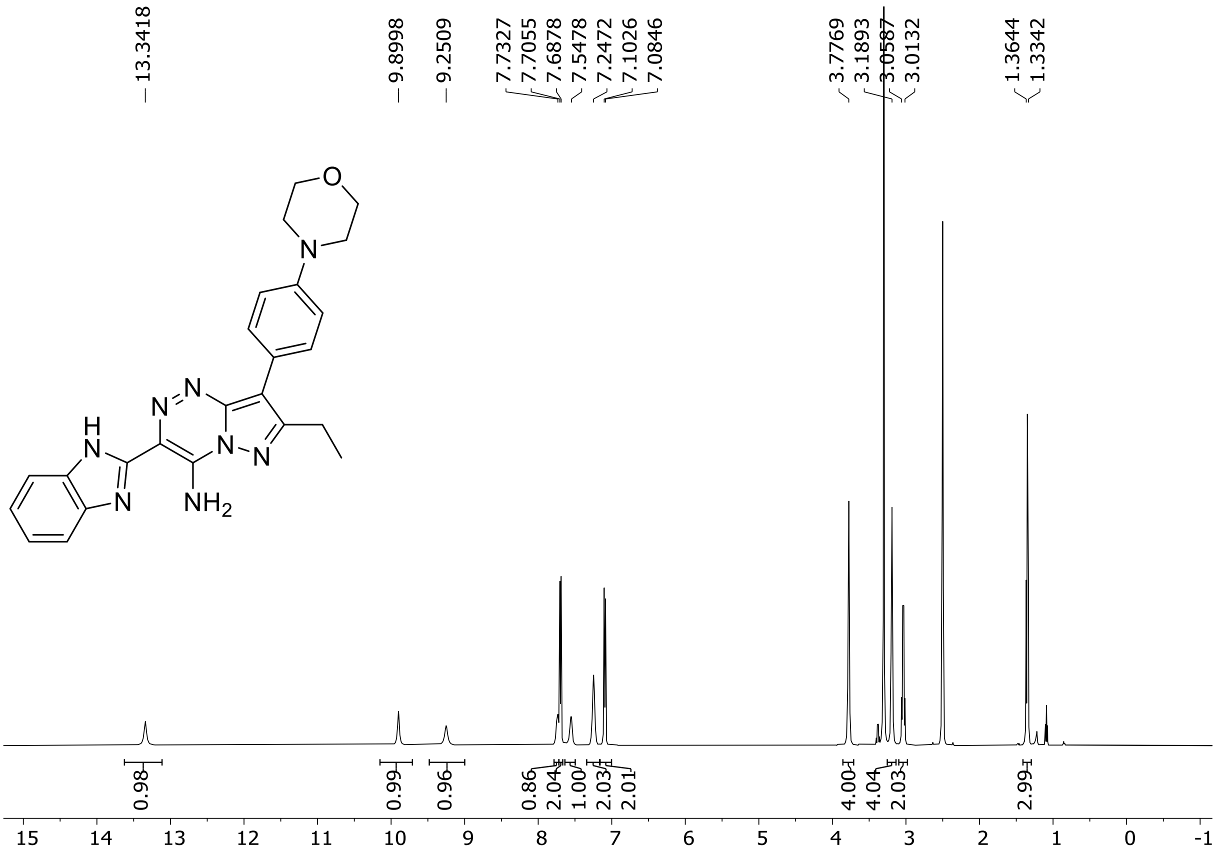

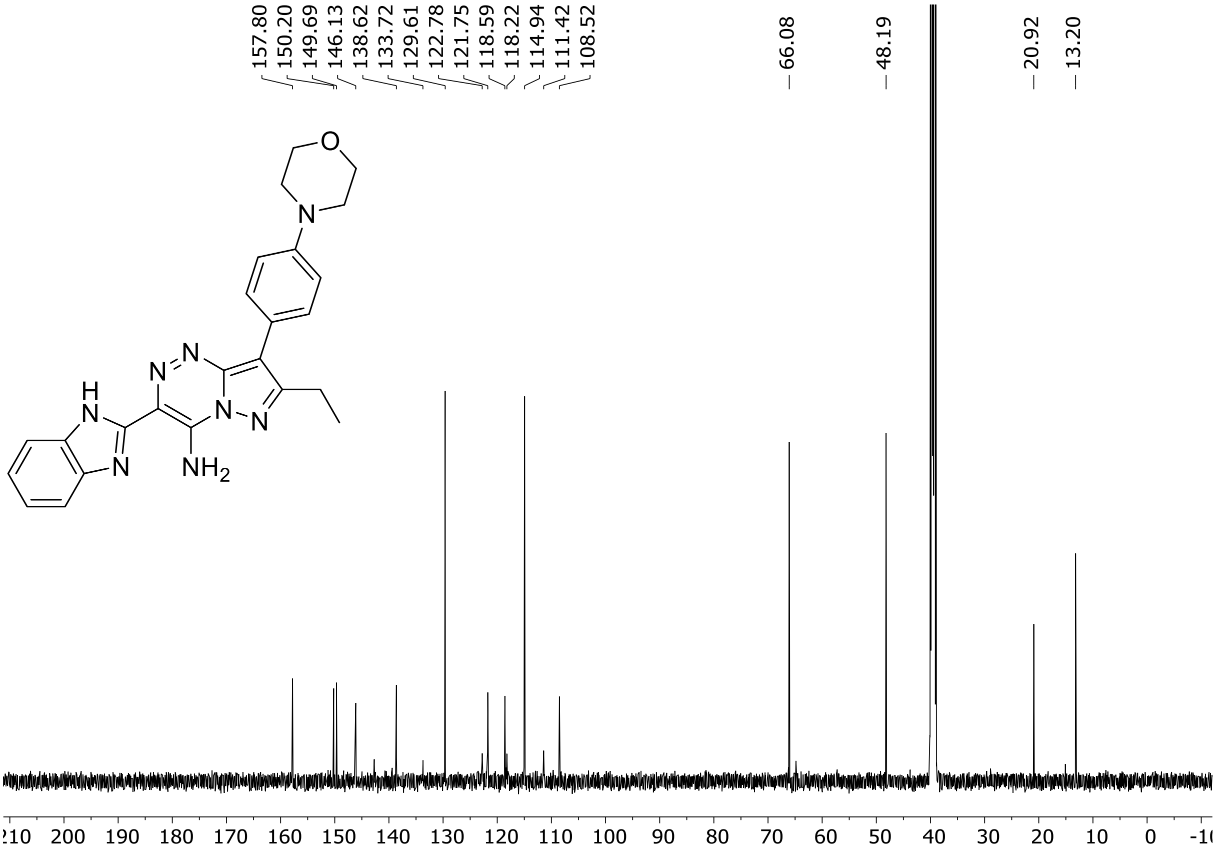

HRMS spectrum of **9**

$C_{24}H_{24}N_8O$ **mono *m/z* 440.2073**

ESI - (MMI)
nitrogen flow 5 L/min, gas temperature 325°C, nebulizer 45 psi, skimmer **65** V, capillary voltage 2500V, fragmentor 35 V, dissolved in methanol

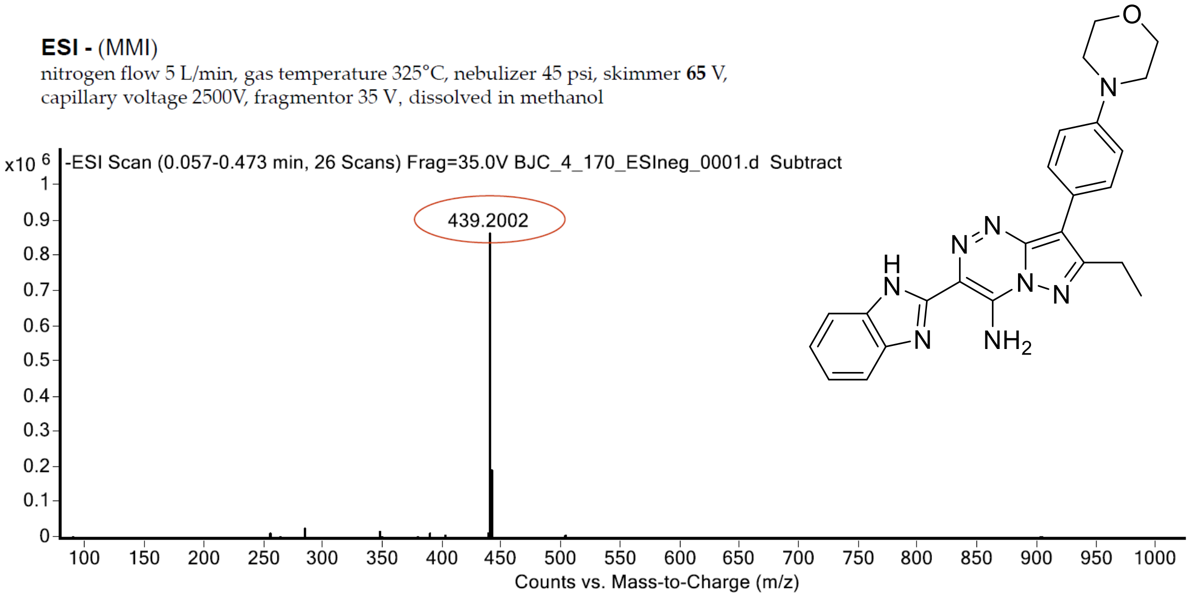

calculated mass: $[M\text{-}H]^-$ = 439.2000 observed: $[M\text{-}H]^-$ = 439.2002 mass accuracy = 0.4 ppm

^{1}H (500 MHz) and ^{13}C NMR (126 MHz) spectra of **10** in DMSO-d_6

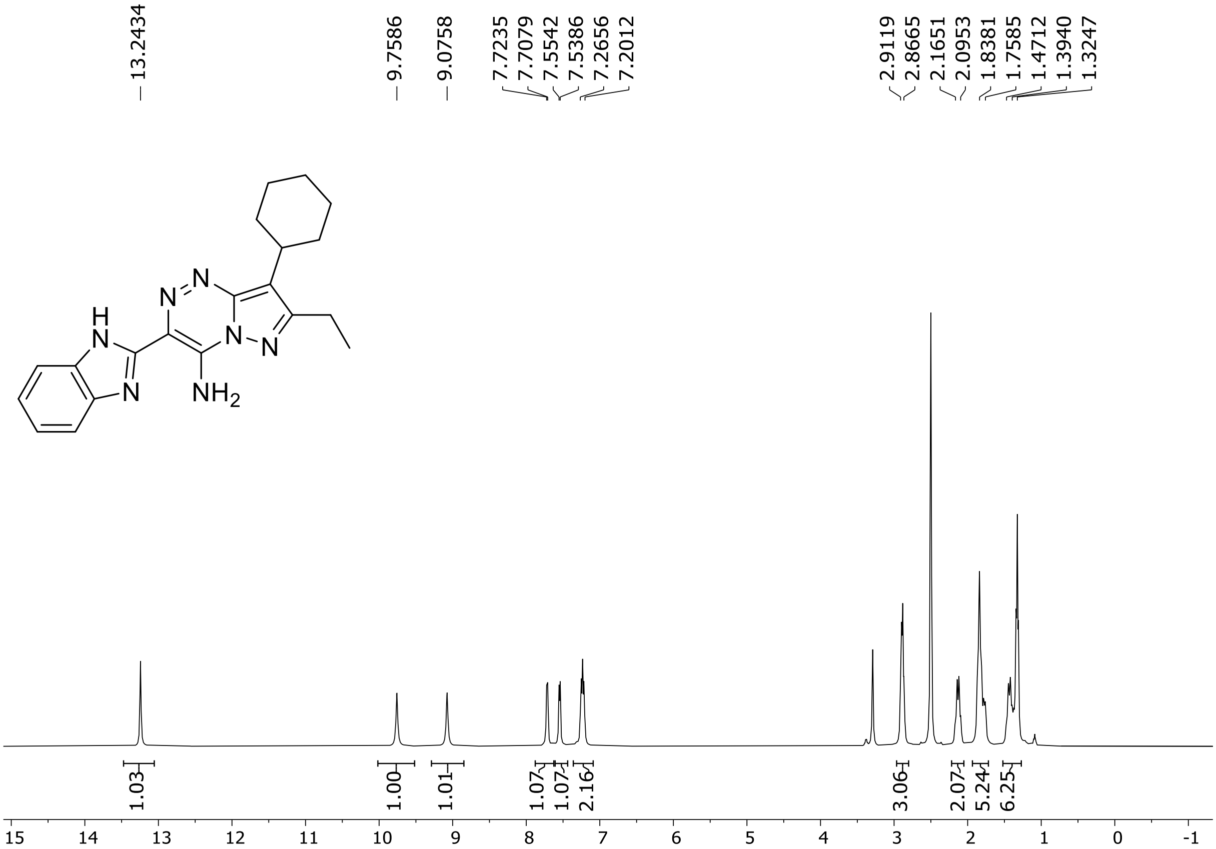

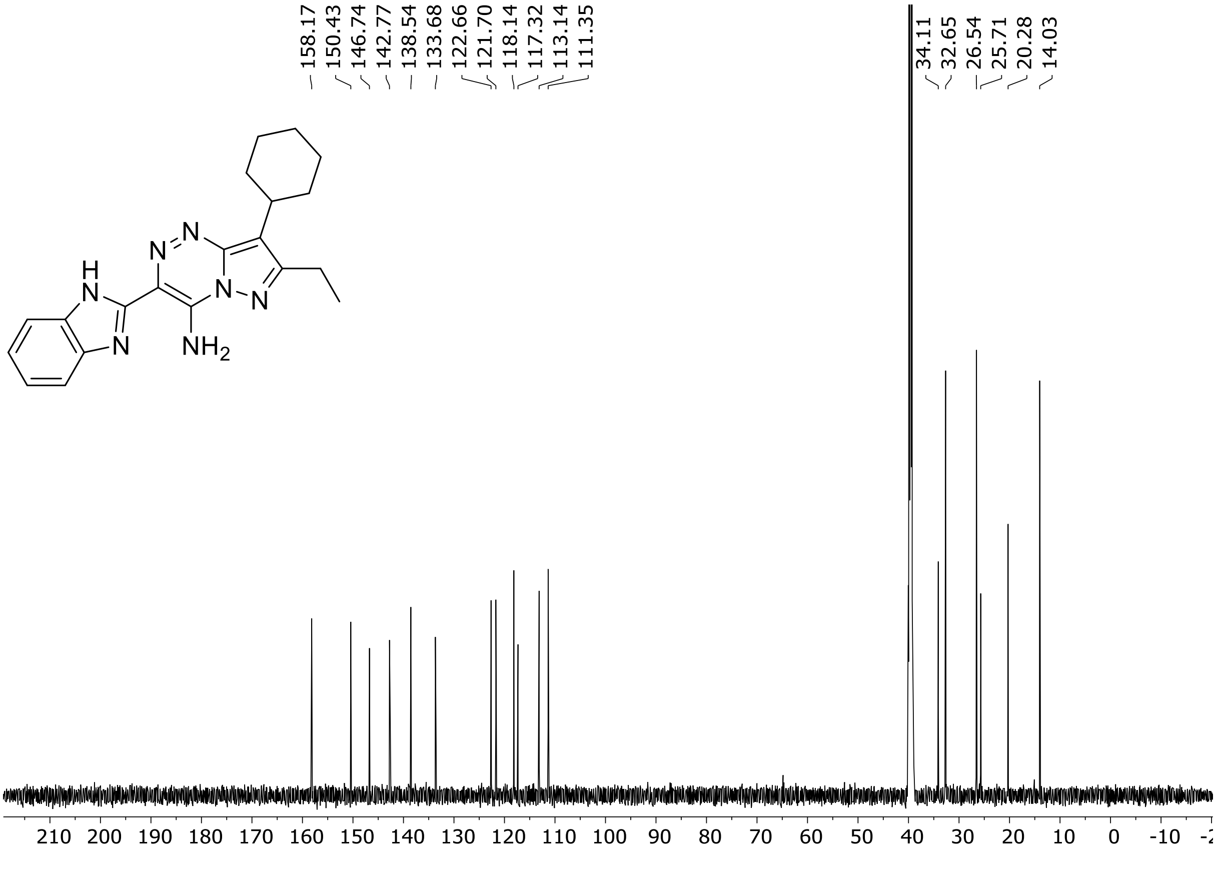

HRMS spectrum of **10**

$C_{20}H_{23}N_7$ **exact mass: 361.2015**

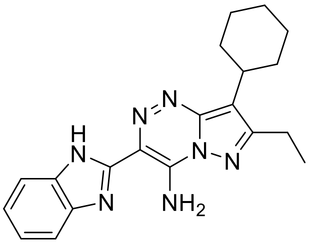

APCI + (MMI)
nitrogen flow 3 L/min, gas temperature 325°C, nebulizer 45 psig, skimmer 65 V, vaporizer 200°C, fragmentor 10 V, dissolved in methanol

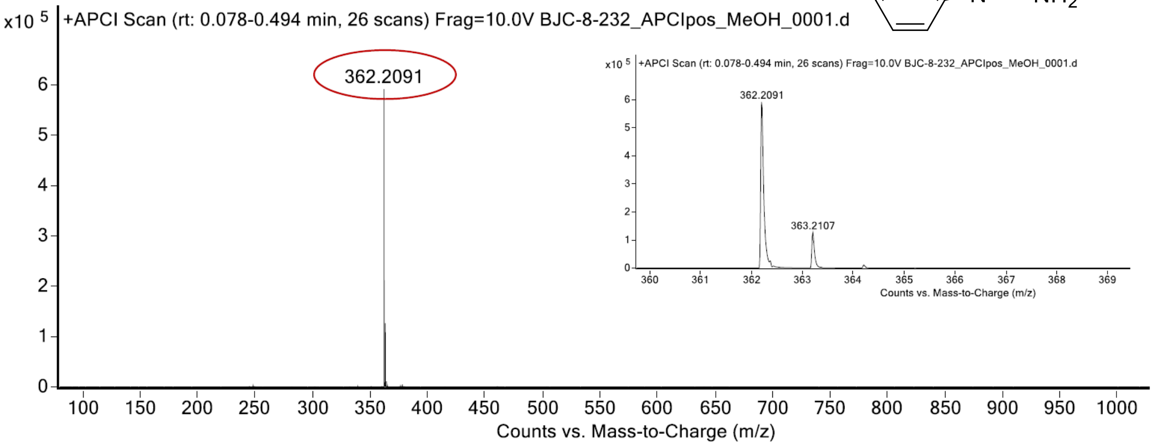

expected mass: $[M+H]^+$ = 362.2088 observed mass: $[M+H]^+$ = 362.2091 mass accuracy = 0.8 ppm

^{1}H (500 MHz) and ^{13}C NMR (126 MHz) spectra of **11** in DMSO-d_6

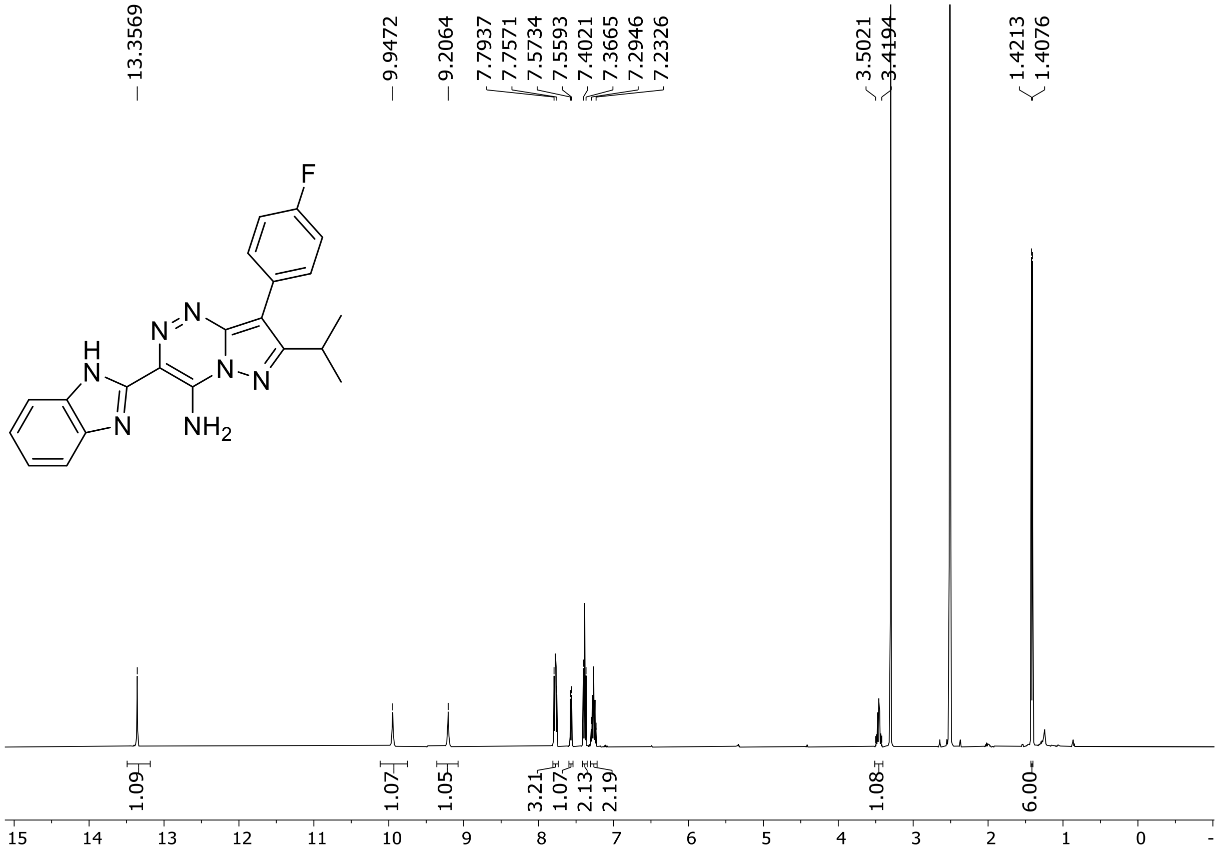

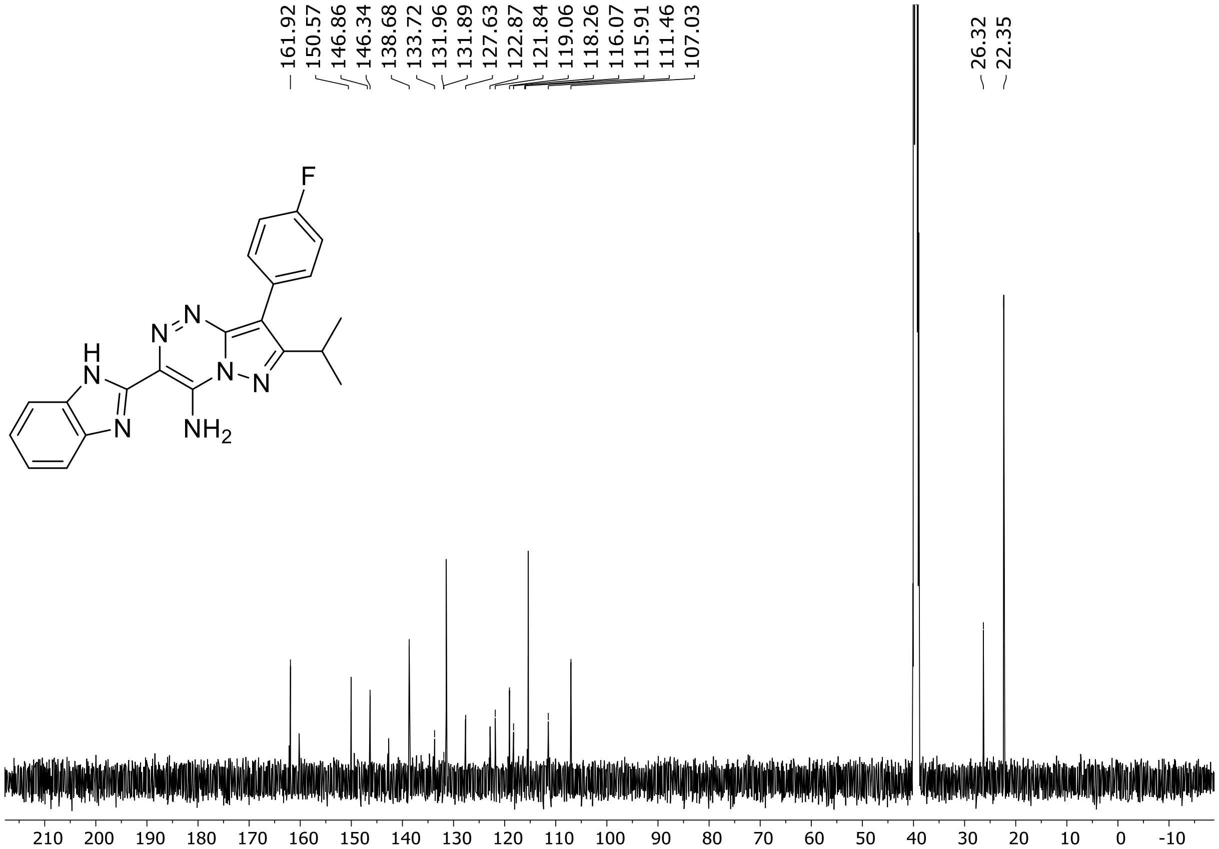

HRMS spectrum of **11**

$C_{21}H_{18}FN_7$ **mono m/z = 387.1602**

ESI - (MMI)
nitrogen flow 5 L/min, gas temperature 300°C, vaporizer 250°C,
nebulizer 45 psi, skimmer **70** V, fragmentor 40 V, dissolved in DMSO

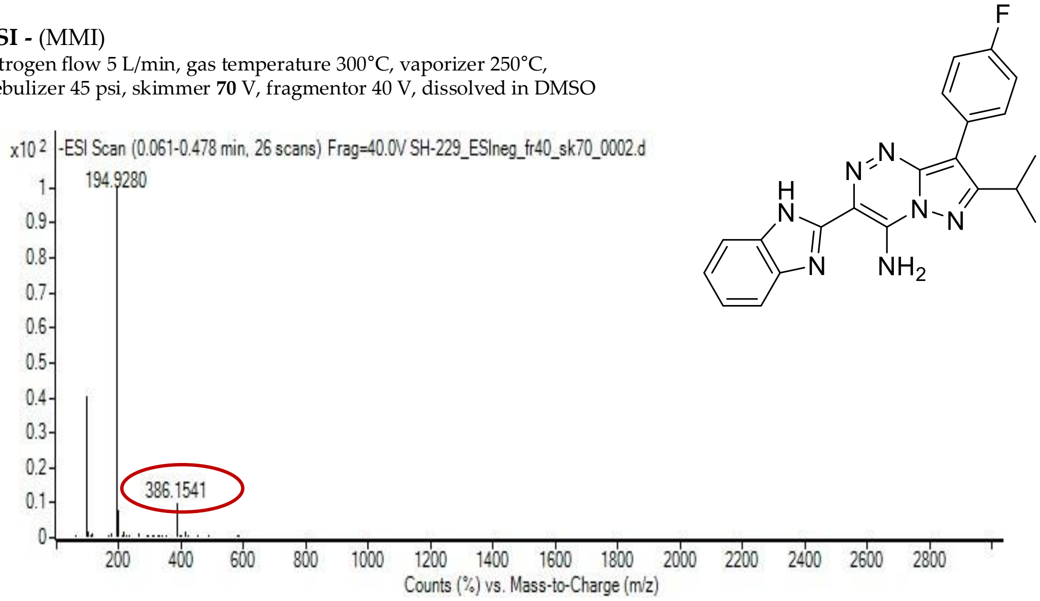

calculated mass: [M-H]$^-$ = 386.1535 observed: [M-H]$^-$ = 386.1541 m ax. mass error = 1.6 ppm

^{1}H (500 MHz) and ^{13}C NMR (126 MHz) spectra of **12** in DMSO-d_6

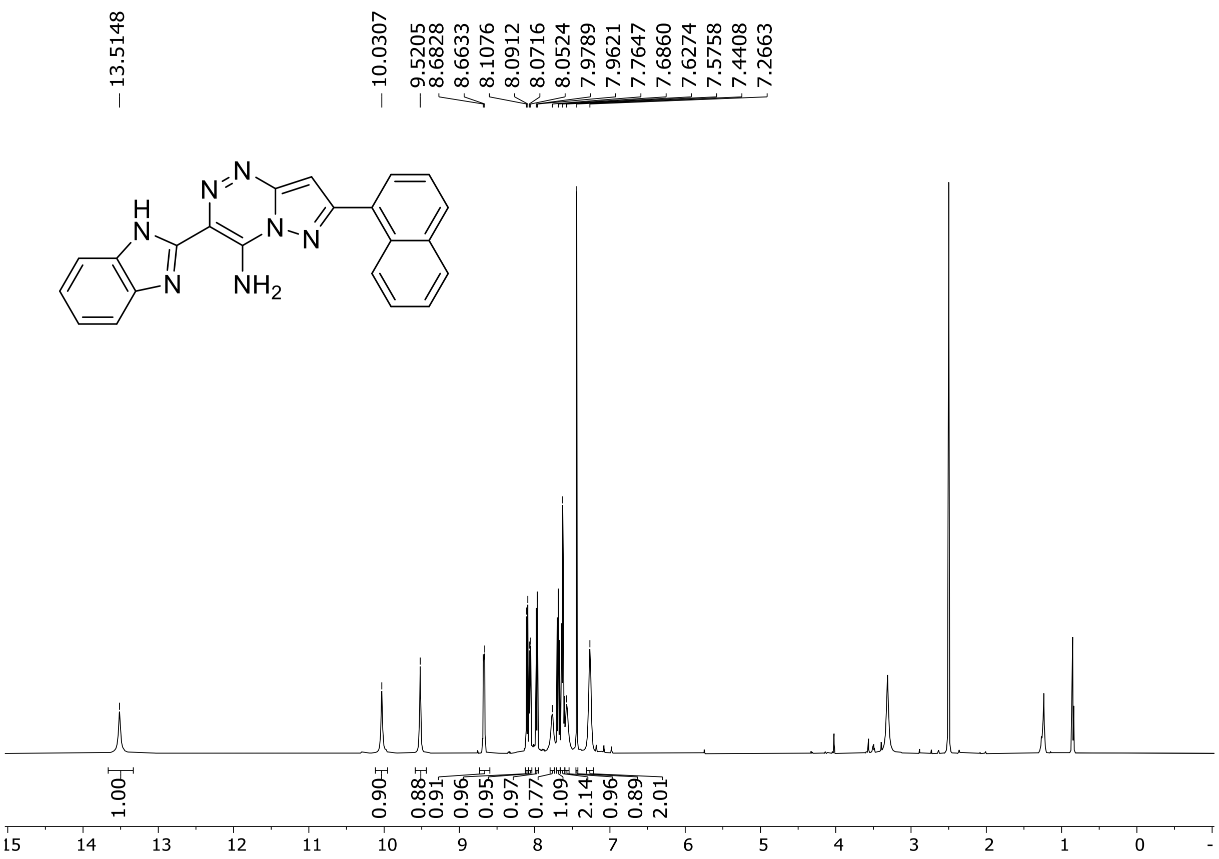

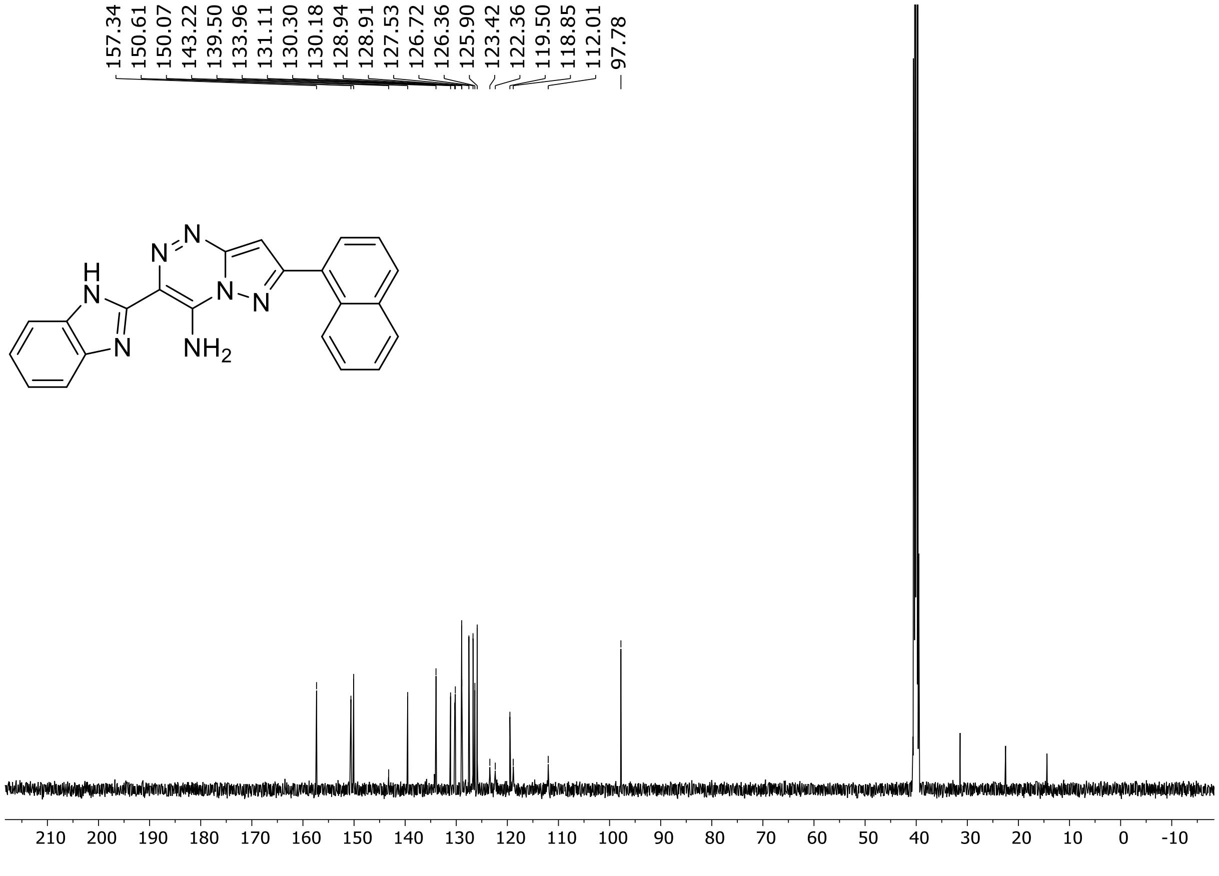

HRMS spectrum of **12**

$C_{22}H_{15}N_7$ **mono m/z = 377.1389**

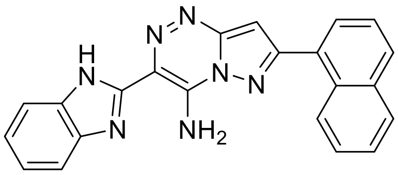

APCI + (MMI)
nitrogen flow 5 L/min, gas temperature 300°C, nebulizer 45 psi, vaporizer 200°C
skimmer **65** V, fragmentor **23** V, dissolved in methanol

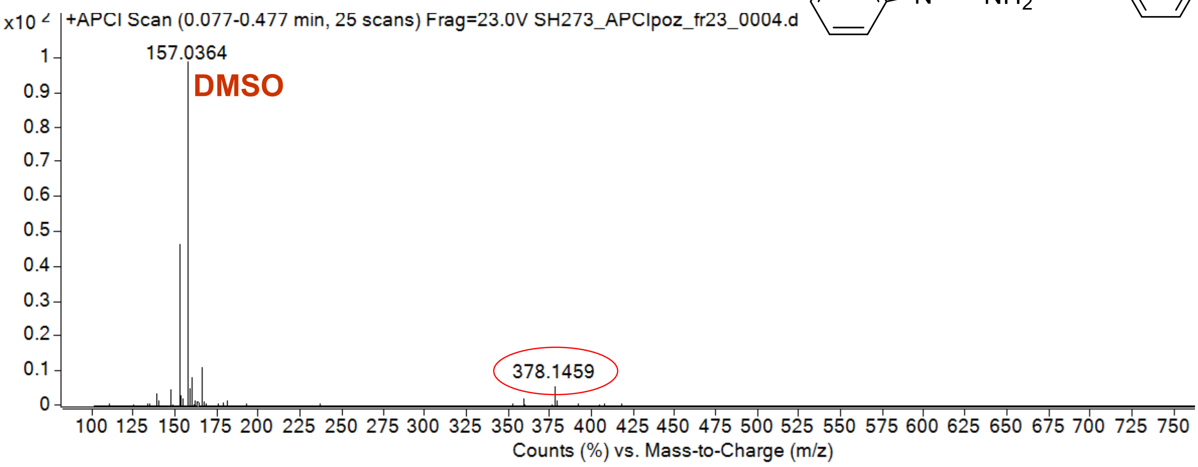

calculated mass: $[M+H]^+$ = 378.1462 observed: $[M+H]^+$ = 378.1459 max. mass error = 0.7 ppm

^{1}H (500 MHz) and ^{13}C NMR (126 MHz) spectra of **13** in DMSO-d_6

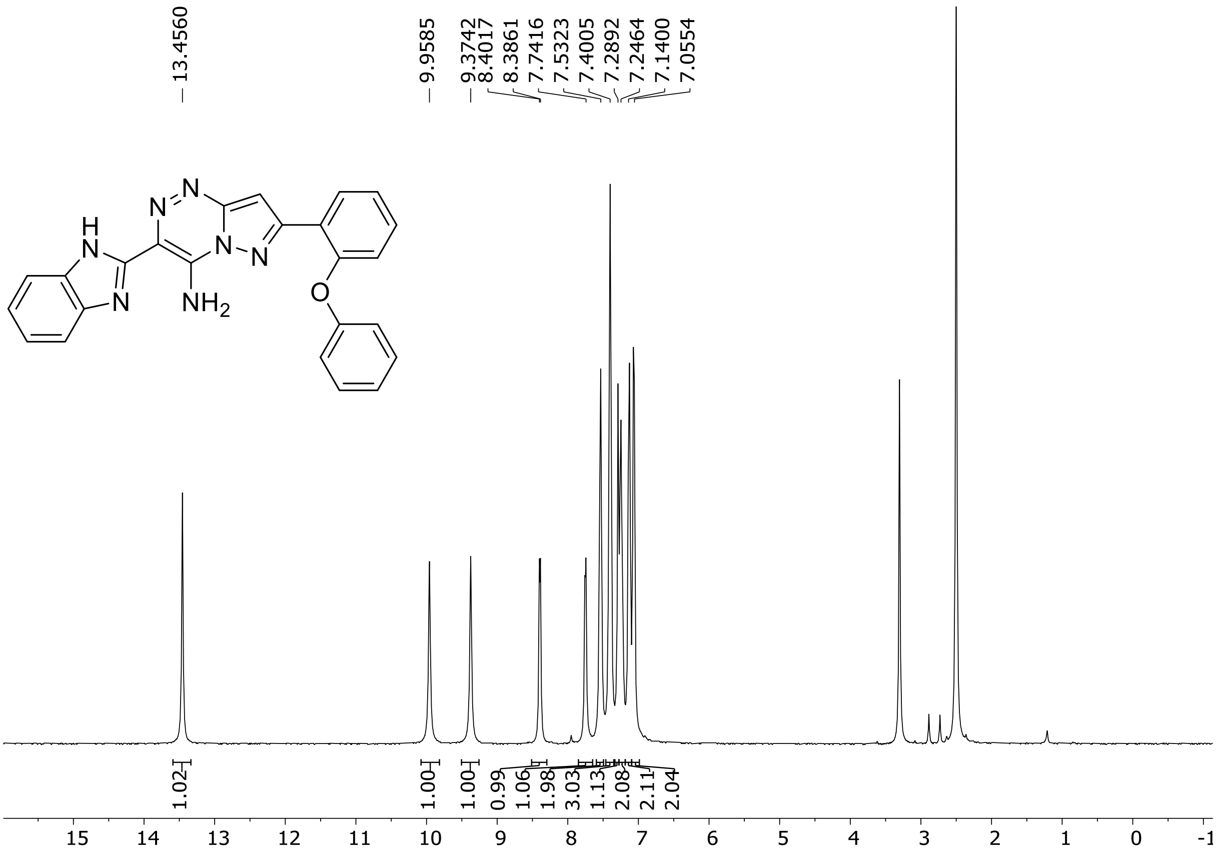

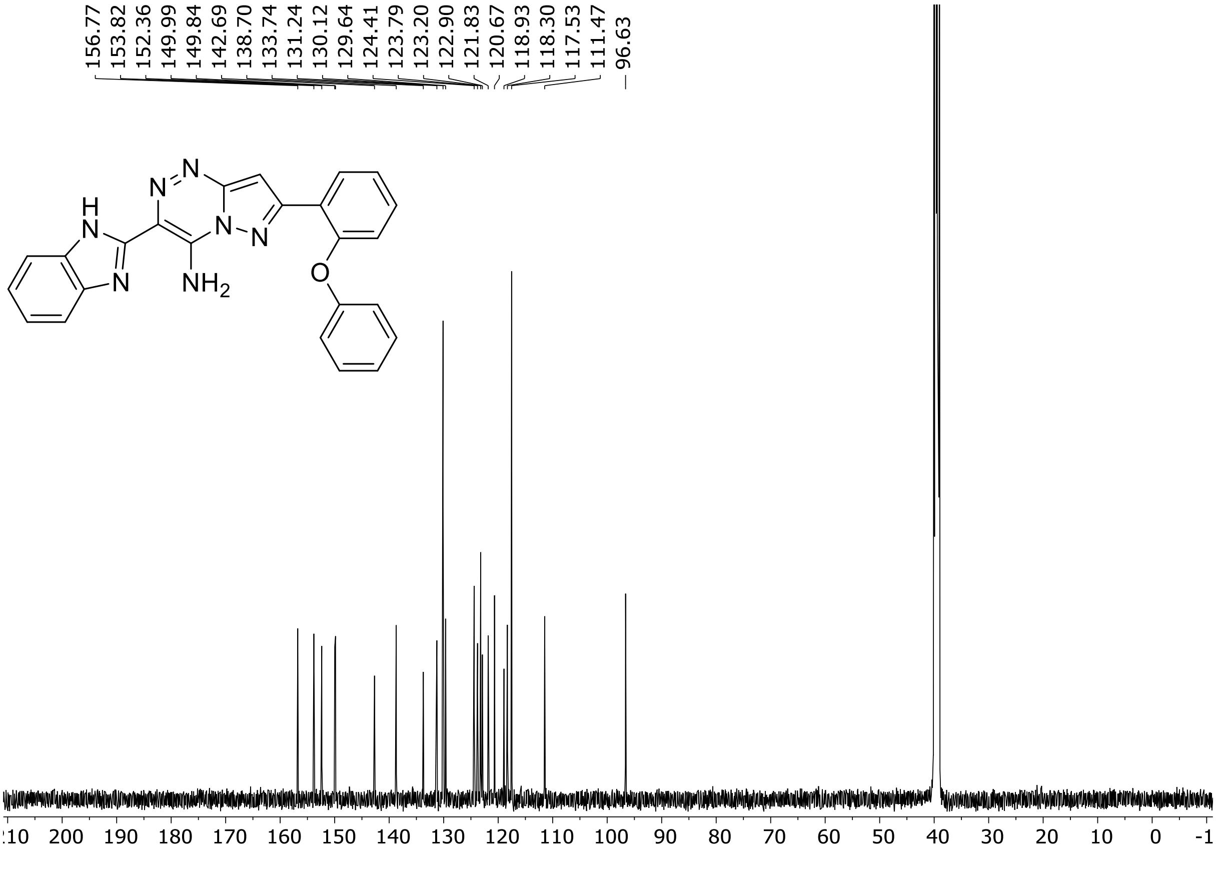

HRMS spectrum of **13**

$C_{24}H_{17}N_7O$ **mono m/z = 419.1495**

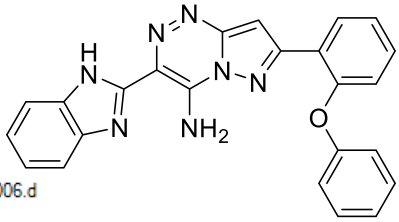

ESI + (MMI)
nitrogen flow 5 L/min, gas temperature 300°C, nebulizer 45 psi, skimmer 60 V, fragmentor 30 V, dissolved in methanol

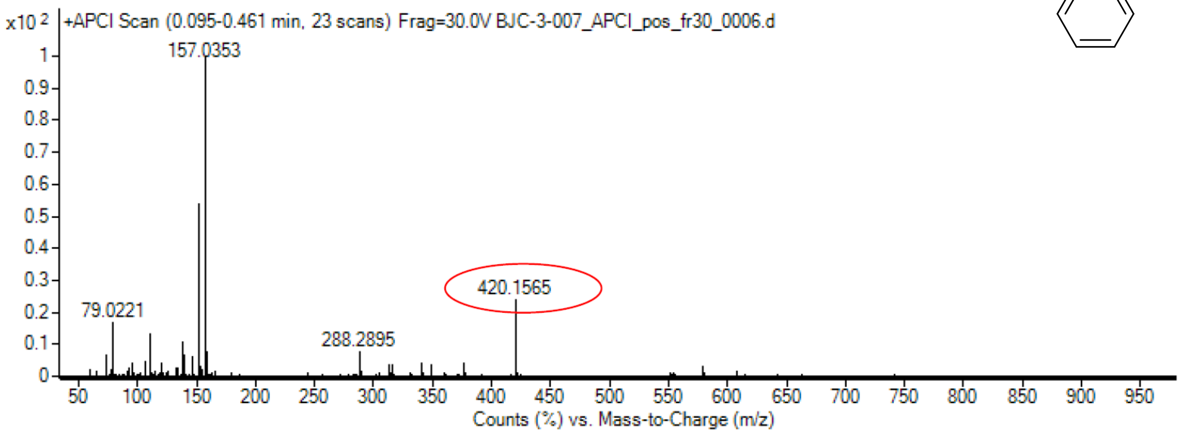

calculated mass: $[M+H]^+$ = 420.1567 observed: $[M+H]^+$ = 420.1565 max. mass error = 0.5 ppm

^{1}H (500 MHz) and ^{13}C NMR (126 MHz) spectra of **14** in DMSO-d_6

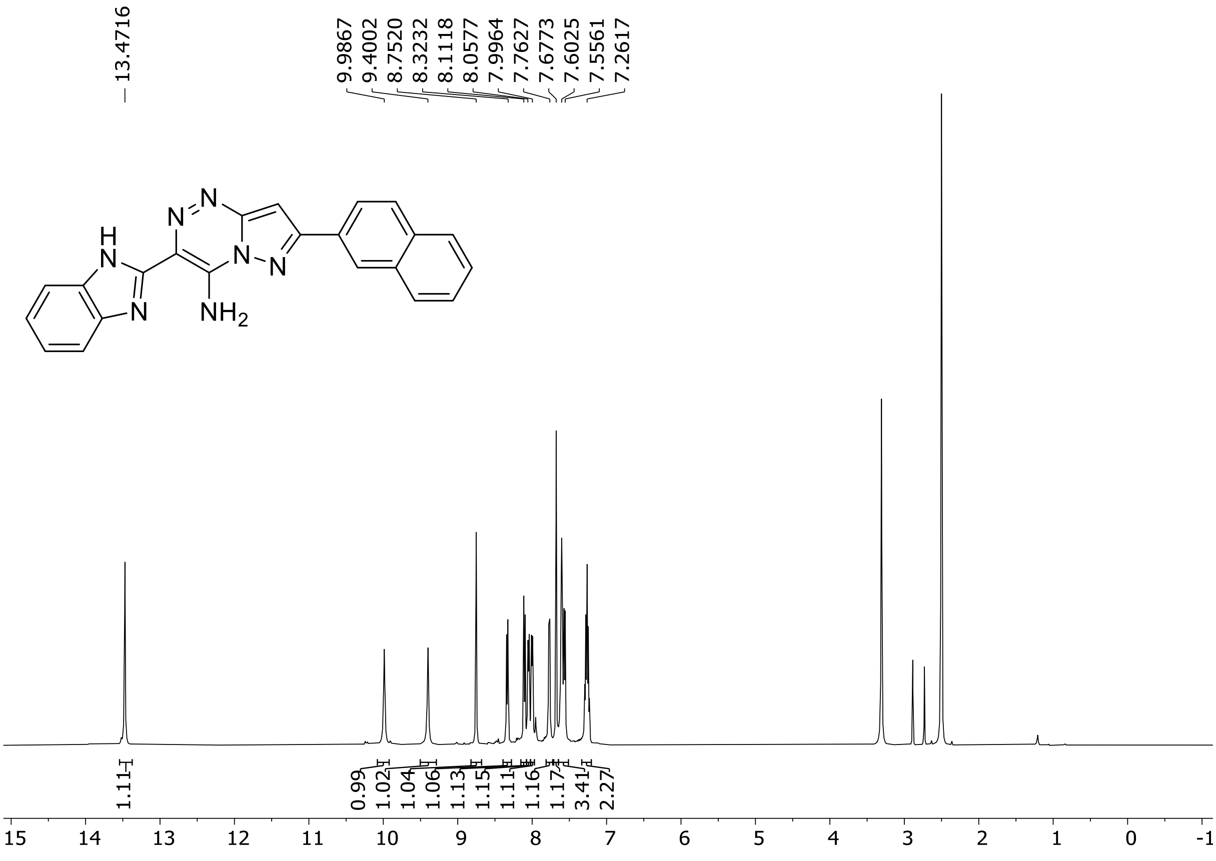

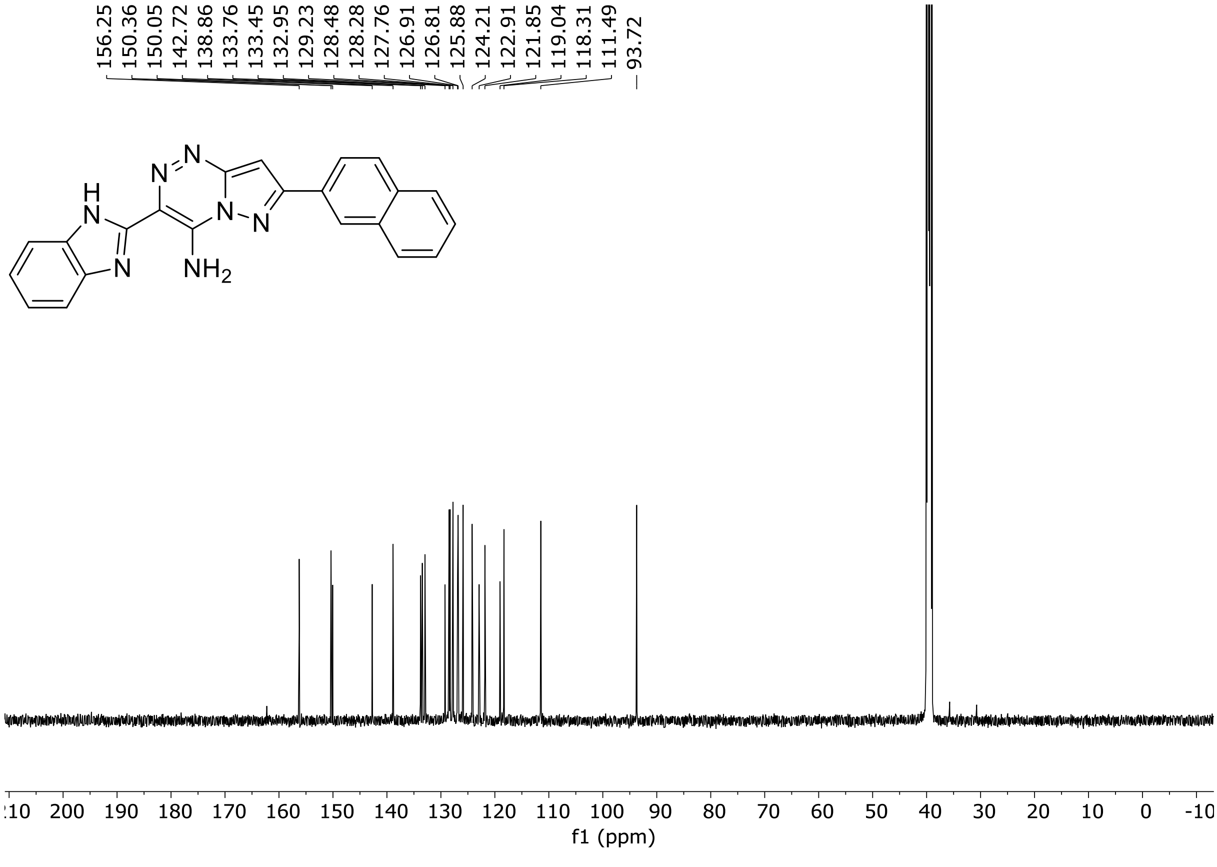

HRMS spectrum of **14**

$C_{22}H_{15}N_7$ **mono m/z = 377.1389**

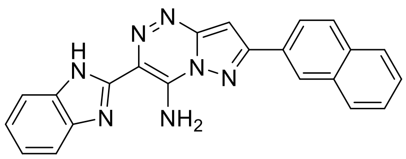

APCI + (MMI)
nitrogen flow 5 L/min, gas temperature 300°C, nebulizer 45 psi, skimmer 60 V, fragmentor 35 V, dissolved in methanol

ZOOM – range of interest

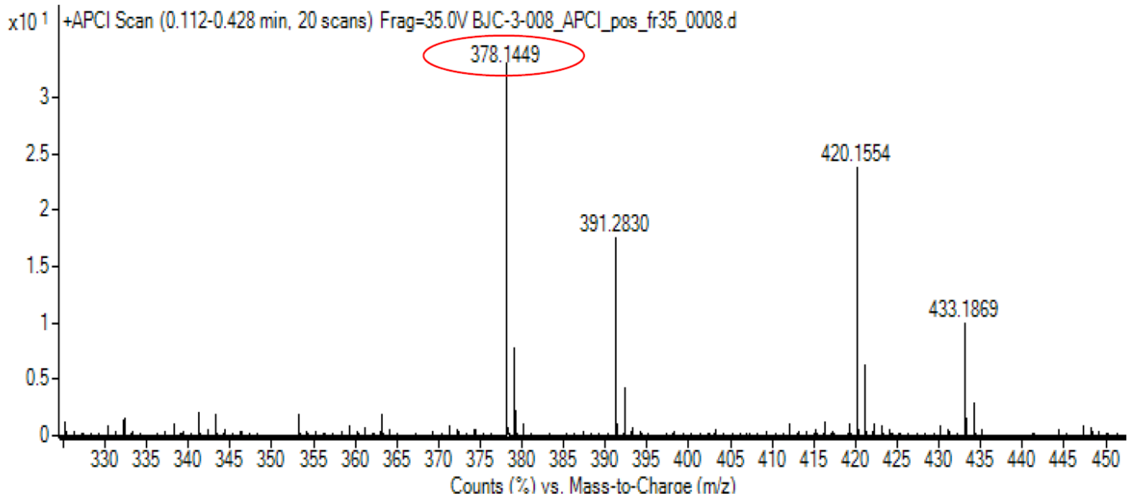

calculated mass: $[M+H]^+$= 378.1462 observed: $[M+H]^+$ = 378.1449 max. mass error = 3.4 ppm

^{1}H (500 MHz) and ^{13}C NMR (126 MHz) spectra of **15** in DMSO-d_6

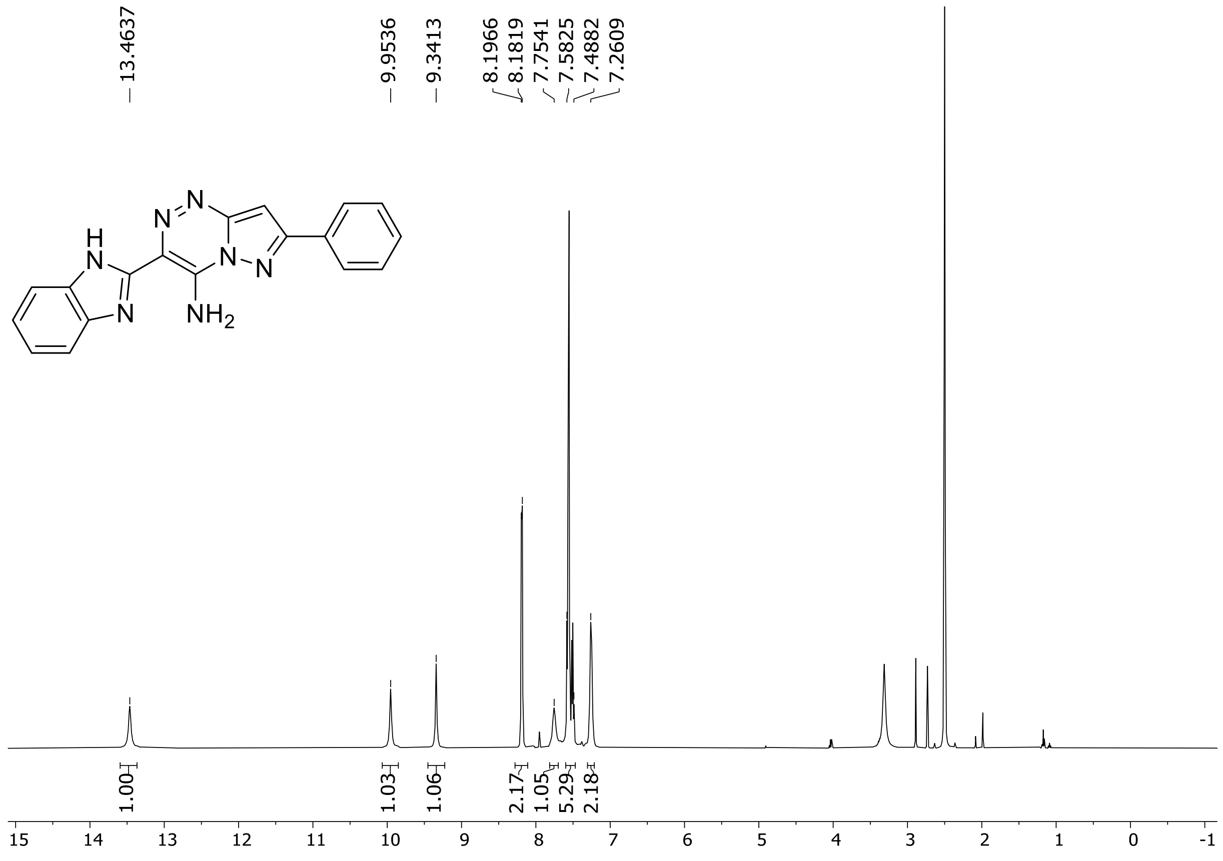

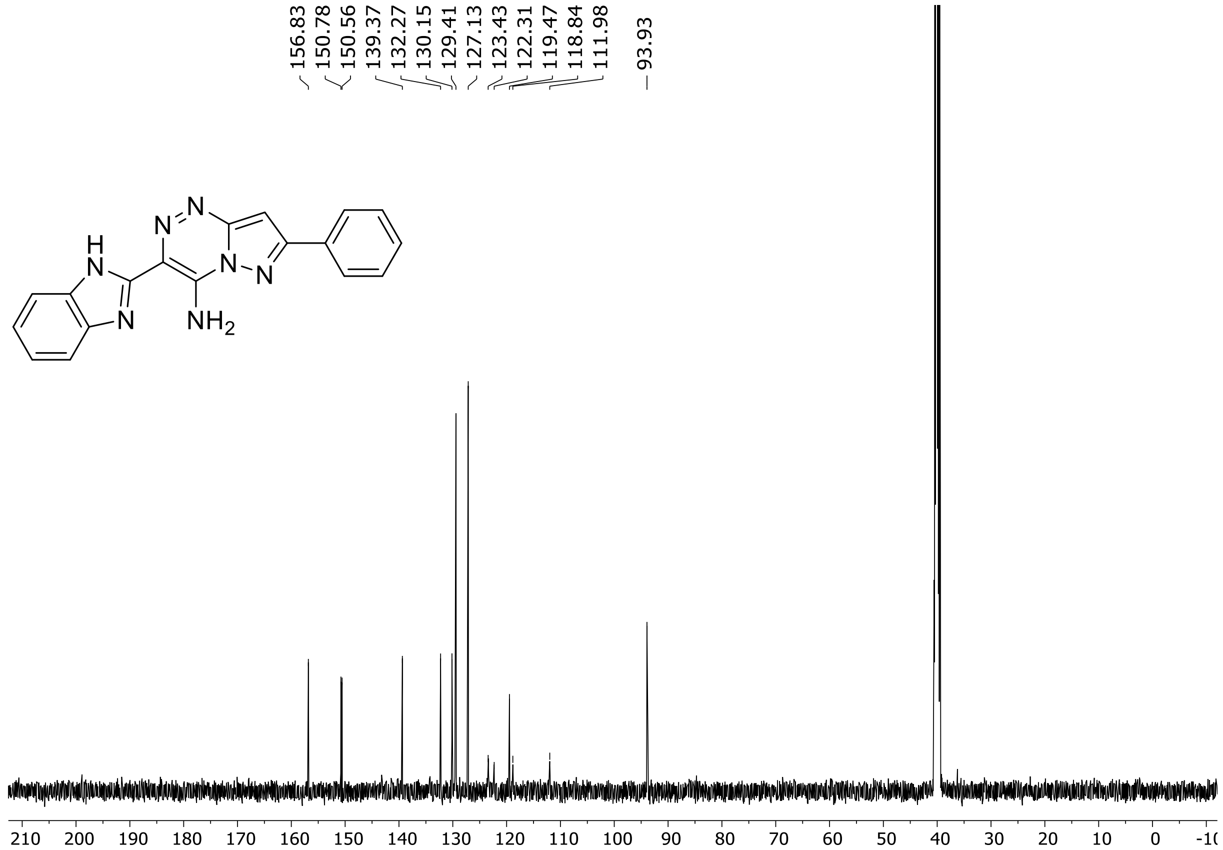

HRMS spectrum of **15**

$C_{18}H_{13}N_7$ **exact mass: 327.1232**

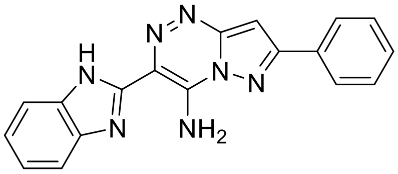

APCI + (MMI)
nitrogen flow 5 L/min, gas temperature 325°C, nebulizer 45 psig, skimmer 65 V, vaporizer 200°C, fragmentor 5 V, dissolved in methanol

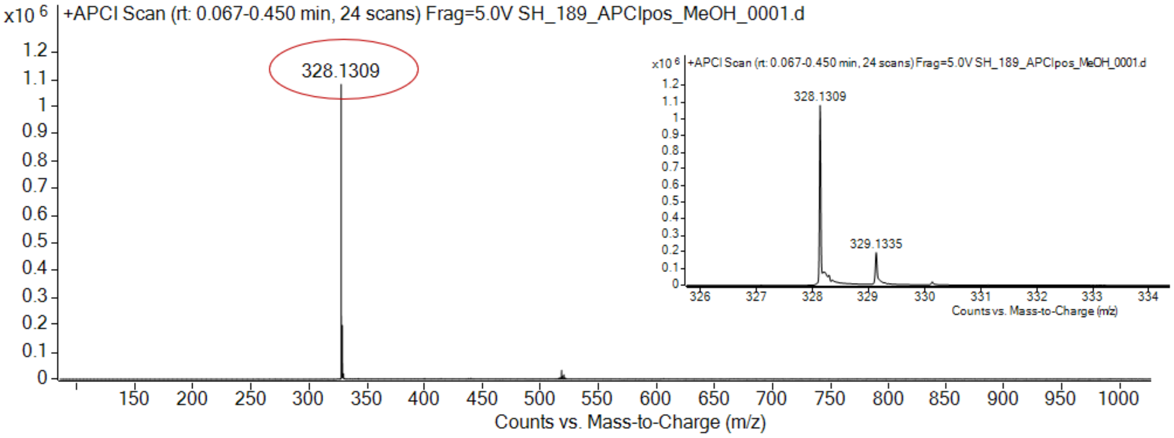

expected mass: $[M+H]^+$ = 328.1305 observed mass : $[M+H]^+$ = 328.1309 mass accuracy = 1.2 ppm

^{1}H (300 MHz) and ^{13}C NMR (126 MHz) spectra of **16** in DMSO-d_6

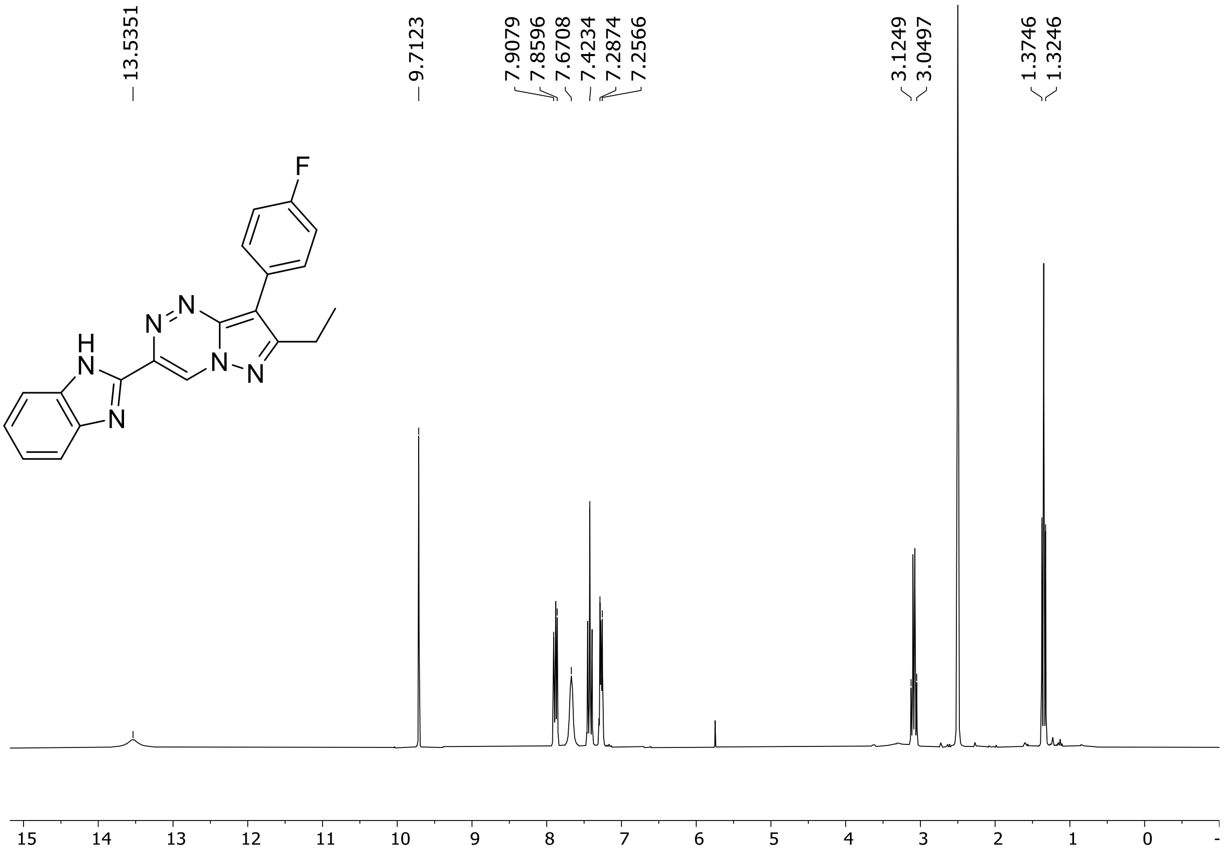

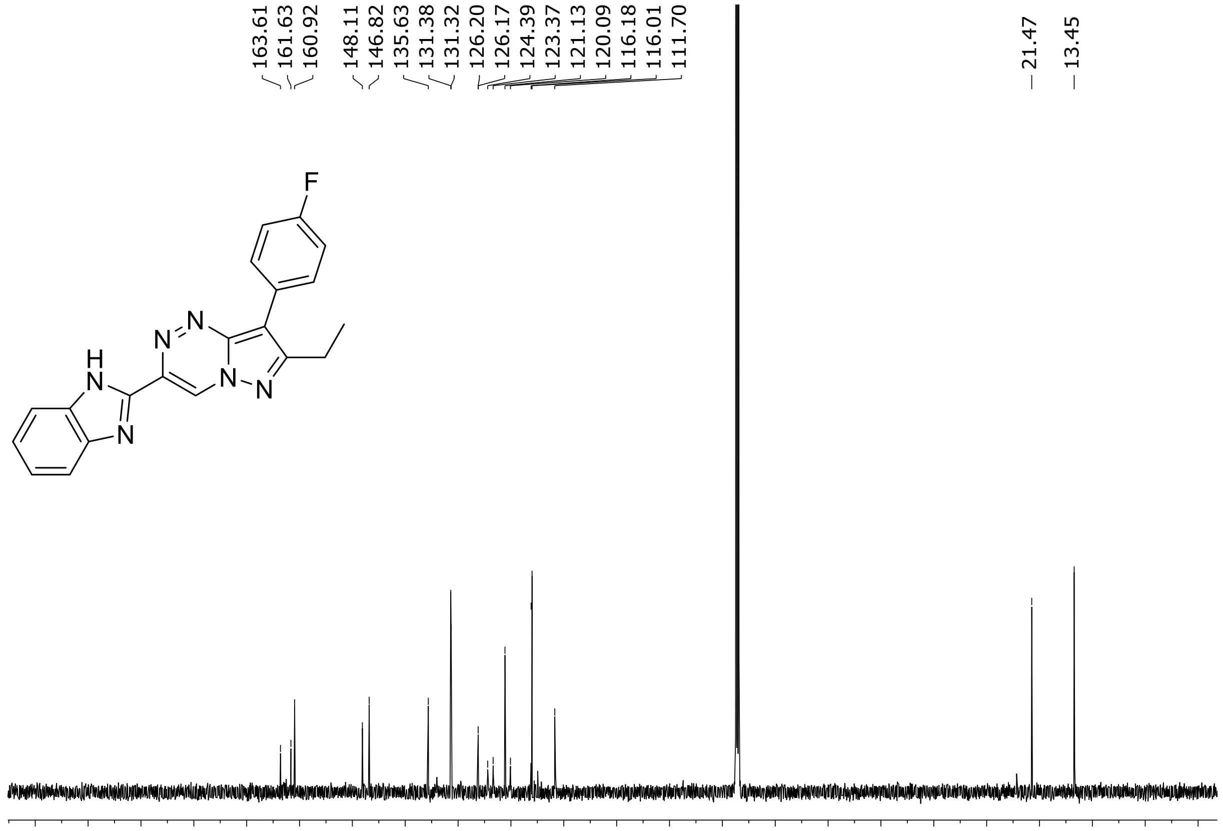

^{19}F (282 MHz) NMR spectrum of **16** DMSO-d_6

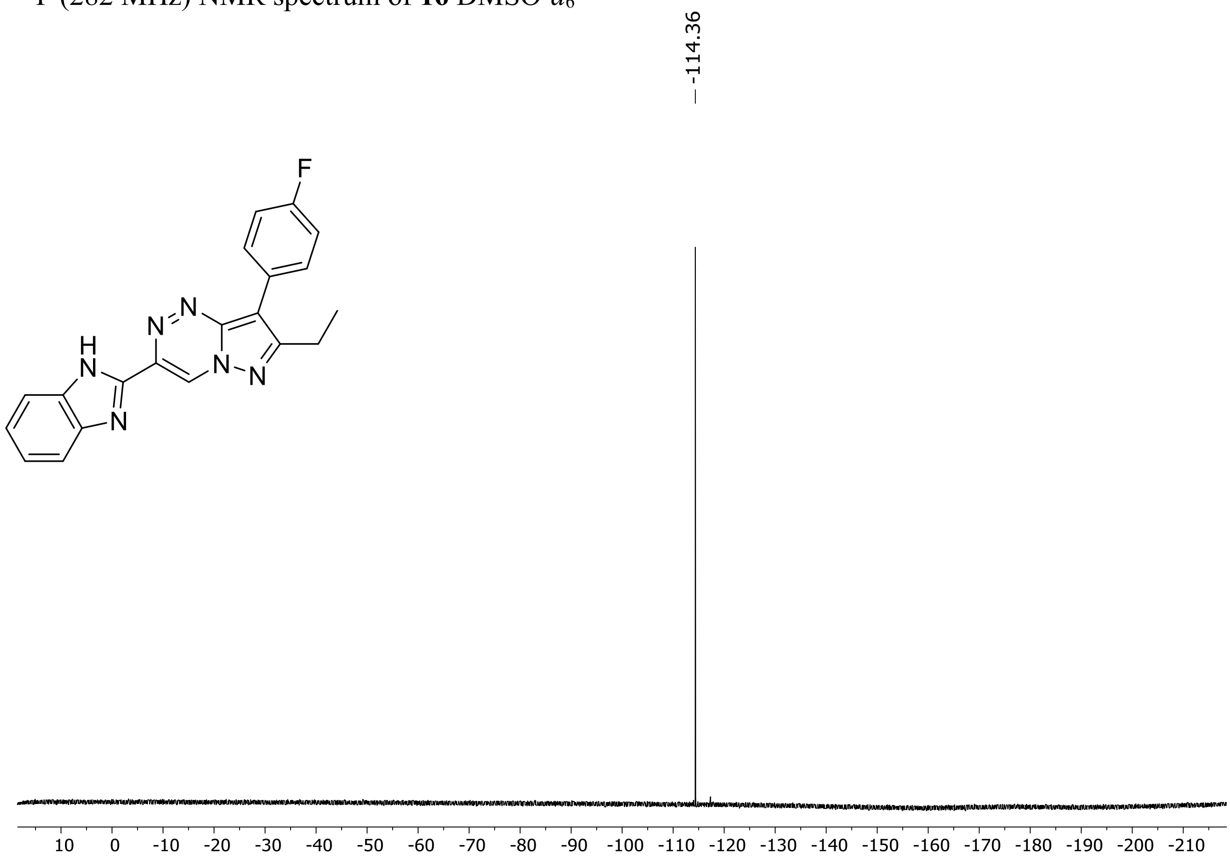

HRMS spectrum of **16**

$C_{20}H_{15}FN_6$ **exact mass: 358.1342**

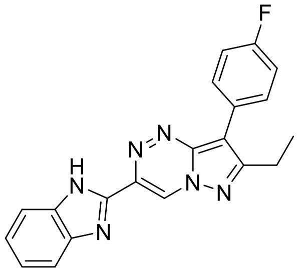

APCI + (MMI)
nitrogen flow 5 L/min, gas temperature 325°C, nebulizer 45 psig, skimmer 65 V, vaporizer 200°C, fragmentor 20 V, dissolved in methanol

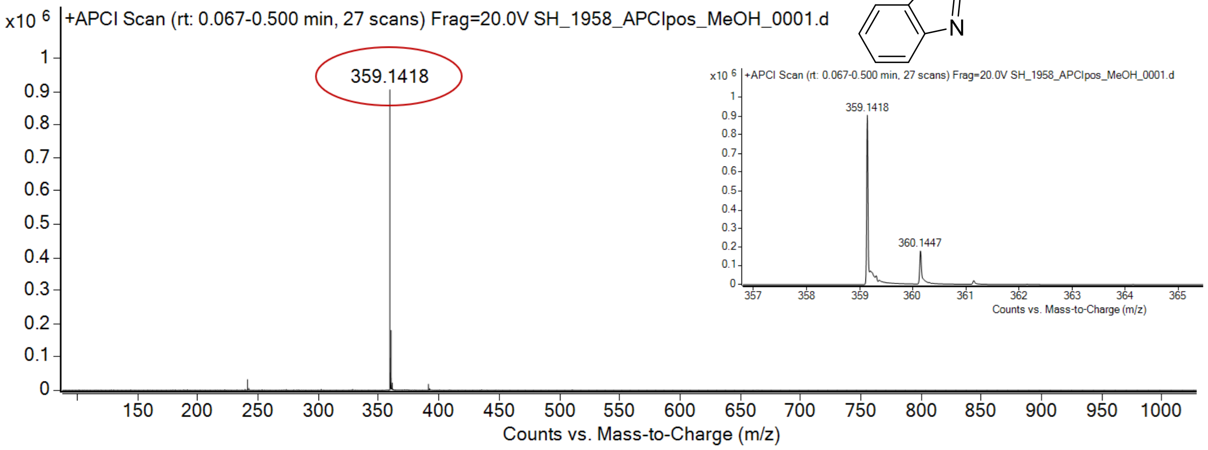

expected mass: [M+H]$^+$ = 359.1415 observed mass : [M+H]$^+$ = 359.1418 mass accuracy = 0.8 ppm

^{1}H (500 MHz) and ^{13}C NMR (126 MHz) spectra of **17** in DMSO-d_6

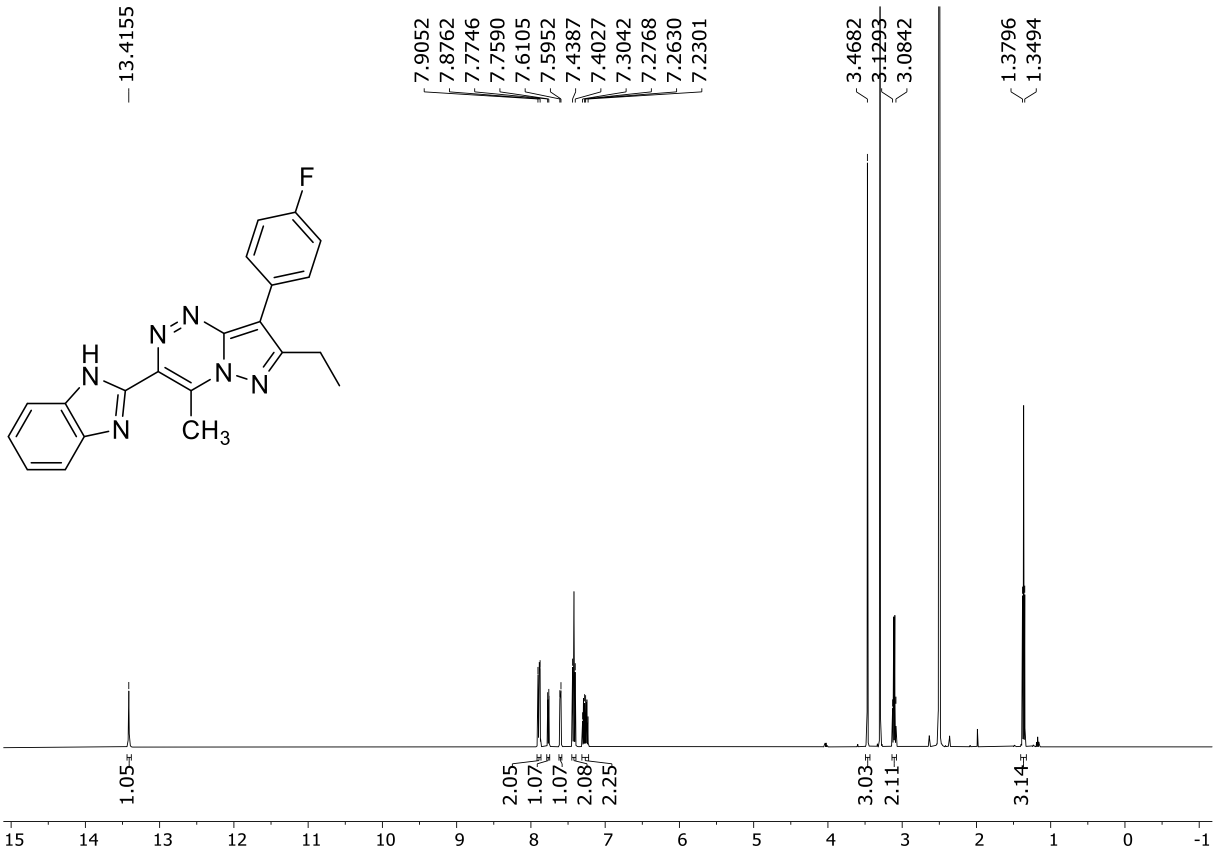

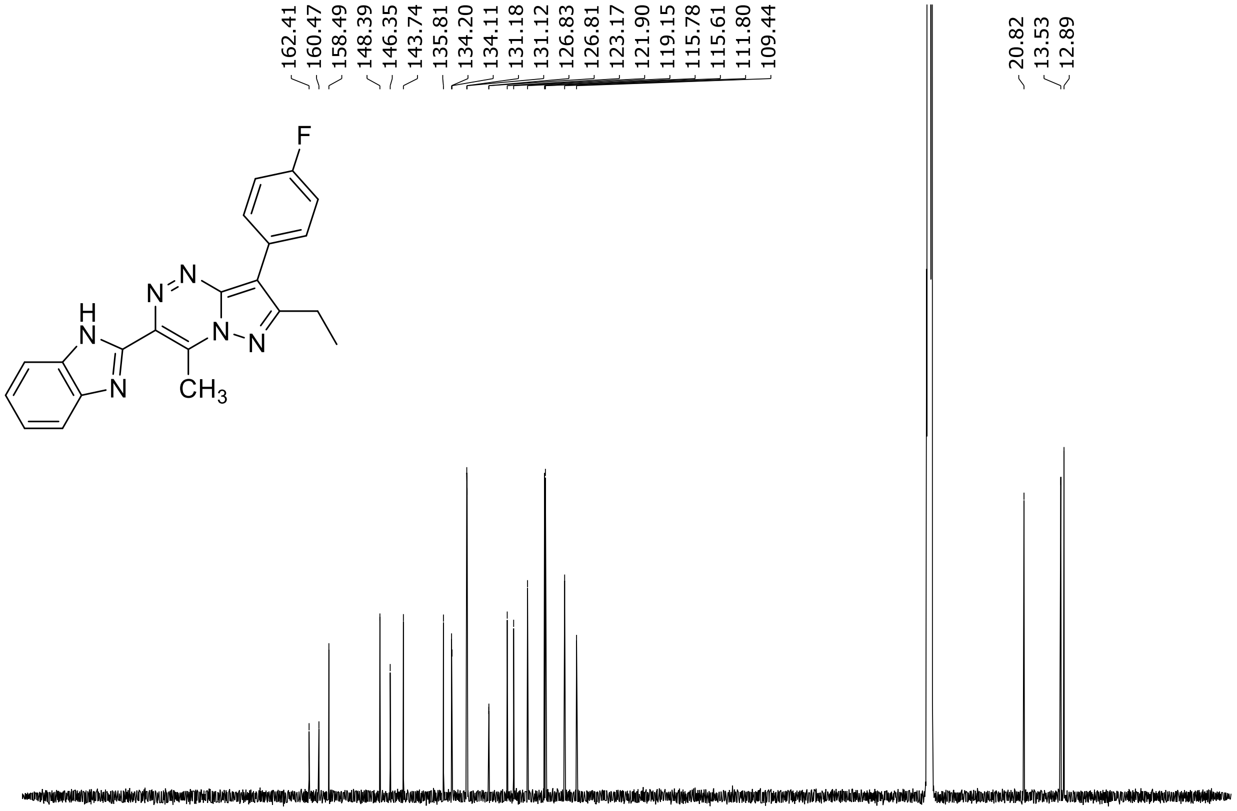

^{19}F (282 MHz) NMR spectrum of **17** DMSO-d_6

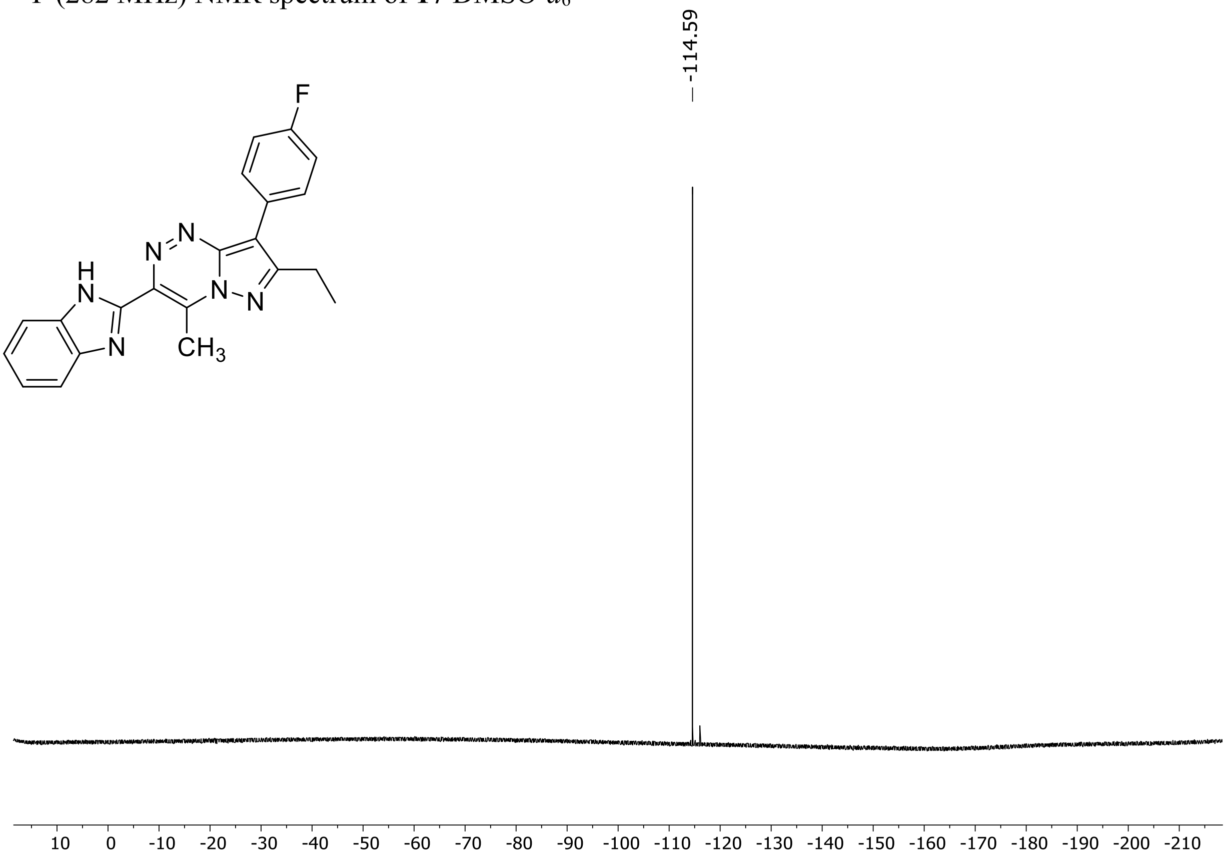

HRMS spectrum of **17**

$C_{21}H_{17}FN_6$ **exact mass: 372.1499**

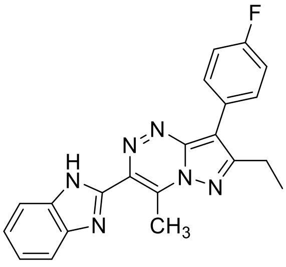

APCI + (MMI)
nitrogen flow 5 L/min, gas temperature 325°C, nebulizer 45 psig, skimmer 65 V, vaporizer 200°C, fragmentor 10 V, dissolved in methanol

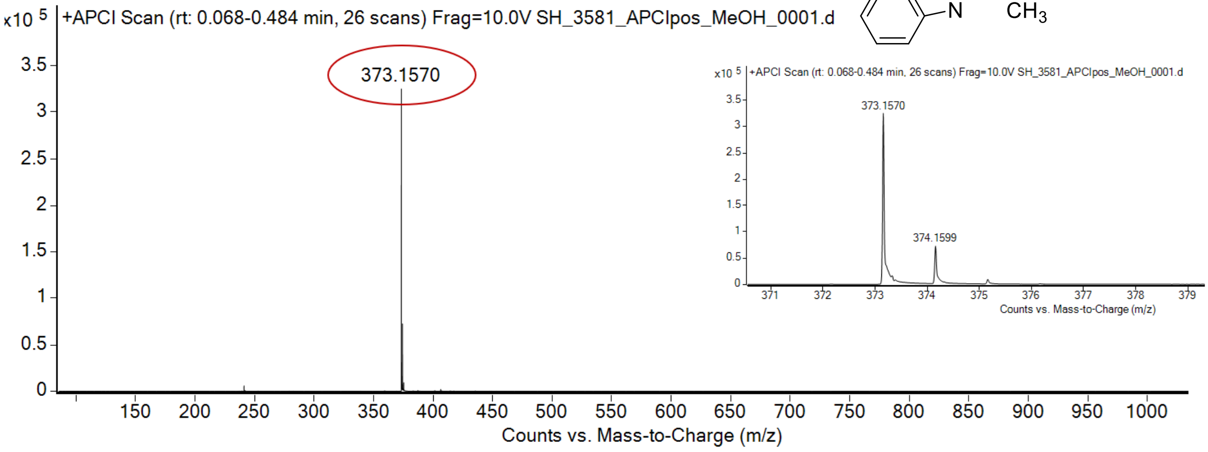

expected mass: $[M+H]^+$ = 373.1571 observed mass : $[M+H]^+$ = 373.1570 mass accuracy = - 0.3 ppm

^{1}H (500 MHz) and ^{13}C NMR (126 MHz) spectra of **18** in DMSO-d_6

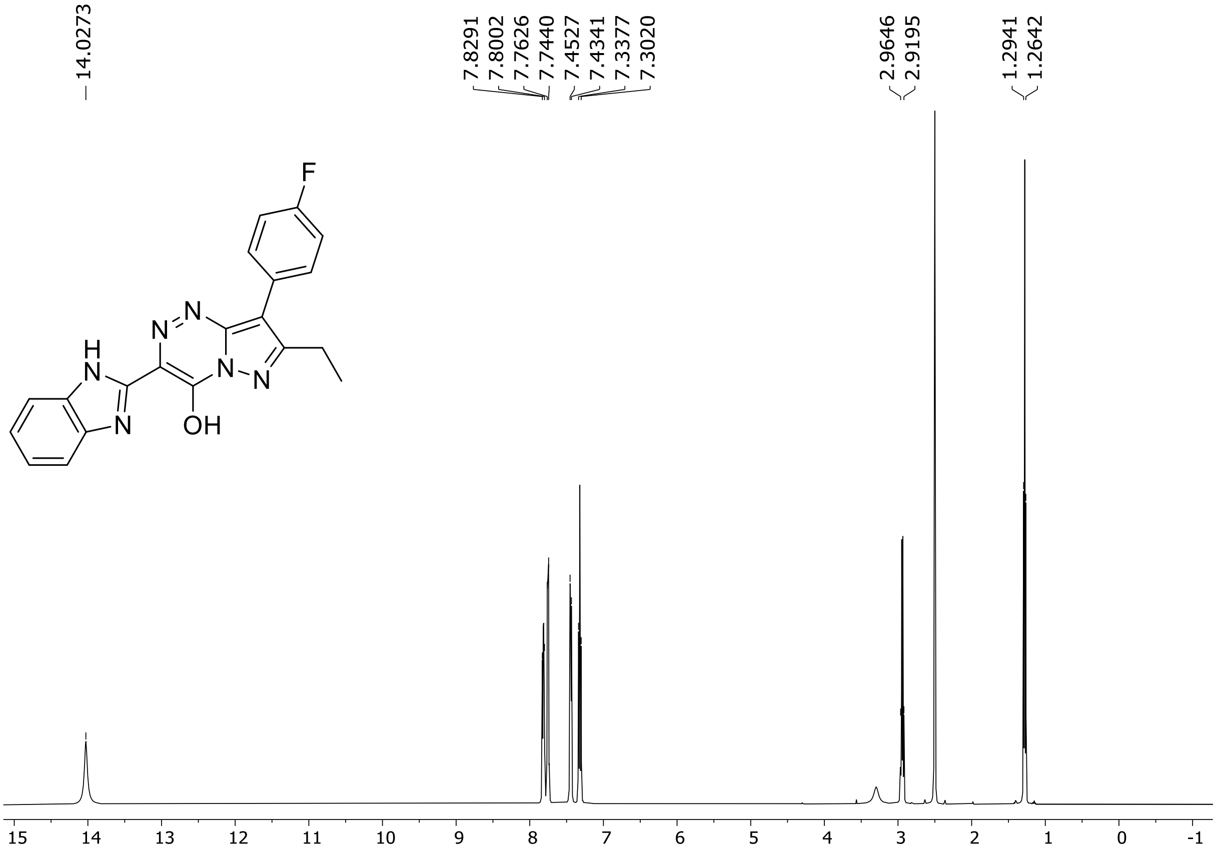

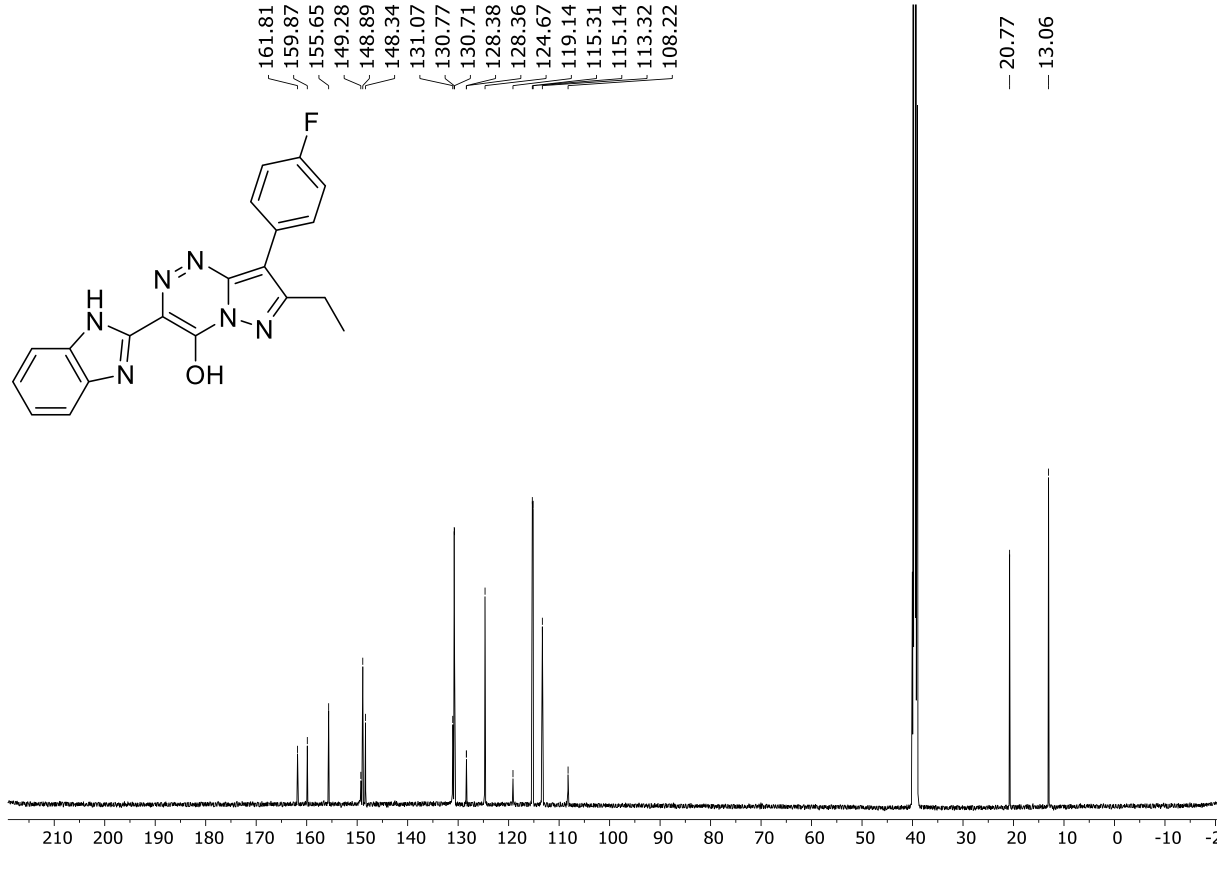

^{19}F (471 MHz) NMR spectrum of **18** DMSO-d_6

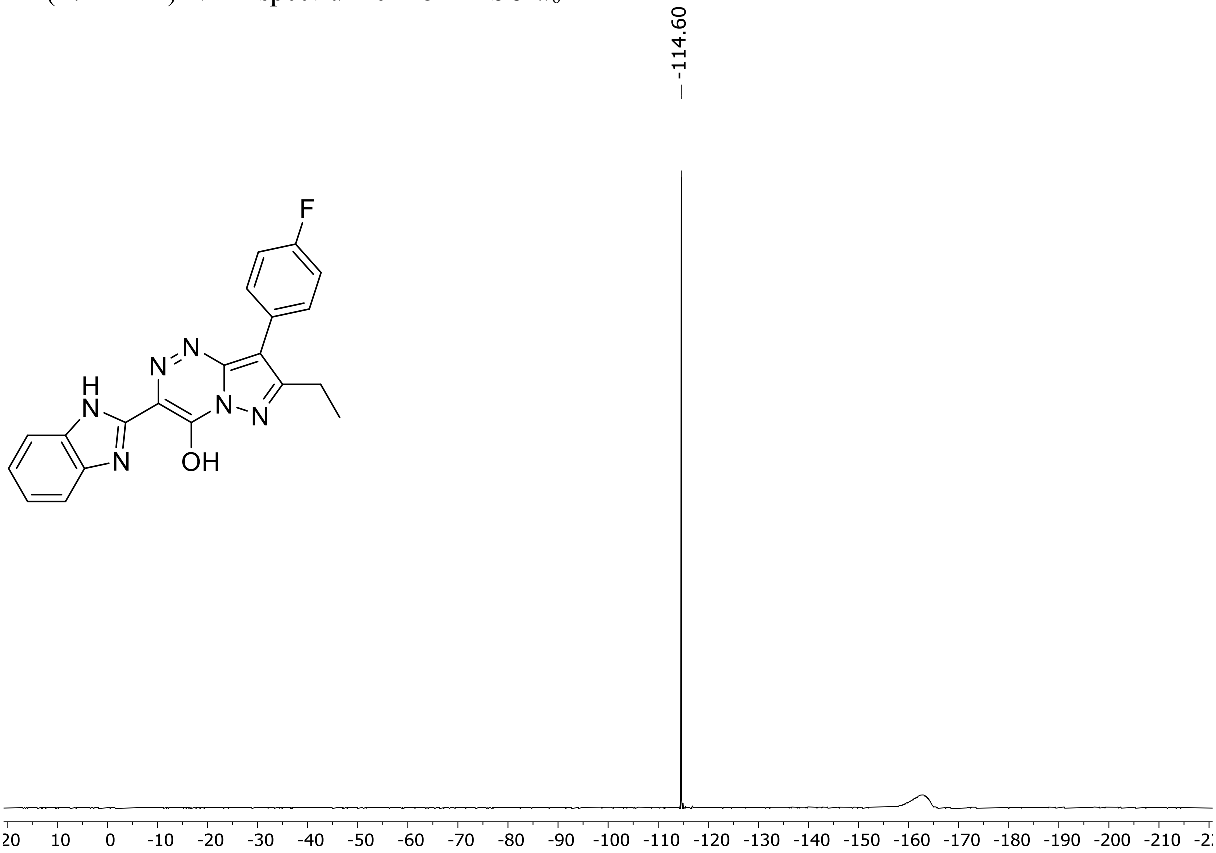

HRMS spectrum of **18**

$C_{20}H_{15}FN_6O$ **mono m/z = 374,1291**

ESI - (MMI)
nitrogen flow 5 L/min, gas temperature 300°C, vaporizer 200°C,
nebulizer 45 psi, Vcap -2000 V, skimmer **60** V, fragmentor **25**V,
dissolved in DMSO

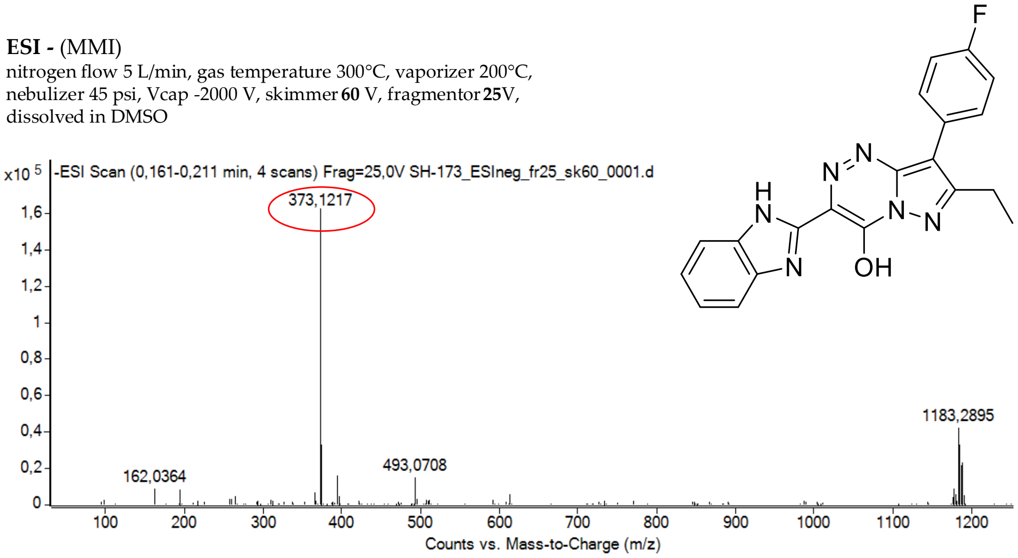

calculated mass:
$[M-H]^-$ = 373,1219 observed: $[M-H]^-$ = 373,1217 max. mass error = 0,5 ppm

^{1}H (500 MHz) and ^{13}C NMR (126 MHz) spectra of **19** in DMSO-d_6

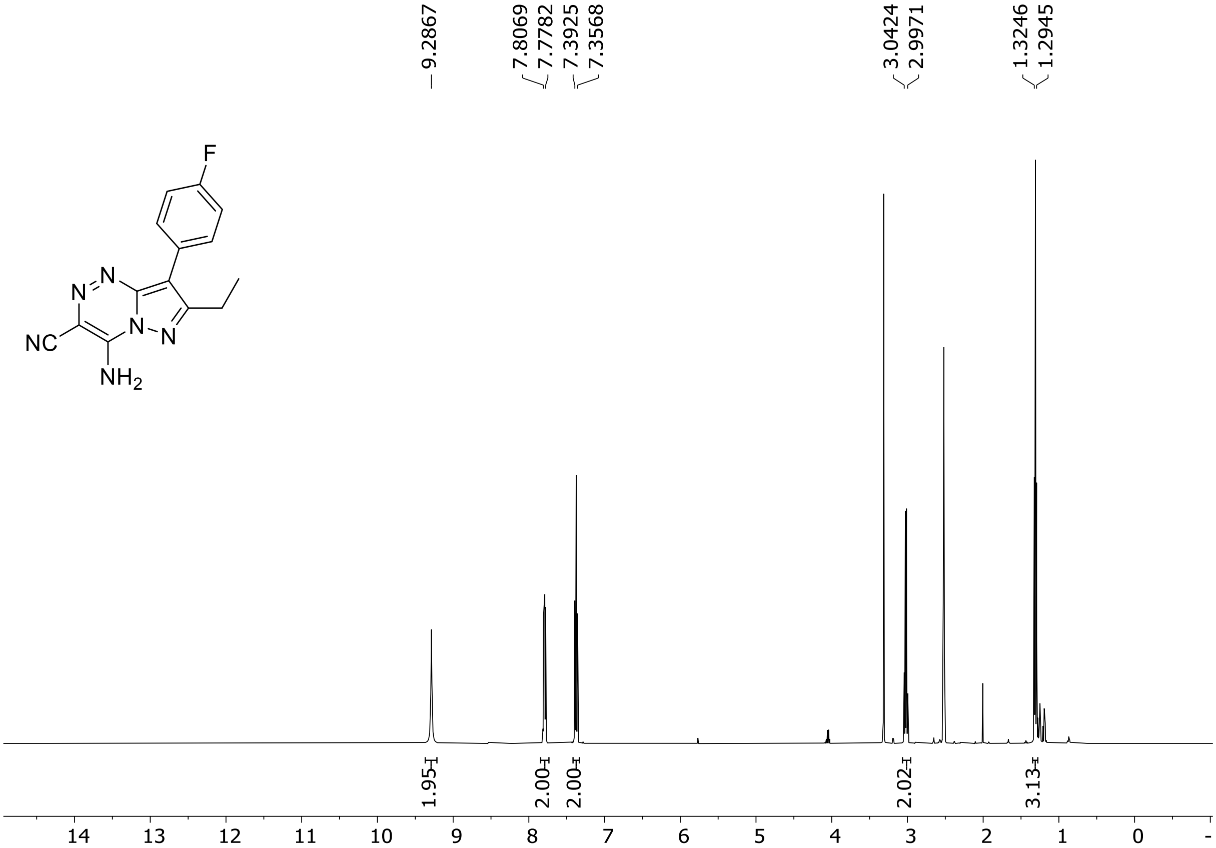

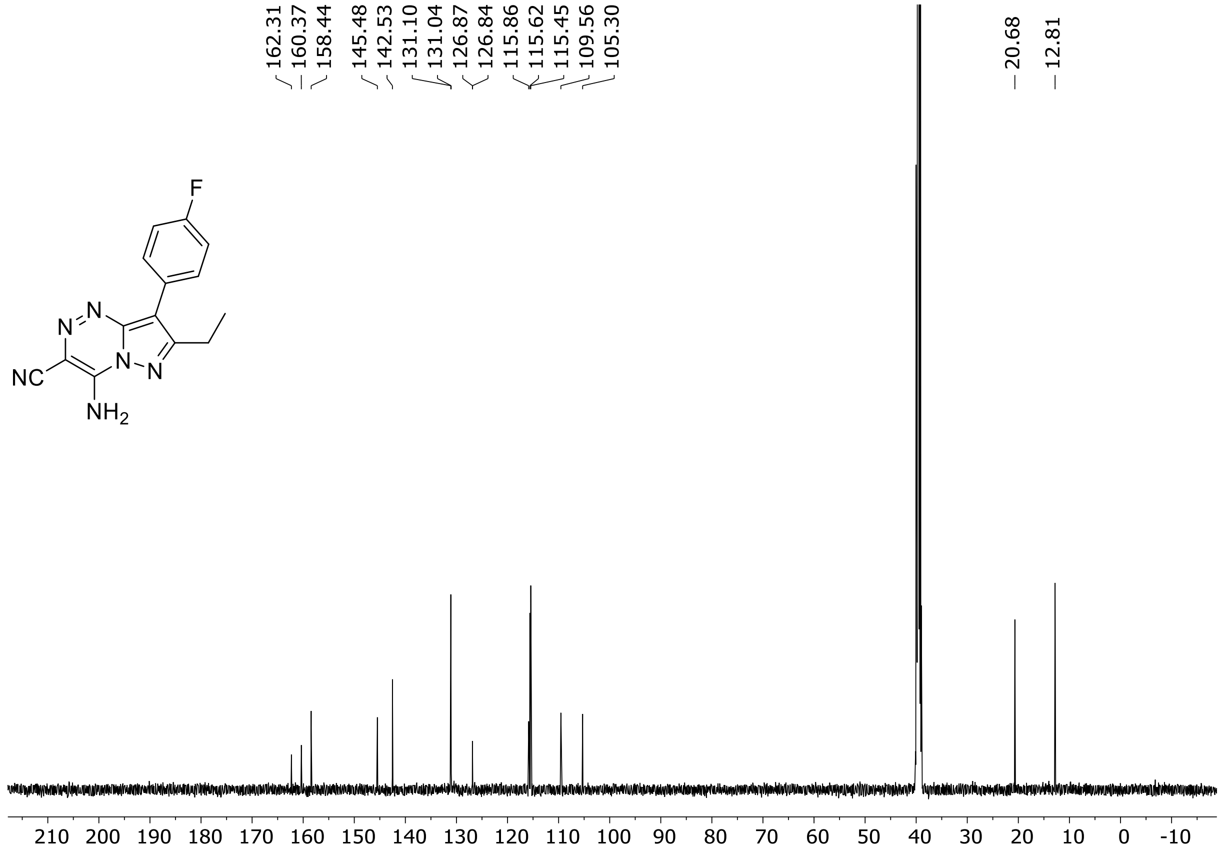

HRMS spectrum of **19**

$C_{14}H_{11}FN_6$ **mono m/z = 282,1029**

ESI - (MMI)
nitrogen flow 5 L/min, gas temperature 300°C, vaporizer 200°C,
nebulizer 30 psi, skimmer **55** V, fragmentor **10** V, dissolved in DMSO

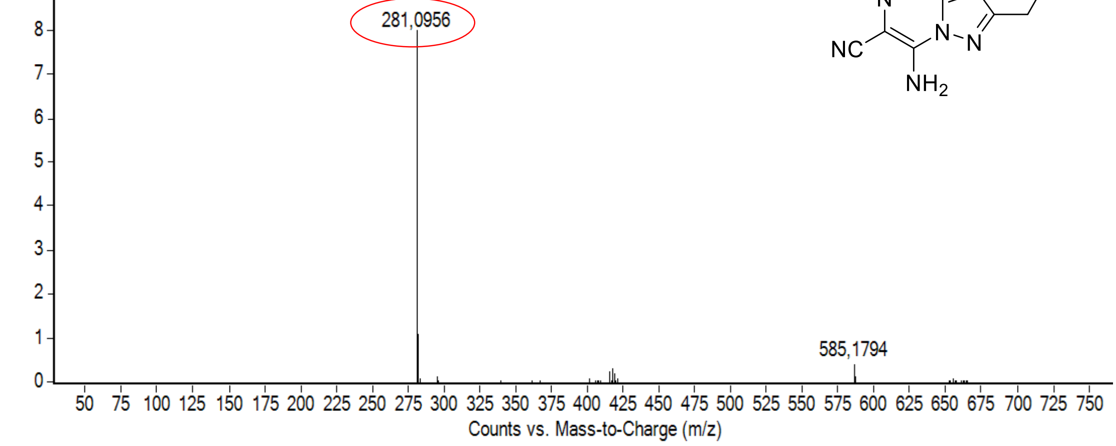

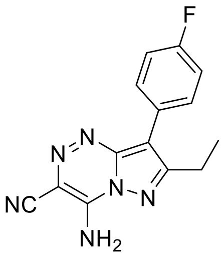

calculated mass: [M-H]$^-$ = 281,0956 observed: [M-H]$^-$ = 281,0956 max. mass error ≤ 0,1 ppm

^{1}H (500 MHz) and ^{13}C NMR (126 MHz) spectra of **20** in DMSO-d_6

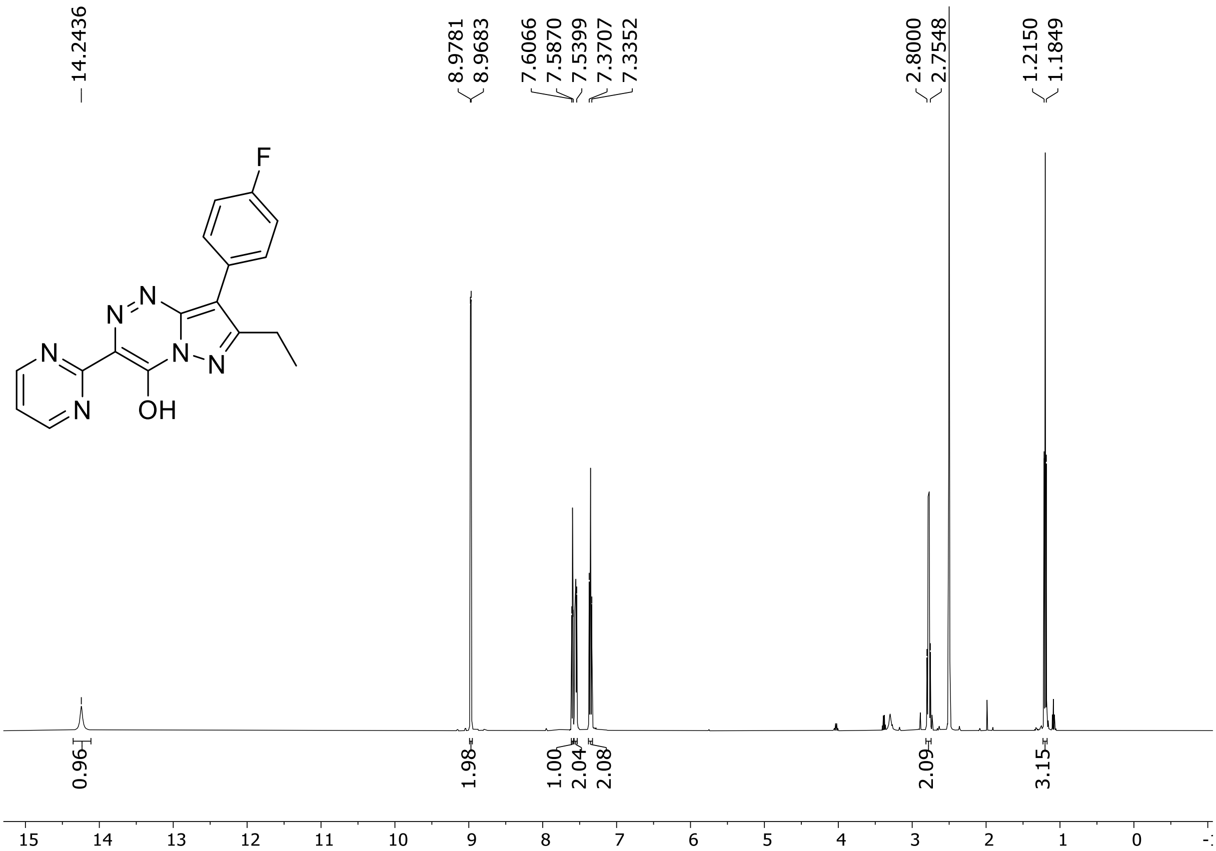

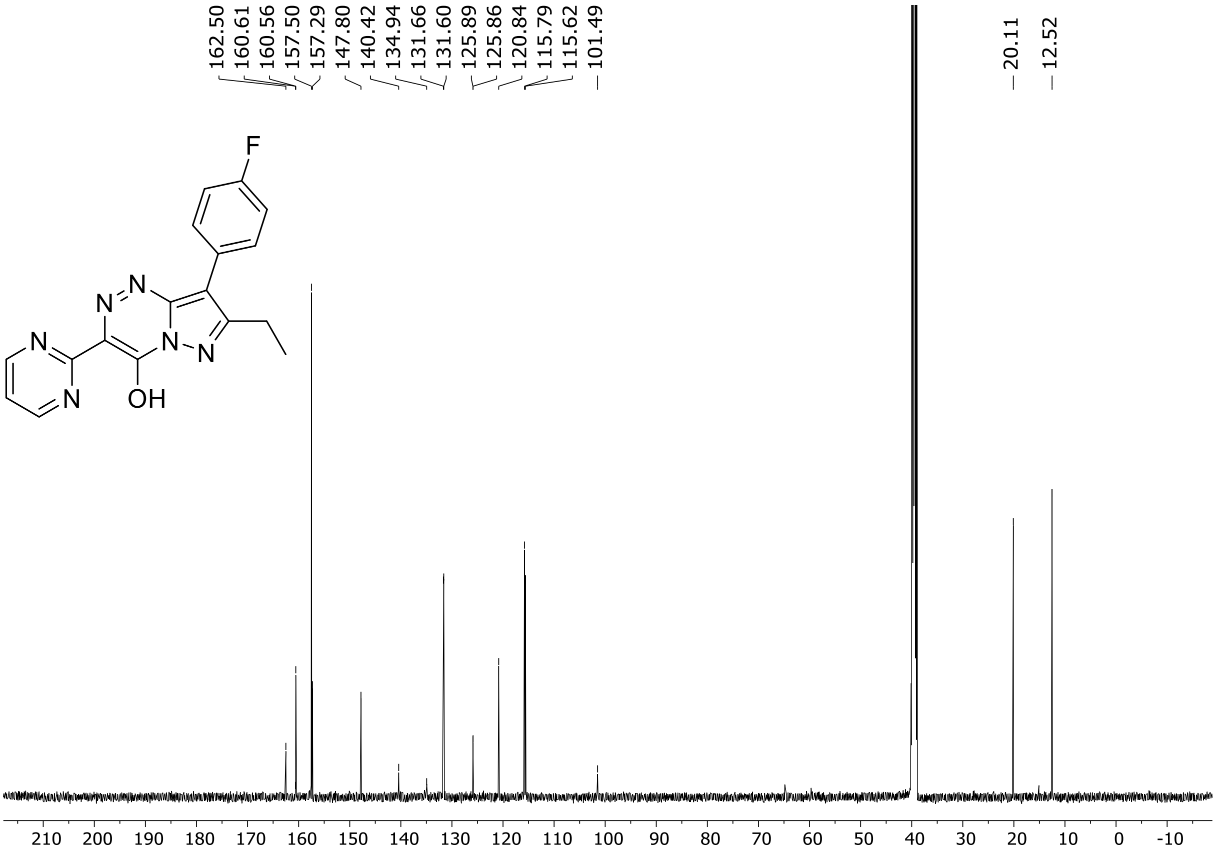

HRMS spectrum of **20**

$C_{17}H_{13}FN_6O$ **exact mass: 336.1135**

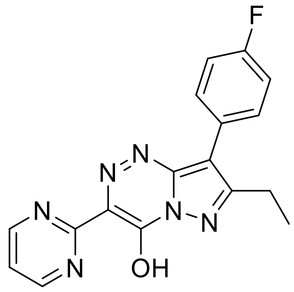

APCI + (MMI)
nitrogen flow 3 L/min, gas temperature 325°C, nebulizer 45 psig, skimmer 65 V, vaporizer 200°C, fragmentor 15 V, dissolved in methanol

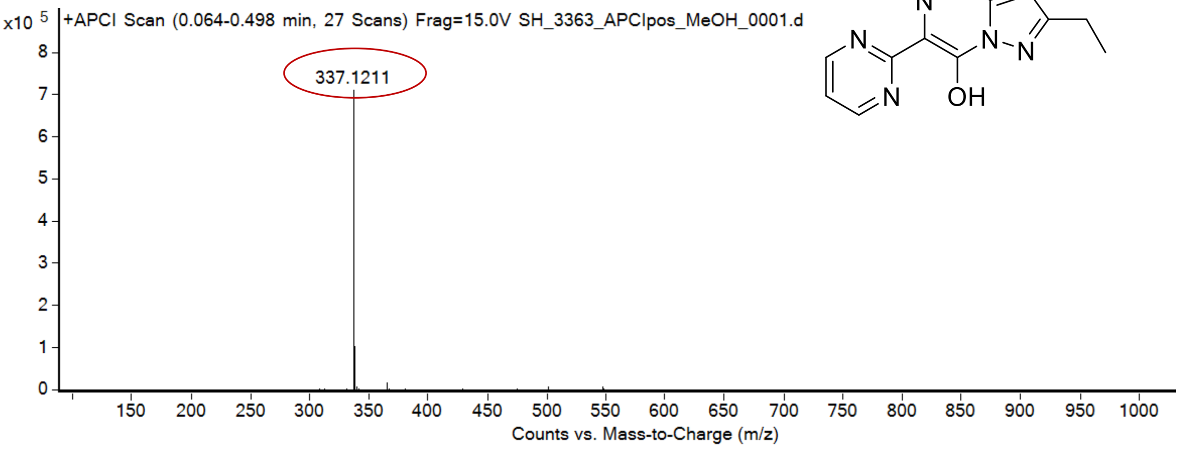

expected mass: $[M+H]^+$ = 337.1208 observed mass: $[M+H]^+$ = 337.1211 mass accuracy = 0.8 ppm

^{1}H (500 MHz) and ^{13}C NMR (126 MHz) spectra of **21** in DMSO-d_6

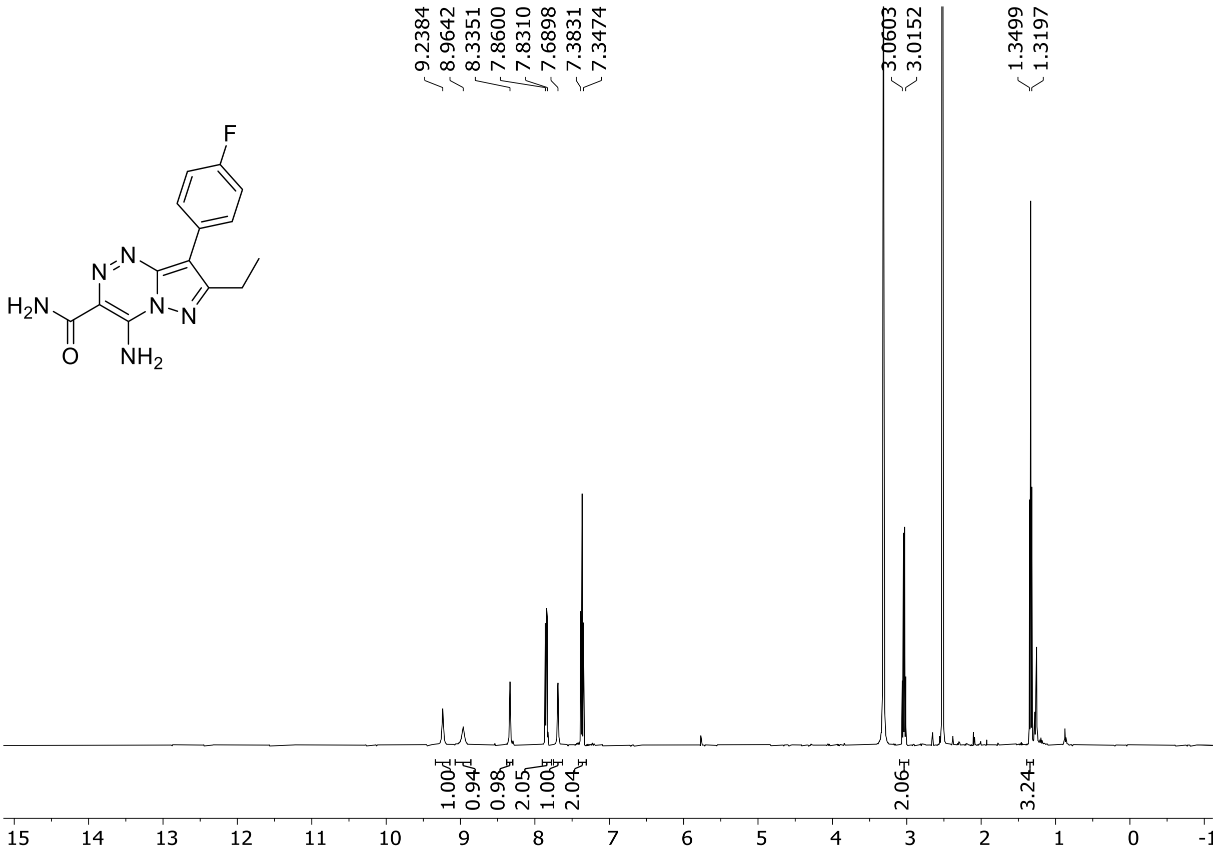

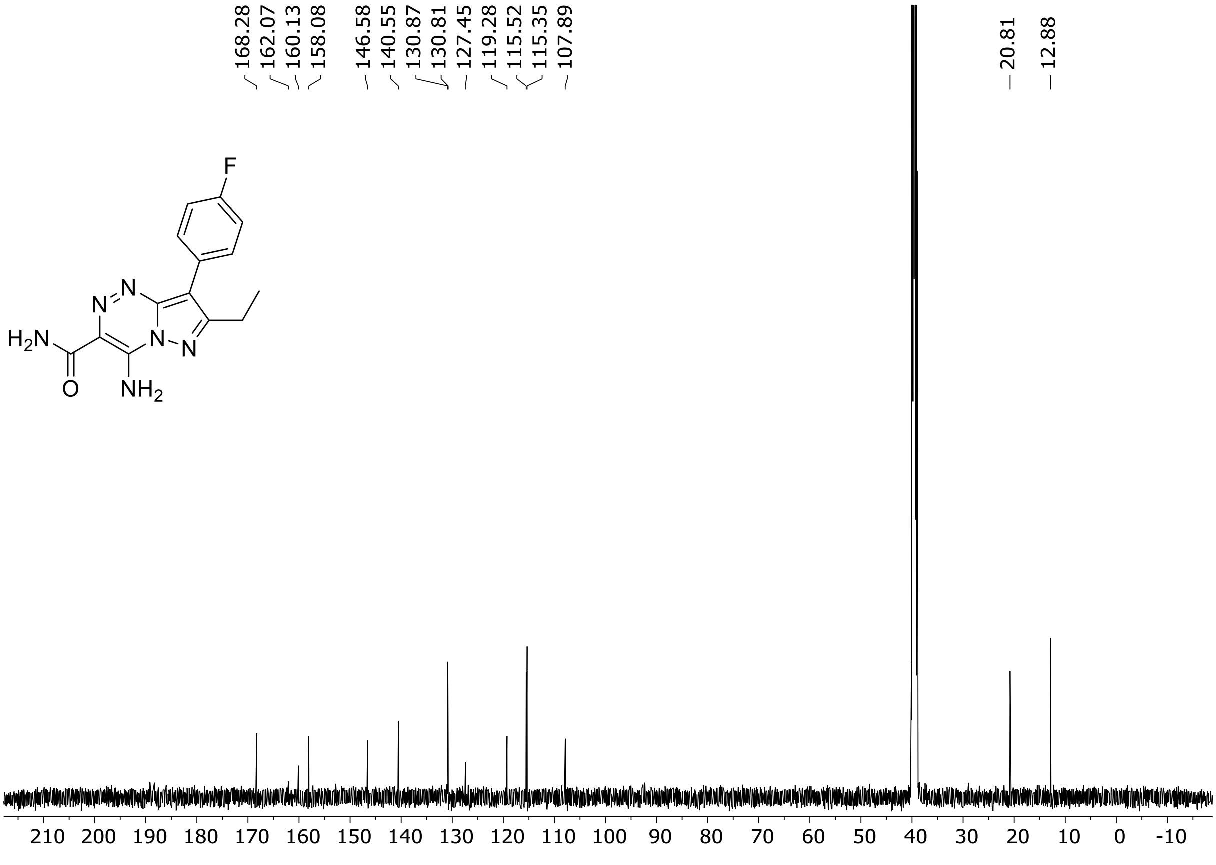

HRMS spectrum of **21**

$C_{14}H_{13}FN_6O$ **mono m/z = 300.1135**

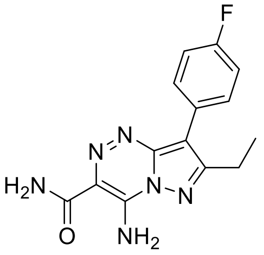

APCI + (MMI)
nitrogen flow 5 L/min, gas temperature 300°C, nebulizer 45 psi, vaporizer 200°C,
skimmer **65** V, fragmentor **25** V, dissolved in methanol

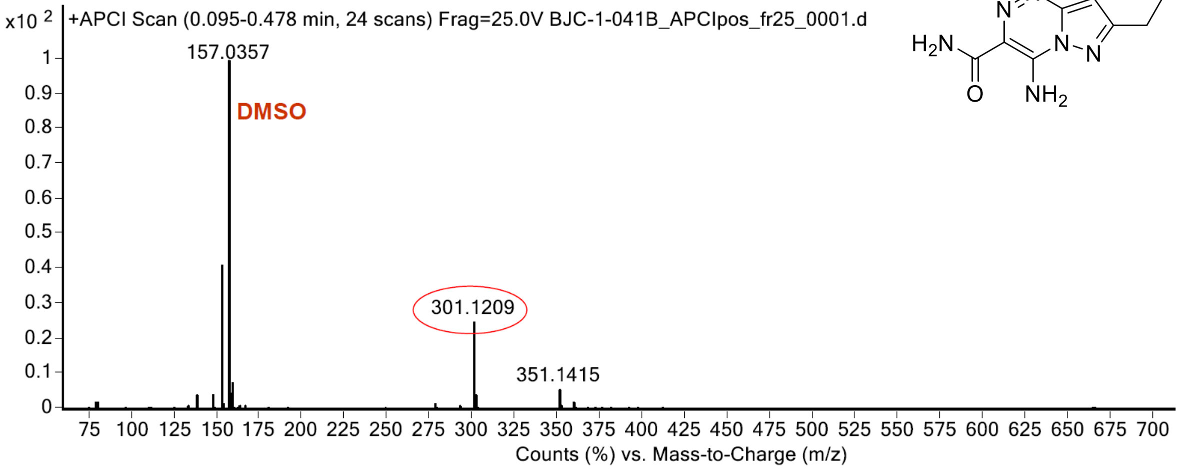

calculated mass: $[M+H]^+$ = 301.1208 observed: $[M+H]^+$ = 301.1209 max. mass error = 0.3 ppm

^{1}H (500 MHz) and ^{13}C NMR (126 MHz) spectra of **22** in DMSO-d_6

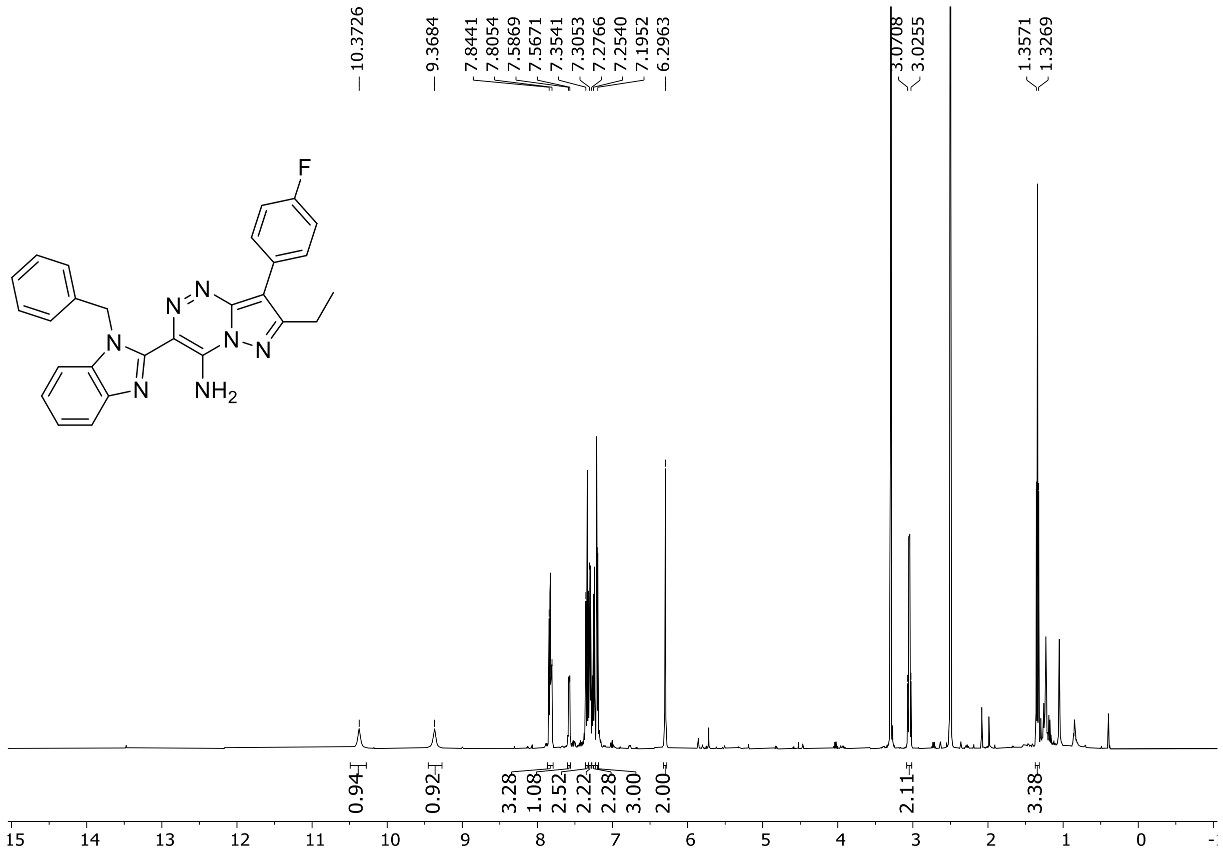

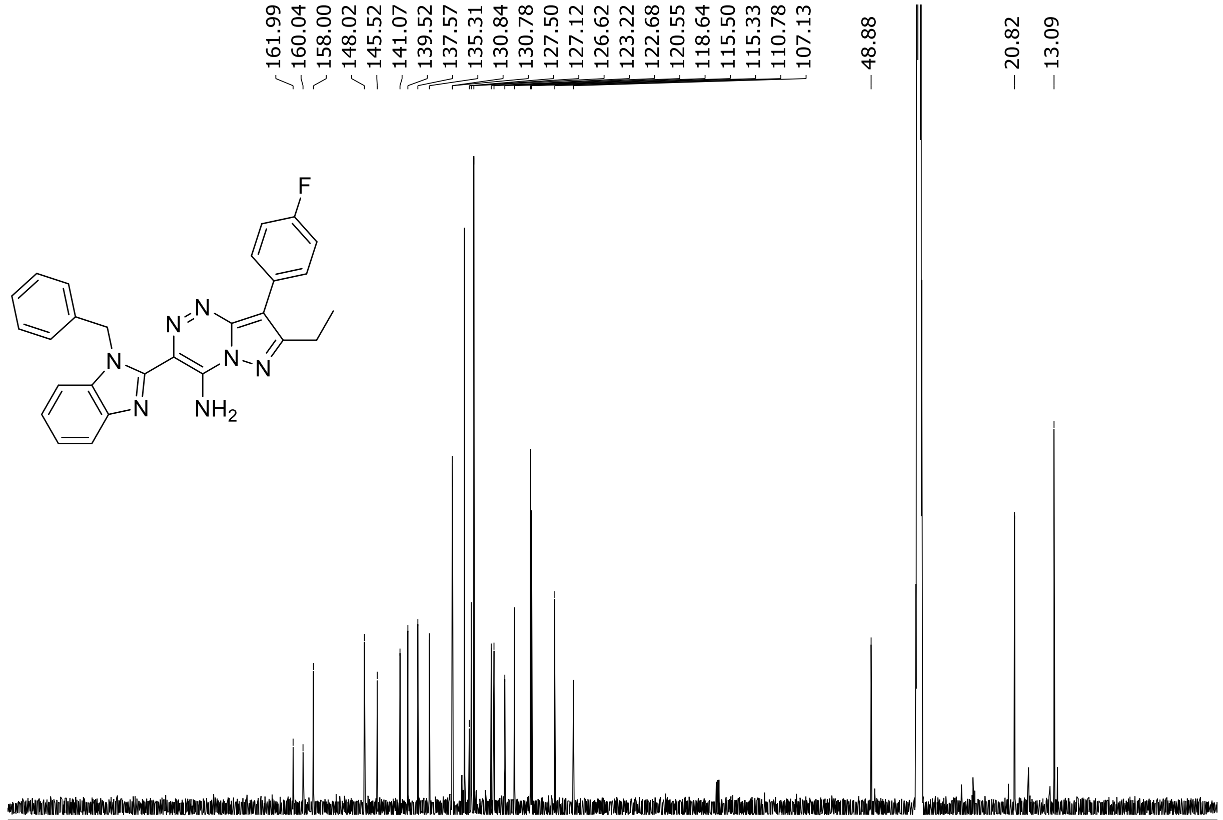

HRMS spectrum of **22**

$C_{27}H_{22}FN_7$ **mono m/z = 463.1921**

APCI + (MMI)
nitrogen flow 5 L/min, gas temperature 300°C, nebulizer 45 psi, vaporizer 200°C
skimmer **65** V, fragmentor **30** V, dissolved in methanol

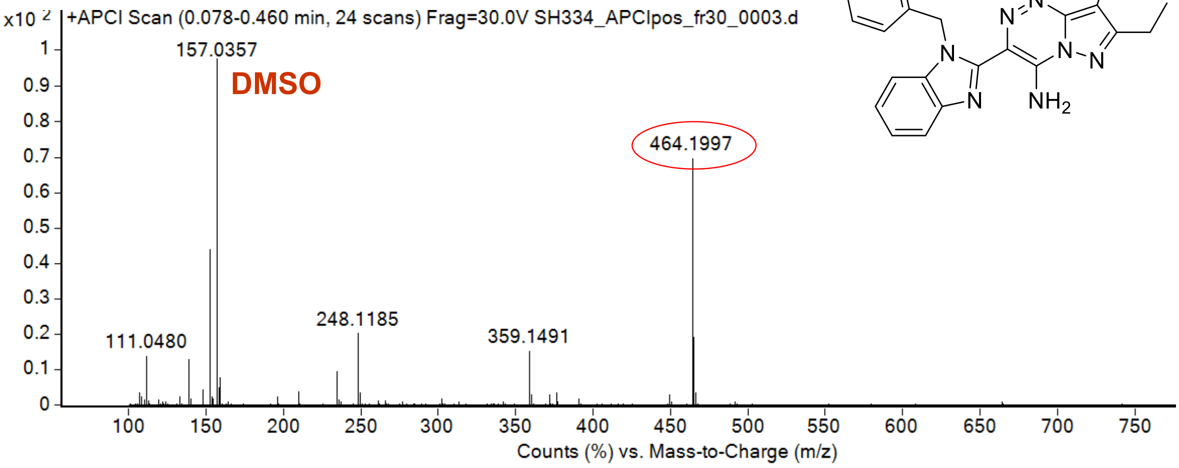

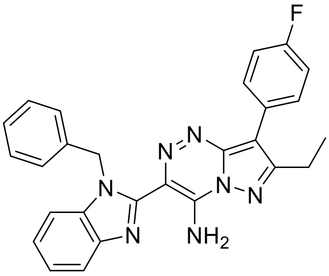

calculated mass: $[M+H]^+ = 464.1993$ observed: $[M+H]^+ = 464.1997$ max. mass error = 0.8 ppm

^{1}H (500 MHz) and ^{13}C NMR (126 MHz) spectra of **23** in DMSO-d_6

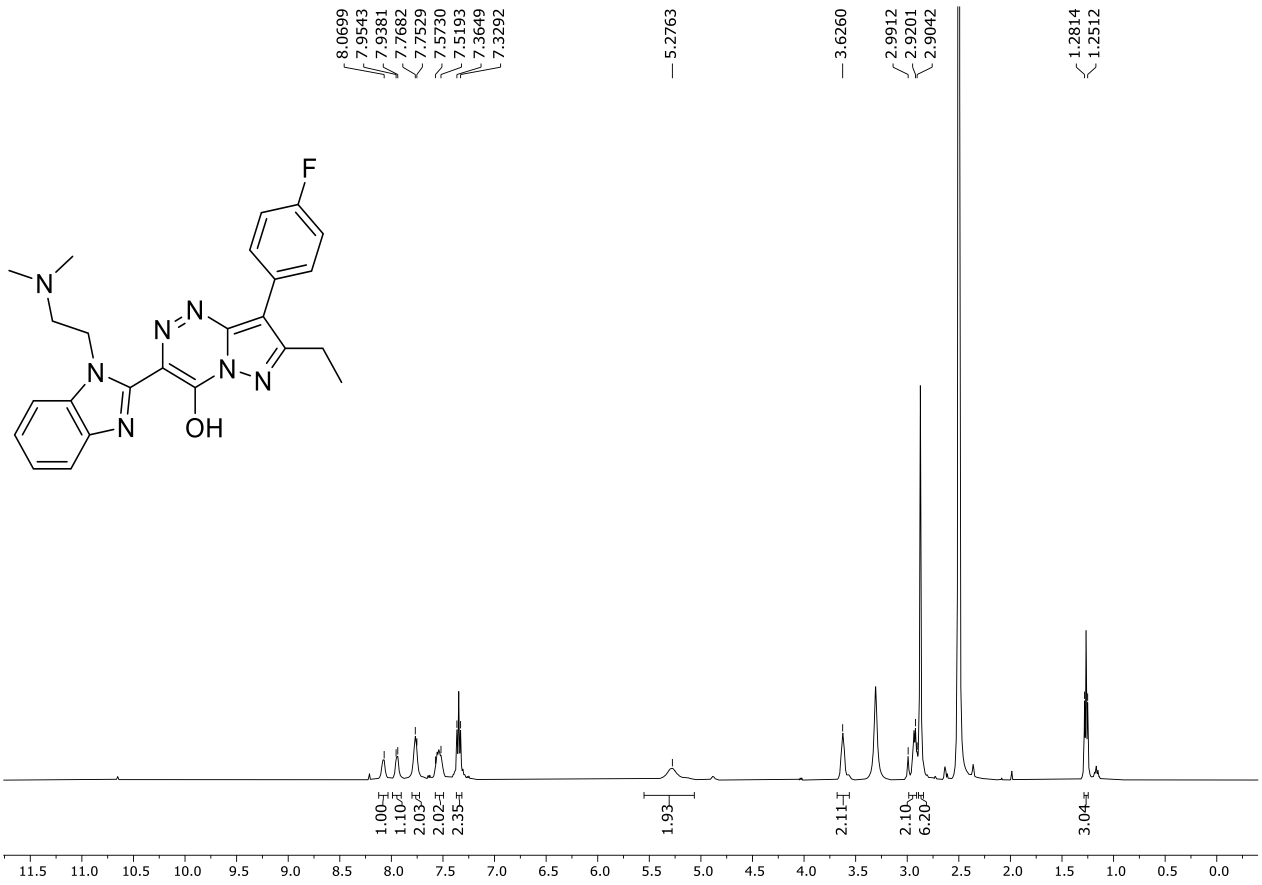

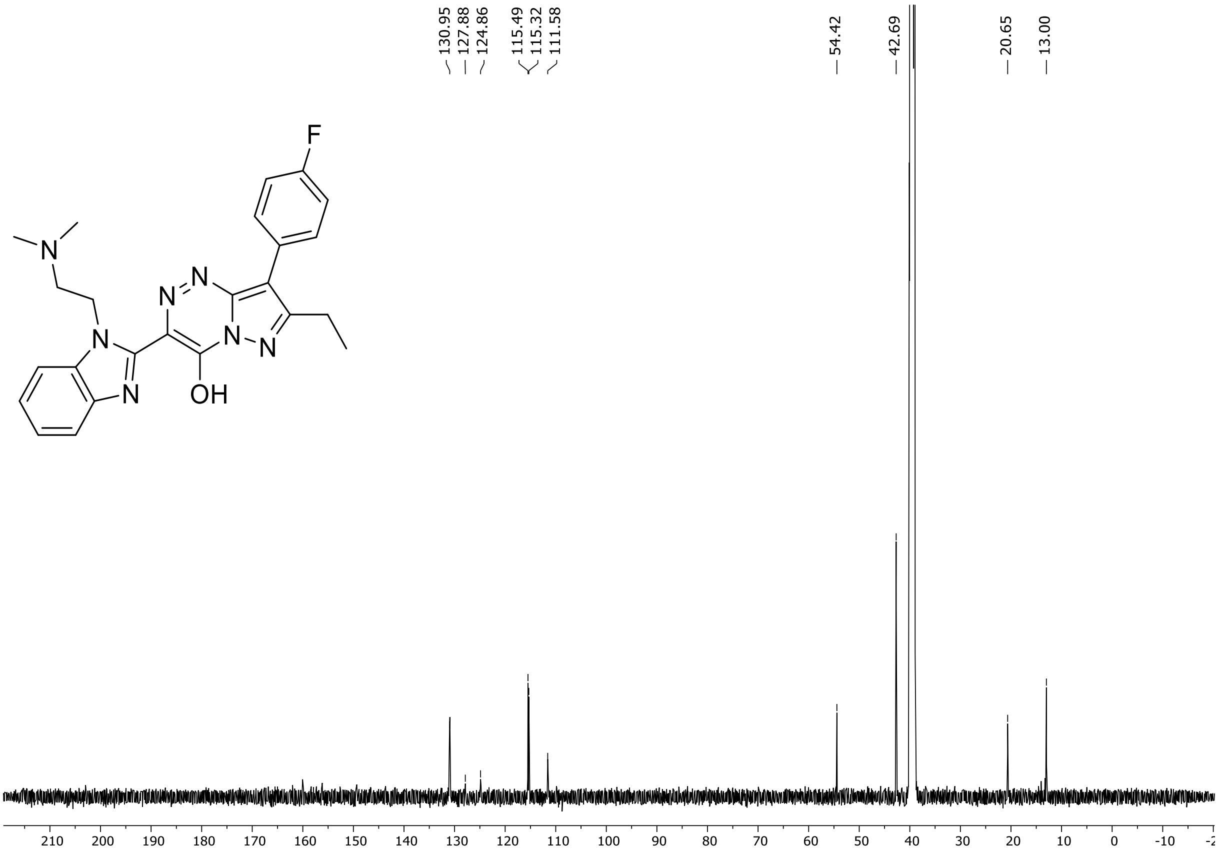

^{19}F (471 MHz) NMR spectrum of **23** in DMSO-d_6

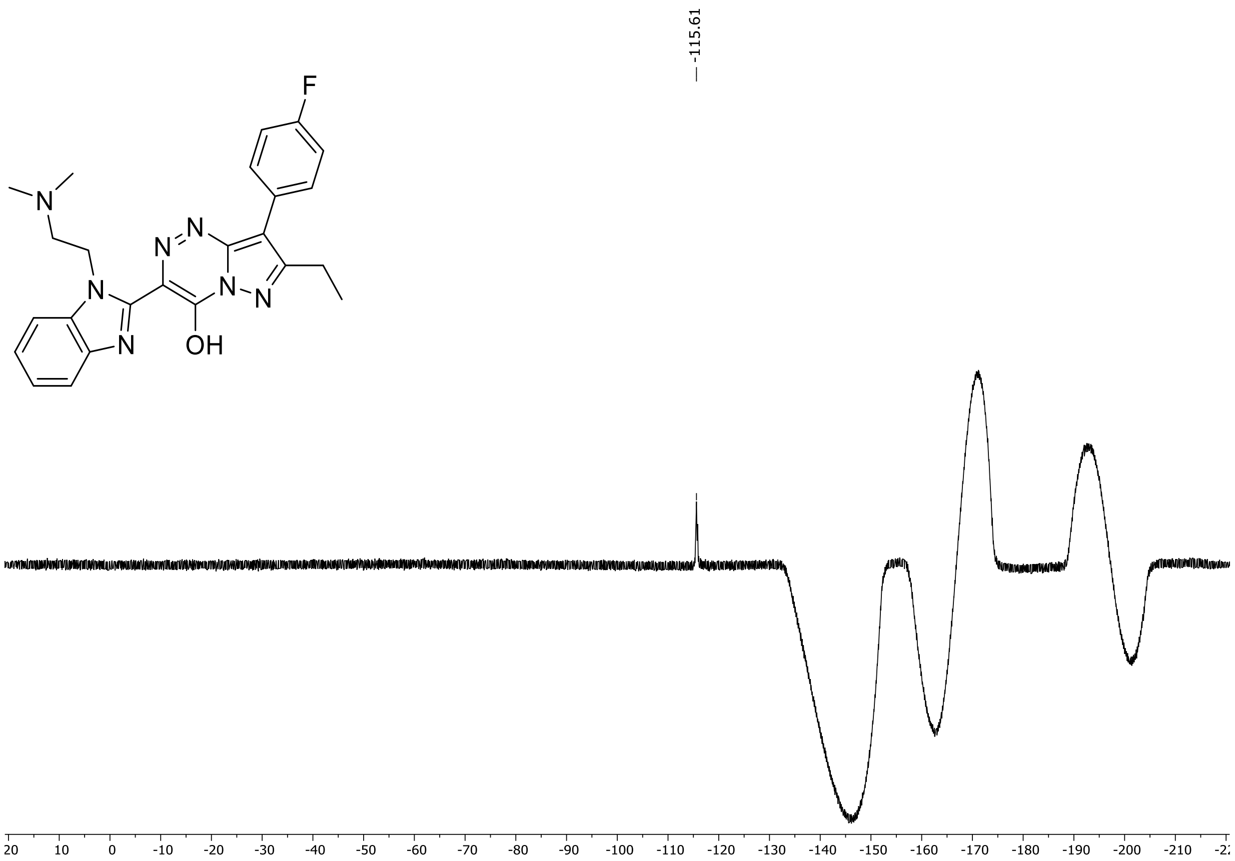

HRMS spectrum of **23**

$C_{24}H_{24}FN_7O$ **exact mass: 445.2026**

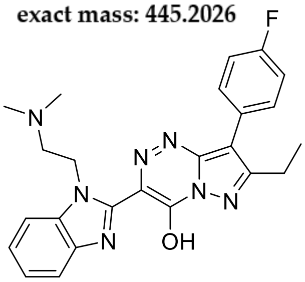

APCI + (MMI)
nitrogen flow 5 L/min, gas temperature 325°C, nebulizer 45 psig, skimmer 65 V, vaporizer 200°C, fragmentor 15 V, dissolved in methanol

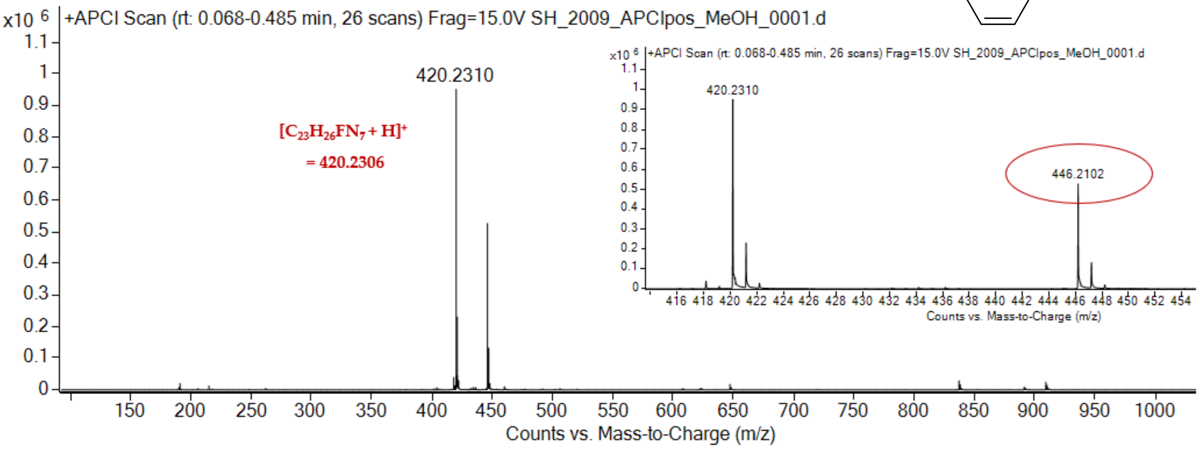

expected mass: $[M+H]^+ = 446.2099$ observed mass : $[M+H]^+ = 446.2102$ mass accuracy = 0.7 ppm

^{1}H (500 MHz) and ^{13}C NMR (126 MHz) spectra of **24** in DMSO-d_6

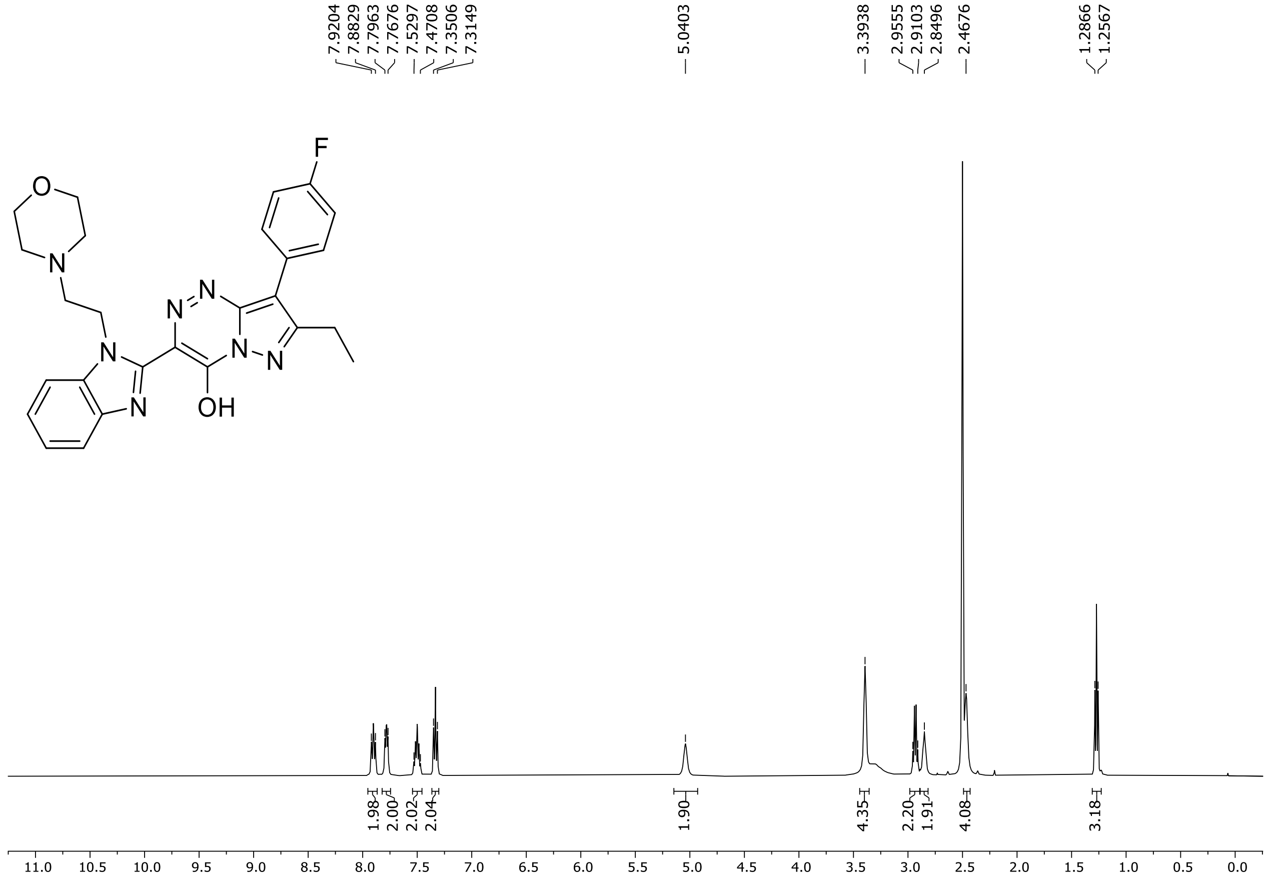

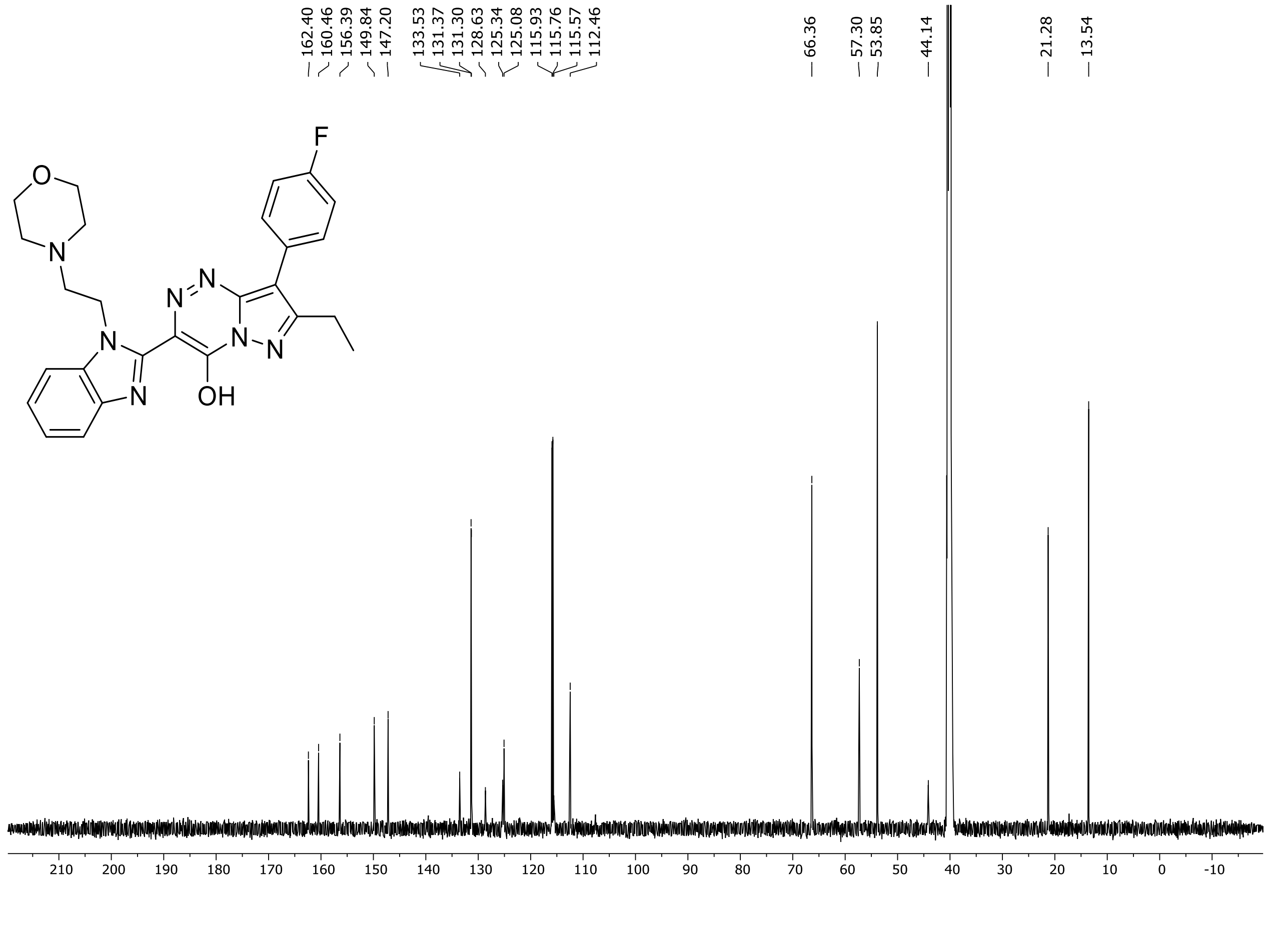

^{19}F (471 MHz) NMR spectrum of **24** in DMSO-d_6

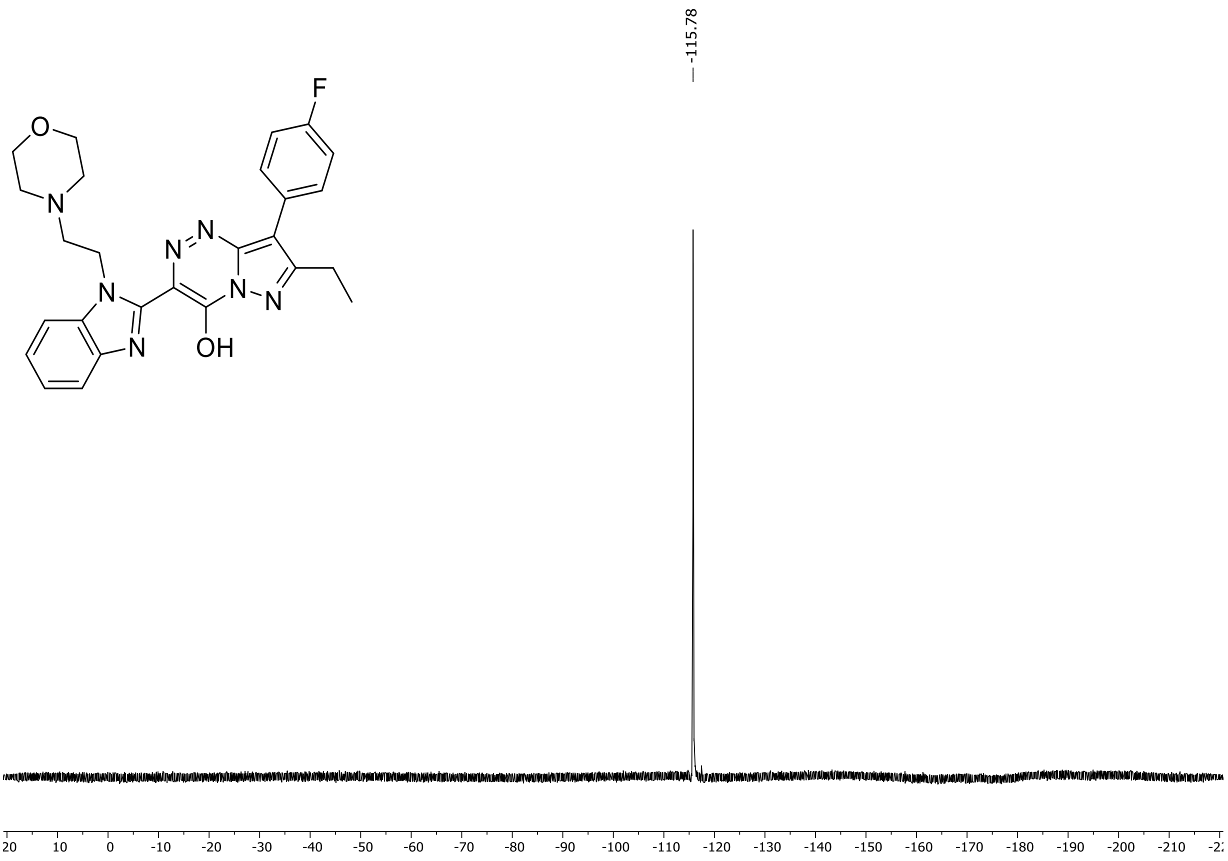

HRMS spectrum of **24**

$C_{26}H_{26}FN_7O_2$ **exact mass: 487.2132**

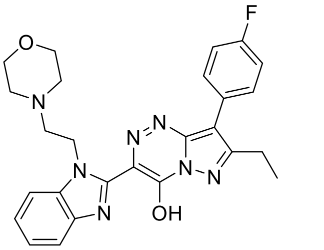

APCI + (MMI)
nitrogen flow 5 L/min, gas temperature 325°C, nebulizer 45 psig, skimmer 65 V, vaporizer 200°C, fragmentor 20 V, dissolved in methanol

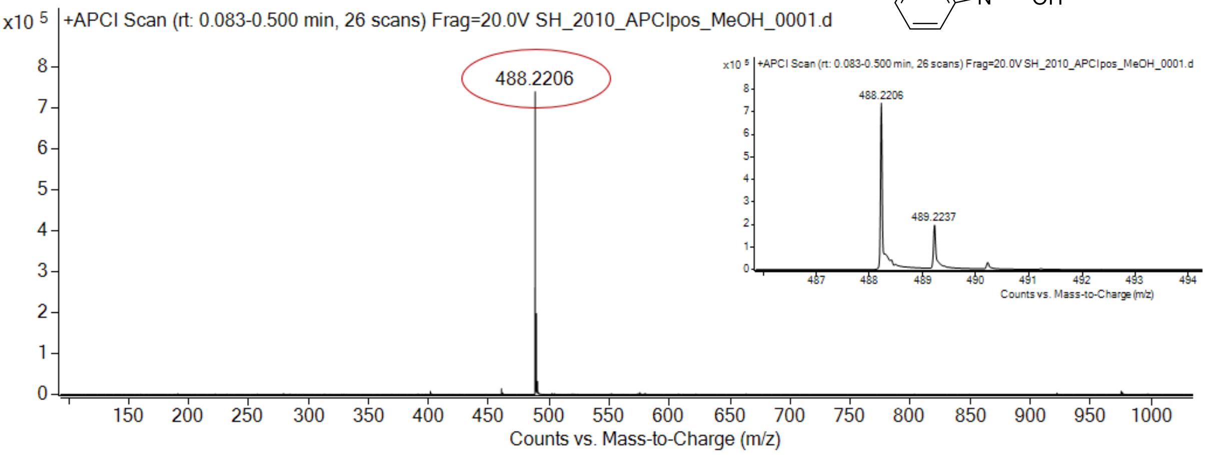

expected mass: $[M+H]^+$ = 488.2205 observed mass : $[M+H]^+$ = 488.2206 mass accuracy = 0.2 ppm

^{1}H (500 MHz) and ^{13}C NMR (126 MHz) spectra of **25** in DMSO-d_6

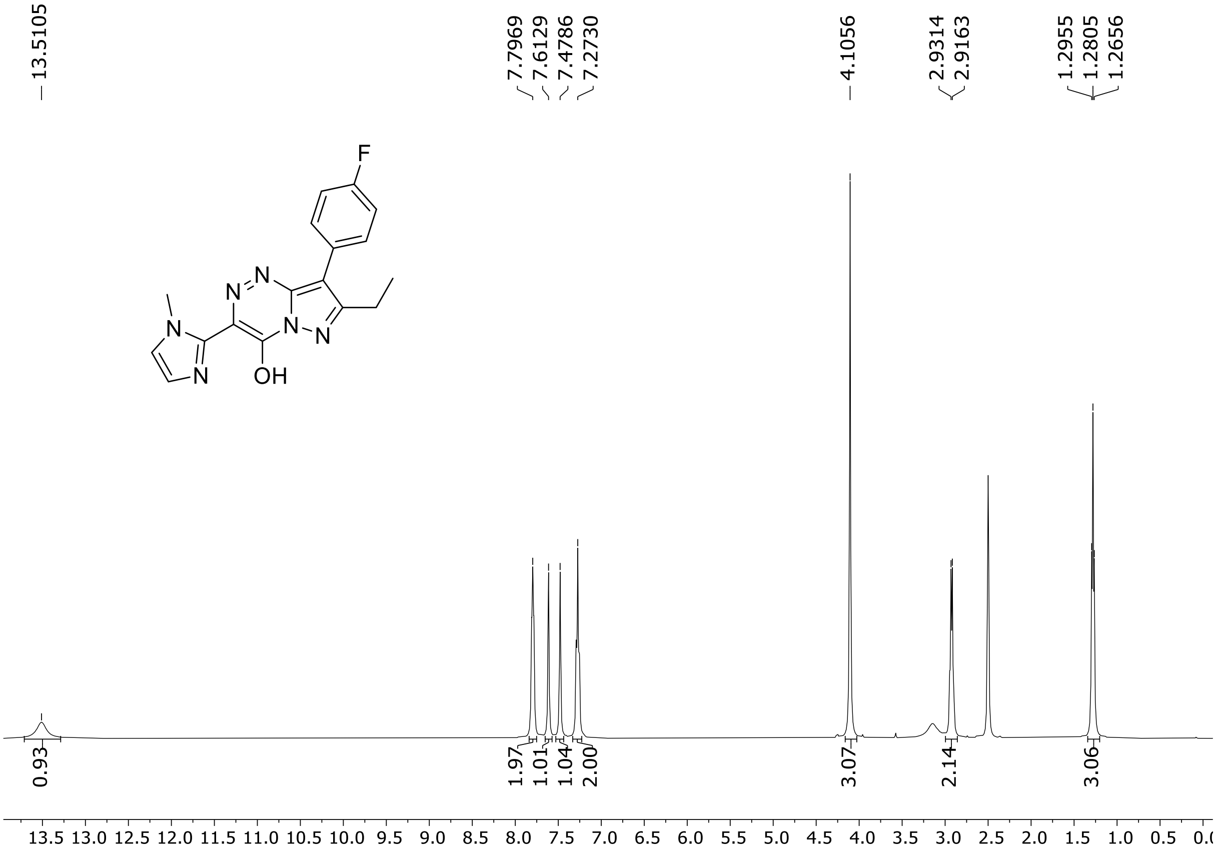

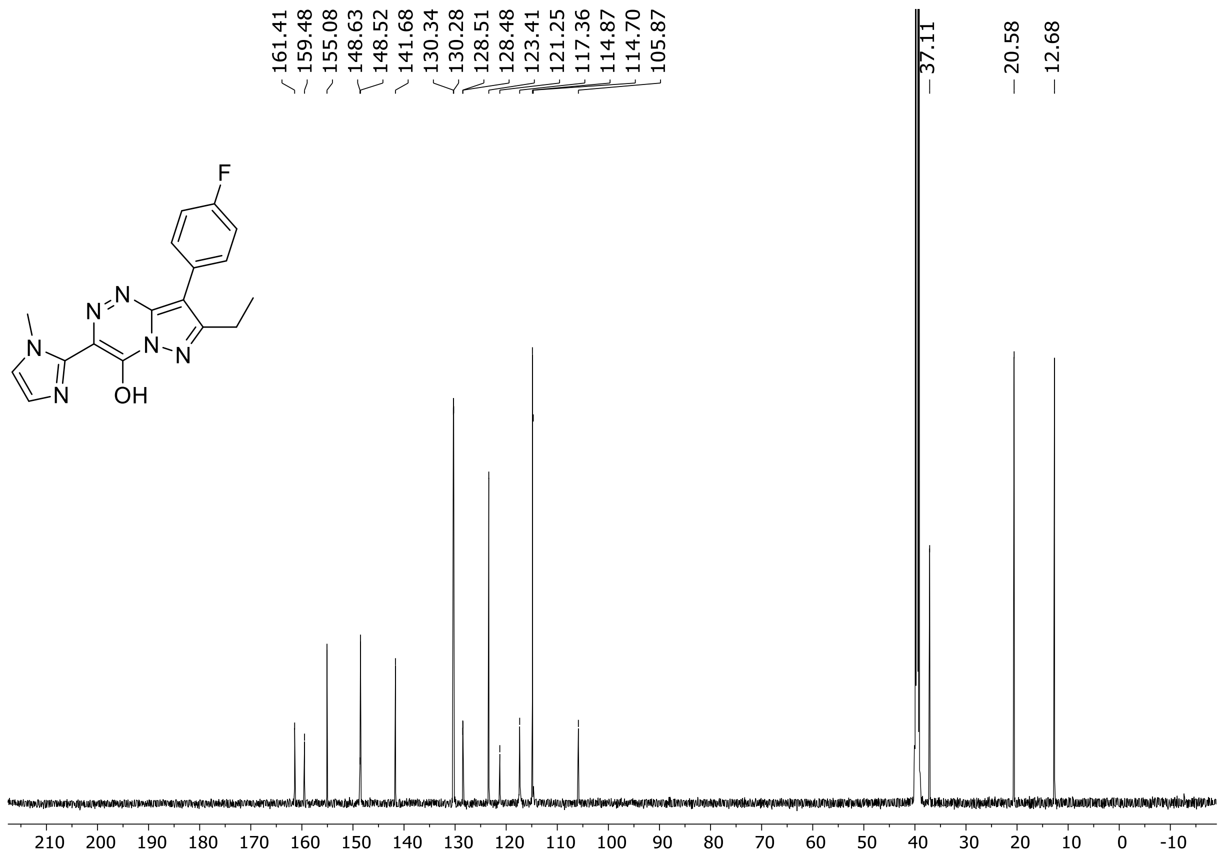

HRMS spectrum of **25**

$C_{17}H_{15}FN_6O$ **exact mass: 338.1291**

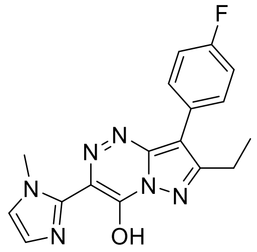

APCI + (MMI)
nitrogen flow 3 L/min, gas temperature 325°C, nebulizer 45 psig, skimmer 65 V, vaporizer 200°C, fragmentor 15 V, dissolved in methanol

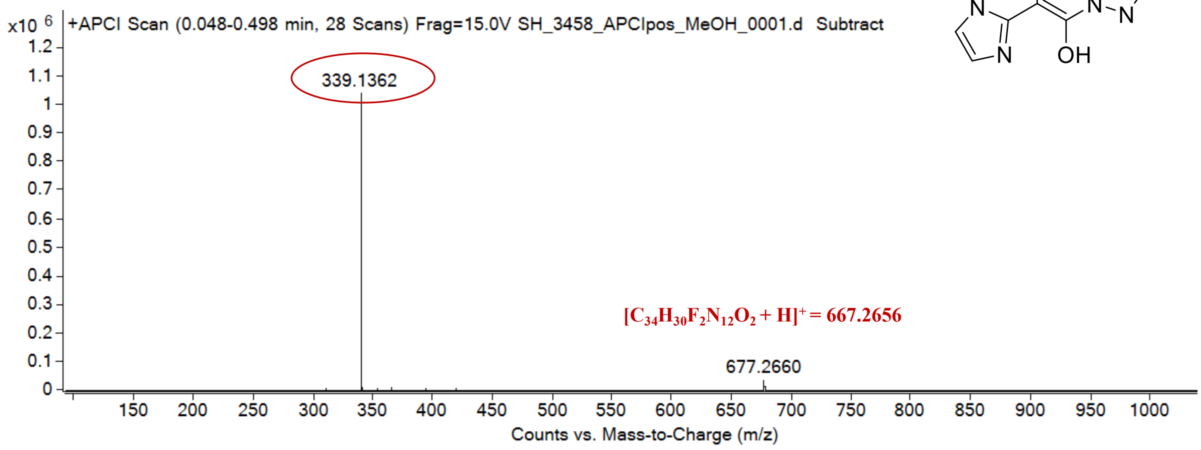

expected mass: $[M+H]^+$ = 339.1364 observed mass: $[M+H]^+$ = 339.1362 mass accuracy = - 0.6 ppm

^{1}H (500 MHz) and ^{13}C NMR (126 MHz) spectra of **26** in MeOD-d_4

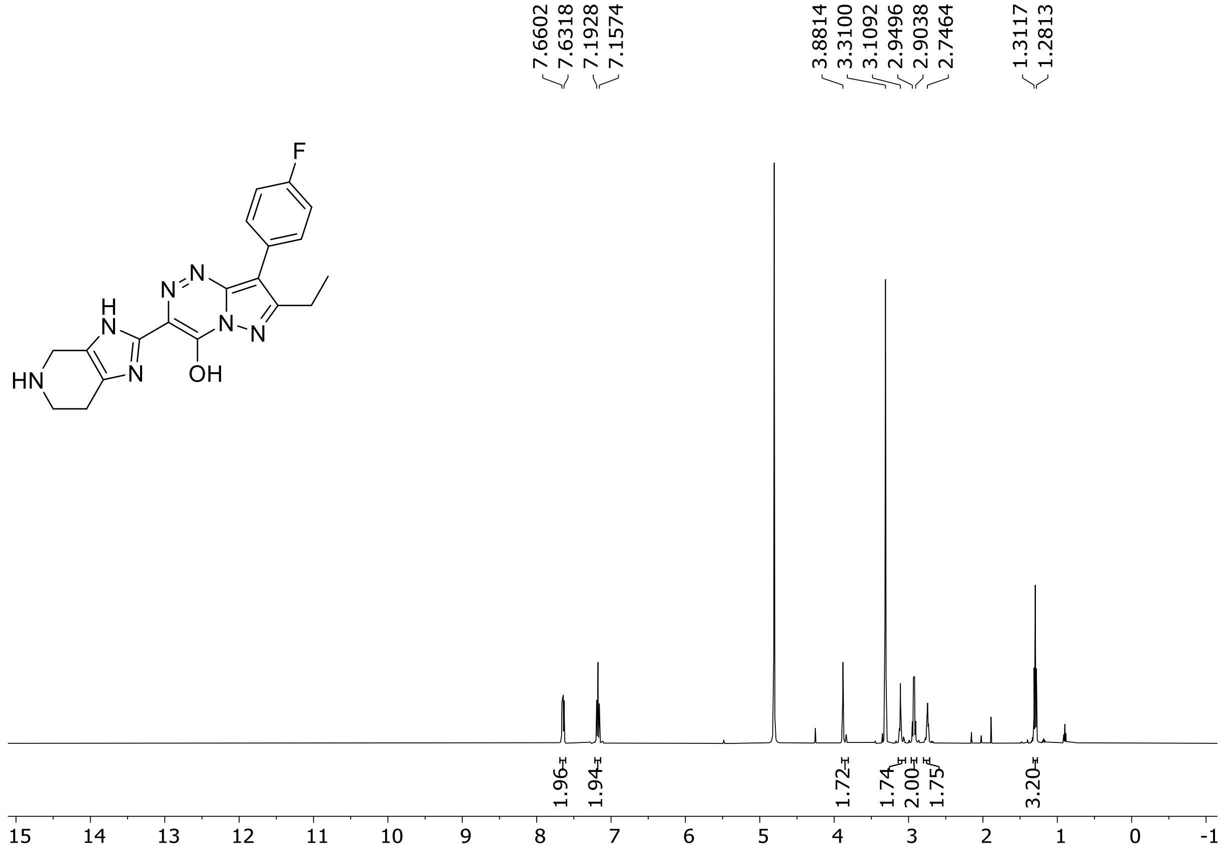

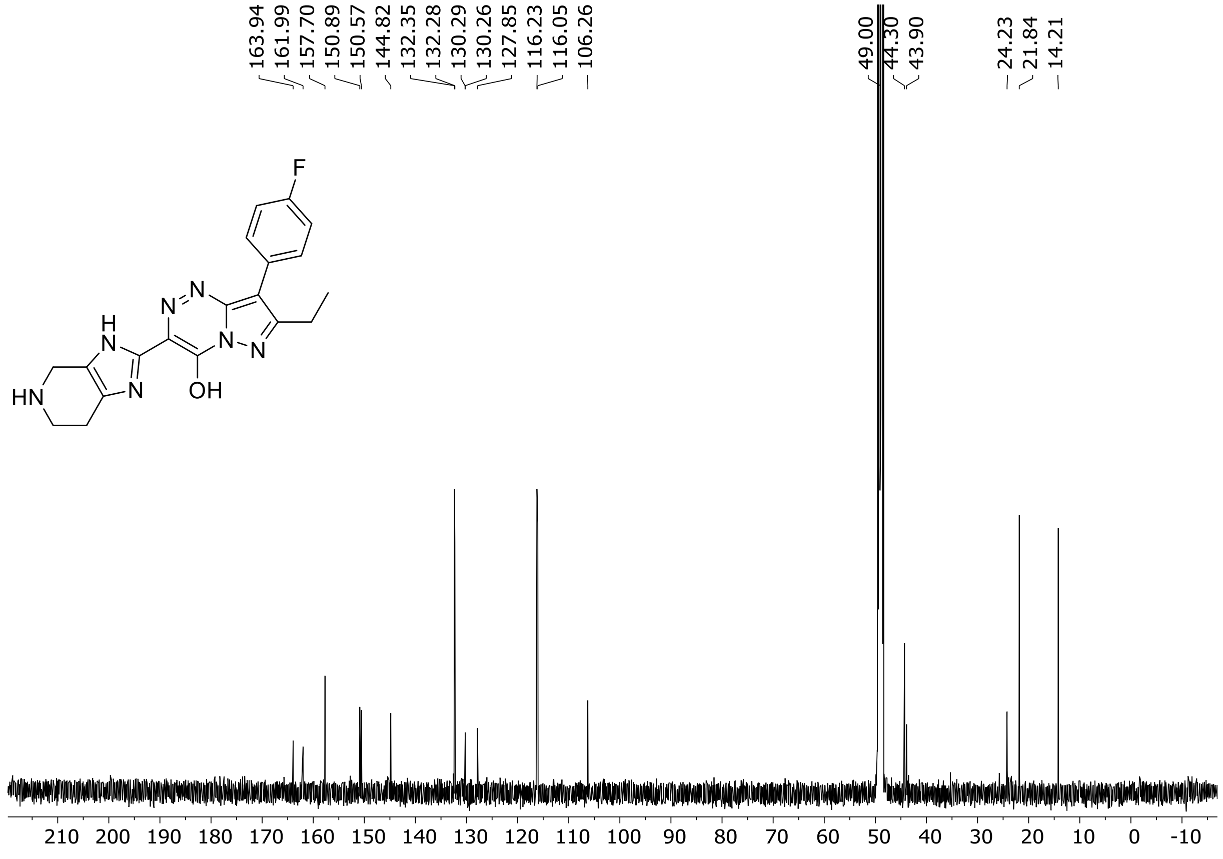

^{19}F (471 MHz) NMR spectrum of **26** in MeOD-d_4

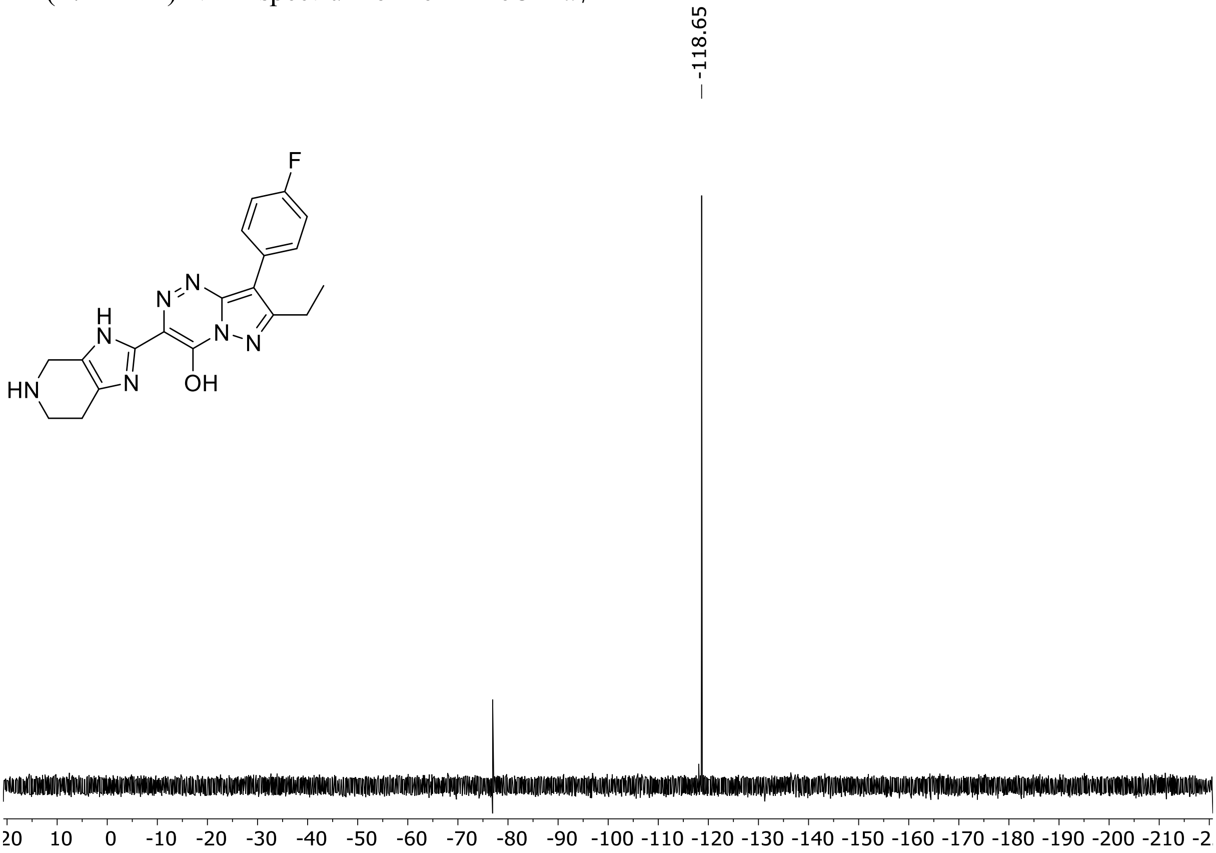

HRMS spectrum of **26**

$C_{19}H_{18}FN_7O$ **exact mass: 379.1557**

ESI - (MMI)
nitrogen flow 3 L/min, gas temperature 325°C, nebulizer 45 psig, skimmer -65 V, Vcap 2500V, fragmentor -110 V, dissolved in methanol

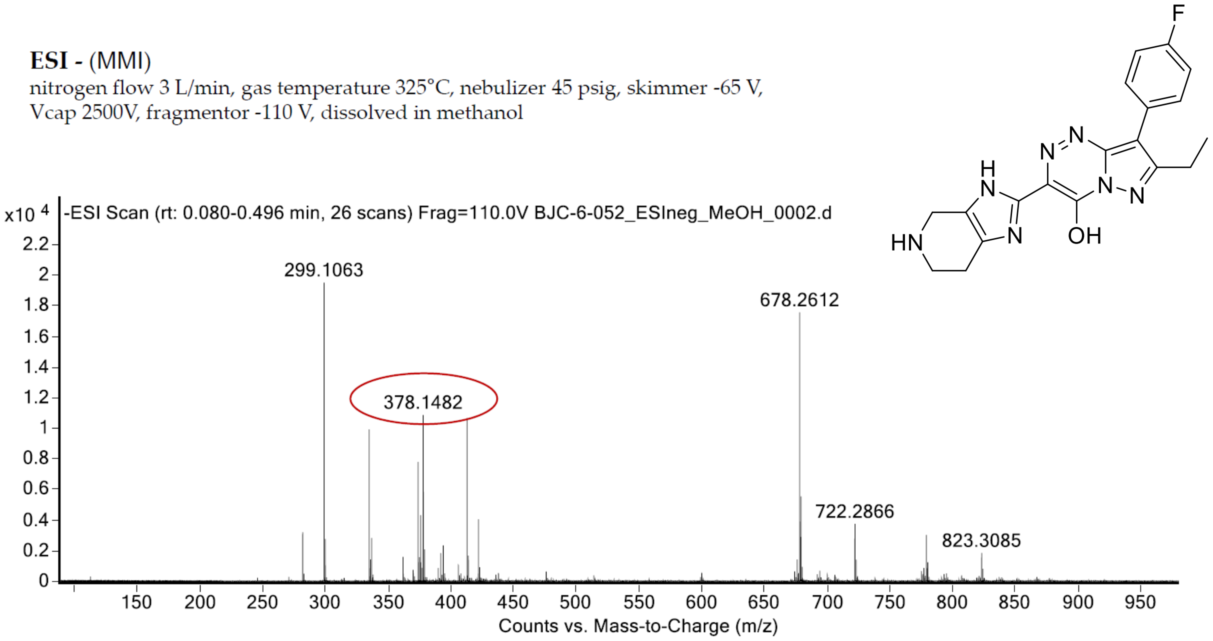

expected mass: [M-H]$^-$ = 378.1484 observed mass: [M-H]$^-$ = 378.1482 mass accuracy = - 0.5 ppm

^{1}H (500 MHz) and ^{13}C NMR (126 MHz) spectra of **27** in DMSO-d_6

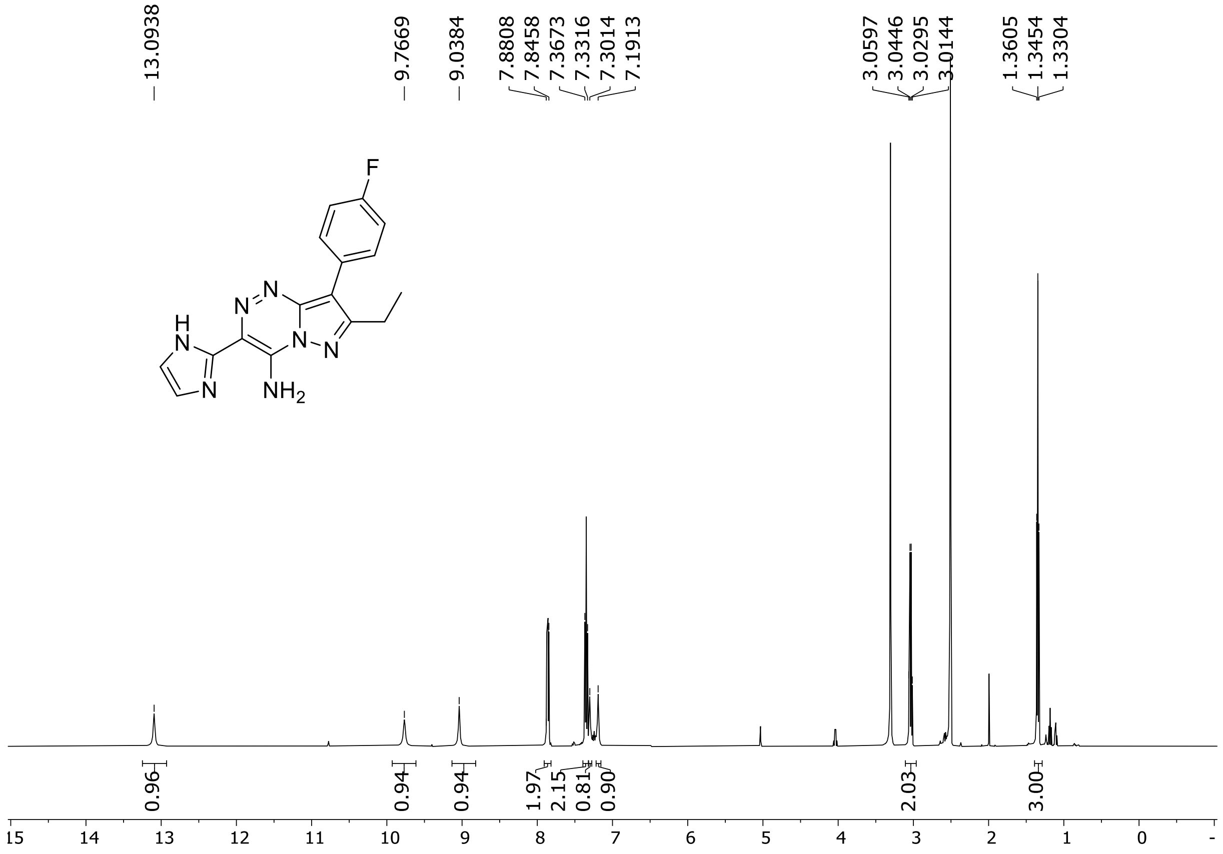

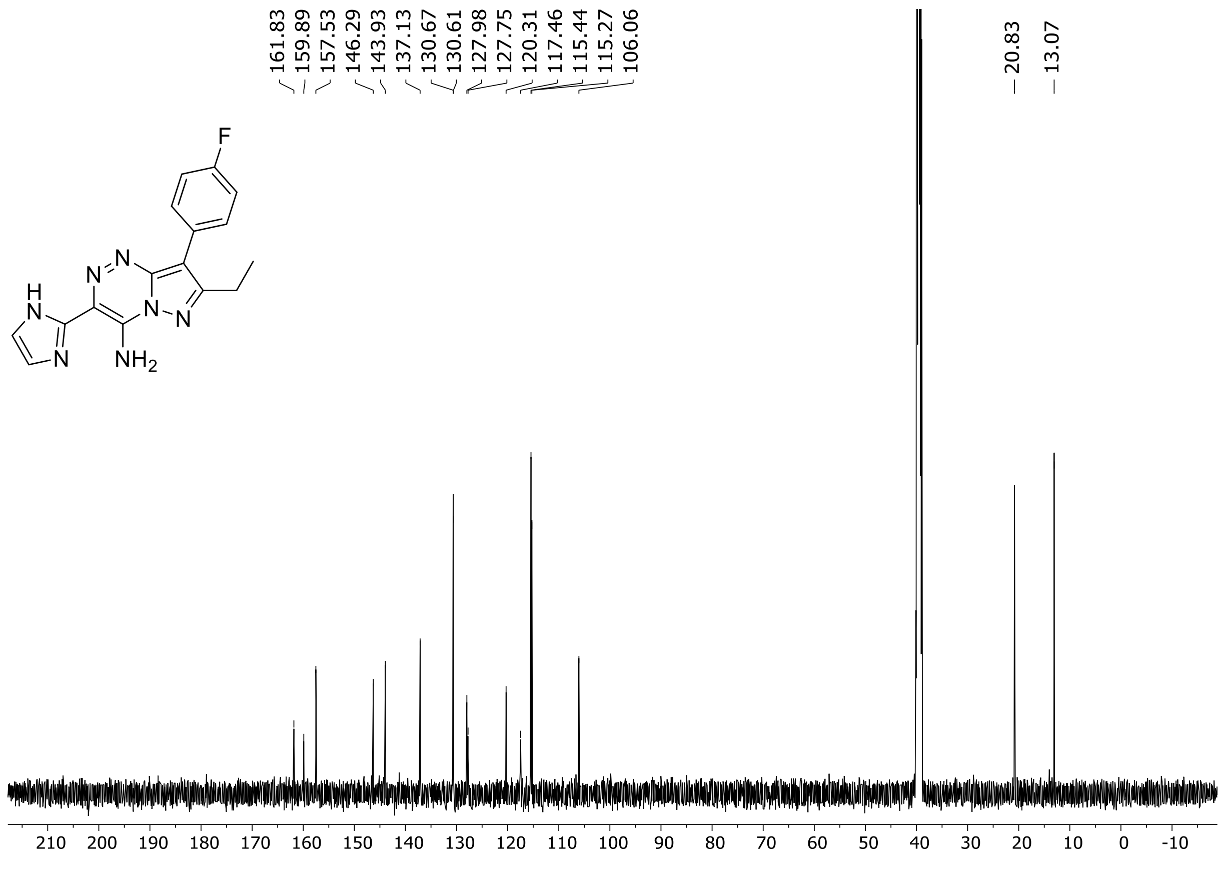

^{19}F (471 MHz) NMR spectrum of **27** DMSO-d_6

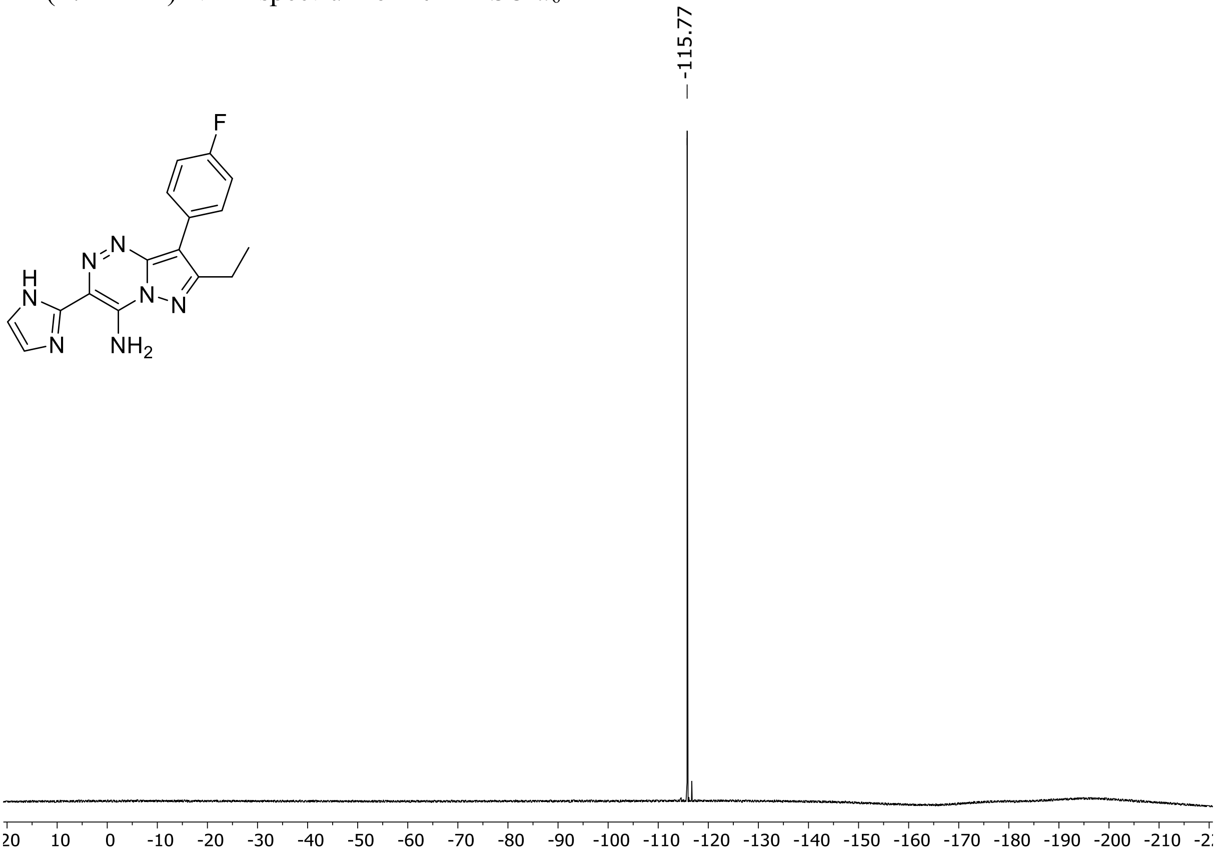

HRMS spectrum of **27**

$C_{16}H_{14}FN_7$ **exact mass: 323.1295**

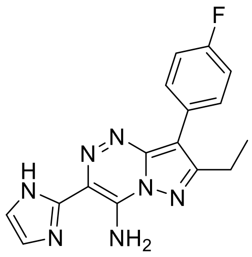

APCI + (MMI)
nitrogen flow 5 L/min, gas temperature 325°C, nebulizer 45 psig, skimmer 65 V, vaporizer 200°C, fragmentor 5 V, dissolved in methanol

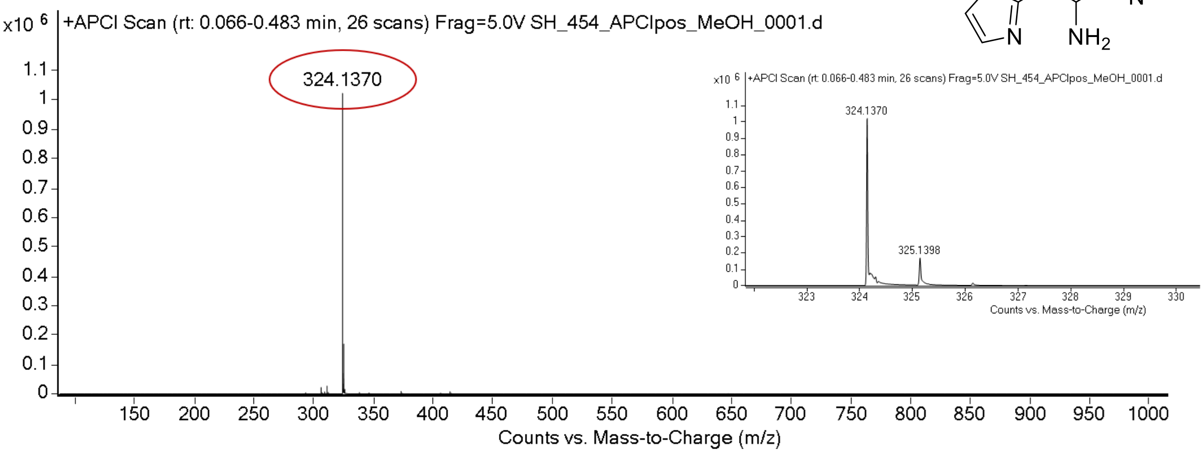

expected mass: [M+H]$^+$ = 324.1367 observed mass : [M+H]$^+$ = 324.1370 mass accuracy = 0.9 ppm

^{1}H (500 MHz) and ^{13}C NMR (126 MHz) spectra of **28** in DMSO-d_6

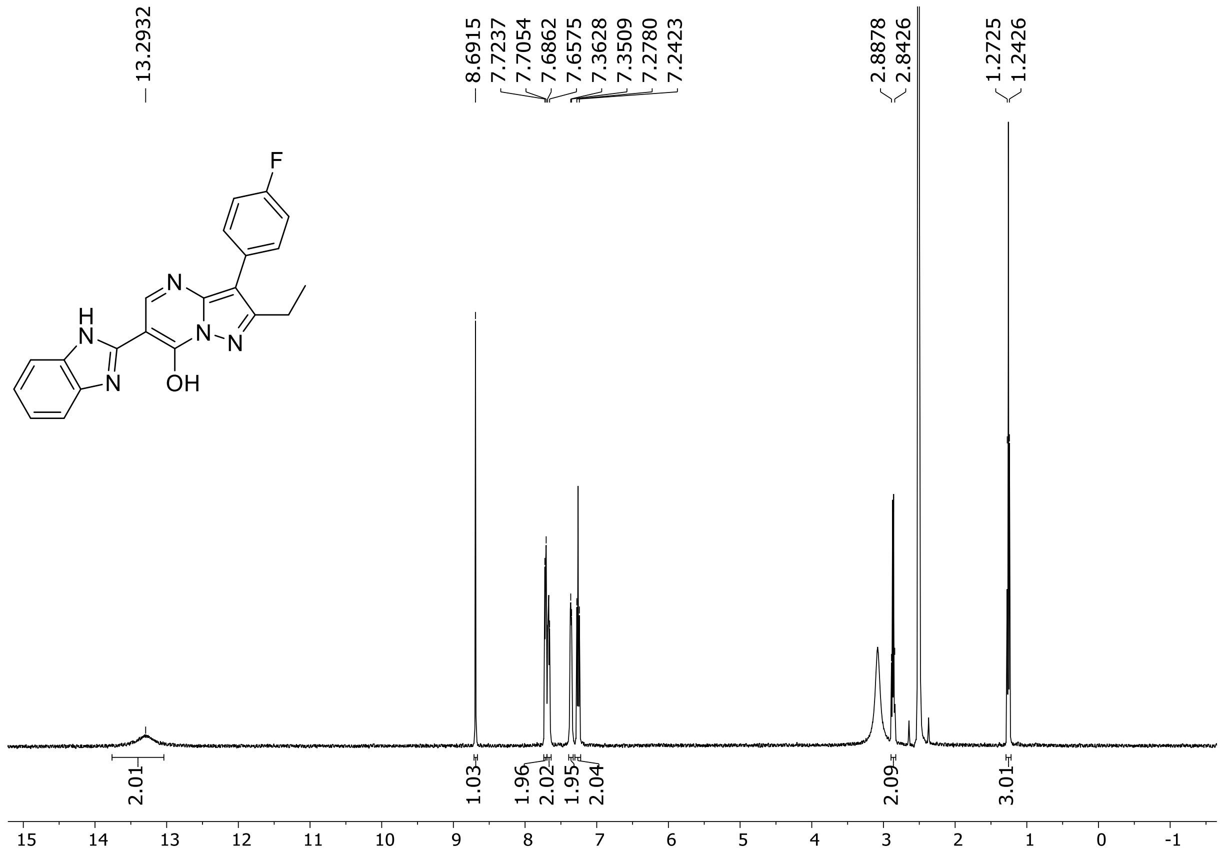

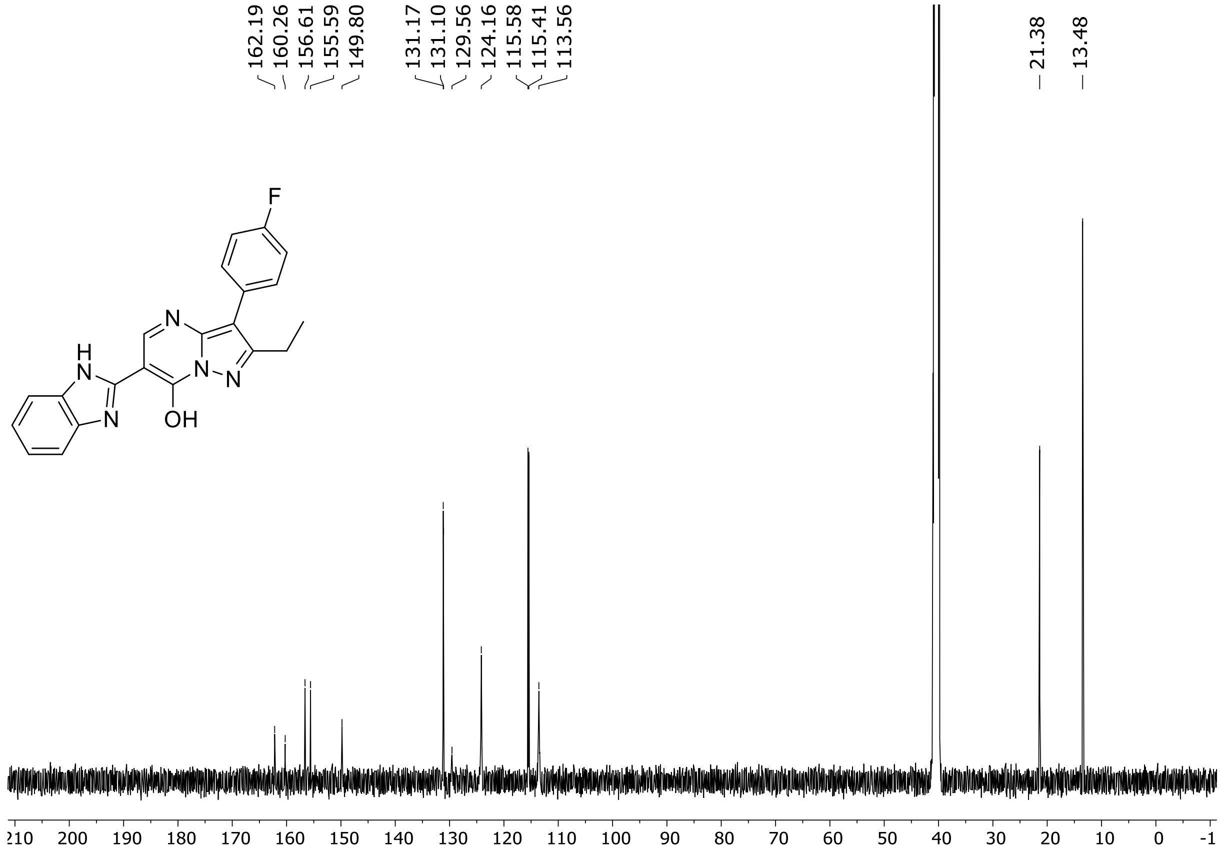

HRMS spectrum of **28**

$C_{21}H_{16}FN_5O$ **exact mass: 373.1339**

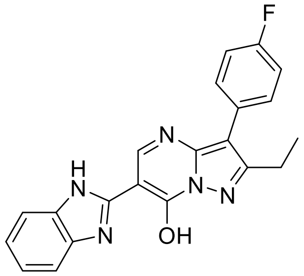

APCI + (MMI)
nitrogen flow 5 L/min, gas temperature 325°C, nebulizer 45 psig, skimmer 65 V, vaporizer 200°C, fragmentor 20 V, dissolved in methanol

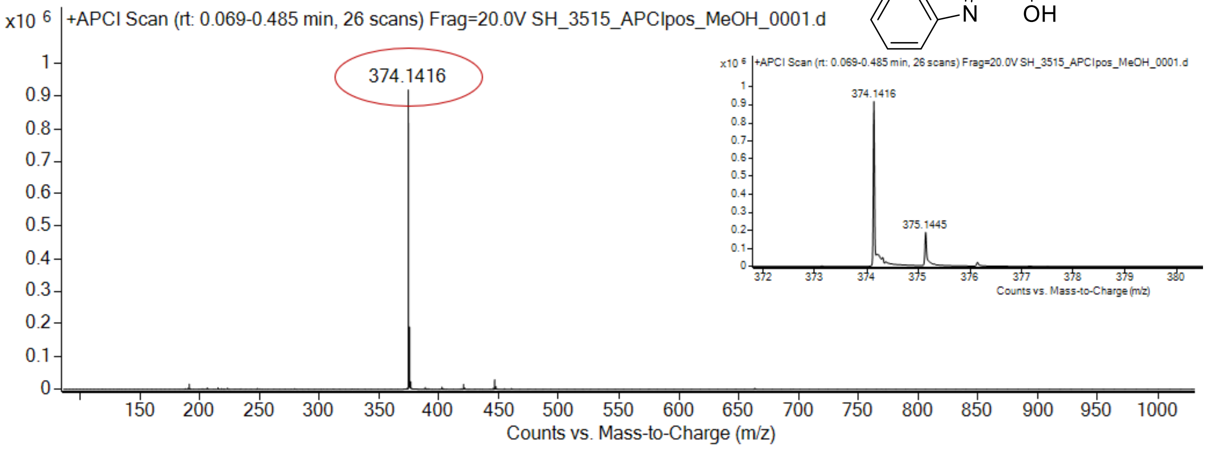

expected mass: $[M+H]^+$ = 374.1412 observed mass : $[M+H]^+$ = 374.1416 mass accuracy = 1.1 ppm

^{1}H NMR, ^{13}C NMR and HRMS spectra of compound 56-117

^{1}H (500 MHz) and ^{13}C NMR (126 MHz) spectra of **56** in DMSO-d_6

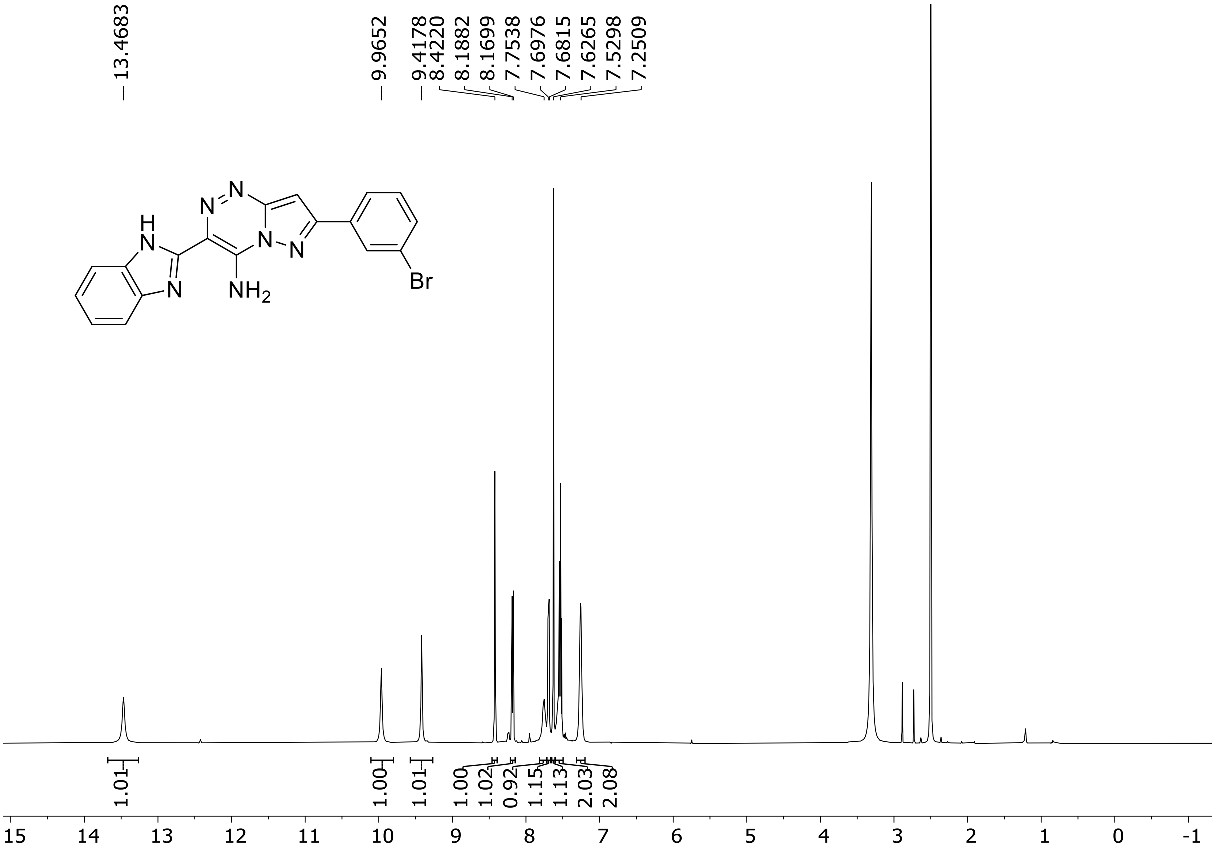

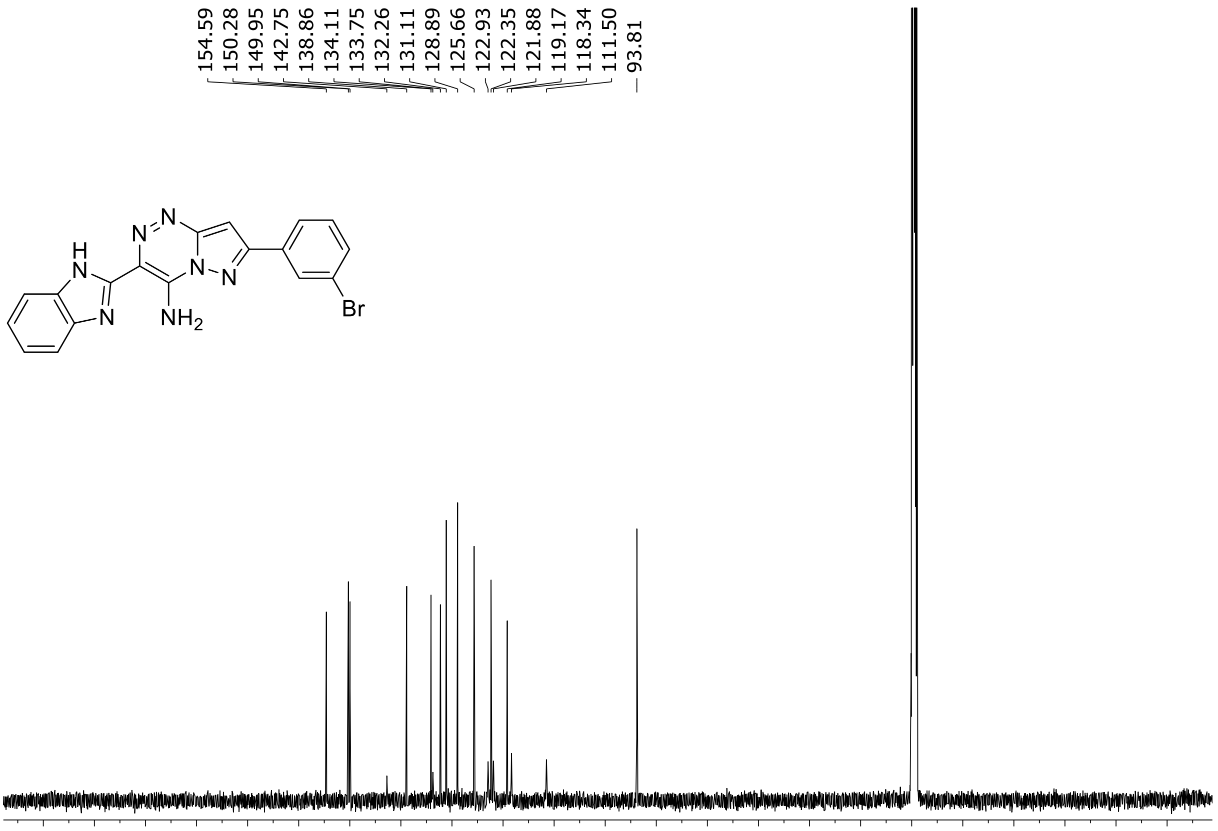

HRMS spectrum of **56**

$C_{18}H_{12}BrN_7$ mono m/z = 405.0338

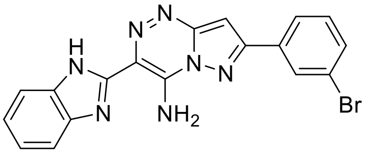

APCI + (MMI)
nitrogen flow 5 L/min, gas temperature 300°C, nebulizer 45 psi, skimmer 60 V, fragmentor 37 V, dissolved in methanol

ZOOM – range of interest

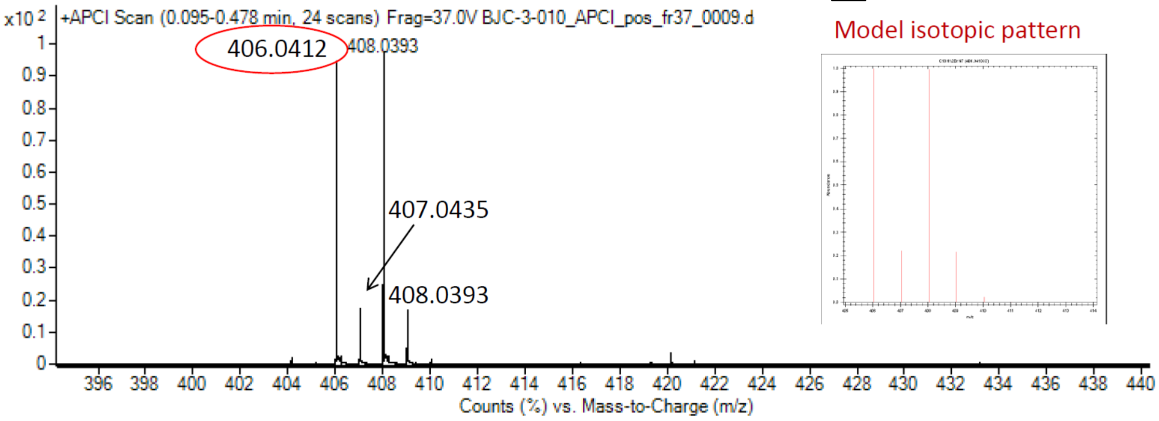

calculated mass: [M+H]+= 406. 0410 observed: $[M+H]^+$ = 406.0412 max. mass error = 0.5 ppm

^{1}H (500 MHz) and ^{13}C NMR (126 MHz) spectra of **57** in DMSO-d_6

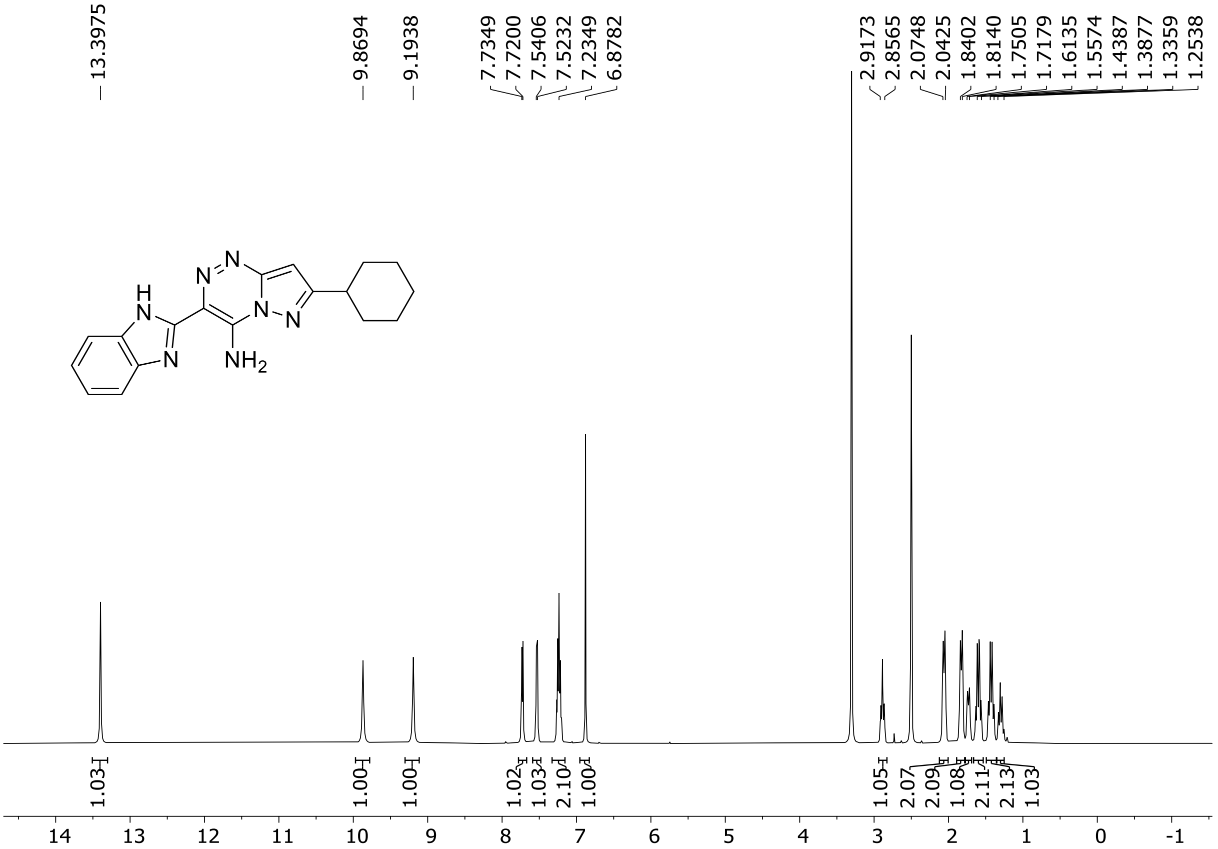

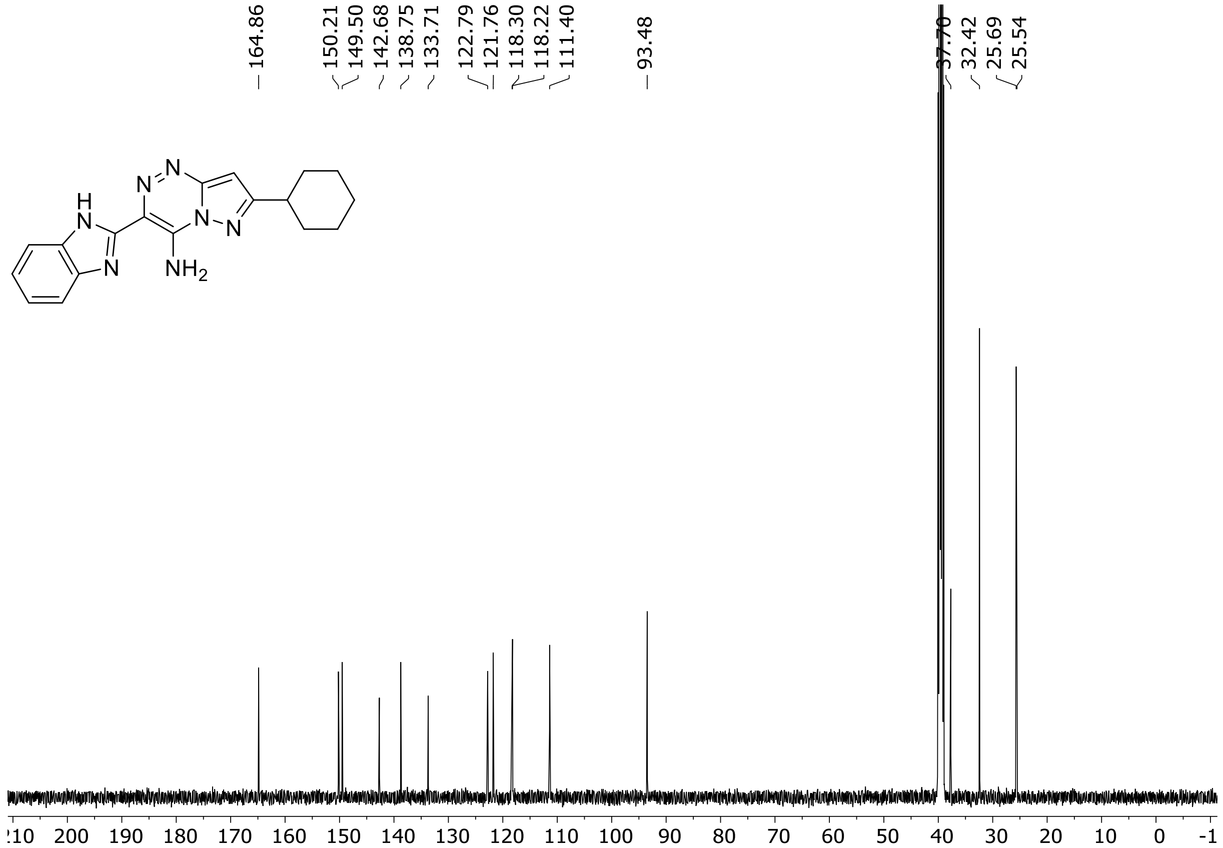

HRMS spectrum of **57**

$C_{18}H_{19}N_7$ mono m/z = 333.1702

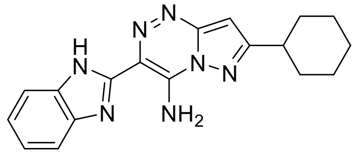

APCI + (MMI)
nitrogen flow 5 L/min, gas temperature 300°C, nebulizer 45 psi,
skimmer 60 V, fragmentor 17 V, dissolved in methanol

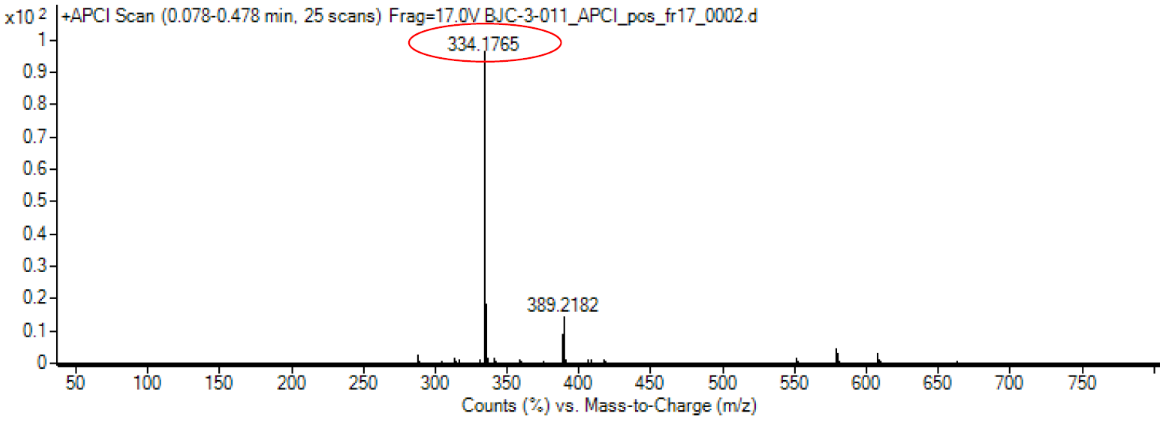

calculated mass: $[M+H]^+$ = 334.1775 observed: $[M+H]^+$ = 334.1765 max. mass error = 3.0 ppm

^{1}H (500 MHz) and ^{13}C NMR (126 MHz) spectra of **58** in DMSO-d_6

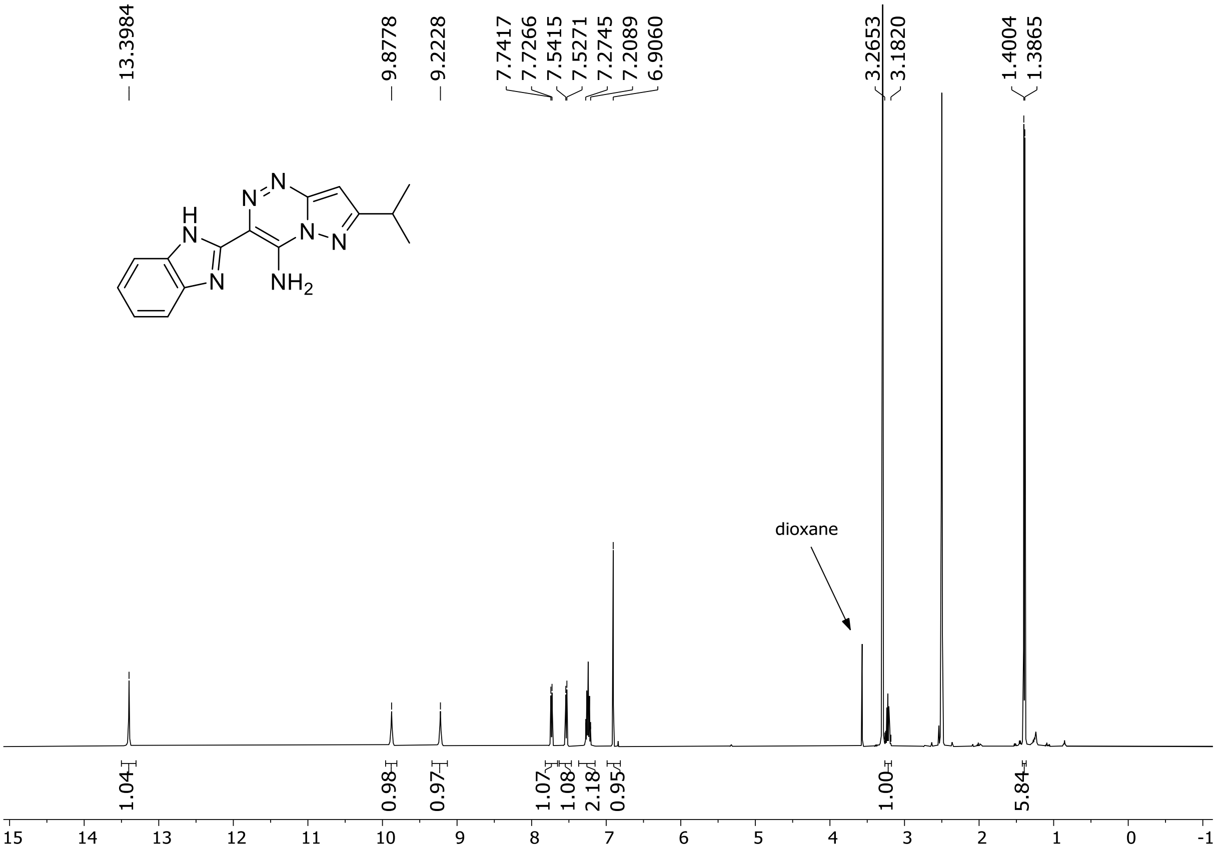

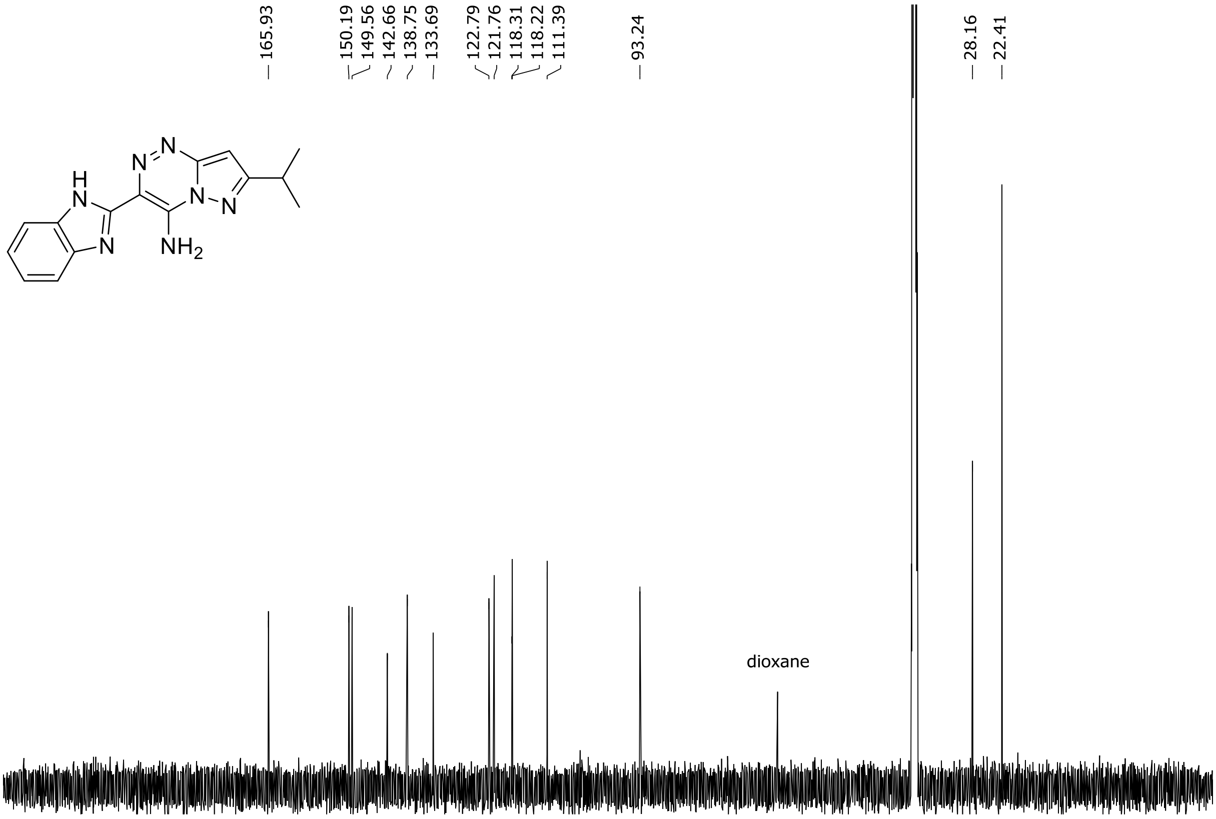

HRMS spectrum of **58**

$C_{15}H_{15}N_7$ **mono m/z = 293.1389**

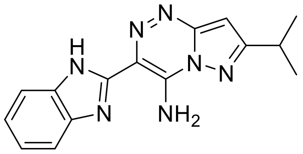

APCI + (MMI)
nitrogen flow 5 L/min, gas temperature 300°C, nebulizer 45 psi, vaporizer 200°C
skimmer **65** V, fragmentor **20** V, dissolved in methanol

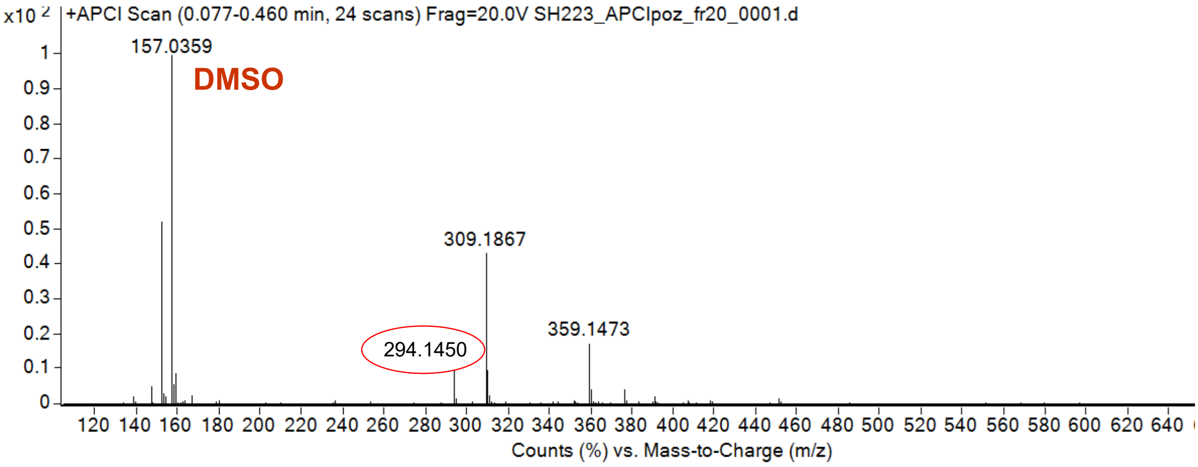

calculated mass: $[M+H]^+$ = 294.1462 observed: $[M+H]^+$ = 294.1450 max. mass error = 4 ppm

^{1}H (500 MHz) and ^{13}C NMR (126 MHz) spectra of **59** in DMSO-d_6

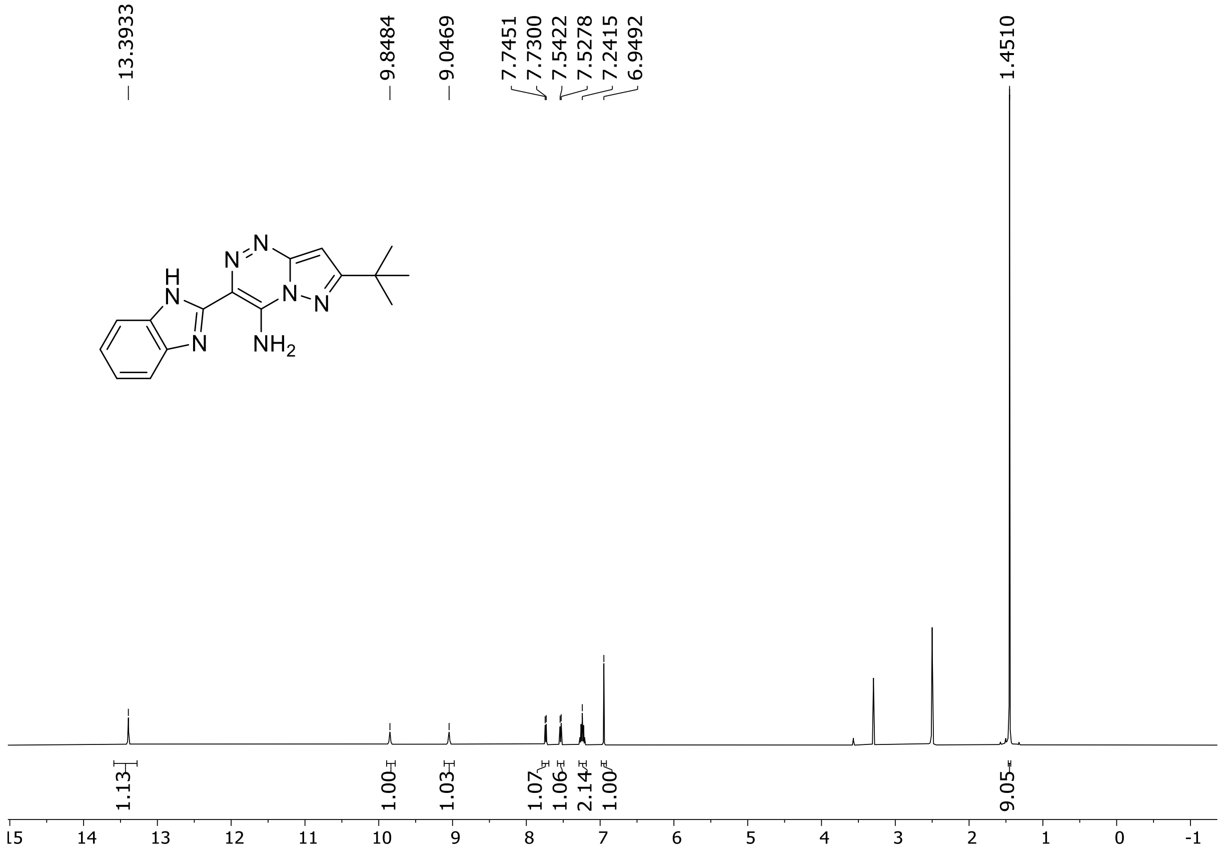

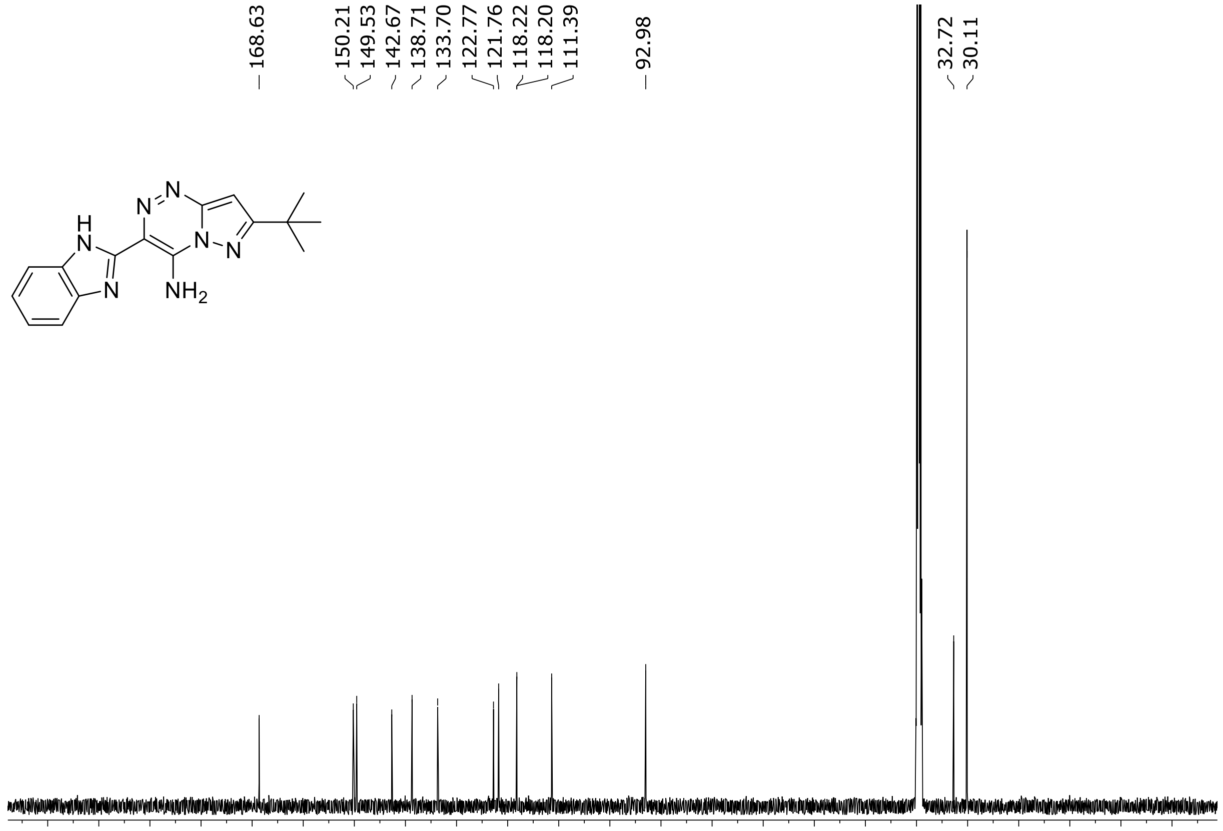

HRMS spectrum of **59**

$C_{16}H_{17}N_7$ **mono m/z = 307.1545**

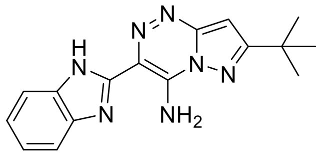

ESI - (MMI)
nitrogen flow 5 L/min, gas temperature 300°C, vaporizer 250°C,
nebulizer 45 psi, skimmer **40** V, fragmentor **40** V, dissolved in DMSO

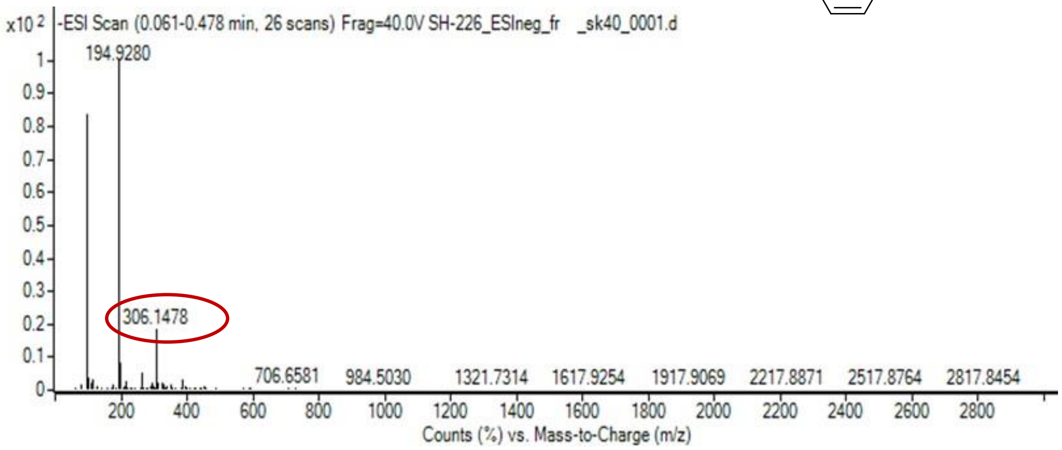

calculated mass: $[M-H]^-$ = 306.1473 observed: $[M-H]^-$ =306.1478 m ax. mass error = 1.6 ppm

^{1}H (500 MHz) and ^{13}C NMR (126 MHz) spectra of **60** in DMSO-d_6

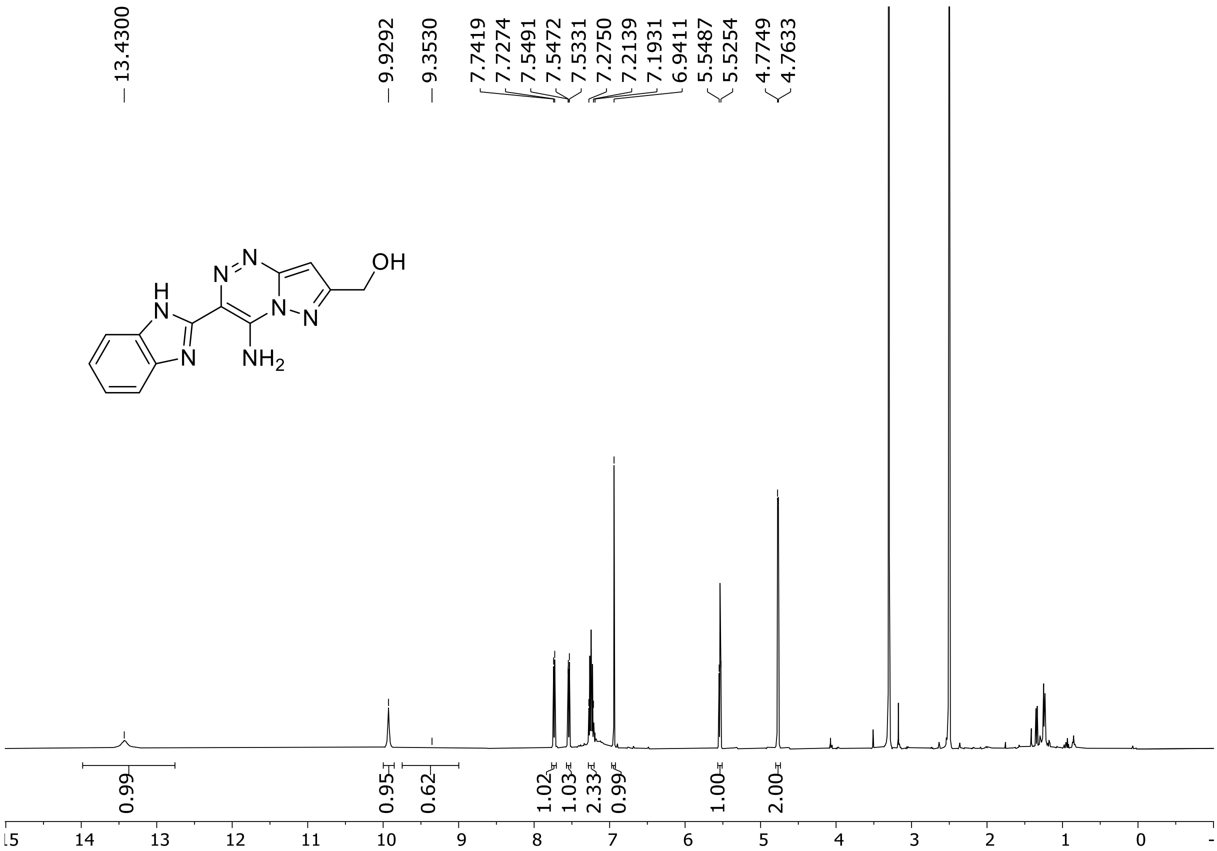

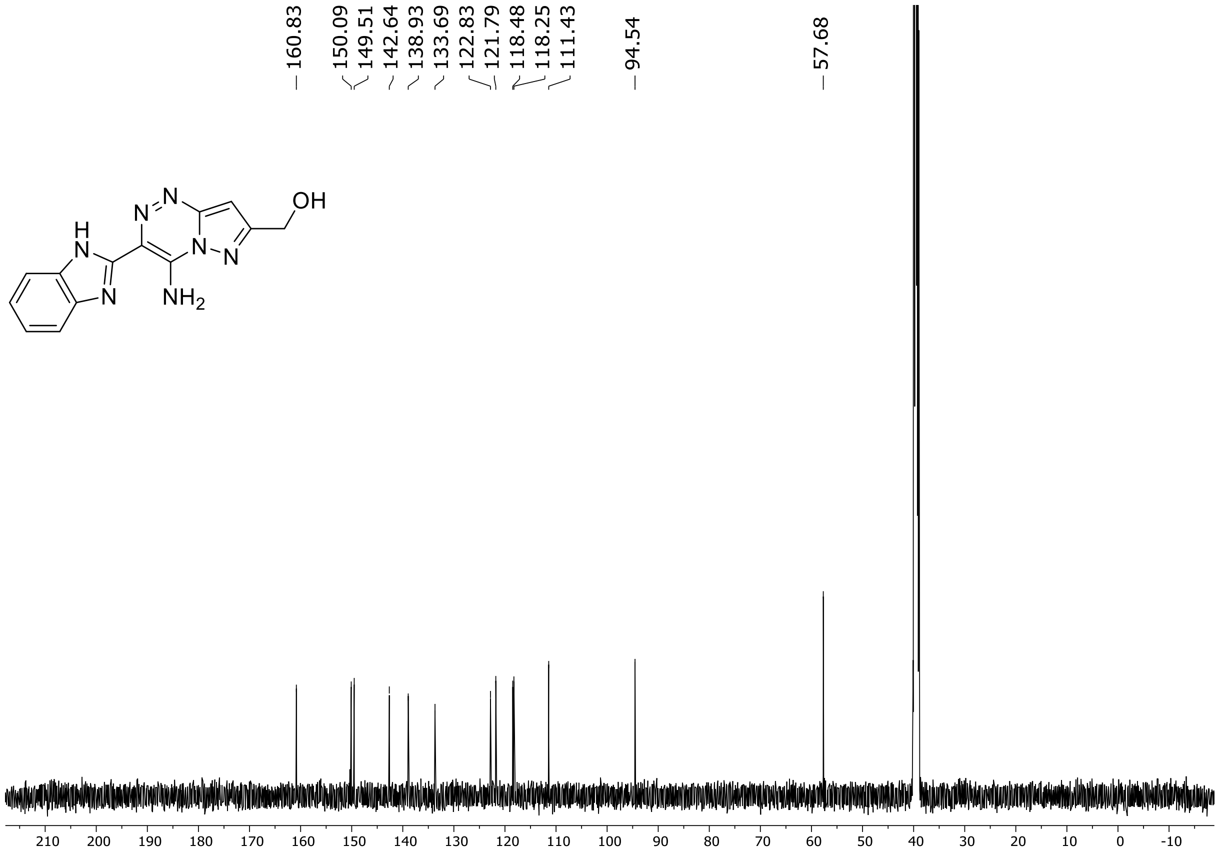

HRMS spectrum of **60**

$C_{13}H_{11}N_7O$ **mono m/z = 281.1025**

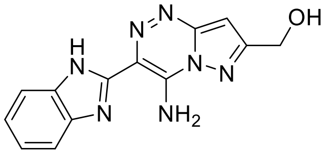

APCI + (MMI)
nitrogen flow 5 L/min, gas temperature 300°C, nebulizer 45 psi, vaporizer 200°C
skimmer **65** V, fragmentor **20** V, dissolved in methanol

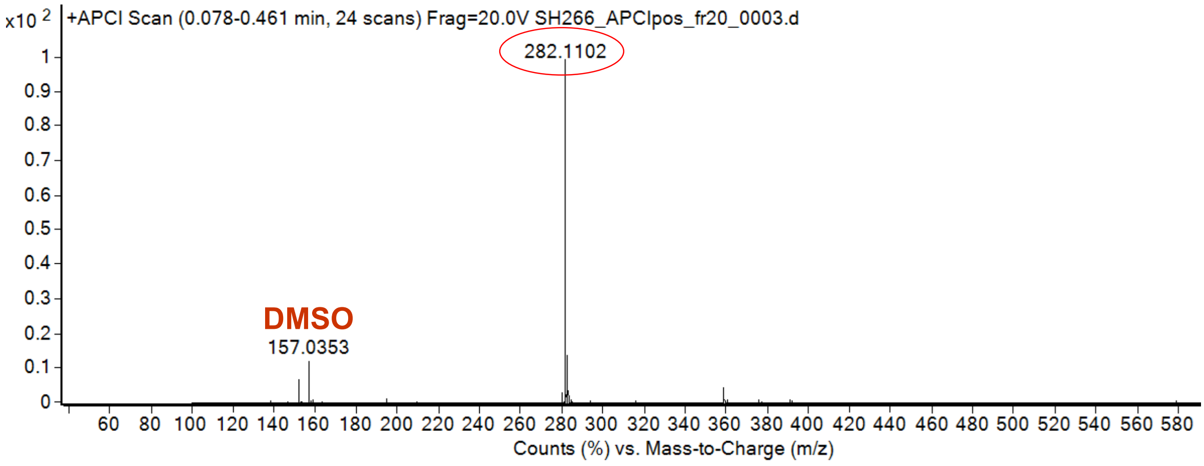

calculated mass: $[M+H]^+$ = 282.1098 observed: $[M+H]^+$ = 282.1102 max. mass error = 1.4 ppm

^{1}H (500 MHz) and ^{13}C NMR (126 MHz) spectra of **61** in DMSO-d_6

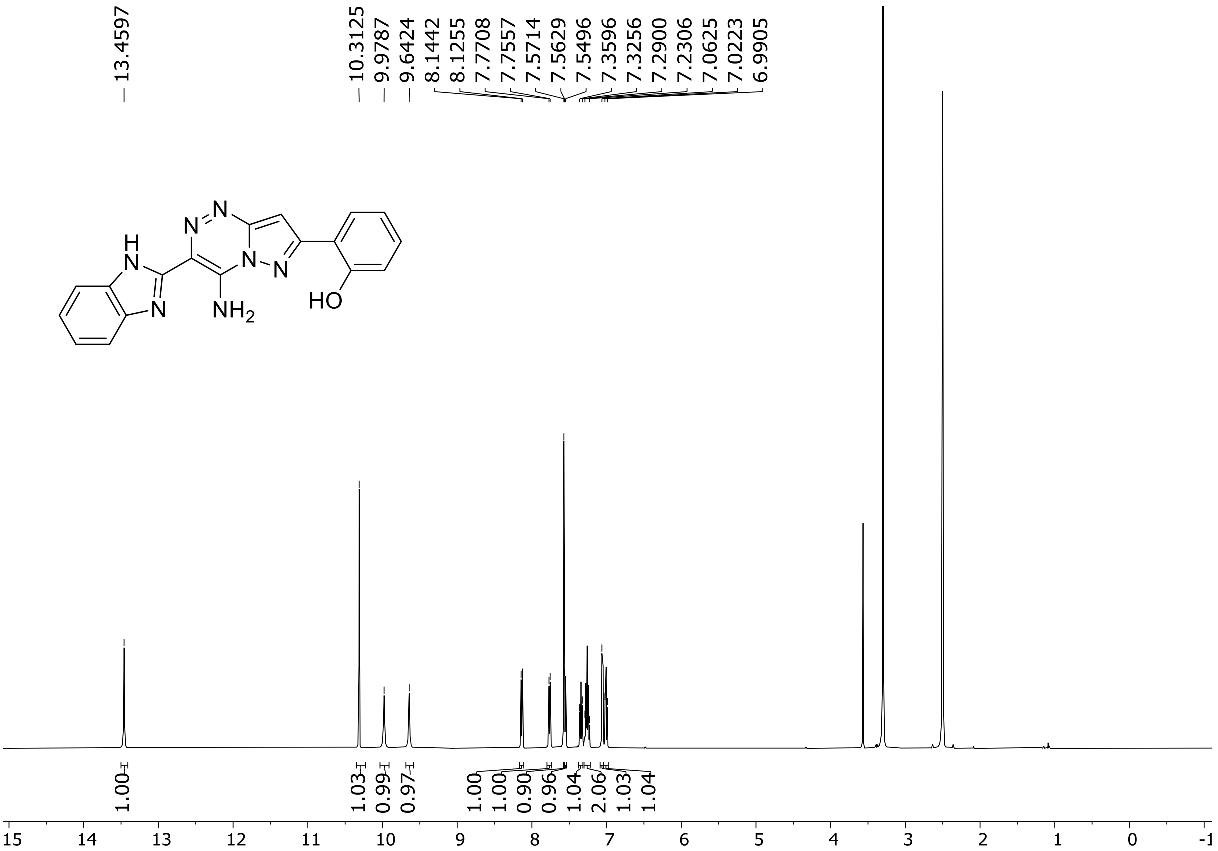

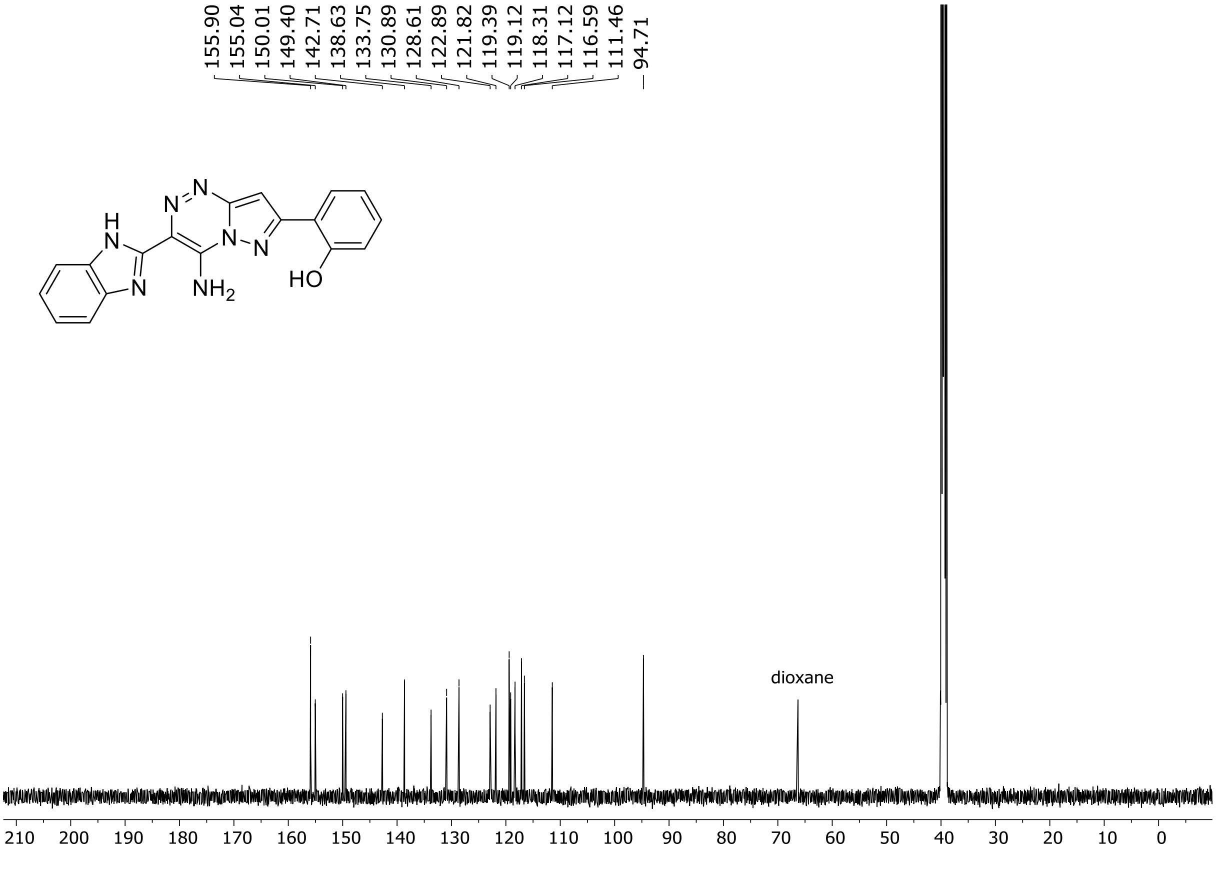

HRMS spectrum of **61**

$C_{18}H_{13}N_7O$ **mono m/z = 343.1182**

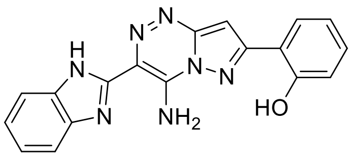

APCI + (MMI)
nitrogen flow 5 L/min, gas temperature 300°C, nebulizer 45 psi, vaporizer 200°C
skimmer **65** V, fragmentor 25 V, dissolved in methanol

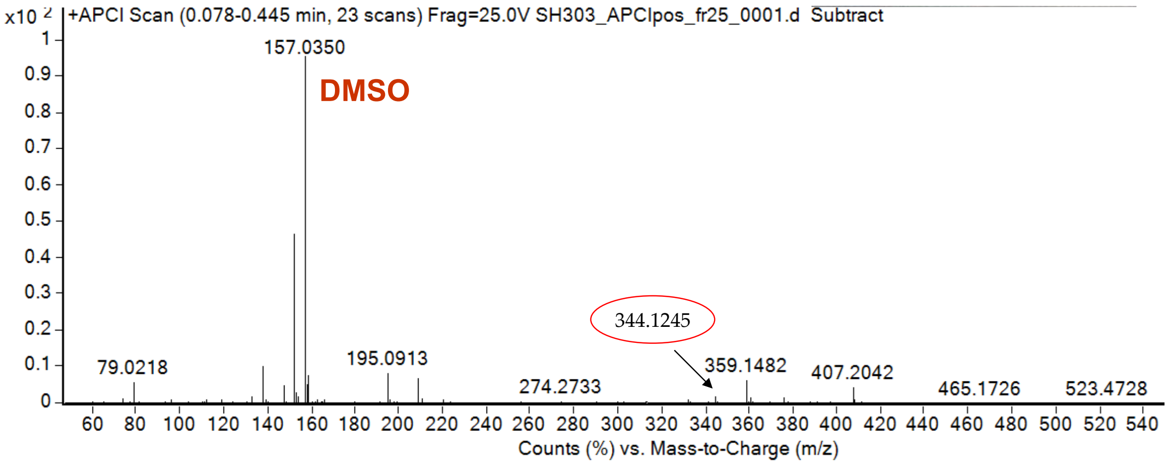

calculated mass: $[M+H]^+$ = 344.1254 observed: $[M+H]^+$ = 344.1245 max. mass error = 2.6 ppm

^{1}H (500 MHz) and ^{13}C NMR (126 MHz) spectra of **62** in DMSO-d_6

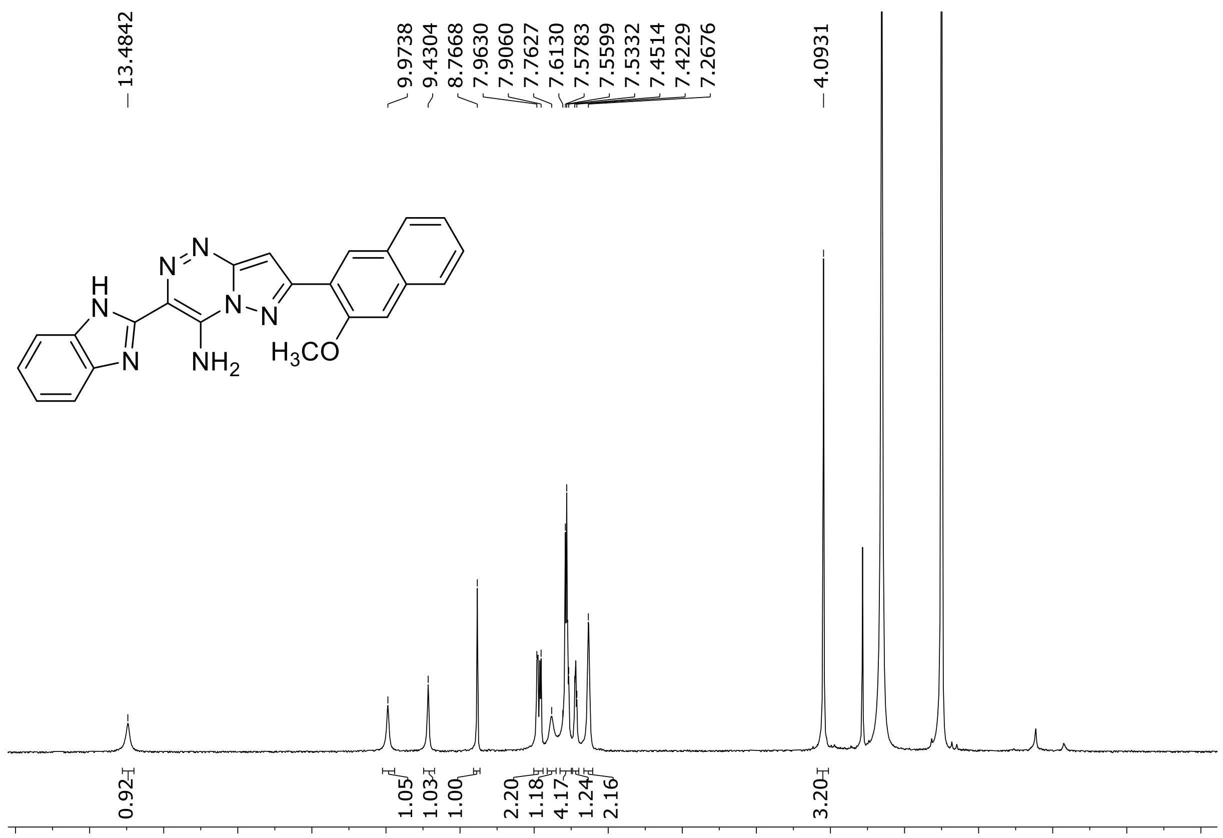

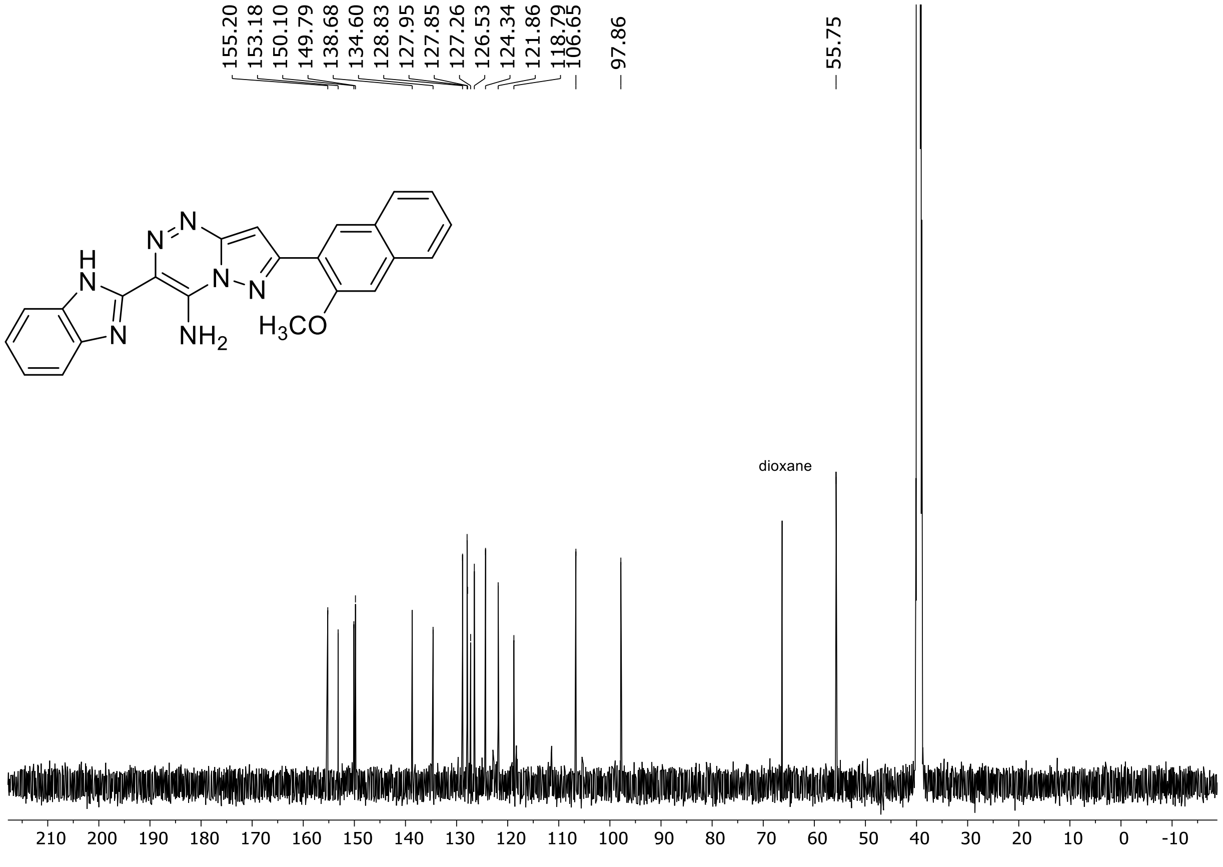

HRMS spectrum of **62**

$C_{23}H_{17}N_7O$ **exact mass: 407.1495**

APCI + (MMI)
nitrogen flow 5 L/min, gas temperature 325°C, nebulizer 45 psig, skimmer 65 V, vaporizer 200°C, fragmentor 5 V, dissolved in methanol

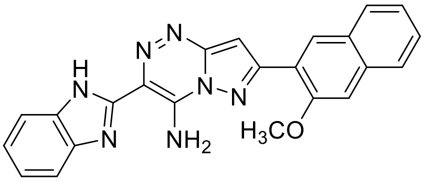

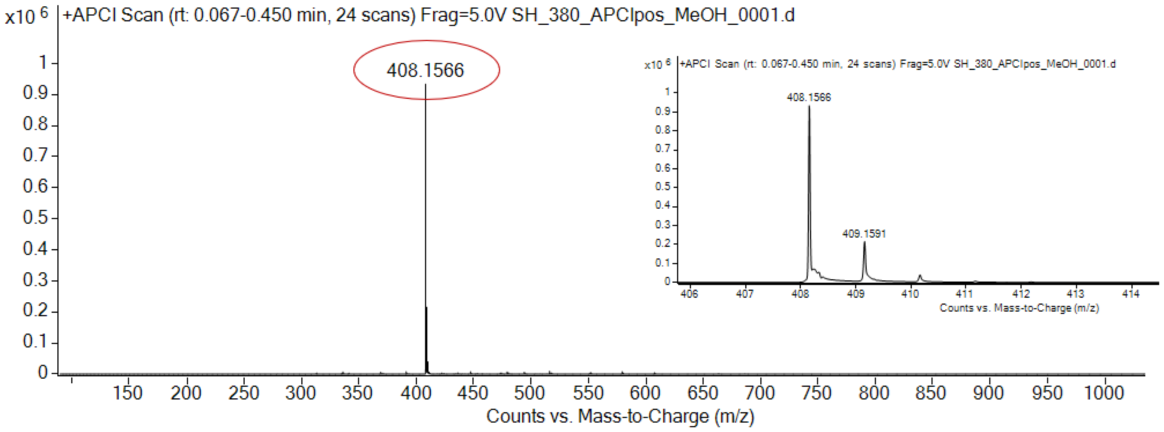

expected mass: $[M+H]^+$ = 408.1567 observed mass : $[M+H]^+$ = 408.1566 mass accuracy = - 0.2 ppm

^{1}H (500 MHz) and ^{13}C NMR (126 MHz) spectra of **63** in DMSO-d_6

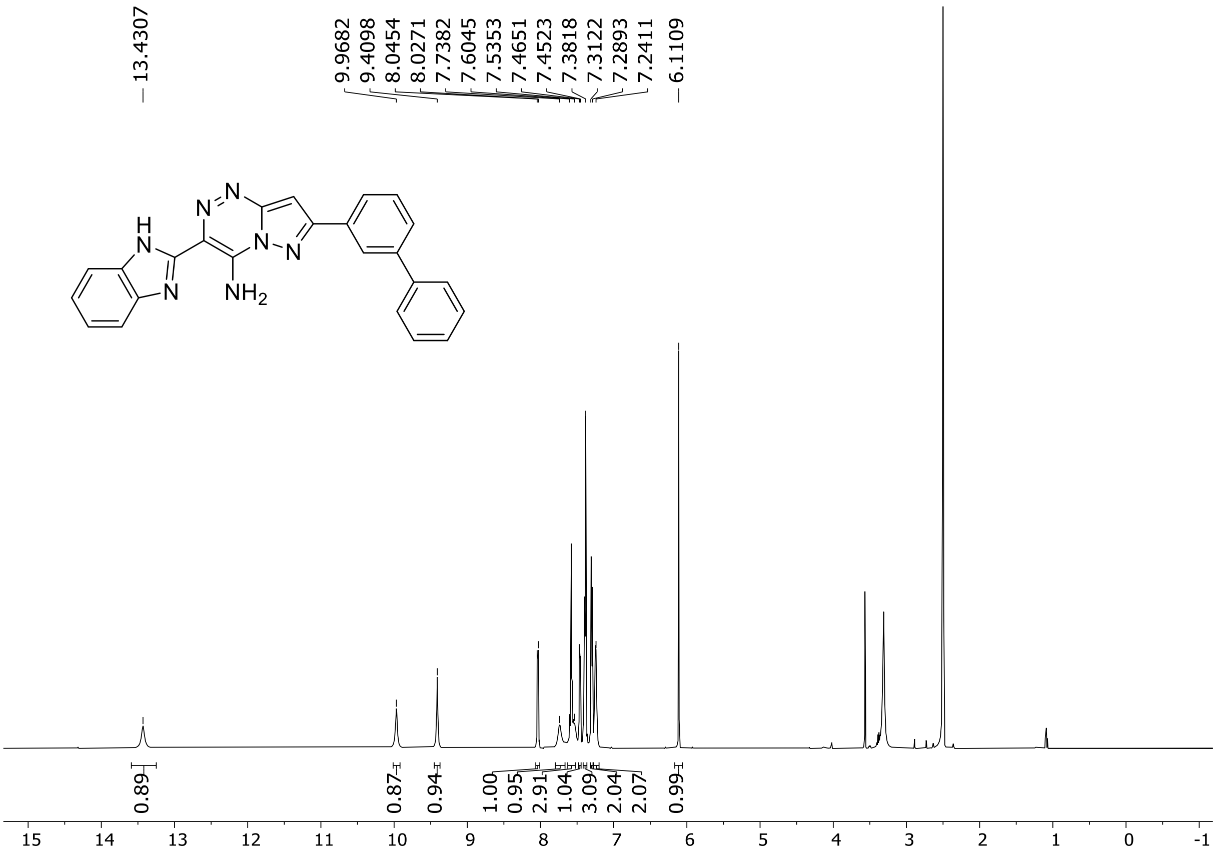

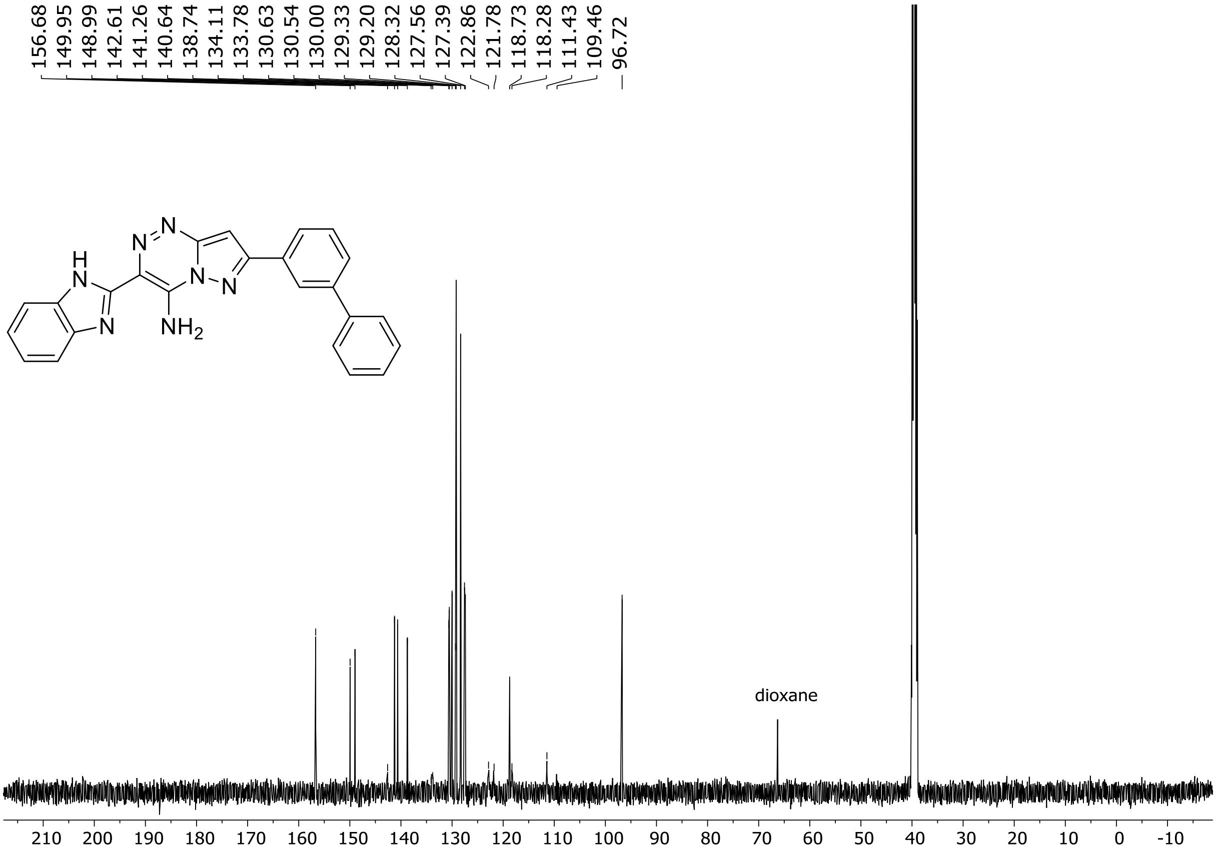

HRMS spectrum of **63**

$C_{24}H_{17}N_7$ **mono m/z = 403.1545**

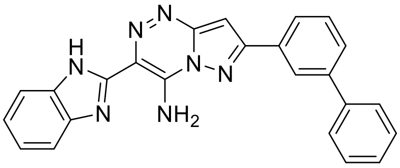

APCI + (MMI)
nitrogen flow 5 L/min, gas temperature 300°C, nebulizer 45 psi, vaporizer 200°C
skimmer **65** V, fragmentor **23** V, dissolved in methanol

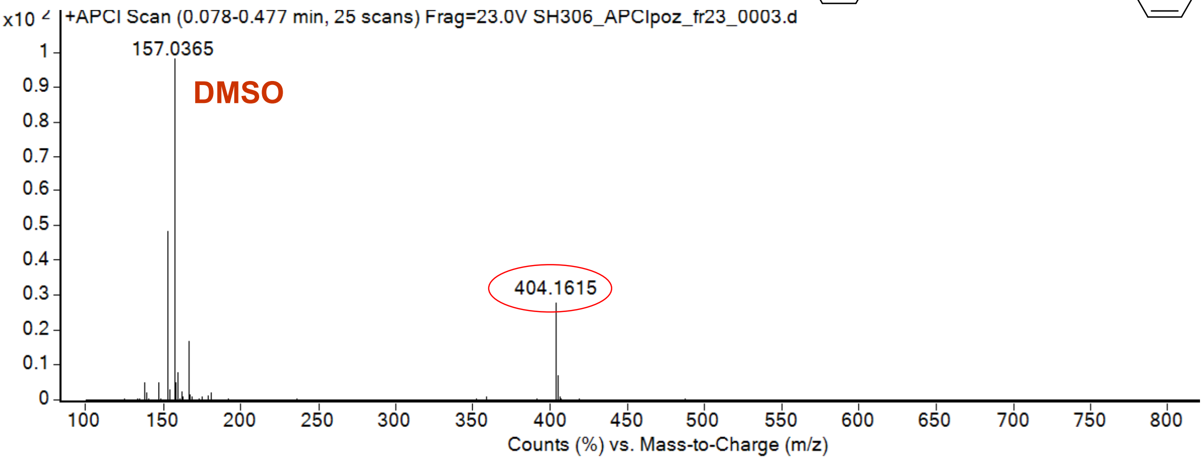

calculated mass: $[M+H]^+$ = 404.1618 observed: $[M+H]^+$ = 404.1615 max. mass error = 0.7 ppm

^1H (500 MHz) and ^{13}C NMR (126 MHz) spectra of **64** in DMSO-d_6

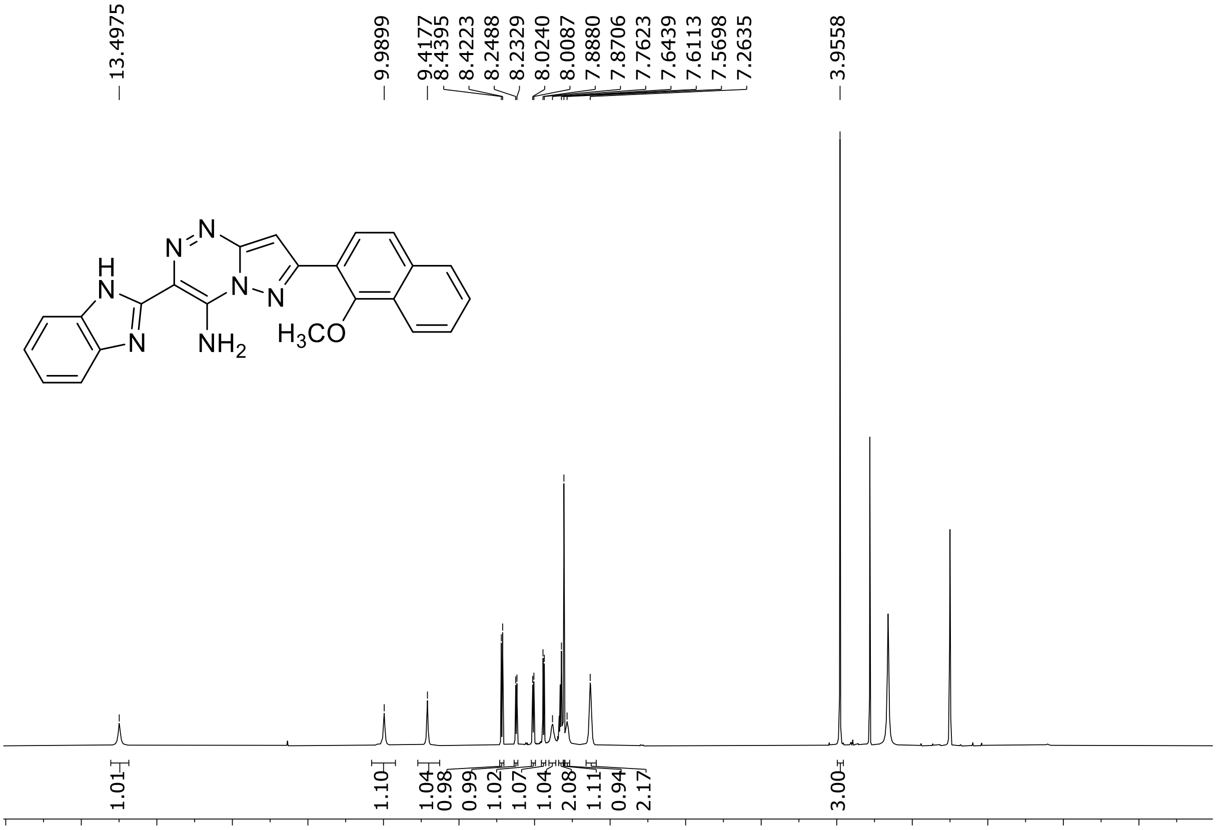

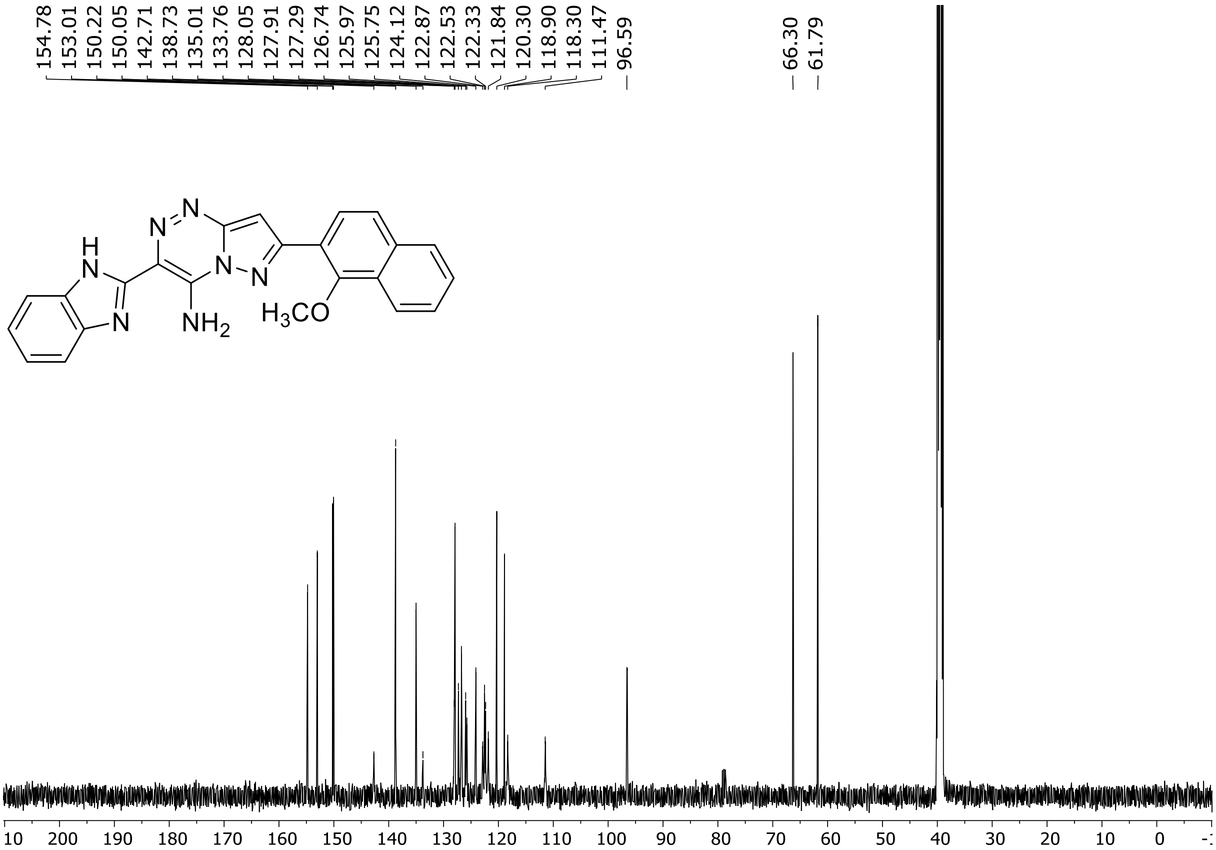

HRMS spectrum of **64**

$C_{23}H_{17}N_7O$ **exact mass: 407.1495**

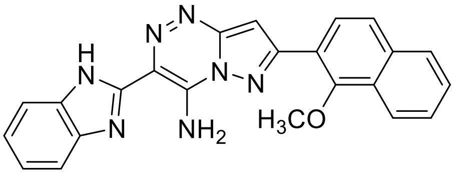

APCI + (MMI)
nitrogen flow 5 L/min, gas temperature 325°C, nebulizer 45 psig, skimmer 65 V, vaporizer 200°C, fragmentor 10 V, dissolved in methanol

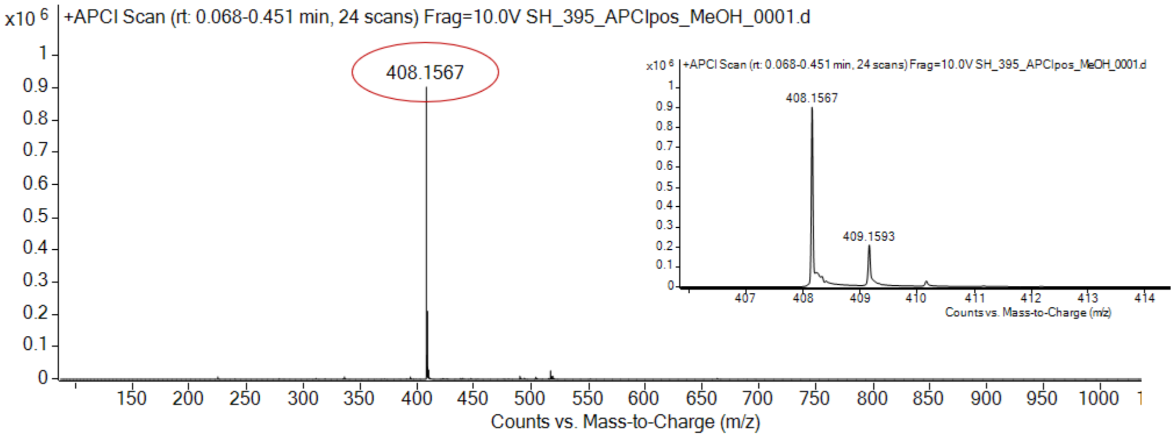

expected mass: [M+H]+ = 408.1567 observed mass : [M+H]+ = 408.1567 mass accuracy < 0.1 ppm

^{1}H (500 MHz) and ^{13}C NMR (126 MHz) spectra of **65** in DMSO-d_6

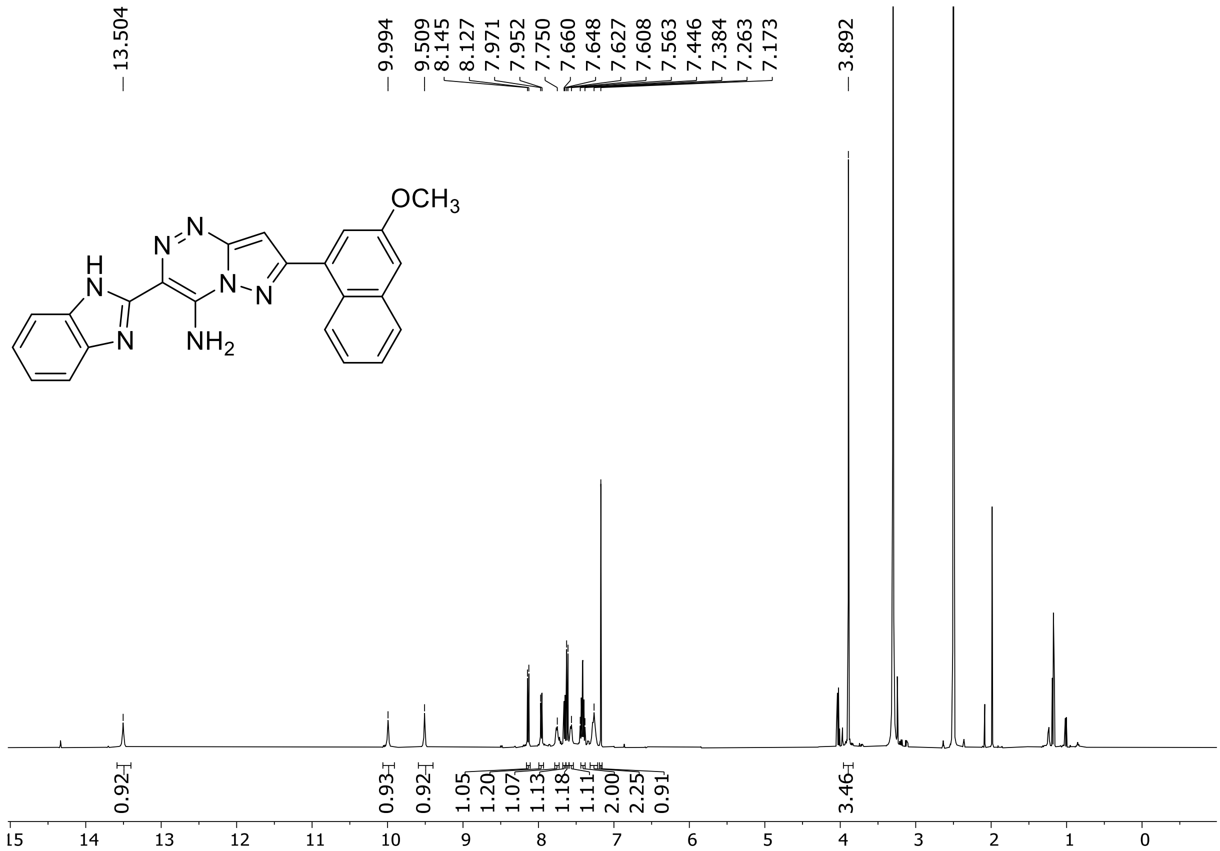

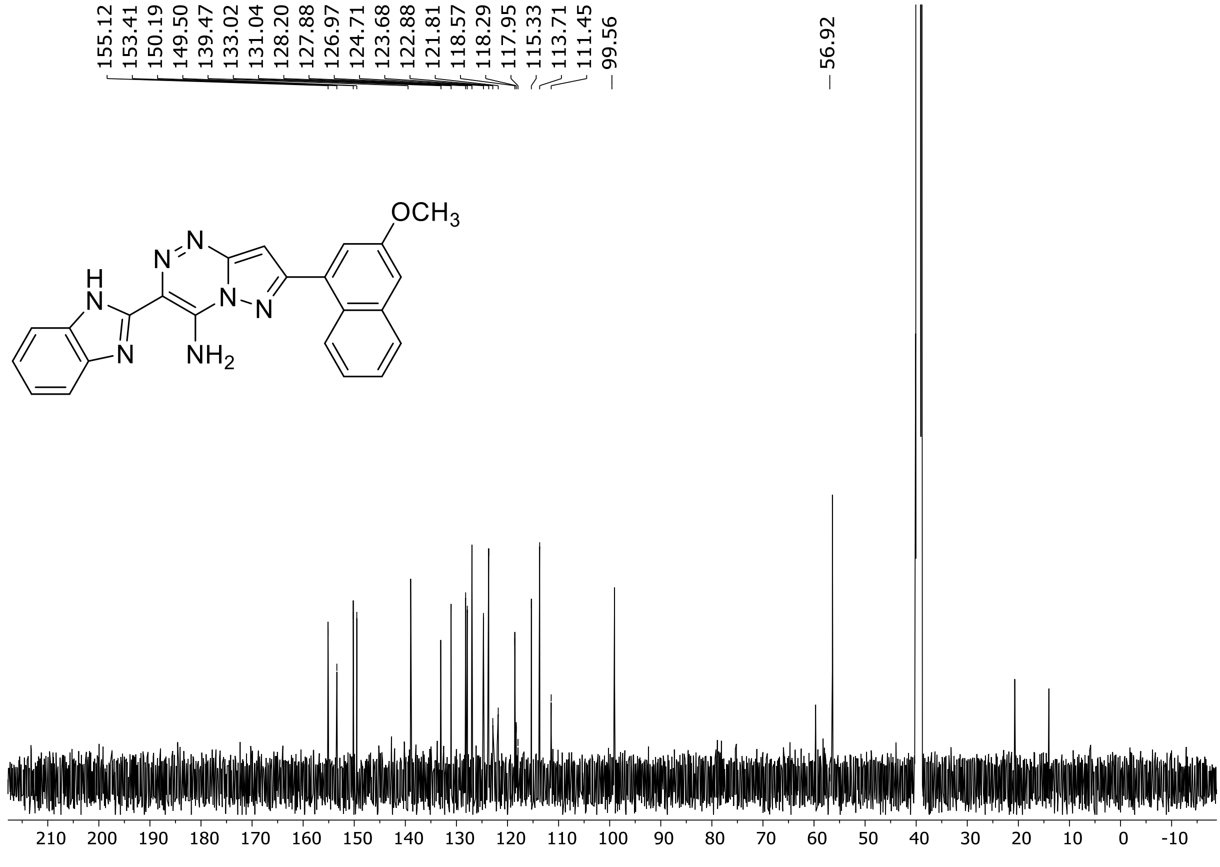

HRMS spectrum of **65**

$C_{23}H_{17}N_7O$ **exact mass: 407.1607**

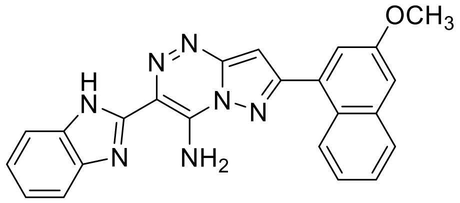

APCI + (MMI)
nitrogen flow 5 L/min, gas temperature 325°C, nebulizer 45 psig, skimmer 65 V, vaporizer 200°C, fragmentor 10 V, dissolved in methanol

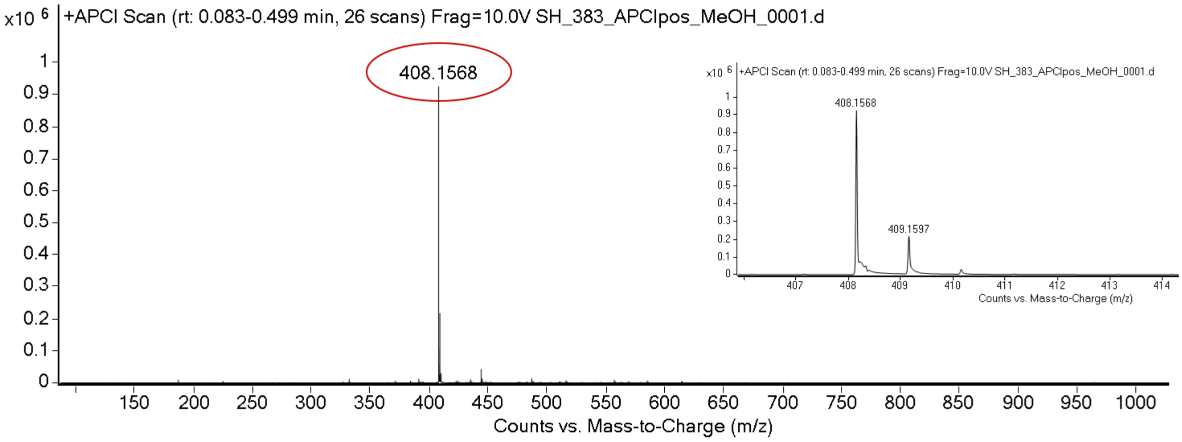

expected mass: [M+H]$^+$ = 408.1567 observed mass : [M+H]$^+$ = 408.1568 mass accuracy = 0.2 ppm

^{1}H (500 MHz) and ^{13}C NMR (126 MHz) spectra of **66** in DMSO-d_6

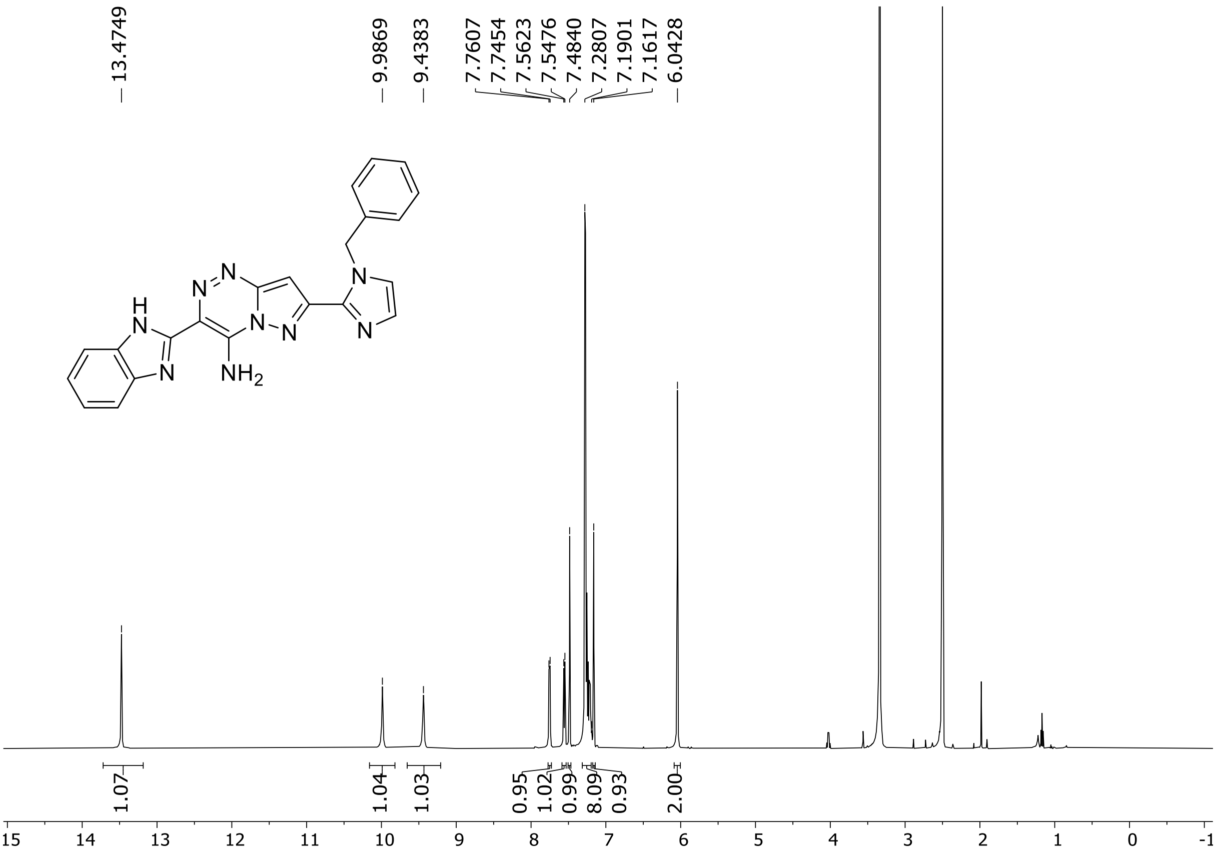

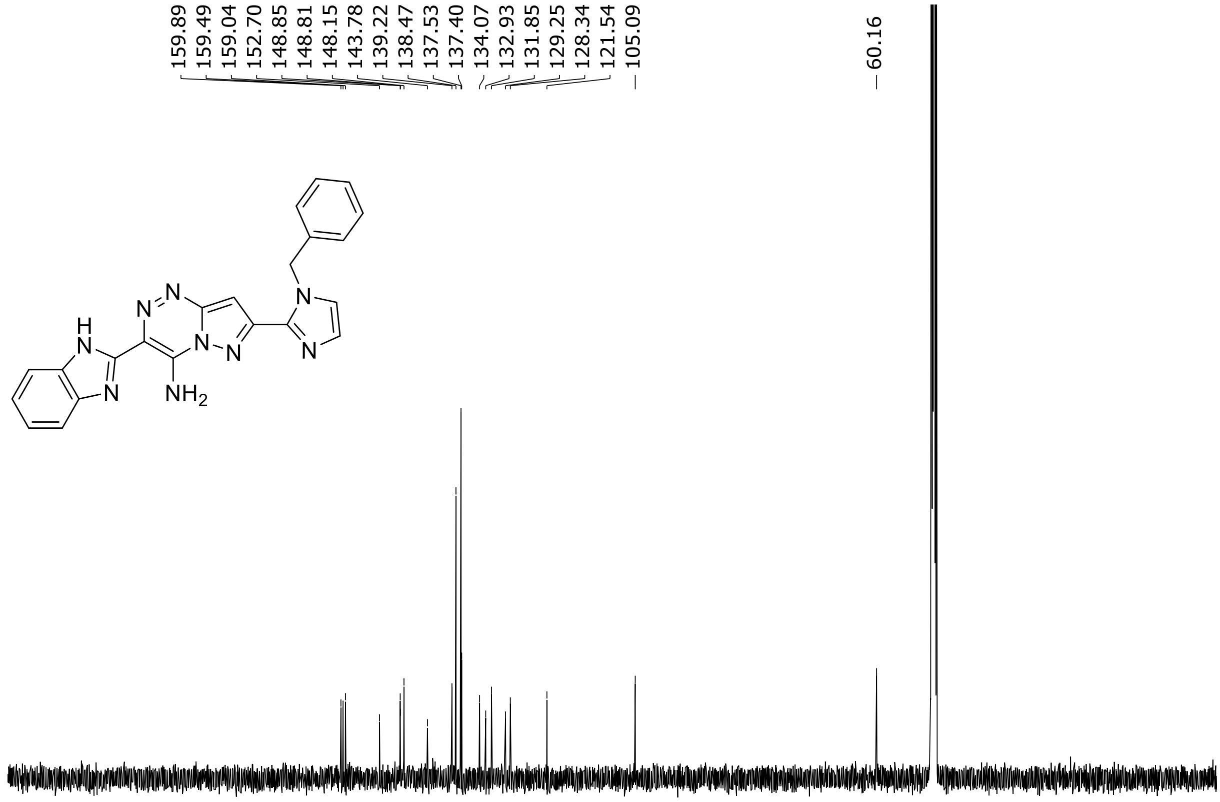

^{1}H (500 MHz) and ^{13}C NMR (126 MHz) spectra of **67** in DMSO-d_6

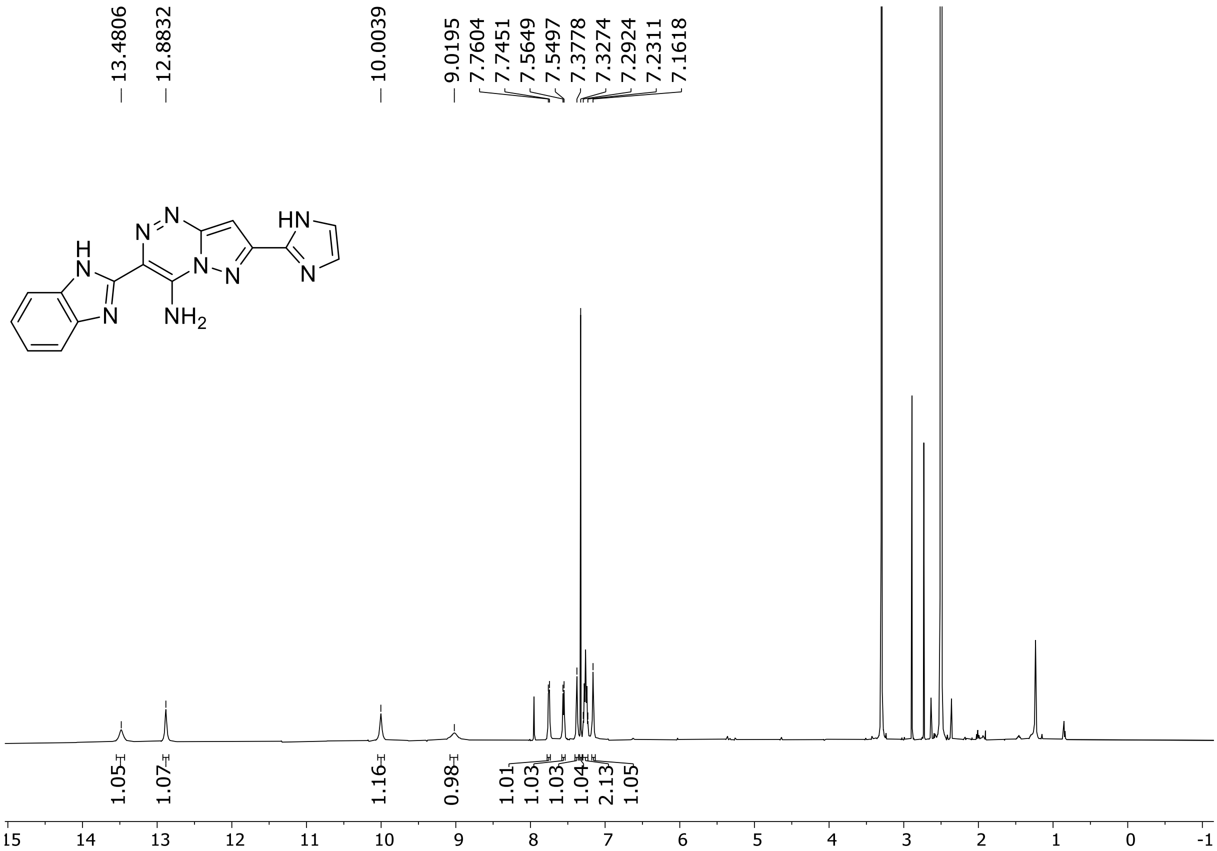

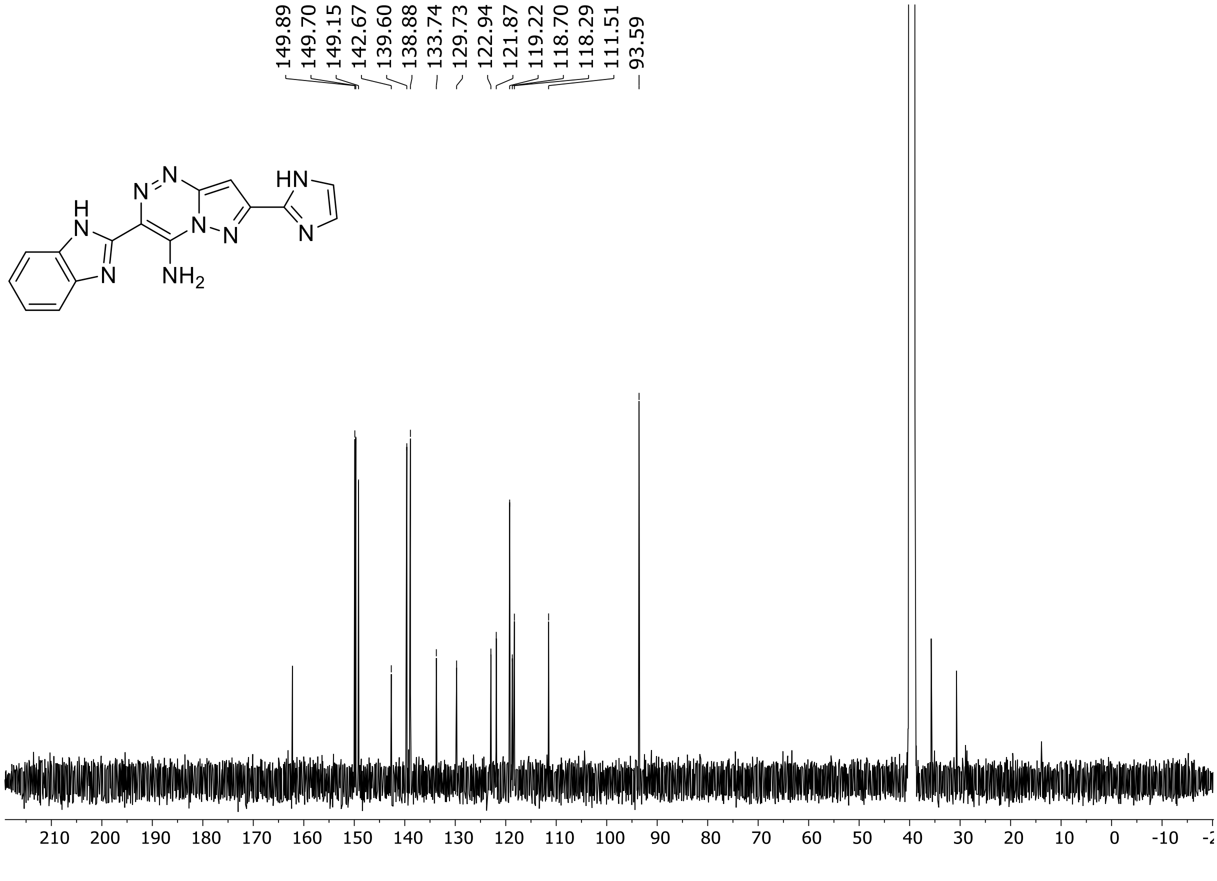

HRMS spectrum of **67**

$C_{15}H_{11}N_9$ **exact mass: 317.1137**

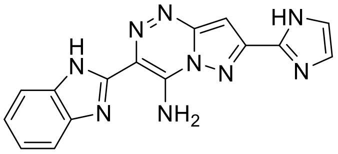

APCI + (MMI)
nitrogen flow 5 L/min, gas temperature 325°C, nebulizer 45 psig, skimmer 65 V, vaporizer 200°C, fragmentor 20 V, dissolved in methanol

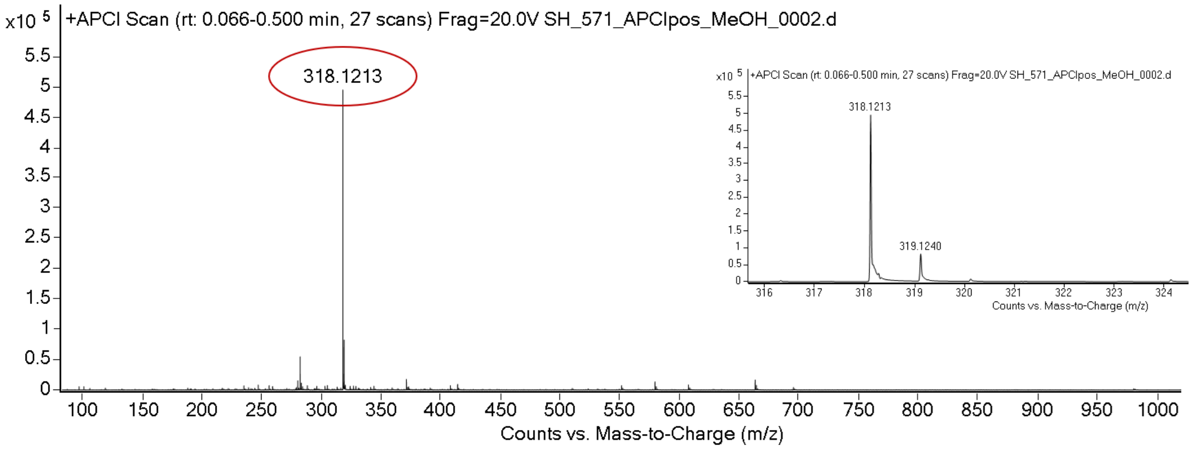

expected mass: $[M+H]^+$ = 318.1210 observed mass : $[M+H]^+$ = 318.1213 mass accuracy = 0.9 ppm

^{1}H (500 MHz) and ^{13}C NMR (126 MHz) spectra of **68** in DMSO-d_6

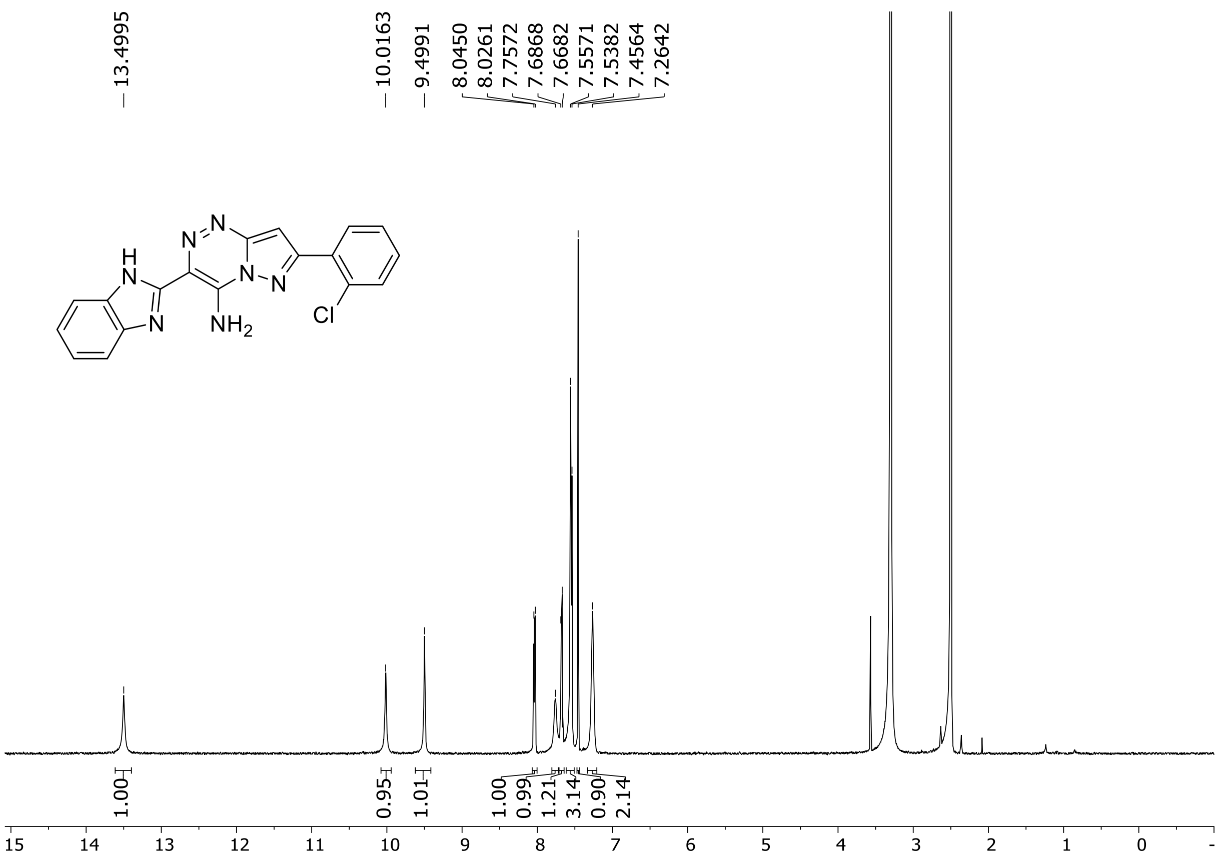

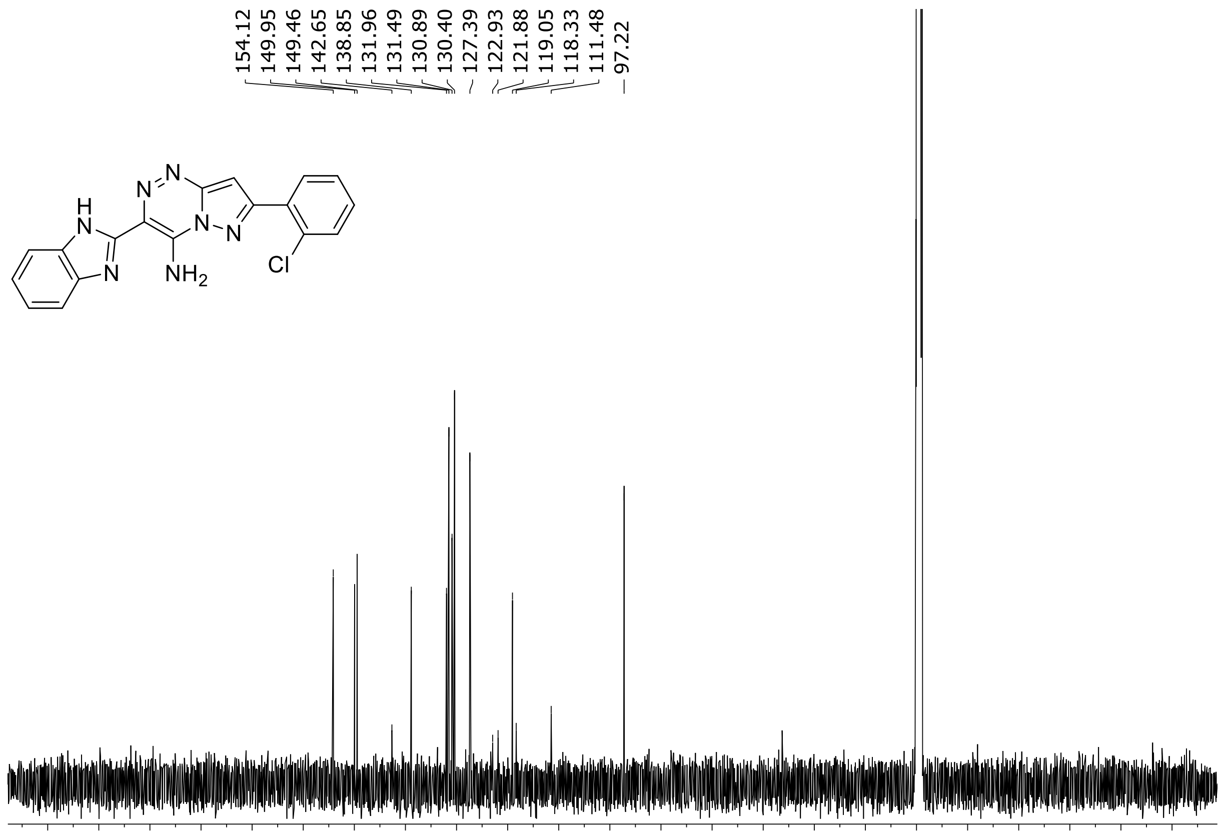

HRMS spectrum of **68**

$C_{18}H_{12}ClN_7$ **mono m/z = 361.0843**

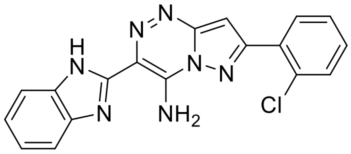

APCI + (MMI)
nitrogen flow 5 L/min, gas temperature 300°C, nebulizer 45 psi, vaporizer 200°C
skimmer **65** V, fragmentor 22 V, dissolved in methanol

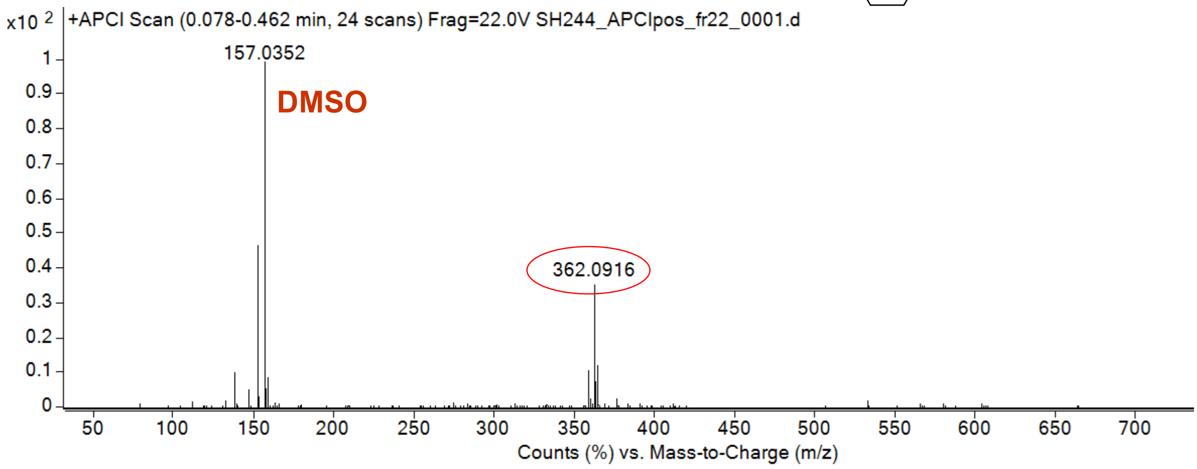

calculated mass: $[M+H]^+$ = 362.0915 observed: $[M+H]^+$ = 362.0916 max. mass error = 0.2 ppm

^{1}H (500 MHz) and ^{13}C NMR (126 MHz) spectra of **69** in DMSO-d_6

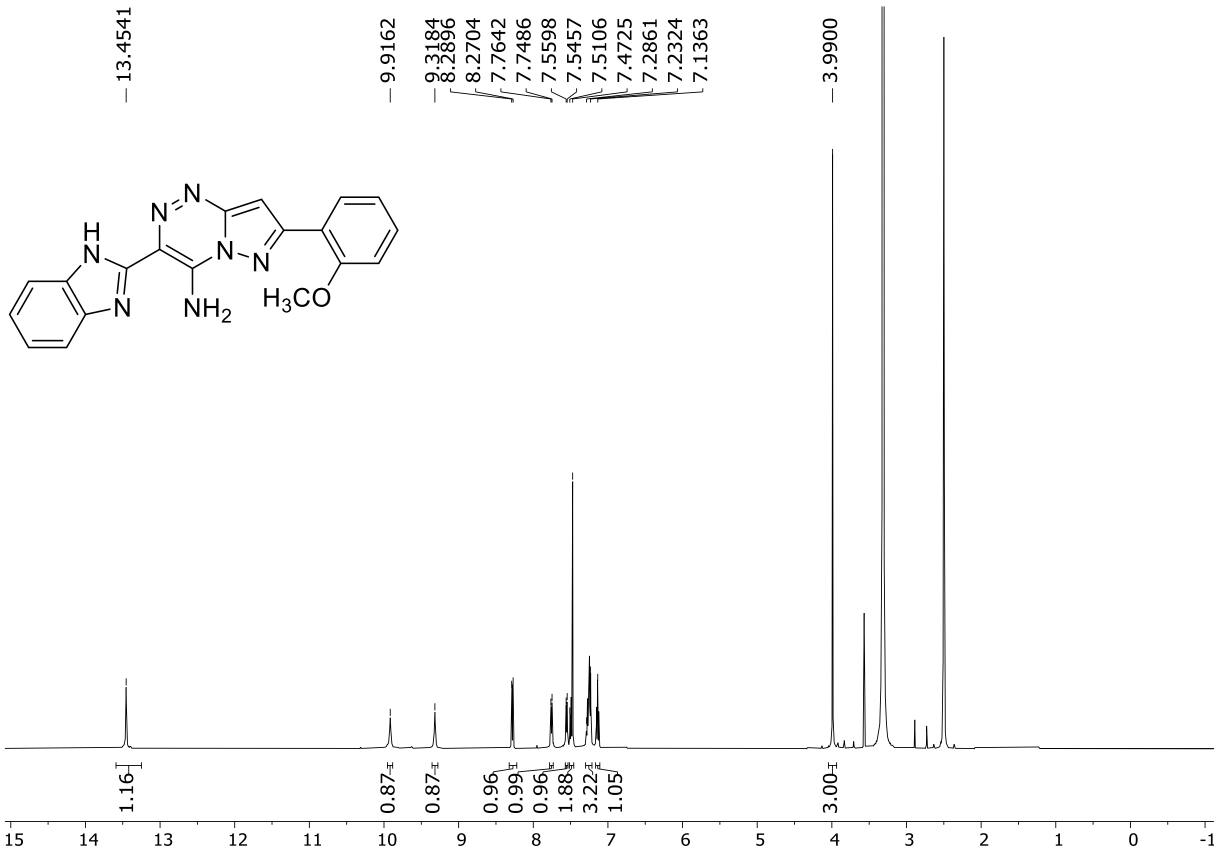

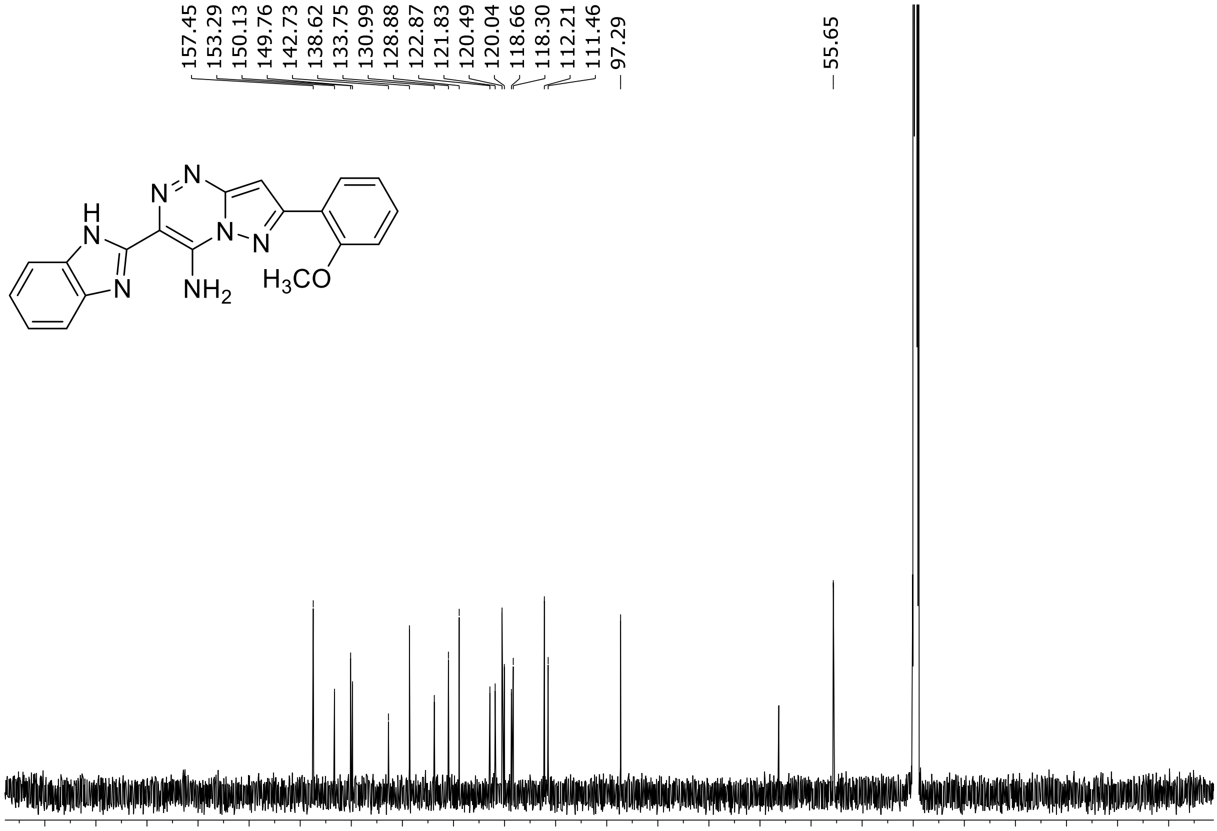

HRMS spectrum of **69**

$C_{19}H_{15}N_7O$ **mono m/z = 357.1338**

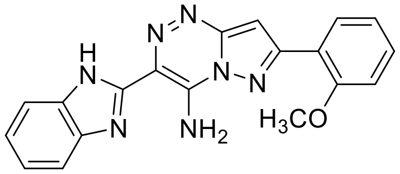

APCI + (MMI)
nitrogen flow 5 L/min, gas temperature 300°C, nebulizer 45 psi, vaporizer 200°C
skimmer **65** V, fragmentor **28** V, dissolved in methanol

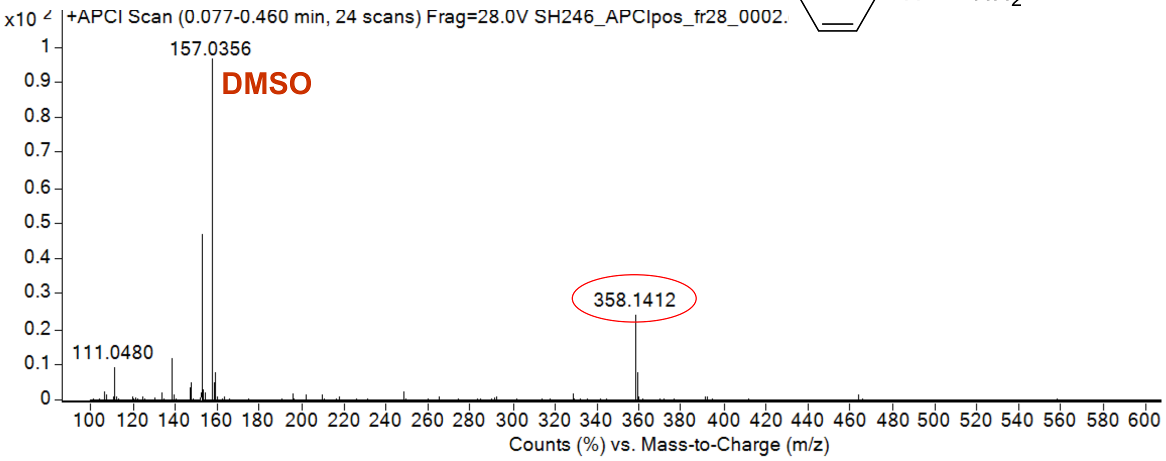

calculated mass: $[M+H]^+$ = 358.1411 observed: $[M+H]^+$ = 358.1412 max. mass error = 0.2 ppm

^{1}H (500 MHz) and ^{13}C NMR (126 MHz) spectra of **70** in DMSO-d_6

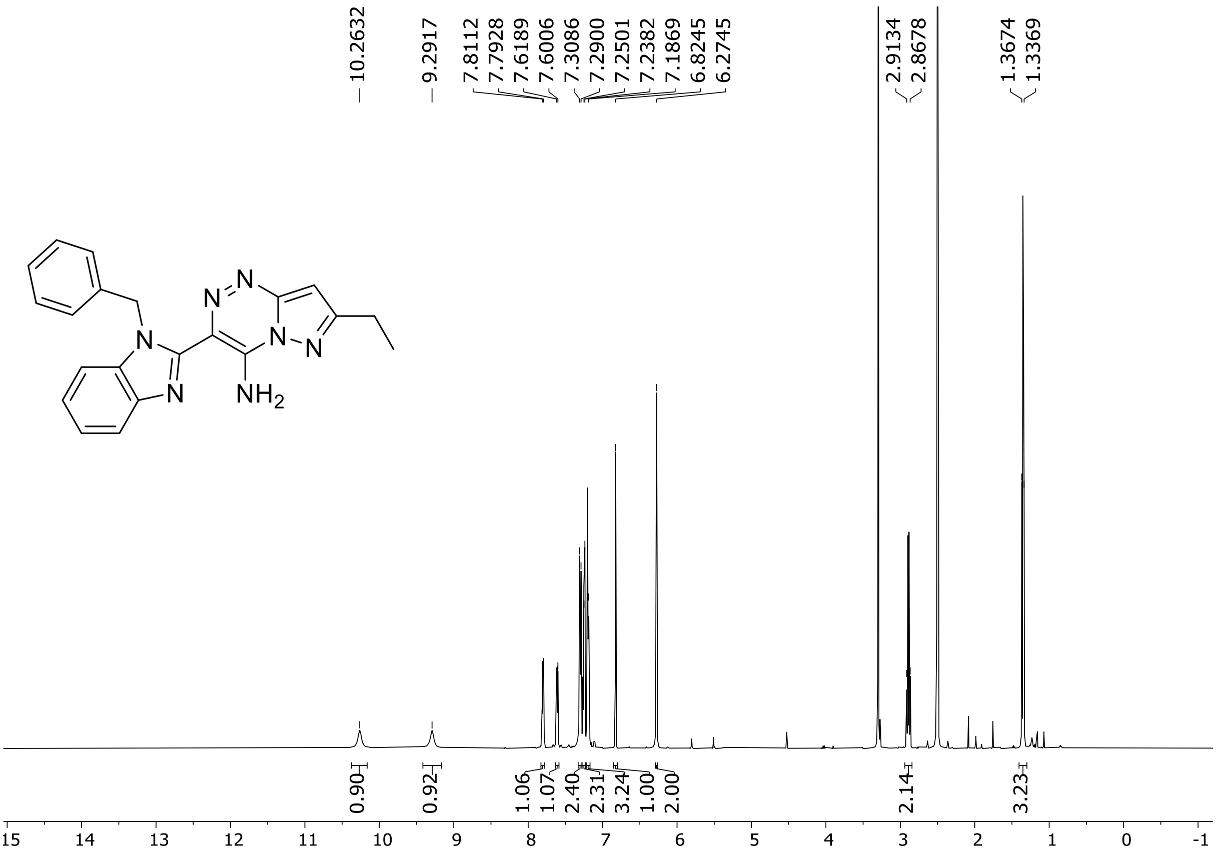

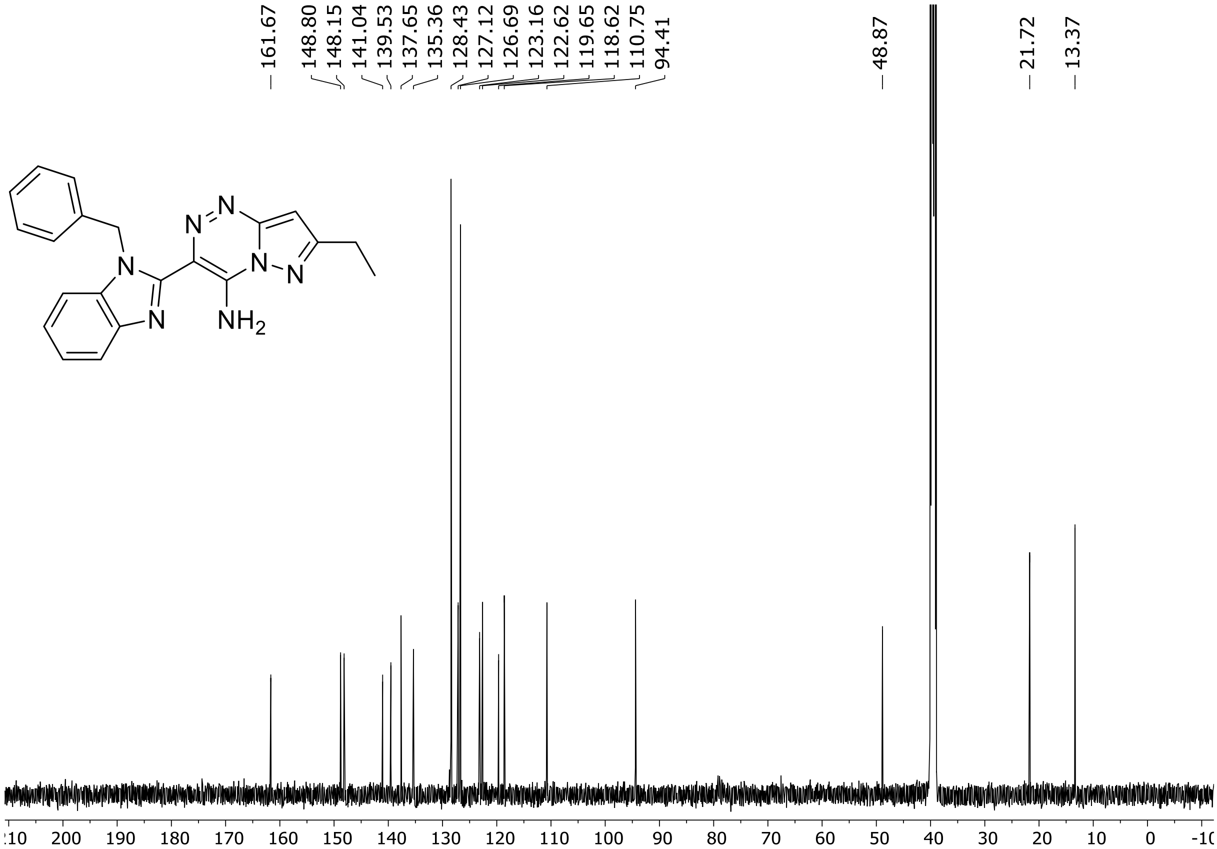

HRMS spectrum of **70**

$C_{21}H_{19}N_7$ **mono m/z = 369.1702**

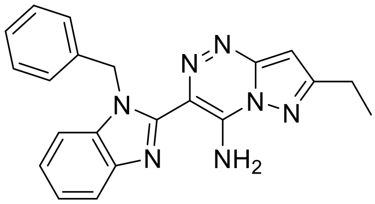

APCI + (MMI)
nitrogen flow 5 L/min, gas temperature 300°C, nebulizer 45 psi, vaporizer 200°C
skimmer **65** V, fragmentor **10** V, dissolved in methanol

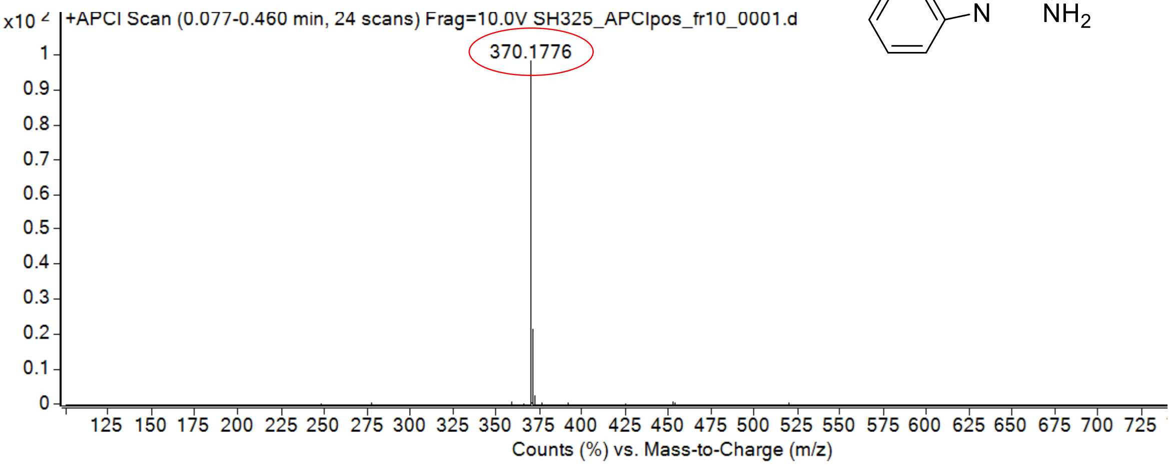

calculated mass: $[M+H]^+$ = 370.1775 observed: $[M+H]^+$ = 370.1776 max. mass error = 0.2 ppm

^{1}H (500 MHz) and ^{13}C NMR (126 MHz) spectra of **71** in DMSO-d_6

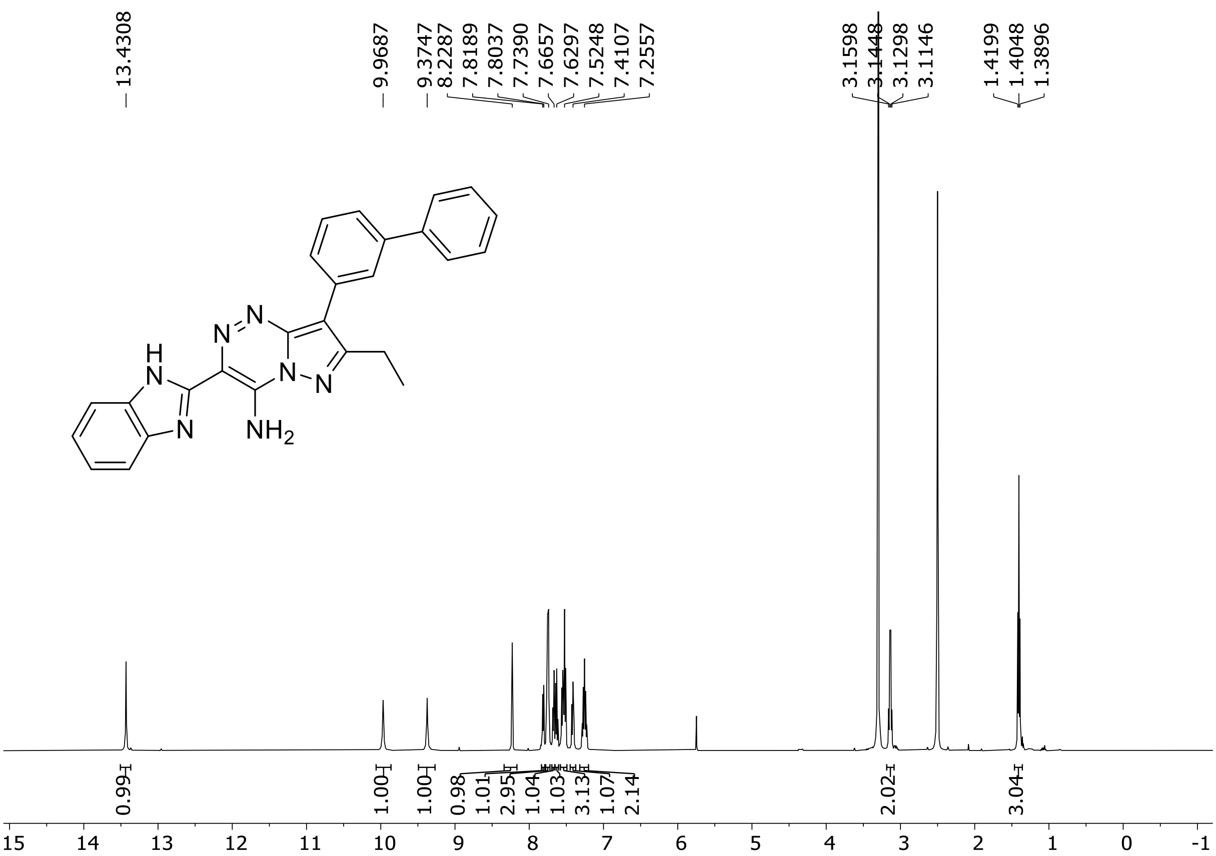

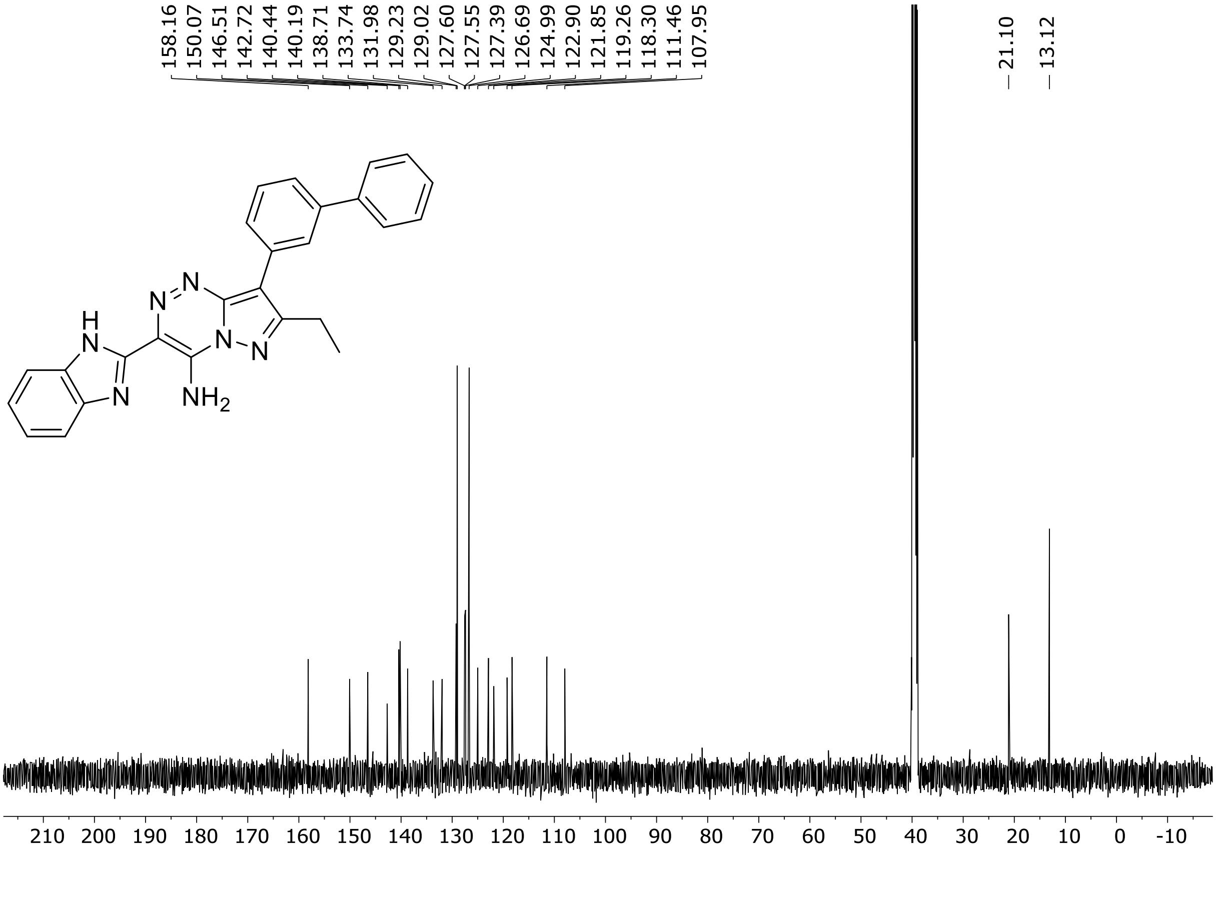

HRMS spectrum of **71**

$C_{26}H_{21}N_7$ mono m/z = 431.49

ESI + (MMI)
nitrogen flow 5 L/min, gas temperature 300°C, vaporizer 200°C, nebulizer 45 psi,
skimmer **65** V, fragmentor 35 V, dissolved in MeOH

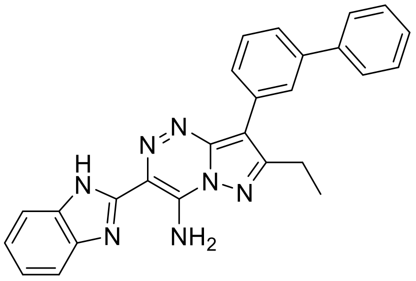

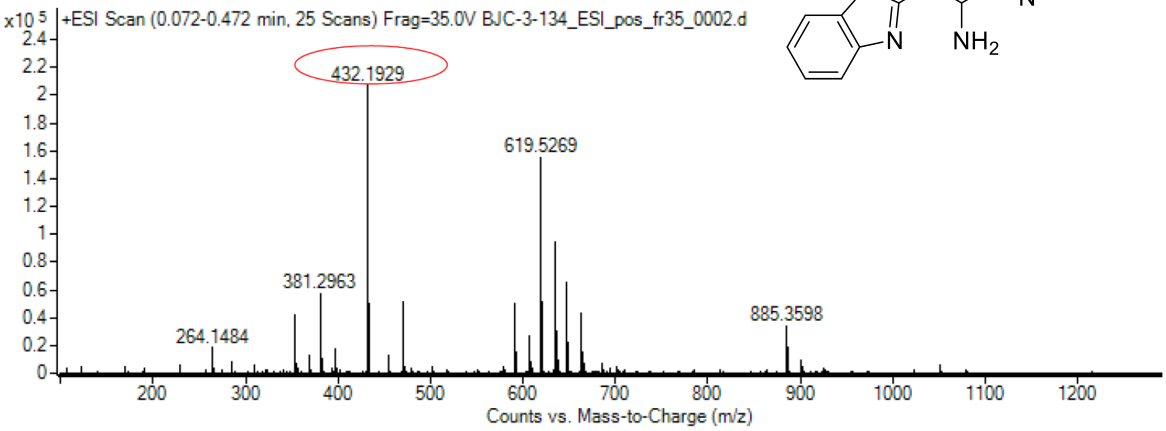

calculated mass: $[M+H]^+$ = 432.1931 observed: $[M+H]^+$ = 432.1929 max. mass error = 0.5 ppm

^{1}H (500 MHz) and ^{13}C NMR (126 MHz) spectra of **72** in DMSO-d_6

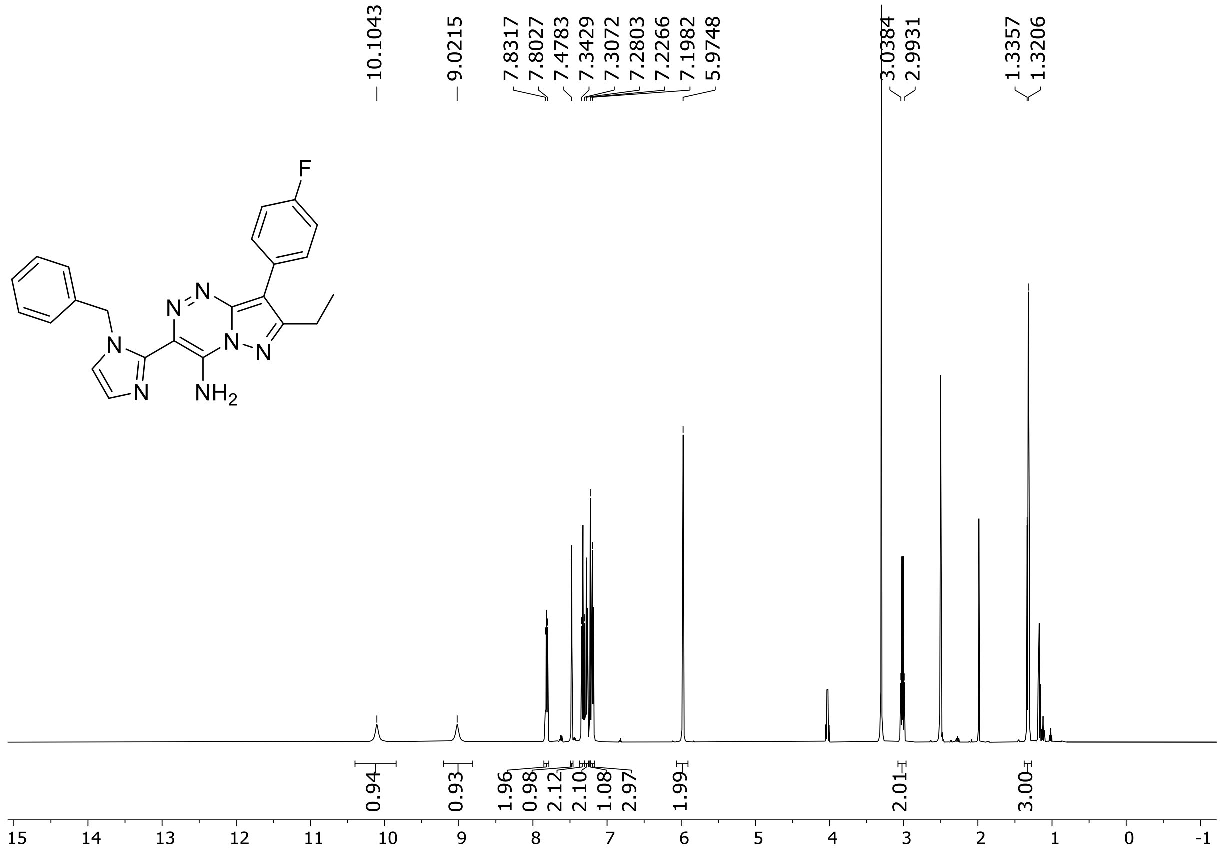

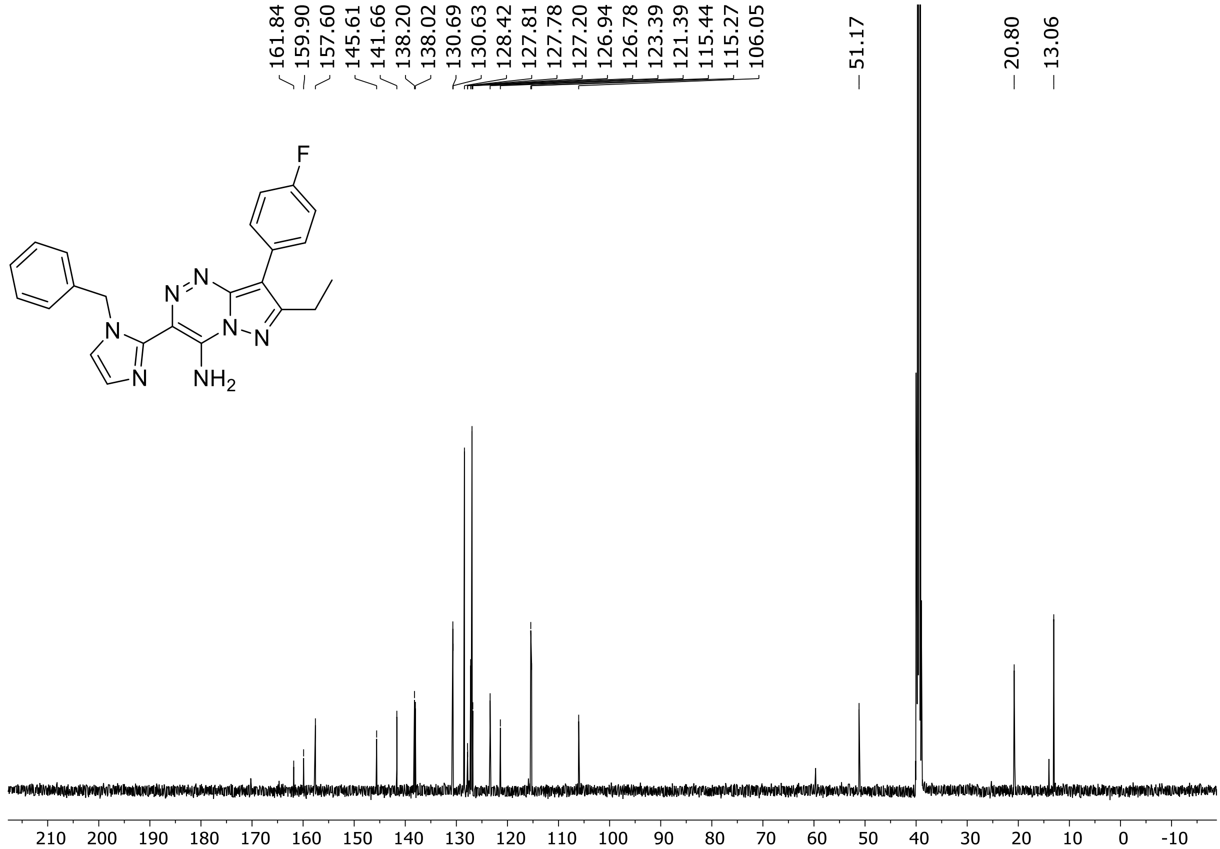

^{19}F (471 MHz) NMR spectrum of **72** DMSO-d_6

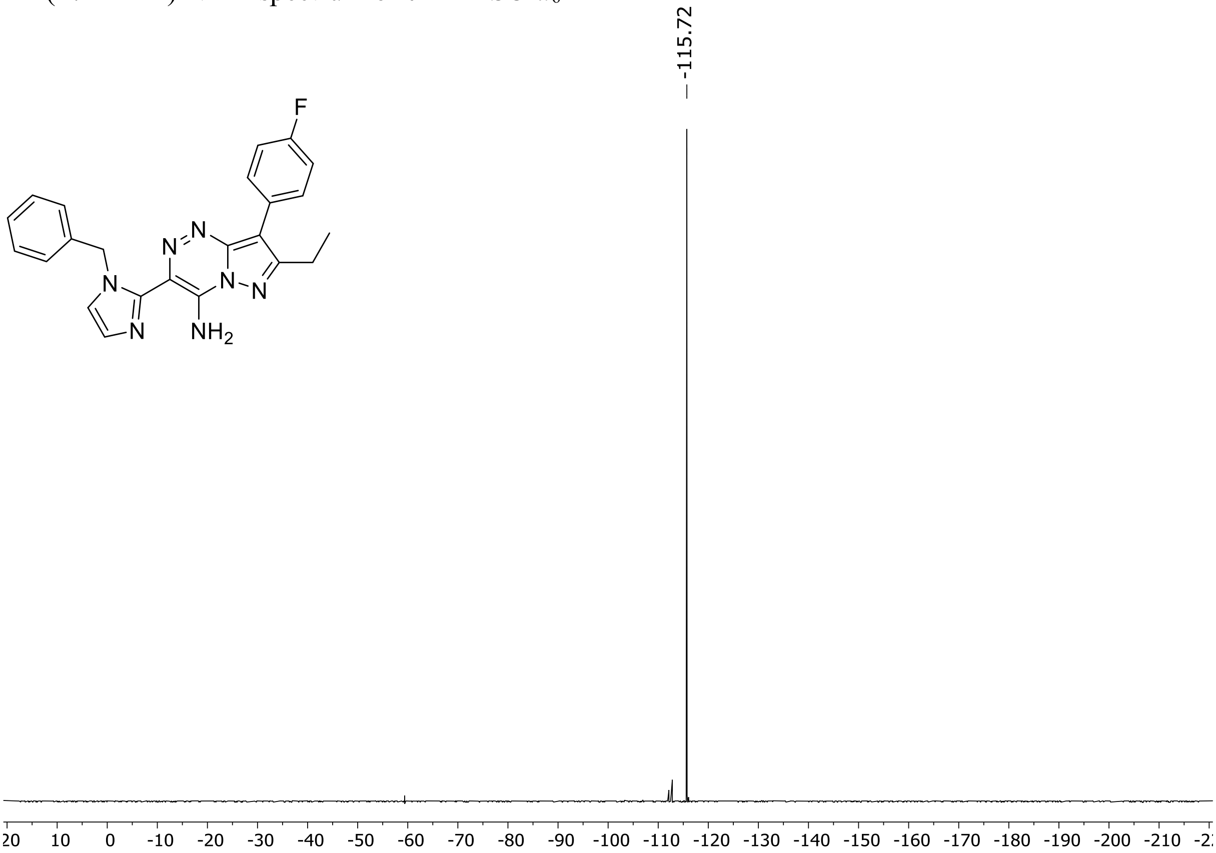

HRMS spectrum of **72**

$C_{23}H_{20}FN_7$ **exact mass: 413.1764**

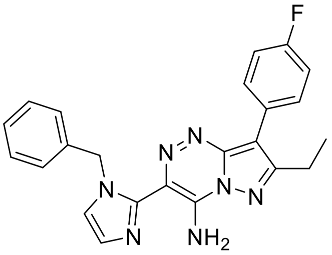

APCI + (MMI)
nitrogen flow 5 L/min, gas temperature 325°C, nebulizer 45 psig, skimmer 65 V, vaporizer 200°C, fragmentor 10 V, dissolved in methanol

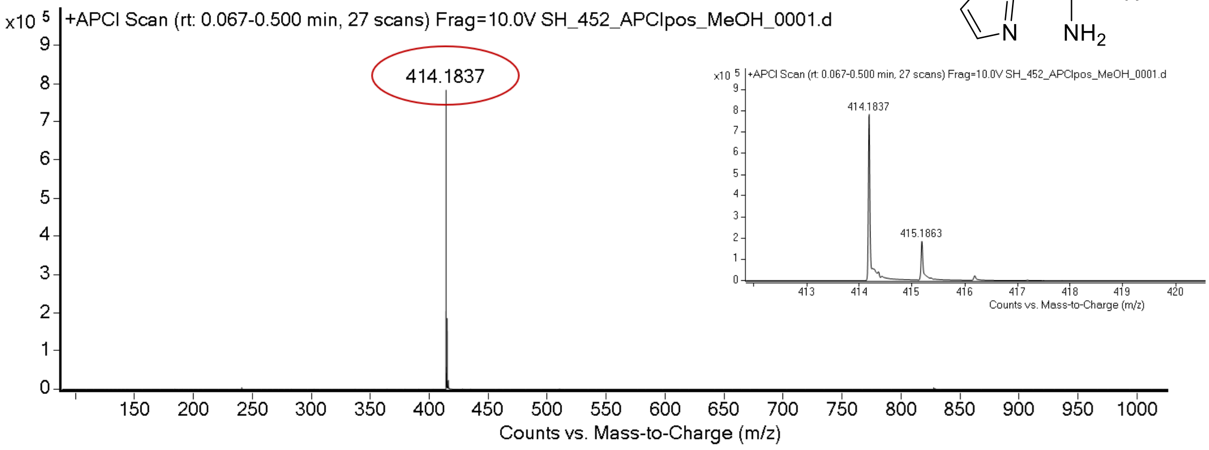

expected mass: [M+H]$^+$ = 414.1837 observed mass : [M+H]$^+$ = 414.1837 mass accuracy < 0.1 ppm

^{1}H (500 MHz) and ^{13}C NMR (126 MHz) spectra of **73** in DMSO-d_6

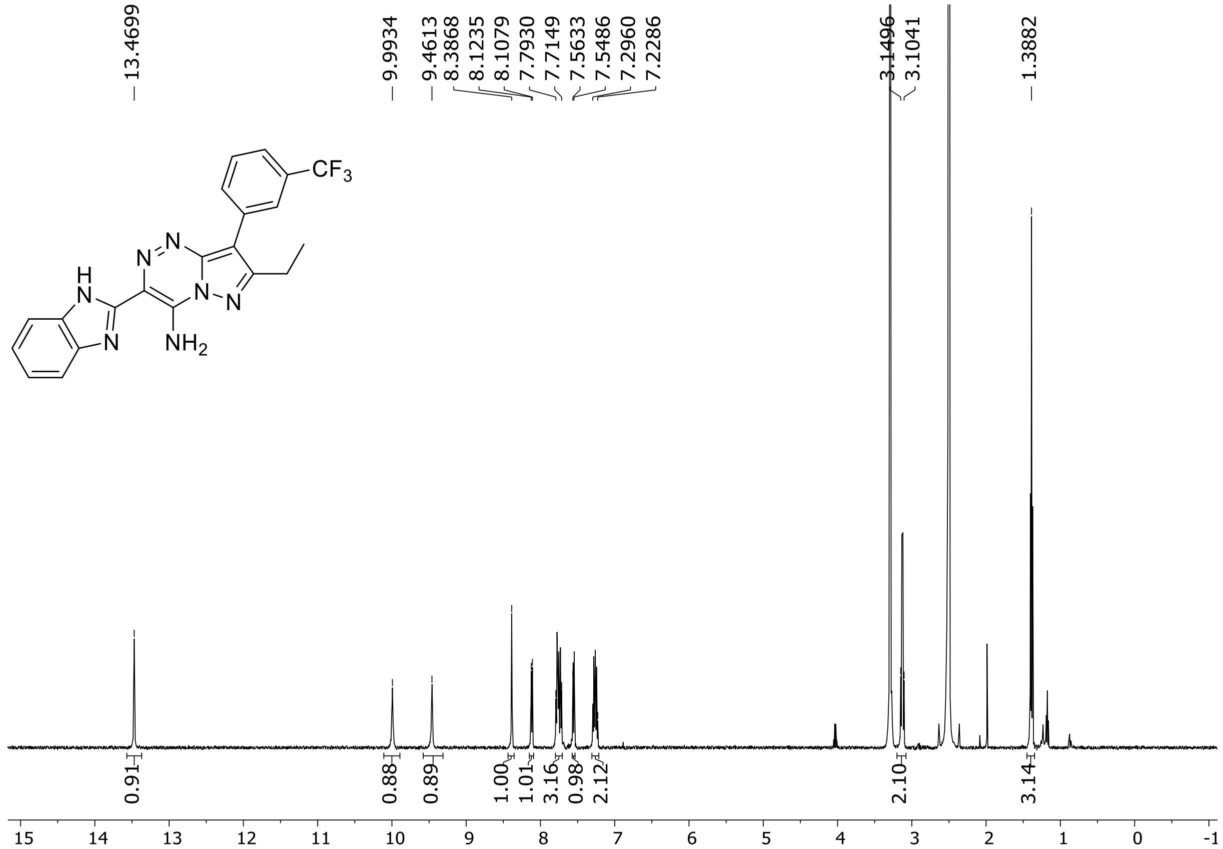

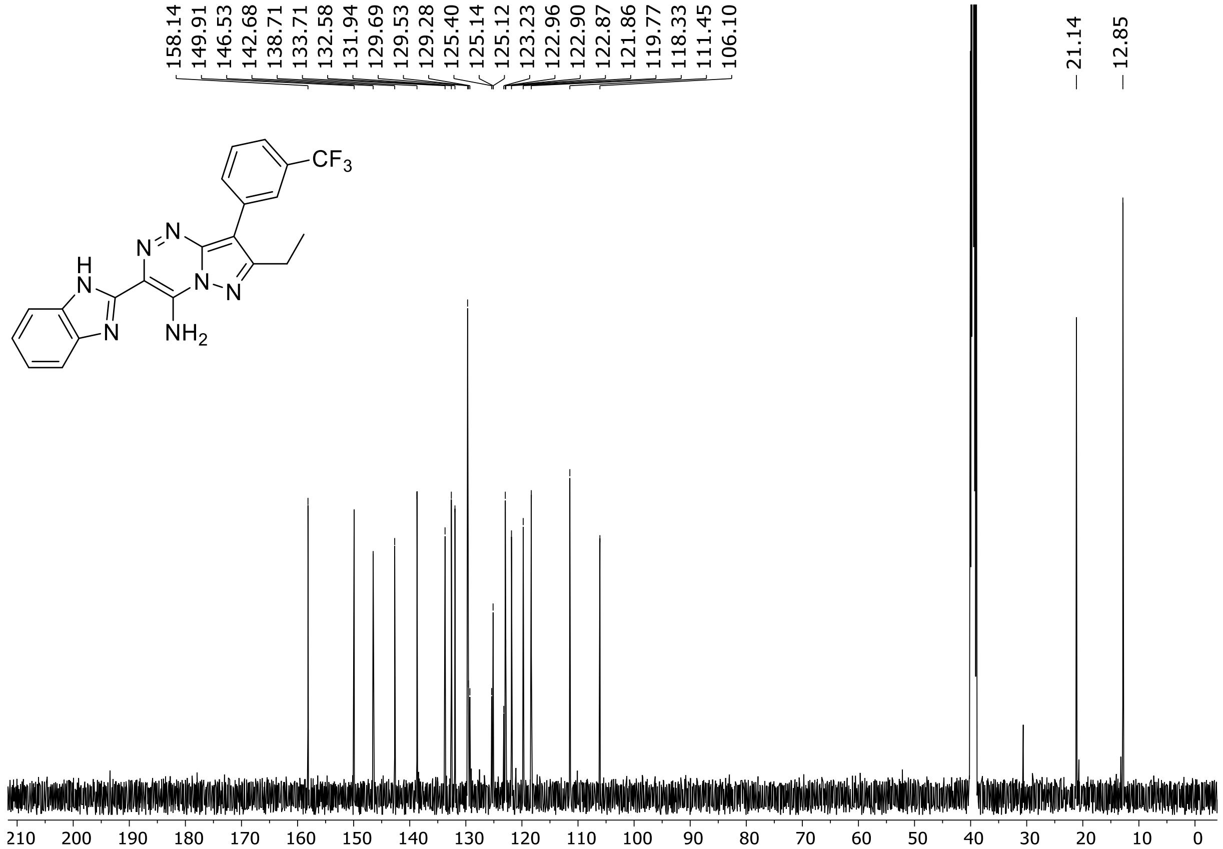

^{19}F (471 MHz) NMR spectrum of **73** DMSO-d_6

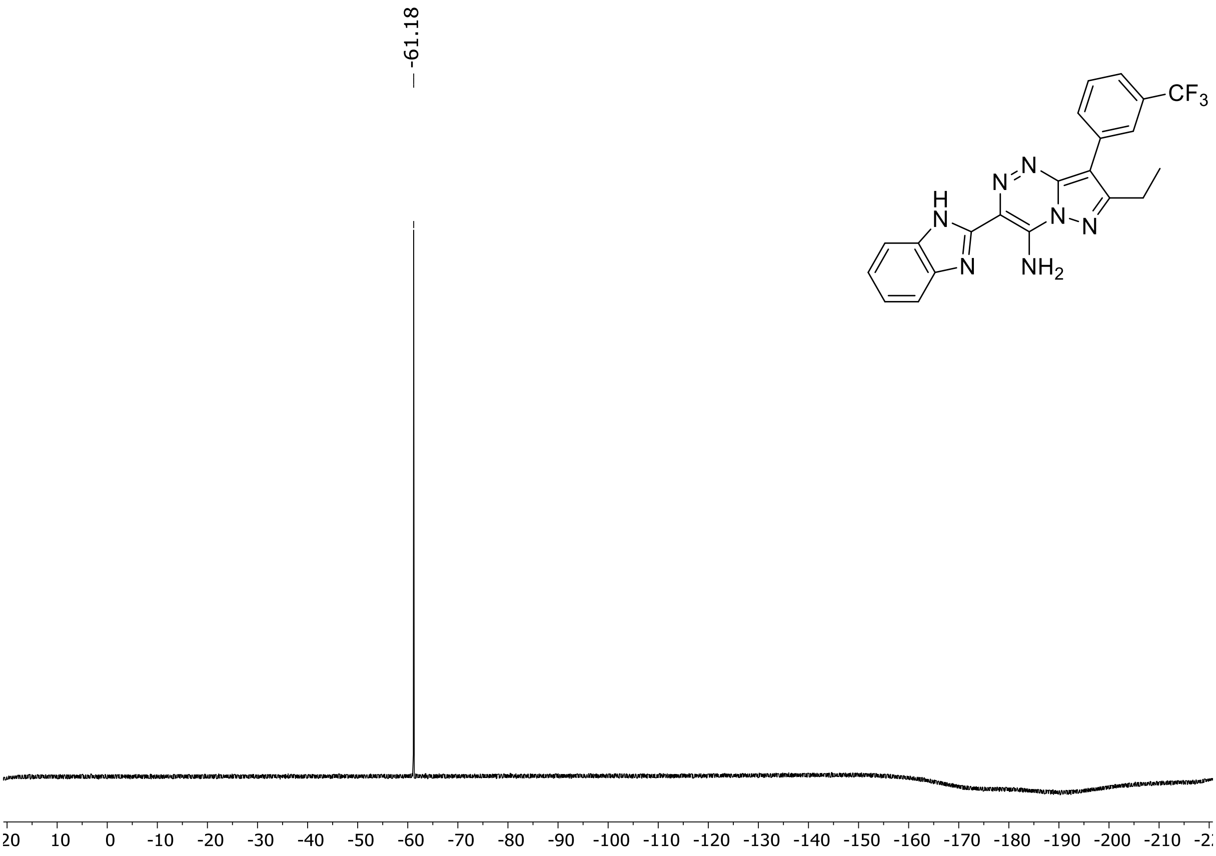

HRMS spectrum of **73**

$C_{21}H_{16}F_3N_7$ **mono *m/z* 423.1419**

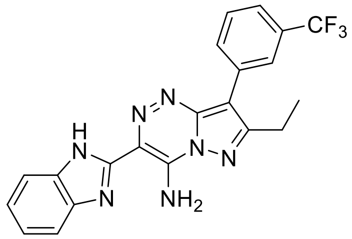

APCI + (MMI)
nitrogen flow 5 L/min, gas temperature 325°C, nebulizer 45 psi, skimmer 65 V, vaporizer 250°C, fragmentor 35 V, dissolved in methanol

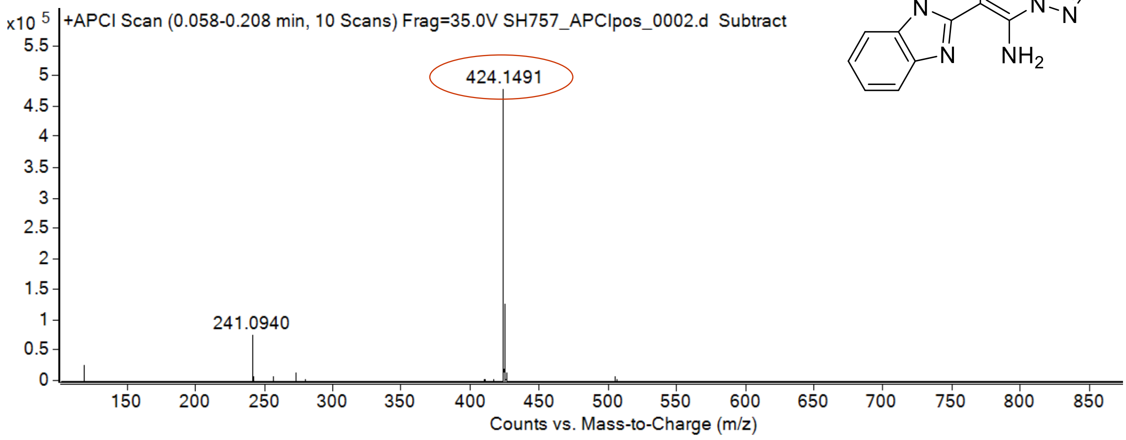

calculated mass: [M+H]$^+$ = 424.1492 observed: [M+H]$^+$ = 424.1491 mass accuracy = - 0.2 ppm

^{1}H (500 MHz) and ^{13}C NMR (126 MHz) spectra of **74** in DMSO-d_6

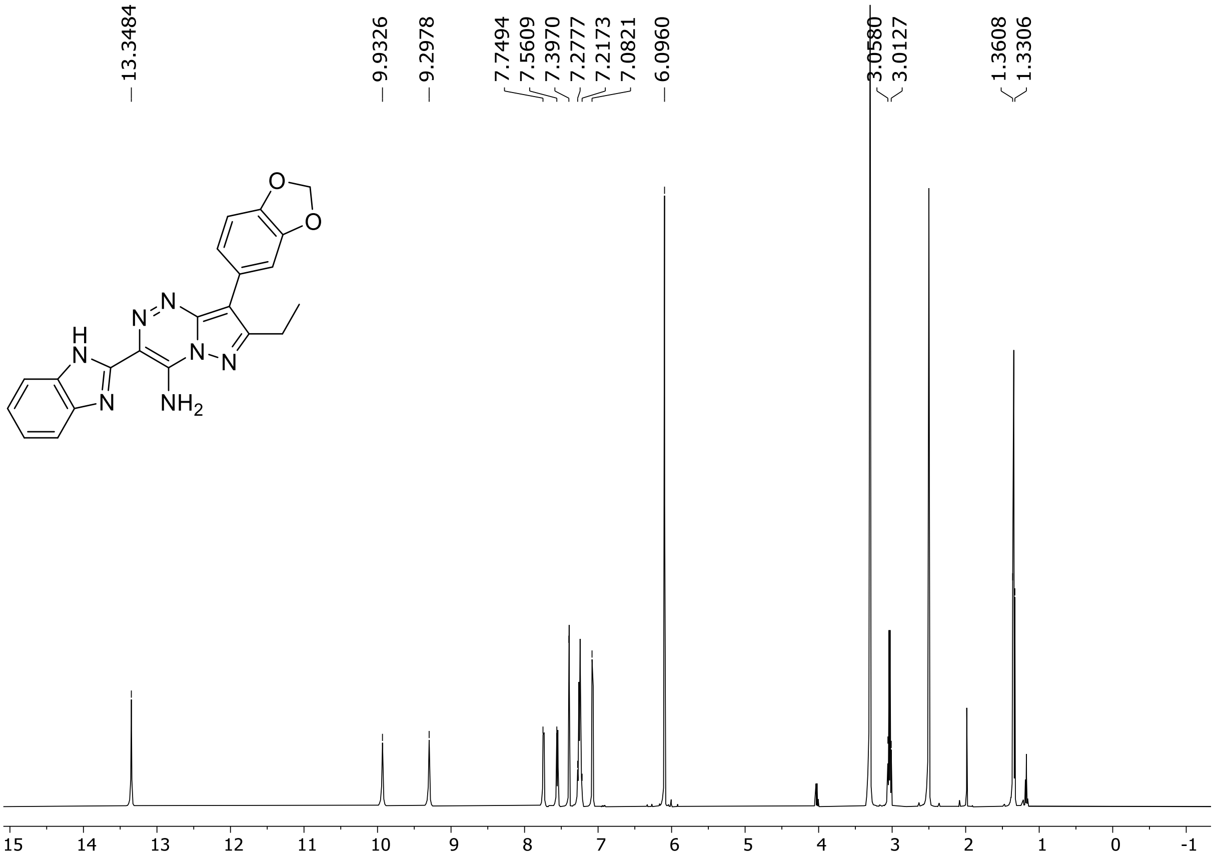

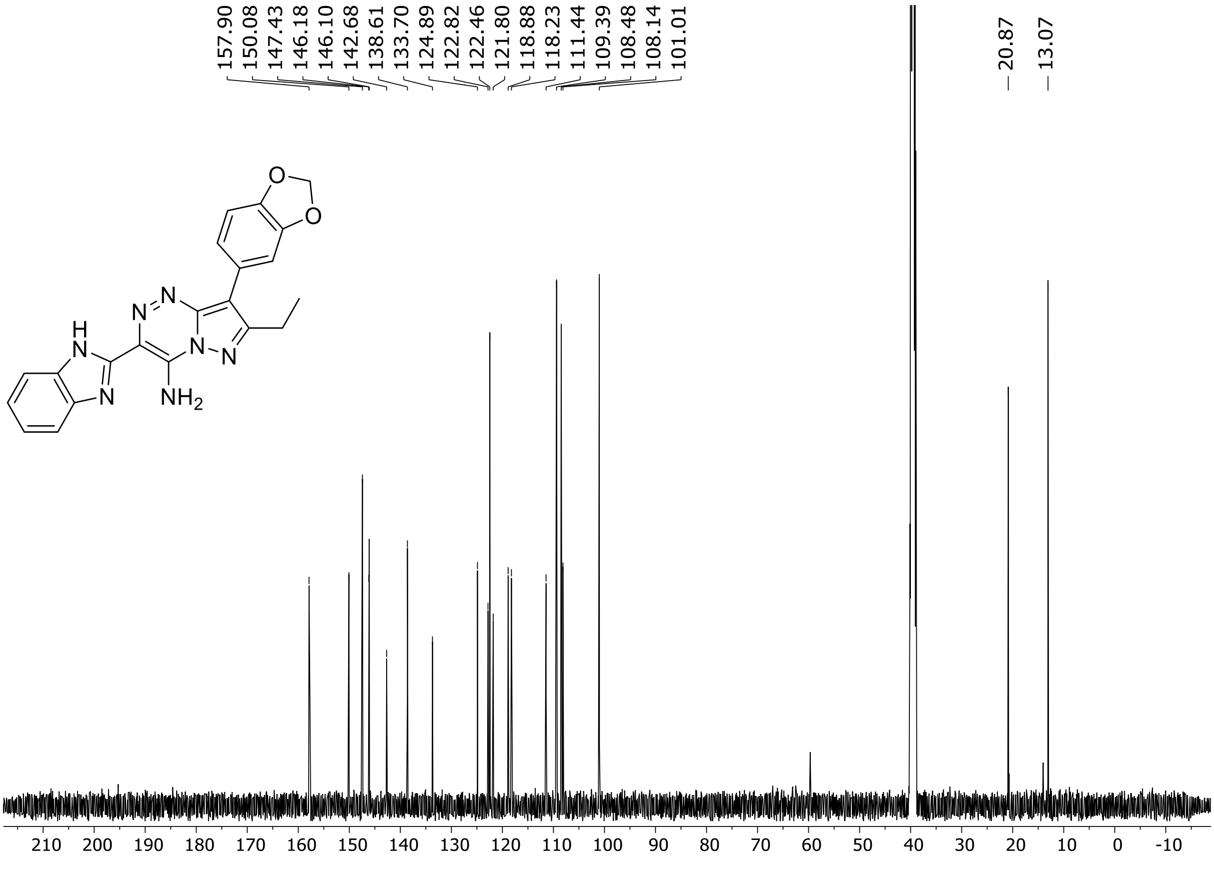

HRMS spectrum of **74**

$C_{21}H_{17}N_7O_2$ **mono *m/z* 399.1444**

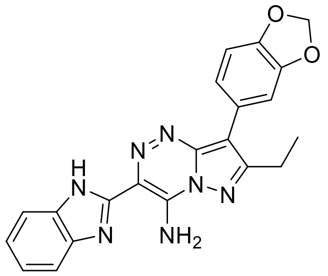

APCI + (MMI)
nitrogen flow 5 L/min, gas temperature 325°C, nebulizer 45 psi, skimmer 65 V, vaporizer 250°C, fragmentor 35 V, dissolved in methanol

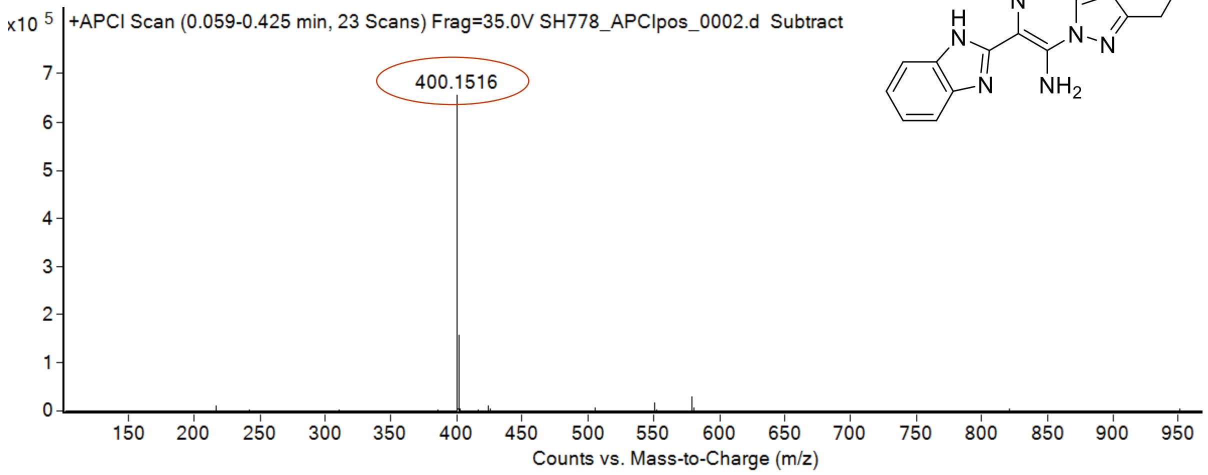

calculated mass: $[M+H]^+$ = 400.1516 observed: $[M+H]^+$ = 400.1516 mass accuracy < 0.1 ppm

^{1}H (500 MHz) and ^{13}C NMR (126 MHz) spectra of **75** in DMSO-d_6

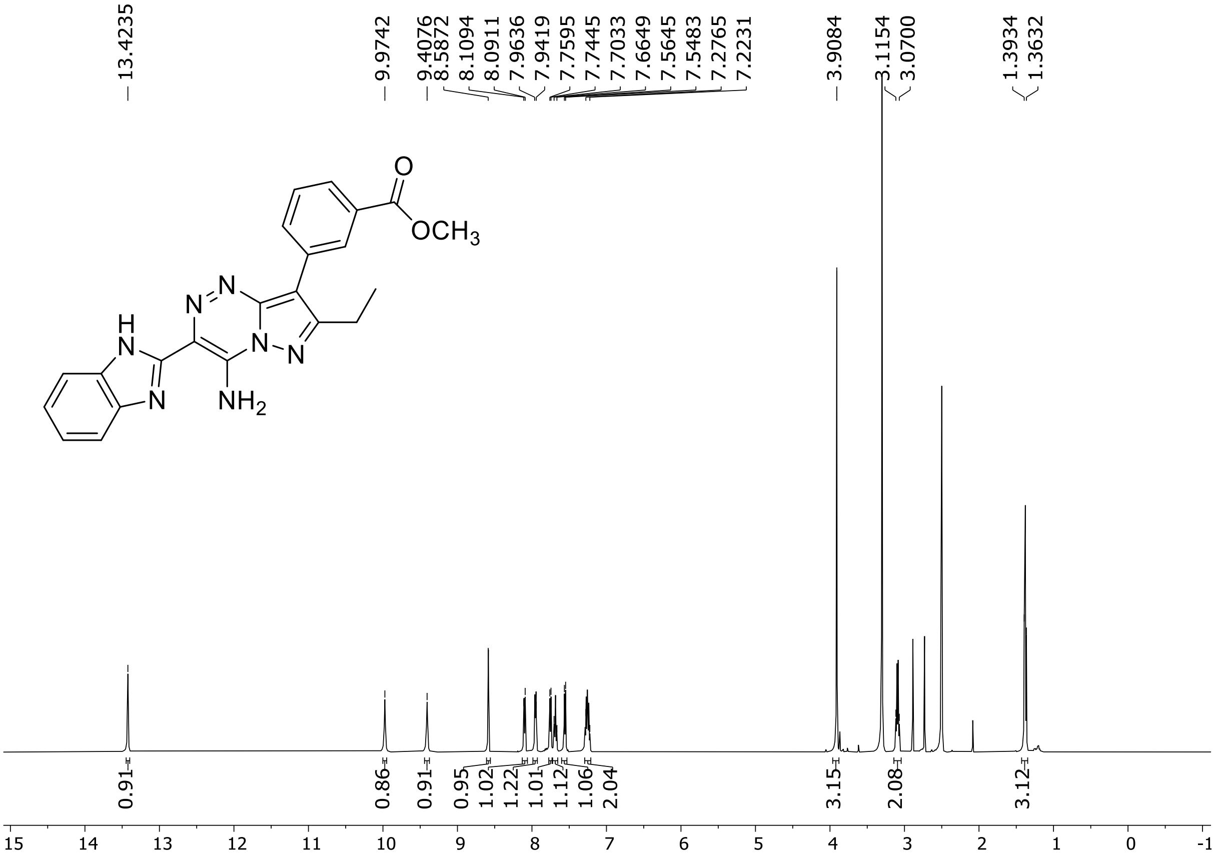

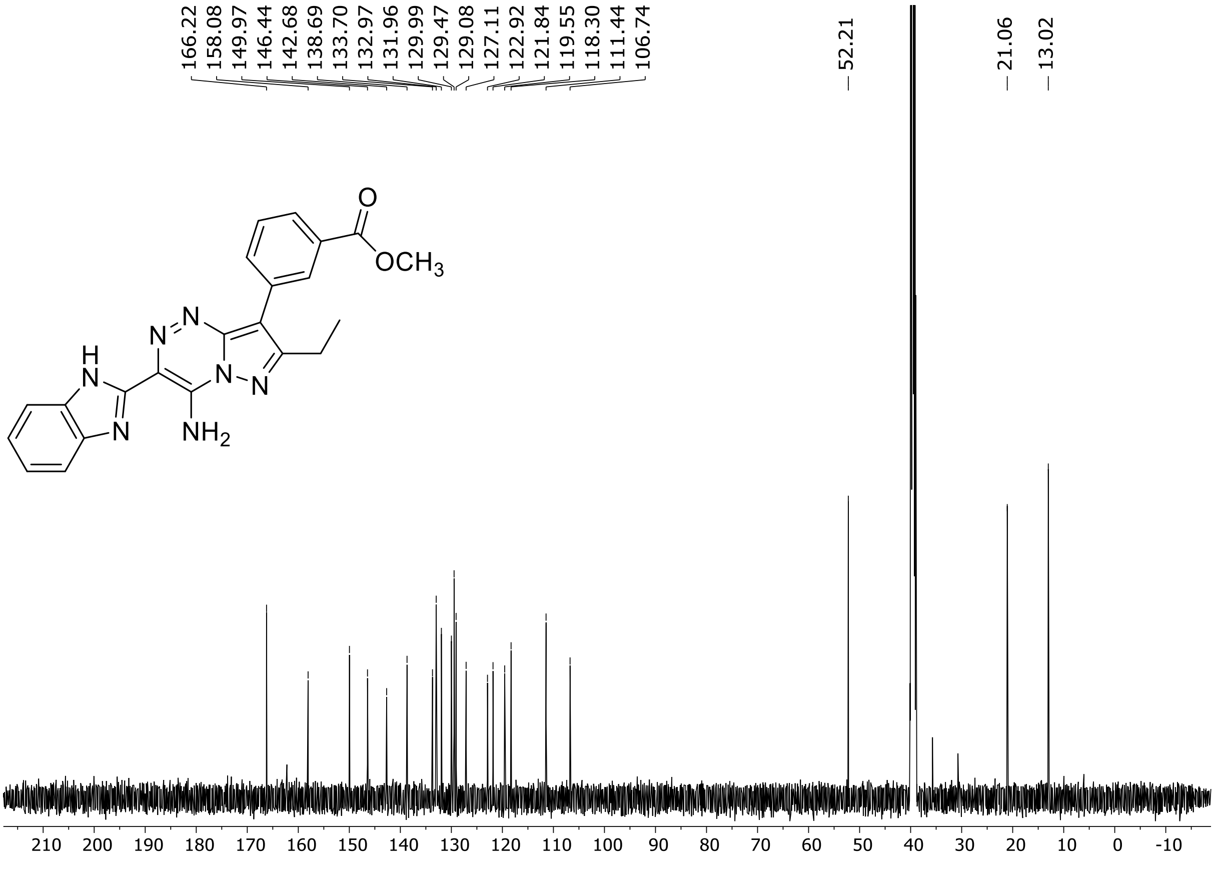

HRMS spectrum of **75**

$C_{22}H_{19}N_7O_2$ mono *m/z* **413.4410**

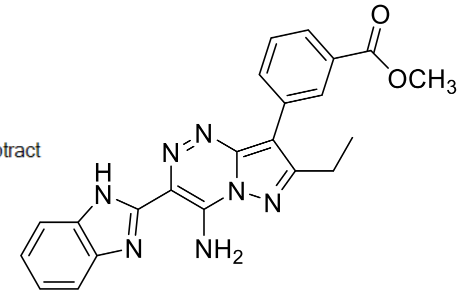

APCI + (MMI)
nitrogen flow 5 L/min, gas temperature 325°C, nebulizer 45 psi, skimmer 65 V, vaporizer 250°C, fragmentor 35 V, dissolved in methanol

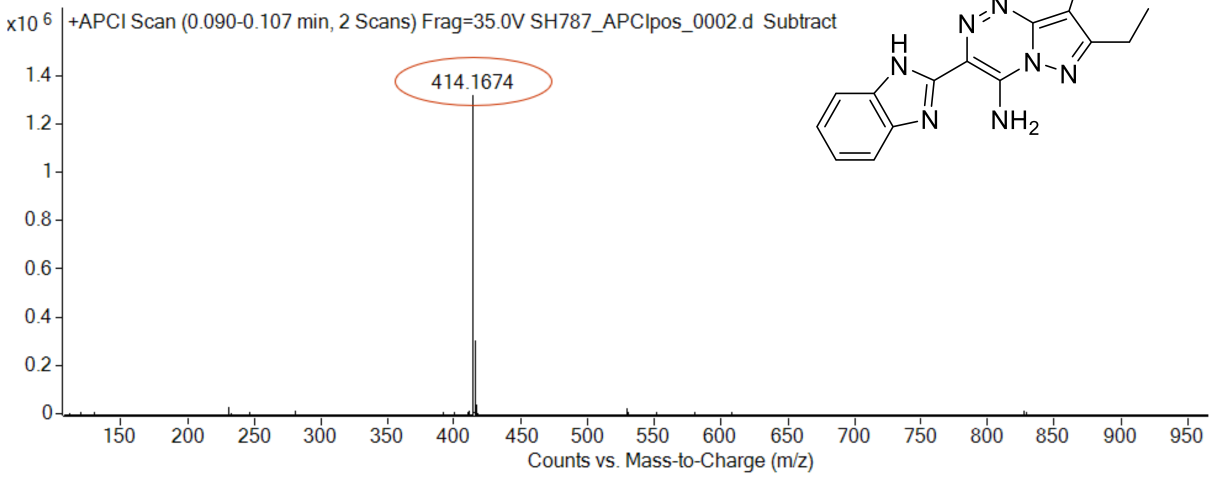

calculated mass: $[M+H]^+$ = 414.1672 observed: $[M+H]^+$ = 414.1674 mass accuracy = 0.2 ppm

^{1}H (500 MHz) and ^{13}C NMR (126 MHz) spectra of **76** in DMSO-d_6

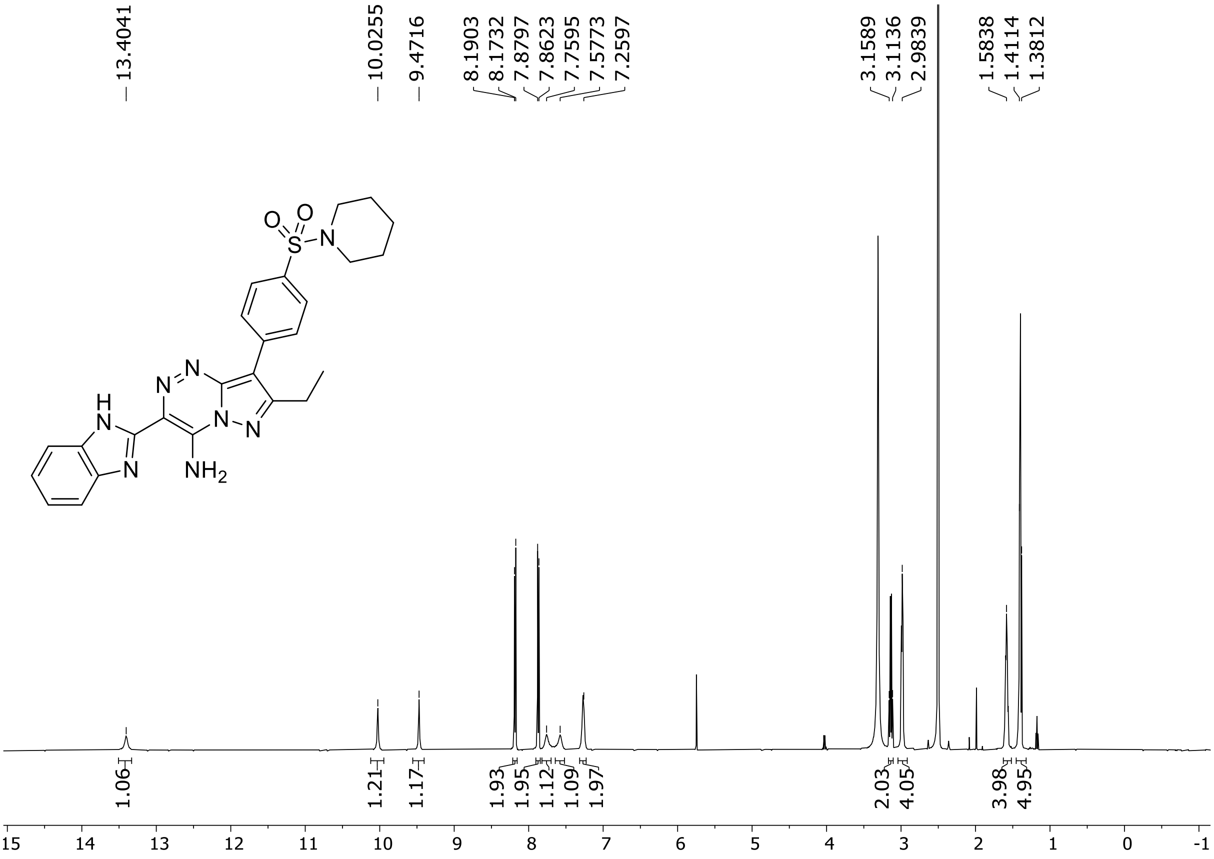

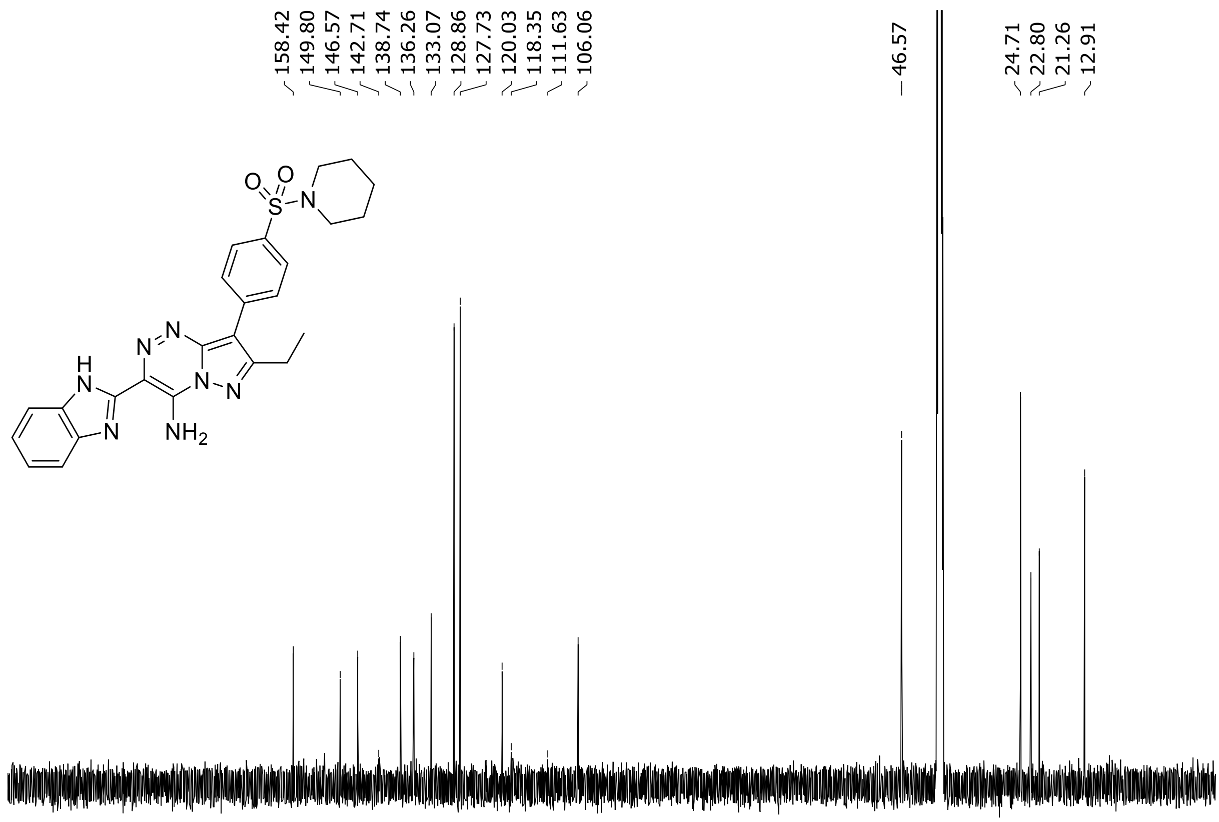

HRMS spectrum of **76**

$C_{25}H_{26}N_8O_2S$ **exact mass: 502.1899**

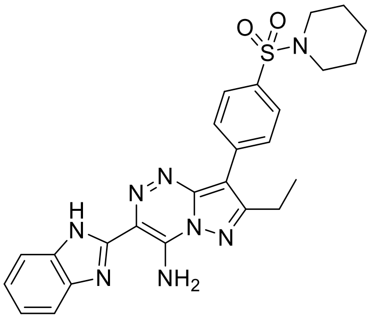

APCI + (MMI)
nitrogen flow 5 L/min, gas temperature 325°C, nebulizer 45 psig, skimmer 65 V, vaporizer 200°C, fragmentor 20 V, dissolved in methanol

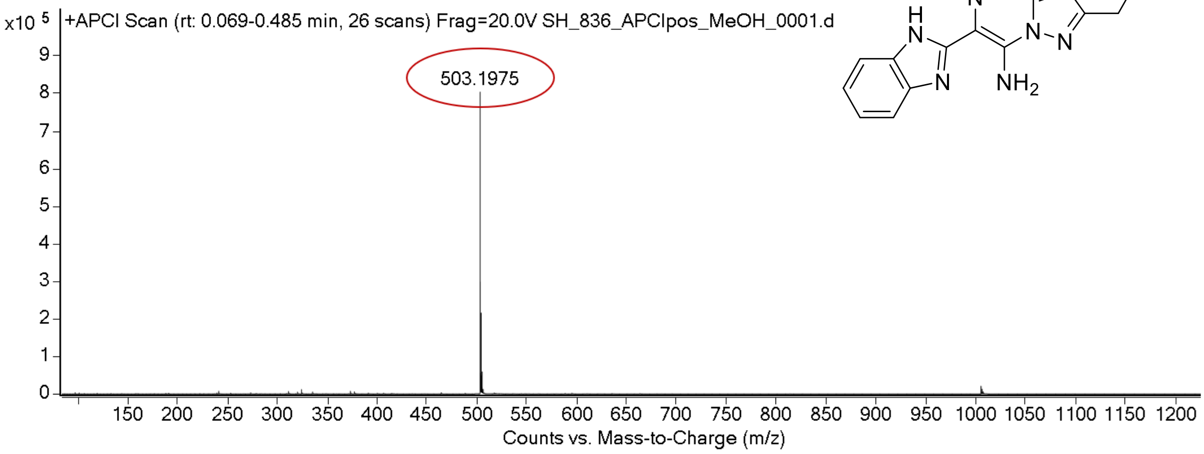

expected mass: $[M+H]^+$ = 503.1972 observed mass : $[M+H]^+$ = 503.1975 mass accuracy = 0.4 ppm

^{1}H (500 MHz) and ^{13}C NMR (126 MHz) spectra of **77** in DMSO-d_6

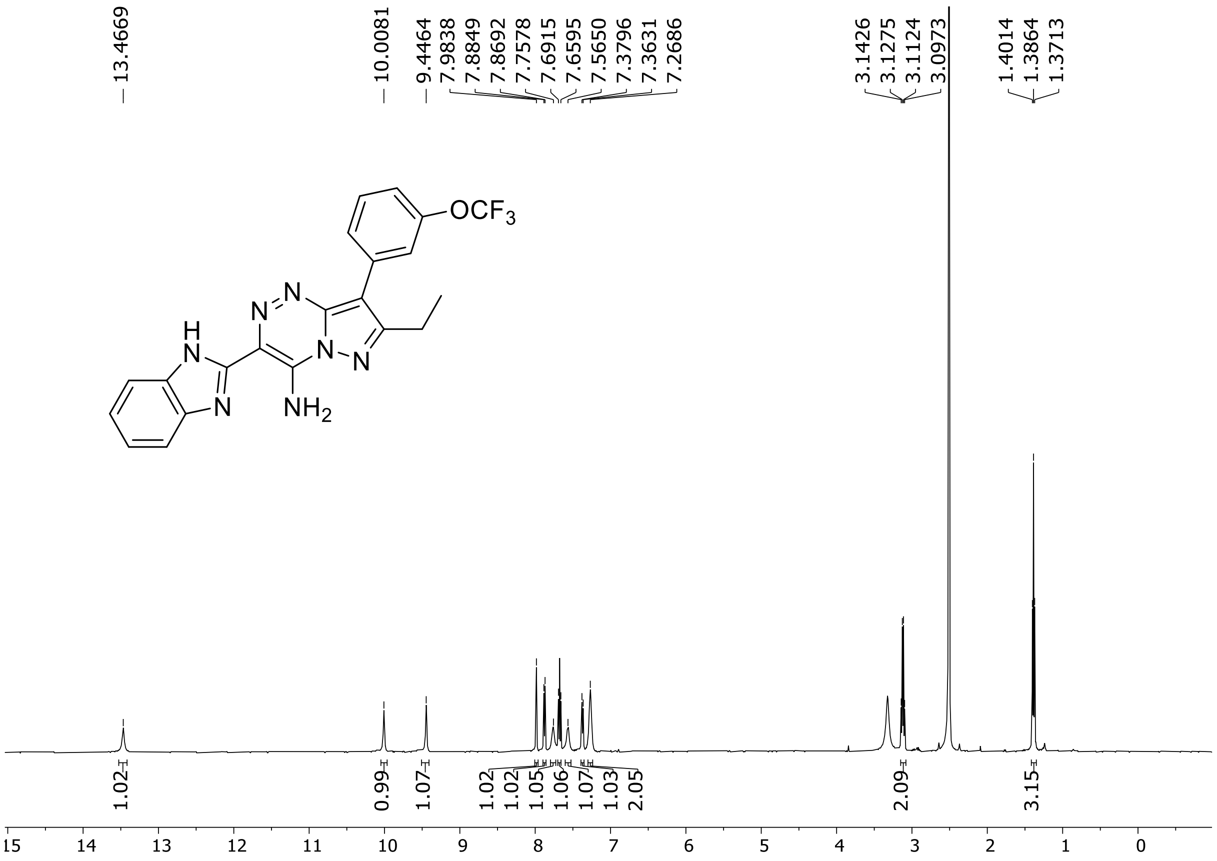

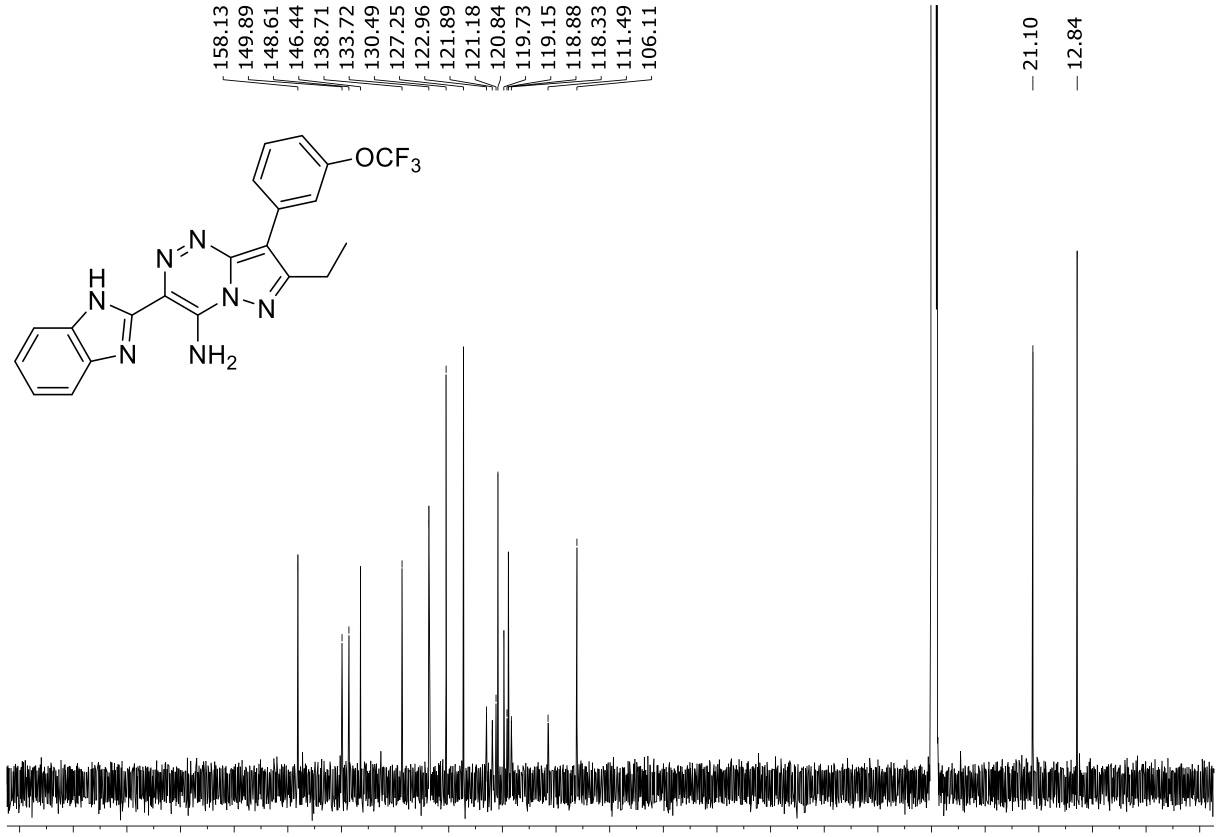

^{19}F (471 MHz) NMR spectrum of **77** DMSO-d_6

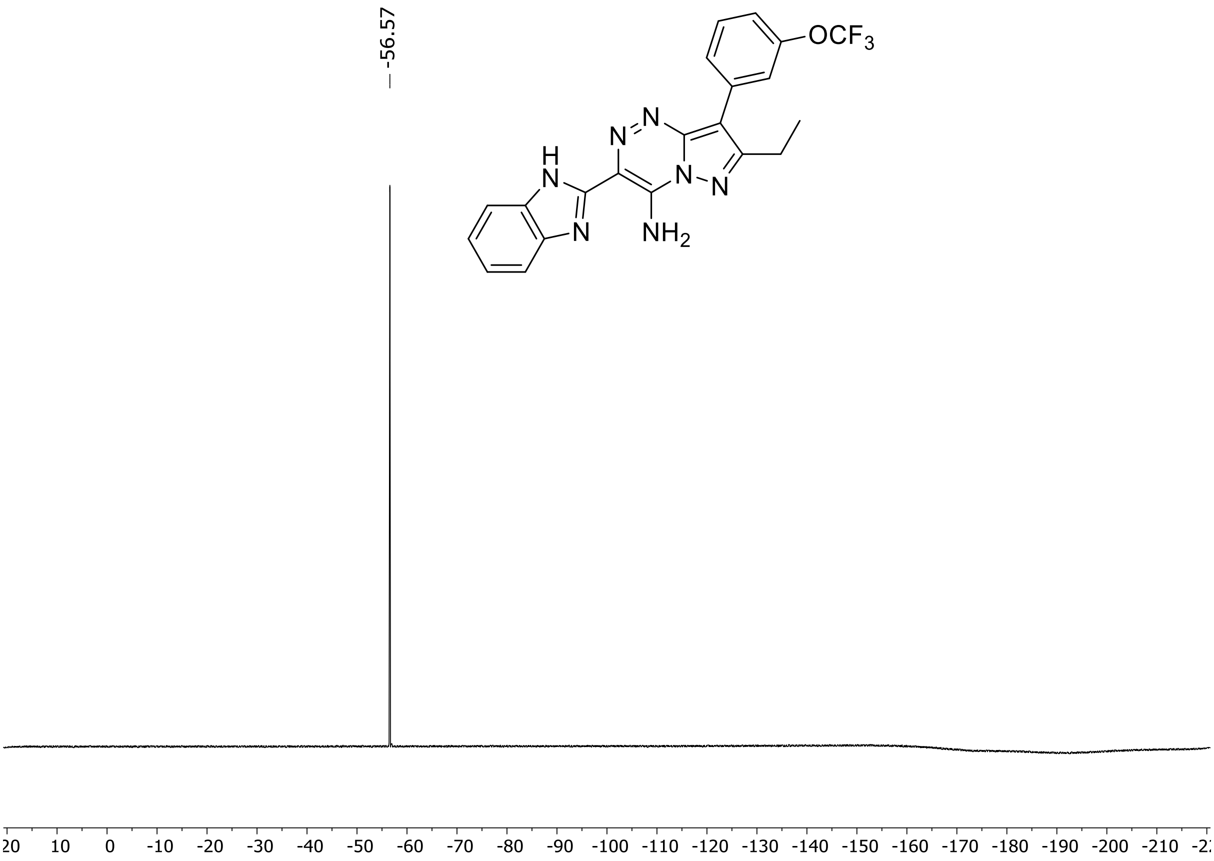

HRMS spectrum of **77**

$C_{21}H_{16}F_3N_7O$ **mono *m/z* 439.1368**

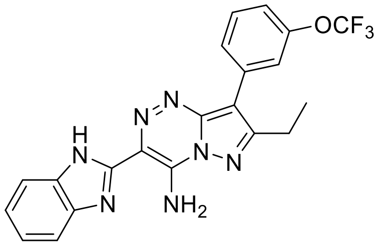

APCI + (MMI)
nitrogen flow 5 L/min, gas temperature 325°C, nebulizer 45 psi, skimmer 65 V, vaporizer 250°C, fragmentor 35 V, dissolved in methanol

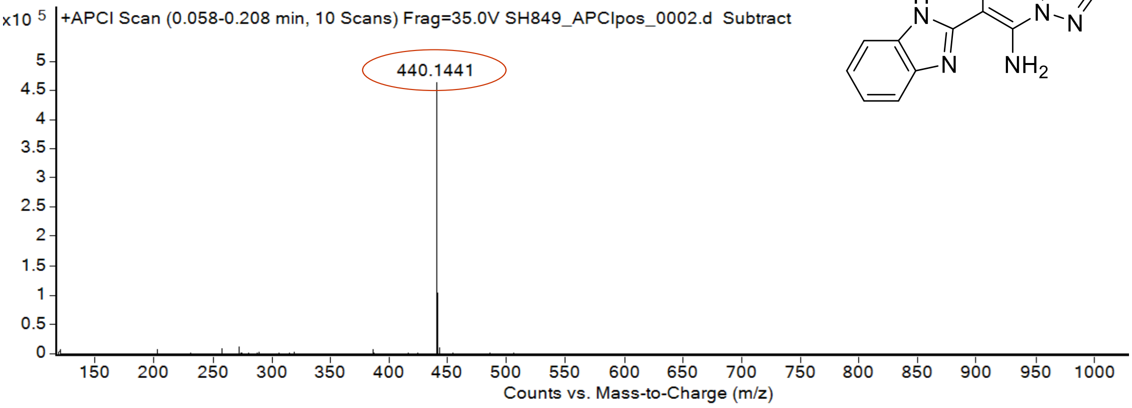

calculated mass: $[M+H]^+$ = 440.1441 observed: $[M+H]^+$ = 440.1441 mass accuracy < 0.1 ppm

^1H (500 MHz) and ^{13}C NMR (126 MHz) spectra of **78** in DMSO-d_6

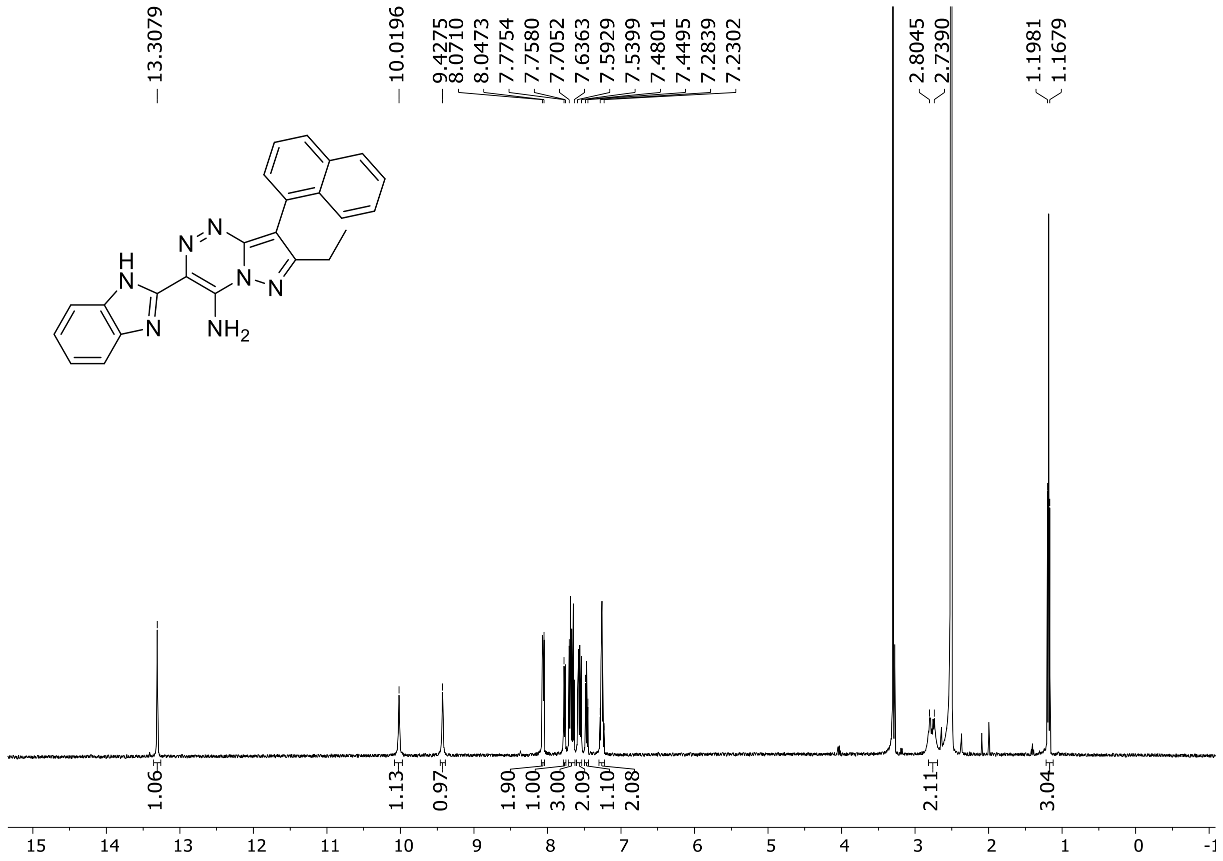

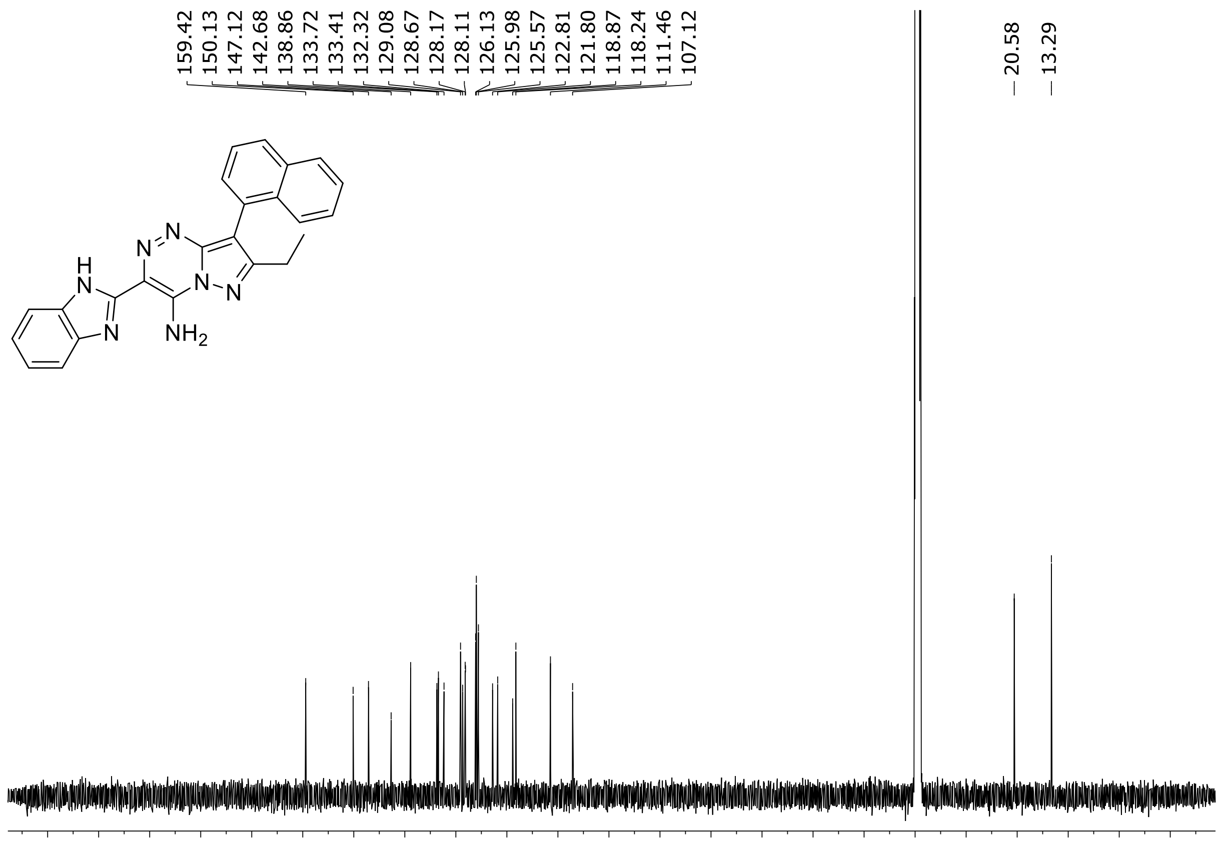

HRMS spectrum of **78**

$C_{24}H_{19}N_7$ **exact mass: 405.1702**

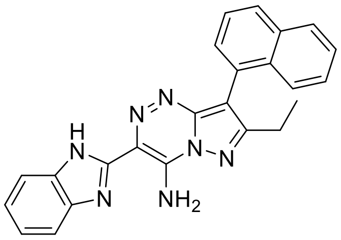

APCI + (MMI)
nitrogen flow 5 L/min, gas temperature 325°C, nebulizer 45 psig, skimmer 65 V, vaporizer 200°C, fragmentor 10 V, dissolved in methanol

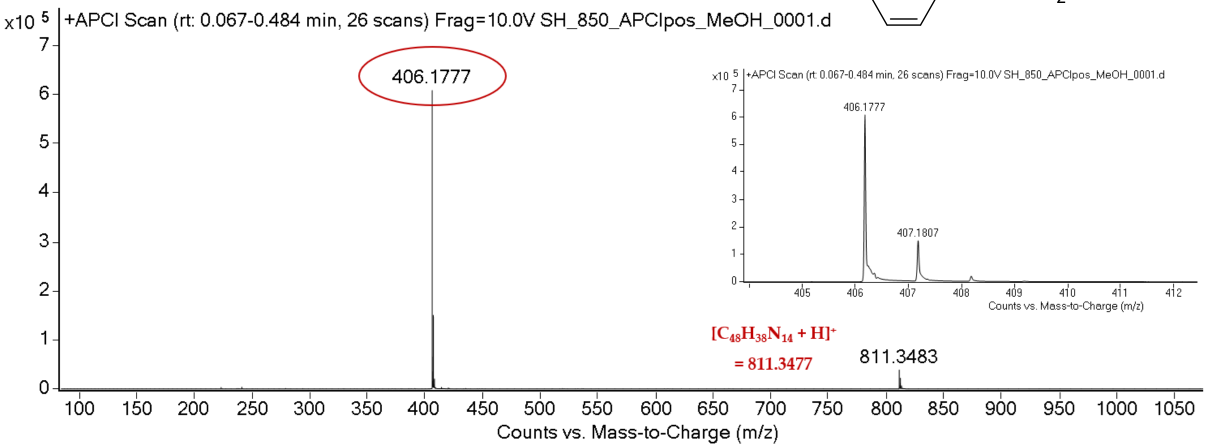

expected mass: $[M+H]^+$ = 406.1775 observed mass : $[M+H]^+$ = 406.1777 mass accuracy = 0.5 ppm

^{1}H (500 MHz) and ^{13}C NMR (126 MHz) spectra of **79** in DMSO-d_6

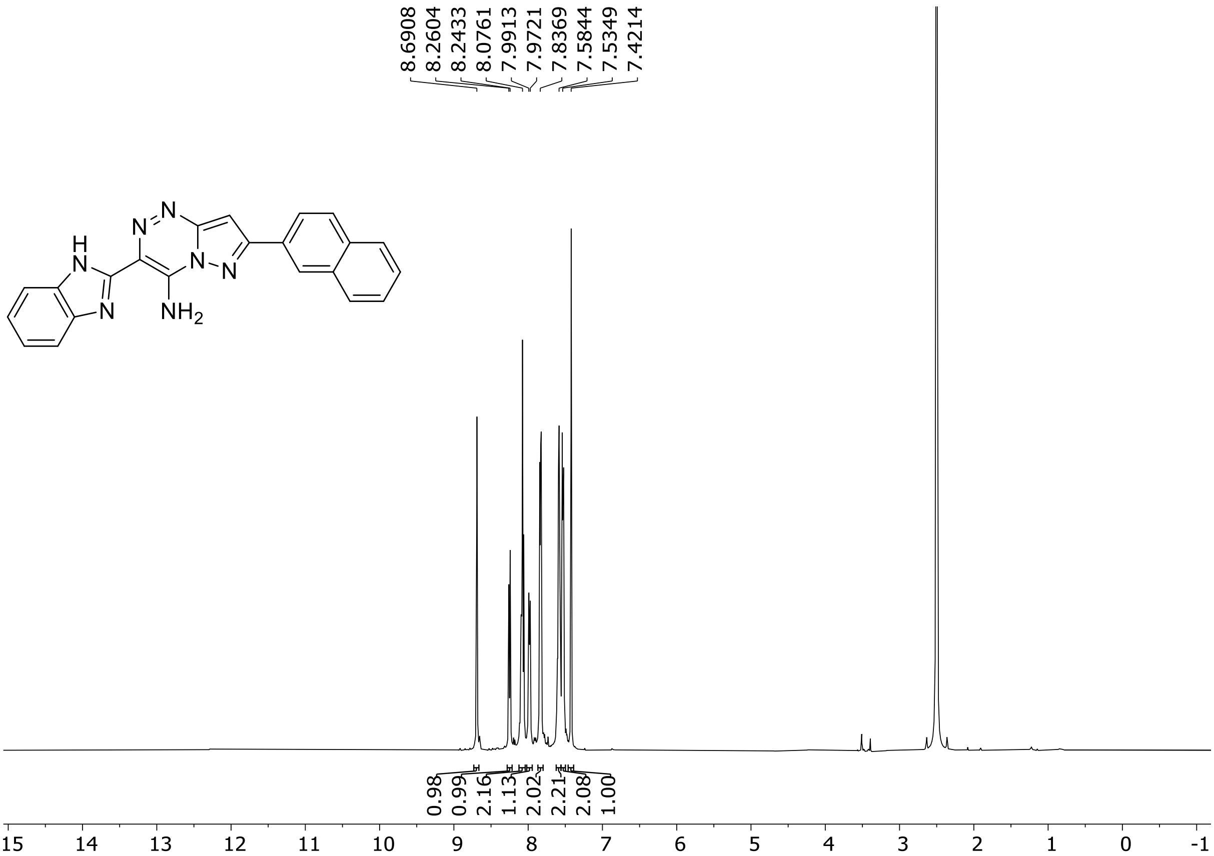

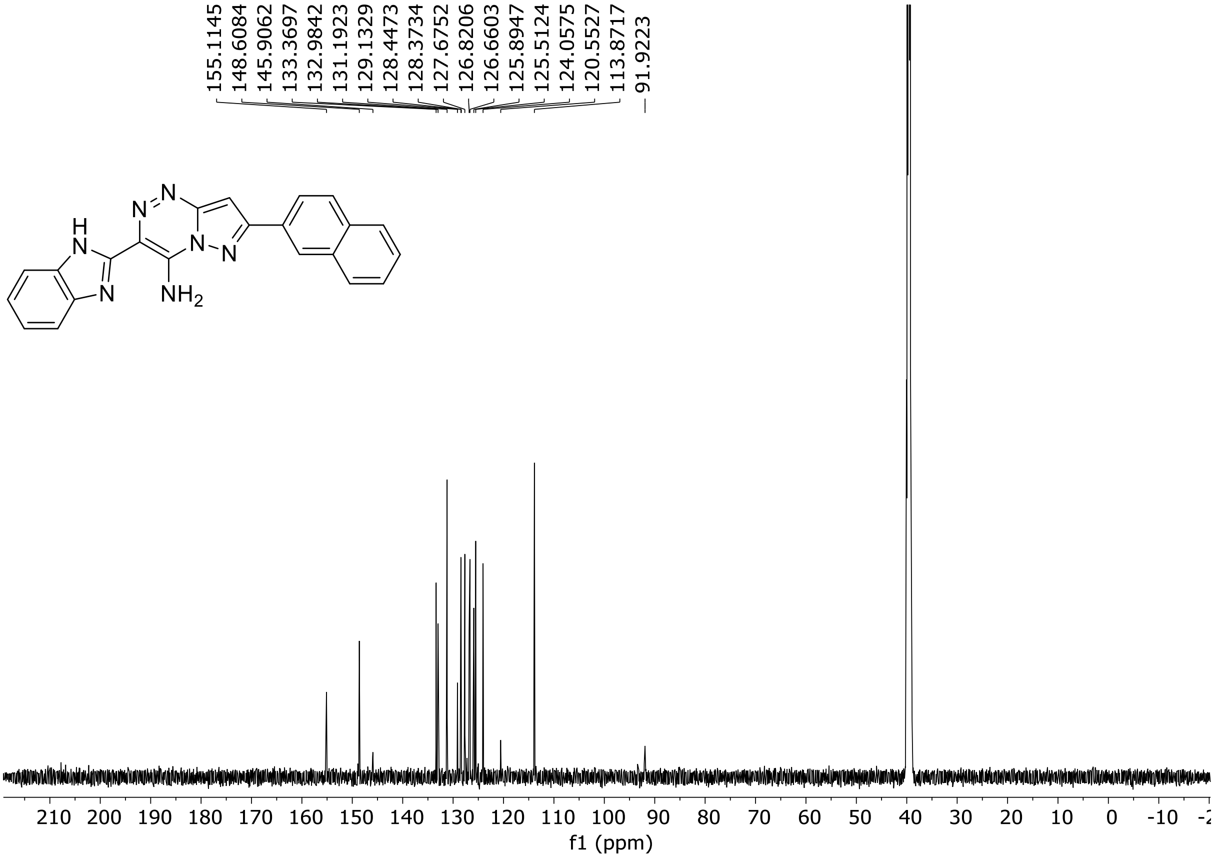

HRMS spectrum of **79**

$C_{22}H_{14}N_6O$ **mono m/z = 378.1229**

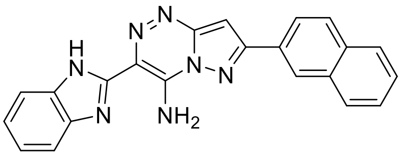

ESI + (MMI)
nitrogen flow 5 L/min, gas temperature 300°C, nebulizer 45 psi,
skimmer 60 V, fragmentor 20 V, dissolved in methanol

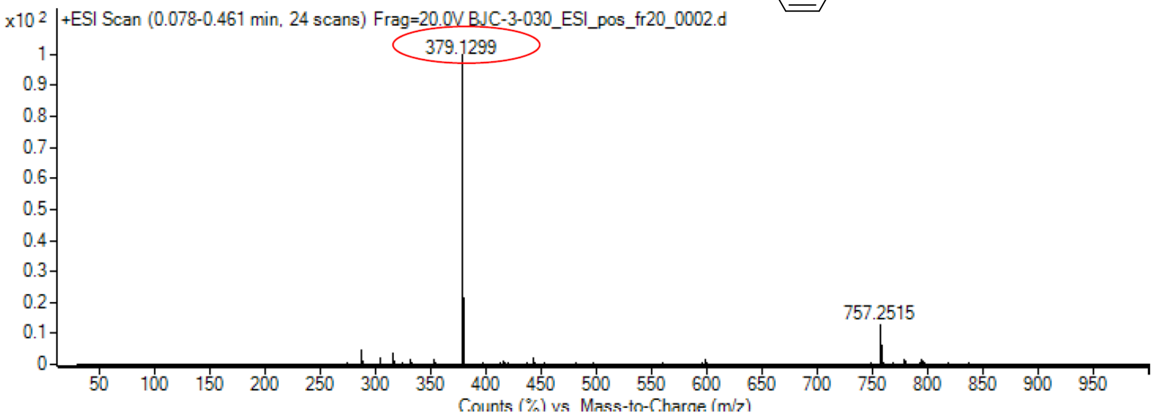

calculated mass: $[M+H]^+$ = 379.1302 observed: $[M+H]^+$ = 379.1299 max. mass error = 0.8ppm

^{1}H (500 MHz) and ^{13}C NMR (126 MHz) spectra of **80** in DMSO-d_6

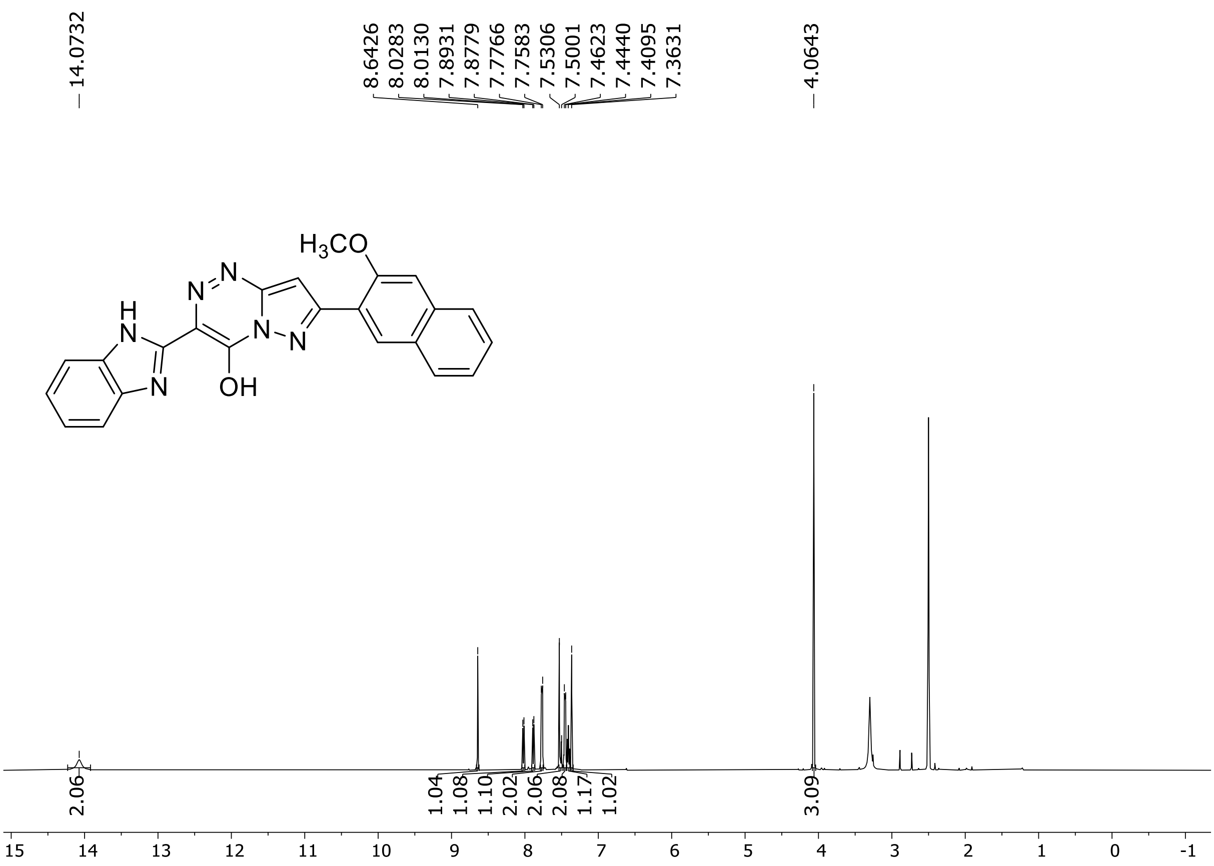

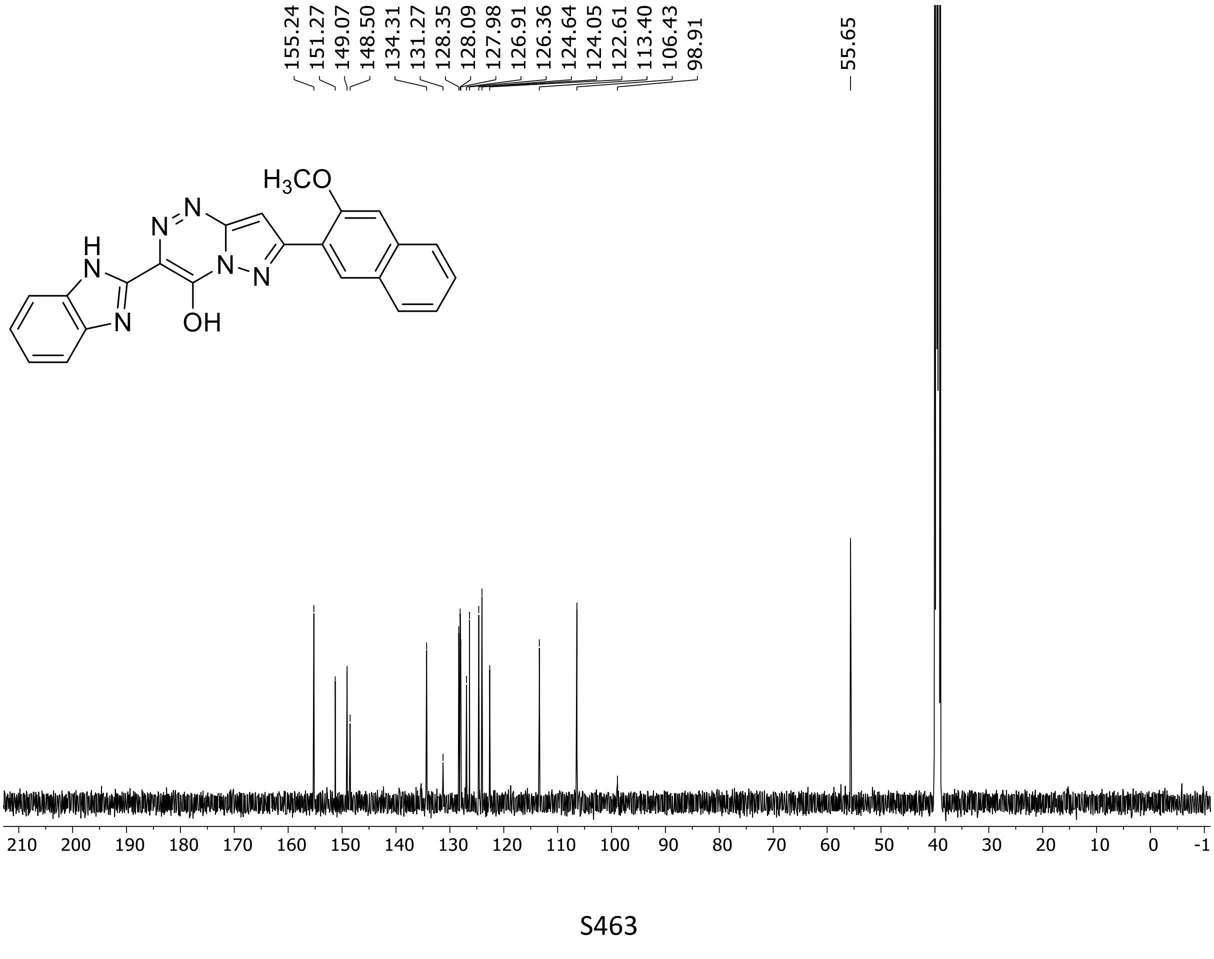

HRMS spectrum of **80**

$C_{23}H_{17}N_7O$ **exact mass: 407.1495**

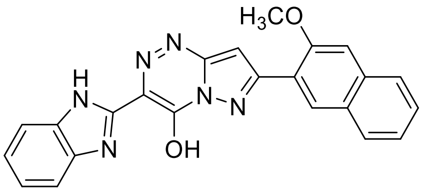

APCI + (MMI)
nitrogen flow 5 L/min, gas temperature 325°C, nebulizer 45 psig, skimmer 65 V, vaporizer 200°C, fragmentor 5 V, dissolved in methanol

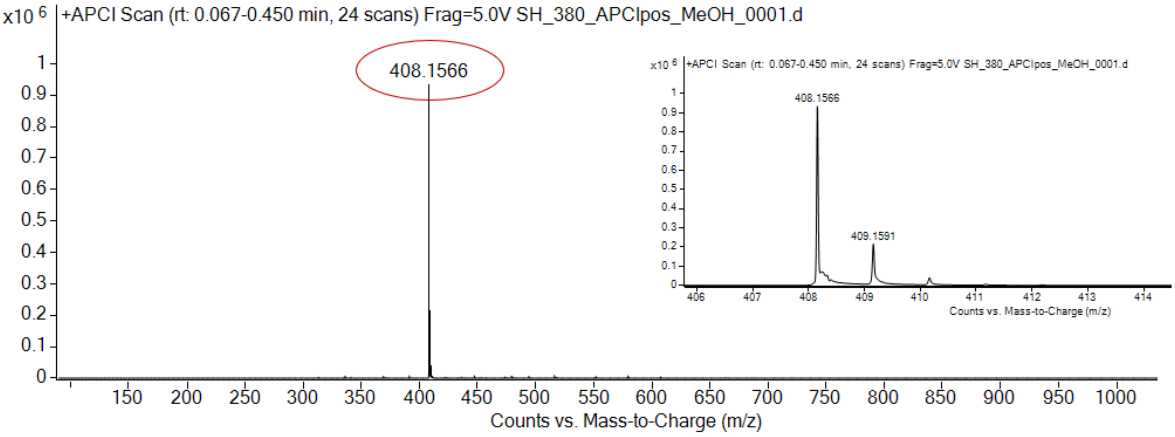

expected mass: $[M+H]^+$ = 408.1567 observed mass : $[M+H]^+$ = 408.1566 mass accuracy = - 0.2 ppm

^{1}H (300 MHz) NMR spectrum of **81** in DMSO-d_6

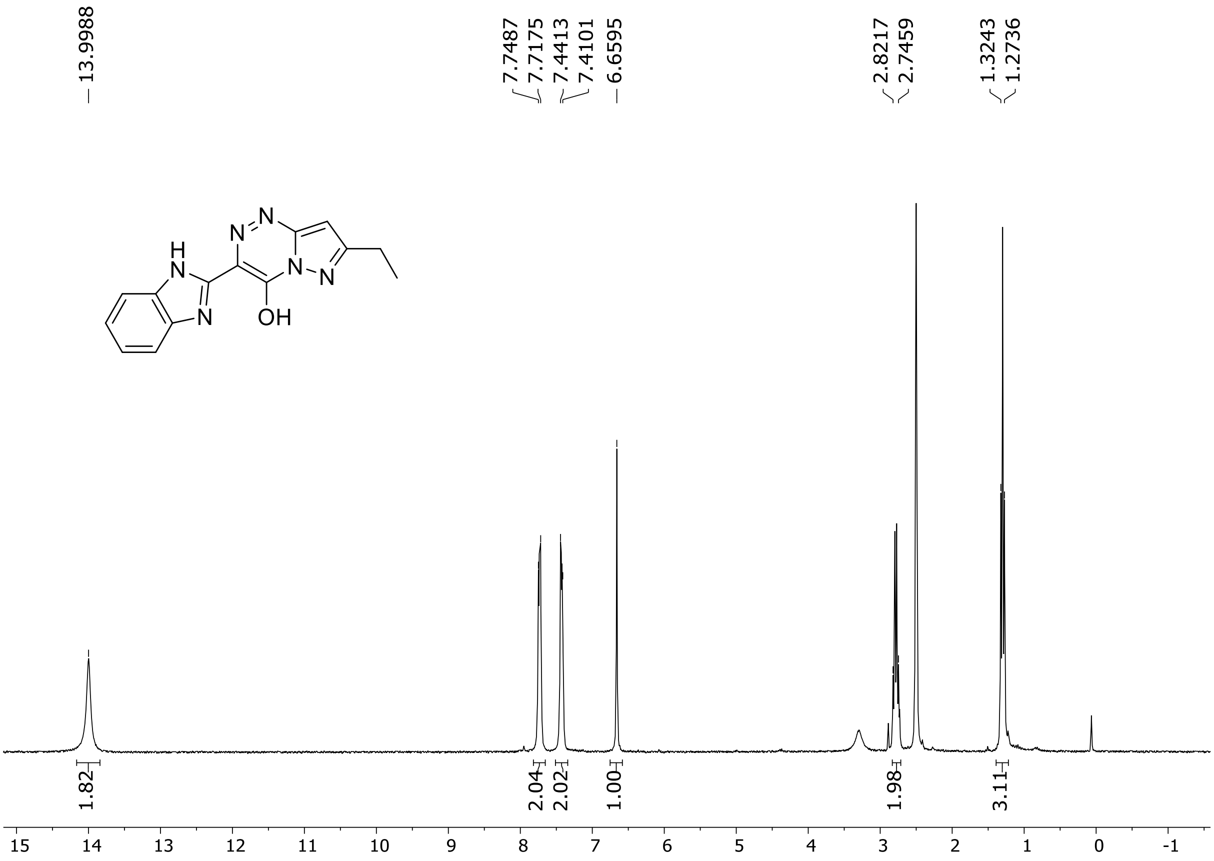

HRMS spectrum of **81**

$C_{14}H_{12}N_6O$ **exact mass: 280.1073**

APCI + (MMI)
nitrogen flow 3 L/min, gas temperature 325°C, nebulizer 45 psig, skimmer 65 V, vaporizer 200°C, fragmentor 20 V, dissolved in methanol

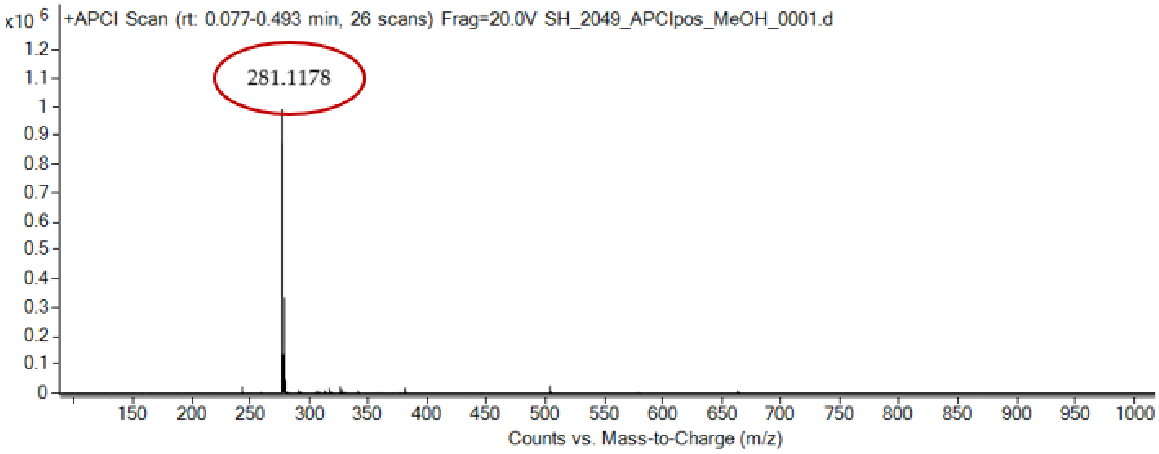

expected mass: $[M+H]^+ = 281.1175$ observed mass: $[M+H]^+ = 281.1178$ mass accuracy < 0.1 ppm

^{1}H (500 MHz) and ^{13}C NMR (126 MHz) spectra of **82** in DMSO-d_6

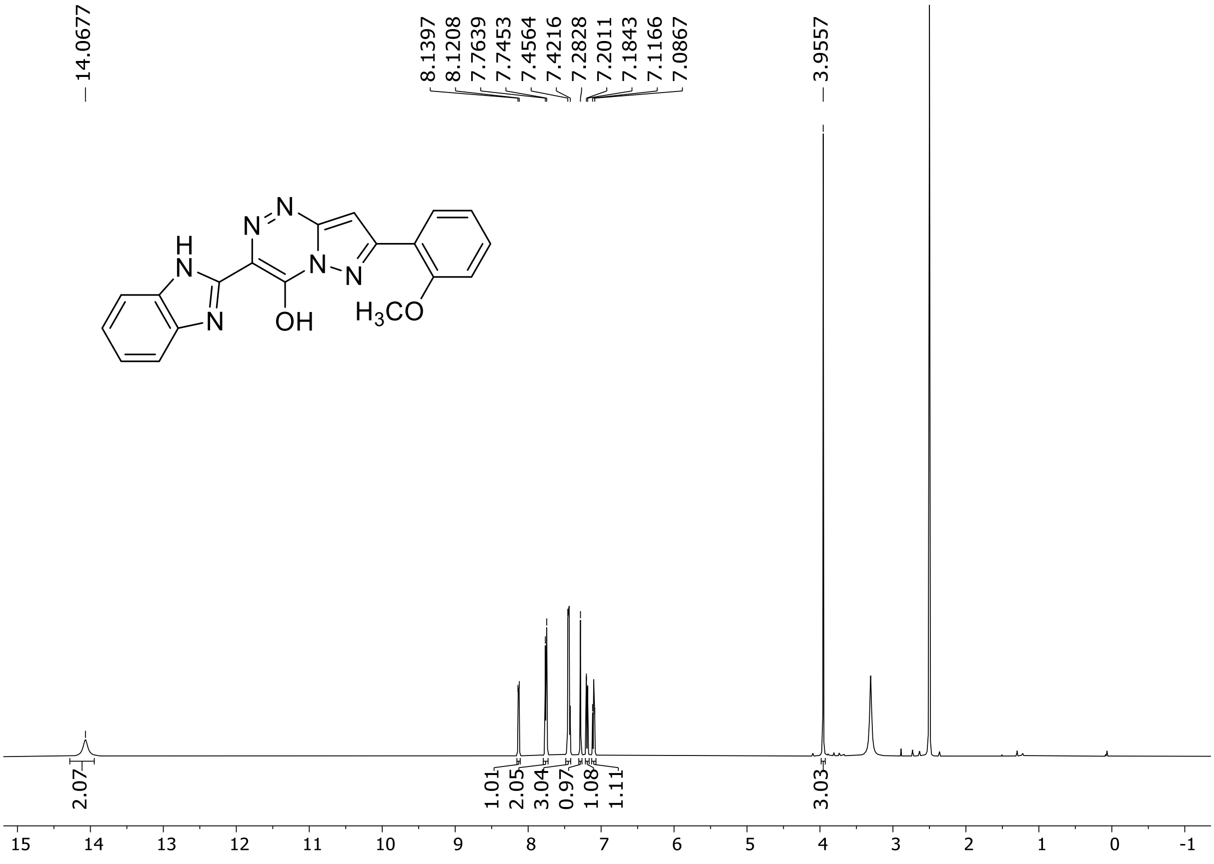

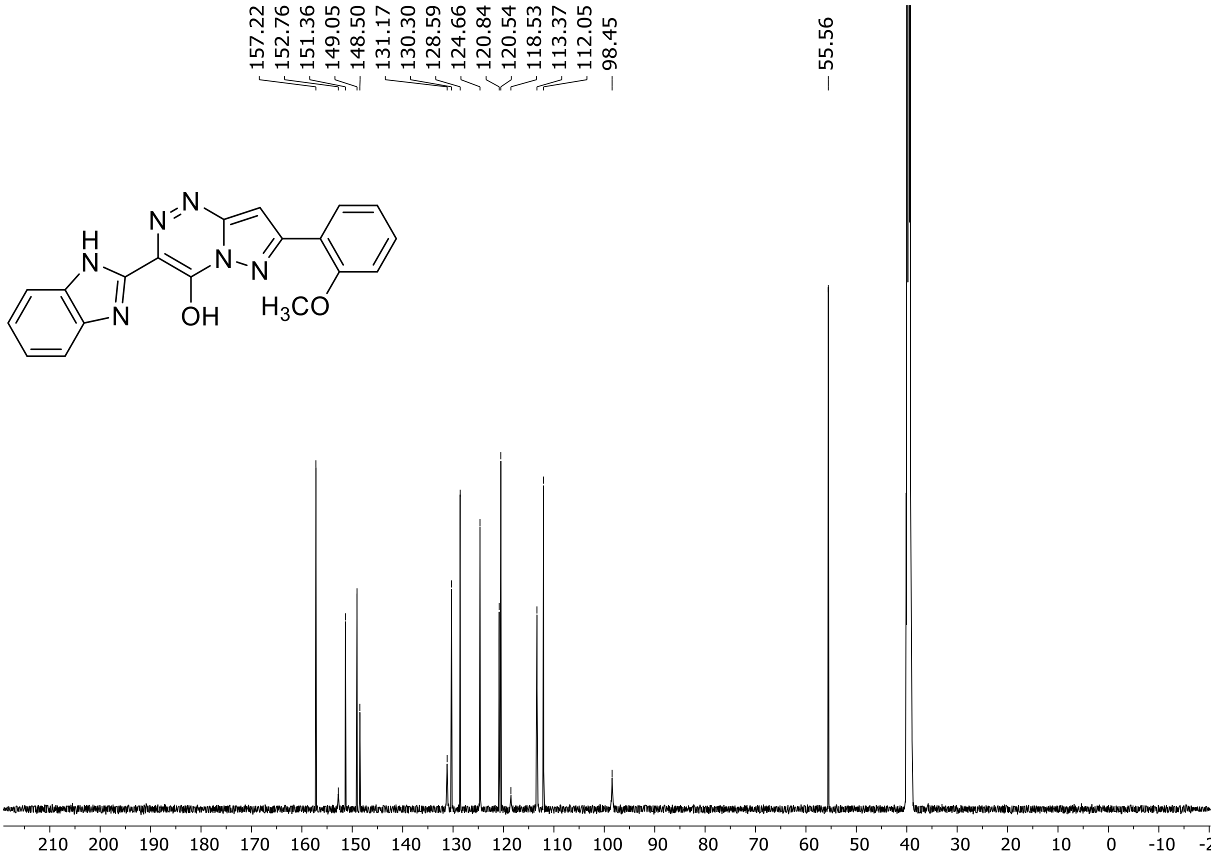

HRMS spectrum of **82**

$C_{19}H_{14}N_6O_2$ **exact mass: 358.1178**

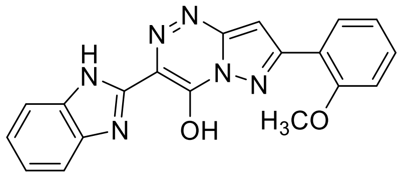

APCI + (MMI)
nitrogen flow 5 L/min, gas temperature 325°C, nebulizer 45 psig, skimmer 65 V, vaporizer 200°C, fragmentor 20 V, dissolved in methanol

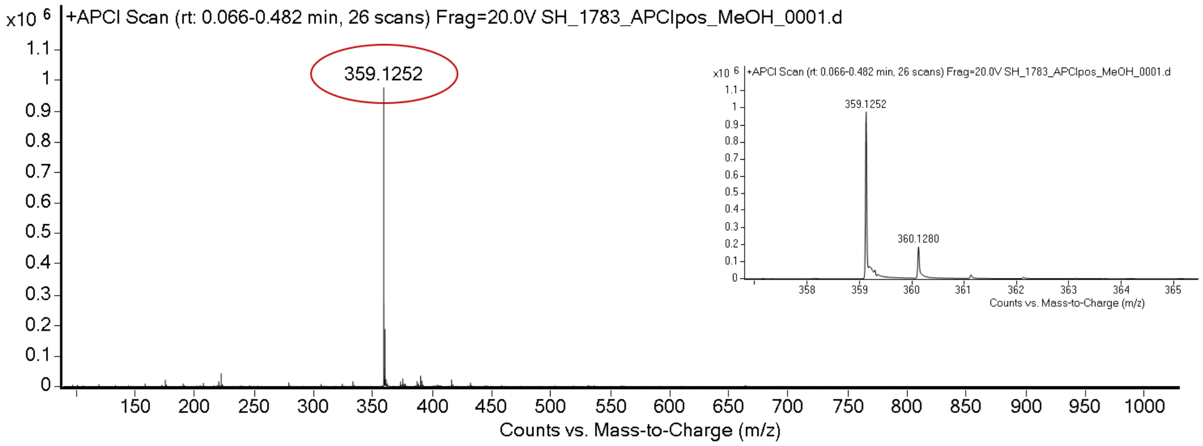

expected mass: [M+H]$^+$ = 359.1251 observed mass : [M+H]$^+$ = 359.1252 mass accuracy = 0.3 ppm

^{1}H (500 MHz) and ^{13}C NMR (126 MHz) spectra of **83** in DMSO-d_6

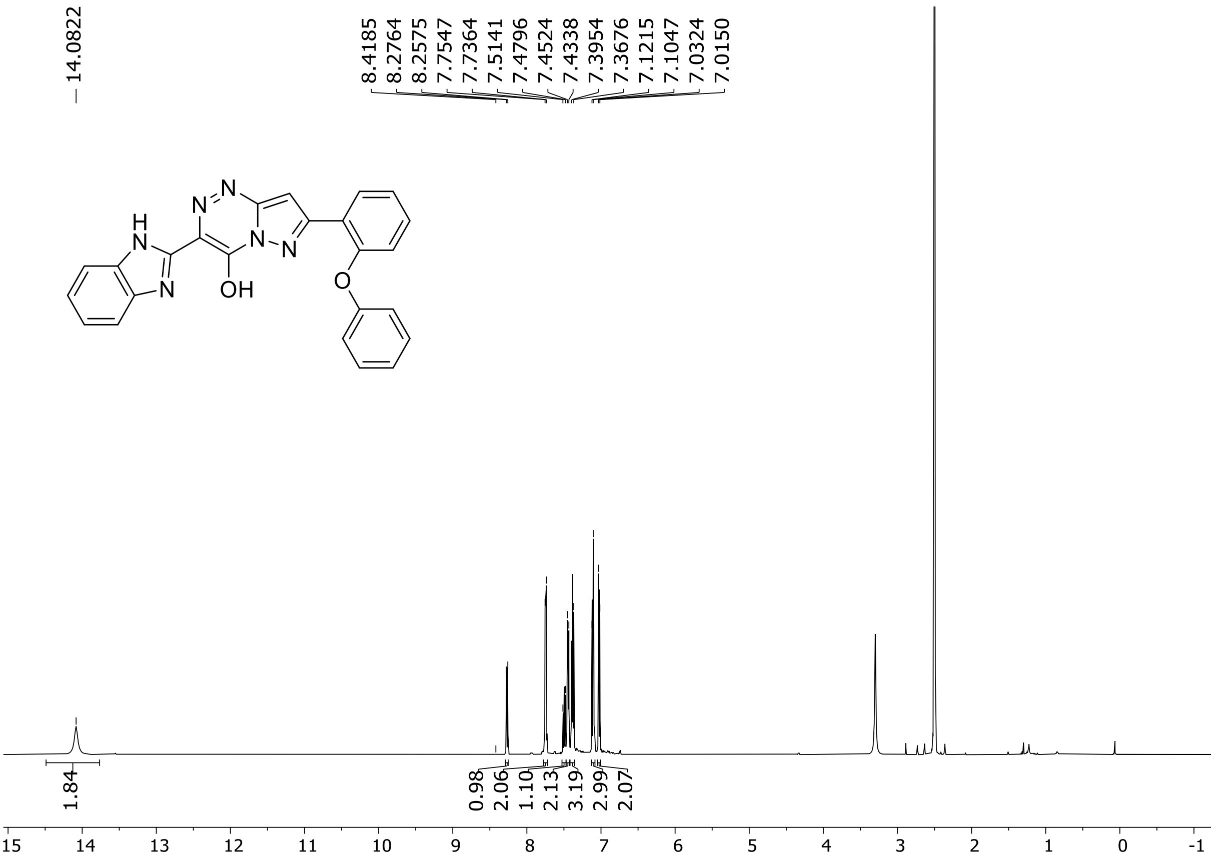

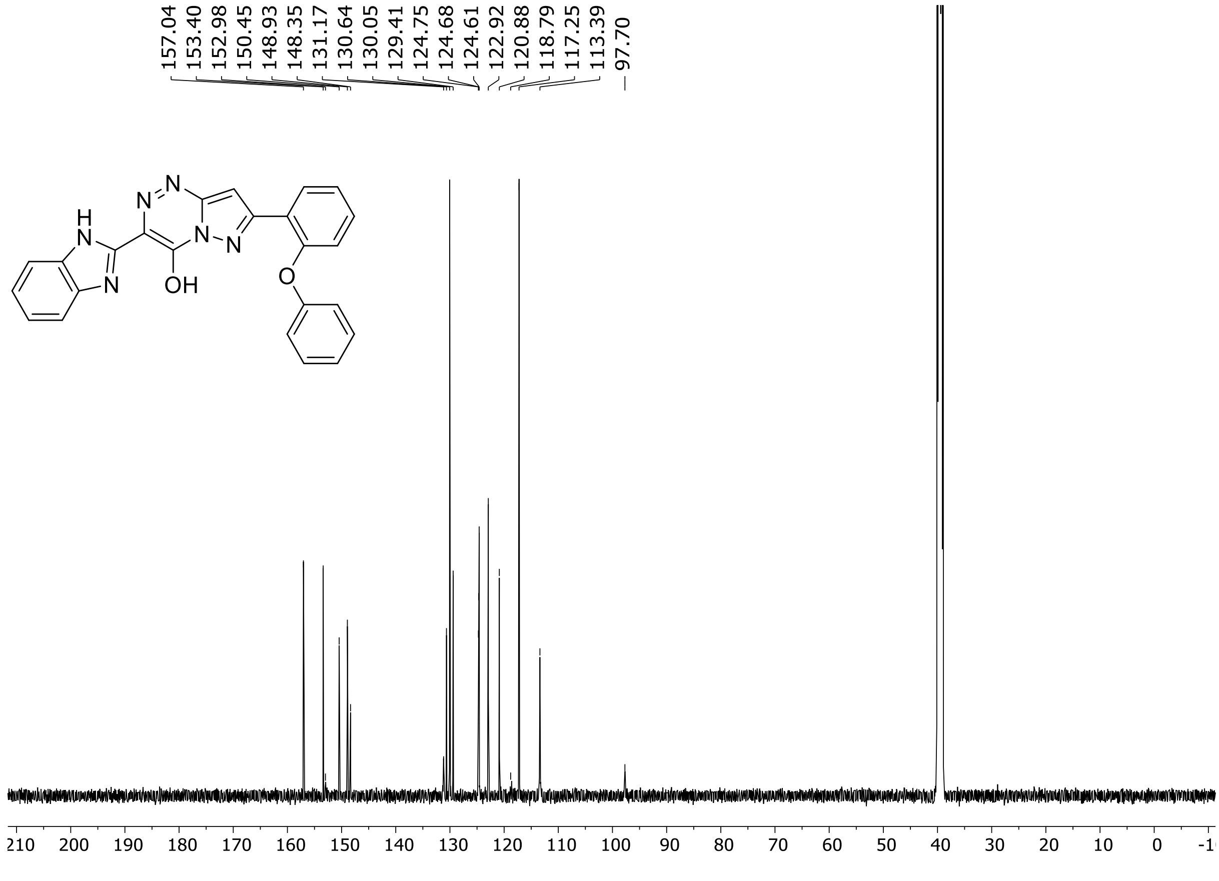

HRMS spectrum of **83**

$C_{24}H_{16}N_6O_2$ **exact mass: 420.1335**

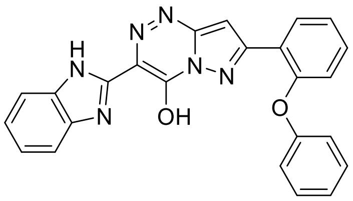

APCI + (MMI)
nitrogen flow 5 L/min, gas temperature 325°C, nebulizer 45 psig, skimmer 65 V, vaporizer 200°C, fragmentor 10 V, dissolved in methanol

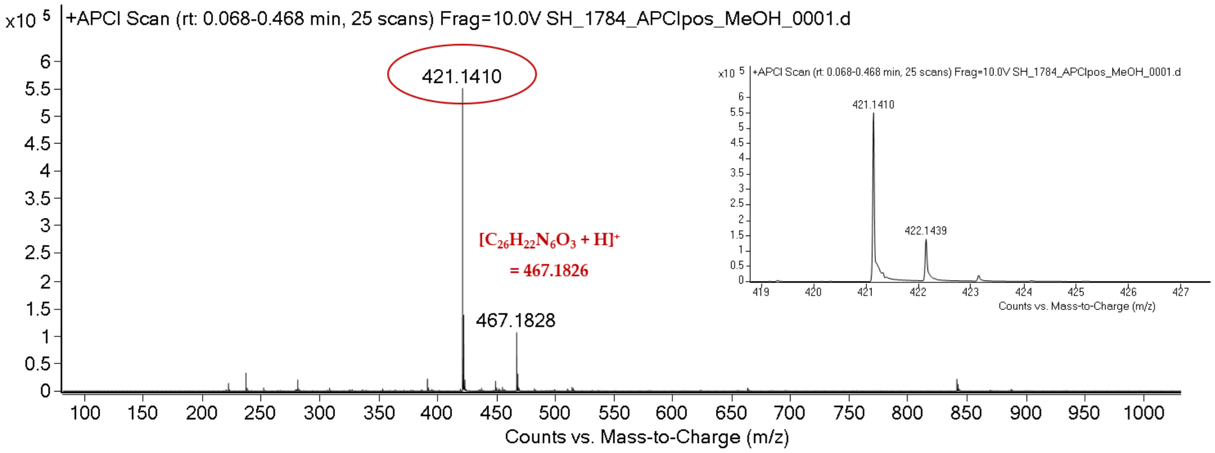

expected mass: $[M+H]^+$ = 421.1408 observed mass : $[M+H]^+$ = 421.1410 mass accuracy = 0.5 ppm

^{1}H (500 MHz) and ^{13}C NMR (126 MHz) spectra of **84** in DMSO-d_6

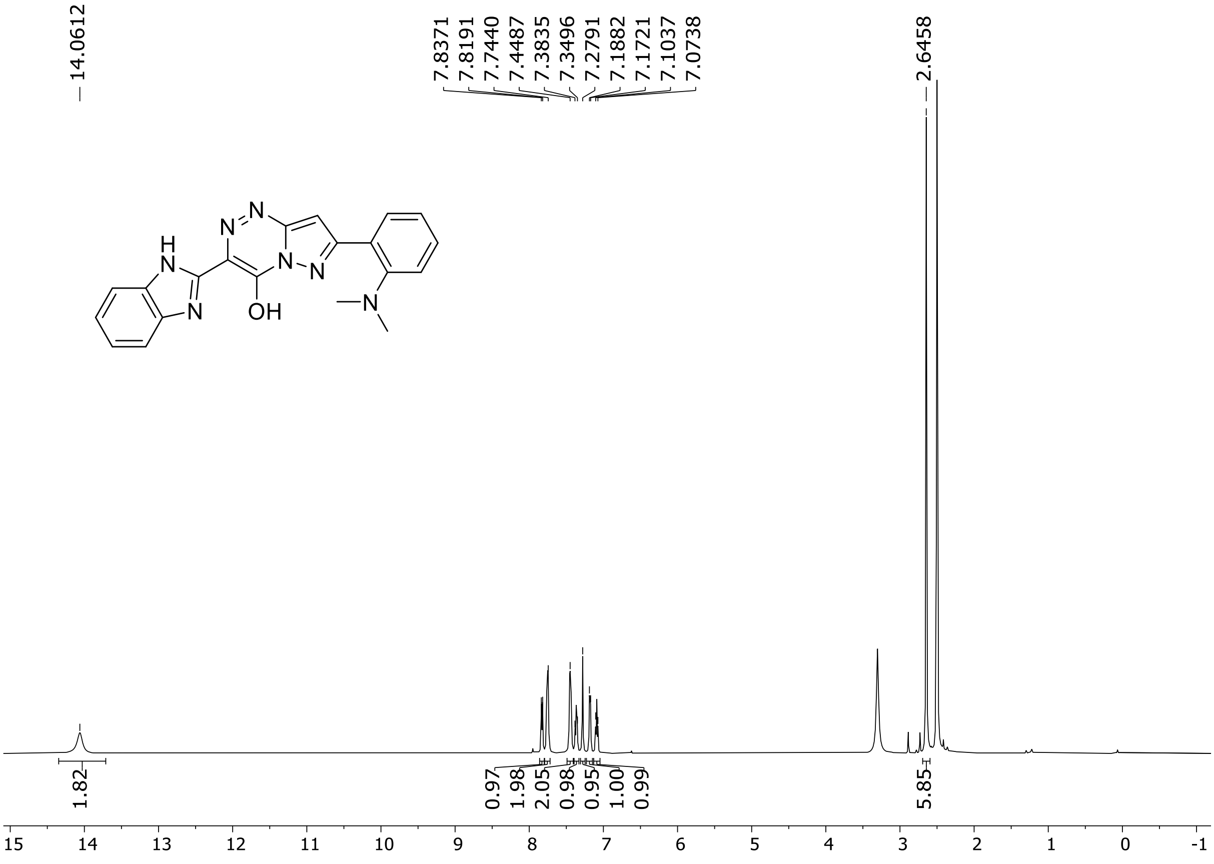

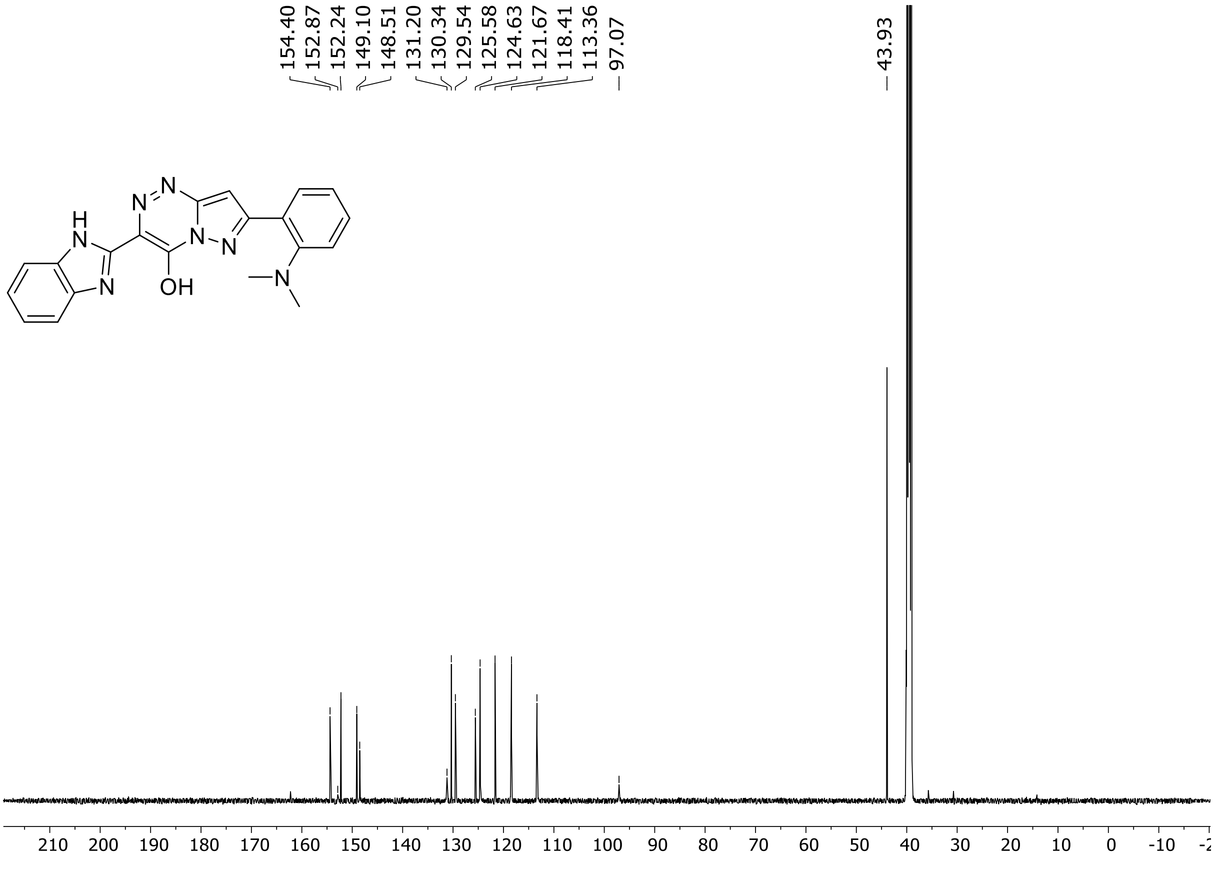

HRMS spectrum of **84**

$C_{20}H_{17}N_7O$ **exact mass: 371.1495**

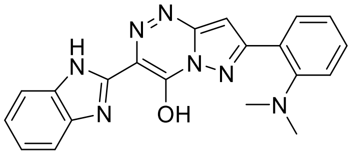

APCI + (MMI)
nitrogen flow 5 L/min, gas temperature 325°C, nebulizer 45 psig, skimmer 65 V, vaporizer 200°C, fragmentor 10 V, dissolved in methanol

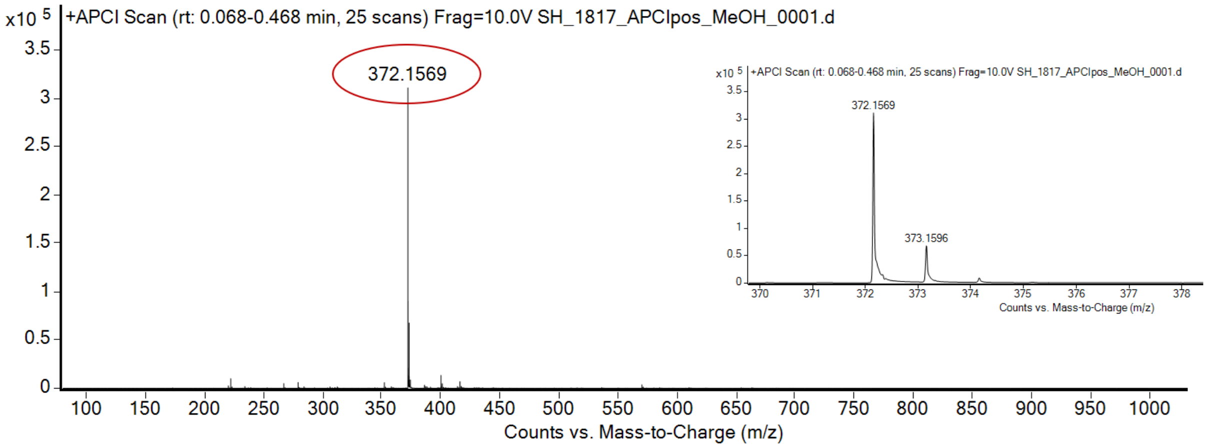

expected mass: $[M+H]^+$ = 372.1567 observed mass : $[M+H]^+$ = 372.1569 mass accuracy = 0.5 ppm

^{1}H (500 MHz) and ^{13}C NMR (126 MHz) spectra of **85** in DMSO-d_6

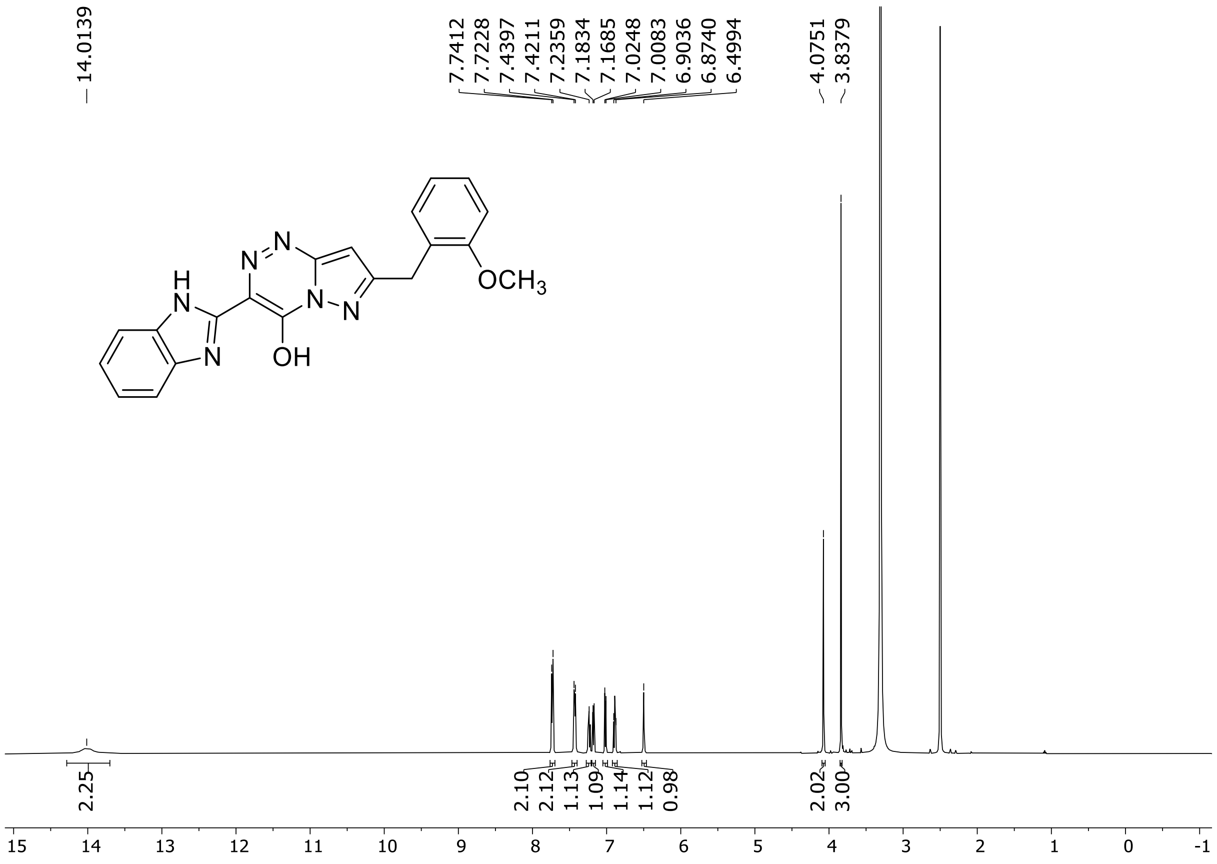

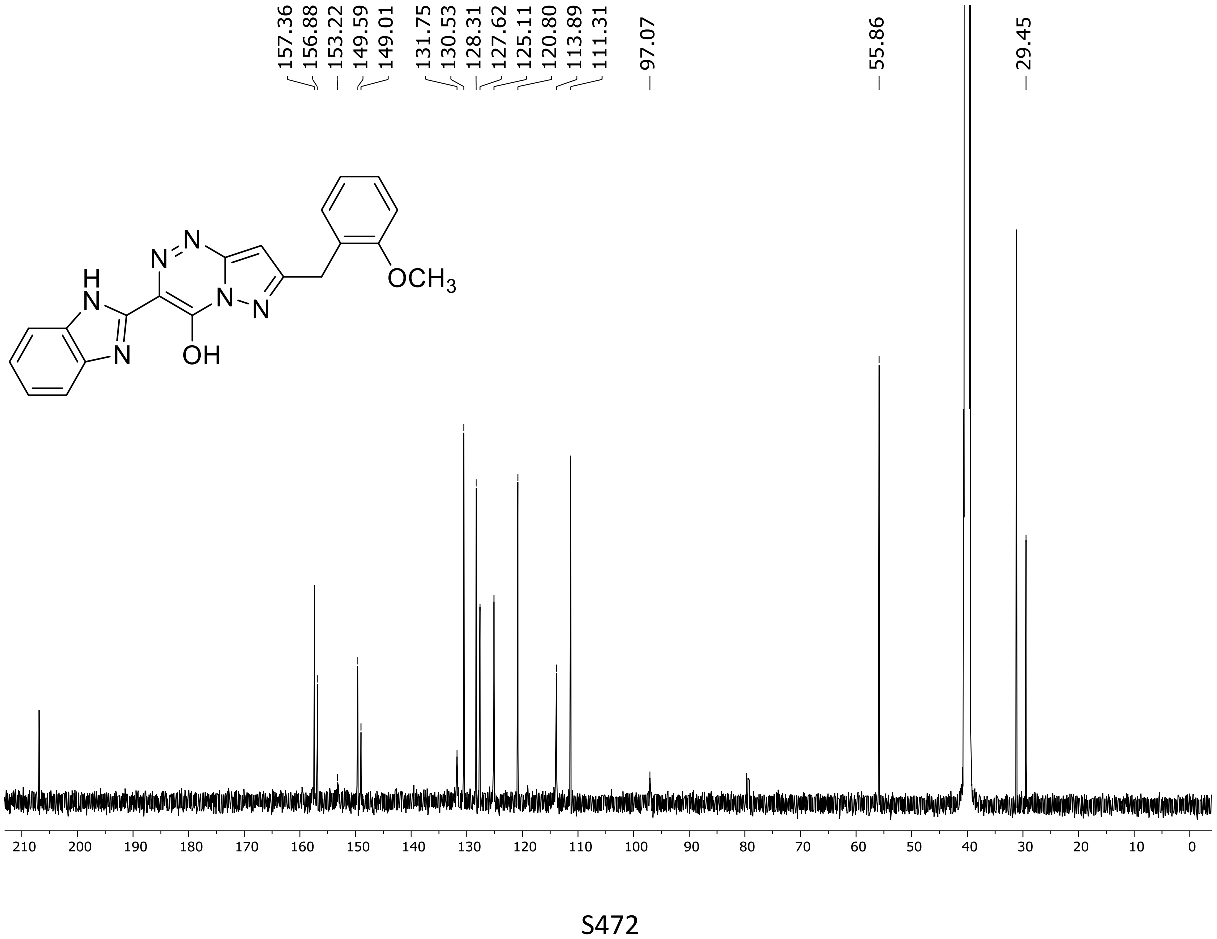

HRMS spectrum of **85**

$C_{20}H_{16}N_6O_2$ **exact mass: 372.1335**

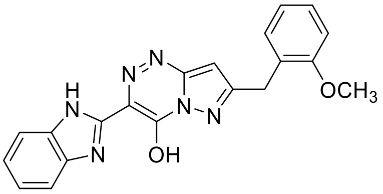

APCI + (MMI)
nitrogen flow 3 L/min, gas temperature 325°C, nebulizer 45 psig, skimmer 65 V, vaporizer 200°C, fragmentor 20 V, dissolved in methanol

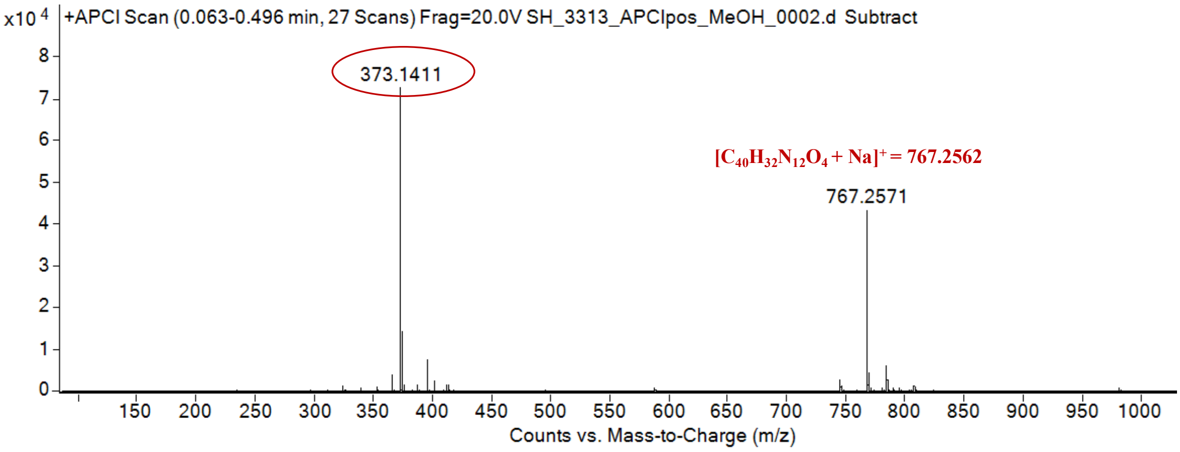

expected mass: $[M+H]^+ = 373.1408$ observed mass: $[M+H]^+ = 373.1411$ mass accuracy = 0.8 ppm

^{1}H (500 MHz) and ^{13}C NMR (126 MHz) spectra of **86** in DMSO-d_6

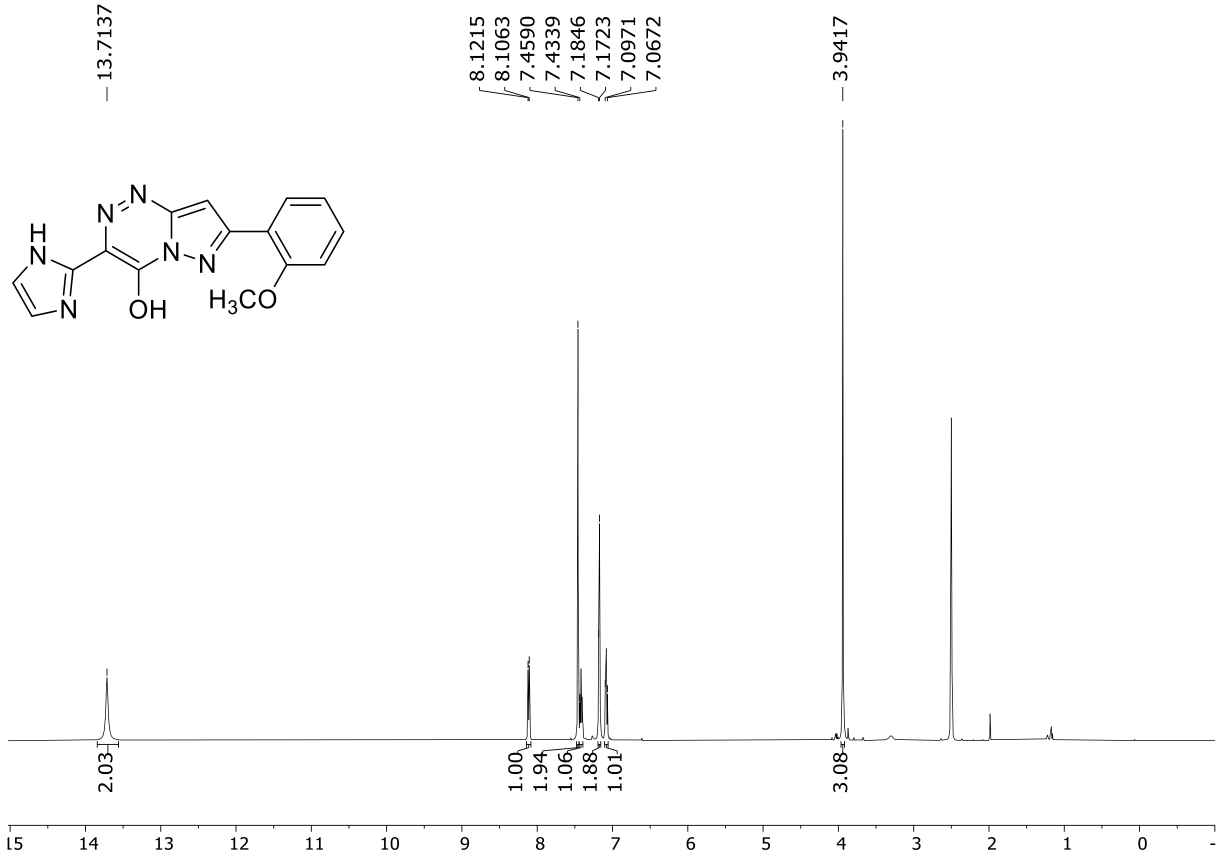

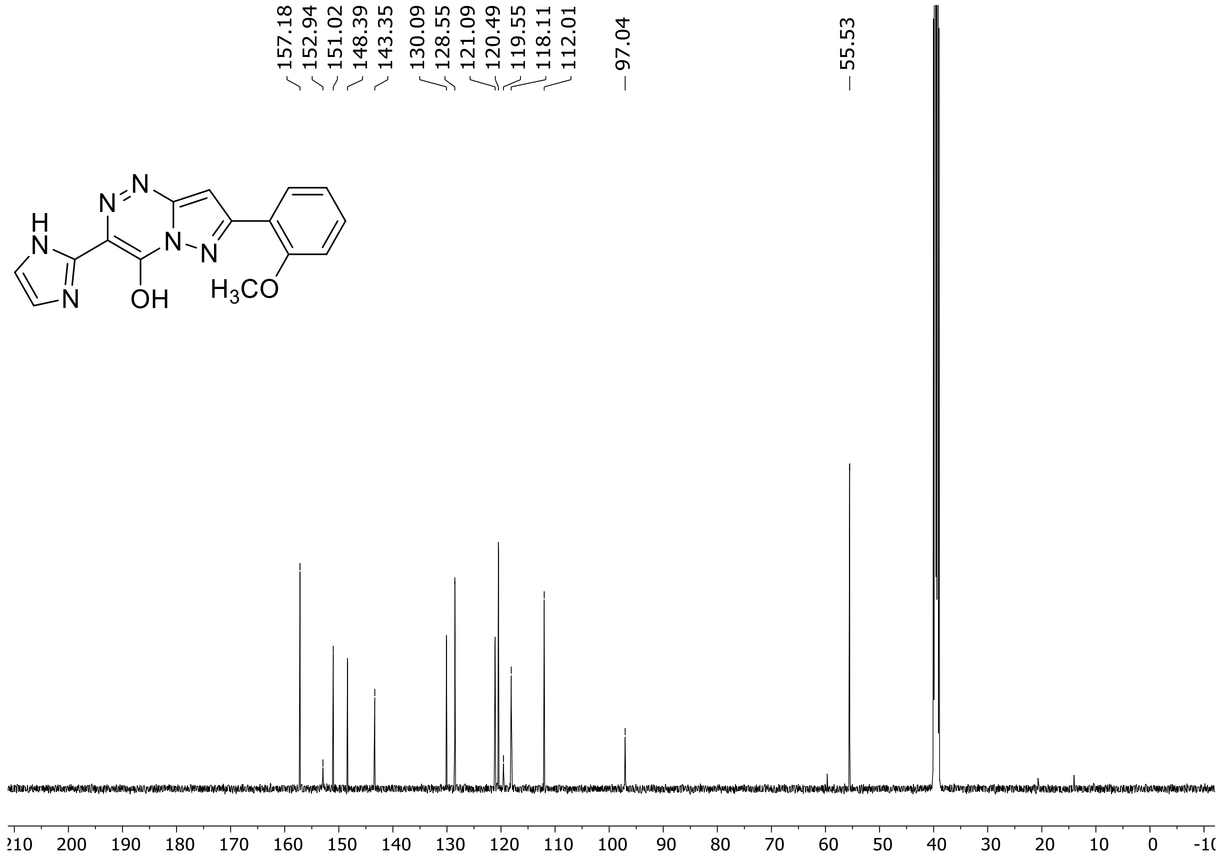

HRMS spectrum of **86**

$C_{15}H_{12}N_6O_2$ **exact mass: 308.1022**

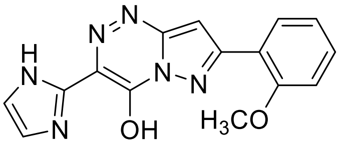

APCI + (MMI)
nitrogen flow 3 L/min, gas temperature 325°C, nebulizer 45 psig, skimmer 65 V, vaporizer 200°C, fragmentor 15 V, dissolved in methanol

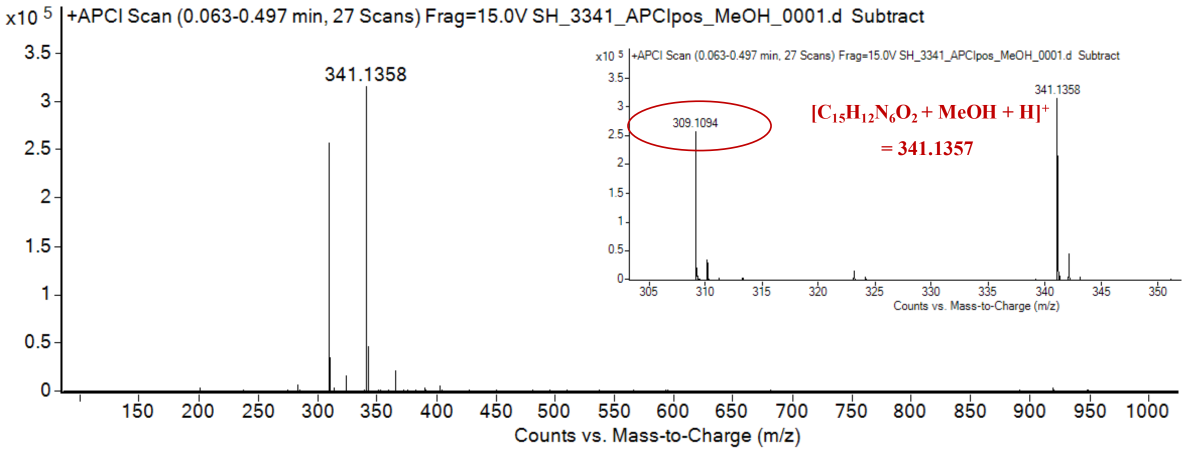

expected mass: $[M+H]^+$ = 309.1095 observed mass: $[M+H]^+$ = 309.1094 mass accuracy = - 0.3 ppm

^{1}H (500 MHz) and ^{13}C NMR (126 MHz) spectra of **87** in DMSO-d_6

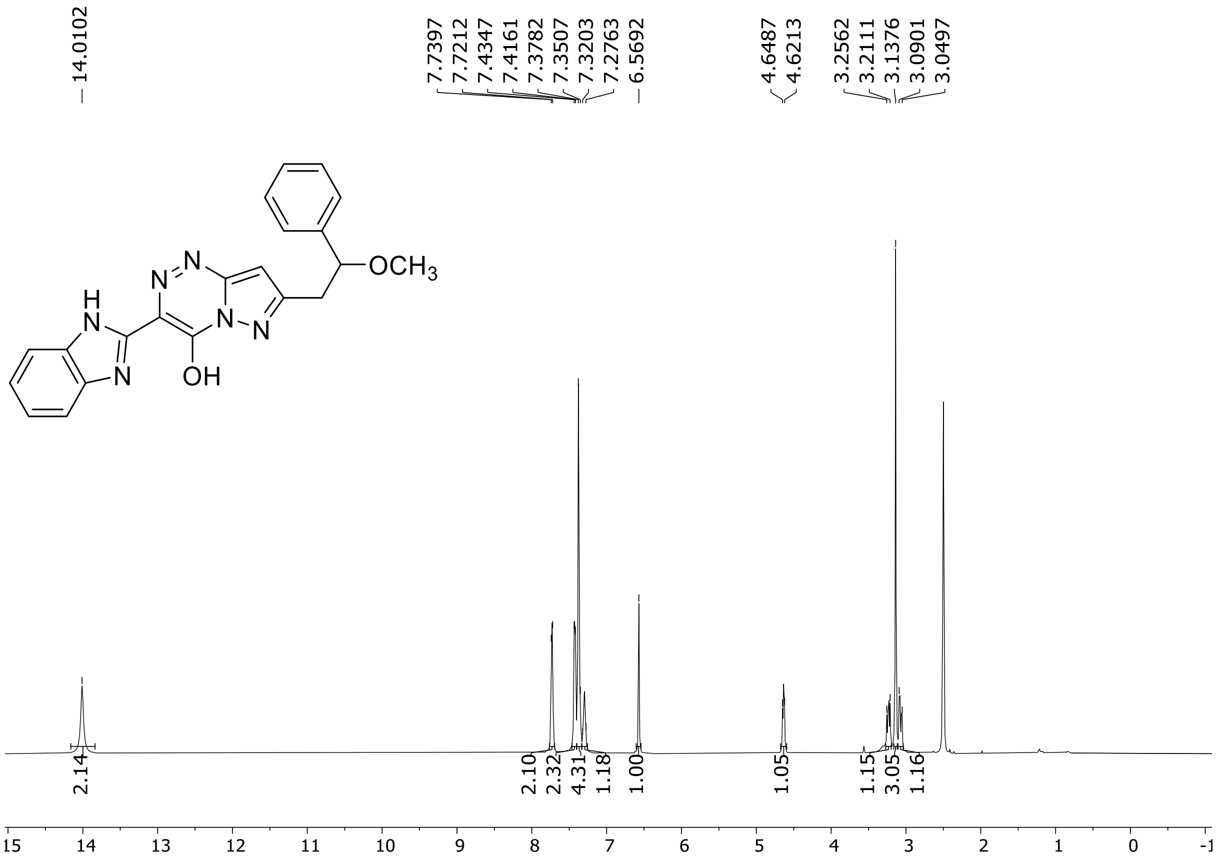

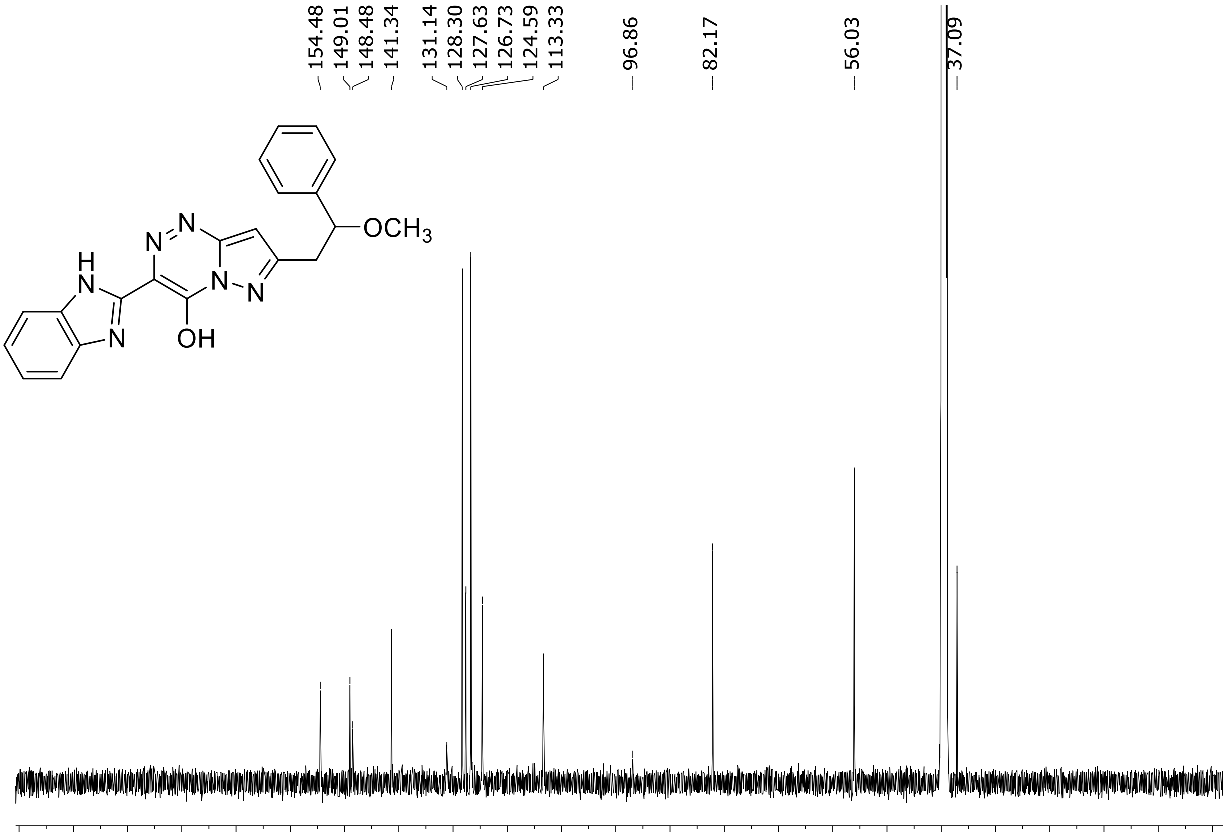

HRMS spectrum of **87**

$C_{21}H_{18}N_6O_2$ **exact mass: 386.1491**

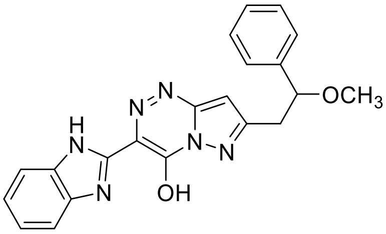

APCI + (MMI)
nitrogen flow 3 L/min, gas temperature 325°C, nebulizer 45 psig, skimmer 65 V, vaporizer 200°C, fragmentor 15 V, dissolved in methanol

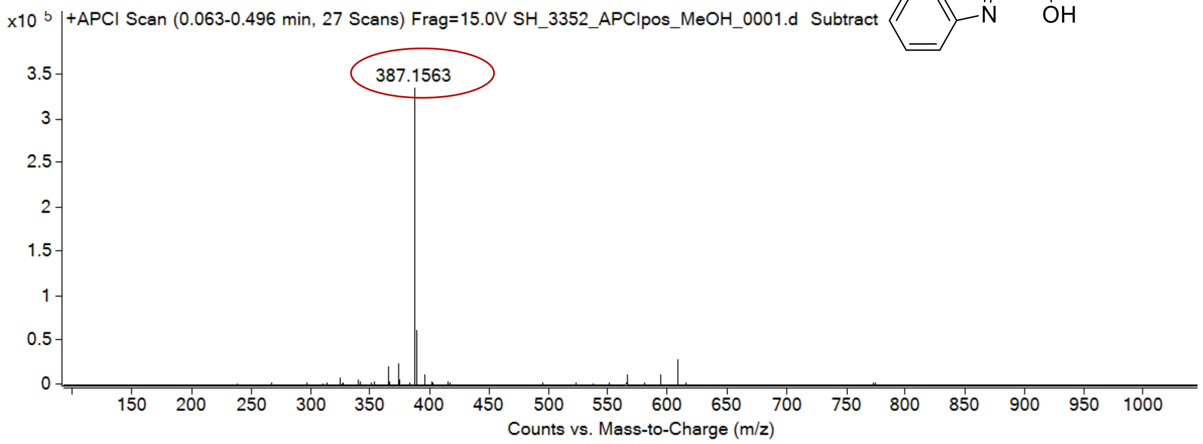

expected mass: $[M+H]^+ = 387.1564$ observed mass: $[M+H]^+ = 387.1563$ mass accuracy = - 0.3 ppm

^{1}H (500 MHz) and ^{13}C NMR (126 MHz) spectra of **88** in DMSO-d_6

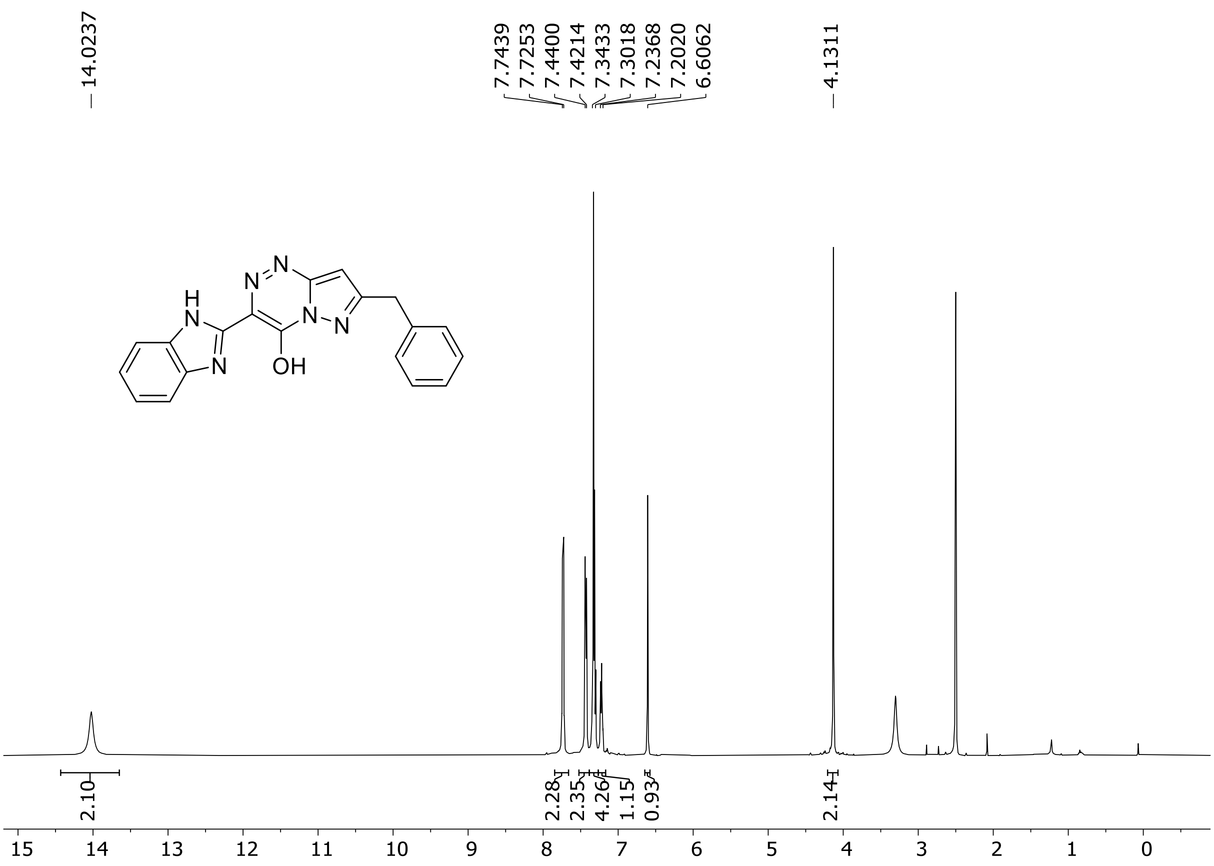

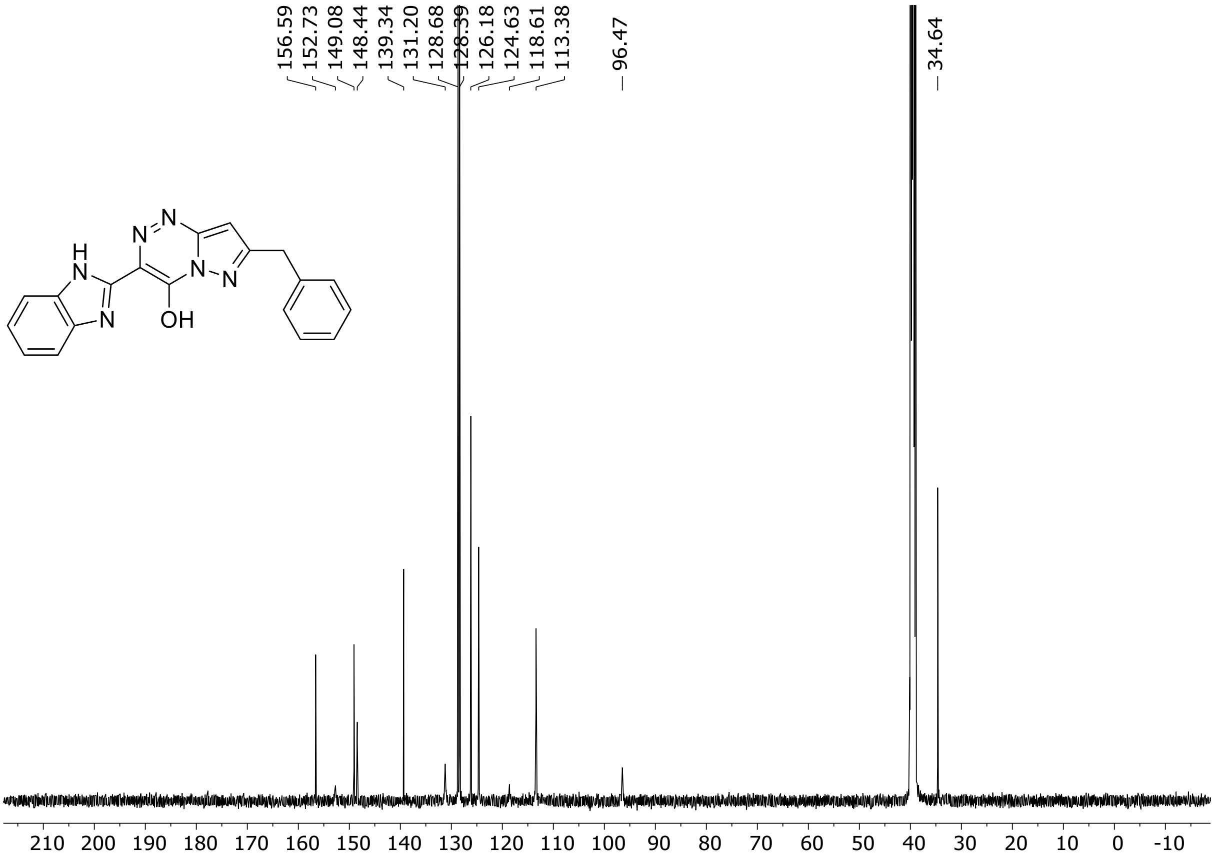

HRMS spectrum of **88**

$C_{19}H_{14}N_6O$ **exact mass: 342.1229**

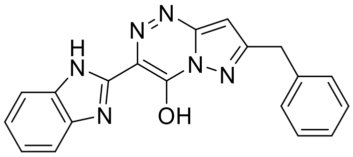

APCI + (MMI)
nitrogen flow 5 L/min, gas temperature 325°C, nebulizer 45 psig, skimmer 65 V, vaporizer 200°C, fragmentor 20 V, dissolved in methanol

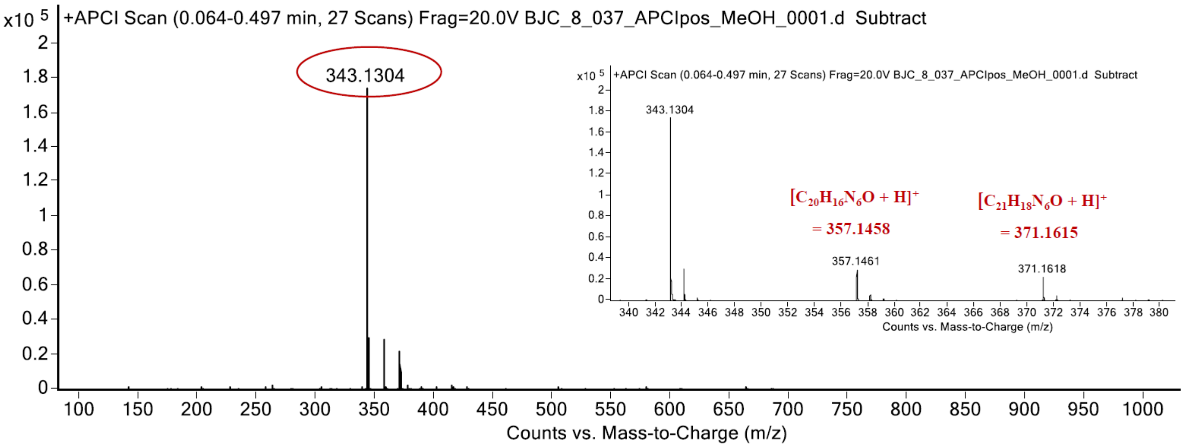

expected mass: $[M+H]^+$ = 343.1302 observed mass: $[M+H]^+$ = 343.1304 mass accuracy = 0.6 ppm

^{1}H (500 MHz) and ^{13}C NMR (126 MHz) spectra of **89** in DMSO-d_6

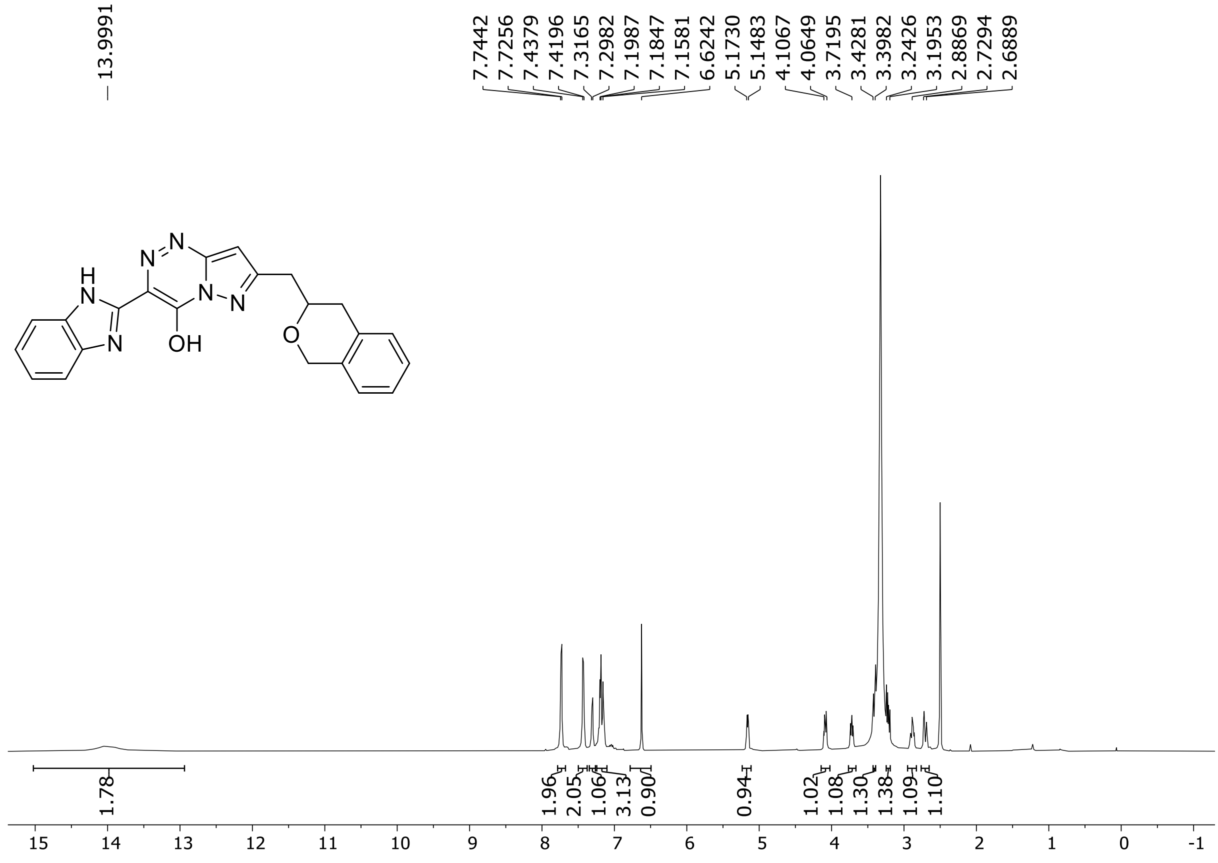

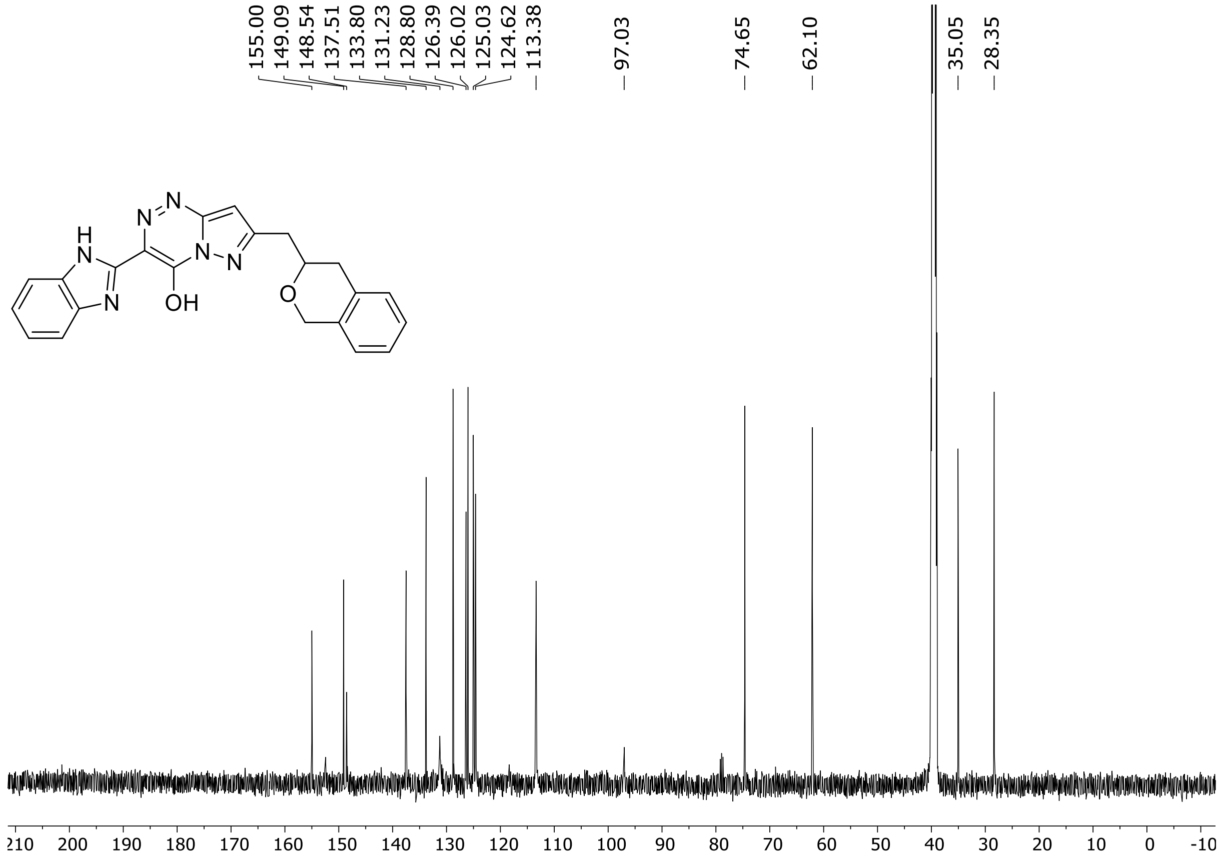

HRMS spectrum of **89**

$C_{22}H_{18}N_6O_2$ **exact mass: 398.1491**

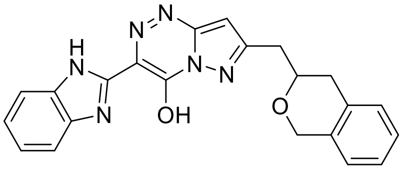

APCI + (MMI)
nitrogen flow 3 L/min, gas temperature 325°C, nebulizer 45 psig, skimmer 65 V, vaporizer 200°C, fragmentor 20 V, dissolved in methanol

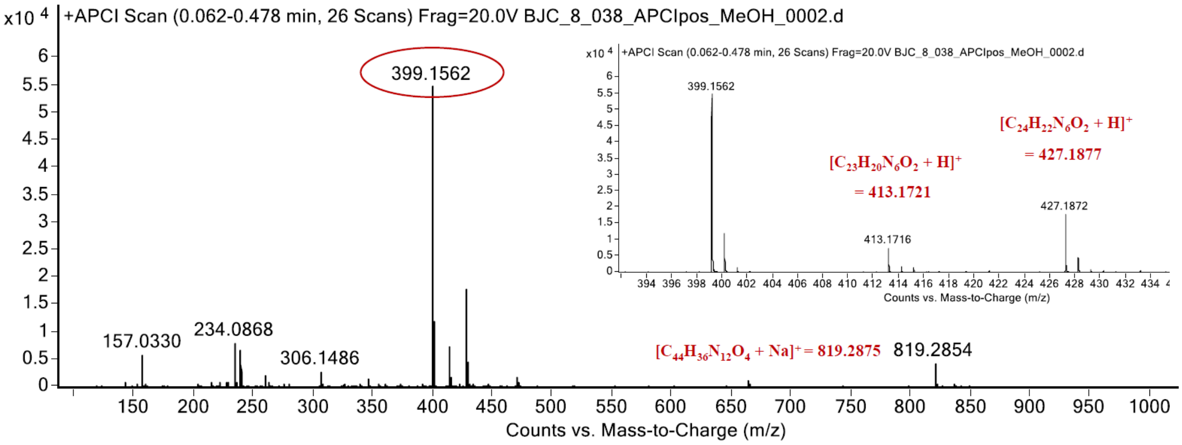

expected mass: $[M+H]^+ = 399.1564$ observed mass: $[M+H]^+ = 399.1562$ mass accuracy = - 0.5 ppm

^{1}H (500 MHz) and ^{13}C NMR (126 MHz) spectra of **90** in DMSO-d_6

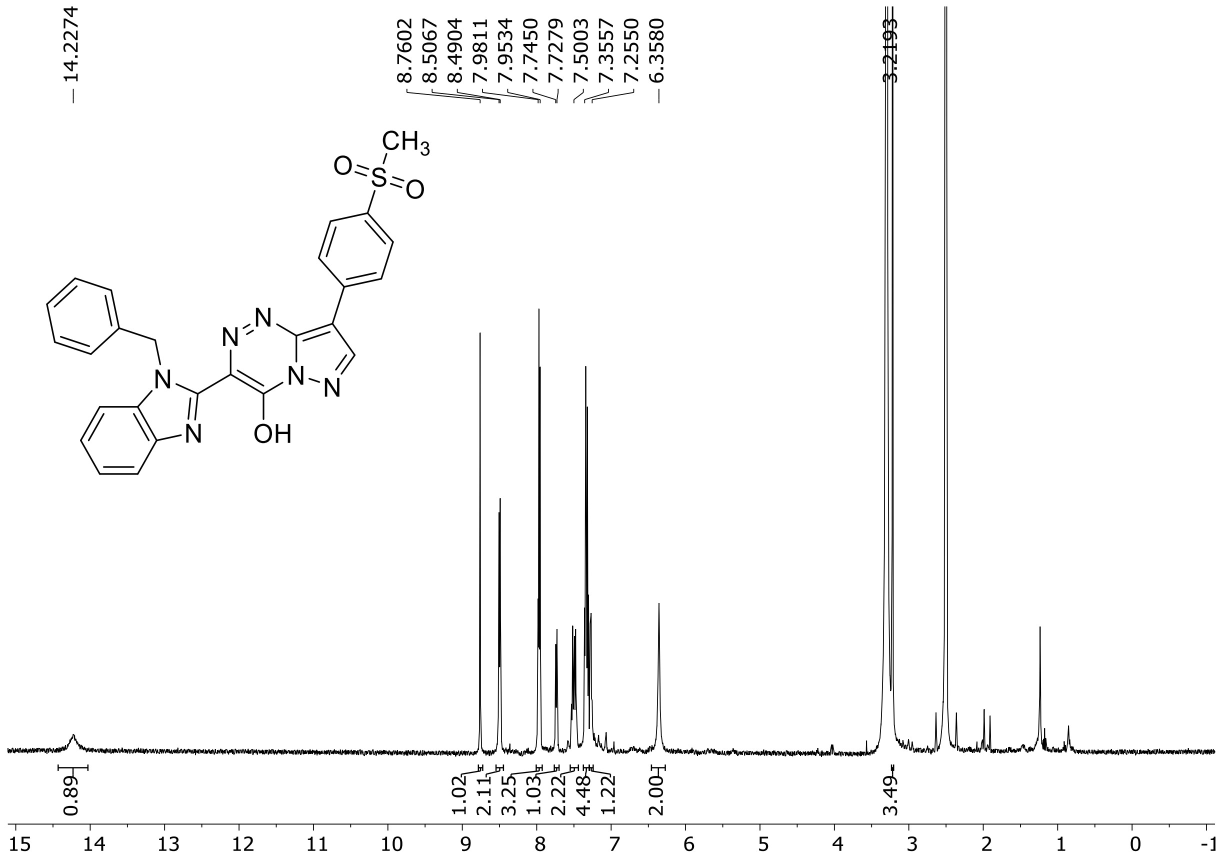

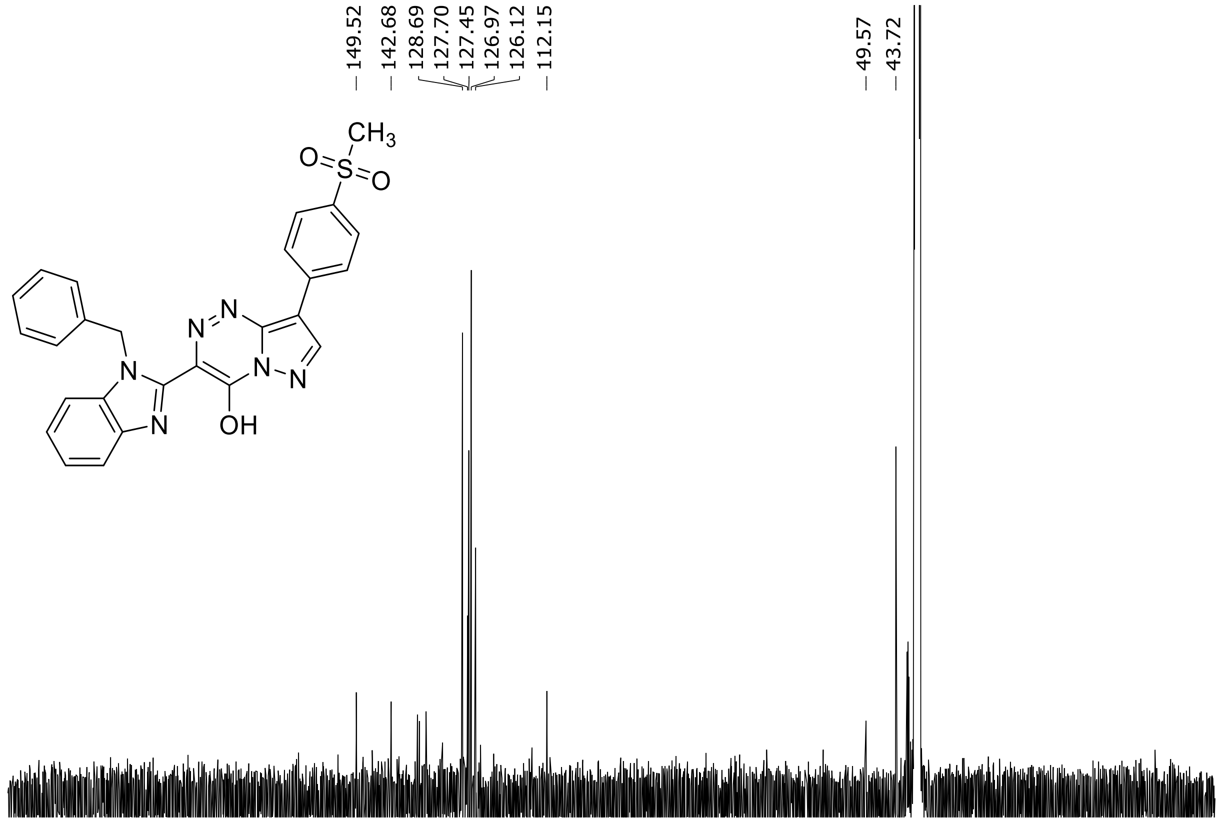

HRMS spectrum of **90**

$C_{26}H_{20}N_6O_3S$ mono m/z = 496.54

ESI + (MMI)

nitrogen flow 5 L/min, gas temperature 300°C, vaporizer 200°C, nebulizer 45 psi, skimmer **65** V, fragmentor 50 V, dissolved in MeOH

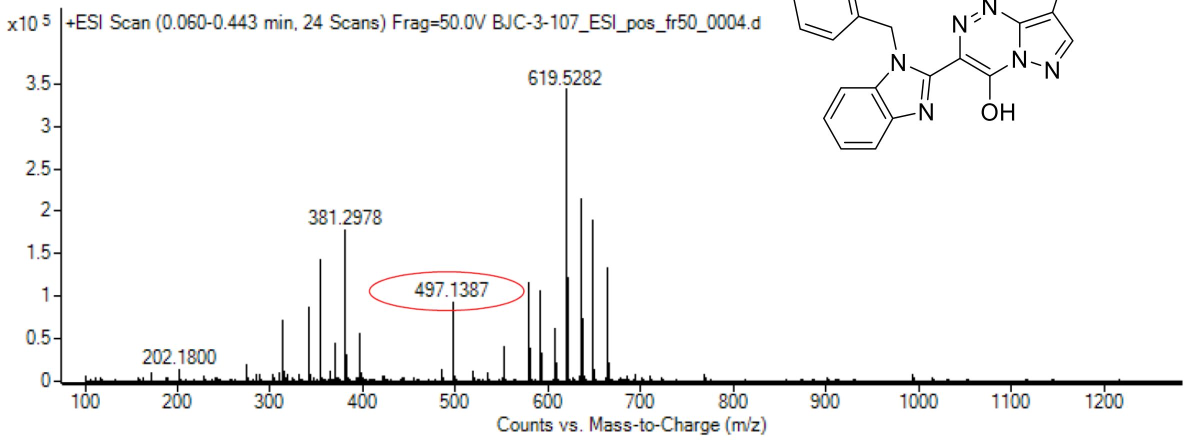

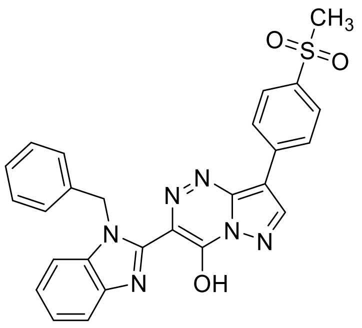

calculated mass: [M+H]$^+$ = 497.1390 observed: [M+H]$^+$ = 497.1387 max. mass error = 0.6 ppm

^{1}H (500 MHz) and ^{13}C NMR (126 MHz) spectra of **91** in DMSO-d_6

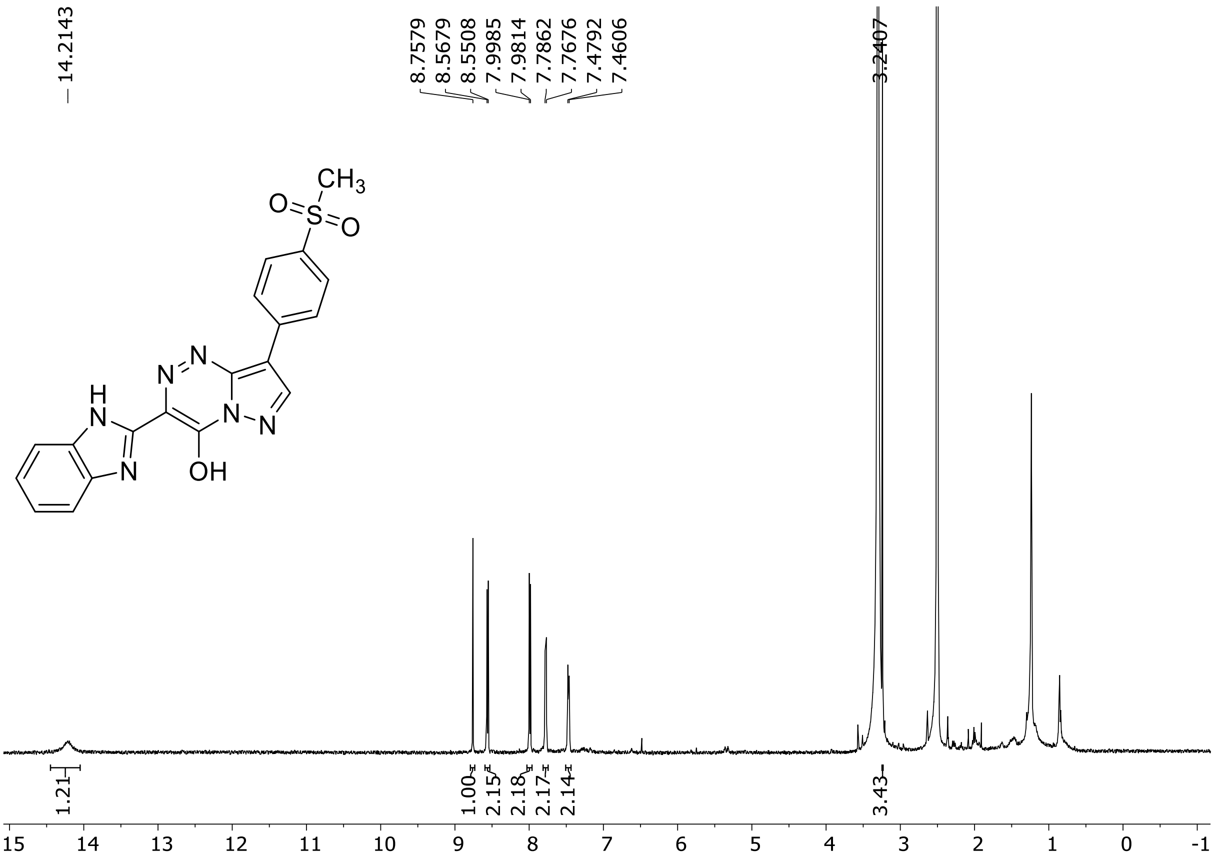

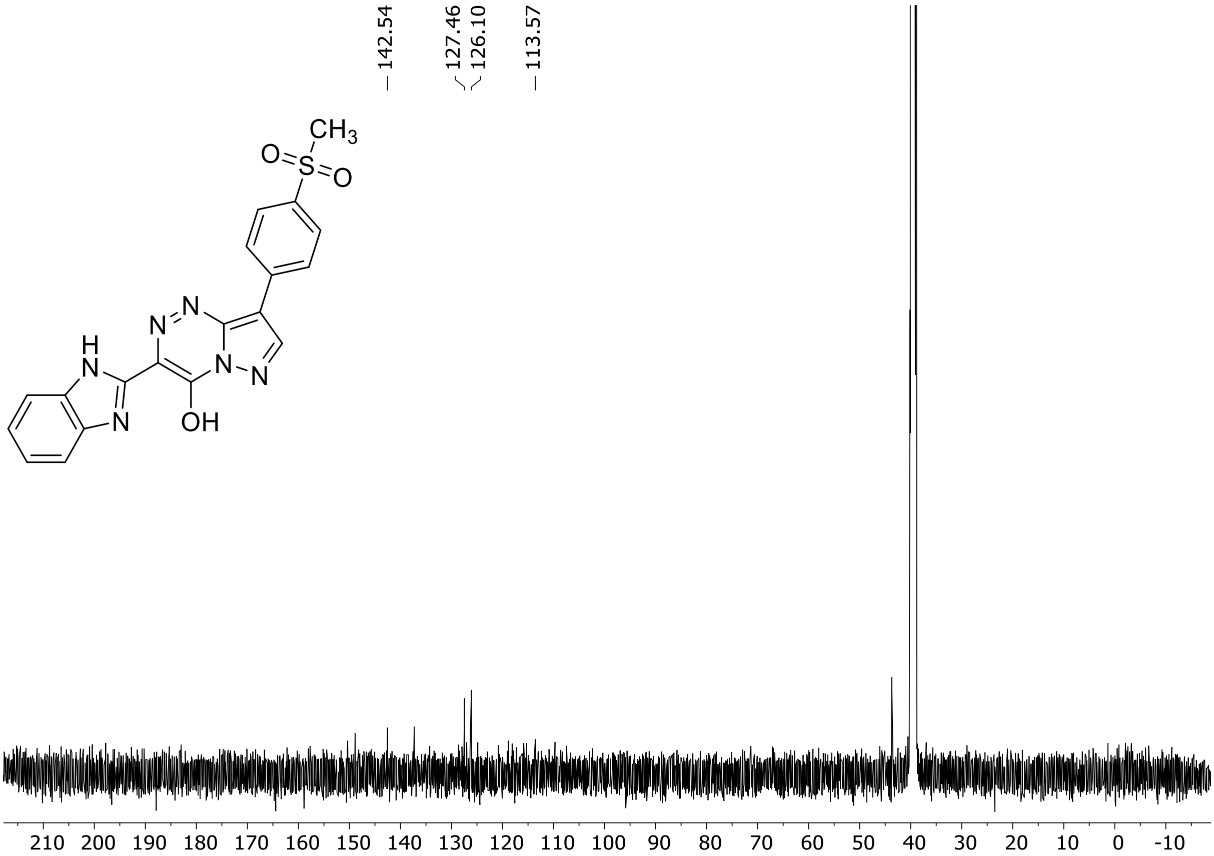

HRMS spectrum of **91**

$C_{19}H_{14}N_6O_3S$ **mono m/z = 406.42**

APCI - (MMI)

nitrogen flow 5 L/min, gas temperature 300°C, vaporizer 200°C, nebulizer 45 psi, skimmer **65** V, fragmentor 80 V, dissolved in MeOH

ZOOM – range of interest

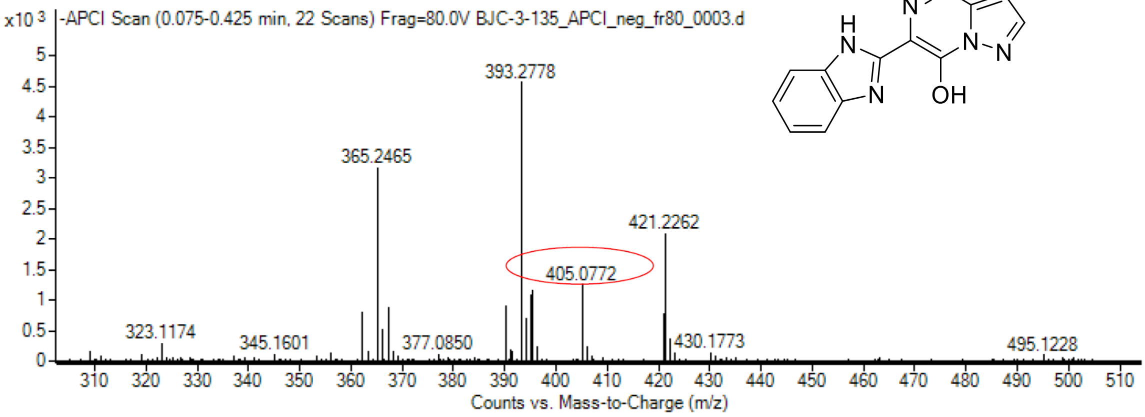

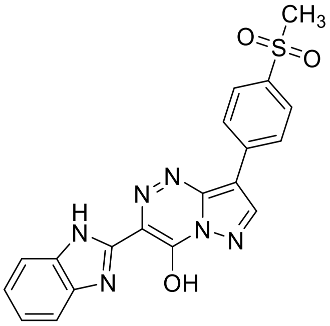

calculated mass: $[M+H]^+$ = 407.0775 observed: $[M+H]^+$ = 405.0772 max. mass error = 0.7 ppm

^{1}H (500 MHz) and ^{13}C NMR (126 MHz) spectra of **92** in DMSO-d_6

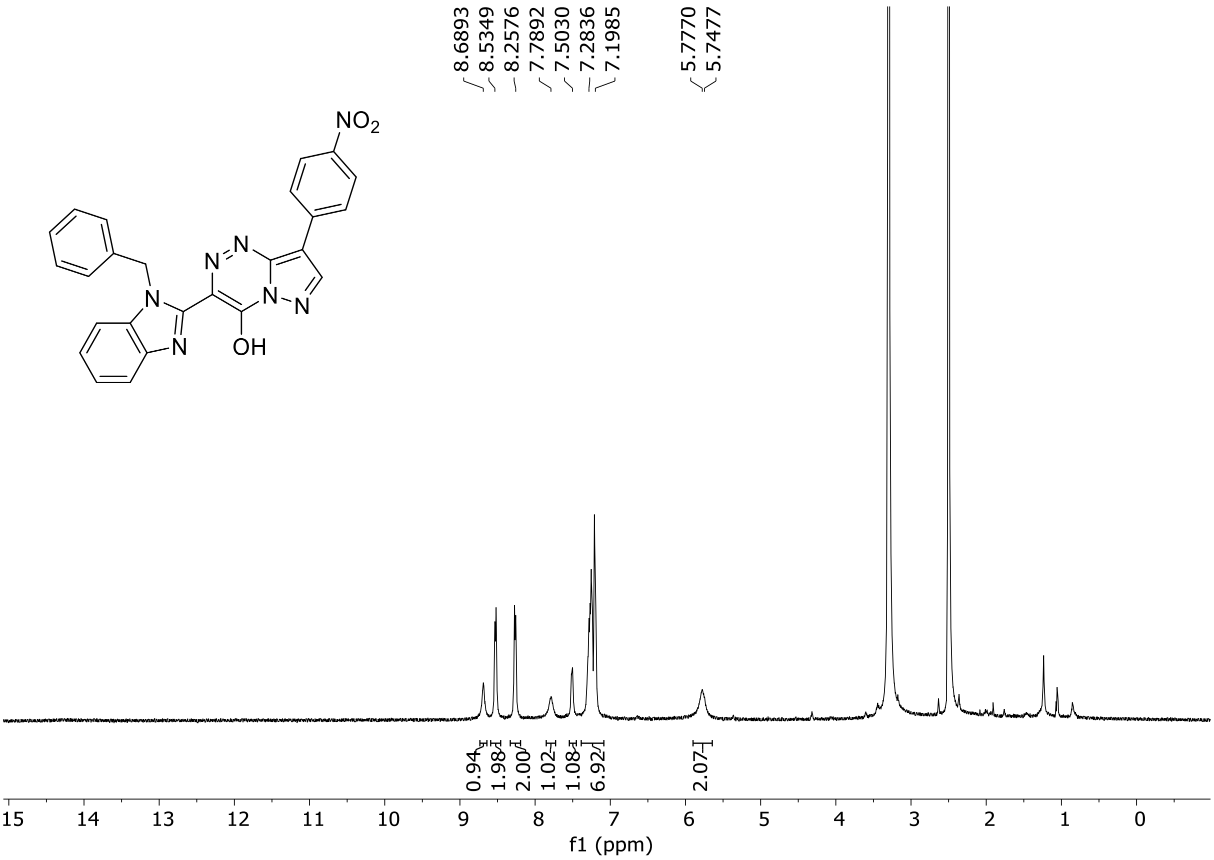

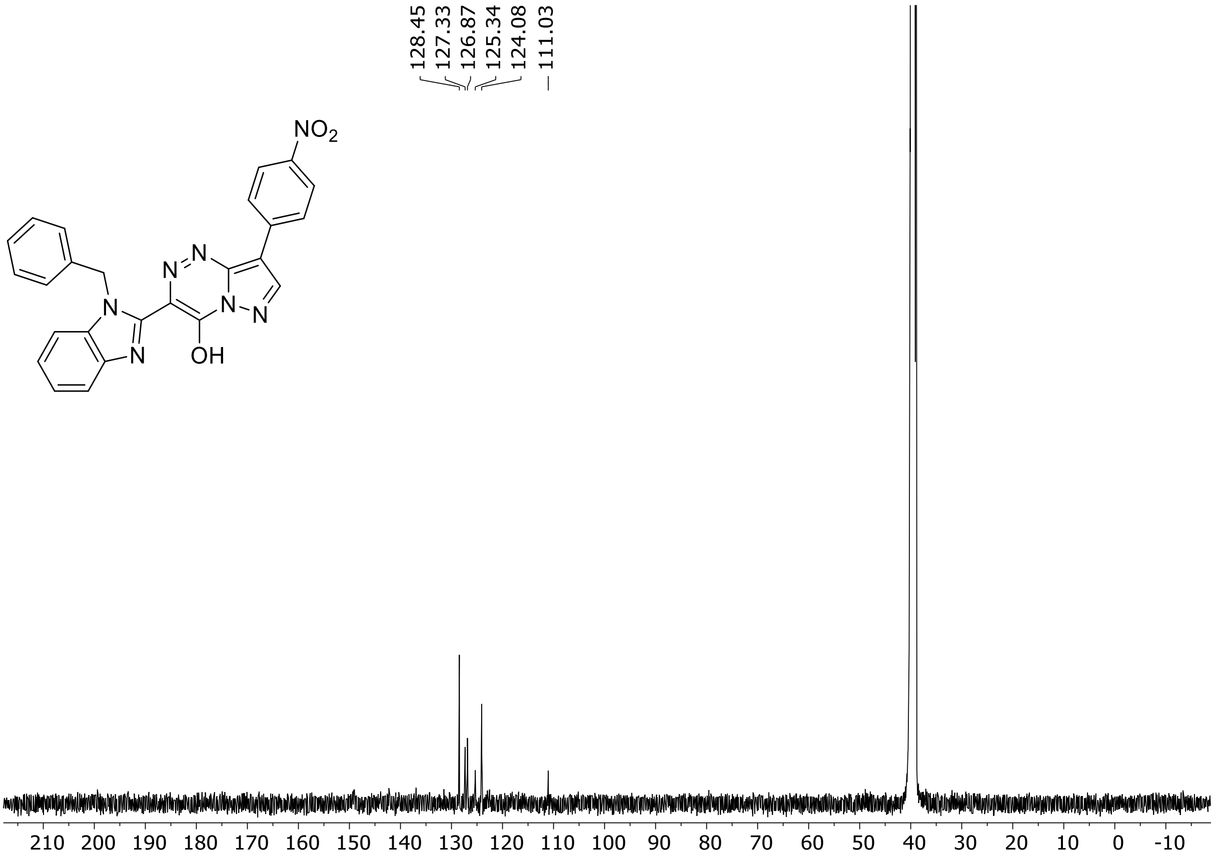

HRMS spectrum of **92**

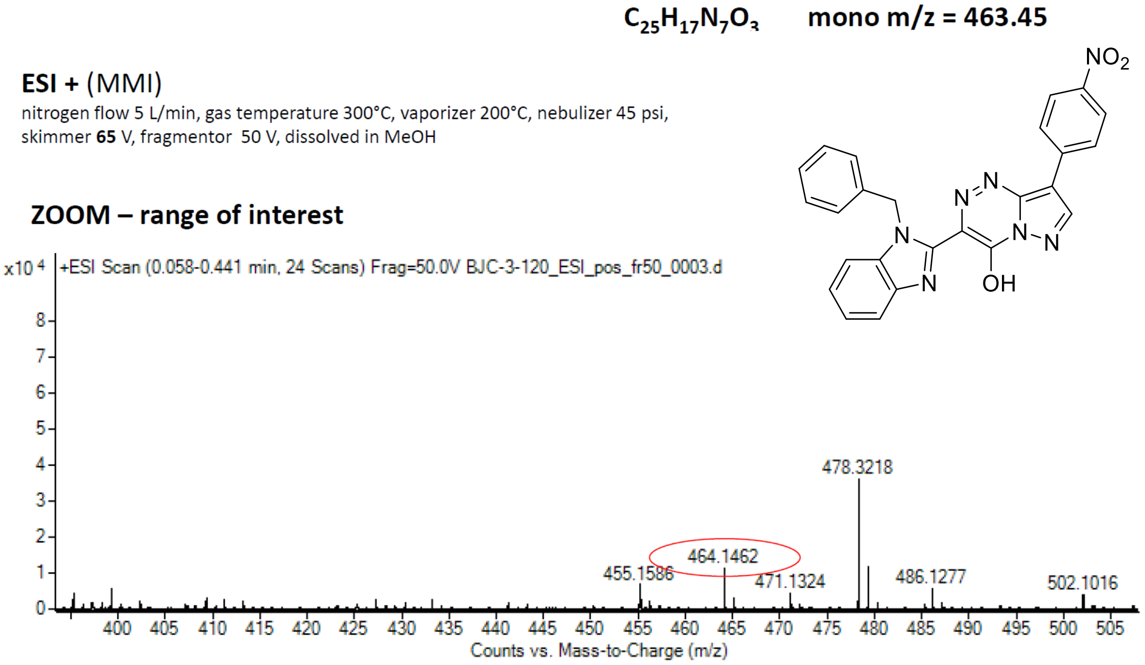

calculated mass: $[M+H]^+$ = 464.1466 observed: $[M+H]^+$ = 464.1462 max. mass error = 0.9 ppm

^{1}H (500 MHz) and ^{19}F (282 MHz) NMR spectra of **93** in DMSO-d_6

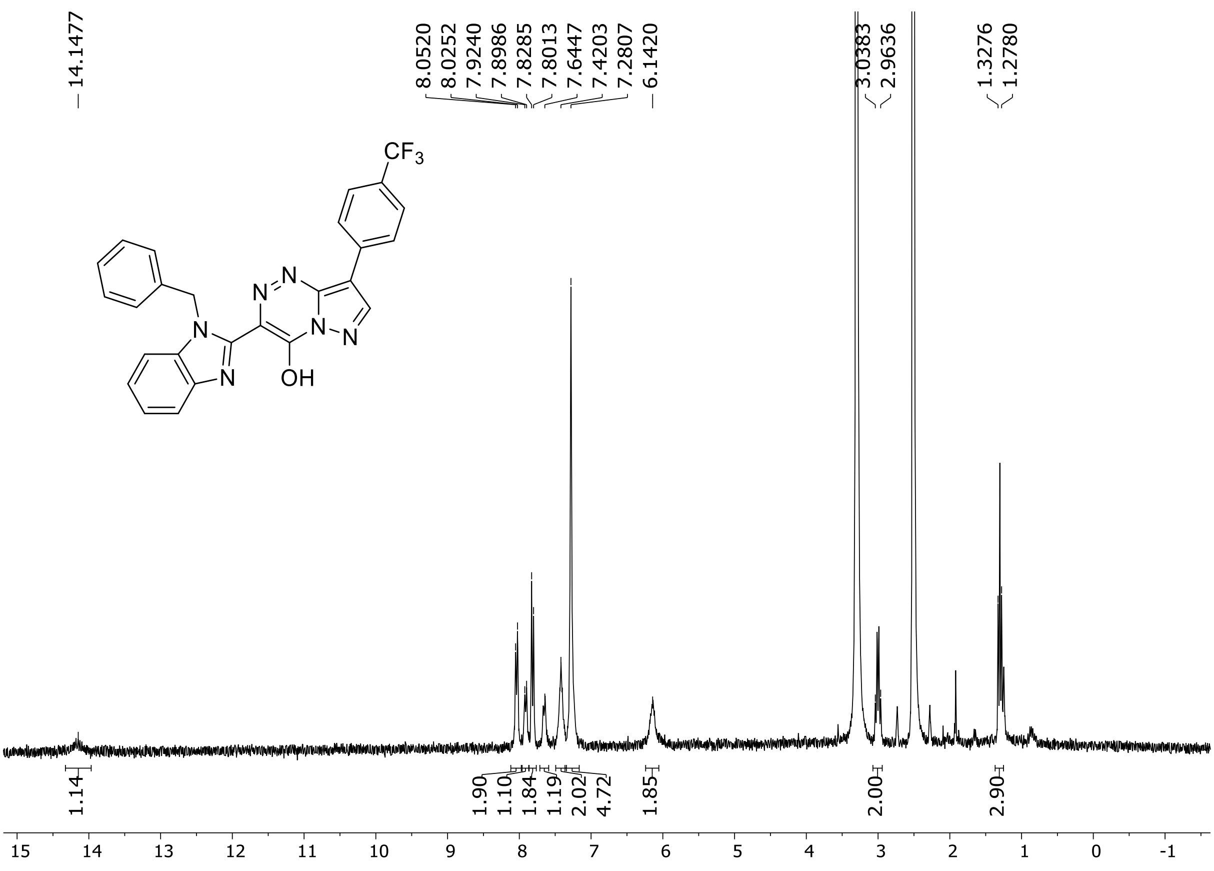

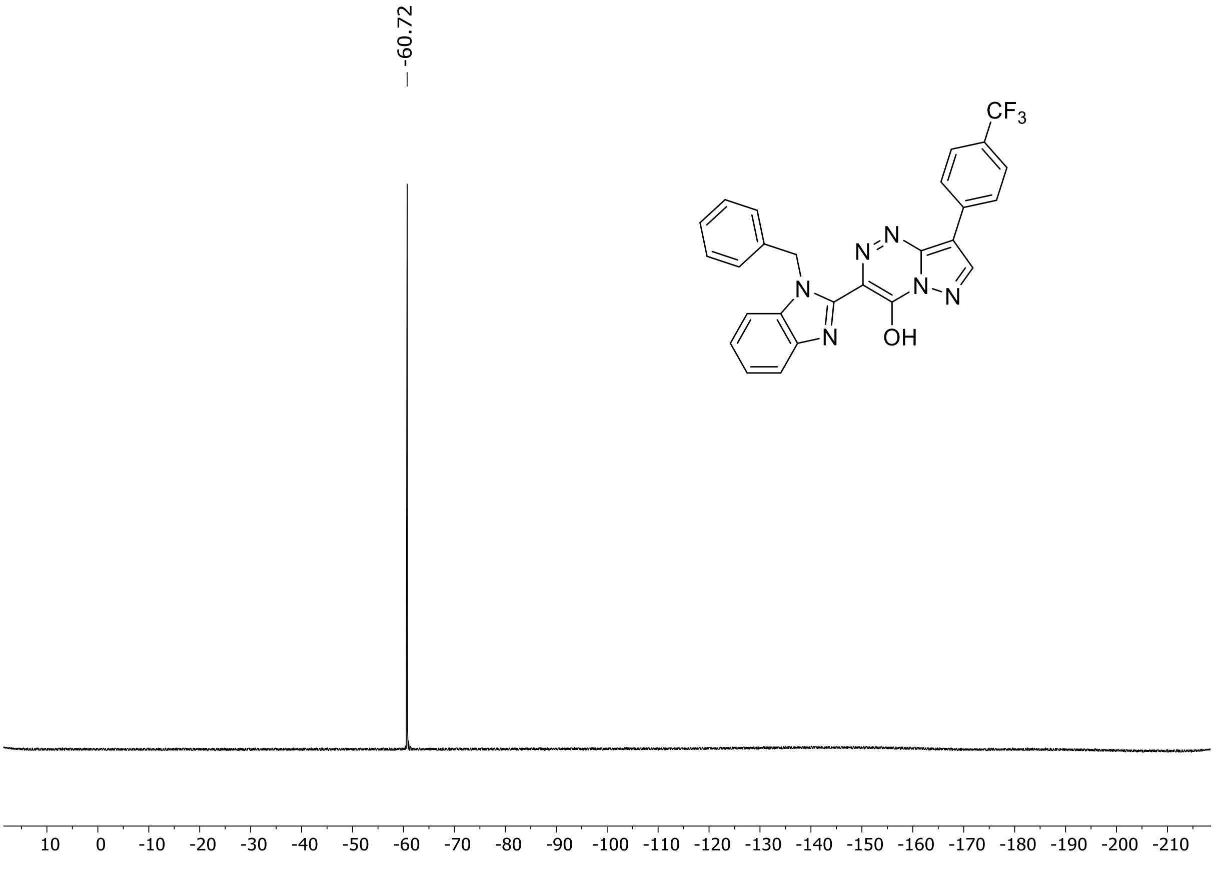

HRMS spectrum of **93**

$C_{28}H_{21}F_3N_6O$ **mono *m/z* 514.1729**

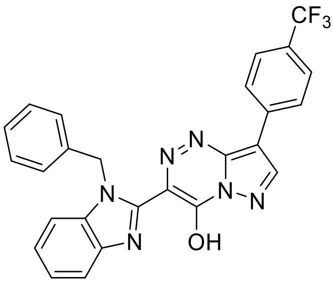

APCI + (MMI)
nitrogen flow 5 L/min, gas temperature 325°C, nebulizer 45 psi, skimmer 65 V, vaporizer 250°C, fragmentor 35 V, dissolved in methanol

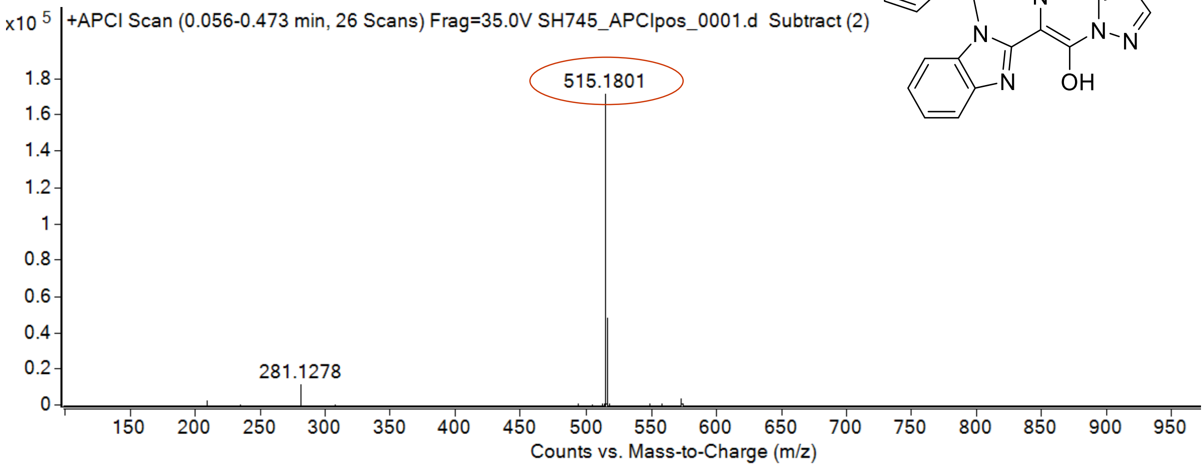

calculated mass: $[M+H]^+$ = 515.1802 observed: $[M+H]^+$ = 515.1801 mass accuracy = - 0.1 ppm

^{1}H (500 MHz) and ^{13}C NMR (126 MHz) spectra of **94** in DMSO-d_6

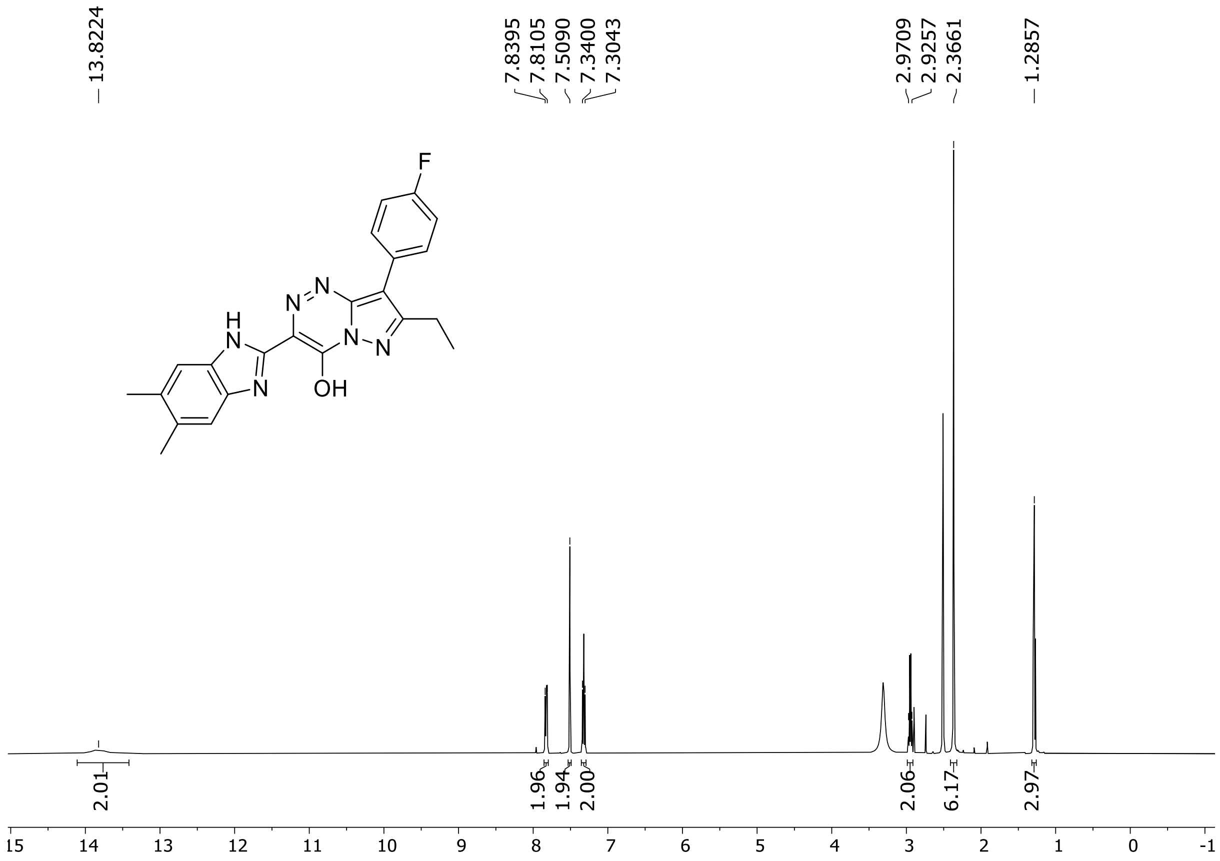

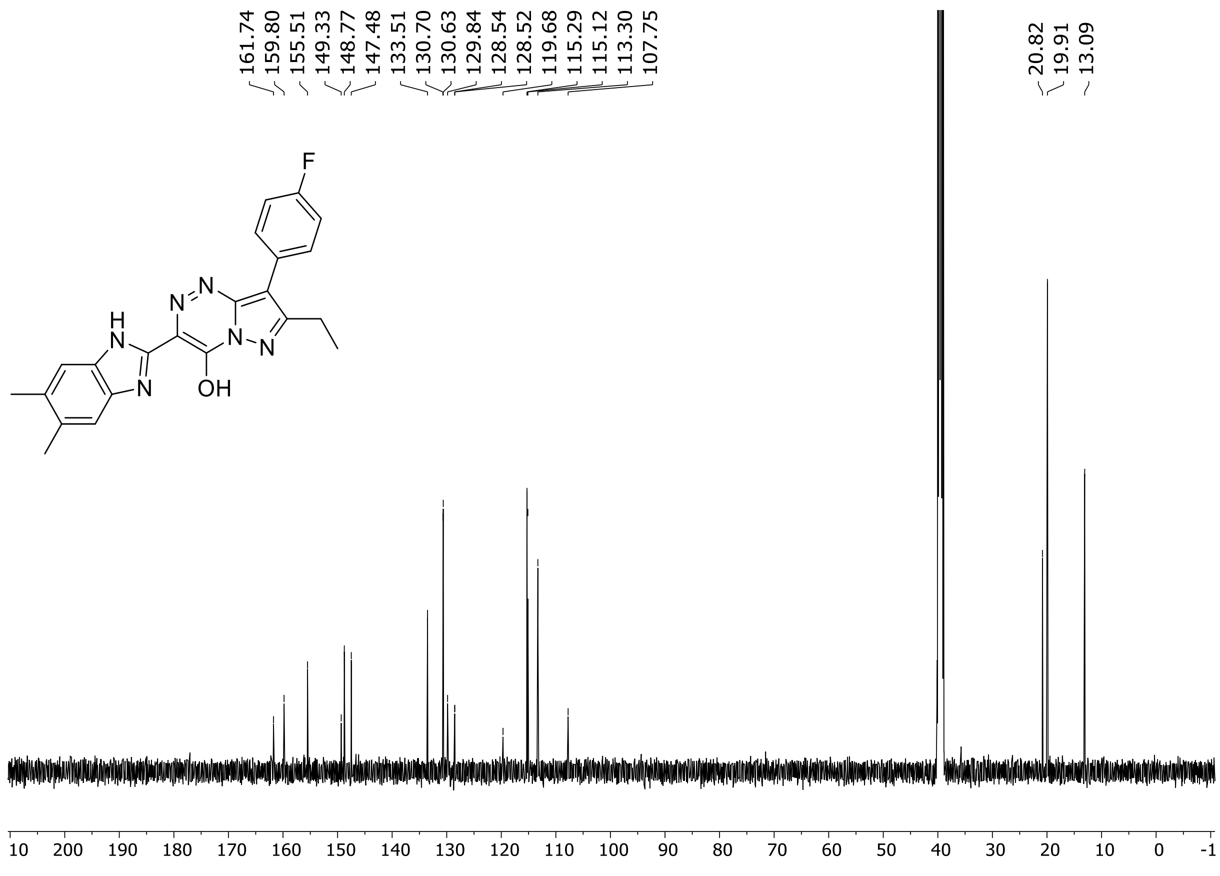

^{19}F (471 MHz) NMR spectrum of **94** DMSO-d_6

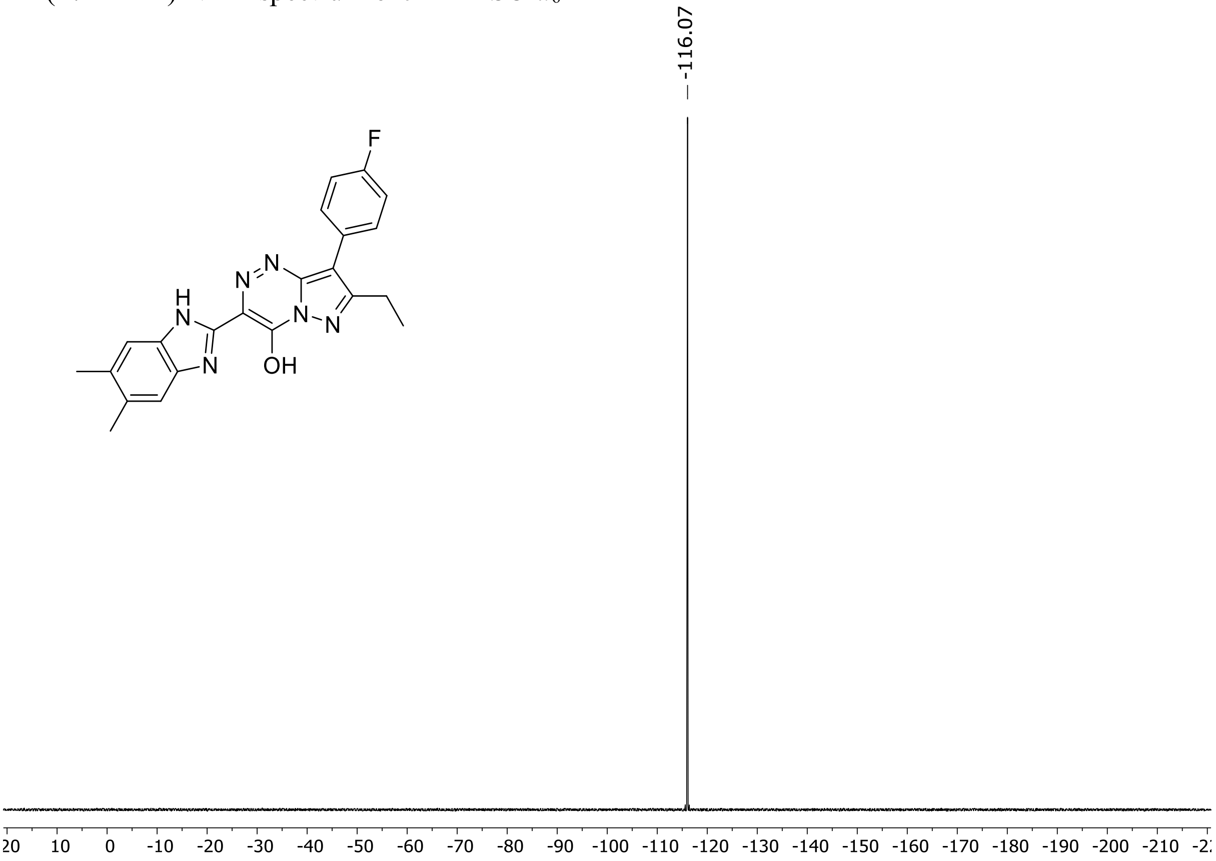

HRMS spectrum of **94**

$C_{22}H_{19}FN_6O$ **exact mass: 402.1604**

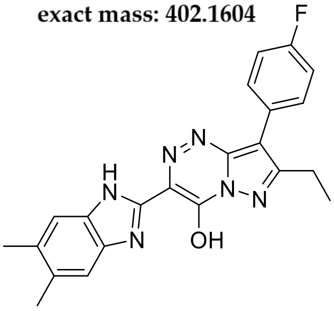

APCI + (MMI)
nitrogen flow 5 L/min, gas temperature 325°C, nebulizer 45 psig, skimmer 65 V, vaporizer 200°C, fragmentor 10 V, dissolved in methanol

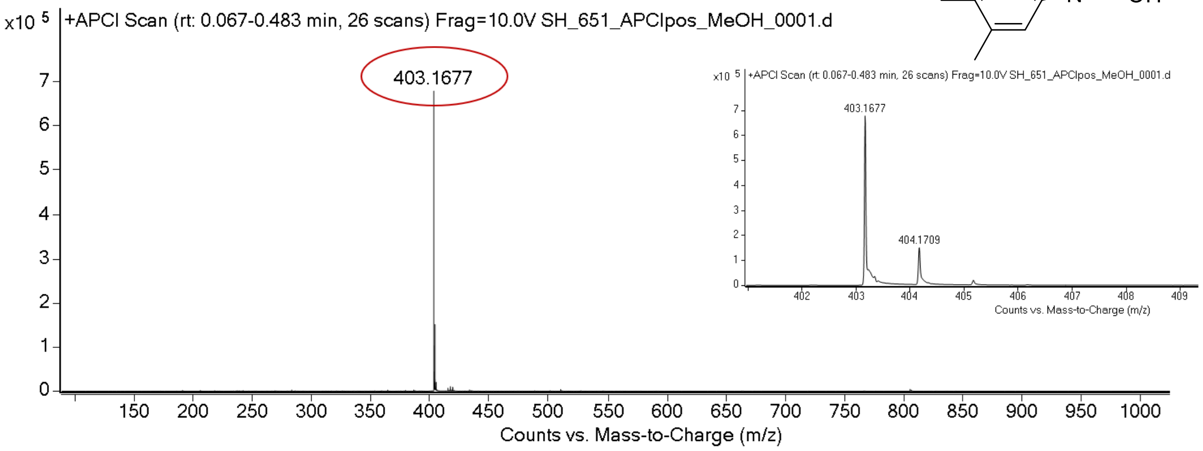

expected mass: [M+H]$^+$ = 403.1677 observed mass : [M+H]$^+$ = 403.1677 mass accuracy < 0.1 ppm

^{1}H (500 MHz) and ^{13}C NMR (126 MHz) spectra of **95** in DMSO-d_6

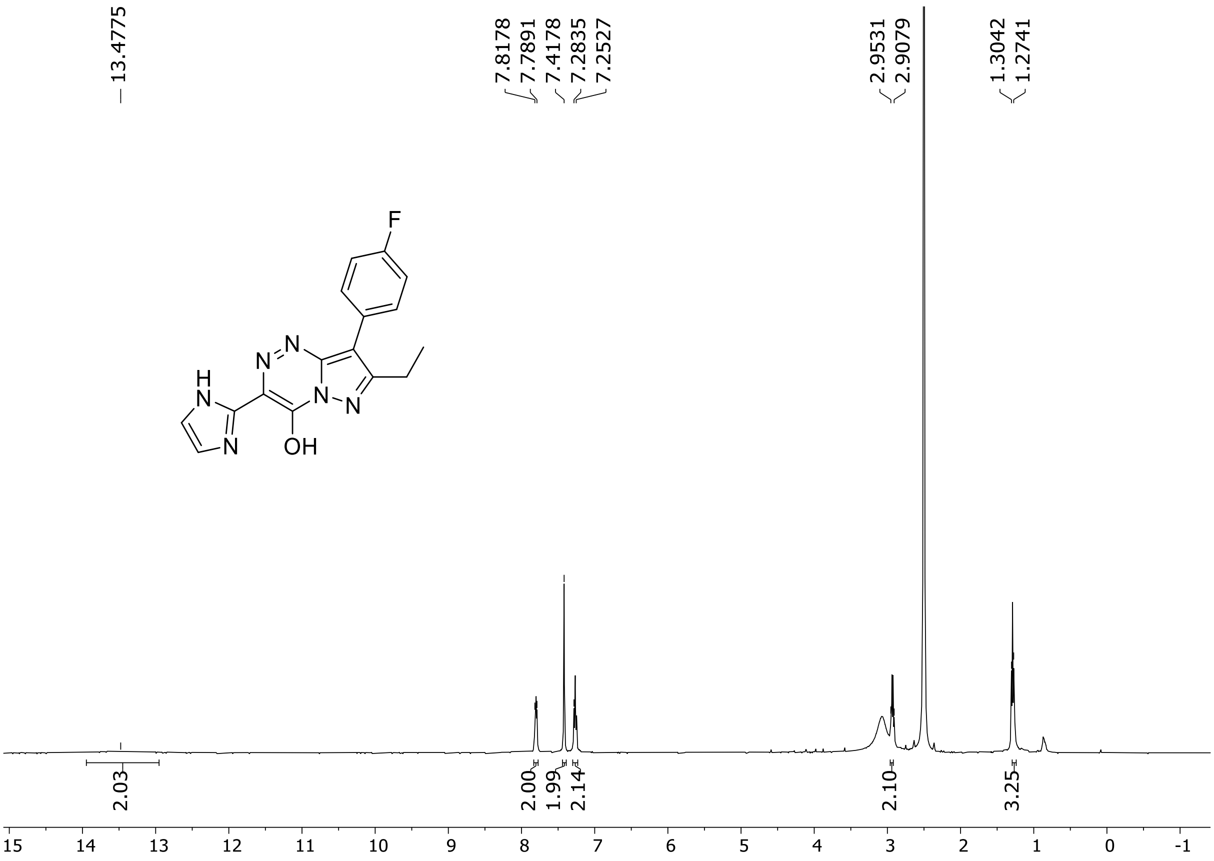

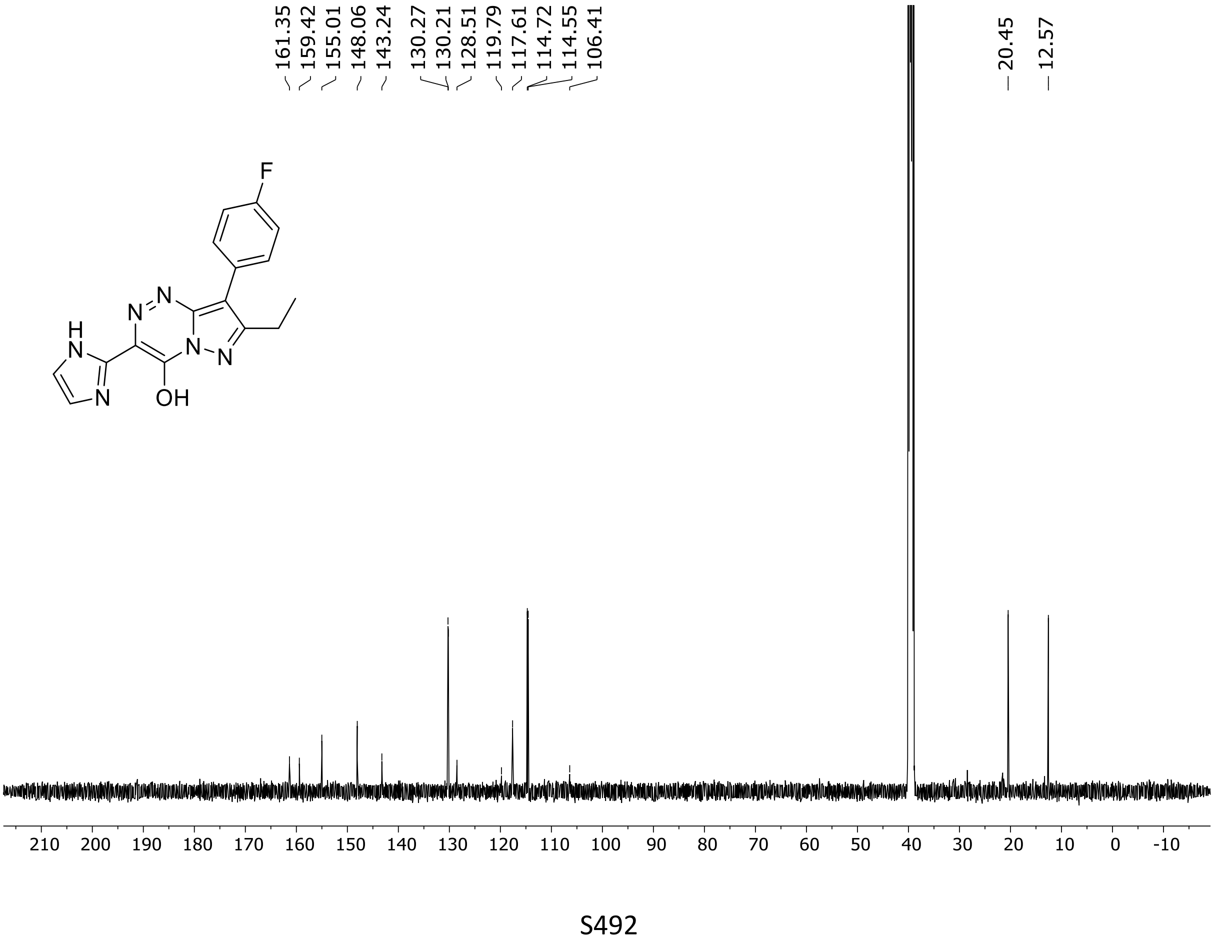

HRMS spectrum of **95**

$C_{16}H_{13}FN_6O$ **exact mass: 324.1135**

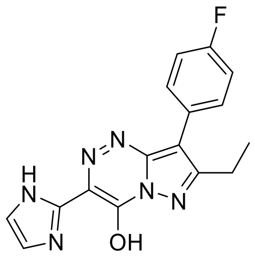

APCI + (MMI)
nitrogen flow 5 L/min, gas temperature 325°C, nebulizer 45 psig, skimmer 65 V, vaporizer 200°C, fragmentor 20 V, dissolved in methanol

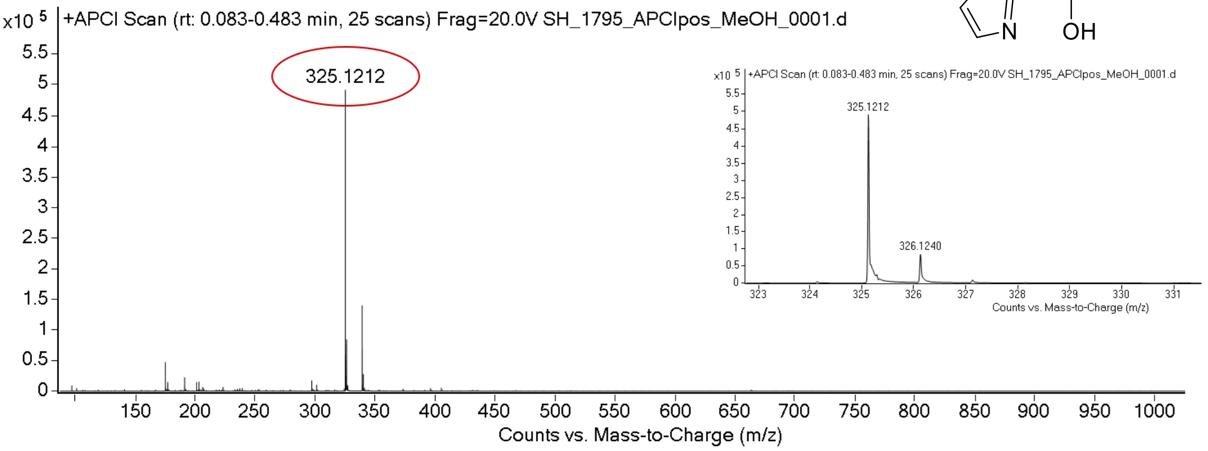

expected mass: $[M+H]^+$ = 325.1208 observed mass : $[M+H]^+$ = 325.1212 mass accuracy = 1.2 ppm

^{1}H (500 MHz) and ^{13}C NMR (126 MHz) spectra of **96** in DMSO-d_6

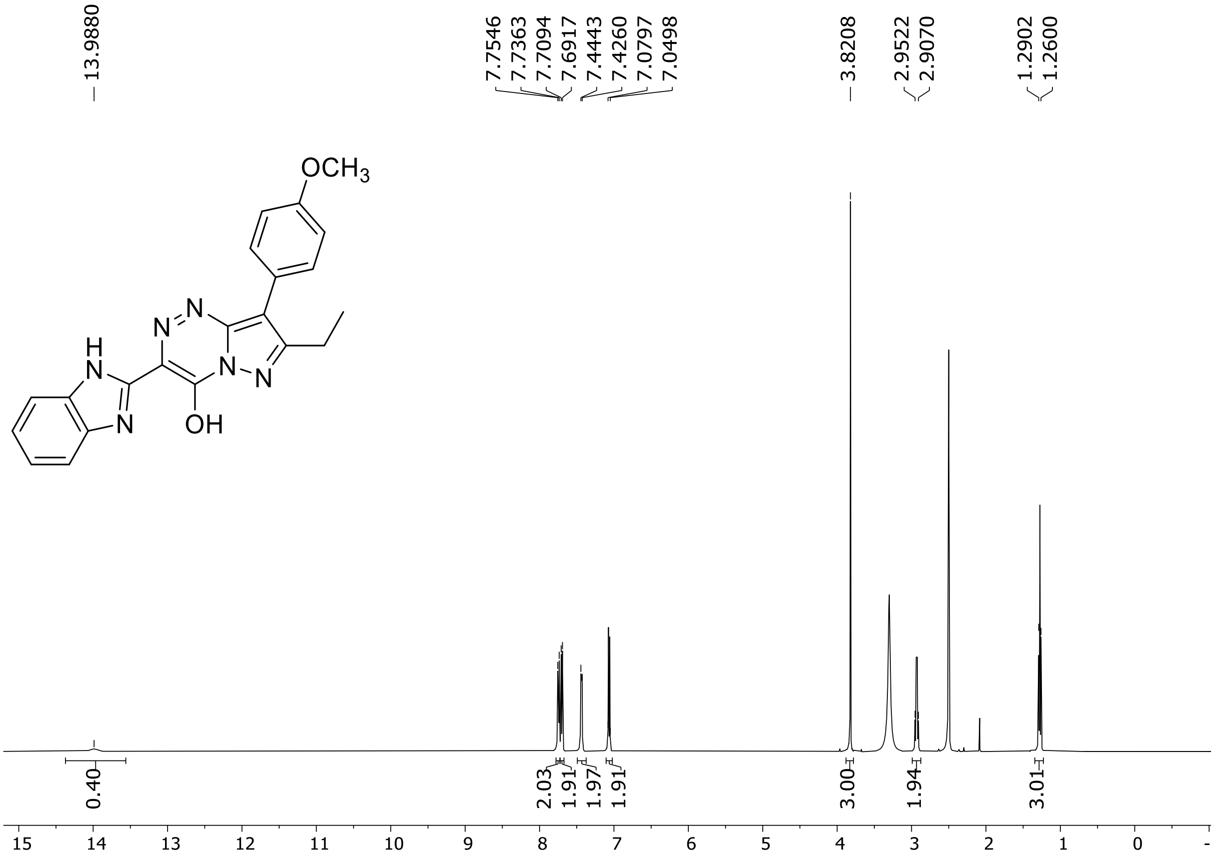

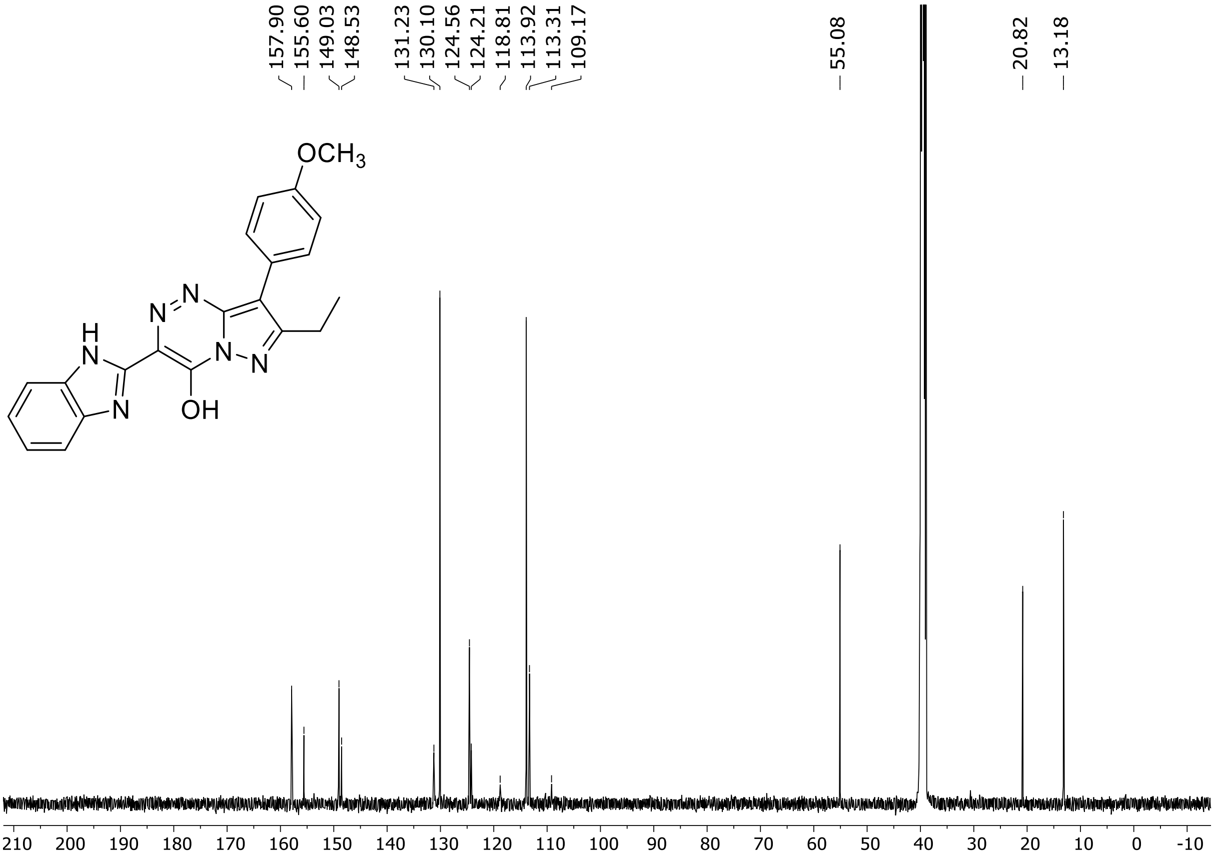

HRMS spectrum of **96**

$C_{21}H_{18}N_6O_2$ **exact mass: 386.1491**

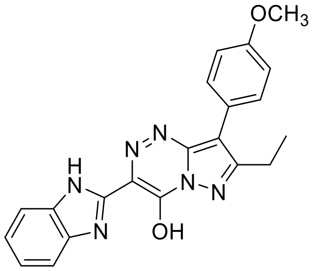

APCI + (MMI)
nitrogen flow 5 L/min, gas temperature 325°C, nebulizer 45 psig, skimmer 65 V, vaporizer 200°C, fragmentor 20 V, dissolved in methanol

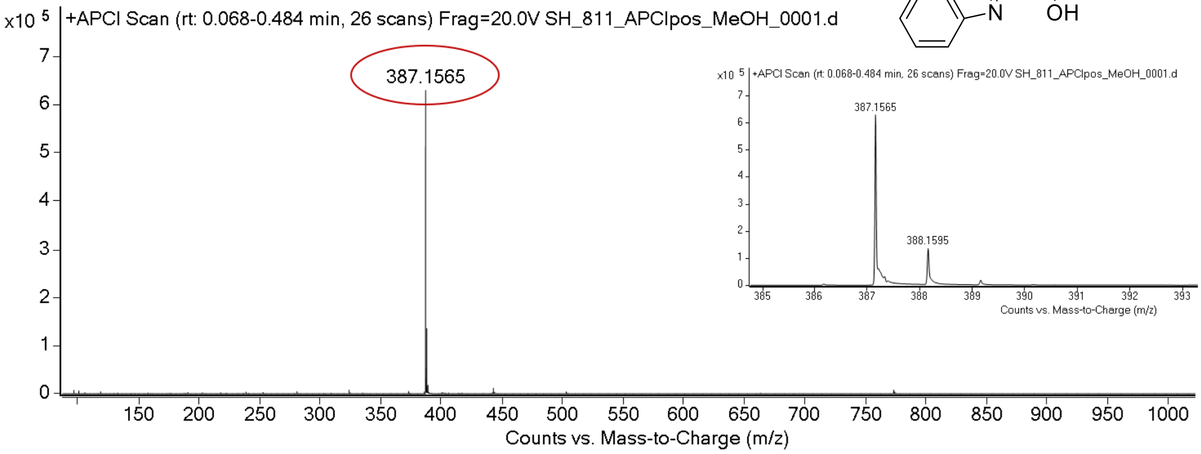

expected mass: $[M+H]^+$ = 387.1564 observed mass : $[M+H]^+$ = 387.1565 mass accuracy = 0.3 ppm

^{1}H (500 MHz) and ^{13}C NMR (126 MHz) spectra of **97** in DMSO-d_6

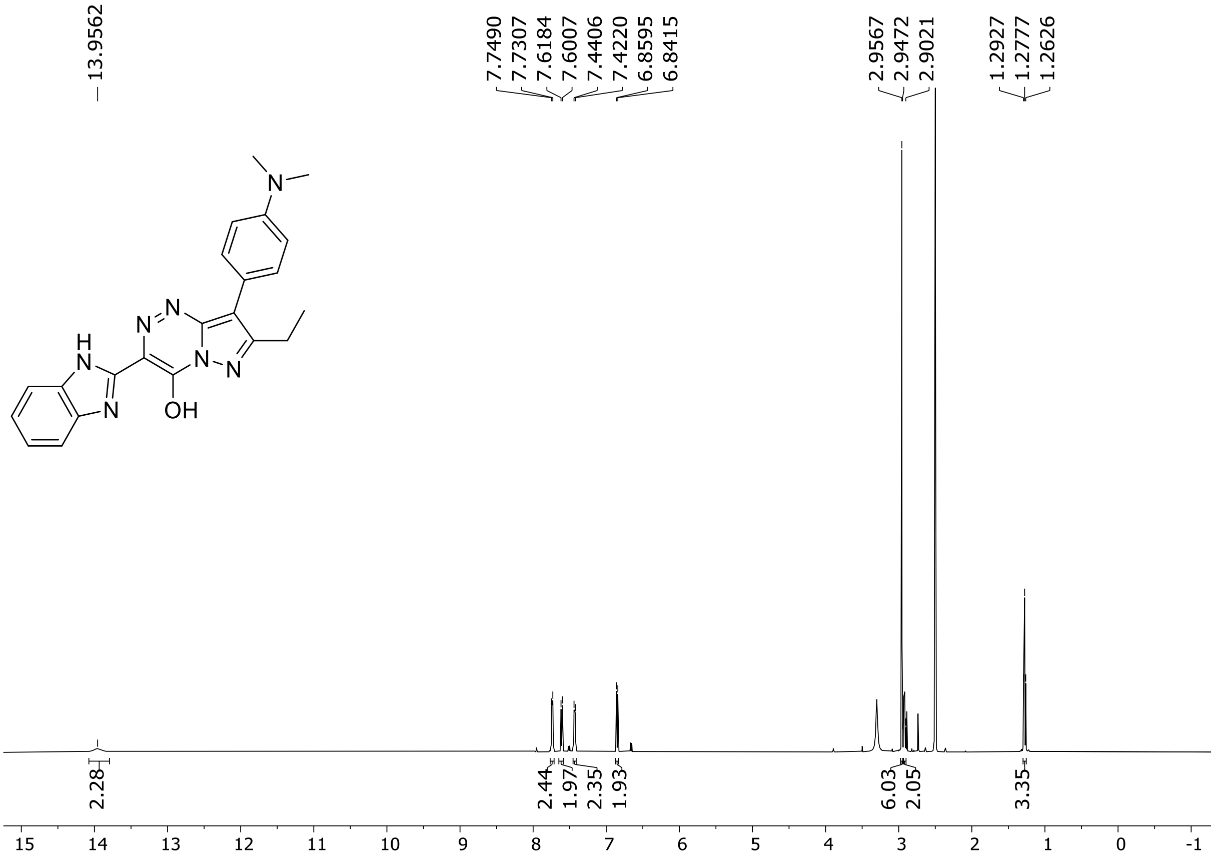

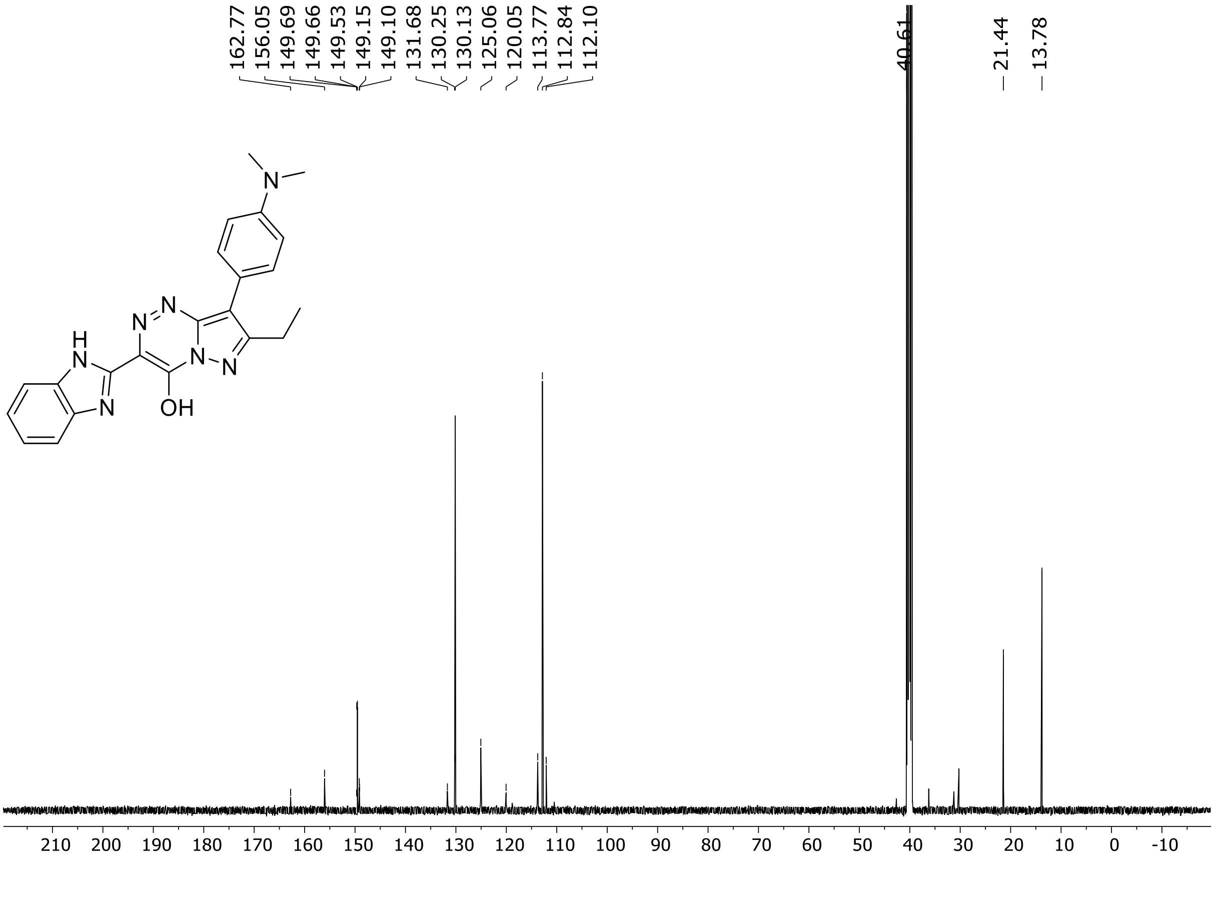

HRMS spectrum of **97**

$C_{22}H_{21}N_7O$ **exact mass: 399.1808**

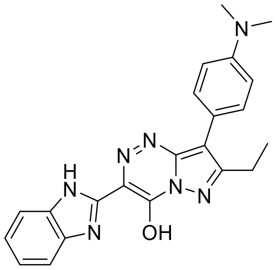

APCI + (MMI)
nitrogen flow 5 L/min, gas temperature 325°C, nebulizer 45 psig, skimmer 65 V, vaporizer 200°C, fragmentor 10 V, dissolved in methanol

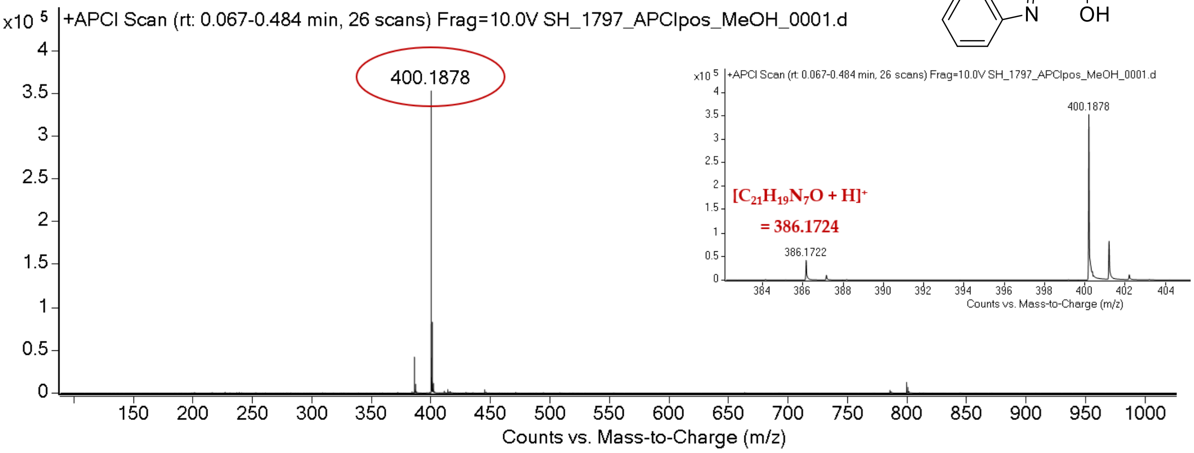

expected mass: $[M+H]^+$ = 400.1880 observed mass : $[M+H]^+$ = 400.1878 mass accuracy = - 0.5 ppm

^{1}H (500 MHz) and ^{13}C NMR (126 MHz) spectra of **98** in DMSO-d_6

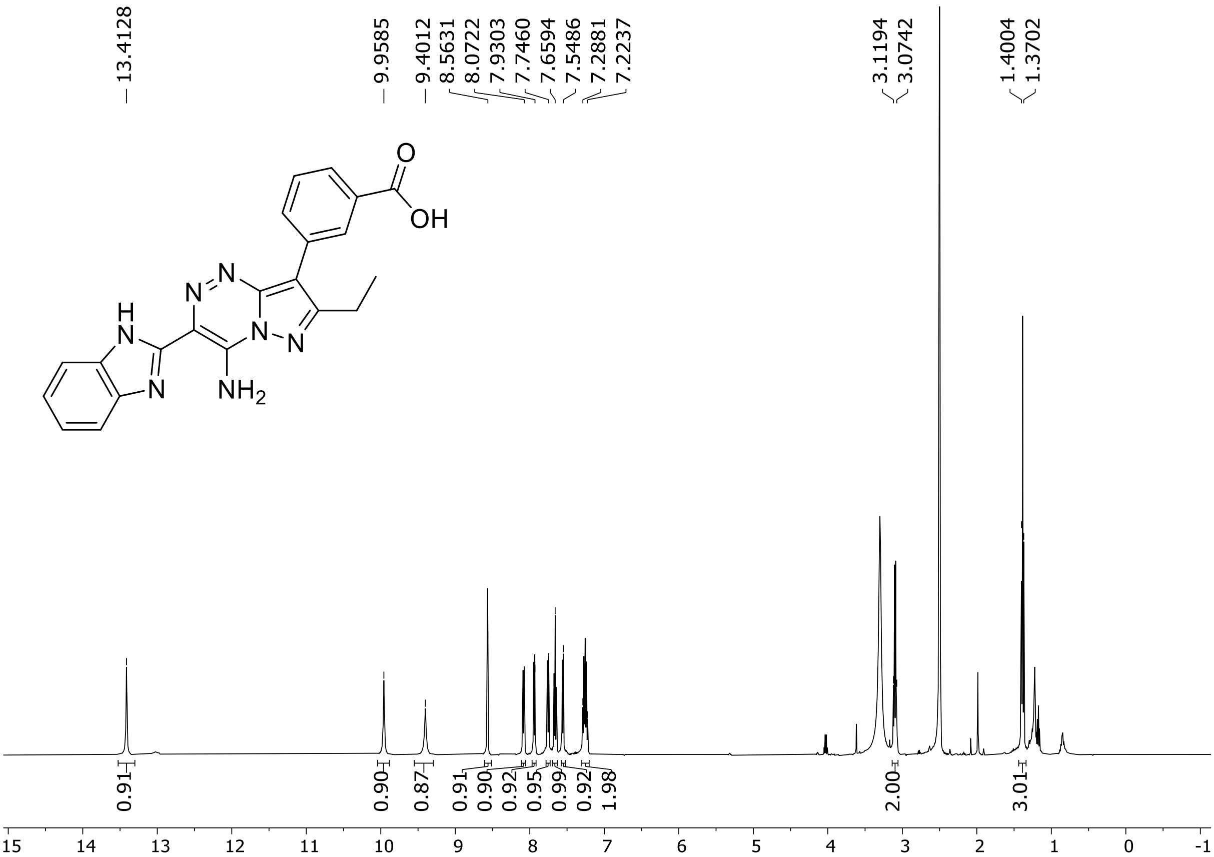

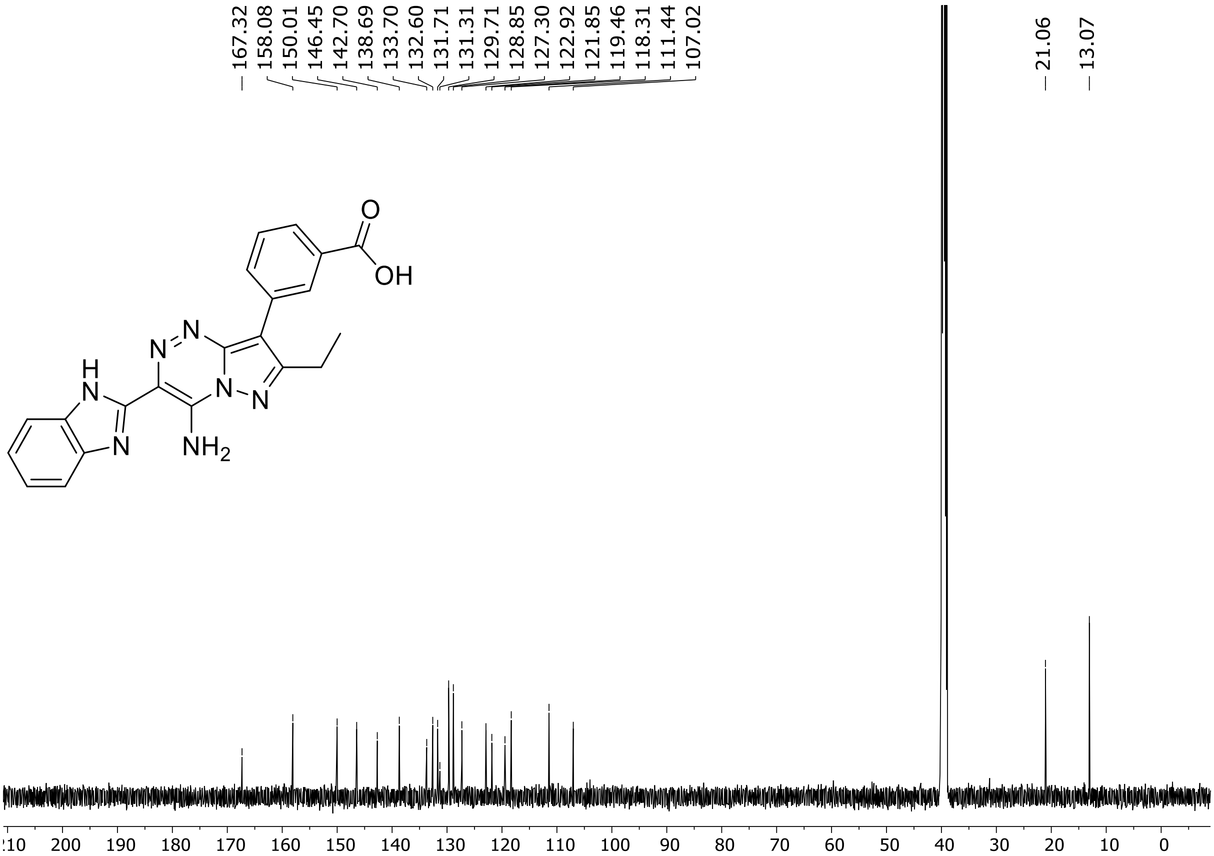

HRMS spectrum of **98**

$C_{21}H_{17}N_7O_2$ exact mass: 399.1444

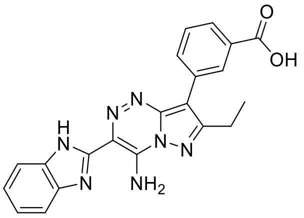

APCI + (MMI)
nitrogen flow 5 L/min, gas temperature 325°C, nebulizer 45 psig, skimmer 65 V, vaporizer 200°C, fragmentor 10 V, dissolved in methanol

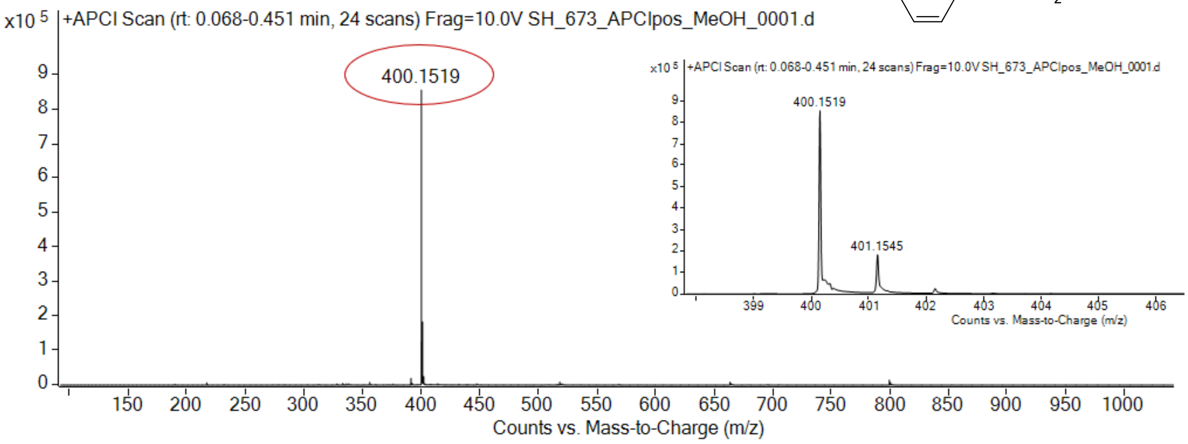

expected mass: $[M+H]^+$ = 400.1516 observed mass : $[M+H]^+$ = 400.1519 mass accuracy = 0.7 ppm

^{1}H (500 MHz) and ^{13}C NMR (126 MHz) spectra of **99** in DMSO-d_6

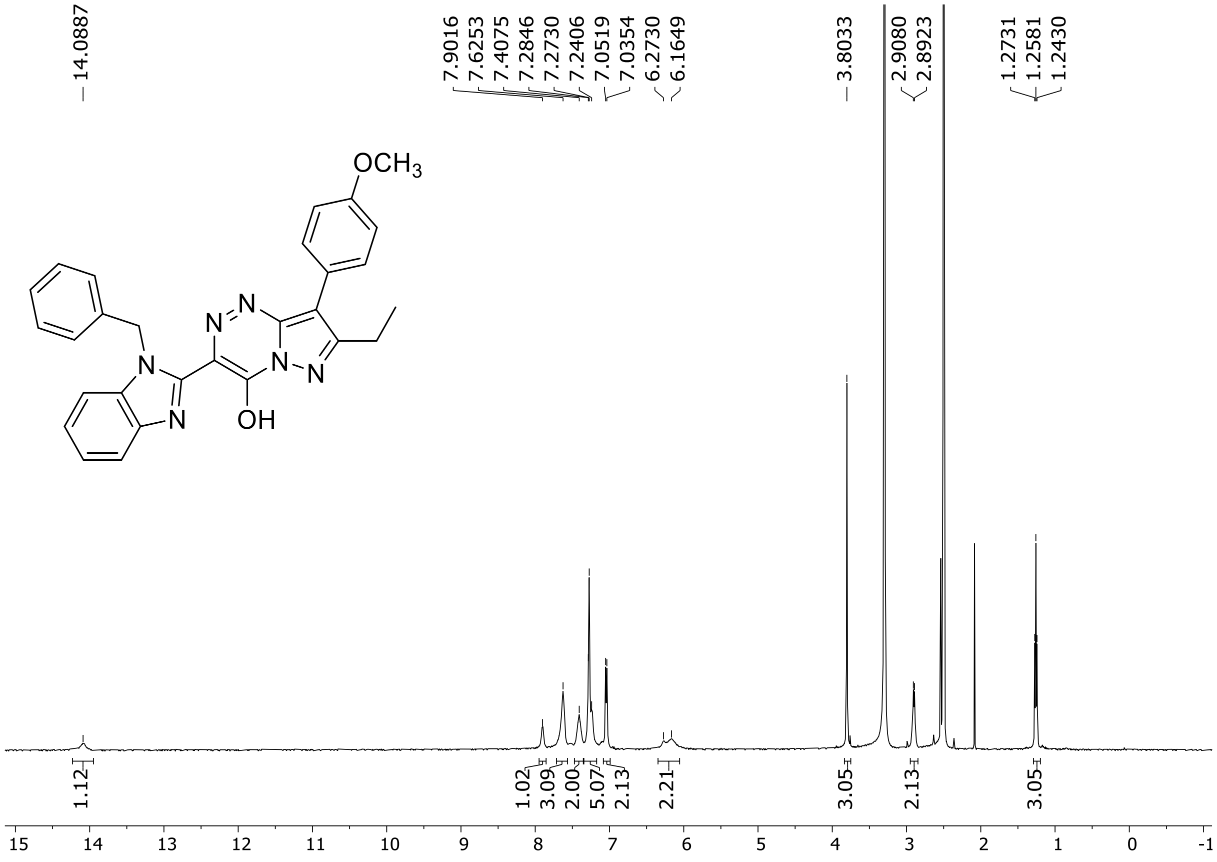

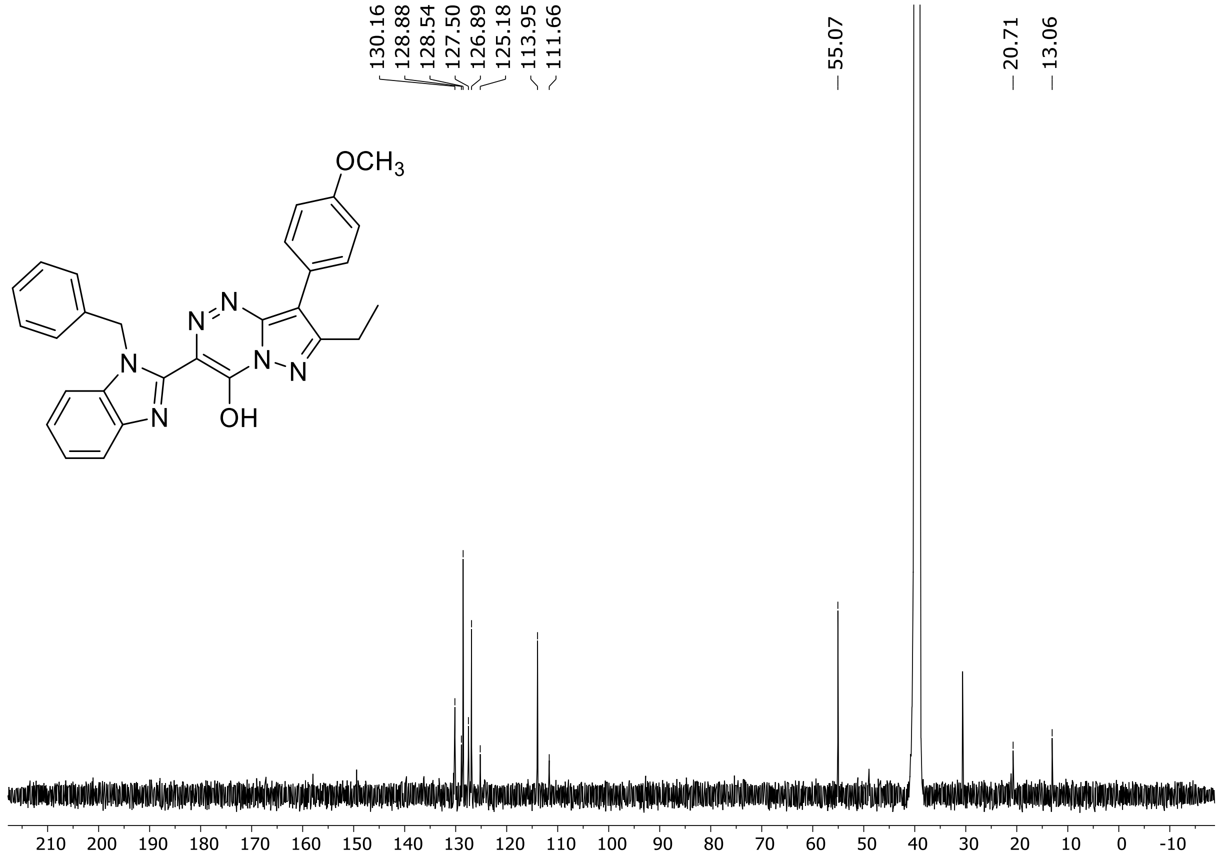

HRMS spectrum of **99**

$C_{28}H_{24}N_6O_2$ **mono *m/z* 476.1961**

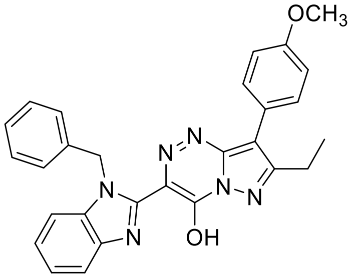

APCI + (MMI)
nitrogen flow 5 L/min, gas temperature 325°C, nebulizer 45 psi, skimmer 65 V, vaporizer 250°C, fragmentor 45 V, dissolved in methanol

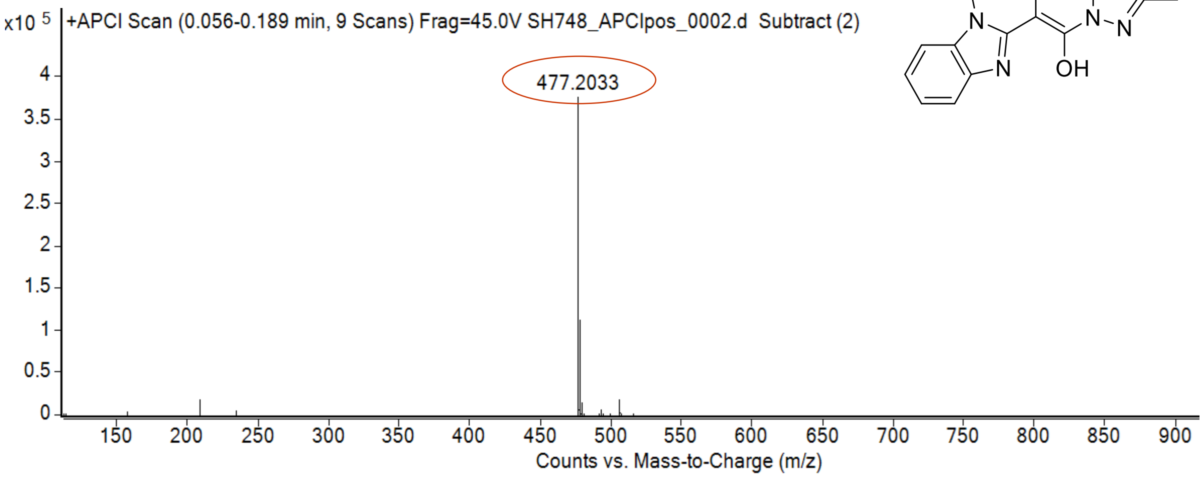

calculated mass: $[M+H]^+ = 477.2034$ observed: $[M+H]^+ = 477.2033$ mass accuracy = - 0.2 ppm

^{1}H (500 MHz) and ^{13}C NMR (126 MHz) spectra of **100** in DMSO-d_6

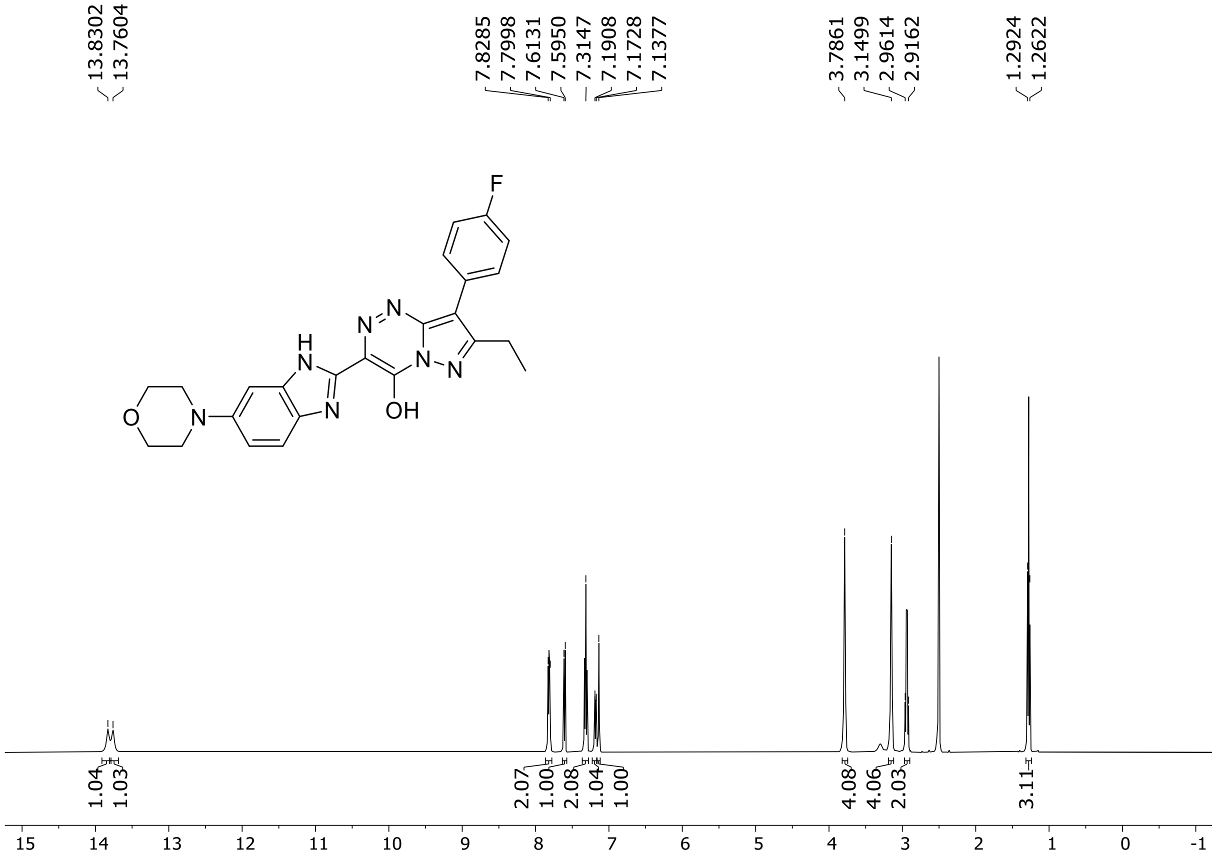

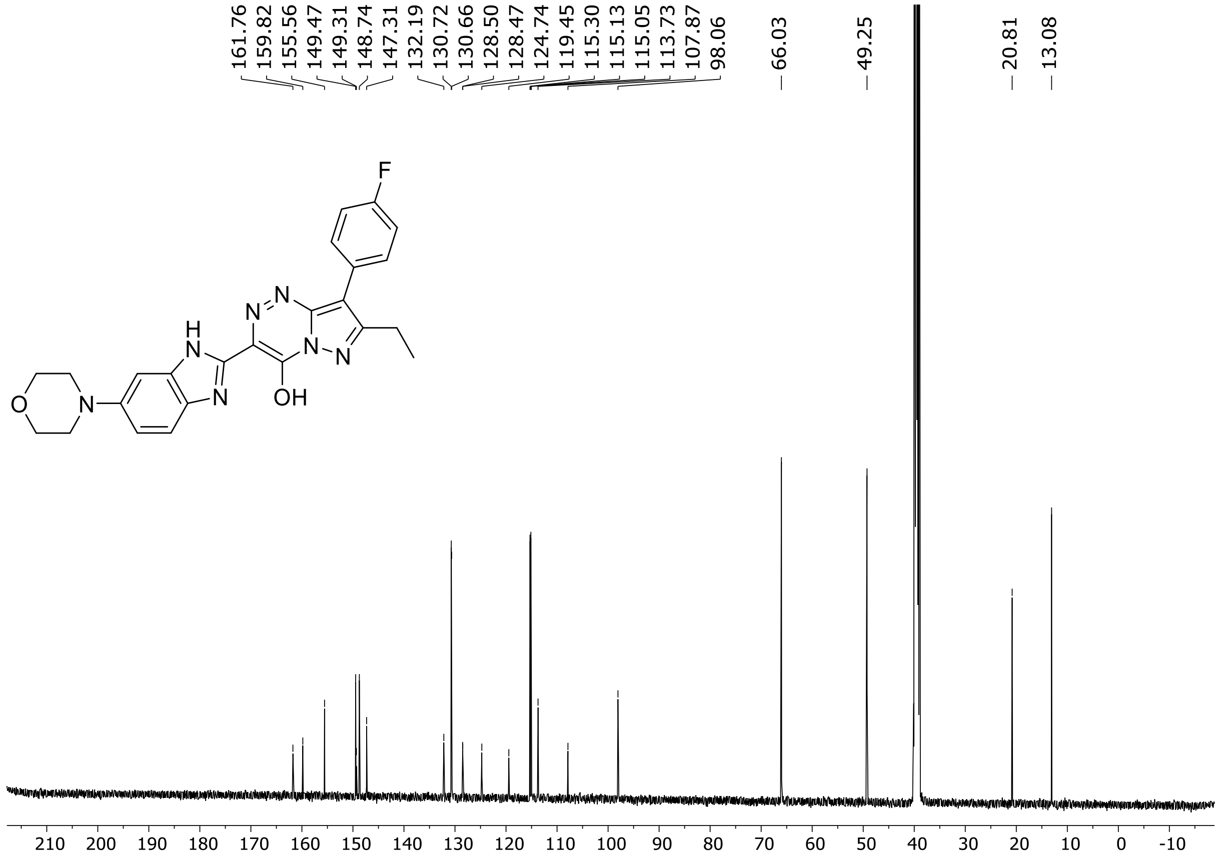

^{19}F (471 MHz) NMR spectrum of **100** in DMSO-d_6

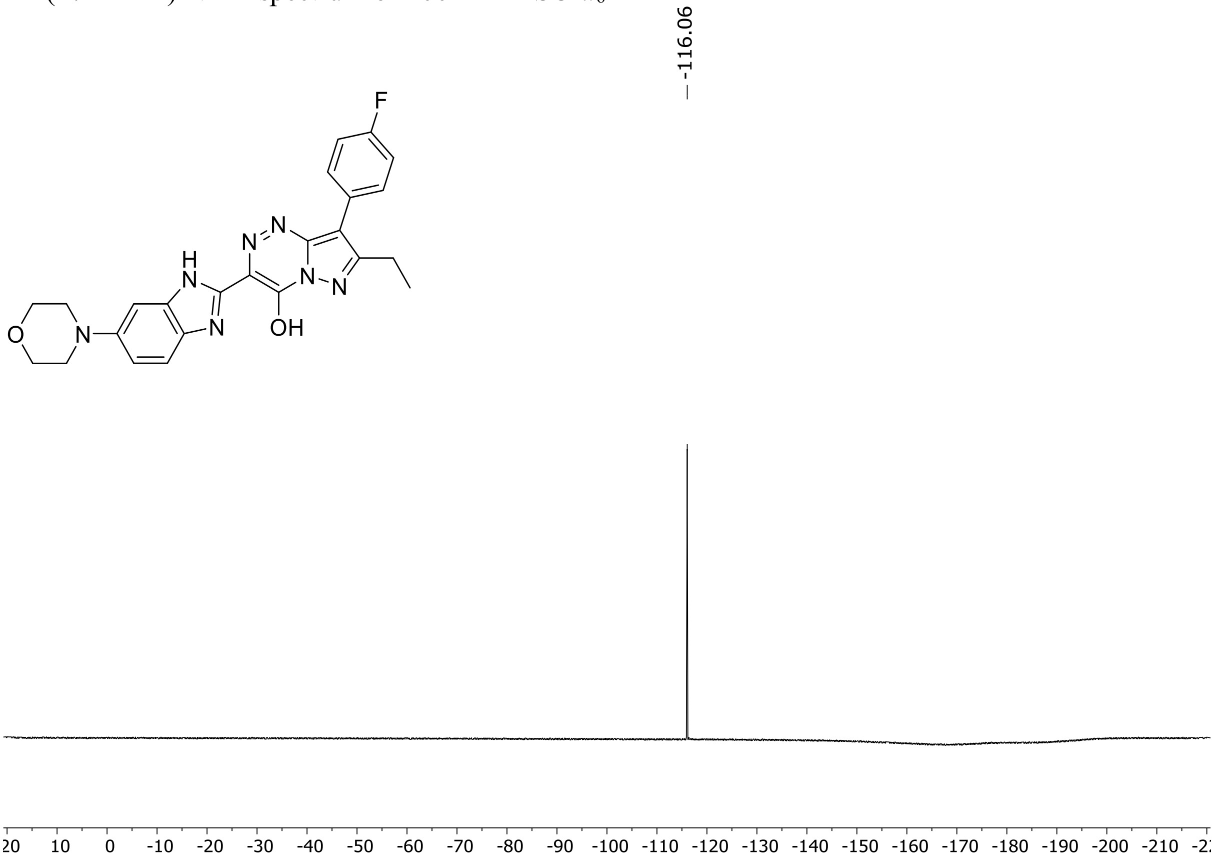

HRMS spectrum of **100**

$C_{24}H_{22}FN_7O_2$ **exact mass: 459.1819**

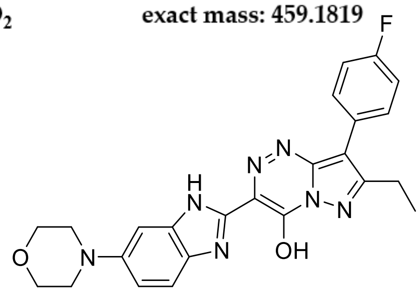

APCI + (MMI)
nitrogen flow 5 L/min, gas temperature 325°C, nebulizer 45 psig, skimmer 65 V, vaporizer 200°C, fragmentor 20 V, dissolved in methanol

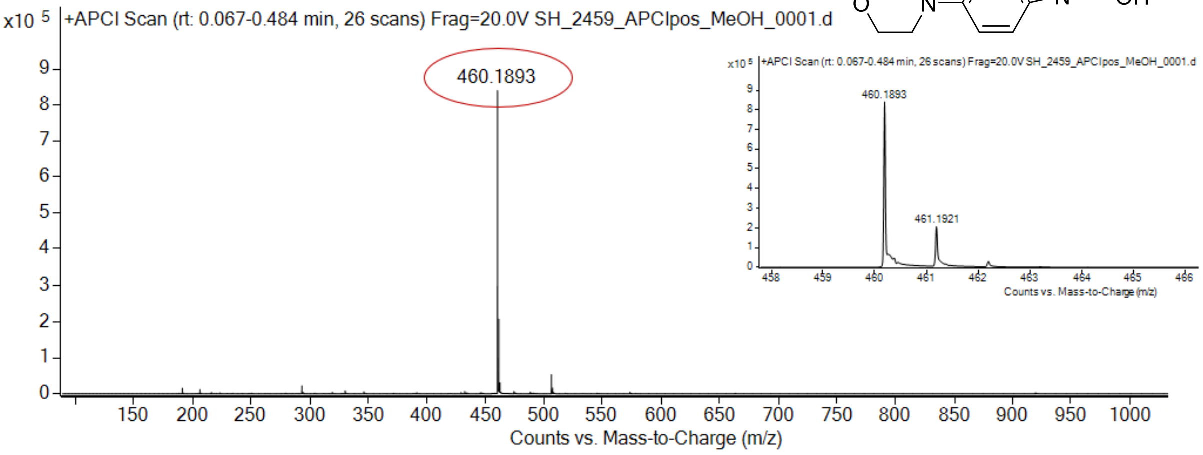

expected mass: [M+H]$^+$ = 460.1892 observed mass : [M+H]$^+$ = 460.1893 mass accuracy = 0.2 ppm

^{1}H (500 MHz) and ^{13}C NMR (126 MHz) spectra of **101** in DMSO-d_6

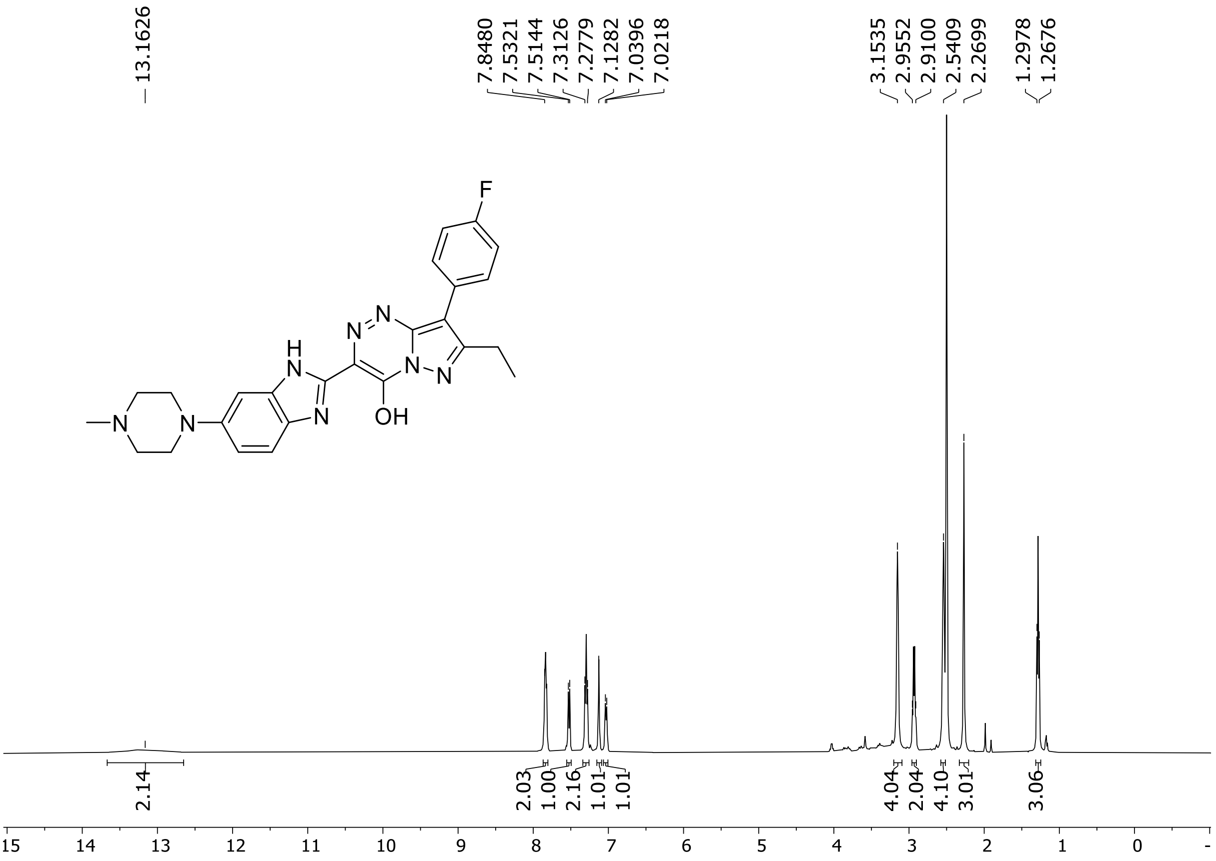

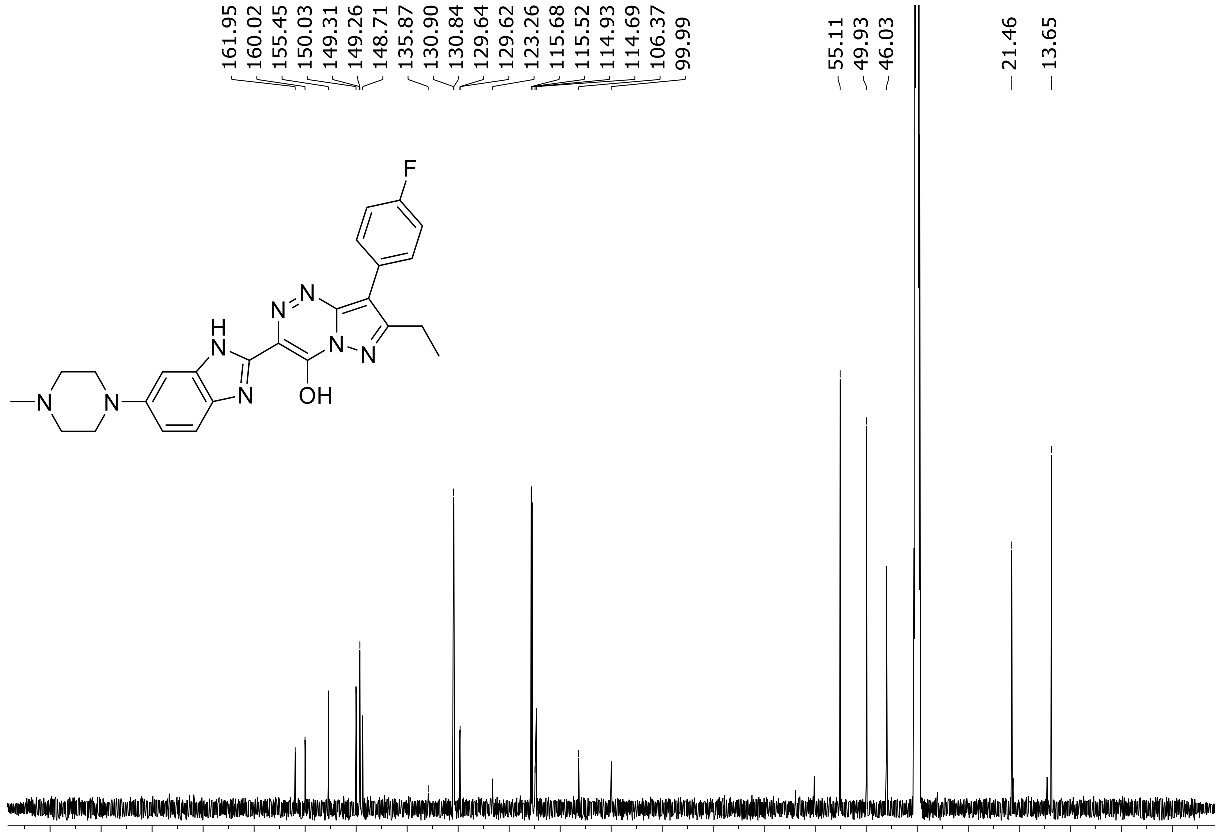

^{19}F (471 MHz) NMR spectrum of **101** in DMSO-d_6

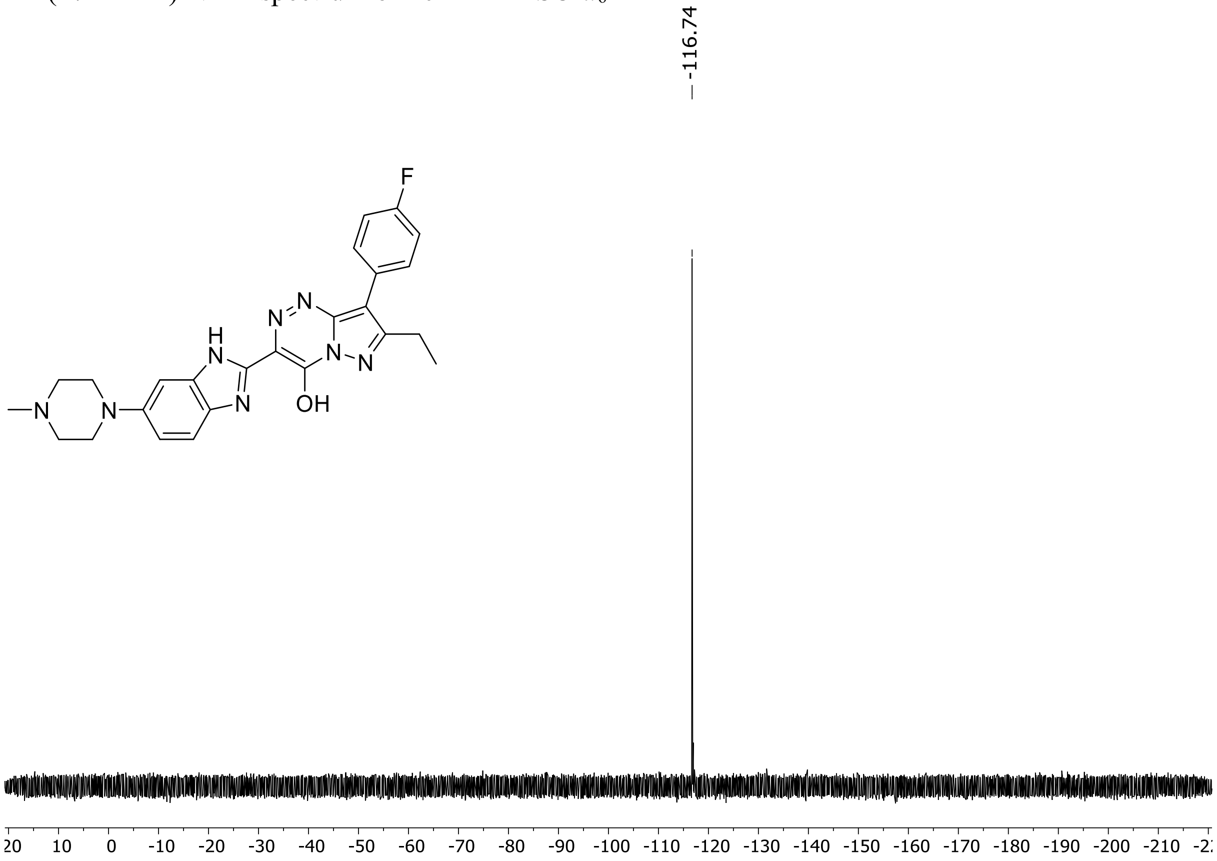

HRMS spectrum of **101**

$C_{25}H_{25}FN_8O$ **exact mass: 472.2135**

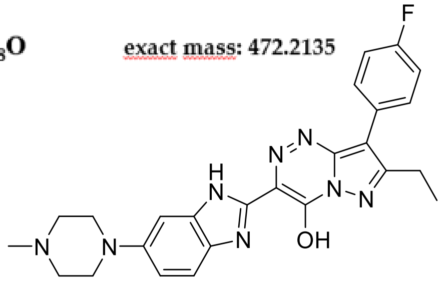

APCI + (MMI)
nitrogen flow 5 L/min, gas temperature 325°C, nebulizer 45 psig, skimmer 65 V, vaporizer 200°C, fragmentor 10 V, dissolved in methanol

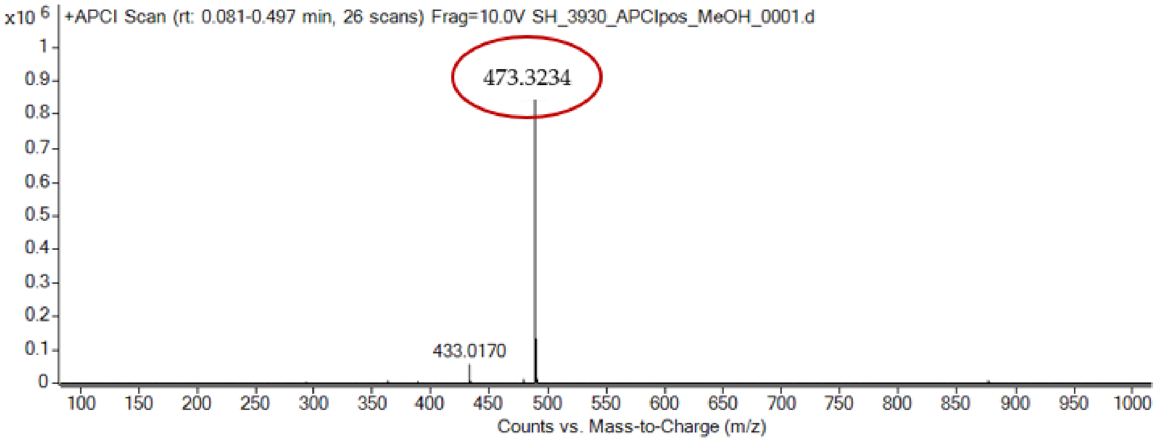

expected mass: $[M+H]^+$ = 473.3223 observed mass: $[M+H]^+$ = 473.3234 mass accuracy = 0.8 ppm

^{1}H (500 MHz) and ^{13}C NMR (126 MHz) spectra of **102** in DMSO-d_6

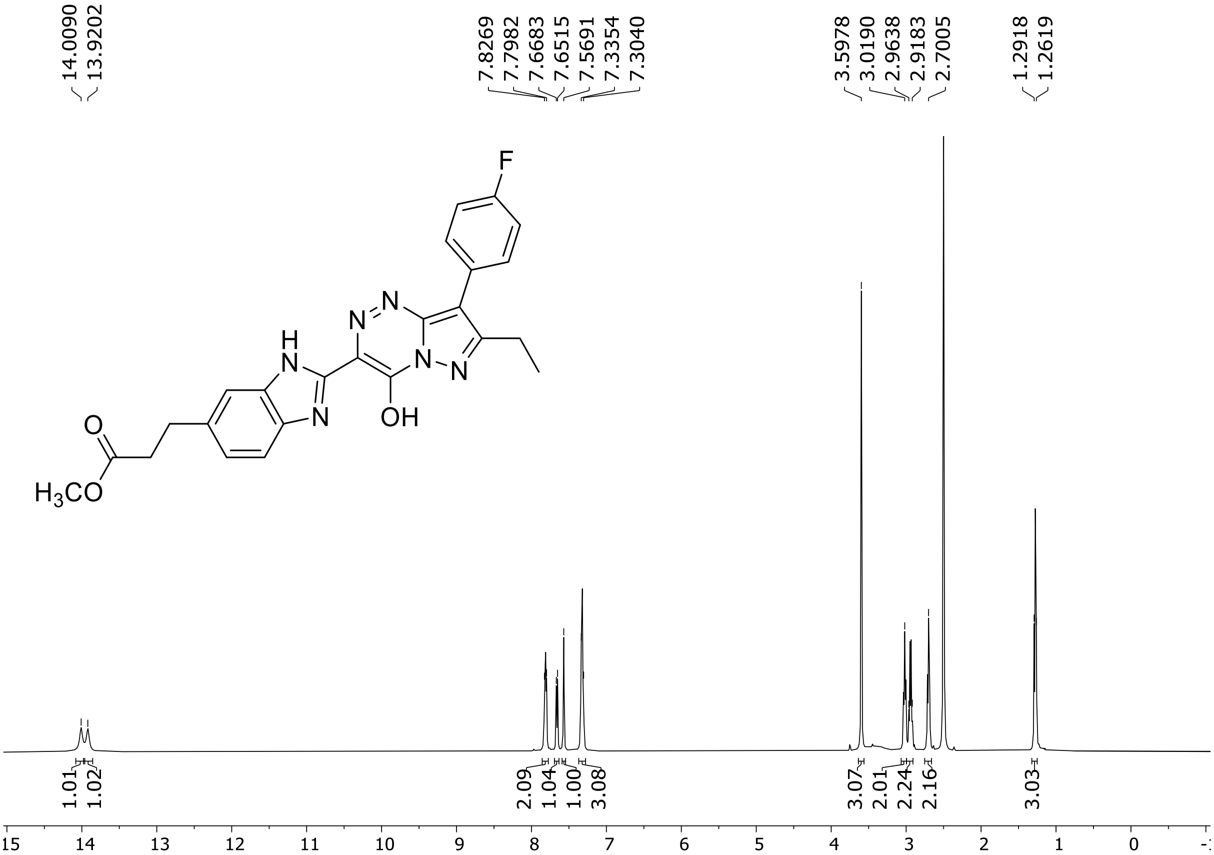

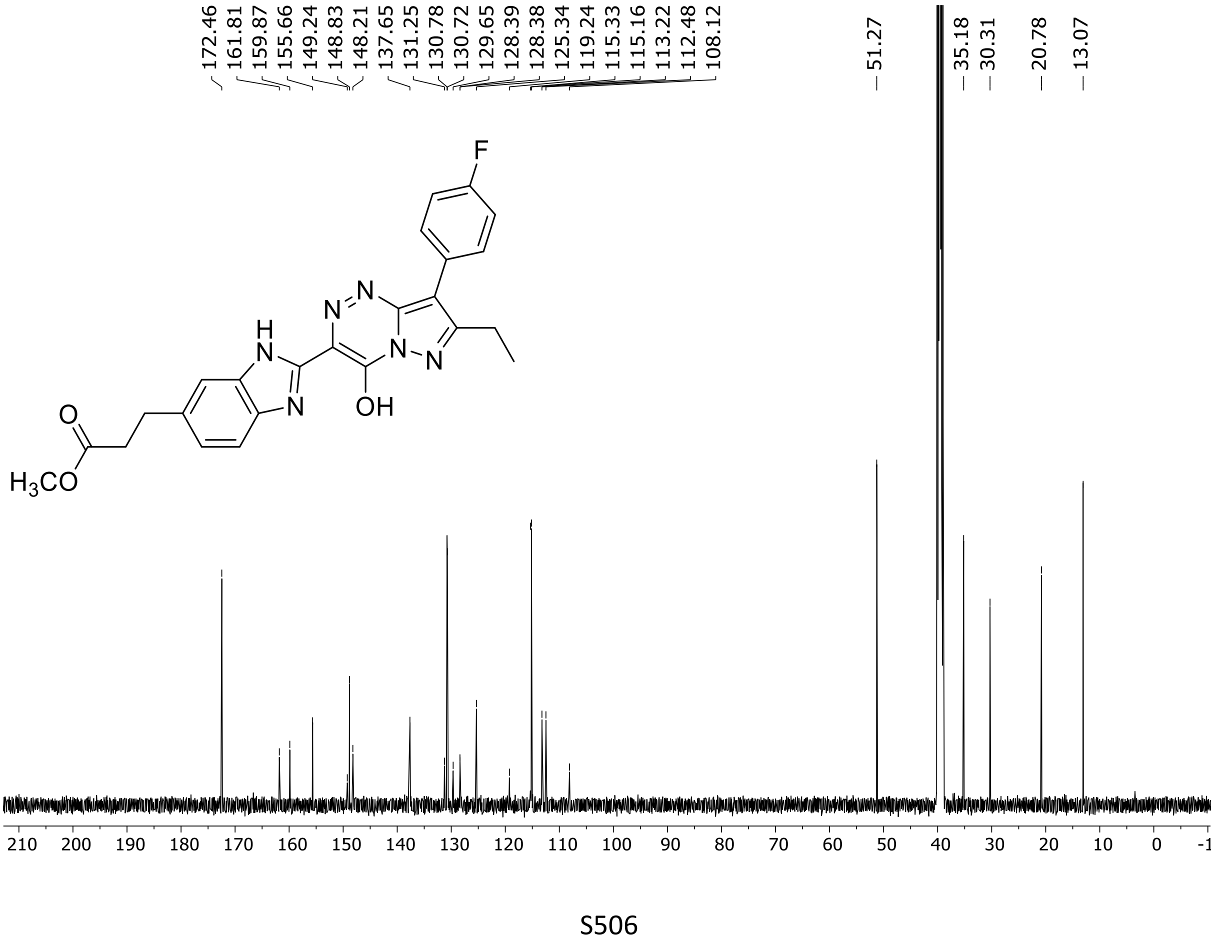

^{19}F (471 MHz) NMR spectrum of **102** in DMSO-d_6

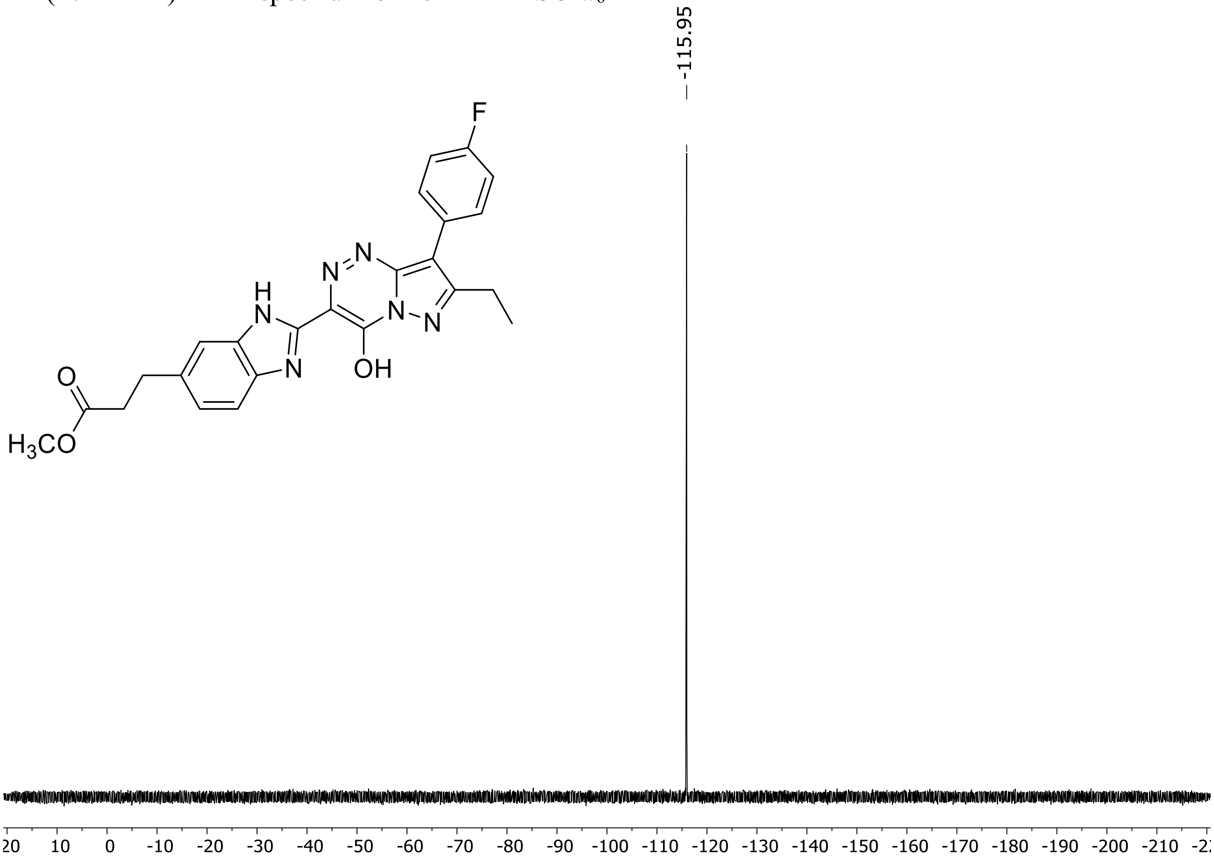

HRMS spectrum of **102**

$C_{24}H_{21}FN_6O_3$ **exact mass: 460.1659**

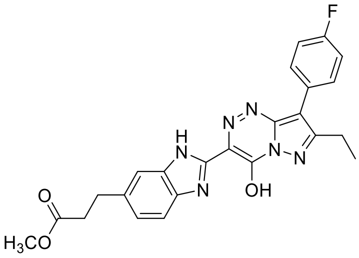

APCI + (MMI)
nitrogen flow 5 L/min, gas temperature 325°C, nebulizer 45 psig, skimmer 65 V, vaporizer 200°C, fragmentor 20 V, dissolved in methanol

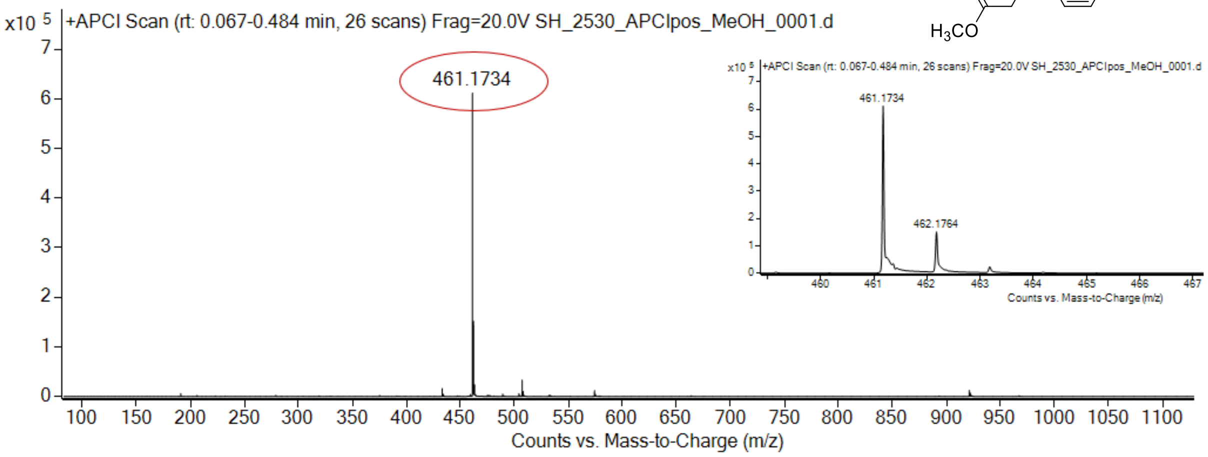

expected mass: $[M+H]^+$ = 461.1732 observed mass : $[M+H]^+$ = 461.1734 mass accuracy = 0.4 ppm

^{1}H (500 MHz) and ^{13}C NMR (126 MHz) spectra of **103** in DMSO-d_6

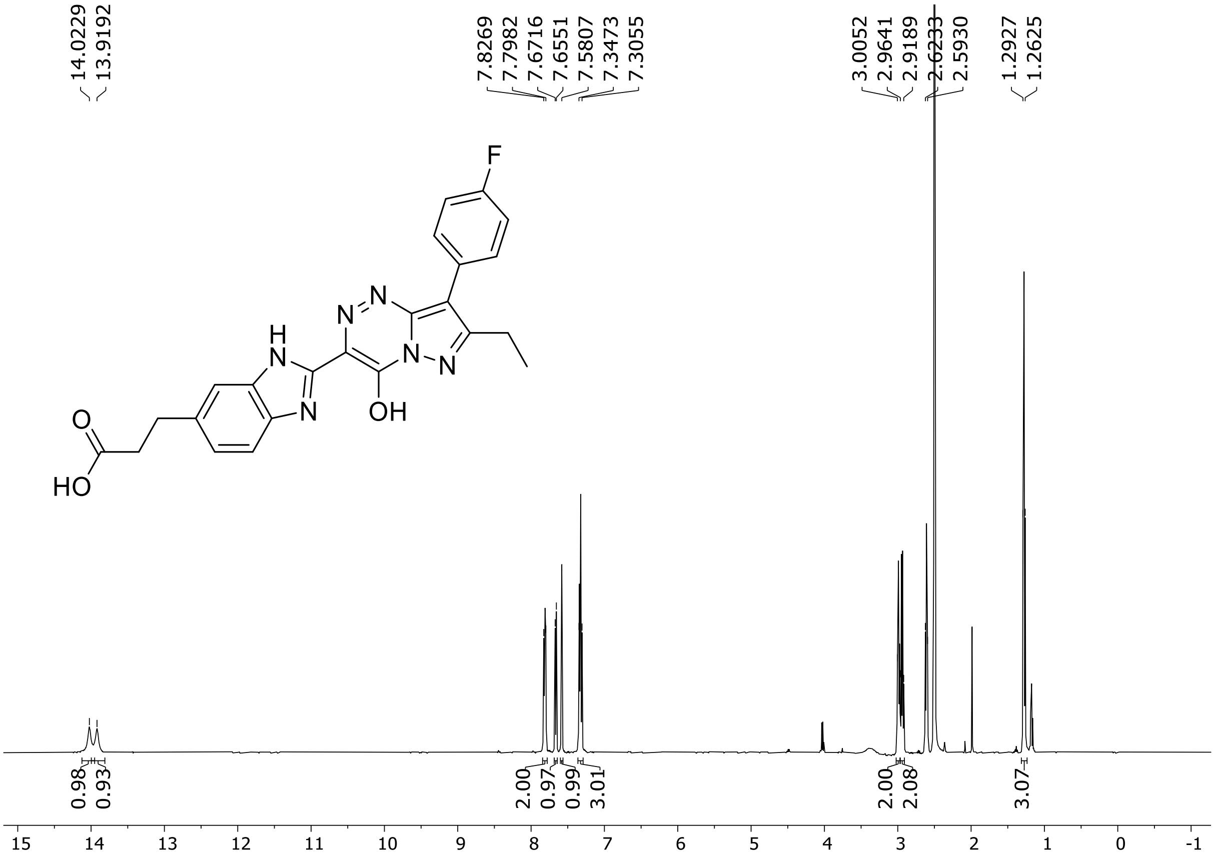

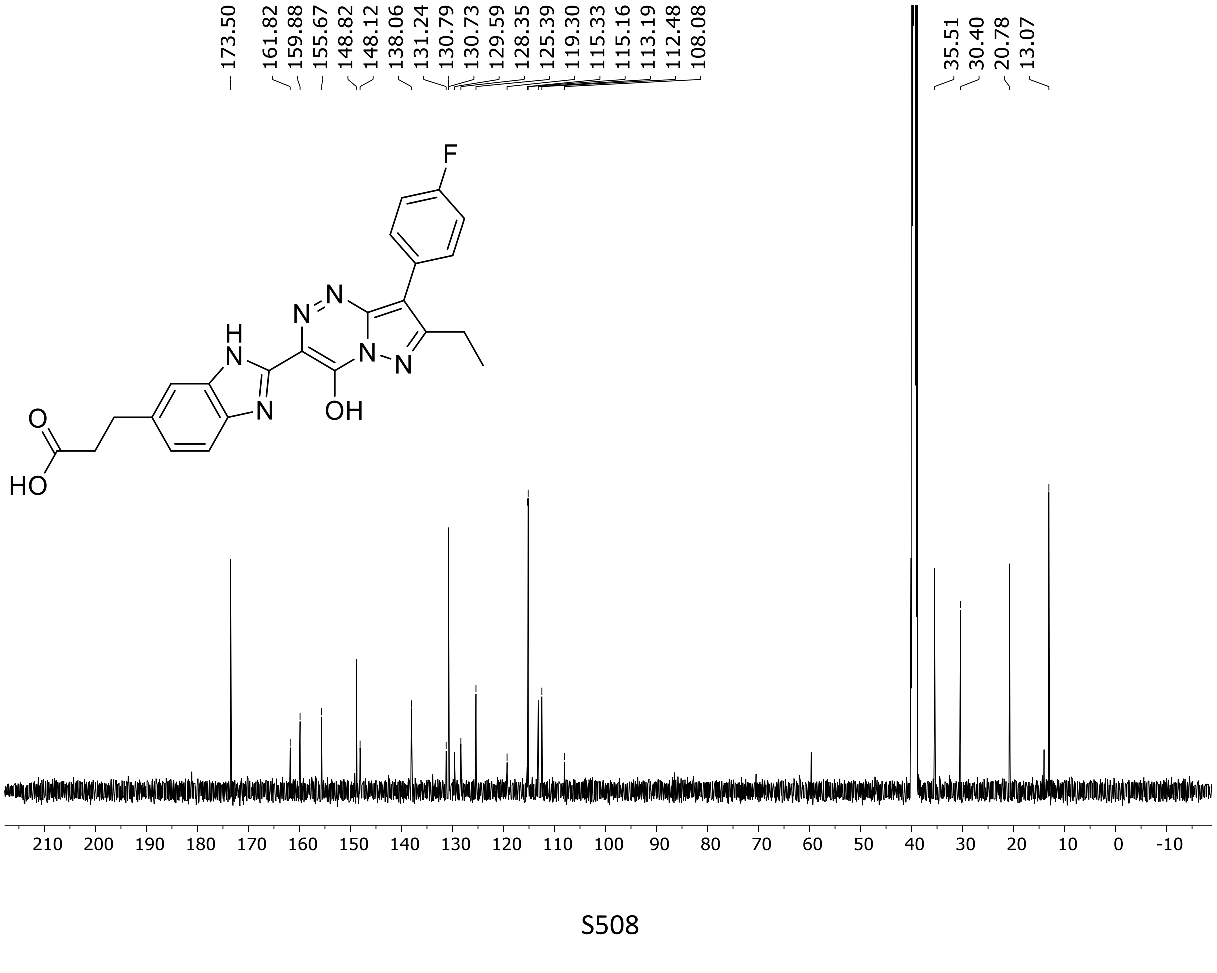

^{19}F (471 MHz) NMR spectrum of **103** in DMSO-d_6

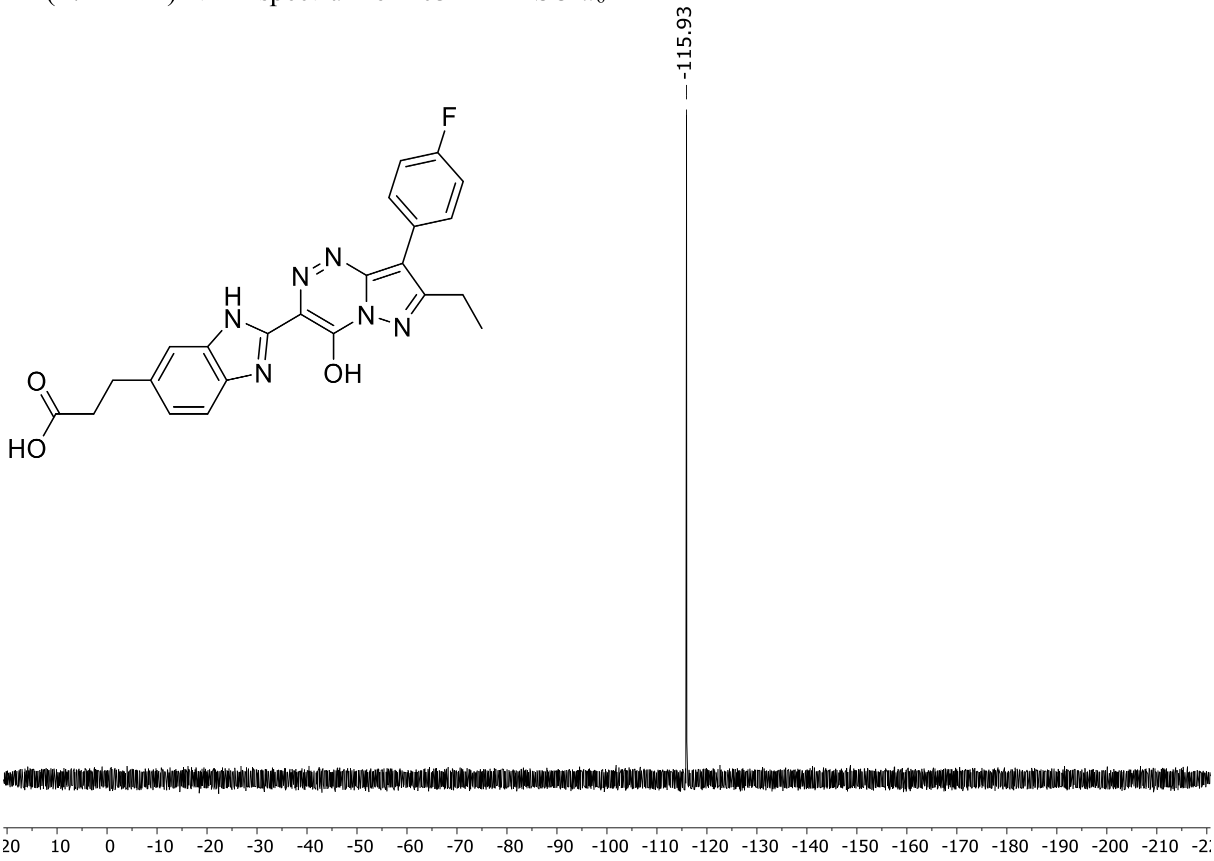

HRMS spectrum of **103**

$C_{23}H_{19}FN_6O_3$ **exact mass: 446.1503**

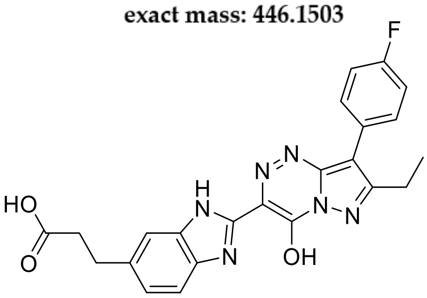

APCI + (MMI)
nitrogen flow 5 L/min, gas temperature 325°C, nebulizer 45 psig, skimmer 65 V, vaporizer 200°C, fragmentor 22 V, dissolved in methanol

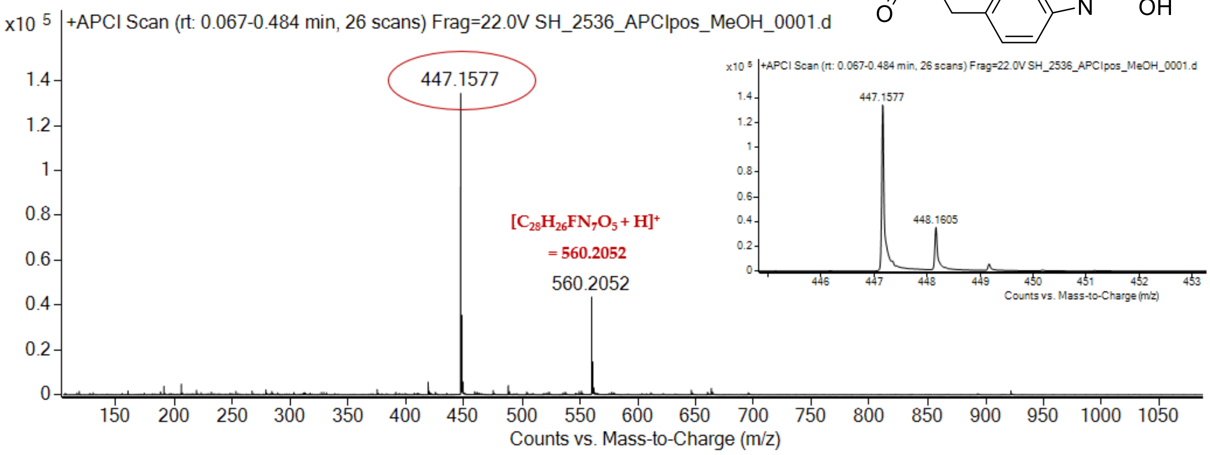

expected mass: $[M+H]^+ = 447.1575$ observed mass : $[M+H]^+ = 447.1577$ mass accuracy = 0.4 ppm

^{1}H (500 MHz) and ^{13}C NMR (126 MHz) spectra of **104** in DMSO-d_6

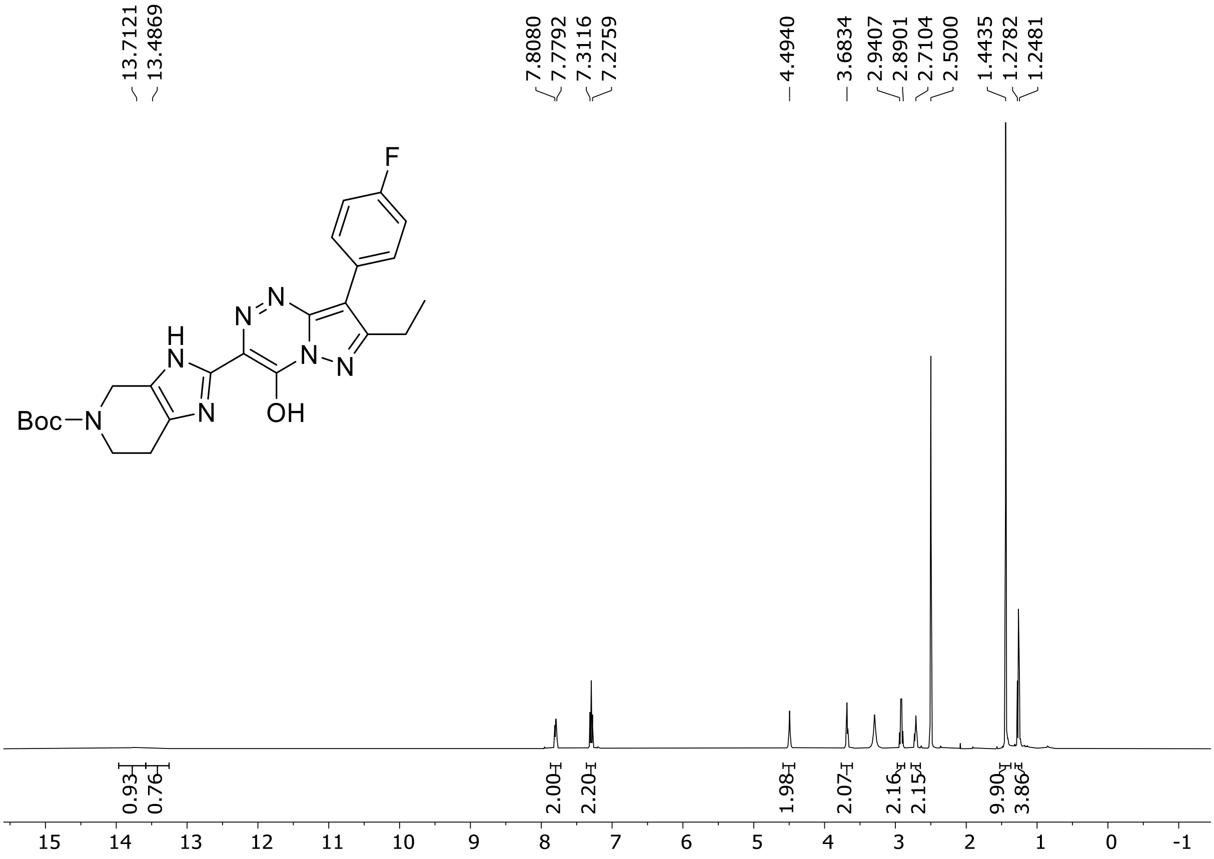

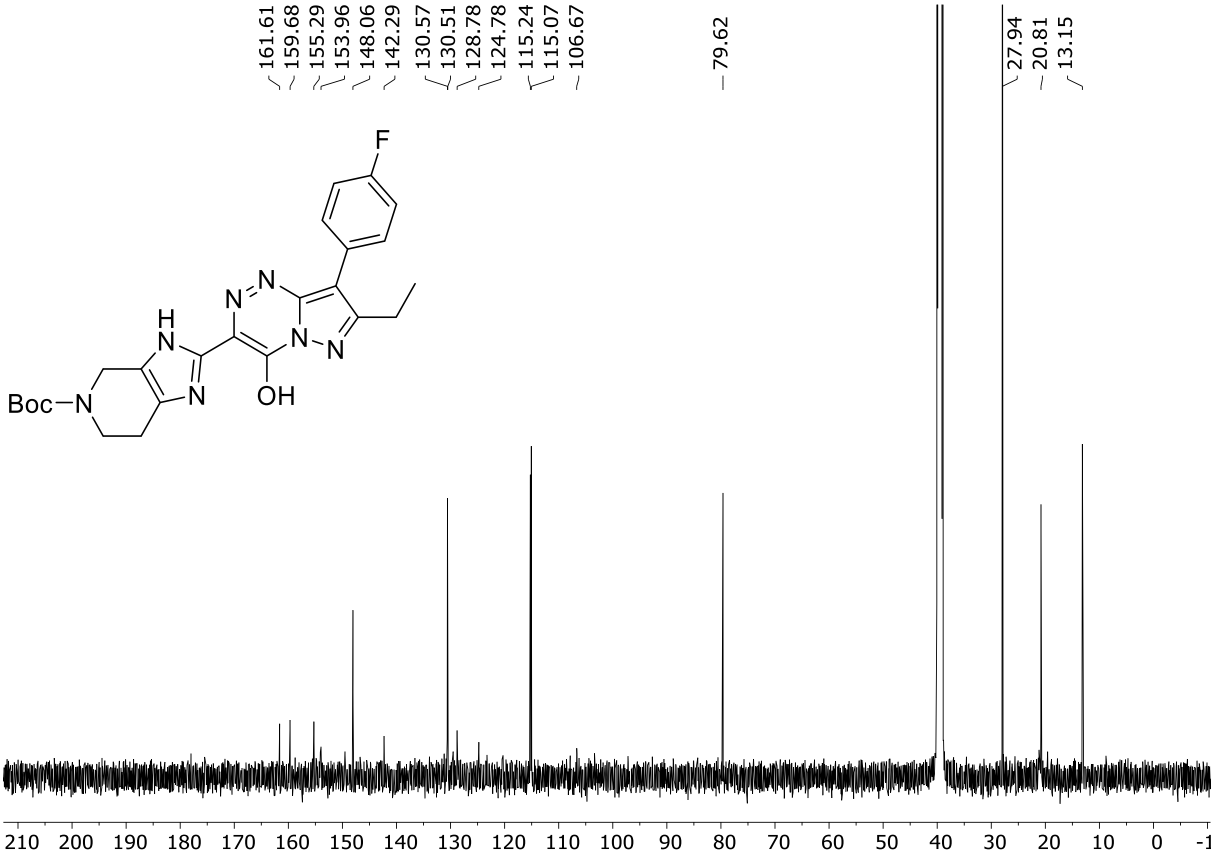

^{19}F (471 MHz) NMR spectrum of **104** in DMSO-d_6

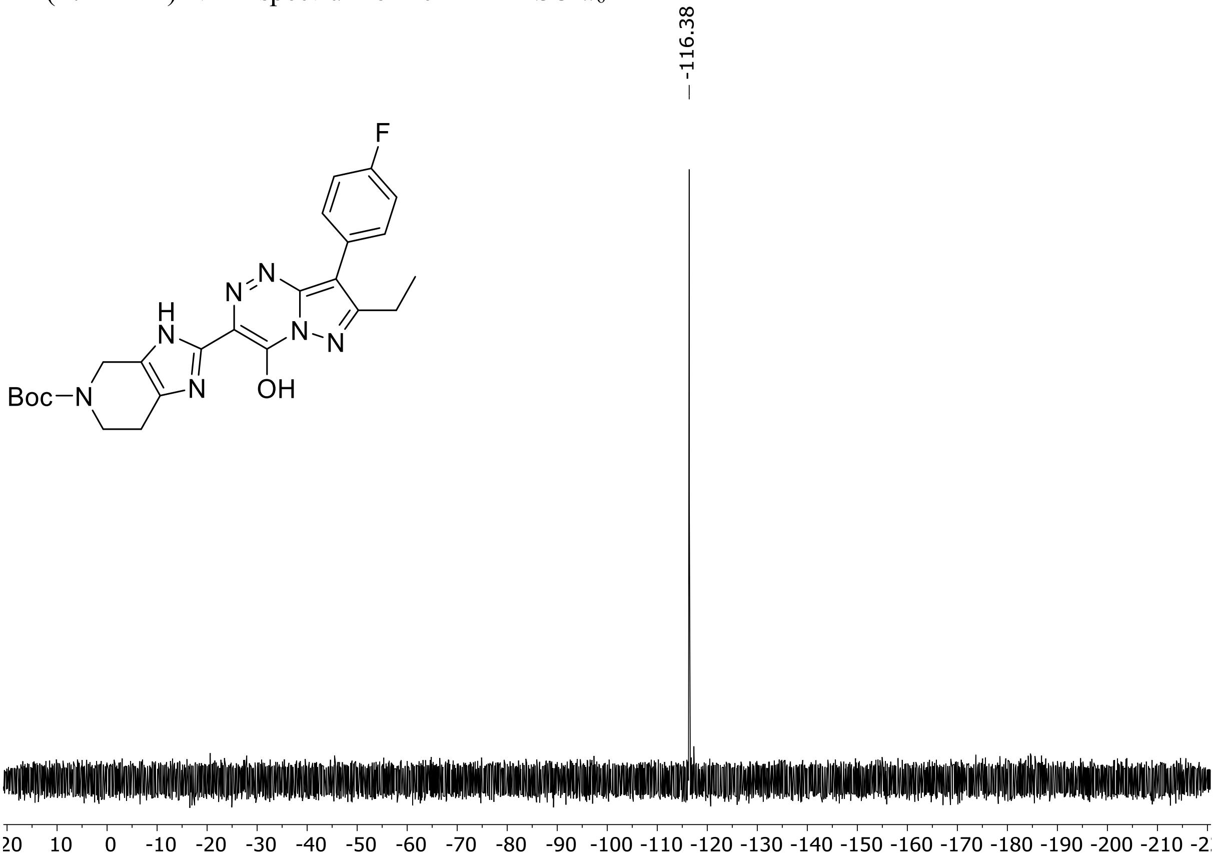

HRMS spectrum of **104**

$C_{24}H_{26}FN_7O_3$ **exact mass: 479.2081**

APCI + (MMI)
nitrogen flow 5 L/min, gas temperature 325°C, nebulizer 45 psig, skimmer 65 V, vaporizer 200°C, fragmentor 30 V, dissolved in methanol

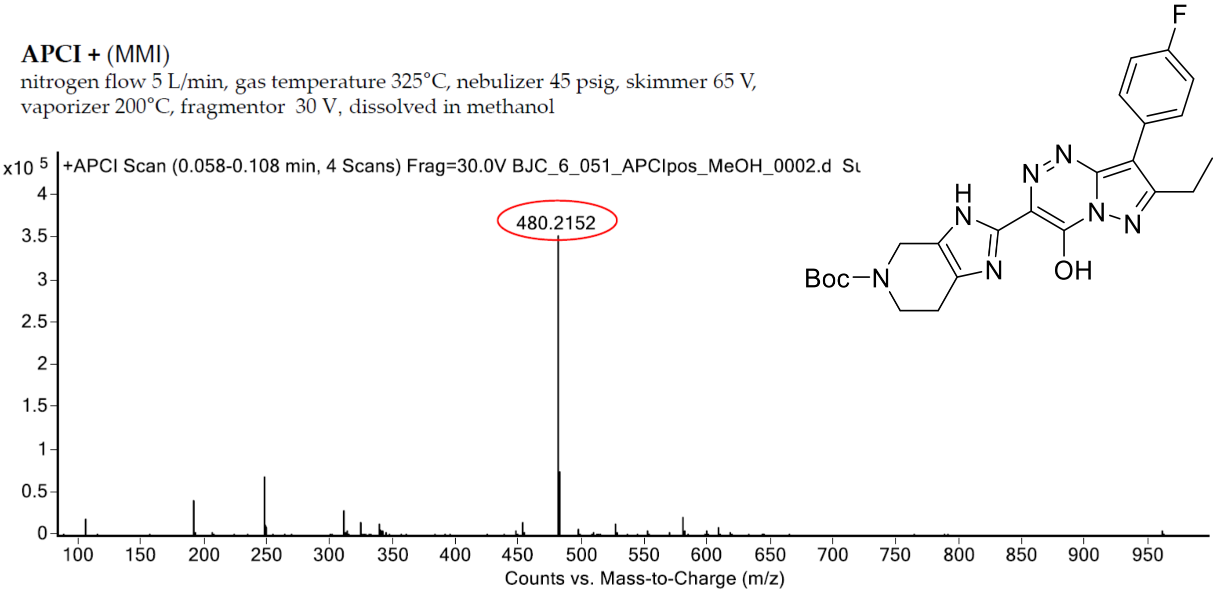

expected mass: $[M+H]^+ = 480.2154$ observed mass: $[M+H]^+ = 480.2152$ mass accuracy = - 0.4 ppm

^{1}H (500 MHz) and ^{13}C NMR (126 MHz) spectra of **105** in DMSO-d_6

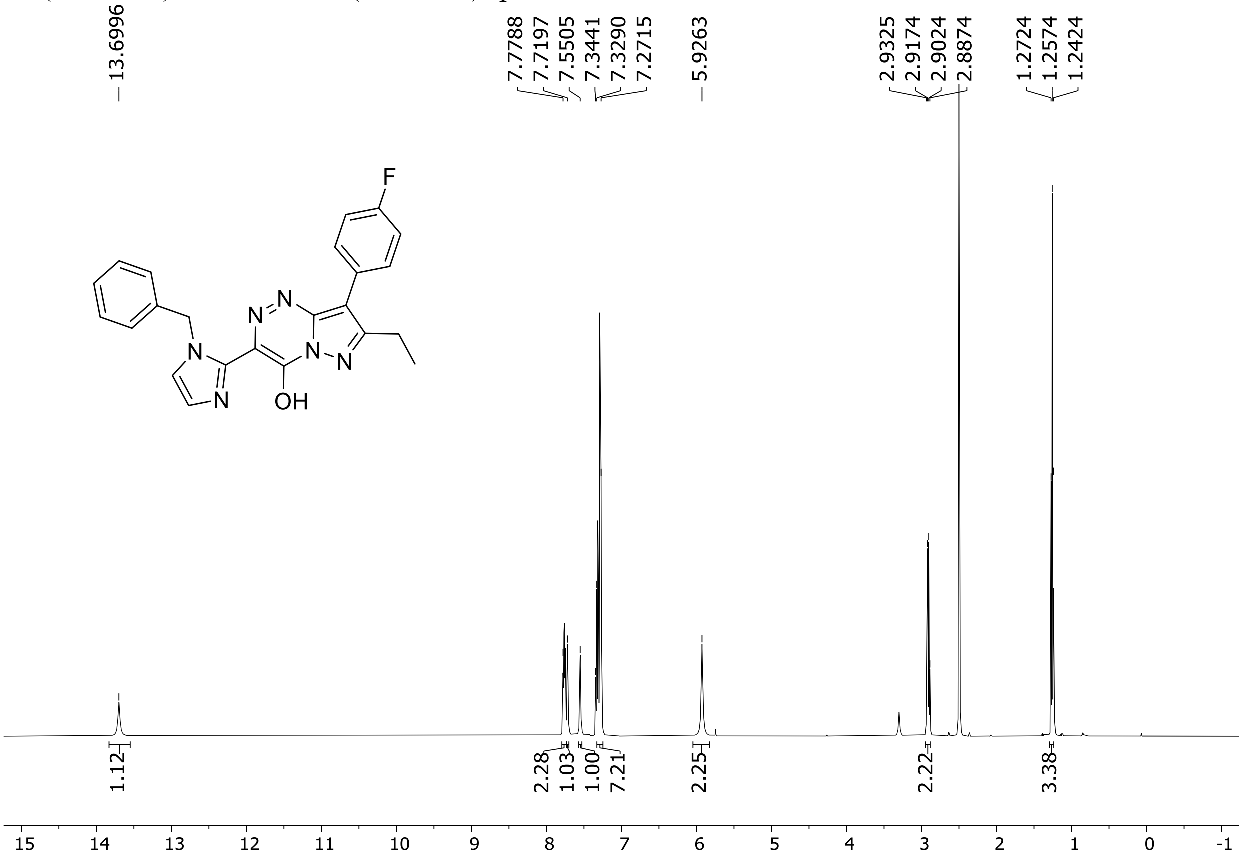

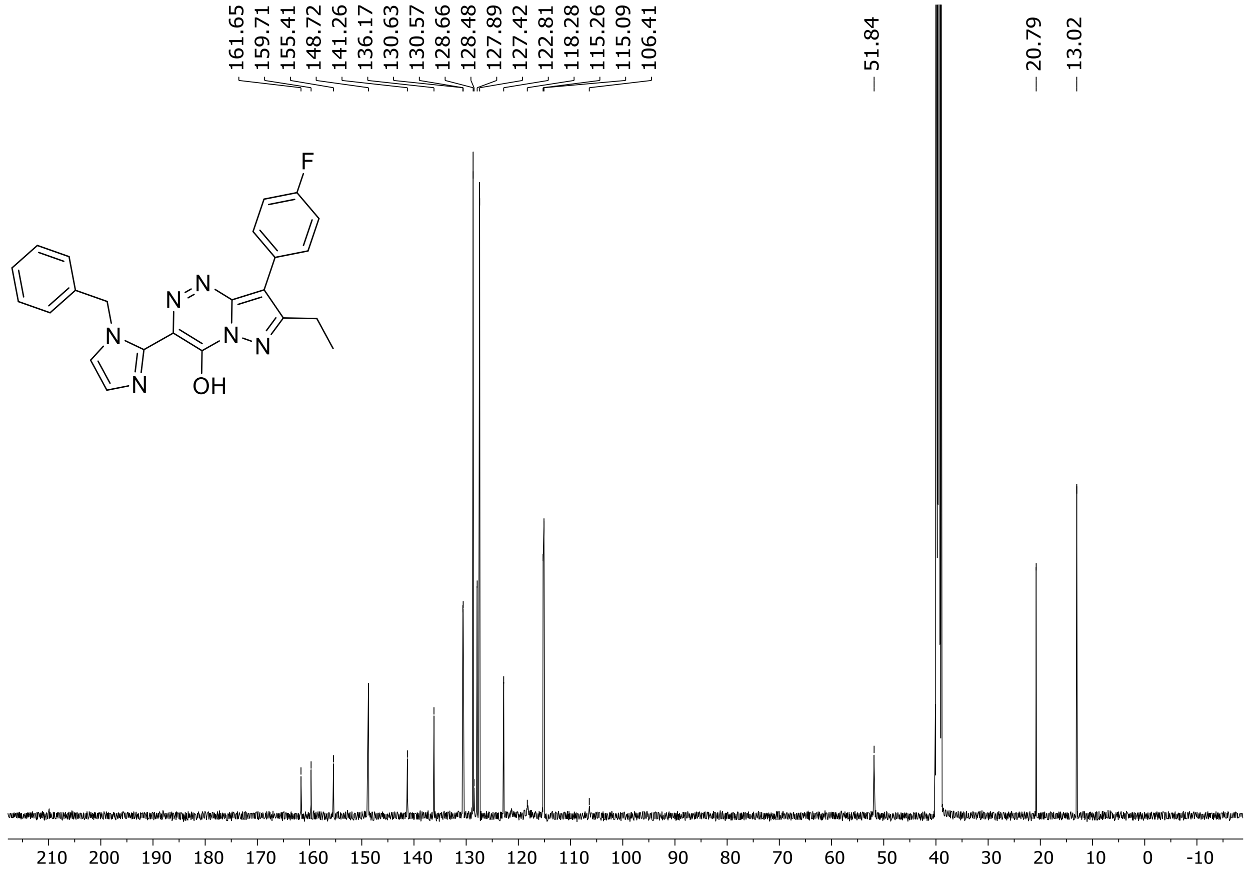

^{19}F (471 MHz) NMR spectrum of **105** in DMSO-d_6

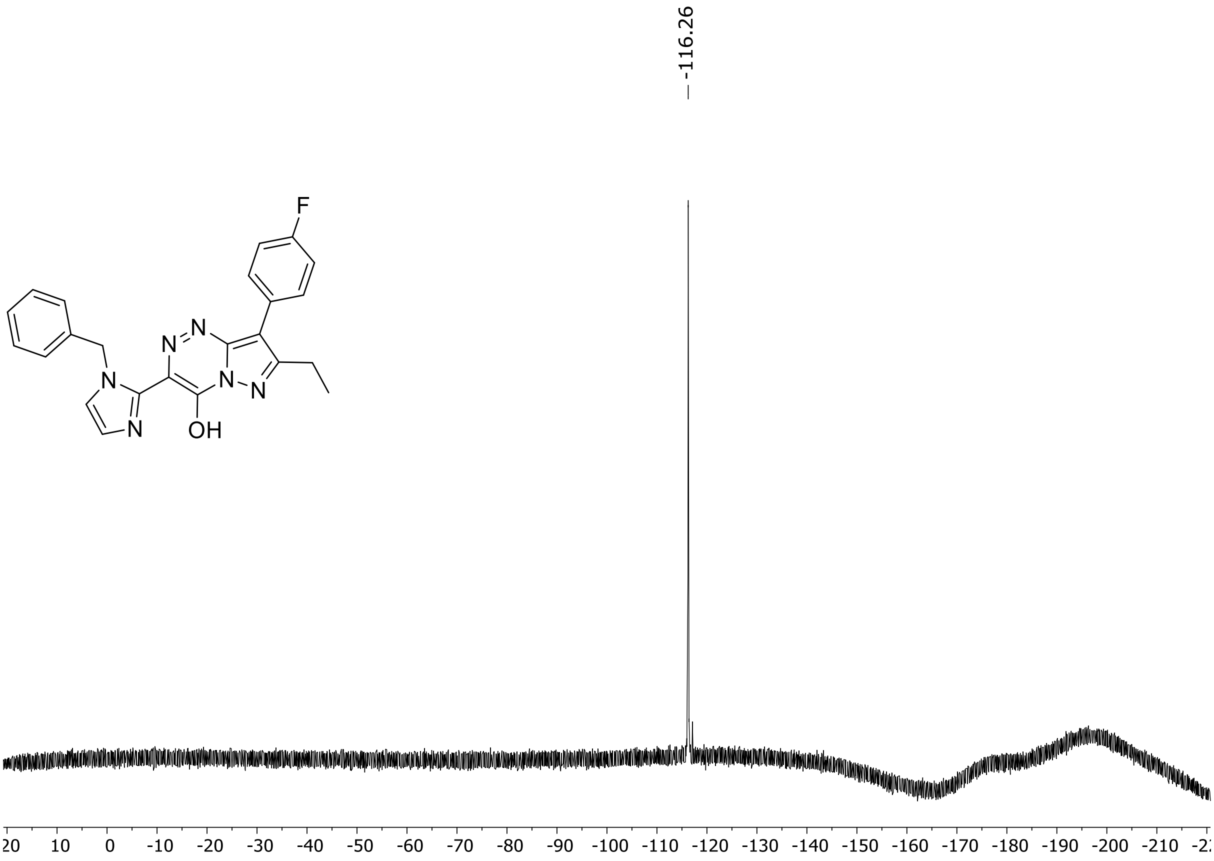

HRMS spectra of **105**

$C_{23}H_{19}FN_6O$ **exact mass: 414.1604**

APCI + (MMI)
nitrogen flow 3 L/min, gas temperature 325°C, nebulizer 45 psig, skimmer 65 V, vaporizer 200°C, fragmentor 15 V, dissolved in methanol

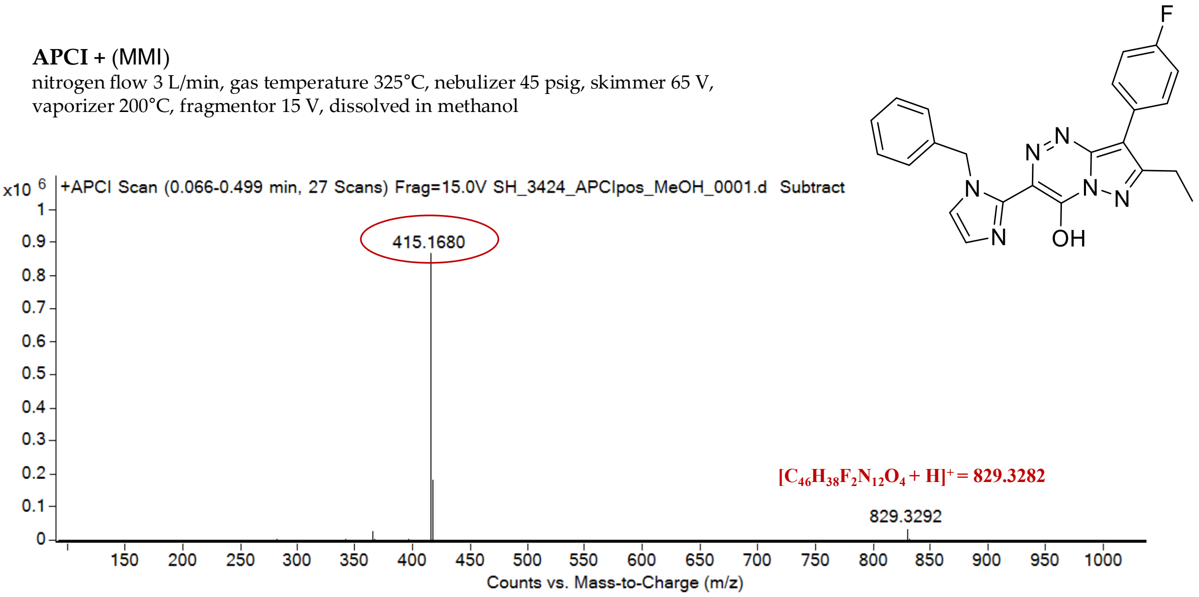

expected mass: $[M+H]^+ = 415.1677$ observed mass: $[M+H]^+ = 415.1680$ mass accuracy = 0.7 ppm

^{1}H (500 MHz) and ^{13}C NMR (126 MHz) spectra of **106** in DMSO-d_6

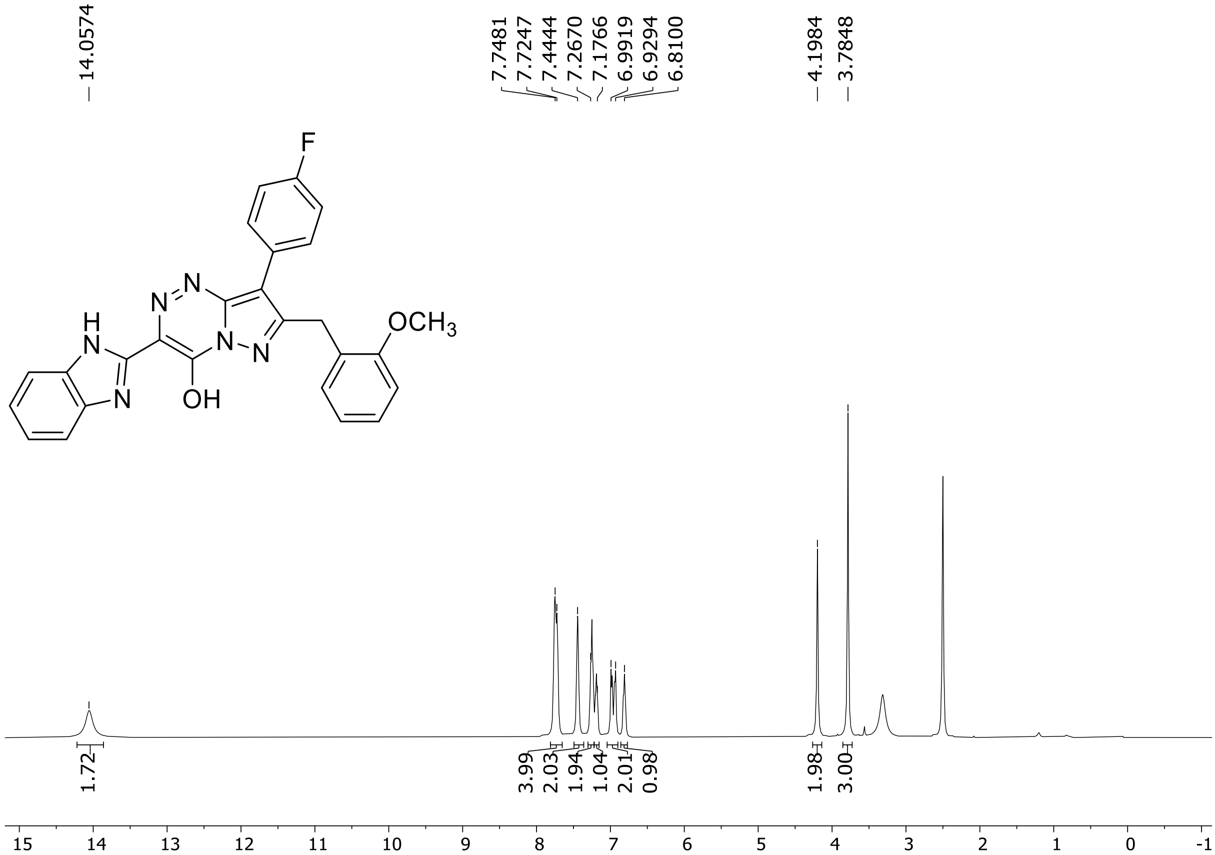

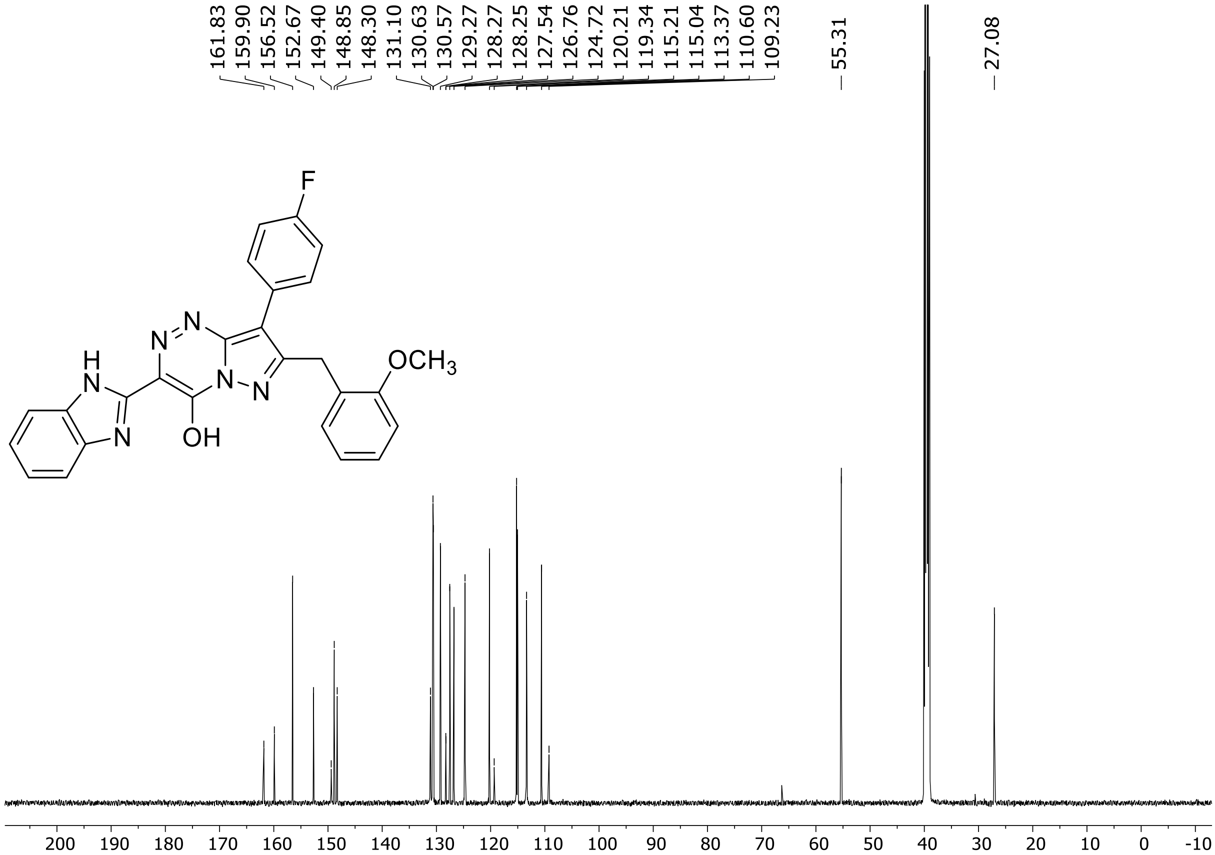

^{19}F (471 MHz) NMR spectrum of **106** in DMSO-d_6

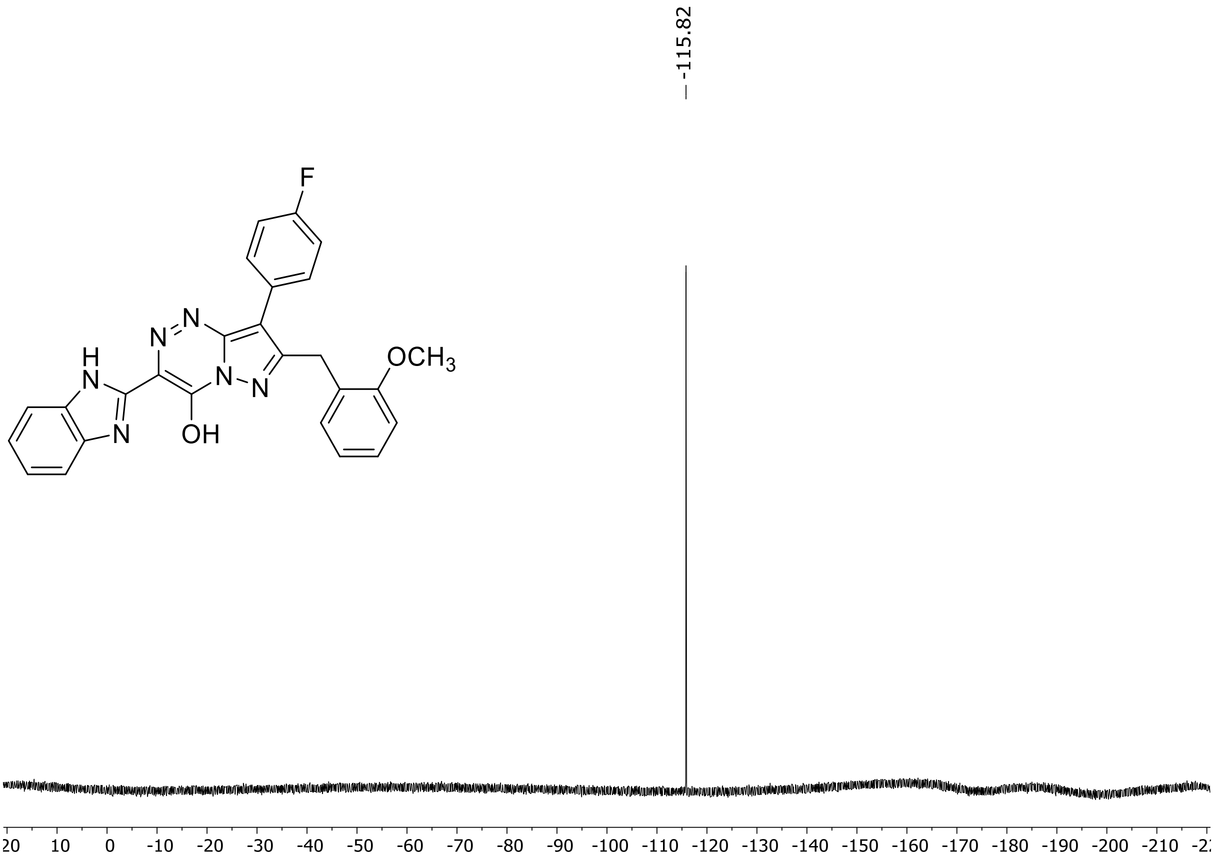

HRMS spectrum of **106**

$C_{26}H_{19}FN_6O_2$ **exact mass: 466.1554**

APCI + (MMI)
nitrogen flow 3 L/min, gas temperature 325°C, nebulizer 45 psig, skimmer 65 V, vaporizer 200°C, fragmentor 20 V, dissolved in methanol

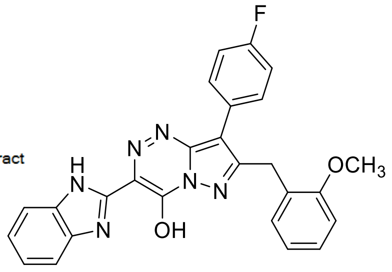

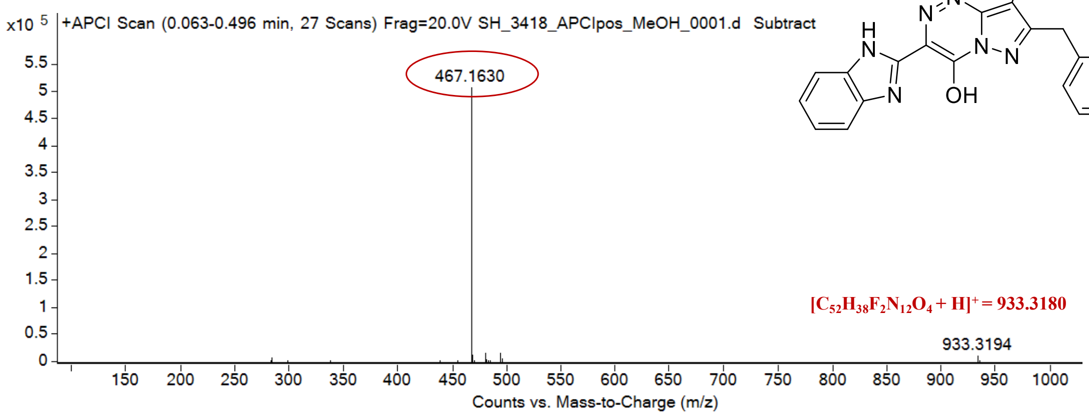

expected mass: $[M+H]^+ = 467.1626$ observed mass: $[M+H]^+ = 467.1630$ mass accuracy = 0.9 ppm

^{1}H (500 MHz) and ^{13}C NMR (126 MHz) spectra of **107** in DMSO-d_6

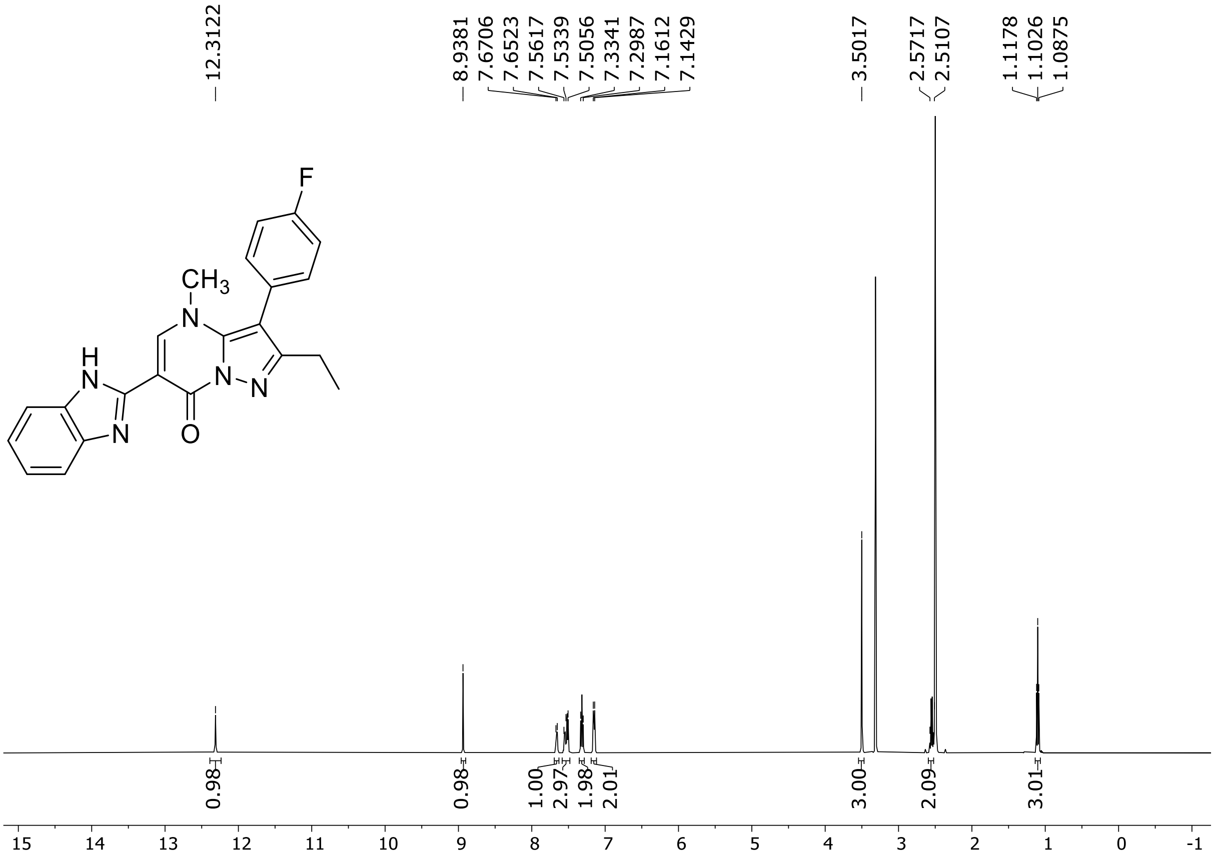

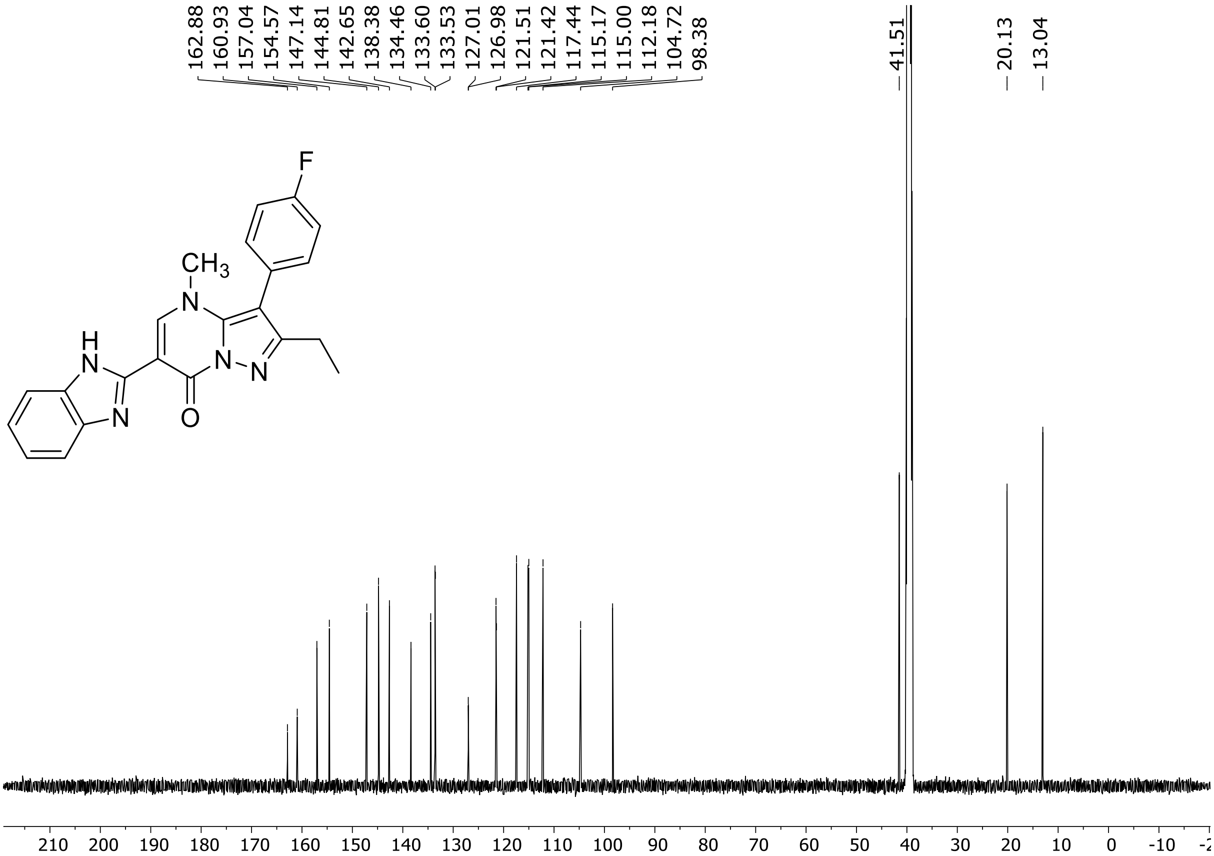

^{19}F (471 MHz) NMR spectrum of **107** in DMSO-d_6

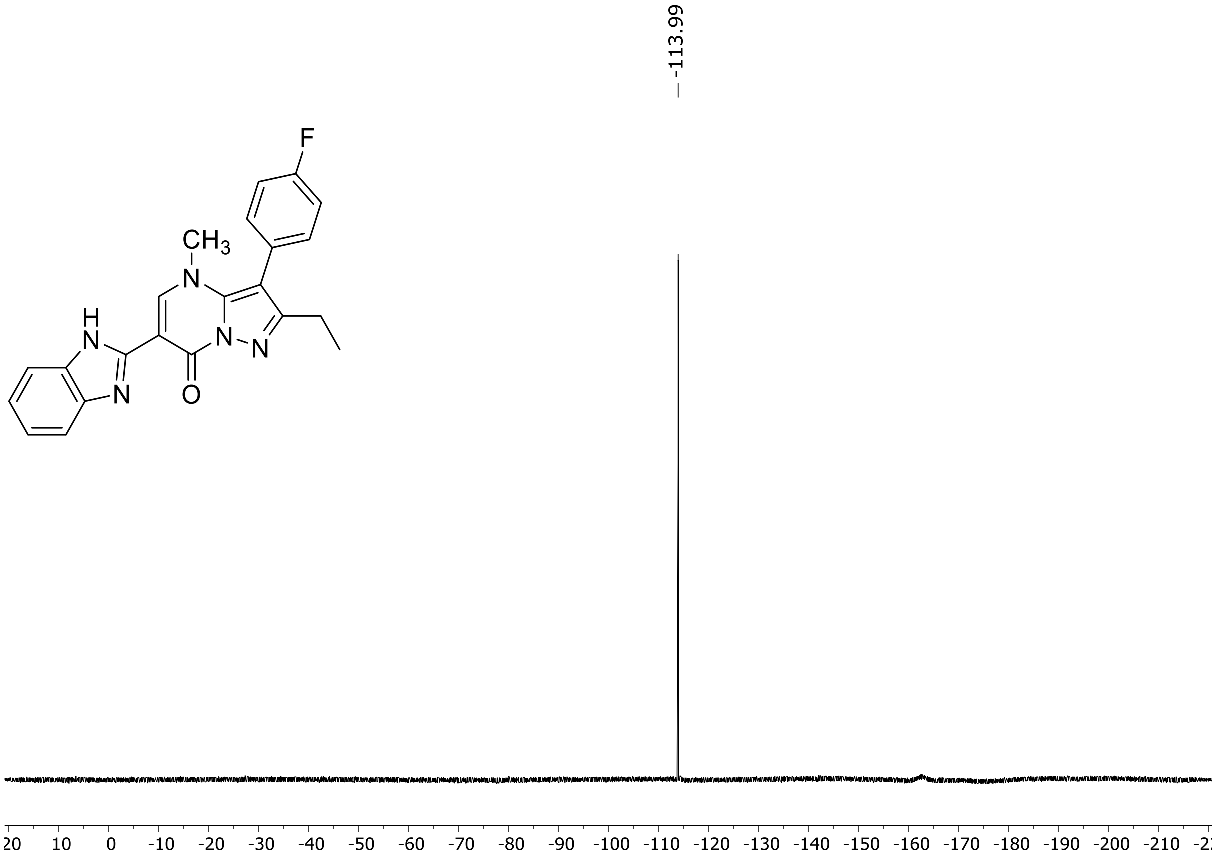

HRMS spectrum of **107**

$C_{22}H_{18}FN_5O$ **exact mass: 387.1495**

APCI + (MMI)
nitrogen flow 5 L/min, gas temperature 325°C, nebulizer 45 psig,
skimmer 65 V, vaporizer 200°C, fragmentor 15 V, dissolved in methanol

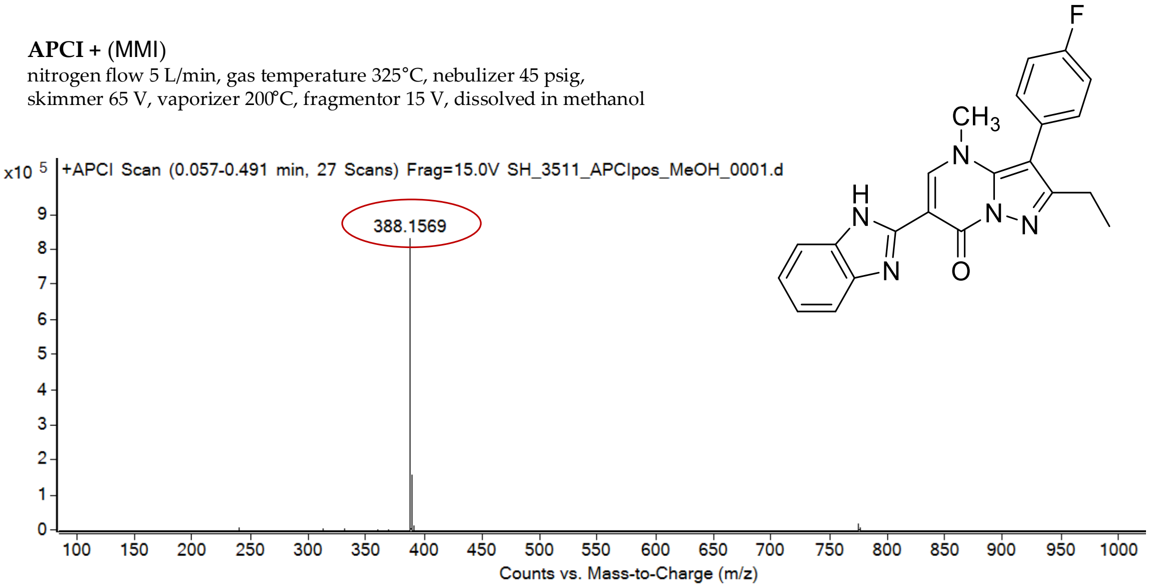

expected mass: $[M+H]^+$ = 388.1568 observed mass: $[M+H]^+$ = 388.1569 mass accuracy = 0.3 ppm

^{1}H (500 MHz) and ^{13}C NMR (126 MHz) spectra of **108** in DMSO-d_6

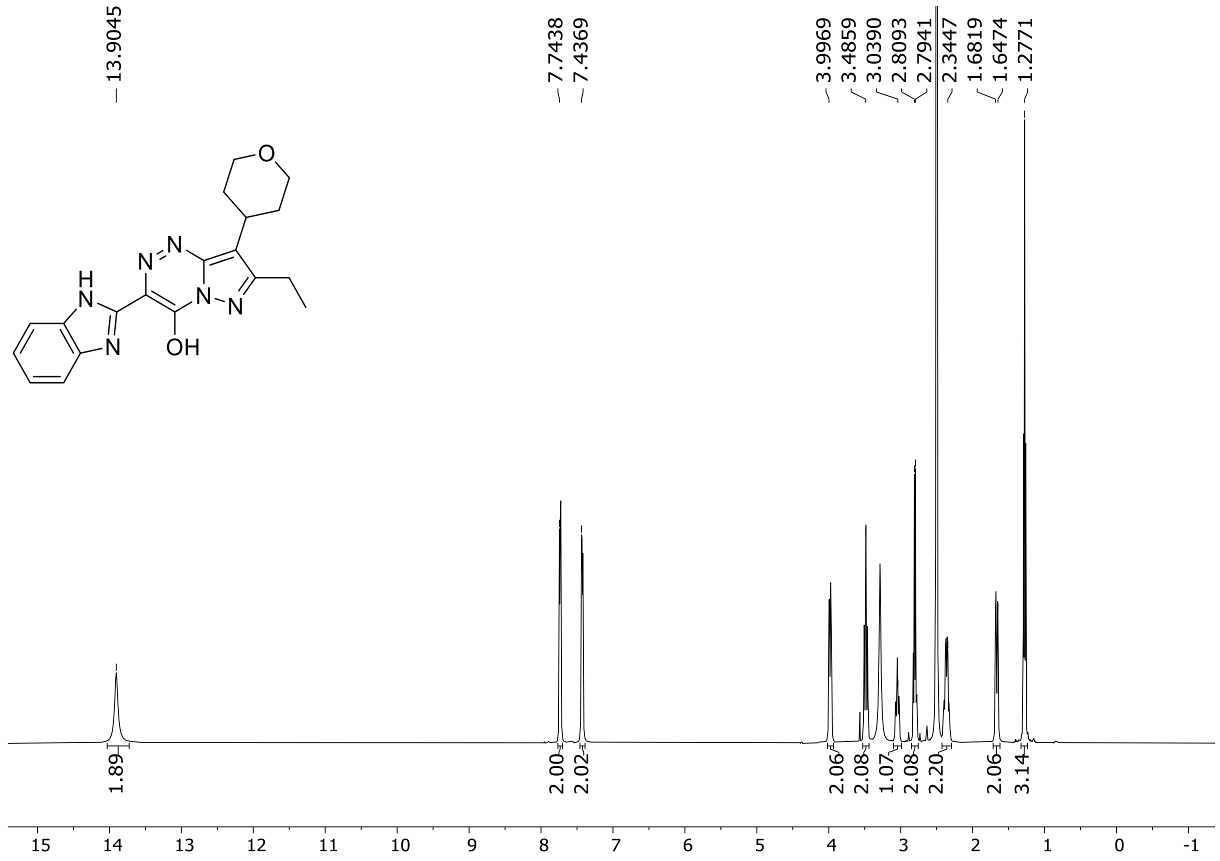

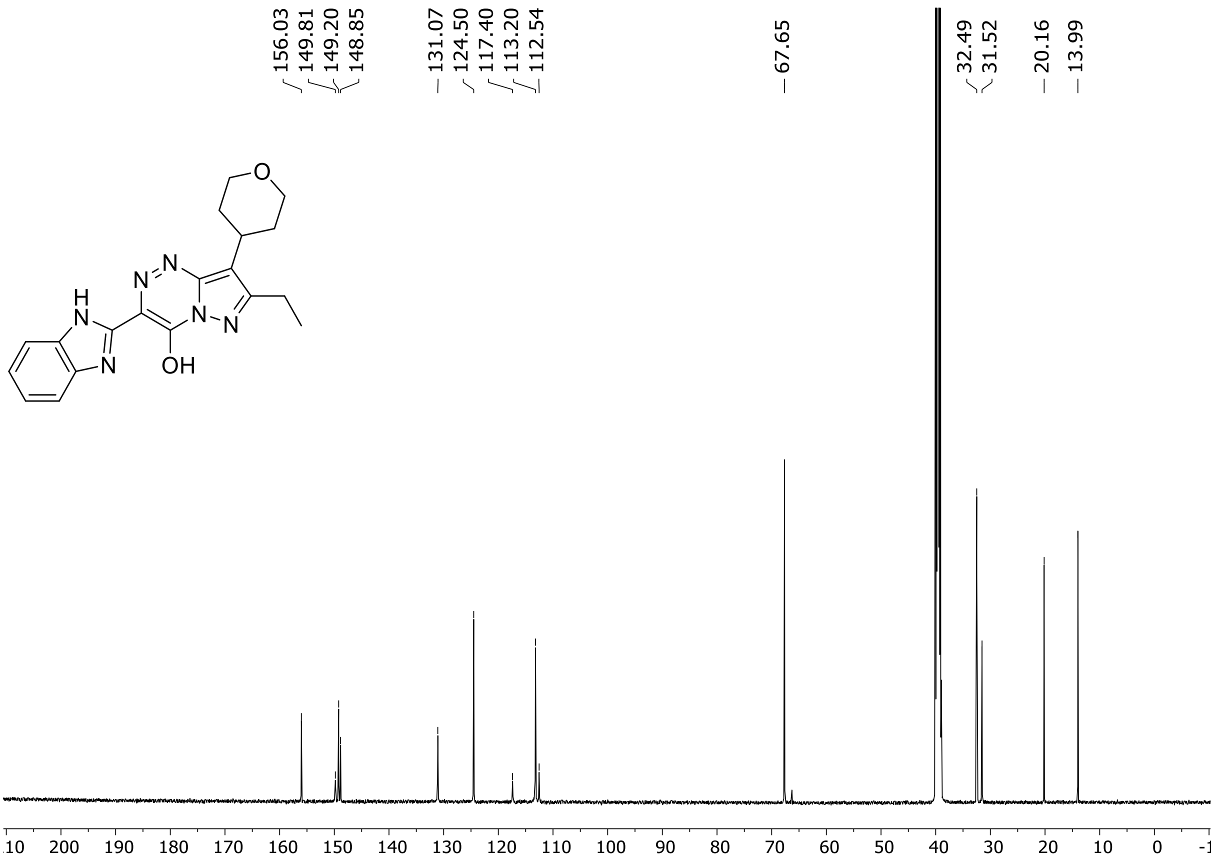

HRMS spectrum of **108**

$C_{19}H_{20}N_6O_2$ **exact mass: 364.1648**

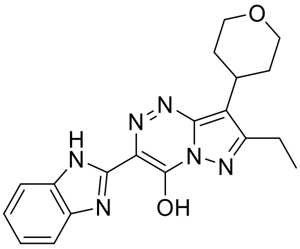

APCI + (MMI)
nitrogen flow 5 L/min, gas temperature 325°C, nebulizer 45 psig, skimmer 65 V, vaporizer 200°C, fragmentor 20 V, dissolved in methanol

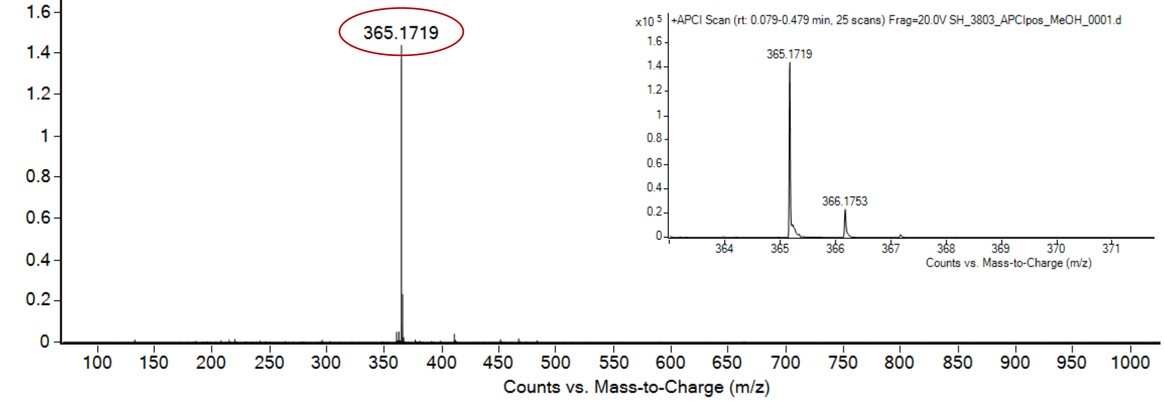

expected mass: $[M+H]^+$ = 365.1721 observed mass : $[M+H]^+$ = 365.1719 mass accuracy = - 0.6 ppm

^{1}H (500 MHz) and ^{13}C NMR (126 MHz) spectra of **109** in DMSO-d_6

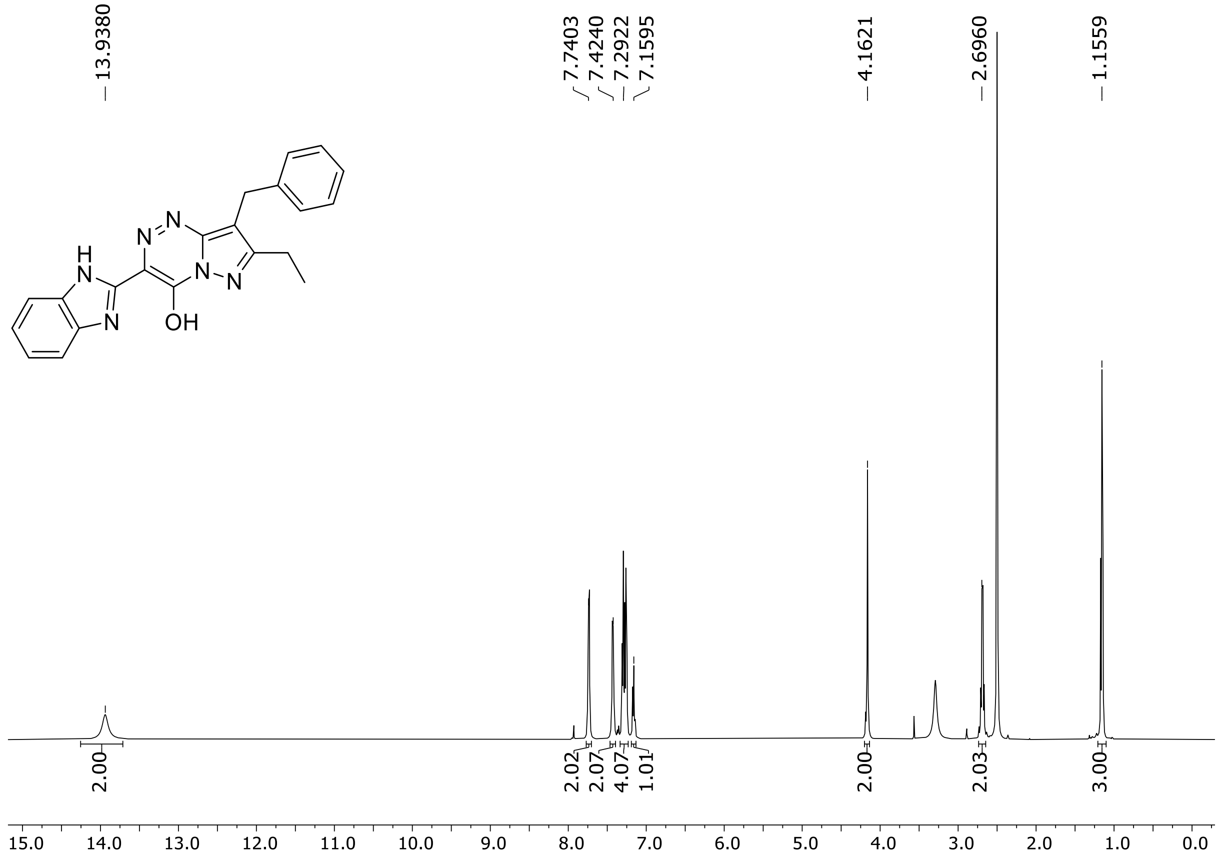

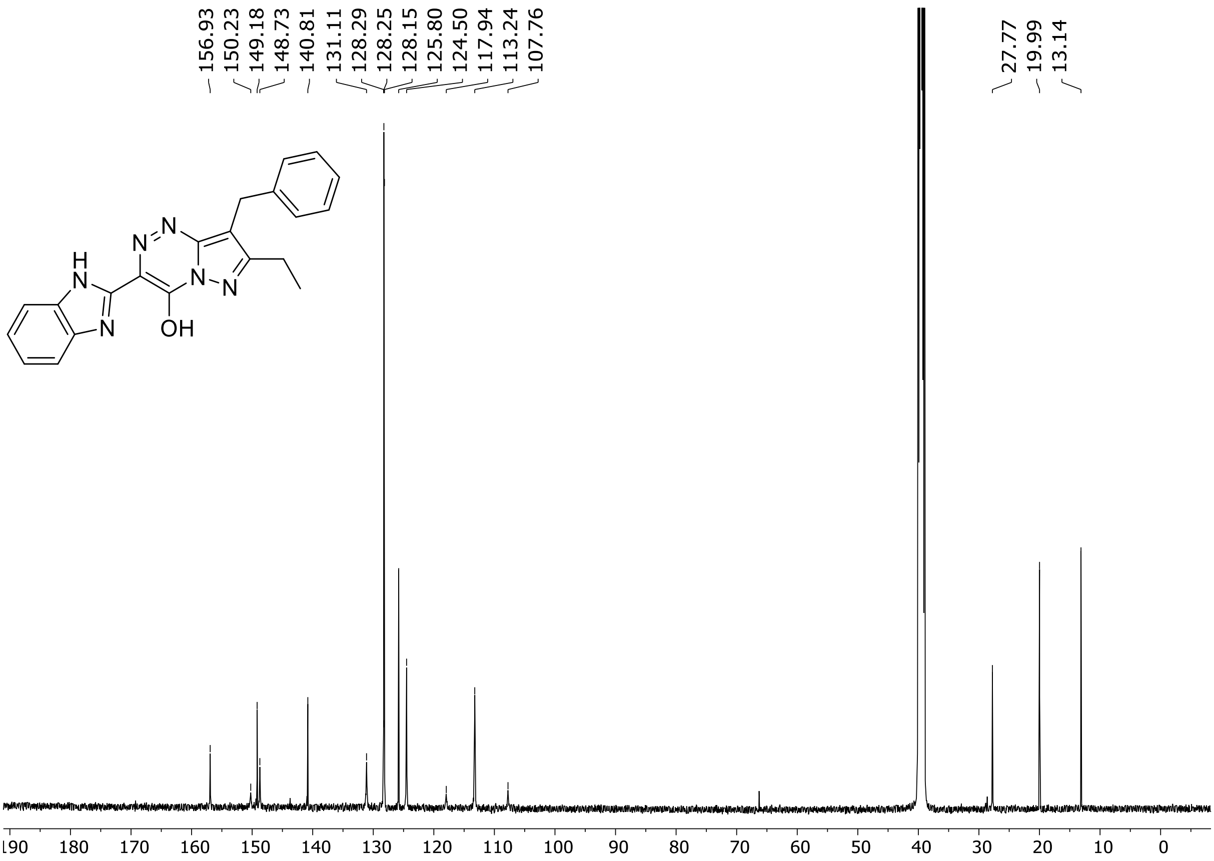

HRMS spectrum of **109**

$C_{21}H_{18}N_6O$ **exact mass: 370.1542**

APCI + (MMI)
nitrogen flow 5 L/min, gas temperature 325°C, nebulizer 45 psig, skimmer 65 V, vaporizer 200°C, fragmentor 20 V, dissolved in methanol

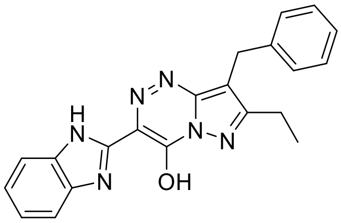

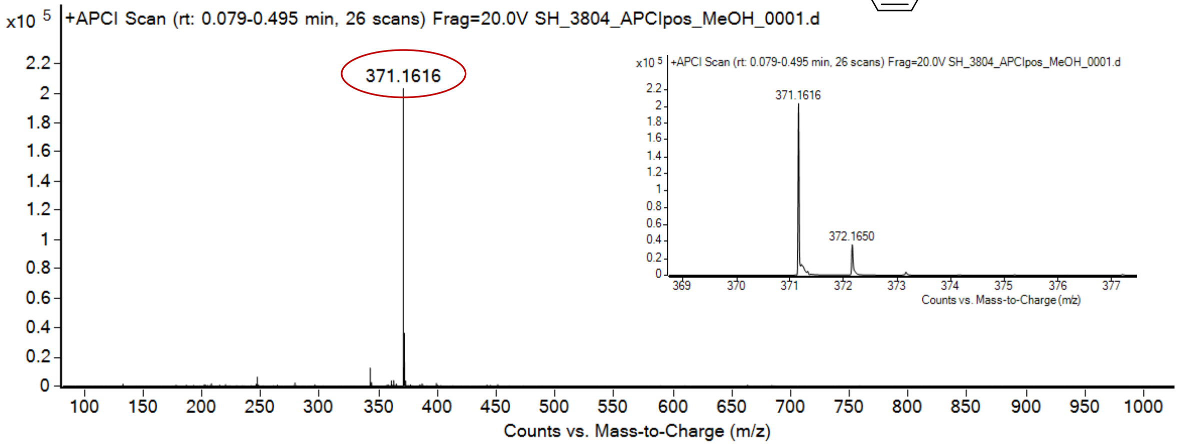

expected mass: $[M+H]^+$ = 371.1615 observed mass : $[M+H]^+$ = 371.1616 mass accuracy = 0.3 ppm

^{1}H (500 MHz) and ^{13}C NMR (126 MHz) spectra of **110** in DMSO-d_6

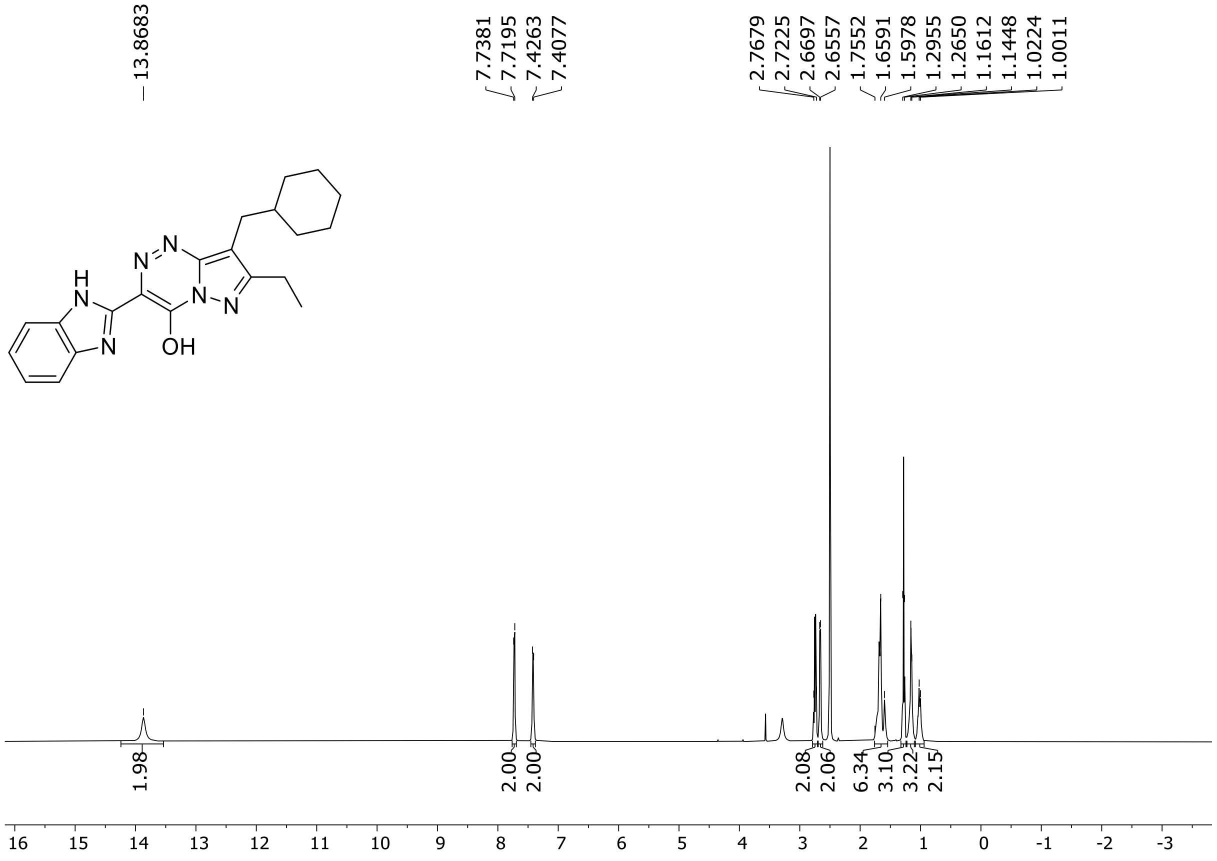

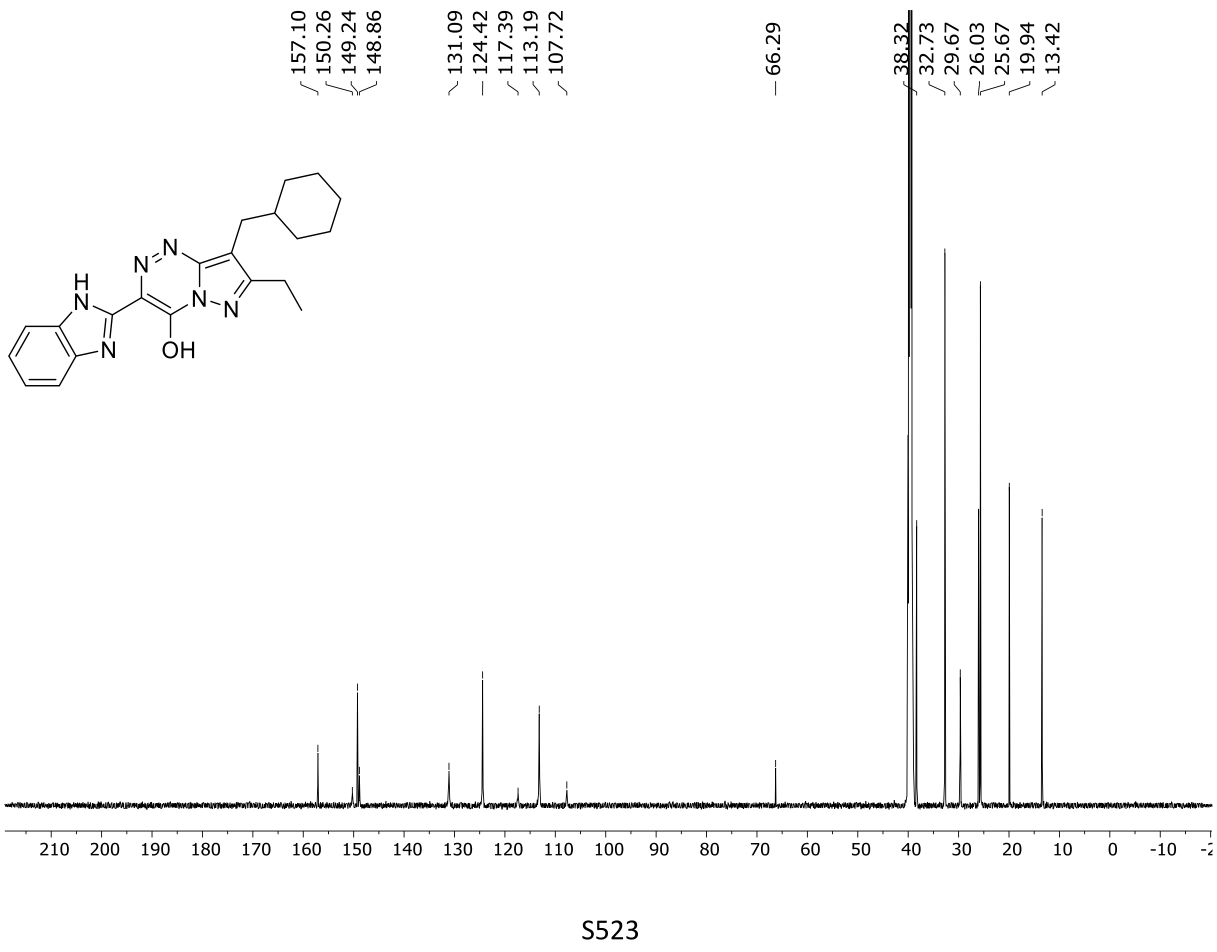

HRMS spectrum of **110**

$C_{21}H_{24}N_6O$ **exact mass: 376.2012**

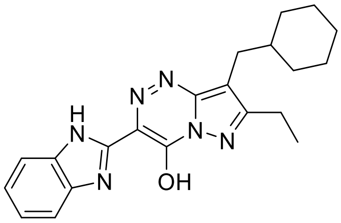

APCI + (MMI)
nitrogen flow 5 L/min, gas temperature 325°C, nebulizer 45 psig, skimmer 65 V, vaporizer 200°C, fragmentor 20 V, dissolved in methanol

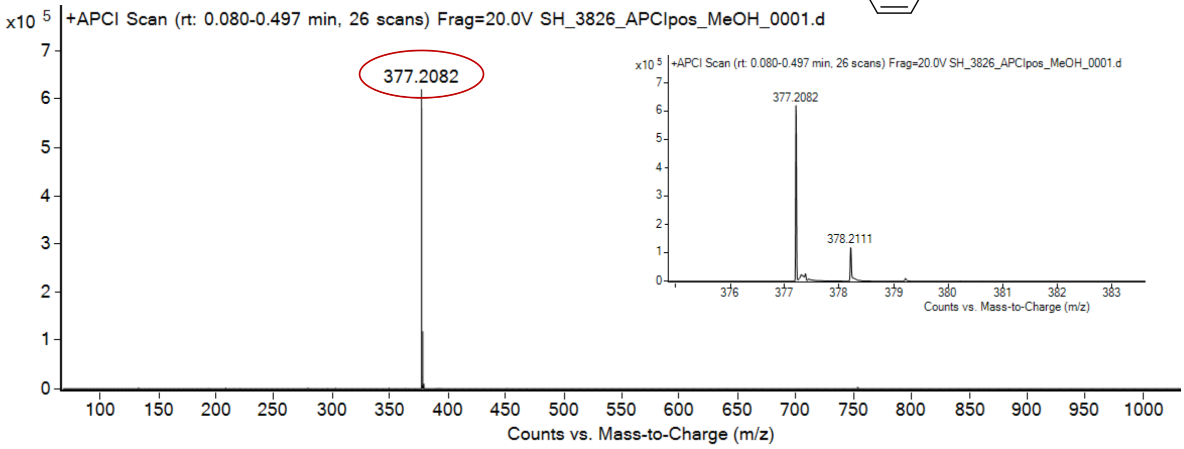

expected mass: $[M+H]^+$ = 377.2084 observed mass : $[M+H]^+$ = 377.2082 mass accuracy = - 0.5 ppm

^{1}H (500 MHz) and ^{13}C NMR (126 MHz) spectra of **111** in DMSO-d_6

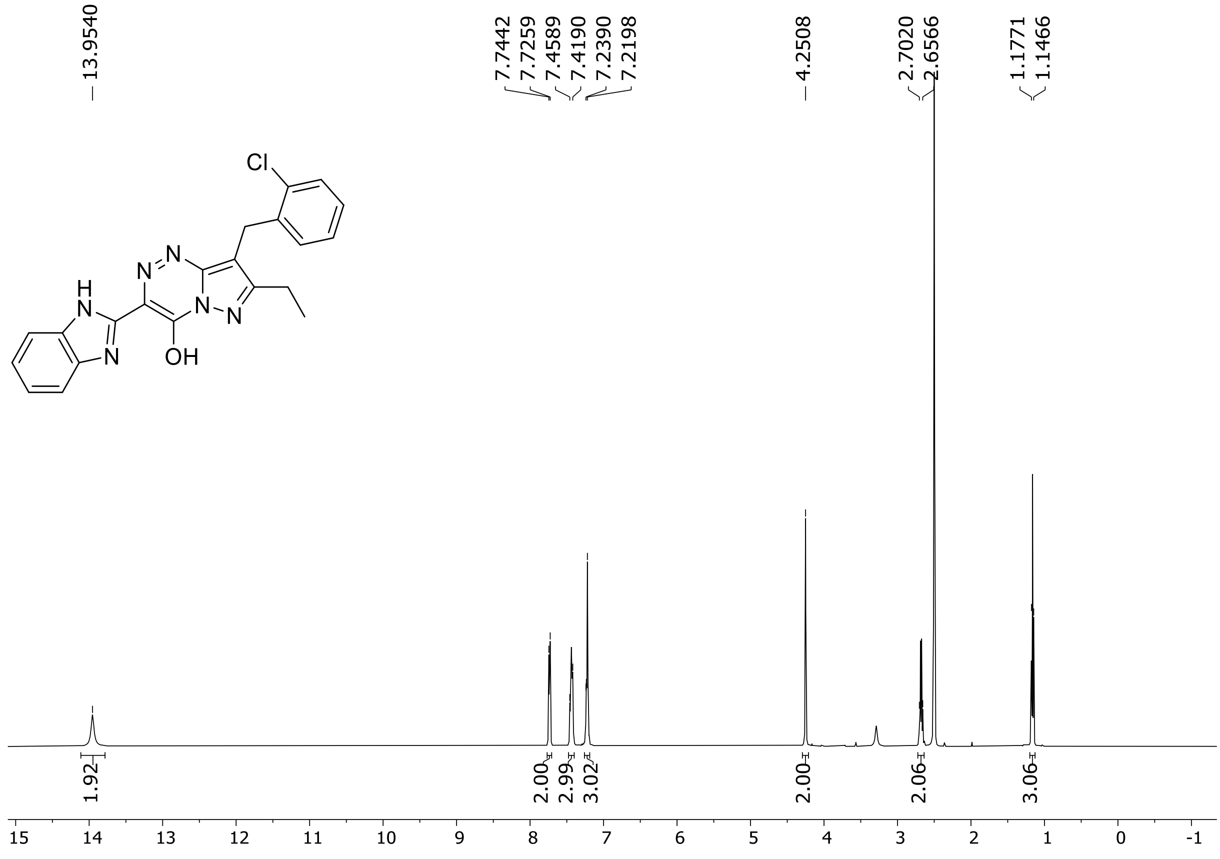

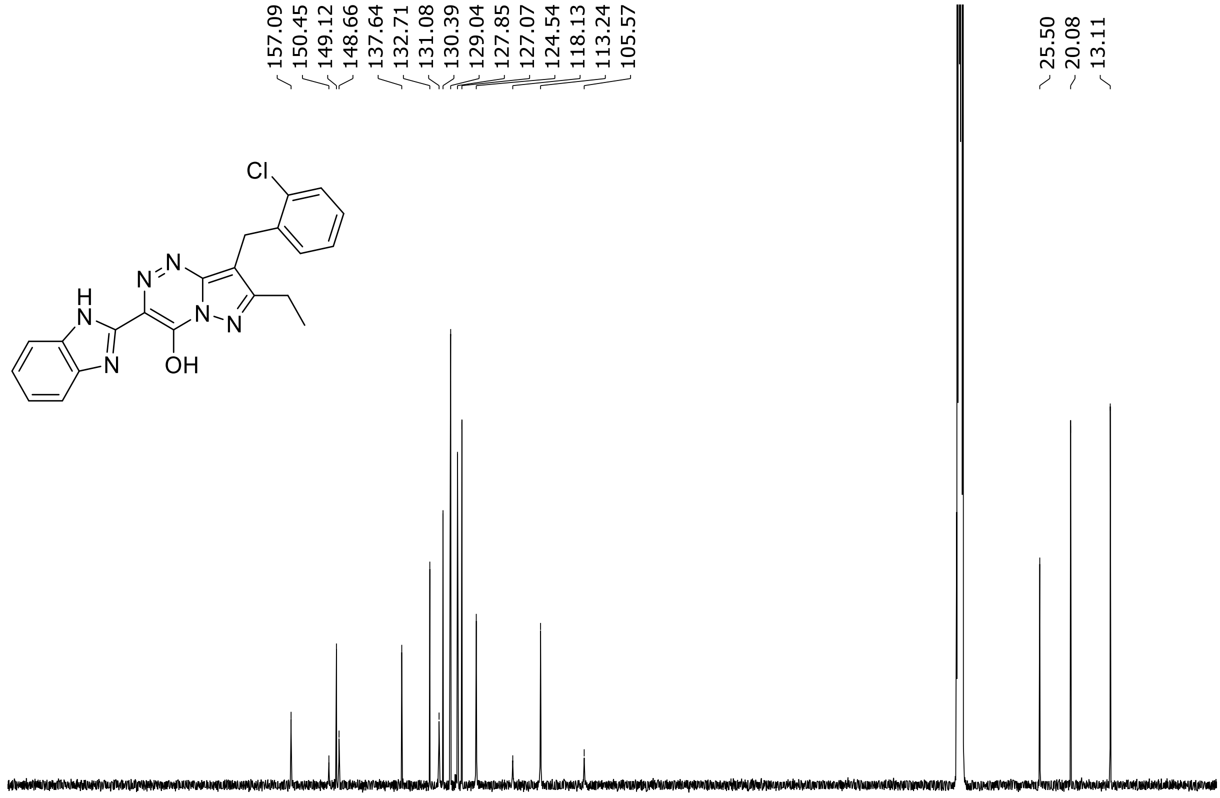

HRMS spectrum of **111**

$C_{21}H_{17}ClN_6O$ exact mass: 404.1152

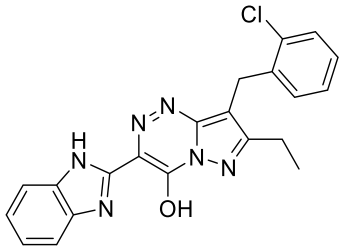

APCI + (MMI)
nitrogen flow 5 L/min, gas temperature 325°C, nebulizer 45 psig, skimmer 65 V, vaporizer 200°C, fragmentor 25 V, dissolved in methanol

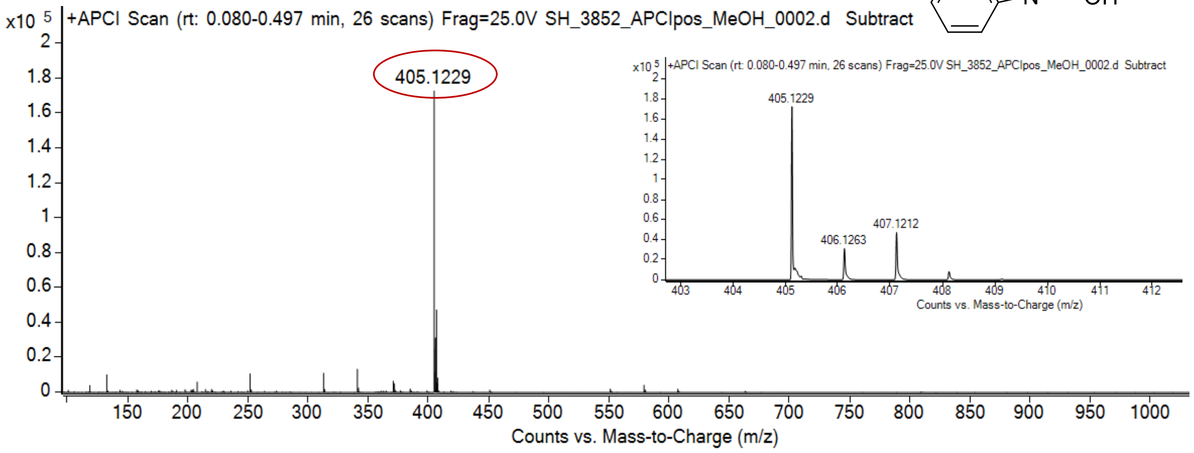

expected mass: [M+H]$^+$ = 405.1225 observed mass : [M+H]$^+$ = 405.1229 mass accuracy = 1.0 ppm

^{1}H (500 MHz) and ^{13}C NMR (126 MHz) spectra of **112** in DMSO-d_6

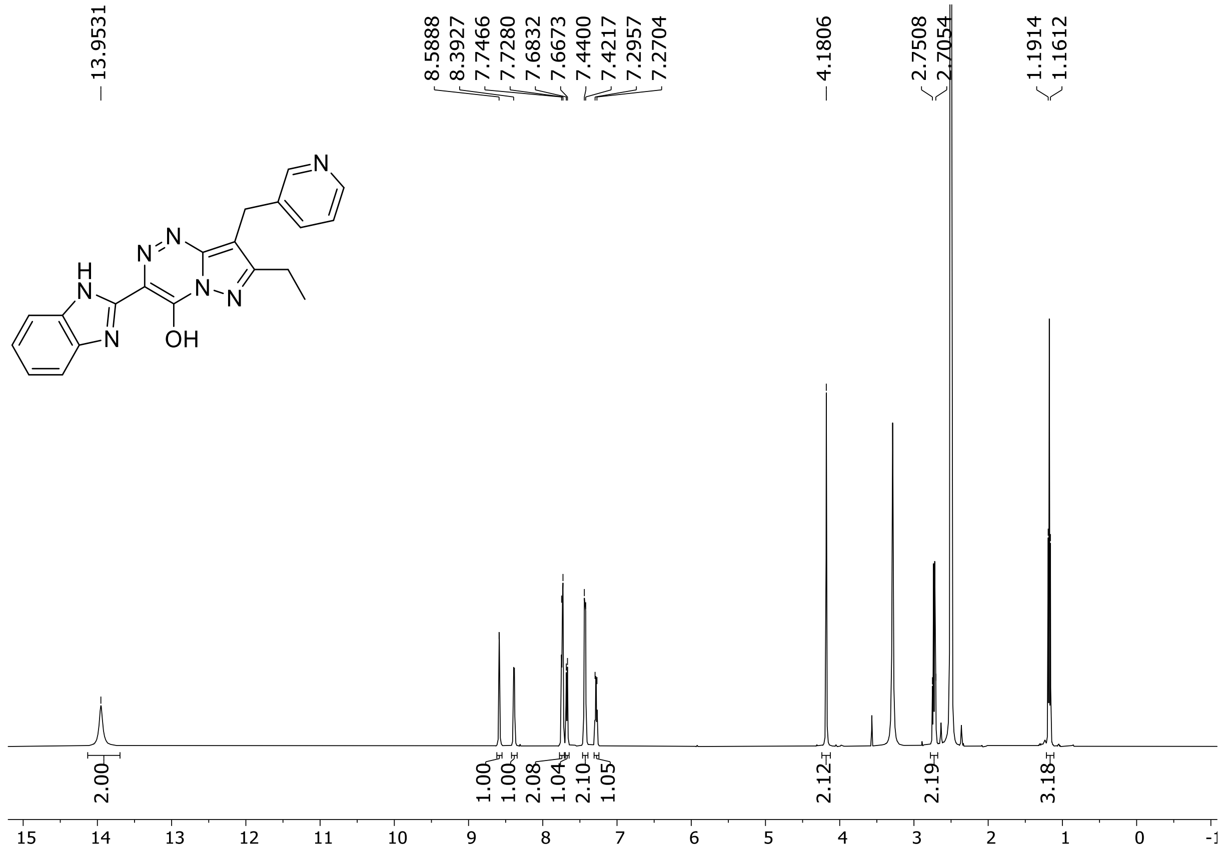

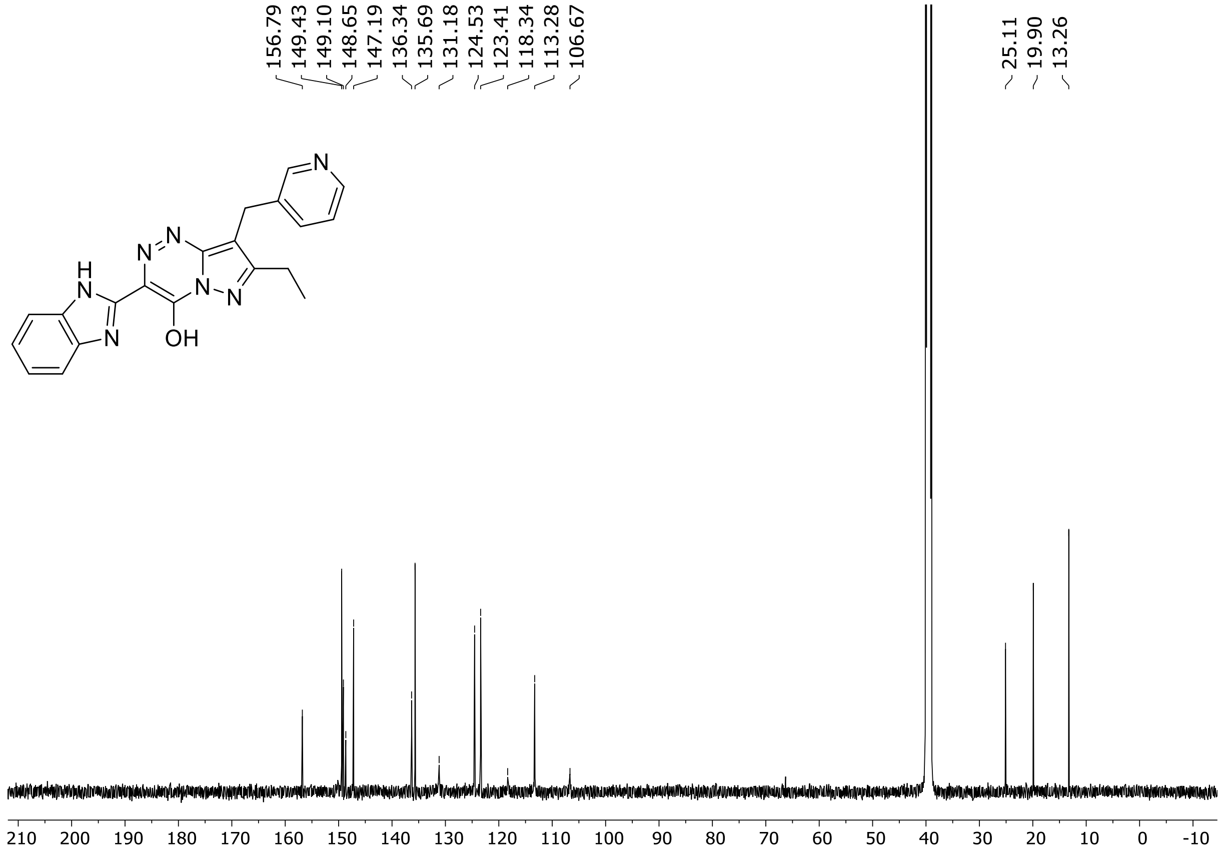

HRMS spectrum of **112**

$C_{20}H_{17}N_7O$ **exact mass: 371.1495**

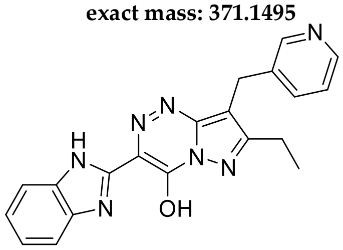

APCI + (MMI)
nitrogen flow 5 L/min, gas temperature 325°C, nebulizer 45 psig, skimmer 65 V, vaporizer 200°C, fragmentor 20 V, dissolved in methanol

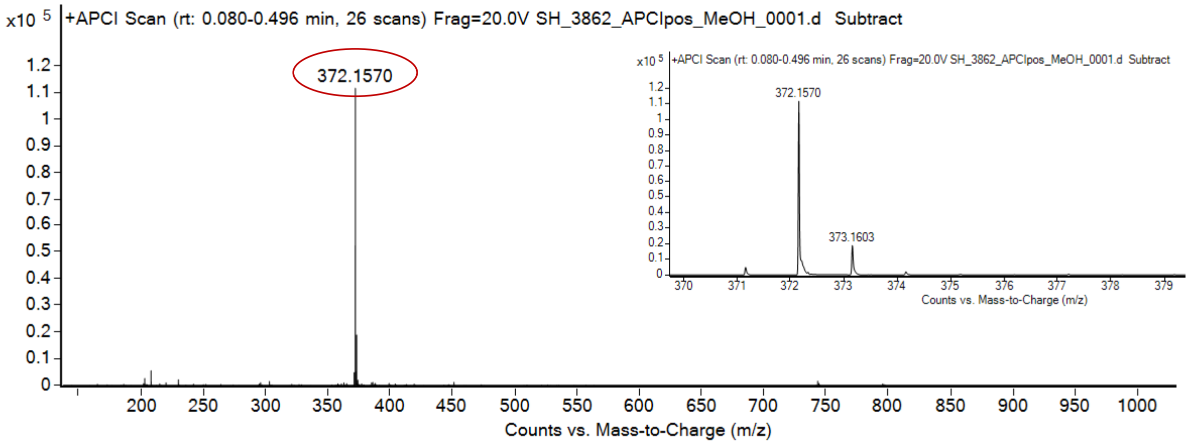

expected mass: [M+H]$^+$ = 372.1567 observed mass : [M+H]$^+$ = 372.1570 mass accuracy = 0.8 ppm

^{1}H (500 MHz) and ^{13}C NMR (126 MHz) spectra of **113** in DMSO-d_6

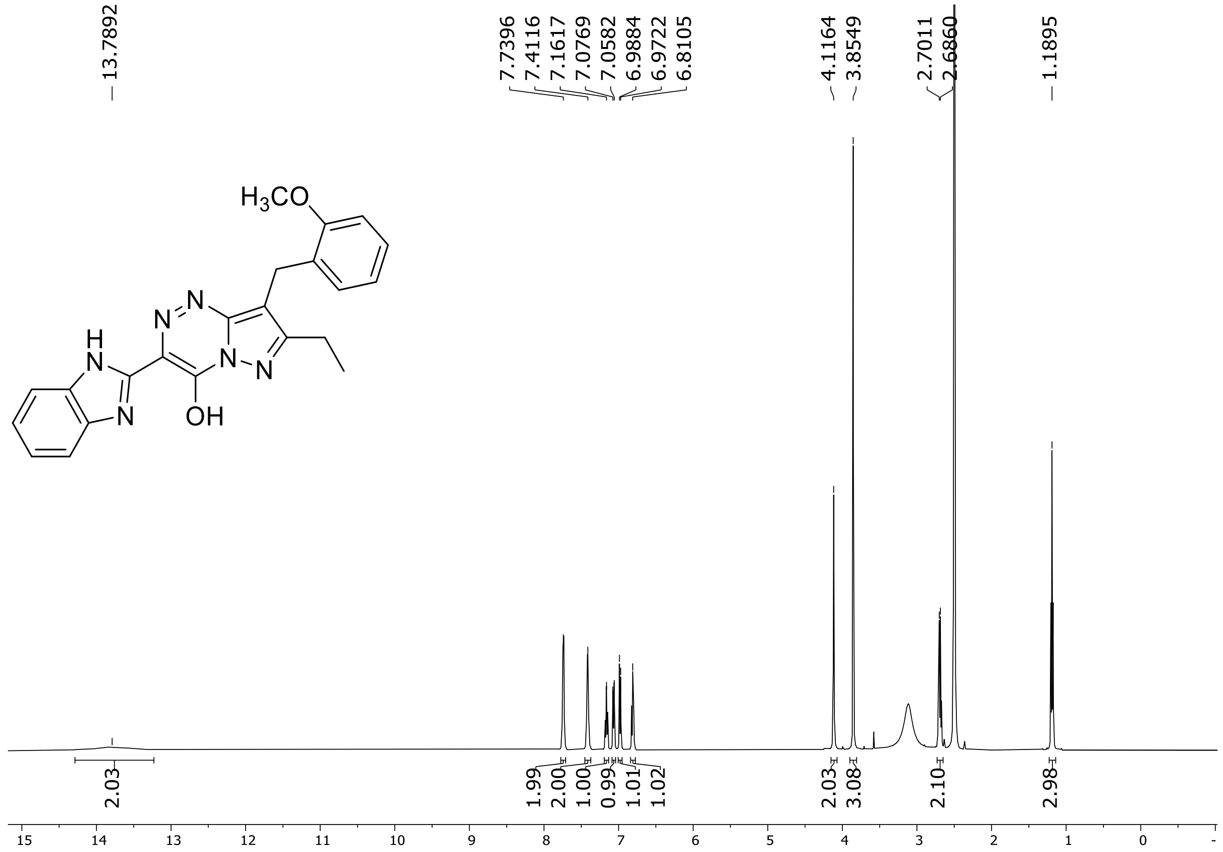

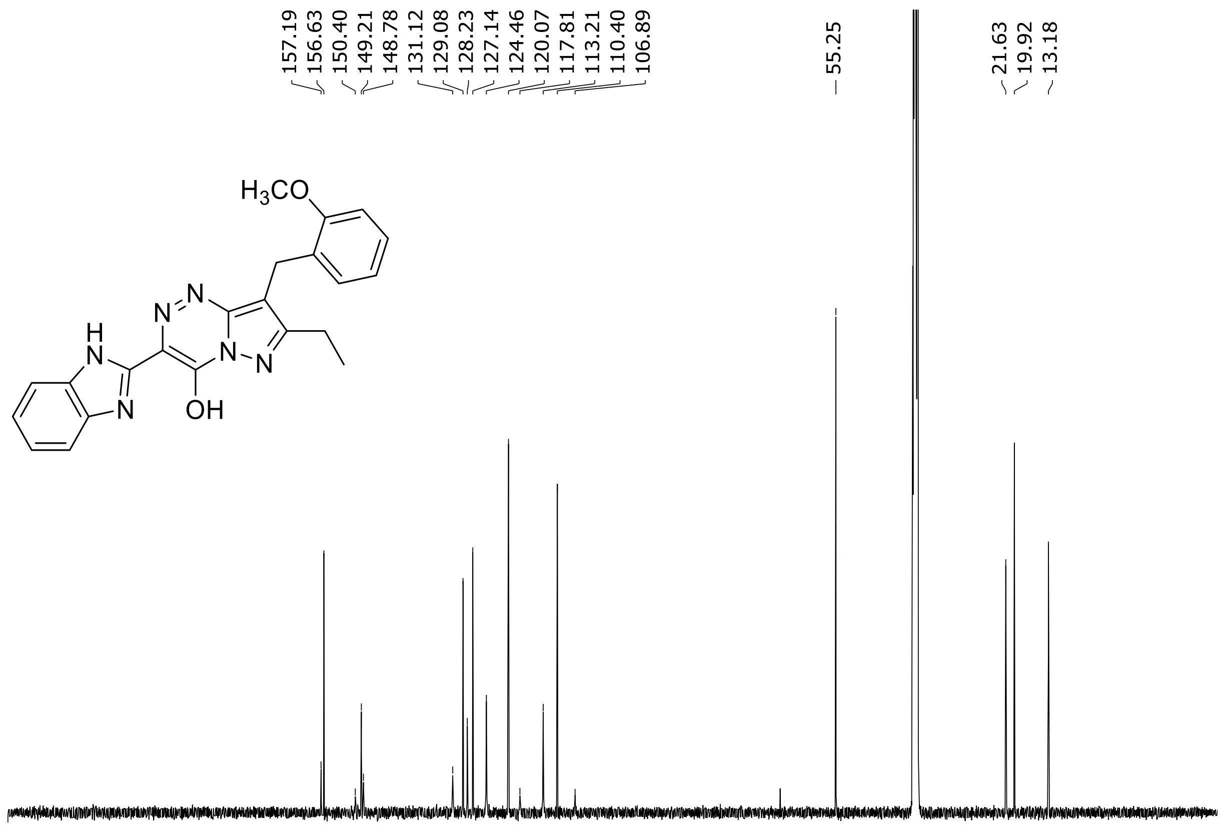

HRMS spectrum of **113**

$C_{22}H_{20}N_6O_2$ **exact mass: 400.1648**

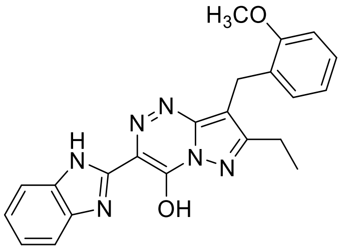

APCI + (MMI)
nitrogen flow 5 L/min, gas temperature 325°C, nebulizer 45 psig, skimmer 65 V, vaporizer 200°C, fragmentor 20 V, dissolved in methanol

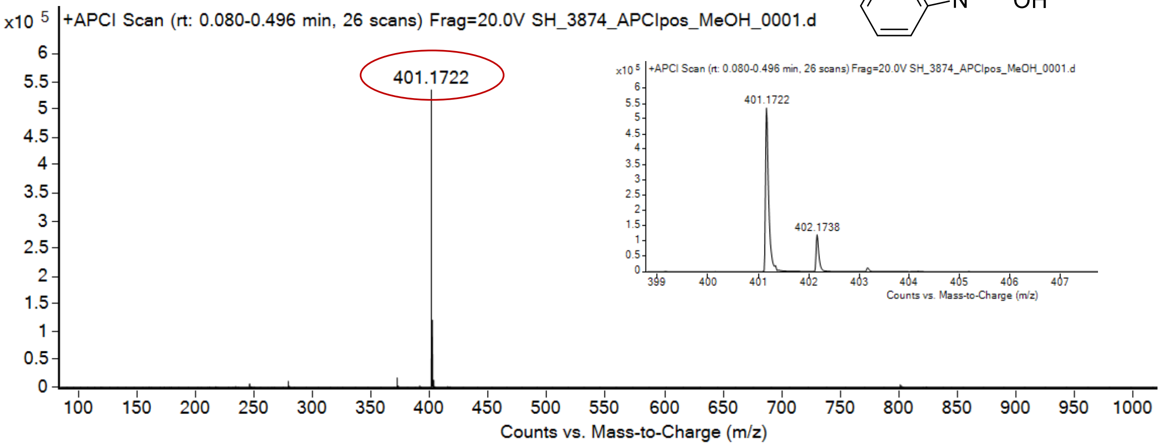

expected mass: $[M+H]^+$ = 401.1721 observed mass: $[M+H]^+$ = 401.1722 mass accuracy = 0.2 ppm

^{1}H (500 MHz) and ^{13}C NMR (126 MHz) spectra of **114** in DMSO-d_6

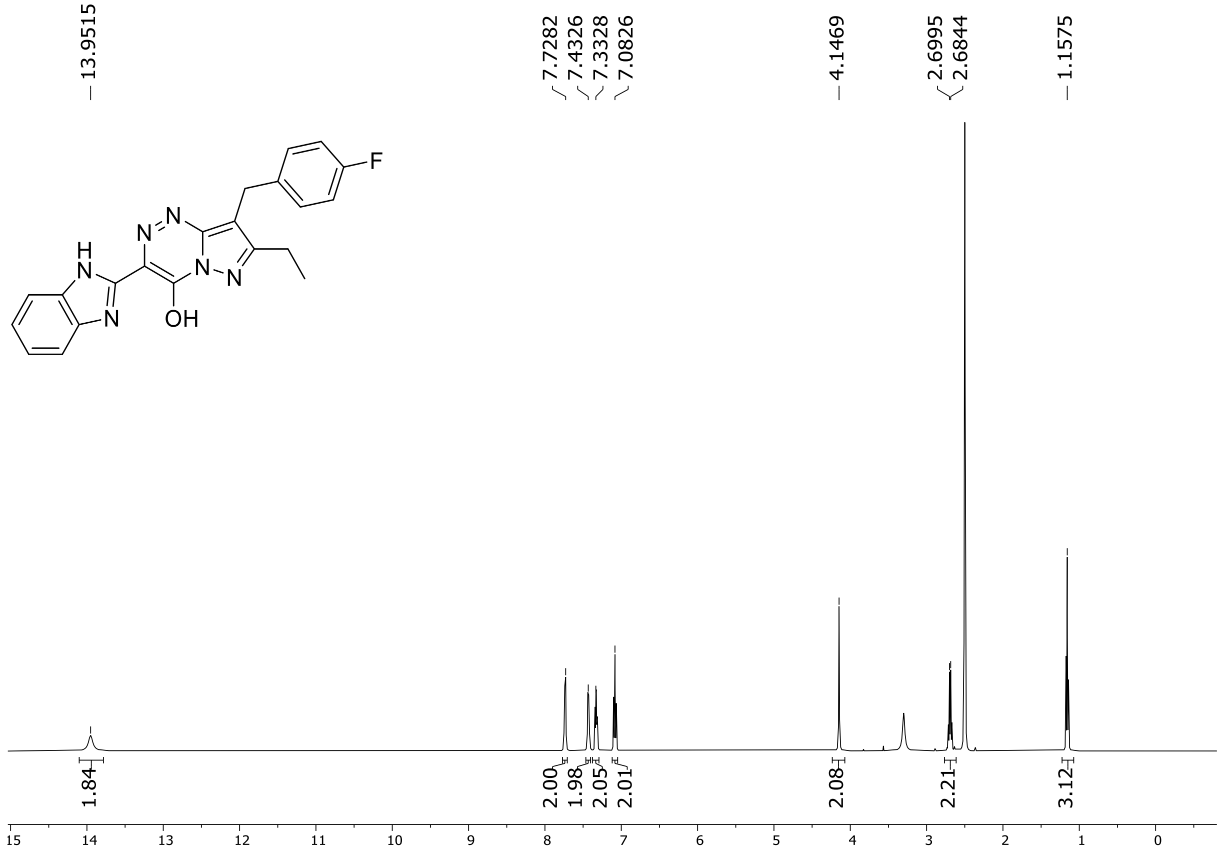

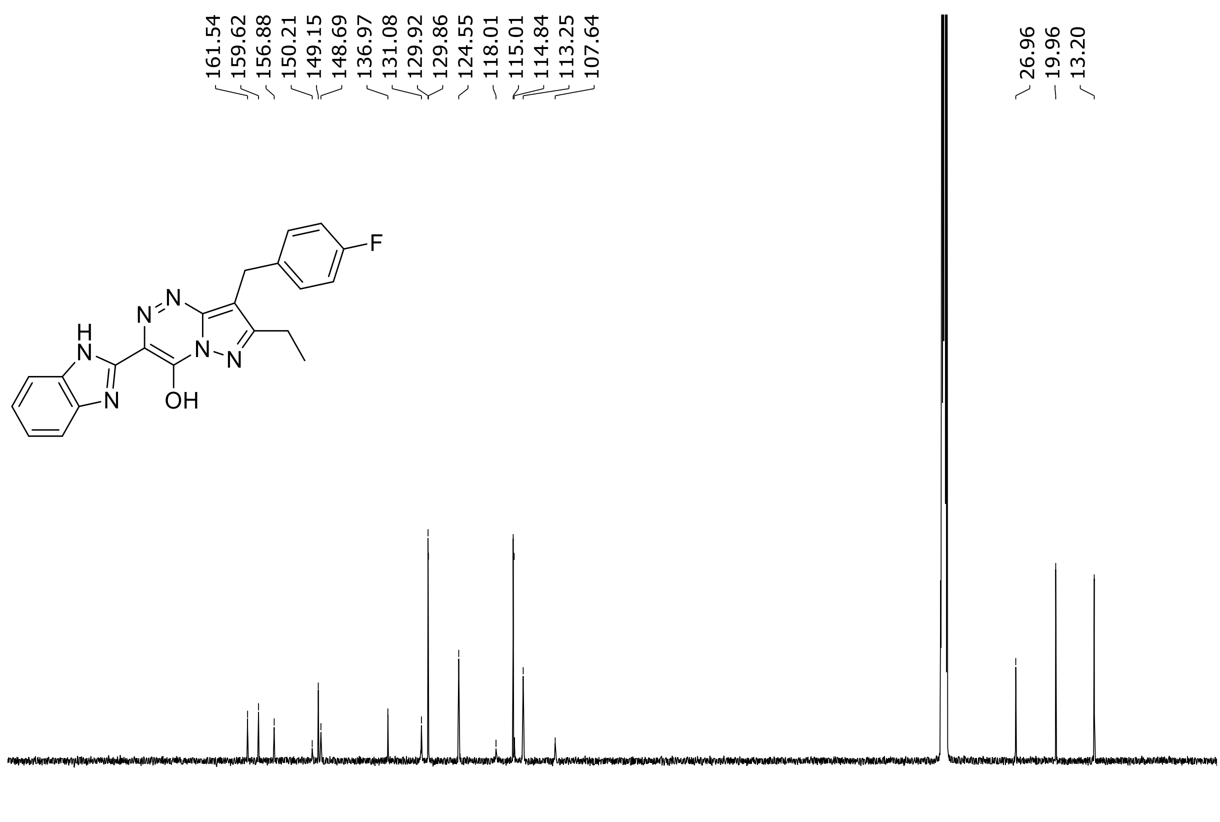

^{19}F (471 MHz) NMR spectrum of **114** in DMSO-d_6

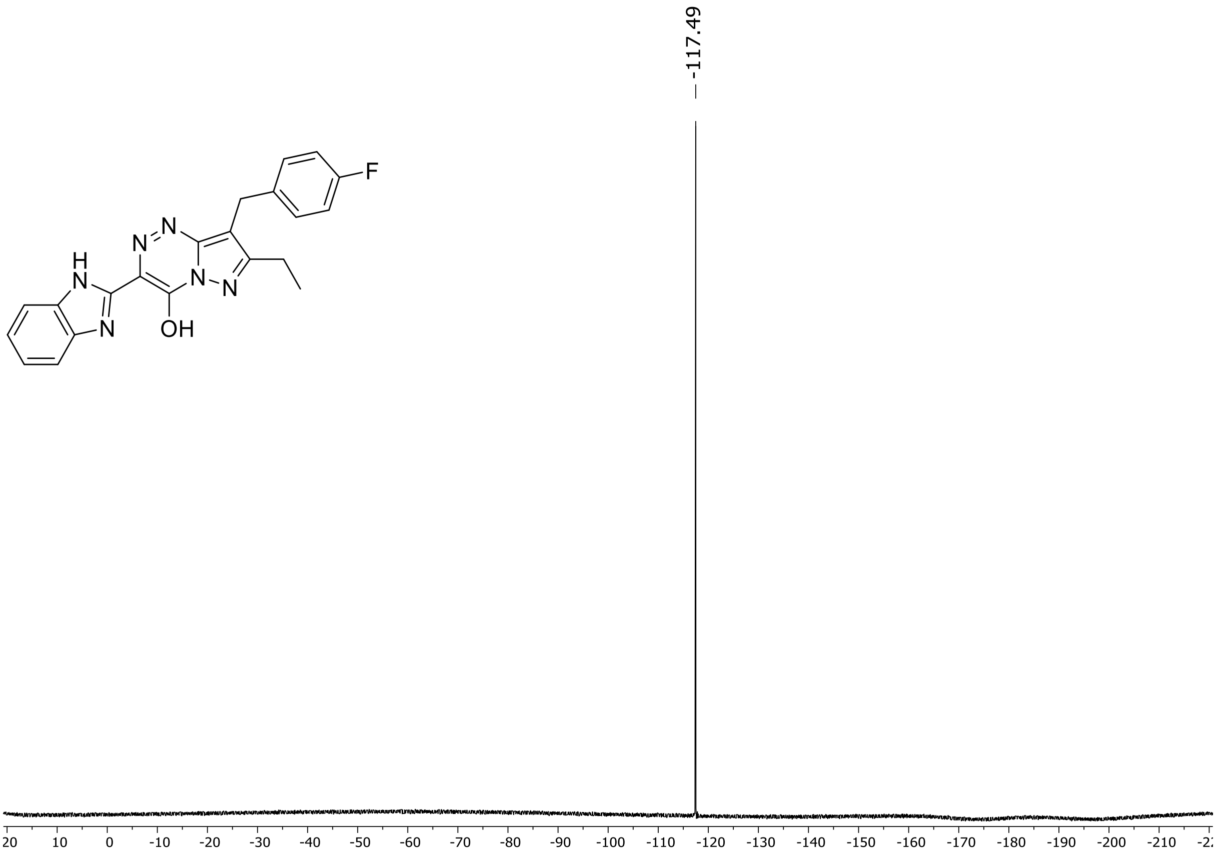

HRMS spectrum of **114**

$C_{21}H_{17}FN_6O$ **exact mass: 388.1448**

APCI + (MMI)
nitrogen flow 5 L/min, gas temperature 325°C, nebulizer 45 psig, skimmer 65 V, vaporizer 200°C, fragmentor 15 V, dissolved in methanol

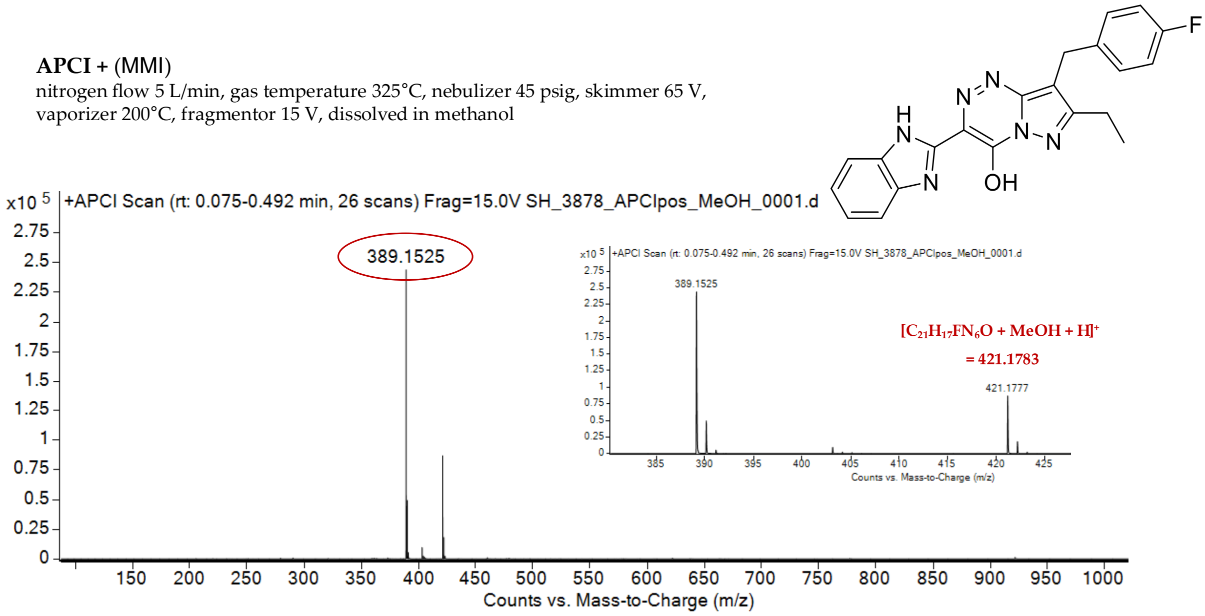

expected mass: $[M+H]^+ = 389.1521$ observed mass: $[M+H]^+ = 389.1525$ mass accuracy = 1.0 ppm

^{1}H (500 MHz) and ^{13}C NMR (126 MHz) spectra of **115** in DMSO-d_6

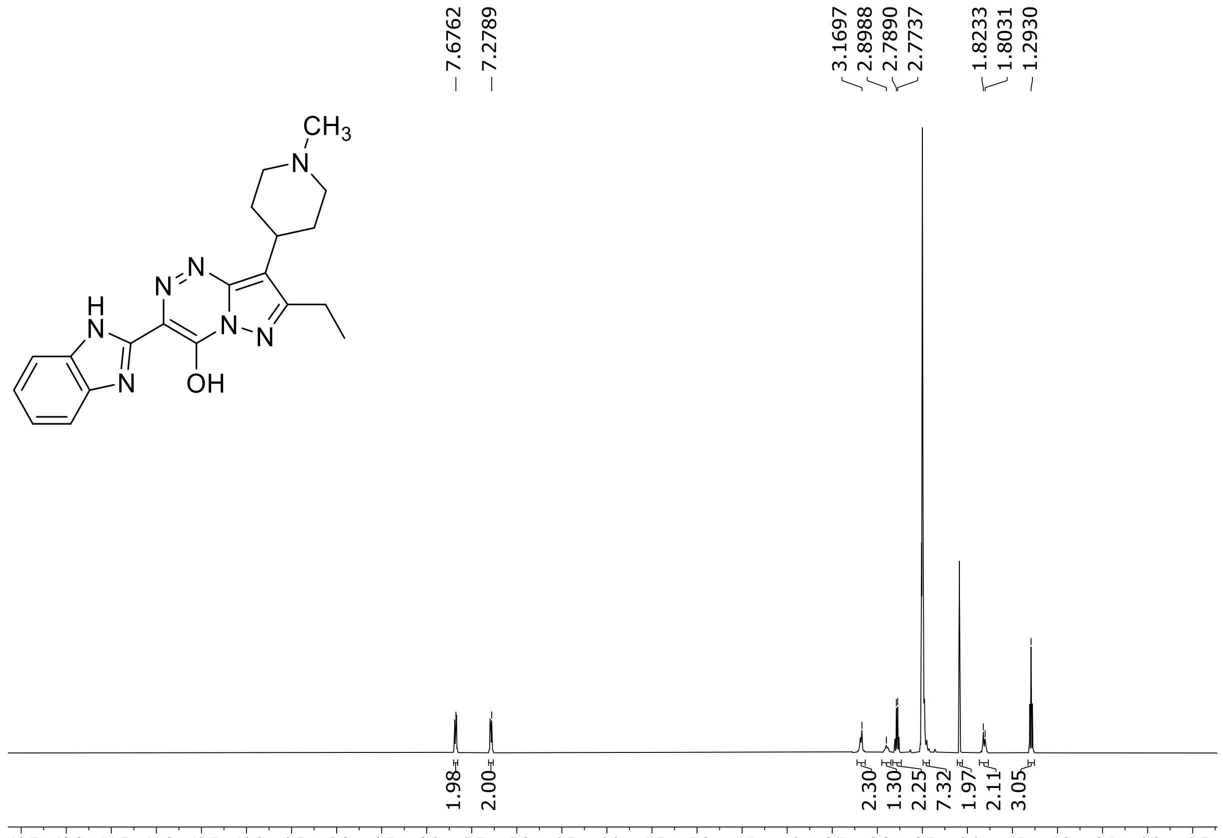

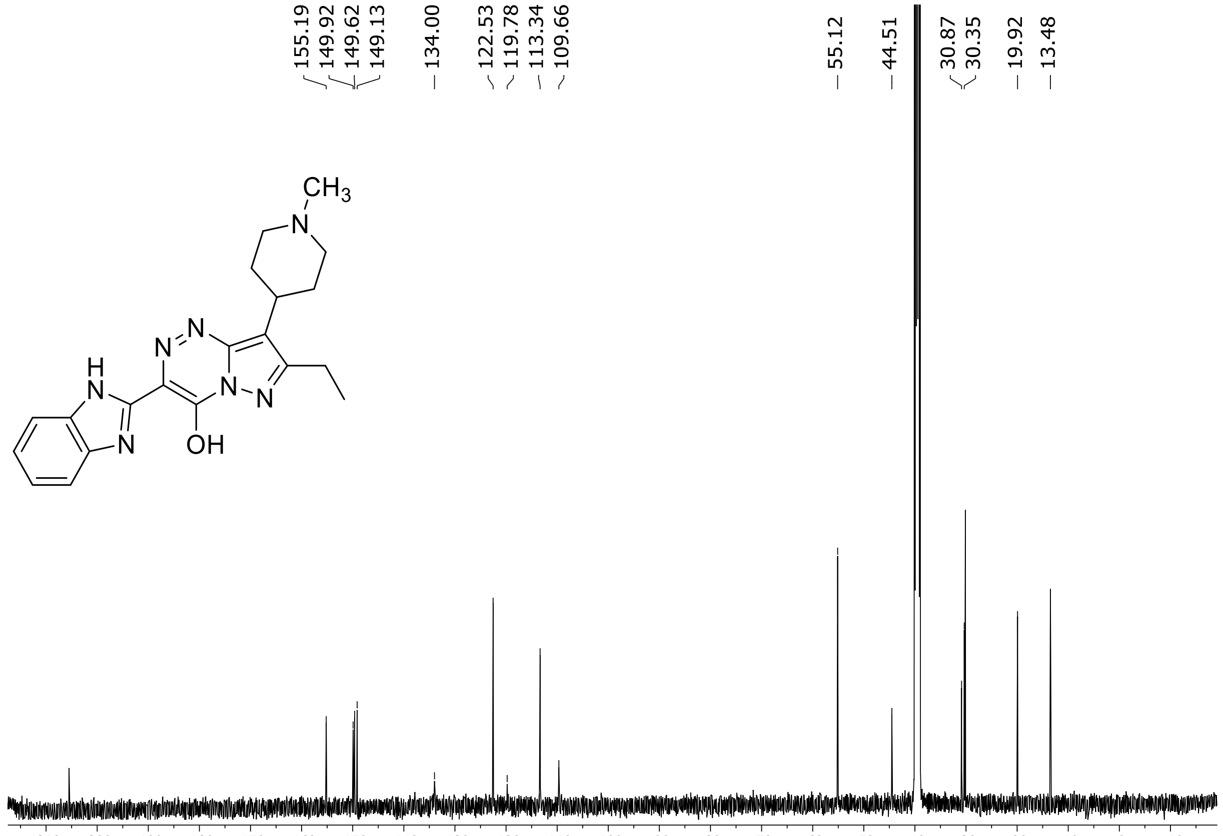

HRMS spectrum of **115**

$C_{20}H_{23}N_7O$ **exact mass: 377.1964**

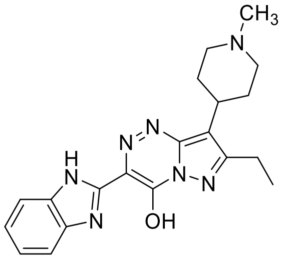

APCI + (MMI)
nitrogen flow 5 L/min, gas temperature 325°C, nebulizer 45 psig, skimmer 65 V, vaporizer 200°C, fragmentor 5 V, dissolved in methanol

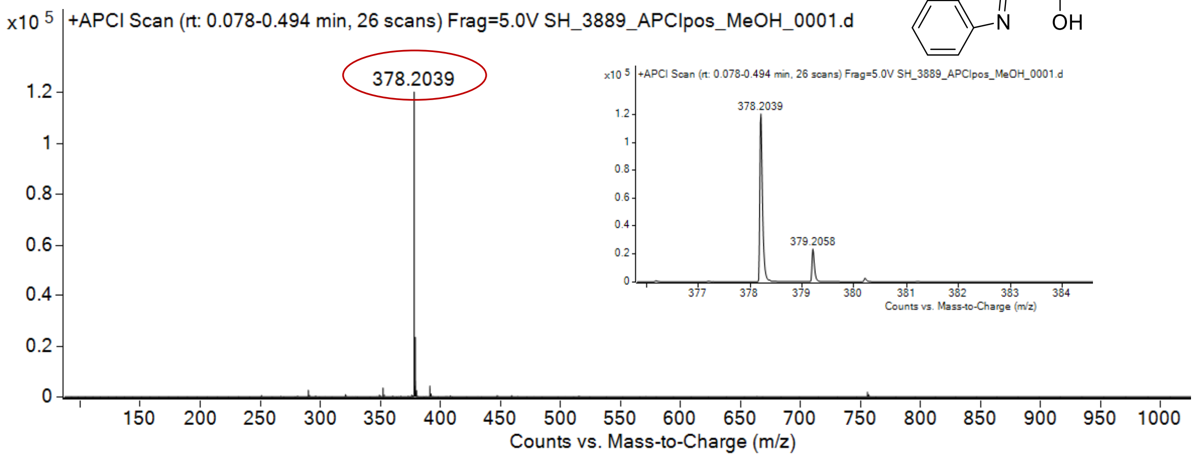

expected mass: $[M+H]^+$ = 378.2037 observed mass: $[M+H]^+$ = 378.2039 mass accuracy = 0.5 ppm

^{1}H (500 MHz) and ^{13}C NMR (126 MHz) spectra of **116** in DMSO-d_6

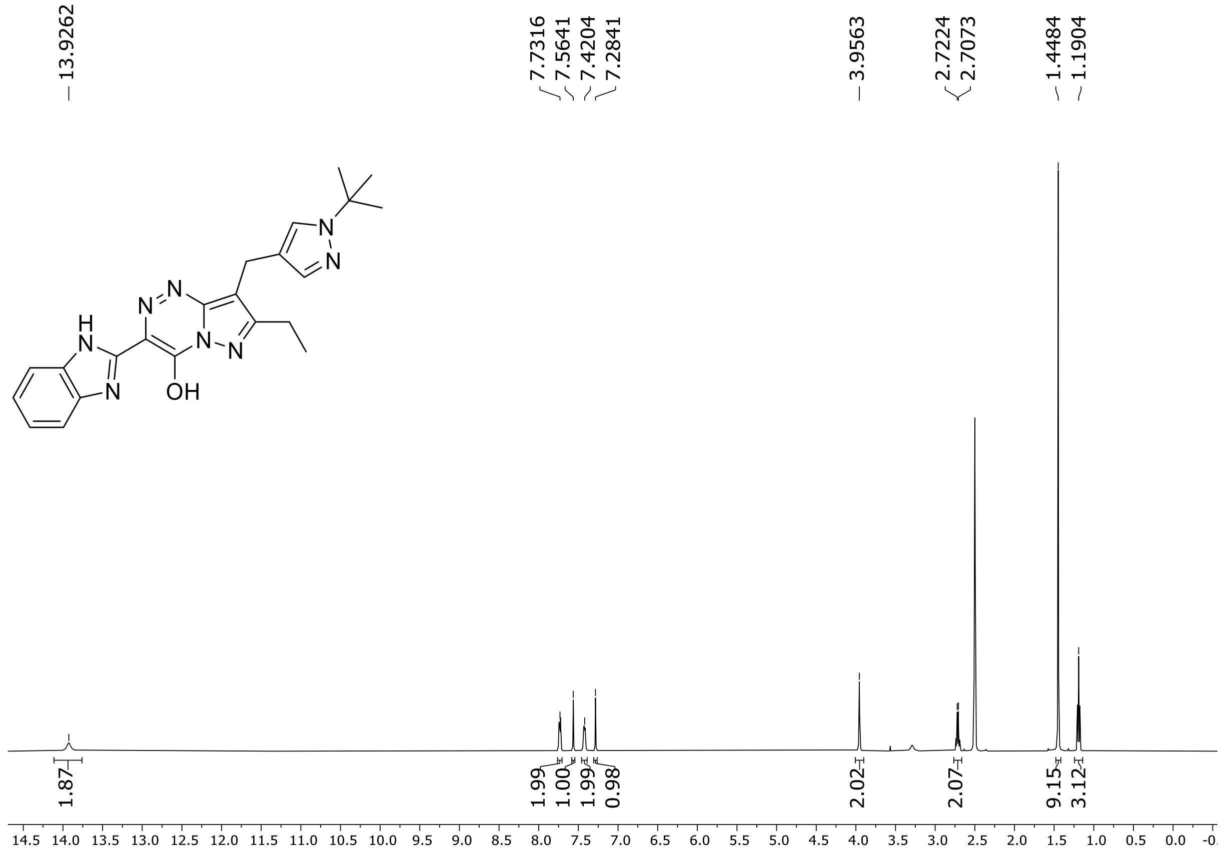

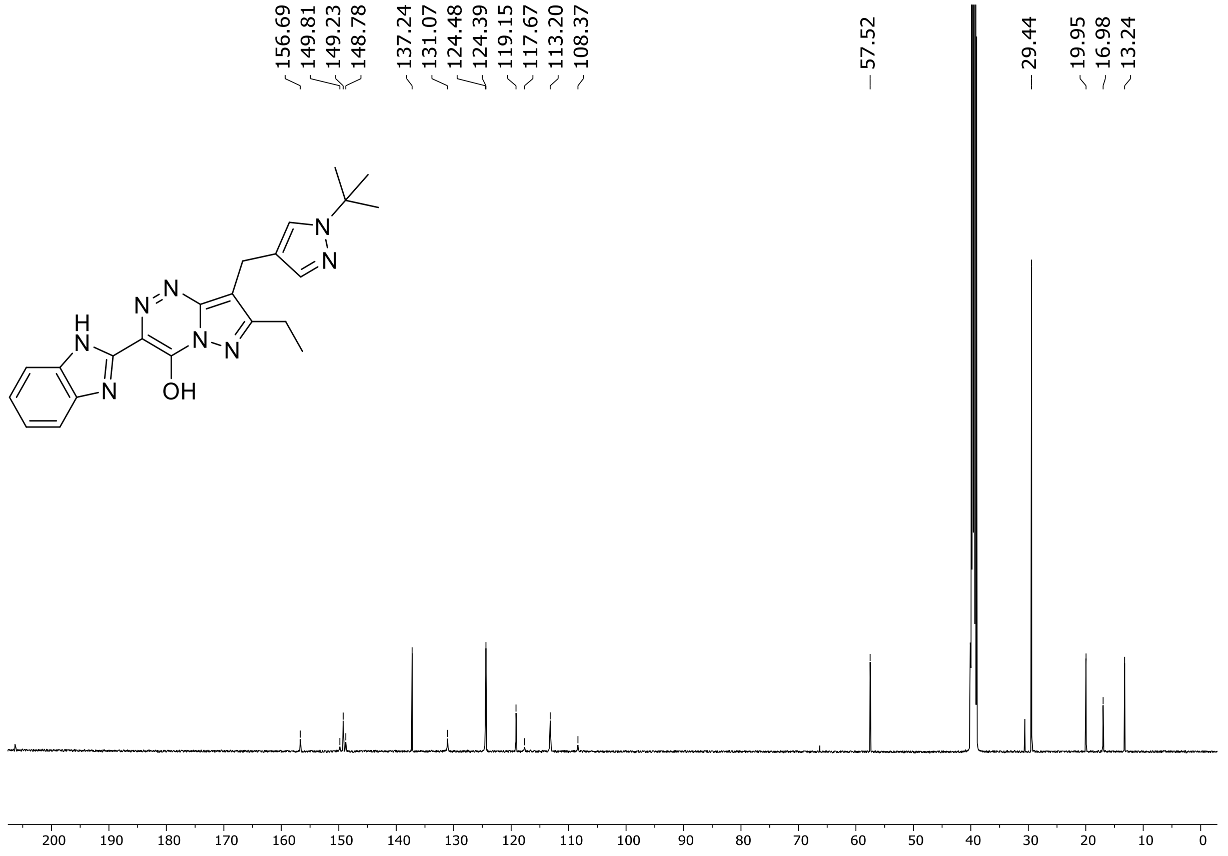

HRMS spectrum of **116**

$C_{22}H_{24}N_8O$ **exact mass: 416.2073**

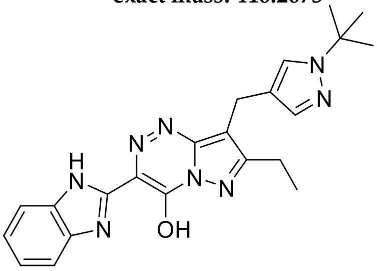

APCI + (MMI)
nitrogen flow 5 L/min, gas temperature 325°C, nebulizer 45 psig, skimmer 65 V, vaporizer 200°C, fragmentor 10 V, dissolved in methanol

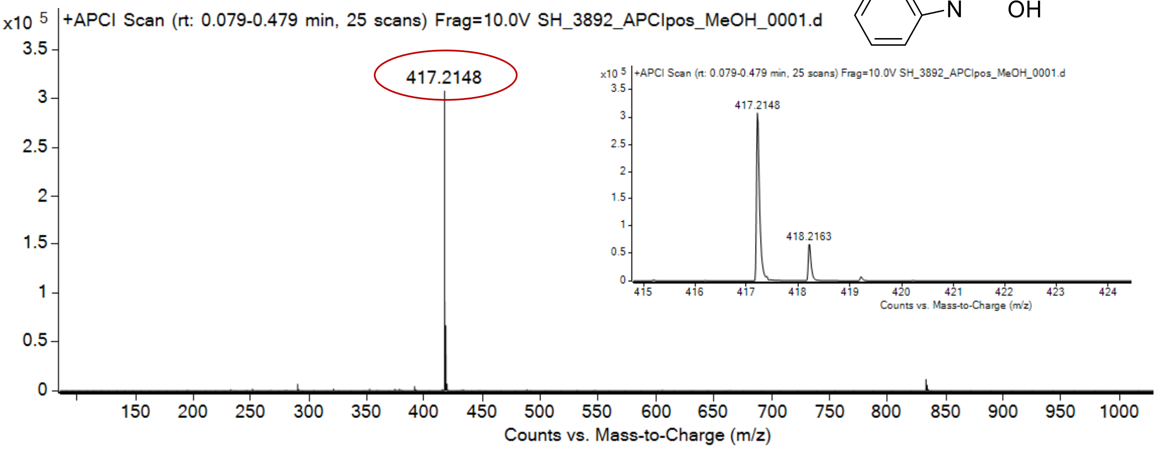

expected mass: [M+H]$^+$ = 417.2146 observed mass: [M+H]$^+$ = 417.2148 mass accuracy = 0.5 ppm

^{1}H (500 MHz) and ^{13}C NMR (126 MHz) spectra of **117** in chloroform-*d*

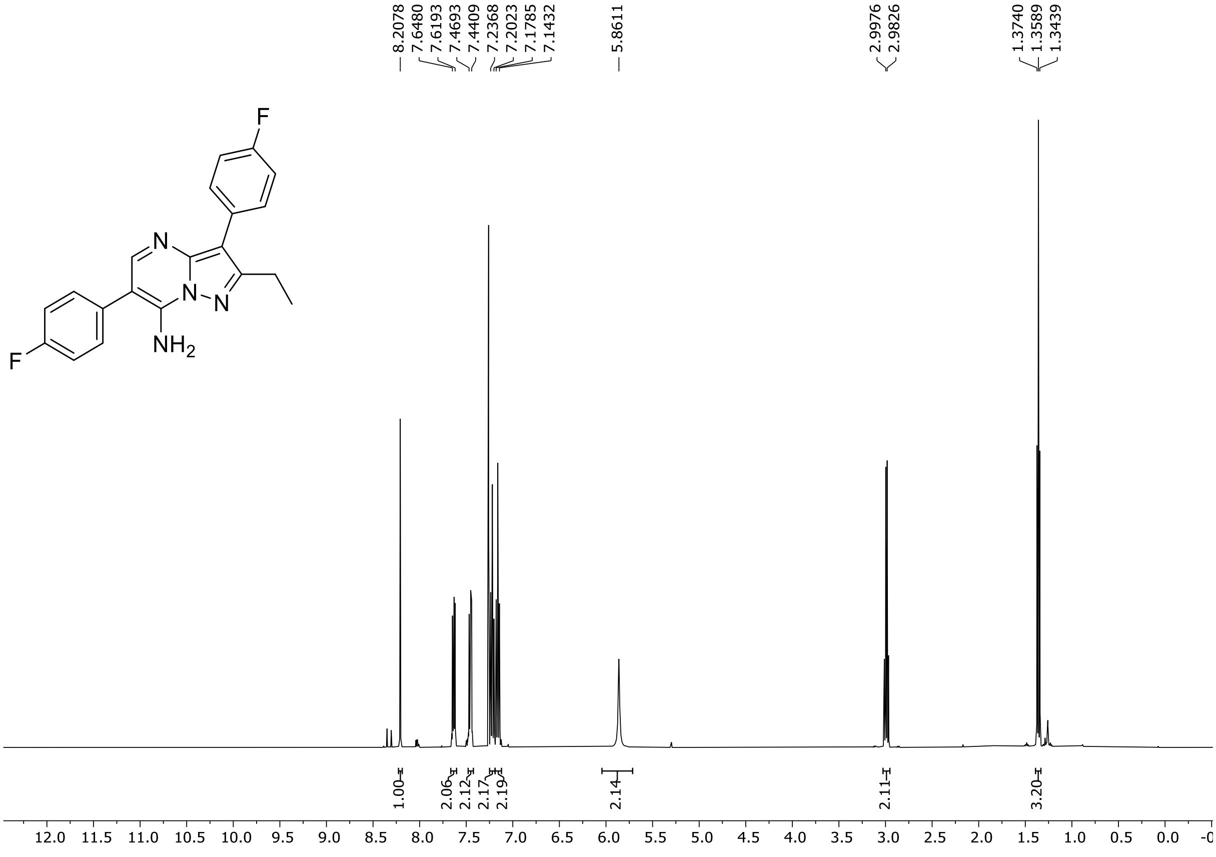

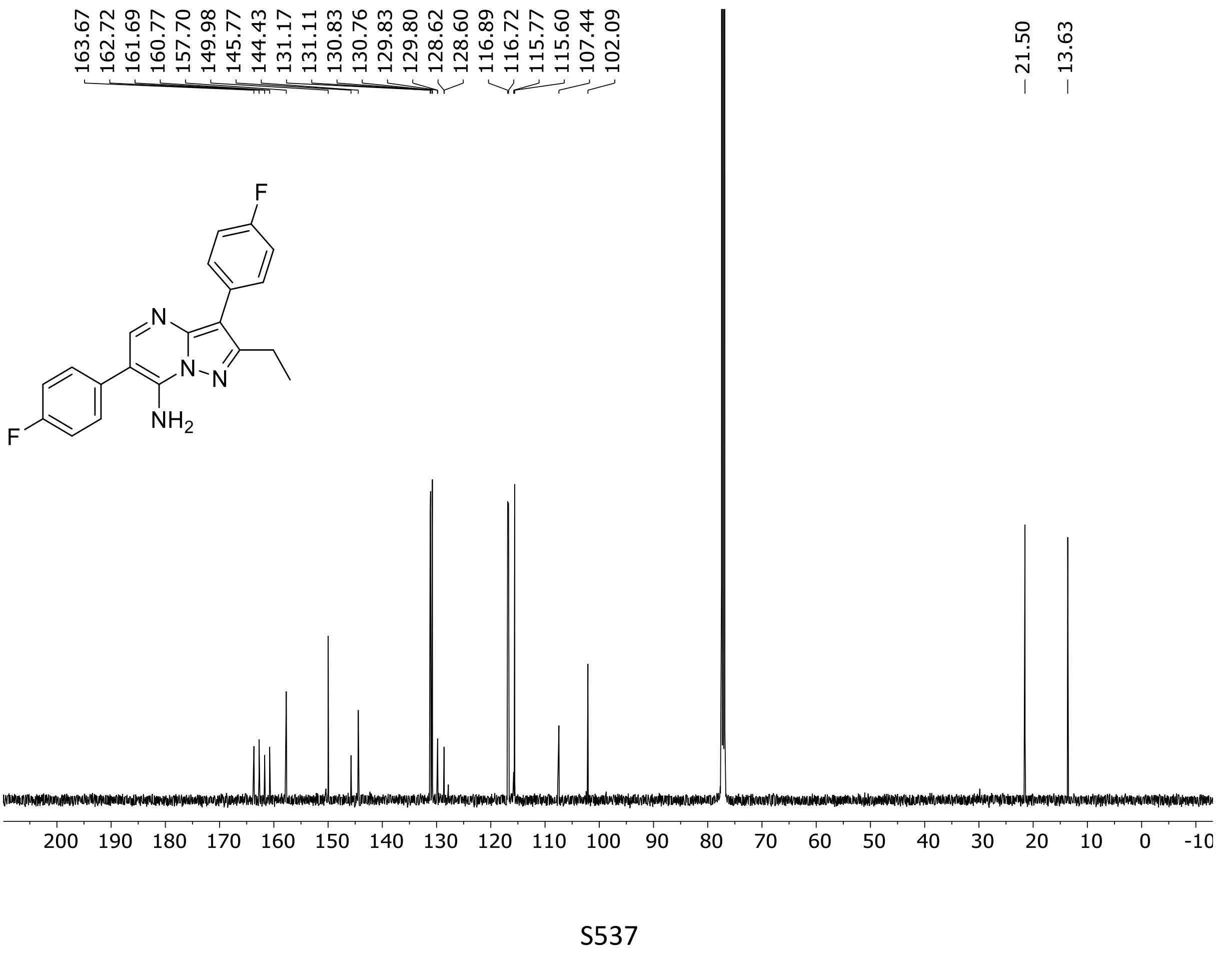

^{19}F (282 MHz) NMR spectrum of **117** chloroform-*d*

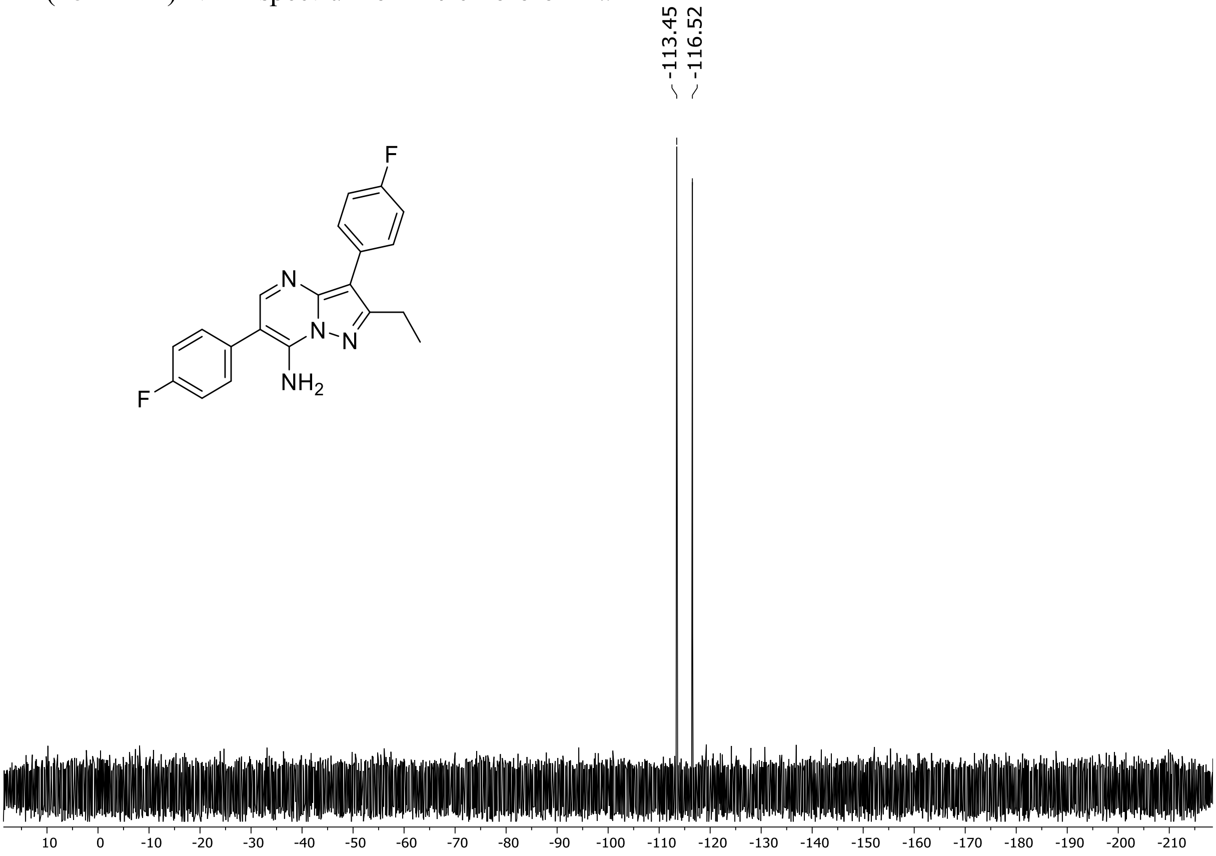

HRMS spectrum of **117**

$C_{20}H_{16}N_4F_2$ **mono m/z = 350.1343**

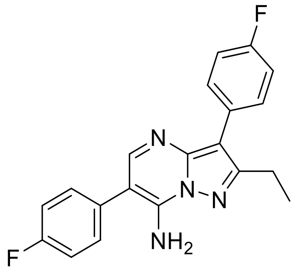

APCI + (MMI)
nitrogen flow 5 L/min, gas temperature 300°C, nebulizer 45 psi, vaporizer 200°C, skimmer **65** V, fragmentor **15** V, dissolved in methanol

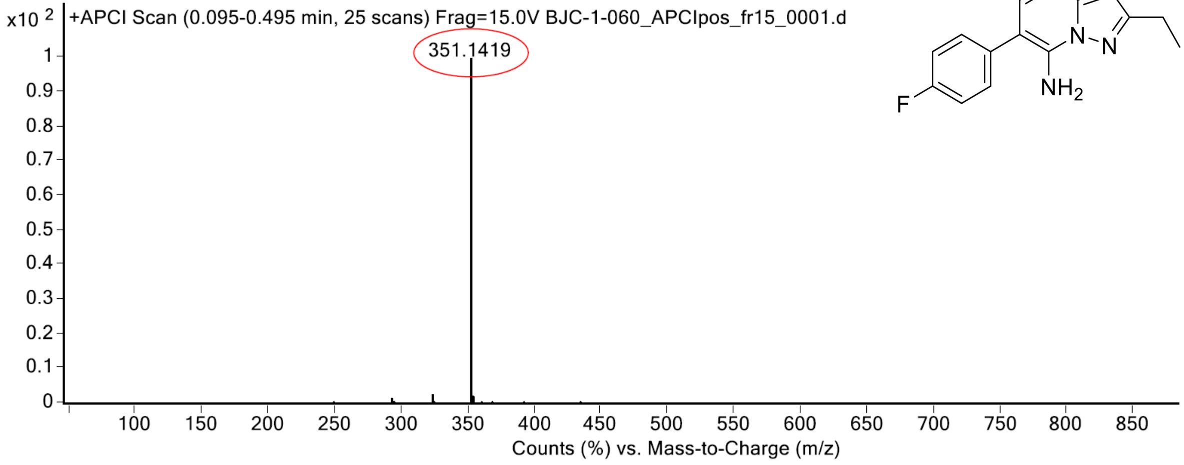

calculated mass: $[M+H]^+$ = 351.1416 observed: $[M+H]^+$ = 351.1419 max. mass error = 0.8 ppm

^{1}H NMR, ^{13}C NMR, HRMS and IR spectra of compound S109-S188

^{1}H (500 MHz) and ^{13}C NMR (126 MHz) spectra of **S109** in chloroform-*d*

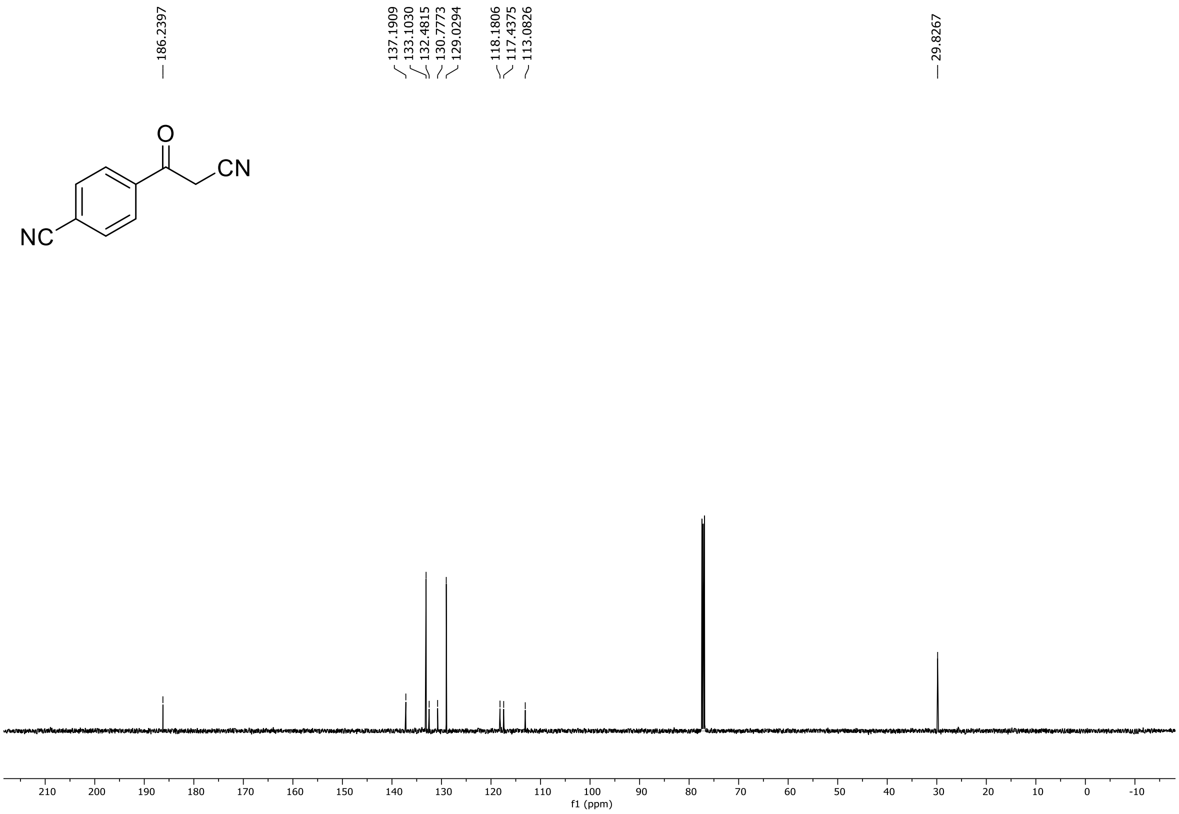

HRMS spectrum of **S109**

NAR-A-107 $C_{10}H_6N_2O$ ***m/z* 170.0480**

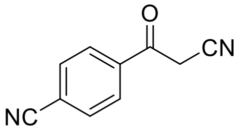

APCI- (MMI)
nitrogen flow 5 L/min, gas temperature 325°C, nebulizer 45 psi, skimmer 65 V, vaporizer 200°C, fragmentor 50 V, dissolved in MeOH

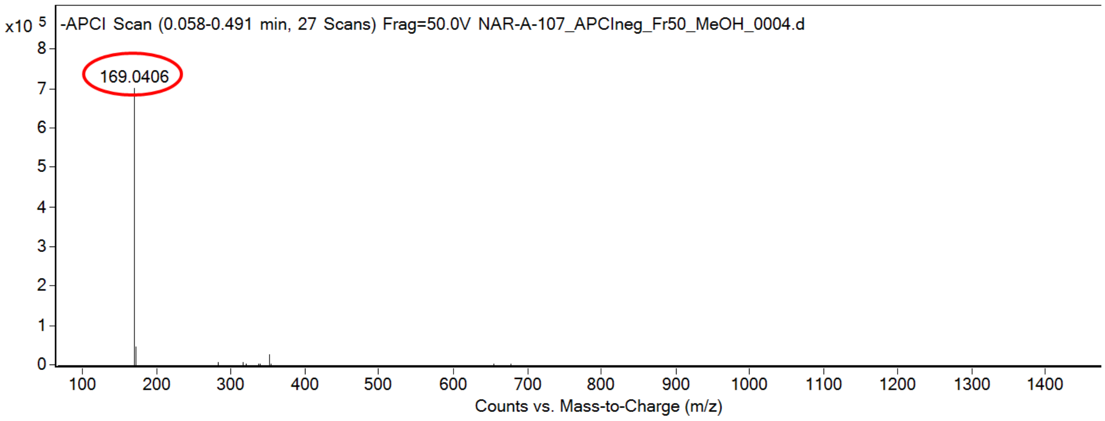

calculated mass: $[M-H]^-$ = 169.0407 observed: $[M-H]^-$ = 169.0406 mass accuracy = -0.6 ppm

FT-IR spectrum (neat) of **S109**

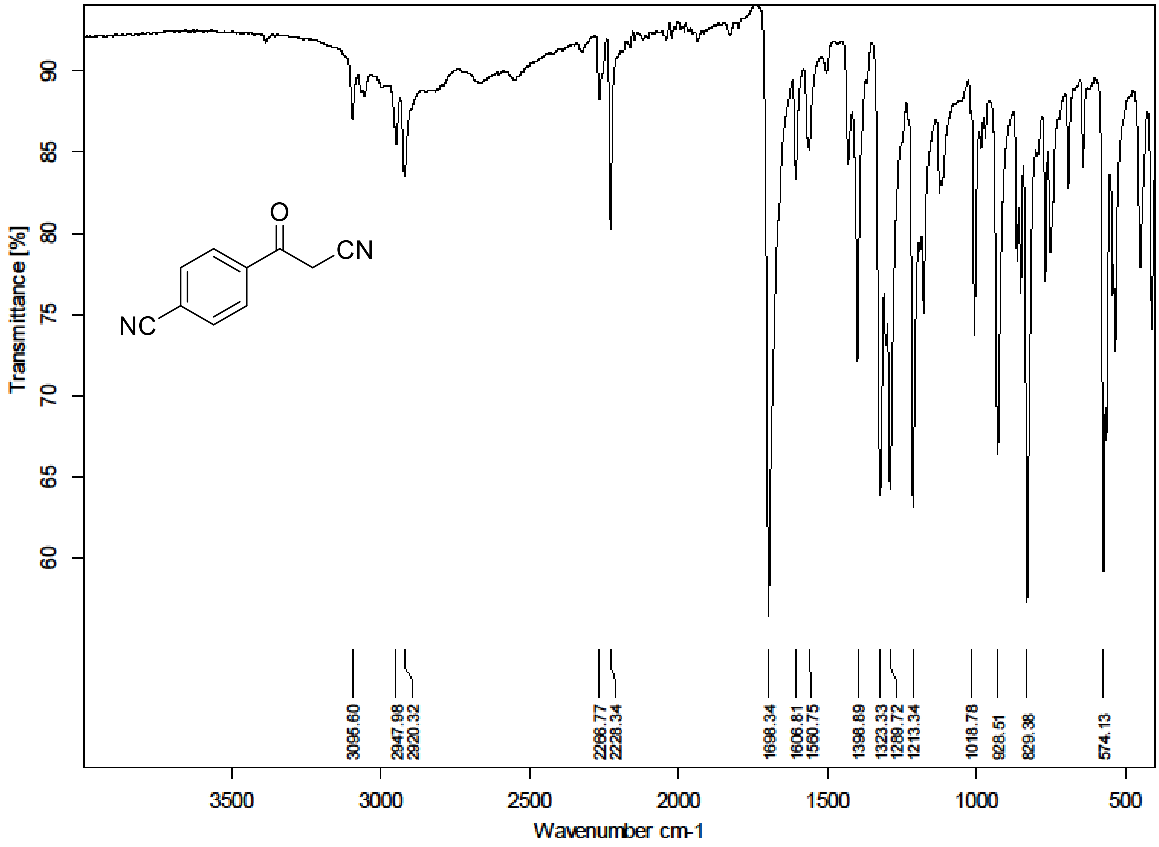

^{1}H (500 MHz) and ^{13}C NMR (126 MHz) spectra of **S110** in chloroform-*d*

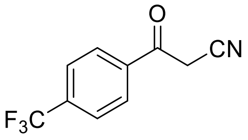

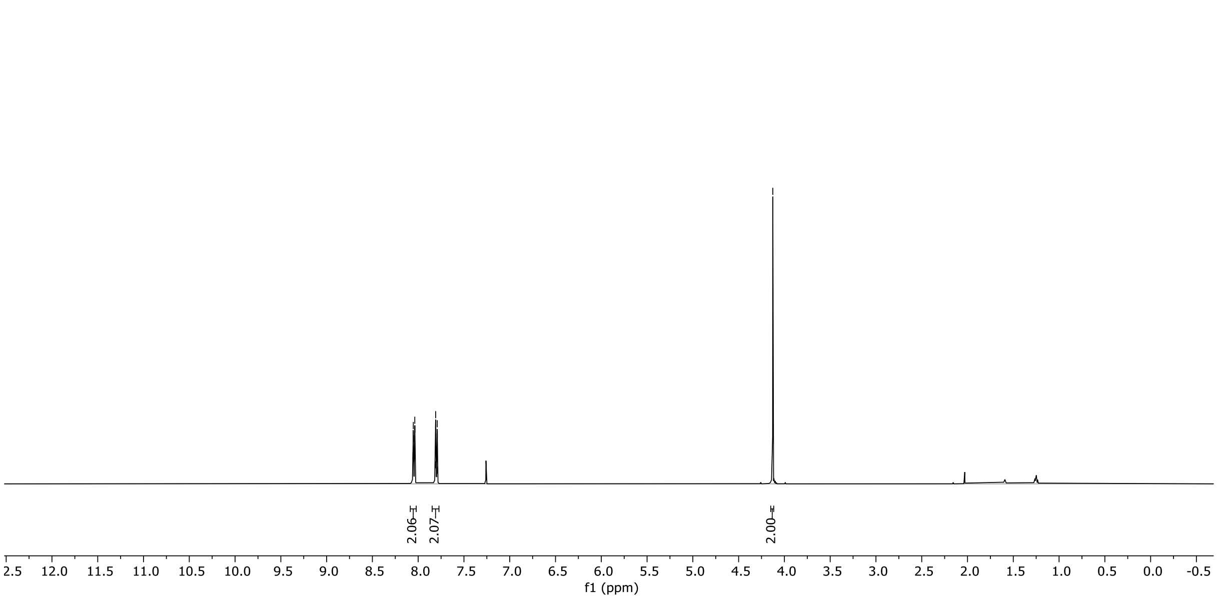

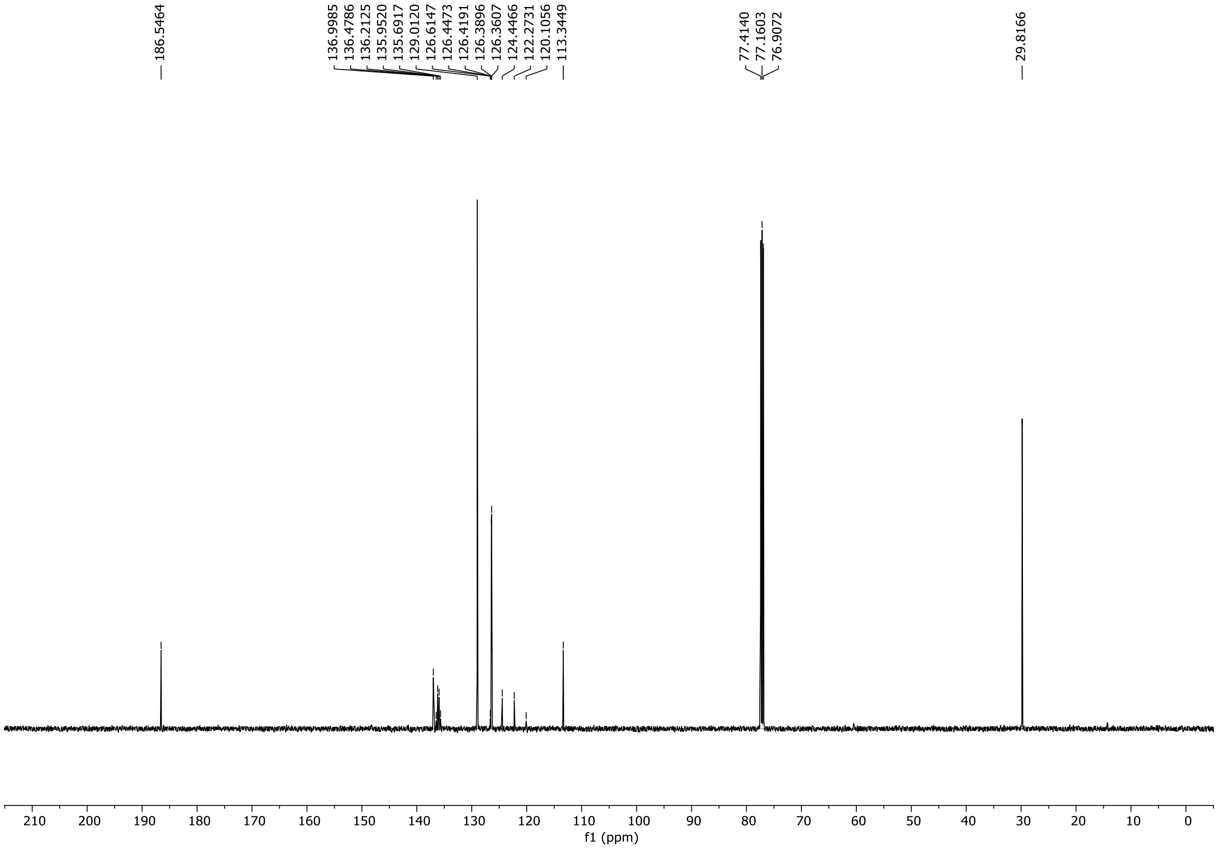

^{19}F NMR (471 MHz) spectrum of **S110** in chloroform-*d*

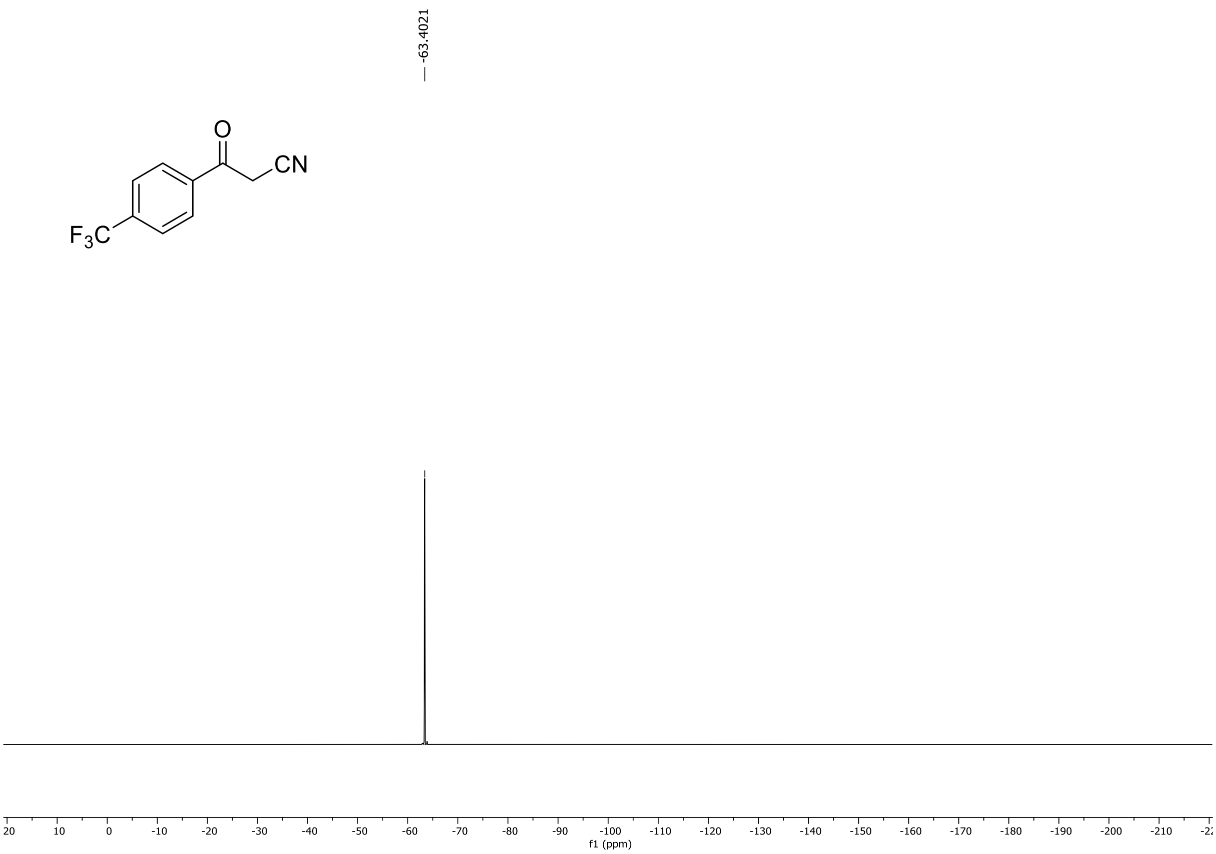

HRMS spectrum of **S110**

NAR-A-110

$C_{10}H_6F_3NO$ ***m/z* 213.0401**

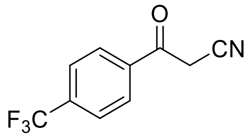

APCI- (MMI)
nitrogen flow 5 L/min, gas temperature 325°C, nebulizer 45 psi, skimmer 65 V, vaporizer 200°C, fragmentor 45 V, dissolved in MeOH

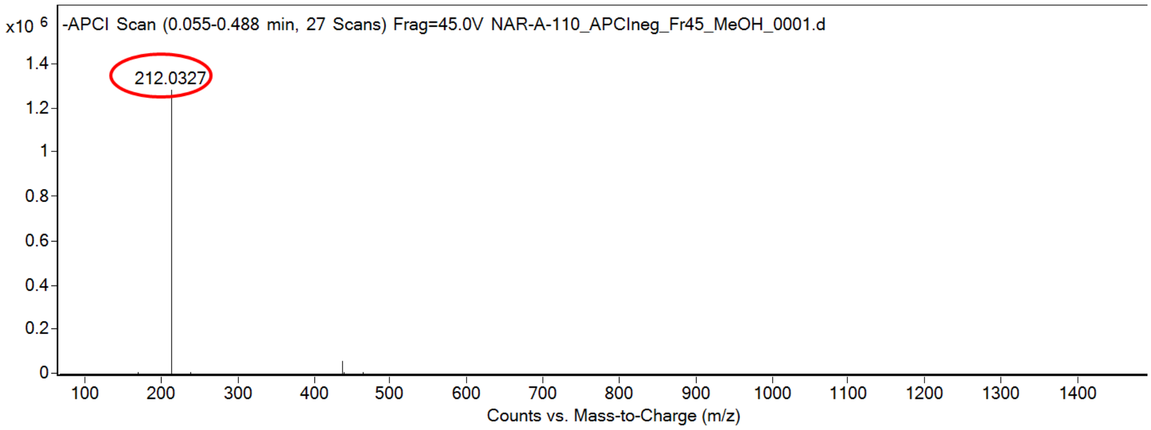

calculated mass: $[M-H]^- = 212.0329$ observed: $[M-H]^- = 212.0327$ mass accuracy = -0.9 ppm

FT-IR spectrum (neat) of **S110**

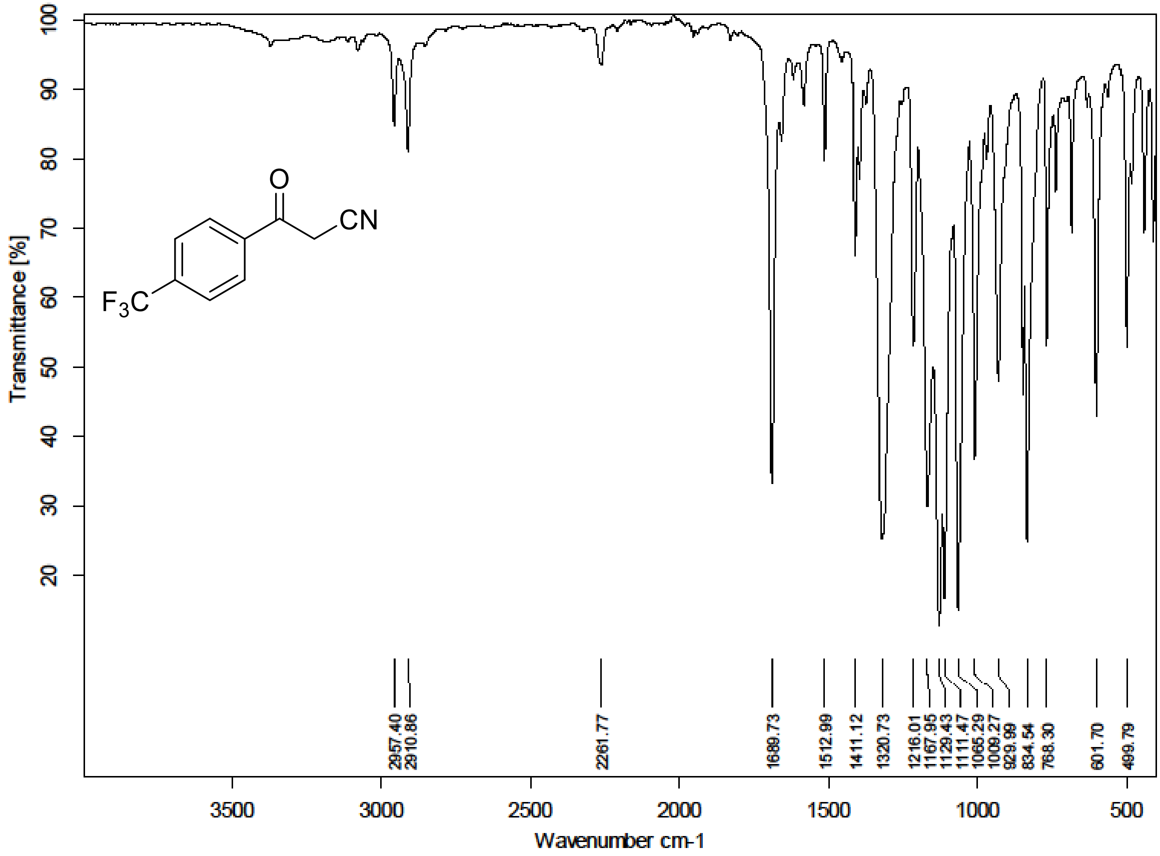

^{1}H (500 MHz) and ^{13}C NMR (126 MHz) spectra of **S111** in chloroform-*d*

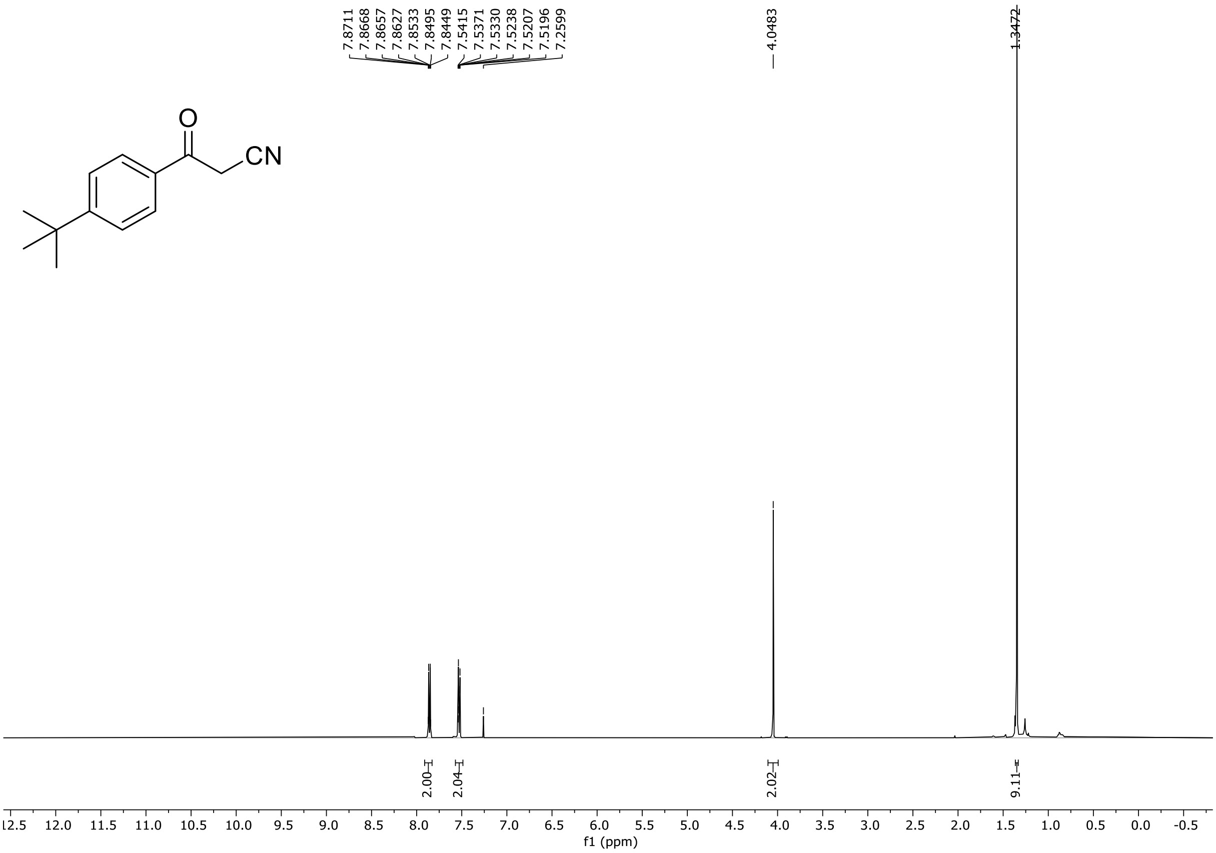

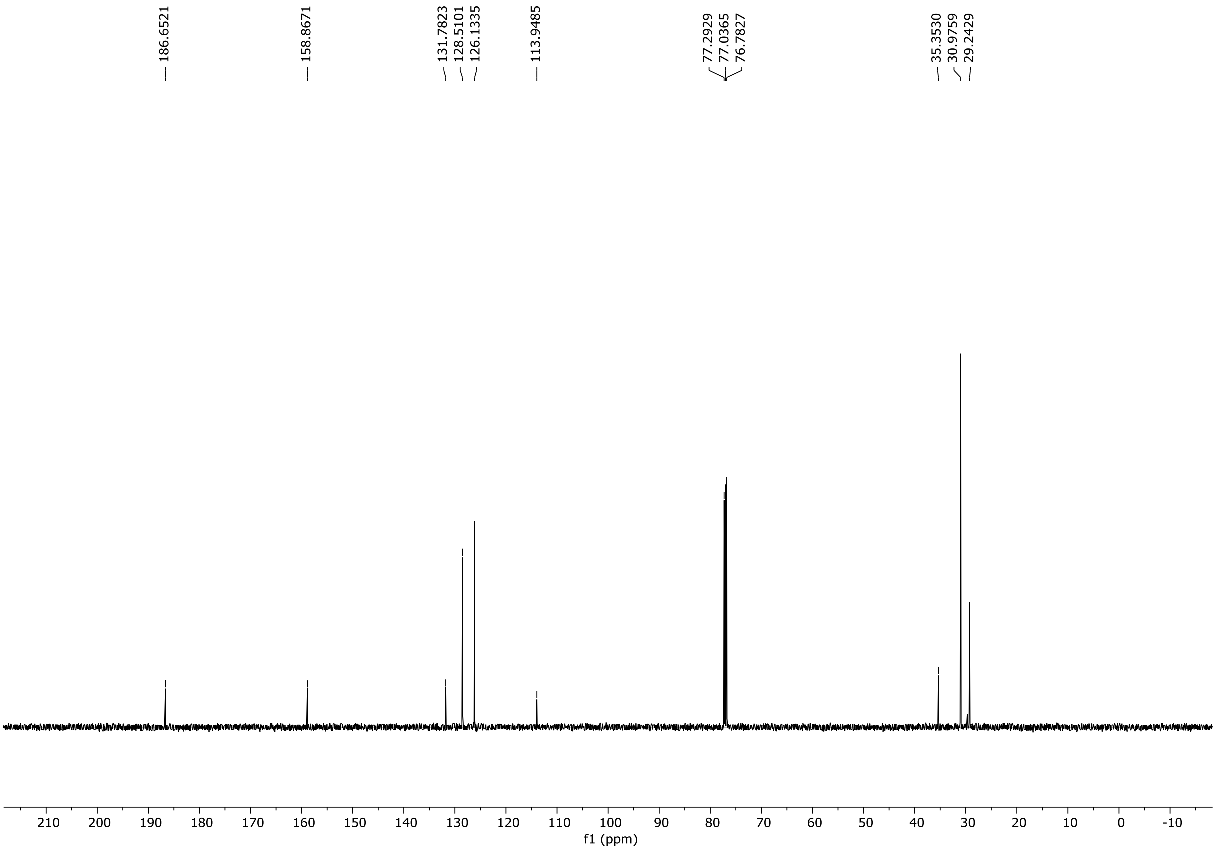

HRMS spectrum of **S111**

NAR-A-108 $C_{13}H_{15}NO$ *m/z* **201.1154**

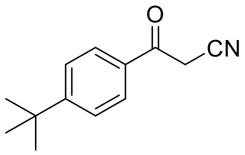

APCI- (MMI)
nitrogen flow 5 L/min, gas temperature 325°C, nebulizer 45 psi, skimmer 65 V, vaporizer 200°C, fragmentor 60 V, dissolved in MeOH

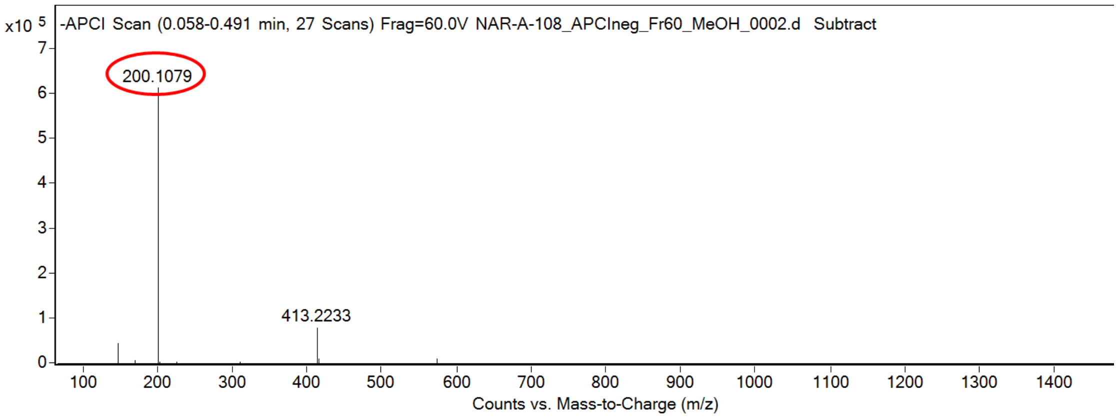

calculated mass: $[M-H]^-$ = 200.1081 observed: $[M-H]^-$ = 200.1079 mass accuracy = -1.0 ppm

FT-IR spectrum (neat) of **S111**

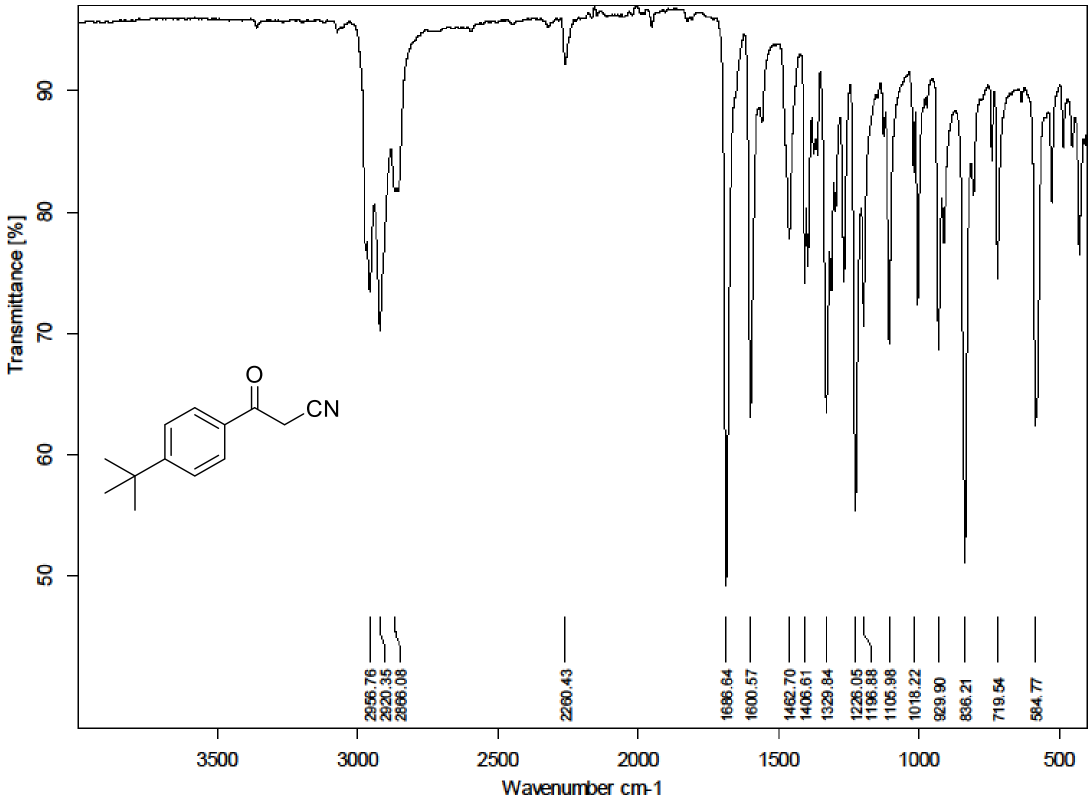

^{1}H (500 MHz) and ^{13}C NMR (126 MHz) spectra of **S112** in chloroform-*d*

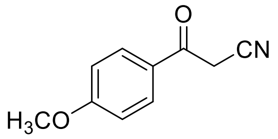

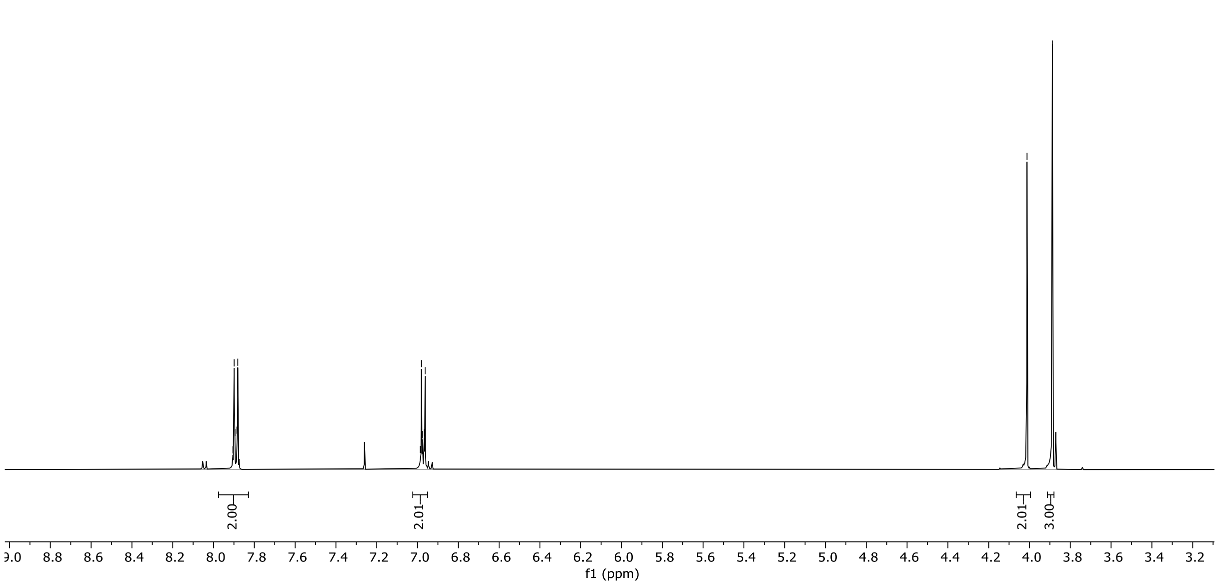

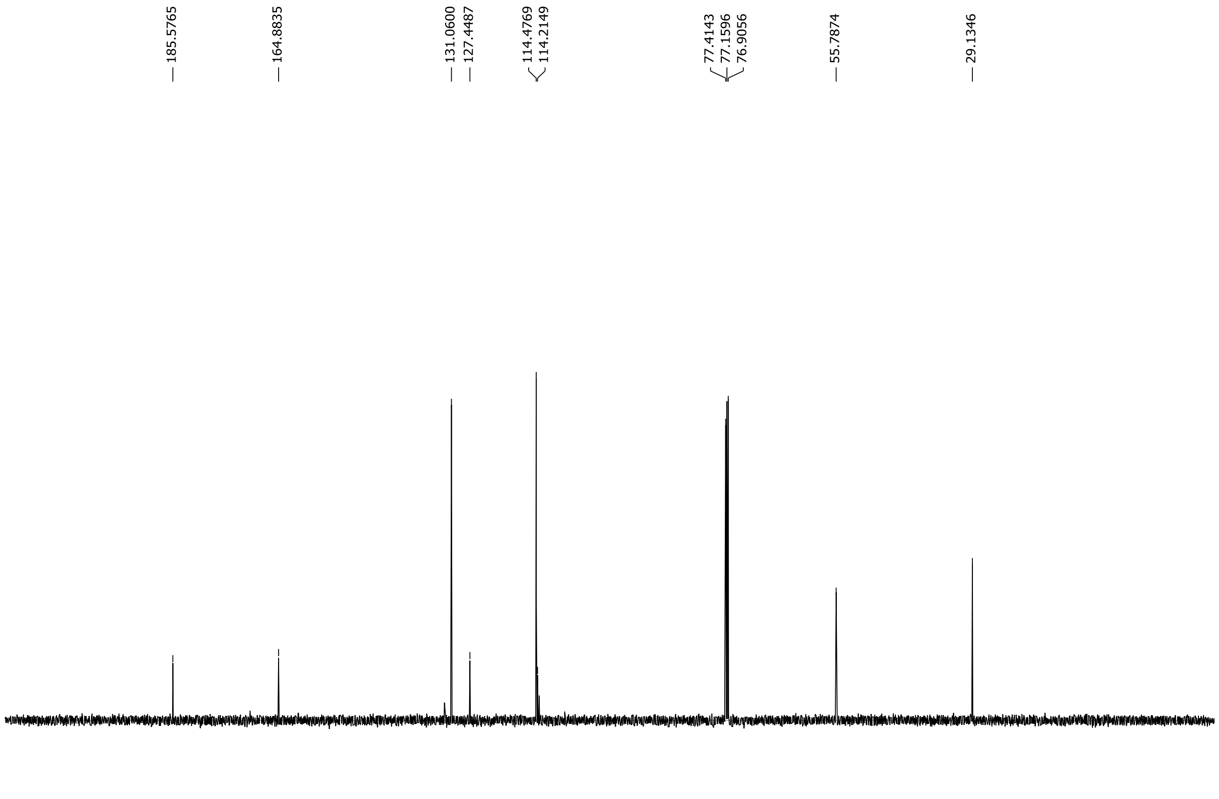

HRMS spectrum of **S112**

NAR-A-112 $C_{10}H_9NO_2$ *m*/*z* **175.0633**

APCI- (MMI)
nitrogen flow 5 L/min, gas temperature 325°C, nebulizer 45 psi, skimmer 65 V, vaporizer 200°C, fragmentor 150 V, dissolved in MeOH

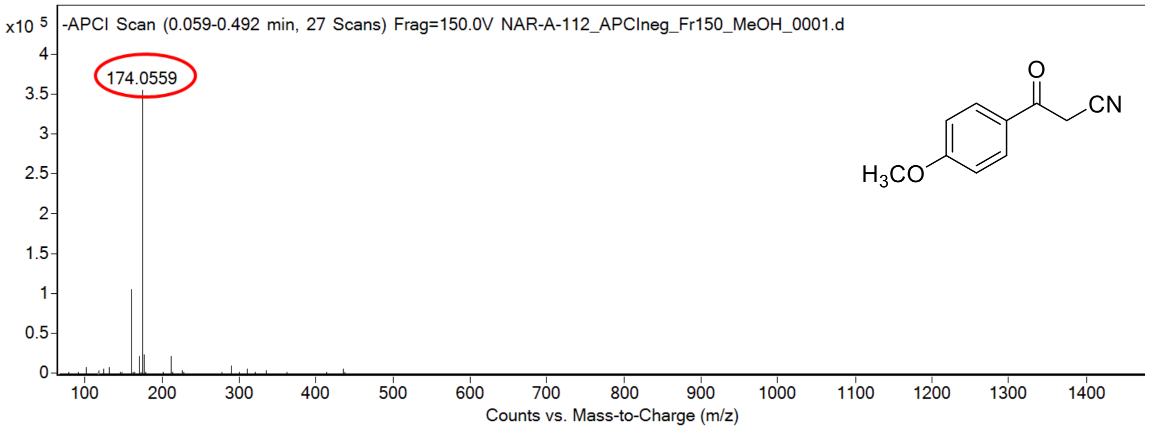

calculated mass: $[M-H]^-$ = 174.0561 observed: $[M-H]^-$ = 174.0559 mass accuracy = -1.1 ppm

FT-IR spectrum (neat) of **S112**

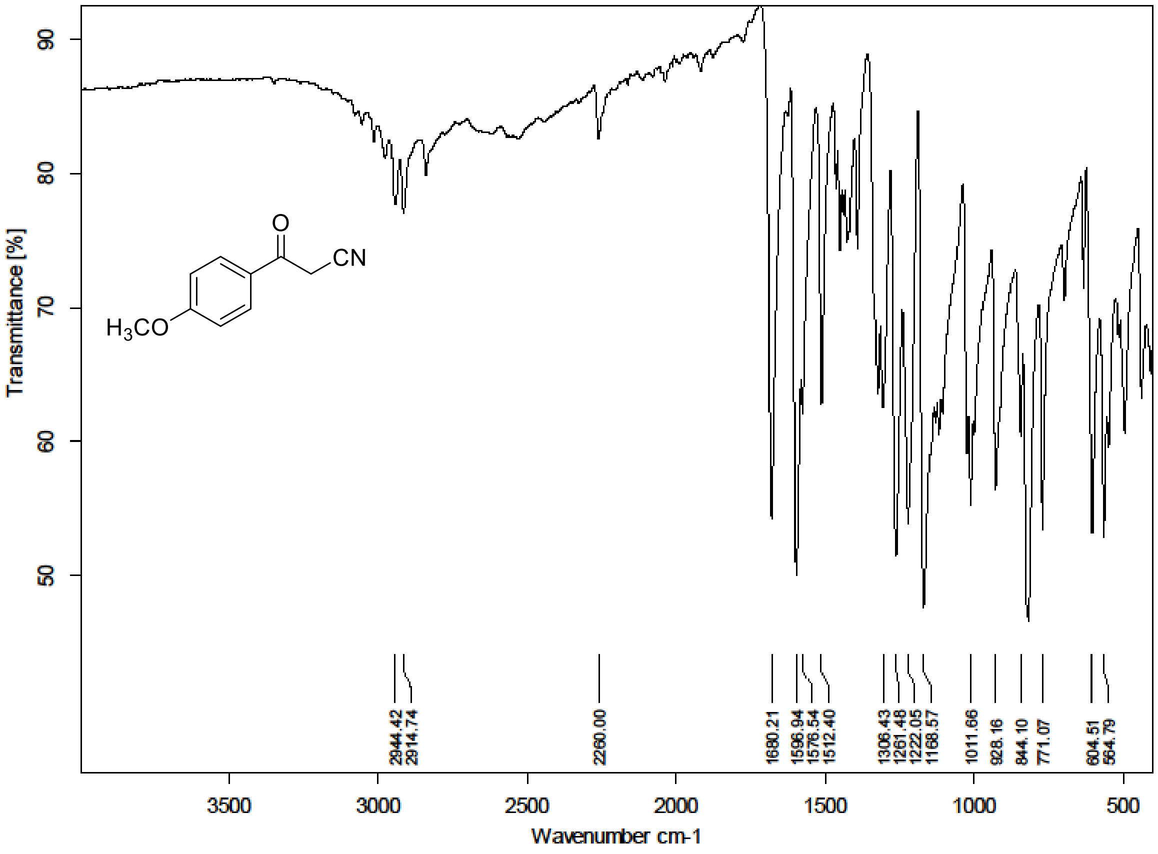

^{1}H (300 MHz) and ^{13}C NMR (75 MHz) spectra of **S113** in chloroform-*d*

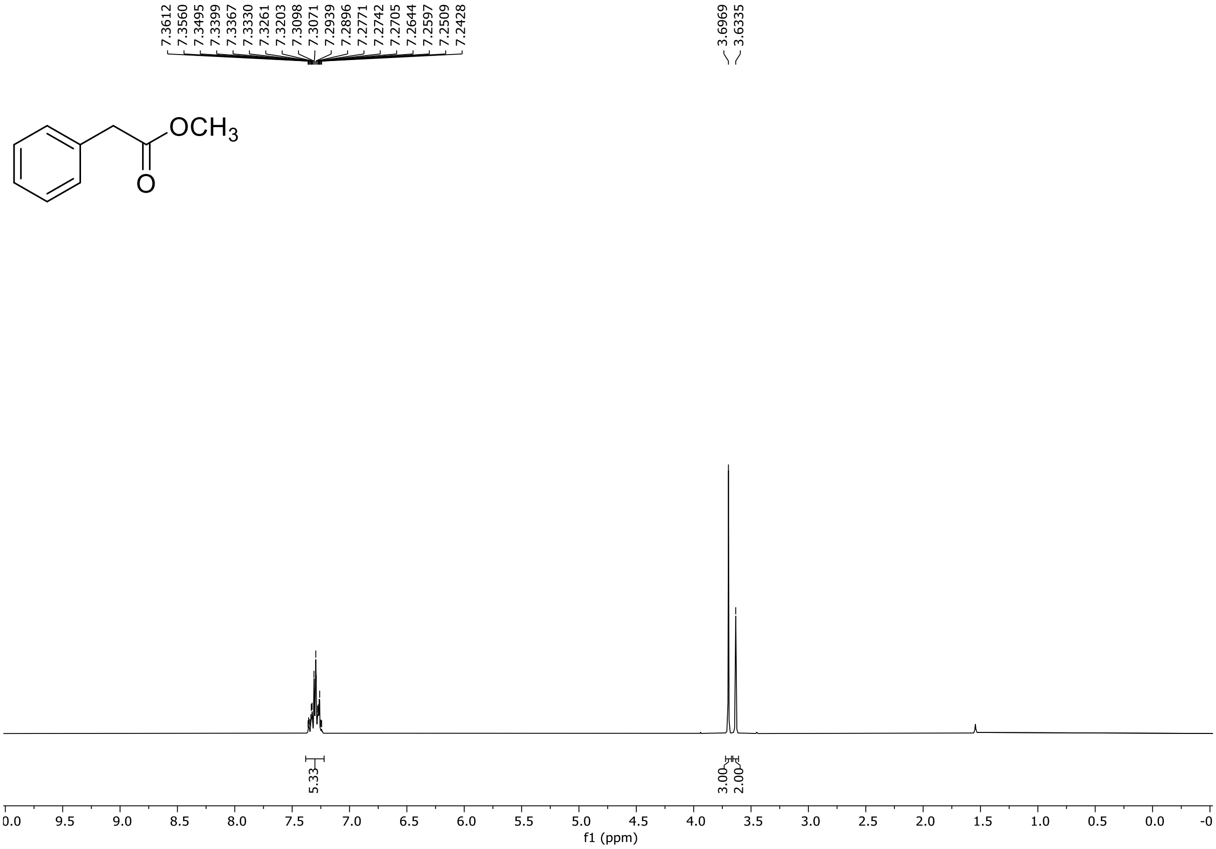

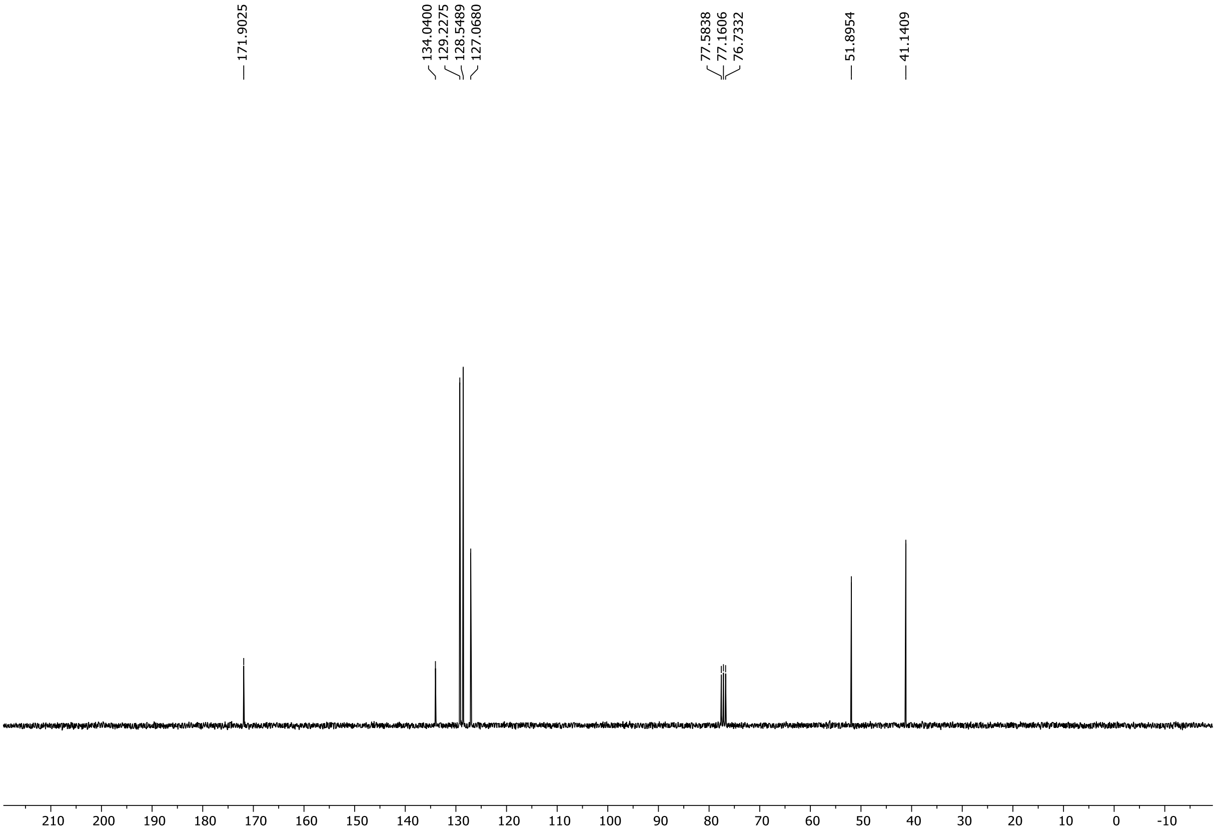

HRMS spectrum of **S113**

NAR-A-182 $C_9H_{10}O_2$ mono *m/z* **150.0681**

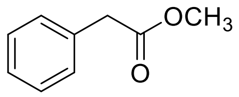

APCI + (MMI)
nitrogen flow 5 L/min, gas temperature 325°C, nebulizer 45 psi, skimmer **65** V,
vaporizer 200°C, fragmentor 50 V, dissolved in methanol

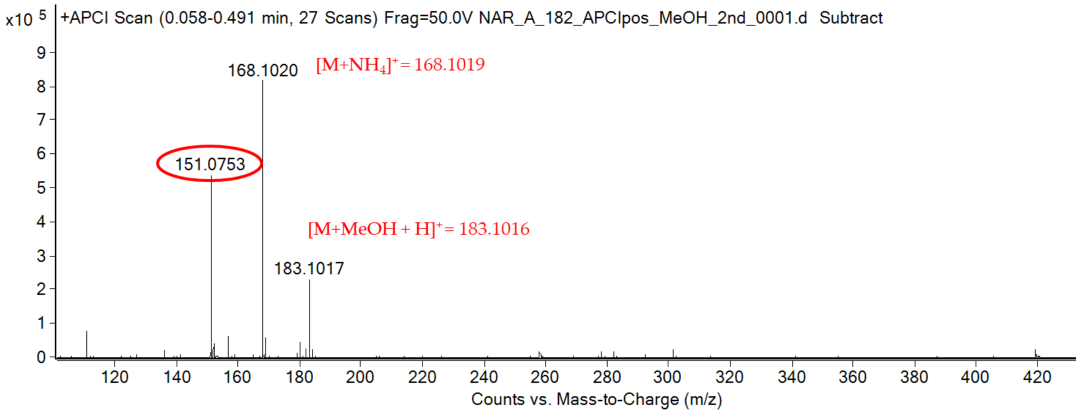

calculated mass: $[M+H]^+ = 151.0754$ observed: $[M+H]^+ = 151.0753$ mass accuracy = - 0.7 ppm

FT-IR spectrum (neat) of **S113**

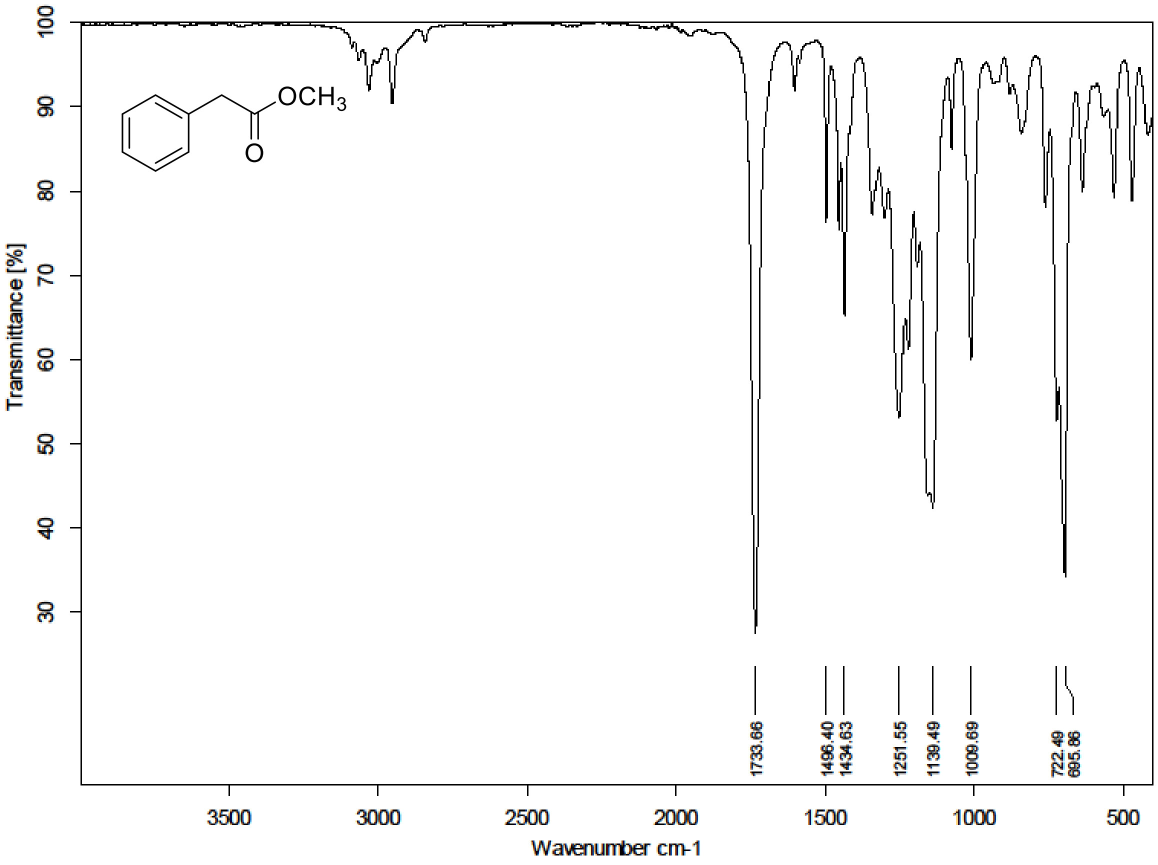

^{1}H (300 MHz) and ^{13}C NMR (75 MHz) spectra of **S114** in chloroform-*d*

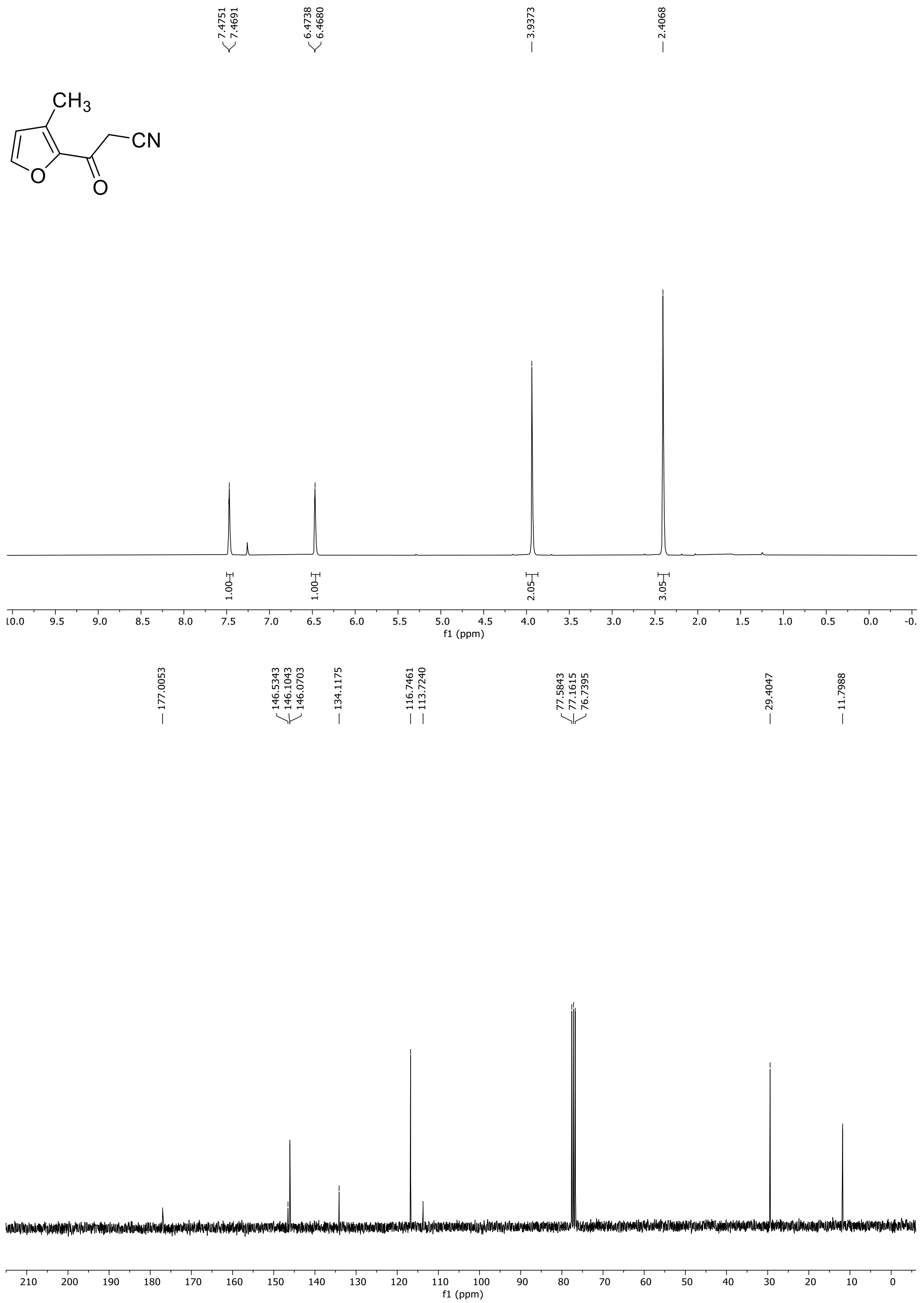

HRMS spectrum of **S114**

NAR-A-174 $C_8H_7NO_2$ mono *m/z* 149.0477

APCI + (MMI)
nitrogen flow 5 L/min, gas temperature 325°C, nebulizer 45 psi, skimmer **65** V, vaporizer 200°C, fragmentor 50 V, dissolved in methanol

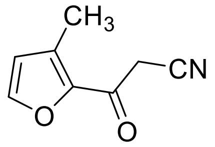

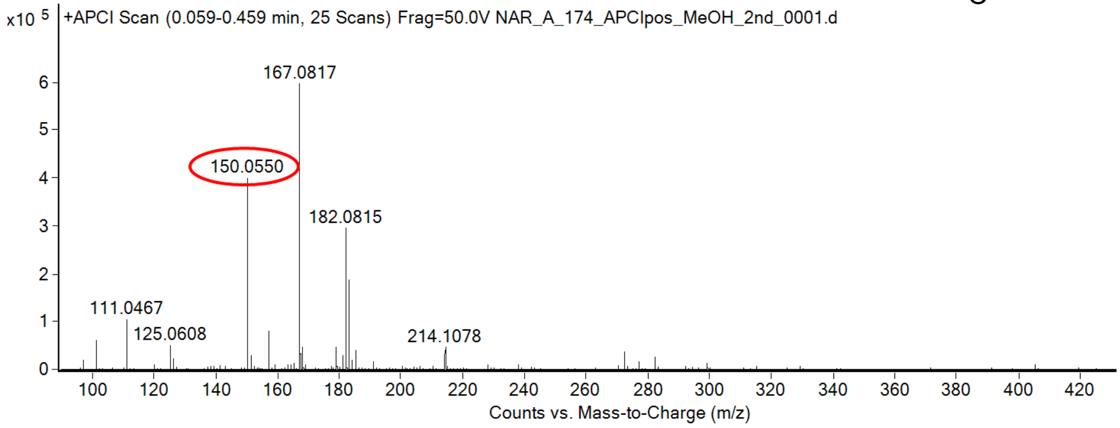

calculated mass: $[M+H]^+ = 150.0550$ observed: $[M+H]^+ = 150.0550$ mass accuracy = < 0.1 ppm

FT-IR spectrum (neat) of **S114**

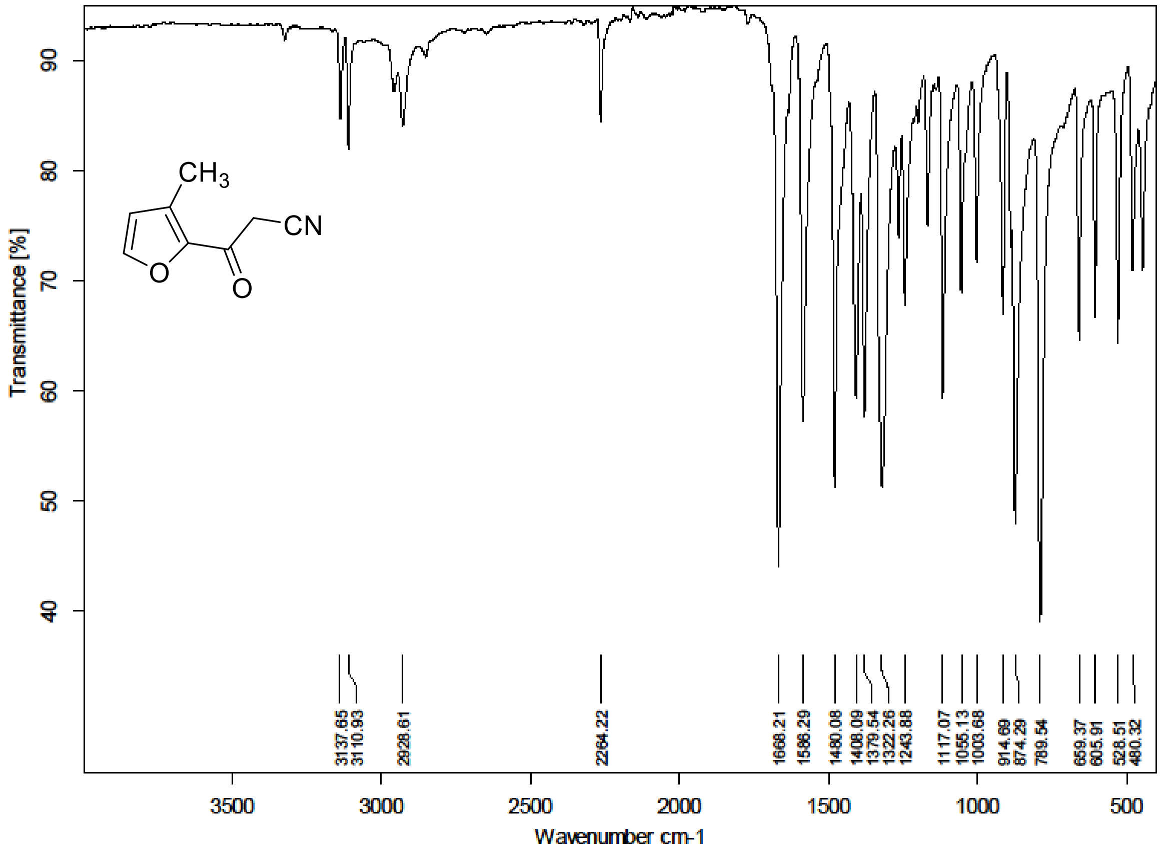

^{1}H (300 MHz) and ^{13}C NMR (75 MHz) spectra of **S115** in chloroform-*d*

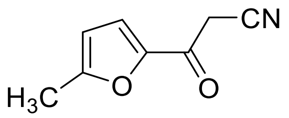

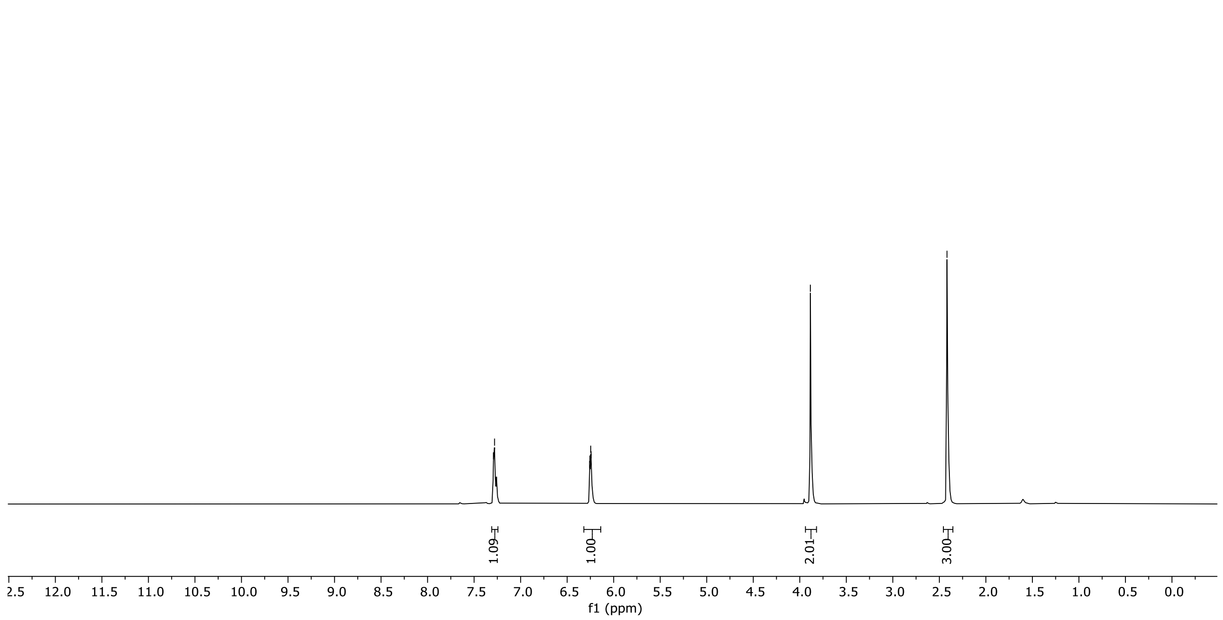

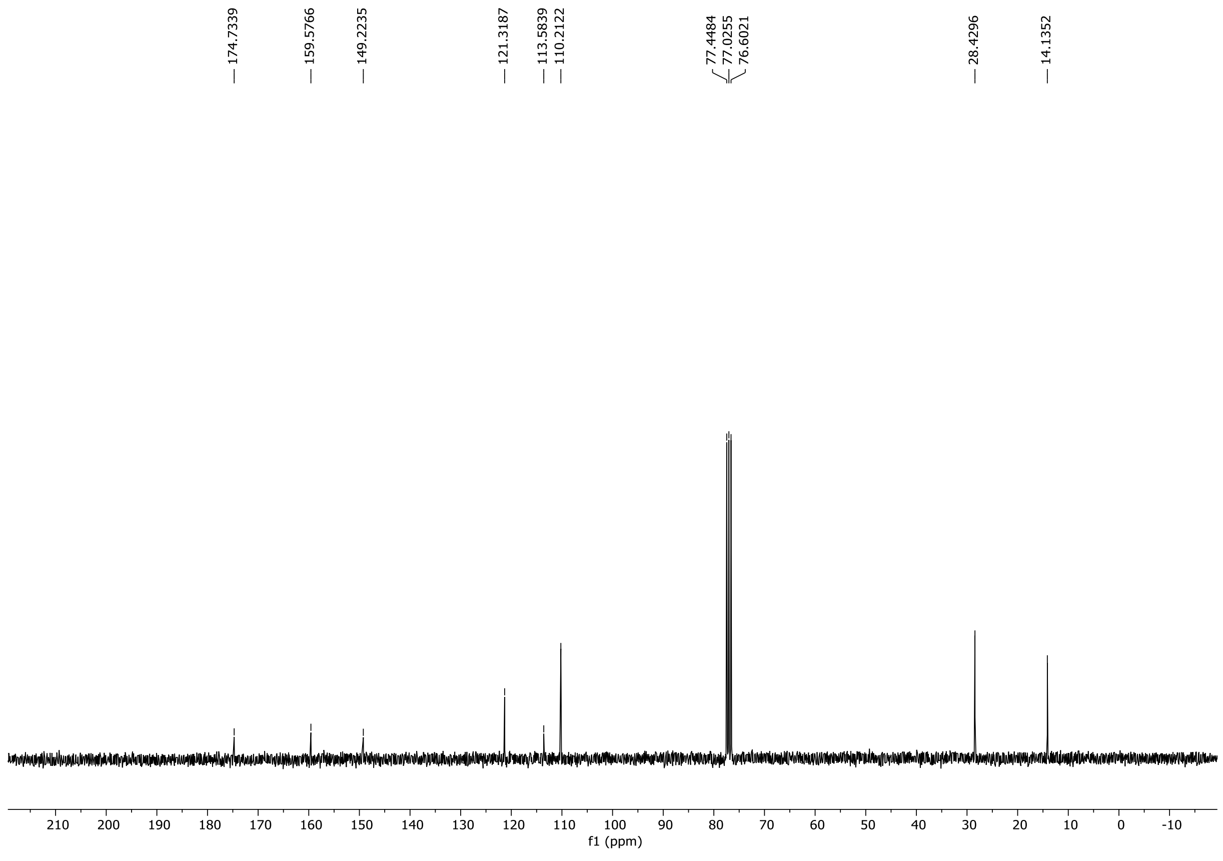

HRMS spectrum of **S115**

NAR-A-173 $C_8H_7NO_2$ mono *m/z* 149.0477

APCI + (MMI)
nitrogen flow 5 L/min, gas temperature 325°C, nebulizer 45 psi, skimmer **65** V, vaporizer 200°C, fragmentor 45 V, dissolved in methanol

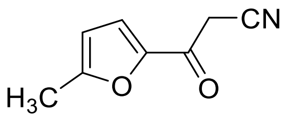

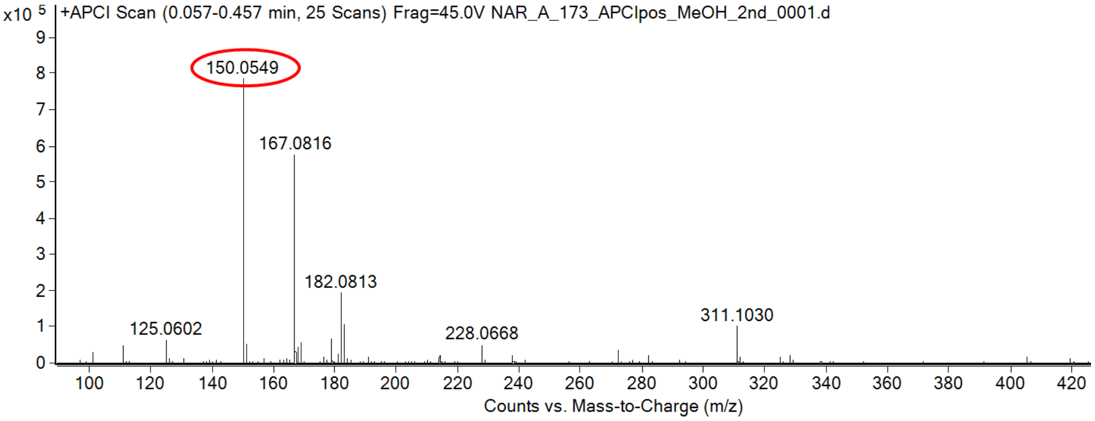

calculated mass: $[M+H]^+ = 150.0550$ observed: $[M+H]^+ = 150.0549$ mass accuracy = - 0.7 ppm

FT-IR spectrum (neat) of **S115**

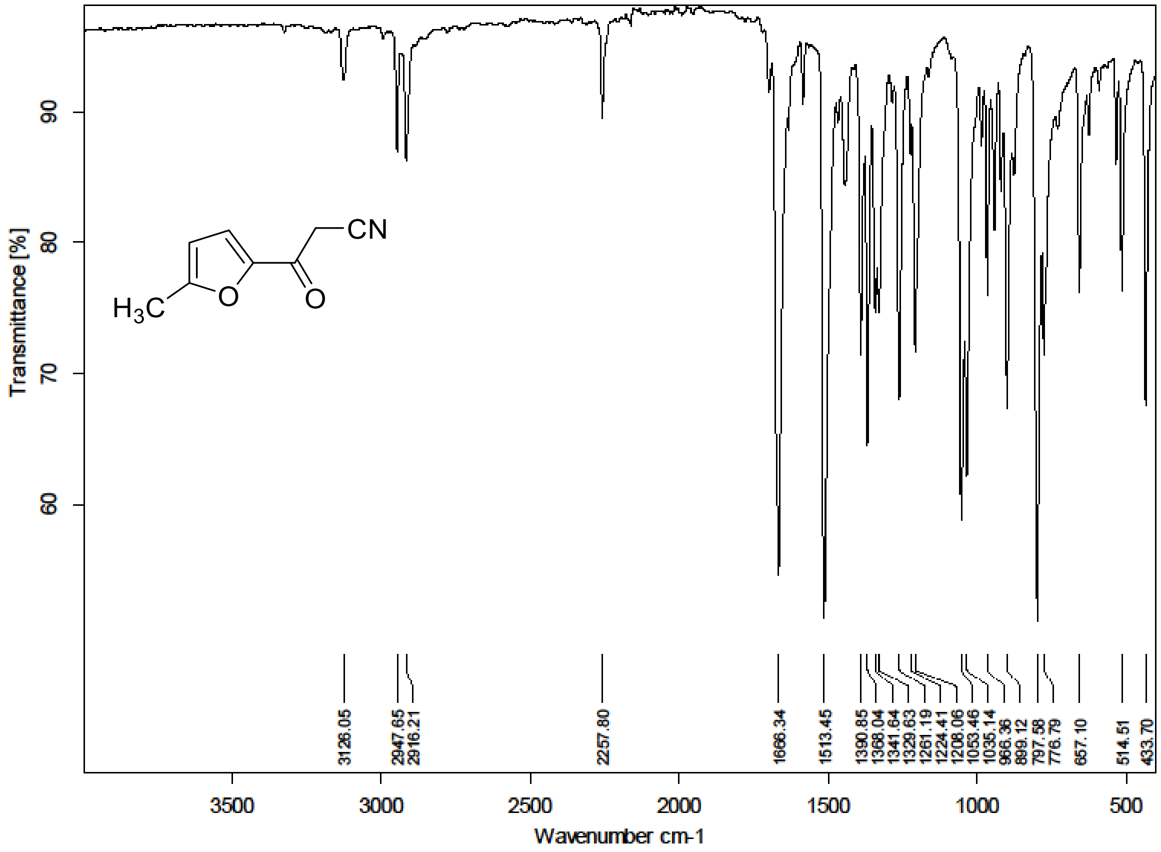

^{1}H (500 MHz) and ^{13}C NMR (126 MHz) spectra of **S116** in chloroform-*d*

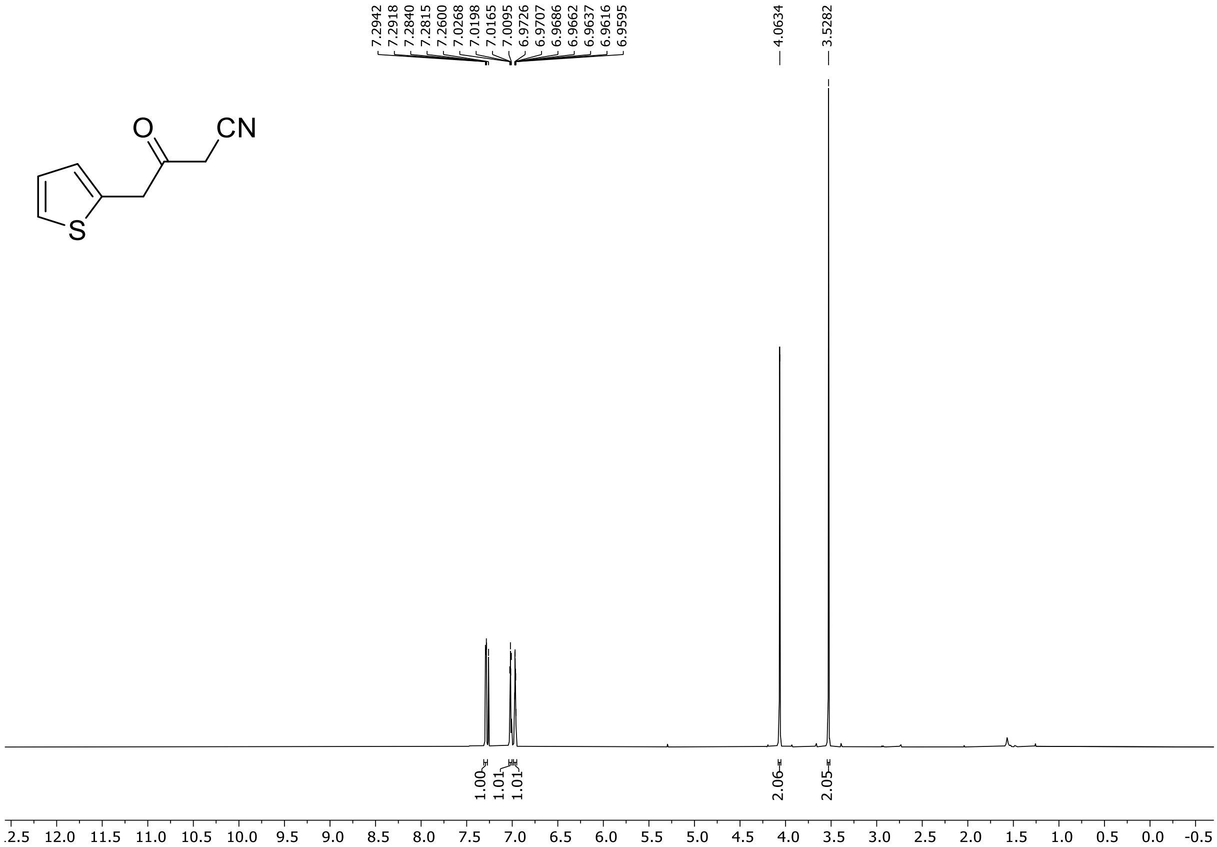

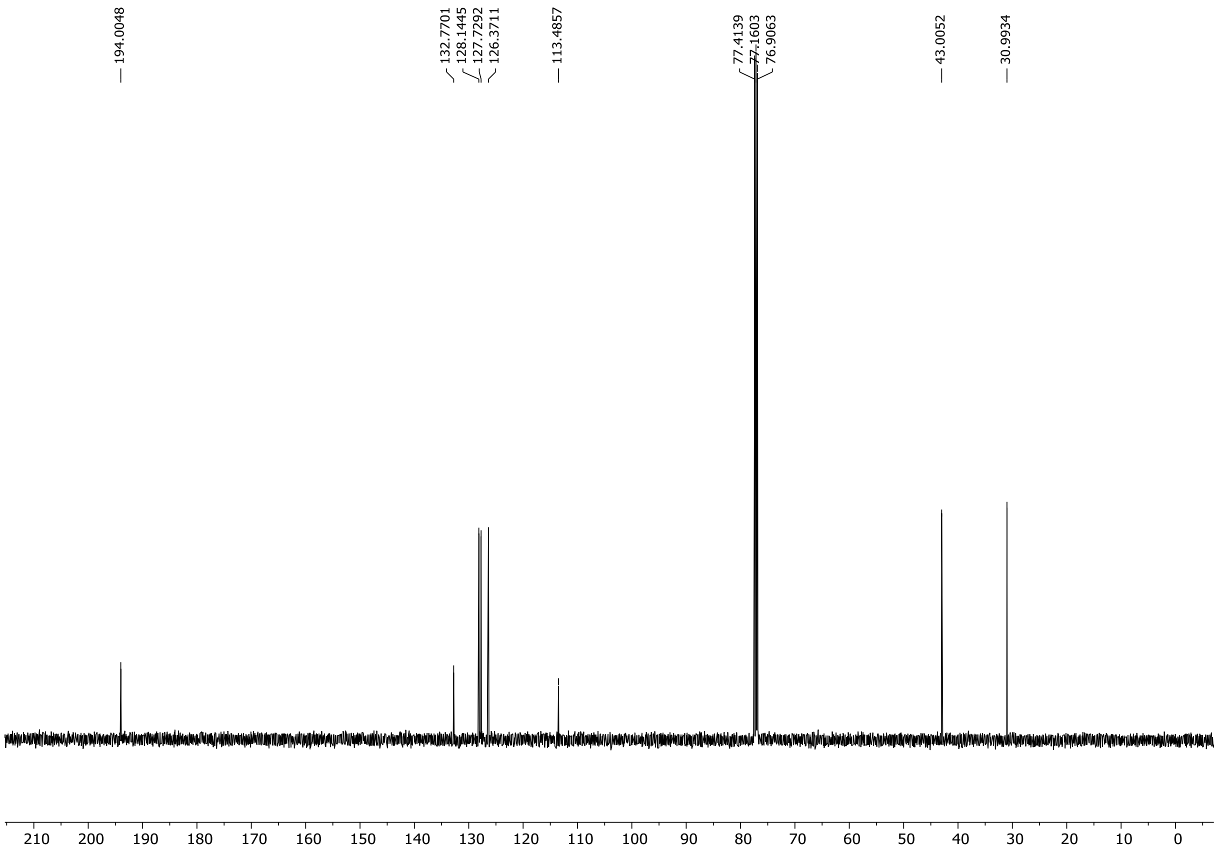

HRMS spectrum of **S116**

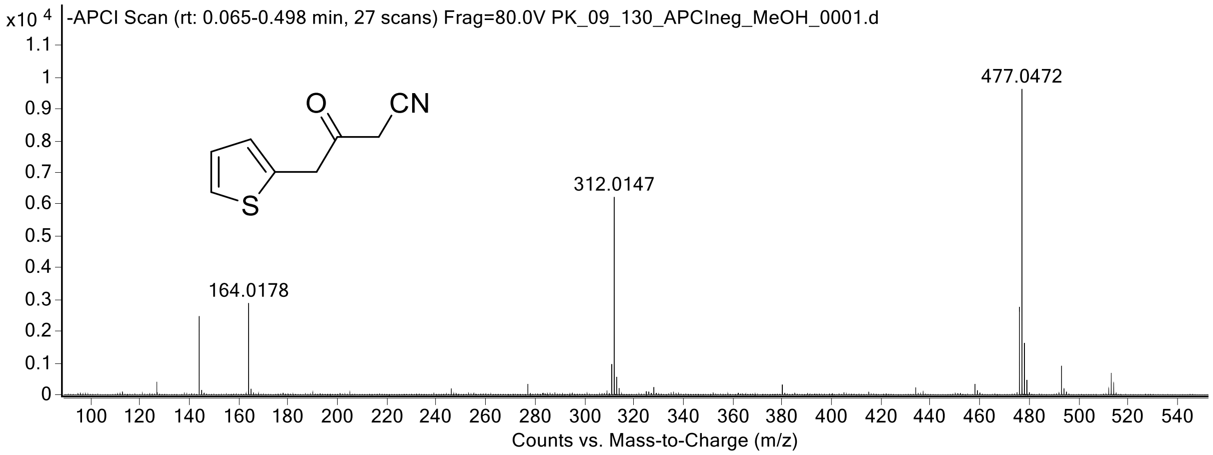

^{1}H (300 MHz) spectrum of **S117** in DMSO-d_6

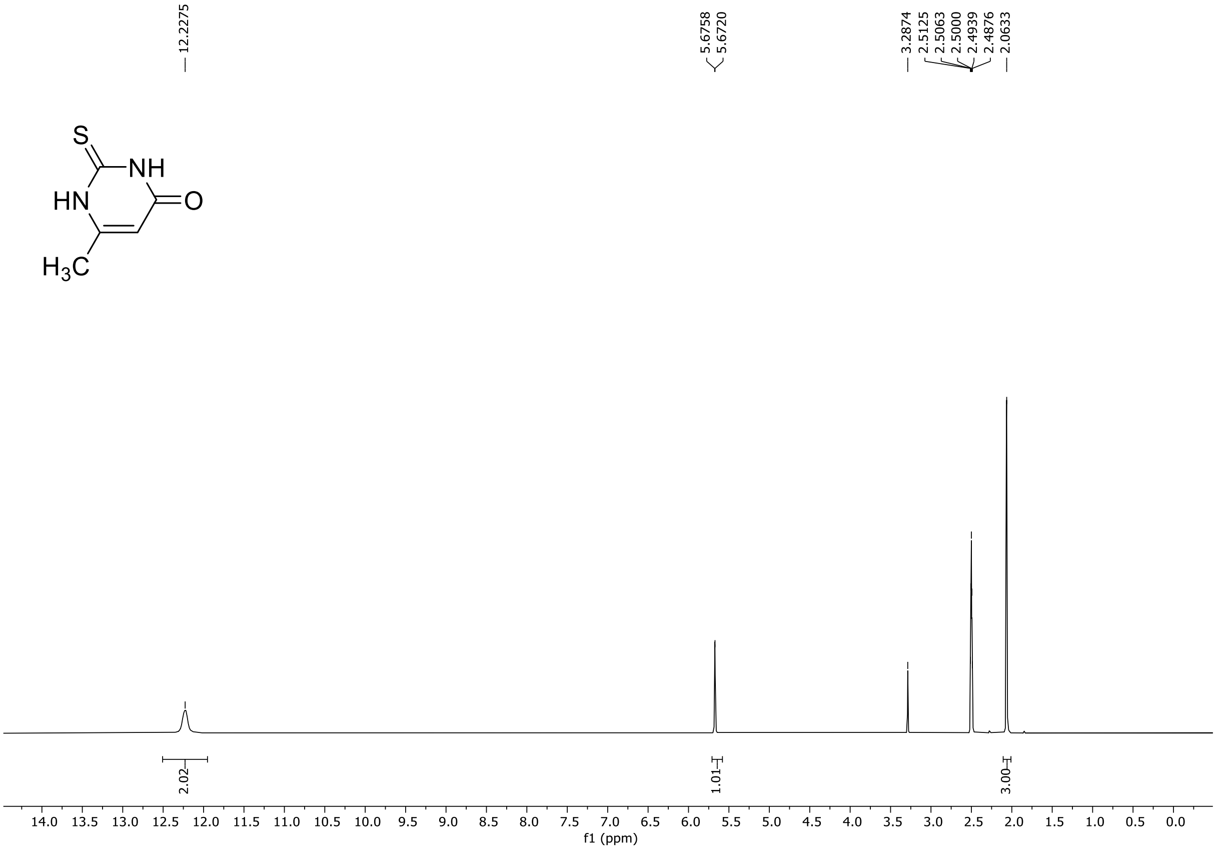

HRMS spectrum of **S117**

NAR-A-127

$C_5H_6N_2OS$

mono *m/z* 142.0201

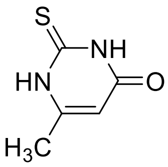

APCI - (MMI)
nitrogen flow 5 L/min, gas temperature 325°C, nebulizer 45 psi, skimmer 65 V, vaporizer 200°C, fragmentor 38 V, dissolved in methanol

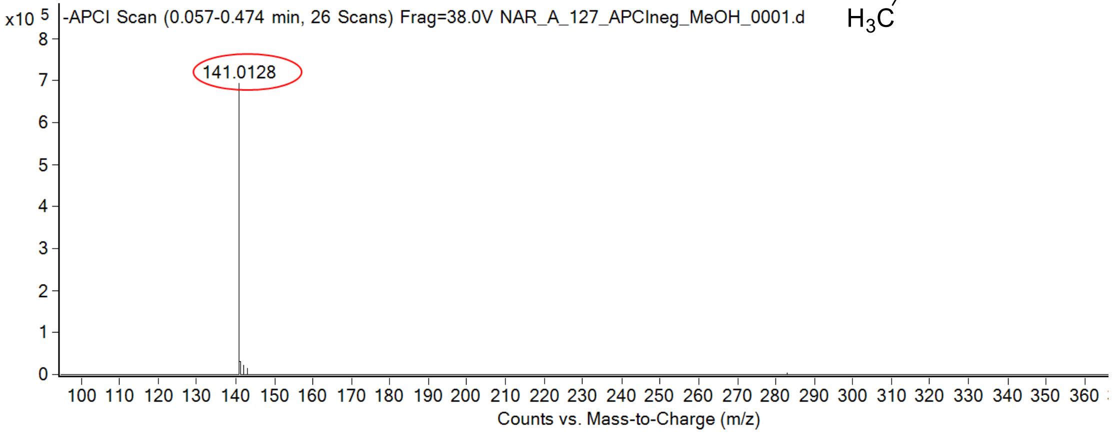

calculated mass: $[M-H]^-$ = 141.0128 observed: $[M-H]^-$ = 141.0128 mass accuracy = < 0.1 ppm

FT-IR spectrum (neat) of **S117**

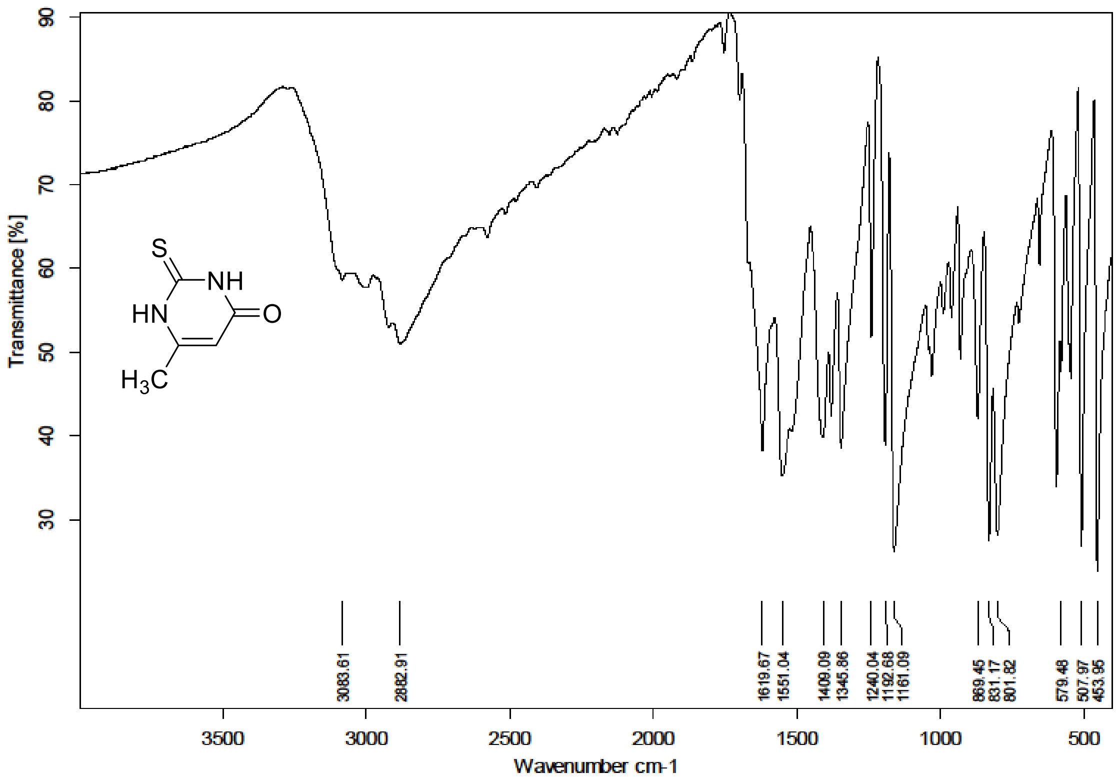

^{1}H (300 MHz) spectrum of **S118** in DMSO-d_6

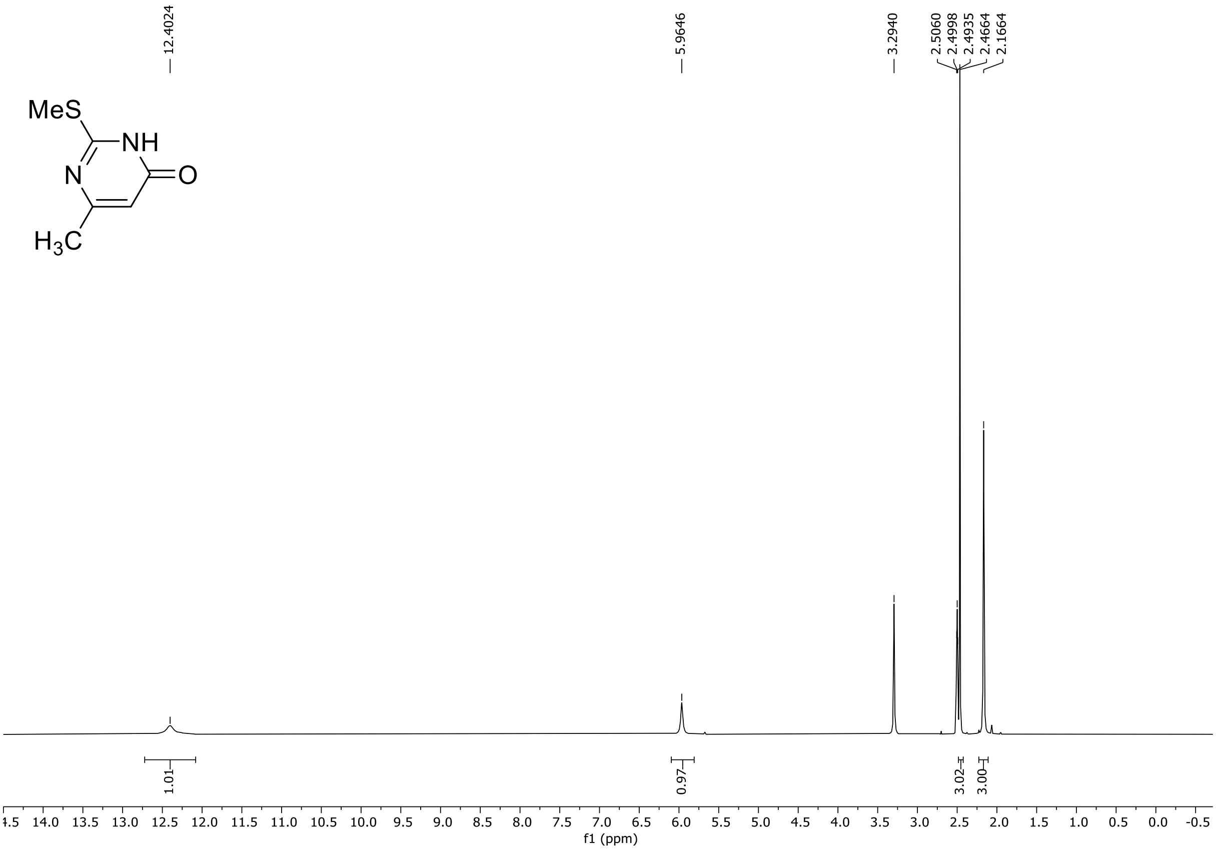

HRMS spectrum of **S118**

NAR-A-130 $C_6H_8N_2OS$ ***m/z* 156.0357**

APCI+ (MMI)
nitrogen flow 5 L/min, gas temperature 325°C, nebulizer 45 psi, skimmer 65 V, vaporizer 200°C, fragmentor 30 V, dissolved in MeOH

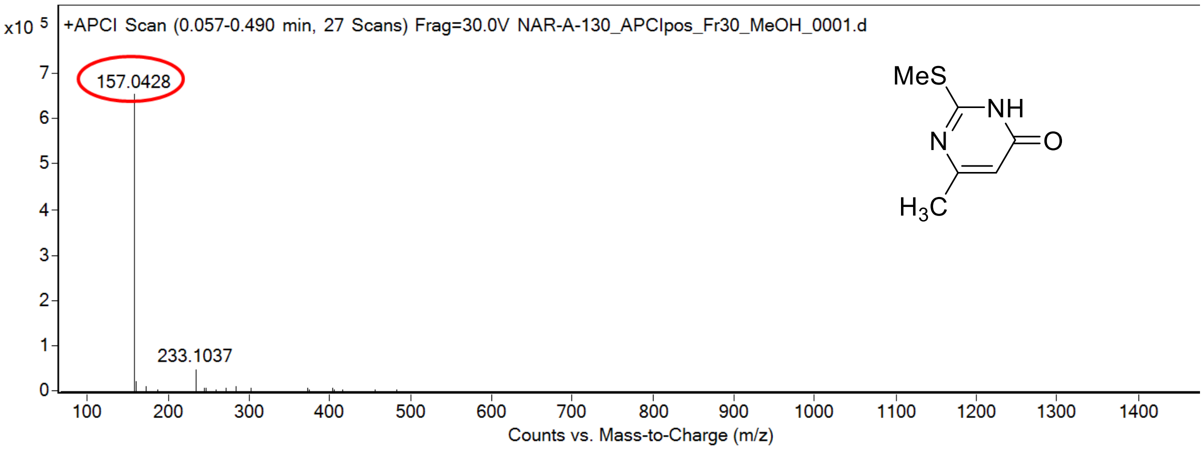

calculated mass: $[M+H]^+ = 157.0430$ observed: $[M+H]^+ = 157.0428$ mass accuracy = -1.3 ppm

FT-IR spectrum (neat) of **S118**

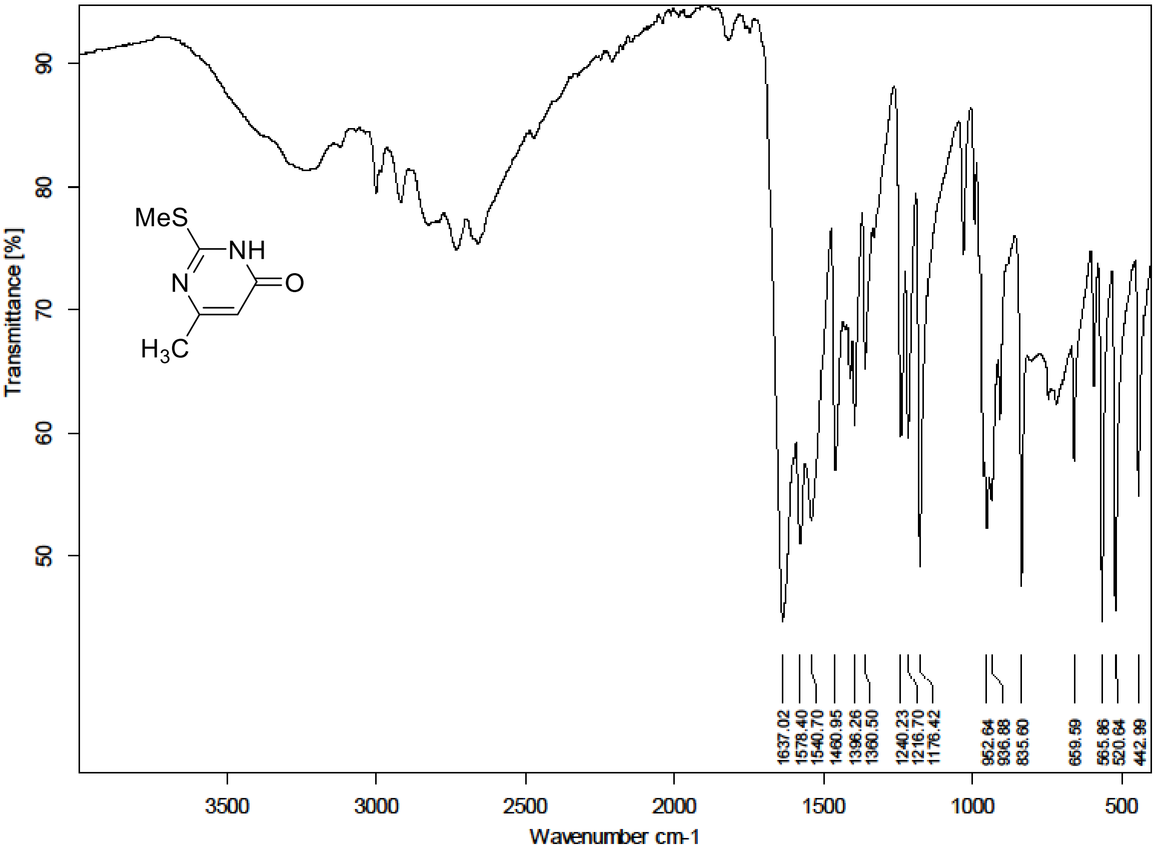

^1H (300 MHz) spectrum of **S119** in DMSO-d_6

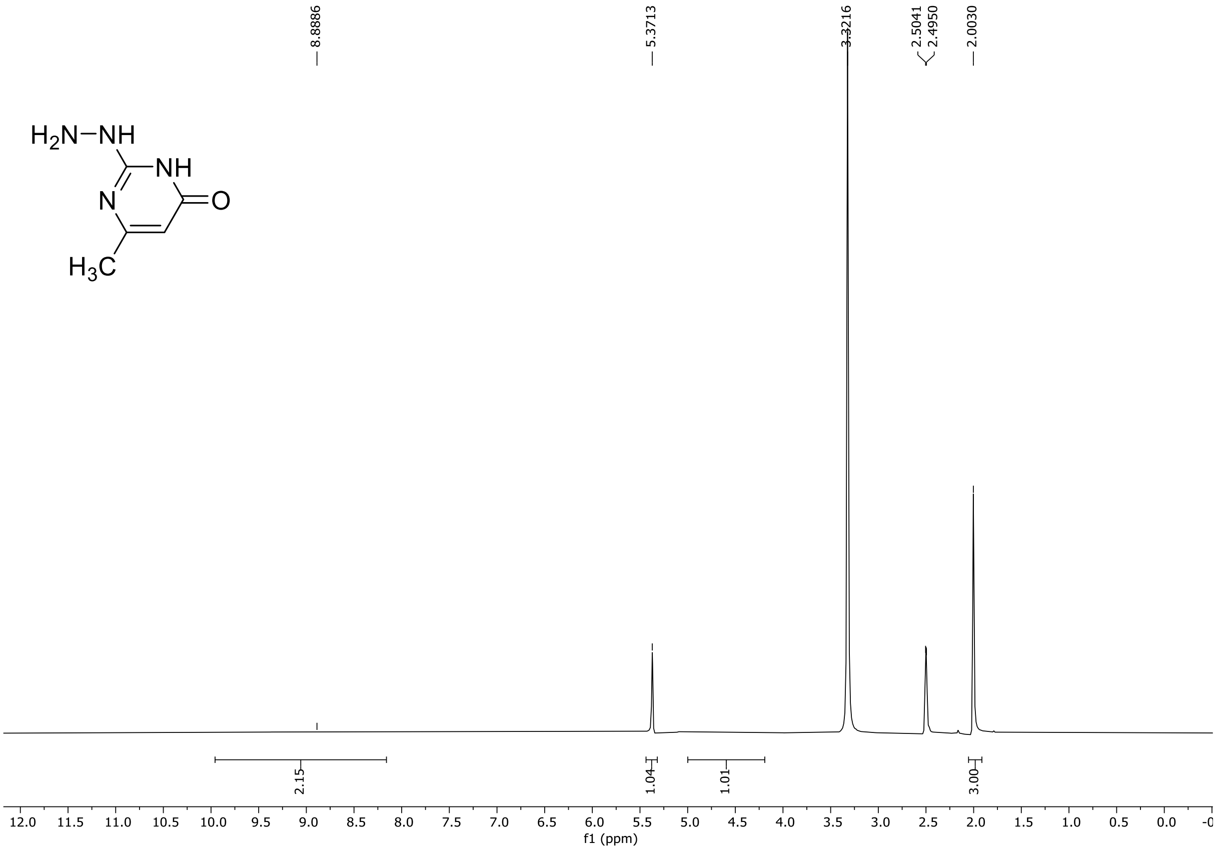

HRMS spectrum of **S119**

NAR-A-133 **$C_5H_8N_4O$** **mono *m/z* 140.0698**

APCI + (MMI)
nitrogen flow 5 L/min, gas temperature 325°C, nebulizer 45 psi, skimmer 65 V, vaporizer 200°C, fragmentor 32 V, dissolved in methanol

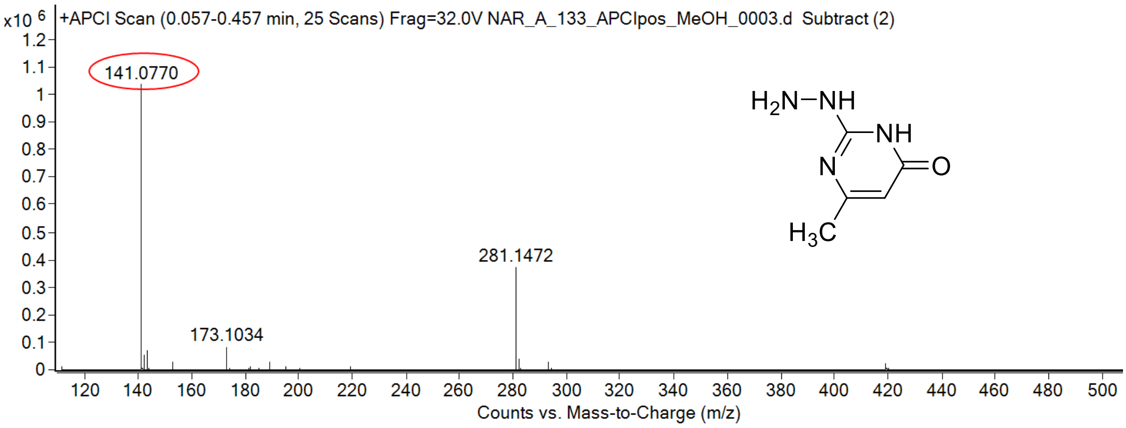

calculated mass: $[M+H]^+$ = 141.0771 observed: $[M+H]^+$ = 141.0770 mass accuracy = - 0.7 ppm

FT-IR spectrum (neat) of **S119**

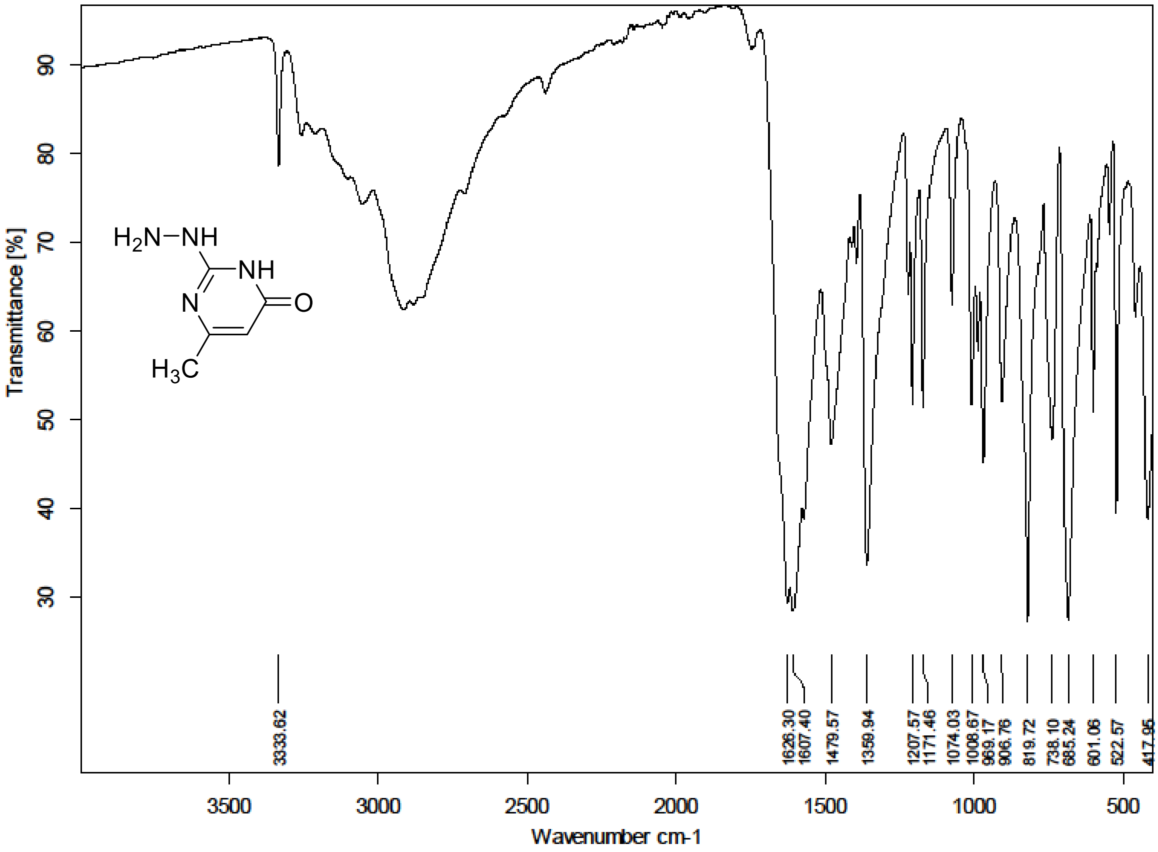

^{1}H (500 MHz) and ^{13}C NMR (126 MHz) spectra of **S120** in DMSO-d_6

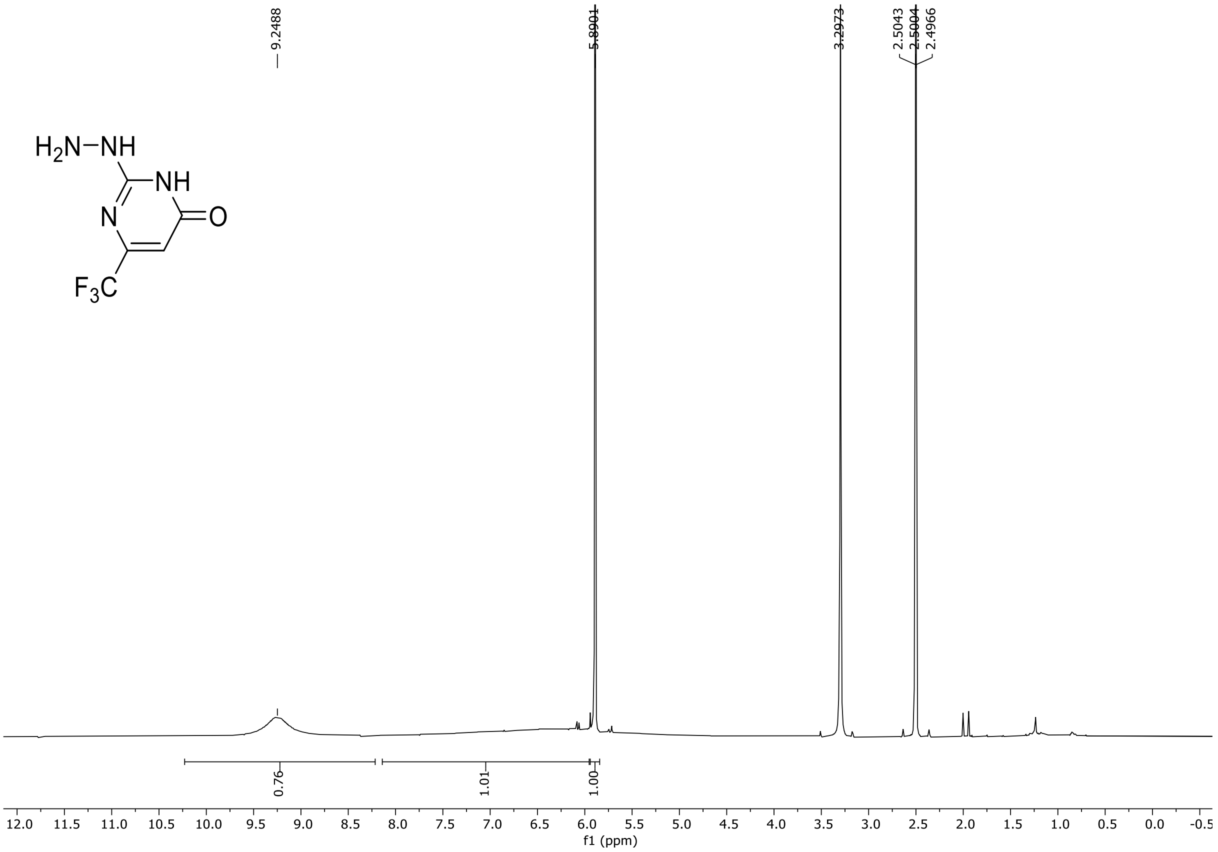

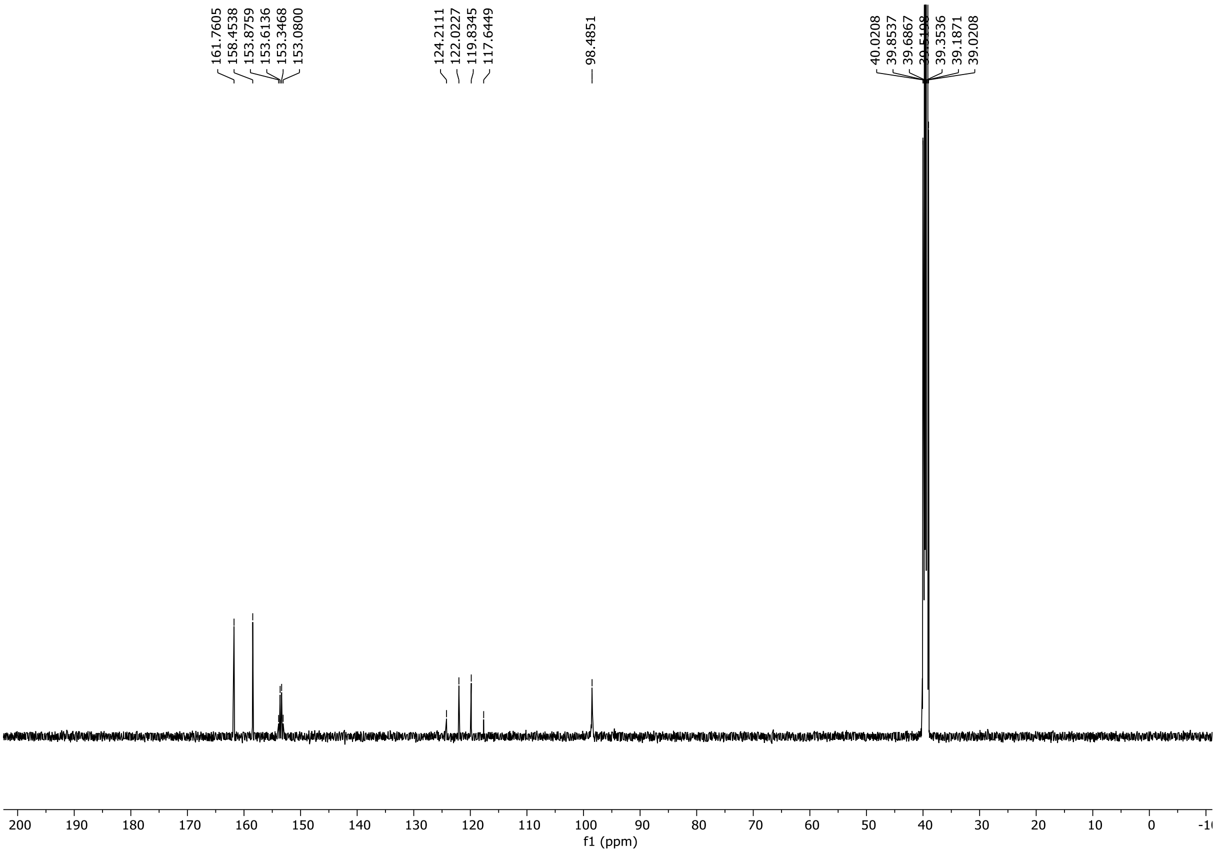

^{19}F NMR (471 MHz) spectrum of **S120** in DMSO-d_6

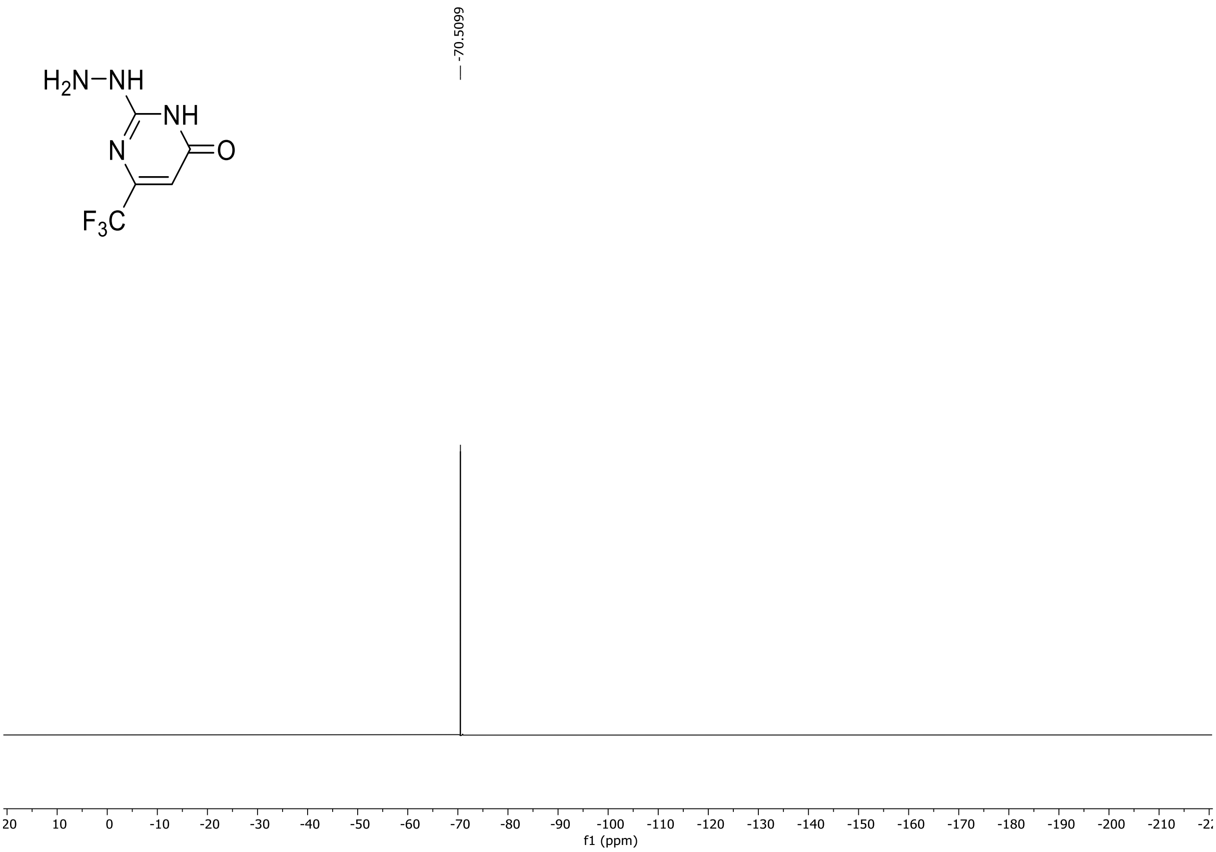

HRMS spectrum of **S120**

NAR-A-89 **$C_5H_5F_3N_4O$** ***m/z* 194.0415**

APCI+ (MMI)
nitrogen flow 5 L/min, gas temperature 325°C, nebulizer 45 psi, skimmer 65 V,
vaporizer 200°C, fragmentor 30 V, dissolved in MeOH

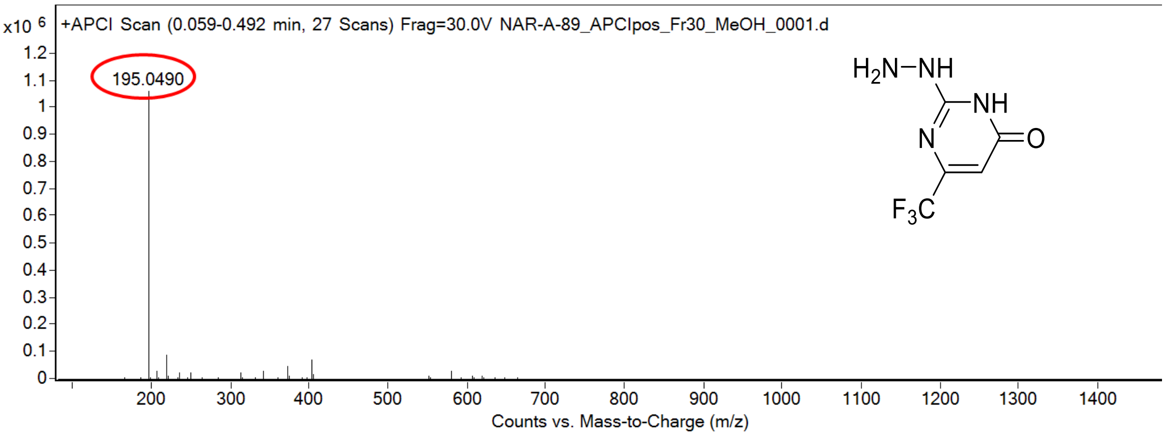

calculated mass: $[M+H]^+$ = 195.0488 observed: $[M+H]^+$ = 195.0490 mass accuracy = +1.0 ppm

FT-IR spectrum (neat) of **S120**

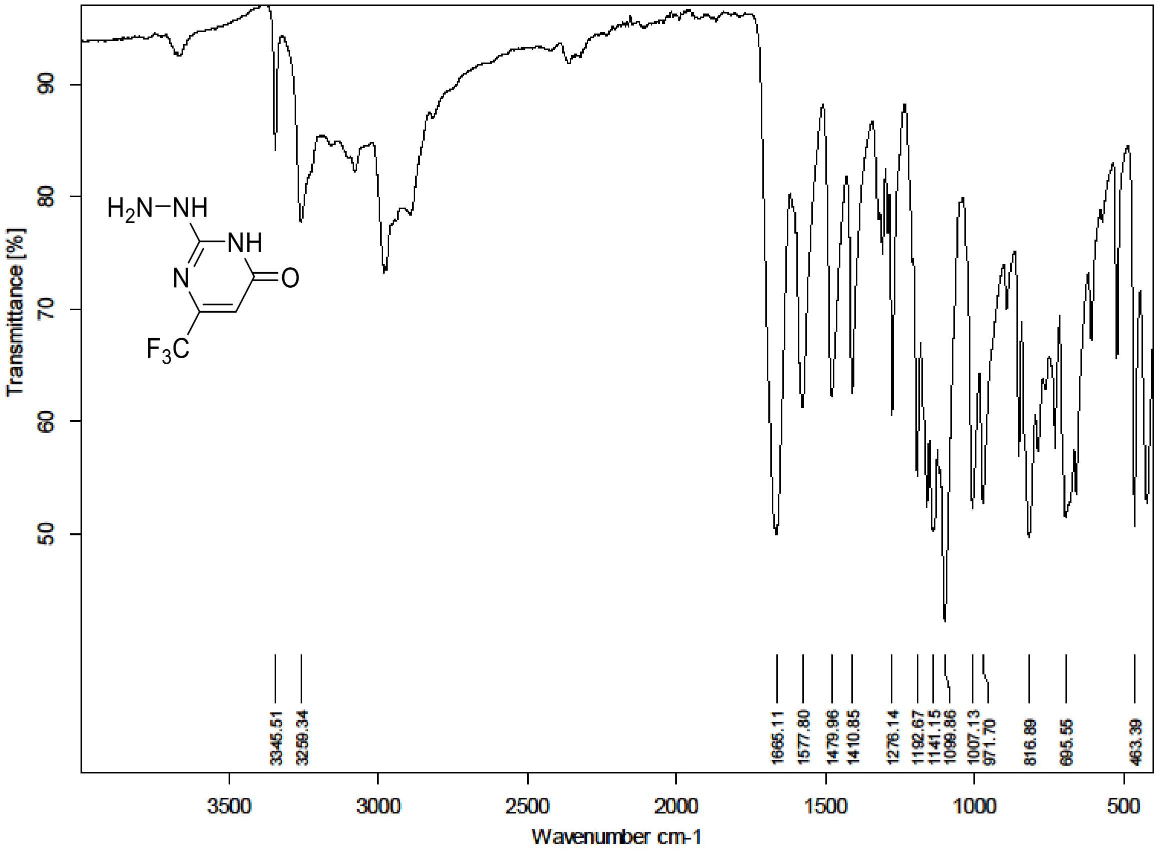

^{1}H (300 MHz) and ^{13}C NMR (75 MHz) spectra of **S121** in DMSO-d_6

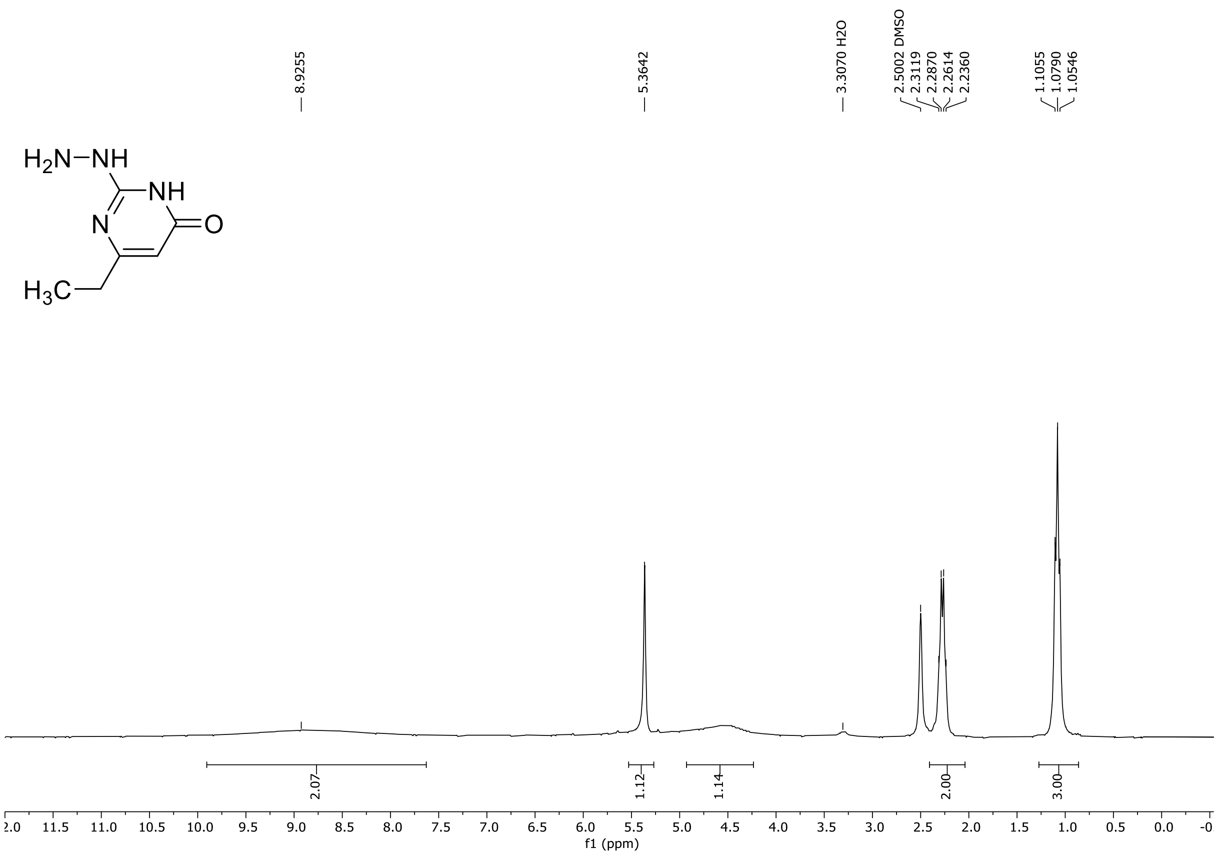

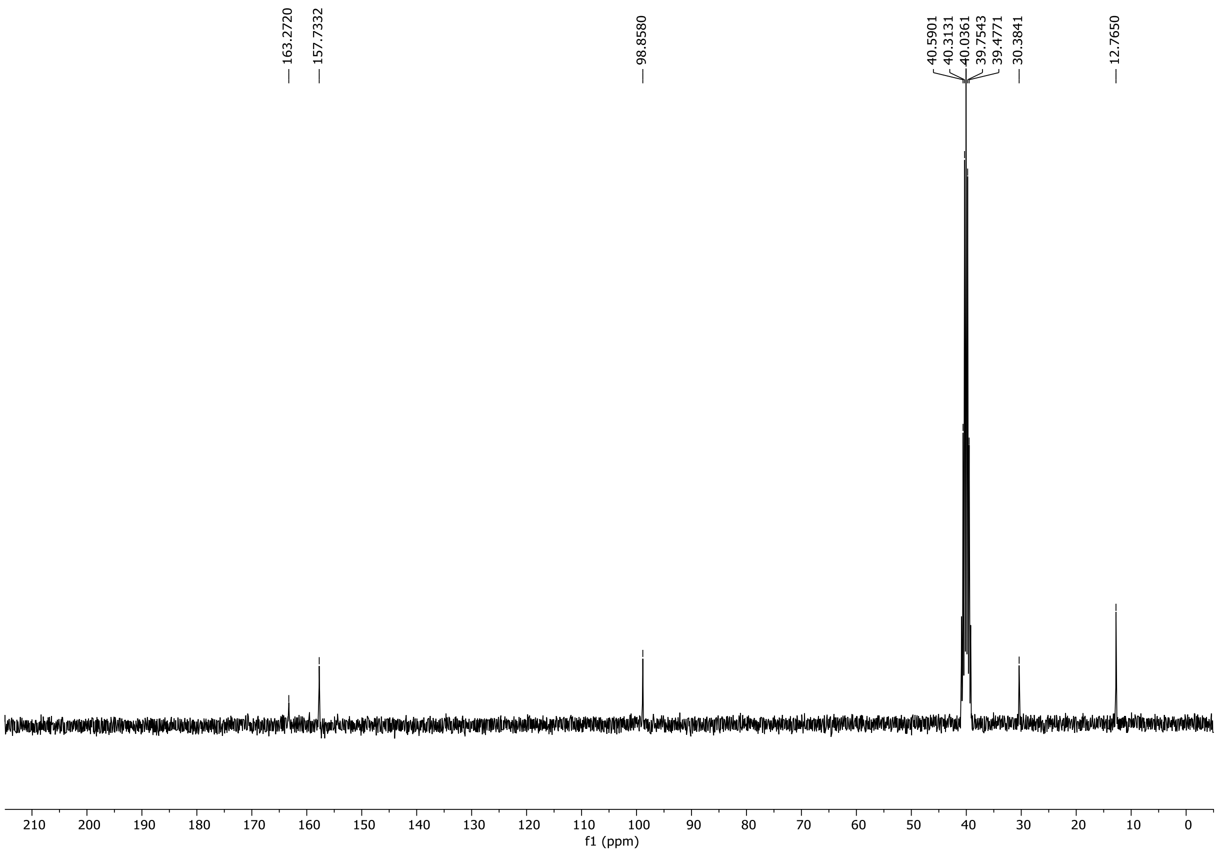

HRMS spectrum of **S121**

NAR-A-164 $C_6H_{10}N_4O$ mono *m/z* **154.0855**

APCI + (MMI)
nitrogen flow 5 L/min, gas temperature 325°C, nebulizer 45 psi, skimmer **65** V, vaporizer 200°C, fragmentor 20 V, dissolved in methanol

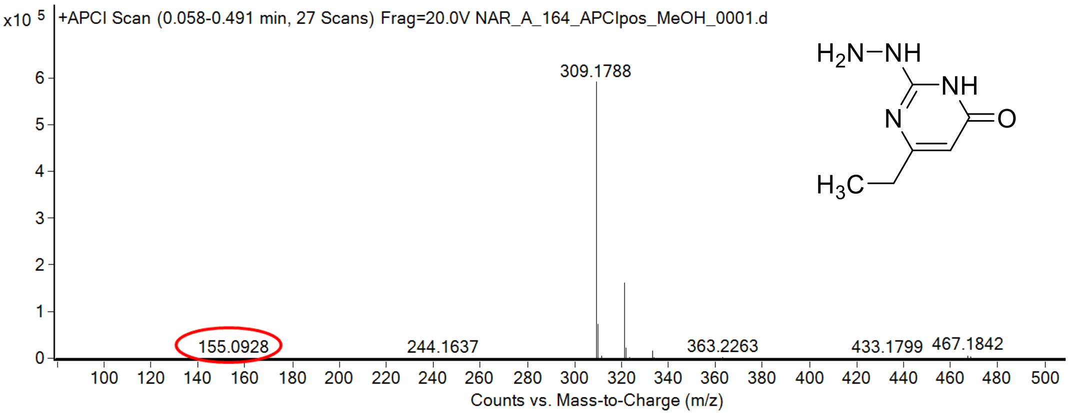

calculated mass: $[M+H]^+$ = 155.0927 observed: $[M+H]^+$ = 155.0928 mass accuracy = 0.6 ppm

FT-IR spectrum (neat) of **S121**

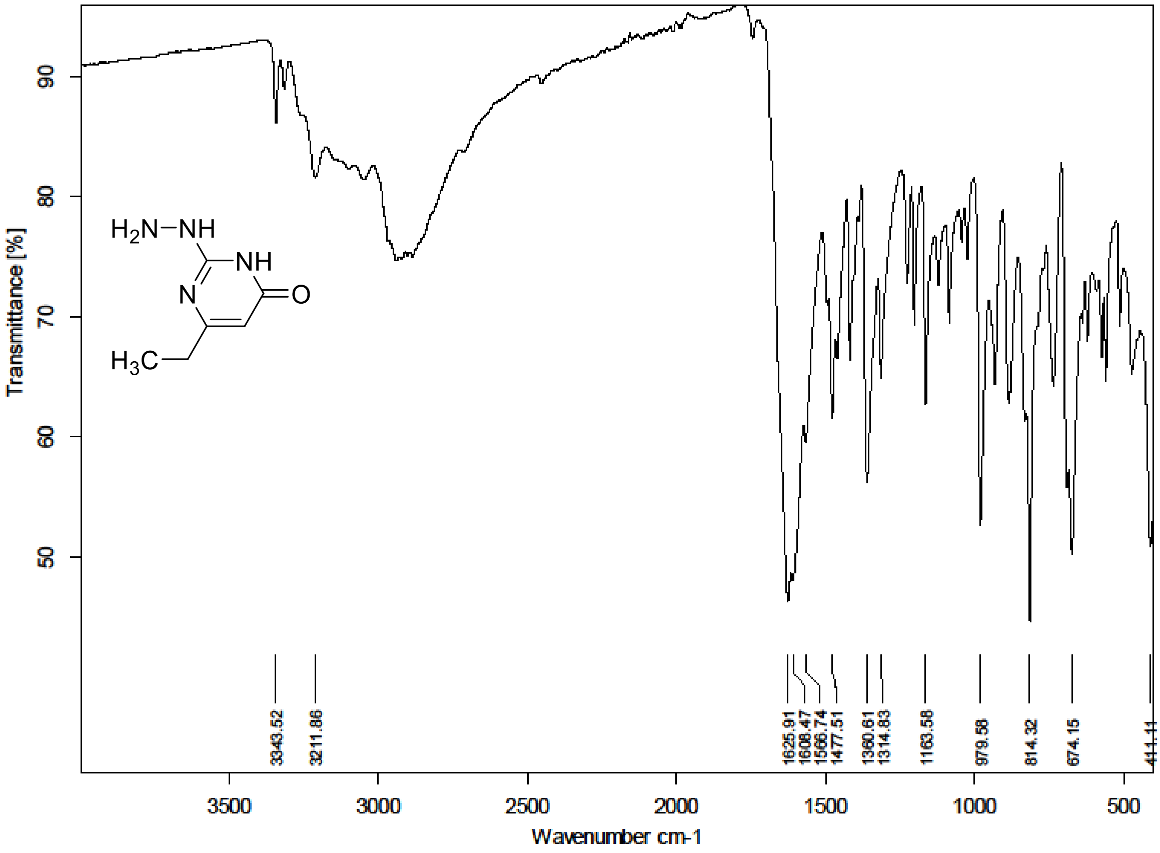

^{1}H (300 MHz) and ^{13}C NMR (75 MHz) spectra of **S122** in Chloroform-*d*

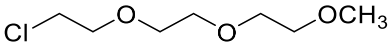

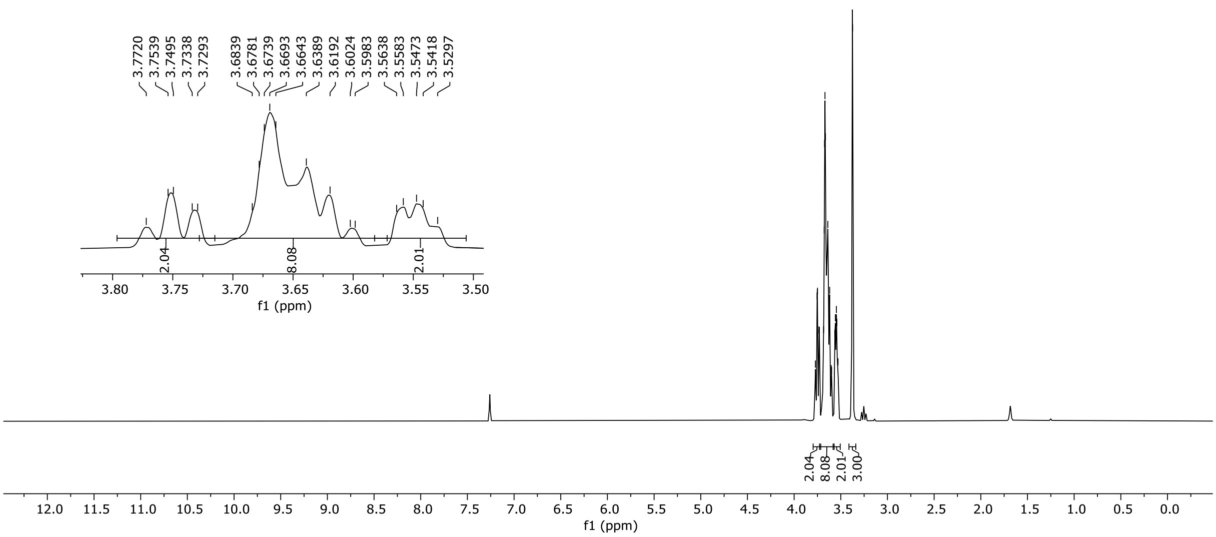

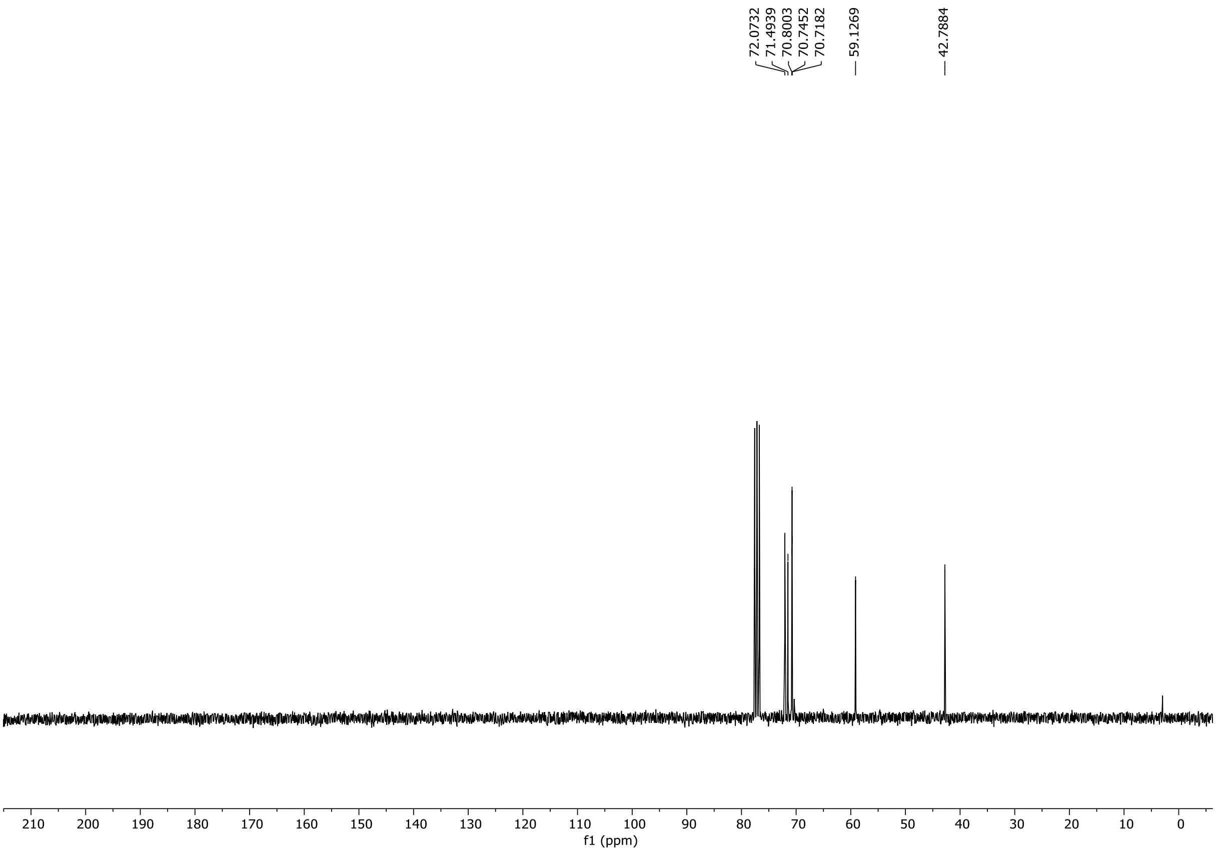

HRMS spectrum of **S122**

NAR-A-185

$C_7H_{15}ClO_3$ mono *m/z* 182.0710

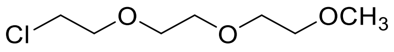

APCI + (MMI)
nitrogen flow 5 L/min, gas temperature 325°C, nebulizer 45 psi, skimmer **65** V, vaporizer 200°C, fragmentor 35 V, dissolved in methanol

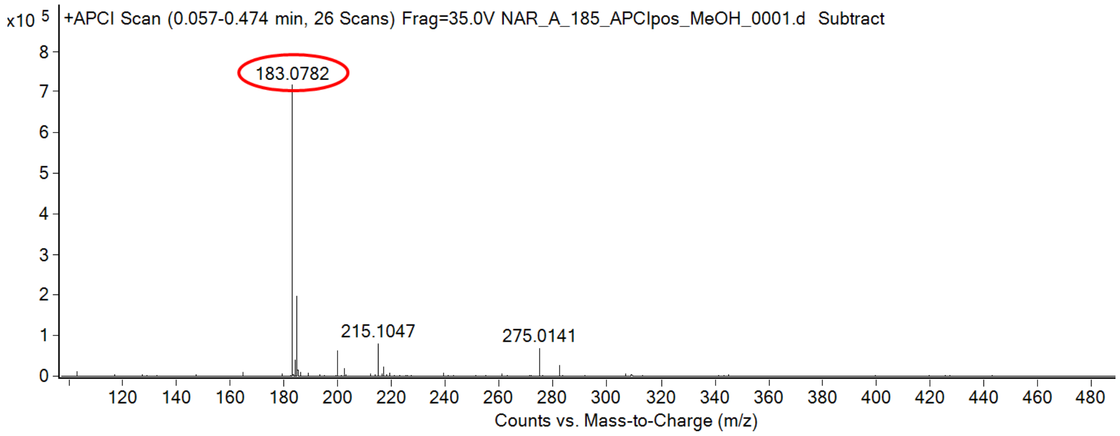

calculated mass: $[M+H]^+$ = 183.0782 observed: $[M+H]^+$ = 183.0782 mass accuracy = < 0.1 ppm

FT-IR spectrum (neat) of **S122**

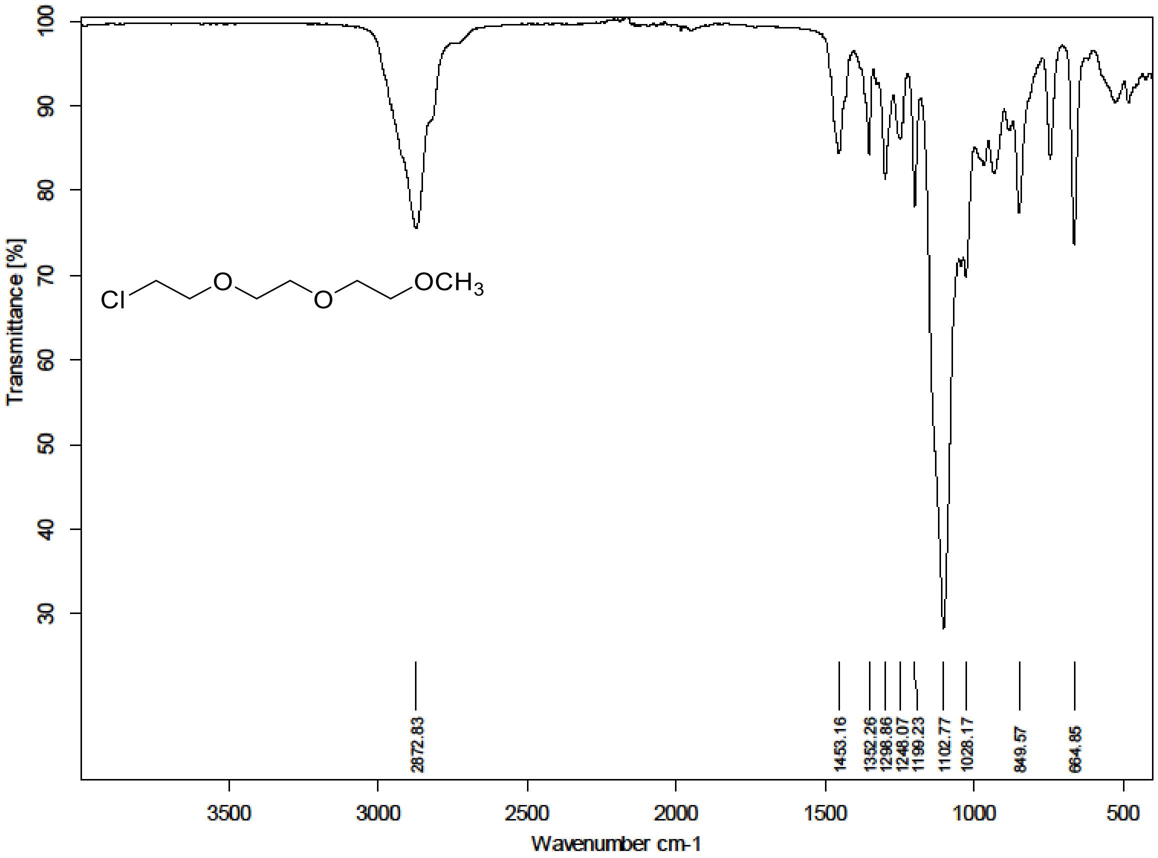

^{1}H (300 MHz) and ^{13}C NMR (75 MHz) spectra of **S123** in Chloroform-*d*

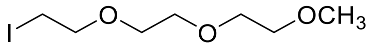

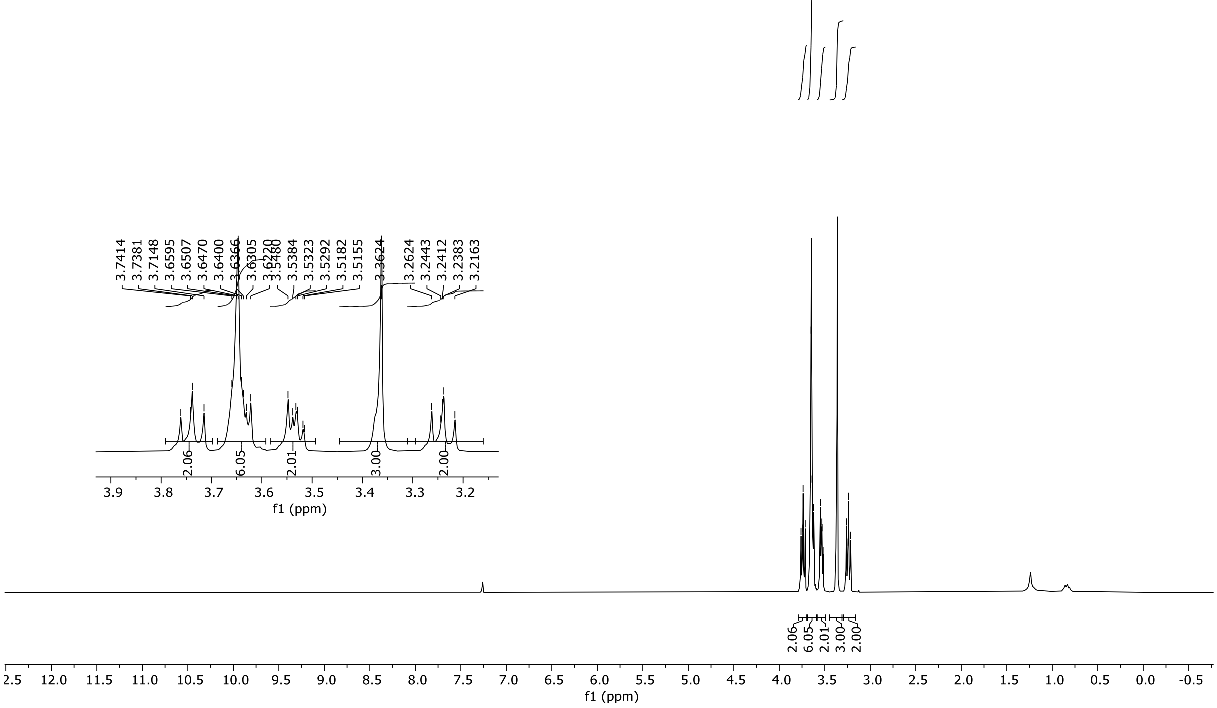

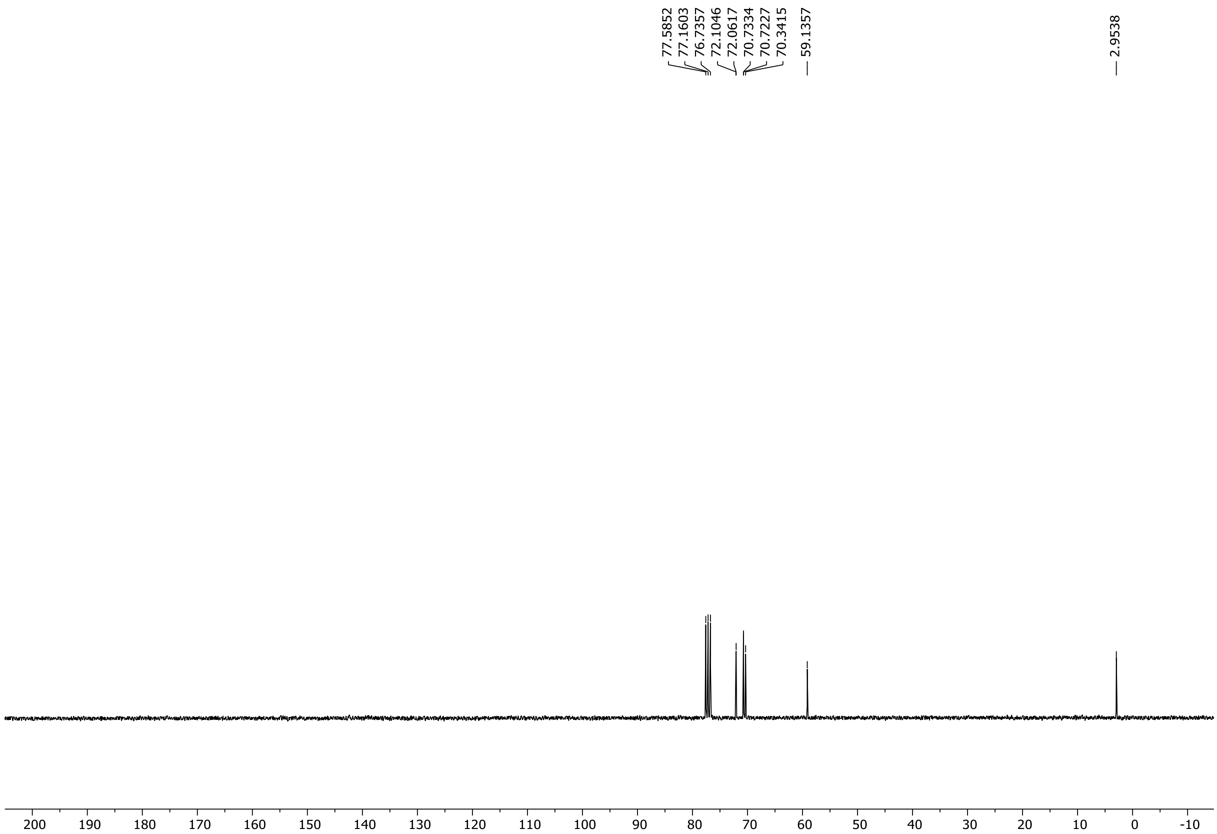

HRMS spectrum of **S123**

NAR-A-212 $C_7H_{15}IO_3$ mono *m/z* 274.0066

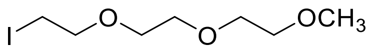

APCI + (MMI)
nitrogen flow 5 L/min, gas temperature 325°C, nebulizer 45 psig, skimmer 65 V, vaporizer 200°C, fragmentor 30 V, dissolved in methanol

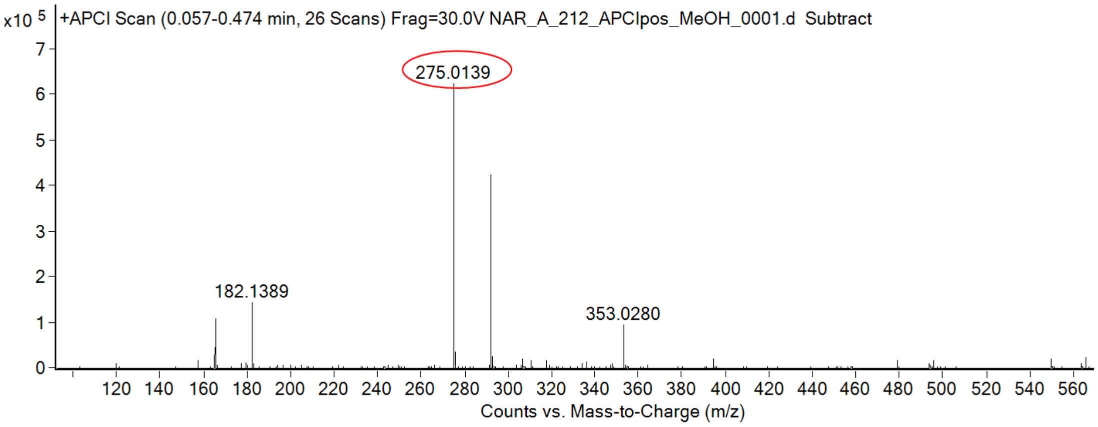

calculated mass: $[M+H]^+$ = 275.0139 observed: $[M+H]^+$ = 275.0139 mass accuracy = < 0.1 ppm

FT-IR spectrum (neat) of **S123**

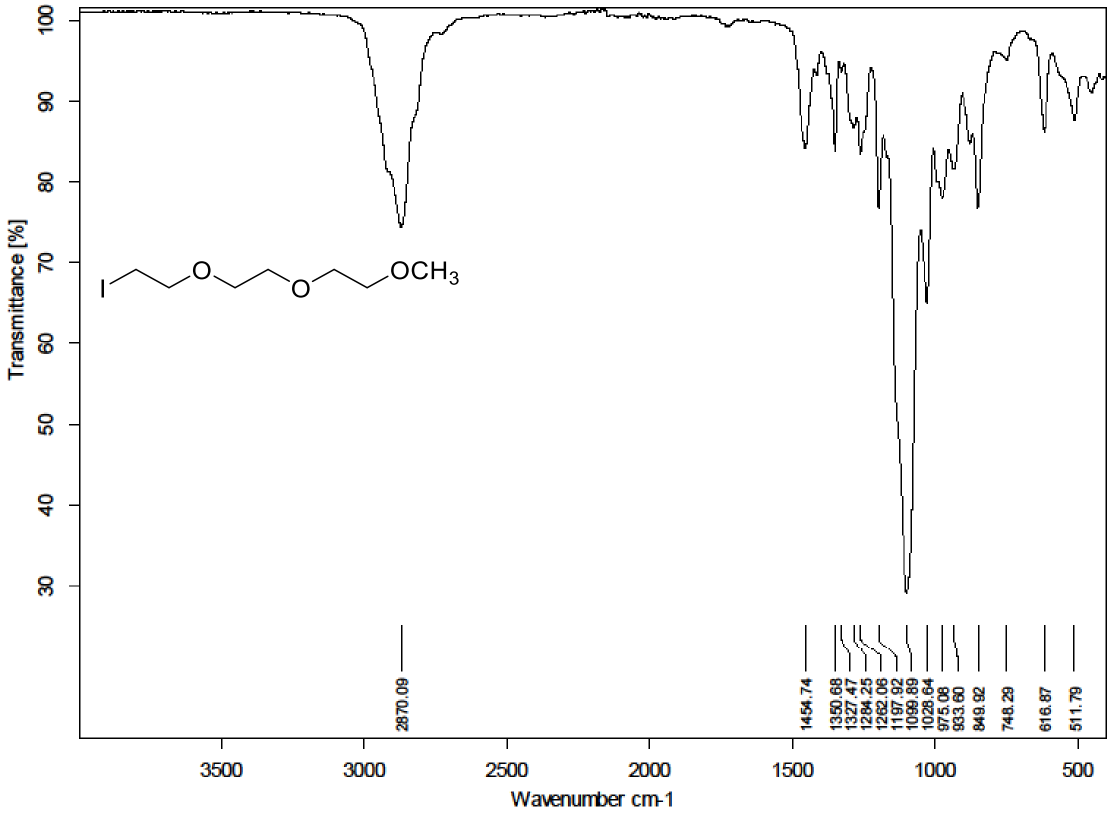

^{1}H (300 MHz) and ^{13}C NMR (75 MHz) spectra of **S124** in Chloroform-*d*

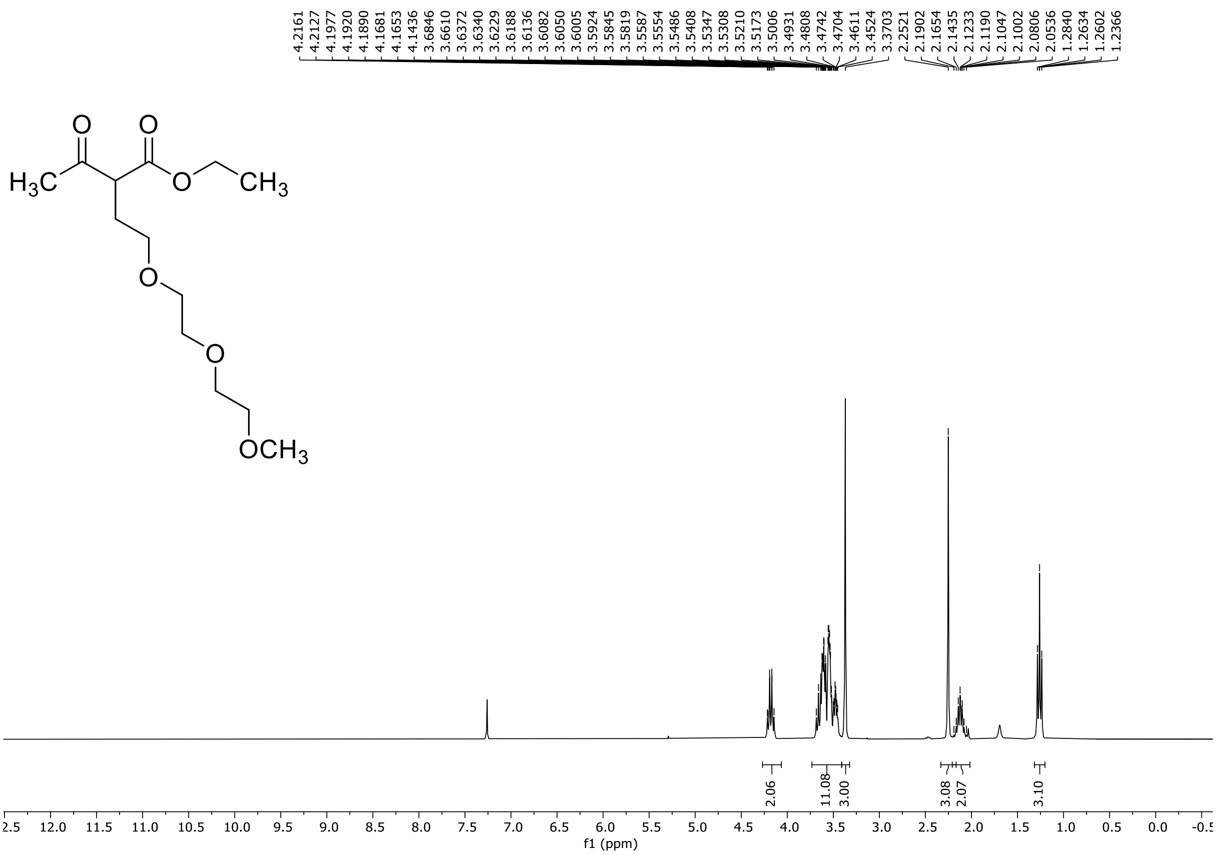

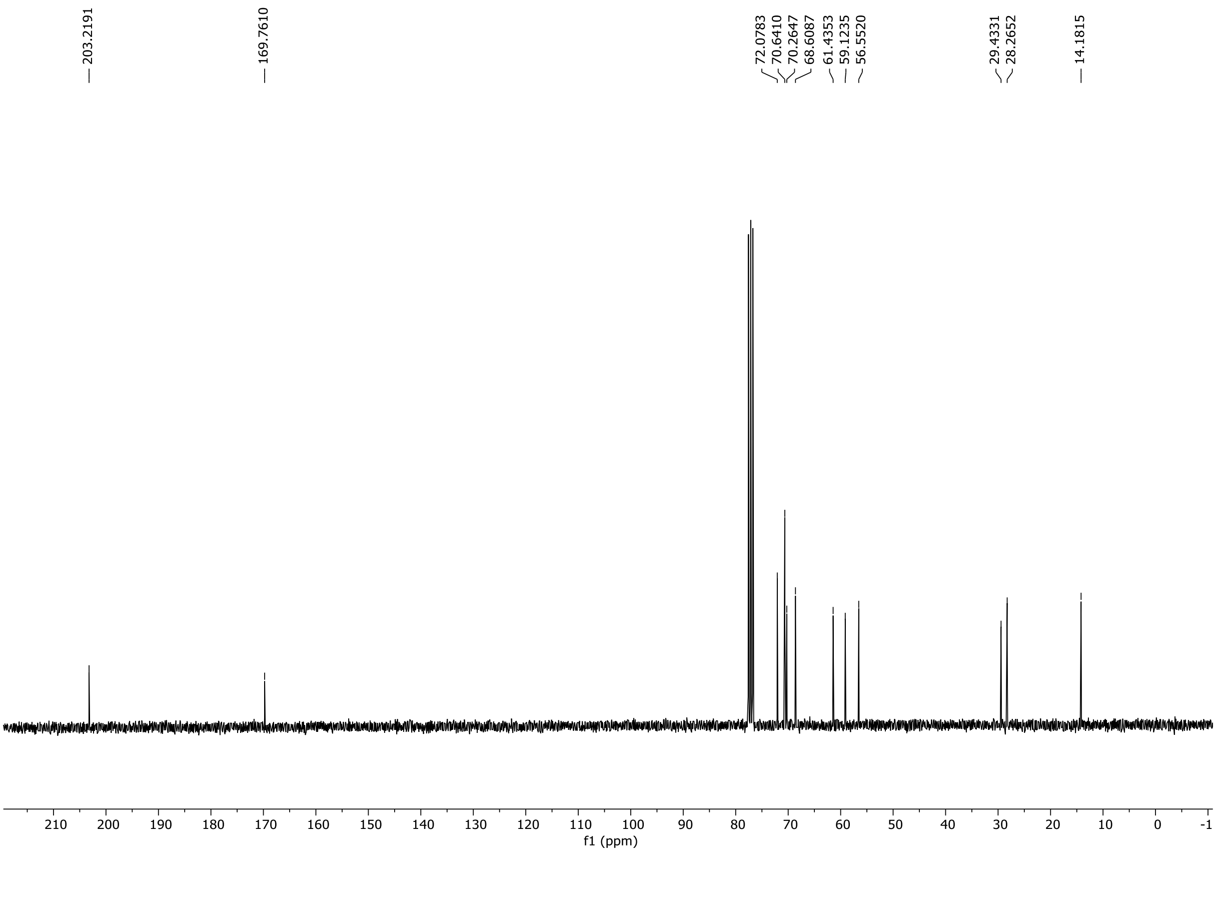

HRMS spectrum of **S124**

NAR-A-203 $C_{13}H_{24}O_6$ mono *m/z* 276.1573

APCI + (MMI)
nitrogen flow 5 L/min, gas temperature 325°C, nebulizer 45 psi, skimmer **65** V, vaporizer 200°C, fragmentor 28 V, dissolved in methanol

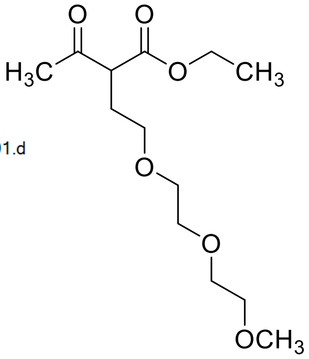

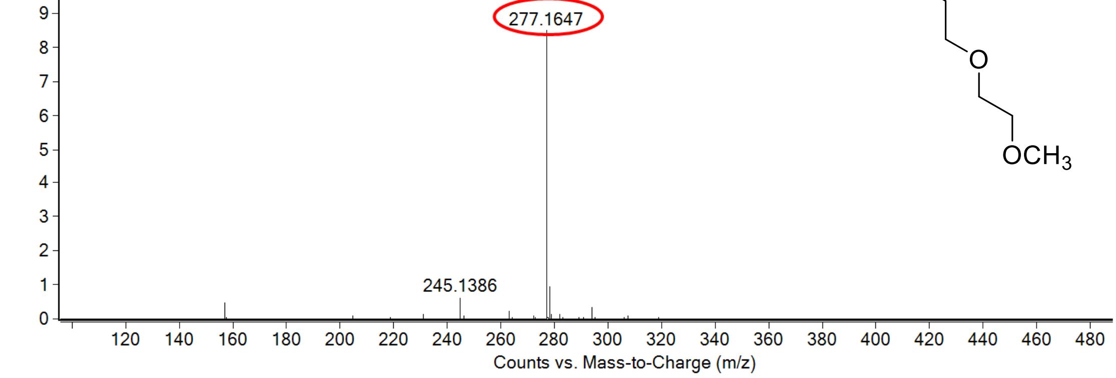

calculated mass: $[M+H]^+ = 277.1646$ observed: $[M+H]^+ = 277.1647$ mass accuracy = 0.4 ppm

Miroslava Bittová

FT-IR spectrum (neat) of **S124**

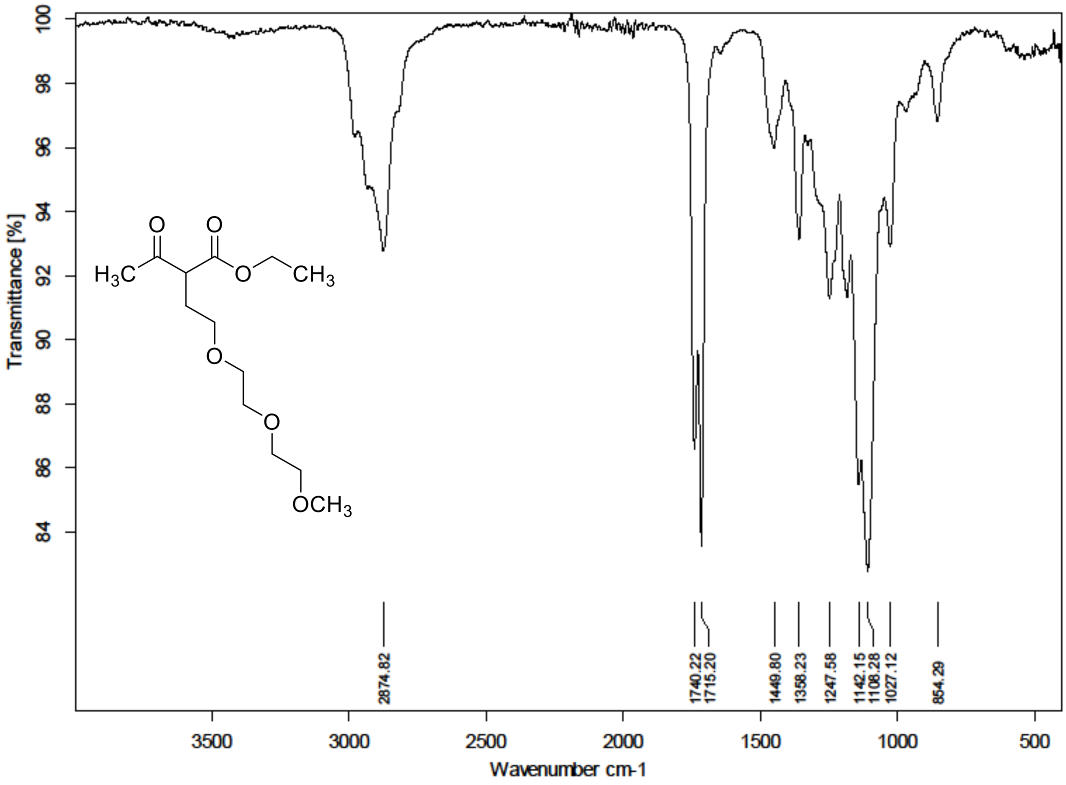

^{1}H (300 MHz) and ^{13}C NMR (75 MHz) spectra of **S125** in Chloroform-*d*

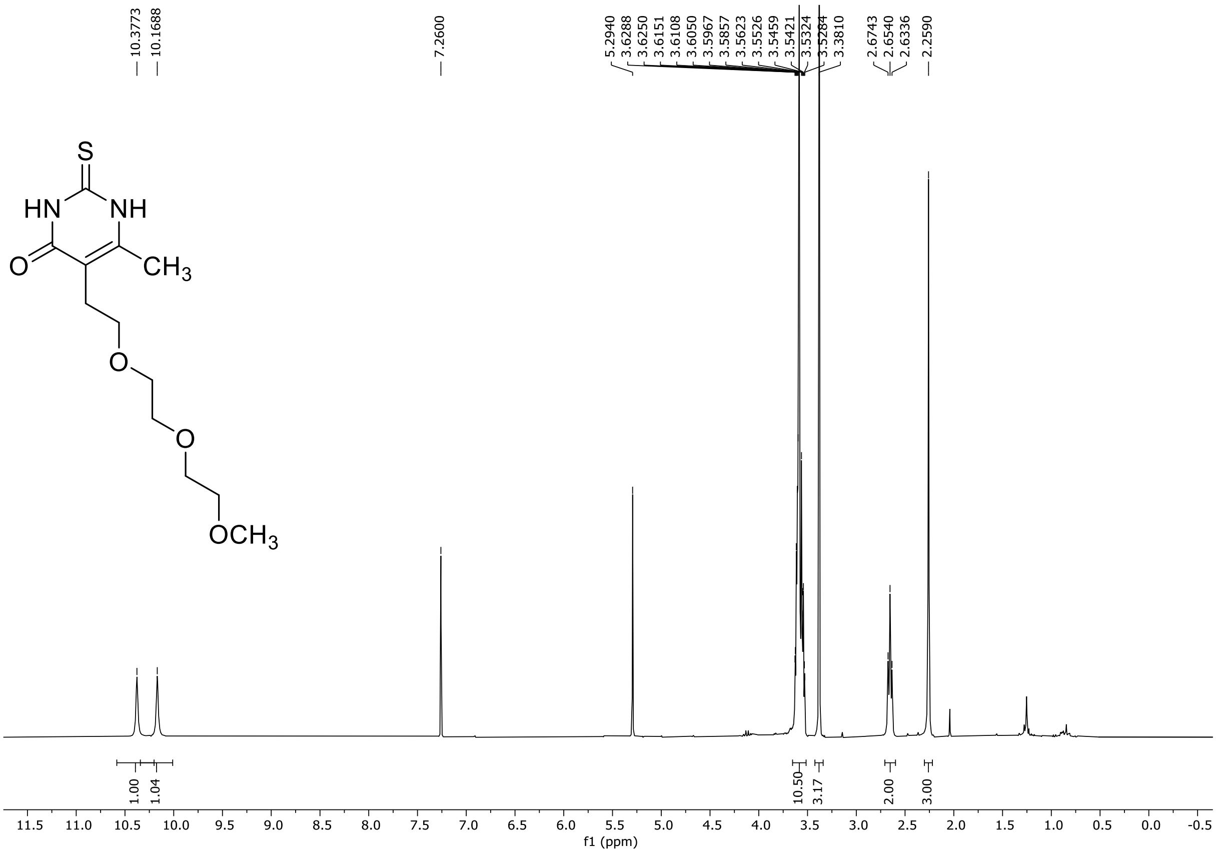

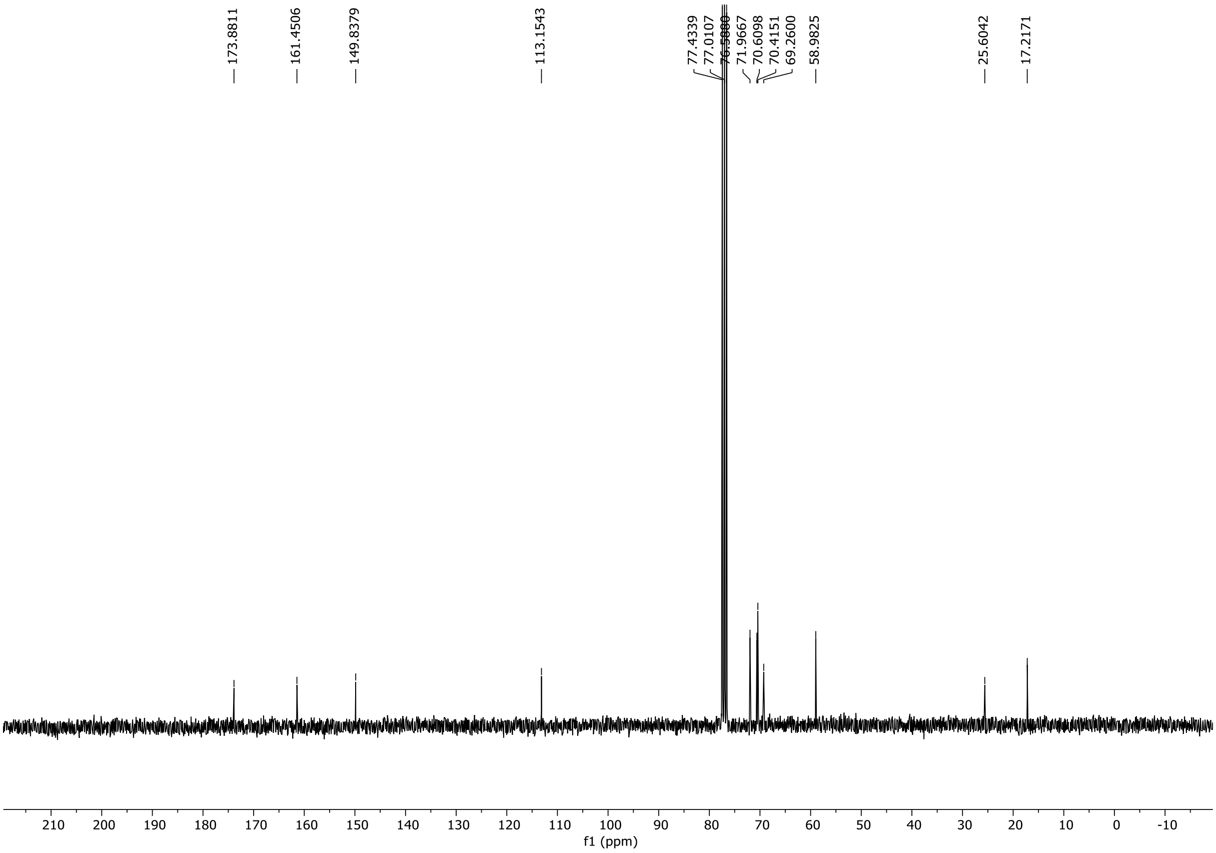

HRMS spectrum of **S125**

NAR-A-213 $C_{12}H_{20}N_2O_4S$ mono *m/z* 288.1144

APCI + (MMI)
nitrogen flow 5 L/min, gas temperature 325°C, nebulizer 45 psig, skimmer 65 V, vaporizer 200°C, fragmentor 22 V, dissolved in methanol

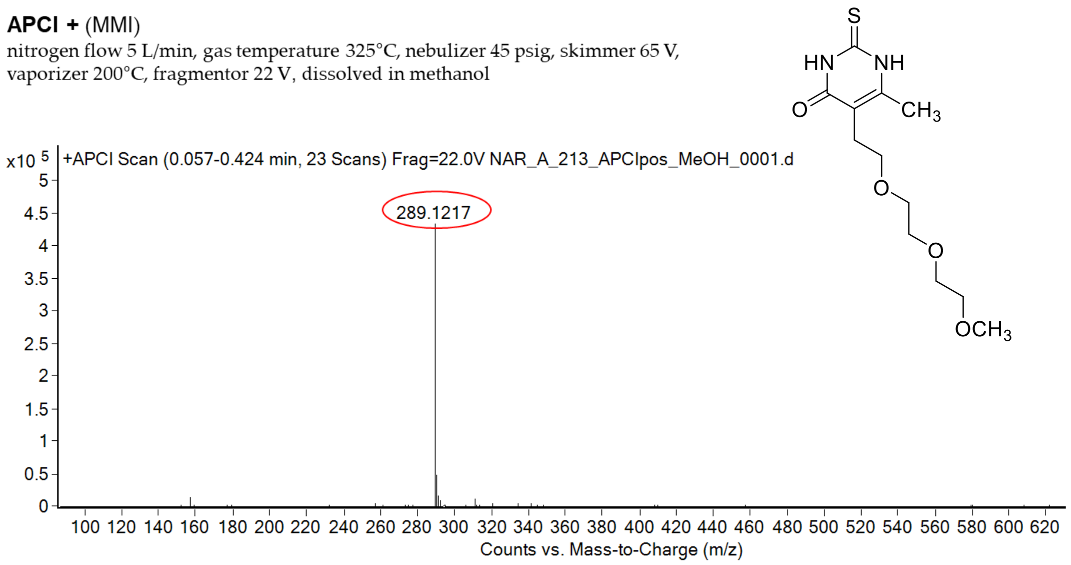

calculated mass: $[M+H]^+$ = 289.1217 observed: $[M+H]^+$ = 289.1217 mass accuracy = < 0.1 ppm

FT-IR spectrum (neat) of **S125**

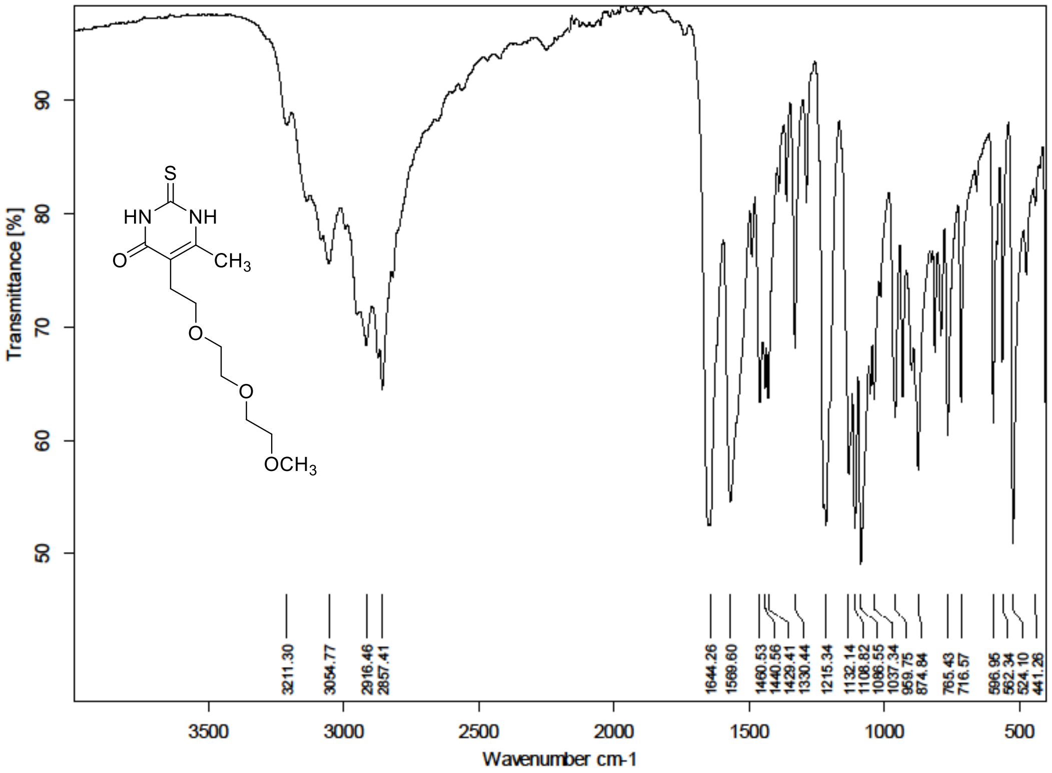

^1H (300 MHz) spectrum of **S126** in DMSO-d_6

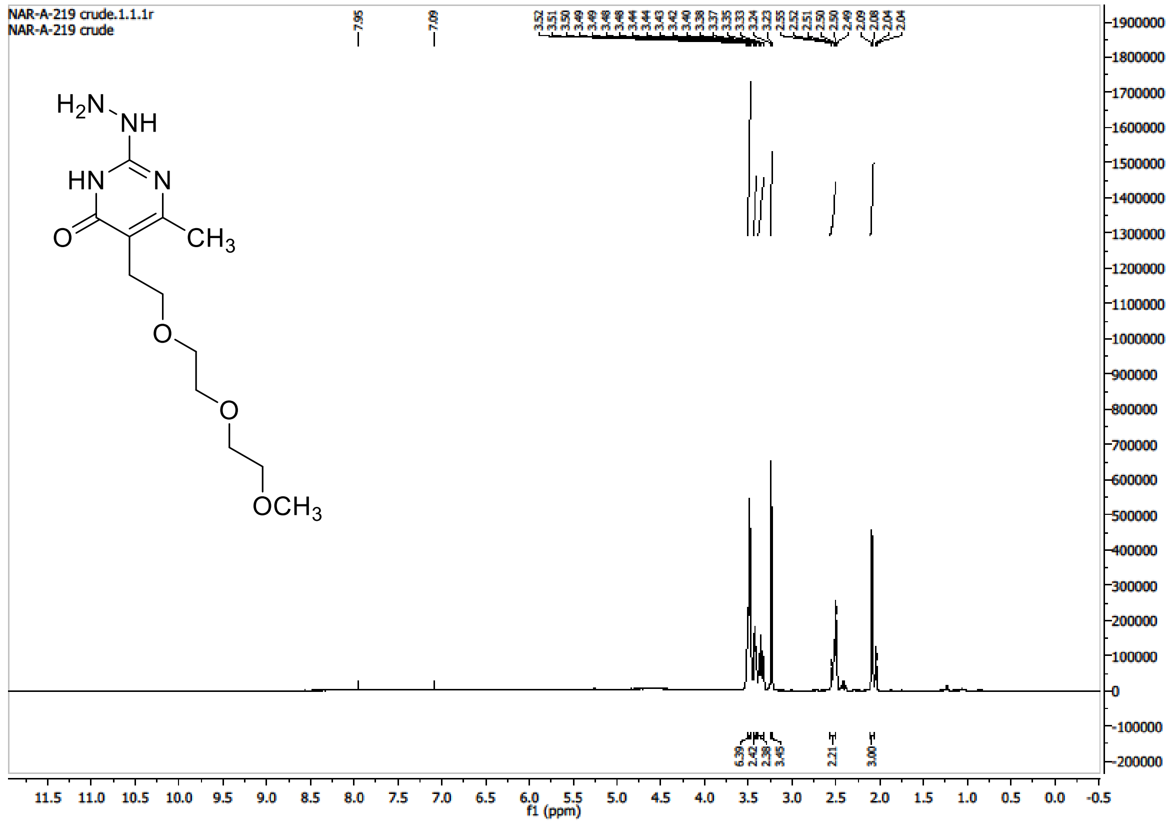

HRMS spectrum of **S126**

NAR-A-219 $C_{12}H_{22}N_4O_4$ mono *m/z* 286.1641

APCI + (MMI)
nitrogen flow 5 L/min, gas temperature 325°C, nebulizer 45 psig, skimmer 65 V, vaporizer 200°C, fragmentor 20 V, dissolved in methanol

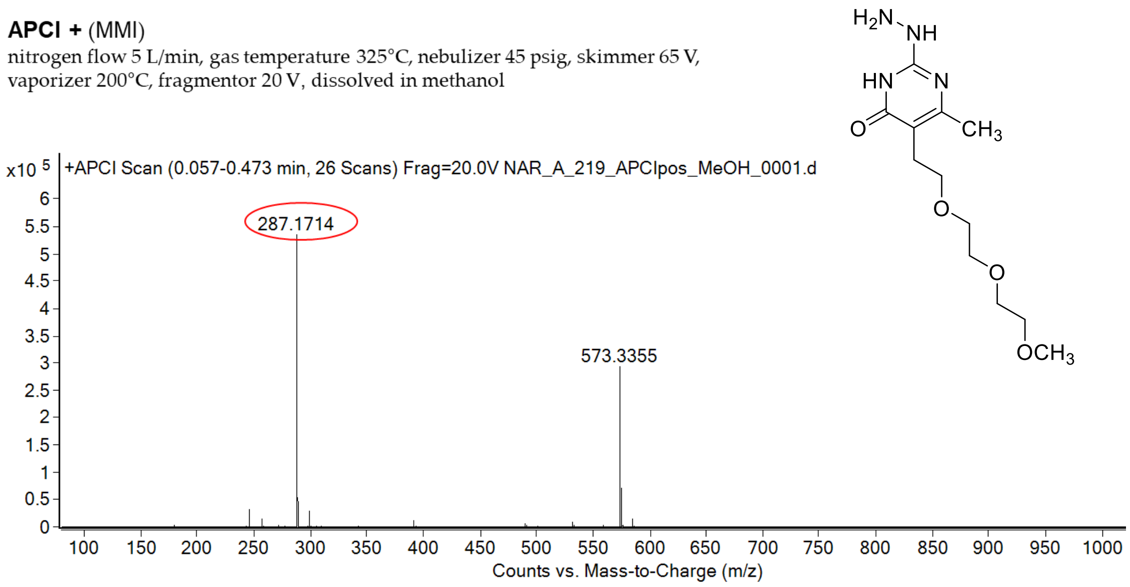

calculated mass: $[M+H]^+ = 287.1714$ observed: $[M+H]^+ = 287.1714$ mass accuracy = < 0.1 ppm

^{1}H (500 MHz) and ^{13}C NMR (126 MHz) spectra of **S127** in Chloroform-*d*

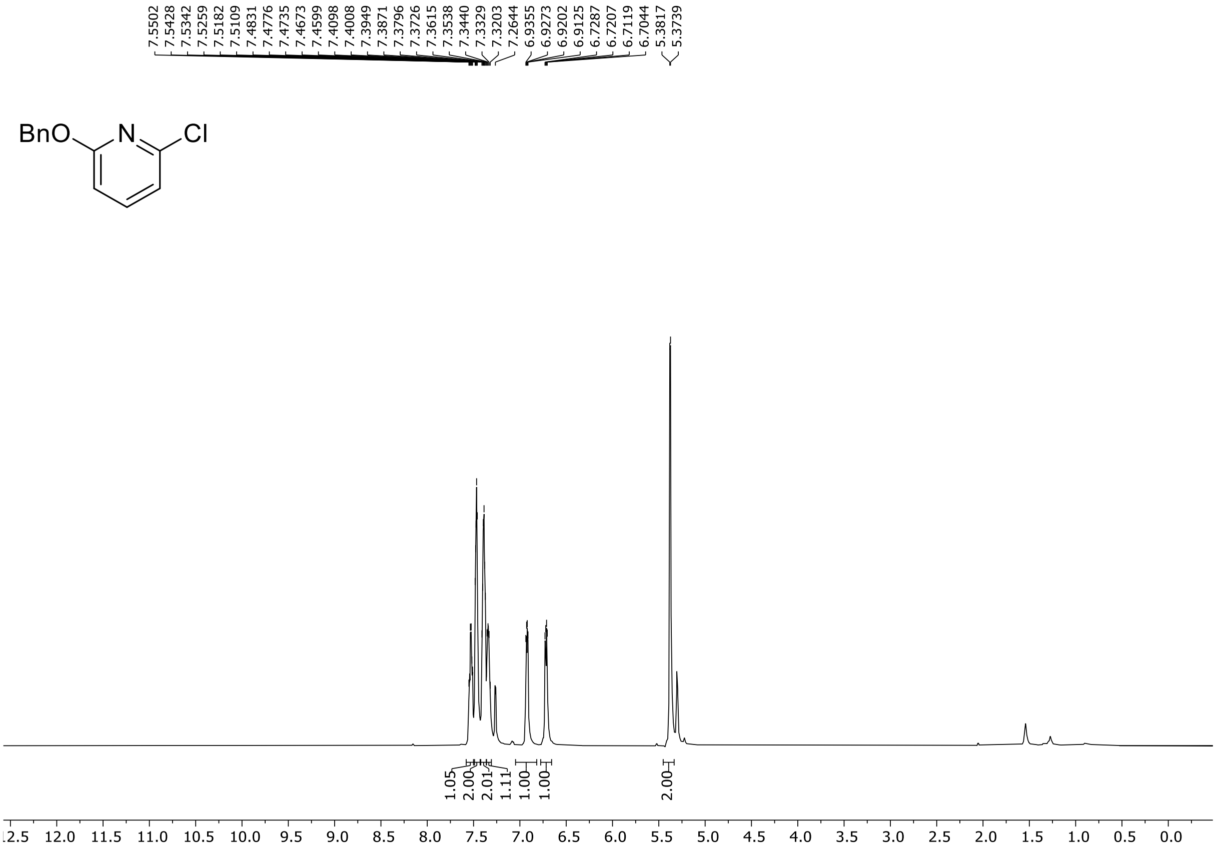

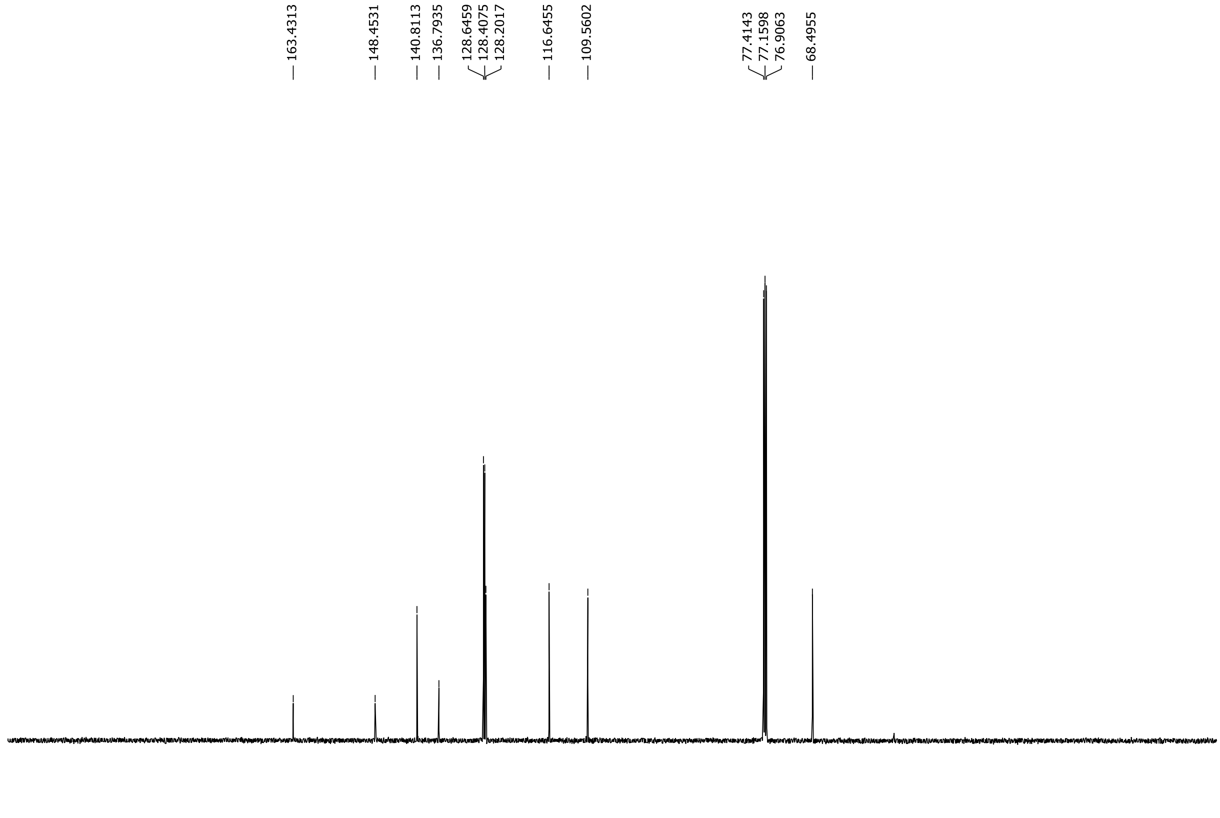

^{1}H (300 MHz) spectrum of **S128** in Chloroform-*d*

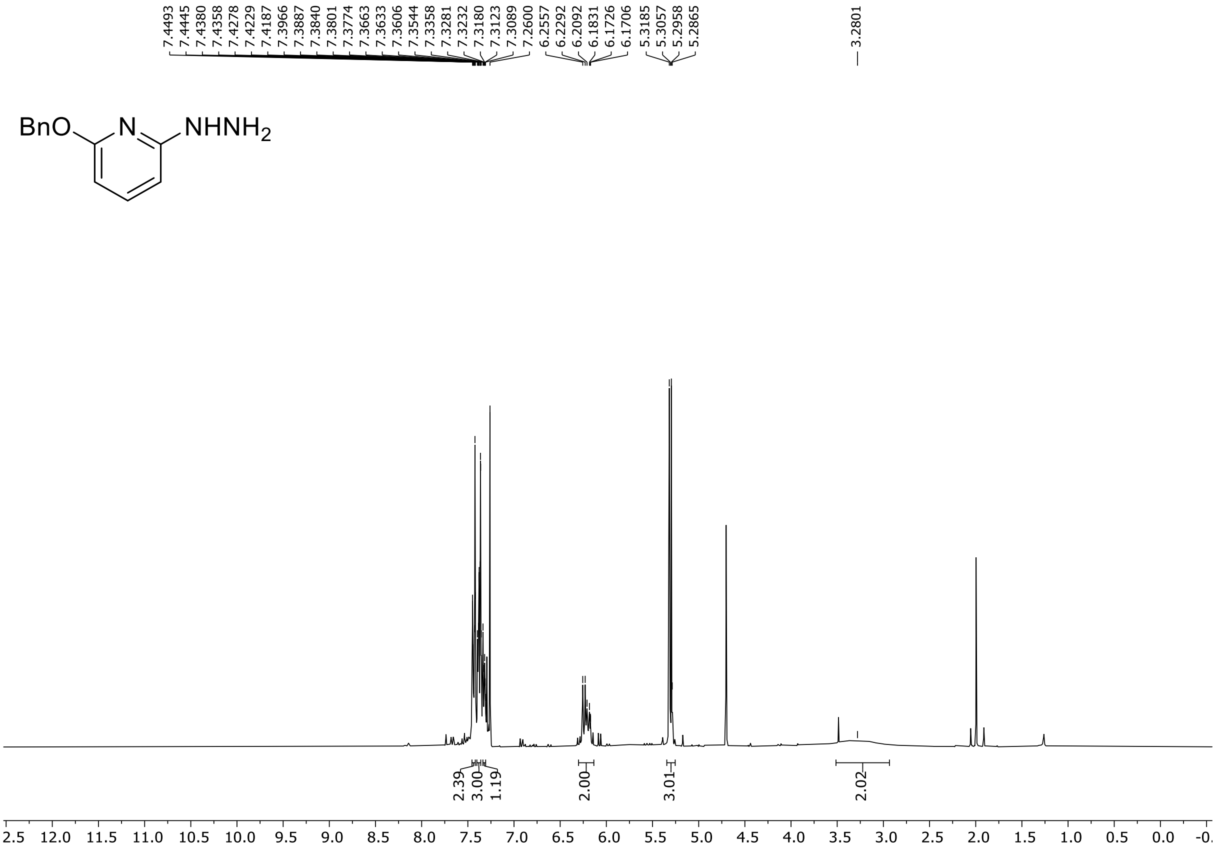

^{1}H (300 MHz) NMR spectrum of **S129** in DMSO-d_6

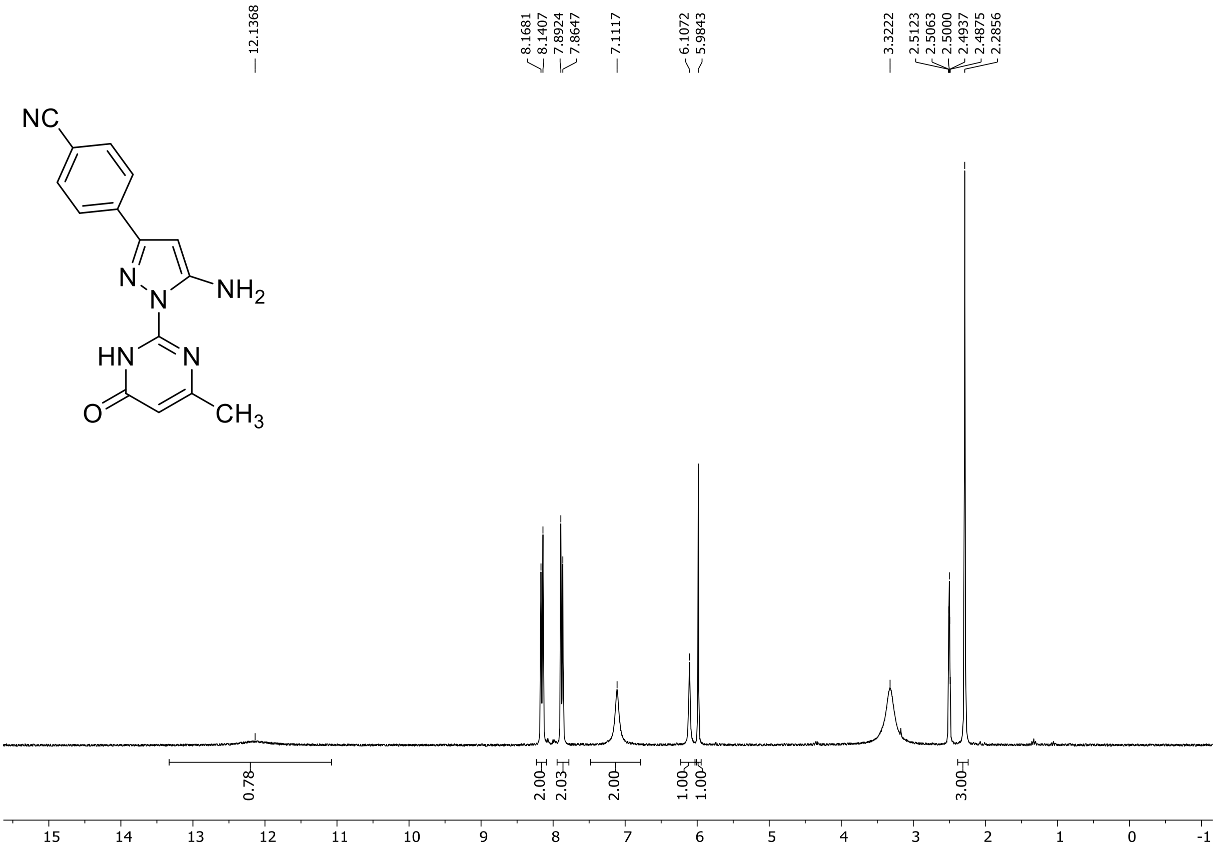

HRMS spectrum of **S129**

NAR-A-114 $C_{15}H_{12}N_6O$ ***m/z* 292.1073**

APCI- (MMI)
nitrogen flow 5 L/min, gas temperature 325°C, nebulizer 45 psi, skimmer 65 V, vaporizer 200°C, fragmentor 60 V, dissolved in DMSO, MeOH

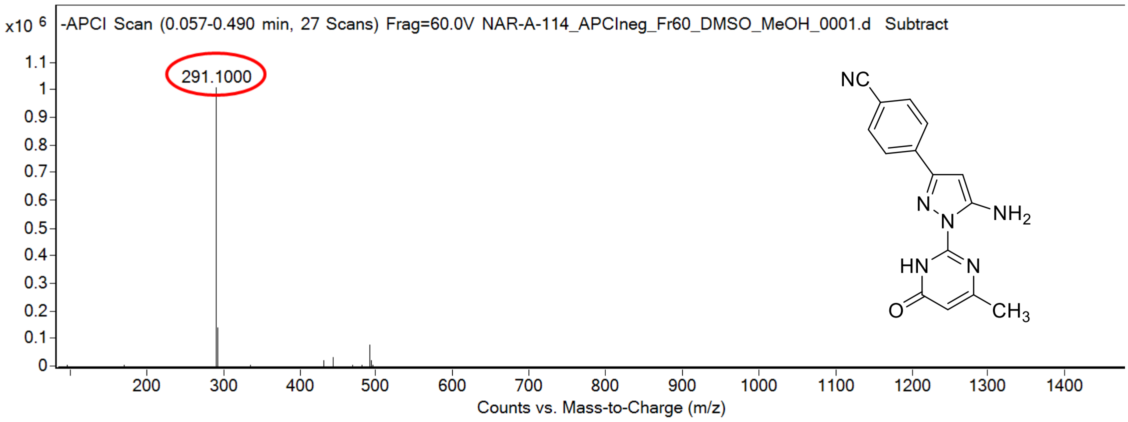

calculated mass: [M-H]$^-$ = 291.1000 observed: [M-H]$^-$ = 291.1000 mass accuracy < 0.1 ppm

FT-IR spectrum (neat) of **S129**

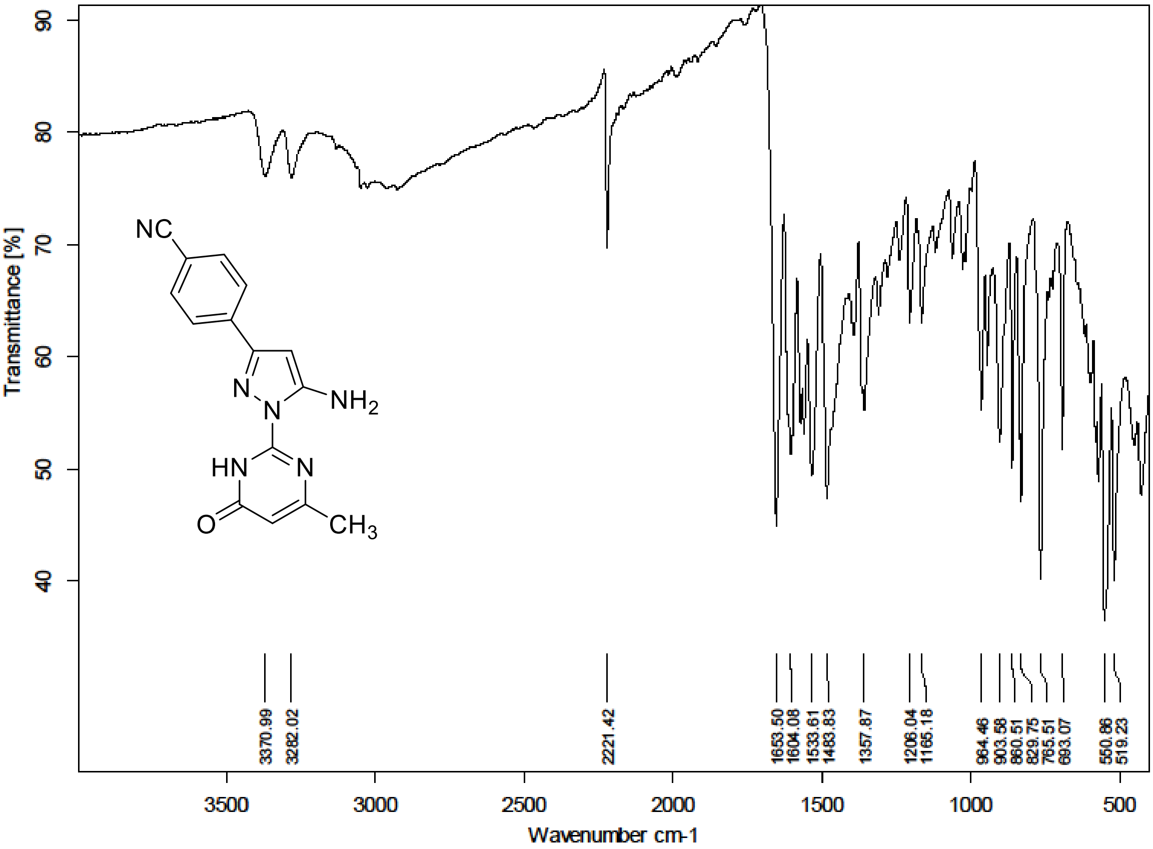

^{1}H (300 MHz) and ^{13}C NMR (75 MHz) spectra of **S130** in Chloroform-*d*

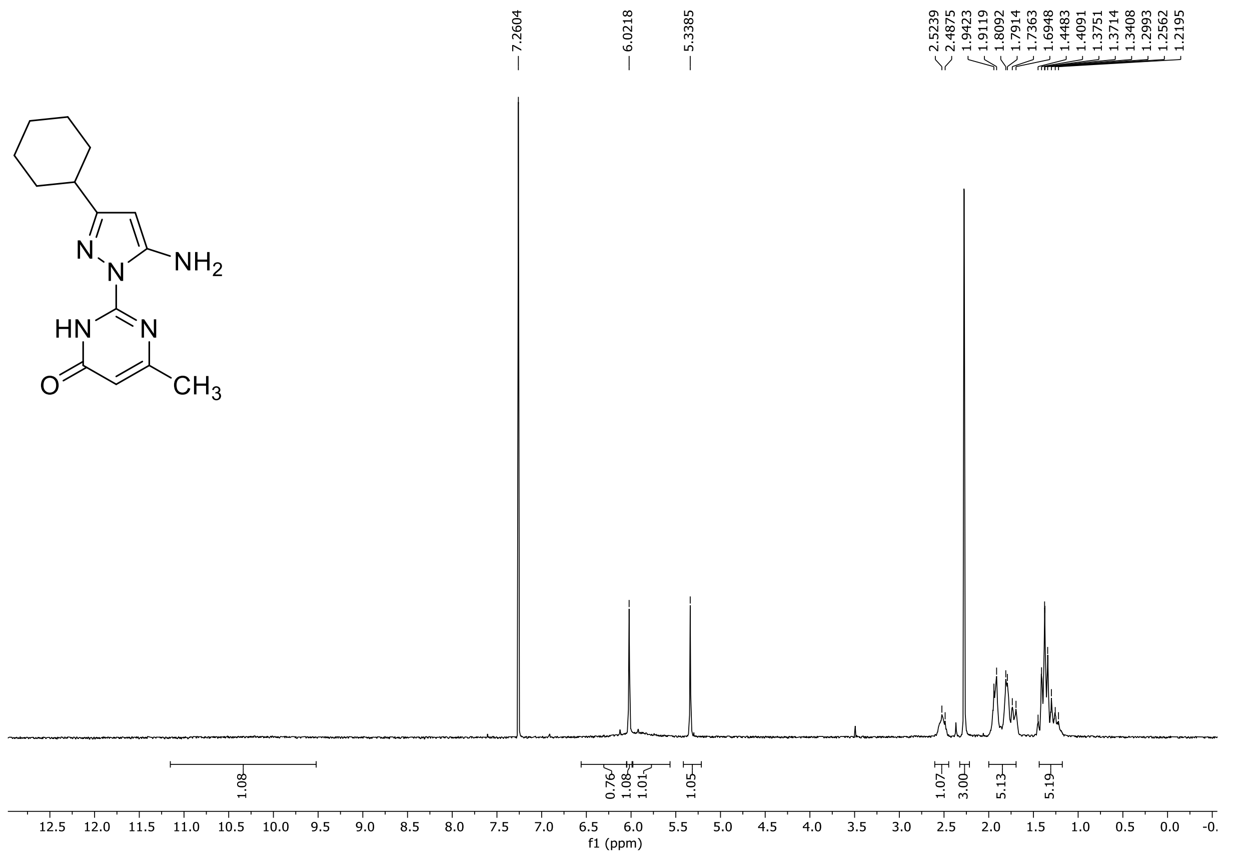

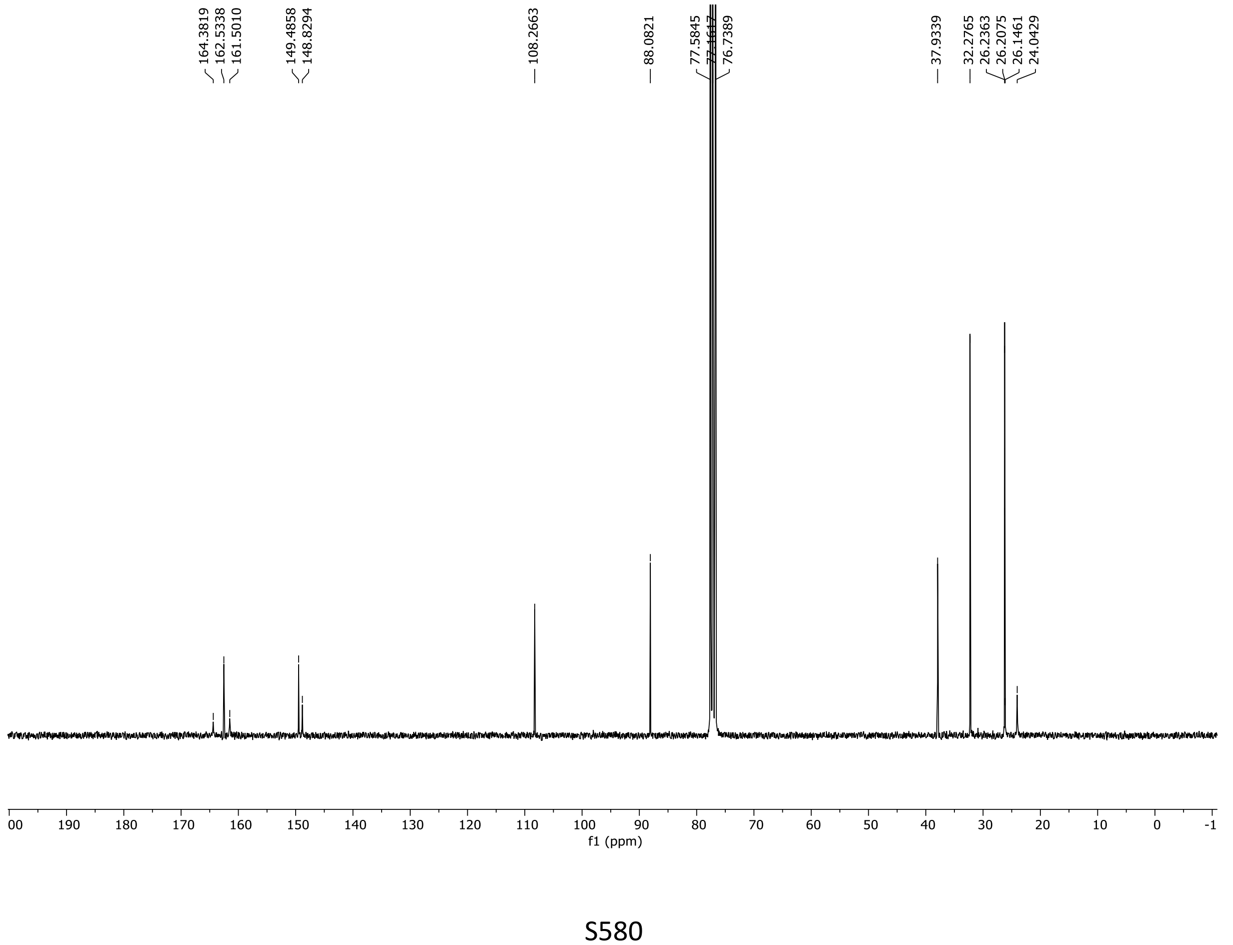

HRMS spectrum of **S130**

NAR-A-116 $C_{14}H_{19}N_5O$ *m/z* **273.1590**

APCI+ (MMI)
nitrogen flow 5 L/min, gas temperature 325°C, nebulizer 45 psi, skimmer 65 V, vaporizer 200°C, fragmentor 20 V, dissolved in DMSO, MeOH

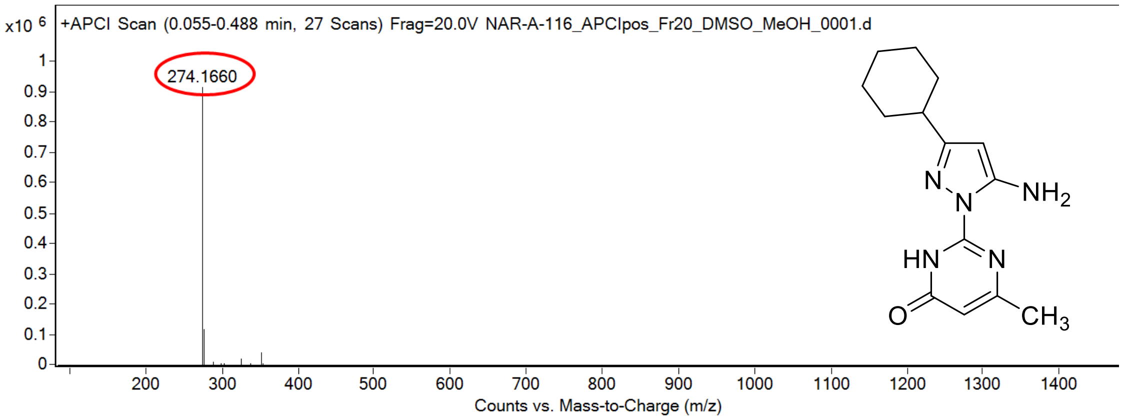

calculated mass: $[M+H]^+$ = 274.1662 observed: $[M+H]^+$ = 274.1660 mass accuracy = -0.7 ppm

FT-IR spectrum (neat) of **S130**

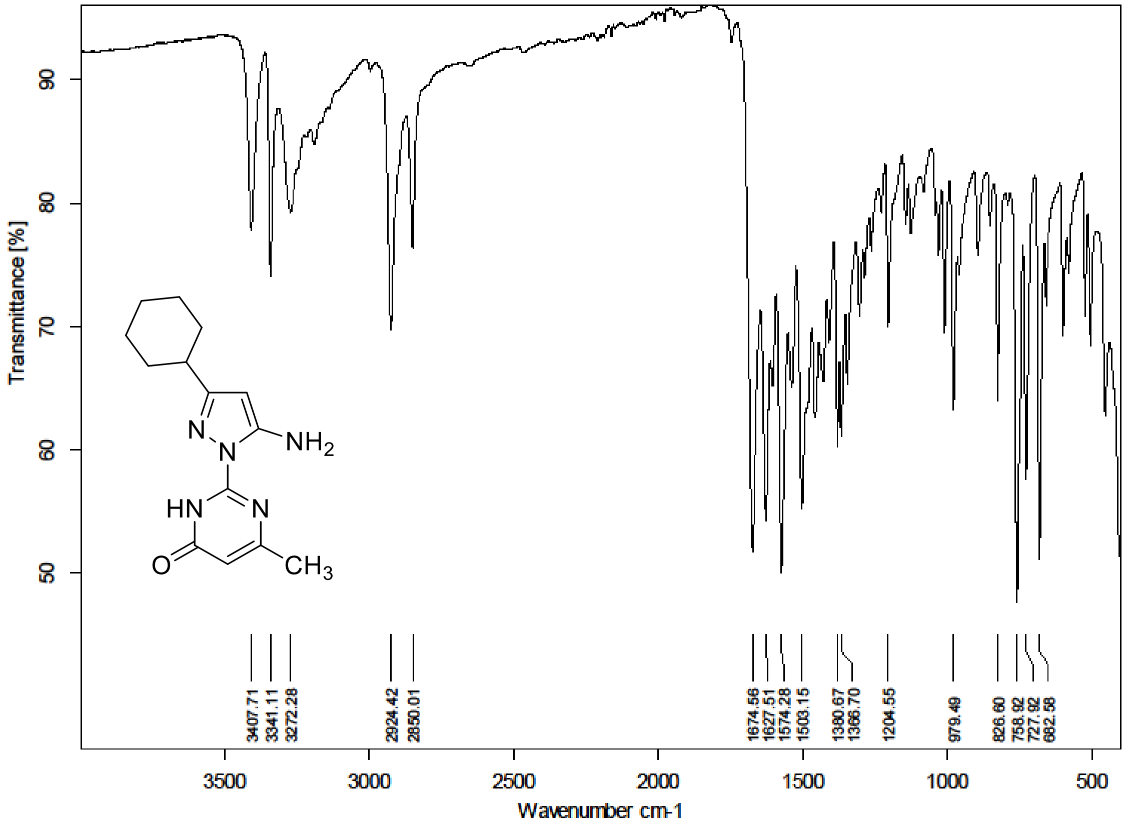

^{1}H (500 MHz) and ^{13}C NMR (126 MHz) spectra of **S131** in DMSO-d_6

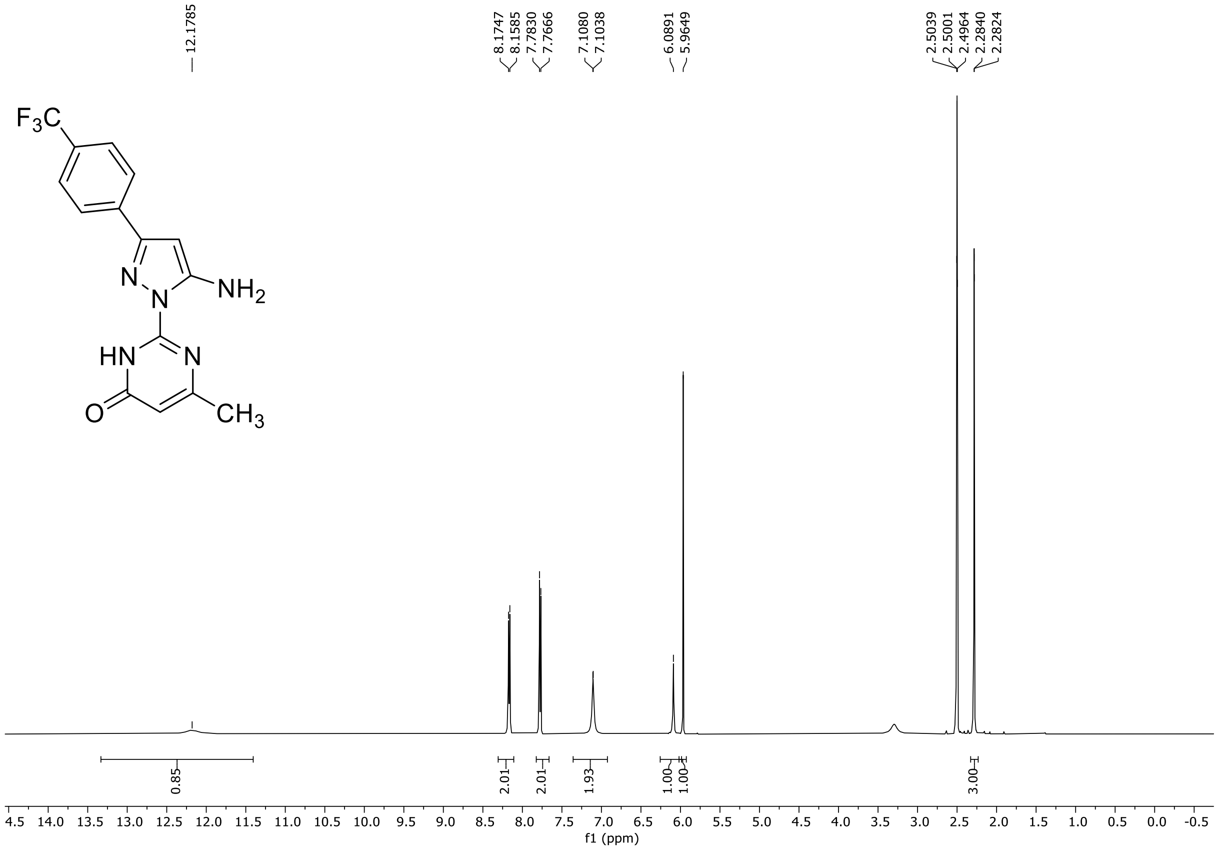

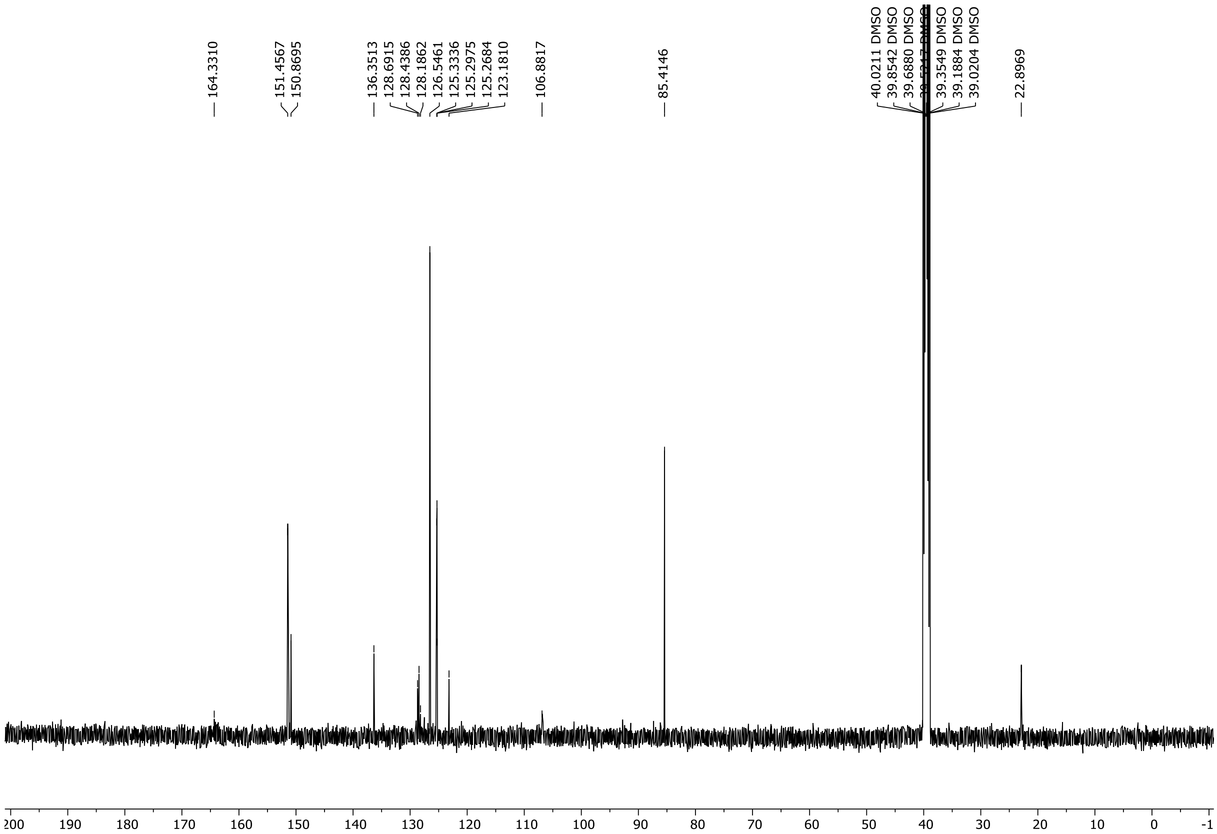

^{19}F NMR (282 MHz) spectrum of **S131** in DMSO-d_6

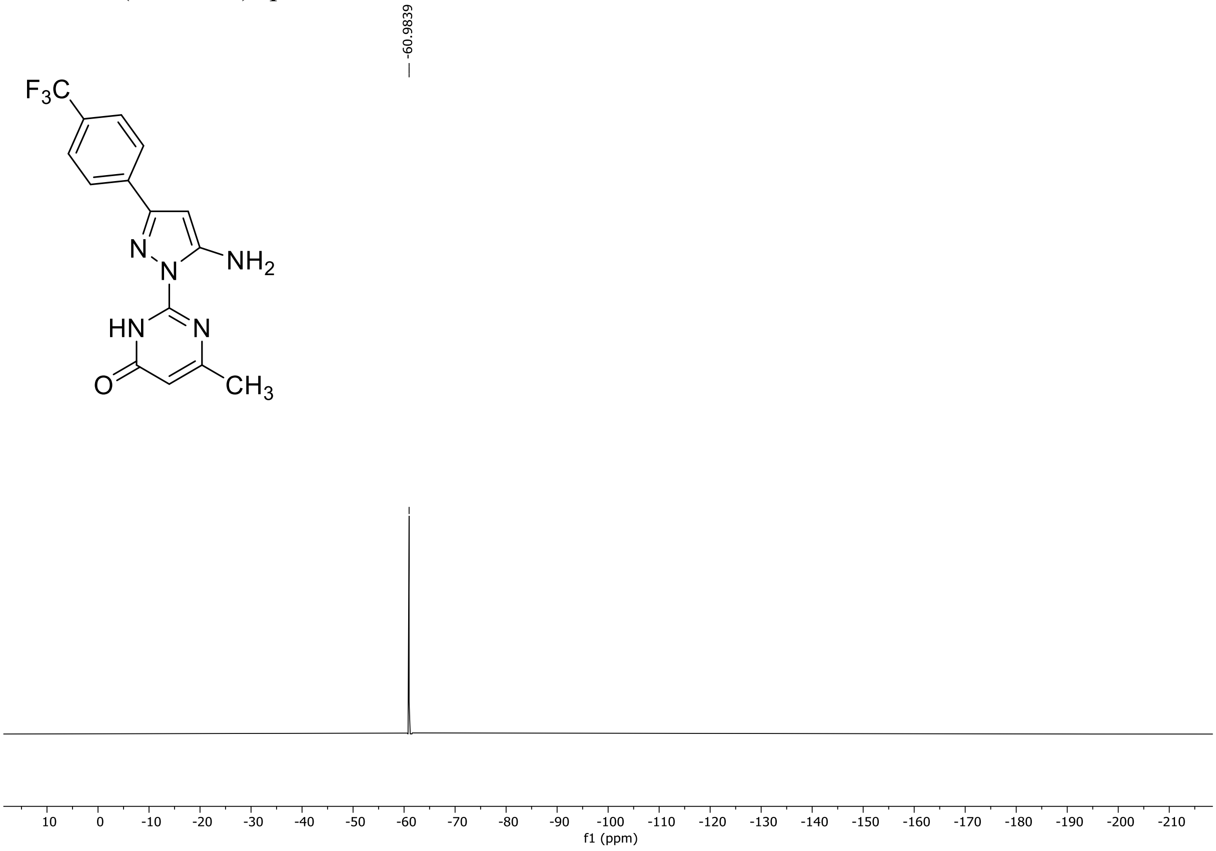

HRMS spectrum of **S131**

NAR-A-117 **$C_{15}H_{12}F_3N_5O$** ***m/z* 335.0994**

APCI+ (MMI)
nitrogen flow 5 L/min, gas temperature 325°C, nebulizer 45 psi, skimmer 65 V,
vaporizer 200°C, fragmentor 30 V, dissolved in DMSO, MeOH

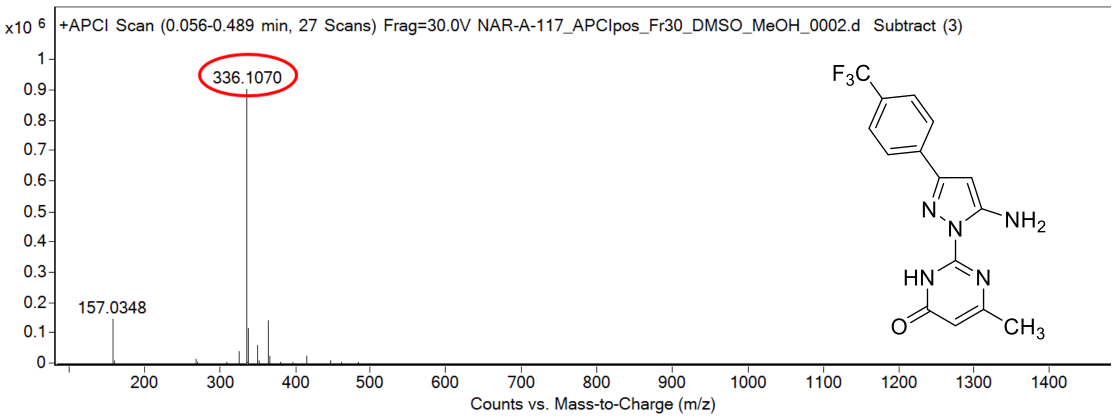

calculated mass: $[M+H]^+$ = 336.1067 observed: $[M+H]^+$ = 336.1070 mass accuracy = +0.9 ppm

FT-IR spectrum (neat) of **S131**

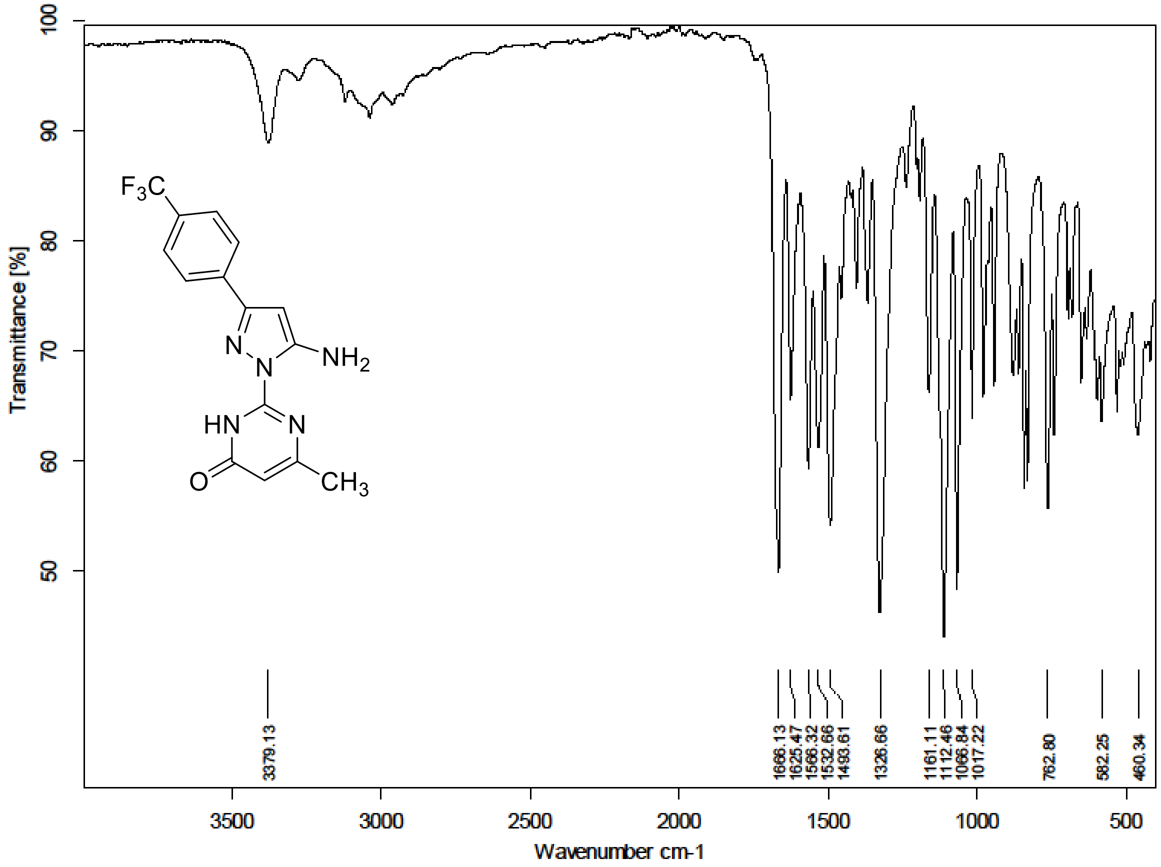

^{1}H (500 MHz) and ^{13}C NMR (126 MHz) spectra of **S132** in DMSO-d_6

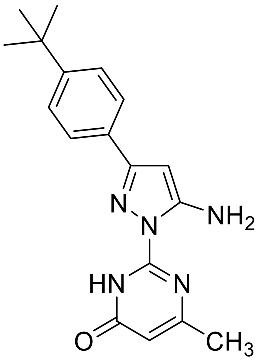

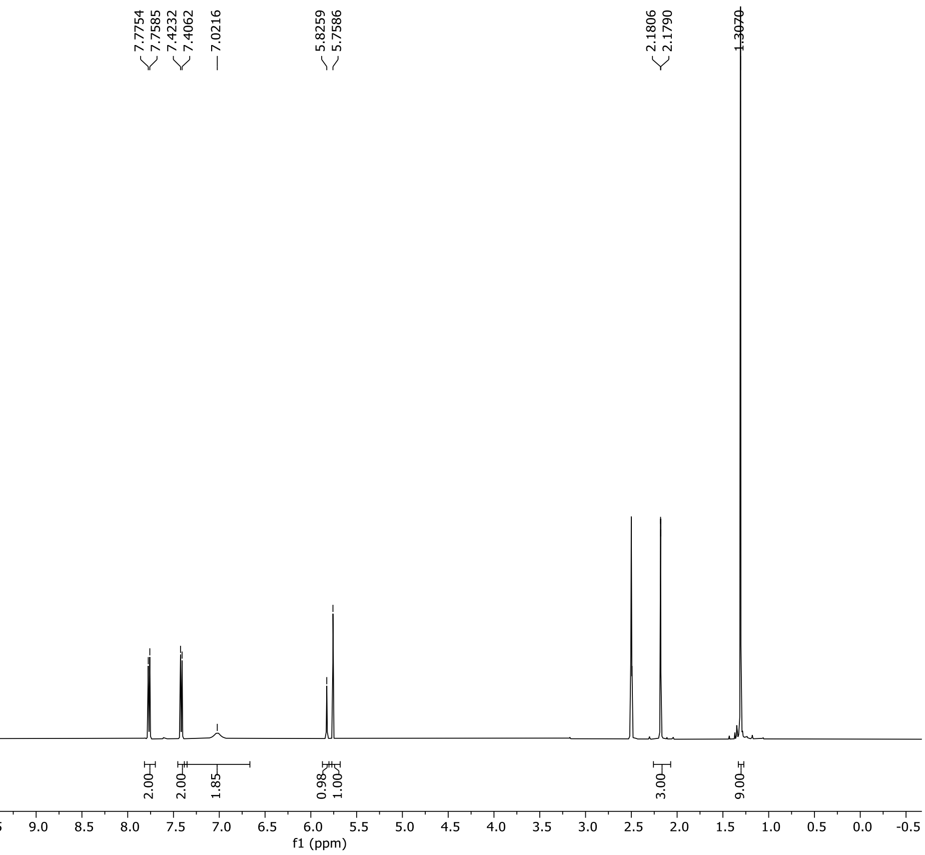

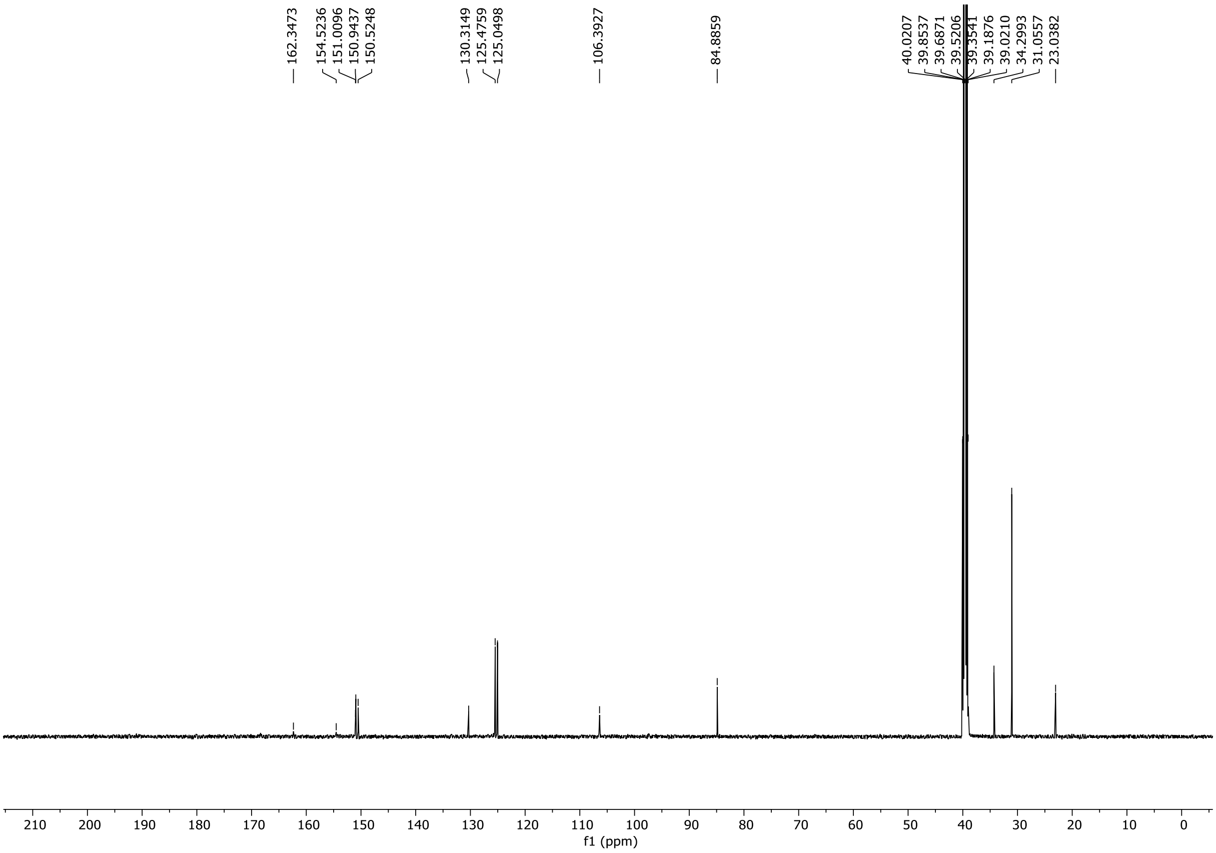

HRMS spectrum of **S132**

NAR-A-115 $C_{28}H_{21}N_5O$ *m/z* **323.1746**

APCI+ (MMI)
nitrogen flow 5 L/min, gas temperature 325°C, nebulizer 45 psi, skimmer 65 V, vaporizer 200°C, fragmentor 15 V, dissolved in DMSO, MeOH

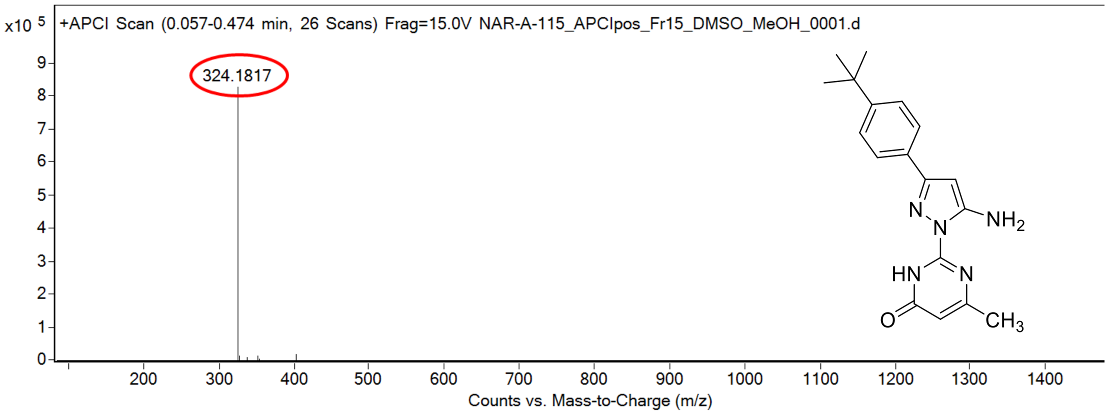

calculated mass: $[M+H]^+$ = 324.1819 observed: $[M+H]^+$ = 324.1817 mass accuracy = -0.6 ppm

FT-IR spectrum (neat) of **S132**

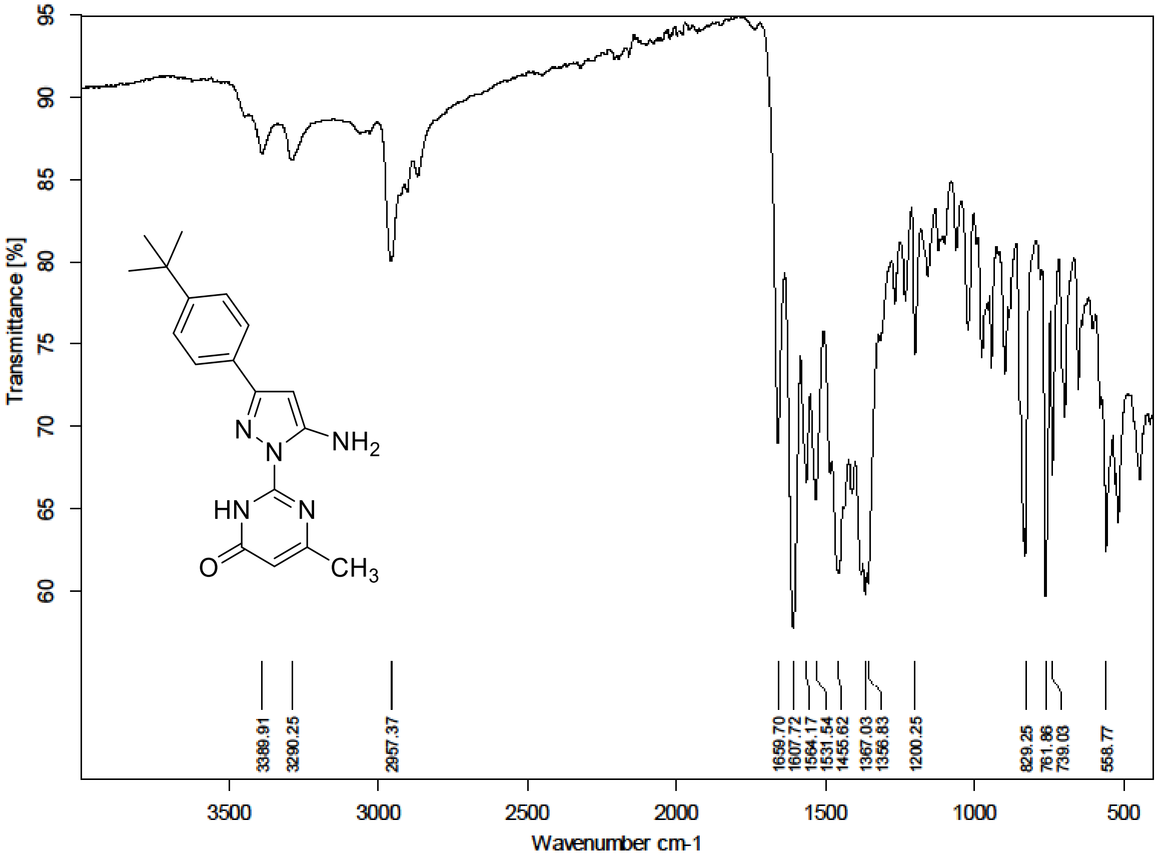

^{1}H (500 MHz) and ^{13}C NMR (126 MHz) spectra of **S133** in DMSO-d_6

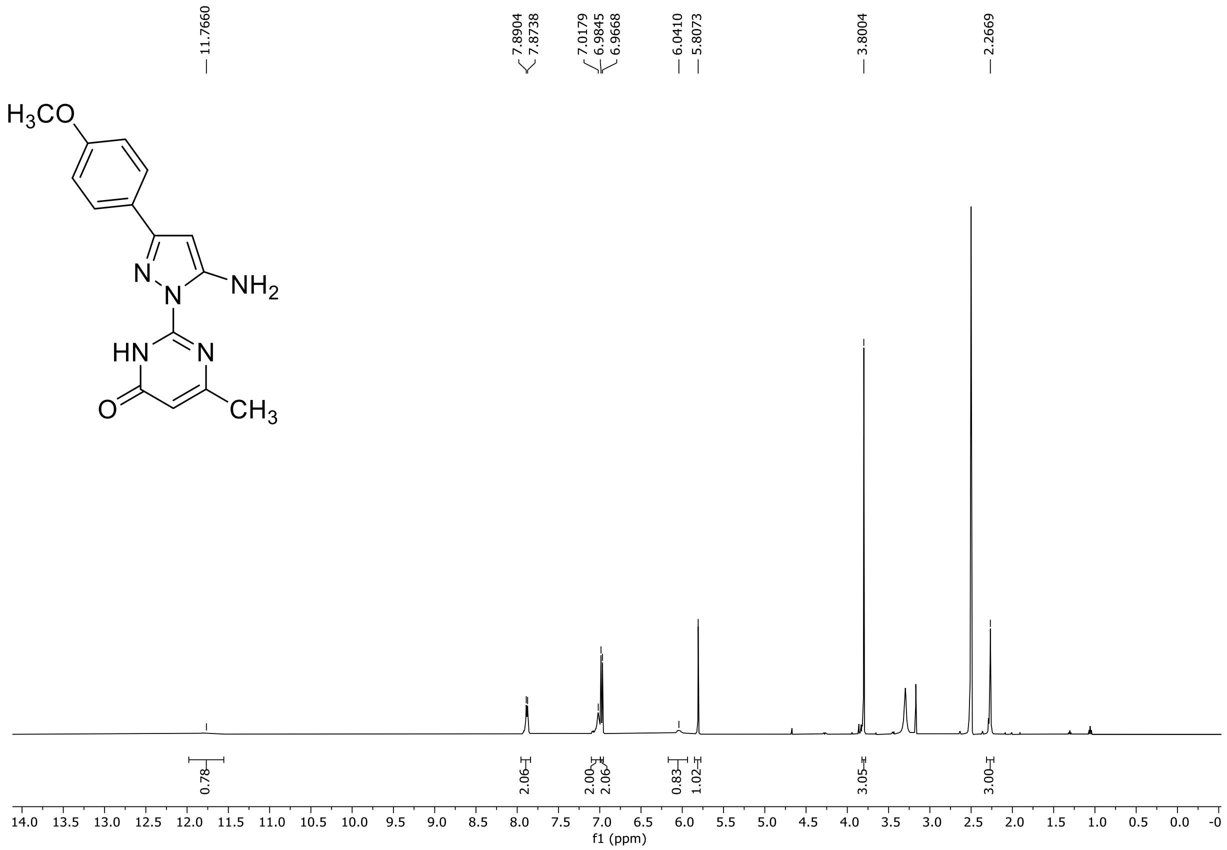

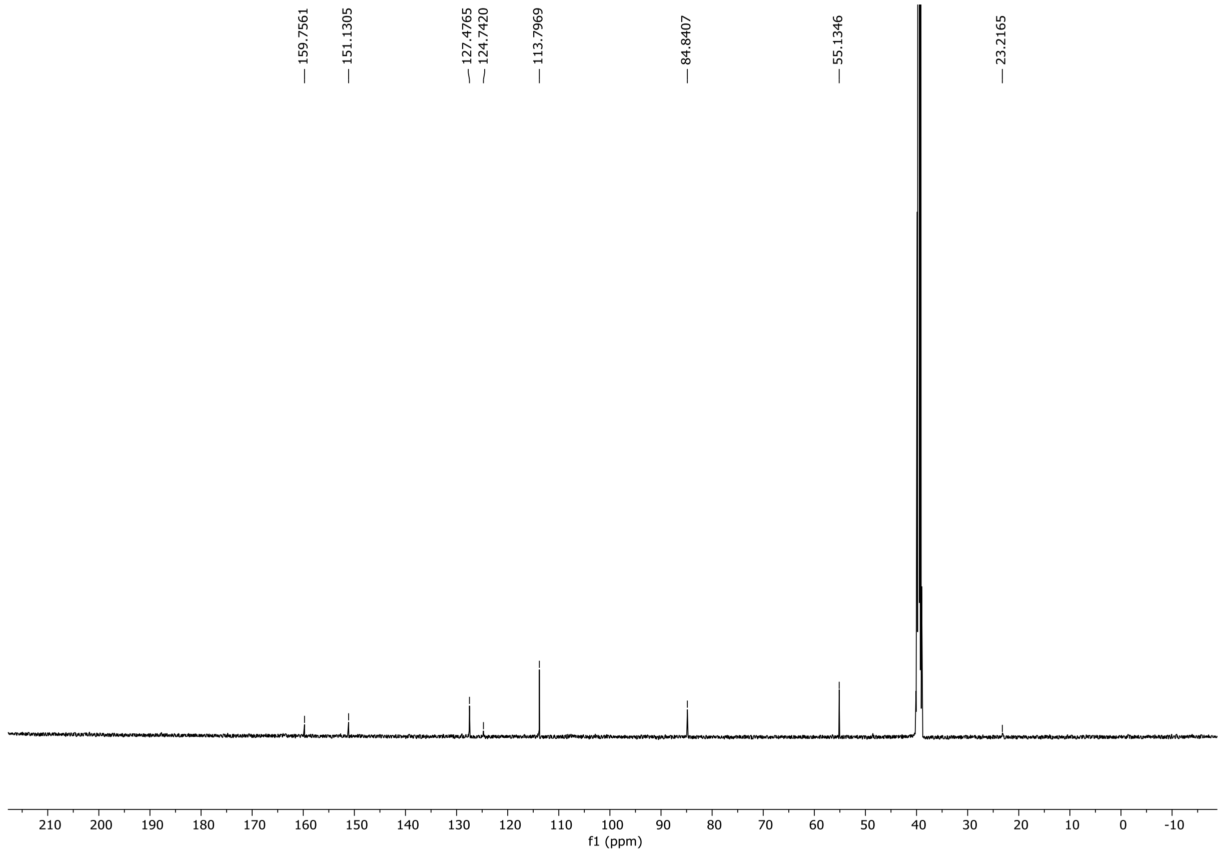

HRMS spectrum of **S133**

NAR-A-119 $C_{15}H_{15}N_5O_2$ *m/z* **297.1226**

APCI+ (MMI)
nitrogen flow 5 L/min, gas temperature 325°C, nebulizer 45 psi, skimmer 65 V, vaporizer 200°C, fragmentor 25 V, dissolved in DMSO, MeOH

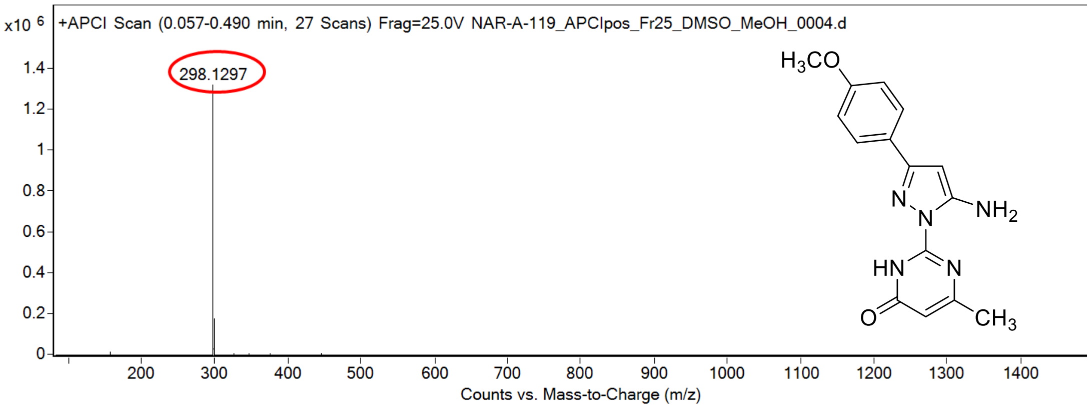

calculated mass: $[M+H]^+$ = 298.1299 observed: $[M+H]^+$ = 298.1297 mass accuracy = -0.7 ppm

FT-IR spectrum (neat) of **S133**

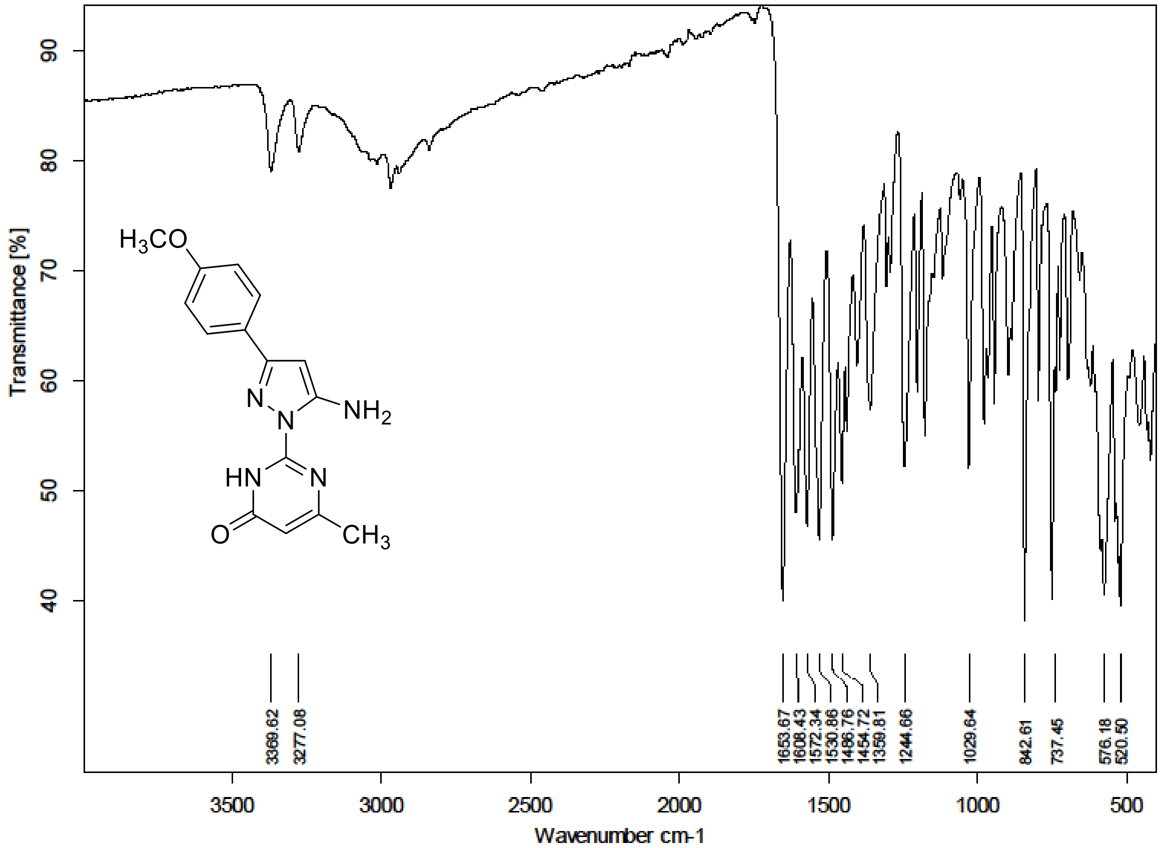

^{1}H (300 MHz) and ^{13}C NMR (75 MHz) spectra of **S134** in DMSO-d_6

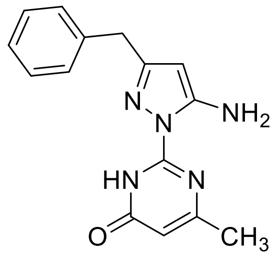

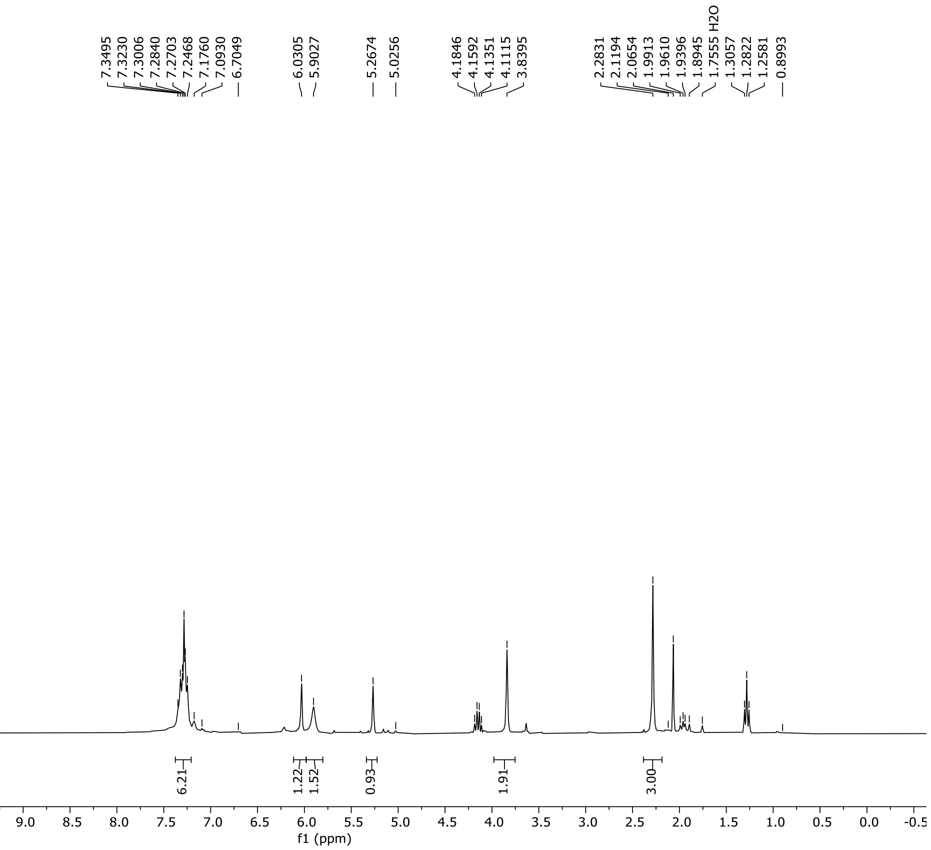

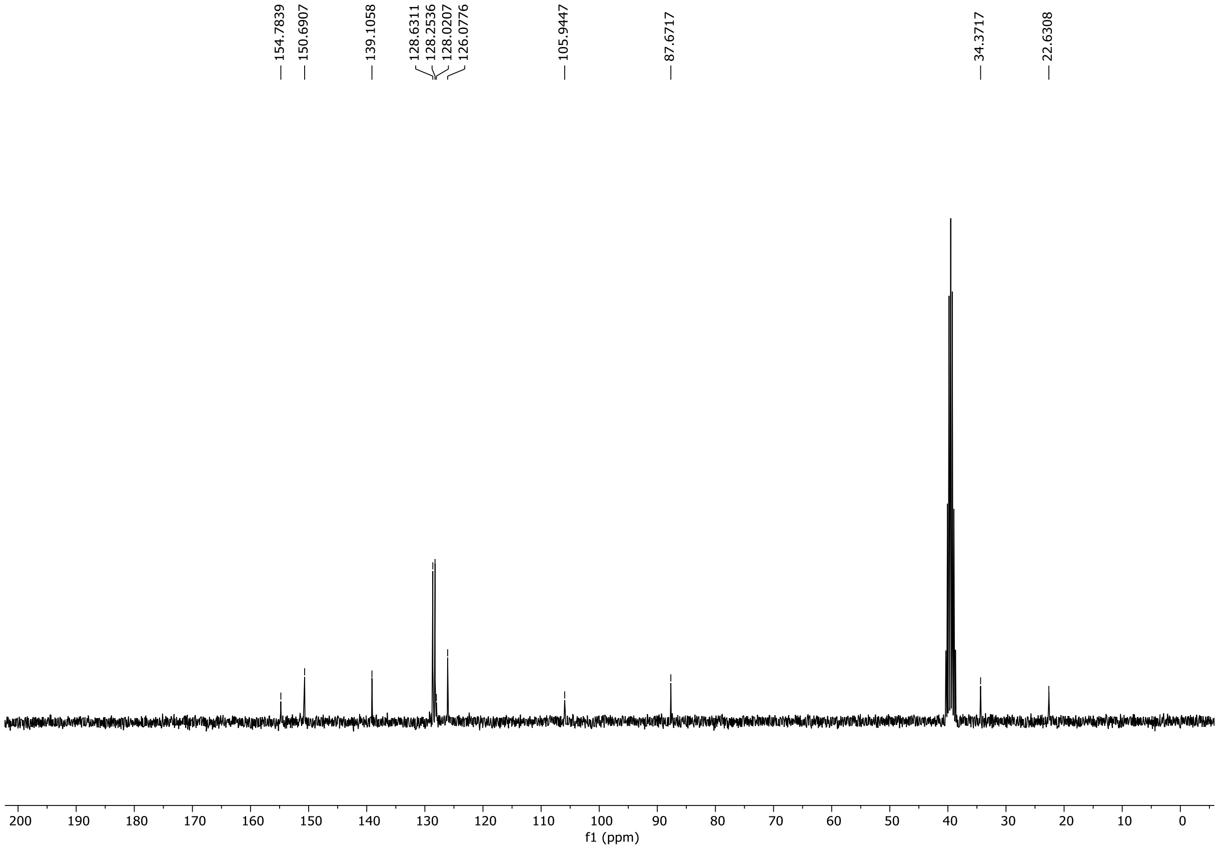

HRMS spectrum of **S134**

NAR-A-184

$C_{15}H_{15}N_5O$

mono *m/z* 281.1277

APCI + (MMI)
nitrogen flow 5 L/min, gas temperature 325°C, nebulizer 45 psi, skimmer **65** V, vaporizer 200°C, fragmentor 25 V, dissolved in methanol

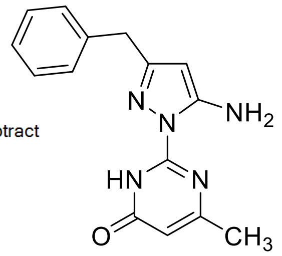

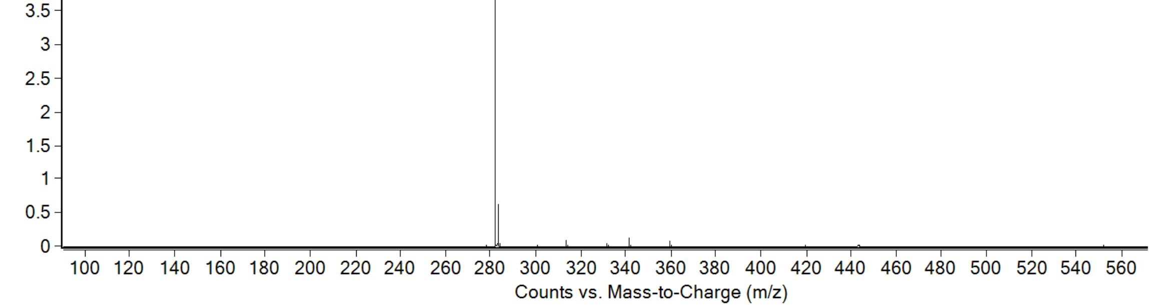

calculated mass: $[M+H]^+$ = 282..1349 observed: $[M+H]^+$ = 282.1349 mass accuracy = < 0.1 ppm

FT-IR spectrum (neat) of **S134**

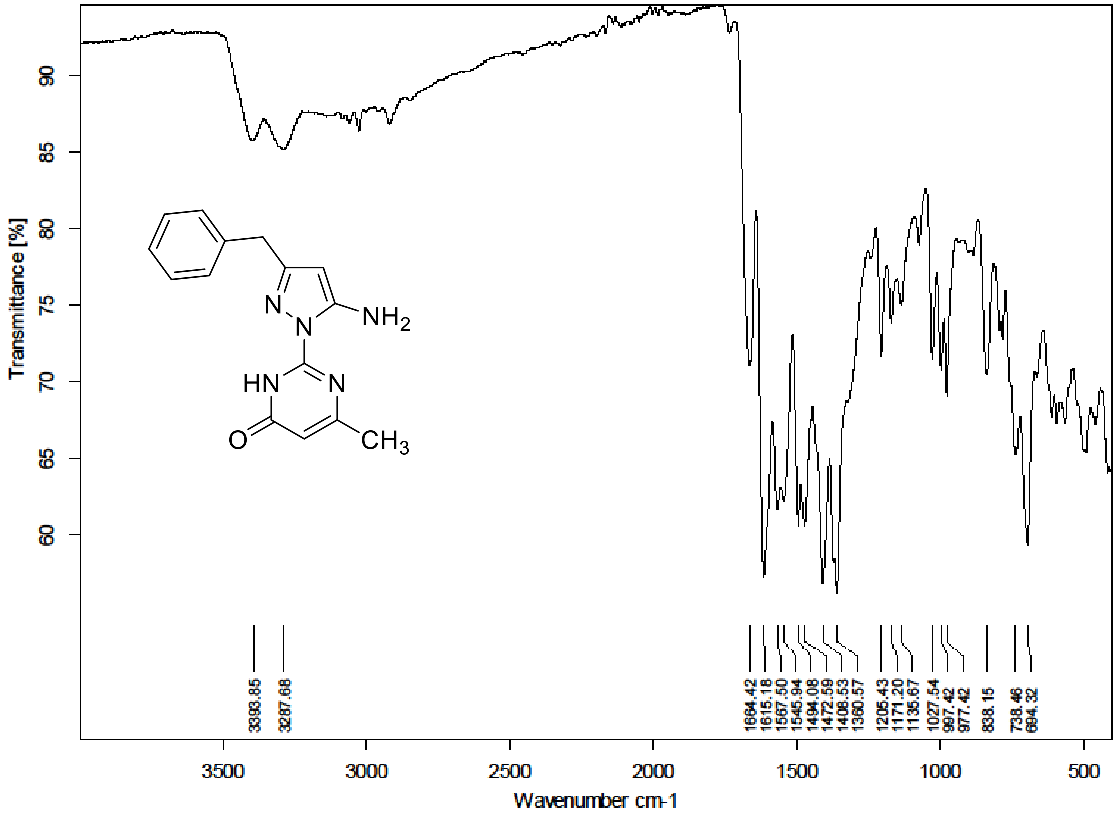

^1H (300 MHz) and ^{13}C NMR (75 MHz) spectra of **S135** in DMSO-d_6

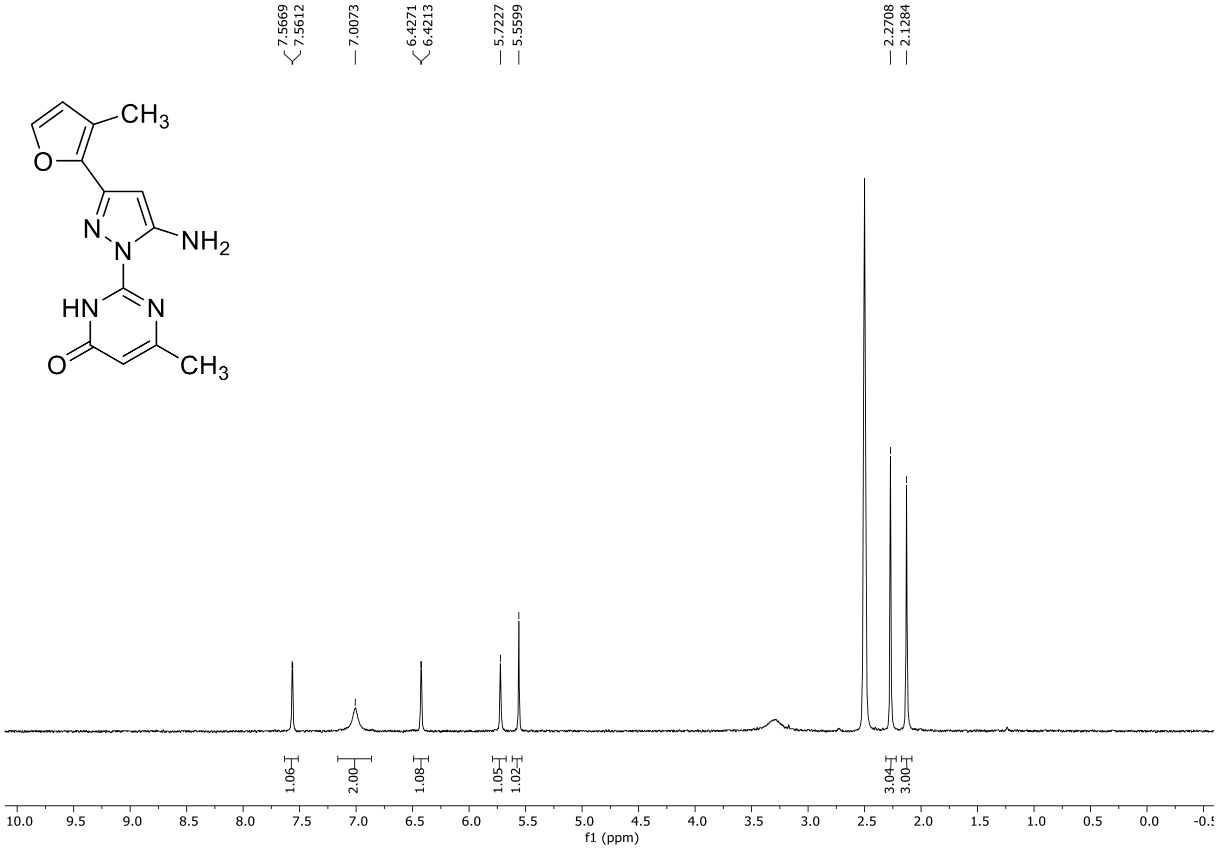

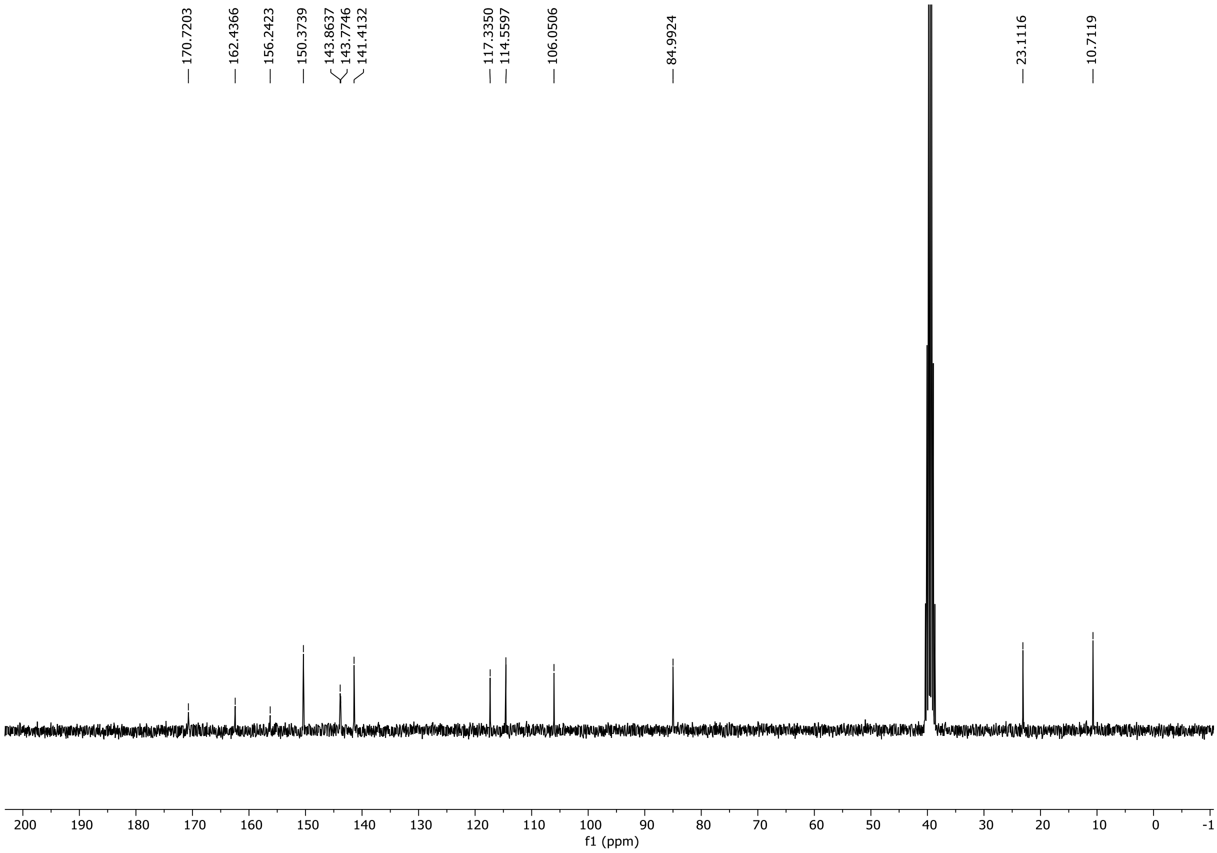

HRMS spectrum of **S135**

NAR-A-178 $C_{13}H_{13}N_5O_2$ mono *m/z* **271.1069**

APCI + (MMI)
nitrogen flow 5 L/min, gas temperature 325°C, nebulizer 45 psi, skimmer **65** V, vaporizer 200°C, fragmentor 23 V, dissolved in methanol

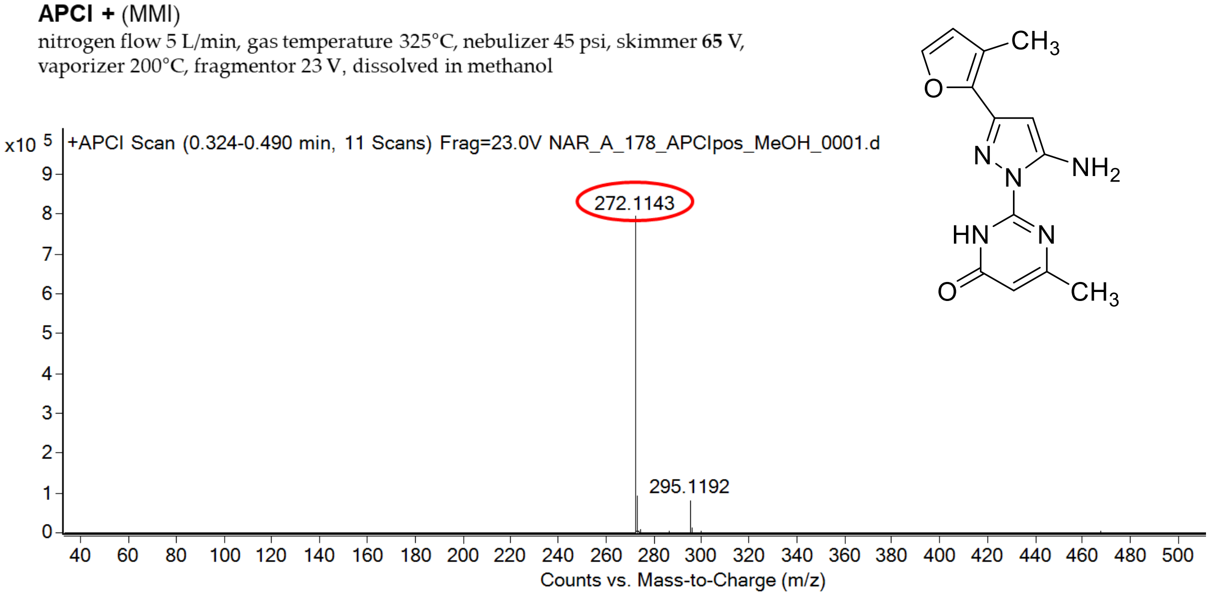

calculated mass: $[M+H]^+$ = 272..1142 observed: $[M+H]^+$ = 272.1143 mass accuracy = 0.4 ppm

FT-IR spectrum (neat) of **S135**

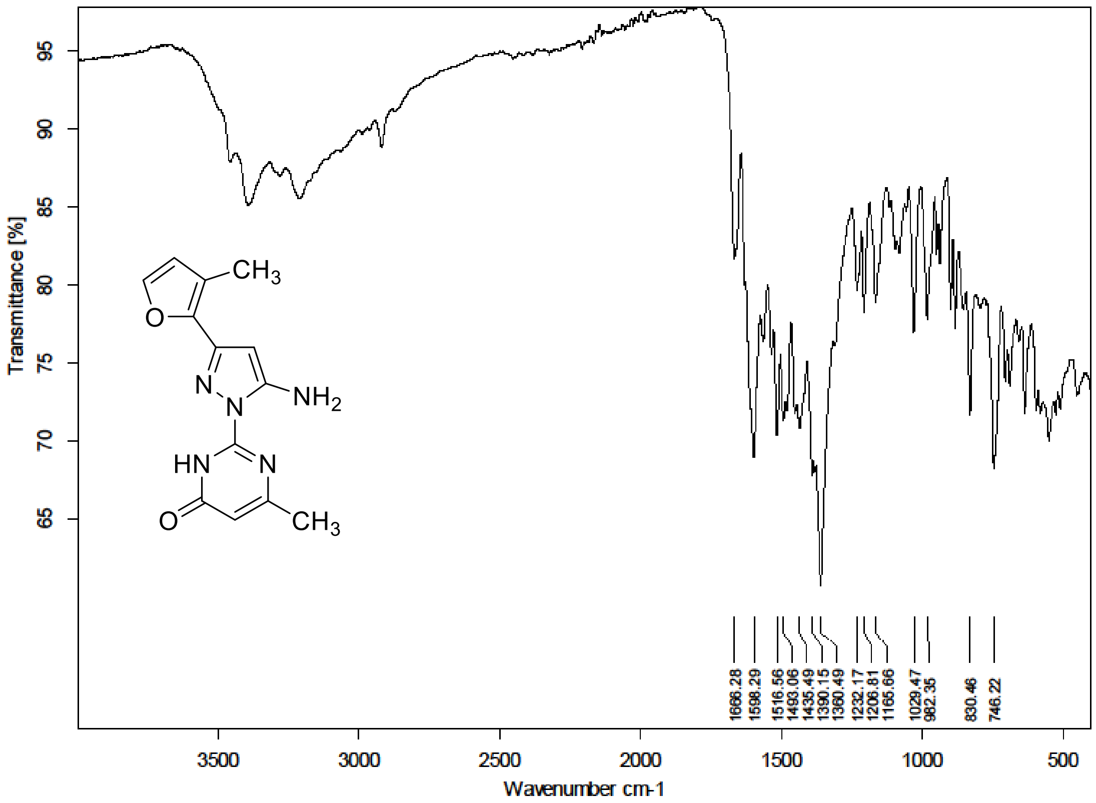

^{1}H (300 MHz) and ^{13}C NMR (75 MHz) spectra of **S136** in DMSO-d_6

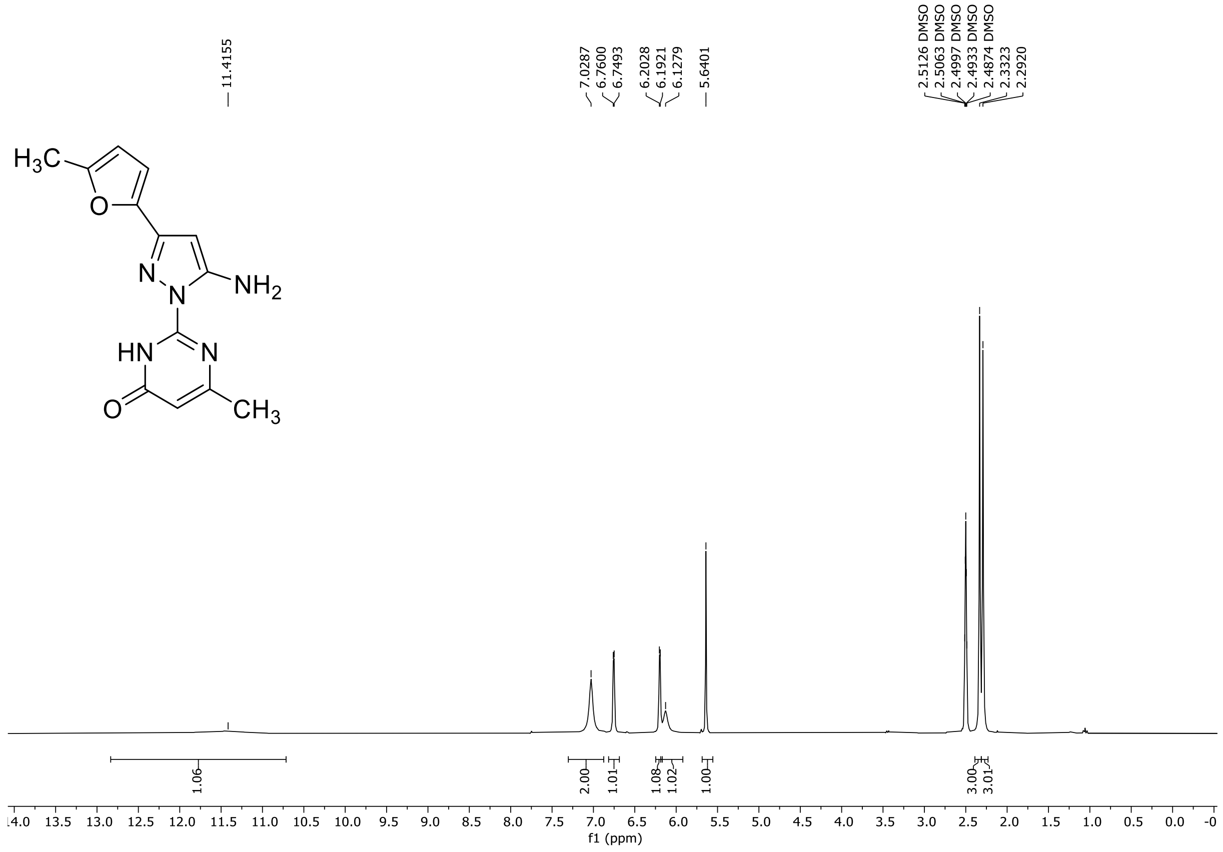

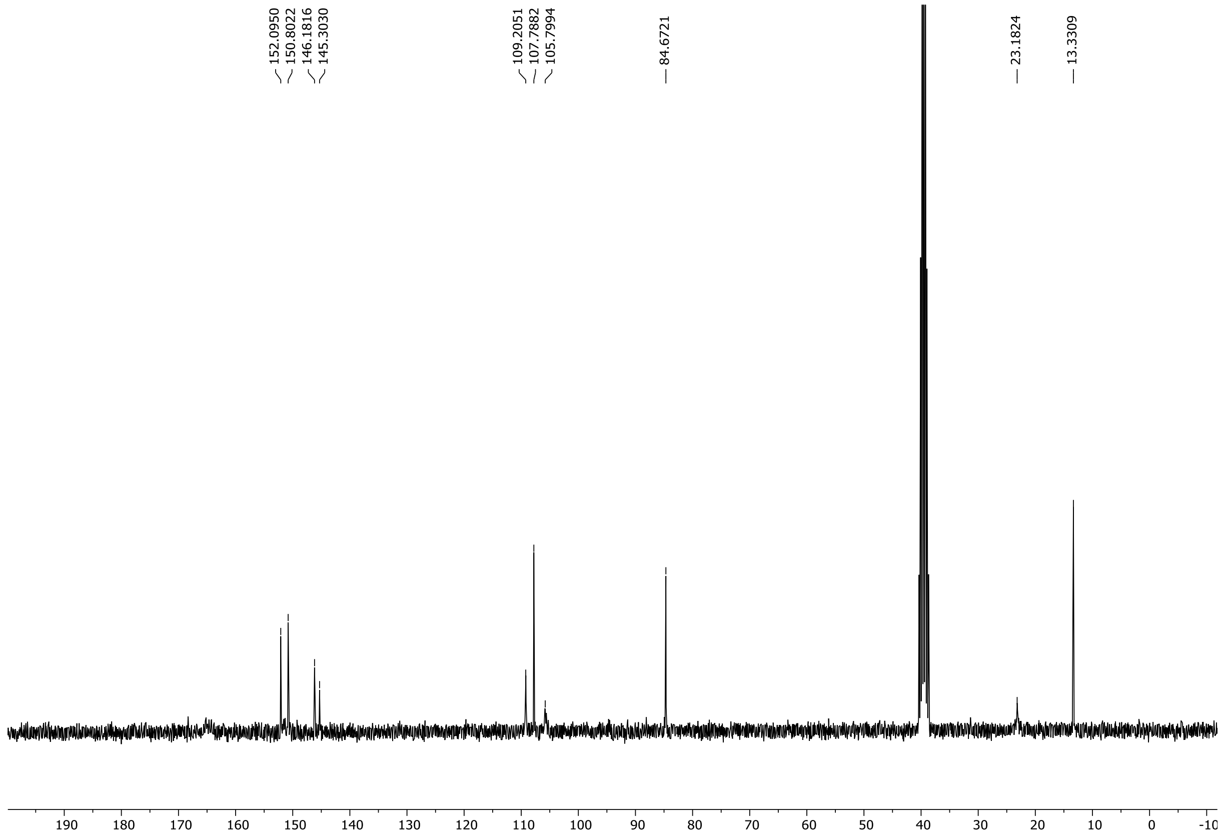

HRMS spectrum of **S136**

NAR-A-177 $C_{13}H_{13}N_5O_2$ mono *m/z* **271.1069**

APCI + (MMI)
nitrogen flow 5 L/min, gas temperature 325°C, nebulizer 45 psi, skimmer **65** V, vaporizer 200°C, fragmentor 20 V, dissolved in methanol

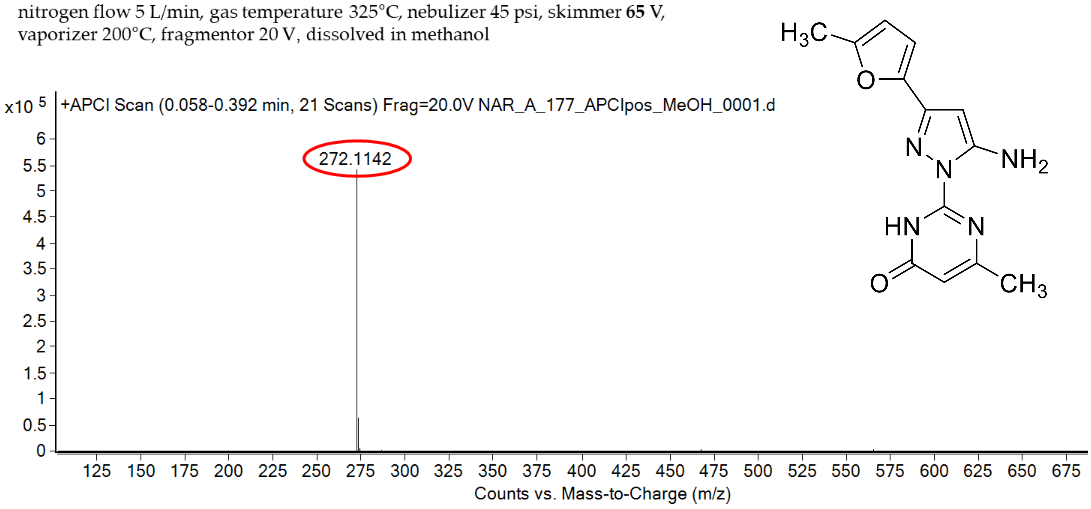

calculated mass: $[M+H]^+$ = 272..1142 observed: $[M+H]^+$ = 272.1142 mass accuracy = < 0.1 ppm

FT-IR spectrum (neat) of **S136**

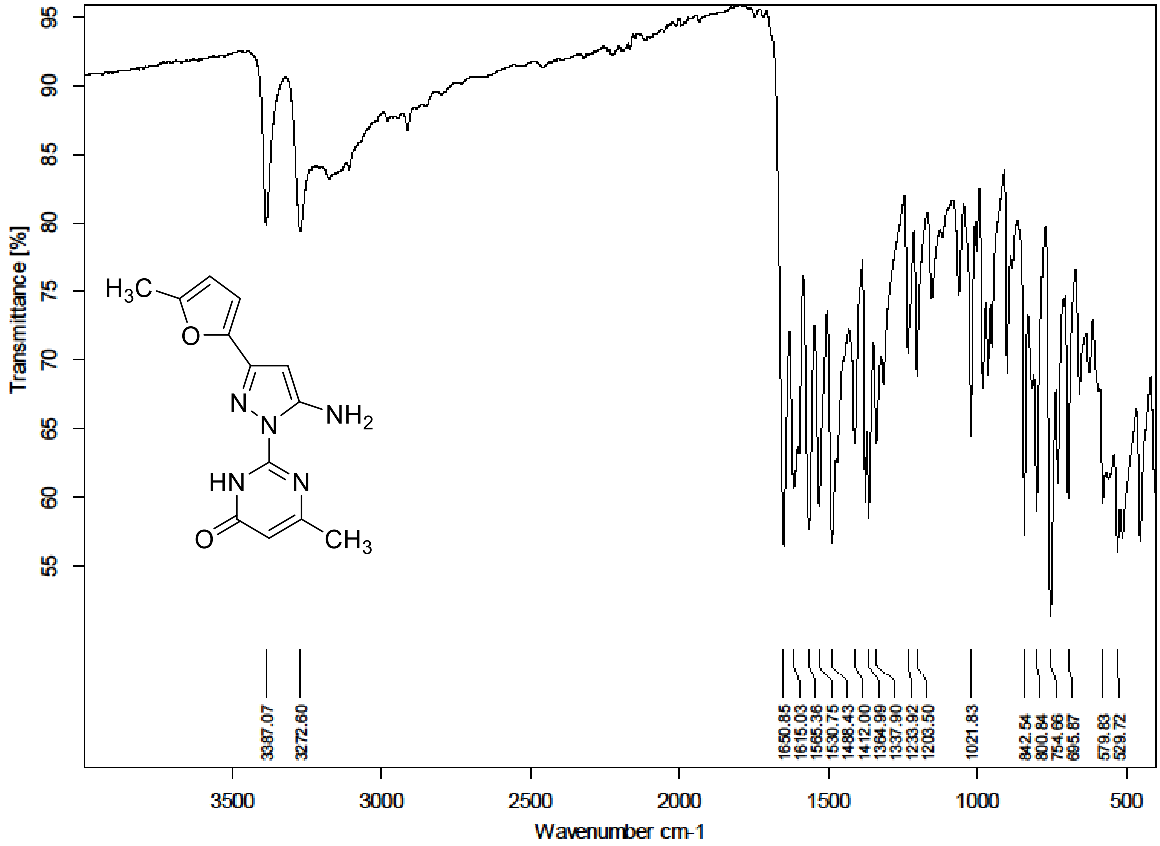

^{1}H (500 MHz) and ^{13}C NMR (126 MHz) spectra of **S137** in DMSO-d_6

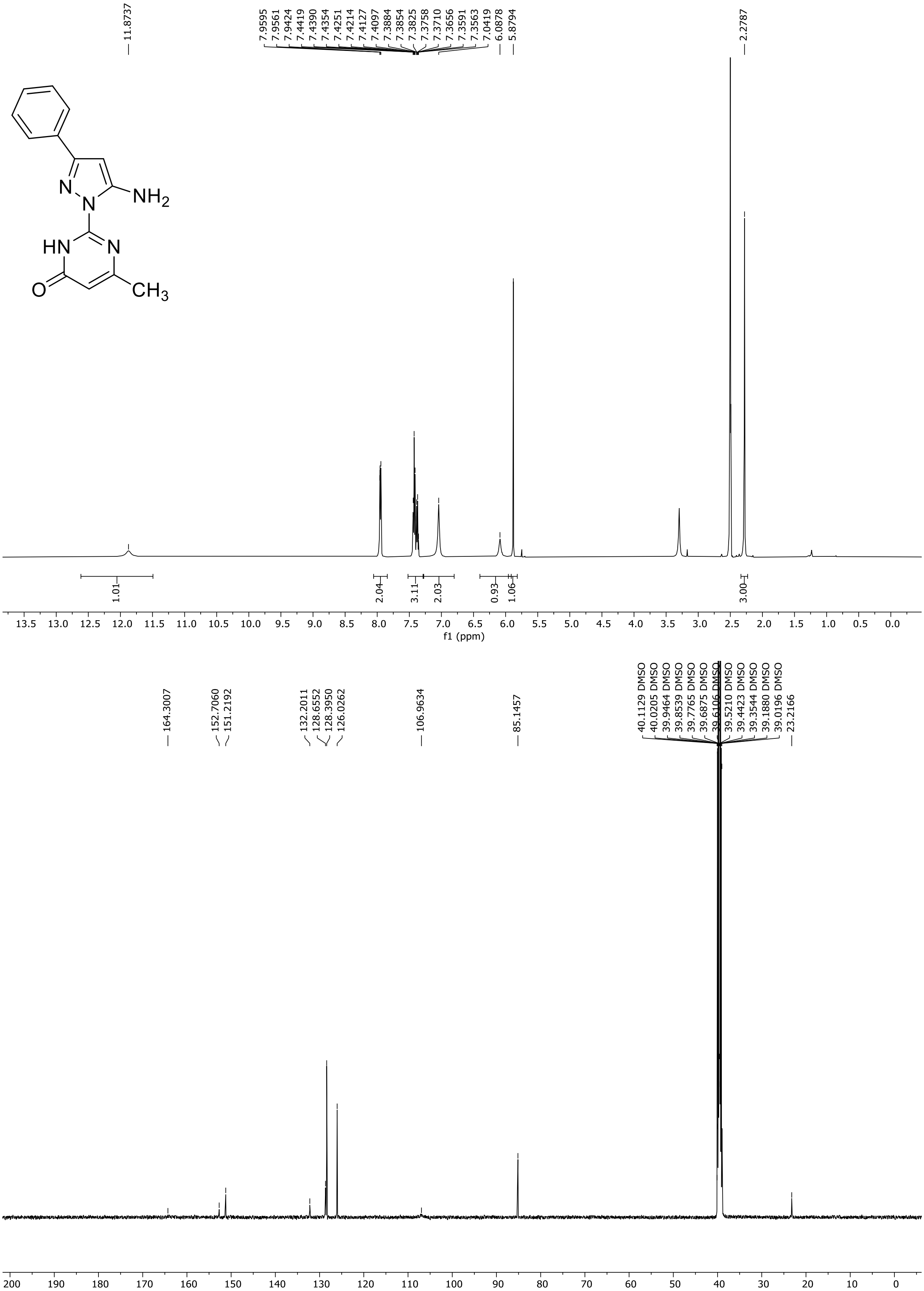

HRMS spectrum of **S137**

NAR-A-1 $C_{14}H_{13}N_5O$ 267.1120 *mono m/z*

APCI + (MMI)
nitrogen flow 5 L/min, gas temperature 325°C, nebulizer 45 psi, skimmer **65** V, vaporizer 200°C, fragmentor 35 V, dissolved in methanol

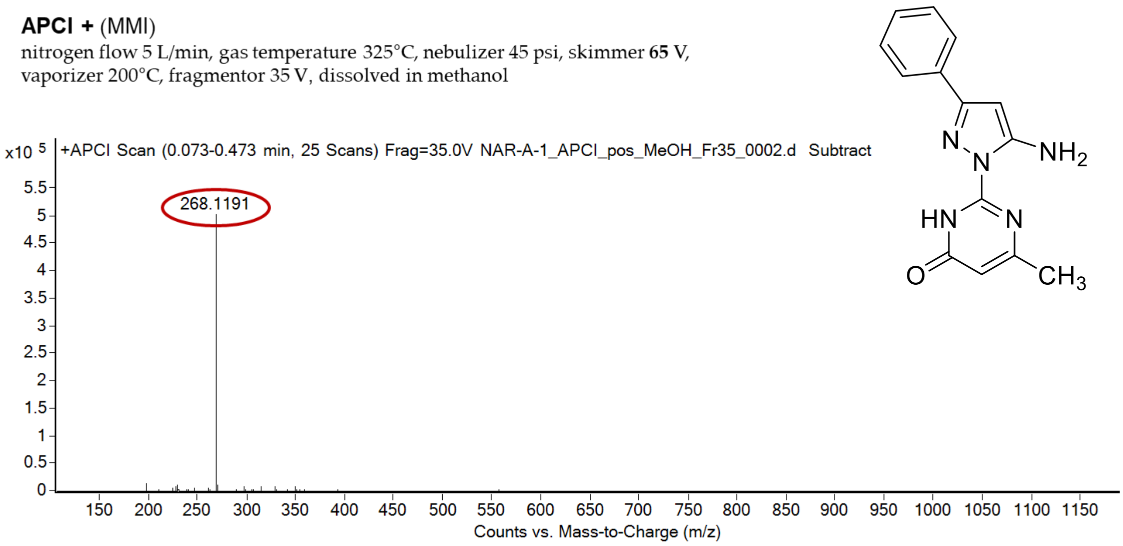

calculated mass: $[M+H]^+$ = 268.1193 observed: $[M+H]^+$ = 268.1193 mass accuracy = 0.7 ppm

FT-IR spectrum (neat) of **S137**

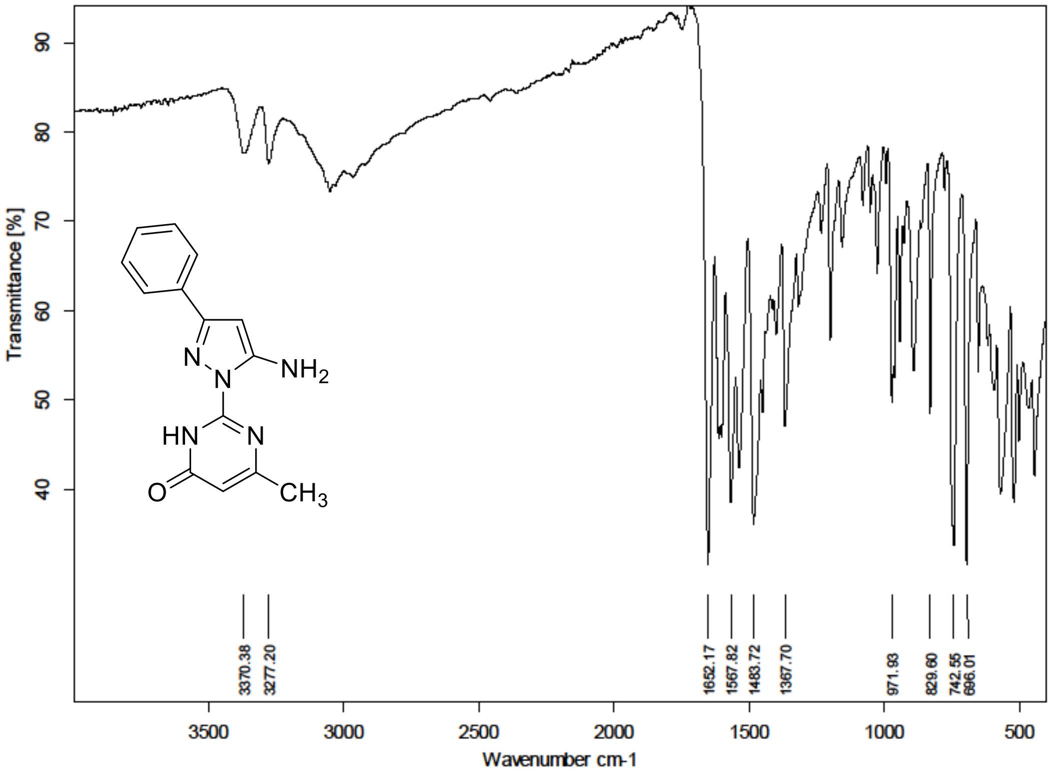

^{1}H (500 MHz) and ^{13}C NMR (126 MHz) spectra of **S138** in DMSO-d_6

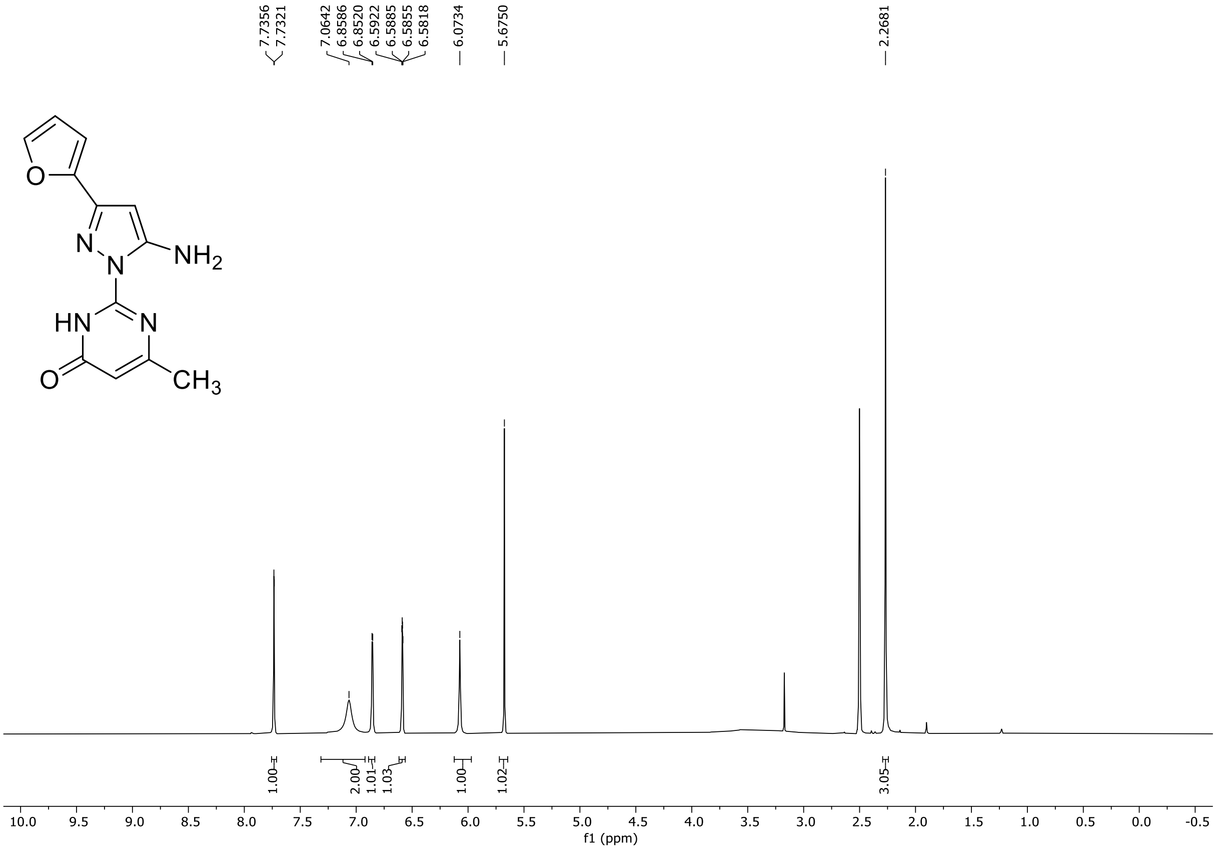

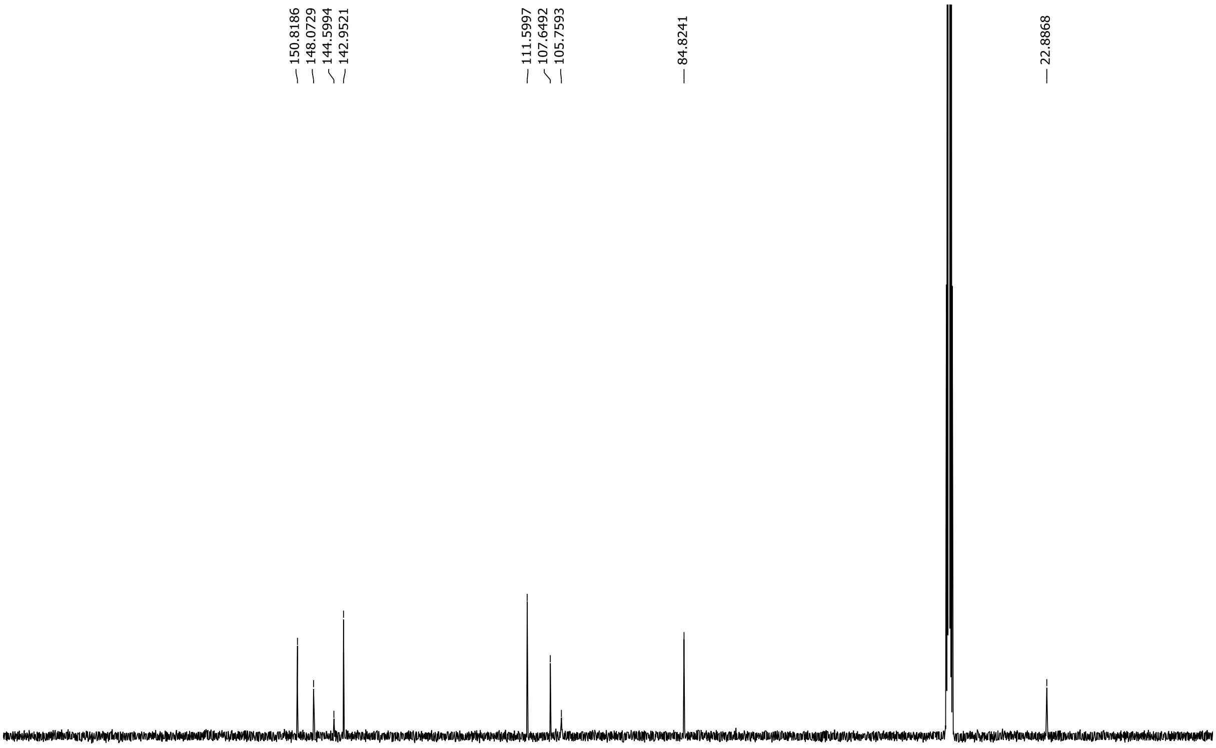

HRMS spectrum of **S138**

NAR-A-33 $C_{12}H_{11}N_5O_2$ mono *m/z 257.0913*

APCI + (MMI)
nitrogen flow 5 L/min, gas temperature 325°C, nebulizer 45 psi, skimmer **65** V, vaporizer 200°C, fragmentor 20 V, dissolved in methanol

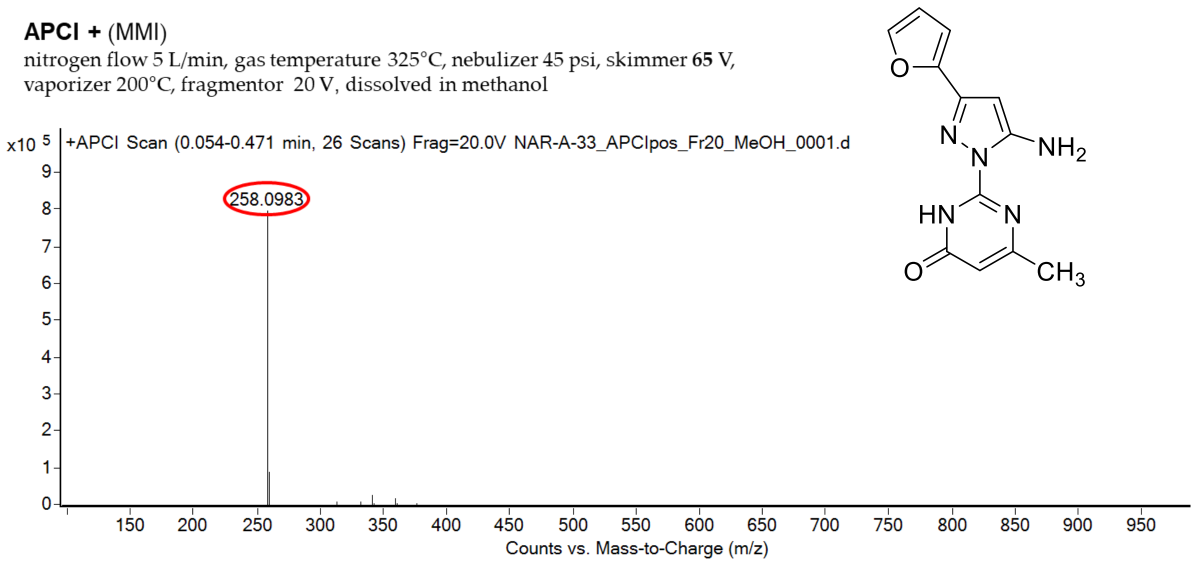

calculated mass: $[M+H]^+$ = 258.0986 observed: $[M+H]^+$ = 258.0983 mass accuracy = -1.2 ppm

FT-IR spectrum (neat) of **S138**

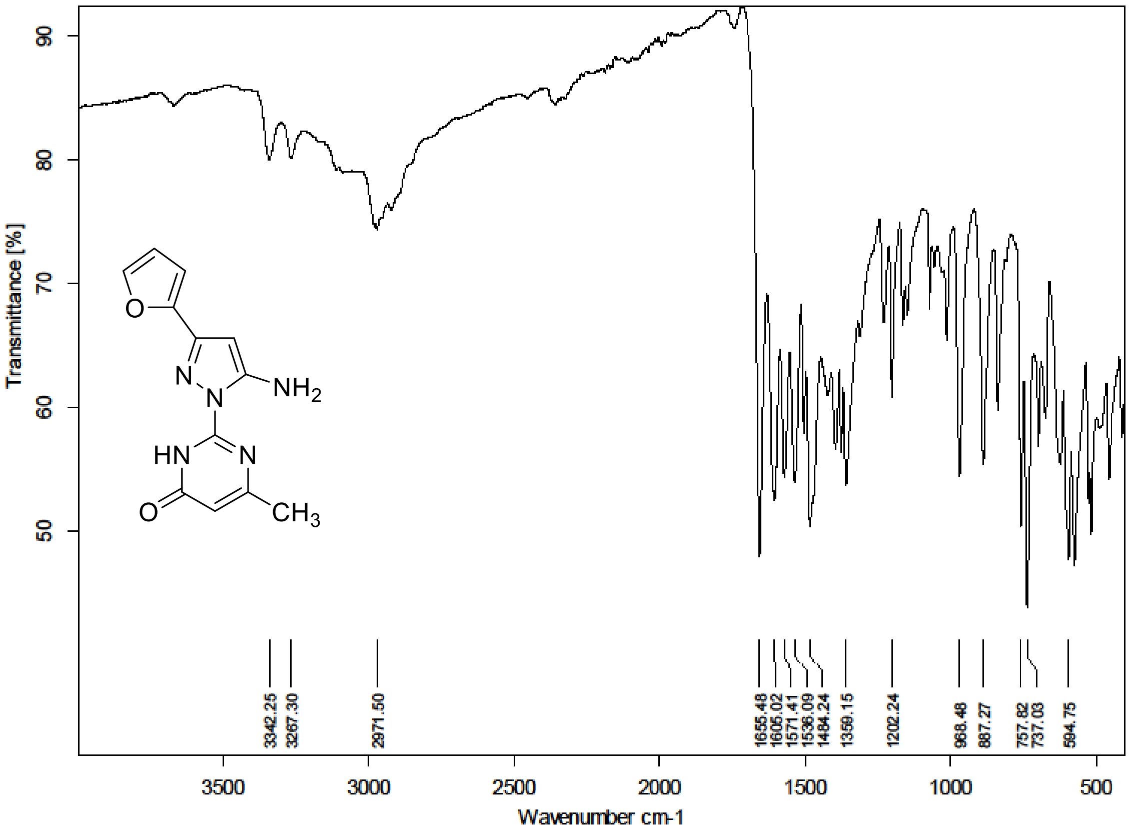

^{1}H (500 MHz) and ^{13}C NMR (126 MHz) spectra of **S140** in DMSO-d_6

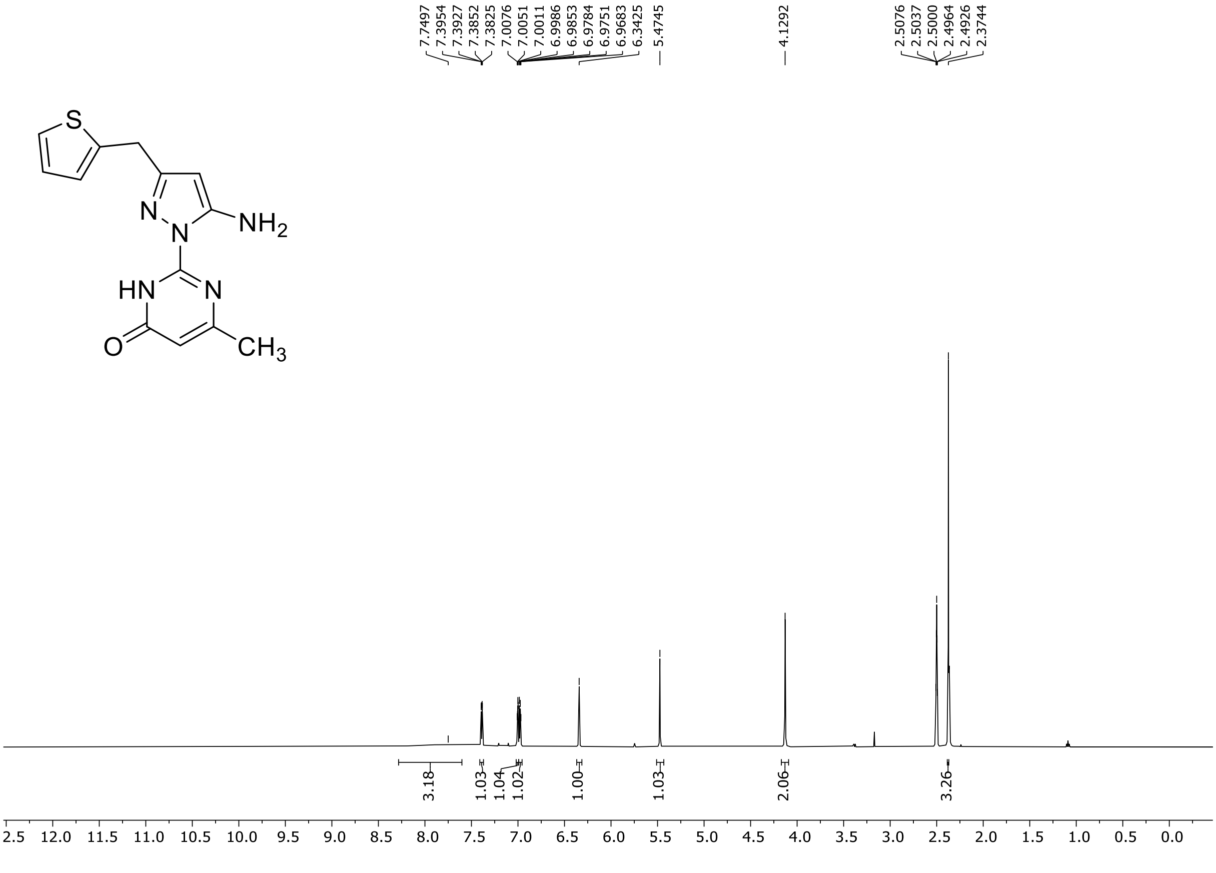

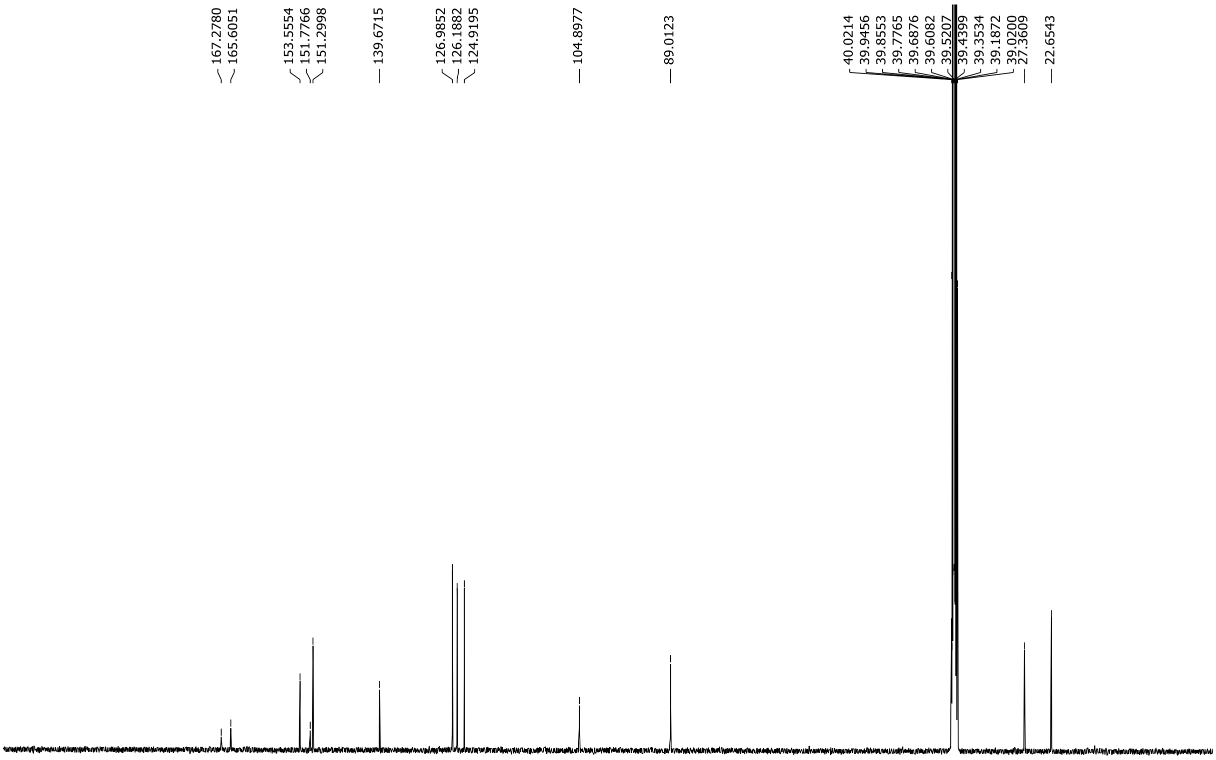

HRMS spectrum of **S140**

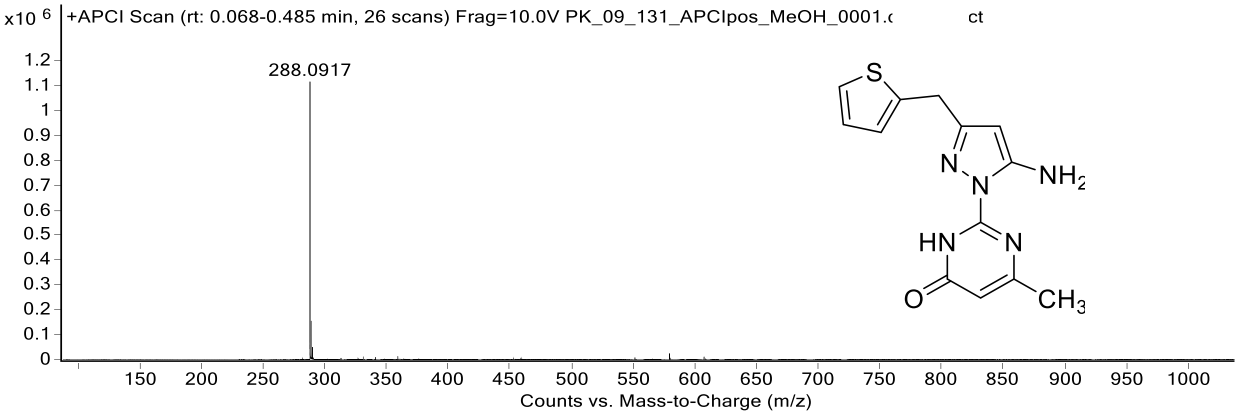

^{1}H (500 MHz) and ^{13}C NMR (126 MHz) spectra of **S141** in DMSO-d_6

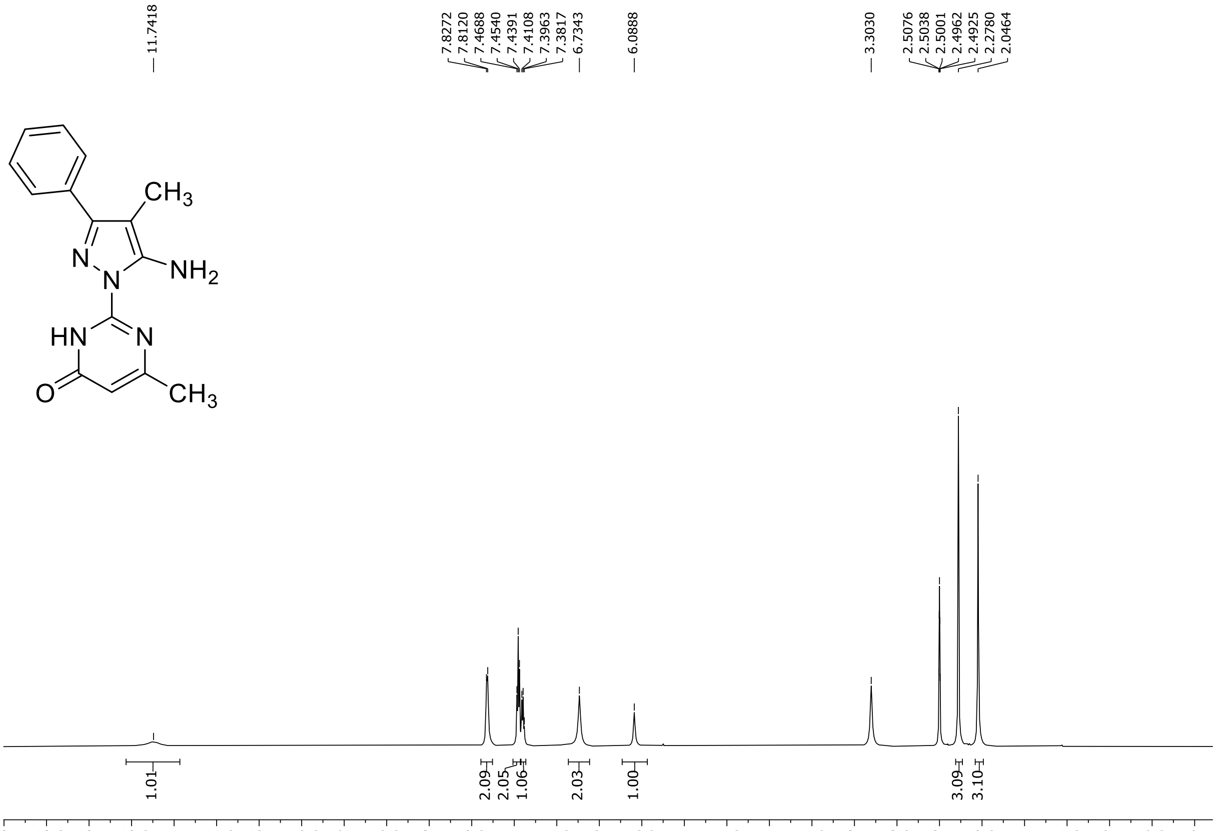

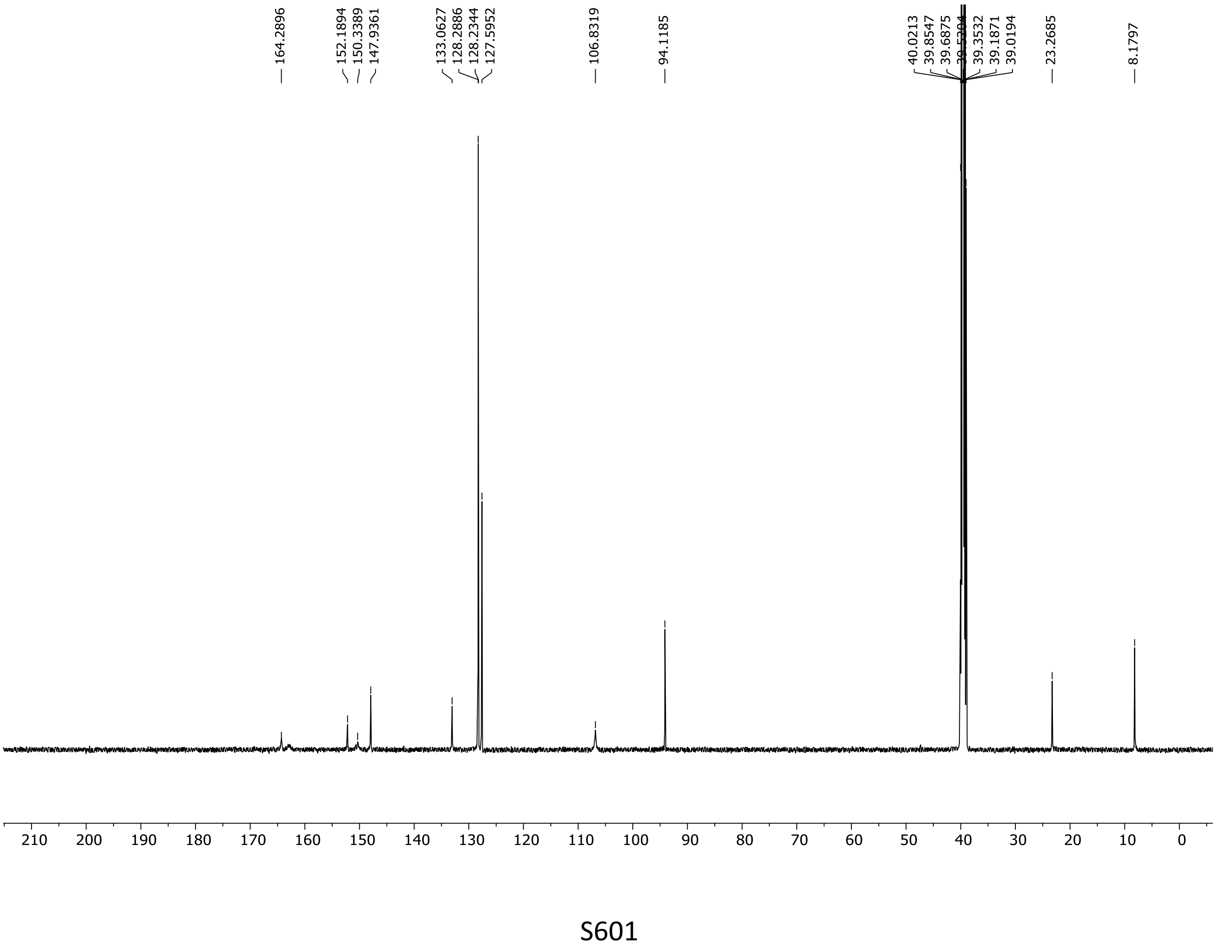

HRMS spectrum of **S141**

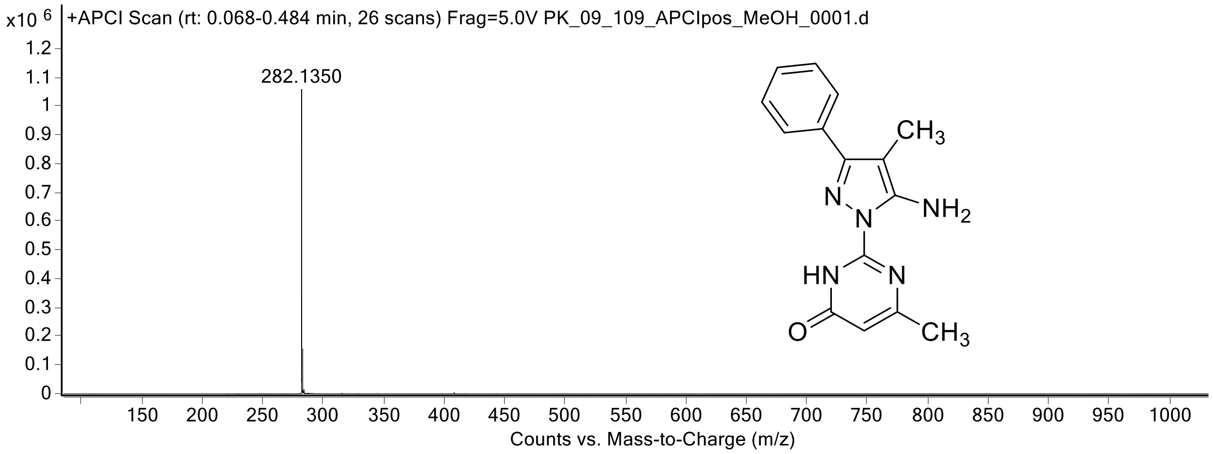

^{1}H (500 MHz) and ^{13}C NMR (126 MHz) spectra of **S142** in DMSO-d_6

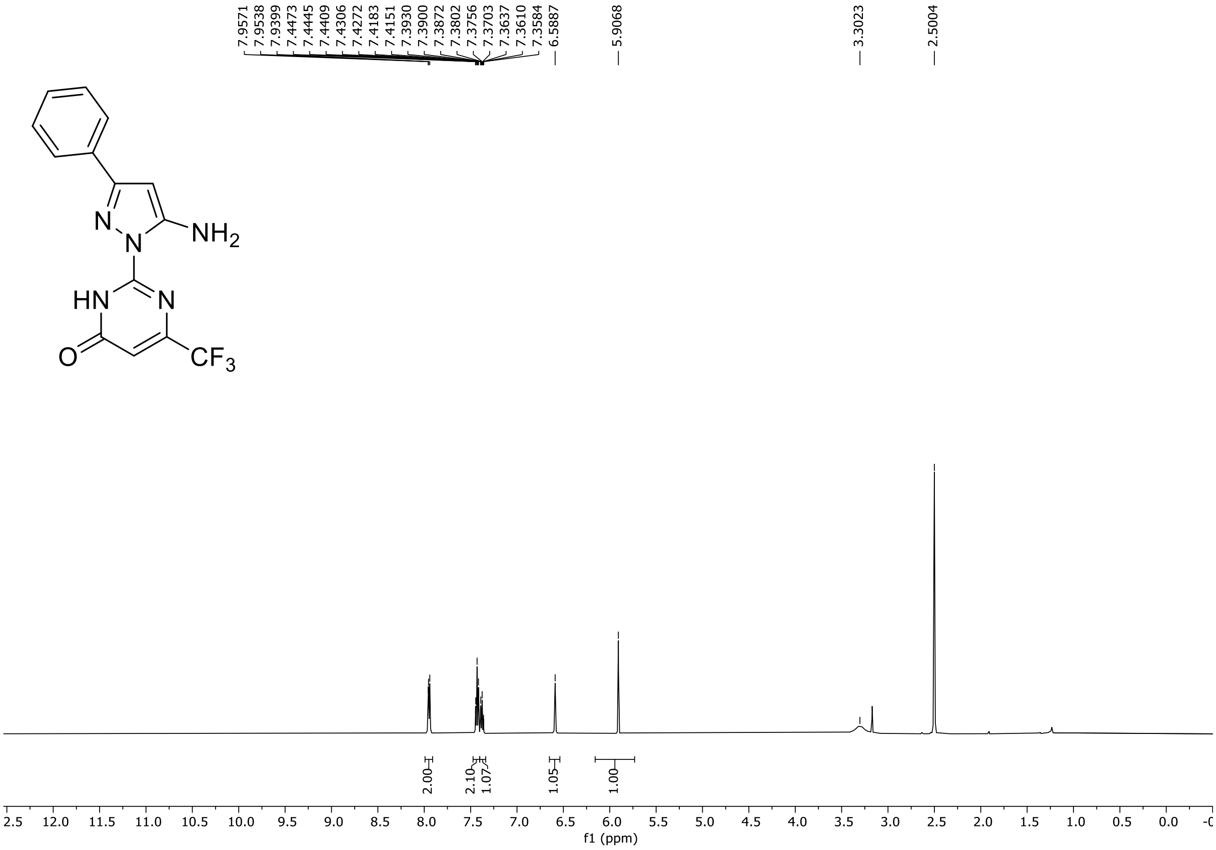

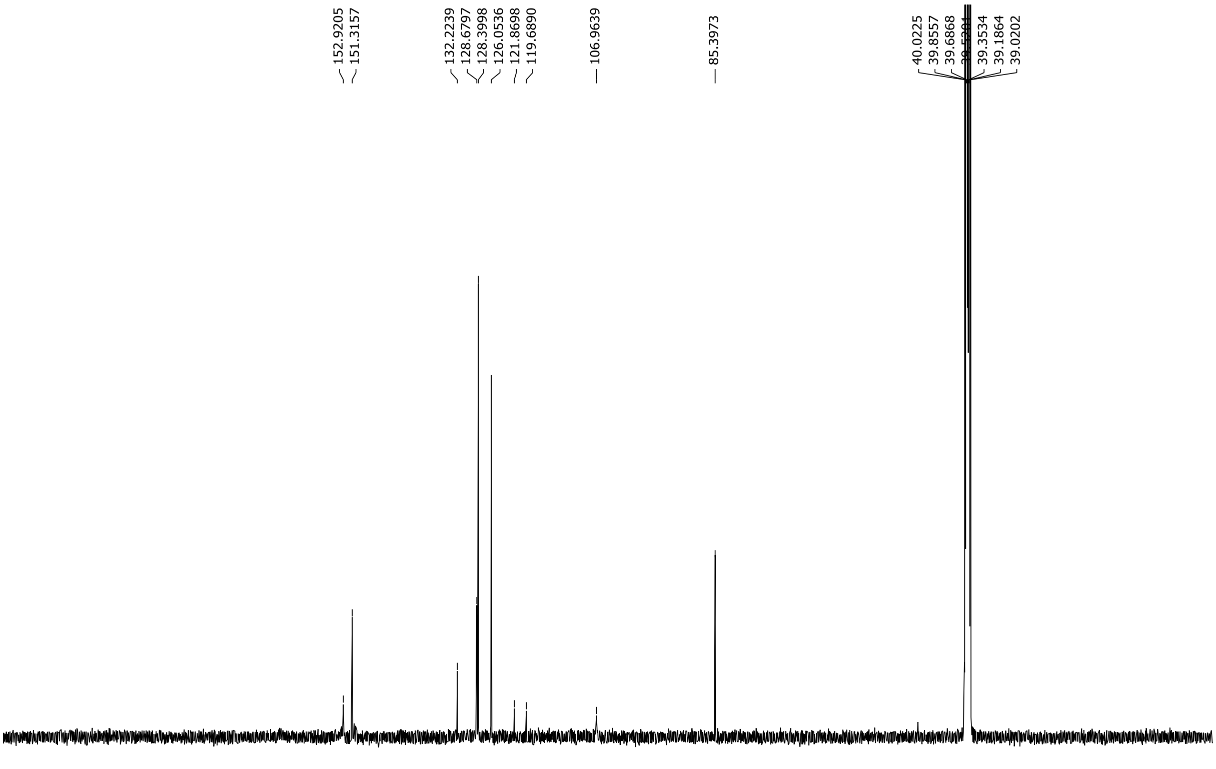

^{19}F NMR (471 MHz) spectrum of **S142** in DMSO-d_6

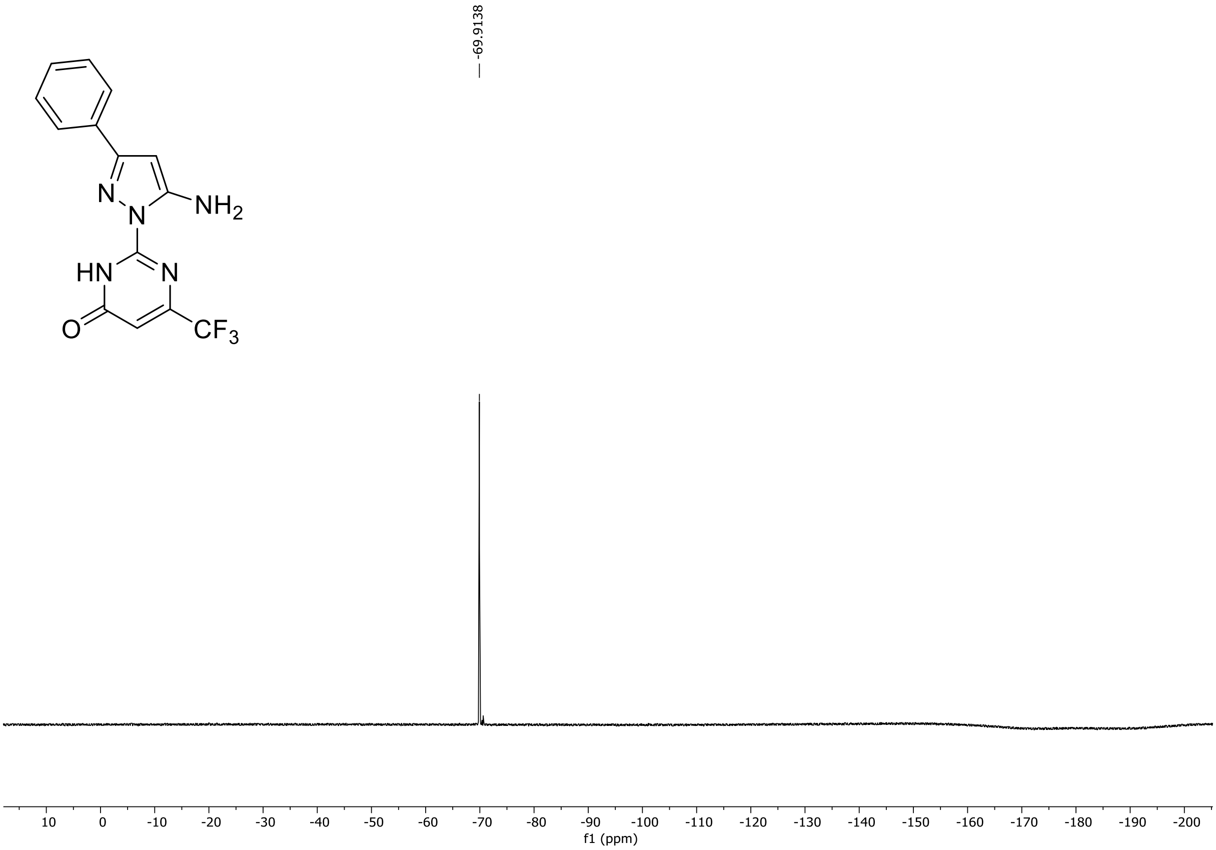

HRMS spectrum of **S142**

NAR-A-90 **$C_{14}H_{10}F_3N_5O$** ***m/z* 321.0837**

APCI+ (MMI)
nitrogen flow 5 L/min, gas temperature 325°C, nebulizer 45 psi, skimmer 65 V, vaporizer 200°C, fragmentor 30 V, dissolved in MeOH

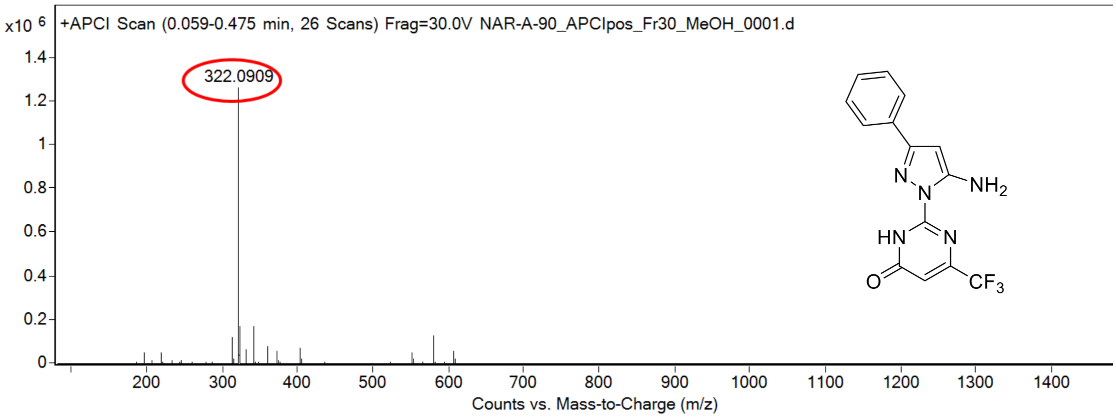

calculated mass: $[M+H]^+$ = 322.0910 observed: $[M+H]^+$ = 322.0909 mass accuracy = -0.3 ppm

FT-IR spectrum (neat) of **S142**

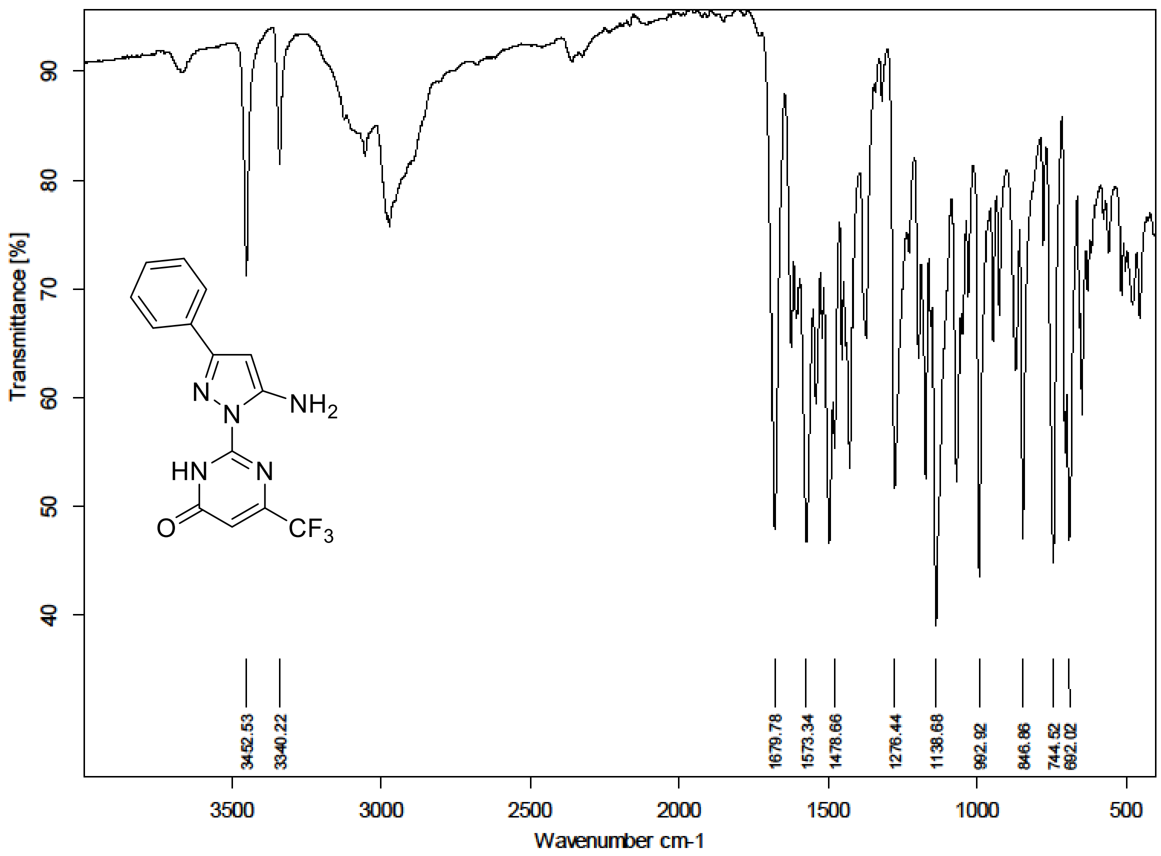

^{1}H (300 MHz) and ^{13}C NMR (75 MHz) spectra of **S143** in DMSO-d_6

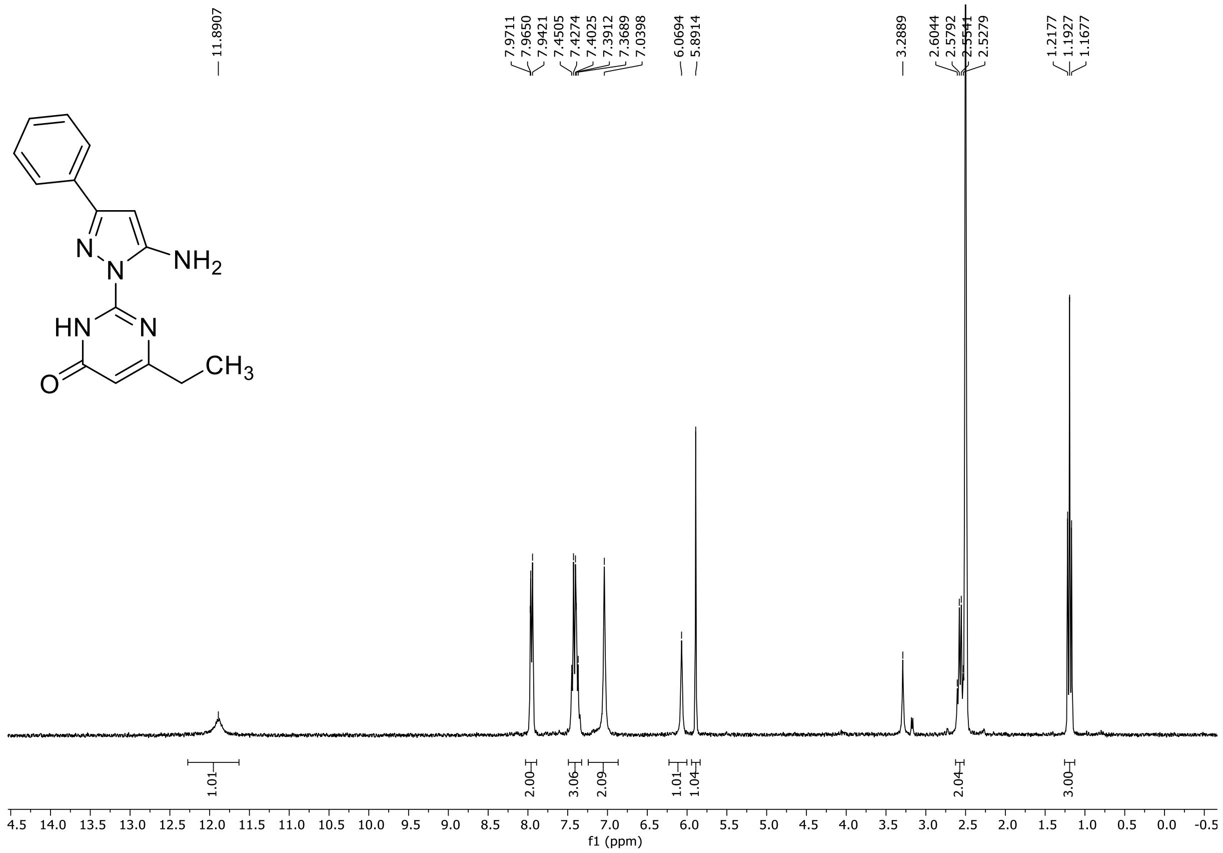

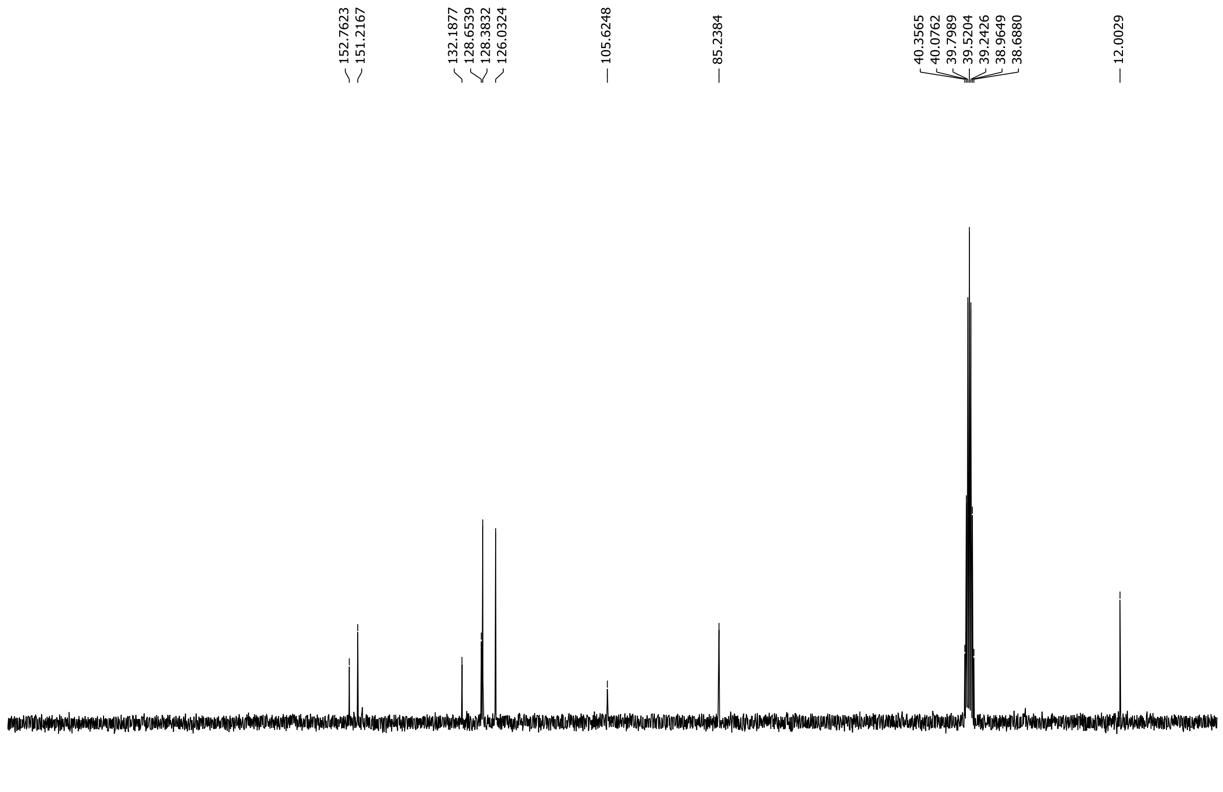

HRMS spectrum of **S143**

NAR-A-167 $C_{15}H_{15}N_5O$ mono *m/z* 281.1277

APCI + (MMI)
nitrogen flow 5 L/min, gas temperature 325°C, nebulizer 45 psi, skimmer **65** V, vaporizer 200°C, fragmentor 20 V, dissolved in methanol

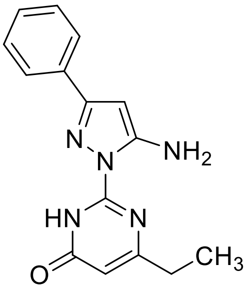

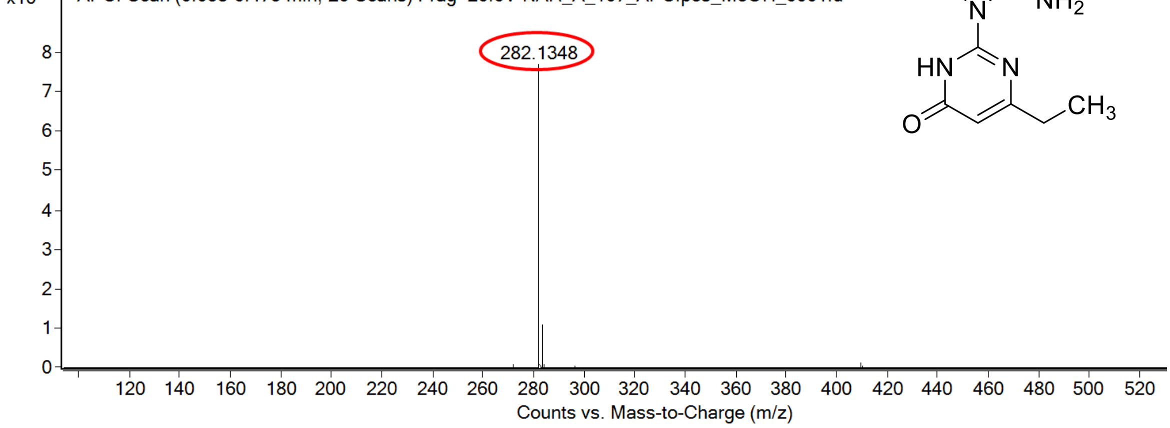

calculated mass: $[M+H]^+$ = 282..1349 observed: $[M+H]^+$ = 282.1348 mass accuracy = - 0.4 ppm

FT-IR spectrum (neat) of **S143**

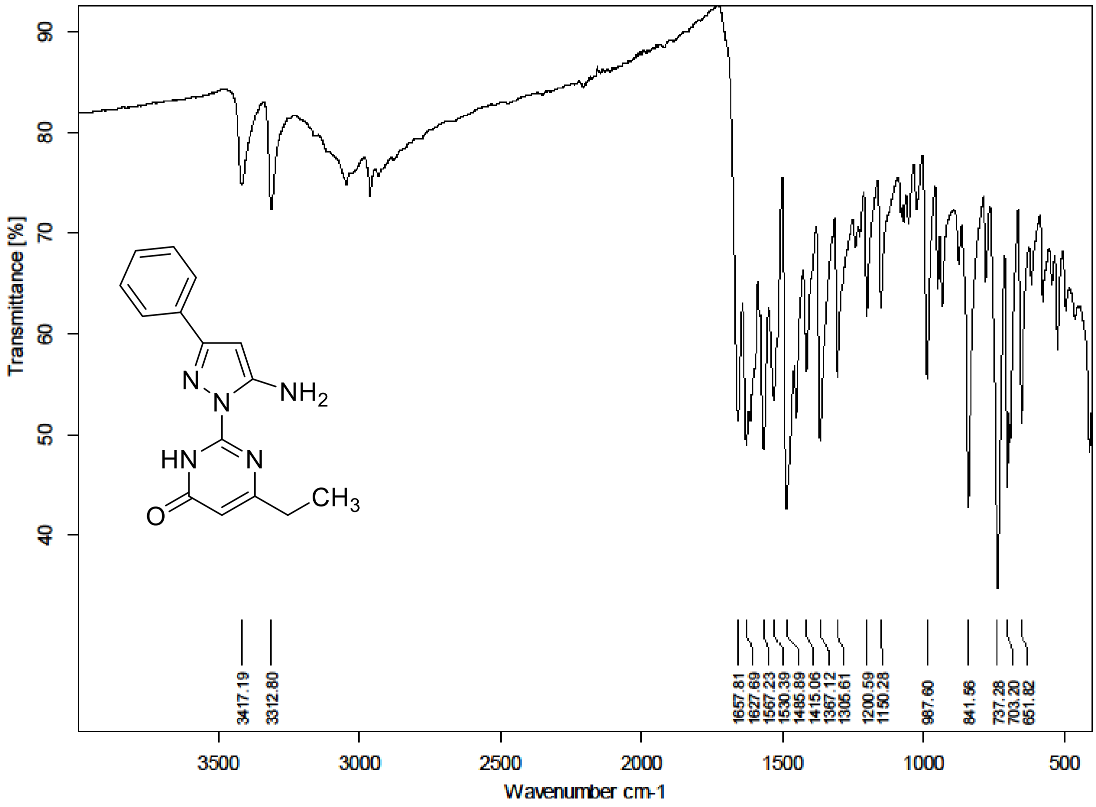

^{1}H (500 MHz) and ^{13}C NMR (126 MHz) spectra of **S144** in DMSO-d_6

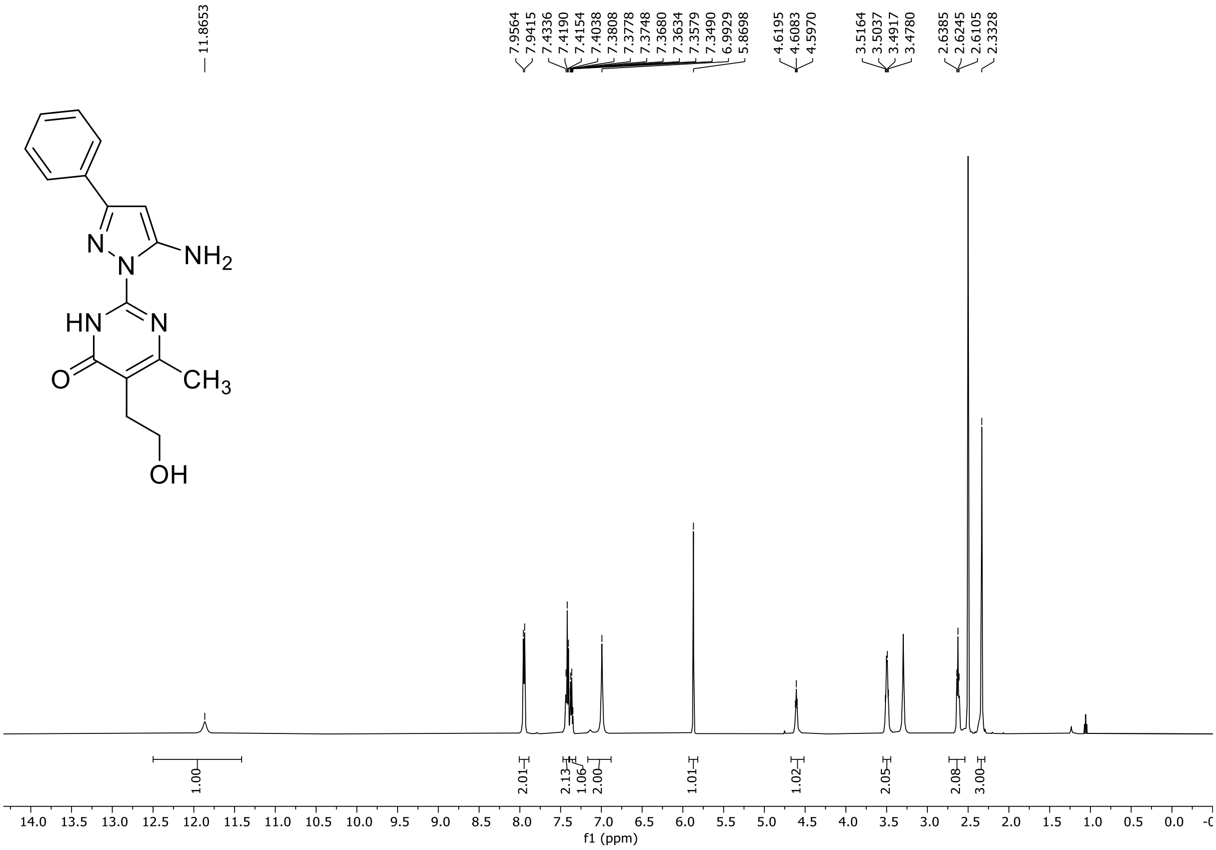

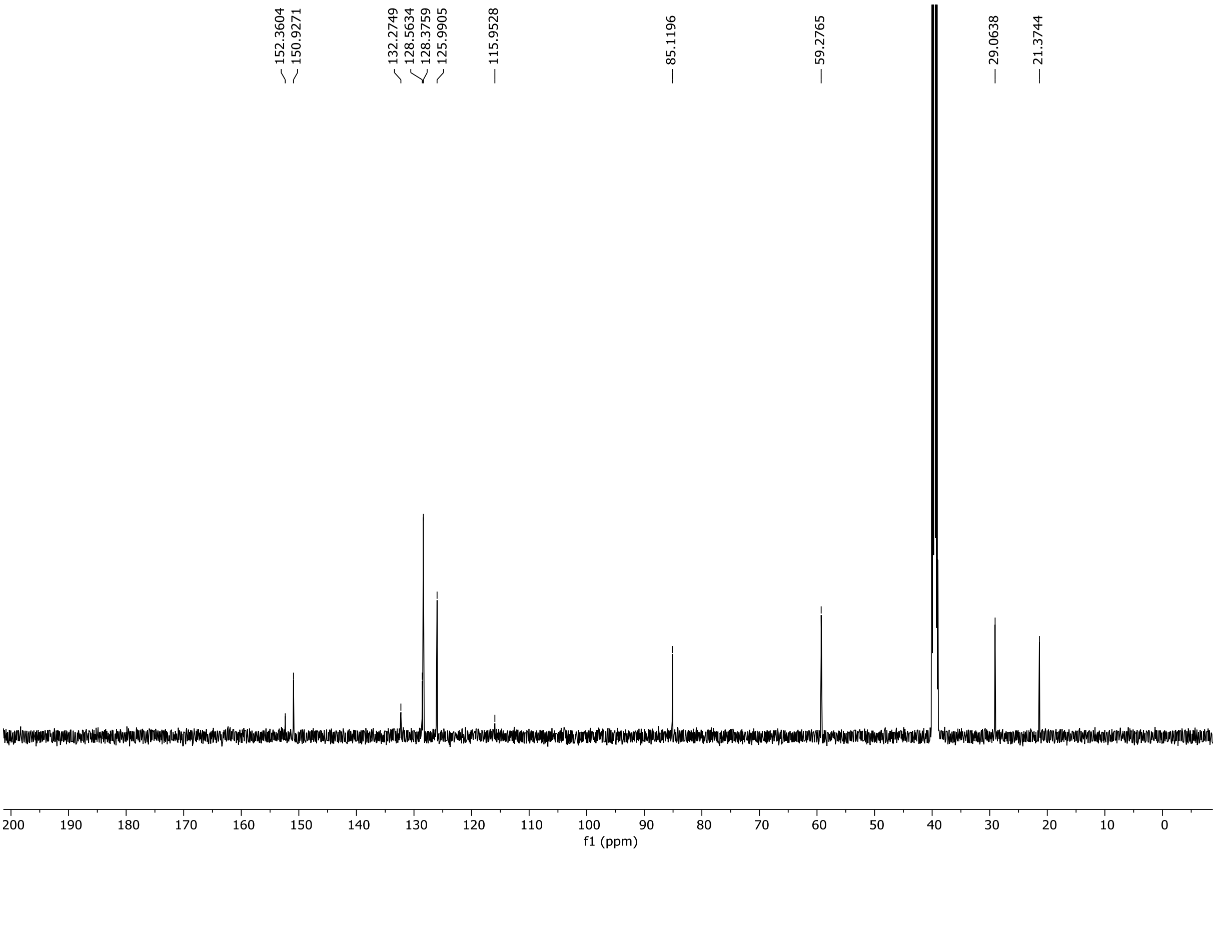

HRMS spectrum of **S144**

NAR-A-15 **$C_{16}H_{17}N_5O_2$** **mono *m/z 311.1382***

APCI + (MMI)
nitrogen flow 5 L/min, gas temperature 325°C, nebulizer 45 psi, skimmer **65** V, vaporizer 200°C, fragmentor 30 V, dissolved in methanol

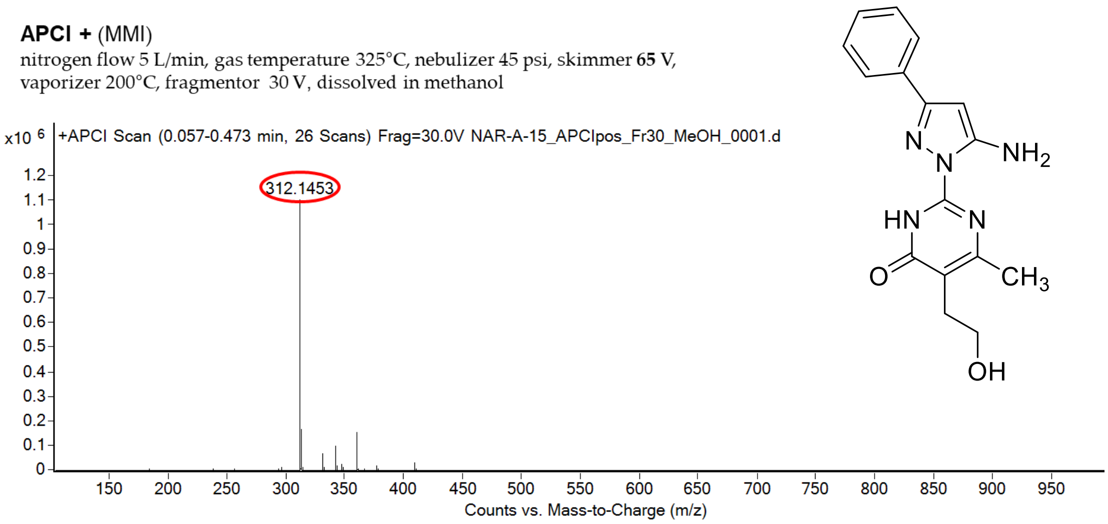

calculated mass: $[M+H]^+$ = 312.1455 observed: $[M+H]^+$ = 312.1453 mass accuracy = -0.6 ppm

FT-IR spectrum (neat) of **S144**

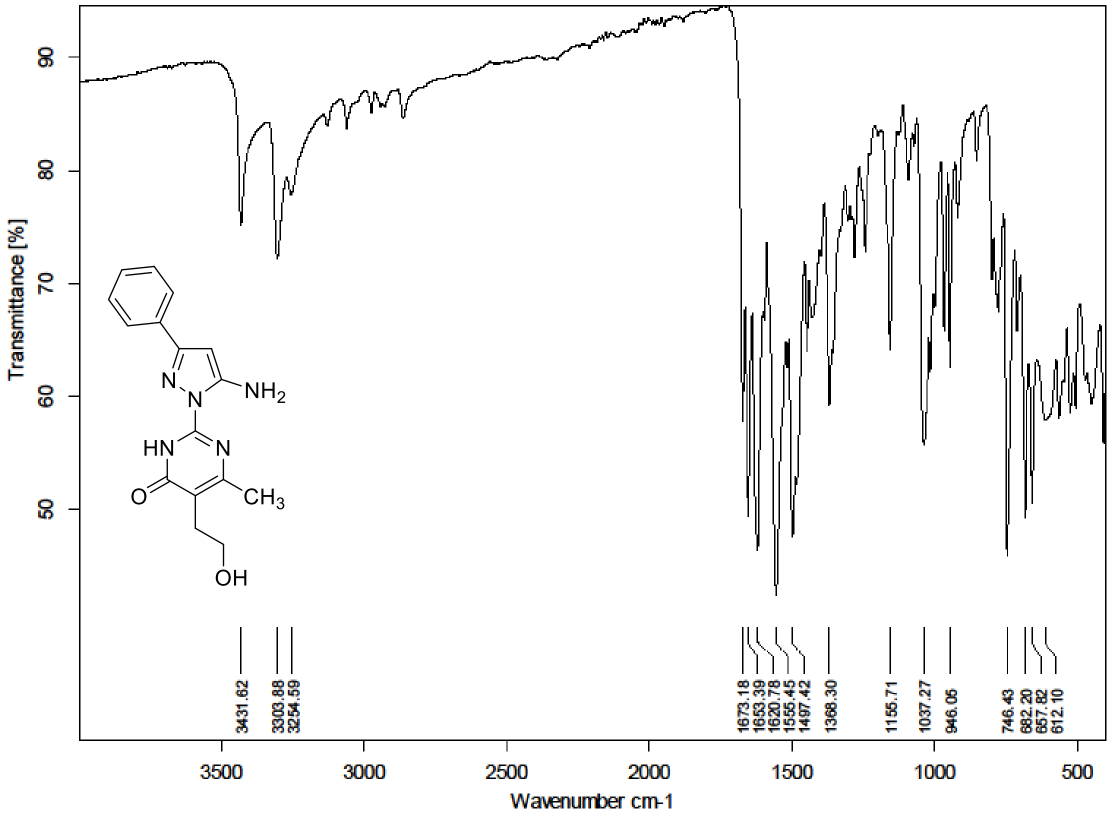

^{1}H (500 MHz) and ^{13}C NMR (126 MHz) spectra of **S145** in Chloroform-*d*

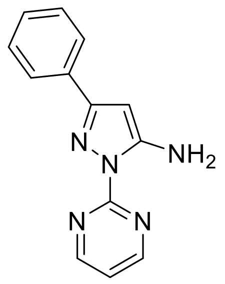

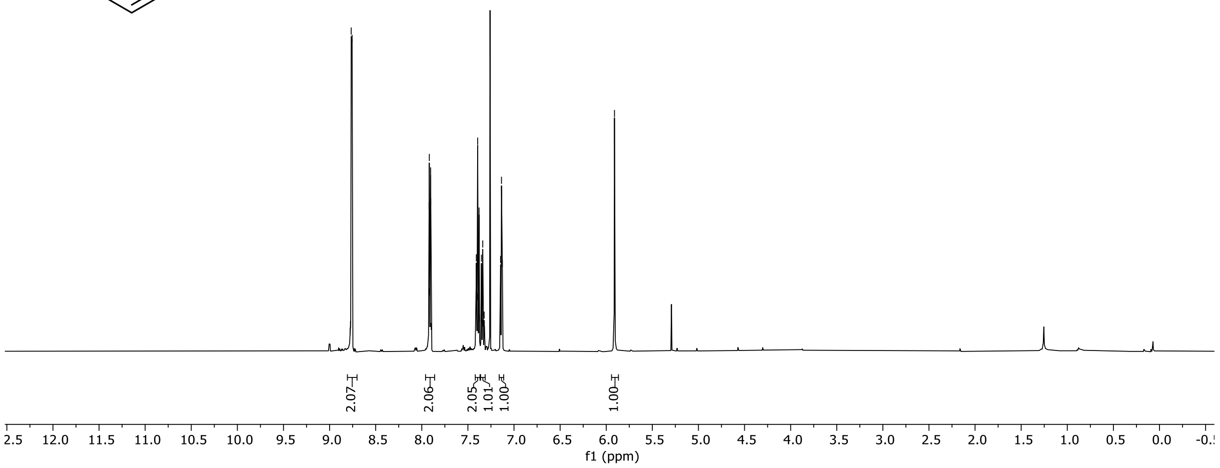

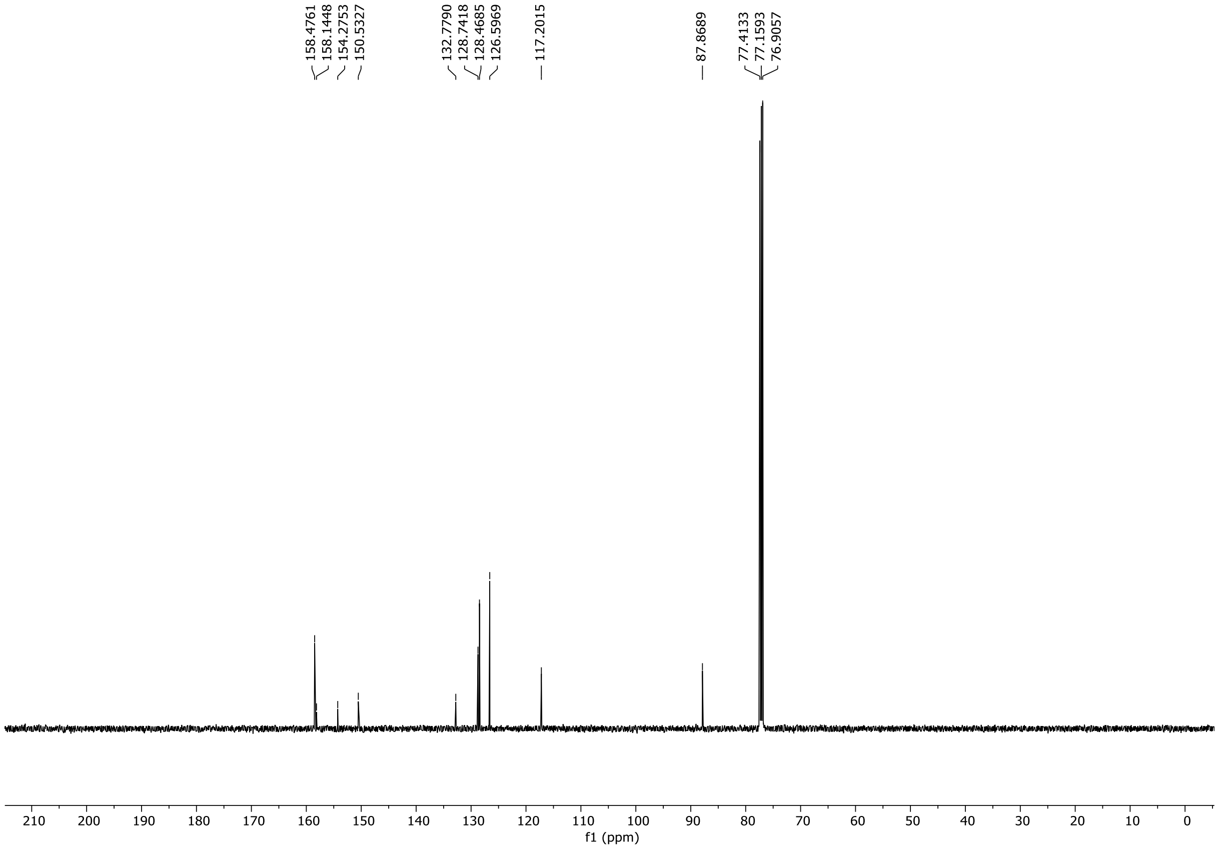

HRMS spectrum of **S145**

NAR-A-9 $C_{13}H_{11}N_5$ *mono m/z* 237.1014

APCI + (MMI)
nitrogen flow 5 L/min, gas temperature 325°C, nebulizer 45 psi, skimmer **65** V, vaporizer 200°C, fragmentor 25 V, dissolved in methanol

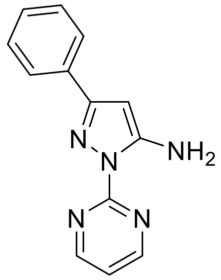

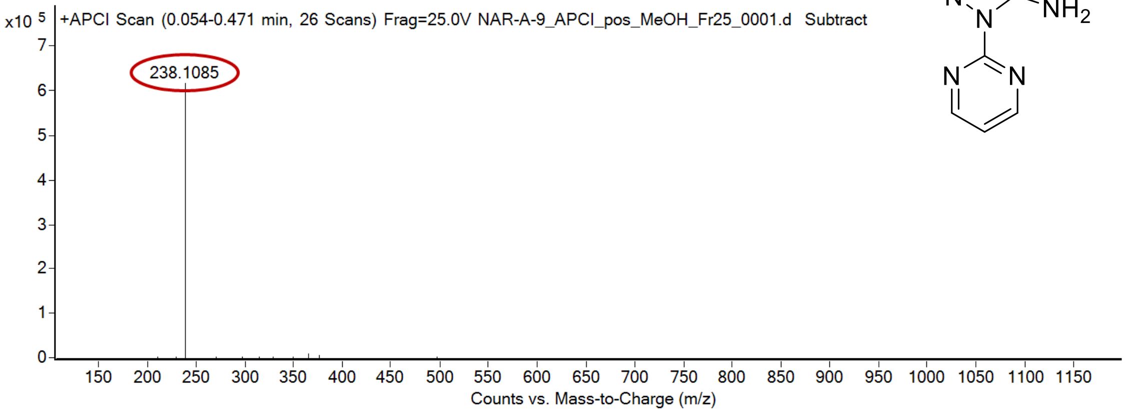

calculated mass: $[M+H]^+$ = 238.1087 observed: $[M+H]^+$ = 238.1085 mass accuracy = 0.8 ppm

FT-IR spectrum (neat) of **S145**

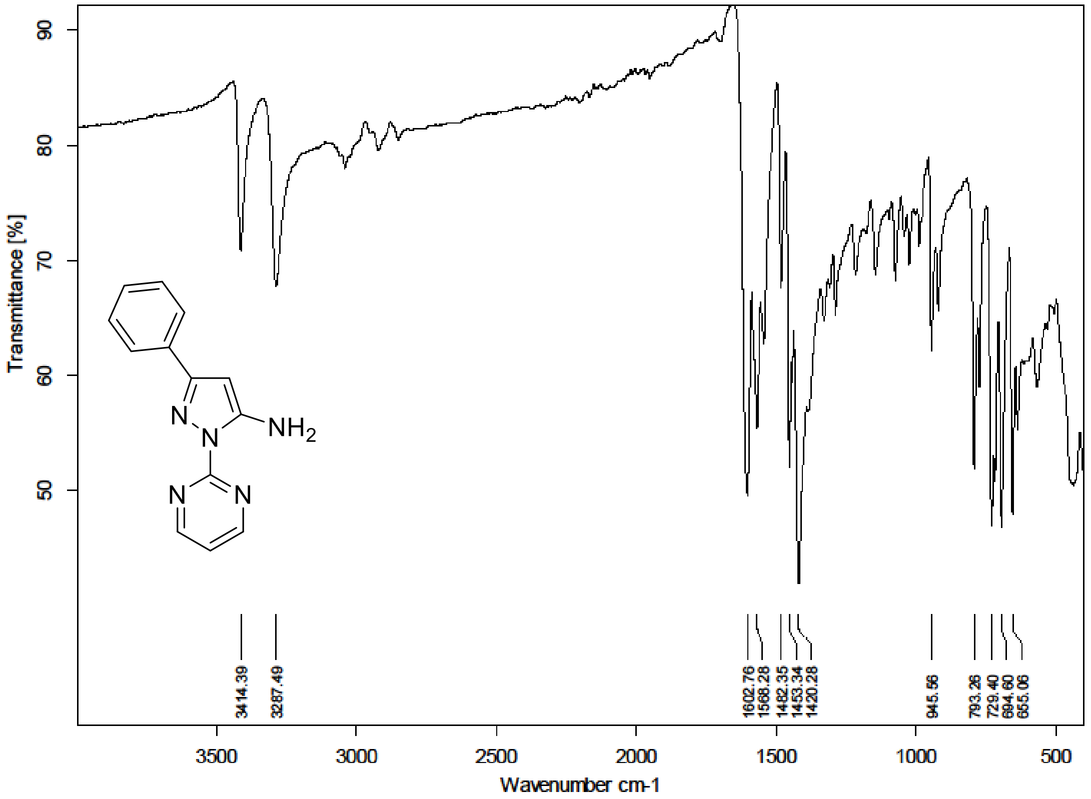

^{1}H (500 MHz) and ^{13}C NMR (126 MHz) spectra of **S146** in Chloroform-*d*

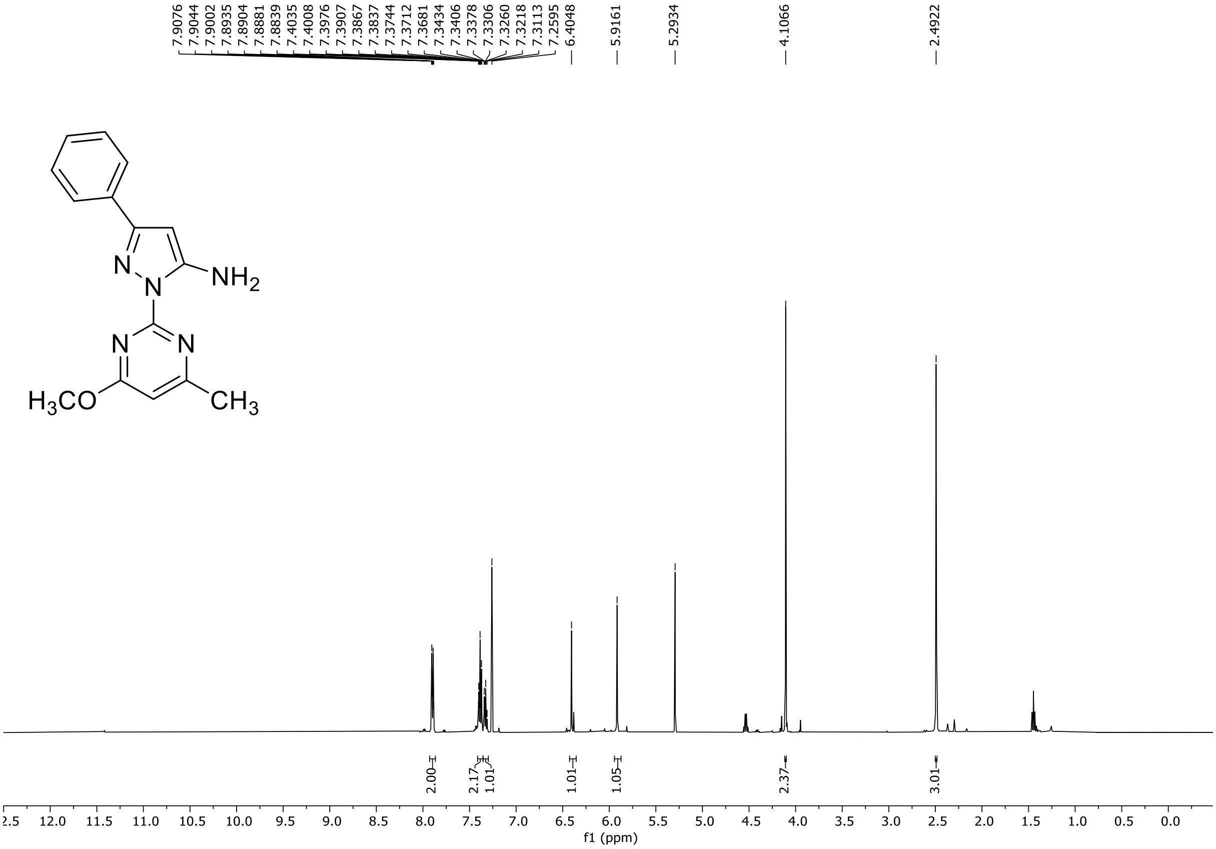

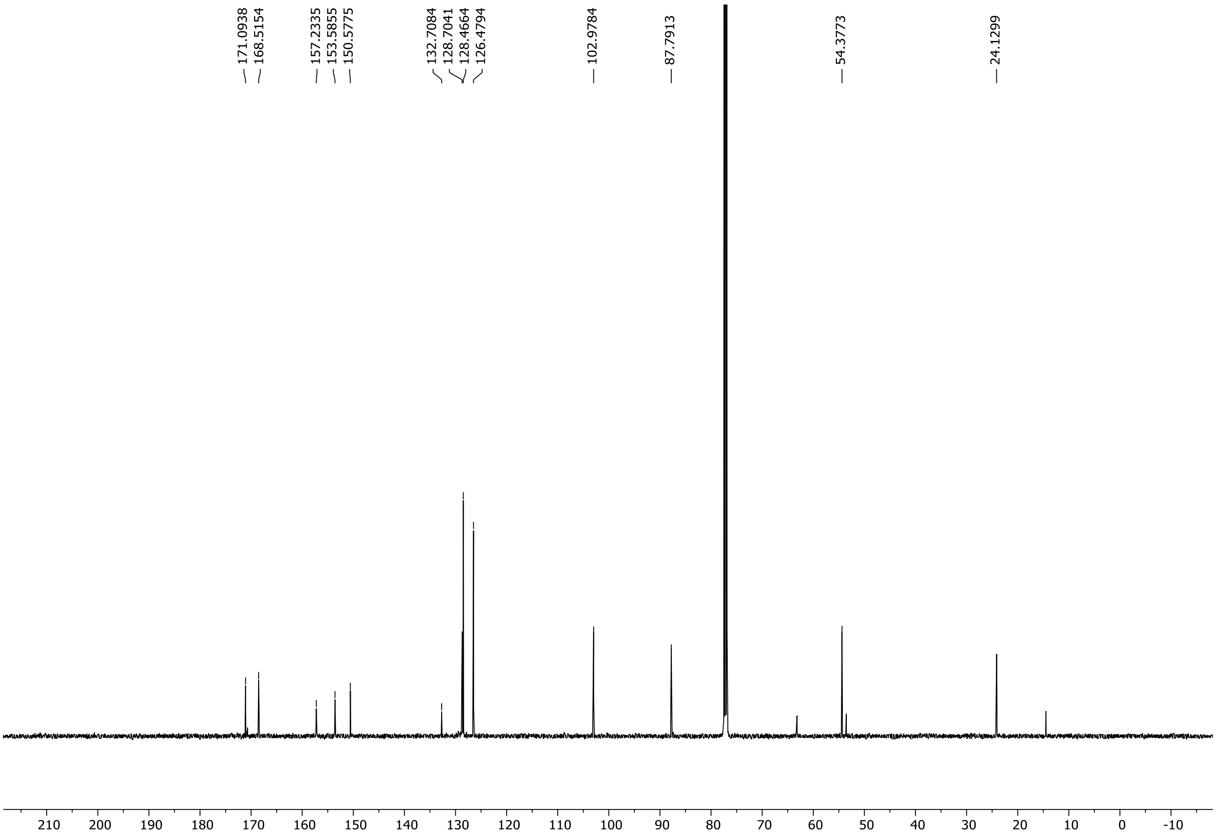

HRMS spectrum of **S146**

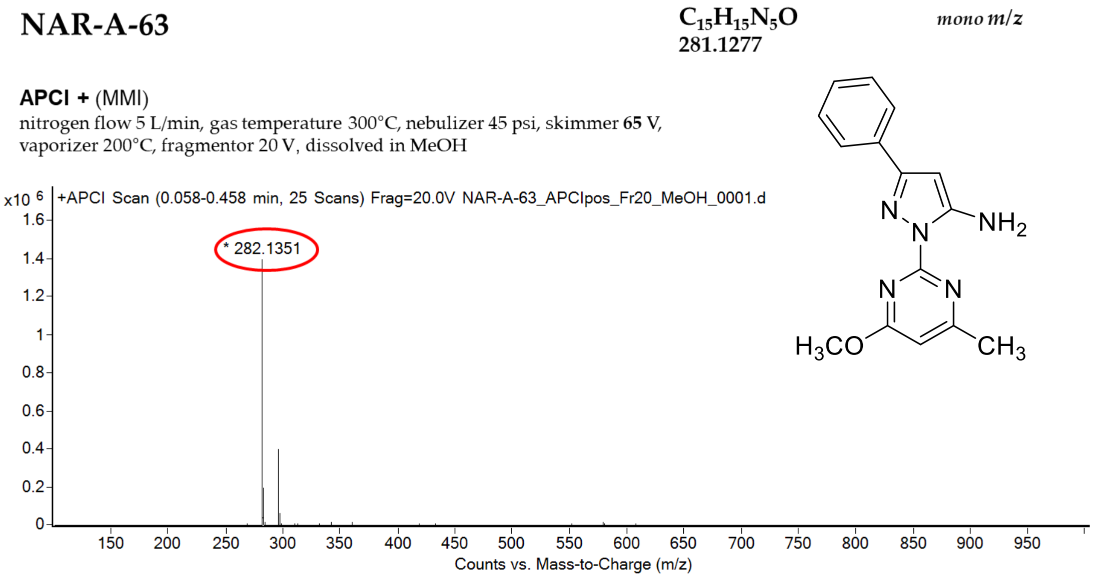

calculated mass: $[M+H]^+$ = 282.1349 observed: $[M+H]^+$ = 282.1351 mass accuracy = 0.4 ppm

FT-IR spectrum (neat) of **S146**

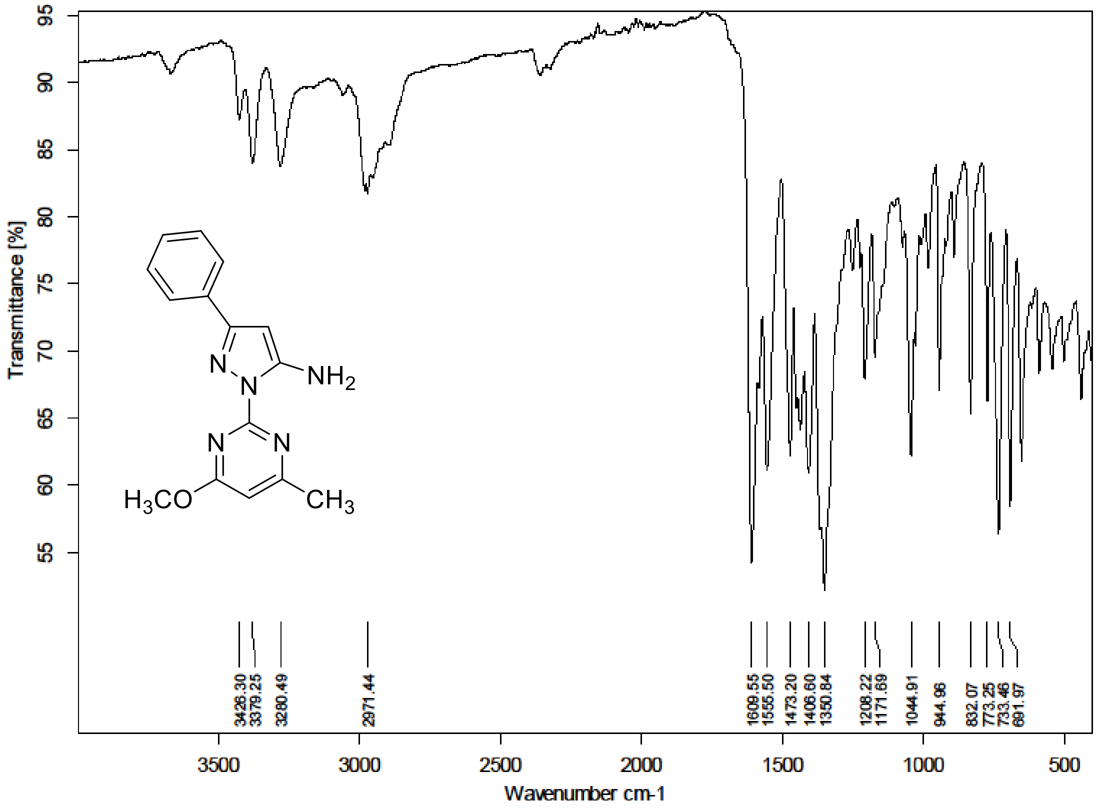

^{1}H (500 MHz) and ^{13}C NMR (126 MHz) spectra of **S147** in DMSO-d_6

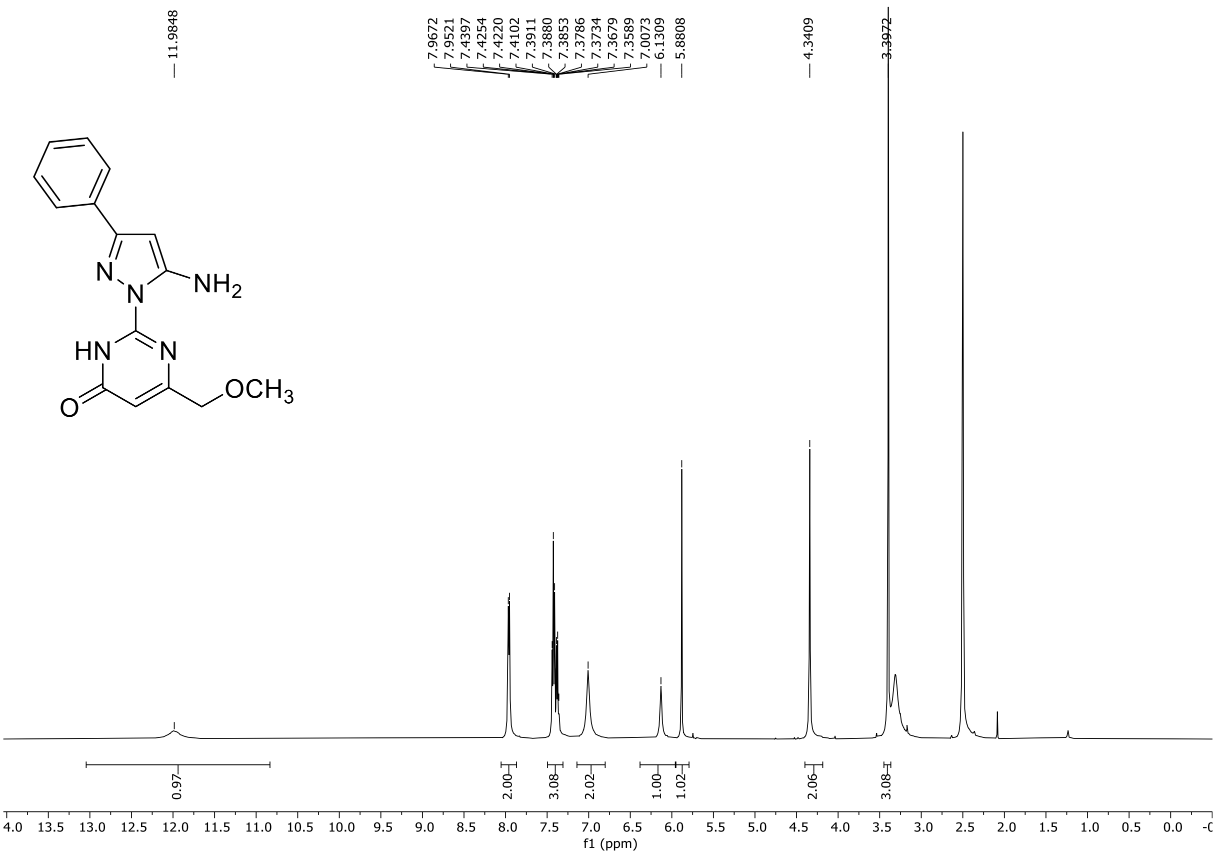

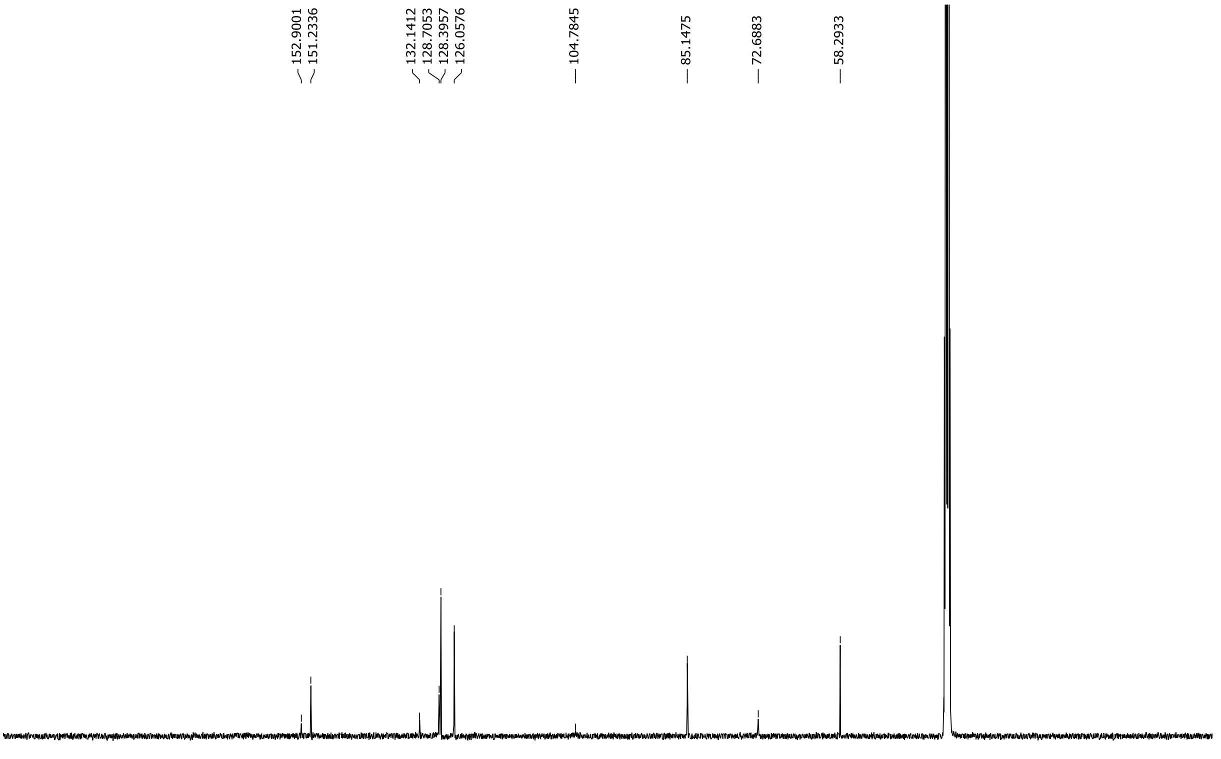

HRMS spectrum of **S147**

NAR-A-79 $C_{15}H_{15}N_5O_2$ *mono m/z* **297.1226**

APCI + (MMI)
nitrogen flow 5 L/min, gas temperature 300°C, nebulizer 45 psi, skimmer **65** V, vaporizer 200°C, fragmentor 20 V, dissolved in MeOH

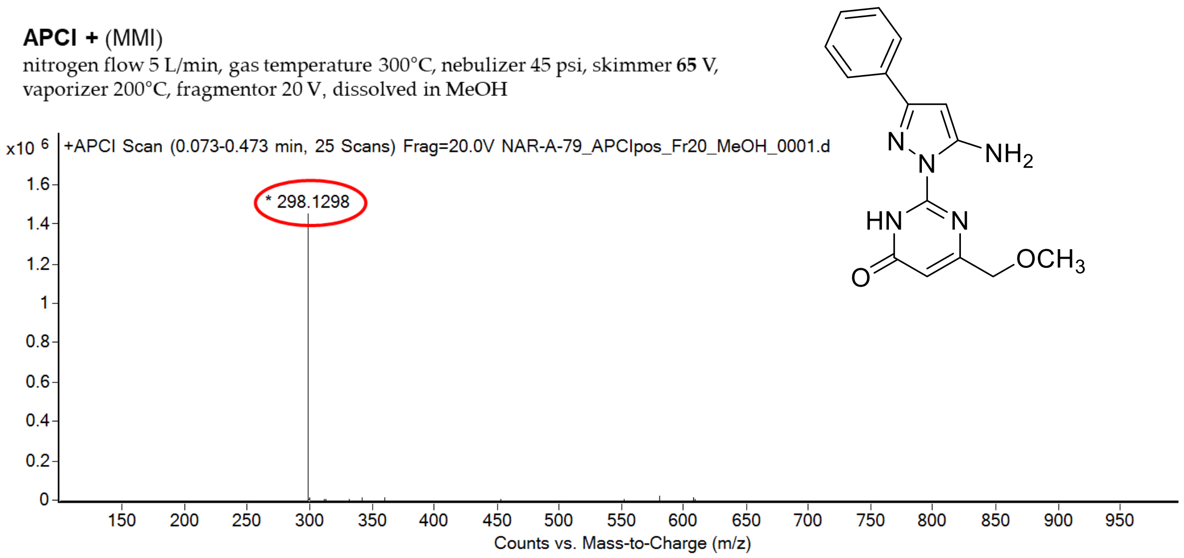

calculated mass: $[M+H]^+$ = 298.1299 observed: $[M+H]^+$ = 298.1298 mass accuracy = -0.3 ppm

FT-IR spectrum (neat) of **S147**

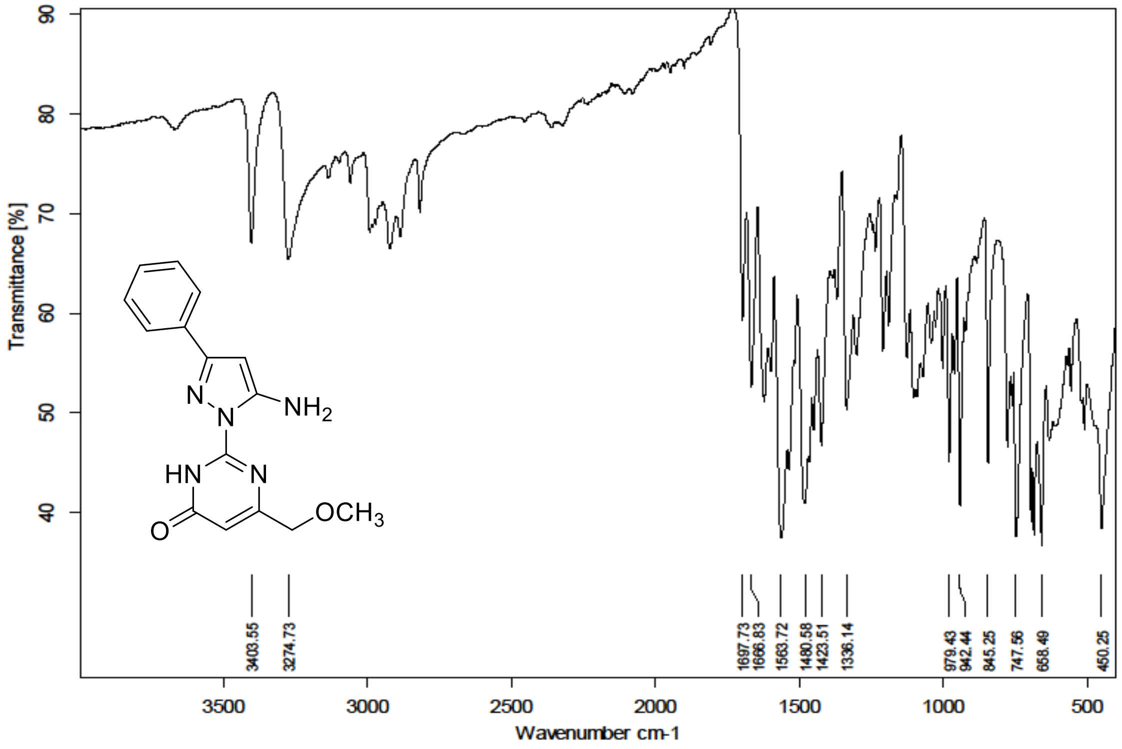

^{1}H (300 MHz) and ^{13}C NMR (75 MHz) spectra of **S148** in Chloroform-*d*

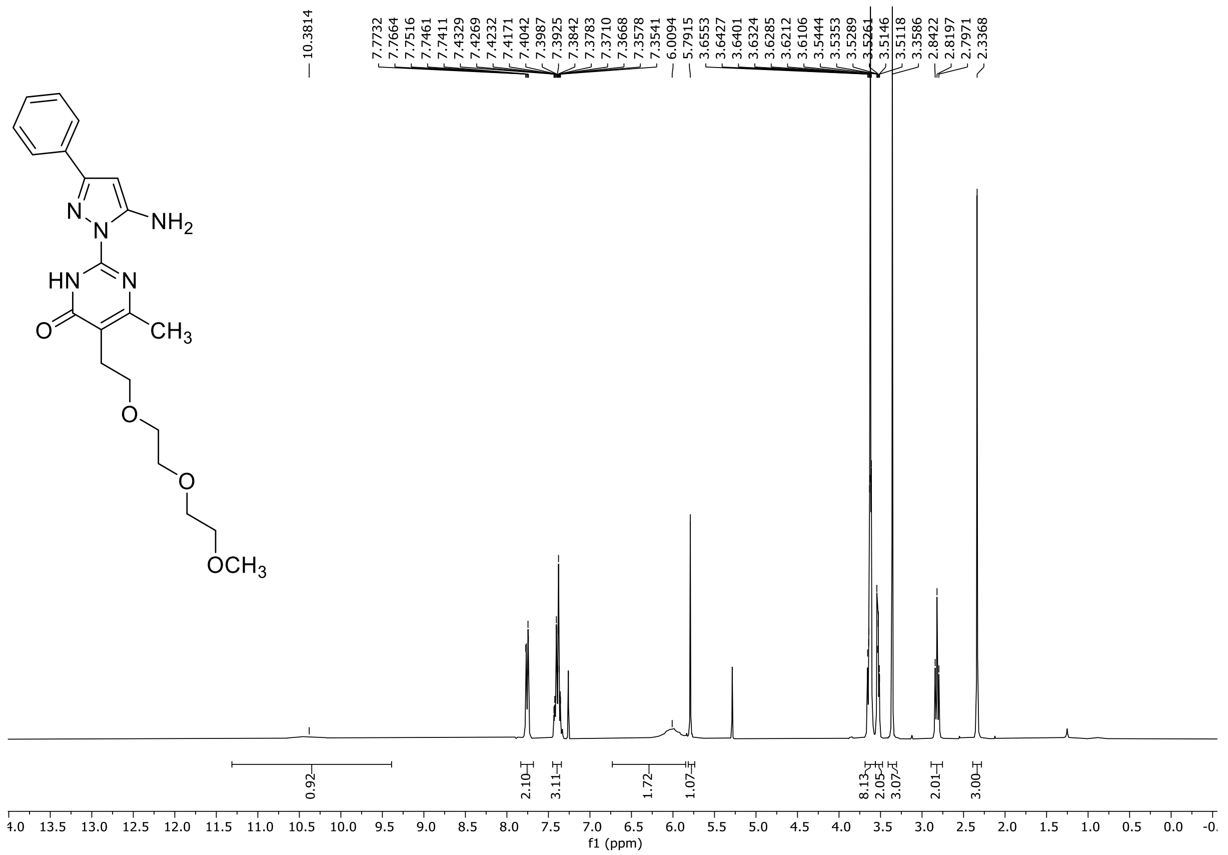

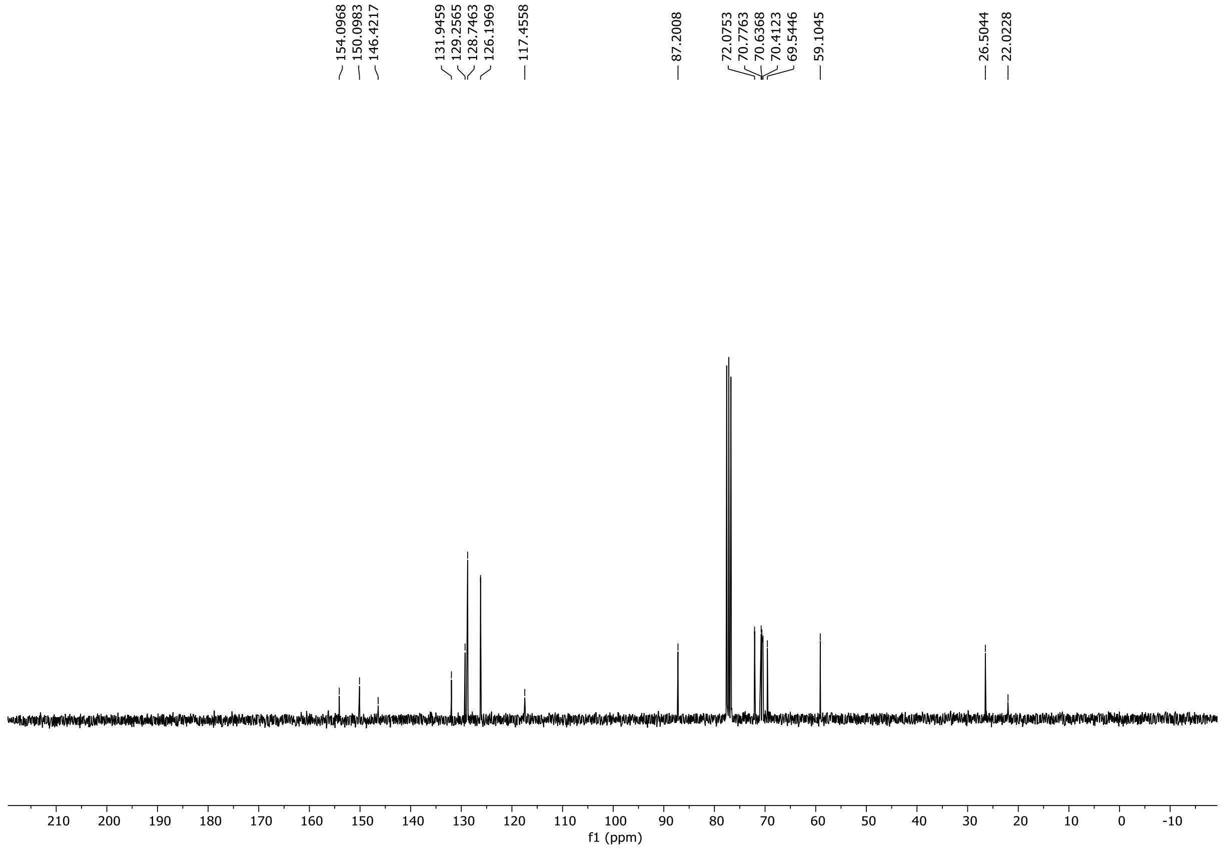

HRMS spectrum of **S148**

NAR-A-220 $C_{21}H_{27}N_5O_4$ mono *m/z* 413.2063

APCI + (MMI)
nitrogen flow 5 L/min, gas temperature 325°C, nebulizer 45 psig, skimmer 65 V, vaporizer 200°C, fragmentor 10 V, dissolved in methanol

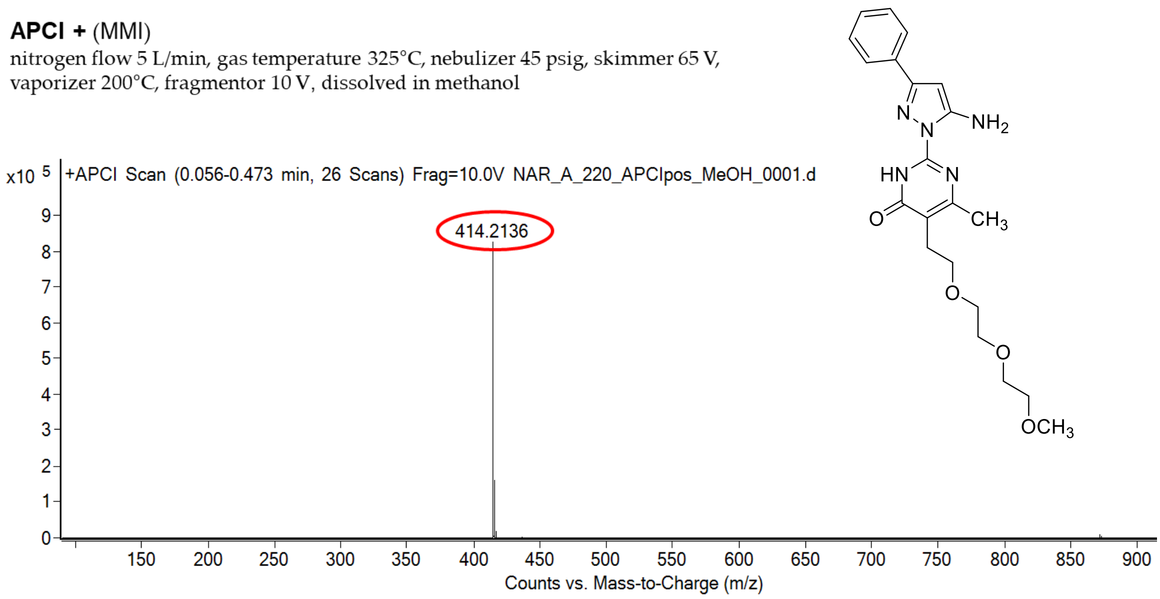

calculated mass: $[M+H]^+$ = 414.2136 observed: $[M+H]^+$ = 414.2136 mass accuracy = < 0.1 ppm

FT-IR spectrum (neat) of **S148**

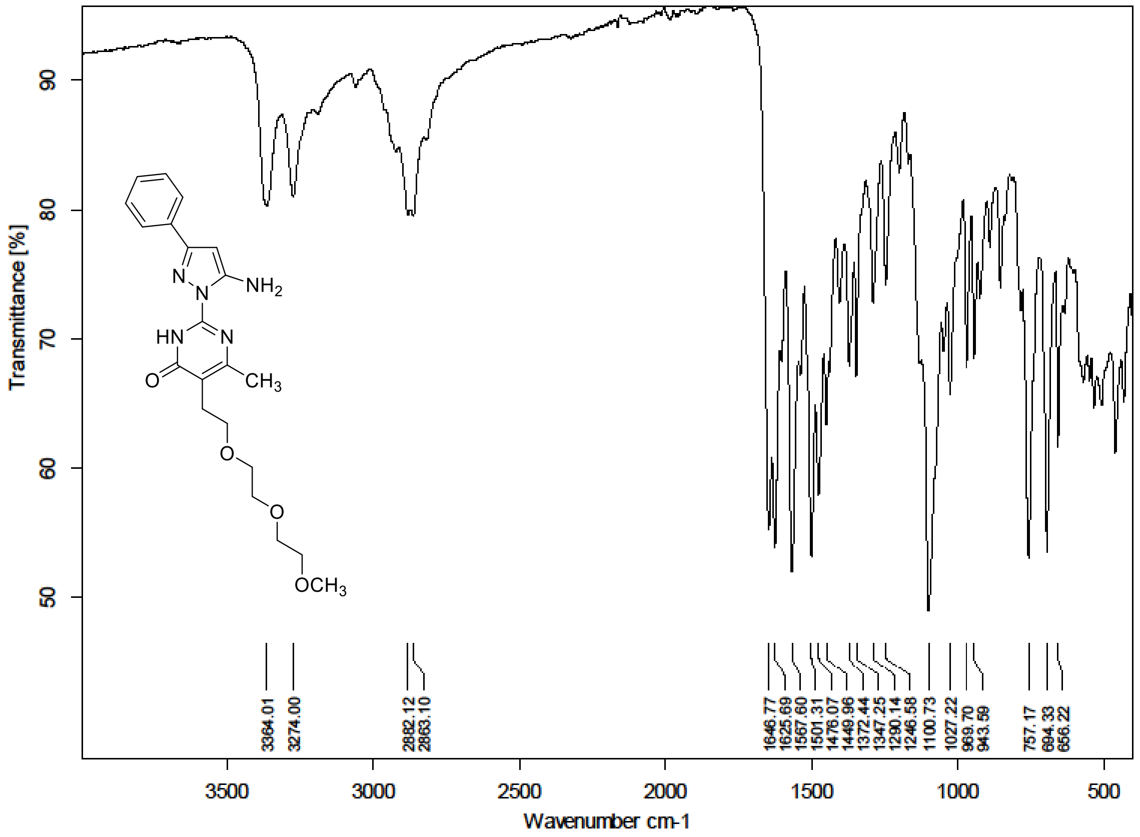

^{1}H (300 MHz) and ^{13}C NMR (75 MHz) spectra of **S149** in DMSO-d_6

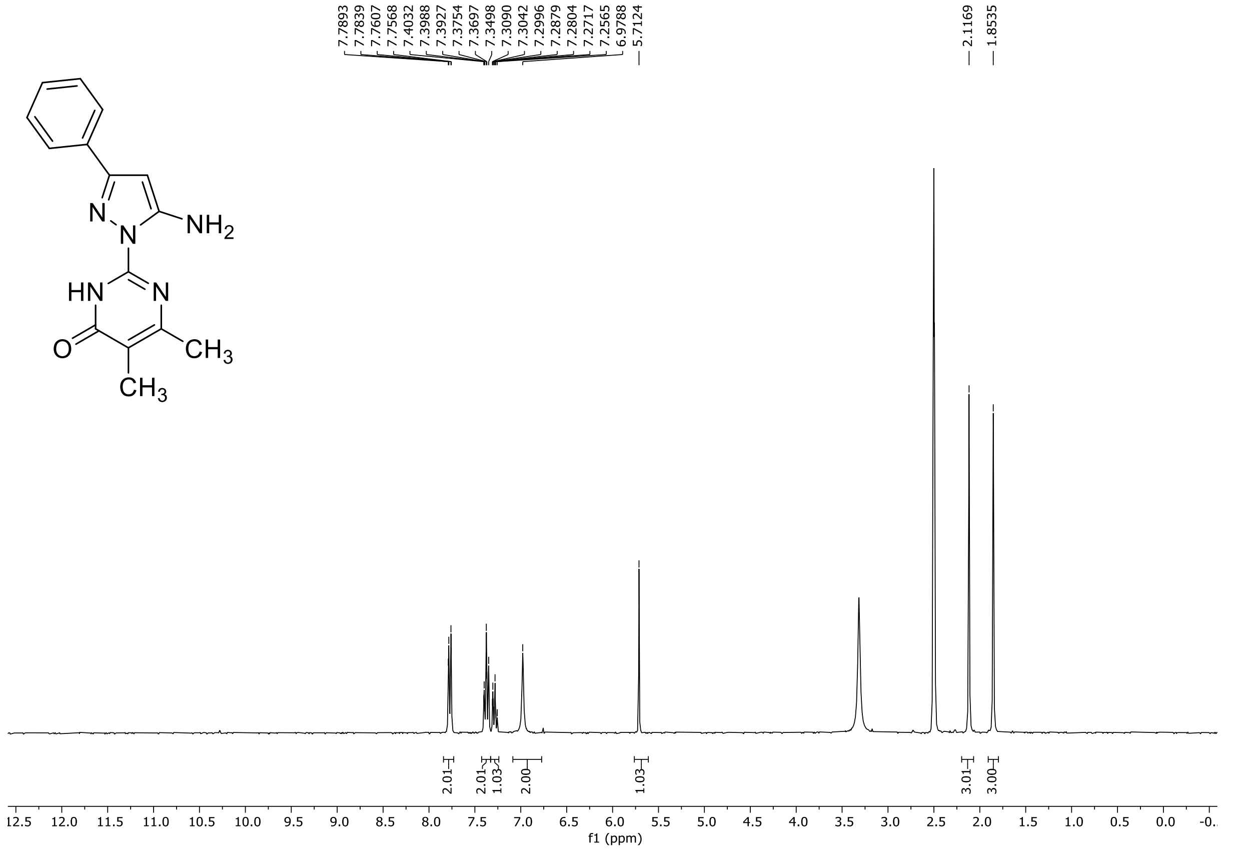

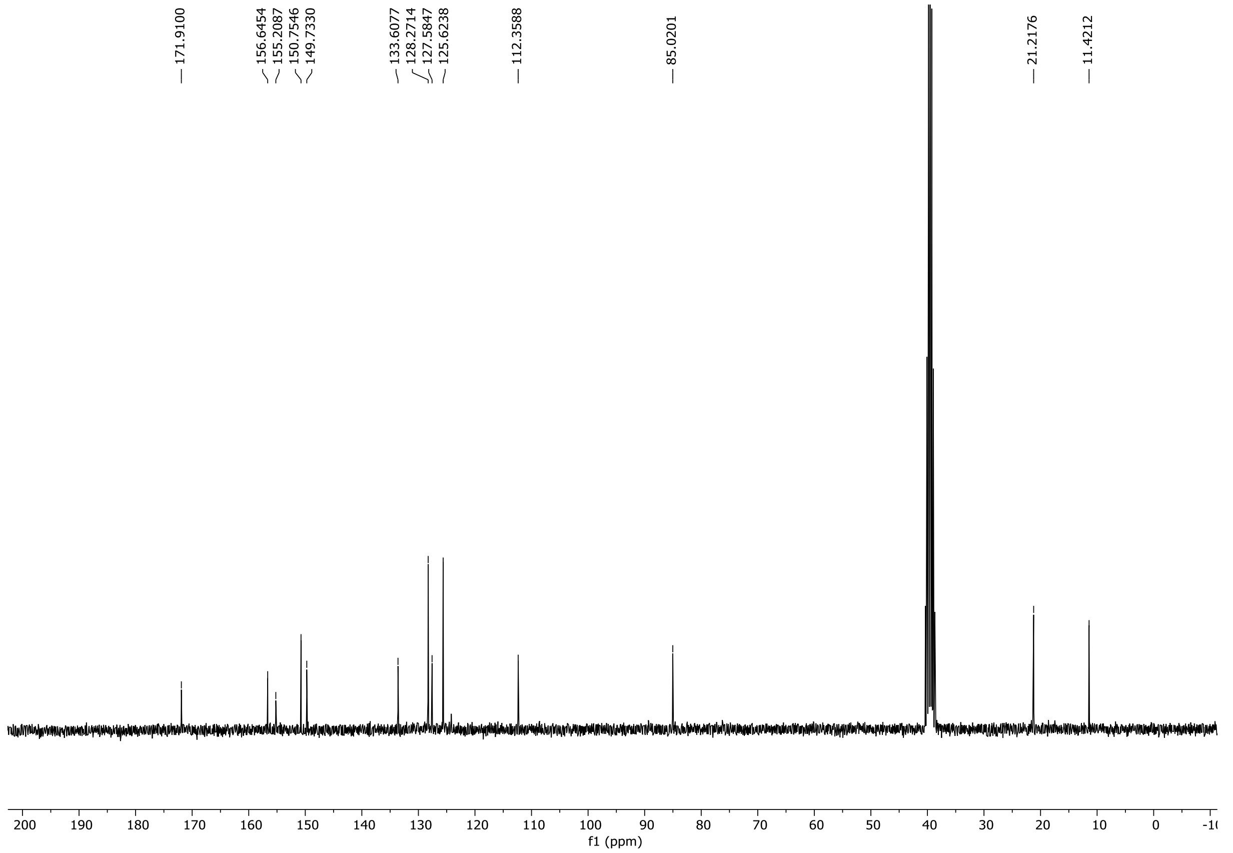

HRMS spectrum of **S149**

NAR-A-222 $C_{15}H_{15}N_5O$ mono *m/z* 281.1277

APCI + (MMI)
nitrogen flow 5 L/min, gas temperature 325°C, nebulizer 45 psig, skimmer 65 V, vaporizer 200°C, fragmentor 24 V, dissolved in methanol

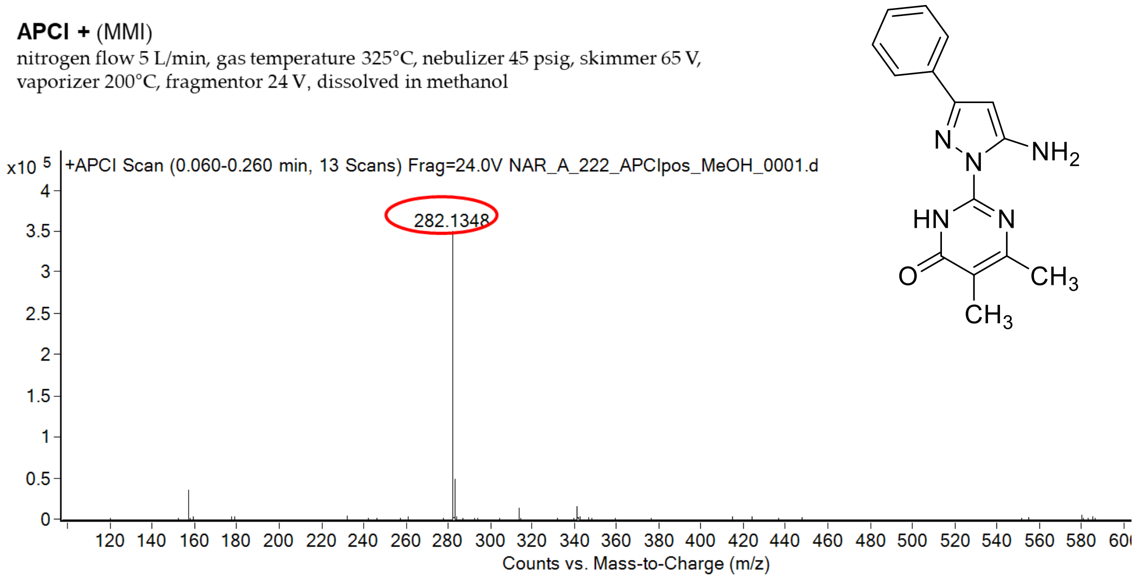

calculated mass: $[M+H]^+$ = 282.1349 observed: $[M+H]^+$ = 282.1348 mass accuracy = - 0.4 ppm

FT-IR spectrum (neat) of **S149**

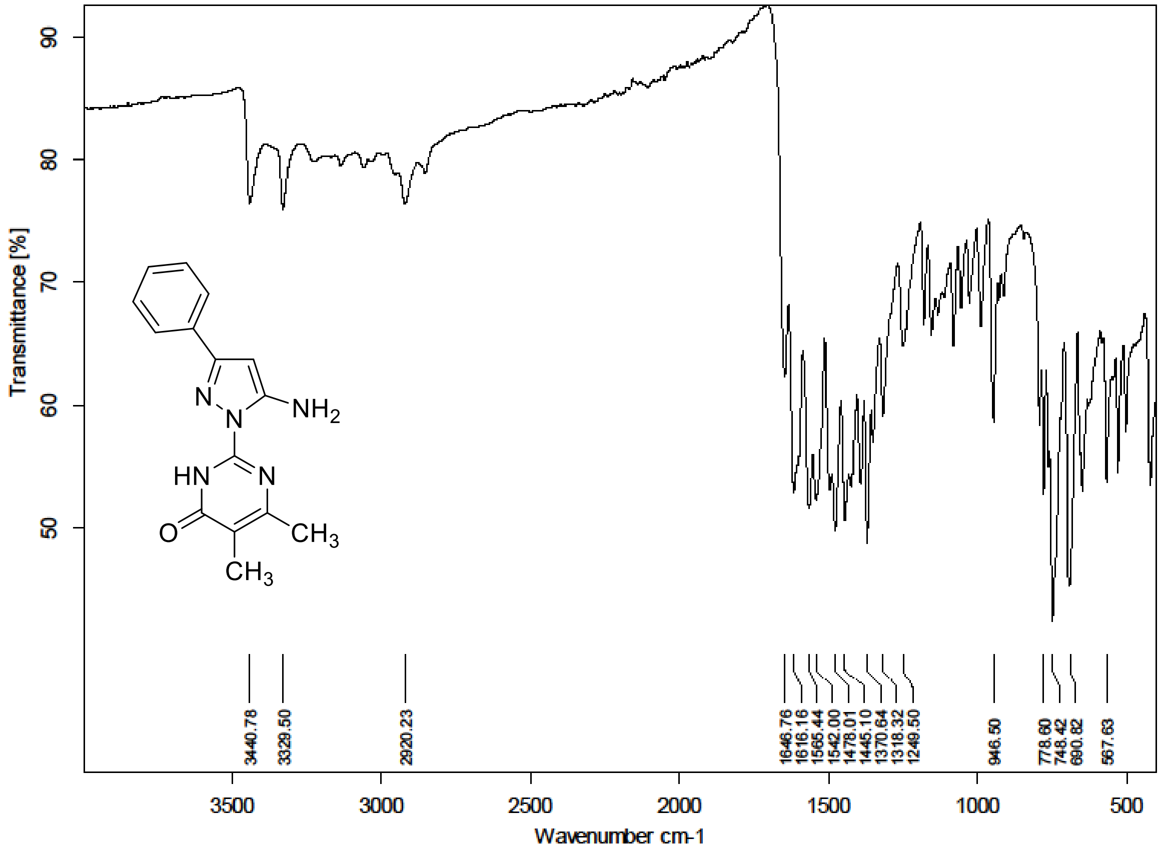

^{1}H (300 MHz) and ^{13}C NMR (75 MHz) spectra of **S150** in Chloroform-*d*

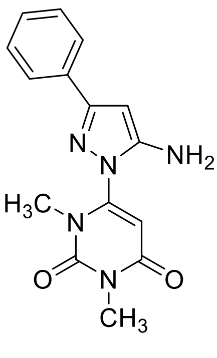

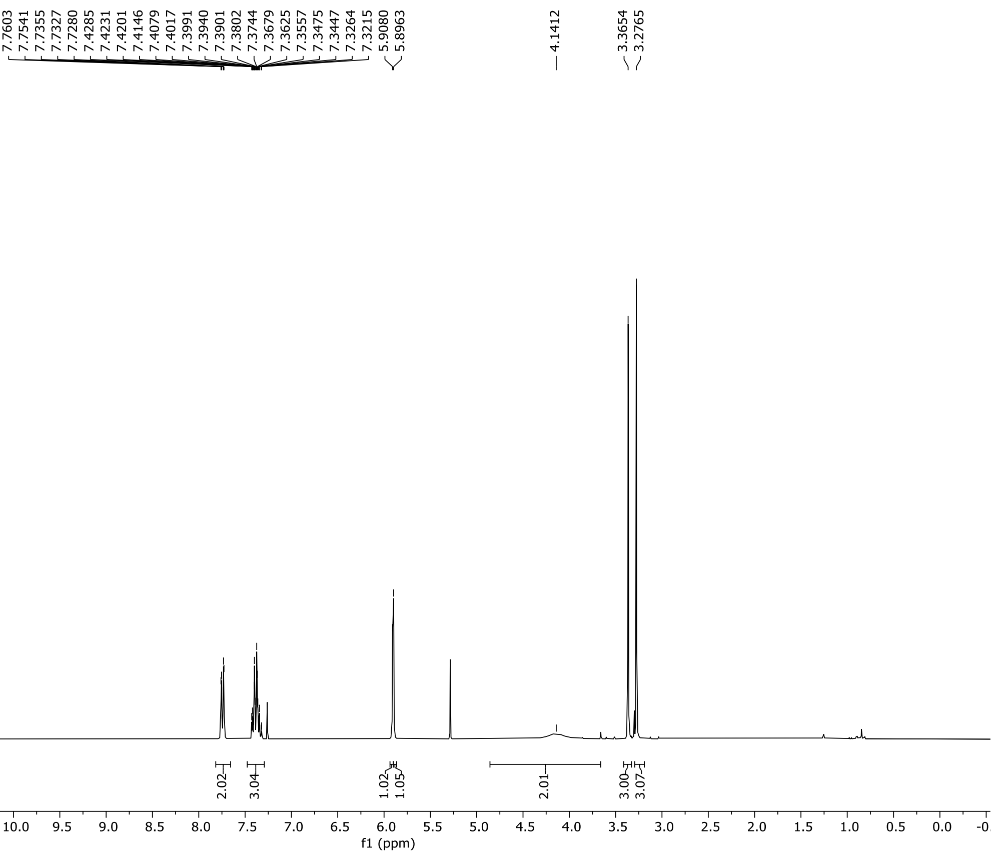

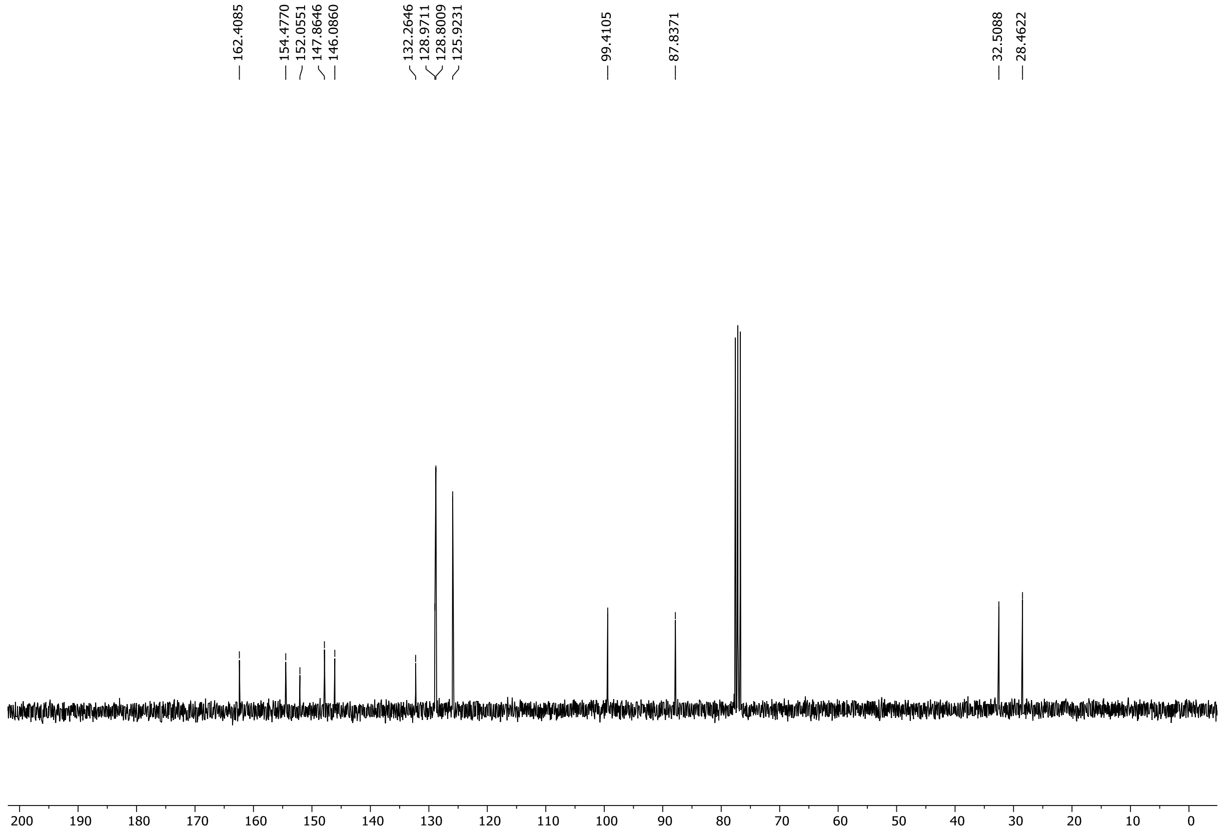

HRMS spectrum of **S150**

NAR-A-223 $C_{15}H_{15}N_5O_2$ mono *m/z* 297.1226

APCI + (MMI)
nitrogen flow 5 L/min, gas temperature 325°C, nebulizer 45 psig, skimmer 65 V, vaporizer 200°C, fragmentor 20 V, dissolved in methanol

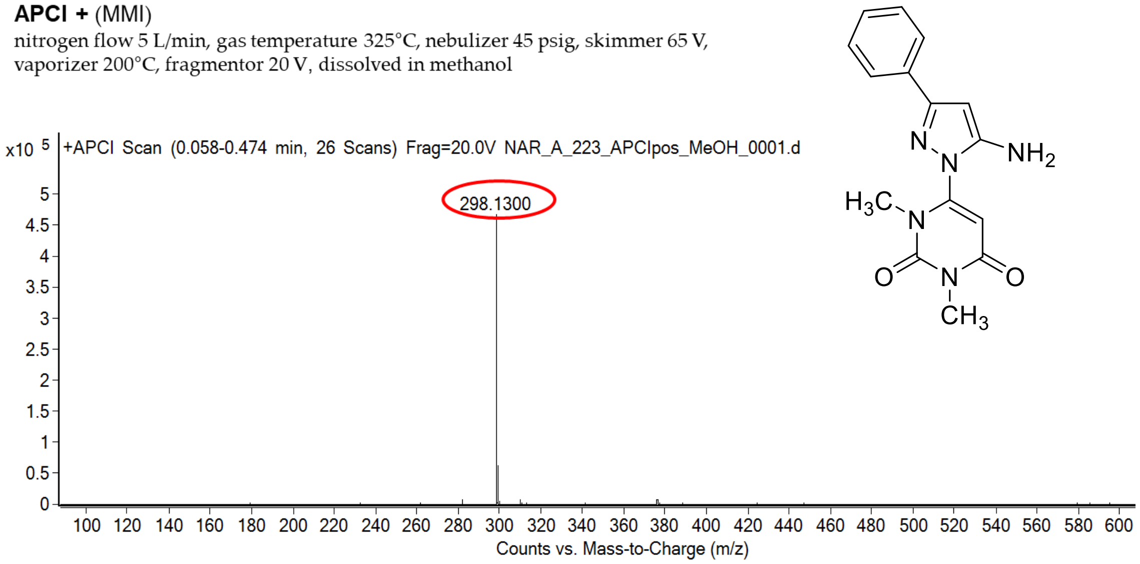

calculated mass: $[M+H]^+$ = 298.1299 observed: $[M+H]^+$ = 298.1300 mass accuracy = 0.3 ppm

FT-IR spectrum (neat) of **S150**

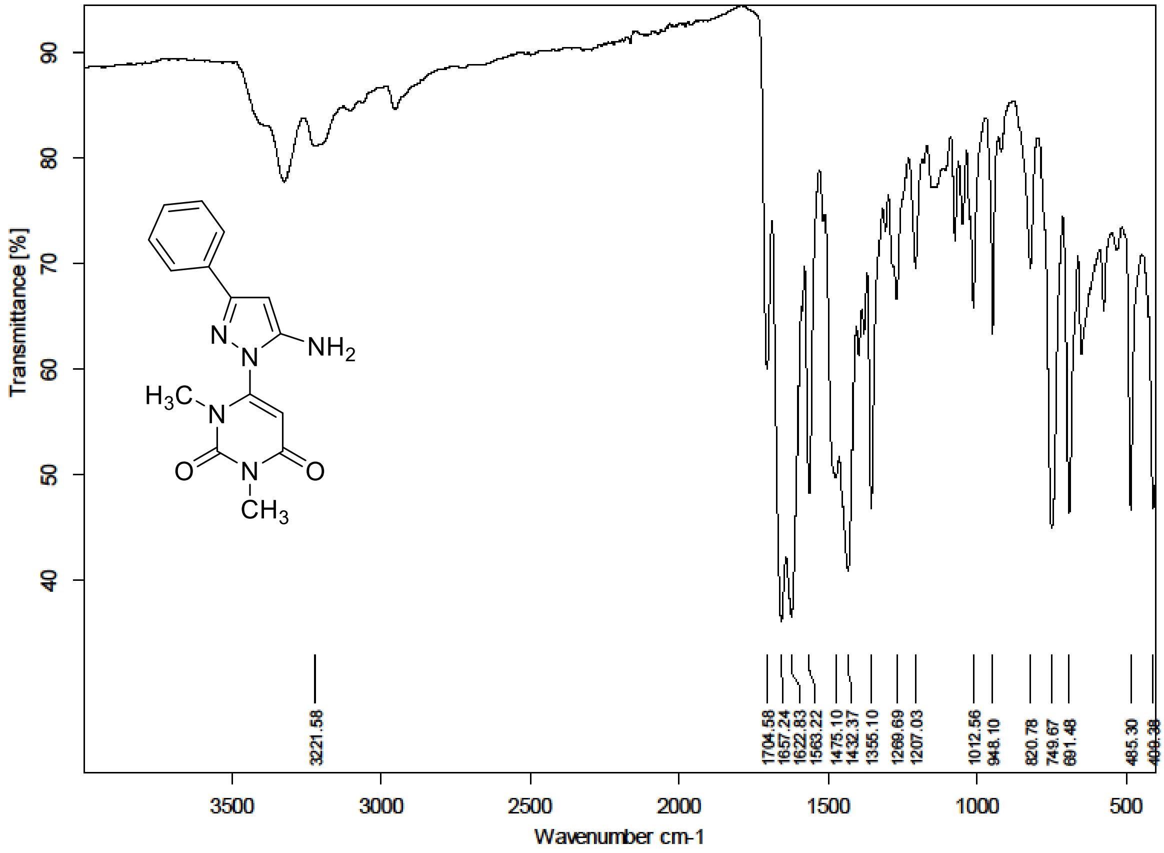

^{1}H (500 MHz) and ^{13}C NMR (126 MHz) spectra of **S151** in Chloroform-*d*

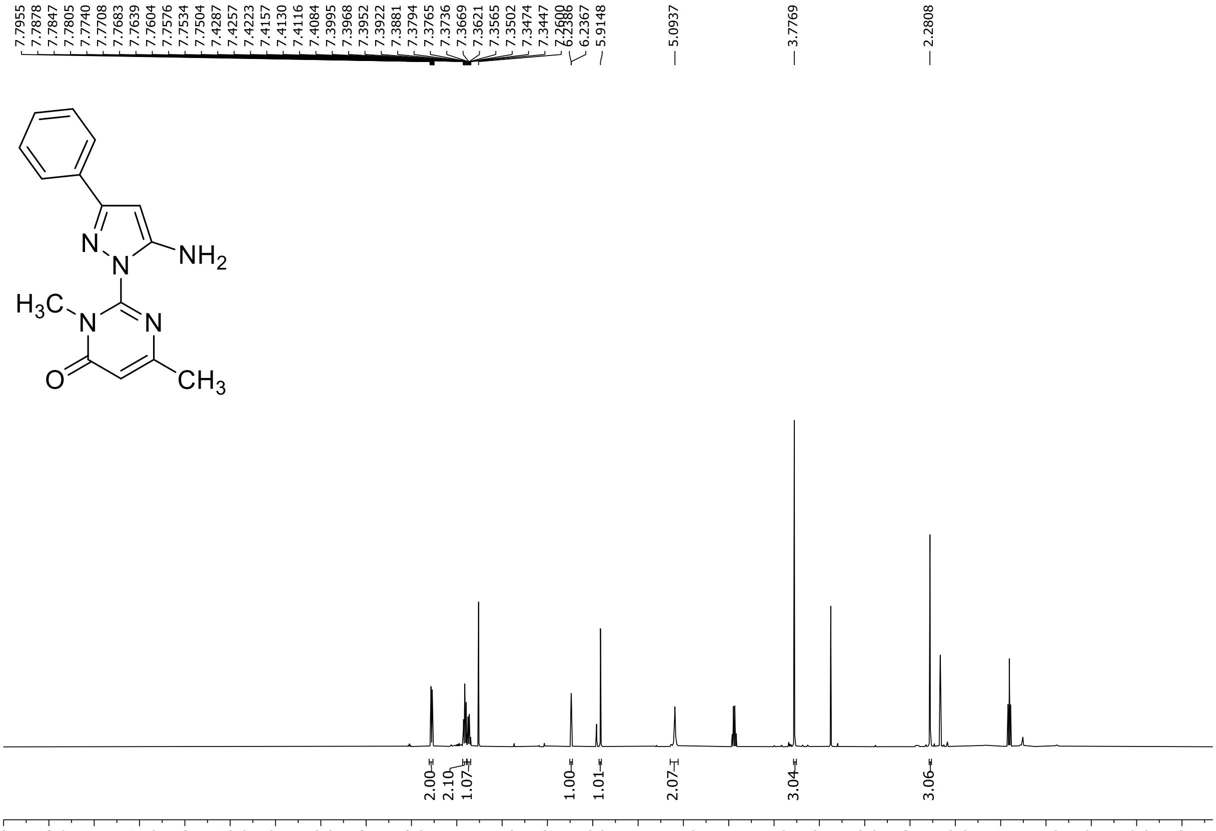

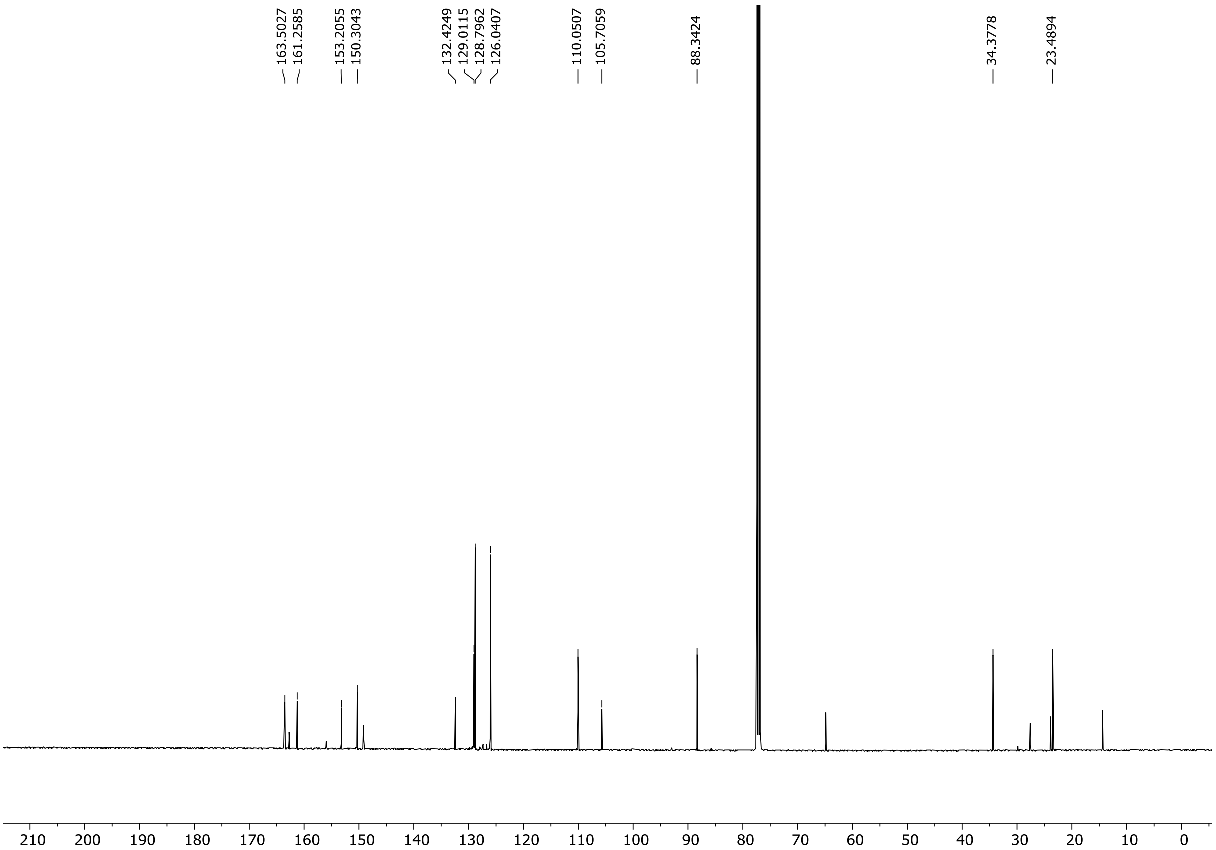

HRMS spectrum of **S151**

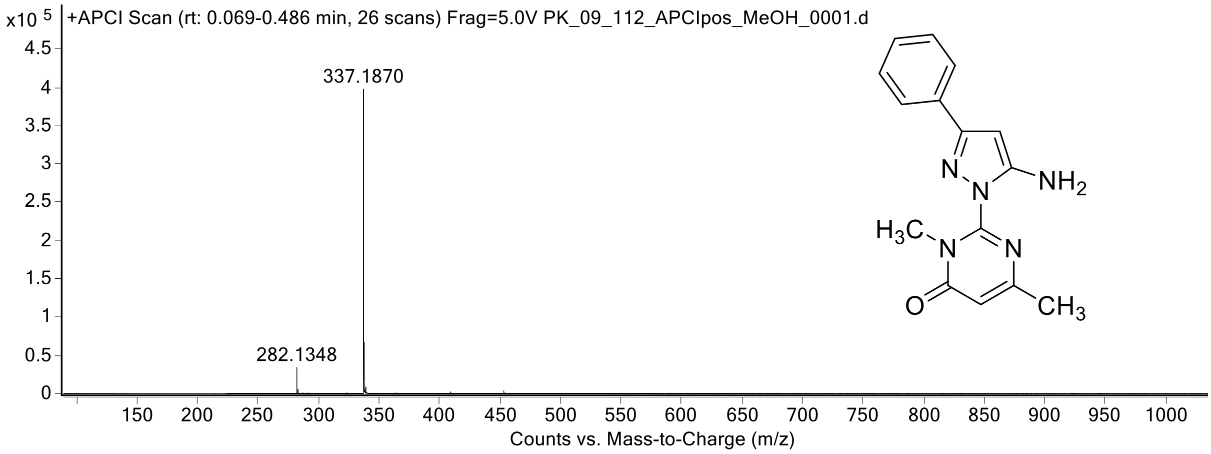

^{1}H (500 MHz) and ^{13}C NMR (126 MHz) spectra of **S152** in Chloroform-*d*

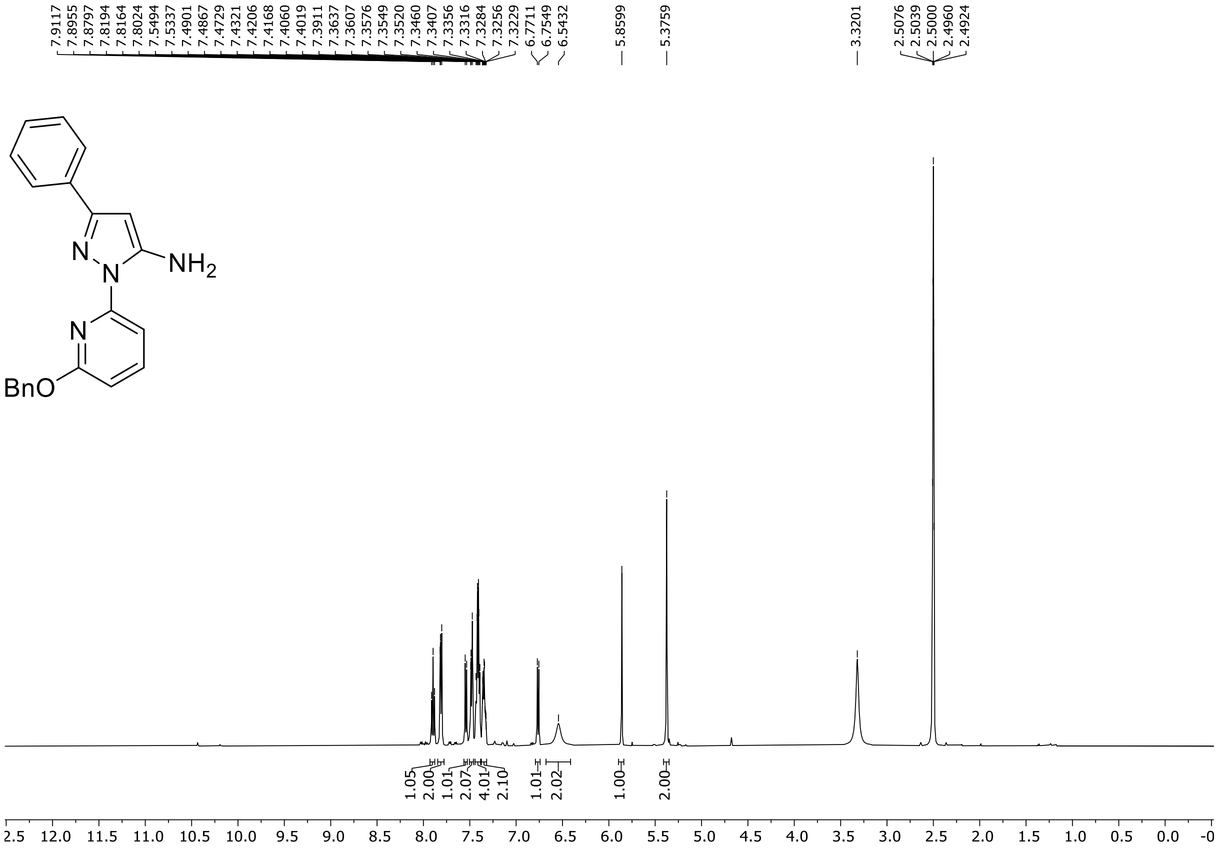

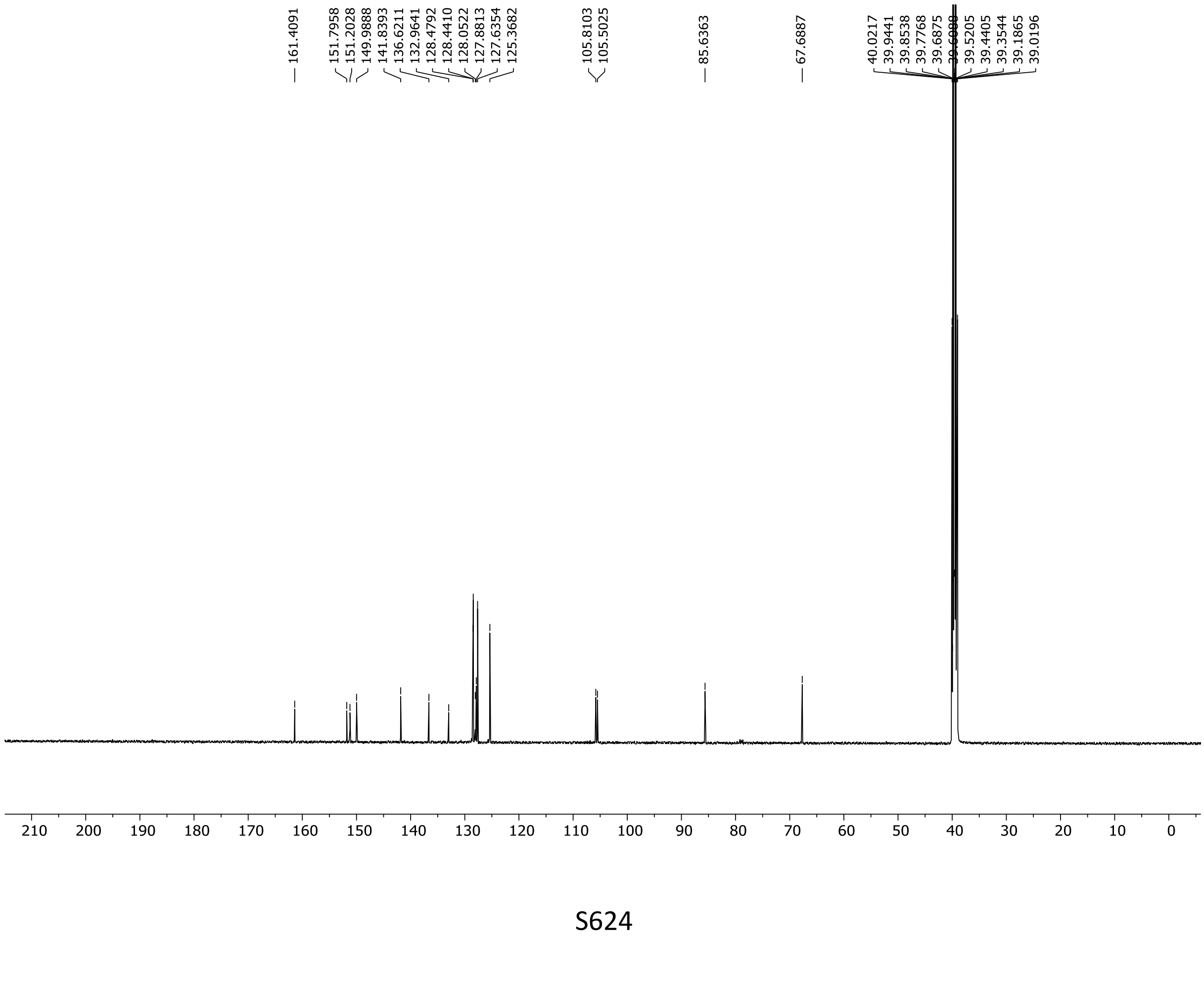

HRMS spectrum of **S152**

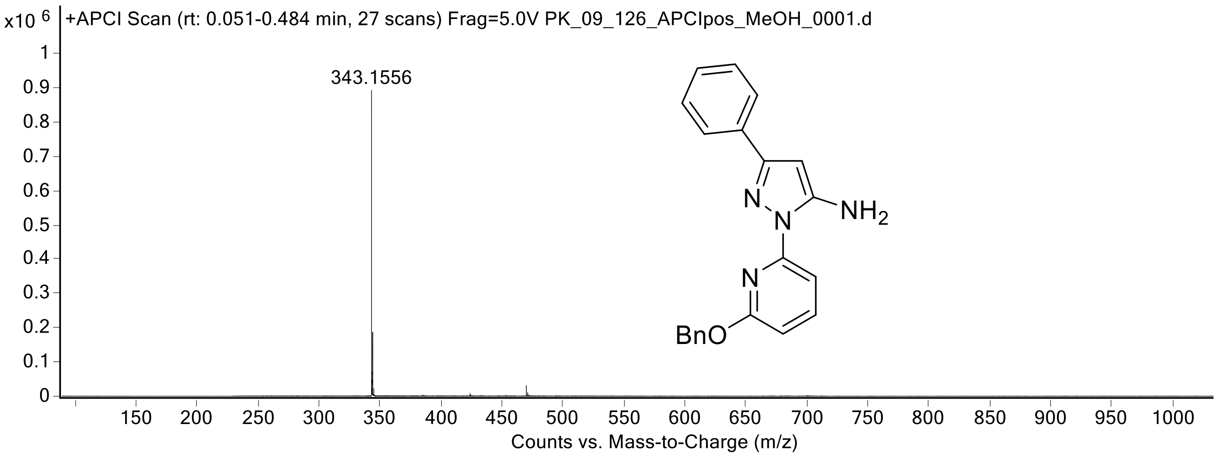

^{1}H (300 MHz) and ^{13}C NMR (126 MHz) spectra of **S153** in DMSO-d_6

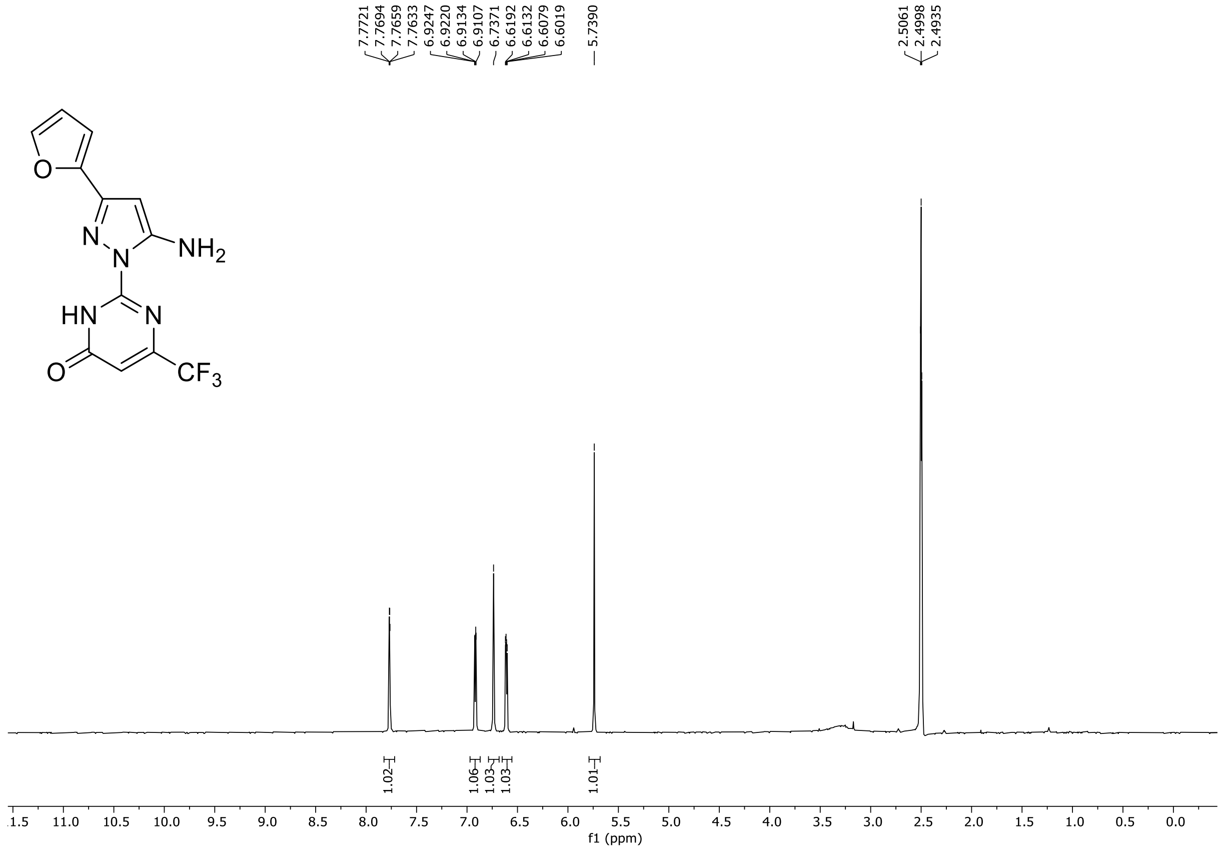

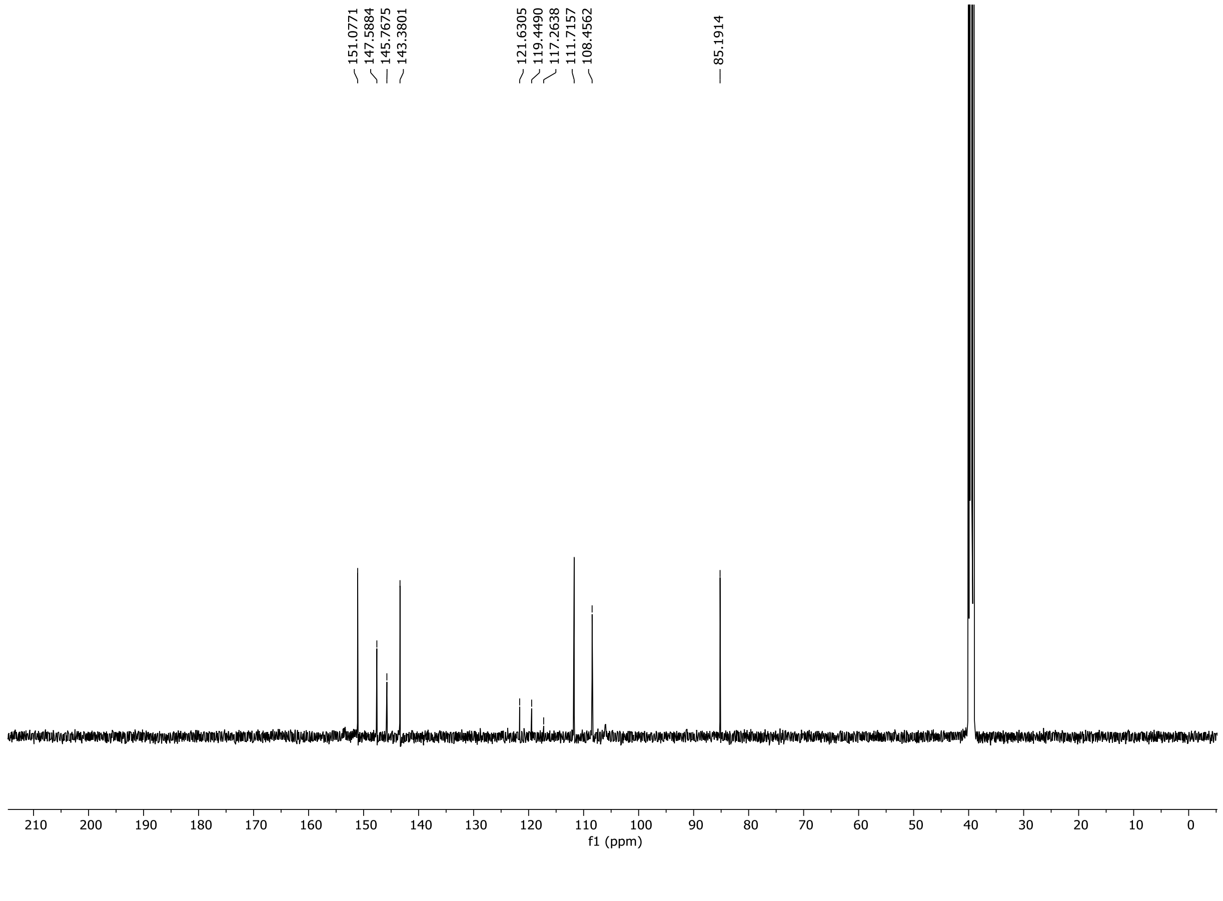

^{19}F NMR (282 MHz) spectrum of **S153** in DMSO-d_6

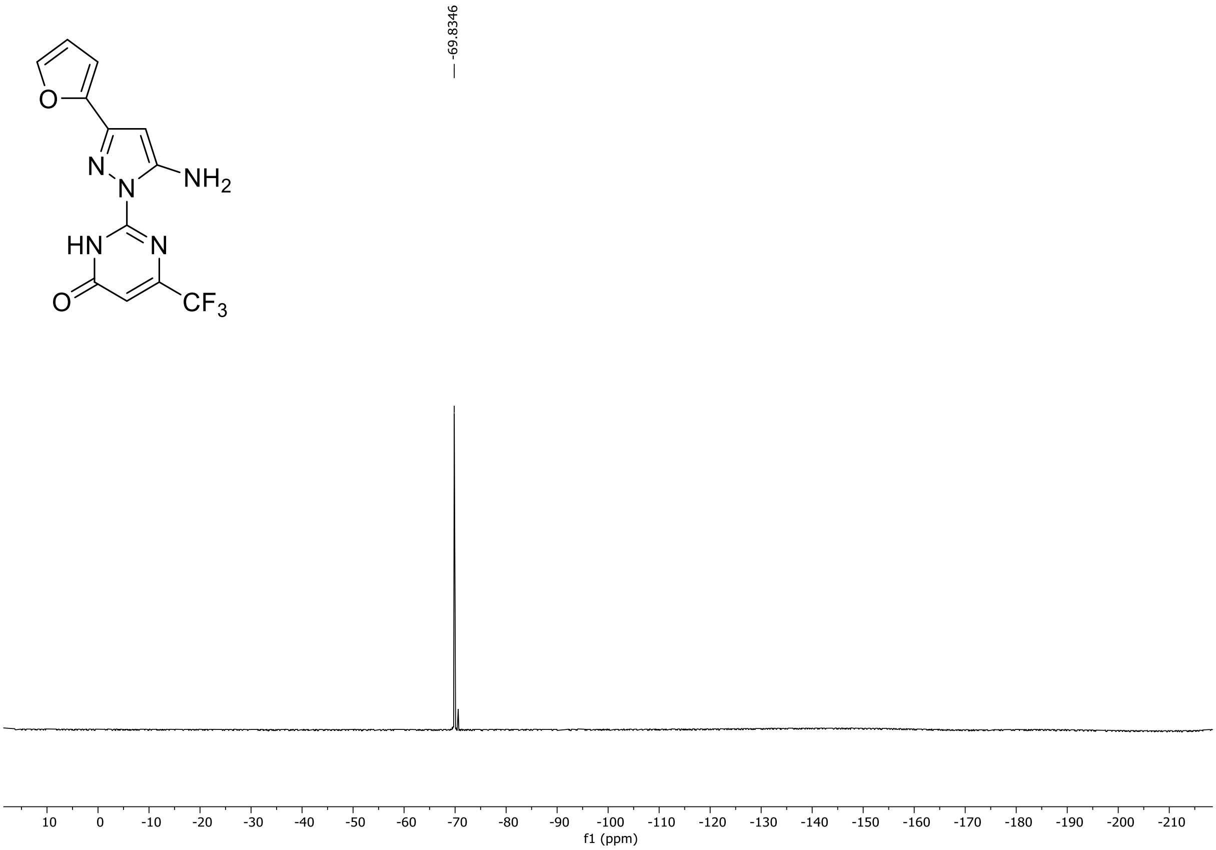

HRMS spectrum of **S153**

NAR-A-102 **$C_{12}H_8F_3N_5O_2$** ***m/z* 311.0630**

APCI+ (MMI)
nitrogen flow 5 L/min, gas temperature 325°C, nebulizer 45 psi, skimmer 65 V, vaporizer 200°C, fragmentor 22 V, dissolved in MeOH

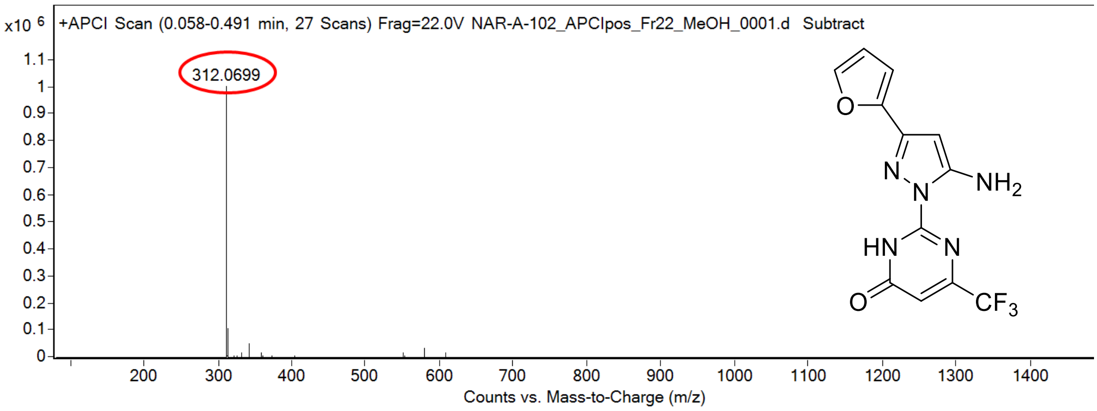

calculated mass: $[M+H]^+ = 312.0703$ observed: $[M+H]^+ = 312.0699$ mass accuracy = -1.3 ppm

FT-IR spectrum (neat) of **S153**

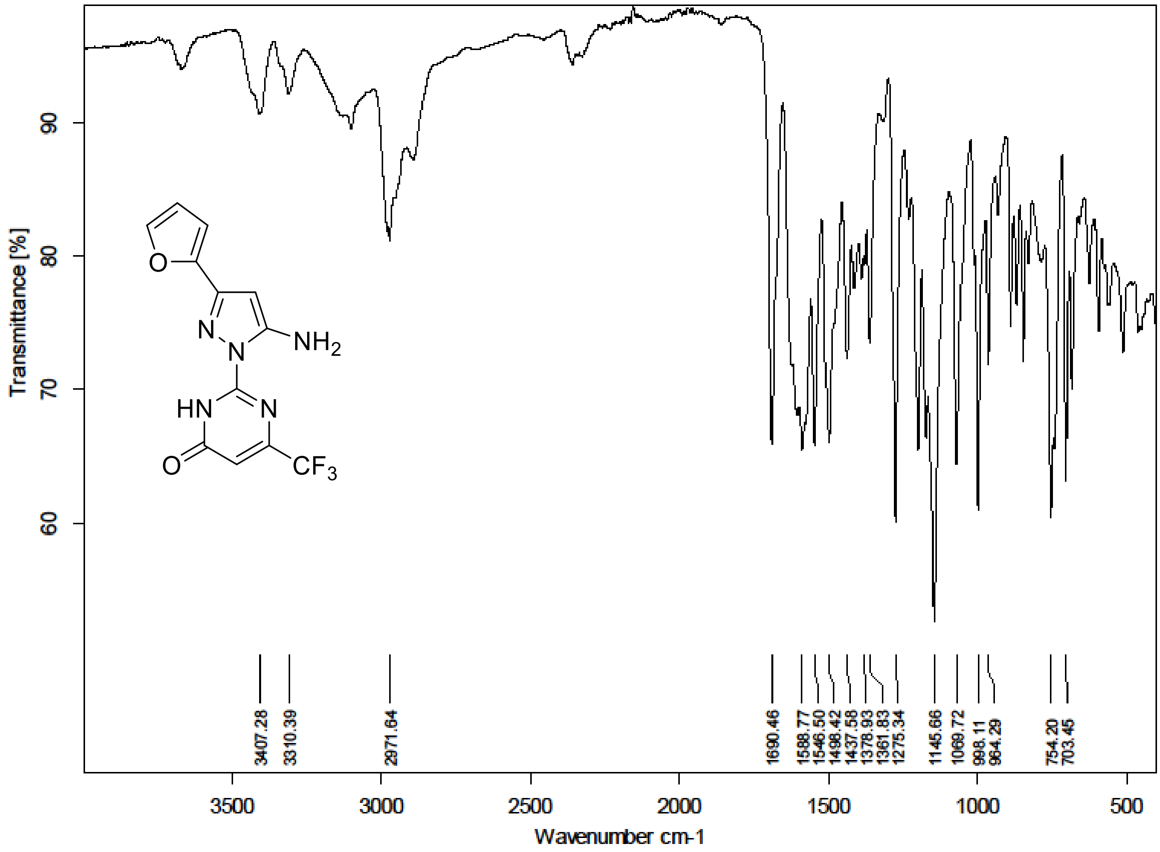

^{1}H (300 MHz) and ^{13}C NMR (126 MHz) spectra of **S154** in DMSO-d_6

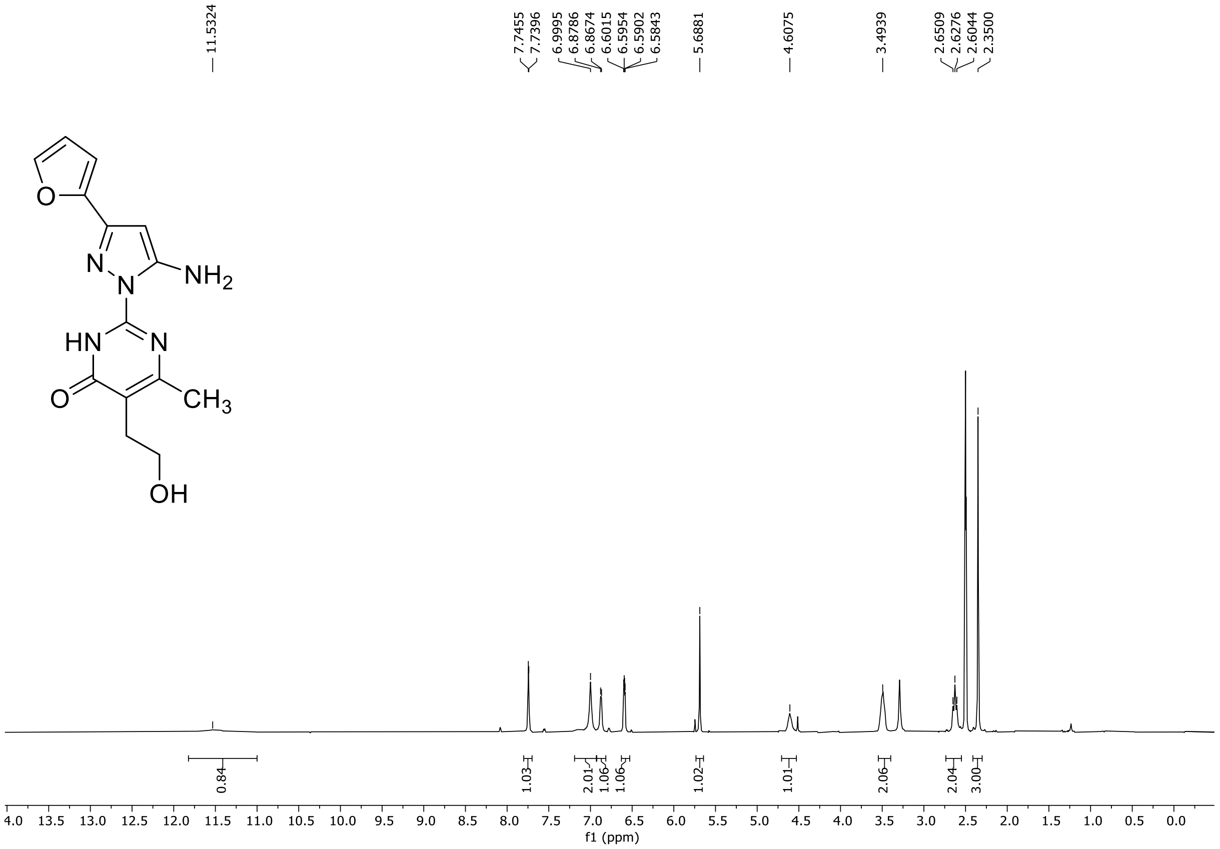

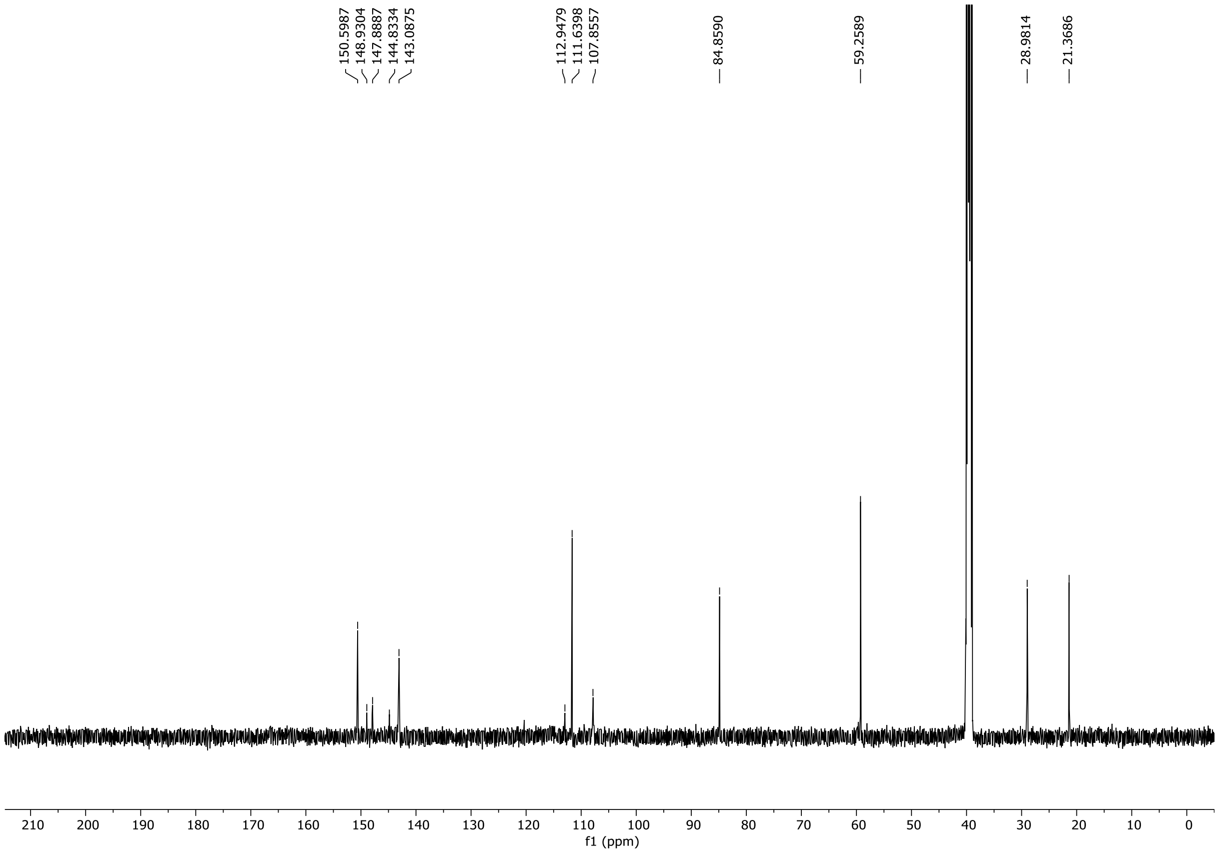

HRMS spectrum of **S154**

NAR-A-101 $C_{14}H_{15}N_5O_3$ *m/z* **301.1175**

APCI+ (MMI)
nitrogen flow 5 L/min, gas temperature 325°C, nebulizer 45 psi, skimmer 65 V, vaporizer 200°C, fragmentor 20 V, dissolved in MeOH

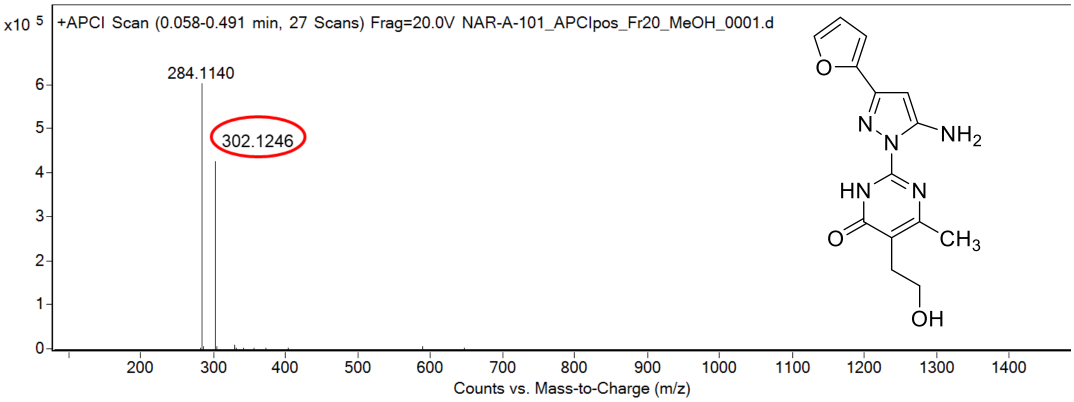

calculated mass: $[M+H]^+$ = 302.1248 observed: $[M+H]^+$ = 302.1246 mass accuracy = -0.7 ppm

FT-IR spectrum (neat) of **S154**

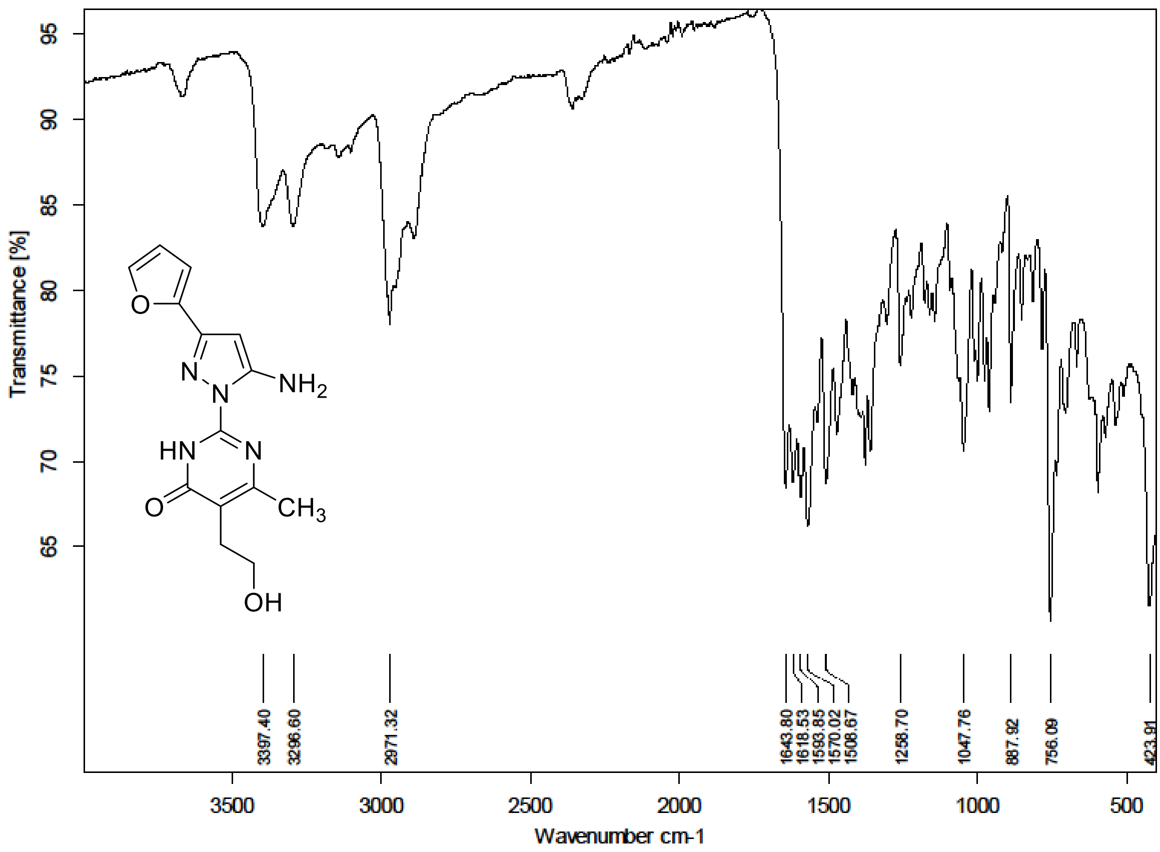

^{1}H (500 MHz) and ^{13}C NMR (126 MHz) spectra of **S155** in Chloroform-*d*

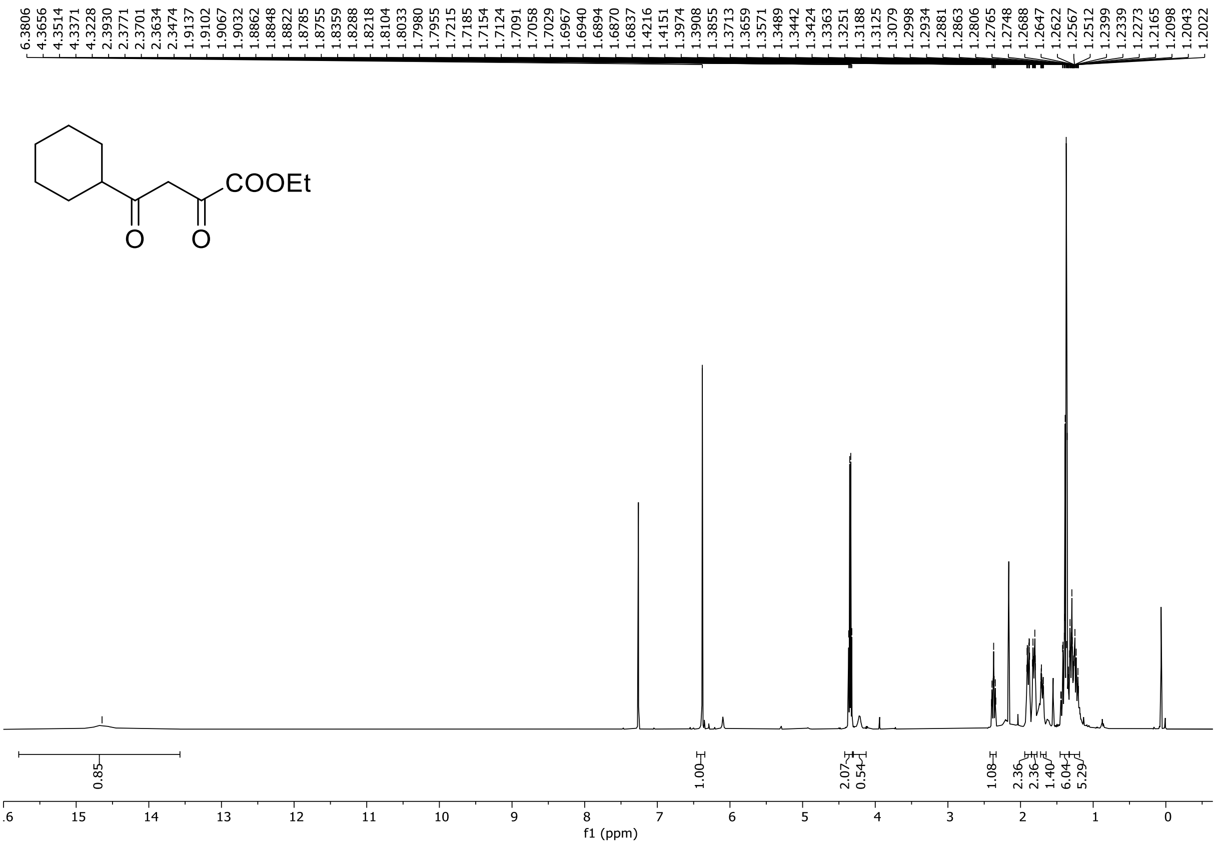

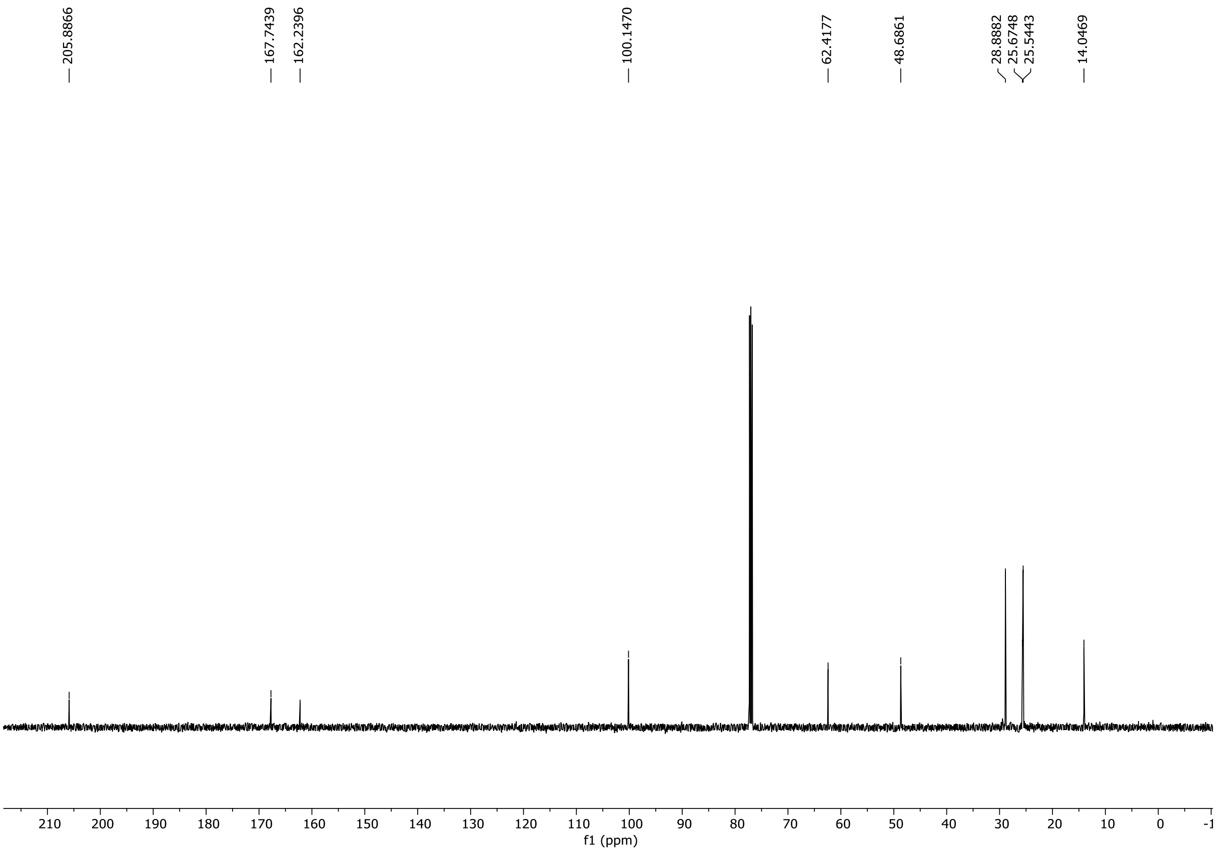

HRMS spectrum of **S155**

NAR-A-5 $C_{12}H_{18}O_4$ *mono m/z* **226.1205**

APCI + (MMI)
nitrogen flow 5 L/min, gas temperature 325°C, nebulizer 45 psi, skimmer **65** V, vaporizer 200°C, fragmentor 30 V, dissolved in methanol

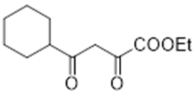

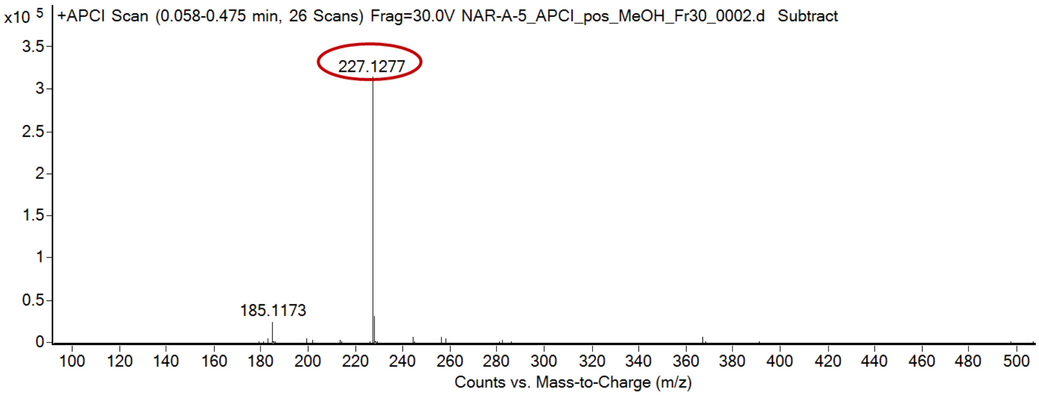

calculated mass: $[M+H]^+$ = 227.1278 observed: $[M+H]^+$ = 227.1277 mass accuracy = - 0.4 ppm

FT-IR spectrum (neat) of **S155**

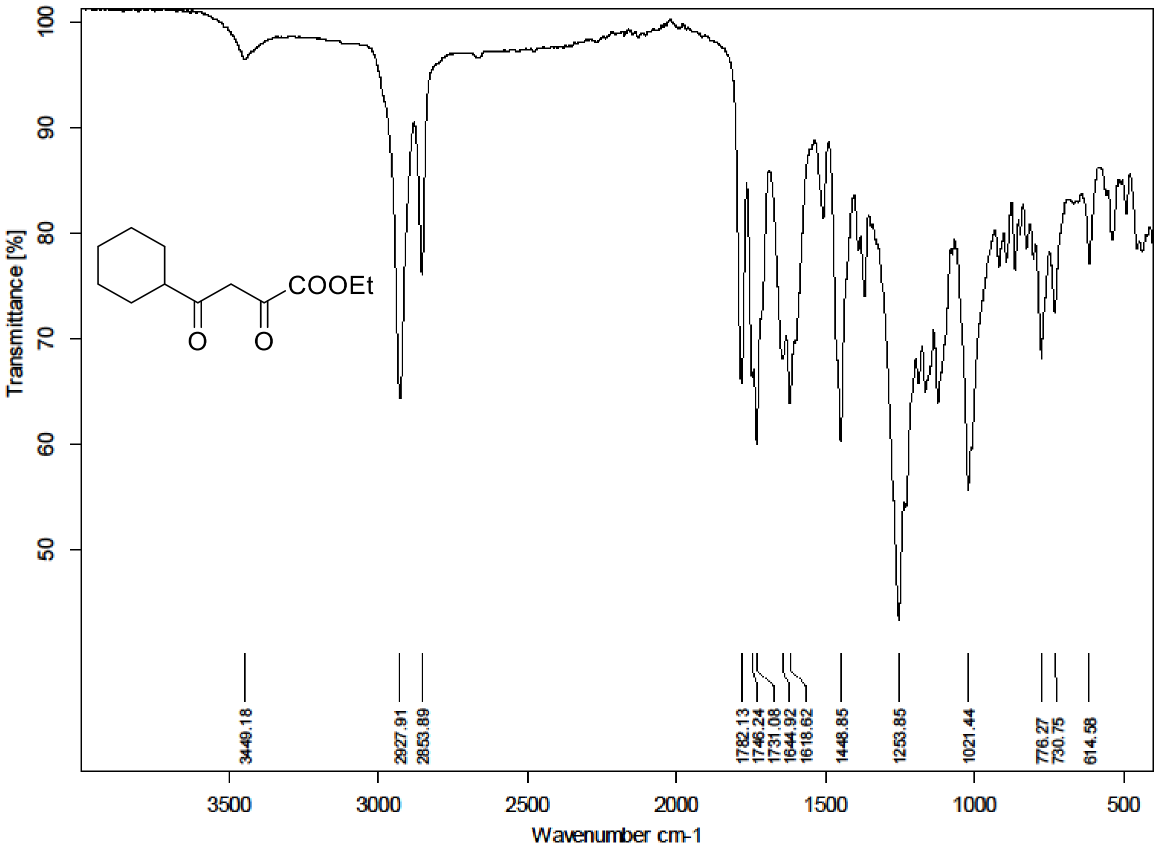

^{1}H (500 MHz) and ^{13}C NMR (126 MHz) spectra of **S156** in Chloroform-*d*

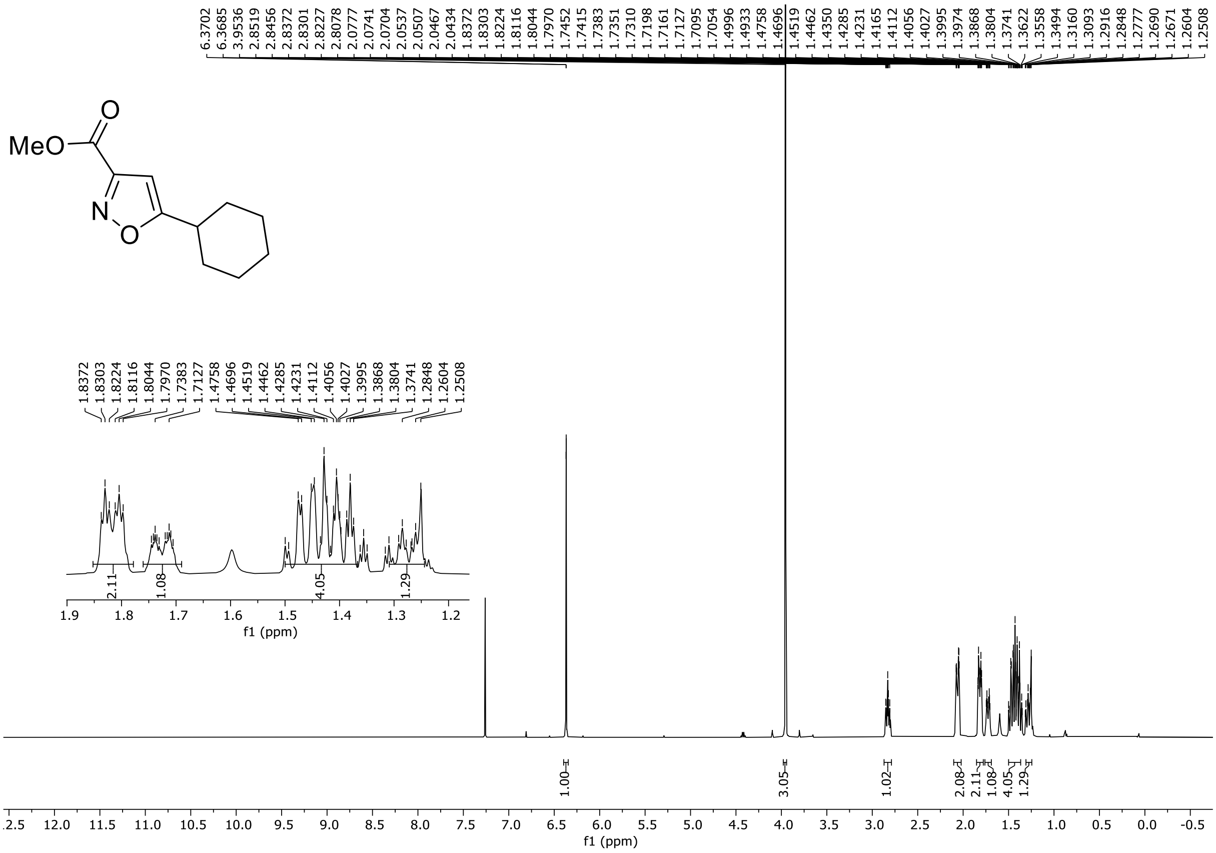

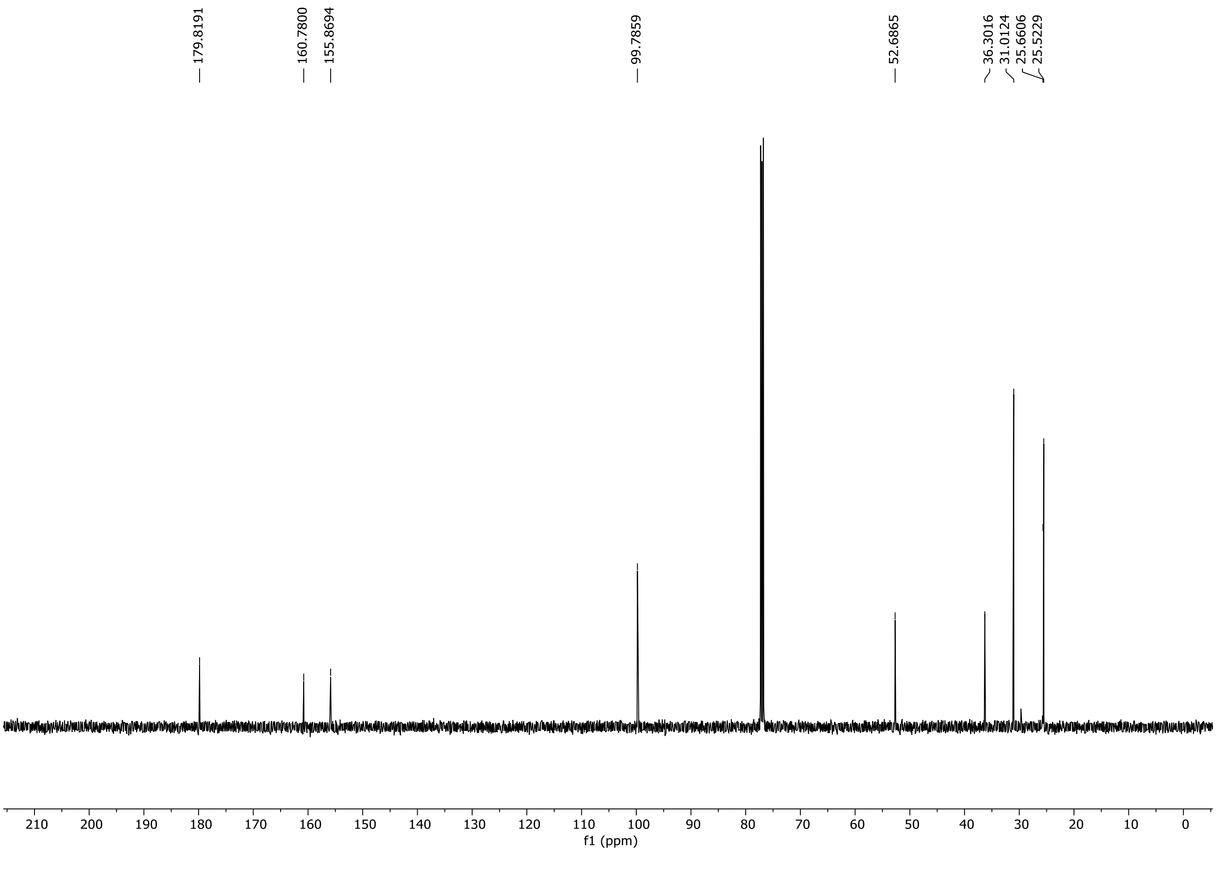

HRMS spectrum of **S156**

NAR-A-6 $C_{11}H_{15}NO_3$ *mono m/z* **209.1052**

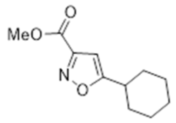

APCI + (MMI)
nitrogen flow 5 L/min, gas temperature 325°C, nebulizer 45 psi, skimmer **65** V, vaporizer 200°C, fragmentor 22 V, dissolved in methanol

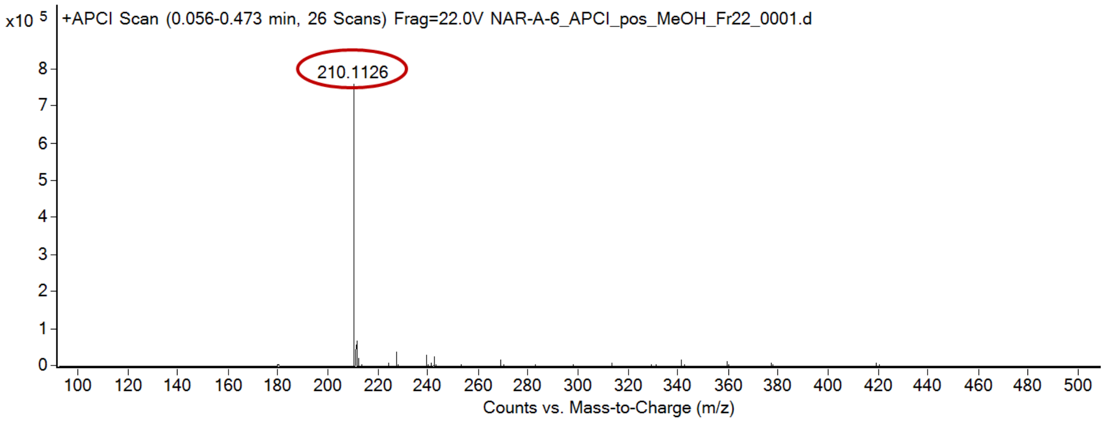

calculated mass: $[M+H]^+ = 210.1125$ observed: $[M+H]^+ = 210.1126$ mass accuracy = 0.5 ppm

FT-IR spectrum (neat) of **S156**

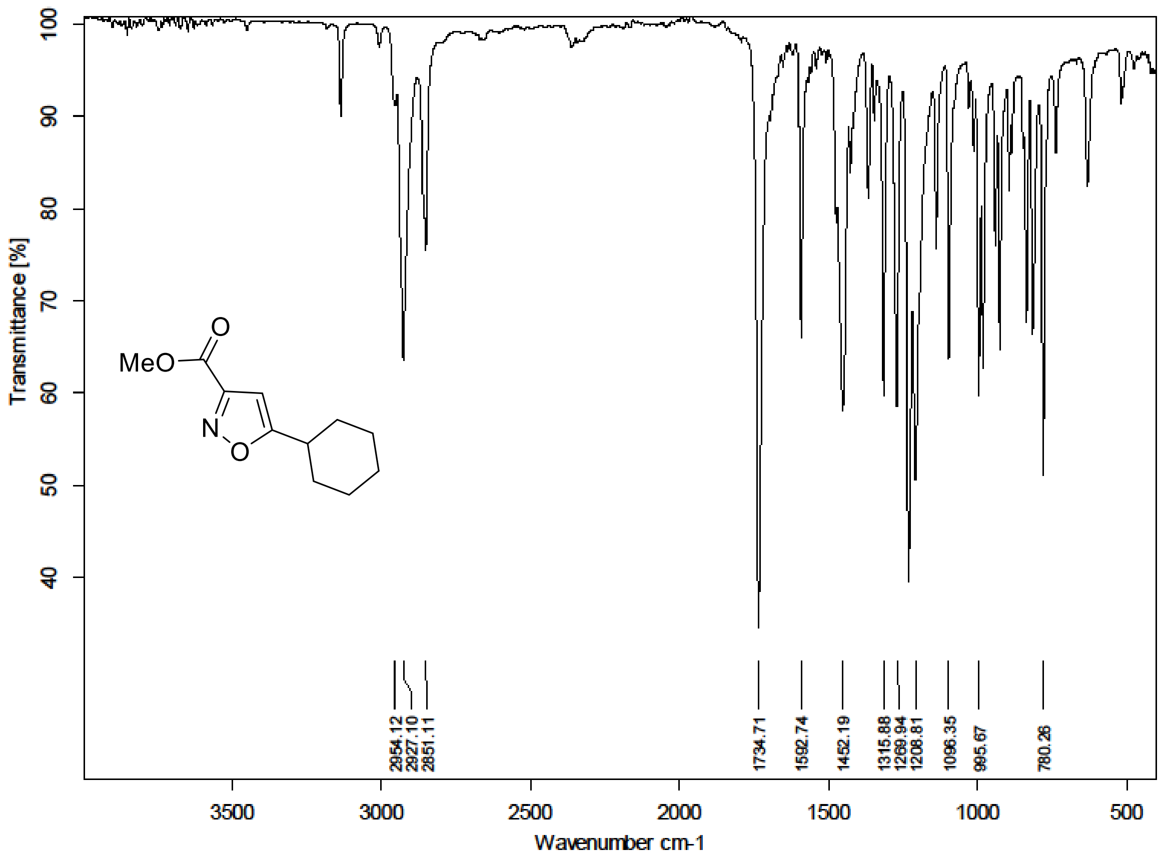

^{1}H (500 MHz) and ^{13}C NMR (126 MHz) spectra of **S157** in Chloroform-*d*

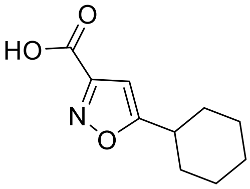

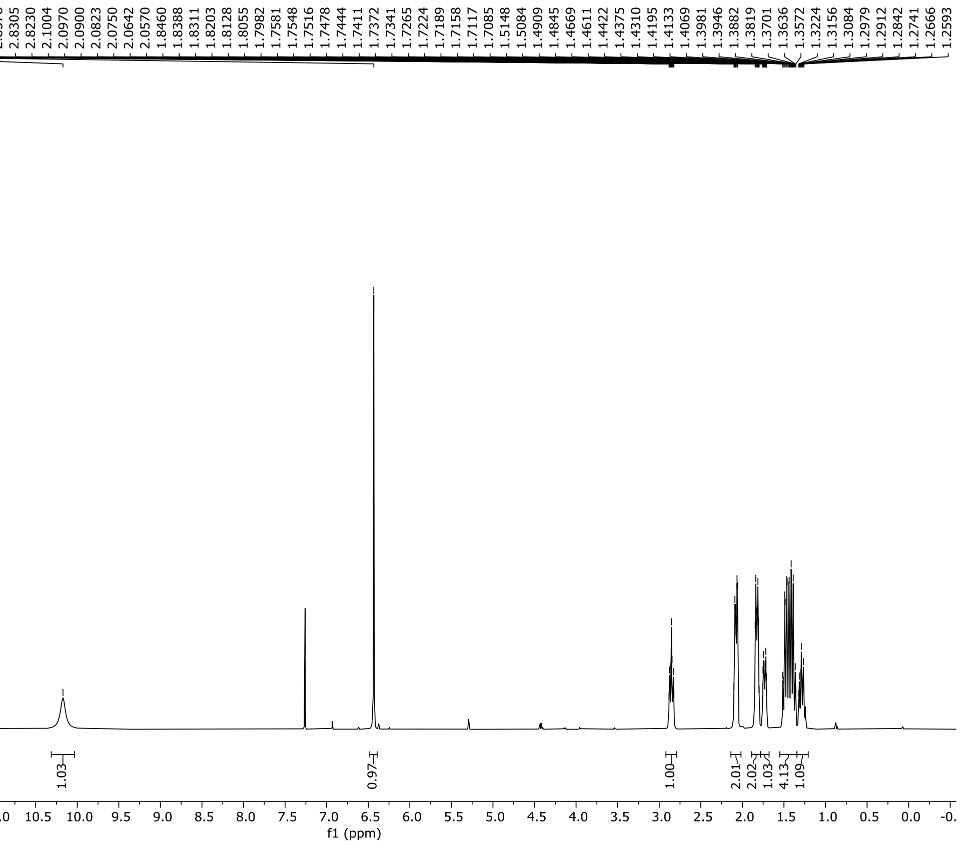

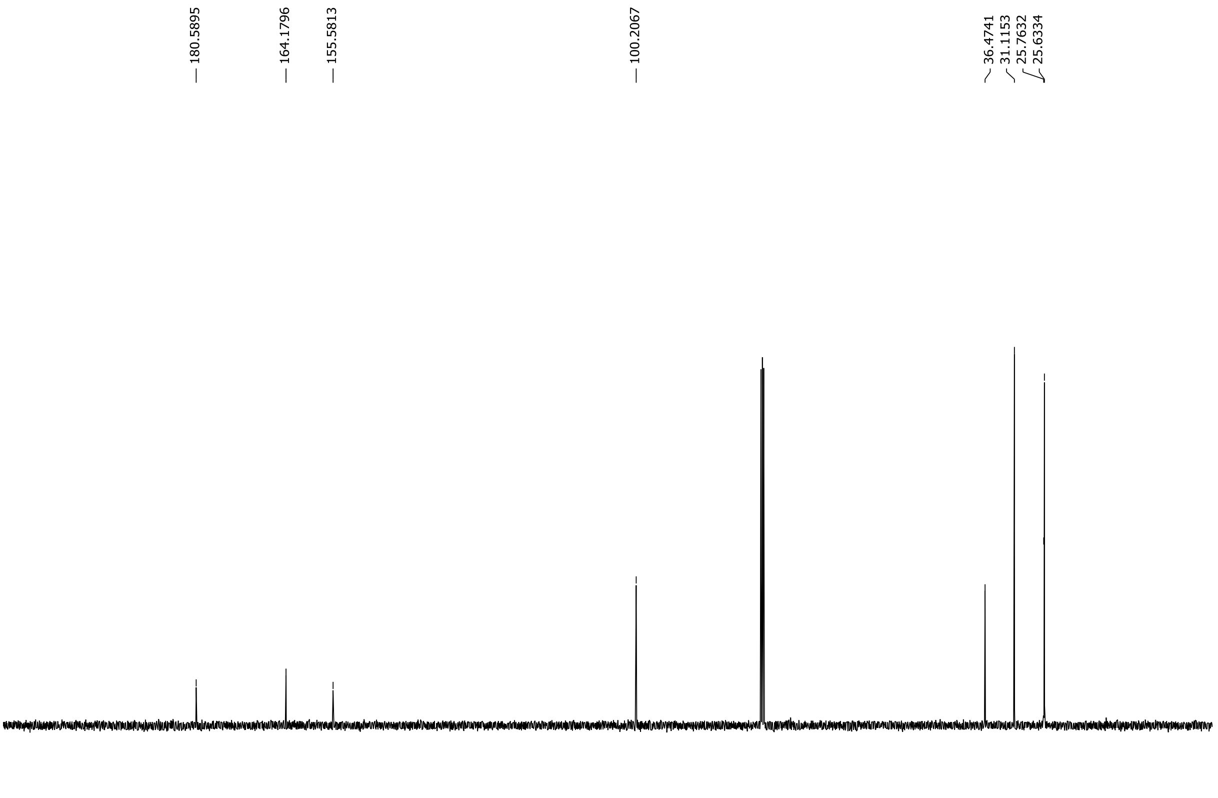

HRMS spectrum of **S157**

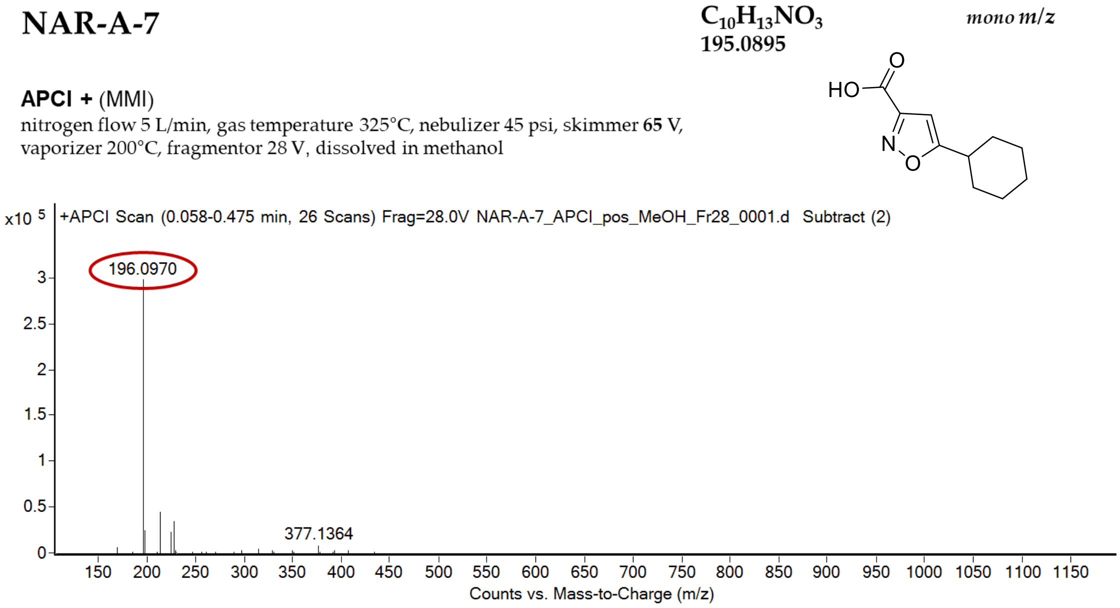

calculated mass: [M+H]$^+$ = 196.0968 observed: [M+H]$^+$ = 196.0970 mass accuracy = 1.0 ppm

FT-IR spectrum (neat) of **S157**

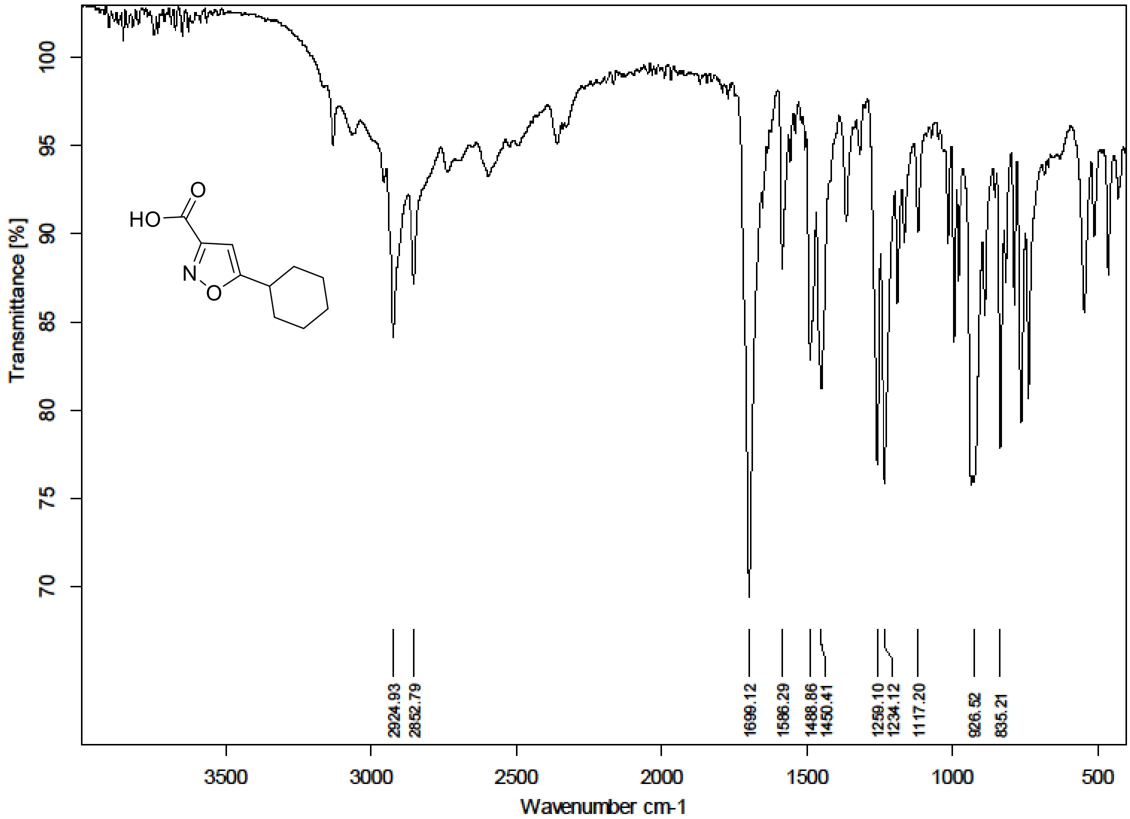

^{1}H (500 MHz) and ^{13}C NMR (126 MHz) spectra of **S159** in Chloroform-*d*

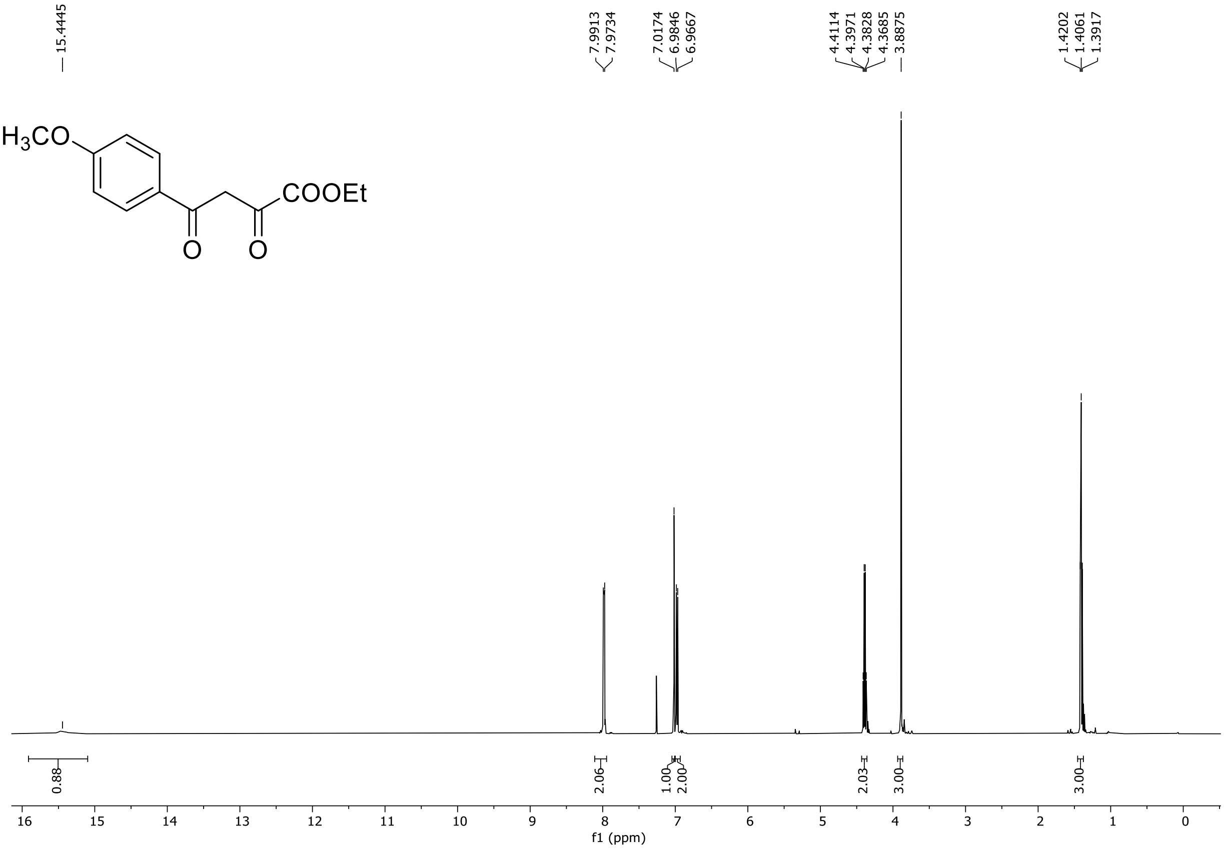

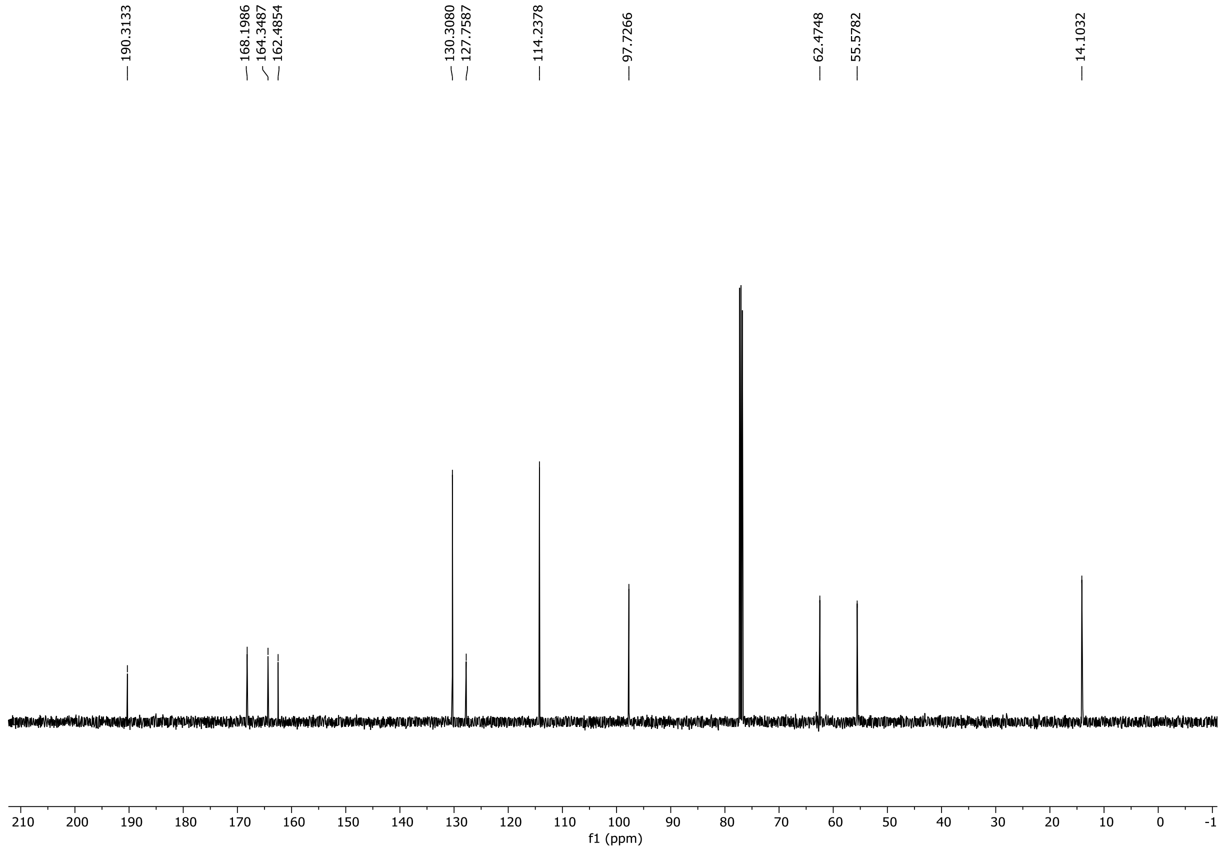

HRMS spectrum of **S159**

NAR-A-19 $C_{13}H_{14}O_5$ mono *m/z 250.0841*

APCI + (MMI)

nitrogen flow 5 L/min, gas temperature 325°C, nebulizer 45 psi, skimmer **65** V, vaporizer 200°C, fragmentor 20 V, dissolved in methanol

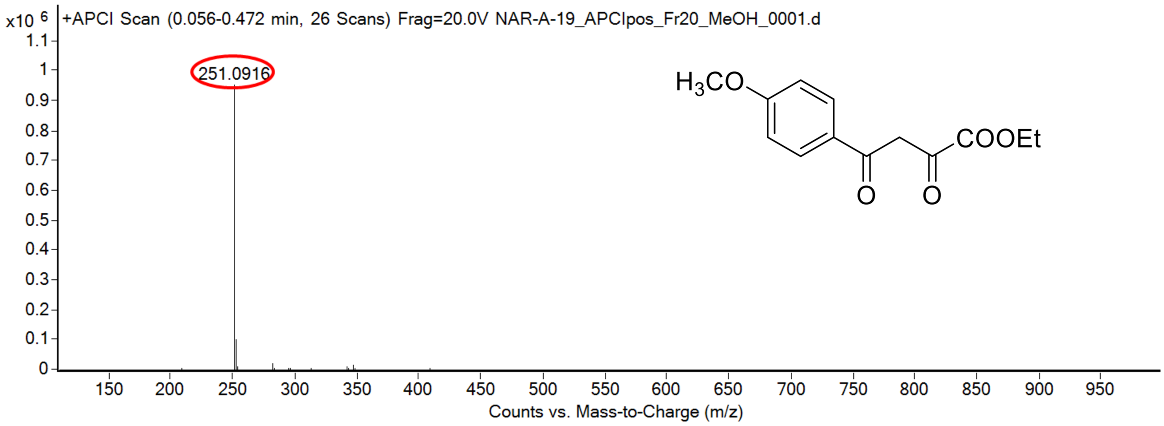

calculated mass: $[M+H]^+ = 251.0914$ observed: $[M+H]^+ = 251.0916$ mass accuracy = 0.8 ppm

FT-IR spectrum (neat) of **S159**

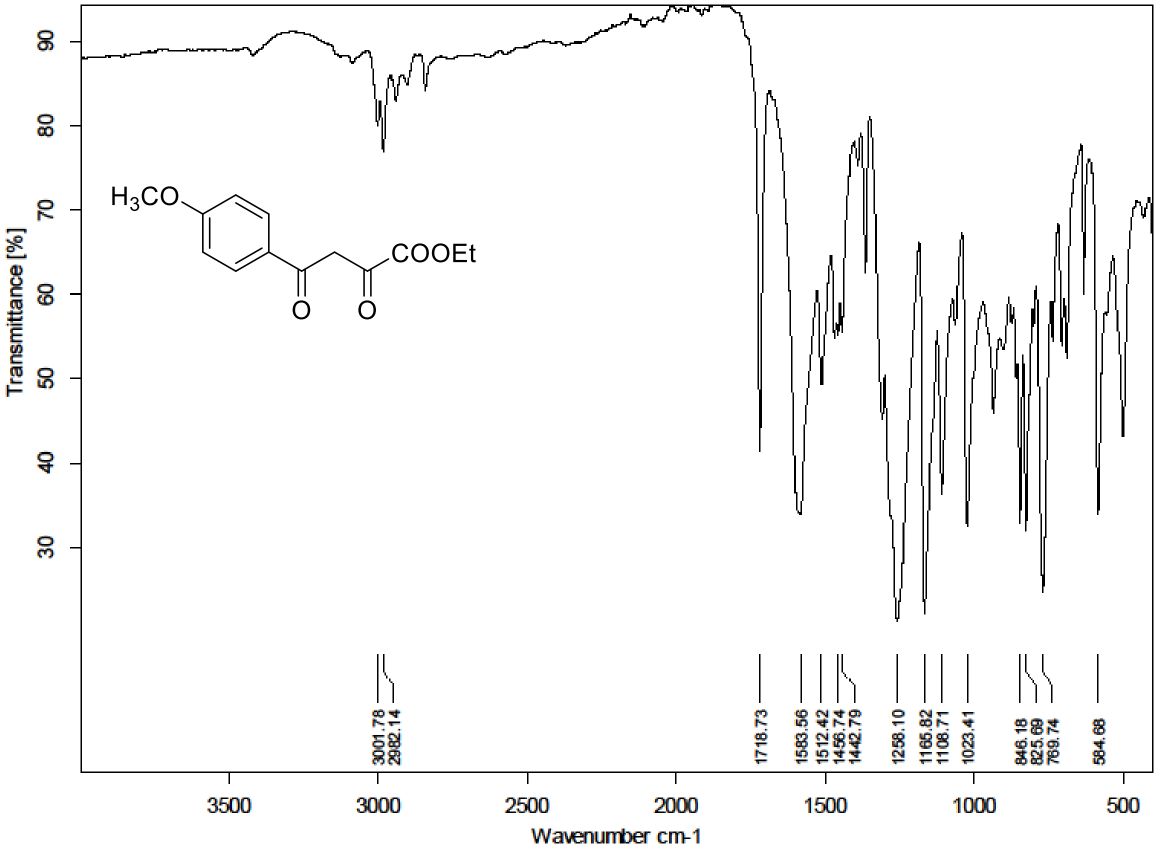

^{1}H (500 MHz) and ^{13}C NMR (126 MHz) spectra of **S160** in Chloroform-*d*

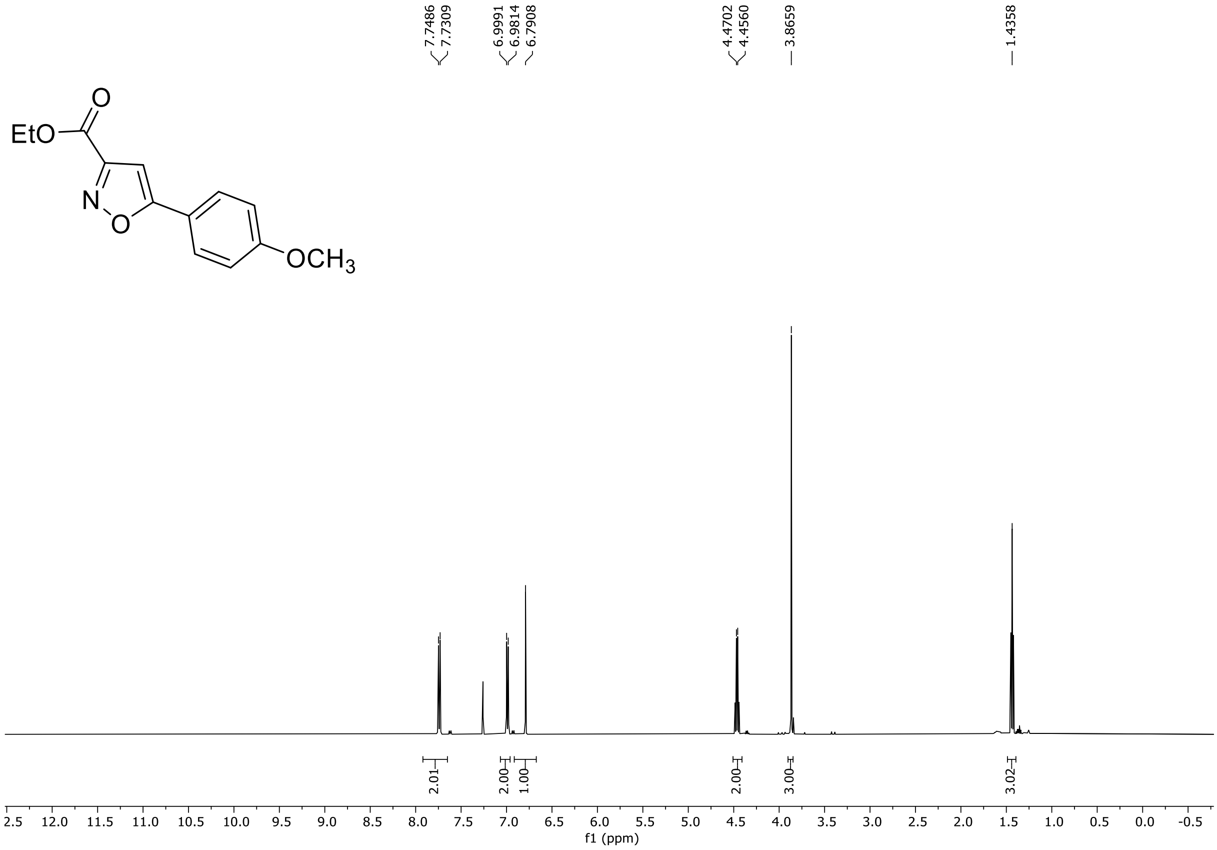

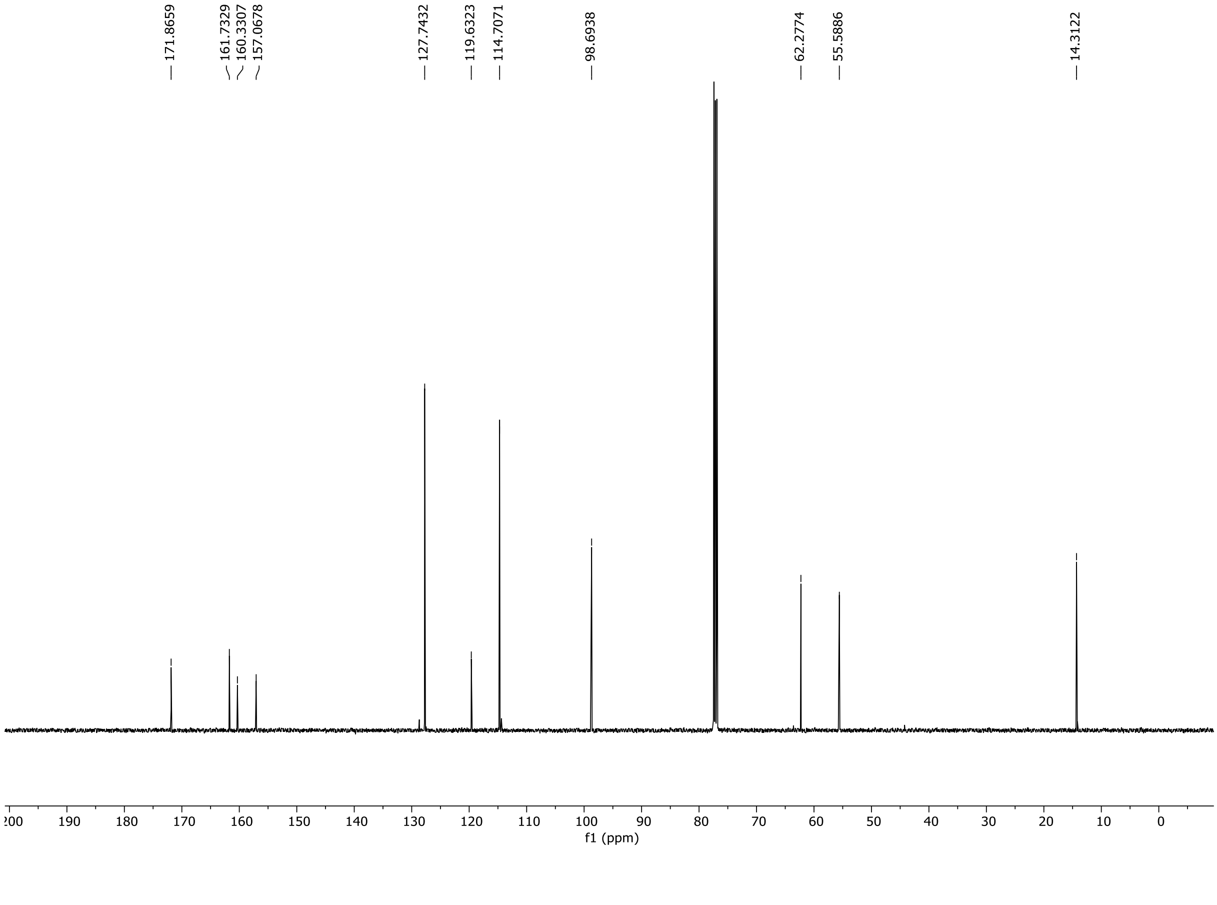

HRMS spectrum of **S160**

NAR-A-25 $C_{13}H_{13}NO_4$ mono *m/z 247.0845*

APCI + (MMI)
nitrogen flow 5 L/min, gas temperature 325°C, nebulizer 45 psi, skimmer **65** V,
vaporizer 200°C, fragmentor 20 V, dissolved in methanol

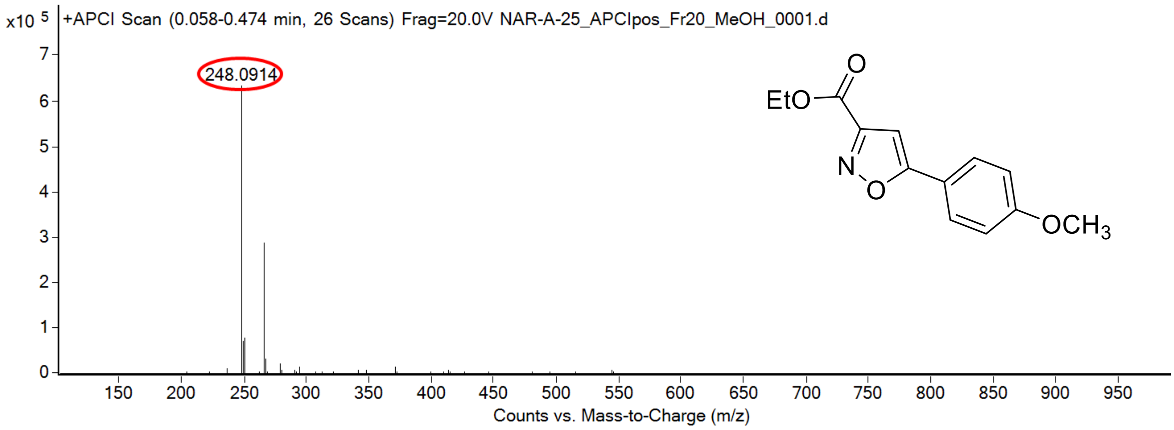

calculated mass: $[M+H]^+$ = 248.0917 observed: $[M+H]^+$ = 248.0914 mass accuracy = -1.2 ppm

FT-IR spectrum (neat) of **S160**

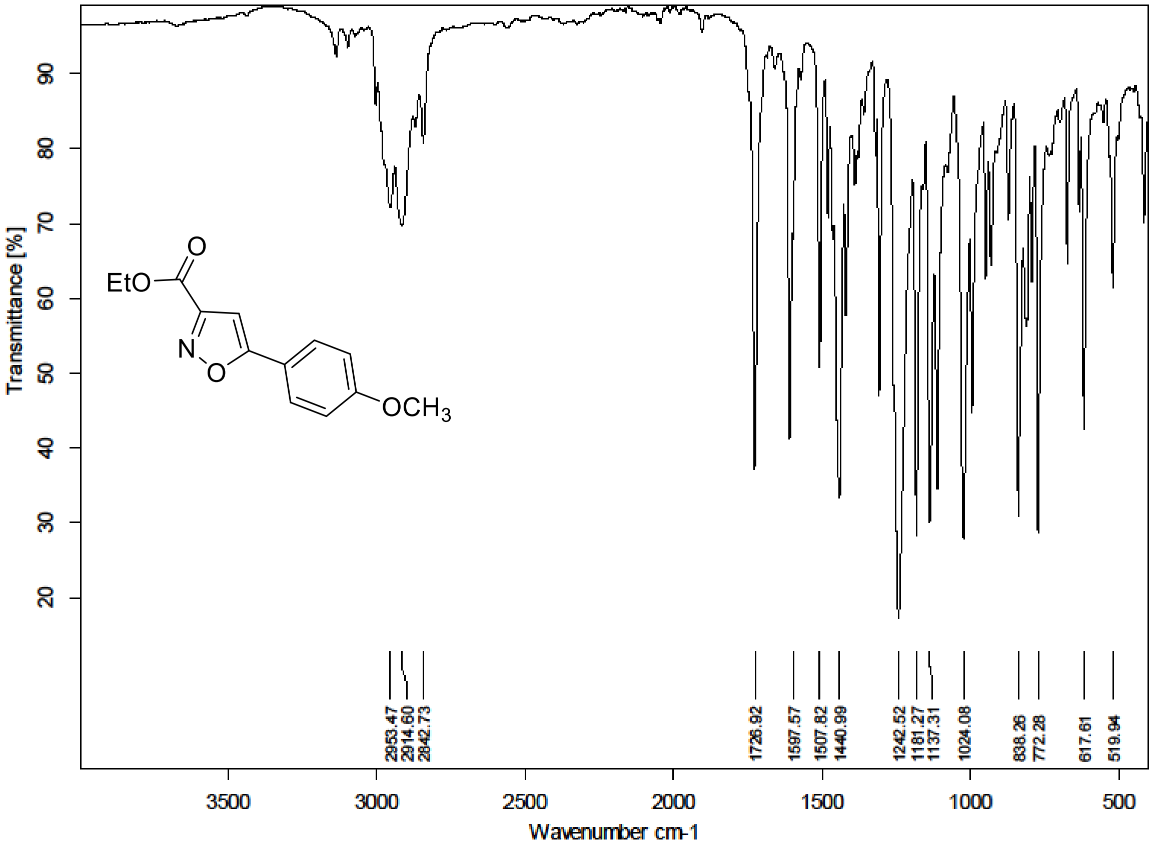

^{1}H (500 MHz) and ^{13}C NMR (126 MHz) spectra of **S161** in Chloroform-*d*

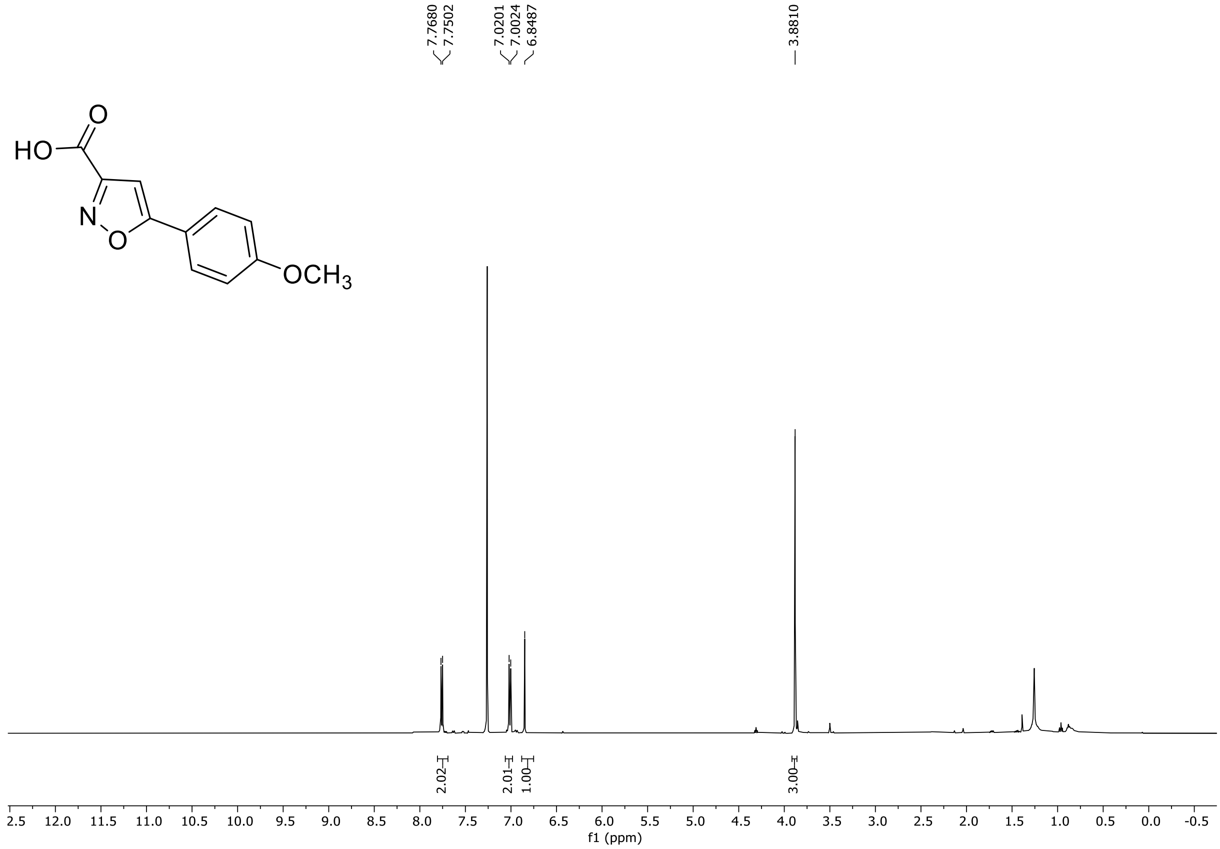

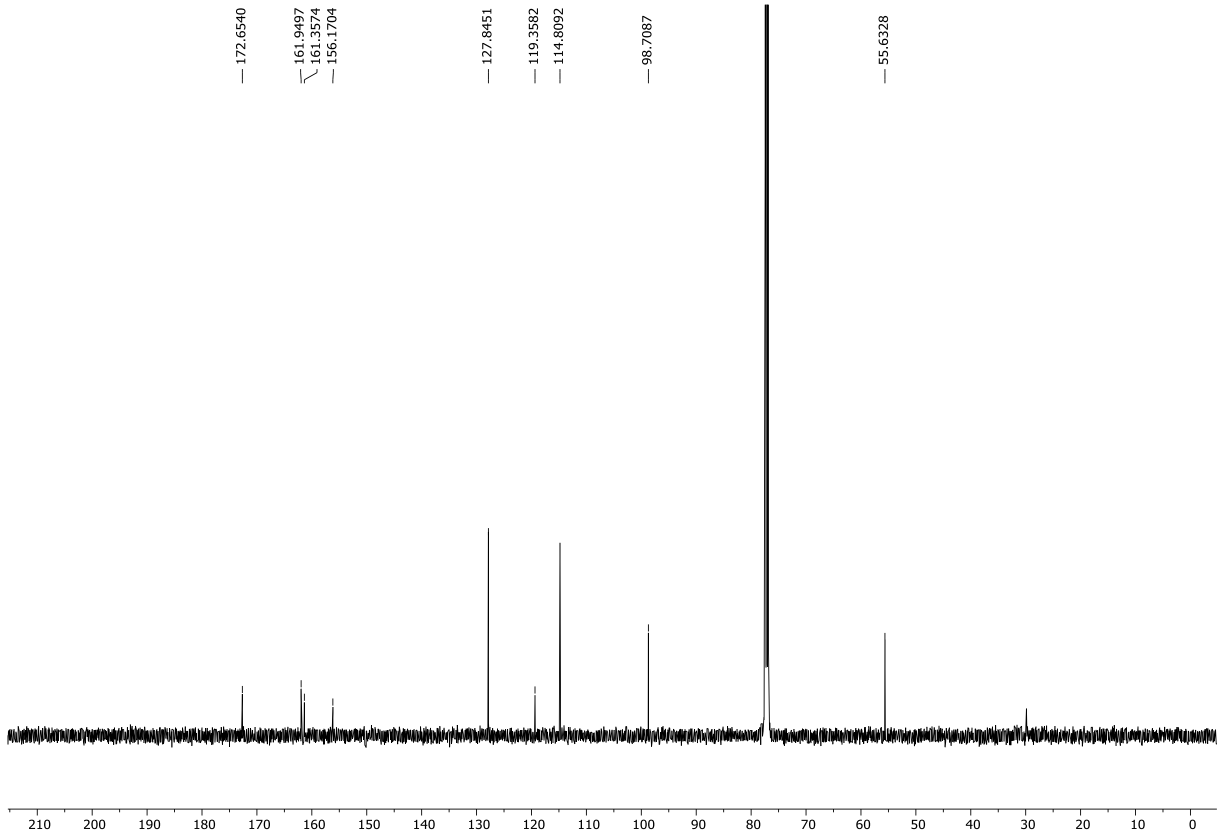

HRMS spectrum of **S161**

NAR-A-30 $C_{11}H_9NO_4$ mono *m/z 219.0532*

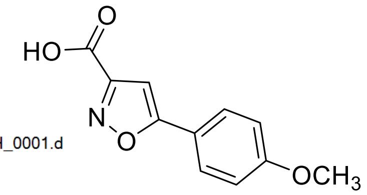

APCI + (MMI)
nitrogen flow 5 L/min, gas temperature 325°C, nebulizer 45 psi, skimmer **65** V, vaporizer 200°C, fragmentor 25 V, dissolved in methanol

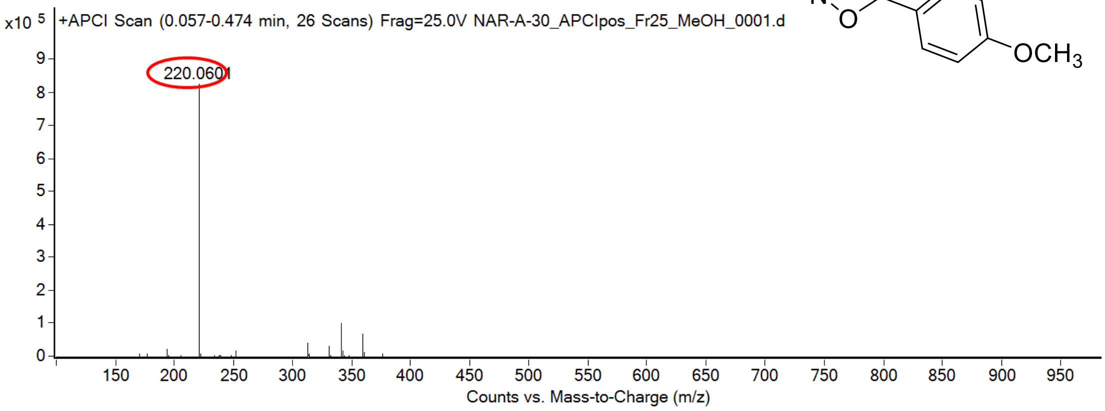

calculated mass: $[M+H]^+ = 220.0604$ observed: $[M+H]^+ = 220.0601$ mass accuracy = -1.3 ppm

FT-IR spectrum (neat) of **S161**

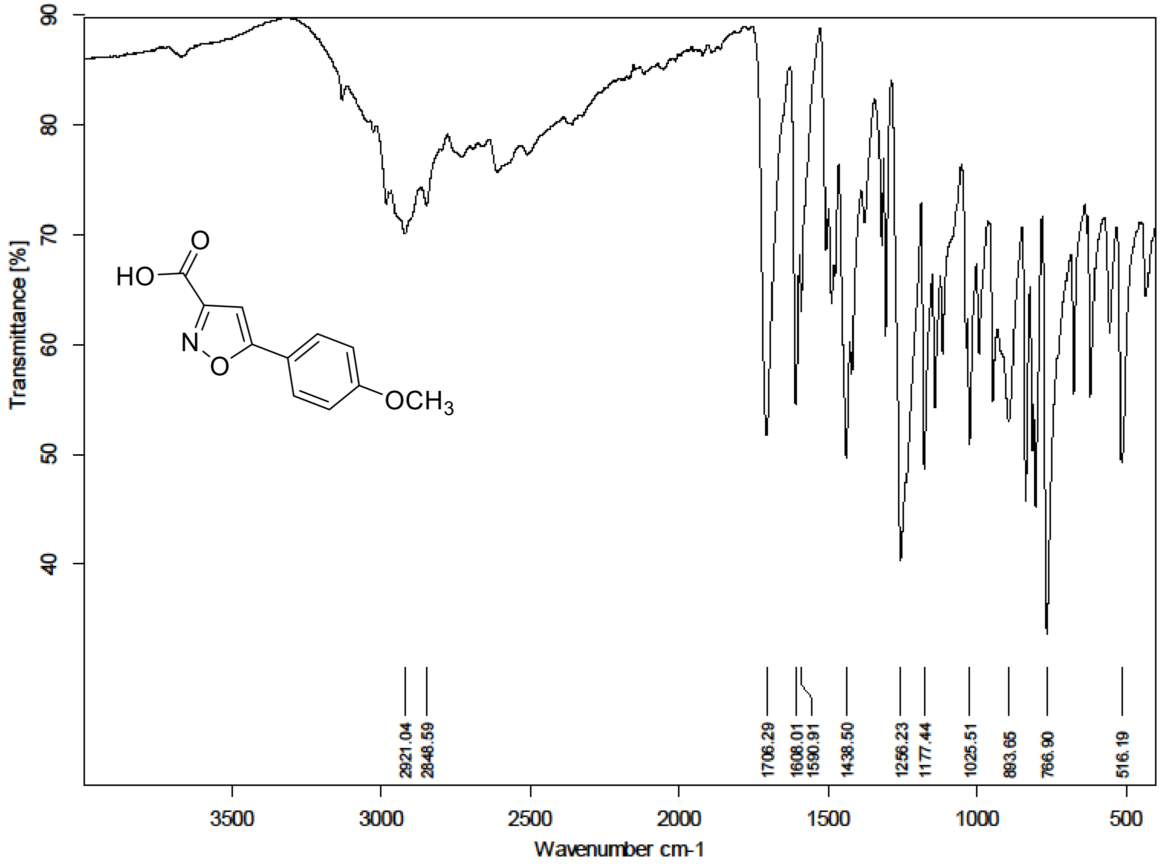

^{1}H (500 MHz) and ^{13}C NMR (126 MHz) spectra of **S163** in Chloroform-*d*

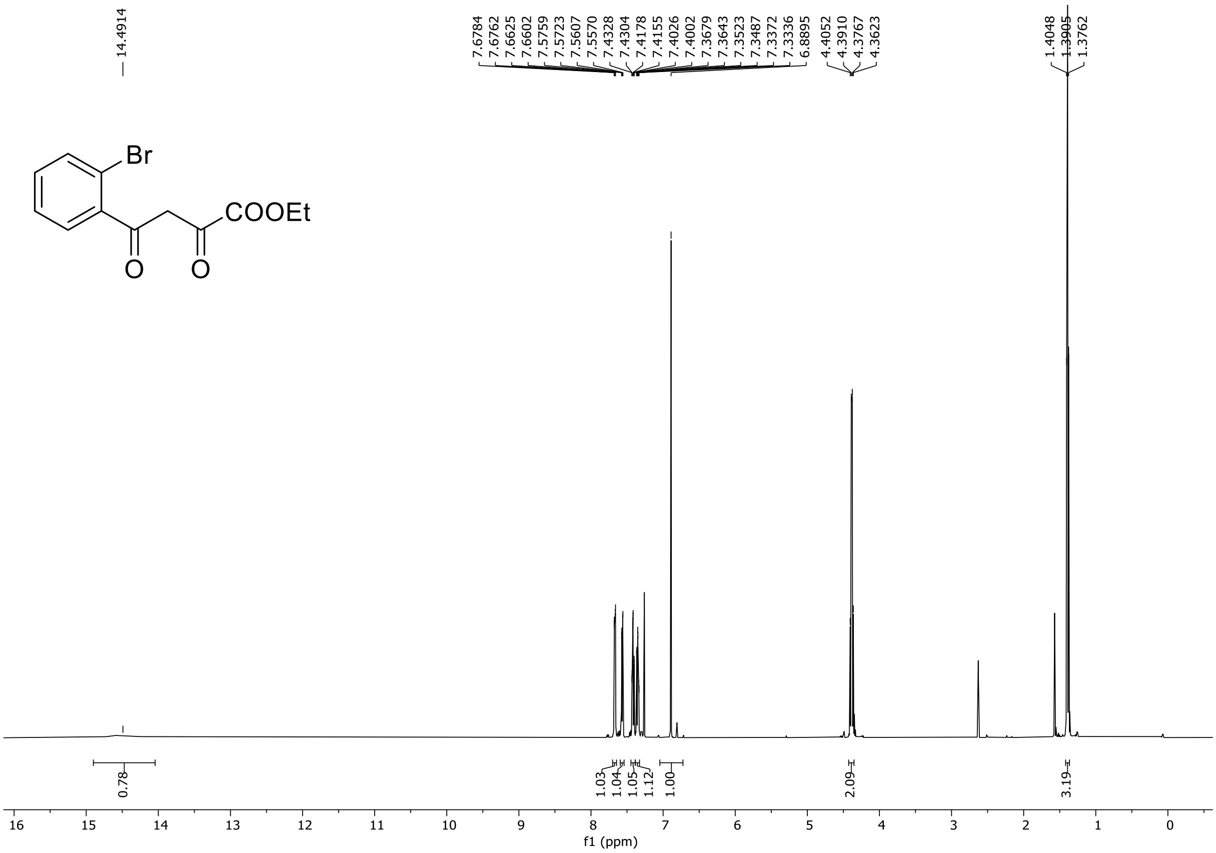

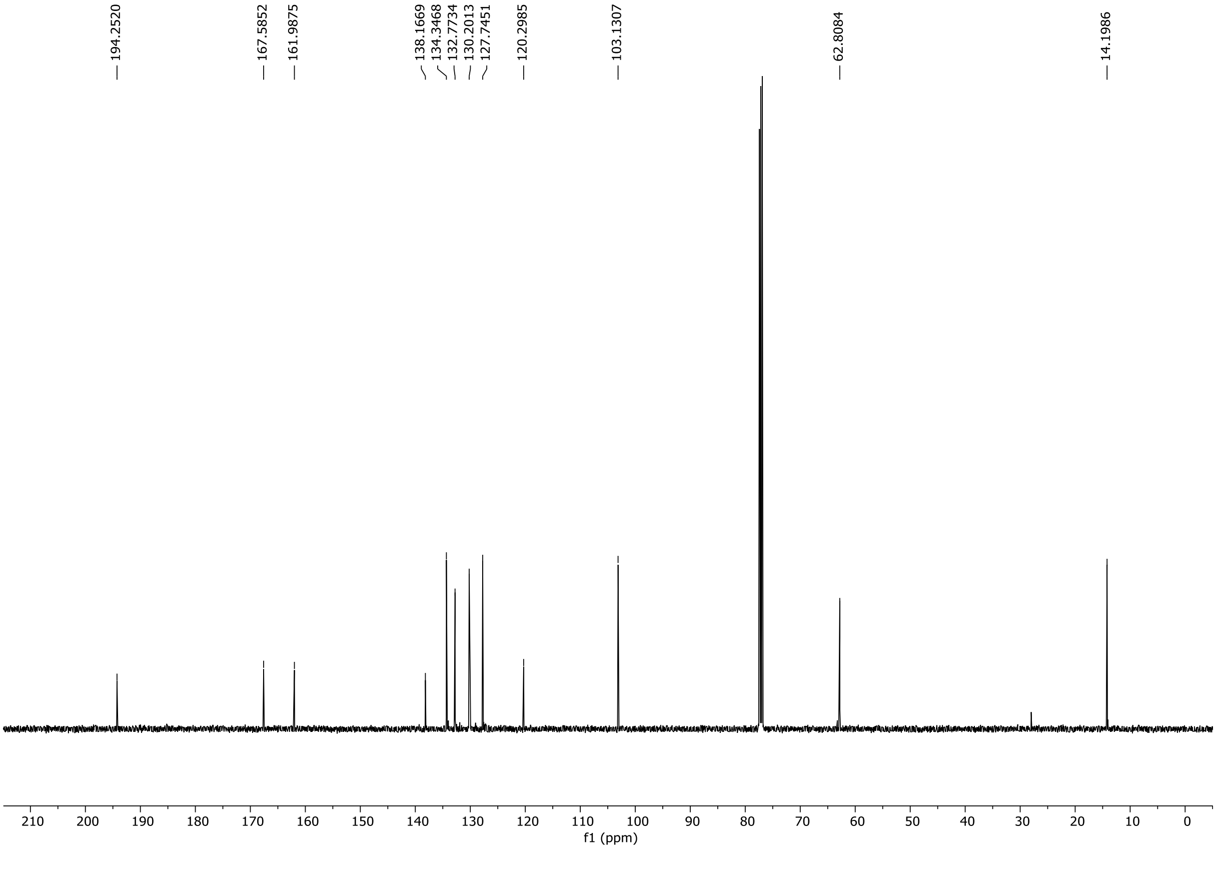

HRMS spectrum of **S163**

NAR-A-21 **$C_{12}H_{11}BrO_4$** mono *m/z 297.9841*

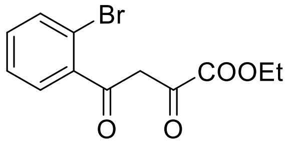

APCI + (MMI)
nitrogen flow 5 L/min, gas temperature 325°C, nebulizer 45 psi, skimmer **65** V, vaporizer 200°C, fragmentor 28 V, dissolved in methanol

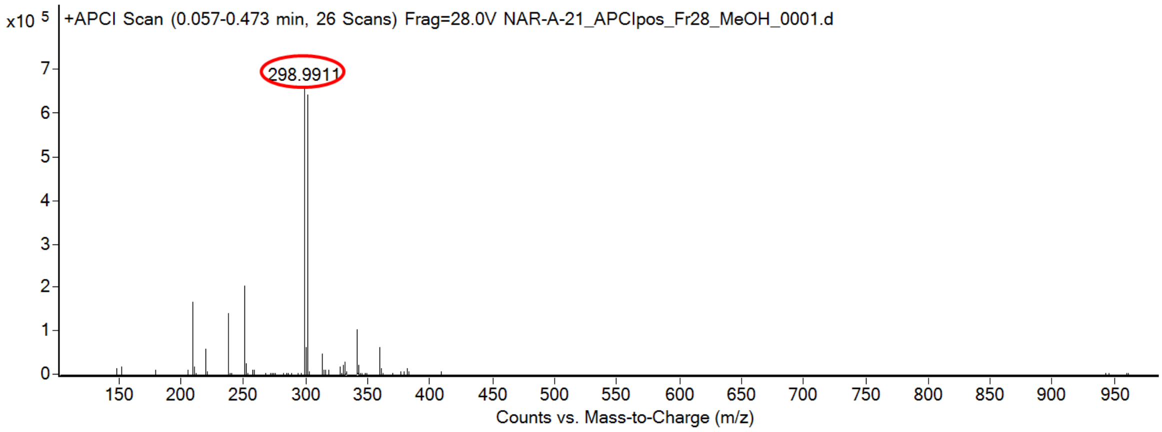

calculated mass: $[M+H]^+$ = 298.9913 observed: $[M+H]^+$ =298.9911 mass accuracy = -0.7 ppm

FT-IR spectrum (neat) of **S163**

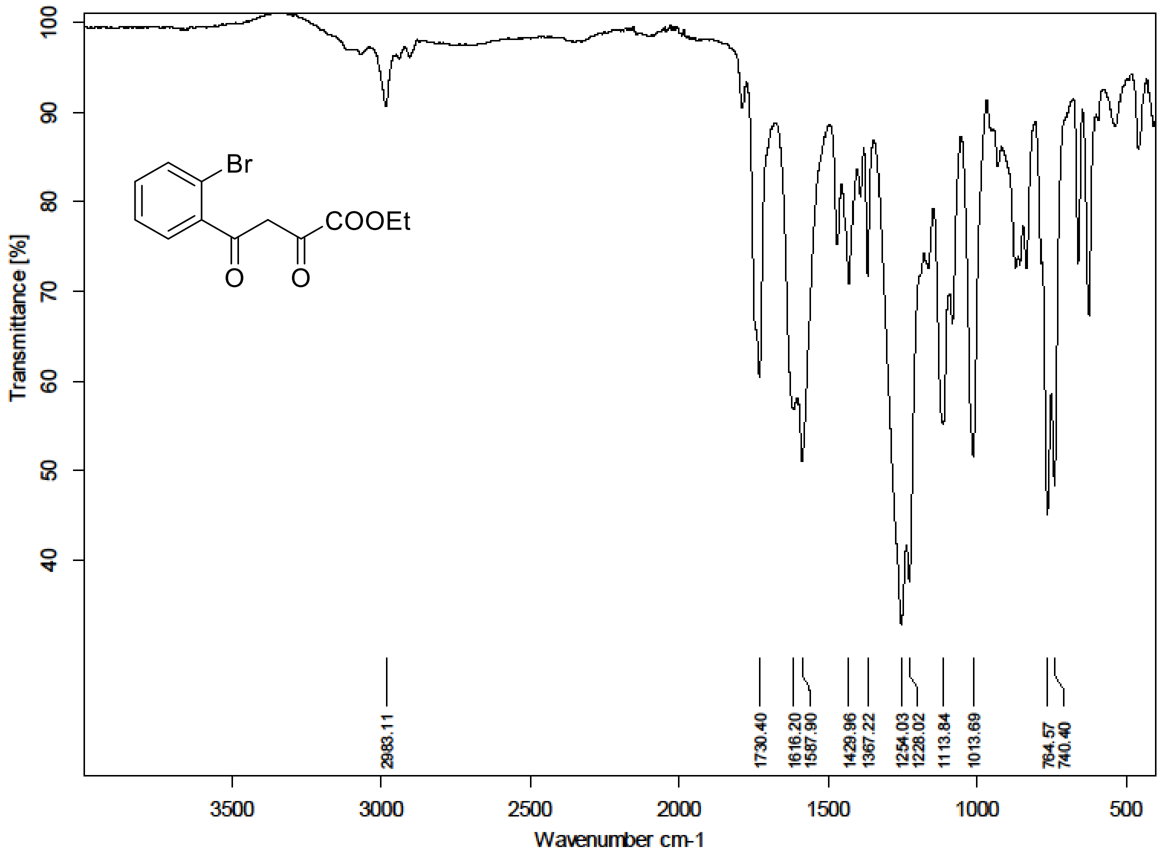

^{1}H (500 MHz) and ^{13}C NMR (126 MHz) spectra of **S164** in Chloroform-*d*

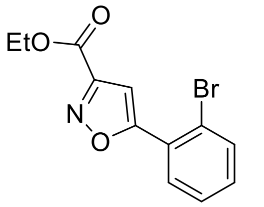

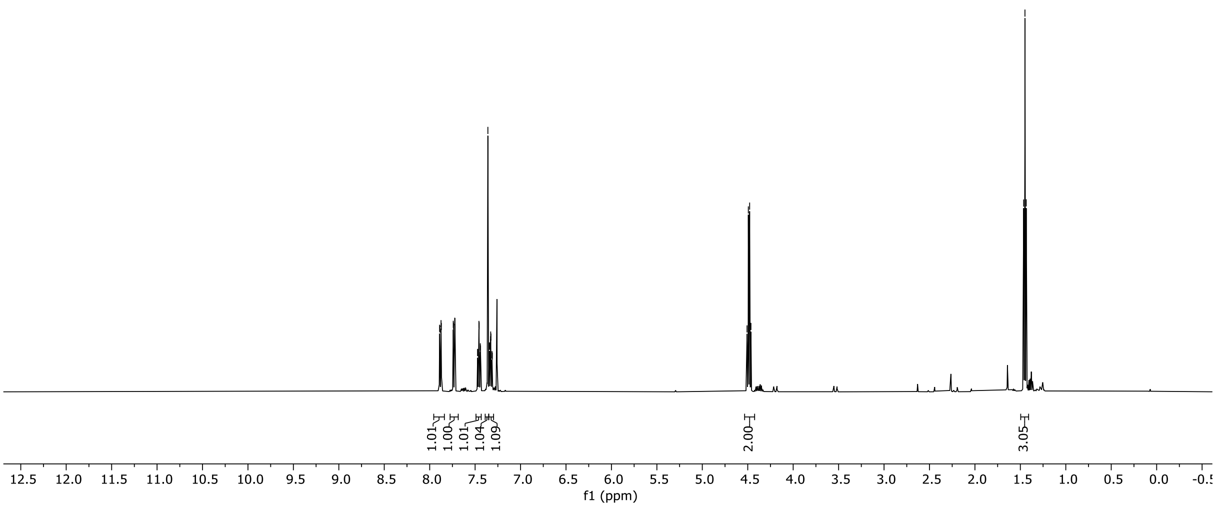

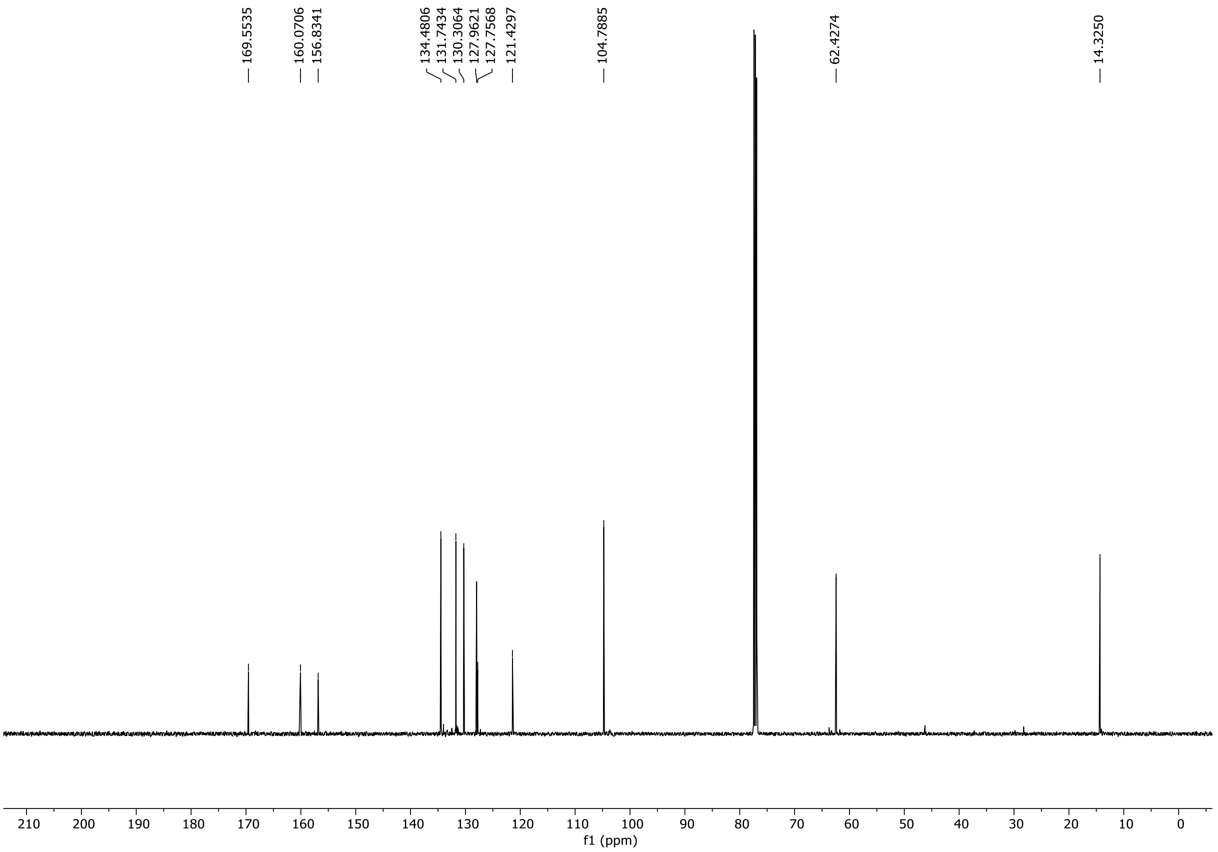

HRMS spectrum of **S164**

NAR-A-26 $C_{12}H_{10}BrNO_3$ mono *m/z 294.9844*

APCI + (MMI)
nitrogen flow 5 L/min, gas temperature 325°C, nebulizer 45 psi, skimmer **65** V, vaporizer 200°C, fragmentor 25 V, dissolved in methanol

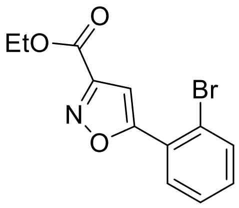

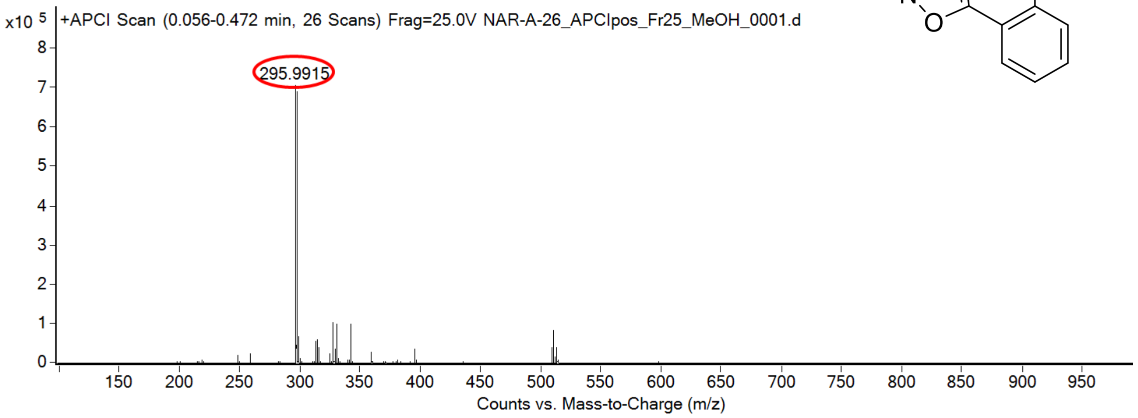

calculated mass: $[M+H]^+$ = 295.9917 observed: $[M+H]^+$ = 295.9915 mass accuracy = -0.7 ppm

FT-IR spectrum (neat) of **S164**

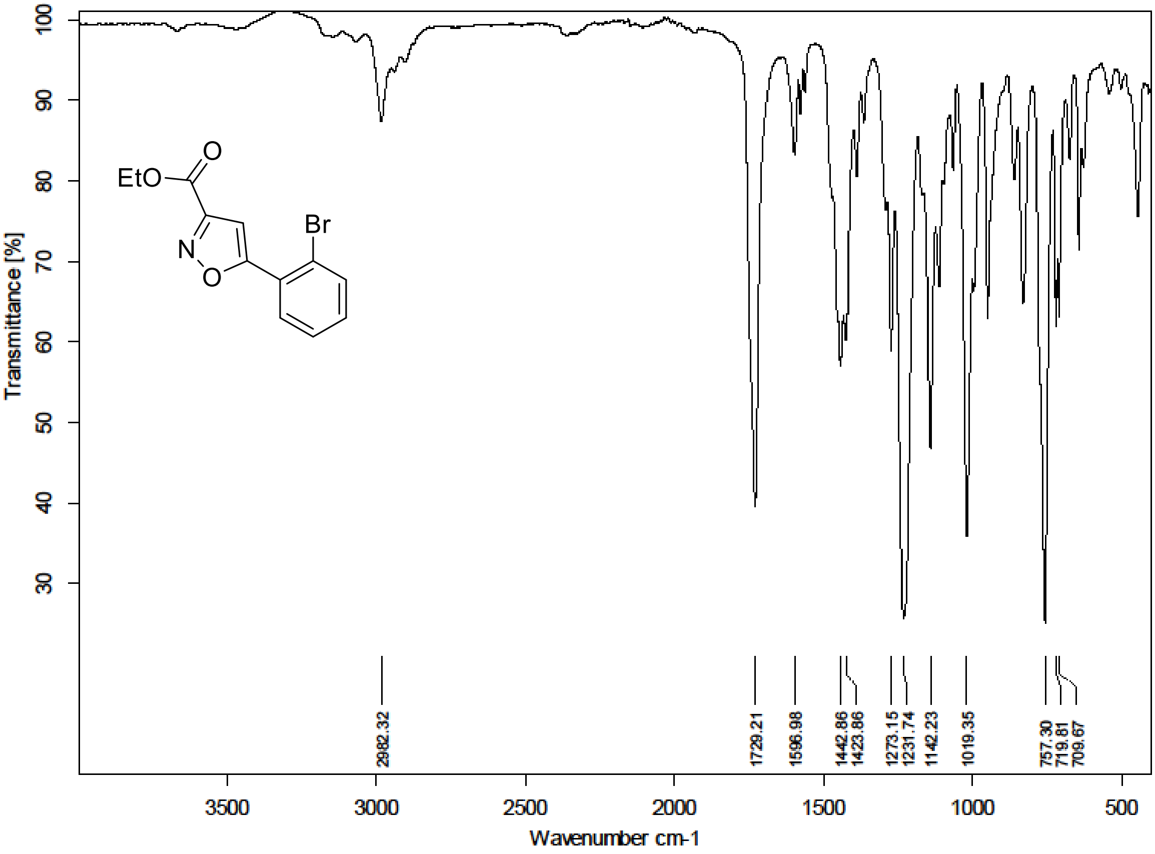

^{1}H (500 MHz) and ^{13}C NMR (126 MHz) spectra of **S165** in Chloroform-*d*

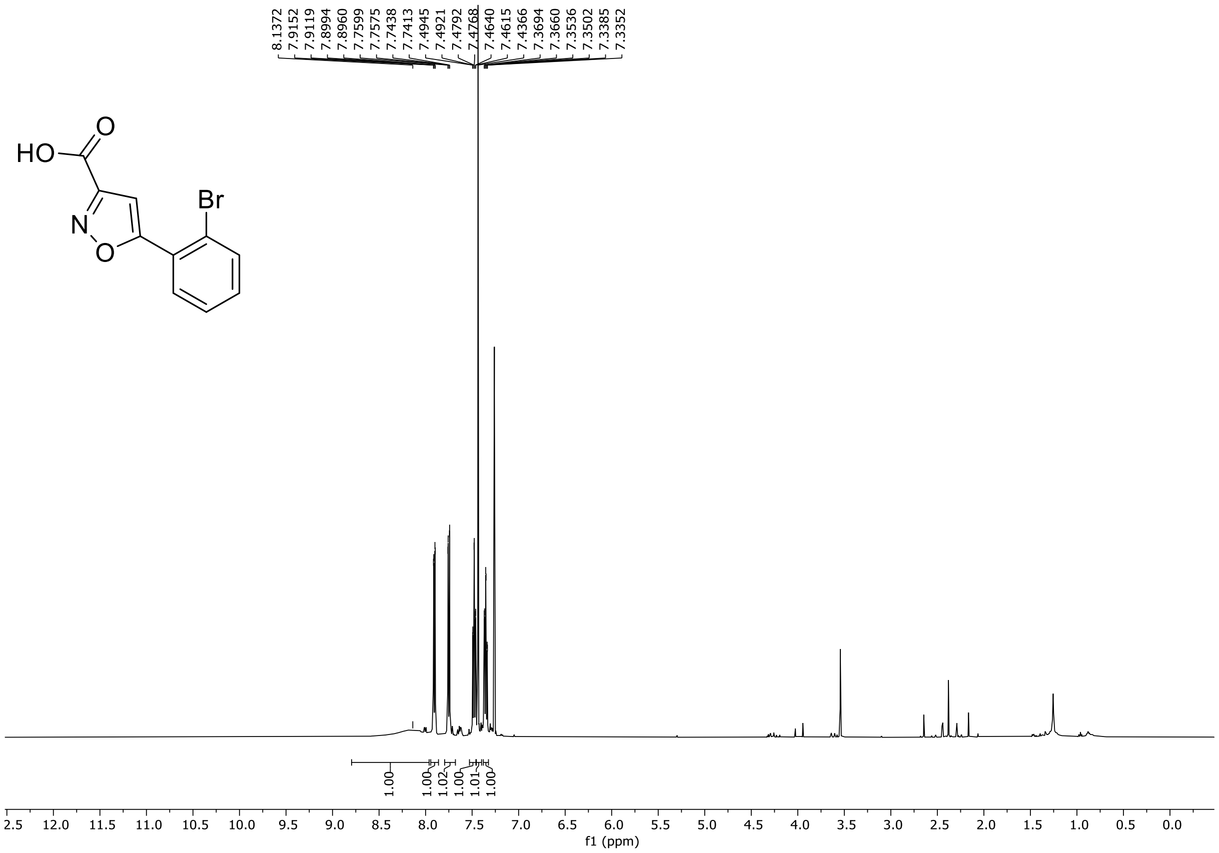

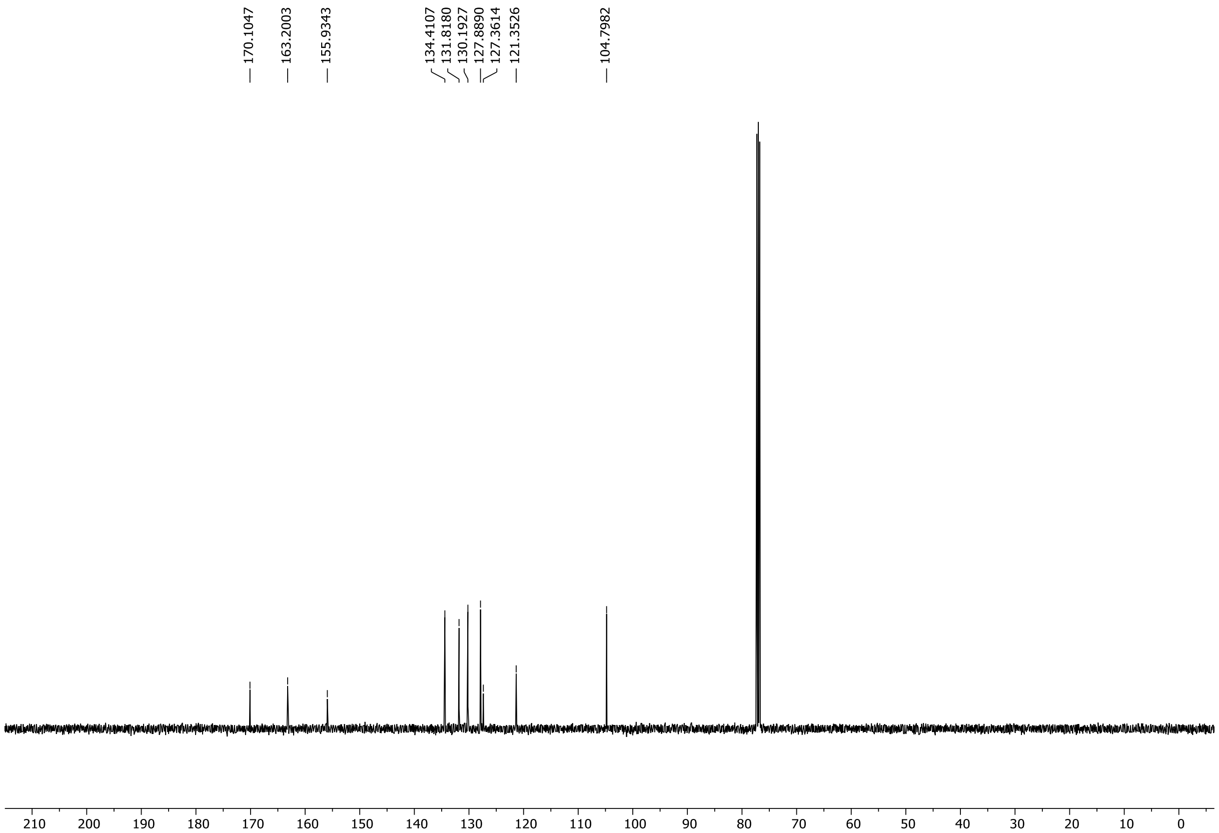

HRMS spectrum of **S165**

NAR-A-31 $C_{10}H_6BrNO_3$ mono *m/z 266.9531*

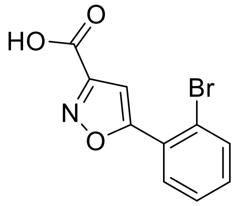

APCI + (MMI)
nitrogen flow 5 L/min, gas temperature 325°C, nebulizer 45 psi, skimmer **65** V, vaporizer 200°C, fragmentor 35 V, dissolved in methanol

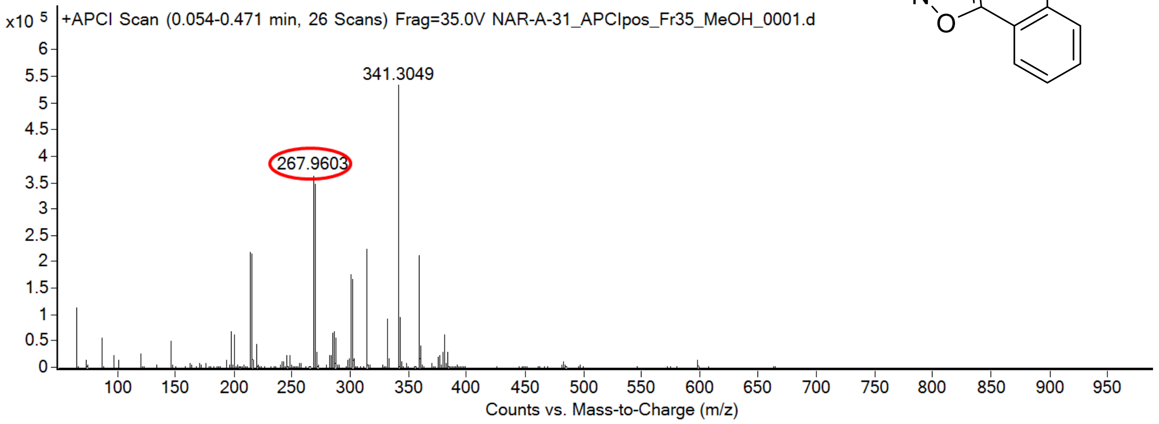

calculated mass: $[M+H]^+ = 267.9604$ observed: $[M+H]^+ = 267.9603$ mass accuracy = -0.3 ppm

FT-IR spectrum (neat) of **S165**

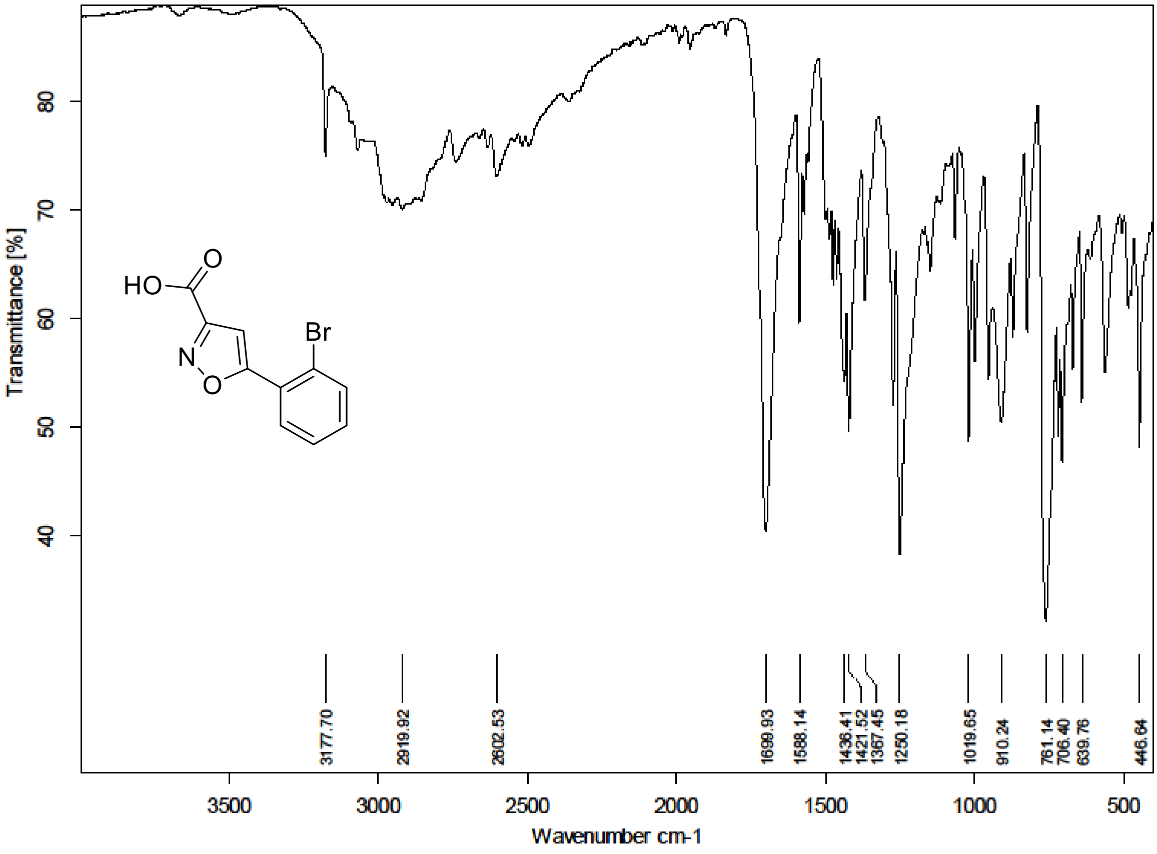

^{1}H (500 MHz) and ^{13}C NMR (126 MHz) spectra of **S167** in Chloroform-*d*

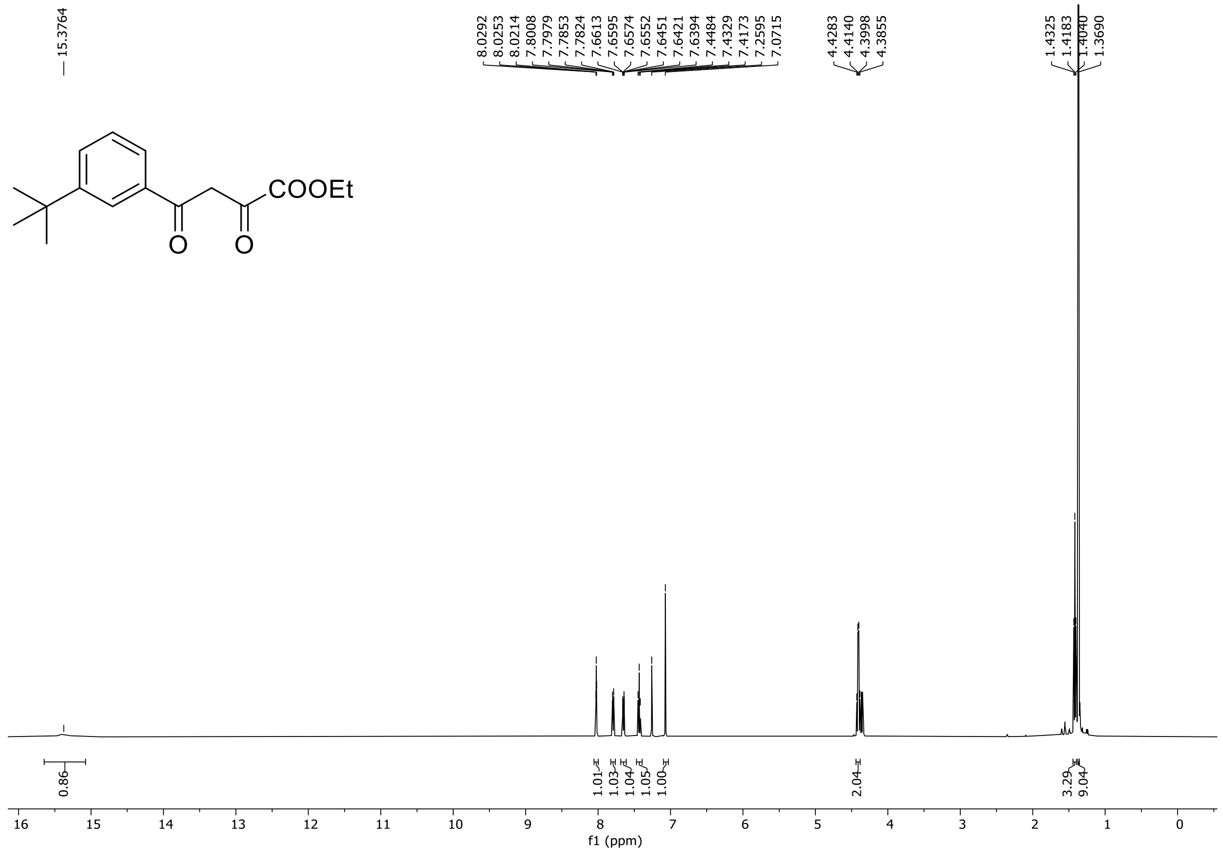

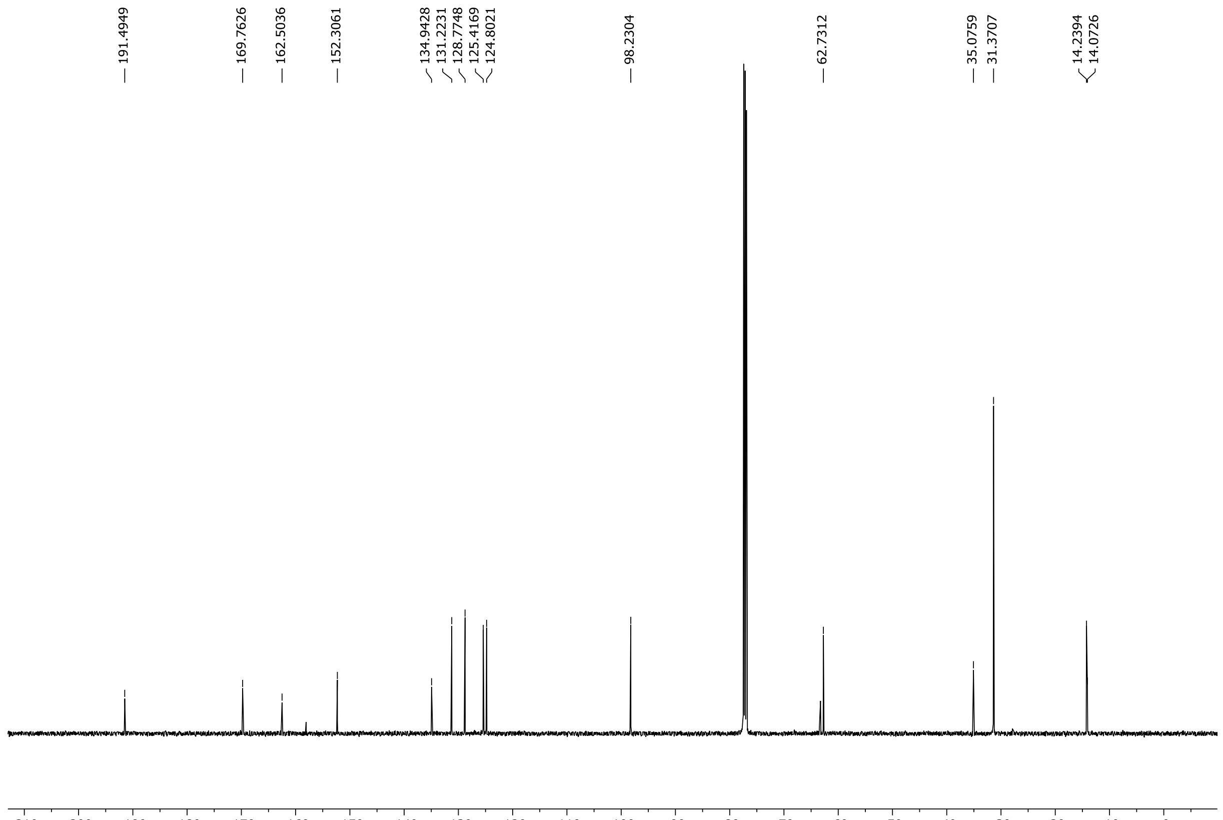

HRMS spectrum of **S167**

NAR-A-48

$C_{16}H_{20}O_4$ *mono m/z* **276.1362**

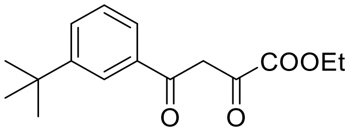

APCI + (MMI)

nitrogen flow 5 L/min, gas temperature 300°C, nebulizer 45 psi, skimmer **65** V, vaporizer 200°C, fragmentor 15 V, dissolved in MeOH

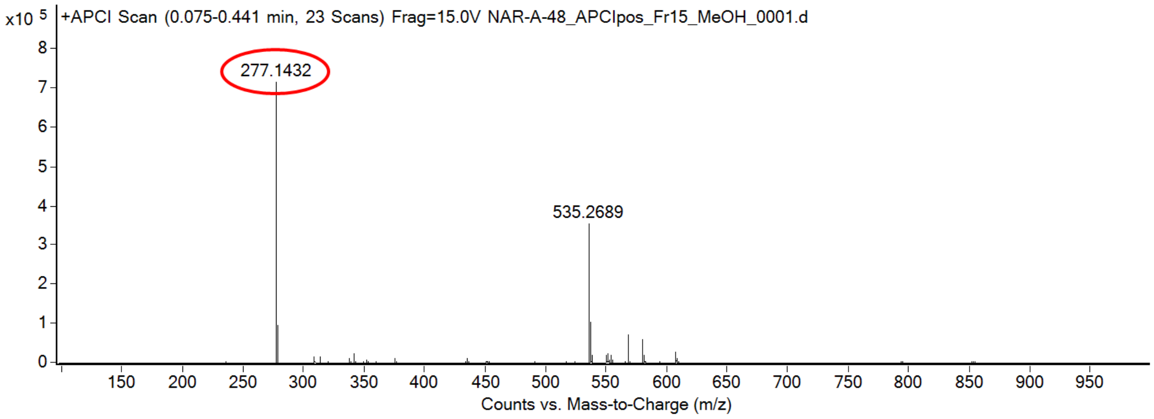

calculated mass: $[M+H]^+$ = 277.1434 observed: $[M+H]^+$ = 277.1432 mass accuracy = -0.7 ppm

FT-IR spectrum (neat) of **S167**

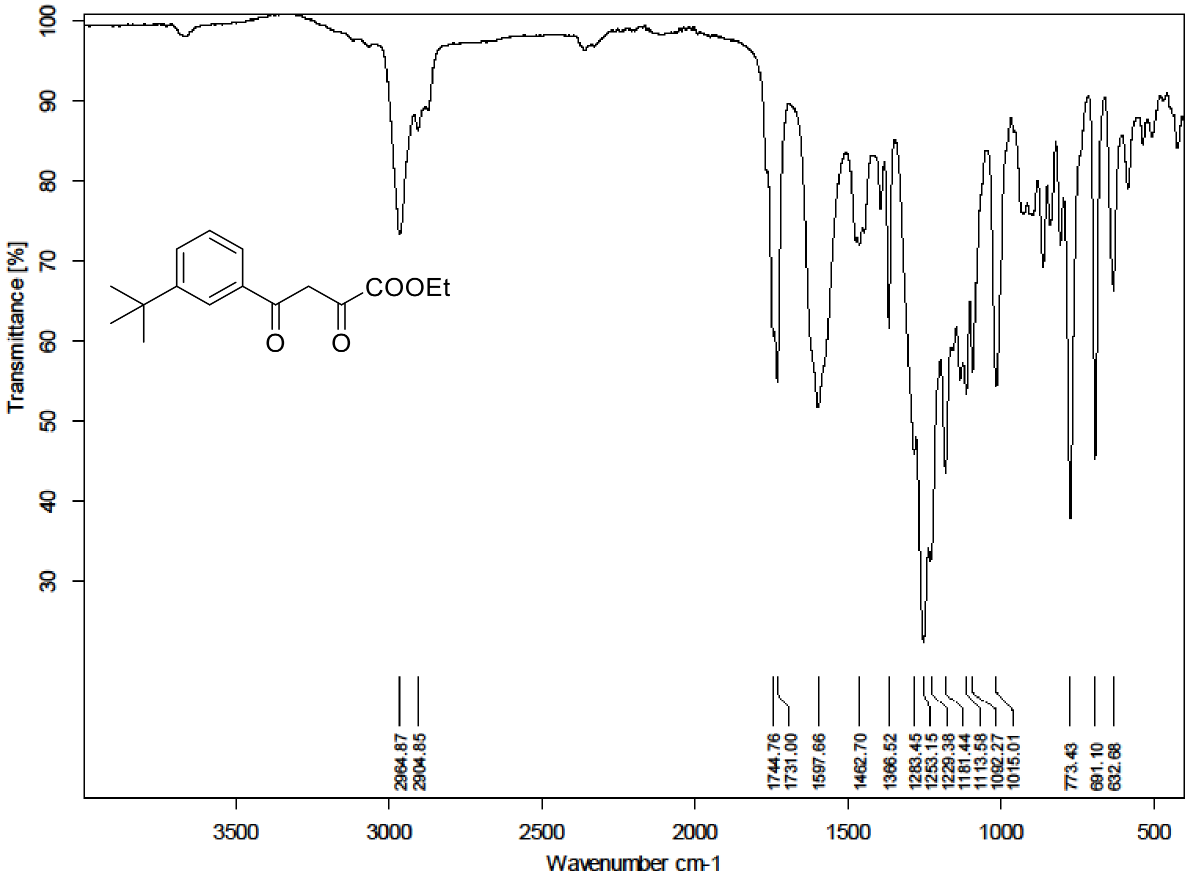

^{1}H (300 MHz) and ^{13}C NMR (126 MHz) spectra of **S168** in Chloroform-*d*

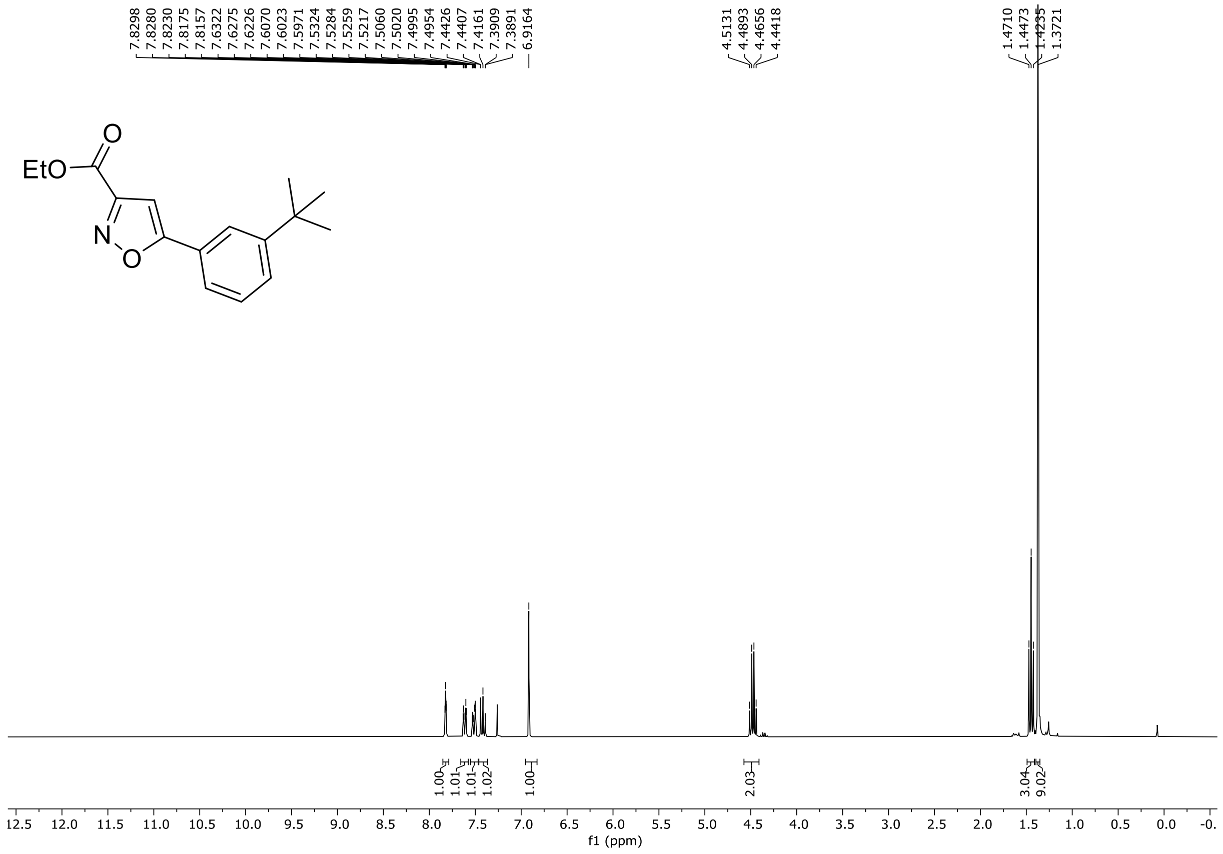

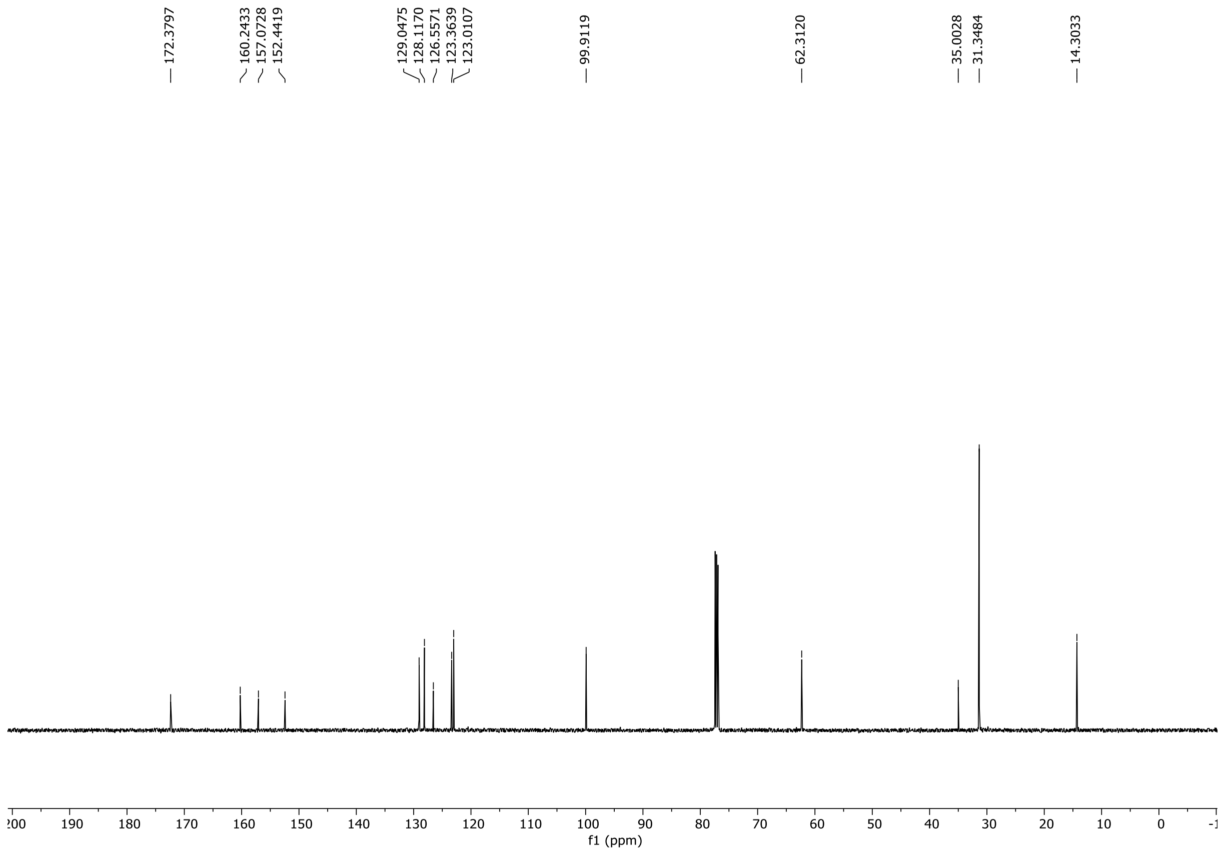

HRMS spectrum of **S168**

NAR-A-51

$C_{16}H_{19}NO_3$ *mono m/z*
273.1365

APCI + (MMI)
nitrogen flow 5 L/min, gas temperature 300°C, nebulizer 45 psi, skimmer **65** V, vaporizer 200°C, fragmentor 15 V, dissolved in MeOH

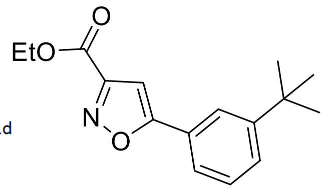

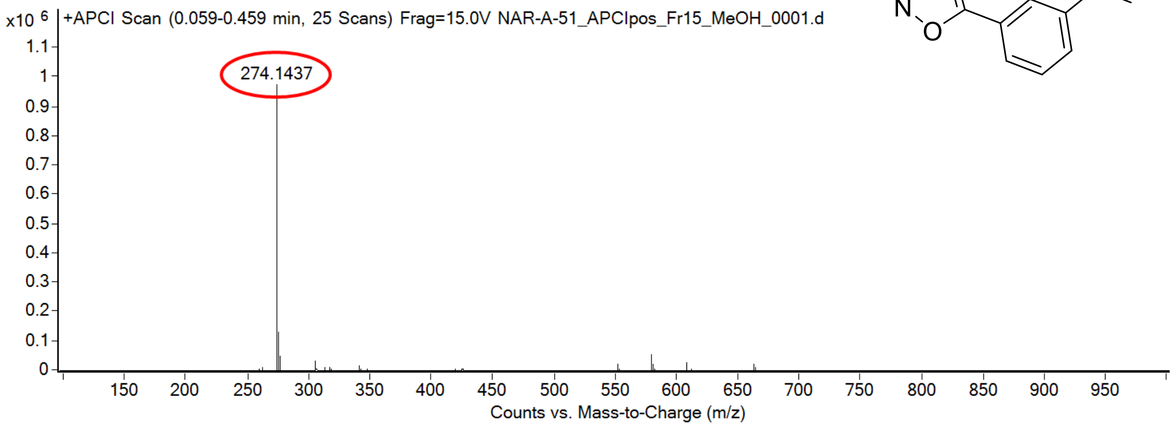

calculated mass: $[M+H]^+$ = 274.1438 observed: $[M+H]^+$ = 274.1437 mass accuracy = -0.4 ppm

FT-IR spectrum (neat) of **S168**

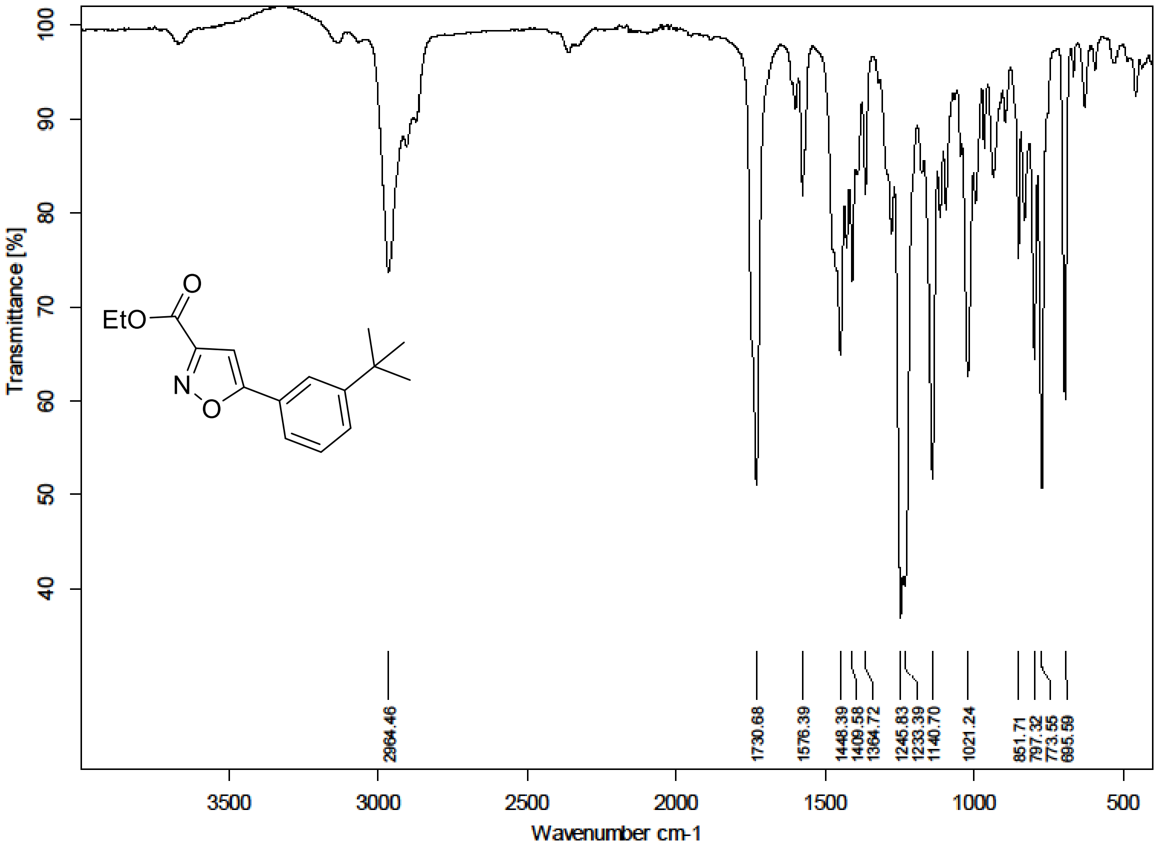

^{1}H (300 MHz) and ^{13}C NMR (126 MHz) spectra of **S169** in DMSO-d_6

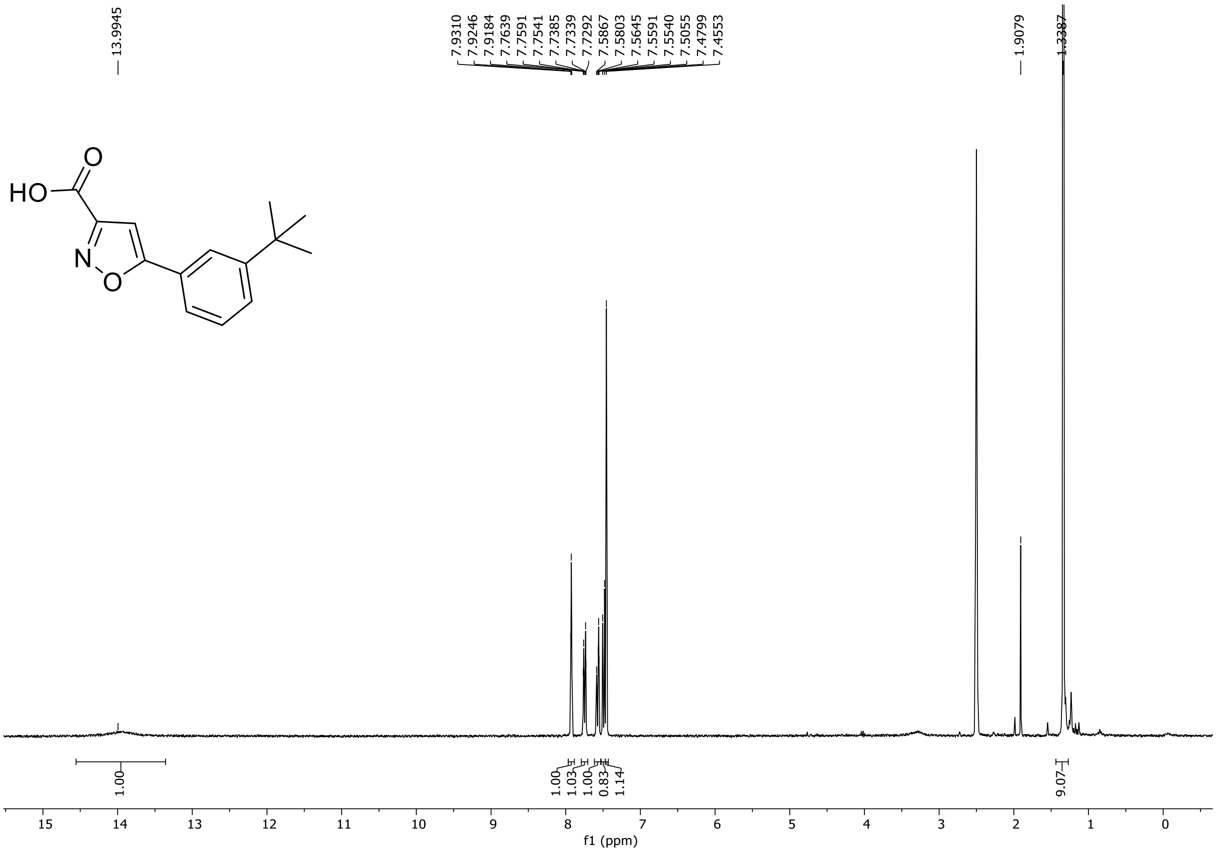

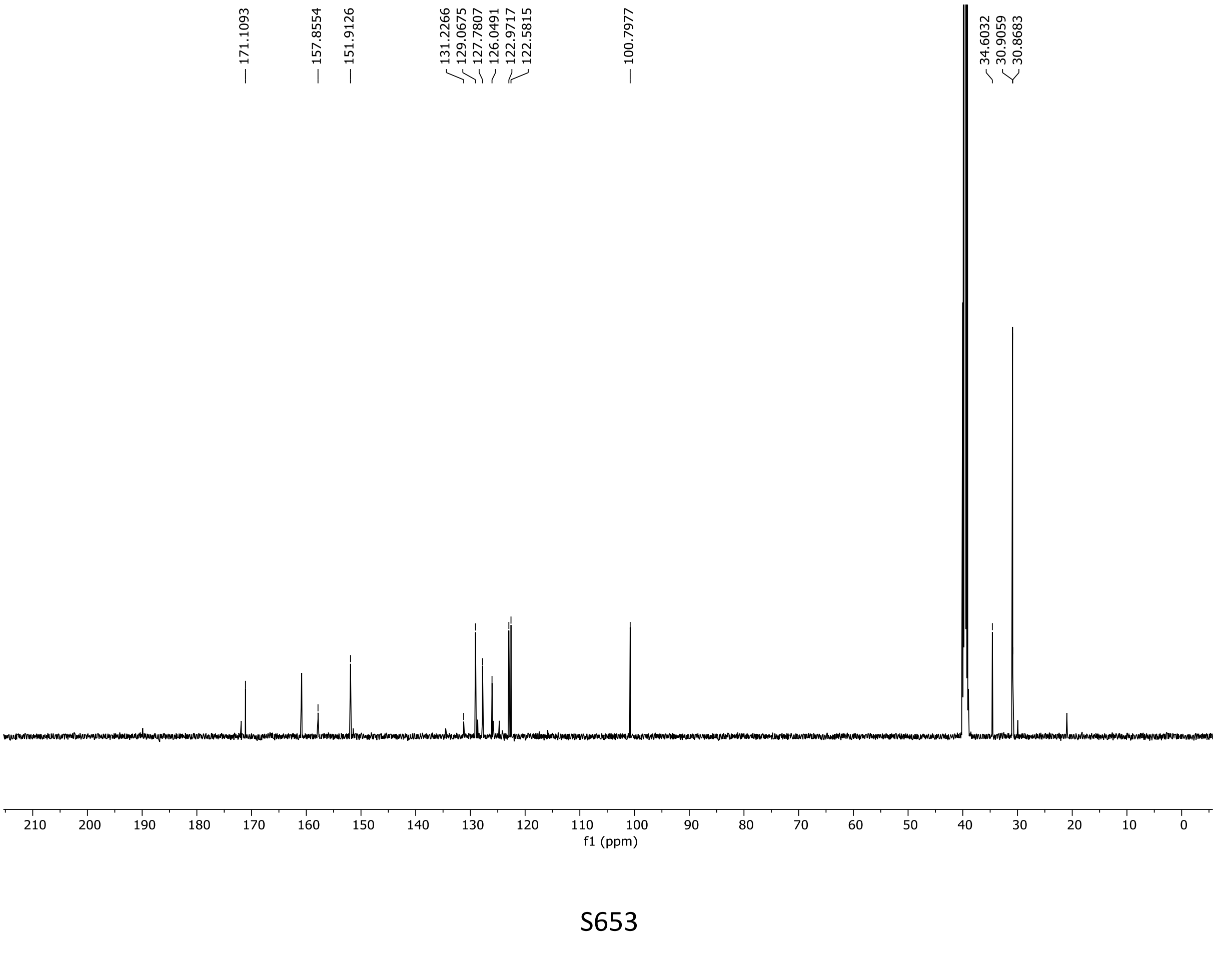

HRMS spectrum of **S169**

NAR-A-81 $C_{14}H_{15}NO_3$ *mono m/z* 245.1052

APCI + (MMI)
nitrogen flow 5 L/min, gas temperature 300°C, nebulizer 45 psi, skimmer **65** V, vaporizer 200°C, fragmentor 20 V, dissolved in MeOH

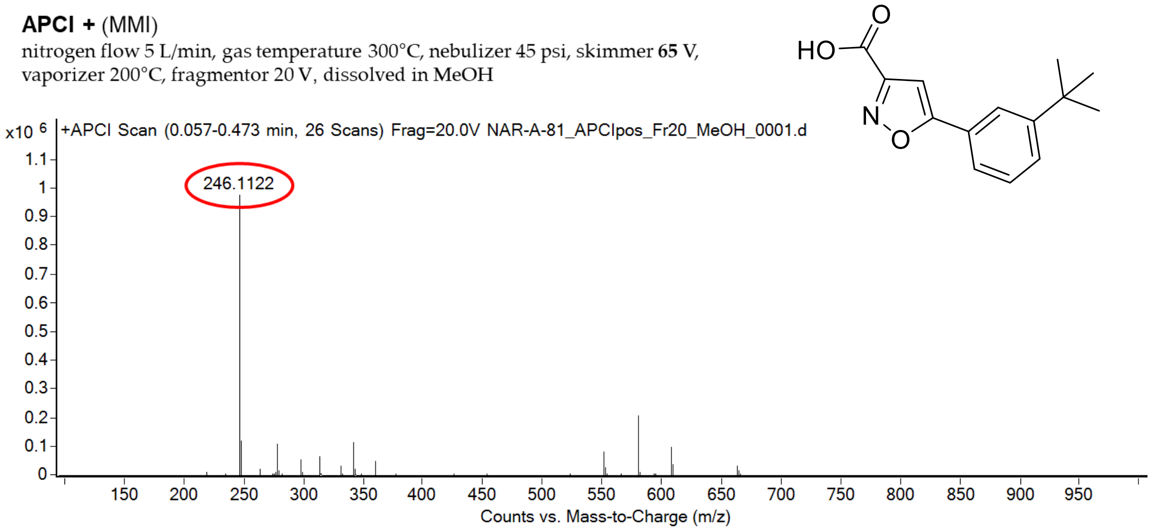

calculated mass: $[M+H]^+$ = 246.1125 observed: $[M+H]^+$ = 246.1122 mass accuracy = -1.2 ppm

FT-IR spectrum (neat) of **S169**

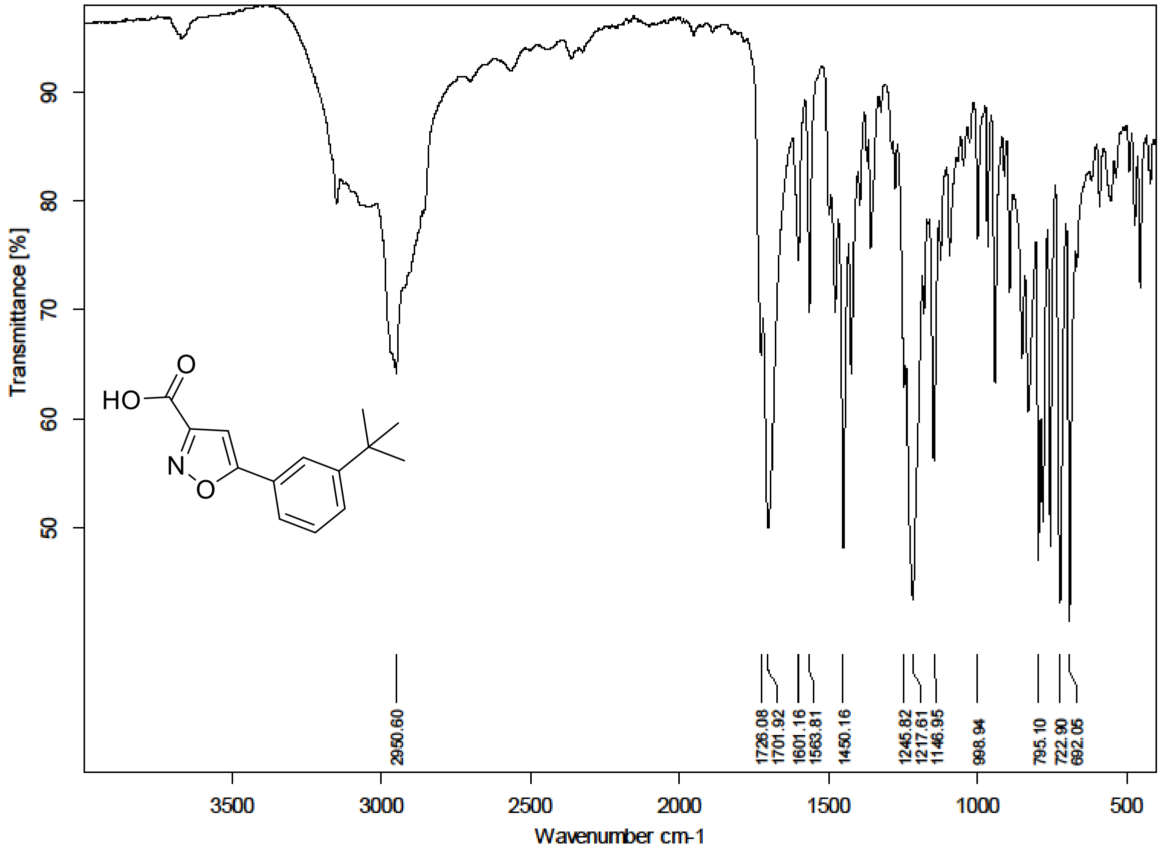

^{1}H (500 MHz) and ^{13}C NMR (126 MHz) spectra of **S171** in Chloroform-*d*

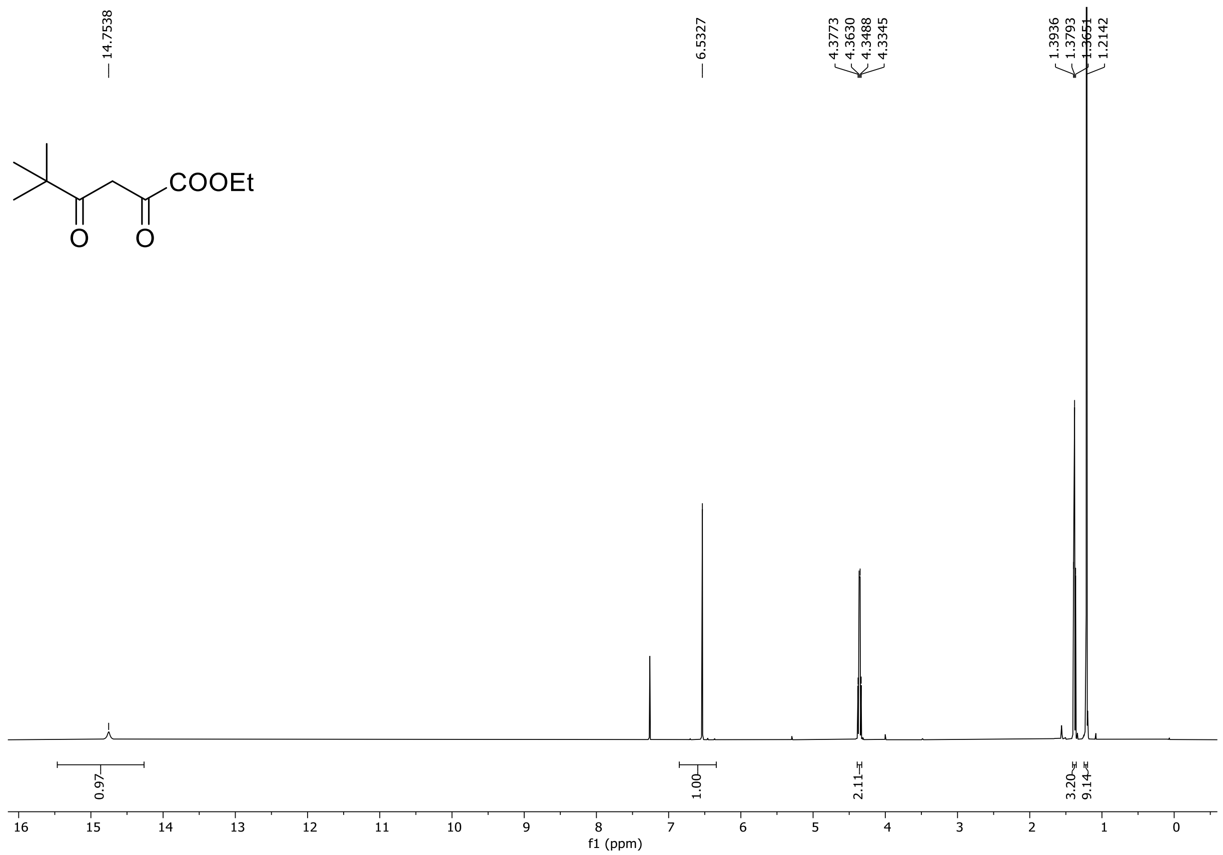

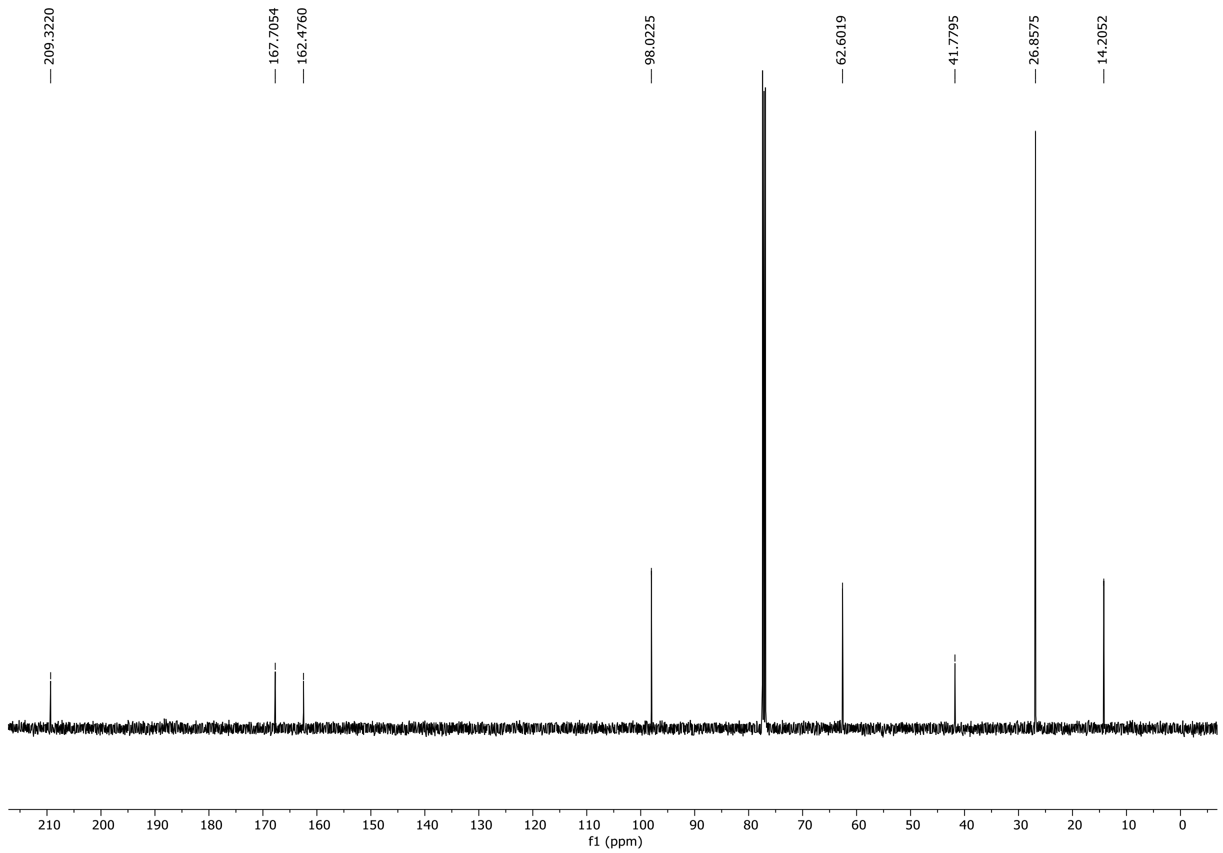

HRMS spectrum of **S171**

NAR-A-18 $C_{10}H_{16}O_4$ mono *m/z 200.1049*

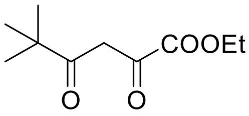

APCI + (MMI)
nitrogen flow 5 L/min, gas temperature 325°C, nebulizer 45 psi, skimmer **65** V, vaporizer 200°C, fragmentor 30 V, dissolved in methanol

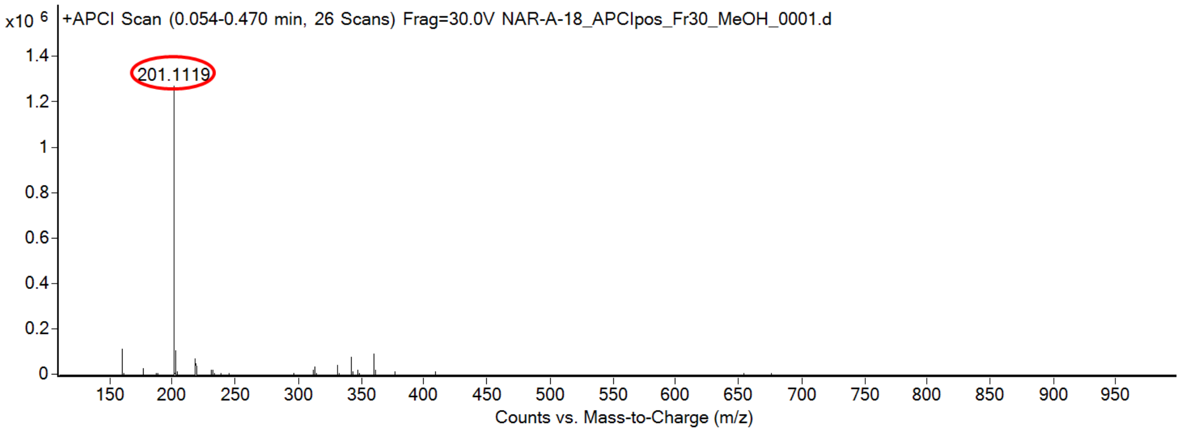

calculated mass: $[M+H]^+ = 201.1121$ observed: $[M+H]^+ = 201.1119$ mass accuracy = -1.0 ppm

FT-IR spectrum (neat) of **S171**

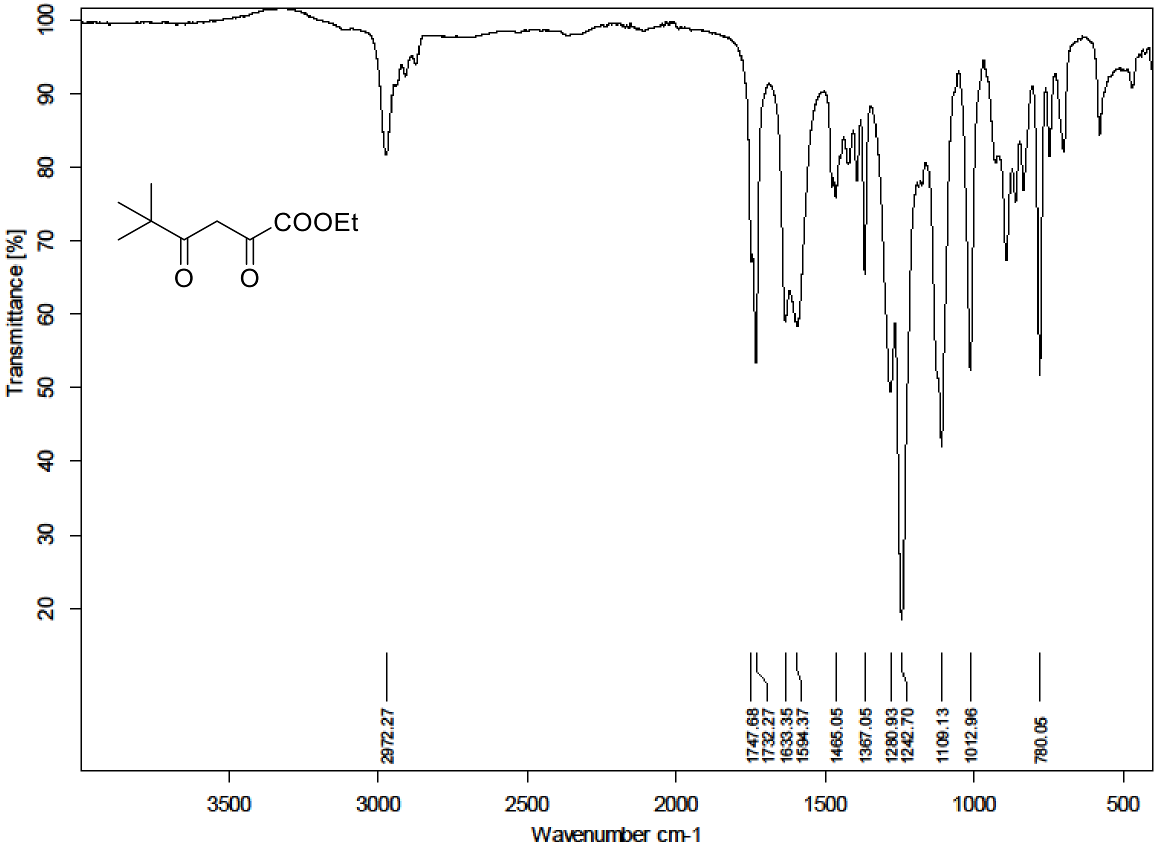

^{1}H (500 MHz) and ^{13}C NMR (126 MHz) spectra of **S172** in Chloroform-*d*

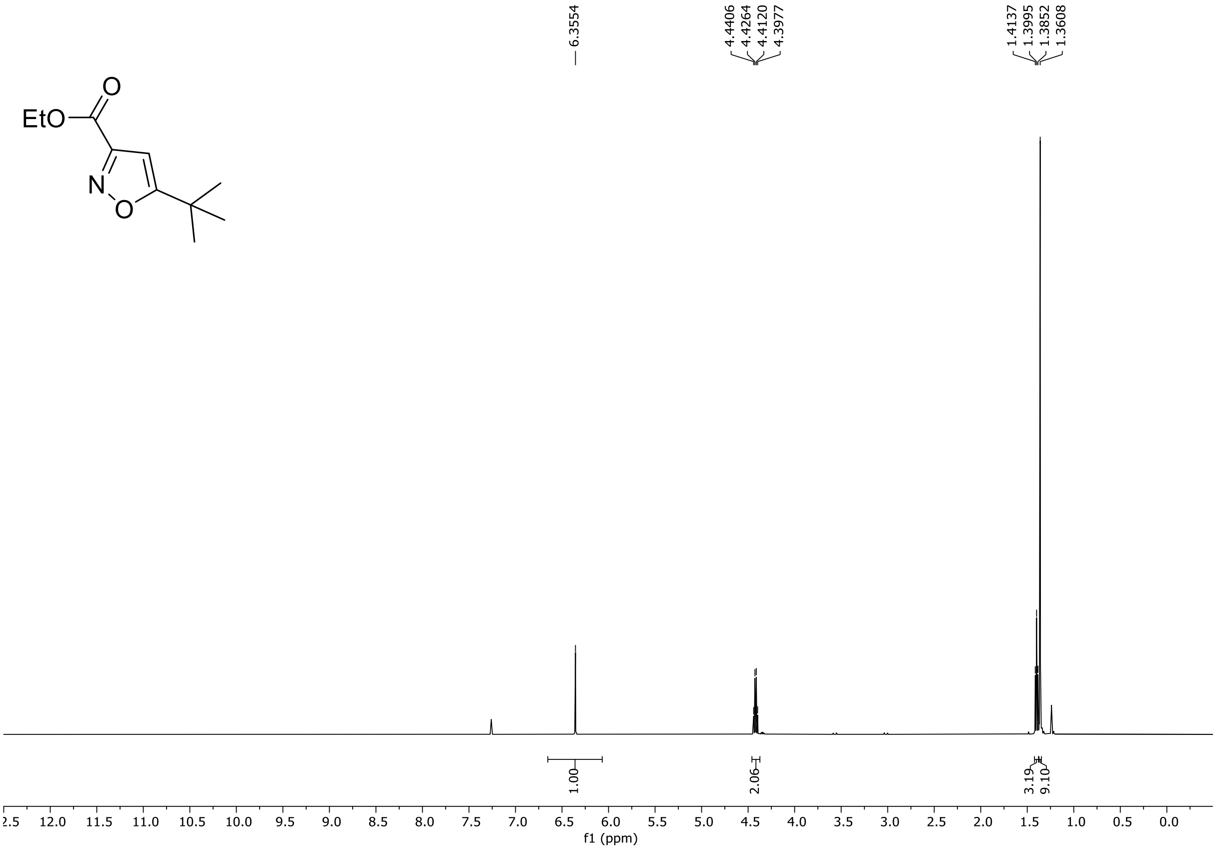

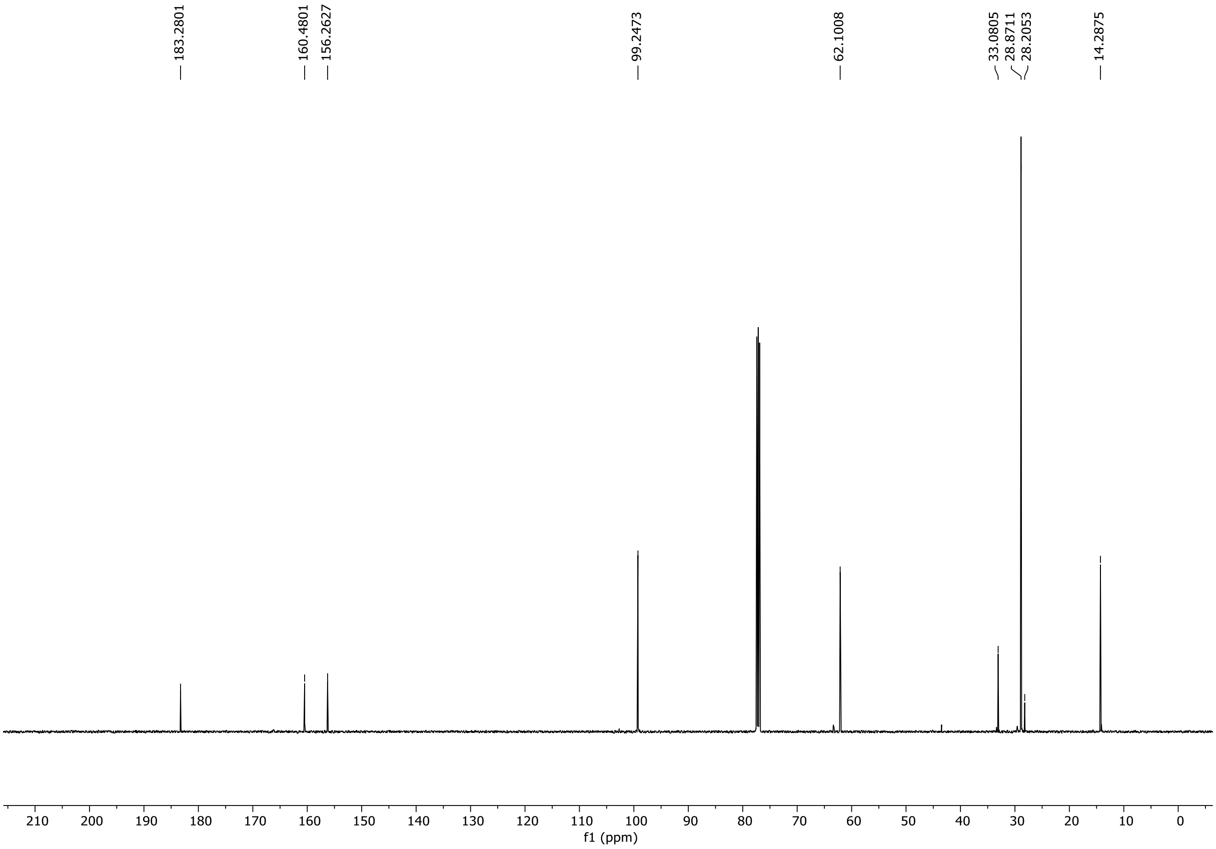

HRMS spectrum of **S172**

NAR-A-24 **$C_{10}H_{15}NO_3$** **mono *m/z 197.1052***

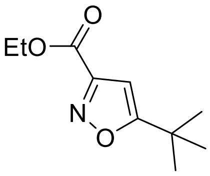

APCI + (MMI)
nitrogen flow 5 L/min, gas temperature 325°C, nebulizer 45 psi, skimmer **65** V, vaporizer 200°C, fragmentor 23 V, dissolved in methanol

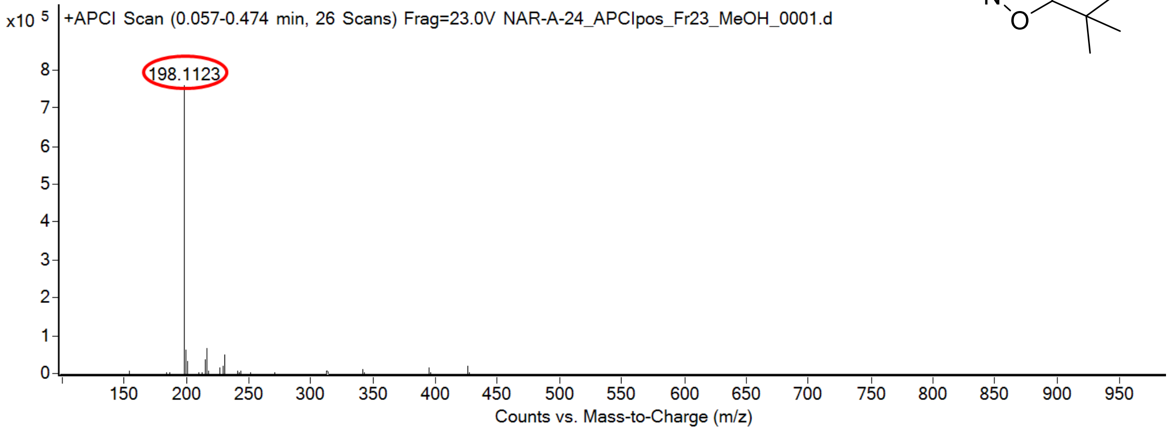

calculated mass: $[M+H]^+$ = 198.1125 observed: $[M+H]^+$ = 198.1123 mass accuracy = -1.0 ppm

FT-IR spectrum (neat) of **S172**

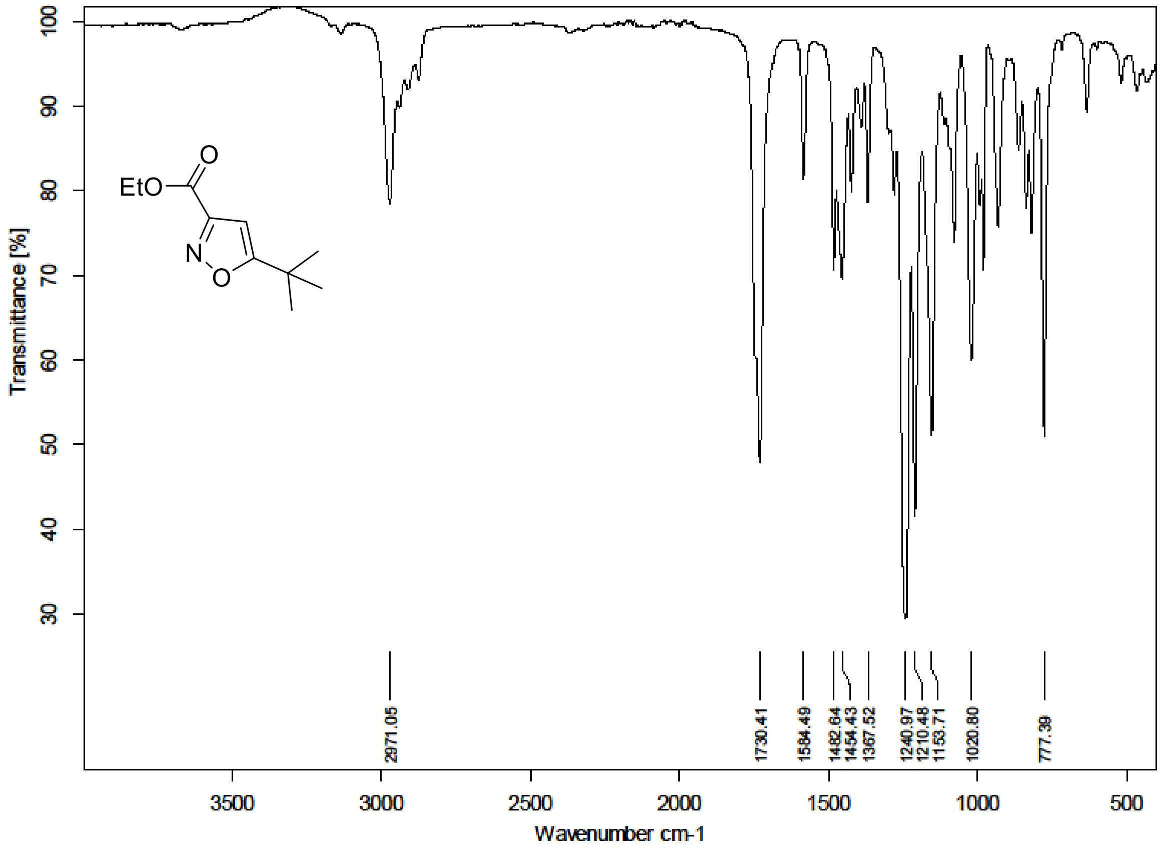

^{1}H (500 MHz) and ^{13}C NMR (126 MHz) spectra of **S173** in Chloroform-*d*

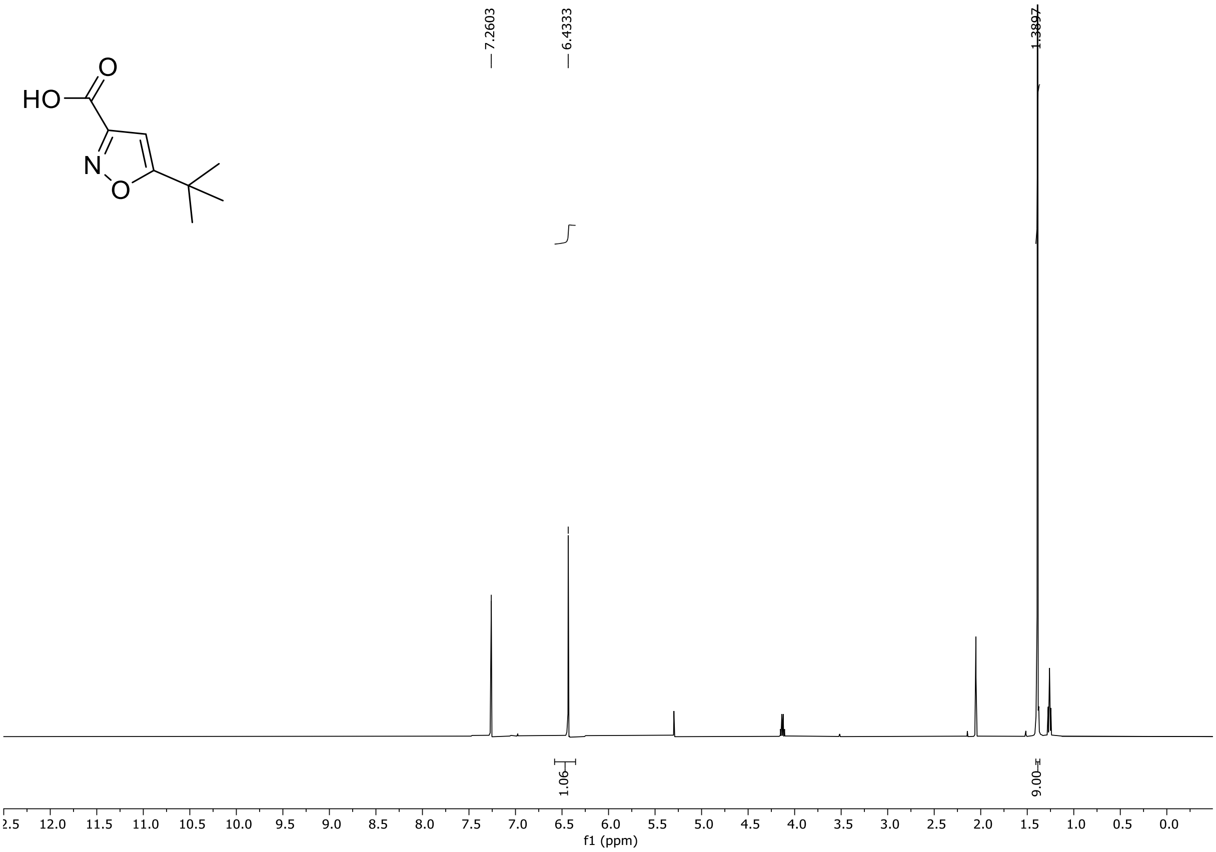

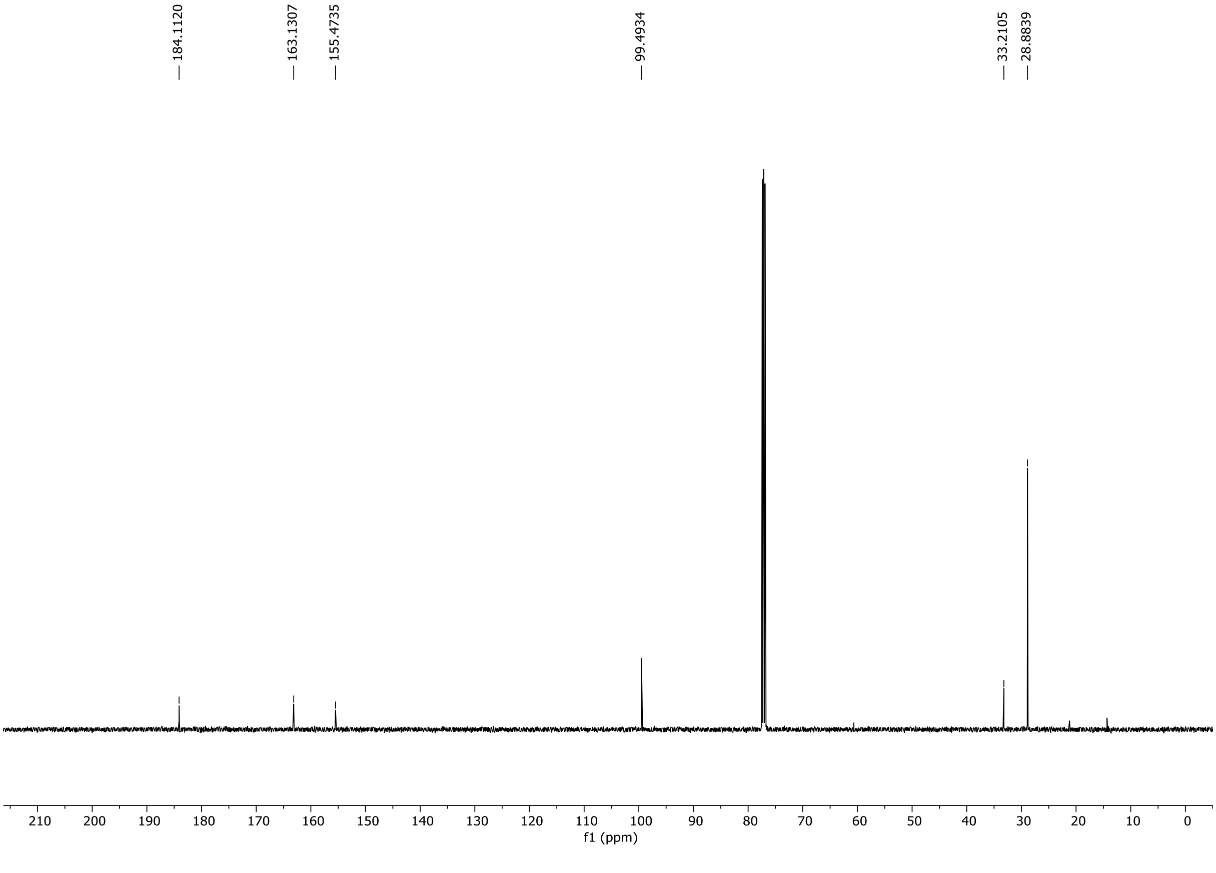

HRMS spectrum of **S173**

NAR-A-29 $C_8H_{11}NO_3$ **mono *m/z 169.0739***

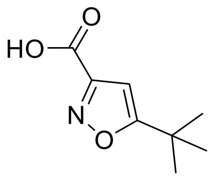

APCI + (MMI)

nitrogen flow 5 L/min, gas temperature 325°C, nebulizer 45 psi, skimmer **65** V, vaporizer 200°C, fragmentor 30 V, dissolved in methanol

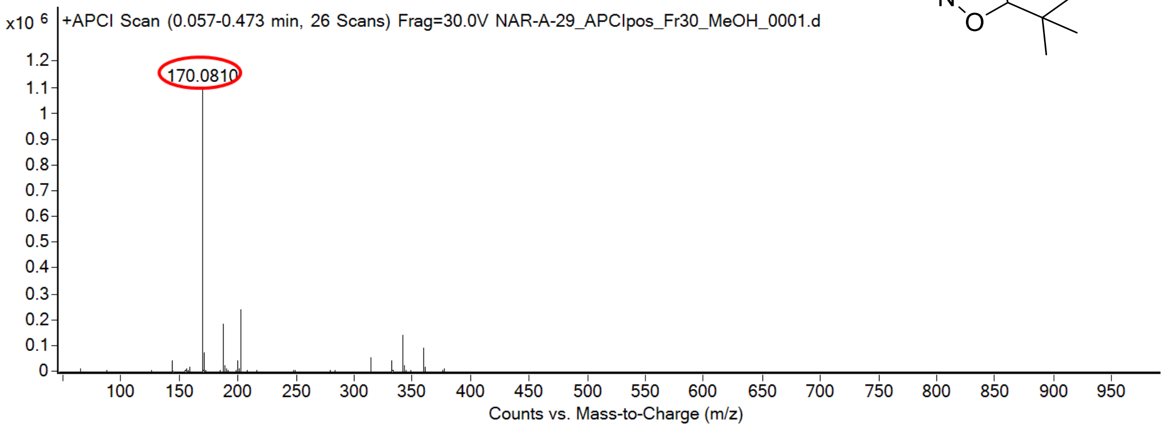

calculated mass: $[M+H]^+ = 170.0812$ observed: $[M+H]^+ = 170.0810$ mass accuracy = -1.2 ppm

FT-IR spectrum (neat) of **S173**

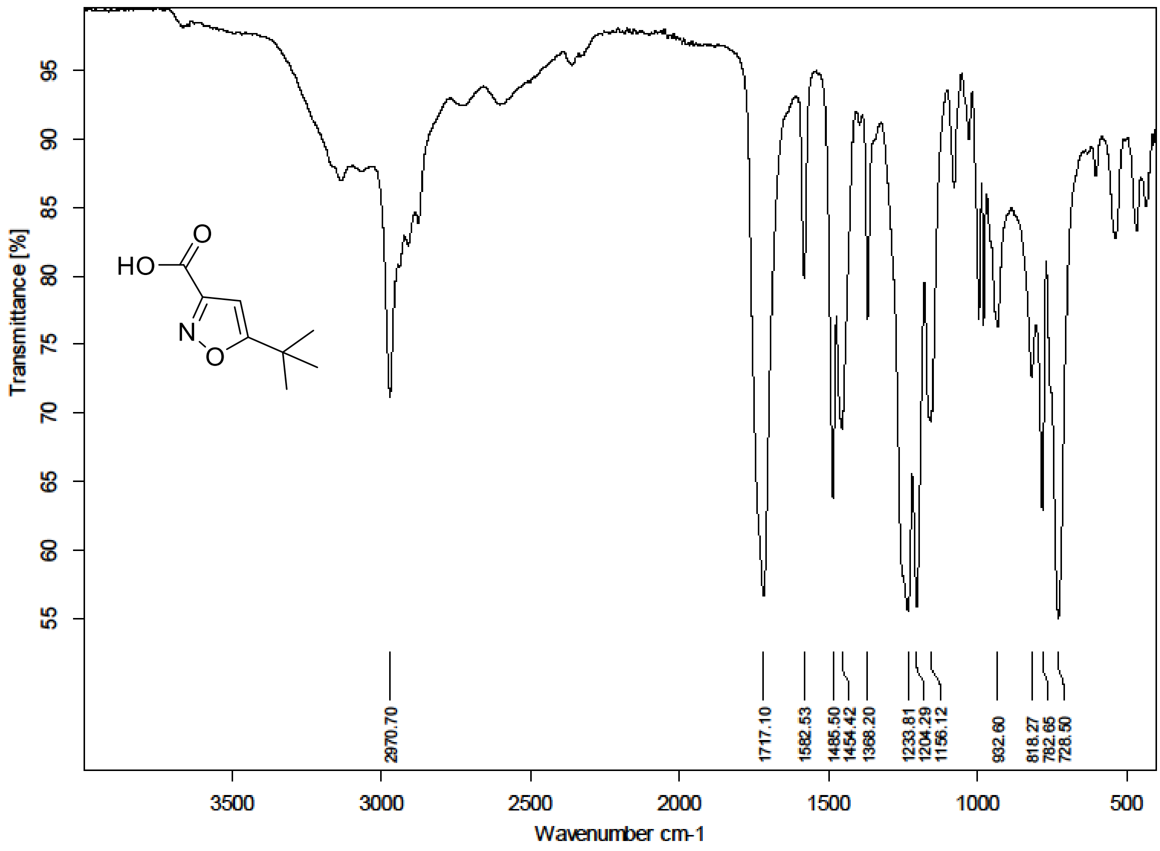

^{1}H (500 MHz) and ^{13}C NMR (126 MHz) spectra of **S174** in Chloroform-*d*

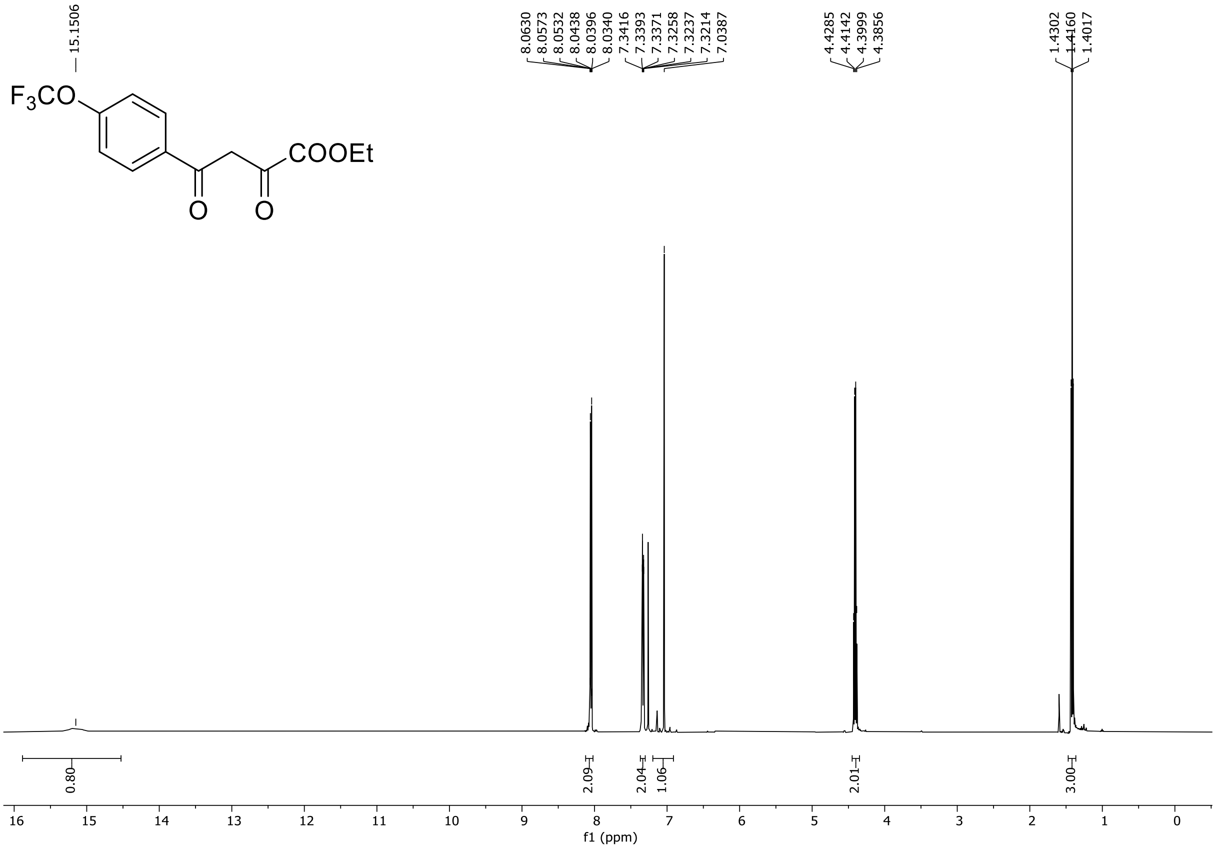

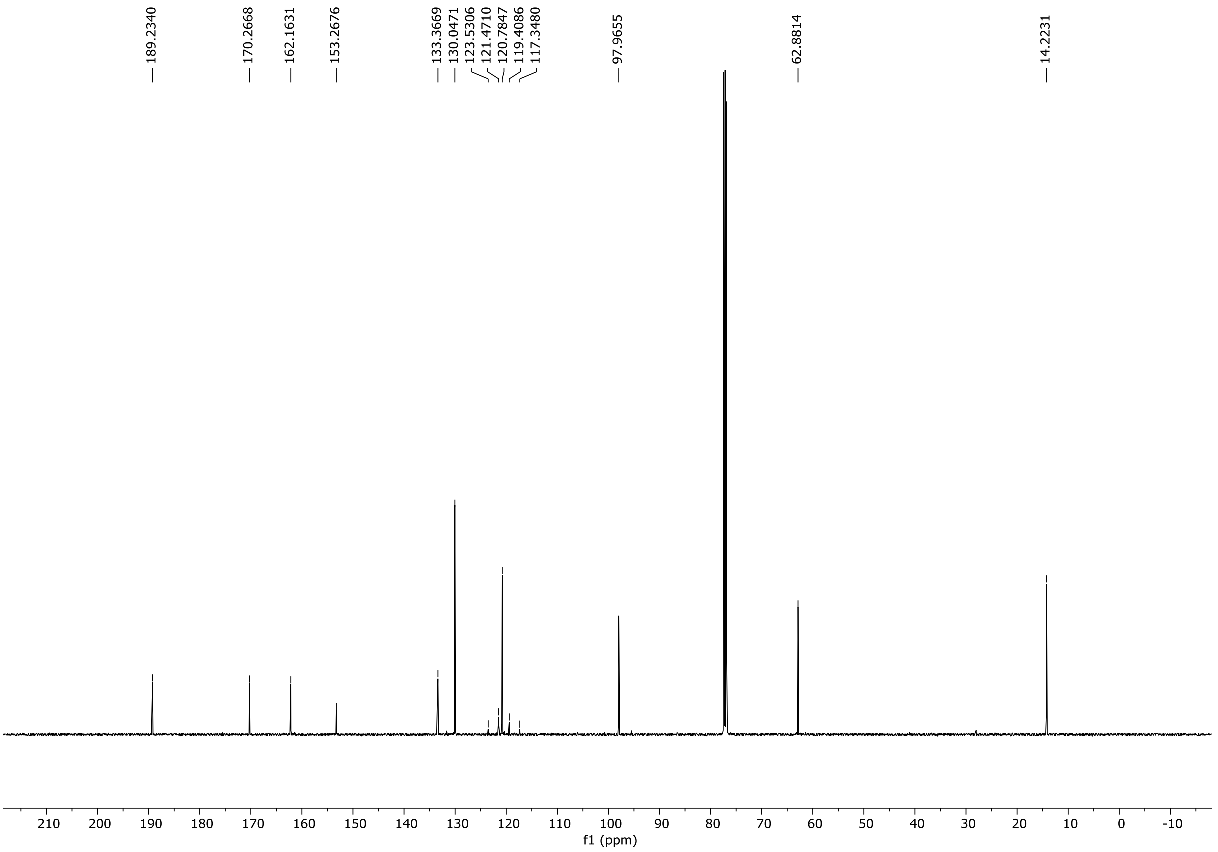

^{19}F NMR (282 MHz) spectrum of **S174** in Chloroform-*d*

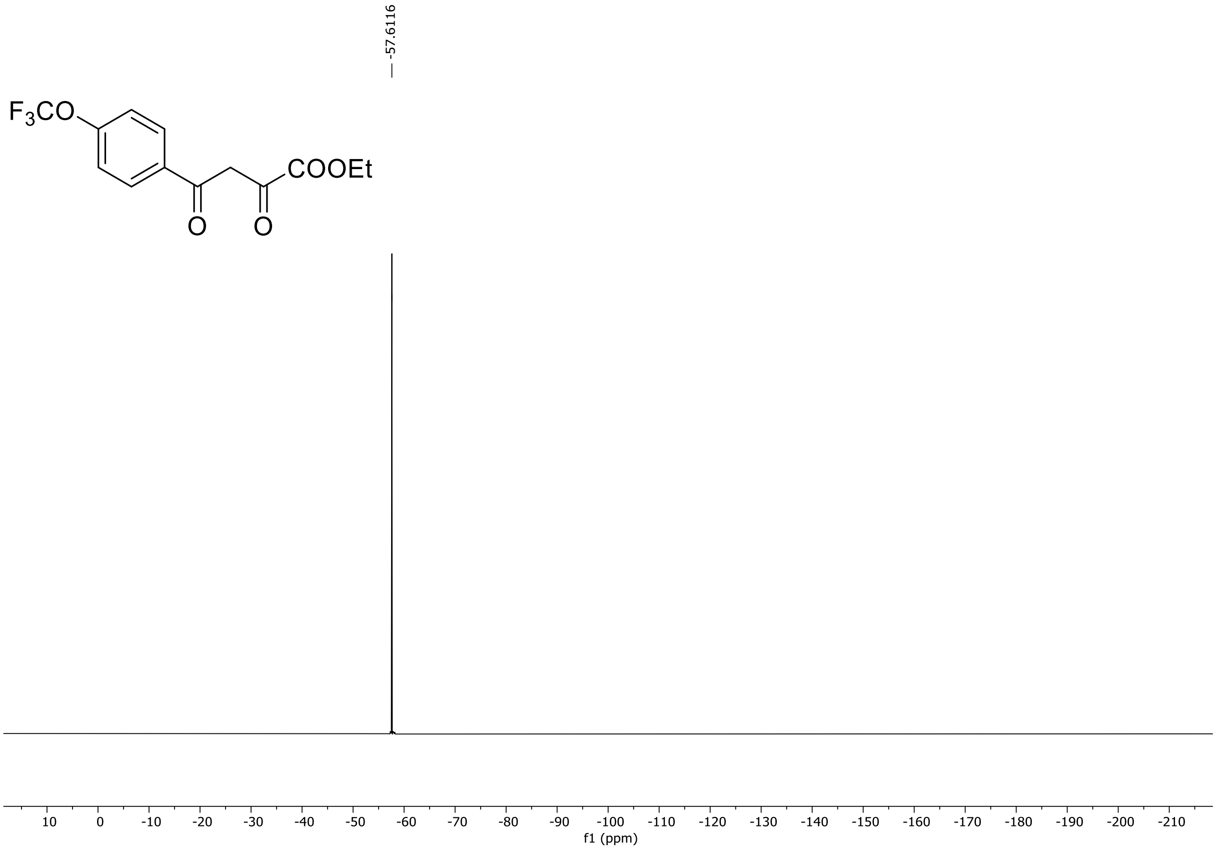

HRMS spectrum of **S174**

NAR-A-49

$C_{13}H_{11}F_3O_5$ *mono m/z*
304.0559

APCI + (MMI)
nitrogen flow 5 L/min, gas temperature 300°C, nebulizer 45 psi, skimmer **65** V,
vaporizer 200°C, fragmentor 20 V, dissolved in MeOH

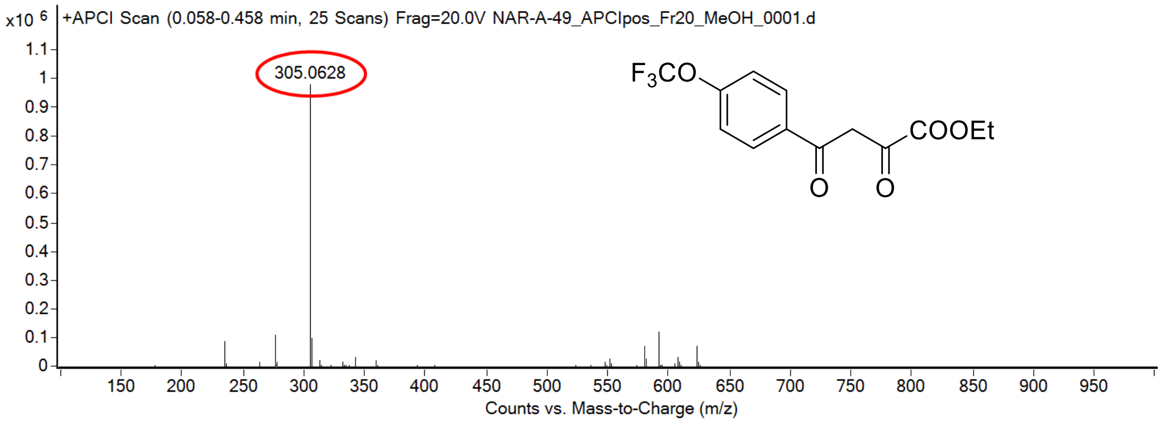

calculated mass: $[M+H]^+$ = 305.0631 observed: $[M+H]^+$ = 305.0628 mass accuracy = -1.0 ppm

FT-IR spectrum (neat) of **S174**

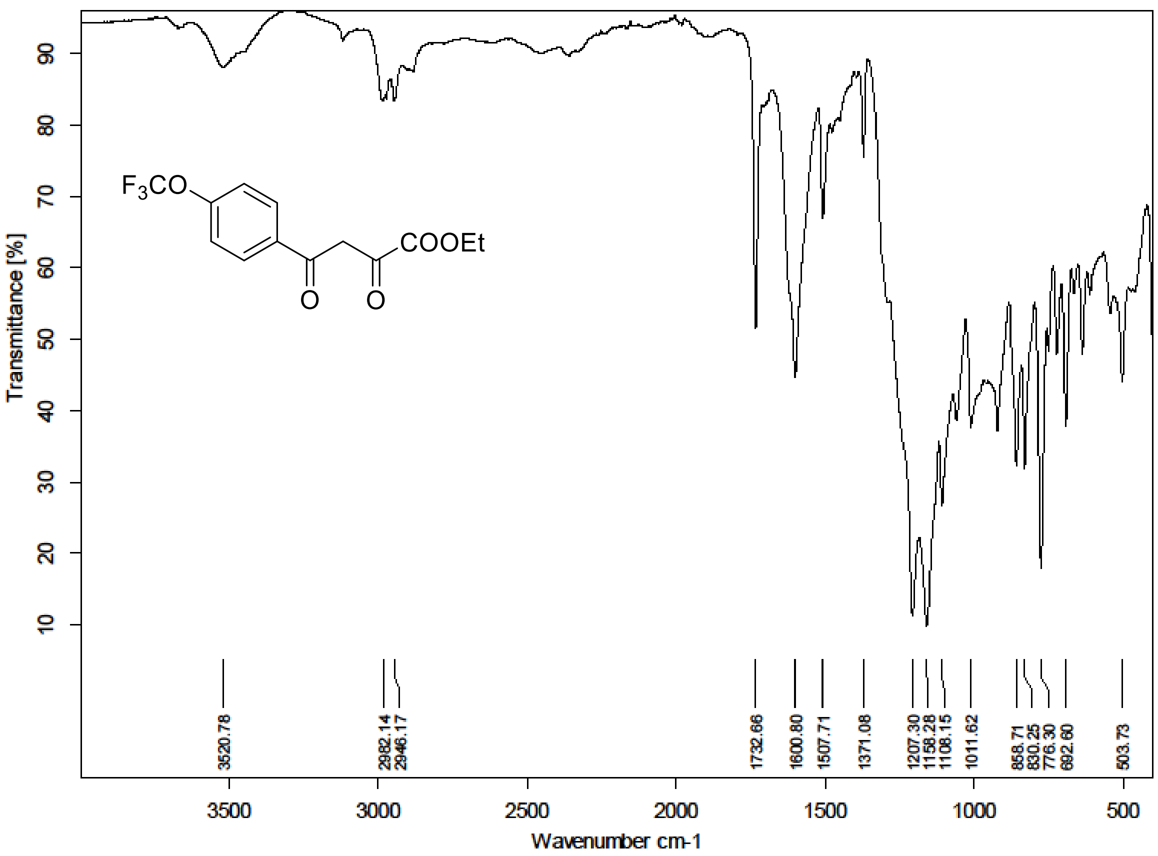

^{1}H (500 MHz) and ^{13}C NMR (126 MHz) spectra of **S175** in Chloroform-*d*

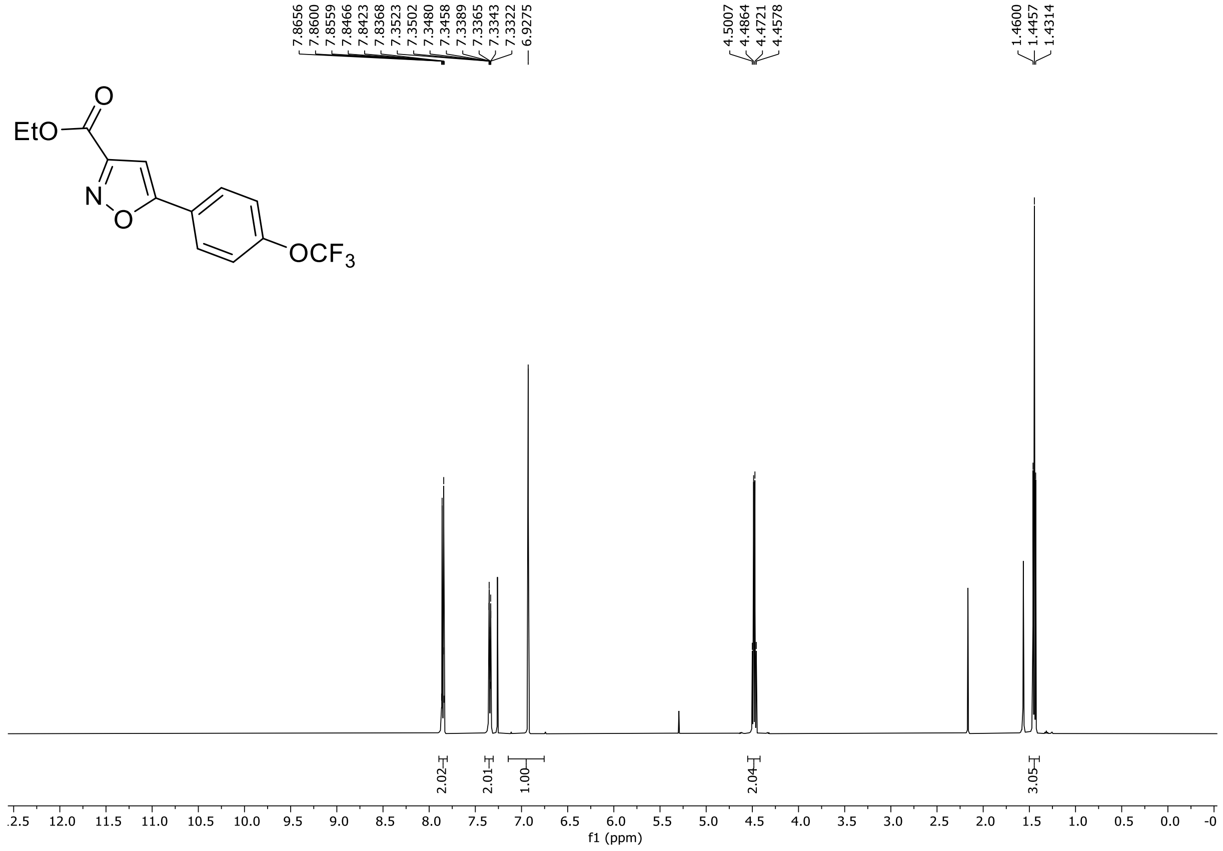

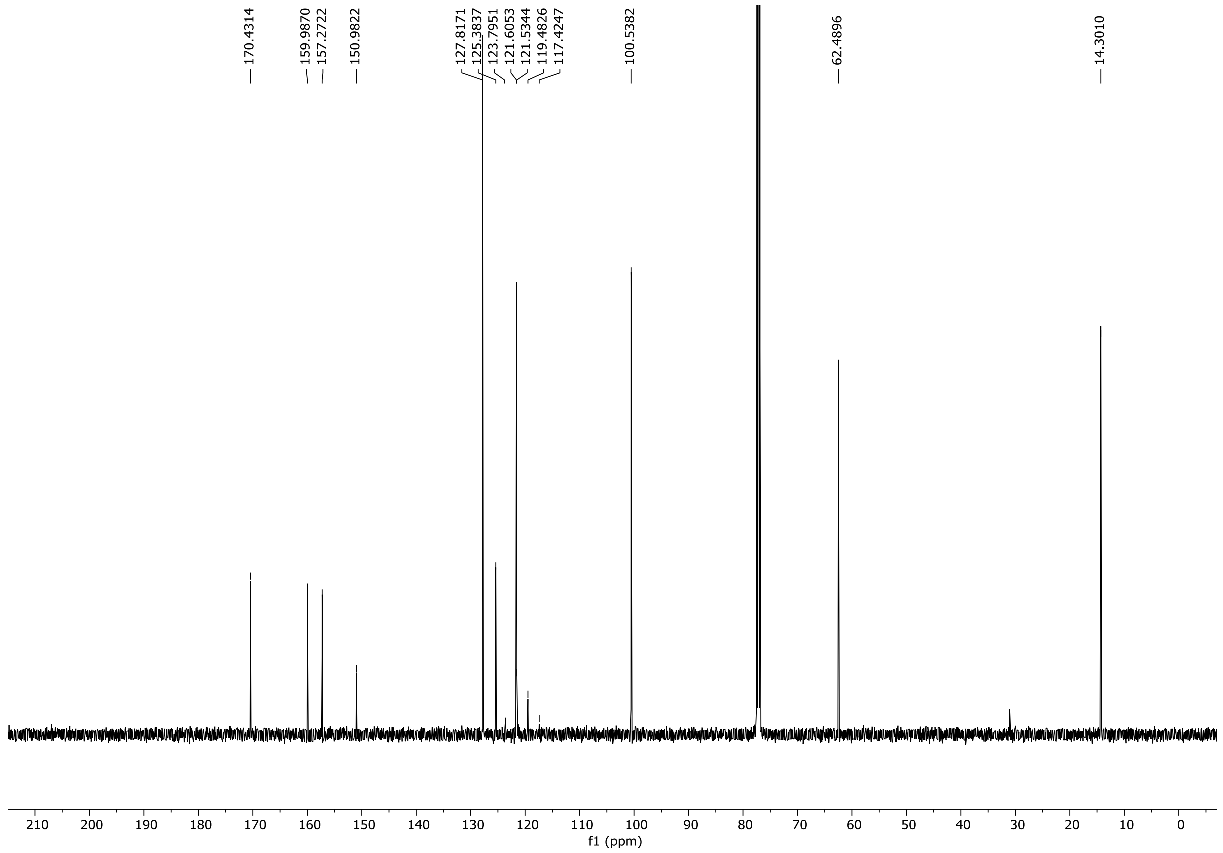

^{19}F NMR (471 MHz) spectrum of **S175** in Chloroform-*d*

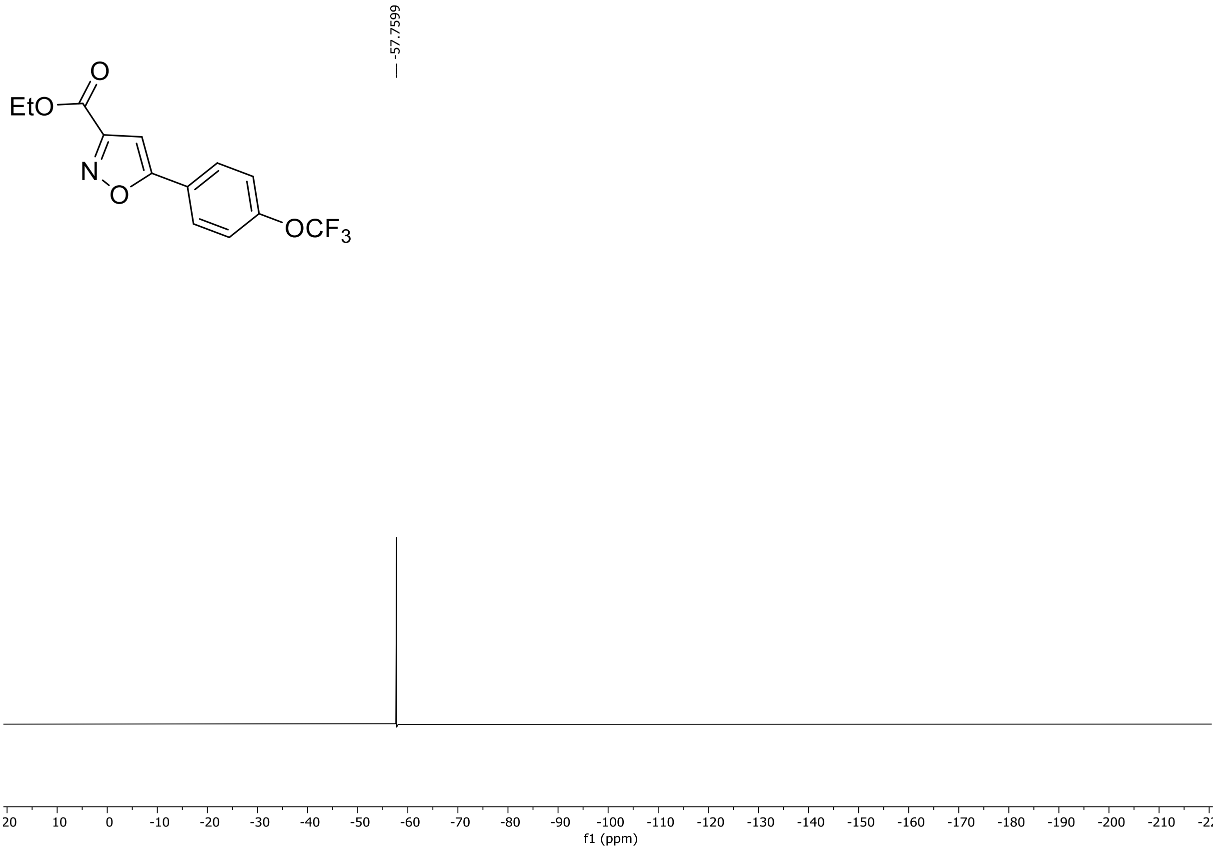

HRMS spectrum of **S175**

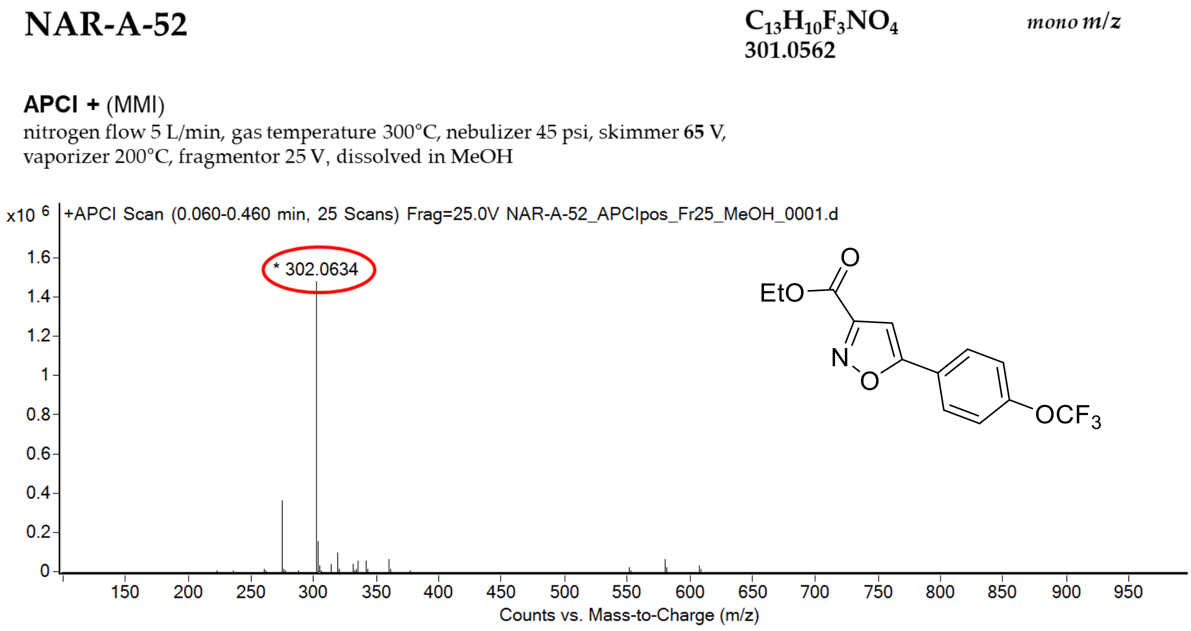

calculated mass: $[M+H]^+$ = 302.0635 observed: $[M+H]^+$ = 302.0634 mass accuracy = -0.3 ppm

FT-IR spectrum (neat) of **S175**

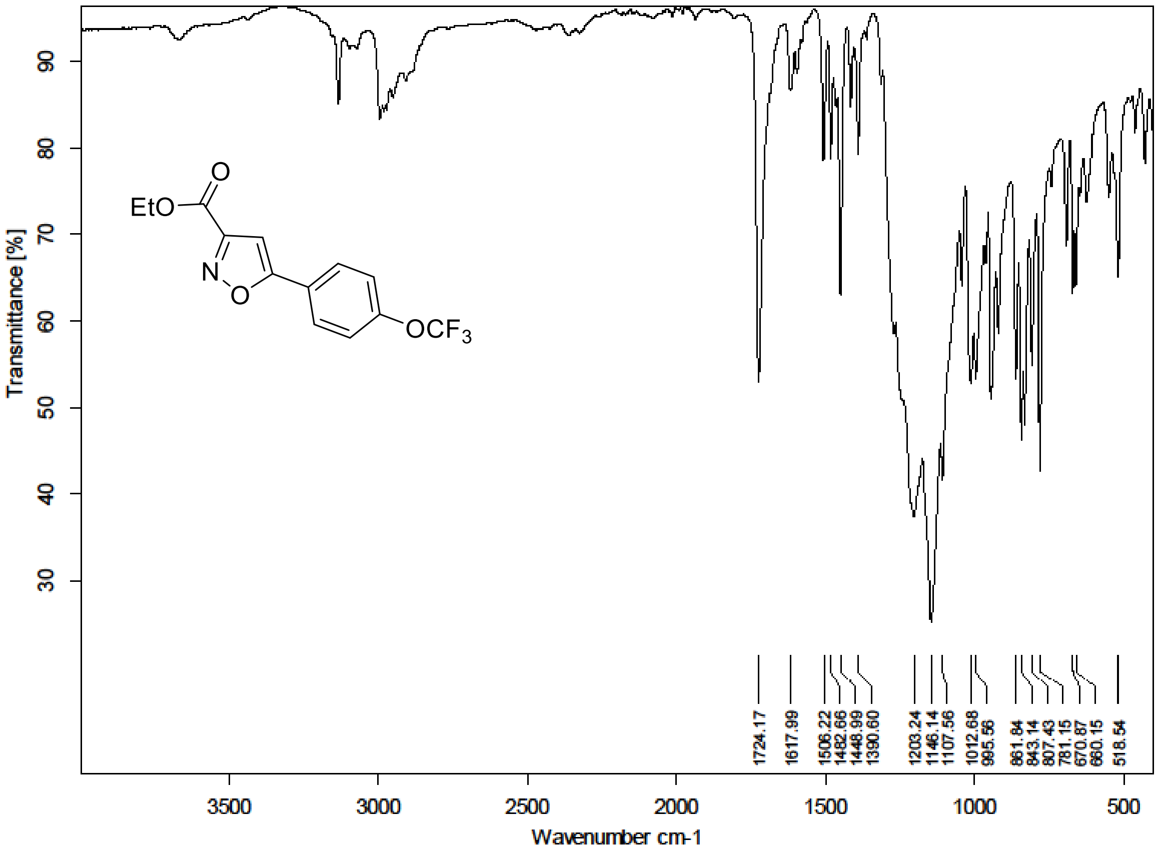

^{1}H (500 MHz) and ^{13}C NMR (126 MHz) spectra of **S176** in DMSO-d_6

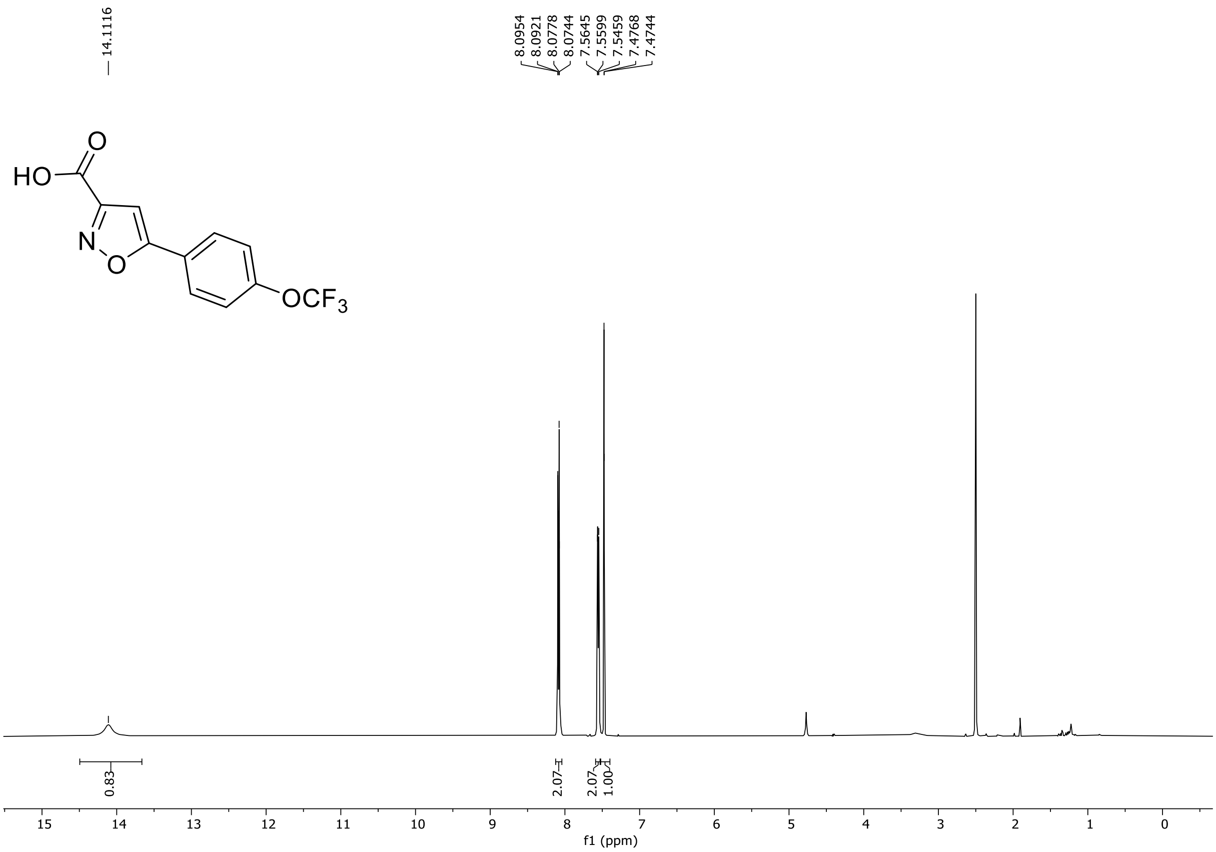

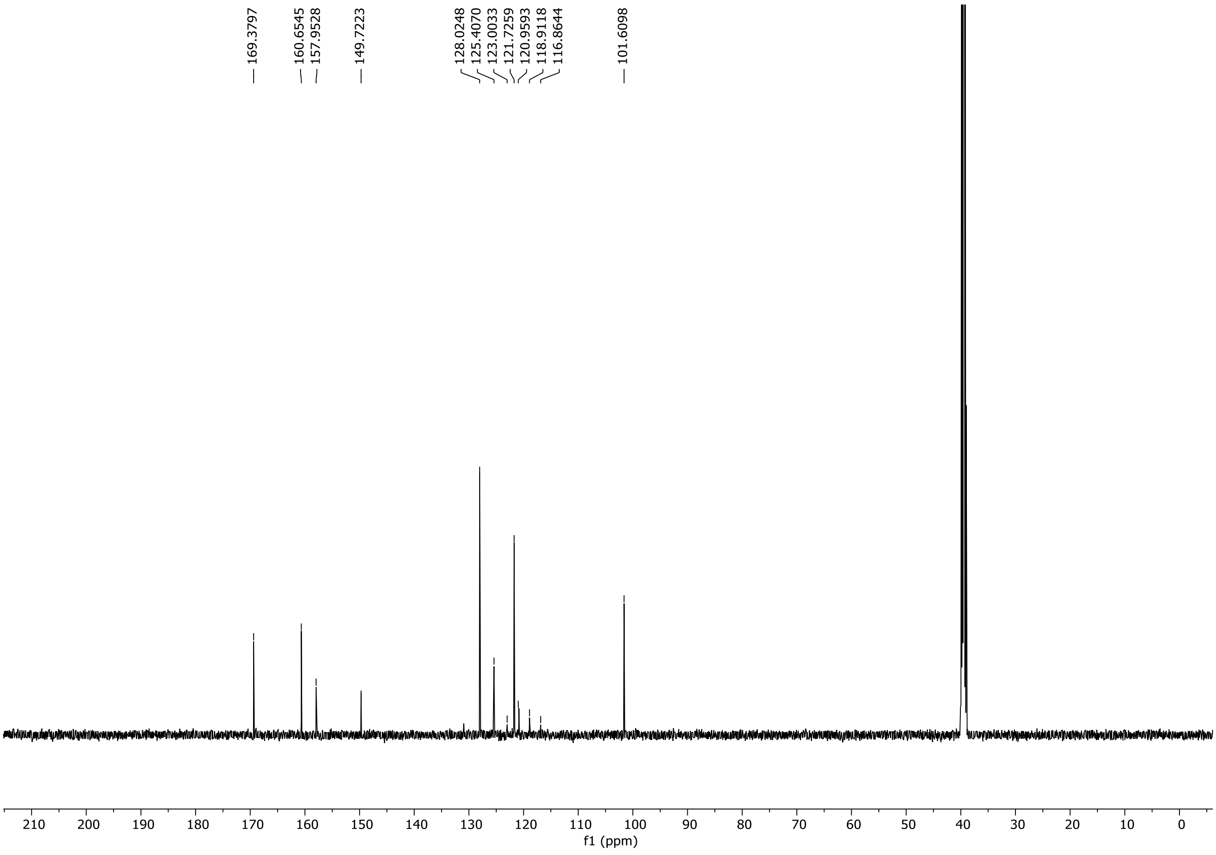

^{19}F NMR (471 MHz) spectrum of **S176** in DMSO-d_6

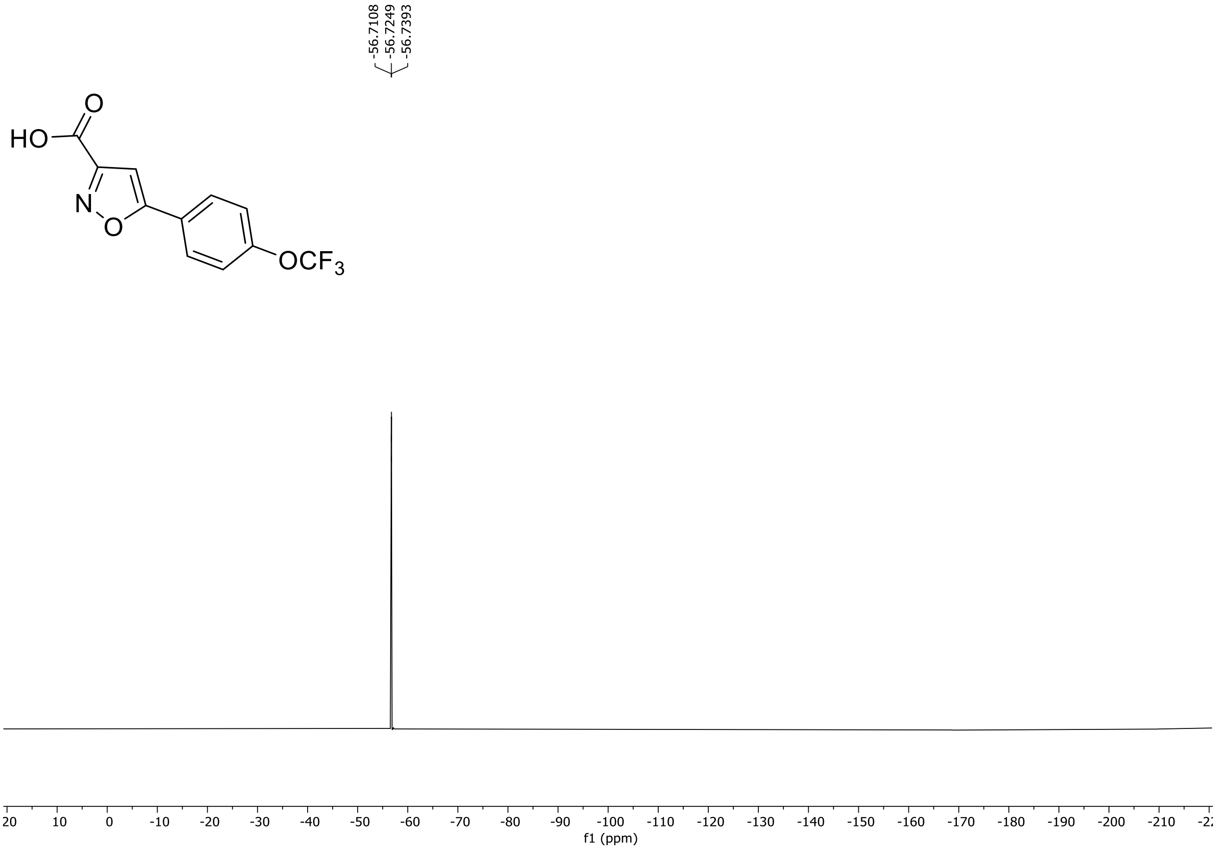

HRMS spectrum of **S176**

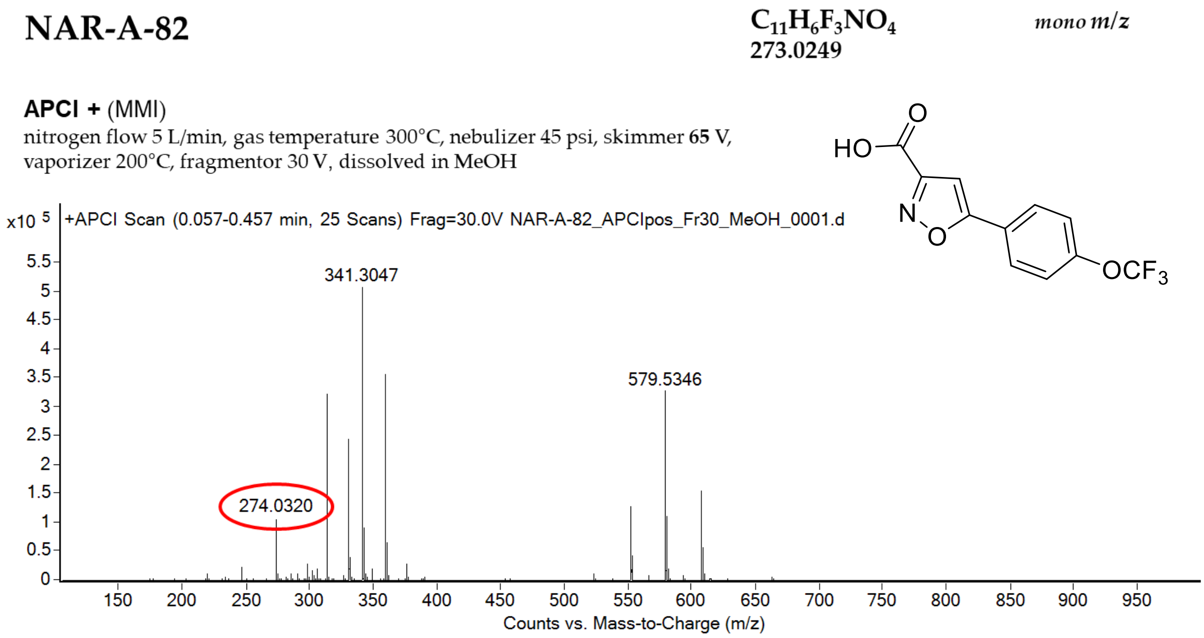

calculated mass: [M+H]$^+$ = 274.0322 observed: [M+H]$^+$ = 274.0320 mass accuracy = -0.7 ppm

FT-IR spectrum (neat) of **S176**

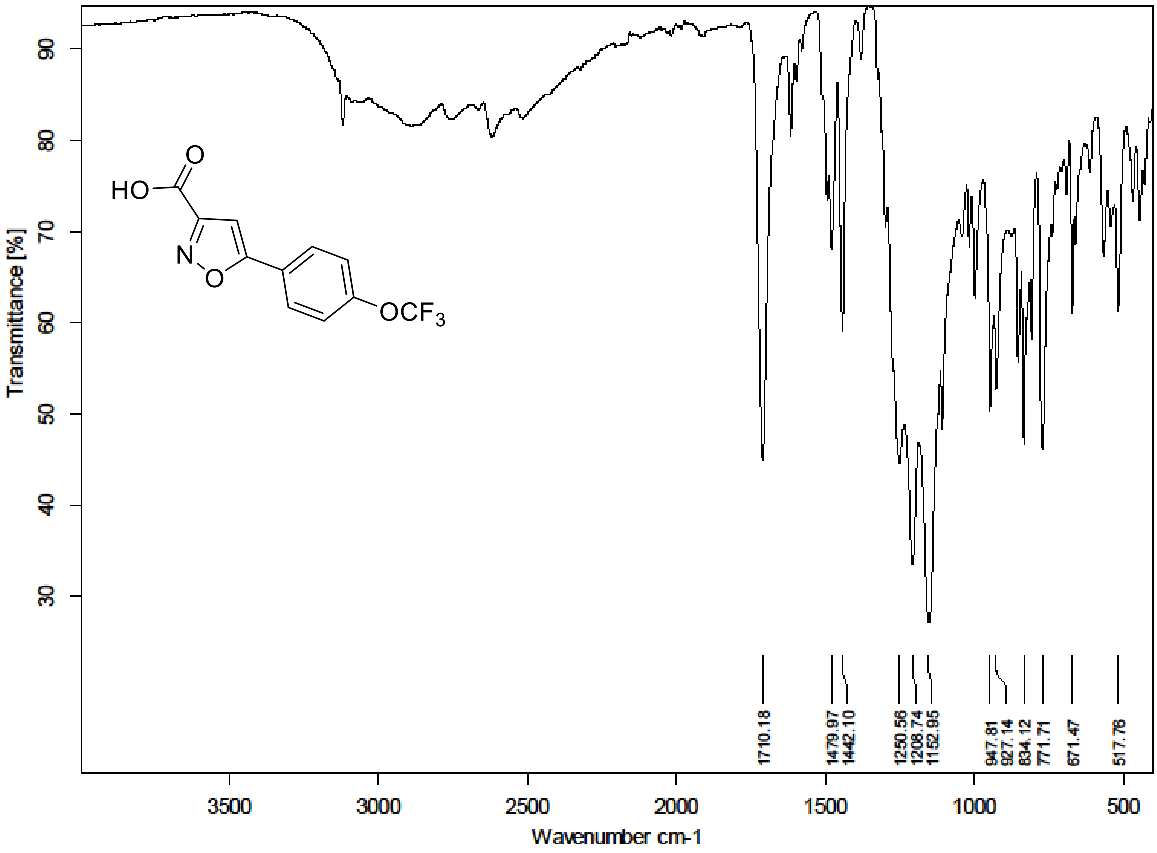

^{1}H (300 MHz) and ^{13}C NMR (75 MHz) spectra of **S185** in DMSO-d_6

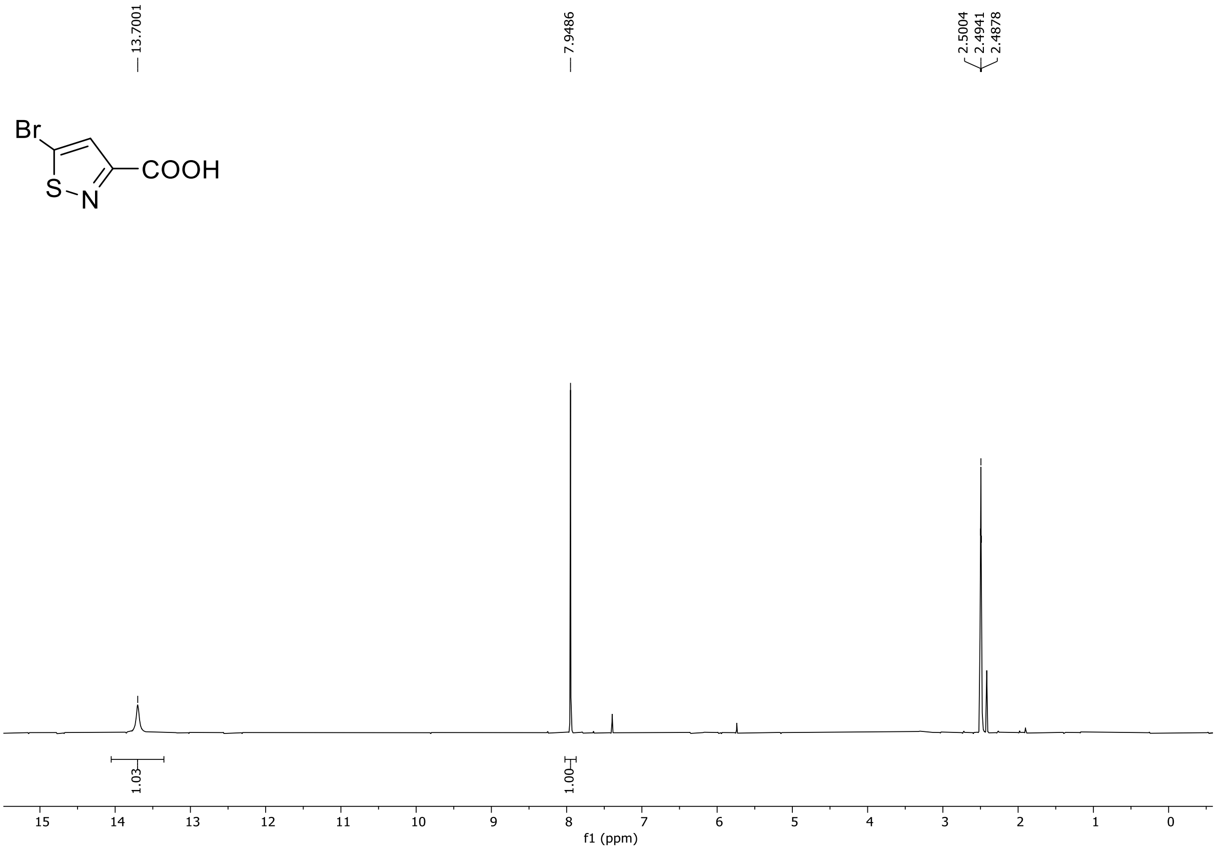

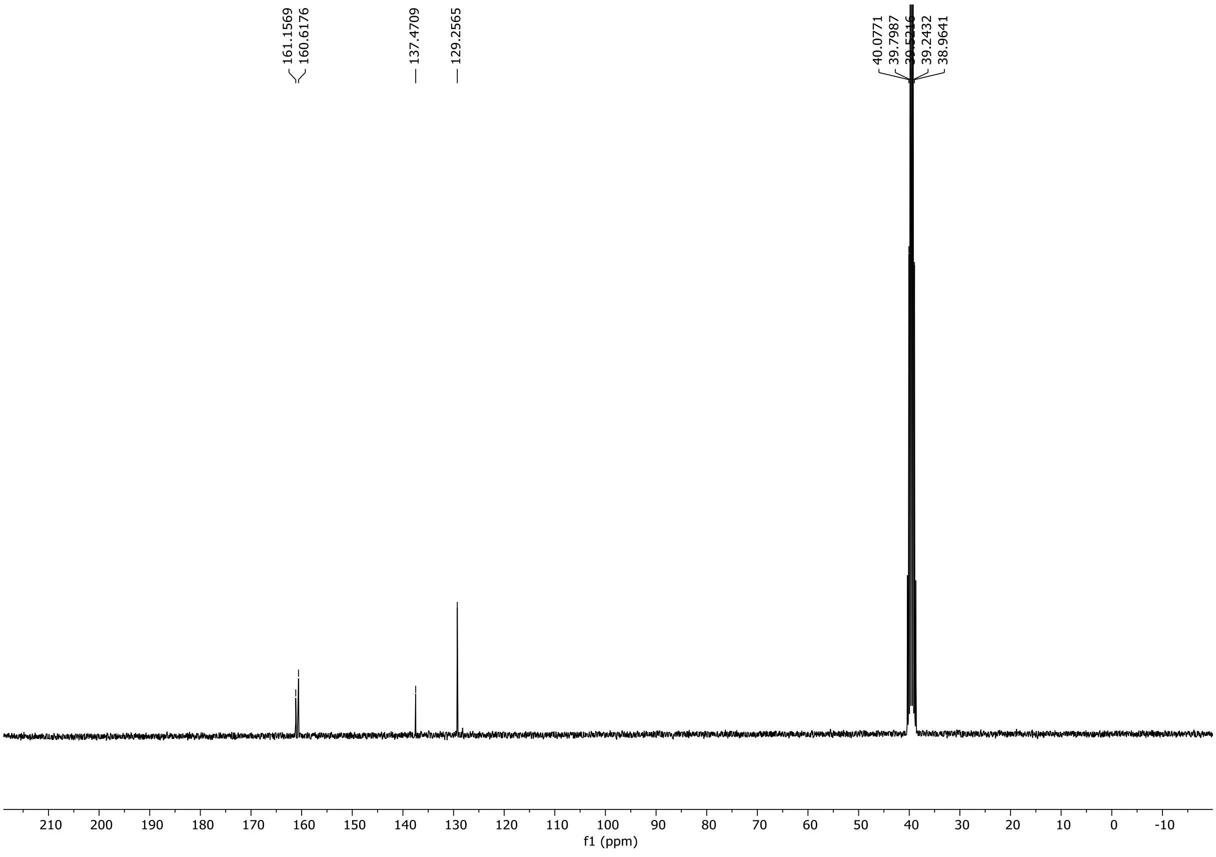

HRMS spectrum of **S185**

NAR-A-136 $C_4H_2BrNO_2S$ $m/z_{100\%}$ **208.8969**

APCI- (MMI)
nitrogen flow 5 L/min, gas temperature 325°C, nebulizer 45 psi, skimmer 65 V, vaporizer 200°C, fragmentor 90 V, dissolved in MeOH

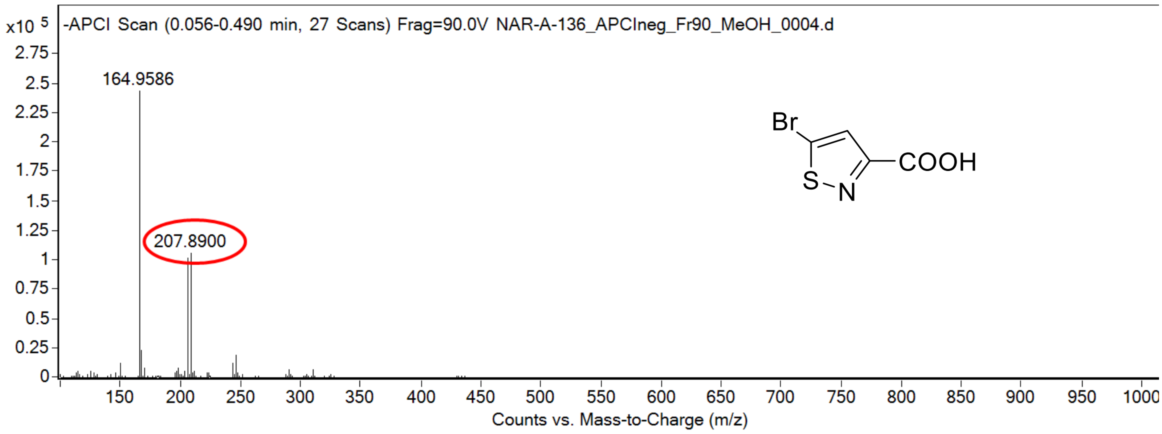

calculated mass: [M-H]- = 207.8896 observed: [M-H]- = 207.8900 mass accuracy = -1.9 ppm

FT-IR spectrum (neat) of **S185**

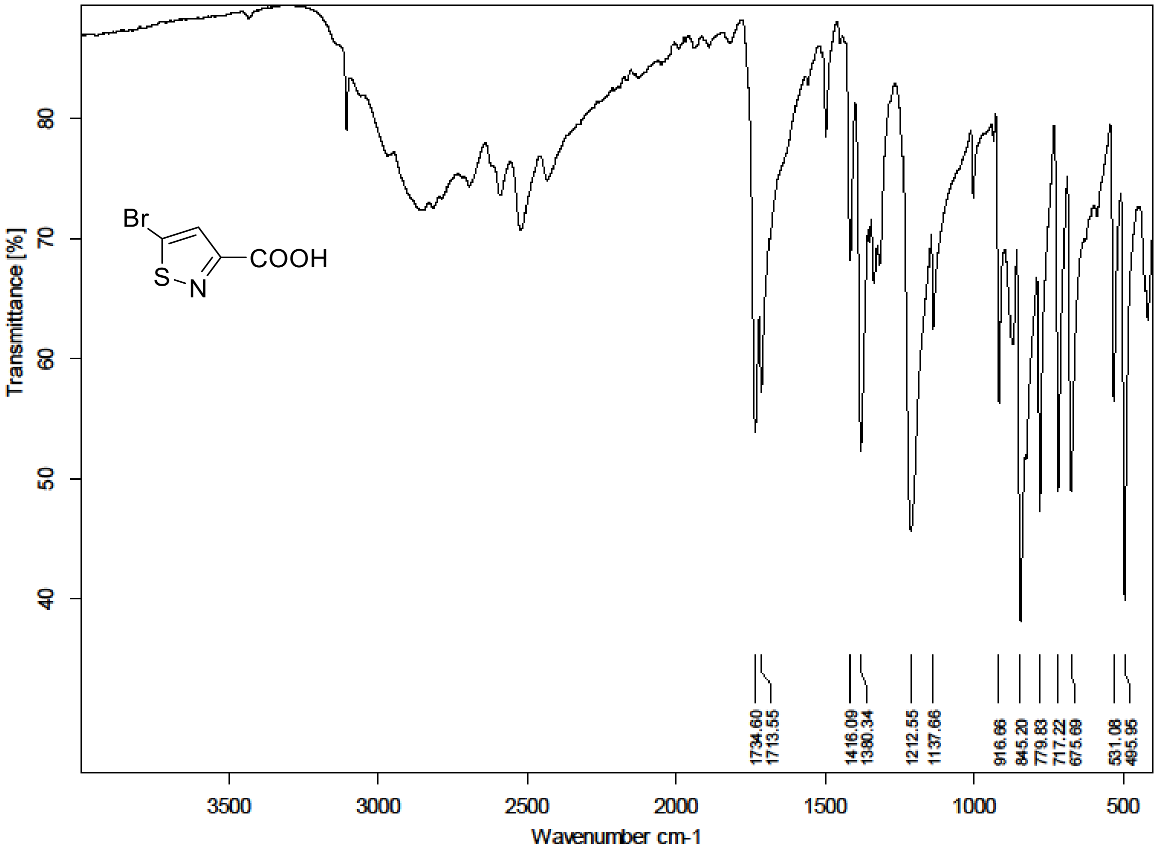

^{1}H (300 MHz) and ^{13}C NMR (75 MHz) spectra of **S186** in Chloroform-*d*

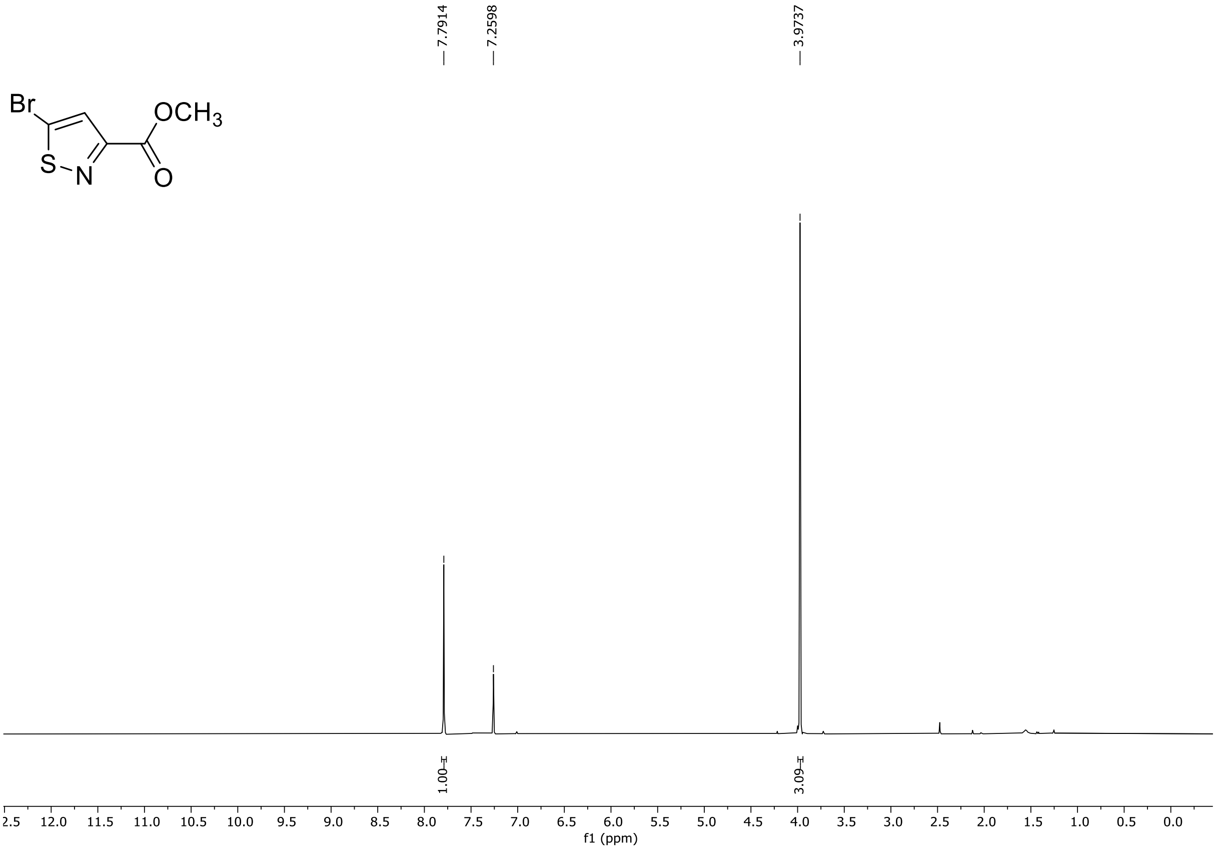

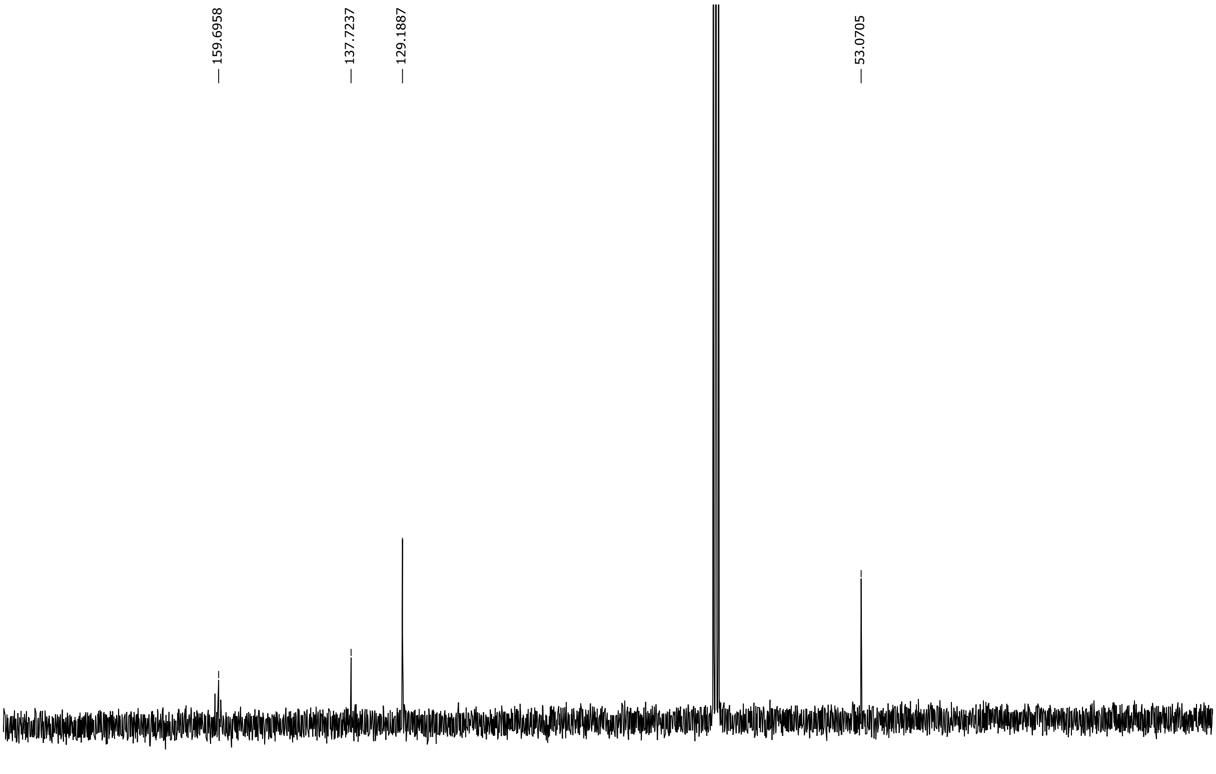

HRMS spectrum of **S186**

NAR-A-144 $C_5H_4BrNO_2S$ $m/z_{100\%}$ **222.9125**

APCI+ (MMI)
nitrogen flow 5 L/min, gas temperature 325°C, nebulizer 45 psi, skimmer 65 V, vaporizer 200°C, fragmentor 35 V, dissolved in MeOH

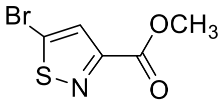

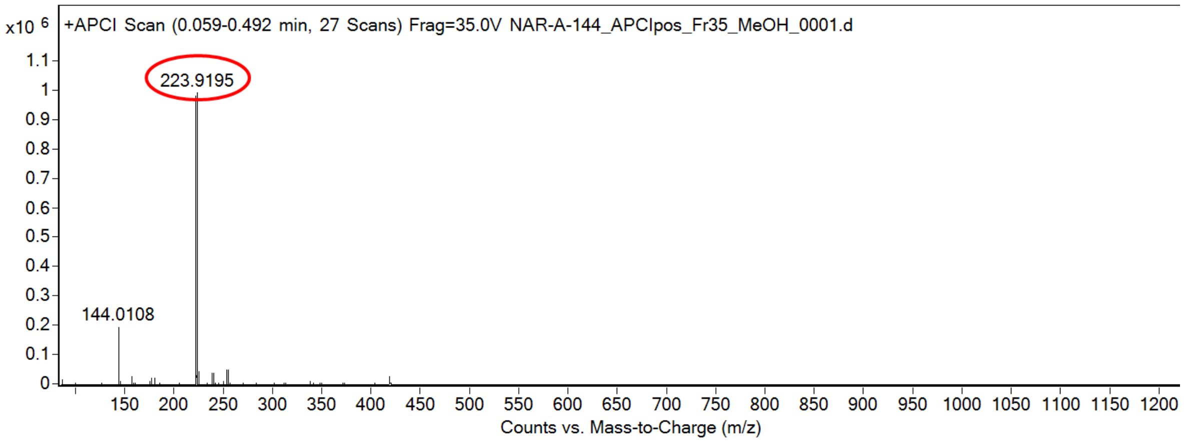

calculated mass: $[M+H]^+ = 223.9198$ observed: $[M+H]^+ = 223.9195$ mass accuracy = -1.3 ppm

FT-IR spectrum (neat) of **S186**

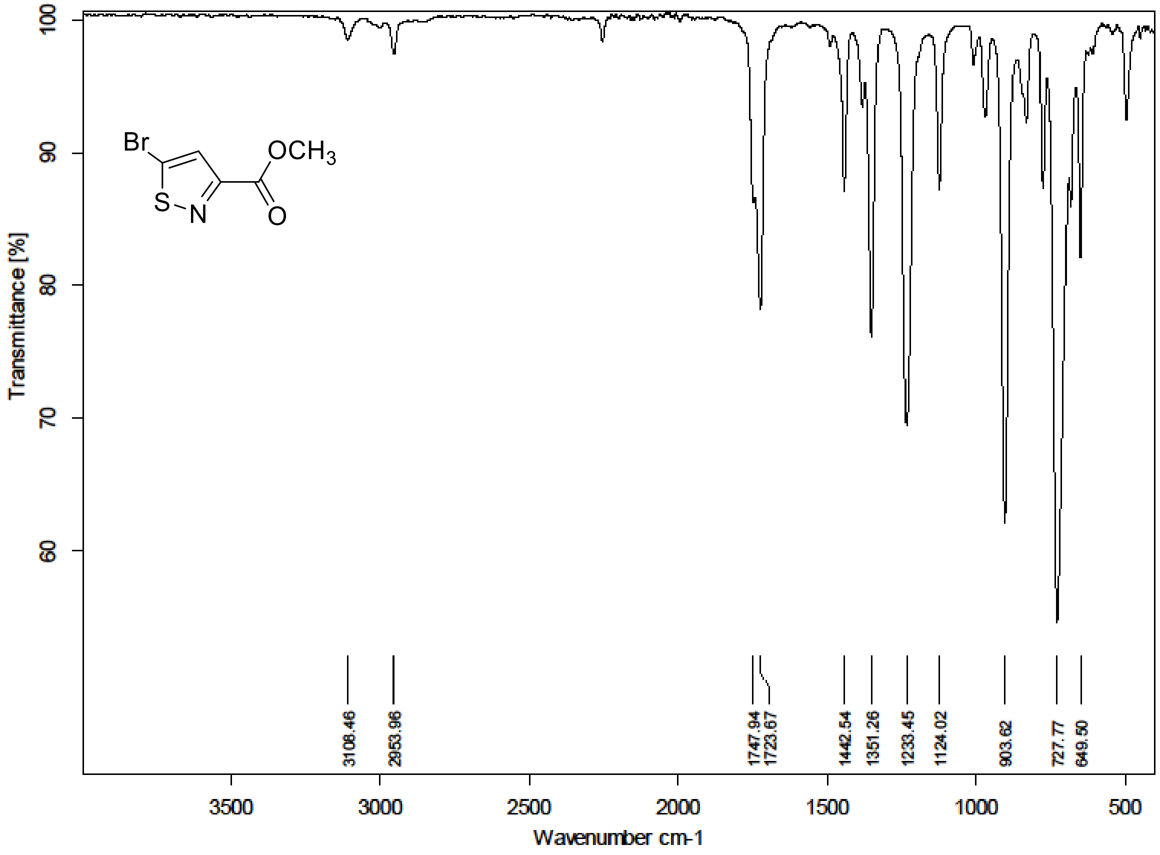

^{1}H (300 MHz) and ^{13}C NMR (75 MHz) spectra of **S187** in Chloroform-*d*

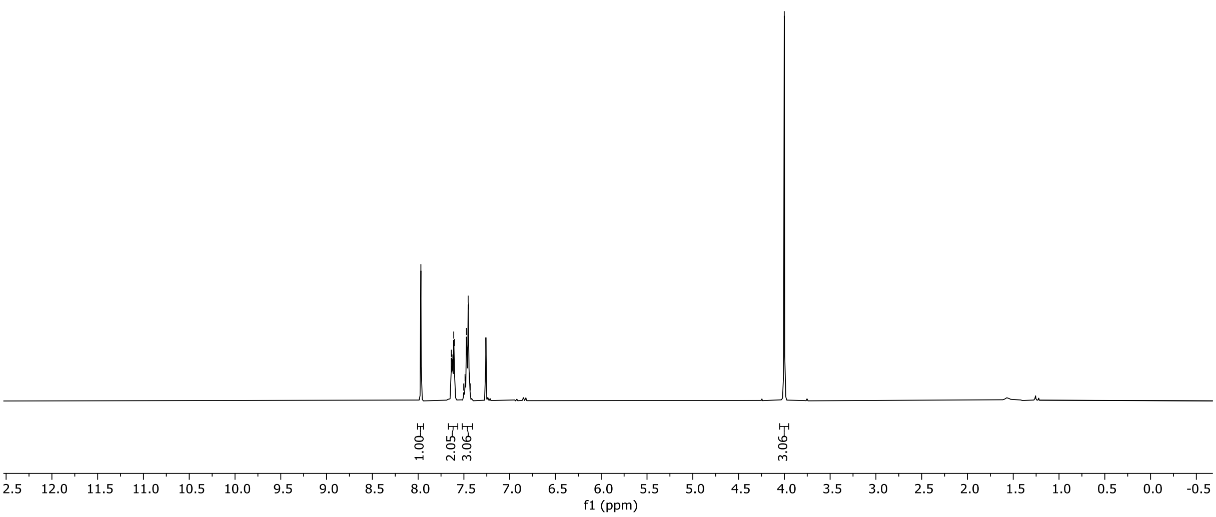

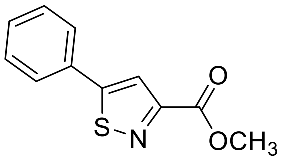

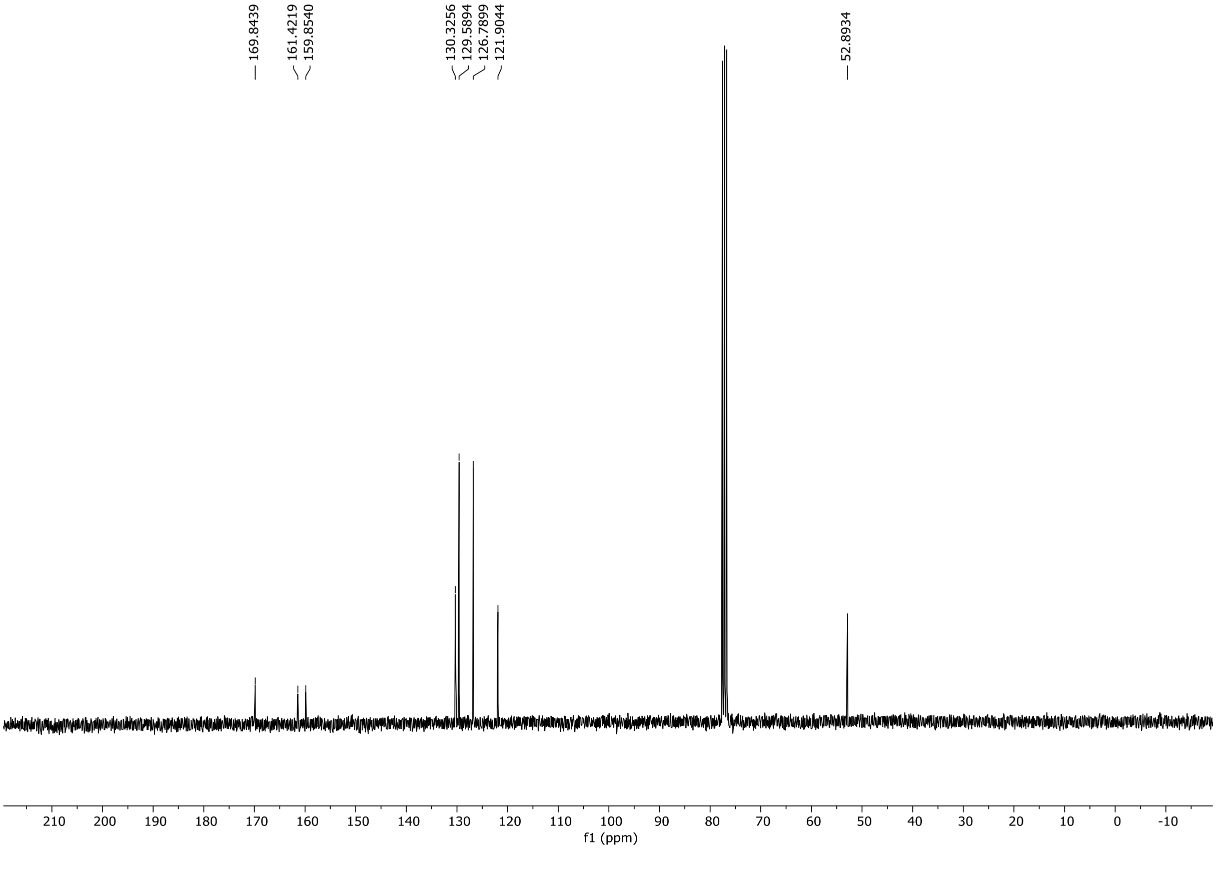

HRMS spectrum of **S187**

NAR-A-145 $C_{11}H_9NO_2S$ *m/z* **219.0354**

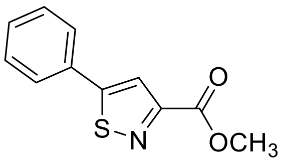

APCI+ (MMI)
nitrogen flow 5 L/min, gas temperature 325°C, nebulizer 45 psi, skimmer 65 V, vaporizer 200°C, fragmentor 25 V, dissolved in MeOH

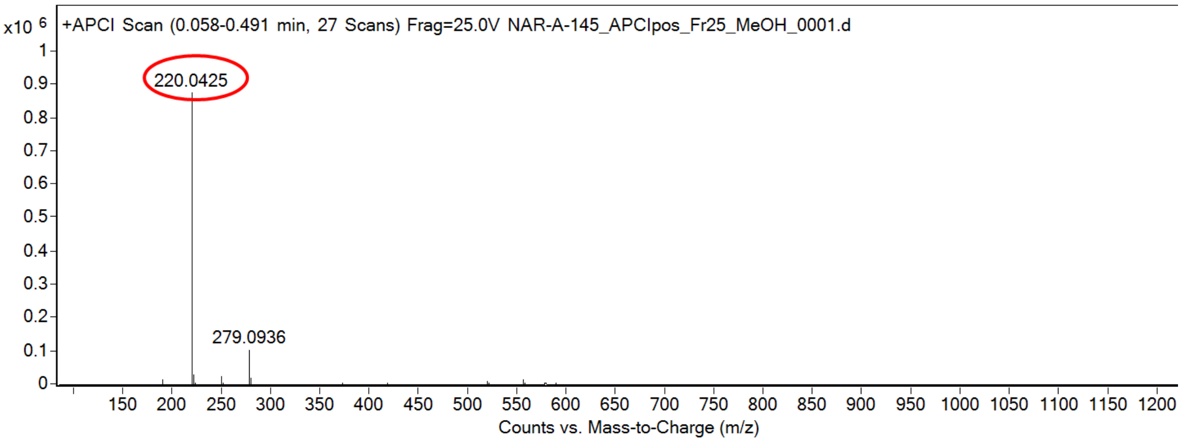

calculated mass: $[M+H]^+$ = 220.0427 observed: $[M+H]^+$ = 220.0425 mass accuracy = -0.9 ppm

FT-IR spectrum (neat) of **S187**

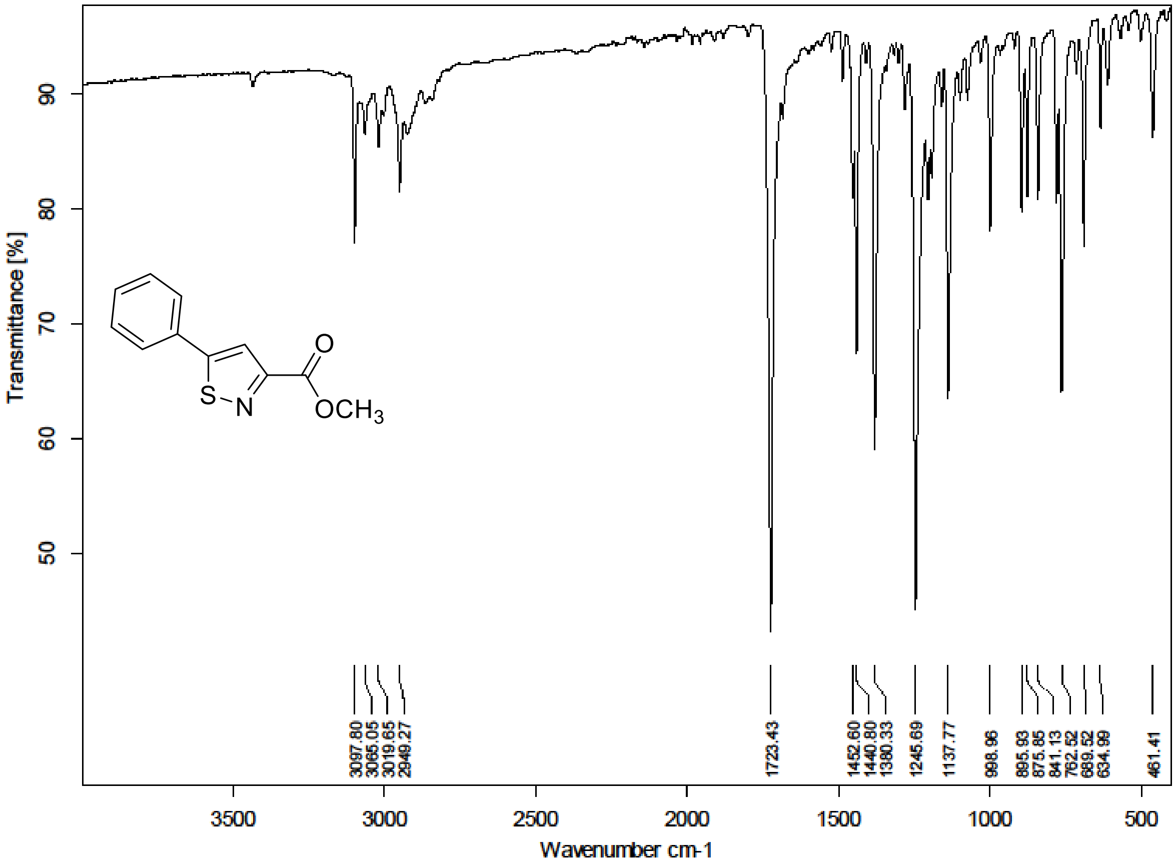

^{1}H (300 MHz) and ^{13}C NMR (75 MHz) spectra of **S188** in Chloroform-*d*

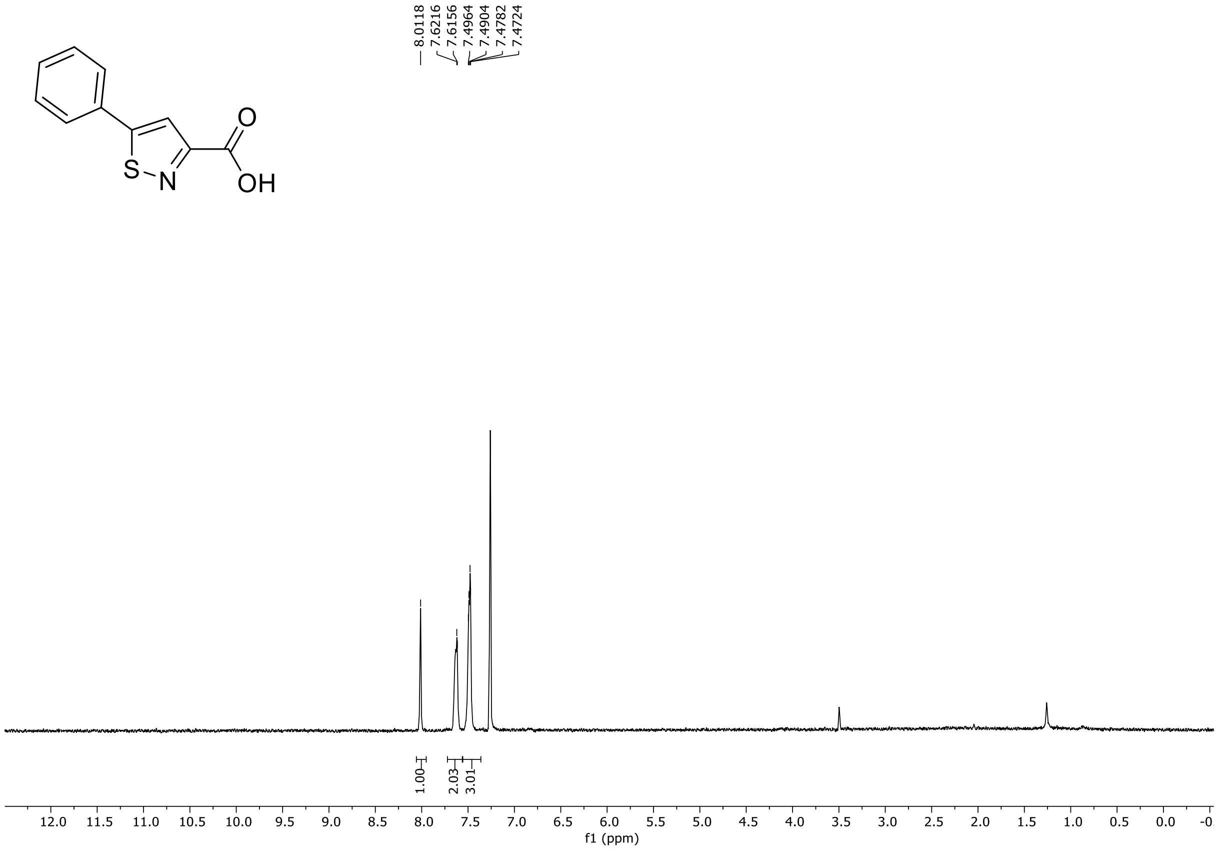

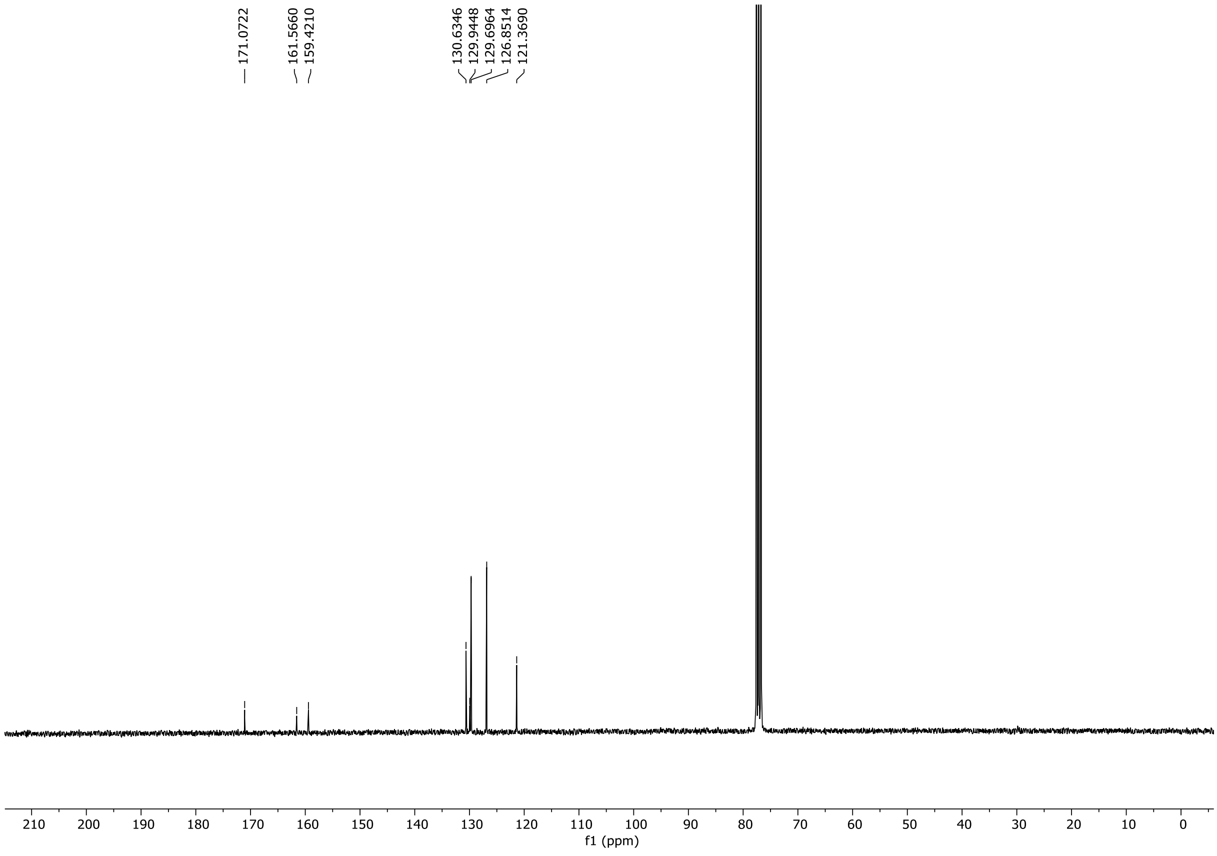

HRMS spectrum of **S188**

NAR-A-195 $C_{10}H_7NO_2S$ mono *m/z* 205.0197

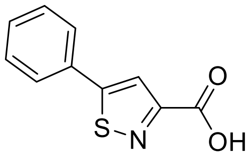

APCI + (MMI)
nitrogen flow 5 L/min, gas temperature 325°C, nebulizer 45 psi, skimmer **65** V, vaporizer 200°C, fragmentor 38 V, dissolved in methanol

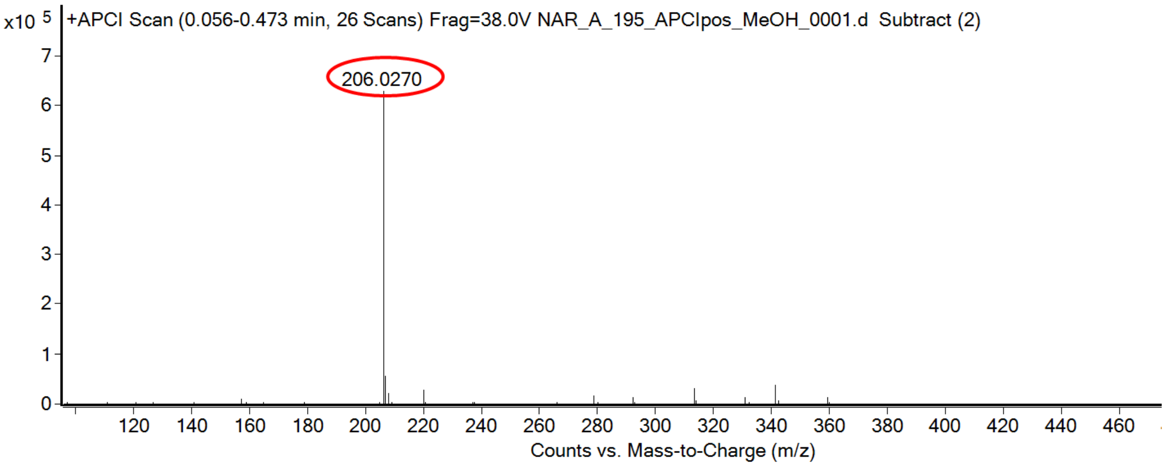

calculated mass: $[M+H]^+$ = 206.0270 observed: $[M+H]^+$ = 206.0270 mass accuracy = < 0.1 ppm

FT-IR spectrum (neat) of **S188**

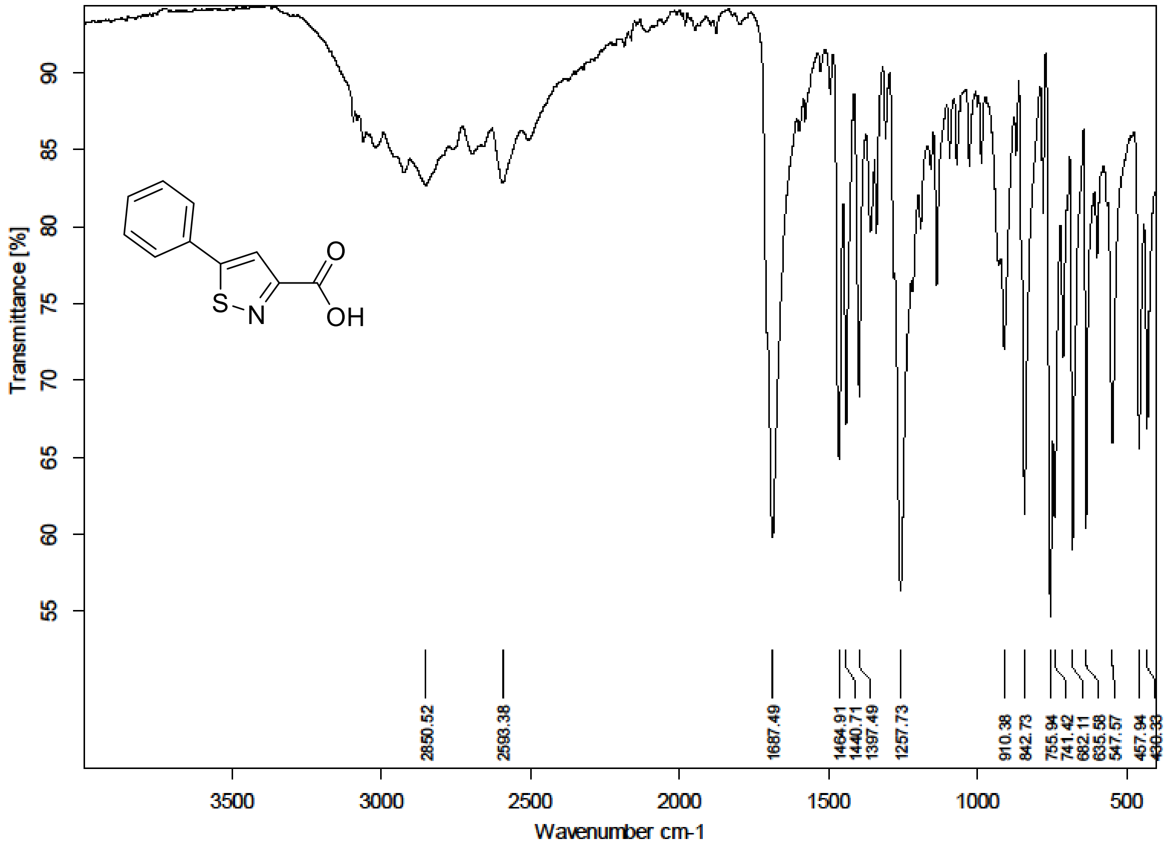

^{1}H NMR, ^{13}C NMR, HRMS and IR spectra of compound 2, S190 and 29-55

^{1}H (500 MHz) and ^{13}C NMR (126 MHz) spectra of **2** in DMSO-d_6

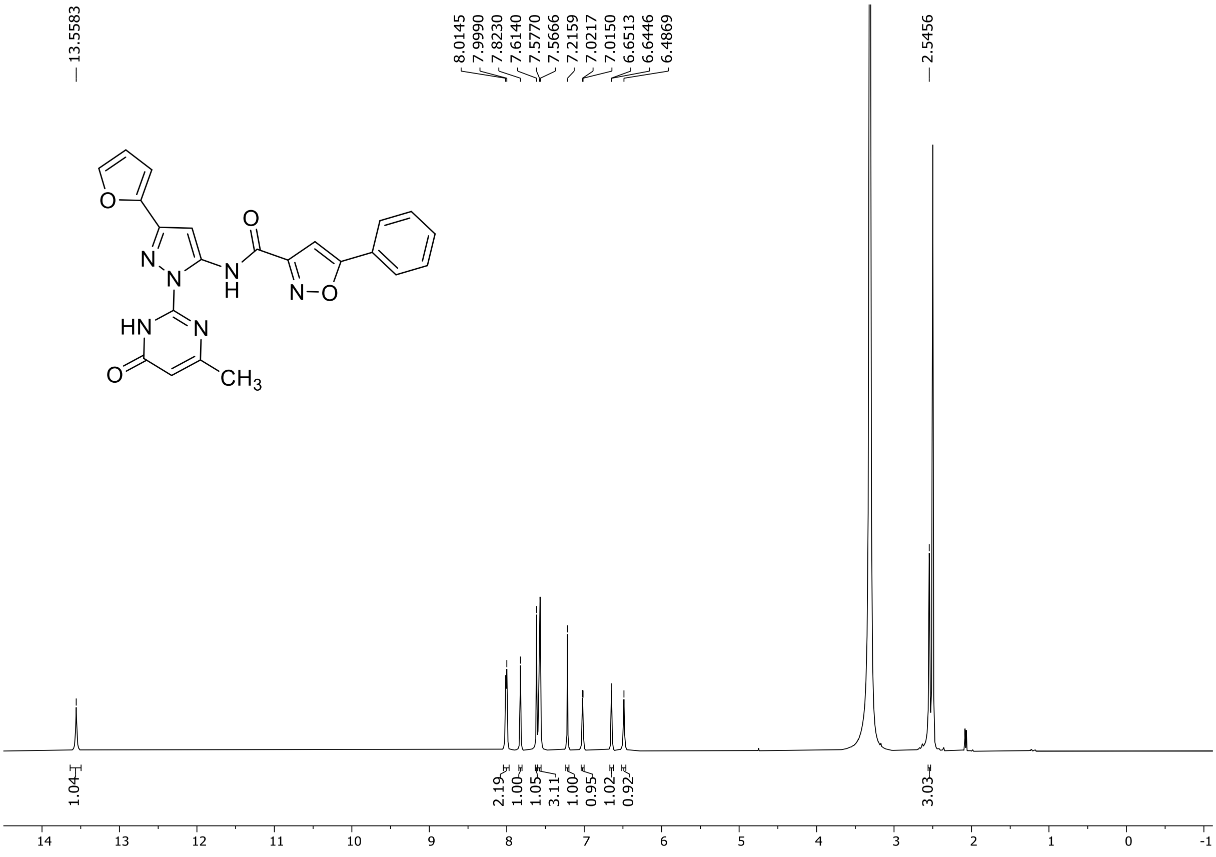

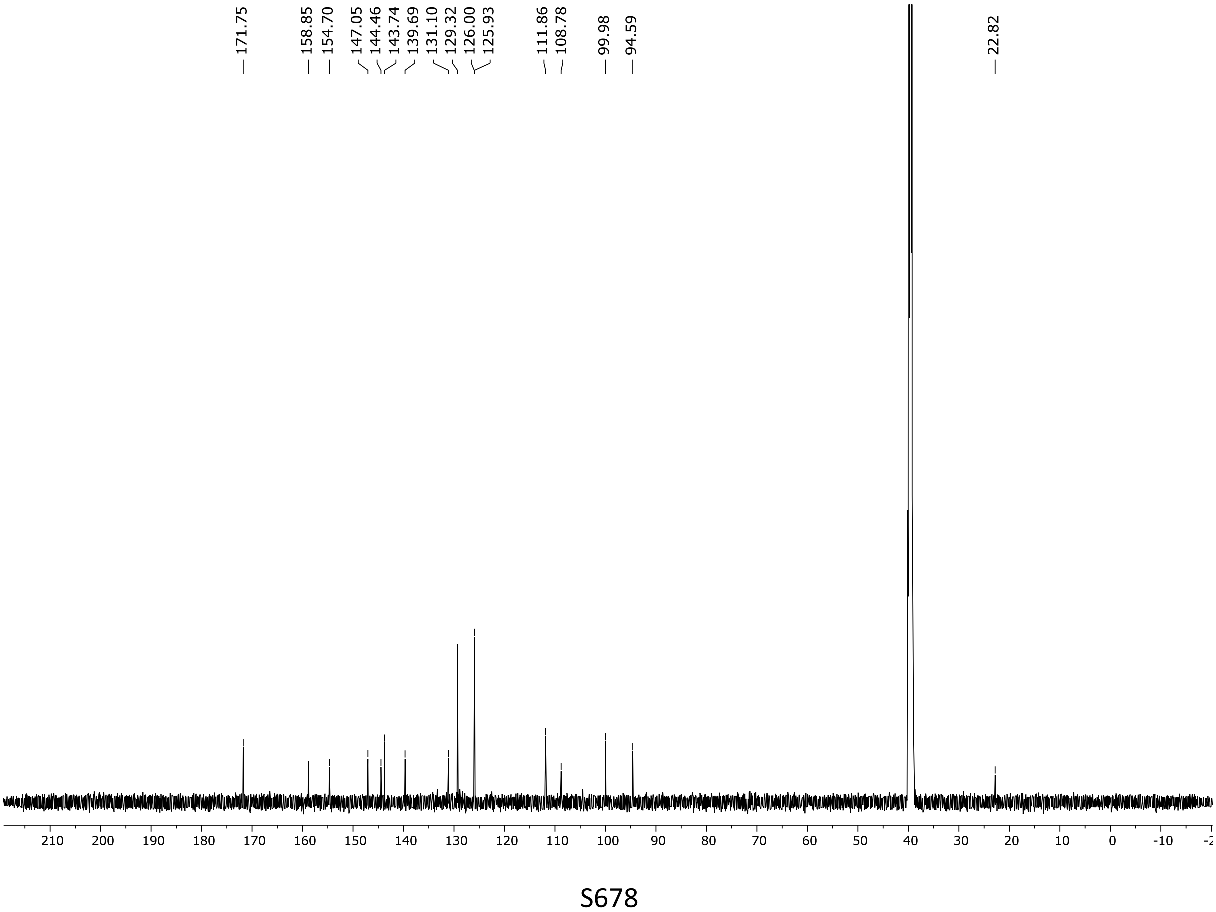

HRMS spectrum of **2**

$C_{22}H_{16}N_6O_4$ **exact mass: 428.1233**

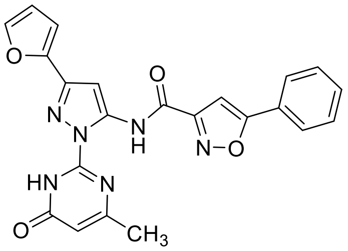

APCI + (MMI)
nitrogen flow 5 L/min, gas temperature 325°C, nebulizer 45 psig, skimmer 65 V, vaporizer 200°C, fragmentor 20 V, dissolved in methanol

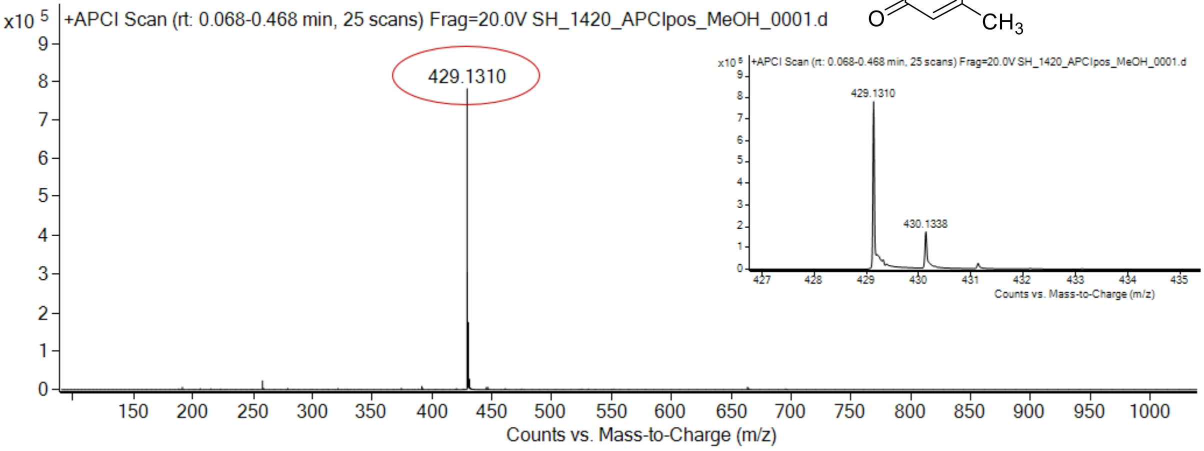

expected mass: $[M+H]^+$ = 429.1306 observed mass : $[M+H]^+$ = 429.1310 mass accuracy = 0.9 ppm

^{1}H (500 MHz) and ^{13}C NMR (126 MHz) spectra of **29** in DMSO-d_6

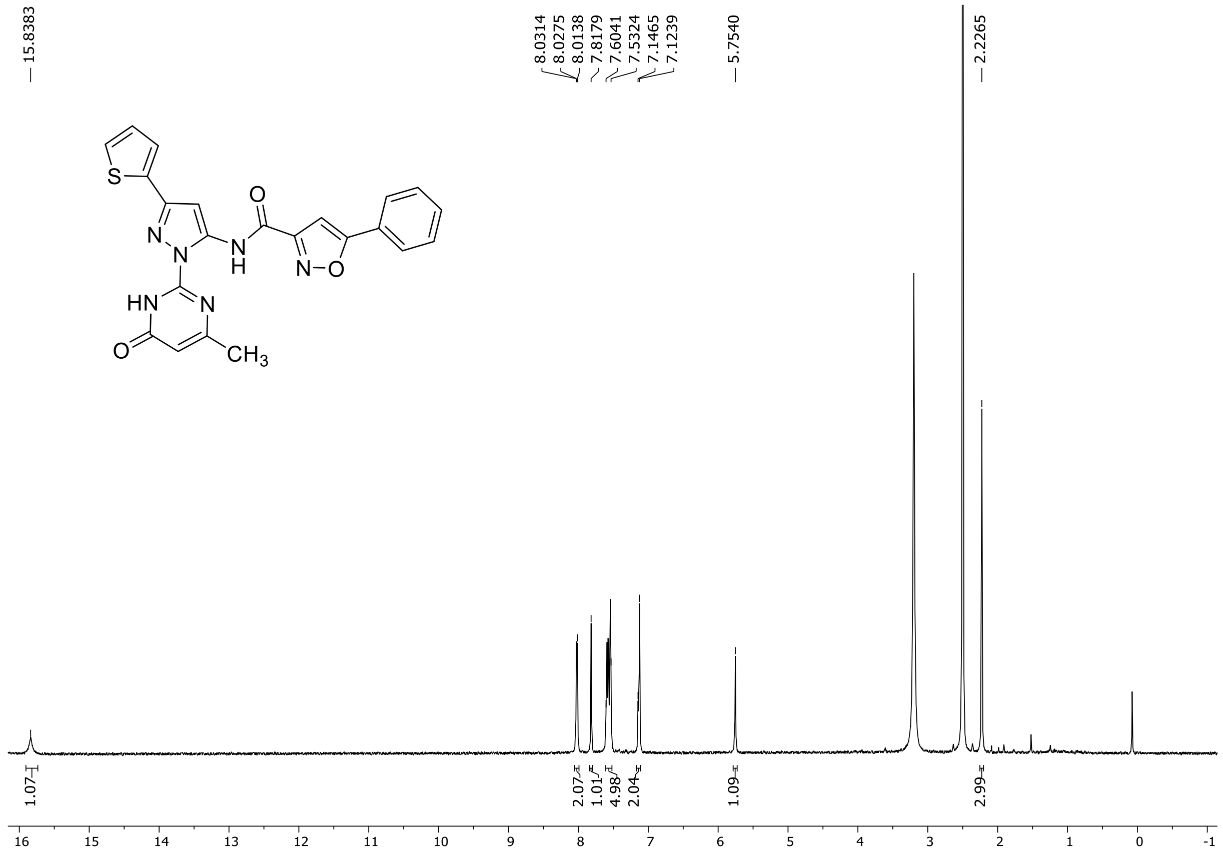

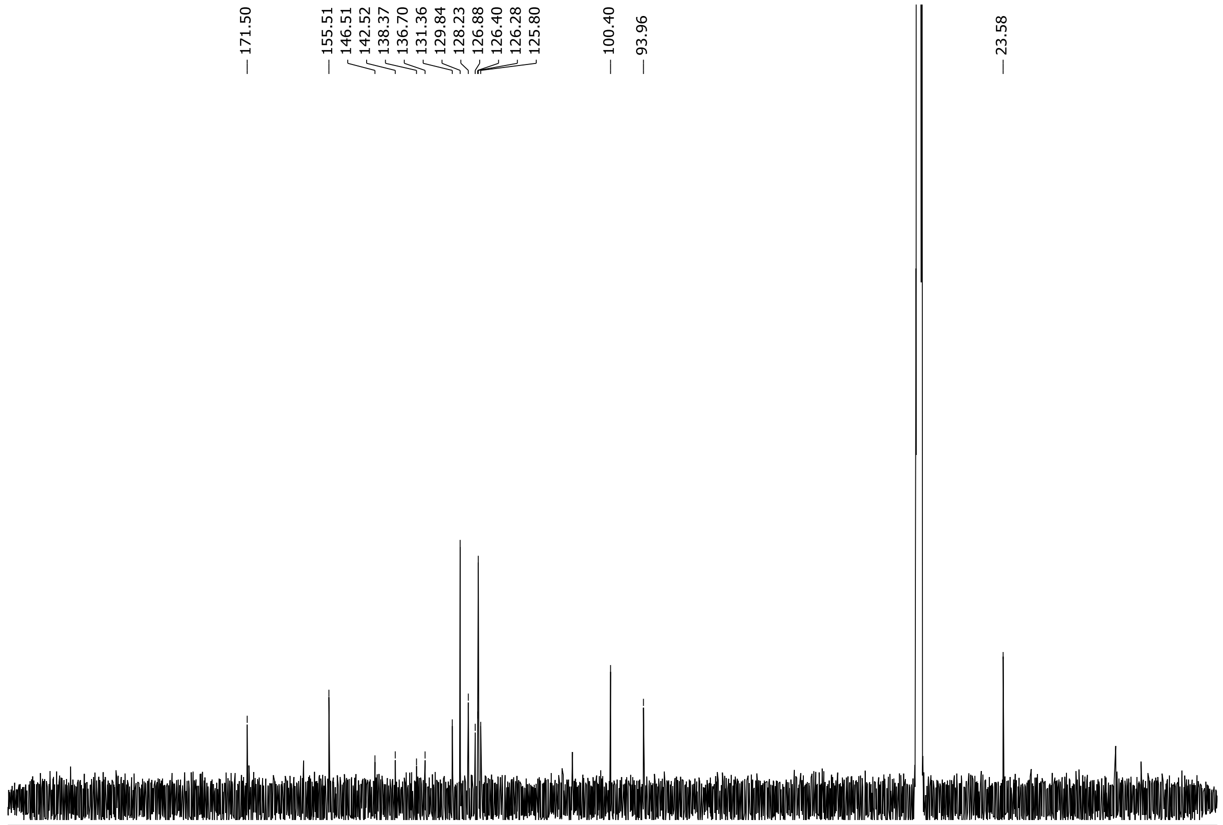

HRMS spectrum of **29**

$C_{22}H_{16}N_6O_3S$ exact mass: 444.1005

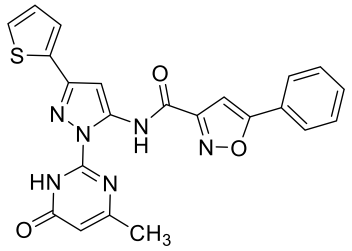

APCI + (MMI)
nitrogen flow 5 L/min, gas temperature 325°C, nebulizer 45 psig, skimmer 65 V, vaporizer 200°C, fragmentor 20 V, dissolved in methanol

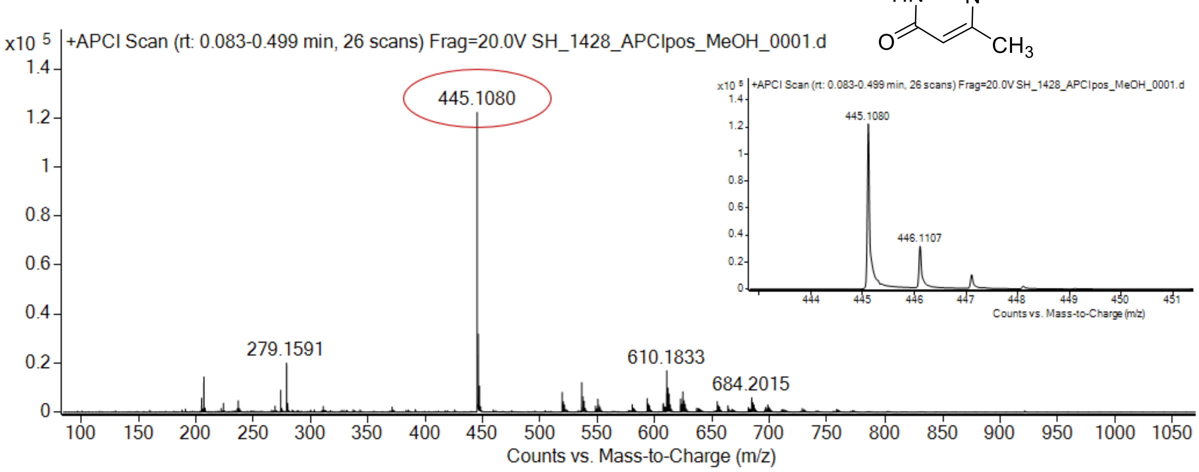

expected mass: $[M+H]^+$ = 445.1077 observed mass : $[M+H]^+$ = 445.1080 mass accuracy = 0.7 ppm

^{1}H (500 MHz) and ^{13}C NMR (126 MHz) spectra of **30** in DMSO-d_6

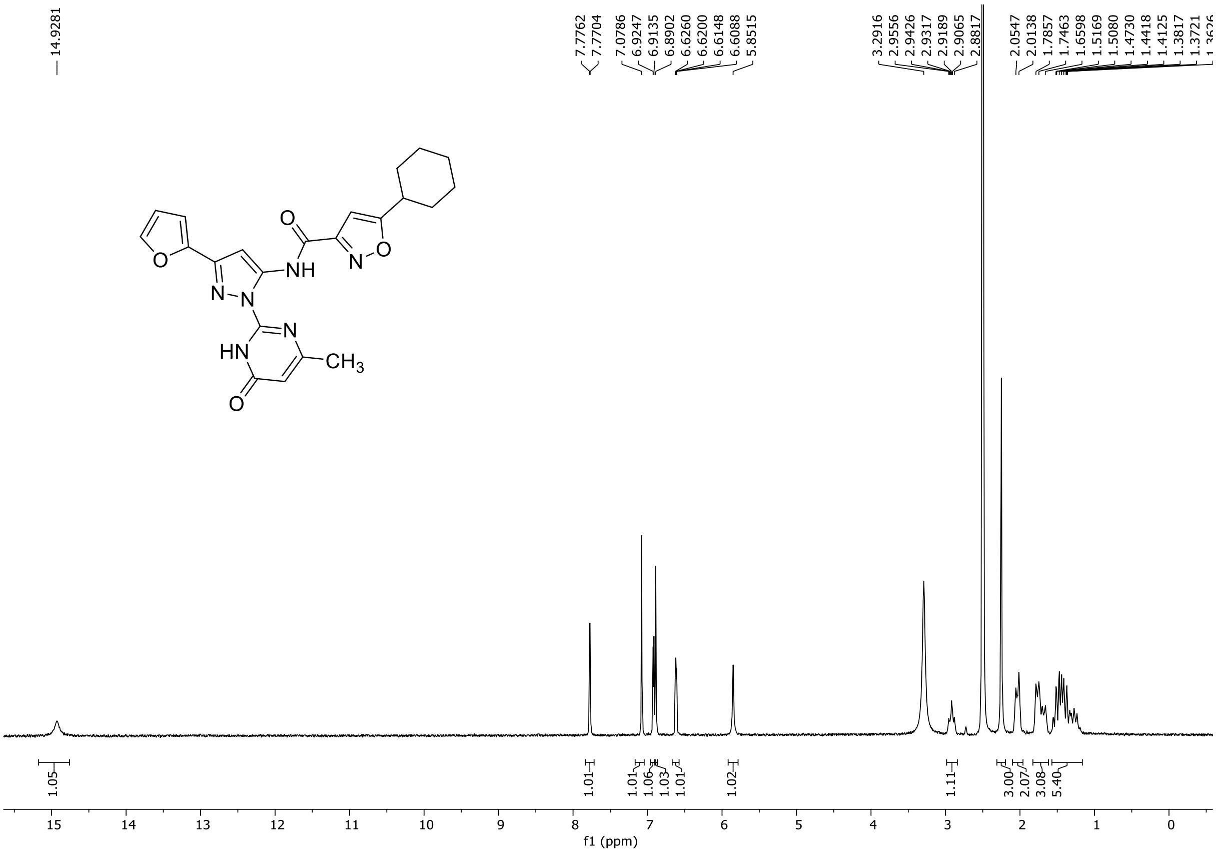

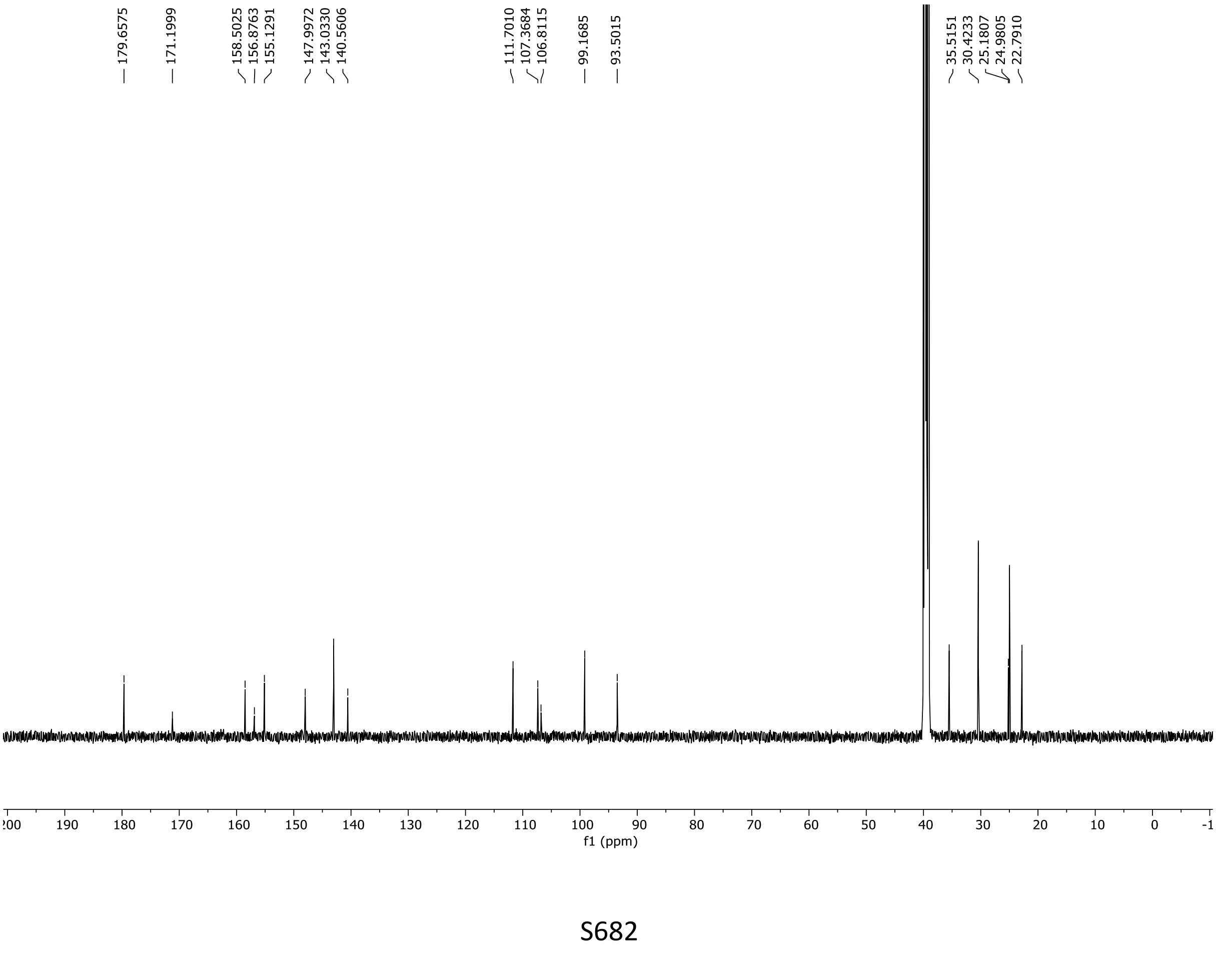

HRMS spectrum of **30**

NAR-A-12 $C_{22}H_{22}N_6O_4$ mono *m/z 434.1703*

APCI + (MMI)
nitrogen flow 5 L/min, gas temperature 325°C, nebulizer 45 psi, skimmer **65** V, vaporizer 200°C, fragmentor 30 V, dissolved in methanol

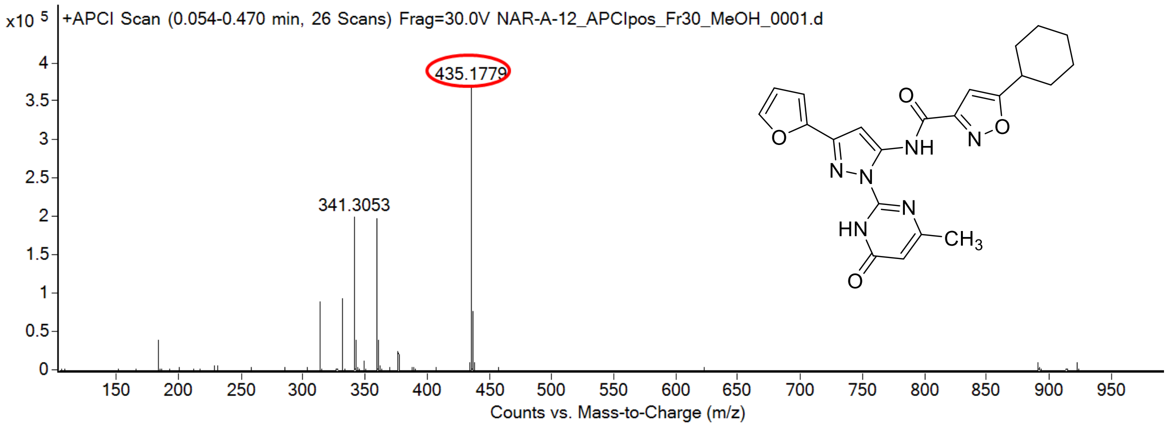

calculated mass: $[M+H]^+ = 435.1775$ observed: $[M+H]^+ = 435.1779$ mass accuracy = 0.9 ppm

FT-IR spectrum (neat) of **30**

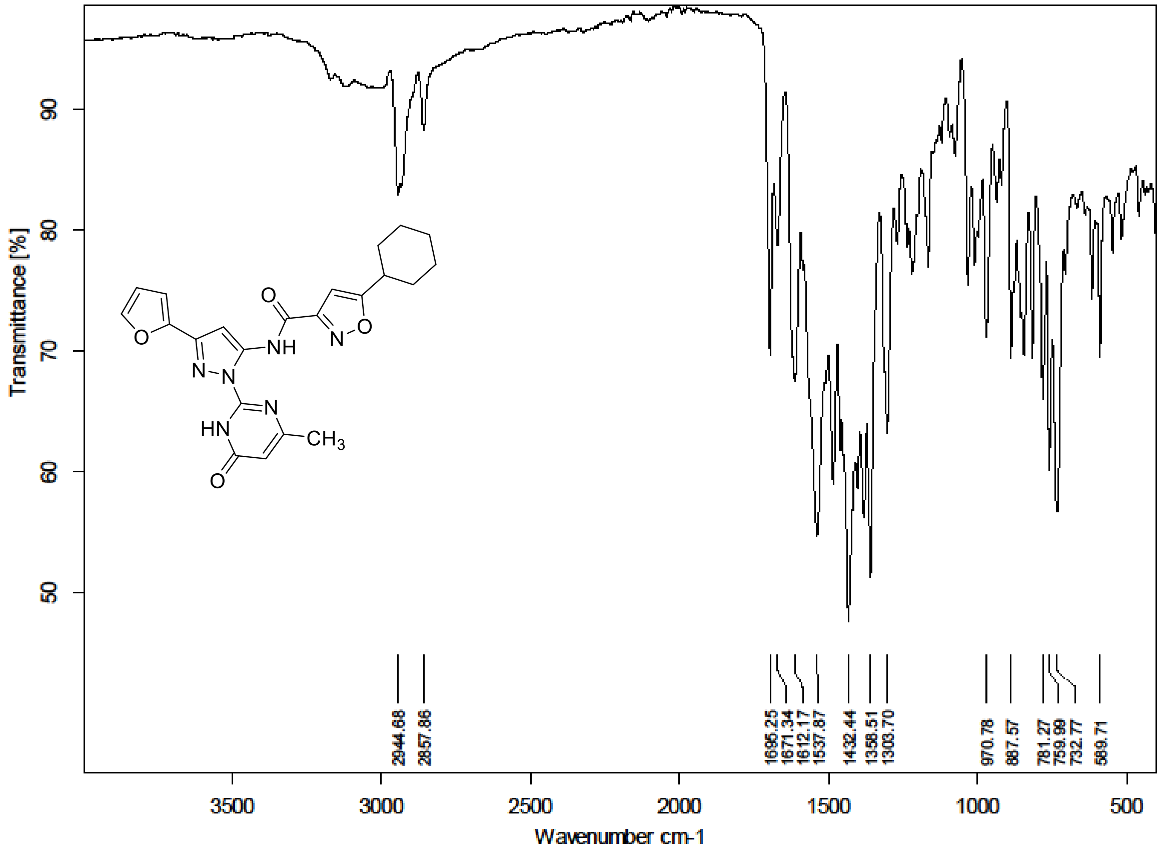

^{1}H NMR (300 MHz) spectrum of **31** in DMSO-d_6

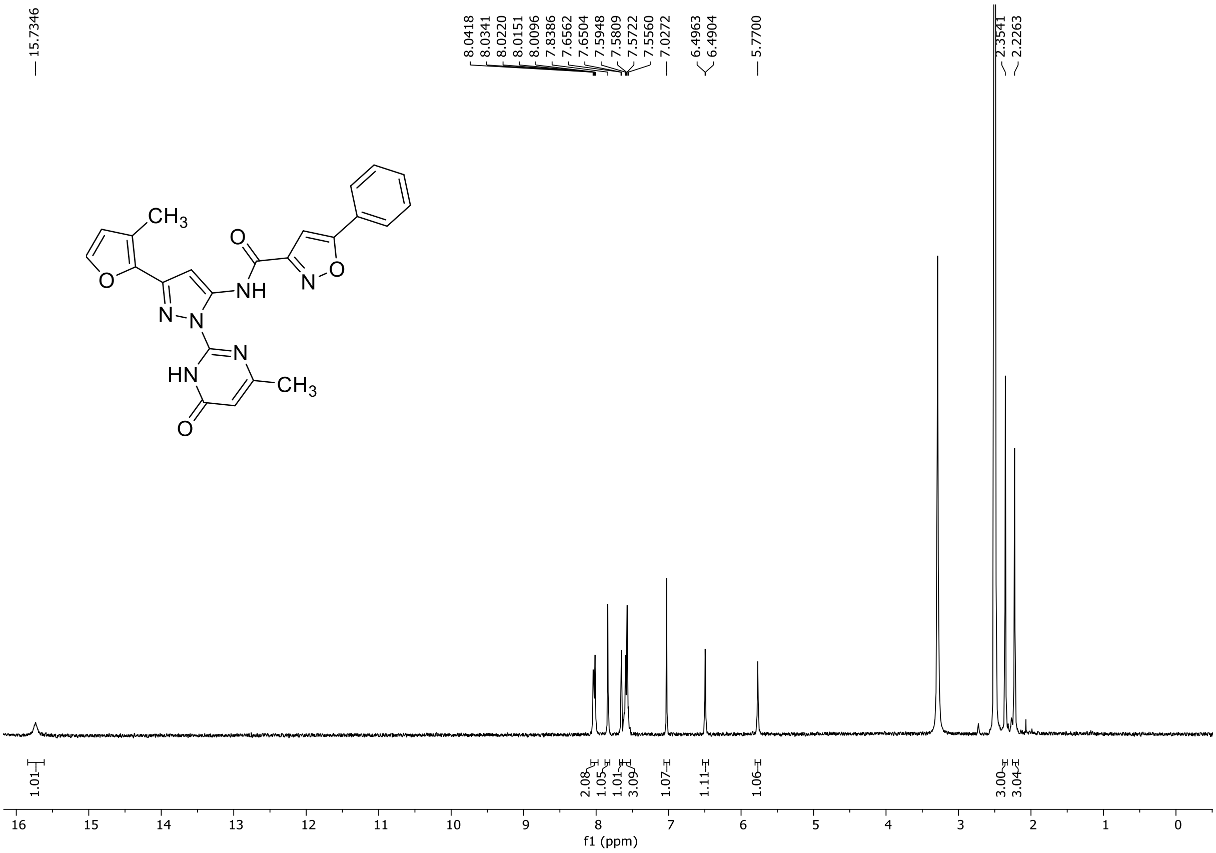

HRMS spectrum of **31**

NAR-A-181 $C_{23}H_{18}N_6O_4$ **mono *m/z* 442.1390**

APCI + (MMI)
nitrogen flow 5 L/min, gas temperature 325°C, nebulizer 45 psi, skimmer **65** V, vaporizer 200°C, fragmentor 25 V, dissolved in methanol

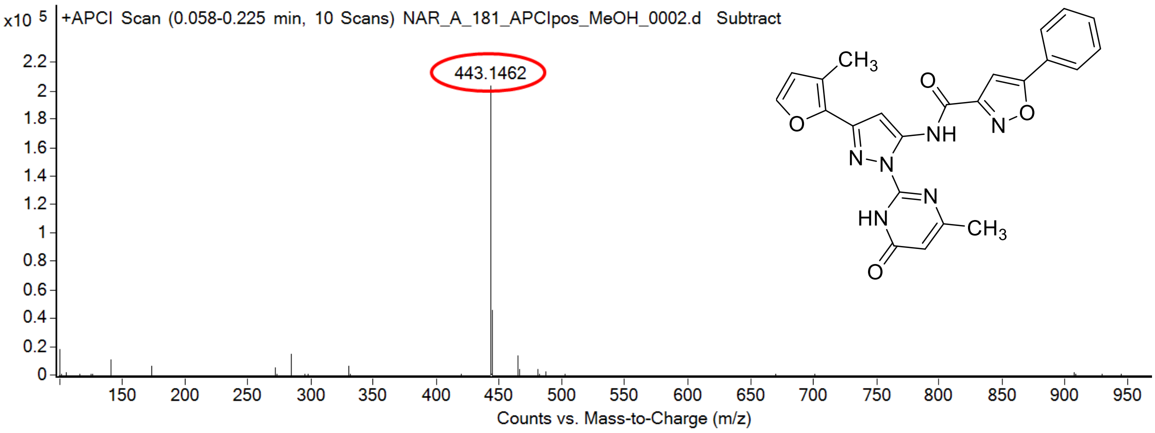

calculated mass: $[M+H]^+$ = 443.1462 observed: $[M+H]^+$ = 443.1462 mass accuracy = < 0.1 ppm

FT-IR spectrum (neat) of **31**

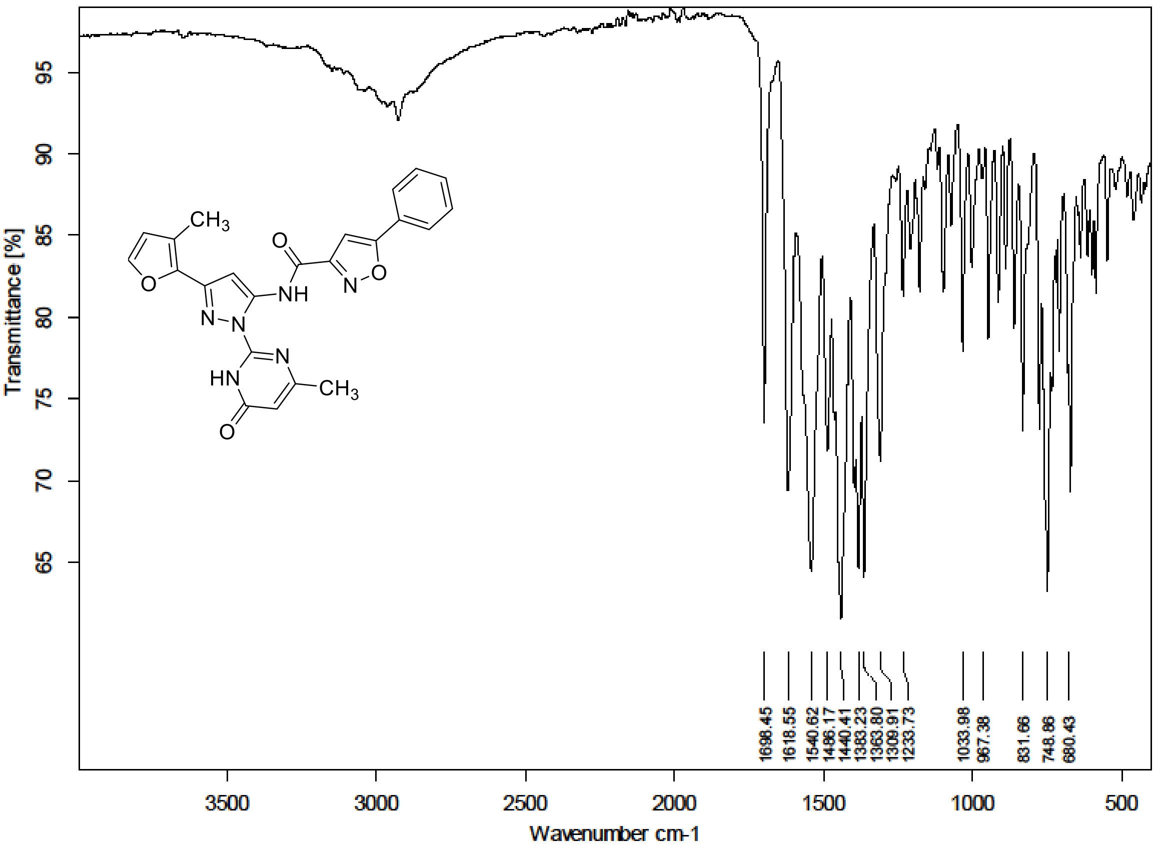

^{1}H (500 MHz) and ^{13}C NMR (126 MHz) spectra of **32** in DMSO-d_6

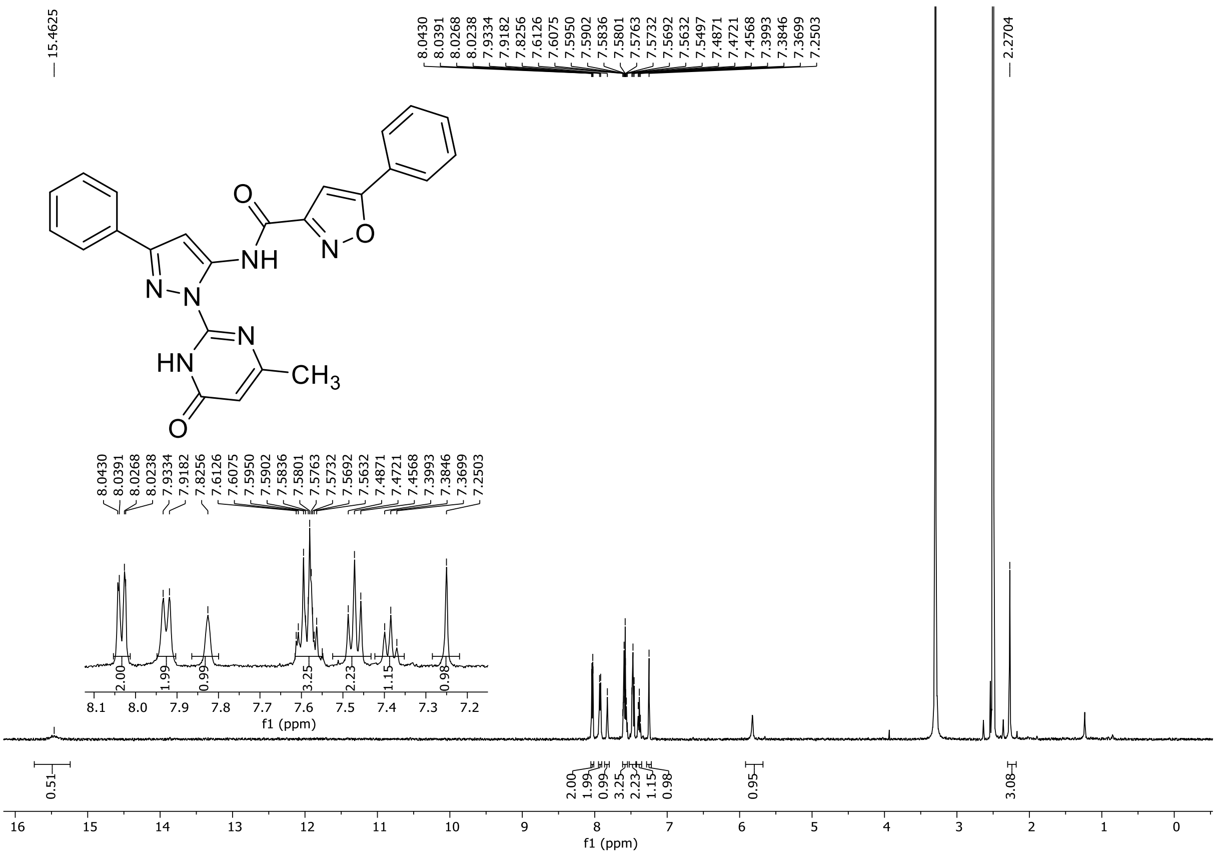

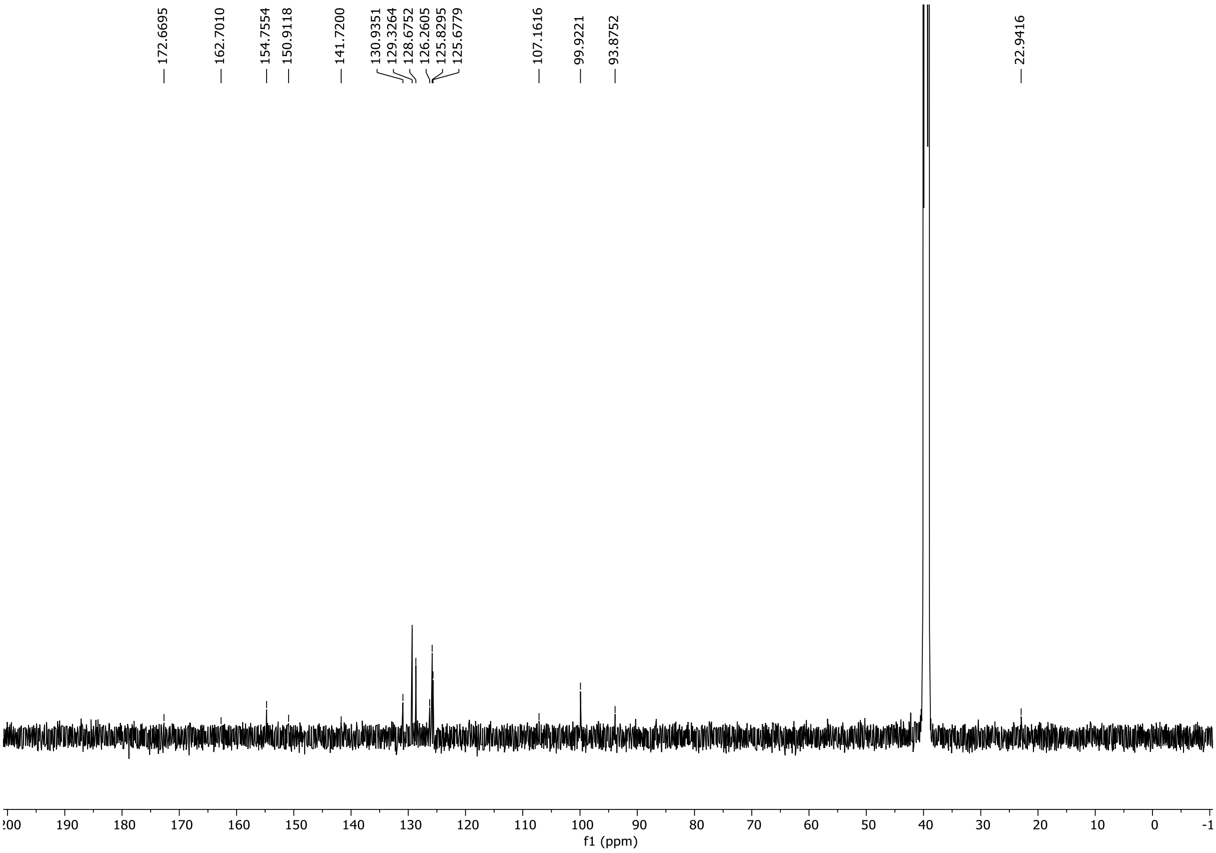

HRMS spectrum of **32**

NAR-A-8 $C_{24}H_{18}N_6O_3$ 438.1440 *mono m/z*

ESI - (MMI)
nitrogen flow 5 L/min, gas temperature 325°C, nebulizer 45 psi, skimmer **65** V, vaporizer 200°C, fragmentor 90 V, dissolved in methanol

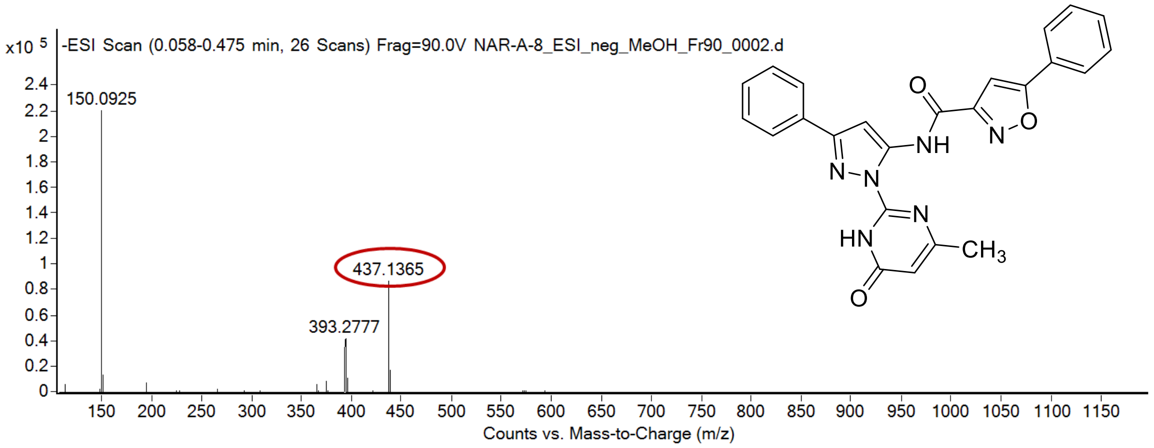

calculated mass: [M-H]⁻ = 437.1368 observed: [M-H]⁻ = 437.1365 mass accuracy = 0.7 ppm

FT-IR spectrum (neat) of **32**

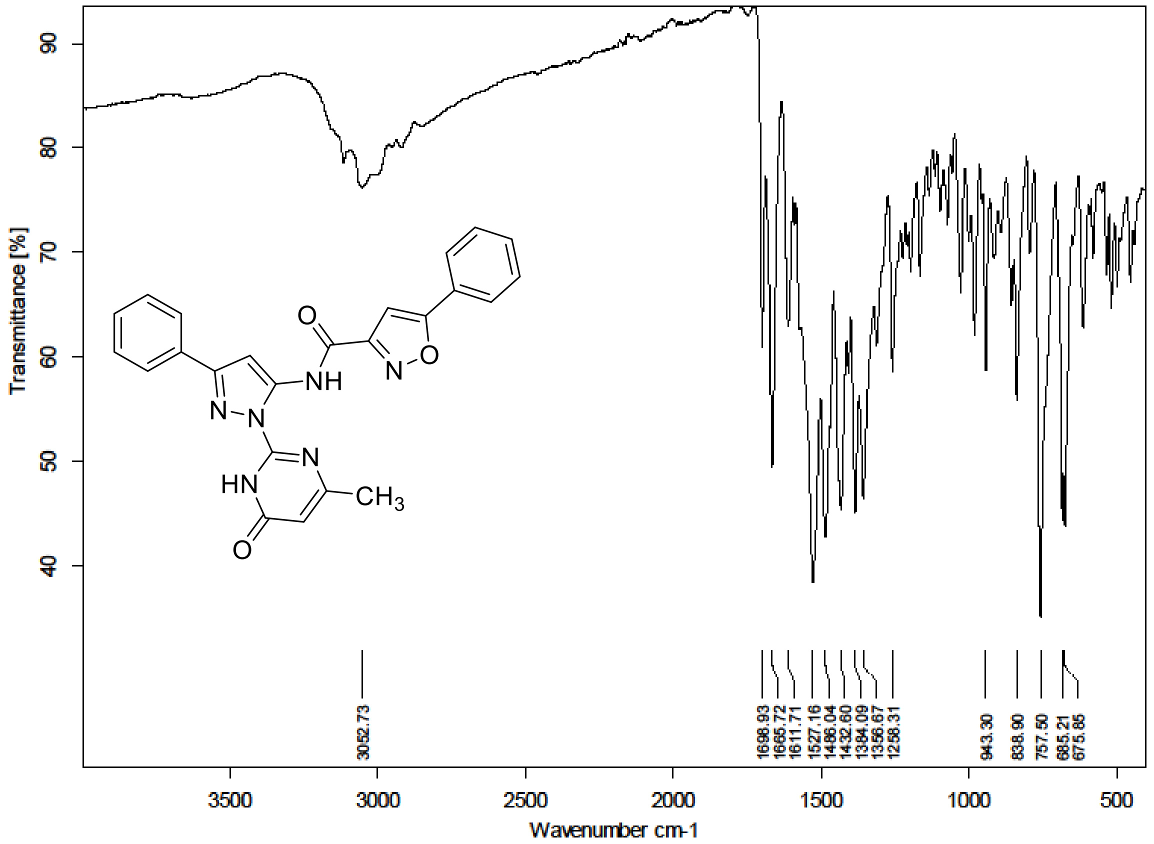

^{1}H (300 MHz) and ^{13}C NMR (176 MHz) spectra of **33** in DMSO-d_6

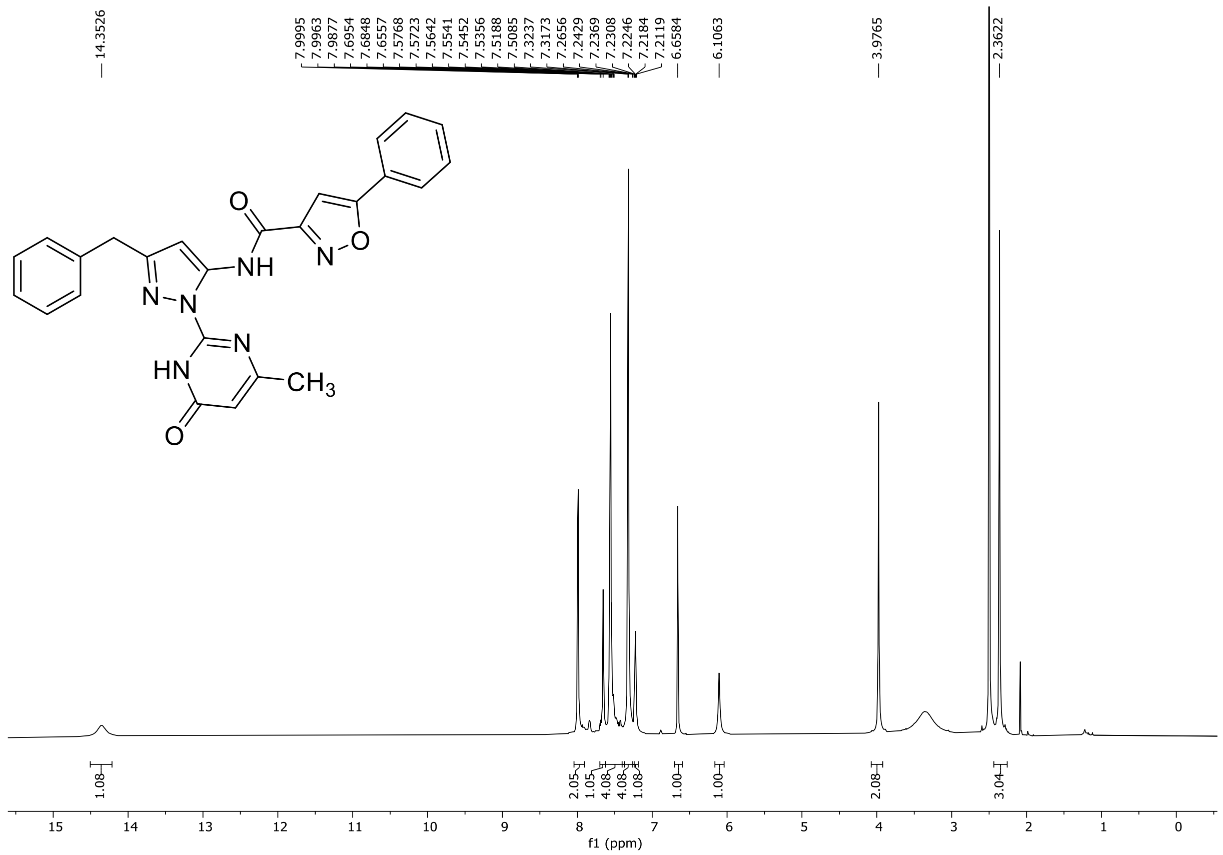

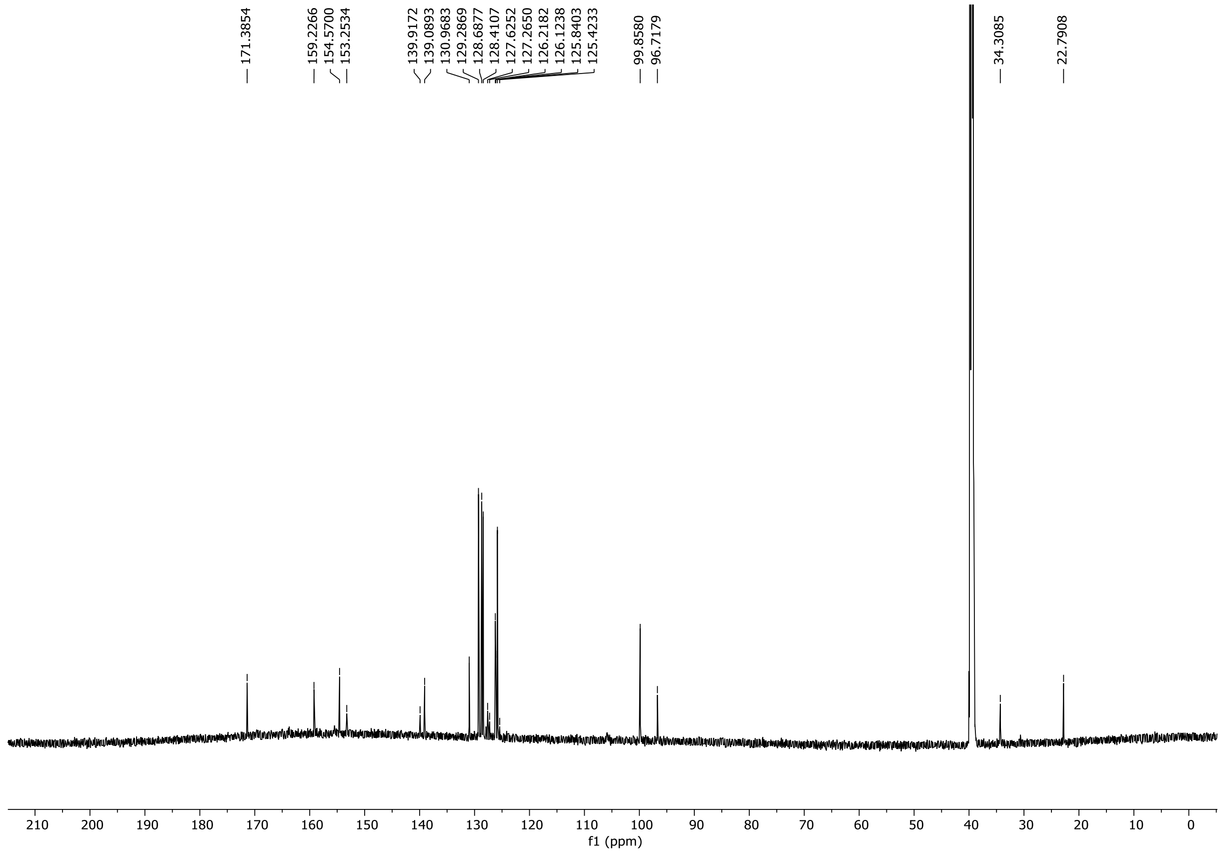

HRMS spectrum of **33**

NAR-A-189 $C_{25}H_{20}N_6O_3$ mono *m/z* 452.1597

APCI + (MMI)
nitrogen flow 5 L/min, gas temperature 325°C, nebulizer 45 psi, skimmer **65** V, vaporizer 200°C, fragmentor 35 V, dissolved in methanol

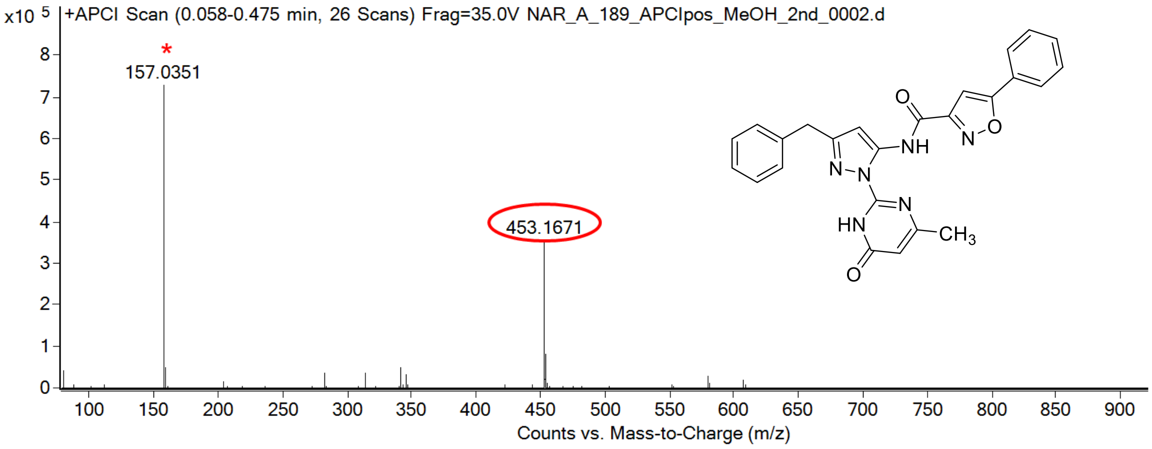

calculated mass: $[M+H]^+$ = 453.1670 observed: $[M+H]^+$ = 453.1671 mass accuracy = 0.2 ppm

* DMSO

FT-IR spectrum (neat) of **33**

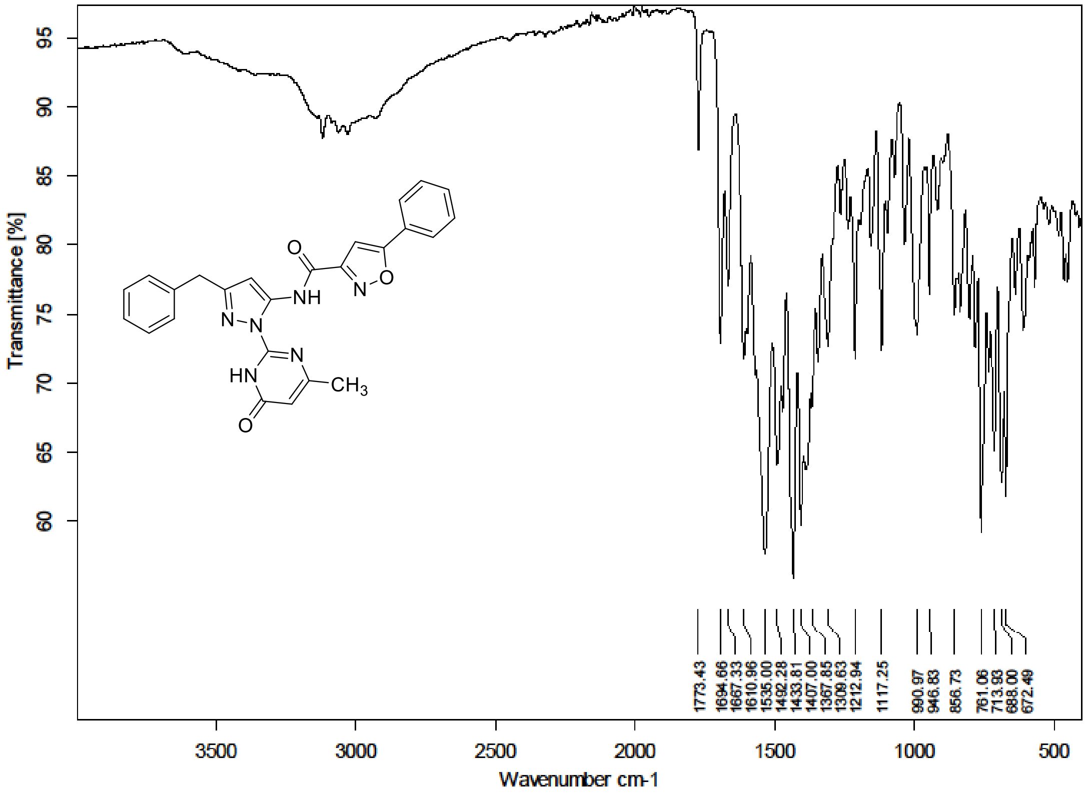

^{1}H NMR (300 MHz) spectrum of **34** in DMSO-d_6

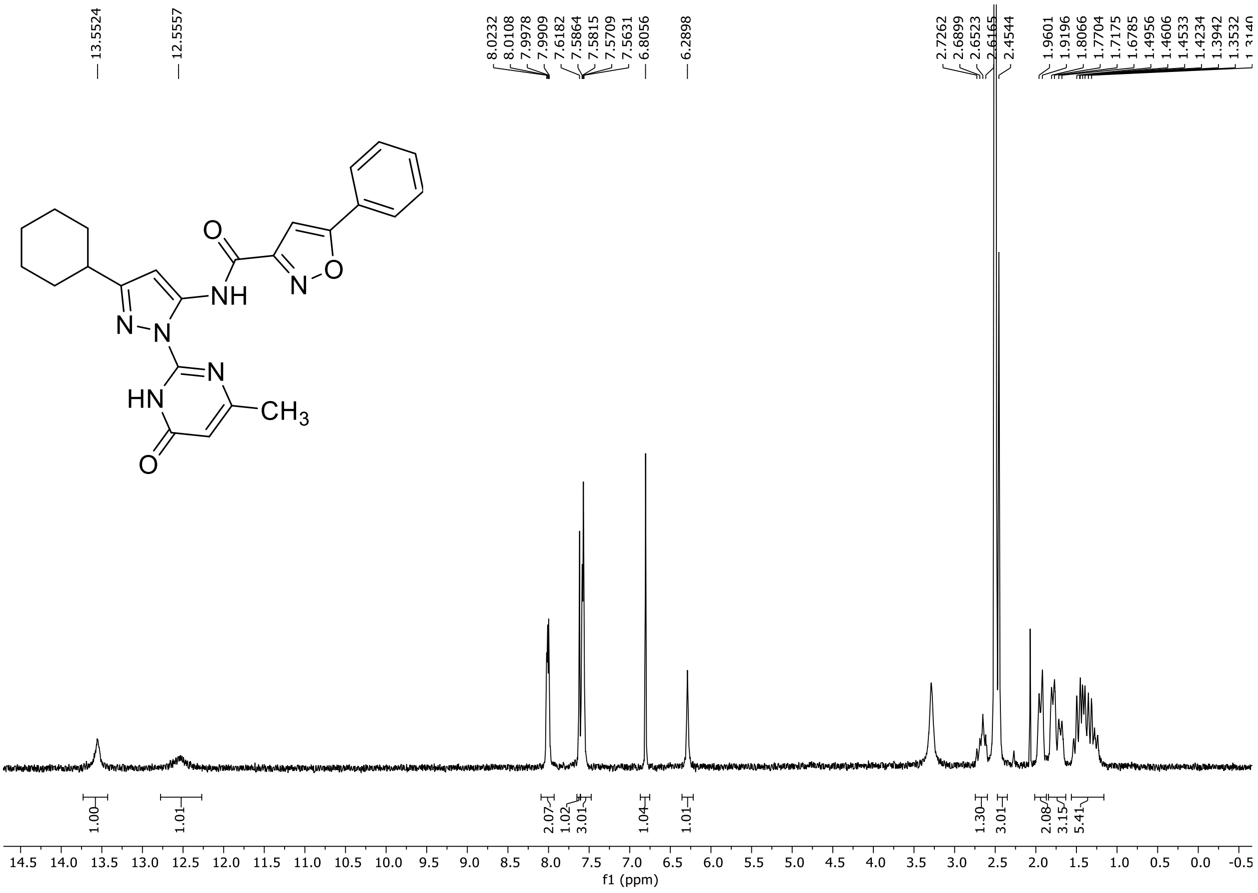

HRMS spectrum of **34**

NAR-A-122 $C_{24}H_{24}N_6O_3$ ***m/z* 444.1910**

ESI- (MMI)
nitrogen flow 5 L/min, gas temperature 325°C, nebulizer 45 psi, skimmer 65 V, fragmentor 100 V, dissolved in DMSO, MeOH

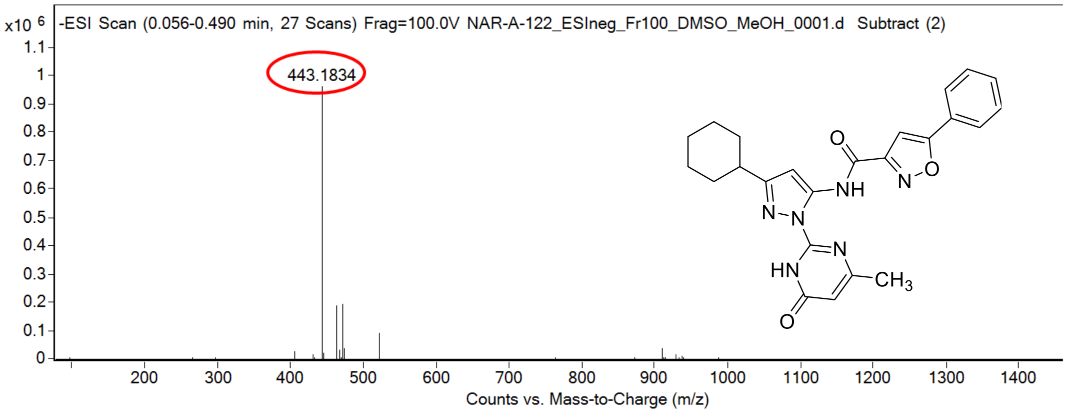

calculated mass: $[M-H]^-$ = 443.1837 observed: $[M-H]^-$ = 443.1834 mass accuracy = -0.7 ppm

FT-IR spectrum (neat) of **34**

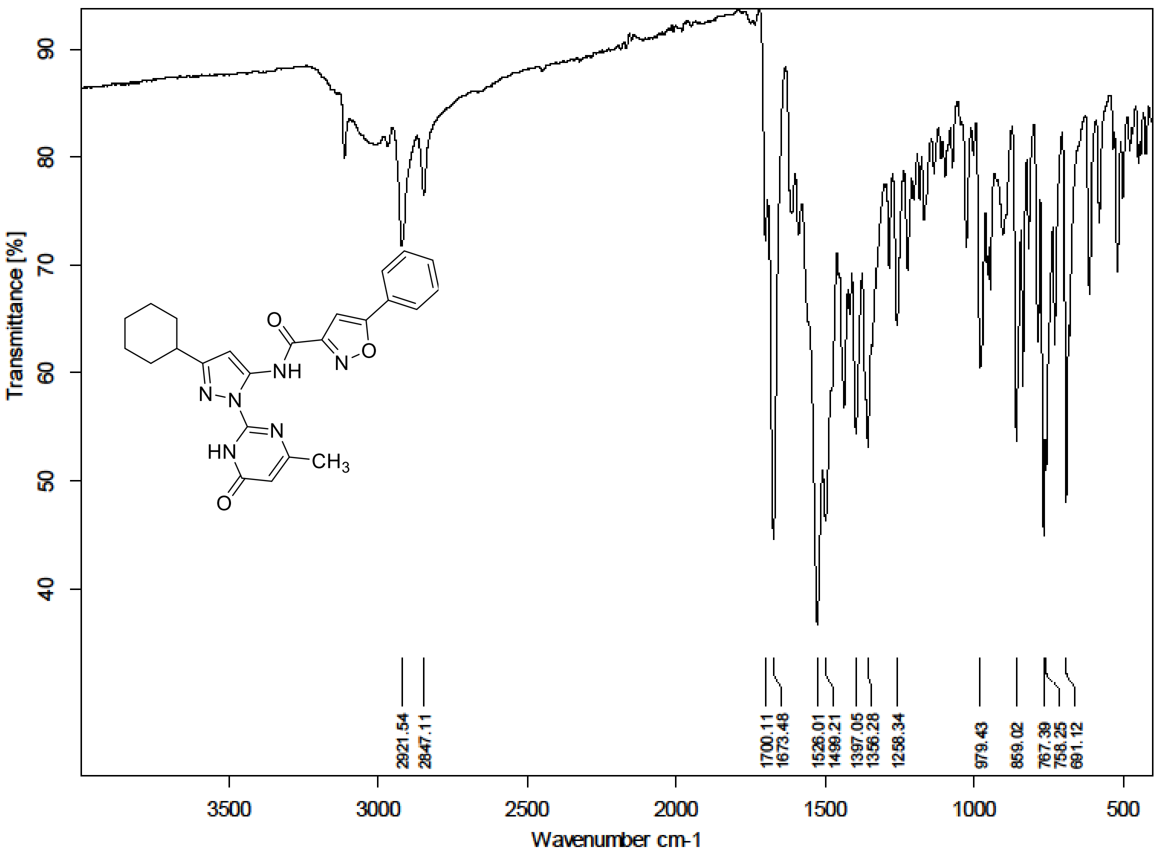

^{1}H (500 MHz) and ^{13}C NMR (126 MHz) spectra of **35** in DMSO-d_6

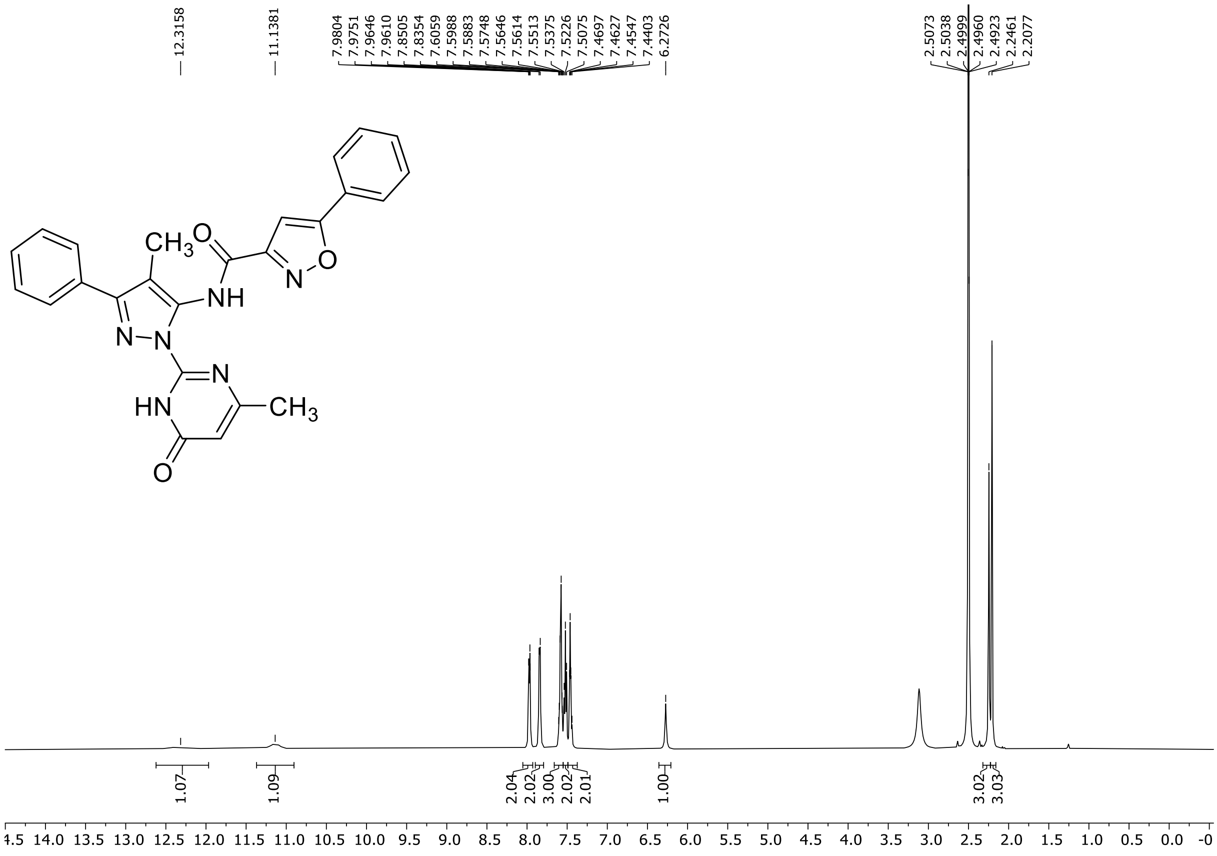

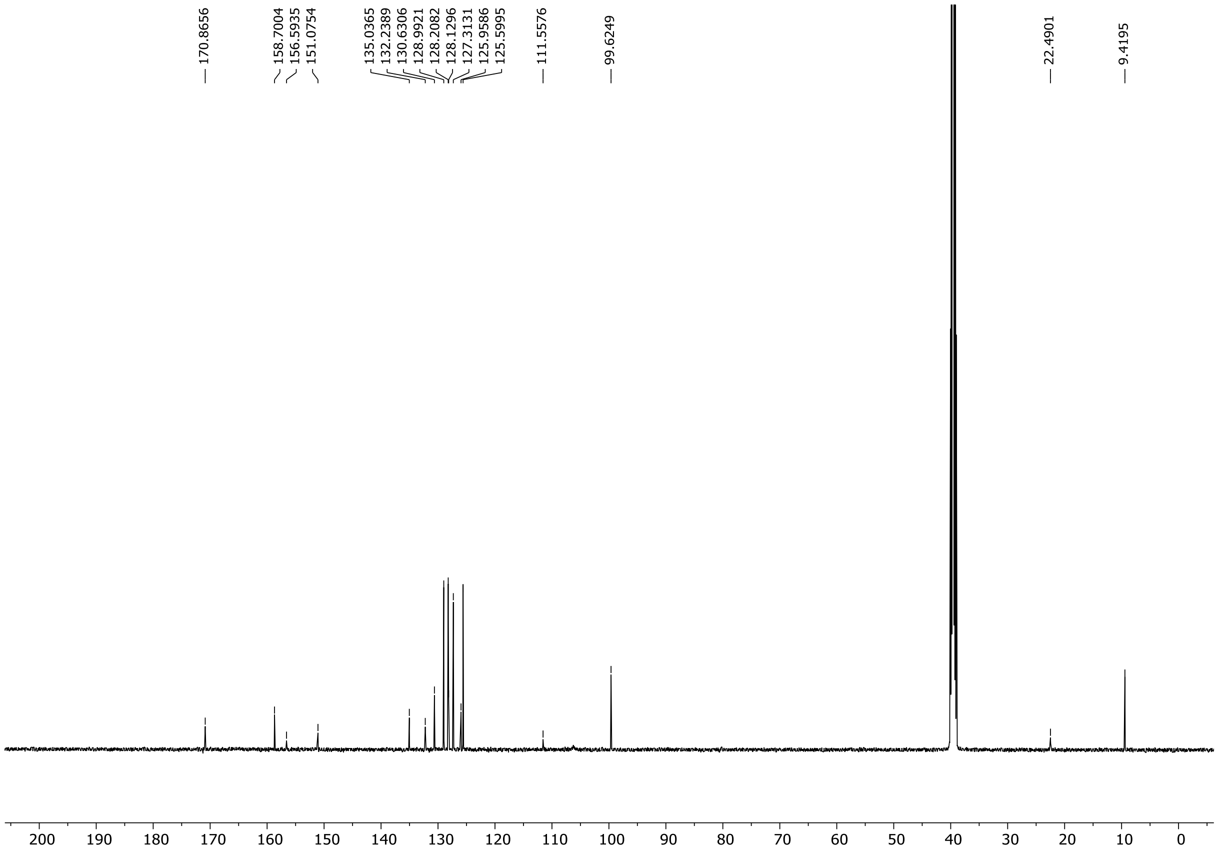

HRMS spectrum of **35**

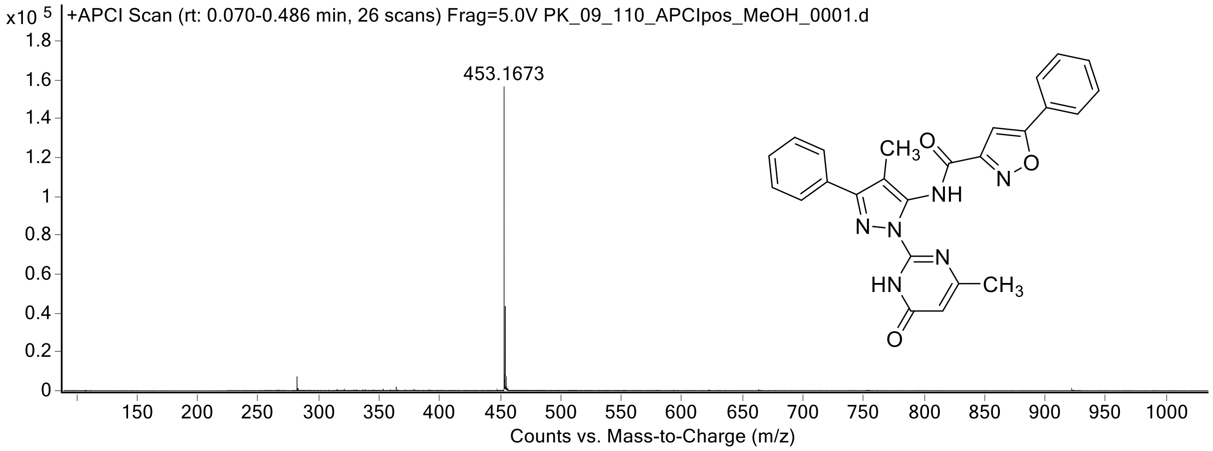

^{1}H (500 MHz) and ^{13}C NMR (126 MHz) spectra of **36** in DMSO-d_6

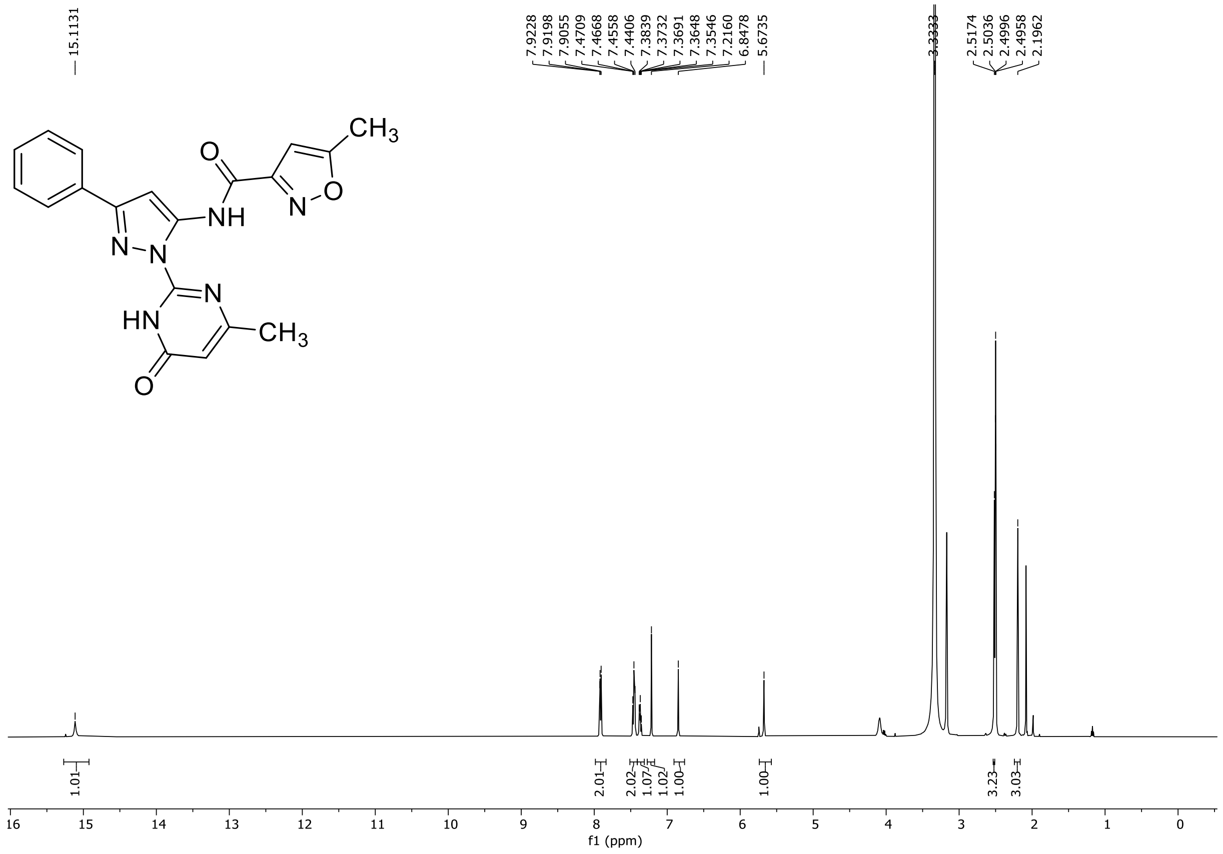

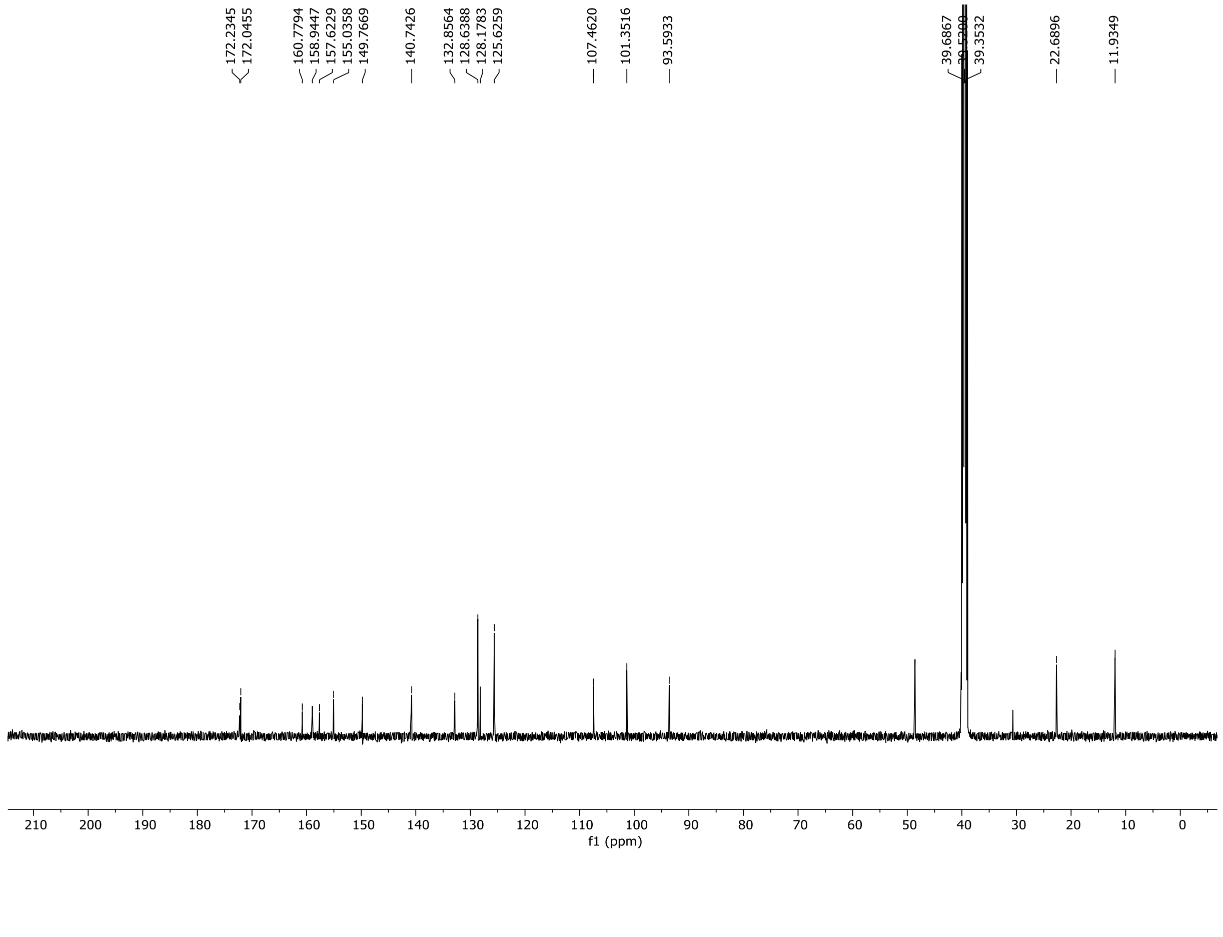

^{1}H NMR (500 MHz) spectrum of **36** in Methanol-d_4

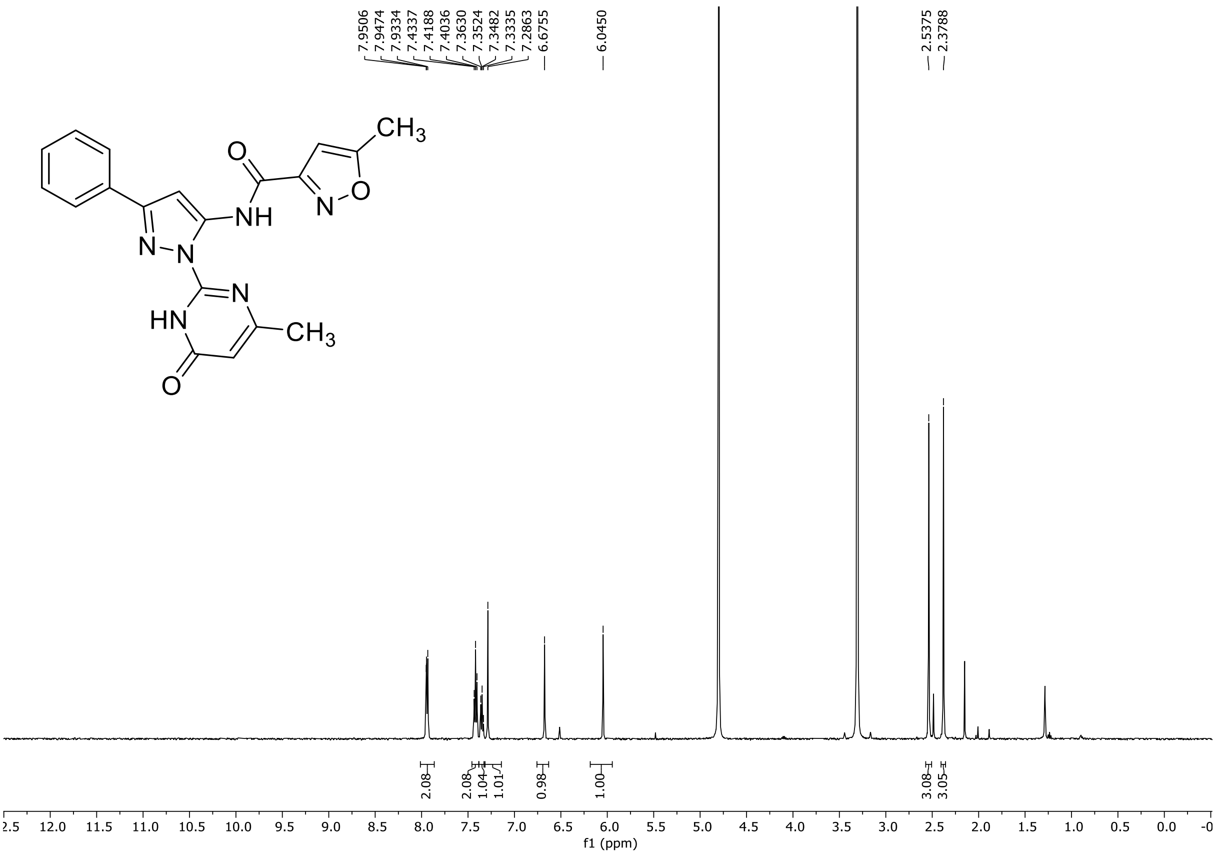

HRMS spectrum of **36**

NAR-A-3 $C_{19}H_{16}N_6O_3$ 376.1284 *mono m/z*

APCI + (MMI)
nitrogen flow 5 L/min, gas temperature 325°C, nebulizer 45 psi, skimmer **65** V, vaporizer 200°C, fragmentor 38 V, dissolved in methanol

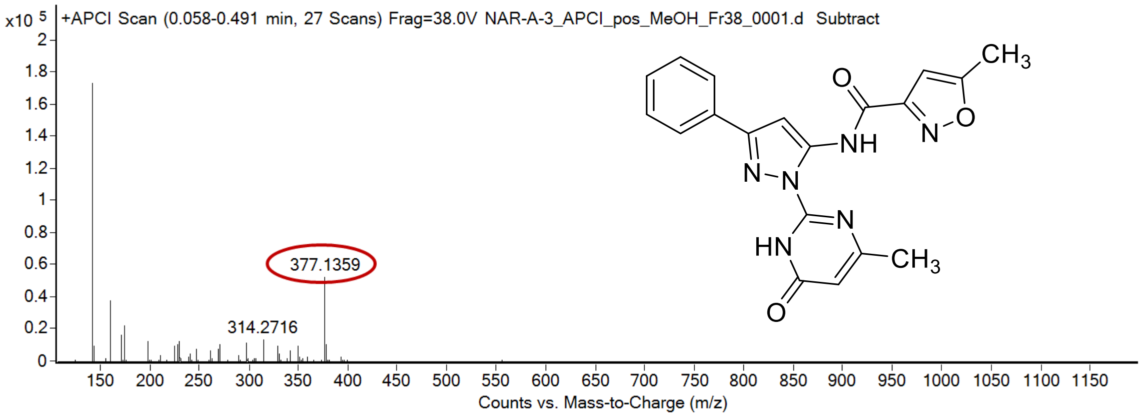

calculated mass: $[M+H]^+$ = 377.1357 observed: $[M+H]^+$ = 377.1359 mass accuracy = 0.5 ppm

FT-IR spectrum (neat) of **36**

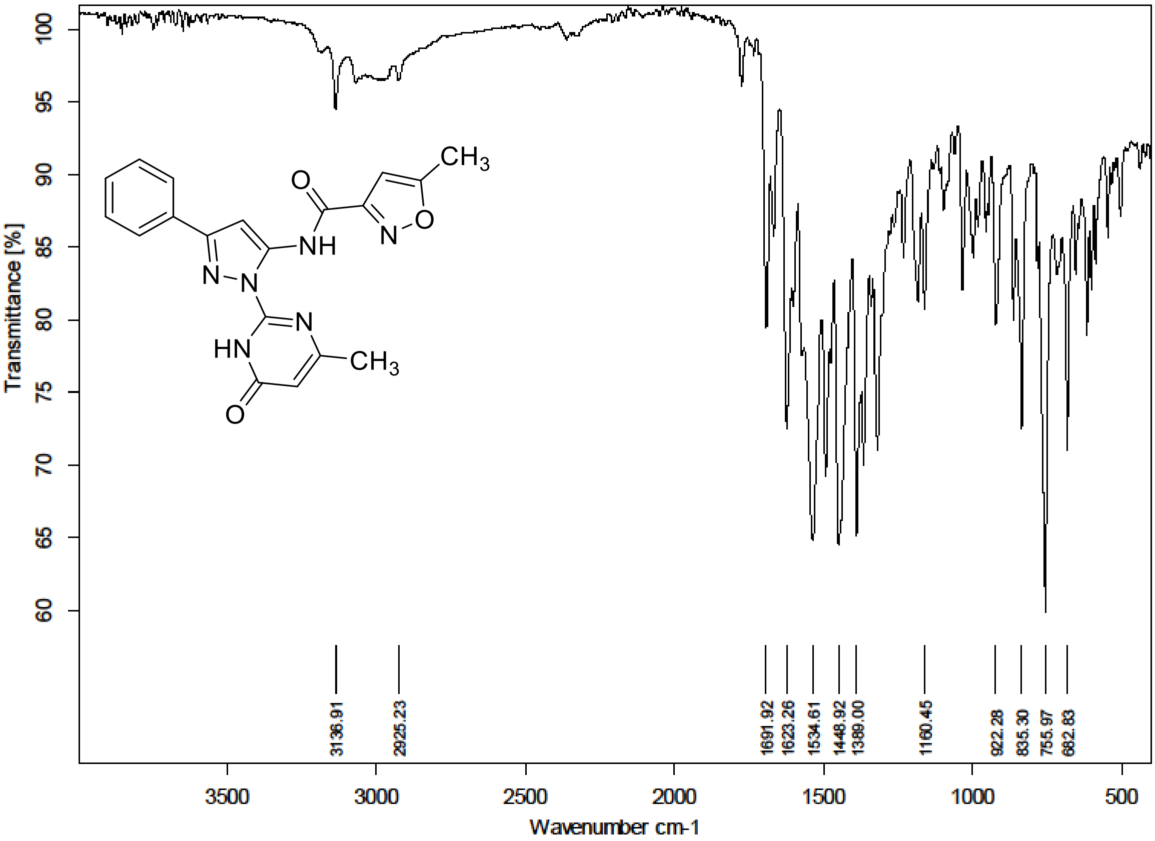

^{1}H NMR (300 MHz) spectrum of **37** in Trifluoroacetic Acid-*d*

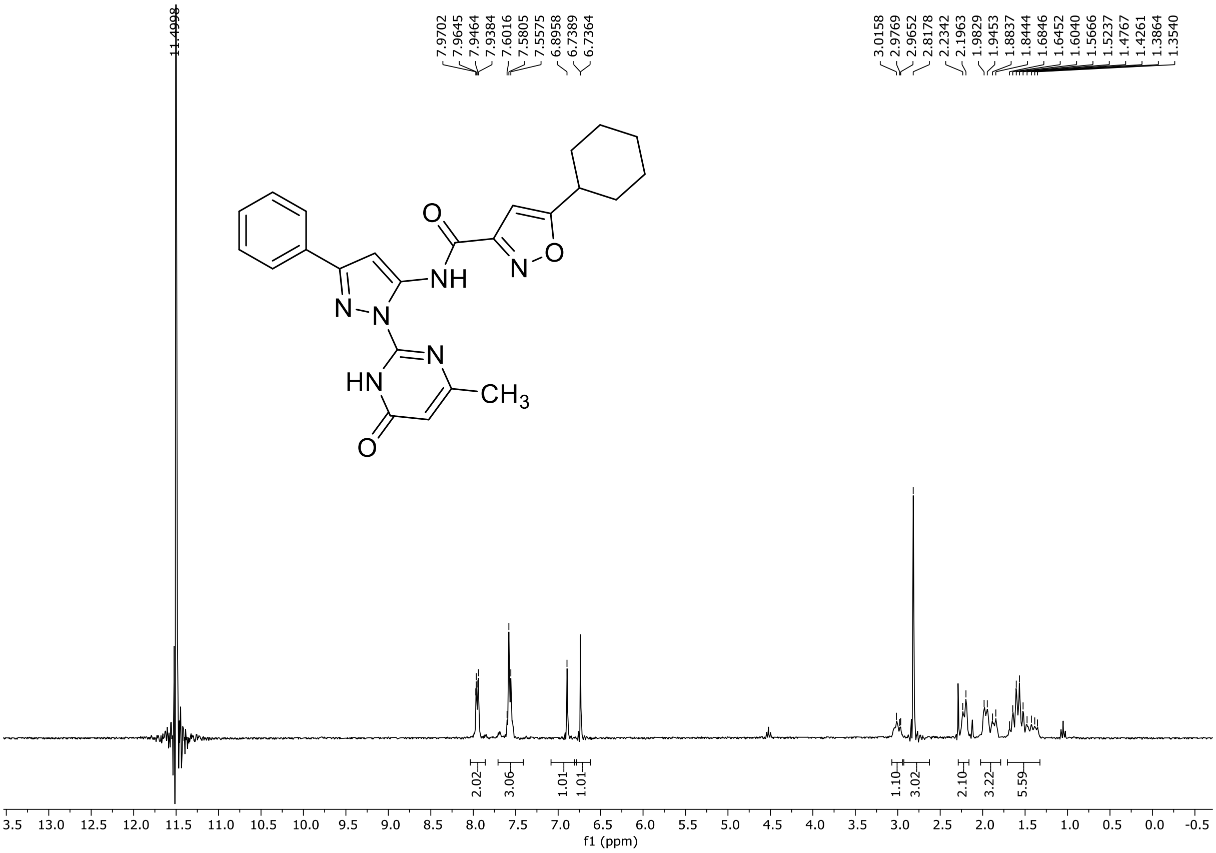

HRMS spectrum of **37**

NAR-A-11 $C_{24}H_{24}N_6O_3$ ***m/z* 444.1910**

ESI- (MMI)
nitrogen flow 5 L/min, gas temperature 325°C, nebulizer 45 psi, skimmer 65 V, fragmentor 150 V, dissolved in DMSO, MeOH

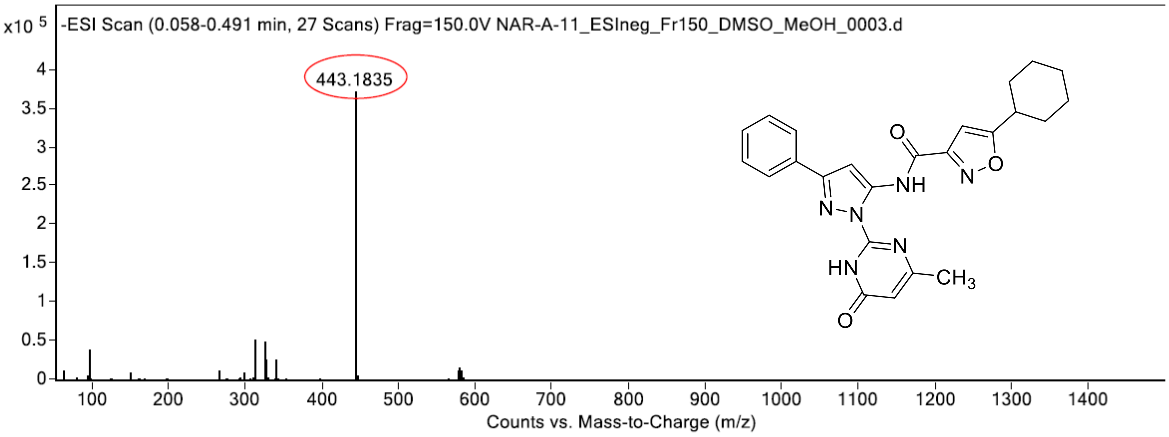

calculated mass: $[M\text{-}H]^{-}$ = 443.1837 observed: $[M\text{-}H]^{-}$ = 443.1835 mass accuracy = -0.5 ppm

FT-IR spectrum (neat) of **37**

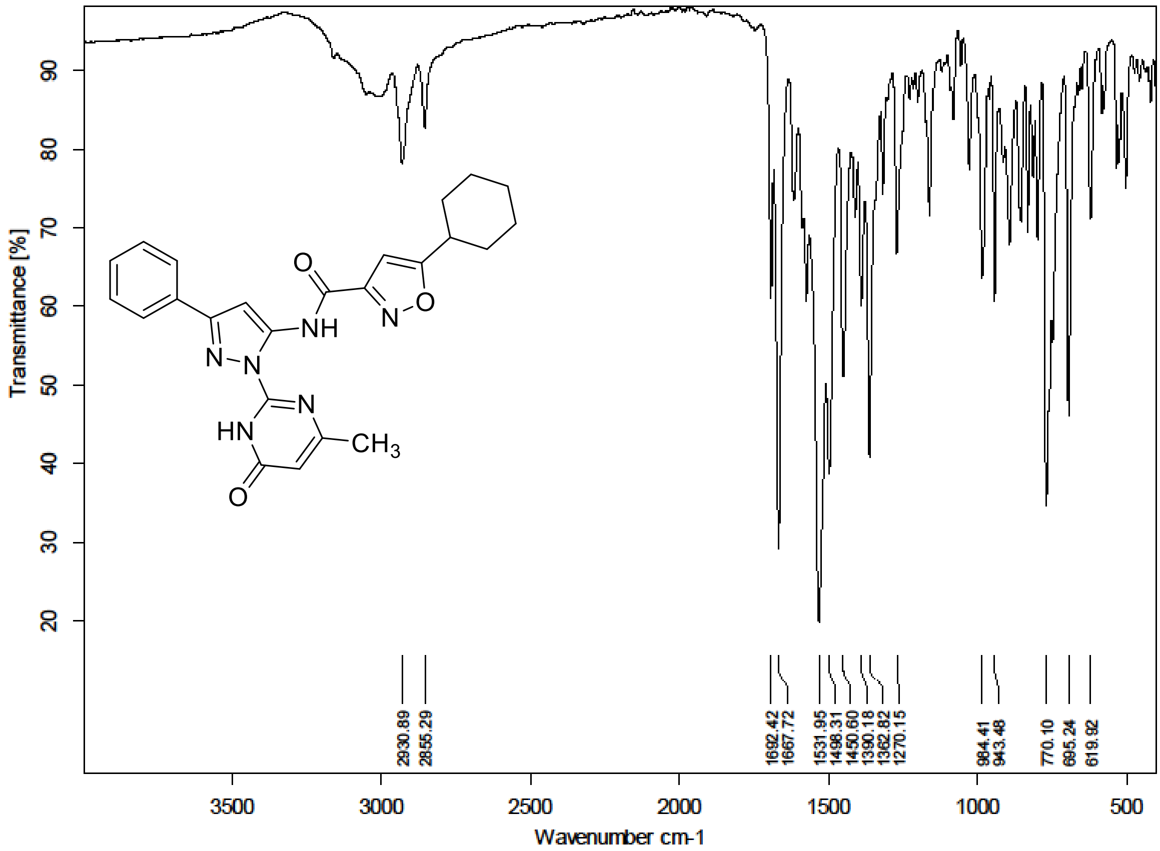

^{1}H NMR (300 MHz) spectrum of **38** in DMSO-d_6

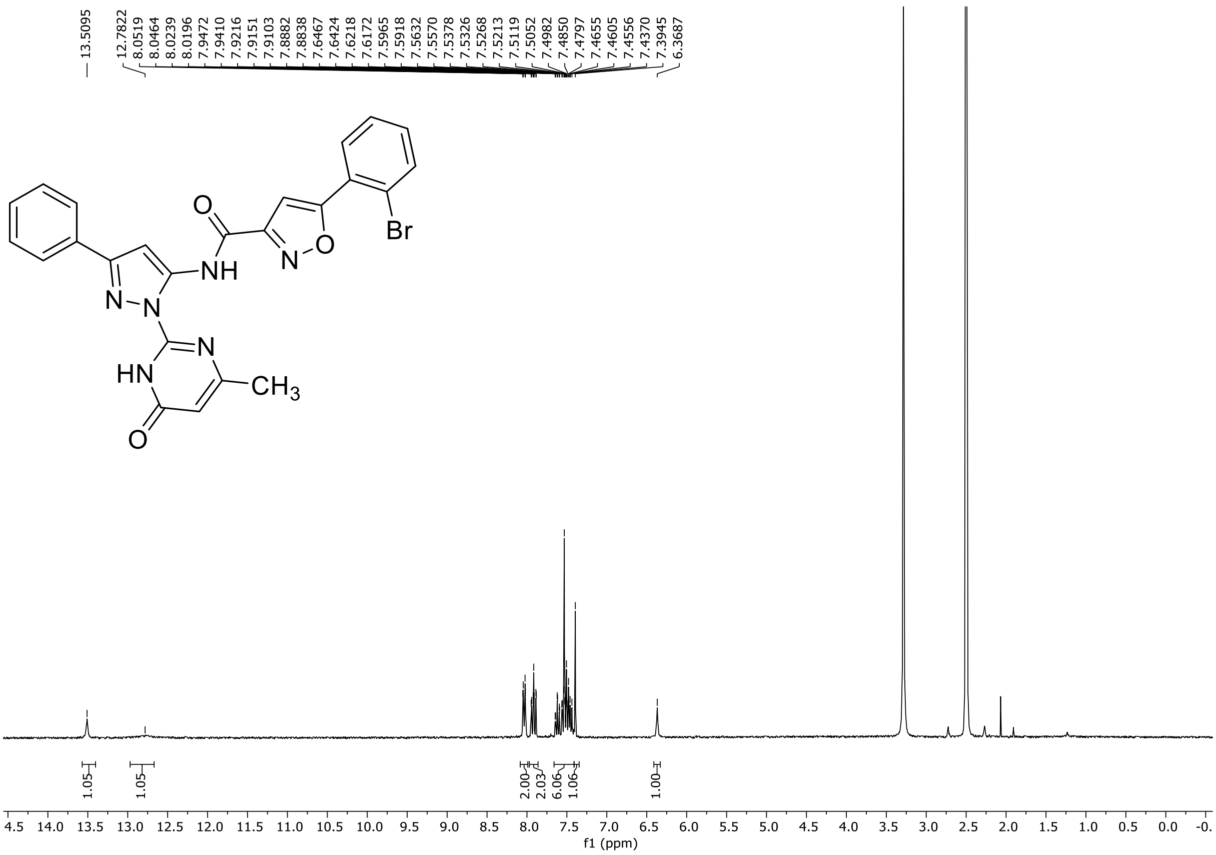

HRMS spectrum of **38**

NAR-A-95 $C_{24}H_{17}BrN_6O_3$ $m/z_{100\%}$ **516.0546**

Mixed Scan (APCI+ESI, both in negative mode) (MMI)
nitrogen flow 5 L/min, gas temperature 325°C, nebulizer 45 psi, skimmer 65 V, vaporizer 200°C, fragmentor 150 V, dissolved in DMSO, MeOH

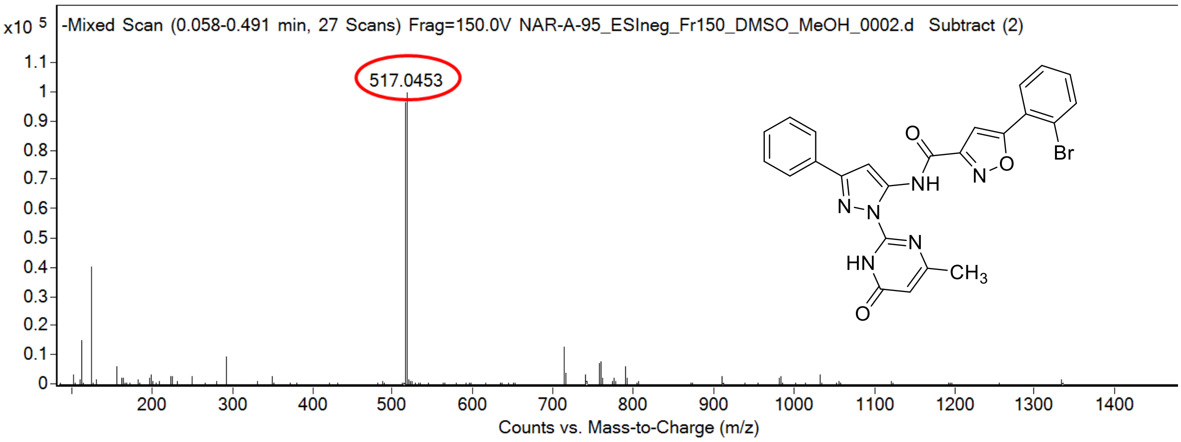

calculated mass: $[M-H]^-$ = 517.0456 observed: $[M-H]^-$ = 517.0453 mass accuracy = -0.6 ppm

FT-IR spectrum (neat) of **38**

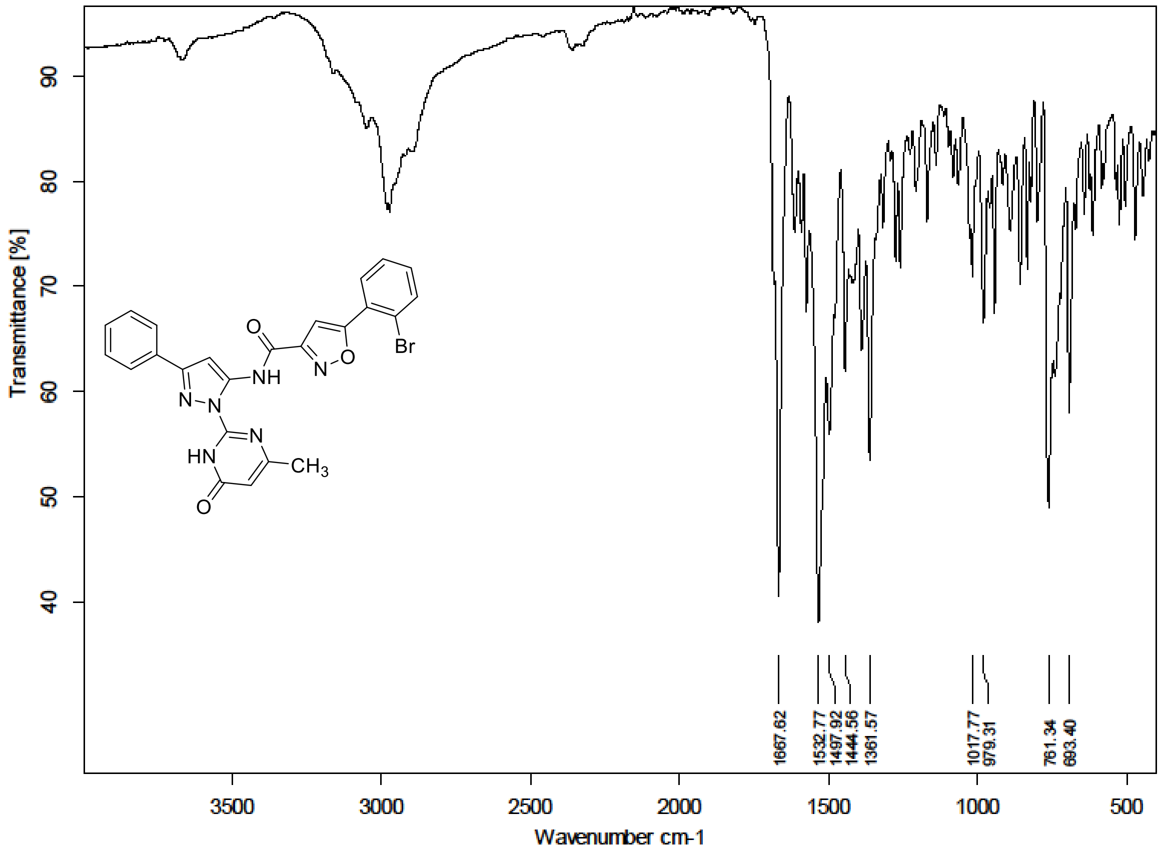

^{1}H NMR (300 MHz) spectrum of **39** in DMSO-d_6

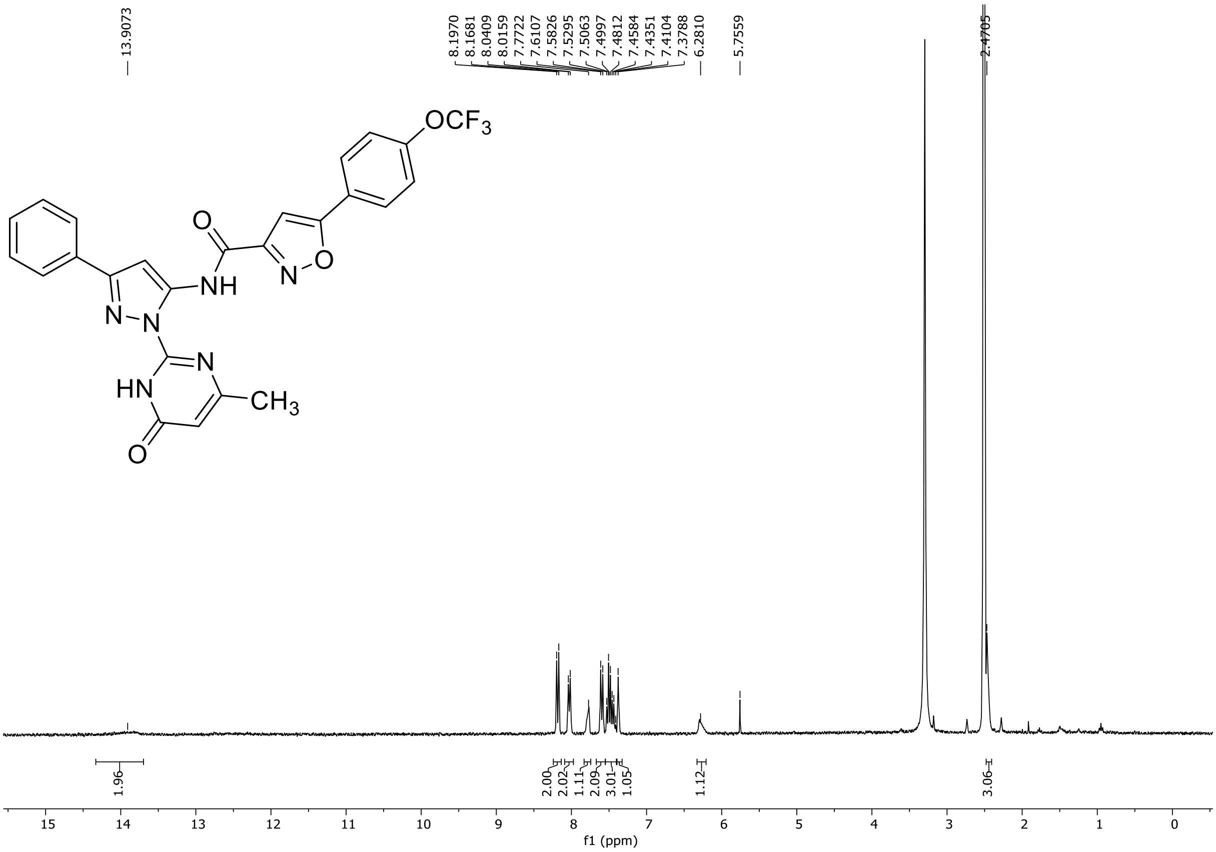

HRMS spectrum of **39**

NAR-A-87 $C_{25}H_{17}F_3N_6O_4$ *m/z* **522.1263**

ESI- (MMI)
nitrogen flow 5 L/min, gas temperature 325°C, nebulizer 45 psi, skimmer 65 V, fragmentor 60 V, dissolved in DMSO, MeOH

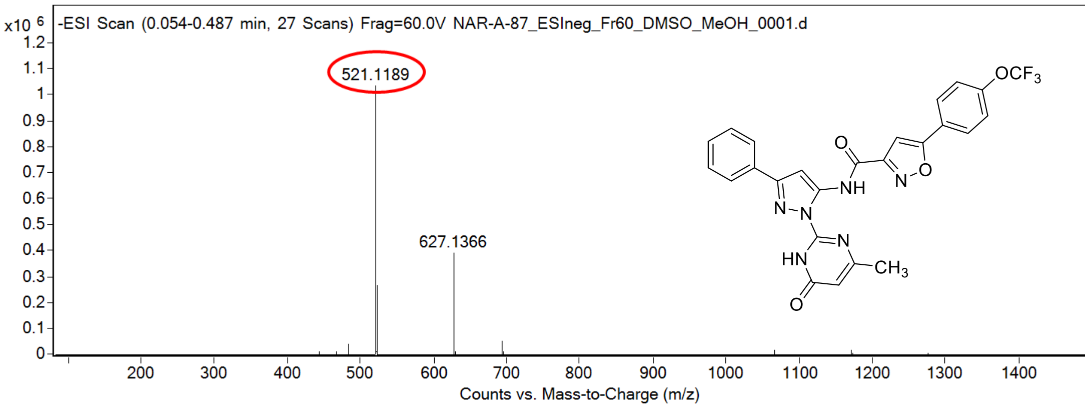

calculated mass: [M-H]$^-$ = 521.1191 observed: [M-H]$^-$ = 521.1189 mass accuracy = -0.4 ppm

FT-IR spectrum (neat) of **39**

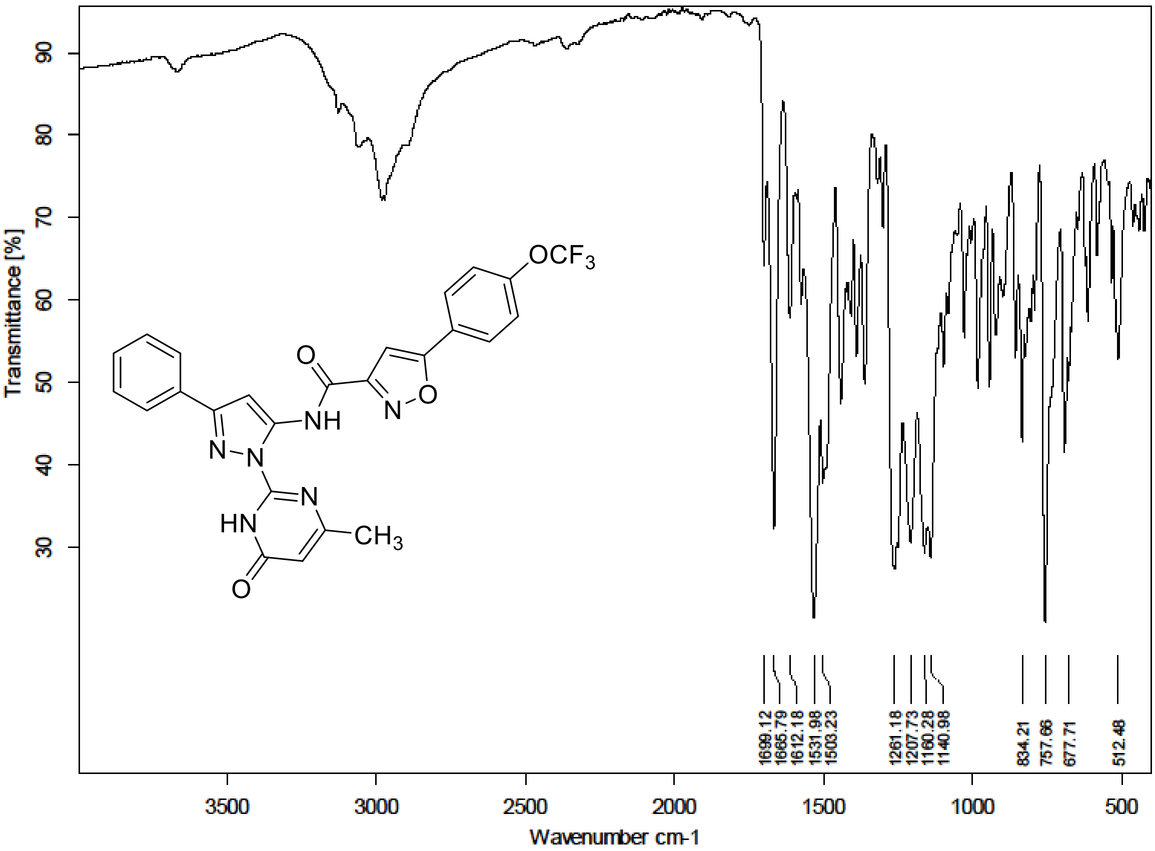

^{1}H NMR (300 MHz) spectrum of **40** in DMSO-d_6

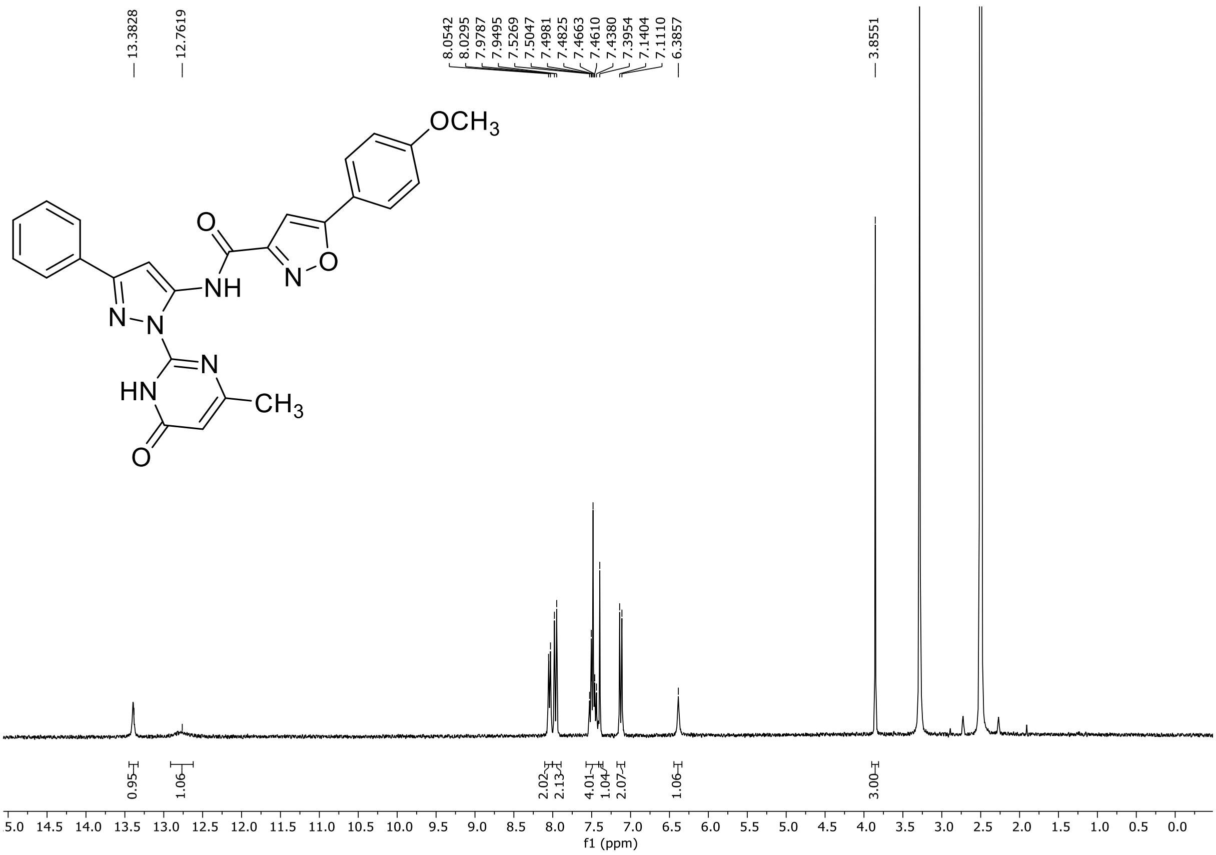

HRMS spectrum of **40**

NAR-A-131-1 $C_{25}H_{20}N_6O_4$ ***m/z*** **468.1546**

ESI- (MMI)
nitrogen flow 5 L/min, gas temperature 325°C, nebulizer 45 psi, skimmer 65 V, fragmentor 120 V, dissolved in DMSO, MeOH

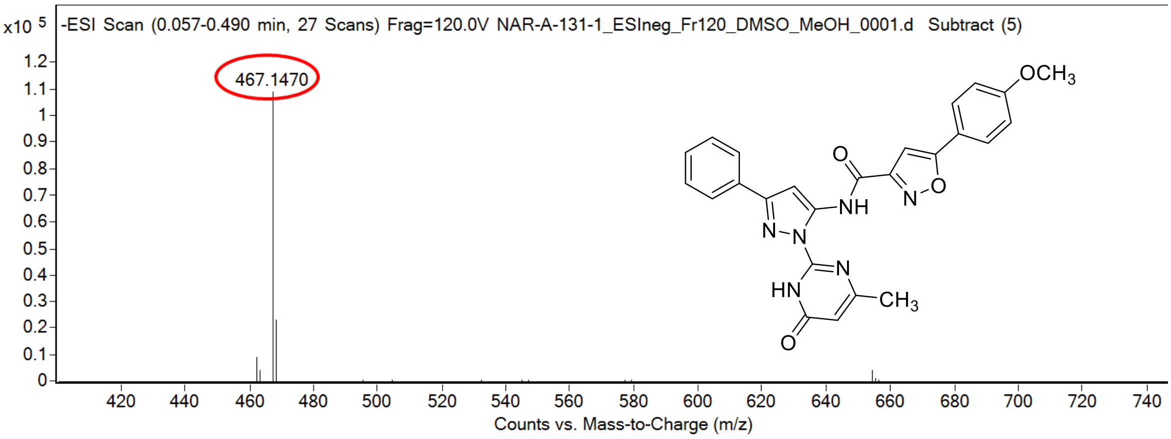

calculated mass: $[M-H]^-$ = 467.1473 observed: $[M-H]^-$ = 467.1470 mass accuracy = -0.6 ppm

FT-IR spectrum (neat) of **40**

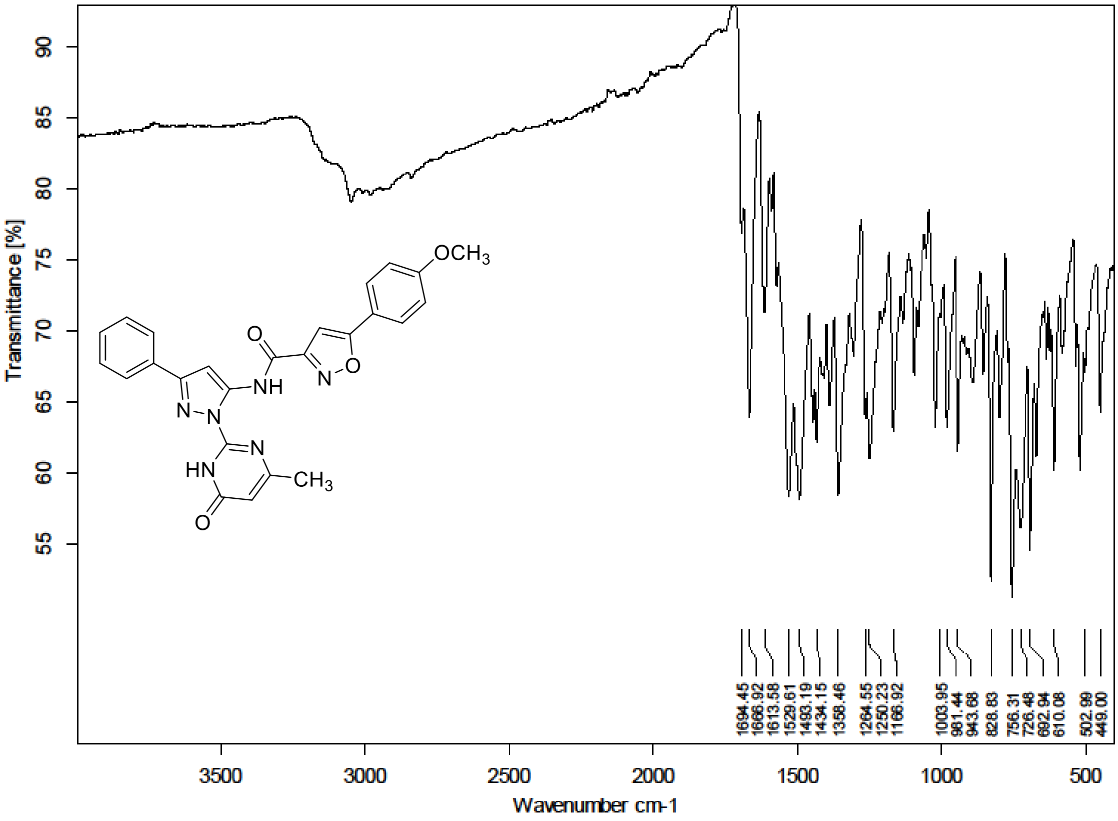

^{1}H NMR (300 MHz) spectrum of **41** in Trifluoroacetic Acid-*d*

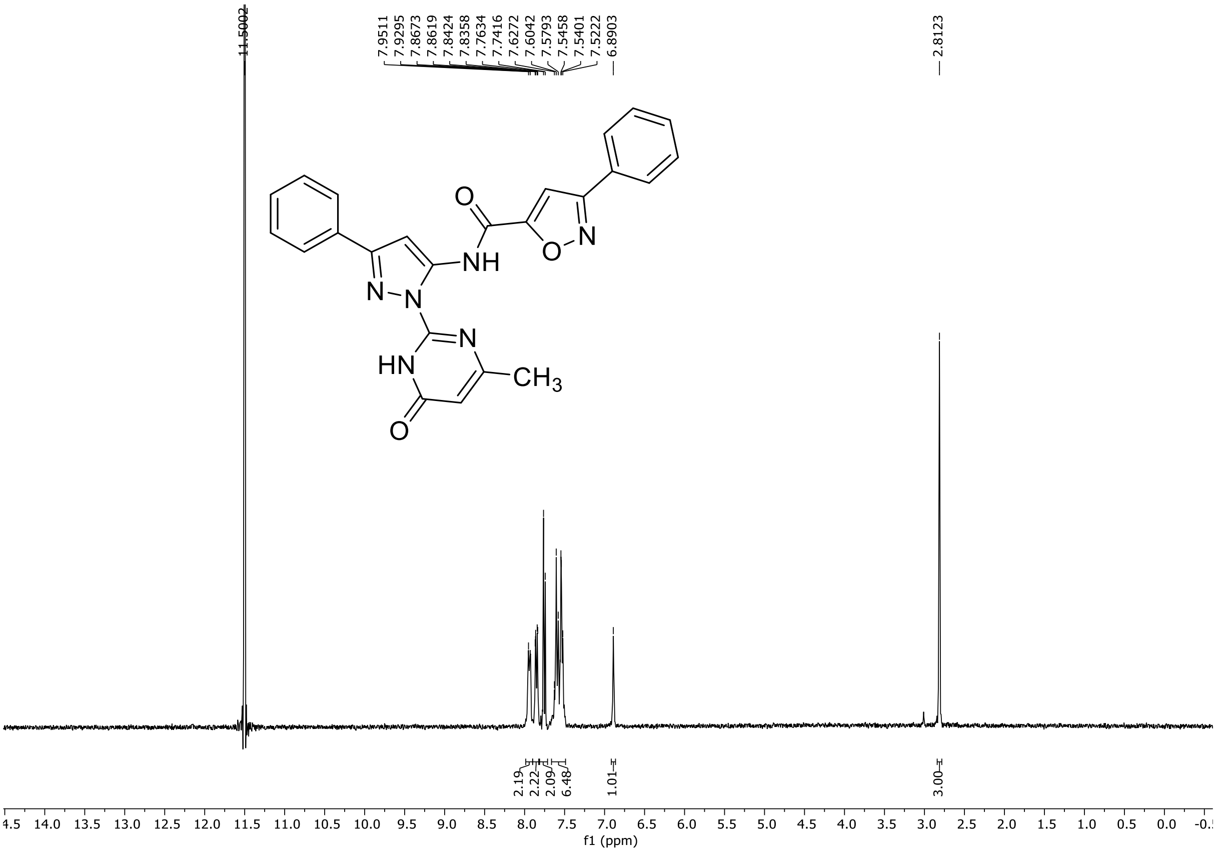

HRMS spectrum of **41**

NAR-A-153 $C_{24}H_{18}N_6O_3$ **mono *m/z* 438.1440**

APCI + (MMI)
nitrogen flow 5 L/min, gas temperature 325°C, nebulizer 45 psi, skimmer 65 V, vaporizer 200°C, fragmentor 33 V, dissolved in methanol / CH_2Cl_2

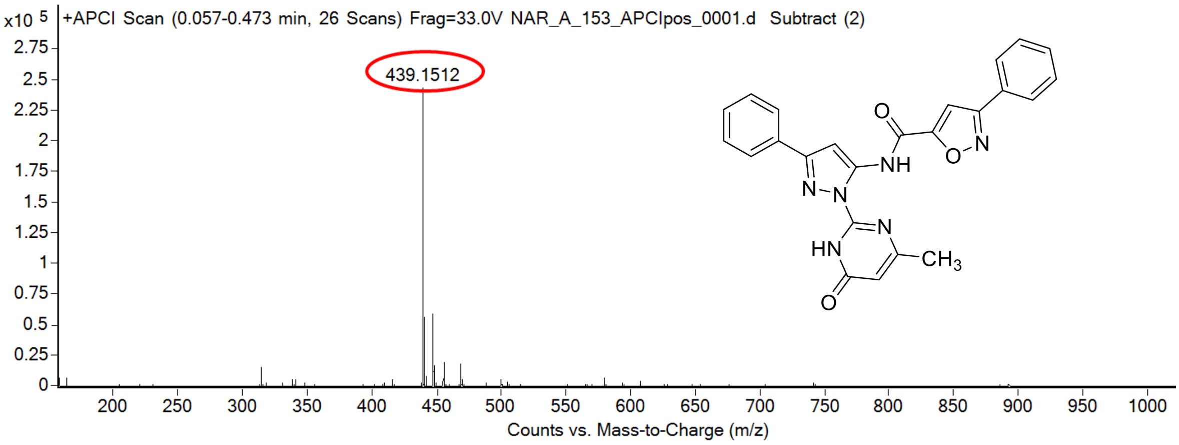

calculated mass: $[M+H]^+ = 439.1513$ observed: $[M+H]^+ = 439.1512$ mass accuracy = - 0.2 ppm

FT-IR spectrum (neat) of **41**

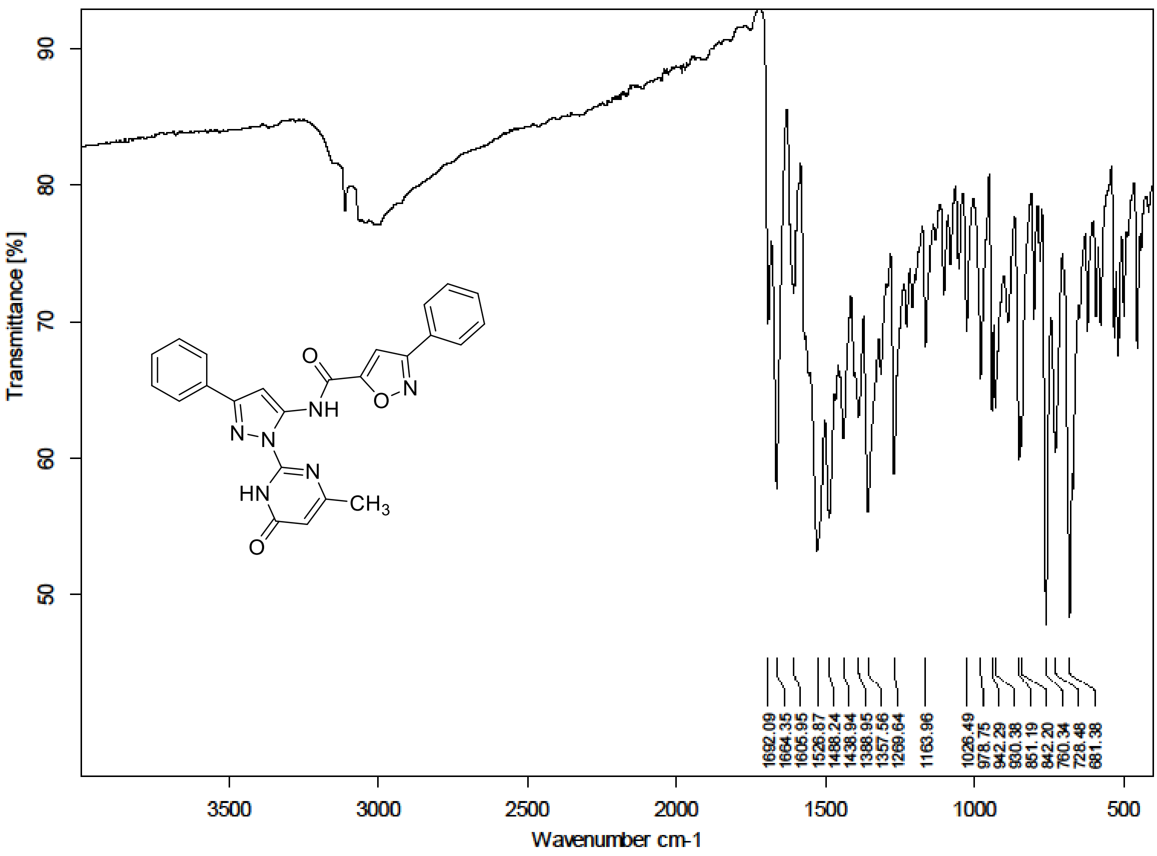

^{1}H (700 MHz) and ^{13}C NMR (176 MHz) spectra of **42** in DMSO-d_6

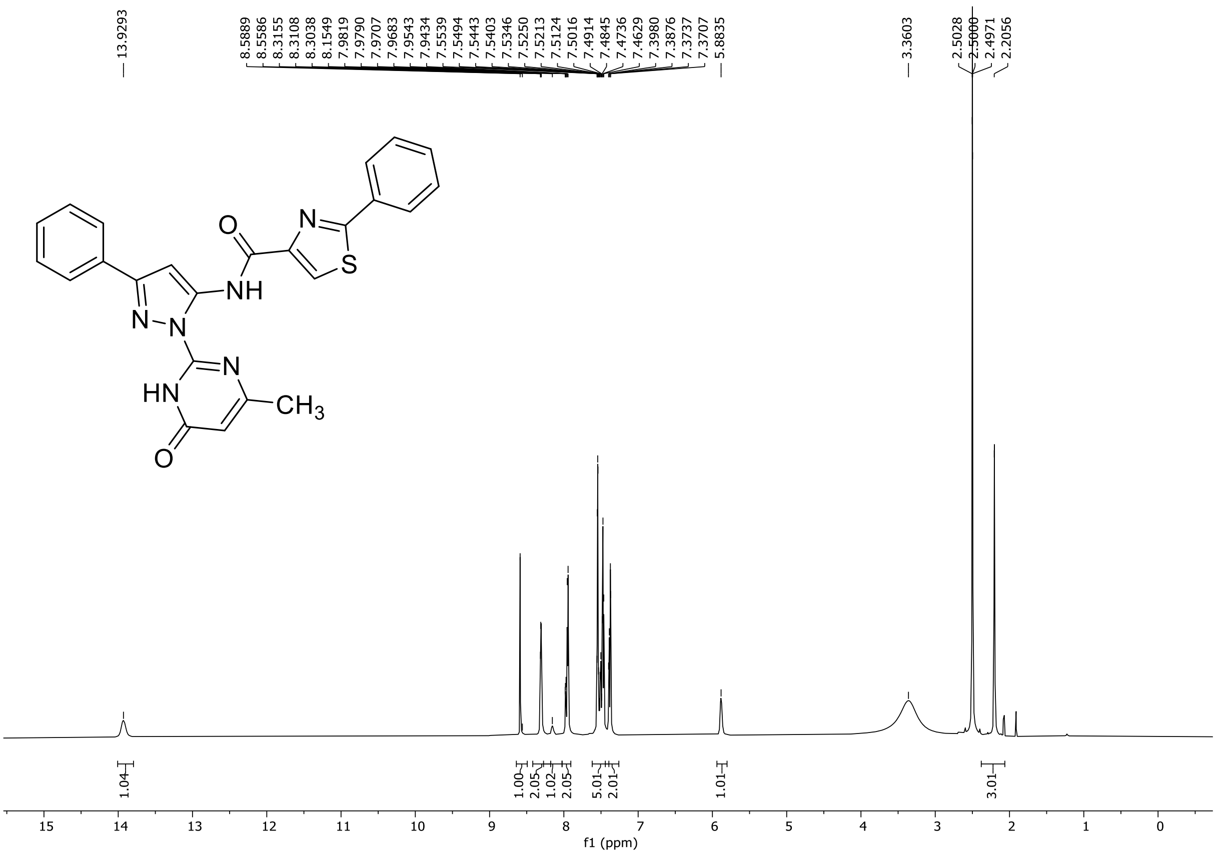

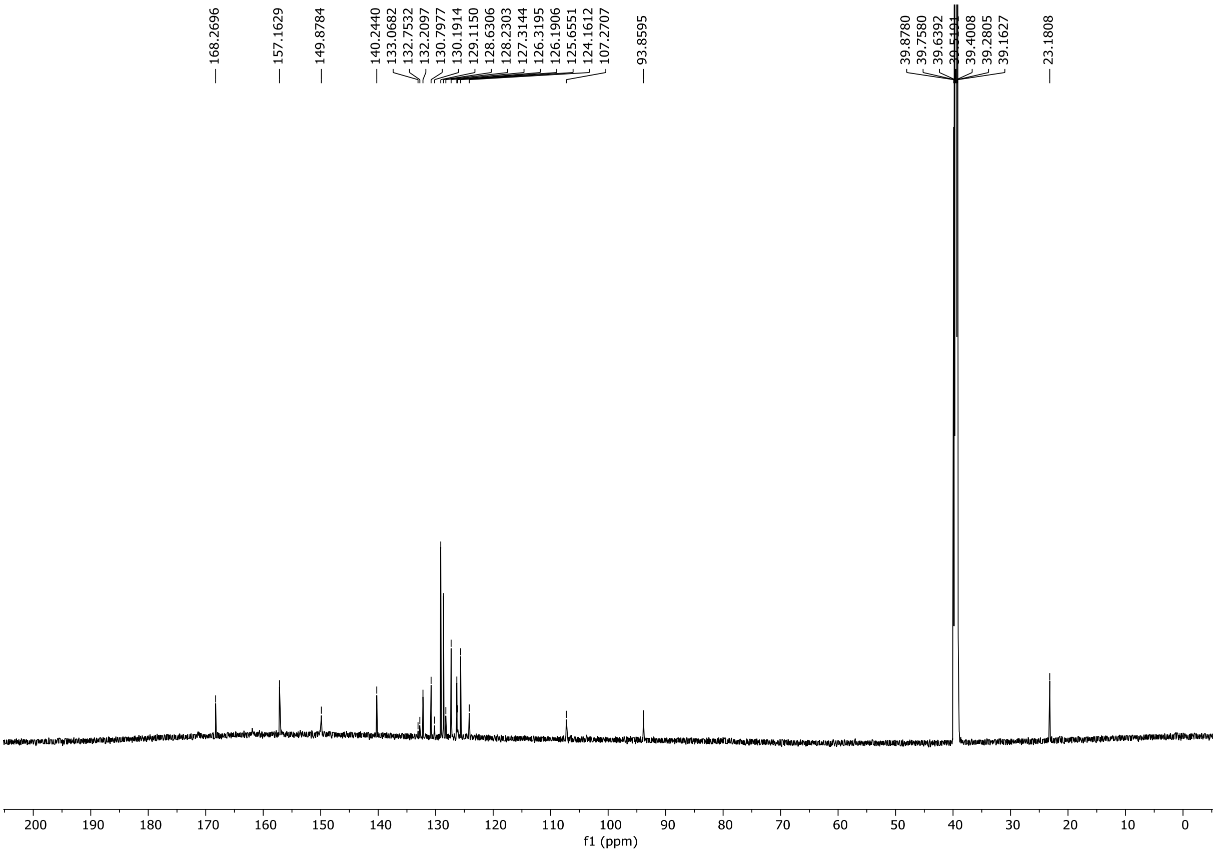

HRMS spectrum of **42**

NAR-A-154 $C_{24}H_{18}N_6O_2S$ mono *m/z* 454.1212

APCI + (MMI)
nitrogen flow 5 L/min, gas temperature 325°C, nebulizer 45 psi, skimmer **65** V, vaporizer 200°C, fragmentor 30 V, dissolved in methanol

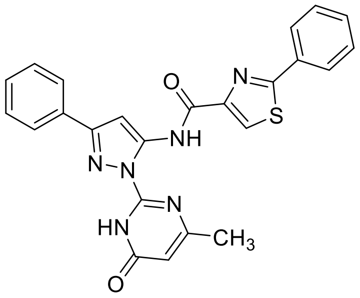

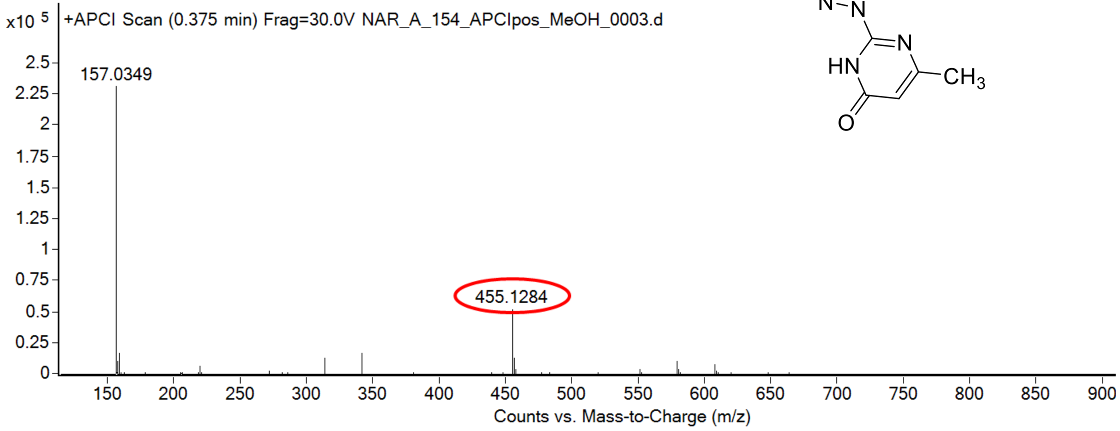

calculated mass: $[M+H]^+ = 455.1285$ observed: $[M+H]^+ = 455.1284$ mass accuracy = - 0.2 ppm

FT-IR spectrum (neat) of **42**

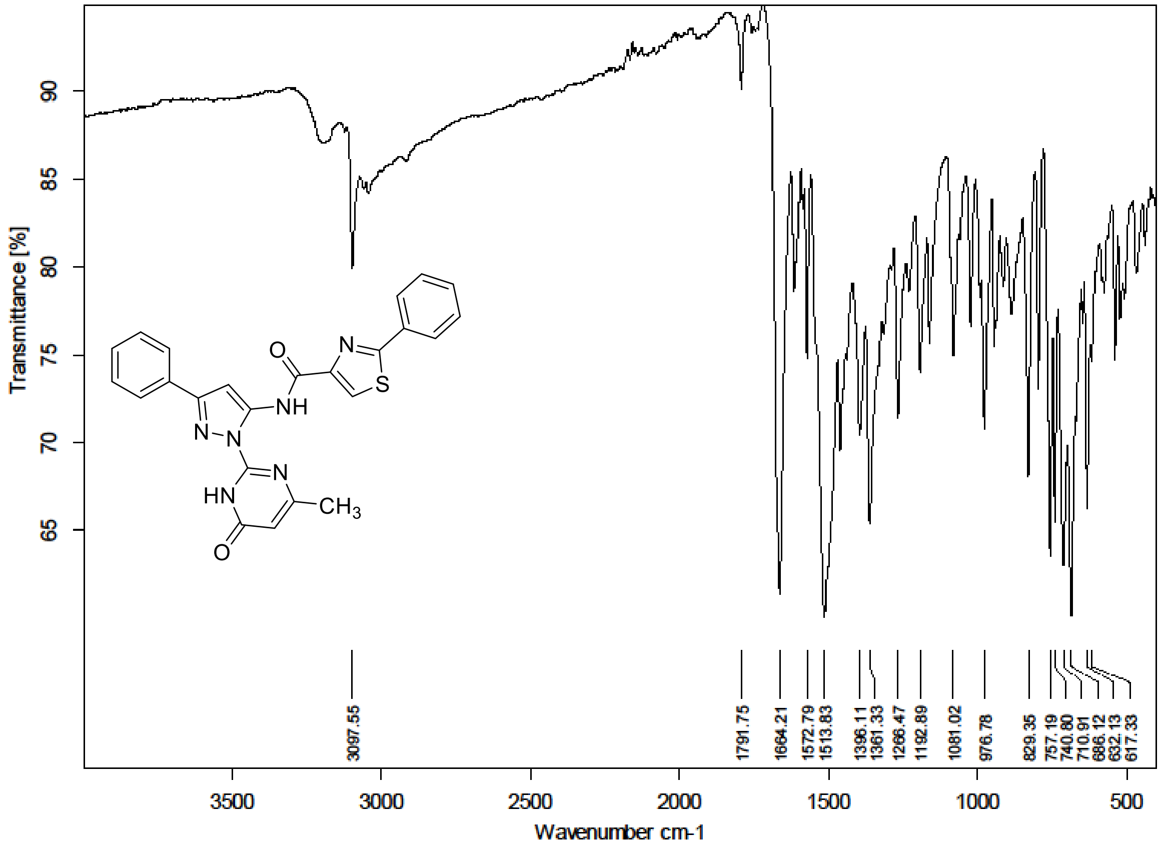

^{1}H NMR (300 MHz) spectrum of **43** in DMSO-d_6

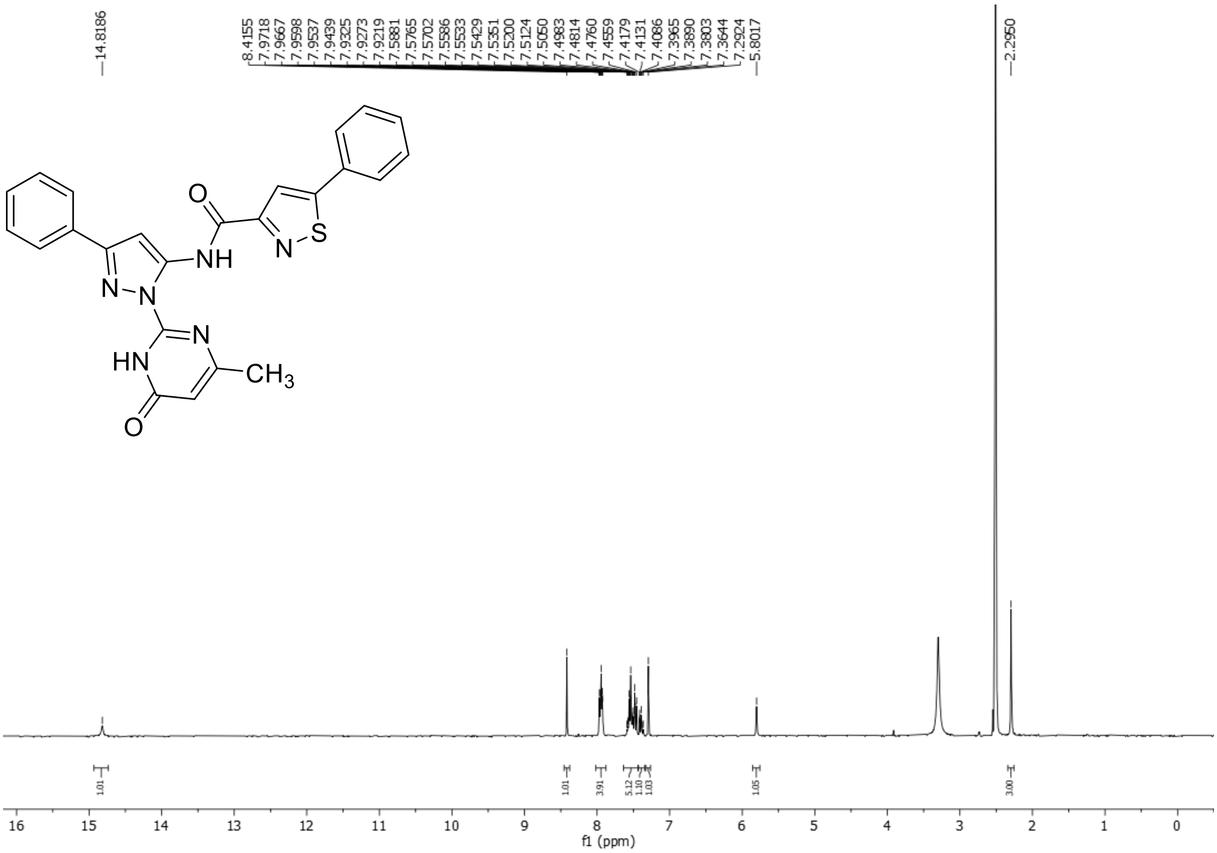

HRMS spectrum of **43**

NAR-A-155 $C_{24}H_{18}N_6O_2S$ mono *m/z* 454.1212

APCI + (MMI)
nitrogen flow 5 L/min, gas temperature 325°C, nebulizer 45 psi, skimmer 65 V, vaporizer 200°C, fragmentor 22 V, dissolved in methanol / CH_2Cl_2

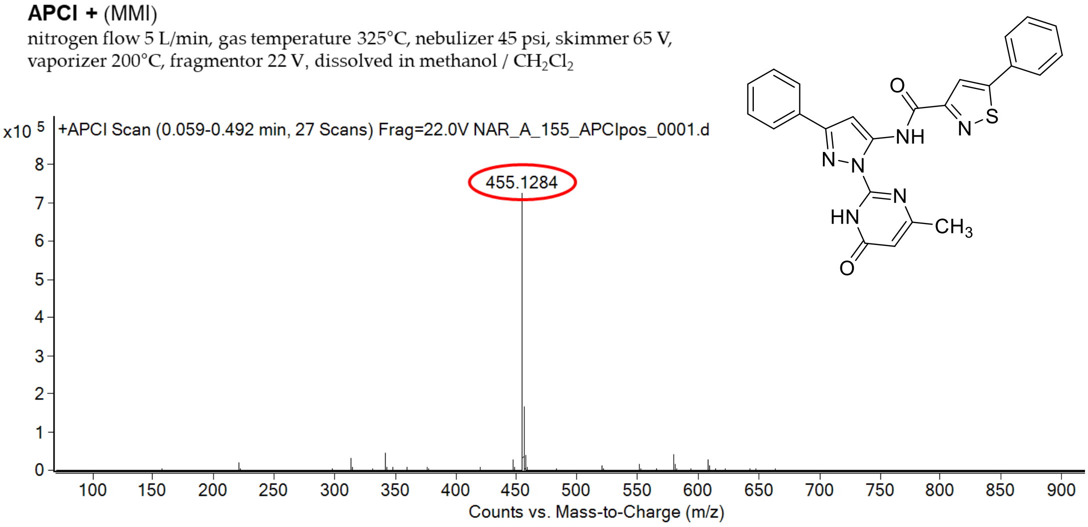

calculated mass: $[M+H]^+ = 455.1285$ observed: $[M+H]^+ = 455.1284$ mass accuracy = - 0.2 ppm

FT-IR spectrum (neat) of **43**

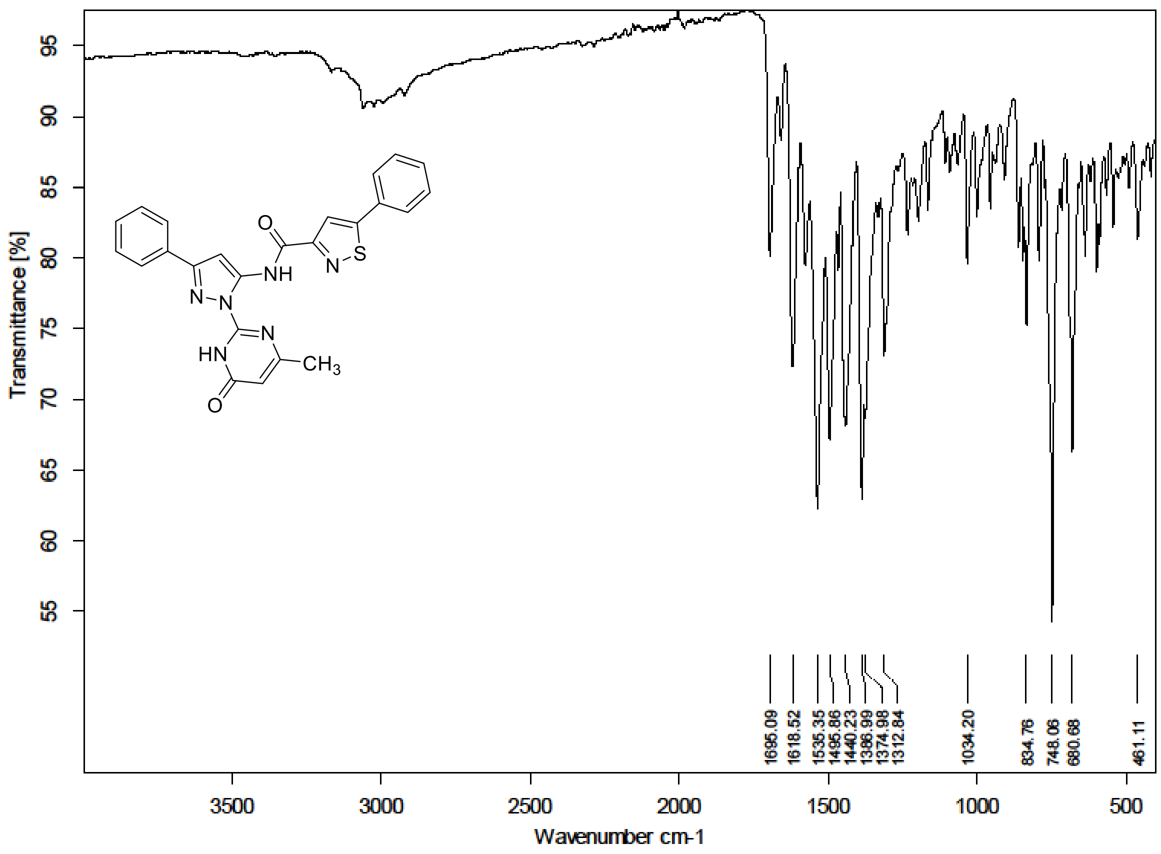

^{1}H (300 MHz) and ^{13}C NMR (126 MHz) spectra of **44** in Chloroform-*d*

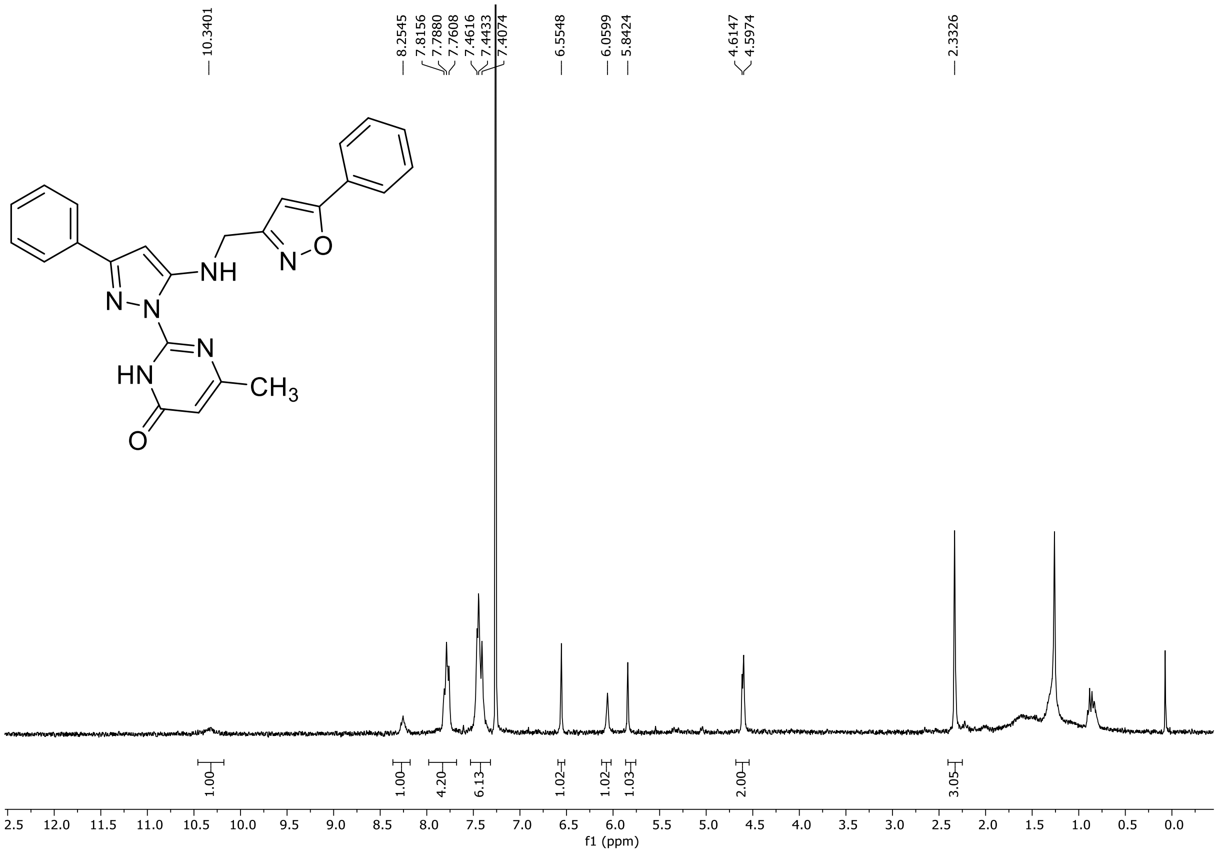

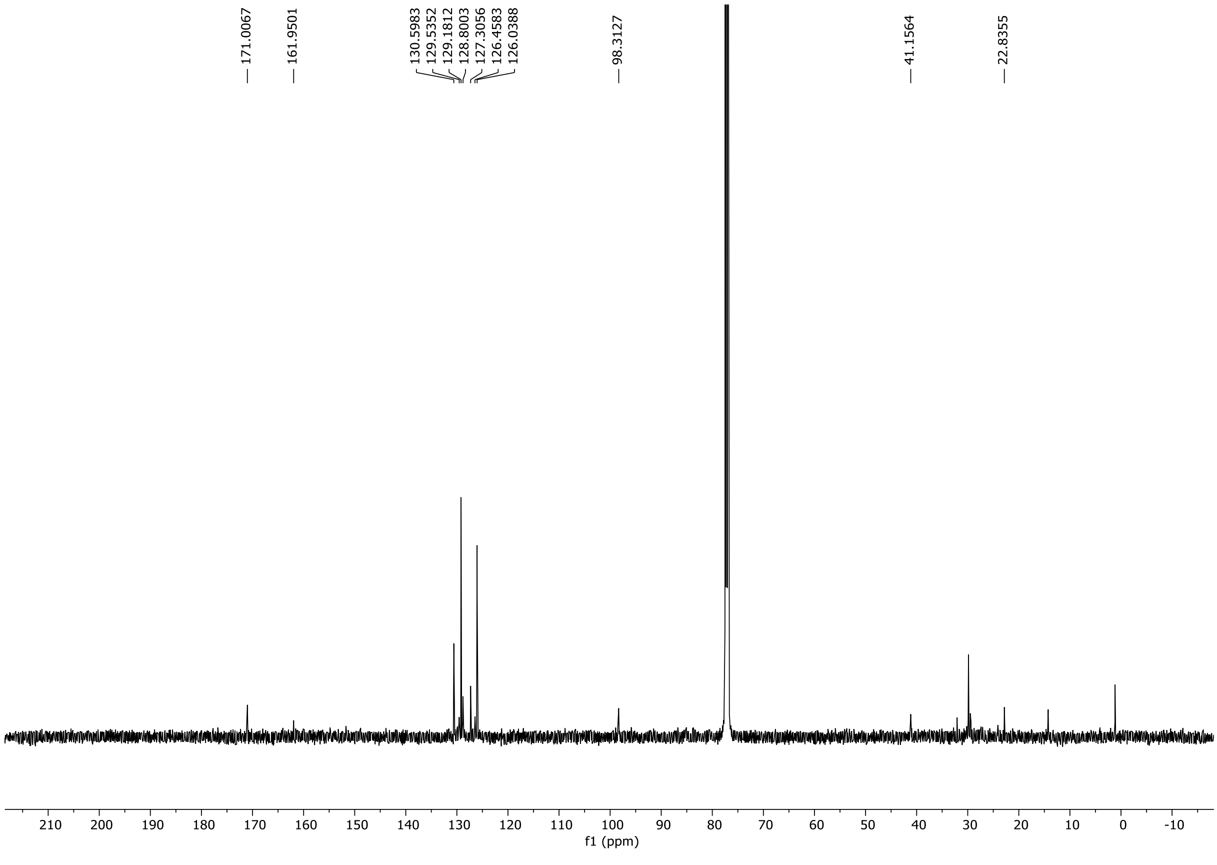

HRMS spectrum of **44**

NAR-A-67-1 $C_{24}H_{20}N_6O_2$ *mono m/z* **424.1648**

APCI + (MMI)
nitrogen flow 5 L/min, gas temperature 300°C, nebulizer 45 psi, skimmer **65** V, vaporizer 200°C, fragmentor 35 V, dissolved in MeOH

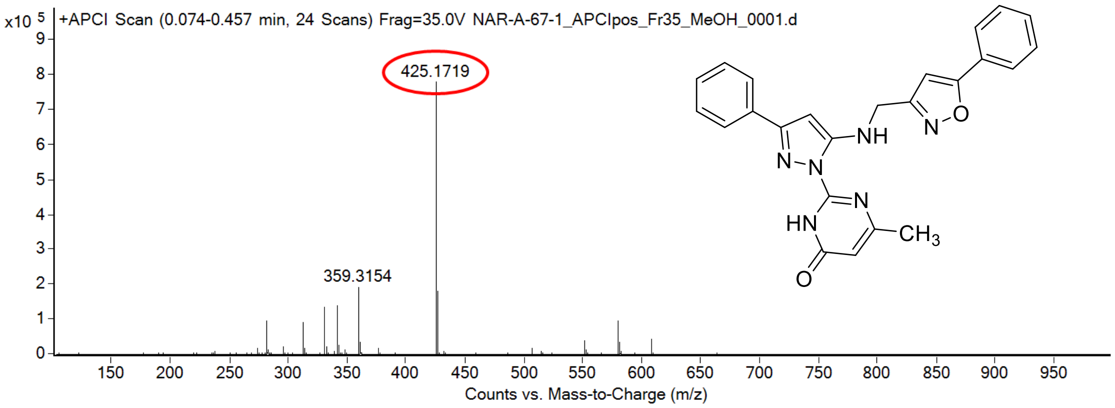

calculated mass: $[M+H]^+$ = 425.1721 observed: $[M+H]^+$ = 425.1719 mass accuracy = -0.5 ppm

FT-IR spectrum (neat) of **44**

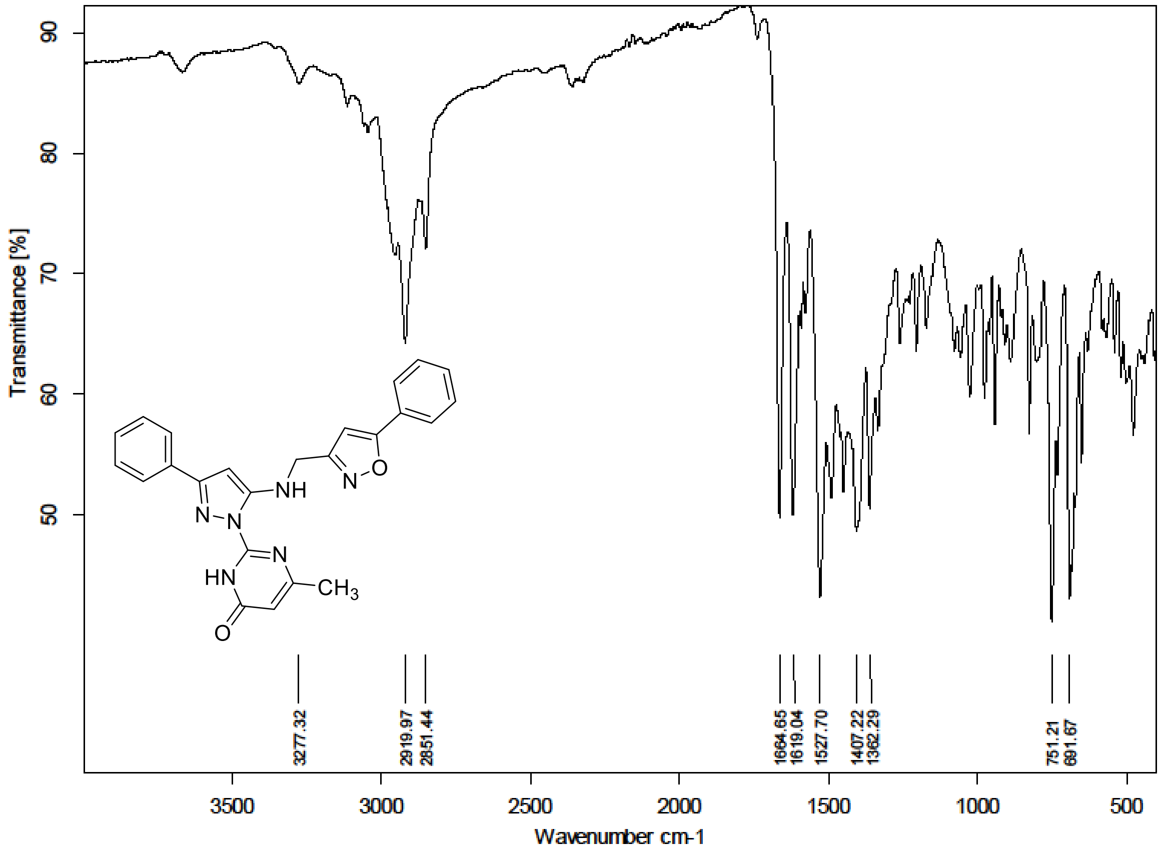

^{1}H NMR (300 MHz) spectrum of **S190** in Chloroform-*d*

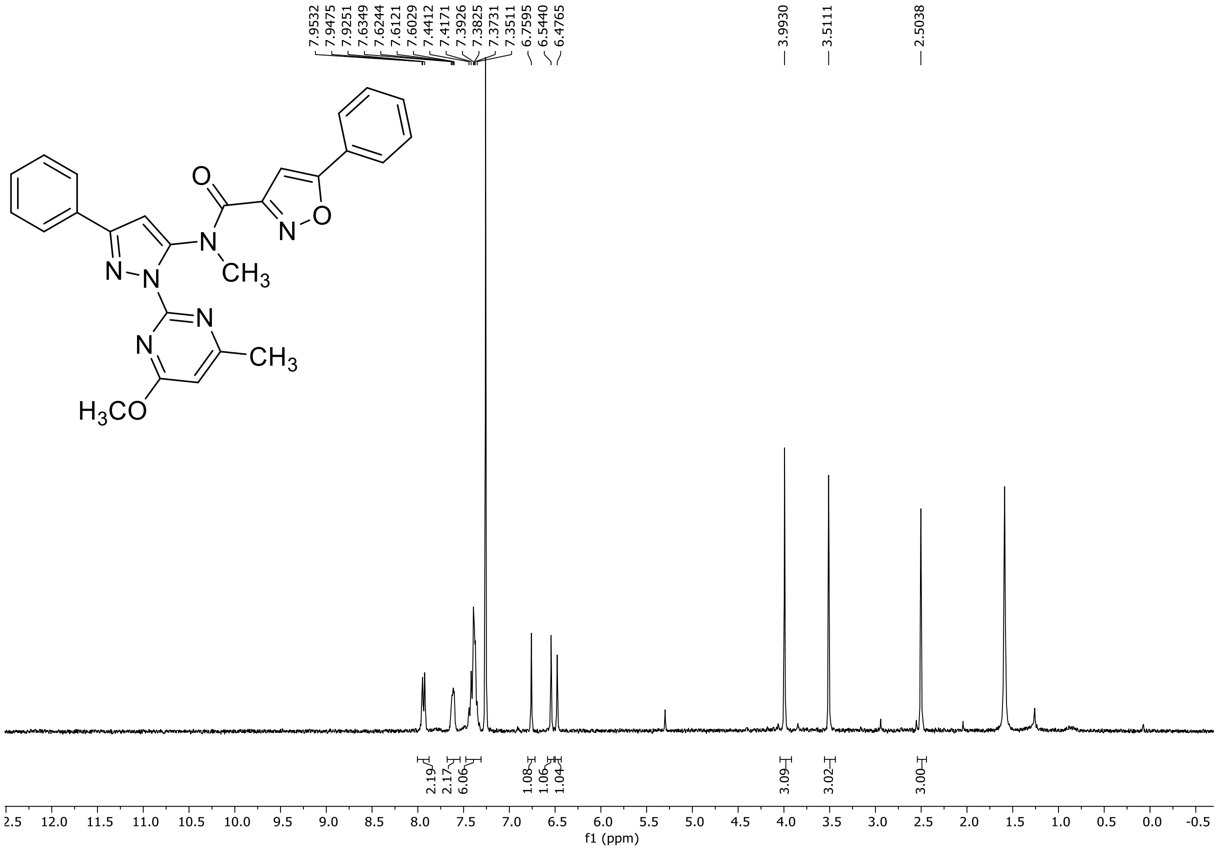

HRMS spectrum of **S190**

NAR-A-165 $C_{26}H_{22}N_6O_3$ mono *m/z* 466.1753

APCI + (MMI)
nitrogen flow 5 L/min, gas temperature 325°C, nebulizer 45 psi, skimmer **65** V, vaporizer 200°C, fragmentor 15 V, dissolved in methanol

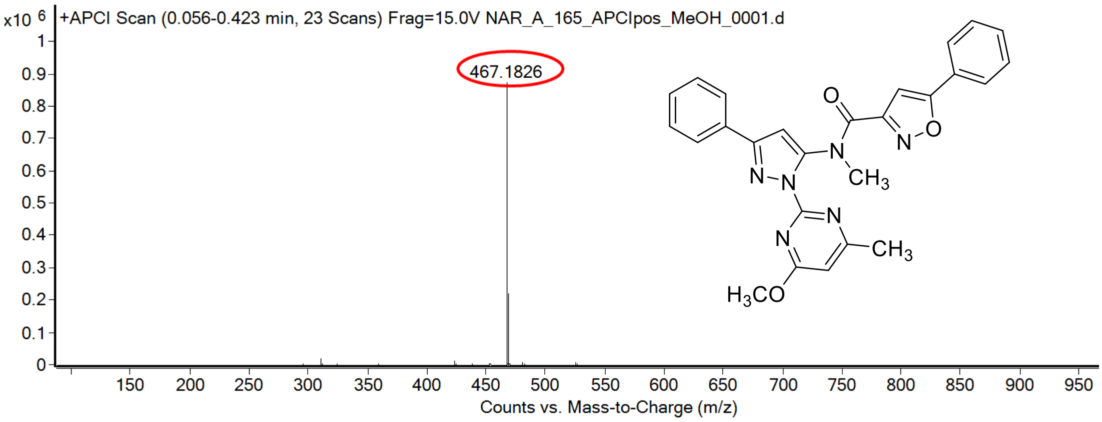

calculated mass: [M+H]$^+$ = 467.1826 observed: [M+H]$^+$ = 467.1826 mass accuracy = < 0.1 ppm

^{1}H (300 MHz) and ^{13}C NMR (75 MHz) spectra of **45** in Chloroform-*d*

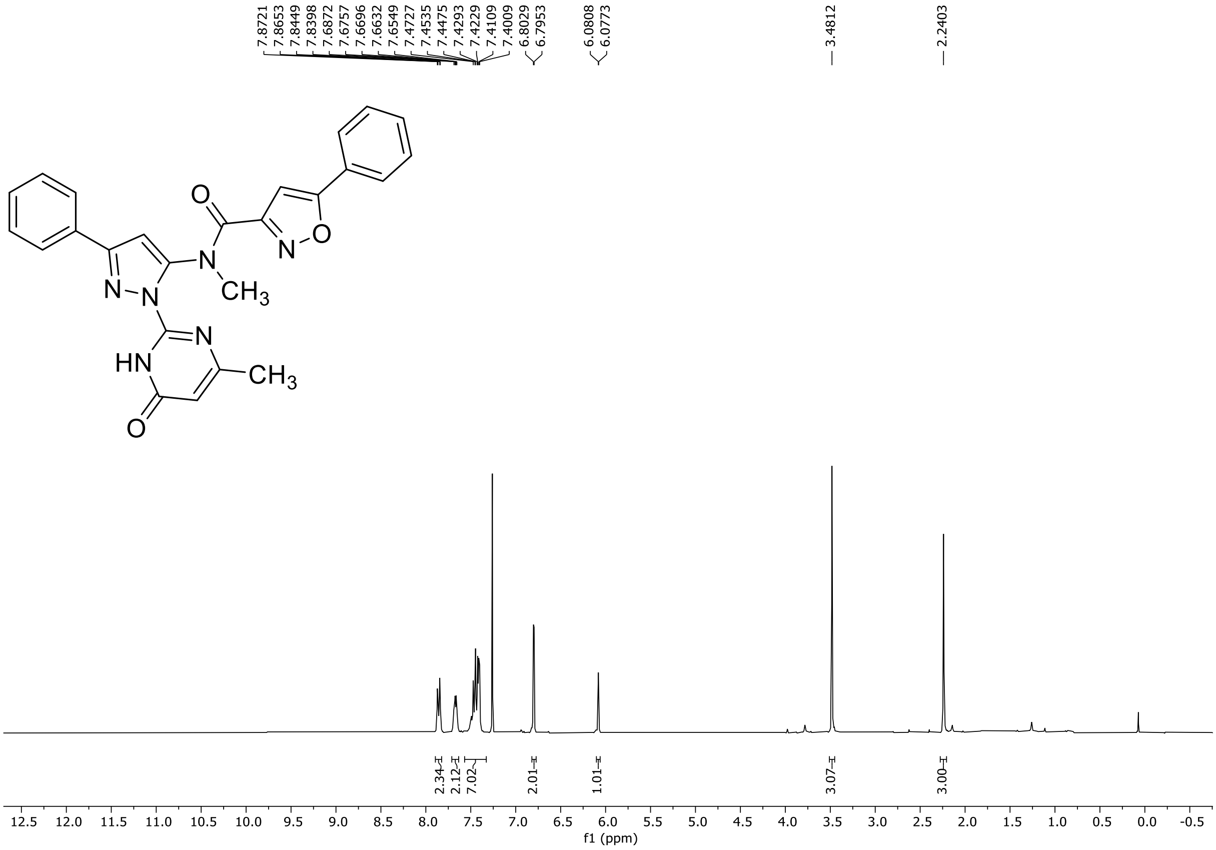

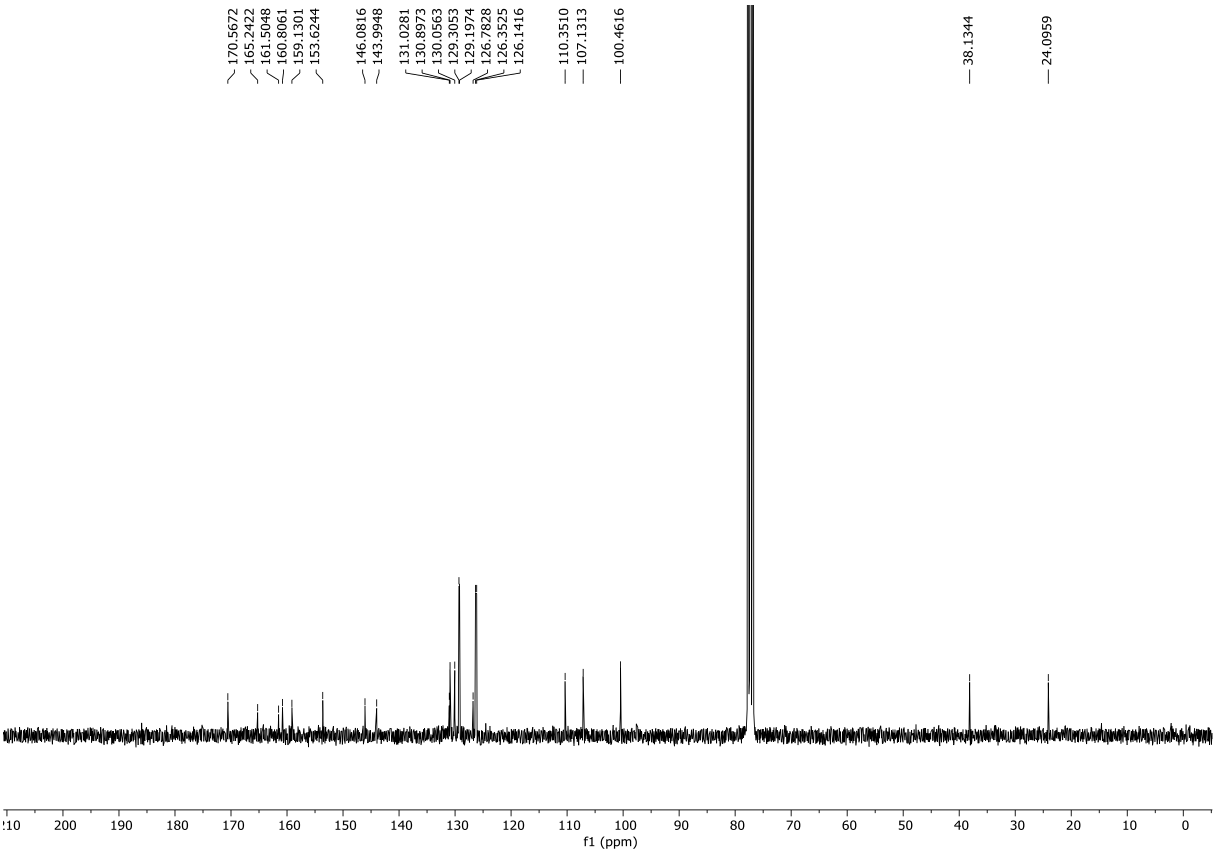

HRMS spectrum of **45**

NAR-A-176 $C_{25}H_{20}N_6O_3$ mono *m/z* 452.1597

APCI + (MMI)
nitrogen flow 5 L/min, gas temperature 325°C, nebulizer 45 psi, skimmer **65** V, vaporizer 200°C, fragmentor 15 V, dissolved in methanol

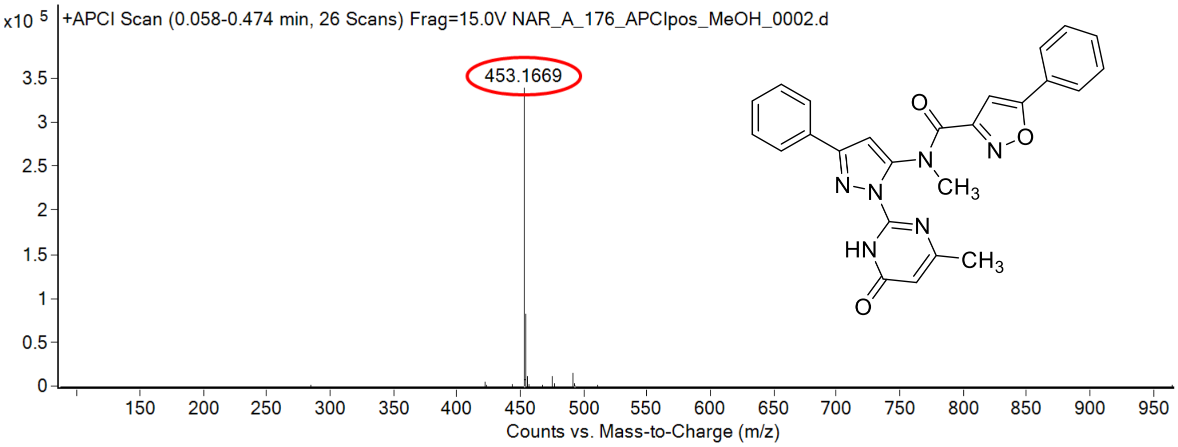

calculated mass: $[M+H]^+$ = 453.1670 observed: $[M+H]^+$ = 453.1669 mass accuracy = - 0.2 ppm

FT-IR spectrum (neat) of **45**

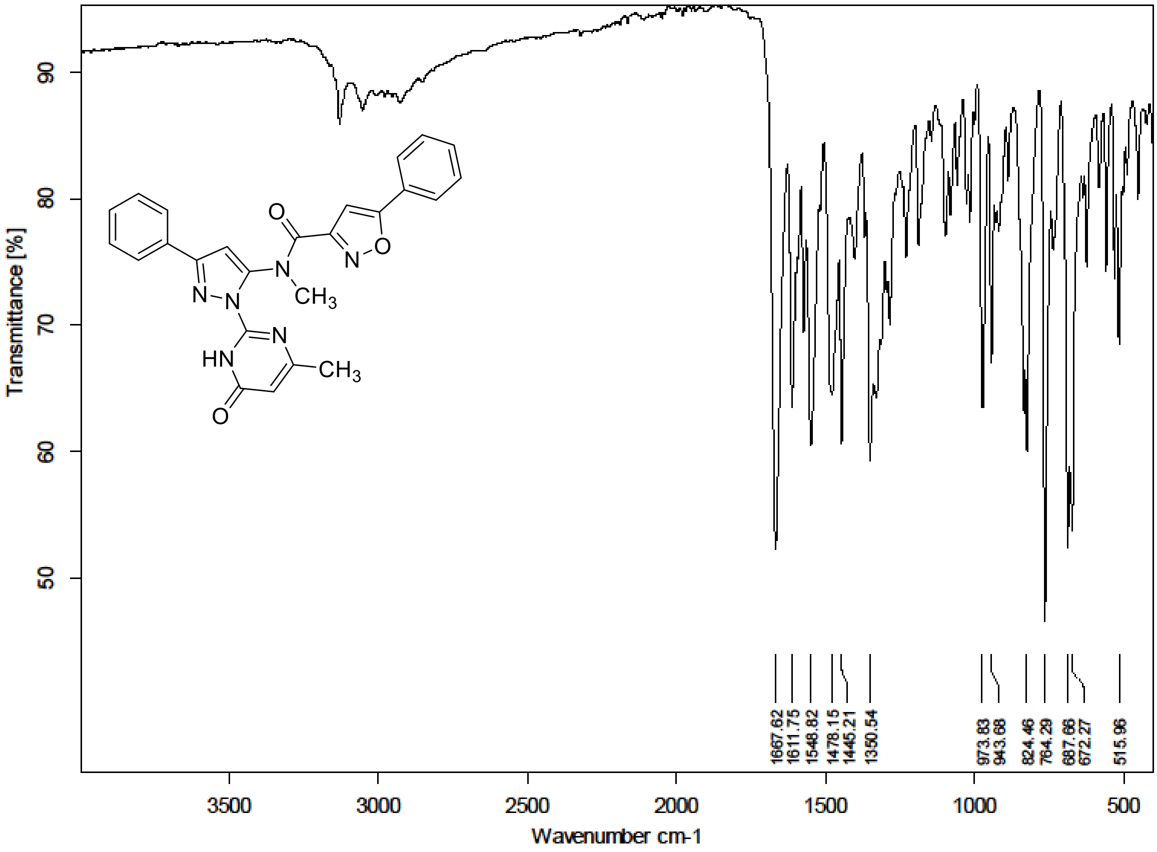

^{1}H (300 MHz) and ^{13}C NMR (126 MHz) spectra of **46** Chloroform-*d*

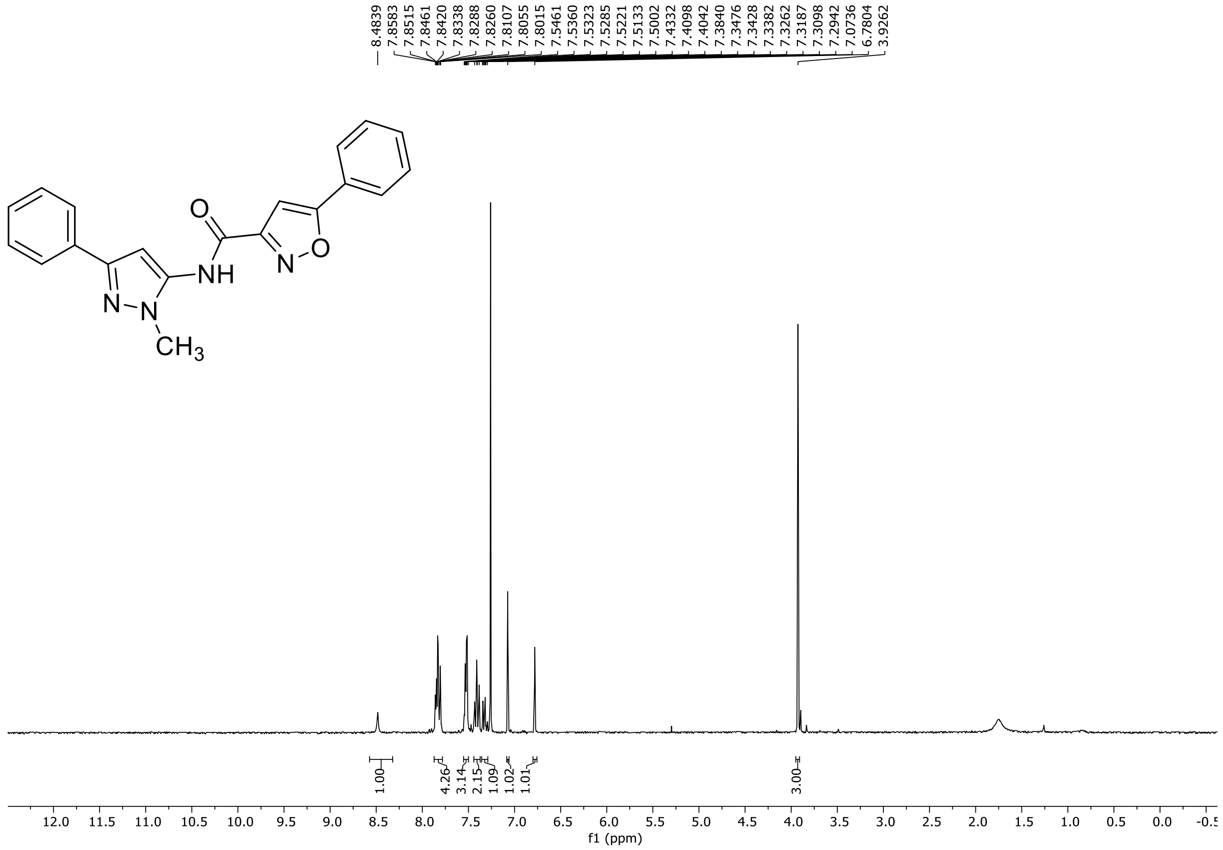

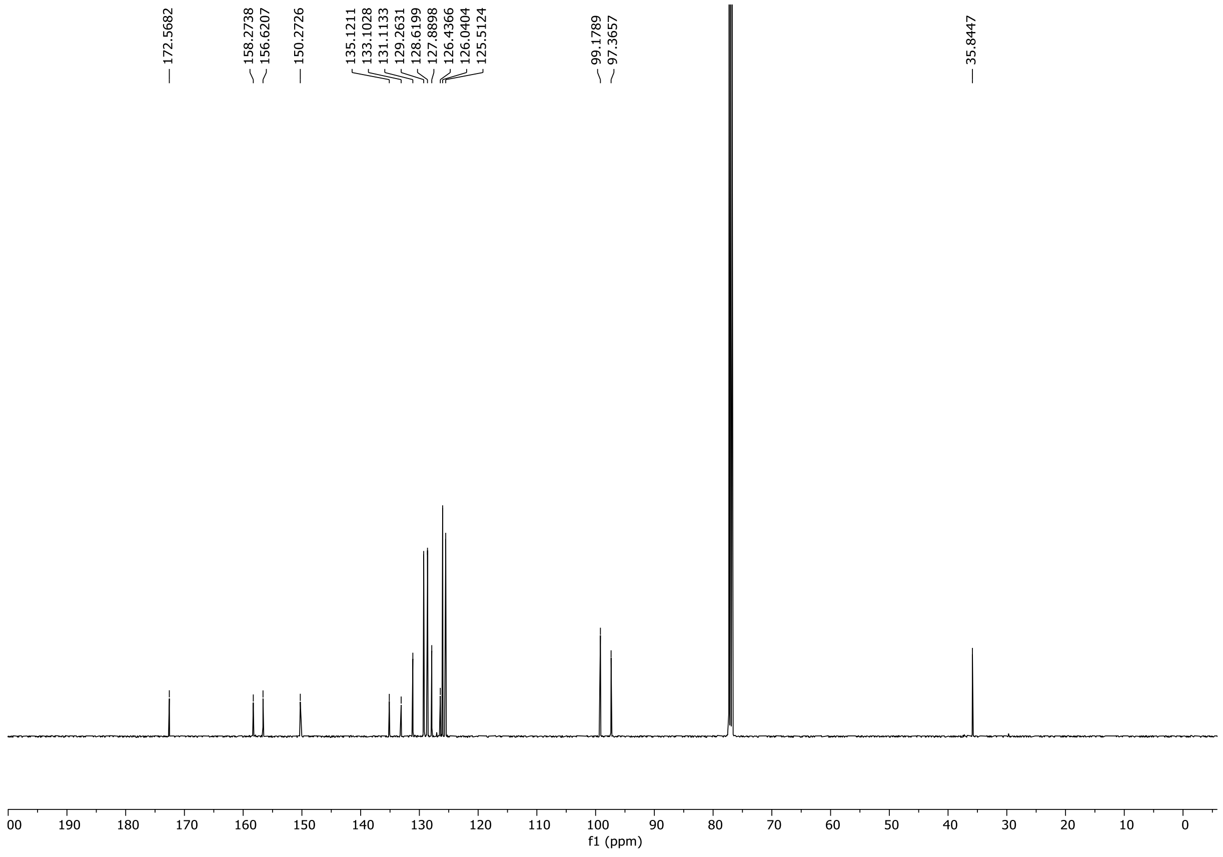

HRMS spectrum of **46**

NAR-A-88 $C_{20}H_{16}N_4O_2$ *m/z* **344.1273**

APCI+ (MMI)
nitrogen flow 5 L/min, gas temperature 325°C, nebulizer 45 psi, skimmer 65 V, vaporizer 200°C, fragmentor 20 V, dissolved in MeOH

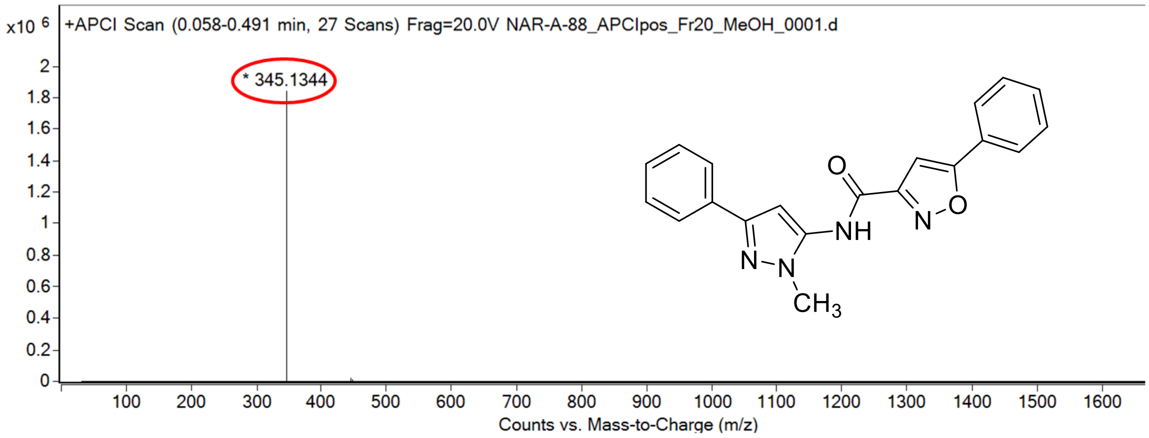

calculated mass: $[M+H]^+$ = 345.1346 observed: $[M+H]^+$ = 345.1344 mass accuracy = -0.6 ppm

FT-IR spectrum (neat) of **46**

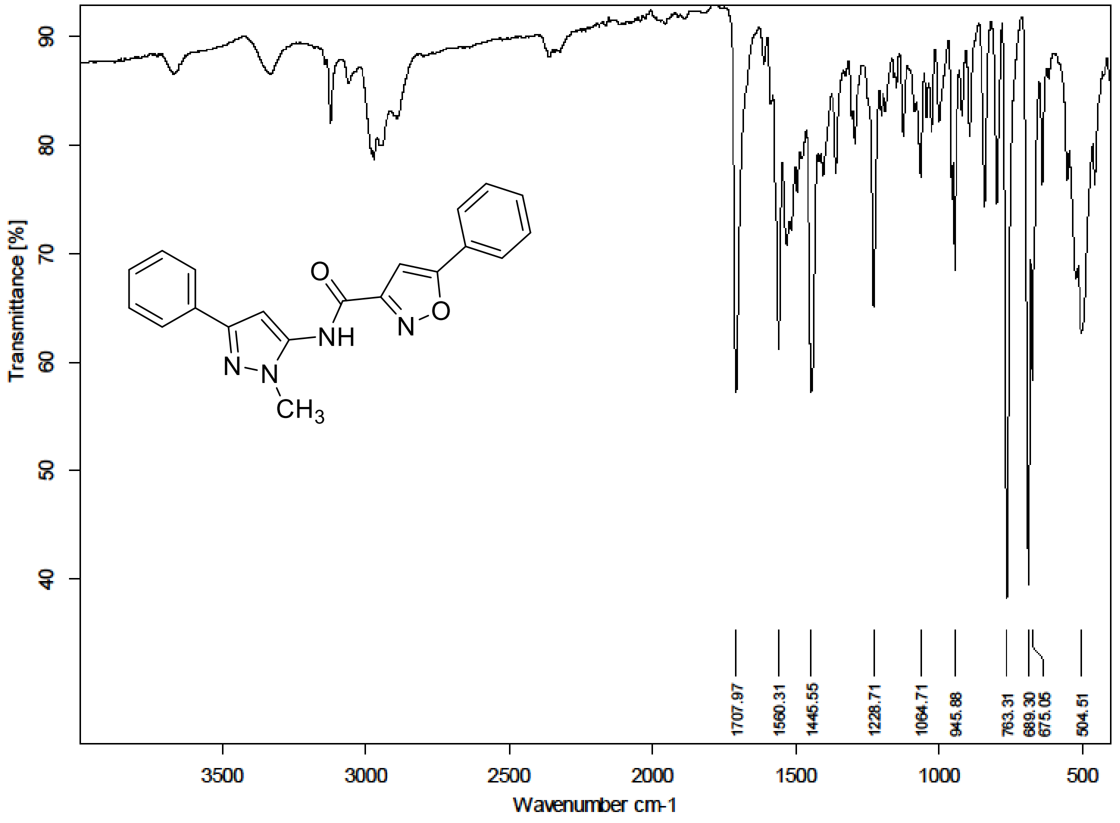

^{1}H (500 MHz) and ^{13}C NMR (126 MHz) spectra of **47** in Chloroform-*d*

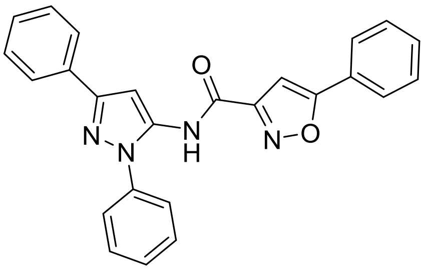

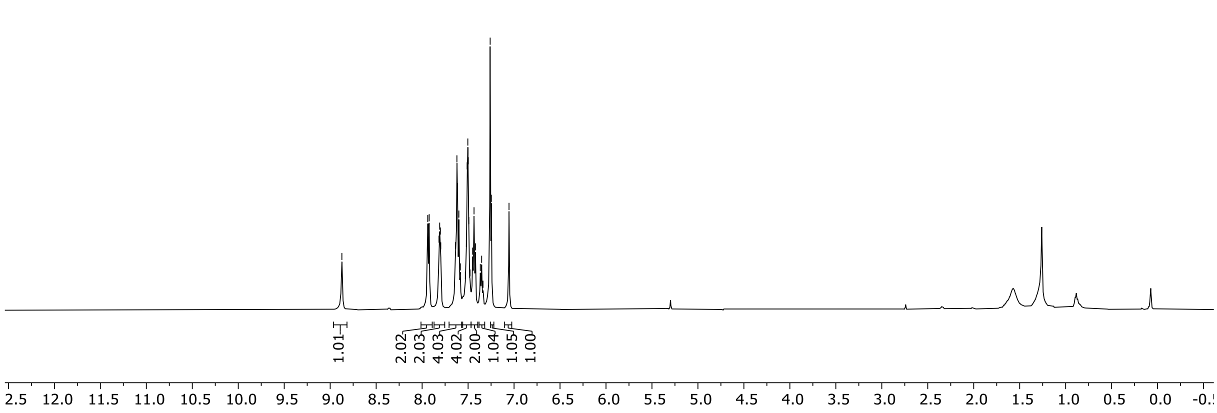

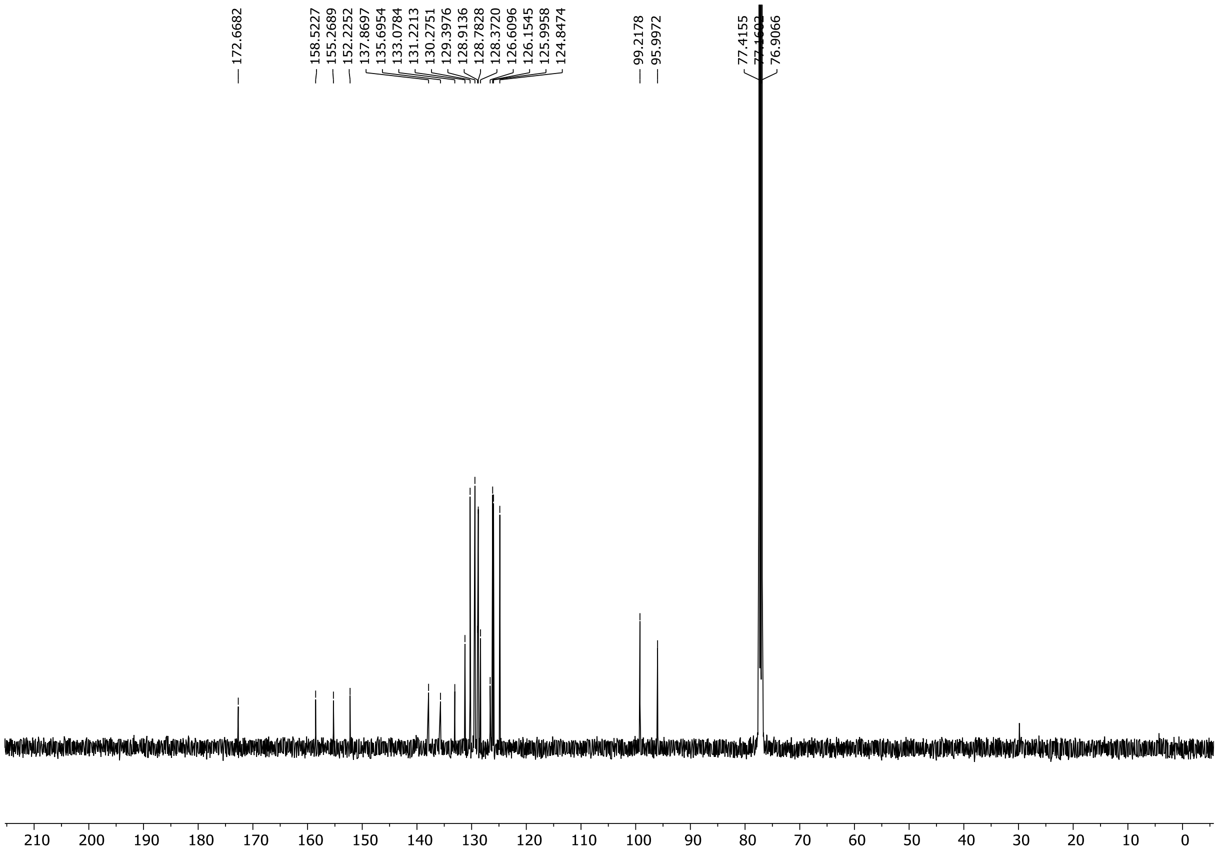

HRMS spectrum of **47**

$C_{25}H_{18}N_4O_2$ **exact mass: 406.1430**

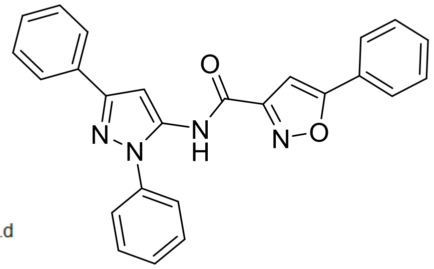

APCI + (MMI)
nitrogen flow 5 L/min, gas temperature 325°C, nebulizer 45 psig, skimmer 65 V, vaporizer 200°C, fragmentor 5 V, dissolved in methanol

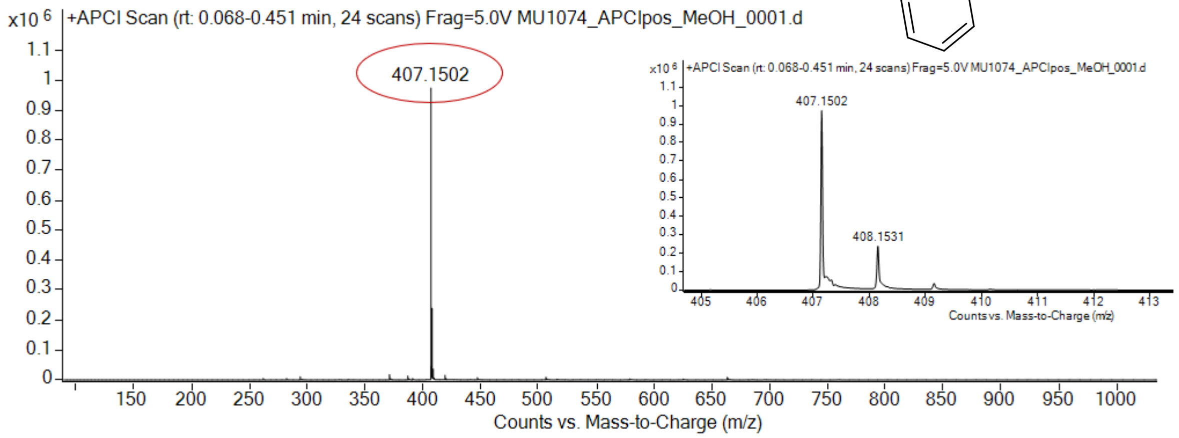

expected mass: $[M+H]^+$ = 407.1503 observed mass : $[M+H]^+$ = 407.1502 mass accuracy = - 0.2 ppm

^{1}H (500 MHz) and ^{13}C NMR (126 MHz) spectra of **48** in Chloroform-*d*

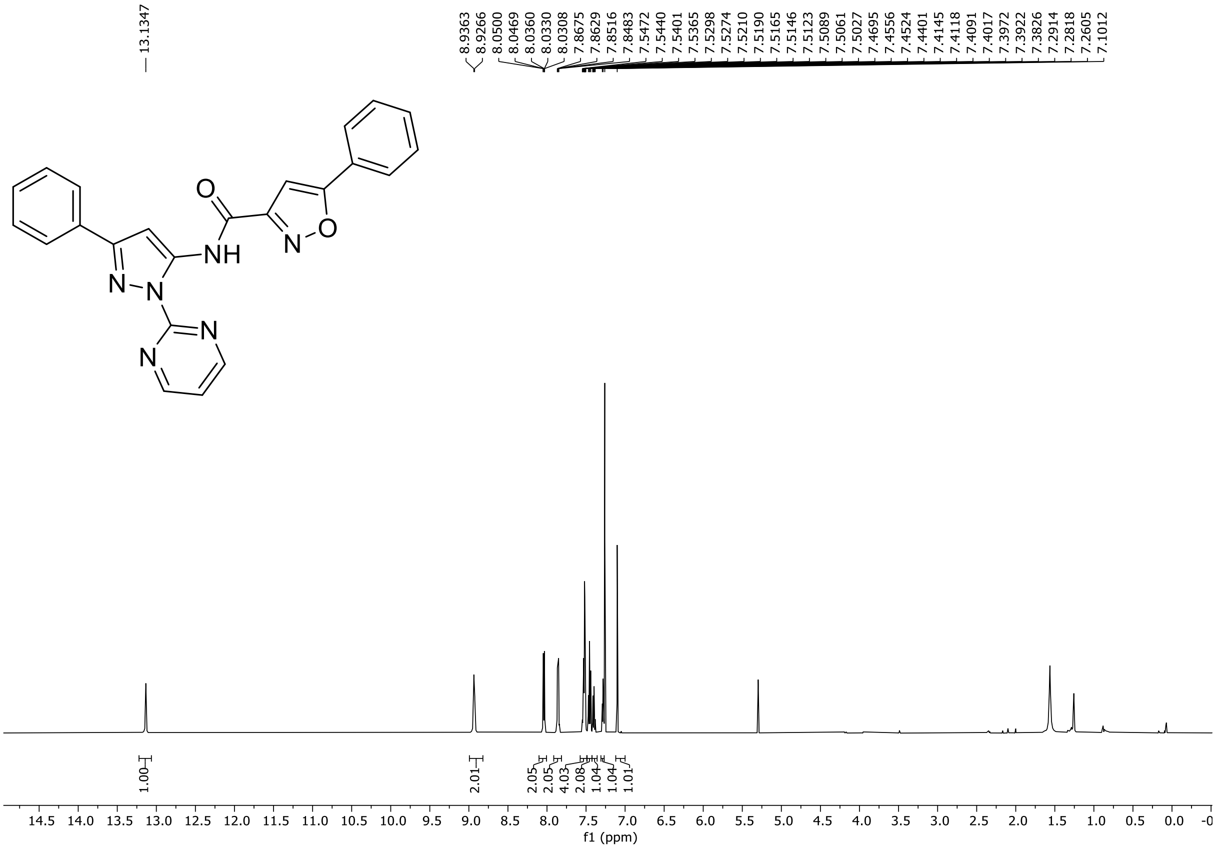

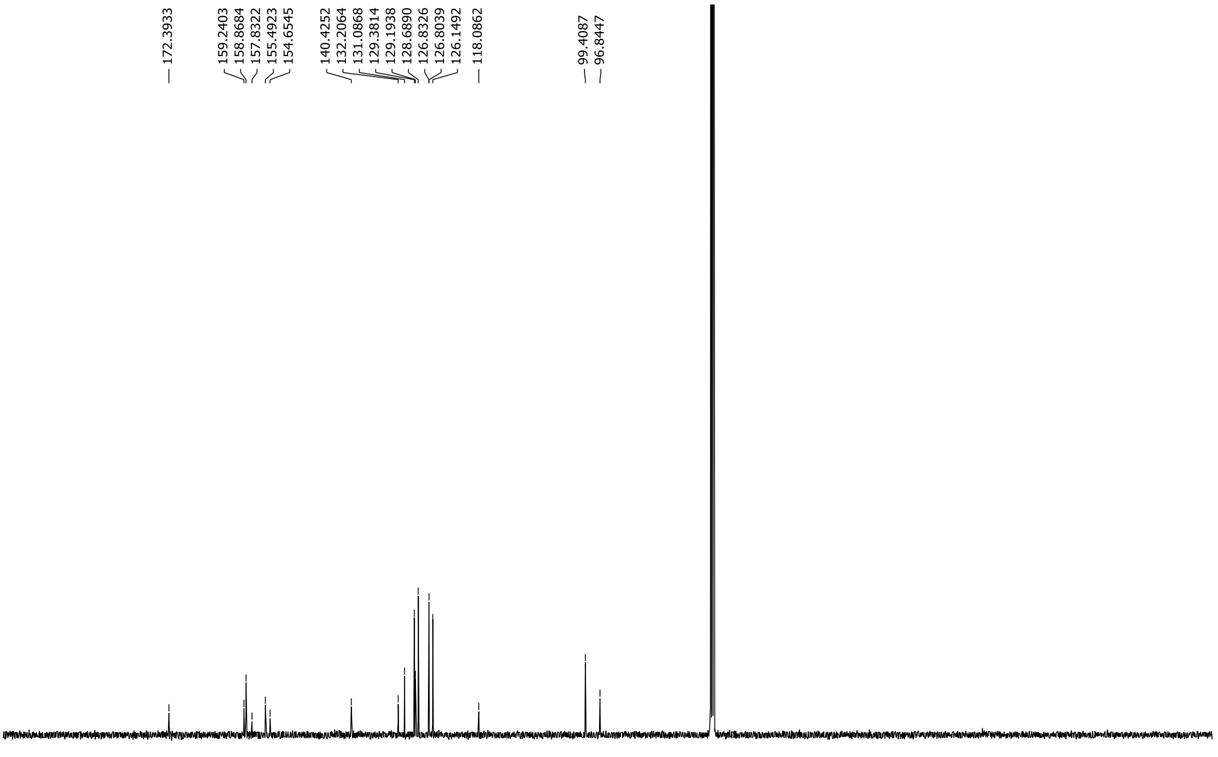

HRMS spectrum of **48**

NAR-A-14 $C_{23}H_{16}N_6O_2$ mono *m/z 408.1335*

APCI + (MMI)
nitrogen flow 5 L/min, gas temperature 325°C, nebulizer 45 psi, skimmer **65** V, vaporizer 200°C, fragmentor 20 V, dissolved in methanol

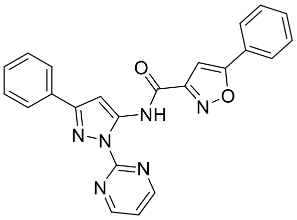

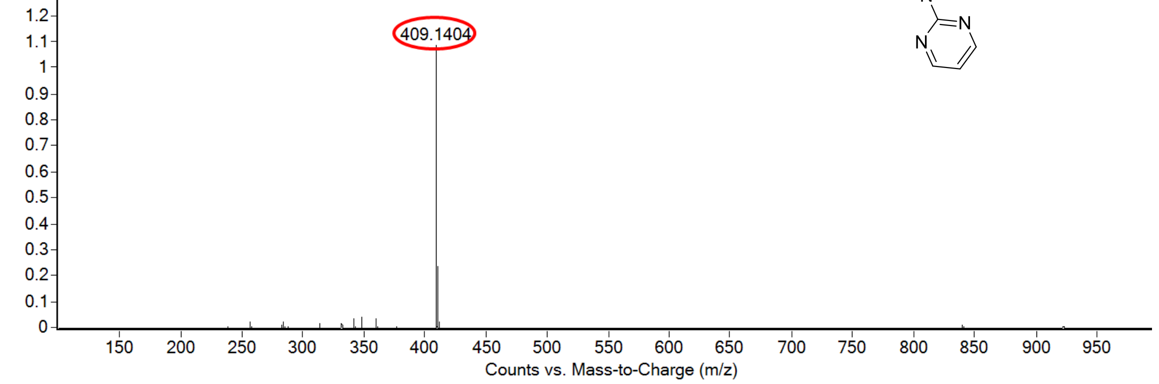

calculated mass: $[M+H]^+$ = 409.1408 observed: $[M+H]^+$ = 409.1404 mass accuracy = -0.9 ppm

FT-IR spectrum (neat) of **48**

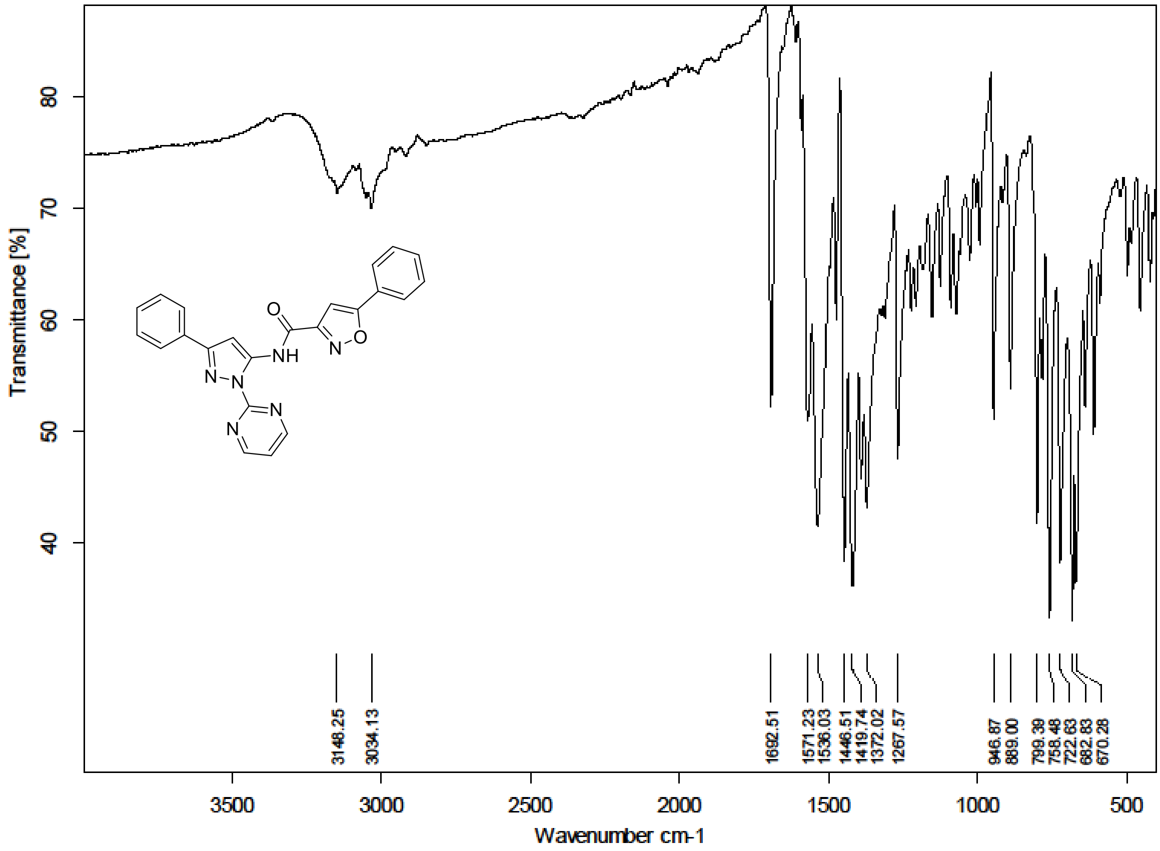

^{1}H (500 MHz) and ^{13}C NMR (126 MHz) spectra of **49** in Chloroform-*d*

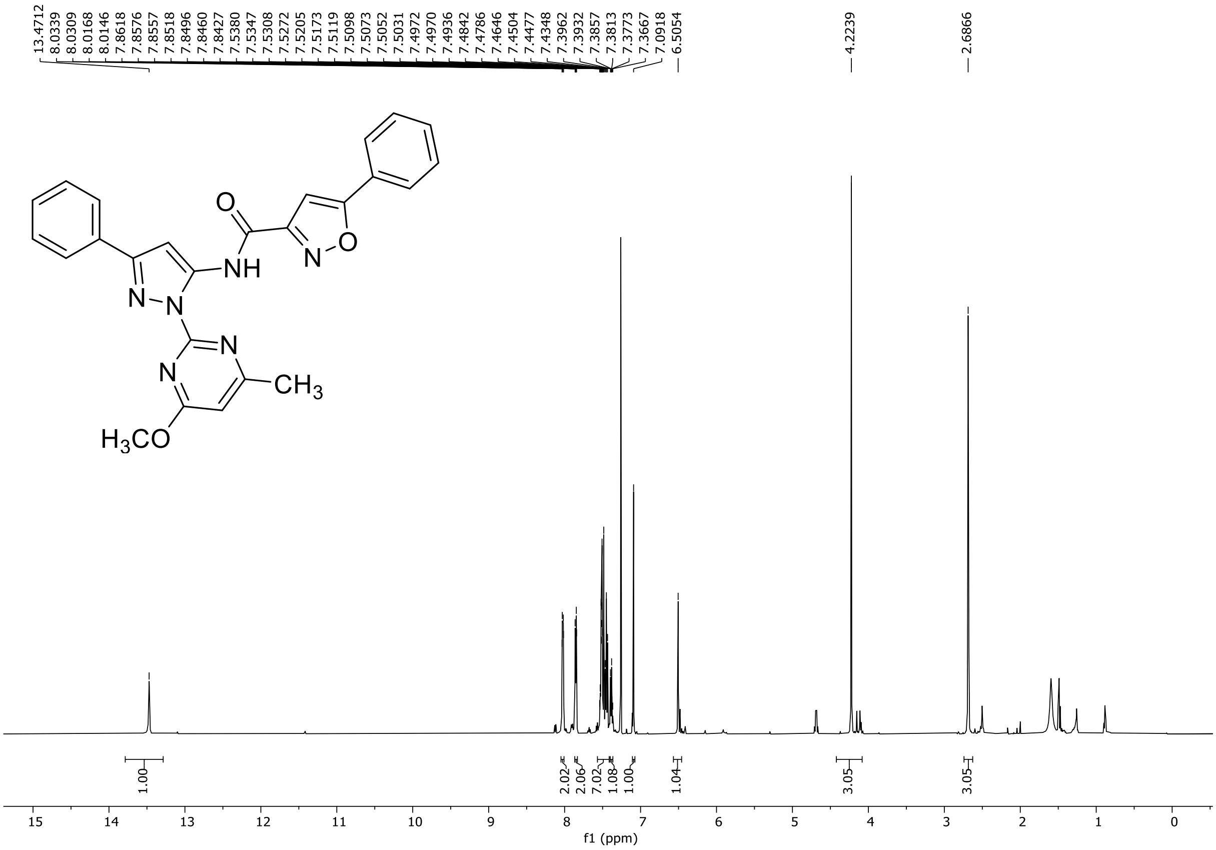

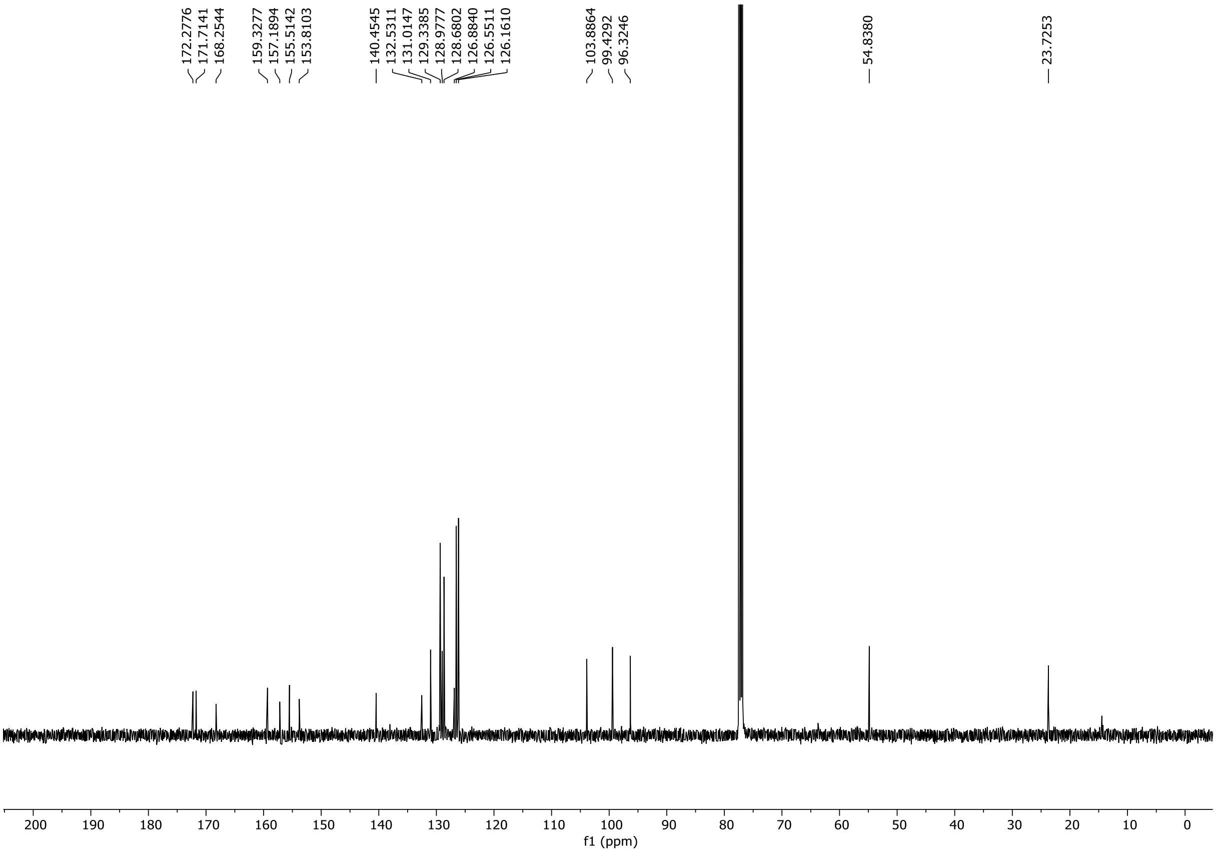

HRMS spectrum of **49**

NAR-A-68 $C_{25}H_{20}N_6O_3$ *mono m/z* 452.1597

APCI + (MMI)
nitrogen flow 5 L/min, gas temperature 300°C, nebulizer 45 psi, skimmer **65** V, vaporizer 200°C, fragmentor 15 V, dissolved in MeOH

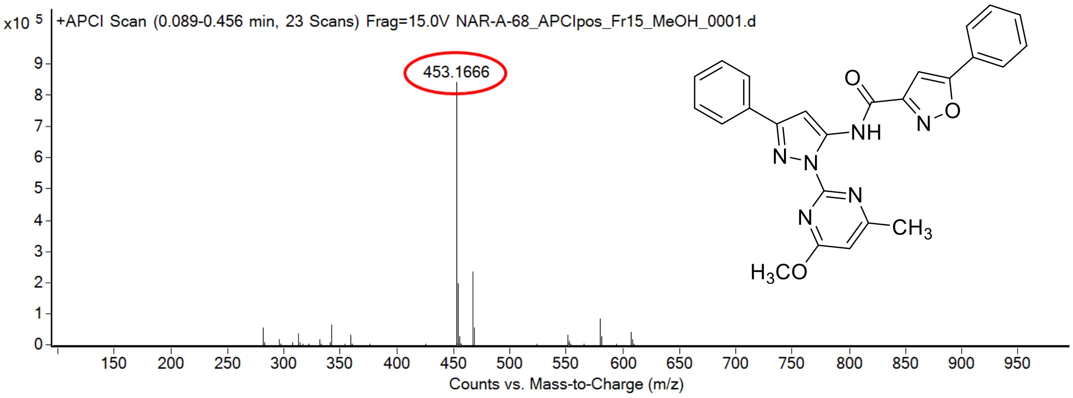

calculated mass: $[M+H]^+$ = 453.1670 observed: $[M+H]^+$ = 453.1666 mass accuracy = -0.9 ppm

FT-IR spectrum (neat) of **49**

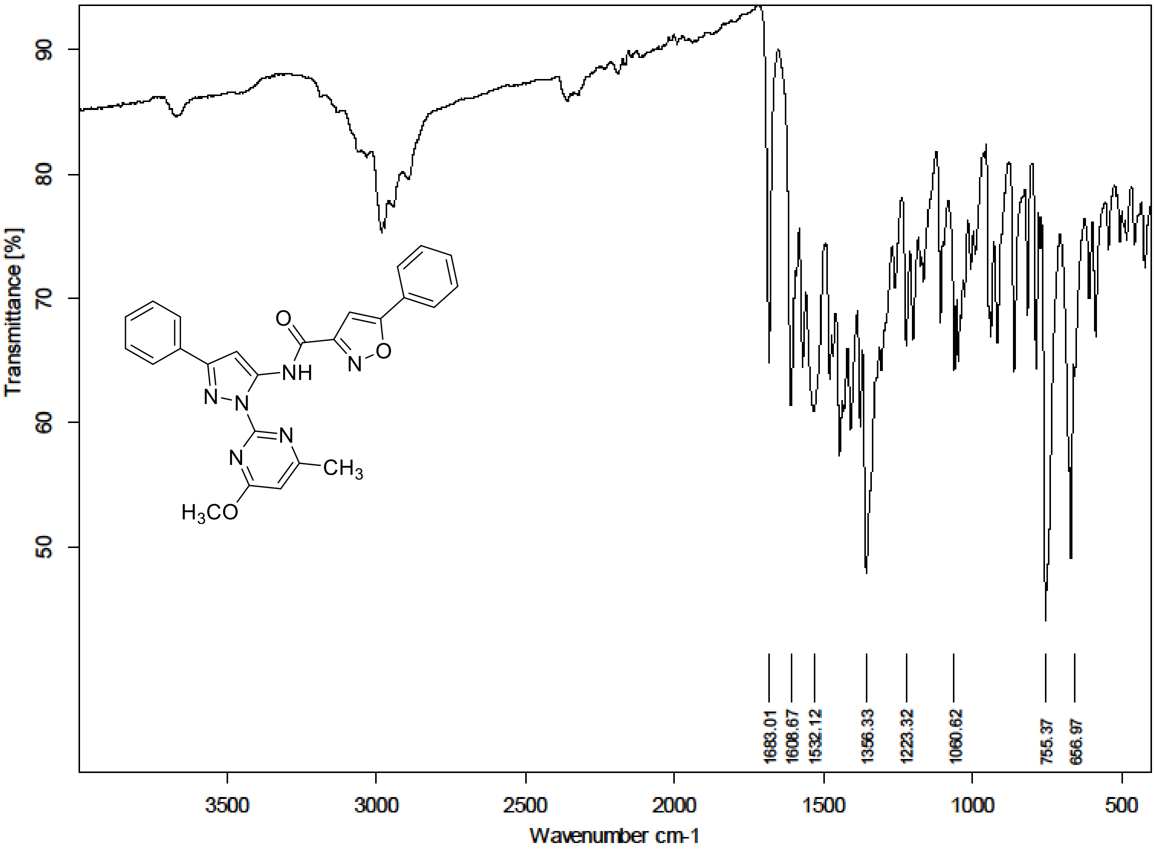

^{1}H NMR (300 MHz) spectrum of **50** DMSO-d_6

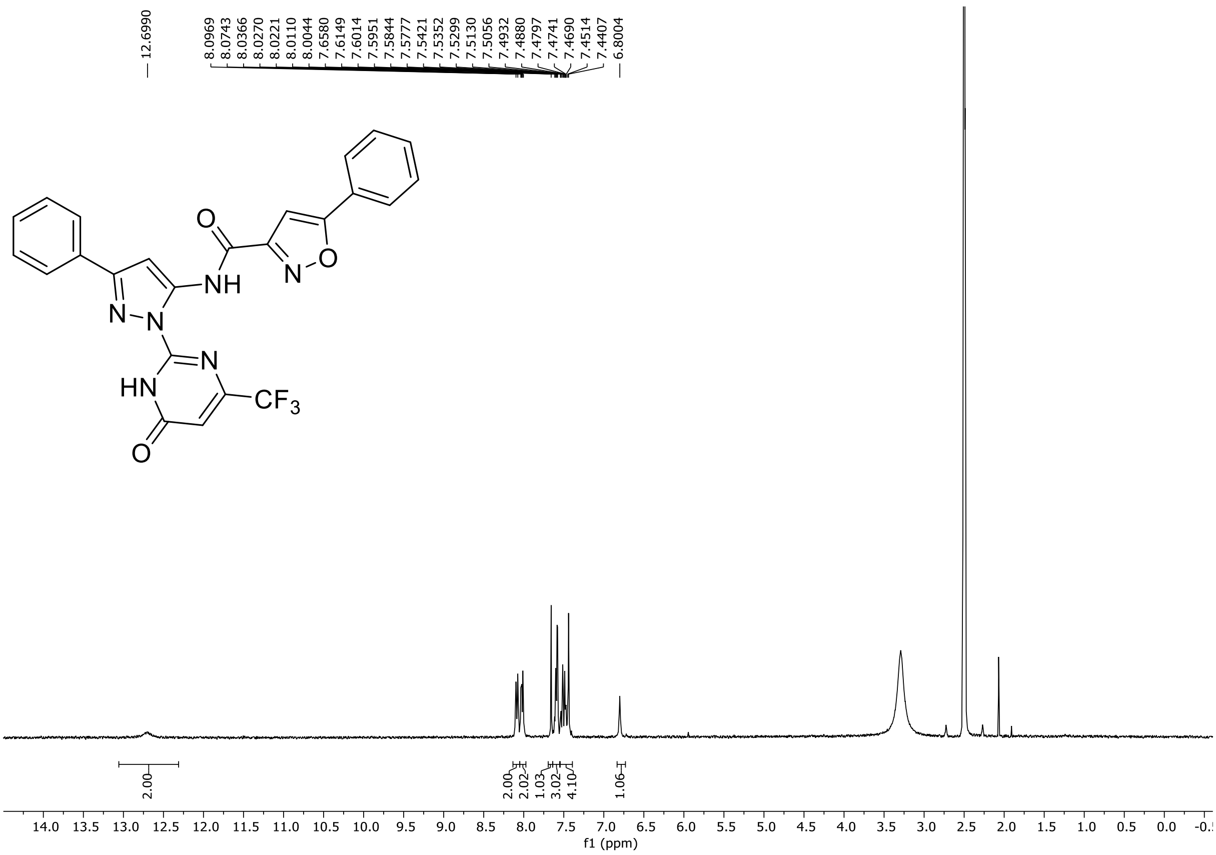

HRMS spectrum of **50**

NAR-A-96 $C_{24}H_{15}F_3N_6O_3$ ***m/z* 492.1158**

ESI- (MMI)
nitrogen flow 5 L/min, gas temperature 325°C, nebulizer 45 psi, skimmer 65 V, fragmentor 60 V, dissolved in DMSO, MeOH

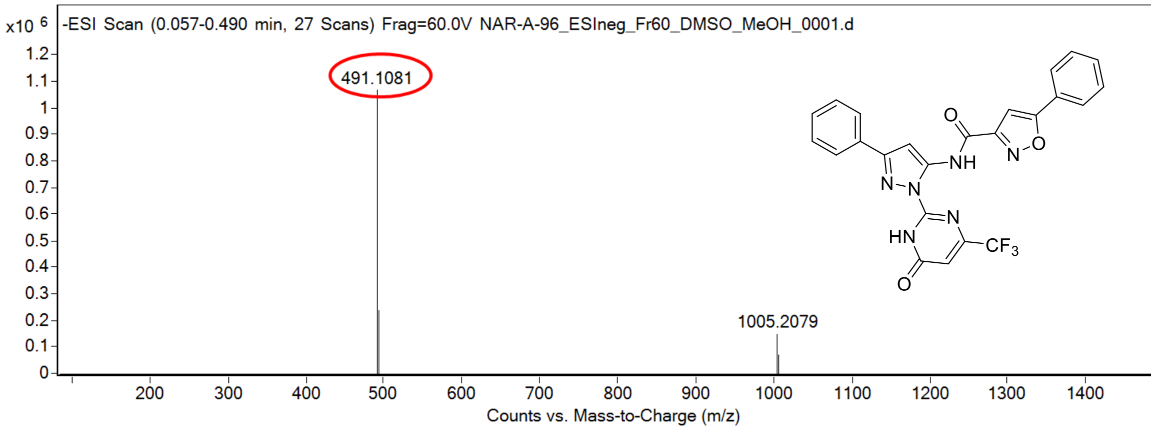

calculated mass: [M-H]⁻ = 491.1085 observed: [M-H]⁻ = 491.1081 mass accuracy = -0.8 ppm

FT-IR spectrum (neat) of **50**

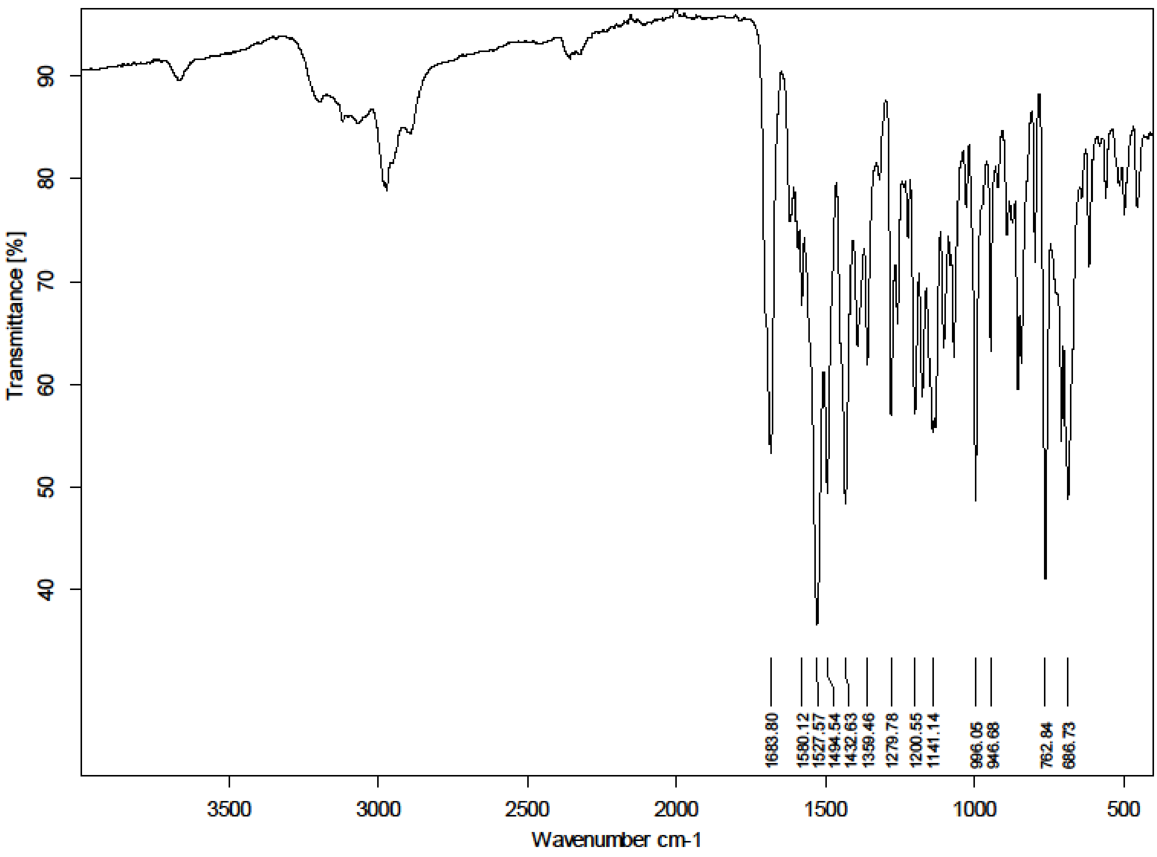

^{1}H NMR (300 MHz) spectrum of **51** DMSO-d_6

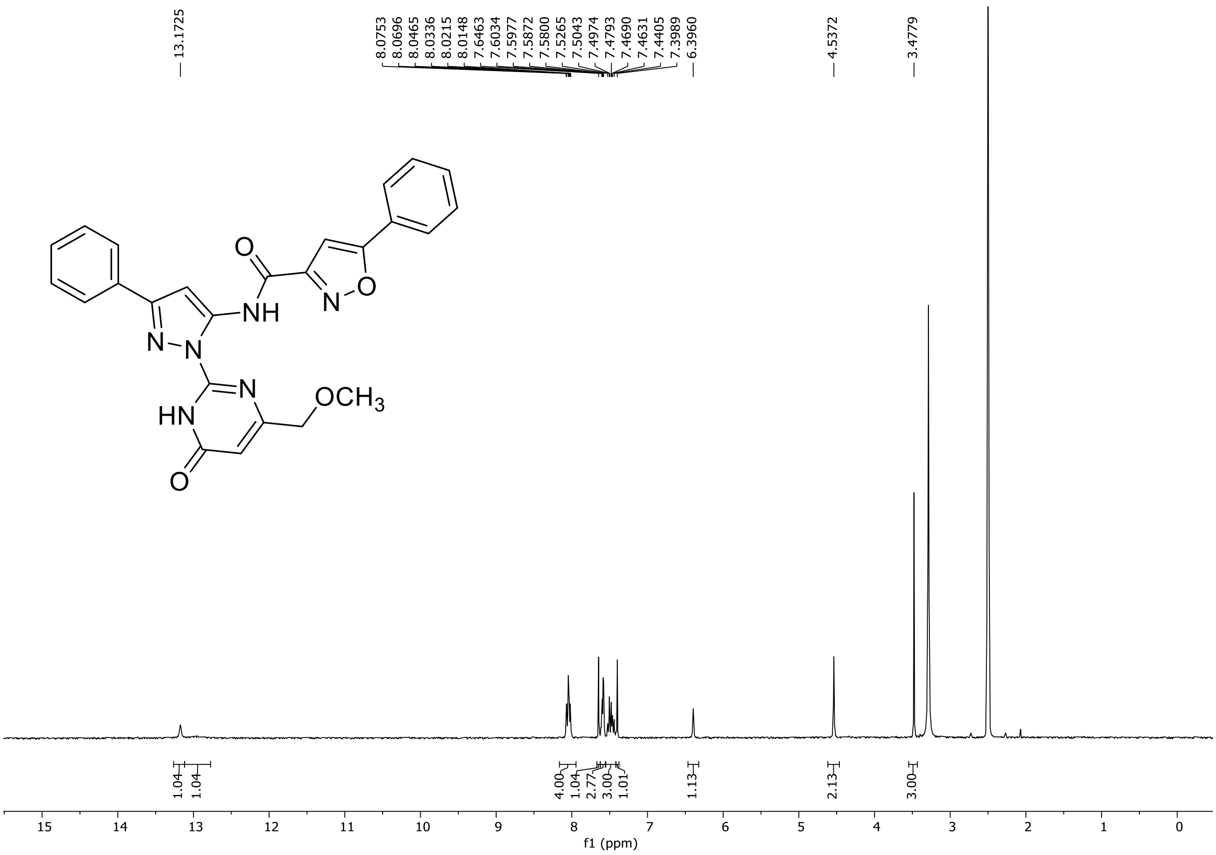

HRMS spectrum of **51**

NAR-A-80 $C_{25}H_{20}N_6O_4$ *m/z* **468.1546**

ESI- (MMI)
nitrogen flow 5 L/min, gas temperature 325°C, nebulizer 45 psi, skimmer 65 V, fragmentor 150 V, dissolved in MeOH

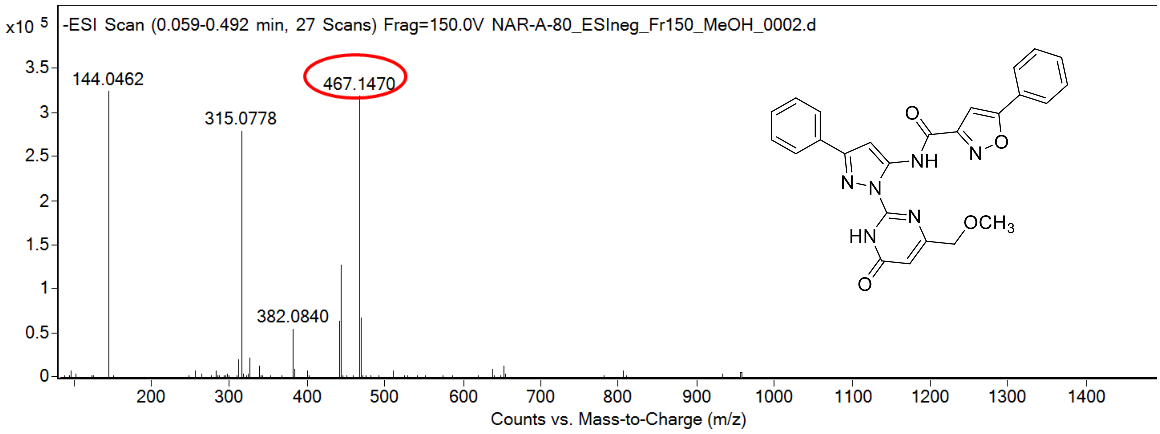

calculated mass: [M-H]$^-$ = 467.1473 observed: [M-H]$^-$ = 467.1470 mass accuracy = -0.6 ppm

FT-IR spectrum (neat) of **51**

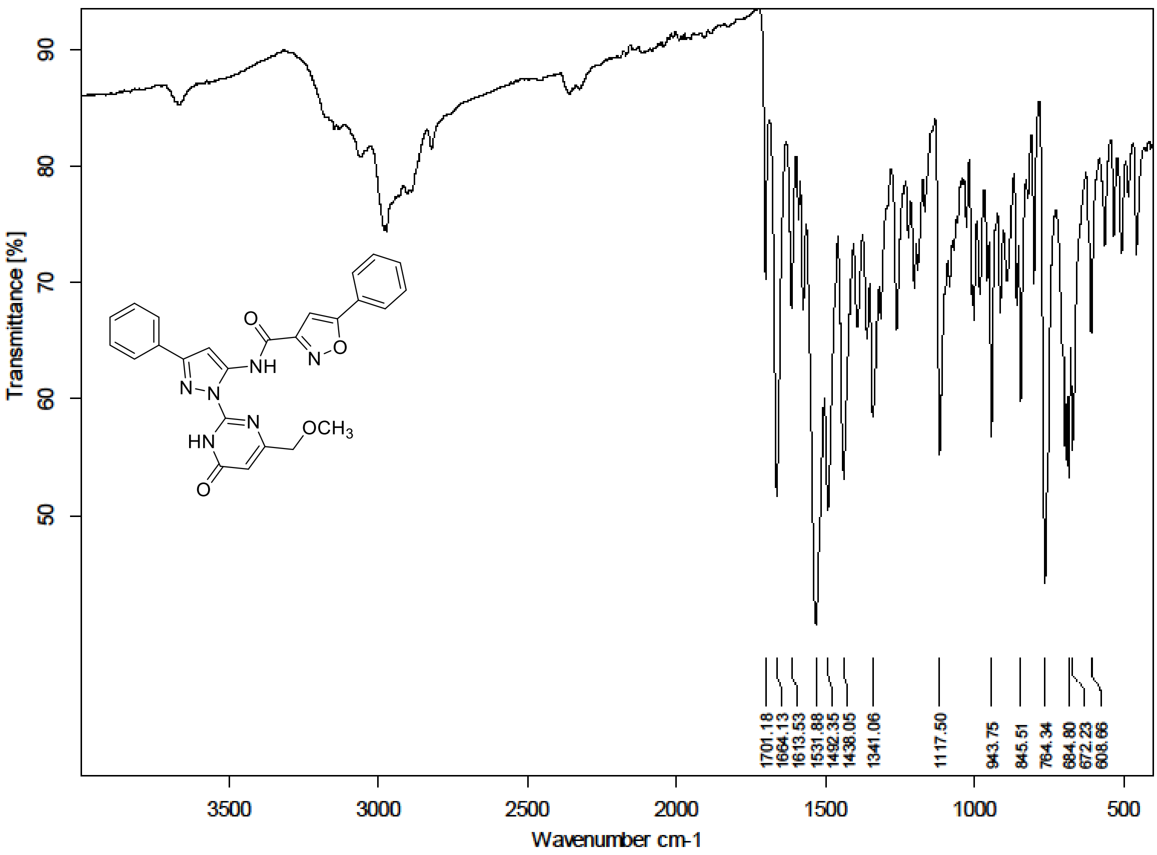

^{1}H NMR (300 MHz) spectrum of **52** in DMSO-d_6 and ^{13}C NMR (75 MHz) spectrum of **52** in Trifluoroacetic Acid-*d*

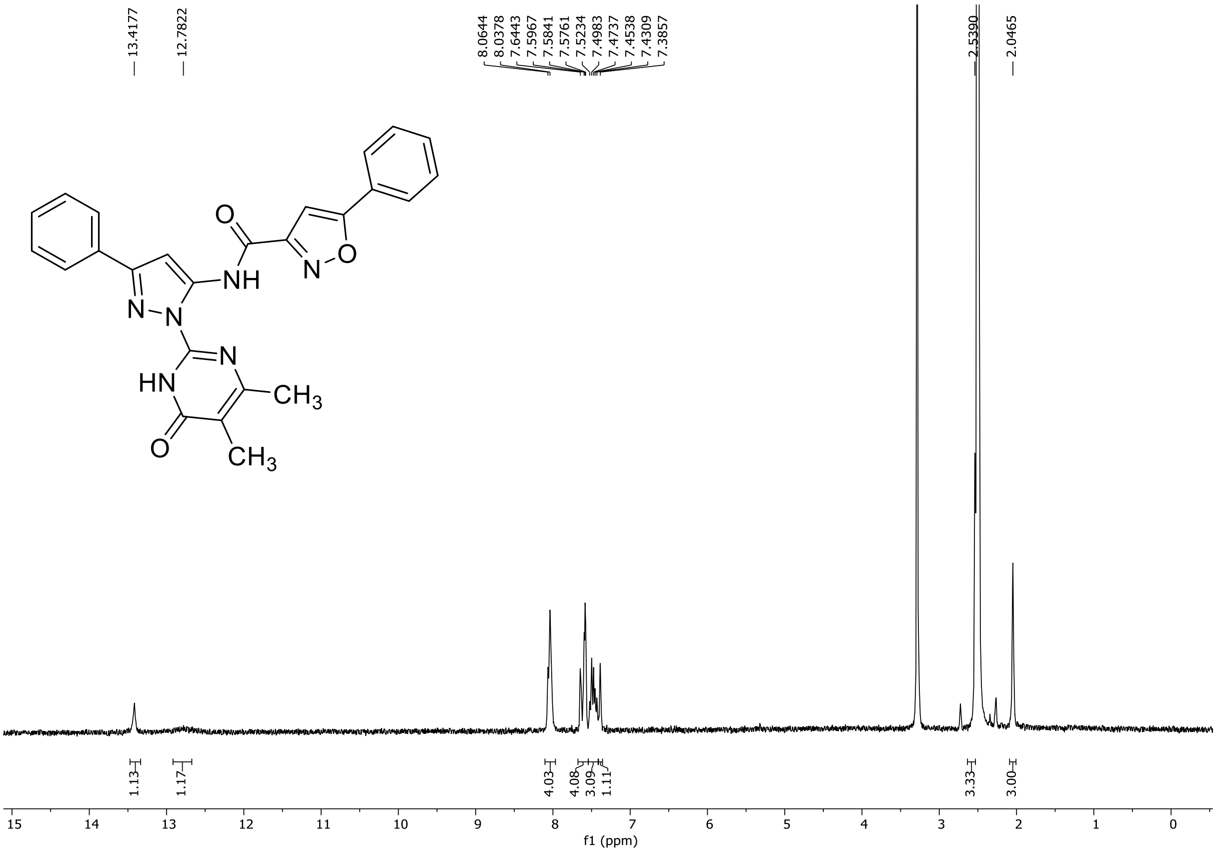

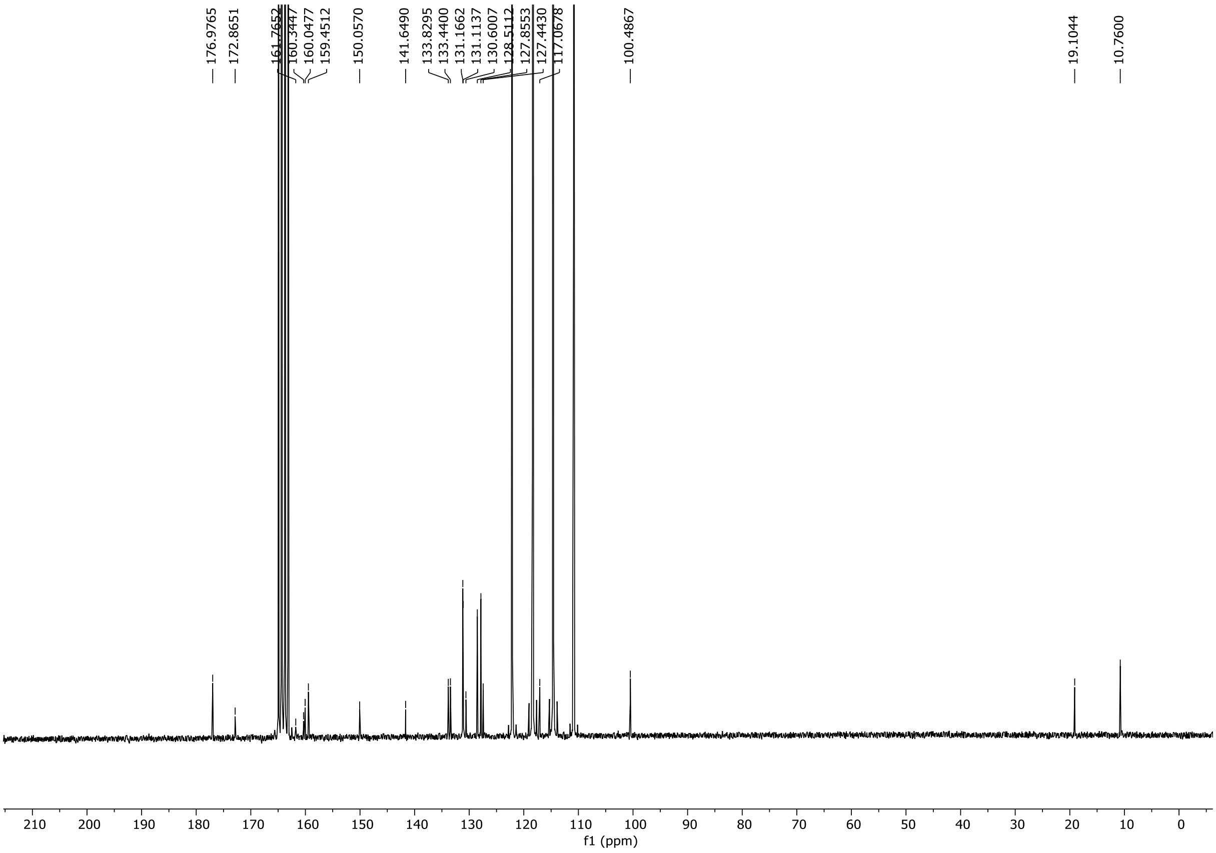

HRMS spectrum of **52**

NAR-A-226 $C_{25}H_{20}N_6O_3$ mono *m/z* 452.1597

ESI - (MMI)
nitrogen flow 5 L/min, gas temperature 325°C, nebulizer 30 psig, skimmer - 65 V, Vcap 2500 V, fragmentor - 50 V, dissolved in methanol

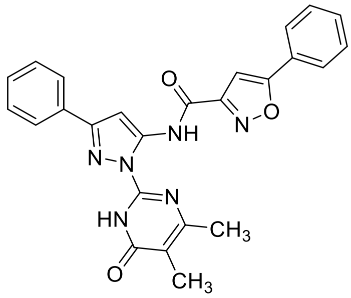

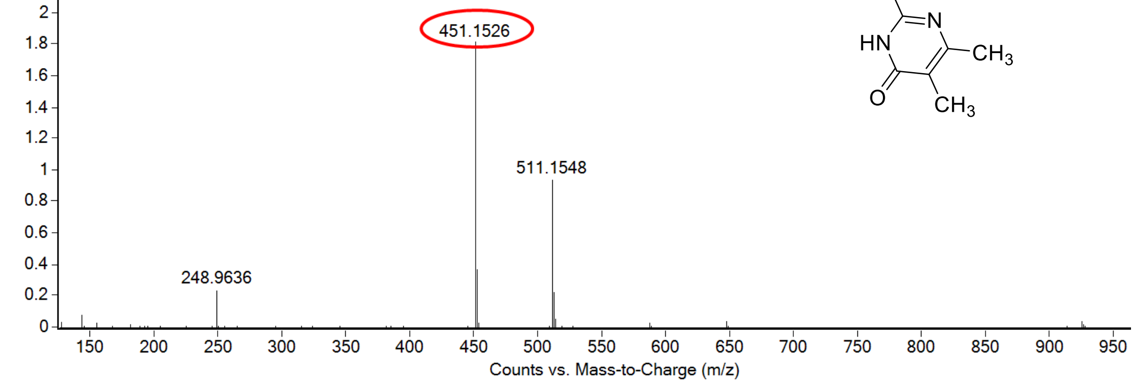

calculated mass: [M-H]$^-$ = 451.1524 observed: [M-H]$^-$ = 451.1526 mass accuracy = 0.4 ppm

FT-IR spectrum (neat) of **52**

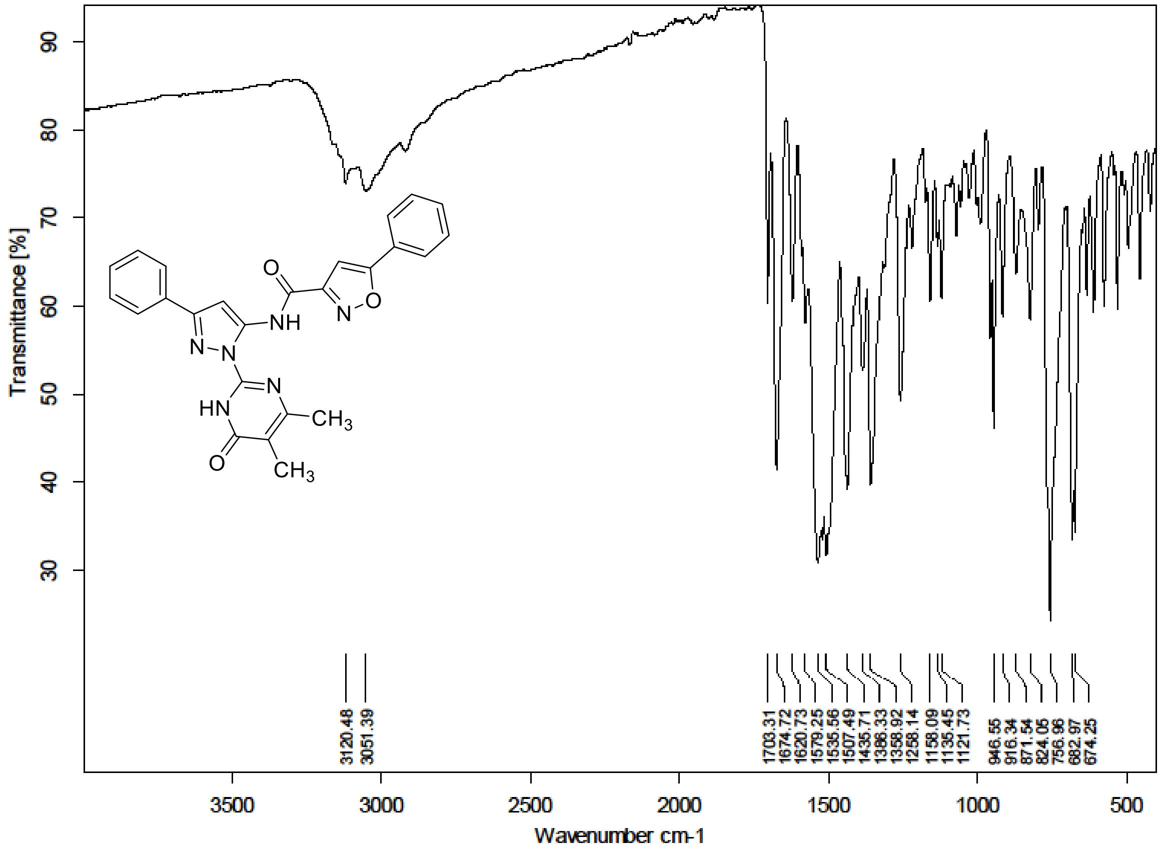

^{1}H (500 MHz) and ^{13}C NMR (126 MHz) spectra of **53** in DMSO-d_6

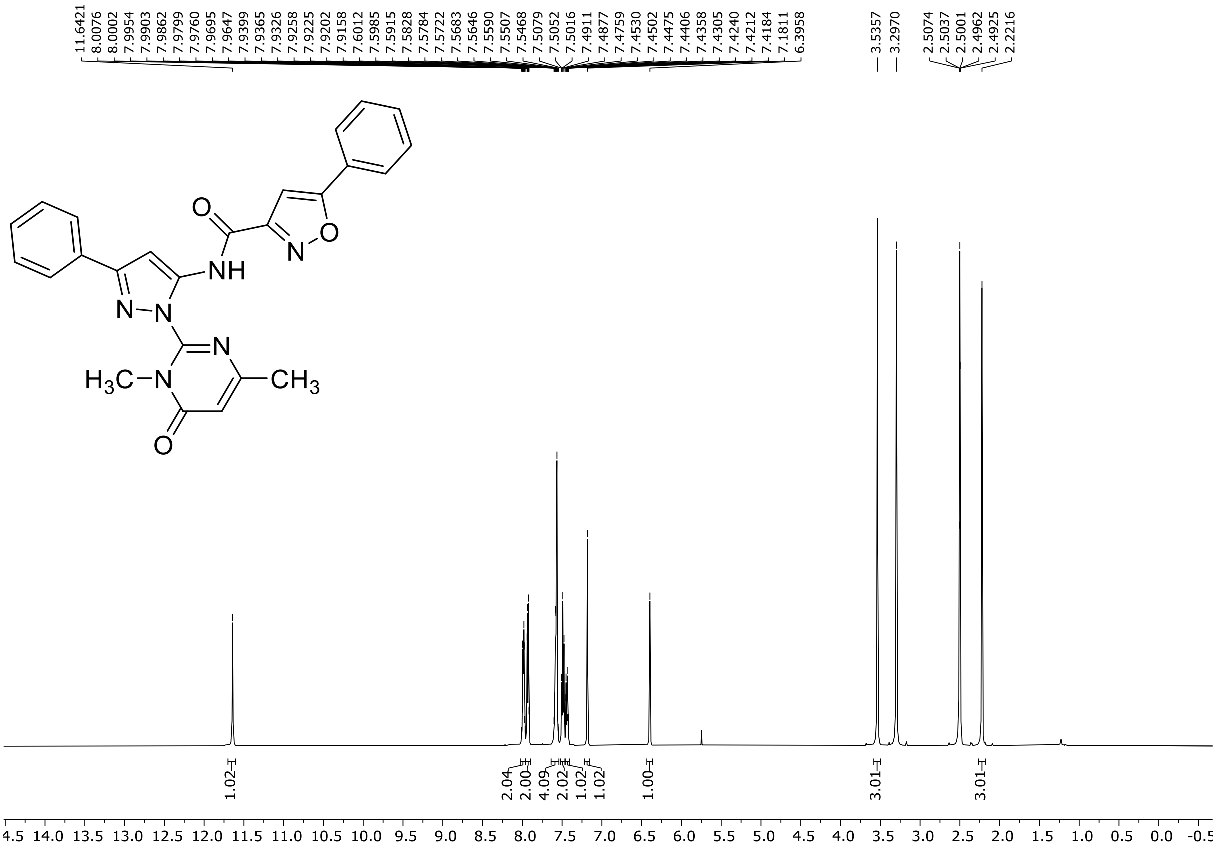

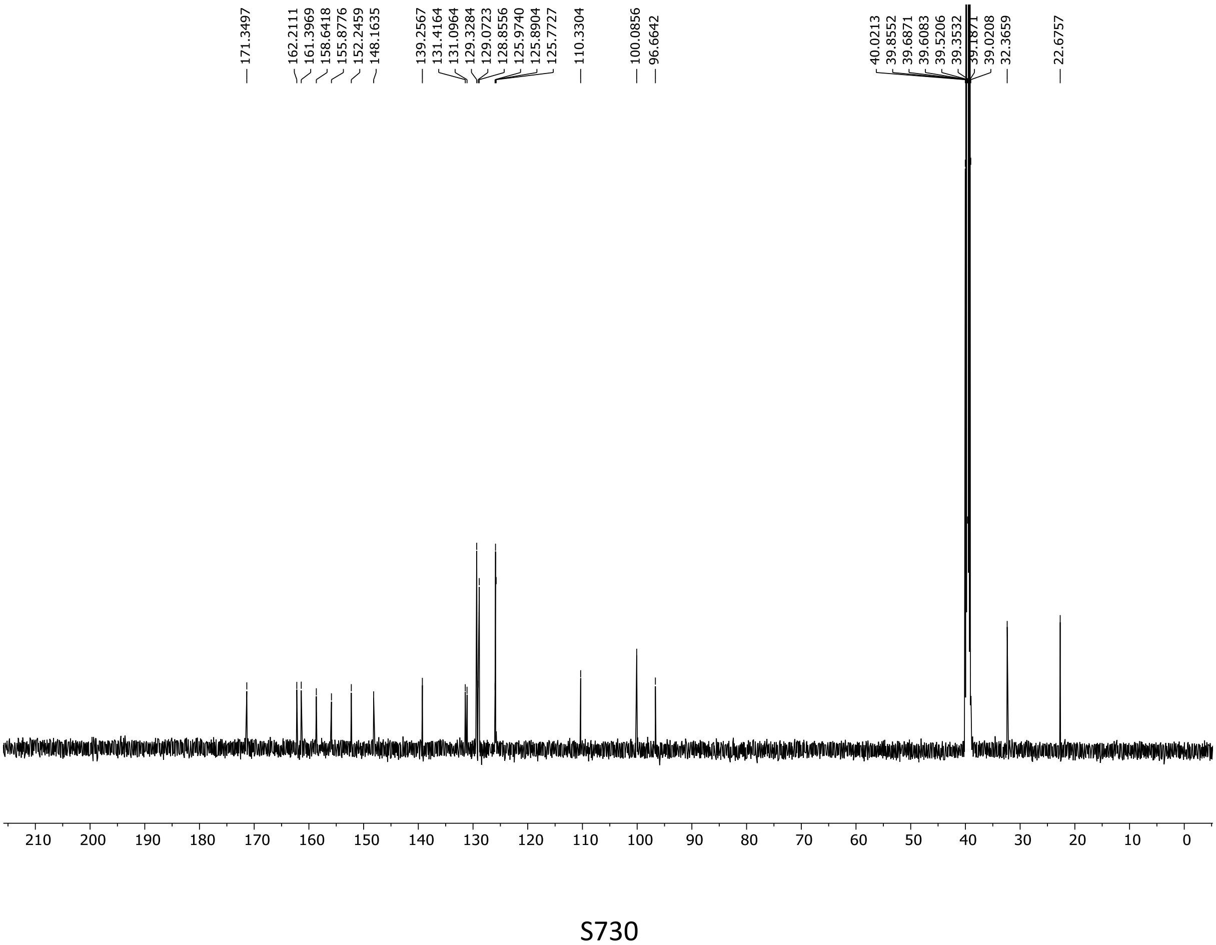

HRMS spectrum of **53**

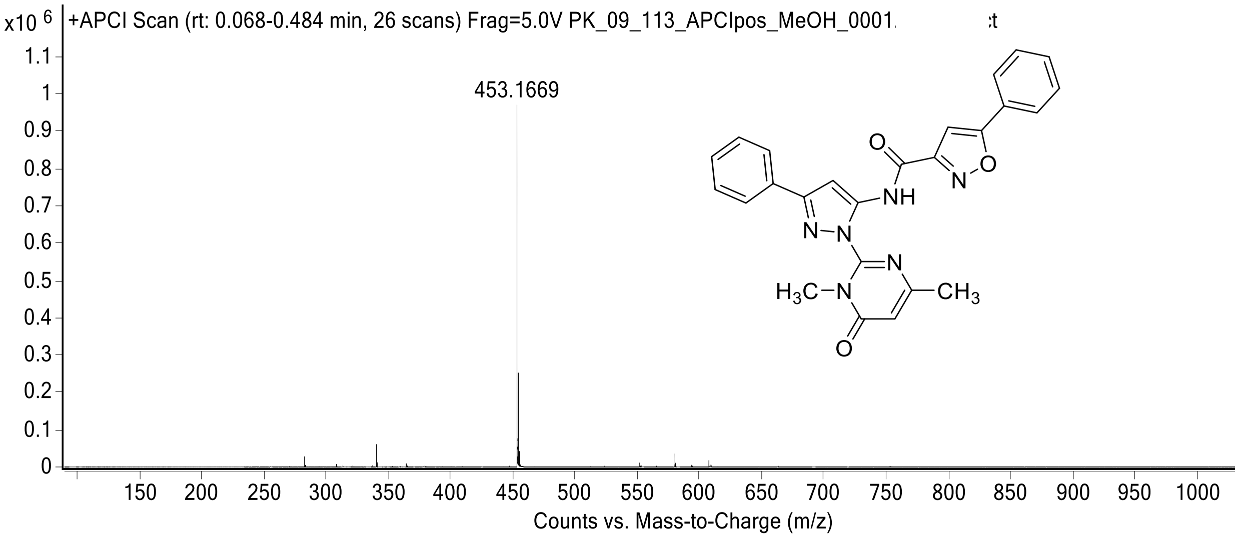

^{1}H (500 MHz) and ^{13}C NMR (126 MHz) spectra of **54** in DMSO-d_6

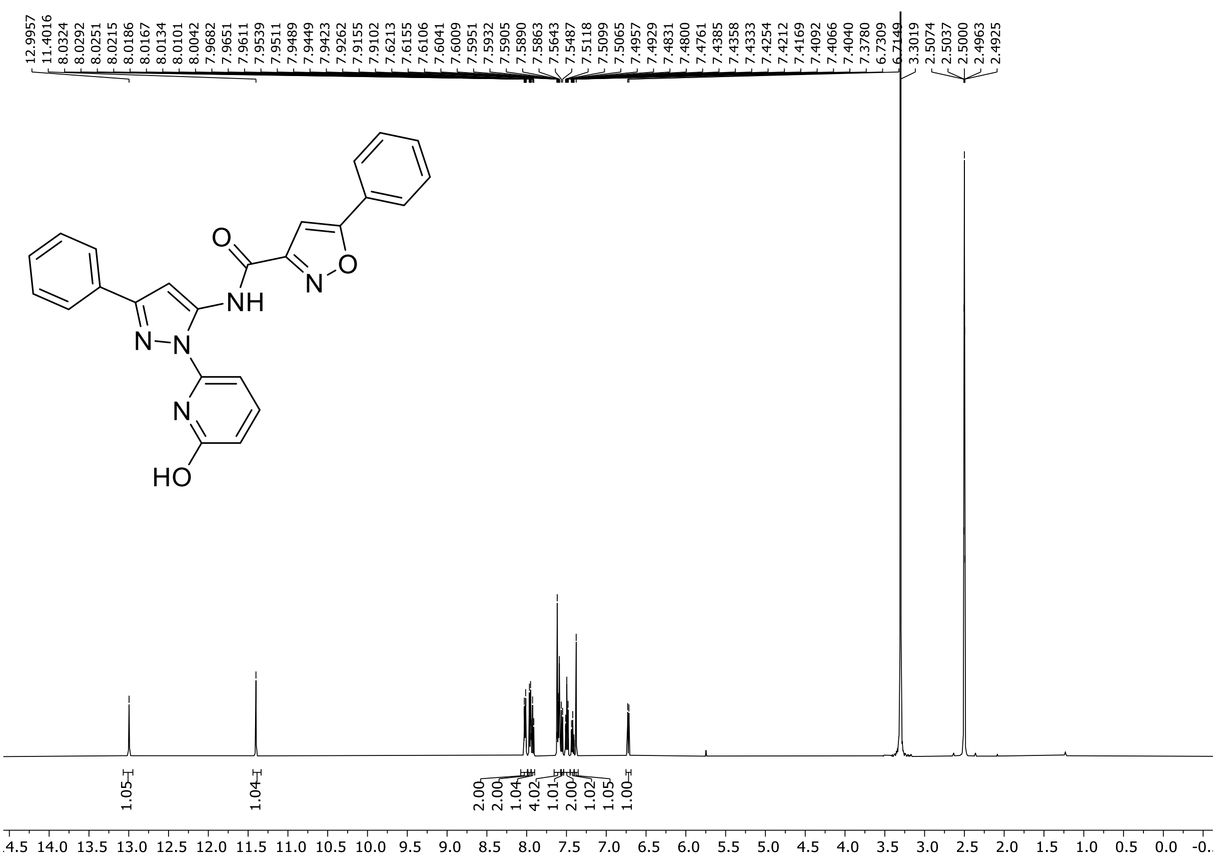

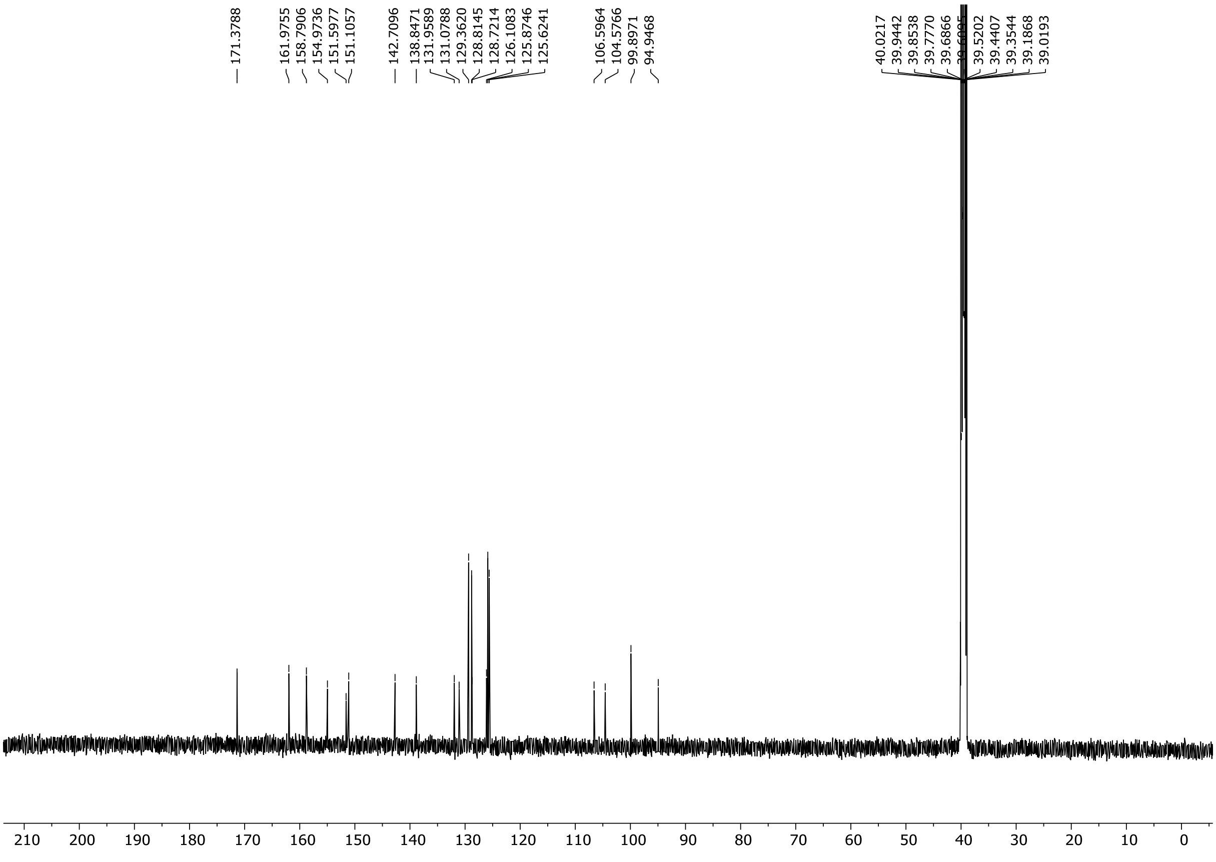

HRMS spectrum of **54**

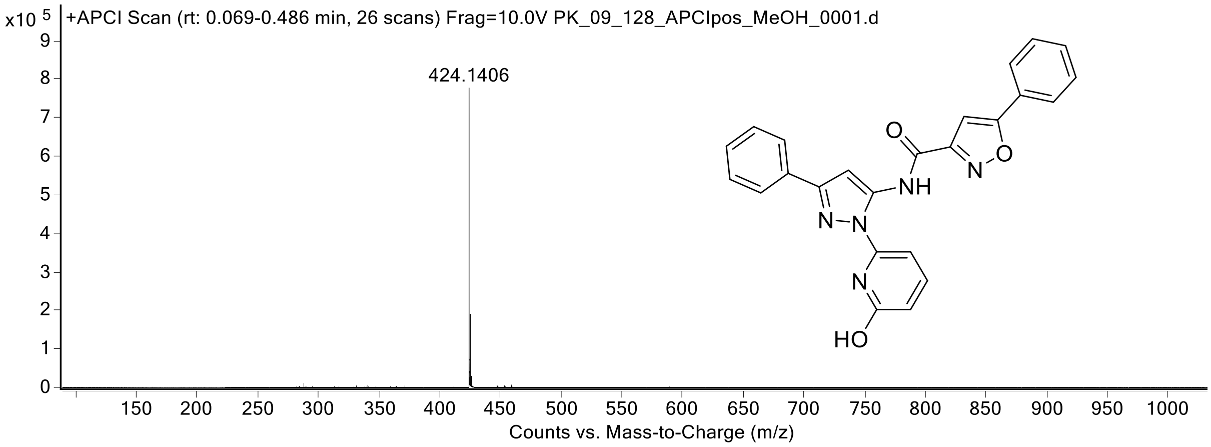

^{1}H NMR (300 MHz) spectrum of **55** DMSO-d_6

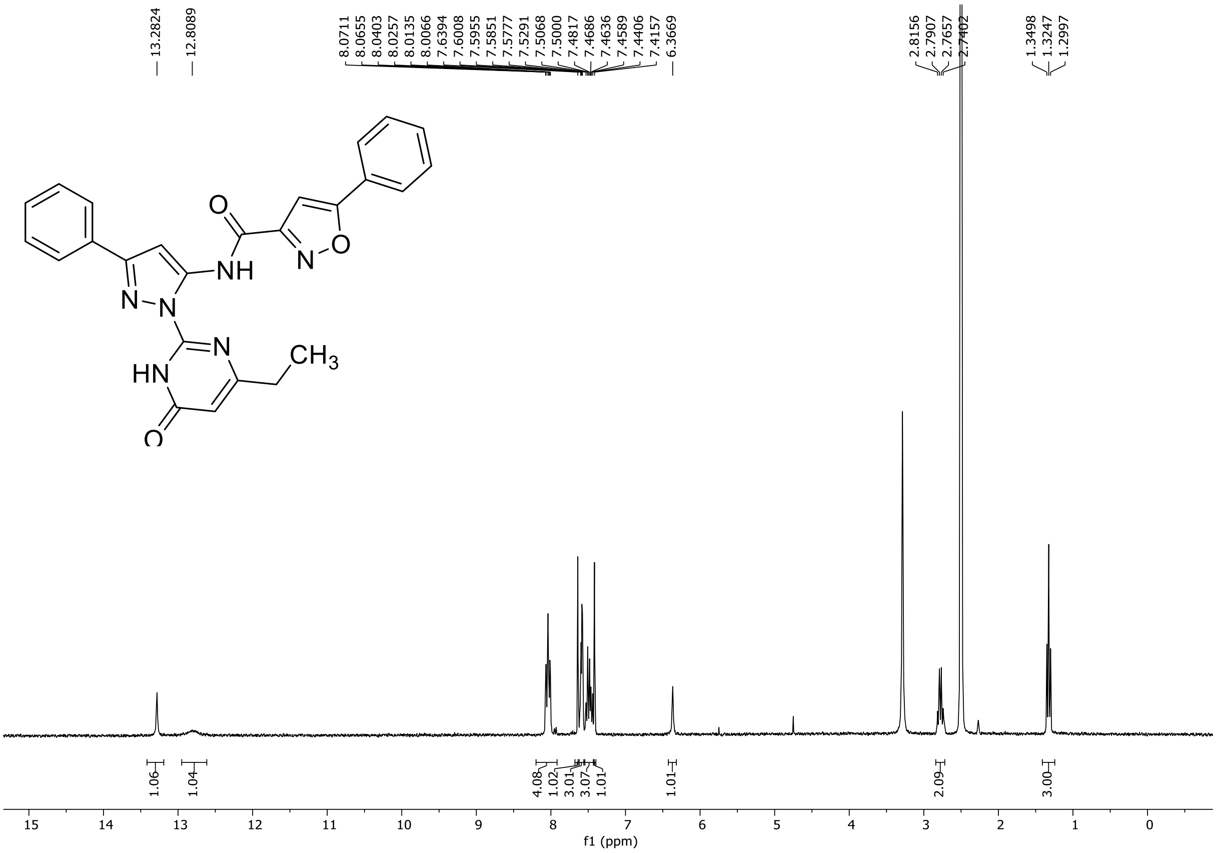

HRMS spectrum of **55**

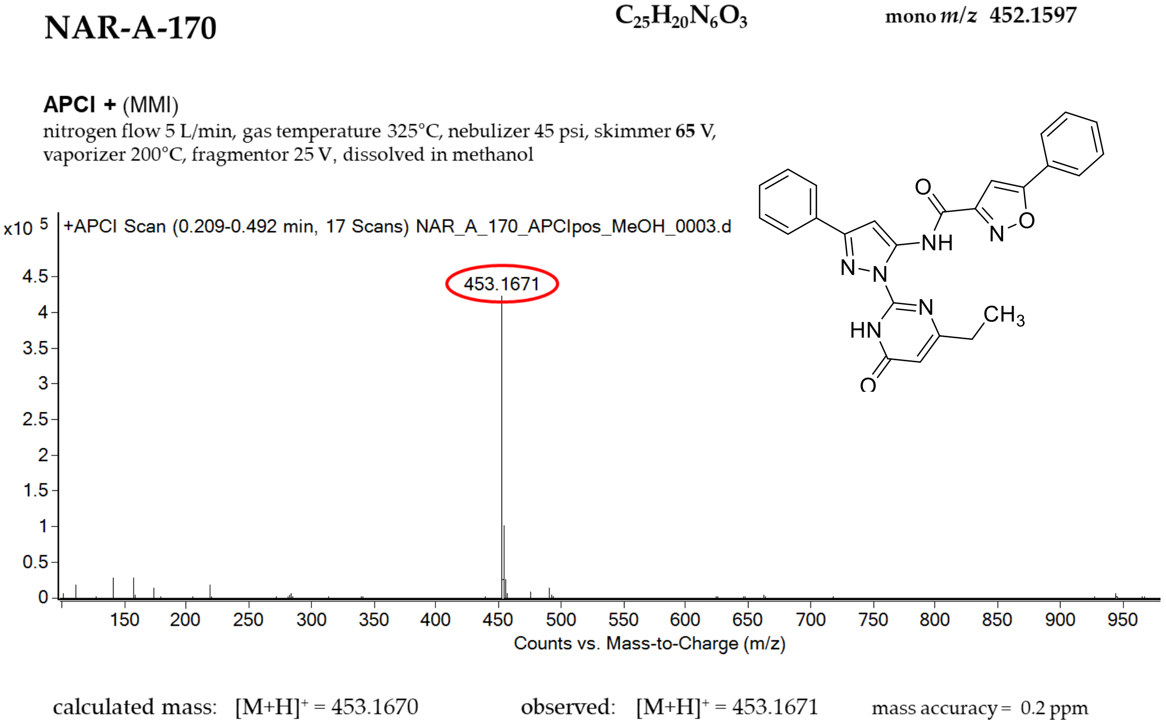

FT-IR spectrum (neat) of **55**

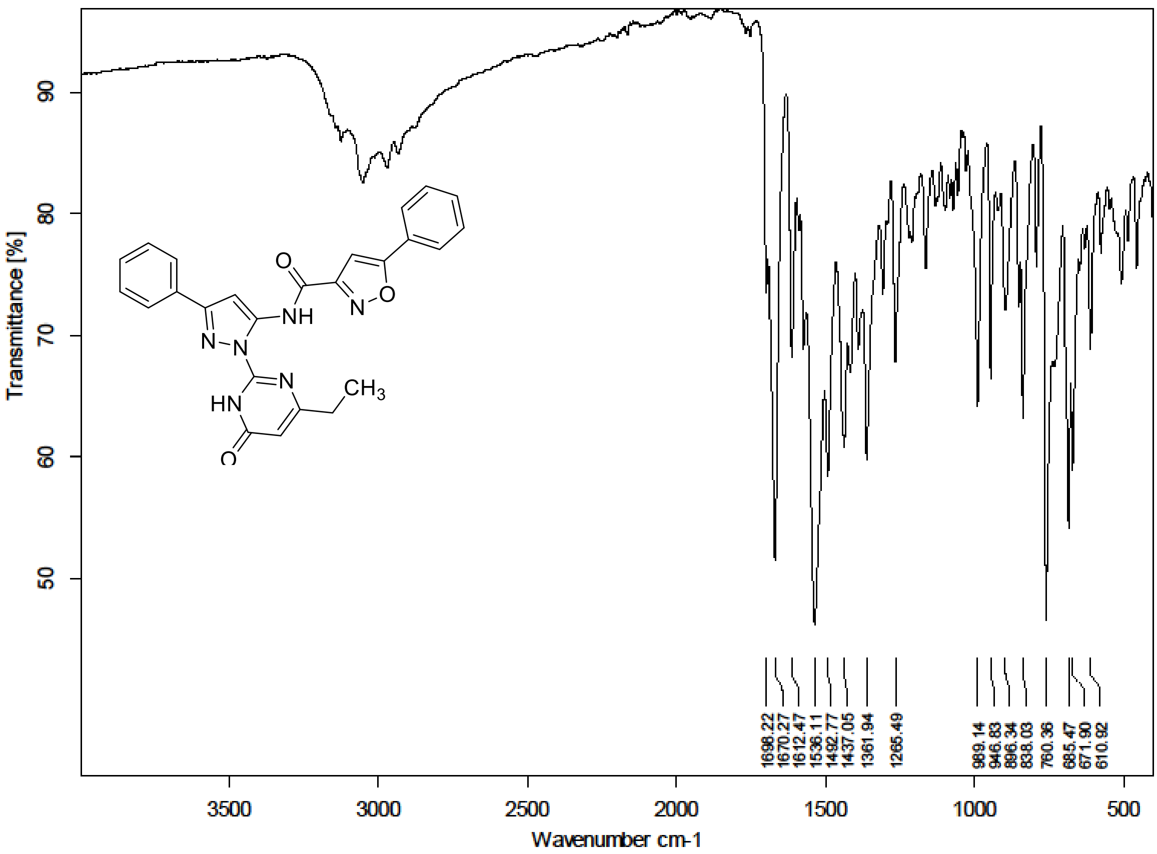

^{1}H NMR, ^{13}C NMR, HRMS and IR spectra of compound 118-141

^{1}H (500 MHz) and ^{13}C NMR (126 MHz) spectra of **118** in DMSO-d_6

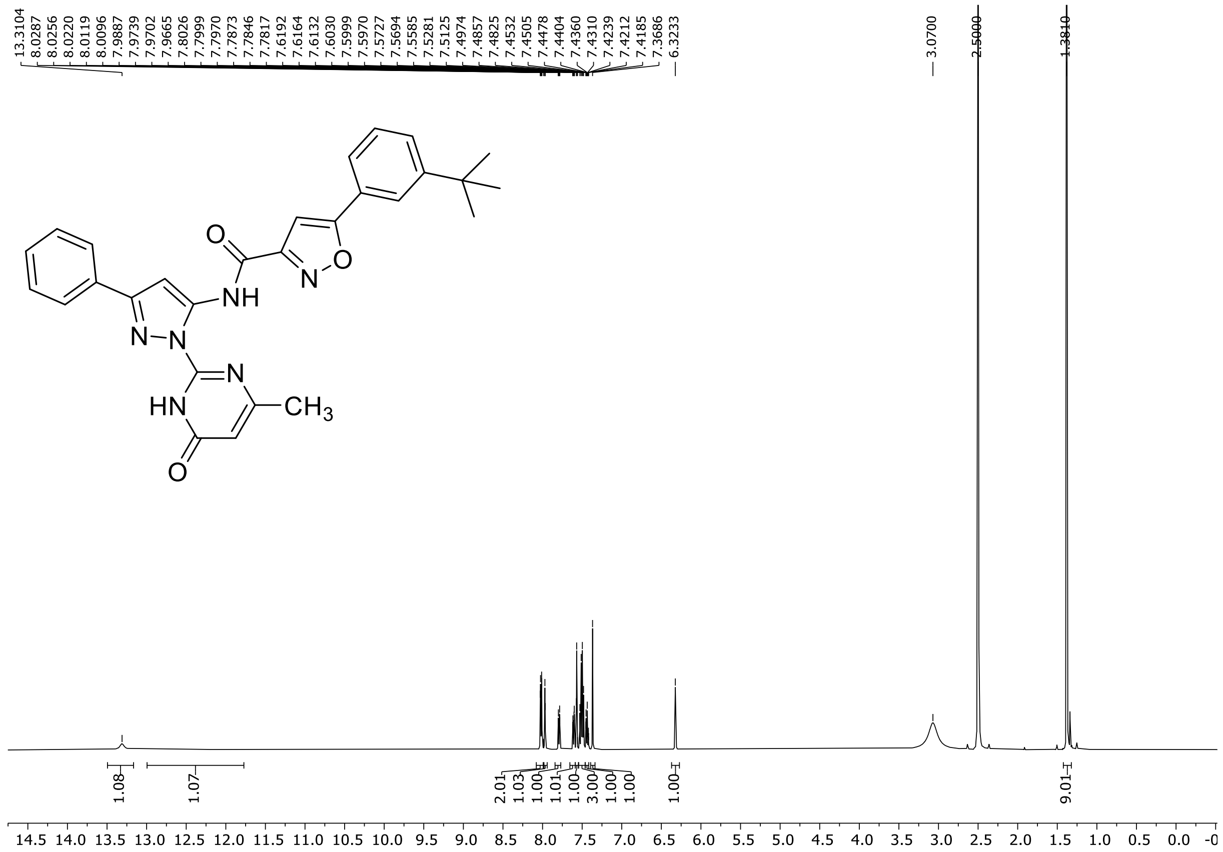

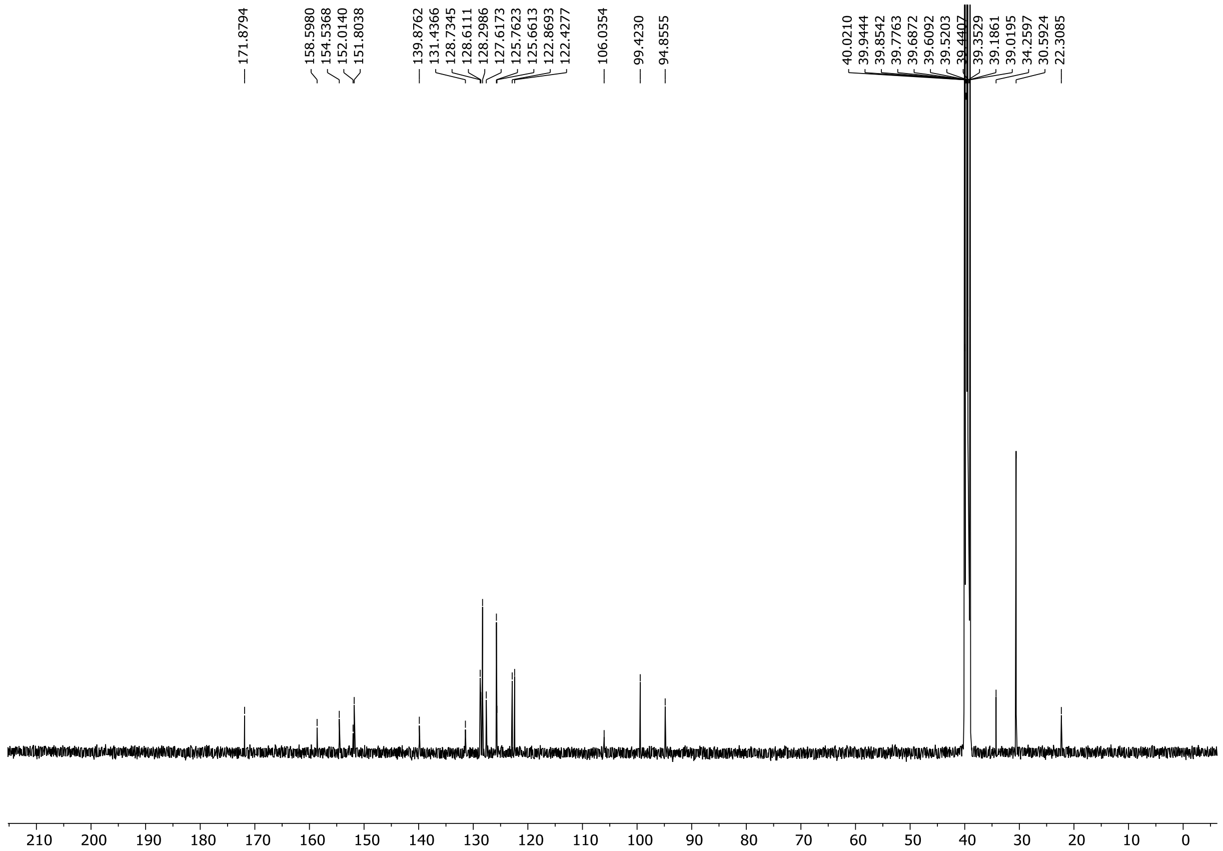

HRMS spectrum of **118**

NAR-A-98 $C_{28}H_{26}N_6O_3$ *m/z* **494.2066**

APCI+ (MMI)
nitrogen flow 5 L/min, gas temperature 325°C, nebulizer 45 psi, skimmer 65 V, vaporizer 200°C, fragmentor 28 V, dissolved in DMSO, MeOH

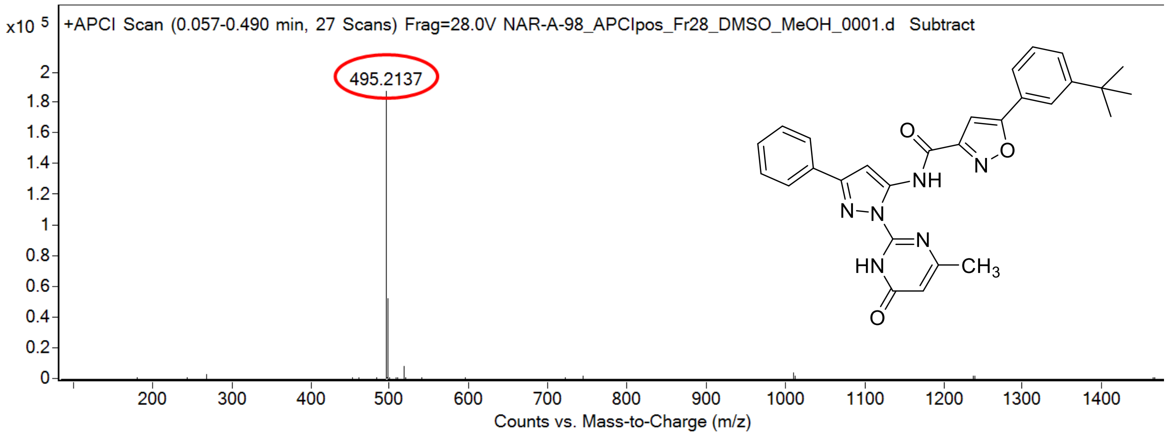

calculated mass: $[M+H]^+ = 495.2139$ observed: $[M+H]^+ = 495.2137$ mass accuracy = -0.4 ppm

FT-IR spectrum (neat) of **118**

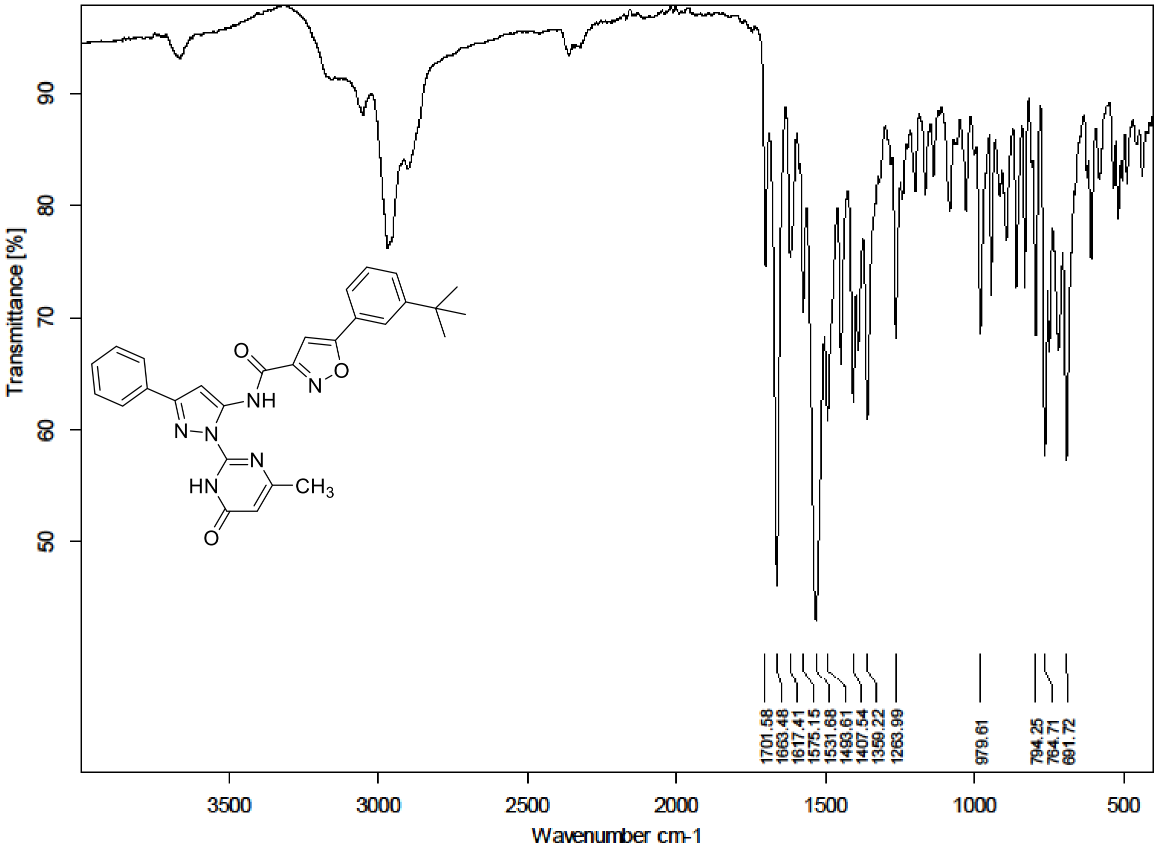

^{1}H NMR (300 MHz) spectrum of **119** in DMSO-d_6

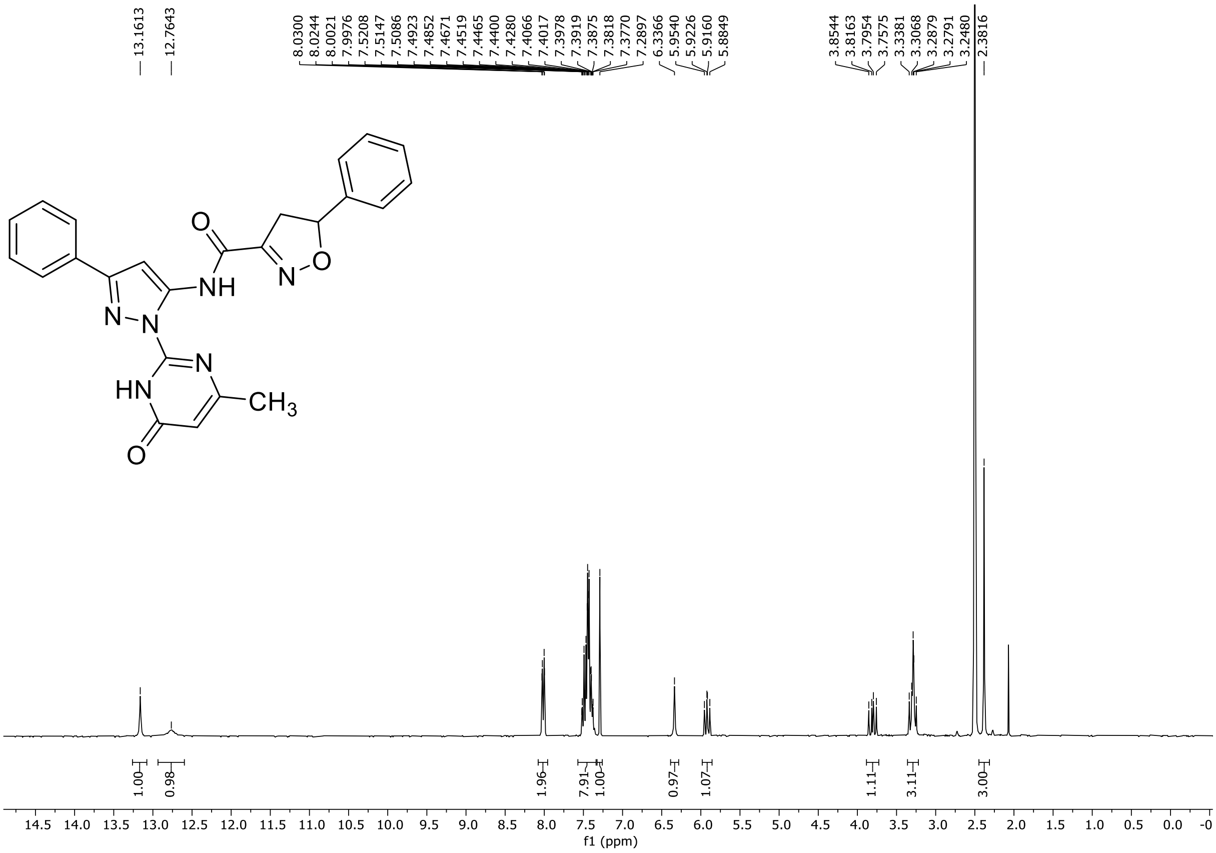

HRMS spectrum of **119**

NAR-A-225 $C_{24}H_{20}N_6O_3$ mono *m/z* 440.1597

ESI - (MMI)
nitrogen flow 5 L/min, gas temperature 325°C, nebulizer 30 psig, skimmer - 65 V, Vcap 2500 V, fragmentor - 90 V, dissolved in methanol

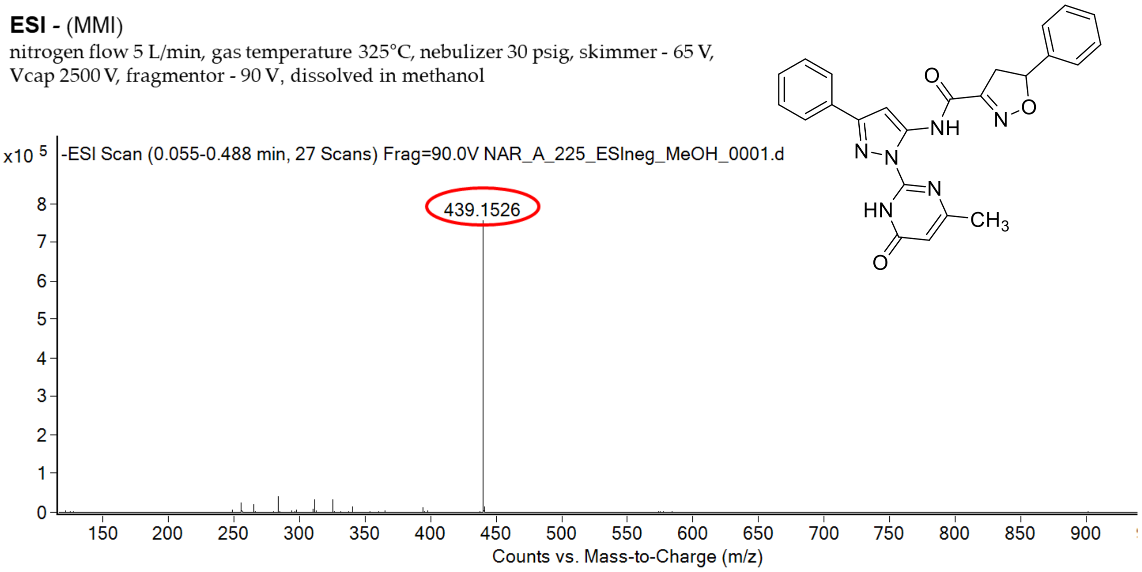

calculated mass: $[M-H]^-$ = 439.1524 observed: $[M-H]^-$ = 439.1526 mass accuracy = 0.5 ppm

FT-IR spectrum (neat) of **119**

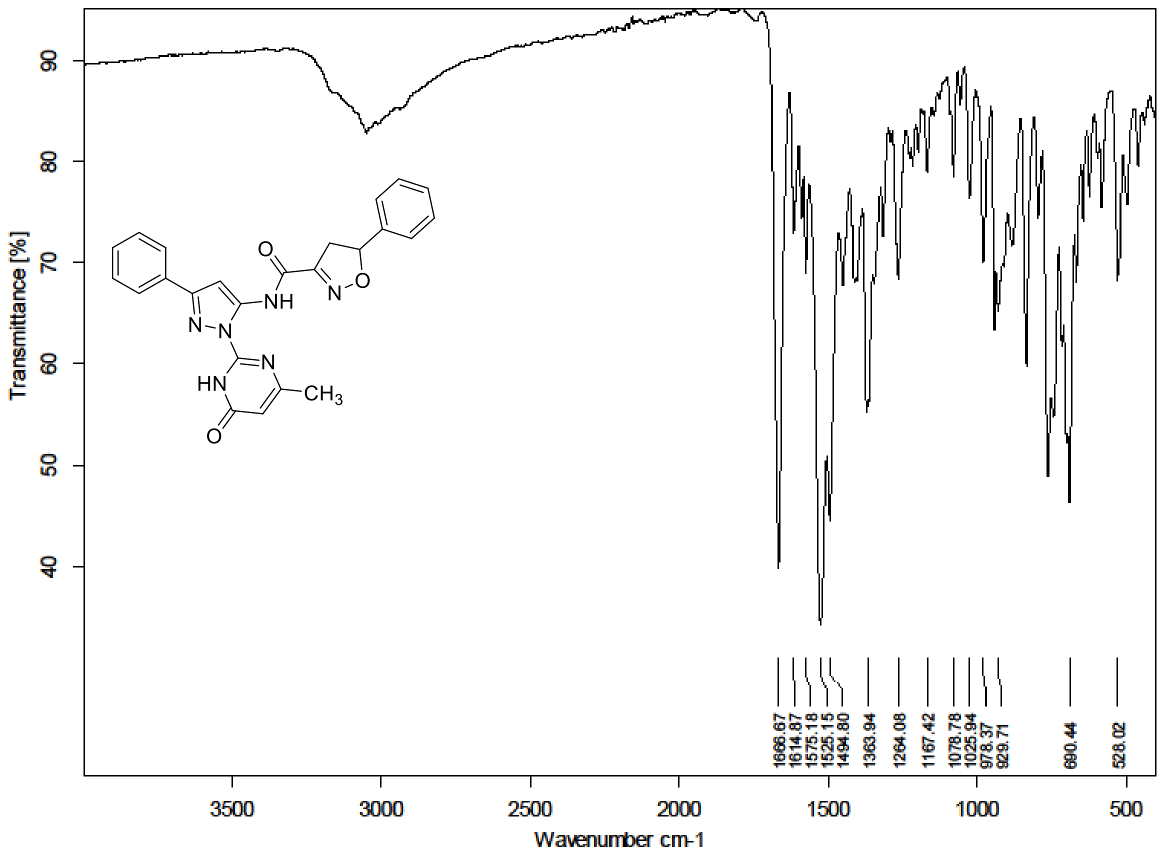

^{1}H NMR (300 MHz) spectrum of **120** in DMSO-d_6

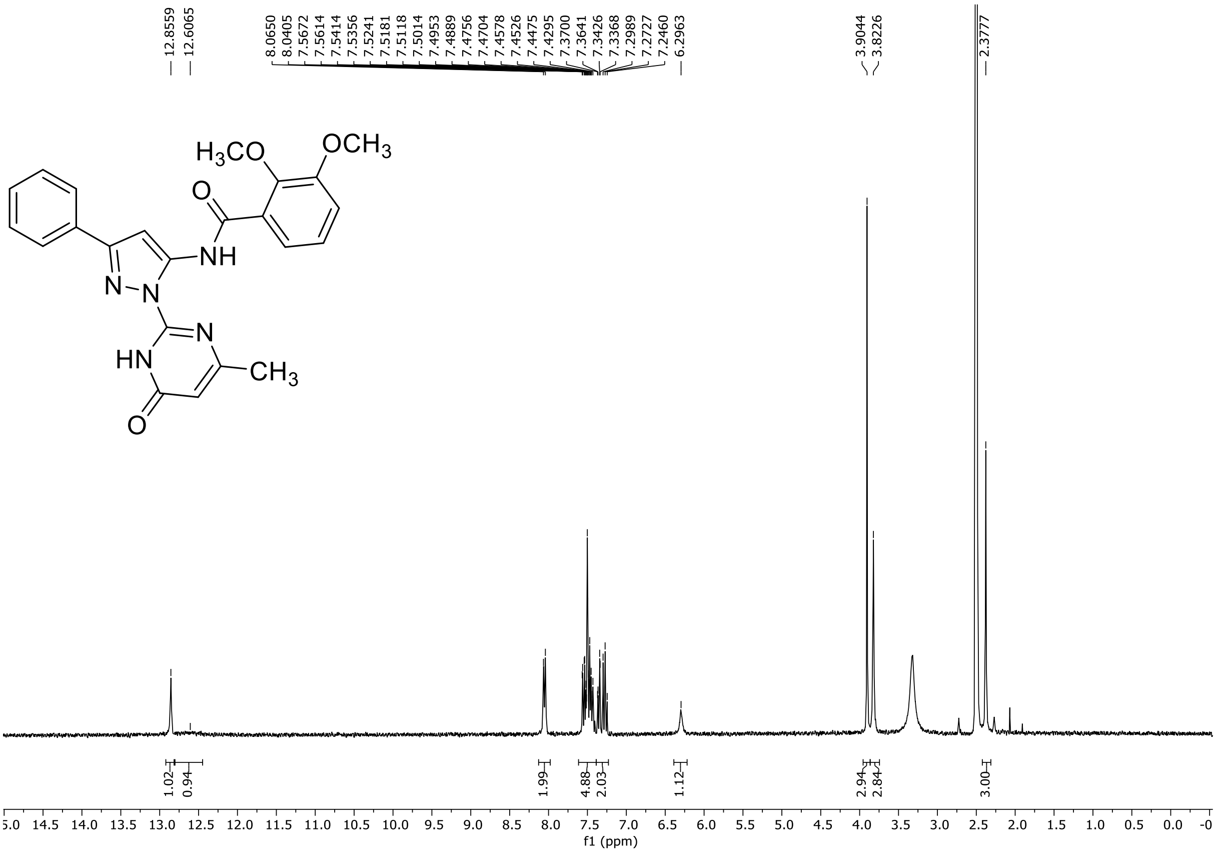

HRMS spectrum of **120**

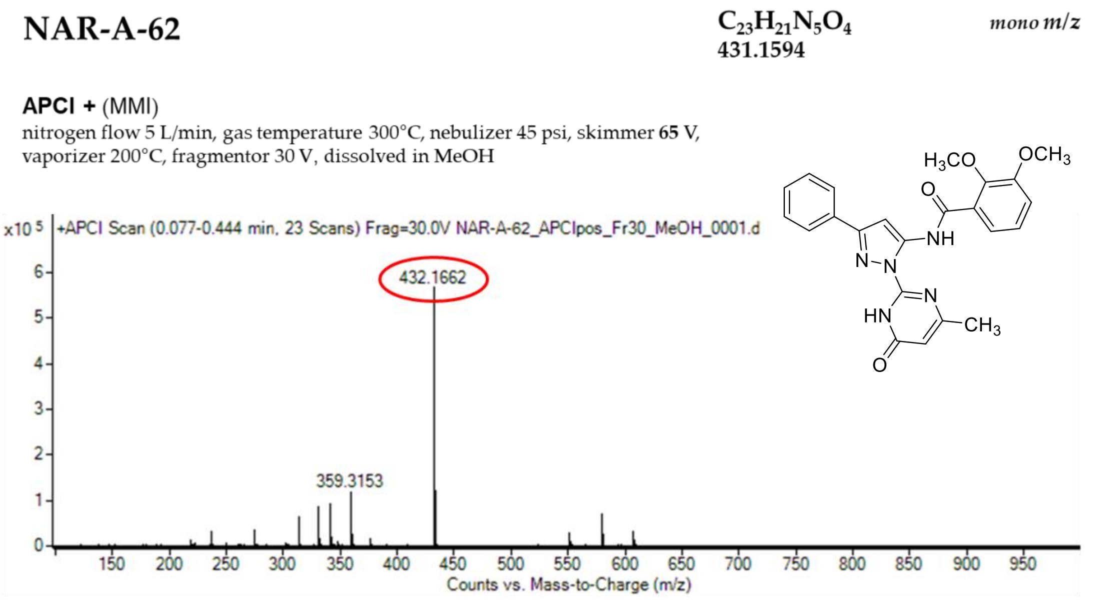

calculated mass: $[M+H]^+ = 432.1666$ observed: $[M+H]^+ = 432.1662$ mass accuracy = -0.9 ppm

FT-IR spectrum (neat) of **120**

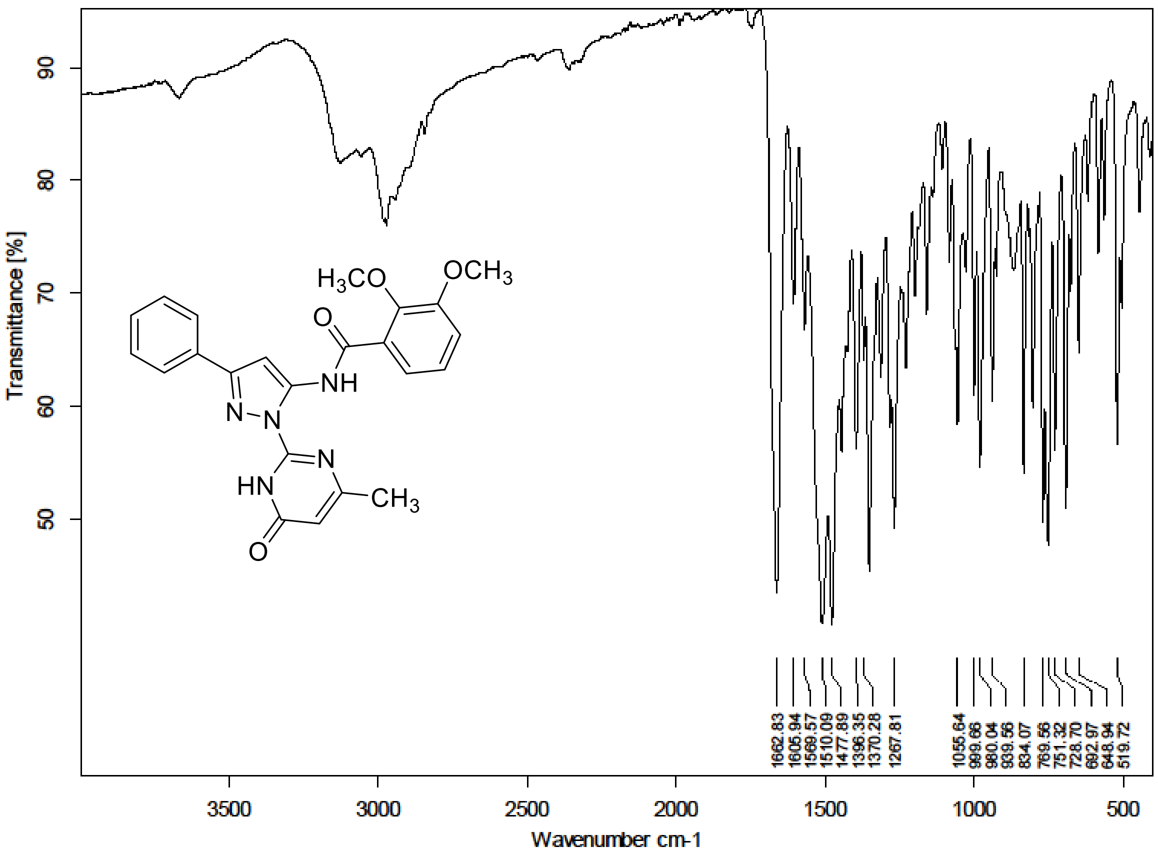

^{1}H NMR (300 MHz) spectrum of **121** in DMSO-d_6

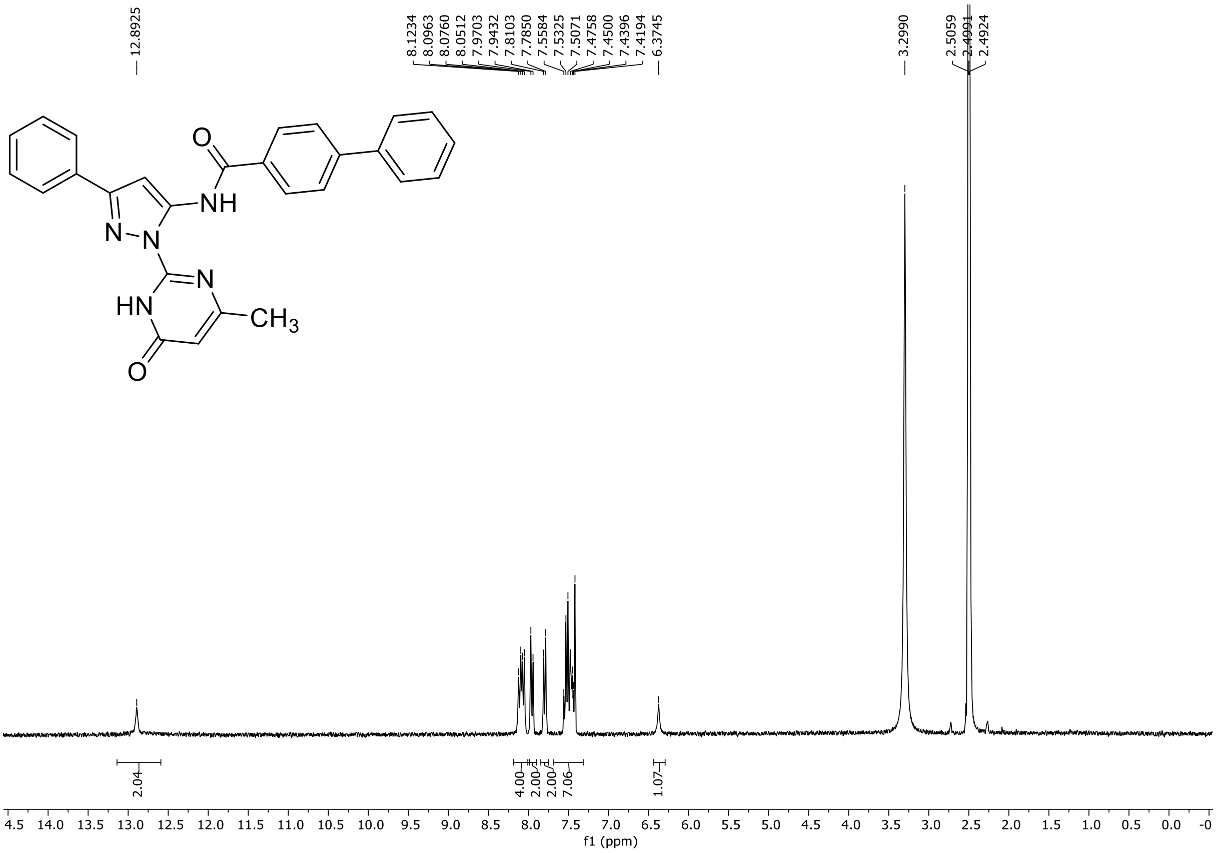

HRMS spectrum of **121**

NAR-A-75 RC

$C_{27}H_{21}N_5O_2$ *mono m/z*

447.1695

APCI + (MMI)

nitrogen flow 5 L/min, gas temperature 300°C, nebulizer 45 psi, skimmer **65** V,
vaporizer 200°C, fragmentor 35 V, dissolved in MeOH

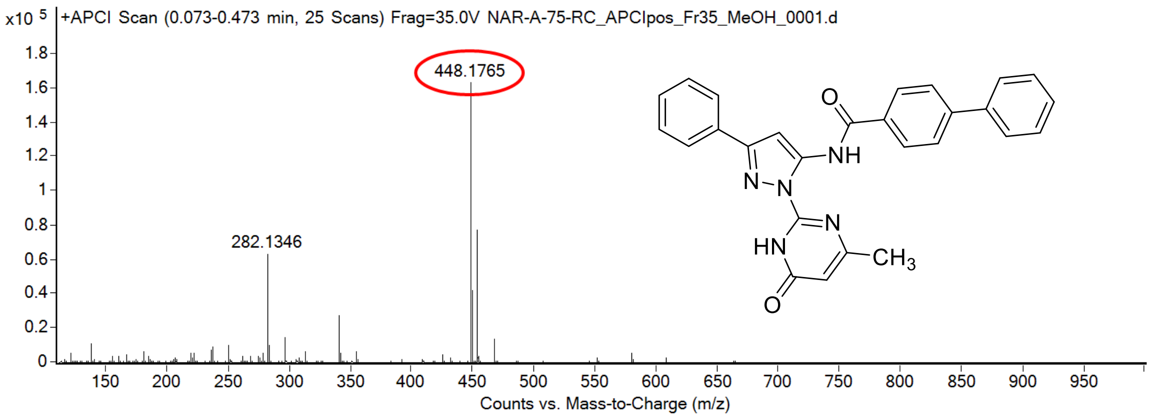

calculated mass: $[M+H]^+$ = 448.1768 observed: $[M+H]^+$ = 448.1765 mass accuracy = -0.7 ppm

FT-IR spectrum (neat) of **121**

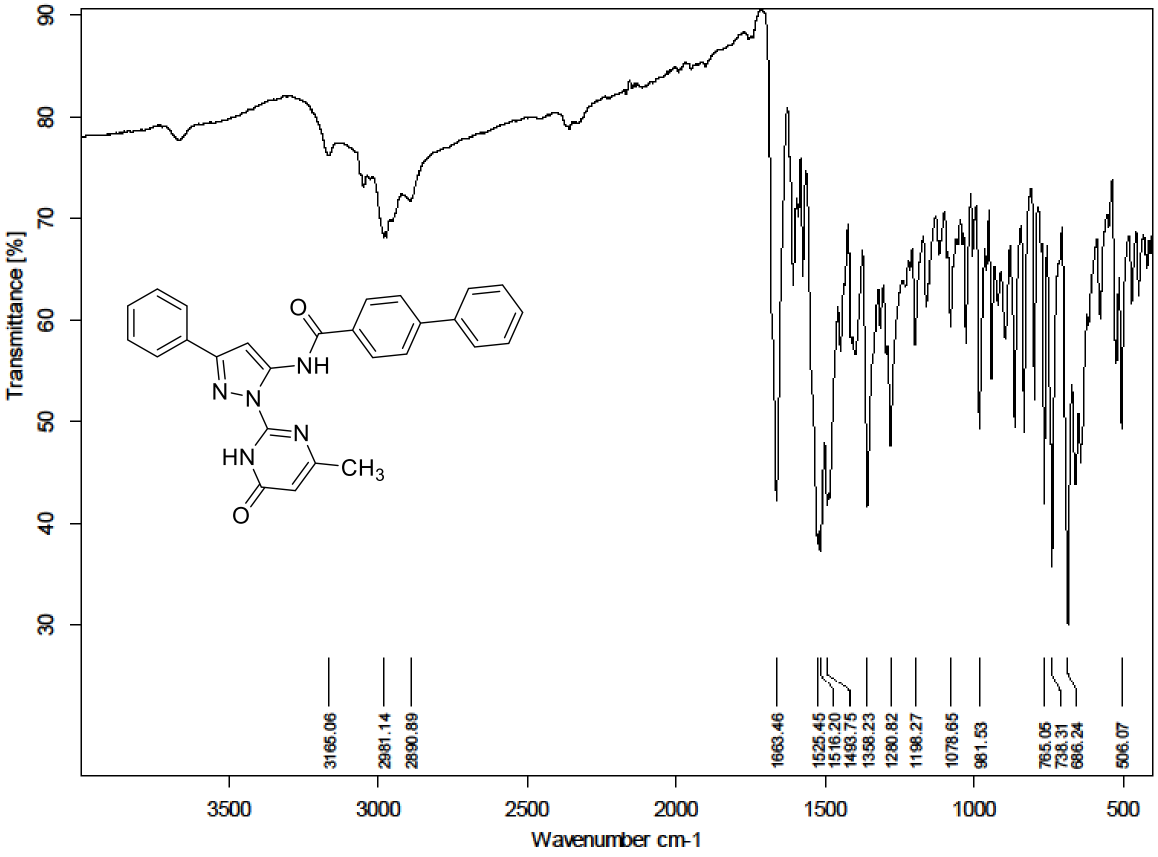

^{1}H NMR (300 MHz) spectrum of **122** in DMSO-d_6

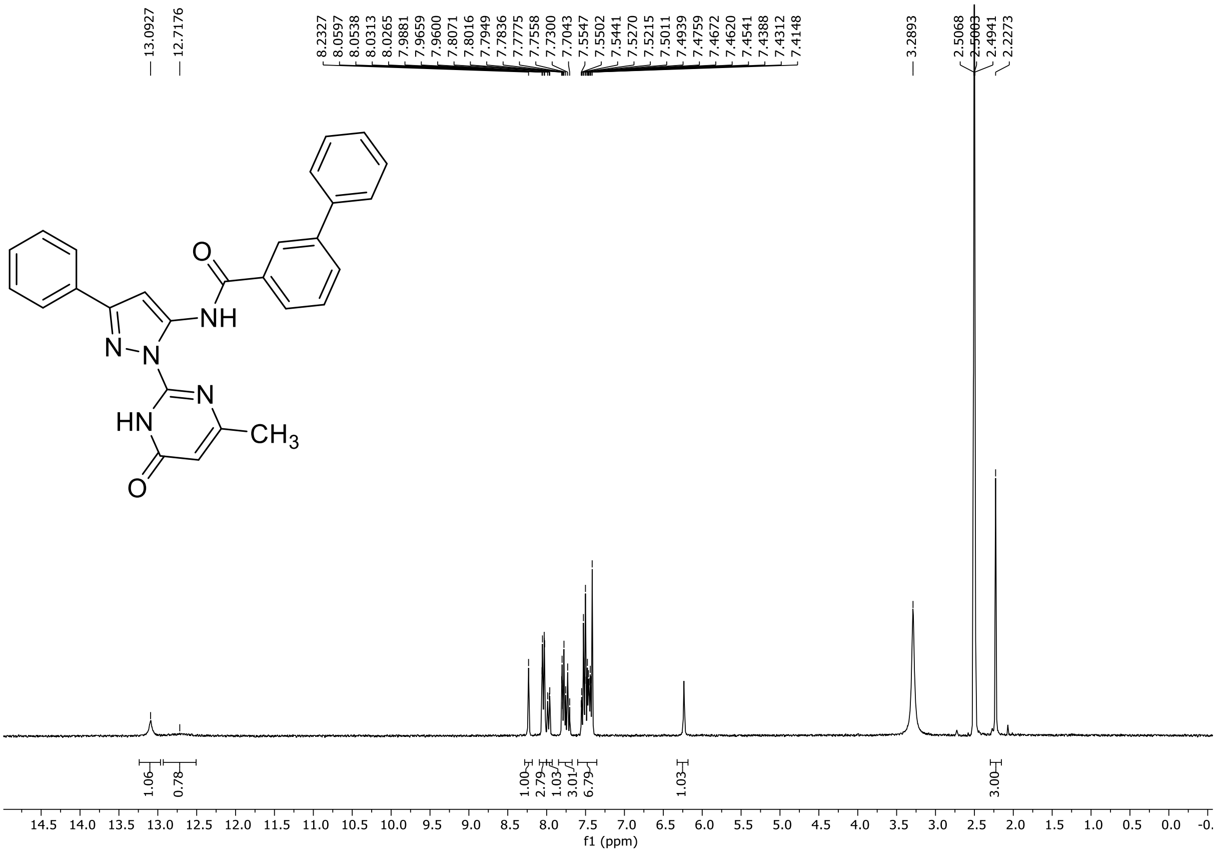

HRMS spectrum of **122**

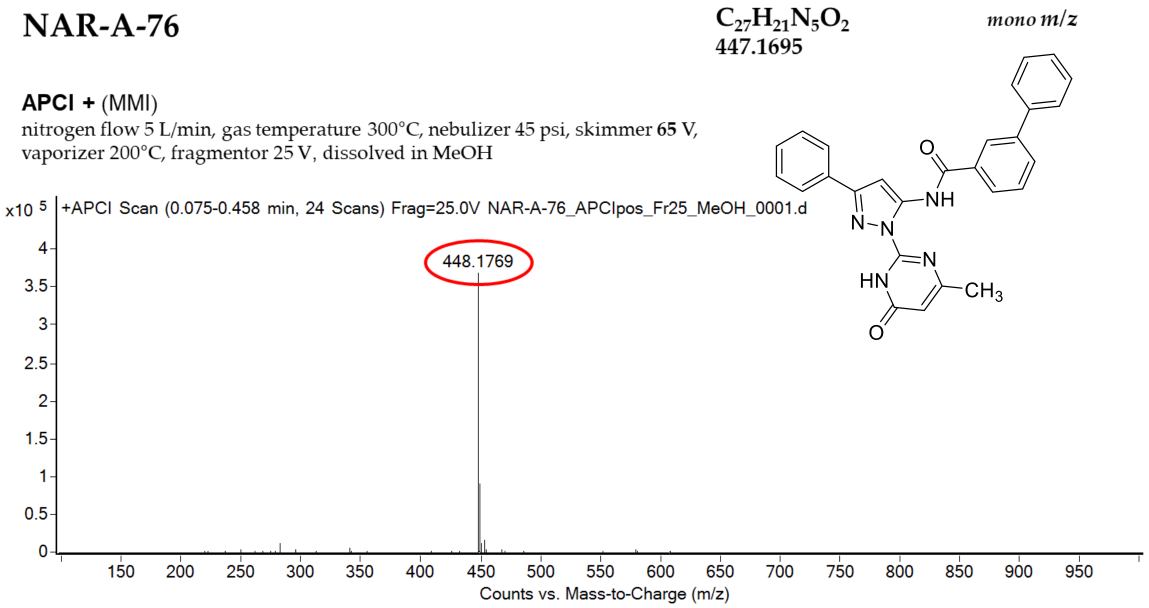

calculated mass: $[M+H]^+$ = 448.1768 observed: $[M+H]^+$ = 448.1769 mass accuracy = 0.2 ppm

FT-IR spectrum (neat) of **122**

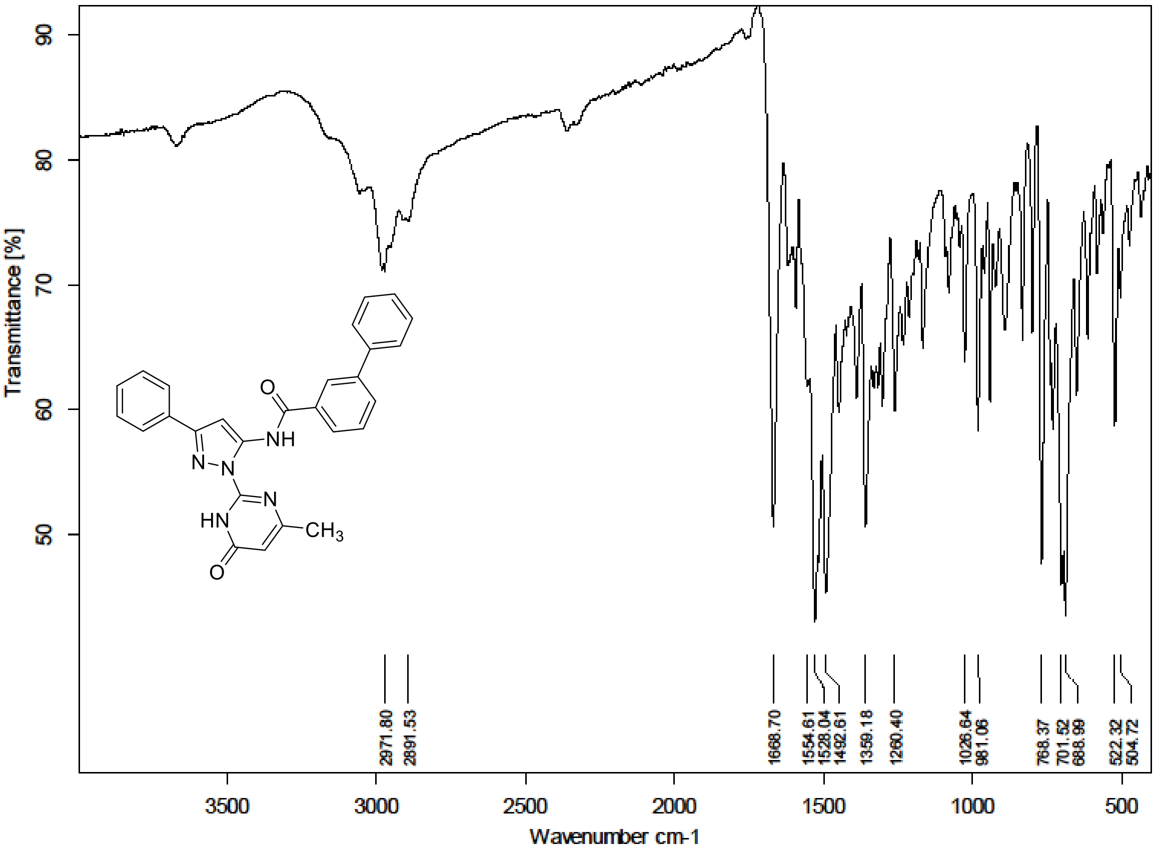

^{1}H (500 MHz) and ^{13}C NMR (126 MHz) spectra of **123** in Methanol-d_4

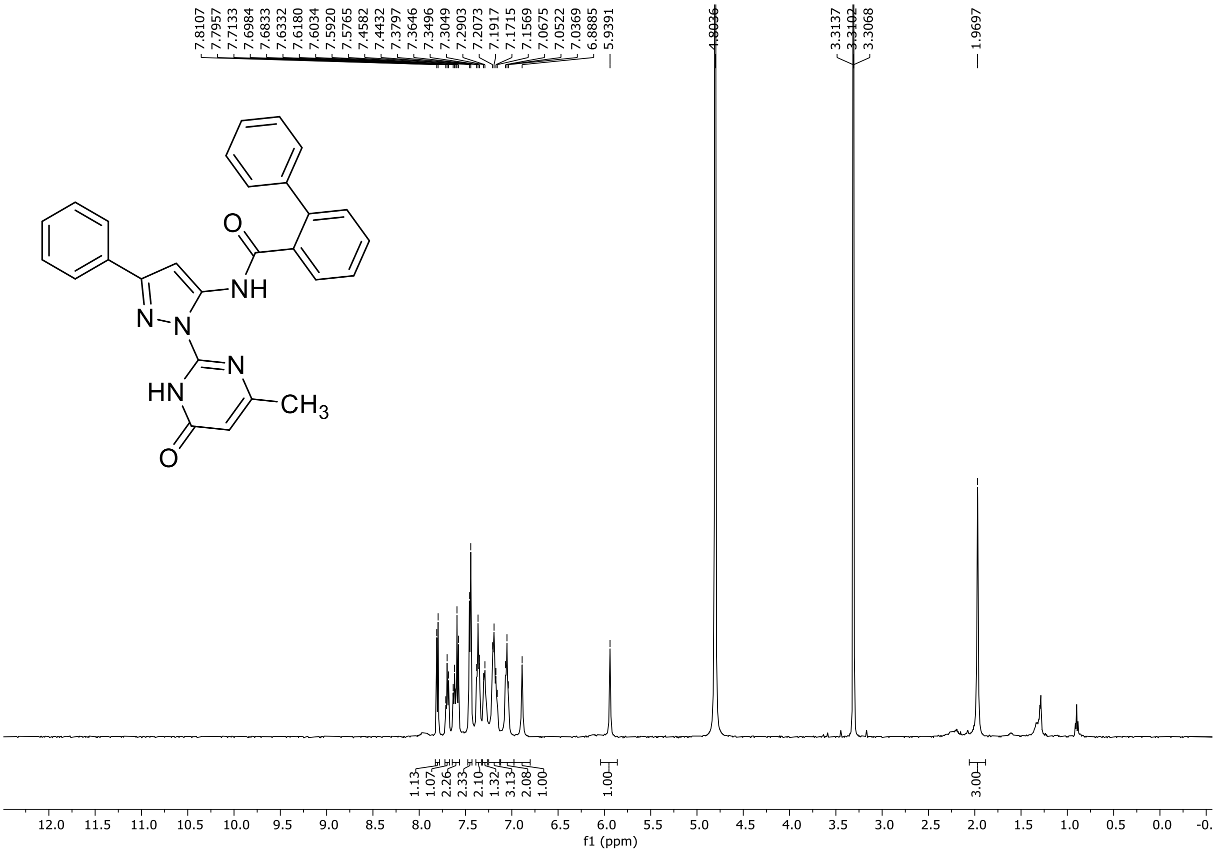

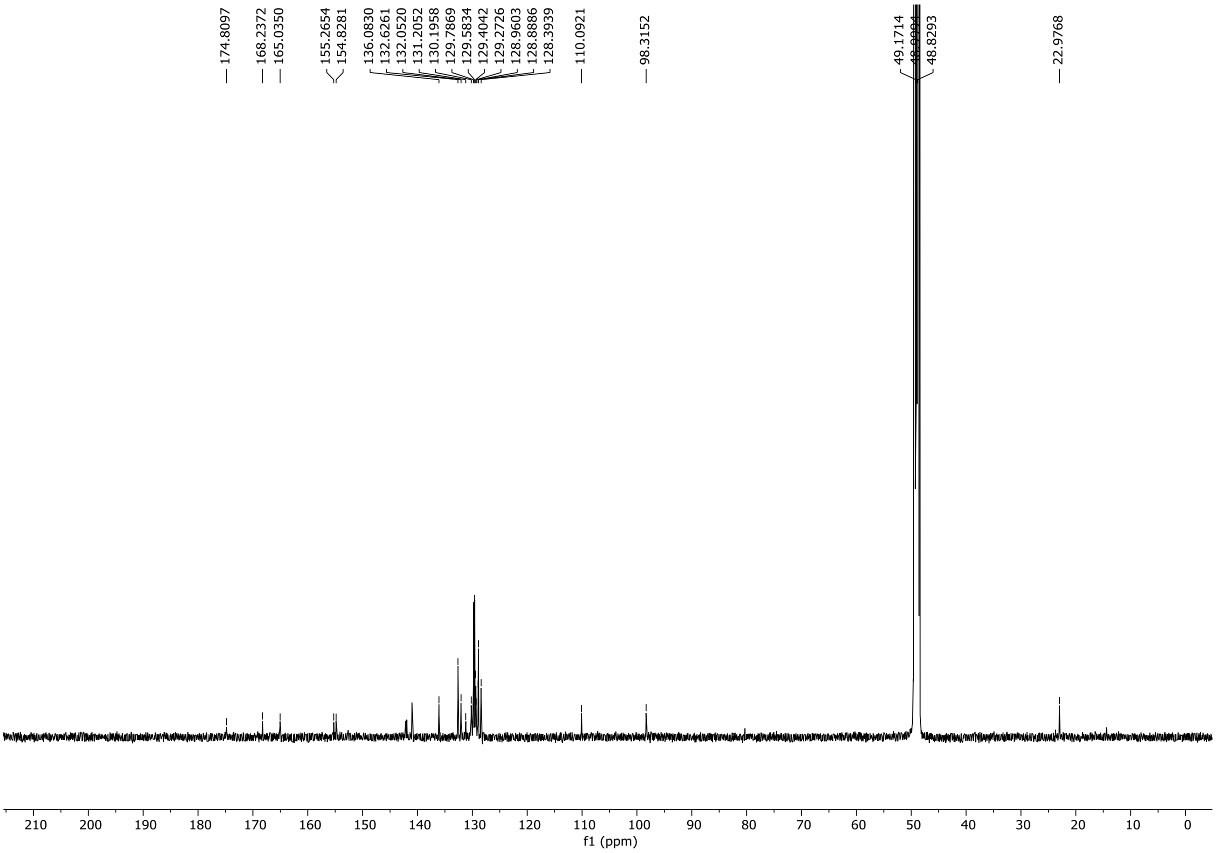

HRMS spectrum of **123**

NAR-A-83-1 $C_{27}H_{21}N_5O_2$ *mono m/z* **447.1695**

APCI + (MMI)
nitrogen flow 5 L/min, gas temperature 300°C, nebulizer 45 psi, skimmer **65** V, vaporizer 200°C, fragmentor 30 V, dissolved in MeOH

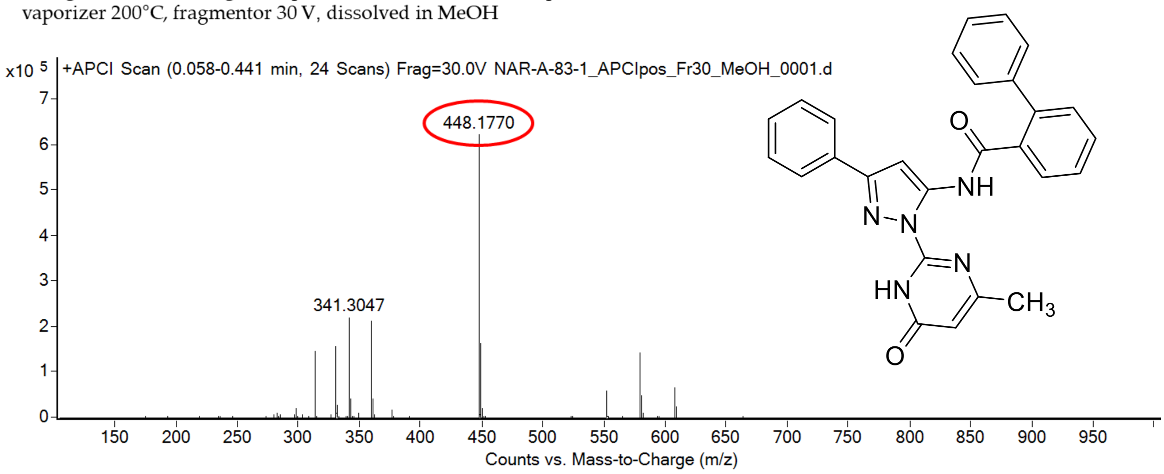

calculated mass: $[M+H]^+ = 448.1768$ observed: $[M+H]^+ = 448.1770$ mass accuracy = 0.4 ppm

FT-IR spectrum (neat) of **123**

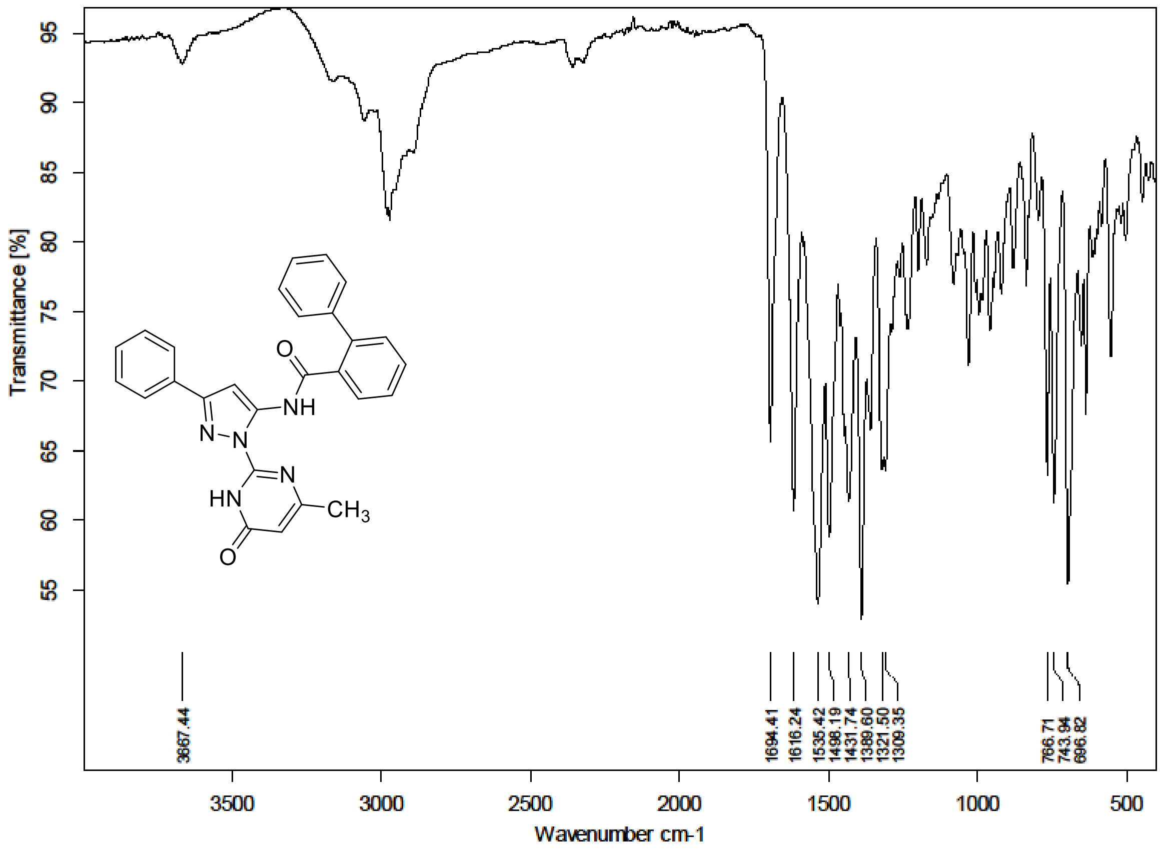

^{1}H (500 MHz) and ^{13}C NMR (126 MHz) spectra of **124** in DMSO-d_6

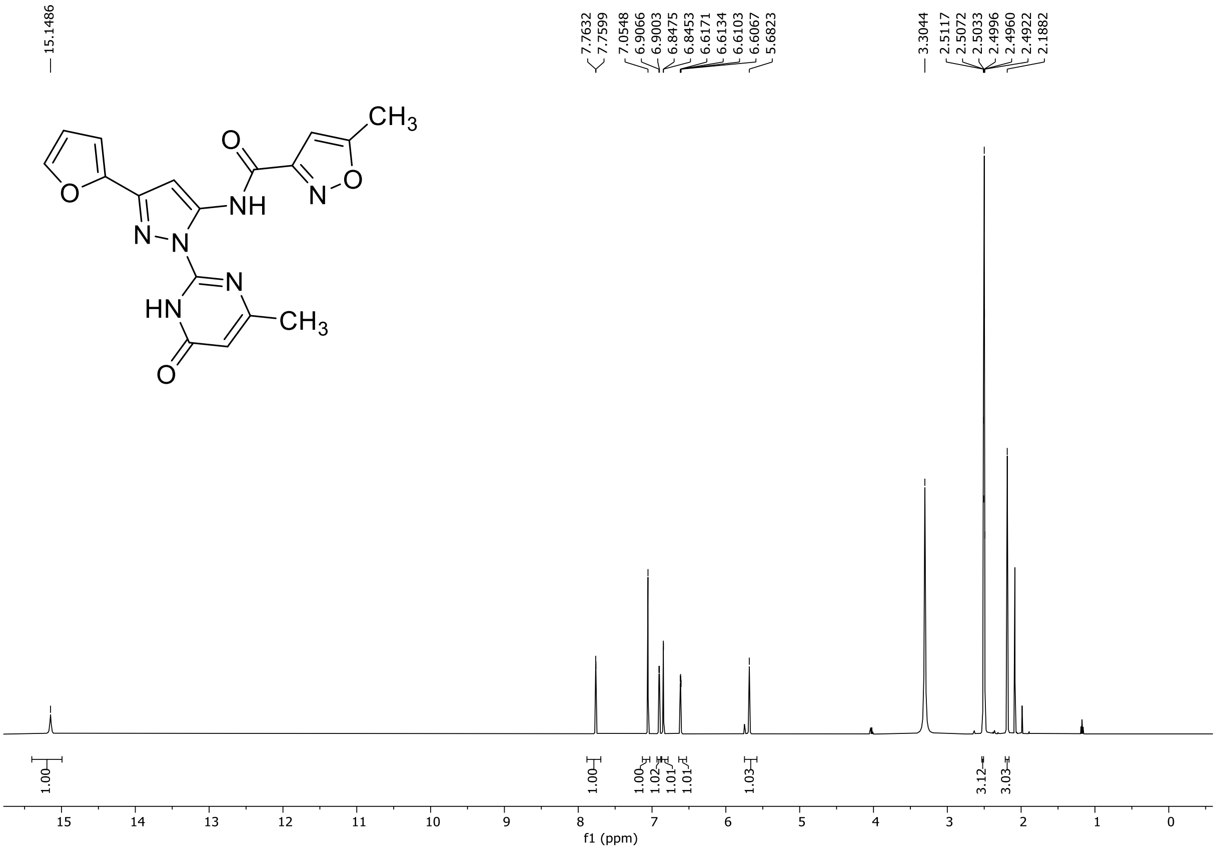

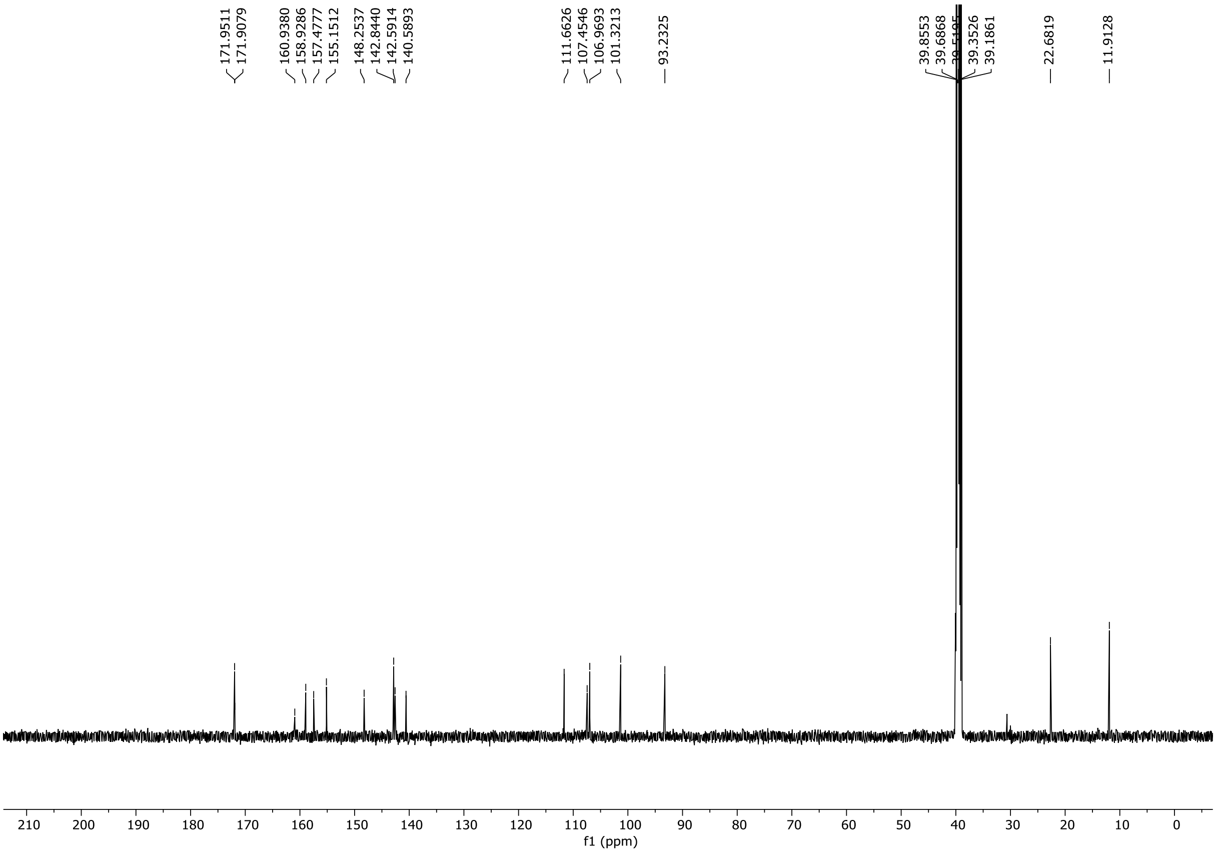

HRMS spectrum of **124**

NAR-A-4 $C_{17}H_{14}N_6O_4$ *mono m/z* **366.1077**

ESI - (MMI)
nitrogen flow 5 L/min, gas temperature 325°C, nebulizer 45 psi, skimmer **65** V, vaporizer 200°C, fragmentor 35 V, dissolved in methanol

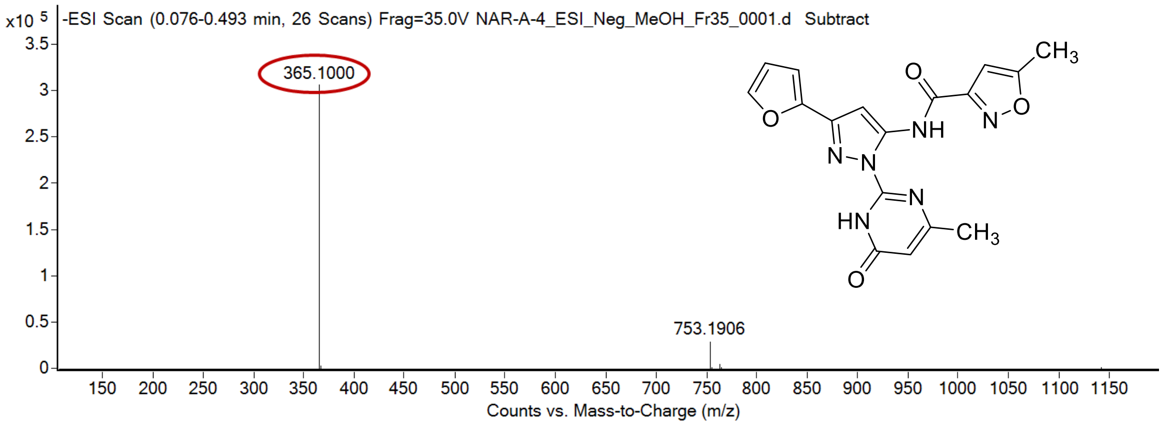

calculated mass: $[M\text{-}H]^-$ = 365.1004 observed: $[M\text{-}H]^-$ = 365.1000 mass accuracy = 1.1 ppm

FT-IR spectrum (neat) of **124**

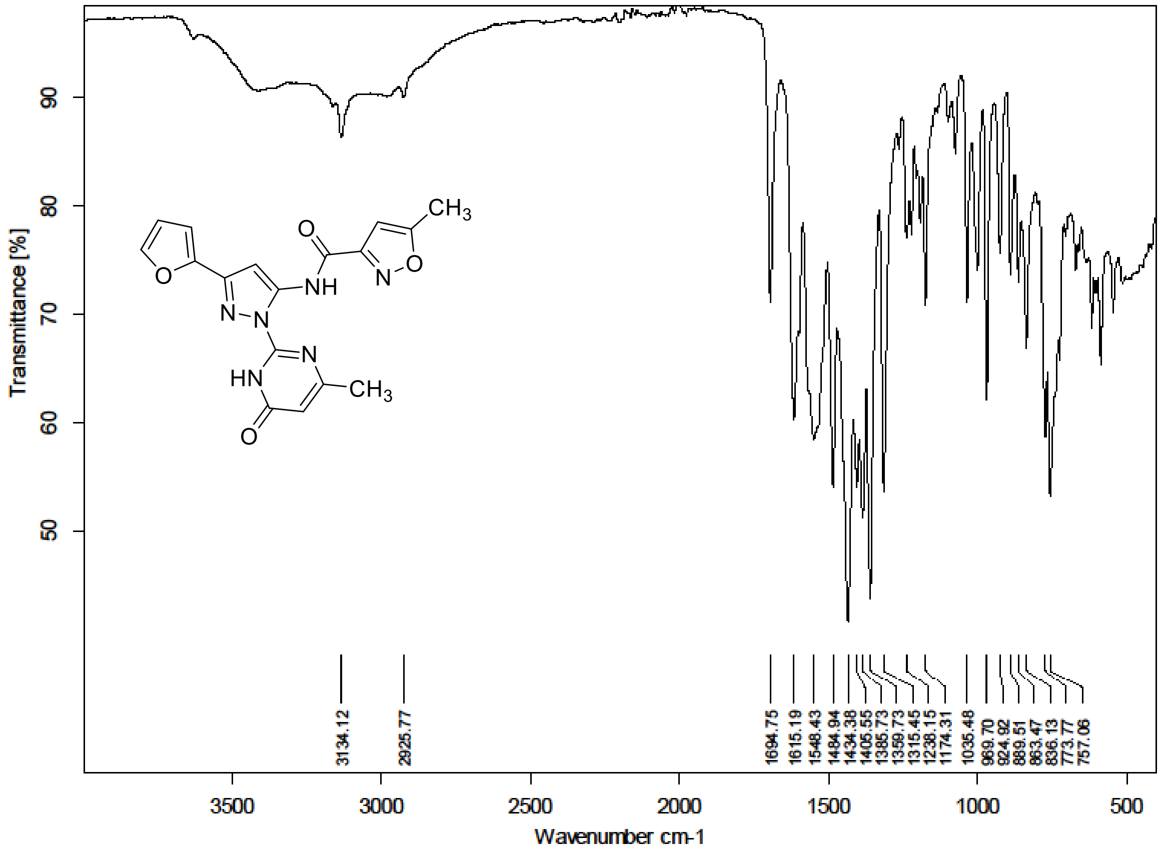

^{1}H (300 MHz) and ^{13}C NMR (176 MHz) spectra of **125** in DMSO-d_6

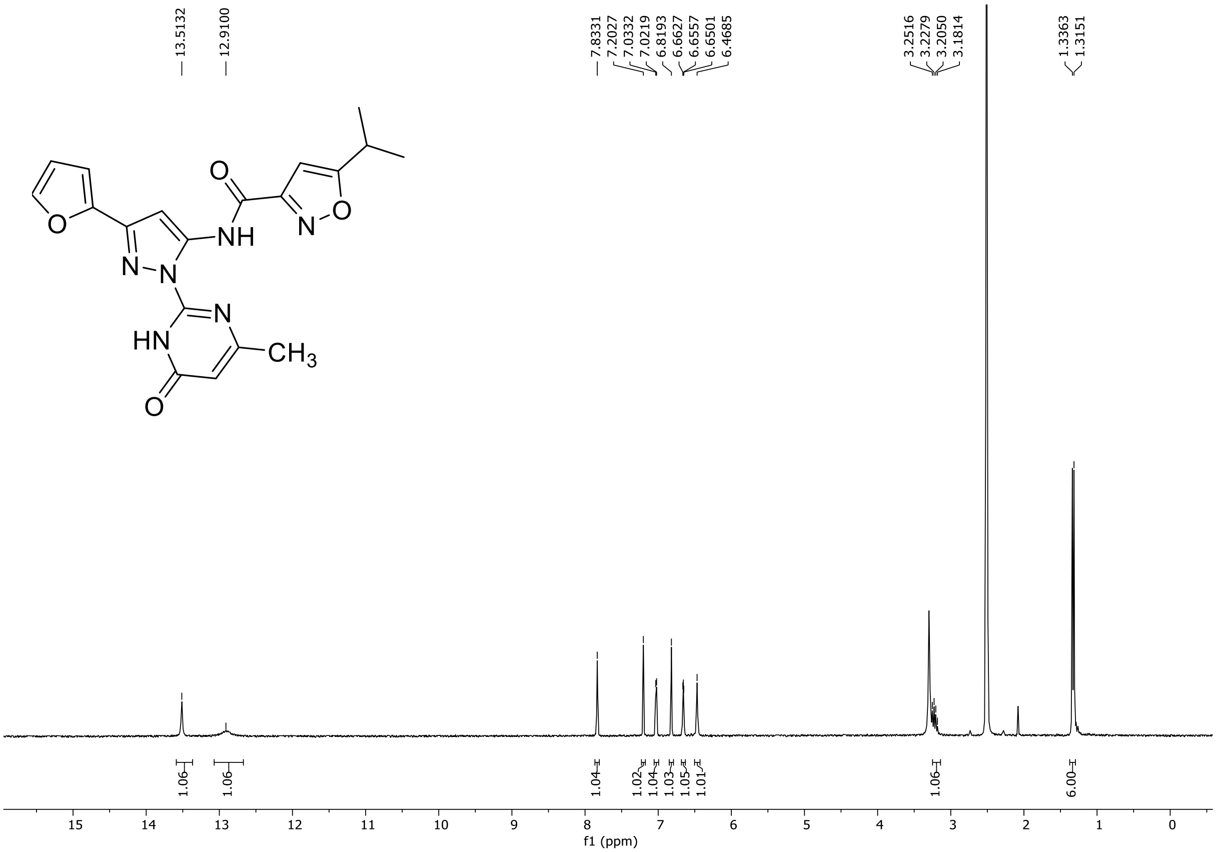

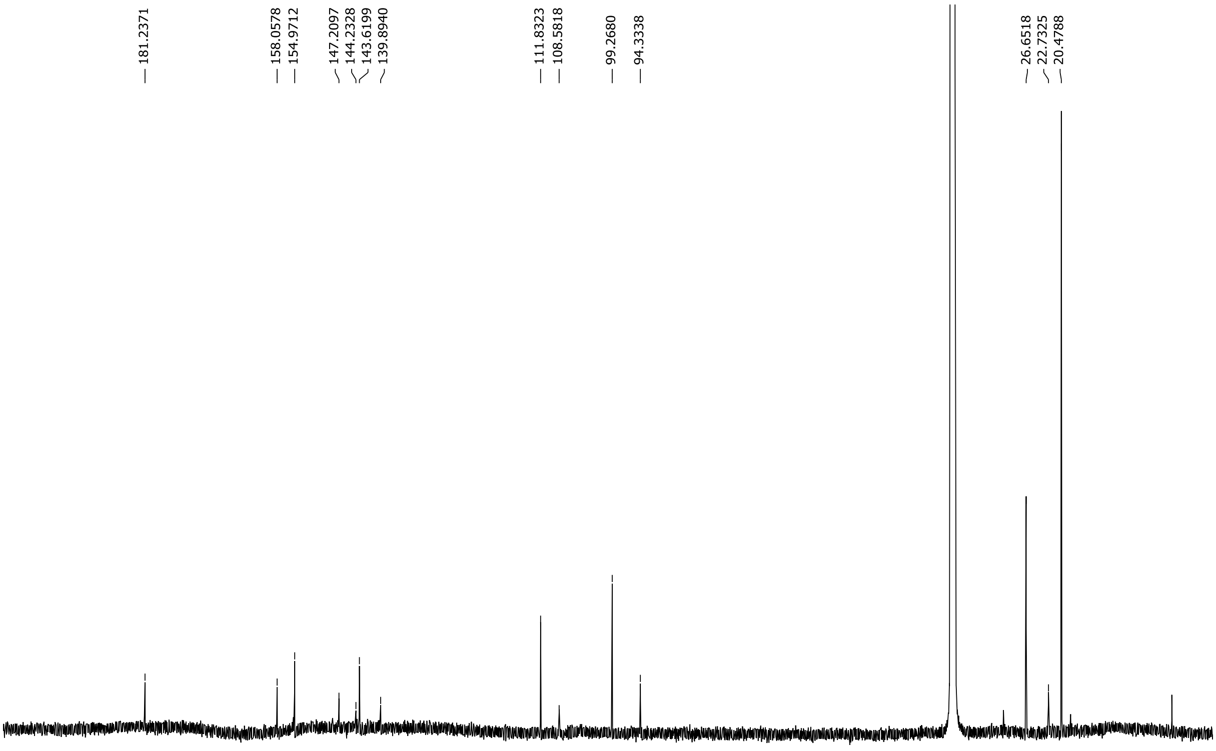

HRMS spectrum of **125**

NAR-A-34 $C_{19}H_{18}N_6O_4$ mono *m/z 394.1390*

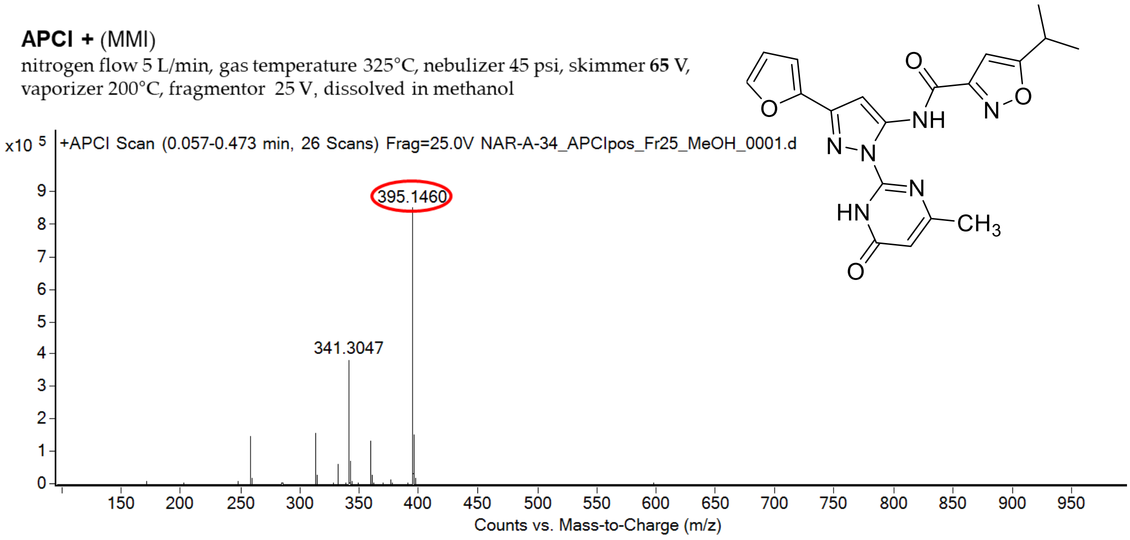

calculated mass: $[M+H]^+ = 395.1462$ observed: $[M+H]^+ = 395.1460$ mass accuracy = -0.5 ppm

FT-IR spectrum (neat) of **125**

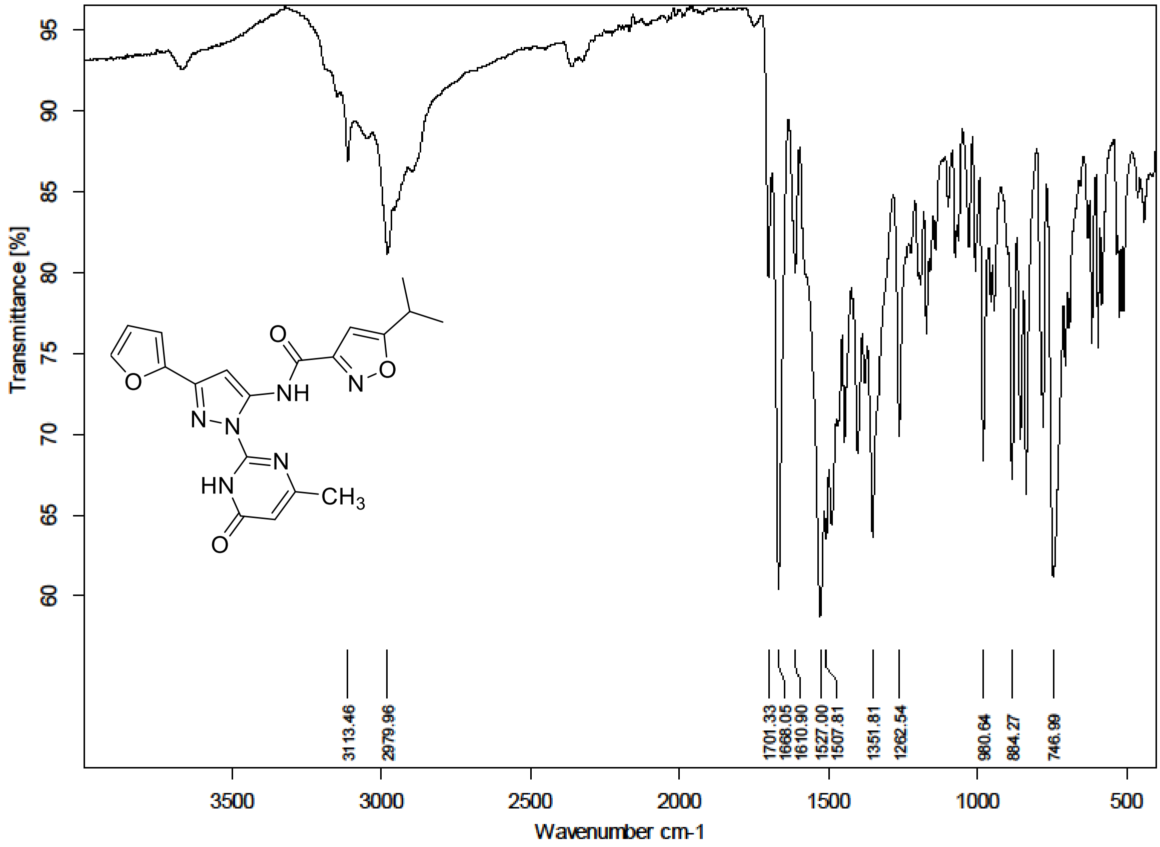

^{1}H (300 MHz) and ^{13}C NMR (126 MHz) spectra of **126** in DMSO-d_6

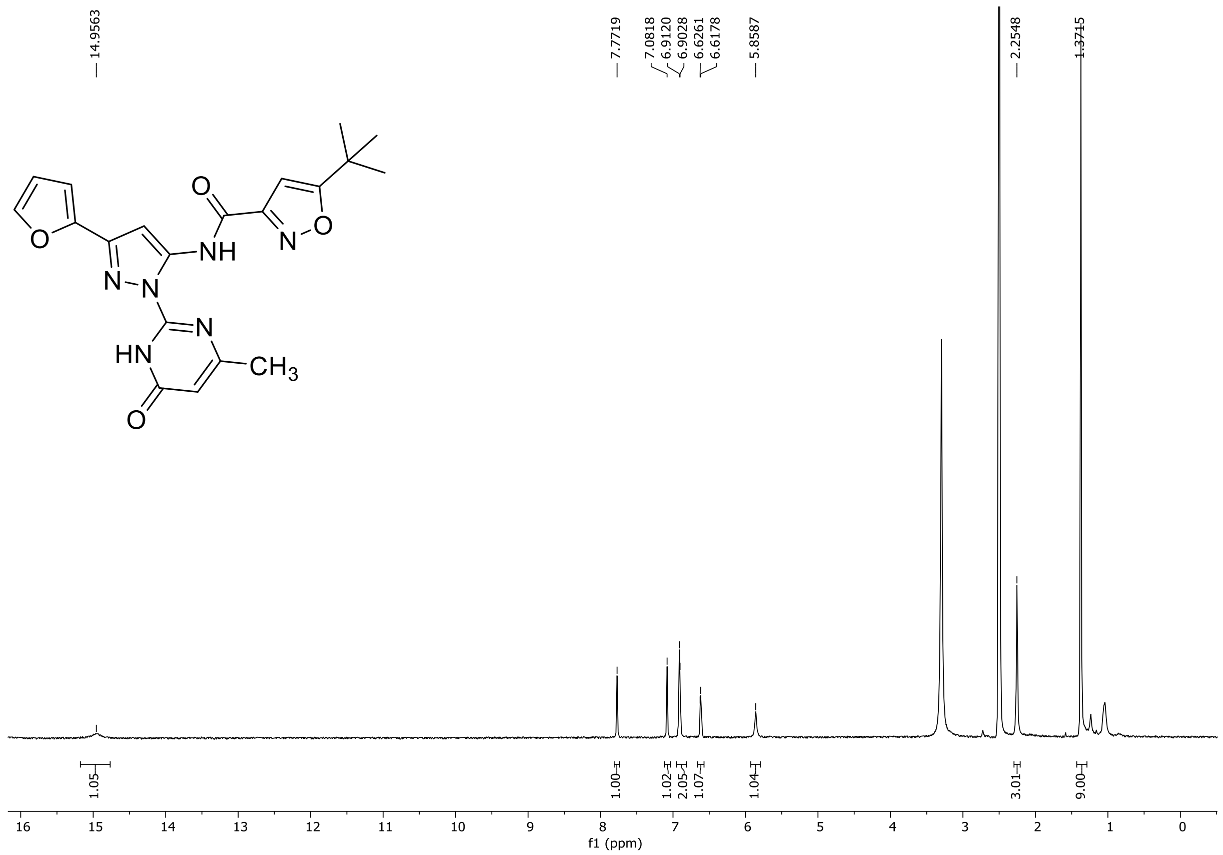

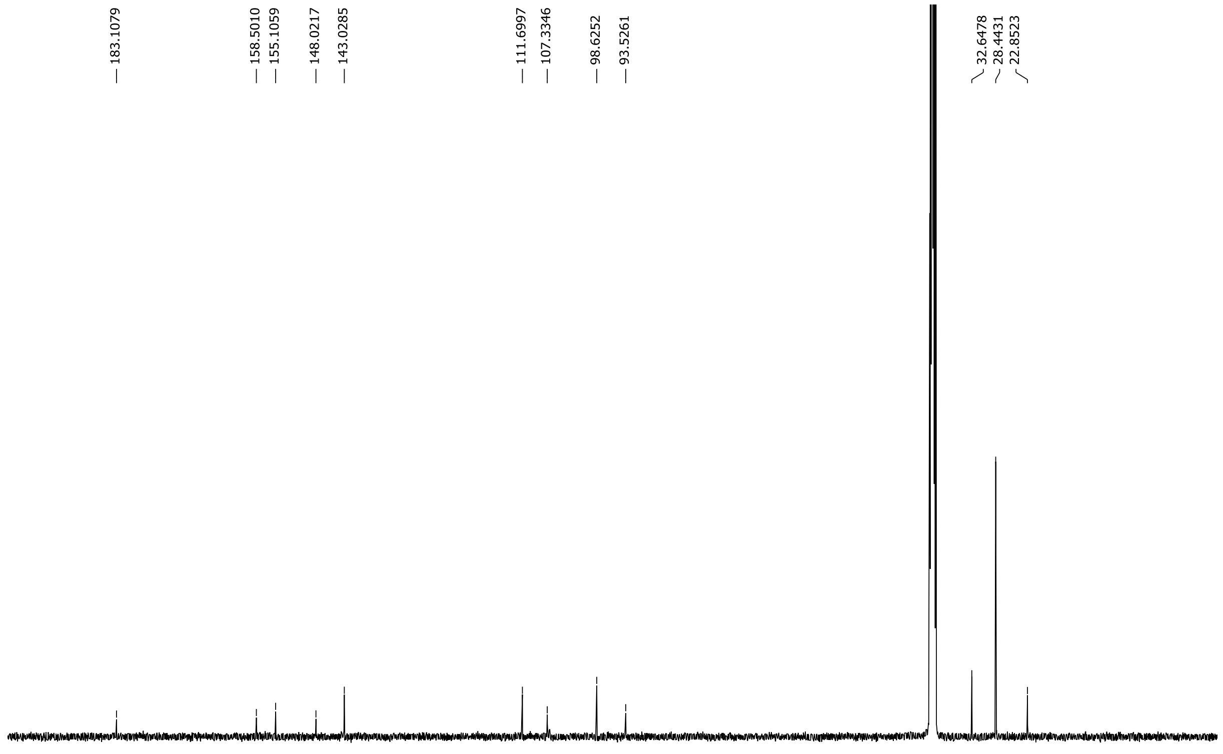

HRMS spectrum of **126**

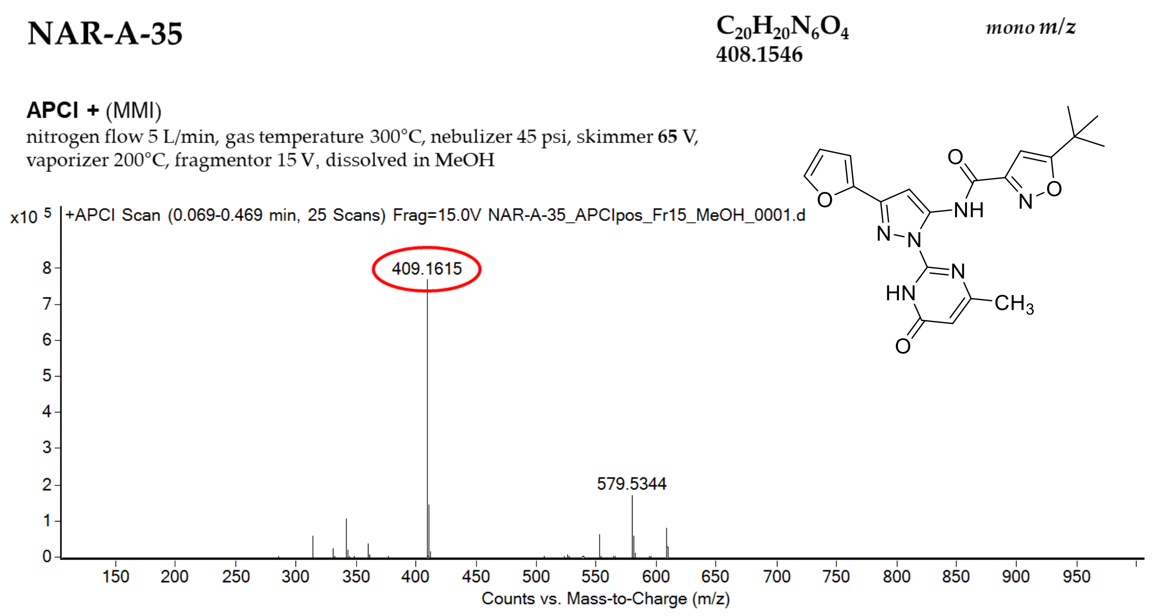

calculated mass: [M+H]+ = 409.1619 observed: [M+H]+ = 409.1615 mass accuracy = -1.0 ppm

FT-IR spectrum (neat) of **126**

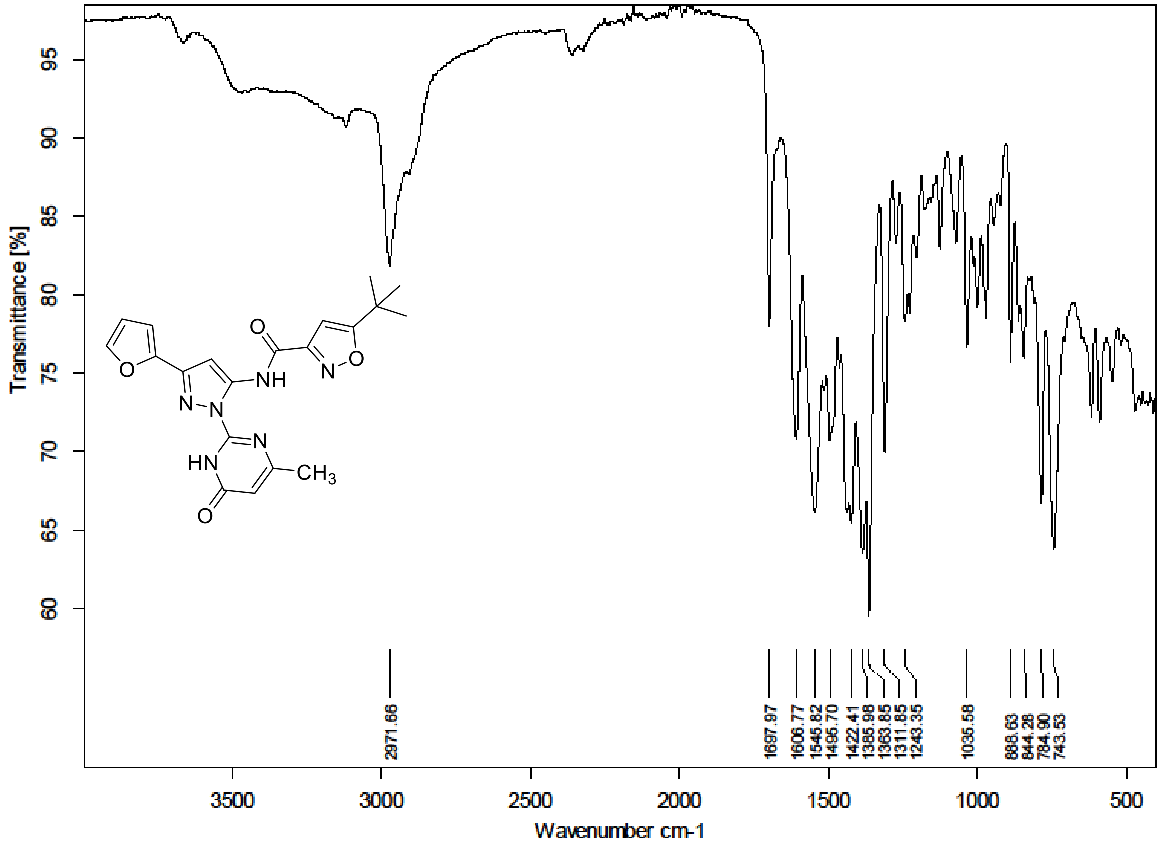

^{1}H (300 MHz) and ^{13}C NMR (75 MHz) spectra of **127** in DMSO-d_6

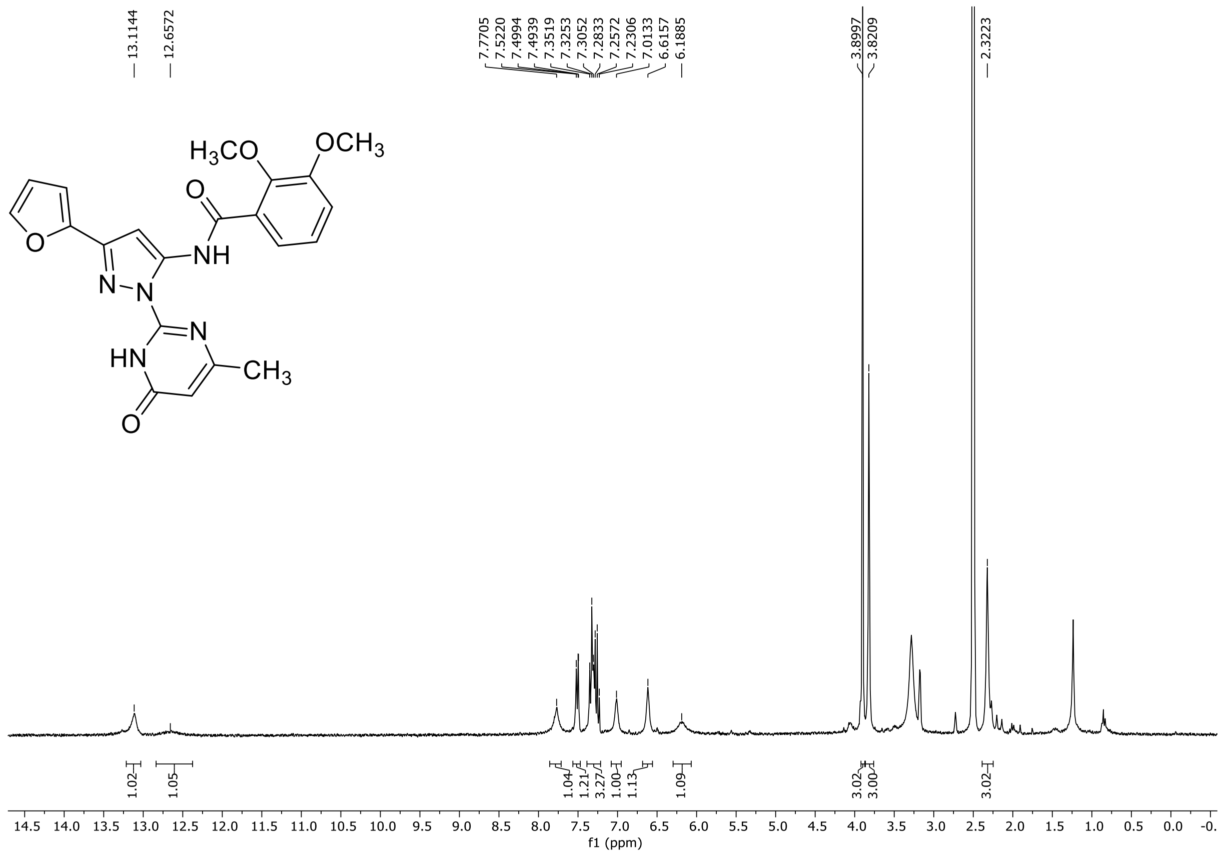

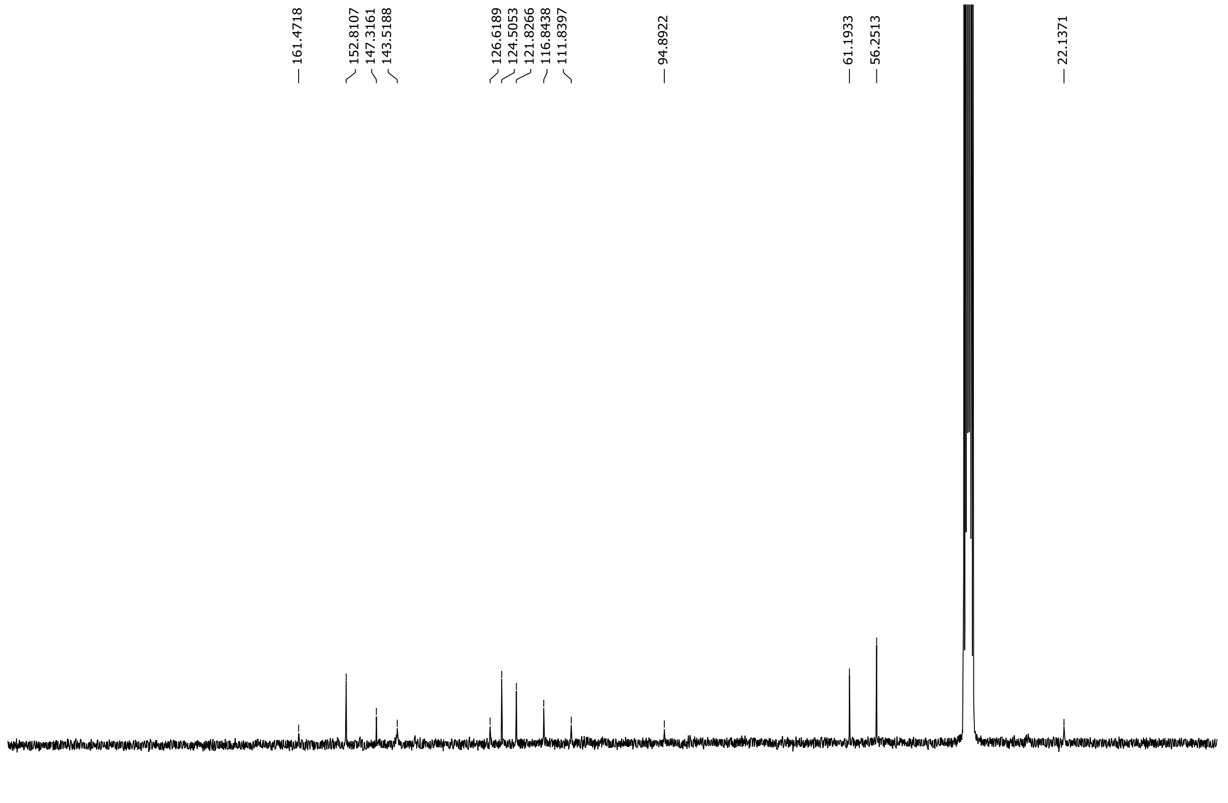

HRMS spectrum of **127**

NAR-A-169 $C_{21}H_{19}N_5O_5$ mono m/z 421.1386

APCI + (MMI)
nitrogen flow 5 L/min, gas temperature 325°C, nebulizer 45 psi, skimmer **65** V, vaporizer 200°C, fragmentor 25 V, dissolved in methanol

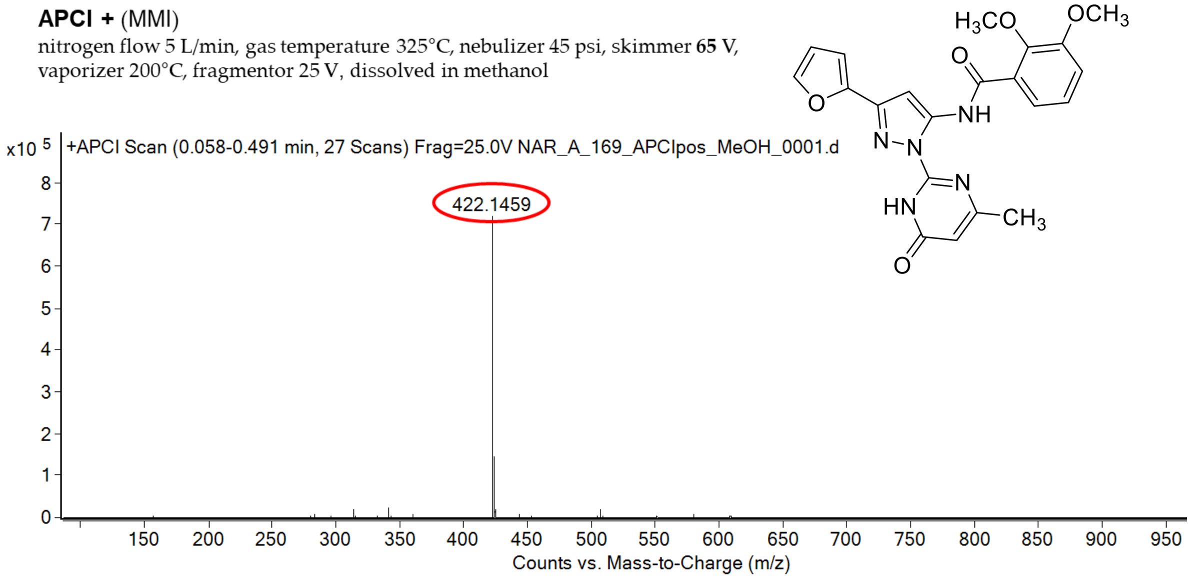

calculated mass: $[M+H]^+$ = 422.1459 observed: $[M+H]^+$ = 422.1459 mass accuracy = < 0.1 ppm

FT-IR spectrum (neat) of **127**

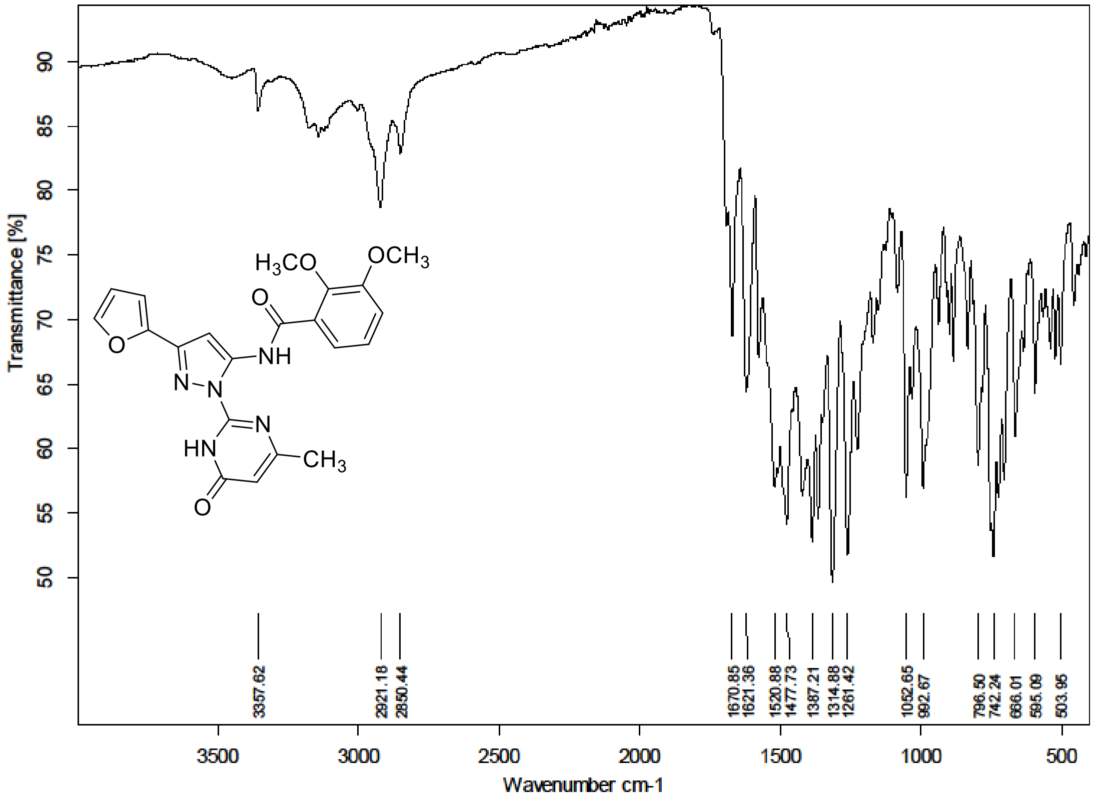

^{1}H NMR (300 MHz) spectrum of **128** in DMSO-d_6

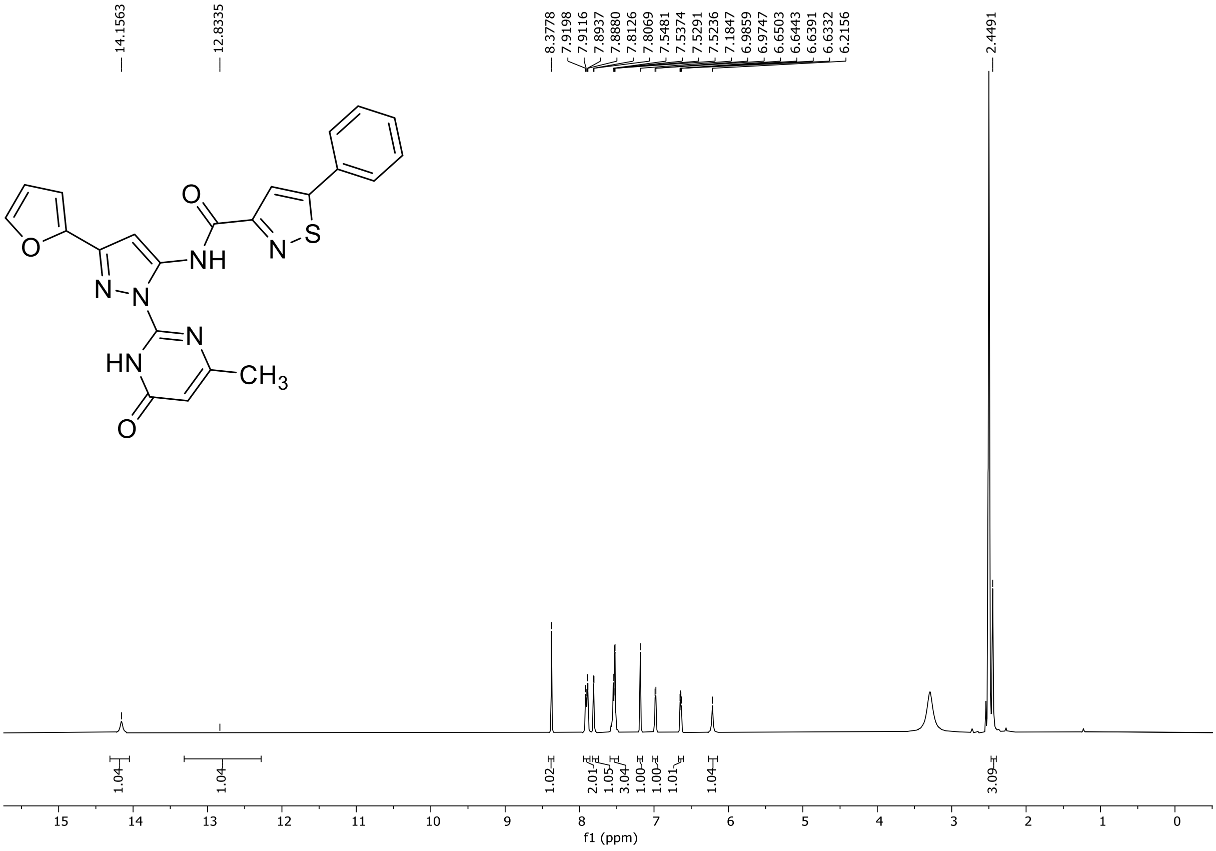

HRMS spectrum of **128**

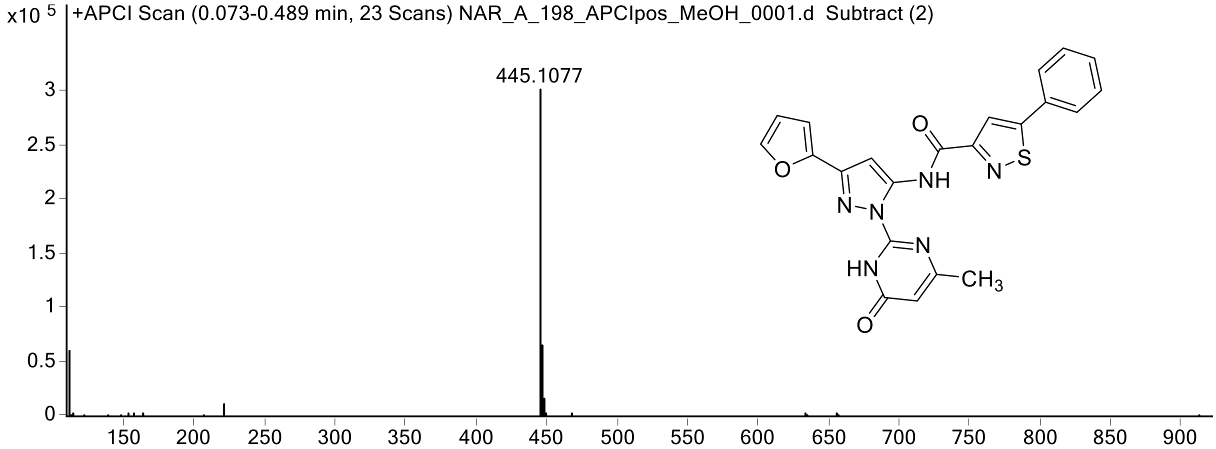

FT-IR spectrum (neat) of **128**

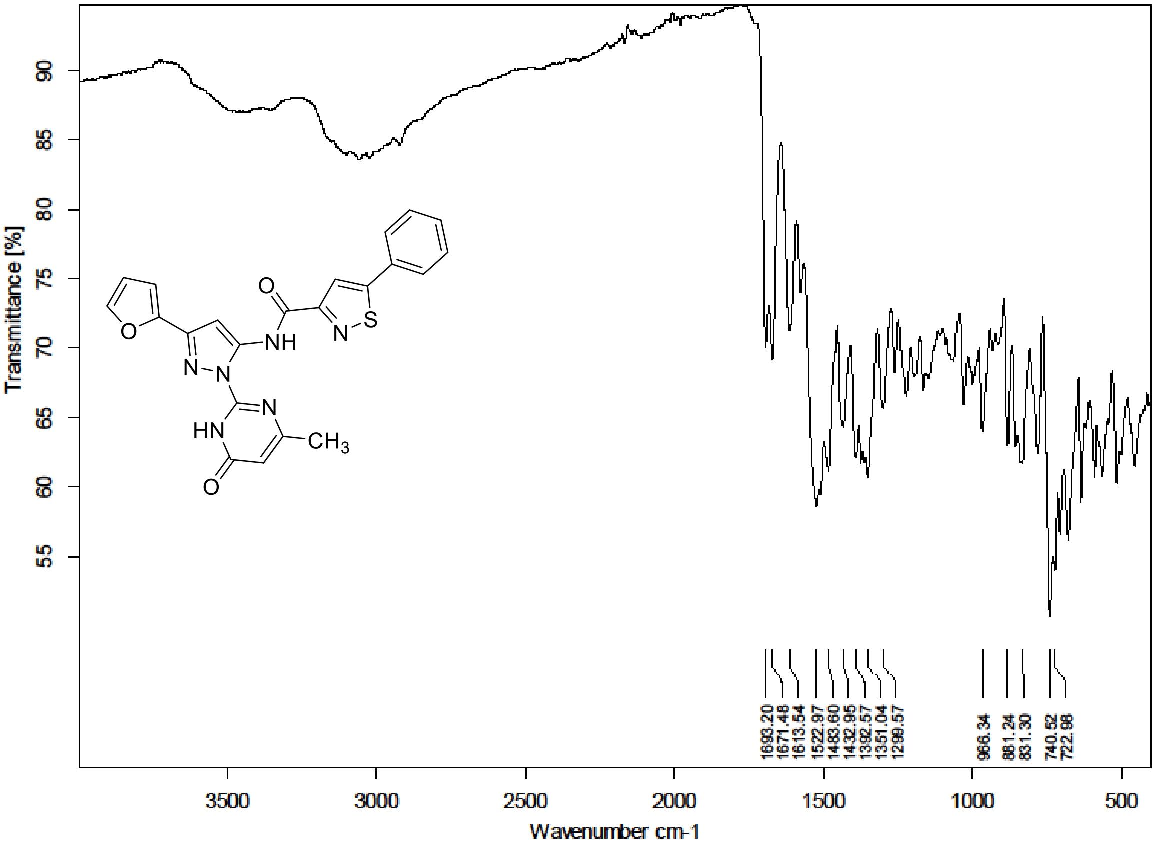

^1H NMR (300 MHz) spectrum of **129** in DMSO-d_6

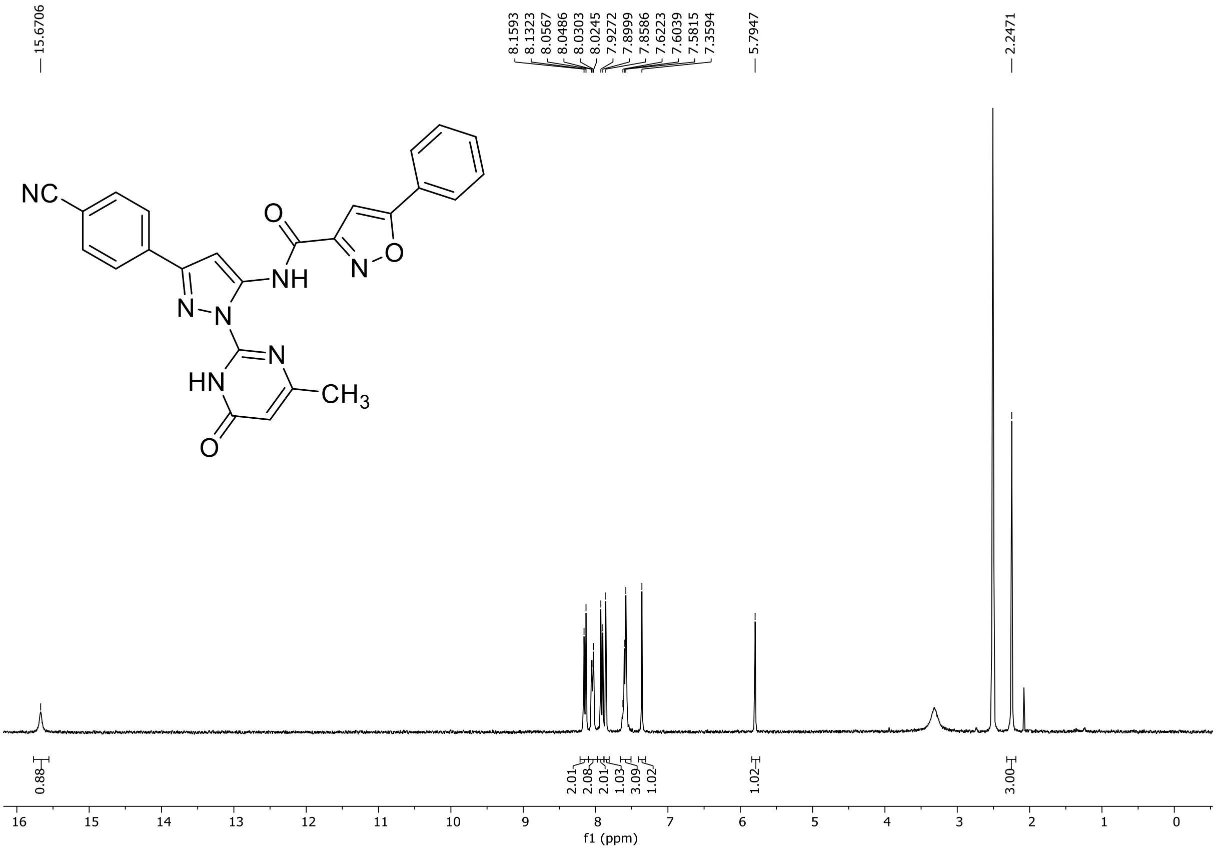

HRMS spectrum of **129**

NAR-A-120 **$C_{25}H_{17}N_7O_3$** ***m/z* 463.1393**

ESI- (MMI)
nitrogen flow 5 L/min, gas temperature 325°C, nebulizer 45 psi, skimmer 65 V, fragmentor 100 V, dissolved in DMSO, MeOH

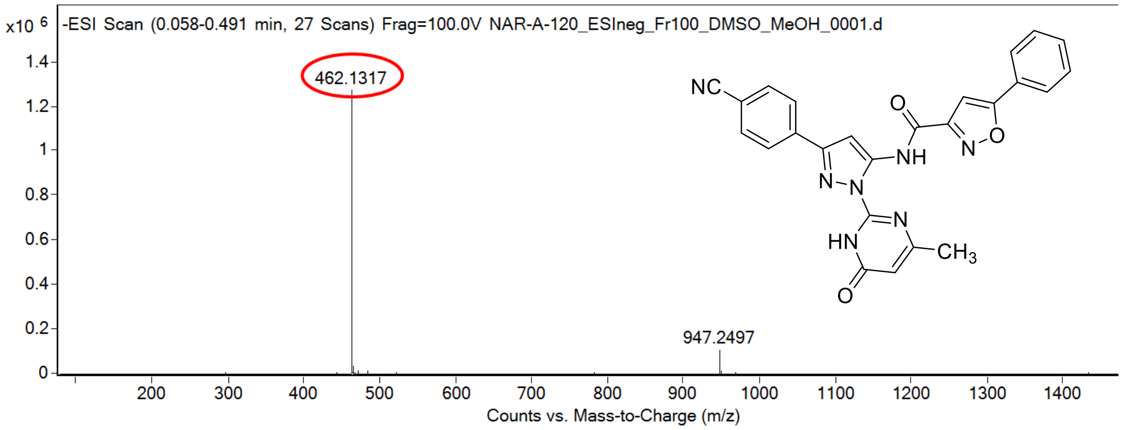

calculated mass: $[M-H]^-$ = 462.1320 observed: $[M-H]^-$ = 462.1317 mass accuracy = -0.6 ppm

FT-IR spectrum (neat) of **129**

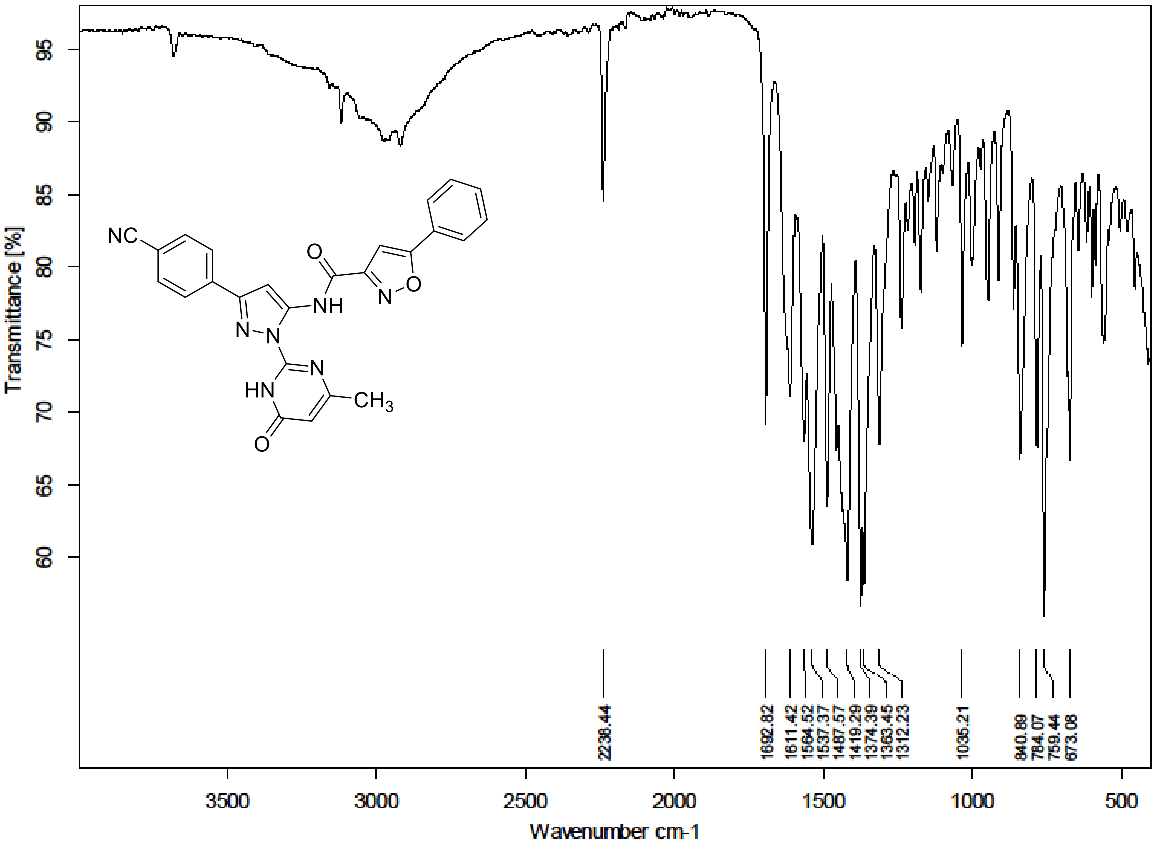

^{1}H NMR (300 MHz) spectrum of **130** in Trifluoroacetic Acid-*d*

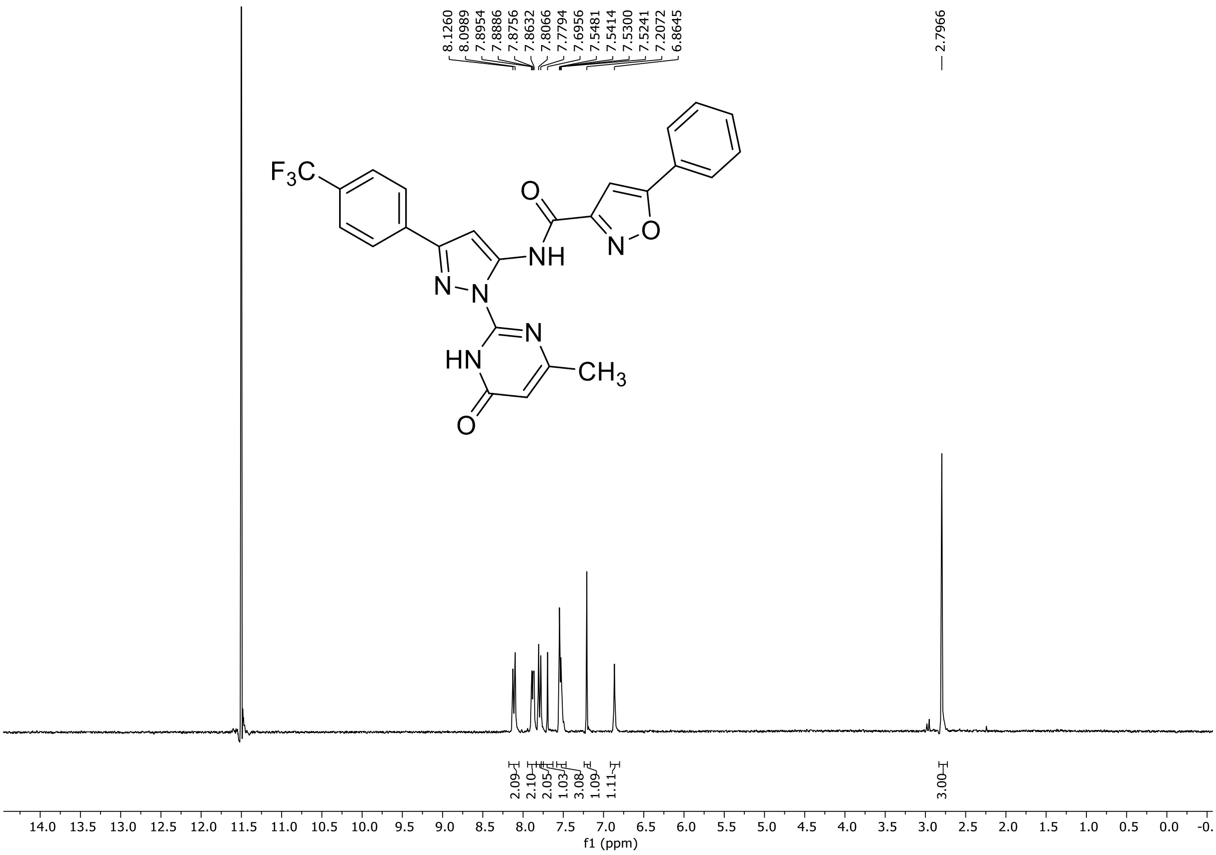

HRMS spectrum of **130**

NAR-A-123 $C_{25}H_{17}F_3N_6O_3$ ***m/z* 506.1314**

ESI- (MMI)
nitrogen flow 5 L/min, gas temperature 325°C, nebulizer 45 psi, skimmer 65 V, fragmentor 150 V, dissolved in DMSO, MeOH

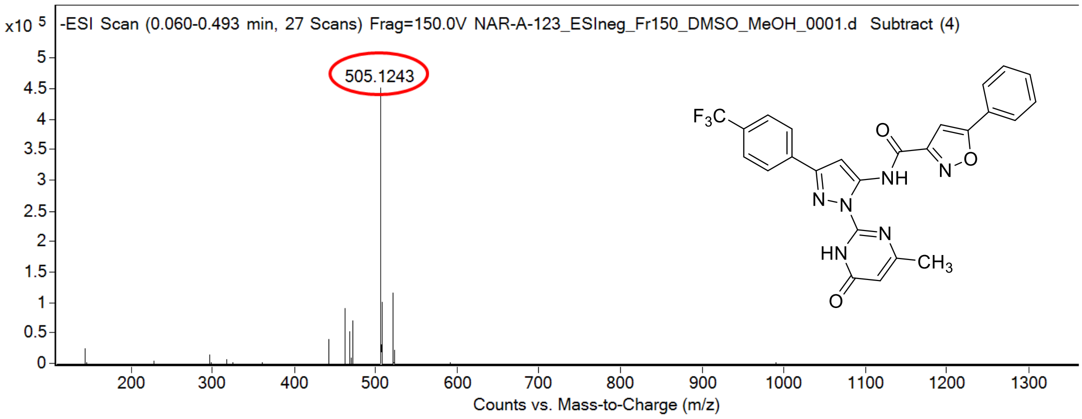

calculated mass: $[M-H]^-$ = 505.1241 observed: $[M-H]^-$ = 505.1243 mass accuracy = +0.4 ppm

FT-IR spectrum (neat) of **130**

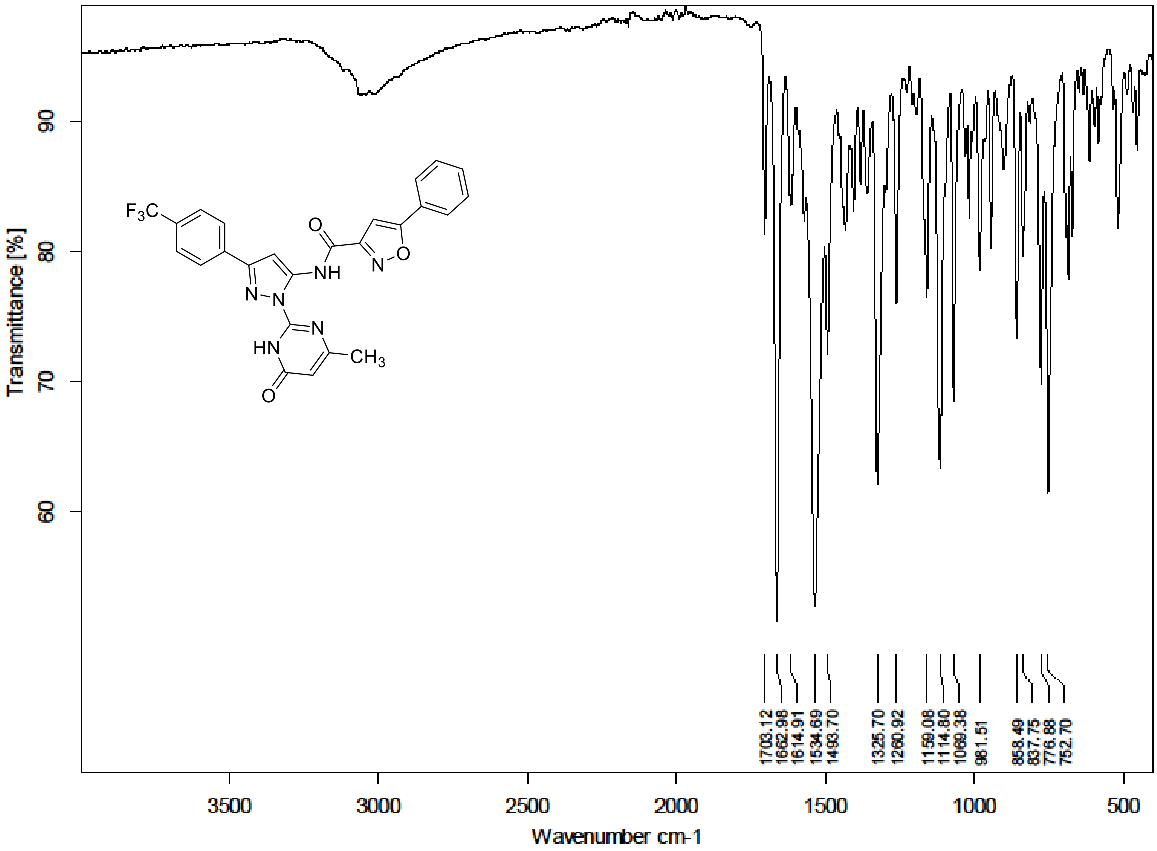

^{1}H NMR (300 MHz) spectrum of **131** in DMSO-d_6

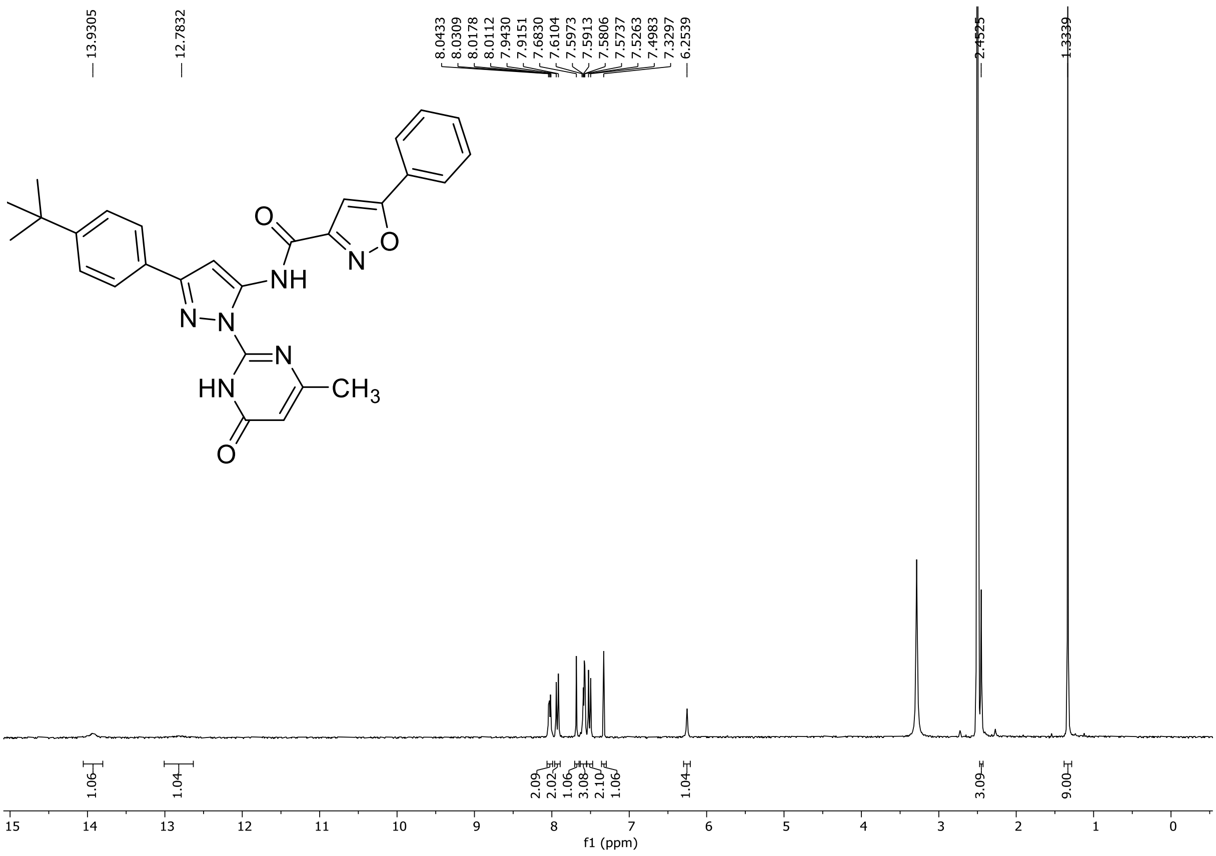

HRMS spectrum of **131**

NAR-A-151 $C_{28}H_{26}N_6O_3$ mono *m/z* **494.2066**

APCI + (MMI)
nitrogen flow 5 L/min, gas temperature 325°C, nebulizer 45 psi, skimmer **65** V, vaporizer 200°C, fragmentor 60 V, dissolved in methanol

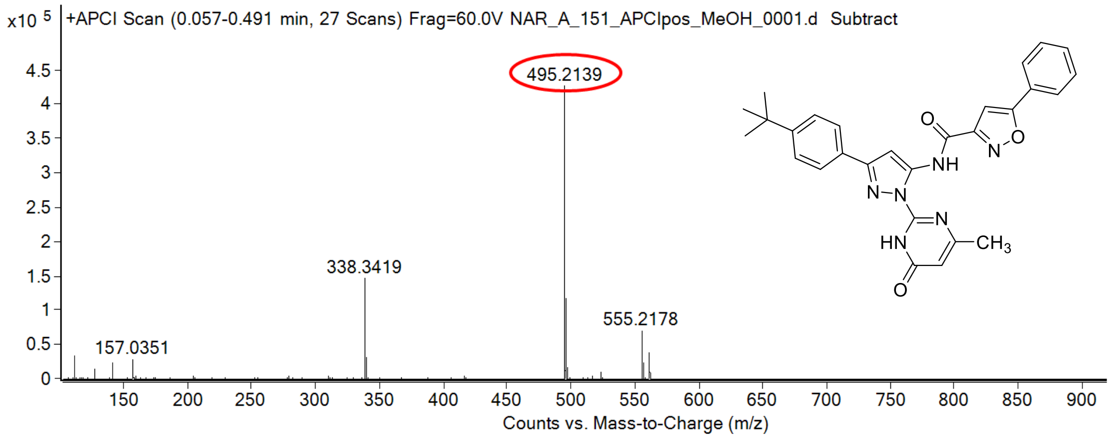

calculated mass: $[M+H]^+ = 495.2139$ observed: $[M+H]^+ = 495.2139$ mass accuracy = < 0.1 ppm

FT-IR spectrum (neat) of **131**

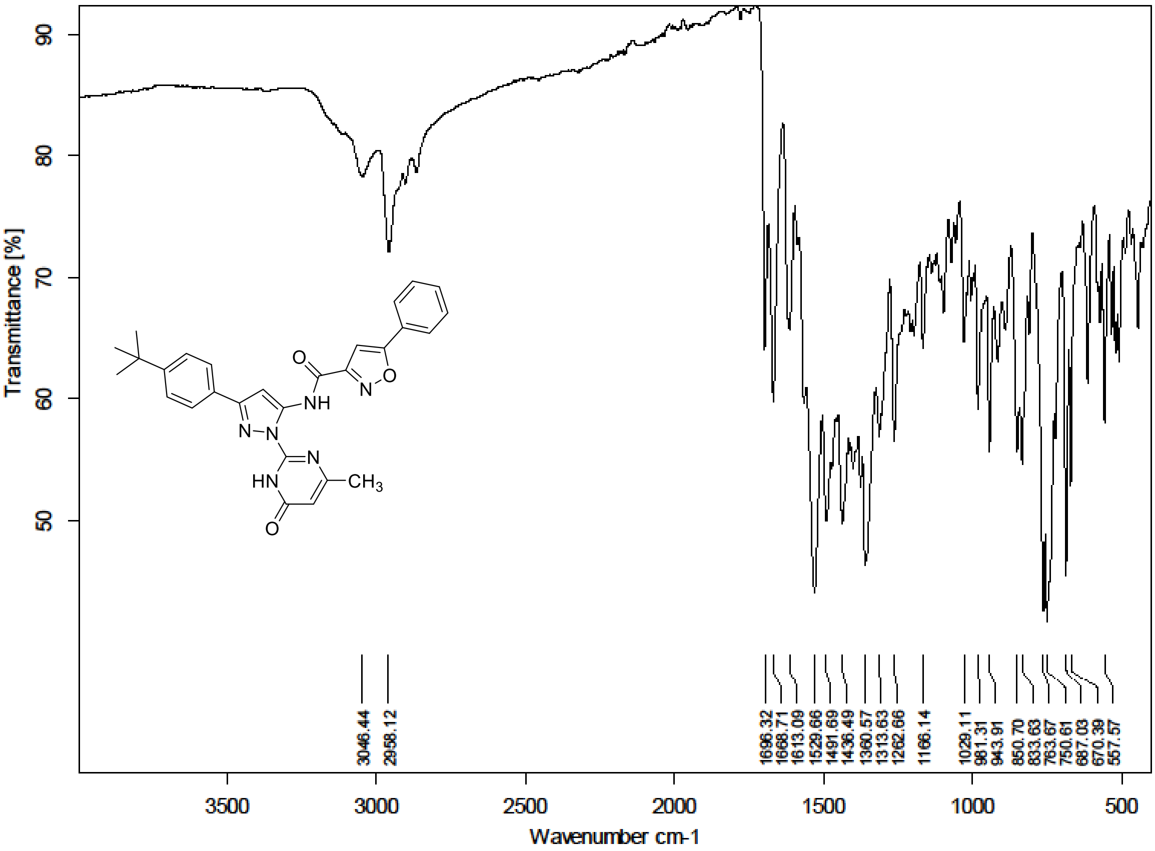

^{1}H NMR (300 MHz) spectrum of **132** in DMSO-d_6

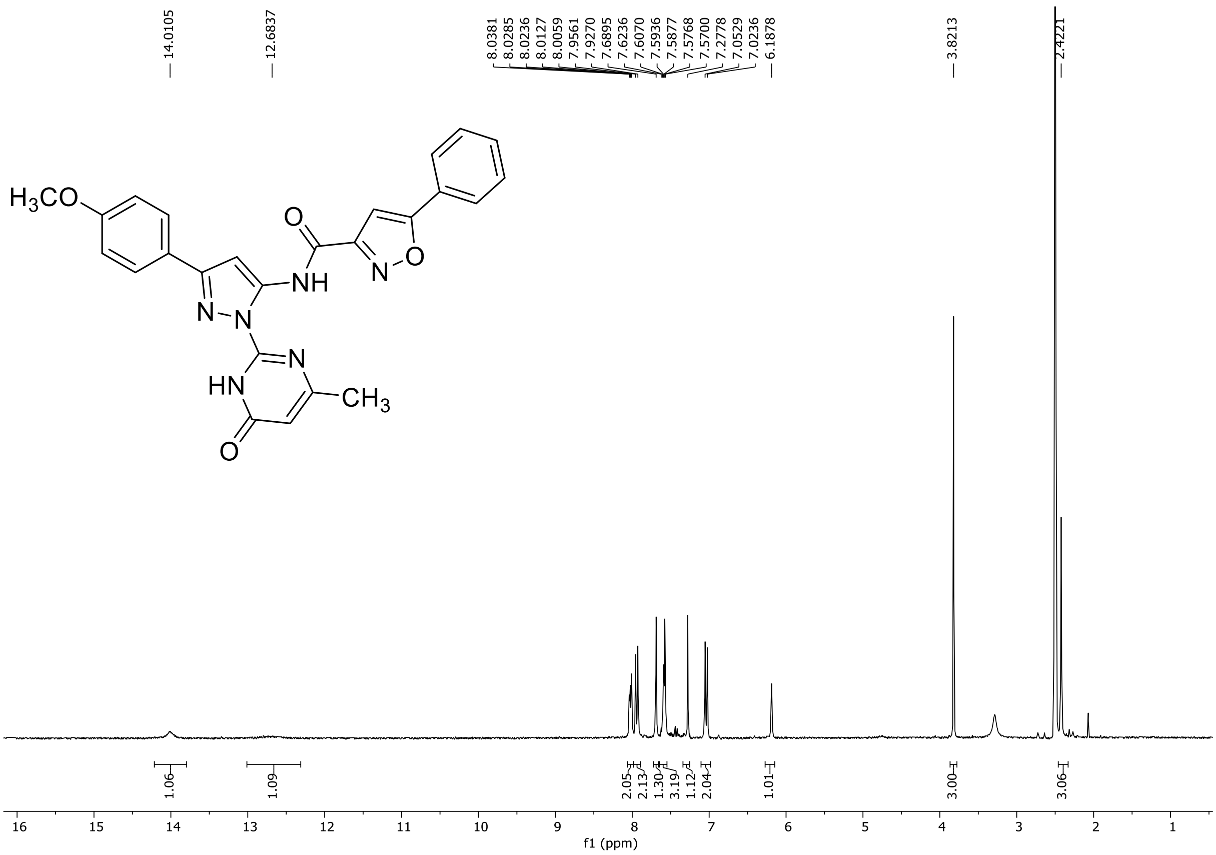

HRMS spectrum of **132**

NAR-A-152 $C_{25}H_{20}N_6O_4$ mono *m/z* **468.1546**

APCI + (MMI)
nitrogen flow 5 L/min, gas temperature 325°C, nebulizer 45 psi, skimmer 65 V, vaporizer 200°C, fragmentor 25 V, dissolved in methanol/ CH_2Cl_2

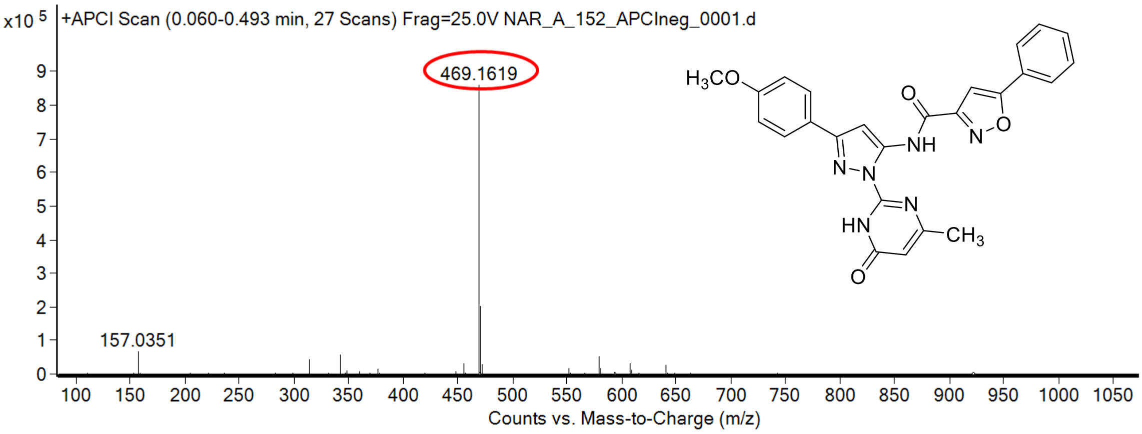

calculated mass: $[M+H]^+ = 469.1619$ observed: $[M+H]^+ = 469.1619$ mass accuracy = < 0.1 ppm

FT-IR spectrum (neat) of **132**

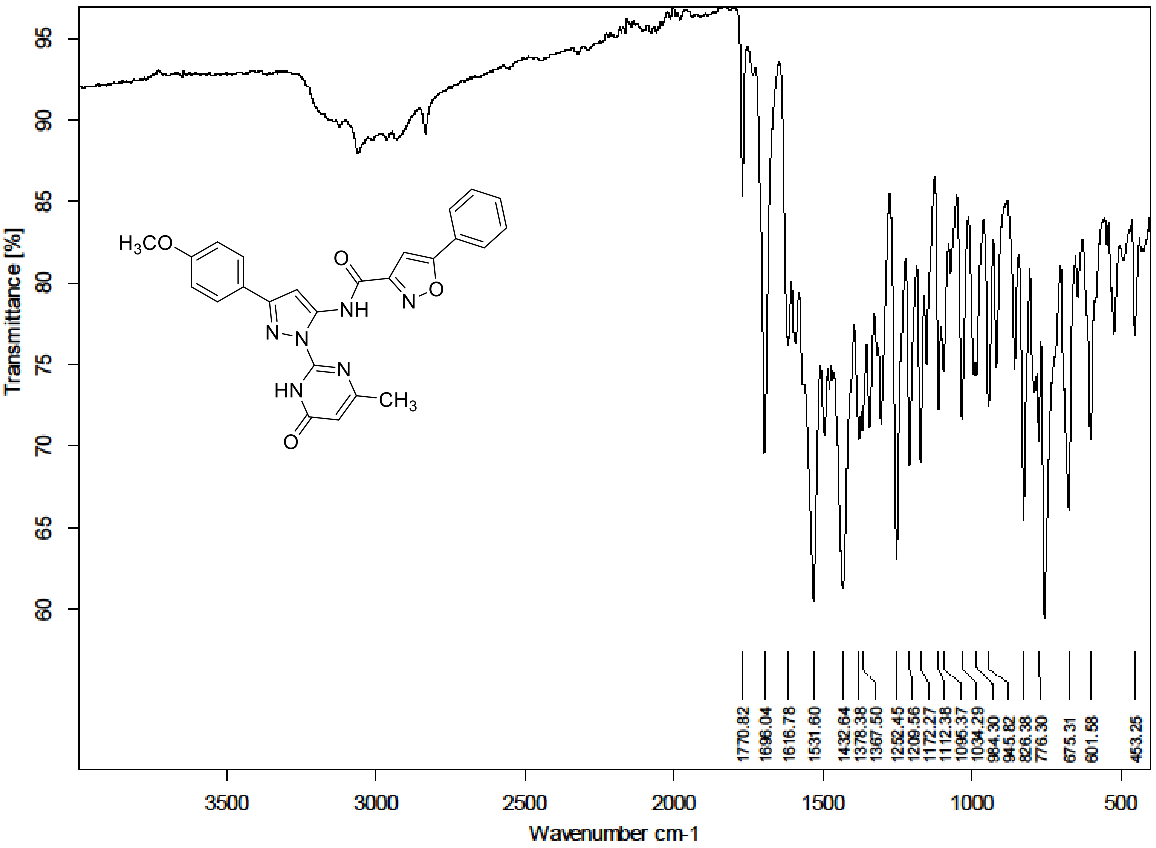

^{1}H NMR (300 MHz) spectrum of **133** in DMSO-d_6

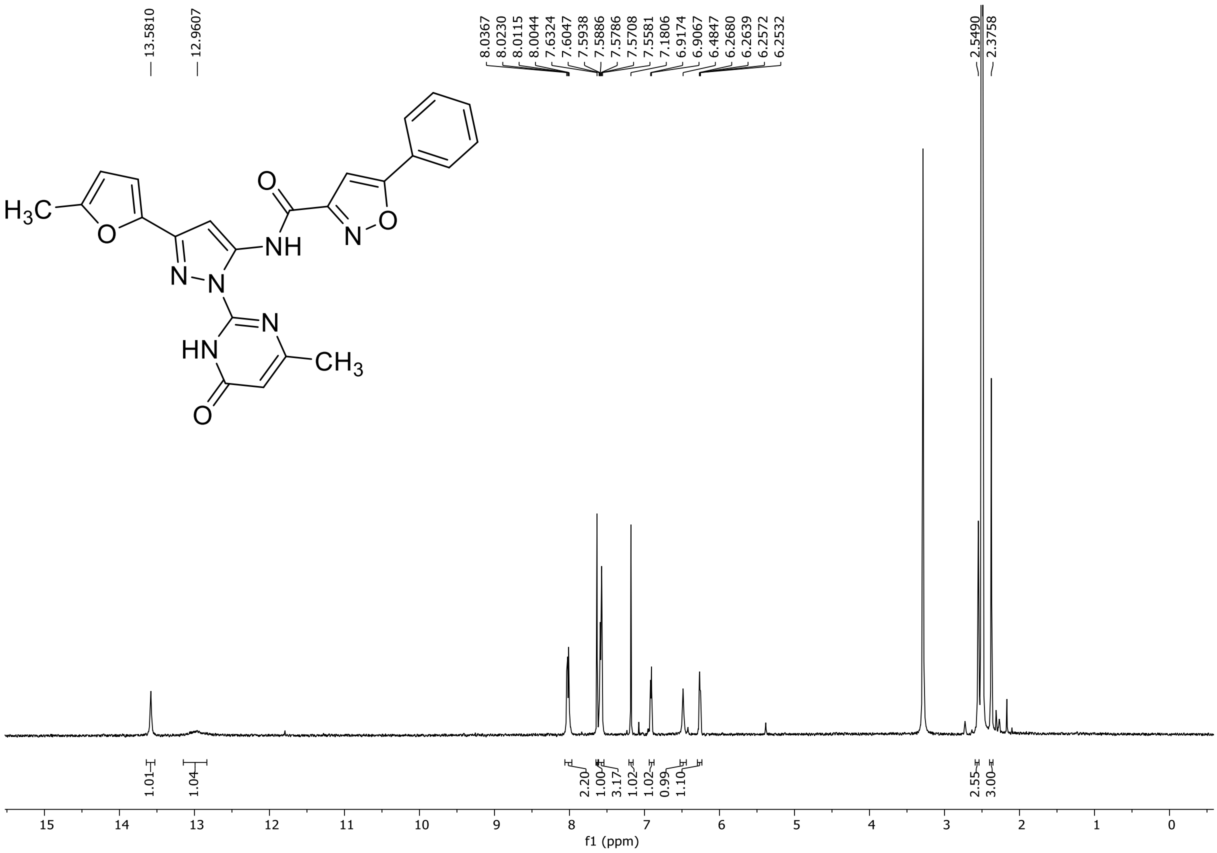

HRMS spectrum of **133**

NAR-A-180 **$C_{23}H_{18}N_6O_4$** **mono *m/z* 442.1390**

APCI + (MMI)
nitrogen flow 5 L/min, gas temperature 325°C, nebulizer 45 psi, skimmer **65** V,
vaporizer 200°C, fragmentor 38 V, dissolved in methanol

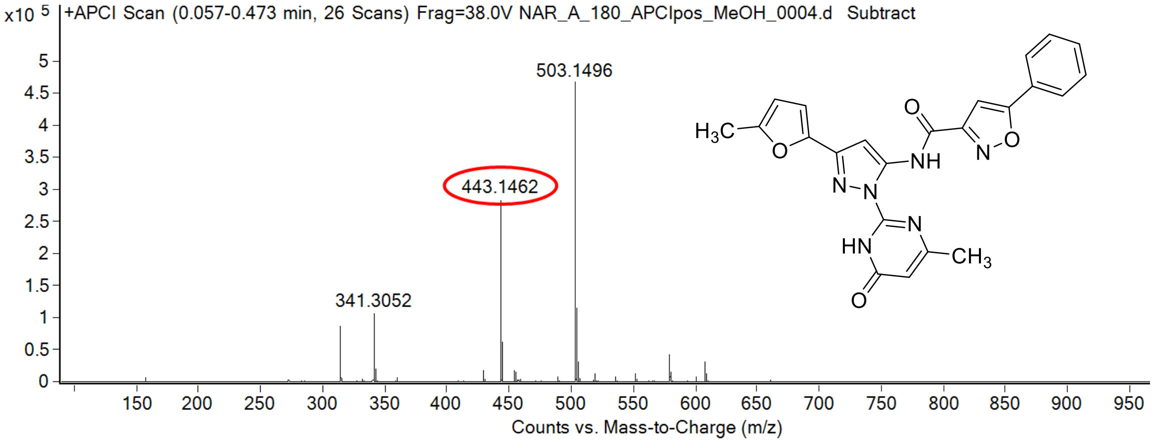

calculated mass: $[M+H]^+ = 443.1462$ observed: $[M+H]^+ = 443.1462$ mass accuracy = < 0.1 ppm

FT-IR spectrum (neat) of **133**

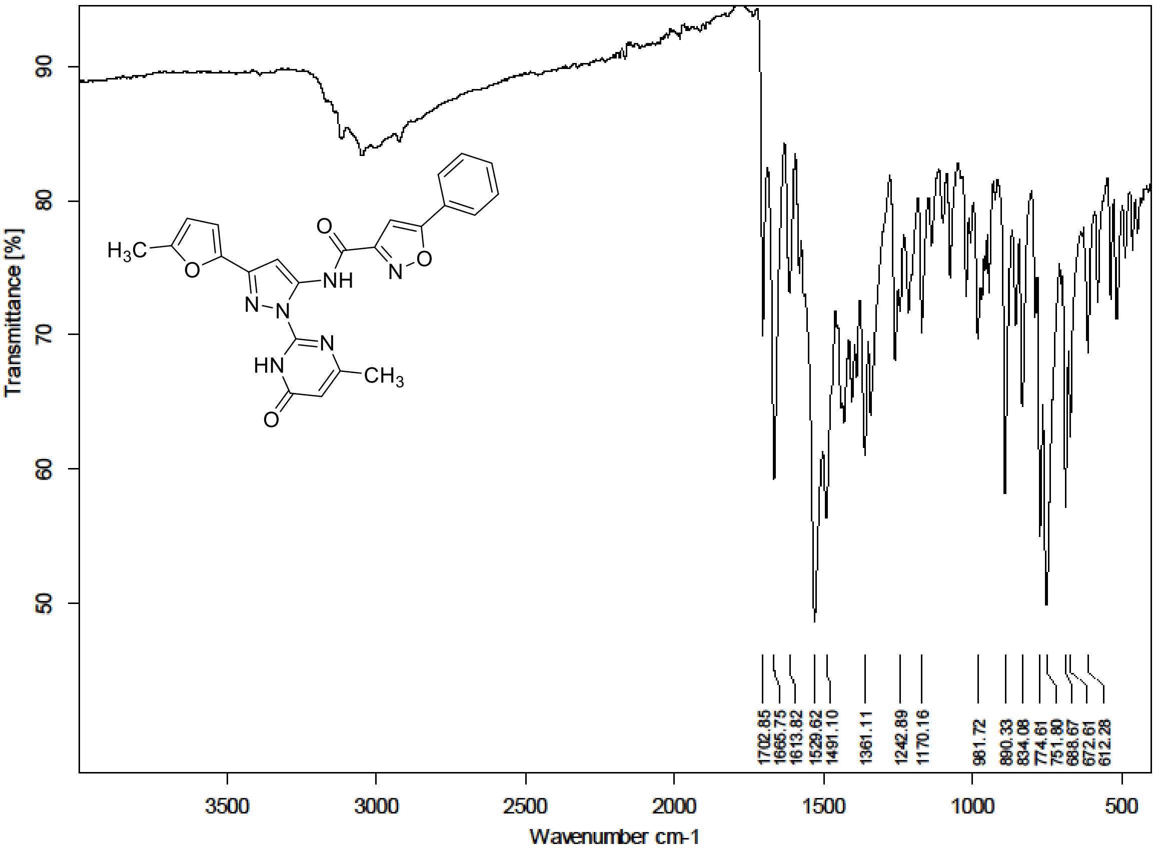

^{1}H (500 MHz) and ^{13}C NMR (126 MHz) spectra of **134** in DMSO-d_6

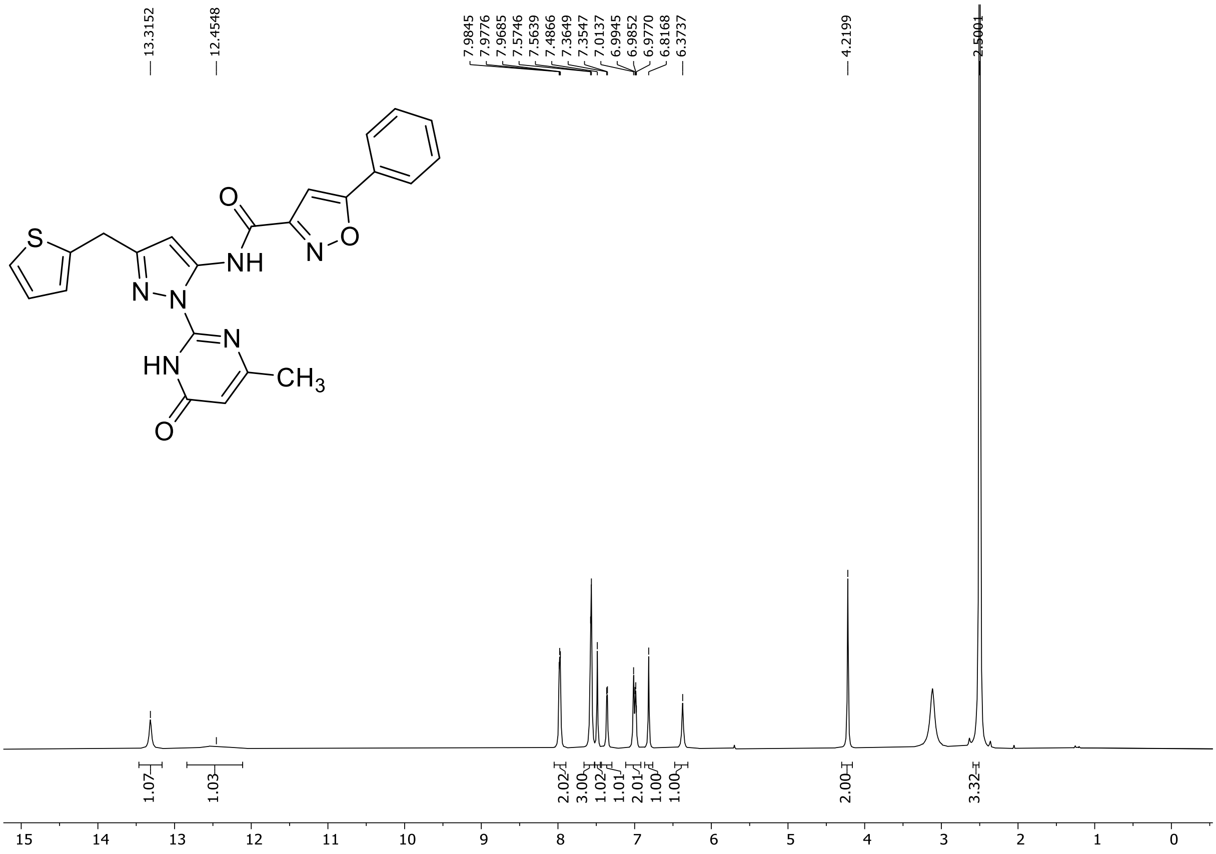

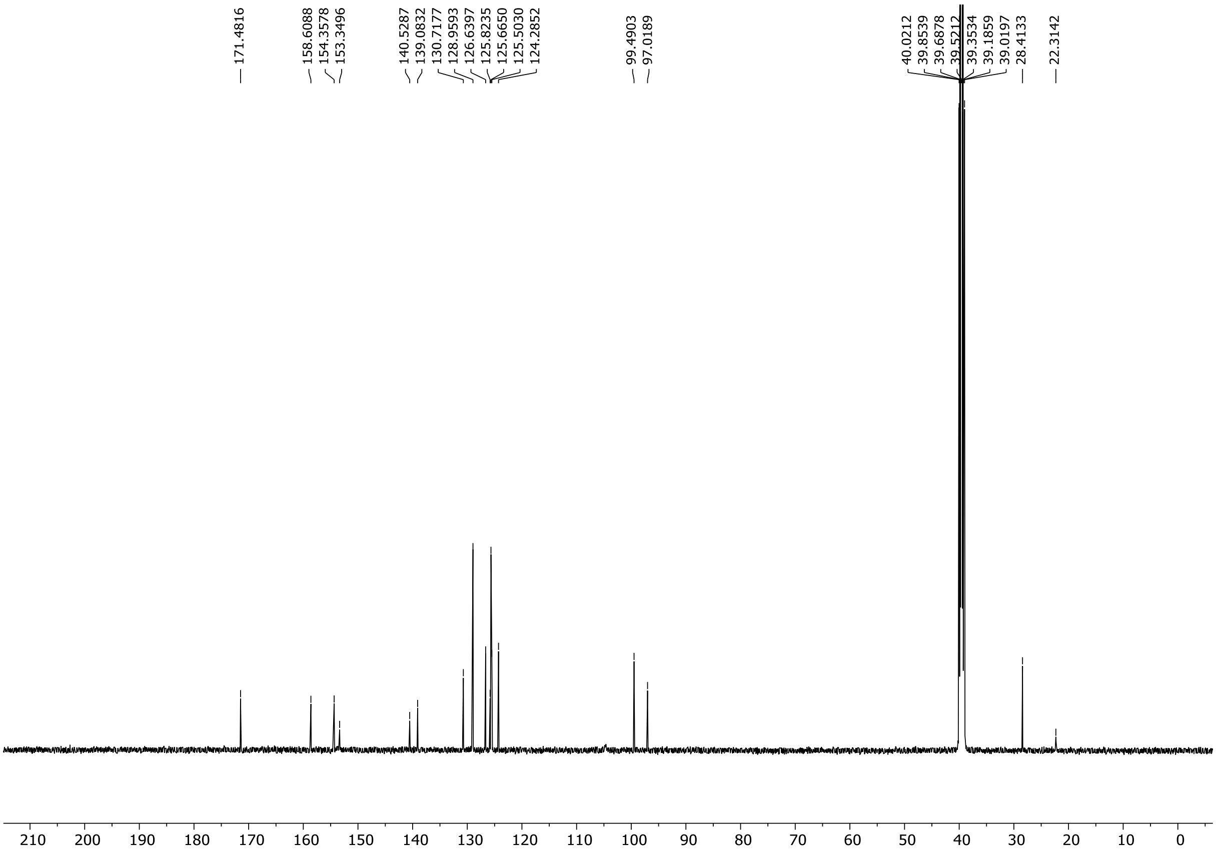

HRMS spectrum of **134**

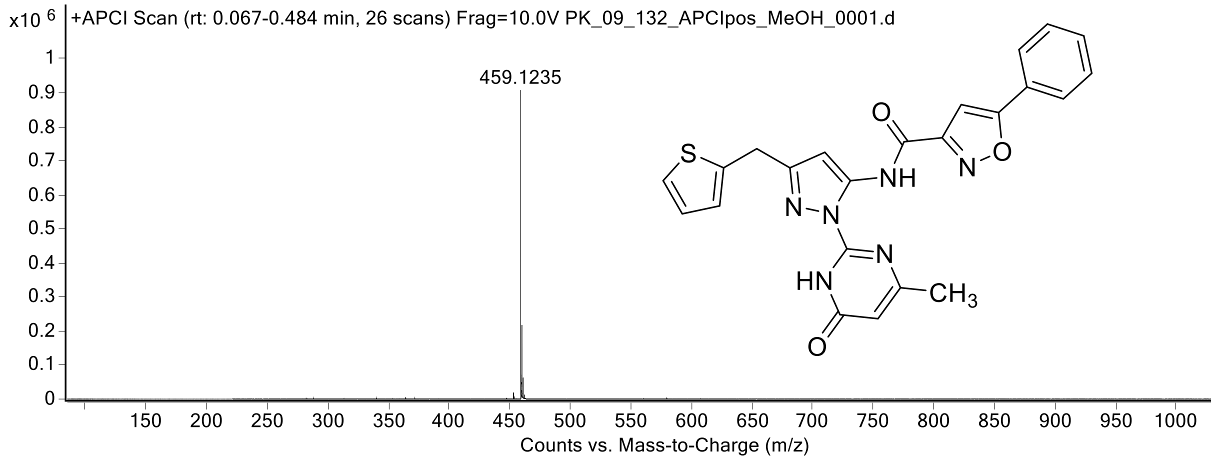

^{1}H (300 MHz) and ^{13}C NMR (75 MHz) spectra of **135** DMSO-d_6

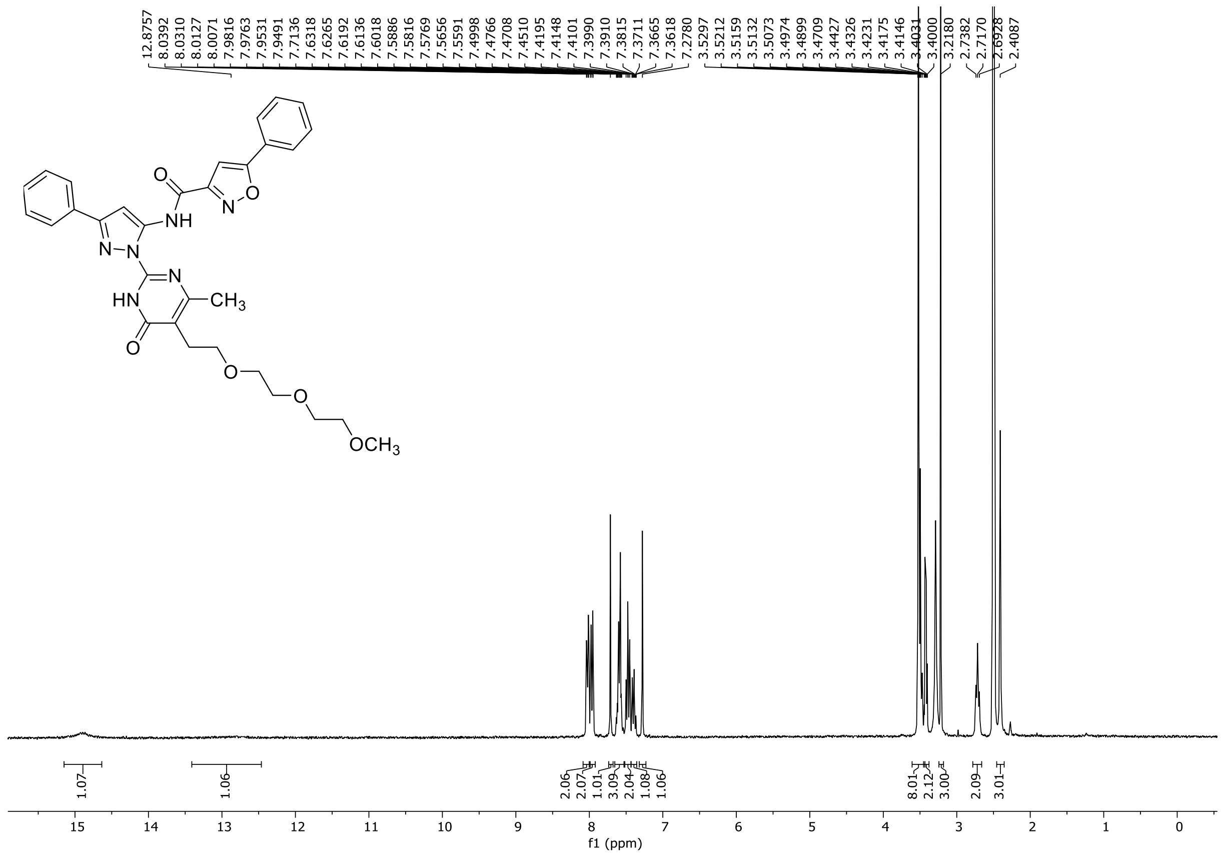

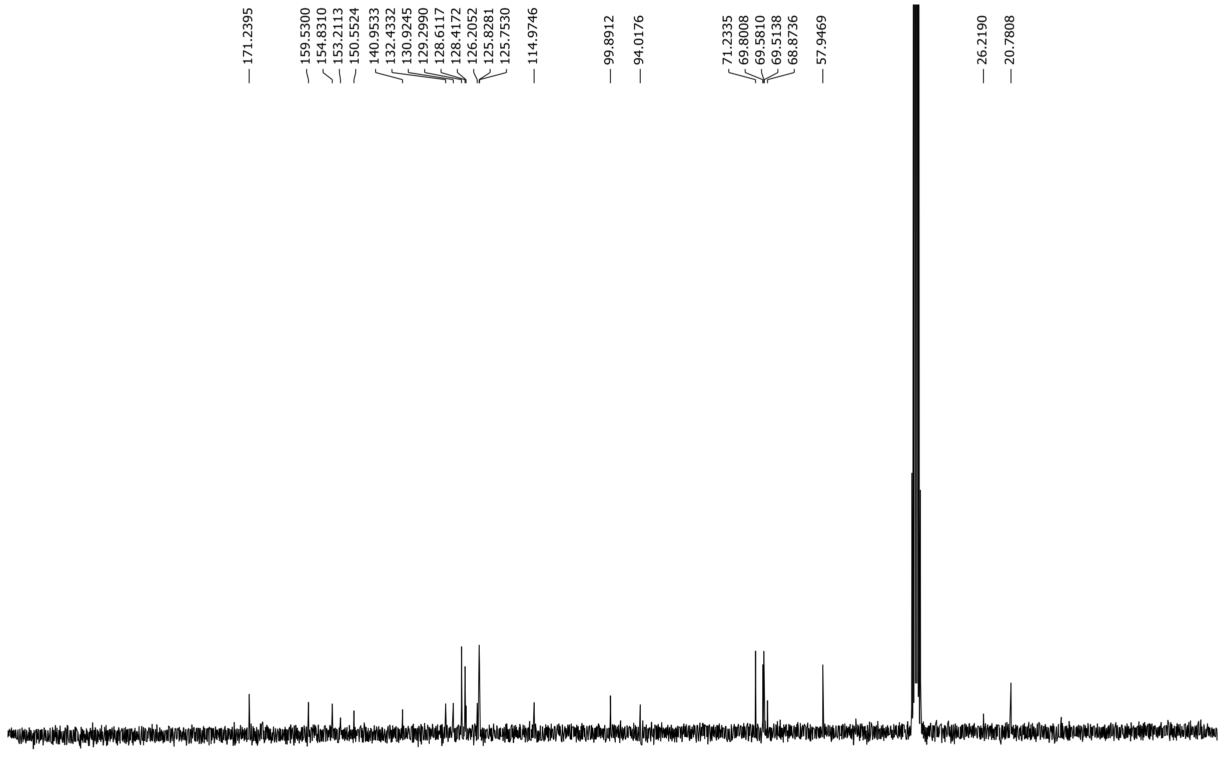

HRMS spectrum of **135**

NAR-A-221 $C_{31}H_{32}N_6O_6$ mono *m/z* 584.2383

APCI + (MMI)
nitrogen flow 5 L/min, gas temperature 325°C, nebulizer 45 psig, skimmer 65 V, vaporizer 200°C, fragmentor 20 V, dissolved in methanol

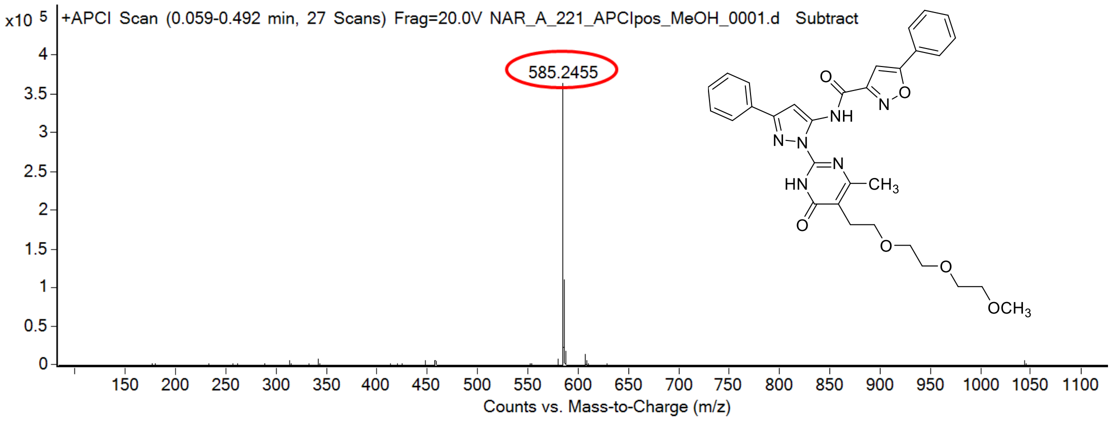

calculated mass: $[M+H]^+$ = 585.2456 observed: $[M+H]^+$ = 585.2455 mass accuracy = - 0.2 ppm

FT-IR spectrum (neat) of **135**

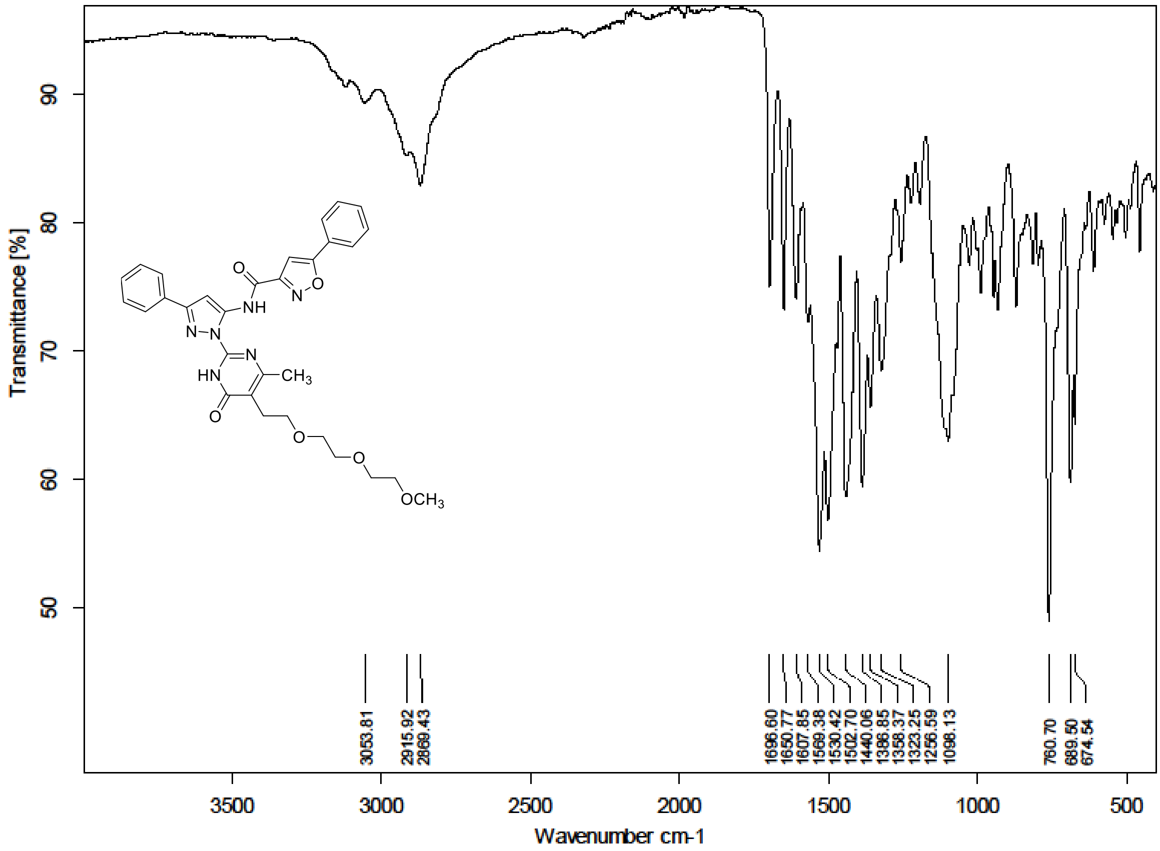

^{1}H NMR (300 MHz) spectrum of **136** in Chloroform-*d*

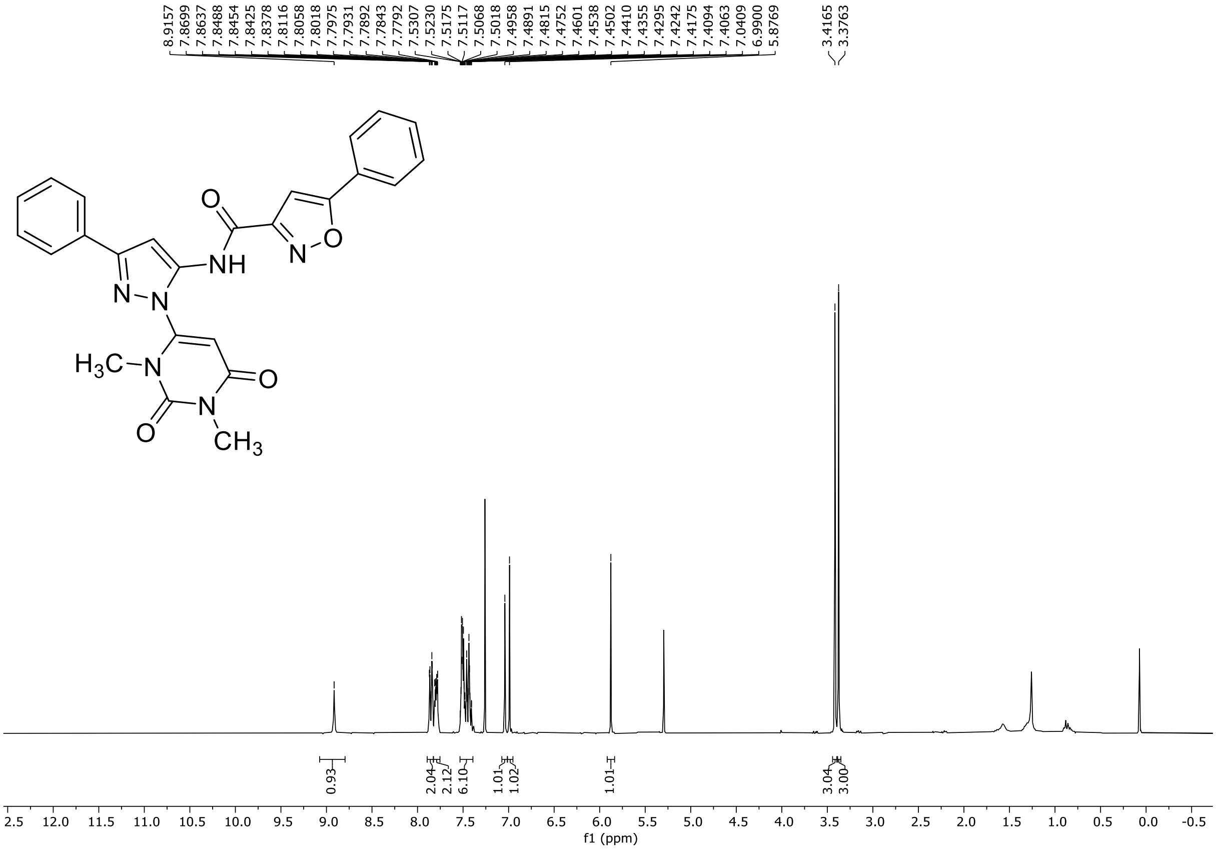

HRMS spectrum of **136**

NAR-A-227 **$C_{25}H_{20}N_6O_4$** **mono *m/z* 468.1546**

APCI + (MMI)
nitrogen flow 5 L/min, gas temperature 325°C, nebulizer 45 psig, skimmer 65 V, vaporizer 200°C, fragmentor 25 V, dissolved in methanol

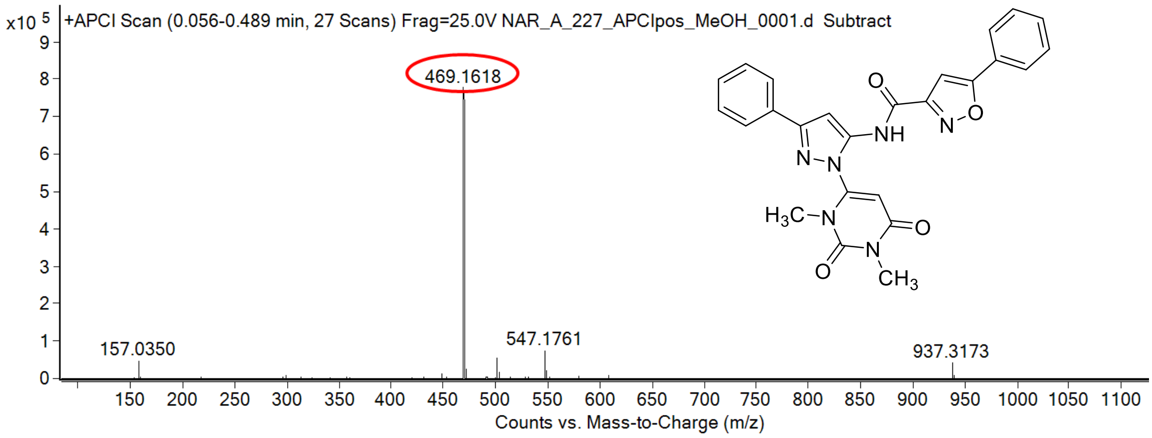

calculated mass: $[M+H]^+$ = 469.1619 observed: $[M+H]^+$ = 469.1618 mass accuracy = - 0.2 ppm

FT-IR spectrum (neat) of **136**

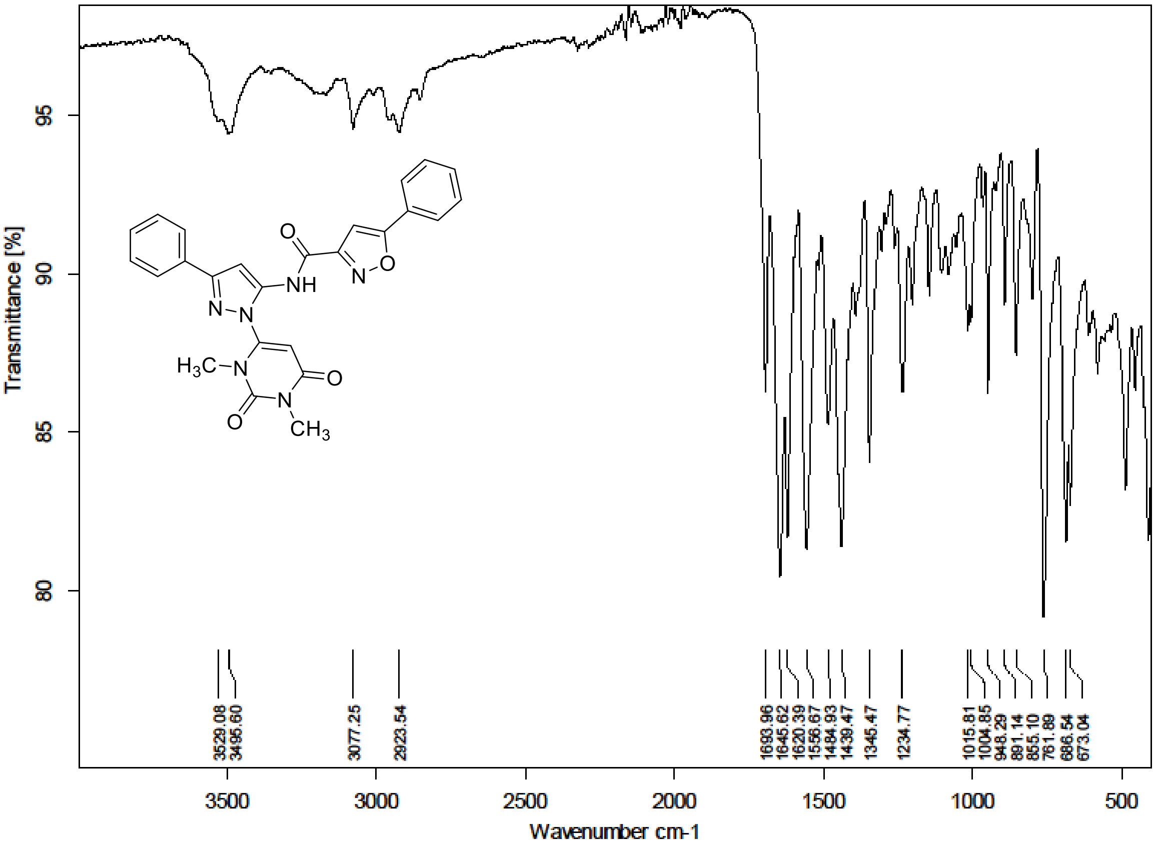

^{1}H (500 MHz) and ^{13}C NMR (126 MHz) spectra of **137** in Chloroform-*d*

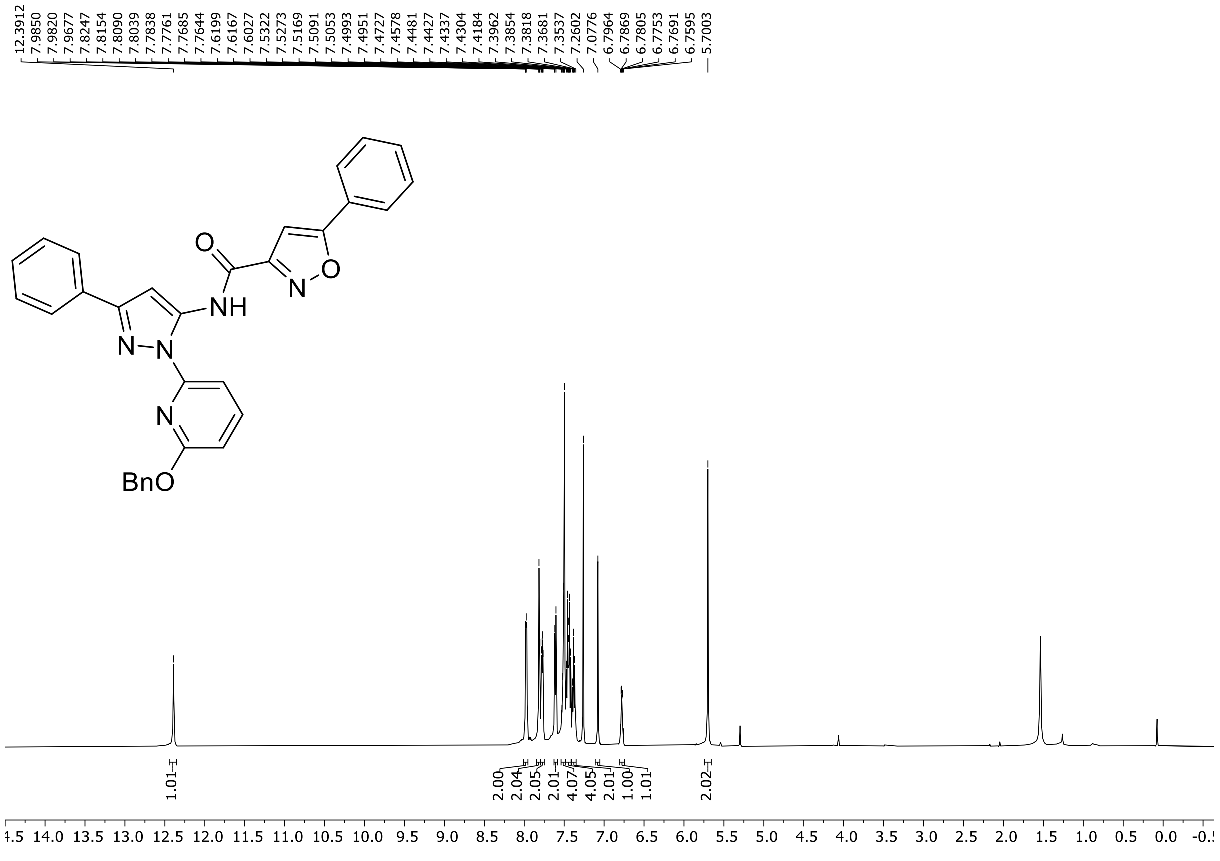

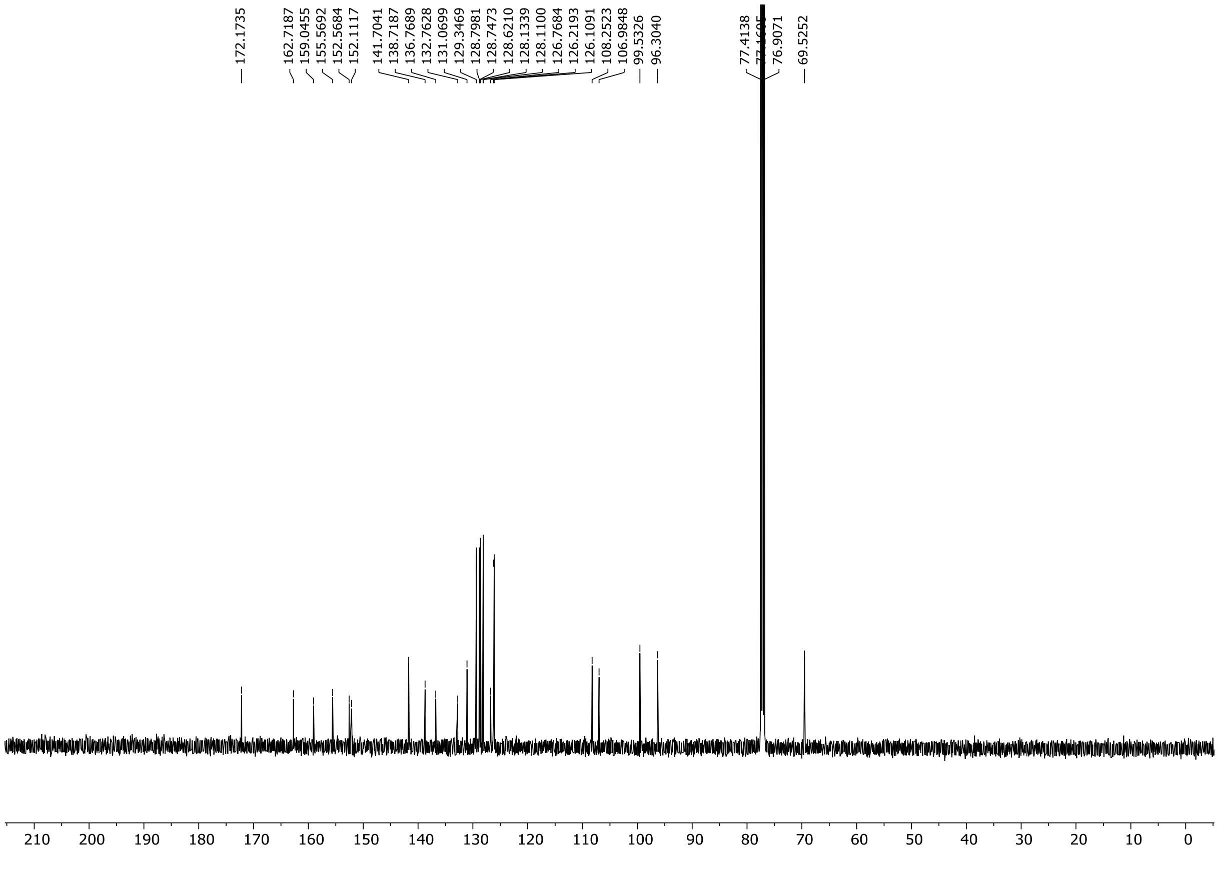

HRMS spectrum of **137**

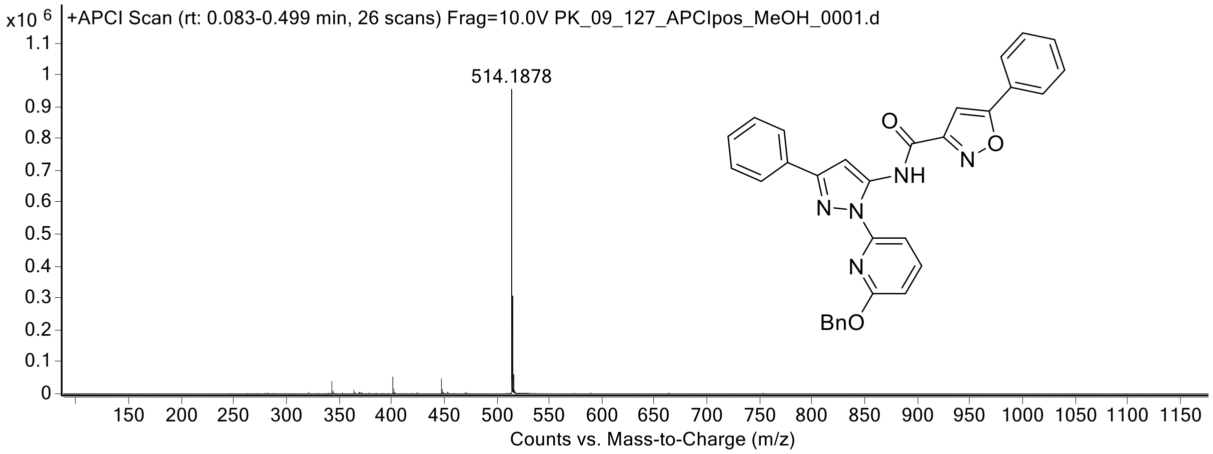

^{1}H (300 MHz) and ^{13}C NMR (126 MHz) spectra of **138** in DMSO-d_6

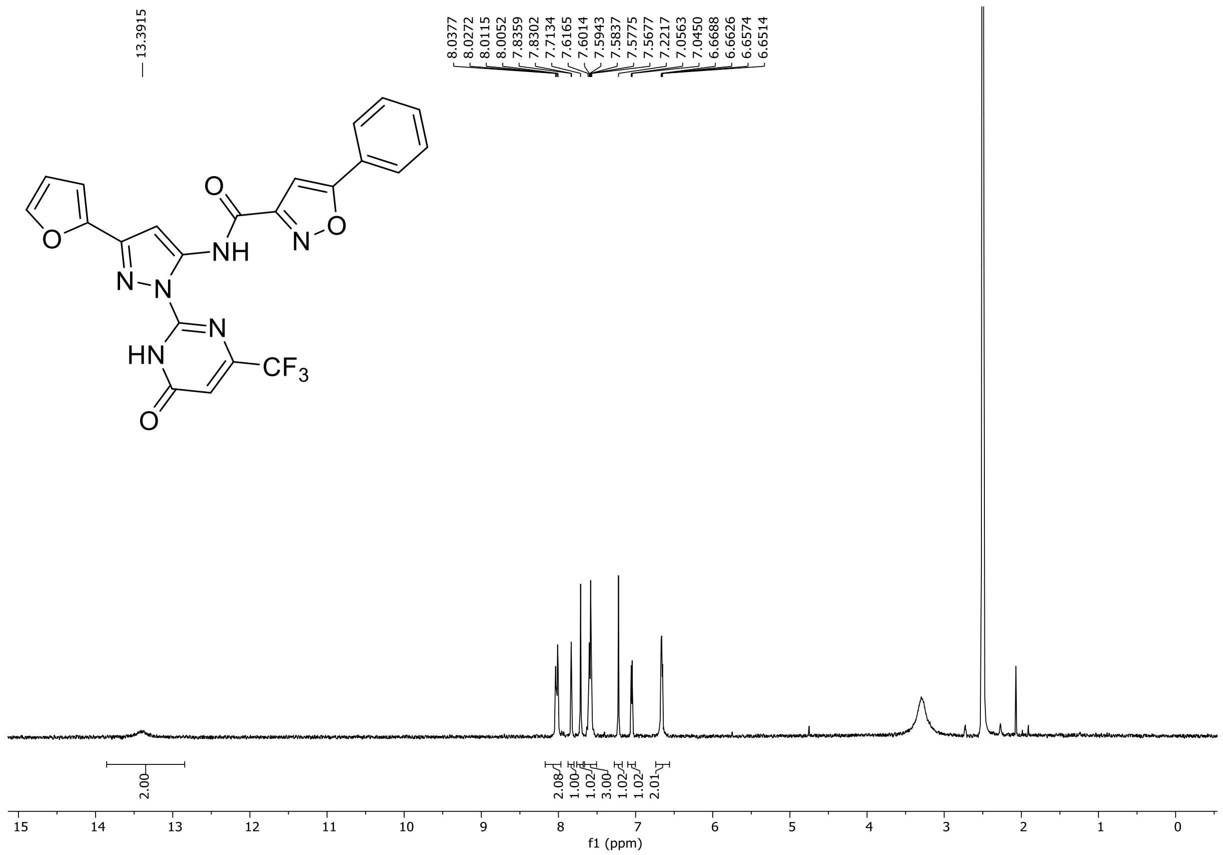

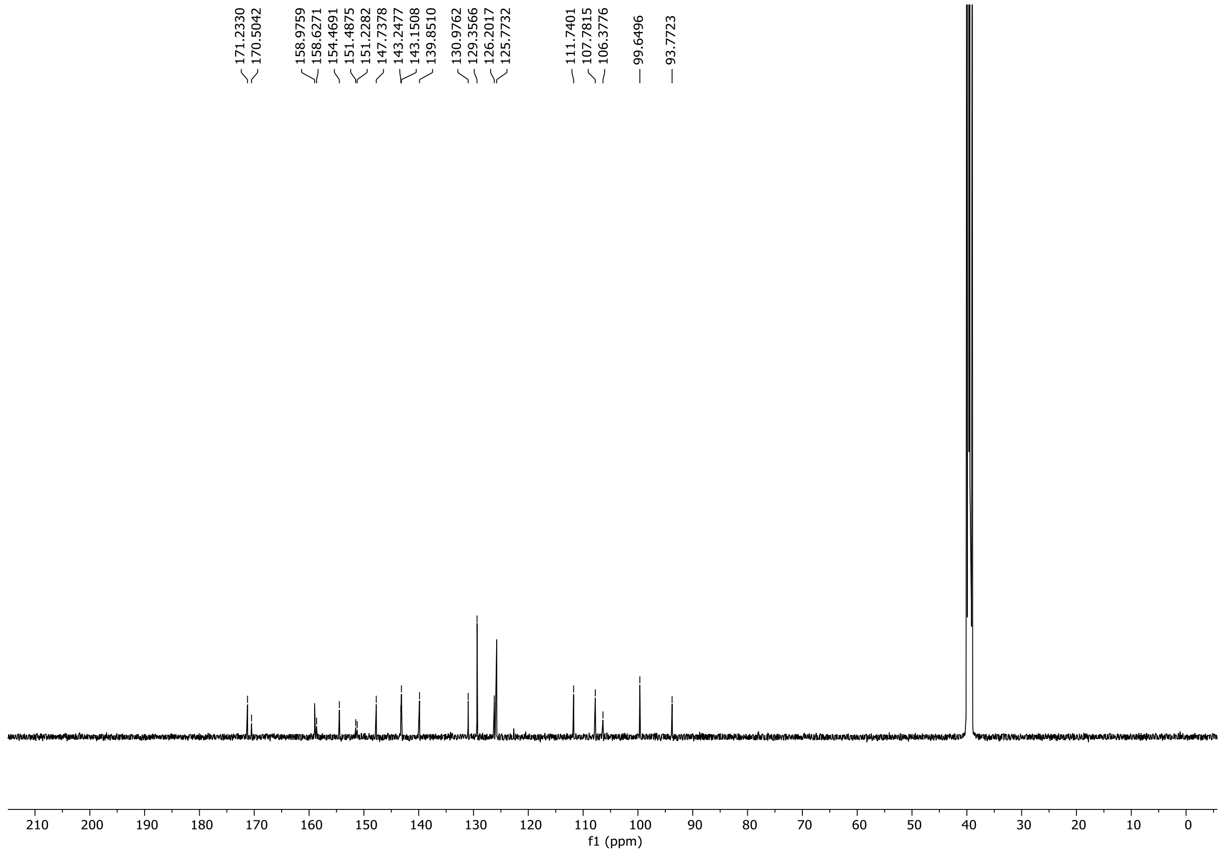

^{19}F NMR (471 MHz) spectrum of **138** in DMSO-d_6

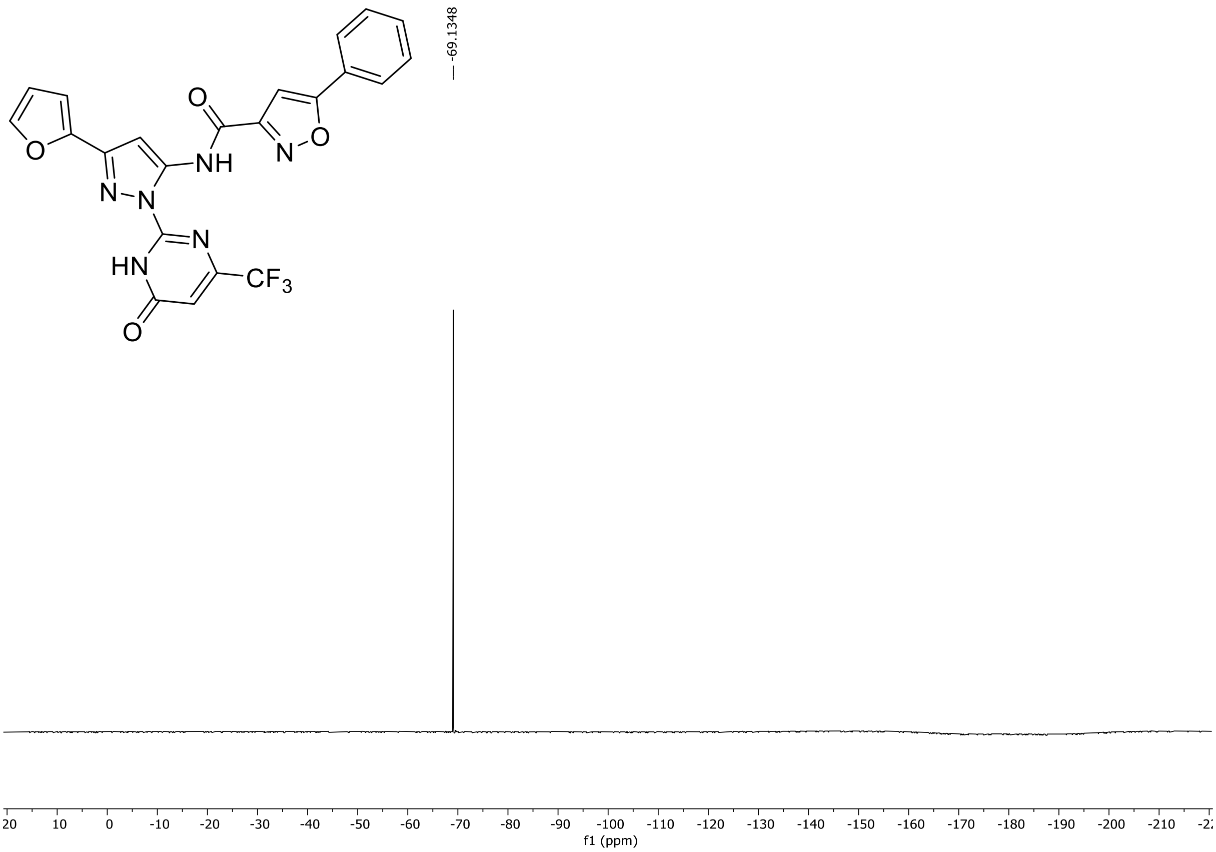

HRMS spectrum of **138**

NAR-A-104 $C_{22}H_{13}F_3N_6O_4$ ***m/z* 482.0950**

ESI- (MMI)
nitrogen flow 5 L/min, gas temperature 325°C, nebulizer 45 psi, skimmer 65 V, fragmentor 30 V, dissolved in DMSO, MeOH

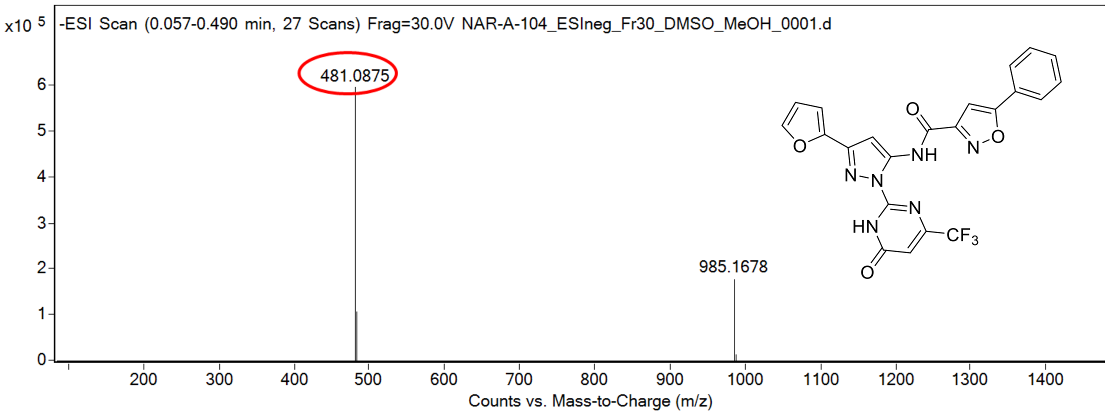

calculated mass: $[M\text{-}H]^-$ = 481.0878 observed: $[M\text{-}H]^-$ = 481.0875 mass accuracy = -0.6 ppm

FT-IR spectrum (neat) of **138**

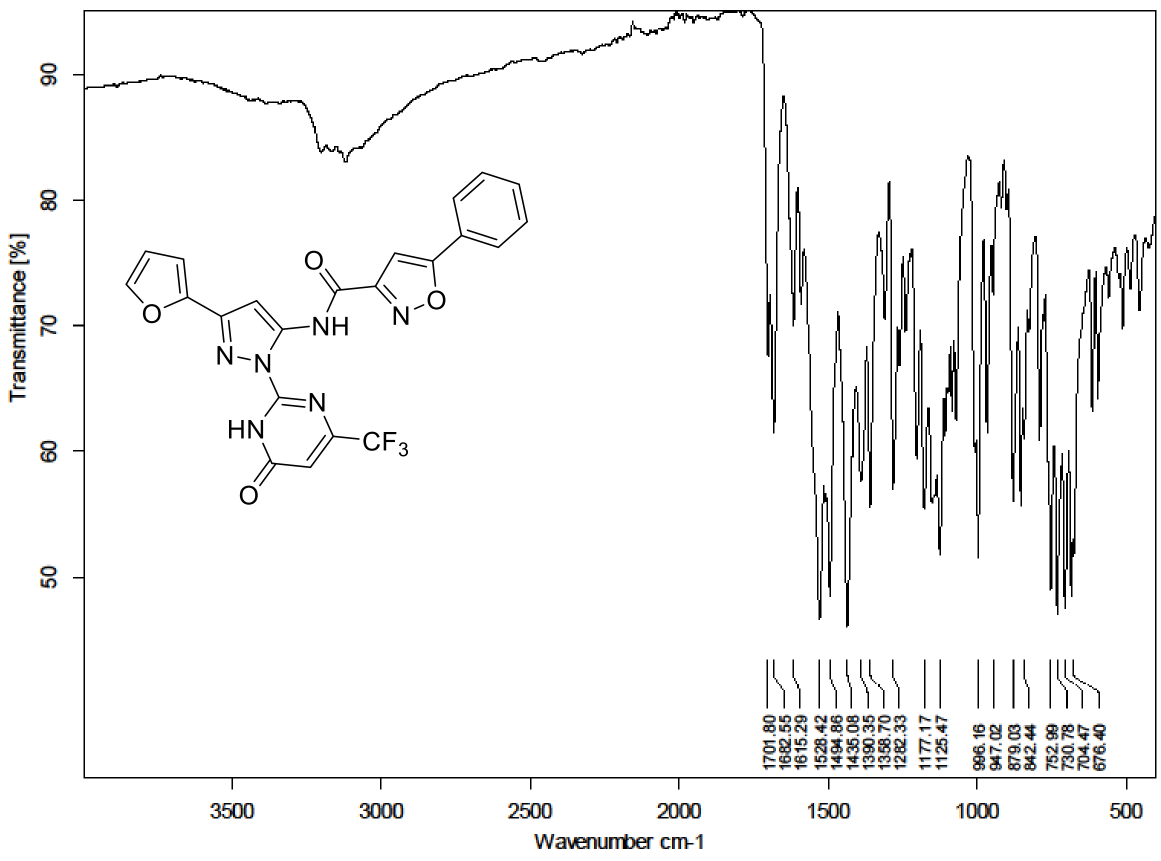

^{1}H (500 MHz) and ^{13}C NMR (126 MHz) spectra of **139** in Chloroform-*d*

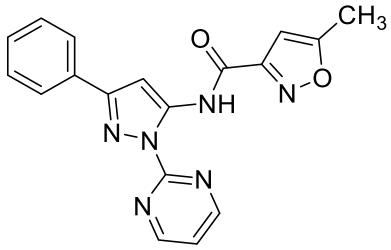

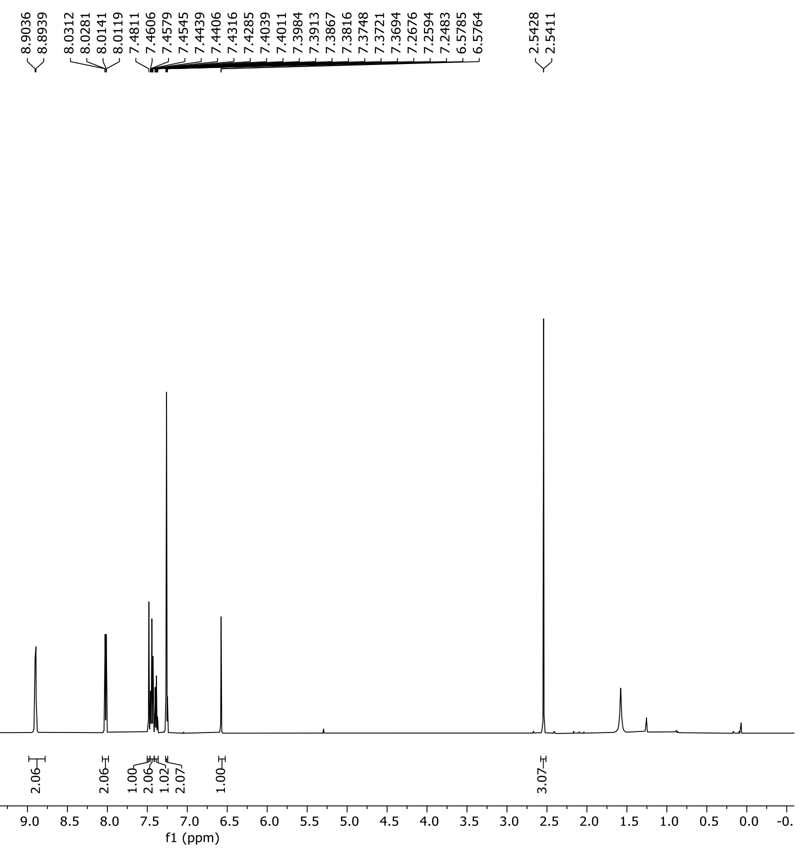

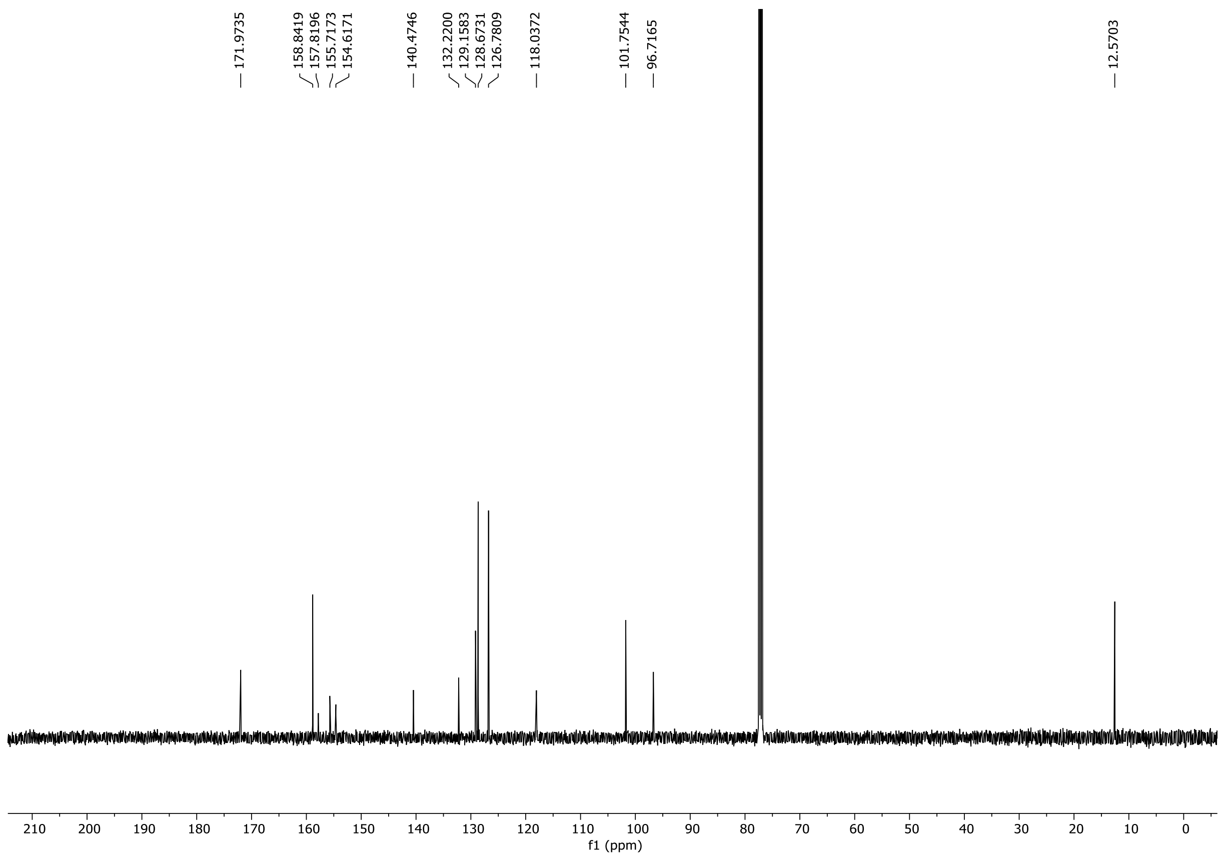

HRMS spectrum of **139**

NAR-A-13 **$C_{18}H_{14}N_6O_2$** mono *m/z 346.1178*

APCI + (MMI)
nitrogen flow 5 L/min, gas temperature 325°C, nebulizer 45 psi, skimmer **65** V,
vaporizer 200°C, fragmentor 25 V, dissolved in methanol

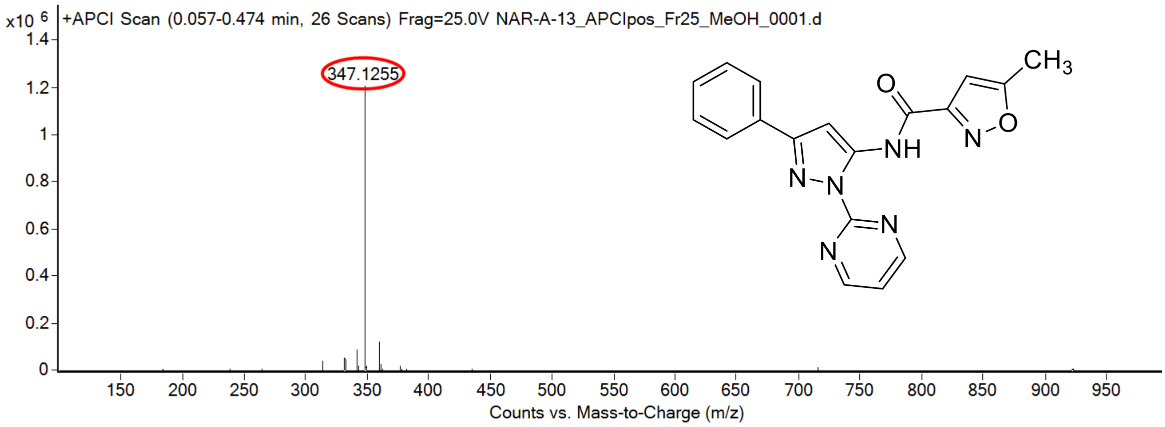

calculated mass: $[M+H]^+$ = 347.1251 observed: $[M+H]^+$ = 347.1255 mass accuracy = 1.1 ppm

FT-IR spectrum (neat) of **139**

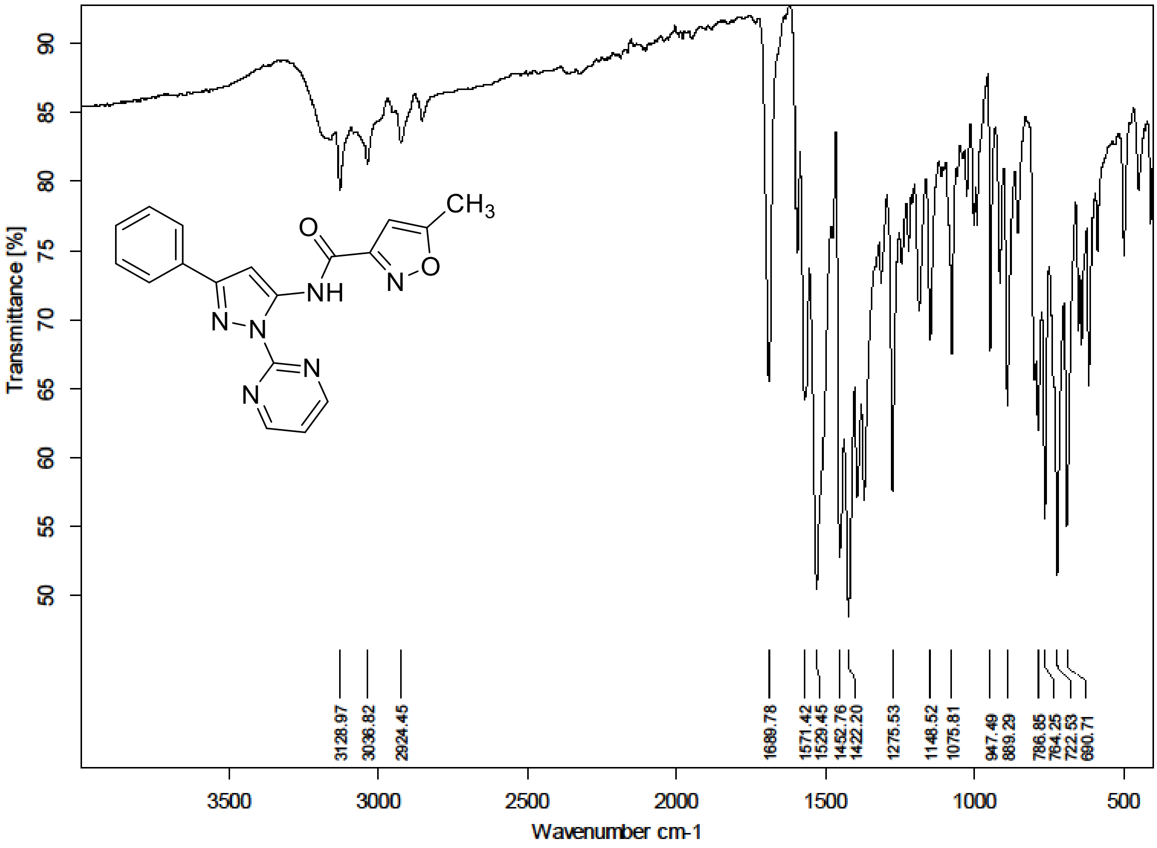

^{1}H (500 MHz) and ^{13}C NMR (126 MHz) spectra of **140** in Chloroform-*d*

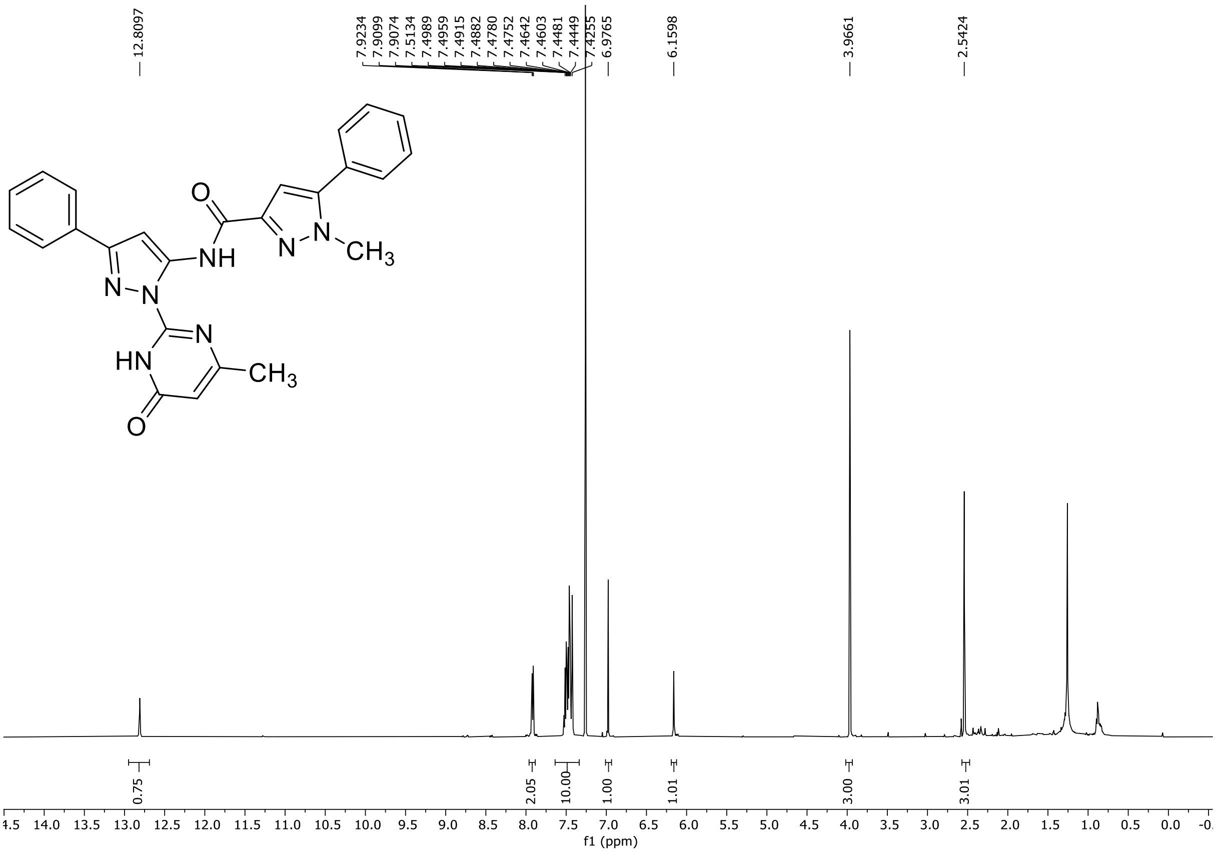

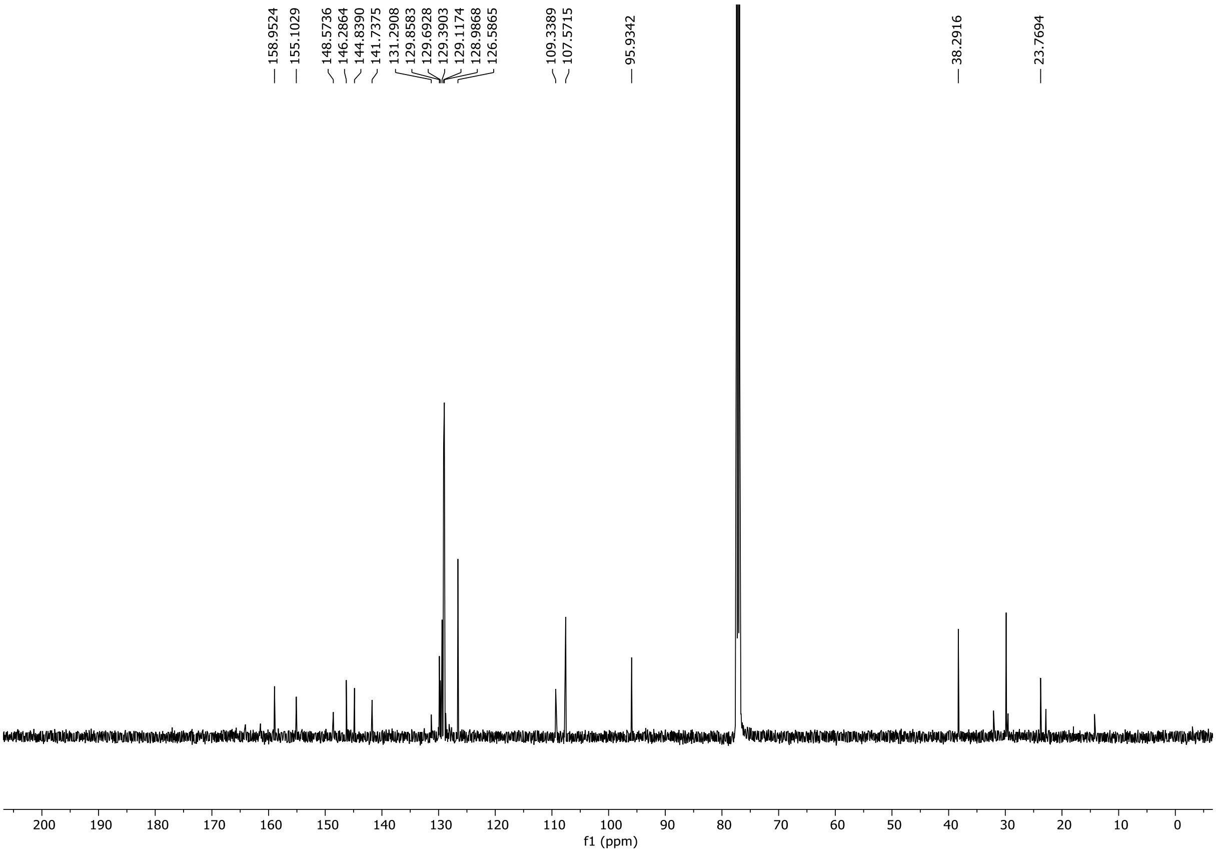

HRMS spectrum of **140**

NAR-A-43

$C_{25}H_{21}N_7O_2$
451.1757

mono m/z

APCI + (MMI)
nitrogen flow 5 L/min, gas temperature 300°C, nebulizer 45 psi, skimmer **65** V, vaporizer 200°C, fragmentor 30 V, dissolved in MeOH

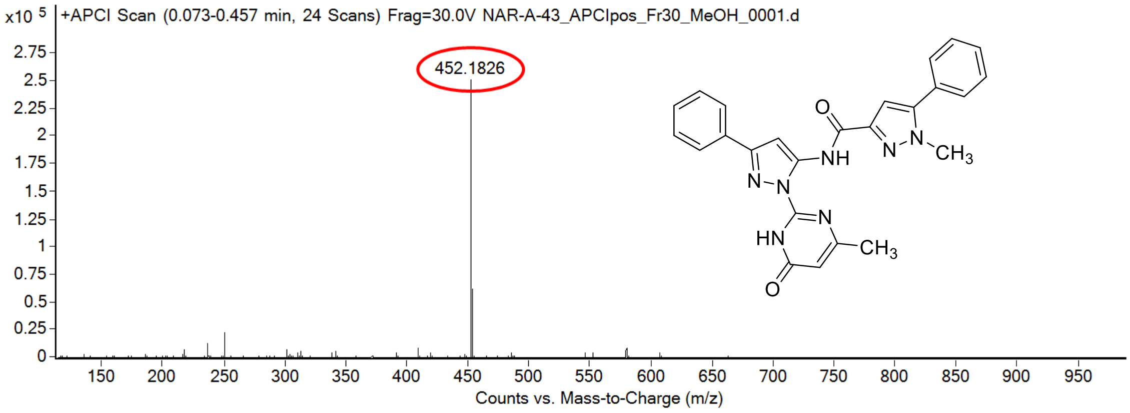

calculated mass: $[M+H]^+$ = 452.1829 observed: $[M+H]^+$ = 452.1826 mass accuracy = -0.7 ppm

FT-IR spectrum (neat) of **140**

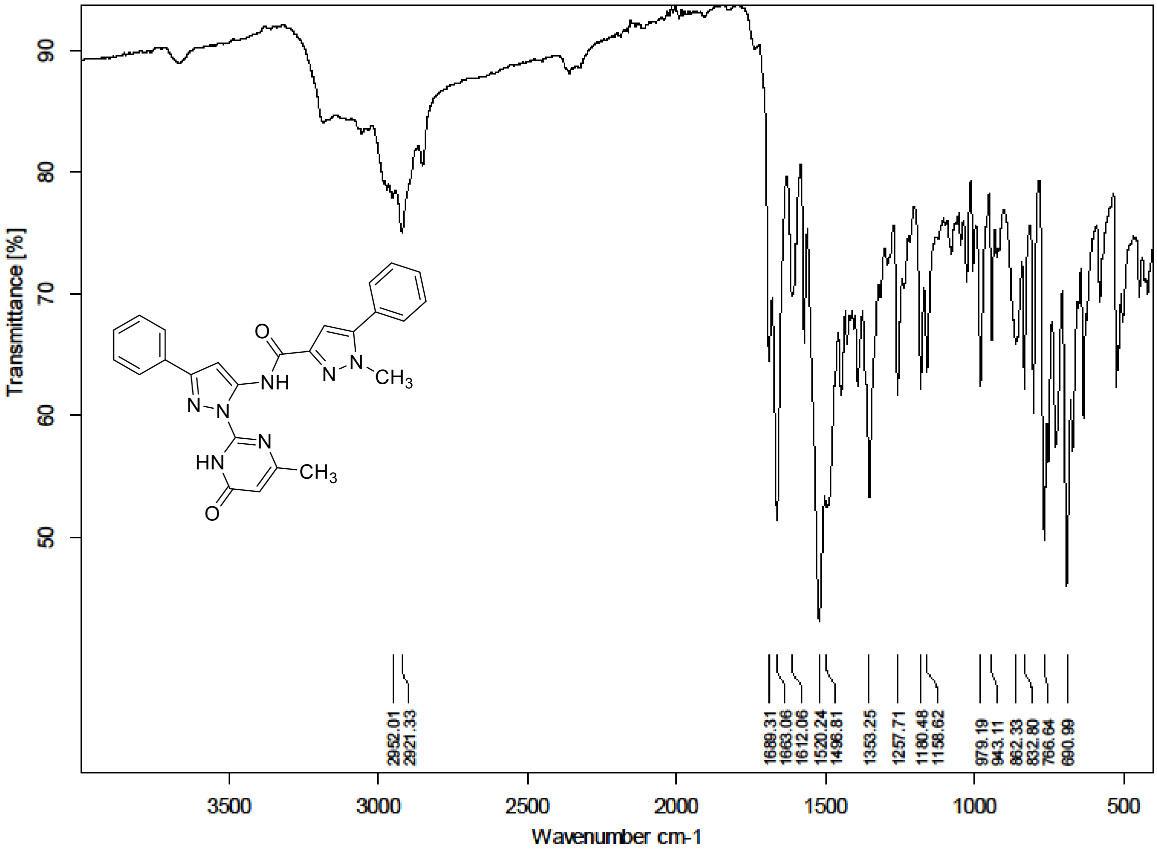

^{1}H (300 MHz) and ^{13}C NMR (176 MHz) spectra of **141** in DMSO-*d*

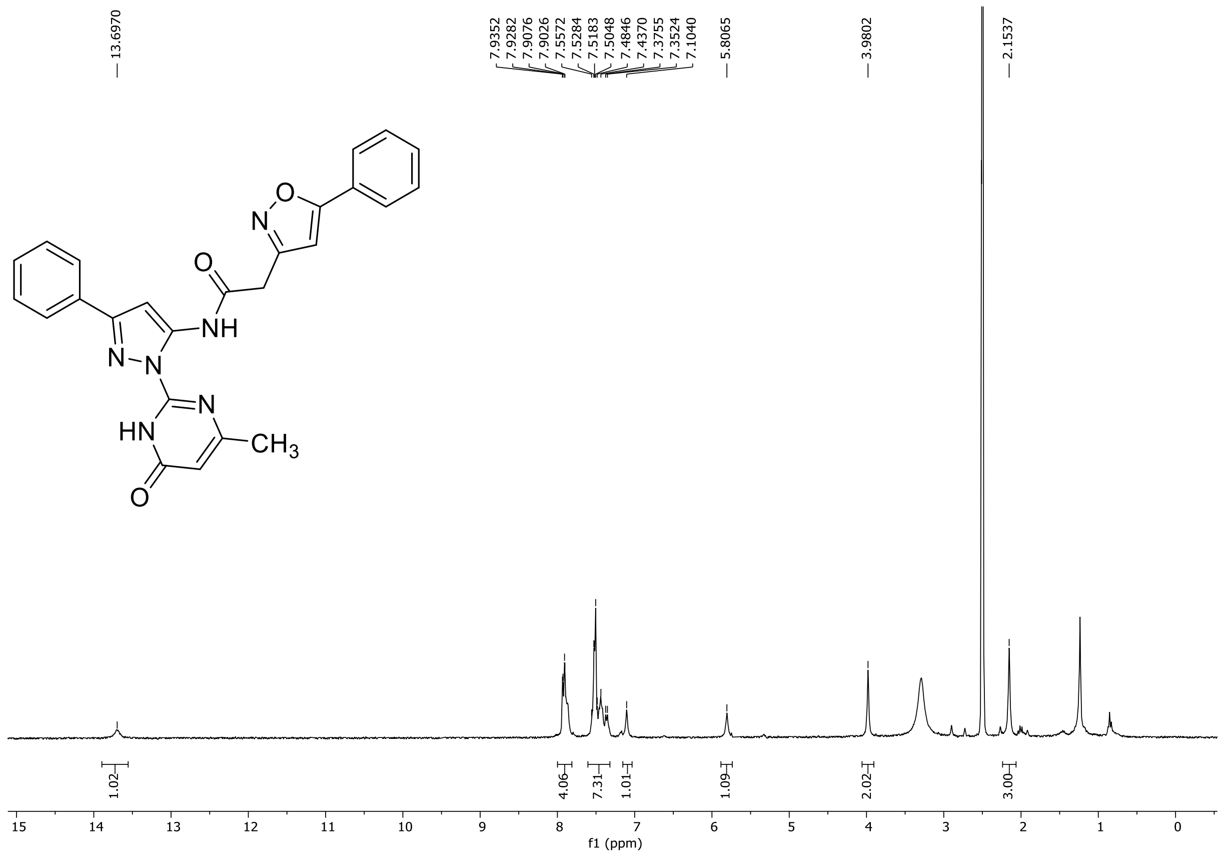

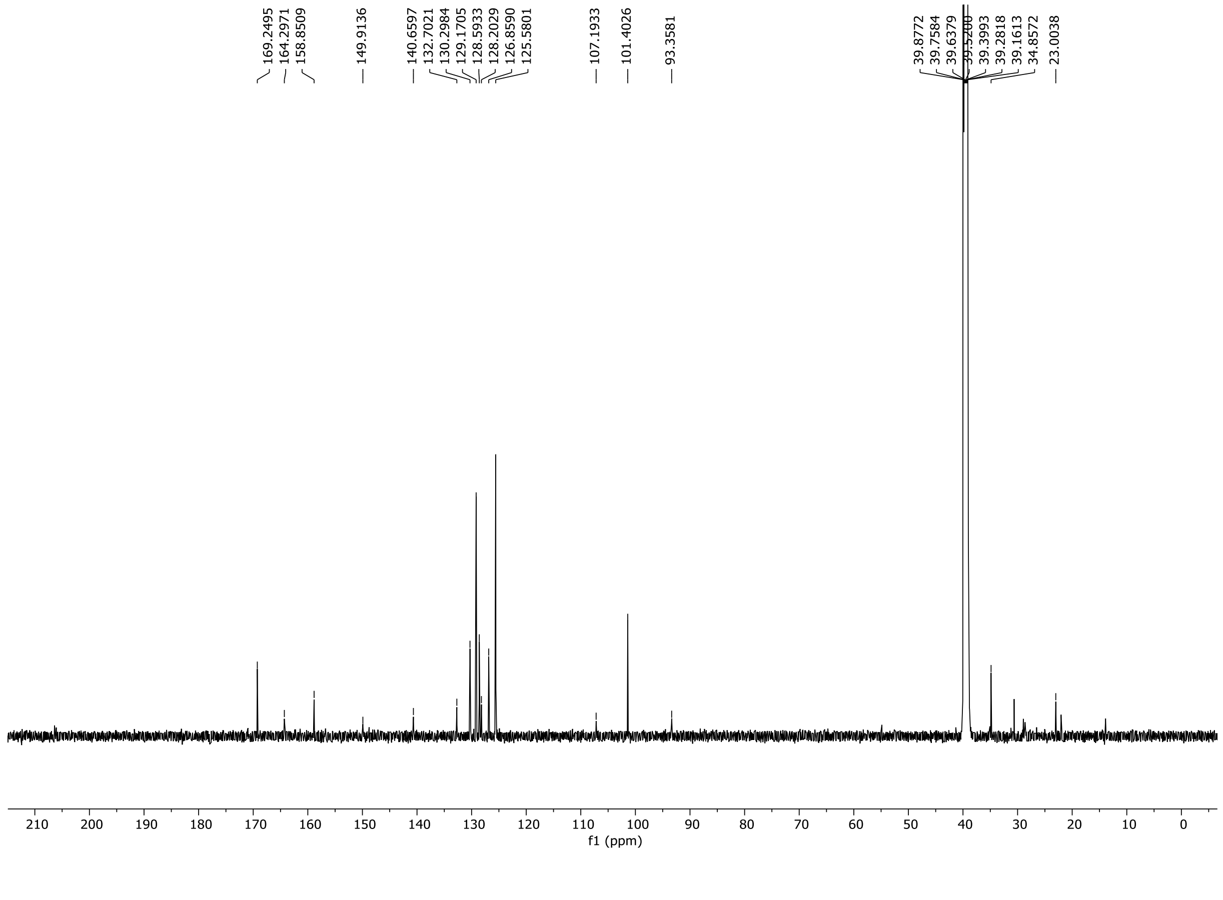

HRMS spectrum of **141**

NAR-A-159

$C_{25}H_{20}N_6O_3$ mono *m/z* 452.1597

APCI + (MMI)
nitrogen flow 5 L/min, gas temperature 325°C, nebulizer 45 psi, skimmer **65** V, vaporizer 200°C, fragmentor 30 V, dissolved in methanol

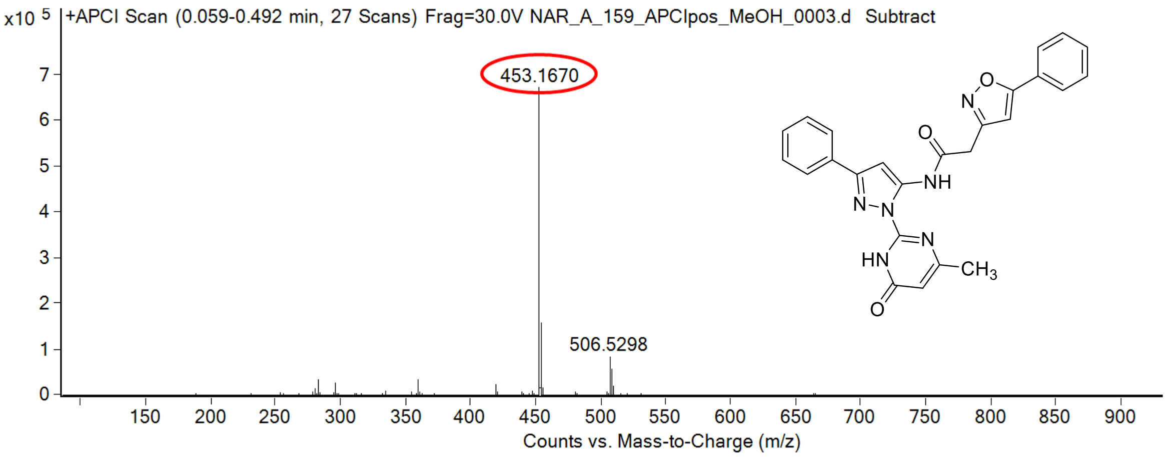

calculated mass: $[M+H]^+$ = 453.1670 observed: $[M+H]^+$ = 453.1670 mass accuracy = < 0.1 ppm

FT-IR spectrum (neat) of **141**

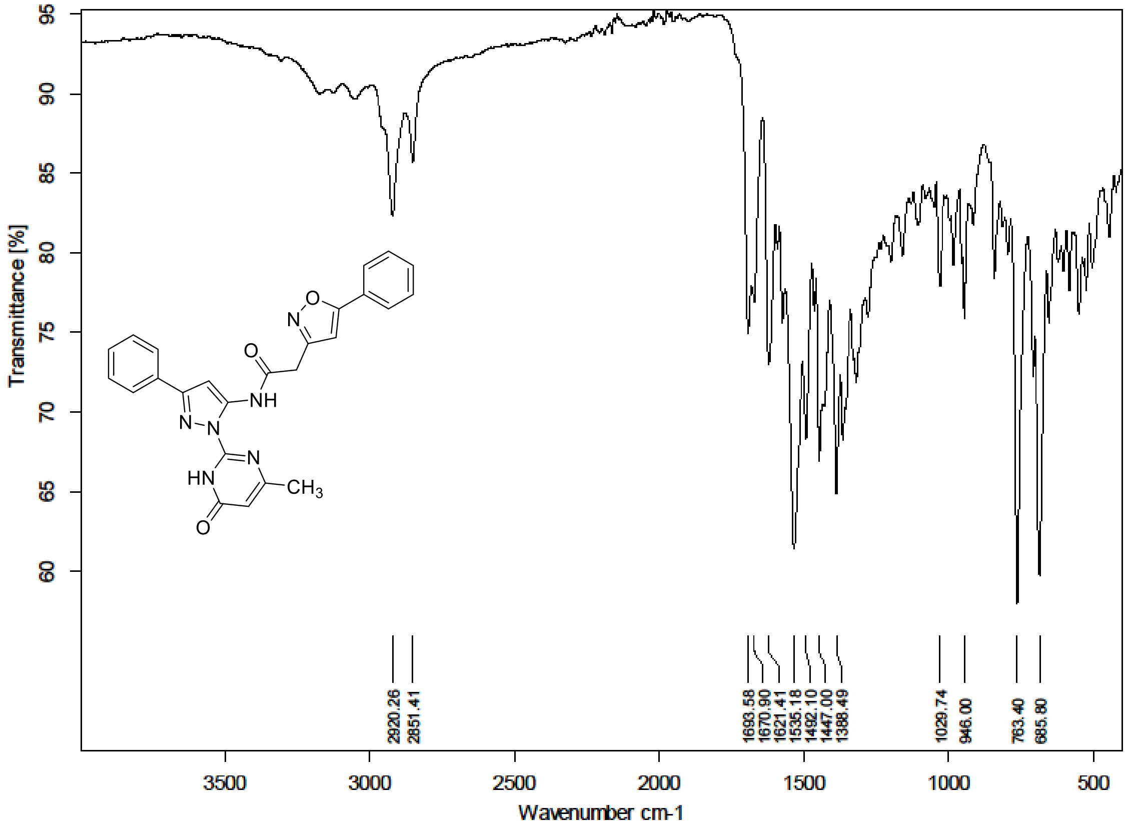

^{1}H NMR, ^{13}C NMR, HRMS and IR spectra of compound S191-S192

^{1}H (500 MHz) and ^{13}C NMR (126 MHz) spectra of **S191** in Chloroform-*d*

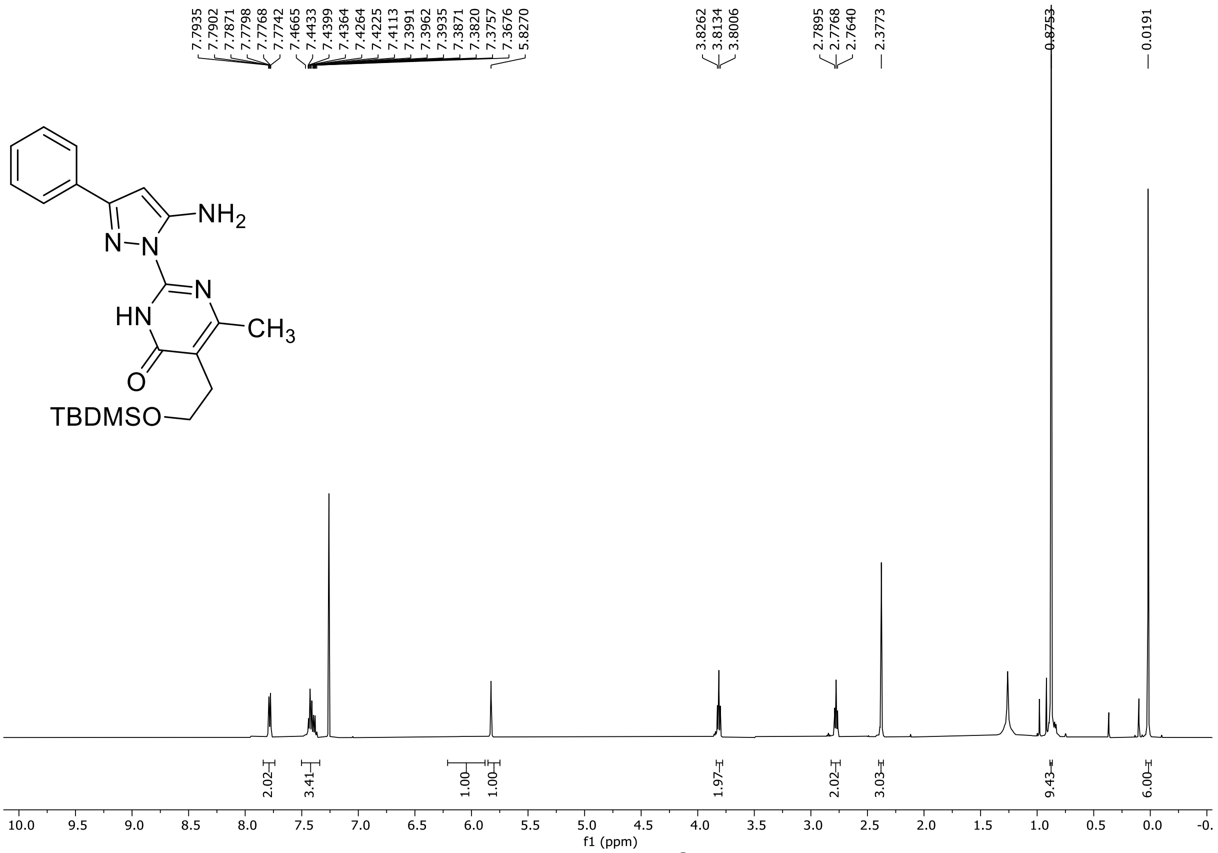

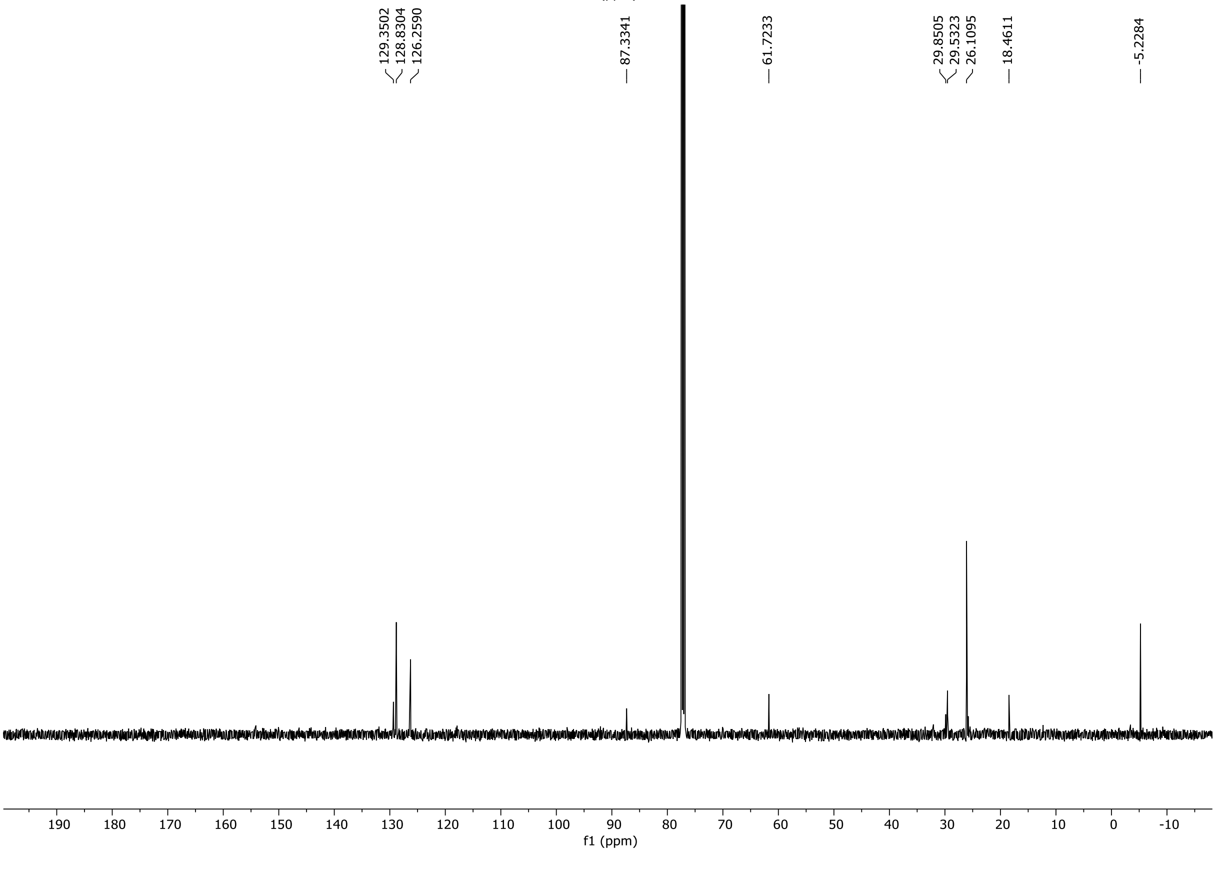

HRMS spectrum of **S191**

NAR-A-22 $C_{22}H_{31}N_5O_2Si$ **mono *m/z 425.2247***

APCI + (MMI)
nitrogen flow 5 L/min, gas temperature 325°C, nebulizer 45 psi, skimmer **65** V,
vaporizer 200°C, fragmentor 25 V, dissolved in methanol

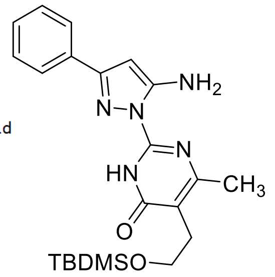

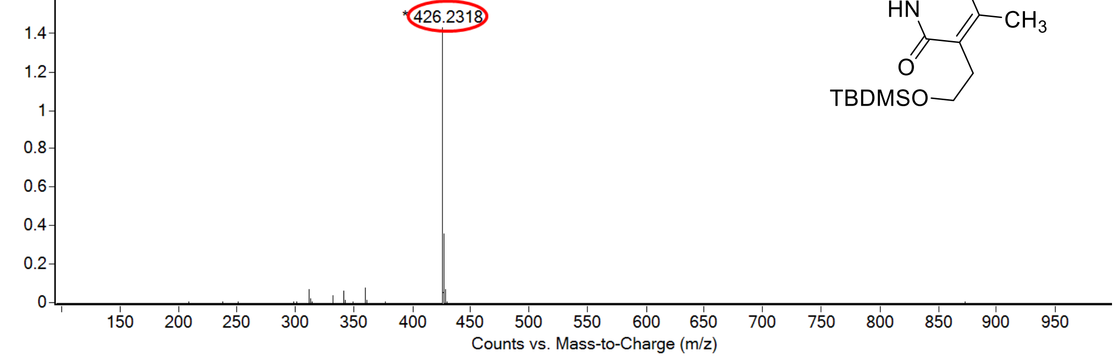

calculated mass: $[M+H]^+ = 426.2320$ observed: $[M+H]^+ = 426.2318$ mass accuracy = -0.5 ppm

FT-IR spectrum (neat) of **S191**

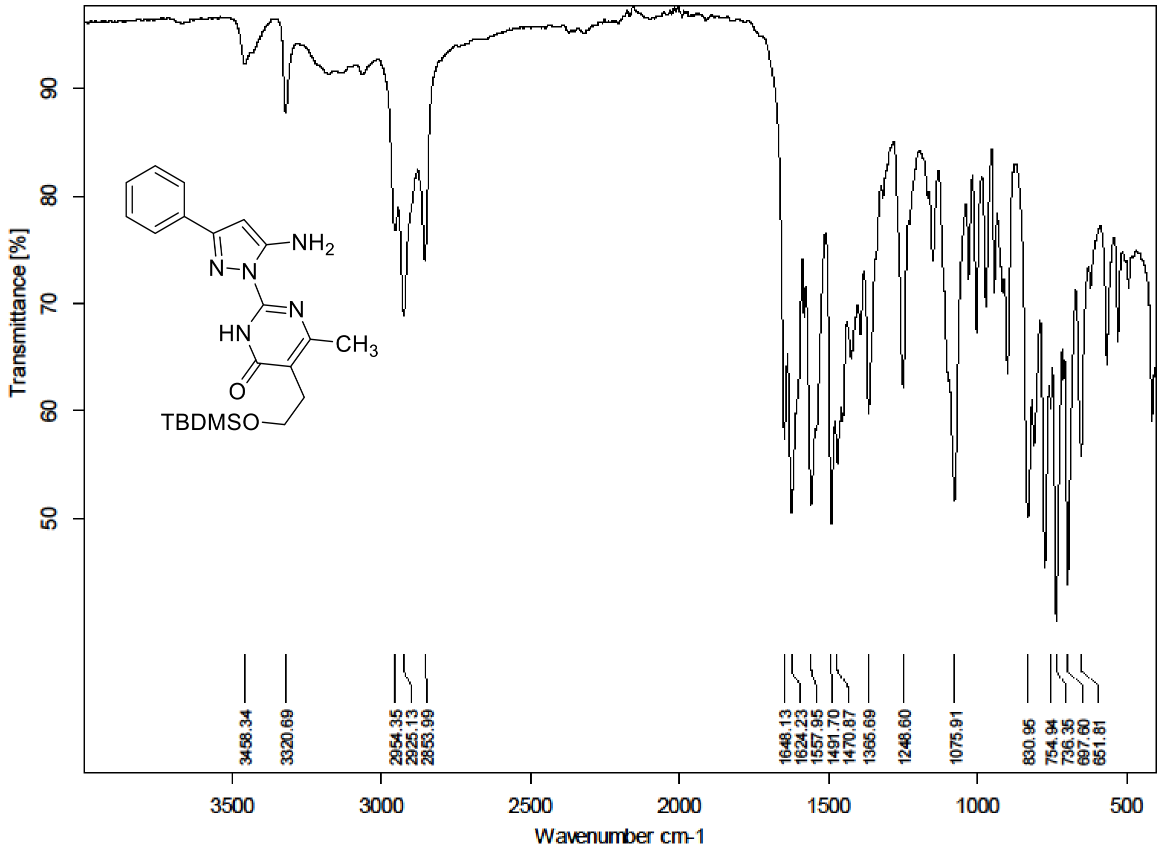

HRMS spectrum of **S192**

NAR-A-28 | $C_{32}H_{36}N_6O_4Si$ | **mono *m/z 596.2567***

APCI + (MMI)
nitrogen flow 5 L/min, gas temperature 325°C, nebulizer 45 psi, skimmer **65** V, vaporizer 200°C, fragmentor 30 V, dissolved in methanol

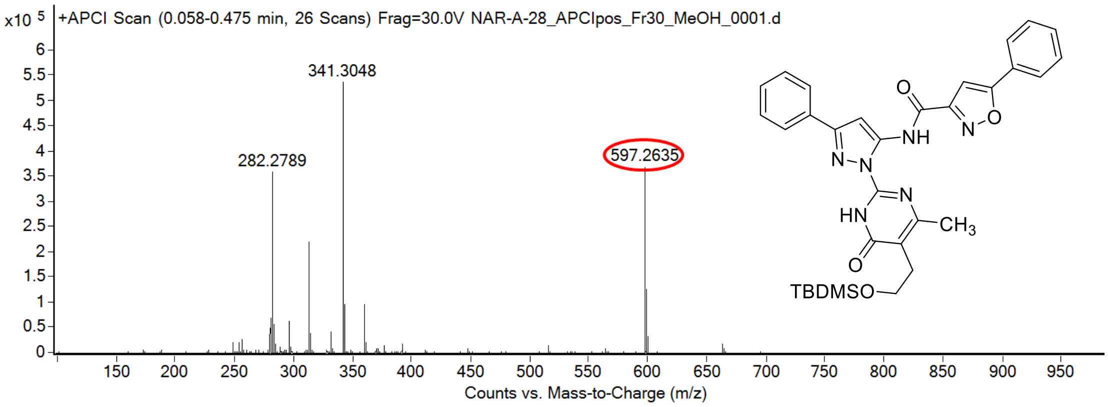

calculated mass: $[M+H]^+$ = 597.2640 observed: $[M+H]^+$ = 597.2635 mass accuracy = -0.8 ppm

^{1}H and ^{13}C NMR HRMS and IR spectra of compound 142

^{1}H (500 MHz) and ^{13}C NMR (126 MHz) spectra of **142** in DMSO-d_6

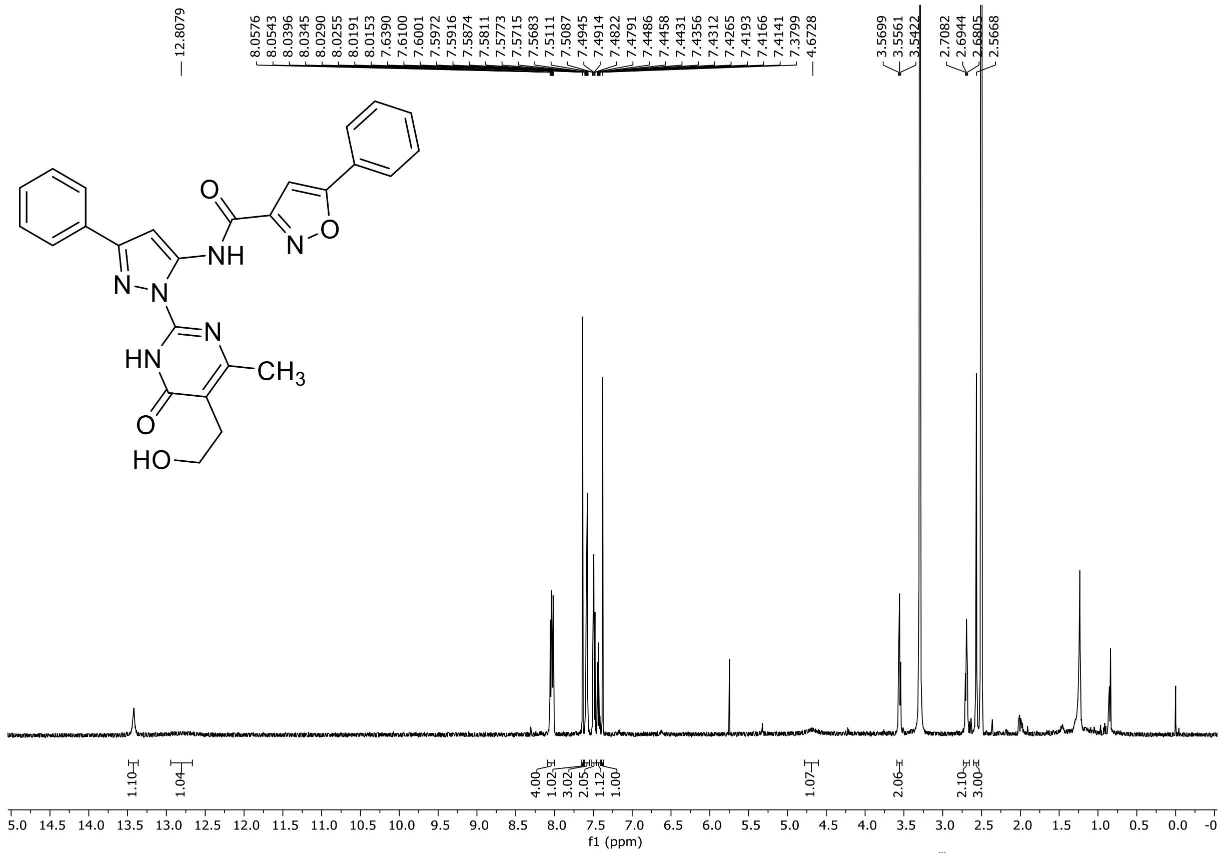

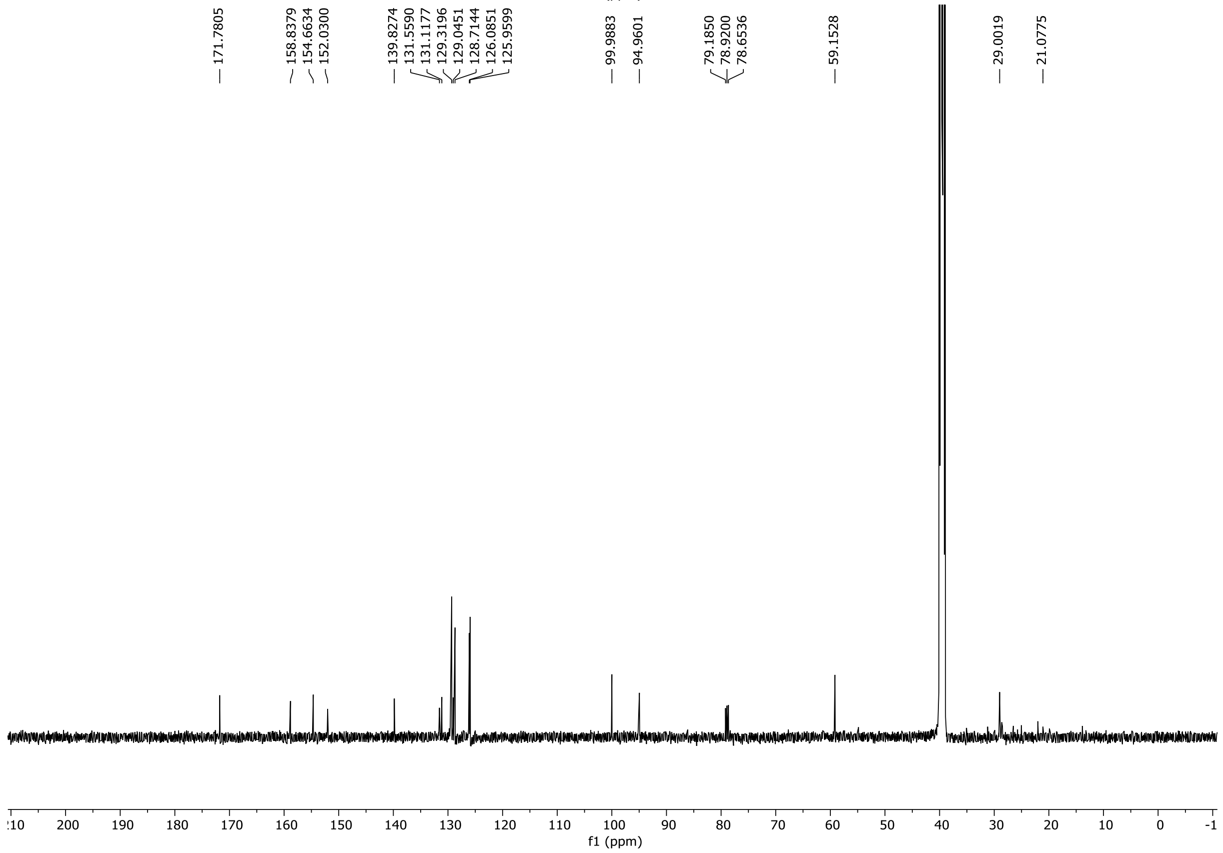

HRMS spectrum of **142**

NAR-A-32 $C_{26}H_{22}N_6O_4$ mono *m/z 482.1703*

APCI + (MMI)
nitrogen flow 5 L/min, gas temperature 325°C, nebulizer 45 psi, skimmer **65** V,
vaporizer 200°C, fragmentor 25 V, dissolved in methanol

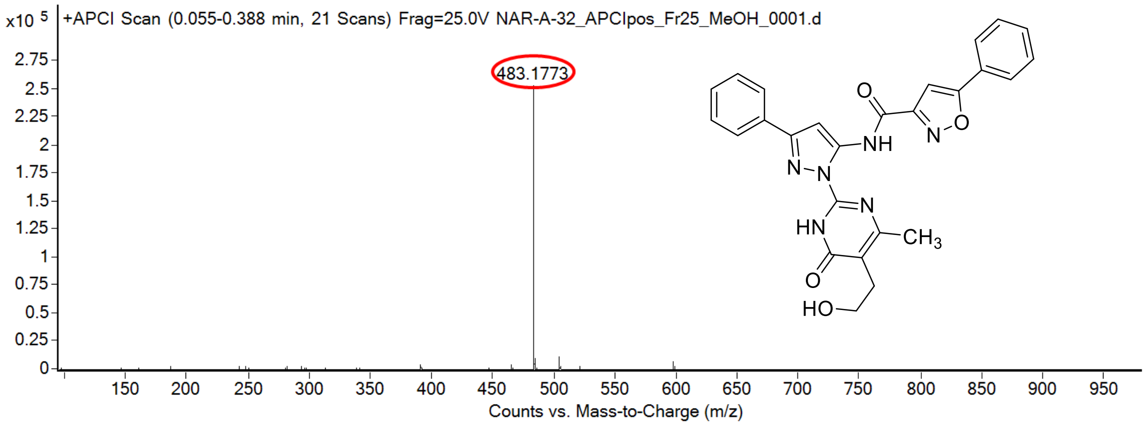

calculated mass: $[M+H]^+$ = 483.1775 observed: $[M+H]^+$ = 483.1773 mass accuracy = -0.4 ppm

FT-IR spectrum (neat) of **142**

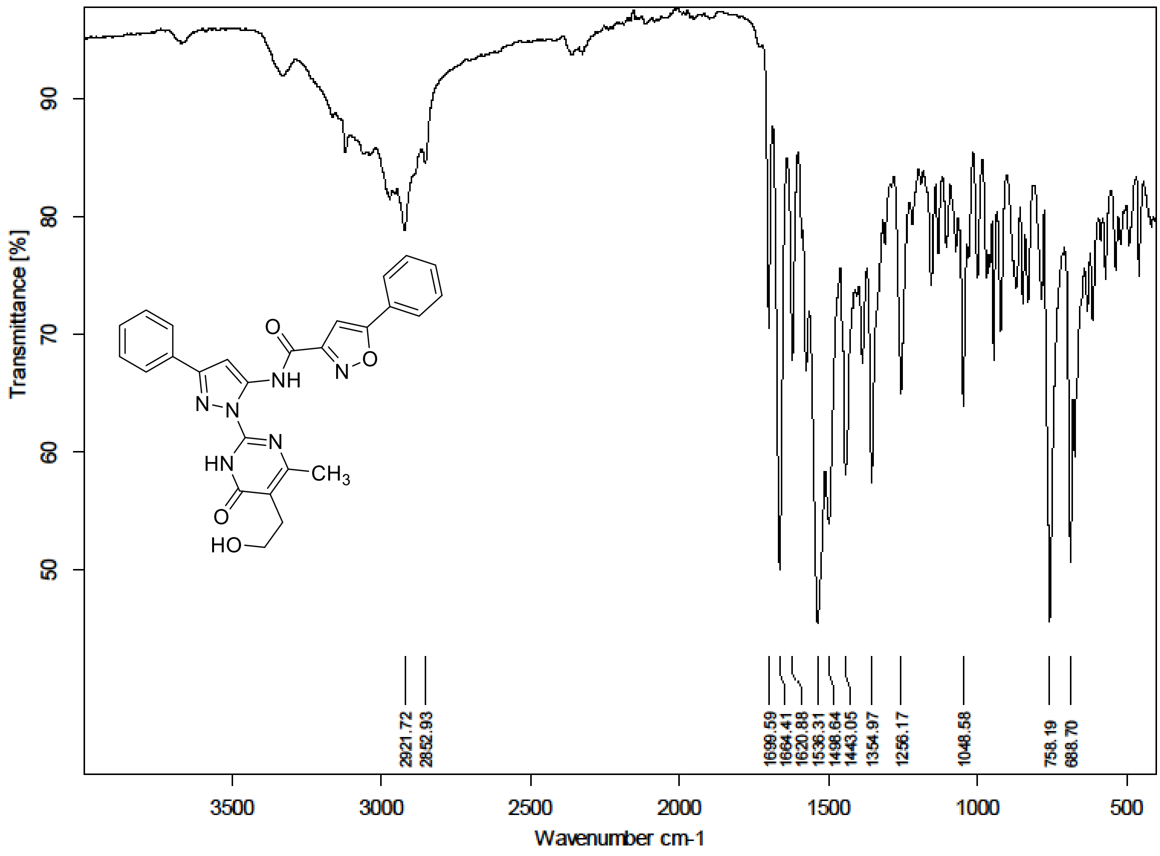

^{1}H and ^{13}C NMR HRMS and IR spectra of compound S193-S194

^{1}H (300 MHz) and ^{13}C NMR (126 MHz) spectra of **S193** in Chloroform-*d*

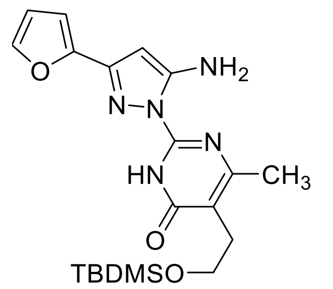

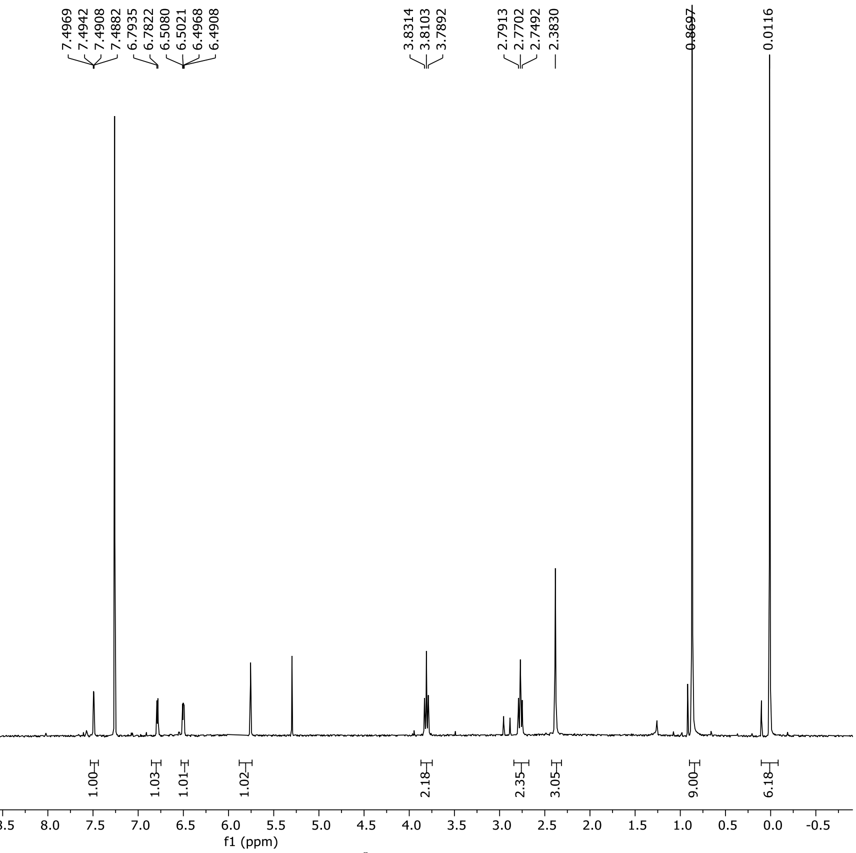

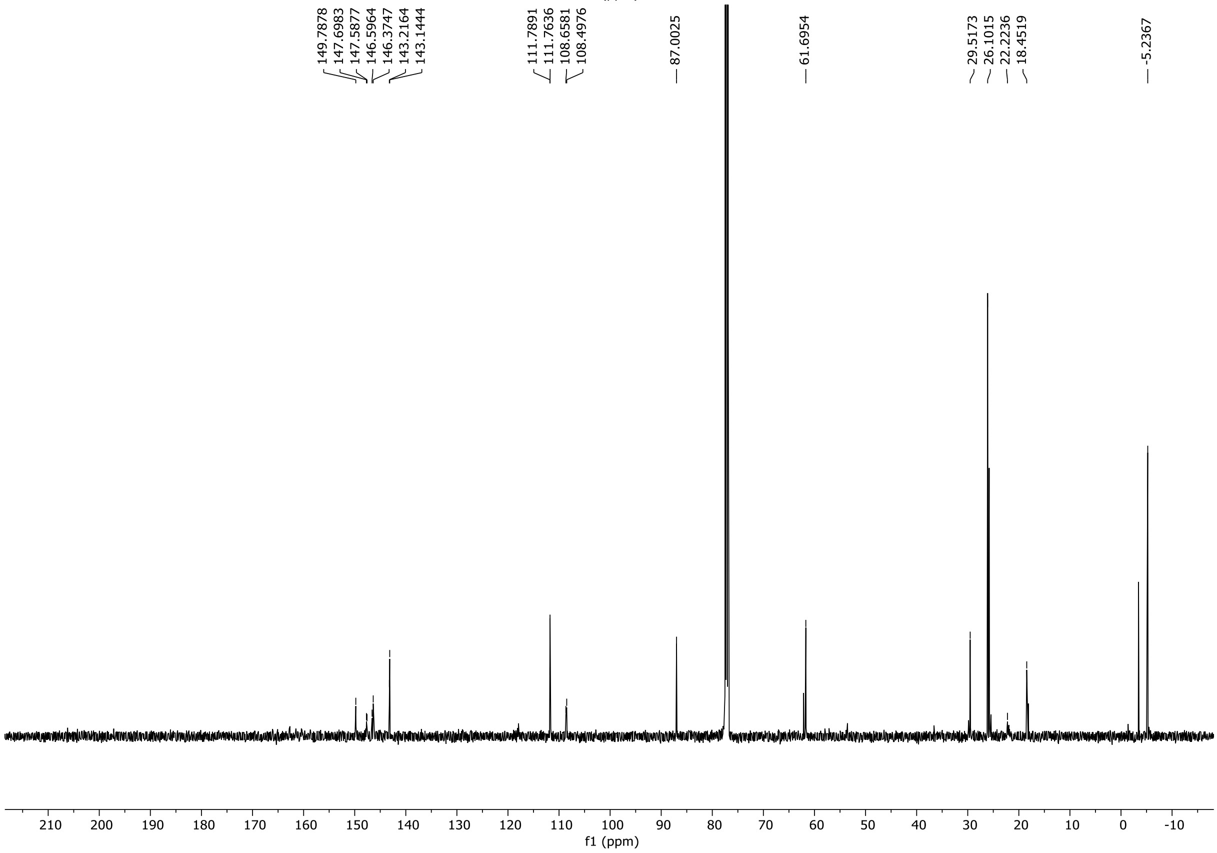

HRMS spectrum of **S193**

NAR-A-103 $C_{20}H_{29}N_5O_3Si$ ***m/z*** **415.2040**

APCI+ (MMI)
nitrogen flow 5 L/min, gas temperature 325°C, nebulizer 45 psi, skimmer 65 V, vaporizer 200°C, fragmentor 10 V, dissolved in MeOH

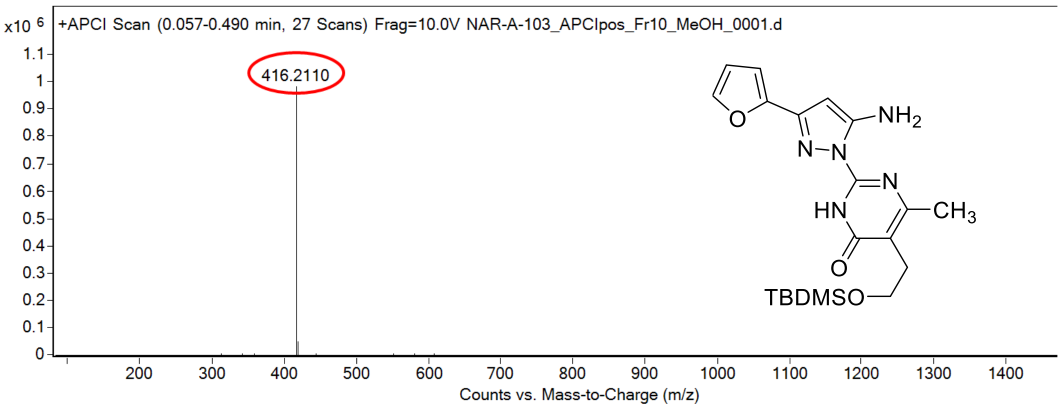

calculated mass: [M+H]+ = 416.2112 observed: [M+H]+ = 416.2110 mass accuracy = -0.5 ppm

FT-IR spectrum (neat) of **S193**

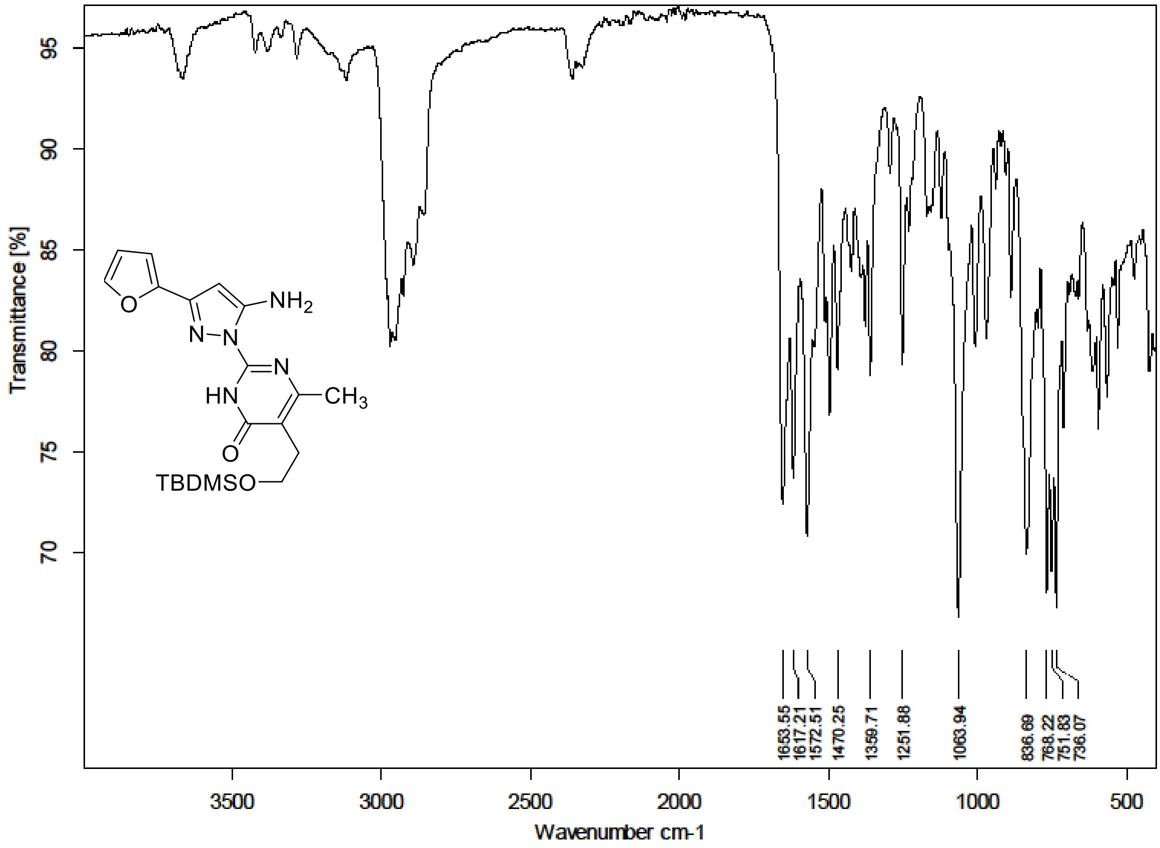

^{1}H (500 MHz) and ^{13}C NMR (126 MHz) spectra of **S194** in Chloroform-*d*

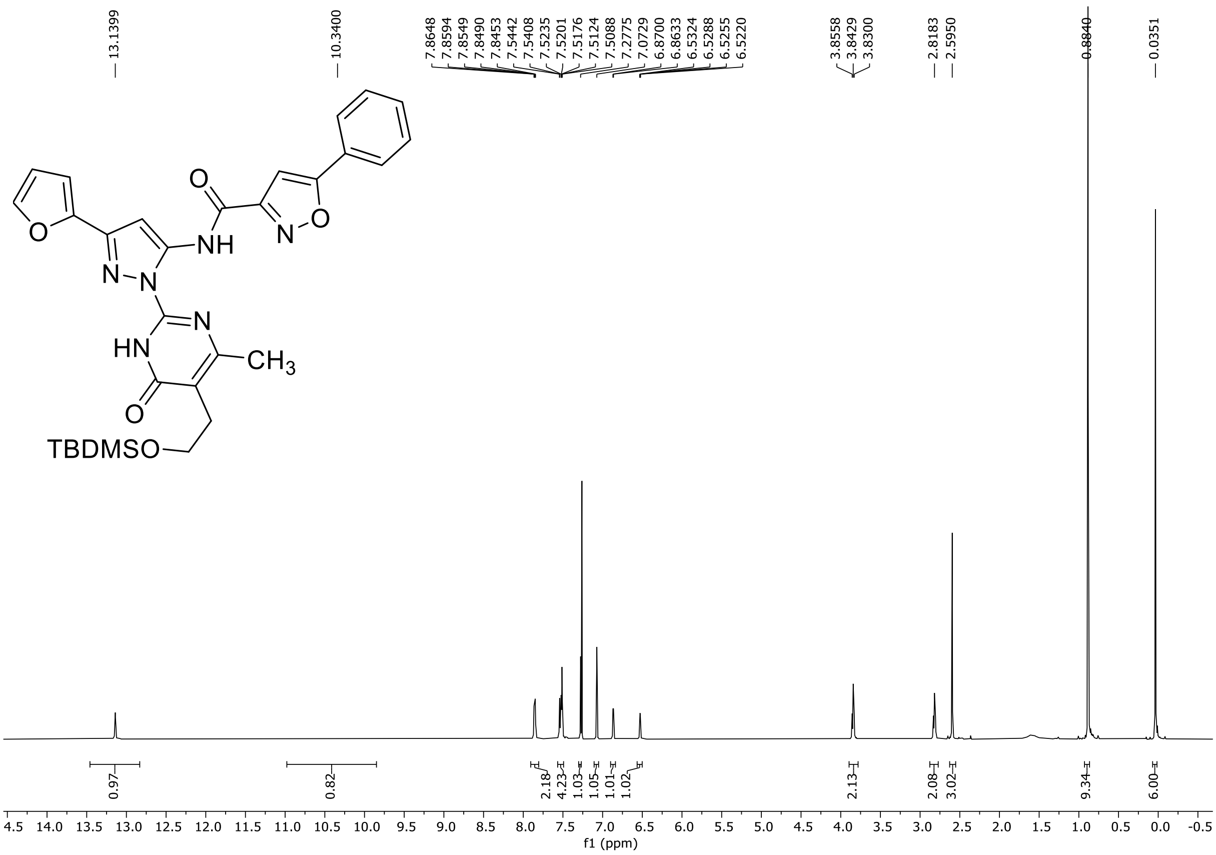

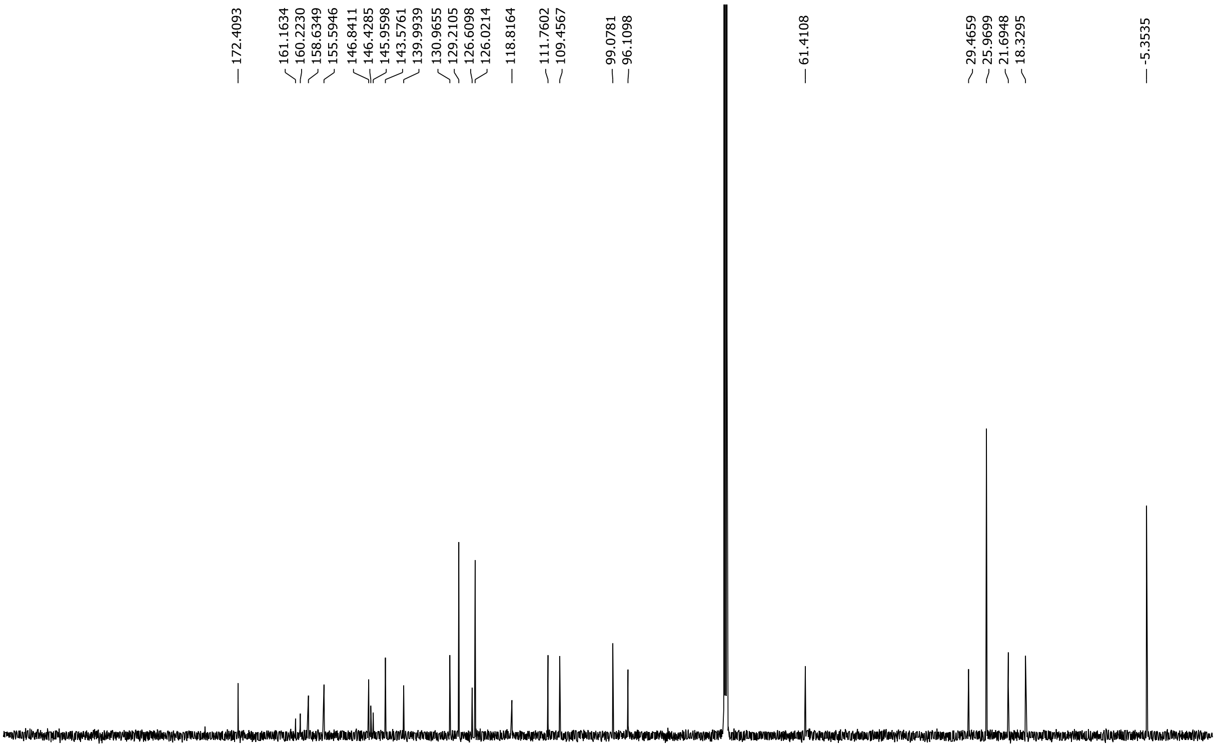

HRMS spectrum of **S194**

NAR-A-105 $C_{30}H_{34}N_6O_5Si$ *m/z* **586.2360**

APCI+ (MMI)
nitrogen flow 5 L/min, gas temperature 325°C, nebulizer 45 psi, skimmer 65 V, vaporizer 200°C, fragmentor 25 V, dissolved in MeOH

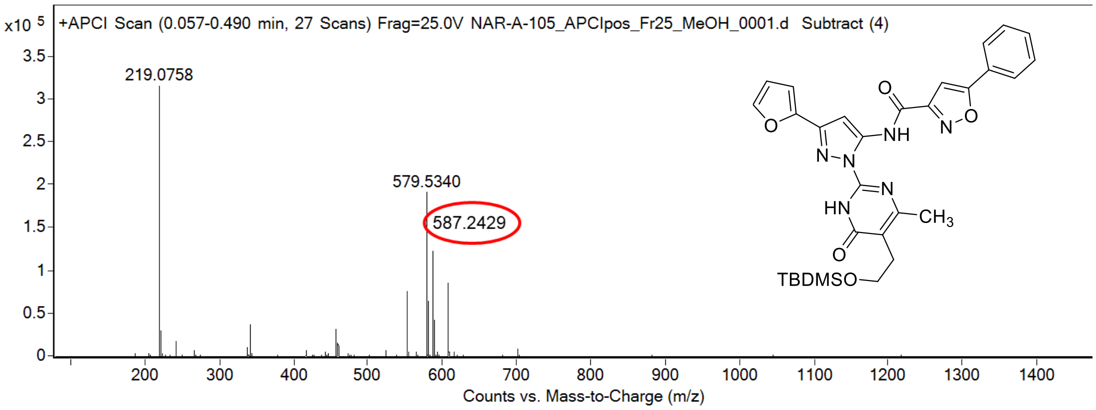

calculated mass: $[M+H]^+$ = 587.2433 observed: $[M+H]^+$ = 587.2429 mass accuracy = -0.7 ppm

FT-IR spectrum (neat) of **S194**

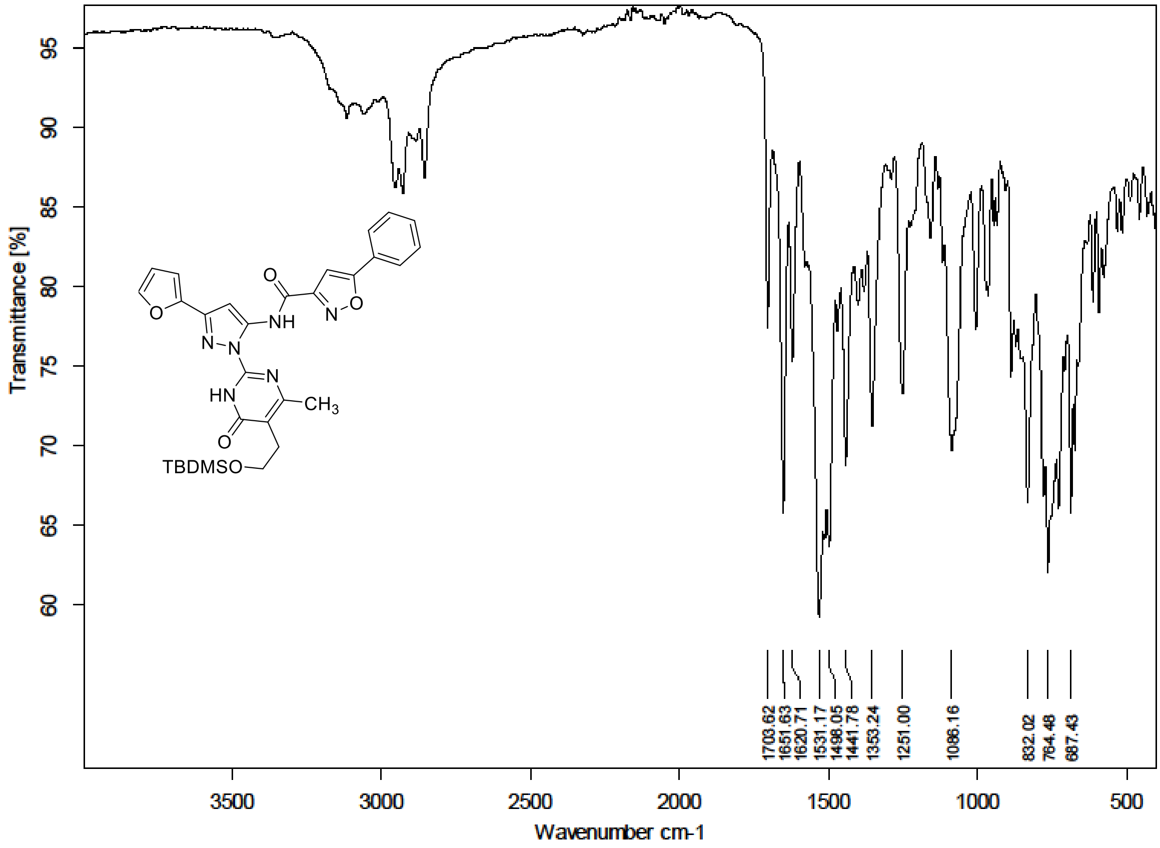

^{1}H and ^{13}C NMR, HRMS and IR spectra of compound 143

^{1}H (300 MHz) and ^{13}C NMR (75 MHz) spectra of **143** in DMSO-d_6

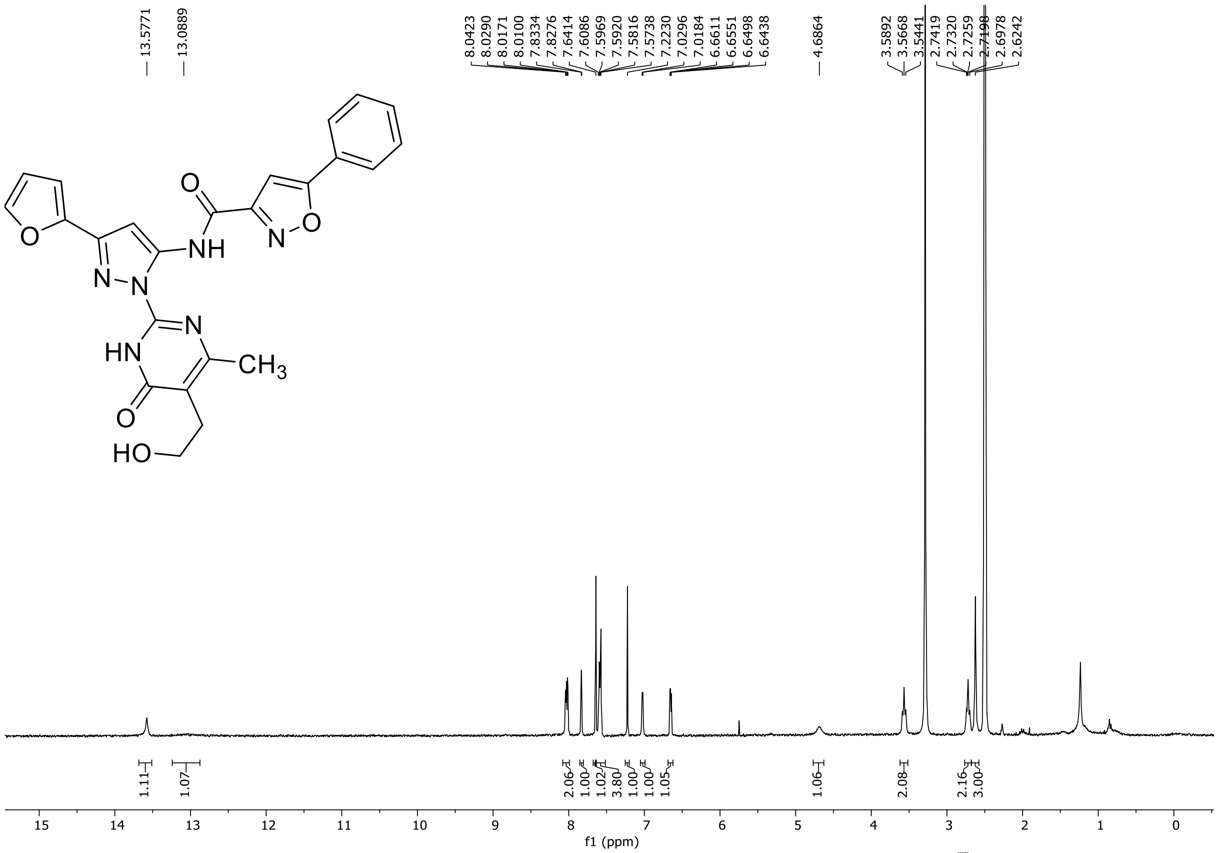

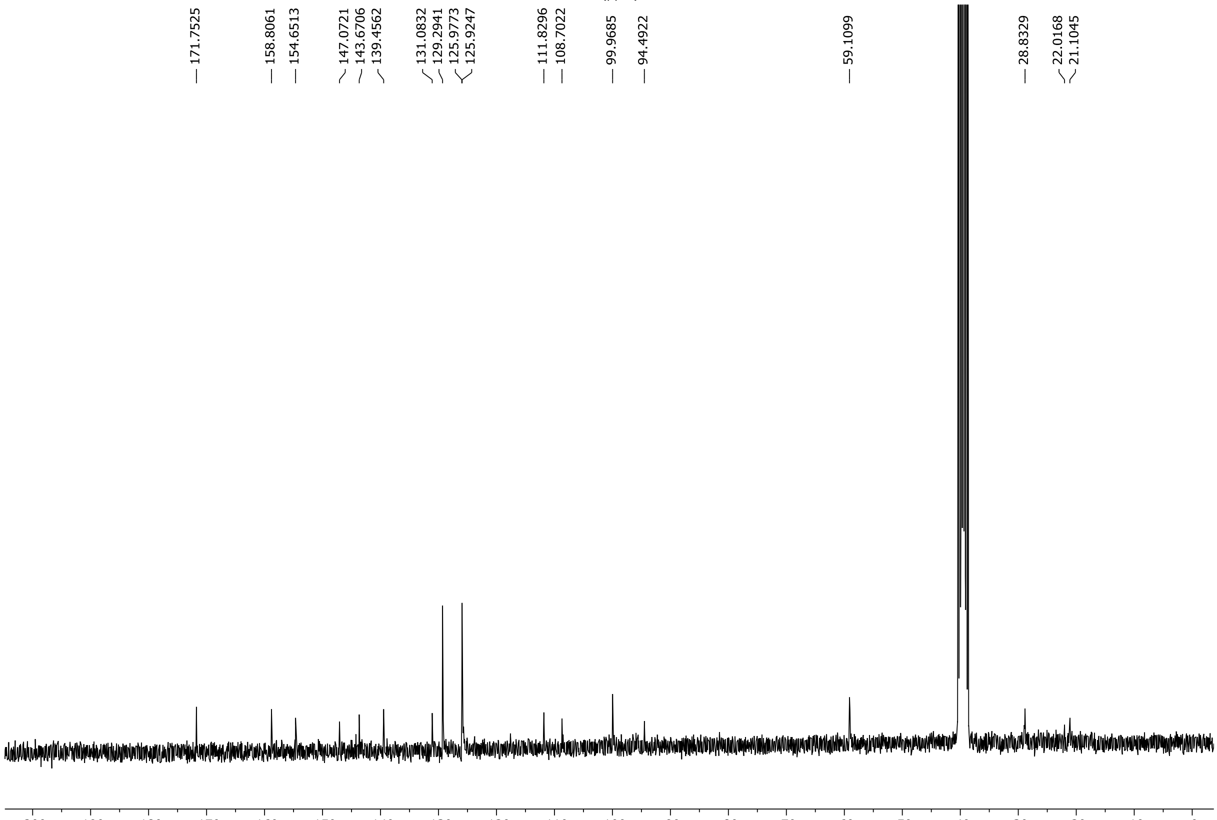

HRMS spectrum of **143**

NAR-A-126 $C_{24}H_{20}N_6O_5$ *m/z* **472.1495**

ESI- (MMI)
nitrogen flow 5 L/min, gas temperature 325°C, nebulizer 45 psi, skimmer 65 V, fragmentor 120 V, dissolved in DMSO, MeOH

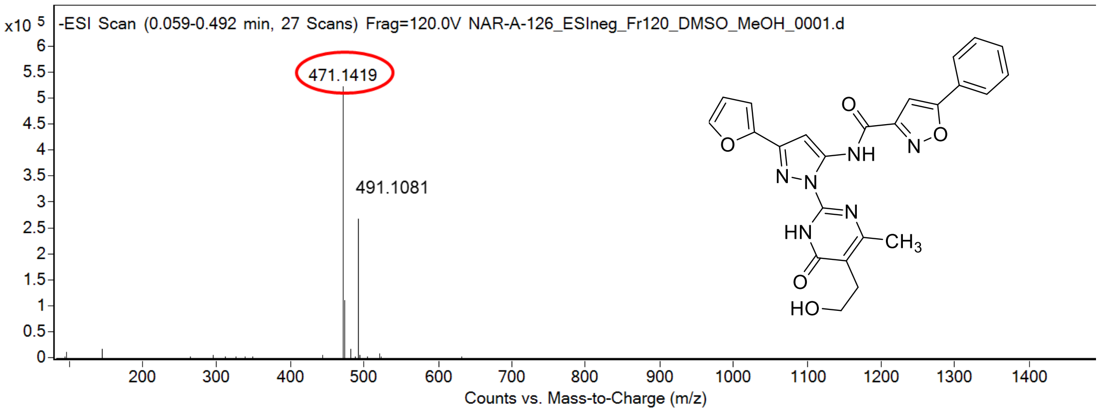

calculated mass: $[M\text{-}H]^-$ = 471.1422 observed: $[M\text{-}H]^-$ = 471.1419 mass accuracy = -0.6 ppm

FT-IR spectrum (neat) of **143**

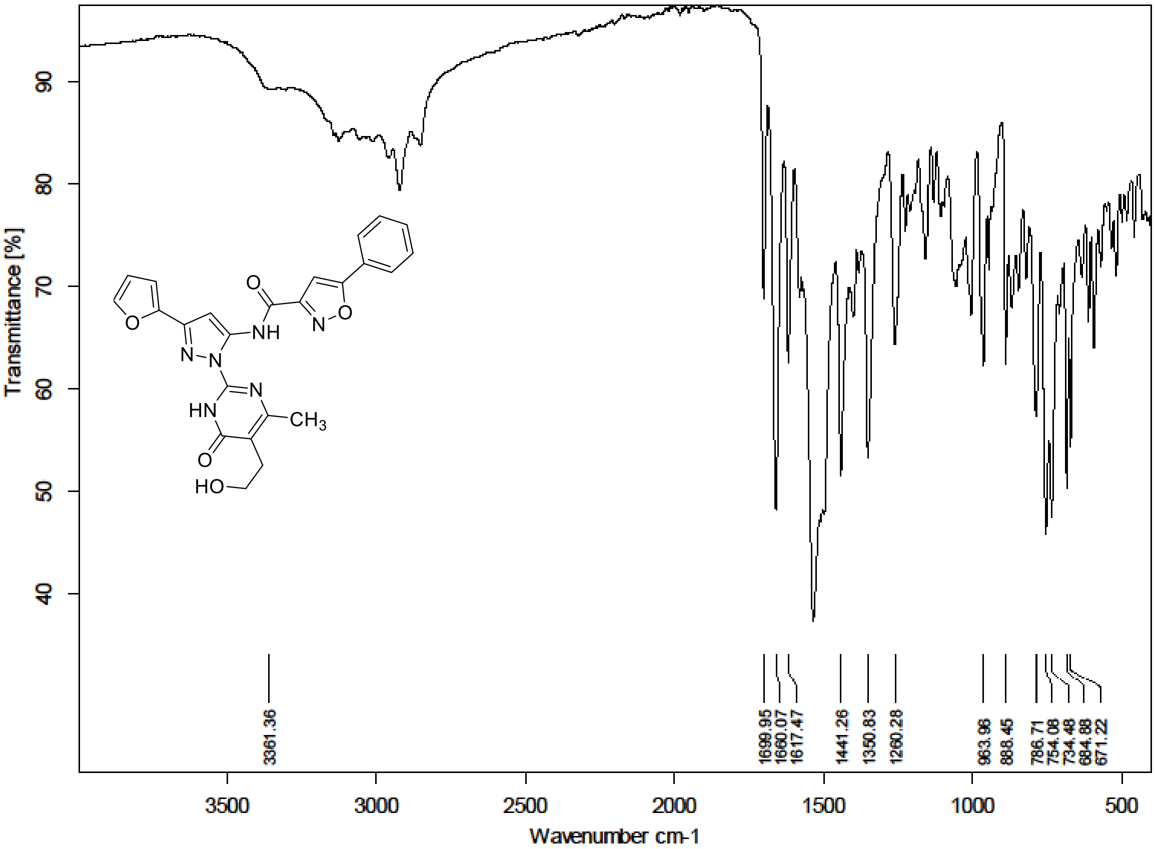

HPLC analytical chromatogram of compound 18

Instrument	: Ultimate 3000 LC Analytical Systems, Thermo Scientific
Column	: Agilent ZORBAX Eclipse Plus C18 (particle size 5 µm, 4.6 × 250 mm)
Sample preparation	: 1 mg of compound **18** dissolved in methanol (1 mL) + 10 µL TFA
Injection volume	: 10 µL
Flow rate	: 1 mL/min
Mobile phase	: water:methanol = 10:90 + 0.1% Trifluoroacetic acid (Isocratic)
Retention times	: 3.68 min.

Chromatogram and Results

Injection Details

Compound Name:	**MU262=18**	*Run Time (min):*	**9.58**
Vial Number:	**129**	*Injection Volume:*	**10.00**
Mobile phase:	**Water:MeOH + 0.1% TFA = 10:90**		
Mobile phase:	**10:90 isocretic**	*Channel:*	**UV_VIS_3**
Column:	**Agilent ZORBAX Eclipse Plus C18, 5um, 4.60 X 25(**	*Wavelength:*	**337**
Instrument Method:	**PK analytical method_Isocratic**	*Flow rate:*	**1 mL/min**
Processing Method:	**PK processing method**	*Dilution Factor:*	**1.0000**
Injection Date/Time:	**25/Jul/25 03:34**	*Sample Weight:*	**1 mg**

Chromatogram

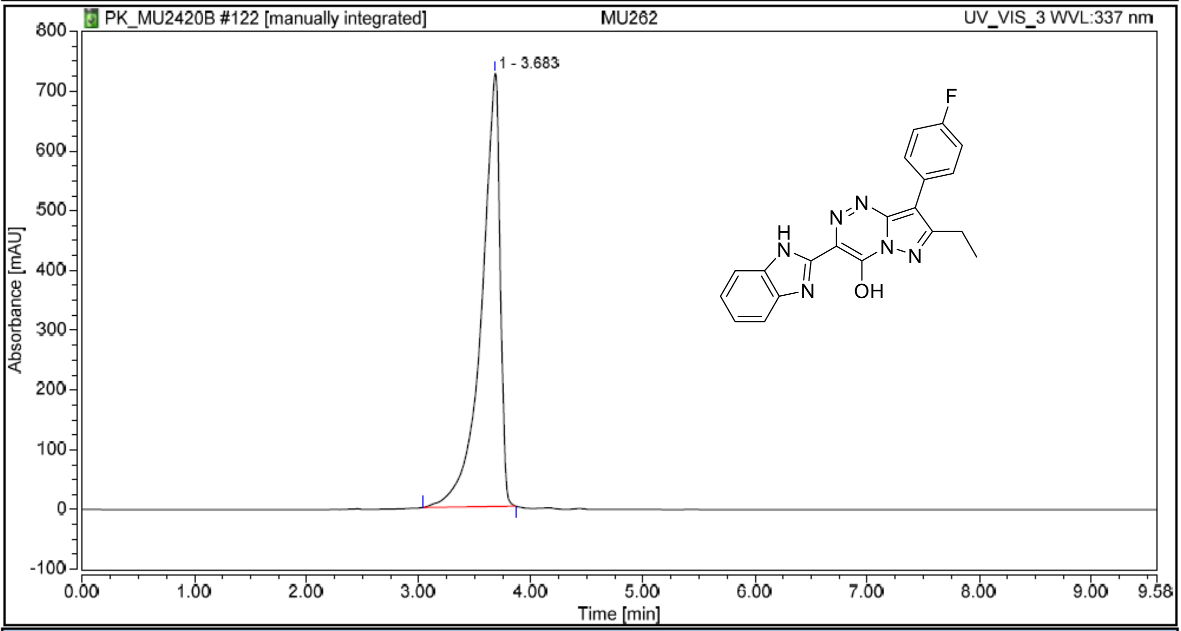

Integration Results

No.	Peak Name	Retention Time min	Area mAU*min	Height mAU	Relative Area %	Relative Height %	Amount n.a.
1		3.683	138.375	725.147	100.00	100.00	n.a.
**Total:**			**138.375**	**725.147**	**100.00**	**100.00**	

HPLC analytical chromatogram of compound 32

Instrument	: Ultimate 3000 LC Analytical Systems, Thermo Scientific
Column	: Agilent ZORBAX Eclipse Plus C18 (particle size 5 µm, 4.6 × 250 mm)
Sample preparation	: 1 mg of compound **32** dissolved in methanol (1 mL) + 10 µL TFA
Injection volume	: 10 µL
Flow rate	: 1 mL/min
Mobile phase	: water:methanol = 10:90 + 0.1% Trifluoroacetic acid (Isocratic)
Retention times	: 2.99 min.

Chromatogram and Results

Injection Details

Compound Name:	MU876=32	*Run Time (min):*	6.20
Vial Number:	136	*Injection Volume:*	10.00
Mobile phase:	Water:MeOH + 0.1% TFA = 10:90		
Mobile phase:	10:90 isocretic	*Channel:*	UV_VIS_1
Column:	Agilent ZORBAX Eclipse Plus C18, 5um, 4.60 X 25(	*Wavelength:*	270
Instrument Method:	PK analytical method_Isocratic	*Flow rate:*	1 mL/min
Processing Method:	PK processing method	*Dilution Factor:*	1.0000
Injection Date/Time:	25/Jul/25 04:53	*Sample Weight:*	1 mg

Chromatogram

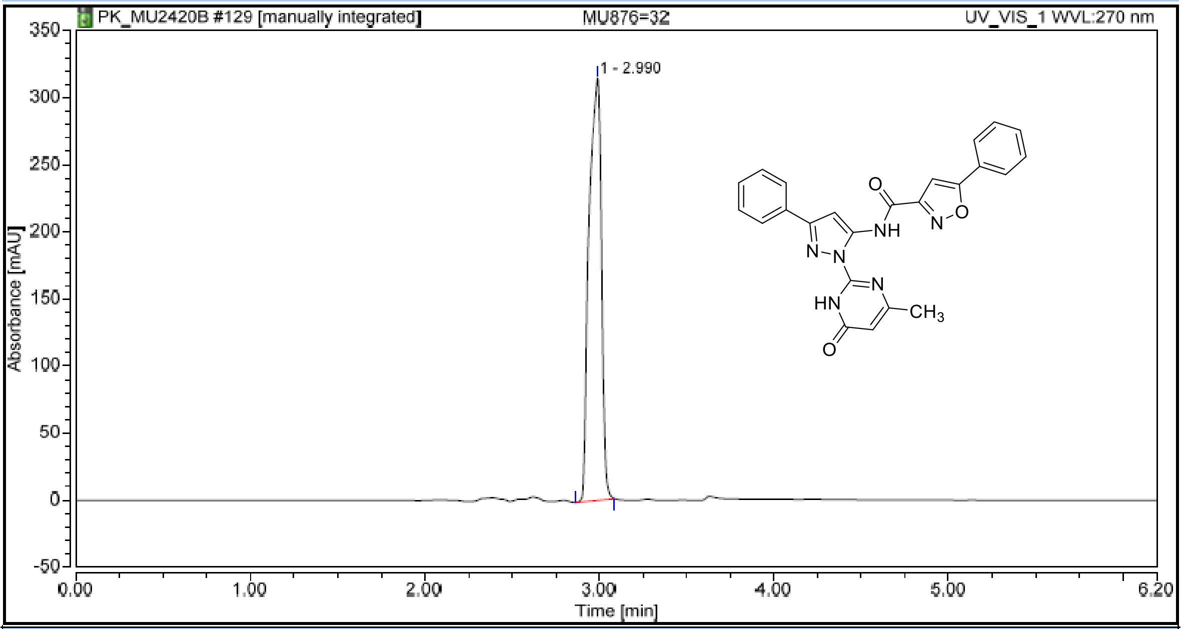

Integration Results

No.	Peak Name	Retention Time min	Area mAU*min	Height mAU	Relative Area %	Relative Height %	Amount n.a.
1		2.990	26.190	314.973	100.00	100.00	n.a.
Total:			26.190	314.973	100.00	100.00	
